# Amyotrophic lateral sclerosis, gene deregulation in the anterior horn of the spinal cord and frontal cortex area 8: implications in frontotemporal lobar degeneration

**DOI:** 10.18632/aging.101195

**Published:** 2017-03-09

**Authors:** Pol Andrés-Benito, Jesús Moreno, Ester Aso, Mónica Povedano, Isidro Ferrer

**Affiliations:** ^1^ Institute of Neuropathology, Pathologic Anatomy Service, Bellvitge University Hospital, IDIBELL, Hospitalet de Llobregat, Spain; ^2^ Service of Neurology, Bellvitge University Hospital, Hospitalet de Llobregat, Spain; ^3^ Department of Pathology and Experimental Therapeutics, University of Barcelona, Spain; ^4^ Institute of Neurosciences, University of Barcelona, Barcelona, Spain; ^5^ Biomedical Network Research Center on Neurodegenerative Diseases (CIBERNED), Institute Carlos III, Hospitalet de Llobregat, Spain

**Keywords:** amyotrophic lateral sclerosis, frontal cortex, spinal cord, frontotemporal lobar degeneration, excitotoxicity, neuroinflammation

## Abstract

Transcriptome arrays identifies 747 genes differentially expressed in the anterior horn of the spinal cord and 2,300 genes differentially expressed in frontal cortex area 8 in a single group of typical sALS cases without frontotemporal dementia compared with age-matched controls. Main up-regulated clusters in the anterior horn are related to inflammation and apoptosis; down-regulated clusters are linked to axoneme structures and protein synthesis. In contrast, up-regulated gene clusters in frontal cortex area 8 involve neurotransmission, synaptic proteins and vesicle trafficking, whereas main down-regulated genes cluster into oligodendrocyte function and myelin-related proteins. RT-qPCR validates the expression of 58 of 66 assessed genes from different clusters. The present results: a. reveal regional differences in de-regulated gene expression between the anterior horn of the spinal cord and frontal cortex area 8 in the same individuals suffering from sALS; b. validate and extend our knowledge about the complexity of the inflammatory response in the anterior horn of the spinal cord; and c. identify for the first time extensive gene up-regulation of neurotransmission and synaptic-related genes, together with significant down-regulation of oligodendrocyte- and myelin-related genes, as important contributors to the pathogenesis of frontal cortex alterations in the sALS/frontotemporal lobar degeneration spectrum complex at stages with no apparent cognitive impairment.

## INTRODUCTION

Amyotrophic lateral sclerosis (ALS) is a progressive age-dependent neurodegenerative disease characterized by degeneration and death of upper (motor cortex) and lower (brain stem and spinal cord) motor neurons, resulting in muscle atrophy, together with variable frontotemporal lobar degeneration (FTLD). ALS may be sporadic (sALS) with unknown cause, in up to 90%-92% of cases, or inherited (fALS), accounting for about 8-10% of cases, most of them transmitted as autosomal dominant but also recessive and X-linked in some families. However, about 13% of sALS cases bear a gene mutation linked to fALS. Main pathological features in sALS are loss of myelin and axons in the pyramidal tracts and anterior spinal roots, chromatolysis of motor neurons, axonal spheroids in the anterior horn, cystatin C-containing Bunina bodies in motor neurons, ubiquitin-immunoreactive TDP-43-positive skein-like and spherical inclusions in motor neurons, and TDP-43 inclusions in oligodendroglial cells. In many cases, the frontal cortex shows cytoplasmic TDP-43-immuno-reactive intracytoplasmic inclusions in neurons and oligodendocytes, and neuropil threads. Neuron loss and spongiosis in the upper cortical layers are usually restricted to cases with severe cognitive impairment and frontotemporal dementia [1, 2].

Several mechanisms have been proposed as contributory factors in the pathogenesis of motor neuron damage in sALS including excitoxicity, mitochondrial and energy metabolism failure, oxidative stress damage, altered glial cells, inflammation, cytoskeletal ab-normalities, alterations in RNA metabolism, and altered TDP-43 metabolism, among others [3-16]. Increased understanding on the pathogenesis of sALS has emerged from the use of transcriptome analysis of the spinal cord and motor cortex [17-26]. Previous transcriptomic studies center in the spinal cord and motor cortex in separate groups of patients, cover a limited number of cases, identify and validate a few genes not coincidental among the different studies. Selection of the sample may account for these differences. Further microarray studies carried out on isolated motor neurons of the spinal cord obtained by laser micro-dissection in sALS cases have revealed up-regulation of genes associated with cell signalling and cell death and down-regulation of genes linked to transcription and composition of the cytoskeleton [27]. Curiously, similar studies performed on samples from individuals bearing mutations linked to ALS show different regulated transcripts, thus suggesting gene expression variants in the spinal cord in fALS [28, 29].

Importantly, no gene expression analyses are available in the frontal cortex area 8 in sALS in spite that frontal alterations are common in this disease. Moreover, ALS and FTLD with TDP inclusions (FTLD-TDP) are within the same disease spectrum [1].

The present study analyzes gene expression in the anterior horn of the spinal cord and frontal cortex area 8 in a series of 18 sALS cases and 23 controls. The main goals of the present study are to analyze and compare gene expression in these two regions, and more specifically to identify altered gene expression and clusters with specific functions in the anterior horn and frontal cortex area 8. Thus, the present study focuses on the pathogenesis of motor neuron damage responsible of altered motor function, and frontal cortex at preclinical stages of cognitive impairment.

## RESULTS

### Microarray analysis

**Figure 1:**
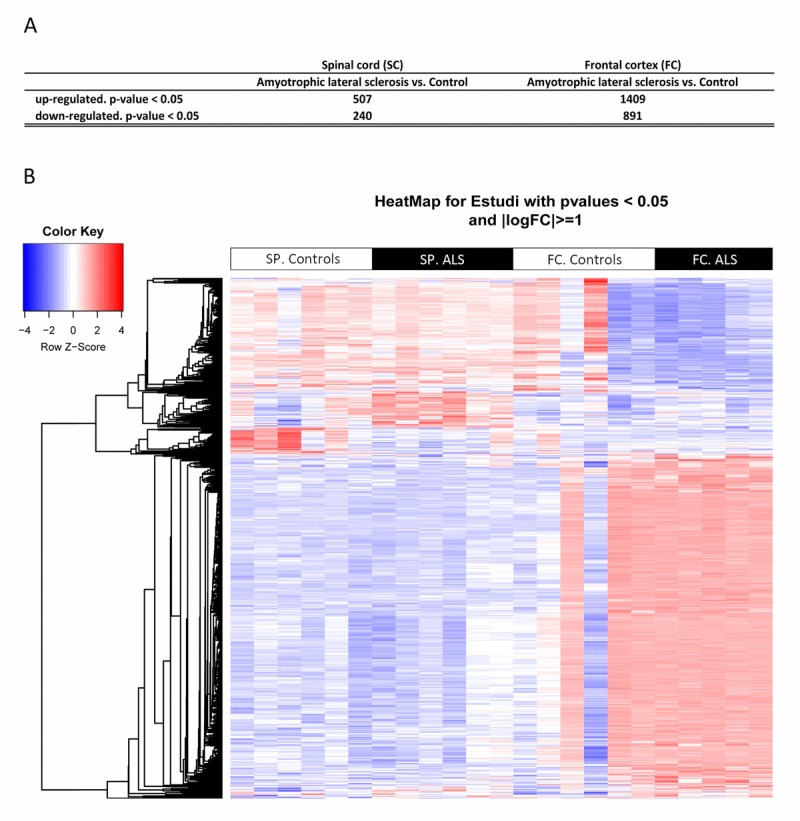
(**A**) Total number of significantly different expressed genes comparing transcriptomic profiles between groups and regions. (**B**) Hierarchical clustering heat map of expression intensities of mRNA array transcripts reflect differential gene expression profiles in the anterior horn of the spinal cord and frontal cortex area 8 in ALS compared with controls. Differences between groups are considered statistically significant at p-value ≤ 0.05. Abbreviations: ALS: amyotrophic lateral sclerosis; FC: frontal cortex area 8; mRNA: messenger RNA; SP: anterior horn of the spinal cord lumbar level

Cofactors age and gender were not relevant for the analysis. 9,563 gene sequences were detected across all samples. Heat map indicates differences in transcripts expression levels between control and ALS cases in the anterior cord of the spinal cord and in frontal cortex area 8 (Figure 1). We identified 747 genes differentially expressed with p-value lower than or equal to 0.05 in the anterior horn of the spinal cord (up: 507 and down: 240) and 2,300 genes differentially expressed in the frontal cortex area 8 (up: 1,409 and down: 891) in sALS (Figure 1).

Supplementary Tables 1 and 2 identify all de-regulated genes. Post-analysis microarray data of differentially ex-pressed genes assessed with enrichment analysis against Go Ontology database are shown in Tables 1 and 2.

**Table 1:** Main significant clusters of altered genes in spinal cord of ALS samples

Cluster	Gene names	Size	Count	Odds Ratio	p-value	Deregulation
Activation of blood coagulation via clotting cascade	*F3, ANO6*	2	2	Inf	0.000574	Up
Antigen processing and presentation of exogenous peptide antigen	*CTSS, FCER1G, FCGR1A, HLA-A, HLA-B, HLA-C, HLA-DMA, HLA-DMB, HLA-DQA1, HLA-DQA2, HLA-DQB1, HLA-DQB2, HLA-DRB1, HLA-DRB5, HLA-F, HLA-G, NCF2, PSMB8, PSMB9, PSMD5, TAP1, IFI30*	165	22	6.58	6.84e-11	Up
Antigen processing and presentation of exogenous peptide antigen via MHC class I	*CTSS, FCER1G, FCGR1A, HLA-A, HLA-B, HLA-C, HLA-F, HLA-G, NCF2, PSMB8, PSMB9, PSMD5, TAP1, IFI30*	75	14	9.66	2.45e-09	Up
Antigen processing and presentation of exogenous peptide antigen via MHC class I, TAP-independent	*CTSS, HLA-A, HLA-B, HLA-C, HLA-F, HLA-G*	9	6	82.7	1.45e-08	Up
Antigen processing and presentation of exogenous peptide antigen via MHC class II	*CTSS, FCER1G, HLA-DMA, HLA-DMB, HLA-DQA1, HLA-DQA2, HLA-DQB1, HLA-DQB2, HLA-DRB1, HLA-DRB5, IFI30*	92	11	5.66	1.24e-05	Up
Antigen processing and presentation of peptide antigen via MHC class I	*CTSS, FCER1G, FCGR1A, HLA-A, HLA-B, HLA-C, HLA-F, HLA-G, NCF2, PSMB8, PSMB9, PSMD5, TAP1, IFI30*	97	14	7.09	7.58e-08	Up
Apoptotic process	*AHR, APOE, FAS, BCL2A1, BCL6, BMP2, BTK, CAMK2D, CASP1, CASP4, TNFSF8, CDKN1A, CTSC, DAB2, NQO1, ECT2, EDN1, F3, FCER1G, HCK, HGF, HIF1A, HMOX1, ICAM1, IFI16, IL1A, ITGA5, JAK3, LMNB1, LYN, MNDA, MYC, NCF2, NOS3, P2RX4, PLAGL1, PLAUR, PLSCR1, PRLR, PSMB8, PSMB9, PSMD5, PTPN2, CCL2, CCL19, SNAI2, STAT1, TEK, TGFB2, TLR2, TLR3, GPR65, YBX3, NOL3, SOCS3, LY86, IKBKE, CHL1, PPP1R15A, RRM2B, SHISA5, TNFRSF12A, ACSL5, FNIP2, DNASE2B, ZMAT3, NOA1, FGD3, IL33, DEDD2, ANO6*	1745	71	1.89	5.22e-06	Up
Apoptotic signaling pathway	*FAS, BCL2A1, BTK, CASP4, CDKN1A, CTSC, ECT2, HGF, HIF1A, HMOX1, ICAM1, IFI16, IL1A, NOS3, P2RX4, PLAUR, PTPN2, SNAI2, TGFB2, TLR3, YBX3, NOL3, IKBKE, PPP1R15A, RRM2B, SHISA5, TNFRSF12A, ACSL5, FNIP2, FGD3, IL33, DEDD2*	596	32	2.43	1.88e-05	Up
Axonemal dynein complex assembly	*DNAH5, DNAI1, TEKT2, ZMYND10, ARMC4, DNAH7, CCDC114, CCDC151, DNAAF1, CCDC39*	21	10	175	8.54e-18	Down
Axoneme	*DNAH5, DNAH9, SPAG6, DNAI1, DCDC2, HYDIN, CFAP46, ARMC4, MNS1, DNAH7, CFAP74, CCDC114, CCDC151, DNAAF1, CFAP54, DNAH2, SPAG17, CFAP221, CCDC39, RSPH4A*	89	20	52.5	1.31e-25	Down
Axoneme assembly	*DNAH5, DNAI1, TEKT2, ZMYND10, HYDIN, CFAP46, ARMC4, DNAH7, CFAP74, RSPH1, CCDC114, CCDC151, DNAAF1, SPAG17, CCDC39, RSPH4A*	42	16	128	5.9e-26	Down
B cell mediated immunity	*FAS, BCL6, BTK, C1QB, C1QC, C7, FCER1G, HLA-DMA, HLA-DQB1, HLA-DRB1, HLA-DRB5, CFI, IL4R, CD226, TLR8*	103	15	7.18	2.28e-08	Up
Cellular protein modification process	*IL12RB1, INS, KCNE1, MAK, CFP, RASA4, TRAK2, MYLK3, NEK5, C17orf97, PPIAL4A*	3527	11	0.473	0.00885	Down
Cellular response to interferon-gamma	*CAMK2D, EDN1, FCGR1A, GBP1, HCK, HLA-A, HLA-B, HLA-C, HLA-DQA1, HLA-DQA2, HLA-DQB1, HLA-DQB2, HLA-DRB1, HLA-DRB5, HLA-F, HLA-G, ICAM1, IRF8, OAS2, PTPN2, CCL2, CCL19, STAT1, SOCS3, IFI30, TRIM38, TRIM5*	126	27	11.9	1.95e-18	Up
Clathrin-coated endocytic vesicle membrane	*FCGR1A, HLA-DQA1, HLA-DQA2, HLA-DQB1, HLA-DQB2, HLA-DRB1, HLA-DRB5*	49	7	7.32	0.000108	Up
Copper ion import	*ATP7B, SLC31A1, STEAP4*	7	3	30.7	0.000446	Up
Cytokine production involved in immune response	*BCL6, BTK, FCER1G, HLA-A, HMOX1, JAK3, SLC11A1, TEK, TGFB2, TLR2, TLR3, TREM1*	69	12	8,81	7,87E-08	Up
Endolysosome membrane	*TLR3, TLR7, TLR8*	4	3	131	4.51e-05	Up
Fc receptor mediated stimulatory signaling pathway	*FCER1G, FCGR1A, FCGR2A, FGR, HCK, ITPR3, LYN, PLSCR1, CD226, MYO1G*	77	10	6.21	1.47e-05	Up
Humoral immune response mediated by circulating immunoglobulin	*C1QB, C1QC, C7, HLA-DQB1, HLA-DRB1, HLA-DRB5, CFI*	46	7	7.42	0.000103	Up
Igg binding	*FCER1G, FCGR1A, FCGR2A, FCGR2B*	10	4	28.3	5.42e-05	Up
Immunoglobulin production	*FAS, BCL6, CD37, HLA-DQB1, HLA-DRB1, HLA-DRB5, IL4R, TNFSF13B, POLM, IL33*	87	10	5.4	4.34e-05	Up
Inner dynein arm assembly	*TEKT2, ZMYND10, DNAH7, DNAAF1, CCDC39*	10	5	182	1.44e-09	Down
Integral component of lumenal side of endoplasmic reticulum membrane	*HLA-A, HLA-B, HLA-C, HLA-DQA1, HLA-DQA2, HLA-DQB1, HLA-DQB2, HLA-DRB1, HLA-DRB5, HLA-F, HLA-G*	28	11	28.8	1.04e-11	Up
Interferon-alpha production	*TLR3, NMI, TLR7, TLR8*	18	4	11.7	0.000764	Up
Interferon-beta biosynthetic process	*TLR3, NMI, TLR7, TLR8*	8	4	41.1	2.12e-05	Up
Interferon-gamma biosynthetic process	*TLR3, EBI3, TLR7, TLR8*	16	4	13.7	0.000472	Up
Interleukin-10 production	*FCER1G, HLA-DRB1, HLA-DRB5, JAK3, TLR2, PDCD1LG2*	42	6	6.87	0.000463	Up
Intrinsic apoptotic signaling pathway	*BCL2A1, CASP4, CDKN1A, HIF1A, HMOX1, IFI16, PLAUR, PTPN2, SNAI2, YBX3, NOL3, IKBKE, PPP1R15A, RRM2B, SHISA5, FNIP2*	284	16	2.49	0.00143	Up
Macrophage activation	*IL4R, SLC11A1, TLR1, SBNO2, CD93, TLR7, TLR8, IL33*	48	8	8.29	1.66e-05	Up
Mast cell cytokine production	*BCL6, FCER1G, HMOX1*	7	3	30.7	0.000446	Up
MHC class II receptor activity	*HLA-DQA1, HLA-DQA2, HLA-DQB1, HLA-DQB2, HLA-DRB1*	11	5	35.4	2.73e-06	Up
MHC protein complex	*HLA-A, HLA-B, HLA-C, HLA-DMA, HLA-DMB, HLA-DQA1, HLA-DQA2, HLA-DQB1, HLA-DQB2, HLA-DRB1, HLA-DRB5, HLA-F, HLA-G*	25	13	48.4	1.34e-15	Up
Microtubule bundle formation	*DNAH5, DNAI1, TEKT2, ZMYND10, HYDIN, CFAP46, ARMC4, DNAH7, CFAP74, RSPH1, CCDC114, CCDC151, DNAAF1, SPAG17, CCDC39, RSPH4A*	63	16	70.7	1.18e-22	Down
Monocyte chemotaxis	*CCR1, LYN, CCL2, CCL19, PLA2G7, ANO6*	49	6	5.75	0.00107	Up
Outer dynein arm assembly	*DNAH5, DNAI1, ZMYND10, ARMC4, CCDC114, CCDC151, DNAAF1*	11	7	325	5.6e-14	Down
Peptide antigen binding	*HLA-A, HLA-B, HLA-C, HLA-DQA1, HLA-DQB1, HLA-DRB1, HLA-DRB5, HLA-F, HLA-G, TAP1*	26	10	26.9	1.57e-10	Up
Platelet-derived growth factor receptor binding	*TYMP, ITGA5, ITGB3, LYN*	12	4	21.2	0.000123	Up
Positive regulation of Fc receptor mediated stimulatory signaling pathway	*LYN, CD226*	2	2	Inf	0.000574	Up
Positive regulation of interleukin-6 production	*FCER1G, TLR1, TLR2, TLR3, TLR7, IL33*	55	6	5.05	0.00197	Up
Positive regulation of interleukin-8 production	*TLR2, TLR3, TLR5, TLR7, TLR8*	42	5	5.56	0.00318	Up
Positive regulation of tumor necrosis factor production	*FCER1G, CCL2, CCL19, TLR1, TLR2, TLR3*	51	6	5.5	0.00133	Up
Protection from natural killer cell mediated cytotoxicity	*HLA-A, HLA-B, TAP1*	5	3	61.5	0.000132	Up
Regulated secretory pathway	*ANXA3, FCER1G, FGR, HCK, HMOX1, IL4R, LYN, STX11, CD300A, RAB11FIP2, RAB11FIP1*	73	11	7.4	1.23e-06	Up
Regulation of apoptotic process	*APOE, FAS, BCL2A1, BCL6, BMP2, BTK, CAMK2D, CASP1, CASP4, CDKN1A, CTSC, DAB2, NQO1, ECT2, EDN1, F3, FCER1G, HCK, HGF, HIF1A, HMOX1, ICAM1, IL1A, ITGA5, JAK3, LYN, MNDA, MYC, NCF2, NOS3, PLAUR, PRLR, PSMB8, PSMB9, PSMD5, PTPN2, CCL2, CCL19, SNAI2, STAT1, TEK, TGFB2, TLR3, YBX3, NOL3, SOCS3, CHL1, RRM2B, TNFRSF12A, ACSL5, ZMAT3, FGD3, DEDD2, ANO6*	1344	54	1.82	0.000117	Up
Regulation of B cell apoptotic process	*BCL6, BTK, LYN*	16	3	9.45	0.00608	Up
Regulation of coagulation	*APOE, EDN1, F3, FCER1G, LYN, NOS3, PLAU, PLAUR, THBD, HPSE, ADAMTS18, ANO6*	85	12	6.87	8.31e-07	Up
Regulation of cytokine biosynthetic process	*CD86, HMOX1, IL1A, TLR1, TLR2, TLR3, NMI, EBI3, TLR7, TLR8*	93	10	5.01	7.72e-05	Up
Regulation of extrinsic apoptotic signaling pathway	*FAS, HMOX1, ICAM1, IL1A, NOS3, SNAI2, TGFB2, NOL3, TNFRSF12A, ACSL5, DEDD2*	155	11	3.17	0.0013	Up
Regulation of Fc receptor mediated stimulatory signaling pathway	*LYN, PLSCR1, CD226*	5	3	61.5	0.000132	Up
Regulation of hemostasis	*APOE, EDN1, F3, FCER1G, LYN, NOS3, PLAU, PLAUR, THBD, HPSE, ADAMTS18, ANO6*	81	12	7.27	4.87e-07	Up
Regulation of leukocyte apoptotic process	*BCL6, BTK, FCER1G, HIF1A, JAK3, LYN, CCL19, TGFB2*	74	8	5.02	0.000386	Up
Regulation of lipid kinase activity	*FGR, LYN, CCL19, TEK, NRBF2*	47	5	4.89	0.0052	Up
Regulation of mast cell activation	*FCER1G, FGR, HMOX1, IL4R, LYN, PLSCR1, CD226, CD300A*	31	8	14.4	4.96e-07	Up
Regulation of mast cell degranulation	*FCER1G, FGR, HMOX1, IL4R, LYN, CD300A*	24	6	13.8	1.71e-05	Up
Regulation of microtubule movement	*DNAH11, ARMC4, DNAAF1, CCDC39*	18	4	51.3	3.03e-06	Down
Regulation of natural killer cell mediated immunity	*HLA-A, HLA-B, PVR, TAP1, CD226*	27	5	9.36	0.000405	Up
Regulation of protein metabolic process	*FOXJ1, INS, CFP, RASA4, NEK5, DTHD1*	2448	6	0.381	0.00803	Down
Regulation of protein modification process	*INS, RASA4*	1641	2	0.192	0.00288	Down
Regulation of T-helper 1 cell differentiation	*HLX, IL4R, JAK3, CCL19*	9	4	32.9	3.74e-05	Up
T cell costimulation	*CD86, HLA-DQA1, HLA-DQA2, HLA-DQB1, HLA-DQB2, HLA-DRB1, HLA-DRB5, LYN, CCL19, TNFSF13B, PDCD1LG2*	71	11	7.65	9.25e-07	Up
TAP binding	*HLA-A, HLA-B, HLA-C, HLA-F, TAP1*	7	5	106	1.34e-07	Up
T-helper 2 cell differentiation	*BCL6, CD86, HLX, IL4R*	14	4	16.4	0.00027	Up

**Table 2:** Main significant clusters of altered genes in frontal cortex of ALS samples

Cluster	Gene names	Size	Count	Odds Ratio	p-value	Deregulation
Adenylate cyclase-inhibiting G-protein coupled receptor signaling pathway	*ADCY1, CHRM1, CHRM3, GNAI3, MCHR1, GRM8, HTR1B, HTR1E, HTR1F, NPY1R, OPRK1, OPRM1, SSTR2*	64	13	3.79	0.000164	Up
Astrocyte differentiation	*ABL1, MAG, NKX2-2, NOTCH1, POU3F2, S100B, TAL1, CNTN2, SOX8*	53	9	5.12	0.000184	Down
Axolemma	*KCNC1, KCNC2, KCNH1, ROBO2, SLC1A2*	14	5	8.26	0.00124	Up
Axon	*DAGLA, CAMK2D, CCK, CHRM1, CHRM3, AP1S1, CTNNA2, DLG4, DRD1, EPHA4, PTK2B, FGF13, GAP43, GARS, GRIA1, GRIK5, GRIN2A, HTR2A, KCNB1, KCNC1, KCNC2, KCNH1, KCNK2, KCNMA1, KCNQ2, KCNQ3, MYH10, NPY1R, NRCAM, NRGN, OPRK1, PAK1, PFN2, MAP2K1, PTPRN2, ROBO2, SCN1A, SCN1B, SCN2A, SCN8A, CCL2, SLC1A2, SNCA, STXBP1, SYN1, KCNAB1, FZD3, GLRA3, PRSS12, CNTNAP1, KCNAB2, NRP1, CDK5R1, BSN, SYT7, SYNGR1, DGKI, NRXN1, HOMER1, KATNB1, SEMA3A, OLFM1, SLC9A6, CPLX1, AAK1, ADGRL1, TPX2, UNC13A, MYCBP2, NCS1, PACSIN1, STMN3, SEPT11, SLC17A7, TBC1D24, NDEL1, LMTK3, MTPN, CNTN4, LRRTM1, HCN1*	358	81	4.6	1.68e-24	Up
Axon extension	*BMPR2, NRCAM, PPP3CB, SLIT1, CDKL5, NRP1, CDK5R1, LHX2, SEMA3A, OLFM1, SLC9A6, BCL11A, ISLR2, NDEL1*	91	14	2.7	0.00176	Up
Axon hillock	*CCK, TPX2, NDEL1*	7	3	11.1	0.00729	Up
Cadherin binding	*CDH13, CTNNA2, TRPC4, CDK5R1, AKAP5, MMP24, PTPRT*	29	7	4.81	0.00167	Up
Calcineurin complex	*ITPR1, PPP3CA, PPP3CB, PPP3R1*	4	4	Inf	1.59e-05	Up
Calcium channel regulator activity	*CACNB2, FKBP1B, ITPR1, PRKCB, STX1A, NRXN1, TSPAN13, HPCAL4, CACNA2D3*	36	9	5.05	0.000281	Up
Calcium ion-dependent exocytosis of neurotransmitter	*CACNA1A, SYT1, SYT5, DOC2A, SYT7, RIMS2, RAB3GAP1, RIMS1, SYT13, SYT12*	28	10	8.25	4.76e-06	Up
Calmodulin binding	*ADCY1, ADD2, ATP2B1, ATP2B2, CACNA1C, CAMK4, CAMK2A, CAMK2B, CAMK2D, GAP43, ITPKA, KCNH1, KCNN1, KCNQ3, MAP2, MYH10, MYO5A, NOS2, NRGN, PDE1B, PPP3CA, PPP3CB, PPP3R1, RGS4, RIT2, RYR2, SLC8A2, SLC8A1, AKAP5, CAMKK2, ARPP21, PLCB1, KCNH5, CAMK1D, CAMK1G, CAMKV, CAMKK1, PNCK, CFAP221, RIIAD1*	176	40	4.57	5.38e-13	Up
Calmodulin-dependent protein kinase activity	*CAMK4, CAMK2A, CAMK2B, CAMK2D, PTK2B, ITPKA, CAMKK2, CAMK1D, CAMK1G, CAMKK1, PNCK*	32	11	7.95	2.02e-06	Up
Camp binding	*PDE2A, PDE4A, PRKAR1B, PRKAR2B, RAPGEF2, RAPGEF4, HCN1*	24	7	6.23	0.000487	Up
Central nervous system neuron axonogenesis	*EPHA4, SCN1B, NR2E1, MYCBP2, PRDM8, ARHGEF28, NDEL1*	29	7	4.71	0.00187	Up
Chloride channel activity	*CLIC2, GABRA1, GABRA2, GABRA3, GABRA4, GABRA5, GABRB2, GABRB3, GABRD, GABRG3, GLRB, SLC26A4, GLRA3, SLC17A7, SLC26A8, ANO5*	78	16	3.93	2.05e-05	Up
Clathrin binding	*SYT1, SYT5, DOC2A, SYT7, SNAP91, HMP19, SYTL2, CEMIP, SYT13, SMAP1, SYT16, SYT12*	56	12	4.14	0.000141	Up
Compact myelin	*MAG, SIRT2, JAM3*	12	3	8.38	0.00957	Down
Cyclin-dependent protein serine/threonine kinase activity	*CDK14, CDKL5, CDKL1, CDK5R1, CDKL2, CDK20*	29	6	3.94	0.008	Up
Cytoskeleton of presynaptic active zone	*BSN, PCLO*	2	2	Inf	0.004	Up
Dendrite	*BMPR2, CACNA1A, CACNA1B, CACNA1C, CCK, CHRM1, CHRM3, CRMP1, DLG3, DLG4, DRD1, EPHA4, EPHA7, PTK2B, FGF13, GABRA5, GRIA1, GRIA2, GRIA3, GRIK5, GRIN2A, GRM1, GRM5, HTR2A, ITPKA, KCNB1, KCNC1, KCNC2, KCND3, KCNH1, KCNJ4, KCNQ3, MAP2, MYH10, NELL2, NRGN, OPRK1, PAK1, PRKAR2B, PRKCG, MAP2K1, RARA, RGS7, SCN8A, CCL2, SLC8A1, CDKL5, SYN1, KCNAB1, FZD3, PRSS12, CDK5R1, BSN, NEURL1, DGKI, HOMER1, CABP1, AKAP5, ARHGAP32, FRMPD4, SEMA3A, BAIAP2, SLC9A6, ARFGEF2, CHL1, PLK2, CPLX1, LZTS1, CPEB3, NCS1, NSMF, SHANK1, IFT57, SEPT11, ANKS1B, SLC4A10, TENM2, DLGAP3, JPH4, PPP1R9B, SHANK3, LMTK3, GRIN3A, SNAP47, CNIH2, HCN1*	406	86	4.24	7.25e-24	Up
Dendrite development	*ADGRB3, CACNA1A, CAMK2B, CTNNA2, DLG4, EPHA4, HPRT1, ITPKA, MAP2, MEF2C, PAK1, PAK3, PPP3CA, CDKL5, NR2E1, NRP1, CDK5R1, NEURL1, AKAP5, RAPGEF2, KIAA0319, SEMA3A, BAIAP2, SLC9A6, PLK2, CIT, LZTS1, CPEB3, NEDD4L, MAPK8IP2, RBFOX2, NGEF, NSMF, SLITRK5, PACSIN1, SHANK1, DCDC2, BCL11A, FEZF2, CAMK1D, SHANK3, GRIN3A, FMN1*	178	43	4.85	1.44e-14	Up
Dendrite extension	*PARK2, SYT1, RIMS2, SLC9A6, RIMS1, UNC13A, NEDD4L, CPNE5*	21	8	9.12	2.53e-05	Up
Dendrite morphogenesis	*ADGRB3, CACNA1A, CAMK2B, CTNNA2, DLG4, EPHA4, HPRT1, ITPKA, MAP2, PAK3, PPP3CA, CDKL5, NR2E1, CDK5R1, AKAP5, RAPGEF2, SEMA3A, BAIAP2, CIT, LZTS1, NEDD4L, MAPK8IP2, RBFOX2, NGEF, NSMF, SLITRK5, SHANK1, DCDC2, SHANK3, FMN1*	109	30	5.73	4E-12	Up
Dendritic shaft	*CACNA1C, DLG3, DRD1, GRM5, HTR2A, MAP2, PRKAR2B, SLC8A1, HOMER1, AKAP5, LZTS1, JPH4, CNIH2*	37	13	8.11	2.07e-07	Up
Dendritic spine development	*CAMK2B, DLG4, EPHA4, ITPKA, MEF2C, PAK1, PAK3, CDK5R1, NEURL1, BAIAP2, SLC9A6, PLK2, CPEB3, NGEF, SHANK1, SHANK3*	58	16	5.68	4.06e-07	Up
Dendritic spine membrane	*ATP2B1, GRIA1, ITGA8, AKAP5, DDN*	9	5	18.6	0.000102	Up
DNA metabolic process	*BMPR2, CDKN2D, CIDEA, DACH1, HGF, IGF1, KCNK2, KPNA2, MAS1, KITLG, ORC4, PAK3, PIK3CA, PRKCG, CHAF1B, CDC7, NPM2, PPARGC1A, PARM1, CHD5, UBE2W, FBXW7, TSPYL2, BCL11B, SLF1, TBRG1, MAEL, XRCC6BP1, ZBED9, KLHDC3, STOX1, KIAA2022*	867	32	0.549	0.000264	Up
Ensheathment of neurons	*MYRF, LPAR1, KCNJ10, KEL, MAG, MAL, NGFR, CLDN11, PMP22, POU3F2, KLK6, CNTN2, QKI, ARHGEF10, OLIG2, NDRG1, SIRT2, PARD3, FA2H, SH3TC2, JAM3, NKX6-2, SERINC5*	101	23	7.53	4.57e-12	Down
Excitatory postsynaptic potential	*DLG4, PTK2B, GRIK5, GRIN2A, GRIN2B, MEF2C, PPP3CA, SNCA, STX1A, DGKI, NRXN1, RIMS2, RAB3GAP1, RIMS1, MAPK8IP2, SHANK1, CELF4, SLC17A7, NETO1, SHANK3*	50	20	9.99	7.46e-12	Up
GABA receptor activity	*GABRA1, GABRA2, GABRA3, GABRA4, GABRA5, GABRB2, GABRB3, GABRD, GABRG3, GABBR2*	22	10	12.6	2.77e-07	Up
GABA receptor binding	*GABRA5, AKAP5, ARFGEF2, JAKMIP1*	14	4	6.03	0.0091	Up
Glial cell development	*MYRF, GSN, KCNJ10, NKX2-2, POU3F2, CNTN2, ARHGEF10, NDRG1, SIRT2, PHGDH, PARD3, FA2H, SH3TC2, NKX6-2*	71	14	6.19	4.84e-07	Down
Glutamate receptor activity	*PTK2B, GRIA1, GRIA2, GRIA3, GRIK5, GRIN2A, GRIN2B, GRM1, GRM5, GRM8, GRIN3A*	27	11	10.4	2.72e-07	Up
Innervation	*GABRA5, GABRB2, GABRB3, PRKCG, NRP1, SEMA3A, UNC13A*	23	7	6.47	0.000412	Up
Inositol phosphate metabolic process	*PTK2B, ITPKA, MAS1, OCRL, SNCA, INPP4B, SYNJ1, PPIP5K1, PLCH1, PLCB1, NUDT11*	65	11	3.02	0.00247	Up
Ionotropic glutamate receptor activity	*PTK2B, GRIA1, GRIA2, GRIA3, GRIK5, GRIN2A, GRIN2B, GRIN3A*	19	8	11	9.08e-06	Up
JNK cascade	*ADORA2B, EPHA4, PTK2B, FGF14, MAP3K9, MAP3K10, GADD45B, PAK1, PARK2, MAPK9, CCL19, MAP2K4, MAP3K6, RB1CC1, RASGRP1, PLCB1, MAPK8IP2, KIAA1804, DUSP19, ZNF675, MAGI3*	185	21	1.9	0.00716	Up
Lipid binding	*ABCA1, ANXA5, APOD, AR, C3, LPAR1, HSD17B10, HIP1, HSPA2, KCNJ2, MAL, MYO1E, NPC1, P2RX7, PLD1, PTGS1, SELL, SNX1, ACOX2, IQGAP1, HIP1R, CYTH1, STARD3, FNBP1, RASGRP3, LDLRAP1, GLTP, ANKFY1, PXK, ADAP2, PARD3, PREX1, WDFY4, PLEKHF1, PRAM1, PAQR8, MVB12B, SNX29, SYTL4, ARAP1, FRMPD2, AMER2, NCF1C, C8orf44-SGK3*	601	44	2.07	2.63e-05	Down
Mrna processing	*LGALS3, CELF2, PPARGC1A, CELF3, CPEB3, RBFOX2, RBFOX1, MTPAP, CELF4, CELF5, SRRM4, LSM11, RBFOX3*	417	13	0.466	0.00202	Up
Myelin maintenance	*MYRF, NDRG1, FA2H, SH3TC2*	11	4	14.2	0.000601	Down
Myelin sheath	*CA2, CNP, CRYAB, GSN, HSPA2, MAG, MOBP, MOG, MYO1D, CLDN11, RDX, CNTN2, NDRG1, SIRT2, PHGDH, GJC2, ERMN, MYH14, JAM3, SERINC5*	156	20	3.77	2.29e-06	Down
Myelination	*MYRF, LPAR1, KCNJ10, KEL, MAG, MAL, NGFR, PMP22, POU3F2, KLK6, CNTN2, QKI, ARHGEF10, OLIG2, NDRG1, SIRT2, PARD3, FA2H, SH3TC2, JAM3, NKX6-2, SERINC5*	98	22	7.38	1.81e-11	Down
Negative regulation of neuron apoptotic process	*CACNA1A, PTK2B, GABRA5, GABRB2, GABRB3, MEF2C, PARK2, PIK3CA, PRKCG, CCL2, SNCB, SNCA, STAR, STXBP1, NRP1, CHL1, PPARGC1A, OXR1, AGAP2*	128	19	2.59	0.000465	Up
Negative regulation of transcription, DNA-templated	*ARNTL, RUNX1T1, CRYM, CYP1B1, DACH1, FGF9, FOXG1, H2AFZ, MEF2C, MAP3K10, TRIM37, PDE2A, RARA, RORB, SATB1, SNCA, SOX5, TBX15, THRB, NR2E1, WNT10B, CDK5R1, LRRFIP1, ZBTB33, BASP1, ZBTB18, KLF12, CPEB3, PLCB1, SATB2, NEDD4L, SIRT5, RBFOX2, ATAD2, TAGLN3, BCL11A, FEZF2, SMYD2, PRDM8, TENM2, MTA3, SCRT1, MAEL, PRICKLE1, EID2, ARX, ZNF675, KCTD1*	1135	48	0.632	0.00083	Up
Neuron apoptotic process	*CACNA1A, EPHA7, PTK2B, GABRA5, GABRB2, GABRB3, GRIK5, KCNB1, MEF2C, PAK3, PARK2, PIK3CA, PRKCG, SCN2A, CCL2, SNCB, SNCA, STAR, STXBP1, NRP1, CDK5R1, CHL1, PPARGC1A, NSMF, OXR1, FBXW7, AGAP2, SDIM1*	206	28	2.35	0.000117	Up
Neuron spine	*DLG4, DRD1, EPHA4, GRIA1, GRM5, ITPKA, MYH10, NRGN, PRKAR2B, SLC8A1, CDK5R1, NEURL1, DGKI, AKAP5, ARHGAP32, FRMPD4, BAIAP2, SLC9A6, ARFGEF2, LZTS1, SHANK1, SEPT11, ANKS1B, TENM2, DLGAP3, PPP1R9B, SHANK3, CNIH2*	104	28	5.57	3.28e-11	Up
Neuronal postsynaptic density	*ADD2, ATP1A1, BMPR2, CAMK2A, CAMK2B, CTNNA2, DLG4, DMTN, GAP43, GRIN2B, MAP2, PAK1, PRKCG, BSN, DGKI, DLGAP1, HOMER1, BAIAP2, CAP2, CNKSR2, CLSTN1, MAPK8IP2, SHANK1, CLSTN2, SHANK3*	64	25	9.69	3.02e-14	Up
Neuron-neuron synaptic transmission	*CA7, CACNA1A, CACNB4, CAMK4, DRD1, PTK2B, GABRA1, GABRB2, GLRB, GRIA1, GRIA2, GRIA3, GRIK5, GRIN2A, GRM1, GRM5, GRM8, HRH2, HTR1B, HTR2A, MEF2C, NPY5R, PAK1, PARK2, PRKCE, PTGS2, SNCA, STXBP1, SYT1, GLRA3, DGKI, DLGAP2, NRXN1, RAB3GAP1, UNC13A, MAPK8IP2, RASD2, TMOD2, SHC3, SLC17A7, SHANK3, GRIN3A, CNIH2*	136	43	7.06	2.63e-19	Up
Neurotransmitter secretion	*CACNA1A, CACNA1B, CAMK2A, GAD1, GLS, GRIK5, MEF2C, PAK1, PARK2, PFN2, SLC1A1, SLC1A2, SNCA, STX1A, STXBP1, SYN1, SYN2, SYT1, SYT5, DOC2A, PPFIA4, PPFIA2, PPFIA3, CADPS, LIN7A, SYNJ1, SYT7, DGKI, BZRAP1, NRXN1, RIMS2, RIMS3, CPLX1, HRH3, ADGRL1, RAB3GAP1, RIMS1, UNC13A, PCLO, SYTL2, SLC17A7, SYT13, SYT16, SYT12, CADPS2, SNAP47*	154	46	6.52	1.93e-19	Up
Node of Ranvier	*KCNQ2, KCNQ3, SCN1A, SCN1B, SCN2A, SCN8A*	15	6	9.92	0.000193	Up
Nucleic acid metabolic process	*ABCA2, ABL1, PARP4, AR, ATM, BMP8B, MYRF, CAPN3, CAT, CBFB, CCNA2, CDKN1C, CENPB, ELF1, EYA4, ERF, FGF1, FGFR2, GDF1, HSD17B10, HDAC1, HIP1, HOXA1, HOXA2, HOXA5, HOXB2, HOXB5, HOXD1, HOXD3, HSPA1A, FOXN2, JUP, SMAD5, SMAD9, MCM7, MEIS1, CIITA, FOXO4, NKX2-2, NOTCH1, YBX1, PBX3, PDE8A, ENPP2, POLR2L, POU3F2, PSEN1, RNH1, RPLP0, RPS5, RXRG, SALL1, SGK1, SOX10, SREBF1, STAT2, SYK, TAL1, TCF12, TRAF1, TRPS1, ZNF3, ZNF69, VEZF1, FZD5, ARHGEF5, HIST1H2AC, HIST1H3E, HIST1H4H, HIST1H4B, RNASET2, CCNE2, QKI, LITAF, ST18, ZNF536, DDX39A, OLIG2, HMG20B, SEMA4D, TXNIP, DMRT2, TCFL5, ATF7, IKZF2, ZNF652, SIRT2, SAMD4A, KANK1, HEY2, BAMBI, ZNF521, ZBTB20, GREM1, CECR2, HIPK2, KLF15, BAZ2B, SLC40A1, SOX8, ZBTB7B, RRNAD1, KLF3, DDIT4, ZNF280D, TRIM62, CHD7, SLF2, ZNF83, SLC2A4RG, OTUD7B, BBX, MAVS, SFMBT2, NCOA5, TP53INP2, ZNF462, ARHGAP22, CREB3L2, CRTC3, TRAK2, BHLHE41, DBF4B, TSC22D4, NKX6-2, ZBTB37, LOXL3, OLIG1, ZSWIM7, GABPB2, CC2D1B, ZBTB12, ZNF844, ZNF326, FRYL, C9orf142, ZNF710, GTF2IRD2B, DBX2, HIST2H4B, ZNF812, TMEM229A, GTF2H2C_2, C8orf44-SGK3*	4679	144	0.718	0.000284	Up
Oligodendrocyte development	*MYRF, GSN, KCNJ10, NKX2-2, CNTN2, FA2H, NKX6-2*	32	7	6.99	0.000187	Down
Oligodendrocyte differentiation	*BOK, MYRF, CNP, GSN, KCNJ10, NKX2-2, NOTCH1, SOX10, CNTN2, OLIG2, SOX8, FA2H, NKX6-2*	75	13	5.27	5.64e-06	Down
Phosphatase activity	*ALPL, ATP1A1, CDKN3, DUSP8, OCRL, PPP2R5D, PPP3CA, PPP3CB, PPP3R1, MAP2K1, PTPN3, PTPN4, PTPRN2, PTPRR, INPP4B, SYNJ1, PPIP5K1, LPPR4, PTPRT, PTP4A3, NT5DC3, PDP1, LPPR3, PTPN5, DUSP19, PPM1L, PPM1J*	254	27	1.81	0.00475	Up
Phosphatidylinositol binding	*HIP1, KCNJ2, MYO1E, PLD1, SNX1, IQGAP1, HIP1R, LDLRAP1, ANKFY1, PXK, ADAP2, PARD3, PLEKHF1, SNX29, ARAP1, FRMPD2, AMER2, NCF1C, C8orf44-SGK3*	187	19	2.92	9.82e-05	Down
Phospholipase C-activating G-protein coupled receptor signaling pathway	*ADRA1B, CCKBR, CHRM1, CHRM3, DRD1, GRM1, GRM5, HRH2, HTR2A, OPRK1, OPRM1, HOMER1, MCHR2*	81	13	2.84	0.00172	Up
Phospholipid binding	*ABCA1, ANXA5, LPAR1, HIP1, KCNJ2, MYO1E, PLD1, SNX1, IQGAP1, HIP1R, LDLRAP1, ANKFY1, PXK, ADAP2, PARD3, PREX1, WDFY4, PLEKHF1, SNX29, SYTL4, ARAP1, FRMPD2, AMER2, NCF1C, C8orf44-SGK3*	332	25	2.1	0.000966	Down
Phospholipid translocation	*ABCA1, P2RX7, ATP10B, ATP11A*	20	4	6.21	0.00667	Down
Positive regulation of RNA metabolic process	*ACVR1B, ARNTL, BMPR2, CAMK4, CAMK2A, CDH13, ETV1, H2AFZ, HGF, IGF1, KRAS, LUM, MEF2C, TRIM37, PPP1R12A, NEUROD2, PARK2, PLAGL1, PPP3CA, PPP3CB, PPP3R1, PRKCB, MAPK9, MAP2K1, RARA, RORB, SOX5, STAT4, THRB, NR2E1, TRAF5, WNT10B, ITGA8, LMO4, LDB2, LHX2, MICAL2, CAMKK2, TBR1, PPARGC1A, MLLT11, CELF3, KLF12, CPEB3, MAPRE3, DDN, PLCB1, SATB2, ATAD2, BCL11A, TESC, FEZF2, FBXW7, DCAF6, CELF4, ARNTL2, ATXN7L3, CAMK1D, MKL2, NEUROD6, BCL11B, CSRNP3, MED12L, RHEBL1, MTPN, SOHLH1*	1455	66	0.678	0.0011	Up
Postsynapse	*ADD2, ATP1A1, BMPR2, CACNA1C, CAMK2A, CAMK2B, CHRM1, CHRM3, CTNNA2, DLG3, DLG4, DRD1, DMTN, EPHA4, EPHA7, PTK2B, GABRA1, GABRA2, GABRA3, GABRA4, GABRA5, GABRB2, GABRB3, GABRD, GABRG3, GAP43, GLRB, GRIA1, GRIA2, GRIA3, GRIK5, GRIN2A, GRIN2B, GRM1, GRM5, ITPKA, ITPR1, KCNB1, KCNC2, KCNJ4, KCNMA1, MAP2, MYH10, NRGN, PAK1, PRKAR2B, PRKCG, SLC8A1, GLRA3, KCNAB2, ITGA8, LIN7A, CDK5R1, BSN, NEURL1, DGKI, DLGAP2, DLGAP1, HOMER1, CABP1, AKAP5, GABBR2, ARHGAP32, FRMPD4, LZTS3, BAIAP2, CAP2, ARFGEF2, LZTS1, CNKSR2, CLSTN1, RIMS1, SYNE1, NCS1, MAPK8IP2, NSMF, PCLO, SHANK1, SEPT11, ANKS1B, TENM2, LRFN2, KCTD16, LRRC7, DLGAP3, CACNG8, CLSTN2, LRRTM4, NETO1, PPP1R9B, SHANK3, CADPS2, GRIN3A, GRASP, CNIH2, LRRTM1, LRRTM3, IQSEC3*	341	98	6.47	7.81e-39	Up
Postsynaptic membrane	*CHRM1, CHRM3, DLG3, DLG4, EPHA4, EPHA7, GABRA1, GABRA2, GABRA3, GABRA4, GABRA5, GABRB2, GABRB3, GABRD, GABRG3, GLRB, GRIA1, GRIA2, GRIA3, GRIK5, GRIN2A, GRIN2B, KCNB1, KCNC2, KCNJ4, KCNMA1, GLRA3, LIN7A, NEURL1, DLGAP2, DLGAP1, HOMER1, CABP1, GABBR2, ARHGAP32, LZTS3, LZTS1, CNKSR2, CLSTN1, SYNE1, NCS1, NSMF, SHANK1, ANKS1B, TENM2, LRFN2, KCTD16, LRRC7, DLGAP3, CACNG8, CLSTN2, LRRTM4, NETO1, SHANK3, CADPS2, GRIN3A, GRASP, CNIH2, LRRTM1, LRRTM3, IQSEC3*	197	61	6.98	1.99e-26	Up
Potassium channel activity	*KCNB1, KCNC1, KCNC2, KCND3, KCNF1, KCNH1, KCNJ3, KCNJ4, KCNJ6, KCNJ9, KCNK2, KCNMA1, KCNN1, KCNQ2, KCNQ3, KCNS1, KCNS2, KCNAB1, KCNAB2, KCNAB3, KCNH4, KCNH3, KCNV1, KCNH5, KCNIP2, KCNQ5, KCNT1, KCNK15, KCNIP4, KCNH7, KCNG3, KCNT2, HCN1*	119	33	5.93	1.53e-13	Up
Presynapse	*DLG4, GABRA2, GRIA1, GRIA2, GRIN2B, ICA1, NPY1R, SNCA, STX1A, SYN1, SYN2, SYT1, SYT5, SLC30A3, FZD3, DOC2A, PPFIA4, PPFIA2, PPFIA3, BSN, SYT7, SYNGR1, DGKI, RIMS2, RIMS3, SV2B, DNM1L, RIMS1, UNC13A, DMXL2, ERC2, PCLO, SVOP, SLC17A7, SYT12, TPRG1L, SYNPR, STXBP5, SCAMP5, SLC6A17, UNC13C*	142	41	6.21	5.89e-17	Up
Presynaptic active zone	*SYN1, FZD3, PPFIA4, PPFIA2, PPFIA3, BSN, DGKI, RIMS2, RIMS3, RIMS1, UNC13A, ERC2, PCLO, SLC17A7, UNC13C*	24	15	25	7.23e-13	Up
Protein kinase C-activating G-protein coupled receptor signaling pathway	*CCK, CHRM1, DGKB, GAP43, GRM1, GRM5, HTR1B, DGKZ, DGKE, DGKI*	32	10	6.74	1.85e-05	Up
Protein lipidation	*ABCA1, ZDHHC9, PIGT, HHATL, ZDHHC14, ZDHHC11, MAP6D1, ATG4C, PIGM, ZDHHC20*	84	10	3.38	0.00152	Down
Regulation of axon guidance	*BMPR2, NRP1, SEMA3A, TBR1, FEZF2*	18	5	5.68	0.00441	Up
Regulation of neuron apoptotic process	*CACNA1A, EPHA7, PTK2B, GABRA5, GABRB2, GABRB3, GRIK5, KCNB1, MEF2C, PAK3, PARK2, PIK3CA, PRKCG, CCL2, SNCB, SNCA, STAR, STXBP1, NRP1, CDK5R1, CHL1, PPARGC1A, NSMF, OXR1, FBXW7, AGAP2*	183	26	2.47	9.7e-05	Up
Regulation of neurotransmitter levels	*DAGLA, CACNA1A, CACNA1B, CAMK2A, DRD1, GABRA2, GAD1, GLS, GRIK5, MEF2C, PAK1, PARK2, PDE1B, PFN2, SLC1A1, SLC1A2, SNCA, STX1A, STXBP1, SYN1, SYN2, SYT1, SYT5, DOC2A, PPFIA4, PPFIA2, PPFIA3, CADPS, LIN7A, SYNJ1, SYT7, DGKI, BZRAP1, NRXN1, RIMS2, RIMS3, CPLX1, HRH3, ADGRL1, RAB3GAP1, RIMS1, UNC13A, PCLO, SYTL2, SLC17A7, SYT13, SYT16, SYT12, CADPS2, SNAP47*	192	50	5.4	3.37e-18	Up
Regulation of postsynaptic membrane potential	*DLG4, PTK2B, FGF14, GABRB3, GRIK5, GRIN2A, GRIN2B, MEF2C, PPP3CA, SNCA, STX1A, DGKI, NRXN1, RIMS2, RAB3GAP1, RIMS1, MAPK8IP2, SHANK1, CELF4, SLC17A7, NETO1, SHANK3*	59	22	8.92	3.58e-12	Up
Regulation of synaptic plasticity	*ATP2B2, CAMK2A, CAMK2B, DLG4, DRD1, PTK2B, FGF14, GRIA1, GRIN2A, GRIN2B, GRM5, HRH2, ITPKA, KCNB1, MEF2C, NEUROD2, NRGN, PAK1, PPP3CB, PTGS2, PTN, SNCA, STAR, STXBP1, NR2E1, PPFIA3, SYNGAP1, SYNGR1, NEURL1, DGKI, RAPGEF2, BAIAP2, PLK2, CPEB3, RAB3GAP1, RIMS1, UNC13A, NSMF, NPTN, JPH3, NETO1, JPH4, SHANK3, SNAP47, CNTN4, LRRTM1*	132	46	8.2	1.48e-22	Up
Regulatory region nucleic acid binding	*ARNTL, ETV1, H2AFZ, HIVEP2, MEF2C, NEUROD2, PLAGL1, RARA, SATB1, SNCA, SOX5, STAT4, TBX15, LMO4, ZBTB33, BASP1, TBR1, KLF12, DDN, BCL11A, FEZF2, ARNTL2, PKNOX2, DMRTC1, NEUROD6, BCL11B, ZNF831, ZNF519, ARX, ZNF675, STOX1, SOHLH1, DMRTC1B*	790	33	0.643	0.00634	Down
Release of cytochrome c from mitochondria	*CCK, IFI6, HGF, IGF1, PARK2, MAPK9, HRK, DNM1L, MLLT11, GGCT*	55	10	3.29	0.00222	Up
SNARE binding	*CACNA1A, STX1A, STXBP1, SYT1, SYT5, DOC2A, NAPG, SYT7, STXBP5L, CPLX1, UNC13A, SYTL2, SYT13, NAPB, SYT16, SYT12, SNAP47, STXBP5*	112	18	2.91	0.000188	Up
Sodium channel activity	*SHROOM2, SCN1A, SCN1B, SCN2A, SCN2B, SCN8A, SCN3B, HCN1*	36	8	4.32	0.00141	Up
Synapse	*ADD2, ATP1A1, ATP2B1, ATP2B2, BMPR2, CACNA1C, CACNB4, CAMK2A, CAMK2B, CAMK2D, CCK, CHRM1, CHRM3, AP1S1, CTNNA2, DLG3, DLG4, DRD1, DMTN, EPHA4, EPHA7, PTK2B, GABRA1, GABRA2, GABRA3, GABRA4, GABRA5, GABRB2, GABRB3, GABRD, GABRG3, GAP43, GLRB, GRIA1, GRIA2, GRIA3, GRIK5, GRIN2A, GRIN2B, GRM1, GRM5, GRM8, ICA1, ITPKA, ITPR1, KCNB1, KCNC2, KCNH1, KCNJ4, KCNMA1, MAP2, MYH10, NPY1R, NRCAM, NRGN, OPRK1, PAK1, PDE2A, PFN2, PRKAR2B, PRKCG, PTPRN2, CCL2, SLC8A1, SNCB, SNCA, STX1A, STXBP1, SYN1, SYN2, SYT1, SYT5, SLC30A3, FZD3, GLRA3, DOC2A, PRSS12, PPFIA4, PPFIA2, KCNAB2, ITGA8, PPFIA3, CADPS, LIN7A, CDK5R1, BSN, WASF1, SYT7, SYNGR1, NEURL1, DGKI, DLGAP2, DLGAP1, NRXN1, HOMER1, CABP1, AKAP5, GABBR2, RAPGEF2, RIMS2, ARHGAP32, FRMPD4, LZTS3, RIMS3, SV2B, DNM1L, OLFM1, BAIAP2, SLC9A6, CAP2, ARFGEF2, CPLX1, LZTS1, AAK1, CPEB3, ADGRL1, CNKSR2, CLSTN1, RIMS1, PDZRN3, UNC13A, NMNAT2, DDN, DMXL2, SYNE1, NCS1, MAPK8IP2, FRRS1L, MYRIP, NSMF, ERC2, CYFIP2, NPTN, PCLO, PACSIN1, SHANK1, NRN1, SVOP, SEPT11, SEPT3, ANKS1B, SLC17A7, TENM2, TBC1D24, LRFN2, KCTD16, LRRC7, DLGAP3, CACNG8, CLSTN2, LRRTM4, NETO1, PPP1R9B, SHANK3, SYT12, CADPS2, PRRT2, GRIN3A, OLFM3, TPRG1L, SYNPR, STXBP5, CBLN4, GRASP, SCAMP5, PHACTR1, CNIH2, LRRTM1, LRRTM3, VWC2, SLC6A17, IQSEC3, UNC13C*	658	173	6.11	1.15e-62	Up
Synapse maturation	*CAMK2B, NEUROD2, NEURL1, NRXN1, ADGRL1, SHANK1*	18	6	7.39	0.000626	Up
Synaptic transmission	*ADCY1, ATP2B2, CA7, CACNA1A, CACNA1B, CACNA1C, CACNB1, CACNB2, CACNB4, CAMK4, CAMK2A, CAMK2B, CAMK2D, CHRM1, CHRM3, DLG3, DLG4, DRD1, EGR3, PTK2B, FGF14, GABRA1, GABRA2, GABRA3, GABRA4, GABRA5, GABRB2, GABRB3, GABRD, GABRG3, GAD1, GLRB, GLS, GNAI3, GRIA1, GRIA2, GRIA3, GRIK5, GRIN2A, GRIN2B, GRM1, GRM5, GRM8, HRH2, HTR1B, HTR1E, HTR1F, HTR2A, ITPKA, KCNB1, KCNC1, KCNC2, KCND3, KCNF1, KCNH1, KCNJ3, KCNJ4, KCNJ6, KCNJ9, KCNK2, KCNMA1, KCNN1, KCNQ2, KCNQ3, KCNS1, KCNS2, KIF5A, MEF2C, MYO5A, NEUROD2, NPY5R, OPRK1, OPRM1, PAK1, PARK2, PFN2, PPP3CA, PPP3CB, PRKCB, PRKCE, PRKCG, PTGS2, PTN, RIT2, SCN1B, SCN2B, CCL2, SLC1A1, SLC1A2, SNCB, SNCA, SSTR2, SSTR4, STAR*,	702	184	6.18	1.54e-66	Up
	*STX1A, STXBP1, SYN1, SYN2, SYT1, SYT5, NR2E1, VIPR1, KCNAB1, GLRA3, DOC2A, PPFIA4, PPFIA2, KCNAB2, PPFIA3, CADPS, LIN7A, SYNGAP1, SYNJ1, BSN, SYT7, SYNGR1, NEURL1, DGKI, KCNAB3, DLGAP2, DLGAP1, BZRAP1, NRXN1, HOMER1, AKAP5, GABBR2, RAPGEF2, RIMS2, RIMS3, SNAP91, CACNG3, BAIAP2, CSPG5, PLK2, CPLX1, HRH3, CPEB3, ADGRL1, CLSTN1, RAB3GAP1, RIMS1, UNC13A, PLCB1, KCNH4, KCNH3, MAPK8IP2, RASD2, NSMF, SLITRK5, KCNV1, NPTN, KCNH5, PCLO, TMOD2, KCNIP2, SHANK1, SHC3, SYTL2, PCDHB13, KCNQ5, CELF4, SLC17A7, JPH3, SYT13, CACNG8, CLSTN2, NETO1, SYT16, CAMKK1, JPH4, PPP1R9B, SHANK3, KCNH7, SYT12, CADPS2, BTBD9, GRIN3A, SNAP47, CNTN4, KCNG3, CNIH2, LRRTM1, HCN1, UNC13C*					
Synaptic transmission, glutamatergic	*CACNA1A, CACNB4, DRD1, PTK2B, GRIA1, GRIA2, GRIA3, GRIK5, GRIN2A, GRM1, GRM5, GRM8, HTR1B, HTR2A, MEF2C, PAK1, PARK2, PTGS2, SYT1, DGKI, NRXN1, RAB3GAP1, UNC13A, MAPK8IP2, SHC3, SLC17A7, SHANK3, GRIN3A, CNIH2*	78	29	8.94	1.42e-15	Up
Synaptic vesicle exocytosis	*GRIK5, PFN2, STX1A, STXBP1, SYN1, SYT1, SYT5, DOC2A, CADPS, SYNJ1, SYT7, RIMS3, CPLX1, ADGRL1, RIMS1, UNC13A, PCLO, SYTL2, SYT13, SYT16, SYT12, CADPS2, SNAP47*	76	23	6.51	1.59e-10	Up
Synaptic vesicle localization	*FGF14, GRIK5, PARK2, PFN2, SH3GL2, SNCA, STX1A, STXBP1, SYN1, SYT1, SYT5, AP3B2, DOC2A, CADPS, LIN7A, SYNJ1, SYT7, NRXN1, RIMS3, CPLX1, ADGRL1, RIMS1, UNC13A, PCLO, PACSIN1, SYTL2, SYT13, SYT16, SYT12, CADPS2, BTBD9, SNAP47*	120	32	5.49	1.97e-12	Up
Synaptic vesicle membrane	*ICA1, STX1A, SYN1, SYN2, SYT1, SYT5, SLC30A3, DOC2A, SYT7, SYNGR1, SV2B, DNM1L, DMXL2, SVOP, SLC17A7, SYT12, SYNPR, SCAMP5, SLC6A17*	55	19	7.94	4.52e-10	Up
Synaptic vesicle priming	*STX1A, STXBP1, CADPS, SYNJ1, CADPS2, SNAP47*	12	6	14.8	4.34e-05	Up
Synaptic vesicle recycling	*FGF14, SH3GL2, SNCA, SYT1, SYT5, SYNJ1, PACSIN1, BTBD9*	29	8	5.64	0.000338	Up
Synaptic vesicle transport	*FGF14, GRIK5, PARK2, PFN2, SH3GL2, SNCA, STX1A, STXBP1, SYN1, SYT1, SYT5, AP3B2, DOC2A, CADPS, LIN7A, SYNJ1, SYT7, RIMS3, CPLX1, ADGRL1, RIMS1, UNC13A, PCLO, PACSIN1, SYTL2, SYT13, SYT16, SYT12, CADPS2, BTBD9, SNAP47*	116	31	5.51	4.11e-12	Up
Syntaxin binding	*CACNA1A, STXBP1, SYT1, SYT5, DOC2A, NAPG, SYT7, STXBP5L, CPLX1, UNC13A, SYTL2, SYT13, NAPB, SYT16, SYT12, SNAP47, STXBP5*	78	17	4.24	4.82e-06	Up
Terminal bouton	*CCK, AP1S1, GRIK5, GRIN2A, KCNC2, KCNMA1, PFN2, PTPRN2, SNCA, STXBP1, SYN1, PRSS12, SYT7, SYNGR1, CPLX1, AAK1, TBC1D24*	61	17	5.8	1.39e-07	Up

Up-regulated genes in ALS anterior horn of the spinal cord cluster into inflammatory responses, metal ion regu-lation and hemostasis; whereas down-regulated genes cluster into neuronal axonal cytoskeleton and apoptosis.

**Figure 2:**
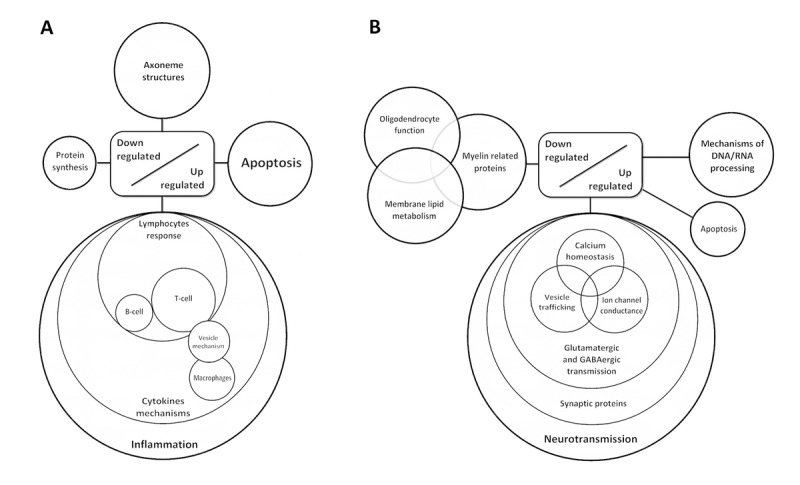
Diagram showing de-regulated gene clusters in the anterior horn of the spinal cord (**A**) and frontal cortex area 8 in ALS (**B**) as revealed by whole transcriptome arrays.

In contrast, clusters of up-regulated genes were involved in neurotransmission, ion channels and ion transport, synapses, maintenance of axons and dendrites, intracellular signaling and synaptic vesicle mechanisms. The majority of down-regulated genes were encoded for proteins associated with myelin and glial cell regulation (Figure 2).

### RT-qPCR validation

Sixty-six genes from different pathways were selected for validation by RT-qPCR.

### Inflammatory gene expression in the anterior horn of the spinal cord

No modifications in the expression levels of glial fibrillary acidic protein gene (*GFAP*) or prostaglandin-endoperoxide synthase 2 gene *(PTGS2*) occurred in ALS when compared with controls (p=0.31 and p=0.55, respectively). However, expression levels of *AIF1* and *CD68* were significantly increased in the anterior horn of the spinal cord in ALS (p=0.044 and p=0.00023, respectively). Gene expression of toll-like receptors (TLRs) *TLR2, TLR and TLR7* was significantly increased in the spinal cord in ALS cases (p=2.48E-05, p=0.00011 and p=0.00074, respectively), but *TLR4* was not (p=0.669). *IL1B* was up-regulated (p=0.005), but *IL6* and *IL6ST* were not (p=0.26 and p=0.76, respectively). In contrast, the expression of *IL10* and its corresponding receptors *IL10RA* and *IL10RB* was increased in ALS (p=0.00046, p=0.022 and p=3.23E-05, respectively). *TNFA* expression was significantly increased whereas a trend was found for *TNFRSF1B* (p=0.04 and p=0.08, respectively). The expression of *CTSC* and *CTSS* was significantly increased in spinal cord in ALS (p=5.82204E-05 and p=0.00014, respectively). Levels of *SLC11A1* were also significantly increased in spinal cord of ALS (p=0.014). *HLA-DRB1*, a protein coding gene for the Major Histocompatibility Complex Class II (MHC-II) DR β1 protein was markedly up-regulated in ALS (p=0.004365).

*PDCD1LG2*, *IFNγ* and *IL33* were significantly up-regulated in the anterior horn of the spinal cord in ALS (p=0.00153, p=0.03 and p=0.0032, respectively).

**Figure 3:**
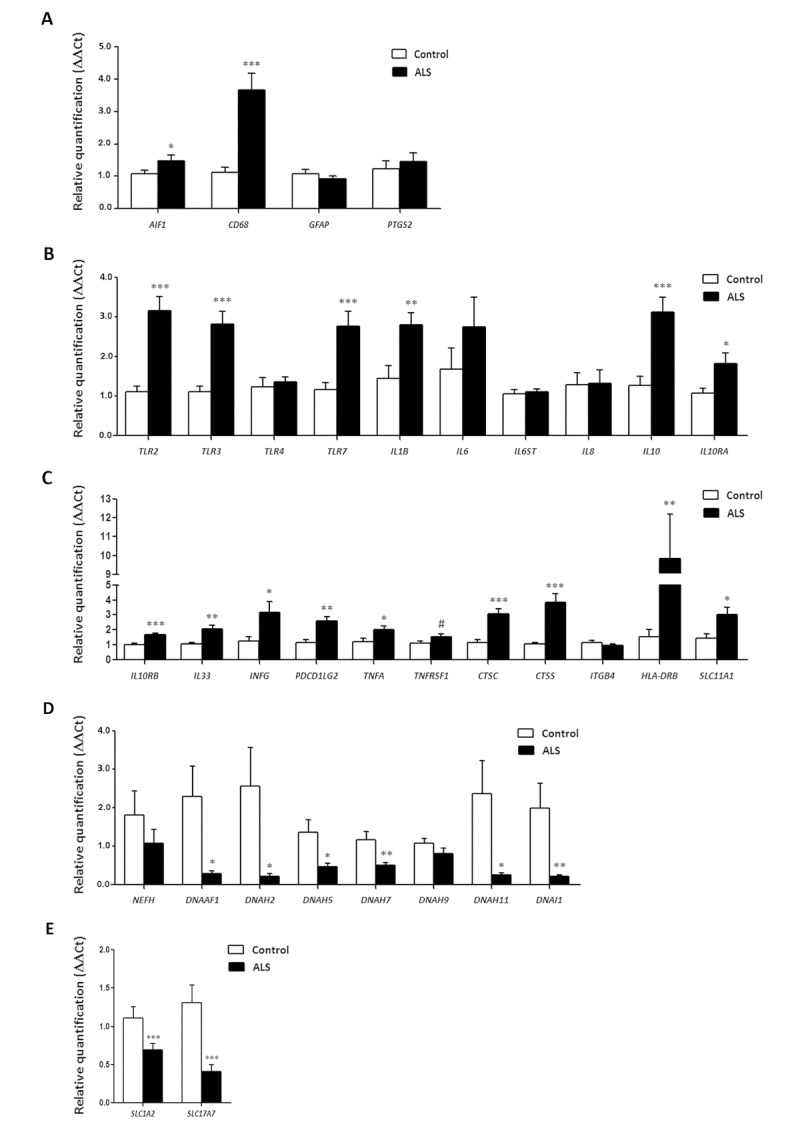
mRNA expression levels of selected deregulated genes identified by microarray analysis in the anterior horn of the spinal cord in ALS determined by TaqMan RT-qPCR assays. (**A**) general glial markers; (**B**-**C**) mediators of the inflammatory response; and (**D**) axolemal components. Up of AIGF1 and CD68, toll-like receptors, cytokines and receptors, chemokines and other mediators of the innate and adaptative inflammatory responses. Axolemal genes, excepting NEFH, which shows a non-significant trend to decrease, are significantly down-regulated. (**E**) glutamate transporter coding genes. The significance level is set at * p < 0.05, ** p < 0.01 and *** p < 0.001.

Finally, *IL8* (interleukin 8) and *ITGB4* (integrin subunit beta 4) expression was similar in control and ALS cases (p=0.92 and p=0.40, respectively) (Figure 3).

### Axonemal gene expression in anterior horn of the spinal cord

No modifications in the expression levels of *NEFH*, which codes for neurofilament heavy polypeptide protein, was seen in ALS when compared with controls (p=0.30). However, *DNAAF1* levels were significantly reduced (p=0.019). Expression of *DNAH2*, *DNAH5*, *DNAH7* and *DNAH11* mRNA was significantly reduced in ALS (p=0.029, p=0.012, p=0.005 and p=0.023, respectively), whereas *DNAH9* mRNA was not altered (p=0.14). *DNAI1* mRNA expression was also significantly reduced in ALS (p=0.0086) (Figure 3).

### SLC1A2 and SC17A7 expression in anterior horn of the spinal cord

SLC1A2 and SLC17A7 expression levels were significantly decreased in the anterior horn of the spinal cord in ALS anterior (p=0.000115 and p=0.000125, respectively). See Figure 3.

### Neurotransmission-related gene expression in frontal cortex area 8

*GRIA1*, which codes for the ionotropic glutamate receptor AMPA 1, and *GRIN2A* and *GRIN2B*, coding for NMDA receptors, were significantly up-regulated (p=0.018, p=0.018 and p=0.029, respectively) in frontal cortex in ALS cases. *GRM5*, which codes for the glutamate metabotropic receptor 5, was also up-regulated (p=0.0079). However, no significant alteration was seen in the expression of *NETO1* (p=0.165).

**Figure 4:**
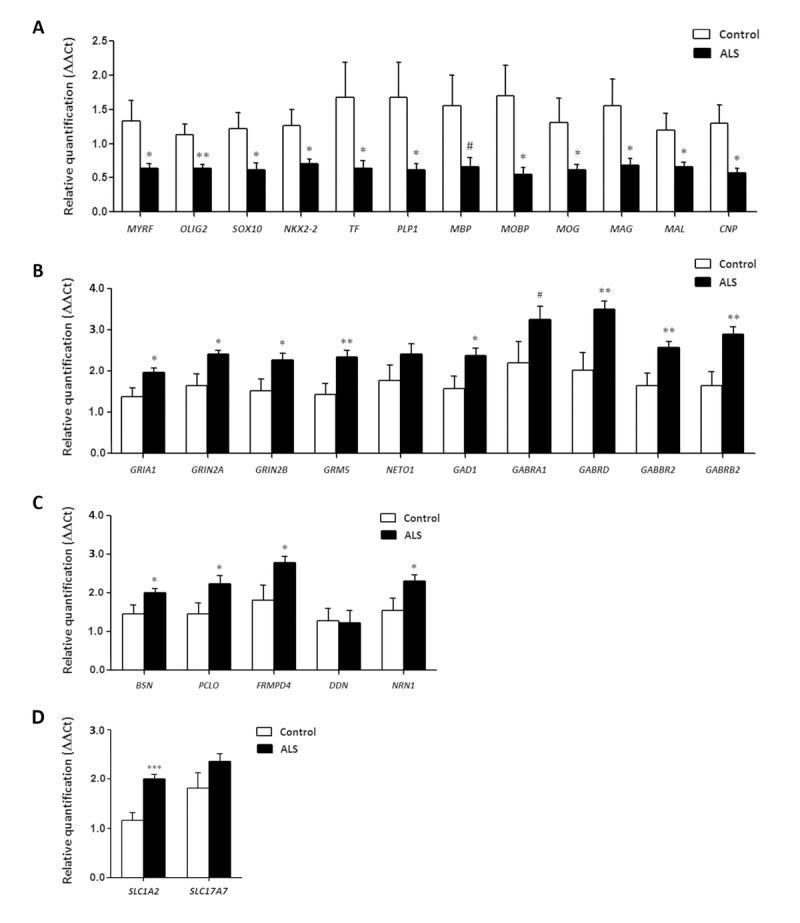
mRNA expression levels of selected deregulated genes identified by microarray analysis in frontal cortex area 8 of ALS cases determined by TaqMan RT-qPCR assays. (**A**) oligodendroglial and myelin-related genes; (**B**) glutamatergic and GABAergic-related genes and corresponding ionotropic and metabotropic receptors; (**C**) genes coding for synaptic cleft proteins. Significant up of genes linked to neurotransmission and synapses, and significant down of genes linked to oligodendroglia and myelination. (**D**) Glutamate transporter coding genes. The significance level is set at * p < 0.05, ** p < 0.01 and *** p < 0.001, and tendencies at # < 0.1.

Regarding the GABAergic system, *GAD1* was up-regulated in ALS (p=0.034). Gene expression of GABA receptors *GABRA1*, *GABRD, GABRB2* was increased (p=0.09, tendency, p=0.006 and p=0.0029, respectively). *GABBR2* mRNA levels were also significantly elevated in the frontal cortex in ALS (p=0.01) (Figure 4).

### Synaptic cleft gene expression in frontal cortex area 8

*BSN*, which codes for Bassoon, a pre-synaptic cytoskeletal matrix, was up-regulated in ALS (p=0.04). mRNA levels of *PCLO*, coding gene for Piccolo protein, and *FRMPD4* were also increased in ALS (p=0.036 and p=0.029, respectively), Finally, *NRN1*, which codes for neuritin 1, but not DDN, which codes for dendrin protein, was up-regulated in the frontal cortex in ALS (p=0.04 and p=0.92, respectively) (Figure 4).

### Myelin- and oligodendrocyte-related gene expression in frontal cortex area 8

Significant decrease in mRNA expression of myelin transcription factor (*MYRF*) (p= 0.028), *OLIG2* (p=0.009), *SOX10* (p=0.02), *NKX2-2* (p=0.032), transferring (TF) (p=0.5), proteolipid protein 1 (*PLP1*) (p=0.040), myelin basic protein (*MBP*) (p=0.061), myelin-associated oligodendrocyte basic protein (*MOBP*) (p=0.019), oligodendrocyte glycoprotein (*MOG*) (p=0.05), Mal T-cell differentiation protein (*MAL*) (p=0.039), myelin associated glycoprotein (*MAG*) (p=0.035), and 2',3'-cyclic nucleotide 3' phosphodiesterase (*CNP1*) (p=0.017) was seen in frontal cortex in ALS cases compared with controls (Figure 4).

### *SLC1A2* and *SLC17A7* expression in frontal cortex area 8

*SLC1A2* expression was significantly increased (p=5.25e-5) whereas *SLC17A7* mRNA showed a non-significant increase (p=0.42) in frontal cortex area 8 in ALS (Figure 4).

### Immunohistochemistry in spinal cord

**Figure 5:**
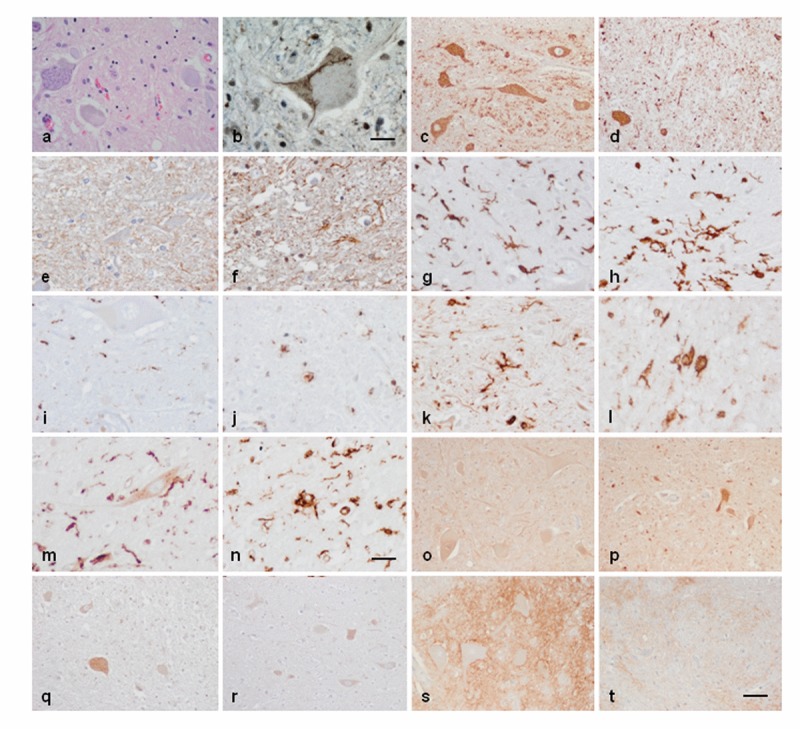
Anterior horn of the spinal cord. Haematoxilin and eosin staining showing damaged neurons in ALS (**a**). Immuno-histochemistry to TDP-43 showing skein-like intracytoplasmic inclusions (**b**), VDAC (**c**, **d**), GFAP (**e**, **f**), IBA-1 (**g**, **h**), CD68 (**i**, **j**), HLA-DRB1 (**k**, **l**), HLA-DRB5 (**m**, n), IL-10 (**o**, **p**), TNF-α (**q**, **r**) and GluT (SLC1A2) (**s**, **t**) in the anterior horn of the lumbar spinal cord in control (**c**, **e**, **g**, **I**, **k**, **m**, **o**, **q**, **s**) and sALS (**a**, **b**, **d**, **f**, **h**, **j**, **l**, **n**, **p**, **r**, **t**) cases. TDP-43-immunoreactive cytoplasmic inclusions are seen in motor neurons in sALS. GFAP is increased in reactive astrocytes; microglial cells have a round, amoeboid morphology as seen with IBA-1, CD-68, HLA-DRB1, and HLA-DRB5 antibodies. VDAC immunoreactivity is decreased whereas IL-10 and TNF-α is increased in remaining motor neurons in sALS. SLC1A2 immunoreactivity is reduced in the membrane of neurons and in neuropil of the anterior horn in sALS. Paraffin sections, slightly counterstained with haematoxylin; a, c-d, o-t, bar in t = 40μm; e-n, bar in = 20μm; bar in b = 10μm

The anterior horn of the spinal cord in ALS cases showed decreased number of neurons and altered morphology of most remaining motor neurons including loss of endoplasmic reticulum (chromatolysis) and axonal ballooning (Figure 5a) and intracytoplasmic TDP-43-immunoreactive inclusions (Figure 5b). Immunohistochemistry was carried out in the lumbar spinal cord in control and sALS cases (Figure 5a and b). VDAC was reduced in a subpopulation of neurons in the anterior horn in ALS, but not in neurons of the Clarke's column and posterior horn, when compared with controls (Figure 5c and d). Increased expression of GFAP was found in reactive astrocytes in the lateral columns and anterior horn of the spinal cord in ALS cases (Figure 5e and f). Marked differences were seen regarding microglial cell markers: IBA-1 and CD68 immunoreactivity was dramatically increased in the pyramidal tracts and anterior horn in ALS; moreover the morphology of microglia was modified in pathological cases with predominance of round, amoeboid microglia (Figure 5g-j). Similar immunoreactivity, distribution and morphology were found in reactive microglia using antibodies against HLA-DRB1 and HLA-DRB5 (Figure 5k-n). In contrast IL-10 and TNF-α immunoreactivity predominated in neurons; immunoreactivity was increased in neurons in ALS cases compared with controls (Figure 5o-r). Finally, GluT (SLC1A2), the transporter of glutamate from the extracellular space at synapses, was expressed in the membrane of neurons and in the neuropil; SLC1A2 immunoreactivity was decreased in neurons and neuropil of the anterior horn in ALS (Figure 5s, t).

### Gel electrophoresis and western blotting in frontal cortex area 8

**Figure 6:**
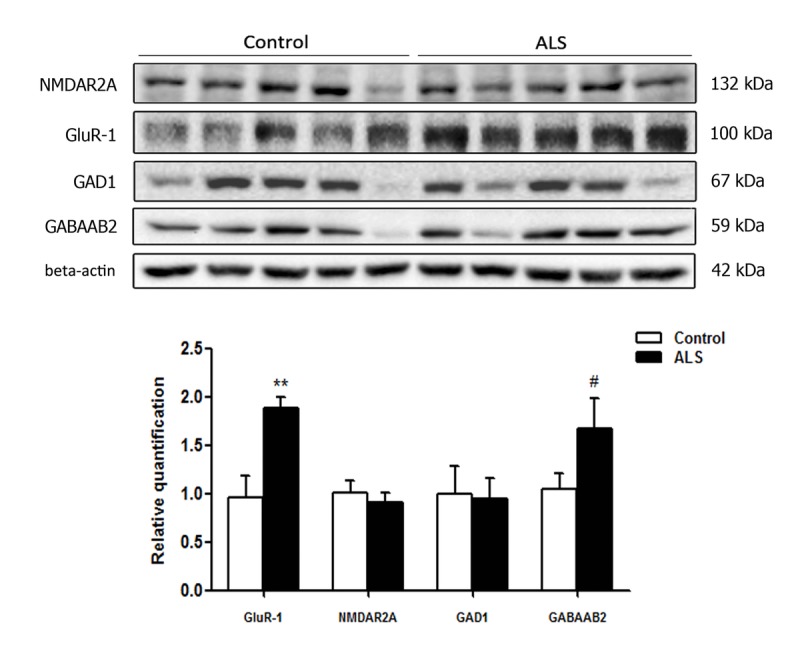
Gel electrophoresis and western blotting to glutamate receptor ionotropic, NMDA 2A (NMDAR2A), α‐amino‐3‐hydroxy‐5‐methyl‐4‐isoxazolepropionic acid receptor 1 (GluR‐1), glutamate decarboxylase 1 (GAD1) and gamma‐aminobutyric acid receptor subunit beta‐2 (GABAAB2) in the frontal cortex area 8 of control and ALS. Significant increased levels of GluR‐1 and a tendency to increased levels of GABAAB2 are seen in ALS when compared with controls. The significance level is set at ** p < 0.01 and tendencies at # < 0.1.

A few tested antibodies were eventually suitable for western blotting studies. No differences in the expression levels of glutamate receptor ionotropic, NMDA 2A (NMDAR2A) and glutamate decarboxylase 1 (GAD1) were observed between control and ALS cases. However, a significant increase in α-amino-3-hydroxy-5-methyl-4-isoxazolepropionic acid receptor 1 (AMPAR GluR-1) ** p < 0.01 and a tendency to increase in the expression of gamma-aminobutyric acid receptor subunit beta-2 (GABAAB2) (# p < 0.1) was found in the frontal cortex in ALS when compared to controls (Figure 6).

## DISCUSSION

Transcriptomic profiles in ALS are region-dependent when comparing the anterior horn of the lumbar spinal cord and frontal cortex area 8 in the same individuals. As an important regional difference related to excitotoxicity, the expression of glutamate transporters is markedly different in the anterior horn of the spinal cord and the frontal cortex area 8. *SLC1A2* and *SLC17A7* mRNA expression is significantly decreased in the anterior horn of the spinal cord, whereas *SLC1A2* is significantly increased in frontal cortex area 8. *SLC1A2* encodes the solute carrier family 1 member 2 or excitatory amino-acid transporter 2 (EAAT2) which clears glutamate from the extracellular space at synapses in the central nervous system. Immunohistochemistry has shown decreased SLC1A2 protein expression in the membrane of neurons and neuropil of the anterior horn in ALS. *SLC17A7* encodes the vesicular glutamate transporter 1 (VGLUT1) which is a vesicle-bound, sodium-dependent phosphate glutamate transporter expressed in the synaptic vesicles. Decreased expression of these proteins is linked to increased excitotoxity which is postulated as primary factor triggering motor neuron degeneration in ALS [30, 31].

Whole transcriptome arrays show that major up-regulated clusters in the anterior horn are related with innate inflammatory and adaptative inflammatory responses. Genes involved in hemostasis and ion transport forms a small up-regulated group. The major group of down-regulated genes is linked to the neuronal cytoskeleton. The majority of significantly differentially up-regulated transcripts in sALS in frontal cortex area 8, as revealed by whole transcriptome arrays, code for proteins linked with neurotransmission, ion channels and ion transport, synapses, and axon and dendrite maintenance, whereas down-regulated genes code for proteins involved in oligodendrocyte development and function, myelin regulation and membrane lipid metabolism.

Altered gene expression as revealed by whole transcriptome arrays has been validated by RT-qPCR in 58 of 66 assessed genes. These observations increase the list of genes which are de-regulated in the anterior spinal cord and provide, for the first time, robust evidence of gene de-regulation in frontal cortex area 8 in sALS. Increased inflammatory response in the anterior horn and increased expression of selected neurotransmitter markers in frontal cortex has been further assessed using immunohistochemistry and western blotting, respectively.

### Inflammation in the anterior horn of the spinal cord

*AIF1* gene codes for the Allograft Inflammatory Factor 1, a protein induced by cytokines and interferon which promotes macrophage and glial activation [32, 33]. *CD68* codes for the macrophage antigen CD68 glycoprotein which is expressed by microglial cells [35-37], the principal resident immune cell population in brain [38, 39]. Microglia pro-inflammatory state activation can be initiated by engagement of germline-encoded pattern-recognition receptors such as Toll-like receptors (TLRs) which are expressed in glial cells [40]. TLR activation, in turn, activates phagocytosis [41-43] and pro-inflammatory responses [44]. Up-regulated interleukins in ALS are *IL1B*, the coding gene for interleukin 1B an important mediator of the inflam-matory response [45], interleukin 10 (encoded by *IL10)* which has pleiotropic effects down-regulating the expression of Th1 cytokines, MHC class II antigens and co-stimulating the production of several molecules by macrophages through the activation of IL10 receptor subunit α and subunit β (encoded by *IL10RA* and *IL10RB*, respectively) [46]. However, IL6 mRNA, which encodes a specific pro-inflammatory cytokine with regenerative and anti-inflammatory activities in particular settings [47-50] is not modified. Tumor Necrosis Factor Receptor Superfamily Member 1A (encoded by *TNFA*) is involved in the regulation of a wide spectrum of biological processes including cell proliferation, cell differentiation, apoptosis, lipid metabolism and coagulation [50, 51]. *CTSC* gene encodes Cathepsin C which is central coordinator of activation of many serine proteinases in immune cells [52]. *CTSS* codes for a protein of the same family, Cathepsin S, which acts as a key protease responsible for the removal of the invariant chain from MHC class II antigens [53]. *SLC11A1* encodes natural resistance-associated macrophage protein 1, which acts as a host resistance to certain pathogens [54].

Major Histocompatibility Complex Class II (MHC-II) DR β1 protein, encoded by *HLA-DRB-1,* plays a central role in the immune system by presenting peptides derived from extracellular proteins [55, 56] and participate in the activation of autophagosomes [57]. *PDCD1LG2* codes for Programmed Cell Death 1 Ligand 2, a protein involved in co-stimulatory signals essential for T-cell proliferation and IFN-γ production [58]. *IFNγ* gene, which codes for the cytokine interferon-*γ*, is key player in antigen-specific immune responses [59]. Finally, interleukin 33, encoded by *IL33*, acts as a chemo-attractant for Th2 cells and functions as an ‘alarm’ that amplifies immune responses during tissue injury [60].

Increased inflammatory response in the anterior horn of the spinal cord has been further documented by immunohistochemistry showing increased expression of IBA-1, the protein encoded by *AIF1*, CD68, and HLA-DRB1 and HLA-DRB5 in reactive microglia. Reactive microglia has a round, amoeboid morphology and is also localized, as expected in the lateral and anterior pyramidal tracts. IL-10 and TNF-α are mainly localized in neurons of the spinal cord, and its expression is increased in remaining motor neurons of the spinal cord in ALS. These findings indicate a parallelism between gene expression and protein expression regarding inflammatory responses of assessed molecules. On the other hand the different localization of microglial markers, and IL-10 and TNF-α in neurons points to a cross-talk between microglia and neurons in the anterior horn of the spinal cord in ALS.

This is in contrast with other markers as glial fibrillary acidic protein and voltage dependent anion channel in which levels of mRNA differ from levels (or intensity) of protein expression. No modifications in the expression of *GFAP* mRNA are observed in the present study, but GFAP immunoreactivity is clearly increased in reactive astrocytes, as already reported in classical neuropathological studies. *VDAC* mRNA is not abnormally regulated in gene arrays; yet VDAC is decreased in motor neurons, but not in neurons of the Clarke's column and neurons of the posterior horn, of the spinal cord in ALS. VDAC immunohistochemistry is in line with observations in human sALS showing deficiencies in mitochondria and energy metabolism [61, 62].

### Reduced expression of axolemal genes in anterior horn of the spinal cord

The expression levels of *NEFH*, which codes for neurofilament heavy polypeptide protein [63], are preserved in ALS. However, *DNAAF1,* which encodes dynein (axonemal) assembly factor 1, and mRNAs encoding several dynein axonemal heavy chains (DHC) are down-regulated thus suggesting impairment of motor ATPases involved in the transport of various cellular cargoes by ‘walking’ along cytoskeletal microtubules towards the minus-end of the microtubule [64-66].

### Up-regulation of neurotransmission-related genes and synaptic cleft genes in frontal cortex

Genes involved in glutamatergic and GABAergic transmission are up-regulated in the frontal cortex in ALS. This applies to genes encoding the ionotropic glutamate receptor AMPA 1 (*GRIA1*), glutamate ionotropic receptor NMDA type subunit 2A (*GRIN2A*), the glutamate ionotropic receptor NMDA type subunit 2B (*GRIN2B)*, and glutamate metabotropic receptor 5 (*GRM5*). Regarding the GABAergic system, *GAD1*, coding for glutamate decarboxylase 1, a rate-limiting enzyme that acts in the decarboxylation of glutamate essential for the conversion reaction of GABA from glutamate [67, 68], is up-regulated, as are *GABRA1*, *GABRD, GABRB2,* which code for different subunits of ionotropic GABA-A receptors. *GABBR2,* which codes for the metabotropic receptor component Gamma-Aminobutyric Acid Type B Receptor Subunit 2 and forms heterodimers with GABBR1, thus resulting in the formation of the G-protein coupled receptor for GABA [69], is also up-regulated in ALS.

In line with increased expression of neurotransmitter-related genes, several genes encoding molecules linked with the synaptic cleft are also up-regulated in ALS. *BSN* codes for Bassoon, a pre-synaptic cytoskeletal matrix (PCM) protein acting as a scaffolding protein and essential for the regulation of neurotransmitter release in a subset of synapses [70, 71]. *PCLO* codes for Piccolo protein, a component of the PCM assembled in the active zone of neurotransmitter release [72, 73]. *FRMPD4* codes for PSD-95-interacting regulator of spine morphogenesis protein which regulates dendritic spine morphogenesis and is required for the maintenance of excitatory synaptic transmission [74]. *DDN* and *NRN1* code for dendrin protein and neuritin 1 protein, respectively which are involved in the remodeling of the postsynaptic cytoskeleton and neuritic outgrowth [75-77].

De-regulation of neurotransmitters and receptors is further supported by the demonstration of significant increase in the levels of GluR-1 and a tendency in those of GABAAB2 in the frontal cortex area 8 in ALS when compared with controls. It is worth stressing that only a few antibodies of the total assessed (eight) were suitable for western blotting.

### Myelin and oligodendrocyte genes in frontal cortex area 8

Myelin transcription factor (encoded by *MYRF*) regulates oligodendrocyte differentiation and is required for central nervous system myelination [78-81]. The basic loop- helix protein OLIG2 mediates motor neuron and oligodendrocyte differentiation [22, 82]. High mobility group protein SOX10 modulates myelin protein transcription [83, 84]. NKX2.2 homeodomain transcription factor is a key regulator of oligodendrocyte differentiation [85]. Transferrin encoded by *TF* participates in the early stages of myelination [86, 87]. Proteolipid protein 1 (encoded by *PLP1*) plays a role in the compaction, stabilization, and maintenance of myelin sheaths, as well as in oligodendrocyte development and axonal survival [88, 89]. Myelin basic protein (encoded by *MBP*) is the second most abundant myelin-associated protein, constituting about 30% of total myelin protein [90]. Myelin-associated oligodendrocyte basic protein (encoded by *MOBP*) constitutes the third most abundant protein in CNS myelin and it acts by compacting and stabilizing myelin sheaths [91]. Myelin oligodendrocyte glycoprotein (encoded by *MOG*) is a cell surface marker of oligodendrocyte maturation [92]. Myelin associated glycoprotein (encoded by *MAG*) is a type I membrane protein and member of the immunoglobulin super-family involved in the process of myelination and certain myelin-neuron cell-cell interactions [93]. Mal T-cell differentiation protein (encoded by *MAL*) is involved in myelin biogenesis [94]. Finally, 2',3'-cyclic nucleotide 3' phosphodiesterase (encoded by *CNP1*) participates in early oligodendrocyte differentiation and myelination [95-97].

### Concluding comments

Results of the present study validate gene expression of individual studies performed in a limited number of samples identifying a limited number of de-regulated genes in the anterior horn of the spinal cord [17, 20, 21, 25]. Present results are more close to those carried out by using laser micro-dissection of anterior horn spinal motor neurons [27] thus reinforcing the consistence of observations in both studies. Whether some changes are related to the variable progression of the disease need further study with a larger number of cases of rapid or slow clinical course. In this line, altered mitochondria, protein degradation and axonal transport predominate in the 129Sv-SOD1(G93A) transgenic mouse with rapidly progressive motor neuron disease, whereas increased immune response is found in the C57-SOD1(G93A) transgenic mouse with more benign course [98].

The most important aspect of the present study is the description of altered gene expression and identification of altered clusters of genes in the frontal cortex area 8 in sALS cases without apparent cognitive impairment. It is worth stressing that altered clusters differ in the spinal cord and frontal cortex in sALS at terminal stages thus providing valuable information of molecular ab-normalities which can also be present within the spectrum of FTLD-TDP. Noteworthy, altered regulation of transcription related to synapses and neuro-transmission covering neurotransmitter receptors, synaptic proteins and ion channels in the frontal cortex in the absence of overt clinical symptoms of cognitive impairment are particularly important to identify early molecular alterations in frontal cortex with the spectrum of ALS/FTLD-TDP.

## MATERIALS AND METHODS

### Tissue collection

Post-mortem fresh-frozen lumbar spinal cord (SC) and frontal cortex (FC) (Brodmann area 8) tissue samples were from the Institute of Neuropathology HUB-ICO-IDIBELL Biobank following the guidelines of Spanish legislation on this matter and the approval of the local ethics committee. The post-mortem interval between death and tissue processing was between 2 and 17 hours. One hemisphere was immediately cut in coronal sections, 1-cm thick, and selected areas of the encephalon were rapidly dissected, frozen on metal plates over dry ice, placed in individual air-tight plastic bags, numbered with water-resistant ink and stored at −80°C until use for biochemical studies. The other hemisphere was fixed by immersion in 4% buffered formalin for 3 weeks for morphologic studies. Transversal sections of the spinal cord were alternatively frozen at −80°C or fixed by immersion in 4% buffered formalin. The whole series included 18 sALS cases and 23 controls. The anterior horn of the spinal cord was examined in 14 sALS (mean age 57 years; 6 men and 8 women) and the frontal cortex area 8 in 15 sALS (mean age 54 years; 11 men and 4 women). Spinal cord and frontal cortex were available in 11 cases. Lumbar anterior spinal cord was dissected on a dry-ice frozen plate under a binocular microscope at a magnification x4. TDP-43-immunoreactive small dystrophic neurites and/or TDP-43-positive granules and/or small cytoplasmic globules in cortical neurons in the contralateral frontal cortex area 8 were observed in 11 of 18 cases, but only abundant in three cases (cases 29, 30 and 31 in Table 3). Spongiosis in the upper cortical layers was found only in one case (case 28 in Table 3). Cases with frontotemporal dementia were not included in the present series. Patients with associated pathology including Alzheimer's disease (excepting neurofibrillary tangle pathology stages I-II of Braak and Braak), Parkinson's disease, tauopathies, vascular diseases, neoplastic diseases affecting the nervous system, metabolic syndrome, hypoxia and prolonged axonal states such as those occurring in intensive care units were excluded. Cases with infectious, inflammatory and autoimmune diseases, either systemic or limited to the nervous system were not included.

**Table 3:** Summary of the fifty six cases analyzed including frontal cortex area 8 of 14 controls and 15 ALS cases, and anterior horn of the spinal cord of 13 controls and 14 ALS cases

						RIN value	
Case	Age	Gender	Diagnosis	PM delay	Initial symptoms	SC	FC
1	49	F	Control	07 h 00 min	-	-	7.2
2	75	F	Control	03 h 00 min	-	-	7.2
3	55	M	Control	05 h 40 min	-	-	7.7
4	59	M	Control	12 h 05 min	-	6.4	-
5	59	M	Control	07 h 05 min	-	-	7.8
6	43	M	Control	05 h 55 min	-	6.6	7.7
7	53	M	Control	07 h 25 min	-	-	5.3
8	56	M	Control	03 h 50 min	-	-	7.6
9	47	M	Control	04 h 55 min	-	5.6	7.7
10	64	F	Control	11 h 20 min	-	6.2	-
11	46	M	Control	15 h 00 min	-	5.9	7.9
12	56	M	Control	07 h 10 min	-	6.1	-
13	71	F	Control	08 h 30 min	-	5.9	-
14	64	F	Control	05 h 00 min	-	7.0	-
15	79	F	Control	06 h 25 min	-	6.7	-
16	75	M	Control	07 h 30 min	-	5.0	-
17	55	M	Control	09 h 45 min	-	5.3	-
18	52	M	Control	03 h 00 min	-	-	8.3
19	52	M	Control	04 h 40 min	-	-	6.3
20	76	M	Control	06 h 30 min	-	6.6	-
21	60	F	Control	11 h 30 min	-	-	7.5
22	51	F	Control	04 h 00 min	-	6.3	7.9
23	54	M	Control	08 h 45 min	-	-	7.0
24	56	M	ALS	10 h 50 min	NA	7.1	-
25	70	M	ALS	03 h 00 min	Respiratory	7.3	7.0
26	77	M	ALS	04 h 30 min	NA	7.4	-
27	56	F	ALS	03 h 45 min	NA	8.2	7.7
28	59	M	ALS	03 h 15 min	NA	7.5	7.7
29	63	F	ALS	13 h 50 min	Bulbar	6.8	8.2
30	59	F	ALS	14 h 15 min	NA	6.4	6.7
31	54	M	ALS	04 h 50 min	Spinal	-	7.8
32	76	M	ALS	12 h 40 min	Spinal	-	7.4
33	64	M	ALS	16 h 30 min	NA	6.3	7.3
34	57	F	ALS	04 h 00 min	Bulbar	6.2	8.6
35	75	F	ALS	04 h 05 min	Bulbar	6.8	6.8
36	79	F	ALS	02 h 10 min	NA	7.0	-
37	57	F	ALS	10 h 00 min	Bulbar	6.5	7.1
38	50	M	ALS	10 h 10 min	Spinal	-	5.9
39	59	F	ALS	02 h 30 min	Spinal	-	7.5
40	46	M	ALS	07 h 00 min	Spinal	7.0	8.0
41	69	F	ALS	17 h 00 min	Spinal	6.4	6.3

Abbreviations: ALS: amyotrophic lateral sclerosis; F: female; M: male; PM: post-mortem delay (hours, minutes); SC: anterior horn of the spinal cord lumbar level; FC: frontal cortex area 8; RIN: RNA integrity number; NA: not available.

Age-matched control cases had not suffered from neurologic or psychiatric diseases, and did not have abnormalities in the neuropathologic examination, excepting sporadic neurofibrillary tangle pathology stages I-II of Braak and Braak. No *C9ORF72*, *SOD1*, *TARDBP* and *FUS* mutations occurred in any case. Table 3 shows a summary of cases.

### Whole-transcriptome array

RNA from frozen anterior horn of the lumbar spinal cord and frontal cortex area 8 was extracted following the instructions of the supplier (RNeasy Mini Kit, Qiagen® GmbH, Hilden, Germany). RNA integrity and 28S/18S ratios were determined with the Agilent Bioanalyzer (Agilent Technologies Inc, Santa Clara, CA, USA) to assess RNA quality, and the RNA concentration was evaluated using a NanoDrop™ Spectrophotometer (Thermo Fisher Scientific). Selected samples were analyzed by microarray hybridization with GeneChip® Human Gene 2.0 ST Array and WT Labeling Kit and microarray 7000G platform from Affymetrix® (Santa Clara, CA, USA). Microarray service was carried out at the High Technology Unit (UAT) at Vall d'Hebron Research Institute (VHIR), Barcelona, Spain.

### Microarray data and statistical analysis

Microarray data quality control, normalization and filtering were performed using bioconductor packages in an R programming environment for genes [99] which enabled data preprocessing for differential gene expression analysis and enrichment analysis. Gene selection was based upon their values using a test for differential expression between two classes (Student's t-test). Genes differentially expressed showed an absolute fold change > 2.0 in combination with a p-value ≤ 0.05.

### RT-qPCR validation

Complementary DNA (cDNA) preparation used High-Capacity cDNA Reverse Transcription kit (Applied Biosystems, Foster City, CA, USA) following the protocol provided by the supplier. Parallel reactions for each RNA sample run in the absence of MultiScribe Reverse Transcriptase to assess the lack of contamination of genomic DNA. TaqMan RT-qPCR assays were performed in duplicate for each gene on cDNA samples in 384-well optical plates using an ABI Prism 7900 Sequence Detection system (Applied Biosystems, Life Technologies, Waltham, MA, USA).

For each 10μL TaqMan reaction, 4.5μL cDNA was mixed with 0.5μL 20x TaqMan Gene Expression Assays and 5μL of 2x TaqMan Universal PCR Master Mix (Applied Biosystems). Table 4 shows identification numbers and names of TaqMan probes. The mean value of one house-keeping gene, hypoxanthine-guanine phosphoribosyltransferase (*HPRT1*), was used as internal control for normalization of spinal cord samples, whereas the mean values of the three house-keeping genes, alanyl-transfer RNA synthase (*AARS*), glucuronidase Beta (*GUS-β*) and X-prolyl amino-peptidase (aminopeptidase P) 1 (*XPNPEP1*) were used as internal controls for normalization of frontal cortex samples [100, 101]. The parameters of the reactions were 50°C for 2 min, 95°C for 10 min, and 40 cycles of 95°C for 15 sec and 60°C for 1 min. Finally, capture of all TaqMan PCR data used the Sequence Detection Software (SDS version 2.2.2, Applied Biosystems). The double-delta cycle threshold (ΔΔCT) method was used to analyze the data; results with T-student test. The significance level was set at * p < 0.05, ** p < 0.01 and *** p < 0.001, and tendencies at # < 0.1. Pearson's correlation method assessed a possible linear association between TDP-43 pathology in frontal cortex area 8 and gene deregulation in the same region; significant correlations were not found.

**Table 4:** Genes, gene symbols and TaqMan probes used for the study of gene expression in the anterior horn of the spinal cord and frontal cortex area 8 in ALS cases and controls including probes for normalization (*AARS*, *GUS-β*, *HPRT-1* and *XPNPEP-1*)

Gene	Gene symbol	Reference
2',3'-Cyclic Nucleotide 3' Phosphodiesterase	*CNP*	Hs00263981_m1
Alanyl-TRNA Synthetase	*AARS*	Hs00609836_m1
Allograft Inflammatory Factor 1	*AIF1*	Hs00741549_g1
Bassoon Presynaptic Cytomatrix Protein	*BSN*	Hs01109152_m1
Cathepsin C	*CTSC*	Hs00175188_m1
Cathepsin S	*CTSS*	Hs00356423_m1
C-X-C Motif Chemokine Ligand 8	*IL8*	Hs00174103_m1
Dendrin	*DDN*	Hs00391784_m1
Dynein (Axonemal) Assembly Factor 1	*DNAAF1*	Hs00698399_m1
Dynein Axonemal Heavy Chain 11	*DNAH11*	Hs00361951_m1
Dynein Axonemal Heavy Chain 2	*DNAH2*	Hs00325838_m1
Dynein Axonemal Heavy Chain 5	*DNAH5*	Hs00292485_m1
Dynein Axonemal Heavy Chain 7	*DNAH7*	Hs00324265_m1
Dynein Axonemal Heavy Chain 9	*DNAH9*	Hs00242096_m1
Dynein Axonemal Intermediate Chain 1	*DNAI1*	Hs00201755_m1
Gamma-Aminobutyric Acid Type A Receptor Alpha 1 Subunit	*GABRA1*	Hs00971228_m1
Gamma-Aminobutyric Acid Type A Receptor Beta 2 Subunit	*GABRB2*	Hs00241451_m1
Gamma-Aminobutyric Acid Type A Receptor Delta Subunit	*GABRD*	Hs00181309_m1
Gamma-Aminobutyric Acid Type B Receptor Subunit 2	*GABBR2*	Hs01554996_m1
Glial Fibrillary Acidic Protein	*GFAP*	Hs00909240_m1
Glutamate Decarboxylase 1	*GAD1*	Hs01065893_m1
Glutamate Ionotropic Receptor AMPA Type Subunit 1	*GRIA1*	Hs00181348_m1
Glutamate Ionotropic Receptor NMDA Type Subunit 2A	*GRIN2A*	Hs00168219_m1
Glutamate Ionotropic Receptor NMDA Type Subunit 2B	*GRIN2B*	Hs01002012_m1
Glutamate Metabotropic Receptor 5	*GRM5*	Hs00168275_m1
Hypoxanthine Phosphoribosyltransferase 1	*HPRT1*	Hs02800695_m1
Integrin Subunit Beta 4	*ITGB4*	Hs00173995_m1
Interferon, Gamma	*IFNG*	Hs00989291_m1
Interleukin 1 Beta	*IL1B*	Hs01555410_m1
Interleukin 10	*IL10*	Hs00961622_m1
Interleukin 10 Receptor Subunit Alpha	*IL10RA*	Hs00155485_m1
Interleukin 10 Receptor Subunit Beta	*IL10RB*	Hs00988697_m1
Interleukin 33	*IL33*	Hs00369211_m1
Interleukin 6	*IL6*	Hs00985639_m1
Interleukin 6 Signal Transducer	*IL6ST*	Hs00174360_m1
Macrophage Antigen CD68	*CD68*	Hs02836816_g1
Major Histocompatibility Complex, Class II, DR Beta 1/4/5	*HLA-DRB*	Hs04192463_mH
Mal T-Cell Differentiation Protein	*MAL*	Hs00360838_m1
Myelin Associated Glycoprotein	*MAG*	Hs01114387_m1
Myelin Basic Protein	*MBP*	Hs00921945_m1
Myelin Oligodendrocyte Glycoprotein	*MOG*	Hs01555268_m1
Myelin Regulatory Factor	*MYRF*	Hs00973739_m1
Myelin-Associated Oligodendrocyte Basic Protein	*MOBP*	Hs01094434_m1
Neuritin 1	*NRN1*	Hs00213192_m1
Neurofilament, Heavy Polypeptide	*NEFH*	Hs00606024_m1
Neuropilin And Tolloid Like 1	*NETO1*	Hs00371151_m1
NK2 Homeobox 2	*NKX2-2*	Hs00159616_m1
Oligodendrocyte Lineage Transcription Factor 2	*OLIG2*	Hs00377820_m1
Piccolo Presynaptic Cytomatrix Protein	*PCLO*	Hs00382694_m1
Programmed Cell Death 1 Ligand 2	*PDCD1LG2*	Hs01057777_m1
Prostaglandin-Endoperoxide Synthase 2	*PTGS2*	Hs00153133_m1
Proteolipid Protein 1	*PLP1*	Hs00166914_m1
PSD-95-Interacting Regulator Of Spine Morphogenesis	*FRMPD4*	Hs01568794_m1
Solute Carrier Family 1 (Glial High Affinity Glutamate Transporter), Member 2 (EAAT-2)	*SLC1A2*	Hs01102423_m1
Solute Carrier Family 11 Member 1	*SLC11A1*	Hs01105516_m1
Solute Carrier Family 17 (Vesicular Glutamate Transporter), Member 7 (VGLUT-1)	*SLC17A7*	Hs00220404_m1
SRY (Sex Determining Region Y)-Box 10	*SOX10*	Hs00366918_m1
Toll Like Receptor 2	*TLR2*	Hs00610101_m1
Toll Like Receptor 3	*TLR3*	Hs01551078_m1
Toll Like Receptor 4	*TLR4*	Hs01060206_m1
Toll Like Receptor 7	*TLR7*	Hs00152971_m1
Transferrin	*TF*	Hs01067777_m1
Tumor Necrosis Factor Receptor Superfamily Member 1A	*TNFRSF1*	Hs01042313_m1
Tumor Necrosis Factor-Alpha	*TNFa*	Hs01113624_g1
X-prolyl aminopepidase P1	*XPNPEP1*	Hs00958026_m1
β-glucuronidase	*GUS-β*	Hs00939627_m1

### Immunohistochemistry

De-waxed sections, 4μm thick, of the lumbar spinal cord from control and ALS cases were processed in parallel for immunohistochemistry. Endogenous peroxidases were blocked by incubation in 10% methanol-1% H_2_O_2_ for 15 min followed by 3% normal horse serum. Then the sections were incubated at 4°C overnight with one of the primary antibodies: rabbit polyclonal antibodies to IBA-1 (019-19749, Wako Chemicals Gmbh, Neuss, GE) were used at a dilution of 1:1,000; VDAC (voltage dependent anion channel, ab15895, Abcam, Cambridge, UK) at 1:100; HLA-DRB1 (GTX104919, GeneTex, Barcelona, Spain) at 1:100; HLA-DRB5 (NBP2, Novusbio, Littleton, Colorado, USA) at 1:100; IL-10 (AP52181PU, ACRIS, ProAlt, Madrid, Spain) at 1:100; and GFAP (glial fibrillary acidic protein, RP014-S, Diagnostic Biosystem, Palex Medica, Sant Cugat, Spain) at 1:400. Mouse monoclonal antibodies to CD68 (ab955, Abcam, Cambridge, UK) and TNF-α (ab1793, Abcam, Cambridge, UK), were used at dilutions of 1:200 and 1:150, respectively. Antibodies to GluT: SLC1A2 (ab1783, Millipore, Billerica, MA, USA) were used at a dilution of 1:100. Following incubation with the primary antibody, the sections were incubated with EnVision + system peroxidase (Dako, Agilent, Santa Clara, CA, USA) for 30 min at room temperature. The peroxidase reaction was visualized with diamino-benzidine and H_2_O_2_. Control of the immunostaining included omission of the primary antibody; no signal was obtained following incubation with only the secondary antibody. Sections were slightly stained with haematoxylin.

### Gel electrophoresis and western blotting

Frozen samples of the somatosensory cortex were homogenized in RIPA lysis buffer composed of 50mM Tris/HCl buffer, pH 7.4 containing 2mM EDTA, 0.2% Nonidet P-40, 1mM PMSF, protease and phosphatase inhibitor cocktail (Roche Molecular Systems, USA). The homogenates were centrifuged for 20 min at 12,000 rpm. Protein concentration was determined with the BCA method (Thermo Scientific). Equal amounts of protein (20μg) for each sample were loaded and separated by electrophoresis on 10% sodium dodecyl sulfate polyacrylamide gel electrophoresis (SDS-PAGE) gels and transferred onto nitrocellulose membranes (Amersham, Freiburg, GE). Non-specific bindings were blocked by incubation in 3% albumin in PBS containing 0.2% Tween for 1 h at room temperature. After washing, membranes were incubated overnight at 4°C with antibodies against glutamate receptor ionotropic, NMDA 2A (NMDAR2A, 130 kDa, rabbit, 1:200, Abcam, Cambridge, UK), α-amino-3-hydroxy-5-methyl-4-isoxazolepropionic acid receptor 1 (AMPAR GluR-1, 100 kDa, rabbit, 1:200, Cell Signaling Technology, Danvers, MA, USA), glutamate decarbo-xylase 1 (GAD1, 67 kDa, rabbit, 1:200, Cell Signaling Technology, Danvers, MA, USA) and gamma-aminobutyric acid receptor subunit beta-2 (GABAAB2, 59 kDa, mouse, 1:1000, Abcam, Cambridge, UK). Protein loading was monitored using an antibody against β-actin (42 kDa, 1:30,000, Sigma). Membranes were incubated for 1 h with appropriate HRP-conjugated secondary antibodies (1:2,000, Dako); the immunoreaction was revealed with a chemilumines-cence reagent (ECL, Amersham). Densitometric quantification was carried out with the ImageLab v4.5.2 software (BioRad), using β-actin for normalization. Seven samples of FC area 8 per group were analyzed.

These antibodies were selected on the basis of a larger screening which included antibodies against proteins whose RNA levels were de-regulated as revealed by RT-qPCR. Only antibodies working for western blotting were eventually assessed. The significance level was set at ** p < 0.01 and tendencies at # < 0.1.

## SUPPLEMENTARY MATERIALS TABLES






