# Supplementary material for: Amyotrophic lateral sclerosis, gene deregulation in the anterior horn of the spinal cord and frontal cortex area 8: implications in frontotemporal lobar degeneration
Source: Aging (Albany NY). 2017 Mar 9;9(3):823–51. doi: 10.18632/aging.101195 (PMC5391234; doi:10.18632/aging.101195)
Supplement: Supplementary file 1 [file aging-09-823-s001.pdf]

Myelin-associated glycoprotein gene mutation causes Pelizaeus-Merzbacher disease-like disorder. Brain. 2015; 138:2521–36. doi: 10.1093/brain/awv204

94. Kim T, Fiedler K, Madison DL, Krueger WH, Pfeiffer SE. Cloning and characterization of MVP17: a developmentally regulated myelin protein in oligodendrocytes. J Neurosci Res. 1995; 42:413–22. doi: 10.1002/jnr.490420316

95. Kasama-Yoshida H, Tohyama Y, Kurihara T, Sakuma M, Kojima H, Tamai Y. A comparative study of 2', 3'-cyclic-nucleotide 3'-phosphodiesterase in vertebrates: cDNA cloning and amino acid sequences for chicken and bullfrog enzymes. J Neurochem. 1997; 69:1335–42. doi: 10.1046/j.1471-4159.1997.69041335.x

96. Kursula P. Structural properties of proteins specific to the myelin sheath. Amino Acids. 2008; 34:175–85. doi: 10.1007/s00726-006-0479-7

97. Lappe-Siefke C, Goebbels S, Gravel M, Nicksch E, Lee J, Braun PE, Griffiths IR, Nave KA. Disruption of Cnp1 uncouples oligodendroglial functions in axonal support and myelination. Nat Genet. 2003; 33:366–74. doi: 10.1038/ng1095

98. Nardo G, Iennaco R, Fusi N, Heath PR, Marino M, Trolese MC, Ferraiuolo L, Lawrence N, Shaw PJ, Bendotti C. Transcriptomic indices of fast and slow disease progression in two mouse models of amyotrophic lateral sclerosis. Brain. 2013; 136:3305–32. doi: 10.1093/brain/awt250

99. Gentleman RC, Carey VJ, Bates DM, Bolstad B, Dettling M, Dudoit S, Ellis B, Gautier L, Ge Y, Gentry J, Hornik K, Hothorn T, Huber W, et al. Bioconductor: open software development for computational biology and bioinformatics. Genome Biol. 2004; 5:R80. doi: 10.1186/gb-2004-5-10-r80

100. Barrachina M, Castaño E, Ferrer I. TaqMan PCR assay in the control of RNA normalization in human post-mortem brain tissue. Neurochem Int. 2006; 49:276–84. doi: 10.1016/j.neuint.2006.01.018

101. Durrenberger PF, Fernando FS, Magliozzi R, Kashefi SN, Bonnert TP, Ferrer I, Seilhean D, Nait-Oumesmar B, Schmitt A, Gebicke-Haerter PJ, Falkai P, Grünblatt E, Palkovits M, et al. Selection of novel reference genes for use in the human central nervous system: a BrainNet Europe Study. Acta Neuropathol. 2012; 124:893–903. doi: 10.1007/s00401-012-1027-z

SUPPLEMENTARY MATERIAL

Please browse the Full text version to see Supplementary Tables 1 and 2 identifying all de-regulated genes.
