# Supplementary material for: Amyotrophic lateral sclerosis, gene deregulation in the anterior horn of the spinal cord and frontal cortex area 8: implications in frontotemporal lobar degeneration
Source: Aging (Albany NY). 2017 Mar 9;9(3):823–51. doi: 10.18632/aging.101195 (PMC5391234; doi:10.18632/aging.101195)
Supplement: Supplementary file 2 [file aging-09-823-s002.pdf]

Gene symbol	logFC	Fold Change	AveExpr
A1BG-AS1	0.295	1.227	3.668
A2M//HDAC1	0.464	1.380	7.771
A2M-AS1	0.332	1.259	4.273
A2ML1	-0.049	0.966	2.737
AACS	0.166	1.122	5.535
AAED1	0.179	1.132	4.270
AAGAB	-0.128	0.915	4.495
AAK1	0.012	1.009	7.244
AASDHPPT	0.104	1.075	5.760
AATK	-0.049	0.966	6.203
ABCA1	0.608	1.524	5.808
ABCA10	-0.051	0.965	2.297
ABCA17P	0.232	1.174	2.505
ABCA5	0.032	1.023	6.465
ABCA6	-0.152	0.900	5.813
ABCA8	0.076	1.054	6.900
ABCA9	-0.173	0.887	5.280
ABCC1	-0.114	0.924	5.556
ABCC11	-0.289	0.819	2.134
ABCC3	0.219	1.164	3.902
ABCC4	0.271	1.207	5.072
ABCC5	-0.226	0.855	6.195
ABCC8	-0.243	0.845	4.582
ABCC9	0.235	1.177	6.267
ABCD2	0.061	1.043	5.017
ABCD4	-0.056	0.962	4.718
ABCG1	0.446	1.362	5.472
ABCG2	-0.437	0.739	5.791
ABCG4	0.257	1.195	4.414
ABHD12B//MIR4454	-0.153	0.899	3.146
ABHD13	0.456	1.372	5.667
ABHD15	0.404	1.323	3.657
ABHD16B	-0.204	0.868	2.402
ABHD17A	-0.221	0.858	2.873
ABHD17B	0.281	1.215	6.296
ABHD3	0.459	1.374	4.498
ABHD4	0.452	1.368	5.625
ABHD5	0.042	1.029	4.976
ABHD8	0.075	1.053	5.807
ABI3	0.434	1.351	4.734
ABI3BP	0.133	1.096	5.402
ABL1	0.039	1.027	7.261
ABLIM2	0.137	1.100	5.351
ABLIM3	-0.096	0.935	6.305
ABRACL	0.027	1.019	3.256
ABTB1	0.031	1.022	3.754
ABTB2	-0.206	0.867	5.749
AC002117.1	-0.095	0.936	4.514
AC002310.12	0.260	1.197	3.805

AC002310.7	-0.516	0.699	2.511
AC002429.5	0.131	1.095	3.586
AC004540.4	-0.168	0.890	2.952
AC004951.6	-0.196	0.873	5.354
AC005220.3	-0.174	0.886	2.299
AC005237.4	-0.059	0.960	2.093
AC005518.2	-0.205	0.868	4.455
AC006003.3	0.148	1.108	3.375
AC006070.12	0.228	1.171	4.592
AC006160.5	-0.199	0.871	3.770
AC006947.1	-0.243	0.845	2.863
AC007036.4	-0.300	0.813	4.274
AC007098.1	-0.071	0.952	2.363
AC007106.1	-1.155	0.449	2.755
AC007383.3	-0.365	0.777	3.021
AC008269.2	0.098	1.070	4.640
AC008277.1	0.198	1.147	3.666
AC008440.10	0.050	1.035	2.785
AC008440.5	0.240	1.181	3.147
AC008746.3	0.147	1.107	2.486
AC009110.1	-0.048	0.967	2.183
AC009262.2	-0.247	0.843	2.875
AC009495.2	-0.035	0.976	2.371
AC009495.3	-0.459	0.728	3.163
AC011196.3	-0.145	0.904	2.444
AC011247.3	-0.060	0.959	2.200
AC011330.5//PPIP5K1	0.207	1.154	3.255
AC011891.5	0.086	1.061	2.313
AC011995.1	-0.180	0.883	3.483
AC012074.2//LOC100131510	0.166	1.122	3.103
AC012442.6	-0.415	0.750	2.917
AC012507.4	-0.110	0.926	2.363
AC012593.1	0.231	1.173	2.322
AC016745.3	-0.367	0.776	3.716
AC017002.2	-0.048	0.967	2.197
AC018685.1	0.045	1.031	2.896
AC018816.3	0.120	1.087	2.564
AC020594.5	0.133	1.096	2.258
AC023481.1	-0.087	0.941	3.740
AC025165.8	-0.419	0.748	2.459
AC026202.3	0.184	1.136	2.534
AC053503.6	0.914	1.884	2.617
AC061961.2	0.267	1.203	3.113
AC067956.1	0.196	1.146	2.470
AC067961.1	0.021	1.015	2.323
AC068490.1	-0.141	0.907	2.391
AC068580.5	0.016	1.011	3.923
AC068580.6	-0.218	0.860	2.394
AC073283.7	0.123	1.089	3.713
AC073834.3	-0.181	0.882	2.601

AC073842.19	0.149	1.109	2.455
AC074183.4	0.021	1.015	3.293
AC074363.1//B3GALT1	-0.056	0.962	3.368
AC078852.2	-0.388	0.764	2.802
AC078883.4	-0.091	0.939	2.737
AC079154.1	-0.125	0.917	2.959
AC079354.5	-0.220	0.859	2.240
AC079779.4	0.107	1.077	2.400
AC079790.2	0.025	1.017	1.519
AC083899.3	-0.082	0.945	5.425
AC084149.2	0.173	1.128	2.577
AC090044.2	-0.131	0.913	4.578
AC091729.8	-0.705	0.613	3.424
AC092168.2	0.292	1.225	3.192
AC092171.4	-0.146	0.904	2.362
AC092661.1	0.209	1.156	2.372
AC092675.3	-0.194	0.874	2.548
AC092835.2	0.081	1.057	5.260
AC093110.3	0.330	1.257	3.305
AC093843.1	-0.153	0.900	1.829
AC102953.6	-0.477	0.718	2.340
AC103564.7	-0.350	0.784	2.259
AC107072.2	-1.112	0.463	3.060
AC109826.1//LOC101927070	0.936	1.913	3.377
AC113617.1	-0.136	0.910	1.834
AC114730.2	0.283	1.217	3.125
AC114730.7	-0.772	0.585	2.324
AC114877.3	0.473	1.388	4.062
AC123886.2	-0.284	0.821	3.154
AC140481.4	0.172	1.127	2.300
AC144449.1//LOC101929231	0.236	1.177	3.858
AC144835.1	0.203	1.151	2.649
AC159540.1//LOC100506123	-0.500	0.707	5.848
AC159540.2	-0.730	0.603	2.940
ACACB	-0.106	0.929	5.301
ACAP1	0.365	1.288	2.364
ACAP2-IT1	0.128	1.093	4.178
ACAT2	0.018	1.013	5.767
ACBD7	-0.306	0.809	6.707
ACCS	0.088	1.063	3.703
ACER2	0.161	1.118	4.122
ACHE	-0.389	0.764	4.853
ACKR1//DARC	0.343	1.269	4.827
ACKR3	-0.219	0.859	3.786
ACLY	-0.042	0.971	8.268
ACOT2	0.529	1.443	4.868
ACOX2	-0.247	0.843	3.930
ACP5	0.326	1.254	2.705
ACPP	0.362	1.285	3.097
ACSBG1	0.133	1.097	5.859

ACSF2	0.057	1.040	3.818
ACSL1	0.583	1.498	7.092
ACSL4	0.320	1.249	7.376
ACSL4	-0.060	0.959	2.346
ACSL5	0.761	1.695	5.348
ACSL6//LOC728637//AC034228.7	-0.115	0.923	3.736
ACSM5	0.354	1.278	4.847
ACSS1	-0.212	0.863	5.329
ACSS3	0.308	1.238	6.228
ACTA1	0.106	1.077	2.688
ACTA2	0.456	1.372	3.404
ACTA2-AS1	-0.179	0.883	3.341
ACTL6A	0.492	1.406	4.024
ACTL6B	-0.082	0.945	4.456
ACTN1	0.764	1.698	4.334
ACTN2	-0.221	0.858	6.026
ACTR10	0.103	1.074	6.153
ACTR1B	-0.144	0.905	6.252
ACTR3B	-0.377	0.770	5.503
ACTR6	0.201	1.150	5.569
ACTRT3	0.143	1.104	3.075
ACVR1B	-0.031	0.979	5.096
ACVR1C	0.348	1.273	4.310
ACVR2A	0.203	1.151	4.720
ACVRL1	0.439	1.355	3.491
ACY3	-0.449	0.733	4.157
ACYP2//LOC101927144	-0.332	0.795	5.007
ADAM11	0.262	1.200	4.601
ADAM12	0.324	1.252	3.774
ADAM1A	0.182	1.134	4.317
ADAM20	0.300	1.231	3.972
ADAM20P1	0.160	1.117	4.607
ADAM22	0.164	1.120	7.233
ADAM23	-0.258	0.836	7.424
ADAM28	0.286	1.220	6.105
ADAM33	-0.543	0.687	2.990
ADAM8	-0.024	0.984	2.849
ADAMTS1	0.157	1.115	5.375
ADAMTS12	0.359	1.283	2.602
ADAMTS14	-0.107	0.929	2.921
ADAMTS15	0.220	1.165	2.679
ADAMTS17	0.034	1.024	3.513
ADAMTS18	1.460	2.750	3.296
ADAMTS19	-0.427	0.744	2.726
ADAMTS2	-0.411	0.752	3.432
ADAMTS3	0.550	1.464	2.648
ADAMTS4	0.167	1.123	5.767
ADAMTS5	0.334	1.261	3.436
ADAMTS7P1	-0.213	0.862	2.741
ADAMTS8	-0.174	0.886	3.976

ADAMTS9	0.348	1.273	6.614
ADAMTS9-AS2	-0.160	0.895	4.541
ADAMTSL2	-0.141	0.907	3.657
ADAMTSL3	-0.424	0.746	4.734
ADAMTSL4	-0.126	0.916	2.276
ADAMTSL5	-0.110	0.926	2.894
ADAP2	0.278	1.212	5.306
ADARB1	0.125	1.090	5.324
ADARB2-AS1	-0.196	0.873	2.074
ADAT2	0.566	1.480	3.677
ADCY1	-0.309	0.807	7.038
ADCY10P1	0.199	1.148	4.277
ADCY7	0.295	1.227	4.582
ADCY8	0.191	1.141	6.761
ADCY9	0.253	1.192	5.023
ADCYAP1	0.346	1.271	3.357
ADCYAP1R1	-0.104	0.930	6.506
ADD3-AS1//RP11-451M19.3	0.186	1.138	2.689
ADH1C	0.416	1.335	2.516
ADIPOR2	-0.122	0.919	5.496
ADIRF	0.101	1.072	5.323
ADM	-0.391	0.763	5.195
ADNP2	0.345	1.271	4.140
ADORA2B	-0.148	0.902	4.531
ADORA3	0.421	1.339	4.862
ADPRHL1	-0.107	0.928	4.183
ADRA1B	-0.396	0.760	3.647
ADRA1D	0.228	1.171	3.429
ADRA2A	0.175	1.129	2.901
ADRA2C	0.055	1.039	2.986
ADRB1	0.327	1.255	4.636
ADRBK1	0.248	1.188	6.521
ADRBK2	-0.100	0.933	7.454
AEBP1//MIR4649	0.054	1.038	4.316
AEN	0.232	1.174	3.450
AES	-0.325	0.799	9.069
AF127936.5	0.451	1.367	3.613
AF131215.3	-0.315	0.804	3.465
AF131215.4	0.267	1.203	2.746
AFAP1L2	0.315	1.244	4.278
AFF2	0.060	1.043	5.749
AFF3	0.155	1.113	5.483
AFMID	0.118	1.086	4.129
AFTPH	0.274	1.209	7.175
AGA	0.307	1.237	5.038
AGAP1-IT1	-0.356	0.781	2.936
AGAP2	0.420	1.338	5.769
AGAP2-AS1	0.294	1.226	3.032
AGAP5//AGAP4//AGAP11//AGAP10//LOC10106058	-0.169	0.890	6.087
AGBL3	-0.090	0.939	2.898

AGBL4	-0.315	0.804	3.982
AGBL4-IT1	-0.042	0.971	1.908
AGFG2	-0.218	0.860	5.941
AGGF1	0.136	1.099	6.930
AGK	0.153	1.112	5.435
AGL	0.320	1.248	5.974
AGMAT	-0.086	0.942	3.122
AGMO	0.209	1.156	2.121
AGPAT4	0.074	1.053	6.837
AGPAT4-IT1	-0.184	0.880	5.273
AGT	0.241	1.182	7.225
AGTRAP//BRAF	0.103	1.074	4.310
AHNAK	-0.094	0.937	7.055
AHNAK2	-0.719	0.608	6.240
AHR	0.805	1.747	7.045
AHSA2	0.571	1.486	4.675
AIF1	0.585	1.500	6.229
AIF1	0.506	1.420	5.891
AIF1	0.529	1.443	6.254
AIF1L	-0.147	0.903	3.813
AIFM3	-0.408	0.754	4.974
AJ006998.2	0.021	1.015	2.176
AJ009632.3	0.106	1.076	1.839
AJAP1	0.235	1.177	6.057
AK5	0.038	1.027	7.739
AKAP11	0.162	1.119	8.000
AKAP3	0.241	1.182	2.506
AKAP5	-0.061	0.959	2.883
AKNA	0.204	1.152	4.214
AKR1C1	0.024	1.017	3.029
AKR7A3	-0.216	0.861	3.669
AKR7L	-0.429	0.743	2.477
AKT3	-0.269	0.830	7.672
AKT3-IT1	-0.240	0.847	3.034
AL121578.2	0.484	1.399	2.257
ALAD	-0.156	0.897	4.924
ALAS1	0.255	1.193	6.079
ALAS2	-1.062	0.479	3.480
ALCAM	0.209	1.156	6.368
ALDH1A1	0.075	1.053	7.244
ALDH1B1	0.060	1.042	4.408
ALDH3B1	-0.099	0.934	3.607
ALDH8A1	0.020	1.014	2.524
ALDH9A1	0.090	1.064	6.813
ALG1//NAGPA-AS1	0.206	1.153	4.703
ALG10	0.447	1.363	3.917
ALG10B//ALG10	0.019	1.013	3.009
ALG1L2	-0.050	0.966	2.467
ALKBH1	0.466	1.381	4.578
ALMS1-IT1	0.067	1.047	2.650

ALOX12P2	0.300	1.231	3.150
ALOX15B	0.337	1.263	3.150
ALOX5	0.609	1.525	5.041
ALOX5AP	0.798	1.738	6.867
ALPK1	0.531	1.445	4.035
ALPL	0.520	1.434	5.542
ALS2	0.154	1.113	5.716
ALS2CR11	-0.371	0.773	3.157
ALX4	-0.015	0.989	2.522
AMER1	0.768	1.703	2.794
AMER2	0.249	1.189	6.424
AMER3	0.326	1.254	3.197
AMICA1	0.202	1.150	2.259
AMIGO1	-0.076	0.949	5.352
AMMECR1	0.263	1.200	2.432
AMN1	-0.180	0.882	6.142
AMPD3//CAND1.11	-0.020	0.986	4.586
AMPH	0.135	1.098	6.617
AMZ2P1	0.397	1.317	3.764
ANAPC1//LOC730268//AC083899.3	0.418	1.337	5.203
ANG//RNASE4	0.485	1.400	4.262
ANGEL2	0.094	1.067	5.366
ANGPT1	0.099	1.071	3.739
ANGPT2	-0.252	0.840	5.994
ANGPTL2	0.108	1.078	5.161
ANGPTL4	0.183	1.136	6.286
ANGPTL6	0.588	1.503	3.520
ANK1	0.030	1.021	5.017
ANK2	0.108	1.078	8.932
ANK3	0.055	1.039	7.720
ANKEF1	-0.072	0.951	2.598
ANKFN1	0.027	1.019	5.117
ANKFY1	0.205	1.153	7.185
ANKLE1	-0.114	0.924	2.710
ANKMY2	0.337	1.263	5.684
ANKRD10-IT1	0.559	1.473	6.178
ANKRD10-IT1	0.549	1.463	6.002
ANKRD13A	0.083	1.059	6.146
ANKRD13C	0.196	1.146	6.641
ANKRD16	-0.057	0.961	5.988
ANKRD18CP//RP11-498P14.5	-0.236	0.849	4.256
ANKRD19P	0.412	1.330	3.840
ANKRD2	0.171	1.126	3.651
ANKRD20A3//ANKRD20A2//ANKRD20A4//ANKRD20	0.171	1.126	6.323
ANKRD20A3//ANKRD20A2//ANKRD20A4//ANKRD20	0.157	1.115	6.553
ANKRD20A5P	0.337	1.263	3.881
ANKRD20A7P	1.098	2.141	2.484
ANKRD22	1.208	2.310	2.265
ANKRD24	0.048	1.034	4.327
ANKRD26	-0.169	0.889	5.397

ANKRD26P3	0.308	1.238	2.669
ANKRD29	-0.232	0.852	5.289
ANKRD30B	0.113	1.082	2.850
ANKRD32	0.131	1.095	4.404
ANKRD33B	0.093	1.067	3.105
ANKRD34A	0.061	1.043	3.568
ANKRD34B	-0.560	0.678	2.378
ANKRD34C	-0.218	0.860	3.527
ANKRD36B	-0.379	0.769	6.793
ANKRD37	-0.059	0.960	5.210
ANKRD40	-0.225	0.855	8.442
ANKRD42	-0.036	0.975	4.462
ANKRD44	0.131	1.095	5.837
ANKRD44-IT1	-0.169	0.889	3.134
ANKRD49	0.245	1.185	4.405
ANKRD50	0.340	1.266	5.504
ANKRD55	0.289	1.222	3.433
ANKS1B	0.059	1.042	6.124
ANKZF1	-0.147	0.903	4.504
ANLN	-0.090	0.939	8.442
ANO3	0.339	1.265	4.259
ANO4	0.048	1.034	5.640
ANO5	-0.143	0.906	6.503
ANO6	0.566	1.480	8.305
ANP32A-IT1	0.335	1.262	4.199
ANP32B	-0.253	0.839	4.529
ANPEP	-0.414	0.751	2.316
ANTXR1	0.027	1.019	6.587
ANTXR2	0.026	1.018	5.055
ANXA1	0.491	1.405	5.307
ANXA11	0.088	1.063	5.057
ANXA13	-0.832	0.562	2.866
ANXA2P2	0.328	1.255	7.708
ANXA2P3	-0.089	0.940	2.088
ANXA2R	0.043	1.030	2.369
ANXA3	0.797	1.738	4.647
ANXA4	0.283	1.217	6.693
ANXA5	0.063	1.045	8.208
ANXA6	0.056	1.040	8.863
AOAH	0.862	1.818	5.110
AOC3	0.055	1.039	2.560
AP000282.2	0.020	1.014	2.396
AP000438.2	-0.017	0.988	2.309
AP000640.10	-0.122	0.919	2.879
AP000688.29	0.487	1.401	5.158
AP001056.1	0.347	1.272	3.046
AP001347.6	-0.525	0.695	2.280
AP001469.7	-0.105	0.930	3.250
AP001476.4	-0.037	0.974	4.224
AP002954.3	0.049	1.035	3.715

AP006216.11	0.270	1.206	4.326
AP006222.2	-0.241	0.846	5.236
AP006621.6	-0.086	0.942	3.993
AP1S1	-0.144	0.905	5.469
AP1S2	0.252	1.191	5.020
AP3B2	-0.172	0.888	5.495
AP3M2	0.205	1.152	5.931
AP4S1	0.077	1.055	3.202
AP5M1	-0.027	0.982	5.865
APBA1	0.092	1.066	6.511
APBB1IP	0.246	1.186	4.668
APCDD1	-0.050	0.966	6.207
API5	0.209	1.156	6.711
APLN	0.591	1.506	5.898
APLNR	0.317	1.246	7.340
APLP1	-0.095	0.936	8.997
APOBEC3C	0.870	1.828	4.571
APOBEC3D	0.115	1.083	3.718
APOBEC3G	-0.019	0.987	3.373
APOBR	0.392	1.312	2.866
APOC1	1.184	2.272	4.538
APOC4-APOC2//APOC2//APOC4	0.996	1.994	3.621
APOD	-0.044	0.970	8.827
APOE	0.381	1.302	4.391
APOL2	0.643	1.561	4.232
APOL4	0.562	1.476	2.110
APOL6	0.444	1.360	5.540
APOO	-0.534	0.691	5.535
APPBP2	0.233	1.175	7.672
APPL2	0.142	1.103	5.772
APRT	0.173	1.127	5.233
AQP1//RP5-877J2.1	0.503	1.417	6.920
AQP11	-0.328	0.797	4.699
AQP4-AS1	0.555	1.469	4.866
AQP9	0.985	1.980	3.361
AR	0.117	1.084	3.621
ARAP1	-0.022	0.985	5.332
ARAP2//LOC101928667	0.137	1.100	6.081
ARC	-0.169	0.889	3.797
ARF3	-0.070	0.953	5.854
ARFGEF2	0.151	1.110	6.998
ARG2	0.163	1.120	4.734
ARHGAP12	-0.094	0.937	7.404
ARHGAP15//LOC101928361	0.639	1.557	2.409
ARHGAP17	0.134	1.097	5.772
ARHGAP18	0.545	1.459	4.715
ARHGAP20	0.348	1.273	4.427
ARHGAP22	0.218	1.163	3.828
ARHGAP22-IT1	0.070	1.050	3.227
ARHGAP23	-0.030	0.980	6.547

ARHGAP24	0.487	1.401	4.769
ARHGAP25	0.727	1.655	3.543
ARHGAP26	0.342	1.268	6.903
ARHGAP26-IT1	0.619	1.536	4.575
ARHGAP27	-0.108	0.928	3.096
ARHGAP28	-0.060	0.959	2.666
ARHGAP30	0.620	1.537	3.204
ARHGAP32	-0.145	0.904	7.579
ARHGAP35	-0.156	0.898	8.140
ARHGAP36	-0.781	0.582	4.599
ARHGAP4	0.136	1.099	4.177
ARHGAP44	0.122	1.088	5.133
ARHGAP6	0.292	1.224	4.249
ARHGDIB	0.166	1.122	3.622
ARHGDIG	-0.167	0.891	3.201
ARHGDIG	-0.038	0.974	5.318
ARHGEF1//LOC100505585	0.035	1.024	4.991
ARHGEF10	-0.123	0.918	6.013
ARHGEF11	0.075	1.053	6.020
ARHGEF12	0.092	1.066	7.885
ARHGEF15	-0.078	0.947	3.091
ARHGEF19	-0.097	0.935	2.411
ARHGEF25	-0.155	0.898	6.050
ARHGEF3	0.191	1.141	4.428
ARHGEF33	-0.244	0.845	3.605
ARHGEF37	-0.227	0.854	5.851
ARHGEF40	0.166	1.122	4.704
ARHGEF5	-0.373	0.772	2.332
ARHGEF6	0.216	1.162	7.664
ARHGEF7-IT1	-0.135	0.911	2.659
ARHGEF9	0.102	1.073	7.535
ARID5A	0.658	1.578	5.304
ARID5B	0.323	1.251	6.475
ARL14EP	0.287	1.220	6.285
ARL15	-0.029	0.980	5.088
ARL4A	-0.484	0.715	4.079
ARL4C	0.304	1.235	6.470
ARL4D	-0.481	0.717	2.935
ARL6	0.185	1.137	4.428
ARL6IP1//RP11-1035H13.3	0.059	1.042	10.236
ARMC3	-1.267	0.415	2.364
ARMC4	-0.674	0.627	1.956
ARMC9	-0.285	0.821	4.566
ARMCX1	0.330	1.257	5.901
ARNT2	-0.177	0.885	7.892
ARNTL	0.242	1.183	5.752
ARNTL2	0.272	1.208	4.095
ARPC1B	0.415	1.334	5.000
ARPP21	0.129	1.093	4.560
ARRB1	-0.213	0.863	4.934

ARRDC2	-0.131	0.913	5.593
ARRDC3	0.019	1.013	8.026
ARRDC4	0.120	1.087	8.005
ARSD	0.073	1.052	4.357
ARSF	-0.550	0.683	2.300
ARSG	0.129	1.093	4.979
ARSJ	0.200	1.149	2.732
ARSK	0.144	1.105	4.186
ASAH1	0.351	1.275	7.151
ASAP1-IT2	0.126	1.092	3.049
ASAP3	0.268	1.204	5.618
ASCC2	0.089	1.064	5.105
ASCL1	-0.161	0.895	6.345
ASGR1	-0.201	0.870	2.924
ASH2L	0.313	1.242	6.358
ASIC1	0.146	1.107	4.004
ASIC2	0.177	1.130	4.540
ASNS	-0.200	0.871	5.361
ASPA	0.152	1.111	7.176
ASPDH	0.159	1.117	4.188
ASPHD2	0.049	1.034	4.480
ASS1	0.330	1.257	6.266
ASTN1	0.201	1.149	7.270
ASUN	0.155	1.114	5.538
ASXL3	-0.061	0.959	4.707
ATAD2	0.036	1.025	4.077
ATCAY	-0.098	0.934	7.165
ATE1-AS1//RP11-500G22.2	-0.079	0.947	4.337
ATF3	0.654	1.574	4.158
ATF5	0.164	1.121	4.074
ATF7//NPFF//RP11-793H13.10	-0.125	0.917	6.106
ATF7IP2	-0.215	0.862	2.849
ATG13	0.151	1.111	5.856
ATG16L1	0.490	1.404	5.412
ATG2B	0.085	1.060	6.561
ATG4C	0.149	1.109	6.307
ATG5	-0.225	0.856	5.901
ATHL1	0.288	1.221	3.507
ATL1	0.085	1.061	5.985
ATL2	0.039	1.027	4.354
ATL3	0.238	1.179	6.874
ATM//NPAT	0.109	1.079	6.375
ATMIN	0.140	1.102	7.928
ATOH8	-0.588	0.665	2.616
ATP10B	-0.027	0.981	6.061
ATP11A	0.135	1.098	6.583
ATP11C	0.382	1.303	5.165
ATP13A2	-0.261	0.834	7.485
ATP13A4	0.038	1.027	5.997
ATP13A5-AS1	-0.025	0.983	2.007

ATP1A1	0.196	1.145	5.125
ATP1A1-AS1	0.223	1.167	4.015
ATP1A3//LOC101927137	-0.355	0.782	4.602
ATP1B1	-0.177	0.885	9.698
ATP2A2	0.242	1.183	8.517
ATP2A3	0.483	1.398	2.600
ATP2B1	0.067	1.048	7.614
ATP2B2	-0.251	0.840	6.237
ATP2B3	-0.436	0.739	4.108
ATP2B4	0.318	1.247	7.781
ATP2C2	-0.389	0.764	2.218
ATP4B	-0.583	0.667	2.060
ATP5A1	0.125	1.091	8.595
ATP5B	0.141	1.103	10.183
ATP5SL	-0.612	0.654	3.648
ATP5SL	0.181	1.133	5.123
ATP6V0A1	0.126	1.091	8.753
ATP6V0C	0.199	1.148	9.554
ATP6V0E1	0.321	1.249	9.537
ATP6V1A	0.178	1.131	7.578
ATP6V1B2	0.117	1.085	8.519
ATP6V1C1	0.279	1.213	8.078
ATP6V1D	-0.183	0.881	5.437
ATP6V1H	0.310	1.239	6.969
ATP7B	0.627	1.544	3.880
ATP8A1	-0.026	0.982	8.915
ATP8A2	-0.462	0.726	6.622
ATP8B1	0.276	1.211	4.107
ATP8B4	0.302	1.233	4.403
ATPAF1	0.062	1.044	6.522
ATR	0.321	1.250	5.225
ATRN	0.103	1.074	6.979
ATRNL1	-0.227	0.855	7.799
ATXN3//RP11-529H20.6	-0.178	0.884	3.301
ATXN7L3	-0.055	0.963	6.897
ATXN7L3B	0.040	1.028	8.034
AURKB	0.022	1.015	2.474
AVIL//PP12719	-0.095	0.936	3.073
AVL9	0.330	1.257	6.726
AVPI1	0.234	1.176	5.751
AZGP1	-0.179	0.883	2.905
B2M	0.432	1.349	8.007
B3GALNT1	0.361	1.285	4.270
B3GALT1	-0.303	0.811	4.751
B3GALT2	0.077	1.055	6.864
B3GAT2	0.171	1.126	6.700
B3GNT4	0.314	1.243	2.366
B3GNT9	0.381	1.302	4.700
B4GALNT1	-0.156	0.897	5.123
B4GALNT3	0.191	1.141	2.462

B4GALT1	0.181	1.134	4.596
B4GALT5	0.791	1.730	7.421
B4GALT6	0.234	1.176	5.965
B9D2	-0.150	0.901	3.724
BAALC-AS1//RP11-318M2.2	0.191	1.141	3.012
BAALCOS//C8orf56	1.095	2.136	3.186
BAAT	-0.081	0.945	2.298
BACE1	0.040	1.028	7.894
BACE2	0.155	1.114	4.239
BACE2-IT1	0.050	1.035	3.281
BACH1-IT3	-0.241	0.846	2.358
BAG2	0.264	1.201	4.615
BAG3	-0.055	0.962	6.218
BAG4	0.170	1.125	4.957
BAI1	-0.330	0.795	4.987
BAI2	-0.050	0.966	5.225
BAI3	0.101	1.072	7.389
BAIAP2	-0.061	0.958	5.026
BAIAP2-AS1	0.201	1.149	4.227
BAIAP2L2	-0.232	0.851	3.296
BAIAP3	0.022	1.016	5.710
BAMBI	-0.176	0.885	5.988
BAP1	0.139	1.101	6.205
BARD1	0.143	1.105	4.529
BARX2	0.385	1.306	1.937
BASP1	0.165	1.121	8.665
BATF3	0.454	1.370	3.318
BAX	0.373	1.295	4.188
BAZ1A	0.528	1.442	4.916
BAZ2B	-0.097	0.935	7.629
BBOX1	0.188	1.139	4.146
BBS10	0.211	1.158	5.264
BBS7	-0.016	0.989	6.834
BBX	0.190	1.141	5.819
BCAN	0.069	1.049	6.658
BCAR3	-0.325	0.798	4.225
BCAS1	-0.323	0.799	6.748
BCAS4	0.182	1.134	4.202
BCAT1	-0.049	0.967	5.739
BCDIN3D-AS1	0.288	1.221	2.731
BCHE	-0.068	0.954	3.069
BCL11A	0.252	1.191	4.255
BCL11B	0.175	1.129	3.522
BCL2	0.244	1.184	6.521
BCL2A1	1.364	2.574	3.559
BCL3//MIR8085	0.084	1.060	4.179
BCL6	0.568	1.482	8.153
BCL6B	-0.171	0.888	2.660
BCL7A	0.108	1.078	5.532
BCL7C	-0.514	0.701	4.179

BCMO1	0.475	1.390	2.451
BCO2	-0.330	0.796	5.061
BCS1L	0.582	1.497	4.242
BDH1	-0.090	0.940	4.398
BDH2	0.018	1.013	4.338
BDNF-AS//BDNF-AS1	-0.236	0.849	3.652
BEAN1	-0.212	0.863	3.345
BEGAIN	-0.351	0.784	2.487
BEND4	-0.038	0.974	2.549
BEND5	-0.141	0.907	4.256
BEND6	0.239	1.180	5.969
BEST1	0.163	1.119	7.087
BEST3	0.212	1.158	3.337
BEX1	-0.194	0.874	8.278
BEX2	-0.275	0.826	4.609
BEX5	-0.107	0.928	4.728
BGN	0.284	1.218	6.883
BHLHA15	-0.340	0.790	3.335
BHLHB9	-0.219	0.859	5.592
BHLHE22	0.169	1.124	2.614
BHLHE23	-0.546	0.685	3.424
BHLHE40	0.203	1.151	7.525
BHLHE41	0.016	1.011	8.257
BHMT2	0.314	1.243	3.378
BICC1	0.064	1.045	4.500
BIN2	0.230	1.173	5.061
BIRC3	0.415	1.333	3.684
BIRC7	0.334	1.261	2.907
BLID	-0.249	0.842	3.195
BLNK	0.105	1.075	4.295
BMP2	0.487	1.402	4.550
BMP2K	0.422	1.340	7.423
BMP3	-0.261	0.834	3.259
BMP4	-0.224	0.856	3.695
BMP5	-0.291	0.817	2.391
BMP6	0.232	1.174	4.246
BMP7	0.021	1.014	4.572
BMP8A	-0.403	0.756	5.005
BMP8B	-0.120	0.920	3.039
BMPER	0.052	1.037	4.588
BMPR2	0.303	1.234	8.766
BMS1//LOC101929959	0.234	1.176	5.999
BMS1P20//LL22NC03-80A10.12	-0.213	0.863	3.620
BMS1P4//BMS1//RP11-574K11.28	-0.429	0.743	3.015
BNC2	0.417	1.335	3.455
BOC	-0.214	0.862	4.358
BOK	-0.062	0.958	6.769
BOLA3-AS1	0.190	1.141	4.014
BRAF//KIAA1549	-0.078	0.947	7.012
BRCC3	0.276	1.210	6.446

BRI3	0.350	1.274	2.387
BRI3	-0.155	0.898	2.880
BRINP1//DBC1	0.198	1.147	7.358
BRINP2//FAM5B	0.026	1.018	5.627
BRINP3//FAM5C	0.265	1.201	6.373
BRMS1L	0.016	1.011	4.297
BRPF3	-0.102	0.932	4.857
BRSK1	-0.071	0.952	6.616
BRSK2	-0.143	0.906	5.625
BSN	-0.120	0.920	5.422
BSPRY	0.429	1.347	2.918
BST1	0.039	1.028	2.977
BST2	0.483	1.397	5.725
BTBD10	-0.091	0.939	5.701
BTBD11	0.287	1.220	4.476
BTBD16	-0.019	0.987	2.431
BTBD17	-0.190	0.876	2.766
BTBD19//PLK3	0.031	1.022	3.155
BTBD3	0.106	1.076	6.467
BTBD6	-0.357	0.781	4.763
BTBD9	-0.063	0.957	5.913
BTG2	0.281	1.215	7.920
BTK	0.893	1.857	4.408
BTRC	-0.034	0.977	7.369
BVES	0.302	1.233	6.506
BVES-AS1	0.293	1.225	2.735
BZRAP1	-0.157	0.897	4.576
BZW2	0.230	1.173	3.927
C10orf10	0.768	1.702	6.564
C10orf105	-0.271	0.828	2.943
C10orf107	0.160	1.118	5.145
C10orf11	0.360	1.283	2.939
C10orf128	0.023	1.016	4.017
C10orf25	0.204	1.152	3.233
C10orf35	-0.225	0.855	2.054
C10orf54	-0.025	0.983	4.029
C10orf85	-0.129	0.915	4.139
C10orf88	0.075	1.053	5.328
C11orf24	0.113	1.081	5.106
C11orf39	-0.113	0.925	3.364
C11orf39	-0.062	0.958	3.344
C11orf70	-0.147	0.903	3.245
C11orf74	0.082	1.058	3.763
C11orf80	0.403	1.322	4.172
C11orf87	0.690	1.613	4.632
C11orf88	-1.464	0.362	1.741
C11orf91	-0.247	0.843	2.728
C11orf97//RP11-867G2.2	-0.822	0.566	2.031
C12orf29	0.283	1.216	6.209
C12orf4	0.158	1.116	6.003

C12orf43	-0.026	0.982	4.883
C12orf45	0.064	1.045	4.062
C12orf55	-1.059	0.480	2.977
C12orf76	0.077	1.055	4.246
C14orf132	-0.219	0.859	6.222
C14orf142	-0.174	0.886	2.980
C14orf2	-0.076	0.949	4.005
C14orf23	0.139	1.101	2.568
C14orf37	-0.066	0.955	5.326
C15orf27	0.144	1.105	2.810
C15orf39	0.279	1.213	3.916
C15orf52	0.140	1.102	3.751
C15orf59	-0.204	0.868	6.031
C15orf61	-0.423	0.746	4.577
C15orf65	-0.092	0.938	4.722
C16orf13	-0.043	0.971	5.806
C16orf45	0.054	1.038	6.956
C16orf52//VWA3A//LOC101930115	0.100	1.072	4.961
C16orf70	0.255	1.193	7.285
C16orf87	0.023	1.016	5.483
C16orf89	-0.103	0.931	4.565
C16orf93	-0.163	0.893	3.127
C17orf100	-0.199	0.871	4.534
C17orf107	-0.349	0.785	3.170
C17orf50	-0.228	0.854	2.751
C17orf51	0.053	1.037	4.460
C17orf62	0.499	1.414	6.266
C17orf75	-0.144	0.905	5.349
C17orf96	0.049	1.035	2.722
C17orf97	-0.544	0.686	4.718
C18orf21	0.253	1.192	3.411
C18orf25	0.239	1.180	5.983
C18orf42	-0.741	0.598	2.703
C18orf54	0.478	1.393	4.303
C19orf12	0.134	1.097	4.055
C19orf18	-0.284	0.821	3.450
C19orf26	-0.373	0.772	3.706
C19orf33//CTB-102L5.4	-0.090	0.940	2.379
C19orf35	0.018	1.013	2.961
C19orf38	0.736	1.666	4.418
C19orf54	-0.016	0.989	3.776
C19orf57	-0.161	0.895	2.847
C19orf81	0.275	1.210	2.351
C19orf82	-0.566	0.676	4.103
C19orf83//AC006132.1	-0.021	0.986	2.722
C1GALT1	0.228	1.171	6.293
C1orf110	0.179	1.132	2.879
C1orf115	0.062	1.044	5.240
C1orf158	-1.413	0.376	1.816
C1orf162	0.474	1.389	5.579

C1orf192	-1.576	0.335	4.436
C1orf194	-0.491	0.712	3.180
C1orf198	0.095	1.068	5.994
C1orf21	-0.355	0.782	5.816
C1orf216	-0.084	0.944	3.563
C1orf220	0.404	1.323	2.522
C1orf226//NOS1AP	-0.271	0.829	2.904
C1orf228	0.055	1.039	3.285
C1orf233	-0.383	0.767	4.361
C1orf54	0.134	1.098	4.495
C1orf61	-0.404	0.756	5.606
C1orf64	0.103	1.074	3.861
C1orf74	0.180	1.133	3.876
C1orf87	-1.178	0.442	2.383
C1orf94	0.324	1.251	2.575
C1orf95	0.337	1.264	3.945
C1QA	0.615	1.532	3.526
C1QB	1.112	2.161	6.710
C1QC	0.959	1.944	7.174
C1QL1	-0.407	0.754	3.998
C1QL2	-0.020	0.987	4.540
C1QL3	0.652	1.572	5.352
C1QL4	0.603	1.519	2.194
C1QTNF4	-0.292	0.817	5.101
C1QTNF6	-0.048	0.967	3.533
C1QTNF8	-0.291	0.818	2.445
C1R//C1RL	0.289	1.221	4.399
C1RL	0.197	1.146	4.122
C1S	0.368	1.291	5.773
C2//CFB//XXbac-BPG116M5.17	0.350	1.275	3.828
C2//CFB//XXbac-BPG116M5.17	0.260	1.197	3.719
C2//CFB//XXbac-BPG116M5.17	0.255	1.194	3.789
C2//CFB//XXbac-BPG116M5.17	0.257	1.195	3.825
C2//XXbac-BPG116M5.17	0.599	1.515	3.770
C20orf197	0.171	1.125	3.436
C20orf26	-0.610	0.655	2.758
C21orf128	0.214	1.160	3.163
C21orf140//AP000322.54	0.380	1.302	2.187
C21orf15//LINC00417//CYP4F29P	-0.217	0.860	3.433
C21orf49	0.015	1.011	2.924
C21orf62	-0.284	0.821	3.993
C21orf88	0.052	1.036	3.836
C21orf91	0.114	1.083	4.614
C22orf24	0.063	1.045	2.390
C22orf39//HIRA	0.032	1.022	4.821
C22orf46	-0.340	0.790	3.820
C2CD2	-0.243	0.845	6.825
C2CD2	0.231	1.174	3.194
C2CD4C	0.024	1.016	3.343
C2CD5	-0.060	0.959	6.657

C2orf15//MRPL30//LOC101927110//RP11-111H13.1	0.289	1.222	3.037
C2orf40	0.049	1.035	3.342
C2orf50	-0.355	0.782	3.203
C2orf68	0.024	1.017	5.684
C2orf69	-0.021	0.985	4.975
C2orf72	0.128	1.093	5.520
C2orf80	0.059	1.042	4.203
C2orf82	-0.119	0.921	3.901
C2orf88	0.404	1.323	3.117
C3	0.719	1.645	8.694
C3AR1	0.428	1.345	3.750
C3orf14	-0.067	0.955	5.037
C3orf17	0.156	1.114	5.372
C3orf33	0.141	1.103	2.255
C3orf35	-0.039	0.973	3.694
C3orf58	0.093	1.066	4.328
C3orf67-AS1//RP11-147N17.1	0.449	1.365	2.456
C3orf80//RP11-431I8.1	-0.473	0.721	3.489
C4A//C4B//C4B_2	0.133	1.097	7.186
C4B//C4A//C4B_2	0.130	1.095	7.480
C4B//C4B_2//C4A	0.126	1.091	7.356
C4B//C4B_2//C4A	0.123	1.089	7.464
C4B//C4B_2//C4A	0.120	1.087	7.513
C4B//C4B_2//C4A	0.119	1.086	7.483
C4B//C4B_2//C4A	0.112	1.081	7.482
C4B//C4B_2//C4A//STK19//DAQB-331I12.15	0.158	1.116	6.703
C4B//C4B_2//C4A//STK19//DAQB-331I12.15	0.120	1.087	6.810
C4orf19	0.415	1.333	2.325
C4orf32	0.159	1.116	3.891
C4orf47	-0.662	0.632	2.827
C4orf50	-0.351	0.784	3.004
C5	0.186	1.137	3.986
C5AR1	0.949	1.930	4.639
C5AR2//GPR77	0.239	1.180	2.983
C5orf22	0.279	1.213	5.808
C5orf27	-0.519	0.698	4.425
C5orf30	0.184	1.136	4.711
C5orf34	-0.039	0.973	2.123
C5orf38	-0.299	0.813	3.137
C5orf49	-0.349	0.785	4.456
C5orf55	0.082	1.059	3.453
C5orf63	0.144	1.105	3.182
C5orf64	-0.060	0.959	3.812
C6orf106	0.129	1.094	6.704
C6orf120	0.272	1.207	5.692
C6orf165//RP3-382I10.7	-0.613	0.654	3.064
C6orf165//RP3-382I10.7	0.185	1.137	5.110
C6orf211	0.494	1.409	4.895
C6orf47	-0.076	0.949	2.573
C6orf48	0.075	1.053	7.378

C6orf48	0.043	1.030	7.564
C6orf48	-0.021	0.985	7.250
C6orf62	0.469	1.384	4.644
C7	1.029	2.041	5.134
C7orf34	-0.212	0.863	2.613
C7orf57	-0.973	0.509	1.764
C7orf60	0.167	1.123	5.462
C7orf61//TSC22D4	-0.356	0.781	3.479
C7orf63	-0.383	0.767	3.254
C7orf65	-0.357	0.781	2.969
C8orf22	-0.397	0.760	2.436
C8orf31	0.021	1.014	2.692
C8orf4	0.478	1.393	2.894
C8orf44-SGK3//SGK3//C8orf44	-0.129	0.914	5.675
C8orf46	-0.043	0.970	5.435
C8orf46	-0.081	0.945	3.198
C8orf58	0.273	1.208	2.475
C8orf88//RP11-122A3.2	-0.191	0.876	3.846
C9orf117	-1.243	0.422	2.395
C9orf129	0.197	1.146	2.841
C9orf142	-0.179	0.883	5.243
C9orf153	-0.226	0.855	2.411
C9orf16	0.113	1.081	3.489
C9orf170	-0.203	0.869	3.128
C9orf172	-0.039	0.973	4.560
C9orf24	-0.704	0.614	3.818
C9orf3	0.060	1.042	4.550
C9orf41	0.204	1.152	5.031
C9orf47//S1PR3	0.635	1.553	3.086
C9orf89	0.282	1.216	4.648
C9orf91	0.055	1.039	5.884
CA10	0.398	1.318	6.510
CA11	-0.072	0.951	7.457
CA13//LOC100507258	0.272	1.208	3.581
CA2	-0.208	0.866	6.047
CA4	-0.440	0.737	3.011
CA5B	0.482	1.397	4.905
CA5BP1	-0.246	0.843	4.624
CA7	-0.446	0.734	2.647
CA8	-0.229	0.853	5.157
CAAP1	0.454	1.370	5.162
CABLES1	-0.190	0.876	5.953
CABLES2	0.214	1.160	4.911
CABP1	-0.028	0.981	3.593
CABP7	-0.508	0.703	4.209
CABYR	-0.018	0.988	5.247
CACNA1A//LOC100507353	0.020	1.014	6.071
CACNA1B	-0.104	0.930	5.409
CACNA1C	0.191	1.142	4.954
CACNA1C-AS2	-0.020	0.986	2.453

CACNA1D	0.059	1.042	5.248
CACNA1E	0.631	1.548	5.147
CACNA1G	0.445	1.361	3.137
CACNA1H	0.312	1.241	3.630
CACNA2D1	0.046	1.032	7.062
CACNA2D2	0.403	1.322	5.088
CACNA2D3	-0.289	0.818	5.671
CACNB1	-0.099	0.933	6.210
CACNB2	-0.402	0.757	5.348
CACNB3	0.052	1.036	3.769
CACNB4	0.137	1.099	6.722
CACNG2	-0.165	0.892	5.740
CACNG3	0.443	1.360	6.867
CACNG4	0.300	1.231	5.726
CACNG5	0.315	1.244	2.314
CACNG8	0.300	1.231	3.831
CADM2	-0.152	0.900	7.285
CADM3	-0.132	0.913	8.080
CADM3-AS1//CTA-134P22.2	-0.072	0.951	5.382
CADPS	0.165	1.121	7.471
CADPS2	0.149	1.109	6.078
CAHM	0.044	1.031	5.175
CALB1	0.517	1.431	6.451
CALB2	-0.088	0.941	6.456
CALCRL	0.691	1.615	5.731
CALHM1	-0.281	0.823	2.962
CALHM2	0.358	1.281	3.979
CALM1//CALM2//CALM3	-0.034	0.977	4.317
CALY	-0.111	0.926	4.939
CAMK1	0.576	1.491	4.374
CAMK1G	-0.394	0.761	4.743
CAMK2A	0.505	1.419	7.727
CAMK2B	0.099	1.071	7.122
CAMK2D	0.514	1.428	6.579
CAMK2G	-0.312	0.805	7.369
CAMK2N1	-0.028	0.981	7.530
CAMK2N2	-0.379	0.769	5.591
CAMK4	-0.075	0.949	4.898
CAMKK1	-0.136	0.910	4.970
CAMKK2	0.126	1.092	5.837
CAMKV	0.692	1.616	5.720
CAMSAP3	-0.132	0.913	3.348
CAMTA1	-0.146	0.904	7.376
CAMTA2	0.129	1.093	6.843
CAND1	0.287	1.220	6.554
CAP2	-0.282	0.823	7.795
CAPG	0.366	1.288	3.239
CAPN13	-0.173	0.887	2.372
CAPN3//RP11-164J13.1	0.125	1.091	7.006
CAPRIN2	0.062	1.044	6.333

CAPS	-0.453	0.730	5.653
CAPS2	-0.226	0.855	4.210
CARD11	-0.176	0.885	2.756
CARD8	0.097	1.070	4.698
CARNS1	0.073	1.052	7.175
CARTPT	-0.072	0.951	5.209
CASC1	-0.382	0.767	2.549
CASC10	-0.052	0.965	4.118
CASC14//RP1-67M12.2//CASC15	-0.277	0.825	3.053
CASD1	0.169	1.124	7.303
CASKIN1	-0.293	0.816	5.843
CASP1	0.726	1.654	2.267
CASP10	0.389	1.310	3.090
CASP4	0.882	1.842	2.880
CASP6	0.410	1.329	2.103
CASQ1	0.029	1.020	4.097
CAT	-0.083	0.944	6.851
CATIP-AS1//AC021016.6	0.055	1.039	2.993
CATSPER2//LOC101930343	-0.321	0.800	3.810
CAV1	0.117	1.084	4.744
CBFB	-0.042	0.971	7.628
CBLN1	0.432	1.349	2.982
CBLN2	0.626	1.543	4.872
CBLN3	-0.020	0.986	3.569
CBLN4	-0.444	0.735	3.479
CBR1	0.172	1.127	5.697
CBR3	-0.036	0.975	2.984
CBX6	-0.125	0.917	6.194
CC2D1B	-0.095	0.937	3.930
CCBE1	-0.144	0.905	2.776
CCDC102B	0.196	1.146	4.842
CCDC103//FAM187A	-0.121	0.920	2.584
CCDC104	-0.188	0.878	5.676
CCDC109B	0.610	1.527	4.650
CCDC11	-0.782	0.582	4.883
CCDC113	-0.550	0.683	5.656
CCDC114	-0.653	0.636	2.622
CCDC122	0.107	1.077	3.710
CCDC132	0.274	1.209	5.414
CCDC135	-0.556	0.680	2.592
CCDC13-AS1	-0.693	0.618	2.975
CCDC14	-0.080	0.946	6.463
CCDC144B//CCDC144CP	0.084	1.060	5.551
CCDC144B//RP11-219A15.1	1.045	2.063	4.080
CCDC144B//RP11-219A15.1	0.681	1.604	3.693
CCDC144CP	0.093	1.067	5.903
CCDC146	-0.544	0.686	4.500
CCDC147-AS1	-0.065	0.956	2.556
CCDC149	0.266	1.202	5.053
CCDC151	-0.788	0.579	3.576

CCDC152	-0.111	0.926	3.985
CCDC159	0.110	1.079	3.516
CCDC160	-0.506	0.704	2.706
CCDC163P	0.179	1.132	3.689
CCDC170	-0.423	0.746	3.671
CCDC173	-0.605	0.657	2.681
CCDC177	0.216	1.161	3.608
CCDC18	-0.110	0.927	2.811
CCDC184//C12orf68	-0.044	0.970	4.200
CCDC19	-0.752	0.594	2.584
CCDC3	0.035	1.024	4.243
CCDC30	-0.530	0.693	3.379
CCDC30	-0.451	0.732	3.094
CCDC30	-0.346	0.787	4.200
CCDC34	-0.303	0.811	2.811
CCDC37	-0.710	0.611	1.824
CCDC39//LOC101928882	-0.766	0.588	2.922
CCDC40	-0.461	0.726	3.190
CCDC6	-0.020	0.986	7.942
CCDC60	-0.769	0.587	1.892
CCDC64	-0.288	0.819	3.201
CCDC65	-0.371	0.773	2.075
CCDC68	-0.161	0.894	2.491
CCDC69	0.132	1.096	3.836
CCDC71	0.674	1.595	4.398
CCDC71L	-0.174	0.886	4.358
CCDC8	-0.276	0.826	3.674
CCDC80	0.615	1.531	4.794
CCDC81	-0.731	0.603	2.104
CCDC85A	0.316	1.245	6.082
CCDC85B	-0.035	0.976	3.951
CCER2//CTC-360G5.1	0.197	1.147	3.283
CCK	0.196	1.145	3.019
CCL19	0.905	1.873	3.308
CCL2	1.729	3.314	7.375
CCL20	0.390	1.311	2.474
CCL3L3//CCL3L1	0.065	1.046	2.932
CCL5	0.291	1.224	3.518
CCNA1	0.104	1.074	3.741
CCNA2	-0.346	0.787	3.797
CCNB1	-0.288	0.819	2.684
CCNB3//BCOR	0.065	1.046	2.293
CCND1	-0.068	0.954	5.011
CCND2-AS1//RP11-264F23.3	0.447	1.363	4.049
CCNDBP1	-0.088	0.941	5.792
CCNE1	0.302	1.233	3.673
CCNE2	0.045	1.031	4.465
CCNF	0.163	1.119	2.933
CCNG2	0.089	1.063	3.604
CCNL1	0.485	1.399	6.530

CCNO	-0.397	0.759	2.170
CCNT2-AS1//AC016725.4	0.125	1.090	4.308
CCP110	-0.048	0.967	7.312
CCR1	1.237	2.357	3.774
CCRL2	0.282	1.216	2.783
CCRN4L	0.408	1.327	4.214
CCSAP	0.070	1.050	5.560
CCSER1	-0.186	0.879	3.099
CCT6P1	-0.564	0.677	3.576
CD109	0.318	1.247	4.848
CD14	0.924	1.897	6.125
CD151	-0.123	0.918	5.107
CD163	0.926	1.900	6.080
CD164	0.138	1.100	7.282
CD177	-0.354	0.782	3.349
CD177//CD177P1//CTC-490G23.5	-0.069	0.953	3.334
CD180	1.127	2.184	4.577
CD200	0.542	1.456	7.505
CD209	0.464	1.379	2.157
CD22	0.365	1.288	6.646
CD226	0.907	1.875	3.580
CD24	-0.416	0.749	4.319
CD274	0.229	1.172	3.262
CD300A	0.839	1.789	4.219
CD300C	0.598	1.514	3.435
CD300LF	1.052	2.074	2.825
CD33	0.725	1.653	3.206
CD34	0.401	1.321	5.680
CD37	0.989	1.985	5.702
CD38	0.219	1.164	4.382
CD4	0.442	1.359	3.716
CD40	-0.119	0.921	3.848
CD44	0.404	1.323	6.118
CD44//RP1-68D18.2	0.457	1.373	3.664
CD48	0.964	1.951	1.680
CD5	-0.203	0.869	2.771
CD53	0.455	1.370	6.268
CD58	0.225	1.169	4.045
CD63	0.134	1.097	5.834
CD68//SNORA67//LOC101928634//RP11-186B7.4	0.744	1.675	8.608
CD69	-0.061	0.958	2.501
CD74	0.363	1.287	6.003
CD81	-0.043	0.971	3.481
CD82	0.103	1.074	4.090
CD83	0.216	1.161	5.804
CD84	0.849	1.802	3.418
CD86	0.877	1.837	4.878
CD9	0.184	1.136	3.975
CD93	0.788	1.727	5.419
CD97	0.735	1.664	4.850

CD99	0.046	1.032	9.192
CD99P1	0.527	1.441	3.883
CDADC1	-0.012	0.992	4.672
CDC14B	0.075	1.053	4.203
CDC40	0.134	1.097	5.563
CDC42EP1	0.173	1.127	4.821
CDC42EP4	0.180	1.133	6.264
CDC42SE2	0.067	1.048	3.304
CDC7	0.462	1.377	5.253
CDCA7L	0.368	1.291	3.282
CDCP1	1.003	2.005	3.380
CDH1	0.231	1.174	3.586
CDH10	0.240	1.181	6.352
CDH13	0.266	1.202	6.425
CDH18	0.092	1.066	4.615
CDH19	0.739	1.669	4.632
CDH20	-0.157	0.897	6.721
CDH22	-0.086	0.942	5.185
CDH26	0.355	1.279	3.255
CDH3	0.541	1.455	3.387
CDH4	0.455	1.371	4.095
CDH5	1.058	2.081	6.304
CDH7	0.040	1.028	3.882
CDH8	0.476	1.391	6.497
CDH9	0.252	1.191	3.412
CDHR1	-0.426	0.744	4.338
CDK14	0.123	1.089	7.809
CDK18	-0.071	0.952	5.930
CDK2	0.349	1.274	4.652
CDK20	0.225	1.169	4.696
CDK2AP2	-0.267	0.831	4.065
CDK5	0.035	1.024	6.231
CDK5R2	-0.374	0.772	4.684
CDK6	0.147	1.108	4.891
CDK7	0.250	1.189	4.739
CDKL1	0.091	1.065	3.739
CDKL2	0.191	1.141	5.315
CDKL5	0.069	1.049	7.043
CDKN1A	0.965	1.951	4.377
CDKN1C	0.170	1.125	4.994
CDKN2AIPNL	-0.204	0.868	5.093
CDKN2B	0.335	1.261	2.581
CDKN2C	0.244	1.184	2.648
CDKN2D	-0.088	0.941	5.186
CDKN3	0.024	1.017	2.289
CDNF	-0.086	0.942	3.388
CDO1	0.067	1.048	6.640
CDON	-0.124	0.918	3.728
CDR2//LOC101060399	0.231	1.174	4.733
CDR2L	-0.333	0.794	5.295

CDRT15	0.320	1.248	3.592
CDS1	-0.409	0.753	5.287
CDYL2	0.602	1.518	4.247
CDYL2	-0.019	0.987	5.667
CEBPA	0.487	1.402	4.468
CEBPB	0.227	1.170	4.646
CEBPD	0.243	1.183	6.707
CECR1	0.430	1.347	4.247
CECR2	-0.157	0.897	4.449
CECR6	0.293	1.226	3.570
CECR7	0.321	1.249	3.488
CELF2	0.441	1.358	7.659
CELF3	-0.107	0.929	4.307
CELF4	0.337	1.263	6.275
CELF5	0.427	1.345	5.031
CELSR3	-0.031	0.979	3.645
CEMIP//KIAA1199	-0.442	0.736	5.461
CEND1	-0.464	0.725	5.733
CENPB	-0.459	0.728	5.531
CENPBD1	0.397	1.317	4.752
CENPJ	0.075	1.053	3.793
CENPP	-0.289	0.819	3.727
CENPV	-0.153	0.899	5.257
CEP170B	-0.303	0.810	5.353
CEP19	0.373	1.295	4.727
CEP192	0.095	1.068	6.167
CEP57L1	0.102	1.073	3.111
CEP76	0.270	1.205	3.854
CEP97	0.034	1.024	6.774
CERCAM	0.082	1.059	6.699
CERKL	-0.386	0.765	2.381
CERS2	0.101	1.072	4.437
CERS6	0.387	1.308	7.244
CES1//LOC100653057	0.327	1.255	3.132
CES1//LOC100653057//LOC100653086//CES1P1//CE	-0.080	0.946	3.625
CES4A//CES8	0.354	1.278	5.301
CFB//XXbac-BPG116M5.17	0.049	1.035	3.561
CFD	0.690	1.613	4.213
CFH	0.084	1.060	4.671
CFI	0.784	1.722	3.717
CFL1P1	-0.025	0.983	2.874
CFL2	0.046	1.032	5.660
CFLAR	0.388	1.309	4.705
CFP	-0.547	0.684	2.363
CGB//CGB5//CGB8//CGB1	0.172	1.126	3.291
CGN	0.039	1.028	2.181
CGREF1	0.126	1.092	4.173
CH25H	1.667	3.176	5.486
CHAC1	0.052	1.037	4.317
CHAD	0.294	1.226	3.924

CHADL	-0.215	0.862	4.777
CHAF1A	-0.254	0.839	3.998
CHAF1B	0.052	1.037	3.619
CHAT	-0.627	0.648	1.965
CHCHD1	0.135	1.098	4.178
CHCHD7	0.108	1.077	2.704
CHD1L	-0.016	0.989	6.109
CHD3	0.052	1.036	6.672
CHD5	-0.125	0.917	5.307
CHD7	-0.203	0.869	5.703
CHDC2	-1.384	0.383	3.029
CHEK2	-0.254	0.838	2.343
CHFR//RNU6-327P	-0.043	0.970	2.484
CHGA	-0.364	0.777	5.141
CHGB	-0.060	0.959	4.481
CHI3L1	0.759	1.692	8.000
CHI3L2	1.210	2.313	4.845
CHIC1	-0.019	0.987	5.882
CHIT1	1.159	2.233	2.675
CHKA	-0.164	0.893	6.140
CHL1	0.552	1.466	6.977
CHL1-AS2	-0.041	0.972	4.252
CHM	0.257	1.195	6.132
CHML	0.249	1.188	4.833
CHN1	0.362	1.285	5.007
CHODL	-0.435	0.740	2.149
CHPF	0.134	1.097	3.890
CHRAC1	0.024	1.017	4.648
CHRD	0.364	1.287	3.532
CHRDL1	0.240	1.181	5.299
CHRM1	-0.323	0.800	2.809
CHRM2	-0.250	0.841	3.814
CHRM3	0.253	1.192	3.331
CHRM4	0.179	1.132	3.355
CHRM5	-0.466	0.724	2.655
CHRNA1	0.537	1.451	2.518
CHRNA4//LOC102723788	-0.030	0.979	3.170
CHRNA6	0.428	1.345	2.850
CHRNA7//CHRFAM7A	-0.249	0.842	2.941
CHRNB2	-0.172	0.887	4.168
CHST1	0.200	1.148	4.806
CHST10	-0.057	0.961	3.434
CHST14	-0.110	0.927	3.480
CHST15	0.272	1.207	6.630
CHST3	0.174	1.128	6.234
CHST6	0.561	1.475	6.084
CHST9	-0.307	0.808	3.426
CHSY3	0.250	1.189	3.404
CHURC1-FNTB//FNTB	-0.126	0.917	6.384
CIART//C1orf51	-0.339	0.790	4.270

CIB1	0.426	1.343	4.692
CIDEA	-0.108	0.928	2.767
CIDEB	0.211	1.158	3.986
CIDECP	-0.145	0.904	4.317
CIITA	0.502	1.416	4.825
CINP	0.421	1.339	3.819
CIRBP	-0.223	0.857	3.400
CIRBP	0.088	1.063	8.323
CIRH1A	0.251	1.190	6.611
CISD1	0.018	1.013	4.133
CISH	0.353	1.277	2.804
CIT//MIR1178	-0.205	0.867	5.328
CITED1	0.061	1.043	3.175
CITED2	0.279	1.214	5.289
CKAP2	0.021	1.015	4.761
CKM	-0.327	0.797	2.595
CKMT1A//CKMT1B	-0.309	0.807	5.884
CKMT2	-0.187	0.879	4.964
CLCA4	0.261	1.198	4.115
CLCF1	0.185	1.137	3.345
CLCN2	-0.054	0.964	5.316
CLCN4	-0.020	0.986	7.469
CLDN10	0.322	1.250	6.256
CLDN11	0.038	1.027	5.205
CLDN15	0.157	1.115	3.124
CLDN16	0.159	1.116	2.564
CLDN19	-0.290	0.818	2.823
CLDN3	-0.350	0.785	3.273
CLDN9	-0.212	0.863	2.677
CLDND1	0.097	1.070	8.345
CLEC18C//CLEC18B	-0.163	0.893	3.106
CLEC2B	0.405	1.324	4.463
CLEC2D	0.072	1.051	3.246
CLEC2L	-0.704	0.614	6.236
CLEC4A	0.879	1.839	2.457
CLEC5A	0.656	1.576	3.134
CLEC7A	0.438	1.354	5.035
CLEC9A	-0.226	0.855	2.414
CLGN	-0.246	0.843	3.394
CLIC1	0.277	1.212	6.481
CLIC1	0.267	1.204	6.483
CLIC1	0.250	1.189	6.498
CLIC1//DDAH2	0.113	1.082	5.558
CLIC1//DDAH2	0.101	1.073	5.539
CLIC2	0.579	1.494	4.643
CLIC3	-0.415	0.750	2.084
CLIC4	0.296	1.228	8.652
CLK2P	0.037	1.026	2.660
CLMP//ASAM	0.186	1.138	4.840
CLN3//RP11-435I10.4	0.282	1.216	5.476

CLN6	0.172	1.126	3.684
CLN8	0.282	1.216	4.469
CLOCK	0.404	1.323	6.982
CLSTN1	-0.297	0.814	8.836
CLSTN2	-0.171	0.888	5.925
CLSTN3	0.049	1.035	5.534
CLTB	-0.231	0.852	6.469
CLTCL1	-0.137	0.910	3.983
CLUL1	1.174	2.257	2.678
CLVS1	-0.077	0.948	4.025
CLYBL	0.314	1.243	2.693
CMAS	0.359	1.282	6.145
CMKLR1	0.442	1.358	4.765
CMTM3	0.308	1.238	4.933
CMTM5	0.076	1.054	6.206
CMTM7	0.210	1.157	3.829
CMYA5	0.253	1.192	4.631
CNDP1	0.203	1.151	9.308
CNEP1R1//TMEM188	0.529	1.443	6.268
CNGA3	0.195	1.144	3.183
CNGB1	-0.022	0.985	2.587
CNIH2	-0.100	0.933	4.570
CNIH3	0.089	1.063	3.217
CNKSR2	-0.029	0.980	7.076
CNKSR3	-0.233	0.851	4.627
CNN2	0.566	1.481	5.254
CNN3	0.382	1.303	8.002
CNP	0.223	1.167	4.582
CNP	0.101	1.073	9.602
CNR1	0.846	1.798	6.099
CNR1	0.698	1.622	6.063
CNRIP1	0.037	1.026	5.364
CNST	0.130	1.095	5.676
CNTFR	-0.676	0.626	6.965
CNTN2	0.096	1.069	7.640
CNTN3	-0.749	0.595	5.526
CNTN4	0.748	1.680	4.555
CNTN5	-0.181	0.882	3.204
CNTN6	0.157	1.115	2.540
CNTNAP1	0.326	1.253	7.341
CNTNAP2	0.144	1.105	7.428
CNTNAP3B//CNTNAP3//CNTNAP3P2	0.685	1.607	4.654
CNTNAP4	-0.044	0.970	7.517
CNTNAP5	0.214	1.160	6.245
COA6//SEC61A1	0.123	1.089	3.986
COA7//SELRC1	-0.056	0.962	4.156
COCH	0.326	1.253	3.360
COG1	0.122	1.088	6.910
COG6	0.452	1.368	6.107
COL10A1	0.125	1.091	2.061

COL12A1	0.110	1.079	4.414
COL14A1	1.218	2.326	4.102
COL15A1	0.088	1.063	2.224
COL16A1	-0.191	0.876	4.634
COL18A1	-0.036	0.975	4.820
COL19A1	0.167	1.123	2.615
COL1A1	0.295	1.227	3.478
COL1A2	0.238	1.179	4.012
COL24A1	-0.283	0.822	2.705
COL25A1	0.264	1.200	3.151
COL26A1	0.020	1.014	4.275
COL27A1	0.404	1.323	2.778
COL28A1	0.127	1.092	3.047
COL3A1	0.333	1.259	2.547
COL4A1	0.952	1.935	4.338
COL4A2	0.577	1.491	4.211
COL4A3	-0.318	0.802	2.290
COL4A3BP	0.350	1.275	7.414
COL4A5	0.186	1.138	5.054
COL5A2	0.119	1.086	4.526
COL6A2	-0.491	0.711	4.260
COL6A3	-0.076	0.949	3.060
COL8A1	0.103	1.074	2.194
COL8A2	0.124	1.090	2.929
COL9A1	0.014	1.009	2.973
COL9A2	-0.147	0.903	4.202
COL9A3	-0.274	0.827	4.194
COLEC12	0.864	1.821	5.659
COLGALT1//GLT25D1	0.344	1.269	6.224
COLQ	-0.470	0.722	3.535
COMMD8	0.215	1.161	4.350
COMT	-0.147	0.903	3.718
COPG2	0.063	1.044	5.045
COPS4	0.090	1.065	6.025
COQ10B	0.472	1.387	5.422
COQ3	0.108	1.077	3.543
CORO1A	0.320	1.248	5.807
CORO2A	-0.314	0.805	3.036
CORO6	0.025	1.018	4.745
COTL1	-0.015	0.990	7.210
COX10	0.338	1.264	5.378
COX11	0.143	1.104	5.698
COX18	0.256	1.194	5.026
COX6A2	0.022	1.015	2.305
COX7A1	-0.062	0.958	6.020
CP	0.651	1.571	6.592
CPA2	-0.493	0.710	2.254
CPAMD8	-0.787	0.579	5.984
CPB2-AS1	0.258	1.196	4.648
CPEB2	0.033	1.023	6.922

CPEB3	0.065	1.046	5.684
CPED1	0.290	1.223	4.408
CPLX1	-0.750	0.595	5.218
CPLX2	-0.054	0.963	3.320
CPLX3//RP11-414J4.2	0.165	1.121	4.546
CPLX3//RP11-414J4.2//LMAN1L	-0.344	0.788	2.746
CPM	0.914	1.884	5.087
CPNE2	-0.013	0.991	7.451
CPNE4	0.665	1.586	4.775
CPNE5	0.533	1.447	4.592
CPNE7	0.070	1.050	3.422
CPNE8	0.307	1.237	4.959
CPNE9	-0.588	0.665	4.143
CPO	0.165	1.121	2.006
CPOX	0.203	1.151	2.320
CPOX	0.055	1.039	5.953
CPPED1	0.241	1.182	6.639
CPQ	0.020	1.014	3.845
CPS1	0.215	1.161	4.710
CPT1C	-0.463	0.725	6.103
CPT2	0.405	1.324	4.781
CPVL	0.562	1.477	2.820
CPXM1	-0.053	0.964	2.507
CPXM2	0.123	1.089	4.287
CR1	0.591	1.507	2.871
CRABP2	-0.253	0.839	2.831
CRADD	-0.172	0.887	3.772
CRB2	0.095	1.068	5.223
CREB3L2	0.098	1.071	4.557
CREB3L3	-0.017	0.988	3.227
CREB3L4	0.271	1.206	2.642
CREG1	0.689	1.612	5.421
CREG2	-0.248	0.842	4.962
CRELD1	0.082	1.059	5.034
CRH	0.049	1.034	2.178
CRHBP	0.295	1.227	5.205
CRHR1-IT1//MGC57346//AC126544.2	0.238	1.179	4.660
CRHR2	-0.107	0.929	3.560
CRIPAK	0.536	1.450	4.291
CRISPLD1	0.711	1.637	5.422
CRISPLD2	0.286	1.219	5.608
CRLF1	-0.209	0.865	4.247
CRMP1	0.161	1.118	4.785
CRNDE	0.085	1.061	3.545
CROCCP2	-0.268	0.830	2.985
CROT	-0.038	0.974	4.582
CRTAC1	-0.316	0.803	4.558
CRTAP	0.162	1.119	7.276
CRTC3	-0.104	0.930	5.409
CRY2	0.391	1.311	5.337

CRYBB1	0.268	1.205	2.680
CRYBG3	-0.101	0.933	6.125
CRYGS	0.413	1.331	3.159
CRYL1	0.018	1.013	6.525
CRYM	0.169	1.125	3.933
CSAD	0.018	1.013	5.433
CSAG1//CSAG3	-0.274	0.827	2.296
CSF1	0.289	1.222	3.714
CSF1R	0.296	1.228	7.734
CSF2RA	0.276	1.211	4.682
CSF2RA	0.082	1.058	4.322
CSF3	-0.361	0.778	3.395
CSF3R	0.831	1.778	3.039
CSMD1	0.152	1.111	5.365
CSMD3	-0.086	0.942	5.840
CSNK1E//RP3-449O17.1	0.284	1.218	4.017
CSNK2A3	0.571	1.485	4.160
CSPG4	0.303	1.234	3.817
CSPG4P1Y//CSPG4P2Y//CSPG4P3Y//DNM1P24//CSP	0.060	1.042	3.348
CSPG5	-0.126	0.916	5.480
CSRNP1	0.080	1.057	5.107
CSRNP2	0.084	1.060	5.420
CSRNP3	0.447	1.364	6.284
CSRP2BP//PET117	-0.022	0.985	4.689
CST4	-0.222	0.858	2.416
CST7	0.048	1.034	4.350
CSTF3	0.210	1.157	5.249
CTA-212D2.2	0.304	1.234	2.237
CTA-797E19.1	-0.051	0.965	3.875
CTAG2	-0.370	0.774	2.608
CTAGE8//CTAGE4//CTAGE9//CTAGE6//CTAGE15	-0.087	0.941	3.731
CTB-118N6.3	-0.233	0.851	2.326
CTB-131B5.2	-0.034	0.977	2.240
CTB-134H23.2//LOC613037//NPIPB9//NPIPB8//NPIP	-0.343	0.788	7.339
CTB-78F1.1	-0.162	0.894	1.981
CTBS	0.557	1.471	5.795
CTC-218H9.1	0.395	1.315	2.017
CTC-255N20.1	0.170	1.125	3.488
CTC-301O7.4	-0.254	0.838	2.761
CTC-304I17.5	-0.150	0.901	3.145
CTC-321K16.1	-0.238	0.848	1.996
CTC-338M12.9	-0.191	0.876	3.501
CTC-340D7.1	-0.073	0.951	2.384
CTC-367F4.1	-0.040	0.972	2.658
CTC-467M3.1	-0.074	0.950	2.987
CTC-467M3.3	-0.212	0.863	2.164
CTC-498M16.4	0.352	1.276	2.861
CTC-552D5.1	-0.190	0.876	1.688
CTC-806A22.1	-0.257	0.837	2.079
CTD-2014B16.3	-0.351	0.784	2.333

CTD-2015H6.3	-0.163	0.893	4.039
CTD-2017C7.2	0.487	1.402	3.368
CTD-2020K17.3	0.170	1.125	3.441
CTD-2026D20.2	-0.725	0.605	3.064
CTD-2033D15.1	0.261	1.198	2.416
CTD-2054N24.1	-0.579	0.670	3.454
CTD-2054N24.2	0.776	1.713	3.004
CTD-2058B24.3	0.025	1.017	3.532
CTD-2152M20.2	-0.021	0.986	2.576
CTD-2175A23.1	0.541	1.455	2.498
CTD-2184D3.6	0.459	1.374	2.691
CTD-2193P3.2	-0.227	0.855	3.980
CTD-2201G16.1	-0.267	0.831	2.961
CTD-2215E18.1	0.150	1.109	2.584
CTD-2223O18.1	-0.388	0.764	2.352
CTD-2269F5.1	-0.474	0.720	3.330
CTD-2277K2.1	0.217	1.162	3.686
CTD-2281E23.2	-0.293	0.816	1.618
CTD-2298J14.2	0.251	1.190	4.124
CTD-2320G14.2	0.148	1.108	2.253
CTD-2369P2.10//FDX1L	0.252	1.191	5.073
CTD-2501E16.2//LOC101928784	-0.109	0.927	2.942
CTD-2531D15.5	0.096	1.069	3.327
CTD-2555A7.2	0.104	1.075	2.685
CTD-2555O16.2	-0.043	0.971	4.427
CTD-2562J17.4	0.056	1.040	2.987
CTD-2562J17.9	0.041	1.029	2.207
CTD-2574D22.2	-0.513	0.701	2.392
CTD-3064C13.1	0.160	1.117	2.275
CTD-3092A11.1	0.181	1.134	4.498
CTD-3138B18.5	0.271	1.207	2.752
CTD-3195I5.5//C17orf85	-0.138	0.909	2.499
CTDSP1	-0.223	0.857	3.626
CTF1	0.071	1.050	4.389
CTGF	-0.145	0.904	5.520
CTH	-0.106	0.929	4.203
CTIF	-0.141	0.907	6.680
CTNNA1	0.281	1.215	6.326
CTNNA2	-0.018	0.988	2.914
CTNNA3	-0.136	0.910	6.741
CTNNAL1	0.397	1.316	4.579
CTNS	0.080	1.057	3.954
CTPS2	0.507	1.421	5.410
CTSC	1.118	2.171	5.139
CTSH	0.187	1.138	4.066
CTSK	0.224	1.168	3.598
CTSLP2//CTSLP7//CTSLP4//CTSLP1//CTSLP6	0.153	1.112	3.948
CTSO	0.397	1.317	5.951
CTSS	0.959	1.944	6.175
CTSV	-0.371	0.773	2.449

CTSW	-0.158	0.896	2.535
CTSZ	0.388	1.309	6.093
CTXN1	-0.387	0.765	5.402
CTXN2	0.142	1.103	2.642
CTXN3	-0.156	0.898	3.183
CUEDC1	-0.096	0.935	5.371
CUL1	0.272	1.208	7.211
CUX2	-0.334	0.793	3.258
CWC22	0.383	1.304	6.066
CWF19L1	0.394	1.314	5.109
CX3CL1	0.390	1.310	6.765
CX3CR1	0.112	1.081	4.346
CXCL10	0.204	1.152	2.558
CXCL11	-0.716	0.609	2.549
CXCL12	0.381	1.302	3.787
CXCL14	-0.125	0.917	6.880
CXCL16	0.825	1.772	6.342
CXCL2	0.641	1.560	2.810
CXCL5	0.180	1.133	3.916
CXCL9	0.230	1.172	1.966
CXCR1	0.579	1.494	2.642
CXCR4	0.201	1.149	7.503
CXorf21	1.048	2.068	2.346
CXorf22	-1.309	0.404	3.814
CXorf28	0.208	1.155	2.453
CXorf30	-1.007	0.498	2.443
CXXC4	-0.286	0.820	4.450
CYB561	-0.046	0.969	4.244
CYB561A3//CYBASC3	-0.117	0.922	2.496
CYB561D2//XXcos-LUCA11.5//XXcos-LUCA11.4	-0.228	0.854	2.453
CYB5A	0.140	1.102	7.284
CYB5R2	0.171	1.126	3.832
CYB5R3	0.111	1.080	6.998
CYBA	0.336	1.263	4.584
CYBB	0.774	1.710	7.387
CYBRD1	-0.017	0.988	7.699
CYFIP2	-0.096	0.936	7.131
CYGB	0.122	1.088	5.220
CYP1B1	0.726	1.654	5.227
CYP20A1	0.220	1.165	4.814
CYP21A1P	-0.567	0.675	3.662
CYP27A1	0.329	1.256	6.988
CYP2B7P//CYP2B7P1	-0.087	0.941	3.062
CYP2D7P//CYP2D6//CYP2D7P1	-0.092	0.938	2.707
CYP2E1	-0.114	0.924	3.222
CYP2J2	-0.087	0.942	4.288
CYP2S1	0.358	1.282	3.549
CYP46A1	-0.161	0.894	5.487
CYP4F11	-0.035	0.976	4.823
CYP4F26P//RP11-255A11.21	0.342	1.268	2.549

CYP4F3	0.156	1.114	2.314
CYP7B1	0.078	1.056	5.797
CYR61	0.268	1.204	4.713
CYSLTR2	-0.180	0.883	2.593
CYTH1	0.232	1.175	5.582
CYTH3	0.092	1.066	2.906
CYTH4	0.664	1.584	3.962
CYTIP	0.143	1.104	2.472
CYTL1	0.400	1.319	4.711
CYYR1	-0.414	0.750	4.017
D2HGDH	-0.320	0.801	3.594
DAAM2	-0.376	0.770	7.492
DAB1	-0.067	0.955	4.225
DAB2//LOC101926921	0.659	1.579	4.354
DACH1	0.096	1.069	4.405
DACH2	-0.019	0.987	1.743
DACT1	0.603	1.519	3.521
DAGLA	0.091	1.065	5.623
DAGLB	0.468	1.384	6.483
DAO	-0.105	0.930	5.270
DAPP1	0.652	1.571	2.120
DAW1	-0.733	0.602	2.402
DBF4B	0.159	1.117	3.587
DBH	-0.080	0.946	2.637
DBI	0.071	1.051	5.400
DBNDD1	-0.164	0.893	5.053
DBP	0.056	1.039	6.050
DBX2	-0.142	0.906	4.453
DCAF12L2	-0.038	0.974	2.317
DCAF13P3	0.081	1.057	2.437
DCAF6	0.130	1.095	5.595
DCC	0.138	1.100	5.233
DCDC1//DCDC5	-0.568	0.674	2.722
DCDC2	-0.702	0.615	2.938
DCHS2	0.079	1.056	4.339
DCLRE1A	0.193	1.143	5.007
DCN	0.137	1.100	3.045
DCP2	0.224	1.168	5.373
DCT	0.380	1.301	3.247
DCTN1-AS1	0.346	1.271	3.796
DCUN1D3	0.238	1.180	4.547
DCX	-0.118	0.921	2.775
DDAH1	0.099	1.071	3.783
DDAH2	-0.079	0.946	5.284
DDAH2	-0.063	0.957	5.299
DDB2	0.668	1.589	4.365
DDIT4	-0.600	0.660	7.493
DDN	0.070	1.050	3.560
DDO	0.080	1.057	3.773
DDR1//MIR4640	0.080	1.057	4.977

DDR1//MIR4640	0.043	1.030	7.114
DDR1//MIR4640	0.037	1.026	7.217
DDR1//MIR4640	0.019	1.013	7.068
DDR1//MIR4640//Error in gsub("^[[:space:]]+|[[:space:]]+$", "", x) :			
DDR2	-0.047	0.968	6.330
DDX1	-0.099	0.934	7.490
DDX10	0.114	1.082	4.951
DDX11L10	0.097	1.069	2.300
DDX11L2	-0.312	0.805	3.052
DDX11L2//DDX11L9//DDX11L10//DDX11L1//DDX11l	-0.164	0.892	3.009
DDX11L5	-0.067	0.955	2.634
DDX12P	0.160	1.118	3.783
DDX20	0.270	1.206	2.849
DDX25	-0.092	0.938	4.463
DDX39A	0.126	1.091	5.058
DDX3Y	0.471	1.386	4.317
DDX55	0.102	1.074	4.448
DDX60	0.404	1.323	4.050
DDX60L	0.457	1.373	3.429
DEDD2	0.645	1.564	5.119
DEF6	0.547	1.461	3.395
DEFA1B//DEFA1//DEFA3	-0.831	0.562	3.054
DEFA1B//DEFA1//DEFA3	-0.759	0.591	3.061
DENND1B	-0.095	0.936	4.261
DENND1C	1.026	2.036	2.921
DENND2A	-0.233	0.851	4.734
DENND2D	0.877	1.837	3.260
DENND3	0.476	1.391	5.322
DENND5A	0.059	1.042	8.167
DENND5B	0.061	1.043	6.649
DENND6B	-0.093	0.937	5.178
DENR	-0.247	0.843	5.518
DEPDC1B	-0.043	0.971	2.779
DEPTOR	0.240	1.181	5.372
DES	0.314	1.244	2.678
DFFB	0.136	1.099	3.468
DFNA5	0.024	1.017	5.553
DFNB31	0.118	1.085	5.512
DGAT2	0.173	1.128	2.809
DGCR10	0.079	1.056	2.403
DGCR11	-0.772	0.586	1.799
DGCR14//TSSK2	-0.021	0.985	4.638
DGCR9//AC000095.10	0.102	1.073	3.143
DGKA	0.363	1.286	4.453
DGKB	0.065	1.046	5.618
DGKE	-0.057	0.961	5.774
DGKG	0.123	1.089	6.141
DGKH	0.171	1.126	4.725
DGKI	-0.297	0.814	5.271
DGKK	0.282	1.216	2.689

DGKZ//DGKZP1	-0.182	0.882	5.549
DHCR24	-0.400	0.758	9.759
DHCR7	-0.139	0.908	6.661
DHH	0.385	1.306	2.291
DHRS11	0.148	1.108	4.349
DHRS3	0.294	1.226	6.141
DHRS4	0.305	1.235	2.721
DHRS4L1//DHRS4L2	0.123	1.089	5.397
DHX57	-0.299	0.813	4.873
DIEXF	0.151	1.110	5.146
DIO2	0.122	1.088	2.101
DIP2A	-0.080	0.946	5.573
DIP2A-IT1	0.098	1.070	2.360
DIP2B	0.131	1.095	8.267
DIRAS1	-0.114	0.924	5.748
DIRAS2	0.423	1.341	6.071
DIRAS3	0.708	1.634	3.383
DISP2	-0.046	0.968	4.369
DKFZP434I0714//MIR4453//RP11-555K12.3	-0.100	0.933	4.047
DKFZp434J0226//AC006262.6	-0.096	0.936	3.326
DKFZP434L187	-0.585	0.667	2.139
DKFZp451B082	0.303	1.234	2.635
DKFZP586I1420	0.437	1.354	5.907
DKFZp667F0711	0.080	1.057	4.629
DKK2	0.196	1.146	3.923
DLD	0.074	1.053	6.273
DLEC1	-0.992	0.503	2.683
DLEU2	0.173	1.127	2.312
DLEU2L	-0.397	0.759	2.962
DLEU7	-0.225	0.856	3.034
DLG2	-0.032	0.978	6.537
DLG3	0.199	1.148	5.155
DLG4	-0.046	0.969	6.250
DLGAP1	0.193	1.143	4.612
DLGAP1-AS1	0.104	1.075	4.385
DLGAP1-AS2	0.267	1.203	2.784
DLGAP1-AS3	0.144	1.105	1.809
DLGAP1-AS5	0.244	1.184	2.255
DLGAP2	0.103	1.074	3.627
DLGAP3	0.041	1.029	3.580
DLGAP4	0.112	1.080	6.497
DLL4	-0.016	0.989	4.115
DLX1	-0.421	0.747	2.875
DLX2	0.018	1.012	1.934
DLX5	-0.555	0.681	2.948
DLX6	-0.140	0.908	2.508
DLX6-AS1	-0.076	0.949	1.860
DMBT1	0.176	1.130	2.480
DMD	-0.131	0.913	6.258
DMKN	-0.240	0.847	2.613

DMRT2	0.459	1.375	2.486
DMRTC1//DMRTC1B	-0.070	0.952	3.587
DMRTC1B//DMRTC1	-0.074	0.950	2.891
DMTN	-0.149	0.902	6.960
DMXL2	0.063	1.045	7.349
DNA2	0.248	1.187	3.523
DNAAF1	-1.239	0.424	3.751
DNAH10	-0.557	0.680	2.975
DNAH10OS	0.211	1.158	4.775
DNAH11	-1.509	0.351	2.265
DNAH2	-1.116	0.462	2.473
DNAH5	-0.551	0.683	2.708
DNAH6	-0.543	0.686	3.371
DNAH7	-0.677	0.625	4.215
DNAH9	-1.241	0.423	3.567
DNAI1	-1.052	0.482	2.735
DNAJA1	0.177	1.131	8.364
DNAJA2	0.188	1.140	7.471
DNAJA4	0.338	1.264	5.626
DNAJB1	0.483	1.397	7.592
DNAJB14	0.190	1.140	5.174
DNAJB2	0.197	1.146	5.161
DNAJC12	-0.062	0.958	6.062
DNAJC19	0.067	1.048	5.046
DNAJC27	-0.257	0.837	5.034
DNAJC28	-0.062	0.958	3.701
DNAJC6	-0.010	0.993	9.207
DNAL4	-0.347	0.786	3.740
DNAPTP3	0.356	1.280	2.873
DNASE1	0.342	1.268	3.274
DNASE1L2	-0.556	0.680	2.761
DNASE2B	1.099	2.142	2.181
DND1	-0.289	0.818	2.861
DNER	0.037	1.026	8.651
DNM1	-0.280	0.823	8.136
DNM1L	0.191	1.142	5.914
DNM1L	-0.057	0.961	3.807
DNM1P35	-0.140	0.907	3.129
DNM1P46	-0.304	0.810	3.009
DNM3	-0.144	0.905	8.080
DNM3-IT1	-0.101	0.932	3.144
DOC2A	0.084	1.060	5.559
DOC2B	0.434	1.351	5.776
DOCK1	-0.184	0.880	7.872
DOCK10	0.222	1.167	8.371
DOCK11	0.720	1.647	5.558
DOCK2	0.462	1.377	5.119
DOCK3	0.091	1.065	7.275
DOCK5	0.063	1.045	6.762
DOCK8	0.372	1.294	4.567

DOK1	-0.103	0.931	2.542
DOK2	0.567	1.481	2.127
DOK3	0.338	1.264	2.849
DOK4	0.155	1.113	4.009
DOK5	0.338	1.264	5.552
DOPEY2	0.290	1.223	5.133
DPCR1	0.294	1.226	3.012
DPF1	-0.108	0.928	4.038
DPF3	-0.165	0.892	4.496
DPH6	0.571	1.486	3.847
DPH6-AS1	0.041	1.029	3.122
DPP10	-0.020	0.986	6.171
DPP10-AS1//AC066593.1	-0.094	0.937	4.069
DPP10-AS2//AC068542.2	-0.021	0.986	2.581
DPP4	0.087	1.062	2.607
DPP6	-0.097	0.935	6.984
DPP8	0.261	1.198	6.069
DPRXP4	0.450	1.366	2.356
DPT	0.142	1.104	2.786
DPY19L1P1//AC018633.4	0.130	1.095	2.240
DPY19L2P1	-0.093	0.938	3.164
DPY19L2P3//AC007276.5	0.224	1.168	2.448
DPY19L2P4	0.209	1.156	2.587
DPYD	0.285	1.218	6.872
DPYD-IT1	0.200	1.148	3.652
DPYSL3	-0.074	0.950	7.216
DPYSL5	0.038	1.027	5.900
DRD1	-0.535	0.690	3.100
DRGX	0.533	1.447	2.754
DRP2	0.323	1.251	5.375
DSC2	0.818	1.763	4.959
DSCAM	-0.331	0.795	5.492
DSEL	-0.124	0.917	6.694
DSN1	0.067	1.047	3.767
DSP	-0.352	0.784	4.514
DSTYK	0.436	1.352	6.228
DTHD1	-2.197	0.218	2.077
DTNA	0.101	1.073	9.309
DTX1	-0.109	0.927	4.871
DTX2	0.425	1.343	3.280
DTX2P1-UPK3BP1-PMS2P11	0.092	1.066	4.129
DTX3L	0.504	1.418	5.519
DTX4	-0.500	0.707	6.406
DUOX1	0.125	1.090	2.271
DUSP1	0.019	1.013	8.171
DUSP10	0.454	1.370	5.467
DUSP14	0.176	1.130	5.378
DUSP15//C20orf57	-0.058	0.961	3.665
DUSP16	0.258	1.196	5.587
DUSP19	0.114	1.082	4.388

DUSP2	0.291	1.223	4.238
DUSP26	-0.016	0.989	6.497
DUSP3	0.058	1.041	6.088
DUSP5	-0.085	0.943	2.950
DUSP7	0.075	1.053	4.676
DUXAP10	0.597	1.512	4.910
DUXAP10//LOC100507460//CTD-2314B22.3//LL22N(	0.157	1.115	3.916
DYNC1I1	-0.368	0.775	7.408
DYNC1LI1	-0.017	0.989	5.449
DYNLL1-AS1	-0.425	0.745	4.324
DYNLRB2	-0.374	0.772	2.638
DYNLT1	-0.216	0.861	5.165
DYNLT3	0.016	1.011	6.913
DZIP1	0.135	1.098	6.645
DZIP3	-0.077	0.948	7.301
E2F3	0.360	1.283	3.671
EBF1	0.631	1.549	3.800
EBF2	0.843	1.794	2.604
EBF3	0.957	1.941	3.085
EBI3	0.871	1.829	2.855
EBLN2	0.139	1.101	3.915
EBLN3//RP11-220I1.1	0.266	1.202	7.065
ECD//RP11-152N13.3	0.403	1.322	6.929
ECE1	-0.027	0.981	6.121
ECEL1	-0.683	0.623	3.221
ECHDC3	0.222	1.167	3.108
ECM2	0.286	1.219	3.931
ECRP	0.944	1.924	4.994
ECSCR	0.302	1.233	4.935
ECT2	0.520	1.434	4.277
EDA2R	0.829	1.777	4.269
EDN1	1.018	2.025	6.437
EDN3	-0.300	0.812	3.624
EDNRA	0.996	1.994	3.996
EDNRB	0.452	1.368	7.814
EEF1A2	-0.485	0.714	7.312
EEF1D//RP11-661A12.7	0.121	1.088	3.568
EEF1DP3	-0.441	0.737	3.290
EEF1G//MIR3654//EEF1GP1//RP11-864I4.1	0.198	1.147	8.629
EEPD1	0.142	1.103	5.276
EFCAB1	-0.934	0.523	2.608
EFCAB12	-1.189	0.439	2.433
EFCAB6	-0.563	0.677	3.884
EFCAB7	-0.063	0.958	4.317
EFEMP1	-0.061	0.959	8.494
EFEMP2	0.122	1.088	4.920
EFHB	-0.486	0.714	2.525
EFHC2	-0.249	0.841	3.543
EFHD1	-0.120	0.920	4.847
EFHD2	0.023	1.016	5.699

EFNA1	0.579	1.493	4.837
EFNA2	-0.636	0.644	3.090
EFNA3//RP11-540D14.8	0.457	1.373	4.749
EFNA5	0.307	1.237	4.388
EFNB2	0.483	1.397	4.676
EFR3A	0.120	1.087	7.567
EFR3B	-0.132	0.913	7.036
EFS	-0.108	0.928	5.187
EGF	0.632	1.550	3.030
EGFEM1P	0.222	1.166	3.097
EGFR	-0.396	0.760	5.759
EGR1	-0.631	0.646	5.072
EGR3	-0.784	0.581	3.614
EGR4	-0.129	0.914	2.317
EHBP1	0.113	1.081	6.461
EHBP1L1	-0.051	0.965	3.598
EHD2	0.065	1.046	5.673
EHD3	0.408	1.327	6.120
EHD4	0.105	1.075	4.978
EHF	-0.416	0.749	3.794
EID2	0.012	1.008	4.379
EID2B	0.377	1.299	3.346
EIF1AD	0.436	1.353	5.588
EIF1AY	0.273	1.208	3.558
EIF4A2//SNORA63//SNORD2//SNORA4//SNORA81//	0.171	1.126	7.665
EIF4B	0.380	1.301	5.487
EIF4E1B	-0.074	0.950	2.895
EIF4EBP1	0.496	1.410	4.449
EIF5A2	-0.532	0.691	5.429
ELAVL2	-0.571	0.673	6.245
ELAVL4	-0.201	0.870	4.566
ELF1	0.254	1.192	7.982
ELF4	0.801	1.743	2.555
ELFN1	0.603	1.519	4.211
ELFN2//RP1-63G5.5	0.309	1.239	3.290
ELK1	0.333	1.259	4.738
ELK3	0.301	1.232	4.434
ELL2	0.193	1.143	8.446
ELMO1	0.247	1.187	7.368
ELMOD1//LOC643923	0.715	1.641	3.605
ELMOD1//LOC643923	0.248	1.188	6.772
ELN	0.018	1.013	3.046
ELOVL1//MIR6734	-0.022	0.985	7.016
ELOVL2	0.260	1.197	7.062
ELOVL4	-0.229	0.853	6.143
ELOVL5	0.204	1.152	6.838
ELP5	0.361	1.285	5.648
EMBP1	-0.423	0.746	3.068
EMC10	0.063	1.045	6.873
EMC3-AS1//AC034193.5	-0.071	0.952	3.586

EMD	0.328	1.255	6.304
EMID1	0.157	1.115	3.835
EMILIN1	-0.032	0.978	3.438
EMILIN2	0.030	1.021	5.541
EMILIN3	0.084	1.060	3.455
EML3	-0.055	0.963	5.231
EML5	-0.173	0.887	4.429
EML6	0.055	1.039	4.747
EMP1	0.887	1.850	5.950
EMP2	0.368	1.290	4.731
EMP3	0.416	1.334	7.612
EMR1	0.784	1.722	2.000
EMR4P	0.808	1.751	2.155
EMX1	-0.161	0.894	2.445
EMX2	0.402	1.321	4.314
EMX2OS	-0.153	0.899	3.143
ENC1	0.512	1.427	4.411
ENDOD1	0.087	1.063	7.143
ENG	0.630	1.547	6.038
ENHO	-0.555	0.681	4.908
ENKUR	-0.240	0.847	4.700
ENO1	0.092	1.066	5.422
ENO1-IT1	0.246	1.186	2.343
ENO2	-0.281	0.823	4.513
ENO4	-0.339	0.791	3.227
ENOSF1	-0.108	0.928	5.737
ENPP1	-0.238	0.848	3.695
ENPP2	0.110	1.079	9.718
ENPP2	-0.080	0.946	5.609
ENPP4	0.205	1.153	7.604
ENPP5	0.340	1.265	6.269
ENPP6	0.091	1.065	6.213
ENTPD1	0.244	1.184	3.650
ENTPD2	-0.338	0.791	4.306
ENTPD3	-0.231	0.852	4.532
ENTPD3-AS1	-0.163	0.893	2.964
ENTPD4	0.331	1.258	3.330
ENTPD5	0.246	1.186	3.804
ENTPD7	0.297	1.228	4.060
EPB41L4B	0.497	1.412	6.009
EPC1//IGHV3-33	-0.512	0.701	2.236
EPCAM	-0.029	0.980	3.570
EPDR1	-0.134	0.911	6.929
EPHA10	-0.137	0.909	2.264
EPHA2	0.396	1.316	3.381
EPHA3	-0.142	0.906	4.300
EPHA4	-0.028	0.981	5.834
EPHA5	0.249	1.189	5.755
EPHA6	-0.249	0.842	2.182
EPHA7	0.186	1.138	4.903

EPHB1	0.543	1.457	3.979
EPHB6	-0.096	0.936	5.178
EPHX1	0.051	1.036	6.585
EPHX2	0.204	1.152	3.879
EPHX4	-0.255	0.838	4.051
EPN2-IT1//EPN2	-0.264	0.833	3.569
EPN3	-0.259	0.836	3.485
EPS8	0.330	1.257	6.173
EPS8L2	-0.401	0.757	3.525
EPSTI1	0.424	1.341	3.419
EPT1	0.158	1.116	6.793
ERAP1	0.176	1.130	5.564
ERAP2	0.558	1.472	2.522
ERBB2	-0.094	0.937	5.157
ERBB2IP	0.148	1.108	8.312
ERBB3	-0.178	0.884	5.572
ERBB4	0.111	1.080	7.111
ERC2	0.038	1.027	5.035
ERC2-IT1	0.053	1.037	2.352
ERF	0.165	1.121	3.907
ERI2	0.261	1.198	4.143
ERICH3//C1orf173	0.140	1.102	4.078
ERLEC1	0.165	1.121	7.188
ERMAP	-0.034	0.977	3.651
ERMARD//C6orf70	0.144	1.105	5.524
ERO1L	0.384	1.305	5.659
ESPNP	-0.731	0.603	2.675
ESRRG	-0.139	0.908	4.106
ESYT3	0.152	1.111	3.808
ETAA1	0.474	1.389	3.745
ETS2	0.056	1.039	7.769
ETV6//INO80D	0.843	1.794	6.444
EVI2A	0.153	1.112	4.069
EVI2B//CTD-2370N5.3	0.729	1.658	3.972
EXOC6	0.158	1.115	5.144
EXOC8	0.260	1.197	5.721
EXOSC3	0.332	1.259	5.896
EXOSC5	0.102	1.073	5.998
EXOSC6	0.051	1.036	6.264
EXTL2	0.158	1.116	4.777
EYA1	-0.181	0.882	4.356
EYA2	-0.015	0.989	5.225
EYA4	-0.429	0.743	4.244
F11R//RP11-544M22.13	0.515	1.429	4.891
F13A1	0.843	1.793	5.968
F2R	0.374	1.296	4.018
F2RL2	-0.268	0.831	2.673
F3	1.094	2.134	7.890
F5	0.056	1.040	4.374
F7	-0.244	0.844	2.350

F8A1//F8A2//F8A3	0.614	1.531	8.091
F8A1//F8A2//F8A3	0.054	1.038	7.733
FA2H	0.161	1.118	7.133
FAAH	-0.215	0.861	5.271
FABP3	-0.163	0.893	6.589
FABP6	-0.253	0.839	4.474
FABP7	0.170	1.125	5.562
FADS2	0.072	1.051	4.477
FADS6	-0.353	0.783	2.618
FAH	0.184	1.136	4.061
FAHD2CP	0.087	1.062	6.766
FAIM2	-0.095	0.936	7.240
FAM101B	-0.768	0.587	5.261
FAM102B	0.609	1.526	6.427
FAM105A	-0.265	0.832	4.975
FAM106A//FAM106CP	-0.141	0.907	2.803
FAM106CP	0.172	1.127	6.211
FAM107A	-0.099	0.934	4.417
FAM110C	-0.109	0.927	3.336
FAM111A	0.209	1.156	3.549
FAM114A1	0.256	1.195	5.426
FAM115A//LOC100294033	0.103	1.074	6.219
FAM117B	0.030	1.021	7.380
FAM118A	-0.052	0.965	3.047
FAM124A	0.288	1.221	4.057
FAM126B	0.217	1.162	5.747
FAM127B	0.228	1.171	4.800
FAM129A	0.474	1.389	5.227
FAM129B	-0.095	0.936	5.993
FAM131A	-0.153	0.899	4.416
FAM133A	-0.253	0.839	4.086
FAM135B	-0.482	0.716	5.215
FAM13A	-0.033	0.977	5.441
FAM13A-AS1	0.415	1.333	4.062
FAM153A//FAM153B//LOC100507387	-0.171	0.888	3.954
FAM154B	-0.407	0.754	2.651
FAM155A	0.252	1.191	5.721
FAM155A-IT1	-0.103	0.931	5.056
FAM160B1	0.185	1.137	6.071
FAM162B	-0.130	0.914	4.538
FAM163A	-0.243	0.845	2.432
FAM163B	0.634	1.551	3.140
FAM163B	0.223	1.167	5.323
FAM169A	0.246	1.186	7.175
FAM171A2	0.386	1.307	5.685
FAM171B	0.121	1.088	9.006
FAM177B	0.365	1.288	2.933
FAM178A	0.128	1.093	5.746
FAM178B	0.199	1.148	2.905
FAM179A	-0.559	0.679	2.957

FAM179B	0.148	1.108	6.405
FAM181A-AS1	-0.417	0.749	2.790
FAM181B	-0.377	0.770	3.224
FAM184A	-0.216	0.861	5.031
FAM189A1	0.201	1.150	4.370
FAM189A2	0.238	1.180	7.379
FAM196A	0.293	1.225	3.099
FAM196B	0.313	1.242	5.408
FAM198A//KRBOX1	0.224	1.168	4.507
FAM198B	0.433	1.350	4.883
FAM19A1	-0.197	0.873	2.407
FAM19A2	-0.095	0.936	2.466
FAM19A4	0.210	1.156	3.623
FAM200A	0.356	1.280	4.312
FAM200B	0.149	1.109	5.052
FAM20A	0.697	1.622	3.325
FAM20B	0.061	1.043	6.762
FAM20C	0.251	1.190	5.645
FAM212B//LOC101928718	-0.141	0.907	3.566
FAM213B	0.189	1.140	4.680
FAM216A	-0.191	0.876	4.596
FAM216B	-1.968	0.256	2.366
FAM217B	0.236	1.178	6.199
FAM218A	-0.020	0.986	4.612
FAM220A//FAM220BP//FAM220CP	0.055	1.039	5.945
FAM221A	0.356	1.280	4.132
FAM224A//FAM224B	-0.123	0.918	2.561
FAM225A//FAM225B	0.379	1.300	4.109
FAM225B	0.323	1.251	2.502
FAM226B//FAM226A//RP1-172N19.5	0.284	1.218	3.085
FAM227A	-0.651	0.637	3.750
FAM228A//RP11-507M3.1	0.024	1.017	4.479
FAM231D//FAM231B//LOC100996721//LOC100996	-0.253	0.839	2.555
FAM26E	0.170	1.125	2.921
FAM26F	0.293	1.225	3.011
FAM27C//FAM27B//FAM182B	0.364	1.287	3.050
FAM27E2//FAM27E3//FAM27E4P	-0.085	0.943	4.939
FAM43A	-0.329	0.796	4.136
FAM46A	0.185	1.137	3.381
FAM46B	0.269	1.205	2.252
FAM46C	-0.212	0.863	4.290
FAM49A	0.449	1.365	7.030
FAM57B//LOC100996332	-0.461	0.726	4.739
FAM63A	-0.018	0.988	5.634
FAM65B	0.161	1.118	5.385
FAM66A	-0.538	0.689	3.931
FAM66B	-0.056	0.962	4.247
FAM66E	0.378	1.300	3.351
FAM69B	0.302	1.233	5.793
FAM69C	0.318	1.247	4.026

FAM76B	0.455	1.371	4.263
FAM78B	0.213	1.159	4.055
FAM81A	-0.126	0.916	4.439
FAM83H	-0.439	0.738	2.502
FAM84A	-0.061	0.959	4.905
FAM84B	-0.267	0.831	4.607
FAM86A//FAM86B2//FAM86KP	0.411	1.329	3.975
FAM86B3P	0.538	1.452	2.435
FAM86C2P	0.266	1.202	2.557
FAM86DP	-0.043	0.970	4.674
FAM86EP	-0.667	0.630	3.691
FAM86HP	-0.399	0.758	3.051
FAM89A//MIR1182	0.171	1.126	4.781
FAM89B//SSSCA1	0.120	1.087	6.915
FAM90A1	0.493	1.407	2.341
FAM90A10P	-0.172	0.888	3.933
FAM99A	-0.084	0.944	2.930
FAM99B	-0.354	0.782	3.075
FANCA	0.407	1.326	2.509
FANCL	-0.015	0.989	5.341
FANCM	-0.243	0.845	2.615
FANK1	-0.497	0.709	2.879
FAP	0.188	1.139	2.978
FAR1-IT1	0.165	1.122	3.678
FAR2	0.391	1.311	4.619
FAR2P2//FAR2P3//AC140481.1	-0.279	0.824	5.069
FAR2P2//FAR2P3//AC140481.1	0.105	1.075	4.664
FARSB	0.196	1.146	7.150
FAS	0.977	1.968	4.831
FASTKD1	0.039	1.028	5.175
FASTKD2	0.410	1.328	4.982
FAT3	0.081	1.058	6.218
FAT4	0.828	1.775	5.675
FATE1	0.177	1.130	1.870
FAXC	-0.091	0.939	5.316
FAXDC2	0.127	1.092	5.001
FBLL1	-0.502	0.706	4.843
FBLN1	-0.142	0.906	4.907
FBLN2	0.446	1.363	4.077
FBLN5	-0.224	0.856	5.328
FBLN7	0.253	1.192	2.673
FBP1	0.925	1.899	3.292
FBXL14	-0.054	0.963	6.063
FBXL15	-0.057	0.962	3.092
FBXL16	-0.177	0.884	6.649
FBXL17	0.190	1.141	6.443
FBXL18	0.049	1.035	3.362
FBXL19	0.218	1.163	4.151
FBXL2	-0.167	0.890	5.231
FBXL20	0.136	1.099	6.684

FBXL7	-0.030	0.979	6.444
FBXO15	0.043	1.031	3.400
FBXO16//ZNF395	0.105	1.075	5.035
FBXO2	-0.137	0.910	5.683
FBXO27	-0.242	0.845	4.530
FBXO28	0.219	1.164	6.160
FBXO3	0.189	1.140	5.832
FBXO31	-0.124	0.918	6.202
FBXO32	-0.105	0.930	7.725
FBXO33	0.310	1.240	5.509
FBXO34	0.076	1.054	4.828
FBXO40	0.107	1.077	2.485
FBXO41//LOC101927826	-0.324	0.799	4.657
FBXO44	-0.080	0.946	4.743
FBXO45	0.616	1.533	4.897
FBXO48	0.497	1.411	4.015
FBXO7	0.084	1.060	7.841
FBXO9	0.211	1.158	5.250
FBXW7	0.174	1.128	7.079
FBXW9	0.210	1.157	2.539
FCAR	0.431	1.348	3.177
FCER1G	1.180	2.266	7.262
FCGBP	0.877	1.836	3.697
FCGR1A//FCGR1B	1.774	3.420	5.193
FCGR1B	0.607	1.524	3.905
FCGR2A	0.866	1.823	3.953
FCGR2B	1.524	2.877	2.285
FCGRT	0.188	1.139	4.487
FCN1	0.382	1.303	2.848
FEM1B	0.259	1.196	7.289
FERMT1	-0.395	0.761	4.397
FERMT3	0.761	1.695	4.254
FEZF2	-0.085	0.943	3.553
FFAR3	-0.350	0.784	2.460
FFAR4	0.408	1.326	2.761
FGD1	0.479	1.394	4.352
FGD3	0.666	1.587	2.647
FGD5	-0.242	0.845	3.321
FGD6	-0.150	0.902	5.385
FGF1	-0.029	0.980	4.146
FGF10	0.069	1.049	3.309
FGF11//RP11-104H15.7//RP11-104H15.10	-0.331	0.795	4.889
FGF12	-0.115	0.924	4.285
FGF13	0.249	1.188	3.866
FGF14	0.661	1.581	5.815
FGF14-AS2//RP11-811P12.4	0.330	1.257	3.397
FGF14-IT1	-0.551	0.683	4.271
FGF17	-0.031	0.979	3.110
FGF18	0.016	1.011	2.817
FGF5	-0.153	0.899	2.730

FGF7	0.632	1.549	2.125
FGF9	-0.461	0.727	4.162
FGFBP2	0.364	1.287	2.275
FGFR1	-0.251	0.840	4.013
FGFR1OP2	0.473	1.388	5.261
FGFR2	0.028	1.020	7.082
FGFR3	-0.455	0.730	6.562
FGL2	0.786	1.724	6.548
FGR	0.543	1.457	3.546
FH	0.170	1.125	5.983
FHDC1	0.063	1.045	2.586
FHL2	0.431	1.348	3.244
FHL3	0.320	1.249	4.352
FHL5	-0.094	0.937	4.667
FHOD3	-0.237	0.848	6.157
FIBIN	0.246	1.186	5.804
FICD	0.357	1.280	3.089
FIG4	0.332	1.259	6.186
FIGNL1	0.093	1.066	3.825
FILIP1	0.059	1.042	3.933
FIRRE//RP11-453F18__B.1	0.344	1.269	2.450
FJX1	0.158	1.116	5.448
FKBP10	0.219	1.164	4.973
FKBP1B	0.353	1.277	3.072
FKBP4	0.047	1.033	6.621
FKBP5//LOC285847	0.071	1.050	5.133
FKSG29	0.122	1.088	3.919
FKSG49	-0.201	0.870	9.278
FKTN	0.225	1.168	5.117
FLG-AS1	0.161	1.118	3.586
FLI1	0.461	1.377	3.763
FLII	0.062	1.044	6.975
FLJ16779	1.082	2.117	2.232
FLJ20021//AP001816.1	0.020	1.014	4.932
FLJ21369	0.348	1.273	3.255
FLJ22184//CTD-3193O13.9	-0.205	0.867	4.076
FLJ27354//RP5-1007M22.2	0.061	1.043	3.882
FLJ31104//CTD-2031P19.3	-0.371	0.773	2.491
FLJ31183	0.383	1.304	2.193
FLJ33360//CTD-2324F15.2	-0.169	0.890	2.379
FLJ33534//AC062028.1	-0.315	0.804	2.347
FLJ35934//RP1-37N7.3	-0.381	0.768	2.657
FLJ37035//RP11-383C5.5	0.106	1.076	2.126
FLJ37201	-0.264	0.833	2.632
FLJ38717	-0.182	0.882	3.181
FLJ41130	0.232	1.174	4.200
FLJ41278//RP11-766N7.3	0.081	1.058	3.466
FLJ41733//AP001046.6	-0.411	0.752	3.327
FLJ42627//RP11-20I23.13	-0.263	0.833	4.578
FLJ43315	-0.108	0.928	2.882

FLJ43681//LOC101930151//AC139099.3	-0.108	0.928	2.745
FLJ44635//NHSL2	0.203	1.151	2.878
FLNA	0.021	1.014	6.235
FLNB	-0.145	0.905	6.351
FLNC	-0.157	0.897	6.718
FLRT2//LOC100506718	-0.323	0.800	4.943
FLRT3	0.545	1.459	4.211
FLT1	0.215	1.161	6.560
FLT3	-0.402	0.757	3.223
FLT3LG//CTD-3148I10.15	0.358	1.282	4.253
FMN1	0.031	1.022	5.219
FMNL2	0.028	1.020	9.652
FMNL3	-0.090	0.940	5.679
FMO2	0.092	1.066	3.128
FMO4	0.299	1.230	3.064
FMOD	0.153	1.112	4.729
FN1	0.474	1.389	7.682
FNBP1	0.036	1.025	8.013
FNBP1L	0.180	1.133	7.107
FNDC3B//LOC101928615	0.432	1.349	6.673
FNDC4	0.136	1.099	6.196
FNDC5	0.124	1.089	4.255
FNDC9	0.393	1.313	4.696
FNIP2	0.492	1.406	6.086
FNTA//RP11-598P20.5	0.073	1.052	6.509
FOCAD	-0.113	0.924	6.589
FOLH1//FOLH1B	0.122	1.088	6.356
FOLH1B	0.115	1.083	2.631
FOLR1	0.074	1.052	2.119
FOLR2	0.285	1.218	3.203
FOS	0.152	1.111	7.068
FOSB	0.305	1.235	3.716
FOSL1	-0.033	0.977	2.696
FOSL2	0.527	1.441	6.033
FOXB1	0.359	1.282	2.051
FOXC1	0.030	1.021	5.794
FOXC2	-0.224	0.856	2.786
FOXD4	-0.115	0.923	4.160
FOXD4L1	-0.115	0.924	3.078
FOXD4L5//FOXD4L4//FOXD4L6//FOXD4L3//FOXD4L1	0.180	1.133	2.490
FOXD4L6//FOXD4L5//FOXD4L3//FOXD4L1//FOXD4L4	0.201	1.150	3.752
FOXF1	-0.258	0.836	4.412
FOXF2	0.177	1.130	4.622
FOXG1	-0.344	0.788	3.405
FOXJ1	-1.388	0.382	3.471
FOXN2	0.201	1.149	6.710
FOXO4	-0.203	0.869	5.668
FOXP1-IT1	-0.411	0.752	3.287
FOXP2	-0.676	0.626	3.330
FOXQ1	0.398	1.318	3.227

FPR1	1.163	2.240	5.205
FPR3	2.061	4.172	3.338
FRAS1	0.149	1.109	3.547
FRAT2	0.064	1.045	4.386
FREM1	0.042	1.030	4.608
FREM2	0.178	1.131	2.856
FREM3	-0.238	0.848	3.043
FRG1//LOC100289097//LOC101930278//LOC10193C	-0.322	0.800	3.401
FRK	-0.148	0.902	3.076
FRMD4B	0.134	1.097	4.936
FRMD8	0.202	1.150	2.887
FRMD8P1	-0.115	0.923	2.689
FRMPD1	-0.030	0.979	2.480
FRMPD2//FRMPD2P1//LOC101930006//FRMPD2P2	-0.633	0.645	5.308
FRMPD2//LOC101930006	-0.698	0.617	3.536
FRMPD3	0.438	1.354	4.669
FRMPD4	0.581	1.495	4.889
FRRS1L	0.173	1.127	6.782
FRS3	-0.016	0.989	3.885
FRY	0.283	1.217	3.482
FRY	0.223	1.167	6.932
FRY-AS1	0.371	1.293	2.591
FRYL	-0.025	0.983	7.654
FRZB	0.295	1.227	5.999
FSD1	-0.177	0.884	5.396
FSTL1	0.336	1.262	5.147
FSTL3	-0.274	0.827	4.502
FSTL4	-0.397	0.760	3.827
FSTL5	-0.119	0.921	5.618
FTL//SRGAP1//GBP4	0.135	1.098	10.983
FUCA1	0.593	1.509	5.262
FUCA2	0.260	1.197	5.152
FUOM	0.529	1.443	2.649
FUT1	0.108	1.078	2.369
FUT10	-0.111	0.926	5.355
FUT4	0.278	1.213	3.682
FXYD1//CTD-2527I21.4	-0.227	0.855	6.939
FXYD4	-0.099	0.934	3.165
FXYD5	0.435	1.352	4.974
FXYD7//CTD-2527I21.4	-0.284	0.821	5.252
FYB	0.754	1.686	5.504
FZD2	0.164	1.121	2.570
FZD3	-0.025	0.983	6.859
FZD4	0.064	1.045	4.599
FZD5	0.061	1.043	3.629
FZD7	-0.136	0.910	3.927
G0S2	0.472	1.387	4.627
G2E3	0.173	1.127	5.089
G3BP2	0.073	1.052	6.647
G6PC3	0.250	1.189	7.914

GAA	0.377	1.299	6.175
GAB2	0.049	1.034	5.018
GAB3	0.710	1.636	3.601
GABARAPL1	-0.015	0.990	6.351
GABBR1	-0.135	0.911	8.488
GABBR1	-0.116	0.923	8.011
GABBR1	-0.104	0.931	8.064
GABBR1	-0.103	0.931	7.895
GABBR1	-0.098	0.934	8.050
GABBR1	-0.089	0.940	7.470
GABBR2	0.209	1.156	7.282
GABPB2	-0.184	0.880	5.594
GABRA1	-0.196	0.873	5.002
GABRA2	0.483	1.398	4.294
GABRA3	0.342	1.267	6.160
GABRA4	0.396	1.316	4.954
GABRA5	0.059	1.042	4.329
GABRB1	0.082	1.059	5.974
GABRB2	0.121	1.087	4.684
GABRB3	0.350	1.274	3.965
GABRD	-0.162	0.893	4.010
GABRE//MIR224//MIR452	0.232	1.174	3.283
GABRG1	0.169	1.124	7.434
GABRG2	-0.238	0.848	6.796
GABRG3	0.391	1.311	2.736
GABRQ	-0.084	0.944	4.212
GAD1	0.168	1.123	4.319
GAD2	0.151	1.110	5.282
GADD45A	1.047	2.067	7.831
GADD45B	0.464	1.379	8.276
GADD45G	-0.719	0.607	4.504
GAL	-0.386	0.765	2.652
GAL3ST2	-0.279	0.824	2.995
GAL3ST3	-0.398	0.759	3.725
GAL3ST4	0.061	1.043	2.579
GALM	0.190	1.141	3.491
GALNT11	0.135	1.098	5.773
GALNT14	0.137	1.100	2.080
GALNT15	0.328	1.256	7.548
GALNT16	0.339	1.265	4.889
GALNT18	0.143	1.104	4.886
GALNT3	0.780	1.717	3.824
GALNT6	0.024	1.017	4.583
GALNT7	0.077	1.055	5.197
GALNT9	-0.598	0.661	5.021
GALNTL6	0.152	1.111	2.423
GALR3	-0.536	0.690	2.269
GANC//RP11-164J13.1//CAPN3	0.122	1.088	3.356
GAP43	0.072	1.052	7.213
GAPT	0.668	1.588	3.356

GAREML	-0.543	0.686	4.898
GARNL3	0.065	1.046	5.409
GARS	0.207	1.154	6.300
GAS2L3	0.784	1.722	3.119
GAS5//SNORD81//SNORD79//SNORD80//SNORD47/	0.398	1.317	4.552
GAS5-AS1//GAS5	0.091	1.065	2.606
GAS6-AS1	0.166	1.122	2.257
GAS6-AS2	0.048	1.034	3.135
GAS7	-0.082	0.945	7.734
GBA2	0.097	1.070	6.075
GBE1	0.090	1.065	5.193
GBP1	0.743	1.674	4.083
GBP2	0.901	1.867	4.176
GBP2	0.756	1.688	5.477
GBP3	1.087	2.124	4.596
GBX2	-0.023	0.984	2.870
GCA	0.592	1.507	5.696
GCC2	0.081	1.058	7.406
GCLM	0.415	1.334	5.140
GCNT1	0.089	1.064	2.980
GCNT4	-0.179	0.883	2.925
GCSHP3//AC007383.4	-0.047	0.968	3.109
GDA	-0.172	0.888	4.772
GDAP1	-0.134	0.912	6.515
GDAP2	0.480	1.394	6.190
GDF1//CERS1	-0.016	0.989	7.142
GDF10	0.045	1.032	4.235
GDF11	-0.362	0.778	6.017
GDF15	0.291	1.223	3.047
GDF7	0.101	1.073	2.446
GDPD1	-0.172	0.888	6.013
GDPD2	0.585	1.500	5.021
GDPD3	-0.484	0.715	2.518
GEM	0.442	1.359	3.704
GEMIN7	-0.151	0.900	3.647
GEMIN8	-0.129	0.914	4.937
GEMIN8P4	0.068	1.048	2.319
GEN1	-0.183	0.881	3.332
GFM2	0.122	1.089	5.481
GFOD1	-0.194	0.874	3.894
GFOD1-AS1	0.203	1.151	2.575
GFOD2	-0.124	0.918	3.989
GFPT2	0.056	1.040	5.403
GFRA1	-0.102	0.932	5.070
GFRA2	0.687	1.610	3.782
GGCT	0.084	1.060	4.737
GGH	0.096	1.068	3.199
GGT2	0.240	1.181	2.551
GGT5	0.404	1.323	4.431
GGTA1P	0.367	1.289	2.592

GHITM	0.054	1.038	8.504
GHR	0.268	1.204	2.703
GHRLOS	-0.033	0.977	2.895
GIMAP4	0.395	1.315	4.877
GIMAP6	0.195	1.145	3.652
GIMAP7	0.303	1.233	4.666
GIMAP8	0.080	1.057	3.971
GINS3	0.065	1.046	4.192
GIPC3	0.265	1.202	3.117
GIT1	-0.136	0.910	6.745
GJA1//IFT140	0.030	1.021	8.955
GJA4	0.573	1.488	2.742
GJB1	-0.067	0.954	3.786
GJB2	-0.550	0.683	3.580
GJB6	-1.048	0.484	4.566
GJC1	0.321	1.249	3.280
GJC2	-0.052	0.965	5.015
GJC3	0.435	1.352	2.696
GJD2	-0.434	0.740	3.391
GK5	-0.076	0.949	5.406
GK-IT1	1.004	2.005	3.845
GLA	0.882	1.843	5.104
GLB1L	-0.126	0.917	3.409
GLB1L3	0.065	1.046	2.403
GLCCI1	0.011	1.008	2.773
GLDC	0.394	1.314	4.868
GLDN	0.286	1.219	6.917
GLI2	-0.162	0.894	3.566
GLI3	-0.174	0.886	3.927
GLIDR//LOC102724238//LOC554249//RP11-111F5.4,	0.130	1.094	4.951
GLIDR//RP11-211N8.2	0.033	1.023	3.664
GLIPR1	0.500	1.414	7.029
GLIPR1L2	-0.615	0.653	4.709
GLIPR2	0.584	1.499	5.667
GLIS3	0.234	1.176	4.919
GLMN	0.079	1.056	3.692
GLP1R	0.408	1.326	2.357
GLP2R	-0.186	0.879	3.008
GLRA1	-0.273	0.828	4.668
GLRA2	0.505	1.419	4.233
GLRA3	0.514	1.428	4.914
GLRB	-0.195	0.874	4.709
GLRX	0.355	1.279	4.697
GLRX2	-0.289	0.818	4.564
GLS	0.142	1.103	6.556
GLS2	0.182	1.134	4.756
GLT1D1	-0.022	0.985	4.943
GLTP	-0.058	0.960	8.323
GLTSCR2//SNORD23	-0.041	0.972	7.087
GLUD1	-0.183	0.881	5.880

GLUD1P7	0.191	1.142	2.865
GLUL	0.572	1.487	8.667
GLYATL2	-0.129	0.914	3.401
GM2A	0.069	1.049	6.202
GMFG	0.507	1.421	6.432
GMIP	0.351	1.275	3.980
GMPR	-0.311	0.806	5.157
GMPS	0.263	1.200	6.728
GNA12	0.128	1.093	5.142
GNA13	0.271	1.207	6.407
GNA14	0.259	1.197	5.424
GNA15	0.553	1.467	3.908
GNAI2	0.305	1.235	4.631
GNAI3//RP5-1160K1.3	0.073	1.052	3.253
GNAL	0.151	1.110	4.039
GNAO1	-0.074	0.950	9.735
GNB1L//C22orf29	-0.256	0.837	3.194
GNB4	0.674	1.595	4.918
GNB5	-0.164	0.893	3.235
GNB5	-0.085	0.943	4.188
GNG12	0.198	1.147	5.459
GNG13	-0.412	0.752	3.669
GNG2	0.233	1.175	2.753
GNG3	-0.403	0.756	8.009
GNG4	-0.037	0.975	4.500
GNG5	0.175	1.129	3.501
GNG5P2	0.133	1.097	5.872
GNL3//SNORD19B	0.359	1.283	5.928
GNLY	0.155	1.114	2.492
GNPDA2	0.396	1.316	5.150
GNPNAT1	0.363	1.286	4.481
GNPTAB	0.269	1.205	5.533
GNRHR2	0.275	1.210	3.010
GOLGA2P1	-0.192	0.875	2.689
GOLGA2P5//GOLGA2B	-0.606	0.657	3.391
GOLGA2P6//MTPAP	-0.677	0.625	2.045
GOLGA2P7//LOC642423//LOC101929479//AC10396	0.087	1.062	3.591
GOLGA6L10//GOLGA6L4//GOLGA6L9//GOLGA6L5P//	-0.296	0.815	5.770
GOLGA6L4//GOLGA6L5P//GOLGA6L17P//GOLGA6L3	-0.262	0.834	5.523
GOLGA7B	-0.504	0.705	4.176
GOLGA7B	0.366	1.289	2.233
GOLGA8A//GOLGA8B//LOC101930583	-0.166	0.891	7.451
GOLGA8A//GOLGA8B//LOC101930583	-0.113	0.925	7.490
GOLGA8EP	-0.031	0.978	3.684
GOLGA8J	-0.098	0.934	3.070
GOLGA8J	-0.067	0.955	3.073
GOLGA8M//GOLGA8J//GOLGA8K//GOLGA8I//GOLG	-0.160	0.895	5.556
GOLGA8O//GOLGA8N//GOLGA8Q//GOLGA8R//GOL	-0.139	0.908	6.517
GOLGA8O//GOLGA8R//GOLGA8N//GOLGA8Q//GOL	-0.121	0.919	5.946
GOLGA8O//GOLGA8R//GOLGA8N//GOLGA8Q//GOL	-0.087	0.941	6.092

GOLGA8S	-0.132	0.912	3.455
GOLIM4	-0.030	0.979	8.350
GOLT1B	0.435	1.352	6.272
GOPC	0.175	1.129	7.188
GORASP1	-0.093	0.938	4.040
GOT1	-0.105	0.930	8.249
GOT2	0.176	1.129	7.789
GPAM	0.039	1.027	5.018
GPATCH1	0.279	1.214	5.970
GPATCH2	0.139	1.101	5.179
GPATCH2L	0.184	1.136	5.122
GPATCH4	-0.188	0.878	3.682
GPC2	-0.289	0.818	2.434
GPC3	0.525	1.439	2.806
GPC4	0.039	1.028	5.379
GPC5	-0.306	0.809	5.463
GPC6	0.154	1.113	5.599
GPCPD1	-0.107	0.928	6.558
GPD1	-0.076	0.949	4.266
GPD1L	0.238	1.180	7.013
GPI	-0.096	0.936	8.480
GPIHBP1	0.047	1.033	5.359
GPLD1	-0.181	0.882	4.308
GPNMB	1.233	2.350	5.228
GPR101	0.027	1.019	3.264
GPR108//MIR6791	0.167	1.123	4.008
GPR116	0.122	1.088	5.759
GPR12	-0.115	0.923	4.202
GPR123	-0.345	0.787	3.264
GPR125	-0.040	0.973	6.387
GPR126	0.210	1.157	3.297
GPR132	-0.174	0.886	2.582
GPR133	-0.116	0.923	2.972
GPR137C	0.204	1.152	5.443
GPR141	0.510	1.424	2.737
GPR146//RP11-449P15.1	-0.497	0.709	2.575
GPR149	0.089	1.064	2.566
GPR150	-0.083	0.944	2.331
GPR155	0.656	1.576	6.330
GPR158	0.124	1.090	7.471
GPR162	-0.122	0.919	5.615
GPR17	0.090	1.064	3.136
GPR176	-0.134	0.911	2.832
GPR179	0.202	1.150	3.424
GPR180	0.395	1.315	5.696
GPR183	0.746	1.677	6.349
GPR20	0.029	1.020	2.798
GPR21//RABGAP1	0.131	1.095	5.205
GPR22	-0.576	0.671	3.876
GPR26	0.487	1.402	3.796

GPR3	0.144	1.105	4.006
GPR34	-0.294	0.815	5.673
GPR37//SEL1L2	-0.119	0.921	8.623
GPR4	0.401	1.320	4.550
GPR45	0.101	1.072	2.542
GPR52	0.098	1.070	3.594
GPR56	0.226	1.170	7.922
GPR6	-0.130	0.914	3.610
GPR62	0.094	1.067	3.573
GPR63	0.127	1.092	3.068
GPR65	1.194	2.288	4.512
GPR83	0.330	1.257	4.085
GPR85	0.129	1.094	4.013
GPR88	-0.252	0.840	3.999
GPR98	-0.230	0.853	4.365
GPRASP1	0.194	1.144	5.853
GPRASP2//ARMCX5//ARMCX5-GPRASP2	0.116	1.084	4.522
GPRC5A	-0.116	0.923	2.334
GPRC5B	0.067	1.047	9.856
GPRIN1	0.372	1.294	4.314
GPSM2	-0.038	0.974	6.659
GPT2	-0.103	0.931	5.960
GPX1	-0.110	0.926	4.873
GPX3	0.291	1.224	3.856
GPX8	0.206	1.154	2.363
GRAMD1B	-0.083	0.944	6.377
GRAMD3	-0.095	0.936	5.501
GRAPL	0.328	1.256	2.392
GRASP	-0.143	0.905	3.731
GRB14	0.191	1.142	3.283
GREB1L	-0.231	0.852	3.774
GREM1	0.141	1.102	5.995
GREM2	-0.361	0.778	4.149
GRHL3	0.587	1.502	3.206
GRIA1	0.232	1.174	5.069
GRIA2	0.213	1.159	6.772
GRIA3	-0.168	0.890	6.672
GRIA4	0.072	1.051	7.020
GRID2	0.227	1.170	4.554
GRIK1	0.077	1.055	4.811
GRIK2	0.189	1.140	6.015
GRIK3	0.052	1.037	5.391
GRIK5	0.031	1.022	6.032
GRIN1	-0.305	0.809	6.872
GRIN2A	0.382	1.303	5.381
GRIN2B	0.419	1.337	5.809
GRIN3A	-0.210	0.865	4.568
GRIP1	-0.023	0.984	4.497
GRIP2	0.172	1.126	3.110
GRIPAP1	-0.176	0.885	6.392

GRM1	0.377	1.299	4.438
GRM2	-0.148	0.903	2.430
GRM3	0.284	1.217	7.632
GRM5	0.478	1.393	6.382
GRM7//LOC100996542	0.202	1.150	3.396
GRM8	-0.030	0.980	2.485
GRN	0.428	1.345	6.920
GRPEL2	0.170	1.125	5.391
GRPR	0.466	1.381	3.847
GRTP1-AS1//LOC101930262//LOC101930007	-0.159	0.895	2.158
GS1-115G20.1	-0.714	0.610	3.297
GS1-259H13.2//ZNF655	-0.091	0.939	2.935
GS1-421I3.4	0.313	1.242	2.293
GS52	-0.190	0.877	2.139
GSDMD	0.019	1.013	2.976
GSKIP	0.198	1.147	4.453
GSN	-0.076	0.949	6.339
GSPT2	0.048	1.034	3.597
GSTA4	0.108	1.078	6.542
GSTM2	-0.027	0.981	5.233
GSTM2P1	-0.548	0.684	2.300
GSTM3	0.289	1.222	5.424
GSTM4	0.465	1.381	4.110
GSTM5	0.130	1.095	5.932
GSTT1	0.280	1.214	4.612
GSTT2	0.197	1.146	4.910
GSTT2//GSTT2B	-0.434	0.740	4.769
GTDC1	0.188	1.140	5.623
GTF2H2C_2//GTF2H2C//GTF2H2//GTF2H2B//GTF2H	-0.272	0.828	6.335
GTF2IRD2B//GTF2IRD2//GTF2IRD2P1	-0.397	0.760	4.843
GTF3C3	0.506	1.420	5.771
GTF3C4	0.153	1.112	5.560
GTPBP8	0.442	1.359	3.434
GUCA1B	0.369	1.292	2.185
GUCY1A3	-0.028	0.981	6.304
GUCY1B3	0.241	1.182	8.037
GUF1	0.277	1.212	6.020
GULP1	-0.050	0.966	3.738
GUSB	0.314	1.243	4.864
GUSBP1//LOC100996497//GUSBP4//LINC00680	0.043	1.030	5.458
GUSBP10	-0.497	0.709	2.078
GUSBP2	-0.145	0.905	4.362
GUSBP3	0.187	1.138	4.946
GUSBP3//SMA4//LOC653080//SMA5//GUSBP2//GUS	0.033	1.023	7.713
GUSBP4//XXbac-BPGBPG55C20.2	0.070	1.049	4.387
GUSBP5	0.197	1.146	3.802
GVINP1	0.426	1.344	2.729
GXYLT2	0.410	1.329	5.202
GYG2	0.035	1.025	5.502
GYPC	0.212	1.158	2.598

GYS1	-0.105	0.930	5.030
H19//MIR675//RPS12	0.270	1.206	2.982
H2AFJ	0.045	1.032	4.475
H2AFX	-0.446	0.734	5.695
H2AFZ	0.071	1.050	4.786
H3F3B//H3F3A//MIR4738	0.161	1.118	8.938
H6PD	-0.156	0.898	3.495
HACE1	0.207	1.155	6.047
HADHB	0.208	1.155	7.335
HAGLR//HOXD-AS1	-0.187	0.878	2.712
HAMP	1.246	2.372	7.735
HAP1	0.412	1.330	4.359
HAPLN2	-0.191	0.876	7.392
HAPLN4//AC138430.4	-0.825	0.564	4.695
HAR1A	-0.432	0.741	2.592
HAR1B	-0.234	0.850	2.654
HARBI1	0.323	1.251	3.803
HAS2	0.580	1.495	3.408
HAUS4//MIR4707//RP11-298I3.5	0.324	1.252	4.153
HAVCR2	0.449	1.365	5.548
HBB	-0.675	0.626	2.471
HBEGF	0.375	1.297	4.895
HBG1	0.389	1.309	2.374
HCAR3//GPR109B	0.311	1.241	3.140
HCCAT5	-0.519	0.698	3.389
HCCS	0.162	1.119	5.662
HCG17	-0.192	0.875	3.813
HCG18	0.188	1.139	3.827
HCG2040054//AC138655.4	-0.514	0.700	2.236
HCG22	-0.346	0.787	2.924
HCG22	-0.342	0.789	2.809
HCG22	-0.278	0.825	2.597
HCG27//MICB	-0.403	0.756	3.380
HCG8	0.448	1.365	3.415
HCG8	0.351	1.275	3.373
HCK	0.938	1.915	5.318
HCLS1	0.430	1.348	5.898
HCN3	0.225	1.169	3.575
HCN4	0.122	1.089	3.616
HCP5	0.907	1.876	4.647
HCP5	0.794	1.733	4.038
HCP5	0.663	1.583	3.775
HCRTR2	0.025	1.018	3.255
HCST	0.532	1.446	4.384
HDAC1	-0.042	0.971	5.529
HDAC7	-0.125	0.917	4.050
HDAC9	0.352	1.276	4.627
HDGFRP3//RP11-382A20.3	-0.049	0.966	7.721
HEATR5A//RP11-176H8.1	0.213	1.159	6.423
HEBP2	0.063	1.045	4.762

HECTD2	0.157	1.115	4.667
HECW1	0.048	1.034	5.025
HECW1-IT1	-0.086	0.942	2.694
HECW2	-0.173	0.887	6.389
HEG1	0.225	1.168	7.778
HELB	0.207	1.154	2.703
HELB	-0.014	0.990	2.299
HELZ2	0.172	1.126	2.304
HENMT1	0.338	1.264	4.329
HEPACAM//HEPN1	-0.372	0.773	7.256
HEPN1	0.084	1.060	4.212
HERC1	0.035	1.025	7.389
HERC2P7	-0.355	0.782	4.604
HERC5	0.226	1.169	3.737
HERC6	0.562	1.477	5.868
HERPUD1	-0.071	0.952	7.209
HES1	-0.206	0.867	5.283
HES4	0.116	1.084	3.448
HEXIM1	-0.356	0.781	5.735
HEY1	0.631	1.549	4.428
HEY2	0.079	1.057	5.828
HFE	0.108	1.078	2.788
HGF	0.822	1.768	4.126
HHATL	0.255	1.193	7.081
HHIP	0.068	1.048	5.376
HHIP-AS1	-0.042	0.971	3.168
HHIPL1	0.441	1.358	4.082
HIBCH	-0.110	0.927	4.770
HID1	0.123	1.089	5.288
HIF1A	0.600	1.516	8.861
HIF1A-AS2	0.374	1.296	3.139
HIF1A-AS2	-0.258	0.836	2.962
HIF3A	-0.480	0.717	4.685
HIGD1B//EFTUD2	-0.321	0.801	4.093
HILPDA	0.120	1.087	7.211
HIP1	0.017	1.012	6.756
HIP1R	-0.557	0.680	5.898
HIPK1-AS1//RP5-1073O3.7	0.399	1.318	2.675
HIPK2	-0.212	0.863	9.837
HIRA//C22orf39	-0.195	0.873	5.910
HIST1H1C	0.313	1.242	5.068
HIST1H1E	0.315	1.244	8.269
HIST1H2AC	-0.098	0.935	6.433
HIST1H2AE	0.041	1.029	4.448
HIST1H2AI//HIST1H2AK//HIST1H2AL//HIST1H2AM//	-0.174	0.887	2.245
HIST1H2AK	-0.336	0.792	4.778
HIST1H2BB	-0.161	0.895	2.399
HIST1H2BD	0.369	1.292	3.829
HIST1H2BF	0.290	1.223	3.695
HIST1H2BG//HIST1H2BF//HIST1H2BE//HIST1H2BI//H	0.240	1.181	3.321

HIST1H2BG//HIST1H2BJ	0.250	1.189	4.682
HIST1H2BH	-0.155	0.898	2.964
HIST1H2BK	0.331	1.258	5.871
HIST1H2BM	-0.254	0.839	4.987
HIST1H2BN	0.440	1.356	2.983
HIST1H2BO	0.066	1.047	2.226
HIST1H3A	0.242	1.183	3.672
HIST1H3E	-0.039	0.973	2.701
HIST1H3I	0.510	1.424	4.535
HIST1H4B//HIST1H4I//HIST1H4A//HIST1H4D//HIST1	-0.568	0.674	3.994
HIST1H4D	-0.528	0.694	5.671
HIST1H4H	-0.145	0.904	4.127
HIST1H4K//HIST1H4J//HIST1H4I//HIST1H4A//HIST1H	0.148	1.108	9.098
HIST1H4L	0.487	1.402	3.451
HIST2H2AA4//HIST2H2AA3	0.230	1.172	7.750
HIST2H2AC	-0.071	0.952	9.673
HIST2H2BF	0.674	1.595	3.891
HIST2H3D	0.580	1.495	4.950
HIST2H3PS2//RP5-998N21.10	-0.127	0.916	4.450
HIST2H4B//HIST2H4A//HIST1H4I//HIST1H4A//HIST1H	0.350	1.275	5.631
HIST3H2BB	-0.608	0.656	4.301
HIVEP1	0.186	1.137	4.990
HIVEP2	0.138	1.100	8.110
HK1	0.017	1.012	6.770
HK2	-0.111	0.926	5.059
HLA-A	0.592	1.507	4.245
HLA-A	1.032	2.045	6.575
HLA-A	0.511	1.426	4.671
HLA-A	0.420	1.338	5.499
HLA-A	0.471	1.387	7.420
HLA-A	0.401	1.320	7.322
HLA-A//HLA-B//HLA-H	0.526	1.440	8.872
HLA-B	0.689	1.612	8.606
HLA-B	0.714	1.640	7.492
HLA-B	0.502	1.417	8.657
HLA-C	0.513	1.427	7.253
HLA-C	0.515	1.429	7.299
HLA-C	0.347	1.272	7.932
HLA-C	0.336	1.262	7.577
HLA-C	0.304	1.235	7.337
HLA-C	0.302	1.233	5.570
HLA-DMA	0.691	1.615	5.666
HLA-DMA	0.608	1.525	5.630
HLA-DMA	0.666	1.587	5.476
HLA-DMB//HLA-DMA	0.623	1.540	5.724
HLA-DMB//HLA-DMA	0.545	1.459	5.708
HLA-DMB//HLA-DMA//XXbac-BPG181M17.5	0.812	1.755	5.745
HLA-DMB//HLA-DMA//XXbac-BPG181M17.5	0.818	1.763	5.743
HLA-DMB//XXbac-BPG181M17.5	0.809	1.752	4.686
HLA-DMB//XXbac-BPG181M17.5	0.781	1.718	5.467

HLA-DMB//XXbac-BPG181M17.5	0.767	1.701	5.454
HLA-DOA	0.631	1.549	4.700
HLA-DOA	0.616	1.532	4.733
HLA-DOA	0.565	1.479	3.331
HLA-DOA	0.573	1.487	3.424
HLA-DOA	0.419	1.337	5.127
HLA-DPA1	0.489	1.404	5.153
HLA-DPA1	0.477	1.391	5.142
HLA-DPA1	0.490	1.404	5.358
HLA-DPA1	0.456	1.372	4.232
HLA-DPA1	0.445	1.362	4.139
HLA-DPB1	0.694	1.618	5.631
HLA-DPB1	0.669	1.589	6.816
HLA-DPB1	0.642	1.561	6.806
HLA-DPB1	0.627	1.544	7.614
HLA-DPB2	0.704	1.628	3.709
HLA-DQA1//LOC100509457	1.135	2.196	2.585
HLA-DQA1//LOC100509457	0.845	1.796	5.233
HLA-DQA1//LOC100509457	0.490	1.404	2.661
HLA-DQA1//LOC100509457	0.412	1.330	2.438
HLA-DQA1//LOC100509457	-0.238	0.848	5.045
HLA-DQA1//LOC100509457	-0.104	0.930	5.723
HLA-DQA2//HLA-DQA1//LOC100509457	1.029	2.040	5.293
HLA-DQA2//HLA-DQA1//LOC100509457	0.994	1.991	4.080
HLA-DQA2//HLA-DQA1//LOC100509457	1.005	2.007	4.914
HLA-DQA2//HLA-DQA1//LOC100509457	0.922	1.895	4.879
HLA-DQA2//HLA-DQA1//LOC100509457	0.909	1.878	4.700
HLA-DQA2//HLA-DQA1//LOC100509457	0.861	1.816	5.572
HLA-DQA2//HLA-DQA1//LOC100509457	0.924	1.897	4.691
HLA-DQB1//LOC101060835	0.717	1.643	4.931
HLA-DQB1-AS1//XXbac-BPG254F23.5	-0.145	0.904	3.357
HLA-DQB2	0.669	1.590	2.819
HLA-DQB2	0.613	1.529	2.778
HLA-DQB2	0.595	1.511	2.789
HLA-DQB2	0.279	1.214	3.048
HLA-DQB2	0.249	1.188	2.824
HLA-DRA	1.046	2.065	8.107
HLA-DRA//HLA-DQA1	0.608	1.524	9.130
HLA-DRA//HLA-DQA1	0.591	1.506	9.349
HLA-DRA//HLA-DQA1	0.559	1.473	9.471
HLA-DRA//HLA-DQA1	0.555	1.469	9.494
HLA-DRB1//LOC100996809	5.230	37.542	4.266
HLA-DRB1//LOC100996809//HLA-DRB3//HLA-DRB5	0.348	1.273	4.118
HLA-DRB1//LOC100996809//HLA-DRB3//HLA-DRB5	0.209	1.156	4.042
HLA-DRB1//LOC100996809//HLA-DRB4//HLA-DRB3/	0.374	1.296	7.156
HLA-DRB3	0.534	1.447	6.330
HLA-DRB3	-0.197	0.872	3.835
HLA-DRB3//HLA-DRB1//LOC100996809//HLA-DRB5/	0.582	1.497	8.158
HLA-DRB4	1.048	2.068	6.699
HLA-DRB4	0.332	1.259	5.181

HLA-DRB5	4.930	30.483	4.521
HLA-DRB6	0.019	1.013	2.598
HLA-E	0.302	1.232	8.630
HLA-E	0.282	1.216	8.663
HLA-F	0.596	1.512	5.111
HLA-F	0.488	1.402	5.482
HLA-F	0.532	1.446	5.114
HLA-F	0.457	1.373	5.196
HLA-G	0.413	1.332	3.581
HLA-G	0.383	1.304	3.998
HLA-G	0.374	1.296	3.846
HLA-L	0.117	1.085	5.280
HLF	-0.137	0.909	6.970
HLX	0.663	1.583	2.521
HMBOX1-IT1	0.131	1.095	2.444
HMCES//C3orf37	0.032	1.022	5.874
HMG20B	-0.112	0.926	4.025
HMGA1//HMGA1P8	0.131	1.095	6.237
HMGB2	0.032	1.022	3.238
HMGCL	0.152	1.111	4.377
HMGCS1	0.032	1.022	6.481
HMGN1//LOC101927733	0.333	1.260	3.959
HMGN2	0.270	1.206	3.141
HMGN5	0.034	1.024	4.527
HMHA1	0.183	1.136	4.486
HMOX1	0.700	1.624	5.164
HMP19	0.065	1.046	3.888
HMSD	0.111	1.080	3.598
HMX3	-0.032	0.978	2.479
HN1	0.385	1.306	5.298
HN1L	0.043	1.030	6.028
HNMT	0.391	1.311	4.523
HNRNPA1L2//MRPS31P4	-0.341	0.789	3.166
HNRNPLL	0.284	1.217	4.341
HNRNPU-AS1	0.308	1.238	6.208
HOGA1	0.062	1.044	2.566
HOMER1	-0.102	0.932	5.935
HOMER3	0.342	1.267	4.629
HOOK1	0.371	1.293	3.404
HOTAIRM1	-0.151	0.900	3.595
HOXA1	0.475	1.390	2.580
HOXA10//HOXA9//MIR196B	-0.663	0.631	2.586
HOXA10-AS//MIR196B//HOXA-AS4	-0.124	0.917	2.916
HOXA2	-0.109	0.928	3.690
HOXA4	0.064	1.045	3.457
HOXA5	-0.065	0.956	4.606
HOXA6	-0.361	0.778	3.705
HOXA7	-0.321	0.800	3.783
HOXB2	0.090	1.064	3.826
HOXB3	0.383	1.304	2.826

HOXB4	0.038	1.027	3.201
HOXB5	0.271	1.206	3.386
HOXB6	0.107	1.077	3.614
HOXB7	-0.218	0.860	2.499
HOXB8	0.531	1.445	3.407
HOXB9	0.217	1.163	3.790
HOXB-AS3	-0.051	0.965	2.494
HOXC10	0.253	1.192	3.553
HOXC6//HOXC4//HOXC5//RP11-834C11.14	-0.079	0.947	2.419
HOXC8	-0.497	0.708	3.914
HOXC9	-0.142	0.906	2.560
HOXC-AS1	-0.245	0.844	2.322
HOXC-AS2	0.127	1.092	3.193
HOXD1	0.055	1.039	3.356
HOXD10	0.333	1.260	2.578
HOXD3//HOXD4//LOC401021	0.307	1.237	3.166
HOXD4	-0.035	0.976	3.294
HOXD8	0.175	1.129	2.992
HP	0.148	1.108	3.528
HPCAL1	-0.080	0.946	3.413
HPCAL4	0.035	1.025	5.978
HPD	0.090	1.064	2.127
HPGDS	0.461	1.376	3.603
HPN	0.022	1.015	3.535
HPN-AS1	0.424	1.342	3.016
HPRT1	-0.048	0.967	6.058
HPS3	0.457	1.372	7.223
HPS5	0.473	1.388	5.754
HPS6	0.110	1.079	2.173
HPSE	0.782	1.720	3.189
HPSE2	-0.025	0.983	5.326
HR	-0.211	0.864	3.478
HRASLS	-0.027	0.981	3.089
HRASLS5	0.394	1.314	5.311
HRH2	0.419	1.337	3.967
HRH3	-0.053	0.964	4.317
HRK	-0.394	0.761	2.328
HS2ST1//RP5-1052I5.2	0.380	1.301	6.049
HS3ST2	-0.065	0.956	3.220
HS3ST3B1	0.430	1.348	2.854
HS3ST4	0.089	1.064	2.259
HS3ST5	0.311	1.240	5.327
HS6ST1	0.361	1.284	3.755
HS6ST2	0.215	1.161	3.114
HSD11B1	0.043	1.030	4.161
HSD11B1L	-0.151	0.901	5.029
HSD11B2	0.192	1.142	2.605
HSD17B1	0.049	1.034	3.486
HSD17B1//RP11-400F19.6	0.515	1.429	5.105
HSD17B10	-0.178	0.884	5.120

HSD17B13	0.579	1.494	2.266
HSD17B14	0.296	1.227	4.760
HSD17B1P1	0.036	1.025	3.568
HSD17B3	0.050	1.035	3.327
HSD17B6	0.081	1.057	3.193
HSD17B7P2	0.219	1.164	5.471
HSD17B8	-0.137	0.909	3.598
HSD3B7	0.233	1.175	3.635
HSDL1	0.306	1.236	7.020
HSF2	0.420	1.338	5.092
HSF4	0.741	1.672	4.169
HSFX2//HSFX1	0.252	1.191	4.793
HSFX2//HSFX1	-0.094	0.937	3.613
HSP90AB3P	-0.198	0.872	5.316
HSP90AB4P	0.064	1.045	2.601
HSPA12A	-0.448	0.733	7.258
HSPA12A//RP11-498B4.5	-0.142	0.906	2.205
HSPA12B	0.303	1.234	3.080
HSPA14	-0.089	0.940	4.410
HSPA1A//HSPA1B//HSPA1L	0.248	1.188	10.751
HSPA1A//HSPA1B//HSPA1L	0.238	1.179	10.836
HSPA1A//HSPA1B//HSPA1L	0.233	1.175	10.322
HSPA1A//HSPA1B//HSPA1L	0.182	1.135	10.031
HSPA1B//HSPA1A//HSPA1L	0.318	1.247	10.094
HSPA1B//HSPA1A//HSPA1L	0.287	1.220	10.116
HSPA1B//HSPA1A//HSPA1L	0.243	1.183	10.505
HSPA1B//HSPA1A//HSPA1L	0.230	1.172	10.198
HSPA1L	0.306	1.236	4.806
HSPA1L	0.197	1.146	4.528
HSPA2	0.073	1.052	8.820
HSPA4L	0.202	1.151	6.997
HSPA5	0.097	1.069	9.479
HSPA6	0.092	1.066	3.039
HSPA7	0.330	1.257	2.580
HSPB1	0.271	1.207	9.423
HSPB3	-0.045	0.970	2.145
HSPB6	0.054	1.038	5.515
HSPB7	-0.170	0.889	2.581
HSPB8	0.207	1.154	5.241
HSPG2	0.392	1.312	4.030
HSPH1	0.352	1.277	8.690
HTATIP2	0.553	1.467	5.338
HTR1A	0.311	1.241	3.286
HTR1B	-0.036	0.976	3.328
HTR1E	-0.077	0.948	3.327
HTR1F	0.351	1.275	2.658
HTR2A	-0.143	0.906	6.275
HTR2C	0.130	1.094	3.909
HTR3B	-0.103	0.931	2.856
HTR4	-0.075	0.949	3.115

HTR5A	0.224	1.168	4.418
HTR5AOS//AC093726.4	-0.285	0.821	3.634
HTRA4	0.223	1.167	2.396
HUNK	0.109	1.078	3.601
HVCN1	0.027	1.019	3.664
HYAL1	-0.257	0.837	2.845
HYDIN//HYDIN2//LOC101930373	-0.691	0.620	3.281
HYDIN//HYDIN2//LOC101930373	-0.403	0.756	4.650
HYI	0.087	1.062	4.727
IBA57-AS1//C1orf148	-0.451	0.732	3.276
ICA1	0.116	1.084	3.588
ICAM1	0.852	1.805	5.086
ICAM2	0.480	1.395	5.649
ICAM3	0.255	1.193	3.133
ICAM5	-0.086	0.942	3.362
ICE1//CTC-501N16.1	0.393	1.313	5.644
ICOSLG	0.185	1.137	4.831
ID1	0.020	1.014	6.316
ID2	0.249	1.188	4.062
ID3	0.401	1.321	4.554
ID4	-0.105	0.930	6.174
IDI2-AS1	0.192	1.142	3.861
IER2	-0.119	0.921	4.546
IER3	0.644	1.563	4.695
IER3	0.620	1.536	4.722
IER3	0.575	1.489	5.144
IER3IP1//RP11-49K24.6	0.069	1.049	5.263
IER5	0.759	1.692	4.913
IER5L//MGAT2	0.735	1.665	3.946
IFI16	0.777	1.713	7.478
IFI27	0.090	1.064	3.648
IFI30//PIK3R2//AC007192.4	1.448	2.729	5.916
IFI35	0.097	1.069	4.789
IFI44	0.186	1.138	3.920
IFI44L	0.616	1.532	4.527
IFI6	0.811	1.754	8.223
IFIH1	0.301	1.232	3.479
IFIT1	0.275	1.210	4.893
IFIT2	0.385	1.306	4.444
IFIT3	0.449	1.365	3.999
IFITM1	0.585	1.500	7.494
IFITM2	0.266	1.202	8.026
IFITM3	0.300	1.231	4.924
IFITM4P	0.349	1.274	3.777
IFITM4P	0.244	1.184	4.468
IFITM4P	0.188	1.139	3.942
IFITM4P	0.132	1.096	3.755
IFITM4P	0.021	1.014	3.030
IFNA14	-0.322	0.800	2.986
IFNGR1	0.139	1.102	5.590

IFRD2	0.063	1.045	3.330
IFT57	0.047	1.033	5.907
IFT80//RP11-432B6.3	0.191	1.142	1.841
IFT80//RP11-432B6.3	-0.017	0.989	6.223
IGANRP	0.189	1.140	3.321
IGDCC4	0.026	1.018	4.283
IGF1	0.035	1.025	3.405
IGF2BP2	-0.064	0.956	2.986
IGFALS	-0.410	0.752	2.901
IGFBP2	-0.100	0.933	3.092
IGFBP3	-0.282	0.823	4.460
IGFBP4	0.710	1.636	5.114
IGFBP5	0.212	1.158	6.747
IGFBP6	-0.351	0.784	4.340
IGFBP7	0.531	1.445	8.443
IGFBPL1	0.613	1.529	2.349
IGFL4	0.428	1.346	3.089
IGFLR1//AD000671.6	0.349	1.274	3.952
IGHA2	0.058	1.041	3.813
IGHD2-15	-0.101	0.933	4.422
IGHG4//IGHG1	-0.432	0.741	2.603
IGHJ1//SKAP2//IGHG1	-0.174	0.886	2.902
IGHJ2//SKAP2//IGHG1//IGHD	0.090	1.064	2.815
IGHJ3//SKAP2//IGHG1//IGHA1//IGH//IGHV1-69//IG[illegible]	-1.044	0.485	3.245
IGHM//IGHG1	-0.393	0.762	2.692
IGHV1-24	-0.094	0.937	2.283
IGIP	-0.114	0.924	6.259
IGKJ5//IGKC	-0.720	0.607	3.249
IGKV1-16//IGKC	-0.073	0.951	2.546
IGKV1-17	-0.284	0.821	2.533
IGKV1D-16//IGKC	-0.085	0.943	3.001
IGKV1OR2-3//IGKV1-12//IGKC	0.436	1.353	2.416
IGKV3-15//IGKC	-0.340	0.790	2.662
IGLJ2	0.134	1.097	4.768
IGLJ4	-0.165	0.892	3.037
IGLL3P	-0.367	0.776	2.650
IGLON5	-0.557	0.680	4.730
IGLV@//IGHG1//IGLC1//IGLJ3//IGLC2	-0.092	0.938	3.067
IGLV2-11	-0.222	0.858	3.012
IGLV2-23//IGLC1	-0.538	0.689	3.328
IGLV2-8	-0.497	0.709	4.182
IGLV3-10//IGLC1	-0.387	0.765	2.653
IGLV3-25	0.336	1.262	2.760
IGLV3-27	0.616	1.532	2.013
IGSF1	0.532	1.446	3.661
IGSF21	0.044	1.031	5.708
IGSF3	-0.338	0.791	4.801
IGSF6	0.749	1.681	4.493
IGSF9B	-0.020	0.986	4.433
IKBIP	0.185	1.137	2.330

IKBKAP	0.212	1.158	7.021
IKBKB	0.131	1.095	5.650
IKBKE	0.615	1.532	3.057
IKZF1	0.722	1.649	3.140
IKZF2	0.284	1.217	3.815
IL10RA	0.516	1.430	4.658
IL10RB//AP000295.9	0.263	1.200	4.641
IL10RB-AS1	0.688	1.611	1.890
IL11RA	-0.013	0.991	4.206
IL12RB1	-0.472	0.721	2.042
IL12RB2	0.037	1.026	2.381
IL13RA1	0.599	1.515	6.730
IL16	0.180	1.133	3.250
IL17D	-0.276	0.826	4.314
IL17RA	0.263	1.200	6.943
IL17RD	-0.035	0.976	4.944
IL18	0.507	1.421	4.610
IL18BP	0.412	1.330	4.462
IL18R1	-0.544	0.686	3.165
IL1A	0.602	1.517	1.985
IL1B	0.667	1.588	2.843
IL1R1	0.469	1.384	5.034
IL1R2	-0.103	0.931	2.450
IL1RAP	0.189	1.140	3.250
IL1RAPL1	0.105	1.075	6.241
IL1RAPL2	-0.035	0.976	1.533
IL1RL1	0.451	1.367	4.158
IL20RA	-0.323	0.799	2.189
IL21R	0.379	1.301	2.237
IL23A//TRBJ2-6	0.362	1.286	3.097
IL2RA	0.300	1.231	2.496
IL2RG	0.441	1.358	3.018
IL32	-0.082	0.945	2.252
IL33	1.078	2.111	4.369
IL34	0.276	1.211	3.814
IL3RA	0.189	1.140	3.616
IL3RA	0.168	1.123	4.152
IL4R	0.916	1.888	5.724
IL6R	0.161	1.118	5.379
IL7R	-0.110	0.926	2.804
ILDR2	-0.459	0.727	5.110
IMPA1	0.271	1.207	6.513
IMPG2	-0.330	0.796	2.856
INF2	-0.026	0.982	5.724
INHBA	0.154	1.112	2.777
INHBA-AS1	-0.076	0.949	1.967
INHBB	-0.085	0.943	4.337
INIP	0.128	1.092	6.085
INO80C	0.277	1.212	3.875
INPP4A	0.292	1.224	7.167

INPP4B	-0.023	0.984	3.743
INPP5A	-0.271	0.829	6.725
INPP5D	0.131	1.095	6.104
INPP5E	0.110	1.079	4.630
INPP5F	0.160	1.117	7.444
INPP5J	0.030	1.021	2.636
INPPL1	0.252	1.191	5.987
INS//IGF2//INS-IGF2	-0.500	0.707	2.340
INSIG1	0.202	1.150	7.295
INSIG2	0.238	1.179	5.679
INSM1	-0.319	0.802	2.952
INTS4L1	-0.138	0.909	2.173
INTS5	0.283	1.217	4.348
INTS7	0.382	1.303	4.889
IP6K1	0.028	1.020	6.472
IP6K3	0.250	1.189	4.731
IPCEF1//CNKSR3	0.221	1.166	5.180
IPO11	0.216	1.162	5.283
IPO13	0.197	1.147	6.487
IPO5P1//RP11-15H20.6	0.117	1.084	3.833
IPO9	0.250	1.189	7.044
IPW//SNORD107//PWARSN//SNORD116-4//SNORD1	0.266	1.202	3.793
IPW//SNORD107//PWARSN//SNORD116-4//SNORD1	-0.089	0.940	4.917
IPW//SNORD107//PWARSN//SNORD116-4//SNORD1	-0.087	0.942	6.241
IPW//SNORD107//PWARSN//SNORD116-4//SNORD1	0.049	1.035	5.867
IPW//SNORD107//PWARSN//SNORD116-4//SNORD1	0.491	1.405	4.076
IPW//SNORD107//PWARSN//SNORD116-4//SNORD1	-0.229	0.853	8.670
IPW//SNORD107//PWARSN//SNORD116-4//SNORD1	0.329	1.256	2.821
IPW//SNORD107//PWARSN//SNORD116-4//SNORD1	-0.236	0.849	8.409
IPW//SNORD107//PWARSN//SNORD116-4//SNORD1	-0.222	0.858	8.709
IPW//SNORD107//PWARSN//SNORD116-4//SNORD1	-0.244	0.844	3.149
IPW//SNORD107//PWARSN//SNORD116-4//SNORD1	0.206	1.154	3.876
IPW//SNORD107//PWARSN//SNORD116-4//SNORD1	-0.279	0.824	2.425
IPW//SNORD107//PWARSN//SNORD116-4//SNORD1	-0.193	0.875	5.150
IPW//SNORD107//PWARSN//SNORD116-4//SNORD1	-0.156	0.897	8.109
IPW//SNORD107//PWARSN//SNORD116-4//SNORD1	-0.157	0.897	9.250
IPW//SNORD107//PWARSN//SNORD116-4//SNORD1	-0.150	0.902	7.559
IPW//SNORD107//PWARSN//SNORD116-4//SNORD1	-0.145	0.904	7.233
IPW//SNORD107//PWARSN//SNORD116-4//SNORD1	-0.104	0.930	5.798
IPW//SNORD107//PWARSN//SNORD116-4//SNORD1	0.118	1.086	3.475
IPW//SNORD107//PWARSN//SNORD116-4//SNORD1	-0.091	0.939	7.336
IPW//SNORD107//PWARSN//SNORD116-4//SNORD1	-0.095	0.936	6.014
IPW//SNORD107//PWARSN//SNORD116-4//SNORD1	0.075	1.053	6.953
IPW//SNORD107//PWARSN//SNORD116-4//SNORD1	-0.067	0.954	6.324
IPW//SNORD107//PWARSN//SNORD116-4//SNORD1	-0.077	0.948	3.353
IPW//SNORD107//PWARSN//SNORD116-4//SNORD1	-0.037	0.974	7.137
IPW//SNORD107//PWARSN//SNORD116-4//SNORD1	-0.164	0.893	5.782
IQCA1	-0.521	0.697	5.119
IQCJ//IQCJ-SCHIP1//SCHIP1	0.313	1.242	3.685
IQGAP1	0.387	1.308	7.515

IQGAP2	0.525	1.439	3.198
IQSEC1	-0.060	0.959	7.182
IQSEC2	0.414	1.332	4.958
IQSEC3	-0.228	0.854	4.400
IQUB	-0.463	0.726	3.907
IRAK1	0.066	1.046	4.836
IRAK1BP1//RP11-173D14.1	0.163	1.119	3.471
IRAK3	0.262	1.199	5.310
IRAK4	0.204	1.152	3.594
IREB2	0.362	1.285	6.534
IRF1	0.314	1.243	3.451
IRF2BP2	0.323	1.251	6.206
IRF7	0.350	1.275	3.247
IRF8	0.696	1.620	4.753
IRF9//RP11-468E2.4	0.203	1.151	5.599
IRF9//RP11-468E2.4//RNF31	-0.285	0.821	2.676
IRGQ	0.311	1.240	5.026
IRS1	-0.042	0.971	3.368
IRS2	0.314	1.243	7.337
IRX1	-0.118	0.922	2.668
IRX2	-0.157	0.897	2.067
IRX3	-0.192	0.875	3.720
IRX5	-0.303	0.811	3.071
ISCA1	0.058	1.041	3.933
ISCU	0.349	1.274	5.419
ISG20	0.159	1.117	2.511
ISLR	0.152	1.111	3.229
ISM1//C20orf82	-0.175	0.886	3.652
ISPD	-0.315	0.804	3.968
ITFG1	0.059	1.041	7.564
ITFG3	0.030	1.021	4.738
ITGA10	-0.375	0.771	2.076
ITGA2	0.108	1.077	5.372
ITGA3	0.381	1.303	5.205
ITGA4	0.507	1.421	2.782
ITGA5	0.559	1.473	4.048
ITGA7	0.145	1.106	5.114
ITGA8	-0.130	0.914	3.926
ITGA9	0.320	1.248	4.953
ITGAL	0.490	1.404	3.688
ITGAM	0.867	1.824	4.800
ITGAV	0.158	1.116	8.925
ITGAX	1.021	2.029	5.152
ITGB1	0.171	1.126	4.326
ITGB2	0.274	1.209	3.499
ITGB2-AS1	0.128	1.093	2.286
ITGB3//RP11-290H9.2	0.922	1.894	3.493
ITGB3BP	0.029	1.020	3.845
ITGB4	-0.039	0.973	7.000
ITGB5	-0.210	0.865	5.476

ITGB8	-0.199	0.871	8.023
ITGBL1	0.800	1.741	5.073
ITIH2	0.528	1.442	3.819
ITM2A	-0.101	0.932	6.409
ITPKA	0.086	1.061	3.092
ITPKB	0.048	1.034	8.172
ITPKB-IT1	-0.302	0.811	3.889
ITPKC	0.567	1.481	5.435
ITPR1-AS1	0.041	1.029	2.171
ITPR2	0.136	1.099	7.405
ITPR3	0.581	1.496	3.995
ITPRIP	0.408	1.327	2.549
ITPRIPL1	0.017	1.012	2.622
ITPRIPL2	0.447	1.364	4.452
ITSN1	0.091	1.065	7.715
JADE3//PHF16	0.042	1.029	4.266
JAG2	0.129	1.094	3.765
JAK3	0.793	1.733	3.864
JAKMIP1	-0.218	0.860	5.237
JAM3	0.071	1.051	7.032
JAZF1	0.362	1.285	3.775
JAZF1-AS1	-0.045	0.969	2.283
JPH1	0.030	1.021	4.744
JPH2	0.486	1.401	2.413
JPH3	-0.032	0.978	5.704
JPH4	0.197	1.146	4.305
JPX	-0.070	0.952	5.444
JUNB	-0.191	0.876	7.887
JUP	-0.136	0.910	4.885
KAL1	0.641	1.559	7.716
KALRN	0.207	1.154	5.381
KANK1//RP11-31F19.1	-0.185	0.880	5.198
KANK2	-0.032	0.978	5.586
KANK3	-0.265	0.832	3.399
KANK4	0.477	1.392	3.384
KATNB1	0.038	1.027	5.460
KB-1107E3.1	0.035	1.025	1.838
KB-1507C5.3	0.332	1.259	4.163
KBTBD12	-0.093	0.938	1.965
KBTBD3	0.229	1.172	4.723
KBTBD6	0.012	1.008	5.594
KBTBD7	0.186	1.138	4.398
KBTBD8	0.341	1.267	3.057
KCNA1	-0.889	0.540	4.718
KCNA2	-0.056	0.962	6.412
KCNA3	0.240	1.181	4.812
KCNA4	0.779	1.716	4.238
KCNA5	0.108	1.078	3.524
KCNA6//GALNT8	-0.578	0.670	5.870
KCNAB1	0.261	1.198	4.524

KCNAB2	0.046	1.032	3.478
KCNAB3//RP11-1099M24.7	0.078	1.055	4.232
KCNB1	0.121	1.088	5.482
KCNB2	0.379	1.301	3.849
KCNC1	-0.053	0.964	4.040
KCNC2	0.199	1.148	5.182
KCNC4	-0.134	0.911	4.319
KCND1	0.374	1.296	3.827
KCND2	-0.072	0.952	6.977
KCND3	0.289	1.222	6.611
KCND3-IT1	-0.302	0.811	2.626
KCNE1	-0.502	0.706	2.615
KCNE4	0.339	1.265	4.446
KCNF1	-0.128	0.915	4.324
KCNG1	-0.422	0.747	2.652
KCNG3	-0.093	0.938	3.179
KCNH1	0.276	1.211	6.521
KCNH1-IT1	0.129	1.093	3.536
KCNH2	-0.063	0.958	3.501
KCNH3	-0.048	0.967	4.003
KCNH4	-0.113	0.925	2.456
KCNH5	0.437	1.354	4.370
KCNH7	0.368	1.291	4.738
KCNH8	0.433	1.350	5.883
KCNIP1	0.373	1.295	4.866
KCNIP2	-0.338	0.791	4.378
KCNIP3	0.022	1.015	5.630
KCNIP4	0.125	1.091	4.316
KCNIP4-IT1	0.211	1.157	4.637
KCNJ10	-0.103	0.931	8.237
KCNJ11	-0.396	0.760	3.512
KCNJ12//KCNJ18//LOC100996843	0.067	1.048	2.287
KCNJ16	-0.457	0.728	5.184
KCNJ2	0.021	1.014	6.282
KCNJ2-AS1	-0.063	0.957	4.394
KCNJ3	0.046	1.033	5.933
KCNJ4	-0.451	0.732	4.334
KCNJ5	0.342	1.268	3.064
KCNJ6	0.640	1.558	4.689
KCNJ8	0.261	1.198	3.138
KCNJ9	-0.041	0.972	6.229
KCNK1	-0.262	0.834	3.293
KCNK10	-0.127	0.916	2.987
KCNK13	-0.079	0.947	2.287
KCNK15	0.155	1.114	2.872
KCNK2	0.118	1.085	4.341
KCNK3	-0.302	0.811	4.952
KCNK4//RP11-783K16.10	-0.332	0.794	3.072
KCNK6	0.349	1.274	3.474
KCNK9	-0.208	0.866	3.525

KCNMA1	0.028	1.020	6.237
KCNMB1	0.063	1.045	3.742
KCNMB2	0.203	1.151	3.573
KCNMB4	0.178	1.132	6.624
KCNN1	-0.258	0.836	4.862
KCNN3	0.218	1.163	6.730
KCNN4	0.222	1.166	3.391
KCNQ1	-0.022	0.985	3.275
KCNQ1DN	-0.604	0.658	2.979
KCNQ1OT1//LOC101927338	-0.406	0.755	3.777
KCNQ2	0.237	1.179	3.943
KCNQ2	0.042	1.029	4.625
KCNQ3	0.367	1.290	6.285
KCNQ5	0.365	1.288	3.908
KCNQ5-IT1	0.068	1.048	2.794
KCNS1	-0.425	0.745	3.739
KCNS2	0.285	1.219	4.103
KCNT1	-0.345	0.787	4.789
KCNT2	0.457	1.372	4.646
KCNV1	-0.411	0.752	3.474
KCTD1	0.027	1.019	5.195
KCTD11	0.186	1.138	4.391
KCTD12	0.152	1.111	7.126
KCTD16	0.402	1.321	4.819
KCTD3	0.026	1.018	7.502
KCTD6	0.632	1.550	3.919
KDELC1	0.490	1.405	4.072
KDM5D	0.883	1.844	4.341
KDM6A	0.293	1.225	5.574
KDR	-0.027	0.981	5.029
KEL	-0.225	0.856	4.479
KGFLP1	-0.538	0.689	4.460
KGFLP2	-0.206	0.867	4.751
KHDRBS2	0.167	1.123	5.053
KHDRBS3	-0.030	0.980	5.771
KHNYN	0.196	1.146	4.734
KIAA0040	0.475	1.390	4.499
KIAA0226L	0.593	1.509	4.201
KIAA0247	0.435	1.352	5.082
KIAA0319	-0.098	0.934	4.835
KIAA0355	0.125	1.090	6.692
KIAA0430	0.043	1.030	7.672
KIAA0513	-0.114	0.924	7.451
KIAA0753	-0.196	0.873	5.899
KIAA0754//RP4-562N20.1	0.051	1.036	4.569
KIAA0825	-0.414	0.751	5.092
KIAA0895	0.046	1.032	3.777
KIAA0922	0.252	1.191	3.906
KIAA1024	-0.043	0.970	4.188
KIAA1045	-0.434	0.740	6.027

KIAA1107	0.106	1.076	7.794
KIAA1161	-0.246	0.843	5.850
KIAA1211L	0.101	1.072	3.892
KIAA1217	-0.465	0.724	4.834
KIAA1279	0.275	1.210	7.841
KIAA1324	0.276	1.211	5.023
KIAA1324L	-0.172	0.887	6.991
KIAA1377	-0.484	0.715	6.604
KIAA1407	-0.319	0.802	4.589
KIAA1456//LOC101927137	-0.101	0.933	4.119
KIAA1462	-0.136	0.910	5.189
KIAA1467	0.088	1.063	6.129
KIAA1468	0.198	1.147	6.618
KIAA1522	-0.444	0.735	2.645
KIAA1524	0.352	1.276	3.032
KIAA1549	-0.238	0.848	5.677
KIAA1549L	0.036	1.025	6.210
KIAA1586	0.274	1.210	6.262
KIAA1598	-0.058	0.961	8.123
KIAA1614-AS1//RP11-46A10.4	0.397	1.317	3.132
KIAA1644	0.253	1.192	4.102
KIAA1671	-0.434	0.740	4.566
KIAA1751//C1orf222	-0.586	0.666	2.017
KIAA1755	0.053	1.037	4.499
KIAA1804//RP5-862P8.2	-0.042	0.972	3.709
KIAA1958	-0.078	0.948	5.910
KIAA2022	0.250	1.189	5.108
KIF13B	-0.050	0.966	7.174
KIF17	0.106	1.076	2.982
KIF19	-0.046	0.969	3.978
KIF1C	-0.468	0.723	7.907
KIF21A	-0.174	0.886	7.703
KIF21B	0.048	1.034	5.306
KIF3A	-0.235	0.849	8.005
KIF3C	-0.204	0.868	6.963
KIF4A	-0.229	0.853	3.182
KIF4B	0.094	1.067	3.009
KIF5A	-0.286	0.820	9.486
KIF5B	-0.269	0.830	9.156
KIF5C	-0.230	0.853	8.877
KIF6	-0.146	0.904	4.076
KIFAP3	-0.078	0.948	8.457
KIFC2	0.223	1.167	3.758
KIR2DL1	-0.464	0.725	2.132
KIRREL3	0.136	1.099	5.388
KIT	0.088	1.063	6.274
KITLG	-0.210	0.865	5.558
KL	0.429	1.346	2.443
KLC1//XRCC3	-0.489	0.713	3.115
KLC2	-0.116	0.922	5.818

KLF10	0.043	1.031	5.343
KLF12	0.144	1.105	6.672
KLF15	-0.348	0.785	4.588
KLF2	-0.449	0.732	5.442
KLF3	0.184	1.136	5.820
KLF4	-0.223	0.857	3.068
KLF6	0.558	1.472	6.911
KLF7-IT1	0.042	1.030	5.382
KLF8	0.372	1.294	3.229
KLF9	0.044	1.031	6.939
KLHDC3	-0.045	0.969	7.564
KLHDC8A//LOC102724743	-0.103	0.931	3.479
KLHDC9	0.029	1.020	4.667
KLHL1	0.257	1.195	3.721
KLHL11	0.268	1.204	5.983
KLHL14	0.180	1.133	2.702
KLHL15	0.303	1.233	5.030
KLHL18	0.321	1.249	5.224
KLHL2	0.049	1.034	7.226
KLHL29	0.210	1.157	4.269
KLHL4	0.237	1.179	5.996
KLHL42	0.049	1.035	4.775
KLHL42//RP11-860B13.3	0.163	1.120	3.486
KLHL6	0.893	1.857	4.006
KLHL7-AS1	0.297	1.228	2.975
KLK5	-0.032	0.978	3.413
KLK6	-0.041	0.972	8.883
KLK7	-0.380	0.768	4.615
KLKB1	-0.237	0.848	2.877
KLRC3//RP11-277P12.6	0.423	1.341	2.696
KNDC1	-0.310	0.807	4.961
KNG1	-0.143	0.906	1.771
KNSTRN	0.079	1.056	4.091
KPNA2	0.539	1.453	6.769
KPNA5	0.219	1.164	6.065
KRAS	0.118	1.085	5.064
KRR1	0.094	1.068	5.924
KRT14	0.281	1.215	2.389
KRT16P2	0.240	1.181	3.980
KRT16P3//KRT16P1//KRT16P2	0.354	1.278	2.354
KRT18P54	0.446	1.363	3.446
KRT222//RP5-1028K7.3	-0.139	0.908	4.896
KRT81	0.295	1.227	2.072
KRT81//RP11-845M18.6	-0.591	0.664	3.703
KRTAP10-9	-0.299	0.813	3.275
KRTAP5-2	-0.028	0.980	3.243
KRTAP6-2	-0.418	0.748	2.640
KRTCAP2//RP11-201K10.3	-0.125	0.917	3.861
KSR2	-0.223	0.857	5.881
KYNU	0.949	1.930	2.767

L1CAM	-0.231	0.852	5.580
L2HGDH	0.279	1.214	3.303
L3MBTL4//CIAO1	-0.103	0.931	4.876
LA16c-23H5.4	0.344	1.269	2.220
LA16c-431H6.6	0.150	1.110	2.792
LACC1	0.417	1.335	5.722
LAIR1	0.703	1.627	4.158
LAMA1	0.476	1.391	4.264
LAMB1	0.200	1.149	4.415
LAMB2	-0.021	0.986	5.429
LAMB2P1//RP11-694I15.7	0.184	1.136	2.793
LAMC1	0.253	1.192	5.538
LAMP1	-0.015	0.990	7.884
LAMP2	0.161	1.118	8.590
LANCL2	0.053	1.038	6.409
LANCL3	0.055	1.039	2.988
LAP3	0.615	1.532	6.008
LAPTM5	0.837	1.786	9.023
LARGE	-0.194	0.874	5.351
LARP6	-0.083	0.944	4.498
LAT2	0.153	1.112	5.038
LATS2	0.119	1.086	4.901
LCA10//U52112.12	0.052	1.037	2.341
LCAT	0.508	1.422	5.464
LCE3A	-0.272	0.828	2.490
LCE3B	-0.475	0.719	3.219
LCLAT1	0.141	1.103	4.764
LCN12	0.188	1.139	3.791
LCP1	0.290	1.222	6.305
LCP2	0.791	1.730	6.426
LCTL	0.125	1.091	3.040
LDB2	0.092	1.066	4.738
LDB3	-0.056	0.962	4.483
LDHA	0.208	1.155	5.112
LDLR	0.192	1.143	6.014
LDLRAD2	-0.456	0.729	2.135
LDLRAD4-AS1	-0.559	0.679	2.255
LDLRAP1	-0.047	0.968	4.629
LDOC1	-0.168	0.890	6.605
LENG8	-0.133	0.912	6.995
LEPREL1	0.341	1.266	4.004
LETM2	-0.288	0.819	4.456
LFNG	-0.209	0.865	3.440
LGALS1	-0.032	0.978	6.000
LGALS3	0.543	1.457	3.244
LGALS3BP	0.072	1.051	6.671
LGALS9B	0.411	1.329	4.604
LGALS9C//LGALS9B	-0.138	0.908	3.078
LGALSL	0.509	1.423	6.576
LGI1	0.064	1.046	7.448

LGI2	0.273	1.208	5.049
LGMN	0.403	1.322	6.319
LGR4	0.128	1.093	6.248
LGR5	0.051	1.036	6.212
LGR6	-0.348	0.786	3.110
LHFPL1	-0.254	0.838	3.303
LHFPL2	0.218	1.163	3.918
LHFPL4	-0.260	0.835	4.243
LHFPL5//RP3-422H11.8	-0.090	0.939	3.282
LHPP	-0.031	0.979	6.146
LHX2	0.249	1.189	4.208
LHX3	-0.731	0.603	2.650
LHX4-AS1//RP5-1180C10.2	-0.228	0.854	3.101
LHX6	-0.150	0.901	3.026
LIAS	0.221	1.165	4.899
LIFR	0.019	1.013	7.413
LILRA1	0.257	1.195	2.849
LILRA2	0.164	1.121	2.828
LILRA4	0.151	1.110	3.369
LILRA6	0.981	1.974	2.414
LILRB3	0.966	1.953	2.949
LILRB4	0.486	1.400	3.636
LIMA1	0.222	1.166	4.773
LIME1//ZGPAT//RP4-583P15.15	-0.144	0.905	2.631
LIMK1	-0.153	0.900	5.690
LIMK2	0.443	1.359	5.862
LIMS2	-0.126	0.916	2.793
LIMS3-LOC440895//LIMS3//LIMS3L//AC108938.5//L	0.418	1.336	4.376
LIN7A	0.310	1.240	3.878
LINC00032	-0.096	0.936	3.436
LINC00086	0.553	1.467	4.576
LINC00087	-0.356	0.781	5.440
LINC00094	-0.115	0.924	5.369
LINC00173	-0.158	0.896	3.065
LINC00173	0.097	1.070	3.792
LINC00202-1	-0.350	0.785	3.863
LINC00271	-0.108	0.928	4.118
LINC00278	0.761	1.694	3.044
LINC00280	-0.481	0.716	2.244
LINC00282	-0.061	0.958	3.693
LINC00294	0.048	1.034	6.360
LINC00298//AC007464.1	-0.133	0.912	2.510
LINC00310	-0.353	0.783	2.737
LINC00315	-0.029	0.980	2.503
LINC00320	0.246	1.186	4.420
LINC00323	-0.318	0.802	2.065
LINC00326	0.045	1.031	1.603
LINC00328	-0.821	0.566	2.840
LINC00339	0.161	1.118	2.441
LINC00342	-0.150	0.901	3.209

LINC00390//RP11-478K15.1	0.025	1.017	2.182
LINC00391	-0.098	0.934	2.349
LINC00403	-0.181	0.882	4.416
LINC00440	-0.480	0.717	1.594
LINC00441	-0.083	0.944	2.236
LINC00443	0.031	1.022	1.857
LINC00467	0.300	1.231	3.562
LINC00471	-0.078	0.947	3.894
LINC00472//RP3-331H24.5	0.372	1.294	3.854
LINC00473	-0.142	0.906	2.407
LINC00476	-0.499	0.708	3.463
LINC00476//LOC101930476	-0.326	0.798	3.048
LINC00484	-0.293	0.816	2.479
LINC00499	-0.695	0.618	2.993
LINC00506	-0.070	0.953	2.819
LINC00507	-0.150	0.901	2.589
LINC00515//AP000223.42	0.322	1.250	3.148
LINC00539//LINC00422	-0.554	0.681	2.571
LINC00566	-0.339	0.791	3.229
LINC00577	-0.066	0.955	2.787
LINC00594	-0.173	0.887	4.491
LINC00599//MIR124-1	-0.295	0.815	4.204
LINC00599//MIR124-1	-0.198	0.871	3.237
LINC00601	0.455	1.371	4.729
LINC00609	-0.239	0.848	4.089
LINC00614	-0.303	0.811	2.790
LINC00621	0.088	1.063	2.698
LINC00622	-0.203	0.868	4.495
LINC00630//LOC102725166//LL0XNC01-237H1.2	-0.044	0.970	5.620
LINC00632	-0.286	0.820	5.780
LINC00633//LOC101928677	-0.832	0.562	2.253
LINC00639	-0.065	0.956	4.021
LINC00641	-0.041	0.972	7.123
LINC00643//RP11-355I22.6	-0.244	0.845	2.478
LINC00665	-0.071	0.952	3.214
LINC00672	0.151	1.111	4.821
LINC00675	1.222	2.332	2.006
LINC00685	0.401	1.320	2.609
LINC00693	0.096	1.069	4.601
LINC00836	0.162	1.119	3.778
LINC00839	-0.254	0.839	3.837
LINC00842	-0.214	0.862	3.546
LINC00862	0.267	1.204	2.365
LINC00877	-0.064	0.957	2.480
LINC00882	-0.047	0.968	4.146
LINC00883	0.302	1.233	4.810
LINC00884	0.015	1.011	3.634
LINC00886	-0.020	0.986	2.389
LINC00888	-0.376	0.770	7.559
LINC00888	0.122	1.088	4.108

LINC00889	0.312	1.242	3.597
LINC00893	0.127	1.092	3.161
LINC00899	-0.340	0.790	3.012
LINC00920	-0.761	0.590	2.696
LINC00936	0.021	1.015	3.725
LINC00950	0.601	1.517	4.773
LINC00951	0.154	1.113	2.303
LINC00957	0.118	1.085	5.094
LINC00961	0.464	1.379	4.546
LINC00963	0.024	1.017	4.058
LINC00969//SDHAP2	-0.627	0.648	5.271
LINC00982	0.061	1.043	3.719
LINC00984	0.509	1.423	5.626
LINC00987	0.104	1.075	4.799
LINC00997	-0.230	0.853	4.568
LINC01000	-0.264	0.833	4.884
LINC01000//LINC01061//LOC101060341	-0.055	0.963	7.091
LINC01000//LINC01061//LOC101060341	-0.052	0.965	6.906
LINC01000//LINC01061//LOC101060341	-0.037	0.975	6.888
LINC01000//LINC01061//LOC729737//LOC729218//l	-0.026	0.982	7.111
LINC01002	0.288	1.221	4.986
LINC01007//RP5-1059M17.1	0.045	1.031	2.848
LINC01018//CTD-2195M18.1	0.043	1.030	2.809
LINC01030//AB015752.4	-0.088	0.941	2.541
LINC01057//RP11-86H7.1	0.796	1.736	2.816
LINC01061	-0.359	0.780	4.680
LINC01063//AC023797.1	-0.105	0.930	3.742
LINC01088//RP11-438E5.1	-0.379	0.769	4.189
LINC01094//RP11-792D21.2	0.285	1.218	6.487
LINC01102//AC013402.2	0.321	1.249	4.218
LINC01105//LOC400940//AC073479.1	0.224	1.168	6.290
LINC01106//LINC01123	-0.087	0.942	4.706
LINC01122//AC007092.1	0.029	1.020	3.298
LINC01123//AC013268.5	0.239	1.180	5.304
LINC01125//AC017099.3	0.122	1.088	3.186
LINC01128//RP11-206L10.11	0.073	1.052	3.818
LINC01137//RP11-422J8.1	-0.101	0.932	2.964
LINC01140//RP5-1052I5.1	-0.060	0.959	2.712
LINC01144	-0.294	0.816	3.309
LINC01149//XXbac-BPG181B23.4	0.682	1.604	2.798
LINC01158//AC018730.1	-0.274	0.827	4.450
LINC01170//RP11-93O7.5	-0.120	0.920	2.588
LINC01197//CTD-2536I1.1	0.244	1.185	2.502
LINC01212//RP11-460N16.1	0.133	1.097	2.677
LINC01234//RP11-438N16.1	-0.263	0.833	3.413
LINC01250//AC019118.2	0.022	1.016	2.001
LINC01252//RP11-434C1.1	-0.098	0.934	4.040
LINC01272//RP11-290F20.3	0.545	1.459	2.425
LINC01277//RP11-439L18.3	0.164	1.120	2.097
LINC01285//RP4-562J12.2	-0.077	0.948	3.742

LINC01338	-0.678	0.625	4.363
LINC01351//RP11-547I7.2	0.310	1.239	2.939
LINC01355//RP5-1057J7.6	0.156	1.114	5.204
LINC01359//RP11-182I10.3	-0.132	0.912	2.546
LINC01377//CTD-2029E14.1	-1.084	0.472	2.729
LINC01422//RP1-90L6.2	0.044	1.031	3.823
LINC-PINT//MIR29A//MIR29B1//AC058791.1	-0.201	0.870	5.821
LINCR-0001//RP11-981G7.2	-0.330	0.795	5.203
LINCR-0002//RP11-197K6.1	0.083	1.059	1.785
LINCR-0003//CTD-2023N9.3	0.074	1.052	2.430
LINGO1	-0.041	0.972	3.826
LINGO2	0.187	1.138	4.702
LINGO4	-0.947	0.519	2.789
LIPA//LOC101926887	0.074	1.052	7.073
LIPE	0.145	1.106	5.907
LIPG	0.028	1.019	3.052
LITAF	0.511	1.425	6.708
LIX1	0.225	1.169	7.348
LL0XNC01-16G2.1	0.182	1.135	2.181
LLGL1	-0.031	0.979	6.823
LLGL2	-0.135	0.910	3.371
LMAN1L//CPLX3//RP11-414J4.2	0.280	1.215	2.471
LMBR1	-0.092	0.938	6.953
LMBRD2	0.286	1.219	7.331
LMLN	0.122	1.088	5.249
LMNA	0.079	1.057	4.603
LMNB1	0.862	1.817	4.148
LMO3	-0.110	0.927	4.901
LMO4	0.329	1.257	6.051
LMO7//RP11-29G8.3	0.043	1.030	4.315
LMOD1	-0.019	0.987	3.595
LMOD1//RP11-307B6.3	0.400	1.319	3.212
LMTK2	-0.127	0.916	6.585
LMX1B	0.368	1.291	2.772
LNP1	0.130	1.094	5.237
LNX1	-0.053	0.964	4.326
LOC100126784//RP11-359E10.1	0.190	1.141	3.740
LOC100127909	-0.415	0.750	2.147
LOC100127910//RP1-79C4.4	0.110	1.079	3.580
LOC100128253//RP5-1063M23.1	0.148	1.108	1.934
LOC100128288	0.118	1.086	3.901
LOC100128343	-0.178	0.884	2.302
LOC100128364	-0.691	0.620	2.342
LOC100128508	-0.022	0.985	4.562
LOC100128554//RP5-944M2.3	0.123	1.089	2.324
LOC100128563	-0.361	0.779	3.616
LOC100129027//AL592528.1	-0.311	0.806	3.315
LOC100129138//RP11-364B6.1	0.117	1.084	2.410
LOC100129223	-0.215	0.861	2.166
LOC100129233	0.300	1.231	2.425

LOC100129361	-0.096	0.936	5.229
LOC100129434//AC007743.1	0.146	1.107	4.330
LOC100129455	-0.160	0.895	2.195
LOC100129473	0.218	1.163	3.083
LOC100129518//SOD2	0.526	1.440	3.153
LOC100129534//RP4-740C4.6	-0.176	0.885	3.348
LOC100129540//RP11-16K12.1//RASGRF1	0.248	1.187	3.356
LOC100129550//RP11-67L2.2	0.067	1.047	4.706
LOC100129697//RP11-830F9.6	0.275	1.210	2.666
LOC100129831	-0.308	0.808	2.697
LOC100129936//CTA-373H7.7	0.204	1.152	3.362
LOC100129973//RP11-430B1.2	0.142	1.104	2.671
LOC100130027	-0.065	0.956	3.253
LOC100130169	0.394	1.314	3.155
LOC100130172//RP11-325L7.1	0.194	1.144	2.460
LOC100130193	-0.066	0.955	2.652
LOC100130285	-0.227	0.855	4.085
LOC100130298//AC022182.3	-0.073	0.951	2.098
LOC100130357//RP1-257A7.4	0.223	1.167	2.258
LOC100130370//RP11-1055B8.3	0.199	1.148	3.331
LOC100130691//AC074286.1//NFE2L2	0.158	1.116	4.311
LOC100130705//RP11-309L24.4	0.236	1.177	2.861
LOC100130876	-0.120	0.920	2.601
LOC100130987	-0.283	0.822	3.218
LOC100130992	0.017	1.012	3.474
LOC100131262	-0.087	0.941	3.501
LOC100131510	-0.158	0.896	2.624
LOC100131564//RP4-717I23.3	-0.126	0.916	4.916
LOC100131802	0.320	1.248	2.328
LOC100131826	0.295	1.227	3.363
LOC100132004//AC125634.1//ANKRD30BP2//ANKRI	0.126	1.091	2.816
LOC100132062//LOC100133331//LINC01001//LOC1(	0.101	1.073	7.928
LOC100132111//AL450992.2	0.067	1.047	2.895
LOC100132215//AC009501.4	0.126	1.091	2.187
LOC100132352	0.014	1.009	4.608
LOC100132731//RP11-723O4.6//KIAA1257	-0.124	0.918	2.872
LOC100132741//LINC00891	0.203	1.151	3.896
LOC100132891//RP11-383H13.1	-0.017	0.988	1.932
LOC100133299	0.605	1.521	3.698
LOC100133331//LOC101929819//LINC01001	-0.209	0.865	5.744
LOC100133445//RP3-395M20.8	0.353	1.277	2.300
LOC100133669//RP11-273G15.2	0.025	1.017	3.590
LOC100134868	-0.347	0.786	4.939
LOC100190986//LOC102725125	-0.216	0.861	8.167
LOC100190986//LOC102725125	-0.190	0.877	8.129
LOC100233156	0.071	1.050	4.757
LOC100270746	-0.112	0.925	4.037
LOC100272216	0.369	1.291	6.269
LOC100272216	0.304	1.234	5.201
LOC100272216	0.189	1.140	5.388

LOC100272216	0.151	1.110	5.405
LOC100272216	-0.124	0.918	4.758
LOC100286906//AC008060.7	0.542	1.456	4.336
LOC100286925//LL22NC03-86G7.1	0.263	1.200	2.590
LOC100287225//RP11-267C16.1	0.191	1.141	3.950
LOC100287497//LOC100287934//LOC101930657//R	-0.020	0.986	4.954
LOC100287704	-0.101	0.933	4.055
LOC100287834	-0.431	0.742	9.111
LOC100287846//LOC286063//RP11-1134I14.8	0.504	1.419	4.302
LOC100287934//LOC101930657//RP11-206L10.9	-0.134	0.911	3.963
LOC100288160	-0.376	0.770	3.305
LOC100288181//CTD-2292P10.4	0.093	1.066	4.561
LOC100288310//RP11-531A24.3	0.482	1.397	2.765
LOC100288842//RP11-27I1.4	0.347	1.272	3.292
LOC100288911//RP11-490M8.1	0.122	1.088	3.607
LOC100289455	0.335	1.261	3.880
LOC100289495	0.133	1.097	3.332
LOC100294341//LOC101929774//RP11-927P21.1//R	-0.399	0.758	3.587
LOC100294362//CTD-2047H16.4	-0.417	0.749	3.489
LOC100379224//AC084219.4	0.158	1.116	4.751
LOC100505501//RP11-25K19.1	0.159	1.117	2.610
LOC100505502	-0.530	0.692	2.426
LOC100505549//ATP8B1	-0.032	0.978	2.626
LOC100505564//RP11-876N24.4	0.415	1.333	2.244
LOC100505570//AP006285.6	0.243	1.183	2.399
LOC100505835//CTD-2081K17.2	0.628	1.546	4.179
LOC100505912	-0.328	0.797	2.682
LOC100505942	-0.405	0.755	4.020
LOC100506022	0.076	1.054	2.761
LOC100506023	0.040	1.028	3.881
LOC100506036//RP11-353K11.1	0.153	1.112	2.283
LOC100506083//RP11-529K1.2	-0.464	0.725	2.614
LOC100506124//TTC21B	0.036	1.025	2.771
LOC100506127//RP11-111M22.2	-0.081	0.945	3.502
LOC100506136//RP11-682N22.1	-0.265	0.832	2.540
LOC100506207//RP11-314C16.1	0.097	1.069	3.785
LOC100506217//RP11-823P9.3	0.303	1.234	2.851
LOC100506258//CTD-2516F10.2	0.331	1.258	3.670
LOC100506276	-0.166	0.891	3.627
LOC100506299//AC011747.7	-0.362	0.778	3.622
LOC100506384//RP4-719C8.1	-0.479	0.718	4.036
LOC100506411//RP6-65G23.3	0.050	1.035	3.034
LOC100506458	-0.133	0.912	2.996
LOC100506497//CTB-113D17.1	-0.027	0.981	2.536
LOC100506504//RP11-181G12.2	-0.291	0.817	2.590
LOC100506514	-0.149	0.902	2.807
LOC100506544//RP4-756G23.5	0.193	1.143	3.511
LOC100506563	0.267	1.203	2.189
LOC100506596	-1.233	0.425	2.289
LOC100506674//RP11-53O19.1	-0.119	0.921	4.746

LOC100506713//AC027763.2	-0.124	0.918	5.286
LOC100506725//AC018647.3	0.090	1.065	3.000
LOC100506731//RP11-497E19.1	0.260	1.198	2.932
LOC100506851//RP1-232L24.3	-0.016	0.989	5.681
LOC100506885//RP4-625H18.2	-0.119	0.921	2.590
LOC100507002	0.061	1.043	3.260
LOC100507006//AC074289.1	0.037	1.026	4.309
LOC100507054//RP11-1398P2.1	0.205	1.153	2.246
LOC100507140//AC007163.3	0.337	1.263	3.071
LOC100507144//RP4-607I7.1	0.229	1.172	2.335
LOC100507194//RP1-40E16.9	0.864	1.820	3.303
LOC100507316//RP13-582O9.5	0.013	1.009	3.402
LOC100507336//RP1-223B1.1	-0.236	0.849	4.245
LOC100507351//RP11-13K12.1	0.290	1.223	3.542
LOC100507395	-0.235	0.849	3.672
LOC100507460	0.412	1.330	3.089
LOC100507494	-0.066	0.955	2.639
LOC100507507//AC093673.5	0.589	1.504	4.264
LOC100507516//RP11-400K9.4	0.322	1.250	3.352
LOC100507520//RP11-1055B8.4	-0.247	0.842	3.669
LOC100507530//CTD-2541M15.3	-0.058	0.961	4.074
LOC100507534//AC140542.2	-0.192	0.875	1.956
LOC100507535//CTD-2521M24.9//MVB12A	0.382	1.303	3.370
LOC100507554//AC098820.3	-0.215	0.862	3.012
LOC100507564//RP5-1024G6.5	0.052	1.036	2.857
LOC100507577//LONP2	0.030	1.021	5.749
LOC100507599//CTA-445C9.14	-0.216	0.861	4.503
LOC100508046//POTEH-AS1//LOC101929572	-0.685	0.622	2.280
LOC100508227	0.300	1.231	4.425
LOC100508782//PPIP5K1	-0.067	0.955	4.238
LOC100630918	0.268	1.204	3.639
LOC100652758	-0.499	0.708	2.576
LOC100652768	0.047	1.033	3.187
LOC100652824//AC079354.1	-0.708	0.612	3.064
LOC100652901	0.219	1.164	2.433
LOC100652999//RP11-793H13.3	0.194	1.144	4.320
LOC100653225//OVOS2//LOC100509445//LOC7287	0.103	1.074	2.662
LOC100653515//CTD-2373H9.6	0.207	1.154	3.425
LOC100653515//CTD-2373H9.6	0.231	1.174	3.564
LOC100996348//RP11-317J10.2	0.085	1.061	2.791
LOC100996385//RP11-826N14.2//RP11-1026M7.2//	-0.141	0.907	4.782
LOC100996385//RP11-826N14.2//RP11-1026M7.2//	0.079	1.057	4.260
LOC100996437//RP4-756H11.3	-0.377	0.770	4.038
LOC100996557//RP11-535M15.1	0.558	1.473	4.861
LOC100996664//CTD-2325P2.4	-0.673	0.627	4.622
LOC101060341//LINC01347//LOC101060183//LINC0	-0.049	0.966	7.974
LOC101060602//AC025627.7	0.290	1.223	2.375
LOC101060691	-0.513	0.701	4.607
LOC101926898//RP11-795J1.1	0.031	1.022	2.415
LOC101926908//RP11-313C15.1	0.588	1.503	2.589

LOC101926964//RP11-436K8.1	0.198	1.147	4.134
LOC101926977//RP11-267M23.4	-0.352	0.783	2.707
LOC101926996//AC003102.3	0.075	1.053	2.035
LOC101927040//RP11-26J3.1	0.148	1.108	2.738
LOC101927043	0.208	1.155	3.108
LOC101927055//RP11-88L24.4	0.505	1.420	3.774
LOC101927057//OMG	-0.232	0.851	3.146
LOC101927063//RP11-310E22.4	-0.340	0.790	3.553
LOC101927085//RP11-363E6.3	-0.024	0.983	4.146
LOC101927184//LOC100507487//RP11-420A23.1	0.027	1.019	3.462
LOC101927226//AP000442.1	0.218	1.163	2.173
LOC101927248	-0.369	0.774	3.655
LOC101927270//LOC100996442//LOC100134822//L	0.027	1.019	6.450
LOC101927286//CTD-2147F2.2	-0.269	0.830	2.517
LOC101927286//CTD-2147F2.2	0.039	1.027	1.949
LOC101927314//RP1-104O17.1//RP1-104O17.2	0.020	1.014	3.462
LOC101927326//LOC100130157//RP3-461P17.10	0.510	1.424	3.529
LOC101927359//RP11-9G1.3	0.121	1.087	4.585
LOC101927397	0.121	1.088	3.102
LOC101927406//AC018799.1	-0.129	0.915	1.594
LOC101927468	0.151	1.111	5.922
LOC101927473	-0.332	0.794	2.809
LOC101927507//ANO7P1	0.505	1.420	2.457
LOC101927531//RP4-809F18.1	-0.241	0.846	1.920
LOC101927532//RP11-552D4.1	0.255	1.193	4.019
LOC101927533//FLJ16124//AC074391.1	-0.253	0.839	2.951
LOC101927621//AC074138.3	0.090	1.064	4.623
LOC101927636//RP11-673E1.1	0.031	1.022	2.987
LOC101927646	1.078	2.111	3.882
LOC101927667//AC012309.5	-0.226	0.855	3.021
LOC101927692//RP11-106M7.1	1.219	2.328	5.327
LOC101927728//RP11-567L7.6	-0.191	0.876	2.441
LOC101927773	0.141	1.103	2.976
LOC101927789	-0.294	0.815	2.788
LOC101927871	-0.254	0.839	1.796
LOC101927885//CTB-78F1.2	-0.147	0.903	1.928
LOC101927895//RP1-43E13.2//LOC284513	0.384	1.305	3.614
LOC101927902//RP11-325F22.2	0.431	1.348	2.656
LOC101927993//RP11-274J16.5	-0.176	0.885	3.098
LOC101928020//AC007038.7	-0.340	0.790	3.803
LOC101928054//RP11-705C15.3	0.886	1.847	3.138
LOC101928058//RP11-439C15.4	-0.136	0.910	4.952
LOC101928062//RP11-320P7.1	-0.086	0.942	2.262
LOC101928067//RP1-156L9.1	0.425	1.343	1.798
LOC101928100//RP11-277P12.20	0.191	1.142	2.299
LOC101928102//LOC102723678//LOC102723709	0.084	1.060	2.503
LOC101928102//RP4-610C12.3	0.081	1.058	2.375
LOC101928111//AC062017.1	-0.274	0.827	3.006
LOC101928161//AC010890.1	0.295	1.227	2.525
LOC101928174//RP11-814P5.1	-0.306	0.809	1.716

LOC101928188//RP11-426C22.4	0.029	1.021	2.333
LOC101928246	0.222	1.166	2.968
LOC101928258	-0.201	0.870	2.061
LOC101928279	-0.310	0.807	2.471
LOC101928290//RP11-695J4.2	0.119	1.086	2.713
LOC101928300//RP11-728G15.1	-0.283	0.822	2.727
LOC101928304//RP11-557H15.4	0.296	1.227	3.348
LOC101928335//RP6-191P20.4	0.064	1.046	2.048
LOC101928370//RP4-575N6.4	-0.124	0.918	3.447
LOC101928387	-0.122	0.919	2.935
LOC101928424//RP11-315O6.1	-0.169	0.889	3.224
LOC101928433//RP1-151F17.1	-0.046	0.969	3.765
LOC101928436//RP11-153F1.1	-0.524	0.696	2.665
LOC101928470//RP11-490D19.6	-0.229	0.853	3.226
LOC101928483//RP11-611D20.2//LOC286254	0.058	1.041	2.646
LOC101928489//RP11-553A21.3	0.063	1.045	3.321
LOC101928545//AC007551.3	-0.065	0.956	4.224
LOC101928564	-1.143	0.453	2.325
LOC101928576//AP001055.6	0.688	1.611	3.051
LOC101928583//RP11-373E16.3	0.118	1.085	2.245
LOC101928633	-0.178	0.884	2.245
LOC101928670//FAM157A	0.612	1.528	4.598
LOC101928670//RP11-206L10.3//RP11-34P13.13//L	0.149	1.109	5.961
LOC101928670//RP11-206L10.3//RP11-34P13.13//L	0.061	1.044	5.953
LOC101928706//LOC101929823//LINC01000//RP4-6	0.074	1.053	7.219
LOC101928706//LOC101929823//RP5-857K21.4//RP	0.634	1.552	4.500
LOC101928737//RP11-482M8.1	-0.034	0.976	2.302
LOC101928738//RP11-353N14.4	-0.154	0.899	3.032
LOC101928762//STX4	0.215	1.161	3.231
LOC101928792	-0.469	0.723	3.907
LOC101928794//MAST4-AS1	-0.260	0.835	2.217
LOC101928817//RP11-356K23.1	-0.520	0.697	2.031
LOC101928829//RP11-452L6.7	-0.145	0.904	4.028
LOC101928832	-0.055	0.963	4.174
LOC101928913//RP11-222A11.1	-0.363	0.778	2.346
LOC101928913//RP11-222A11.1	0.219	1.164	4.564
LOC101928979	0.190	1.141	3.304
LOC101928989//RP11-179A16.1	-0.623	0.649	3.182
LOC101929008//LOC101929038//LOC101930130//L	0.507	1.421	7.479
LOC101929038//LOC101926894//LINC01000//LINC0	0.184	1.136	7.667
LOC101929054	-0.189	0.877	2.943
LOC101929066//CTD-2547L16.1	0.157	1.115	3.305
LOC101929108//RP11-259O2.1	-0.145	0.904	2.464
LOC101929118//RP11-10L7.1	1.042	2.059	2.652
LOC101929132	0.204	1.152	2.887
LOC101929179//RP11-143M1.3	-0.026	0.982	2.078
LOC101929237//RP11-87E22.2	-0.174	0.886	2.074
LOC101929289	-0.448	0.733	3.150
LOC101929302//RP11-509J21.3	0.160	1.117	4.751
LOC101929307	0.395	1.315	2.269

LOC101929331//RP11-344B5.2	0.350	1.274	2.987
LOC101929514	-0.109	0.927	2.106
LOC101929515//LINC00951//RP11-552E20.4	0.147	1.107	2.337
LOC101929544	-0.133	0.912	2.164
LOC101929587//LOC101927777//LOC101927805//A	0.149	1.109	3.532
LOC101929595//RP11-286E11.1	0.188	1.139	2.644
LOC101929625	-0.264	0.833	2.424
LOC101929634//RP11-982M15.8	-0.407	0.754	4.287
LOC101929643	0.089	1.063	3.236
LOC101929668//LOC100507419//RP11-473M20.7	-0.409	0.753	2.674
LOC101929679	-0.502	0.706	2.463
LOC101929761//RP11-195F19.9//LOC730098	0.469	1.384	4.479
LOC101929777//WNT3	-0.153	0.900	4.874
LOC101929787	-0.549	0.683	3.942
LOC101929920//LOC101930237//AB015752.3	0.242	1.183	2.147
LOC101929949//RP11-982M15.6	0.340	1.266	3.031
LOC101929964//LINC01184//CTC-228N24.3	0.141	1.102	3.320
LOC101930100//LOC101928669//LOC101927648//L	-0.028	0.981	6.777
LOC101930275//RP11-806O11.1	0.091	1.065	3.918
LOC101930276	0.330	1.257	3.788
LOC101930277//RP11-166O4.1	0.260	1.198	4.016
LOC101930305//RP11-341G23.4	-0.411	0.752	3.381
LOC101930415//RP11-732M18.3//TULP4	-0.122	0.919	8.127
LOC101930421//RP11-809C18.3	-0.139	0.908	5.280
LOC101930532//LOC101930156//RP11-195C7.1	-0.386	0.765	4.213
LOC101930595//RP11-326C3.2//ATHL1	-0.344	0.788	2.748
LOC101930599//LENG8-AS1	0.169	1.125	4.116
LOC101930660//RP11-380O24.1	0.379	1.300	2.461
LOC101930660//RP11-380O24.1	-0.225	0.856	3.933
LOC101930660//RP11-380O24.1	-0.024	0.984	4.606
LOC102546299//CTC-340A15.2	0.058	1.041	2.003
LOC102577424//RP11-375B1.3	-0.059	0.960	4.305
LOC102723350//RP11-262I2.2	0.402	1.322	2.294
LOC102723392	-0.053	0.964	2.001
LOC102723403	0.190	1.141	2.429
LOC102723466//RP11-441F2.2	-0.033	0.977	3.520
LOC102723493//RP11-798K3.2	0.586	1.501	2.515
LOC102723521//RP11-513I15.6	-0.040	0.973	4.403
LOC102723544//RP11-283I3.4	-0.914	0.531	2.562
LOC102723619//RP11-686G8.2	-0.023	0.984	3.213
LOC102723630//LOC100505874	1.036	2.050	5.201
LOC102723676	0.204	1.152	3.108
LOC102723678//LOC102723709//RP11-374M1.5//L(	-0.027	0.981	3.428
LOC102723724//RP3-460G2.2	-0.065	0.956	3.029
LOC102723736//RP1-153P14.5	0.207	1.154	2.654
LOC102723779//RP11-326I11.3	-0.284	0.821	3.911
LOC102723817//RP11-100E13.1	-0.550	0.683	2.410
LOC102723828//RP11-734I18.1	-0.128	0.915	2.182
LOC102723854//AC016735.2	-0.045	0.970	2.362
LOC102723894//AP003774.6	0.166	1.122	2.880

LOC102723895//RP11-886D15.1	-0.060	0.959	1.502
LOC102723899//CDK14//CLDN12//AC006153.3//CTE	0.068	1.048	4.468
LOC102723927//AC093642.3	-0.120	0.920	2.525
LOC102724094//RP11-395B7.4	-0.163	0.893	2.507
LOC102724129	0.396	1.316	2.508
LOC102724155	0.121	1.087	2.805
LOC102724156	-0.641	0.641	3.729
LOC102724312	0.181	1.134	2.242
LOC102724314//FAM95B1//LOC101928261	-0.091	0.939	4.194
LOC102724351	0.186	1.137	2.254
LOC102724425	0.438	1.355	3.349
LOC102724530	-0.314	0.804	2.500
LOC102724532//LOC100506325//RP11-6N17.4	-0.035	0.976	4.484
LOC102724596//RP11-1079K10.2	-0.359	0.780	2.784
LOC102724596//RP11-1079K10.3	-0.240	0.847	2.465
LOC102724640	-0.129	0.914	2.824
LOC102724646	0.346	1.271	2.430
LOC102724679//AP000343.2	0.289	1.222	4.454
LOC102724687	0.224	1.168	2.628
LOC102724731	0.385	1.306	3.273
LOC102724768	-0.248	0.842	3.743
LOC102724814//RP11-66N24.3	0.336	1.262	2.810
LOC102724834	0.281	1.215	2.773
LOC102724842//RP11-597D13.8	-0.271	0.829	2.500
LOC102724883	0.681	1.603	4.148
LOC102724970//LOC100133616	-0.017	0.988	2.759
LOC102725086//AC090952.5	-0.042	0.971	2.270
LOC102725236//BAGE2//RP11-763B22.9//AC02761Z	0.038	1.026	4.596
LOC102725258	0.326	1.253	2.856
LOC102725378	0.460	1.376	2.370
LOC102725394	0.380	1.301	5.251
LOC102725417	-0.546	0.685	4.704
LOC102725458	0.291	1.224	2.970
LOC103344931//RP11-57H14.4	-0.136	0.910	5.268
LOC145474	0.611	1.527	3.139
LOC150776//AC093838.4	0.293	1.225	5.316
LOC151760//RP11-553A10.1	-0.195	0.874	3.484
LOC153684//AC025171.1	0.432	1.349	2.677
LOC154761//RP11-61L23.2	0.541	1.455	4.012
LOC166994	0.528	1.442	3.319
LOC220729//AC024560.3	0.221	1.166	7.262
LOC257396//CTD-2366F13.1	0.186	1.138	3.716
LOC283045	0.134	1.097	2.839
LOC283335//RP11-983P16.4	0.028	1.020	4.620
LOC283588//RPS6KA5	0.124	1.090	4.278
LOC283692//RP11-752G15.3	0.122	1.088	2.861
LOC283693//RP11-752G15.9	-0.020	0.986	2.395
LOC283731//RP11-247C2.2	-0.253	0.839	2.138
LOC284023//AC025335.1	0.017	1.012	3.824
LOC284395//CTC-525D6.1	0.223	1.167	2.727

LOC284412	-0.111	0.926	4.086
LOC284578	0.233	1.175	3.631
LOC284648//RP11-317P15.4	0.691	1.615	2.089
LOC284926	-0.277	0.825	2.606
LOC284930//RP11-191L9.4	-0.078	0.948	2.238
LOC285484//RP11-586D19.1	0.148	1.108	2.840
LOC285696//AC091878.1	0.368	1.290	3.592
LOC285857	-0.527	0.694	2.260
LOC286367	-0.175	0.886	3.743
LOC286437	-0.078	0.947	4.559
LOC374443//RP11-705C15.2//RP11-705C15.5	0.581	1.496	3.705
LOC374890//AC011516.2	0.196	1.145	2.324
LOC388572//LOC101060494//LOC101926894//LOC1	0.317	1.246	5.018
LOC388849//AC006547.14	0.384	1.305	3.218
LOC389607	0.170	1.125	3.015
LOC389705	0.234	1.176	3.569
LOC389831//LOC102724951	-0.165	0.892	4.708
LOC389834	-0.100	0.933	2.874
LOC390705//RP11-17M15.2	-0.373	0.772	2.886
LOC390937//CTD-2575K13.6	-0.252	0.840	2.119
LOC399491//RP11-958N24.2	-0.129	0.914	3.705
LOC399715//RP11-563J2.2	0.235	1.177	3.719
LOC399900	-0.029	0.980	3.185
LOC400043//RP11-834C11.4	0.324	1.252	3.741
LOC400553//RP11-863P13.4	-0.087	0.941	2.714
LOC400558//RP11-46C24.3	-0.509	0.703	2.598
LOC400622//RP11-13K12.2	-0.236	0.849	2.131
LOC400706	0.682	1.605	4.019
LOC400743//RP11-380J14.1	-0.376	0.771	2.370
LOC400927//RP3-449O17.1	-0.589	0.665	5.539
LOC400940//AC073479.1	-0.465	0.724	3.125
LOC401134//RP11-707A18.1	0.179	1.132	3.770
LOC401320//LOC101928623//AC005154.6	0.124	1.090	5.157
LOC401357	-0.383	0.767	2.436
LOC401357	-0.291	0.817	2.405
LOC407835//RP11-286H14.4	-0.267	0.831	2.834
LOC414300//RP11-61E11.2	-0.023	0.984	2.465
LOC440040//RP11-707M1.1	0.244	1.184	2.477
LOC440149	-0.163	0.893	4.085
LOC440300	0.358	1.282	2.177
LOC440896	-0.063	0.957	2.404
LOC441081//LOC101929573//LOC101060368//LOC1	0.182	1.134	6.943
LOC441081//LOC101929573//LOC101060368//LOC1	0.071	1.051	6.405
LOC441081//LOC102725466//LOC166994	-0.070	0.953	3.858
LOC441204	-0.253	0.839	3.710
LOC441268//RP11-481A20.11	-0.346	0.787	7.343
LOC613037//LOC101929910//RP11-347C12.1//NPIP	-0.205	0.868	9.656
LOC642236//RP11-764K9.4	-0.165	0.892	2.647
LOC642361//LOC439994//RP11-182L21.6	0.140	1.102	3.213
LOC642776//RP11-761E20.1	-0.034	0.977	3.706

LOC642846//RP11-22B23.1	0.126	1.091	2.318
LOC642852	-0.431	0.742	4.149
LOC642934	-0.206	0.867	3.193
LOC643355//RP11-88H9.2	0.201	1.149	2.376
LOC644172//RP11-259G18.2//RP11-707O23.5	0.547	1.461	5.208
LOC644450	0.210	1.157	4.773
LOC644669//AP005901.1	0.217	1.162	2.174
LOC644794//RP11-458F8.4	-0.156	0.897	4.335
LOC645321//RP11-849I19.1	-0.350	0.784	5.155
LOC645513//RP11-33B1.1	0.431	1.349	5.661
LOC645513//RP11-33B1.1	0.265	1.201	5.152
LOC645752//RP11-114H24.2	0.749	1.680	2.711
LOC646214	-0.590	0.665	2.413
LOC646241//RP11-454P21.1	0.142	1.104	2.069
LOC646719	-0.209	0.865	5.216
LOC646778//RP11-549B18.1	0.219	1.164	2.647
LOC647264//LOC101927016	0.194	1.144	4.157
LOC647859//RP11-195E2.1	-2.029	0.245	4.286
LOC650226//RP13-492C18.2	-0.806	0.572	3.343
LOC651337	0.294	1.226	2.735
LOC652276//AC141586.5	0.112	1.081	4.982
LOC653513	-0.210	0.864	5.918
LOC654342	-0.109	0.927	5.818
LOC727896	0.079	1.056	8.585
LOC728084//RP11-13A1.1	0.072	1.051	1.814
LOC728392//NLRP1	0.081	1.058	9.756
LOC728485//CTD-2162K18.4	-0.028	0.981	3.200
LOC728554//RP11-423H2.1	-0.401	0.757	5.776
LOC728613//RP11-43F13.1	0.169	1.125	4.124
LOC728730//AC007246.3	-0.175	0.886	3.032
LOC728739//RP11-554D14.2	0.232	1.175	2.287
LOC728752//CTD-2630F21.1	0.084	1.060	3.773
LOC729305//RP11-469N6.1	-0.067	0.954	2.282
LOC729658	-0.166	0.891	3.209
LOC729737	0.198	1.147	5.825
LOC729739//MIR7975//CTD-2311M21.2	-0.342	0.789	2.484
LOC729770//CTD-2308L22.1	-0.089	0.940	2.372
LOC729870	-0.256	0.838	2.188
LOC729911//RP11-17L5.4	0.226	1.170	2.935
LOC729970//RP4-639F20.1	0.516	1.430	4.255
LOC730081//RP5-884G6.2	0.199	1.148	2.925
LOC730183//RP11-146F11.1	-0.317	0.803	3.158
LOC81691//AC004381.6	0.184	1.136	2.903
LOC90784//POLR1A	-0.377	0.770	4.347
LOC93432//RP11-1220K2.2	0.354	1.278	2.528
LOC93432//RP11-1220K2.2	-0.155	0.898	3.389
LOC93432//RP11-1220K2.2	-0.185	0.880	2.168
LOC93622//AC093323.3	0.055	1.039	4.028
LONRF1	0.375	1.297	5.461
LOX	-0.103	0.931	2.609

LOXL1	0.250	1.189	3.014
LOXL2	0.700	1.625	4.520
LOXL3	0.123	1.089	3.901
LPAR1	-0.166	0.891	8.463
LPAR3	-0.151	0.901	2.292
LPAR4	0.292	1.224	3.623
LPAR5	0.508	1.422	3.554
LPAR6	0.580	1.495	2.723
LPCAT2	0.309	1.239	7.050
LPCAT3	0.436	1.353	5.758
LPCAT4	0.045	1.031	6.008
LPEQ6126	-0.033	0.978	3.066
LPGAT1	0.054	1.038	6.975
LPHN1	-0.229	0.853	7.190
LPHN2//LOC101927458	-0.099	0.934	5.328
LPHN3	-0.141	0.907	7.098
LPL	0.456	1.372	3.490
LPP	0.235	1.177	3.569
LPP-AS2	0.191	1.142	4.275
LPPR1	0.085	1.060	4.981
LPPR4//RP4-788L13.1	0.235	1.177	5.972
LPPR5	0.302	1.233	4.004
LPXN	0.044	1.031	5.112
LRAT//LOC101927965	0.390	1.310	2.665
LRCH4	0.294	1.226	3.254
LRFN1	0.146	1.106	3.481
LRFN2	-0.310	0.807	3.530
LRG1	0.173	1.127	3.555
LRIG3	0.250	1.189	3.932
LRP10	0.077	1.055	4.895
LRP11	0.242	1.183	4.778
LRP12	0.054	1.038	5.433
LRP1B	-0.116	0.923	6.929
LRP2	-0.056	0.962	6.489
LRP2BP	-0.731	0.602	5.169
LRP3	0.345	1.270	4.638
LRP5	-0.149	0.902	4.536
LRP8	-0.118	0.922	5.168
LRRC10B	0.142	1.103	3.100
LRRC16A	0.192	1.142	6.552
LRRC2	-0.289	0.818	3.435
LRRC24	-0.246	0.843	3.190
LRRC25	0.468	1.383	2.599
LRRC32	0.632	1.550	4.315
LRRC37A2	-0.143	0.906	6.276
LRRC37A3	-0.548	0.684	4.775
LRRC37A6P	0.465	1.380	3.611
LRRC37B//LOC101929841	-0.070	0.953	4.725
LRRC38	0.041	1.029	2.390
LRRC39	0.310	1.240	3.870

LRRC3B	0.337	1.264	3.594
LRRC4	-0.226	0.855	4.420
LRRC40	0.230	1.173	5.444
LRRC46	-0.970	0.510	2.088
LRRC48	-0.972	0.510	3.259
LRRC49	-0.122	0.919	3.997
LRRC4C	0.297	1.228	6.515
LRRC53	0.313	1.242	1.899
LRRC55	0.871	1.829	4.774
LRRC6	-0.352	0.783	4.843
LRRC63	-0.092	0.938	4.263
LRRC7	0.278	1.212	1.758
LRRC7	0.093	1.066	5.759
LRRC71	-0.913	0.531	2.409
LRRC73	-0.376	0.770	3.700
LRRC8B	0.335	1.262	6.388
LRRC9	-1.163	0.447	2.082
LRRCC1	0.116	1.084	4.855
LRRFIP1	0.094	1.067	5.274
LRRK1	0.251	1.190	4.174
LRRK2	0.441	1.358	5.969
LRRN1	0.018	1.013	4.897
LRRN2	0.351	1.275	4.390
LRRN3	0.209	1.156	4.482
LRRTM1	0.383	1.304	4.679
LRRTM2	0.160	1.117	7.272
LRRTM3//RP11-68P3.2	0.186	1.137	6.297
LRRTM4	0.069	1.049	5.519
LRTM2	-0.055	0.963	3.419
LSM11	0.504	1.419	4.017
LSM7	-0.070	0.953	4.340
LSMEM2	-0.069	0.953	3.117
LSP1	-0.165	0.892	2.931
LSR	-0.400	0.758	4.138
LSS	-0.151	0.901	6.877
LST1	0.822	1.768	3.478
LST1	0.720	1.648	3.637
LST1	0.730	1.658	3.527
LST1	0.701	1.626	3.633
LST1	0.673	1.594	3.855
LST1	0.691	1.614	3.564
LTB4R	0.552	1.466	2.412
LTBP1	0.100	1.071	5.644
LTBP2	0.439	1.356	3.990
LTBP3	-0.233	0.851	5.913
LTBP4	0.214	1.160	4.724
LTBR	0.333	1.260	6.085
LTF	-0.457	0.728	4.217
LTN1	0.216	1.161	6.147
LUM	0.945	1.925	3.045

LURAP1L	1.023	2.032	5.127
LUZP2	0.064	1.045	7.175
LY6E	-0.125	0.917	3.666
LY6H	-0.039	0.974	3.479
LY75-CD302//LY75//CD302	0.608	1.524	4.657
LY86	0.960	1.946	6.828
LY86-AS1	-0.046	0.968	2.368
LYAR	0.265	1.202	3.085
LYL1	0.416	1.335	3.739
LYN	0.698	1.623	5.840
LYNX1	-0.179	0.884	4.015
LYPD1	-0.645	0.639	4.312
LYPD5	0.269	1.205	5.475
LYPD8//XX-CR54.3	0.277	1.212	1.792
LYSMD1	0.276	1.211	4.686
LYSMD2	-0.333	0.794	4.160
LYSMD4	-0.015	0.989	3.697
LYST	0.548	1.462	3.261
LYVE1	0.479	1.394	6.937
LYZ	0.848	1.799	4.831
LZTFL1	-0.193	0.875	3.530
LZTS1	0.783	1.721	4.032
LZTS2	-0.019	0.987	4.566
MAATS1	-0.716	0.609	4.015
MAB21L1//MIR548F5	0.391	1.312	2.582
MAB21L2	0.156	1.114	3.728
MAD2L1	0.185	1.137	2.767
MADCAM1	-0.158	0.896	2.254
MADD	-0.068	0.954	6.467
MAEL	0.098	1.070	2.151
MAFB	0.947	1.928	4.694
MAFF	-0.235	0.850	3.591
MAFG-AS1	-0.338	0.791	2.482
MAFIP	-0.037	0.975	4.370
MAG	-0.079	0.947	8.093
MAGED1	0.028	1.019	7.210
MAGEE1	0.022	1.015	5.154
MAGEE2	-0.094	0.937	3.504
MAGEH1	0.226	1.170	6.314
MAGEL2	-0.039	0.973	2.578
MAGI1//MAGI1-IT1	-0.086	0.942	3.953
MAGI2-AS3	0.101	1.072	5.978
MAGI2-IT1	-0.066	0.955	3.168
MAGI3	0.313	1.242	6.335
MAGOH//MAGOHB	0.355	1.279	2.743
MAGT1	0.095	1.068	6.022
MAK	-0.524	0.695	1.998
MAL	-0.040	0.973	8.484
MAL2	0.083	1.059	3.710
MALAT1	0.087	1.062	9.692

MALL	0.453	1.369	3.534
MAMDC2	-0.223	0.857	3.877
MAMDC2-AS1//RP11-195E11.2	0.200	1.149	2.551
MAMDC4	0.037	1.026	3.012
MAML2	0.071	1.051	5.737
MAN1A1	0.331	1.258	5.924
MAN1A2	-0.034	0.977	7.963
MAN1B1-AS1	0.048	1.034	3.120
MAN1C1	0.237	1.178	6.306
MAN2A1	0.117	1.085	7.123
MAN2B1	0.417	1.335	4.842
MANEAL	-0.300	0.812	4.100
MANF	-0.043	0.971	4.560
MANSC1	0.118	1.085	5.317
MAOB	0.361	1.284	7.345
MAP1A	-0.300	0.812	8.590
MAP1LC3B2	0.592	1.507	3.914
MAP2	-0.190	0.877	8.295
MAP2K1	0.087	1.062	7.753
MAP2K4	0.026	1.018	4.312
MAP2K6	-0.199	0.871	5.618
MAP3K10	0.293	1.225	5.550
MAP3K11	0.179	1.132	4.132
MAP3K14	0.341	1.266	3.923
MAP3K6	0.247	1.187	4.602
MAP3K8	0.063	1.045	4.799
MAP3K8	-0.061	0.958	4.665
MAP3K9	-0.328	0.796	4.495
MAP4	-0.201	0.870	7.361
MAP4K1	0.054	1.038	2.341
MAP4K2	-0.032	0.978	4.840
MAP4K4	-0.172	0.887	8.473
MAP4K5	-0.067	0.955	7.647
MAP6	-0.273	0.828	6.469
MAP6D1	0.325	1.252	5.625
MAP7	-0.038	0.974	8.246
MAP7D2	-0.812	0.570	6.614
MAP7D3	-0.187	0.878	5.011
MAP9	0.080	1.057	5.534
MAPK12	-0.070	0.953	2.489
MAPK13	0.390	1.311	3.765
MAPK6	0.168	1.124	4.672
MAPK7	-0.030	0.980	2.705
MAPK8	-0.020	0.986	3.880
MAPK8IP1	0.278	1.213	4.182
MAPK8IP2	-0.470	0.722	5.822
MAPK9	0.206	1.153	6.000
MAPKAPK2	-0.084	0.943	6.424
MAPKAPK3	0.362	1.285	4.912
MAPRE3	0.148	1.108	4.850

MAPT-IT1	-0.603	0.658	3.730
MARC2	0.173	1.127	5.526
MARCH10	-0.498	0.708	2.485
MARCH11	-0.054	0.963	2.998
MARCH3	0.468	1.383	5.555
MARCH4	0.274	1.209	4.003
MARCKS	-0.123	0.918	8.048
MARCKSL1	0.085	1.061	6.776
MARCO	0.422	1.340	2.585
MARK1	0.080	1.057	5.831
MARK4	-0.037	0.975	5.738
MAS1	-0.193	0.875	2.850
MASP1	0.231	1.174	5.154
MAST1	-0.606	0.657	5.544
MAST3	0.168	1.123	7.377
MAST4	-0.076	0.949	4.551
MASTL	0.493	1.407	4.000
MAT2A	0.064	1.045	7.689
MATK	0.169	1.124	4.032
MATN1-AS1	0.211	1.158	2.750
MATN2//LOC100506558	0.247	1.186	3.418
MATN3	-0.157	0.897	3.362
MAVS	-0.035	0.976	6.062
MB21D1	0.708	1.633	2.335
MB21D2	0.368	1.291	4.863
MBD6	-0.067	0.955	3.720
MBLAC1	-0.157	0.897	4.074
MBLAC2	-0.082	0.945	5.020
MBNL3	0.337	1.263	3.753
MBOAT1	0.236	1.178	5.089
MBOAT7	0.244	1.184	5.169
MBP	0.061	1.043	6.846
MBTPS1	0.230	1.173	5.914
MBTPS2	0.472	1.387	6.865
MC4R	0.334	1.261	2.780
MCAM//MIR6756	0.119	1.086	3.767
MCAT	-0.197	0.872	4.790
MCC	0.068	1.048	5.576
MCEMP1//C19orf59	0.373	1.295	2.344
MCF2	-0.101	0.933	4.552
MCF2L-AS1	-0.319	0.802	3.126
MCHR1	-0.359	0.780	3.948
MCHR2	-0.141	0.907	2.676
MCL1	0.283	1.217	7.345
MCM4	0.181	1.134	5.516
MCM5	0.409	1.328	4.476
MCM6	0.712	1.638	4.963
MCM8	0.538	1.452	4.311
MCTP1	0.235	1.177	5.141
MCTP2	0.390	1.310	2.891

MCU	-0.251	0.840	4.909
MDFIC	-0.014	0.990	3.093
MDGA2	0.055	1.039	5.269
MDH1B	-0.191	0.876	3.315
MDK	0.463	1.379	4.695
MDM1	0.249	1.189	3.989
MDM4	0.050	1.035	6.325
MEAF6	0.116	1.083	6.489
MED12L	-0.128	0.915	5.725
MED15P9//MED15P2	-0.615	0.653	2.732
MED31	0.145	1.106	5.499
MEDAG	0.433	1.350	3.128
MEF2C	0.204	1.152	4.465
MEF2D	0.123	1.089	6.691
MEG8//SNORD112	-0.166	0.891	4.375
MEGF10	-0.099	0.934	7.556
MEIS1	-0.052	0.964	3.932
MEIS3P1	0.040	1.028	4.301
MEOX2	-0.113	0.925	2.936
MEPE	0.082	1.058	2.116
MESP2	0.226	1.170	2.946
MEST	0.066	1.047	4.238
MET	-0.189	0.877	4.879
METAP1	0.373	1.295	4.927
METRN	0.044	1.031	6.660
METRNL	0.416	1.334	4.238
METTL1	0.213	1.159	2.952
METTL10//RP11-12J10.3	0.174	1.128	5.188
METTL15	0.226	1.169	3.958
METTL16//LOC284009	-0.254	0.838	3.134
METTL23	0.511	1.425	4.634
METTL25	0.097	1.069	3.171
METTL2A	0.425	1.343	5.875
METTL7A	0.022	1.015	8.258
METTL7B	1.113	2.163	4.493
MEX3B	0.130	1.094	2.221
MFAP3	0.329	1.256	5.266
MFAP3	0.020	1.014	3.506
MFAP3//PCNXL2//LOC101927169	0.019	1.014	4.101
MFAP4	0.322	1.250	4.561
MFAP5	0.142	1.103	2.609
MFNG	0.208	1.155	3.414
MFSD2A	-0.176	0.885	4.262
MFSD3	0.016	1.011	3.761
MFSD4	0.186	1.137	5.998
MFSD7	0.395	1.315	3.630
MFSD9	-0.211	0.864	3.494
MGAT1//LOC102724833	0.060	1.042	4.671
MGAT4A	0.751	1.682	6.242
MGAT4C	0.186	1.138	4.486

MGAT5B	-0.248	0.842	4.388
MGC12916//RP11-214O1.2	0.242	1.183	2.496
MGC16025	-0.061	0.959	2.867
MGC16142	0.097	1.070	2.730
MGC16275//CTD-2514K5.2	-0.031	0.979	4.706
MGC24103	0.605	1.521	2.037
MGC27345//RBM28	-0.108	0.928	4.657
MGC2889	0.066	1.047	2.365
MGC57346//CRHR1-IT1//AC126544.2//CRHR1	0.268	1.204	5.033
MGC70870	0.107	1.077	4.620
MGC72080	-0.297	0.814	5.932
MGLL	-0.116	0.923	3.855
MGME1	0.130	1.094	4.641
MGP	0.234	1.176	5.401
MGST1	0.166	1.122	5.003
MGST3	0.601	1.516	3.776
MICA	0.392	1.312	4.665
MICA	0.308	1.238	4.671
MICAL2	-0.126	0.917	4.100
MICALL2	-0.019	0.987	3.454
MICU1	0.136	1.099	6.104
MICU3	-0.214	0.862	4.857
MID1IP1	-0.112	0.925	6.898
MIDN	0.203	1.151	6.472
MIF4GD	0.579	1.494	4.721
MINOS1P1	-0.334	0.793	2.926
MIPEPP3	0.225	1.169	2.480
MIR106B	0.022	1.016	2.550
MIR1180	0.145	1.105	2.675
MIR1184-1//MIR1184-2//MIR1184-3	0.384	1.305	4.995
MIR1200	-0.284	0.821	4.117
MIR1208	-0.495	0.709	2.518
MIR1226	-0.600	0.660	2.398
MIR1227	0.224	1.168	3.591
MIR1231	-0.169	0.889	3.584
MIR1238	-0.021	0.986	5.043
MIR124-2HG//LINC00966	0.170	1.125	4.664
MIR1243	0.278	1.212	3.253
MIR1250	0.032	1.023	3.340
MIR1254-1	-0.286	0.820	3.623
MIR1260A	-0.143	0.905	2.433
MIR1270	0.318	1.247	2.756
MIR1282	0.015	1.011	5.229
MIR1290	0.270	1.206	3.748
MIR1299	-0.021	0.986	6.526
MIR134	0.073	1.052	3.054
MIR137//MIR137HG//MIR2682	0.024	1.017	2.855
MIR138-1	0.087	1.062	2.350
MIR139	-0.298	0.813	3.550
MIR147A	0.100	1.071	2.351

MIR149	-0.318	0.802	2.458
MIR151A	-0.304	0.810	2.996
MIR151B	-0.328	0.797	3.046
MIR1538	-0.726	0.605	3.808
MIR1587	0.828	1.775	2.323
MIR181A1HG	0.288	1.221	4.644
MIR181B1	0.635	1.553	2.538
MIR181C	-0.158	0.896	2.893
MIR185	-0.047	0.968	4.107
MIR191	-0.178	0.884	2.312
MIR1910	0.333	1.259	4.004
MIR1913	-0.081	0.946	3.760
MIR197//GNAI3	-0.242	0.846	2.862
MIR21//VMP1	0.992	1.989	5.018
MIR210HG	-0.370	0.774	2.935
MIR219A2//MIR219B//RP11-339B21.12	0.605	1.521	4.903
MIR219B	-0.139	0.908	2.945
MIR2278	-0.261	0.834	2.320
MIR22HG//MIR22	0.035	1.024	3.173
MIR23B//C9orf3//MIR24-1	0.180	1.133	2.936
MIR23C	0.136	1.099	1.682
MIR25	0.362	1.285	2.117
MIR2682//MIR137HG//MIR137	0.041	1.029	2.256
MIR27B//C9orf3//MIR24-1	0.694	1.618	2.103
MIR29C//MIR29B2//C1orf132	0.157	1.115	3.336
MIR3065	-0.092	0.938	2.700
MIR3120	0.170	1.125	3.931
MIR3132	0.095	1.068	2.960
MIR3135B	0.173	1.128	2.376
MIR3137	0.168	1.123	3.666
MIR3139	0.054	1.038	2.424
MIR3143	0.213	1.159	4.160
MIR3147	0.153	1.112	1.910
MIR3151	-0.578	0.670	3.437
MIR3155A//MIR3155B	0.302	1.233	2.764
MIR3157	0.159	1.117	2.932
MIR3161	0.950	1.931	2.874
MIR3175	0.488	1.403	4.487
MIR3180-4	-0.264	0.833	3.509
MIR3180-4//MIR3180-5	-0.099	0.934	2.617
MIR3192	-0.037	0.975	3.357
MIR3198-1	0.088	1.063	3.208
MIR31HG	-0.535	0.690	2.609
MIR32	0.342	1.268	2.766
MIR324	-0.099	0.934	3.473
MIR326	-0.085	0.943	3.994
MIR338//MIR3065	0.738	1.668	3.488
MIR339	-0.437	0.739	4.006
MIR33B	0.019	1.013	3.367
MIR3619	-0.446	0.734	4.682

MIR3622A	-0.427	0.744	2.454
MIR3622B//MIR3622A	-0.083	0.944	3.004
MIR3650	-0.074	0.950	3.636
MIR3661	0.146	1.106	3.984
MIR3662	0.049	1.035	2.781
MIR3667	0.121	1.088	3.215
MIR3671	0.687	1.610	2.147
MIR3689A	-0.744	0.597	3.485
MIR3689B	-0.291	0.817	2.442
MIR3689F	-0.412	0.752	5.855
MIR3691	-0.195	0.874	2.323
MIR370	0.037	1.026	1.906
MIR375	-0.204	0.868	3.901
MIR378E	-0.239	0.847	2.671
MIR378I	-0.670	0.628	3.631
MIR3916//RP11-488L18.4	0.431	1.348	5.051
MIR3936	-0.552	0.682	4.078
MIR3939	0.671	1.592	2.879
MIR3942	0.151	1.110	2.312
MIR3944	-0.131	0.913	3.184
MIR3972	0.429	1.346	2.412
MIR3978	0.627	1.544	3.776
MIR412	0.344	1.269	2.116
MIR421	0.297	1.228	5.320
MIR4288	0.538	1.452	3.262
MIR4292	-0.184	0.880	2.390
MIR4294	0.744	1.674	2.584
MIR4295	0.041	1.029	2.864
MIR4300	-0.307	0.808	2.219
MIR4312	-0.237	0.848	2.911
MIR4314	-0.110	0.926	2.503
MIR4316	-0.207	0.866	3.722
MIR4324	-0.838	0.559	3.689
MIR4420	-0.165	0.892	2.038
MIR4441	0.350	1.275	4.812
MIR4450	-1.022	0.492	4.678
MIR4451	0.362	1.285	3.167
MIR4454	0.109	1.078	6.165
MIR4461	-0.830	0.563	6.289
MIR4476	0.040	1.028	2.866
MIR4481	-0.033	0.978	4.105
MIR4482	0.247	1.187	3.083
MIR4486	0.167	1.123	2.542
MIR4492	-0.682	0.623	3.884
MIR4497	-0.527	0.694	2.867
MIR4500	0.257	1.195	2.562
MIR4500HG	-0.047	0.968	2.581
MIR4502	-0.127	0.916	2.613
MIR4516//LOC102724992//LOC101927469	-0.111	0.926	3.937
MIR4518	-0.154	0.899	3.207

MIR4519	-0.470	0.722	3.351
MIR4522	-0.550	0.683	2.324
MIR4525	0.027	1.019	6.348
MIR4530	-0.177	0.884	2.930
MIR4533	-0.541	0.687	4.825
MIR4534	-0.553	0.682	6.730
MIR4638	0.561	1.475	3.154
MIR4644	-0.051	0.965	3.463
MIR4646	0.231	1.174	2.752
MIR4655	0.240	1.181	2.235
MIR4663	0.084	1.060	2.679
MIR4668	1.127	2.185	1.924
MIR4673	-0.650	0.637	4.829
MIR4677	0.578	1.493	4.228
MIR4686	0.406	1.325	3.053
MIR4689	-0.268	0.830	2.797
MIR4697HG//MIR4697	0.243	1.183	4.215
MIR4705	0.110	1.079	1.506
MIR4710	-0.460	0.727	2.711
MIR4730	0.567	1.481	3.502
MIR4742	0.457	1.373	3.332
MIR4746	-0.040	0.972	2.623
MIR4748	-0.206	0.867	3.998
MIR4756	-0.335	0.793	3.492
MIR4757	-0.375	0.771	2.273
MIR4780	-0.031	0.978	2.391
MIR486-1	-0.054	0.963	2.687
MIR502	-0.064	0.957	3.115
MIR513A2	-0.238	0.848	3.052
MIR516B1	-0.132	0.912	2.245
MIR539	-0.400	0.758	4.292
MIR548AL	0.214	1.160	2.713
MIR548F2	0.640	1.558	3.029
MIR548H2	0.439	1.355	3.657
MIR548I2	-0.250	0.841	2.955
MIR548I2//MIR548I1//MIR548I3	-0.356	0.782	3.336
MIR548I2//MIR548I1//MIR548I3	-0.326	0.798	3.379
MIR548L	-0.054	0.963	2.473
MIR548V	0.293	1.225	5.193
MIR550A1//MIR550B1	0.250	1.189	2.669
MIR550A3	0.347	1.272	3.110
MIR552	-0.206	0.867	2.265
MIR570	-0.922	0.528	6.440
MIR571	0.498	1.413	3.848
MIR573	0.581	1.496	2.532
MIR574	0.052	1.037	5.026
MIR575	-0.383	0.767	5.244
MIR577	0.156	1.114	2.431
MIR579	0.968	1.957	4.046
MIR585	0.100	1.072	2.183

MIR587	-0.152	0.900	2.868
MIR597	0.251	1.190	3.972
MIR600HG//MIR600	-0.027	0.982	4.799
MIR601	-0.494	0.710	2.784
MIR604	0.383	1.304	3.453
MIR606	-0.418	0.748	3.354
MIR628	0.248	1.188	2.440
MIR637//DAPK3	0.658	1.578	3.651
MIR642A//MIR642B	-0.291	0.817	2.639
MIR644A	0.342	1.267	4.301
MIR645	-0.160	0.895	3.723
MIR646	0.239	1.180	3.056
MIR647	-0.037	0.974	4.935
MIR648	-0.531	0.692	2.248
MIR654	0.027	1.019	2.020
MIR662	-0.450	0.732	2.637
MIR663B	-0.442	0.736	4.296
MIR6735//SZT2-AS1	0.233	1.175	2.623
MIR7-3HG	0.213	1.159	4.109
MIR744	0.176	1.130	2.824
MIR760	0.085	1.061	3.429
MIR7641-2	-0.053	0.964	2.171
MIR765	0.230	1.173	2.369
MIR770//MEG3	0.620	1.537	4.197
MIR877	-0.901	0.535	2.924
MIR877	-0.628	0.647	3.230
MIR92B	-0.293	0.816	4.261
MIR9-3	0.222	1.166	2.129
MIR941-1//DNAJC5//MIR941-4	-0.262	0.834	5.444
MIR941-1//MIR941-2//MIR941-3//MIR941-4//DNAJ(	-0.303	0.810	4.188
MIRLET7A1	-0.440	0.737	2.284
MIRLET7D	0.302	1.233	2.352
MITF	-0.087	0.942	2.940
MKKS	0.210	1.157	6.194
MKL2	0.543	1.457	8.113
MKNK2	-0.071	0.952	5.496
MKRN2	0.301	1.232	6.354
MKRN3	0.285	1.218	3.419
MKX	0.076	1.054	4.186
MLC1	0.020	1.014	8.797
MLIP	0.176	1.130	3.438
MLKL	0.995	1.993	4.635
MLLT11	-0.115	0.924	8.193
MLLT3	-0.156	0.898	5.838
MMAA	0.410	1.329	4.035
MMAB	-0.176	0.885	5.751
MMD	0.727	1.656	7.006
MMD2	-0.273	0.827	5.289
MME	0.347	1.272	2.683
MMEL1	0.022	1.016	3.344

MMP14	0.148	1.108	4.859
MMP16	0.196	1.146	5.975
MMP17	-0.198	0.872	3.748
MMP2	0.224	1.168	4.464
MMP24	0.111	1.080	4.156
MMP24-AS1	0.415	1.334	2.197
MMP28	0.140	1.102	3.027
MMP8	-0.541	0.687	2.580
MMRN1	-0.169	0.889	2.707
MMS22L	0.261	1.198	2.607
MN1	-0.174	0.886	4.439
MNDA	0.678	1.600	4.080
MNS1	-0.635	0.644	5.072
MOAP1	-0.053	0.964	6.370
MOB3A	0.034	1.024	7.466
MOB3B	-0.497	0.708	2.726
MOB3B	-0.086	0.942	7.565
MOBP	-0.324	0.799	8.161
MOCS2	0.280	1.214	8.190
MOCS3	-0.082	0.945	3.490
MOG	0.232	1.175	8.090
MOG	0.207	1.154	8.141
MOGS	0.086	1.061	3.911
MOK	-0.430	0.742	2.927
MORC3	0.302	1.233	4.495
MORC4	0.327	1.254	4.567
MORN1	-0.052	0.965	3.534
MORN3	0.290	1.223	2.939
MORN4	0.248	1.188	4.227
MOSPD1	0.657	1.577	4.301
MOSPD2	0.159	1.116	5.905
MOXD1	0.059	1.042	4.200
MPC2	0.056	1.040	7.186
MPEG1	0.288	1.221	4.572
MPP3	-0.123	0.918	4.286
MPP6	-0.295	0.815	7.310
MPP7	-0.069	0.953	3.754
MPPED1	0.452	1.368	4.697
MPPED2	-0.081	0.945	3.749
MPRIP-AS1	0.018	1.013	2.643
MPZ	-0.191	0.876	4.068
MPZL2	0.697	1.622	3.267
MR1	0.344	1.269	3.938
MRC1	0.063	1.044	4.205
MRC1	0.055	1.039	4.473
MRC2	-0.138	0.908	4.262
MREG	0.222	1.167	3.780
MRFAP1L1	0.205	1.153	6.983
MRGBP	0.155	1.113	4.069
MRGPRE	0.196	1.146	3.484

MRI1	0.091	1.065	4.991
MRO	-0.149	0.902	6.608
MRPL1	0.063	1.045	3.866
MRPL15	0.266	1.202	6.263
MRPL30//LOC101927110//RP11-111H13.1	0.071	1.051	6.064
MRPL30//LOC101927110//RP11-111H13.1	0.097	1.070	5.297
MRPL35	0.163	1.119	6.210
MRPL42	0.250	1.189	4.573
MRPL45	0.483	1.398	6.234
MRPL46//RP11-97O12.7	0.177	1.131	5.725
MRPL50	0.479	1.394	6.363
MRPS12	0.148	1.108	3.663
MRPS17//RP11-15K19.2	0.041	1.029	4.894
MRPS21	-0.044	0.970	9.556
MRPS30	0.240	1.181	6.551
MRPS31P5	0.252	1.191	4.104
MRPS36	0.021	1.015	2.647
MRRF	0.075	1.053	4.367
MRS2	0.315	1.244	5.533
MRVI1	0.112	1.081	6.954
MS4A14	1.007	2.009	3.430
MS4A4A	0.423	1.341	3.089
MS4A6A	1.027	2.038	3.790
MS4A7	0.813	1.757	5.267
MSANTD4	0.238	1.179	5.601
MSH2	0.195	1.145	5.891
MSH5//MSH5-SAPCD1//SAPCD1	0.014	1.010	3.274
MSI2	-0.132	0.913	7.352
MSLN	0.465	1.381	2.695
MSMO1	0.142	1.104	7.149
MSN	0.124	1.090	9.050
MSR1	0.883	1.844	2.492
MSRA	0.128	1.093	3.763
MST1L	0.075	1.053	2.540
MSTN	0.347	1.272	2.531
MSX1	-0.486	0.714	3.136
MT1A	0.632	1.549	4.342
MT1CP	-0.025	0.983	6.753
MT1E	0.146	1.106	4.804
MT1F	-0.159	0.895	6.892
MT1G	-0.997	0.501	5.649
MT1JP	-0.361	0.779	2.633
MT1L//NUTF2//MT1M	0.291	1.224	3.777
MT1M	-0.256	0.837	7.883
MT1X	-0.050	0.966	9.610
MTA3	0.103	1.074	3.841
MTCL1//SOGA2	-0.040	0.973	4.421
MTERF1//MTERF	0.363	1.286	3.979
MTFMT	0.123	1.089	4.428
MTFP1//RP4-539M6.19	-0.251	0.840	3.313

MTHFD1//CTD-2555O16.4	-0.216	0.861	3.320
MTHFD2	0.524	1.438	4.689
MTHFSD	0.276	1.211	3.984
MTMR10	-0.028	0.981	8.709
MTMR11	0.588	1.503	4.592
MTMR12	0.094	1.068	6.442
MTMR4	-0.025	0.983	7.135
MTMR7	-0.050	0.966	5.930
MTMR9	0.154	1.112	5.306
MTMR9LP	-0.026	0.982	3.252
MTO1	0.259	1.197	4.387
MTPAP	0.176	1.130	6.112
MTPN	0.185	1.137	4.111
MTRNR2L2	-0.719	0.608	3.826
MTRNR2L8//MIR4485	-0.409	0.753	6.693
MTSS1	-0.025	0.983	6.509
MTSS1L	-0.301	0.811	7.979
MT-TA	-0.191	0.876	10.177
MT-TD	-0.635	0.644	3.118
MT-TG	-0.890	0.540	6.800
MT-TH	-0.437	0.739	7.912
MT-TI	-0.351	0.784	5.115
MT-TL2	-0.727	0.604	6.515
MT-TM	-0.457	0.728	9.248
MT-TN	-0.200	0.871	8.010
MT-TP	-1.591	0.332	3.842
MT-TQ	-0.236	0.849	8.604
MT-TR	-0.780	0.582	6.044
MT-TS1//UIMC1	-0.640	0.642	7.823
MT-TS2	-0.324	0.799	9.637
MT-TT	-0.896	0.537	3.760
MT-TW	-0.497	0.709	4.821
MTUS1	-0.200	0.871	5.525
MTUS2	0.194	1.144	3.929
MTX3	-0.080	0.946	6.037
MUC1	0.025	1.018	3.607
MUC20	-0.615	0.653	3.389
MUM1L1	-0.121	0.920	3.662
MURC	0.273	1.208	3.831
MVB12B	-0.157	0.897	6.693
MVD	-0.202	0.869	5.388
MVK	-0.022	0.985	4.233
MVP	0.474	1.389	5.722
MX2	1.057	2.081	3.273
MXD1	0.557	1.471	6.176
MXRA7	0.141	1.103	6.017
MXRA8	0.038	1.027	4.810
MYBPC1	0.096	1.069	2.657
MYBPH	0.084	1.060	2.348
MYC	0.676	1.597	3.297

MYCBP2	0.051	1.036	7.914
MYCN	-0.165	0.892	3.947
MYD88	0.404	1.323	3.317
MYH10	-0.300	0.812	6.663
MYH11	-0.241	0.846	5.811
MYH14	-0.300	0.812	5.096
MYL12A	0.470	1.385	3.852
MYL3	0.269	1.205	3.265
MYL5	0.017	1.012	3.103
MYLIP	0.612	1.528	5.375
MYLK	-0.049	0.966	5.173
MYLK3	-0.736	0.601	3.176
MYLK4	-0.298	0.813	3.080
MYO15B	-0.183	0.881	4.379
MYO16	0.380	1.301	4.765
MYO1A	-1.351	0.392	4.032
MYO1B	0.164	1.120	5.252
MYO1C	0.207	1.154	5.678
MYO1D	-0.166	0.892	6.730
MYO1E	0.076	1.054	6.643
MYO1F	0.142	1.103	5.236
MYO1G	1.047	2.066	3.129
MYO5A	-0.090	0.939	8.324
MYO5C	-0.165	0.892	4.747
MYO9A	0.122	1.089	7.145
MYO9B	0.150	1.110	7.282
MYOF	0.718	1.645	5.798
MYOM2	-0.083	0.944	3.626
MYOZ1	0.271	1.207	2.734
MYRF	-0.157	0.897	6.428
MYRIP	0.086	1.061	5.215
MYSM1	-0.056	0.962	6.714
MYT1L	0.199	1.148	6.029
MZT1	0.416	1.334	3.964
N4BP2L1	0.885	1.847	5.140
N4BP2L2-IT2	-0.051	0.965	6.105
N4BP3//RP11-889L3.3	-0.615	0.653	2.570
NAA30	0.125	1.091	6.080
NAALAD2	0.082	1.058	3.720
NABP1	0.939	1.918	3.740
NACA	0.203	1.151	3.889
NACAD	-0.262	0.834	5.983
NACC1	0.165	1.121	3.740
NACC2	-0.314	0.804	7.157
NAF1	0.021	1.014	5.036
NAG20	-0.274	0.827	3.033
NAGA	0.517	1.431	5.487
NAGLU//RP11-400F19.8	0.417	1.335	4.700
NAGPA	0.199	1.148	4.966
NAIP//RP11-1319K7.1	0.519	1.433	6.497

NAMPT//NAMPTL	0.732	1.661	8.312
NAP1L2	-0.023	0.984	7.003
NAP1L3	0.030	1.021	7.160
NAPA-AS1	0.248	1.188	3.916
NAPB	0.042	1.029	7.547
NAPEPLD	0.210	1.157	6.461
NAPG	0.059	1.041	7.864
NAPSB	0.443	1.359	4.046
NASP	-0.023	0.984	5.823
NAT14	0.080	1.057	4.124
NAT8L	-0.480	0.717	7.254
NAV2	-0.063	0.957	6.815
NAV2//RP1-34H18.1	0.379	1.300	4.350
NAV2-AS1	-0.179	0.883	2.984
NAV2-AS2	0.081	1.058	3.245
NAV2-IT1	-0.320	0.801	2.470
NBEA	0.157	1.115	7.125
NBPF1//NBPF11//NBPF15	-0.156	0.897	5.881
NBPF11//NBPF12//NBPF10//NBPF9//NBPF14//NBPF	-0.097	0.935	7.889
NBPF11//NBPF12//NBPF15//NBPF8//NBPF26//NBPF	0.022	1.015	7.592
NBPF12	0.065	1.046	5.718
NBPF14//NBPF10//NBPF26	-0.086	0.942	6.810
NBPF19//AC023137.2	0.131	1.095	2.622
NBPF20	0.297	1.229	7.869
NBPF20	-0.190	0.876	6.368
NBPF20//NBPF14//NBPF8//NBPF23//NBPF15//NBPF	-0.142	0.906	8.348
NBPF25P//NOTCH2NL//LOC100996763	0.261	1.198	5.426
NBR2	0.030	1.021	3.829
NCALD	0.158	1.116	3.012
NCAM2	0.029	1.020	8.416
NCAN	0.375	1.297	6.677
NCAPD2	0.061	1.043	5.329
NCBP2-AS2	0.037	1.026	5.541
NCDN	0.200	1.148	6.455
NCEH1	0.050	1.035	5.827
NCF1C	-0.057	0.961	3.402
NCF2	0.998	1.997	4.979
NCF4	0.545	1.459	4.483
NCK1-AS1//RP11-85F14.5	0.022	1.016	3.510
NCK2	0.386	1.307	6.697
NCKAP1	0.170	1.125	9.218
NCKAP1L	0.473	1.388	5.501
NCKAP5-IT1	-0.333	0.794	2.983
NCKIPSD	0.075	1.054	5.620
NCMAP	0.237	1.179	3.419
NCOA5	-0.078	0.948	6.060
NCOA7	-0.072	0.951	7.321
NCRNA00185//TTTY14	0.172	1.126	2.041
NCRNA00185//TTTY14//LOC102723889	0.521	1.435	3.963
NCS1	-0.324	0.799	7.348

ND6	-0.502	0.706	8.506
NDE1	-0.070	0.953	7.450
NDEL1	0.046	1.032	7.734
NDFIP1	0.042	1.029	7.192
NDFIP2	0.075	1.053	6.328
NDN	0.030	1.021	4.023
NDNF	-0.046	0.969	3.216
NDP	0.666	1.586	5.194
NDRG1	-0.166	0.891	5.802
NDRG2	-0.283	0.822	6.408
NDRG3	0.128	1.093	7.248
NDRG4	-0.334	0.793	7.610
NDST1	-0.022	0.985	6.750
NDST3	-0.035	0.976	3.819
NDST4	0.290	1.223	2.309
NDUFA5	0.039	1.027	4.343
NDUFA6-AS1	0.058	1.041	2.954
NDUFA9//RP11-500M8.7	0.143	1.104	7.133
NDUFAF1	0.305	1.236	3.799
NDUFAF5	0.147	1.108	3.684
NDUFAF7	0.151	1.110	5.250
NDUFB5	0.165	1.121	4.771
NEAT1//MIR612	-0.708	0.612	3.756
NEAT1//MIR612	0.042	1.030	9.809
NEBL-AS1	-0.353	0.783	2.950
NECAB1	-0.147	0.903	7.615
NECAB2	-0.126	0.917	5.470
NECAB3	-0.182	0.881	4.472
NECAP1	0.040	1.028	7.131
NECAP2	0.166	1.122	5.476
NEDD1	0.288	1.221	5.501
NEDD4L	-0.036	0.976	6.402
NEFH	-1.443	0.368	8.367
NEFL	-0.965	0.512	10.027
NEFM	-1.041	0.486	9.736
NEGR1	0.158	1.115	7.098
NEGR1-IT1	-0.126	0.916	2.165
NEK10	-0.144	0.905	3.163
NEK3	0.243	1.184	4.519
NEK5//LOC100506859	-0.802	0.574	2.967
NEK7	-0.126	0.917	6.538
NELL1	0.071	1.051	4.078
NELL2	-0.442	0.736	5.369
NENF	-0.055	0.962	5.597
NES	0.267	1.204	5.297
NET1	-0.283	0.822	6.177
NETO1	-0.124	0.917	5.139
NETO2	0.600	1.516	6.126
NEURL1B	0.233	1.175	2.943
NEURL4	-0.252	0.840	4.950

NEUROD1	-0.095	0.936	1.823
NEUROD2	-0.222	0.858	3.574
NF2	0.155	1.113	6.236
NFAM1	0.423	1.341	3.524
NFATC1	0.041	1.029	4.131
NFATC2	0.178	1.131	4.452
NFATC4	0.064	1.045	3.322
NFE2L2	0.418	1.336	6.800
NFE2L3	0.231	1.173	2.136
NFIA-AS2//RP4-668G5.1	0.252	1.191	2.896
NFIL3	0.664	1.585	5.813
NFKB2	0.367	1.289	3.336
NFKBIA	-0.016	0.989	7.231
NFKBIE	0.489	1.403	3.257
NFKBIZ	0.325	1.253	4.709
NGB	0.213	1.159	2.893
NGEF	0.292	1.225	4.580
NGFR	-0.314	0.804	3.681
NGRN	0.090	1.064	8.900
NGRN	0.032	1.023	4.256
NHLRC1	0.239	1.181	2.630
NHLRC3	0.438	1.355	5.475
NHS	-0.178	0.884	4.132
NHSL1	-0.159	0.896	4.236
NHSL2	0.100	1.072	3.444
NICN1	0.165	1.121	6.297
NID1	0.214	1.160	4.891
NID2	0.361	1.284	3.942
NIM1K//NIM1	0.020	1.014	4.672
NINJ1	0.253	1.191	5.347
NINJ2	0.111	1.080	4.055
NINL	-0.132	0.912	4.295
NIPA2	0.236	1.178	5.156
NIPAL2	0.389	1.309	4.769
NIPAL3	-0.032	0.978	6.974
NIPAL4	-0.085	0.943	3.833
NIPSNAP1	0.263	1.200	5.548
NIPSNAP3B	0.137	1.100	4.007
NKAIN1	0.161	1.118	3.697
NKAIN2	-0.032	0.978	8.380
NKAIN4	0.548	1.462	2.953
NKD1	-0.390	0.763	5.448
NKG7	0.017	1.012	3.108
NKIRAS1	0.042	1.029	4.446
NKRF	0.177	1.131	3.963
NKX2-2	-0.301	0.812	5.745
NKX3-1	0.229	1.172	3.111
NKX6-1	-0.614	0.653	2.693
NKX6-2	-0.182	0.881	6.861
NLE1	-0.111	0.926	4.463

NLGN1	0.256	1.194	5.443
NLGN4Y-AS1	-0.171	0.888	2.537
NLK	0.145	1.106	8.637
NLRC5	0.377	1.299	3.097
NLRP1	0.027	1.019	3.283
NLRP2	-0.357	0.781	2.531
NLRP3	0.064	1.045	4.240
NMB	-0.062	0.958	4.593
NME5	-0.078	0.948	3.559
NME9	-0.235	0.850	3.438
NMI	0.527	1.441	3.217
NMNAT2	-0.404	0.756	6.681
NMRK1	0.582	1.497	3.850
NMT2	0.202	1.150	4.497
NMUR2	0.230	1.172	2.513
NNAT	-0.834	0.561	5.312
NNMT//LOC101928916	0.465	1.380	3.063
NNT	0.234	1.176	7.511
NOA1	0.580	1.495	5.758
NOD1	0.374	1.296	4.401
NOD2	0.129	1.093	2.104
NOL3	0.544	1.458	4.459
NOL4	0.280	1.214	5.731
NOL6	0.213	1.159	4.426
NOMO1	0.153	1.112	7.257
NOMO2//NOMO3//NOMO1	0.208	1.155	9.272
NOMO2//NOMO3//NOMO1	0.179	1.132	9.309
NOP14-AS1	-0.227	0.854	3.890
NOS1AP	-0.304	0.810	4.237
NOS2	0.455	1.370	2.429
NOS3	0.677	1.599	3.793
NOSTRIN	-0.036	0.975	3.066
NOTCH1	-0.298	0.814	6.517
NOTCH2NL	-0.102	0.932	7.181
NOTCH2NL	0.024	1.017	5.764
NOTCH3	0.623	1.541	4.127
NOV	-0.287	0.820	4.722
NOVA2	-0.033	0.978	4.894
NOXA1	0.257	1.195	3.700
NOXO1	-0.140	0.908	2.652
NPAS1	-0.183	0.881	2.313
NPAS3	0.028	1.020	5.750
NPB	-0.209	0.865	2.442
NPC1	0.156	1.114	7.543
NPC2//MIR4709	0.195	1.144	8.661
NPFF//RP11-793H13.10	-0.141	0.907	3.725
NPFFR1	0.451	1.367	3.005
NPIPB11//NPIPB5//NPIPB3//NPIPB4//NPIPA5//LOC1	-0.301	0.812	9.019
NPIPB3//NPIPB5//NPIPB4	-0.201	0.870	7.464
NPIPB4//LOC613037//NPIPB5//NPIPA5//NPIPB3//NI	-0.216	0.861	8.616

NPIPB5//NPIPB3//NPIPB4//LOC613037//NPIPA5//LC	-0.188	0.878	9.092
NPIPB5//NPIPB4//LOC613037//NPIPA5//NPIPB3//NI	-0.222	0.857	8.918
NPIPB6	-0.136	0.910	5.592
NPIPB8//NPIPB9//NPIPB6//NPIPB15//NPIPB7//CTB-:	-0.302	0.811	7.508
NPL	0.021	1.015	5.531
NPM2	-0.083	0.944	4.550
NPM3	0.646	1.564	3.310
NPNT	-0.396	0.760	3.266
NPPA	-0.321	0.801	2.661
NPPC	0.565	1.479	5.628
NPR3	-0.255	0.838	2.661
NPTN	-0.137	0.909	8.338
NPTX1	0.118	1.085	5.611
NPTX2	-0.336	0.792	5.365
NPTXR	-0.116	0.923	5.147
NPW	-0.596	0.662	2.890
NPY	0.157	1.115	3.934
NPY1R	0.712	1.638	4.752
NPY2R	0.499	1.413	3.220
NPY5R	0.022	1.016	2.830
NQO1	0.598	1.513	5.950
NQO2//RP1-90J20.11	0.154	1.113	4.668
NR1D1	-0.055	0.963	6.338
NR1D2	0.093	1.067	6.067
NR2E1	-0.096	0.936	3.091
NR2F1-AS1	0.297	1.228	4.516
NR2F2	0.034	1.024	2.612
NR2F6	0.319	1.248	5.236
NR3C2	0.049	1.034	5.703
NR4A1	-0.841	0.558	4.106
NR4A2	-0.090	0.939	4.763
NR4A3	-0.673	0.627	3.400
NRBF2	0.579	1.494	5.779
NRBP2//MIR6845	-0.112	0.925	6.422
NRCAM	0.131	1.095	8.430
NREP	0.101	1.072	2.733
NRG1	-0.511	0.702	4.108
NRG1-IT1	-0.177	0.885	2.926
NRG1-IT2	0.226	1.170	2.225
NRG1-IT3	-0.124	0.918	2.250
NRG3	0.191	1.142	5.228
NRG4	-0.579	0.670	3.302
NRGN	0.145	1.106	6.031
NRIP2	0.513	1.427	4.749
NRN1	0.166	1.122	7.810
NRP1	-0.043	0.970	5.816
NRP2	0.448	1.364	4.552
NRSN1	0.172	1.127	4.821
NRSN2	0.240	1.181	5.332
NRXN1	0.356	1.280	6.909

NRXN3	0.043	1.030	6.897
NS3BP	-0.051	0.965	3.831
NSF//LOC101930324	-0.190	0.877	8.140
NSF//NSFP1//LOC101930324	-0.066	0.955	8.276
NSG1	-0.034	0.976	3.073
NSMF//MIR7114	-0.080	0.946	7.221
NSUN5P1	0.322	1.250	4.826
NT5C	-0.066	0.955	4.490
NT5C1A	0.070	1.050	3.677
NT5DC1	0.138	1.100	6.590
NT5DC3	-0.147	0.903	5.706
NT5E	0.210	1.156	4.481
NTM-IT	-0.072	0.951	3.020
NTN1	-0.066	0.956	4.458
NTN3	-0.025	0.983	2.350
NTN4	0.341	1.266	5.350
NTNG1	0.221	1.165	4.977
NTNG2	0.209	1.156	4.879
NTSR2	0.261	1.198	6.119
NUAK1	0.235	1.177	6.655
NUAK2	0.380	1.301	3.316
NUDT1	0.487	1.402	3.448
NUDT10	-0.126	0.917	4.018
NUDT11	0.087	1.062	4.594
NUDT13	-0.275	0.826	2.753
NUDT16P1//NUDT16P	0.302	1.233	3.720
NUDT19	0.353	1.277	4.295
NUDT21	0.034	1.024	8.210
NUDT3	-0.035	0.976	9.258
NUDT4	0.709	1.635	2.790
NUDT4P1//NUDT4P2	0.586	1.501	2.737
NUDT9P1	-0.021	0.986	2.742
NUFIP1	0.298	1.229	4.924
NUMBL	0.272	1.208	5.093
NUP155	0.382	1.303	6.014
NUPR1	0.069	1.049	7.437
NUPR1L	-0.075	0.950	2.565
NUSAP1	-0.068	0.954	2.318
NWD1	-0.582	0.668	4.263
NWD2//KIAA1239	0.315	1.244	3.044
NXNL2	-0.143	0.905	2.419
NXPE3	0.334	1.261	6.832
NXPH1	0.135	1.098	4.981
NXPH2	-0.326	0.798	3.064
NXPH3	0.455	1.371	4.241
NXT1	0.310	1.239	3.551
NYAP1	-0.397	0.759	4.129
NYAP2	0.275	1.210	4.100
NYNRIN	0.256	1.194	3.664
OAF	-0.088	0.941	3.843

OARD1	0.050	1.035	4.837
OAS1	0.527	1.441	3.342
OAS2	0.807	1.749	3.940
OAS3	0.399	1.319	3.023
OASL	0.394	1.314	2.237
OBFC1	0.642	1.561	5.324
OBP2A	-0.179	0.883	2.741
OCA2	-0.141	0.907	2.707
OCIAD2	0.269	1.205	3.007
OCLM	0.458	1.374	2.307
OCLN	-0.540	0.688	2.900
OCRL	0.192	1.142	6.606
ODF2L	-0.164	0.893	5.392
ODF3B	-0.129	0.915	3.083
OGDHL	-0.566	0.676	6.297
OGFRL1	0.189	1.140	9.038
OGN	-0.105	0.930	5.037
OIP5-AS1	0.089	1.064	4.982
OLA1	0.109	1.078	5.818
OLFM1	-0.327	0.797	4.594
OLFM2	0.035	1.025	6.587
OLFM3	-0.114	0.924	3.841
OLFM4	0.133	1.097	2.355
OLFML1	-0.292	0.817	2.659
OLFML2A	-1.186	0.439	2.815
OLFML2B	0.486	1.401	4.028
OLFML3	0.391	1.311	3.470
OLIG1	-0.362	0.778	6.322
OLIG2	-0.252	0.840	4.611
OLR1	0.753	1.685	5.591
OMD	-0.640	0.642	3.845
OMG//LOC101927057	-0.044	0.970	6.592
OPA1	0.224	1.168	6.877
OPALIN	-0.068	0.954	6.749
OPCML	0.159	1.116	4.236
OPCML	0.204	1.152	5.959
OPCML-IT2	-0.118	0.921	4.059
OPLAH	-0.293	0.816	3.667
OPN3	0.297	1.228	4.227
OPN4	-0.034	0.977	2.689
OPRD1	0.240	1.181	3.515
OPRK1	-0.217	0.860	3.190
OPRL1	0.177	1.130	4.043
OPRM1	0.265	1.201	3.409
OR2A1-AS1//LOC101928605//RP4-545C24.1//RP4-7[illegible]	-0.461	0.727	1.958
OR2A9P//OR2A20P//OR2A1-AS1//LOC101928605//[illegible]	0.156	1.114	3.704
OR2AK2	0.131	1.095	2.980
OR2L13	0.091	1.065	4.548
OR2L1P	-0.308	0.808	5.958
OR2L2	-0.156	0.898	2.915

OR2L3	0.192	1.142	2.833
OR2L5	-0.088	0.941	3.220
OR2L8	-0.319	0.801	4.498
OR2M4	-0.343	0.788	2.642
OR2T12	-0.119	0.921	3.376
OR6W1P	0.034	1.023	3.361
OR7A5	-0.388	0.764	2.562
OR7E47P	0.113	1.081	2.531
OR7E91P	-0.344	0.788	3.536
OR9A2	-0.152	0.900	2.894
ORAI1	0.244	1.184	3.459
ORAI3	-0.221	0.858	3.375
ORC4	0.038	1.027	5.284
ORC5	0.044	1.031	5.812
ORC6	0.232	1.174	2.910
ORMDL2	-0.156	0.898	4.568
OSBPL10	0.089	1.064	2.823
OSBPL11	0.581	1.496	6.941
OSBPL3	-0.165	0.892	5.332
OSBPL6	-0.167	0.891	5.667
OSCAR	0.166	1.122	2.683
OSCP1	-0.200	0.870	4.235
OSER1-AS1	0.121	1.088	3.270
OSGIN2	0.216	1.162	4.851
OSM	0.342	1.267	2.029
OSMR	0.875	1.834	5.789
OSTF1	0.573	1.488	7.661
OSTM1-AS1	-0.174	0.886	2.463
OTOGL	-0.018	0.988	2.580
OTOS	0.206	1.153	3.569
OTUB2	-0.153	0.899	2.931
OTUD3	0.209	1.156	4.987
OTUD7A	-0.296	0.815	5.999
OTUD7B	-0.073	0.950	7.194
OVGP1	0.246	1.186	3.964
OVOL3	0.238	1.180	2.499
OXCT1	-0.084	0.943	7.328
OXCT2	0.129	1.094	2.546
OXGR1	-0.169	0.889	2.013
OXR1	-0.395	0.760	6.363
OXR1//RP11-227D13.2	0.210	1.157	7.349
OXT	-0.114	0.924	3.024
OXTR	0.324	1.252	3.898
P2RX1	0.211	1.157	2.888
P2RX3	-0.310	0.807	2.318
P2RX4	0.696	1.620	3.051
P2RX6	-0.314	0.804	3.845
P2RX6P	-0.115	0.924	1.982
P2RX7	0.137	1.100	6.426
P2RY1	-0.130	0.914	5.034

P2RY13	0.044	1.031	4.963
P2RY14	-0.374	0.772	3.947
P4HA1	0.025	1.018	6.985
PABPC1L	0.458	1.374	2.699
PABPC1L2A//PABPC1L2B//RP11-493K23.3	-0.060	0.959	1.598
PABPC1L2B//PABPC1L2A	-0.098	0.934	2.766
PABPC5	0.149	1.108	3.721
PACRG	-0.325	0.798	3.990
PACRG-AS1	-0.141	0.907	2.836
PACS2	-0.169	0.890	6.154
PACSIN1	-0.427	0.744	5.998
PACSIN3	0.025	1.017	5.458
PADI2	0.271	1.207	8.633
PAFAH1B1	-0.071	0.952	8.606
PAFAH1B3	0.247	1.187	4.523
PAGR1//CTD-2574D22.6	-0.017	0.988	5.093
PAH//LOC101929036	-0.293	0.816	2.123
PAIP2//CTB-43P18.1	0.018	1.012	5.622
PAIP2B//VPS36	-0.574	0.672	8.307
PAK1	0.102	1.073	7.128
PAK2	0.091	1.065	6.200
PAK3	-0.047	0.968	5.693
PAK7	0.460	1.376	4.566
PALD1	-0.165	0.892	4.276
PALM2-AKAP2//AKAP2//PALM2	-0.091	0.939	4.798
PALMD	0.082	1.059	5.052
PAM	0.244	1.185	6.914
PAMR1	0.075	1.054	5.936
PANK1	-0.032	0.978	2.684
PANX1	0.250	1.189	4.948
PANX2	-0.726	0.605	4.790
PAPD7	-0.121	0.919	5.520
PAPLN	-0.168	0.890	3.495
PAPPA	-0.361	0.779	2.880
PAPSS2	0.194	1.144	4.708
PAQR4	-0.084	0.943	6.284
PAQR5	-0.131	0.913	4.660
PAQR6	-0.633	0.645	8.355
PAQR8	0.093	1.067	4.954
PARD3	-0.289	0.818	5.664
PARD3B	-0.205	0.867	5.319
PARD6A	-0.327	0.797	4.930
PARD6G	-0.281	0.823	2.146
PARD6G-AS1//AC139100.2	-0.033	0.977	3.565
PARG	-0.065	0.956	5.166
PARK2	0.380	1.301	5.857
PARM1	0.211	1.158	7.229
PARP12	0.429	1.346	4.807
PARP14	0.644	1.563	5.989
PARP2	0.213	1.159	4.595

PARP4	0.132	1.096	7.510
PARP9	0.610	1.526	4.865
PART1	0.088	1.063	2.763
PARVG	0.788	1.726	3.471
PAX2	0.034	1.024	2.374
PAX3	0.184	1.136	3.839
PAX8	0.422	1.340	2.055
PAX8-AS1//AC016683.6	0.281	1.215	2.294
PAXBP1-AS1	0.325	1.252	3.204
PBLD	0.044	1.031	5.069
PBX1	-0.402	0.757	4.231
PBXIP1	-0.208	0.866	5.439
PCBD2	-0.253	0.839	3.551
PCBP3	-0.349	0.785	3.438
PCBP4	-0.063	0.957	6.528
PCDH1	0.138	1.101	5.224
PCDH10	-0.308	0.808	6.956
PCDH11Y//PCDH11X	0.419	1.337	3.810
PCDH15	0.169	1.124	4.540
PCDH18	0.721	1.648	5.650
PCDH19	-0.094	0.937	4.385
PCDH7	0.171	1.126	5.994
PCDH8	0.583	1.497	4.683
PCDH9	0.012	1.008	8.556
PCDHB10//PCDHB9	0.313	1.242	5.965
PCDHB12	0.383	1.304	5.763
PCDHB13	1.132	2.192	4.035
PCDHB14	0.582	1.497	5.244
PCDHB15	0.471	1.386	4.900
PCDHB16	0.528	1.441	4.026
PCDHB18	0.267	1.203	4.884
PCDHB19P//AC005752.8	0.434	1.351	4.046
PCDHB2	0.047	1.033	3.895
PCDHB4	0.831	1.779	5.757
PCDHB6	0.378	1.300	4.899
PCDHB7	0.203	1.151	4.494
PCDHB8	0.321	1.250	2.469
PCDHB9	0.041	1.029	3.954
PCDHGB8P	0.015	1.010	4.445
PCDP1	-0.620	0.651	5.094
PCED1B-AS1	0.602	1.517	3.061
PCK2	0.530	1.443	2.665
PCLO	0.141	1.103	5.550
PCMT1	-0.031	0.979	6.088
PCOLCE	0.327	1.255	3.768
PCOLCE2	-0.183	0.881	2.163
PCP2	-0.176	0.885	2.198
PCP4	-0.205	0.867	3.320
PCP4L1	0.354	1.278	5.586
PCSK1	0.480	1.395	4.771

PCSK1N	-0.048	0.967	6.031
PCSK2	-0.360	0.779	6.295
PCSK6//LOC100507472	0.038	1.027	5.366
PCTP	0.488	1.402	2.114
PCYOX1	0.201	1.149	6.597
PCYOX1L	0.157	1.115	4.805
PDCD1LG2	1.145	2.212	3.519
PDCD6IPP2	-0.099	0.934	3.951
PDCL3P4	-0.321	0.801	4.974
PDDC1	-0.214	0.862	5.715
PDE10A	0.075	1.053	5.195
PDE10A	-0.026	0.982	2.309
PDE1A	-0.049	0.967	6.150
PDE1B	0.081	1.057	5.742
PDE1C	0.472	1.387	5.185
PDE2A	-0.185	0.880	4.025
PDE3A	0.093	1.067	4.638
PDE3B	0.310	1.240	4.734
PDE4A	0.067	1.048	4.857
PDE4B	0.245	1.185	5.436
PDE4D	0.068	1.048	3.808
PDE5A	0.094	1.067	3.565
PDE6B	0.440	1.357	5.029
PDE7B	-0.196	0.873	6.531
PDE8A	0.162	1.119	6.457
PDE8B	0.059	1.042	6.706
PDE9A	-0.070	0.953	4.952
PDGFB	-0.361	0.779	4.511
PDGFD	-0.839	0.559	3.514
PDGFRA//FIP1L1	-0.236	0.849	6.188
PDGFRL	-0.127	0.916	4.380
PDIA2	-0.195	0.874	4.901
PDIA3P1	-0.470	0.722	5.860
PDIA4	-0.178	0.884	5.913
PDIK1L	0.073	1.052	3.896
PDK1	-0.030	0.980	6.183
PDK3	-0.050	0.966	5.677
PDK4	-0.025	0.983	7.349
PDLIM1	0.059	1.041	5.234
PDLIM3	0.041	1.029	4.492
PDLIM4	0.440	1.356	3.509
PDLIM5	0.063	1.045	4.853
PDP1	0.262	1.199	3.902
PDP2	0.281	1.215	4.663
PDPK1//LOC101060747//PDPK2	-0.029	0.980	7.401
PDPN	0.418	1.336	4.430
PDPR	-0.149	0.902	6.211
PDS5B	0.143	1.104	7.555
PDSS2	0.208	1.155	2.575
PDXP//SH3BP1	0.079	1.056	6.356

PDYN	0.625	1.542	2.232
PDZD2	-0.024	0.984	5.387
PDZD4	-0.307	0.808	6.296
PDZD7	0.020	1.014	3.307
PDZRN3	-0.268	0.831	4.594
PEBP4	0.385	1.306	3.535
PEG10	0.238	1.179	7.631
PEG3//ZIM2	0.060	1.042	6.394
PEG3-AS1	-0.200	0.870	3.791
PELI1	0.376	1.297	6.911
PELI3	0.219	1.164	4.937
PENK	0.105	1.075	2.500
PER1//MIR6883	-0.231	0.852	6.198
PERP	-0.079	0.947	4.066
PEX1	0.323	1.251	5.884
PEX10	0.115	1.083	2.677
PEX11B	0.204	1.152	6.360
PEX11G	-0.515	0.700	3.809
PEX12	0.356	1.279	5.098
PEX5L	0.098	1.070	7.577
PEX5L-AS1	0.175	1.129	3.351
PFDN4	0.037	1.026	4.745
PFKFB2	0.370	1.292	5.900
PFKFB3	0.310	1.240	7.743
PFKM	-0.149	0.902	4.389
PFKP	-0.298	0.813	7.553
PFN1	0.037	1.026	8.636
PGAM2	0.387	1.308	5.665
PGAM4	-0.560	0.678	3.427
PGAM5	-0.020	0.987	4.563
PGAP1	0.160	1.117	6.517
PGAP3	0.513	1.427	3.874
PGBD2	0.305	1.235	3.454
PGBD4	0.020	1.014	5.039
PGBD5	0.150	1.110	5.903
PGM2	0.323	1.251	5.997
PGM2L1	0.247	1.187	6.009
PGP	0.039	1.027	5.622
PGPEP1//FKBP8	-0.144	0.905	6.079
PHACTR4	0.059	1.042	6.690
PHC1	-0.350	0.785	6.698
PHC2	0.062	1.044	5.607
PHEX	-0.038	0.974	2.192
PHF14	-0.042	0.971	5.993
PHGDH	0.047	1.033	5.797
PHKA1	0.423	1.340	5.378
PHKA2	0.331	1.258	5.451
PHKG1	-0.189	0.877	3.822
PHLDA3	-0.263	0.834	4.310
PHLDB1//MIR6716	0.159	1.117	4.335

PHLDB2	-0.089	0.940	2.736
PHLPP1	0.070	1.050	8.135
PHLPP2	0.145	1.106	6.015
PHOSPHO2-KLHL23//PHOSPHO2//KLHL23	0.068	1.048	3.960
PHPT1	-0.145	0.904	4.991
PHTF1	-0.365	0.776	4.752
PHTF2	0.353	1.277	5.028
PHYHD1	-0.222	0.857	4.740
PHYHIP	-0.187	0.879	6.339
PHYHIPL	0.036	1.025	7.626
PHYKPL	0.170	1.125	4.782
PI15	0.958	1.943	3.496
PI16	-0.579	0.669	4.042
PI4K2A//RP11-548K23.11	0.309	1.239	7.120
PI4K2B//SEPSECS-AS1	-0.438	0.738	4.245
PI4K2B//SEPSECS-AS1	0.130	1.094	3.868
PI4KA	-0.073	0.951	7.887
PI4KA//PI4KAP2//PI4KAP1	-0.085	0.943	6.586
PI4KA//PI4KAP2//PI4KAP1	-0.095	0.936	7.818
PIANP	-0.115	0.923	5.131
PID1	-0.436	0.739	3.347
PIEZO1	0.053	1.037	5.055
PIEZO2	0.266	1.202	5.575
PIFO	-0.175	0.886	3.851
PIGBOS1//RP11-139H15.1	0.064	1.045	4.015
PIGF	0.282	1.216	4.116
PIGM	-0.039	0.973	6.235
PIGN	0.195	1.145	5.822
PIGT	0.047	1.033	7.360
PIGZ	-0.210	0.864	4.724
PIH1D2	-0.333	0.794	2.286
PIK3AP1	0.875	1.834	5.456
PIK3CA	0.242	1.182	6.923
PIK3CB	0.256	1.194	6.144
PIK3CG	0.621	1.538	5.266
PIK3R1	0.077	1.055	8.515
PIK3R2//AC007192.4	-0.061	0.959	5.379
PIK3R3//LOC102724021//RP11-322N21.2	0.233	1.175	5.198
PIK3R5	0.244	1.184	3.804
PIK3R6	0.228	1.171	2.297
PILRA	0.600	1.516	2.913
PIM1	0.481	1.396	5.723
PIM2	0.369	1.291	4.277
PIN4P1	0.047	1.033	2.320
PIP4K2A	0.099	1.071	8.654
PIP4K2B	-0.019	0.987	8.427
PIP4K2C	0.075	1.053	6.180
PIP5K1B	-0.088	0.941	4.386
PIPOX	0.255	1.193	4.398
PIR-FIGF//PIR//FIGF	0.223	1.167	3.658

PIRT	0.907	1.875	5.963
PITPNB	0.158	1.116	6.640
PITPNM1	-0.023	0.984	5.408
PITPNM2	0.054	1.038	4.503
PITPNM3	0.039	1.027	4.607
PITRM1-AS1	-0.357	0.781	2.376
PIWIL4	0.179	1.132	4.005
PJA2	0.083	1.059	8.300
PKDCC	0.026	1.018	3.434
PKI55//RP11-566E18.3	0.060	1.043	6.403
PKIA	0.374	1.296	5.417
PKNOX2	0.177	1.131	4.610
PKP2	-0.081	0.945	4.346
PKP4	-0.396	0.760	8.625
PLA1A	0.359	1.282	3.836
PLA2G15	0.377	1.299	5.215
PLA2G16	0.017	1.012	8.115
PLA2G4A	0.165	1.121	4.853
PLA2G4C	0.222	1.166	5.388
PLA2G4C	0.048	1.034	3.619
PLA2G7	1.507	2.842	5.337
PLA2R1	0.161	1.118	3.553
PLAC8	-0.297	0.814	4.058
PLAGL1	0.623	1.540	4.833
PLAT	0.195	1.145	5.926
PLAU	0.473	1.388	2.478
PLAUR	0.614	1.530	4.887
PLBD1	0.756	1.689	4.158
PLCB1	0.142	1.104	7.656
PLCB1-IT1//LOC100128668	-0.019	0.987	2.279
PLCB2	0.219	1.164	4.836
PLCB3	-0.014	0.990	5.197
PLCB4	-0.101	0.932	5.978
PLCD1	-0.024	0.984	4.552
PLCD3	-0.017	0.988	6.819
PLCG2	0.471	1.386	4.703
PLCH1	0.139	1.101	4.495
PLCL1	-0.137	0.909	6.892
PLCXD1	0.519	1.433	4.545
PLCXD3	0.118	1.086	5.875
PLD1	-0.031	0.979	6.126
PLD2	0.197	1.146	5.748
PLD3	0.066	1.047	6.278
PLD4	0.352	1.276	3.868
PLD5	-0.183	0.881	5.293
PLEC	-0.197	0.873	6.186
PLEK	0.391	1.311	4.310
PLEKHA1	0.261	1.198	6.403
PLEKHA4	0.105	1.076	3.902
PLEKHA6	0.286	1.219	3.356

PLEKHA6	-0.277	0.825	6.913
PLEKHA8P1//RP11-139E19.1	0.177	1.130	3.764
PLEKHB1	-0.132	0.912	5.773
PLEKHF1	-0.177	0.884	4.251
PLEKHG2	0.216	1.161	2.837
PLEKHG3	0.145	1.106	4.644
PLEKHG5	-0.017	0.989	3.560
PLEKHH2	-0.031	0.979	4.788
PLEKHM3	0.035	1.025	5.617
PLEKHO2//AC069368.3	0.489	1.404	5.830
PLIN1	-0.659	0.633	4.277
PLIN2	0.459	1.375	3.507
PLIN3	0.258	1.196	6.112
PLIN4	-0.222	0.857	3.413
PLK2	0.350	1.274	4.106
PLK3	0.705	1.630	4.580
PLK5	-0.051	0.965	2.928
PLLP	0.317	1.246	7.947
PLN	-0.361	0.779	2.597
PLOD1	-0.206	0.867	6.696
PLOD2	0.126	1.092	6.951
PLOD3	0.083	1.059	5.372
PLP2	0.148	1.108	5.579
PLS1	0.302	1.233	3.250
PLSCR1	1.024	2.034	5.563
PLSCR4	0.419	1.337	6.783
PLTP	-0.021	0.986	8.217
PLVAP	0.104	1.075	3.041
PLXDC2	0.637	1.555	5.581
PLXDC2	0.293	1.225	9.067
PLXNA1	-0.233	0.851	5.629
PLXNA2	0.470	1.385	5.904
PLXNA3	-0.088	0.941	4.653
PLXNA4	-0.267	0.831	4.998
PLXNB1	0.048	1.034	6.289
PLXNB2	-0.069	0.953	6.457
PLXNB3	0.017	1.012	5.231
PLXND1	-0.093	0.938	5.652
PMP2	0.105	1.076	10.309
PMP22	0.118	1.085	6.682
PMS2	0.192	1.143	5.473
PMS2CL	0.377	1.299	5.408
PMS2P1	-0.468	0.723	4.464
PMS2P9	0.058	1.041	3.262
PNCK	0.084	1.060	3.654
PNKD	0.099	1.071	4.545
PNLDC1	0.091	1.065	2.323
PNMA1	0.109	1.078	7.936
PNMA2	-0.138	0.909	6.853
PNMA3	-0.281	0.823	4.524

PNMA5	0.384	1.305	3.501
PNMA6A//PNMA6C//PNMA6D	0.203	1.151	3.878
PNMA6A//PNMA6C//PNMA6D	0.225	1.169	3.703
PNMA6A//PNMA6C//PNMA6D	0.185	1.137	3.887
PNMA6A//PNMA6C//PNMA6D	0.149	1.109	3.711
PNMAL1	0.228	1.171	6.083
PNMAL2	-0.204	0.868	4.571
PNOC	0.236	1.178	3.003
PNP	0.381	1.302	4.669
POC1B//POC1B-GALNT4//GALNT4	0.294	1.226	4.805
PODN	0.253	1.191	4.026
PODNL1	-0.085	0.943	3.080
PODXL2	-0.296	0.814	6.705
POLD1	0.260	1.198	3.264
POLM	0.537	1.451	3.713
POLR2B	0.330	1.257	6.651
POLR2G	0.105	1.075	5.567
POLR2L	-0.262	0.834	5.061
POLR3A	0.081	1.058	6.517
POLR3B	-0.048	0.967	5.491
POLR3F	0.241	1.182	5.027
POM121L10P	-0.355	0.782	2.287
POM121L1P	-0.127	0.916	2.797
POM121L8P	0.034	1.024	2.745
POM121L9P	-0.126	0.916	2.664
POMGNT1	0.193	1.143	6.458
POMK//SGK196	0.318	1.246	6.589
PON3	-0.568	0.675	2.880
POPDC3	0.157	1.115	4.515
PORCN	0.267	1.203	5.479
POSTN	0.624	1.541	2.993
POTEF	0.045	1.032	3.033
POU3F1	0.144	1.105	3.115
POU3F2	-0.621	0.650	6.224
POU4F1	0.044	1.031	2.841
POU5F1P4	-0.626	0.648	3.612
POU5F2	-0.514	0.700	2.546
POU6F1	0.181	1.134	5.864
POU6F2	0.103	1.074	3.291
PP12613	-0.162	0.894	1.668
PP13	-0.407	0.754	3.036
PP13	0.022	1.015	3.031
PP13439	-0.359	0.780	2.973
PPAPDC1A	-0.017	0.988	6.322
PPAPDC2	0.201	1.149	3.394
PPARGC1A	-0.403	0.756	3.367
PPARGC1B	0.035	1.024	2.893
PPAT	0.229	1.172	4.436
PPBP	0.277	1.212	2.319
PPDPF	-0.346	0.787	7.126

PPEF1	-0.374	0.772	4.565
PPFIA3	-0.162	0.894	3.806
PPFIA4	-0.154	0.899	4.317
PPFIBP1	-0.224	0.856	6.751
PPFIBP2	0.047	1.033	4.726
PPIAL4B	-0.170	0.889	2.352
PPIAL4B//PPIAL4C//PPIAL4D//PPIAL4F//PPIAL4E//PP	-0.446	0.734	3.043
PPIAL4B//PPIAL4C//PPIAL4E	-0.941	0.521	2.333
PPIAP30	0.165	1.121	3.162
PPID	0.245	1.185	6.272
PPIL6	-0.138	0.909	3.928
PPIP5K1	-0.176	0.885	3.775
PPIP5K1//LOC100508782//AC011330.5	0.240	1.181	7.854
PPIP5K1//LOC100508782//AC011330.5	0.181	1.133	6.517
PPIP5K1//LOC100508782//AC011330.5	0.161	1.118	6.490
PPIP5K1//LOC100508782//AC011330.5	0.179	1.132	7.578
PPIP5K1//LOC100508782//AC011330.5	0.098	1.070	7.149
PPIP5K1//LOC100508782//AC011330.5	0.050	1.035	5.831
PPIP5K1//LOC100508782//AC011330.5	0.047	1.033	5.215
PPIP5K1//LOC100508782//AC011330.5	-0.056	0.962	3.485
PPIP5K1//LOC100508782//AC011330.5	-0.048	0.967	6.794
PPIP5K1//LOC100508782//AC011330.5	0.038	1.027	5.874
PPIP5K1//LOC100508782//AC011330.5	-0.039	0.973	7.629
PPIP5K1//LOC100508782//AC011330.5	0.036	1.025	8.357
PPIP5K1//LOC100508782//AC011330.5	0.033	1.023	6.429
PPIP5K1//LOC100508782//AC011330.5	0.027	1.019	4.568
PPIP5K2	-0.021	0.985	5.986
PPL	0.192	1.143	3.431
PPM1E	-0.345	0.787	4.195
PPM1H	-0.083	0.944	6.067
PPM1J	0.196	1.145	3.071
PPM1K	0.149	1.109	7.058
PPM1L	0.044	1.031	5.025
PPME1	0.086	1.062	6.340
PPP1R12A	0.087	1.062	6.518
PPP1R13B	-0.216	0.861	5.191
PPP1R14C	-0.161	0.894	3.662
PPP1R15A	0.683	1.606	4.514
PPP1R16B	0.295	1.227	6.901
PPP1R18	0.402	1.321	4.626
PPP1R18	0.338	1.264	5.700
PPP1R18	0.337	1.263	4.674
PPP1R18	0.309	1.239	4.731
PPP1R18	0.195	1.145	4.659
PPP1R1A	0.110	1.079	5.837
PPP1R1B	-0.068	0.954	5.467
PPP1R1C	0.275	1.210	3.303
PPP1R32	-0.191	0.876	3.755
PPP1R3B	0.384	1.305	2.939
PPP1R3C	-0.343	0.788	7.013

PPP1R9A	-0.069	0.953	6.638
PPP1R9B	-0.055	0.963	7.080
PPP2R2B//RNA5SP196	-0.136	0.910	6.441
PPP2R2B-IT1	-0.438	0.738	4.537
PPP2R3A	0.064	1.045	5.188
PPP2R3B//LOC102725016	-0.094	0.937	3.544
PPP2R5D	0.024	1.017	6.099
PPP2R5E	0.173	1.128	6.618
PPP3CA	0.174	1.128	8.269
PPP3CB	0.110	1.080	6.532
PPP3CB-AS1//RP11-137L10.6	-0.218	0.860	3.522
PPP3R1//RP11-474G23.1	-0.040	0.972	7.987
PPP4R1L	0.064	1.046	2.651
PPP4R4	-0.109	0.927	5.132
PPP5D1	-0.092	0.938	2.663
PPTC7	0.027	1.019	5.526
PPWD1	0.429	1.346	5.865
PRAM1	0.325	1.253	3.549
PRB1	0.075	1.053	3.993
PRC1	0.094	1.067	4.384
PRDM1	0.119	1.086	3.686
PRDM10	0.316	1.245	5.314
PRDM11//LOC101060179	-0.087	0.942	3.833
PRDM2	-0.053	0.964	5.909
PRDM4	0.297	1.229	5.305
PRDM5	-0.344	0.788	4.674
PRDM6	-0.386	0.765	2.802
PRDM8	-0.146	0.904	4.195
PRELP	-0.050	0.966	5.301
PREP	-0.048	0.967	5.925
PREPL	0.151	1.110	8.870
PREX1	0.102	1.073	7.450
PRF1	-0.543	0.686	2.171
PRG4	-0.740	0.599	1.728
PRICKLE1	0.039	1.027	5.480
PRICKLE2	0.015	1.011	6.921
PRICKLE4//TOMM6	-0.329	0.796	2.844
PRIMA1	-0.117	0.922	4.914
PRKAA2	0.474	1.389	5.653
PRKACB	0.153	1.112	7.585
PRKAG2-AS1	0.130	1.094	2.667
PRKAR1B	-0.376	0.771	5.512
PRKAR1B	-0.195	0.874	3.052
PRKAR2B	0.137	1.100	6.204
PRKCB	0.165	1.121	5.200
PRKCE	0.032	1.023	4.428
PRKCG	0.415	1.334	4.438
PRKCI	0.163	1.120	6.028
PRKCQ	0.222	1.167	4.462
PRKCQ-AS1	0.271	1.206	2.816

PRKCSH	-0.096	0.936	8.186
PRKD1	-0.351	0.784	4.640
PRKD3	0.261	1.198	4.874
PRKG2	0.169	1.124	3.376
PRKX	-0.065	0.956	4.381
PRKXP1//RNU6-322P	0.370	1.292	2.902
PRKY	0.427	1.344	3.151
PRLR	0.766	1.700	1.924
PRMT6	0.043	1.030	3.811
PRMT8	-0.207	0.866	4.280
PROCR	0.339	1.265	4.467
PRODH//LOC102724788	-0.432	0.741	4.718
PROK2	0.415	1.333	2.749
PROKR2	-0.134	0.911	2.847
PRORSD1P	-0.258	0.836	2.254
PROS1	0.267	1.203	4.992
PRPF19	0.052	1.037	6.646
PRPH2	-0.127	0.916	3.298
PRPS2	0.476	1.391	6.775
PRR11	0.030	1.021	3.262
PRR16	0.055	1.039	3.028
PRR18	0.109	1.078	5.256
PRR24	0.222	1.166	5.053
PRR34-AS1//RP6-109B7.3	-0.200	0.870	4.399
PRR5L	0.121	1.088	3.761
PRRG1	-0.241	0.846	6.442
PRRG3	0.174	1.128	3.256
PRRG4	0.347	1.271	3.595
PRRT1//LOC100507547	-0.087	0.942	3.995
PRRT1//LOC100507547	-0.089	0.940	3.736
PRRT1//LOC100507547	-0.042	0.972	3.862
PRRT2	-0.021	0.985	4.365
PRRT3	-0.157	0.897	4.499
PRRT3-AS1	0.066	1.047	4.230
PRRX1	0.701	1.625	4.782
PRSS12	-0.022	0.985	2.768
PRSS23	0.970	1.959	3.071
PRSS23//CTD-2005H7.1	0.117	1.085	2.187
PRSS35	-0.481	0.716	2.682
PRTFDC1	0.061	1.043	6.574
PRTG	0.026	1.018	4.316
PSD	0.069	1.049	4.907
PSD2	0.036	1.025	7.183
PSD4	0.243	1.183	2.883
PSEN1	0.191	1.141	5.136
PSMB10//CTC-479C5.12	0.050	1.035	5.007
PSMB8	0.591	1.507	5.437
PSMB8	0.537	1.451	5.435
PSMB8-AS1//XXbac-BPG246D15.8	0.457	1.373	3.556
PSMB9	0.651	1.570	3.032

PSMB9	0.609	1.526	2.690
PSMB9	0.702	1.627	3.488
PSMB9//PSMB8-AS1//XXbac-BPG246D15.8	0.611	1.527	3.669
PSMB9//PSMB8-AS1//XXbac-BPG246D15.8	0.639	1.557	3.442
PSMD12	-0.020	0.986	7.240
PSMD3	0.291	1.224	3.542
PSMD5	0.461	1.376	5.263
PSMD5-AS1	-0.173	0.887	4.894
PSMG3	-0.115	0.923	5.727
PSRC1	-0.067	0.954	4.851
PSTPIP2	0.888	1.850	3.903
PTAFR	0.562	1.476	3.651
PTBP1//MIR4745	0.240	1.181	7.309
PTCH1	-0.372	0.773	4.978
PTCH2	-0.583	0.667	3.455
PTCHD1	0.591	1.507	5.300
PTCHD2	0.343	1.269	3.269
PTCHD4	0.440	1.357	3.799
PTCRA	-0.387	0.765	2.835
PTDSS1	0.181	1.133	6.781
PTGDR	-1.009	0.497	2.661
PTGDS	-0.097	0.935	8.315
PTGER4	0.151	1.110	2.435
PTGES	0.201	1.149	2.703
PTGES3L-AARSD1//PTGES3L//AARSD1	0.172	1.127	3.755
PTGFRN	0.263	1.200	5.548
PTGIS	0.043	1.030	3.346
PTGR1	0.243	1.183	6.451
PTGS1	0.360	1.283	3.752
PTGS2	0.067	1.048	4.117
PTH1R	0.110	1.079	3.848
PTH2R	-0.147	0.903	2.483
PTK2B	0.084	1.060	5.771
PTMA//MIR1244-1//MIR1244-2//MIR1244-3//LOC72	-0.173	0.887	5.709
PTMS	-0.325	0.798	5.606
PTN	0.374	1.296	4.868
PTN	0.035	1.025	8.215
PTP4A3	0.597	1.512	5.690
PTPLAD2	0.446	1.362	3.380
PTPN2	0.551	1.465	4.893
PTPN20B//PTPN20A	0.039	1.027	2.836
PTPN3	0.357	1.281	3.842
PTPN4	-0.092	0.938	5.781
PTPN5	-0.176	0.885	5.503
PTPN6	0.381	1.303	4.696
PTPRC	0.639	1.557	5.689
PTPRCAP	-0.048	0.967	3.262
PTPRD	0.183	1.135	7.632
PTPRD-AS1//RP11-75C9.1	-0.448	0.733	4.391
PTPRD-AS2//RP11-87N24.2	0.318	1.247	2.156

PTPRH	-0.086	0.942	3.295
PTPRJ	0.321	1.250	6.406
PTPRK	0.122	1.088	6.761
PTPRM	-0.013	0.991	7.264
PTPRN	-0.131	0.913	5.566
PTPRN2	-0.112	0.925	5.717
PTPRO	0.336	1.263	4.514
PTPRR	0.294	1.226	3.514
PTPRT	0.171	1.126	5.690
PTRF	-0.263	0.833	5.538
PTTG1IP	0.213	1.159	7.292
PTTG2	-0.015	0.989	3.064
PURB//MIR4657	0.319	1.247	5.926
PURG	-0.100	0.933	5.100
PUS10	0.050	1.035	4.149
PUS7	0.167	1.123	4.302
PUS7L	0.601	1.517	5.374
PVALB	-0.678	0.625	3.301
PVR	0.591	1.506	5.585
PVRL2	0.413	1.332	3.901
PWAR1	0.506	1.420	5.322
PWAR4//SNORD115-23	0.157	1.115	2.247
PWAR5	-0.198	0.872	8.716
PWARSN//IPW//SNORD107//SNORD116-4//SNORD1	-0.266	0.832	8.053
PWP2	0.274	1.209	4.694
PXDC1	0.217	1.162	5.981
PXDN	0.261	1.198	3.917
PXK	0.124	1.090	8.100
PXN	0.185	1.137	4.234
PYCARD	0.452	1.368	3.423
PYCR1	0.152	1.111	3.594
PYCR2//MIR6741//RP4-559A3.7	-0.263	0.833	4.469
PYGL	0.895	1.860	4.940
PYGM	0.123	1.089	5.251
PYROXD2//MIR1287	0.112	1.081	3.892
PYY2	-0.023	0.984	2.527
QDPR	-0.045	0.970	8.226
QKI	-0.048	0.968	8.957
QPCT	0.077	1.055	3.852
QPRT//LOC101929880	0.281	1.215	5.334
QRICH2	0.237	1.179	3.896
QRSL1	0.482	1.396	5.665
QSOX1//FLJ23867	-0.102	0.931	5.413
R3HCC1	-0.080	0.946	5.193
R3HDM1	-0.043	0.971	7.477
RAB11B-AS1//RP11-886P16.3	0.295	1.227	3.736
RAB11FIP1	0.489	1.403	2.419
RAB11FIP2	0.527	1.441	7.299
RAB11FIP4	-0.379	0.769	7.574
RAB11FIP5	-0.176	0.885	4.112

RAB13	-0.109	0.928	4.147
RAB15	0.111	1.080	4.674
RAB20	0.357	1.281	4.510
RAB24//MXD3	0.436	1.353	4.768
RAB26	0.081	1.058	4.764
RAB27A	0.436	1.353	4.424
RAB27B	0.817	1.761	5.397
RAB29//RAB7L1	0.552	1.466	6.018
RAB32	0.457	1.372	3.510
RAB34//NARR	-0.189	0.877	3.940
RAB37	-0.101	0.933	2.426
RAB39A	0.624	1.541	4.287
RAB39B	0.227	1.171	5.793
RAB3A	-0.461	0.726	5.719
RAB3B	-0.177	0.885	4.672
RAB3C	-0.180	0.882	4.747
RAB3D	-0.441	0.736	5.540
RAB3GAP1	0.104	1.075	7.380
RAB3IL1	0.579	1.494	3.873
RAB40A	0.137	1.099	2.978
RAB40C	0.028	1.020	4.864
RAB42	0.766	1.700	2.094
RAB4A//SPHAR	0.090	1.065	6.575
RAB6A//WTH3DI	0.254	1.193	8.834
RAB6B	-0.128	0.915	8.299
RAB6C-AS1//AC079776.7	0.952	1.934	4.967
RAB9B	-0.258	0.836	4.520
RAC3	-0.145	0.904	4.240
RAD1	0.131	1.095	4.093
RAD50	0.053	1.038	7.479
RAD51-AS1	0.106	1.077	4.236
RAD51C	0.288	1.221	4.848
RAD51D	0.311	1.241	5.487
RAD54B//FSBP	0.079	1.056	4.355
RAE1	0.172	1.127	4.345
RAET1E-AS1//RP11-244K5.1//RP11-244K5.8	-0.526	0.694	2.742
RAI14	-0.260	0.835	4.144
RAI2	0.030	1.021	3.544
RALB	-0.036	0.976	6.562
RALGDS	0.304	1.235	7.680
RALYL	-0.191	0.876	4.775
RAMP1	-0.158	0.896	5.427
RAMP2	0.379	1.301	5.062
RAMP2-AS1//CTD-3193K9.7	0.059	1.042	4.457
RAMP3	0.158	1.116	4.898
RANBP3L	0.305	1.236	7.651
RANBP6	0.131	1.095	6.146
RAP1B	0.356	1.280	4.045
RAP1GAP	0.079	1.056	5.508
RAP1GAP2	-0.119	0.921	5.226

RAP2A	0.028	1.020	7.551
RAP2B	0.223	1.167	4.612
RAPGEF2	0.220	1.165	6.894
RAPGEF4	0.155	1.114	6.815
RAPGEF4-AS1	0.256	1.194	2.328
RAPGEFL1	-0.265	0.832	3.855
RAPH1	0.115	1.083	6.285
RARA//BCOR	0.106	1.076	3.954
RARB	0.167	1.123	4.246
RARG	-0.113	0.925	3.036
RARRES2	-0.230	0.852	5.083
RARRES3	0.548	1.462	4.875
RASA1	0.067	1.048	6.932
RASA2	0.470	1.385	4.989
RASA4//RASA4B	-0.300	0.812	3.672
RASA4//RASA4B//AC004985.12//RP11-514P8.6//RP1	0.126	1.091	4.878
RASA4//RASA4B//LOC102725198//RASA4CP//RASA4	-0.091	0.939	5.163
RASA4//RASA4B//POLR2J4//AC004985.12//RP11-514	-0.388	0.764	7.582
RASA4//RASA4B//POLR2J4//AC004985.12//RP11-514	-0.280	0.823	5.453
RASA4//RASA4B//RASA4CP//LOC102725198//RASA4	-0.350	0.784	6.278
RASA4//RASA4B//RASA4CP//LOC102725198//RASA4	-0.031	0.978	6.159
RASA4//RASA4B//RP11-514P8.6//RP11-514P8.8	-0.316	0.804	7.907
RASA4//RASA4B//RP11-514P8.6//RP11-514P8.8	-0.096	0.936	6.173
RASA4//RASA4B//RP11-514P8.6//RP11-514P8.8//PC	-0.233	0.851	5.841
RASA4//RASA4CP//LOC102725198//RASA4B//RP11-5	-0.647	0.639	6.574
RASA4B//RASA4	0.488	1.403	3.954
RASA4B//RASA4	0.214	1.160	4.296
RASA4B//RASA4//RP11-514P8.8//RASA4CP//RP11-5	-0.228	0.854	6.560
RASAL1	0.282	1.216	3.843
RASAL2	0.021	1.015	6.895
RASAL3	0.199	1.148	3.985
RASD1	0.362	1.285	7.383
RASD2	0.118	1.085	5.024
RASGEF1A	0.465	1.380	5.344
RASGEF1B	0.138	1.101	4.164
RASGRF2	0.103	1.074	7.384
RASGRF2-AS1//CTC-459I6.1	-0.050	0.966	2.420
RASGRP1	-0.031	0.979	4.790
RASGRP2	0.125	1.091	2.545
RASGRP3	0.104	1.075	5.535
RASGRP4	0.430	1.347	3.401
RASIP1	0.118	1.085	3.420
RASL10A	-0.089	0.940	3.271
RASL10B	-0.186	0.879	5.868
RASL11B	-0.211	0.864	3.729
RASL12	0.068	1.048	4.558
RASSF1	0.426	1.344	3.064
RASSF2	-0.132	0.913	9.236
RASSF3	0.309	1.239	4.440
RASSF4	0.120	1.087	6.472

RASSF8	-0.097	0.935	5.851
RASSF9	-0.313	0.805	3.014
RB1CC1	0.086	1.061	6.888
RBBP5	0.386	1.306	6.434
RBBP9	0.321	1.250	5.832
RBCK1	0.061	1.043	5.673
RBFOX1	0.199	1.148	5.493
RBFOX2	-0.074	0.950	5.351
RBFOX3	0.344	1.269	4.648
RBM11	-0.014	0.991	4.616
RBM24	-0.358	0.780	2.242
RBM3	-0.028	0.981	5.045
RBM38	-0.488	0.713	3.900
RBM47	0.687	1.610	2.898
RBM48	0.185	1.137	4.812
RBM5-AS1	0.075	1.053	2.699
RBMS3	-0.144	0.905	5.058
RBP4	0.103	1.074	5.313
RBP7	0.431	1.348	3.513
RBPMS	0.351	1.276	3.813
RCAN2	-0.087	0.941	7.878
RCAN3	0.211	1.157	4.634
RCHY1	0.068	1.048	4.733
RCN3	-0.250	0.841	3.354
RCOR2	-0.455	0.729	2.989
RCSD1	0.188	1.140	3.017
RCVRN	0.080	1.057	2.353
RDH10	0.981	1.974	4.698
RDX	0.150	1.109	6.169
REC8	0.047	1.033	3.970
RECQL	0.618	1.534	5.777
REEP1	-0.455	0.730	3.288
REEP2	-0.220	0.858	5.961
REEP3	0.049	1.035	7.366
REEP4	0.095	1.068	3.743
REEP5	0.139	1.101	7.909
REL	0.252	1.191	6.012
RELA	0.223	1.167	5.809
RELL1	0.148	1.108	7.082
RELL2	-0.228	0.854	5.144
RELN	0.304	1.235	5.684
RENBP	0.316	1.245	3.698
REPIN1	0.312	1.241	4.311
REPS2	-0.228	0.854	7.377
RERG	-0.208	0.865	3.836
RET//KIF5B	-0.063	0.957	4.188
RETSAT	0.093	1.067	7.032
REXO1//MIR1909	-0.089	0.940	4.915
RFC3	0.310	1.239	4.389
RFFL//RAD51D//RAD51L3-RFFL	0.073	1.052	5.090

RFK	-0.050	0.966	3.798
RFNG	-0.139	0.908	4.891
RFPL1S	0.350	1.274	4.651
RFT1	0.385	1.306	5.475
RFTN1	0.423	1.341	3.544
RFTN2	0.238	1.179	8.079
RFX1	-0.387	0.764	3.566
RFX2	-0.414	0.751	4.937
RFX4	-0.059	0.960	6.555
RGAG4	0.103	1.074	4.499
RGCC	0.030	1.021	7.568
RGL3	-0.233	0.851	3.856
RGMA	-0.139	0.908	4.362
RGPD4	0.212	1.158	3.329
RGPD6//RGPD8//RGPD5//RGPD4//RGPD3//RGPD1//	0.129	1.094	7.143
RGPD6//RGPD8//RGPD5//RGPD4//RGPD3//RGPD1//	0.118	1.085	7.471
RGPD6//RGPD8//RGPD5//RGPD4//RGPD3//RGPD1//	0.107	1.077	8.470
RGR	-0.249	0.842	2.887
RGS1	-0.086	0.942	7.146
RGS10	0.493	1.407	3.925
RGS11	0.057	1.040	5.390
RGS14	-0.095	0.936	3.540
RGS16	1.080	2.114	4.917
RGS17	0.091	1.065	4.288
RGS19	0.513	1.427	5.066
RGS2	0.222	1.167	6.838
RGS20	0.370	1.292	3.106
RGS22	-0.790	0.578	2.010
RGS4	-0.252	0.840	5.053
RGS6	-0.743	0.598	4.762
RGS7	-0.175	0.886	5.510
RGS7BP	-0.544	0.686	6.231
RGS8	0.100	1.072	4.082
RGS9	0.033	1.023	4.908
RHBDD2	0.190	1.141	6.595
RHBDF1	-0.023	0.984	3.333
RHBDF2	0.609	1.526	5.545
RHBDL1	0.055	1.039	3.305
RHBDL2	-0.190	0.877	4.268
RHBDL3	-0.223	0.857	4.197
RHCG	-0.043	0.970	2.388
RHOB	-0.195	0.874	9.291
RHOBTB2	-0.224	0.856	5.372
RHOF	0.065	1.046	3.588
RHOG	0.221	1.166	2.717
RHOJ	0.213	1.159	4.905
RHOT1	0.240	1.181	7.535
RHOU//DUSP5P1	0.176	1.129	5.147
RHPN2	-0.056	0.962	5.297
RIBC2	-0.486	0.714	2.243

RIIAD1	-0.068	0.954	2.576
RILP	-0.032	0.978	3.676
RIMBP2	-0.104	0.930	5.400
RIMBP2//RP11-117L5.4	0.284	1.217	2.870
RIMKLA	-0.506	0.704	5.098
RIMS1	-0.474	0.720	5.540
RIMS2	0.263	1.200	5.488
RIMS3	0.149	1.109	6.109
RIMS4	0.096	1.069	3.743
RIN1	-0.277	0.825	2.580
RIN3	0.111	1.080	2.982
RINT1	0.198	1.147	4.762
RIPK2	0.354	1.278	5.534
RIPPLY2	-0.376	0.770	3.142
RITA1//C12orf52	0.102	1.074	4.784
RLBP1	0.322	1.250	2.836
RLF	0.227	1.170	6.848
RLIM	0.207	1.155	7.353
RLN3	-0.116	0.923	2.966
RMDN1	0.332	1.258	4.898
RMND1	0.121	1.088	5.125
RMND5A	0.283	1.217	6.477
RMRP//RP11-331F9.10	-0.211	0.864	10.953
RMST	-0.442	0.736	4.984
RN7SK	-0.319	0.802	11.098
RN7SKP105	0.199	1.148	3.803
RN7SKP106	-0.153	0.899	1.873
RN7SKP111	-0.408	0.754	6.019
RN7SKP134	0.017	1.012	2.341
RN7SKP154	-0.235	0.850	2.655
RN7SKP173	-0.358	0.780	3.104
RN7SKP174	-0.173	0.887	2.816
RN7SKP207	-0.214	0.862	2.913
RN7SKP231	-0.304	0.810	5.644
RN7SKP232	-0.730	0.603	2.492
RN7SKP233	-0.377	0.770	3.136
RN7SKP25	-0.556	0.680	3.883
RN7SKP262	-0.340	0.790	2.309
RN7SKP40	0.050	1.035	2.839
RN7SKP5	0.339	1.265	4.208
RN7SKP53	-0.355	0.782	3.262
RN7SKP55	-0.434	0.740	6.015
RN7SKP56	0.593	1.508	4.569
RN7SKP62	0.043	1.030	2.901
RN7SKP65	0.170	1.125	2.645
RN7SKP7	-0.017	0.988	4.456
RN7SKP75	0.383	1.304	2.654
RN7SKP80	-0.128	0.915	4.011
RN7SKP86	-0.478	0.718	2.544
RNA5-8SP2	-0.255	0.838	3.375

RNA5-8SP4	-0.083	0.944	2.772
RNA5-8SP6	0.096	1.069	3.436
RNA5SP111	0.794	1.734	3.324
RNA5SP113	0.216	1.161	3.838
RNA5SP122	-0.329	0.796	3.101
RNA5SP132	-0.059	0.960	5.958
RNA5SP134	-0.844	0.557	2.463
RNA5SP145	-0.037	0.974	2.809
RNA5SP150	-0.505	0.704	5.774
RNA5SP153	-0.088	0.941	3.218
RNA5SP156	0.183	1.135	3.752
RNA5SP161	-0.486	0.714	3.599
RNA5SP174	-0.239	0.847	3.268
RNA5SP176	0.242	1.182	2.223
RNA5SP180	-0.135	0.911	2.254
RNA5SP182	0.366	1.289	3.039
RNA5SP19	-0.081	0.945	5.250
RNA5SP190	-0.194	0.874	2.450
RNA5SP191	-0.097	0.935	3.175
RNA5SP195	-0.084	0.943	4.510
RNA5SP199	-0.323	0.800	9.273
RNA5SP199	-0.286	0.820	9.939
RNA5SP20	-0.079	0.947	2.265
RNA5SP207	0.036	1.026	2.482
RNA5SP218	-0.235	0.850	2.427
RNA5SP237	0.026	1.018	4.277
RNA5SP242	-0.529	0.693	2.831
RNA5SP248	0.155	1.113	2.580
RNA5SP25	-0.541	0.687	2.550
RNA5SP256	-0.231	0.852	3.064
RNA5SP259	-0.102	0.932	2.636
RNA5SP260	0.137	1.100	4.065
RNA5SP289	-0.087	0.942	2.674
RNA5SP290	-0.591	0.664	3.352
RNA5SP294	0.110	1.080	2.737
RNA5SP300	0.179	1.132	2.288
RNA5SP33	0.258	1.196	4.226
RNA5SP341	0.086	1.061	2.380
RNA5SP343	-0.147	0.903	2.267
RNA5SP344	0.786	1.724	2.549
RNA5SP345	-0.331	0.795	3.366
RNA5SP361	-0.504	0.705	3.247
RNA5SP366	-0.251	0.840	2.802
RNA5SP370	0.042	1.030	2.971
RNA5SP372	0.110	1.079	2.191
RNA5SP383	0.149	1.109	3.233
RNA5SP395	0.344	1.269	3.633
RNA5SP399	-0.358	0.780	4.599
RNA5SP403	-0.243	0.845	4.663
RNA5SP405	0.024	1.017	3.014

RNA5SP406	0.086	1.062	2.719
RNA5SP408	-0.169	0.890	2.647
RNA5SP409	-0.276	0.826	3.467
RNA5SP415	-0.120	0.920	2.648
RNA5SP425	-0.019	0.987	3.538
RNA5SP428	0.179	1.132	4.838
RNA5SP434	0.234	1.176	3.729
RNA5SP435	0.689	1.613	4.000
RNA5SP441	-0.367	0.775	2.739
RNA5SP450	0.360	1.283	2.706
RNA5SP455	-0.328	0.796	2.591
RNA5SP473	-0.426	0.744	4.220
RNA5SP474	0.130	1.094	3.395
RNA5SP478	0.145	1.106	2.609
RNA5SP486	-0.019	0.987	2.866
RNA5SP488	-0.356	0.781	3.114
RNA5SP497	-0.109	0.927	1.986
RNA5SP513	0.163	1.120	2.419
RNA5SP55	-0.450	0.732	3.113
RNA5SP60	-0.377	0.770	4.978
RNA5SP64	-0.322	0.800	2.872
RNA5SP65	-0.193	0.875	3.948
RNA5SP68	0.290	1.223	2.386
RNA5SP69	-0.409	0.753	2.457
RNA5SP82	0.302	1.233	2.727
RNA5SP85	-0.439	0.738	6.222
RNA5SP93	0.097	1.069	3.372
RNA5SP99	-0.360	0.779	4.633
RNASE1	-0.062	0.958	5.614
RNASE2	0.354	1.278	5.822
RNASE3	0.217	1.162	3.402
RNASE6	0.457	1.373	5.815
RNASEL	0.502	1.416	4.165
RNASET2	0.448	1.364	5.408
RND1	0.552	1.466	5.108
RND2	-0.321	0.800	7.959
RND3	0.370	1.292	3.581
RNF11	0.179	1.132	8.372
RNF111	0.308	1.238	6.246
RNF112	0.109	1.079	3.930
RNF122	0.791	1.731	4.228
RNF125//RP11-53I6.2	-0.180	0.883	4.091
RNF126	-0.059	0.960	4.900
RNF128	0.063	1.044	3.027
RNF13	0.028	1.020	8.090
RNF135	0.098	1.070	3.479
RNF139-AS1	0.082	1.059	3.109
RNF144A-AS1	-0.077	0.948	2.831
RNF144B	0.342	1.268	5.233
RNF145	0.266	1.202	5.957

RNF149	0.473	1.388	5.380
RNF150	0.593	1.508	4.676
RNF157	-0.298	0.814	7.294
RNF165	0.109	1.078	4.100
RNF175	-0.479	0.717	4.143
RNF182	0.330	1.257	4.364
RNF185-AS1	0.427	1.345	2.644
RNF19B	0.450	1.366	6.226
RNF2	0.223	1.167	5.182
RNF213	0.120	1.087	6.601
RNF216P1	0.213	1.159	3.336
RNF219	-0.190	0.877	5.542
RNF219-AS1	0.839	1.789	5.161
RNF219-AS1	0.230	1.173	5.319
RNF220	-0.079	0.946	5.538
RNF32	0.096	1.069	2.988
RNF41	0.104	1.075	6.568
RNF43	0.161	1.118	3.208
RNF5P1	-0.069	0.953	3.365
RNF6	0.121	1.088	6.623
RNF8	-0.069	0.953	4.660
RNFT2	0.094	1.067	5.888
RNGTT	0.298	1.229	5.585
RNH1	0.156	1.114	6.242
RNMT	0.220	1.165	5.814
RNMTL1	0.277	1.211	4.465
RNPC3	-0.213	0.863	7.070
RNPEPL1	0.022	1.015	4.391
RNPS1//MIR572	0.451	1.367	3.903
RNU105B	-0.089	0.940	4.358
RNU11	-0.350	0.785	9.485
RNU1-103P	0.107	1.077	3.296
RNU1-106P	0.681	1.603	2.135
RNU1-112P	-0.148	0.903	2.402
RNU1-119P	-0.362	0.778	2.520
RNU1-119P//RNU1-78P	-0.381	0.768	5.830
RNU1-132P	-0.050	0.966	3.903
RNU1-136P	-0.193	0.875	4.799
RNU1-138P	-0.577	0.670	2.686
RNU1-13P	-0.300	0.812	4.864
RNU1-14P	-0.387	0.765	3.943
RNU1-150P	-0.022	0.985	2.578
RNU1-16P	0.392	1.312	4.423
RNU1-18P	-0.798	0.575	3.674
RNU1-19P	-0.279	0.824	4.932
RNU12	-0.569	0.674	6.189
RNU1-23P	0.241	1.182	3.128
RNU1-24P	0.850	1.803	3.320
RNU1-38P	-0.340	0.790	7.243
RNU1-41P//RNU1-48P	0.295	1.227	4.197

RNU1-42P	-0.773	0.585	3.881
RNU1-44P	0.357	1.281	3.127
RNU1-47P	0.018	1.012	2.585
RNU1-51P	0.020	1.014	1.986
RNU1-56P	0.017	1.012	3.587
RNU1-58P	-0.091	0.939	2.519
RNU1-59P	0.330	1.257	4.391
RNU1-74P	-0.188	0.878	2.734
RNU1-82P	-0.116	0.923	2.471
RNU1-83P	-0.608	0.656	8.129
RNU1-87P	-0.145	0.905	4.866
RNU1-91P	0.187	1.138	4.498
RNU1-94P	-0.639	0.642	3.906
RNU2-58P	0.349	1.274	2.875
RNU2-7P	-0.105	0.930	2.779
RNU4-24P	-0.222	0.858	3.142
RNU4-32P//MMAB	0.352	1.276	4.667
RNU4-33P//RNU4-87P	0.441	1.357	3.182
RNU4-61P	-0.530	0.693	2.790
RNU4-62P	0.496	1.411	4.265
RNU4-78P	-0.085	0.943	5.598
RNU4-82P	-0.342	0.789	2.286
RNU4-87P	0.855	1.809	3.626
RNU4ATAC	-0.300	0.812	10.048
RNU4ATAC11P	-0.304	0.810	6.015
RNU4ATAC16P	0.586	1.501	2.795
RNU4ATAC18P	0.332	1.259	2.958
RNU5B-1	0.952	1.935	4.253
RNU5D-1	-0.629	0.647	3.074
RNU5E-1	-0.358	0.780	7.511
RNU5E-9P	-0.288	0.819	5.344
RNU5F-1	0.285	1.218	2.310
RNU6-1003P	0.743	1.674	1.764
RNU6-1005P	-0.053	0.964	5.646
RNU6-1010P	-0.727	0.604	2.330
RNU6-1013P	-0.262	0.834	2.659
RNU6-1014P	0.115	1.083	5.915
RNU6-1016P	-0.408	0.753	5.408
RNU6-1017P	-0.089	0.940	4.872
RNU6-1052P	0.100	1.072	2.552
RNU6-1053P	0.503	1.417	2.650
RNU6-1054P	0.648	1.567	3.555
RNU6-1064P	-0.292	0.817	2.945
RNU6-1067P	-0.398	0.759	4.397
RNU6-1073P	0.221	1.165	2.501
RNU6-1080P	-0.365	0.777	3.043
RNU6-1087P	0.191	1.142	2.765
RNU6-108P	0.150	1.110	2.930
RNU6-1099P	-0.232	0.851	4.815
RNU6-1105P	-0.760	0.590	2.102

RNU6-1112P	-0.620	0.650	2.986
RNU6-1122P	-0.192	0.876	2.427
RNU6-1123P	0.084	1.060	5.057
RNU6-1141P	0.529	1.442	2.120
RNU6-1162P	0.631	1.549	2.082
RNU6-1181P	-0.352	0.783	3.842
RNU6-1184P	-0.444	0.735	3.893
RNU6-118P	-0.248	0.842	7.024
RNU6-119P	-1.020	0.493	3.546
RNU6-1226P	0.025	1.017	4.210
RNU6-126P	-0.498	0.708	2.183
RNU6-1274P	-0.546	0.685	2.033
RNU6-1279P	-0.272	0.828	3.270
RNU6-1284P	-0.188	0.878	3.759
RNU6-1295P	0.380	1.301	2.455
RNU6-1298P	0.048	1.034	2.878
RNU6-1305P	0.136	1.099	2.646
RNU6-130P	-0.203	0.869	4.069
RNU6-1319P//RNU6-747P//RNU6-241P//RNU6-177P	0.134	1.097	4.080
RNU6-1320P//RNU6-1269P//RNU6-599P//RNU6-119	0.314	1.243	4.003
RNU6-1320P//RNU6-1269P//RNU6-599P//RNU6-119	0.229	1.172	4.366
RNU6-1320P//RNU6-1269P//RNU6-599P//RNU6-119	0.228	1.171	4.112
RNU6-1327P	-0.431	0.742	2.527
RNU6-1340P	-0.222	0.857	2.754
RNU6-135P	-0.052	0.965	3.314
RNU6-137P	-0.340	0.790	3.592
RNU6-143P	-0.414	0.751	3.121
RNU6-165P	-0.191	0.876	2.874
RNU6-171P	-0.478	0.718	3.614
RNU6-176P	-0.168	0.890	2.980
RNU6-177P	0.457	1.373	2.901
RNU6-178P	-0.320	0.801	3.522
RNU6-188P	0.316	1.245	4.036
RNU6-195P	-0.290	0.818	2.536
RNU6-196P	0.281	1.215	4.020
RNU6-202P	-0.113	0.925	2.236
RNU6-204P	-0.843	0.557	1.643
RNU6-208P	-0.493	0.711	2.442
RNU6-220P	0.047	1.033	2.632
RNU6-226P	0.589	1.504	4.054
RNU6-252P	-0.460	0.727	2.985
RNU6-271P	-0.659	0.633	1.780
RNU6-283P//RNU6-683P	-0.022	0.985	3.423
RNU6-28P//RNU6-738P//APBA3	-0.281	0.823	3.787
RNU6-294P	0.336	1.262	2.790
RNU6-313P	0.367	1.290	2.774
RNU6-321P	-0.059	0.960	3.943
RNU6-326P	-0.019	0.987	2.506
RNU6-331P	-0.151	0.901	2.636
RNU6-341P	0.218	1.163	4.601

RNU6-353P	-0.508	0.703	5.686
RNU6-359P	-0.474	0.720	2.548
RNU6-360P	-0.367	0.775	2.607
RNU6-367P	-1.020	0.493	3.551
RNU6-371P	0.367	1.290	3.264
RNU6-377P	0.089	1.064	4.223
RNU6-386P	0.137	1.100	2.725
RNU6-395P	-0.361	0.778	3.455
RNU6-398P	0.209	1.156	2.662
RNU6-411P	-0.591	0.664	5.458
RNU6-418P	-0.112	0.925	1.908
RNU6-429P	0.397	1.317	2.907
RNU6-431P	-0.289	0.818	3.515
RNU6-433P	0.184	1.136	3.635
RNU6-439P	0.063	1.044	4.326
RNU6-441P	0.474	1.389	3.130
RNU6-447P//RNU6-1118P//RNU6-1076P//RNU6-35[illegible]	0.246	1.186	4.910
RNU6-457P	-0.353	0.783	2.835
RNU6-466P	-0.199	0.871	5.075
RNU6-469P	0.927	1.901	2.576
RNU6-482P	0.367	1.290	3.968
RNU6-522P	-0.387	0.765	2.654
RNU6-530P	-0.456	0.729	4.869
RNU6-548P	-0.029	0.980	2.006
RNU6-552P	-0.154	0.899	3.551
RNU6-560P	-0.898	0.537	4.390
RNU6-564P	0.309	1.239	2.651
RNU6-572P	-0.415	0.750	2.645
RNU6-580P	-0.752	0.594	5.715
RNU6-59P	-0.635	0.644	2.755
RNU6-605P	0.134	1.097	2.760
RNU6-606P	-0.050	0.966	3.675
RNU6-611P	-0.329	0.796	4.163
RNU6-618P	-0.238	0.848	2.744
RNU6-621P	-0.215	0.862	2.330
RNU6-622P	0.302	1.233	3.504
RNU6-623P	0.175	1.129	2.496
RNU6-652P	-0.422	0.746	2.822
RNU6-653P	0.240	1.181	2.469
RNU6-654P	-0.114	0.924	3.218
RNU6-680P	-0.290	0.818	2.332
RNU6-700P	-0.510	0.702	2.538
RNU6-701P	0.198	1.147	2.652
RNU6-702P	-0.532	0.692	5.919
RNU6-703P	-0.234	0.850	2.381
RNU6-711P	-0.480	0.717	4.087
RNU6-729P	0.090	1.064	5.280
RNU6-731P	0.166	1.122	2.481
RNU6-742P	0.031	1.022	3.811
RNU6-748P	0.430	1.348	2.321

RNU6-758P	-0.307	0.809	5.493
RNU6-759P	-0.843	0.558	2.839
RNU6-760P	-0.479	0.718	5.378
RNU6-767P	0.540	1.453	2.989
RNU6-787P	-0.311	0.806	4.439
RNU6-798P	-0.430	0.742	2.762
RNU6-801P	-0.304	0.810	3.959
RNU6-817P	-0.244	0.844	4.689
RNU6-818P	-0.180	0.882	3.638
RNU6-823P	0.127	1.092	2.651
RNU6-831P	-0.276	0.826	2.003
RNU6-856P	0.568	1.483	2.392
RNU6-876P	-0.432	0.741	2.804
RNU6-878P	-0.462	0.726	3.569
RNU6-882P	0.068	1.048	2.509
RNU6-883P	-0.168	0.890	4.811
RNU6-892P	-0.427	0.744	4.024
RNU6-909P	-0.377	0.770	2.879
RNU6-912P//RNU6-1319P//RNU6-747P//RNU6-241P	-0.442	0.736	3.085
RNU6-925P	-0.882	0.543	3.614
RNU6-928P	-0.627	0.648	2.218
RNU6-936P	0.181	1.133	3.694
RNU6-942P	0.623	1.540	2.914
RNU6-945P	-0.668	0.629	2.062
RNU6-946P	-0.197	0.872	4.808
RNU6-954P	0.183	1.135	1.731
RNU6-957P	-0.183	0.881	2.387
RNU6-979P	-0.475	0.720	3.941
RNU6-984P	-0.195	0.874	3.120
RNU6-992P	-0.277	0.825	2.550
RNU6-999P	-0.023	0.984	2.306
RNU6ATAC//RNU6ATAC2P	-0.466	0.724	7.236
RNU6ATAC10P	-0.462	0.726	4.442
RNU6ATAC11P	-0.222	0.858	2.668
RNU6ATAC24P//PPP1R2	0.518	1.432	3.683
RNU6ATAC27P	0.037	1.026	2.859
RNU6ATAC2P//RNU6ATAC	-0.531	0.692	6.460
RNU6ATAC36P	0.038	1.026	4.054
RNU6ATAC5P	-0.504	0.705	2.943
RNU7-13P//RNU7-41P	0.270	1.206	2.248
RNU7-151P	-0.905	0.534	1.946
RNU7-156P	0.176	1.129	2.915
RNU7-170P	-0.479	0.717	2.475
RNU7-181P	-0.024	0.983	2.830
RNU7-187P	0.551	1.465	3.356
RNU7-193P	-0.448	0.733	2.981
RNU7-194P	-0.036	0.976	2.579
RNU7-35P	0.170	1.125	4.548
RNU7-57P//RNU7-189P	-0.116	0.922	2.604
RNU7-77P	0.526	1.440	2.951

RNU7-80P	0.475	1.390	2.262
RNU7-96P	0.489	1.403	2.291
RNVU1-14	-0.052	0.965	2.716
RNVU1-15	-0.377	0.770	6.023
RNVU1-15	-0.262	0.834	5.616
RNVU1-3	-0.041	0.972	2.800
RNVU1-6	-0.106	0.929	2.429
RNVU1-8	-0.321	0.801	2.317
RNY3P2	0.439	1.356	1.971
RNY3P9	0.537	1.451	2.557
RNY4P13	-0.218	0.860	1.996
RNY4P18	-0.393	0.762	4.974
ROBO2	0.538	1.452	6.263
ROCK2	0.016	1.011	6.663
ROM1	-0.062	0.958	4.318
ROPN1L	-1.469	0.361	3.668
ROPN1L//RP11-1C1.7	-0.296	0.814	2.781
RORB	0.199	1.148	6.389
ROS1//SLC34A2	-0.372	0.773	2.584
RP1-102E24.8	-0.077	0.948	2.859
RP11-1008C21.2	-0.292	0.817	2.813
RP11-102L12.2	0.045	1.032	4.232
RP11-1049A21.2	0.297	1.228	2.154
RP11-1049H7.2	0.049	1.035	3.727
RP11-1079K10.4	-0.132	0.913	3.843
RP11-108K3.4	0.132	1.096	3.485
RP11-108M9.2//RP5-875O13.6	-0.527	0.694	3.083
RP11-10A14.4//LOC102724880	0.166	1.122	2.372
RP11-10J21.4	0.488	1.402	2.638
RP11-10K16.1	0.149	1.108	4.209
RP11-10L12.4	0.198	1.147	3.988
RP11-110I1.12	-0.060	0.959	3.840
RP11-110I1.13	0.210	1.156	3.147
RP11-1114A5.4	0.369	1.291	3.516
RP11-111F5.1//RP11-204M4.1	-0.250	0.841	3.237
RP11-1143G9.4	0.759	1.692	2.025
RP11-116N8.1	-0.059	0.960	5.871
RP11-118B22.4	-0.316	0.803	5.185
RP1-111C20.3	0.480	1.395	2.325
RP11-11N9.4	0.212	1.158	3.908
RP11-120J1.1	-0.420	0.747	3.592
RP11-121A14.3//NEK6	0.097	1.069	3.081
RP11-122C5.3	0.343	1.269	3.168
RP11-122K13.12	0.400	1.320	4.315
RP11-123O10.4	-0.434	0.740	3.574
RP11-124D2.3	-0.282	0.822	2.843
RP11-124N14.3	0.445	1.361	4.640
RP11-124N2.1	-0.198	0.872	2.474
RP11-134G8.7	0.135	1.098	2.647
RP11-134G8.8	-0.047	0.968	2.625

RP11-13E1.5	-0.261	0.835	3.347
RP11-143A22.1	0.076	1.054	2.844
RP11-144G6.4//RP11-96C23.10//RP11-464F9.21	0.104	1.075	3.002
RP11-144G6.4//RP11-96C23.10//RP11-464F9.21	0.043	1.030	2.799
RP11-14I17.2	-0.742	0.598	3.388
RP11-154D6.1//LINC00472//RP3-331H24.5	-0.075	0.949	3.541
RP11-156E6.1	0.070	1.050	5.843
RP11-158A14.1	-0.479	0.717	3.091
RP11-158J3.2	0.066	1.047	2.281
RP11-159F24.6	0.358	1.282	2.341
RP11-164J13.1	0.065	1.046	3.313
RP11-165J3.6	-0.104	0.930	2.522
RP11-166B2.1	-0.357	0.781	3.924
RP11-169E6.1	0.330	1.257	2.143
RP11-169K16.4	-0.329	0.796	3.621
RP11-16D22.2	-0.190	0.877	2.443
RP11-16P20.3	0.209	1.156	3.253
RP11-171A24.3	-0.287	0.819	2.608
RP11-174M13.2	0.030	1.021	1.914
RP11-181B11.1	-0.019	0.987	1.715
RP11-184A2.2	0.029	1.020	3.501
RP11-190P13.2	-0.079	0.947	2.821
RP11-196D18.1	-0.150	0.901	2.474
RP11-196G18.22	0.019	1.013	3.954
RP11-1C8.4	0.123	1.089	2.596
RP11-203E8.1	-0.187	0.878	2.137
RP11-203F10.5	-0.055	0.962	3.577
RP11-206L10.4	-0.340	0.790	2.719
RP11-207C16.4	-0.029	0.980	3.049
RP11-212D3.4	0.159	1.116	2.352
RP11-216M21.7	-0.038	0.974	3.895
RP11-229P13.15	-0.231	0.852	2.761
RP11-22P4.1	0.654	1.573	2.829
RP11-236B18.5	-0.804	0.573	2.384
RP11-238K6.2	-0.118	0.921	2.214
RP11-244F12.2	0.019	1.013	2.238
RP11-244N9.4//XPA	0.286	1.219	4.182
RP11-247L20.4	-0.038	0.974	2.538
RP11-250B2.3	0.109	1.078	3.486
RP11-253I19.3	0.263	1.200	4.082
RP11-257K9.8	-0.278	0.825	4.210
RP11-261C10.7	-0.174	0.887	4.278
RP11-263K19.4	-0.187	0.879	4.852
RP11-264A11.1	-0.123	0.919	2.998
RP11-272L13.3	0.425	1.343	3.549
RP11-272P10.2	-0.122	0.919	2.503
RP11-274H2.3	0.040	1.028	2.478
RP11-274J7.2	0.527	1.441	4.113
RP11-275H4.1	0.280	1.215	2.462
RP11-276M12.1	-0.015	0.989	2.366

RP1-127H14.3	-0.214	0.862	2.559
RP11-281A20.2	-0.069	0.954	2.077
RP11-281O15.4	-0.145	0.905	3.174
RP11-282K24.3	-0.126	0.916	4.588
RP11-283C24.1	-0.023	0.984	7.689
RP11-283G6.3	0.318	1.246	4.719
RP11-284M14.1	0.124	1.090	4.524
RP11-286E11.2	-0.044	0.970	2.672
RP11-288G11.3	-0.348	0.785	5.981
RP11-288I21.1	-0.509	0.703	2.872
RP11-290D2.3	0.128	1.093	2.967
RP11-293M10.6	-0.330	0.796	3.469
RP11-295K2.3	0.477	1.392	2.870
RP11-305F18.1	-0.287	0.820	3.104
RP11-305L7.3	0.190	1.141	3.010
RP11-30J20.1	-0.308	0.808	2.080
RP11-30K9.4	-0.216	0.861	2.421
RP11-313F23.4	0.142	1.103	2.382
RP11-314N13.3	0.128	1.093	3.773
RP11-314P15.2	0.209	1.156	3.297
RP11-315E17.1	0.242	1.183	3.990
RP11-315I14.2//LOC101929563	-0.415	0.750	2.898
RP11-316M1.12	0.353	1.277	3.838
RP11-317G6.1	-0.026	0.982	3.059
RP11-318M2.3	0.015	1.010	4.897
RP11-319G6.3	0.267	1.203	2.527
RP11-320M2.1	-0.337	0.792	2.504
RP11-320P7.2	0.215	1.161	5.920
RP11-324O2.6	0.051	1.036	2.421
RP11-326C3.7	0.145	1.106	2.362
RP11-326I11.4	0.439	1.356	4.785
RP11-32B5.7	-0.341	0.790	3.495
RP11-334E6.3	-0.140	0.908	2.159
RP11-334J6.6	0.264	1.201	3.469
RP11-337L12.1	0.025	1.017	2.049
RP11-33B1.3	0.180	1.133	4.783
RP11-342M1.3	0.369	1.292	2.594
RP11-344N10.5	0.198	1.147	3.178
RP11-348F1.3	-0.066	0.955	2.745
RP11-350F4.2	0.116	1.084	4.216
RP11-353N4.5	0.796	1.737	2.604
RP11-355I22.2	-0.045	0.970	2.559
RP11-356M20.3	0.354	1.278	2.922
RP11-359J14.2	0.057	1.040	4.011
RP11-370I10.6//LOC102723866	-0.745	0.596	4.218
RP11-374A22.1	-0.290	0.818	3.748
RP11-379B18.5//LOC101927056	0.228	1.171	3.124
RP11-380G5.2	0.327	1.254	2.563
RP11-381K20.2//LOC101928005	-0.133	0.912	4.712
RP11-383B4.4	0.217	1.162	2.737

RP11-385D13.1	0.026	1.018	2.673
RP11-386G11.3	0.062	1.044	3.312
RP11-386G11.5	-0.130	0.914	1.679
RP11-386G21.2	0.073	1.052	3.030
RP11-388P9.2	-0.146	0.904	3.975
RP11-389O22.1	-0.477	0.718	3.665
RP11-38P22.2	0.208	1.155	3.718
RP11-395I14.2	0.079	1.056	2.432
RP11-396B14.2	-0.088	0.941	3.169
RP11-397D12.4	-0.294	0.815	3.802
RP11-398K22.12	0.101	1.072	3.399
RP11-3D4.2	-0.598	0.661	3.175
RP11-402N8.1//RP11-449H15.2	0.784	1.722	3.228
RP11-402N8.1//RP11-449H15.2	0.716	1.643	4.712
RP11-402N8.1//RP11-449H15.2	0.520	1.434	2.419
RP11-407G23.3	0.160	1.118	4.111
RP11-417L19.2	-0.699	0.616	2.337
RP11-418I22.2//LOC101927026	-0.385	0.766	2.509
RP11-430H10.4	0.217	1.163	2.058
RP11-432J22.2//LOC102724037	0.032	1.023	4.110
RP11-432M8.12	-0.021	0.985	2.459
RP11-432M8.3//RP11-432M8.4//RP11-432M8.5//RP	0.316	1.245	3.139
RP11-432M8.3//RP11-432M8.4//RP11-432M8.5//RP	0.078	1.056	2.979
RP11-432M8.3//RP11-432M8.4//RP11-432M8.5//RP	0.039	1.028	2.995
RP11-436H11.5	-0.027	0.982	4.309
RP11-436I9.5	-0.209	0.865	5.141
RP11-445H22.4	0.039	1.027	2.635
RP11-445N18.5	-0.276	0.826	2.790
RP11-44N11.1	-0.402	0.757	3.055
RP11-463O12.3	-0.540	0.688	3.257
RP11-464F9.21	-0.728	0.604	2.222
RP11-466L17.1	0.222	1.167	3.059
RP11-467H10.2	-0.168	0.890	2.589
RP11-467K18.2	0.445	1.361	2.332
RP11-468H14.2	0.218	1.163	2.216
RP11-469A15.2	0.620	1.537	2.615
RP1-146A15.1	-0.090	0.939	4.492
RP11-473I1.9	0.096	1.069	6.801
RP11-47I22.1	-0.252	0.840	3.656
RP11-480I12.5	-0.129	0.914	2.204
RP11-484O2.1	0.326	1.254	3.098
RP11-486G15.2	0.085	1.060	2.669
RP11-486O12.2	-0.547	0.685	2.862
RP11-486O13.4	0.032	1.023	3.349
RP11-488C13.6	-0.275	0.826	3.120
RP11-489O18.1	-0.238	0.848	3.778
RP11-490H24.5	-0.116	0.922	4.184
RP11-495K9.7	-0.509	0.703	2.277
RP11-495O11.1	-0.258	0.836	2.626
RP11-496D24.2	-0.104	0.930	2.649

RP11-497E19.2	-0.067	0.955	2.320
RP11-498J9.2	-0.243	0.845	3.017
RP11-49O14.2	-0.086	0.942	3.078
RP11-503E24.2	0.641	1.560	4.577
RP11-504P24.2	0.331	1.258	4.842
RP11-505E24.3	0.212	1.159	2.458
RP11-506M12.1	0.075	1.053	2.552
RP11-507B12.2//LOC101928828	-0.229	0.853	2.605
RP11-507K2.3	0.082	1.059	2.965
RP11-509E10.1	0.027	1.019	3.400
RP11-509J21.2	-0.476	0.719	4.006
RP11-513D5.5	0.135	1.098	2.266
RP11-513I15.6	-0.134	0.912	4.913
RP11-514F3.5	-0.336	0.792	2.788
RP11-51G5.1	0.109	1.079	2.348
RP11-521D12.1	0.149	1.109	2.173
RP11-527D7.1	-0.158	0.896	2.996
RP11-527F13.1	-0.170	0.889	2.211
RP11-531A24.5	0.331	1.258	4.271
RP11-536K7.3	0.371	1.293	2.805
RP11-539G18.2	0.133	1.097	3.919
RP11-539I5.1	-0.513	0.701	5.818
RP11-543C4.1	0.092	1.066	2.429
RP11-556E13.1	0.676	1.597	2.715
RP11-556G22.3	-0.096	0.936	2.700
RP11-561I11.3	-0.177	0.884	3.686
RP11-561O23.8	-0.212	0.863	4.416
RP11-562L8.1	-0.056	0.962	1.488
RP11-563N6.6	0.304	1.235	2.731
RP11-56G10.2	0.043	1.030	3.953
RP11-574K11.5	-0.026	0.982	3.916
RP11-579E24.2	-0.183	0.881	2.496
RP11-57H14.2	-0.290	0.818	2.391
RP11-582J16.4	0.422	1.340	4.814
RP11-586K2.1	-0.101	0.932	3.100
RP11-588P7.1	-0.148	0.903	4.903
RP11-589M4.1	0.094	1.068	2.512
RP11-594C13.1	0.118	1.085	2.239
RP11-597D13.7	0.079	1.056	5.309
RP11-5L12.1	-0.082	0.945	2.253
RP11-5N11.2	-0.480	0.717	3.121
RP11-5N11.3	-0.448	0.733	1.886
RP11-5N11.7	-0.462	0.726	2.563
RP11-603J24.17	0.163	1.119	3.767
RP11-617F23.1	0.208	1.155	5.582
RP11-624C23.1	-0.018	0.988	2.126
RP11-624G17.3	-0.270	0.829	2.596
RP11-631M21.1//AP005530.2	-0.088	0.941	3.227
RP11-638I2.2	-0.026	0.982	2.475
RP11-642D21.1	-0.318	0.802	3.107

RP11-644F5.11	-0.359	0.780	3.898
RP11-64B16.3	-0.339	0.791	3.285
RP11-64K12.4	0.253	1.192	3.159
RP11-653B10.1	0.131	1.095	1.812
RP11-660L16.2	-0.055	0.963	3.409
RP11-661A12.5	-0.125	0.917	3.897
RP11-666A20.4	-0.204	0.868	3.638
RP11-66B24.4	0.071	1.051	2.810
RP11-66N11.8	-0.404	0.756	2.289
RP11-672F9.1	-0.053	0.964	4.211
RP11-673E1.4	-0.391	0.763	4.880
RP11-675F6.3	0.030	1.021	2.816
RP11-680F8.4	0.252	1.191	4.163
RP11-685N10.1	0.158	1.116	2.865
RP11-688I9.4	-0.296	0.814	4.998
RP11-691N7.6	0.146	1.106	2.680
RP11-692D12.1	-0.195	0.874	3.741
RP11-696P8.2	-0.300	0.812	3.299
RP11-69A21.2	-0.353	0.783	2.502
RP11-701H24.3	-0.675	0.627	3.421
RP11-706O15.1	-0.563	0.677	2.524
RP11-706O15.7	-0.370	0.774	2.460
RP11-707G14.1	0.260	1.197	5.520
RP11-707G14.7	0.085	1.061	2.177
RP11-707P17.2	-0.119	0.921	2.929
RP11-70C1.3	-0.368	0.775	2.835
RP11-70J12.1	0.623	1.540	4.097
RP11-711K1.7	-0.114	0.924	2.178
RP11-712L6.7	0.063	1.044	2.106
RP11-716H6.1	-0.187	0.878	2.862
RP11-723D22.2	0.447	1.364	3.026
RP11-728F11.4	0.195	1.145	2.795
RP11-736K20.5	0.018	1.013	2.675
RP11-737F9.2	-0.083	0.944	3.233
RP11-739P1.2	0.320	1.249	4.587
RP11-73E17.2	0.492	1.406	2.209
RP11-745L13.2	0.115	1.083	6.193
RP11-745L13.2	-0.022	0.985	2.756
RP11-760H22.2	0.342	1.268	3.071
RP11-762H8.4	0.632	1.550	3.639
RP11-768G7.2	0.126	1.091	4.778
RP11-769N19.2	-0.026	0.982	2.250
RP11-774O3.3	-0.249	0.842	4.953
RP11-776H12.1	0.021	1.015	3.094
RP11-77P16.4	-0.254	0.839	2.818
RP11-77P6.2	-0.056	0.962	3.123
RP11-783K16.10//KCNK4	-0.503	0.706	2.542
RP11-785H5.2	-0.159	0.895	4.412
RP11-798M19.3	0.252	1.191	2.150
RP11-815J21.1	0.144	1.105	2.407

RP11-815J21.2	0.073	1.052	2.673
RP11-819C21.1	0.296	1.227	3.533
RP11-81K13.1	0.018	1.013	2.197
RP11-82L18.2	0.222	1.167	3.227
RP11-839G9.1	0.154	1.113	2.005
RP11-841O20.2	0.503	1.417	4.592
RP11-85M11.2	-0.074	0.950	2.420
RP11-85O21.5	0.104	1.075	2.632
RP11-861E21.2	0.094	1.067	2.920
RP11-862L9.3	0.173	1.128	3.437
RP11-864N7.2	-0.188	0.878	8.365
RP11-867G2.8	-0.216	0.861	2.854
RP11-867G23.12	-0.695	0.618	2.176
RP11-867G23.8//LOC102724064	0.048	1.034	3.129
RP11-86H7.7//LOC102723542	0.332	1.259	3.436
RP1-187B23.1	-0.034	0.977	2.369
RP11-883G14.1	0.222	1.166	2.714
RP11-897M7.1	-0.246	0.843	3.664
RP11-90D4.3	0.136	1.099	2.409
RP11-90J19.1	-0.252	0.840	6.744
RP11-950C14.3	0.279	1.213	3.342
RP11-956J14.1	0.580	1.495	2.173
RP11-966I7.1	0.212	1.158	2.097
RP11-977B10.2	0.681	1.603	2.771
RP11-989F5.3	0.225	1.169	4.155
RP11-99H8.1	0.402	1.321	2.327
RP11-9L18.3	-0.257	0.837	2.970
RP11-9M16.2	0.054	1.038	3.009
RP1-20N2.6//FUCA2	0.208	1.155	2.736
RP1-212P9.2	-0.079	0.947	1.915
RP1-239B22.5	-0.121	0.920	2.175
RP1-249F5.3	-0.104	0.930	2.276
RP1-251M9.2	-0.365	0.777	1.718
RP1-269M15.3	-0.085	0.943	3.720
RP1-293L6.1	0.122	1.089	3.302
RP13-216E22.4	-0.393	0.761	4.766
RP13-317D12.3	-0.288	0.819	3.563
RP13-401N8.1	0.159	1.117	3.301
RP13-578N3.3	-0.340	0.790	1.870
RP13-635I23.3	-0.027	0.981	2.315
RP1-45C12.1	-0.276	0.826	2.826
RP1-46F2.3	0.287	1.220	2.192
RP1-5O6.4	0.252	1.191	4.266
RP1-69D17.4	0.140	1.102	2.337
RP1-8B1.4//LOC102725160	0.283	1.217	3.231
RP1-90G24.10	-0.072	0.951	4.293
RP1-90G24.6	-0.208	0.866	1.732
RP1-97G4.1	0.177	1.131	2.746
RP2	0.417	1.335	5.244
RP3-325F22.5	-0.297	0.814	2.719

RP3-368A4.5	0.120	1.087	6.971
RP3-377H17.2	0.047	1.033	3.006
RP3-380B8.4	-0.091	0.939	2.333
RP3-395P12.2	-0.045	0.969	2.314
RP3-404F18.5	-0.439	0.737	3.911
RP3-405J10.3	0.085	1.061	3.055
RP3-418C23.2//LOC101927314	-0.170	0.889	3.413
RP3-428L16.1	-0.313	0.805	2.240
RP3-462E2.3	-0.073	0.951	3.388
RP3-508I15.18//RP3-508I15.19	-0.280	0.823	4.282
RP3-510D11.1	-0.029	0.980	3.051
RP3-525N10.2	0.110	1.079	7.264
RP4-537K23.4	-0.247	0.843	2.769
RP4-541C22.5	-0.129	0.915	1.918
RP4-564M11.2	0.053	1.038	3.143
RP4-565E6.1	-0.118	0.922	7.305
RP4-580N22.2	-0.017	0.988	2.797
RP4-613B23.1//LOC101928323	-0.307	0.808	4.395
RP4-630C24.3	-0.437	0.739	4.729
RP4-647C14.3	-0.252	0.840	2.939
RP4-666F24.3	-1.457	0.364	1.918
RP4-758J18.7	-0.127	0.916	3.597
RP4-771M4.3	0.177	1.130	2.809
RP4-782G3.1	-0.207	0.866	3.983
RP4-784A16.5	0.116	1.084	3.408
RP4-791M13.3	-0.301	0.812	7.501
RP4-803J11.2	0.147	1.107	2.348
RP4-809F18.2	-0.240	0.847	1.660
RP5-1031D4.2	-0.142	0.906	2.396
RP5-1050D4.2	0.020	1.014	3.094
RP5-1056L3.1	0.041	1.029	2.966
RP5-1104E15.6	-0.181	0.882	2.887
RP5-1157M23.2//LOC101929054	-0.077	0.948	3.264
RP5-1160K1.8	0.061	1.043	2.674
RP5-1172N10.2	-0.793	0.577	2.797
RP5-1177I5.3	0.259	1.197	2.381
RP5-827C21.2	-0.431	0.742	2.757
RP5-857K21.1	0.157	1.115	2.937
RP5-884C9.2	-0.027	0.981	3.093
RP5-940J5.3	-0.191	0.876	3.090
RP5-965F6.2	0.504	1.418	3.256
RP5-968J1.1	-0.258	0.837	2.774
RP6-109B7.4	0.345	1.270	2.850
RPARP-AS1//RP11-18I14.10	-0.028	0.981	3.882
RPE65	0.580	1.494	3.571
RPGR	-0.166	0.891	4.550
RPGRIP1L	0.210	1.156	4.873
RPH3A	-0.061	0.958	5.867
RPL12	0.272	1.207	2.582
RPL12//RPL12P4//NBEAL1//RPL12P8//AC011737.2	-0.057	0.961	9.898

RPL13//SNORD68	-0.037	0.975	3.725
RPL13A//SNORD35A//SNORD34//SNORD33//SNORD	0.111	1.080	2.974
RPL13AP20//RP11-392P7.2	-0.042	0.972	7.186
RPL19	-0.045	0.969	8.464
RPL19P12	-0.088	0.941	3.346
RPL23AP32	0.284	1.218	5.765
RPL23AP53	0.287	1.220	4.469
RPL23AP60//XX-C2158C6.2//AL672183.2//AC138031	0.332	1.259	5.582
RPL23AP7//RPL23AP82//AC002055.4	-0.066	0.955	3.415
RPL39	0.171	1.126	4.088
RPLP0	0.193	1.143	3.139
RPP14	0.592	1.508	4.440
RPP30	0.202	1.150	4.411
RPP40	-0.060	0.959	3.041
RPRM	-0.076	0.949	3.546
RPRML	0.341	1.267	2.703
RPS15A	0.647	1.566	4.939
RPS15AP10	0.334	1.261	3.208
RPS19	-0.129	0.914	8.146
RPS2//SNORA64//AC005363.9	0.301	1.232	3.371
RPS2//SNORA64//RPS2P55//RPS2P46//RPS2P5//RPS	0.207	1.154	9.475
RPS2//SNORA64//RPS2P7//RPS2P46//RPS2P5//RP3-	0.174	1.128	10.794
RPS25	0.494	1.409	5.080
RPS26//LOC100996747//LOC101929876	0.156	1.114	3.171
RPS27	0.103	1.074	10.423
RPS5	-0.062	0.958	8.619
RPS6KA2-IT1	0.050	1.035	4.401
RPS6KA6	0.060	1.042	4.596
RPS6KC1	0.147	1.107	6.056
RPS6KL1	-0.058	0.961	4.290
RPSAP52	0.735	1.665	2.185
RPSAP58	-0.559	0.679	2.158
RPUSD1	0.093	1.067	3.996
RRAD	-0.247	0.843	2.720
RRAGB	0.573	1.488	5.179
RRAS	0.457	1.372	6.426
RRBP1	0.220	1.165	6.778
RREB1	0.286	1.220	5.178
RRH	0.127	1.092	2.448
RRM2B	0.628	1.546	5.949
RRN3	0.510	1.424	5.696
RRN3P2	0.433	1.350	3.239
RRN3P3//LOC101060405//RP11-21M24.1	0.055	1.039	4.424
RRNAD1	0.166	1.122	3.852
RRP12	-0.276	0.826	4.848
RRP7B	0.294	1.226	3.069
RSAD2	0.504	1.419	2.659
RSC1A1//DDI2	0.462	1.378	5.579
RSPH1	-0.760	0.591	3.721
RSPH3	-0.341	0.790	5.519

RSPH4A	-1.428	0.372	2.936
RSPO2	0.168	1.124	3.821
RSPO3	0.139	1.101	2.748
RTCA	0.078	1.056	5.284
RTF1	0.105	1.075	7.159
RTKN	0.027	1.019	5.907
RTKN2	0.126	1.091	1.920
RTN1	-0.390	0.763	7.437
RTN2	-0.383	0.767	4.283
RTN4IP1	0.196	1.146	4.000
RTN4RL1	-0.106	0.929	4.439
RTN4RL2	-0.043	0.970	2.474
RTP1	0.172	1.127	1.938
RTP4	0.125	1.091	3.009
RTP5//CXXC11	0.380	1.302	3.057
RTTN	0.130	1.094	5.847
RUNDC1	0.080	1.057	5.489
RUNDC3A	-0.254	0.838	7.695
RUNDC3B	-0.159	0.895	4.293
RUNX1-IT1	0.309	1.239	2.608
RUNX1T1	0.063	1.045	4.538
RUNX2	0.429	1.347	2.741
RUSC1	-0.136	0.910	3.950
RUSC2	-0.011	0.992	6.591
RWDD2A	0.102	1.073	5.391
RWDD2B	0.172	1.126	3.281
RXFP1	-0.108	0.928	2.785
RXRG	-0.164	0.892	4.425
RYR3	0.035	1.025	6.445
S100A1	-0.374	0.772	4.044
S100A10	0.317	1.245	3.344
S100A11	0.429	1.346	4.012
S100A12	0.135	1.098	4.126
S100A16	0.208	1.155	4.555
S100A3	0.119	1.086	2.429
S100A6	0.041	1.029	4.850
S100A8	0.033	1.023	4.901
S100A9	0.459	1.375	5.300
S100B	-0.189	0.877	10.047
S100PBP	0.140	1.102	3.812
S1PR5	-0.150	0.901	5.321
SAE1	-0.089	0.940	3.460
SALL2	-0.273	0.828	6.064
SALL3	-0.183	0.881	3.980
SAMD1	-0.358	0.781	5.210
SAMD11	0.156	1.114	3.820
SAMD12	-0.211	0.864	5.598
SAMD15	-0.379	0.769	3.025
SAMD5	-0.090	0.939	2.933
SAMD9	0.582	1.497	3.854

SAMD9L	0.761	1.694	4.919
SAMHD1	0.210	1.156	7.676
SAP25//LRCH4	0.305	1.235	3.201
SAPCD1//MSH5-SAPCD1	-0.112	0.925	3.174
SAPCD1-AS1//XXbac-BPG32J3.18	-0.099	0.934	2.377
SASH1	-0.144	0.905	8.037
SASH3	0.547	1.461	4.113
SAT1	0.253	1.192	7.847
SATB2	-0.231	0.852	6.541
SATL1	0.090	1.064	2.750
SBK1	-0.183	0.881	4.124
SBNO1	-0.105	0.930	7.469
SBNO2	0.594	1.510	4.782
SBSPON	0.614	1.531	6.021
SCAI	0.124	1.090	5.646
SCAMP1	0.103	1.074	7.740
SCAMP2	-0.023	0.984	6.101
SCAMP5	-0.106	0.929	7.671
SCARA3	0.273	1.209	7.427
SCARNA1	-0.192	0.876	2.169
SCARNA10	-0.463	0.725	8.615
SCARNA11	-0.757	0.592	6.856
SCARNA14	-0.027	0.981	3.203
SCARNA2	-0.514	0.700	5.708
SCARNA20	-0.609	0.656	4.436
SCARNA21	0.237	1.179	5.237
SCARNA22	-0.284	0.821	5.650
SCARNA27	-0.516	0.699	5.216
SCARNA3	-0.634	0.644	6.747
SCARNA4	-0.214	0.862	7.685
SCARNA5	-0.145	0.904	8.099
SCARNA6	-0.413	0.751	7.408
SCARNA7	-0.623	0.650	7.772
SCARNA8	-0.608	0.656	4.759
SCART1//RP11-108K14.4	-0.067	0.955	2.695
SCCPDH	-0.079	0.947	7.533
SCD	0.054	1.038	10.462
SCFD2	0.151	1.110	6.677
SCG2	0.311	1.241	5.299
SCG3	-0.041	0.972	7.889
SCG5	0.113	1.082	4.919
SCIMP	0.995	1.993	4.037
SCIN	0.377	1.298	5.881
SCML1	0.336	1.262	3.498
SCML2	0.032	1.022	2.302
SCN11A	0.328	1.255	2.374
SCN1A	0.154	1.113	6.693
SCN1B	-0.079	0.947	6.831
SCN2A	0.298	1.230	5.957
SCN3A	0.221	1.166	6.065

SCN3B	0.728	1.656	5.589
SCN4B	-0.701	0.615	4.726
SCN7A	0.621	1.538	3.828
SCN8A	-0.265	0.832	5.392
SCN8A	-0.106	0.929	6.601
SCN9A	-0.047	0.968	3.898
SCNN1D	-0.268	0.831	3.476
SCOC-AS1//RP11-425I13.3	-0.203	0.869	2.775
SCRT1	-0.226	0.855	3.359
SCRT2	-0.153	0.899	2.961
SCUBE1	-0.346	0.787	2.886
SCUBE2	0.245	1.185	3.819
SCX//SCXB	0.381	1.302	2.771
SDAD1	0.015	1.011	6.412
SDC4//ROS1	0.088	1.063	8.065
SDCBP	0.445	1.361	5.143
SDCBP2//FKBP1A-SDCBP2	-0.054	0.963	3.802
SDCBP2-AS1	0.275	1.210	3.443
SDF2L1	0.385	1.306	4.246
SDHAP2//LINC00969	-0.479	0.717	4.665
SDHAP2//LINC00969	-0.980	0.507	4.890
SDHAP2//LINC00969	-0.311	0.806	4.410
SDHAP2//LINC00969//LOC102725383	-0.496	0.709	3.917
SDHC//RP11-122G18.5	-0.370	0.774	4.655
SDIM1	0.109	1.079	2.019
SDPR	-0.707	0.613	5.365
SDR16C5	-0.532	0.691	2.618
SDS	-0.210	0.864	3.240
SDSL	0.207	1.154	4.855
SEC11A	0.268	1.204	4.661
SEC11C	-0.117	0.922	6.898
SEC14L1P1	0.477	1.392	2.925
SEC14L5	0.032	1.022	6.002
SEC16B//RP4-798P15.3	-0.463	0.726	2.552
SEC16B//RP4-798P15.3	0.095	1.068	2.527
SEC16B//RP4-798P15.3	0.061	1.043	3.211
SEC23A	0.257	1.195	6.459
SEC24D	0.465	1.380	5.004
SECTM1	0.778	1.715	4.280
SEH1L	0.426	1.343	7.851
SELE	0.189	1.140	2.840
SELENBP1	0.249	1.189	4.908
SELL	-0.067	0.955	4.967
SELPLG	0.390	1.311	5.167
SEMA3A	0.122	1.088	4.739
SEMA3B//MIR6872	-0.217	0.860	6.835
SEMA3B-AS1//U73167.7	0.307	1.238	3.210
SEMA3C	-0.028	0.981	5.955
SEMA3D	0.077	1.055	4.942
SEMA3E	0.189	1.140	5.108

SEMA3F	0.050	1.036	3.025
SEMA3G	0.631	1.548	3.293
SEMA4B	-0.206	0.867	4.236
SEMA4D	-0.027	0.982	5.951
SEMA4F	-0.155	0.898	4.548
SEMA4G	0.183	1.135	3.499
SEMA5A	0.217	1.162	6.526
SEMA5B	-0.055	0.963	4.002
SEMA6A	0.117	1.084	6.096
SEMA6B	0.323	1.251	5.379
SEMA6C	-0.060	0.960	2.544
SEMA6D	0.229	1.172	6.870
SENP5	0.067	1.047	6.856
SEPHS2	0.237	1.179	5.290
SEPN1	-0.156	0.897	7.040
SEPP1	-0.047	0.968	4.850
SEPSECS	0.033	1.023	5.822
SEPT10	-0.121	0.920	6.167
SEPT11	-0.091	0.939	6.045
SEPT12	-0.025	0.983	2.612
SEPT3	0.041	1.029	9.240
SEPT4	-0.046	0.968	7.136
SEPT5-GP1BB//GP1BB//SEPT5	0.053	1.037	5.393
SEPT6	0.394	1.314	5.135
SEPT9	-0.014	0.991	6.935
SERAC1	0.330	1.257	5.098
SERGEF	-0.067	0.955	4.367
SERHL	0.397	1.317	2.408
SERINC2	0.203	1.151	3.230
SERINC5	0.249	1.188	6.890
SERP2	-0.081	0.946	4.568
SERPINA1	1.110	2.159	2.710
SERPINA3//RP11-986E7.7	0.743	1.673	5.019
SERPINA5	0.221	1.165	2.445
SERPINB1	0.552	1.466	5.535
SERPINB6	0.183	1.135	5.453
SERPINB8	0.537	1.451	3.458
SERPINB9	0.234	1.176	6.070
SERPINB9P1//RP11-420G6.4	0.060	1.042	3.427
SERPIND1	-0.603	0.658	2.846
SERPINE1	0.180	1.133	4.291
SERPINF1	0.729	1.657	6.405
SERPING1	0.112	1.081	6.374
SERPINH1	0.777	1.713	5.473
SERPINI1	0.067	1.048	8.433
SERPINI2	0.026	1.018	2.971
SERTAD4	0.054	1.038	3.462
SERTAD4-AS1	-0.087	0.941	3.823
SERTM1	0.199	1.148	4.553
SESN1	0.077	1.055	6.723

SESN2	0.194	1.144	4.575
SETBP1	0.135	1.098	4.613
SETDB2	0.141	1.102	4.969
SEZ6	0.350	1.274	3.939
SEZ6L	-0.230	0.853	6.413
SEZ6L2	-0.175	0.886	6.633
SFMBT2	-0.237	0.849	6.534
SFN	0.901	1.868	4.245
SFR1	0.132	1.096	3.669
SFRP1	-0.420	0.748	4.223
SFRP2	-0.585	0.666	5.544
SFRP4	0.150	1.109	2.931
SFRP5	-0.095	0.936	3.379
SFT2D2	0.344	1.270	6.747
SGCZ	0.525	1.438	3.802
SGK1	0.166	1.122	6.468
SGK2	0.204	1.152	4.459
SGK223//PRAGMIN	0.391	1.311	4.267
SGK494	0.032	1.023	4.444
SGMS2	-0.148	0.903	3.431
SGPP2	-0.558	0.679	4.886
SGSM1	0.079	1.056	5.091
SGSM2	0.025	1.018	6.680
SGTB	0.226	1.169	7.721
SH2B2	0.294	1.226	3.185
SH2D5	-0.058	0.960	3.047
SH3BGR	0.194	1.144	4.925
SH3BGRL2	-0.169	0.890	8.616
SH3BGRL3	-0.155	0.898	5.093
SH3BP1	0.023	1.016	2.605
SH3BP2	0.229	1.172	3.438
SH3BP4	-0.111	0.926	4.450
SH3BP5-AS1	-0.258	0.836	3.826
SH3D21	0.103	1.074	3.844
SH3GL1	-0.047	0.968	3.133
SH3GL2	0.159	1.117	7.047
SH3PXD2B	0.168	1.124	5.465
SH3RF2	-0.039	0.974	2.118
SH3TC1	0.136	1.099	3.085
SHANK1	0.246	1.186	4.431
SHANK2	-0.298	0.813	2.275
SHANK3	0.208	1.155	5.457
SHB//RP11-613M10.9	0.164	1.120	4.909
SHC1	0.219	1.164	5.744
SHC3	0.053	1.038	6.587
SHC4	0.318	1.247	5.350
SHD	-0.295	0.815	3.120
SHISA2	0.178	1.131	3.631
SHISA3	-0.018	0.987	2.754
SHISA5	0.502	1.416	3.446

SHISA7	0.218	1.163	4.415
SHISA9	0.064	1.046	4.770
SHKBP1	0.252	1.191	5.219
SHMT1//MIR6778	0.432	1.349	4.167
SHROOM1	-0.232	0.852	4.872
SHROOM2	-0.093	0.938	3.075
SHROOM3	-0.102	0.932	3.631
SHROOM4	0.123	1.089	4.958
SIAH3	-0.142	0.907	3.268
SIDT1	0.662	1.582	4.666
SIDT2	0.062	1.044	6.211
SIGLEC10	0.614	1.531	5.213
SIGLEC11	0.270	1.206	2.317
SIGLEC14	1.049	2.069	3.153
SIGLEC7	0.791	1.730	3.626
SIGLEC8	0.801	1.742	4.363
SIGLEC9	1.187	2.278	4.027
SIK1	-0.381	0.768	5.212
SIK3-IT1	0.407	1.326	3.837
SIN3B	-0.038	0.974	6.214
SIPA1L1	0.138	1.101	6.683
SIRPA	0.080	1.057	7.787
SIRPB2	0.863	1.818	3.788
SIRT2	-0.021	0.985	5.615
SIRT3	-0.167	0.890	4.022
SIRT4	-0.079	0.947	1.992
SIRT5	0.090	1.064	5.006
SIX4	-0.044	0.970	2.766
SIX5	0.337	1.263	2.226
SKAP2	-0.030	0.979	6.412
SLA	0.593	1.508	4.911
SLAIN1	-0.063	0.957	7.776
SLAMF8	1.571	2.971	4.386
SLBP	0.038	1.027	5.776
SLC10A4	-0.887	0.541	3.653
SLC10A5	-0.104	0.930	2.763
SLC11A1	1.285	2.437	4.777
SLC12A4	0.332	1.259	6.218
SLC12A5	-0.189	0.877	6.183
SLC12A8	0.139	1.101	3.258
SLC12A9	-0.088	0.941	3.480
SLC13A3	0.148	1.108	5.129
SLC13A4	-0.776	0.584	3.605
SLC13A5	-0.410	0.753	3.932
SLC14A1	-0.337	0.792	9.047
SLC15A2	0.334	1.260	5.644
SLC15A3	0.438	1.355	3.918
SLC16A10	0.462	1.378	4.225
SLC16A12	-0.680	0.624	2.373
SLC16A14	0.254	1.192	4.077

SLC16A3//MIR6787	0.431	1.348	5.227
SLC16A4	0.360	1.283	4.842
SLC16A6	-0.284	0.821	4.195
SLC16A7	0.166	1.122	4.531
SLC16A9	0.016	1.011	5.381
SLC17A5	0.395	1.315	5.835
SLC17A6	0.202	1.150	5.175
SLC17A7	-0.765	0.589	5.603
SLC17A8	0.255	1.193	2.429
SLC18A3	-0.775	0.584	3.371
SLC19A2	0.049	1.035	5.431
SLC1A1	0.102	1.073	5.933
SLC1A2	-0.336	0.792	9.287
SLC1A5	0.929	1.904	4.644
SLC1A6	0.019	1.013	2.687
SLC22A10	-0.143	0.906	1.773
SLC22A15	0.259	1.197	5.450
SLC22A4	0.103	1.074	3.547
SLC22A6	0.180	1.133	3.422
SLC22A9	-0.173	0.887	2.736
SLC24A1	-0.187	0.878	3.122
SLC24A2	-0.011	0.992	9.042
SLC24A3	0.189	1.140	5.111
SLC24A4	-0.372	0.773	4.168
SLC25A1	-0.243	0.845	5.905
SLC25A12	-0.227	0.855	6.238
SLC25A13	-0.104	0.930	5.431
SLC25A14	0.062	1.044	5.344
SLC25A16//LINC00849	-0.328	0.797	3.413
SLC25A18	0.192	1.142	6.631
SLC25A19	0.759	1.692	4.559
SLC25A22	0.135	1.098	4.421
SLC25A23	-0.095	0.937	7.742
SLC25A27	-0.123	0.918	7.077
SLC25A29	-0.061	0.959	6.243
SLC25A3	-0.989	0.504	4.121
SLC25A32	0.299	1.231	5.670
SLC25A37	0.149	1.109	6.174
SLC25A4	0.122	1.088	8.151
SLC25A40	0.314	1.243	5.440
SLC25A44	0.195	1.145	6.021
SLC25A46	0.116	1.083	6.117
SLC26A4	0.158	1.116	2.817
SLC26A4-AS1	0.236	1.178	3.706
SLC26A8	0.315	1.244	3.070
SLC27A1	0.165	1.121	5.719
SLC27A6	-0.381	0.768	3.679
SLC29A1	0.690	1.614	4.925
SLC29A2	-0.134	0.912	4.606
SLC29A3	0.238	1.179	4.822

SLC29A4	-0.925	0.527	4.754
SLC2A12	-0.311	0.806	4.855
SLC2A13	0.391	1.311	5.826
SLC2A3	0.586	1.501	5.126
SLC2A4RG	-0.181	0.882	4.357
SLC2A5	0.422	1.340	4.204
SLC30A3	-0.256	0.837	3.226
SLC30A4	0.106	1.077	4.956
SLC30A9	0.150	1.110	7.228
SLC31A1	0.485	1.399	6.105
SLC31A2	0.240	1.181	6.861
SLC32A1	-0.226	0.855	4.180
SLC35A1//C6orf165//RP3-382I10.7	0.447	1.363	5.625
SLC35B2//MIR4647	0.131	1.095	5.146
SLC35B4	0.148	1.108	6.315
SLC35D1	0.462	1.377	4.698
SLC35E2	-0.054	0.963	4.744
SLC35F1	0.469	1.384	7.097
SLC35F3	0.032	1.022	4.939
SLC36A1	-0.013	0.991	4.773
SLC36A4	0.293	1.225	5.465
SLC37A2	0.629	1.547	2.964
SLC37A3	0.310	1.240	5.011
SLC38A10	-0.133	0.912	4.834
SLC38A11	-0.082	0.945	3.073
SLC38A3	-0.112	0.925	6.746
SLC38A6	0.105	1.075	4.389
SLC38A8	0.131	1.095	2.613
SLC39A10	-0.078	0.947	5.294
SLC39A11	0.126	1.091	5.888
SLC39A12	-0.214	0.862	5.272
SLC39A14	0.350	1.275	5.709
SLC39A3	-0.096	0.936	3.477
SLC39A8	0.073	1.052	4.803
SLC40A1	0.232	1.175	4.136
SLC41A2	0.134	1.098	5.702
SLC43A3	0.735	1.664	3.903
SLC44A2	-0.053	0.964	8.210
SLC44A3	0.887	1.850	4.021
SLC44A5	0.251	1.190	3.898
SLC45A3	-0.040	0.973	4.977
SLC45A4	-0.207	0.866	4.815
SLC46A1	-0.057	0.961	4.869
SLC47A1	-0.245	0.844	3.420
SLC47A2	-0.326	0.798	2.891
SLC4A10	0.205	1.152	5.719
SLC4A2	-0.152	0.900	3.252
SLC4A3	-0.189	0.877	5.133
SLC4A4	0.245	1.185	8.761
SLC4A5//RP11-287D1.3	0.434	1.351	4.222

SLC4A7	0.126	1.091	5.797
SLC4A8	-0.110	0.927	3.980
SLC50A1	0.333	1.260	4.082
SLC51B	-0.058	0.961	2.767
SLC5A11	0.259	1.197	6.596
SLC5A5	-0.208	0.865	2.209
SLC5A6	0.079	1.057	3.769
SLC5A7	-1.011	0.496	3.494
SLC6A1	-0.093	0.937	8.567
SLC6A11	-0.098	0.935	6.164
SLC6A13	-0.729	0.603	3.348
SLC6A15	-0.329	0.796	5.218
SLC6A17	0.180	1.133	6.072
SLC6A20	-0.601	0.659	3.103
SLC6A5	-0.508	0.703	3.553
SLC6A6	0.595	1.511	5.695
SLC6A7	0.482	1.397	3.918
SLC6A9	-0.074	0.950	6.319
SLC7A10	-0.280	0.824	4.177
SLC7A11	-0.031	0.979	7.824
SLC7A14	-0.586	0.666	6.085
SLC7A2	0.265	1.202	5.847
SLC7A3	-0.262	0.834	1.890
SLC7A5	-0.182	0.881	7.849
SLC7A6	0.372	1.294	4.640
SLC7A7	0.281	1.215	2.366
SLC7A8	-0.320	0.801	4.148
SLC8A1	0.174	1.128	4.831
SLC8A2	0.275	1.210	5.910
SLC8A3	-0.203	0.869	3.317
SLC8B1	0.156	1.114	4.481
SLC9A1	0.485	1.400	5.313
SLC9A2	-0.302	0.811	2.270
SLC9A3R1	0.286	1.220	7.578
SLC9A5	0.218	1.163	3.096
SLC9A6	0.196	1.146	8.944
SLC9A7	0.076	1.054	6.525
SLC9A7	0.052	1.037	5.616
SLC9A7P1	0.409	1.328	4.563
SLC9A9	0.267	1.203	7.077
SLC9B2	0.100	1.072	6.169
SLCO1A2	0.025	1.018	6.031
SLCO1C1	1.038	2.054	5.629
SLCO2A1	0.640	1.559	3.726
SLCO2B1	0.476	1.391	5.378
SLCO3A1	0.102	1.073	6.280
SLCO4A1	0.256	1.195	4.266
SLCO4C1	-0.078	0.947	2.560
SLED1//RP11-701P16.1	0.518	1.432	2.882
SLFN11	0.303	1.234	4.128

SLFN12	0.522	1.436	2.327
SLFN5	0.571	1.485	6.107
SLIT1//ARHGAP19-SLIT1//ARHGAP19	0.472	1.387	5.338
SLIT2	-0.440	0.737	5.477
SLIT2-IT1	-0.210	0.864	3.195
SLIT3	-0.385	0.766	5.332
SLITRK1	-0.079	0.947	6.369
SLITRK3	-0.096	0.936	5.324
SLITRK4	0.088	1.063	3.440
SLITRK5	0.881	1.842	5.441
SLITRK5	0.030	1.021	4.917
SLPI	-0.502	0.706	4.156
SLU7	0.186	1.137	5.759
SMA5//SMA4//GUSBP3//LOC653080//GUSBP2//GUS	-0.058	0.960	7.674
SMAD5	-0.419	0.748	2.142
SMAD9	-0.125	0.917	6.802
SMAGP	-0.118	0.921	3.317
SMAP1	0.020	1.014	6.489
SMG1P7//RP11-296I10.6	0.050	1.035	3.699
SMIM10	-0.244	0.844	4.836
SMIM13	0.199	1.148	7.121
SMIM17	0.068	1.048	5.133
SMIM18	-0.288	0.819	2.197
SMIM24//C19orf77	0.163	1.120	2.713
SMIM3	0.032	1.022	4.238
SMIM5	0.260	1.198	2.759
SMIM6	0.134	1.097	3.576
SMIM8	-0.121	0.920	4.716
SMKR1	-0.502	0.706	3.394
SMOC1	-0.107	0.929	5.590
SMOX	0.056	1.039	6.355
SMPD3	0.316	1.245	3.520
SMYD2	-0.179	0.884	6.382
SMYD3	0.143	1.104	4.517
SMYD3-IT1	-0.414	0.751	4.781
SNAI1	-0.022	0.985	2.995
SNAI2	0.681	1.603	4.470
SNAP23	0.288	1.221	7.427
SNAP25	-0.307	0.809	9.944
SNAP25-AS1	0.129	1.094	2.241
SNAP47//LOC100130093	-0.072	0.952	3.870
SNAR-B2//SNAR-B1	-1.217	0.430	5.001
SNAR-C2//SNAR-C4//SNAR-C5//SNAR-C1//SNAR-C3/	-1.465	0.362	4.502
SNAR-C2//SNAR-C4//SNAR-C5//SNAR-C1//SNAR-C3/	-1.499	0.354	5.329
SNAR-C2//SNAR-C5//SNAR-C1	-0.664	0.631	4.369
SNAR-D	-0.755	0.593	3.357
SNAR-F	-1.064	0.478	3.089
SNAR-G1	-0.105	0.930	4.176
SNAR-G2	-0.314	0.804	2.860
SNAR-H	-1.093	0.469	1.894

SNCA	-0.345	0.787	6.617
SNCAIP	-0.336	0.792	3.531
SNCB	-0.100	0.933	6.651
SNCG	-0.640	0.642	6.206
SNED1	-0.495	0.710	4.619
SNHG10//SCARNA13	-0.421	0.747	4.483
SNHG12//SNORA44//SNORA61//SNORA16A	0.105	1.076	4.482
SNHG15//SNORA9//LOC101928927	0.421	1.339	3.958
SNHG18//CTD-2001E22.2	0.586	1.501	3.227
SNHG24//RP11-909M7.3	-0.245	0.844	5.274
SNHG7//SNORA17//SNORA43	0.243	1.184	4.527
SNN	0.150	1.110	8.153
SNORA10//RPS2//SNORA64	-0.109	0.927	6.654
SNORA11B	-0.257	0.837	3.791
SNORA13	-0.151	0.901	5.429
SNORA14A	-0.156	0.898	5.874
SNORA14B	-0.622	0.650	6.899
SNORA15	-0.312	0.806	3.963
SNORA16B	0.170	1.125	3.752
SNORA21//RPL23	-0.107	0.928	6.775
SNORA22	-0.304	0.810	8.990
SNORA23	-0.195	0.873	8.947
SNORA24//SNHG8	0.216	1.161	5.642
SNORA26	-0.474	0.720	4.420
SNORA28	0.250	1.189	3.892
SNORA31	1.303	2.468	2.244
SNORA33	-0.291	0.818	8.874
SNORA34//MIR1291	-0.123	0.918	9.183
SNORA35	-0.151	0.901	4.515
SNORA36B//MIR664A	-0.867	0.548	3.298
SNORA36C	-0.208	0.865	2.412
SNORA37	-0.324	0.799	8.356
SNORA38	-0.443	0.736	4.638
SNORA38	-0.212	0.864	3.216
SNORA38B	0.093	1.066	9.049
SNORA45B	-0.739	0.599	8.224
SNORA46	-0.590	0.664	7.665
SNORA47	-0.290	0.818	10.675
SNORA48//EIF4A1//SNORA67//SNORD10	-0.078	0.947	11.099
SNORA54	-0.518	0.698	9.137
SNORA55	-0.062	0.958	4.927
SNORA57//METTL12	-0.164	0.892	9.140
SNORA59B//SNORA59A	-0.128	0.915	6.744
SNORA5A//TBRG4	-0.023	0.984	5.507
SNORA5C//TBRG4	-0.246	0.843	4.158
SNORA60	-0.713	0.610	8.740
SNORA64//RPS2	-0.098	0.934	6.986
SNORA65	-0.253	0.839	7.104
SNORA66//RPL5	0.053	1.038	3.442
SNORA68//RPL18A	-0.285	0.821	9.855

SNORA70D	0.094	1.067	2.815
SNORA70E	-0.883	0.542	3.284
SNORA70G	0.344	1.270	3.750
SNORA71A	0.250	1.189	5.564
SNORA71B//SNHG17	-0.062	0.958	8.138
SNORA71C	0.104	1.075	5.699
SNORA71D	-0.177	0.885	6.503
SNORA72//RPL30	-0.597	0.661	7.426
SNORA74A	-0.367	0.775	3.397
SNORA74B	-0.466	0.724	6.468
SNORA75	-0.668	0.629	9.566
SNORA76A	-0.279	0.824	8.677
SNORA76C//RP11-329L6.1	-0.596	0.662	6.309
SNORA77	-0.148	0.903	4.105
SNORA79	-0.135	0.911	2.971
SNORA7B	-0.164	0.892	7.154
SNORA80A	-0.537	0.689	6.314
SNORA80B	-0.317	0.803	3.895
SNORA80E	-0.882	0.543	6.747
SNORA84//MIR3651	0.160	1.117	5.361
SNORA9//SNHG15//LOC101928927	0.225	1.169	6.048
SNORD10//EIF4A1//SNORA67//SNORA48	0.132	1.096	8.373
SNORD100//SNORA33	-0.131	0.913	9.728
SNORD103A//SNORD103B	-0.138	0.909	3.366
SNORD104	0.080	1.057	6.621
SNORD109B//SNORD109A//RP11-701H24.8	-0.102	0.932	6.434
SNORD111	0.596	1.511	5.441
SNORD113-1	0.469	1.384	4.211
SNORD113-3	0.184	1.136	3.753
SNORD113-4//SNHG23//AL132709.5	-0.438	0.738	5.577
SNORD113-5	0.068	1.048	2.194
SNORD113-8	0.384	1.305	3.510
SNORD113-9	-0.090	0.940	3.897
SNORD114-1	-0.425	0.745	8.583
SNORD114-10	0.394	1.314	1.814
SNORD114-11	0.231	1.174	3.108
SNORD114-12	-0.212	0.863	9.262
SNORD114-13	-0.269	0.830	3.010
SNORD114-14	-0.214	0.862	5.376
SNORD114-15	-0.245	0.844	4.988
SNORD114-16	0.271	1.207	3.702
SNORD114-17	0.067	1.048	3.997
SNORD114-2	-0.029	0.980	3.395
SNORD114-20	-0.242	0.846	3.131
SNORD114-21	0.038	1.027	5.691
SNORD114-22	-0.137	0.909	4.686
SNORD114-23	0.695	1.618	2.337
SNORD114-26//SNHG24//RP11-909M7.3	0.216	1.162	4.605
SNORD114-28	-0.176	0.885	6.652
SNORD114-3	-0.116	0.923	8.943

SNORD114-30	0.227	1.170	3.039
SNORD114-31	-0.064	0.957	2.942
SNORD114-4	0.600	1.516	1.991
SNORD114-6	-0.051	0.966	2.335
SNORD114-7	-0.174	0.887	2.711
SNORD114-9	-0.043	0.970	4.802
SNORD115-10//SNORD115-21	0.579	1.494	5.423
SNORD115-15//SNORD115-21	-0.240	0.847	3.764
SNORD115-17//SNORD115-18//SNORD115-19//SNO	-0.095	0.936	9.964
SNORD115-20//IPW//SNORD107//PWARSN//SNORD	-0.119	0.921	10.523
SNORD115-21	-0.186	0.879	2.392
SNORD115-25	-0.030	0.979	7.977
SNORD115-27	-0.191	0.876	7.359
SNORD115-3	0.344	1.270	2.935
SNORD115-30	0.285	1.218	7.912
SNORD115-31	-0.248	0.842	6.843
SNORD115-32	-0.048	0.967	4.979
SNORD115-38	-0.058	0.961	4.928
SNORD115-4//SNORD115-6//SNORD115-42//SNORD	0.020	1.014	11.172
SNORD115-40	0.128	1.093	4.167
SNORD115-41	0.158	1.116	2.298
SNORD115-44//SNORD115-1//SNORD115-4//SNORD	-0.022	0.985	10.604
SNORD115-45	1.012	2.017	3.669
SNORD115-5//SNORD115-9//SNORD115-11//SNORD	-0.030	0.980	11.040
SNORD115-5//SNORD115-9//SNORD115-11//SNORD	-0.031	0.979	11.025
SNORD115-5//SNORD115-9//SNORD115-11//SNORD	-0.041	0.972	10.981
SNORD115-8	0.636	1.554	2.541
SNORD116-1	-0.278	0.825	7.871
SNORD116-14//SNRPN	-0.360	0.779	9.539
SNORD116-16	-0.022	0.985	2.215
SNORD116-20//SNORD116-19//SNORD116-17//SNO	-0.464	0.725	7.432
SNORD116-21//SNORD116-19//SNORD116-17//SNO	0.545	1.459	6.446
SNORD116-23	-0.448	0.733	5.106
SNORD116-25	-0.031	0.978	4.066
SNORD116-26	-0.325	0.798	6.203
SNORD116-27	-0.346	0.787	5.203
SNORD116-29	-0.455	0.729	6.987
SNORD116-30	-0.191	0.876	6.211
SNORD117	-0.164	0.892	8.351
SNORD117	-0.129	0.914	8.189
SNORD12	-0.147	0.903	6.133
SNORD123	0.570	1.484	4.488
SNORD124	0.409	1.327	2.396
SNORD125//MIR3653	-0.306	0.809	3.250
SNORD126	-0.127	0.916	2.976
SNORD12B	0.071	1.050	5.062
SNORD12C	-0.148	0.902	4.299
SNORD13P2	-0.139	0.908	5.201
SNORD13P3	0.179	1.132	7.005
SNORD14A	-0.213	0.863	4.475

SNORD14E	0.811	1.754	5.153
SNORD15B	-0.133	0.912	9.129
SNORD17	-0.519	0.698	7.683
SNORD18A//RPL4//SNORD16//SNORD18B//SNORD1	-0.121	0.919	2.779
SNORD18C//RPL4//SNORD16//SNORD18A//SNORD1	1.197	2.293	4.214
SNORD2//EIF4A2//SNORA63//SNORA4//SNORA81//	-0.099	0.934	5.976
SNORD20	-0.699	0.616	1.890
SNORD23//GLTSCR2	-0.273	0.828	2.765
SNORD29//SNORD31//SNORD30//SNORD28//SNORI	0.427	1.344	3.856
SNORD35B//RPS11	0.145	1.106	4.860
SNORD37	0.279	1.213	4.016
SNORD3C	-0.185	0.880	8.050
SNORD3D//RP11-160E2.6	-0.400	0.758	5.839
SNORD42A//RPL23A	-0.410	0.753	9.067
SNORD4B//RPL23A//SNORD42A	0.147	1.107	5.548
SNORD51	0.426	1.344	4.977
SNORD56B	0.524	1.438	2.296
SNORD58A//RPL17//SNORD58B//SNORD58C	0.212	1.159	4.603
SNORD58C//RPL17//SNORD58B//SNORD58A	-0.037	0.974	5.924
SNORD59B	0.295	1.227	3.780
SNORD60	-0.237	0.848	6.697
SNORD61//RBMX//LOC101928747	-0.260	0.835	8.668
SNORD62A//SNORD62B	-0.104	0.930	5.802
SNORD66	-0.305	0.809	6.034
SNORD67	-0.520	0.697	5.078
SNORD7	0.725	1.653	2.711
SNORD70	-0.069	0.953	2.939
SNORD71	-0.035	0.976	11.202
SNORD75	0.120	1.087	2.644
SNORD82	-0.191	0.876	8.829
SNORD83A	-0.017	0.988	2.617
SNORD85	0.033	1.023	2.383
SNORD88A	0.034	1.024	4.974
SNORD88B//C19orf48//SNORD88C	0.024	1.017	3.784
SNORD88C//C19orf48	0.374	1.296	2.598
SNORD9	-0.336	0.792	8.082
SNORD90	-0.167	0.891	6.119
SNORD91A	-0.493	0.710	3.477
SNORD91B	-0.581	0.668	7.377
SNORD92	0.203	1.151	4.766
SNORD94	-0.387	0.764	8.106
SNORD96B	0.275	1.210	2.527
SNORD99	0.421	1.339	2.048
SNPH	-0.185	0.879	5.991
SNRPN//IPW//SNORD107//PWARSN//SNORD116-4/,	0.280	1.214	4.959
SNRPN//SNURF	-0.099	0.934	9.522
SNTA1	-0.218	0.860	6.186
SNTB2	0.638	1.556	6.383
SNTG1	0.088	1.063	5.512
SNURF//IPW//SNORD107//PWARSN//SNORD116-4/,	0.055	1.039	6.420

SNX1	-0.130	0.914	6.133
SNX10	0.039	1.028	4.741
SNX16	0.081	1.058	3.951
SNX18	0.165	1.121	4.592
SNX20	0.182	1.135	2.445
SNX29//LOC101929304//AC007601.2	0.256	1.194	7.125
SNX32	0.568	1.483	3.233
SNX33	0.160	1.117	5.028
SNX4	0.388	1.308	6.512
SOAT1	0.703	1.628	6.124
SOBP	-0.384	0.766	6.509
SOCS3	1.325	2.506	6.432
SOCS6	0.318	1.246	5.546
SOCS7	0.083	1.059	6.300
SOD2//LOC100129518	0.247	1.187	4.162
SOD3	0.257	1.195	4.245
SOGA3//KIAA0408//RP3-403A15.5	-0.281	0.823	6.121
SOHLH1	-0.210	0.864	2.652
SORBS2	-0.174	0.886	5.287
SORCS1	0.633	1.551	5.011
SORCS3	0.199	1.148	5.364
SOS1-IT1	0.155	1.113	2.866
SOSTDC1	0.250	1.189	2.884
SOWAHA	-0.115	0.923	4.705
SOWAHB	0.184	1.136	3.499
SOWAHC	0.467	1.382	2.912
SOX1	-0.206	0.867	2.886
SOX13	-0.173	0.887	4.343
SOX18	0.259	1.196	2.563
SOX2-OT	-0.069	0.953	3.948
SOX5	0.053	1.037	5.125
SOX8	-0.065	0.956	5.787
SOX9-AS1//AC005152.2	0.108	1.078	2.813
SP1	0.300	1.231	6.846
SP100	0.375	1.297	4.771
SP110	0.530	1.444	5.076
SP9	0.029	1.020	3.419
SPAG17	-1.173	0.443	1.964
SPAG5	0.036	1.026	3.344
SPAG6	-1.214	0.431	2.477
SPAG8	-0.745	0.597	2.797
SPARC	-0.277	0.825	6.921
SPATA13//RP11-307N16.6	0.230	1.173	6.134
SPATA17	-0.514	0.700	2.231
SPATA18	-0.442	0.736	3.542
SPATA2L	0.523	1.437	3.011
SPATA31C1	0.124	1.090	2.420
SPATA41	-0.144	0.905	2.146
SPATA6	0.344	1.269	3.967
SPATA6	0.091	1.065	4.941

SPATA6L	-0.413	0.751	3.528
SPATA7	-0.174	0.886	4.741
SPATS2	-0.180	0.883	4.069
SPDYA	0.181	1.133	4.125
SPDYE6//SPDYE2B//SPDYE2	-0.176	0.885	2.730
SPEG//LOC100996693	0.240	1.181	3.670
SPHKAP	0.373	1.295	4.769
SPI1	0.435	1.352	5.303
SPIN1	0.024	1.017	8.535
SPIN3	0.018	1.012	4.403
SPINT1	0.125	1.090	2.514
SPINT2	0.225	1.169	5.793
SPIRE2	-0.604	0.658	4.072
SPN//LOC101929889	0.546	1.460	3.356
SPOCD1	0.141	1.103	2.243
SPOCK1	0.242	1.182	6.833
SPOCK2	0.093	1.067	6.969
SPON1	-0.277	0.825	6.611
SPP1	0.163	1.119	10.916
SPPL2C//IMP5	0.237	1.179	2.967
SPRED2	0.085	1.061	4.477
SPRN	0.158	1.115	3.922
SPRR2F	-0.295	0.815	2.107
SPRY1	0.254	1.193	4.472
SPRY3	-0.230	0.853	4.649
SPRY3	-0.158	0.896	4.137
SPTB	-0.195	0.873	4.636
SPTBN2	-0.253	0.839	5.175
SPTBN4	-0.351	0.784	5.042
SPTLC2	0.038	1.027	6.869
SPTLC3	0.603	1.519	4.957
SPTSSA	0.218	1.163	5.713
SPTSSB	-0.150	0.901	3.539
SPX//C12orf39	-0.017	0.989	3.257
SQLE	-0.042	0.971	6.750
SQRDL//RP11-96O20.4	0.699	1.624	4.602
SQSTM1	-0.466	0.724	8.059
SRD5A1	0.207	1.154	2.488
SREBF1	0.346	1.271	4.993
SRGAP1	0.220	1.164	6.219
SRGAP2	0.068	1.048	7.319
SRGAP2//SRGAP2B//SRGAP2C	-0.223	0.857	7.162
SRGN	0.375	1.297	9.004
SRL	0.103	1.074	2.178
SRP14-AS1	-0.082	0.945	3.549
SRP68	0.328	1.255	7.240
SRPK1	0.219	1.164	5.338
SRPK2	-0.021	0.986	7.507
SRPX	0.243	1.183	5.748
SRPX2	0.122	1.088	3.602

SRSF12	0.301	1.232	3.412
SS18L2	0.359	1.282	4.998
SSBP3	-0.462	0.726	4.663
SSBP4	-0.074	0.950	5.124
SSC5D	0.033	1.023	2.849
SSFA2	0.174	1.128	7.046
SSH3	0.179	1.132	4.907
SSSCA1	0.041	1.029	5.658
SST	0.550	1.464	6.793
SSTR2	0.093	1.067	4.880
SSTR3	-0.043	0.971	2.144
SSTR4	0.154	1.113	2.209
SSX2IP	0.235	1.177	4.340
ST18	-0.086	0.942	6.029
ST3GAL1	0.370	1.292	4.249
ST3GAL2	0.075	1.053	3.882
ST3GAL6-AS1	-0.120	0.920	2.784
ST6GAL1	0.233	1.176	4.273
ST6GAL2	-0.339	0.791	3.535
ST6GALNAC2	0.227	1.170	4.757
ST6GALNAC3	0.202	1.150	5.932
ST6GALNAC5	0.128	1.093	3.629
ST6GALNAC6	0.179	1.132	6.853
ST8SIA3	-0.037	0.975	6.004
ST8SIA4	0.597	1.513	4.788
ST8SIA5	0.032	1.023	5.802
ST8SIA6	0.450	1.366	3.296
STAB1	0.372	1.294	5.072
STAC2	-0.433	0.741	4.510
STAG3	0.032	1.022	3.025
STAMBPL1	0.255	1.193	5.261
STAR	0.096	1.069	3.109
STARD10	0.120	1.087	4.456
STARD13	-0.300	0.812	3.921
STARD3	-0.113	0.925	6.052
STARD4	-0.086	0.942	5.982
STARD8	0.087	1.062	3.494
STARD9	0.038	1.027	5.500
STAT1	0.518	1.432	7.361
STAT2	0.158	1.116	6.118
STAT3	0.309	1.239	8.600
STAT4	0.503	1.417	4.037
STAT6	0.436	1.353	4.617
STAU2	0.015	1.010	6.038
STC1	0.961	1.947	3.931
STC2	0.049	1.034	3.032
STEAP1B	0.110	1.079	2.272
STEAP2	0.507	1.421	5.739
STEAP3	0.589	1.504	5.279
STEAP4	1.156	2.228	2.991

STH	-0.128	0.915	4.543
STIL	0.126	1.091	3.494
STK10	0.163	1.120	5.074
STK24	0.198	1.147	4.578
STK32A	0.046	1.032	5.702
STK33	-0.169	0.890	4.488
STMN2	-0.755	0.593	8.996
STMN3	-0.157	0.897	6.579
STMN4	0.170	1.125	6.380
STOM	0.244	1.184	8.829
STOML1	-0.232	0.852	4.569
STON1-GTF2A1L//STON1//GTF2A1L	0.165	1.121	4.166
STOX1	0.103	1.074	4.661
STOX2	-0.131	0.913	6.123
STPG1	0.103	1.074	2.646
STRBP	-0.056	0.962	5.317
STRC	0.258	1.196	3.458
STRIP2	0.240	1.181	4.418
STX11	0.496	1.411	3.099
STX1B	-0.352	0.784	8.049
STX2	-0.136	0.910	5.837
STX4	0.240	1.181	5.692
STXBP2	0.474	1.389	3.720
STXBP3	0.183	1.135	7.085
STXBP5	0.069	1.049	6.345
STXBP5-AS1	0.260	1.197	2.486
STXBP5L	-0.059	0.960	6.955
STXBP6	-0.174	0.887	4.742
STYK1	0.162	1.119	3.179
STYX	0.221	1.166	5.650
SUCLA2	-0.141	0.907	6.792
SUCLG2	0.321	1.249	5.738
SUDS3	-0.164	0.892	5.459
SULF1	0.248	1.188	4.457
SULT1A2	-0.061	0.959	2.435
SULT1B1	0.395	1.315	2.258
SULT1C4	0.033	1.023	5.261
SULT4A1	-0.224	0.856	5.892
SUMO4	0.525	1.439	5.816
SUN2	-0.444	0.735	3.494
SUN2	-0.343	0.788	6.355
SUPV3L1	0.259	1.196	5.928
SUSD1	0.124	1.090	5.105
SUSD2	-0.179	0.884	4.849
SUSD4	0.130	1.094	4.732
SUSD5	-0.017	0.988	4.466
SUV39H2	0.047	1.033	3.193
SV2A	0.184	1.136	7.502
SV2B	-0.189	0.877	6.627
SV2C	-0.787	0.580	5.849

SVEP1	0.047	1.033	3.597
SVIL	0.157	1.115	4.821
SVOP	0.247	1.187	4.940
SYCE1	-0.222	0.857	3.006
SYDE1	-0.112	0.926	3.439
SYDE2	0.667	1.588	4.084
SYK	0.344	1.269	4.020
SYN1	-0.137	0.909	7.087
SYN2	-0.604	0.658	6.939
SYN3	0.084	1.060	4.304
SYNDIG1L//TMEM90A	-0.263	0.833	2.929
SYNE1	-0.332	0.795	6.685
SYNE3	0.115	1.083	4.191
SYNGAP1	-0.363	0.778	5.560
SYNGAP1	-0.245	0.844	5.584
SYNGR1	-0.031	0.979	4.952
SYNGR2	0.232	1.175	6.853
SYNGR3	0.055	1.039	5.264
SYNJ2	-0.107	0.928	7.467
SYNJ2-IT1	0.140	1.102	2.355
SYNPO	-0.089	0.940	4.606
SYNPR	0.372	1.294	2.245
SYP	-0.366	0.776	5.512
SYP-AS1	-0.020	0.986	5.279
SYPL1	0.030	1.021	7.031
SYT1	0.086	1.061	4.700
SYT10	0.278	1.212	4.350
SYT12	-0.078	0.947	2.887
SYT13	0.077	1.055	4.542
SYT14	0.152	1.111	5.530
SYT16	0.111	1.080	4.374
SYT17	0.139	1.101	6.014
SYT2	-1.077	0.474	5.961
SYT3	-0.137	0.910	4.235
SYT4	-0.209	0.865	7.506
SYT5	0.036	1.025	5.664
SYT6	0.109	1.079	3.287
SYT7	0.189	1.140	4.035
SYTL2	0.196	1.146	5.245
SYTL3	1.039	2.055	4.419
SYTL4	-0.272	0.828	4.543
SYTL5	0.450	1.366	3.672
TAB1//LOC100506472	-0.326	0.798	3.228
TAB3-AS2	0.232	1.174	3.736
TAC1	0.536	1.450	3.284
TAC3	0.411	1.329	3.700
TACC2	-0.051	0.966	4.153
TACR1	-0.238	0.848	3.653
TACR2	-0.182	0.881	3.054
TACR3	0.339	1.265	2.833

TADA1	0.137	1.100	4.715
TAF1D//SNORA8//SNORA1//SNORA18//SNORA40//S	0.569	1.483	6.145
TAF4B	-0.127	0.916	5.178
TAF5	0.181	1.134	4.437
TAGAP	0.952	1.934	3.423
TAGLN	0.059	1.042	5.023
TAGLN2	0.510	1.424	2.879
TAGLN3	-0.267	0.831	6.386
TAL1	-0.121	0.919	2.694
TALDO1	0.041	1.029	5.122
TANC1	0.090	1.065	6.402
TAP1//PSMB8-AS1	0.681	1.603	4.608
TAP1//PSMB8-AS1	0.668	1.589	4.822
TAPBPL	0.078	1.056	3.795
TAS2R10	-0.015	0.989	2.561
TAS2R13	-0.034	0.977	3.442
TAS2R14	-0.111	0.926	4.239
TAS2R19	-0.208	0.866	2.727
TAS2R20	0.221	1.166	3.073
TAS2R3	0.398	1.318	4.775
TAS2R31//TAS2R45	-0.154	0.899	3.809
TAS2R4	0.368	1.290	4.193
TBC1D12	0.067	1.048	6.320
TBC1D2	0.376	1.297	5.653
TBC1D22B	0.257	1.195	5.595
TBC1D24	0.144	1.105	5.603
TBC1D2B	0.295	1.227	4.550
TBC1D30	0.554	1.469	5.302
TBC1D4	-0.037	0.975	5.122
TBC1D9	0.471	1.386	6.046
TBKBP1	-0.270	0.829	4.613
TBPL1	0.054	1.038	4.735
TBR1	0.273	1.208	3.143
TBRG1	0.296	1.227	5.326
TBX10	-0.106	0.929	2.605
TBX15	-0.152	0.900	3.033
TBX3	-0.301	0.812	3.646
TBXAS1	0.394	1.314	3.544
TC2N	0.603	1.519	5.326
TCEA3	0.101	1.073	3.907
TCEAL5	-0.365	0.776	5.209
TCEB3-AS1//RP5-886K2.3	0.530	1.444	4.039
TCEB3CL2//TCEB3C//LOC101060464//LOC10192985	-0.314	0.804	2.660
TCERG1L	0.023	1.016	3.766
TCF12	0.120	1.087	6.651
TCF19	0.212	1.158	2.894
TCFL5	-0.463	0.726	6.518
TCIRG1	0.174	1.128	4.539
TCN2	0.130	1.095	3.989
TCP10L//LINC00846	0.184	1.136	3.633

TCP11L1	0.064	1.046	4.855
TCP11L2	0.121	1.087	5.698
TCTE1	-0.562	0.677	2.555
TCTEX1D1	-1.001	0.500	2.843
TDH	0.244	1.184	2.887
TDO2	0.049	1.034	1.702
TDRD9	0.152	1.111	2.490
TDRKH	-0.045	0.969	5.436
TDRP	-0.319	0.802	4.680
TEAD2	0.126	1.091	3.429
TEAD3	0.127	1.092	3.002
TEF	-0.092	0.938	4.188
TEK	0.857	1.811	5.152
TEKT1	-1.562	0.339	2.458
TEKT2	-0.606	0.657	2.291
TEKT4	0.092	1.066	2.682
TEKT4P2//MAFIP//LOC100233156	-0.187	0.878	4.451
TENC1	-0.114	0.924	5.940
TENM1	0.406	1.325	5.751
TENM2	0.411	1.330	4.743
TENM3	-0.150	0.901	5.265
TENM4//ODZ4	-0.111	0.926	2.449
TEP1	0.169	1.124	4.686
TERC	-0.220	0.859	5.601
TERF2IP	0.302	1.233	3.220
TES	0.122	1.088	2.560
TESC	-0.078	0.947	3.363
TESK2	0.113	1.082	5.406
TEX10	0.397	1.317	5.375
TEX26	-0.464	0.725	2.263
TEX29	0.062	1.044	3.149
TEX30	-0.018	0.987	3.892
TEX9	-0.498	0.708	3.241
TF	0.034	1.024	9.937
TFB2M	0.431	1.348	4.975
TFEC	0.419	1.337	2.130
TFPI	-0.285	0.821	2.597
TFPT	0.251	1.190	7.010
TFR2	0.050	1.035	2.607
TFRC	0.264	1.201	6.156
TGFB1	0.070	1.050	5.654
TGFB1I1	0.352	1.276	4.587
TGFB2	0.626	1.543	7.469
TGFB2-AS1//RP11-224O19.2	0.698	1.622	2.177
TGFB3	-0.337	0.792	4.927
TGFBI	0.919	1.890	3.997
TGFBR1	0.495	1.410	6.431
TGFBR2	0.434	1.351	6.441
TGFBR3L	0.029	1.020	4.017
TGIF1	0.194	1.144	3.046

TGM2	0.110	1.079	5.901
THAP10	-0.099	0.934	4.059
THAP9	0.257	1.195	3.240
THAP9-AS1	0.646	1.565	4.397
THBD	0.898	1.864	3.609
THBS1	0.787	1.725	5.555
THBS2	0.647	1.566	7.091
THBS4	-0.102	0.932	5.278
THEM6	0.362	1.285	4.153
THEMIS	0.175	1.129	2.395
THEMIS2	0.716	1.642	5.230
THNSL2	-0.169	0.889	4.226
THPO	0.160	1.117	2.814
THRB	0.095	1.068	5.246
THRB-IT1	-0.039	0.974	2.512
THSD1	-0.053	0.964	3.586
THSD4	-0.118	0.921	3.873
THUMPD2	0.456	1.371	4.021
THY1	-0.274	0.827	3.631
THYN1	0.050	1.035	5.199
TIA1	0.194	1.144	5.822
TIAM2	0.309	1.238	3.511
TIFA	0.212	1.158	3.228
TIGD2	0.038	1.027	2.900
TIMELESS	0.342	1.267	3.314
TIMM22	0.153	1.112	6.086
TIMM8A	0.023	1.016	4.594
TIMP1	0.419	1.337	6.705
TIMP2	-0.022	0.985	7.751
TIMP3	0.050	1.035	7.601
TIMP4	-0.704	0.614	5.236
TIPARP	0.504	1.418	7.210
TIPRL	0.393	1.313	5.869
TJAP1	0.044	1.031	5.206
TJP2	0.131	1.095	7.171
TK2	0.216	1.162	2.898
TLCD2	0.246	1.186	2.926
TLE1	-0.250	0.841	5.098
TLE2	-0.326	0.798	5.016
TLL1	-0.227	0.854	3.663
TLL2	-0.034	0.977	3.310
TLN1	0.049	1.034	6.876
TLN2	0.104	1.075	6.314
TLR1	0.933	1.909	4.220
TLR10	0.636	1.554	3.516
TLR2	1.052	2.073	5.295
TLR3	0.945	1.925	4.425
TLR4	0.349	1.274	6.039
TLR5	0.778	1.715	4.873
TLR6	0.317	1.246	3.145

TLR7	0.937	1.914	4.147
TLR8	0.925	1.898	2.090
TM2D2	0.454	1.370	4.348
TM4SF1	0.705	1.630	6.690
TM4SF18	0.390	1.311	3.647
TM6SF1	0.696	1.619	5.910
TMBIM1//MIR6513	0.158	1.116	3.764
TMBIM4//RP11-745O10.4	0.020	1.014	4.682
TMC1	0.170	1.125	2.380
TMC6	0.360	1.283	5.321
TMC7	0.278	1.212	6.849
TMC8	0.250	1.189	3.449
TMCC1-AS1//AL449209.1	-0.332	0.794	4.170
TMCC3	0.272	1.208	5.658
TMCO4	0.512	1.426	2.746
TMCO6	0.374	1.296	3.759
TMED8	0.458	1.374	6.114
TMEFF2	-0.246	0.843	5.590
TMEM104	-0.107	0.929	5.347
TMEM106A	-0.078	0.947	4.369
TMEM108	0.080	1.057	2.842
TMEM119	0.407	1.326	3.965
TMEM125	-0.293	0.816	2.612
TMEM132B	0.273	1.208	4.854
TMEM132D	-0.101	0.932	3.848
TMEM132E	0.367	1.290	5.117
TMEM139	0.196	1.146	3.468
TMEM139//AC073342.12	-0.551	0.682	4.204
TMEM140	0.656	1.575	5.589
TMEM144	0.387	1.308	6.780
TMEM145	0.054	1.038	4.900
TMEM14A	-0.027	0.981	7.129
TMEM150C	0.273	1.208	6.778
TMEM151A	-0.128	0.915	6.001
TMEM151B//RP11-444E17.6	-0.306	0.809	2.154
TMEM155	-0.103	0.931	2.984
TMEM156	0.554	1.469	3.825
TMEM158	0.643	1.561	4.245
TMEM160	-0.016	0.989	4.763
TMEM163	0.156	1.115	5.920
TMEM165	0.134	1.097	7.445
TMEM168	0.252	1.191	5.417
TMEM169	0.162	1.119	3.084
TMEM17	0.103	1.074	2.810
TMEM170B	0.225	1.169	5.598
TMEM173	0.383	1.304	3.001
TMEM176A	0.431	1.348	4.256
TMEM176B	0.441	1.357	5.808
TMEM177	0.198	1.147	4.725
TMEM178B	-0.264	0.833	5.781

TMEM179	-0.322	0.800	3.556
TMEM179	-0.409	0.753	4.389
TMEM179B//MIR6748	0.194	1.144	4.957
TMEM181	0.025	1.018	6.666
TMEM183B//RP11-651P23.4	0.048	1.034	5.776
TMEM185A	0.420	1.338	4.532
TMEM196	-0.040	0.973	4.624
TMEM198	-0.219	0.859	3.437
TMEM198B	0.310	1.239	5.405
TMEM2	0.438	1.355	5.443
TMEM204	0.297	1.229	4.303
TMEM206	0.054	1.038	5.148
TMEM220	0.122	1.088	3.948
TMEM229A	-0.025	0.983	3.751
TMEM229B	-0.253	0.839	2.988
TMEM232	-0.543	0.686	3.949
TMEM235	-0.239	0.847	3.745
TMEM240	-0.148	0.902	3.746
TMEM241	-0.132	0.913	4.068
TMEM242	-0.128	0.915	5.584
TMEM25	0.045	1.032	4.552
TMEM254-AS1	-0.150	0.901	2.409
TMEM255A	0.475	1.389	6.551
TMEM255B	0.164	1.121	3.527
TMEM257	0.202	1.150	4.915
TMEM30B	-0.623	0.649	2.428
TMEM35	0.138	1.100	5.040
TMEM38A	-0.082	0.945	5.258
TMEM38B	0.335	1.261	5.276
TMEM44	-0.494	0.710	3.302
TMEM44-AS1	0.050	1.036	3.400
TMEM45A	0.338	1.264	2.775
TMEM51	0.427	1.345	2.933
TMEM56//TMEM56-RWDD3//RWDD3//RP11-57H12	0.063	1.045	5.266
TMEM59L	-0.289	0.819	6.330
TMEM60	0.376	1.297	4.654
TMEM61	0.033	1.023	2.292
TMEM63C	-0.046	0.969	5.792
TMEM65	0.238	1.179	6.905
TMEM67	-0.468	0.723	4.277
TMEM70	0.558	1.472	3.186
TMEM71	0.885	1.846	2.326
TMEM72-AS1	0.018	1.013	2.768
TMEM74B	0.381	1.302	3.162
TMEM81	-0.031	0.979	3.076
TMEM87A	0.162	1.119	7.126
TMEM88	-0.198	0.872	3.331
TMEM88B	-0.060	0.959	6.518
TMEM98	0.412	1.330	6.465
TMEM9B-AS1	-0.272	0.828	4.828

TMIE	-0.199	0.871	3.946
TMOD1	-0.133	0.912	7.913
TMOD2	0.040	1.028	8.610
TMPRSS3	0.050	1.035	2.536
TMPRSS5	0.404	1.323	3.926
TMSB4X	0.244	1.184	5.326
TMSB4XP4	0.040	1.028	4.348
TMSB4Y	0.062	1.044	2.674
TMTC1	0.107	1.077	6.189
TMTC3	0.367	1.289	6.648
TMTC4	0.286	1.219	6.227
TMX3	0.293	1.226	6.055
TNC	0.584	1.499	5.863
TNFAIP2	0.431	1.348	4.348
TNFAIP3	0.383	1.304	5.136
TNFAIP6	0.730	1.659	3.513
TNFAIP8	0.327	1.254	2.198
TNFAIP8L1	0.131	1.095	3.211
TNFAIP8L3	0.152	1.111	4.226
TNFRSF10C//LOC254896	0.210	1.157	3.218
TNFRSF10D	0.354	1.278	3.242
TNFRSF11A	-0.225	0.855	3.343
TNFRSF11B	0.357	1.281	4.103
TNFRSF12A	0.709	1.635	5.414
TNFRSF13C	-0.227	0.854	2.456
TNFRSF14	0.526	1.440	3.469
TNFRSF1A	0.387	1.307	5.557
TNFRSF1B	0.887	1.850	6.924
TNFRSF21	-0.075	0.949	7.681
TNFRSF25	-0.220	0.858	3.438
TNFSF10	0.305	1.236	4.997
TNFSF13B	1.049	2.069	3.813
TNFSF14	0.162	1.119	2.769
TNFSF8	0.955	1.939	2.601
TNKS	-0.055	0.962	6.799
TNNC1	-0.178	0.884	2.348
TNNT1	-0.051	0.966	2.379
TNNT2	-0.044	0.970	2.399
TNS1	-0.336	0.792	4.777
TNS3	-0.071	0.952	5.303
TNXB	-0.406	0.755	4.426
TNXB	-0.138	0.909	4.100
TNXB//TNXA	0.093	1.067	4.335
TNXB//TNXA	-0.067	0.954	4.641
TNXB//TNXA	-0.044	0.970	4.513
TOB2	0.069	1.049	5.096
TOB2P1	-0.465	0.724	2.635
TOLLIP	-0.052	0.964	5.808
TOM1L1	0.054	1.038	6.078
TOMM20	0.039	1.027	6.539

TOMM34	0.234	1.176	6.618
TOMM40L	0.116	1.083	4.274
TOMM70A	0.175	1.129	6.493
TOR1A	0.400	1.319	6.492
TOX	0.092	1.065	6.481
TOX2//C20orf100	0.080	1.057	3.610
TP53	0.336	1.262	4.964
TP53I3	-0.019	0.987	3.567
TP53INP1	0.217	1.163	4.184
TP53INP2	-0.614	0.653	7.811
TP53RK	0.450	1.366	4.339
TP53TG5	0.547	1.461	4.318
TPBG	0.093	1.067	2.735
TPBGL	-0.099	0.933	3.117
TPGS1	0.078	1.055	3.053
TPI1	0.082	1.058	4.367
TPM2	-0.095	0.936	3.965
TPMT	0.257	1.195	6.387
TPPP	0.136	1.099	8.980
TPPP3	-0.256	0.838	7.821
TPRG1L	-0.048	0.967	7.471
TPSB2//TPSAB1	0.448	1.364	2.542
TPSD1	-0.541	0.687	2.161
TPST1	0.599	1.514	5.041
TPT1//LOC101928826	-0.406	0.755	7.722
TPTE2P1	-0.106	0.929	4.739
TPTE2P5	0.073	1.052	3.209
TPX2	0.222	1.167	2.743
TRADD	0.234	1.176	4.069
TRAF1	0.376	1.297	4.081
TRAF3IP3	-0.082	0.945	2.685
TRAF5	-0.104	0.930	3.327
TRAFD1	0.166	1.122	5.084
TRAJ11	-0.485	0.715	2.800
TRAJ32//YME1L1	0.427	1.344	2.308
TRAJ59	-1.020	0.493	2.737
TRAK2	-0.600	0.660	7.677
TRAM1	0.537	1.451	2.492
TRAM1L1	-0.237	0.849	2.410
TRANK1	0.506	1.421	5.375
TRAPPC13//CTC-534A2.2	0.153	1.112	4.765
TRAPPC2	-0.296	0.815	2.437
TRAPPC6B	0.186	1.138	6.912
TRAV1-1	-0.842	0.558	2.982
TRAV38-1//YME1L1	0.045	1.032	3.670
TRAV38-2DV8//YME1L1	0.325	1.253	3.313
TRBC2//IL23A	0.166	1.122	5.188
TRBJ2-1//IL23A	0.281	1.215	2.681
TRBJ2-5//EPC1	-0.071	0.952	2.263
TRBJ2-7//IL23A	-0.079	0.947	3.456

TRBV20OR9-2	0.166	1.122	3.037
TRBV23-1	-0.222	0.857	3.521
TRBV7-1//TRBV7-2	-0.088	0.941	2.945
TRDN	-0.065	0.956	3.697
TREM1	0.867	1.824	3.254
TREM2	0.668	1.589	5.456
TREML1	0.091	1.065	2.450
TRERF1	0.349	1.273	4.454
TRGJ2//TRGJ1//TARP	-0.205	0.867	2.280
TRGJP1//TARP	0.304	1.234	2.730
TRGV9//TARP	0.213	1.159	2.302
TRH	0.233	1.176	3.001
TRHDE	0.387	1.307	4.042
TRHDE-AS1	0.341	1.267	3.132
TRIB1	0.907	1.875	4.300
TRIL	0.038	1.027	6.247
TRIM17	0.126	1.091	3.003
TRIM21	0.472	1.387	3.546
TRIM22	0.488	1.403	5.857
TRIM23	0.170	1.125	6.071
TRIM32	0.098	1.070	4.286
TRIM36	0.021	1.015	4.060
TRIM37	0.028	1.019	7.967
TRIM38	0.708	1.634	5.065
TRIM47	0.275	1.210	4.922
TRIM5	0.518	1.432	3.213
TRIM52	0.089	1.064	5.138
TRIM56	0.169	1.124	5.136
TRIM59	0.297	1.228	3.706
TRIM61	0.385	1.306	2.824
TRIM62	0.055	1.039	4.589
TRIM65	0.143	1.104	4.947
TRIM66	-0.506	0.704	3.784
TRIM66	-0.471	0.721	4.043
TRIM9	0.063	1.045	6.619
TRIO	0.103	1.074	6.167
TRIOBP//NOL12	0.194	1.144	3.161
TRIP10	0.208	1.155	4.797
TRIQK	0.201	1.150	3.477
TRMT12	0.168	1.124	4.550
TRMT61B	-0.058	0.961	4.131
TRNP1	-0.511	0.702	4.485
TRO	0.071	1.050	3.392
TRPC3	0.448	1.364	2.347
TRPC4	0.169	1.124	3.601
TRPC5	0.117	1.085	4.338
TRPC6	-0.165	0.892	2.333
TRPM4	0.155	1.113	4.080
TRPM6	0.128	1.093	4.031
TRPS1	-0.031	0.979	6.464

TRPV5	-0.318	0.802	3.391
TRUB1	0.148	1.108	5.020
TRUB2	0.159	1.117	6.312
TSC22D1	-0.028	0.980	4.952
TSC22D2	0.540	1.453	5.713
TSC22D4	-0.214	0.862	4.887
TSEN54	-0.329	0.796	3.631
TSHR	-0.119	0.921	2.763
TSHZ2	0.325	1.253	5.580
TSHZ3	-0.039	0.973	4.671
TSKU	-0.480	0.717	3.432
TSPAN11	0.231	1.174	3.090
TSPAN13	-0.236	0.849	6.738
TSPAN14	0.284	1.217	3.467
TSPAN15	0.121	1.087	5.590
TSPAN17	0.393	1.313	4.952
TSPAN18	-0.304	0.810	3.825
TSPAN33	0.594	1.509	6.137
TSPAN5	0.115	1.083	5.187
TSPAN6	0.203	1.151	5.306
TSPAN7//RP5-972B16.2	-0.021	0.986	6.882
TSPAN8	-0.039	0.973	3.008
TSPAN9	0.205	1.153	4.154
TSPO	0.213	1.159	3.841
TSPY26P	-0.270	0.829	3.762
TSPYL1	-0.083	0.944	7.123
TSPYL2	-0.110	0.927	7.385
TSPYL4	0.040	1.028	7.694
TSPYL5	-0.353	0.783	4.842
TSSC1-IT1	0.238	1.179	2.621
TST	-0.184	0.881	5.957
TSTD1	-0.041	0.972	3.495
TTBK1	-0.083	0.944	5.341
TTBK2	0.077	1.055	7.421
TTC18	-0.513	0.701	4.502
TTC19	0.162	1.119	6.035
TTC21B//LOC100506124	-0.297	0.814	3.316
TTC21B//LOC100506124	0.075	1.054	6.001
TTC25	-0.562	0.677	3.387
TTC26	-0.474	0.720	3.685
TTC30B	-0.101	0.932	2.429
TTC33	0.336	1.262	5.784
TTC34	-0.052	0.965	2.653
TTC38	0.491	1.406	5.009
TTC39A	-0.094	0.937	2.952
TTC39B	0.101	1.073	4.748
TTC39C	-0.104	0.930	4.971
TTC3P1	-0.023	0.984	5.066
TTC40	-0.636	0.643	2.616
TTC5	0.144	1.105	3.510

TTC7A	0.518	1.432	5.278
TTC7B	0.100	1.072	6.044
TTC9	0.133	1.097	5.444
TTC9B	0.391	1.311	3.141
TTF1	0.382	1.303	4.949
TTF2	0.086	1.062	3.457
TTLL4	0.130	1.094	5.910
TTLL7	0.175	1.129	7.527
TTLL7-IT1	-0.247	0.843	4.598
TTLL9	-0.172	0.888	4.437
TTN	-0.282	0.823	4.153
TTN-AS1	0.184	1.136	4.620
TTPA	0.498	1.412	3.569
TTPAL	-0.175	0.886	4.630
TTR	-0.029	0.980	3.506
TTTY10	0.421	1.339	2.997
TTTY15	0.986	1.981	4.857
TTYH1	-0.118	0.921	5.683
TTYH2	0.024	1.017	7.825
TUB	-0.228	0.854	6.769
TUBA3C//TUBA3D	0.027	1.019	2.381
TUBA3FP	-0.278	0.825	2.419
TUBA4B	-0.977	0.508	2.461
TUBA8	0.214	1.160	3.371
TUBB1	0.188	1.139	2.466
TUBB2A	-0.120	0.920	7.602
TUBB2B	0.307	1.237	4.925
TUBB6	0.485	1.400	4.733
TUBGCP4	0.394	1.314	6.621
TUNAR//LINC00617	0.218	1.163	3.731
TUSC2	-0.084	0.944	4.624
TUSC3	0.033	1.023	5.790
TVP23A	0.217	1.162	3.794
TXLNB	0.494	1.409	4.199
TXLNGY//TXLNG2P	0.860	1.815	3.987
TXLNGY//TXLNG2P	0.855	1.808	4.067
TXNDC16	0.089	1.064	5.371
TXNIP	0.104	1.075	8.580
TYK2	0.109	1.078	4.675
TYMP//SCO2	0.431	1.348	4.439
TYMS	0.178	1.131	3.644
TYROBP	0.834	1.783	8.375
TYRP1	-0.282	0.822	2.630
TYW3	0.193	1.143	4.378
U91328.19	-0.109	0.927	2.438
UAP1	0.234	1.176	5.823
UAP1L1	-0.015	0.990	4.568
UBA7//MIR5193	0.645	1.563	4.516
UBALD1	-0.281	0.823	5.369
UBALD2	0.142	1.103	6.088

UBC	0.184	1.136	6.758
UBE2E2	-0.075	0.949	5.648
UBE2MP1	-0.027	0.981	6.058
UBE2O	-0.256	0.838	6.329
UBE2Q2L//RP11-182J1.16	-0.159	0.896	2.845
UBE2QL1	-0.319	0.802	5.480
UBE2W//RP11-463D19.2	0.399	1.319	4.285
UBL7-AS1	0.058	1.041	2.497
UBLCP1	0.296	1.228	6.870
UBP1	-0.061	0.959	3.496
UBQLN2	0.208	1.155	7.618
UBR3	0.137	1.099	7.444
UBTD1	-0.239	0.847	4.246
UBXN10	-0.542	0.687	4.637
UBXN10-AS1//RP3-340N1.5	-0.048	0.967	3.081
UBXN2B	0.681	1.604	5.152
UCHL1	-0.207	0.867	7.102
UCHL5	0.257	1.195	5.069
UCN	0.157	1.115	3.490
UCP2	0.287	1.220	3.662
UG0898H09	0.174	1.128	6.257
UGCG	1.072	2.103	6.060
UGDH-AS1	0.023	1.016	3.416
UGP2	0.367	1.290	4.159
UGT8	0.059	1.042	9.349
UHRF1BP1L	0.362	1.285	6.404
ULK1	-0.135	0.911	5.047
ULK4	-0.480	0.717	3.782
ULK4P1//RP13-395E19.3//ULK4P3//ULK4P2//CHRFA	0.303	1.233	5.372
ULK4P3	0.808	1.750	3.037
ULK4P3//ULK4P1//RP13-395E19.3//CHRFAM7A//ULI	0.418	1.336	5.583
UNC13A	-0.077	0.948	6.083
UNC13C	0.535	1.449	6.023
UNC13D	-0.036	0.975	3.982
UNC5A	-0.287	0.819	3.924
UNC5B	-0.136	0.910	6.311
UNC5CL//RP1-34B21.5	0.192	1.142	2.589
UNC5D	-0.050	0.966	4.758
UNC79	0.040	1.028	5.864
UNC80	-0.115	0.923	6.503
UNC93B1	0.336	1.262	4.053
UNC93B3//UNC93B1//UNC93B7//UNC93B2//UNC93	0.419	1.337	4.223
UPF2	0.171	1.126	5.921
UPP2	0.143	1.104	3.478
UPRT	0.329	1.256	3.775
UQCRB	0.304	1.235	3.832
UQCRC2	0.158	1.116	8.615
UQCRFS1	-0.128	0.915	4.660
UQCRHL//RP11-169K16.7	-0.085	0.943	6.254
USH1C	-0.451	0.731	3.477

USP11	0.063	1.045	7.602
USP17L15//USP17L6P//USP17L9P//USP17L12//USP1	-0.079	0.946	4.568
USP17L15//USP17L6P//USP17L9P//USP17L12//USP1	-0.049	0.966	4.435
USP17L15//USP17L6P//USP17L9P//USP17L12//USP1	0.021	1.015	4.602
USP17L5	0.301	1.232	4.290
USP25	0.242	1.183	6.672
USP27X	0.224	1.168	3.713
USP28	0.304	1.235	4.674
USP32	0.345	1.270	7.579
USP32P2	0.466	1.381	3.661
USP33	0.033	1.023	6.136
USP43	-0.351	0.784	3.126
USP53	0.058	1.041	6.652
USP54	-0.042	0.971	7.779
USP9Y	0.793	1.733	5.172
UTP14A	0.592	1.507	3.235
UTP23	0.265	1.201	5.378
UTRN	0.108	1.078	6.937
UTS2	-0.693	0.619	2.285
UTY	1.056	2.079	3.510
VAC14-AS1	-0.234	0.850	4.115
VAMP1	-0.744	0.597	7.972
VAMP3	0.175	1.129	7.659
VAMP4	0.178	1.131	6.450
VAMP5	-0.020	0.986	5.275
VAMP8	0.695	1.619	4.699
VANGL2	-0.259	0.835	4.877
VASH1	0.039	1.027	5.492
VASP	0.190	1.141	5.666
VAT1	0.240	1.181	6.576
VAT1L	-0.320	0.801	7.969
VAV1	0.413	1.331	4.883
VAV3	-0.102	0.932	2.509
VAX1	0.045	1.032	2.393
VCAM1	0.268	1.204	5.033
VCAN	0.107	1.077	6.898
VCAN-AS1	0.099	1.071	1.877
VCL	0.784	1.722	6.110
VCPIP1	0.357	1.281	6.558
VCPKMT//METTL21D	0.107	1.077	4.022
VCX2//VCX	0.521	1.435	3.470
VDAC3	-0.046	0.969	8.659
VEGFA	-0.324	0.799	5.518
VEGFC	0.294	1.226	3.252
VEZF1	0.021	1.015	6.217
VGF	0.249	1.189	4.633
VGLL3	-0.109	0.927	2.993
VIM	0.344	1.269	6.136
VIM-AS1	0.213	1.159	5.032
VIP	0.061	1.043	3.672

VIPR1	-0.153	0.900	3.709
VMAC	-0.148	0.903	4.601
VMO1	0.476	1.391	2.623
VN1R1	0.114	1.082	4.111
VNN1	0.155	1.113	2.409
VNN2	0.711	1.637	2.516
VPS11	0.294	1.226	5.916
VPS13A	0.093	1.067	6.139
VPS33A//RP11-512M8.5	0.135	1.098	4.260
VPS33B	0.250	1.189	5.364
VPS35	0.331	1.258	8.852
VPS36	0.163	1.120	6.224
VRK1	0.061	1.043	3.446
VRK2	0.183	1.135	4.186
VSIG1	0.239	1.180	2.578
VSIG4	0.277	1.212	6.044
VSNL1	-0.302	0.811	2.481
VSTM2A	-0.196	0.873	5.823
VSTM2A-OT1//GS1-18A18.1	0.214	1.160	3.198
VSTM2B	-0.077	0.948	6.069
VSTM2L	0.507	1.421	4.765
VSTM5	-0.256	0.837	2.711
VSX1	-0.174	0.886	2.851
VTA1	0.204	1.152	6.956
VTRNA1-1	-0.226	0.855	5.430
VTRNA1-2	-0.749	0.595	2.010
VTRNA1-3	0.106	1.076	3.889
VTRNA2-1	-0.114	0.924	4.591
VWA1	0.193	1.143	5.171
VWA3A	-1.253	0.419	4.222
VWA5A	0.456	1.372	5.507
VWA5B1	-0.658	0.634	1.943
VWA5B2	-0.249	0.841	3.393
VWA7	-0.221	0.858	3.355
VWA7	-0.118	0.921	3.055
VWC2L	-0.400	0.758	3.635
VWF	0.103	1.074	5.970
WAC-AS1	0.046	1.032	4.917
WAS	0.437	1.354	4.799
WASF1	-0.095	0.936	6.429
WASF2	0.110	1.079	8.447
WASH3P	-0.607	0.657	3.081
WASH3P//ABC7-42389800N19.1//WASH2P//LOC100	-0.631	0.646	5.681
WASH5P	0.238	1.180	5.572
WASL	0.221	1.166	7.659
WBP11P1	-0.147	0.903	2.119
WBP1L	0.099	1.071	7.468
WBP2NL	0.345	1.271	2.855
WBP5	-0.134	0.911	6.357
WBSCR17	0.329	1.256	5.236

WDFY2	0.163	1.119	6.696
WDFY3-AS1	0.678	1.600	5.013
WDFY4	0.359	1.283	3.307
WDHD1	0.324	1.252	3.657
WDR11	0.093	1.067	6.681
WDR11-AS1	0.061	1.043	3.731
WDR16	-1.596	0.331	3.971
WDR17	0.375	1.297	6.436
WDR19	-0.050	0.966	6.075
WDR3	0.382	1.303	5.776
WDR36	0.398	1.318	6.460
WDR37	-0.061	0.958	6.253
WDR38	-1.063	0.479	2.822
WDR47	0.278	1.212	7.450
WDR49	-0.436	0.739	6.582
WDR52	-0.760	0.591	4.419
WDR54	0.196	1.145	4.049
WDR5B	0.488	1.403	4.481
WDR63	-1.185	0.440	3.021
WDR65	-0.614	0.653	2.291
WDR7	0.146	1.107	7.554
WDR86-AS1	-0.078	0.947	2.936
WDR92//RP11-474G23.1	0.043	1.031	4.742
WDR96	-1.307	0.404	3.877
WEE1	0.125	1.090	3.012
WFDC1	0.106	1.076	4.368
WHAMMP1	0.070	1.050	4.366
WIF1	-0.796	0.576	4.038
WIPF1	0.282	1.216	4.684
WIPF2	-0.084	0.944	6.072
WIPF3	0.607	1.523	4.693
WIPI1	0.254	1.193	6.531
WISP1	-0.257	0.837	2.432
WNK1	-0.075	0.950	8.467
WNK2	0.031	1.021	5.425
WNK3	-0.096	0.936	4.407
WNT10B	0.023	1.016	3.501
WNT3//LOC101929777	0.481	1.396	3.763
WNT5A-AS1	-0.222	0.857	3.317
WNT7A	-0.303	0.811	3.811
WNT9A	0.034	1.024	3.057
WSB1	0.138	1.101	8.719
WSCD1//LOC339166	-0.147	0.903	5.455
WSCD2	0.055	1.039	2.893
WWC1	-0.352	0.784	5.839
WWTR1	0.062	1.044	4.472
XAF1	0.490	1.405	6.405
XBP1	0.258	1.196	6.243
XG//XGY2	0.391	1.311	2.394
XGY2//XG//SRY//RPS4Y1	-0.036	0.975	2.583

XIST	-1.341	0.395	3.852
XKR4	0.530	1.444	4.083
XKR4	0.305	1.235	5.149
XKR6	0.131	1.095	3.873
XKR6//AF131215.2	-0.065	0.956	3.295
XKR7	-0.054	0.964	2.291
XKR8	-0.160	0.895	2.838
XKR9	-0.098	0.934	2.894
XPC	0.079	1.056	5.895
XPR1	0.180	1.133	7.494
XRCC4	0.410	1.328	4.457
XRCC6BP1	0.509	1.423	2.837
XRRA1	-0.141	0.907	4.838
XXbac-B476C20.13	-0.065	0.956	4.147
XXbac-B476C20.14	-0.192	0.875	2.503
XXbac-BPG116M5.15	0.253	1.192	4.202
XXbac-BPG116M5.17//C2//CFB	0.261	1.198	3.907
XXbac-BPG154L12.4	-0.301	0.812	3.434
XXbac-BPG55C20.7	0.454	1.370	2.353
YAP1	0.076	1.054	5.306
YARS2	-0.060	0.960	4.766
YBX1	-0.042	0.972	8.285
YBX3	0.693	1.616	4.962
YBX3P1//CSDAP1	0.292	1.224	2.479
YEATS4	0.297	1.229	4.993
YIF1A	-0.074	0.950	4.914
YIPF4	0.298	1.229	6.677
YIPF5	0.255	1.193	7.430
YJEFN3//CTC-260F20.3	0.141	1.102	4.113
YME1L1//TRAJ17//TRAV20//TRAC//TRDV2	0.089	1.064	3.614
YME1L1//TRAV12-2	-0.239	0.847	3.253
YOD1	0.124	1.089	4.039
YPEL1	-0.226	0.855	3.708
YPEL3	-0.113	0.925	7.615
YPEL4	0.397	1.316	3.892
YTHDF1	0.638	1.556	6.893
YWHAB	0.044	1.031	6.829
YWHAG	-0.177	0.884	9.525
YWHAH	0.265	1.201	4.644
YY2	-0.117	0.922	3.143
ZADH2	0.066	1.047	5.793
ZAK//AC013461.1	0.242	1.183	5.264
ZBBX	-1.406	0.377	3.249
ZBED8//C5orf54	0.059	1.041	3.918
ZBED9//SCAND3	-0.340	0.790	2.975
ZBTB12	-0.393	0.761	3.156
ZBTB16	-0.275	0.826	3.406
ZBTB16	-0.068	0.954	5.897
ZBTB18	-0.139	0.908	6.038
ZBTB20	-0.199	0.871	6.414

ZBTB20//MIR568	0.580	1.494	3.820
ZBTB24	0.405	1.324	5.650
ZBTB33	0.552	1.467	4.921
ZBTB37	0.152	1.111	5.823
ZBTB39	-0.208	0.866	4.521
ZBTB40-IT1	0.340	1.266	3.675
ZBTB44	0.237	1.178	6.969
ZBTB6	0.373	1.295	5.132
ZBTB7B	0.122	1.089	4.765
ZBTB7C	0.110	1.079	3.983
ZBTB8A//RP1-27O5.3	-0.106	0.929	3.740
ZC2HC1A	0.113	1.082	6.509
ZC3H12A//MIR6732	-0.097	0.935	3.934
ZC3H12B	0.204	1.152	5.248
ZC3H8	-0.295	0.815	4.956
ZC3HAV1	0.311	1.241	6.638
ZCCHC12	0.218	1.163	4.470
ZCCHC24	-0.060	0.959	6.897
ZCWPW1	-0.271	0.829	4.354
ZDBF2	0.164	1.121	6.157
ZDHHC11	-0.062	0.958	3.772
ZDHHC12	0.125	1.090	3.718
ZDHHC13	0.110	1.079	4.491
ZDHHC14	-0.137	0.910	6.637
ZDHHC20	-0.160	0.895	7.162
ZDHHC20-IT1	-0.185	0.879	4.022
ZDHHC21	0.242	1.183	6.600
ZDHHC23	0.127	1.092	3.406
ZDHHC8P1	-0.485	0.714	2.951
ZDHHC9	0.133	1.097	8.342
ZEB2-AS1	0.211	1.158	2.680
ZER1	-0.040	0.972	7.289
ZFAND1	0.250	1.190	5.138
ZFAS1	0.094	1.067	7.020
ZFP1	0.493	1.408	6.019
ZFP14	-0.022	0.985	4.207
ZFP28	0.076	1.054	4.165
ZFP36	0.350	1.274	7.914
ZFP36L1	-0.180	0.882	4.540
ZFP36L2	0.311	1.240	6.473
ZFP69B	0.123	1.089	4.288
ZFP82	-0.156	0.898	4.940
ZFP90	0.069	1.049	3.576
ZFP92	0.162	1.119	3.087
ZFY	0.326	1.254	2.779
ZFYVE16	-0.046	0.969	7.030
ZFYVE28	0.172	1.127	2.889
ZFYVE28	0.095	1.068	3.184
ZFYVE9	-0.093	0.938	6.738
ZHX2	-0.024	0.983	5.691

ZIC1	0.621	1.538	5.310
ZIC2	0.057	1.041	3.888
ZIC5	-0.118	0.922	4.724
ZIK1	0.023	1.016	3.822
ZKSCAN2	0.297	1.229	4.897
ZKSCAN3	0.216	1.162	4.132
ZMAT1	0.265	1.202	5.760
ZMAT3	0.404	1.323	7.308
ZMAT4	0.169	1.125	4.781
ZMYM6NB//ZMYM6//RP11-244H3.4	0.521	1.435	6.006
ZMYND10	-0.664	0.631	2.332
ZMYND15	0.087	1.062	2.885
ZMYND8	-0.264	0.833	6.423
ZNF100	0.444	1.360	5.212
ZNF114	0.380	1.301	4.115
ZNF117	0.053	1.037	4.634
ZNF132	0.764	1.698	3.636
ZNF134	0.215	1.161	5.502
ZNF138	-0.034	0.976	6.107
ZNF14	-0.101	0.932	4.109
ZNF140	0.222	1.166	3.220
ZNF154	-0.073	0.951	4.332
ZNF17	0.045	1.032	4.481
ZNF184	0.561	1.476	5.379
ZNF185	0.061	1.043	3.386
ZNF189	0.152	1.111	5.645
ZNF192P1	0.283	1.217	4.067
ZNF200	0.442	1.359	3.336
ZNF204P	-0.101	0.932	2.807
ZNF208	0.318	1.246	2.626
ZNF215	0.107	1.077	1.921
ZNF217	0.161	1.118	3.962
ZNF22	0.044	1.031	3.405
ZNF224	0.769	1.704	4.027
ZNF225	0.584	1.499	3.429
ZNF227	0.437	1.354	5.154
ZNF229//CTC-512J12.4//ZFP28	0.238	1.179	4.351
ZNF23//AC010547.9	0.085	1.061	4.005
ZNF230	0.926	1.900	3.082
ZNF232	0.592	1.508	3.146
ZNF234	0.350	1.274	4.141
ZNF239	-0.087	0.942	2.946
ZNF248	-0.081	0.945	5.664
ZNF25	0.454	1.370	7.603
ZNF253	0.154	1.113	4.593
ZNF256	0.228	1.171	3.697
ZNF267	0.671	1.592	4.200
ZNF274	0.705	1.630	3.335
ZNF276	-0.038	0.974	4.557
ZNF280B	0.175	1.129	5.011

ZNF280C	0.305	1.236	5.633
ZNF280D//LOC145783	0.336	1.262	2.303
ZNF280D//LOC145783	-0.055	0.962	2.813
ZNF280D//LOC145783	0.020	1.014	4.920
ZNF284//ZNF223//AC084219.2	0.862	1.818	4.028
ZNF285	0.477	1.392	3.141
ZNF3	-0.031	0.978	3.826
ZNF300	0.506	1.420	3.013
ZNF302	0.349	1.274	5.790
ZNF304	0.057	1.040	4.364
ZNF311	0.147	1.107	2.232
ZNF320	0.183	1.135	4.274
ZNF322	0.332	1.259	4.552
ZNF324B	0.154	1.113	3.333
ZNF326	-0.322	0.800	6.753
ZNF329	0.351	1.275	3.874
ZNF33B	-0.121	0.919	3.092
ZNF34	-0.154	0.899	3.728
ZNF346-IT1	-0.350	0.785	2.206
ZNF350	0.479	1.394	4.051
ZNF350-AS1//HCCAT3	-0.102	0.932	3.061
ZNF365	-0.071	0.952	2.640
ZNF37BP	0.180	1.133	5.663
ZNF382	0.418	1.336	4.155
ZNF383	0.532	1.445	4.322
ZNF385A	-0.311	0.806	6.743
ZNF385B	-0.193	0.875	3.479
ZNF385D	0.133	1.097	2.202
ZNF395//FBXO16	-0.631	0.646	4.250
ZNF404	0.305	1.235	4.905
ZNF416	0.051	1.036	2.965
ZNF417	-0.182	0.881	5.032
ZNF418	0.396	1.316	4.080
ZNF419	0.222	1.166	3.556
ZNF420	0.170	1.125	5.280
ZNF43	0.443	1.360	4.488
ZNF436	0.988	1.983	5.518
ZNF440	0.515	1.429	4.882
ZNF441	0.247	1.187	4.037
ZNF443	0.233	1.176	2.573
ZNF449	0.408	1.327	4.535
ZNF451	0.037	1.026	5.987
ZNF454	0.166	1.122	4.762
ZNF461	0.448	1.364	3.398
ZNF462	-0.259	0.836	6.223
ZNF468	0.276	1.210	5.111
ZNF48	-0.642	0.641	2.373
ZNF480	0.329	1.256	4.792
ZNF483	0.372	1.295	5.707
ZNF486	0.264	1.201	3.349

ZNF487	-0.209	0.865	4.274
ZNF488	0.190	1.141	4.244
ZNF502	0.172	1.127	3.353
ZNF503//RP11-399K21.11	0.163	1.120	3.529
ZNF510	0.062	1.044	5.120
ZNF512//RP11-158I13.2	0.335	1.261	6.380
ZNF518A	0.429	1.347	5.200
ZNF518B	0.273	1.208	4.713
ZNF519	0.117	1.084	4.177
ZNF521	0.160	1.117	5.548
ZNF534	0.323	1.251	2.538
ZNF536	0.121	1.087	6.515
ZNF542P//ZNF542//LOC283788	0.213	1.159	3.787
ZNF547	0.201	1.149	4.653
ZNF549	0.226	1.170	3.702
ZNF554	0.075	1.053	4.135
ZNF557	0.487	1.402	4.032
ZNF558	0.715	1.641	4.270
ZNF563	0.263	1.200	2.583
ZNF567	0.068	1.048	3.754
ZNF569	0.387	1.307	3.911
ZNF571	-0.203	0.869	3.233
ZNF572	-0.121	0.919	2.527
ZNF581	0.274	1.209	4.161
ZNF582	0.037	1.026	3.779
ZNF582-AS1//AC006116.25	0.019	1.013	3.235
ZNF584	0.144	1.105	4.277
ZNF587B	0.023	1.016	3.728
ZNF594	0.261	1.198	4.904
ZNF596	-0.036	0.975	2.160
ZNF597	0.527	1.441	5.270
ZNF607//CTD-2528L19.4	0.329	1.256	4.948
ZNF611	0.214	1.160	4.079
ZNF613	0.141	1.103	3.744
ZNF615	0.449	1.365	4.267
ZNF616	-0.113	0.925	2.603
ZNF624	0.566	1.481	3.879
ZNF626	0.203	1.151	3.852
ZNF638//ZNF638-IT1	0.520	1.434	4.671
ZNF649	0.164	1.120	4.599
ZNF652	0.133	1.097	6.978
ZNF658//ZNF658B//CTD-2173L22.4	0.437	1.353	4.950
ZNF667	0.100	1.072	4.506
ZNF667-AS1//LOC100288114	0.073	1.052	7.459
ZNF669	0.373	1.295	3.786
ZNF674-AS1	0.563	1.478	3.455
ZNF675	0.495	1.409	2.534
ZNF681	0.302	1.233	3.631
ZNF689	0.138	1.100	3.029
ZNF69	0.095	1.068	2.893

ZNF691	0.305	1.236	3.075
ZNF697	-0.221	0.858	3.464
ZNF699	0.357	1.281	3.851
ZNF702P	0.603	1.519	2.981
ZNF708	0.254	1.193	4.744
ZNF711	0.433	1.350	4.867
ZNF714	0.525	1.439	2.644
ZNF718//ZNF595	0.629	1.546	4.052
ZNF721//ABCA11P	0.289	1.222	4.590
ZNF724P	0.720	1.648	2.795
ZNF727P//RP11-3N2.13	-0.157	0.897	2.920
ZNF727P//RP11-3N2.13	0.035	1.025	4.465
ZNF749	0.123	1.089	3.310
ZNF75D	0.203	1.151	3.398
ZNF764	-0.035	0.976	3.474
ZNF770	0.215	1.161	6.360
ZNF771	-0.361	0.779	2.799
ZNF774	-0.195	0.873	4.119
ZNF776	0.118	1.085	5.235
ZNF780A	0.168	1.124	4.065
ZNF780B	0.397	1.317	5.807
ZNF781//ZFP30	-0.107	0.929	3.898
ZNF783	0.140	1.102	5.837
ZNF786	0.025	1.018	4.862
ZNF787	-0.029	0.980	4.128
ZNF789	-0.475	0.720	3.367
ZNF790-AS1	-0.115	0.923	3.115
ZNF791	0.296	1.228	6.429
ZNF804A	-0.100	0.933	3.891
ZNF804B	-0.257	0.837	3.919
ZNF812//ZNF562	0.464	1.379	3.179
ZNF815P	-0.126	0.917	3.522
ZNF821	0.174	1.128	4.635
ZNF826P	0.369	1.291	3.348
ZNF829	0.321	1.249	2.346
ZNF83	0.321	1.249	5.389
ZNF831	-0.074	0.950	2.810
ZNF835	0.029	1.020	3.815
ZNF836	0.372	1.294	4.668
ZNF843	-0.062	0.958	2.378
ZNF844	0.060	1.043	4.660
ZNF850	-0.332	0.795	3.826
ZNF852	0.054	1.038	3.042
ZNF91	-0.107	0.928	5.433
ZNF91	-0.089	0.940	2.930
ZNF93	0.209	1.156	3.909
ZNRF2P1	0.127	1.092	4.007
ZRANB2-AS2	-0.102	0.931	3.492
ZRANB3	0.201	1.150	3.652
ZSCAN12	0.251	1.190	3.026

ZSCAN12P1	0.447	1.363	3.359
ZSCAN16	0.240	1.181	2.841
ZSCAN22	0.428	1.345	3.670
ZSCAN23	0.169	1.124	3.850
ZSCAN31	-0.020	0.986	3.964
ZSWIM1	0.356	1.280	3.712
ZSWIM7	-0.602	0.659	3.682
ZUFSP	0.283	1.217	4.366
ZWILCH	0.457	1.373	3.347
ZXDB	0.142	1.104	5.899
ZYG11B	0.202	1.151	7.983

780	5.85278262	0.050182172
SNORA11//MAGED2	-0.014998046	9.550254344
RP11-71N10.1	-0.016711987	3.118586349
RHEBL1	-0.015028476	2.216141506
SLC35G5//SLC35G4P//SLC35G6	-0.016110757	2.987849167
HCN1	-0.03953936	6.265702166
SHISA6	0.023759574	6.950188196
SPOCK3	-0.011778532	6.362986672
ARX	-0.01856777	3.5346982
ITPR1	0.024818946	6.217928352
RNU6ATAC9P	-0.012323506	3.111856899
CTB-25B13.12	-0.011443064	2.342889335
LINC00662//CTC-459F4.2	0.010770652	3.611333179
ETNPPL	0.01877229	7.690721139
SNAP91	-0.019297383	7.348728347
STXBP1	-0.01702518	9.599699483
RP11-360F5.3	-0.01577745	2.908954244
RNF208	-0.011124132	2.744564418
PPFIA2	0.012920483	7.134271872
DAPK1	-0.014855179	7.126569082
FAT1	-0.011154128	6.981369541
FDX1L	0.010414561	2.952535116
AP1S3	0.011974807	2.25978026
PRPH	0.022495751	2.646853683
SALL1	-0.011144467	6.244469085
FBXO27	-0.011548806	5.198070049
SLC27A2	-0.01933673	2.599426948
ADSSL1	0.009626678	4.219355968
RP11-678G14.3	0.011254194	3.024828047
RNU6-447P//RNU6-1118P//RNU6-1076P//RNU6-35	0.013355066	5.059195997
CDH12	-0.018946266	3.07624155
TSC22D1-AS1	0.00993969	4.251417688
ZXDA	0.011612631	3.892447441
AC093388.3	0.010537703	5.548901329
FDFT1	0.011838851	5.673285125
RNF113B	-0.010782788	2.369629403
LOC101929709//RP11-37B2.1	-0.012147253	5.136947732

RP11-605F22.2	-0.013303311	1.992268382
SLC52A2//GPR172A	0.012913881	2.70194573
ABT1	0.010048771	4.190873425
ISOC1	-0.009823254	7.034227315
LINC01061//LOC729218//LOC101926918//RP11-384	-0.010685613	6.785527601
C6orf48	-0.009393101	8.646682506
TMEM200A	0.00996321	2.736402787
SOX10	0.013243885	5.405575382
DLAT	-0.008520089	6.716206362
WLS	0.009224658	6.557502063
LRRC57	0.008983394	5.329475669
CYP26B1	0.012348778	2.291541755
RP11-161H23.5//RP11-386G11.10	0.010720385	7.897715556
EDIL3	-0.011443333	9.316007823
HMGCR	-0.011534926	7.102154553
LOC100506114//CTD-2545M3.8	-0.012285855	2.605064153
RIT2	-0.015508032	4.323061354
CAMSAP2	-0.008233961	8.704972117
TMTC2	0.014478904	6.151450353
RP11-458D21.1//RP4-791M13.3	0.01586992	4.154293475
ZNF223	-0.010588173	3.776249801
LOC100130093//SNAP47//JMJD4	0.010317344	3.103424556
RNY4P19	0.015895837	4.39077591
SPATA13//RP11-309I15.1//RP11-307N16.6	0.010815406	3.557995216
SEC14L6	0.013635743	2.790288698
RP11-436D23.1	-0.011906821	2.510304398
RP3-323P24.3	-0.012068436	3.508946708
RP11-958J22.1	-0.014757238	2.703365548
CNNM1	-0.017277889	4.435665339
UBE2T	0.01472271	4.03543568
GUSBP3//SMA4//LOC653080//SMA5//GUSBP9//RP1	-0.011611309	7.302078021
DNAH17	-0.008951861	2.637799092
GSK3A	0.008778734	6.364809357
MRTO4	0.00936268	4.93683276
NPIPB3	0.012036795	8.293394809
HEYL	-0.01431899	2.950902493
HIST1H3F	-0.015886533	4.157506876
RTN3	-0.007415536	9.054498643
COPZ2	0.010620362	4.576501008
ANGPTL1	-0.011259821	3.058888985
VAMP2//RP11-599B13.6	0.007562291	7.770861384
HAAO	0.010389706	2.657956303
CDC42EP2	-0.012173343	3.883954626
DNAH17-AS1//RP11-559N14.5	0.013893245	2.457409351
RYR2	0.018587224	5.986950073
TPCN1	0.009698389	5.849874374
SLC35D2	-0.016198393	5.449272727
MYL9	-0.015558346	6.420879203
LINC00461//MIR9-2	0.009382253	5.578513422
RNU6ATAC6P	-0.009599691	2.011001864

PFN2	-0.007522297	7.579309739
RTN4R	-0.010147405	3.171125192
VPS37D	-0.011108013	2.753426466
CA14	-0.018162517	4.550308935
LOC100130950//RP11-333E1.1	0.010687315	3.664755301
PIP5K1C	-0.011916682	6.848915248
PLEKHH1	0.019254217	7.103170608
ZNF233	0.009306921	2.038145687
CNN1	0.014208359	4.599997107
NAP1L5	-0.013458194	7.617949806
LOC100129129//AF131216.5	0.010525639	4.10185052
RNU6-1283P	-0.012411314	2.944726759
RNF130	-0.009114133	6.863480908
PRX	-0.018745631	3.24804739
SIAH2	-0.009323158	5.515491051
ICA1L	-0.007506466	5.106957609
PGLS	-0.009005907	4.734534814
ERMN	-0.014357736	8.471827169
RP11-270M14.5	0.010526322	2.569151442
C21orf37	-0.025360613	4.392107379
TMEM221	-0.012154166	2.971677769
GANC	0.009402251	5.515513935
MLXIPL	0.009752592	3.732471407
LUCAT1	-0.014858004	2.744207745
AP001631.9	0.008980939	3.083024305
CCDC110	-0.012150218	2.4687675
SNAI3-AS1	0.009625935	4.49646089
AIM1	0.00950365	2.344091527
GAB1	0.010268771	5.829886633
ADAM15	0.00851043	4.414217921
MTMR6	-0.007222576	6.483023564
PRKCDBP	-0.008545975	3.46079518
STX1A	-0.010850438	4.267115481
USP40	0.008748385	5.400009878
LAMP5	-0.024333265	7.096703968
GUCY1A2	-0.014601731	5.447902131
CLVS2	-0.018554771	5.237699802
C6orf48	-0.00656294	7.237891733
CTD-2647L4.5	-0.009611904	4.172888976
CACNA1I	0.014254753	4.275154775
LDLRAD3	-0.008613135	5.837807296
OSGIN1	-0.008260978	3.141664641
MYOM1	0.0099665	4.940360882
ISLR2	-0.011126069	2.370569856
ROCK1P1	-0.017685957	1.410698544
PPAP2C	-0.011953747	6.575488152
SLC4A1AP	0.006705118	5.650773618
TPD52L1	-0.007979206	5.625924961
RNA5SP129	-0.010815133	3.286778303
TPRN	-0.008501657	2.976011567

SCAMP4	0.007673808	4.356357239
ZDHHC22	-0.008112099	3.962423857
SLN	0.01110311	2.55498209
TNRC6C-AS1	-0.013865635	5.361223449
BTBD8	0.008895526	4.900524726
PRB2	-0.011641445	2.938503594
CRYAB	-0.00834509	7.988554933
RBP1	-0.006472967	3.300584947
NUTM2D	0.008415937	3.937480259
DNM2	-0.007972633	5.492939175
MACROD2	0.006511747	2.989405523
LPPR3	-0.015826656	4.514919837
GPR61	-0.009413239	2.918209809
TMX4	-0.00766605	7.452679979
HOXD4//HOXD3//LOC401021	0.011710263	3.614534612
RN7SKP283	-0.008499984	2.673594613
TSSK3	0.010519766	3.212150481
FAM222A	0.01089034	3.860882738
GDAP1L1	0.012090664	3.586680951
RNA5SP125	-0.009010705	2.969975156
PLEKHM2	-0.006784895	7.917270303
CAMK1D	0.010832961	6.976248885
IDH3A	-0.005965619	3.295522561
MPLKIP	0.005928808	4.720912894
CBR3-AS1	-0.007782089	2.144508948
RNU1-122P	0.009607698	4.252580118
TANC2	-0.00571815	7.451813501
CRTC1	-0.009118106	5.573454017
PDE7A	0.006695218	4.593809734
KIF3B	0.006702687	7.224758796
SLC44A1	0.009272578	8.912711277
LINC01315//Z83851.1	-0.00810251	3.982516949
SH3TC2	0.013394909	4.977077288
PLEKHG1	-0.009327083	6.545615633
SPRYD3	0.00701785	6.741014811
DUSP8//LOC101927562	0.009287335	4.653502972
LOC101928590//RP11-806K15.1	0.00797044	2.026634665
PQLC3	0.007352689	4.725421327
LOC101929302//RP11-509J21.1	-0.010681371	4.090458244
FLJ25917//RP5-1158E12.3	0.009840756	1.776566167
ZNF391	-0.008006214	3.593925432
SNORA70B	-0.008904567	2.90024231
SMO	0.006215357	4.679359092
PLCE1	-0.005271486	6.108145595
EML1	-0.006302488	5.549620635
RP11-946L20.2//LOC101927459	0.01277714	3.703904449
SLC38A5	-0.010664815	3.213900428
ADD2	-0.011708357	6.151262315
TCEB3C//LOC101929870//LOC101060464//LOC1019	-0.00777087	3.439537564
RNA5SP217	-0.010318788	3.373130878

OR7C1	0.011358198	2.718519257
LMTK3	0.008891461	5.177536026
SNX22	0.008199691	4.299521243
LOC101927699//RP11-38L15.3	0.010546898	5.445907323
FKBP11	-0.006827706	4.14425275
CUEDC1	-0.007991152	3.809891533
PCYT1B	0.004961256	6.348202763
PCDH20//LOC101926951	-0.006773223	2.132199512
NPAP1	0.007780908	2.778961728
RP11-326C3.11	0.006989936	4.085852953
LRFN5	0.012118639	5.491679709
GBP4	0.01176897	4.390470018
RP11-627D16.1	-0.017015619	4.71158525
HS6ST3	-0.012714641	6.228476712
LONRF2	-0.007998484	7.861756688
VWC2	-0.007888259	4.732491258
DLG5	0.005195571	6.512943598
DLL1	-0.007259471	4.837303742
SDHAP1//SDHAP2	0.008961908	5.068910571
ADAMTS16	0.007378149	2.365100598
MDH1	0.006510463	7.025527208
ZNF710	-0.004985336	3.237248326
IGHV4-34//SKAP2//IGHG1	0.007465816	2.571853792
RP1-122P22.2	-0.006414308	3.25881977
TIMM23B	-0.006438441	4.777112035
FGD2	0.006707387	3.83218281
CLVS2	-0.012135548	5.82604226
LOC102724851	0.007612614	5.558363805
MEG3	0.017641665	6.140043279
ITFG2//LOC100507424	-0.005681017	3.066728918
TMCC2	0.005630948	3.392240073
MIR127	0.006885512	3.259241022
SLC22A3	0.006406054	3.410105669
LOC100133106	0.005358943	3.623909875
SAMD4A	0.005630104	5.367832343
XK	-0.006399821	4.459953629
TARBP1	0.006875155	4.951087415
RIMBP3	0.00622355	2.92203745
CLMN	-0.007985967	5.469792992
SNORD115-1//SNORD115-13//SNORD115-16//IPW//	-0.006341295	10.60255084
SATB1	0.005172918	5.850189422
NCKAP5	-0.007328551	5.262081744
SACS	0.004218716	7.227665096
POMZP3	0.005661502	3.550045768
LOC100128340	0.008888053	2.916646296
FOXD4L6	0.006500077	3.416647005
PARS2	-0.005496637	3.104991941
CCL4L2//CCL4L1//CCL4	-0.007706587	2.189324622
GGA3	-0.004191496	5.56533925
MIR1229	-0.008952919	3.278986841

SCN2B	0.009318407	6.113876968
ENTPD6	0.005223171	6.102441172
SHANK2	-0.005754116	2.545877518
NEURL1//NEURL	0.007310989	4.575693788
C6orf48	0.004534578	7.127938736
SSSCA1-AS1	0.00544528	3.997339797
PPIP5K1//AC011330.5	0.004599077	6.998382577
ABCA2	-0.006347673	9.157623562
DAPK2	-0.005074056	3.542136567
LOC100131541	0.007112871	3.861565717
LINC00341//SYNE3	0.006568081	3.834001121
RNU6-216P	-0.006295235	3.973011186
ALOX12B	0.006328751	1.994004919
LMCD1	0.005123355	4.613346262
LAMTOR3	-0.004221714	5.836192429
P2RY12	0.008890506	5.572125789
HIST2H2BE	0.006301771	5.039650763
ANAPC1P1	0.005594439	2.793540309
DIAPH1	-0.004622047	5.398704058
GPR153	-0.006654542	3.522933782
SSTR1	-0.0071983	3.554003304
PHACTR1	-0.00861485	3.780985384
GUSBP9//GUSBP2//GUSBP3//SMA4//SMA5//LOC65	-0.005260582	8.675741464
STS	0.006941113	6.937128175
RNU7-40P	0.008115389	3.288999725
MICALL1	0.005622585	4.137715753
FAR1	0.004547707	7.839952937
ME3	0.00465963	5.299904139
TRPM3	0.005951719	7.403903817
ZNF814	0.004121037	3.779870322
UNC5C	-0.004388248	5.563307734
CYP4X1	-0.008644025	4.597591437
MMP24-AS1//EDEM2//RP4-614O4.11	0.004188832	5.211498582
CREB5//LOC401317	-0.005466263	3.713962942
PBX3	0.005669995	4.8006012
MCM7	0.004164807	4.472481454
OCEL1	-0.005051962	3.939638777
PGM5	-0.004134631	2.837567466
LINC01354//RP5-855F14.1	0.006802024	5.399266714
LOC100133050	0.004569679	2.413704382
DOK6	0.010297035	6.190014136
NCR3LG1	-0.007125559	4.191263318
LOC101927300//RP11-441O15.3	-0.003959977	3.08530883
RPL29P2//RP11-199F11.1	-0.003177698	3.756795522
FAM83D	-0.005940972	2.302410922
RNA5SP427	0.005119403	2.484993907
AP000473.5	0.00461457	3.946241612
SNORD114-27	0.004047119	4.491759621
SRRM4	-0.0074498	4.606291052
TMEM63A	-0.00666005	7.885317156

CTAGE6	0.004218705	3.332229045
ZFHX3	-0.003484586	5.5412803
PCNXL2	0.003103169	6.861239766
CLEC18B	0.004650244	3.267079015
GPSM3	-0.003436596	3.070750761
RNU6-316P	-0.005461395	2.890632488
TSPAN2	-0.004257287	4.01383126
RP1-167F1.2//LOC102724609	-0.003492046	2.529867284
ASB2	0.003229366	2.115255468
SPRYD7	0.002547128	5.691241582
MT2A	0.003233719	6.778996671
SGIP1	0.00242385	6.55549285
CCM2L	-0.003190066	3.524892312
LAYN	0.003297049	3.690270534
SRRM3	-0.003676101	4.270853666
SLC38A1	-0.002889372	9.187548601
AQP5	-0.003553435	2.681502401
YME1L1	-0.002853765	3.827387644
MEG3	-0.003668892	5.175962781
B3GNT1	0.003022831	6.555856974
RP11-405A12.2	0.003272266	2.766978901
CTC-458G6.2	-0.003901582	3.155224262
LAMC3	0.002655525	2.879556933
FAM215A	0.003717906	3.10165808
SNORD114-25	0.007729405	4.152230749
PPP1R14A	0.004963851	5.765757557
NKAIN3	-0.002665071	5.906162625
UACA	-0.002996205	5.26096464
LOC101928113	0.006026873	4.406933667
RN7SKP16	0.003114015	3.925493146
LOC100289473//RP4-673D20.3	-0.002465327	4.212059973
PPP1R13L	-0.002711208	3.793701349
RNF141	0.002439634	6.385623793
LZTS3//RP5-1187M17.10	-0.003069666	4.832617671
CRABP1	0.004231004	4.843532694
CENPBD1P1//RPL23AP79	0.002193655	4.281267535
HIST2H2BA	0.002149622	6.632873881
MYPOP	-0.002448675	3.615426574
OR14I1	0.005896932	2.508332886
RNU6-1262P	-0.003575072	3.222427183
LOC100506713//AC027763.2	-0.002599398	5.507207711
NT5C3B	0.002069221	6.518462116
INA	-0.006637249	7.951214635
C17orf58	-0.002076764	3.340483716
CTD-2555K7.2	-0.00275172	2.555809879
RNU6-1057P	0.002895955	4.231807532
SUSD3	-0.002578588	3.571078523
FMO5	-0.002437987	3.18421414
ARPC5L	-0.001958558	4.871754154
ZNF527	0.002028789	2.612668567

RP11-229C3.2	0.003058509	2.878316149
EFNB3	-0.004148648	4.4736849
TRGJP2//TARP//TRGC2	0.005794003	4.75387662
MRAP2	0.003626955	4.421854725
CEP85	0.001983242	5.355805932
ZNF429	-0.002063064	5.704744135
CTD-2538C1.2	-0.002007713	3.036667518
LRRC1	0.002656377	5.580392067
RP11-164P12.4	0.002107273	1.952484035
C6orf57	0.00185312	3.919012893
SYNPO2	-0.001723001	6.258681692
MYO5B	-0.002158852	3.737446121
EXTL1	0.002472955	3.162376842
IDS	-0.001678936	8.598974871
PJA1	0.001530801	5.578718218
AC015936.3	0.002161289	5.908799123
LUZP1	-0.002136495	5.638097435
SOX9	0.001433046	8.204496326
GFAP	0.002135355	10.92945578
FLVCR2	0.001454035	2.944147863
RP11-555M1.3	-0.002165683	2.146929421
TGFA	-0.002017121	4.529506822
MSH5//MSH5-SAPCD1//SAPCD1	-0.001524288	4.085795322
CTC-329D1.3	0.001636031	3.177087512
MIR657	-0.001906399	3.076478035
UQCRH//UQCRHL	0.001118726	4.050134757
C10orf90	-0.002069453	4.6857254
NRIP3	0.002722462	5.398524787
GPR37L1	0.001545504	6.898093773
CCKBR	-0.001736369	3.073769314
TMEM130	0.003272487	6.640273254
SEL1L3	0.001530782	4.847979795
SYBU	0.002081532	4.82538603
SYNJ1	-0.001328998	7.210031881
SPR	-0.001460404	4.710183485
OAF	0.001187439	2.039617107
LOC101928344//LINC01347//LOC729218//LOC4411	0.001236144	6.627184629
PLXDC1	-0.000992227	5.386081738
FAM115C//LOC101928422	0.001088396	3.340158193
FYCO1	-0.000862948	5.974937197
CDK5R1	0.001700954	5.454525544
GCSHP3//NDUFS1	-0.00087605	6.290813966
IGSF11	0.000888767	5.410485094
LINC01289//RP11-579E24.1	0.001104834	1.594460992
BTG3	0.00087272	3.726410107
LOC100128751	0.00114923	2.340364255
RNU6-529P	-0.000827596	5.861770822
MCF2L2	-0.000759109	6.129622258
SC22CB-1E7.1	-0.00069829	5.696131518
TESPA1	-0.002175014	4.46549981

HIST2H2BC	-0.000811841	5.058907665
TECTA	0.000479939	3.551925623
RP11-259K15.2	0.00109015	3.446640222
SEMA4C	-0.000361216	5.028911745
AKAP12	-0.00060585	5.889865675
NEUROD6	-0.001071312	3.535413792
SNORA2A	0.000611599	2.471588555
SLC25A48	-0.000399376	4.020108072
TRIM74	0.000224095	3.449921019
TRIP6//MIR6875	0.000154402	5.443096048
ETV1	-4.59E-05	4.323143532
IMPACT	-4.29E-05	6.061625496
ARHGEF28	-2.82E-05	4.96917731
LINC01166//RP13-137A17.4	3.03E-05	3.779673714
GRHL1	2.09E-05	1.979045015
AASS	1.01E-05	6.841998306

t	P.Value	adj.P.Val	B	logFC validation	ALS.SP
1.010	0.322	0.950	-5.128	0.295	3.683
1.600	0.122	0.784	-4.493	0.464	8.326
1.257	0.220	0.882	-4.893	0.332	4.486
-0.186	0.854	0.999	-5.559	-0.049	2.795
0.643	0.526	0.992	-5.391	0.166	5.150
0.737	0.468	0.983	-5.335	0.179	4.535
-0.623	0.539	0.994	-5.402	-0.128	4.067
0.053	0.958	0.999	-5.573	0.012	6.730
0.486	0.631	0.999	-5.469	0.104	5.251
-0.192	0.849	0.999	-5.557	-0.049	6.404
1.721	0.098	0.757	-4.334	0.608	6.835
-0.198	0.844	0.999	-5.556	-0.051	2.051
0.810	0.426	0.972	-5.285	0.232	2.472
0.138	0.891	0.999	-5.565	0.032	6.153
-0.243	0.810	0.999	-5.548	-0.152	6.283
0.175	0.862	0.999	-5.560	0.076	7.316
-0.490	0.628	0.999	-5.467	-0.173	5.242
-0.559	0.581	0.999	-5.435	-0.114	5.779
-1.238	0.227	0.889	-4.912	-0.289	2.213
0.440	0.664	0.999	-5.488	0.219	5.413
0.999	0.327	0.954	-5.138	0.271	5.518
-1.081	0.290	0.932	-5.065	-0.226	5.637
-0.904	0.375	0.963	-5.216	-0.243	4.248
0.739	0.467	0.983	-5.333	0.235	6.310
0.153	0.880	0.999	-5.564	0.061	4.337
-0.258	0.798	0.999	-5.544	-0.056	4.949
1.976	0.059	0.663	-3.970	0.446	5.358
-1.369	0.183	0.841	-4.771	-0.437	5.665
0.720	0.478	0.984	-5.345	0.257	3.848
-0.513	0.613	0.999	-5.457	-0.153	2.413
2.469	0.021	0.517	-3.177	0.456	5.517
1.500	0.146	0.804	-4.618	0.404	4.090
-1.000	0.327	0.954	-5.137	-0.204	2.572
-0.900	0.376	0.963	-5.219	-0.221	2.908
1.026	0.315	0.945	-5.115	0.281	6.204
1.739	0.094	0.753	-4.310	0.459	4.725
1.518	0.141	0.802	-4.596	0.452	6.128
0.161	0.873	0.999	-5.562	0.042	5.338
0.214	0.832	0.999	-5.553	0.075	5.255
1.292	0.208	0.868	-4.855	0.434	5.584
0.345	0.733	0.999	-5.521	0.133	6.414
0.202	0.842	0.999	-5.556	0.039	7.532
0.374	0.712	0.999	-5.512	0.137	4.829
-0.392	0.699	0.999	-5.506	-0.096	6.610
0.095	0.925	0.999	-5.570	0.027	3.038
0.138	0.891	0.999	-5.565	0.031	3.425
-0.544	0.591	0.999	-5.443	-0.206	6.215
-0.349	0.730	0.999	-5.520	-0.095	4.621
0.939	0.357	0.962	-5.188	0.260	3.908

-2.081	0.048	0.630	-3.811	-0.516	2.340
0.442	0.662	0.999	-5.487	0.131	3.453
-0.541	0.593	0.999	-5.444	-0.168	2.660
-0.807	0.427	0.973	-5.287	-0.196	5.317
-0.562	0.579	0.999	-5.434	-0.174	2.326
-0.176	0.861	0.999	-5.560	-0.059	2.429
-0.433	0.669	0.999	-5.491	-0.205	4.395
0.544	0.591	0.999	-5.443	0.148	3.251
0.892	0.381	0.964	-5.225	0.228	4.644
-0.812	0.425	0.972	-5.284	-0.199	3.771
-0.976	0.339	0.957	-5.158	-0.243	2.898
-0.913	0.370	0.963	-5.209	-0.300	4.123
-0.261	0.796	0.999	-5.544	-0.071	1.919
-3.958	0.001	0.301	-0.347	-1.155	3.048
-1.402	0.173	0.828	-4.734	-0.365	2.880
0.290	0.774	0.999	-5.536	0.098	3.965
0.733	0.470	0.983	-5.337	0.198	3.634
0.179	0.860	0.999	-5.560	0.050	2.818
0.903	0.375	0.963	-5.216	0.240	3.171
0.563	0.578	0.999	-5.433	0.147	2.833
-0.071	0.944	0.999	-5.572	-0.048	2.399
-0.923	0.365	0.963	-5.201	-0.247	2.540
-0.150	0.882	0.999	-5.564	-0.035	2.646
-1.868	0.073	0.698	-4.128	-0.459	2.727
-0.485	0.632	0.999	-5.469	-0.145	2.057
-0.266	0.792	0.999	-5.542	-0.060	2.452
0.794	0.435	0.973	-5.296	0.207	3.016
0.186	0.854	0.999	-5.559	0.086	2.585
-0.527	0.603	0.999	-5.451	-0.180	2.846
0.653	0.520	0.990	-5.386	0.166	2.886
-1.547	0.134	0.793	-4.560	-0.415	2.749
-0.399	0.693	0.999	-5.503	-0.110	2.061
0.556	0.583	0.999	-5.437	0.231	2.465
-1.445	0.161	0.820	-4.684	-0.367	3.315
-0.181	0.858	0.999	-5.559	-0.048	2.123
0.181	0.858	0.999	-5.559	0.045	2.764
0.419	0.679	0.999	-5.496	0.120	2.689
0.505	0.618	0.999	-5.461	0.133	2.374
-0.173	0.864	0.999	-5.561	-0.087	4.274
-1.768	0.089	0.738	-4.270	-0.419	2.102
0.588	0.562	0.996	-5.421	0.184	2.744
1.655	0.110	0.767	-4.421	0.914	3.139
1.080	0.290	0.932	-5.066	0.267	2.954
0.557	0.582	0.999	-5.436	0.196	1.551
0.076	0.940	0.999	-5.571	0.021	1.912
-0.289	0.775	0.999	-5.537	-0.141	1.372
0.065	0.949	0.999	-5.572	0.016	4.121
-0.842	0.408	0.969	-5.262	-0.218	2.584
0.516	0.610	0.999	-5.456	0.123	3.950
-0.638	0.529	0.992	-5.394	-0.181	2.449

0.511	0.614	0.999	-5.458	0.149	2.223
0.072	0.943	0.999	-5.572	0.021	3.233
-0.205	0.839	0.999	-5.555	-0.056	2.734
-1.588	0.125	0.786	-4.508	-0.388	2.669
-0.404	0.690	0.999	-5.501	-0.091	2.924
-0.416	0.681	0.999	-5.497	-0.125	2.402
-0.970	0.341	0.957	-5.163	-0.220	1.885
0.468	0.643	0.999	-5.476	0.107	2.232
0.091	0.928	0.999	-5.570	0.025	0.984
-0.263	0.795	0.999	-5.543	-0.082	5.036
0.561	0.580	0.999	-5.434	0.173	2.835
-0.388	0.702	0.999	-5.507	-0.131	4.862
-2.819	0.009	0.446	-2.556	-0.705	3.358
1.038	0.309	0.943	-5.104	0.292	3.567
-0.606	0.550	0.994	-5.411	-0.146	2.128
0.474	0.639	0.999	-5.474	0.209	1.522
-0.701	0.490	0.986	-5.357	-0.194	2.649
0.416	0.681	0.999	-5.497	0.081	4.966
1.126	0.271	0.919	-5.024	0.330	3.633
-0.471	0.642	0.999	-5.476	-0.153	1.138
-1.986	0.058	0.660	-3.955	-0.477	2.295
-1.251	0.222	0.885	-4.899	-0.350	2.008
-2.173	0.039	0.612	-3.666	-1.112	2.824
3.573	0.001	0.301	-1.112	0.936	4.136
-0.415	0.682	0.999	-5.497	-0.136	1.222
0.973	0.340	0.957	-5.160	0.283	3.597
-2.659	0.013	0.498	-2.845	-0.772	1.993
1.475	0.153	0.813	-4.648	0.473	3.755
-0.936	0.358	0.963	-5.191	-0.284	2.512
0.444	0.660	0.999	-5.486	0.172	2.534
0.879	0.388	0.964	-5.235	0.236	4.120
0.347	0.731	0.999	-5.520	0.203	2.756
-1.415	0.169	0.825	-4.718	-0.500	5.314
-2.323	0.029	0.567	-3.423	-0.730	2.504
-0.469	0.643	0.999	-5.476	-0.106	5.480
1.363	0.185	0.841	-4.778	0.365	2.953
0.425	0.674	0.999	-5.494	0.128	3.597
0.066	0.948	0.999	-5.572	0.018	5.744
-1.134	0.268	0.917	-5.016	-0.306	6.505
0.416	0.681	0.999	-5.497	0.088	3.987
0.634	0.532	0.992	-5.396	0.161	4.009
-1.167	0.254	0.913	-4.984	-0.389	4.832
0.954	0.349	0.960	-5.175	0.343	5.067
-0.807	0.427	0.973	-5.287	-0.219	3.929
-0.157	0.877	0.999	-5.563	-0.042	8.384
2.097	0.046	0.630	-3.787	0.529	5.156
-1.038	0.309	0.943	-5.104	-0.247	4.027
1.301	0.205	0.866	-4.846	0.326	3.260
0.827	0.416	0.970	-5.273	0.362	3.422
0.500	0.622	0.999	-5.463	0.133	6.698

0.285	0.778	0.999	-5.538	0.057	4.155
1.595	0.123	0.785	-4.500	0.583	7.904
1.604	0.121	0.784	-4.488	0.320	7.246
-0.242	0.811	0.999	-5.548	-0.060	2.112
3.096	0.005	0.359	-2.038	0.761	5.591
-0.398	0.694	0.999	-5.503	-0.115	3.384
1.335	0.194	0.853	-4.809	0.354	5.839
-0.916	0.369	0.963	-5.206	-0.212	4.984
1.287	0.210	0.868	-4.861	0.308	6.575
0.383	0.705	0.999	-5.509	0.106	2.570
1.205	0.239	0.901	-4.946	0.456	3.930
-0.678	0.504	0.988	-5.371	-0.179	2.672
2.029	0.053	0.653	-3.891	0.492	4.495
-0.215	0.831	0.999	-5.553	-0.082	3.588
2.049	0.051	0.642	-3.860	0.764	4.842
-0.693	0.494	0.987	-5.362	-0.221	5.653
0.524	0.605	0.999	-5.452	0.103	5.828
-0.626	0.537	0.993	-5.400	-0.144	5.916
-1.140	0.265	0.916	-5.010	-0.377	4.555
1.057	0.300	0.941	-5.087	0.201	5.358
0.599	0.555	0.995	-5.415	0.143	2.965
-0.121	0.905	0.999	-5.567	-0.031	4.608
1.213	0.237	0.899	-4.939	0.348	3.706
1.014	0.320	0.950	-5.125	0.203	4.430
1.803	0.083	0.724	-4.221	0.439	3.862
-1.039	0.309	0.943	-5.104	-0.449	4.155
-1.345	0.191	0.849	-4.798	-0.332	4.992
0.729	0.473	0.984	-5.340	0.262	3.766
1.313	0.201	0.860	-4.833	0.324	4.170
0.866	0.395	0.964	-5.245	0.182	3.987
1.152	0.260	0.914	-4.999	0.300	3.990
0.535	0.597	0.999	-5.447	0.160	4.201
0.687	0.498	0.987	-5.365	0.164	6.806
-0.559	0.581	0.999	-5.435	-0.258	6.452
0.676	0.505	0.988	-5.372	0.286	6.930
-1.853	0.076	0.704	-4.151	-0.543	2.782
-0.111	0.913	0.999	-5.568	-0.024	3.122
0.562	0.579	0.999	-5.434	0.157	5.667
0.974	0.339	0.957	-5.159	0.359	3.162
-0.366	0.718	0.999	-5.514	-0.107	2.993
0.567	0.576	0.999	-5.432	0.220	3.310
0.123	0.903	0.999	-5.567	0.034	3.528
2.091	0.047	0.630	-3.796	1.460	4.397
-1.732	0.096	0.754	-4.319	-0.427	2.023
-1.299	0.206	0.867	-4.848	-0.411	3.254
1.612	0.119	0.781	-4.478	0.550	2.318
0.389	0.701	0.999	-5.507	0.167	6.441
1.504	0.145	0.803	-4.613	0.334	3.976
-0.679	0.503	0.988	-5.370	-0.213	2.709
-0.612	0.546	0.994	-5.408	-0.174	3.446

1.019	0.318	0.949	-5.121	0.348	7.183
-0.511	0.614	0.999	-5.458	-0.160	4.534
-0.596	0.557	0.995	-5.417	-0.141	3.387
-1.375	0.181	0.840	-4.765	-0.424	4.748
-0.389	0.701	0.999	-5.507	-0.126	2.525
-0.438	0.665	0.999	-5.489	-0.110	2.839
0.870	0.392	0.964	-5.241	0.278	5.809
0.505	0.618	0.999	-5.461	0.125	4.940
-0.710	0.484	0.985	-5.351	-0.196	2.297
2.427	0.023	0.542	-3.248	0.566	3.909
-0.612	0.546	0.994	-5.408	-0.309	5.738
0.783	0.441	0.977	-5.304	0.199	4.519
1.106	0.279	0.922	-5.043	0.295	4.967
0.776	0.445	0.979	-5.309	0.191	7.275
0.905	0.374	0.963	-5.215	0.253	4.824
0.875	0.390	0.964	-5.238	0.346	3.365
-0.373	0.712	0.999	-5.512	-0.104	6.448
0.666	0.512	0.989	-5.378	0.186	2.833
0.961	0.346	0.957	-5.170	0.416	3.006
-0.445	0.660	0.999	-5.486	-0.122	5.658
0.459	0.650	0.999	-5.480	0.101	5.972
-0.965	0.344	0.957	-5.167	-0.391	5.917
1.381	0.179	0.838	-4.757	0.345	3.985
-0.534	0.598	0.999	-5.447	-0.148	4.291
1.292	0.208	0.868	-4.855	0.421	5.429
-0.348	0.731	0.999	-5.520	-0.107	3.669
-1.194	0.243	0.904	-4.957	-0.396	3.093
0.815	0.423	0.972	-5.282	0.228	3.365
0.576	0.570	0.997	-5.427	0.175	2.588
0.176	0.862	0.999	-5.560	0.055	3.135
0.842	0.408	0.969	-5.262	0.327	3.938
1.114	0.276	0.921	-5.035	0.248	6.414
-0.318	0.753	0.999	-5.529	-0.100	6.783
0.197	0.846	0.999	-5.557	0.054	5.233
0.965	0.344	0.957	-5.167	0.232	3.763
-1.360	0.186	0.843	-4.781	-0.325	8.727
1.483	0.150	0.811	-4.638	0.451	3.795
-1.042	0.307	0.942	-5.101	-0.315	3.243
0.956	0.348	0.959	-5.174	0.267	2.671
1.132	0.268	0.917	-5.018	0.315	4.460
0.124	0.902	0.999	-5.567	0.060	5.211
0.660	0.515	0.990	-5.381	0.155	5.157
0.493	0.627	0.999	-5.466	0.118	4.150
1.450	0.159	0.820	-4.678	0.274	6.925
1.085	0.288	0.929	-5.062	0.307	5.397
-1.310	0.202	0.862	-4.836	-0.356	2.664
0.771	0.448	0.979	-5.312	0.420	4.767
1.228	0.231	0.894	-4.922	0.294	2.841
-0.610	0.548	0.994	-5.409	-0.169	6.033
-0.380	0.707	0.999	-5.510	-0.090	2.751

-0.901	0.376	0.963	-5.218	-0.315	3.074
-0.162	0.873	0.999	-5.562	-0.042	1.593
-0.870	0.392	0.964	-5.241	-0.218	6.126
0.810	0.425	0.972	-5.285	0.136	6.639
0.758	0.456	0.981	-5.321	0.153	5.160
1.366	0.184	0.841	-4.775	0.320	5.944
-0.361	0.721	0.999	-5.516	-0.086	2.641
0.581	0.566	0.997	-5.424	0.209	2.112
0.218	0.829	0.999	-5.553	0.074	7.276
-0.506	0.617	0.999	-5.460	-0.184	5.695
0.903	0.375	0.963	-5.216	0.241	7.741
0.326	0.747	0.999	-5.527	0.103	4.888
-0.377	0.709	0.999	-5.511	-0.094	7.677
-1.527	0.139	0.799	-4.585	-0.719	5.890
2.987	0.006	0.393	-2.243	0.805	7.733
2.367	0.026	0.556	-3.350	0.571	4.958
1.125	0.271	0.919	-5.025	0.585	7.126
1.093	0.285	0.927	-5.055	0.506	6.718
1.042	0.308	0.942	-5.101	0.529	7.116
-0.544	0.591	0.999	-5.443	-0.147	4.163
-1.533	0.138	0.795	-4.577	-0.408	4.783
0.053	0.958	0.999	-5.573	0.021	1.024
0.333	0.742	0.999	-5.524	0.106	1.424
0.789	0.438	0.974	-5.300	0.235	5.354
0.127	0.900	0.999	-5.567	0.038	6.758
0.876	0.390	0.964	-5.237	0.162	7.711
0.974	0.339	0.957	-5.159	0.241	2.547
-0.114	0.910	0.999	-5.568	-0.061	1.710
0.830	0.415	0.970	-5.271	0.204	4.711
0.084	0.934	0.999	-5.571	0.024	3.027
-0.804	0.429	0.973	-5.290	-0.216	3.512
-1.718	0.098	0.757	-4.338	-0.429	2.276
-1.300	0.206	0.867	-4.847	-0.269	7.091
-0.814	0.423	0.972	-5.282	-0.240	2.758
1.450	0.159	0.820	-4.677	0.484	2.082
-0.773	0.447	0.979	-5.311	-0.156	5.177
1.193	0.244	0.904	-4.958	0.255	5.966
-1.801	0.084	0.724	-4.224	-1.062	3.254
0.774	0.446	0.979	-5.310	0.209	6.427
0.246	0.808	0.999	-5.547	0.075	7.800
0.226	0.823	0.999	-5.551	0.060	4.192
-0.357	0.724	0.999	-5.517	-0.099	3.937
0.077	0.940	0.999	-5.571	0.020	2.380
0.399	0.693	0.999	-5.503	0.090	7.069
0.737	0.468	0.983	-5.334	0.206	4.630
1.902	0.069	0.684	-4.079	0.447	4.012
0.066	0.948	0.999	-5.572	0.019	2.901
-0.143	0.888	0.999	-5.565	-0.050	2.602
2.197	0.037	0.611	-3.628	0.466	4.459
0.216	0.831	0.999	-5.553	0.067	2.926

1.290	0.209	0.868	-4.858	0.300	2.969
0.709	0.485	0.985	-5.352	0.337	3.808
1.511	0.143	0.803	-4.604	0.609	5.937
1.776	0.088	0.734	-4.258	0.798	7.773
1.418	0.169	0.825	-4.715	0.531	4.755
1.925	0.066	0.676	-4.046	0.520	5.852
0.843	0.407	0.969	-5.262	0.154	5.396
-1.258	0.220	0.881	-4.892	-0.371	3.529
-0.058	0.954	0.999	-5.572	-0.015	2.472
2.989	0.006	0.393	-2.240	0.768	2.885
0.934	0.359	0.963	-5.192	0.249	6.467
1.280	0.212	0.870	-4.868	0.326	2.682
0.742	0.465	0.983	-5.331	0.202	2.568
-0.183	0.856	0.999	-5.559	-0.076	4.658
1.017	0.319	0.950	-5.123	0.263	2.249
-0.981	0.336	0.957	-5.154	-0.180	5.657
-0.076	0.940	0.999	-5.571	-0.020	4.787
0.243	0.810	0.999	-5.548	0.135	5.735
1.453	0.159	0.819	-4.674	0.397	3.724
2.161	0.040	0.617	-3.686	0.418	5.081
1.806	0.083	0.724	-4.217	0.485	4.989
0.469	0.643	0.999	-5.476	0.094	5.083
0.303	0.764	0.999	-5.533	0.099	4.223
-0.614	0.545	0.994	-5.407	-0.252	6.150
0.334	0.741	0.999	-5.524	0.108	5.806
0.435	0.668	0.999	-5.490	0.183	6.880
2.016	0.055	0.655	-3.910	0.588	3.978
0.066	0.948	0.999	-5.572	0.030	4.775
0.630	0.535	0.993	-5.399	0.108	8.597
0.268	0.791	0.999	-5.542	0.055	7.212
-0.269	0.791	0.999	-5.542	-0.072	2.381
0.092	0.928	0.999	-5.570	0.027	5.511
1.111	0.277	0.921	-5.038	0.205	7.555
-0.450	0.656	0.999	-5.484	-0.114	2.768
1.549	0.134	0.793	-4.558	0.337	5.424
2.065	0.049	0.639	-3.836	0.559	6.536
1.563	0.131	0.791	-4.540	0.549	6.306
0.331	0.743	0.999	-5.525	0.083	6.298
1.046	0.306	0.942	-5.097	0.196	6.403
-0.295	0.770	0.999	-5.535	-0.057	5.672
-0.629	0.535	0.993	-5.399	-0.236	3.968
1.413	0.170	0.825	-4.721	0.412	3.711
0.605	0.551	0.994	-5.412	0.171	3.288
0.590	0.561	0.996	-5.420	0.171	6.086
0.512	0.613	0.999	-5.458	0.157	6.353
0.843	0.407	0.969	-5.262	0.337	3.485
1.712	0.099	0.757	-4.346	1.098	2.248
3.405	0.002	0.301	-1.443	1.208	3.224
0.161	0.874	0.999	-5.562	0.048	3.663
-0.816	0.422	0.972	-5.281	-0.169	5.000

0.774	0.446	0.979	-5.310	0.308	2.758
-0.534	0.598	0.999	-5.447	-0.232	4.418
0.246	0.808	0.999	-5.547	0.113	2.522
0.464	0.646	0.999	-5.478	0.131	3.869
0.277	0.784	0.999	-5.540	0.093	2.675
0.198	0.844	0.999	-5.556	0.061	2.976
-1.795	0.085	0.726	-4.232	-0.560	2.060
-0.592	0.559	0.996	-5.419	-0.218	2.466
-1.366	0.184	0.841	-4.774	-0.379	6.541
-0.160	0.874	0.999	-5.562	-0.059	5.339
-1.046	0.306	0.942	-5.097	-0.225	8.648
-0.118	0.907	0.999	-5.568	-0.036	3.923
0.523	0.605	0.999	-5.452	0.131	6.175
-0.490	0.628	0.999	-5.467	-0.169	3.263
1.070	0.295	0.936	-5.076	0.245	4.215
1.390	0.177	0.835	-4.747	0.340	5.183
1.126	0.271	0.919	-5.024	0.289	3.461
0.212	0.833	0.999	-5.554	0.059	5.481
-0.574	0.571	0.997	-5.428	-0.147	4.525
-0.224	0.824	0.999	-5.552	-0.090	8.851
0.623	0.539	0.994	-5.402	0.339	3.147
0.169	0.867	0.999	-5.561	0.048	5.134
-0.519	0.609	0.999	-5.455	-0.143	5.847
2.339	0.028	0.563	-3.396	0.566	9.128
1.211	0.237	0.899	-4.940	0.335	4.562
-1.169	0.253	0.913	-4.982	-0.253	4.838
-1.155	0.259	0.913	-4.995	-0.414	2.364
0.108	0.915	0.999	-5.569	0.027	7.302
0.136	0.893	0.999	-5.566	0.026	5.417
1.564	0.130	0.790	-4.539	0.491	6.525
0.334	0.741	0.999	-5.524	0.088	5.106
-2.202	0.037	0.610	-3.620	-0.832	3.101
1.259	0.220	0.880	-4.891	0.328	8.610
-0.401	0.692	0.999	-5.502	-0.089	1.672
0.172	0.865	0.999	-5.561	0.043	2.487
2.653	0.014	0.498	-2.856	0.797	4.795
1.136	0.267	0.917	-5.014	0.283	7.087
0.250	0.805	0.999	-5.546	0.063	8.737
0.186	0.854	0.999	-5.559	0.056	8.477
3.370	0.002	0.301	-1.510	0.862	6.054
0.197	0.845	0.999	-5.557	0.055	2.766
0.054	0.958	0.999	-5.573	0.020	2.322
-0.078	0.938	0.999	-5.571	-0.017	1.993
-0.401	0.692	0.999	-5.502	-0.122	2.826
1.266	0.217	0.876	-4.884	0.487	5.326
1.040	0.308	0.943	-5.103	0.347	3.868
-1.683	0.105	0.764	-4.385	-0.525	2.038
-0.381	0.707	0.999	-5.509	-0.105	3.260
-0.147	0.884	0.999	-5.564	-0.037	4.357
0.113	0.911	0.999	-5.568	0.049	4.088

0.920	0.366	0.963	-5.203	0.270	3.992
-0.927	0.363	0.963	-5.198	-0.241	4.934
-0.285	0.778	0.999	-5.538	-0.086	4.353
-0.548	0.589	0.999	-5.441	-0.144	4.981
0.966	0.343	0.957	-5.166	0.252	5.048
-0.500	0.621	0.999	-5.463	-0.172	4.811
0.937	0.358	0.962	-5.190	0.205	5.626
0.384	0.704	0.999	-5.508	0.077	2.913
-0.147	0.884	0.999	-5.564	-0.027	5.486
0.314	0.756	0.999	-5.530	0.092	5.863
0.619	0.541	0.994	-5.404	0.246	5.317
-0.170	0.867	0.999	-5.561	-0.050	6.323
1.134	0.268	0.917	-5.016	0.209	6.500
1.955	0.062	0.667	-4.002	0.591	7.017
0.686	0.499	0.987	-5.366	0.317	9.065
-0.386	0.703	0.999	-5.508	-0.095	9.169
2.034	0.053	0.653	-3.883	0.870	5.810
0.378	0.709	0.999	-5.510	0.115	4.436
-0.088	0.931	0.999	-5.570	-0.019	3.639
1.724	0.097	0.757	-4.329	0.392	3.603
2.401	0.024	0.543	-3.293	1.184	6.133
3.512	0.002	0.301	-1.232	0.996	4.481
-0.188	0.852	0.999	-5.558	-0.044	9.194
2.246	0.034	0.595	-3.548	0.381	4.928
2.627	0.014	0.498	-2.900	0.643	4.322
1.902	0.069	0.684	-4.079	0.562	2.533
1.591	0.124	0.786	-4.505	0.444	6.274
-1.868	0.074	0.698	-4.129	-0.534	4.877
1.226	0.232	0.895	-4.925	0.233	7.407
0.612	0.546	0.994	-5.408	0.142	6.008
0.729	0.473	0.984	-5.340	0.173	5.544
0.813	0.424	0.972	-5.283	0.503	8.563
-1.431	0.165	0.824	-4.699	-0.328	4.147
1.658	0.110	0.767	-4.418	0.555	5.511
1.801	0.084	0.724	-4.224	0.985	3.797
0.499	0.622	0.999	-5.463	0.117	4.032
-0.107	0.916	0.999	-5.569	-0.022	5.638
0.586	0.563	0.997	-5.422	0.137	5.961
-0.623	0.539	0.994	-5.402	-0.169	3.657
-0.312	0.757	0.999	-5.530	-0.070	5.543
0.830	0.414	0.970	-5.271	0.151	6.631
0.492	0.627	0.999	-5.467	0.163	4.448
-0.383	0.705	0.999	-5.509	-0.094	7.602
2.201	0.037	0.610	-3.622	0.639	3.253
0.539	0.594	0.999	-5.445	0.134	6.074
2.337	0.028	0.563	-3.400	0.545	5.442
0.881	0.387	0.964	-5.233	0.348	3.936
0.750	0.460	0.983	-5.326	0.218	4.217
0.238	0.814	0.999	-5.549	0.070	3.516
-0.103	0.919	0.999	-5.569	-0.030	6.840

1.831	0.079	0.713	-4.181	0.487	5.383
2.492	0.020	0.516	-3.138	0.727	4.053
1.721	0.098	0.757	-4.335	0.342	6.826
1.695	0.102	0.764	-4.369	0.619	4.890
-0.456	0.652	0.999	-5.482	-0.108	3.271
-0.209	0.836	0.999	-5.555	-0.060	2.700
2.142	0.042	0.619	-3.716	0.620	4.032
-0.490	0.629	0.999	-5.467	-0.145	6.787
-0.822	0.419	0.972	-5.277	-0.156	7.703
-1.319	0.199	0.857	-4.827	-0.781	5.038
0.641	0.527	0.992	-5.392	0.136	4.682
0.316	0.755	0.999	-5.530	0.122	4.213
1.308	0.203	0.862	-4.838	0.292	4.824
0.691	0.496	0.987	-5.363	0.166	4.055
-0.616	0.543	0.994	-5.406	-0.167	2.956
-0.100	0.921	0.999	-5.569	-0.038	4.771
0.130	0.898	0.999	-5.566	0.035	5.459
-0.558	0.582	0.999	-5.436	-0.123	6.318
0.302	0.765	0.999	-5.533	0.075	5.753
0.453	0.654	0.999	-5.483	0.092	7.627
-0.313	0.757	0.999	-5.530	-0.078	3.219
-0.378	0.709	0.999	-5.510	-0.097	2.567
-0.426	0.674	0.999	-5.493	-0.155	5.265
0.771	0.448	0.979	-5.312	0.191	4.369
-0.984	0.335	0.957	-5.151	-0.244	3.382
-0.673	0.507	0.988	-5.374	-0.227	6.240
0.736	0.469	0.983	-5.335	0.166	5.307
-1.466	0.155	0.817	-4.659	-0.373	2.341
0.945	0.353	0.961	-5.183	0.216	8.155
-0.491	0.628	0.999	-5.467	-0.135	2.355
0.335	0.741	0.999	-5.524	0.102	6.931
2.565	0.017	0.500	-3.011	0.658	5.975
1.222	0.233	0.898	-4.929	0.323	6.268
1.457	0.157	0.817	-4.669	0.287	6.053
-0.124	0.902	0.999	-5.567	-0.029	4.682
-1.318	0.199	0.857	-4.827	-0.484	3.286
0.774	0.446	0.979	-5.310	0.304	6.449
-1.240	0.226	0.888	-4.910	-0.481	2.095
0.559	0.581	0.999	-5.435	0.185	3.718
0.322	0.750	0.999	-5.528	0.059	9.926
-3.298	0.003	0.331	-1.651	-1.267	1.960
-2.780	0.010	0.456	-2.627	-0.674	1.895
-1.038	0.309	0.943	-5.104	-0.285	3.862
1.763	0.090	0.742	-4.276	0.330	5.727
-0.780	0.443	0.979	-5.306	-0.177	7.454
0.959	0.347	0.958	-5.172	0.242	5.381
0.702	0.489	0.986	-5.356	0.272	3.453
1.433	0.164	0.824	-4.698	0.415	5.572
0.308	0.761	0.999	-5.532	0.129	3.416
-0.920	0.366	0.963	-5.203	-0.213	4.475

-0.367	0.717	0.999	-5.514	-0.131	5.816
0.057	0.955	0.999	-5.572	0.019	8.381
0.340	0.737	0.999	-5.523	0.120	9.065
0.243	0.810	0.999	-5.548	0.073	4.588
-1.824	0.080	0.718	-4.191	-0.550	1.932
0.491	0.627	0.999	-5.467	0.129	4.695
0.875	0.390	0.964	-5.238	0.200	2.623
0.661	0.515	0.990	-5.381	0.144	3.954
1.585	0.125	0.786	-4.512	0.351	7.816
0.396	0.695	0.999	-5.504	0.126	3.340
1.433	0.164	0.824	-4.697	0.268	6.257
0.403	0.691	0.999	-5.502	0.089	5.378
-0.602	0.553	0.994	-5.414	-0.161	6.364
-0.847	0.405	0.968	-5.259	-0.201	2.574
1.687	0.104	0.764	-4.380	0.313	6.212
0.575	0.570	0.997	-5.427	0.146	3.851
0.385	0.703	0.999	-5.508	0.177	3.807
-0.835	0.411	0.970	-5.267	-0.200	4.995
0.347	0.731	0.999	-5.520	0.152	7.333
0.583	0.565	0.997	-5.424	0.159	3.690
0.141	0.889	0.999	-5.565	0.049	3.814
1.054	0.302	0.941	-5.090	0.330	6.461
0.769	0.449	0.979	-5.314	0.201	6.996
0.617	0.543	0.994	-5.405	0.155	5.756
-0.280	0.781	0.999	-5.539	-0.061	4.209
0.171	0.866	0.999	-5.561	0.036	3.729
-0.234	0.817	0.999	-5.549	-0.098	6.187
-0.197	0.846	0.999	-5.557	-0.079	4.300
1.796	0.084	0.726	-4.230	0.654	5.102
0.672	0.508	0.988	-5.375	0.164	4.417
-0.514	0.612	0.999	-5.457	-0.125	6.371
-0.847	0.405	0.968	-5.258	-0.215	2.432
0.749	0.461	0.983	-5.327	0.151	5.643
1.887	0.071	0.694	-4.102	0.490	5.273
0.456	0.653	0.999	-5.482	0.085	6.232
0.605	0.550	0.994	-5.412	0.149	6.142
-1.104	0.280	0.922	-5.044	-0.225	5.388
1.213	0.236	0.899	-4.938	0.288	4.079
0.367	0.717	0.999	-5.514	0.085	5.426
0.154	0.879	0.999	-5.563	0.039	3.805
1.011	0.322	0.950	-5.128	0.238	7.315
0.448	0.658	0.999	-5.485	0.109	6.522
0.756	0.457	0.981	-5.322	0.140	7.619
-1.809	0.082	0.722	-4.212	-0.588	2.669
-0.058	0.954	0.999	-5.572	-0.027	6.439
0.491	0.628	0.999	-5.467	0.135	6.887
1.716	0.098	0.757	-4.340	0.382	5.582
-1.058	0.300	0.941	-5.087	-0.261	7.149
0.129	0.898	0.999	-5.566	0.038	6.006
-0.114	0.910	0.999	-5.568	-0.025	1.697

1.021	0.317	0.948	-5.119	0.196	4.935
0.894	0.380	0.964	-5.224	0.223	3.975
-0.773	0.447	0.979	-5.311	-0.355	3.741
-0.952	0.350	0.961	-5.178	-0.177	9.230
1.260	0.219	0.880	-4.890	0.242	8.198
1.897	0.069	0.687	-4.087	0.483	2.936
0.165	0.870	0.999	-5.562	0.067	6.709
-0.507	0.616	0.999	-5.460	-0.251	5.092
-0.763	0.452	0.981	-5.317	-0.436	2.653
1.447	0.160	0.820	-4.681	0.318	7.729
-1.473	0.153	0.813	-4.651	-0.389	1.771
-1.985	0.058	0.661	-3.958	-0.583	1.971
0.659	0.516	0.990	-5.382	0.125	8.341
0.712	0.483	0.985	-5.350	0.141	9.849
-2.032	0.053	0.653	-3.886	-0.612	3.460
0.897	0.378	0.963	-5.221	0.181	4.896
0.631	0.534	0.992	-5.398	0.126	8.353
1.035	0.310	0.943	-5.107	0.199	9.335
1.497	0.147	0.804	-4.621	0.321	9.963
0.592	0.559	0.996	-5.419	0.178	7.021
0.480	0.635	0.999	-5.472	0.117	8.058
1.052	0.303	0.941	-5.091	0.279	7.558
-0.985	0.334	0.957	-5.150	-0.183	5.020
1.163	0.256	0.913	-4.987	0.310	6.504
2.261	0.033	0.594	-3.525	0.627	3.894
-0.121	0.905	0.999	-5.567	-0.026	8.625
-0.865	0.395	0.964	-5.246	-0.462	5.532
0.837	0.410	0.970	-5.266	0.276	3.913
1.050	0.304	0.941	-5.093	0.302	4.809
0.354	0.727	0.999	-5.518	0.062	6.161
1.639	0.114	0.775	-4.443	0.321	5.082
0.552	0.586	0.999	-5.439	0.103	6.677
-0.461	0.649	0.999	-5.479	-0.227	6.459
-0.609	0.548	0.994	-5.409	-0.178	3.389
-0.185	0.855	0.999	-5.559	-0.055	6.321
0.186	0.854	0.999	-5.558	0.040	7.569
0.078	0.939	0.999	-5.571	0.022	2.811
-0.338	0.738	0.999	-5.523	-0.095	3.014
1.669	0.108	0.764	-4.404	0.330	6.510
0.832	0.413	0.970	-5.270	0.234	5.760
-0.510	0.614	0.999	-5.458	-0.179	2.930
1.968	0.060	0.663	-3.983	0.432	8.634
1.545	0.135	0.793	-4.562	0.361	4.227
-0.698	0.492	0.986	-5.359	-0.303	3.568
0.266	0.792	0.999	-5.542	0.077	5.961
0.544	0.591	0.999	-5.443	0.171	7.275
1.007	0.324	0.951	-5.132	0.314	1.834
1.532	0.138	0.795	-4.578	0.381	5.247
-0.342	0.735	0.999	-5.522	-0.156	4.349
0.758	0.455	0.981	-5.321	0.191	2.434

0.756	0.457	0.981	-5.322	0.181	5.042
3.944	0.001	0.301	-0.374	0.791	7.563
0.462	0.648	0.999	-5.479	0.234	5.125
-0.576	0.569	0.997	-5.427	-0.150	3.555
0.576	0.570	0.997	-5.427	0.191	3.170
4.133	0.000	0.257	0.004	1.095	3.924
-0.349	0.730	0.999	-5.520	-0.081	2.013
0.141	0.889	0.999	-5.565	0.040	8.203
0.579	0.568	0.997	-5.425	0.155	4.769
0.178	0.860	0.999	-5.560	0.050	3.641
-0.932	0.360	0.963	-5.194	-0.241	2.227
1.053	0.302	0.941	-5.091	0.264	4.493
-0.167	0.869	0.999	-5.561	-0.055	6.996
0.725	0.475	0.984	-5.342	0.170	4.431
-0.929	0.362	0.963	-5.196	-0.330	4.383
-0.179	0.859	0.999	-5.560	-0.050	4.664
0.353	0.727	0.999	-5.519	0.101	6.614
-0.183	0.856	0.999	-5.559	-0.061	4.560
0.776	0.445	0.979	-5.309	0.201	4.122
-0.896	0.379	0.963	-5.222	-0.232	3.077
0.061	0.952	0.999	-5.572	0.022	5.460
-0.594	0.558	0.995	-5.418	-0.176	6.347
0.539	0.594	0.999	-5.445	0.139	5.950
0.598	0.555	0.995	-5.416	0.143	4.918
1.652	0.111	0.767	-4.426	0.385	2.656
0.369	0.716	0.999	-5.513	0.165	8.116
1.697	0.102	0.764	-4.367	0.454	3.466
1.523	0.140	0.801	-4.589	0.373	4.624
2.086	0.047	0.630	-3.803	0.528	5.488
-0.433	0.668	0.999	-5.490	-0.097	7.775
0.764	0.452	0.980	-5.317	0.188	4.725
0.803	0.430	0.973	-5.290	0.211	5.000
-0.074	0.942	0.999	-5.571	-0.016	6.314
0.852	0.402	0.967	-5.255	0.190	6.030
0.271	0.789	0.999	-5.541	0.069	6.718
-1.446	0.161	0.820	-4.682	-0.325	4.299
-1.160	0.257	0.913	-4.991	-0.323	7.092
0.758	0.456	0.981	-5.321	0.182	3.855
-0.185	0.854	0.999	-5.559	-0.049	5.354
1.335	0.194	0.853	-4.809	0.288	2.498
-0.264	0.794	0.999	-5.543	-0.068	2.927
0.538	0.596	0.999	-5.446	0.252	3.385
0.409	0.686	0.999	-5.499	0.175	2.949
1.127	0.270	0.919	-5.023	0.244	6.887
3.299	0.003	0.331	-1.649	1.364	4.678
0.356	0.725	0.999	-5.518	0.084	4.555
2.080	0.048	0.630	-3.812	0.568	8.810
-0.564	0.578	0.999	-5.433	-0.171	2.507
0.448	0.658	0.999	-5.485	0.108	5.051
-2.249	0.034	0.595	-3.545	-0.514	4.205

1.471	0.154	0.814	-4.653	0.475	2.586
-1.262	0.219	0.878	-4.888	-0.330	4.795
2.347	0.027	0.559	-3.383	0.582	4.426
-0.438	0.665	0.999	-5.489	-0.090	3.881
0.076	0.940	0.999	-5.571	0.018	4.630
-0.911	0.371	0.963	-5.211	-0.236	3.625
-0.879	0.388	0.964	-5.235	-0.212	2.949
-1.086	0.288	0.929	-5.061	-0.351	1.891
-0.141	0.889	0.999	-5.565	-0.038	2.161
-0.526	0.604	0.999	-5.451	-0.141	3.545
0.741	0.465	0.983	-5.332	0.239	5.438
0.350	0.729	0.999	-5.519	0.163	7.894
0.546	0.590	0.999	-5.442	0.212	3.877
-0.481	0.635	0.999	-5.471	-0.194	7.427
-1.013	0.320	0.950	-5.126	-0.275	4.054
-0.329	0.745	0.999	-5.526	-0.107	4.079
0.968	0.342	0.957	-5.164	0.284	7.106
-1.161	0.256	0.913	-4.990	-0.340	3.405
-0.834	0.412	0.970	-5.268	-0.219	4.845
0.678	0.504	0.988	-5.371	0.169	2.419
-2.684	0.013	0.498	-2.800	-0.546	3.404
0.855	0.401	0.965	-5.253	0.203	7.932
0.073	0.943	0.999	-5.572	0.016	8.676
1.431	0.165	0.824	-4.700	0.314	3.855
0.209	0.836	0.999	-5.554	0.064	4.423
0.773	0.447	0.979	-5.311	0.230	5.542
1.271	0.215	0.875	-4.878	0.415	3.888
1.347	0.190	0.849	-4.796	0.334	3.187
-0.609	0.548	0.994	-5.410	-0.249	3.264
0.230	0.820	0.999	-5.550	0.105	4.773
2.114	0.045	0.630	-3.759	0.487	4.964
1.871	0.073	0.698	-4.125	0.422	8.059
-0.865	0.395	0.964	-5.246	-0.261	2.912
-0.593	0.559	0.996	-5.418	-0.224	3.296
-0.479	0.636	0.999	-5.472	-0.291	2.109
0.629	0.535	0.993	-5.399	0.232	4.335
0.070	0.945	0.999	-5.572	0.021	4.874
-1.847	0.077	0.707	-4.160	-0.403	5.105
-0.379	0.708	0.999	-5.510	-0.120	3.109
0.194	0.848	0.999	-5.557	0.052	4.080
1.603	0.121	0.784	-4.489	0.303	8.498
1.160	0.257	0.913	-4.991	0.234	5.826
-0.677	0.504	0.988	-5.371	-0.213	3.229
-1.194	0.244	0.904	-4.958	-0.429	2.807
1.448	0.160	0.820	-4.680	0.417	4.231
-0.912	0.370	0.963	-5.209	-0.214	4.871
-0.161	0.873	0.999	-5.562	-0.062	7.291
0.726	0.475	0.984	-5.342	0.190	3.663
-0.424	0.675	0.999	-5.494	-0.078	6.575
1.470	0.154	0.815	-4.654	0.276	6.272

1.866	0.074	0.698	-4.131	0.350	2.860
-0.597	0.556	0.995	-5.416	-0.155	2.947
0.626	0.537	0.993	-5.401	0.198	7.221
0.069	0.945	0.999	-5.572	0.026	4.863
0.967	0.343	0.957	-5.165	0.265	6.030
0.075	0.940	0.999	-5.571	0.016	4.005
-0.466	0.645	0.999	-5.477	-0.102	4.458
-0.295	0.771	0.999	-5.535	-0.071	6.050
-0.673	0.507	0.988	-5.374	-0.143	5.187
-0.241	0.811	0.999	-5.548	-0.120	4.371
1.569	0.129	0.789	-4.532	0.429	3.054
0.153	0.880	0.999	-5.564	0.039	3.272
1.812	0.082	0.722	-4.208	0.483	6.329
-0.417	0.680	0.999	-5.497	-0.091	5.109
0.931	0.360	0.963	-5.194	0.287	4.401
-0.077	0.939	0.999	-5.571	-0.019	2.553
-0.716	0.480	0.984	-5.348	-0.190	2.787
0.095	0.925	0.999	-5.570	0.031	3.626
0.492	0.627	0.999	-5.467	0.106	6.041
-1.504	0.145	0.803	-4.613	-0.357	4.352
-0.258	0.798	0.999	-5.544	-0.063	5.285
1.056	0.301	0.941	-5.089	0.281	8.731
2.610	0.015	0.498	-2.931	0.893	5.376
-0.183	0.856	0.999	-5.559	-0.034	6.864
0.857	0.400	0.964	-5.252	0.302	7.080
0.883	0.385	0.964	-5.231	0.293	2.892
-0.450	0.657	0.999	-5.484	-0.157	4.183
0.819	0.421	0.972	-5.279	0.230	3.616
1.688	0.104	0.764	-4.378	0.768	7.659
-0.568	0.575	0.999	-5.431	-0.271	3.145
0.585	0.564	0.997	-5.423	0.160	5.765
1.343	0.191	0.850	-4.800	0.360	3.433
0.053	0.958	0.999	-5.573	0.023	4.410
0.805	0.428	0.973	-5.289	0.204	3.444
-0.802	0.430	0.973	-5.291	-0.225	1.561
-0.079	0.937	0.999	-5.571	-0.025	4.395
-0.465	0.646	0.999	-5.478	-0.129	4.260
0.303	0.764	0.999	-5.533	0.075	4.990
0.472	0.641	0.999	-5.475	0.113	5.321
-0.275	0.786	0.999	-5.540	-0.113	3.206
-0.202	0.841	0.999	-5.556	-0.062	3.173
-0.470	0.642	0.999	-5.476	-0.147	3.357
0.355	0.726	0.999	-5.518	0.082	3.448
1.737	0.095	0.754	-4.312	0.403	4.059
0.867	0.394	0.964	-5.244	0.690	3.429
-3.390	0.002	0.301	-1.472	-1.464	1.403
-0.863	0.396	0.964	-5.247	-0.247	2.675
-2.234	0.035	0.597	-3.569	-0.822	1.263
1.621	0.117	0.776	-4.466	0.283	6.012
0.695	0.494	0.987	-5.361	0.158	5.632

-0.132	0.896	0.999	-5.566	-0.026	4.584
0.289	0.775	0.999	-5.537	0.064	3.799
-3.028	0.006	0.393	-2.166	-1.059	2.752
0.332	0.743	0.999	-5.525	0.077	4.018
-0.792	0.436	0.973	-5.298	-0.219	6.018
-0.709	0.485	0.985	-5.352	-0.174	2.762
-0.348	0.731	0.999	-5.520	-0.076	3.608
0.320	0.751	0.999	-5.528	0.139	1.388
-0.221	0.827	0.999	-5.552	-0.066	4.899
0.384	0.704	0.999	-5.508	0.144	2.311
1.265	0.218	0.876	-4.885	0.279	4.372
0.655	0.518	0.990	-5.384	0.140	4.092
-0.724	0.476	0.984	-5.343	-0.204	5.724
-1.532	0.138	0.795	-4.578	-0.423	3.783
-0.342	0.735	0.999	-5.522	-0.092	4.450
-0.215	0.832	0.999	-5.553	-0.043	5.461
0.258	0.798	0.999	-5.544	0.054	6.427
0.469	0.643	0.999	-5.476	0.100	4.425
0.996	0.329	0.955	-5.141	0.255	7.126
0.085	0.933	0.999	-5.571	0.023	5.116
-0.419	0.679	0.999	-5.496	-0.103	4.765
-0.716	0.480	0.984	-5.347	-0.163	2.858
-0.849	0.404	0.968	-5.257	-0.199	4.081
-1.185	0.247	0.906	-4.966	-0.349	2.793
-1.003	0.325	0.954	-5.135	-0.228	2.841
0.178	0.860	0.999	-5.560	0.053	3.855
2.008	0.056	0.656	-3.923	0.499	6.759
-0.617	0.543	0.994	-5.405	-0.144	4.719
0.154	0.879	0.999	-5.563	0.049	2.178
-2.082	0.048	0.630	-3.809	-0.544	4.139
1.155	0.259	0.913	-4.996	0.253	3.123
1.187	0.246	0.906	-4.964	0.239	5.755
-2.192	0.038	0.611	-3.636	-0.741	1.832
1.636	0.114	0.775	-4.446	0.478	4.588
0.661	0.515	0.990	-5.381	0.134	3.833
-0.965	0.344	0.957	-5.167	-0.284	3.585
-1.465	0.155	0.817	-4.660	-0.373	3.340
-0.286	0.777	0.999	-5.537	-0.090	2.560
0.077	0.939	0.999	-5.571	0.018	3.302
1.888	0.071	0.694	-4.100	0.736	5.145
-0.054	0.958	0.999	-5.573	-0.016	4.148
-0.621	0.540	0.994	-5.403	-0.161	2.925
0.975	0.339	0.957	-5.158	0.275	2.459
-2.265	0.032	0.591	-3.518	-0.566	3.762
-0.083	0.935	0.999	-5.571	-0.021	2.975
0.839	0.409	0.970	-5.265	0.228	6.539
0.552	0.586	0.999	-5.439	0.179	3.253
0.106	0.917	0.999	-5.569	0.062	3.837
-3.400	0.002	0.301	-1.453	-1.413	1.508
1.034	0.311	0.943	-5.108	0.474	6.454

-3.191	0.004	0.340	-1.857	-1.576	4.047
-1.727	0.096	0.756	-4.326	-0.491	3.003
0.333	0.742	0.999	-5.525	0.095	6.425
-1.400	0.174	0.828	-4.736	-0.355	5.389
-0.327	0.747	0.999	-5.526	-0.084	3.032
1.531	0.138	0.796	-4.580	0.404	2.438
-1.157	0.258	0.913	-4.994	-0.271	3.201
0.203	0.841	0.999	-5.556	0.055	3.419
-1.228	0.231	0.894	-4.923	-0.383	3.763
0.625	0.537	0.993	-5.401	0.134	4.881
-1.385	0.178	0.837	-4.753	-0.404	5.494
0.375	0.711	0.999	-5.511	0.103	3.883
0.869	0.393	0.964	-5.242	0.180	3.631
-2.420	0.023	0.543	-3.260	-1.178	2.294
1.267	0.217	0.876	-4.882	0.324	3.075
1.359	0.186	0.843	-4.783	0.337	3.743
1.510	0.144	0.803	-4.606	0.615	4.372
2.317	0.029	0.570	-3.432	1.112	7.793
2.173	0.039	0.612	-3.667	0.959	8.236
-1.223	0.233	0.897	-4.928	-0.407	3.902
-0.059	0.954	0.999	-5.572	-0.020	3.804
0.841	0.408	0.969	-5.263	0.652	3.805
1.964	0.061	0.663	-3.988	0.603	2.726
-0.676	0.505	0.988	-5.372	-0.292	4.641
-0.170	0.867	0.999	-5.561	-0.048	3.668
-1.185	0.247	0.906	-4.966	-0.291	2.463
0.939	0.357	0.962	-5.188	0.289	5.253
0.729	0.473	0.984	-5.339	0.197	4.562
1.289	0.209	0.868	-4.859	0.368	6.694
1.332	0.195	0.853	-4.812	0.350	4.256
0.963	0.345	0.957	-5.168	0.260	4.120
0.960	0.346	0.957	-5.171	0.255	4.189
0.933	0.360	0.963	-5.193	0.257	4.237
1.907	0.068	0.684	-4.072	0.599	4.313
0.710	0.484	0.985	-5.351	0.171	3.697
-2.470	0.021	0.517	-3.174	-0.610	2.473
0.559	0.581	0.999	-5.435	0.214	2.731
1.673	0.107	0.764	-4.399	0.380	2.148
-0.687	0.498	0.987	-5.366	-0.217	3.440
0.057	0.955	0.999	-5.573	0.015	3.010
-0.919	0.367	0.963	-5.204	-0.284	4.286
0.243	0.810	0.999	-5.548	0.052	4.020
0.297	0.769	0.999	-5.535	0.114	4.987
0.161	0.873	0.999	-5.562	0.063	1.693
0.122	0.904	0.999	-5.567	0.032	4.674
-1.361	0.186	0.842	-4.780	-0.340	4.066
-1.297	0.207	0.868	-4.851	-0.243	7.001
0.735	0.469	0.983	-5.336	0.231	3.415
0.100	0.921	0.999	-5.569	0.024	3.152
-0.311	0.759	0.999	-5.531	-0.060	6.133

1.011	0.322	0.950	-5.128	0.289	2.806
0.208	0.837	0.999	-5.555	0.049	3.882
-1.182	0.248	0.908	-4.969	-0.355	2.732
0.086	0.932	0.999	-5.571	0.024	6.040
-0.109	0.914	0.999	-5.569	-0.021	4.644
0.546	0.590	0.999	-5.442	0.128	5.301
0.108	0.915	0.999	-5.569	0.059	3.006
-0.387	0.702	0.999	-5.507	-0.119	4.209
1.703	0.101	0.763	-4.358	0.404	3.681
1.713	0.099	0.757	-4.345	0.719	9.728
1.368	0.183	0.841	-4.772	0.428	4.467
-0.290	0.774	0.999	-5.536	-0.067	4.496
0.677	0.504	0.988	-5.371	0.156	5.219
0.716	0.481	0.984	-5.348	0.141	1.982
-0.155	0.878	0.999	-5.563	-0.039	3.429
0.407	0.687	0.999	-5.500	0.093	4.141
1.816	0.081	0.720	-4.202	0.449	2.260
-0.695	0.494	0.987	-5.361	-0.473	1.798
0.327	0.746	0.999	-5.526	0.133	8.470
0.313	0.757	0.999	-5.530	0.130	8.770
0.306	0.762	0.999	-5.532	0.126	8.654
0.296	0.770	0.999	-5.535	0.123	8.768
0.290	0.774	0.999	-5.536	0.120	8.800
0.277	0.784	0.999	-5.540	0.119	8.800
0.264	0.794	0.999	-5.543	0.112	8.788
0.425	0.675	0.999	-5.494	0.158	7.878
0.313	0.757	0.999	-5.530	0.120	7.980
1.600	0.122	0.784	-4.493	0.415	2.613
0.652	0.520	0.990	-5.386	0.159	3.933
-2.307	0.030	0.570	-3.449	-0.662	2.726
-0.930	0.361	0.963	-5.195	-0.351	2.252
0.645	0.525	0.992	-5.390	0.186	4.191
1.545	0.135	0.793	-4.563	0.949	6.113
0.631	0.534	0.992	-5.398	0.239	3.441
1.320	0.199	0.857	-4.825	0.279	5.473
-0.930	0.361	0.963	-5.195	-0.519	5.351
0.700	0.490	0.986	-5.358	0.184	4.263
-0.181	0.858	0.999	-5.559	-0.039	1.781
-1.313	0.201	0.860	-4.833	-0.299	3.560
-0.886	0.384	0.964	-5.230	-0.349	4.166
0.313	0.757	0.999	-5.530	0.082	3.436
0.578	0.568	0.997	-5.426	0.144	3.505
-0.126	0.901	0.999	-5.567	-0.060	3.898
0.635	0.531	0.992	-5.396	0.129	6.341
1.321	0.198	0.857	-4.825	0.272	5.576
-1.704	0.101	0.763	-4.357	-0.613	2.495
0.498	0.623	0.999	-5.464	0.185	4.640
2.052	0.051	0.641	-3.855	0.494	4.636
-0.247	0.807	0.999	-5.547	-0.076	2.834
0.322	0.750	0.999	-5.528	0.075	7.854

0.194	0.848	0.999	-5.557	0.043	8.013
-0.113	0.911	0.999	-5.568	-0.021	7.613
1.544	0.135	0.793	-4.563	0.469	4.752
2.849	0.009	0.441	-2.501	1.029	6.322
-0.795	0.434	0.973	-5.296	-0.212	2.573
-3.114	0.005	0.354	-2.004	-0.973	1.652
0.665	0.512	0.990	-5.379	0.167	5.176
-1.218	0.235	0.899	-4.933	-0.356	3.427
-1.511	0.143	0.803	-4.604	-0.383	2.894
-1.506	0.145	0.803	-4.611	-0.357	3.008
-0.736	0.469	0.983	-5.335	-0.397	2.848
0.080	0.937	0.999	-5.571	0.021	2.761
1.680	0.105	0.764	-4.389	0.478	3.099
-0.560	0.581	0.999	-5.435	-0.129	5.724
-0.236	0.816	0.999	-5.549	-0.043	5.039
-0.234	0.817	0.999	-5.550	-0.081	3.021
1.193	0.244	0.904	-4.958	0.273	2.578
-0.723	0.476	0.984	-5.343	-0.191	3.938
-2.407	0.024	0.543	-3.283	-1.243	2.072
0.553	0.585	0.999	-5.439	0.197	2.218
-0.876	0.390	0.964	-5.237	-0.179	5.433
-0.817	0.421	0.972	-5.280	-0.226	2.437
0.392	0.698	0.999	-5.505	0.113	3.213
-0.758	0.456	0.981	-5.321	-0.203	3.043
-0.169	0.867	0.999	-5.561	-0.039	4.214
-2.809	0.009	0.446	-2.572	-0.704	3.325
0.245	0.808	0.999	-5.547	0.060	4.774
0.800	0.431	0.973	-5.292	0.204	4.732
2.755	0.011	0.468	-2.672	0.635	3.958
1.391	0.177	0.835	-4.747	0.282	5.058
0.165	0.870	0.999	-5.562	0.055	5.243
1.173	0.252	0.911	-4.978	0.398	5.937
-0.163	0.872	0.999	-5.562	-0.072	6.534
1.293	0.208	0.868	-4.855	0.272	3.468
-0.656	0.518	0.990	-5.384	-0.208	6.015
-1.185	0.247	0.906	-4.966	-0.440	2.314
1.403	0.173	0.828	-4.732	0.482	4.998
-0.879	0.388	0.964	-5.235	-0.246	4.523
-0.890	0.382	0.964	-5.226	-0.446	1.383
-0.906	0.374	0.963	-5.214	-0.229	4.881
1.871	0.073	0.698	-4.125	0.454	5.091
-0.505	0.618	0.999	-5.461	-0.190	5.345
0.892	0.381	0.964	-5.225	0.214	4.590
-0.059	0.954	0.999	-5.572	-0.028	2.260
-1.139	0.265	0.916	-5.011	-0.508	4.449
-0.080	0.937	0.999	-5.571	-0.018	4.890
0.056	0.956	0.999	-5.573	0.020	5.342
-0.241	0.812	0.999	-5.548	-0.104	4.976
0.576	0.570	0.997	-5.427	0.191	4.659
-0.077	0.939	0.999	-5.571	-0.020	2.479

0.225	0.824	0.999	-5.551	0.059	4.865
1.140	0.265	0.916	-5.010	0.631	4.376
1.491	0.148	0.806	-4.629	0.445	3.069
1.089	0.286	0.929	-5.058	0.312	3.577
0.189	0.852	0.999	-5.558	0.046	6.422
0.964	0.344	0.957	-5.167	0.403	5.016
-0.805	0.428	0.973	-5.289	-0.289	4.566
-0.256	0.800	0.999	-5.545	-0.099	5.463
-0.849	0.404	0.968	-5.258	-0.402	4.142
0.162	0.873	0.999	-5.562	0.052	3.233
0.372	0.713	0.999	-5.512	0.137	5.993
-0.293	0.772	0.999	-5.536	-0.165	4.542
0.652	0.520	0.990	-5.386	0.443	5.291
1.140	0.265	0.916	-5.010	0.300	5.854
1.041	0.308	0.942	-5.102	0.315	2.388
0.578	0.568	0.997	-5.426	0.300	3.130
-0.605	0.551	0.994	-5.412	-0.152	6.627
-0.261	0.797	0.999	-5.544	-0.132	7.787
-0.250	0.805	0.999	-5.546	-0.072	5.121
0.390	0.700	0.999	-5.506	0.165	6.998
0.335	0.741	0.999	-5.524	0.149	5.167
0.162	0.872	0.999	-5.562	0.044	5.356
0.926	0.363	0.963	-5.198	0.517	6.413
-0.108	0.915	0.999	-5.569	-0.088	6.156
2.370	0.026	0.556	-3.344	0.691	6.482
-0.993	0.330	0.956	-5.143	-0.281	2.382
1.586	0.125	0.786	-4.511	0.358	4.417
-0.148	0.884	0.999	-5.564	-0.034	3.818
-0.258	0.798	0.999	-5.544	-0.111	4.268
2.226	0.035	0.598	-3.581	0.576	4.405
-0.527	0.603	0.999	-5.451	-0.394	3.010
0.773	0.447	0.979	-5.311	0.505	6.368
0.254	0.801	0.999	-5.545	0.099	6.384
2.650	0.014	0.498	-2.861	0.514	6.453
-1.022	0.317	0.948	-5.119	-0.312	6.969
-0.106	0.916	0.999	-5.569	-0.028	6.917
-0.866	0.394	0.964	-5.244	-0.379	5.339
-0.159	0.875	0.999	-5.563	-0.075	3.551
-0.343	0.734	0.999	-5.522	-0.136	4.001
0.304	0.763	0.999	-5.533	0.126	4.809
1.058	0.300	0.941	-5.086	0.692	4.986
-0.421	0.678	0.999	-5.495	-0.132	2.744
-0.654	0.519	0.990	-5.385	-0.146	6.968
0.483	0.633	0.999	-5.470	0.129	6.350
1.435	0.164	0.824	-4.696	0.287	6.337
-0.610	0.547	0.994	-5.409	-0.282	6.569
1.509	0.144	0.803	-4.607	0.366	3.944
-0.581	0.566	0.997	-5.424	-0.173	2.276
0.294	0.771	0.999	-5.535	0.125	7.584
0.289	0.775	0.999	-5.537	0.062	6.074

-1.306	0.203	0.862	-4.840	-0.453	6.577
-0.949	0.352	0.961	-5.180	-0.226	3.843
-0.625	0.537	0.993	-5.401	-0.176	2.879
0.479	0.636	0.999	-5.472	0.097	5.079
0.156	0.877	0.999	-5.563	0.073	7.852
-0.119	0.907	0.999	-5.568	-0.072	5.302
-1.207	0.239	0.901	-4.944	-0.382	2.280
-0.188	0.853	0.999	-5.558	-0.052	4.060
-0.885	0.385	0.964	-5.231	-0.277	2.543
0.829	0.415	0.970	-5.272	0.169	6.894
-0.816	0.422	0.972	-5.281	-0.293	5.223
2.791	0.010	0.453	-2.606	0.726	2.996
1.640	0.114	0.775	-4.442	0.389	3.508
3.226	0.003	0.340	-1.790	0.882	3.757
1.687	0.104	0.764	-4.380	0.410	2.493
0.073	0.943	0.999	-5.572	0.029	3.136
-0.325	0.748	0.999	-5.527	-0.083	7.014
0.240	0.813	0.999	-5.548	0.055	3.324
-1.249	0.223	0.886	-4.902	-0.321	3.501
0.484	0.633	0.999	-5.470	0.117	5.171
-0.185	0.855	0.999	-5.559	-0.042	7.911
1.348	0.190	0.849	-4.794	0.432	3.143
0.946	0.353	0.961	-5.182	0.626	3.930
-0.081	0.936	0.999	-5.571	-0.020	3.762
-0.860	0.398	0.964	-5.249	-0.444	1.892
0.697	0.492	0.986	-5.360	0.172	5.827
-0.137	0.892	0.999	-5.566	-0.036	2.819
-0.565	0.577	0.999	-5.433	-0.125	5.671
-0.438	0.665	0.999	-5.489	-0.095	4.163
-0.341	0.736	0.999	-5.522	-0.144	2.033
0.767	0.450	0.980	-5.315	0.196	4.844
-0.461	0.649	0.999	-5.480	-0.121	2.254
-0.878	0.388	0.964	-5.236	-0.188	5.287
2.180	0.039	0.611	-3.656	0.610	5.403
-2.210	0.036	0.607	-3.607	-0.782	4.745
-2.086	0.047	0.630	-3.803	-0.550	5.048
-2.112	0.045	0.630	-3.762	-0.653	2.689
0.342	0.735	0.999	-5.522	0.107	3.916
1.423	0.167	0.825	-4.710	0.274	5.131
-2.160	0.041	0.617	-3.687	-0.556	2.460
-1.945	0.063	0.670	-4.016	-0.693	2.794
-0.331	0.743	0.999	-5.525	-0.080	6.621
0.298	0.768	0.999	-5.534	0.084	5.426
2.266	0.032	0.591	-3.516	1.045	4.215
1.659	0.109	0.766	-4.416	0.681	3.644
0.340	0.737	0.999	-5.523	0.093	5.745
-2.309	0.029	0.570	-3.445	-0.544	4.330
-0.211	0.835	0.999	-5.554	-0.065	2.049
1.467	0.155	0.816	-4.657	0.266	4.876
-2.661	0.013	0.498	-2.841	-0.788	3.200

-0.415	0.681	0.999	-5.497	-0.111	3.981
0.452	0.655	0.999	-5.483	0.110	3.721
-1.792	0.085	0.728	-4.236	-0.506	2.606
0.583	0.565	0.997	-5.423	0.179	4.200
-1.576	0.128	0.789	-4.524	-0.423	3.782
-1.844	0.077	0.707	-4.163	-0.605	2.410
0.487	0.631	0.999	-5.469	0.216	2.610
-0.380	0.707	0.999	-5.510	-0.110	2.876
-0.137	0.892	0.999	-5.566	-0.044	3.918
-2.362	0.026	0.556	-3.358	-0.752	2.584
0.098	0.922	0.999	-5.570	0.035	3.626
-1.684	0.105	0.764	-4.384	-0.530	2.874
-1.604	0.121	0.784	-4.487	-0.451	2.807
-1.441	0.162	0.822	-4.688	-0.346	3.760
-1.096	0.284	0.925	-5.052	-0.303	2.334
-1.932	0.065	0.673	-4.036	-0.710	1.806
-3.119	0.005	0.353	-1.993	-0.766	2.429
-1.759	0.091	0.743	-4.282	-0.461	3.146
-0.093	0.927	0.999	-5.570	-0.020	7.618
-2.558	0.017	0.500	-3.023	-0.769	1.794
-0.941	0.355	0.962	-5.186	-0.288	2.576
-1.689	0.104	0.764	-4.376	-0.371	1.621
-0.628	0.536	0.993	-5.399	-0.161	1.881
0.463	0.647	0.999	-5.479	0.132	4.113
2.610	0.015	0.498	-2.931	0.674	4.636
-0.612	0.546	0.994	-5.408	-0.174	4.383
-1.217	0.235	0.899	-4.934	-0.276	3.856
1.969	0.060	0.663	-3.981	0.615	5.808
-2.541	0.018	0.501	-3.052	-0.731	2.071
1.136	0.267	0.917	-5.014	0.316	6.273
-0.148	0.884	0.999	-5.564	-0.035	3.629
0.662	0.514	0.990	-5.380	0.197	3.363
0.405	0.689	0.999	-5.501	0.196	1.868
2.953	0.007	0.403	-2.308	0.905	3.991
2.907	0.008	0.418	-2.394	1.729	9.398
1.092	0.285	0.927	-5.055	0.390	3.006
0.202	0.842	0.999	-5.556	0.065	3.154
0.862	0.397	0.964	-5.248	0.291	4.407
0.253	0.802	0.999	-5.545	0.104	3.216
-0.995	0.329	0.955	-5.141	-0.346	3.630
-1.192	0.244	0.904	-4.959	-0.288	2.217
0.237	0.815	0.999	-5.549	0.065	2.511
-0.249	0.805	0.999	-5.546	-0.068	5.420
1.625	0.117	0.776	-4.461	0.447	4.328
-0.499	0.622	0.999	-5.464	-0.088	5.344
1.246	0.224	0.886	-4.904	0.302	3.245
0.111	0.912	0.999	-5.568	0.045	4.760
0.640	0.528	0.992	-5.393	0.163	2.603
0.384	0.704	0.999	-5.508	0.089	3.370
2.057	0.050	0.640	-3.847	0.485	6.923

-1.423	0.167	0.825	-4.709	-0.397	1.699
0.511	0.613	0.999	-5.458	0.125	4.051
-0.189	0.852	0.999	-5.558	-0.048	6.933
3.038	0.005	0.392	-2.148	1.237	4.815
1.164	0.255	0.913	-4.987	0.282	3.076
1.633	0.115	0.776	-4.451	0.408	4.113
0.363	0.720	0.999	-5.515	0.070	5.047
-0.532	0.599	0.999	-5.448	-0.186	2.402
-2.148	0.042	0.619	-3.707	-0.564	3.326
1.121	0.273	0.921	-5.029	0.318	5.740
1.956	0.062	0.667	-4.001	0.924	7.159
-0.514	0.612	0.999	-5.457	-0.123	5.451
1.383	0.179	0.838	-4.755	0.926	7.261
0.575	0.570	0.997	-5.427	0.138	7.576
-0.709	0.485	0.985	-5.352	-0.354	3.108
-0.108	0.915	0.999	-5.569	-0.069	3.371
2.518	0.019	0.501	-3.092	1.127	5.467
1.932	0.065	0.673	-4.036	0.542	7.091
1.451	0.159	0.820	-4.676	0.464	2.581
0.806	0.428	0.973	-5.288	0.365	7.178
2.508	0.019	0.507	-3.109	0.907	4.516
-1.021	0.317	0.948	-5.119	-0.416	4.439
0.880	0.387	0.964	-5.234	0.229	3.226
2.062	0.050	0.639	-3.841	0.839	5.119
2.011	0.055	0.656	-3.919	0.598	4.090
3.550	0.002	0.301	-1.159	1.052	3.935
2.346	0.027	0.559	-3.384	0.725	3.977
1.233	0.229	0.891	-4.917	0.401	5.812
2.536	0.018	0.501	-3.061	0.989	7.198
0.578	0.568	0.997	-5.426	0.219	4.712
1.656	0.110	0.767	-4.420	0.442	4.388
-0.493	0.627	0.999	-5.466	-0.119	4.084
0.724	0.476	0.984	-5.343	0.404	7.711
0.965	0.344	0.957	-5.167	0.457	4.824
3.627	0.001	0.301	-1.006	0.964	2.583
-0.794	0.434	0.973	-5.296	-0.203	2.883
1.561	0.131	0.791	-4.543	0.455	6.965
1.113	0.276	0.921	-5.035	0.225	4.458
0.753	0.459	0.982	-5.324	0.134	6.228
1.952	0.062	0.667	-4.007	0.744	9.728
-0.141	0.889	0.999	-5.565	-0.061	3.405
0.967	0.343	0.957	-5.165	0.363	7.047
-0.252	0.803	0.999	-5.546	-0.043	3.835
0.390	0.700	0.999	-5.506	0.103	4.383
0.799	0.432	0.973	-5.293	0.216	5.810
2.024	0.054	0.655	-3.898	0.849	4.383
2.062	0.050	0.639	-3.840	0.877	5.987
0.685	0.500	0.987	-5.367	0.184	4.321
2.548	0.017	0.500	-3.040	0.788	6.239
2.253	0.033	0.595	-3.538	0.735	5.475

0.142	0.888	0.999	-5.565	0.046	10.215
2.243	0.034	0.596	-3.554	0.527	4.534
-0.064	0.950	0.999	-5.572	-0.012	4.331
0.166	0.870	0.999	-5.562	0.075	4.308
0.512	0.613	0.999	-5.457	0.134	5.065
0.566	0.577	0.999	-5.432	0.173	5.305
0.821	0.420	0.972	-5.278	0.180	6.929
0.362	0.721	0.999	-5.516	0.067	3.007
1.348	0.190	0.849	-4.794	0.462	5.055
1.178	0.250	0.910	-4.973	0.368	3.362
3.605	0.001	0.301	-1.048	1.003	4.233
0.653	0.519	0.990	-5.385	0.231	3.806
0.603	0.552	0.994	-5.413	0.240	5.573
0.582	0.566	0.997	-5.424	0.266	5.631
0.198	0.845	0.999	-5.556	0.092	3.611
1.448	0.160	0.820	-4.680	0.739	5.040
-0.628	0.536	0.993	-5.399	-0.157	6.741
-0.200	0.843	0.999	-5.556	-0.086	4.979
1.431	0.165	0.824	-4.700	0.355	3.703
1.595	0.123	0.785	-4.499	0.541	3.583
1.879	0.072	0.698	-4.113	0.455	3.914
4.252	0.000	0.256	0.243	1.058	6.738
0.101	0.920	0.999	-5.569	0.040	3.008
1.417	0.169	0.825	-4.716	0.476	5.817
0.394	0.697	0.999	-5.505	0.252	1.774
-1.491	0.148	0.806	-4.628	-0.426	3.650
0.305	0.763	0.999	-5.532	0.123	7.068
-0.175	0.862	0.999	-5.560	-0.071	6.585
1.418	0.168	0.825	-4.715	0.349	5.088
0.827	0.416	0.970	-5.273	0.225	4.343
-0.903	0.375	0.963	-5.216	-0.267	4.160
0.141	0.889	0.999	-5.565	0.035	5.712
-1.132	0.268	0.917	-5.018	-0.374	3.850
0.576	0.570	0.997	-5.427	0.147	4.840
1.247	0.224	0.886	-4.903	0.250	4.577
0.348	0.731	0.999	-5.520	0.091	3.347
0.833	0.413	0.970	-5.269	0.191	4.834
0.254	0.801	0.999	-5.545	0.069	6.323
2.818	0.009	0.446	-2.556	0.965	5.372
0.467	0.644	0.999	-5.477	0.170	5.505
-1.084	0.289	0.930	-5.063	-0.204	4.575
1.381	0.179	0.838	-4.757	0.335	2.900
0.904	0.374	0.963	-5.216	0.244	2.723
-0.339	0.738	0.999	-5.523	-0.088	4.738
0.106	0.917	0.999	-5.569	0.024	1.988
-0.316	0.755	0.999	-5.529	-0.086	3.577
0.234	0.817	0.999	-5.550	0.067	6.192
-0.548	0.589	0.999	-5.441	-0.124	3.235
0.790	0.437	0.973	-5.299	0.231	4.349
-1.172	0.252	0.911	-4.979	-0.333	5.471

1.269	0.216	0.876	-4.880	0.320	3.773
-0.821	0.419	0.972	-5.277	-0.409	4.149
1.454	0.158	0.819	-4.673	0.602	3.721
-0.057	0.955	0.999	-5.572	-0.019	5.101
1.772	0.088	0.734	-4.264	0.487	5.130
0.926	0.363	0.963	-5.198	0.227	5.065
0.702	0.489	0.986	-5.356	0.243	7.503
1.477	0.152	0.813	-4.646	0.430	4.947
-0.471	0.642	0.999	-5.475	-0.157	4.754
1.048	0.305	0.942	-5.096	0.293	3.229
1.176	0.251	0.910	-4.975	0.321	3.540
1.629	0.116	0.776	-4.456	0.441	7.498
-0.256	0.800	0.999	-5.545	-0.107	3.500
0.647	0.523	0.990	-5.389	0.337	5.336
0.929	0.362	0.963	-5.196	0.427	4.237
-0.105	0.917	0.999	-5.569	-0.031	3.213
-0.793	0.435	0.973	-5.297	-0.442	5.614
-1.425	0.167	0.825	-4.707	-0.464	4.732
-2.054	0.051	0.641	-3.852	-0.459	5.800
1.752	0.092	0.746	-4.292	0.397	4.621
0.358	0.723	0.999	-5.517	0.075	3.520
-1.095	0.284	0.926	-5.053	-0.289	3.363
-0.694	0.494	0.987	-5.361	-0.153	4.691
-1.010	0.322	0.950	-5.129	-0.303	4.780
1.107	0.279	0.922	-5.042	0.373	4.235
0.360	0.722	0.999	-5.516	0.095	6.033
0.428	0.672	0.999	-5.493	0.102	2.847
1.297	0.206	0.868	-4.850	0.270	3.661
0.158	0.876	0.999	-5.563	0.034	6.532
0.199	0.844	0.999	-5.556	0.082	7.158
-1.314	0.201	0.860	-4.831	-0.386	1.947
0.335	0.740	0.999	-5.524	0.101	4.855
1.428	0.166	0.825	-4.703	0.387	6.906
1.077	0.292	0.933	-5.069	0.327	3.627
-0.302	0.765	0.999	-5.533	-0.080	3.982
1.215	0.236	0.899	-4.936	0.354	5.133
0.165	0.870	0.999	-5.562	0.049	3.902
1.966	0.060	0.663	-3.985	0.690	5.007
0.188	0.852	0.999	-5.558	0.084	4.865
2.235	0.035	0.597	-3.567	0.784	4.845
-0.124	0.903	0.999	-5.567	-0.025	2.527
0.195	0.847	0.999	-5.557	0.046	5.543
1.694	0.103	0.764	-4.370	0.388	5.145
-2.208	0.037	0.608	-3.611	-0.547	2.293
0.558	0.582	0.999	-5.436	0.172	3.523
0.159	0.875	0.999	-5.563	0.039	2.159
0.457	0.652	0.999	-5.481	0.126	3.628
3.877	0.001	0.301	-0.509	1.667	7.323
0.145	0.886	0.999	-5.565	0.052	4.116
0.814	0.423	0.972	-5.283	0.294	4.099

-0.483	0.633	0.999	-5.470	-0.215	5.266
-1.006	0.324	0.951	-5.132	-0.254	3.735
0.186	0.854	0.999	-5.559	0.052	3.100
-2.020	0.054	0.655	-3.905	-0.627	1.887
0.527	0.603	0.999	-5.451	0.135	3.904
0.460	0.650	0.999	-5.480	0.108	2.554
-0.067	0.947	0.999	-5.572	-0.016	6.320
0.151	0.881	0.999	-5.564	0.052	6.229
-0.201	0.843	0.999	-5.556	-0.125	3.998
-0.664	0.513	0.990	-5.379	-0.203	5.914
-4.601	0.000	0.256	0.941	-1.384	2.942
-0.849	0.404	0.968	-5.257	-0.254	2.409
-0.142	0.888	0.999	-5.565	-0.043	2.739
-0.647	0.524	0.990	-5.389	-0.364	3.955
-0.124	0.902	0.999	-5.567	-0.060	3.832
1.039	0.309	0.943	-5.104	0.759	9.044
1.809	0.082	0.722	-4.213	1.210	7.020
-0.093	0.927	0.999	-5.570	-0.019	5.457
3.695	0.001	0.301	-0.871	1.159	3.612
-0.639	0.529	0.992	-5.394	-0.164	5.989
2.212	0.036	0.606	-3.603	0.552	6.834
-0.179	0.860	0.999	-5.560	-0.041	3.966
1.192	0.244	0.904	-4.959	0.257	5.723
0.868	0.394	0.964	-5.243	0.249	4.171
0.937	0.358	0.962	-5.190	0.362	4.104
-1.351	0.189	0.847	-4.791	-0.435	2.084
0.595	0.557	0.995	-5.417	0.134	4.183
0.093	0.926	0.999	-5.570	0.024	4.845
1.195	0.243	0.904	-4.956	0.364	3.567
0.500	0.622	0.999	-5.463	0.240	4.712
-0.738	0.467	0.983	-5.334	-0.323	1.803
-0.660	0.515	0.990	-5.382	-0.250	3.408
0.589	0.561	0.996	-5.420	0.253	2.469
0.537	0.596	0.999	-5.446	0.179	2.896
-0.589	0.561	0.996	-5.420	-0.466	3.000
1.733	0.095	0.754	-4.318	0.537	2.787
-0.116	0.909	0.999	-5.568	-0.030	3.003
1.820	0.081	0.718	-4.197	0.428	2.840
-0.675	0.506	0.988	-5.373	-0.249	2.651
-0.487	0.631	0.999	-5.469	-0.172	3.322
0.574	0.571	0.997	-5.428	0.200	4.269
-0.266	0.793	0.999	-5.542	-0.057	3.049
-0.476	0.638	0.999	-5.473	-0.110	3.753
1.129	0.269	0.918	-5.020	0.272	6.547
0.482	0.634	0.999	-5.471	0.174	6.785
1.505	0.145	0.803	-4.611	0.561	7.066
-0.908	0.372	0.963	-5.213	-0.307	3.454
0.666	0.511	0.989	-5.378	0.250	2.973
-0.398	0.694	0.999	-5.503	-0.126	6.833
-1.271	0.215	0.875	-4.878	-0.339	3.732

1.910	0.068	0.683	-4.068	0.426	5.168
-0.469	0.643	0.999	-5.476	-0.108	2.417
0.841	0.408	0.970	-5.263	0.211	4.440
-0.548	0.588	0.999	-5.441	-0.145	4.088
1.464	0.155	0.817	-4.660	0.502	5.727
1.390	0.177	0.835	-4.748	0.421	3.926
-0.838	0.410	0.970	-5.266	-0.223	2.947
0.324	0.749	0.999	-5.527	0.088	8.469
1.266	0.217	0.876	-4.883	0.251	6.401
0.075	0.941	0.999	-5.571	0.018	3.826
1.148	0.262	0.914	-5.002	0.353	3.333
-0.579	0.568	0.997	-5.425	-0.205	4.576
0.234	0.817	0.999	-5.549	0.061	2.986
0.721	0.478	0.984	-5.345	0.279	5.055
0.080	0.937	0.999	-5.571	0.021	4.470
-1.125	0.271	0.919	-5.025	-0.327	2.554
-0.495	0.625	0.999	-5.465	-0.309	5.030
-0.692	0.495	0.987	-5.362	-0.187	5.023
0.517	0.610	0.999	-5.455	0.261	4.510
0.800	0.431	0.973	-5.292	0.185	3.630
-0.171	0.866	0.999	-5.561	-0.054	5.637
-0.114	0.910	0.999	-5.568	-0.020	7.062
1.151	0.261	0.914	-5.000	0.322	5.868
0.090	0.929	0.999	-5.570	0.038	5.696
0.665	0.512	0.989	-5.378	0.157	3.393
0.722	0.477	0.984	-5.344	0.159	2.380
-1.060	0.299	0.941	-5.085	-0.290	2.971
-1.523	0.140	0.801	-4.590	-0.350	3.316
-0.854	0.401	0.966	-5.253	-0.212	2.273
0.301	0.766	0.999	-5.533	0.097	8.753
-0.513	0.613	0.999	-5.457	-0.163	3.214
1.151	0.260	0.914	-4.999	0.405	5.294
0.303	0.765	0.999	-5.533	0.072	3.578
-1.711	0.099	0.758	-4.348	-0.704	5.291
3.622	0.001	0.301	-1.015	0.879	3.009
1.299	0.206	0.867	-4.848	0.656	4.218
1.018	0.319	0.950	-5.122	0.438	6.082
-0.715	0.481	0.984	-5.348	-0.226	2.475
-0.744	0.464	0.983	-5.330	-0.246	2.935
1.178	0.250	0.910	-4.974	0.277	7.256
1.148	0.262	0.914	-5.002	0.267	7.255
1.038	0.309	0.943	-5.104	0.250	7.244
0.574	0.571	0.997	-5.428	0.113	6.213
0.504	0.618	0.999	-5.461	0.101	6.190
1.492	0.148	0.805	-4.627	0.579	5.021
-1.943	0.063	0.671	-4.019	-0.415	2.130
0.961	0.346	0.957	-5.170	0.296	9.175
0.111	0.912	0.999	-5.568	0.037	2.752
0.552	0.586	0.999	-5.439	0.186	5.199
1.252	0.222	0.885	-4.898	0.282	5.819

0.663	0.514	0.990	-5.380	0.172	3.656
1.196	0.243	0.904	-4.956	0.282	4.814
1.919	0.066	0.681	-4.055	0.404	6.878
-1.330	0.196	0.853	-4.815	-0.297	8.194
-0.316	0.754	0.999	-5.529	-0.171	4.724
0.107	0.916	0.999	-5.569	0.049	4.867
-1.017	0.319	0.950	-5.122	-0.231	6.179
-0.531	0.600	0.999	-5.449	-0.137	4.317
3.371	0.002	0.301	-1.510	1.174	2.889
-0.197	0.845	0.999	-5.557	-0.077	3.279
1.231	0.230	0.893	-4.920	0.314	2.802
1.636	0.114	0.775	-4.447	0.359	5.878
1.498	0.147	0.804	-4.621	0.442	5.485
1.186	0.247	0.906	-4.965	0.308	5.622
0.154	0.879	0.999	-5.563	0.076	6.851
0.609	0.548	0.994	-5.410	0.210	4.431
1.185	0.247	0.906	-4.966	0.253	5.010
0.444	0.661	0.999	-5.486	0.203	9.518
2.398	0.024	0.543	-3.298	0.529	6.325
0.480	0.635	0.999	-5.471	0.195	3.545
-0.063	0.950	0.999	-5.572	-0.022	2.224
-0.303	0.764	0.999	-5.533	-0.100	4.114
0.400	0.693	0.999	-5.503	0.089	2.856
-0.067	0.947	0.999	-5.572	-0.029	5.940
-0.734	0.470	0.983	-5.337	-0.233	4.758
1.682	0.105	0.764	-4.387	0.566	5.871
1.418	0.169	0.825	-4.715	0.382	8.646
0.558	0.582	0.999	-5.436	0.223	4.973
0.288	0.776	0.999	-5.537	0.101	10.036
1.581	0.126	0.786	-4.517	0.846	5.398
1.243	0.225	0.886	-4.907	0.698	5.375
0.171	0.866	0.999	-5.561	0.037	5.007
0.467	0.644	0.999	-5.477	0.130	5.069
-2.520	0.018	0.501	-3.089	-0.676	6.495
0.259	0.798	0.999	-5.544	0.096	8.066
-2.190	0.038	0.611	-3.639	-0.749	4.249
1.690	0.103	0.764	-4.375	0.748	4.104
-0.457	0.652	0.999	-5.481	-0.181	2.005
0.495	0.625	0.999	-5.465	0.157	2.247
1.017	0.319	0.950	-5.123	0.326	7.292
0.365	0.718	0.999	-5.515	0.144	6.828
3.220	0.004	0.340	-1.801	0.685	4.960
-0.130	0.898	0.999	-5.566	-0.044	7.879
0.525	0.604	0.999	-5.452	0.214	5.340
0.437	0.666	0.999	-5.489	0.123	3.621
-0.261	0.796	0.999	-5.544	-0.056	3.635
1.027	0.314	0.944	-5.114	0.326	3.438
0.512	0.613	0.999	-5.458	0.122	6.610
2.240	0.034	0.597	-3.559	0.452	5.984
0.524	0.605	0.999	-5.452	0.125	1.820

0.346	0.732	0.999	-5.521	0.110	4.522
2.999	0.006	0.393	-2.222	1.218	6.002
0.382	0.706	0.999	-5.509	0.088	2.646
-0.686	0.499	0.987	-5.366	-0.191	4.924
-0.124	0.902	0.999	-5.567	-0.036	5.449
0.715	0.481	0.984	-5.348	0.167	2.361
1.272	0.215	0.875	-4.877	0.295	3.982
0.769	0.449	0.979	-5.314	0.238	4.461
-1.041	0.308	0.942	-5.102	-0.283	2.051
0.825	0.417	0.970	-5.275	0.264	3.138
0.069	0.946	0.999	-5.572	0.020	3.624
1.978	0.059	0.663	-3.967	0.404	3.275
0.400	0.693	0.999	-5.503	0.127	3.335
1.121	0.273	0.921	-5.028	0.333	2.983
2.690	0.013	0.495	-2.790	0.952	5.172
1.965	0.061	0.663	-3.987	0.577	4.822
-1.229	0.231	0.894	-4.922	-0.318	2.407
1.889	0.071	0.694	-4.099	0.350	7.193
0.420	0.678	0.999	-5.495	0.186	5.468
0.300	0.767	0.999	-5.534	0.119	3.901
-1.876	0.072	0.698	-4.117	-0.491	4.517
-0.310	0.759	0.999	-5.531	-0.076	3.375
0.387	0.702	0.999	-5.507	0.103	2.807
0.453	0.654	0.999	-5.483	0.124	3.381
0.051	0.960	0.999	-5.573	0.014	3.158
-0.570	0.574	0.999	-5.430	-0.147	4.501
-1.093	0.285	0.927	-5.054	-0.274	4.390
2.664	0.013	0.498	-2.836	0.864	6.330
1.480	0.151	0.813	-4.642	0.344	6.677
-1.635	0.114	0.775	-4.447	-0.470	3.097
1.127	0.270	0.919	-5.023	0.215	4.142
-0.729	0.473	0.984	-5.339	-0.147	3.937
0.255	0.801	0.999	-5.545	0.063	4.761
0.474	0.640	0.999	-5.474	0.090	5.706
2.035	0.053	0.653	-3.882	0.472	5.359
0.464	0.647	0.999	-5.478	0.108	3.176
0.829	0.415	0.970	-5.272	0.320	5.986
-0.724	0.476	0.984	-5.343	-0.314	2.431
0.081	0.936	0.999	-5.571	0.025	4.364
-0.061	0.952	0.999	-5.572	-0.015	7.773
1.916	0.067	0.682	-4.059	0.338	5.234
0.673	0.507	0.988	-5.373	0.143	5.480
1.100	0.282	0.924	-5.048	0.256	4.980
0.087	0.931	0.999	-5.571	0.022	2.480
-0.184	0.855	0.999	-5.559	-0.062	5.477
1.320	0.199	0.857	-4.825	0.651	7.683
-1.294	0.207	0.868	-4.853	-0.493	2.494
-1.338	0.193	0.853	-4.806	-0.787	7.261
0.612	0.546	0.994	-5.408	0.258	4.643
0.140	0.889	0.999	-5.565	0.033	6.882

0.188	0.852	0.999	-5.558	0.065	4.842
1.156	0.258	0.913	-4.994	0.290	4.821
-2.053	0.051	0.641	-3.854	-0.750	4.358
-0.131	0.897	0.999	-5.566	-0.054	2.450
0.547	0.589	0.999	-5.441	0.165	4.886
-1.178	0.250	0.910	-4.973	-0.344	2.941
2.411	0.024	0.543	-3.275	0.914	5.681
-0.051	0.960	0.999	-5.573	-0.013	7.784
1.105	0.280	0.922	-5.044	0.665	4.122
1.240	0.226	0.888	-4.910	0.533	3.949
0.254	0.802	0.999	-5.545	0.070	2.983
1.170	0.253	0.913	-4.981	0.307	4.872
-1.526	0.140	0.799	-4.586	-0.588	3.392
0.662	0.514	0.990	-5.380	0.165	1.793
0.605	0.551	0.994	-5.412	0.203	2.263
0.173	0.864	0.999	-5.561	0.055	6.119
0.743	0.464	0.983	-5.330	0.241	6.905
0.084	0.934	0.999	-5.571	0.020	4.132
0.493	0.626	0.999	-5.466	0.215	5.004
-1.990	0.058	0.660	-3.950	-0.463	5.414
1.404	0.173	0.828	-4.732	0.405	5.043
1.621	0.117	0.776	-4.466	0.562	3.546
-0.167	0.869	0.999	-5.561	-0.053	2.672
0.337	0.739	0.999	-5.523	0.123	5.351
1.600	0.122	0.784	-4.493	0.591	3.489
-0.907	0.373	0.963	-5.214	-0.253	2.671
-0.744	0.464	0.983	-5.330	-0.172	3.727
0.503	0.619	0.999	-5.462	0.095	5.635
0.471	0.642	0.999	-5.476	0.098	4.960
-0.065	0.949	0.999	-5.572	-0.017	2.946
1.143	0.264	0.915	-5.007	0.271	2.982
2.864	0.008	0.437	-2.472	0.689	5.878
-0.353	0.727	0.999	-5.519	-0.248	3.294
0.406	0.688	0.999	-5.501	0.082	4.739
0.120	0.905	0.999	-5.567	0.049	1.694
0.941	0.356	0.962	-5.187	0.295	4.414
1.035	0.311	0.943	-5.107	0.238	4.421
-0.384	0.704	0.999	-5.508	-0.107	3.031
2.245	0.034	0.595	-3.551	0.536	4.737
1.802	0.084	0.724	-4.222	0.711	6.320
0.922	0.366	0.963	-5.202	0.286	6.013
-0.550	0.588	0.999	-5.440	-0.209	3.775
0.623	0.539	0.994	-5.402	0.161	4.300
0.177	0.861	0.999	-5.560	0.085	4.582
-0.999	0.327	0.954	-5.138	-0.268	2.870
-0.178	0.860	0.999	-5.560	-0.038	4.326
-1.470	0.154	0.814	-4.653	-0.316	4.003
0.858	0.399	0.964	-5.251	0.162	7.686
-0.430	0.671	0.999	-5.492	-0.104	5.517
1.604	0.121	0.784	-4.488	0.391	5.347

0.673	0.507	0.988	-5.374	0.268	2.817
-0.345	0.733	0.999	-5.521	-0.101	6.428
1.773	0.088	0.734	-4.263	0.413	3.105
0.065	0.949	0.999	-5.572	0.018	6.957
0.248	0.806	0.999	-5.546	0.169	2.347
0.078	0.939	0.999	-5.571	0.018	5.683
-0.949	0.352	0.961	-5.180	-0.274	2.239
1.176	0.250	0.910	-4.975	0.289	4.367
0.880	0.387	0.964	-5.234	0.296	8.434
0.803	0.430	0.973	-5.290	0.276	5.490
0.260	0.797	0.999	-5.544	0.082	4.950
-0.706	0.487	0.985	-5.354	-0.361	3.197
2.348	0.027	0.559	-3.381	0.831	3.947
0.421	0.677	0.999	-5.495	0.152	4.609
-0.239	0.813	0.999	-5.548	-0.086	5.182
0.962	0.345	0.957	-5.169	0.284	4.037
1.748	0.093	0.749	-4.297	0.571	4.109
1.278	0.213	0.871	-4.870	0.303	4.236
0.258	0.799	0.999	-5.544	0.060	3.117
-0.511	0.614	0.999	-5.458	-0.126	4.990
0.300	0.767	0.999	-5.534	0.080	5.588
0.457	0.652	0.999	-5.481	0.084	5.108
1.176	0.251	0.910	-4.975	0.447	5.526
-0.113	0.911	0.999	-5.568	-0.022	4.229
-0.832	0.413	0.970	-5.269	-0.222	2.427
0.136	0.893	0.999	-5.566	0.048	4.986
1.037	0.310	0.943	-5.106	0.210	5.066
0.889	0.383	0.964	-5.227	0.304	2.201
-0.209	0.836	0.999	-5.554	-0.051	4.024
-1.382	0.179	0.838	-4.757	-0.370	2.589
-0.446	0.660	0.999	-5.486	-0.087	4.000
-0.725	0.475	0.984	-5.342	-0.233	2.372
-0.118	0.907	0.999	-5.568	-0.034	2.487
-1.538	0.137	0.795	-4.572	-0.343	7.438
-0.385	0.703	0.999	-5.508	-0.162	1.141
1.486	0.150	0.810	-4.635	0.557	6.267
1.166	0.254	0.913	-4.985	0.395	1.669
0.775	0.446	0.979	-5.310	0.170	4.068
-0.888	0.383	0.964	-5.228	-0.254	2.777
-0.493	0.626	0.999	-5.466	-0.150	2.673
-1.031	0.312	0.943	-5.110	-0.238	1.592
-0.741	0.466	0.983	-5.332	-0.191	3.527
-0.248	0.806	0.999	-5.547	-0.073	1.873
-0.122	0.904	0.999	-5.567	-0.040	2.360
-0.268	0.791	0.999	-5.542	-0.074	2.474
-0.677	0.504	0.988	-5.371	-0.212	1.804
1.158	0.258	0.913	-4.993	0.352	2.727
-0.444	0.661	0.999	-5.486	-0.190	0.937
-0.859	0.398	0.964	-5.250	-0.257	1.636
-1.398	0.174	0.828	-4.738	-0.351	2.242

-0.612	0.546	0.994	-5.408	-0.163	3.466
1.496	0.147	0.804	-4.623	0.487	3.729
0.442	0.662	0.999	-5.487	0.170	3.124
-2.013	0.055	0.656	-3.915	-0.725	2.663
0.870	0.393	0.964	-5.242	0.261	2.564
-1.653	0.111	0.767	-4.424	-0.579	3.202
2.402	0.024	0.543	-3.291	0.776	3.434
0.073	0.942	0.999	-5.572	0.025	3.443
-0.074	0.942	0.999	-5.572	-0.021	2.426
0.997	0.328	0.954	-5.139	0.541	3.071
1.751	0.092	0.747	-4.293	0.459	2.311
-1.086	0.288	0.929	-5.061	-0.227	3.592
-0.935	0.359	0.963	-5.191	-0.267	3.063
0.553	0.585	0.999	-5.438	0.150	2.680
-1.530	0.139	0.796	-4.581	-0.388	2.332
-1.854	0.075	0.704	-4.149	-0.474	2.670
0.697	0.492	0.986	-5.360	0.217	3.300
-1.052	0.303	0.941	-5.092	-0.293	1.177
0.586	0.563	0.997	-5.422	0.251	3.415
0.682	0.501	0.988	-5.368	0.148	2.077
1.113	0.276	0.921	-5.036	0.252	4.878
-0.409	0.686	0.999	-5.499	-0.109	3.034
0.406	0.688	0.999	-5.501	0.096	3.134
0.340	0.737	0.999	-5.523	0.104	2.321
-0.153	0.879	0.999	-5.563	-0.043	4.687
0.248	0.806	0.999	-5.547	0.056	2.683
0.180	0.859	0.999	-5.559	0.041	1.993
-2.113	0.045	0.630	-3.761	-0.513	2.152
0.693	0.495	0.987	-5.362	0.160	2.098
0.619	0.541	0.994	-5.404	0.181	4.541
1.045	0.306	0.942	-5.098	0.271	2.707
-0.526	0.603	0.999	-5.451	-0.138	2.166
-1.029	0.313	0.944	-5.112	-0.223	3.816
0.276	0.785	0.999	-5.540	0.071	4.636
-0.392	0.698	0.999	-5.506	-0.145	5.708
-0.474	0.639	0.999	-5.474	-0.106	3.743
-0.597	0.556	0.995	-5.416	-0.141	6.391
1.294	0.207	0.868	-4.853	0.281	6.652
-0.071	0.944	0.999	-5.572	-0.018	2.158
-0.271	0.788	0.999	-5.541	-0.136	7.569
1.244	0.225	0.886	-4.907	0.397	4.796
0.350	0.729	0.999	-5.519	0.080	4.173
2.084	0.048	0.630	-3.807	0.507	5.778
2.962	0.007	0.400	-2.291	1.118	6.089
0.715	0.481	0.984	-5.348	0.187	4.327
0.869	0.393	0.964	-5.242	0.224	3.583
0.687	0.498	0.987	-5.365	0.153	4.284
1.498	0.147	0.804	-4.620	0.397	6.374
2.800	0.010	0.447	-2.590	0.959	7.364
-1.386	0.178	0.837	-4.752	-0.371	2.349

-0.558	0.582	0.999	-5.436	-0.158	2.856
1.522	0.141	0.801	-4.591	0.388	6.812
-0.911	0.371	0.963	-5.210	-0.387	4.554
0.343	0.734	0.999	-5.521	0.142	1.852
-0.276	0.785	0.999	-5.540	-0.156	1.973
-0.332	0.742	0.999	-5.525	-0.096	5.739
1.347	0.190	0.849	-4.796	0.272	7.001
-0.728	0.473	0.984	-5.340	-0.334	2.262
2.018	0.054	0.655	-3.908	0.383	5.964
1.567	0.130	0.789	-4.535	0.394	4.988
1.128	0.270	0.918	-5.022	0.390	6.710
0.210	0.835	0.999	-5.554	0.112	4.726
0.390	0.699	0.999	-5.506	0.204	3.057
-2.028	0.053	0.653	-3.892	-0.716	2.470
1.443	0.161	0.820	-4.686	0.381	4.523
-0.182	0.857	0.999	-5.559	-0.125	5.560
2.525	0.018	0.501	-3.079	0.825	7.499
1.801	0.084	0.724	-4.224	0.641	3.184
0.661	0.514	0.990	-5.381	0.180	3.996
0.615	0.544	0.994	-5.406	0.230	2.542
1.475	0.153	0.813	-4.648	0.579	3.028
0.548	0.589	0.999	-5.441	0.201	9.075
3.330	0.003	0.320	-1.589	1.048	3.077
-3.949	0.001	0.301	-0.364	-1.309	3.799
0.403	0.691	0.999	-5.502	0.208	2.741
-3.504	0.002	0.301	-1.247	-1.007	2.431
-1.008	0.323	0.950	-5.131	-0.286	3.776
-0.145	0.886	0.999	-5.565	-0.046	3.989
-0.551	0.586	0.999	-5.439	-0.117	2.718
-0.867	0.394	0.964	-5.244	-0.228	2.061
0.592	0.559	0.996	-5.418	0.140	7.579
0.654	0.519	0.990	-5.385	0.171	4.106
0.544	0.591	0.999	-5.443	0.111	7.492
0.979	0.337	0.957	-5.155	0.336	5.256
1.520	0.141	0.801	-4.594	0.774	8.443
-0.064	0.950	0.999	-5.572	-0.017	7.954
-0.379	0.708	0.999	-5.510	-0.096	6.408
0.356	0.725	0.999	-5.517	0.122	4.993
1.732	0.096	0.754	-4.319	0.726	5.817
0.873	0.391	0.964	-5.240	0.220	4.958
-2.481	0.020	0.517	-3.155	-0.567	3.484
0.898	0.377	0.963	-5.220	0.329	7.829
-0.210	0.836	0.999	-5.554	-0.087	2.448
-0.280	0.782	0.999	-5.539	-0.092	2.590
-0.507	0.617	0.999	-5.460	-0.114	2.944
-0.303	0.765	0.999	-5.533	-0.087	4.451
1.404	0.172	0.828	-4.731	0.358	3.830
-0.527	0.603	0.999	-5.451	-0.161	4.800
-0.116	0.908	0.999	-5.568	-0.035	5.027
0.717	0.480	0.984	-5.347	0.342	2.241

0.306	0.762	0.999	-5.532	0.156	2.877
0.197	0.846	0.999	-5.557	0.078	5.917
0.745	0.463	0.983	-5.329	0.268	5.045
-0.623	0.539	0.994	-5.402	-0.180	2.299
0.951	0.351	0.961	-5.178	0.232	5.808
0.443	0.662	0.999	-5.487	0.092	2.621
2.021	0.054	0.655	-3.902	0.664	4.618
0.468	0.644	0.999	-5.477	0.143	3.092
1.012	0.321	0.950	-5.127	0.400	5.333
-1.434	0.164	0.824	-4.697	-0.414	3.960
-1.380	0.180	0.838	-4.758	-0.320	3.624
-0.873	0.391	0.964	-5.239	-0.376	7.981
-0.220	0.828	0.999	-5.552	-0.067	3.566
2.584	0.016	0.500	-2.977	0.659	5.198
0.340	0.737	0.999	-5.522	0.096	4.206
-0.072	0.943	0.999	-5.572	-0.019	1.174
2.528	0.018	0.501	-3.075	0.603	3.783
0.269	0.790	0.999	-5.542	0.091	5.110
1.744	0.093	0.751	-4.302	0.468	6.468
-0.139	0.891	0.999	-5.565	-0.105	6.843
2.517	0.019	0.501	-3.094	0.652	2.689
-1.972	0.060	0.663	-3.976	-0.733	2.048
0.664	0.513	0.990	-5.379	0.159	3.697
-0.263	0.794	0.999	-5.543	-0.080	2.298
0.354	0.726	0.999	-5.518	0.071	5.967
-0.634	0.532	0.992	-5.396	-0.164	4.758
0.231	0.819	0.999	-5.550	0.056	5.818
-0.520	0.608	0.999	-5.454	-0.142	4.976
-0.158	0.876	0.999	-5.563	-0.038	2.127
0.259	0.798	0.999	-5.544	0.081	2.371
0.454	0.654	0.999	-5.482	0.130	5.119
0.438	0.665	0.999	-5.489	0.138	4.427
-2.276	0.032	0.589	-3.501	-0.568	2.583
-2.389	0.025	0.547	-3.312	-0.702	2.549
0.286	0.778	0.999	-5.538	0.079	4.460
0.936	0.358	0.963	-5.190	0.193	4.643
0.570	0.574	0.999	-5.430	0.137	3.547
1.034	0.311	0.943	-5.108	0.224	5.065
1.551	0.134	0.791	-4.555	0.380	3.257
1.399	0.174	0.828	-4.737	0.346	3.389
1.116	0.275	0.921	-5.033	0.238	4.403
-0.431	0.670	0.999	-5.491	-0.118	2.306
0.423	0.676	0.999	-5.494	0.099	3.425
-0.371	0.714	0.999	-5.513	-0.079	5.869
-0.299	0.768	0.999	-5.534	-0.063	5.889
2.428	0.023	0.542	-3.247	0.668	5.268
-1.581	0.126	0.786	-4.517	-0.600	8.042
0.090	0.929	0.999	-5.570	0.070	1.730
0.229	0.821	0.999	-5.551	0.080	4.089
0.308	0.761	0.999	-5.532	0.080	5.614

0.161	0.873	0.999	-5.562	0.043	7.826
0.138	0.891	0.999	-5.565	0.037	7.922
0.070	0.945	0.999	-5.572	0.019	7.767
-0.166	0.870	0.999	-5.562	-0.047	7.063
-0.556	0.583	0.999	-5.437	-0.099	7.101
0.584	0.564	0.997	-5.423	0.114	4.696
0.345	0.733	0.999	-5.521	0.097	2.290
-0.863	0.396	0.964	-5.247	-0.312	3.038
-0.671	0.508	0.988	-5.375	-0.164	3.047
-0.236	0.815	0.999	-5.549	-0.067	2.610
0.533	0.599	0.999	-5.448	0.160	4.060
1.198	0.242	0.904	-4.953	0.270	2.663
-0.280	0.782	0.999	-5.539	-0.092	3.682
0.594	0.558	0.995	-5.418	0.126	5.427
0.458	0.651	0.999	-5.481	0.471	4.068
0.377	0.709	0.999	-5.511	0.102	4.404
1.403	0.173	0.828	-4.733	0.404	4.587
2.138	0.042	0.622	-3.722	0.457	3.960
2.217	0.036	0.601	-3.595	0.645	5.254
1.514	0.142	0.802	-4.600	0.547	4.022
-1.581	0.126	0.786	-4.518	-0.831	3.006
-1.461	0.156	0.817	-4.665	-0.759	3.038
-0.429	0.671	0.999	-5.492	-0.095	3.952
3.373	0.002	0.301	-1.505	1.026	3.639
-0.880	0.387	0.964	-5.234	-0.233	5.064
3.682	0.001	0.301	-0.897	0.877	4.238
1.670	0.107	0.764	-4.403	0.476	5.843
0.265	0.793	0.999	-5.543	0.059	8.414
0.262	0.795	0.999	-5.543	0.061	6.185
-0.373	0.712	0.999	-5.512	-0.093	4.940
-1.112	0.277	0.921	-5.037	-0.247	5.118
-0.130	0.898	0.999	-5.566	-0.043	2.824
1.098	0.282	0.924	-5.050	0.240	5.479
0.645	0.525	0.992	-5.390	0.314	2.881
0.639	0.529	0.992	-5.394	0.136	3.228
0.105	0.918	0.999	-5.569	0.024	5.876
0.373	0.712	0.999	-5.512	0.118	5.862
0.794	0.434	0.973	-5.296	0.173	2.496
0.203	0.841	0.999	-5.556	0.079	1.904
-2.788	0.010	0.453	-2.611	-0.772	1.757
-0.087	0.931	0.999	-5.571	-0.021	4.399
0.194	0.848	0.999	-5.557	0.102	2.148
1.334	0.194	0.853	-4.810	0.363	4.404
0.155	0.878	0.999	-5.563	0.065	4.719
-0.226	0.823	0.999	-5.551	-0.057	5.080
0.645	0.525	0.992	-5.390	0.123	5.869
0.552	0.586	0.999	-5.439	0.171	4.166
-0.856	0.400	0.964	-5.252	-0.297	4.168
0.793	0.435	0.973	-5.297	0.282	2.837

-0.555	0.584	0.999	-5.437	-0.182	4.891
-1.438	0.163	0.824	-4.692	-0.400	9.934
-0.475	0.639	0.999	-5.474	-0.139	6.879
1.431	0.165	0.824	-4.699	0.385	2.699
0.521	0.607	0.999	-5.454	0.148	3.731
0.979	0.337	0.957	-5.155	0.294	6.832
1.101	0.281	0.922	-5.047	0.305	2.911
0.492	0.627	0.999	-5.466	0.123	5.279
-1.312	0.201	0.861	-4.834	-0.299	4.504
0.716	0.481	0.984	-5.348	0.151	4.933
0.487	0.630	0.999	-5.469	0.122	1.849
-0.329	0.745	0.999	-5.526	-0.080	5.666
0.291	0.773	0.999	-5.536	0.098	2.484
0.544	0.591	0.999	-5.443	0.131	8.356
-0.271	0.788	0.999	-5.541	-0.114	4.971
0.861	0.398	0.964	-5.249	0.423	5.072
1.779	0.087	0.732	-4.254	0.708	3.654
-0.157	0.877	0.999	-5.563	-0.046	3.754
-0.393	0.698	0.999	-5.505	-0.100	3.776
-0.321	0.751	0.999	-5.528	-0.096	3.201
-1.167	0.254	0.913	-4.984	-0.585	2.020
1.005	0.324	0.952	-5.133	0.303	2.896
1.652	0.111	0.767	-4.426	0.437	6.059
0.161	0.873	0.999	-5.562	0.080	5.336
0.714	0.482	0.984	-5.349	0.196	3.834
0.392	0.698	0.999	-5.506	0.074	5.917
-3.451	0.002	0.301	-1.353	-0.992	2.596
0.584	0.564	0.997	-5.423	0.173	2.309
-1.148	0.262	0.914	-5.003	-0.397	2.601
-0.778	0.444	0.979	-5.307	-0.225	2.571
-0.136	0.893	0.999	-5.566	-0.032	5.962
0.522	0.606	0.999	-5.453	0.199	4.381
-0.117	0.908	0.999	-5.568	-0.046	5.572
0.448	0.658	0.999	-5.485	0.193	3.689
0.418	0.680	0.999	-5.496	0.104	4.630
0.892	0.381	0.964	-5.225	0.267	2.981
0.421	0.677	0.999	-5.495	0.144	1.307
0.982	0.335	0.957	-5.152	0.244	2.551
0.213	0.833	0.999	-5.554	0.103	2.799
0.080	0.937	0.999	-5.571	0.041	2.629
0.412	0.684	0.999	-5.498	0.112	6.087
-0.057	0.955	0.999	-5.572	-0.016	4.091
-0.951	0.351	0.961	-5.178	-0.421	2.034
0.075	0.941	0.999	-5.571	0.018	1.560
-1.865	0.074	0.699	-4.134	-0.555	2.272
-0.554	0.584	0.999	-5.438	-0.140	2.196
-0.289	0.775	0.999	-5.537	-0.076	1.370
0.421	0.678	0.999	-5.495	0.176	3.030
-0.699	0.491	0.986	-5.358	-0.131	5.839
-0.971	0.341	0.957	-5.161	-0.240	2.336

1.587	0.125	0.786	-4.510	0.459	2.589
-0.351	0.728	0.999	-5.519	-0.070	3.279
-0.364	0.719	0.999	-5.515	-0.074	2.565
-0.351	0.728	0.999	-5.519	-0.149	6.083
0.252	0.803	0.999	-5.546	0.063	6.872
1.001	0.327	0.954	-5.137	0.248	3.440
-2.314	0.029	0.570	-3.437	-1.239	3.147
-2.019	0.054	0.655	-3.905	-0.557	2.892
0.751	0.460	0.983	-5.325	0.211	4.488
-2.893	0.008	0.422	-2.418	-1.509	1.953
-3.386	0.002	0.301	-1.479	-1.116	2.087
-2.103	0.046	0.630	-3.777	-0.551	2.555
-1.717	0.098	0.757	-4.339	-0.543	2.892
-2.070	0.049	0.636	-3.827	-0.677	3.403
-3.196	0.004	0.340	-1.848	-1.241	3.398
-3.604	0.001	0.301	-1.050	-1.052	2.335
0.702	0.489	0.986	-5.357	0.177	8.209
1.037	0.310	0.943	-5.106	0.188	7.252
1.280	0.212	0.870	-4.868	0.338	5.452
1.289	0.209	0.868	-4.859	0.483	8.062
0.901	0.376	0.963	-5.218	0.190	4.942
0.885	0.385	0.964	-5.230	0.197	5.414
-0.285	0.778	0.999	-5.538	-0.062	5.744
0.364	0.719	0.999	-5.515	0.067	4.747
-1.247	0.224	0.886	-4.903	-0.257	4.496
-0.293	0.772	0.999	-5.536	-0.062	3.226
-0.055	0.956	0.999	-5.573	-0.010	8.792
-1.495	0.147	0.804	-4.623	-0.347	3.374
1.412	0.170	0.825	-4.722	0.356	3.096
1.340	0.192	0.851	-4.803	0.342	3.407
-2.303	0.030	0.570	-3.455	-0.556	2.358
2.584	0.016	0.500	-2.977	1.099	2.882
-1.183	0.248	0.907	-4.968	-0.289	2.970
0.165	0.871	0.999	-5.562	0.037	8.966
-0.417	0.681	0.999	-5.497	-0.280	6.843
0.822	0.419	0.972	-5.277	0.191	5.610
-0.213	0.833	0.999	-5.554	-0.057	3.353
-0.439	0.664	0.999	-5.488	-0.140	2.827
-0.959	0.346	0.958	-5.171	-0.304	2.923
-0.718	0.479	0.984	-5.346	-0.144	7.650
-0.322	0.750	0.999	-5.528	-0.101	3.109
0.157	0.877	0.999	-5.563	0.084	4.397
1.213	0.237	0.899	-4.939	0.434	5.746
-0.767	0.450	0.980	-5.315	-0.184	8.065
0.671	0.508	0.988	-5.375	0.222	8.814
2.891	0.008	0.422	-2.423	0.720	6.228
1.545	0.135	0.793	-4.563	0.462	6.029
0.412	0.684	0.999	-5.499	0.091	6.628
0.167	0.869	0.999	-5.562	0.063	7.032
1.048	0.305	0.942	-5.096	0.372	5.346

-0.529	0.601	0.999	-5.450	-0.103	2.789
2.304	0.030	0.570	-3.455	0.567	2.566
1.205	0.239	0.901	-4.946	0.338	3.377
0.497	0.624	0.999	-5.464	0.155	3.655
1.410	0.171	0.826	-4.724	0.338	5.436
1.224	0.232	0.897	-4.927	0.290	5.074
0.822	0.419	0.972	-5.277	0.294	3.191
-0.355	0.726	0.999	-5.518	-0.108	3.484
-0.746	0.463	0.983	-5.329	-0.165	4.654
1.982	0.059	0.663	-3.962	0.571	4.303
0.138	0.891	0.999	-5.565	0.041	3.016
-0.071	0.944	0.999	-5.572	-0.020	5.721
-0.283	0.780	0.999	-5.538	-0.094	3.246
-0.071	0.944	0.999	-5.572	-0.021	2.608
0.299	0.768	0.999	-5.534	0.087	2.482
-0.367	0.716	0.999	-5.514	-0.097	6.305
1.367	0.184	0.841	-4.774	0.261	5.845
1.362	0.185	0.842	-4.779	0.450	2.757
0.529	0.601	0.999	-5.450	0.142	3.068
0.425	0.675	0.999	-5.494	0.130	2.214
-0.210	0.836	0.999	-5.554	-0.093	2.132
1.120	0.273	0.921	-5.029	0.224	2.338
0.590	0.560	0.996	-5.420	0.209	1.960
0.970	0.341	0.957	-5.162	0.285	7.289
0.384	0.705	0.999	-5.508	0.200	3.959
-0.298	0.768	0.999	-5.534	-0.074	7.878
0.115	0.909	0.999	-5.568	0.038	6.512
-1.055	0.301	0.941	-5.089	-0.535	1.975
1.617	0.118	0.779	-4.471	0.533	3.250
0.544	0.591	0.999	-5.443	0.323	5.033
2.560	0.017	0.500	-3.020	0.818	5.878
-1.139	0.265	0.916	-5.011	-0.331	5.068
-0.461	0.649	0.999	-5.480	-0.124	6.735
0.248	0.806	0.999	-5.546	0.067	3.547
-0.623	0.539	0.994	-5.402	-0.352	4.525
2.148	0.042	0.619	-3.707	0.436	6.035
-4.085	0.000	0.270	-0.093	-2.197	1.625
0.510	0.614	0.999	-5.458	0.101	9.755
-0.478	0.637	0.999	-5.473	-0.109	4.569
1.909	0.068	0.684	-4.070	0.425	3.575
0.343	0.734	0.999	-5.521	0.092	4.345
2.144	0.042	0.619	-3.713	0.504	6.127
-1.673	0.107	0.764	-4.398	-0.500	6.277
0.434	0.668	0.999	-5.490	0.125	2.228
0.063	0.950	0.999	-5.572	0.019	9.033
1.676	0.106	0.764	-4.394	0.454	5.875
0.599	0.555	0.995	-5.415	0.176	5.436
-0.231	0.819	0.999	-5.550	-0.058	3.889
1.082	0.289	0.931	-5.064	0.258	5.685
0.317	0.754	0.999	-5.529	0.114	3.566

1.053	0.302	0.941	-5.091	0.291	4.517
-0.075	0.941	0.999	-5.571	-0.016	6.107
0.260	0.797	0.999	-5.544	0.058	5.843
-0.308	0.761	0.999	-5.532	-0.085	2.751
0.383	0.705	0.999	-5.509	0.075	4.400
1.740	0.094	0.753	-4.308	0.597	5.036
0.410	0.685	0.999	-5.499	0.157	3.498
-1.535	0.137	0.795	-4.575	-0.368	6.779
-0.085	0.933	0.999	-5.571	-0.017	5.147
-1.652	0.111	0.767	-4.426	-0.425	4.138
-1.720	0.098	0.757	-4.335	-0.374	2.639
-0.954	0.349	0.960	-5.176	-0.216	5.422
0.069	0.946	0.999	-5.572	0.016	6.437
0.570	0.574	0.999	-5.430	0.135	6.450
-0.382	0.705	0.999	-5.509	-0.077	6.857
1.554	0.133	0.791	-4.551	0.360	3.635
1.810	0.082	0.722	-4.211	0.631	4.828
2.766	0.010	0.466	-2.651	0.843	3.548
1.602	0.122	0.784	-4.491	0.957	4.581
2.545	0.017	0.500	-3.045	0.871	3.734
0.450	0.657	0.999	-5.484	0.139	4.087
1.196	0.243	0.904	-4.955	0.266	6.939
1.855	0.075	0.704	-4.148	0.403	6.895
-0.130	0.898	0.999	-5.566	-0.027	6.502
-1.485	0.150	0.811	-4.636	-0.683	3.066
0.768	0.449	0.979	-5.314	0.222	3.595
0.768	0.450	0.980	-5.314	0.286	4.792
1.105	0.280	0.922	-5.044	0.944	5.902
0.905	0.374	0.963	-5.215	0.302	5.520
2.253	0.033	0.595	-3.538	0.520	4.173
1.966	0.060	0.663	-3.985	0.829	5.067
2.285	0.031	0.585	-3.486	1.018	6.711
-1.052	0.303	0.941	-5.091	-0.300	3.340
3.242	0.003	0.340	-1.759	0.996	4.562
1.100	0.282	0.923	-5.048	0.452	8.575
-1.065	0.297	0.937	-5.080	-0.485	6.496
0.439	0.664	0.999	-5.488	0.121	3.767
-1.870	0.073	0.698	-4.126	-0.441	2.937
1.046	0.305	0.942	-5.097	0.198	9.039
0.540	0.594	0.999	-5.445	0.142	5.448
-2.488	0.020	0.517	-3.145	-0.934	2.357
-2.703	0.012	0.490	-2.765	-1.189	1.857
-3.166	0.004	0.340	-1.905	-0.563	3.355
-0.248	0.806	0.999	-5.546	-0.063	3.716
-0.201	0.842	0.999	-5.556	-0.061	8.817
0.601	0.553	0.994	-5.414	0.122	5.423
-1.500	0.146	0.804	-4.618	-0.486	2.520
-0.893	0.380	0.964	-5.224	-0.249	3.311
-0.410	0.685	0.999	-5.499	-0.120	5.040
0.093	0.927	0.999	-5.570	0.023	5.321

1.652	0.111	0.767	-4.426	0.579	5.443
-2.412	0.024	0.543	-3.274	-0.636	2.946
1.105	0.279	0.922	-5.043	0.457	4.155
0.735	0.469	0.983	-5.336	0.307	3.485
1.147	0.262	0.914	-5.003	0.483	4.162
0.445	0.660	0.999	-5.486	0.120	7.018
-0.584	0.564	0.997	-5.423	-0.132	6.722
-0.310	0.759	0.999	-5.531	-0.108	5.558
2.982	0.006	0.393	-2.253	0.632	3.617
0.747	0.462	0.983	-5.328	0.222	3.255
-1.308	0.203	0.862	-4.839	-0.396	5.315
-2.003	0.056	0.658	-3.930	-0.631	4.702
-2.076	0.048	0.633	-3.819	-0.784	2.636
-0.496	0.624	0.999	-5.465	-0.129	2.006
0.497	0.623	0.999	-5.464	0.113	6.177
-0.197	0.845	0.999	-5.557	-0.051	3.884
0.296	0.770	0.999	-5.535	0.065	5.996
1.153	0.260	0.914	-4.998	0.408	5.472
0.410	0.685	0.999	-5.499	0.105	5.388
-1.251	0.222	0.885	-4.899	-0.416	4.107
0.052	0.959	0.999	-5.573	0.012	3.923
1.427	0.166	0.825	-4.705	0.377	2.946
2.415	0.023	0.543	-3.268	0.436	5.465
0.458	0.651	0.999	-5.481	0.273	3.512
0.944	0.354	0.962	-5.184	0.171	7.396
1.087	0.287	0.929	-5.060	0.380	5.956
-0.336	0.740	0.999	-5.524	-0.074	2.656
1.449	0.160	0.820	-4.678	0.496	5.099
-1.786	0.086	0.731	-4.245	-0.532	4.484
-1.005	0.324	0.952	-5.133	-0.571	4.765
-0.416	0.681	0.999	-5.497	-0.201	3.465
1.111	0.277	0.921	-5.037	0.254	8.561
2.714	0.012	0.488	-2.746	0.801	3.326
2.151	0.041	0.619	-3.702	0.603	4.336
1.519	0.141	0.801	-4.595	0.309	3.086
0.906	0.374	0.963	-5.214	0.333	4.373
1.194	0.244	0.904	-4.957	0.301	5.061
0.727	0.474	0.984	-5.341	0.193	8.564
0.755	0.457	0.981	-5.323	0.247	7.766
2.670	0.013	0.498	-2.825	0.715	3.724
0.507	0.617	0.999	-5.460	0.248	6.334
0.077	0.939	0.999	-5.571	0.018	3.348
-0.052	0.959	0.999	-5.573	-0.022	7.583
1.001	0.326	0.954	-5.137	0.260	7.378
-0.497	0.623	0.999	-5.464	-0.229	5.196
0.752	0.459	0.982	-5.324	0.204	7.131
1.752	0.092	0.746	-4.292	0.361	5.573
-1.564	0.130	0.790	-4.539	-0.423	2.886
0.298	0.768	0.999	-5.534	0.063	7.137
-0.221	0.827	0.999	-5.552	-0.071	3.322

1.287	0.210	0.868	-4.861	0.328	6.682
0.635	0.531	0.992	-5.396	0.157	3.690
-0.123	0.903	0.999	-5.567	-0.032	3.942
0.065	0.948	0.999	-5.572	0.030	6.543
0.307	0.762	0.999	-5.532	0.084	3.541
-0.270	0.790	0.999	-5.542	-0.055	5.687
-0.730	0.472	0.984	-5.339	-0.173	3.866
0.197	0.846	0.999	-5.557	0.055	4.233
2.254	0.033	0.595	-3.536	0.887	7.105
1.677	0.106	0.764	-4.393	0.368	5.170
1.465	0.155	0.817	-4.660	0.416	8.565
2.017	0.054	0.655	-3.908	0.784	2.690
1.957	0.062	0.666	-3.999	0.808	2.928
-0.562	0.579	0.999	-5.434	-0.161	2.195
0.790	0.437	0.973	-5.299	0.402	3.578
-0.395	0.696	0.999	-5.504	-0.153	2.501
0.771	0.448	0.979	-5.312	0.512	3.078
0.334	0.741	0.999	-5.524	0.087	7.293
2.427	0.023	0.542	-3.247	0.630	6.589
-1.374	0.182	0.840	-4.766	-0.555	4.799
-0.856	0.400	0.964	-5.252	-0.240	4.996
0.389	0.700	0.999	-5.507	0.092	5.678
1.030	0.313	0.944	-5.112	0.246	2.674
-1.083	0.289	0.931	-5.063	-0.281	3.935
-1.250	0.223	0.886	-4.900	-0.339	3.289
-0.298	0.768	0.999	-5.534	-0.108	6.095
-0.743	0.464	0.983	-5.331	-0.238	3.878
0.293	0.772	0.999	-5.536	0.110	10.180
-0.142	0.888	0.999	-5.565	-0.080	6.229
0.777	0.444	0.979	-5.308	0.205	7.666
1.230	0.230	0.894	-4.921	0.340	5.797
0.182	0.857	0.999	-5.559	0.091	6.795
1.131	0.269	0.918	-5.019	0.244	4.084
-1.268	0.216	0.876	-4.881	-0.338	4.684
-0.458	0.651	0.999	-5.481	-0.231	3.477
-0.663	0.514	0.990	-5.380	-0.163	2.961
1.199	0.242	0.904	-4.952	0.331	3.961
1.013	0.321	0.950	-5.126	0.246	4.137
1.280	0.212	0.870	-4.869	0.297	4.037
1.228	0.231	0.894	-4.923	0.497	5.583
-1.508	0.144	0.803	-4.608	-0.512	1.976
-0.098	0.922	0.999	-5.570	-0.029	3.468
-0.459	0.650	0.999	-5.480	-0.134	6.670
-0.586	0.563	0.997	-5.422	-0.137	1.800
1.735	0.095	0.754	-4.315	0.396	3.764
-0.539	0.595	0.999	-5.445	-0.142	3.970
-0.054	0.957	0.999	-5.573	-0.028	4.776
0.771	0.448	0.979	-5.312	0.249	5.520
-0.794	0.434	0.973	-5.296	-0.249	1.325
0.467	0.644	0.999	-5.477	0.186	4.266

2.327	0.028	0.566	-3.416	0.543	3.875
-0.188	0.852	0.999	-5.558	-0.096	4.085
0.211	0.835	0.999	-5.554	0.051	6.880
0.820	0.420	0.972	-5.278	0.204	4.230
-0.536	0.597	0.999	-5.446	-0.255	2.690
-0.914	0.369	0.963	-5.208	-0.264	3.472
-0.977	0.338	0.957	-5.157	-0.259	2.975
1.414	0.170	0.825	-4.720	0.330	6.629
-1.730	0.096	0.754	-4.321	-0.401	3.714
1.715	0.099	0.757	-4.341	0.424	3.870
0.646	0.524	0.991	-5.389	0.158	6.430
0.628	0.536	0.993	-5.400	0.176	5.747
1.691	0.103	0.764	-4.374	0.558	3.067
-0.430	0.671	0.999	-5.492	-0.094	5.568
0.608	0.548	0.994	-5.410	0.148	8.688
-0.408	0.686	0.999	-5.500	-0.178	5.769
0.492	0.627	0.999	-5.466	0.111	6.725
0.100	0.921	0.999	-5.569	0.038	4.119
0.203	0.841	0.999	-5.556	0.053	2.176
0.687	0.498	0.987	-5.365	0.165	4.180
0.909	0.372	0.963	-5.212	0.261	4.291
0.273	0.787	0.999	-5.541	0.140	3.085
0.825	0.417	0.970	-5.275	0.165	6.918
-0.178	0.860	0.999	-5.560	-0.034	3.952
0.533	0.599	0.999	-5.448	0.144	5.752
1.397	0.175	0.828	-4.739	0.384	5.622
-2.344	0.027	0.559	-3.388	-0.731	2.517
-0.432	0.670	0.999	-5.491	-0.139	3.505
0.481	0.635	0.999	-5.471	0.152	3.508
2.167	0.040	0.616	-3.677	0.474	3.821
0.226	0.823	0.999	-5.551	0.056	7.578
3.751	0.001	0.301	-0.759	0.843	7.210
0.359	0.722	0.999	-5.516	0.153	4.348
2.284	0.031	0.585	-3.487	0.729	4.824
0.612	0.546	0.994	-5.408	0.158	4.781
1.135	0.267	0.917	-5.015	0.260	5.458
1.728	0.096	0.756	-4.325	0.332	5.700
0.376	0.710	0.999	-5.511	0.102	5.907
0.230	0.820	0.999	-5.550	0.051	6.008
0.677	0.505	0.988	-5.371	0.158	4.263
-0.633	0.532	0.992	-5.396	-0.181	3.732
-0.057	0.955	0.999	-5.573	-0.015	5.532
-1.609	0.120	0.784	-4.482	-0.429	4.432
1.784	0.087	0.731	-4.248	0.515	5.429
1.606	0.121	0.784	-4.486	0.843	6.554
1.475	0.153	0.813	-4.648	0.374	4.447
-0.613	0.546	0.994	-5.408	-0.268	2.885
3.017	0.006	0.393	-2.187	1.094	8.840
0.128	0.899	0.999	-5.567	0.056	5.096
-0.910	0.372	0.963	-5.211	-0.244	2.009

2.119	0.044	0.630	-3.751	0.614	7.987
0.283	0.780	0.999	-5.538	0.054	7.450
0.341	0.736	0.999	-5.522	0.161	7.969
-0.852	0.402	0.967	-5.255	-0.215	5.164
-0.378	0.708	0.999	-5.510	-0.163	6.143
-0.837	0.410	0.970	-5.266	-0.253	4.014
0.615	0.544	0.994	-5.407	0.170	5.245
0.308	0.761	0.999	-5.532	0.072	4.882
-1.184	0.247	0.907	-4.967	-0.353	1.976
0.845	0.406	0.969	-5.260	0.184	4.502
0.258	0.799	0.999	-5.544	0.087	7.189
-0.495	0.625	0.999	-5.465	-0.095	6.809
-2.931	0.007	0.411	-2.348	-0.768	4.981
1.964	0.061	0.663	-3.989	0.609	6.099
-0.929	0.362	0.963	-5.196	-0.265	4.932
-0.569	0.574	0.999	-5.430	-0.141	2.453
0.465	0.646	0.999	-5.478	0.172	6.164
-0.369	0.715	0.999	-5.513	-0.099	4.415
-0.357	0.724	0.999	-5.517	-0.109	3.213
0.911	0.371	0.963	-5.210	0.209	4.100
1.205	0.240	0.901	-4.947	0.256	6.016
0.456	0.652	0.999	-5.481	0.103	5.828
0.121	0.904	0.999	-5.567	0.030	6.894
-0.204	0.840	0.999	-5.555	-0.052	3.093
0.928	0.362	0.963	-5.197	0.288	4.316
0.859	0.398	0.964	-5.250	0.217	5.118
0.769	0.449	0.979	-5.313	0.228	4.952
1.441	0.162	0.822	-4.688	0.474	5.945
-0.423	0.676	0.999	-5.494	-0.095	6.597
-0.671	0.508	0.988	-5.375	-0.153	4.001
-0.890	0.382	0.964	-5.227	-0.253	3.670
-1.758	0.091	0.743	-4.283	-0.482	4.427
-0.151	0.881	0.999	-5.564	-0.033	5.162
1.581	0.126	0.786	-4.517	0.415	4.174
-0.393	0.697	0.999	-5.505	-0.171	2.965
-1.562	0.131	0.791	-4.542	-0.407	2.175
0.698	0.491	0.986	-5.359	0.252	5.031
-0.287	0.777	0.999	-5.537	-0.103	4.548
0.916	0.368	0.963	-5.206	0.185	5.840
-0.347	0.731	0.999	-5.520	-0.130	3.466
-0.848	0.404	0.968	-5.258	-0.243	2.036
1.937	0.064	0.673	-4.029	0.634	3.003
0.413	0.683	0.999	-5.498	0.223	4.254
1.239	0.227	0.888	-4.911	0.246	6.859
1.145	0.263	0.914	-5.006	0.386	5.179
0.528	0.602	0.999	-5.450	0.121	8.596
1.234	0.229	0.890	-4.916	0.365	3.344
0.541	0.594	0.999	-5.444	0.128	5.842
0.910	0.371	0.963	-5.211	0.199	3.280
-1.937	0.064	0.673	-4.028	-0.559	2.977

0.681	0.502	0.988	-5.369	0.148	6.006
-1.492	0.148	0.805	-4.627	-0.417	2.694
-1.469	0.154	0.815	-4.655	-0.377	3.407
-1.008	0.323	0.950	-5.130	-0.216	4.529
0.748	0.462	0.983	-5.328	0.201	3.891
0.623	0.539	0.994	-5.402	0.238	8.238
1.071	0.294	0.935	-5.074	0.293	3.128
1.285	0.211	0.868	-4.863	0.313	5.415
0.871	0.392	0.964	-5.241	0.224	4.862
1.818	0.081	0.719	-4.200	0.433	5.335
-0.600	0.554	0.995	-5.415	-0.197	1.589
-0.318	0.753	0.999	-5.529	-0.095	1.788
0.706	0.487	0.985	-5.354	0.210	3.601
1.904	0.068	0.684	-4.077	0.356	4.216
0.704	0.488	0.985	-5.355	0.149	4.784
2.556	0.017	0.500	-3.026	0.697	4.069
0.285	0.778	0.999	-5.538	0.061	6.379
0.818	0.421	0.972	-5.280	0.251	6.221
-0.368	0.716	0.999	-5.514	-0.141	2.876
0.793	0.435	0.973	-5.297	0.189	4.533
-0.861	0.397	0.964	-5.248	-0.191	4.082
-3.427	0.002	0.301	-1.399	-1.968	1.810
1.102	0.281	0.922	-5.046	0.236	5.710
-0.083	0.935	0.999	-5.571	-0.020	4.449
0.232	0.818	0.999	-5.550	0.055	5.539
1.308	0.203	0.862	-4.839	0.356	4.274
-0.483	0.633	0.999	-5.470	-0.123	2.270
1.196	0.243	0.904	-4.956	0.379	4.797
0.912	0.371	0.963	-5.210	0.323	2.886
1.110	0.278	0.922	-5.039	0.284	3.011
-1.886	0.071	0.694	-4.103	-0.651	3.541
0.085	0.933	0.999	-5.571	0.024	4.223
-0.610	0.547	0.994	-5.409	-0.253	2.305
0.468	0.644	0.999	-5.477	0.170	3.096
0.995	0.329	0.955	-5.142	0.293	3.498
1.452	0.159	0.820	-4.675	0.364	3.050
-0.230	0.820	0.999	-5.550	-0.085	5.045
-1.109	0.278	0.922	-5.040	-0.329	3.944
0.878	0.388	0.964	-5.236	0.185	3.787
1.069	0.295	0.936	-5.076	0.269	2.480
-0.559	0.581	0.999	-5.435	-0.212	5.052
1.583	0.126	0.786	-4.515	0.449	6.655
-1.389	0.177	0.835	-4.748	-0.461	4.388
-0.055	0.956	0.999	-5.573	-0.018	6.092
0.469	0.643	0.999	-5.476	0.161	4.997
-0.982	0.336	0.957	-5.153	-0.538	3.251
-0.160	0.874	0.999	-5.563	-0.056	3.977
1.074	0.293	0.934	-5.072	0.378	3.551
1.090	0.286	0.929	-5.057	0.302	5.490
1.119	0.274	0.921	-5.031	0.318	3.962

1.725	0.097	0.757	-4.328	0.455	4.476
0.603	0.552	0.994	-5.413	0.213	3.362
-0.378	0.709	0.999	-5.511	-0.126	3.609
-1.515	0.142	0.802	-4.600	-0.439	2.255
-0.153	0.880	0.999	-5.564	-0.061	4.289
-1.026	0.315	0.945	-5.115	-0.267	4.650
1.665	0.108	0.766	-4.409	0.411	4.396
2.145	0.042	0.619	-3.712	0.538	2.909
0.980	0.336	0.957	-5.154	0.266	2.913
-0.168	0.868	0.999	-5.561	-0.043	4.411
-2.396	0.024	0.543	-3.301	-0.667	3.285
-0.981	0.336	0.957	-5.154	-0.399	2.461
0.864	0.396	0.964	-5.246	0.171	5.261
0.504	0.619	0.999	-5.461	0.120	6.638
1.515	0.142	0.802	-4.599	0.493	2.700
-0.692	0.495	0.987	-5.362	-0.172	4.043
-0.314	0.756	0.999	-5.530	-0.084	3.004
-1.280	0.212	0.870	-4.869	-0.354	3.014
1.462	0.156	0.817	-4.664	0.407	2.802
-0.057	0.955	0.999	-5.572	-0.015	5.460
-0.970	0.341	0.957	-5.162	-0.243	2.252
-1.793	0.085	0.728	-4.235	-0.497	2.799
0.735	0.469	0.983	-5.336	0.188	2.889
0.484	0.633	0.999	-5.470	0.165	3.887
1.205	0.239	0.901	-4.946	0.391	4.106
-1.065	0.297	0.937	-5.080	-0.279	4.449
0.405	0.689	0.999	-5.501	0.105	4.363
0.858	0.399	0.964	-5.250	0.196	6.851
2.619	0.015	0.498	-2.916	0.977	5.584
0.220	0.828	0.999	-5.552	0.039	4.766
1.894	0.070	0.689	-4.092	0.410	4.745
0.242	0.811	0.999	-5.548	0.081	5.686
3.206	0.004	0.340	-1.828	0.828	6.115
0.604	0.551	0.994	-5.413	0.177	1.665
-0.204	0.840	0.999	-5.555	-0.091	4.261
0.365	0.718	0.999	-5.515	0.127	5.312
-1.292	0.208	0.868	-4.856	-0.502	4.196
-0.613	0.545	0.994	-5.408	-0.142	5.382
1.796	0.085	0.726	-4.231	0.446	4.774
-1.004	0.325	0.952	-5.134	-0.224	5.632
0.884	0.385	0.964	-5.231	0.253	2.272
3.126	0.004	0.353	-1.981	0.925	4.373
-0.267	0.792	0.999	-5.542	-0.054	5.752
-0.247	0.807	0.999	-5.547	-0.057	2.682
-0.319	0.752	0.999	-5.529	-0.177	5.429
0.851	0.403	0.967	-5.256	0.190	6.159
0.169	0.867	0.999	-5.561	0.049	3.109
0.718	0.479	0.984	-5.346	0.218	3.797
-0.639	0.529	0.992	-5.393	-0.167	4.590
0.704	0.488	0.985	-5.355	0.136	6.312

-0.139	0.891	0.999	-5.565	-0.030	6.700
0.144	0.887	0.999	-5.565	0.043	3.360
0.348	0.731	0.999	-5.520	0.105	4.476
-0.620	0.541	0.994	-5.404	-0.137	6.043
-0.992	0.331	0.956	-5.144	-0.242	3.982
1.161	0.256	0.913	-4.989	0.219	5.822
0.947	0.352	0.961	-5.181	0.189	5.501
-0.534	0.598	0.999	-5.448	-0.124	5.828
-0.392	0.698	0.999	-5.505	-0.105	8.115
1.113	0.276	0.921	-5.035	0.310	4.885
0.235	0.816	0.999	-5.549	0.076	4.144
0.297	0.769	0.999	-5.535	0.107	1.817
-0.843	0.407	0.969	-5.261	-0.324	3.620
-0.334	0.741	0.999	-5.524	-0.080	4.478
2.845	0.009	0.441	-2.507	0.616	4.732
2.094	0.046	0.630	-3.790	0.497	3.909
0.365	0.718	0.999	-5.515	0.084	8.056
0.922	0.365	0.963	-5.201	0.211	4.902
0.456	0.652	0.999	-5.481	0.174	6.023
0.712	0.483	0.985	-5.350	0.210	2.605
1.212	0.237	0.899	-4.939	0.431	3.736
2.100	0.046	0.630	-3.782	1.180	8.592
1.473	0.153	0.813	-4.650	0.877	4.603
3.393	0.002	0.301	-1.467	1.774	6.008
2.054	0.051	0.641	-3.852	0.607	4.246
2.348	0.027	0.559	-3.381	0.866	4.929
3.840	0.001	0.301	-0.582	1.524	3.503
0.889	0.382	0.964	-5.227	0.188	5.085
1.394	0.176	0.831	-4.743	0.382	3.210
1.321	0.199	0.857	-4.825	0.259	7.070
-1.206	0.239	0.901	-4.945	-0.395	4.271
2.417	0.023	0.543	-3.265	0.761	4.971
-0.164	0.871	0.999	-5.562	-0.085	2.206
-1.002	0.326	0.954	-5.136	-0.350	2.260
0.947	0.353	0.961	-5.181	0.408	2.499
1.970	0.060	0.663	-3.980	0.479	4.174
2.335	0.028	0.563	-3.402	0.666	3.497
-1.045	0.306	0.942	-5.098	-0.242	3.050
-0.585	0.564	0.997	-5.422	-0.150	5.152
-0.099	0.922	0.999	-5.570	-0.029	4.507
0.218	0.829	0.999	-5.553	0.069	3.399
-1.155	0.259	0.913	-4.996	-0.331	5.129
-0.239	0.813	0.999	-5.549	-0.115	3.238
0.664	0.513	0.990	-5.379	0.249	3.193
1.420	0.168	0.825	-4.713	0.661	5.025
1.059	0.300	0.941	-5.086	0.330	3.920
-1.574	0.128	0.789	-4.526	-0.551	3.658
-0.111	0.912	0.999	-5.568	-0.031	2.880
0.068	0.946	0.999	-5.572	0.016	2.545
-0.604	0.551	0.994	-5.412	-0.153	2.175

1.739	0.094	0.753	-4.309	0.632	2.940
-0.977	0.338	0.957	-5.157	-0.461	2.974
1.042	0.307	0.942	-5.101	0.364	2.923
-1.461	0.156	0.817	-4.665	-0.251	4.231
1.501	0.146	0.804	-4.617	0.473	5.504
0.089	0.929	0.999	-5.570	0.028	7.535
-1.447	0.160	0.820	-4.681	-0.455	6.635
2.222	0.036	0.600	-3.588	0.786	7.583
2.528	0.018	0.501	-3.074	0.543	4.090
0.794	0.434	0.973	-5.296	0.170	5.826
0.227	0.823	0.999	-5.551	0.063	2.420
1.310	0.202	0.862	-4.836	0.431	3.255
1.281	0.212	0.870	-4.867	0.320	4.951
-0.251	0.804	0.999	-5.546	-0.094	4.474
-0.712	0.483	0.985	-5.350	-0.237	5.632
1.143	0.264	0.915	-5.008	0.246	6.291
1.910	0.068	0.683	-4.069	0.357	3.544
1.586	0.125	0.786	-4.511	0.332	5.957
0.391	0.699	0.999	-5.506	0.093	3.524
0.225	0.824	0.999	-5.551	0.059	3.758
1.588	0.125	0.786	-4.508	0.344	2.858
0.568	0.575	0.999	-5.431	0.158	5.529
1.057	0.301	0.941	-5.088	0.219	5.458
1.086	0.288	0.929	-5.061	0.353	2.486
0.171	0.865	0.999	-5.561	0.047	6.530
0.220	0.828	0.999	-5.552	0.071	5.228
0.490	0.629	0.999	-5.468	0.122	4.140
-0.898	0.378	0.963	-5.220	-0.201	9.411
0.876	0.389	0.964	-5.237	0.225	5.086
0.591	0.560	0.996	-5.419	0.161	3.599
1.854	0.076	0.704	-4.149	0.461	4.302
0.322	0.750	0.999	-5.528	0.062	7.310
4.251	0.000	0.256	0.241	1.082	3.086
0.066	0.948	0.999	-5.572	0.020	4.327
1.134	0.268	0.917	-5.016	0.348	3.436
-0.553	0.585	0.999	-5.439	-0.205	3.365
0.205	0.839	0.999	-5.555	0.061	3.865
-1.481	0.151	0.813	-4.641	-0.371	2.359
1.845	0.077	0.707	-4.162	0.383	2.129
-0.633	0.533	0.992	-5.397	-0.169	2.184
-1.133	0.268	0.917	-5.017	-0.315	1.786
-1.686	0.104	0.764	-4.381	-0.381	2.750
0.456	0.652	0.999	-5.482	0.106	1.947
-1.082	0.290	0.931	-5.065	-0.264	2.357
-0.730	0.472	0.984	-5.339	-0.182	2.967
0.611	0.547	0.994	-5.409	0.232	3.661
0.196	0.846	0.999	-5.557	0.081	2.961
-1.564	0.130	0.790	-4.539	-0.411	3.326
-1.028	0.314	0.944	-5.113	-0.263	3.992
-0.389	0.701	0.999	-5.507	-0.108	3.000

-0.454	0.654	0.999	-5.482	-0.108	2.485
0.829	0.415	0.970	-5.272	0.203	2.734
0.087	0.931	0.999	-5.571	0.021	6.914
-0.606	0.550	0.994	-5.412	-0.145	6.805
-0.449	0.657	0.999	-5.484	-0.157	7.888
-0.842	0.408	0.969	-5.262	-0.323	4.000
1.495	0.147	0.805	-4.624	0.545	3.624
0.826	0.417	0.970	-5.274	0.215	6.527
-1.324	0.197	0.855	-4.821	-0.402	3.050
1.671	0.107	0.764	-4.400	0.358	4.754
0.104	0.918	0.999	-5.569	0.031	4.800
0.103	0.919	0.999	-5.569	0.028	9.990
-0.349	0.730	0.999	-5.520	-0.090	5.944
0.322	0.750	0.999	-5.528	0.092	3.476
0.845	0.406	0.969	-5.260	0.299	3.582
0.399	0.693	0.999	-5.503	0.153	5.391
1.678	0.106	0.764	-4.391	0.474	8.294
0.130	0.897	0.999	-5.566	0.036	8.361
0.843	0.407	0.969	-5.262	0.180	7.560
2.147	0.042	0.619	-3.707	0.432	7.162
0.370	0.715	0.999	-5.513	0.136	6.295
0.409	0.686	0.999	-5.499	0.124	3.783
0.950	0.351	0.961	-5.179	0.393	4.330
2.418	0.023	0.543	-3.264	0.492	6.050
0.327	0.747	0.999	-5.526	0.073	6.807
-0.466	0.645	0.999	-5.477	-0.113	6.037
0.222	0.826	0.999	-5.552	0.122	6.735
0.428	0.672	0.999	-5.492	0.115	2.840
0.257	0.799	0.999	-5.545	0.074	2.562
0.945	0.354	0.961	-5.183	0.285	3.455
0.404	0.690	0.999	-5.502	0.152	8.185
0.920	0.367	0.963	-5.203	0.305	3.804
-0.109	0.914	0.999	-5.569	-0.033	2.591
1.520	0.141	0.801	-4.593	0.527	6.468
1.504	0.145	0.803	-4.613	0.359	2.666
0.101	0.921	0.999	-5.569	0.030	5.968
-0.651	0.521	0.990	-5.387	-0.224	2.896
-0.272	0.788	0.999	-5.541	-0.115	3.826
-0.359	0.722	0.999	-5.516	-0.115	2.925
0.576	0.570	0.997	-5.427	0.180	2.227
0.812	0.424	0.972	-5.284	0.201	3.602
-0.930	0.361	0.963	-5.195	-0.258	4.376
0.648	0.523	0.990	-5.388	0.177	4.453
-0.444	0.661	0.999	-5.486	-0.344	1.154
-4.388	0.000	0.256	0.515	-1.388	3.124
0.775	0.445	0.979	-5.309	0.201	6.851
-0.759	0.455	0.981	-5.320	-0.203	5.655
-1.432	0.165	0.824	-4.699	-0.411	3.206
-2.364	0.026	0.556	-3.355	-0.676	2.474
1.581	0.126	0.786	-4.517	0.398	3.395

2.166	0.040	0.616	-3.678	1.163	6.495
4.360	0.000	0.256	0.458	2.061	5.507
0.398	0.694	0.999	-5.503	0.149	2.830
0.244	0.810	0.999	-5.547	0.064	4.294
0.140	0.890	0.999	-5.565	0.042	5.271
0.605	0.551	0.994	-5.412	0.178	2.479
-0.412	0.684	0.999	-5.499	-0.238	1.645
-1.214	0.236	0.899	-4.937	-0.322	3.259
-0.529	0.601	0.999	-5.450	-0.148	2.974
0.362	0.720	0.999	-5.515	0.134	5.257
0.780	0.443	0.979	-5.306	0.202	3.122
-0.334	0.741	0.999	-5.524	-0.115	2.618
-0.114	0.910	0.999	-5.568	-0.030	2.221
-1.590	0.124	0.786	-4.506	-0.633	4.386
-1.715	0.099	0.757	-4.342	-0.698	3.690
1.583	0.126	0.786	-4.515	0.438	5.437
0.772	0.447	0.979	-5.311	0.581	3.380
0.418	0.679	0.999	-5.496	0.173	5.988
-0.091	0.928	0.999	-5.570	-0.016	3.536
1.116	0.275	0.921	-5.033	0.283	3.060
0.842	0.408	0.969	-5.262	0.223	6.656
1.351	0.189	0.847	-4.791	0.371	2.890
-0.091	0.928	0.999	-5.570	-0.025	7.704
0.627	0.536	0.993	-5.400	0.295	6.109
-0.613	0.545	0.994	-5.408	-0.177	4.842
1.742	0.094	0.753	-4.306	0.336	5.630
-1.068	0.296	0.936	-5.078	-0.274	4.692
-0.967	0.343	0.957	-5.165	-0.397	2.761
-0.442	0.663	0.999	-5.487	-0.119	5.084
0.693	0.495	0.987	-5.362	0.135	11.382
2.112	0.045	0.630	-3.763	0.593	5.653
1.059	0.300	0.941	-5.086	0.260	5.631
2.257	0.033	0.594	-3.531	0.529	3.243
0.457	0.651	0.999	-5.481	0.108	2.071
-0.483	0.633	0.999	-5.470	-0.111	5.058
1.142	0.264	0.916	-5.009	0.278	3.913
-1.135	0.267	0.917	-5.015	-0.227	7.280
-0.342	0.736	0.999	-5.522	-0.099	2.989
1.696	0.102	0.764	-4.368	0.435	5.891
-0.797	0.433	0.973	-5.294	-0.284	4.332
1.968	0.060	0.663	-3.983	0.754	6.663
0.635	0.531	0.992	-5.395	0.164	2.716
-0.083	0.935	0.999	-5.571	-0.025	6.257
0.229	0.821	0.999	-5.551	0.064	4.273
0.256	0.800	0.999	-5.545	0.061	3.756
-0.379	0.708	0.999	-5.510	-0.136	4.347
1.247	0.224	0.886	-4.903	0.472	5.462
0.738	0.467	0.983	-5.334	0.173	4.961
0.376	0.710	0.999	-5.511	0.073	6.253
1.132	0.268	0.917	-5.018	0.250	8.274

2.197	0.037	0.611	-3.628	0.377	6.678
0.174	0.863	0.999	-5.560	0.049	5.385
2.623	0.015	0.498	-2.908	0.710	4.404
-0.061	0.951	0.999	-5.572	-0.015	6.163
-0.553	0.585	0.999	-5.439	-0.135	7.991
-0.488	0.630	0.999	-5.468	-0.116	7.548
-0.438	0.665	0.999	-5.489	-0.104	7.603
-0.430	0.671	0.999	-5.492	-0.103	7.434
-0.418	0.679	0.999	-5.496	-0.098	7.591
-0.367	0.717	0.999	-5.514	-0.089	7.015
0.303	0.765	0.999	-5.533	0.209	6.099
-0.847	0.405	0.968	-5.259	-0.184	5.583
-0.263	0.795	0.999	-5.543	-0.196	2.694
1.290	0.209	0.868	-4.858	0.483	3.265
0.718	0.479	0.984	-5.346	0.342	5.312
0.580	0.567	0.997	-5.425	0.396	3.219
0.099	0.922	0.999	-5.570	0.059	2.799
0.279	0.782	0.999	-5.539	0.082	5.269
0.166	0.869	0.999	-5.562	0.121	2.584
1.164	0.255	0.913	-4.987	0.350	3.323
-0.263	0.795	0.999	-5.543	-0.162	2.372
0.775	0.445	0.979	-5.309	0.232	3.685
0.595	0.557	0.995	-5.417	0.169	7.438
-0.313	0.757	0.999	-5.530	-0.238	5.370
1.210	0.238	0.900	-4.941	0.391	2.233
-0.149	0.883	0.999	-5.564	-0.084	4.509
0.380	0.707	0.999	-5.510	0.168	3.531
0.242	0.810	0.999	-5.548	0.151	4.114
1.906	0.068	0.684	-4.074	1.047	8.603
1.256	0.221	0.882	-4.894	0.464	9.097
-1.333	0.194	0.853	-4.811	-0.719	4.428
-1.183	0.248	0.907	-4.968	-0.386	2.506
-0.907	0.373	0.963	-5.214	-0.279	2.941
-1.820	0.081	0.718	-4.197	-0.398	3.293
0.229	0.820	0.999	-5.550	0.061	2.848
0.733	0.470	0.983	-5.337	0.190	3.679
0.568	0.575	0.999	-5.431	0.135	5.506
0.530	0.601	0.999	-5.449	0.137	1.845
0.770	0.448	0.979	-5.312	0.328	8.790
1.072	0.294	0.935	-5.074	0.339	4.777
0.533	0.599	0.999	-5.448	0.143	4.792
3.112	0.005	0.354	-2.007	0.780	4.775
0.070	0.945	0.999	-5.572	0.024	4.750
0.310	0.759	0.999	-5.531	0.077	5.343
-1.371	0.183	0.840	-4.769	-0.598	3.867
0.405	0.689	0.999	-5.501	0.152	1.932
-2.174	0.039	0.612	-3.664	-0.536	1.958
0.333	0.742	0.999	-5.525	0.122	3.648
0.163	0.872	0.999	-5.562	0.072	6.645
1.590	0.124	0.786	-4.506	0.668	4.350

-1.599	0.122	0.784	-4.495	-0.543	4.861
0.362	0.721	0.999	-5.516	0.065	5.090
0.797	0.433	0.973	-5.294	0.207	6.124
2.313	0.029	0.570	-3.439	0.784	3.782
1.704	0.101	0.763	-4.357	0.398	4.954
0.304	0.764	0.999	-5.533	0.091	2.412
0.680	0.503	0.988	-5.370	0.166	2.511
0.150	0.882	0.999	-5.564	0.048	2.455
-0.366	0.718	0.999	-5.514	-0.082	7.313
0.487	0.630	0.999	-5.468	0.097	5.748
0.349	0.730	0.999	-5.520	0.090	5.299
2.090	0.047	0.630	-3.796	0.743	5.297
1.658	0.110	0.767	-4.418	0.901	5.334
1.623	0.117	0.776	-4.464	0.756	6.655
3.277	0.003	0.340	-1.692	1.087	5.664
-0.098	0.922	0.999	-5.570	-0.023	3.179
1.994	0.057	0.659	-3.944	0.592	5.937
0.435	0.667	0.999	-5.490	0.081	7.096
1.691	0.103	0.764	-4.374	0.415	5.318
0.377	0.709	0.999	-5.511	0.089	2.653
-0.407	0.687	0.999	-5.500	-0.179	1.782
-0.227	0.822	0.999	-5.551	-0.047	2.805
-0.199	0.844	0.999	-5.556	-0.172	2.701
-0.534	0.598	0.999	-5.447	-0.134	5.886
2.098	0.046	0.630	-3.784	0.480	6.124
-0.079	0.938	0.999	-5.571	-0.016	7.620
0.199	0.844	0.999	-5.556	0.045	3.989
-1.457	0.157	0.817	-4.669	-0.362	5.964
0.887	0.383	0.964	-5.229	0.291	3.709
0.321	0.751	0.999	-5.528	0.101	2.473
-0.683	0.501	0.988	-5.368	-0.172	5.449
2.598	0.015	0.500	-2.951	0.585	5.535
-1.758	0.091	0.743	-4.283	-0.484	2.680
1.518	0.141	0.802	-4.595	0.442	4.101
-0.594	0.558	0.995	-5.418	-0.151	3.334
-0.522	0.606	0.999	-5.453	-0.129	5.150
0.284	0.778	0.999	-5.538	0.068	2.134
-0.688	0.498	0.987	-5.365	-0.183	3.033
0.626	0.537	0.993	-5.401	0.122	5.172
-0.524	0.605	0.999	-5.452	-0.194	3.069
0.693	0.495	0.987	-5.362	0.203	2.310
-0.394	0.697	0.999	-5.505	-0.124	3.511
0.173	0.864	0.999	-5.561	0.056	6.035
-0.364	0.719	0.999	-5.515	-0.102	4.854
1.415	0.169	0.825	-4.719	0.687	3.346
0.339	0.737	0.999	-5.523	0.084	4.575
0.487	0.630	0.999	-5.469	0.096	2.883
0.841	0.408	0.970	-5.263	0.240	2.761
1.042	0.307	0.942	-5.101	0.404	5.312
1.280	0.212	0.870	-4.868	0.367	2.615

0.253	0.802	0.999	-5.545	0.054	8.099
1.074	0.293	0.934	-5.072	0.268	2.988
-0.152	0.880	0.999	-5.564	-0.033	2.654
1.465	0.155	0.817	-4.660	0.395	5.350
0.734	0.470	0.983	-5.336	0.195	3.894
0.956	0.348	0.959	-5.174	0.303	4.976
0.256	0.800	0.999	-5.545	0.080	4.277
0.270	0.789	0.999	-5.541	0.065	4.398
0.795	0.434	0.973	-5.296	0.265	3.379
-0.583	0.565	0.997	-5.423	-0.136	6.280
0.095	0.925	0.999	-5.570	0.030	9.200
2.013	0.055	0.656	-3.916	0.573	3.137
-0.152	0.880	0.999	-5.564	-0.067	4.311
-1.193	0.244	0.904	-4.959	-0.550	3.444
-1.837	0.078	0.710	-4.173	-1.048	3.719
1.072	0.294	0.935	-5.074	0.321	3.005
-0.156	0.877	0.999	-5.563	-0.052	5.572
1.026	0.315	0.945	-5.115	0.435	3.343
-1.484	0.150	0.811	-4.637	-0.434	2.705
-0.283	0.779	0.999	-5.538	-0.076	5.566
3.391	0.002	0.301	-1.471	1.004	4.623
2.982	0.006	0.393	-2.252	0.882	5.730
-0.472	0.641	0.999	-5.475	-0.126	3.521
0.227	0.822	0.999	-5.551	0.065	1.767
0.058	0.954	0.999	-5.572	0.011	2.302
1.313	0.201	0.860	-4.833	0.394	4.792
0.714	0.482	0.984	-5.349	0.286	7.387
-0.632	0.533	0.992	-5.397	-0.162	3.777
-0.845	0.406	0.969	-5.261	-0.174	3.529
0.520	0.608	0.999	-5.454	0.130	4.983
0.124	0.903	0.999	-5.567	0.033	3.806
1.593	0.124	0.786	-4.503	0.500	7.213
-1.743	0.094	0.751	-4.304	-0.615	4.377
1.756	0.091	0.745	-4.287	0.584	6.378
0.839	0.409	0.970	-5.265	0.234	5.631
0.319	0.753	0.999	-5.529	0.079	3.124
1.637	0.114	0.775	-4.445	0.408	2.490
-0.402	0.691	0.999	-5.502	-0.186	2.267
-0.309	0.760	0.999	-5.531	-0.273	5.465
1.036	0.310	0.943	-5.106	0.505	4.173
0.733	0.470	0.983	-5.337	0.514	4.069
-0.400	0.693	0.999	-5.503	-0.195	3.598
0.980	0.336	0.957	-5.154	0.355	4.977
-1.364	0.185	0.841	-4.777	-0.289	4.094
0.474	0.640	0.999	-5.474	0.142	5.859
0.389	0.701	0.999	-5.507	0.182	3.929
-0.060	0.953	0.999	-5.572	-0.022	4.141
-0.162	0.872	0.999	-5.562	-0.058	8.869
-0.224	0.825	0.999	-5.552	-0.041	7.527
-0.669	0.509	0.989	-5.376	-0.183	5.995

0.763	0.452	0.980	-5.317	0.191	2.841
1.890	0.070	0.692	-4.097	0.572	9.650
-0.304	0.764	0.999	-5.533	-0.129	3.630
0.329	0.745	0.999	-5.526	0.069	6.596
1.222	0.233	0.898	-4.929	0.507	7.181
1.191	0.245	0.905	-4.960	0.351	4.265
-0.864	0.396	0.964	-5.246	-0.311	5.875
1.409	0.171	0.826	-4.726	0.263	6.460
0.625	0.538	0.994	-5.401	0.128	5.515
1.167	0.254	0.913	-4.984	0.271	6.978
0.816	0.422	0.972	-5.281	0.259	5.734
1.580	0.127	0.786	-4.518	0.553	4.452
1.407	0.172	0.828	-4.728	0.305	5.029
0.324	0.749	0.999	-5.527	0.073	3.116
0.381	0.706	0.999	-5.509	0.151	3.533
-0.392	0.699	0.999	-5.506	-0.074	9.281
-0.976	0.338	0.957	-5.157	-0.256	2.823
2.969	0.006	0.400	-2.278	0.674	5.419
-0.649	0.522	0.990	-5.388	-0.164	2.619
-0.199	0.844	0.999	-5.556	-0.085	3.158
0.795	0.434	0.973	-5.296	0.198	6.218
-1.115	0.275	0.921	-5.034	-0.412	2.879
0.932	0.360	0.963	-5.194	0.233	2.664
-0.807	0.427	0.973	-5.287	-0.403	6.843
-0.131	0.897	0.999	-5.566	-0.037	4.472
0.673	0.507	0.988	-5.374	0.175	3.756
0.472	0.641	0.999	-5.475	0.133	6.365
1.629	0.116	0.776	-4.456	0.359	5.843
0.528	0.602	0.999	-5.450	0.155	2.618
1.612	0.119	0.781	-4.478	0.396	4.792
1.750	0.092	0.748	-4.294	0.363	4.303
1.331	0.195	0.853	-4.813	0.269	5.347
1.192	0.244	0.904	-4.959	0.275	2.772
-0.606	0.550	0.994	-5.412	-0.192	2.603
-2.184	0.039	0.611	-3.649	-0.606	3.295
-2.406	0.024	0.543	-3.284	-0.677	1.811
0.207	0.837	0.999	-5.555	0.087	3.620
-1.313	0.201	0.860	-4.832	-0.296	5.890
-1.123	0.272	0.920	-5.027	-0.262	5.647
-1.620	0.118	0.776	-4.467	-0.504	3.575
1.074	0.293	0.934	-5.072	0.366	1.937
-0.765	0.451	0.980	-5.316	-0.166	7.676
-0.507	0.616	0.999	-5.460	-0.113	7.731
-0.122	0.904	0.999	-5.567	-0.031	3.864
-0.296	0.769	0.999	-5.535	-0.098	3.200
-0.228	0.821	0.999	-5.551	-0.067	3.223
-0.709	0.485	0.985	-5.352	-0.160	5.802
-0.554	0.584	0.999	-5.438	-0.139	6.875
-0.535	0.598	0.999	-5.447	-0.121	6.226
-0.374	0.712	0.999	-5.512	-0.087	6.343

-0.389	0.700	0.999	-5.507	-0.132	3.333
-0.153	0.879	0.999	-5.563	-0.030	8.807
2.000	0.056	0.658	-3.934	0.435	6.279
0.876	0.389	0.964	-5.237	0.175	6.813
-0.381	0.706	0.999	-5.509	-0.093	4.193
-0.347	0.732	0.999	-5.520	-0.105	7.452
0.975	0.339	0.957	-5.158	0.176	7.515
0.171	0.866	0.999	-5.561	0.039	4.676
1.410	0.171	0.826	-4.724	0.279	5.749
0.733	0.471	0.983	-5.337	0.139	4.919
0.868	0.393	0.964	-5.243	0.184	4.742
-0.753	0.458	0.982	-5.324	-0.188	3.350
-1.304	0.204	0.863	-4.843	-0.289	2.604
1.683	0.105	0.764	-4.385	0.525	3.525
0.192	0.849	0.999	-5.558	0.039	5.683
-1.238	0.227	0.889	-4.913	-0.306	5.026
0.574	0.571	0.997	-5.428	0.154	5.761
-0.427	0.673	0.999	-5.493	-0.107	6.327
-0.192	0.849	0.999	-5.558	-0.076	4.983
1.009	0.323	0.950	-5.130	0.238	6.938
-0.373	0.712	0.999	-5.512	-0.096	8.074
0.094	0.926	0.999	-5.570	0.047	5.788
-0.606	0.550	0.994	-5.411	-0.181	3.686
2.943	0.007	0.407	-2.326	1.233	6.597
0.105	0.917	0.999	-5.569	0.027	3.606
0.822	0.419	0.972	-5.277	0.167	4.386
0.651	0.521	0.990	-5.387	0.122	5.516
-0.284	0.779	0.999	-5.538	-0.115	3.287
-1.458	0.157	0.817	-4.668	-0.345	2.945
-0.162	0.872	0.999	-5.562	-0.040	6.603
0.742	0.465	0.983	-5.331	0.210	3.714
-0.656	0.518	0.990	-5.383	-0.174	2.791
-0.469	0.643	0.999	-5.476	-0.116	3.136
0.919	0.367	0.963	-5.204	0.204	4.991
2.423	0.023	0.543	-3.255	0.510	3.451
-1.637	0.114	0.775	-4.445	-0.497	2.370
0.260	0.797	0.999	-5.544	0.089	2.552
-0.307	0.762	0.999	-5.532	-0.083	1.643
2.227	0.035	0.598	-3.580	0.656	6.188
0.328	0.746	0.999	-5.526	0.124	6.934
-0.322	0.750	0.999	-5.528	-0.122	4.944
0.251	0.804	0.999	-5.546	0.090	3.185
-0.492	0.627	0.999	-5.467	-0.134	2.415
0.948	0.352	0.961	-5.181	0.202	3.363
1.662	0.109	0.766	-4.413	0.395	5.510
1.512	0.143	0.803	-4.603	0.746	8.005
0.098	0.922	0.999	-5.570	0.029	2.568
0.344	0.734	0.999	-5.521	0.131	4.353
-0.847	0.405	0.968	-5.259	-0.576	2.085
0.957	0.348	0.959	-5.173	0.487	2.821

0.491	0.627	0.999	-5.467	0.144	4.194
-0.764	0.452	0.980	-5.316	-0.294	6.009
-0.294	0.772	0.999	-5.536	-0.119	8.871
1.248	0.224	0.886	-4.902	0.401	4.811
0.397	0.695	0.999	-5.504	0.101	2.261
0.381	0.707	0.999	-5.509	0.098	3.232
1.056	0.301	0.941	-5.088	0.226	8.283
-0.473	0.640	0.999	-5.475	-0.130	3.419
0.277	0.784	0.999	-5.540	0.094	4.009
0.274	0.786	0.999	-5.540	0.127	1.798
3.413	0.002	0.301	-1.426	1.194	5.752
0.619	0.542	0.994	-5.404	0.330	3.436
0.354	0.726	0.999	-5.518	0.129	3.547
-0.856	0.400	0.964	-5.252	-0.252	3.623
-0.470	0.642	0.999	-5.476	-0.230	2.787
0.758	0.456	0.981	-5.321	0.194	5.367
0.415	0.682	0.999	-5.497	0.116	3.997
-0.342	0.735	0.999	-5.522	-0.116	2.500
0.280	0.782	0.999	-5.539	0.067	10.080
0.996	0.329	0.955	-5.141	0.372	3.895
-0.163	0.872	0.999	-5.562	-0.038	6.798
-0.485	0.632	0.999	-5.470	-0.103	6.187
-0.510	0.615	0.999	-5.459	-0.110	5.141
0.741	0.466	0.983	-5.332	0.291	4.181
0.648	0.523	0.990	-5.388	0.206	2.885
-0.254	0.801	0.999	-5.545	-0.083	5.766
-0.342	0.735	0.999	-5.522	-0.095	5.628
0.836	0.411	0.970	-5.266	0.328	2.492
-0.580	0.567	0.997	-5.425	-0.143	3.479
0.516	0.610	0.999	-5.456	0.191	2.637
-0.757	0.456	0.981	-5.321	-0.231	3.639
0.407	0.687	0.999	-5.500	0.141	5.962
-1.030	0.313	0.944	-5.112	-0.361	3.725
1.164	0.256	0.913	-4.987	0.587	4.539
0.523	0.605	0.999	-5.452	0.232	4.268
0.714	0.482	0.984	-5.349	0.213	5.903
-0.618	0.542	0.994	-5.405	-0.168	5.953
0.335	0.740	0.999	-5.524	0.072	6.588
0.985	0.334	0.957	-5.150	0.227	4.447
0.198	0.845	0.999	-5.556	0.077	4.061
0.531	0.600	0.999	-5.449	0.189	5.451
0.229	0.821	0.999	-5.551	0.052	5.165
0.126	0.901	0.999	-5.567	0.031	5.838
-0.392	0.699	0.999	-5.506	-0.305	5.708
0.680	0.503	0.988	-5.370	0.382	4.107
0.640	0.528	0.992	-5.393	0.419	4.379
-0.742	0.465	0.983	-5.331	-0.210	3.879
-0.087	0.931	0.999	-5.571	-0.023	4.131
0.650	0.521	0.990	-5.387	0.172	2.829
-0.947	0.352	0.961	-5.181	-0.176	5.993

0.931	0.361	0.963	-5.194	0.377	3.592
-0.356	0.725	0.999	-5.518	-0.148	1.643
1.180	0.249	0.909	-4.971	0.284	7.124
0.938	0.357	0.962	-5.188	0.478	5.532
0.619	0.542	0.994	-5.405	0.202	3.058
-0.135	0.893	0.999	-5.566	-0.030	2.113
1.632	0.115	0.776	-4.452	0.428	7.514
0.604	0.551	0.994	-5.412	0.170	5.082
1.209	0.238	0.901	-4.943	0.466	3.762
-0.805	0.428	0.973	-5.289	-0.159	2.434
-2.640	0.014	0.498	-2.877	-0.714	3.044
-0.283	0.780	0.999	-5.538	-0.091	2.796
1.274	0.214	0.874	-4.875	0.313	2.411
-0.667	0.511	0.989	-5.377	-0.190	1.729
0.086	0.932	0.999	-5.571	0.019	3.286
1.021	0.317	0.948	-5.119	0.198	4.203
-0.245	0.808	0.999	-5.547	-0.076	6.967
0.225	0.824	0.999	-5.551	0.048	3.359
0.516	0.610	0.999	-5.456	0.108	6.308
-0.092	0.928	0.999	-5.570	-0.027	5.532
-1.954	0.062	0.667	-4.004	-0.548	2.132
1.047	0.305	0.942	-5.096	0.289	5.583
1.929	0.065	0.674	-4.040	0.465	4.235
0.252	0.803	0.999	-5.546	0.130	6.258
0.468	0.644	0.999	-5.476	0.280	4.835
0.312	0.757	0.999	-5.530	0.197	4.816
-0.907	0.373	0.963	-5.214	-0.434	4.392
1.088	0.287	0.929	-5.059	0.188	5.386
-1.136	0.267	0.917	-5.014	-0.272	6.325
-1.660	0.109	0.766	-4.415	-0.397	4.711
2.449	0.022	0.530	-3.211	0.506	5.696
0.809	0.426	0.973	-5.286	0.153	5.277
1.646	0.112	0.771	-4.434	0.442	3.454
1.317	0.200	0.858	-4.829	0.369	2.286
-0.111	0.912	0.999	-5.568	-0.028	5.773
0.704	0.488	0.985	-5.355	0.241	7.298
1.096	0.284	0.925	-5.052	0.277	5.831
-0.153	0.880	0.999	-5.564	-0.050	3.025
1.325	0.197	0.855	-4.820	0.314	5.267
0.166	0.869	0.999	-5.562	0.043	5.443
-1.800	0.084	0.724	-4.226	-0.497	2.032
-0.415	0.681	0.999	-5.497	-0.145	4.456
0.515	0.611	0.999	-5.456	0.187	5.171
0.119	0.906	0.999	-5.568	0.033	7.978
0.226	0.823	0.999	-5.551	0.070	4.244
0.864	0.396	0.964	-5.246	0.197	3.506
1.674	0.106	0.764	-4.396	0.426	2.944
1.566	0.130	0.789	-4.536	0.410	5.601
0.098	0.922	0.999	-5.570	0.035	6.647
0.894	0.380	0.964	-5.223	0.212	3.265

-0.454	0.654	0.999	-5.482	-0.105	5.254
0.845	0.406	0.969	-5.260	0.270	3.149
0.159	0.875	0.999	-5.563	0.045	4.829
-2.121	0.044	0.630	-3.749	-0.446	5.679
0.341	0.736	0.999	-5.522	0.071	4.553
0.580	0.567	0.997	-5.425	0.161	9.294
-0.631	0.534	0.992	-5.398	-0.156	3.803
1.133	0.268	0.917	-5.017	0.207	5.845
1.013	0.321	0.950	-5.126	0.208	7.750
-0.635	0.531	0.992	-5.396	-0.187	2.888
1.988	0.058	0.660	-3.953	1.246	9.037
1.023	0.316	0.947	-5.118	0.412	4.818
-0.363	0.720	0.999	-5.515	-0.191	8.212
-1.283	0.211	0.869	-4.865	-0.825	3.395
-1.822	0.080	0.718	-4.195	-0.432	2.203
-0.674	0.506	0.988	-5.373	-0.234	1.955
1.447	0.160	0.820	-4.681	0.323	3.753
2.552	0.017	0.500	-3.033	0.580	4.077
1.456	0.158	0.818	-4.671	0.324	4.657
1.387	0.178	0.837	-4.751	0.449	6.388
-2.487	0.020	0.517	-3.146	-0.675	2.645
1.329	0.196	0.854	-4.816	0.375	5.448
1.596	0.123	0.785	-4.498	0.389	2.694
1.120	0.273	0.921	-5.029	0.311	3.683
-2.166	0.040	0.616	-3.678	-0.519	3.603
0.610	0.547	0.994	-5.409	0.162	5.229
-0.891	0.381	0.964	-5.226	-0.192	3.427
0.590	0.561	0.996	-5.420	0.188	3.483
-2.100	0.046	0.630	-3.781	-0.514	2.040
-0.980	0.336	0.957	-5.154	-0.346	3.035
-0.959	0.347	0.958	-5.172	-0.342	2.890
-0.820	0.420	0.972	-5.278	-0.278	2.712
-1.415	0.169	0.825	-4.719	-0.403	3.244
1.684	0.105	0.764	-4.384	0.448	3.692
1.155	0.259	0.913	-4.995	0.351	3.548
2.502	0.019	0.511	-3.120	0.938	6.315
1.116	0.275	0.921	-5.033	0.430	6.783
0.987	0.333	0.957	-5.149	0.225	3.497
0.378	0.708	0.999	-5.510	0.122	3.631
3.288	0.003	0.336	-1.671	0.907	5.550
2.983	0.006	0.393	-2.251	0.794	4.769
2.268	0.032	0.591	-3.513	0.663	4.479
0.093	0.927	0.999	-5.570	0.025	3.164
1.340	0.192	0.851	-4.803	0.532	5.126
-0.191	0.850	0.999	-5.558	-0.042	5.791
-0.622	0.539	0.994	-5.402	-0.125	4.296
1.686	0.104	0.764	-4.381	0.352	4.423
-0.262	0.795	0.999	-5.543	-0.049	7.356
0.937	0.357	0.962	-5.189	0.213	6.680
0.285	0.778	0.999	-5.538	0.063	5.081

0.573	0.572	0.998	-5.429	0.157	4.267
0.108	0.915	0.999	-5.569	0.048	4.073
-0.239	0.813	0.999	-5.548	-0.086	2.220
-0.808	0.427	0.973	-5.287	-0.173	5.632
0.686	0.499	0.987	-5.366	0.225	8.186
0.835	0.412	0.970	-5.268	0.207	2.987
-0.066	0.948	0.999	-5.572	-0.014	2.573
0.861	0.397	0.964	-5.248	0.172	2.695
1.355	0.187	0.845	-4.787	0.338	3.833
-1.402	0.173	0.828	-4.733	-0.372	7.397
0.288	0.775	0.999	-5.537	0.084	4.599
0.197	0.846	0.999	-5.557	0.035	6.913
-0.992	0.331	0.956	-5.144	-0.355	4.396
1.103	0.280	0.922	-5.045	0.226	4.146
1.923	0.066	0.676	-4.049	0.562	5.668
-0.285	0.778	0.999	-5.538	-0.071	7.425
-0.811	0.425	0.972	-5.284	-0.206	5.699
0.430	0.671	0.999	-5.492	0.116	3.569
-1.445	0.161	0.820	-4.684	-0.356	5.276
2.362	0.026	0.556	-3.358	0.631	4.816
0.251	0.804	0.999	-5.546	0.079	6.366
0.520	0.608	0.999	-5.454	0.108	3.133
2.467	0.021	0.517	-3.179	0.822	4.358
0.621	0.540	0.994	-5.403	0.255	7.864
0.169	0.867	0.999	-5.561	0.068	5.252
-0.165	0.870	0.999	-5.562	-0.042	2.870
1.827	0.080	0.716	-4.187	0.441	3.974
-0.433	0.669	0.999	-5.491	-0.110	4.794
0.522	0.606	0.999	-5.453	0.123	5.001
2.701	0.012	0.491	-2.769	0.600	9.413
0.926	0.363	0.963	-5.198	0.374	3.747
-0.596	0.557	0.995	-5.417	-0.258	3.322
-1.516	0.142	0.802	-4.599	-0.480	4.635
-1.289	0.209	0.868	-4.859	-0.321	3.740
0.243	0.810	0.999	-5.548	0.120	8.120
0.054	0.957	0.999	-5.573	0.017	7.056
-1.898	0.069	0.687	-4.086	-0.557	5.886
1.381	0.179	0.838	-4.757	0.399	2.722
-0.819	0.421	0.972	-5.279	-0.212	10.046
-0.873	0.391	0.964	-5.239	-0.195	5.595
1.223	0.233	0.897	-4.928	0.313	5.223
1.255	0.221	0.882	-4.895	0.315	8.583
-0.403	0.691	0.999	-5.502	-0.098	6.607
0.104	0.918	0.999	-5.569	0.041	4.321
-0.484	0.633	0.999	-5.470	-0.174	2.174
-0.737	0.468	0.983	-5.334	-0.336	4.766
-0.394	0.697	0.999	-5.505	-0.161	2.452
1.078	0.291	0.933	-5.069	0.369	4.026
0.660	0.516	0.990	-5.382	0.290	4.061
0.727	0.474	0.984	-5.341	0.240	3.436

0.424	0.675	0.999	-5.494	0.250	5.144
-0.347	0.731	0.999	-5.520	-0.155	3.129
1.088	0.287	0.929	-5.059	0.331	6.032
-0.484	0.632	0.999	-5.470	-0.254	5.257
1.151	0.261	0.914	-5.000	0.440	3.351
0.211	0.835	0.999	-5.554	0.066	2.493
0.616	0.544	0.994	-5.406	0.242	3.387
-0.078	0.938	0.999	-5.571	-0.039	2.781
0.783	0.441	0.977	-5.304	0.510	5.812
-2.013	0.055	0.656	-3.915	-0.568	3.986
-1.545	0.135	0.793	-4.562	-0.528	5.496
-0.406	0.688	0.999	-5.501	-0.145	4.169
0.634	0.532	0.992	-5.396	0.148	9.023
1.233	0.229	0.891	-4.918	0.487	4.244
0.617	0.543	0.994	-5.405	0.230	8.430
-0.372	0.713	0.999	-5.512	-0.071	10.010
2.187	0.038	0.611	-3.644	0.674	4.240
1.681	0.105	0.764	-4.387	0.580	5.216
-0.429	0.672	0.999	-5.492	-0.127	4.694
0.981	0.336	0.957	-5.154	0.350	5.634
-1.527	0.139	0.799	-4.585	-0.608	4.249
0.872	0.391	0.964	-5.240	0.186	4.748
0.448	0.658	0.999	-5.485	0.138	7.615
0.064	0.950	0.999	-5.572	0.017	6.412
-0.263	0.794	0.999	-5.543	-0.111	5.752
2.158	0.041	0.617	-3.691	0.592	4.598
2.109	0.045	0.630	-3.767	1.032	7.550
2.083	0.048	0.630	-3.808	0.511	5.385
1.848	0.076	0.707	-4.158	0.420	6.177
1.779	0.087	0.732	-4.254	0.471	8.159
1.691	0.103	0.764	-4.374	0.401	7.887
2.193	0.038	0.611	-3.634	0.526	9.664
2.736	0.011	0.481	-2.706	0.689	9.470
2.695	0.012	0.492	-2.780	0.714	8.421
2.023	0.054	0.655	-3.900	0.502	9.468
2.080	0.048	0.630	-3.812	0.513	7.876
2.000	0.056	0.658	-3.934	0.515	7.948
1.448	0.160	0.820	-4.680	0.347	8.590
1.423	0.167	0.825	-4.710	0.336	8.245
1.382	0.179	0.838	-4.756	0.304	7.938
1.279	0.213	0.871	-4.870	0.302	6.249
2.153	0.041	0.619	-3.698	0.691	6.773
2.074	0.048	0.634	-3.822	0.608	6.588
2.011	0.055	0.656	-3.918	0.666	6.547
1.854	0.076	0.704	-4.149	0.623	6.863
1.556	0.132	0.791	-4.549	0.545	6.791
2.228	0.035	0.598	-3.578	0.812	6.971
2.222	0.036	0.600	-3.589	0.818	6.981
2.156	0.041	0.618	-3.693	0.809	5.857
2.011	0.055	0.656	-3.918	0.781	6.650

1.966	0.060	0.663	-3.986	0.767	6.628
1.989	0.058	0.660	-3.951	0.631	5.800
1.986	0.058	0.660	-3.955	0.616	5.840
1.883	0.071	0.696	-4.107	0.565	4.377
1.831	0.079	0.713	-4.182	0.573	4.478
1.455	0.158	0.819	-4.672	0.419	6.071
1.427	0.166	0.825	-4.704	0.489	6.145
1.380	0.180	0.838	-4.758	0.477	6.140
1.371	0.183	0.840	-4.769	0.490	6.379
1.363	0.185	0.842	-4.778	0.456	5.226
1.220	0.234	0.899	-4.931	0.445	5.178
1.995	0.057	0.659	-3.942	0.694	6.789
1.948	0.063	0.668	-4.012	0.669	7.982
1.925	0.066	0.676	-4.045	0.642	7.926
1.782	0.087	0.731	-4.251	0.627	8.774
2.240	0.034	0.597	-3.558	0.704	4.553
3.331	0.003	0.320	-1.586	1.135	3.781
1.964	0.061	0.663	-3.988	0.845	6.420
1.705	0.101	0.763	-4.356	0.490	3.477
1.543	0.135	0.794	-4.565	0.412	3.144
-0.494	0.625	0.999	-5.465	-0.238	5.703
-0.224	0.825	0.999	-5.552	-0.104	6.438
2.844	0.009	0.441	-2.510	1.029	6.650
2.821	0.009	0.446	-2.552	0.994	5.435
2.817	0.009	0.446	-2.559	1.005	6.315
2.694	0.012	0.492	-2.782	0.922	6.177
2.653	0.014	0.498	-2.855	0.909	6.019
2.640	0.014	0.498	-2.878	0.861	6.854
2.633	0.014	0.498	-2.891	0.924	5.994
2.220	0.036	0.600	-3.591	0.717	5.961
-0.484	0.632	0.999	-5.470	-0.145	3.472
2.577	0.016	0.500	-2.989	0.669	3.717
2.518	0.019	0.501	-3.093	0.613	3.571
2.477	0.020	0.517	-3.162	0.595	3.567
1.123	0.272	0.919	-5.026	0.279	3.604
1.024	0.315	0.946	-5.116	0.249	3.339
1.873	0.073	0.698	-4.122	1.046	9.681
1.476	0.152	0.813	-4.646	0.608	10.363
1.408	0.171	0.827	-4.727	0.591	10.587
1.350	0.189	0.848	-4.792	0.559	10.689
1.333	0.194	0.853	-4.811	0.555	10.702
3.410	0.002	0.301	-1.433	5.230	7.668
1.121	0.273	0.921	-5.028	0.348	4.779
0.694	0.494	0.987	-5.361	0.209	4.654
1.198	0.242	0.904	-4.953	0.374	8.133
1.684	0.105	0.764	-4.384	0.534	7.384
-0.288	0.775	0.999	-5.537	-0.197	4.628
1.635	0.115	0.775	-4.448	0.582	9.397
1.821	0.081	0.718	-4.196	1.048	7.862
0.508	0.616	0.999	-5.459	0.332	5.519

3.246	0.003	0.340	-1.752	4.930	7.960
0.091	0.928	0.999	-5.570	0.019	3.053
1.267	0.217	0.876	-4.882	0.302	9.349
1.199	0.242	0.904	-4.952	0.282	9.366
2.729	0.011	0.484	-2.718	0.596	5.823
2.552	0.017	0.500	-3.034	0.488	6.185
2.395	0.024	0.543	-3.302	0.532	5.852
2.279	0.031	0.588	-3.496	0.457	5.861
2.250	0.033	0.595	-3.542	0.413	4.114
2.132	0.043	0.626	-3.732	0.383	4.499
2.027	0.053	0.653	-3.894	0.374	4.372
0.425	0.675	0.999	-5.494	0.117	5.567
-0.264	0.794	0.999	-5.543	-0.137	5.949
3.265	0.003	0.340	-1.714	0.663	3.026
0.442	0.662	0.999	-5.487	0.131	2.657
0.152	0.881	0.999	-5.564	0.032	5.587
-0.523	0.605	0.999	-5.452	-0.112	4.286
0.454	0.654	0.999	-5.482	0.131	6.401
0.112	0.911	0.999	-5.568	0.032	3.523
0.643	0.526	0.992	-5.391	0.152	4.691
0.103	0.918	0.999	-5.569	0.032	6.359
1.245	0.225	0.886	-4.905	0.333	4.122
0.902	0.376	0.963	-5.217	0.270	3.342
0.150	0.882	0.999	-5.564	0.034	4.926
0.649	0.522	0.990	-5.388	0.183	4.915
2.245	0.034	0.595	-3.551	0.700	5.937
0.229	0.821	0.999	-5.551	0.065	3.486
0.354	0.726	0.999	-5.518	0.111	4.134
-0.121	0.904	0.999	-5.567	-0.032	2.444
1.537	0.137	0.795	-4.572	0.385	5.224
0.086	0.932	0.999	-5.571	0.043	6.859
1.600	0.122	0.784	-4.493	0.391	5.023
-0.885	0.385	0.964	-5.230	-0.341	2.609
1.406	0.172	0.828	-4.729	0.284	4.213
1.164	0.255	0.913	-4.987	0.308	6.333
0.208	0.837	0.999	-5.555	0.062	2.941
-0.296	0.770	0.999	-5.535	-0.102	4.874
1.346	0.190	0.849	-4.796	0.342	5.234
0.574	0.571	0.997	-5.428	0.371	2.165
-0.393	0.698	0.999	-5.505	-0.151	4.559
1.880	0.072	0.698	-4.111	0.475	3.399
-2.227	0.035	0.598	-3.580	-0.663	2.850
-0.459	0.650	0.999	-5.480	-0.124	3.395
-0.174	0.863	0.999	-5.560	-0.109	4.986
0.105	0.917	0.999	-5.569	0.064	4.496
-0.097	0.924	0.999	-5.570	-0.065	5.972
-1.112	0.276	0.921	-5.036	-0.361	4.251
-1.186	0.247	0.906	-4.965	-0.321	4.680
0.217	0.830	0.999	-5.553	0.090	4.878
1.054	0.302	0.941	-5.090	0.383	3.900

0.112	0.912	0.999	-5.568	0.038	3.948
0.484	0.633	0.999	-5.470	0.271	4.645
0.348	0.731	0.999	-5.520	0.107	4.778
-0.851	0.403	0.967	-5.256	-0.218	3.170
1.539	0.136	0.795	-4.570	0.531	4.778
0.624	0.538	0.994	-5.402	0.217	4.940
-0.170	0.867	0.999	-5.561	-0.051	2.857
0.859	0.399	0.964	-5.250	0.253	4.949
-0.410	0.686	0.999	-5.499	-0.079	3.087
-2.199	0.037	0.611	-3.624	-0.497	5.075
-0.718	0.479	0.984	-5.347	-0.142	3.591
-0.868	0.393	0.964	-5.243	-0.245	3.147
0.487	0.631	0.999	-5.469	0.127	3.633
0.137	0.892	0.999	-5.566	0.055	3.805
0.828	0.415	0.970	-5.272	0.333	3.081
0.750	0.460	0.983	-5.326	0.307	4.099
-0.094	0.926	0.999	-5.570	-0.035	4.037
0.689	0.497	0.987	-5.364	0.175	3.949
0.171	0.865	0.999	-5.561	0.148	5.229
-0.357	0.724	0.999	-5.517	-0.080	3.099
0.092	0.928	0.999	-5.570	0.035	5.252
0.374	0.712	0.999	-5.512	0.090	2.525
1.034	0.311	0.943	-5.108	0.461	4.070
0.080	0.937	0.999	-5.571	0.022	3.678
1.366	0.184	0.841	-4.775	0.424	3.143
-0.176	0.862	0.999	-5.560	-0.048	5.419
2.006	0.056	0.657	-3.926	0.457	7.636
1.905	0.068	0.684	-4.075	0.473	6.204
0.464	0.647	0.999	-5.478	0.110	2.024
2.412	0.024	0.543	-3.274	0.782	3.797
-0.061	0.952	0.999	-5.572	-0.025	5.929
-0.839	0.410	0.970	-5.265	-0.211	3.251
-0.106	0.916	0.999	-5.569	-0.027	2.446
1.308	0.203	0.862	-4.838	0.394	5.768
1.581	0.126	0.786	-4.517	0.419	3.935
-0.118	0.907	0.999	-5.568	-0.053	3.472
-1.599	0.122	0.784	-4.494	-0.394	1.907
1.784	0.087	0.731	-4.248	0.380	6.034
-0.181	0.858	0.999	-5.559	-0.065	2.554
1.050	0.304	0.941	-5.093	0.430	3.182
0.297	0.769	0.999	-5.535	0.089	1.792
0.949	0.352	0.961	-5.180	0.311	5.420
1.387	0.178	0.837	-4.751	0.361	3.765
0.764	0.452	0.980	-5.317	0.215	2.876
0.152	0.880	0.999	-5.564	0.043	3.658
-0.565	0.577	0.999	-5.432	-0.151	4.562
0.648	0.523	0.990	-5.388	0.192	2.441
0.170	0.866	0.999	-5.561	0.049	3.453
2.239	0.034	0.597	-3.561	0.515	5.126
-0.858	0.399	0.964	-5.250	-0.178	5.315

2.489	0.020	0.517	-3.142	0.579	2.416
1.431	0.165	0.824	-4.700	0.296	5.295
0.129	0.899	0.999	-5.567	0.036	3.459
0.138	0.892	0.999	-5.565	0.050	3.697
0.327	0.746	0.999	-5.526	0.081	3.039
0.925	0.364	0.963	-5.199	0.219	5.897
-0.529	0.602	0.999	-5.450	-0.137	3.957
1.021	0.317	0.948	-5.119	0.233	3.961
1.167	0.254	0.913	-4.984	0.306	6.667
1.696	0.102	0.764	-4.368	0.420	4.841
2.676	0.013	0.498	-2.814	0.741	4.546
0.971	0.341	0.957	-5.162	0.252	4.618
-0.284	0.778	0.999	-5.538	-0.094	3.009
-0.519	0.608	0.999	-5.454	-0.198	4.899
0.182	0.857	0.999	-5.559	0.064	2.535
-1.433	0.164	0.824	-4.698	-0.448	6.442
-0.635	0.531	0.992	-5.395	-0.142	1.815
1.142	0.264	0.916	-5.008	0.303	3.189
-0.430	0.671	0.999	-5.492	-0.089	4.026
0.830	0.414	0.970	-5.271	0.248	11.252
0.800	0.431	0.973	-5.292	0.238	11.329
0.737	0.468	0.983	-5.334	0.233	10.847
0.587	0.563	0.996	-5.422	0.182	10.515
0.978	0.338	0.957	-5.156	0.318	10.669
0.909	0.372	0.963	-5.212	0.287	10.679
0.815	0.423	0.972	-5.282	0.243	11.025
0.778	0.444	0.979	-5.307	0.230	10.709
1.202	0.241	0.902	-4.949	0.306	5.022
0.775	0.446	0.979	-5.310	0.197	4.707
0.190	0.851	0.999	-5.558	0.073	9.350
0.721	0.477	0.984	-5.344	0.202	6.467
0.372	0.713	0.999	-5.512	0.097	9.678
0.240	0.812	0.999	-5.548	0.092	3.471
0.807	0.427	0.973	-5.287	0.330	3.274
0.755	0.457	0.981	-5.322	0.271	9.810
-0.101	0.920	0.999	-5.569	-0.045	1.239
0.178	0.860	0.999	-5.560	0.054	6.341
-0.748	0.461	0.983	-5.327	-0.170	2.740
0.856	0.400	0.964	-5.252	0.207	5.765
1.855	0.075	0.704	-4.148	0.392	4.501
1.023	0.316	0.947	-5.117	0.352	8.334
1.753	0.092	0.746	-4.291	0.553	5.542
0.602	0.553	0.994	-5.414	0.311	2.353
-0.094	0.926	0.999	-5.570	-0.036	2.604
-0.145	0.886	0.999	-5.565	-0.077	1.930
0.989	0.332	0.957	-5.147	0.351	2.049
-0.255	0.801	0.999	-5.545	-0.143	4.709
0.210	0.836	0.999	-5.554	0.130	4.008
-0.213	0.833	0.999	-5.554	-0.103	1.614
-0.299	0.767	0.999	-5.534	-0.075	2.389

0.487	0.631	0.999	-5.469	0.224	3.602
-0.628	0.535	0.993	-5.399	-0.285	2.410
0.928	0.362	0.963	-5.197	0.223	2.731
0.264	0.794	0.999	-5.543	0.109	2.626
0.108	0.915	0.999	-5.569	0.027	4.114
-0.929	0.362	0.963	-5.196	-0.257	2.797
-2.658	0.013	0.498	-2.845	-0.691	3.070
-1.375	0.181	0.840	-4.764	-0.403	4.744
0.382	0.706	0.999	-5.509	0.087	5.087
-1.875	0.073	0.698	-4.119	-0.451	3.146
0.348	0.731	0.999	-5.520	0.116	2.882
2.639	0.014	0.498	-2.879	0.852	6.055
1.764	0.090	0.741	-4.275	0.480	5.991
0.981	0.336	0.957	-5.154	0.255	3.599
-0.158	0.876	0.999	-5.563	-0.086	2.348
1.935	0.064	0.673	-4.032	0.393	5.474
0.640	0.528	0.992	-5.393	0.185	5.188
0.061	0.952	0.999	-5.572	0.020	6.577
0.946	0.353	0.961	-5.182	0.249	4.237
1.262	0.218	0.877	-4.887	0.401	5.369
-0.422	0.676	0.999	-5.495	-0.105	6.281
0.942	0.355	0.962	-5.186	0.192	3.638
-0.562	0.579	0.999	-5.434	-0.119	4.770
1.916	0.067	0.682	-4.059	0.644	5.542
1.715	0.099	0.757	-4.342	0.620	5.584
1.662	0.109	0.766	-4.413	0.575	6.017
0.305	0.763	0.999	-5.533	0.069	5.117
2.017	0.055	0.655	-3.909	0.759	5.225
1.941	0.064	0.671	-4.022	0.735	4.533
2.823	0.009	0.446	-2.549	0.777	8.396
0.389	0.701	0.999	-5.507	0.090	3.912
2.540	0.018	0.501	-3.055	1.448	7.413
0.334	0.741	0.999	-5.524	0.097	5.199
0.584	0.565	0.997	-5.423	0.186	4.466
1.645	0.112	0.771	-4.435	0.616	5.461
1.774	0.088	0.734	-4.261	0.811	8.584
1.245	0.225	0.886	-4.905	0.301	3.793
0.941	0.356	0.962	-5.186	0.275	4.573
1.462	0.156	0.817	-4.663	0.385	4.807
1.265	0.217	0.876	-4.884	0.449	4.124
1.799	0.084	0.724	-4.226	0.585	8.322
0.657	0.517	0.990	-5.383	0.266	8.824
1.007	0.324	0.951	-5.132	0.300	5.454
1.090	0.286	0.929	-5.057	0.349	4.312
0.652	0.521	0.990	-5.386	0.244	4.966
0.505	0.618	0.999	-5.461	0.188	4.391
0.394	0.697	0.999	-5.505	0.132	4.262
0.070	0.945	0.999	-5.572	0.021	3.413
-1.272	0.215	0.875	-4.877	-0.322	2.710
0.598	0.555	0.995	-5.416	0.139	5.957

0.263	0.795	0.999	-5.543	0.063	3.556
0.232	0.818	0.999	-5.550	0.047	5.628
0.520	0.608	0.999	-5.454	0.191	1.041
-0.072	0.943	0.999	-5.572	-0.017	5.740
0.501	0.621	0.999	-5.462	0.189	3.636
0.108	0.915	0.999	-5.569	0.026	4.861
0.152	0.880	0.999	-5.564	0.035	3.143
-0.275	0.785	0.999	-5.540	-0.064	3.392
-1.727	0.096	0.756	-4.326	-0.410	2.900
-0.354	0.726	0.999	-5.518	-0.100	2.918
-0.696	0.493	0.986	-5.360	-0.282	4.410
1.412	0.170	0.825	-4.722	0.710	5.730
0.626	0.537	0.993	-5.401	0.212	7.297
-1.015	0.320	0.950	-5.125	-0.351	4.576
2.299	0.030	0.573	-3.462	0.531	8.974
2.070	0.049	0.636	-3.828	0.613	2.247
1.560	0.131	0.791	-4.543	0.428	2.713
1.196	0.243	0.904	-4.956	0.349	4.471
0.184	0.855	0.999	-5.559	0.058	3.984
-0.333	0.742	0.999	-5.525	-0.101	4.164
-1.842	0.077	0.707	-4.166	-0.432	2.557
-0.543	0.592	0.999	-5.443	-0.174	3.013
0.315	0.755	0.999	-5.530	0.090	2.901
-1.990	0.058	0.660	-3.950	-1.044	2.995
-1.009	0.323	0.950	-5.129	-0.393	2.637
-0.318	0.753	0.999	-5.529	-0.094	2.087
-0.590	0.560	0.996	-5.420	-0.114	5.870
-2.112	0.045	0.630	-3.762	-0.720	3.099
-0.256	0.800	0.999	-5.545	-0.073	2.619
-0.973	0.340	0.957	-5.160	-0.284	2.504
-0.274	0.787	0.999	-5.541	-0.085	3.355
1.376	0.181	0.840	-4.763	0.436	2.629
-1.364	0.185	0.841	-4.777	-0.340	2.782
0.507	0.617	0.999	-5.460	0.134	4.961
-0.541	0.593	0.999	-5.444	-0.165	3.028
-0.997	0.328	0.954	-5.140	-0.367	2.475
-1.229	0.230	0.894	-4.922	-0.557	4.246
-0.229	0.820	0.999	-5.550	-0.092	2.942
-0.669	0.509	0.989	-5.376	-0.222	2.837
-2.067	0.049	0.638	-3.833	-0.538	3.288
-2.047	0.051	0.644	-3.864	-0.497	4.217
-1.465	0.155	0.817	-4.660	-0.387	2.513
0.951	0.351	0.961	-5.178	0.336	2.966
1.768	0.089	0.738	-4.270	0.616	2.536
1.834	0.079	0.711	-4.178	0.532	4.285
0.165	0.870	0.999	-5.562	0.044	5.339
-1.324	0.197	0.855	-4.821	-0.338	4.543
2.080	0.048	0.630	-3.812	0.749	5.350
-0.080	0.937	0.999	-5.571	-0.020	4.208
0.708	0.486	0.985	-5.353	0.185	2.525

1.099	0.282	0.924	-5.049	0.212	6.804
0.592	0.559	0.996	-5.419	0.131	6.049
2.223	0.035	0.600	-3.587	0.615	3.620
2.842	0.009	0.441	-2.514	0.722	3.796
1.038	0.309	0.943	-5.104	0.284	4.107
1.600	0.122	0.784	-4.493	0.516	5.631
1.106	0.279	0.922	-5.043	0.263	5.040
2.307	0.030	0.570	-3.450	0.688	2.549
-0.053	0.958	0.999	-5.573	-0.013	4.421
-2.234	0.035	0.597	-3.569	-0.472	2.041
0.083	0.935	0.999	-5.571	0.037	1.367
1.987	0.058	0.660	-3.955	0.599	7.587
0.915	0.369	0.963	-5.207	0.180	3.717
-1.061	0.299	0.940	-5.084	-0.276	4.301
0.865	0.395	0.964	-5.246	0.263	7.418
-0.147	0.884	0.999	-5.564	-0.035	5.216
1.458	0.157	0.817	-4.669	0.507	5.542
1.581	0.126	0.786	-4.518	0.412	4.839
-2.076	0.048	0.633	-3.818	-0.544	2.974
2.150	0.041	0.619	-3.703	0.602	2.521
1.930	0.065	0.674	-4.039	0.667	3.239
1.540	0.136	0.795	-4.568	0.469	5.743
-0.236	0.815	0.999	-5.549	-0.103	2.484
0.749	0.461	0.983	-5.326	0.189	3.269
0.405	0.689	0.999	-5.501	0.105	5.784
-0.118	0.907	0.999	-5.568	-0.035	0.974
1.013	0.321	0.950	-5.127	0.451	4.200
-1.406	0.172	0.828	-4.729	-0.323	2.289
1.477	0.152	0.813	-4.645	0.379	2.638
0.794	0.434	0.973	-5.296	0.362	3.490
0.886	0.384	0.964	-5.229	0.300	3.095
1.436	0.163	0.824	-4.694	0.441	3.671
-0.392	0.699	0.999	-5.506	-0.082	2.533
3.439	0.002	0.301	-1.376	1.078	5.030
0.755	0.457	0.981	-5.322	0.276	3.623
0.734	0.469	0.983	-5.336	0.189	3.795
0.637	0.530	0.992	-5.394	0.168	4.396
2.757	0.011	0.468	-2.669	0.916	6.608
0.840	0.409	0.970	-5.264	0.161	5.757
-0.378	0.708	0.999	-5.510	-0.110	3.082
-1.407	0.172	0.828	-4.728	-0.459	4.204
1.219	0.234	0.899	-4.932	0.271	6.288
-1.504	0.145	0.803	-4.613	-0.330	2.449
-0.107	0.916	0.999	-5.569	-0.026	5.978
0.708	0.486	0.985	-5.353	0.154	3.184
-0.239	0.813	0.999	-5.548	-0.076	1.580
-0.326	0.747	0.999	-5.526	-0.085	4.370
0.620	0.541	0.994	-5.404	0.128	5.851
1.210	0.237	0.899	-4.941	0.277	3.753
1.205	0.239	0.901	-4.946	0.292	6.910

-0.101	0.921	0.999	-5.569	-0.023	3.216
-1.458	0.157	0.817	-4.668	-0.271	6.284
0.464	0.647	0.999	-5.478	0.131	6.757
0.437	0.666	0.999	-5.489	0.110	4.451
0.633	0.532	0.992	-5.397	0.160	7.280
0.137	0.892	0.999	-5.566	0.030	2.346
1.307	0.203	0.862	-4.840	0.252	6.456
-2.205	0.037	0.610	-3.615	-0.500	2.350
0.771	0.448	0.979	-5.312	0.202	7.573
0.974	0.339	0.957	-5.159	0.238	5.359
-1.176	0.250	0.910	-4.975	-0.319	2.677
-0.346	0.732	0.999	-5.521	-0.138	1.735
1.192	0.244	0.904	-4.959	0.283	4.240
1.958	0.061	0.665	-3.997	0.382	4.771
0.152	0.881	0.999	-5.564	0.028	6.153
0.604	0.552	0.994	-5.413	0.250	5.270
0.439	0.665	0.999	-5.488	0.221	4.221
1.114	0.276	0.921	-5.035	0.216	4.996
0.886	0.384	0.964	-5.229	0.197	6.716
0.551	0.586	0.999	-5.439	0.117	3.583
1.181	0.249	0.908	-4.970	0.250	6.805
0.675	0.506	0.988	-5.373	0.266	3.774
-0.232	0.819	0.999	-5.550	-0.089	4.717
-0.377	0.709	0.999	-5.511	-0.087	5.933
0.163	0.872	0.999	-5.562	0.049	5.654
1.621	0.118	0.776	-4.467	0.491	3.914
-0.888	0.383	0.964	-5.228	-0.229	8.415
0.886	0.384	0.964	-5.230	0.329	2.874
-0.885	0.385	0.964	-5.230	-0.236	8.143
-0.880	0.387	0.964	-5.234	-0.222	8.426
-0.831	0.414	0.970	-5.270	-0.244	2.664
0.805	0.428	0.973	-5.289	0.206	3.814
-0.787	0.439	0.975	-5.301	-0.279	2.141
-0.728	0.474	0.984	-5.340	-0.193	5.274
-0.612	0.546	0.994	-5.408	-0.156	7.874
-0.606	0.550	0.994	-5.411	-0.157	9.019
-0.575	0.570	0.997	-5.427	-0.150	7.334
-0.535	0.597	0.999	-5.447	-0.145	6.907
-0.413	0.683	0.999	-5.498	-0.104	5.561
0.384	0.704	0.999	-5.508	0.118	3.294
-0.351	0.729	0.999	-5.519	-0.091	7.113
-0.311	0.759	0.999	-5.531	-0.095	6.198
0.300	0.767	0.999	-5.534	0.075	6.668
-0.293	0.772	0.999	-5.536	-0.067	6.053
-0.282	0.781	0.999	-5.539	-0.077	3.397
-0.136	0.893	0.999	-5.566	-0.037	6.844
-0.536	0.597	0.999	-5.447	-0.164	5.865
-2.180	0.039	0.611	-3.655	-0.521	4.524
1.379	0.180	0.839	-4.760	0.313	3.388
1.403	0.173	0.828	-4.732	0.387	7.959

1.847	0.077	0.707	-4.160	0.525	4.079
-0.246	0.808	0.999	-5.547	-0.060	6.667
1.213	0.236	0.899	-4.938	0.414	4.561
-0.502	0.620	0.999	-5.462	-0.228	3.669
-1.644	0.113	0.773	-4.436	-0.463	3.401
0.327	0.747	0.999	-5.526	0.066	5.267
0.716	0.481	0.984	-5.348	0.163	3.294
0.879	0.388	0.964	-5.235	0.262	5.724
0.770	0.449	0.979	-5.313	0.204	3.928
1.700	0.101	0.764	-4.362	0.362	6.374
1.103	0.280	0.922	-5.045	0.314	3.764
1.161	0.257	0.913	-4.990	0.323	6.530
1.598	0.123	0.785	-4.496	0.350	3.738
2.060	0.050	0.639	-3.843	0.696	5.315
0.874	0.390	0.964	-5.238	0.203	6.048
-1.036	0.310	0.943	-5.106	-0.285	2.571
1.420	0.168	0.825	-4.713	0.311	4.928
-0.163	0.872	0.999	-5.562	-0.042	3.027
1.186	0.247	0.906	-4.966	0.314	7.588
-0.373	0.713	0.999	-5.512	-0.118	3.338
-0.656	0.518	0.990	-5.384	-0.157	2.348
-0.683	0.501	0.988	-5.368	-0.192	4.135
-1.153	0.260	0.914	-4.998	-0.303	3.321
0.332	0.743	0.999	-5.525	0.058	3.622
1.692	0.103	0.764	-4.373	0.349	5.266
0.440	0.664	0.999	-5.488	0.159	2.739
0.657	0.517	0.990	-5.383	0.152	3.423
-0.522	0.606	0.999	-5.453	-0.175	3.888
-1.473	0.153	0.813	-4.650	-0.315	3.353
0.265	0.793	0.999	-5.543	0.059	7.133
0.081	0.936	0.999	-5.571	0.030	5.411
-1.438	0.163	0.824	-4.692	-0.375	2.106
0.226	0.823	0.999	-5.551	0.108	5.562
1.444	0.161	0.820	-4.684	0.381	5.444
1.775	0.088	0.734	-4.260	0.507	3.183
2.343	0.027	0.559	-3.390	0.559	4.656
0.744	0.464	0.983	-5.330	0.145	5.734
-0.390	0.700	0.999	-5.506	-0.130	3.651
1.038	0.309	0.943	-5.105	0.320	4.731
1.252	0.222	0.885	-4.898	0.490	4.416
2.811	0.009	0.446	-2.569	0.867	5.594
0.867	0.394	0.964	-5.244	0.158	9.295
2.330	0.028	0.566	-3.411	1.021	6.552
0.784	0.441	0.977	-5.303	0.171	4.673
0.882	0.386	0.964	-5.232	0.274	4.097
0.523	0.606	0.999	-5.453	0.128	2.529
2.709	0.012	0.488	-2.755	0.922	4.201
0.105	0.917	0.999	-5.569	0.029	3.745
-0.121	0.905	0.999	-5.567	-0.039	7.819
-0.883	0.385	0.964	-5.232	-0.210	5.927

-0.744	0.464	0.983	-5.330	-0.199	8.218
2.058	0.050	0.640	-3.846	0.800	6.187
1.252	0.222	0.885	-4.898	0.528	4.761
-0.397	0.695	0.999	-5.504	-0.101	6.434
0.171	0.865	0.999	-5.561	0.086	2.183
0.181	0.858	0.999	-5.559	0.048	8.880
-0.857	0.400	0.964	-5.252	-0.302	4.502
1.975	0.059	0.663	-3.972	0.567	5.942
0.110	0.914	0.999	-5.569	0.041	1.734
0.701	0.490	0.986	-5.357	0.136	7.800
2.177	0.039	0.611	-3.660	0.581	4.613
1.659	0.109	0.766	-4.416	0.408	3.034
0.058	0.955	0.999	-5.572	0.017	2.748
1.995	0.057	0.659	-3.942	0.447	4.887
0.416	0.681	0.999	-5.497	0.091	7.510
0.135	0.894	0.999	-5.566	0.042	4.604
0.488	0.630	0.999	-5.468	0.129	3.726
2.235	0.035	0.597	-3.567	0.793	4.581
-0.571	0.573	0.999	-5.429	-0.218	4.351
0.265	0.793	0.999	-5.543	0.071	7.167
1.622	0.117	0.776	-4.464	0.362	3.709
-0.155	0.878	0.999	-5.563	-0.045	1.567
0.088	0.931	0.999	-5.570	0.030	4.223
1.967	0.060	0.663	-3.984	0.486	3.129
-0.060	0.953	0.999	-5.572	-0.032	4.967
0.490	0.628	0.999	-5.467	0.197	3.546
-0.262	0.795	0.999	-5.543	-0.070	5.563
-0.684	0.500	0.987	-5.367	-0.191	8.600
-0.492	0.627	0.999	-5.467	-0.136	4.985
2.780	0.010	0.456	-2.626	0.641	8.640
0.555	0.584	0.999	-5.437	0.207	4.741
-0.688	0.498	0.987	-5.365	-0.185	5.384
-0.142	0.888	0.999	-5.565	-0.032	6.056
-1.040	0.308	0.943	-5.103	-0.265	3.397
1.075	0.293	0.934	-5.071	0.477	3.879
0.174	0.863	0.999	-5.560	0.038	5.133
0.104	0.918	0.999	-5.569	0.035	1.283
1.090	0.286	0.929	-5.057	0.332	4.376
-0.332	0.743	0.999	-5.525	-0.093	1.240
1.183	0.248	0.907	-4.968	0.229	4.449
0.053	0.959	0.999	-5.573	0.012	5.195
0.724	0.476	0.984	-5.343	0.186	4.091
1.234	0.229	0.890	-4.916	0.341	2.739
-1.619	0.118	0.777	-4.469	-0.889	3.319
-0.130	0.898	0.999	-5.566	-0.056	5.196
0.676	0.505	0.988	-5.372	0.240	4.311
1.476	0.152	0.813	-4.646	0.779	3.492
0.299	0.768	0.999	-5.534	0.108	4.109
-2.101	0.046	0.630	-3.780	-0.578	5.096
0.936	0.358	0.963	-5.190	0.261	3.964

0.128	0.899	0.999	-5.567	0.046	2.698
0.231	0.819	0.999	-5.550	0.078	3.787
0.259	0.798	0.999	-5.544	0.121	4.619
0.733	0.470	0.983	-5.337	0.379	3.194
-0.107	0.916	0.999	-5.569	-0.053	3.063
0.276	0.785	0.999	-5.540	0.199	3.668
-0.396	0.696	0.999	-5.504	-0.134	4.092
1.183	0.248	0.907	-4.968	0.374	4.442
-0.222	0.826	0.999	-5.552	-0.072	6.489
0.888	0.383	0.964	-5.228	0.289	5.989
-0.983	0.335	0.957	-5.152	-0.302	2.359
-2.139	0.042	0.621	-3.720	-0.502	2.547
0.835	0.411	0.970	-5.267	0.339	5.195
-0.324	0.749	0.999	-5.527	-0.128	3.893
-1.379	0.180	0.839	-4.760	-0.422	2.219
-0.207	0.838	0.999	-5.555	-0.093	2.330
0.568	0.575	0.999	-5.431	0.276	5.658
0.418	0.680	0.999	-5.496	0.129	3.177
-0.218	0.830	0.999	-5.553	-0.063	3.433
-0.082	0.936	0.999	-5.571	-0.048	2.516
-0.354	0.726	0.999	-5.518	-0.113	1.840
0.895	0.379	0.964	-5.223	0.437	3.492
0.691	0.496	0.987	-5.363	0.368	3.390
0.984	0.335	0.957	-5.151	0.433	6.036
1.348	0.190	0.849	-4.795	0.373	4.941
-0.858	0.399	0.964	-5.251	-0.338	3.470
0.064	0.949	0.999	-5.572	0.022	5.066
0.248	0.806	0.999	-5.547	0.125	3.356
0.387	0.702	0.999	-5.507	0.211	3.620
-0.336	0.740	0.999	-5.524	-0.103	8.461
-1.634	0.115	0.775	-4.449	-0.396	3.064
0.297	0.769	0.999	-5.535	0.067	1.997
-0.930	0.361	0.963	-5.195	-0.457	5.287
0.074	0.941	0.999	-5.571	0.021	6.317
-0.178	0.860	0.999	-5.560	-0.063	4.372
0.107	0.915	0.999	-5.569	0.046	4.791
-0.826	0.417	0.970	-5.274	-0.451	3.181
1.319	0.199	0.857	-4.827	0.342	3.525
0.964	0.344	0.957	-5.168	0.640	3.859
0.974	0.339	0.957	-5.159	0.261	3.342
-0.144	0.886	0.999	-5.565	-0.041	5.620
-1.046	0.306	0.942	-5.097	-0.262	2.520
-0.451	0.656	0.999	-5.483	-0.127	2.691
-0.320	0.751	0.999	-5.528	-0.079	2.631
0.670	0.509	0.989	-5.376	0.155	3.018
0.425	0.675	0.999	-5.494	0.118	4.708
-0.780	0.443	0.979	-5.306	-0.302	4.619
-1.370	0.183	0.840	-4.770	-0.332	2.672
1.493	0.148	0.805	-4.627	0.349	3.842
-0.607	0.549	0.994	-5.411	-0.208	2.869

0.081	0.936	0.999	-5.571	0.028	5.707
0.250	0.805	0.999	-5.546	0.063	4.397
0.898	0.378	0.963	-5.221	0.203	3.284
0.524	0.605	0.999	-5.452	0.178	6.508
-0.600	0.554	0.995	-5.415	-0.258	3.965
0.697	0.492	0.986	-5.360	0.218	7.114
0.902	0.376	0.963	-5.218	0.222	3.670
-0.091	0.928	0.999	-5.570	-0.022	3.544
-1.958	0.061	0.665	-3.997	-0.604	3.062
-0.978	0.338	0.957	-5.156	-0.406	3.759
0.414	0.683	0.999	-5.498	0.237	3.021
0.101	0.920	0.999	-5.569	0.042	3.850
0.938	0.357	0.962	-5.189	0.367	5.614
0.650	0.521	0.990	-5.387	0.365	2.667
0.157	0.877	0.999	-5.563	0.068	1.948
-0.753	0.459	0.982	-5.324	-0.425	2.396
0.537	0.596	0.999	-5.446	0.285	3.360
-0.687	0.498	0.987	-5.365	-0.345	3.835
1.281	0.212	0.870	-4.867	0.457	4.463
-0.591	0.560	0.996	-5.419	-0.411	1.896
0.088	0.930	0.999	-5.570	0.027	4.628
0.858	0.399	0.964	-5.251	0.186	4.850
0.705	0.487	0.985	-5.355	0.152	7.550
0.745	0.463	0.983	-5.329	0.402	3.807
0.118	0.907	0.999	-5.568	0.026	7.772
2.550	0.017	0.500	-3.036	0.632	3.868
1.795	0.085	0.726	-4.232	0.490	4.074
0.835	0.411	0.970	-5.267	0.883	4.273
1.100	0.282	0.923	-5.048	0.293	5.697
-0.070	0.945	0.999	-5.572	-0.027	5.140
-0.621	0.540	0.994	-5.403	-0.225	4.683
-0.861	0.398	0.964	-5.249	-0.538	3.764
-0.589	0.561	0.996	-5.420	-0.206	3.979
0.429	0.672	0.999	-5.492	0.167	3.802
-0.095	0.925	0.999	-5.570	-0.030	5.205
0.899	0.377	0.963	-5.220	0.196	5.027
1.438	0.163	0.824	-4.692	0.475	5.204
2.855	0.009	0.441	-2.489	0.593	4.588
1.996	0.057	0.659	-3.941	0.435	5.627
-0.230	0.820	0.999	-5.550	-0.098	4.110
0.527	0.603	0.999	-5.451	0.125	6.863
0.236	0.816	0.999	-5.549	0.043	7.255
-0.302	0.765	0.999	-5.533	-0.114	6.765
-0.829	0.415	0.970	-5.272	-0.196	5.485
0.198	0.844	0.999	-5.556	0.051	4.980
-1.414	0.170	0.825	-4.720	-0.414	4.414
0.187	0.853	0.999	-5.558	0.046	3.467
0.990	0.332	0.957	-5.146	0.252	3.796
-0.142	0.888	0.999	-5.565	-0.043	3.884
-0.810	0.425	0.972	-5.285	-0.434	4.806

0.420	0.678	0.999	-5.495	0.106	7.045
-0.872	0.392	0.964	-5.240	-0.246	5.597
0.170	0.867	0.999	-5.561	0.101	2.507
-1.373	0.182	0.840	-4.766	-0.465	4.157
1.344	0.191	0.850	-4.799	0.275	7.650
0.762	0.453	0.981	-5.318	0.276	4.272
-0.621	0.540	0.994	-5.403	-0.172	6.847
-1.614	0.119	0.780	-4.475	-0.484	5.921
-1.309	0.203	0.862	-4.838	-0.319	4.706
-0.423	0.676	0.999	-5.494	-0.101	3.835
-0.499	0.622	0.999	-5.463	-0.136	4.738
0.280	0.782	0.999	-5.539	0.088	5.427
1.151	0.261	0.914	-5.000	0.198	6.356
-1.692	0.103	0.764	-4.373	-0.444	2.049
1.220	0.234	0.899	-4.931	0.352	3.022
-0.742	0.465	0.983	-5.331	-0.238	5.263
0.062	0.951	0.999	-5.572	0.036	5.136
1.195	0.243	0.904	-4.957	0.274	5.880
-0.219	0.828	0.999	-5.553	-0.058	8.217
1.533	0.138	0.795	-4.577	0.397	3.177
0.592	0.559	0.996	-5.419	0.253	3.104
-1.886	0.071	0.694	-4.103	-0.434	4.251
-2.440	0.022	0.537	-3.226	-0.586	1.579
0.144	0.887	0.999	-5.565	0.053	4.737
-0.172	0.865	0.999	-5.561	-0.042	3.246
-0.311	0.759	0.999	-5.531	-0.078	5.957
0.538	0.595	0.999	-5.445	0.250	4.267
-0.155	0.878	0.999	-5.563	-0.050	7.553
0.357	0.724	0.999	-5.517	0.106	2.656
-0.138	0.891	0.999	-5.565	-0.046	4.220
-1.600	0.122	0.784	-4.493	-0.468	8.213
-0.746	0.463	0.983	-5.329	-0.174	7.375
0.166	0.869	0.999	-5.562	0.048	4.872
-0.946	0.353	0.961	-5.182	-0.235	7.373
-0.600	0.554	0.995	-5.415	-0.204	6.317
-0.840	0.409	0.970	-5.264	-0.229	3.040
0.387	0.702	0.999	-5.507	0.094	2.936
-0.706	0.487	0.985	-5.354	-0.286	8.474
-1.366	0.184	0.841	-4.775	-0.269	9.285
-1.300	0.206	0.867	-4.848	-0.230	8.373
-0.463	0.647	0.999	-5.479	-0.146	4.256
-0.330	0.744	0.999	-5.525	-0.078	7.864
0.684	0.500	0.987	-5.367	0.223	3.240
-1.902	0.069	0.684	-4.080	-0.464	2.024
0.515	0.611	0.999	-5.456	0.136	5.030
0.307	0.761	0.999	-5.532	0.088	5.930
-0.539	0.595	0.999	-5.445	-0.210	4.665
1.638	0.114	0.775	-4.444	0.429	2.542
-1.764	0.090	0.741	-4.275	-0.489	2.860
-0.418	0.679	0.999	-5.496	-0.116	5.260

0.188	0.852	0.999	-5.558	0.043	5.607
0.691	0.496	0.987	-5.363	0.144	6.465
-1.157	0.258	0.913	-4.994	-0.348	4.701
-1.118	0.274	0.921	-5.031	-0.449	5.556
0.849	0.404	0.968	-5.258	0.184	6.084
-0.729	0.473	0.984	-5.340	-0.223	3.232
2.854	0.009	0.441	-2.490	0.558	7.550
0.128	0.899	0.999	-5.567	0.042	5.910
1.316	0.200	0.859	-4.830	0.372	3.073
0.169	0.867	0.999	-5.561	0.044	6.963
-0.183	0.856	0.999	-5.559	-0.045	6.915
-0.352	0.728	0.999	-5.519	-0.103	3.515
0.103	0.919	0.999	-5.569	0.029	4.488
0.511	0.614	0.999	-5.458	0.257	3.999
1.324	0.198	0.856	-4.821	0.268	5.590
0.737	0.468	0.983	-5.334	0.180	2.547
1.414	0.170	0.825	-4.720	0.303	4.842
1.457	0.157	0.817	-4.669	0.321	4.987
0.241	0.812	0.999	-5.548	0.049	6.859
0.617	0.542	0.994	-5.405	0.210	3.942
0.811	0.425	0.972	-5.284	0.237	5.840
0.283	0.779	0.999	-5.538	0.049	4.443
0.488	0.630	0.999	-5.468	0.163	2.985
2.355	0.027	0.559	-3.370	0.893	5.170
1.218	0.235	0.899	-4.933	0.297	3.162
-0.118	0.907	0.999	-5.568	-0.032	3.317
-0.075	0.941	0.999	-5.571	-0.041	9.785
-0.860	0.398	0.964	-5.249	-0.380	3.419
-0.913	0.370	0.963	-5.209	-0.237	2.688
1.197	0.242	0.904	-4.954	0.423	2.955
-0.966	0.343	0.957	-5.166	-0.310	4.541
-0.393	0.697	0.999	-5.505	-0.143	1.297
0.366	0.718	0.999	-5.514	0.079	3.840
2.547	0.017	0.500	-3.041	0.539	6.711
1.103	0.281	0.922	-5.045	0.219	5.777
0.577	0.569	0.997	-5.427	0.118	4.731
0.516	0.610	0.999	-5.456	0.094	5.590
0.961	0.345	0.957	-5.170	0.281	2.783
0.921	0.366	0.963	-5.202	0.240	4.104
1.099	0.282	0.924	-5.049	0.354	2.654
1.352	0.188	0.847	-4.790	0.446	3.843
-0.266	0.793	0.999	-5.542	-0.139	3.548
0.988	0.332	0.957	-5.147	0.295	2.491
-2.583	0.016	0.500	-2.979	-0.591	3.521
-1.050	0.304	0.941	-5.094	-0.299	3.194
-0.101	0.920	0.999	-5.569	-0.028	2.573
-1.694	0.103	0.764	-4.371	-0.418	2.667
-0.694	0.494	0.987	-5.362	-0.125	4.093
-0.658	0.516	0.990	-5.382	-0.223	5.338
3.057	0.005	0.387	-2.112	0.949	3.683

-0.418	0.680	0.999	-5.496	-0.231	4.691
1.219	0.234	0.899	-4.932	0.279	3.084
-0.509	0.616	0.999	-5.459	-0.103	4.466
1.262	0.218	0.877	-4.887	0.344	2.570
0.397	0.695	0.999	-5.504	0.150	3.040
1.130	0.269	0.918	-5.020	0.417	5.868
1.914	0.067	0.682	-4.063	0.703	5.099
2.014	0.055	0.656	-3.913	0.476	4.864
0.489	0.629	0.999	-5.468	0.200	4.658
-0.109	0.914	0.999	-5.569	-0.021	5.811
0.604	0.551	0.994	-5.412	0.184	2.635
1.167	0.254	0.913	-4.984	0.253	5.890
-0.067	0.947	0.999	-5.572	-0.015	8.211
0.664	0.513	0.990	-5.379	0.161	8.795
0.263	0.794	0.999	-5.543	0.053	6.008
0.233	0.817	0.999	-5.550	0.055	2.641
2.530	0.018	0.501	-3.071	0.615	6.657
1.806	0.083	0.724	-4.216	0.837	10.127
-0.758	0.455	0.981	-5.320	-0.194	4.671
-0.331	0.743	0.999	-5.525	-0.083	4.479
0.686	0.499	0.987	-5.366	0.153	5.402
0.580	0.567	0.997	-5.425	0.119	5.226
0.201	0.843	0.999	-5.556	0.052	2.541
2.343	0.027	0.559	-3.389	0.508	6.267
-1.034	0.311	0.943	-5.108	-0.272	2.419
-1.286	0.210	0.868	-4.862	-0.475	3.302
0.722	0.477	0.984	-5.344	0.141	4.485
0.803	0.430	0.973	-5.290	0.188	4.267
0.856	0.400	0.964	-5.252	0.290	6.971
3.579	0.001	0.301	-1.101	0.791	7.000
0.375	0.711	0.999	-5.511	0.125	3.824
0.220	0.828	0.999	-5.552	0.092	3.917
-0.118	0.907	0.999	-5.568	-0.056	5.008
0.796	0.433	0.973	-5.295	0.208	5.317
0.627	0.536	0.993	-5.400	0.192	6.402
-1.977	0.059	0.663	-3.969	-0.456	2.100
-2.136	0.043	0.623	-3.725	-0.559	2.224
-0.123	0.903	0.999	-5.567	-0.047	5.116
-0.848	0.404	0.968	-5.258	-0.168	6.142
-0.534	0.598	0.999	-5.447	-0.133	7.257
1.212	0.237	0.899	-4.939	0.341	4.639
-1.243	0.225	0.886	-4.908	-0.288	4.086
-0.903	0.375	0.963	-5.217	-0.209	3.635
-0.141	0.889	0.999	-5.565	-0.032	6.477
2.007	0.056	0.656	-3.925	0.543	3.664
0.197	0.845	0.999	-5.557	0.072	7.445
1.371	0.183	0.840	-4.769	0.411	5.220
-0.476	0.638	0.999	-5.473	-0.138	3.279
1.861	0.075	0.702	-4.140	0.509	6.250
0.204	0.840	0.999	-5.555	0.064	6.923

0.801	0.430	0.973	-5.291	0.273	4.806
1.571	0.129	0.789	-4.529	0.403	6.465
0.478	0.637	0.999	-5.472	0.128	6.537
0.098	0.923	0.999	-5.570	0.051	6.863
-0.851	0.403	0.967	-5.256	-0.348	3.138
-1.132	0.268	0.917	-5.018	-0.254	2.917
0.915	0.369	0.963	-5.207	0.218	4.325
-0.786	0.439	0.976	-5.302	-0.260	3.476
-0.301	0.766	0.999	-5.534	-0.090	2.633
-0.148	0.884	0.999	-5.564	-0.031	6.399
0.388	0.701	0.999	-5.507	0.249	2.630
-2.585	0.016	0.500	-2.975	-0.731	2.332
-0.912	0.370	0.963	-5.209	-0.228	2.953
-0.472	0.641	0.999	-5.475	-0.150	2.584
1.075	0.293	0.934	-5.071	0.221	4.713
0.074	0.942	0.999	-5.572	0.019	7.606
0.760	0.454	0.981	-5.320	0.257	3.290
0.550	0.587	0.999	-5.440	0.164	3.042
0.343	0.735	0.999	-5.522	0.151	3.698
2.579	0.016	0.500	-2.985	0.981	3.219
2.991	0.006	0.393	-2.237	0.966	4.045
1.782	0.087	0.731	-4.250	0.486	4.095
0.986	0.334	0.957	-5.149	0.222	5.092
-0.446	0.660	0.999	-5.486	-0.144	2.584
-0.423	0.676	0.999	-5.495	-0.153	5.272
1.570	0.129	0.789	-4.531	0.443	6.864
-0.520	0.607	0.999	-5.454	-0.126	2.922
1.687	0.104	0.764	-4.379	0.418	4.948
0.949	0.352	0.961	-5.180	0.310	3.340
-0.299	0.767	0.999	-5.534	-0.096	3.607
1.602	0.122	0.784	-4.491	0.553	4.353
-0.731	0.472	0.984	-5.338	-0.356	4.572
-0.513	0.612	0.999	-5.457	-0.115	5.027
-0.443	0.662	0.999	-5.487	-0.158	3.070
0.358	0.724	0.999	-5.517	0.097	4.097
-1.312	0.202	0.861	-4.835	-0.350	3.356
-0.448	0.658	0.999	-5.485	-0.108	4.271
1.684	0.105	0.764	-4.383	0.761	3.666
-1.599	0.122	0.784	-4.495	-0.481	2.143
-0.294	0.771	0.999	-5.536	-0.061	3.387
0.220	0.827	0.999	-5.552	0.048	5.893
-0.496	0.624	0.999	-5.465	-0.133	2.283
-1.431	0.165	0.824	-4.700	-0.353	2.627
-0.112	0.912	0.999	-5.568	-0.029	2.539
0.748	0.462	0.983	-5.328	0.246	4.367
-0.648	0.523	0.990	-5.388	-0.318	2.439
0.158	0.876	0.999	-5.563	0.045	1.291
-1.325	0.197	0.855	-4.820	-0.821	2.602
0.633	0.533	0.992	-5.397	0.161	2.202
-0.444	0.661	0.999	-5.486	-0.150	3.323

0.060	0.953	0.999	-5.572	0.025	1.684
-0.336	0.740	0.999	-5.524	-0.098	2.152
-0.438	0.665	0.999	-5.489	-0.181	3.392
-1.836	0.078	0.710	-4.174	-0.480	0.862
-0.298	0.768	0.999	-5.534	-0.083	2.400
0.092	0.928	0.999	-5.570	0.031	1.426
1.326	0.197	0.855	-4.818	0.300	3.943
-0.308	0.760	0.999	-5.532	-0.078	3.662
1.400	0.174	0.828	-4.736	0.372	3.865
-0.562	0.579	0.999	-5.434	-0.142	2.009
-1.217	0.235	0.899	-4.934	-0.499	2.975
-1.215	0.236	0.899	-4.936	-0.326	2.829
-1.147	0.262	0.914	-5.003	-0.293	2.437
-1.094	0.284	0.926	-5.054	-0.695	2.209
-0.276	0.785	0.999	-5.540	-0.070	2.402
-0.212	0.833	0.999	-5.554	-0.150	0.992
1.285	0.211	0.869	-4.864	0.322	2.983
-2.257	0.033	0.594	-3.531	-0.554	2.341
-1.285	0.210	0.868	-4.863	-0.339	3.185
-0.218	0.830	0.999	-5.553	-0.066	2.336
-0.429	0.672	0.999	-5.492	-0.173	3.613
-0.802	0.430	0.973	-5.291	-0.295	3.577
-0.400	0.693	0.999	-5.503	-0.198	2.180
0.963	0.345	0.957	-5.169	0.455	5.298
-0.419	0.679	0.999	-5.496	-0.239	4.563
-0.793	0.435	0.973	-5.297	-0.303	2.450
0.252	0.803	0.999	-5.546	0.088	2.238
-0.369	0.715	0.999	-5.513	-0.203	3.298
-0.245	0.808	0.999	-5.547	-0.044	5.261
-0.871	0.392	0.964	-5.241	-0.286	5.158
-2.323	0.029	0.567	-3.423	-0.832	1.990
-0.117	0.907	0.999	-5.568	-0.065	4.480
-0.200	0.843	0.999	-5.556	-0.041	6.639
-0.962	0.345	0.957	-5.169	-0.244	1.919
-0.280	0.782	0.999	-5.539	-0.071	2.975
0.721	0.478	0.984	-5.345	0.151	4.366
4.764	0.000	0.256	1.264	1.222	2.775
1.715	0.099	0.757	-4.342	0.401	2.985
0.397	0.695	0.999	-5.504	0.096	4.906
0.355	0.725	0.999	-5.518	0.162	4.455
-0.722	0.477	0.984	-5.344	-0.254	3.498
-0.656	0.518	0.990	-5.383	-0.214	3.532
0.799	0.432	0.973	-5.293	0.267	2.359
-0.260	0.797	0.999	-5.544	-0.064	2.657
-0.237	0.814	0.999	-5.549	-0.047	3.809
1.211	0.237	0.899	-4.940	0.302	4.719
0.059	0.953	0.999	-5.572	0.015	3.370
-0.077	0.939	0.999	-5.571	-0.020	2.760
-1.847	0.076	0.707	-4.158	-0.376	7.760
0.517	0.609	0.999	-5.455	0.122	3.851

0.612	0.546	0.994	-5.408	0.312	2.498
0.460	0.650	0.999	-5.480	0.127	3.189
-1.035	0.310	0.943	-5.107	-0.340	3.132
-2.066	0.049	0.638	-3.834	-0.761	2.356
0.061	0.951	0.999	-5.572	0.021	3.015
2.506	0.019	0.507	-3.112	0.601	4.560
0.561	0.580	0.999	-5.434	0.154	2.084
0.637	0.530	0.992	-5.394	0.118	4.736
1.820	0.081	0.718	-4.197	0.464	4.705
0.090	0.929	0.999	-5.570	0.024	4.377
-2.005	0.056	0.657	-3.927	-0.627	5.149
0.257	0.799	0.999	-5.544	0.061	3.250
1.396	0.175	0.829	-4.740	0.509	5.976
0.281	0.781	0.999	-5.539	0.104	5.015
-0.816	0.422	0.972	-5.281	-0.230	4.394
-0.938	0.357	0.962	-5.188	-0.264	4.814
-0.209	0.836	0.999	-5.554	-0.055	7.222
-0.207	0.837	0.999	-5.555	-0.052	7.027
-0.152	0.880	0.999	-5.564	-0.037	7.068
-0.110	0.913	0.999	-5.569	-0.026	7.284
0.922	0.365	0.963	-5.201	0.288	5.389
0.076	0.940	0.999	-5.571	0.045	1.771
0.159	0.875	0.999	-5.563	0.043	2.363
-0.325	0.748	0.999	-5.527	-0.088	2.525
2.801	0.010	0.447	-2.588	0.796	3.482
-0.964	0.344	0.957	-5.168	-0.359	4.829
-0.386	0.703	0.999	-5.508	-0.105	3.813
-0.792	0.436	0.973	-5.298	-0.379	5.101
0.930	0.361	0.963	-5.195	0.285	7.627
1.112	0.276	0.921	-5.036	0.321	3.885
0.637	0.530	0.992	-5.395	0.224	6.601
-0.177	0.861	0.999	-5.560	-0.087	4.082
0.097	0.924	0.999	-5.570	0.029	2.861
0.450	0.656	0.999	-5.484	0.239	4.406
0.458	0.651	0.999	-5.481	0.122	3.302
0.263	0.794	0.999	-5.543	0.073	3.485
-0.325	0.748	0.999	-5.527	-0.101	3.309
-0.214	0.833	0.999	-5.554	-0.060	2.415
-1.110	0.277	0.922	-5.039	-0.294	3.159
2.060	0.050	0.639	-3.843	0.682	3.127
-1.400	0.174	0.828	-4.736	-0.274	3.931
-0.405	0.689	0.999	-5.501	-0.120	2.758
0.641	0.527	0.992	-5.392	0.244	1.916
0.460	0.650	0.999	-5.480	0.133	2.948
-0.852	0.402	0.967	-5.255	-0.263	2.968
0.065	0.949	0.999	-5.572	0.022	1.462
-0.297	0.769	0.999	-5.535	-0.098	3.429
2.027	0.053	0.653	-3.894	0.545	2.934
0.680	0.503	0.988	-5.369	0.164	2.080
-0.194	0.848	0.999	-5.557	-0.077	3.808

-1.343	0.191	0.850	-4.800	-0.678	4.347
1.175	0.251	0.910	-4.976	0.310	2.614
0.610	0.547	0.994	-5.409	0.156	5.226
-0.407	0.688	0.999	-5.500	-0.132	2.666
-1.387	0.178	0.837	-4.751	-1.084	3.208
0.120	0.905	0.999	-5.567	0.044	3.925
-0.727	0.474	0.984	-5.341	-0.201	5.568
-1.110	0.278	0.922	-5.039	-0.330	4.870
0.235	0.816	0.999	-5.549	0.083	1.237
0.120	0.906	0.999	-5.568	0.074	1.165
-0.143	0.888	0.999	-5.565	-0.041	3.253
0.373	0.713	0.999	-5.512	0.187	3.869
-1.869	0.073	0.698	-4.127	-0.947	2.909
0.243	0.810	0.999	-5.548	0.074	7.240
0.325	0.748	0.999	-5.527	0.145	6.528
0.060	0.952	0.999	-5.572	0.028	3.485
1.465	0.155	0.817	-4.660	0.511	7.436
0.675	0.506	0.988	-5.373	0.225	7.824
0.711	0.484	0.985	-5.351	0.182	1.971
-0.096	0.925	0.999	-5.570	-0.031	7.320
-0.511	0.614	0.999	-5.458	-0.135	3.349
0.865	0.395	0.964	-5.245	0.280	3.245
-0.476	0.638	0.999	-5.473	-0.092	6.580
1.253	0.222	0.884	-4.897	0.286	6.965
0.620	0.541	0.994	-5.404	0.122	4.942
0.352	0.728	0.999	-5.519	0.079	5.069
3.001	0.006	0.393	-2.217	0.862	4.599
-0.336	0.740	0.999	-5.524	-0.110	4.043
0.886	0.384	0.964	-5.230	0.329	5.645
0.133	0.895	0.999	-5.566	0.043	3.420
-0.057	0.955	0.999	-5.572	-0.019	3.698
1.368	0.184	0.841	-4.773	0.400	3.334
-0.363	0.720	0.999	-5.515	-0.127	5.687
1.297	0.207	0.868	-4.851	0.368	3.329
0.439	0.664	0.999	-5.488	0.130	4.760
-0.110	0.913	0.999	-5.569	-0.053	3.156
0.707	0.486	0.985	-5.353	0.190	3.805
-1.818	0.081	0.719	-4.201	-0.415	2.124
0.394	0.697	0.999	-5.505	0.110	3.809
0.423	0.676	0.999	-5.494	0.148	1.509
0.477	0.637	0.999	-5.473	0.118	3.931
-0.720	0.478	0.984	-5.345	-0.178	2.410
-1.994	0.057	0.659	-3.944	-0.691	1.966
-0.073	0.942	0.999	-5.572	-0.022	4.526
0.417	0.680	0.999	-5.497	0.123	2.092
-1.431	0.165	0.824	-4.700	-0.361	3.564
-1.539	0.136	0.795	-4.570	-0.311	3.491
0.438	0.665	0.999	-5.489	0.117	2.515
-1.028	0.314	0.944	-5.113	-0.215	1.727
1.022	0.317	0.948	-5.118	0.300	2.378

-0.411	0.685	0.999	-5.499	-0.096	4.975
0.478	0.637	0.999	-5.473	0.146	4.742
-0.874	0.390	0.964	-5.239	-0.160	2.685
0.512	0.613	0.999	-5.458	0.218	3.135
1.117	0.274	0.921	-5.032	0.526	3.177
-0.765	0.451	0.980	-5.316	-0.176	2.996
0.853	0.402	0.966	-5.254	0.248	3.615
0.286	0.777	0.999	-5.537	0.067	4.475
1.107	0.279	0.922	-5.042	0.275	2.651
-1.240	0.226	0.888	-4.910	-0.308	2.636
0.678	0.504	0.988	-5.371	0.204	3.020
0.493	0.626	0.999	-5.466	0.142	2.437
-0.246	0.808	0.999	-5.547	-0.065	2.948
1.276	0.213	0.873	-4.872	0.394	3.061
0.714	0.482	0.984	-5.349	0.194	2.230
-0.287	0.777	0.999	-5.537	-0.066	2.838
-0.973	0.340	0.957	-5.160	-0.227	4.171
-0.332	0.742	0.999	-5.525	-0.073	2.437
0.761	0.454	0.981	-5.319	0.223	2.159
0.750	0.460	0.983	-5.326	0.199	3.495
0.576	0.570	0.997	-5.427	0.158	4.508
0.673	0.507	0.988	-5.374	0.236	2.315
-0.393	0.698	0.999	-5.505	-0.120	2.471
-1.255	0.221	0.882	-4.894	-0.283	2.847
0.055	0.957	0.999	-5.573	0.017	3.214
-0.298	0.768	0.999	-5.534	-0.087	3.355
-0.516	0.611	0.999	-5.456	-0.158	2.353
-0.408	0.687	0.999	-5.500	-0.126	5.110
1.128	0.270	0.918	-5.021	0.320	2.242
1.126	0.271	0.919	-5.024	0.295	2.910
0.416	0.681	0.999	-5.497	0.126	2.449
0.448	0.658	0.999	-5.485	0.101	8.237
0.241	0.811	0.999	-5.548	0.067	2.570
0.485	0.632	0.999	-5.470	0.126	1.869
0.052	0.959	0.999	-5.573	0.014	4.399
-0.439	0.665	0.999	-5.488	-0.124	2.629
0.774	0.446	0.979	-5.310	0.203	4.017
-0.063	0.950	0.999	-5.572	-0.017	1.531
2.119	0.044	0.630	-3.752	0.605	3.915
-0.888	0.383	0.964	-5.228	-0.209	5.898
1.463	0.156	0.817	-4.662	0.353	2.761
0.077	0.939	0.999	-5.571	0.025	3.183
-1.345	0.191	0.849	-4.798	-0.347	4.739
-0.784	0.440	0.977	-5.303	-0.216	8.351
-0.669	0.510	0.989	-5.376	-0.190	8.353
0.145	0.886	0.999	-5.565	0.071	4.859
-0.389	0.700	0.999	-5.507	-0.112	3.991
1.198	0.242	0.904	-4.954	0.369	6.763
0.922	0.366	0.963	-5.202	0.304	5.743
0.688	0.498	0.987	-5.365	0.189	5.869

0.518	0.609	0.999	-5.455	0.151	5.875
-0.359	0.723	0.999	-5.517	-0.124	5.136
0.955	0.349	0.960	-5.175	0.542	4.624
1.073	0.294	0.935	-5.073	0.263	2.559
0.587	0.562	0.996	-5.421	0.191	4.080
-0.075	0.941	0.999	-5.571	-0.020	4.549
-0.370	0.714	0.999	-5.513	-0.101	4.187
-1.155	0.259	0.913	-4.995	-0.431	9.067
1.953	0.062	0.667	-4.005	0.504	4.130
-0.626	0.537	0.993	-5.401	-0.134	3.589
-1.514	0.143	0.802	-4.601	-0.376	3.054
0.305	0.763	0.999	-5.532	0.093	4.689
1.355	0.187	0.845	-4.786	0.482	2.131
1.283	0.211	0.869	-4.865	0.347	3.410
0.415	0.682	0.999	-5.497	0.122	3.003
1.148	0.262	0.914	-5.003	0.335	3.678
0.611	0.547	0.994	-5.409	0.133	3.210
-1.607	0.120	0.784	-4.484	-0.399	3.687
-1.658	0.110	0.767	-4.418	-0.417	3.392
0.764	0.452	0.980	-5.317	0.158	4.343
0.810	0.426	0.972	-5.285	0.159	2.425
-2.061	0.050	0.639	-3.842	-0.530	2.283
-0.126	0.901	0.999	-5.567	-0.032	2.823
1.946	0.063	0.670	-4.015	0.415	2.754
0.561	0.580	0.999	-5.434	0.243	2.749
1.058	0.300	0.941	-5.087	0.628	5.483
-1.218	0.235	0.899	-4.933	-0.328	2.554
-1.432	0.164	0.824	-4.699	-0.405	3.882
0.315	0.755	0.999	-5.530	0.076	2.973
0.158	0.876	0.999	-5.563	0.040	3.645
0.553	0.585	0.999	-5.439	0.153	2.323
-1.584	0.126	0.786	-4.514	-0.464	2.451
0.152	0.881	0.999	-5.564	0.036	2.569
-0.383	0.705	0.999	-5.509	-0.081	2.994
-0.921	0.366	0.963	-5.203	-0.265	2.180
0.364	0.719	0.999	-5.515	0.097	3.946
0.883	0.386	0.964	-5.232	0.303	2.922
1.135	0.267	0.917	-5.015	0.331	3.938
-0.605	0.551	0.994	-5.412	-0.166	3.221
-1.426	0.166	0.825	-4.706	-0.362	3.566
-1.287	0.210	0.868	-4.861	-0.479	3.715
0.118	0.907	0.999	-5.568	0.050	2.298
-0.520	0.607	0.999	-5.454	-0.133	2.740
-0.074	0.942	0.999	-5.572	-0.027	2.373
-1.228	0.231	0.894	-4.923	-0.291	2.665
-0.548	0.589	0.999	-5.441	-0.149	2.601
0.638	0.530	0.992	-5.394	0.193	3.675
0.939	0.357	0.962	-5.188	0.267	1.951
-3.717	0.001	0.301	-0.827	-1.233	1.622
-0.460	0.649	0.999	-5.480	-0.119	4.179

-0.510	0.615	0.999	-5.459	-0.124	5.357
0.338	0.738	0.999	-5.523	0.090	3.010
0.546	0.590	0.999	-5.442	0.260	1.921
-0.053	0.958	0.999	-5.573	-0.016	5.523
-0.334	0.741	0.999	-5.524	-0.119	2.727
0.256	0.800	0.999	-5.545	0.061	3.477
0.124	0.902	0.999	-5.567	0.037	4.633
0.877	0.389	0.964	-5.237	0.205	2.583
0.828	0.415	0.970	-5.272	0.337	2.931
0.611	0.547	0.994	-5.409	0.229	3.113
2.553	0.017	0.500	-3.032	0.864	3.941
0.051	0.960	0.999	-5.573	0.013	3.393
-0.381	0.706	0.999	-5.509	-0.236	4.983
0.626	0.537	0.993	-5.400	0.290	2.751
-0.697	0.492	0.986	-5.360	-0.235	2.996
1.677	0.106	0.764	-4.393	0.412	3.091
-0.224	0.825	0.999	-5.552	-0.066	2.645
1.959	0.061	0.665	-3.996	0.589	4.543
0.704	0.488	0.985	-5.355	0.322	3.582
-0.984	0.334	0.957	-5.151	-0.247	3.543
-0.220	0.827	0.999	-5.552	-0.058	4.004
-0.812	0.424	0.972	-5.284	-0.192	1.483
1.734	0.095	0.754	-4.317	0.382	3.760
-0.665	0.512	0.989	-5.378	-0.215	3.170
0.186	0.854	0.999	-5.559	0.052	3.003
0.122	0.904	0.999	-5.567	0.030	5.612
-0.714	0.482	0.984	-5.349	-0.216	3.967
-2.735	0.011	0.481	-2.707	-0.685	2.289
0.995	0.329	0.955	-5.141	0.300	5.086
-0.184	0.856	0.999	-5.559	-0.067	3.583
1.143	0.264	0.915	-5.008	0.268	3.553
-1.810	0.082	0.722	-4.211	-0.499	2.470
0.163	0.872	0.999	-5.562	0.047	3.463
-2.025	0.054	0.655	-3.897	-0.708	2.690
0.540	0.594	0.999	-5.445	0.219	2.438
0.726	0.475	0.984	-5.342	0.194	4.648
0.246	0.807	0.999	-5.547	0.103	2.681
0.779	0.443	0.979	-5.307	0.207	3.586
0.664	0.513	0.990	-5.379	0.231	3.820
0.347	0.732	0.999	-5.520	0.085	2.662
-0.307	0.761	0.999	-5.532	-0.141	3.567
0.168	0.868	0.999	-5.561	0.079	3.156
-1.013	0.321	0.950	-5.126	-0.377	3.955
2.090	0.047	0.630	-3.797	0.558	4.707
-2.430	0.023	0.542	-3.243	-0.673	4.357
-0.203	0.841	0.999	-5.556	-0.049	8.151
1.135	0.267	0.917	-5.015	0.290	2.406
-1.545	0.135	0.793	-4.563	-0.513	4.408
0.089	0.930	0.999	-5.570	0.031	2.304
1.614	0.119	0.780	-4.475	0.588	2.476

0.535	0.597	0.999	-5.447	0.198	4.476
-1.345	0.191	0.849	-4.798	-0.352	2.581
0.293	0.772	0.999	-5.536	0.075	1.569
0.514	0.612	0.999	-5.457	0.148	3.390
0.713	0.482	0.985	-5.349	0.208	3.300
1.625	0.117	0.776	-4.461	0.505	3.492
-0.790	0.437	0.973	-5.299	-0.232	2.523
-1.391	0.176	0.834	-4.746	-0.340	3.012
-0.108	0.915	0.999	-5.569	-0.024	4.426
0.091	0.929	0.999	-5.570	0.027	3.600
0.999	0.327	0.954	-5.138	0.218	2.559
-1.094	0.284	0.926	-5.053	-0.369	3.257
0.109	0.914	0.999	-5.569	0.027	6.593
-0.738	0.468	0.983	-5.334	-0.269	1.554
0.160	0.874	0.999	-5.563	0.039	1.766
0.071	0.944	0.999	-5.572	0.020	3.207
1.680	0.105	0.764	-4.388	0.510	3.659
0.391	0.699	0.999	-5.506	0.121	4.167
0.289	0.775	0.999	-5.537	0.121	3.576
-0.538	0.595	0.999	-5.446	-0.129	1.003
0.455	0.653	0.999	-5.482	0.151	6.056
-1.419	0.168	0.825	-4.714	-0.332	2.349
2.016	0.055	0.655	-3.910	0.505	2.980
-0.611	0.547	0.994	-5.409	-0.241	1.224
0.648	0.523	0.990	-5.388	0.255	4.581
-0.898	0.378	0.963	-5.221	-0.253	2.898
0.420	0.678	0.999	-5.496	0.090	4.256
0.090	0.929	0.999	-5.570	0.031	2.329
2.317	0.029	0.570	-3.432	1.078	4.774
-0.892	0.381	0.964	-5.225	-0.226	2.893
3.174	0.004	0.340	-1.889	1.219	6.722
-0.430	0.671	0.999	-5.492	-0.191	1.280
0.500	0.621	0.999	-5.463	0.141	3.025
-1.116	0.275	0.921	-5.033	-0.294	2.816
-0.752	0.459	0.982	-5.325	-0.254	1.278
-0.517	0.610	0.999	-5.455	-0.147	1.472
1.566	0.130	0.789	-4.537	0.384	4.036
1.698	0.102	0.764	-4.365	0.431	3.191
-0.550	0.587	0.999	-5.440	-0.176	3.156
-1.342	0.192	0.850	-4.801	-0.340	3.610
3.027	0.006	0.393	-2.169	0.886	4.052
-0.312	0.758	0.999	-5.531	-0.136	5.027
-0.290	0.774	0.999	-5.537	-0.086	2.216
1.105	0.280	0.922	-5.044	0.425	1.682
0.659	0.516	0.990	-5.382	0.191	2.381
0.159	0.875	0.999	-5.563	0.084	2.615
0.206	0.838	0.999	-5.555	0.081	2.594
-1.125	0.271	0.919	-5.025	-0.274	3.042
0.985	0.334	0.957	-5.150	0.295	2.660
-0.929	0.362	0.963	-5.196	-0.306	0.894

0.078	0.938	0.999	-5.571	0.029	2.707
0.931	0.361	0.963	-5.194	0.222	2.808
-0.896	0.379	0.963	-5.222	-0.201	1.737
-1.355	0.188	0.846	-4.787	-0.310	1.855
0.469	0.643	0.999	-5.476	0.119	3.130
-0.594	0.558	0.995	-5.418	-0.283	1.724
0.875	0.390	0.964	-5.238	0.296	3.640
0.148	0.883	0.999	-5.564	0.064	1.271
-0.470	0.643	0.999	-5.476	-0.124	3.477
-0.431	0.670	0.999	-5.491	-0.122	2.391
-0.703	0.489	0.986	-5.356	-0.169	3.419
-0.221	0.827	0.999	-5.552	-0.046	3.292
-1.375	0.181	0.840	-4.764	-0.524	1.983
-0.873	0.391	0.964	-5.240	-0.229	3.080
0.200	0.843	0.999	-5.556	0.058	2.816
0.223	0.825	0.999	-5.552	0.063	3.369
-0.292	0.773	0.999	-5.536	-0.065	4.386
-3.366	0.002	0.301	-1.518	-1.143	2.099
2.930	0.007	0.411	-2.351	0.688	3.545
0.404	0.689	0.999	-5.501	0.118	2.551
-0.428	0.672	0.999	-5.492	-0.178	1.992
1.718	0.098	0.757	-4.338	0.612	5.012
0.582	0.566	0.997	-5.424	0.149	6.273
0.252	0.803	0.999	-5.546	0.061	6.249
0.337	0.739	0.999	-5.523	0.074	7.515
1.599	0.122	0.784	-4.495	0.634	4.667
-0.162	0.872	0.999	-5.562	-0.034	2.016
-0.497	0.623	0.999	-5.464	-0.154	3.179
0.696	0.493	0.986	-5.360	0.215	3.597
-1.173	0.252	0.910	-4.978	-0.469	3.908
-1.153	0.260	0.914	-4.998	-0.260	2.300
-1.471	0.154	0.814	-4.653	-0.520	2.003
-0.512	0.613	0.999	-5.458	-0.145	3.758
-0.106	0.917	0.999	-5.569	-0.055	4.849
-1.217	0.235	0.899	-4.934	-0.363	2.461
0.497	0.624	0.999	-5.464	0.219	5.556
0.517	0.610	0.999	-5.456	0.190	2.827
-1.535	0.137	0.795	-4.575	-0.623	3.367
1.580	0.127	0.786	-4.519	0.507	7.998
0.723	0.476	0.984	-5.343	0.184	7.924
-0.849	0.404	0.968	-5.257	-0.189	3.119
0.513	0.612	0.999	-5.457	0.157	3.202
-0.640	0.528	0.992	-5.393	-0.145	2.218
2.611	0.015	0.498	-2.929	1.042	3.196
0.608	0.549	0.994	-5.410	0.204	2.738
-0.104	0.918	0.999	-5.569	-0.026	1.568
-0.512	0.613	0.999	-5.458	-0.174	1.594
-1.377	0.181	0.840	-4.763	-0.448	2.896
0.450	0.657	0.999	-5.484	0.160	5.296
1.476	0.152	0.813	-4.646	0.395	2.541

1.471	0.154	0.814	-4.652	0.350	3.634
-0.499	0.622	0.999	-5.463	-0.109	1.439
0.449	0.657	0.999	-5.484	0.147	1.953
-0.450	0.657	0.999	-5.484	-0.133	1.619
0.549	0.588	0.999	-5.440	0.149	3.042
0.613	0.545	0.994	-5.407	0.188	2.789
-0.718	0.479	0.984	-5.346	-0.264	1.525
-1.850	0.076	0.706	-4.154	-0.407	4.289
0.321	0.751	0.999	-5.528	0.089	3.417
-1.684	0.104	0.764	-4.383	-0.409	2.595
-2.038	0.052	0.652	-3.878	-0.502	2.257
1.867	0.074	0.698	-4.130	0.469	4.678
-0.261	0.796	0.999	-5.544	-0.153	4.682
-1.321	0.199	0.857	-4.825	-0.549	4.153
0.910	0.371	0.963	-5.211	0.242	2.080
1.353	0.188	0.847	-4.789	0.340	3.130
0.478	0.637	0.999	-5.473	0.141	3.282
-0.072	0.943	0.999	-5.572	-0.028	6.894
0.292	0.773	0.999	-5.536	0.091	4.337
0.631	0.534	0.992	-5.398	0.330	4.285
0.872	0.392	0.964	-5.240	0.260	4.129
-1.310	0.202	0.862	-4.836	-0.411	3.284
-0.612	0.546	0.994	-5.408	-0.122	7.651
-0.340	0.737	0.999	-5.522	-0.139	5.064
-1.526	0.139	0.799	-4.586	-0.386	3.979
-1.447	0.160	0.820	-4.681	-0.344	2.802
0.671	0.508	0.988	-5.375	0.169	4.292
1.192	0.244	0.904	-4.959	0.379	2.767
-0.910	0.372	0.963	-5.211	-0.225	3.966
-0.093	0.926	0.999	-5.570	-0.024	4.728
0.224	0.824	0.999	-5.552	0.058	1.635
-0.181	0.858	0.999	-5.559	-0.059	3.617
1.583	0.126	0.786	-4.514	0.402	1.975
-0.159	0.875	0.999	-5.563	-0.053	1.605
0.692	0.495	0.987	-5.363	0.190	2.263
-0.125	0.901	0.999	-5.567	-0.033	3.599
2.603	0.015	0.500	-2.943	0.586	3.065
-0.146	0.885	0.999	-5.564	-0.040	4.165
-2.935	0.007	0.411	-2.340	-0.914	2.211
-0.076	0.940	0.999	-5.571	-0.023	2.976
1.572	0.128	0.789	-4.528	1.036	5.752
0.577	0.569	0.997	-5.426	0.204	2.741
-0.089	0.930	0.999	-5.570	-0.027	3.839
-0.174	0.863	0.999	-5.560	-0.065	3.368
0.757	0.456	0.981	-5.321	0.207	2.652
-0.873	0.391	0.964	-5.240	-0.284	3.842
-1.912	0.067	0.682	-4.065	-0.550	1.485
-0.431	0.670	0.999	-5.491	-0.128	2.402
-0.190	0.851	0.999	-5.558	-0.045	2.525
0.624	0.538	0.994	-5.402	0.166	2.894

-0.218	0.829	0.999	-5.553	-0.060	0.845
0.178	0.860	0.999	-5.560	0.068	3.685
-0.452	0.655	0.999	-5.483	-0.120	2.346
-0.511	0.614	0.999	-5.458	-0.163	2.173
1.129	0.269	0.918	-5.021	0.396	2.956
0.494	0.626	0.999	-5.466	0.121	2.654
-2.665	0.013	0.498	-2.834	-0.641	3.075
0.686	0.499	0.987	-5.366	0.181	2.483
-0.315	0.756	0.999	-5.530	-0.091	4.186
0.673	0.507	0.988	-5.374	0.186	2.066
1.447	0.160	0.820	-4.682	0.438	3.038
-1.250	0.223	0.886	-4.900	-0.314	1.951
-0.133	0.895	0.999	-5.566	-0.035	4.726
-0.702	0.489	0.986	-5.356	-0.359	2.538
-0.574	0.571	0.997	-5.428	-0.240	2.207
-0.452	0.655	0.999	-5.483	-0.129	2.646
0.754	0.458	0.982	-5.324	0.346	3.130
0.771	0.448	0.979	-5.312	0.289	4.314
0.859	0.399	0.964	-5.250	0.224	2.612
1.069	0.295	0.936	-5.076	0.385	3.471
-0.832	0.413	0.970	-5.269	-0.248	3.676
1.332	0.195	0.853	-4.812	0.336	3.111
0.439	0.664	0.999	-5.488	0.281	1.166
-0.686	0.499	0.987	-5.366	-0.271	2.036
2.755	0.011	0.468	-2.672	0.681	4.222
-0.064	0.949	0.999	-5.572	-0.017	2.582
-0.158	0.876	0.999	-5.563	-0.042	1.990
0.141	0.889	0.999	-5.565	0.038	4.623
1.000	0.327	0.954	-5.137	0.326	3.153
1.563	0.131	0.791	-4.540	0.460	2.978
0.910	0.372	0.963	-5.211	0.380	5.038
-2.589	0.016	0.500	-2.969	-0.546	4.703
0.994	0.330	0.955	-5.142	0.291	3.050
-0.590	0.561	0.996	-5.420	-0.136	4.896
2.399	0.024	0.543	-3.296	0.611	3.350
1.152	0.260	0.914	-4.999	0.293	5.506
-0.722	0.477	0.984	-5.344	-0.195	3.255
1.509	0.144	0.803	-4.607	0.432	3.009
1.103	0.281	0.922	-5.045	0.541	4.445
1.447	0.160	0.820	-4.682	0.528	3.918
1.118	0.274	0.921	-5.031	0.221	6.877
0.695	0.493	0.987	-5.361	0.186	3.541
0.447	0.658	0.999	-5.485	0.134	2.559
0.093	0.927	0.999	-5.570	0.028	4.942
0.544	0.591	0.999	-5.443	0.124	3.804
0.459	0.650	0.999	-5.480	0.122	3.121
-0.070	0.944	0.999	-5.572	-0.020	2.361
-0.833	0.412	0.970	-5.269	-0.253	1.833
0.071	0.944	0.999	-5.572	0.017	3.624
0.892	0.381	0.964	-5.225	0.223	2.626

-0.306	0.762	0.999	-5.532	-0.111	3.985
0.533	0.599	0.999	-5.448	0.233	2.771
2.988	0.006	0.393	-2.242	0.691	2.773
-0.974	0.339	0.957	-5.159	-0.277	2.315
-0.210	0.836	0.999	-5.554	-0.078	1.582
0.500	0.622	0.999	-5.463	0.148	2.252
0.792	0.436	0.973	-5.298	0.368	2.599
-2.035	0.053	0.653	-3.882	-0.527	2.082
-0.710	0.484	0.985	-5.351	-0.175	3.242
-0.315	0.755	0.999	-5.530	-0.078	4.451
1.910	0.068	0.683	-4.068	0.581	4.565
0.633	0.533	0.992	-5.397	0.196	2.221
1.175	0.251	0.910	-4.977	0.317	5.426
1.534	0.138	0.795	-4.576	0.384	3.404
0.711	0.484	0.985	-5.351	0.170	3.279
0.644	0.525	0.992	-5.390	0.234	3.439
-0.412	0.684	0.999	-5.498	-0.165	4.755
-0.193	0.849	0.999	-5.557	-0.100	2.807
-1.337	0.193	0.853	-4.806	-0.373	2.899
-0.875	0.390	0.964	-5.238	-0.252	2.335
-0.225	0.824	0.999	-5.551	-0.129	3.330
0.668	0.510	0.989	-5.377	0.235	4.359
-0.095	0.925	0.999	-5.570	-0.029	3.155
0.902	0.376	0.963	-5.217	0.324	4.925
-0.337	0.739	0.999	-5.523	-0.087	2.491
-2.395	0.024	0.543	-3.303	-0.509	2.562
-0.968	0.342	0.957	-5.164	-0.236	1.672
1.734	0.095	0.754	-4.317	0.682	3.568
-1.291	0.209	0.868	-4.857	-0.376	2.295
-2.175	0.039	0.612	-3.664	-0.589	5.518
-1.228	0.231	0.894	-4.923	-0.465	3.055
0.580	0.567	0.997	-5.425	0.179	3.869
0.441	0.663	0.999	-5.487	0.124	5.261
-1.490	0.149	0.806	-4.630	-0.383	2.502
-0.954	0.349	0.960	-5.176	-0.291	2.367
-0.954	0.349	0.960	-5.175	-0.267	2.741
-0.100	0.921	0.999	-5.569	-0.023	2.152
0.461	0.649	0.999	-5.479	0.244	1.703
-0.424	0.675	0.999	-5.494	-0.163	4.155
1.259	0.220	0.880	-4.890	0.358	2.156
-0.241	0.811	0.999	-5.548	-0.063	2.195
0.502	0.620	0.999	-5.462	0.182	7.564
0.197	0.845	0.999	-5.557	0.071	6.932
-0.206	0.838	0.999	-5.555	-0.070	4.257
-1.072	0.294	0.935	-5.074	-0.253	3.423
-1.159	0.257	0.913	-4.991	-0.346	7.396
-0.907	0.373	0.963	-5.213	-0.205	9.768
-0.477	0.637	0.999	-5.473	-0.165	2.614
0.381	0.707	0.999	-5.509	0.140	3.106
-0.138	0.891	0.999	-5.565	-0.034	3.527

0.519	0.608	0.999	-5.455	0.126	2.551
-1.985	0.058	0.661	-3.957	-0.431	3.682
-0.298	0.768	0.999	-5.534	-0.206	3.953
0.679	0.503	0.988	-5.370	0.201	2.081
1.510	0.144	0.803	-4.606	0.547	5.654
0.406	0.688	0.999	-5.500	0.210	5.036
0.812	0.425	0.972	-5.284	0.217	2.050
-0.444	0.661	0.999	-5.486	-0.156	4.566
-1.527	0.139	0.799	-4.585	-0.350	5.338
1.286	0.210	0.868	-4.862	0.431	5.608
0.943	0.354	0.962	-5.184	0.265	4.981
1.671	0.107	0.764	-4.401	0.749	3.483
-2.154	0.041	0.619	-3.697	-0.590	2.019
0.520	0.608	0.999	-5.454	0.142	1.824
-1.002	0.326	0.954	-5.136	-0.209	4.870
0.635	0.531	0.992	-5.395	0.219	2.170
0.731	0.471	0.984	-5.338	0.194	4.285
-3.805	0.001	0.301	-0.653	-2.029	3.725
-1.542	0.136	0.795	-4.567	-0.806	3.336
1.199	0.242	0.904	-4.952	0.294	3.176
0.474	0.640	0.999	-5.474	0.112	4.733
-0.540	0.594	0.999	-5.445	-0.210	5.825
-0.342	0.735	0.999	-5.522	-0.109	5.588
0.287	0.776	0.999	-5.537	0.079	8.354
0.173	0.864	0.999	-5.561	0.072	0.997
0.333	0.742	0.999	-5.525	0.081	10.013
-0.099	0.922	0.999	-5.570	-0.028	2.958
-1.399	0.174	0.828	-4.737	-0.401	5.237
0.515	0.611	0.999	-5.456	0.169	4.028
-0.802	0.430	0.973	-5.291	-0.175	2.653
0.771	0.448	0.979	-5.312	0.232	2.039
0.330	0.744	0.999	-5.525	0.084	3.603
-0.220	0.827	0.999	-5.552	-0.067	1.979
-0.395	0.696	0.999	-5.505	-0.166	3.226
0.757	0.456	0.981	-5.322	0.198	6.199
-1.141	0.265	0.916	-5.010	-0.342	2.385
-0.258	0.799	0.999	-5.544	-0.089	1.994
-1.055	0.301	0.941	-5.089	-0.256	1.959
0.627	0.537	0.993	-5.400	0.226	2.292
1.673	0.107	0.764	-4.398	0.516	4.817
0.929	0.362	0.963	-5.196	0.199	2.724
-1.264	0.218	0.877	-4.886	-0.317	3.078
0.892	0.381	0.964	-5.225	0.184	2.726
-1.652	0.111	0.767	-4.426	-0.377	3.843
1.247	0.224	0.886	-4.903	0.354	2.651
-0.687	0.499	0.987	-5.366	-0.155	3.426
-0.519	0.608	0.999	-5.455	-0.185	2.313
0.228	0.822	0.999	-5.551	0.055	3.718
1.588	0.125	0.786	-4.508	0.375	5.517
-0.474	0.640	0.999	-5.474	-0.103	2.840

1.150	0.261	0.914	-5.000	0.250	3.521
2.398	0.024	0.543	-3.298	0.700	5.627
0.539	0.594	0.999	-5.445	0.123	4.321
-0.503	0.619	0.999	-5.461	-0.166	8.996
-0.363	0.720	0.999	-5.515	-0.151	1.621
0.777	0.445	0.979	-5.308	0.292	3.695
1.454	0.158	0.819	-4.673	0.508	4.148
1.875	0.073	0.698	-4.119	0.580	3.345
0.908	0.372	0.963	-5.212	0.309	7.838
2.117	0.044	0.630	-3.755	0.436	6.278
0.141	0.889	0.999	-5.565	0.045	5.451
-0.107	0.916	0.999	-5.569	-0.033	3.104
0.293	0.772	0.999	-5.536	0.054	6.653
-0.942	0.355	0.962	-5.185	-0.229	6.630
-0.216	0.831	0.999	-5.553	-0.099	4.321
-0.714	0.482	0.984	-5.349	-0.141	6.630
1.369	0.183	0.841	-4.771	0.456	3.812
1.040	0.308	0.943	-5.102	0.235	4.084
0.638	0.530	0.992	-5.394	0.191	4.638
0.249	0.805	0.999	-5.546	0.085	4.854
0.421	0.678	0.999	-5.495	0.235	4.979
0.637	0.530	0.992	-5.394	0.302	2.921
0.154	0.879	0.999	-5.563	0.044	5.201
1.085	0.288	0.929	-5.062	0.390	3.719
0.974	0.339	0.957	-5.159	0.294	3.625
0.469	0.643	0.999	-5.476	0.146	3.032
-0.766	0.451	0.980	-5.315	-0.310	2.570
0.518	0.609	0.999	-5.455	0.173	3.868
0.944	0.354	0.961	-5.184	0.250	4.284
0.360	0.722	0.999	-5.516	0.077	5.476
1.202	0.240	0.902	-4.949	0.242	4.541
0.221	0.827	0.999	-5.552	0.054	4.826
-0.557	0.583	0.999	-5.437	-0.116	6.514
-0.092	0.927	0.999	-5.570	-0.056	6.899
-2.224	0.035	0.600	-3.585	-0.731	4.852
1.239	0.227	0.888	-4.911	0.345	4.433
-0.568	0.575	0.999	-5.431	-0.149	4.741
-0.489	0.629	0.999	-5.468	-0.118	4.869
0.393	0.697	0.999	-5.505	0.142	2.710
0.824	0.418	0.971	-5.275	0.192	6.403
-0.754	0.458	0.982	-5.323	-0.289	2.509
-0.975	0.339	0.957	-5.159	-0.246	2.913
1.540	0.136	0.795	-4.568	0.468	3.256
1.714	0.099	0.757	-4.343	0.632	4.542
-0.495	0.625	0.999	-5.465	-0.143	6.155
-1.497	0.147	0.804	-4.621	-0.548	4.421
1.734	0.095	0.754	-4.317	0.465	4.014
-0.266	0.792	0.999	-5.542	-0.070	4.494
0.172	0.865	0.999	-5.561	0.041	2.279
1.146	0.262	0.914	-5.004	0.310	3.385

1.331	0.195	0.853	-4.813	0.337	3.366
-0.612	0.546	0.994	-5.408	-0.226	3.721
0.929	0.362	0.963	-5.196	0.230	5.192
-2.838	0.009	0.441	-2.520	-0.970	1.895
-2.883	0.008	0.427	-2.437	-0.972	3.052
-0.506	0.617	0.999	-5.460	-0.122	3.493
1.288	0.210	0.868	-4.860	0.297	6.202
0.742	0.465	0.983	-5.331	0.313	1.542
2.477	0.020	0.517	-3.163	0.871	5.743
-1.457	0.157	0.817	-4.669	-0.352	4.361
-0.203	0.841	0.999	-5.556	-0.092	4.757
0.945	0.354	0.961	-5.183	0.278	1.510
0.196	0.846	0.999	-5.557	0.093	4.436
-4.236	0.000	0.256	0.211	-0.913	2.141
-0.999	0.327	0.954	-5.138	-0.376	3.076
1.045	0.306	0.942	-5.098	0.335	5.956
-3.063	0.005	0.386	-2.100	-1.163	1.657
0.395	0.696	0.999	-5.504	0.116	5.308
0.406	0.688	0.999	-5.501	0.094	5.091
0.905	0.374	0.963	-5.215	0.251	3.881
1.547	0.134	0.793	-4.559	0.441	6.204
0.066	0.948	0.999	-5.572	0.018	4.532
1.416	0.169	0.825	-4.717	0.351	4.274
0.569	0.574	0.999	-5.430	0.209	3.715
1.089	0.287	0.929	-5.058	0.383	3.976
0.462	0.648	0.999	-5.479	0.160	6.279
0.564	0.578	0.999	-5.433	0.186	5.394
0.166	0.869	0.999	-5.562	0.069	4.489
-0.165	0.870	0.999	-5.562	-0.055	2.723
1.553	0.133	0.791	-4.552	0.504	3.636
-0.317	0.754	0.999	-5.529	-0.070	4.575
-0.263	0.795	0.999	-5.543	-0.069	3.214
-0.675	0.506	0.988	-5.373	-0.165	3.139
-1.643	0.113	0.774	-4.438	-0.400	4.119
-0.506	0.617	0.999	-5.460	-0.151	7.183
3.056	0.005	0.387	-2.114	0.822	4.250
2.656	0.014	0.498	-2.850	0.720	4.375
2.604	0.015	0.500	-2.942	0.730	4.274
2.583	0.016	0.500	-2.979	0.701	4.373
2.528	0.018	0.501	-3.075	0.673	4.602
2.415	0.023	0.543	-3.269	0.691	4.309
1.872	0.073	0.698	-4.124	0.552	2.829
0.418	0.679	0.999	-5.496	0.100	5.997
1.953	0.062	0.667	-4.006	0.439	4.763
-1.155	0.259	0.913	-4.995	-0.233	6.278
0.642	0.527	0.992	-5.392	0.214	5.100
1.409	0.171	0.826	-4.725	0.333	6.790
-0.650	0.521	0.990	-5.387	-0.457	5.160
0.862	0.397	0.964	-5.248	0.216	6.051
2.870	0.008	0.434	-2.461	0.945	3.721

3.628	0.001	0.301	-1.005	1.023	5.383
0.210	0.835	0.999	-5.554	0.064	7.782
-0.463	0.647	0.999	-5.479	-0.125	3.409
-0.128	0.899	0.999	-5.567	-0.039	3.063
2.435	0.022	0.540	-3.235	0.608	5.263
2.246	0.034	0.595	-3.549	0.960	7.987
-0.102	0.920	0.999	-5.569	-0.046	1.422
0.959	0.347	0.958	-5.171	0.265	3.281
1.931	0.065	0.673	-4.037	0.416	4.190
2.159	0.041	0.617	-3.689	0.698	6.661
-0.738	0.467	0.983	-5.334	-0.179	3.640
-2.107	0.045	0.630	-3.771	-0.645	4.198
1.201	0.241	0.902	-4.950	0.269	5.060
0.684	0.500	0.988	-5.367	0.277	1.276
1.327	0.197	0.855	-4.818	0.276	4.528
-1.319	0.199	0.857	-4.827	-0.333	3.553
-0.060	0.953	0.999	-5.572	-0.015	3.367
1.803	0.083	0.724	-4.222	0.548	3.601
0.996	0.329	0.955	-5.141	0.479	7.560
1.732	0.096	0.754	-4.319	0.848	5.939
-0.929	0.362	0.963	-5.196	-0.193	3.019
1.681	0.105	0.764	-4.387	0.783	3.582
-0.076	0.940	0.999	-5.571	-0.019	4.868
-3.258	0.003	0.340	-1.729	-0.716	3.619
1.042	0.307	0.942	-5.101	0.391	3.085
0.210	0.835	0.999	-5.554	0.156	4.694
0.795	0.434	0.973	-5.295	0.185	2.508
-0.607	0.549	0.994	-5.411	-0.158	1.878
-0.301	0.766	0.999	-5.533	-0.068	5.965
0.426	0.674	0.999	-5.493	0.098	1.753
3.175	0.004	0.340	-1.887	0.947	5.498
-1.042	0.307	0.942	-5.101	-0.235	3.716
-1.077	0.292	0.933	-5.069	-0.338	2.210
-0.110	0.913	0.999	-5.569	-0.037	4.545
-0.152	0.881	0.999	-5.564	-0.079	8.834
0.112	0.912	0.999	-5.568	0.028	7.115
0.072	0.943	0.999	-5.572	0.022	4.627
-0.232	0.818	0.999	-5.550	-0.094	2.790
1.118	0.274	0.921	-5.031	0.226	6.001
-0.167	0.869	0.999	-5.561	-0.039	2.185
-0.420	0.678	0.999	-5.495	-0.086	3.546
0.413	0.683	0.999	-5.498	0.101	5.973
-0.196	0.847	0.999	-5.557	-0.066	3.118
1.133	0.268	0.917	-5.017	0.313	6.127
1.404	0.172	0.828	-4.731	0.355	2.878
0.397	0.695	0.999	-5.504	0.095	6.368
-2.312	0.029	0.570	-3.441	-0.524	1.989
-0.104	0.918	0.999	-5.569	-0.040	8.965
0.144	0.887	0.999	-5.565	0.083	2.141
0.392	0.698	0.999	-5.505	0.087	9.983

1.477	0.152	0.813	-4.646	0.453	3.994
-0.734	0.470	0.983	-5.336	-0.223	3.717
0.950	0.351	0.961	-5.179	0.200	2.954
0.151	0.881	0.999	-5.564	0.037	3.263
0.346	0.733	0.999	-5.521	0.071	6.221
0.977	0.338	0.957	-5.157	0.331	6.025
-0.162	0.873	0.999	-5.562	-0.034	7.452
0.170	0.866	0.999	-5.561	0.048	3.144
0.762	0.453	0.981	-5.318	0.237	6.946
0.326	0.747	0.999	-5.527	0.117	7.543
1.574	0.128	0.789	-4.526	0.417	5.541
-1.429	0.165	0.825	-4.703	-0.300	3.583
-0.172	0.865	0.999	-5.561	-0.043	4.814
0.565	0.577	0.999	-5.433	0.118	4.929
1.356	0.187	0.845	-4.785	0.361	8.064
-1.255	0.221	0.882	-4.895	-0.300	8.088
1.714	0.099	0.757	-4.344	0.592	4.550
-0.473	0.640	0.999	-5.475	-0.190	7.542
0.322	0.750	0.999	-5.528	0.087	7.324
0.099	0.922	0.999	-5.570	0.026	3.651
-0.862	0.397	0.964	-5.248	-0.199	5.278
1.109	0.278	0.922	-5.039	0.293	5.221
0.685	0.500	0.987	-5.367	0.179	4.482
1.487	0.150	0.810	-4.634	0.341	4.325
1.124	0.272	0.919	-5.026	0.247	5.181
0.160	0.874	0.999	-5.563	0.063	5.111
-0.120	0.906	0.999	-5.568	-0.061	5.235
-0.833	0.413	0.970	-5.269	-0.328	3.582
-1.024	0.316	0.946	-5.117	-0.201	7.520
0.217	0.830	0.999	-5.553	0.054	2.533
-0.130	0.898	0.999	-5.566	-0.032	4.496
-0.654	0.519	0.990	-5.385	-0.172	8.690
-0.285	0.778	0.999	-5.538	-0.067	7.723
-1.032	0.312	0.943	-5.110	-0.273	6.026
1.272	0.215	0.875	-4.877	0.325	5.966
-0.145	0.886	0.999	-5.565	-0.038	8.551
-1.136	0.267	0.917	-5.014	-0.812	5.528
-0.896	0.379	0.963	-5.222	-0.187	5.419
0.300	0.767	0.999	-5.534	0.080	5.059
-0.199	0.844	0.999	-5.556	-0.070	2.456
1.295	0.207	0.868	-4.853	0.390	3.357
0.901	0.376	0.963	-5.218	0.168	4.368
-0.119	0.906	0.999	-5.568	-0.030	2.810
-0.086	0.932	0.999	-5.571	-0.020	3.508
0.972	0.340	0.957	-5.161	0.278	4.383
-1.354	0.188	0.847	-4.788	-0.470	5.023
0.892	0.381	0.964	-5.225	0.206	5.636
-0.406	0.688	0.999	-5.501	-0.084	6.833
1.690	0.104	0.764	-4.376	0.362	5.436
0.587	0.563	0.996	-5.422	0.148	4.542

-2.237	0.034	0.597	-3.564	-0.603	3.466
0.800	0.431	0.973	-5.292	0.173	5.217
-1.820	0.081	0.718	-4.197	-0.498	2.303
-0.178	0.860	0.999	-5.560	-0.054	2.382
1.271	0.216	0.875	-4.878	0.468	6.571
0.657	0.517	0.990	-5.383	0.274	3.649
-0.631	0.534	0.992	-5.398	-0.123	8.441
0.365	0.718	0.999	-5.515	0.085	7.069
1.624	0.117	0.776	-4.462	0.422	3.035
0.319	0.752	0.999	-5.529	0.080	5.378
-0.168	0.868	0.999	-5.561	-0.037	5.514
-0.311	0.758	0.999	-5.531	-0.193	1.450
0.686	0.499	0.987	-5.366	0.231	6.185
-1.428	0.166	0.825	-4.703	-0.606	4.680
0.600	0.554	0.995	-5.414	0.168	6.625
-0.298	0.768	0.999	-5.534	-0.076	3.991
1.760	0.091	0.743	-4.281	0.493	3.994
0.264	0.794	0.999	-5.543	0.064	7.869
0.594	0.558	0.995	-5.418	0.169	3.631
0.771	0.448	0.979	-5.312	0.211	3.013
1.337	0.193	0.853	-4.807	0.247	3.940
-0.458	0.651	0.999	-5.481	-0.157	3.510
-0.157	0.876	0.999	-5.563	-0.035	6.376
2.635	0.014	0.498	-2.887	0.708	2.872
1.307	0.203	0.862	-4.839	0.368	4.630
-0.307	0.761	0.999	-5.532	-0.067	3.901
-0.662	0.514	0.990	-5.380	-0.157	3.760
-0.389	0.700	0.999	-5.507	-0.082	4.570
0.950	0.351	0.961	-5.179	0.337	4.259
0.449	0.657	0.999	-5.484	0.236	6.167
1.202	0.240	0.902	-4.949	0.244	5.019
0.278	0.783	0.999	-5.539	0.061	7.167
1.236	0.228	0.890	-4.914	0.230	5.690
1.803	0.083	0.724	-4.221	0.472	6.783
0.820	0.420	0.972	-5.278	0.334	2.213
0.362	0.721	0.999	-5.516	0.119	3.987
-0.666	0.512	0.989	-5.378	-0.197	4.280
0.297	0.769	0.999	-5.535	0.068	5.878
0.852	0.402	0.967	-5.255	0.373	2.566
-0.278	0.784	0.999	-5.540	-0.101	3.907
-0.963	0.345	0.957	-5.168	-0.319	2.814
-1.145	0.263	0.914	-5.005	-0.359	3.365
-0.198	0.845	0.999	-5.557	-0.141	0.936
1.359	0.186	0.843	-4.782	0.283	7.861
0.760	0.454	0.981	-5.320	0.181	5.319
1.071	0.295	0.936	-5.075	0.409	5.063
2.895	0.008	0.422	-2.415	0.712	5.180
2.565	0.017	0.500	-3.010	0.538	4.361
0.667	0.511	0.989	-5.377	0.235	4.444
1.381	0.179	0.838	-4.757	0.390	2.922

-1.218	0.235	0.899	-4.933	-0.251	4.418
-0.054	0.957	0.999	-5.573	-0.014	3.676
0.202	0.841	0.999	-5.556	0.055	5.261
-0.675	0.506	0.988	-5.372	-0.191	3.102
1.938	0.064	0.673	-4.027	0.463	5.304
0.891	0.382	0.964	-5.226	0.249	4.041
0.229	0.821	0.999	-5.551	0.050	6.560
0.641	0.527	0.992	-5.392	0.116	6.227
-0.490	0.628	0.999	-5.467	-0.128	5.038
-2.410	0.024	0.543	-3.277	-0.615	2.559
0.807	0.428	0.973	-5.288	0.145	5.146
0.875	0.390	0.964	-5.238	0.433	3.647
0.478	0.637	0.999	-5.472	0.204	3.695
0.565	0.577	0.999	-5.433	0.123	6.362
-0.515	0.611	0.999	-5.456	-0.166	3.981
-0.335	0.741	0.999	-5.524	-0.099	7.718
-0.182	0.857	0.999	-5.559	-0.052	4.061
0.128	0.899	0.999	-5.567	0.040	4.201
-0.372	0.713	0.999	-5.512	-0.113	2.970
0.148	0.884	0.999	-5.564	0.082	0.996
0.834	0.412	0.970	-5.268	0.226	3.050
0.228	0.822	0.999	-5.551	0.066	3.700
-0.489	0.629	0.999	-5.468	-0.189	4.410
1.943	0.063	0.671	-4.020	0.373	4.742
0.158	0.876	0.999	-5.563	0.044	7.261
1.461	0.156	0.817	-4.665	0.416	4.875
0.823	0.418	0.972	-5.276	0.213	3.140
0.822	0.419	0.972	-5.277	0.174	4.970
0.975	0.339	0.957	-5.159	0.226	3.833
-1.051	0.303	0.941	-5.093	-0.254	2.896
2.117	0.044	0.630	-3.754	0.511	4.888
0.486	0.631	0.999	-5.469	0.097	2.849
2.102	0.046	0.630	-3.778	0.425	5.828
0.088	0.930	0.999	-5.570	0.022	8.426
3.447	0.002	0.301	-1.361	1.113	5.449
0.609	0.548	0.994	-5.410	0.130	2.030
1.537	0.137	0.795	-4.572	0.329	5.147
0.053	0.958	0.999	-5.573	0.020	3.891
0.060	0.952	0.999	-5.572	0.019	3.592
1.114	0.276	0.921	-5.035	0.322	5.220
0.433	0.669	0.999	-5.491	0.142	3.091
0.794	0.435	0.973	-5.297	0.208	3.882
-0.683	0.501	0.988	-5.368	-0.176	4.689
0.068	0.946	0.999	-5.572	0.016	3.516
0.398	0.694	0.999	-5.503	0.186	4.966
1.660	0.109	0.766	-4.416	0.395	4.075
-0.874	0.391	0.964	-5.239	-0.211	3.085
0.291	0.773	0.999	-5.536	0.060	5.012
3.587	0.001	0.301	-1.085	0.751	6.449
0.675	0.506	0.988	-5.372	0.186	4.187

-0.498	0.623	0.999	-5.464	-0.248	3.129
0.805	0.428	0.973	-5.289	0.242	2.798
-0.182	0.857	0.999	-5.559	-0.061	2.862
0.357	0.724	0.999	-5.517	0.097	2.354
-0.081	0.936	0.999	-5.571	-0.031	4.909
1.772	0.088	0.734	-4.264	0.605	2.795
-0.446	0.660	0.999	-5.486	-0.108	4.391
0.238	0.814	0.999	-5.549	0.066	1.735
1.288	0.210	0.868	-4.860	0.268	4.687
0.236	0.815	0.999	-5.549	0.107	5.076
-1.149	0.262	0.914	-5.002	-0.297	6.104
-0.500	0.621	0.999	-5.463	-0.116	3.475
0.546	0.590	0.999	-5.442	0.130	4.889
0.608	0.548	0.994	-5.410	0.234	6.021
0.739	0.466	0.983	-5.333	0.166	5.381
2.425	0.023	0.542	-3.252	0.601	4.017
1.637	0.114	0.775	-4.446	0.392	5.126
1.286	0.210	0.868	-4.862	0.308	5.070
-0.339	0.738	0.999	-5.523	-0.126	3.236
-0.080	0.937	0.999	-5.571	-0.019	3.720
0.738	0.467	0.983	-5.334	0.136	5.789
-0.820	0.420	0.972	-5.278	-0.214	4.125
-0.450	0.657	0.999	-5.484	-0.112	7.253
0.891	0.381	0.964	-5.226	0.203	6.924
2.265	0.032	0.591	-3.518	0.579	5.357
-0.967	0.343	0.957	-5.165	-0.334	2.652
0.865	0.395	0.964	-5.246	0.225	2.476
0.087	0.931	0.999	-5.571	0.022	2.610
0.437	0.666	0.999	-5.489	0.145	2.843
1.844	0.077	0.707	-4.164	0.384	4.813
-0.677	0.504	0.988	-5.371	-0.284	3.969
-1.860	0.075	0.702	-4.140	-0.495	2.358
-1.919	0.066	0.681	-4.056	-0.600	2.253
0.827	0.416	0.970	-5.273	0.224	3.688
-0.454	0.654	0.999	-5.482	-0.169	3.650
-0.065	0.948	0.999	-5.572	-0.021	5.648
0.343	0.734	0.999	-5.521	0.170	3.571
0.589	0.561	0.996	-5.420	0.278	3.014
0.104	0.918	0.999	-5.569	0.032	3.345
-0.820	0.420	0.972	-5.279	-0.286	3.592
-0.449	0.657	0.999	-5.484	-0.143	2.253
1.255	0.221	0.882	-4.894	0.318	2.676
0.058	0.954	0.999	-5.572	0.015	5.691
0.676	0.505	0.988	-5.372	0.270	3.615
-0.068	0.946	0.999	-5.572	-0.021	6.501
0.247	0.807	0.999	-5.547	0.073	2.945
0.087	0.931	0.999	-5.571	0.024	2.406
0.337	0.739	0.999	-5.523	0.087	2.267
-1.181	0.249	0.908	-4.970	-0.298	3.724
0.293	0.772	0.999	-5.536	0.100	2.325

-0.919	0.367	0.963	-5.204	-0.318	2.355
-0.758	0.456	0.981	-5.321	-0.304	2.913
-1.208	0.238	0.901	-4.943	-0.328	2.943
-3.236	0.003	0.340	-1.770	-0.726	3.469
1.662	0.109	0.766	-4.413	0.828	2.565
0.998	0.328	0.954	-5.139	0.288	4.629
1.507	0.144	0.803	-4.609	0.635	2.276
-0.576	0.570	0.997	-5.427	-0.158	2.800
-0.189	0.851	0.999	-5.558	-0.047	4.228
-0.613	0.546	0.994	-5.408	-0.178	2.107
1.347	0.190	0.849	-4.795	0.333	4.317
-0.291	0.773	0.999	-5.536	-0.081	3.789
-0.742	0.465	0.983	-5.331	-0.242	2.799
2.917	0.007	0.412	-2.375	0.992	5.374
-0.937	0.358	0.962	-5.190	-0.370	2.979
1.216	0.235	0.899	-4.935	0.605	5.255
-0.435	0.667	0.999	-5.490	-0.139	2.720
-1.014	0.320	0.950	-5.126	-0.261	2.123
0.153	0.880	0.999	-5.564	0.035	2.992
0.539	0.594	0.999	-5.445	0.180	2.684
0.559	0.581	0.999	-5.436	0.136	1.358
1.198	0.242	0.904	-4.953	0.362	2.203
0.145	0.886	0.999	-5.565	0.041	1.842
1.773	0.088	0.734	-4.263	0.694	2.031
0.427	0.673	0.999	-5.493	0.157	2.715
-0.343	0.734	0.999	-5.521	-0.092	2.679
0.436	0.666	0.999	-5.489	0.170	3.712
0.385	0.704	0.999	-5.508	0.095	3.194
0.482	0.634	0.999	-5.471	0.173	2.550
0.459	0.650	0.999	-5.480	0.168	4.008
0.191	0.850	0.999	-5.558	0.054	2.344
0.614	0.545	0.994	-5.407	0.213	4.180
0.445	0.660	0.999	-5.486	0.153	2.580
-2.331	0.028	0.566	-3.410	-0.578	3.312
0.928	0.362	0.963	-5.197	0.302	2.970
0.471	0.642	0.999	-5.476	0.159	2.950
3.627	0.001	0.301	-1.006	0.950	3.218
1.994	0.057	0.659	-3.944	0.488	4.717
-1.031	0.312	0.943	-5.111	-0.264	3.515
-0.293	0.772	0.999	-5.536	-0.099	2.591
-0.125	0.902	0.999	-5.567	-0.037	3.298
0.338	0.738	0.999	-5.523	0.088	3.085
-1.532	0.138	0.795	-4.578	-0.535	1.646
1.240	0.226	0.888	-4.910	0.342	2.745
-0.358	0.723	0.999	-5.517	-0.099	3.332
-0.310	0.759	0.999	-5.531	-0.085	3.939
2.528	0.018	0.501	-3.074	0.738	3.792
-1.452	0.159	0.820	-4.676	-0.437	3.984
0.051	0.960	0.999	-5.573	0.019	3.324
-1.462	0.156	0.817	-4.664	-0.446	4.477

-1.470	0.154	0.815	-4.654	-0.427	2.397
-0.232	0.818	0.999	-5.550	-0.083	3.064
-0.242	0.811	0.999	-5.548	-0.074	3.552
0.648	0.523	0.990	-5.388	0.146	3.745
0.146	0.885	0.999	-5.564	0.049	2.672
0.476	0.638	0.999	-5.473	0.121	3.322
2.315	0.029	0.570	-3.436	0.687	2.101
-2.463	0.021	0.520	-3.187	-0.744	3.305
-1.076	0.292	0.934	-5.070	-0.291	2.232
-1.602	0.122	0.784	-4.491	-0.412	6.001
-0.675	0.506	0.988	-5.373	-0.195	2.494
0.151	0.881	0.999	-5.564	0.037	1.480
-0.897	0.378	0.963	-5.221	-0.204	4.092
-0.819	0.421	0.972	-5.279	-0.239	2.670
-2.098	0.046	0.630	-3.784	-0.670	3.344
1.070	0.295	0.936	-5.075	0.431	5.345
-1.875	0.073	0.698	-4.119	-0.552	3.713
1.567	0.130	0.789	-4.535	0.671	3.126
0.480	0.635	0.999	-5.471	0.151	2.277
-0.555	0.584	0.999	-5.437	-0.131	3.357
1.595	0.123	0.785	-4.500	0.429	2.588
2.045	0.052	0.645	-3.867	0.627	3.926
1.292	0.208	0.868	-4.856	0.344	2.541
0.962	0.345	0.957	-5.170	0.297	5.110
1.843	0.077	0.707	-4.164	0.538	3.281
-0.471	0.642	0.999	-5.476	-0.184	2.307
1.725	0.097	0.757	-4.328	0.744	2.834
0.136	0.893	0.999	-5.566	0.041	2.806
-0.714	0.482	0.984	-5.349	-0.307	2.338
-0.811	0.425	0.972	-5.285	-0.237	3.192
-0.431	0.670	0.999	-5.491	-0.110	2.408
-0.643	0.526	0.992	-5.391	-0.207	3.895
-2.352	0.027	0.559	-3.375	-0.838	3.237
-0.587	0.562	0.996	-5.421	-0.165	2.347
1.128	0.270	0.918	-5.022	0.350	4.980
-2.053	0.051	0.641	-3.853	-1.022	4.572
0.816	0.422	0.972	-5.281	0.362	4.388
0.380	0.707	0.999	-5.510	0.109	5.946
-2.108	0.045	0.630	-3.769	-0.830	6.173
0.126	0.900	0.999	-5.567	0.040	2.947
-0.125	0.901	0.999	-5.567	-0.033	4.402
0.989	0.332	0.957	-5.147	0.247	3.012
0.560	0.580	0.999	-5.435	0.167	2.657
-2.508	0.019	0.507	-3.109	-0.682	3.533
-1.539	0.136	0.795	-4.570	-0.527	2.884
0.673	0.507	0.988	-5.374	0.257	2.182
-0.140	0.890	0.999	-5.565	-0.047	1.927
-0.395	0.696	0.999	-5.505	-0.127	2.452
-0.297	0.769	0.999	-5.535	-0.111	3.958
-0.362	0.720	0.999	-5.516	-0.154	3.221

-1.147	0.262	0.914	-5.004	-0.470	3.214
-1.497	0.147	0.804	-4.622	-0.550	1.971
0.107	0.916	0.999	-5.569	0.027	6.562
-0.347	0.732	0.999	-5.520	-0.177	2.759
-2.244	0.034	0.595	-3.552	-0.541	4.575
-1.679	0.106	0.764	-4.391	-0.553	6.472
2.218	0.036	0.601	-3.594	0.561	3.338
-0.142	0.888	0.999	-5.565	-0.051	3.303
0.648	0.523	0.990	-5.388	0.231	3.032
0.800	0.431	0.973	-5.292	0.240	2.525
0.256	0.800	0.999	-5.545	0.084	2.511
3.207	0.004	0.340	-1.825	1.127	3.184
-2.810	0.009	0.446	-2.571	-0.650	4.601
1.809	0.082	0.722	-4.213	0.578	4.368
1.428	0.166	0.825	-4.704	0.406	3.292
-0.830	0.414	0.970	-5.271	-0.268	2.743
0.850	0.403	0.968	-5.257	0.243	3.907
0.304	0.764	0.999	-5.533	0.110	1.085
-1.387	0.178	0.837	-4.750	-0.460	2.803
1.436	0.163	0.824	-4.694	0.567	3.590
0.978	0.338	0.957	-5.156	0.457	3.416
-0.099	0.922	0.999	-5.570	-0.040	2.732
-0.825	0.417	0.970	-5.275	-0.206	4.023
-0.983	0.335	0.957	-5.151	-0.335	3.336
-1.630	0.116	0.776	-4.455	-0.375	2.049
-0.101	0.920	0.999	-5.569	-0.031	2.637
-0.201	0.842	0.999	-5.556	-0.054	2.676
-0.244	0.810	0.999	-5.548	-0.064	3.250
-0.606	0.550	0.994	-5.412	-0.238	2.926
-0.418	0.680	0.999	-5.496	-0.132	2.107
-1.582	0.126	0.786	-4.516	-0.400	4.039
0.887	0.383	0.964	-5.229	0.214	2.666
2.359	0.026	0.557	-3.363	0.640	2.924
1.506	0.145	0.803	-4.610	0.439	3.725
-0.540	0.594	0.999	-5.444	-0.250	3.027
-0.924	0.365	0.963	-5.200	-0.356	3.268
-0.803	0.429	0.973	-5.290	-0.326	3.355
-0.181	0.858	0.999	-5.559	-0.054	2.373
0.623	0.539	0.994	-5.402	0.293	5.459
0.901	0.376	0.963	-5.218	0.250	2.701
0.984	0.334	0.957	-5.151	0.347	3.209
-0.766	0.451	0.980	-5.315	-0.206	2.089
-2.816	0.009	0.446	-2.561	-0.922	6.110
1.972	0.060	0.663	-3.977	0.498	3.952
2.190	0.038	0.611	-3.639	0.581	2.650
0.215	0.831	0.999	-5.553	0.052	5.439
-1.075	0.292	0.934	-5.071	-0.383	5.501
0.379	0.708	0.999	-5.510	0.156	2.432
2.469	0.021	0.517	-3.177	0.968	4.441
0.421	0.677	0.999	-5.495	0.100	2.059

-0.531	0.600	0.999	-5.449	-0.152	2.740
0.966	0.343	0.957	-5.166	0.251	4.091
-0.078	0.938	0.999	-5.571	-0.027	3.837
-1.537	0.137	0.795	-4.572	-0.494	2.535
1.046	0.306	0.942	-5.098	0.383	4.272
-1.289	0.209	0.868	-4.859	-0.418	3.225
0.734	0.470	0.983	-5.337	0.248	1.794
1.535	0.137	0.795	-4.575	0.658	4.219
-0.896	0.379	0.963	-5.222	-0.291	2.575
0.833	0.413	0.970	-5.269	0.342	4.669
-0.460	0.649	0.999	-5.480	-0.160	3.419
0.803	0.430	0.973	-5.291	0.239	3.131
-0.161	0.873	0.999	-5.562	-0.037	5.121
-1.560	0.131	0.791	-4.544	-0.531	2.033
0.072	0.943	0.999	-5.572	0.027	1.474
-1.663	0.109	0.766	-4.412	-0.450	2.436
-1.568	0.129	0.789	-4.534	-0.442	4.213
0.851	0.403	0.967	-5.256	0.233	2.805
0.475	0.639	0.999	-5.474	0.213	2.934
0.638	0.529	0.992	-5.394	0.176	2.397
0.331	0.744	0.999	-5.525	0.085	3.197
-0.165	0.870	0.999	-5.562	-0.053	2.623
0.942	0.355	0.962	-5.185	0.230	2.690
1.104	0.280	0.922	-5.044	0.620	3.577
-2.283	0.031	0.585	-3.488	-0.901	2.782
-1.808	0.083	0.722	-4.214	-0.628	3.160
-1.074	0.293	0.934	-5.072	-0.293	4.487
0.602	0.553	0.994	-5.413	0.222	1.892
-1.104	0.280	0.922	-5.045	-0.262	5.680
-1.212	0.237	0.899	-4.939	-0.303	4.499
-1.106	0.279	0.922	-5.043	-0.440	2.098
1.183	0.248	0.907	-4.969	0.302	2.311
-0.355	0.726	0.999	-5.518	-0.087	2.968
1.118	0.274	0.921	-5.031	0.210	5.765
1.209	0.238	0.900	-4.942	0.543	7.671
-0.209	0.836	0.999	-5.555	-0.071	5.541
1.627	0.116	0.776	-4.458	0.301	6.209
0.963	0.345	0.957	-5.168	0.285	3.659
0.279	0.782	0.999	-5.539	0.076	3.977
0.105	0.918	0.999	-5.569	0.020	9.132
0.318	0.753	0.999	-5.529	0.176	2.848
3.326	0.003	0.320	-1.596	0.995	5.432
-0.294	0.771	0.999	-5.535	-0.115	7.507
-0.795	0.434	0.973	-5.296	-0.156	5.314
2.076	0.048	0.633	-3.819	0.410	3.975
-0.773	0.447	0.979	-5.311	-0.176	5.879
2.637	0.014	0.498	-2.883	0.727	6.955
-0.884	0.385	0.964	-5.231	-0.273	5.477
0.761	0.454	0.981	-5.319	0.347	3.318
0.106	0.916	0.999	-5.569	0.022	3.632

0.353	0.727	0.999	-5.518	0.148	5.744
0.782	0.442	0.978	-5.305	0.196	5.643
-0.754	0.458	0.982	-5.323	-0.198	3.242
0.779	0.443	0.979	-5.307	0.224	5.339
0.261	0.796	0.999	-5.544	0.111	3.563
1.444	0.161	0.820	-4.684	0.415	2.497
0.503	0.619	0.999	-5.462	0.140	2.897
-1.027	0.314	0.944	-5.114	-0.541	2.185
-0.464	0.646	0.999	-5.478	-0.169	2.837
1.048	0.305	0.942	-5.096	0.261	2.811
-0.592	0.559	0.996	-5.419	-0.174	4.161
2.106	0.045	0.630	-3.772	0.678	4.754
-2.484	0.020	0.517	-3.150	-0.635	4.865
-0.178	0.860	0.999	-5.560	-0.053	5.691
0.167	0.869	0.999	-5.561	0.034	7.754
-1.632	0.115	0.776	-4.452	-0.497	2.778
-0.253	0.803	0.999	-5.545	-0.086	8.028
-0.671	0.508	0.988	-5.375	-0.324	8.788
1.534	0.138	0.795	-4.576	0.280	8.018
-0.275	0.786	0.999	-5.540	-0.082	2.688
0.472	0.641	0.999	-5.475	0.232	8.711
0.416	0.681	0.999	-5.497	0.207	8.765
0.329	0.745	0.999	-5.526	0.086	3.885
-1.578	0.127	0.788	-4.522	-0.430	2.667
1.292	0.208	0.868	-4.856	0.302	4.327
1.236	0.228	0.890	-4.914	0.327	5.008
-0.222	0.826	0.999	-5.552	-0.052	3.470
1.125	0.271	0.919	-5.025	0.290	2.930
0.829	0.415	0.970	-5.272	0.248	3.935
2.306	0.030	0.570	-3.451	0.657	4.223
0.508	0.616	0.999	-5.459	0.159	6.118
0.113	0.911	0.999	-5.568	0.059	2.982
0.242	0.811	0.999	-5.548	0.056	6.802
0.830	0.414	0.970	-5.271	0.288	5.111
-0.482	0.634	0.999	-5.471	-0.123	4.129
-1.270	0.216	0.876	-4.879	-0.295	6.969
-0.199	0.844	0.999	-5.556	-0.069	2.885
0.811	0.425	0.972	-5.285	0.452	3.830
-0.278	0.783	0.999	-5.539	-0.081	3.236
0.074	0.942	0.999	-5.572	0.018	2.795
-0.295	0.771	0.999	-5.535	-0.191	5.441
2.083	0.048	0.630	-3.808	0.697	3.954
1.569	0.129	0.789	-4.533	0.344	4.392
0.151	0.882	0.999	-5.564	0.063	4.003
0.135	0.893	0.999	-5.566	0.055	4.271
-0.498	0.623	0.999	-5.464	-0.138	4.770
0.866	0.395	0.964	-5.245	0.222	3.792
1.033	0.312	0.943	-5.109	0.205	6.691
0.639	0.529	0.992	-5.393	0.155	3.927
0.782	0.442	0.978	-5.305	0.196	3.717

0.329	0.745	0.999	-5.526	0.091	5.123
-0.557	0.582	0.999	-5.436	-0.149	6.740
0.272	0.788	0.999	-5.541	0.063	3.523
1.246	0.224	0.886	-4.905	0.266	6.013
0.395	0.696	0.999	-5.504	0.071	5.767
0.391	0.699	0.999	-5.506	0.097	5.047
0.712	0.483	0.985	-5.350	0.163	5.856
1.147	0.262	0.914	-5.004	0.250	4.403
2.613	0.015	0.498	-2.925	0.483	6.196
0.900	0.377	0.963	-5.219	0.177	5.509
2.002	0.056	0.658	-3.931	0.479	6.086
0.674	0.506	0.988	-5.373	0.148	3.455
0.211	0.834	0.999	-5.554	0.041	4.570
-0.230	0.820	0.999	-5.550	-0.044	9.222
1.174	0.251	0.910	-4.977	0.240	6.175
0.988	0.332	0.957	-5.147	0.252	4.179
0.094	0.926	0.999	-5.570	0.021	2.314
0.257	0.799	0.999	-5.545	0.075	4.301
1.524	0.140	0.801	-4.589	0.315	5.323
0.477	0.638	0.999	-5.473	0.112	7.450
2.472	0.021	0.517	-3.171	1.007	4.335
1.139	0.266	0.916	-5.011	0.423	3.848
2.924	0.007	0.412	-2.362	1.027	4.956
1.934	0.064	0.673	-4.032	0.813	6.335
1.327	0.196	0.855	-4.817	0.238	5.339
0.917	0.368	0.963	-5.205	0.195	5.556
0.058	0.954	0.999	-5.572	0.014	3.731
-0.559	0.581	0.999	-5.435	-0.132	7.652
1.486	0.150	0.810	-4.635	0.465	3.002
0.472	0.641	0.999	-5.475	0.142	7.178
0.442	0.662	0.999	-5.487	0.124	9.723
3.441	0.002	0.301	-1.372	0.883	3.487
0.392	0.698	0.999	-5.505	0.128	3.618
0.319	0.753	0.999	-5.529	0.075	2.724
0.942	0.355	0.962	-5.186	0.347	2.498
-1.934	0.064	0.673	-4.033	-0.486	3.103
0.992	0.331	0.956	-5.144	0.632	4.716
-0.098	0.923	0.999	-5.570	-0.025	7.103
0.246	0.807	0.999	-5.547	0.146	5.158
-0.314	0.756	0.999	-5.530	-0.159	7.074
-2.158	0.041	0.617	-3.691	-0.997	4.546
-0.821	0.419	0.972	-5.278	-0.361	2.599
0.798	0.432	0.973	-5.293	0.291	4.193
-0.774	0.446	0.979	-5.310	-0.256	7.907
-0.173	0.864	0.999	-5.561	-0.050	9.792
0.455	0.653	0.999	-5.482	0.103	3.472
-0.201	0.842	0.999	-5.556	-0.040	4.101
1.412	0.170	0.825	-4.722	0.363	3.940
0.562	0.579	0.999	-5.434	0.123	4.135
-0.653	0.519	0.990	-5.385	-0.251	3.306

-0.803	0.430	0.973	-5.290	-0.216	3.548
1.676	0.106	0.764	-4.394	0.524	5.654
1.073	0.294	0.935	-5.073	0.276	4.001
-0.085	0.933	0.999	-5.571	-0.028	9.229
2.072	0.049	0.635	-3.825	0.588	5.107
0.508	0.616	0.999	-5.459	0.094	6.129
-0.142	0.888	0.999	-5.565	-0.025	6.748
-0.198	0.845	0.999	-5.557	-0.050	5.533
0.761	0.454	0.981	-5.319	0.154	5.021
-0.091	0.928	0.999	-5.570	-0.026	3.401
1.287	0.210	0.868	-4.861	0.259	4.194
0.959	0.347	0.958	-5.172	0.176	5.744
0.826	0.417	0.970	-5.274	0.185	4.096
-2.184	0.038	0.611	-3.648	-0.719	3.673
-1.645	0.112	0.771	-4.434	-0.409	6.818
-0.103	0.919	0.999	-5.569	-0.025	6.509
-1.479	0.152	0.813	-4.643	-0.301	8.275
-0.731	0.472	0.984	-5.338	-0.191	10.277
-1.062	0.299	0.940	-5.083	-0.635	2.349
-1.747	0.093	0.750	-4.298	-0.890	6.362
-1.344	0.191	0.850	-4.798	-0.437	7.932
-0.575	0.571	0.997	-5.428	-0.351	4.909
-1.772	0.089	0.734	-4.264	-0.727	6.065
-1.387	0.178	0.837	-4.751	-0.457	9.131
-0.540	0.594	0.999	-5.444	-0.200	8.229
-4.507	0.000	0.256	0.752	-1.591	3.372
-0.622	0.540	0.994	-5.403	-0.236	8.839
-1.566	0.130	0.789	-4.537	-0.780	5.689
-1.959	0.061	0.665	-3.996	-0.640	7.782
-1.125	0.271	0.919	-5.025	-0.324	9.748
-2.247	0.034	0.595	-3.547	-0.896	3.244
-1.552	0.133	0.791	-4.553	-0.497	4.446
-0.714	0.482	0.984	-5.349	-0.200	5.603
0.456	0.652	0.999	-5.482	0.194	3.406
-0.333	0.742	0.999	-5.525	-0.080	5.629
0.110	0.913	0.999	-5.569	0.025	4.120
-1.868	0.074	0.698	-4.129	-0.615	2.938
-0.227	0.822	0.999	-5.551	-0.121	2.215
1.046	0.306	0.942	-5.097	0.273	3.645
-0.496	0.624	0.999	-5.465	-0.157	7.102
-0.758	0.456	0.981	-5.321	-0.202	5.490
-0.073	0.942	0.999	-5.572	-0.022	4.376
1.257	0.220	0.881	-4.892	0.474	6.561
3.494	0.002	0.301	-1.268	1.057	4.262
1.832	0.079	0.713	-4.181	0.557	6.484
0.491	0.628	0.999	-5.467	0.141	6.278
0.140	0.890	0.999	-5.565	0.038	5.545
0.396	0.695	0.999	-5.504	0.096	2.550
0.290	0.774	0.999	-5.536	0.084	2.510
2.336	0.028	0.563	-3.401	0.676	3.979

0.221	0.827	0.999	-5.552	0.051	7.377
-0.616	0.543	0.994	-5.406	-0.165	3.893
1.653	0.111	0.767	-4.424	0.404	3.750
-0.901	0.376	0.963	-5.218	-0.300	5.889
-0.598	0.555	0.995	-5.415	-0.241	5.745
-1.276	0.214	0.873	-4.873	-0.300	5.153
1.879	0.072	0.698	-4.113	0.470	4.421
0.798	0.433	0.973	-5.294	0.269	3.969
0.065	0.949	0.999	-5.572	0.017	3.377
2.923	0.007	0.412	-2.363	0.612	5.289
-0.165	0.870	0.999	-5.562	-0.049	5.769
-2.275	0.032	0.589	-3.502	-0.736	3.208
-0.879	0.388	0.964	-5.235	-0.298	2.509
-0.661	0.515	0.990	-5.381	-0.183	4.626
1.362	0.185	0.842	-4.779	0.380	4.632
-2.371	0.026	0.556	-3.342	-1.351	4.358
0.466	0.646	0.999	-5.478	0.164	4.843
0.949	0.352	0.961	-5.180	0.207	6.147
-0.401	0.692	0.999	-5.502	-0.166	7.065
0.242	0.811	0.999	-5.548	0.076	7.147
0.429	0.672	0.999	-5.492	0.142	5.828
2.093	0.047	0.630	-3.793	1.047	4.159
-0.353	0.727	0.999	-5.519	-0.090	7.489
-0.499	0.622	0.999	-5.463	-0.165	5.571
0.699	0.491	0.986	-5.358	0.122	6.879
0.516	0.611	0.999	-5.456	0.150	7.825
2.127	0.043	0.628	-3.739	0.718	6.846
-0.250	0.804	0.999	-5.546	-0.083	3.216
1.070	0.295	0.936	-5.076	0.271	3.119
-0.446	0.660	0.999	-5.486	-0.157	6.853
0.313	0.757	0.999	-5.530	0.086	4.721
-0.297	0.769	0.999	-5.535	-0.056	6.375
0.358	0.723	0.999	-5.517	0.199	4.833
1.629	0.116	0.776	-4.456	0.416	3.706
3.630	0.001	0.301	-1.000	0.885	5.198
-0.165	0.871	0.999	-5.562	-0.051	6.212
-1.516	0.142	0.802	-4.598	-0.615	2.179
0.618	0.542	0.994	-5.405	0.125	5.691
0.291	0.773	0.999	-5.536	0.082	3.286
3.825	0.001	0.301	-0.611	0.939	4.421
0.877	0.389	0.964	-5.236	0.203	4.182
-0.911	0.371	0.963	-5.210	-0.262	6.240
0.511	0.614	0.999	-5.458	0.165	3.804
-1.413	0.170	0.825	-4.721	-0.314	7.283
0.105	0.918	0.999	-5.569	0.021	4.699
-0.827	0.416	0.970	-5.273	-0.274	2.486
2.157	0.041	0.617	-3.691	0.517	6.157
1.790	0.086	0.729	-4.239	0.417	5.234
0.748	0.462	0.983	-5.327	0.199	4.604
1.475	0.153	0.813	-4.648	0.519	7.112

1.785	0.086	0.731	-4.247	0.732	9.315
-0.059	0.953	0.999	-5.572	-0.023	6.039
0.104	0.918	0.999	-5.569	0.030	6.378
1.032	0.312	0.943	-5.110	0.248	4.190
0.082	0.935	0.999	-5.571	0.042	6.541
1.036	0.310	0.943	-5.106	0.210	5.823
0.287	0.776	0.999	-5.537	0.059	7.542
0.791	0.436	0.973	-5.299	0.443	4.496
-0.092	0.927	0.999	-5.570	-0.023	6.001
0.314	0.756	0.999	-5.530	0.080	4.055
-1.786	0.086	0.731	-4.245	-0.480	6.688
-0.247	0.807	0.999	-5.547	-0.063	7.266
1.289	0.209	0.868	-4.859	0.379	3.925
-0.691	0.496	0.987	-5.363	-0.179	3.152
0.334	0.741	0.999	-5.524	0.081	3.523
-1.111	0.277	0.921	-5.037	-0.320	2.556
0.459	0.650	0.999	-5.480	0.157	6.372
-0.565	0.577	0.999	-5.432	-0.156	6.311
-0.493	0.626	0.999	-5.466	-0.097	8.190
0.115	0.909	0.999	-5.568	0.022	7.909
0.254	0.802	0.999	-5.545	0.065	5.905
-0.416	0.681	0.999	-5.497	-0.086	7.121
0.307	0.761	0.999	-5.532	0.131	2.752
0.947	0.353	0.961	-5.182	0.297	8.414
-0.602	0.553	0.994	-5.414	-0.190	6.542
-0.678	0.504	0.988	-5.371	-0.142	8.592
0.772	0.447	0.979	-5.311	0.261	5.682
0.108	0.915	0.999	-5.569	0.030	3.983
0.655	0.519	0.990	-5.385	0.158	2.485
0.159	0.875	0.999	-5.563	0.029	8.083
1.102	0.281	0.922	-5.046	0.375	6.455
0.249	0.805	0.999	-5.546	0.061	5.557
0.207	0.838	0.999	-5.555	0.037	5.097
0.688	0.498	0.987	-5.365	0.200	5.929
0.113	0.911	0.999	-5.568	0.050	5.091
-0.157	0.876	0.999	-5.563	-0.057	3.460
2.629	0.014	0.498	-2.897	0.998	6.017
1.675	0.106	0.764	-4.396	0.545	5.151
0.077	0.939	0.999	-5.571	0.022	3.623
1.796	0.085	0.726	-4.231	0.386	6.565
0.818	0.421	0.972	-5.280	0.170	8.878
1.148	0.262	0.914	-5.003	0.473	6.303
-0.759	0.455	0.981	-5.320	-0.333	2.908
0.349	0.730	0.999	-5.520	0.075	5.123
0.435	0.667	0.999	-5.490	0.237	4.274
-0.306	0.762	0.999	-5.532	-0.078	5.877
-0.326	0.747	0.999	-5.526	-0.072	6.883
0.472	0.641	0.999	-5.475	0.172	1.806
0.714	0.482	0.984	-5.349	0.521	3.851
-0.727	0.474	0.984	-5.341	-0.324	6.531

-1.975	0.059	0.663	-3.972	-0.502	8.598
-0.192	0.849	0.999	-5.558	-0.070	7.868
0.218	0.829	0.999	-5.553	0.046	7.428
0.218	0.829	0.999	-5.553	0.042	6.812
0.288	0.776	0.999	-5.537	0.075	5.662
0.139	0.891	0.999	-5.565	0.030	3.722
-0.134	0.895	0.999	-5.566	-0.046	3.766
2.548	0.017	0.500	-3.040	0.666	5.233
-0.599	0.555	0.995	-5.415	-0.166	6.003
-1.163	0.256	0.913	-4.988	-0.283	6.391
0.540	0.594	0.999	-5.445	0.128	6.773
-0.970	0.342	0.957	-5.163	-0.334	7.040
-0.112	0.911	0.999	-5.568	-0.022	7.098
-0.075	0.941	0.999	-5.571	-0.035	2.570
0.890	0.382	0.964	-5.226	0.290	2.409
0.207	0.838	0.999	-5.555	0.039	3.943
0.143	0.887	0.999	-5.565	0.058	2.724
0.804	0.429	0.973	-5.289	0.143	6.868
1.327	0.196	0.855	-4.818	0.305	3.558
0.626	0.537	0.993	-5.401	0.147	3.280
0.846	0.406	0.968	-5.260	0.151	4.963
0.688	0.498	0.987	-5.365	0.165	4.345
-1.913	0.067	0.682	-4.063	-0.708	3.908
0.105	0.917	0.999	-5.569	0.042	10.588
-1.360	0.186	0.843	-4.782	-0.353	2.719
-0.484	0.633	0.999	-5.470	-0.147	6.808
-0.306	0.762	0.999	-5.532	-0.126	4.860
-0.741	0.465	0.983	-5.332	-0.182	4.180
0.136	0.893	0.999	-5.566	0.040	6.579
0.845	0.406	0.969	-5.260	0.166	5.957
1.376	0.181	0.840	-4.763	0.288	5.923
-0.094	0.926	0.999	-5.570	-0.036	5.499
-1.778	0.087	0.732	-4.255	-1.443	7.636
-1.191	0.245	0.905	-4.961	-0.965	9.080
-1.611	0.120	0.782	-4.479	-1.041	8.889
0.519	0.608	0.999	-5.454	0.158	6.386
-0.468	0.644	0.999	-5.477	-0.126	1.808
-0.409	0.686	0.999	-5.500	-0.144	2.400
0.644	0.526	0.992	-5.391	0.243	4.852
-2.366	0.026	0.556	-3.350	-0.802	2.795
-0.562	0.579	0.999	-5.434	-0.126	6.718
0.162	0.873	0.999	-5.562	0.071	3.121
-0.910	0.371	0.963	-5.211	-0.442	3.853
-0.262	0.795	0.999	-5.543	-0.055	5.887
1.121	0.273	0.921	-5.029	0.267	5.951
-1.304	0.204	0.863	-4.843	-0.283	6.308
-0.229	0.821	0.999	-5.551	-0.124	3.432
1.517	0.142	0.802	-4.597	0.600	5.668
0.924	0.364	0.963	-5.200	0.233	2.904
-1.195	0.243	0.904	-4.956	-0.252	4.490

-0.340	0.737	0.999	-5.523	-0.095	1.464
-0.405	0.689	0.999	-5.501	-0.222	2.152
0.760	0.455	0.981	-5.320	0.155	5.887
1.913	0.067	0.682	-4.063	0.423	3.939
0.178	0.860	0.999	-5.560	0.041	4.559
0.709	0.485	0.985	-5.352	0.178	4.998
0.297	0.769	0.999	-5.535	0.064	3.647
1.946	0.063	0.670	-4.015	0.418	7.254
0.787	0.439	0.975	-5.301	0.231	2.585
0.756	0.457	0.981	-5.322	0.252	3.539
1.479	0.151	0.813	-4.643	0.664	6.515
1.613	0.119	0.781	-4.477	0.367	3.752
-0.063	0.950	0.999	-5.572	-0.016	7.631
2.161	0.040	0.617	-3.686	0.489	3.335
1.432	0.164	0.824	-4.699	0.325	5.162
0.786	0.439	0.975	-5.302	0.213	2.491
0.741	0.466	0.983	-5.332	0.292	3.998
-0.970	0.341	0.957	-5.163	-0.314	4.154
0.415	0.681	0.999	-5.497	0.090	8.584
0.131	0.897	0.999	-5.566	0.032	4.117
1.146	0.263	0.914	-5.005	0.239	2.306
1.640	0.114	0.775	-4.442	0.438	5.699
-0.656	0.518	0.990	-5.384	-0.178	3.508
-0.709	0.485	0.985	-5.352	-0.159	4.390
0.350	0.730	0.999	-5.520	0.100	3.335
0.741	0.466	0.983	-5.332	0.165	6.059
0.802	0.430	0.973	-5.291	0.214	5.300
1.595	0.123	0.785	-4.500	0.361	4.502
0.073	0.943	0.999	-5.572	0.020	4.332
1.036	0.310	0.943	-5.106	0.253	5.848
0.382	0.706	0.999	-5.509	0.111	4.412
-0.534	0.598	0.999	-5.447	-0.132	4.519
1.096	0.284	0.925	-5.052	0.236	4.918
0.938	0.357	0.962	-5.189	0.389	4.097
-0.128	0.899	0.999	-5.567	-0.032	6.734
-0.167	0.869	0.999	-5.562	-0.085	4.273
1.169	0.253	0.913	-4.982	0.263	5.218
0.330	0.744	0.999	-5.525	0.137	3.317
0.742	0.465	0.983	-5.331	0.161	3.379
-0.120	0.905	0.999	-5.567	-0.032	8.458
2.288	0.031	0.584	-3.481	0.548	3.452
-1.373	0.182	0.840	-4.767	-0.390	5.664
0.069	0.946	0.999	-5.572	0.017	3.500
0.233	0.818	0.999	-5.550	0.042	4.125
0.769	0.449	0.979	-5.314	0.177	3.509
-0.833	0.413	0.970	-5.269	-0.301	6.025
0.862	0.397	0.964	-5.248	0.229	3.184
-3.041	0.005	0.392	-2.142	-0.614	2.755
-0.436	0.666	0.999	-5.489	-0.182	7.360
-0.538	0.595	0.999	-5.445	-0.111	4.052

1.016	0.319	0.950	-5.123	0.256	4.964
-0.649	0.522	0.990	-5.388	-0.171	2.442
0.530	0.601	0.999	-5.449	0.145	7.832
1.791	0.085	0.729	-4.238	0.377	3.632
0.099	0.922	0.999	-5.570	0.027	3.695
-1.457	0.158	0.817	-4.669	-0.357	2.122
0.212	0.834	0.999	-5.554	0.064	4.619
-0.204	0.840	0.999	-5.555	-0.062	5.202
-0.229	0.821	0.999	-5.551	-0.078	3.073
-0.757	0.456	0.981	-5.322	-0.235	3.064
2.085	0.047	0.630	-3.805	0.527	3.608
-0.733	0.470	0.983	-5.337	-0.404	5.312
2.558	0.017	0.500	-3.023	0.582	3.864
0.999	0.327	0.954	-5.138	0.202	4.307
0.802	0.430	0.973	-5.291	0.230	2.857
-1.713	0.099	0.757	-4.345	-0.834	4.233
1.291	0.209	0.868	-4.857	0.465	3.924
1.095	0.284	0.926	-5.053	0.234	7.159
2.651	0.014	0.498	-2.858	0.580	5.779
1.469	0.154	0.815	-4.655	0.374	5.030
0.522	0.606	0.999	-5.453	0.129	2.506
2.280	0.031	0.587	-3.494	0.544	4.964
0.761	0.454	0.981	-5.319	0.280	4.972
0.898	0.378	0.963	-5.221	0.213	4.257
0.771	0.448	0.979	-5.312	0.153	6.992
1.060	0.299	0.941	-5.084	0.208	8.990
0.936	0.358	0.963	-5.190	0.179	9.018
-0.856	0.400	0.964	-5.252	-0.227	3.896
-1.266	0.217	0.876	-4.883	-0.304	3.771
1.457	0.157	0.817	-4.669	0.455	2.194
2.442	0.022	0.536	-3.223	0.677	4.119
-0.140	0.889	0.999	-5.565	-0.036	3.132
-1.245	0.225	0.886	-4.906	-0.298	6.719
-0.460	0.649	0.999	-5.480	-0.102	7.434
0.078	0.938	0.999	-5.571	0.024	6.243
2.266	0.032	0.591	-3.517	0.623	4.387
-0.404	0.690	0.999	-5.501	-0.287	4.044
-0.145	0.886	0.999	-5.565	-0.033	4.414
0.878	0.388	0.964	-5.236	0.257	3.598
-0.554	0.584	0.999	-5.438	-0.140	2.750
-0.766	0.451	0.980	-5.315	-0.183	2.025
0.116	0.908	0.999	-5.568	0.028	6.010
-0.775	0.445	0.979	-5.309	-0.209	2.661
0.492	0.627	0.999	-5.466	0.156	7.889
0.628	0.535	0.993	-5.399	0.195	9.443
-0.683	0.501	0.988	-5.368	-0.141	3.911
1.571	0.129	0.789	-4.530	0.451	3.371
-1.317	0.200	0.858	-4.829	-0.301	9.102
-0.832	0.413	0.970	-5.269	-0.201	7.693
-0.903	0.375	0.963	-5.216	-0.216	8.722

-0.826	0.417	0.970	-5.274	-0.188	9.228
-0.984	0.334	0.957	-5.151	-0.222	9.000
-0.414	0.682	0.999	-5.498	-0.136	5.706
-1.382	0.179	0.838	-4.756	-0.302	7.575
0.074	0.942	0.999	-5.572	0.021	5.939
-0.241	0.811	0.999	-5.548	-0.083	3.822
2.777	0.010	0.457	-2.631	0.646	3.478
-1.368	0.183	0.841	-4.772	-0.396	2.904
-0.939	0.357	0.962	-5.188	-0.321	2.385
1.218	0.235	0.899	-4.933	0.565	6.074
-0.909	0.372	0.963	-5.212	-0.255	2.405
-0.441	0.663	0.999	-5.487	-0.137	7.655
0.220	0.828	0.999	-5.552	0.118	4.311
-0.558	0.582	0.999	-5.436	-0.336	4.800
-0.295	0.771	0.999	-5.535	-0.116	4.187
-3.213	0.004	0.340	-1.815	-0.596	2.855
0.436	0.666	0.999	-5.489	0.157	3.647
1.463	0.156	0.817	-4.662	0.712	4.438
1.566	0.130	0.789	-4.536	0.499	2.682
0.081	0.936	0.999	-5.571	0.022	2.554
2.161	0.040	0.617	-3.685	0.598	6.328
0.429	0.672	0.999	-5.492	0.154	4.115
-0.197	0.845	0.999	-5.557	-0.055	5.918
0.461	0.649	0.999	-5.480	0.093	5.645
-0.163	0.872	0.999	-5.562	-0.096	1.599
1.404	0.173	0.828	-4.732	0.297	4.358
0.130	0.897	0.999	-5.566	0.034	2.772
1.374	0.182	0.840	-4.765	0.319	5.179
0.222	0.826	0.999	-5.552	0.049	5.336
-2.944	0.007	0.407	-2.324	-0.841	4.047
-0.241	0.812	0.999	-5.548	-0.090	5.147
-1.608	0.120	0.784	-4.483	-0.673	3.638
2.245	0.034	0.595	-3.550	0.579	5.944
-0.448	0.658	0.999	-5.485	-0.112	6.886
0.629	0.535	0.993	-5.399	0.131	8.095
0.413	0.683	0.999	-5.498	0.101	2.217
-1.177	0.250	0.910	-4.974	-0.511	3.500
-0.593	0.559	0.996	-5.418	-0.177	2.526
0.707	0.486	0.985	-5.353	0.226	2.003
-0.465	0.646	0.999	-5.478	-0.124	1.924
0.908	0.372	0.963	-5.212	0.191	5.035
-1.923	0.066	0.676	-4.049	-0.579	2.749
0.199	0.844	0.999	-5.556	0.145	4.238
1.988	0.058	0.660	-3.953	0.513	4.739
0.332	0.743	0.999	-5.525	0.166	6.586
-0.138	0.891	0.999	-5.565	-0.043	5.637
2.019	0.054	0.655	-3.906	0.448	5.006
0.411	0.685	0.999	-5.499	0.172	3.873
0.719	0.479	0.984	-5.346	0.240	5.047
0.988	0.333	0.957	-5.148	0.356	6.113

0.135	0.894	0.999	-5.566	0.043	6.551
-0.137	0.892	0.999	-5.566	-0.051	3.727
-0.442	0.662	0.999	-5.487	-0.190	7.241
-0.160	0.874	0.999	-5.563	-0.066	7.391
-0.111	0.913	0.999	-5.568	-0.034	2.783
-0.272	0.788	0.999	-5.541	-0.080	7.092
1.033	0.312	0.943	-5.109	0.322	4.969
-0.300	0.766	0.999	-5.534	-0.066	4.791
0.194	0.848	0.999	-5.557	0.070	3.488
0.521	0.607	0.999	-5.454	0.138	6.713
-0.459	0.650	0.999	-5.480	-0.147	5.055
0.767	0.450	0.980	-5.315	0.210	4.631
-0.260	0.797	0.999	-5.544	-0.072	3.298
-0.237	0.815	0.999	-5.549	-0.066	5.194
-0.092	0.928	0.999	-5.570	-0.025	2.527
1.186	0.247	0.906	-4.965	0.341	4.915
0.744	0.464	0.983	-5.330	0.221	4.661
0.814	0.423	0.972	-5.282	0.209	5.036
0.905	0.374	0.963	-5.215	0.261	6.262
0.838	0.410	0.970	-5.265	0.235	6.047
1.169	0.253	0.913	-4.982	0.380	3.757
2.169	0.040	0.616	-3.673	0.487	3.952
-0.452	0.655	0.999	-5.483	-0.126	3.417
0.167	0.869	0.999	-5.562	0.087	3.549
-1.101	0.281	0.922	-5.047	-0.275	2.714
1.419	0.168	0.825	-4.714	0.302	4.192
1.449	0.160	0.820	-4.678	0.353	4.313
0.195	0.847	0.999	-5.557	0.034	7.883
-0.162	0.873	0.999	-5.562	-0.035	8.933
2.358	0.026	0.557	-3.364	0.709	2.779
2.347	0.027	0.559	-3.382	0.586	2.728
-0.074	0.942	0.999	-5.572	-0.021	2.472
1.332	0.195	0.853	-4.812	0.298	4.828
1.049	0.304	0.942	-5.095	0.272	5.025
1.654	0.110	0.767	-4.423	0.382	5.927
0.200	0.843	0.999	-5.556	0.069	8.402
-0.213	0.833	0.999	-5.554	-0.075	2.137
-0.253	0.802	0.999	-5.545	-0.068	2.429
-1.885	0.071	0.694	-4.104	-0.582	3.989
0.658	0.516	0.990	-5.382	0.315	2.201
-0.542	0.593	0.999	-5.444	-0.143	2.505
1.360	0.186	0.842	-4.781	0.334	6.824
0.483	0.633	0.999	-5.470	0.135	4.339
-0.969	0.342	0.957	-5.163	-0.326	2.492
1.452	0.159	0.820	-4.676	0.455	4.262
1.414	0.170	0.825	-4.720	0.310	3.936
-1.268	0.216	0.876	-4.881	-0.397	3.620
0.577	0.569	0.997	-5.426	0.275	3.633
0.964	0.344	0.957	-5.167	0.256	3.794
-0.345	0.733	0.999	-5.521	-0.088	3.560

0.194	0.848	0.999	-5.557	0.050	5.146
2.009	0.055	0.656	-3.921	0.527	3.965
2.990	0.006	0.393	-2.238	0.807	4.639
1.568	0.129	0.789	-4.533	0.399	3.360
1.621	0.118	0.776	-4.466	0.394	2.544
2.381	0.025	0.548	-3.325	0.642	5.718
-0.648	0.523	0.990	-5.388	-0.179	2.838
-0.389	0.701	0.999	-5.507	-0.141	1.982
0.980	0.336	0.957	-5.154	0.269	2.887
1.854	0.076	0.704	-4.150	0.458	2.480
-2.180	0.039	0.611	-3.656	-0.540	2.630
0.867	0.394	0.964	-5.244	0.192	6.196
-0.691	0.496	0.987	-5.363	-0.164	5.088
-0.428	0.672	0.999	-5.493	-0.129	3.443
-1.163	0.256	0.913	-4.988	-0.566	5.396
0.884	0.385	0.964	-5.231	0.189	9.689
-0.198	0.845	0.999	-5.556	-0.105	5.396
0.457	0.652	0.999	-5.481	0.089	4.502
0.589	0.561	0.996	-5.420	0.109	5.507
-0.993	0.330	0.956	-5.143	-0.327	3.602
0.107	0.916	0.999	-5.569	0.035	6.413
-0.218	0.829	0.999	-5.553	-0.114	2.391
0.424	0.675	0.999	-5.494	0.133	2.128
-0.980	0.337	0.957	-5.154	-0.292	2.571
-3.433	0.002	0.301	-1.387	-1.186	2.243
1.852	0.076	0.704	-4.151	0.486	4.426
1.321	0.198	0.857	-4.824	0.391	3.781
-1.082	0.289	0.931	-5.064	-0.362	6.334
-0.827	0.416	0.970	-5.273	-0.252	4.493
1.744	0.093	0.751	-4.302	0.753	6.870
-1.319	0.199	0.857	-4.826	-0.640	3.661
-0.156	0.878	0.999	-5.563	-0.044	6.024
1.281	0.212	0.870	-4.867	0.224	6.669
-0.162	0.873	0.999	-5.562	-0.068	6.406
0.471	0.642	0.999	-5.476	0.159	3.663
0.446	0.659	0.999	-5.485	0.204	4.760
-0.284	0.778	0.999	-5.538	-0.118	3.106
-1.066	0.297	0.937	-5.079	-0.293	3.888
0.793	0.435	0.973	-5.297	0.297	3.506
-0.134	0.895	0.999	-5.566	-0.034	2.400
0.683	0.501	0.988	-5.368	0.240	3.152
-0.642	0.526	0.992	-5.391	-0.217	2.369
0.654	0.519	0.990	-5.385	0.177	3.687
0.903	0.375	0.963	-5.216	0.265	3.006
-1.732	0.096	0.754	-4.319	-0.461	2.045
0.610	0.548	0.994	-5.409	0.156	4.263
0.464	0.647	0.999	-5.478	0.131	2.858
0.307	0.762	0.999	-5.532	0.091	4.229
-0.777	0.445	0.979	-5.308	-0.308	5.694
-0.381	0.706	0.999	-5.509	-0.156	2.444

0.643	0.526	0.992	-5.391	0.192	3.081
-0.208	0.837	0.999	-5.555	-0.088	2.861
-0.885	0.385	0.964	-5.230	-0.319	4.382
-1.008	0.323	0.950	-5.131	-0.343	2.276
-0.444	0.661	0.999	-5.486	-0.119	3.541
0.096	0.924	0.999	-5.570	0.034	3.749
-0.678	0.504	0.988	-5.371	-0.388	2.175
0.348	0.731	0.999	-5.520	0.113	2.775
-1.363	0.185	0.841	-4.778	-0.344	3.498
-0.290	0.774	0.999	-5.536	-0.152	3.593
0.908	0.373	0.963	-5.213	0.244	3.835
-0.948	0.352	0.961	-5.181	-0.221	3.652
0.181	0.858	0.999	-5.559	0.038	4.823
0.261	0.796	0.999	-5.544	0.044	5.490
0.825	0.417	0.970	-5.275	0.232	2.651
-0.672	0.508	0.988	-5.374	-0.156	4.746
0.350	0.729	0.999	-5.519	0.089	2.492
2.104	0.046	0.630	-3.775	0.581	7.571
-0.573	0.572	0.998	-5.429	-0.165	4.842
-0.643	0.526	0.992	-5.391	-0.167	4.912
0.565	0.577	0.999	-5.433	0.166	2.909
-0.882	0.386	0.964	-5.232	-0.200	3.694
0.493	0.626	0.999	-5.466	0.121	3.168
0.797	0.433	0.973	-5.294	0.216	5.333
1.372	0.182	0.840	-4.768	0.342	2.554
2.094	0.047	0.630	-3.791	0.875	6.733
2.491	0.020	0.516	-3.138	0.573	7.546
-0.604	0.551	0.994	-5.412	-0.174	2.065
-0.052	0.959	0.999	-5.573	-0.018	1.839
0.698	0.491	0.986	-5.359	0.206	3.732
-0.608	0.549	0.994	-5.410	-0.153	2.709
0.905	0.374	0.963	-5.215	0.209	4.661
-1.551	0.133	0.791	-4.555	-0.296	5.534
-0.270	0.789	0.999	-5.541	-0.073	7.664
0.980	0.336	0.957	-5.154	0.246	3.975
1.000	0.327	0.954	-5.137	0.238	2.485
-0.372	0.713	0.999	-5.512	-0.084	6.730
0.430	0.671	0.999	-5.492	0.129	2.666
-0.550	0.587	0.999	-5.440	-0.169	1.534
-1.224	0.232	0.897	-4.927	-0.395	5.421
0.640	0.528	0.992	-5.393	0.210	6.694
-0.311	0.758	0.999	-5.531	-0.114	2.843
0.982	0.336	0.957	-5.153	0.324	4.087
0.863	0.396	0.964	-5.247	0.211	3.315
-1.405	0.172	0.828	-4.730	-0.310	2.399
2.807	0.010	0.446	-2.576	0.696	3.528
-1.213	0.237	0.899	-4.938	-0.314	3.603
-0.260	0.797	0.999	-5.544	-0.115	1.342
0.413	0.683	0.999	-5.498	0.137	6.573
-0.433	0.669	0.999	-5.491	-0.130	4.886

0.097	0.924	0.999	-5.570	0.044	5.186
-1.572	0.128	0.789	-4.528	-0.374	3.342
0.067	0.947	0.999	-5.572	0.025	7.333
1.627	0.116	0.776	-4.458	0.458	3.125
-0.189	0.852	0.999	-5.558	-0.060	0.967
-0.212	0.834	0.999	-5.554	-0.098	1.977
0.540	0.594	0.999	-5.445	0.149	3.696
-1.332	0.195	0.853	-4.812	-0.325	3.701
-0.472	0.641	0.999	-5.475	-0.141	2.602
-0.712	0.483	0.985	-5.350	-0.169	6.549
-0.740	0.466	0.983	-5.332	-0.427	4.694
0.083	0.935	0.999	-5.571	0.025	5.858
0.681	0.502	0.988	-5.369	0.271	9.657
-0.403	0.690	0.999	-5.502	-0.071	8.237
0.956	0.348	0.959	-5.174	0.247	4.718
-0.086	0.932	0.999	-5.571	-0.017	5.402
-1.246	0.224	0.886	-4.904	-0.293	1.838
0.069	0.945	0.999	-5.572	0.018	5.463
-1.613	0.119	0.781	-4.477	-0.574	8.274
0.287	0.776	0.999	-5.537	0.102	6.317
0.384	0.704	0.999	-5.508	0.091	6.422
-0.102	0.919	0.999	-5.569	-0.047	4.707
0.836	0.411	0.970	-5.267	0.460	3.496
-0.721	0.478	0.984	-5.345	-0.165	4.575
-0.438	0.665	0.999	-5.489	-0.091	4.250
0.329	0.745	0.999	-5.526	0.082	5.269
0.972	0.340	0.957	-5.161	0.244	6.836
0.271	0.789	0.999	-5.541	0.075	6.631
-0.143	0.887	0.999	-5.565	-0.032	2.370
0.860	0.398	0.964	-5.249	0.250	4.469
-1.620	0.118	0.776	-4.467	-0.726	4.075
-0.677	0.505	0.988	-5.371	-0.121	5.128
-0.639	0.528	0.992	-5.393	-0.168	3.387
-1.028	0.314	0.944	-5.113	-0.361	3.230
0.733	0.471	0.983	-5.337	0.194	4.845
-0.269	0.790	0.999	-5.542	-0.084	6.637
-0.449	0.658	0.999	-5.485	-0.131	4.537
-1.771	0.089	0.735	-4.266	-0.633	8.932
0.315	0.755	0.999	-5.530	0.093	5.231
-1.424	0.167	0.825	-4.708	-0.289	5.847
-0.927	0.363	0.963	-5.198	-0.205	5.756
-1.119	0.274	0.921	-5.030	-0.327	4.571
-1.163	0.256	0.913	-4.988	-0.281	2.199
-0.114	0.910	0.999	-5.568	-0.033	3.329
-0.298	0.768	0.999	-5.534	-0.065	4.841
1.462	0.156	0.817	-4.663	0.380	5.470
0.356	0.725	0.999	-5.517	0.211	6.246
1.720	0.098	0.757	-4.335	0.429	5.443
2.362	0.026	0.556	-3.357	0.644	6.732
0.953	0.350	0.961	-5.177	0.213	4.276

0.429	0.672	0.999	-5.492	0.132	8.049
2.719	0.012	0.488	-2.737	0.610	5.549
0.182	0.857	0.999	-5.559	0.088	1.649
2.877	0.008	0.431	-2.448	0.788	4.473
0.118	0.907	0.999	-5.568	0.034	2.642
0.626	0.537	0.993	-5.400	0.184	4.657
1.278	0.213	0.871	-4.871	0.422	2.549
0.928	0.362	0.963	-5.197	0.281	2.499
1.050	0.304	0.941	-5.093	0.325	3.387
0.186	0.854	0.999	-5.558	0.044	5.370
-1.343	0.191	0.850	-4.800	-0.402	3.939
-0.895	0.379	0.964	-5.223	-0.208	5.815
-1.015	0.320	0.950	-5.125	-0.253	3.641
-1.334	0.194	0.853	-4.810	-0.349	3.177
-0.226	0.823	0.999	-5.551	-0.063	6.810
0.681	0.502	0.988	-5.369	0.138	5.013
-1.186	0.247	0.906	-4.965	-0.308	5.981
0.726	0.475	0.984	-5.341	0.419	2.963
0.583	0.565	0.997	-5.423	0.169	4.639
3.152	0.004	0.346	-1.932	0.721	6.335
-0.225	0.824	0.999	-5.551	-0.094	3.304
0.497	0.624	0.999	-5.464	0.171	5.486
0.951	0.351	0.961	-5.178	0.583	3.986
0.051	0.960	0.999	-5.573	0.012	8.210
1.227	0.231	0.894	-4.924	0.313	5.963
1.322	0.198	0.857	-4.823	0.383	5.842
4.310	0.000	0.256	0.359	1.132	4.365
2.188	0.038	0.611	-3.642	0.582	5.154
1.835	0.078	0.710	-4.175	0.471	5.142
1.759	0.091	0.743	-4.283	0.528	4.404
1.085	0.288	0.929	-5.062	0.267	4.790
1.697	0.102	0.764	-4.366	0.434	4.156
0.165	0.870	0.999	-5.562	0.047	3.627
2.191	0.038	0.611	-3.638	0.831	6.377
1.009	0.323	0.950	-5.130	0.378	4.996
0.657	0.517	0.990	-5.383	0.203	4.783
1.337	0.193	0.853	-4.807	0.321	2.495
0.153	0.880	0.999	-5.564	0.041	3.966
0.055	0.956	0.999	-5.573	0.015	4.572
-2.407	0.024	0.543	-3.282	-0.620	4.356
1.928	0.065	0.675	-4.042	0.602	3.635
2.142	0.042	0.619	-3.716	0.530	3.368
0.282	0.780	0.999	-5.539	0.141	4.615
-0.163	0.872	0.999	-5.562	-0.031	5.687
1.314	0.201	0.860	-4.832	0.327	4.234
-0.795	0.434	0.973	-5.296	-0.183	2.362
-0.668	0.510	0.989	-5.377	-0.176	2.390
-0.470	0.642	0.999	-5.476	-0.205	2.764
0.639	0.529	0.992	-5.393	0.354	5.197
0.847	0.405	0.968	-5.259	0.480	4.053

-0.182	0.857	0.999	-5.559	-0.048	5.723
-0.605	0.550	0.994	-5.412	-0.360	5.008
0.092	0.927	0.999	-5.570	0.038	6.116
1.950	0.062	0.667	-4.009	0.488	2.271
1.056	0.301	0.941	-5.088	0.201	6.340
0.708	0.486	0.985	-5.353	0.157	4.627
3.193	0.004	0.340	-1.853	1.145	4.520
-0.387	0.702	0.999	-5.507	-0.099	3.579
-0.813	0.424	0.972	-5.283	-0.321	4.787
-1.052	0.303	0.941	-5.092	-0.214	5.880
0.233	0.818	0.999	-5.550	0.075	4.475
-0.104	0.918	0.999	-5.569	-0.026	2.019
-0.111	0.913	0.999	-5.568	-0.049	5.300
0.203	0.841	0.999	-5.556	0.081	4.799
1.538	0.136	0.795	-4.571	0.472	5.239
-0.418	0.679	0.999	-5.496	-0.185	2.682
0.446	0.660	0.999	-5.486	0.093	4.987
1.184	0.248	0.907	-4.967	0.310	4.713
0.257	0.800	0.999	-5.545	0.067	4.419
1.140	0.265	0.916	-5.011	0.245	5.784
0.293	0.772	0.999	-5.536	0.068	3.507
0.293	0.772	0.999	-5.536	0.094	3.409
1.269	0.216	0.876	-4.880	0.440	5.691
-0.798	0.432	0.973	-5.294	-0.196	6.265
0.521	0.607	0.999	-5.454	0.162	6.772
0.264	0.794	0.999	-5.543	0.059	6.294
-0.310	0.759	0.999	-5.531	-0.070	5.153
-1.448	0.160	0.820	-4.680	-0.361	4.488
-1.689	0.104	0.764	-4.377	-0.839	3.053
-0.830	0.414	0.970	-5.271	-0.236	6.121
-0.418	0.679	0.999	-5.496	-0.127	4.282
-0.398	0.694	0.999	-5.503	-0.195	4.975
-1.496	0.147	0.804	-4.622	-0.470	5.939
-0.655	0.519	0.990	-5.384	-0.178	6.179
0.342	0.735	0.999	-5.522	0.073	3.525
-0.100	0.921	0.999	-5.569	-0.030	6.206
-0.158	0.876	0.999	-5.563	-0.050	4.977
-0.074	0.942	0.999	-5.572	-0.025	7.678
0.159	0.875	0.999	-5.563	0.059	5.617
0.186	0.854	0.999	-5.559	0.041	5.037
1.497	0.147	0.804	-4.621	0.440	4.296
0.295	0.770	0.999	-5.535	0.063	5.171
0.739	0.467	0.983	-5.333	0.262	3.112
1.274	0.214	0.874	-4.874	0.281	4.452
-0.146	0.885	0.999	-5.564	-0.029	6.943
1.418	0.169	0.825	-4.716	0.418	5.304
-0.616	0.543	0.994	-5.406	-0.149	6.308
0.592	0.559	0.996	-5.419	0.143	7.034
0.792	0.436	0.973	-5.298	0.208	2.538
0.260	0.797	0.999	-5.544	0.079	5.819

1.904	0.068	0.684	-4.076	0.625	2.862
-0.103	0.919	0.999	-5.569	-0.024	5.060
-0.866	0.395	0.964	-5.245	-0.307	5.732
0.073	0.943	0.999	-5.572	0.020	3.254
-0.837	0.410	0.970	-5.266	-0.268	4.089
1.510	0.144	0.803	-4.606	0.385	3.710
0.780	0.442	0.979	-5.306	0.238	7.368
0.195	0.847	0.999	-5.557	0.060	5.881
-0.699	0.491	0.986	-5.358	-0.200	3.171
1.704	0.101	0.763	-4.356	0.376	7.333
1.069	0.295	0.936	-5.077	0.219	4.598
0.256	0.800	0.999	-5.545	0.105	2.844
-0.817	0.422	0.972	-5.280	-0.231	6.383
-0.300	0.767	0.999	-5.534	-0.079	4.063
1.256	0.221	0.882	-4.894	0.323	5.830
0.516	0.610	0.999	-5.456	0.115	3.005
1.070	0.295	0.936	-5.076	0.204	5.992
-2.209	0.037	0.608	-3.610	-0.515	3.640
1.621	0.117	0.776	-4.466	0.356	5.102
0.480	0.635	0.999	-5.472	0.098	7.281
0.666	0.512	0.989	-5.378	0.175	3.207
0.172	0.865	0.999	-5.561	0.037	4.456
1.365	0.184	0.841	-4.775	0.370	6.260
1.216	0.235	0.899	-4.935	0.310	8.576
-0.643	0.526	0.992	-5.391	-0.149	4.103
-1.163	0.256	0.913	-4.988	-0.298	7.340
0.182	0.857	0.999	-5.559	0.037	9.064
1.000	0.327	0.954	-5.137	0.387	6.799
-2.272	0.032	0.590	-3.507	-0.560	2.918
-0.085	0.933	0.999	-5.571	-0.020	4.246
0.719	0.478	0.984	-5.345	0.160	6.296
1.905	0.068	0.684	-4.075	0.513	3.862
1.366	0.184	0.841	-4.774	0.305	3.501
0.117	0.907	0.999	-5.568	0.020	4.572
0.486	0.631	0.999	-5.469	0.150	5.259
1.187	0.246	0.906	-4.965	0.323	6.201
0.498	0.623	0.999	-5.464	0.247	5.227
0.163	0.872	0.999	-5.562	0.039	5.313
-0.780	0.442	0.979	-5.306	-0.144	6.404
0.278	0.783	0.999	-5.539	0.059	6.943
-1.716	0.099	0.757	-4.341	-0.350	6.826
0.280	0.782	0.999	-5.539	0.062	5.938
-0.184	0.855	0.999	-5.559	-0.038	2.509
-0.221	0.827	0.999	-5.552	-0.042	5.659
0.200	0.843	0.999	-5.556	0.047	6.183
1.834	0.078	0.711	-4.177	0.423	5.891
1.412	0.170	0.825	-4.722	0.331	5.834
-0.655	0.519	0.990	-5.384	-0.189	4.052
-1.005	0.324	0.952	-5.133	-0.263	4.498
0.514	0.612	0.999	-5.457	0.159	4.691

-0.277	0.784	0.999	-5.540	-0.089	2.595
0.285	0.778	0.999	-5.538	0.070	8.243
0.612	0.546	0.994	-5.408	0.145	5.433
0.296	0.770	0.999	-5.535	0.068	3.749
-0.732	0.471	0.983	-5.337	-0.145	5.221
-1.661	0.109	0.766	-4.414	-0.365	4.085
1.652	0.111	0.767	-4.426	0.353	4.951
-0.720	0.478	0.984	-5.345	-0.222	5.429
-0.349	0.730	0.999	-5.520	-0.187	5.339
0.135	0.894	0.999	-5.566	0.036	7.556
0.686	0.499	0.987	-5.366	0.170	5.175
1.417	0.169	0.825	-4.716	0.958	4.020
-1.451	0.159	0.820	-4.677	-0.579	4.245
1.519	0.141	0.801	-4.594	0.309	7.007
-1.491	0.148	0.806	-4.629	-0.438	4.070
0.528	0.602	0.999	-5.450	0.130	4.215
-0.223	0.826	0.999	-5.552	-0.073	7.211
-0.291	0.773	0.999	-5.536	-0.085	6.103
-0.288	0.776	0.999	-5.537	-0.095	7.249
-0.318	0.753	0.999	-5.529	-0.115	4.403
-1.808	0.083	0.722	-4.214	-0.436	2.955
0.198	0.844	0.999	-5.556	0.053	5.609
0.515	0.611	0.999	-5.456	0.266	6.077
-0.596	0.556	0.995	-5.416	-0.175	4.259
0.221	0.827	0.999	-5.552	0.064	4.095
1.119	0.274	0.921	-5.030	0.282	4.280
-0.142	0.888	0.999	-5.565	-0.039	6.337
0.741	0.466	0.983	-5.332	0.195	5.791
0.214	0.832	0.999	-5.553	0.047	7.664
-0.699	0.491	0.986	-5.358	-0.210	4.157
-1.394	0.176	0.831	-4.743	-0.333	2.274
2.621	0.015	0.498	-2.912	0.875	6.366
1.261	0.219	0.878	-4.888	0.242	6.762
1.398	0.174	0.828	-4.738	0.256	5.857
1.652	0.111	0.767	-4.426	0.621	5.864
0.390	0.700	0.999	-5.506	0.077	8.159
-0.290	0.774	0.999	-5.536	-0.061	4.983
1.133	0.268	0.917	-5.017	0.233	5.028
1.009	0.323	0.950	-5.129	0.244	4.414
0.948	0.352	0.961	-5.180	0.228	2.771
2.361	0.026	0.557	-3.360	0.600	3.724
1.460	0.157	0.817	-4.666	0.481	6.290
1.453	0.159	0.820	-4.675	0.369	4.258
0.175	0.863	0.999	-5.560	0.047	2.249
0.311	0.759	0.999	-5.531	0.099	8.969
-0.095	0.925	0.999	-5.570	-0.019	8.051
0.307	0.761	0.999	-5.532	0.075	5.628
-0.262	0.796	0.999	-5.543	-0.088	3.562
0.902	0.376	0.963	-5.217	0.255	4.484
0.787	0.439	0.975	-5.301	0.223	3.867

1.843	0.077	0.707	-4.165	0.907	7.521
0.803	0.429	0.973	-5.290	0.158	6.413
-0.078	0.938	0.999	-5.571	-0.023	5.198
0.212	0.834	0.999	-5.554	0.054	4.321
0.069	0.945	0.999	-5.572	0.039	3.633
-1.497	0.147	0.804	-4.621	-0.357	2.363
0.698	0.492	0.986	-5.359	0.179	4.184
0.410	0.686	0.999	-5.499	0.083	7.952
0.106	0.916	0.999	-5.569	0.026	3.178
0.178	0.860	0.999	-5.560	0.060	5.779
1.584	0.126	0.786	-4.513	0.374	5.253
0.523	0.606	0.999	-5.453	0.177	4.344
-0.143	0.887	0.999	-5.565	-0.081	4.269
-1.567	0.130	0.789	-4.535	-0.396	8.815
1.458	0.157	0.817	-4.668	0.359	4.168
1.364	0.185	0.841	-4.776	0.377	5.259
0.062	0.951	0.999	-5.572	0.017	8.501
0.587	0.562	0.996	-5.421	0.165	5.160
0.775	0.445	0.979	-5.309	0.222	5.609
0.166	0.870	0.999	-5.562	0.048	3.841
3.778	0.001	0.301	-0.706	1.507	6.065
0.440	0.664	0.999	-5.488	0.161	3.703
-0.702	0.489	0.986	-5.356	-0.297	4.075
2.647	0.014	0.498	-2.865	0.623	5.017
0.731	0.472	0.984	-5.338	0.195	6.211
2.534	0.018	0.501	-3.064	0.473	3.089
2.102	0.046	0.630	-3.778	0.614	5.817
2.511	0.019	0.506	-3.104	0.756	5.086
0.342	0.736	0.999	-5.522	0.142	6.761
-0.064	0.949	0.999	-5.572	-0.019	1.793
0.724	0.476	0.984	-5.343	0.219	5.562
-0.078	0.939	0.999	-5.571	-0.014	5.521
-0.326	0.747	0.999	-5.527	-0.101	5.449
-0.093	0.926	0.999	-5.570	-0.024	5.080
-0.062	0.951	0.999	-5.572	-0.017	7.709
1.377	0.181	0.840	-4.762	0.471	5.377
0.274	0.787	0.999	-5.541	0.139	3.563
-0.501	0.621	0.999	-5.462	-0.137	6.899
1.633	0.115	0.776	-4.450	0.519	4.333
0.379	0.708	0.999	-5.510	0.118	5.914
-0.090	0.929	0.999	-5.570	-0.031	6.410
0.704	0.488	0.985	-5.355	0.197	6.370
0.321	0.751	0.999	-5.528	0.066	6.009
0.928	0.362	0.963	-5.197	0.352	4.632
-0.733	0.470	0.983	-5.337	-0.183	4.993
-1.116	0.275	0.921	-5.034	-0.197	6.444
1.001	0.326	0.954	-5.137	0.391	4.938
1.169	0.253	0.913	-4.982	0.261	5.847
0.341	0.736	0.999	-5.522	0.105	4.725
1.124	0.272	0.919	-5.026	0.286	3.626

-1.085	0.288	0.929	-5.062	-0.277	6.675
0.637	0.530	0.992	-5.395	0.177	4.051
-0.468	0.644	0.999	-5.477	-0.132	6.163
-0.788	0.438	0.975	-5.301	-0.177	4.403
0.901	0.376	0.963	-5.218	0.216	3.141
0.509	0.615	0.999	-5.459	0.145	4.974
-0.065	0.949	0.999	-5.572	-0.017	3.098
-0.115	0.910	0.999	-5.568	-0.031	5.015
0.181	0.858	0.999	-5.559	0.035	5.068
2.298	0.030	0.573	-3.463	0.489	6.363
-1.760	0.091	0.743	-4.281	-0.659	4.233
1.668	0.108	0.764	-4.405	0.459	4.021
0.737	0.468	0.983	-5.334	0.258	6.785
-0.924	0.364	0.963	-5.200	-0.222	3.568
0.707	0.486	0.985	-5.353	0.350	3.044
1.952	0.062	0.667	-4.007	0.705	5.625
-0.188	0.853	0.999	-5.558	-0.051	2.735
0.802	0.430	0.973	-5.291	0.317	8.135
-0.915	0.369	0.963	-5.207	-0.361	2.285
-0.768	0.450	0.980	-5.314	-0.206	6.690
0.499	0.622	0.999	-5.463	0.126	7.294
0.292	0.773	0.999	-5.536	0.083	5.859
0.530	0.601	0.999	-5.449	0.148	6.289
1.111	0.277	0.922	-5.038	0.302	2.990
2.806	0.010	0.446	-2.579	1.024	6.521
1.808	0.083	0.722	-4.214	0.419	7.211
-0.078	0.938	0.999	-5.571	-0.021	8.702
0.288	0.776	0.999	-5.537	0.104	2.984
2.518	0.019	0.501	-3.092	0.637	6.088
1.245	0.225	0.886	-4.906	0.293	9.560
-0.925	0.364	0.963	-5.199	-0.233	5.189
1.426	0.166	0.825	-4.705	0.470	5.475
-0.403	0.690	0.999	-5.502	-0.088	4.964
-0.749	0.461	0.983	-5.326	-0.267	4.255
0.188	0.853	0.999	-5.558	0.048	6.716
-0.379	0.708	0.999	-5.510	-0.069	6.863
0.052	0.959	0.999	-5.573	0.017	5.687
-0.416	0.681	0.999	-5.497	-0.093	6.010
0.397	0.694	0.999	-5.504	0.105	10.894
0.409	0.686	0.999	-5.499	0.118	7.101
0.722	0.477	0.984	-5.344	0.192	5.074
1.513	0.143	0.802	-4.602	0.377	5.341
-2.007	0.056	0.656	-3.924	-0.468	4.439
0.185	0.855	0.999	-5.559	0.058	3.355
0.280	0.782	0.999	-5.539	0.084	3.242
0.423	0.676	0.999	-5.494	0.099	4.372
0.324	0.748	0.999	-5.527	0.091	1.880
0.565	0.577	0.999	-5.432	0.109	7.607
-0.409	0.686	0.999	-5.499	-0.138	6.328
-0.703	0.489	0.986	-5.356	-0.281	3.800

1.494	0.148	0.805	-4.625	0.384	3.501
0.789	0.438	0.974	-5.300	0.203	3.515
0.743	0.464	0.983	-5.330	0.225	3.503
0.726	0.475	0.984	-5.342	0.185	3.516
0.594	0.557	0.995	-5.417	0.149	3.361
0.722	0.477	0.984	-5.344	0.228	5.578
-0.568	0.575	0.999	-5.431	-0.204	3.707
0.711	0.484	0.985	-5.351	0.236	3.023
1.514	0.142	0.802	-4.600	0.381	4.954
1.193	0.244	0.904	-4.958	0.294	5.066
0.818	0.421	0.972	-5.280	0.253	4.410
-0.350	0.730	0.999	-5.520	-0.085	3.434
-1.270	0.216	0.876	-4.879	-0.296	6.356
1.145	0.263	0.914	-5.005	0.260	3.658
2.663	0.013	0.498	-2.838	0.537	4.358
1.670	0.107	0.764	-4.402	0.330	6.501
0.431	0.670	0.999	-5.492	0.105	5.773
-1.336	0.194	0.853	-4.808	-0.262	5.201
0.353	0.727	0.999	-5.519	0.081	6.045
-0.230	0.820	0.999	-5.550	-0.048	5.112
1.163	0.256	0.913	-4.988	0.241	4.752
-1.293	0.208	0.868	-4.855	-0.355	2.311
-0.462	0.648	0.999	-5.479	-0.127	2.732
0.141	0.889	0.999	-5.565	0.034	3.002
-0.359	0.723	0.999	-5.517	-0.126	3.590
0.922	0.365	0.963	-5.202	0.193	6.817
1.402	0.173	0.828	-4.734	0.318	6.212
-2.012	0.055	0.656	-3.916	-0.568	2.874
0.689	0.497	0.987	-5.364	0.157	4.077
1.228	0.231	0.894	-4.923	0.267	5.163
1.498	0.147	0.804	-4.620	0.624	3.471
0.154	0.879	0.999	-5.563	0.045	3.070
0.546	0.590	0.999	-5.442	0.144	3.140
-2.904	0.008	0.418	-2.398	-0.621	5.892
0.098	0.923	0.999	-5.570	0.044	3.012
-2.327	0.028	0.566	-3.415	-0.626	3.181
-1.893	0.070	0.689	-4.093	-0.514	2.305
0.810	0.426	0.972	-5.285	0.181	5.669
0.357	0.724	0.999	-5.517	0.103	2.590
-0.486	0.631	0.999	-5.469	-0.162	1.138
-1.572	0.128	0.789	-4.529	-0.407	2.713
0.051	0.960	0.999	-5.573	0.022	3.010
-1.032	0.312	0.943	-5.110	-0.359	2.901
-0.059	0.953	0.999	-5.572	-0.017	6.835
1.096	0.283	0.925	-5.052	0.201	3.124
-1.754	0.092	0.745	-4.289	-0.403	2.700
0.155	0.878	0.999	-5.563	0.035	2.582
1.041	0.308	0.942	-5.102	0.229	4.197
0.430	0.671	0.999	-5.492	0.277	2.875
-1.179	0.249	0.910	-4.972	-0.346	7.399

-0.736	0.469	0.983	-5.335	-0.374	4.756
-0.519	0.608	0.999	-5.454	-0.162	3.073
-0.383	0.705	0.999	-5.509	-0.154	3.541
-1.028	0.314	0.944	-5.113	-0.224	6.268
0.188	0.852	0.999	-5.558	0.047	4.862
-0.540	0.594	0.999	-5.445	-0.170	2.283
-1.896	0.069	0.687	-4.088	-0.446	2.619
-2.624	0.015	0.498	-2.907	-0.941	1.841
0.590	0.560	0.996	-5.420	0.165	3.232
0.926	0.363	0.963	-5.199	0.245	5.973
-0.653	0.520	0.990	-5.385	-0.138	3.664
-0.686	0.499	0.987	-5.366	-0.176	3.811
0.970	0.341	0.957	-5.162	0.240	7.464
0.832	0.413	0.970	-5.270	0.181	6.155
0.810	0.426	0.972	-5.285	0.161	6.129
0.799	0.432	0.973	-5.293	0.179	7.259
0.434	0.668	0.999	-5.490	0.098	6.789
0.275	0.785	0.999	-5.540	0.050	5.349
0.227	0.822	0.999	-5.551	0.047	4.873
-0.226	0.823	0.999	-5.551	-0.056	2.925
-0.222	0.826	0.999	-5.552	-0.048	6.448
0.201	0.843	0.999	-5.556	0.038	5.452
-0.172	0.865	0.999	-5.561	-0.039	7.260
0.168	0.868	0.999	-5.561	0.036	7.962
0.161	0.874	0.999	-5.562	0.033	6.056
0.065	0.949	0.999	-5.572	0.027	3.773
-0.093	0.926	0.999	-5.570	-0.021	5.680
0.525	0.604	0.999	-5.452	0.192	2.832
-0.978	0.337	0.957	-5.156	-0.345	3.223
-0.426	0.674	0.999	-5.493	-0.083	5.369
0.735	0.469	0.983	-5.336	0.196	3.084
0.638	0.529	0.992	-5.394	0.149	6.751
0.215	0.832	0.999	-5.553	0.044	4.563
0.404	0.690	0.999	-5.501	0.086	5.936
0.452	0.655	0.999	-5.483	0.087	6.168
-0.918	0.367	0.963	-5.204	-0.216	4.600
-0.391	0.699	0.999	-5.506	-0.161	2.969
2.311	0.029	0.570	-3.443	0.683	5.271
1.108	0.278	0.922	-5.041	0.295	6.312
1.568	0.129	0.789	-4.534	0.402	5.261
1.520	0.141	0.801	-4.594	0.338	6.320
1.356	0.187	0.845	-4.785	0.337	5.344
1.324	0.197	0.855	-4.821	0.309	5.386
0.764	0.452	0.980	-5.317	0.195	5.256
0.383	0.705	0.999	-5.509	0.110	6.131
-0.239	0.813	0.999	-5.548	-0.068	5.366
1.076	0.292	0.934	-5.070	0.275	3.477
-0.712	0.483	0.985	-5.350	-0.191	3.878
1.397	0.175	0.828	-4.739	0.384	3.520
-1.364	0.185	0.841	-4.776	-0.343	7.040

-0.387	0.702	0.999	-5.507	-0.069	6.230
-0.223	0.825	0.999	-5.552	-0.055	6.690
-0.362	0.720	0.999	-5.516	-0.136	5.760
-0.885	0.385	0.964	-5.230	-0.438	4.563
0.245	0.808	0.999	-5.547	0.064	5.194
-0.430	0.671	0.999	-5.492	-0.094	3.753
0.117	0.908	0.999	-5.568	0.024	5.617
0.921	0.366	0.963	-5.202	0.173	6.299
0.474	0.639	0.999	-5.474	0.174	7.505
0.442	0.662	0.999	-5.487	0.110	6.019
-1.133	0.268	0.917	-5.017	-0.218	3.758
-0.134	0.895	0.999	-5.566	-0.040	7.311
0.222	0.826	0.999	-5.552	0.064	2.933
-0.321	0.751	0.999	-5.528	-0.109	4.513
-0.328	0.746	0.999	-5.526	-0.092	2.677
0.111	0.913	0.999	-5.568	0.027	5.137
1.604	0.121	0.784	-4.488	0.429	5.902
0.964	0.344	0.957	-5.168	0.325	4.304
0.288	0.776	0.999	-5.537	0.075	3.731
0.363	0.719	0.999	-5.515	0.094	4.296
0.400	0.693	0.999	-5.503	0.119	4.247
1.641	0.113	0.775	-4.440	0.316	5.131
-0.331	0.744	0.999	-5.525	-0.087	3.497
-0.283	0.780	0.999	-5.538	-0.053	5.553
1.426	0.166	0.825	-4.705	0.297	5.100
-1.340	0.192	0.851	-4.803	-0.344	4.818
-1.661	0.109	0.766	-4.415	-0.386	2.777
-0.546	0.590	0.999	-5.442	-0.146	3.539
-0.123	0.903	0.999	-5.567	-0.050	5.552
-0.212	0.833	0.999	-5.554	-0.048	5.444
0.673	0.507	0.988	-5.374	0.151	8.370
0.387	0.702	0.999	-5.507	0.102	7.936
-1.758	0.091	0.743	-4.284	-0.543	2.230
-1.159	0.257	0.913	-4.992	-0.740	1.775
0.110	0.913	0.999	-5.568	0.039	4.778
0.055	0.957	0.999	-5.573	0.015	6.628
-1.147	0.262	0.914	-5.003	-0.329	2.835
-0.268	0.791	0.999	-5.542	-0.117	5.644
1.444	0.161	0.820	-4.685	0.474	5.100
0.887	0.384	0.964	-5.229	0.153	7.257
0.459	0.650	0.999	-5.480	0.130	2.236
-0.965	0.344	0.957	-5.167	-0.376	4.692
-0.714	0.482	0.984	-5.349	-0.195	2.734
0.380	0.707	0.999	-5.510	0.137	5.492
0.489	0.629	0.999	-5.468	0.165	4.511
0.103	0.919	0.999	-5.569	0.032	3.732
0.576	0.570	0.997	-5.427	0.415	3.420
0.727	0.474	0.984	-5.340	0.163	5.704
0.776	0.445	0.979	-5.309	0.222	4.297
0.873	0.391	0.964	-5.239	0.271	2.586

-0.500	0.622	0.999	-5.463	-0.096	8.447
-1.936	0.064	0.673	-4.030	-0.351	4.768
0.988	0.333	0.957	-5.148	0.261	5.396
0.587	0.563	0.996	-5.421	0.169	2.757
-0.150	0.882	0.999	-5.564	-0.065	4.874
0.767	0.451	0.980	-5.315	0.370	2.992
1.047	0.305	0.942	-5.097	0.427	3.205
2.302	0.030	0.570	-3.457	0.766	2.501
0.173	0.864	0.999	-5.561	0.043	3.678
-0.502	0.620	0.999	-5.462	-0.207	3.029
1.265	0.218	0.876	-4.885	0.339	5.077
-1.555	0.133	0.791	-4.550	-0.432	4.738
0.962	0.345	0.957	-5.169	0.415	3.150
-0.356	0.725	0.999	-5.517	-0.134	2.077
-1.171	0.253	0.913	-4.980	-0.258	1.905
1.000	0.327	0.954	-5.138	0.267	5.250
0.253	0.802	0.999	-5.545	0.052	6.350
-0.340	0.737	0.999	-5.522	-0.127	2.737
1.591	0.124	0.786	-4.504	0.476	6.428
0.119	0.906	0.999	-5.568	0.030	3.437
0.235	0.816	0.999	-5.549	0.055	2.753
0.293	0.772	0.999	-5.536	0.109	5.334
0.779	0.443	0.979	-5.307	0.222	5.233
-0.792	0.436	0.973	-5.298	-0.200	4.369
0.365	0.718	0.999	-5.515	0.121	4.421
-0.866	0.394	0.964	-5.244	-0.241	6.549
0.416	0.681	0.999	-5.497	0.174	2.661
0.927	0.363	0.963	-5.198	0.347	4.279
-0.369	0.715	0.999	-5.513	-0.087	3.690
-0.350	0.730	0.999	-5.520	-0.089	3.249
-0.178	0.860	0.999	-5.560	-0.042	3.560
-0.058	0.954	0.999	-5.572	-0.021	3.802
-0.515	0.611	0.999	-5.456	-0.157	3.868
0.256	0.800	0.999	-5.545	0.066	4.700
2.653	0.014	0.498	-2.855	0.701	5.319
-0.081	0.936	0.999	-5.571	-0.022	2.263
4.159	0.000	0.257	0.057	0.970	3.616
0.443	0.662	0.999	-5.487	0.117	2.517
-2.095	0.046	0.630	-3.790	-0.481	2.237
0.252	0.803	0.999	-5.546	0.061	6.768
0.063	0.951	0.999	-5.572	0.026	4.707
0.172	0.864	0.999	-5.561	0.069	4.051
0.108	0.915	0.999	-5.569	0.036	7.404
0.889	0.383	0.964	-5.228	0.243	3.378
0.698	0.492	0.986	-5.359	0.191	5.327
0.253	0.802	0.999	-5.545	0.050	5.377
2.393	0.024	0.543	-3.305	0.591	6.075
2.219	0.036	0.601	-3.593	0.537	6.027
2.181	0.039	0.611	-3.654	0.457	4.063
3.457	0.002	0.301	-1.340	0.651	3.716

3.446	0.002	0.301	-1.362	0.609	3.335
3.413	0.002	0.301	-1.427	0.702	4.250
3.241	0.003	0.340	-1.760	0.611	4.309
3.202	0.004	0.340	-1.836	0.639	4.105
-0.097	0.924	0.999	-5.570	-0.020	6.818
1.062	0.298	0.940	-5.083	0.291	3.705
2.313	0.029	0.570	-3.439	0.461	5.190
-0.491	0.628	0.999	-5.467	-0.173	4.676
-0.506	0.617	0.999	-5.460	-0.115	5.390
-0.253	0.803	0.999	-5.545	-0.067	4.999
3.181	0.004	0.340	-1.876	0.888	4.869
1.649	0.112	0.768	-4.429	0.562	4.238
1.155	0.259	0.913	-4.995	0.240	7.797
-1.493	0.148	0.805	-4.626	-0.372	4.767
-2.119	0.044	0.630	-3.752	-0.583	3.198
2.261	0.033	0.594	-3.525	0.591	5.417
1.399	0.174	0.828	-4.737	0.343	3.189
1.274	0.214	0.874	-4.875	0.440	3.534
-1.530	0.138	0.796	-4.581	-0.387	2.708
0.770	0.448	0.979	-5.313	0.181	6.518
-2.115	0.045	0.630	-3.758	-1.009	2.384
-0.461	0.648	0.999	-5.479	-0.097	8.542
0.472	0.641	0.999	-5.475	0.151	2.785
0.738	0.467	0.983	-5.334	0.201	2.668
0.804	0.429	0.973	-5.289	0.172	3.553
1.015	0.320	0.950	-5.124	0.263	5.534
0.175	0.863	0.999	-5.560	0.043	3.513
0.925	0.364	0.963	-5.199	0.243	6.245
1.045	0.306	0.942	-5.098	0.360	4.409
0.191	0.850	0.999	-5.558	0.067	4.248
0.530	0.601	0.999	-5.449	0.110	4.172
-0.397	0.695	0.999	-5.504	-0.147	1.662
0.183	0.857	0.999	-5.559	0.084	4.983
-0.555	0.584	0.999	-5.437	-0.173	6.009
-1.048	0.304	0.942	-5.095	-0.325	5.380
1.622	0.117	0.776	-4.464	0.374	4.820
0.158	0.876	0.999	-5.563	0.035	8.020
2.177	0.039	0.611	-3.660	0.597	5.975
1.790	0.086	0.729	-4.240	0.446	3.873
2.327	0.028	0.566	-3.416	0.551	5.310
0.109	0.914	0.999	-5.569	0.039	2.541
0.791	0.436	0.973	-5.299	0.357	3.517
-0.399	0.693	0.999	-5.503	-0.092	5.028
-0.318	0.753	0.999	-5.529	-0.176	4.546
1.350	0.189	0.848	-4.792	0.381	5.261
1.695	0.102	0.764	-4.369	0.639	6.792
-0.211	0.834	0.999	-5.554	-0.048	3.522
0.796	0.433	0.973	-5.295	0.183	7.445
-1.532	0.138	0.795	-4.579	-0.448	4.015
1.274	0.214	0.874	-4.875	0.318	1.732

-0.232	0.818	0.999	-5.550	-0.086	3.566
1.606	0.121	0.784	-4.486	0.321	6.295
0.615	0.544	0.994	-5.407	0.122	6.249
-0.056	0.955	0.999	-5.573	-0.013	7.005
-0.298	0.768	0.999	-5.534	-0.131	4.879
-0.249	0.805	0.999	-5.546	-0.112	4.777
0.970	0.341	0.957	-5.163	0.336	3.877
0.687	0.498	0.987	-5.366	0.294	2.865
0.321	0.751	0.999	-5.528	0.171	4.578
-0.988	0.333	0.957	-5.148	-0.263	6.102
0.930	0.361	0.963	-5.195	0.213	7.784
-0.057	0.955	0.999	-5.572	-0.015	3.011
1.559	0.132	0.791	-4.546	0.319	5.701
-0.466	0.645	0.999	-5.478	-0.100	4.781
0.209	0.836	0.999	-5.555	0.050	3.991
0.898	0.378	0.963	-5.220	0.167	4.094
2.425	0.023	0.542	-3.252	0.601	5.371
-1.637	0.114	0.775	-4.445	-0.678	2.639
2.614	0.015	0.498	-2.924	0.591	5.610
2.004	0.056	0.657	-3.929	0.413	4.336
1.158	0.258	0.913	-4.992	0.506	4.852
0.602	0.553	0.994	-5.414	0.157	2.129
-0.707	0.486	0.985	-5.353	-0.198	8.210
-0.881	0.387	0.964	-5.233	-0.266	7.497
1.053	0.303	0.941	-5.091	0.274	4.779
1.025	0.315	0.946	-5.116	0.217	6.492
0.986	0.334	0.957	-5.150	0.261	4.554
0.398	0.694	0.999	-5.503	0.124	8.274
0.954	0.349	0.960	-5.176	0.185	4.745
1.028	0.314	0.944	-5.113	0.452	4.281
0.678	0.504	0.988	-5.371	0.152	3.948
-1.221	0.233	0.898	-4.930	-0.263	4.685
2.527	0.018	0.501	-3.076	0.895	5.860
0.281	0.781	0.999	-5.539	0.123	5.756
0.436	0.666	0.999	-5.489	0.112	4.353
-0.078	0.939	0.999	-5.571	-0.023	2.657
-0.177	0.861	0.999	-5.560	-0.045	8.190
-0.176	0.862	0.999	-5.560	-0.048	9.259
0.323	0.749	0.999	-5.527	0.077	3.527
0.997	0.328	0.954	-5.140	0.281	5.693
0.818	0.421	0.972	-5.279	0.237	3.848
1.997	0.057	0.659	-3.939	0.482	5.801
-0.467	0.645	0.999	-5.477	-0.102	5.621
-0.327	0.746	0.999	-5.526	-0.080	5.332
-0.124	0.902	0.999	-5.567	-0.043	6.556
0.836	0.411	0.970	-5.267	0.295	4.021
2.064	0.050	0.639	-3.837	0.489	2.556
2.574	0.016	0.500	-2.995	0.527	7.182
-2.032	0.053	0.653	-3.887	-0.379	6.949
-0.706	0.487	0.985	-5.354	-0.176	3.851

-0.456	0.652	0.999	-5.482	-0.109	4.700
0.288	0.775	0.999	-5.537	0.111	4.118
1.379	0.180	0.839	-4.760	0.357	5.212
2.402	0.024	0.543	-3.291	0.436	4.710
0.221	0.827	0.999	-5.552	0.081	4.086
1.651	0.111	0.767	-4.427	0.436	4.990
1.458	0.157	0.817	-4.668	0.817	4.548
2.748	0.011	0.474	-2.685	0.552	6.695
1.735	0.095	0.754	-4.315	0.457	4.015
-0.917	0.368	0.963	-5.205	-0.189	4.195
-0.431	0.670	0.999	-5.491	-0.101	2.058
1.941	0.063	0.671	-4.022	0.624	4.197
0.967	0.343	0.957	-5.165	0.227	5.375
-1.145	0.263	0.914	-5.006	-0.461	4.808
-0.332	0.743	0.999	-5.525	-0.177	3.983
-0.363	0.719	0.999	-5.515	-0.180	3.791
-1.607	0.121	0.784	-4.484	-0.441	5.456
0.630	0.534	0.993	-5.398	0.104	7.084
2.568	0.017	0.500	-3.005	0.579	4.315
0.606	0.550	0.994	-5.411	0.137	2.791
0.132	0.896	0.999	-5.566	0.028	4.573
1.668	0.108	0.764	-4.405	0.766	3.227
0.498	0.623	0.999	-5.464	0.090	6.259
1.402	0.173	0.828	-4.733	0.254	8.516
-0.346	0.732	0.999	-5.521	-0.128	7.607
2.188	0.038	0.611	-3.643	0.952	5.081
-0.761	0.454	0.981	-5.319	-0.258	3.783
-0.521	0.607	0.999	-5.453	-0.145	3.998
0.562	0.579	0.999	-5.434	0.131	3.820
0.240	0.812	0.999	-5.548	0.053	7.260
0.386	0.703	0.999	-5.508	0.106	4.334
1.358	0.186	0.843	-4.783	0.288	4.523
1.162	0.256	0.913	-4.988	0.311	5.178
0.374	0.712	0.999	-5.512	0.079	4.051
0.869	0.393	0.964	-5.242	0.172	4.138
-2.212	0.036	0.606	-3.605	-0.526	2.388
-1.138	0.266	0.917	-5.012	-0.260	4.426
0.131	0.897	0.999	-5.566	0.030	3.378
-0.161	0.874	0.999	-5.562	-0.036	6.251
1.159	0.257	0.913	-4.992	0.304	8.243
-0.516	0.610	0.999	-5.456	-0.191	3.892
-0.751	0.459	0.983	-5.325	-0.158	5.860
1.060	0.299	0.941	-5.085	0.379	5.595
0.285	0.778	0.999	-5.538	0.059	4.818
0.412	0.684	0.999	-5.499	0.158	5.513
0.864	0.395	0.964	-5.246	0.305	7.333
0.588	0.562	0.996	-5.421	0.131	5.604
1.306	0.203	0.862	-4.841	0.356	4.400
0.210	0.836	0.999	-5.554	0.079	4.789
-0.258	0.799	0.999	-5.544	-0.119	4.418

0.132	0.896	0.999	-5.566	0.028	7.185
0.871	0.392	0.964	-5.241	0.223	4.811
0.993	0.330	0.956	-5.144	0.220	6.382
0.384	0.704	0.999	-5.508	0.155	5.573
0.873	0.391	0.964	-5.240	0.256	1.822
-0.652	0.520	0.990	-5.386	-0.265	3.114
0.499	0.622	0.999	-5.463	0.115	5.968
0.432	0.669	0.999	-5.491	0.106	3.953
0.472	0.641	0.999	-5.475	0.167	3.501
-0.445	0.660	0.999	-5.486	-0.113	3.223
-1.271	0.215	0.875	-4.878	-0.230	5.522
1.805	0.083	0.724	-4.218	0.548	5.532
0.375	0.711	0.999	-5.511	0.067	6.581
2.180	0.039	0.611	-3.655	0.470	5.026
-1.013	0.321	0.950	-5.126	-0.300	3.792
0.519	0.608	0.999	-5.454	0.126	5.205
-0.368	0.716	0.999	-5.514	-0.091	5.359
-1.444	0.161	0.820	-4.685	-0.388	7.784
-1.190	0.245	0.905	-4.961	-0.280	5.693
-1.377	0.181	0.840	-4.761	-0.350	6.219
-0.126	0.901	0.999	-5.567	-0.031	6.338
-1.146	0.262	0.914	-5.004	-0.316	8.224
-0.387	0.702	0.999	-5.507	-0.096	6.541
-1.056	0.301	0.941	-5.088	-0.233	6.056
-2.283	0.031	0.585	-3.489	-0.647	6.394
1.386	0.178	0.837	-4.752	0.488	4.194
0.753	0.458	0.982	-5.324	0.214	4.736
-0.831	0.414	0.970	-5.270	-0.228	6.853
1.078	0.291	0.933	-5.069	0.282	3.408
0.088	0.931	0.999	-5.571	0.021	6.541
0.718	0.479	0.984	-5.346	0.199	4.450
0.857	0.400	0.964	-5.251	0.362	7.708
0.290	0.774	0.999	-5.536	0.118	4.277
1.867	0.074	0.698	-4.130	0.465	5.536
0.485	0.632	0.999	-5.469	0.138	4.346
0.308	0.761	0.999	-5.532	0.103	6.776
-0.196	0.846	0.999	-5.557	-0.050	2.387
-0.072	0.943	0.999	-5.572	-0.031	3.724
0.487	0.630	0.999	-5.469	0.125	2.278
0.237	0.814	0.999	-5.549	0.104	6.115
1.383	0.179	0.838	-4.755	0.430	3.994
0.532	0.599	0.999	-5.448	0.118	3.251
-0.305	0.763	0.999	-5.533	-0.089	3.066
-0.684	0.501	0.988	-5.368	-0.186	5.599
-0.596	0.556	0.995	-5.416	-0.211	2.771
0.253	0.802	0.999	-5.545	0.068	5.361
1.785	0.086	0.731	-4.247	0.426	3.470
-0.334	0.741	0.999	-5.524	-0.132	9.925
1.284	0.211	0.869	-4.865	0.309	5.009
0.458	0.651	0.999	-5.481	0.120	6.795

-0.466	0.645	0.999	-5.478	-0.097	6.091
-0.863	0.396	0.964	-5.247	-0.313	3.645
0.432	0.669	0.999	-5.491	0.086	6.488
1.759	0.091	0.743	-4.281	0.386	6.150
1.156	0.259	0.913	-4.995	0.321	5.913
0.274	0.786	0.999	-5.540	0.061	5.925
0.381	0.707	0.999	-5.509	0.199	4.517
-0.263	0.795	0.999	-5.543	-0.074	4.550
0.601	0.553	0.994	-5.414	0.344	3.664
-0.052	0.959	0.999	-5.573	-0.014	4.682
-1.166	0.254	0.913	-4.985	-0.358	1.653
-0.084	0.933	0.999	-5.571	-0.028	5.357
-2.107	0.045	0.630	-3.770	-0.488	3.869
3.014	0.006	0.393	-2.194	0.687	3.653
0.874	0.391	0.964	-5.239	0.185	4.538
0.249	0.805	0.999	-5.546	0.075	2.862
-0.553	0.585	0.999	-5.438	-0.144	5.101
0.238	0.814	0.999	-5.549	0.103	4.186
0.949	0.351	0.961	-5.180	0.431	4.114
1.270	0.216	0.876	-4.879	0.351	4.313
-0.382	0.705	0.999	-5.509	-0.087	7.035
0.716	0.480	0.984	-5.348	0.211	4.821
0.353	0.727	0.999	-5.518	0.068	4.408
-1.137	0.266	0.917	-5.013	-0.250	3.468
-1.886	0.071	0.694	-4.102	-0.455	2.211
0.585	0.563	0.997	-5.422	0.188	3.306
0.252	0.803	0.999	-5.546	0.080	2.111
3.395	0.002	0.301	-1.462	0.981	5.831
0.501	0.620	0.999	-5.462	0.150	6.565
0.206	0.838	0.999	-5.555	0.047	4.256
2.617	0.015	0.498	-2.919	0.618	6.064
-1.577	0.127	0.788	-4.522	-0.455	2.823
-0.773	0.447	0.979	-5.311	-0.220	5.539
0.154	0.878	0.999	-5.563	0.049	7.767
0.462	0.648	0.999	-5.479	0.095	4.172
0.757	0.456	0.981	-5.321	0.139	7.589
0.988	0.333	0.957	-5.148	0.252	6.426
0.974	0.339	0.957	-5.159	0.223	6.186
0.527	0.603	0.999	-5.451	0.148	7.618
-0.686	0.499	0.987	-5.366	-0.228	4.722
0.642	0.527	0.992	-5.392	0.304	5.449
1.247	0.224	0.886	-4.903	0.316	4.375
1.314	0.201	0.860	-4.832	0.312	4.291
-1.108	0.278	0.922	-5.040	-0.228	6.606
-0.800	0.431	0.973	-5.293	-0.208	3.436
-0.151	0.881	0.999	-5.564	-0.063	4.425
0.496	0.624	0.999	-5.465	0.093	7.387
-0.374	0.712	0.999	-5.512	-0.089	4.637
1.206	0.239	0.901	-4.945	0.310	4.446
0.281	0.781	0.999	-5.539	0.073	5.303

-0.255	0.801	0.999	-5.545	-0.050	3.408
-0.523	0.605	0.999	-5.452	-0.139	5.006
0.648	0.523	0.990	-5.388	0.350	3.720
1.554	0.133	0.791	-4.551	0.385	5.563
1.306	0.203	0.862	-4.841	0.423	3.586
0.792	0.436	0.973	-5.298	0.238	8.320
-1.723	0.097	0.757	-4.331	-0.387	3.569
-1.876	0.072	0.698	-4.117	-0.414	5.254
-0.243	0.810	0.999	-5.548	-0.059	7.210
0.409	0.686	0.999	-5.500	0.103	4.223
0.096	0.924	0.999	-5.570	0.030	7.481
-0.908	0.372	0.963	-5.212	-0.233	4.132
-0.605	0.550	0.994	-5.412	-0.139	4.677
1.056	0.301	0.941	-5.088	0.212	3.095
0.721	0.478	0.984	-5.344	0.129	6.879
0.657	0.517	0.990	-5.383	0.118	7.193
0.603	0.552	0.994	-5.413	0.107	8.173
-0.575	0.571	0.997	-5.428	-0.249	1.868
-0.166	0.869	0.999	-5.562	-0.086	8.748
1.409	0.171	0.826	-4.725	0.493	4.616
0.229	0.821	0.999	-5.551	0.057	5.501
-0.365	0.718	0.999	-5.515	-0.095	3.328
2.399	0.024	0.543	-3.296	1.080	5.993
0.366	0.718	0.999	-5.514	0.091	4.021
1.701	0.101	0.764	-4.361	0.513	5.612
0.655	0.518	0.990	-5.384	0.222	7.503
1.734	0.095	0.754	-4.316	0.370	3.041
-3.452	0.002	0.301	-1.351	-0.790	1.917
-0.381	0.706	0.999	-5.509	-0.252	3.227
-2.166	0.040	0.616	-3.678	-0.743	3.954
-0.374	0.712	0.999	-5.512	-0.175	4.287
-0.907	0.373	0.963	-5.214	-0.544	4.628
0.258	0.798	0.999	-5.544	0.100	3.868
0.111	0.912	0.999	-5.568	0.033	5.315
0.885	0.385	0.964	-5.231	0.190	6.348
-0.098	0.922	0.999	-5.570	-0.023	3.608
1.969	0.060	0.663	-3.981	0.609	6.359
0.192	0.849	0.999	-5.557	0.055	2.810
-0.451	0.656	0.999	-5.484	-0.190	4.442
-0.843	0.407	0.969	-5.262	-0.223	3.745
-0.210	0.835	0.999	-5.554	-0.043	2.001
-0.879	0.388	0.964	-5.235	-0.195	9.653
-0.715	0.481	0.984	-5.349	-0.224	4.758
0.262	0.795	0.999	-5.543	0.065	3.343
0.638	0.529	0.992	-5.394	0.221	3.106
0.801	0.431	0.973	-5.292	0.213	4.564
1.372	0.182	0.840	-4.768	0.240	7.241
0.706	0.486	0.985	-5.354	0.176	5.274
-0.167	0.868	0.999	-5.561	-0.056	5.636
-1.699	0.102	0.764	-4.363	-0.486	2.099

-0.217	0.830	0.999	-5.553	-0.068	2.206
-0.124	0.902	0.999	-5.567	-0.032	3.510
-0.289	0.775	0.999	-5.537	-0.104	5.021
0.997	0.328	0.954	-5.140	0.284	2.872
-1.204	0.240	0.902	-4.947	-0.506	3.953
-1.158	0.258	0.913	-4.992	-0.474	4.319
0.603	0.552	0.994	-5.413	0.263	4.468
0.268	0.791	0.999	-5.542	0.149	5.064
0.244	0.810	0.999	-5.547	0.096	2.832
-1.085	0.288	0.929	-5.062	-0.277	2.375
0.349	0.730	0.999	-5.520	0.111	3.365
1.027	0.314	0.944	-5.114	0.198	4.582
1.688	0.104	0.764	-4.378	0.354	5.971
-1.591	0.124	0.786	-4.504	-0.376	2.680
0.367	0.717	0.999	-5.514	0.102	4.452
0.946	0.353	0.961	-5.182	0.322	2.661
1.109	0.278	0.922	-5.040	0.227	6.657
1.054	0.302	0.941	-5.090	0.207	7.190
-0.461	0.649	0.999	-5.479	-0.116	3.028
1.517	0.142	0.802	-4.597	0.332	5.320
0.601	0.553	0.994	-5.414	0.121	4.864
1.416	0.169	0.825	-4.718	0.283	6.195
-1.287	0.210	0.868	-4.861	-0.211	11.217
-1.473	0.153	0.813	-4.650	-0.442	5.075
-1.441	0.162	0.822	-4.689	-0.319	11.233
0.576	0.570	0.997	-5.427	0.199	3.720
-0.396	0.695	0.999	-5.504	-0.153	1.180
-1.582	0.126	0.786	-4.515	-0.408	6.125
0.074	0.941	0.999	-5.571	0.017	2.130
-0.502	0.620	0.999	-5.462	-0.235	2.702
-0.876	0.389	0.964	-5.237	-0.358	2.964
-0.668	0.510	0.989	-5.377	-0.173	2.852
-0.896	0.379	0.963	-5.222	-0.214	3.029
-1.131	0.269	0.918	-5.019	-0.304	5.749
-3.138	0.004	0.352	-1.958	-0.730	2.154
-0.697	0.492	0.986	-5.359	-0.377	3.185
-1.965	0.061	0.663	-3.987	-0.556	3.699
-1.444	0.161	0.820	-4.685	-0.340	2.351
0.153	0.880	0.999	-5.564	0.050	2.925
0.602	0.553	0.994	-5.413	0.339	5.079
-0.903	0.375	0.963	-5.217	-0.355	2.498
-1.897	0.069	0.687	-4.086	-0.434	6.068
1.216	0.235	0.899	-4.935	0.593	5.094
0.169	0.867	0.999	-5.561	0.043	3.030
0.456	0.652	0.999	-5.481	0.170	2.765
-0.066	0.948	0.999	-5.572	-0.017	4.560
1.158	0.258	0.913	-4.992	0.383	2.645
-0.458	0.651	0.999	-5.481	-0.128	3.817
-1.076	0.292	0.934	-5.070	-0.478	2.342
-0.604	0.552	0.994	-5.413	-0.255	3.535

-0.260	0.797	0.999	-5.544	-0.083	2.893
0.284	0.779	0.999	-5.538	0.096	3.757
2.135	0.043	0.624	-3.727	0.794	3.206
0.535	0.598	0.999	-5.447	0.216	4.148
-0.998	0.328	0.954	-5.139	-0.329	3.070
-0.293	0.772	0.999	-5.536	-0.059	6.252
-2.373	0.026	0.556	-3.340	-0.844	2.229
-0.098	0.923	0.999	-5.570	-0.037	3.091
-1.524	0.140	0.801	-4.588	-0.505	6.297
-0.197	0.846	0.999	-5.557	-0.088	3.437
0.535	0.597	0.999	-5.447	0.183	3.867
-2.107	0.045	0.630	-3.771	-0.486	3.163
-1.215	0.236	0.899	-4.936	-0.239	3.508
0.692	0.495	0.987	-5.362	0.242	2.536
-0.385	0.704	0.999	-5.508	-0.135	1.944
1.307	0.203	0.862	-4.840	0.366	3.166
-0.210	0.836	0.999	-5.554	-0.081	5.596
-0.783	0.441	0.977	-5.304	-0.194	2.492
-0.173	0.864	0.999	-5.561	-0.097	2.124
-0.276	0.785	0.999	-5.540	-0.084	4.658
-1.363	0.185	0.841	-4.778	-0.323	9.500
-1.169	0.253	0.913	-4.982	-0.286	10.139
-0.246	0.808	0.999	-5.547	-0.079	2.421
0.142	0.888	0.999	-5.565	0.036	2.863
-0.697	0.492	0.986	-5.359	-0.235	2.651
0.074	0.942	0.999	-5.572	0.026	4.515
-2.086	0.047	0.630	-3.803	-0.529	2.815
0.574	0.571	0.998	-5.428	0.155	2.639
-1.533	0.138	0.795	-4.577	-0.541	2.200
-0.784	0.441	0.977	-5.304	-0.231	2.966
-0.329	0.745	0.999	-5.526	-0.102	2.299
0.300	0.767	0.999	-5.534	0.137	4.146
-0.323	0.749	0.999	-5.527	-0.087	2.804
-1.872	0.073	0.698	-4.123	-0.591	3.529
0.233	0.818	0.999	-5.550	0.110	2.573
0.409	0.686	0.999	-5.499	0.179	2.024
0.966	0.343	0.957	-5.166	0.258	4.273
0.263	0.794	0.999	-5.543	0.086	2.566
-0.397	0.695	0.999	-5.504	-0.147	1.963
2.276	0.032	0.589	-3.499	0.786	2.973
-1.102	0.281	0.922	-5.046	-0.331	3.298
-1.865	0.074	0.698	-4.133	-0.504	3.418
-0.701	0.490	0.986	-5.357	-0.251	2.300
0.168	0.868	0.999	-5.561	0.042	3.175
0.296	0.769	0.999	-5.535	0.110	2.170
0.463	0.648	0.999	-5.479	0.149	3.344
0.945	0.354	0.961	-5.183	0.344	4.193
-1.207	0.239	0.901	-4.944	-0.358	4.737
-0.794	0.435	0.973	-5.296	-0.243	4.803
0.072	0.943	0.999	-5.572	0.024	3.116

0.280	0.782	0.999	-5.539	0.086	2.796
-0.463	0.648	0.999	-5.479	-0.169	3.021
-0.766	0.451	0.980	-5.316	-0.276	3.058
-0.315	0.755	0.999	-5.530	-0.120	2.559
-0.061	0.952	0.999	-5.572	-0.019	3.340
0.669	0.510	0.989	-5.376	0.179	5.000
0.828	0.415	0.970	-5.273	0.234	3.917
1.674	0.107	0.764	-4.397	0.689	4.155
-1.273	0.215	0.874	-4.876	-0.367	2.869
0.970	0.341	0.957	-5.163	0.360	3.183
-1.308	0.203	0.862	-4.839	-0.328	2.716
-1.804	0.083	0.724	-4.220	-0.426	4.366
0.367	0.717	0.999	-5.514	0.130	3.605
0.518	0.609	0.999	-5.455	0.145	2.829
-0.071	0.944	0.999	-5.572	-0.019	2.909
-1.249	0.223	0.886	-4.901	-0.356	3.001
-0.364	0.719	0.999	-5.515	-0.109	1.648
0.513	0.613	0.999	-5.457	0.163	2.331
-1.553	0.133	0.791	-4.552	-0.450	3.205
-1.482	0.151	0.812	-4.640	-0.377	4.742
-0.719	0.479	0.984	-5.346	-0.322	2.742
-0.651	0.521	0.990	-5.386	-0.193	3.947
1.041	0.308	0.942	-5.102	0.290	2.693
-1.237	0.228	0.889	-4.914	-0.409	2.114
0.848	0.404	0.968	-5.258	0.302	2.858
-1.108	0.278	0.922	-5.041	-0.439	6.303
0.400	0.692	0.999	-5.503	0.097	3.602
-1.045	0.306	0.942	-5.098	-0.360	4.897
-0.201	0.842	0.999	-5.556	-0.062	5.645
0.463	0.648	0.999	-5.479	0.354	6.697
0.334	0.741	0.999	-5.524	0.217	3.941
1.033	0.312	0.943	-5.109	0.457	6.768
2.086	0.047	0.630	-3.803	0.502	4.301
1.665	0.108	0.766	-4.408	0.448	6.029
1.336	0.194	0.853	-4.808	0.552	5.010
-1.044	0.306	0.942	-5.099	-0.321	8.494
1.332	0.195	0.853	-4.812	0.370	3.827
1.013	0.321	0.950	-5.126	0.179	8.093
1.612	0.119	0.781	-4.478	0.308	6.104
0.416	0.681	0.999	-5.497	0.109	3.631
2.229	0.035	0.598	-3.576	0.791	5.177
-0.399	0.693	0.999	-5.503	-0.180	4.276
-0.215	0.832	0.999	-5.553	-0.059	4.988
0.246	0.808	0.999	-5.547	0.063	2.819
0.121	0.905	0.999	-5.567	0.028	8.395
0.305	0.763	0.999	-5.533	0.098	3.952
0.339	0.738	0.999	-5.523	0.082	2.886
-0.186	0.854	0.999	-5.558	-0.077	1.710
1.120	0.273	0.921	-5.029	0.342	5.791
1.413	0.170	0.825	-4.721	0.266	5.774

1.601	0.122	0.784	-4.491	0.473	5.677
2.593	0.016	0.500	-2.960	0.593	4.862
-1.124	0.272	0.919	-5.026	-0.298	6.681
0.254	0.802	0.999	-5.545	0.109	3.340
-1.061	0.299	0.940	-5.084	-0.479	2.875
1.371	0.182	0.840	-4.769	0.330	4.356
1.280	0.212	0.870	-4.868	0.427	2.838
2.304	0.030	0.570	-3.454	0.450	6.172
1.051	0.303	0.941	-5.093	0.223	4.871
0.462	0.648	0.999	-5.479	0.120	7.198
0.756	0.456	0.981	-5.322	0.213	3.444
-0.814	0.423	0.972	-5.283	-0.190	5.033
2.606	0.015	0.500	-2.939	0.839	5.956
0.694	0.494	0.987	-5.361	0.230	6.028
-0.269	0.790	0.999	-5.542	-0.079	5.636
0.374	0.712	0.999	-5.512	0.096	3.175
0.517	0.609	0.999	-5.455	0.104	6.188
0.558	0.582	0.999	-5.436	0.161	3.014
-0.256	0.800	0.999	-5.545	-0.069	3.212
0.563	0.578	0.999	-5.433	0.121	6.327
-0.351	0.729	0.999	-5.519	-0.069	4.289
0.394	0.697	0.999	-5.505	0.094	5.439
1.616	0.119	0.779	-4.472	0.298	5.323
0.692	0.495	0.987	-5.362	0.156	6.644
0.944	0.354	0.961	-5.184	0.220	5.507
1.352	0.188	0.847	-4.790	0.277	4.315
-0.935	0.359	0.963	-5.191	-0.213	6.681
0.104	0.918	0.999	-5.569	0.022	4.699
1.106	0.279	0.922	-5.042	0.451	4.167
-0.200	0.843	0.999	-5.556	-0.089	3.656
-0.930	0.361	0.963	-5.195	-0.350	10.157
0.243	0.810	0.999	-5.548	0.107	3.655
1.666	0.108	0.766	-4.408	0.681	2.825
-0.476	0.638	0.999	-5.473	-0.148	2.485
-1.577	0.127	0.788	-4.523	-0.362	2.640
-1.482	0.151	0.811	-4.639	-0.381	6.124
-0.184	0.855	0.999	-5.559	-0.050	4.197
-0.798	0.433	0.973	-5.294	-0.193	5.095
-1.571	0.129	0.789	-4.530	-0.577	2.838
-0.983	0.335	0.957	-5.152	-0.300	5.033
-1.133	0.268	0.917	-5.017	-0.387	4.185
-0.098	0.922	0.999	-5.570	-0.022	2.825
1.347	0.190	0.849	-4.796	0.392	4.748
-2.970	0.006	0.400	-2.275	-0.798	3.714
-1.139	0.265	0.916	-5.011	-0.279	5.164
-2.007	0.056	0.656	-3.924	-0.569	6.577
0.787	0.439	0.975	-5.302	0.241	2.922
2.061	0.050	0.639	-3.842	0.850	3.268
-1.130	0.269	0.918	-5.020	-0.340	7.602
0.518	0.609	0.999	-5.455	0.295	3.897

-2.820	0.009	0.446	-2.554	-0.773	4.276
1.027	0.314	0.944	-5.114	0.357	3.731
0.067	0.947	0.999	-5.572	0.018	2.639
0.053	0.958	0.999	-5.573	0.020	2.565
0.069	0.946	0.999	-5.572	0.017	3.740
-0.327	0.746	0.999	-5.526	-0.091	2.468
0.866	0.395	0.964	-5.244	0.330	5.509
-0.689	0.497	0.987	-5.365	-0.188	2.848
-0.317	0.754	0.999	-5.529	-0.116	2.797
-2.107	0.045	0.630	-3.771	-0.608	8.670
-0.534	0.598	0.999	-5.448	-0.145	5.070
0.776	0.445	0.979	-5.309	0.187	4.820
-1.990	0.058	0.660	-3.950	-0.639	3.762
1.297	0.206	0.868	-4.851	0.349	3.006
-0.317	0.754	0.999	-5.529	-0.105	2.493
-0.736	0.469	0.983	-5.335	-0.222	3.155
1.153	0.260	0.914	-4.998	0.352	4.896
1.623	0.117	0.776	-4.463	0.441	3.441
-1.694	0.103	0.764	-4.370	-0.530	2.847
1.266	0.217	0.876	-4.883	0.496	5.108
-0.307	0.761	0.999	-5.532	-0.085	5.732
-1.213	0.236	0.899	-4.938	-0.342	2.209
2.573	0.016	0.500	-2.996	0.855	3.780
-1.528	0.139	0.798	-4.584	-0.300	10.305
-0.754	0.458	0.982	-5.324	-0.304	6.189
1.196	0.243	0.904	-4.955	0.586	3.016
0.737	0.468	0.983	-5.334	0.332	3.424
2.682	0.013	0.498	-2.804	0.952	4.904
-0.999	0.327	0.954	-5.138	-0.629	2.920
-0.791	0.436	0.973	-5.298	-0.358	7.508
-1.129	0.270	0.918	-5.021	-0.288	5.558
0.680	0.503	0.988	-5.370	0.285	2.656
2.597	0.016	0.500	-2.954	0.743	1.935
-0.183	0.856	0.999	-5.559	-0.053	5.790
-1.819	0.081	0.719	-4.199	-0.727	2.236
-0.633	0.532	0.992	-5.396	-0.262	2.859
0.164	0.871	0.999	-5.562	0.115	7.280
-1.143	0.264	0.915	-5.008	-0.408	5.489
-0.422	0.677	0.999	-5.495	-0.089	5.116
0.223	0.826	0.999	-5.552	0.100	2.827
1.129	0.270	0.918	-5.021	0.503	2.953
1.659	0.109	0.766	-4.416	0.648	4.064
-0.981	0.336	0.957	-5.153	-0.292	3.011
-1.621	0.117	0.776	-4.466	-0.398	4.463
0.810	0.425	0.972	-5.285	0.221	2.554
-1.424	0.167	0.825	-4.708	-0.365	2.912
0.674	0.506	0.988	-5.373	0.191	3.020
0.415	0.682	0.999	-5.497	0.150	3.247
-0.653	0.520	0.990	-5.385	-0.232	4.646
-2.994	0.006	0.393	-2.231	-0.760	1.971

-1.825	0.080	0.717	-4.190	-0.620	2.848
-0.661	0.515	0.990	-5.381	-0.192	2.345
0.255	0.801	0.999	-5.545	0.084	5.070
1.227	0.231	0.894	-4.923	0.529	2.099
2.272	0.032	0.590	-3.506	0.631	2.548
-0.860	0.398	0.964	-5.249	-0.352	4.048
-0.993	0.330	0.956	-5.144	-0.444	3.833
-1.058	0.300	0.941	-5.086	-0.248	7.139
-4.032	0.000	0.289	-0.198	-1.020	3.269
0.087	0.932	0.999	-5.571	0.025	4.338
-1.207	0.239	0.901	-4.944	-0.498	2.035
-1.977	0.059	0.663	-3.969	-0.546	2.140
-0.675	0.506	0.988	-5.373	-0.272	3.120
-0.459	0.650	0.999	-5.480	-0.188	3.793
1.018	0.318	0.950	-5.122	0.380	2.722
0.181	0.858	0.999	-5.559	0.048	3.063
0.392	0.698	0.999	-5.505	0.136	2.503
-0.698	0.491	0.986	-5.359	-0.203	4.073
0.468	0.644	0.999	-5.477	0.134	4.443
1.031	0.312	0.943	-5.110	0.314	3.960
0.695	0.494	0.987	-5.361	0.229	4.280
0.741	0.466	0.983	-5.332	0.228	4.000
-0.905	0.374	0.963	-5.215	-0.431	2.134
-0.690	0.496	0.987	-5.364	-0.222	2.859
-0.155	0.878	0.999	-5.563	-0.052	3.339
-1.093	0.285	0.927	-5.055	-0.340	3.298
-0.975	0.339	0.957	-5.158	-0.414	2.580
-0.769	0.449	0.979	-5.313	-0.191	2.946
-1.972	0.060	0.663	-3.977	-0.478	3.227
-0.499	0.622	0.999	-5.464	-0.168	3.324
1.341	0.192	0.851	-4.802	0.457	3.270
-1.331	0.195	0.853	-4.813	-0.320	3.651
0.908	0.372	0.963	-5.212	0.316	4.254
-0.840	0.409	0.970	-5.264	-0.290	2.557
0.633	0.532	0.992	-5.397	0.281	4.182
-0.332	0.743	0.999	-5.525	-0.113	2.145
-2.369	0.026	0.556	-3.345	-0.843	1.727
-1.876	0.072	0.698	-4.118	-0.493	2.346
0.173	0.864	0.999	-5.561	0.047	2.864
1.410	0.171	0.826	-4.724	0.589	3.667
-1.836	0.078	0.710	-4.174	-0.460	2.842
-1.996	0.057	0.659	-3.940	-0.659	1.818
-0.083	0.934	0.999	-5.571	-0.022	3.484
-0.854	0.401	0.966	-5.254	-0.281	3.997
0.833	0.413	0.970	-5.269	0.336	2.740
1.016	0.319	0.950	-5.124	0.367	3.441
-0.135	0.894	0.999	-5.566	-0.059	3.930
-0.068	0.947	0.999	-5.572	-0.019	2.922
-0.581	0.566	0.997	-5.424	-0.151	2.395
0.621	0.540	0.994	-5.403	0.218	4.622

-2.362	0.026	0.556	-3.357	-0.508	5.839
-1.656	0.110	0.767	-4.421	-0.474	2.409
-1.186	0.247	0.906	-4.966	-0.367	2.327
-3.431	0.002	0.301	-1.391	-1.020	3.151
1.086	0.288	0.929	-5.061	0.367	3.785
0.273	0.787	0.999	-5.541	0.089	4.427
0.515	0.611	0.999	-5.456	0.137	2.923
-1.511	0.143	0.803	-4.604	-0.361	3.558
0.724	0.476	0.984	-5.342	0.209	2.929
-0.870	0.392	0.964	-5.241	-0.591	6.184
-0.461	0.649	0.999	-5.480	-0.112	1.540
1.058	0.300	0.941	-5.086	0.397	3.200
-0.778	0.444	0.979	-5.307	-0.289	3.391
0.434	0.668	0.999	-5.490	0.184	3.800
0.242	0.811	0.999	-5.548	0.063	4.441
1.562	0.131	0.791	-4.542	0.474	3.606
0.744	0.464	0.983	-5.330	0.246	5.311
-0.640	0.528	0.992	-5.393	-0.353	2.296
-0.441	0.663	0.999	-5.488	-0.199	5.453
2.154	0.041	0.619	-3.696	0.927	2.907
1.190	0.245	0.905	-4.961	0.367	4.135
-0.881	0.387	0.964	-5.233	-0.387	2.625
-1.178	0.250	0.910	-4.973	-0.456	4.562
-0.128	0.899	0.999	-5.567	-0.029	1.700
-0.555	0.584	0.999	-5.437	-0.154	3.376
-2.541	0.018	0.501	-3.053	-0.898	3.975
0.933	0.360	0.963	-5.193	0.309	2.775
-1.311	0.202	0.861	-4.835	-0.415	2.685
-2.554	0.017	0.500	-3.029	-0.752	5.892
-2.266	0.032	0.591	-3.517	-0.635	2.686
0.398	0.694	0.999	-5.504	0.134	2.822
-0.148	0.883	0.999	-5.564	-0.050	3.866
-1.129	0.270	0.918	-5.021	-0.329	4.262
-0.745	0.463	0.983	-5.329	-0.238	2.551
-0.603	0.552	0.994	-5.413	-0.215	2.513
1.042	0.307	0.942	-5.101	0.302	3.823
0.598	0.555	0.995	-5.415	0.175	2.361
-0.983	0.335	0.957	-5.152	-0.422	2.747
0.529	0.602	0.999	-5.450	0.240	2.193
-0.443	0.662	0.999	-5.487	-0.114	3.335
-0.878	0.388	0.964	-5.236	-0.290	2.202
-1.348	0.190	0.849	-4.795	-0.510	2.289
0.549	0.588	0.999	-5.440	0.198	2.626
-1.740	0.094	0.753	-4.308	-0.532	5.978
-0.691	0.496	0.987	-5.363	-0.234	2.448
-1.975	0.059	0.663	-3.972	-0.480	3.946
0.218	0.830	0.999	-5.553	0.090	5.062
0.529	0.601	0.999	-5.450	0.166	2.688
0.134	0.894	0.999	-5.566	0.031	4.161
1.304	0.204	0.863	-4.843	0.430	2.600

-0.962	0.345	0.957	-5.169	-0.307	6.286
-1.838	0.078	0.710	-4.172	-0.843	2.501
-1.232	0.229	0.892	-4.919	-0.479	5.303
1.219	0.234	0.899	-4.932	0.540	3.275
-1.142	0.264	0.916	-5.009	-0.311	4.384
-1.155	0.259	0.913	-4.996	-0.430	2.746
-0.909	0.372	0.963	-5.212	-0.304	3.993
-0.834	0.412	0.970	-5.268	-0.244	4.533
-0.518	0.609	0.999	-5.455	-0.180	3.896
0.357	0.724	0.999	-5.517	0.127	2.917
-0.601	0.553	0.994	-5.414	-0.276	1.354
1.669	0.107	0.764	-4.403	0.568	2.684
-0.900	0.377	0.963	-5.219	-0.432	2.312
-1.536	0.137	0.795	-4.573	-0.462	3.616
0.244	0.809	0.999	-5.547	0.068	2.663
-0.367	0.716	0.999	-5.514	-0.168	5.217
-1.462	0.156	0.817	-4.664	-0.427	3.754
-1.418	0.168	0.825	-4.715	-0.377	2.900
-1.326	0.197	0.855	-4.819	-0.442	2.962
-3.426	0.002	0.301	-1.402	-0.882	3.465
-2.088	0.047	0.630	-3.800	-0.627	2.025
0.556	0.583	0.999	-5.437	0.181	4.079
1.291	0.209	0.868	-4.857	0.623	3.235
-1.485	0.150	0.811	-4.636	-0.668	1.250
-0.616	0.543	0.994	-5.406	-0.197	4.974
0.649	0.522	0.990	-5.388	0.183	1.444
-0.363	0.720	0.999	-5.515	-0.183	2.752
-0.940	0.356	0.962	-5.187	-0.475	3.956
-0.767	0.450	0.980	-5.315	-0.195	2.963
-1.018	0.318	0.950	-5.121	-0.277	2.415
-0.083	0.935	0.999	-5.571	-0.023	2.161
-1.590	0.124	0.786	-4.506	-0.466	7.405
-1.179	0.249	0.910	-4.972	-0.462	4.405
-0.897	0.378	0.963	-5.221	-0.222	2.753
1.790	0.086	0.729	-4.240	0.518	3.654
0.124	0.902	0.999	-5.567	0.037	3.145
-2.003	0.056	0.658	-3.931	-0.531	6.523
0.133	0.895	0.999	-5.566	0.038	3.899
-1.699	0.102	0.764	-4.364	-0.504	2.668
0.667	0.511	0.989	-5.377	0.270	2.631
-2.518	0.019	0.501	-3.092	-0.905	1.671
0.243	0.810	0.999	-5.548	0.176	1.567
-1.695	0.102	0.764	-4.369	-0.479	2.323
-0.059	0.953	0.999	-5.572	-0.024	3.156
1.216	0.235	0.899	-4.935	0.551	3.423
-1.575	0.128	0.789	-4.525	-0.448	2.962
-0.110	0.913	0.999	-5.569	-0.036	2.727
0.584	0.564	0.997	-5.423	0.170	4.762
-0.367	0.717	0.999	-5.514	-0.116	2.426
1.546	0.135	0.793	-4.561	0.526	3.128

1.531	0.138	0.796	-4.580	0.475	2.564
1.331	0.195	0.853	-4.813	0.489	2.707
-0.160	0.874	0.999	-5.562	-0.052	3.493
-1.151	0.261	0.914	-5.000	-0.377	6.362
-0.874	0.390	0.964	-5.239	-0.262	5.954
-0.149	0.883	0.999	-5.564	-0.041	2.859
-0.378	0.709	0.999	-5.511	-0.106	2.683
-1.225	0.232	0.895	-4.925	-0.321	2.435
1.176	0.251	0.910	-4.976	0.439	2.492
2.204	0.037	0.610	-3.617	0.537	3.104
-0.678	0.504	0.988	-5.371	-0.218	2.274
-0.962	0.345	0.957	-5.169	-0.393	5.301
1.876	0.072	0.698	-4.117	0.538	6.015
0.066	0.948	0.999	-5.572	0.016	6.194
-0.284	0.779	0.999	-5.538	-0.062	4.680
-2.919	0.007	0.412	-2.371	-1.469	2.897
-0.957	0.348	0.959	-5.173	-0.296	2.643
0.402	0.691	0.999	-5.502	0.199	5.246
-0.821	0.419	0.972	-5.277	-0.372	1.377
-0.247	0.807	0.999	-5.547	-0.077	3.096
-1.187	0.246	0.906	-4.964	-0.292	2.571
0.217	0.830	0.999	-5.553	0.045	3.997
1.330	0.196	0.853	-4.815	0.297	2.523
0.160	0.875	0.999	-5.563	0.049	3.649
-0.509	0.615	0.999	-5.459	-0.132	3.811
0.416	0.681	0.999	-5.497	0.132	3.619
-1.589	0.125	0.786	-4.507	-0.527	2.705
0.656	0.518	0.990	-5.384	0.166	2.482
1.681	0.105	0.764	-4.387	0.488	2.798
0.565	0.577	0.999	-5.432	0.149	4.345
0.656	0.518	0.990	-5.384	0.198	3.902
-0.244	0.809	0.999	-5.547	-0.060	3.662
0.698	0.492	0.986	-5.359	0.210	3.239
1.066	0.296	0.937	-5.079	0.369	3.604
-0.637	0.530	0.992	-5.395	-0.250	3.037
1.574	0.128	0.789	-4.527	0.759	2.869
-0.100	0.921	0.999	-5.570	-0.059	6.481
-0.731	0.472	0.984	-5.338	-0.316	5.325
2.122	0.044	0.630	-3.746	0.480	2.752
0.456	0.652	0.999	-5.482	0.212	4.375
-1.195	0.243	0.904	-4.956	-0.420	3.412
0.379	0.708	0.999	-5.510	0.097	3.398
0.866	0.395	0.964	-5.245	0.343	4.326
1.504	0.145	0.803	-4.612	0.400	4.772
-1.227	0.231	0.894	-4.924	-0.434	3.034
-0.932	0.360	0.963	-5.193	-0.282	2.972
1.429	0.165	0.825	-4.702	0.445	5.436
-0.724	0.475	0.984	-5.342	-0.198	2.219
0.513	0.613	0.999	-5.457	0.135	2.655
-0.172	0.865	0.999	-5.561	-0.047	2.524

-1.078	0.291	0.933	-5.068	-0.261	3.405
0.299	0.768	0.999	-5.534	0.076	2.976
0.330	0.744	0.999	-5.526	0.104	3.375
0.138	0.891	0.999	-5.565	0.043	3.127
-1.622	0.117	0.776	-4.464	-0.742	3.006
-0.317	0.754	0.999	-5.529	-0.075	3.816
0.308	0.761	0.999	-5.532	0.070	5.281
-1.305	0.204	0.863	-4.842	-0.479	3.516
0.138	0.892	0.999	-5.565	0.066	1.320
1.031	0.312	0.943	-5.110	0.358	2.561
0.209	0.836	0.999	-5.554	0.065	3.586
-0.406	0.688	0.999	-5.500	-0.104	2.128
-0.906	0.374	0.963	-5.214	-0.357	3.877
1.496	0.147	0.804	-4.623	0.330	2.011
-1.385	0.178	0.837	-4.753	-0.329	3.620
-0.467	0.644	0.999	-5.477	-0.190	3.075
0.797	0.433	0.973	-5.294	0.209	3.668
-0.714	0.482	0.984	-5.349	-0.287	1.643
0.107	0.915	0.999	-5.569	0.030	1.585
-0.062	0.951	0.999	-5.572	-0.019	1.235
0.077	0.940	0.999	-5.571	0.029	2.881
-0.258	0.798	0.999	-5.544	-0.079	2.851
-0.606	0.550	0.994	-5.411	-0.150	2.545
0.054	0.957	0.999	-5.573	0.019	3.539
0.404	0.690	0.999	-5.501	0.123	2.235
-0.696	0.493	0.986	-5.360	-0.187	1.296
-0.149	0.883	0.999	-5.564	-0.055	4.189
-1.210	0.238	0.900	-4.941	-0.340	2.798
-0.110	0.913	0.999	-5.569	-0.029	3.125
0.616	0.543	0.994	-5.406	0.159	2.512
-0.154	0.879	0.999	-5.563	-0.038	3.678
-0.883	0.386	0.964	-5.232	-0.231	2.662
2.471	0.021	0.517	-3.173	0.654	3.512
-2.494	0.020	0.515	-3.133	-0.804	2.025
-0.408	0.686	0.999	-5.500	-0.118	2.015
0.078	0.939	0.999	-5.571	0.019	1.862
0.814	0.423	0.972	-5.282	0.286	4.542
-0.111	0.913	0.999	-5.568	-0.038	1.925
0.417	0.680	0.999	-5.496	0.109	3.614
1.041	0.308	0.942	-5.102	0.263	3.832
-0.983	0.335	0.957	-5.152	-0.278	4.064
-0.559	0.581	0.999	-5.435	-0.174	4.339
-0.867	0.394	0.964	-5.244	-0.187	5.051
-0.453	0.654	0.999	-5.483	-0.123	3.062
0.742	0.465	0.983	-5.331	0.425	3.011
-0.445	0.660	0.999	-5.486	-0.122	2.273
0.108	0.915	0.999	-5.569	0.040	2.566
1.571	0.129	0.789	-4.530	0.527	4.725
0.538	0.595	0.999	-5.445	0.280	2.094
-0.058	0.954	0.999	-5.572	-0.015	2.408

-0.904	0.374	0.963	-5.215	-0.214	2.159
-0.192	0.850	0.999	-5.558	-0.069	2.008
-0.623	0.539	0.994	-5.402	-0.145	2.729
-0.370	0.715	0.999	-5.513	-0.126	4.840
-0.064	0.950	0.999	-5.572	-0.023	8.265
1.244	0.225	0.886	-4.907	0.318	4.766
0.506	0.617	0.999	-5.460	0.124	4.764
-0.118	0.907	0.999	-5.568	-0.044	2.803
-0.826	0.417	0.970	-5.274	-0.348	6.194
-2.007	0.056	0.656	-3.924	-0.509	2.672
0.435	0.668	0.999	-5.490	0.128	3.119
-1.086	0.288	0.929	-5.061	-0.330	2.687
1.769	0.089	0.737	-4.268	0.477	2.837
-0.805	0.428	0.973	-5.289	-0.287	2.681
0.588	0.562	0.996	-5.421	0.190	3.344
-0.887	0.384	0.964	-5.229	-0.308	1.486
-0.753	0.459	0.982	-5.324	-0.216	2.221
0.485	0.632	0.999	-5.469	0.142	2.311
0.532	0.599	0.999	-5.448	0.128	3.966
0.480	0.635	0.999	-5.472	0.209	2.655
0.552	0.586	0.999	-5.439	0.242	4.238
-1.097	0.283	0.924	-5.051	-0.415	1.899
1.213	0.237	0.899	-4.939	0.353	4.375
-0.086	0.932	0.999	-5.571	-0.026	2.490
0.056	0.956	0.999	-5.573	0.015	5.208
0.780	0.443	0.979	-5.306	0.267	2.630
-1.115	0.275	0.921	-5.034	-0.337	2.352
0.239	0.813	0.999	-5.548	0.215	6.608
0.181	0.858	0.999	-5.559	0.051	2.109
0.396	0.696	0.999	-5.504	0.145	2.606
1.826	0.080	0.717	-4.188	0.439	5.093
-0.997	0.328	0.954	-5.140	-0.341	3.153
-0.410	0.685	0.999	-5.499	-0.140	1.676
0.864	0.396	0.964	-5.246	0.264	3.821
0.086	0.932	0.999	-5.571	0.025	1.701
0.669	0.510	0.989	-5.376	0.180	4.535
1.722	0.097	0.757	-4.332	0.369	3.024
0.706	0.487	0.985	-5.354	0.198	2.978
-0.214	0.832	0.999	-5.553	-0.066	2.323
0.486	0.631	0.999	-5.469	0.116	4.028
1.992	0.057	0.660	-3.947	0.796	3.107
-0.144	0.887	0.999	-5.565	-0.045	2.007
0.979	0.337	0.957	-5.155	0.354	3.234
0.210	0.835	0.999	-5.554	0.057	3.630
-2.452	0.022	0.528	-3.206	-0.745	3.786
-0.737	0.468	0.983	-5.335	-0.290	3.342
0.762	0.453	0.981	-5.318	0.228	3.171
1.087	0.287	0.929	-5.060	0.327	2.744
-0.493	0.626	0.999	-5.466	-0.133	4.599
0.713	0.482	0.984	-5.349	0.217	2.764

0.099	0.922	0.999	-5.570	0.026	2.897
0.142	0.888	0.999	-5.565	0.062	2.432
-0.420	0.678	0.999	-5.495	-0.130	1.133
0.263	0.795	0.999	-5.543	0.073	2.743
-0.641	0.527	0.992	-5.392	-0.146	3.406
-1.740	0.094	0.753	-4.308	-0.477	3.405
0.514	0.612	0.999	-5.457	0.208	3.726
0.268	0.791	0.999	-5.542	0.079	2.373
-0.351	0.729	0.999	-5.519	-0.088	3.295
-1.345	0.191	0.849	-4.798	-0.294	4.027
0.414	0.682	0.999	-5.498	0.101	3.145
-2.202	0.037	0.610	-3.620	-0.598	2.768
1.868	0.074	0.698	-4.129	0.784	3.223
1.521	0.141	0.801	-4.592	0.716	4.774
1.199	0.242	0.904	-4.953	0.520	2.467
0.628	0.536	0.993	-5.399	0.160	3.892
-2.647	0.014	0.498	-2.865	-0.699	1.914
-1.538	0.137	0.795	-4.571	-0.385	2.312
0.734	0.469	0.983	-5.336	0.217	1.933
0.132	0.896	0.999	-5.566	0.032	3.949
-0.096	0.925	0.999	-5.570	-0.021	2.539
1.083	0.289	0.931	-5.063	0.316	3.294
0.198	0.845	0.999	-5.557	0.078	2.965
0.103	0.919	0.999	-5.569	0.039	2.967
-0.097	0.924	0.999	-5.570	-0.027	4.448
-0.721	0.478	0.984	-5.345	-0.209	5.359
0.170	0.867	0.999	-5.561	0.039	2.267
-0.435	0.667	0.999	-5.490	-0.276	2.323
-1.302	0.205	0.865	-4.845	-0.402	2.856
-2.144	0.042	0.619	-3.712	-0.540	2.944
-1.555	0.132	0.791	-4.550	-0.728	2.173
0.796	0.433	0.973	-5.295	0.222	3.534
-0.711	0.483	0.985	-5.351	-0.168	2.708
1.653	0.111	0.767	-4.424	0.445	2.392
0.926	0.363	0.963	-5.198	0.218	2.123
2.706	0.012	0.488	-2.759	0.620	3.463
-0.225	0.824	0.999	-5.551	-0.090	4.196
0.428	0.672	0.999	-5.492	0.096	6.442
-0.503	0.620	0.999	-5.462	-0.252	3.965
-0.486	0.631	0.999	-5.469	-0.129	2.012
1.075	0.293	0.934	-5.071	0.326	3.246
0.300	0.766	0.999	-5.534	0.085	2.356
-1.964	0.061	0.663	-3.988	-0.547	2.526
0.098	0.923	0.999	-5.570	0.032	3.094
-1.293	0.208	0.868	-4.855	-0.275	3.245
-0.769	0.449	0.979	-5.313	-0.238	3.824
-0.534	0.598	0.999	-5.447	-0.116	3.857
-2.121	0.044	0.630	-3.749	-0.509	2.174
-1.010	0.322	0.950	-5.129	-0.258	2.279
-0.286	0.777	0.999	-5.538	-0.104	2.703

-0.246	0.808	0.999	-5.547	-0.067	2.125
-0.956	0.348	0.959	-5.174	-0.243	3.086
-0.226	0.823	0.999	-5.551	-0.086	3.186
2.470	0.021	0.517	-3.174	0.641	4.733
1.245	0.225	0.886	-4.906	0.331	5.133
0.681	0.502	0.988	-5.369	0.212	2.368
0.291	0.774	0.999	-5.536	0.075	2.459
-0.992	0.331	0.956	-5.144	-0.229	2.873
0.353	0.727	0.999	-5.519	0.082	2.726
0.075	0.941	0.999	-5.571	0.027	2.903
-1.115	0.275	0.921	-5.034	-0.476	4.376
0.566	0.577	0.999	-5.432	0.135	2.181
-0.452	0.655	0.999	-5.483	-0.134	4.647
-1.057	0.301	0.941	-5.088	-0.336	2.977
0.288	0.776	0.999	-5.537	0.109	1.859
0.337	0.739	0.999	-5.523	0.149	2.103
-0.684	0.500	0.988	-5.367	-0.158	2.695
-0.620	0.541	0.994	-5.404	-0.170	1.931
1.267	0.217	0.876	-4.882	0.331	4.348
1.562	0.131	0.791	-4.541	0.371	3.208
0.640	0.528	0.992	-5.393	0.133	4.225
-2.068	0.049	0.638	-3.831	-0.513	5.078
0.357	0.724	0.999	-5.517	0.092	2.388
2.190	0.038	0.611	-3.639	0.676	3.512
-0.371	0.714	0.999	-5.513	-0.096	2.179
-0.460	0.649	0.999	-5.480	-0.177	3.542
-0.516	0.610	0.999	-5.456	-0.212	3.687
-0.170	0.866	0.999	-5.561	-0.056	0.886
0.809	0.426	0.973	-5.286	0.304	3.223
0.157	0.876	0.999	-5.563	0.043	3.532
-0.100	0.921	0.999	-5.570	-0.026	3.572
-0.578	0.569	0.997	-5.426	-0.183	1.714
-1.050	0.304	0.941	-5.094	-0.290	2.298
1.553	0.133	0.791	-4.552	0.422	5.091
-0.335	0.741	0.999	-5.524	-0.101	2.566
-0.304	0.764	0.999	-5.533	-0.148	4.415
0.324	0.749	0.999	-5.527	0.094	2.518
0.290	0.774	0.999	-5.537	0.118	1.868
0.151	0.882	0.999	-5.564	0.079	5.635
-0.230	0.820	0.999	-5.550	-0.082	2.492
-1.739	0.094	0.753	-4.309	-0.480	2.788
-1.400	0.174	0.828	-4.735	-0.448	1.255
-1.547	0.134	0.793	-4.560	-0.462	2.004
0.627	0.536	0.993	-5.400	0.163	3.915
0.763	0.453	0.981	-5.318	0.208	5.732
-0.064	0.949	0.999	-5.572	-0.018	1.662
-1.117	0.274	0.921	-5.032	-0.270	2.657
-0.148	0.884	0.999	-5.564	-0.088	2.750
-0.083	0.935	0.999	-5.571	-0.026	2.220
-0.876	0.389	0.964	-5.237	-0.318	2.506

-1.587	0.125	0.786	-4.510	-0.359	3.963
-1.482	0.151	0.811	-4.639	-0.339	3.428
0.973	0.340	0.957	-5.160	0.253	3.054
0.463	0.648	0.999	-5.479	0.131	1.251
-0.208	0.837	0.999	-5.555	-0.055	3.374
-0.505	0.618	0.999	-5.461	-0.125	4.171
-0.665	0.512	0.989	-5.378	-0.204	3.553
0.196	0.846	0.999	-5.557	0.071	1.994
-1.679	0.106	0.764	-4.391	-0.404	1.978
-0.176	0.862	0.999	-5.560	-0.053	4.023
-1.115	0.275	0.921	-5.034	-0.391	3.701
0.078	0.939	0.999	-5.571	0.030	2.023
0.636	0.530	0.992	-5.395	0.252	4.454
0.370	0.714	0.999	-5.513	0.158	2.969
-0.760	0.454	0.981	-5.319	-0.296	4.585
0.512	0.613	0.999	-5.457	0.146	2.713
-0.774	0.446	0.979	-5.310	-0.195	3.494
-0.962	0.345	0.957	-5.169	-0.300	2.877
-1.087	0.288	0.929	-5.060	-0.353	2.367
-1.669	0.108	0.764	-4.403	-0.675	2.805
-1.677	0.106	0.764	-4.393	-0.563	1.743
-0.968	0.342	0.957	-5.165	-0.370	2.226
1.059	0.300	0.941	-5.086	0.260	5.290
0.345	0.733	0.999	-5.521	0.085	2.044
-0.354	0.727	0.999	-5.518	-0.119	2.821
-1.499	0.146	0.804	-4.619	-0.368	2.758
1.390	0.177	0.835	-4.748	0.623	5.015
-0.431	0.670	0.999	-5.491	-0.114	1.780
0.240	0.813	0.999	-5.548	0.063	1.854
-0.726	0.475	0.984	-5.341	-0.187	2.886
1.739	0.094	0.753	-4.310	0.447	3.034
0.801	0.431	0.973	-5.292	0.195	2.525
0.089	0.930	0.999	-5.570	0.018	2.424
-0.292	0.772	0.999	-5.536	-0.083	3.329
1.031	0.312	0.943	-5.111	0.320	4.645
2.001	0.056	0.658	-3.934	0.492	2.478
0.441	0.663	0.999	-5.487	0.115	6.117
-0.053	0.958	0.999	-5.573	-0.022	2.627
1.043	0.307	0.942	-5.100	0.342	3.264
2.958	0.007	0.400	-2.298	0.632	3.528
0.480	0.635	0.999	-5.472	0.126	4.953
-0.089	0.930	0.999	-5.570	-0.026	1.937
-0.991	0.331	0.957	-5.145	-0.249	4.463
0.067	0.947	0.999	-5.572	0.021	3.099
-0.717	0.480	0.984	-5.347	-0.254	2.538
-0.233	0.818	0.999	-5.550	-0.056	2.793
-1.439	0.162	0.824	-4.690	-0.503	2.210
-0.471	0.642	0.999	-5.475	-0.159	4.584
0.982	0.336	0.957	-5.153	0.252	1.846
0.445	0.660	0.999	-5.486	0.144	2.698

0.263	0.795	0.999	-5.543	0.073	3.207
1.572	0.128	0.789	-4.528	0.296	3.296
0.065	0.948	0.999	-5.572	0.018	1.563
0.916	0.368	0.963	-5.206	0.222	3.171
0.533	0.599	0.999	-5.448	0.154	1.504
1.495	0.147	0.804	-4.623	0.503	4.513
-0.322	0.750	0.999	-5.528	-0.074	1.928
0.376	0.710	0.999	-5.511	0.104	2.243
0.257	0.800	0.999	-5.545	0.094	3.149
0.482	0.634	0.999	-5.471	0.173	3.757
-0.851	0.403	0.967	-5.255	-0.188	8.621
-0.715	0.481	0.984	-5.348	-0.216	2.270
-3.123	0.004	0.353	-1.986	-0.695	2.123
0.194	0.848	0.999	-5.557	0.048	2.844
1.024	0.315	0.946	-5.116	0.332	3.673
-0.108	0.914	0.999	-5.569	-0.034	2.712
0.906	0.374	0.963	-5.214	0.222	3.023
-1.150	0.261	0.914	-5.001	-0.246	3.969
0.404	0.690	0.999	-5.501	0.136	2.146
-0.966	0.343	0.957	-5.166	-0.252	6.898
1.045	0.306	0.942	-5.098	0.279	3.312
1.925	0.066	0.676	-4.047	0.580	3.148
0.501	0.620	0.999	-5.462	0.212	1.237
2.346	0.027	0.559	-3.385	0.681	3.170
0.928	0.362	0.963	-5.197	0.225	4.070
1.587	0.125	0.786	-4.509	0.402	2.646
-0.932	0.360	0.963	-5.194	-0.257	2.728
0.194	0.848	0.999	-5.557	0.054	3.295
0.689	0.497	0.987	-5.364	0.208	2.585
-0.235	0.816	0.999	-5.549	-0.079	1.347
-0.344	0.734	0.999	-5.521	-0.121	1.519
-0.304	0.763	0.999	-5.533	-0.104	1.571
-1.057	0.301	0.941	-5.088	-0.365	0.982
-0.145	0.886	0.999	-5.565	-0.085	2.048
0.455	0.653	0.999	-5.482	0.122	2.971
-1.104	0.280	0.922	-5.044	-0.393	4.656
-1.024	0.315	0.946	-5.116	-0.288	3.505
0.432	0.669	0.999	-5.491	0.159	3.108
-0.681	0.502	0.988	-5.369	-0.340	0.678
-0.105	0.917	0.999	-5.569	-0.027	2.472
-1.168	0.254	0.913	-4.983	-0.276	2.877
0.752	0.459	0.982	-5.325	0.287	1.688
0.814	0.423	0.972	-5.283	0.252	4.326
0.513	0.612	0.999	-5.457	0.140	2.218
1.065	0.297	0.937	-5.080	0.283	2.811
-0.181	0.858	0.999	-5.559	-0.072	3.716
-0.552	0.586	0.999	-5.439	-0.208	0.944
0.559	0.581	0.999	-5.435	0.177	2.623
1.627	0.116	0.776	-4.458	0.417	5.752
-1.112	0.277	0.921	-5.037	-0.297	2.624

0.513	0.612	0.999	-5.457	0.120	6.887
0.161	0.873	0.999	-5.562	0.047	2.793
-0.346	0.732	0.999	-5.521	-0.091	2.067
-0.161	0.873	0.999	-5.562	-0.045	2.134
-1.730	0.096	0.755	-4.322	-0.439	4.218
0.315	0.755	0.999	-5.530	0.085	3.113
-0.685	0.499	0.987	-5.366	-0.170	3.365
-1.166	0.254	0.913	-4.985	-0.313	1.834
-0.268	0.791	0.999	-5.542	-0.073	3.146
-1.080	0.290	0.932	-5.066	-0.280	4.344
-0.112	0.911	0.999	-5.568	-0.029	3.239
0.425	0.674	0.999	-5.493	0.110	7.124
-0.810	0.425	0.972	-5.285	-0.247	2.551
-0.335	0.740	0.999	-5.524	-0.129	1.250
0.133	0.896	0.999	-5.566	0.053	2.254
-0.438	0.665	0.999	-5.489	-0.118	7.436
-0.083	0.935	0.999	-5.571	-0.017	3.213
-1.065	0.297	0.937	-5.080	-0.307	4.490
-0.862	0.397	0.964	-5.248	-0.437	4.813
-0.865	0.395	0.964	-5.246	-0.252	2.686
-3.845	0.001	0.301	-0.573	-1.457	1.586
-0.512	0.613	0.999	-5.457	-0.127	3.603
0.503	0.619	0.999	-5.462	0.177	2.688
-0.489	0.629	0.999	-5.468	-0.207	2.764
0.316	0.755	0.999	-5.529	0.116	2.825
-1.125	0.271	0.919	-5.024	-0.301	7.256
0.652	0.520	0.990	-5.386	0.147	2.188
-0.864	0.396	0.964	-5.247	-0.240	1.120
-0.515	0.611	0.999	-5.456	-0.142	2.215
0.077	0.939	0.999	-5.571	0.020	2.815
0.160	0.874	0.999	-5.563	0.041	2.669
-0.741	0.465	0.983	-5.332	-0.181	2.942
-0.264	0.794	0.999	-5.543	-0.077	3.429
0.221	0.827	0.999	-5.552	0.061	2.816
-3.259	0.003	0.340	-1.726	-0.793	2.439
0.940	0.356	0.962	-5.187	0.259	2.582
-1.351	0.189	0.847	-4.791	-0.431	2.250
0.498	0.623	0.999	-5.464	0.157	2.915
-0.109	0.914	0.999	-5.569	-0.027	2.674
-0.740	0.466	0.983	-5.332	-0.191	3.187
1.678	0.106	0.764	-4.392	0.504	3.507
-0.751	0.460	0.983	-5.325	-0.258	2.347
1.331	0.195	0.853	-4.814	0.345	2.882
-0.099	0.922	0.999	-5.570	-0.028	3.498
1.202	0.241	0.902	-4.950	0.580	4.396
-0.643	0.526	0.992	-5.391	-0.166	4.606
0.679	0.504	0.988	-5.370	0.210	4.506
-0.134	0.894	0.999	-5.566	-0.061	4.834
1.096	0.284	0.925	-5.052	0.272	2.840
-0.314	0.756	0.999	-5.530	-0.057	10.242

-0.154	0.879	0.999	-5.563	-0.037	3.966
0.478	0.637	0.999	-5.472	0.111	3.316
-0.194	0.848	0.999	-5.557	-0.042	7.462
-0.245	0.808	0.999	-5.547	-0.045	8.764
-0.295	0.771	0.999	-5.535	-0.088	3.069
0.940	0.356	0.962	-5.187	0.284	5.759
0.899	0.377	0.963	-5.220	0.287	4.308
1.140	0.265	0.916	-5.010	0.332	5.478
-0.173	0.864	0.999	-5.561	-0.066	2.563
0.832	0.413	0.970	-5.270	0.171	4.505
1.031	0.312	0.943	-5.110	0.193	3.624
2.326	0.028	0.567	-3.418	0.592	4.239
1.051	0.303	0.941	-5.092	0.202	4.191
-0.242	0.810	0.999	-5.548	-0.060	2.647
-0.348	0.731	0.999	-5.520	-0.076	3.208
0.907	0.373	0.963	-5.214	0.341	2.260
2.113	0.045	0.630	-3.761	0.647	5.275
1.267	0.217	0.876	-4.882	0.334	3.488
-0.578	0.569	0.997	-5.426	-0.129	8.549
0.974	0.339	0.957	-5.159	0.301	3.931
0.881	0.387	0.964	-5.233	0.207	9.921
0.836	0.411	0.970	-5.267	0.174	11.167
1.927	0.065	0.675	-4.043	0.494	5.247
0.665	0.512	0.989	-5.378	0.156	3.007
0.603	0.552	0.994	-5.413	0.103	10.810
-0.332	0.743	0.999	-5.525	-0.062	8.951
0.194	0.847	0.999	-5.557	0.050	4.458
0.174	0.863	0.999	-5.560	0.060	3.885
0.831	0.414	0.970	-5.271	0.147	5.698
-0.235	0.816	0.999	-5.549	-0.058	3.954
1.984	0.058	0.662	-3.959	0.735	2.633
-2.122	0.044	0.630	-3.747	-0.559	1.970
0.395	0.696	0.999	-5.505	0.093	3.681
-0.754	0.458	0.982	-5.323	-0.247	2.955
2.662	0.013	0.498	-2.839	0.573	5.148
2.000	0.056	0.658	-3.935	0.457	6.989
0.871	0.392	0.964	-5.241	0.220	7.395
1.362	0.185	0.842	-4.779	0.286	5.820
0.529	0.601	0.999	-5.450	0.127	2.353
2.651	0.014	0.498	-2.859	0.628	6.122
1.906	0.068	0.684	-4.074	0.510	5.511
1.004	0.325	0.952	-5.134	0.433	3.627
0.167	0.869	0.999	-5.562	0.055	4.015
0.755	0.457	0.981	-5.323	0.166	4.123
-1.039	0.309	0.943	-5.103	-0.276	4.534
0.435	0.668	0.999	-5.490	0.294	2.985
1.716	0.098	0.757	-4.341	0.504	2.854
2.076	0.048	0.633	-3.818	0.462	5.620
-2.333	0.028	0.565	-3.406	-0.760	3.382
-1.555	0.132	0.791	-4.549	-0.341	5.138

-3.420	0.002	0.301	-1.412	-1.428	2.520
0.387	0.702	0.999	-5.507	0.168	2.835
0.393	0.697	0.999	-5.505	0.139	1.978
0.366	0.717	0.999	-5.514	0.078	5.049
0.410	0.685	0.999	-5.499	0.105	6.861
0.079	0.938	0.999	-5.571	0.027	6.273
0.364	0.719	0.999	-5.515	0.126	1.438
-1.485	0.150	0.811	-4.636	-0.390	6.634
-1.068	0.296	0.936	-5.078	-0.383	3.510
0.918	0.367	0.963	-5.205	0.196	3.733
-0.219	0.829	0.999	-5.553	-0.106	3.551
-0.120	0.906	0.999	-5.568	-0.043	2.091
0.512	0.613	0.999	-5.458	0.172	1.529
0.463	0.647	0.999	-5.479	0.125	3.204
1.436	0.163	0.824	-4.694	0.380	3.219
0.419	0.679	0.999	-5.496	0.130	6.199
0.336	0.739	0.999	-5.524	0.080	5.105
-0.692	0.496	0.987	-5.363	-0.254	6.775
-0.489	0.629	0.999	-5.468	-0.159	3.611
0.955	0.349	0.960	-5.175	0.309	3.090
0.240	0.812	0.999	-5.548	0.063	4.180
1.895	0.070	0.688	-4.090	0.429	3.217
-0.581	0.566	0.997	-5.424	-0.136	3.690
-0.054	0.958	0.999	-5.573	-0.011	6.055
0.421	0.677	0.999	-5.495	0.102	4.907
0.506	0.617	0.999	-5.460	0.172	2.977
-0.178	0.860	0.999	-5.560	-0.108	1.058
-0.849	0.404	0.968	-5.257	-0.164	4.576
0.145	0.886	0.999	-5.565	0.035	6.733
-1.690	0.103	0.764	-4.376	-0.374	4.278
1.289	0.209	0.868	-4.859	0.317	4.268
1.384	0.179	0.838	-4.754	0.429	4.745
0.169	0.867	0.999	-5.561	0.135	4.353
0.813	0.424	0.972	-5.283	0.208	5.095
0.357	0.724	0.999	-5.517	0.119	2.753
0.218	0.829	0.999	-5.553	0.041	5.347
0.057	0.955	0.999	-5.572	0.033	5.099
0.742	0.465	0.983	-5.331	0.459	5.929
-0.514	0.612	0.999	-5.457	-0.189	10.731
0.539	0.594	0.999	-5.445	0.140	3.953
-0.426	0.674	0.999	-5.493	-0.150	5.664
-0.403	0.691	0.999	-5.502	-0.089	3.178
-1.059	0.300	0.941	-5.086	-0.273	6.138
-0.624	0.538	0.994	-5.401	-0.183	4.168
-1.862	0.074	0.701	-4.138	-0.358	5.354
0.576	0.570	0.997	-5.427	0.156	4.520
-0.966	0.343	0.957	-5.166	-0.211	4.880
-1.577	0.127	0.788	-4.523	-0.379	2.616
-0.328	0.746	0.999	-5.526	-0.090	2.492
2.740	0.011	0.480	-2.699	0.582	4.461

2.636	0.014	0.498	-2.885	0.761	5.688
0.916	0.368	0.963	-5.206	0.210	7.946
1.045	0.306	0.942	-5.098	0.305	3.545
-0.420	0.678	0.999	-5.496	-0.112	3.221
-0.365	0.718	0.999	-5.515	-0.099	1.910
-0.609	0.548	0.994	-5.409	-0.144	8.215
1.781	0.087	0.731	-4.251	0.547	4.897
0.939	0.357	0.962	-5.188	0.253	8.323
-0.549	0.588	0.999	-5.440	-0.231	5.595
0.283	0.780	0.999	-5.538	0.090	2.390
-0.517	0.610	0.999	-5.455	-0.183	3.507
-0.559	0.581	0.999	-5.435	-0.105	6.998
2.129	0.043	0.628	-3.737	0.594	5.553
1.016	0.319	0.950	-5.124	0.614	8.460
0.415	0.681	0.999	-5.497	0.124	4.733
0.499	0.622	0.999	-5.464	0.103	7.383
-0.093	0.926	0.999	-5.570	-0.023	6.588
-0.357	0.724	0.999	-5.517	-0.106	6.820
0.916	0.368	0.963	-5.206	0.273	8.150
-0.859	0.399	0.964	-5.250	-0.192	2.408
-1.748	0.093	0.749	-4.297	-0.463	8.818
-2.182	0.039	0.611	-3.652	-0.757	7.366
-0.073	0.942	0.999	-5.572	-0.027	4.047
-2.144	0.042	0.619	-3.713	-0.514	5.769
-1.550	0.134	0.792	-4.557	-0.609	4.651
0.790	0.437	0.973	-5.299	0.237	5.616
-1.053	0.302	0.941	-5.091	-0.284	5.927
-1.637	0.114	0.775	-4.445	-0.516	5.658
-2.164	0.040	0.616	-3.680	-0.634	7.207
-0.587	0.562	0.996	-5.421	-0.214	8.623
-0.249	0.806	0.999	-5.546	-0.145	8.377
-2.018	0.054	0.655	-3.908	-0.413	7.529
-2.464	0.021	0.520	-3.185	-0.623	8.002
-2.019	0.054	0.655	-3.906	-0.608	5.145
-0.243	0.810	0.999	-5.548	-0.067	2.831
-0.331	0.743	0.999	-5.525	-0.079	7.770
0.183	0.856	0.999	-5.559	0.054	10.968
0.574	0.571	0.997	-5.428	0.151	6.547
0.800	0.431	0.973	-5.292	0.311	5.268
-0.135	0.894	0.999	-5.566	-0.041	7.315
0.455	0.653	0.999	-5.482	0.113	4.434
2.455	0.021	0.527	-3.201	0.995	5.122
0.746	0.463	0.983	-5.329	0.377	6.902
1.483	0.150	0.811	-4.638	0.336	3.574
0.161	0.873	0.999	-5.562	0.032	2.022
0.954	0.349	0.960	-5.176	0.328	2.600
0.295	0.770	0.999	-5.535	0.154	5.831
-0.239	0.813	0.999	-5.548	-0.079	6.281
0.499	0.622	0.999	-5.463	0.298	4.696
0.594	0.558	0.995	-5.418	0.221	5.445

1.323	0.198	0.857	-4.822	0.728	4.900
-1.755	0.091	0.745	-4.287	-0.701	3.931
1.308	0.203	0.862	-4.839	0.621	4.540
-0.401	0.692	0.999	-5.502	-0.265	4.096
-0.174	0.863	0.999	-5.560	-0.106	5.348
-0.135	0.893	0.999	-5.566	-0.047	4.120
-1.032	0.312	0.943	-5.110	-0.268	3.170
-0.712	0.483	0.985	-5.350	-0.203	2.694
-0.418	0.680	0.999	-5.496	-0.226	2.363
-0.620	0.541	0.994	-5.404	-0.153	2.749
-1.176	0.251	0.910	-4.975	-0.346	2.829
0.904	0.375	0.963	-5.216	0.245	4.485
0.984	0.335	0.957	-5.151	0.381	3.049
0.079	0.938	0.999	-5.571	0.015	6.055
0.222	0.826	0.999	-5.552	0.088	8.465
1.823	0.080	0.718	-4.194	0.445	5.707
-0.216	0.831	0.999	-5.553	-0.054	3.518
1.068	0.295	0.936	-5.077	0.275	3.468
1.146	0.263	0.914	-5.004	0.385	4.655
-1.433	0.164	0.824	-4.697	-0.479	4.469
-1.330	0.196	0.853	-4.815	-0.980	4.663
-1.011	0.322	0.950	-5.128	-0.311	4.369
-1.523	0.140	0.801	-4.590	-0.496	3.800
-1.590	0.124	0.786	-4.506	-0.370	4.116
0.429	0.672	0.999	-5.492	0.109	1.909
-2.083	0.048	0.630	-3.808	-0.707	4.985
-1.547	0.134	0.793	-4.559	-0.532	1.806
-0.824	0.418	0.971	-5.275	-0.210	3.168
0.829	0.415	0.970	-5.272	0.207	5.104
1.288	0.210	0.868	-4.860	0.268	5.100
-0.527	0.603	0.999	-5.451	-0.117	7.266
1.193	0.244	0.904	-4.959	0.477	2.714
0.085	0.933	0.999	-5.571	0.032	5.883
-1.207	0.239	0.901	-4.944	-0.463	2.409
0.374	0.711	0.999	-5.512	0.095	2.434
0.296	0.770	0.999	-5.535	0.061	3.033
1.263	0.218	0.877	-4.887	0.257	6.252
1.625	0.117	0.776	-4.461	0.465	5.330
1.738	0.095	0.754	-4.311	0.778	5.154
1.717	0.098	0.757	-4.340	0.426	7.558
0.228	0.821	0.999	-5.551	0.189	2.861
0.941	0.356	0.962	-5.186	0.249	5.214
-0.192	0.849	0.999	-5.557	-0.067	5.373
1.191	0.245	0.905	-4.960	0.390	5.966
0.485	0.632	0.999	-5.470	0.122	4.288
-0.476	0.638	0.999	-5.473	-0.217	7.381
0.876	0.389	0.964	-5.237	0.307	3.644
-0.107	0.916	0.999	-5.569	-0.028	5.675
0.263	0.794	0.999	-5.543	0.077	5.561
0.792	0.436	0.973	-5.298	0.189	5.301

0.181	0.858	0.999	-5.559	0.050	3.004
2.383	0.025	0.548	-3.323	0.631	3.613
-0.921	0.366	0.963	-5.203	-0.206	4.369
-0.082	0.935	0.999	-5.571	-0.027	6.498
-0.571	0.573	0.999	-5.429	-0.155	3.830
0.706	0.487	0.985	-5.354	0.183	3.651
0.977	0.338	0.957	-5.157	0.217	6.899
-0.206	0.839	0.999	-5.555	-0.055	3.603
0.396	0.696	0.999	-5.504	0.117	6.455
0.902	0.375	0.963	-5.217	0.323	4.963
-0.255	0.800	0.999	-5.545	-0.060	2.268
1.130	0.269	0.918	-5.020	0.229	6.622
0.373	0.712	0.999	-5.512	0.067	6.463
1.295	0.207	0.868	-4.852	0.237	5.130
-0.764	0.452	0.980	-5.317	-0.156	7.410
-0.139	0.891	0.999	-5.565	-0.047	5.369
0.129	0.899	0.999	-5.567	0.033	5.955
-0.360	0.722	0.999	-5.516	-0.121	6.803
-0.424	0.675	0.999	-5.494	-0.091	5.770
-0.098	0.922	0.999	-5.570	-0.025	2.752
0.111	0.913	0.999	-5.568	0.041	8.593
-0.164	0.871	0.999	-5.562	-0.046	7.191
0.136	0.893	0.999	-5.566	0.053	4.908
1.896	0.070	0.687	-4.088	0.394	5.021
-0.075	0.941	0.999	-5.571	-0.014	7.308
1.657	0.110	0.767	-4.419	0.330	4.934
-0.317	0.754	0.999	-5.529	-0.067	4.034
1.019	0.318	0.949	-5.121	0.397	2.630
1.037	0.310	0.943	-5.105	0.203	3.633
0.961	0.346	0.957	-5.170	0.249	7.175
-0.379	0.708	0.999	-5.510	-0.081	4.103
2.694	0.012	0.492	-2.782	1.110	3.770
1.153	0.260	0.914	-4.997	0.743	6.369
0.698	0.491	0.986	-5.359	0.221	2.945
1.604	0.121	0.784	-4.488	0.552	6.187
0.847	0.405	0.968	-5.259	0.183	5.933
1.674	0.106	0.764	-4.396	0.537	3.832
0.940	0.356	0.962	-5.187	0.234	6.310
0.193	0.848	0.999	-5.557	0.060	3.608
-1.410	0.171	0.826	-4.724	-0.603	2.642
0.271	0.788	0.999	-5.541	0.180	5.382
1.268	0.216	0.876	-4.881	0.729	6.081
0.436	0.666	0.999	-5.489	0.112	7.120
1.961	0.061	0.665	-3.993	0.777	6.546
0.229	0.820	0.999	-5.550	0.067	7.713
0.067	0.947	0.999	-5.572	0.026	3.613
0.217	0.830	0.999	-5.553	0.054	3.196
-0.305	0.763	0.999	-5.532	-0.087	3.935
0.368	0.716	0.999	-5.514	0.199	3.432
0.349	0.730	0.999	-5.520	0.077	6.360

0.650	0.521	0.990	-5.387	0.194	4.748
0.522	0.606	0.999	-5.453	0.135	4.437
0.670	0.509	0.989	-5.375	0.141	4.763
1.135	0.267	0.917	-5.015	0.350	3.606
-0.709	0.485	0.985	-5.352	-0.230	5.733
-0.522	0.606	0.999	-5.453	-0.175	5.898
-1.196	0.243	0.904	-4.955	-0.237	6.736
1.786	0.086	0.731	-4.245	0.901	5.104
0.408	0.687	0.999	-5.500	0.132	3.699
-1.861	0.075	0.702	-4.139	-0.420	3.775
-1.239	0.227	0.888	-4.912	-0.585	5.902
0.631	0.534	0.993	-5.398	0.150	3.635
-0.198	0.845	0.999	-5.556	-0.095	4.003
1.539	0.136	0.795	-4.569	0.344	7.122
1.538	0.137	0.795	-4.571	0.525	3.802
0.467	0.644	0.999	-5.477	0.166	6.961
0.423	0.676	0.999	-5.494	0.204	5.164
1.367	0.184	0.841	-4.773	0.391	4.078
0.137	0.892	0.999	-5.566	0.032	4.032
-0.638	0.530	0.992	-5.394	-0.148	3.621
-1.357	0.187	0.845	-4.785	-0.558	4.188
0.233	0.818	0.999	-5.550	0.079	4.838
0.098	0.922	0.999	-5.570	0.025	6.426
0.793	0.435	0.973	-5.297	0.226	7.248
1.307	0.203	0.862	-4.839	0.294	3.577
-0.209	0.836	0.999	-5.554	-0.058	2.649
0.657	0.517	0.990	-5.383	0.194	5.243
-0.576	0.570	0.997	-5.427	-0.169	8.151
-0.812	0.424	0.972	-5.284	-0.155	5.355
0.090	0.929	0.999	-5.570	0.023	2.745
0.939	0.357	0.962	-5.188	0.229	4.051
-0.479	0.636	0.999	-5.472	-0.111	4.535
-0.887	0.384	0.964	-5.229	-0.258	3.749
0.311	0.759	0.999	-5.531	0.103	3.923
-0.175	0.862	0.999	-5.560	-0.047	3.160
0.310	0.759	0.999	-5.531	0.159	6.457
0.663	0.513	0.990	-5.380	0.168	5.859
-0.152	0.880	0.999	-5.564	-0.039	1.783
0.597	0.556	0.995	-5.416	0.136	3.514
0.488	0.630	0.999	-5.468	0.246	3.711
-1.333	0.195	0.853	-4.812	-0.298	2.343
0.498	0.623	0.999	-5.464	0.208	4.873
0.647	0.523	0.990	-5.389	0.164	5.111
0.984	0.335	0.957	-5.151	0.219	6.150
0.112	0.911	0.999	-5.568	0.053	5.586
0.849	0.404	0.968	-5.257	0.318	5.608
-1.043	0.307	0.942	-5.100	-0.295	2.637
0.556	0.583	0.999	-5.437	0.178	3.917
-0.073	0.942	0.999	-5.572	-0.018	2.931
2.147	0.042	0.619	-3.707	0.502	3.787

0.832	0.413	0.970	-5.270	0.218	4.140
0.173	0.864	0.999	-5.561	0.064	4.241
0.983	0.335	0.957	-5.152	0.252	5.691
1.858	0.075	0.703	-4.143	0.432	4.575
-0.855	0.401	0.965	-5.253	-0.232	5.016
-0.392	0.699	0.999	-5.506	-0.093	2.599
-0.369	0.715	0.999	-5.513	-0.102	3.713
0.352	0.728	0.999	-5.519	0.123	5.355
-0.342	0.735	0.999	-5.522	-0.142	2.659
2.181	0.039	0.611	-3.654	0.662	4.718
0.283	0.780	0.999	-5.538	0.062	6.522
1.417	0.169	0.825	-4.716	0.614	6.020
1.098	0.283	0.924	-5.050	0.270	2.455
2.393	0.024	0.543	-3.305	1.049	4.026
1.897	0.069	0.687	-4.087	0.791	4.472
2.453	0.021	0.527	-3.204	0.801	5.377
2.564	0.017	0.500	-3.013	1.187	4.990
-0.987	0.333	0.957	-5.148	-0.381	5.498
1.082	0.289	0.931	-5.064	0.407	4.078
-0.179	0.859	0.999	-5.560	-0.038	5.947
0.574	0.571	0.997	-5.428	0.138	6.406
0.400	0.692	0.999	-5.503	0.080	7.532
3.102	0.005	0.357	-2.026	0.863	4.565
-0.082	0.936	0.999	-5.571	-0.021	6.013
-0.870	0.392	0.964	-5.242	-0.167	3.621
-0.328	0.746	0.999	-5.526	-0.079	1.632
0.429	0.671	0.999	-5.492	0.090	4.637
-0.161	0.873	0.999	-5.562	-0.044	2.469
1.334	0.194	0.853	-4.810	0.337	2.535
-0.153	0.880	0.999	-5.563	-0.030	6.786
1.763	0.090	0.742	-4.277	0.593	5.738
-0.199	0.844	0.999	-5.556	-0.063	8.031
3.020	0.006	0.393	-2.182	1.571	5.736
0.165	0.870	0.999	-5.562	0.038	5.541
-1.477	0.152	0.813	-4.645	-0.887	3.151
-0.396	0.695	0.999	-5.504	-0.104	2.080
2.144	0.042	0.619	-3.712	1.285	6.370
1.383	0.179	0.838	-4.755	0.332	6.907
-0.261	0.796	0.999	-5.544	-0.189	5.081
0.508	0.616	0.999	-5.459	0.139	3.136
-0.425	0.674	0.999	-5.494	-0.088	3.685
0.379	0.708	0.999	-5.510	0.148	5.440
-1.244	0.225	0.886	-4.906	-0.776	3.135
-1.094	0.284	0.926	-5.054	-0.410	3.104
-0.663	0.513	0.990	-5.380	-0.337	9.396
1.257	0.220	0.882	-4.893	0.334	5.485
1.950	0.062	0.667	-4.009	0.438	4.653
2.052	0.051	0.641	-3.856	0.462	3.985
-1.781	0.087	0.731	-4.252	-0.680	2.036
0.836	0.411	0.970	-5.267	0.254	3.558

1.173	0.252	0.910	-4.978	0.431	5.992
1.413	0.170	0.825	-4.721	0.360	5.656
-0.854	0.401	0.966	-5.254	-0.284	3.680
0.618	0.542	0.994	-5.405	0.166	4.316
0.050	0.960	0.999	-5.573	0.016	5.644
1.636	0.114	0.775	-4.447	0.395	5.879
0.292	0.773	0.999	-5.536	0.202	4.542
-0.901	0.376	0.963	-5.218	-0.765	2.591
1.107	0.279	0.922	-5.041	0.255	2.362
-1.814	0.082	0.721	-4.206	-0.775	3.429
0.189	0.852	0.999	-5.558	0.049	5.373
0.315	0.755	0.999	-5.530	0.102	5.435
-1.164	0.255	0.913	-4.987	-0.336	8.537
2.613	0.015	0.498	-2.925	0.929	5.728
0.062	0.951	0.999	-5.572	0.019	2.142
-0.489	0.629	0.999	-5.468	-0.143	1.303
1.009	0.323	0.950	-5.130	0.259	5.544
0.395	0.696	0.999	-5.505	0.103	4.042
0.580	0.567	0.997	-5.425	0.180	3.536
-0.609	0.548	0.994	-5.410	-0.173	2.353
-0.665	0.512	0.989	-5.378	-0.187	3.297
-0.059	0.954	0.999	-5.572	-0.011	8.428
0.619	0.541	0.994	-5.404	0.189	4.699
-1.123	0.272	0.919	-5.026	-0.372	4.344
-1.042	0.307	0.942	-5.101	-0.243	6.308
-0.656	0.518	0.990	-5.384	-0.227	5.370
-0.285	0.778	0.999	-5.538	-0.104	5.661
0.254	0.801	0.999	-5.545	0.062	4.852
-1.475	0.153	0.813	-4.648	-0.328	3.062
0.667	0.511	0.989	-5.378	0.192	6.735
2.233	0.035	0.597	-3.570	0.759	4.987
0.454	0.653	0.999	-5.482	0.135	3.893
-0.447	0.658	0.999	-5.485	-0.095	7.328
-0.611	0.547	0.994	-5.409	-0.123	6.628
-0.220	0.828	0.999	-5.552	-0.061	6.679
-2.556	0.017	0.500	-3.025	-0.989	3.226
1.331	0.195	0.853	-4.814	0.299	5.422
0.554	0.585	0.999	-5.438	0.149	6.506
0.566	0.576	0.999	-5.432	0.122	7.633
1.158	0.258	0.913	-4.992	0.314	5.111
0.982	0.336	0.957	-5.153	0.195	5.766
0.648	0.523	0.990	-5.388	0.116	5.796
0.467	0.645	0.999	-5.477	0.158	2.165
0.415	0.682	0.999	-5.497	0.236	2.520
0.994	0.330	0.956	-5.143	0.315	2.646
0.772	0.447	0.979	-5.312	0.165	6.014
-1.009	0.323	0.950	-5.130	-0.381	4.396
3.131	0.004	0.353	-1.972	0.690	5.136
-0.503	0.619	0.999	-5.462	-0.134	4.872
0.905	0.374	0.963	-5.215	0.238	5.036

-2.349	0.027	0.559	-3.380	-0.925	4.356
-0.892	0.381	0.964	-5.225	-0.311	3.729
0.866	0.394	0.964	-5.244	0.391	4.999
1.234	0.229	0.890	-4.917	0.586	5.788
-0.883	0.385	0.964	-5.232	-0.181	4.535
1.297	0.206	0.868	-4.850	0.422	4.842
-0.806	0.428	0.973	-5.288	-0.256	2.353
0.474	0.640	0.999	-5.474	0.106	4.381
0.722	0.477	0.984	-5.344	0.150	6.942
2.064	0.049	0.639	-3.837	0.485	6.264
0.610	0.547	0.994	-5.409	0.240	7.311
-0.551	0.587	0.999	-5.439	-0.226	3.444
1.939	0.064	0.672	-4.025	0.447	5.402
0.502	0.620	0.999	-5.462	0.131	5.444
0.816	0.422	0.972	-5.281	0.148	5.969
1.787	0.086	0.731	-4.243	0.462	4.860
-0.183	0.857	0.999	-5.559	-0.054	4.359
1.844	0.077	0.707	-4.163	0.469	6.656
0.114	0.910	0.999	-5.568	0.032	4.375
-0.053	0.958	0.999	-5.573	-0.013	4.412
1.289	0.209	0.868	-4.859	0.293	5.414
2.035	0.053	0.653	-3.881	0.629	3.769
1.370	0.183	0.840	-4.770	0.310	4.951
-0.579	0.568	0.997	-5.425	-0.133	4.995
-0.278	0.783	0.999	-5.539	-0.082	2.242
-0.373	0.713	0.999	-5.512	-0.112	6.936
0.507	0.617	0.999	-5.460	0.105	4.147
0.487	0.630	0.999	-5.468	0.131	2.489
-0.260	0.797	0.999	-5.544	-0.078	4.558
0.501	0.621	0.999	-5.463	0.126	6.113
-0.947	0.353	0.961	-5.181	-0.214	4.567
0.819	0.421	0.972	-5.279	0.350	6.148
-0.395	0.696	0.999	-5.505	-0.096	3.189
0.348	0.731	0.999	-5.520	0.073	5.132
0.776	0.445	0.979	-5.309	0.232	4.635
0.585	0.563	0.997	-5.422	0.134	5.312
3.183	0.004	0.340	-1.872	0.735	4.682
-0.203	0.841	0.999	-5.556	-0.053	8.631
3.204	0.004	0.340	-1.833	0.887	4.765
0.705	0.487	0.985	-5.354	0.251	3.600
-0.081	0.936	0.999	-5.571	-0.040	5.469
-0.640	0.528	0.992	-5.393	-0.207	4.060
-0.234	0.817	0.999	-5.550	-0.057	5.091
-0.529	0.601	0.999	-5.450	-0.245	3.252
-0.833	0.413	0.970	-5.269	-0.326	3.489
0.312	0.757	0.999	-5.531	0.205	4.319
-0.772	0.447	0.979	-5.311	-0.152	3.437
-0.721	0.477	0.984	-5.344	-0.189	4.800
0.774	0.446	0.979	-5.310	0.245	9.114
1.361	0.185	0.842	-4.780	0.434	4.644

0.604	0.551	0.994	-5.412	0.126	5.457
-0.407	0.688	0.999	-5.500	-0.110	3.413
1.420	0.168	0.825	-4.713	0.333	4.529
-0.252	0.803	0.999	-5.546	-0.058	3.070
0.551	0.586	0.999	-5.439	0.259	7.365
-0.668	0.510	0.989	-5.377	-0.208	2.085
0.350	0.729	0.999	-5.519	0.079	3.556
-1.187	0.246	0.906	-4.964	-1.011	4.065
-0.375	0.711	0.999	-5.511	-0.093	8.165
-0.235	0.816	0.999	-5.549	-0.098	6.081
-2.343	0.027	0.559	-3.390	-0.729	2.885
-0.662	0.514	0.990	-5.380	-0.329	3.899
0.374	0.711	0.999	-5.512	0.180	4.701
-1.513	0.143	0.802	-4.602	-0.601	2.539
-0.528	0.602	0.999	-5.450	-0.508	4.270
1.624	0.117	0.776	-4.462	0.595	6.137
0.865	0.395	0.964	-5.246	0.482	3.054
-0.208	0.837	0.999	-5.555	-0.074	7.004
-0.654	0.519	0.990	-5.385	-0.280	3.782
-0.135	0.894	0.999	-5.566	-0.031	8.078
-1.778	0.087	0.732	-4.255	-0.586	5.580
0.559	0.581	0.999	-5.435	0.265	6.380
-0.920	0.366	0.963	-5.203	-0.262	1.265
-0.651	0.521	0.990	-5.387	-0.182	8.005
1.484	0.150	0.811	-4.637	0.372	4.702
1.074	0.293	0.934	-5.072	0.281	2.892
-0.893	0.380	0.964	-5.224	-0.320	3.687
0.622	0.539	0.994	-5.403	0.174	4.253
0.566	0.576	0.999	-5.432	0.275	4.863
-0.800	0.431	0.973	-5.292	-0.203	2.803
0.729	0.473	0.984	-5.339	0.156	4.882
1.781	0.087	0.731	-4.252	0.485	5.419
-1.054	0.302	0.941	-5.090	-0.302	1.902
1.211	0.237	0.899	-4.940	0.286	7.999
0.731	0.472	0.984	-5.338	0.218	2.893
0.689	0.497	0.987	-5.364	0.196	8.347
0.241	0.811	0.999	-5.548	0.076	6.097
0.131	0.897	0.999	-5.566	0.052	5.033
1.110	0.277	0.922	-5.038	0.409	4.187
0.897	0.378	0.963	-5.221	0.267	7.704
0.373	0.713	0.999	-5.512	0.100	5.712
0.080	0.937	0.999	-5.571	0.025	6.116
3.209	0.004	0.340	-1.823	1.038	5.682
2.311	0.029	0.570	-3.443	0.640	4.025
1.708	0.100	0.761	-4.352	0.476	6.155
0.427	0.673	0.999	-5.493	0.102	6.575
0.783	0.441	0.977	-5.304	0.256	4.232
-0.195	0.847	0.999	-5.557	-0.078	1.686
1.352	0.188	0.847	-4.790	0.518	3.776
1.376	0.181	0.840	-4.763	0.303	4.608

2.181	0.039	0.611	-3.654	0.522	2.894
1.816	0.081	0.720	-4.203	0.571	6.625
1.496	0.147	0.804	-4.623	0.472	4.893
-1.009	0.323	0.950	-5.130	-0.440	4.469
-0.601	0.553	0.994	-5.414	-0.210	2.641
-1.089	0.287	0.929	-5.058	-0.385	4.775
-0.284	0.779	0.999	-5.538	-0.079	5.313
-0.251	0.804	0.999	-5.546	-0.096	4.418
0.180	0.859	0.999	-5.559	0.088	2.256
3.126	0.004	0.353	-1.980	0.881	5.295
0.095	0.925	0.999	-5.570	0.030	4.598
-0.640	0.528	0.992	-5.393	-0.502	4.678
1.008	0.323	0.950	-5.131	0.186	5.452
-0.215	0.831	0.999	-5.553	-0.058	7.909
-1.059	0.300	0.941	-5.086	-0.419	1.977
-0.426	0.673	0.999	-5.493	-0.125	7.417
-0.379	0.708	0.999	-5.510	-0.118	3.274
0.112	0.911	0.999	-5.568	0.020	6.211
0.193	0.849	0.999	-5.557	0.050	3.735
-1.087	0.288	0.929	-5.060	-0.244	5.307
0.984	0.334	0.957	-5.151	0.199	6.713
0.137	0.892	0.999	-5.566	0.068	4.629
-0.909	0.372	0.963	-5.212	-0.288	1.730
0.456	0.652	0.999	-5.481	0.163	2.465
0.118	0.907	0.999	-5.568	0.032	4.474
0.681	0.502	0.988	-5.369	0.260	3.150
0.349	0.730	0.999	-5.520	0.134	3.878
-0.562	0.579	0.999	-5.434	-0.121	4.182
-1.580	0.127	0.786	-4.518	-0.502	2.692
-0.317	0.754	0.999	-5.529	-0.107	6.007
0.214	0.832	0.999	-5.554	0.056	6.867
1.148	0.262	0.914	-5.003	0.316	3.124
-0.541	0.593	0.999	-5.444	-0.179	5.487
0.667	0.511	0.989	-5.377	0.143	4.267
-1.700	0.101	0.764	-4.362	-0.414	4.384
-0.088	0.931	0.999	-5.571	-0.022	3.249
2.150	0.041	0.619	-3.704	0.681	4.872
1.247	0.224	0.886	-4.903	0.288	7.819
-0.528	0.602	0.999	-5.450	-0.307	8.740
0.556	0.583	0.999	-5.437	0.129	1.957
-0.370	0.715	0.999	-5.513	-0.072	3.454
-3.177	0.004	0.340	-1.884	-1.217	4.640
-3.537	0.002	0.301	-1.184	-1.465	3.991
-2.785	0.010	0.455	-2.618	-1.499	4.741
-1.778	0.087	0.732	-4.255	-0.664	4.274
-2.229	0.035	0.598	-3.577	-0.755	3.047
-1.836	0.078	0.710	-4.175	-1.064	2.710
-0.482	0.634	0.999	-5.471	-0.105	4.390
-1.340	0.192	0.851	-4.804	-0.314	2.910
-2.519	0.018	0.501	-3.090	-1.093	1.601

-1.814	0.082	0.721	-4.205	-0.345	5.854
-1.321	0.198	0.857	-4.824	-0.336	3.332
-0.190	0.851	0.999	-5.558	-0.100	5.551
-1.560	0.131	0.791	-4.544	-0.640	5.881
-1.706	0.100	0.762	-4.354	-0.495	4.748
-1.859	0.075	0.703	-4.142	-0.421	4.681
0.335	0.740	0.999	-5.524	0.105	5.098
1.705	0.101	0.763	-4.356	0.421	4.368
1.661	0.109	0.766	-4.414	0.586	3.727
-0.500	0.622	0.999	-5.463	-0.245	4.698
1.177	0.250	0.910	-4.974	0.243	5.064
0.580	0.567	0.997	-5.425	0.150	8.011
-0.360	0.722	0.999	-5.516	-0.109	7.558
-0.808	0.427	0.973	-5.287	-0.257	4.148
-0.359	0.723	0.999	-5.517	-0.151	6.209
-0.414	0.683	0.999	-5.498	-0.156	6.036
-1.943	0.063	0.671	-4.019	-0.622	7.314
-1.383	0.179	0.838	-4.755	-0.312	4.049
0.528	0.602	0.999	-5.450	0.170	3.756
-0.563	0.578	0.999	-5.433	-0.107	7.318
-1.279	0.213	0.871	-4.869	-0.304	9.187
-0.708	0.486	0.985	-5.353	-0.195	9.593
0.860	0.398	0.964	-5.249	0.216	6.320
-1.945	0.063	0.670	-4.016	-0.474	4.717
0.897	0.378	0.963	-5.221	0.250	4.403
4.438	0.000	0.256	0.614	1.303	3.111
-0.792	0.436	0.973	-5.298	-0.291	9.937
-0.661	0.515	0.990	-5.381	-0.123	9.444
-0.316	0.754	0.999	-5.529	-0.151	5.125
-2.796	0.010	0.450	-2.597	-0.867	3.176
-0.659	0.516	0.990	-5.382	-0.208	2.416
-1.307	0.203	0.862	-4.840	-0.324	8.956
-1.372	0.182	0.840	-4.767	-0.443	4.915
-0.474	0.640	0.999	-5.474	-0.212	3.706
0.192	0.850	0.999	-5.558	0.093	10.265
-2.037	0.052	0.652	-3.878	-0.739	9.025
-1.607	0.121	0.784	-4.484	-0.590	8.446
-1.532	0.138	0.795	-4.578	-0.290	10.843
-0.456	0.652	0.999	-5.481	-0.078	11.446
-1.508	0.144	0.803	-4.608	-0.518	9.733
-0.265	0.793	0.999	-5.543	-0.062	5.478
-0.724	0.476	0.984	-5.343	-0.164	9.770
-0.671	0.508	0.988	-5.375	-0.128	7.111
-0.083	0.934	0.999	-5.571	-0.023	5.911
-0.930	0.361	0.963	-5.195	-0.246	4.400
-2.306	0.030	0.570	-3.451	-0.713	9.016
-0.434	0.668	0.999	-5.490	-0.098	7.754
-1.374	0.182	0.840	-4.765	-0.253	7.599
0.198	0.845	0.999	-5.556	0.053	3.657
-1.544	0.135	0.793	-4.563	-0.285	10.180

0.351	0.728	0.999	-5.519	0.094	2.931
-1.933	0.065	0.673	-4.035	-0.883	3.250
1.116	0.275	0.921	-5.033	0.344	4.084
1.235	0.228	0.890	-4.916	0.250	6.137
-0.223	0.825	0.999	-5.552	-0.062	8.863
0.467	0.645	0.999	-5.477	0.104	6.217
-0.675	0.506	0.988	-5.373	-0.177	7.186
-1.627	0.116	0.776	-4.459	-0.597	8.260
-1.445	0.161	0.820	-4.684	-0.367	3.502
-1.027	0.314	0.944	-5.114	-0.466	7.253
-1.423	0.167	0.825	-4.709	-0.668	10.110
-1.331	0.195	0.853	-4.813	-0.279	9.165
-1.851	0.076	0.705	-4.153	-0.596	7.130
-0.662	0.514	0.990	-5.380	-0.148	4.316
-0.466	0.645	0.999	-5.478	-0.135	2.992
-0.597	0.556	0.995	-5.416	-0.164	7.758
-2.144	0.042	0.619	-3.712	-0.537	6.739
-1.135	0.267	0.917	-5.015	-0.317	4.074
-2.664	0.013	0.498	-2.835	-0.882	7.185
0.874	0.390	0.964	-5.239	0.160	5.880
1.173	0.252	0.911	-4.978	0.225	6.735
0.630	0.535	0.993	-5.399	0.132	9.042
-0.361	0.721	0.999	-5.516	-0.131	10.331
-0.552	0.586	0.999	-5.439	-0.138	3.564
0.280	0.782	0.999	-5.539	0.080	7.528
-0.264	0.794	0.999	-5.543	-0.102	6.218
2.384	0.025	0.548	-3.320	0.596	5.946
0.787	0.439	0.975	-5.301	0.469	3.617
0.415	0.682	0.999	-5.497	0.184	3.184
-1.074	0.293	0.934	-5.072	-0.438	4.886
0.216	0.831	0.999	-5.553	0.068	1.879
0.692	0.495	0.987	-5.362	0.384	3.128
-0.221	0.827	0.999	-5.552	-0.090	3.427
-0.839	0.410	0.970	-5.265	-0.425	8.225
0.949	0.352	0.961	-5.180	0.394	1.581
0.439	0.664	0.999	-5.488	0.231	2.624
-0.538	0.595	0.999	-5.446	-0.212	9.085
-0.531	0.600	0.999	-5.449	-0.269	2.469
-0.562	0.579	0.999	-5.434	-0.214	5.170
-0.540	0.594	0.999	-5.445	-0.245	4.401
0.484	0.632	0.999	-5.470	0.271	3.046
0.136	0.893	0.999	-5.566	0.067	3.435
-0.053	0.958	0.999	-5.573	-0.029	2.575
-0.320	0.752	0.999	-5.528	-0.242	2.362
0.079	0.937	0.999	-5.571	0.038	5.355
-0.279	0.783	0.999	-5.539	-0.137	4.105
1.474	0.153	0.813	-4.649	0.695	2.194
0.395	0.696	0.999	-5.505	0.216	4.295
-0.307	0.761	0.999	-5.532	-0.176	6.251
-0.266	0.792	0.999	-5.542	-0.116	8.631

0.482	0.634	0.999	-5.471	0.227	2.861
-0.127	0.900	0.999	-5.567	-0.064	2.380
1.614	0.119	0.780	-4.475	0.600	1.795
-0.088	0.931	0.999	-5.571	-0.051	1.925
-0.332	0.743	0.999	-5.525	-0.174	1.928
-0.072	0.943	0.999	-5.572	-0.043	4.341
1.148	0.262	0.914	-5.003	0.579	5.470
-0.631	0.534	0.992	-5.398	-0.240	3.347
-0.265	0.793	0.999	-5.543	-0.095	9.772
-0.354	0.727	0.999	-5.518	-0.119	10.340
-0.547	0.589	0.999	-5.441	-0.186	2.011
-0.075	0.941	0.999	-5.571	-0.030	7.899
-0.449	0.657	0.999	-5.484	-0.191	7.121
0.666	0.512	0.989	-5.378	0.344	2.495
0.503	0.619	0.999	-5.461	0.285	7.493
-0.562	0.579	0.999	-5.434	-0.248	6.628
-0.094	0.926	0.999	-5.570	-0.048	4.611
-0.108	0.915	0.999	-5.569	-0.058	4.468
0.067	0.947	0.999	-5.572	0.020	11.029
0.304	0.763	0.999	-5.533	0.128	3.904
0.434	0.668	0.999	-5.490	0.158	2.176
-0.064	0.950	0.999	-5.572	-0.022	10.429
1.869	0.073	0.698	-4.128	1.012	3.420
-0.099	0.922	0.999	-5.570	-0.030	10.904
-0.103	0.919	0.999	-5.569	-0.031	10.891
-0.149	0.883	0.999	-5.564	-0.041	10.839
1.667	0.108	0.765	-4.406	0.636	2.431
-1.017	0.319	0.950	-5.123	-0.278	7.769
-1.505	0.145	0.803	-4.611	-0.360	9.512
-0.071	0.944	0.999	-5.572	-0.022	2.111
-1.951	0.062	0.667	-4.007	-0.464	7.364
0.813	0.424	0.972	-5.283	0.545	6.660
-1.499	0.146	0.804	-4.619	-0.448	4.918
-0.089	0.929	0.999	-5.570	-0.031	3.699
-0.993	0.330	0.956	-5.144	-0.325	5.881
-0.969	0.342	0.957	-5.164	-0.346	4.995
-1.400	0.174	0.828	-4.736	-0.455	6.631
-0.545	0.590	0.999	-5.442	-0.191	5.966
-0.937	0.358	0.962	-5.190	-0.164	8.589
-0.735	0.469	0.983	-5.336	-0.129	8.473
-0.643	0.526	0.992	-5.391	-0.147	6.570
1.344	0.191	0.850	-4.799	0.570	5.262
1.506	0.145	0.803	-4.611	0.409	2.574
-0.879	0.388	0.964	-5.235	-0.306	3.561
-0.452	0.655	0.999	-5.483	-0.127	3.142
0.294	0.771	0.999	-5.536	0.071	5.418
-0.613	0.545	0.994	-5.407	-0.148	4.890
-0.422	0.677	0.999	-5.495	-0.139	5.411
0.718	0.479	0.984	-5.346	0.179	7.404
-0.667	0.511	0.989	-5.377	-0.213	4.698

2.259	0.033	0.594	-3.528	0.811	6.128
-0.692	0.495	0.987	-5.362	-0.133	9.501
-1.831	0.079	0.713	-4.182	-0.519	8.290
-0.337	0.739	0.999	-5.523	-0.121	2.902
3.166	0.004	0.340	-1.905	1.197	4.807
-0.423	0.676	0.999	-5.494	-0.099	6.382
-2.715	0.012	0.488	-2.744	-0.699	1.911
-0.753	0.459	0.982	-5.324	-0.273	2.780
1.734	0.095	0.754	-4.316	0.427	3.888
0.670	0.509	0.989	-5.376	0.145	5.283
0.898	0.377	0.963	-5.220	0.279	4.295
-0.652	0.520	0.990	-5.386	-0.185	8.284
-1.879	0.072	0.698	-4.112	-0.400	5.954
-2.386	0.025	0.548	-3.318	-0.410	9.250
0.726	0.474	0.984	-5.341	0.147	5.906
1.551	0.133	0.791	-4.555	0.426	5.567
1.401	0.173	0.828	-4.734	0.524	2.818
0.930	0.361	0.963	-5.195	0.212	5.158
-0.158	0.876	0.999	-5.563	-0.037	6.393
1.116	0.275	0.921	-5.033	0.295	3.934
-1.192	0.244	0.904	-4.959	-0.237	7.018
-1.215	0.236	0.899	-4.936	-0.260	8.900
-0.323	0.750	0.999	-5.528	-0.104	6.317
-1.032	0.312	0.943	-5.109	-0.305	6.653
-2.231	0.035	0.598	-3.573	-0.520	5.105
2.730	0.011	0.484	-2.716	0.725	3.010
-0.297	0.769	0.999	-5.535	-0.069	3.116
-0.142	0.888	0.999	-5.565	-0.035	11.462
0.341	0.736	0.999	-5.522	0.120	2.990
-0.804	0.429	0.973	-5.290	-0.191	9.282
-0.055	0.957	0.999	-5.573	-0.017	3.082
0.117	0.908	0.999	-5.568	0.033	2.603
0.113	0.911	0.999	-5.568	0.034	5.508
0.089	0.929	0.999	-5.570	0.024	4.006
1.561	0.131	0.791	-4.543	0.374	3.103
-1.597	0.123	0.785	-4.497	-0.336	8.193
-0.704	0.488	0.985	-5.355	-0.167	6.261
-1.558	0.132	0.791	-4.546	-0.493	3.240
-2.641	0.014	0.498	-2.875	-0.581	7.352
0.762	0.453	0.981	-5.318	0.203	5.252
-1.884	0.071	0.696	-4.107	-0.387	8.339
0.954	0.349	0.960	-5.176	0.275	2.511
1.410	0.171	0.826	-4.725	0.421	2.600
-0.400	0.693	0.999	-5.503	-0.185	5.318
0.762	0.453	0.981	-5.318	0.280	4.607
-0.396	0.695	0.999	-5.504	-0.099	9.037
-0.836	0.411	0.970	-5.267	-0.218	6.117
2.346	0.027	0.559	-3.385	0.638	6.451
0.233	0.818	0.999	-5.550	0.088	4.478
0.163	0.872	0.999	-5.562	0.055	5.725

-0.576	0.570	0.997	-5.427	-0.130	6.359
0.103	0.919	0.999	-5.569	0.039	4.237
0.348	0.730	0.999	-5.520	0.081	3.636
0.629	0.535	0.993	-5.399	0.165	4.847
0.589	0.561	0.996	-5.420	0.182	2.931
0.918	0.367	0.963	-5.204	0.256	7.410
1.433	0.164	0.824	-4.697	0.568	2.798
0.820	0.420	0.972	-5.278	0.160	5.407
1.413	0.170	0.825	-4.721	0.388	6.535
3.005	0.006	0.393	-2.210	0.703	6.661
-1.978	0.059	0.663	-3.968	-0.384	6.009
2.652	0.014	0.498	-2.857	1.325	7.712
1.342	0.192	0.850	-4.801	0.318	5.610
0.383	0.705	0.999	-5.509	0.083	5.965
0.828	0.416	0.970	-5.273	0.247	4.635
0.946	0.353	0.961	-5.183	0.257	4.677
-1.176	0.251	0.910	-4.975	-0.281	5.591
-0.757	0.456	0.981	-5.321	-0.210	2.035
-0.487	0.630	0.999	-5.468	-0.174	4.680
1.556	0.132	0.791	-4.549	0.633	4.690
0.803	0.430	0.973	-5.290	0.199	4.949
0.423	0.676	0.999	-5.494	0.155	2.798
0.667	0.511	0.989	-5.377	0.250	2.128
-0.332	0.743	0.999	-5.525	-0.115	4.014
0.452	0.655	0.999	-5.483	0.184	2.724
1.870	0.073	0.698	-4.125	0.467	3.472
-0.698	0.491	0.986	-5.359	-0.206	2.508
-0.827	0.416	0.970	-5.273	-0.173	4.561
0.830	0.414	0.970	-5.271	0.259	2.832
-0.274	0.787	0.999	-5.541	-0.069	3.874
0.237	0.815	0.999	-5.549	0.053	4.863
-0.210	0.836	0.999	-5.554	-0.065	5.918
0.394	0.697	0.999	-5.505	0.108	3.055
1.523	0.140	0.801	-4.590	0.300	7.313
1.498	0.146	0.804	-4.620	0.375	5.384
1.746	0.093	0.750	-4.300	0.530	5.743
0.103	0.919	0.999	-5.569	0.029	3.045
-2.932	0.007	0.411	-2.346	-1.173	1.869
0.124	0.903	0.999	-5.567	0.036	2.977
-3.399	0.002	0.301	-1.454	-1.214	2.070
-2.582	0.016	0.500	-2.981	-0.745	2.623
-0.914	0.370	0.963	-5.208	-0.277	7.656
0.783	0.441	0.977	-5.304	0.230	6.619
-1.756	0.091	0.745	-4.286	-0.514	2.090
-1.052	0.303	0.941	-5.092	-0.442	3.506
2.131	0.043	0.626	-3.733	0.523	3.337
0.454	0.654	0.999	-5.483	0.124	2.283
-0.540	0.594	0.999	-5.445	-0.144	1.792
1.116	0.275	0.921	-5.033	0.344	4.262
0.314	0.756	0.999	-5.530	0.091	5.171

-1.445	0.161	0.820	-4.683	-0.413	3.028
-0.896	0.379	0.963	-5.222	-0.174	4.315
-0.700	0.491	0.986	-5.358	-0.180	3.402
0.562	0.579	0.999	-5.434	0.181	4.639
-0.569	0.574	0.999	-5.430	-0.176	2.645
0.952	0.350	0.961	-5.177	0.240	3.918
0.741	0.466	0.983	-5.332	0.373	3.944
1.668	0.108	0.764	-4.405	0.435	5.934
0.143	0.887	0.999	-5.565	0.024	8.212
0.073	0.943	0.999	-5.572	0.018	3.820
0.525	0.604	0.999	-5.452	0.125	2.756
0.784	0.440	0.976	-5.303	0.225	5.539
-1.595	0.123	0.785	-4.500	-0.604	3.454
1.426	0.166	0.825	-4.706	0.546	4.183
0.597	0.556	0.995	-5.416	0.141	2.595
1.131	0.269	0.918	-5.019	0.242	6.636
0.340	0.737	0.999	-5.523	0.093	7.183
-0.916	0.368	0.963	-5.206	-0.277	6.321
0.444	0.661	0.999	-5.486	0.163	11.506
0.929	0.362	0.963	-5.196	0.237	3.186
0.366	0.717	0.999	-5.514	0.085	4.200
0.347	0.732	0.999	-5.520	0.158	3.241
-1.099	0.282	0.924	-5.049	-0.295	2.268
0.843	0.407	0.969	-5.262	0.254	4.515
-0.910	0.372	0.963	-5.211	-0.230	4.192
-0.492	0.627	0.999	-5.466	-0.158	3.754
-0.558	0.582	0.999	-5.436	-0.195	4.152
-0.638	0.529	0.992	-5.394	-0.253	4.254
-1.138	0.266	0.917	-5.012	-0.351	4.416
0.134	0.894	0.999	-5.566	0.038	7.027
1.548	0.134	0.793	-4.559	0.603	5.848
0.999	0.327	0.954	-5.138	0.218	6.066
-0.313	0.757	0.999	-5.530	-0.150	2.451
-0.050	0.960	0.999	-5.573	-0.017	3.421
-0.130	0.898	0.999	-5.566	-0.042	6.815
1.880	0.072	0.698	-4.112	0.699	5.372
-2.033	0.053	0.653	-3.884	-0.466	8.435
0.652	0.520	0.990	-5.386	0.207	2.520
1.294	0.208	0.868	-4.854	0.346	5.707
0.868	0.394	0.964	-5.243	0.220	6.804
0.349	0.730	0.999	-5.520	0.068	7.630
-0.739	0.466	0.983	-5.333	-0.223	7.640
1.010	0.322	0.950	-5.129	0.375	9.923
0.373	0.712	0.999	-5.512	0.103	2.198
-0.323	0.749	0.999	-5.528	-0.082	3.724
1.689	0.104	0.764	-4.377	0.328	7.039
0.932	0.360	0.963	-5.194	0.219	5.040
-0.123	0.903	0.999	-5.567	-0.021	7.086
0.634	0.532	0.992	-5.396	0.243	6.638
0.592	0.559	0.996	-5.418	0.122	4.408

1.301	0.205	0.866	-4.846	0.301	3.101
1.522	0.141	0.801	-4.591	0.359	4.871
-2.383	0.025	0.548	-3.323	-0.462	4.187
-0.229	0.820	0.999	-5.550	-0.074	4.934
0.137	0.892	0.999	-5.566	0.033	3.050
0.874	0.391	0.964	-5.239	0.174	7.424
0.850	0.403	0.968	-5.257	0.179	5.288
0.184	0.856	0.999	-5.559	0.041	5.410
0.863	0.396	0.964	-5.247	0.550	7.142
0.213	0.833	0.999	-5.554	0.093	4.211
-0.137	0.892	0.999	-5.566	-0.043	1.611
0.486	0.632	0.999	-5.469	0.154	2.020
0.620	0.541	0.994	-5.404	0.235	3.485
-0.179	0.859	0.999	-5.560	-0.086	6.520
1.631	0.115	0.776	-4.454	0.370	4.306
0.314	0.756	0.999	-5.530	0.075	3.661
-0.426	0.673	0.999	-5.493	-0.120	2.692
0.954	0.349	0.960	-5.176	0.233	4.814
-1.144	0.263	0.915	-5.006	-0.339	2.490
0.598	0.555	0.995	-5.416	0.227	6.085
0.773	0.447	0.979	-5.311	0.202	6.258
0.221	0.826	0.999	-5.552	0.128	2.383
0.840	0.409	0.970	-5.264	0.179	6.662
-0.065	0.949	0.999	-5.572	-0.037	4.510
2.195	0.038	0.611	-3.631	0.597	5.283
0.074	0.942	0.999	-5.572	0.032	5.162
1.512	0.143	0.803	-4.603	0.450	3.460
0.862	0.397	0.964	-5.248	0.372	5.712
-1.032	0.312	0.943	-5.109	-0.433	3.621
0.125	0.901	0.999	-5.567	0.032	3.188
1.010	0.322	0.950	-5.129	0.255	5.094
0.334	0.741	0.999	-5.524	0.096	2.840
0.581	0.566	0.997	-5.424	0.120	4.192
-1.291	0.208	0.868	-4.857	-0.300	3.967
-0.505	0.618	0.999	-5.461	-0.113	6.192
-0.307	0.761	0.999	-5.532	-0.086	5.613
0.338	0.738	0.999	-5.523	0.087	3.552
0.147	0.885	0.999	-5.564	0.038	5.628
2.088	0.047	0.630	-3.801	0.518	7.617
0.604	0.551	0.994	-5.412	0.158	6.538
1.559	0.131	0.791	-4.544	0.309	9.061
1.016	0.319	0.950	-5.123	0.503	3.089
1.804	0.083	0.724	-4.219	0.436	4.982
0.071	0.944	0.999	-5.572	0.015	5.525
1.638	0.114	0.775	-4.444	0.961	4.827
0.142	0.888	0.999	-5.565	0.049	3.346
0.466	0.645	0.999	-5.477	0.110	2.102
1.371	0.182	0.840	-4.768	0.507	5.314
1.577	0.127	0.788	-4.522	0.589	6.575
3.536	0.002	0.301	-1.186	1.156	3.969

-0.324	0.748	0.999	-5.527	-0.128	4.311
0.603	0.552	0.994	-5.413	0.126	3.198
0.614	0.545	0.994	-5.407	0.163	5.668
0.947	0.352	0.961	-5.181	0.198	4.414
0.116	0.909	0.999	-5.568	0.046	6.512
-0.624	0.538	0.994	-5.401	-0.169	4.738
-1.264	0.218	0.876	-4.885	-0.755	7.947
-0.570	0.574	0.999	-5.430	-0.157	6.264
0.496	0.624	0.999	-5.465	0.170	6.459
1.241	0.226	0.888	-4.909	0.244	9.428
-1.003	0.325	0.954	-5.135	-0.232	4.136
0.418	0.680	0.999	-5.496	0.165	5.605
0.315	0.755	0.999	-5.530	0.103	3.816
-0.700	0.490	0.986	-5.358	-0.131	5.701
0.597	0.556	0.995	-5.416	0.103	2.182
-0.198	0.845	0.999	-5.557	-0.056	4.488
1.235	0.228	0.890	-4.916	0.258	3.280
0.776	0.445	0.979	-5.308	0.240	4.050
2.142	0.042	0.619	-3.716	0.496	3.675
-0.610	0.547	0.994	-5.409	-0.352	7.157
-0.692	0.495	0.987	-5.362	-0.136	6.071
1.168	0.254	0.913	-4.983	0.240	6.105
1.533	0.138	0.795	-4.578	0.474	4.278
0.787	0.439	0.975	-5.301	0.183	7.354
0.230	0.820	0.999	-5.550	0.069	5.495
1.128	0.270	0.918	-5.022	0.260	2.269
-0.158	0.876	0.999	-5.563	-0.059	6.037
-0.902	0.376	0.963	-5.217	-0.174	4.201
0.322	0.750	0.999	-5.528	0.162	2.155
0.842	0.408	0.969	-5.262	0.221	5.694
-0.742	0.465	0.983	-5.331	-0.141	6.279
1.463	0.156	0.817	-4.662	0.321	6.209
-0.785	0.440	0.976	-5.303	-0.164	5.014
0.964	0.344	0.957	-5.167	0.248	4.773
-0.220	0.828	0.999	-5.552	-0.061	2.340
1.605	0.121	0.784	-4.487	0.395	2.533
0.127	0.900	0.999	-5.567	0.033	5.575
-0.376	0.710	0.999	-5.511	-0.224	4.752
1.719	0.098	0.757	-4.336	0.525	6.147
-1.700	0.102	0.764	-4.363	-0.444	3.607
-1.217	0.235	0.899	-4.934	-0.343	6.372
1.403	0.173	0.828	-4.733	0.259	5.713
0.406	0.688	0.999	-5.500	0.124	4.764
-0.409	0.686	0.999	-5.500	-0.179	5.616
0.299	0.767	0.999	-5.534	0.130	4.002
-0.072	0.943	0.999	-5.572	-0.017	4.069
0.199	0.844	0.999	-5.556	0.047	2.983
0.438	0.665	0.999	-5.489	0.184	6.734
-0.321	0.751	0.999	-5.528	-0.189	4.753
-1.165	0.255	0.913	-4.986	-0.787	5.279

0.168	0.868	0.999	-5.561	0.047	3.381
0.655	0.518	0.990	-5.384	0.157	5.466
0.520	0.608	0.999	-5.454	0.247	3.724
-0.705	0.487	0.985	-5.354	-0.222	2.448
-0.519	0.608	0.999	-5.454	-0.112	3.631
2.439	0.022	0.537	-3.228	0.667	4.078
1.107	0.279	0.922	-5.041	0.344	4.701
-0.233	0.818	0.999	-5.550	-0.137	5.669
-0.916	0.369	0.963	-5.207	-0.604	5.449
0.203	0.841	0.999	-5.556	0.084	3.533
-0.963	0.345	0.957	-5.168	-0.263	2.994
-1.186	0.247	0.906	-4.966	-0.332	6.102
0.403	0.690	0.999	-5.502	0.115	4.611
-1.282	0.211	0.870	-4.866	-0.363	4.882
-0.860	0.398	0.964	-5.249	-0.245	4.978
-0.142	0.888	0.999	-5.565	-0.031	4.558
0.705	0.487	0.985	-5.355	0.232	7.481
0.120	0.905	0.999	-5.567	0.055	4.702
-0.294	0.771	0.999	-5.535	-0.107	7.941
0.435	0.667	0.999	-5.490	0.140	2.404
-0.278	0.784	0.999	-5.540	-0.089	4.297
0.979	0.337	0.957	-5.155	0.372	1.530
-1.166	0.255	0.913	-4.985	-0.366	4.693
-0.069	0.945	0.999	-5.572	-0.020	4.791
0.097	0.924	0.999	-5.570	0.030	7.585
0.165	0.870	0.999	-5.562	0.086	3.330
0.775	0.445	0.979	-5.309	0.278	4.201
-0.319	0.752	0.999	-5.529	-0.078	2.631
0.166	0.869	0.999	-5.562	0.077	3.688
0.624	0.538	0.994	-5.401	0.152	5.135
0.231	0.819	0.999	-5.550	0.111	3.288
0.522	0.606	0.999	-5.453	0.139	5.596
-1.378	0.180	0.839	-4.760	-1.077	5.010
-0.367	0.717	0.999	-5.514	-0.137	3.673
-0.281	0.781	0.999	-5.539	-0.209	6.427
0.090	0.929	0.999	-5.570	0.036	4.850
0.381	0.707	0.999	-5.509	0.109	3.483
0.374	0.711	0.999	-5.512	0.189	3.048
0.902	0.375	0.963	-5.217	0.196	5.026
2.709	0.012	0.488	-2.754	1.039	5.521
-0.848	0.405	0.968	-5.258	-0.272	5.006
0.874	0.391	0.964	-5.239	0.450	3.294
-1.175	0.251	0.910	-4.977	-0.326	3.150
0.865	0.395	0.964	-5.246	0.232	3.612
1.053	0.302	0.941	-5.091	0.536	3.557
0.905	0.374	0.963	-5.215	0.411	4.057
-0.216	0.831	0.999	-5.553	-0.051	3.870
-0.957	0.348	0.959	-5.173	-0.238	3.297
-0.650	0.522	0.990	-5.387	-0.182	2.855
1.081	0.290	0.932	-5.066	0.339	2.833

0.671	0.508	0.988	-5.375	0.137	4.420
2.552	0.017	0.500	-3.033	0.569	6.218
-0.543	0.592	0.999	-5.443	-0.127	4.675
0.880	0.387	0.964	-5.234	0.181	4.135
2.623	0.015	0.498	-2.907	0.952	4.377
0.180	0.859	0.999	-5.559	0.059	5.517
2.029	0.053	0.653	-3.891	0.510	3.644
-0.667	0.511	0.989	-5.377	-0.267	5.186
-0.538	0.596	0.999	-5.446	-0.121	2.734
0.184	0.856	0.999	-5.559	0.041	5.415
0.289	0.775	0.999	-5.537	0.090	6.963
3.051	0.005	0.387	-2.123	0.681	5.299
3.049	0.005	0.387	-2.127	0.668	5.523
0.375	0.711	0.999	-5.511	0.078	4.228
-0.056	0.956	0.999	-5.573	-0.015	2.105
-0.104	0.918	0.999	-5.569	-0.034	3.327
-0.271	0.788	0.999	-5.541	-0.111	3.961
-0.742	0.465	0.983	-5.331	-0.208	2.613
0.622	0.540	0.994	-5.403	0.221	3.298
1.554	0.133	0.791	-4.551	0.398	4.735
-0.458	0.651	0.999	-5.481	-0.154	3.777
1.236	0.228	0.890	-4.914	0.368	4.171
0.197	0.845	0.999	-5.557	0.067	6.763
1.277	0.213	0.873	-4.872	0.376	6.220
1.160	0.257	0.913	-4.991	0.257	5.478
0.427	0.673	0.999	-5.493	0.144	5.198
1.207	0.239	0.901	-4.944	0.295	4.903
1.496	0.147	0.804	-4.622	0.554	4.917
-0.132	0.896	0.999	-5.566	-0.037	4.749
1.720	0.098	0.757	-4.335	0.471	5.988
-1.062	0.298	0.940	-5.083	-0.270	4.246
0.220	0.827	0.999	-5.552	0.054	4.262
0.432	0.669	0.999	-5.491	0.273	1.778
1.629	0.116	0.776	-4.456	0.296	5.120
-0.409	0.686	0.999	-5.499	-0.106	2.526
-0.557	0.583	0.999	-5.437	-0.152	2.747
-1.157	0.258	0.913	-4.993	-0.301	3.559
1.337	0.193	0.853	-4.806	0.394	4.267
1.243	0.225	0.886	-4.907	0.603	5.839
0.444	0.661	0.999	-5.486	0.101	4.449
-1.457	0.158	0.817	-4.670	-0.365	4.657
2.268	0.032	0.591	-3.513	0.530	4.335
-1.553	0.133	0.791	-4.552	-0.314	2.758
0.063	0.950	0.999	-5.572	0.023	3.107
0.563	0.578	0.999	-5.433	0.120	6.841
0.875	0.390	0.964	-5.238	0.212	3.246
-2.057	0.050	0.640	-3.848	-0.463	6.496
0.834	0.412	0.970	-5.268	0.174	4.971
0.580	0.567	0.997	-5.425	0.130	4.351
0.592	0.559	0.996	-5.419	0.184	3.488

0.350	0.729	0.999	-5.519	0.064	4.514
0.433	0.669	0.999	-5.491	0.121	5.946
-2.240	0.034	0.597	-3.558	-0.562	2.295
-2.484	0.020	0.517	-3.150	-1.001	2.271
0.853	0.402	0.966	-5.254	0.244	3.429
0.175	0.862	0.999	-5.560	0.049	1.256
0.507	0.617	0.999	-5.460	0.152	2.543
-0.203	0.841	0.999	-5.556	-0.045	5.100
-1.312	0.201	0.861	-4.834	-0.319	4.318
0.426	0.673	0.999	-5.493	0.126	3.686
0.490	0.628	0.999	-5.467	0.127	3.349
-0.374	0.712	0.999	-5.512	-0.092	3.646
3.379	0.002	0.301	-1.493	0.857	5.227
-3.298	0.003	0.331	-1.651	-1.562	2.054
-2.568	0.017	0.500	-3.005	-0.606	2.212
0.362	0.720	0.999	-5.516	0.092	2.880
-0.379	0.708	0.999	-5.510	-0.187	4.497
-0.500	0.622	0.999	-5.463	-0.114	6.250
1.227	0.231	0.894	-4.924	0.406	5.582
0.876	0.390	0.964	-5.238	0.411	4.105
-0.430	0.671	0.999	-5.492	-0.150	4.720
-0.387	0.702	0.999	-5.507	-0.111	2.606
0.662	0.514	0.990	-5.380	0.169	5.049
-0.944	0.354	0.961	-5.184	-0.220	5.752
0.877	0.389	0.964	-5.236	0.302	3.378
0.410	0.685	0.999	-5.499	0.122	2.790
-0.241	0.812	0.999	-5.548	-0.078	2.881
0.395	0.696	0.999	-5.505	0.113	5.736
2.181	0.039	0.611	-3.653	0.397	5.281
-1.761	0.090	0.743	-4.279	-0.464	2.403
0.198	0.845	0.999	-5.556	0.062	2.307
-0.104	0.918	0.999	-5.569	-0.018	3.495
-2.470	0.021	0.517	-3.175	-0.498	3.269
0.080	0.937	0.999	-5.571	0.034	10.570
1.837	0.078	0.710	-4.173	0.431	4.831
1.952	0.062	0.667	-4.006	0.419	2.583
-1.158	0.258	0.913	-4.993	-0.285	2.696
1.087	0.287	0.929	-5.060	0.251	6.685
0.156	0.877	0.999	-5.563	0.050	2.163
1.041	0.308	0.942	-5.101	0.264	6.273
0.278	0.783	0.999	-5.539	0.070	6.279
1.167	0.254	0.913	-4.984	0.352	5.202
2.128	0.043	0.628	-3.738	0.626	8.030
2.370	0.026	0.556	-3.345	0.698	2.721
-0.911	0.371	0.963	-5.210	-0.337	4.918
2.842	0.009	0.441	-2.513	0.919	5.156
1.668	0.108	0.764	-4.404	0.495	6.996
1.759	0.091	0.743	-4.283	0.434	7.060
0.088	0.931	0.999	-5.571	0.029	3.689
0.921	0.366	0.963	-5.202	0.194	3.521

0.337	0.739	0.999	-5.523	0.110	6.141
-0.376	0.710	0.999	-5.511	-0.099	3.454
0.823	0.418	0.972	-5.276	0.257	3.200
2.580	0.016	0.500	-2.983	0.646	4.646
2.499	0.019	0.512	-3.124	0.898	4.452
2.046	0.051	0.644	-3.865	0.787	6.434
2.253	0.033	0.595	-3.537	0.647	8.042
-0.359	0.723	0.999	-5.517	-0.102	5.817
1.478	0.152	0.813	-4.645	0.362	4.223
0.736	0.468	0.983	-5.335	0.175	2.313
1.774	0.088	0.734	-4.261	0.716	6.265
-0.455	0.653	0.999	-5.482	-0.169	4.389
0.571	0.573	0.999	-5.430	0.160	2.909
0.219	0.829	0.999	-5.553	0.095	4.310
-0.114	0.910	0.999	-5.568	-0.039	1.959
-0.210	0.835	0.999	-5.554	-0.053	3.282
-0.442	0.662	0.999	-5.487	-0.118	4.075
2.324	0.028	0.567	-3.420	0.456	3.976
-0.707	0.486	0.985	-5.353	-0.274	2.997
0.232	0.818	0.999	-5.550	0.050	4.961
0.809	0.426	0.973	-5.286	0.194	5.735
0.839	0.410	0.970	-5.265	0.309	2.947
0.767	0.450	0.980	-5.315	0.212	3.159
0.170	0.866	0.999	-5.561	0.038	2.559
1.254	0.221	0.882	-4.895	0.342	3.667
0.598	0.555	0.995	-5.416	0.153	5.748
0.115	0.909	0.999	-5.568	0.023	4.204
1.422	0.167	0.825	-4.710	0.419	7.660
-0.103	0.919	0.999	-5.569	-0.022	8.186
0.218	0.829	0.999	-5.553	0.050	7.933
-1.713	0.099	0.757	-4.345	-0.704	5.551
1.622	0.117	0.776	-4.465	0.504	7.917
1.626	0.117	0.776	-4.460	0.393	5.541
0.129	0.899	0.999	-5.567	0.044	5.405
0.371	0.714	0.999	-5.513	0.131	8.079
0.875	0.390	0.964	-5.238	0.216	2.833
0.960	0.346	0.957	-5.171	0.246	3.224
-1.164	0.255	0.913	-4.987	-0.250	4.664
-1.614	0.119	0.780	-4.475	-0.326	5.141
-1.012	0.321	0.950	-5.127	-0.227	3.085
-0.099	0.922	0.999	-5.570	-0.034	3.345
0.216	0.831	0.999	-5.553	0.049	7.397
0.567	0.576	0.999	-5.431	0.104	5.976
2.522	0.018	0.501	-3.085	0.933	4.952
1.725	0.097	0.757	-4.328	0.636	3.744
2.407	0.024	0.543	-3.282	1.052	6.302
2.547	0.017	0.500	-3.042	0.945	5.430
1.055	0.301	0.941	-5.089	0.349	6.447
2.179	0.039	0.611	-3.657	0.778	5.575
1.104	0.280	0.922	-5.044	0.317	3.531

2.395	0.024	0.543	-3.302	0.937	5.097
2.566	0.017	0.500	-3.009	0.925	2.759
2.050	0.051	0.642	-3.859	0.454	4.310
2.590	0.016	0.500	-2.967	0.705	7.070
1.539	0.136	0.795	-4.570	0.390	3.728
2.710	0.012	0.488	-2.754	0.696	6.145
0.489	0.629	0.999	-5.468	0.158	4.402
0.084	0.934	0.999	-5.571	0.020	4.926
0.818	0.421	0.972	-5.280	0.170	2.760
0.942	0.355	0.962	-5.185	0.360	6.109
0.649	0.522	0.990	-5.387	0.278	7.271
0.882	0.386	0.964	-5.233	0.250	3.920
-1.359	0.186	0.843	-4.783	-0.332	3.629
0.897	0.378	0.963	-5.221	0.272	6.164
1.964	0.061	0.663	-3.989	0.512	3.247
1.623	0.117	0.776	-4.463	0.374	4.122
1.923	0.066	0.676	-4.049	0.458	5.935
-1.078	0.291	0.933	-5.068	-0.246	4.861
-0.413	0.683	0.999	-5.498	-0.107	5.791
-0.315	0.756	0.999	-5.530	-0.078	4.791
0.271	0.789	0.999	-5.541	0.080	2.473
1.234	0.229	0.890	-4.917	0.407	4.450
-1.157	0.258	0.913	-4.994	-0.293	2.600
0.764	0.452	0.980	-5.317	0.273	3.935
-0.250	0.804	0.999	-5.546	-0.101	2.905
1.245	0.225	0.886	-4.905	0.367	6.069
0.453	0.654	0.999	-5.483	0.196	4.447
-1.573	0.128	0.789	-4.528	-0.551	4.252
1.258	0.220	0.880	-4.891	0.656	6.389
0.977	0.338	0.957	-5.157	0.387	7.293
0.183	0.856	0.999	-5.559	0.054	4.564
-0.135	0.893	0.999	-5.566	-0.027	6.799
0.897	0.378	0.963	-5.221	0.273	6.646
-0.466	0.645	0.999	-5.477	-0.128	6.240
-0.982	0.335	0.957	-5.152	-0.306	1.643
-0.177	0.861	0.999	-5.560	-0.103	1.547
1.672	0.107	0.764	-4.399	0.554	4.468
1.855	0.075	0.704	-4.148	0.643	4.381
-0.064	0.949	0.999	-5.572	-0.016	4.519
0.635	0.531	0.992	-5.395	0.156	6.134
0.326	0.747	0.999	-5.527	0.134	7.853
0.950	0.351	0.961	-5.179	0.252	5.530
0.542	0.593	0.999	-5.444	0.162	2.755
0.502	0.620	0.999	-5.462	0.103	2.572
1.055	0.302	0.941	-5.089	0.225	5.189
1.414	0.170	0.825	-4.720	0.383	3.450
1.717	0.098	0.757	-4.340	0.431	5.126
1.628	0.116	0.776	-4.457	0.441	6.658
0.930	0.361	0.963	-5.195	0.198	4.484
-0.727	0.474	0.984	-5.341	-0.264	5.065

-1.515	0.142	0.802	-4.600	-0.322	3.133
-1.503	0.145	0.804	-4.614	-0.409	3.943
0.732	0.471	0.983	-5.337	0.194	5.241
0.125	0.901	0.999	-5.567	0.025	6.319
0.197	0.845	0.999	-5.557	0.048	5.212
1.688	0.104	0.764	-4.378	0.420	4.351
-0.085	0.933	0.999	-5.571	-0.040	3.528
-0.890	0.382	0.964	-5.226	-0.219	3.146
1.102	0.281	0.922	-5.046	0.310	5.756
1.811	0.082	0.722	-4.210	0.438	5.453
0.995	0.329	0.955	-5.142	0.297	4.228
0.140	0.889	0.999	-5.565	0.054	5.537
0.516	0.611	0.999	-5.456	0.122	4.203
-0.110	0.913	0.999	-5.569	-0.025	3.420
-0.944	0.354	0.962	-5.184	-0.253	2.785
-2.100	0.046	0.630	-3.781	-0.543	3.345
-0.734	0.469	0.983	-5.336	-0.239	3.743
-0.381	0.706	0.999	-5.509	-0.148	2.967
-0.471	0.642	0.999	-5.475	-0.132	3.512
-0.651	0.521	0.990	-5.387	-0.128	5.096
0.192	0.849	0.999	-5.557	0.045	4.235
-0.535	0.597	0.999	-5.447	-0.150	2.242
1.116	0.275	0.921	-5.033	0.475	7.600
0.690	0.497	0.987	-5.364	0.164	3.789
0.734	0.470	0.983	-5.336	0.202	4.595
-1.557	0.132	0.791	-4.547	-0.623	2.196
0.288	0.776	0.999	-5.537	0.138	4.134
-0.295	0.771	0.999	-5.535	-0.082	4.907
1.391	0.177	0.835	-4.747	0.335	5.577
-1.904	0.068	0.684	-4.077	-0.494	2.801
0.181	0.858	0.999	-5.559	0.050	3.562
1.330	0.196	0.853	-4.815	0.338	3.002
1.603	0.121	0.784	-4.489	0.427	3.383
0.375	0.711	0.999	-5.511	0.063	4.896
-0.718	0.479	0.984	-5.346	-0.289	5.348
1.840	0.078	0.709	-4.169	0.376	4.568
0.144	0.886	0.999	-5.565	0.033	2.672
-0.161	0.874	0.999	-5.562	-0.046	5.525
1.079	0.291	0.933	-5.067	0.238	6.748
-1.917	0.067	0.682	-4.058	-0.468	3.789
2.724	0.012	0.485	-2.727	0.558	3.241
3.142	0.004	0.352	-1.950	0.885	3.106
0.066	0.948	0.999	-5.572	0.018	2.638
1.153	0.260	0.914	-4.998	0.381	2.862
-0.119	0.906	0.999	-5.568	-0.031	2.704
0.611	0.546	0.994	-5.408	0.162	7.426
-0.557	0.583	0.999	-5.436	-0.198	3.245
-0.170	0.866	0.999	-5.561	-0.060	6.817
1.037	0.309	0.943	-5.105	0.412	6.934
-0.736	0.469	0.983	-5.335	-0.272	5.058

-0.751	0.460	0.983	-5.325	-0.199	3.904
-0.531	0.600	0.999	-5.449	-0.133	8.019
0.162	0.872	0.999	-5.562	0.040	8.123
0.162	0.872	0.999	-5.562	0.050	2.386
1.079	0.291	0.933	-5.067	0.404	4.300
1.019	0.318	0.949	-5.121	0.244	5.653
0.131	0.897	0.999	-5.566	0.040	4.641
0.143	0.888	0.999	-5.565	0.062	2.459
0.489	0.629	0.999	-5.468	0.107	6.026
1.971	0.060	0.663	-3.977	0.367	6.513
0.842	0.408	0.969	-5.263	0.286	6.501
1.352	0.188	0.847	-4.790	0.293	5.871
1.170	0.253	0.913	-4.981	0.584	7.357
1.746	0.093	0.750	-4.300	0.431	4.900
1.013	0.321	0.950	-5.126	0.383	6.007
2.724	0.012	0.485	-2.727	0.730	3.788
1.504	0.145	0.803	-4.612	0.327	2.644
0.351	0.728	0.999	-5.519	0.131	2.475
0.586	0.563	0.997	-5.422	0.152	4.035
0.821	0.419	0.972	-5.277	0.210	3.471
1.350	0.189	0.848	-4.793	0.354	3.345
-0.910	0.371	0.963	-5.211	-0.225	3.120
1.202	0.241	0.902	-4.949	0.357	4.454
2.116	0.044	0.630	-3.756	0.709	6.210
-0.672	0.508	0.988	-5.374	-0.227	2.449
1.730	0.096	0.754	-4.321	0.526	3.853
1.812	0.082	0.722	-4.209	0.387	6.314
2.047	0.051	0.644	-3.863	0.887	8.152
-0.241	0.812	0.999	-5.548	-0.075	7.891
-0.826	0.417	0.970	-5.274	-0.220	3.182
0.799	0.432	0.973	-5.293	0.305	5.638
3.703	0.001	0.301	-0.854	1.049	4.609
0.738	0.467	0.983	-5.333	0.162	3.111
2.916	0.007	0.412	-2.376	0.955	3.471
-0.278	0.784	0.999	-5.540	-0.055	6.431
-0.616	0.543	0.994	-5.406	-0.178	2.628
-0.207	0.837	0.999	-5.555	-0.051	1.927
-0.159	0.875	0.999	-5.563	-0.044	2.010
-1.241	0.226	0.888	-4.909	-0.336	5.175
-0.395	0.696	0.999	-5.505	-0.071	5.622
-1.600	0.122	0.784	-4.493	-0.406	4.372
-0.590	0.560	0.996	-5.420	-0.138	4.175
0.384	0.704	0.999	-5.508	0.093	4.745
-0.274	0.787	0.999	-5.541	-0.067	4.907
-0.191	0.850	0.999	-5.558	-0.044	4.750
0.276	0.785	0.999	-5.540	0.069	5.361
-1.713	0.099	0.757	-4.345	-0.465	2.554
-0.238	0.814	0.999	-5.549	-0.052	5.396
0.166	0.869	0.999	-5.562	0.054	6.469
0.181	0.858	0.999	-5.559	0.039	6.251

0.814	0.423	0.972	-5.282	0.234	6.106
0.550	0.587	0.999	-5.440	0.116	3.968
0.979	0.337	0.957	-5.155	0.175	6.168
1.935	0.064	0.673	-4.032	0.400	6.408
0.315	0.756	0.999	-5.530	0.092	5.608
0.272	0.788	0.999	-5.541	0.080	3.171
1.240	0.227	0.888	-4.911	0.336	5.426
-0.072	0.943	0.999	-5.572	-0.019	3.638
0.816	0.422	0.972	-5.281	0.217	4.472
-1.583	0.126	0.786	-4.515	-0.614	8.222
1.898	0.069	0.687	-4.085	0.450	4.416
1.474	0.153	0.813	-4.650	0.547	4.714
0.331	0.743	0.999	-5.525	0.093	2.578
-0.309	0.760	0.999	-5.531	-0.099	2.713
0.283	0.779	0.999	-5.538	0.078	2.987
0.313	0.757	0.999	-5.530	0.082	4.386
-0.377	0.709	0.999	-5.511	-0.095	4.123
0.910	0.371	0.963	-5.211	0.257	6.124
0.677	0.505	0.988	-5.371	0.136	8.649
-0.655	0.518	0.990	-5.384	-0.256	8.206
-0.179	0.859	0.999	-5.560	-0.048	6.944
1.274	0.214	0.874	-4.874	0.448	2.791
-2.258	0.033	0.594	-3.530	-0.541	2.009
2.084	0.047	0.630	-3.806	0.599	5.602
-1.552	0.133	0.791	-4.554	-0.406	8.307
-0.285	0.778	0.999	-5.538	-0.106	3.666
0.264	0.794	0.999	-5.543	0.073	2.860
0.675	0.506	0.988	-5.372	0.222	2.459
0.878	0.388	0.964	-5.236	0.234	4.237
1.151	0.261	0.914	-5.000	0.376	4.349
-0.297	0.769	0.999	-5.535	-0.082	3.018
-0.488	0.630	0.999	-5.468	-0.104	2.919
0.637	0.530	0.992	-5.394	0.166	5.371
-2.432	0.022	0.541	-3.240	-0.485	2.815
1.423	0.167	0.825	-4.709	0.427	2.547
-1.990	0.058	0.660	-3.950	-1.020	2.214
-2.468	0.021	0.517	-3.179	-0.600	7.817
2.083	0.048	0.630	-3.808	0.537	3.042
-1.229	0.231	0.894	-4.922	-0.237	1.970
1.403	0.173	0.828	-4.733	0.506	5.253
0.610	0.547	0.994	-5.409	0.153	4.475
-1.073	0.293	0.935	-5.073	-0.296	2.005
0.763	0.453	0.981	-5.318	0.186	6.449
-2.965	0.007	0.400	-2.285	-0.842	2.455
0.156	0.877	0.999	-5.563	0.045	3.862
0.838	0.410	0.970	-5.265	0.325	3.354
0.451	0.656	0.999	-5.483	0.166	5.164
1.015	0.320	0.950	-5.125	0.281	2.885
-0.224	0.824	0.999	-5.551	-0.071	2.544
-0.219	0.828	0.999	-5.553	-0.079	3.853

0.612	0.546	0.994	-5.408	0.166	3.282
-0.615	0.544	0.994	-5.407	-0.222	3.412
-0.339	0.737	0.999	-5.523	-0.088	2.961
-0.222	0.826	0.999	-5.552	-0.065	4.774
2.893	0.008	0.422	-2.419	0.867	4.197
1.786	0.086	0.731	-4.245	0.668	6.608
0.305	0.763	0.999	-5.533	0.091	2.864
1.006	0.324	0.952	-5.132	0.349	4.183
-0.545	0.591	0.999	-5.442	-0.205	2.783
0.499	0.622	0.999	-5.464	0.304	2.977
0.799	0.432	0.973	-5.293	0.213	2.519
0.884	0.385	0.964	-5.231	0.233	3.090
0.740	0.466	0.983	-5.332	0.387	2.653
0.900	0.377	0.963	-5.219	0.341	2.594
3.718	0.001	0.301	-0.826	0.907	4.725
0.110	0.913	0.999	-5.569	0.038	6.798
0.441	0.663	0.999	-5.488	0.126	2.735
1.668	0.108	0.764	-4.405	0.472	3.934
1.833	0.079	0.712	-4.179	0.488	6.688
0.842	0.408	0.969	-5.263	0.170	5.565
0.390	0.700	0.999	-5.506	0.098	3.759
0.094	0.926	0.999	-5.570	0.021	3.722
0.092	0.928	0.999	-5.570	0.028	7.268
2.204	0.037	0.610	-3.617	0.708	5.911
0.989	0.332	0.957	-5.147	0.275	5.330
2.496	0.019	0.514	-3.129	0.518	3.753
0.434	0.668	0.999	-5.490	0.089	4.877
0.762	0.453	0.981	-5.318	0.169	5.552
0.733	0.471	0.983	-5.337	0.297	3.693
1.326	0.197	0.855	-4.819	0.385	2.904
0.199	0.844	0.999	-5.556	0.055	4.651
0.577	0.569	0.997	-5.426	0.143	5.214
-1.663	0.109	0.766	-4.411	-0.506	3.510
-1.411	0.171	0.826	-4.724	-0.471	4.143
0.310	0.759	0.999	-5.531	0.063	6.348
0.457	0.652	0.999	-5.481	0.103	6.017
0.922	0.365	0.963	-5.202	0.194	3.534
0.926	0.363	0.963	-5.198	0.208	5.203
0.746	0.463	0.983	-5.329	0.201	3.269
0.755	0.457	0.981	-5.323	0.168	4.089
-0.262	0.796	0.999	-5.543	-0.058	3.779
-1.428	0.166	0.825	-4.703	-0.511	3.580
0.334	0.741	0.999	-5.524	0.071	3.102
1.372	0.182	0.840	-4.768	0.448	2.171
0.508	0.616	0.999	-5.459	0.169	3.206
0.209	0.836	0.999	-5.554	0.117	3.567
-0.652	0.520	0.990	-5.386	-0.165	1.971
0.628	0.536	0.993	-5.399	0.155	4.371
0.366	0.717	0.999	-5.514	0.128	4.356
-0.145	0.886	0.999	-5.565	-0.031	6.675

-0.751	0.459	0.983	-5.325	-0.318	3.694
0.689	0.497	0.987	-5.364	0.148	4.534
0.796	0.434	0.973	-5.295	0.159	6.039
-0.138	0.892	0.999	-5.566	-0.028	4.582
1.844	0.077	0.707	-4.164	0.540	5.239
-0.778	0.444	0.979	-5.307	-0.214	5.327
-1.419	0.168	0.825	-4.714	-0.329	3.751
-0.349	0.730	0.999	-5.520	-0.119	3.237
1.425	0.166	0.825	-4.707	0.325	6.043
-0.167	0.869	0.999	-5.561	-0.039	4.344
-1.858	0.075	0.703	-4.144	-0.480	3.517
0.940	0.356	0.962	-5.187	0.231	3.281
-0.443	0.662	0.999	-5.487	-0.236	5.433
1.002	0.326	0.954	-5.136	0.284	3.112
0.307	0.762	0.999	-5.532	0.121	6.100
1.712	0.099	0.757	-4.347	0.393	4.774
-1.068	0.296	0.936	-5.077	-0.304	3.857
2.982	0.006	0.393	-2.254	0.594	6.729
0.549	0.588	0.999	-5.440	0.115	4.754
0.927	0.363	0.963	-5.197	0.203	5.748
-0.072	0.943	0.999	-5.572	-0.021	6.171
-0.106	0.916	0.999	-5.569	-0.039	3.554
1.080	0.290	0.932	-5.067	0.205	3.982
0.828	0.416	0.970	-5.273	0.213	4.319
-1.136	0.267	0.917	-5.014	-0.270	3.368
-0.261	0.796	0.999	-5.544	-0.083	6.451
-0.446	0.660	0.999	-5.486	-0.110	6.874
0.176	0.862	0.999	-5.560	0.040	7.158
-1.654	0.111	0.767	-4.424	-0.353	4.283
0.895	0.379	0.964	-5.223	0.238	2.714
-0.594	0.558	0.995	-5.418	-0.184	6.003
-0.127	0.900	0.999	-5.567	-0.041	3.025
-0.378	0.708	0.999	-5.510	-0.083	4.976
0.360	0.722	0.999	-5.516	0.077	7.005
-1.783	0.087	0.731	-4.249	-0.513	4.442
0.813	0.424	0.972	-5.283	0.162	5.800
-1.248	0.223	0.886	-4.902	-0.297	2.988
0.402	0.691	0.999	-5.502	0.075	5.709
-2.074	0.049	0.634	-3.822	-0.562	3.018
-1.923	0.066	0.676	-4.049	-0.474	3.348
-0.387	0.702	0.999	-5.507	-0.101	2.251
1.497	0.147	0.804	-4.621	0.336	5.343
-0.220	0.827	0.999	-5.552	-0.052	2.958
1.981	0.059	0.663	-3.963	0.491	5.530
-0.340	0.737	0.999	-5.523	-0.094	2.716
0.327	0.746	0.999	-5.526	0.101	4.477
-0.396	0.695	0.999	-5.504	-0.104	4.558
-0.090	0.929	0.999	-5.570	-0.023	4.674
-2.666	0.013	0.498	-2.832	-0.636	2.313
0.674	0.507	0.988	-5.373	0.144	3.270

1.976	0.059	0.663	-3.971	0.518	5.923
0.490	0.628	0.999	-5.467	0.100	5.663
0.241	0.812	0.999	-5.548	0.133	5.124
0.989	0.332	0.957	-5.146	0.391	2.543
1.715	0.099	0.757	-4.342	0.382	4.874
0.283	0.780	0.999	-5.538	0.086	3.694
0.505	0.618	0.999	-5.461	0.130	6.358
0.736	0.469	0.983	-5.335	0.175	7.313
-0.675	0.506	0.988	-5.372	-0.247	4.255
-0.583	0.565	0.997	-5.423	-0.172	4.676
-0.528	0.602	0.999	-5.450	-0.282	4.094
0.646	0.524	0.991	-5.389	0.184	4.675
1.296	0.207	0.868	-4.851	0.498	3.635
-0.813	0.424	0.972	-5.283	-0.175	4.239
-0.064	0.950	0.999	-5.572	-0.029	3.560
0.956	0.348	0.959	-5.174	0.421	2.988
0.890	0.382	0.964	-5.226	0.986	4.635
-0.543	0.592	0.999	-5.443	-0.118	5.932
0.065	0.948	0.999	-5.572	0.024	8.226
-0.900	0.377	0.963	-5.219	-0.228	6.491
0.101	0.920	0.999	-5.569	0.027	2.509
-0.914	0.369	0.963	-5.208	-0.278	2.088
-3.163	0.004	0.340	-1.910	-0.977	2.137
0.594	0.558	0.995	-5.418	0.214	2.866
0.428	0.672	0.999	-5.493	0.188	2.821
-0.489	0.629	0.999	-5.468	-0.120	8.055
1.111	0.277	0.921	-5.038	0.307	5.425
1.567	0.130	0.789	-4.535	0.485	5.362
1.684	0.105	0.764	-4.384	0.394	6.347
0.646	0.524	0.991	-5.390	0.218	3.405
-0.399	0.693	0.999	-5.503	-0.084	4.277
0.097	0.924	0.999	-5.570	0.033	5.270
0.826	0.417	0.970	-5.274	0.217	3.526
1.639	0.114	0.775	-4.442	0.494	4.923
0.973	0.340	0.957	-5.160	0.860	3.970
0.903	0.375	0.963	-5.216	0.855	3.988
0.423	0.676	0.999	-5.495	0.089	5.009
0.285	0.778	0.999	-5.538	0.104	9.047
0.479	0.636	0.999	-5.472	0.109	4.940
2.169	0.040	0.616	-3.673	0.431	4.935
0.405	0.689	0.999	-5.501	0.178	3.839
2.051	0.051	0.641	-3.857	0.834	9.596
-0.978	0.337	0.957	-5.156	-0.282	2.349
1.017	0.319	0.950	-5.122	0.193	4.166
-0.517	0.610	0.999	-5.455	-0.109	2.095
0.827	0.416	0.970	-5.273	0.234	5.965
-0.059	0.953	0.999	-5.572	-0.015	4.870
1.912	0.067	0.682	-4.065	0.645	5.232
-1.210	0.237	0.899	-4.941	-0.281	4.964
0.541	0.593	0.999	-5.444	0.142	6.747

0.634	0.532	0.992	-5.396	0.184	7.540
-0.380	0.707	0.999	-5.510	-0.075	5.253
-0.121	0.905	0.999	-5.567	-0.027	5.844
-1.059	0.300	0.941	-5.085	-0.256	5.719
-0.475	0.639	0.999	-5.474	-0.159	2.645
-0.611	0.546	0.994	-5.408	-0.319	4.246
1.908	0.068	0.684	-4.071	0.399	4.087
0.191	0.850	0.999	-5.558	0.058	2.584
1.397	0.175	0.828	-4.739	0.296	6.665
-0.297	0.769	0.999	-5.535	-0.061	3.188
1.081	0.290	0.932	-5.066	0.208	7.407
0.718	0.480	0.984	-5.347	0.137	7.130
-1.086	0.288	0.929	-5.061	-0.239	4.459
-1.822	0.080	0.718	-4.194	-0.542	4.149
-0.182	0.857	0.999	-5.559	-0.048	2.954
2.461	0.021	0.521	-3.190	0.681	5.343
-0.836	0.411	0.970	-5.267	-0.207	6.644
1.329	0.196	0.854	-4.815	0.257	4.874
0.636	0.531	0.992	-5.395	0.157	3.393
0.773	0.446	0.979	-5.310	0.287	4.347
0.626	0.537	0.993	-5.401	0.174	6.128
4.167	0.000	0.257	0.071	1.072	6.524
0.074	0.941	0.999	-5.571	0.023	3.573
1.822	0.080	0.718	-4.194	0.367	3.868
0.138	0.891	0.999	-5.565	0.059	9.948
1.554	0.133	0.791	-4.552	0.362	6.179
-0.615	0.544	0.994	-5.406	-0.135	4.646
-1.844	0.077	0.707	-4.163	-0.480	3.582
1.076	0.292	0.934	-5.070	0.303	6.057
2.434	0.022	0.540	-3.236	0.808	3.889
1.431	0.165	0.824	-4.700	0.418	6.347
-0.134	0.894	0.999	-5.566	-0.077	4.830
0.910	0.372	0.963	-5.211	0.535	5.348
-0.124	0.903	0.999	-5.567	-0.036	4.383
-0.590	0.560	0.996	-5.420	-0.287	2.684
-0.494	0.626	0.999	-5.466	-0.136	6.544
0.727	0.474	0.984	-5.341	0.192	2.818
-0.102	0.920	0.999	-5.569	-0.050	3.714
0.128	0.900	0.999	-5.567	0.040	5.052
-0.273	0.787	0.999	-5.541	-0.115	5.731
1.397	0.175	0.828	-4.739	0.336	4.501
1.863	0.074	0.700	-4.136	0.419	4.739
0.741	0.466	0.983	-5.332	0.171	5.680
0.517	0.609	0.999	-5.455	0.143	3.069
1.490	0.149	0.806	-4.630	0.329	3.656
1.394	0.176	0.831	-4.743	0.304	3.744
0.917	0.368	0.963	-5.205	0.158	8.341
-0.527	0.603	0.999	-5.451	-0.128	4.339
-0.333	0.742	0.999	-5.525	-0.085	5.736
-1.288	0.209	0.868	-4.860	-0.451	3.818

0.311	0.759	0.999	-5.531	0.063	7.242
-0.300	0.767	0.999	-5.534	-0.079	4.476
-0.184	0.855	0.999	-5.559	-0.049	4.322
0.080	0.937	0.999	-5.571	0.021	4.550
1.029	0.313	0.944	-5.112	0.301	4.281
1.198	0.242	0.904	-4.953	0.242	6.432
0.686	0.499	0.987	-5.366	0.224	3.176
1.287	0.210	0.868	-4.861	0.304	4.589
1.826	0.080	0.717	-4.189	0.345	7.451
1.288	0.209	0.868	-4.860	0.466	3.687
0.188	0.852	0.999	-5.558	0.033	5.795
-1.187	0.246	0.906	-4.964	-0.351	2.441
0.251	0.804	0.999	-5.546	0.058	6.974
-0.148	0.884	0.999	-5.564	-0.042	8.080
0.712	0.483	0.985	-5.350	0.793	4.761
2.405	0.024	0.543	-3.285	0.592	3.498
1.248	0.223	0.886	-4.902	0.265	5.148
0.472	0.641	0.999	-5.475	0.108	7.483
-1.558	0.132	0.791	-4.546	-0.693	2.498
1.067	0.296	0.936	-5.078	1.056	3.690
-0.799	0.432	0.973	-5.293	-0.234	4.204
-2.104	0.046	0.630	-3.775	-0.744	7.192
0.654	0.519	0.990	-5.385	0.175	8.122
0.725	0.475	0.984	-5.342	0.178	6.306
-0.093	0.927	0.999	-5.570	-0.020	5.758
2.041	0.052	0.649	-3.873	0.695	5.690
-1.096	0.284	0.925	-5.052	-0.259	5.100
0.163	0.872	0.999	-5.562	0.039	5.785
0.825	0.417	0.970	-5.275	0.190	6.110
1.029	0.314	0.944	-5.113	0.240	7.313
-0.844	0.407	0.969	-5.261	-0.320	8.119
1.293	0.208	0.868	-4.854	0.413	5.649
-0.451	0.656	0.999	-5.484	-0.102	2.101
0.159	0.875	0.999	-5.563	0.045	2.095
0.725	0.475	0.984	-5.342	0.268	5.772
0.334	0.741	0.999	-5.524	0.107	7.507
0.362	0.720	0.999	-5.516	0.099	2.535
2.841	0.009	0.441	-2.515	0.784	6.537
1.691	0.103	0.764	-4.374	0.357	6.363
0.491	0.628	0.999	-5.467	0.107	3.581
1.754	0.092	0.745	-4.289	0.521	3.818
-0.239	0.813	0.999	-5.548	-0.046	8.313
-0.634	0.532	0.992	-5.396	-0.324	5.837
1.163	0.256	0.913	-4.988	0.294	3.520
0.097	0.924	0.999	-5.570	0.021	6.400
0.671	0.509	0.988	-5.375	0.249	4.426
-0.435	0.667	0.999	-5.490	-0.109	3.258
1.552	0.133	0.791	-4.554	0.344	6.879
0.706	0.487	0.985	-5.354	0.213	5.903
0.146	0.885	0.999	-5.564	0.061	2.696

-0.378	0.709	0.999	-5.510	-0.153	2.853
-0.533	0.598	0.999	-5.448	-0.148	4.885
2.099	0.046	0.630	-3.783	0.476	3.248
0.619	0.542	0.994	-5.405	0.114	3.833
0.344	0.734	0.999	-5.521	0.155	2.706
1.677	0.106	0.764	-4.393	0.711	3.106
1.268	0.216	0.876	-4.881	0.294	5.847
0.372	0.713	0.999	-5.512	0.093	5.664
0.612	0.546	0.994	-5.408	0.135	4.064
1.159	0.257	0.913	-4.992	0.250	5.231
1.729	0.096	0.755	-4.323	0.331	8.650
0.749	0.461	0.983	-5.327	0.163	5.864
0.287	0.776	0.999	-5.537	0.061	3.125
0.512	0.613	0.999	-5.458	0.183	4.497
0.820	0.420	0.972	-5.278	0.239	2.343
0.504	0.619	0.999	-5.461	0.277	6.474
-0.640	0.528	0.992	-5.393	-0.302	1.238
-0.277	0.784	0.999	-5.540	-0.196	4.238
0.379	0.708	0.999	-5.510	0.214	1.947
-0.372	0.713	0.999	-5.512	-0.077	5.570
1.118	0.274	0.921	-5.032	0.507	4.125
-1.015	0.320	0.950	-5.124	-0.256	2.135
-0.708	0.485	0.985	-5.353	-0.174	3.028
1.010	0.322	0.950	-5.128	0.204	6.721
-0.446	0.659	0.999	-5.485	-0.226	6.690
-2.640	0.014	0.498	-2.879	-0.749	1.968
0.263	0.795	0.999	-5.543	0.106	4.456
-0.240	0.812	0.999	-5.548	-0.114	5.576
0.581	0.566	0.997	-5.424	0.193	5.580
-3.510	0.002	0.301	-1.236	-1.253	3.663
1.650	0.111	0.768	-4.428	0.456	5.895
-2.388	0.025	0.547	-3.314	-0.658	1.891
-0.808	0.427	0.973	-5.287	-0.249	2.940
-0.781	0.442	0.978	-5.305	-0.221	2.874
-0.422	0.677	0.999	-5.495	-0.118	2.581
-0.942	0.355	0.962	-5.185	-0.400	2.516
0.392	0.698	0.999	-5.506	0.103	6.293
0.232	0.818	0.999	-5.550	0.046	4.624
1.553	0.133	0.791	-4.552	0.437	5.591
-0.328	0.746	0.999	-5.526	-0.095	5.589
0.458	0.651	0.999	-5.481	0.110	8.964
-2.545	0.017	0.500	-3.046	-0.607	2.950
-1.799	0.084	0.724	-4.226	-0.631	5.459
0.998	0.328	0.954	-5.139	0.238	5.823
1.002	0.326	0.954	-5.135	0.221	7.264
-0.640	0.528	0.992	-5.393	-0.147	2.027
0.570	0.574	0.999	-5.430	0.099	7.887
1.193	0.244	0.904	-4.958	0.345	3.157
-0.682	0.501	0.988	-5.368	-0.134	6.585
0.582	0.566	0.997	-5.424	0.329	4.349

0.679	0.503	0.988	-5.370	0.163	7.037
3.034	0.006	0.393	-2.156	0.678	5.223
1.320	0.199	0.857	-4.825	0.359	3.820
1.155	0.259	0.913	-4.996	0.324	3.479
0.358	0.723	0.999	-5.517	0.093	7.016
0.195	0.847	0.999	-5.557	0.061	3.980
-3.433	0.002	0.301	-1.388	-1.596	3.322
1.142	0.264	0.916	-5.008	0.375	5.943
-0.237	0.815	0.999	-5.549	-0.050	5.752
1.815	0.081	0.720	-4.204	0.382	5.682
1.877	0.072	0.698	-4.115	0.398	6.306
-0.281	0.781	0.999	-5.539	-0.061	5.789
-2.858	0.008	0.441	-2.484	-1.063	2.714
1.057	0.300	0.941	-5.087	0.278	6.855
-1.205	0.240	0.901	-4.947	-0.436	7.363
-3.243	0.003	0.340	-1.757	-0.760	4.185
0.908	0.373	0.963	-5.213	0.196	3.755
1.774	0.088	0.734	-4.261	0.488	4.687
-2.578	0.016	0.500	-2.987	-1.185	2.692
-2.238	0.034	0.597	-3.563	-0.614	2.015
0.790	0.437	0.973	-5.299	0.146	7.159
-0.316	0.755	0.999	-5.530	-0.078	3.085
0.198	0.845	0.999	-5.556	0.043	4.493
-2.561	0.017	0.500	-3.018	-1.307	3.552
0.652	0.520	0.990	-5.386	0.125	3.418
0.354	0.726	0.999	-5.518	0.106	3.754
0.208	0.837	0.999	-5.555	0.070	4.774
-1.366	0.184	0.841	-4.774	-0.796	2.801
0.788	0.438	0.975	-5.301	0.282	5.354
-0.428	0.672	0.999	-5.493	-0.084	5.716
1.475	0.153	0.813	-4.648	0.607	4.212
1.145	0.263	0.914	-5.005	0.254	6.939
-1.064	0.297	0.938	-5.081	-0.257	2.596
-0.365	0.718	0.999	-5.515	-0.075	8.671
0.096	0.924	0.999	-5.570	0.031	5.247
-0.407	0.687	0.999	-5.500	-0.096	3.880
0.073	0.942	0.999	-5.572	0.023	2.892
1.478	0.152	0.813	-4.644	0.481	3.957
-1.218	0.235	0.899	-4.933	-0.222	3.608
-0.992	0.331	0.956	-5.144	-0.303	3.585
0.121	0.904	0.999	-5.567	0.034	3.096
0.466	0.645	0.999	-5.477	0.138	9.012
-0.672	0.508	0.988	-5.375	-0.147	5.590
0.190	0.851	0.999	-5.558	0.055	2.297
-1.942	0.063	0.671	-4.021	-0.352	6.034
0.275	0.786	0.999	-5.540	0.062	5.175
1.622	0.117	0.776	-4.465	0.490	6.760
1.092	0.285	0.927	-5.056	0.258	6.569
1.516	0.142	0.802	-4.599	0.391	3.011
-0.117	0.908	0.999	-5.568	-0.036	3.089

-0.698	0.491	0.986	-5.359	-1.341	4.192
1.293	0.208	0.868	-4.854	0.530	3.408
0.804	0.429	0.973	-5.289	0.305	4.530
0.549	0.588	0.999	-5.440	0.131	3.556
-0.220	0.827	0.999	-5.552	-0.065	2.945
-0.195	0.847	0.999	-5.557	-0.054	2.050
-0.745	0.463	0.983	-5.329	-0.160	3.003
-0.381	0.707	0.999	-5.509	-0.098	2.483
0.315	0.756	0.999	-5.530	0.079	6.103
0.929	0.362	0.963	-5.196	0.180	7.292
1.539	0.136	0.795	-4.570	0.410	4.632
1.959	0.061	0.665	-3.997	0.509	2.610
-0.553	0.585	0.999	-5.438	-0.141	4.660
-0.269	0.790	0.999	-5.542	-0.065	3.894
-0.818	0.421	0.972	-5.280	-0.192	2.241
0.634	0.532	0.992	-5.396	0.253	4.568
0.919	0.367	0.963	-5.204	0.261	4.336
-1.097	0.283	0.924	-5.050	-0.301	3.297
1.797	0.084	0.726	-4.229	0.454	2.559
0.392	0.698	0.999	-5.506	0.076	5.754
-0.300	0.766	0.999	-5.534	-0.060	4.403
-0.198	0.844	0.999	-5.556	-0.042	8.737
2.094	0.046	0.630	-3.790	0.693	5.775
1.001	0.326	0.954	-5.136	0.292	2.894
1.179	0.249	0.910	-4.972	0.297	4.846
-0.357	0.724	0.999	-5.517	-0.074	5.303
1.431	0.165	0.824	-4.700	0.298	6.495
1.273	0.215	0.874	-4.876	0.255	7.239
0.504	0.619	0.999	-5.461	0.141	3.642
0.240	0.812	0.999	-5.548	0.089	4.071
-0.947	0.353	0.961	-5.182	-0.239	3.483
0.346	0.732	0.999	-5.521	0.124	4.194
-0.797	0.433	0.973	-5.294	-0.226	3.160
-0.527	0.603	0.999	-5.451	-0.113	7.205
1.597	0.123	0.785	-4.497	0.397	3.522
2.958	0.007	0.400	-2.297	0.638	7.342
0.229	0.821	0.999	-5.551	0.044	6.460
-0.595	0.557	0.995	-5.417	-0.177	8.940
1.068	0.295	0.936	-5.077	0.265	4.287
-0.405	0.689	0.999	-5.501	-0.117	3.127
0.309	0.760	0.999	-5.531	0.066	5.487
0.969	0.342	0.957	-5.163	0.242	5.674
-2.251	0.033	0.595	-3.541	-1.406	1.752
0.289	0.775	0.999	-5.537	0.059	3.577
-0.785	0.440	0.976	-5.303	-0.340	1.895
-1.695	0.102	0.764	-4.368	-0.393	2.897
-0.664	0.513	0.990	-5.379	-0.275	3.594
-0.209	0.836	0.999	-5.554	-0.068	6.021
-0.588	0.562	0.996	-5.421	-0.139	5.314
-0.717	0.480	0.984	-5.347	-0.199	6.656

1.376	0.181	0.840	-4.764	0.580	3.823
1.879	0.072	0.698	-4.114	0.405	5.525
2.826	0.009	0.446	-2.542	0.552	5.004
0.609	0.548	0.994	-5.410	0.152	6.084
-0.846	0.405	0.968	-5.259	-0.208	4.245
1.103	0.280	0.922	-5.045	0.340	3.976
1.281	0.212	0.870	-4.868	0.237	6.771
1.798	0.084	0.726	-4.228	0.373	5.026
0.516	0.611	0.999	-5.456	0.122	5.216
0.445	0.660	0.999	-5.486	0.110	4.357
-0.454	0.653	0.999	-5.482	-0.106	3.481
0.501	0.621	0.999	-5.463	0.113	6.195
-0.447	0.659	0.999	-5.485	-0.097	4.191
0.808	0.427	0.973	-5.287	0.204	4.768
-1.069	0.295	0.936	-5.076	-0.295	4.370
1.172	0.252	0.911	-4.979	0.311	7.127
0.388	0.701	0.999	-5.507	0.218	3.760
-0.237	0.815	0.999	-5.549	-0.060	7.323
-1.211	0.237	0.899	-4.940	-0.271	4.476
0.588	0.562	0.996	-5.421	0.164	5.854
-0.195	0.847	0.999	-5.557	-0.062	3.677
0.494	0.625	0.999	-5.465	0.125	4.068
0.422	0.677	0.999	-5.495	0.110	4.051
-0.631	0.534	0.992	-5.398	-0.137	6.904
-0.506	0.618	0.999	-5.460	-0.160	7.258
-0.471	0.642	0.999	-5.475	-0.185	4.006
1.013	0.321	0.950	-5.126	0.242	6.263
0.298	0.768	0.999	-5.534	0.127	2.687
-1.668	0.108	0.764	-4.405	-0.485	2.319
0.494	0.625	0.999	-5.465	0.133	8.506
0.868	0.393	0.964	-5.243	0.211	2.953
-0.207	0.837	0.999	-5.555	-0.040	6.742
1.216	0.235	0.899	-4.935	0.250	4.913
0.518	0.609	0.999	-5.455	0.094	7.392
2.058	0.050	0.640	-3.846	0.493	5.727
-0.082	0.935	0.999	-5.571	-0.022	3.829
0.346	0.732	0.999	-5.521	0.076	3.910
0.815	0.423	0.972	-5.282	0.350	8.908
-0.803	0.430	0.973	-5.290	-0.180	5.101
0.908	0.373	0.963	-5.213	0.311	7.485
0.328	0.745	0.999	-5.526	0.123	3.717
-0.689	0.497	0.987	-5.364	-0.156	4.466
0.274	0.786	0.999	-5.540	0.069	3.291
0.557	0.582	0.999	-5.436	0.162	2.775
0.764	0.452	0.980	-5.317	0.326	2.624
-0.142	0.888	0.999	-5.565	-0.046	7.243
0.640	0.528	0.992	-5.393	0.172	3.088
0.333	0.742	0.999	-5.525	0.095	3.363
-0.411	0.684	0.999	-5.499	-0.093	6.136
-0.087	0.932	0.999	-5.571	-0.024	5.809

2.133	0.043	0.625	-3.730	0.621	5.876
0.224	0.824	0.999	-5.551	0.057	3.711
-0.453	0.655	0.999	-5.483	-0.118	4.595
0.104	0.918	0.999	-5.569	0.023	3.557
1.488	0.149	0.809	-4.633	0.297	4.672
0.723	0.476	0.984	-5.343	0.216	4.000
0.718	0.480	0.984	-5.347	0.265	6.004
2.177	0.039	0.611	-3.660	0.404	7.192
0.447	0.659	0.999	-5.485	0.169	3.785
2.030	0.053	0.653	-3.889	0.521	6.544
-2.308	0.030	0.570	-3.448	-0.664	2.140
0.413	0.683	0.999	-5.498	0.087	3.197
-1.403	0.173	0.828	-4.733	-0.264	5.955
1.648	0.112	0.770	-4.431	0.444	5.280
1.370	0.183	0.840	-4.770	0.380	4.495
0.205	0.840	0.999	-5.555	0.053	4.541
3.408	0.002	0.301	-1.437	0.764	3.593
1.067	0.296	0.936	-5.078	0.215	5.254
-0.155	0.878	0.999	-5.563	-0.034	5.758
-0.403	0.691	0.999	-5.502	-0.101	3.661
0.732	0.471	0.984	-5.338	0.222	3.148
-0.312	0.758	0.999	-5.531	-0.073	4.024
0.180	0.858	0.999	-5.559	0.045	4.142
1.959	0.061	0.665	-3.996	0.561	4.941
0.253	0.802	0.999	-5.545	0.061	3.729
0.697	0.492	0.986	-5.360	0.152	5.377
0.894	0.380	0.964	-5.224	0.283	3.872
2.029	0.053	0.653	-3.890	0.442	3.287
-0.242	0.811	0.999	-5.548	-0.101	1.756
0.710	0.484	0.985	-5.352	0.318	2.561
0.386	0.702	0.999	-5.507	0.107	1.535
0.718	0.479	0.984	-5.346	0.161	4.396
0.174	0.863	0.999	-5.560	0.044	3.601
2.713	0.012	0.488	-2.748	0.769	4.040
2.591	0.016	0.500	-2.964	0.584	3.421
1.843	0.077	0.707	-4.164	0.437	5.249
0.844	0.407	0.969	-5.261	0.238	4.403
0.341	0.736	0.999	-5.522	0.085	3.827
2.874	0.008	0.432	-2.454	0.926	3.293
1.970	0.060	0.663	-3.979	0.592	3.390
1.417	0.169	0.825	-4.716	0.350	4.082
-0.314	0.756	0.999	-5.530	-0.087	2.617
-0.366	0.718	0.999	-5.514	-0.081	5.108
1.646	0.112	0.771	-4.433	0.454	7.222
0.638	0.529	0.992	-5.394	0.154	4.498
0.862	0.397	0.964	-5.248	0.228	3.293
2.194	0.038	0.611	-3.633	0.671	4.369
3.259	0.003	0.340	-1.726	0.705	3.792
-0.172	0.865	0.999	-5.561	-0.038	4.829
0.591	0.559	0.996	-5.419	0.175	4.515

1.150	0.261	0.914	-5.001	0.305	5.501
1.431	0.165	0.824	-4.699	0.336	2.200
-0.199	0.844	0.999	-5.556	-0.055	2.532
0.072	0.943	0.999	-5.572	0.020	4.738
2.535	0.018	0.501	-3.063	0.862	4.007
1.520	0.141	0.801	-4.593	0.477	3.407
-0.133	0.895	0.999	-5.566	-0.031	3.719
2.126	0.044	0.629	-3.741	0.506	3.358
1.461	0.156	0.817	-4.665	0.349	5.815
0.242	0.811	0.999	-5.548	0.057	4.078
0.562	0.579	0.999	-5.434	0.147	2.505
0.720	0.478	0.984	-5.345	0.183	4.248
1.566	0.130	0.789	-4.536	0.332	4.471
0.540	0.594	0.999	-5.444	0.154	3.421
-1.494	0.148	0.805	-4.625	-0.322	6.893
1.641	0.113	0.775	-4.440	0.351	3.799
-0.392	0.698	0.999	-5.506	-0.121	2.778
-0.660	0.515	0.990	-5.382	-0.154	3.411
-1.267	0.217	0.876	-4.883	-0.350	1.786
2.027	0.053	0.653	-3.893	0.479	4.150
-0.413	0.683	0.999	-5.498	-0.102	3.170
-0.354	0.726	0.999	-5.518	-0.071	2.028
0.859	0.398	0.964	-5.250	0.180	5.361
1.912	0.067	0.682	-4.065	0.418	3.872
2.292	0.031	0.580	-3.475	0.532	4.376
-1.474	0.153	0.813	-4.650	-0.311	7.081
-0.372	0.713	0.999	-5.512	-0.193	2.170
0.487	0.630	0.999	-5.469	0.133	1.783
-2.480	0.020	0.517	-3.158	-0.631	4.103
1.202	0.241	0.902	-4.949	0.305	4.854
0.191	0.850	0.999	-5.558	0.051	2.524
-0.802	0.430	0.973	-5.291	-0.182	4.682
1.837	0.078	0.710	-4.173	0.396	3.970
0.938	0.357	0.962	-5.189	0.222	3.471
0.903	0.375	0.963	-5.217	0.170	5.039
1.436	0.163	0.824	-4.694	0.443	4.435
3.626	0.001	0.301	-1.007	0.988	6.236
1.972	0.060	0.663	-3.977	0.515	4.751
0.983	0.335	0.957	-5.151	0.247	3.919
0.708	0.486	0.985	-5.353	0.233	2.600
1.259	0.220	0.880	-4.891	0.408	4.538
0.159	0.875	0.999	-5.563	0.037	5.682
0.709	0.485	0.985	-5.352	0.166	4.349
2.122	0.044	0.630	-3.747	0.448	3.235
-1.049	0.304	0.942	-5.095	-0.259	6.292
0.985	0.334	0.957	-5.150	0.276	4.909
-2.620	0.015	0.498	-2.913	-0.642	1.895
1.066	0.297	0.937	-5.080	0.329	4.945
1.511	0.143	0.803	-4.604	0.372	5.253
0.822	0.419	0.972	-5.276	0.264	3.359

-0.701	0.490	0.986	-5.357	-0.209	3.852
0.570	0.574	0.999	-5.430	0.190	4.662
0.547	0.589	0.999	-5.441	0.172	3.314
0.715	0.481	0.984	-5.348	0.163	3.865
0.364	0.719	0.999	-5.515	0.062	4.756
1.692	0.103	0.764	-4.373	0.335	6.220
1.510	0.144	0.803	-4.606	0.429	5.412
1.222	0.233	0.898	-4.929	0.273	4.343
0.411	0.685	0.999	-5.499	0.117	3.679
0.693	0.495	0.987	-5.362	0.160	5.760
1.203	0.240	0.902	-4.948	0.323	2.664
0.328	0.746	0.999	-5.526	0.121	6.790
1.103	0.280	0.922	-5.045	0.213	3.481
0.870	0.393	0.964	-5.242	0.201	4.405
0.760	0.454	0.981	-5.319	0.226	3.519
0.302	0.765	0.999	-5.533	0.075	3.839
1.735	0.095	0.754	-4.315	0.487	3.830
3.108	0.005	0.354	-2.015	0.715	4.483
0.708	0.485	0.985	-5.352	0.263	2.728
0.283	0.780	0.999	-5.538	0.068	3.235
1.579	0.127	0.787	-4.520	0.387	3.808
-0.703	0.489	0.986	-5.356	-0.203	2.928
-0.409	0.686	0.999	-5.499	-0.121	2.288
0.882	0.386	0.964	-5.233	0.274	4.360
0.159	0.875	0.999	-5.563	0.037	3.562
0.096	0.925	0.999	-5.570	0.019	2.913
0.549	0.588	0.999	-5.440	0.144	4.126
0.094	0.926	0.999	-5.570	0.023	3.519
1.015	0.320	0.950	-5.124	0.261	4.795
-0.148	0.884	0.999	-5.564	-0.036	1.835
2.476	0.020	0.517	-3.164	0.527	5.175
1.521	0.141	0.801	-4.592	0.329	4.723
0.964	0.344	0.957	-5.168	0.214	3.892
0.489	0.629	0.999	-5.468	0.141	3.592
1.939	0.064	0.672	-4.025	0.449	4.159
-0.408	0.687	0.999	-5.500	-0.113	2.200
2.381	0.025	0.548	-3.326	0.566	4.120
0.916	0.368	0.963	-5.206	0.203	3.596
1.414	0.170	0.825	-4.720	0.520	4.979
0.651	0.521	0.990	-5.386	0.164	4.648
0.480	0.635	0.999	-5.472	0.133	7.177
1.718	0.098	0.757	-4.337	0.437	4.641
0.500	0.622	0.999	-5.463	0.100	4.067
0.353	0.727	0.999	-5.519	0.073	7.166
1.456	0.158	0.818	-4.671	0.373	3.642
2.287	0.031	0.584	-3.482	0.563	3.461
1.932	0.065	0.673	-4.035	0.495	2.274
1.177	0.250	0.910	-4.974	0.302	3.062
0.556	0.583	0.999	-5.437	0.138	2.818
0.389	0.700	0.999	-5.507	0.095	3.117

1.174	0.251	0.910	-4.977	0.305	3.214
-0.921	0.366	0.963	-5.202	-0.221	3.036
1.428	0.166	0.825	-4.703	0.357	3.790
1.745	0.093	0.750	-4.301	0.603	2.792
0.659	0.516	0.990	-5.382	0.254	4.939
1.791	0.085	0.729	-4.238	0.433	4.548
1.420	0.168	0.825	-4.712	0.525	2.610
2.758	0.011	0.468	-2.666	0.629	4.091
1.182	0.248	0.907	-4.969	0.289	4.487
2.198	0.037	0.611	-3.627	0.720	3.128
-0.408	0.686	0.999	-5.500	-0.157	2.607
0.145	0.886	0.999	-5.565	0.035	4.229
0.533	0.598	0.999	-5.448	0.123	3.082
0.903	0.375	0.963	-5.216	0.203	3.226
-0.127	0.900	0.999	-5.567	-0.035	3.300
0.951	0.351	0.961	-5.178	0.215	6.004
-1.583	0.126	0.786	-4.515	-0.361	2.332
-0.840	0.409	0.970	-5.264	-0.195	3.691
0.576	0.570	0.997	-5.427	0.118	4.862
0.596	0.556	0.995	-5.416	0.168	3.739
1.915	0.067	0.682	-4.061	0.397	5.696
-0.518	0.609	0.999	-5.455	-0.107	3.538
0.533	0.599	0.999	-5.448	0.140	6.246
0.085	0.933	0.999	-5.571	0.025	4.433
-0.098	0.922	0.999	-5.570	-0.029	4.133
-2.020	0.054	0.655	-3.904	-0.475	3.029
-0.399	0.693	0.999	-5.503	-0.115	2.959
1.504	0.145	0.803	-4.612	0.296	6.290
-0.225	0.824	0.999	-5.551	-0.100	3.182
-0.656	0.518	0.990	-5.384	-0.257	2.982
1.326	0.197	0.855	-4.819	0.464	3.866
-0.556	0.583	0.999	-5.437	-0.126	3.202
0.716	0.481	0.984	-5.348	0.174	4.405
1.122	0.272	0.920	-5.027	0.369	3.065
1.314	0.201	0.860	-4.832	0.321	2.413
1.343	0.191	0.850	-4.800	0.321	5.692
-0.222	0.826	0.999	-5.552	-0.074	2.186
0.146	0.885	0.999	-5.564	0.029	3.497
1.241	0.226	0.888	-4.910	0.372	4.340
-0.238	0.814	0.999	-5.549	-0.062	2.521
0.208	0.837	0.999	-5.555	0.060	4.616
-1.341	0.192	0.851	-4.802	-0.332	3.686
0.233	0.818	0.999	-5.550	0.054	2.772
-0.400	0.693	0.999	-5.503	-0.107	4.933
-0.297	0.769	0.999	-5.535	-0.089	2.239
0.812	0.424	0.972	-5.284	0.209	3.849
0.548	0.589	0.999	-5.441	0.127	3.824
-0.384	0.705	0.999	-5.508	-0.102	3.259
0.964	0.344	0.957	-5.168	0.201	3.400
1.101	0.281	0.923	-5.047	0.251	2.782

1.429	0.165	0.825	-4.702	0.447	3.438
0.863	0.396	0.964	-5.247	0.240	2.772
1.703	0.101	0.763	-4.358	0.428	3.937
0.661	0.515	0.990	-5.381	0.169	3.704
-0.071	0.944	0.999	-5.572	-0.020	3.893
1.710	0.100	0.758	-4.349	0.356	3.566
-2.760	0.011	0.468	-2.663	-0.602	3.352
1.322	0.198	0.857	-4.823	0.283	4.200
1.317	0.200	0.858	-4.828	0.457	3.032
0.594	0.558	0.995	-5.418	0.142	5.503
0.916	0.369	0.963	-5.207	0.202	7.519

0.960374239	0.999213026	-5.57282326	0.014674354	6.537221884	6.52254753
-0.050151745	0.960398245	0.999213026	-5.572824623	-0.014998046	10.16682289
-0.050069457	0.960463166	0.999213026	-5.572828305	-0.016711987	3.283409073
-0.049979405	0.960534213	0.999213026	-5.572832327	-0.015028476	1.74449385
-0.049423438	0.960972854	0.999213026	-5.572857	-0.016110757	3.085275222
-0.049323339	0.96105183	0.999213026	-5.572861413	-0.03953936	4.524989932
0.049062564	0.961257579	0.999213026	-5.572872868	0.023759574	7.88958042
-0.049056849	0.961262088	0.999213026	-5.572873118	-0.011778532	6.330150766
-0.048880259	0.961401417	0.999213026	-5.57288084	-0.01856777	2.860478027
0.048244454	0.961903077	0.999213026	-5.572908409	0.024818946	5.216544741
-0.048239478	0.961907003	0.999213026	-5.572908624	-0.012323506	3.360427854
-0.048167447	0.961963838	0.999213026	-5.572911724	-0.011443064	2.425285873
0.04811063	0.962008668	0.999213026	-5.572914166	0.010770652	3.211924672
0.047695293	0.962336385	0.999213026	-5.572931932	0.01877229	7.586085982
-0.047613733	0.962400741	0.999213026	-5.572935402	-0.019297383	6.488941498
-0.047517987	0.96247629	0.999213026	-5.572939469	-0.01702518	8.851773069
-0.047481453	0.962505117	0.999213026	-5.572941018	-0.01577745	3.080050135
-0.047460424	0.96252171	0.999213026	-5.57294191	-0.011124132	2.381272113
0.047397845	0.962571089	0.999213026	-5.57294456	0.012920483	6.576592121
-0.047168066	0.962752399	0.999213026	-5.572954261	-0.014855179	6.852030622
-0.047111421	0.962797096	0.999213026	-5.572956645	-0.011154128	7.304539332
0.046953607	0.962921623	0.999213026	-5.572963273	0.010414561	2.743303322
0.046937809	0.962934089	0.999213026	-5.572963935	0.011974807	1.999484809
0.046750869	0.9630816	0.999213026	-5.572971755	0.022495751	3.525034611
-0.046666447	0.963148216	0.999213026	-5.572975276	-0.011144467	6.619019703
-0.046579351	0.963216943	0.999213026	-5.572978901	-0.011548806	4.826533795
-0.046513356	0.96326902	0.999213026	-5.572981644	-0.01933673	1.701953423
0.046325699	0.963417099	0.999213026	-5.572989423	0.009626678	4.471277959
0.046097126	0.963597467	0.999213026	-5.572998854	0.011254194	3.475844811
0.046060731	0.963626188	0.999213026	-5.573000352	0.013355066	5.370564784
-0.045903345	0.963750383	0.999213026	-5.573006814	-0.018946266	2.282117318
0.045818058	0.963817685	0.999213026	-5.573010306	0.00993969	4.007458307
0.045773596	0.96385277	0.999213026	-5.573012124	0.011612631	3.647066706
0.045394935	0.964151583	0.999213026	-5.573027537	0.010537703	5.39442115
0.045349914	0.964187111	0.999213026	-5.573029361	0.011838851	5.701286065
-0.045314244	0.964215259	0.999213026	-5.573030805	-0.010782788	2.592932363
-0.045237819	0.96427557	0.999213026	-5.573033895	-0.012147253	5.238042905

-0.045128928	0.9643615	0.999213026	-5.573038288	-0.013303311	1.407300986
0.044978883	0.964479908	0.999213026	-5.573044324	0.012913881	2.924009566
0.044939631	0.964510884	0.999213026	-5.5730459	0.010048771	3.940418121
-0.044604942	0.964775008	0.999213026	-5.57305928	-0.009823254	7.190499656
-0.044460931	0.964888657	0.999213026	-5.573065007	-0.010685613	6.925826504
-0.044252052	0.965053499	0.999213026	-5.57307328	-0.009393101	9.087925163
0.044133379	0.965147153	0.999213026	-5.573077963	0.00996321	2.455266254
0.0440172	0.96523884	0.999213026	-5.573082535	0.013243885	5.913899494
-0.043876574	0.96534982	0.999213026	-5.573088054	-0.008520089	6.405489712
0.043731138	0.965464598	0.999213026	-5.573093742	0.009224658	6.949041043
0.043700343	0.965488901	0.999213026	-5.573094945	0.008983394	5.019274016
0.043367053	0.965751935	0.999213026	-5.5731079	0.012348778	1.943146883
0.043272144	0.965826838	0.999213026	-5.573111572	0.010720385	8.249608022
-0.043132256	0.96593724	0.999213026	-5.573116968	-0.011443333	9.437091001
-0.043019379	0.966026326	0.999213026	-5.57312131	-0.011534926	6.761357814
-0.042451781	0.966474292	0.999213026	-5.573142969	-0.012285855	2.937884854
-0.042330953	0.966569655	0.999213026	-5.573147543	-0.015508032	3.580315231
-0.041808118	0.966982306	0.999213026	-5.573167184	-0.008233961	8.349498447
0.041759944	0.967020328	0.999213026	-5.573168981	0.014478904	6.266751145
0.041659771	0.967099391	0.999213026	-5.573172712	0.01586992	3.632281638
-0.04140284	0.967302181	0.999213026	-5.57318224	-0.010588173	3.193907933
0.041268526	0.967408192	0.999213026	-5.573187198	0.010317344	2.961108657
0.041148407	0.967503001	0.999213026	-5.573191618	0.015895837	4.029904809
0.041141028	0.967508825	0.999213026	-5.573191889	0.010815406	3.856671513
0.041099446	0.967541645	0.999213026	-5.573193416	0.013635743	2.910202596
-0.041058502	0.967573962	0.999213026	-5.573194917	-0.011906821	1.920121105
-0.041034292	0.96759307	0.999213026	-5.573195805	-0.012068436	3.483076725
-0.04101015	0.967612125	0.999213026	-5.573196689	-0.014757238	2.119279107
-0.040957669	0.967653548	0.999213026	-5.57319861	-0.017277889	3.571388856
0.040946354	0.967662479	0.999213026	-5.573199024	0.01472271	3.152459582
-0.040781321	0.96779274	0.999213026	-5.573205045	-0.011611309	7.536880147
-0.040769704	0.967801909	0.999213026	-5.573205468	-0.008951861	3.023880167
0.040659668	0.96788876	0.999213026	-5.573209469	0.008778734	6.005284329
0.040514837	0.968003076	0.999213026	-5.573214718	0.00936268	4.665053993
0.040443289	0.96805955	0.999213026	-5.573217304	0.012036795	8.540584526
-0.040340191	0.968140927	0.999213026	-5.573221022	-0.01431899	3.196850904
-0.040227338	0.968230003	0.999213026	-5.573225082	-0.015886533	4.481624145
-0.040177057	0.968269691	0.999213026	-5.573226887	-0.007415536	8.627398808
0.040036497	0.968380638	0.999213026	-5.573231921	0.010620362	5.227489191
-0.039994724	0.96841361	0.999213026	-5.573233413	-0.011259821	3.141600527
0.039799382	0.9685678	0.999213026	-5.573240373	0.007562291	7.335699247
0.039777031	0.968585443	0.999213026	-5.573241167	0.010389706	2.854418335
-0.039699757	0.968646438	0.999213026	-5.573243909	-0.012173343	3.963437281
0.039681438	0.968660897	0.999213026	-5.573244558	0.013893245	3.080914299
0.039641931	0.968692082	0.999213026	-5.573245957	0.018587224	4.806618771
0.039623108	0.968706939	0.999213026	-5.573246623	0.009698389	5.981572625
-0.03957972	0.968741187	0.999213026	-5.573248158	-0.016198393	5.820854129
-0.03953902	0.968773314	0.999213026	-5.573249595	-0.015558346	6.86533249
0.039482386	0.968818017	0.999213026	-5.573251593	0.009382253	5.368796627
-0.039294363	0.968966432	0.999213026	-5.573258207	-0.009599691	1.771947942

-0.039046846	0.96916181	0.999213026	-5.573266864	-0.007522297	7.250841197
-0.0390313	0.969174082	0.999213026	-5.573267406	-0.010147405	2.7794444
-0.03890812	0.969271315	0.999213026	-5.573271692	-0.011108013	2.77451556
-0.03887717	0.969295746	0.999213026	-5.573272767	-0.018162517	5.02492689
0.038863667	0.969306404	0.999213026	-5.573273236	0.010687315	3.460161269
-0.038827243	0.969335156	0.999213026	-5.573274499	-0.011916682	6.358805582
0.038662429	0.969465255	0.999213026	-5.573280201	0.019254217	7.681673791
0.038262164	0.969781214	0.999213026	-5.573293948	0.009306921	1.715057537
0.038132241	0.969883773	0.999213026	-5.57329838	0.014208359	4.574036971
-0.038040009	0.96995658	0.999213026	-5.573301517	-0.013458194	7.048684332
0.037886828	0.9700775	0.999213026	-5.573306709	0.010525639	4.540012447
-0.037864809	0.970094881	0.999213026	-5.573307454	-0.012411314	2.974759562
-0.037850396	0.970106259	0.999213026	-5.573307941	-0.009114133	7.033280069
-0.037707604	0.970218979	0.999213026	-5.573312758	-0.018745631	3.682812193
-0.037516821	0.970369583	0.999213026	-5.573319166	-0.009323158	5.30417642
-0.037458746	0.970415428	0.999213026	-5.57332111	-0.007506466	4.849435147
-0.037335485	0.970512731	0.999213026	-5.573325226	-0.009005907	5.078918002
-0.03716165	0.970649959	0.999213026	-5.573331008	-0.014357736	8.90158351
0.037072417	0.970720401	0.999213026	-5.573333966	0.010526322	2.622933682
-0.036912625	0.970846544	0.999213026	-5.573339244	-0.025360613	5.635389313
-0.036867169	0.970882428	0.999213026	-5.573340741	-0.012154166	2.766279603
0.036798757	0.970936434	0.999213026	-5.573342992	0.009402251	5.818706875
0.036587863	0.971102921	0.999213026	-5.573349902	0.009752592	3.601564506
-0.036518116	0.971157981	0.999213026	-5.573352178	-0.014858004	3.360806928
0.036394555	0.971255525	0.999213026	-5.573356201	0.008980939	3.246994364
-0.036345725	0.971294073	0.999213026	-5.573357786	-0.012150218	2.014264463
0.036277678	0.971347792	0.999213026	-5.573359993	0.009625935	4.003828394
0.036128468	0.971465585	0.999213026	-5.573364816	0.00950365	2.672214556
0.036023936	0.971548108	0.999213026	-5.573368184	0.010268771	5.852904181
0.036009672	0.971559369	0.999213026	-5.573368643	0.00851043	4.740854896
-0.035877794	0.97166348	0.999213026	-5.573372875	-0.007222576	6.09934972
-0.035599091	0.971883504	0.999213026	-5.57338177	-0.008545975	3.712399181
-0.03539758	0.972042591	0.999213026	-5.573388157	-0.010850438	3.447836508
0.035327116	0.972098219	0.999213026	-5.573390382	0.008748385	5.700946178
-0.035292357	0.972125661	0.999213026	-5.573391478	-0.024333265	6.521264552
-0.035166467	0.972225048	0.999213026	-5.573395439	-0.014601731	4.634913898
-0.035105248	0.972273378	0.999213026	-5.573397359	-0.018554771	3.880225442
-0.035021123	0.972339793	0.999213026	-5.573399993	-0.00656294	7.617181011
-0.034516967	0.972737818	0.999213026	-5.573415646	-0.009611904	4.564323553
0.034426326	0.972809378	0.999213026	-5.573418436	0.014254753	3.694700192
-0.03439509	0.972834039	0.999213026	-5.573419396	-0.008613135	6.25047284
-0.034328635	0.972886505	0.999213026	-5.573421435	-0.008260978	3.396914087
0.034192545	0.972993948	0.999213026	-5.573425598	0.0099665	5.475746668
-0.03381239	0.973294084	0.999213026	-5.573437141	-0.011126069	1.765898152
-0.03361297	0.973451529	0.999213026	-5.573443144	-0.017685957	0.969319665
-0.0335282	0.973518456	0.999213026	-5.573445686	-0.011953747	6.908509929
0.03338099	0.973634683	0.999213026	-5.573450083	0.006705118	5.249017646
-0.033092721	0.97386228	0.999213026	-5.573458639	-0.007979206	5.204166969
-0.032950313	0.973974715	0.999213026	-5.573462838	-0.010815133	3.486317459
-0.032535475	0.974302247	0.999213026	-5.573474967	-0.008501657	2.933581742

0.032226473	0.974546221	0.999213026	-5.573483902	0.007673808	4.798499753
-0.032220793	0.974550705	0.999213026	-5.573484066	-0.008112099	3.414388542
0.03196409	0.974753388	0.999213026	-5.573491422	0.01110311	2.072769988
-0.031946381	0.97476737	0.999213026	-5.573491927	-0.013865635	5.836186949
0.03180221	0.974881202	0.999213026	-5.573496031	0.008895526	4.858648084
-0.031622267	0.975023281	0.999213026	-5.573501127	-0.011641445	2.550315595
-0.031545925	0.975083558	0.999213026	-5.57350328	-0.00834509	8.608830809
-0.031545432	0.975083947	0.999213026	-5.573503294	-0.006472967	3.694077267
0.031527752	0.975097907	0.999213026	-5.573503791	0.008415937	4.01731421
-0.031519314	0.975104569	0.999213026	-5.573504029	-0.007972633	5.904130684
0.031385452	0.975210264	0.999213026	-5.573507789	0.006511747	2.693382169
-0.031378941	0.975215405	0.999213026	-5.573507971	-0.015826656	3.954613335
-0.031323729	0.975258998	0.999213026	-5.573509517	-0.009413239	2.26856341
-0.0311943	0.975361193	0.999213026	-5.57351313	-0.00766605	7.131229742
0.031191345	0.975363527	0.999213026	-5.573513212	0.011710263	4.253800431
-0.031164863	0.975384436	0.999213026	-5.573513949	-0.008499984	2.549864347
0.031109273	0.975428329	0.999213026	-5.573515495	0.010519766	3.319048493
0.031041809	0.975481598	0.999213026	-5.573517367	0.01089034	4.186153556
0.030958135	0.975547666	0.999213026	-5.573519683	0.012090664	2.840642531
-0.030702177	0.975749768	0.999213026	-5.57352673	-0.009010705	2.949733901
-0.030588849	0.975839252	0.999213026	-5.573529831	-0.006784895	7.618171157
0.030588066	0.975839871	0.999213026	-5.573529853	0.010832961	6.348217746
-0.03048668	0.975919925	0.999213026	-5.573532617	-0.005965619	2.872940322
0.030404051	0.975985169	0.999213026	-5.573534864	0.005928808	4.409507997
-0.030057262	0.976258996	0.999213026	-5.573544225	-0.007782089	1.959311262
0.029999428	0.976304663	0.999213026	-5.573545776	0.009607698	4.210843603
-0.029972942	0.976325576	0.999213026	-5.573546485	-0.00571815	7.099295875
-0.029921717	0.976366025	0.999213026	-5.573547855	-0.009118106	5.074515875
0.029852721	0.976420505	0.999213026	-5.573549697	0.006695218	4.355042653
0.029817451	0.976448355	0.999213026	-5.573550636	0.006702687	6.948813262
0.029636389	0.976591325	0.999213026	-5.573555443	0.009272578	9.288658286
-0.029608729	0.976613166	0.999213026	-5.573556174	-0.00810251	4.404773088
0.029518359	0.976684524	0.999213026	-5.57355856	0.013394909	5.374692775
-0.029266095	0.976883718	0.999213026	-5.573565181	-0.009327083	6.914560862
0.029229027	0.976912989	0.999213026	-5.573566149	0.00701785	6.330382969
0.029011305	0.977084909	0.999213026	-5.573571811	0.009287335	4.073460038
0.028989047	0.977102485	0.999213026	-5.573572387	0.00797044	1.623649989
0.028687937	0.977340254	0.999213026	-5.573580141	0.007352689	4.979763709
-0.028684748	0.977342772	0.999213026	-5.573580223	-0.010681371	4.511859592
0.028569717	0.977433606	0.999213026	-5.573583164	0.009840756	1.260696216
-0.028501151	0.977487749	0.999213026	-5.573584911	-0.008006214	3.307716397
-0.028400175	0.977567485	0.999213026	-5.573587476	-0.008904567	3.02217354
0.02828229	0.977660573	0.999213026	-5.57359046	0.006215357	5.052591743
-0.028059231	0.977836713	0.999213026	-5.573596071	-0.005271486	6.480521235
-0.02792356	0.977943848	0.999213026	-5.573599462	-0.006302488	5.347667245
0.02777565	0.978060646	0.999213026	-5.57360314	0.01277714	3.474152006
-0.027646987	0.978162247	0.999213026	-5.573606324	-0.010664815	3.33945066
-0.027349442	0.978397211	0.999213026	-5.573613631	-0.011708357	5.525817597
-0.027136414	0.978565434	0.999213026	-5.573618813	-0.00777087	3.382988147
-0.02688806	0.978761555	0.999213026	-5.573624804	-0.010318788	3.08193769

0.026861533	0.978782503	0.999213026	-5.57362544	0.011358198	2.565870326
0.026795518	0.978834634	0.999213026	-5.573627022	0.008891461	4.68201139
0.026749065	0.978871317	0.999213026	-5.573628132	0.008199691	4.527703222
0.026600247	0.978988838	0.999213026	-5.573631677	0.010546898	4.885560342
-0.026566324	0.979015627	0.999213026	-5.573632482	-0.006827706	3.668128387
-0.02629191	0.97923233	0.999213026	-5.573638959	-0.007991152	4.196064901
0.026240009	0.979273316	0.999213026	-5.573640176	0.004961256	5.987206774
-0.026099035	0.979384644	0.999213026	-5.573643471	-0.006773223	1.68308318
0.026079342	0.979400195	0.999213026	-5.573643929	0.007780908	2.96422372
0.025885058	0.979553623	0.999213026	-5.573648437	0.006989936	4.112939646
0.025743101	0.979665727	0.999213026	-5.573651709	0.012118639	4.454611347
0.025710078	0.979691806	0.999213026	-5.573652468	0.01176897	4.896169714
-0.025666151	0.979726496	0.999213026	-5.573653476	-0.017015619	6.052749671
-0.025613489	0.979768084	0.999213026	-5.573654681	-0.012714641	5.092570306
-0.02561051	0.979770436	0.999213026	-5.57365475	-0.007998484	7.174694731
-0.025343508	0.979981292	0.999213026	-5.573660824	-0.007888259	4.203194142
0.025241083	0.980062179	0.999213026	-5.573663137	0.005195571	6.836887071
-0.025161815	0.980124779	0.999213026	-5.573664921	-0.007259471	4.822885305
0.025080133	0.980189285	0.999213026	-5.573666754	0.008961908	4.978781917
0.024986187	0.980263477	0.999213026	-5.573668854	0.007378149	2.046566067
0.024865308	0.980358938	0.999213026	-5.573671544	0.006510463	6.45697781
-0.024725526	0.980469328	0.999213026	-5.573674639	-0.004985336	3.520533319
0.024577515	0.980586217	0.999213026	-5.573677897	0.007465816	2.789038548
-0.02454736	0.980610032	0.999213026	-5.573678559	-0.006414308	3.444935259
-0.024421524	0.980709409	0.999213026	-5.57368131	-0.006438441	4.81817424
0.024307234	0.980799668	0.999213026	-5.573683797	0.006707387	4.131490679
-0.024240532	0.980852346	0.999213026	-5.573685243	-0.012135548	4.597482078
0.024062427	0.980993003	0.999213026	-5.573689084	0.007612614	5.557302301
0.023927667	0.981099429	0.999213026	-5.573691971	0.017641665	5.384054927
-0.023106298	0.98174811	0.999213026	-5.57370922	-0.005681017	3.359722552
0.022910171	0.981903004	0.999213026	-5.57371325	0.005630948	3.572304483
0.022788002	0.98199949	0.999213026	-5.573715743	0.006885512	3.198626567
0.022767272	0.982015862	0.999213026	-5.573716164	0.006406054	3.380705342
0.022729023	0.982046069	0.999213026	-5.573716941	0.005358943	3.658549095
0.02246719	0.982252858	0.999213026	-5.573722225	0.005630104	6.044728361
-0.022464811	0.982254737	0.999213026	-5.573722273	-0.006399821	4.046951389
0.022458378	0.982259818	0.999213026	-5.573722402	0.006875155	4.819121829
0.022403934	0.982302816	0.999213026	-5.573723493	0.00622355	2.971294945
-0.022307111	0.982379285	0.999213026	-5.573725425	-0.007985967	5.498996504
-0.022223311	0.982445468	0.999213026	-5.573727092	-0.006341295	10.4654414
0.022072898	0.982564262	0.999213026	-5.573730066	0.005172918	5.361049006
-0.021555134	0.982973185	0.999213026	-5.573740152	-0.007328551	5.413764198
0.021455898	0.983051561	0.999213026	-5.573742058	0.004218716	6.765808821
0.021428666	0.983073069	0.999213026	-5.57374258	0.005661502	3.41389155
0.021363803	0.983124297	0.999213026	-5.573743819	0.008888053	2.78337252
0.021317282	0.983161039	0.999213026	-5.573744705	0.006500077	3.481683613
-0.021191078	0.983260715	0.999213026	-5.573747101	-0.005496637	2.89194228
-0.02111596	0.983320043	0.999213026	-5.57374852	-0.007706587	2.548866059
-0.021090175	0.983340408	0.999213026	-5.573749006	-0.004191496	5.272013268
-0.021051232	0.983371165	0.999213026	-5.573749738	-0.008952919	3.663820794

0.020984402	0.983423948	0.999213026	-5.573750993	0.009318407	5.040875113
0.020961427	0.983442093	0.999213026	-5.573751423	0.005223171	5.757168393
-0.020831103	0.983545023	0.999213026	-5.573753855	-0.005754116	2.200552475
0.020660145	0.983680047	0.999213026	-5.573757022	0.007310989	3.953068669
0.020618367	0.983713044	0.999213026	-5.573757792	0.004534578	7.552948145
0.020227685	0.984021609	0.999213026	-5.573764917	0.00544528	3.911955182
0.020180299	0.984059035	0.999213026	-5.573765772	0.004599077	6.720741583
-0.020064262	0.984150683	0.999213026	-5.573767857	-0.006347673	9.604799345
-0.020051017	0.984161145	0.999213026	-5.573768094	-0.005074056	3.590774487
0.020025463	0.984181328	0.999213026	-5.573768552	0.007112871	4.276758914
0.01996006	0.984232984	0.999213026	-5.573769719	0.006568081	4.317633264
-0.0196671	0.98446437	0.999213026	-5.573774903	-0.006295235	4.011155506
0.019476085	0.984615238	0.999213026	-5.573778241	0.006328751	1.53899037
0.01944812	0.984637326	0.999213026	-5.573778727	0.005123355	5.078720371
-0.019439707	0.984643971	0.999213026	-5.573778873	-0.004221714	5.377357041
0.019421247	0.984658551	0.999213026	-5.573779194	0.008890506	5.576733269
0.019320909	0.984737801	0.999213026	-5.573780929	0.006301771	5.278733605
0.019191791	0.984839782	0.999213026	-5.573783149	0.005594439	2.887324363
-0.019160767	0.984864286	0.999213026	-5.573783681	-0.004622047	5.174567518
-0.019038963	0.984960491	0.999213026	-5.573785758	-0.006654542	3.229747625
-0.018840604	0.985117161	0.999213026	-5.573789113	-0.0071983	2.904915449
-0.018813536	0.985138541	0.999213026	-5.573789568	-0.00861485	2.824690468
-0.01868309	0.985241572	0.999213026	-5.573791752	-0.005260582	8.972284111
0.018520541	0.98536996	0.999213026	-5.573794452	0.006941113	6.387858742
0.018466291	0.985412809	0.999213026	-5.573795348	0.008115389	3.665971367
0.018273248	0.985565283	0.999213026	-5.573798515	0.005622585	4.442898634
0.018263852	0.985572705	0.999213026	-5.573798668	0.004547707	7.95468645
0.018158885	0.985655612	0.999213026	-5.573800375	0.00465963	5.009259436
0.017767621	0.985964652	0.999213026	-5.573806652	0.005951719	7.776212902
0.017609625	0.986089446	0.999213026	-5.573809147	0.004121037	3.536220902
-0.017297108	0.98633629	0.999213026	-5.573814018	-0.004388248	5.415503644
-0.017140604	0.986459906	0.999213026	-5.573816425	-0.008644025	3.728986921
0.017006756	0.986565628	0.999213026	-5.573818465	0.004188832	5.505035827
-0.016930956	0.9866255	0.999213026	-5.573819614	-0.005466263	3.748626401
0.016863626	0.986678682	0.999213026	-5.57382063	0.005669995	5.47021519
0.016853394	0.986686763	0.999213026	-5.573820784	0.004164807	4.695280128
-0.016820867	0.986712455	0.999213026	-5.573821273	-0.005051962	3.946666754
-0.016422615	0.987027022	0.999213026	-5.573827184	-0.004134631	3.041250142
0.016330722	0.987099606	0.999213026	-5.573828528	0.006802024	6.037797633
0.016209057	0.987195706	0.999213026	-5.573830296	0.004569679	2.576953463
0.016206253	0.987197921	0.999213026	-5.573830336	0.010297035	5.363165384
-0.015731998	0.987572525	0.999213026	-5.5738371	-0.007125559	3.486300701
-0.015373421	0.987855758	0.999213026	-5.57384208	-0.003959977	2.989957963
-0.015358742	0.987867354	0.999213026	-5.573842281	-0.003177698	4.060669893
-0.015299496	0.987914151	0.999213026	-5.573843092	-0.005940972	1.89675702
0.015220781	0.987976327	0.999213026	-5.573844165	0.005119403	2.682385518
0.015147013	0.988034595	0.999213026	-5.573845165	0.00461457	3.858413263
0.015146424	0.988035061	0.999213026	-5.573845173	0.004047119	4.527103508
-0.015141065	0.988039294	0.999213026	-5.573845245	-0.0074498	3.70142429
-0.014773673	0.988329493	0.999213026	-5.573850153	-0.00666005	8.534533663

0.014482929	0.988559151	0.999213026	-5.573853951	0.004218705	3.492660697
-0.014457107	0.988579548	0.999213026	-5.573854284	-0.003484586	5.852279219
0.014050183	0.988900976	0.999213026	-5.573859464	0.003103169	6.323755373
0.013478244	0.989352754	0.999213026	-5.573866494	0.004650244	3.419926841
-0.013227411	0.989550889	0.999213026	-5.573869485	-0.003436596	3.331488547
-0.013133264	0.989625257	0.999213026	-5.573870593	-0.005461395	2.194707654
-0.013091169	0.989658508	0.999213026	-5.573871086	-0.004257287	3.956004624
-0.013012356	0.989720764	0.999213026	-5.573872004	-0.003492046	2.781480563
0.013010384	0.989722322	0.999213026	-5.573872027	0.003229366	2.038190637
0.012874675	0.98982952	0.999213026	-5.573873596	0.002547128	5.325472482
0.012726175	0.989946822	0.999213026	-5.573875293	0.003233719	7.100569859
0.012547434	0.990088013	0.999213026	-5.57387731	0.00242385	6.150467053
-0.012467805	0.990150914	0.999213026	-5.5738782	-0.003190066	3.400219652
0.012429873	0.990180877	0.999213026	-5.573878621	0.003297049	3.653826767
-0.012404684	0.990200774	0.999213026	-5.573878901	-0.003676101	3.947741925
-0.012399128	0.990205162	0.999213026	-5.573878962	-0.002889372	9.470389522
-0.012245043	0.990326878	0.999213026	-5.573880658	-0.003553435	2.768131274
-0.012125718	0.990421135	0.999213026	-5.573881956	-0.002853765	4.054666615
-0.012101965	0.990439898	0.999213026	-5.573882213	-0.003668892	4.801095924
0.011924999	0.990579688	0.999213026	-5.573884112	0.003022831	6.11792827
0.011532228	0.990889948	0.999213026	-5.573888225	0.003272266	2.480815956
-0.011447736	0.99095669	0.999213026	-5.573889092	-0.003901582	3.403659839
0.011229492	0.991129088	0.999213026	-5.573891302	0.002655525	3.088729072
0.011054821	0.991267066	0.999213026	-5.57389304	0.003717906	3.148998864
0.011052933	0.991268557	0.999213026	-5.573893059	0.007729405	3.671657113
0.01094613	0.991352925	0.999213026	-5.573894108	0.004963851	6.347380419
-0.010877781	0.991406916	0.999213026	-5.573894774	-0.002665071	5.683599041
-0.010738152	0.991517214	0.999213026	-5.573896122	-0.002996205	5.782741954
0.010667907	0.991572703	0.999213026	-5.573896793	0.006026873	4.693965218
0.010664922	0.991575061	0.999213026	-5.573896821	0.003114015	4.299281635
-0.010424584	0.991764913	0.999213026	-5.573899085	-0.002465327	3.980740838
-0.010394793	0.991788446	0.999213026	-5.573899361	-0.002711208	4.209158442
0.010312811	0.991853207	0.999213026	-5.573900119	0.002439634	6.61346501
-0.010178429	0.991959361	0.999213026	-5.573901349	-0.003069666	4.274114778
0.010161429	0.991972789	0.999213026	-5.573901503	0.004231004	5.070071927
0.009819997	0.992242501	0.999213026	-5.57390455	0.002193655	4.026054085
0.009625777	0.992395923	0.999213026	-5.573906236	0.002149622	6.924375845
-0.008885767	0.992980492	0.999213026	-5.573912352	-0.002448675	3.371633874
0.008803054	0.993045832	0.999213026	-5.573913006	0.005896932	0.984801541
-0.008760725	0.99307927	0.999213026	-5.573913338	-0.003575072	3.278062845
-0.008652797	0.993164527	0.999213026	-5.573914177	-0.002599398	5.57730343
0.008461305	0.993315797	0.999213026	-5.57391564	0.002069221	6.169811113
-0.008398859	0.993365127	0.999213026	-5.57391611	-0.006637249	6.748976322
-0.008320925	0.993426691	0.999213026	-5.573916692	-0.002076764	3.070624171
-0.008310052	0.99343528	0.999213026	-5.573916773	-0.00275172	2.559858284
0.008285762	0.993454468	0.999213026	-5.573916953	0.002895955	4.180241397
-0.008264857	0.993470982	0.999213026	-5.573917107	-0.002578588	3.91118791
-0.008218563	0.993507552	0.999213026	-5.573917448	-0.002437987	3.424190532
-0.008206505	0.993517078	0.999213026	-5.573917536	-0.001958558	4.599538621
0.008138062	0.993571144	0.999213026	-5.573918036	0.002028789	2.235690412

0.008114669	0.993589624	0.999213026	-5.573918206	0.003058509	2.107462942
-0.008003115	0.993677747	0.999213026	-5.573919008	-0.004148648	3.665760972
0.0079646	0.993708172	0.999213026	-5.573919283	0.005794003	4.619310681
0.007953858	0.993716658	0.999213026	-5.573919359	0.003626955	3.623736067
0.007936506	0.993730365	0.999213026	-5.573919483	0.001983242	5.240372998
-0.007882022	0.993773405	0.999213026	-5.573919867	-0.002063064	5.249867607
-0.007851174	0.993797773	0.999213026	-5.573920084	-0.002007713	2.72533383
0.00783876	0.99380758	0.999213026	-5.573920171	0.002656377	5.836037571
0.007715102	0.993905264	0.999213026	-5.57392103	0.002107273	1.365173624
0.007595665	0.993999615	0.999213026	-5.573921846	0.00185312	3.543375306
-0.007544332	0.994040166	0.999213026	-5.573922193	-0.001723001	6.491362736
-0.007305891	0.994228525	0.999213026	-5.573923774	-0.002158852	3.391795171
0.007281643	0.99424768	0.999213026	-5.573923932	0.002472955	2.603367487
-0.007099495	0.99439157	0.999213026	-5.573925102	-0.001678936	8.165078765
0.007024628	0.994450712	0.999213026	-5.573925574	0.001530801	5.219736586
0.006986113	0.994481137	0.999213026	-5.573925815	0.002161289	6.487216371
-0.006902512	0.994547179	0.999213026	-5.573926333	-0.002136495	5.090540118
0.00659756	0.99478808	0.999213026	-5.573928172	0.001433046	8.492157611
0.006414441	0.994932738	0.999213026	-5.573929236	0.002135355	11.63145902
0.006393977	0.994948904	0.999213026	-5.573929353	0.001454035	3.34201317
-0.006321634	0.995006053	0.999213026	-5.573929763	-0.002165683	1.744600528
-0.006309969	0.995015267	0.999213026	-5.573929829	-0.002017121	4.759965528
-0.005892739	0.995344865	0.999213026	-5.573932102	-0.001524288	4.590815588
0.005718692	0.995482357	0.999213026	-5.573933005	0.001636031	3.277730363
-0.005492787	0.995660815	0.999213026	-5.573934136	-0.001906399	3.328717702
0.005440524	0.995702102	0.999213026	-5.573934391	0.001118726	3.776625395
-0.005296454	0.995815913	0.999213026	-5.573935081	-0.002069453	4.952964812
0.00527917	0.995829566	0.999213026	-5.573935163	0.002722462	4.956631196
0.005248493	0.9958538	0.999213026	-5.573935307	0.001545504	7.051679768
-0.005236486	0.995863286	0.999213026	-5.573935363	-0.001736369	2.071316485
0.005185698	0.995903406	0.999213026	-5.5739356	0.003272487	5.788679789
0.005069041	0.995995563	0.999213026	-5.573936134	0.001530782	4.546899757
0.00497593	0.996069118	0.999213026	-5.573936552	0.002081532	4.350637744
-0.004971441	0.996072664	0.999213026	-5.573936572	-0.001328998	6.414062509
-0.004955944	0.996084906	0.999213026	-5.57393664	-0.001460404	4.662406307
0.004844602	0.996172863	0.999213026	-5.573937127	0.001187439	1.924965077
0.004832308	0.996182575	0.999213026	-5.573937181	0.001236144	6.937820273
-0.004392785	0.996529787	0.999450985	-5.573938991	-0.000992227	4.953386016
0.004161916	0.996712168	0.999450985	-5.573939873	0.001088396	3.429429252
-0.004135062	0.996733382	0.999450985	-5.573939972	-0.000862948	6.450257425
0.003880537	0.99693445	0.999547784	-5.573940883	0.001700954	4.534896112
-0.003579912	0.997171937	0.999549032	-5.573941885	-0.00087605	5.754065476
0.003575219	0.997175645	0.999549032	-5.5739419	0.000888767	5.501694491
0.003369146	0.997338438	0.999549032	-5.573942539	0.001104834	1.135704306
0.00328972	0.997401182	0.999549032	-5.573942775	0.00087272	4.129286024
0.003217338	0.997458363	0.999549032	-5.573942985	0.00114923	2.575834099
-0.003011342	0.997621096	0.999607347	-5.573943558	-0.000827596	6.117659531
-0.002841916	0.997754938	0.999615915	-5.573944001	-0.000759109	5.40459527
-0.002735851	0.997838727	0.999615915	-5.573944265	-0.00069829	6.051416951
-0.0024707	0.998048191	0.999649188	-5.573944881	-0.002175014	2.428333891

-0.002429133	0.998081029	0.999649188	-5.573944972	-0.000811841	4.916138996
0.002265793	0.998210064	0.999673715	-5.573945315	0.000479939	3.278396359
0.002051471	0.998379374	0.999738567	-5.573945728	0.00109015	3.916992454
-0.001632968	0.998709984	0.999837305	-5.573946416	-0.000361216	5.273636947
-0.001622781	0.998718031	0.999837305	-5.573946431	-0.00060585	5.483327906
-0.001529567	0.998791669	0.999837305	-5.573946562	-0.001071312	2.01747852
0.001386117	0.998904992	0.999846073	-5.573946749	0.000611599	2.694378744
-0.001048017	0.999172084	0.999970213	-5.573947117	-0.000399376	3.980233728
0.000691024	0.999454103	0.999970213	-5.573947394	0.000224095	3.545616122
0.000538167	0.999574857	0.999970213	-5.573947478	0.000154402	6.002654614
-0.000207357	0.999836192	0.999970213	-5.573947588	-4.59E-05	4.132577119
-0.000203475	0.999839258	0.999970213	-5.573947588	-4.29E-05	6.342864724
-9.70E-05	0.999923357	0.999970213	-5.573947603	-2.82E-05	4.778549035
9.36E-05	0.999926065	0.999970213	-5.573947603	3.03E-05	3.486491954
7.09E-05	0.999943968	0.999970213	-5.573947605	2.09E-05	1.55505361
3.77E-05	0.999970213	0.999970213	-5.573947606	1.01E-05	7.172064835

CTL.SP	07.ALS.SP.M	08.ALS.SP.M	09.ALS.SP.F	10.ALS.SP.M	11.ALS.SP.F
3.388	3.144	3.524	3.951	4.117	3.252
7.861	8.787	8.223	8.525	8.479	7.939
4.154	4.682	4.942	3.870	4.873	4.326
2.844	2.428	3.335	2.643	2.740	2.491
4.984	4.809	4.780	5.367	4.849	5.567
4.357	4.498	4.790	5.078	4.584	3.893
4.196	4.229	3.965	4.077	3.620	4.204
6.717	6.153	6.811	6.730	6.754	7.065
5.147	5.079	5.282	5.462	4.883	5.368
6.453	6.339	6.280	6.666	5.894	6.678
6.227	7.291	7.433	5.768	7.319	6.314
2.102	2.263	1.958	1.611	1.753	1.960
2.241	2.298	2.849	1.939	3.048	2.478
6.121	5.599	6.150	6.007	6.116	6.426
6.435	5.302	7.024	6.616	6.071	6.221
7.240	7.263	7.812	6.882	7.271	7.180
5.415	4.987	5.132	5.078	5.072	5.499
5.894	5.558	5.773	5.982	6.394	5.378
2.502	2.288	1.984	2.791	2.247	1.776
5.194	6.256	5.308	6.023	6.328	4.828
5.247	5.452	5.557	6.089	5.761	5.053
5.863	5.469	5.655	5.389	5.613	5.903
4.491	4.367	4.252	4.852	3.747	4.230
6.075	6.073	6.749	6.714	6.993	5.656
4.276	3.243	4.307	5.022	3.633	4.606
5.006	5.213	5.353	5.118	5.020	4.361
4.912	5.475	5.796	4.914	5.568	5.076
6.102	6.488	4.828	6.150	5.127	6.280
3.591	3.949	3.020	4.397	3.402	4.443
2.566	2.790	2.399	2.031	1.819	3.026
5.061	5.459	5.559	5.566	5.534	5.253
3.686	4.219	4.761	4.066	4.201	3.804
2.777	2.467	2.372	2.899	2.611	2.233
3.129	3.512	2.376	3.181	2.393	3.311
5.923	5.517	6.052	6.388	6.386	6.365
4.266	5.307	5.159	4.501	4.937	3.924
5.676	6.044	6.414	6.697	6.691	5.472
5.296	5.117	5.639	5.490	5.702	5.107
5.181	4.957	5.117	5.240	4.982	5.821
5.150	6.493	5.179	6.346	5.902	4.536
6.281	6.087	7.215	6.509	6.810	5.644
7.493	7.934	7.525	7.407	7.690	7.283
4.692	4.337	4.412	4.763	4.232	5.648
6.706	6.694	6.588	6.767	6.293	6.763
3.011	3.668	3.270	2.882	3.030	2.868
3.394	3.538	3.689	3.025	3.201	3.667
6.422	6.069	6.650	6.021	5.996	6.483
4.716	4.259	4.221	4.674	4.490	5.135
3.648	3.931	3.800	4.498	4.093	3.187

2.857	2.494	2.353	1.912	1.919	3.006
3.322	2.761	3.697	3.724	3.040	4.284
2.828	2.403	2.496	2.742	2.653	2.960
5.513	5.335	5.007	5.120	5.600	5.334
2.501	1.890	3.258	1.900	2.316	2.491
2.488	2.417	2.079	3.559	1.439	2.802
4.600	3.906	4.955	3.554	3.944	5.188
3.104	3.344	2.760	2.959	3.334	2.890
4.416	5.156	4.262	3.916	4.759	4.594
3.969	3.478	3.904	3.877	4.054	3.659
3.141	2.954	2.437	2.642	2.858	2.651
4.423	3.704	4.695	4.360	3.796	3.798
1.989	1.976	1.963	1.746	1.970	1.977
4.203	3.196	3.883	3.154	2.877	2.832
3.245	2.283	3.370	2.902	3.632	2.755
3.867	3.345	3.949	3.064	3.716	4.876
3.435	3.187	4.183	3.981	3.712	3.068
2.768	2.673	3.221	2.447	3.889	3.009
2.931	3.672	3.459	3.207	3.100	3.065
2.686	3.349	3.153	2.778	2.490	2.756
2.447	1.194	2.757	1.910	1.722	3.313
2.787	2.241	2.902	2.913	2.705	2.380
2.681	2.064	2.990	2.527	2.675	2.789
3.186	2.712	2.465	2.887	2.982	2.445
2.202	2.171	2.024	1.741	1.544	2.713
2.512	2.192	2.408	2.339	2.224	3.051
2.809	2.916	3.358	2.754	2.973	3.051
2.500	2.214	1.822	4.387	3.597	1.774
3.027	3.207	2.743	3.143	2.843	2.589
2.720	3.138	2.924	2.494	2.366	3.286
3.164	3.368	2.710	2.414	2.766	2.703
2.171	2.218	2.163	1.750	1.962	2.530
2.235	2.164	2.213	2.607	2.301	2.467
3.681	3.564	3.180	3.276	3.027	3.493
2.170	2.312	1.804	1.257	1.694	3.281
2.720	2.573	2.871	2.402	3.486	2.452
2.569	2.187	3.707	2.529	2.796	2.397
2.241	2.708	2.033	2.286	2.082	2.568
4.361	3.171	5.129	3.199	4.694	4.628
2.521	1.835	2.045	2.258	2.114	2.028
2.560	2.481	3.001	2.462	3.689	2.403
2.225	2.397	2.893	3.984	2.637	3.160
2.687	3.100	3.138	2.518	3.339	2.480
1.355	1.981	1.269	1.429	1.794	1.117
1.891	1.740	1.966	1.423	2.304	2.222
1.513	1.586	1.148	1.241	1.607	1.296
4.105	4.923	4.025	4.122	3.714	3.959
2.802	2.879	2.721	2.821	2.874	2.352
3.827	3.852	3.951	4.168	4.329	4.008
2.629	2.975	2.703	1.561	2.527	2.502

2.074	2.515	2.475	2.996	1.710	1.588
3.212	3.733	3.252	2.730	3.178	3.252
2.790	2.274	3.062	2.683	2.449	2.751
3.056	2.989	2.848	2.814	2.303	2.340
3.015	2.735	3.076	2.692	3.051	3.211
2.528	2.243	2.304	2.377	2.235	2.956
2.105	1.555	1.583	1.884	1.916	2.051
2.125	1.807	2.125	2.481	2.154	1.912
0.959	1.523	0.892	0.870	0.978	1.003
5.119	5.222	5.481	5.266	4.015	5.166
2.662	2.661	3.482	2.764	2.209	2.796
4.993	5.047	4.923	5.203	4.456	4.696
4.063	3.605	3.218	2.973	3.578	3.570
3.275	3.120	4.467	3.342	3.925	3.256
2.275	2.227	2.335	1.902	1.987	2.248
1.313	1.235	1.162	1.712	1.582	1.285
2.843	2.461	3.302	2.110	2.960	2.634
4.885	4.833	4.682	5.324	4.909	5.348
3.303	3.225	4.161	4.272	3.197	3.341
1.290	1.224	0.895	0.861	1.450	0.961
2.772	2.054	2.590	2.063	2.250	2.728
2.358	1.804	2.124	2.168	2.124	1.841
3.936	2.903	3.208	2.499	1.955	3.759
3.200	4.499	3.340	4.277	4.046	4.404
1.358	0.877	1.068	0.988	1.482	1.629
3.314	3.757	3.934	2.974	4.237	3.569
2.765	2.184	2.525	2.097	1.836	1.702
3.282	2.976	3.754	3.955	3.469	4.279
2.796	2.587	2.328	1.967	2.529	2.924
2.362	3.417	3.070	1.325	2.192	3.350
3.885	3.998	4.232	3.842	4.245	3.986
2.553	2.408	2.212	1.684	4.065	3.722
5.814	4.326	5.269	4.111	5.637	6.230
3.234	2.614	2.699	2.242	2.970	2.304
5.587	5.379	5.772	5.389	5.573	5.344
2.588	4.003	2.654	3.278	2.753	2.849
3.469	3.023	3.971	3.549	3.510	3.422
5.725	5.440	5.543	6.844	4.909	5.980
6.811	5.714	6.817	6.391	6.424	6.900
3.899	3.841	3.598	4.235	4.094	4.123
3.848	4.681	3.561	4.446	3.821	3.405
5.221	3.964	4.504	5.009	4.370	5.481
4.723	6.546	5.185	4.763	4.833	4.563
4.148	4.141	4.053	3.681	4.459	3.719
8.426	8.401	7.713	8.994	8.131	8.520
4.628	5.088	4.920	5.583	5.138	5.579
4.274	3.579	3.906	3.810	4.882	3.922
2.934	3.201	2.868	3.500	3.419	3.701
3.060	4.225	2.518	4.496	3.929	2.970
6.565	6.375	7.189	6.876	6.860	6.390

4.099	4.399	4.174	4.308	4.124	4.008
7.321	7.830	8.561	7.990	7.933	7.175
6.926	7.179	7.515	7.388	7.095	6.881
2.173	2.478	2.554	1.661	1.825	2.399
4.830	6.020	5.298	5.558	5.533	5.492
3.499	2.473	3.223	3.775	3.052	3.787
5.485	5.951	5.724	6.047	6.005	5.592
5.196	4.649	4.794	4.705	5.217	5.060
6.267	6.407	6.926	6.135	7.055	6.395
2.463	2.261	2.489	2.714	3.150	2.340
3.474	5.228	3.657	4.806	3.527	3.177
2.852	2.569	2.690	2.511	2.676	2.624
4.004	4.417	4.728	4.527	4.629	4.609
3.670	2.937	2.827	3.920	3.293	4.242
4.078	5.500	4.534	4.961	5.451	4.466
5.874	5.377	6.126	5.626	5.972	5.411
5.726	5.536	5.646	5.952	5.604	6.194
6.061	5.620	5.595	6.062	5.462	6.538
4.933	4.056	4.740	4.966	3.951	4.553
5.157	4.824	5.430	5.552	5.538	5.399
2.823	2.938	3.117	2.704	3.432	2.738
4.639	4.494	4.342	4.750	4.584	4.564
3.358	3.561	3.420	3.693	3.628	3.881
4.227	4.118	4.470	4.585	4.566	4.379
3.423	4.263	3.853	4.012	3.278	3.870
4.604	4.209	3.807	3.995	3.755	5.010
5.324	4.618	5.421	5.117	5.162	4.891
3.503	3.284	3.339	4.063	3.668	3.656
3.846	4.052	3.627	4.563	4.624	4.321
3.805	3.783	3.661	3.808	4.482	3.994
3.690	3.903	4.360	3.417	4.370	3.434
4.041	3.226	4.815	4.232	4.216	3.736
6.642	6.100	7.316	6.718	6.852	6.949
6.710	5.427	6.383	6.851	5.506	7.298
6.643	6.815	7.263	7.321	7.526	5.763
3.324	2.825	2.985	2.112	3.081	3.120
3.146	3.705	3.189	2.587	3.419	2.795
5.510	6.127	5.705	5.587	5.329	5.374
2.803	3.681	3.841	2.800	3.113	3.311
3.100	3.327	3.048	2.124	3.344	3.232
3.090	4.274	3.123	4.264	4.020	2.303
3.493	3.571	4.083	2.909	3.435	3.878
2.937	4.666	4.032	2.513	4.990	5.299
2.450	1.769	1.913	1.900	1.839	2.308
3.665	3.923	3.205	3.607	2.719	2.988
1.768	2.212	2.453	1.505	2.840	2.174
6.274	6.529	6.545	6.583	5.959	6.699
3.642	3.729	4.155	3.943	4.129	4.389
2.923	3.064	2.562	3.303	2.157	3.451
3.620	3.882	2.950	3.142	3.164	3.588

6.836	7.029	7.871	6.970	6.784	6.802
4.695	4.696	4.319	4.198	5.448	3.824
3.528	3.372	3.381	3.723	2.870	3.625
5.172	5.317	5.079	4.397	4.811	4.338
2.651	3.697	2.217	2.910	2.116	1.423
2.949	3.189	2.177	3.419	2.692	3.002
5.531	6.973	5.043	6.258	5.745	5.831
4.816	4.413	4.909	5.149	4.669	5.204
2.493	2.292	1.834	2.664	3.200	2.214
3.343	3.721	3.709	3.915	3.963	3.973
6.047	5.913	4.642	5.634	5.519	6.289
4.320	4.398	5.121	4.619	4.507	4.106
4.673	5.500	4.570	4.855	5.538	4.621
7.084	6.561	7.535	7.489	7.332	7.038
4.571	5.109	4.897	4.971	4.837	4.427
3.019	2.636	3.643	4.163	3.417	2.522
6.552	5.833	6.652	6.004	6.329	6.799
2.647	2.688	3.552	2.685	3.068	2.922
2.590	4.502	3.028	1.433	3.814	2.427
5.780	5.254	5.951	5.630	5.474	5.687
5.872	6.504	6.039	5.771	6.412	5.745
6.308	7.037	5.004	6.126	6.969	5.190
3.640	3.146	4.240	4.050	4.094	4.106
4.439	4.417	4.391	4.514	4.223	4.417
5.008	6.055	4.940	6.254	5.717	4.794
3.777	3.145	3.467	3.573	3.203	4.371
3.488	3.071	3.411	3.195	2.389	2.800
3.137	3.295	3.258	3.453	2.893	3.948
2.413	1.606	2.928	2.414	1.806	3.170
3.080	3.924	2.700	2.684	3.897	2.865
3.610	3.739	3.545	4.330	3.702	4.294
6.166	6.556	6.218	6.501	6.292	6.644
6.884	6.791	6.791	6.473	6.858	6.470
5.179	5.559	5.294	4.972	5.875	4.724
3.531	4.357	3.802	3.602	3.577	3.797
9.052	8.840	8.317	8.699	8.418	9.068
3.344	4.317	3.036	4.727	3.761	3.267
3.558	2.984	3.131	3.117	2.926	3.583
2.404	2.682	2.772	2.426	2.774	2.670
4.146	5.240	4.663	4.378	3.954	4.202
5.150	4.890	4.272	5.339	4.613	6.128
5.002	4.916	5.245	4.895	5.124	5.248
4.031	4.192	3.988	3.966	4.196	4.261
6.651	6.941	6.903	6.842	7.154	6.918
5.090	5.313	5.433	5.218	5.468	5.376
3.020	2.149	2.693	2.367	2.770	3.344
4.346	4.545	4.501	4.943	3.885	5.037
2.547	3.160	2.770	2.953	3.242	2.319
6.201	6.258	6.422	5.370	5.992	5.951
2.841	2.781	2.226	2.709	2.692	2.770

3.389	2.684	2.907	2.888	3.091	3.251
1.636	1.608	1.095	1.573	1.596	1.802
6.344	5.815	6.653	6.686	5.957	6.052
6.503	6.416	6.794	6.612	6.844	6.548
5.007	5.108	5.138	5.230	4.959	5.170
5.624	5.450	6.147	6.209	5.909	5.859
2.727	2.815	2.510	2.923	2.840	2.058
1.903	1.900	1.814	2.588	2.105	1.559
7.202	6.930	7.741	7.608	6.851	7.061
5.878	5.781	6.017	5.596	5.311	5.434
7.500	7.354	7.986	7.786	7.658	7.909
4.785	5.207	4.963	4.959	5.035	4.785
7.771	7.707	8.132	7.252	7.695	7.533
6.609	5.655	5.362	6.190	5.907	6.446
6.928	8.039	7.408	7.613	7.842	7.441
4.387	4.763	5.634	4.685	5.050	4.712
6.541	8.255	6.351	7.533	7.578	6.580
6.212	7.760	6.022	7.069	7.066	6.255
6.588	8.171	6.376	7.519	7.500	6.614
4.310	4.478	4.257	4.583	3.795	3.662
5.191	4.484	4.740	3.970	5.155	5.242
1.003	0.818	0.817	1.245	1.527	0.536
1.318	1.286	1.242	1.384	1.074	1.595
5.119	5.051	4.825	4.883	4.675	6.436
6.720	6.377	6.554	6.366	6.493	7.270
7.549	7.330	8.014	7.928	7.813	7.642
2.305	2.691	2.063	2.524	3.377	2.532
1.771	1.580	1.422	2.093	1.263	2.194
4.506	5.201	5.098	4.441	4.928	3.908
3.003	2.545	3.780	2.518	3.604	2.895
3.728	3.104	3.565	3.726	2.817	4.126
2.705	2.500	2.061	2.149	1.718	2.391
7.359	6.475	7.454	6.791	7.044	7.359
2.998	2.029	3.580	2.177	2.976	2.478
1.598	1.957	1.700	1.601	1.726	2.228
5.333	4.996	5.423	5.258	5.203	5.088
5.712	5.580	5.438	6.384	6.034	6.136
4.315	3.563	2.567	4.652	3.556	2.564
6.218	6.039	6.876	6.515	6.394	6.189
7.725	7.946	7.778	8.149	7.287	8.006
4.132	4.836	3.838	4.807	4.195	3.509
4.036	4.938	3.795	4.184	3.559	4.011
2.359	2.068	3.216	2.038	2.501	2.095
6.979	7.016	7.165	7.256	7.519	6.755
4.425	5.084	3.797	4.742	4.844	4.886
3.565	4.209	3.819	3.837	4.343	4.463
2.881	2.194	2.882	3.251	2.689	3.194
2.653	3.253	3.059	2.821	1.756	2.408
3.994	4.179	4.804	4.668	4.611	3.992
2.859	2.685	2.358	2.832	2.731	3.361

2.669	3.063	3.351	2.651	2.757	3.394
3.470	4.122	4.969	2.950	5.304	2.212
5.328	6.858	5.708	6.404	5.928	5.265
6.975	8.573	7.845	8.224	8.223	6.814
4.224	4.581	4.330	5.197	5.657	3.868
5.332	6.994	5.348	5.989	5.775	5.585
5.242	5.162	5.360	5.304	5.437	5.516
3.899	3.360	4.000	3.292	3.529	3.887
2.487	2.830	1.680	2.317	2.676	2.862
2.117	2.345	3.108	2.388	2.882	3.107
6.218	5.933	6.424	6.464	6.532	6.749
2.356	2.435	2.503	2.839	2.537	2.913
2.366	4.164	2.139	2.216	2.574	2.202
4.734	3.417	4.459	4.681	4.484	5.276
1.986	3.122	2.022	2.008	1.841	2.370
5.837	5.489	5.849	5.886	5.523	5.735
4.808	5.186	4.795	4.636	4.864	4.616
5.600	4.853	5.713	5.781	4.414	6.642
3.328	3.342	3.925	3.733	4.113	3.313
4.663	4.771	4.882	5.394	5.202	5.061
4.504	5.507	4.754	5.151	5.459	4.247
4.989	4.922	4.907	5.206	5.186	5.009
4.124	4.423	4.170	3.811	4.818	3.839
6.401	6.414	6.306	5.857	5.559	6.317
5.697	6.101	5.500	5.226	5.875	5.931
6.697	6.870	7.229	6.215	7.521	6.896
3.390	4.330	3.590	5.233	4.262	3.031
4.745	4.091	4.338	5.108	3.522	5.665
8.489	7.972	8.812	8.547	8.585	8.675
7.157	6.636	7.287	7.060	6.769	7.755
2.453	3.032	2.690	2.216	2.621	1.929
5.484	4.678	5.722	5.095	5.776	5.358
7.349	7.296	7.788	7.641	7.681	7.483
2.882	3.248	2.658	3.081	2.415	2.572
5.087	5.067	5.248	5.481	5.327	5.491
5.977	6.194	7.124	6.538	6.520	6.159
5.757	5.619	6.958	6.078	6.526	6.143
6.215	6.434	5.957	6.415	6.189	6.338
6.207	6.087	6.575	6.448	6.446	6.285
5.728	5.297	5.351	5.634	5.608	6.138
4.204	2.173	4.330	4.178	3.579	4.694
3.299	3.112	3.636	3.884	3.336	3.933
3.117	3.558	2.655	3.240	3.565	3.441
5.915	6.226	6.160	5.393	5.874	6.266
6.195	6.463	6.541	5.528	6.175	6.477
3.148	3.588	3.201	2.943	2.325	4.732
1.150	2.808	3.592	1.583	0.919	1.895
2.016	3.871	3.099	4.002	3.424	2.527
3.615	3.850	3.426	3.703	4.511	3.190
5.169	4.217	4.993	5.210	4.866	5.246

2.450	2.598	2.072	2.120	2.067	3.745
4.650	3.027	4.027	5.191	3.315	5.563
2.409	1.958	2.733	1.798	2.309	2.806
3.738	2.724	4.098	3.918	3.864	4.613
2.582	2.551	2.408	3.087	2.751	3.263
2.915	3.149	2.932	3.368	2.391	2.931
2.619	2.168	1.949	2.439	1.327	2.176
2.684	1.898	2.503	2.669	2.333	2.688
6.920	5.689	6.926	6.873	5.758	6.995
5.397	6.552	5.249	4.807	6.068	4.620
8.874	8.389	8.957	8.562	8.694	8.884
3.959	4.040	3.381	3.552	3.448	4.726
6.045	6.244	6.247	5.884	6.517	6.222
3.432	3.406	3.466	2.599	3.954	2.507
3.970	3.967	4.270	4.444	4.375	3.922
4.843	4.744	4.818	5.149	4.862	5.772
3.172	3.183	3.403	3.418	2.829	3.757
5.421	5.264	5.549	5.008	5.188	5.843
4.672	4.337	5.003	4.315	4.935	4.162
8.941	8.532	9.104	8.741	8.947	8.940
2.808	3.326	2.844	2.862	2.969	3.402
5.086	4.595	4.672	4.628	4.920	6.055
5.990	5.008	5.914	6.072	6.145	6.193
8.562	9.161	9.060	9.452	9.700	8.737
4.227	4.626	4.916	4.337	5.131	4.047
5.090	4.616	5.230	4.884	4.558	5.045
2.778	4.276	2.208	1.984	1.799	1.963
7.275	6.894	7.601	7.450	7.493	7.302
5.392	5.561	5.386	5.135	5.777	5.233
6.034	7.026	6.337	6.581	6.877	6.485
5.018	5.362	4.939	5.496	5.570	4.914
3.933	3.771	3.141	3.250	3.624	2.125
8.282	8.701	8.772	8.723	8.623	8.560
1.761	1.556	1.574	1.716	1.733	1.418
2.444	2.685	2.322	2.078	2.991	2.881
3.997	5.592	4.958	4.224	4.061	5.065
6.804	7.399	7.060	7.094	7.101	7.076
8.674	8.945	9.027	8.982	8.720	8.291
8.420	8.360	8.210	8.691	8.157	8.853
5.192	6.538	5.808	6.407	6.737	5.170
2.710	3.122	2.567	2.809	2.573	2.612
2.302	2.190	2.325	2.232	2.651	2.213
2.010	2.346	2.026	2.176	1.490	2.407
2.948	2.545	2.750	3.756	3.072	2.093
4.839	4.086	5.895	5.458	5.441	4.949
3.520	3.748	3.640	3.938	4.307	3.521
2.562	1.993	2.162	1.500	1.504	2.536
3.365	2.976	3.555	3.195	3.290	3.545
4.394	4.421	4.328	4.216	4.497	4.594
4.039	3.463	3.889	3.729	3.796	5.057

3.722	3.405	3.726	4.479	3.671	4.283
5.175	4.389	4.653	5.046	5.210	5.155
4.439	5.157	4.051	3.850	4.651	4.602
5.125	4.992	4.724	4.902	5.084	5.120
4.796	4.618	5.556	5.023	5.094	4.774
4.983	4.901	4.611	4.796	3.968	5.351
5.422	5.143	5.406	5.339	6.113	5.905
2.836	2.397	2.932	2.662	3.049	3.269
5.513	4.878	5.866	5.808	5.440	5.439
5.772	5.429	5.740	5.683	5.608	6.295
5.072	6.447	4.269	6.327	5.483	5.010
6.373	6.364	6.556	6.018	5.959	6.796
6.291	6.359	6.530	6.647	6.534	6.411
6.426	7.240	7.399	7.394	7.050	6.382
8.748	9.402	8.894	9.421	9.429	8.712
9.264	9.208	9.294	9.105	9.016	9.259
4.940	6.318	6.229	6.862	6.447	4.501
4.321	5.236	3.874	4.929	5.266	3.794
3.658	3.594	3.491	3.815	3.869	3.659
3.212	4.302	3.488	3.125	3.925	3.095
4.949	7.288	6.942	5.263	7.292	4.533
3.485	4.841	4.539	4.850	4.650	4.094
9.238	9.469	9.326	9.071	8.986	9.175
4.547	5.036	5.390	4.527	4.939	4.733
3.679	4.356	4.762	4.217	4.619	3.588
1.972	1.459	3.276	2.623	2.441	1.912
5.831	6.052	5.821	6.385	6.529	6.127
5.411	4.182	5.116	5.034	4.184	5.827
7.174	7.009	7.484	7.500	7.427	7.542
5.867	5.669	6.370	6.195	6.056	5.863
5.371	6.502	4.895	5.719	5.579	5.510
8.060	8.530	8.576	8.816	8.638	8.650
4.475	3.580	4.627	4.115	3.765	4.757
4.956	4.807	5.026	5.419	6.532	6.170
2.812	6.231	4.035	3.488	4.722	1.997
3.915	3.523	4.293	4.027	4.426	3.997
5.660	5.909	5.836	5.351	5.659	5.732
5.824	5.467	6.315	5.854	6.026	5.881
3.826	3.301	3.421	3.885	3.712	3.683
5.613	5.288	5.551	5.663	5.499	5.566
6.480	6.661	6.550	6.543	6.722	6.658
4.285	4.268	4.285	5.212	4.443	4.272
7.695	7.290	7.761	7.973	7.581	7.618
2.614	4.099	3.513	3.314	4.030	2.038
5.941	5.849	6.326	6.433	6.114	5.835
4.897	5.923	5.582	5.047	5.846	5.023
3.587	4.255	2.999	4.401	3.729	4.155
3.999	3.955	4.202	4.011	4.719	4.421
3.446	3.463	3.048	3.221	3.775	3.588
6.870	6.944	6.894	6.785	6.662	6.847

4.896	5.228	5.277	5.553	5.726	4.771
3.326	4.968	2.963	4.877	4.160	3.943
6.484	6.764	6.967	7.029	6.586	6.632
4.271	4.100	6.035	5.025	4.835	3.785
3.379	2.617	3.153	3.544	3.225	3.880
2.760	3.240	3.313	2.850	2.365	2.066
3.412	4.749	4.010	3.947	4.395	3.543
6.932	6.219	6.998	6.795	6.784	6.764
7.859	7.365	7.697	7.551	7.460	8.197
5.819	3.537	5.386	5.762	3.509	5.523
4.546	5.097	4.281	5.154	4.710	4.489
4.091	3.815	3.714	4.500	3.624	4.983
4.532	5.351	5.138	4.915	4.985	4.188
3.888	4.027	4.323	4.428	4.253	3.687
3.122	2.839	3.406	2.622	3.013	2.830
4.810	4.457	4.932	4.384	4.230	5.350
5.425	5.798	5.470	5.525	5.411	5.113
6.441	5.982	6.282	6.342	6.654	6.568
5.678	5.626	5.480	5.752	5.237	6.071
7.536	7.454	7.586	7.492	7.543	7.731
3.298	3.370	3.643	3.000	2.614	3.484
2.664	3.011	2.339	2.783	2.359	2.591
5.421	5.129	4.641	5.510	4.954	5.490
4.178	4.096	4.285	4.232	5.063	4.455
3.626	3.106	3.712	2.866	3.445	3.997
6.467	6.351	6.566	6.229	6.106	6.145
5.141	5.642	5.585	4.845	5.463	5.061
2.713	2.369	1.659	3.314	2.566	2.544
7.939	7.805	8.591	8.257	8.412	7.775
2.490	1.620	2.431	2.189	2.386	2.890
6.829	6.319	6.932	7.062	6.636	7.260
5.317	5.921	6.393	5.562	5.895	5.489
5.946	6.053	6.279	6.197	6.466	6.020
5.766	5.446	6.062	6.258	5.975	6.250
4.711	4.579	4.417	4.756	4.680	4.714
3.770	3.696	3.253	3.185	3.145	3.199
6.145	6.561	6.459	5.996	6.670	6.415
2.575	1.922	1.274	2.918	1.423	2.399
3.532	3.695	3.313	4.064	3.605	3.470
9.866	9.664	10.059	9.988	9.855	10.098
3.227	1.828	2.265	1.369	1.856	1.616
2.569	1.590	1.995	1.855	1.820	1.825
4.147	3.484	4.049	3.781	3.633	4.081
5.398	5.789	5.708	5.788	5.460	5.698
7.631	7.071	7.575	7.607	7.423	7.487
5.139	5.402	5.586	5.414	5.408	5.481
3.180	3.206	3.514	3.484	3.112	3.701
5.157	6.703	5.416	5.943	5.404	4.994
3.288	2.953	3.176	3.313	2.974	3.955
4.688	4.057	4.999	4.306	4.190	4.658

5.946	5.962	6.339	5.508	5.804	5.227
8.362	8.836	8.578	8.026	8.997	7.905
8.945	9.082	9.708	8.953	9.669	9.102
4.516	5.030	4.200	4.337	4.648	4.772
2.482	1.428	2.146	1.475	2.218	2.260
4.566	4.807	4.144	4.576	4.327	4.796
2.423	2.576	3.473	2.634	2.356	2.437
3.810	4.047	3.832	4.053	4.291	3.918
7.465	7.548	8.130	7.841	7.988	7.710
3.214	3.526	3.849	2.582	2.797	3.000
5.989	6.414	6.125	6.249	6.688	6.211
5.289	5.801	5.040	5.475	5.671	5.113
6.525	5.823	6.318	6.344	6.501	6.881
2.776	2.193	2.378	2.708	2.540	2.616
5.899	5.919	5.958	6.369	6.256	6.352
3.704	3.436	4.195	3.789	3.307	4.037
3.630	2.585	3.710	3.851	3.377	4.675
5.195	4.619	4.798	5.432	4.934	5.087
7.180	6.510	7.357	6.939	7.263	7.982
3.531	4.083	3.496	3.415	3.923	3.686
3.765	4.376	3.513	3.641	3.627	3.576
6.131	7.196	5.986	6.615	6.568	6.524
6.796	6.605	7.318	6.998	6.555	7.182
5.601	5.377	6.204	5.902	5.974	5.579
4.270	4.110	4.329	3.895	4.123	4.341
3.693	3.714	3.572	3.703	4.062	3.671
6.285	5.477	6.207	6.099	5.278	6.931
4.379	3.570	5.111	4.046	4.709	4.704
4.448	6.671	4.984	4.707	5.924	3.949
4.253	4.842	3.907	4.408	5.391	3.871
6.496	5.993	6.663	6.455	6.667	6.138
2.647	1.850	1.887	2.248	2.836	2.566
5.491	5.564	5.715	5.450	5.635	5.746
4.784	5.341	5.220	5.392	5.512	5.015
6.147	5.543	6.476	6.234	6.289	6.390
5.993	5.885	6.312	6.307	6.094	6.044
5.613	4.956	5.380	5.387	5.611	5.544
3.791	4.292	3.940	4.612	3.704	3.837
5.341	4.703	5.310	5.600	5.237	5.942
3.766	3.774	3.928	3.998	3.778	3.616
7.077	7.446	7.146	7.634	7.643	7.220
6.413	6.560	6.570	6.554	6.782	6.355
7.479	7.350	7.358	7.651	7.531	7.827
3.257	2.946	2.971	2.365	3.041	2.255
6.466	5.915	6.784	6.744	6.329	6.180
6.752	6.808	6.935	6.896	6.885	6.809
5.200	5.365	5.941	5.667	5.670	5.133
7.410	6.902	6.746	7.265	6.934	7.518
5.968	5.273	6.378	5.925	6.216	6.359
1.722	1.988	1.620	1.423	1.400	1.989

4.739	4.990	4.814	4.921	4.949	4.854
3.752	4.508	3.989	3.937	3.622	3.967
4.096	2.662	3.372	4.384	2.364	4.768
9.406	8.636	9.414	9.499	8.817	9.556
7.955	8.114	8.365	8.117	8.033	8.338
2.453	3.688	2.288	2.573	3.518	3.098
6.641	6.777	6.781	6.695	6.435	6.977
5.343	3.987	4.756	5.459	4.315	6.113
3.089	1.308	2.318	2.842	1.643	3.526
7.411	7.621	7.742	7.857	7.679	7.618
2.160	1.785	2.417	1.680	0.964	1.930
2.555	1.823	1.911	1.861	1.804	2.157
8.216	8.340	8.154	8.566	8.124	8.444
9.708	9.813	9.689	10.089	9.614	9.995
4.072	3.132	3.515	3.600	3.534	3.380
4.715	4.804	4.897	4.793	5.143	5.016
8.227	8.040	8.451	8.585	8.314	8.418
9.136	9.337	9.196	9.562	9.360	9.335
9.642	10.047	10.079	10.016	10.141	9.659
6.844	6.763	6.688	7.375	6.625	7.284
7.941	7.932	7.725	8.297	7.964	8.273
7.279	7.250	7.479	7.674	7.590	7.667
5.202	4.720	5.186	4.825	5.011	5.222
6.194	6.605	6.226	6.299	6.699	6.628
3.268	4.495	4.024	3.341	4.335	3.737
8.651	8.266	8.723	8.338	8.554	8.875
5.994	3.931	4.670	6.132	5.022	6.674
3.637	4.812	4.675	3.397	3.606	3.638
4.507	4.976	4.681	5.047	5.048	4.611
6.099	5.628	6.336	6.313	6.006	6.470
4.761	5.019	5.129	4.889	4.756	5.299
6.574	6.630	6.581	6.727	6.498	6.888
6.686	6.163	6.566	6.659	6.660	6.437
3.567	3.506	3.816	2.681	3.537	3.690
6.376	5.902	6.246	6.059	6.148	6.769
7.529	7.429	7.254	7.662	7.411	7.898
2.790	3.443	2.402	2.990	2.899	1.983
3.109	2.876	3.261	2.263	3.070	3.441
6.181	6.477	6.354	6.442	6.532	6.665
5.526	6.155	6.309	5.826	6.007	5.111
3.109	3.785	2.142	2.719	2.635	2.668
8.202	8.839	8.560	8.845	8.446	8.367
3.866	3.865	4.010	4.224	4.487	4.319
3.870	2.140	3.626	3.795	3.224	4.209
5.883	5.644	6.266	5.231	6.255	6.195
7.103	6.582	8.013	7.401	7.366	7.110
1.521	1.836	2.277	1.579	1.531	1.572
4.867	5.149	5.609	5.509	5.433	4.706
4.505	3.840	3.717	4.352	4.215	5.122
2.243	3.014	2.032	2.518	2.695	1.883

4.861	5.918	4.753	5.192	5.259	4.435
6.772	7.323	7.320	7.649	7.746	7.681
4.892	4.614	4.788	5.107	4.215	5.944
3.705	3.301	3.688	3.346	3.610	3.493
2.979	2.717	3.284	2.940	3.270	3.451
2.829	3.736	4.084	3.475	4.642	3.567
2.094	2.043	1.521	2.211	1.895	2.197
8.163	8.157	8.667	8.397	7.879	8.187
4.613	5.367	4.569	4.927	4.988	4.377
3.591	3.842	3.994	3.244	3.941	3.581
2.468	2.624	2.446	1.363	2.786	2.192
4.230	4.643	5.150	3.911	4.687	4.429
7.051	7.151	7.389	6.779	7.865	6.377
4.261	4.348	4.246	4.391	4.340	4.827
4.713	4.074	3.905	4.219	4.217	5.022
4.714	4.420	4.808	4.705	4.384	4.832
6.514	6.077	6.487	6.405	6.223	7.044
4.621	4.844	4.386	4.591	4.730	4.126
3.921	3.862	3.909	4.222	4.007	4.514
3.309	2.545	3.556	2.996	3.277	2.631
5.438	5.166	5.232	5.219	4.315	6.201
6.523	6.593	6.574	6.405	6.546	6.245
5.812	5.685	5.797	5.955	6.055	6.510
4.775	4.171	5.138	5.212	5.009	4.389
2.271	2.644	2.126	3.133	2.157	2.853
7.951	8.483	7.839	8.010	7.971	8.109
3.012	3.129	2.800	3.928	3.321	3.593
4.252	4.993	4.916	5.128	5.172	3.393
4.960	5.637	5.610	5.484	5.607	5.331
7.872	7.428	8.019	7.491	7.852	7.857
4.537	4.037	4.985	4.447	5.055	4.949
4.789	4.530	4.967	5.092	5.732	5.035
6.330	5.813	6.143	6.357	6.237	6.808
5.840	5.719	6.227	5.859	6.156	6.053
6.649	6.235	7.488	6.439	6.593	6.824
4.624	4.042	4.320	4.197	4.374	4.617
7.415	7.059	7.182	7.043	6.867	7.347
3.673	3.623	3.571	4.028	3.647	4.319
5.403	5.189	4.720	5.804	5.308	5.632
2.210	2.286	2.306	2.459	2.405	2.603
2.995	2.401	3.123	2.688	3.058	3.442
3.133	3.111	3.209	3.255	2.882	3.721
2.775	2.973	3.125	2.329	2.425	3.768
6.644	6.499	7.065	6.797	6.916	6.806
3.315	6.326	3.964	5.748	4.403	3.903
4.471	4.498	4.712	4.166	5.159	4.097
8.242	8.944	9.154	8.760	9.188	8.179
2.679	2.506	2.450	2.441	2.342	2.502
4.942	4.676	4.672	5.257	4.983	5.404
4.718	4.191	4.486	3.878	3.812	4.650

2.111	2.065	2.848	1.776	2.599	3.339
5.125	4.504	5.105	4.351	5.401	4.370
3.844	4.563	4.290	4.358	4.597	4.375
3.971	3.542	3.893	3.944	3.295	4.538
4.611	4.385	4.886	5.023	4.420	4.598
3.861	3.374	3.856	3.560	3.547	3.598
3.161	2.559	2.832	2.967	3.133	3.204
2.242	2.377	1.391	2.160	1.513	1.519
2.199	2.141	1.581	2.469	1.902	2.362
3.686	3.212	3.399	3.762	3.707	3.019
5.199	5.169	5.550	5.464	5.035	5.883
7.732	8.227	8.053	7.676	7.859	7.727
3.664	4.575	3.062	3.996	4.538	3.844
7.621	6.902	6.749	7.695	6.588	8.142
4.329	3.198	4.088	4.188	3.585	4.772
4.186	3.952	3.990	4.052	3.095	4.601
6.822	7.761	6.799	7.328	7.225	7.020
3.745	3.340	3.462	3.708	3.104	3.678
5.065	4.672	5.084	4.836	4.533	5.082
2.250	1.929	2.002	2.429	2.416	2.356
3.950	3.646	3.164	3.418	3.082	3.257
7.729	8.256	7.810	7.882	8.096	7.839
8.660	8.867	8.659	9.083	8.710	8.511
3.541	4.100	3.740	3.626	4.596	3.248
4.359	4.888	4.473	4.167	4.548	3.837
5.312	6.743	4.999	5.875	5.935	4.978
3.474	3.702	4.533	3.524	4.101	3.584
2.853	3.435	2.479	3.469	2.987	3.166
3.512	2.484	4.121	2.701	3.675	3.180
4.668	5.715	3.594	6.243	5.701	4.284
4.476	5.015	5.198	4.812	4.624	4.895
7.637	8.240	7.838	8.422	8.291	7.763
3.174	2.151	2.895	2.479	3.185	3.388
3.521	4.039	3.350	2.873	3.777	3.095
2.400	3.901	1.824	1.475	1.128	1.325
4.103	4.277	4.534	4.465	3.176	5.337
4.854	4.855	4.709	5.049	4.779	4.667
5.508	5.315	4.860	5.366	4.985	4.797
3.229	2.955	2.754	3.415	2.983	2.971
4.027	4.476	4.099	4.230	3.618	3.820
8.195	8.414	8.329	8.621	8.429	8.568
5.592	5.726	5.632	6.079	5.712	5.911
3.442	3.001	3.246	3.113	2.916	3.002
3.236	1.757	3.071	3.092	3.254	2.541
3.814	4.544	4.405	3.971	4.559	3.752
5.086	4.908	4.890	5.062	5.392	4.337
7.353	7.485	7.708	6.716	7.546	7.290
3.473	3.848	3.344	4.208	4.241	2.815
6.653	6.191	6.660	6.515	6.367	6.754
5.996	6.587	5.901	6.311	6.200	6.469

2.510	3.142	2.711	3.054	3.060	2.622
3.102	2.242	3.323	3.166	2.319	3.596
7.023	6.562	7.343	7.522	6.905	7.433
4.838	4.624	4.927	4.729	3.850	5.280
5.765	5.581	6.143	5.935	5.527	6.425
3.989	3.790	4.291	3.998	4.022	3.902
4.560	4.685	4.271	4.129	4.573	4.575
6.121	5.523	6.156	5.927	5.837	6.365
5.330	4.836	5.161	5.090	4.972	5.428
4.491	3.852	3.983	4.623	3.822	5.019
2.625	3.220	2.842	3.122	2.984	3.195
3.232	3.799	2.970	3.338	3.737	2.816
5.847	7.306	5.940	5.820	6.652	5.874
5.200	4.996	5.109	5.422	4.942	5.003
4.113	4.115	3.703	4.777	4.104	4.635
2.572	2.587	2.377	2.466	3.099	2.393
2.978	2.503	2.390	2.666	3.349	2.892
3.595	4.672	3.498	3.849	3.844	3.057
5.936	5.312	6.358	6.122	5.752	6.247
4.709	4.652	4.327	4.235	4.442	3.967
5.348	5.194	5.209	5.019	5.144	5.577
8.450	8.755	9.223	8.706	8.566	8.394
4.483	5.852	5.171	5.680	6.312	4.727
6.898	6.563	6.833	6.573	6.940	7.181
6.778	7.340	7.205	7.328	7.192	6.759
2.600	3.385	3.468	2.621	2.908	2.249
4.340	4.126	3.629	4.477	3.713	4.637
3.386	3.974	3.712	4.029	3.265	3.285
6.891	8.105	7.433	7.742	8.217	7.050
3.417	2.517	3.842	2.582	3.383	3.265
5.604	4.900	5.995	5.810	5.710	6.231
3.074	3.354	3.516	3.957	3.563	2.709
4.387	4.660	5.185	4.220	4.735	3.993
3.240	3.713	3.468	2.904	3.997	3.373
1.787	0.934	1.137	2.048	1.231	2.191
4.420	4.933	4.679	4.944	4.805	3.537
4.389	3.472	4.902	4.540	4.150	4.096
4.915	4.277	4.520	5.294	5.006	5.420
5.209	5.333	4.924	5.290	5.351	5.305
3.319	2.702	3.141	2.473	3.618	3.136
3.235	3.315	3.213	2.974	2.979	3.279
3.504	2.739	3.134	3.158	3.668	3.007
3.366	2.922	3.446	3.741	3.324	3.473
3.656	4.197	4.183	3.933	3.891	3.927
2.739	2.173	2.870	3.176	2.683	4.649
2.868	1.252	1.055	1.410	1.251	1.366
2.922	3.861	2.186	2.626	2.377	2.769
2.085	1.359	1.362	0.974	0.938	1.277
5.730	5.762	6.030	6.183	6.126	6.044
5.474	5.613	5.187	5.739	5.773	5.758

4.609	4.669	4.467	4.540	4.382	4.790
3.735	3.798	3.724	3.239	3.627	4.060
3.811	2.042	2.803	2.489	2.791	2.803
3.941	4.113	3.884	4.203	3.380	4.329
6.237	5.554	6.267	5.962	5.923	6.277
2.937	2.666	2.519	2.907	2.655	3.027
3.684	3.585	3.507	3.723	3.485	3.669
1.250	1.237	1.668	1.504	1.339	1.337
4.965	4.264	4.918	5.431	4.244	4.948
2.168	1.848	2.744	2.418	1.862	2.852
4.093	4.914	4.839	4.129	4.029	4.368
3.952	4.525	3.720	4.032	4.108	4.326
5.927	4.941	5.655	5.527	5.185	6.966
4.206	3.815	4.043	3.124	3.343	4.401
4.542	4.224	4.496	4.399	4.468	4.620
5.504	6.082	5.083	5.369	5.566	5.249
6.374	6.280	6.111	6.623	6.154	6.602
4.325	4.315	4.149	4.454	4.360	4.481
6.871	7.263	7.066	7.350	7.117	7.266
5.093	4.914	5.046	5.650	5.045	4.992
4.869	4.790	5.217	4.236	5.073	4.366
3.021	2.970	2.486	3.045	2.492	3.286
4.280	3.488	3.926	4.439	3.960	4.588
3.142	3.173	2.593	2.376	3.018	2.289
3.069	3.148	3.027	2.897	2.466	2.817
3.803	3.666	3.924	3.001	3.760	4.007
6.260	7.366	6.492	6.781	7.001	6.477
4.863	4.138	4.705	4.620	4.293	5.432
2.129	2.421	1.737	1.692	2.541	2.602
4.682	3.931	3.800	4.008	3.811	4.578
2.870	2.567	3.556	3.149	3.406	2.862
5.516	5.569	5.530	5.844	5.750	5.838
2.573	1.099	1.228	2.279	1.442	1.885
4.110	4.146	4.514	5.024	4.861	4.686
3.700	3.852	3.655	3.614	4.230	3.796
3.869	3.426	3.591	4.356	3.524	3.282
3.713	4.108	2.901	3.269	3.185	3.375
2.650	2.845	2.770	2.646	3.012	2.110
3.284	3.385	3.481	2.979	3.936	3.225
4.409	5.926	4.396	5.938	5.805	4.469
4.164	4.419	4.355	4.855	3.923	3.684
3.086	2.365	2.566	3.261	2.745	3.535
2.184	2.781	2.343	2.590	1.958	2.911
4.328	3.925	4.149	3.958	4.127	3.511
2.996	3.853	2.809	2.667	2.915	3.062
6.311	6.527	6.517	6.652	6.365	6.691
3.074	2.378	3.348	3.868	3.477	3.396
3.775	3.915	3.621	4.003	3.647	4.027
2.921	1.119	1.264	1.069	1.590	1.180
5.980	7.232	6.419	6.288	6.875	5.744

5.623	2.762	4.231	2.340	4.675	4.348
3.494	2.168	3.135	2.944	2.913	3.135
6.330	5.841	6.616	6.554	6.529	6.485
5.744	4.580	5.912	5.383	5.691	5.120
3.116	3.160	2.863	3.002	2.672	3.069
2.034	2.373	3.098	2.040	2.280	2.120
3.472	3.218	3.511	3.509	3.573	2.805
3.364	2.913	2.750	3.568	3.499	3.873
4.146	3.258	3.722	3.618	3.645	3.997
4.747	4.514	5.026	5.191	5.057	5.048
5.898	5.036	5.488	5.481	5.416	6.061
3.780	4.764	2.873	3.926	4.278	3.415
3.450	3.584	3.520	4.133	3.203	3.393
3.472	1.780	2.008	2.306	2.462	1.945
2.751	2.781	2.513	3.740	3.820	2.935
3.406	3.900	3.527	3.881	2.977	4.196
3.757	5.639	3.660	5.004	4.826	3.461
6.681	8.746	7.499	8.265	8.255	6.795
7.278	8.996	7.977	8.737	8.637	7.468
4.309	4.857	3.248	4.318	3.619	4.017
3.823	3.278	3.749	4.300	3.576	4.122
3.153	3.173	3.084	3.792	2.614	4.991
2.122	3.208	2.169	4.263	2.483	2.135
4.933	4.490	3.758	5.254	3.509	5.624
3.716	3.719	4.348	2.800	4.224	3.482
2.754	2.807	2.364	2.622	2.327	2.808
4.965	5.912	4.761	5.212	5.622	5.094
4.364	4.609	4.526	5.033	4.600	4.075
6.326	7.130	6.409	6.791	7.168	6.151
3.906	4.848	3.838	4.527	4.630	3.942
3.860	4.802	3.583	4.377	4.501	3.714
3.934	4.867	3.725	4.448	4.577	3.765
3.980	4.970	3.745	4.471	4.673	3.788
3.714	5.199	3.812	4.876	4.409	3.903
3.527	3.801	4.063	3.679	4.013	3.104
3.084	2.488	2.544	2.166	2.362	2.494
2.518	3.095	2.728	2.054	2.566	2.871
1.768	1.800	2.281	2.303	2.487	2.066
3.657	3.436	3.400	3.046	3.378	3.205
2.994	2.496	3.584	3.258	2.775	2.647
4.570	4.117	4.189	4.490	4.277	4.052
3.968	4.143	3.829	3.988	4.268	3.913
4.873	4.600	5.061	5.160	5.089	5.131
1.630	1.722	1.670	1.817	1.353	1.828
4.643	4.991	4.465	3.925	4.873	4.899
4.406	4.398	4.104	4.073	4.147	3.983
7.244	7.158	7.349	6.840	7.367	7.020
3.184	3.539	3.102	3.049	4.787	2.838
3.129	3.153	2.980	3.087	2.900	3.149
6.192	6.030	6.302	5.899	6.286	6.179

2.517	2.894	2.912	2.925	2.695	3.009
3.833	3.402	3.827	4.280	4.271	3.646
3.087	2.363	2.465	2.159	2.876	3.114
6.016	6.216	6.148	5.743	5.939	6.014
4.665	4.058	4.672	4.757	4.557	4.959
5.174	4.706	4.777	5.178	5.192	6.114
2.947	2.162	2.219	3.384	2.316	4.218
4.329	4.688	4.181	4.301	3.928	4.294
3.277	3.447	3.808	3.529	3.663	4.101
9.010	10.276	9.596	10.168	9.983	9.008
4.040	5.223	4.547	5.098	4.863	3.561
4.563	4.025	4.752	4.483	4.218	4.822
5.063	4.898	4.839	5.449	5.107	5.530
1.840	1.724	1.809	2.204	1.758	2.270
3.468	3.211	3.393	3.791	2.910	3.130
4.048	4.003	3.939	4.090	4.678	4.180
1.811	2.021	2.261	2.512	1.917	2.489
2.270	1.283	2.320	1.382	1.500	2.346
8.337	8.736	7.584	8.893	9.012	8.261
8.640	9.041	7.892	9.191	9.306	8.575
8.529	8.916	7.772	9.094	9.214	8.423
8.644	9.016	7.892	9.199	9.339	8.535
8.680	9.061	8.005	9.223	9.313	8.533
8.682	9.086	7.906	9.215	9.377	8.557
8.676	9.049	7.915	9.226	9.354	8.537
7.720	8.106	7.093	8.316	8.419	7.678
7.860	8.197	7.196	8.431	8.542	7.732
2.198	2.209	3.423	2.024	2.541	2.421
3.775	4.319	3.686	4.583	3.972	3.332
3.388	3.479	2.183	2.491	3.028	2.539
2.602	1.843	1.171	2.187	3.107	2.153
4.006	3.672	4.314	4.071	4.774	4.013
5.164	7.088	7.552	5.492	6.739	4.190
3.202	4.366	4.106	3.638	3.105	2.425
5.194	5.219	5.129	5.693	5.226	5.790
5.870	6.398	6.029	5.883	5.613	4.369
4.079	4.032	3.615	4.248	4.389	4.620
1.821	1.568	1.466	1.813	2.081	1.681
3.859	3.484	3.772	3.540	3.427	3.396
4.515	3.531	4.119	4.133	4.205	4.380
3.353	3.079	3.424	3.456	3.198	3.854
3.361	3.381	3.439	3.440	4.074	3.963
3.959	3.583	4.280	3.991	3.539	4.025
6.212	6.312	6.343	6.395	6.184	6.478
5.304	5.607	5.568	6.020	5.347	5.684
3.108	2.137	2.159	2.371	2.698	2.976
4.455	3.920	4.430	3.994	4.558	5.494
4.142	4.667	4.195	4.992	4.711	4.826
2.910	1.827	3.116	3.651	2.504	2.476
7.779	8.072	7.953	8.026	7.794	7.771

7.970	8.187	8.125	8.209	7.957	7.871
7.634	7.909	7.587	7.696	7.559	7.511
4.283	4.229	4.947	5.454	4.876	3.714
5.293	7.667	5.094	6.345	6.720	5.908
2.785	2.569	3.147	2.526	2.873	2.220
2.625	1.159	1.498	1.879	1.378	2.043
5.009	5.044	5.464	4.942	5.439	5.145
3.783	2.564	4.196	3.243	3.614	3.418
3.277	2.652	3.130	2.570	2.880	2.579
3.365	3.561	3.224	2.875	2.962	2.734
3.245	2.396	3.498	2.332	4.575	1.753
2.740	2.484	3.137	2.823	2.595	2.600
2.621	3.446	2.438	2.619	3.603	3.615
5.853	5.334	5.783	6.006	5.948	5.696
5.082	4.845	5.405	5.311	4.778	4.891
3.102	2.684	3.119	3.842	2.682	2.237
2.305	2.744	2.356	2.689	3.321	2.260
4.129	4.226	3.775	3.290	4.507	3.603
3.316	2.231	1.641	1.520	1.920	1.808
2.021	2.019	1.622	2.641	1.439	2.669
5.612	5.986	5.512	5.351	5.476	5.374
2.663	2.491	2.421	2.118	2.632	1.970
3.100	3.125	3.521	3.425	3.484	2.731
3.245	2.804	2.966	2.711	3.113	3.377
4.253	4.316	4.484	4.287	4.173	4.141
4.029	3.050	3.239	3.310	3.039	3.357
4.715	4.489	4.994	4.489	4.842	4.723
4.528	4.783	4.584	4.465	4.889	4.839
3.324	4.142	4.263	3.967	3.749	3.806
4.776	5.135	5.146	5.214	5.127	5.126
5.188	5.242	4.811	5.578	5.199	5.216
5.539	5.297	5.724	5.983	5.799	6.545
6.606	6.251	6.687	6.467	6.257	6.914
3.195	2.921	3.551	3.631	3.913	3.142
6.223	5.605	6.409	5.563	5.938	6.470
2.754	2.735	1.649	2.324	1.788	3.328
4.516	4.553	4.951	5.313	5.117	4.965
4.768	4.974	4.404	4.306	3.815	5.326
1.830	1.652	1.328	1.557	1.610	1.278
5.110	4.172	5.187	5.229	5.015	4.821
4.637	5.058	5.231	5.303	5.383	4.595
5.535	4.756	5.831	5.031	5.086	5.618
4.377	4.711	4.643	4.813	4.673	4.357
2.288	1.796	2.112	1.983	2.226	3.074
4.957	4.537	3.296	4.023	4.165	5.764
4.908	4.547	4.729	5.200	4.655	5.205
5.321	5.442	4.866	5.106	4.562	6.165
5.080	4.644	5.183	4.841	4.603	5.325
4.468	4.529	4.504	4.749	4.440	4.785
2.499	2.354	3.067	2.527	2.487	2.487

4.806	4.670	4.605	4.700	4.786	5.136
3.745	3.670	4.281	3.875	3.183	5.385
2.624	3.171	2.739	2.959	2.365	3.184
3.266	3.379	3.060	3.827	2.750	4.336
6.376	5.755	6.428	6.268	6.108	6.985
4.613	4.713	4.573	4.901	4.157	5.794
4.855	3.774	4.320	4.517	4.059	5.218
5.562	4.977	4.901	5.732	5.343	5.956
4.545	3.768	3.883	4.216	3.607	4.626
3.181	3.178	2.877	3.389	3.160	3.515
5.857	5.171	5.941	6.133	5.778	6.470
4.707	3.607	4.042	4.883	3.156	5.716
4.847	4.102	5.349	5.596	4.055	6.322
5.554	5.739	5.774	5.473	5.417	6.570
2.074	2.265	2.227	1.588	2.304	2.320
2.830	3.140	2.426	3.119	2.706	3.690
6.779	5.946	6.501	6.737	6.228	7.206
7.919	7.218	7.948	7.722	7.593	8.037
5.192	5.077	5.129	4.999	4.810	5.245
6.834	6.799	6.694	7.213	6.867	7.069
5.018	4.976	5.257	4.694	5.269	5.135
5.312	5.027	5.467	5.321	5.284	5.606
5.895	5.518	6.084	6.439	5.138	7.564
6.244	4.498	5.749	6.593	4.818	7.391
5.790	6.527	7.126	6.386	7.183	5.850
2.662	2.872	2.438	2.223	2.125	2.731
4.059	4.990	4.015	4.741	4.560	3.841
3.852	3.893	3.607	3.784	3.569	4.087
4.379	3.474	4.107	4.305	3.435	5.139
3.829	4.848	3.902	4.369	4.150	4.470
3.404	1.422	3.016	3.224	2.115	4.052
5.863	5.910	5.909	6.193	5.651	7.183
6.284	5.888	6.034	6.427	5.476	6.865
5.939	6.245	6.418	6.290	6.592	6.520
7.281	6.496	7.067	7.126	6.318	7.397
6.945	6.084	6.716	6.884	6.799	7.381
5.718	4.585	4.713	5.505	4.289	6.391
3.626	2.553	3.241	3.576	3.454	4.299
4.137	3.597	3.761	3.875	3.828	4.626
4.682	5.167	4.707	4.719	4.357	5.135
4.293	4.522	4.164	5.168	3.871	6.146
2.876	2.704	2.717	2.736	2.327	2.894
7.115	6.500	6.975	7.044	6.529	7.395
6.222	5.975	5.998	6.594	6.289	6.784
6.050	5.798	6.315	6.476	6.439	6.458
6.851	5.640	6.504	6.853	6.405	7.101
3.578	4.762	3.946	4.017	4.168	3.558
2.449	1.902	2.109	1.889	2.347	2.521
7.459	7.640	7.688	7.306	7.482	7.804
6.012	5.701	6.090	6.079	5.827	6.078

7.031	6.670	6.540	6.033	7.311	6.304
4.068	3.642	4.067	3.309	3.508	4.252
3.054	2.880	3.429	3.695	2.883	2.352
4.982	5.301	5.234	5.348	5.344	4.671
7.779	7.763	8.027	7.981	7.513	7.839
5.374	3.656	5.489	6.158	4.941	5.226
2.663	1.708	2.588	1.469	2.143	2.668
4.111	3.984	4.098	3.674	4.249	4.538
2.820	1.520	1.926	2.558	2.988	3.241
6.725	6.541	6.883	6.973	6.617	7.135
5.516	4.457	5.243	5.091	4.757	5.764
2.270	3.098	2.875	3.394	3.562	2.453
3.119	3.294	4.211	3.525	3.381	3.087
2.875	4.661	3.868	3.848	2.928	3.442
2.082	2.723	2.029	2.549	3.403	1.894
3.108	2.737	3.621	3.789	3.278	2.677
7.097	7.188	7.284	6.832	7.397	6.799
3.269	3.386	2.920	3.190	3.914	3.436
3.822	3.640	3.248	3.790	2.690	3.794
5.054	6.210	5.020	4.833	5.425	4.852
7.953	7.916	8.032	7.870	7.901	7.909
2.710	2.308	3.414	2.898	2.823	3.910
3.305	3.521	3.981	3.554	3.346	4.471
3.782	3.857	3.495	4.639	3.808	3.571
2.336	1.455	1.391	2.264	1.205	2.354
5.655	5.356	6.012	5.923	5.719	5.952
2.855	3.088	2.354	3.365	2.544	3.150
5.795	5.671	5.808	5.846	5.452	5.713
4.257	4.119	4.007	4.400	4.214	4.393
2.177	1.569	1.850	2.360	1.595	2.807
4.648	4.621	4.823	5.459	4.565	4.694
2.376	2.299	1.909	1.732	1.585	3.094
5.476	4.885	5.324	5.330	5.158	5.399
4.793	5.527	5.472	5.731	5.679	5.111
5.527	4.880	4.894	4.158	4.808	4.600
5.598	4.445	4.979	5.320	4.712	5.310
3.341	2.650	2.713	2.325	2.255	2.325
3.809	3.707	3.805	3.453	4.323	3.660
4.857	4.657	5.327	4.885	5.417	5.303
3.016	2.577	2.472	1.634	2.823	2.532
3.487	2.658	2.757	3.207	2.823	3.005
6.701	6.264	6.767	6.961	6.957	6.384
5.342	5.822	6.179	5.582	4.829	4.553
3.170	4.183	5.262	4.530	2.648	4.292
2.963	3.701	4.485	3.782	3.426	2.976
5.652	5.663	6.453	5.239	5.429	5.559
4.874	3.521	4.199	4.408	4.316	4.658
2.115	1.768	1.867	2.020	1.861	2.465
4.610	4.844	4.823	4.981	4.824	4.893
3.988	3.134	3.552	3.552	3.444	2.637

4.092	3.628	4.419	4.099	3.864	3.834
3.611	3.950	4.095	3.565	3.648	3.620
3.111	2.196	2.370	2.978	2.817	2.659
4.021	4.253	4.408	4.490	4.604	3.713
4.205	3.158	3.949	3.489	3.784	3.644
3.015	1.815	2.458	2.127	2.307	2.768
2.395	2.149	2.466	3.084	2.339	2.641
2.986	2.940	3.087	2.349	3.929	2.948
3.962	3.863	3.715	4.049	3.889	4.518
3.336	2.084	2.708	2.349	2.382	2.555
3.591	4.303	3.614	3.785	3.076	3.253
3.404	2.692	2.640	3.292	2.152	3.081
3.258	2.408	2.919	3.057	1.857	3.116
4.106	3.866	3.734	3.290	3.250	4.078
2.637	2.213	2.306	1.906	2.615	2.156
2.516	2.082	1.304	1.546	1.701	1.566
3.195	2.193	2.157	2.394	2.587	2.746
3.608	2.521	2.923	3.524	3.120	3.420
7.638	7.279	7.600	7.688	7.744	7.732
2.563	1.737	1.982	1.779	1.391	1.494
2.863	2.523	2.411	2.306	2.244	3.150
1.992	1.360	1.260	1.557	1.740	1.613
2.042	2.274	1.703	2.010	1.902	1.533
3.980	4.062	4.432	4.409	4.267	3.975
3.963	4.796	3.920	5.166	4.693	4.357
4.557	4.746	4.272	3.828	4.638	4.751
4.132	4.400	3.707	3.640	3.892	3.895
5.193	5.881	6.106	6.247	5.858	5.376
2.802	2.373	2.739	2.113	1.990	1.227
5.956	6.474	6.306	6.217	6.720	5.940
3.664	4.318	3.631	3.677	3.601	3.450
3.166	3.692	3.133	3.051	3.181	3.943
1.673	1.974	1.622	1.593	1.061	2.247
3.086	4.774	3.433	4.878	4.010	3.745
7.669	8.667	9.845	9.526	9.427	8.960
2.616	3.608	3.294	2.637	4.573	1.904
3.089	3.299	2.895	4.846	2.500	3.041
4.115	5.683	4.416	3.846	4.558	4.679
3.112	2.704	3.156	3.260	2.591	3.894
3.976	3.245	4.345	3.670	3.907	2.914
2.505	1.926	2.120	2.349	1.762	2.723
2.446	2.391	2.780	3.014	2.122	2.673
5.488	6.150	4.831	5.784	5.165	5.598
3.881	3.956	3.941	4.945	4.311	3.975
5.432	5.210	5.321	5.272	5.468	5.300
2.943	2.978	3.211	3.380	2.903	3.138
4.715	4.133	5.275	4.350	5.066	4.869
2.441	2.129	2.760	2.889	2.513	2.326
3.281	3.443	3.564	3.543	3.587	3.060
6.439	7.198	7.196	6.788	6.826	6.595

2.096	1.740	1.866	1.637	1.590	1.723
3.926	3.828	3.840	3.696	3.791	4.914
6.981	6.248	7.062	6.652	6.828	7.401
3.577	5.759	3.095	6.088	4.853	4.613
2.794	3.551	3.390	2.748	3.389	3.212
3.705	3.994	4.603	3.877	4.392	3.924
4.976	4.652	5.327	4.923	4.878	5.154
2.588	1.953	2.754	1.960	2.222	3.032
3.889	3.308	2.492	3.603	3.129	3.547
5.422	5.207	5.829	5.773	6.258	6.004
6.235	8.031	5.949	7.667	7.493	7.021
5.575	6.142	5.259	5.759	5.742	5.326
6.335	8.104	7.099	7.293	7.983	6.110
7.438	7.736	7.864	7.525	7.523	7.303
3.462	5.333	3.121	2.771	2.537	2.374
3.440	6.651	2.887	3.243	2.284	2.308
4.340	6.982	4.263	5.735	6.857	5.057
6.549	6.450	6.898	7.220	7.204	7.266
2.118	2.845	2.390	1.824	2.920	3.109
6.813	7.195	7.406	6.786	7.351	7.009
3.609	4.672	5.542	4.705	5.076	3.050
4.855	3.653	4.256	3.378	4.865	5.124
2.997	3.706	3.366	2.998	2.531	3.079
4.279	6.158	4.414	5.340	5.692	4.692
3.492	5.129	4.384	4.124	4.416	3.465
2.882	4.125	3.846	4.251	4.632	3.040
3.252	4.532	3.833	4.409	4.846	3.152
5.411	6.564	5.064	6.247	5.187	6.837
6.209	7.857	7.507	7.076	7.788	6.548
4.493	3.945	4.294	5.500	4.654	4.780
3.945	5.172	4.075	4.721	5.143	3.572
4.203	4.360	4.165	4.150	4.481	3.456
7.308	7.571	8.209	7.767	8.063	7.452
4.367	3.557	5.343	4.884	5.290	4.656
1.619	2.621	2.963	1.452	3.653	2.261
3.086	3.795	2.785	2.926	2.437	2.663
6.510	7.783	6.695	7.333	7.252	6.359
4.233	4.825	4.365	4.416	4.592	4.590
6.094	6.533	6.075	6.405	6.406	6.093
8.983	10.492	9.497	9.821	10.127	9.133
3.466	4.738	2.987	3.867	2.605	3.352
6.683	7.355	6.924	7.504	7.331	6.479
3.878	3.856	3.889	3.846	3.905	3.843
4.279	4.429	4.083	4.448	4.612	4.505
5.595	6.052	5.227	5.697	5.889	6.006
3.534	4.659	4.895	4.518	5.037	2.802
5.110	6.265	6.136	6.351	6.726	4.867
4.137	4.332	4.349	4.473	4.487	4.164
5.451	6.680	6.415	6.403	6.409	5.428
4.740	6.094	5.597	5.622	5.299	4.996

10.169	10.223	10.864	9.865	10.611	9.890
4.007	4.918	4.487	4.174	4.927	4.329
4.343	4.142	4.117	4.319	4.288	4.251
4.232	2.925	5.743	4.148	4.548	3.762
4.931	5.403	4.824	5.325	5.098	4.816
5.132	5.416	5.119	5.615	5.365	5.151
6.750	6.947	7.156	6.873	7.174	6.823
2.940	2.662	3.267	2.996	3.120	2.960
4.593	4.724	4.692	5.004	4.934	5.175
2.994	3.389	3.129	3.065	3.985	3.159
3.230	4.694	4.585	3.616	4.960	3.524
3.575	4.547	3.211	2.880	3.467	3.908
5.333	4.643	5.841	5.520	5.787	5.915
5.366	4.911	5.256	5.500	5.000	6.569
3.519	3.087	2.953	3.593	2.947	4.497
4.301	5.628	4.372	4.353	4.749	4.579
6.897	6.263	7.416	6.692	6.771	6.610
5.066	5.389	5.849	4.466	4.914	4.649
3.348	3.155	3.835	3.971	4.152	3.417
3.043	3.322	3.450	3.778	2.818	3.869
3.459	4.260	3.730	3.496	3.994	4.161
5.680	7.009	6.485	6.370	6.197	7.371
2.968	2.841	2.157	3.253	2.704	3.352
5.341	4.995	5.880	5.651	5.249	6.380
1.522	1.257	1.052	1.844	1.476	2.414
4.076	3.592	3.335	4.201	2.890	4.362
6.946	6.324	6.948	7.065	6.964	7.523
6.656	6.610	6.987	6.845	6.374	6.296
4.739	5.437	4.732	5.718	5.122	4.632
4.118	4.300	3.985	4.415	4.081	4.749
4.427	4.935	4.382	3.936	4.806	4.117
5.677	5.569	5.878	5.927	5.513	5.610
4.224	3.711	3.799	4.572	2.427	4.305
4.692	5.085	4.592	5.088	4.502	4.697
4.327	4.415	4.365	4.783	4.815	4.763
3.256	3.005	3.983	3.165	3.281	3.275
4.644	4.422	4.912	5.095	4.686	4.888
6.253	5.784	6.234	6.479	6.466	6.433
4.408	5.610	5.891	5.720	5.659	4.482
5.336	5.764	5.860	5.463	5.664	5.225
4.779	4.366	4.410	4.886	4.383	4.988
2.565	2.373	2.968	3.237	3.535	3.059
2.479	2.700	3.125	2.784	3.370	2.546
4.827	5.077	4.507	4.745	4.323	4.846
1.964	2.066	1.932	1.817	2.079	2.133
3.663	3.039	3.363	3.922	3.484	4.012
6.125	5.810	6.438	6.452	6.014	5.950
3.359	3.115	3.004	3.252	3.772	3.255
4.118	4.400	3.811	4.767	4.090	4.440
5.805	5.305	5.730	5.409	5.365	5.354

3.453	4.114	3.769	4.304	3.922	3.660
4.557	2.727	4.422	4.190	4.028	4.651
3.118	3.319	3.910	3.509	3.457	4.362
5.121	5.034	4.807	5.249	5.018	5.381
4.643	5.563	5.206	5.543	5.694	4.426
4.838	5.308	5.078	5.238	5.359	4.495
7.260	8.145	7.743	7.287	7.790	6.564
4.517	5.273	4.906	5.505	5.107	4.433
4.911	3.874	5.193	4.497	4.823	4.804
2.936	3.252	2.895	3.724	3.422	3.116
3.219	3.689	3.035	3.129	4.111	3.790
7.056	7.474	7.621	7.153	7.856	7.327
3.606	3.387	3.635	3.181	3.575	4.074
4.999	4.742	5.072	5.633	4.067	5.960
3.810	3.796	3.956	3.936	3.945	4.881
3.243	3.007	3.013	3.614	3.395	3.157
6.056	7.613	5.104	4.823	5.684	5.238
5.196	4.301	4.527	4.637	4.178	5.624
6.259	6.055	5.947	5.677	5.504	6.101
4.224	4.416	4.286	4.618	4.861	4.845
3.446	3.300	3.678	3.126	3.301	3.944
3.652	3.462	3.183	2.910	3.323	4.011
4.844	4.850	4.639	4.135	4.461	5.257
5.084	4.155	4.786	4.752	4.630	5.168
3.862	4.689	3.972	3.762	4.054	4.656
5.939	5.463	6.788	5.780	6.159	6.027
2.746	2.304	3.194	3.641	2.964	2.316
3.391	3.565	3.314	3.514	3.858	3.665
6.498	6.060	6.818	6.337	6.418	6.916
7.076	6.944	7.096	7.020	6.759	7.278
2.333	1.506	2.137	1.595	2.126	1.721
4.755	5.087	5.007	4.892	4.737	4.716
6.518	6.385	7.123	6.864	7.147	6.862
3.300	3.375	3.410	4.380	4.102	3.333
4.062	4.404	3.391	4.555	4.333	3.834
4.779	4.802	4.997	5.286	5.276	5.219
3.853	4.719	3.379	3.905	4.389	3.476
4.316	6.783	5.206	4.230	5.726	4.126
4.781	6.537	3.918	4.729	5.121	3.919
4.061	5.471	4.342	5.382	5.747	4.156
2.552	2.406	2.226	2.502	2.644	3.131
5.497	5.269	5.363	5.647	5.370	5.797
4.757	5.482	5.183	5.039	5.351	4.864
2.840	2.502	2.974	2.035	2.263	1.820
3.351	3.408	3.955	3.454	3.895	3.239
2.120	1.538	1.991	2.553	1.926	2.704
3.502	3.646	3.525	3.876	3.190	3.696
5.656	7.222	8.270	7.124	8.299	6.296
4.064	3.232	3.856	4.103	4.538	4.452
3.806	3.663	4.370	4.053	4.288	4.181

5.481	5.683	5.501	5.103	5.189	5.185
3.989	4.073	3.805	3.537	3.149	4.240
3.047	2.901	2.738	3.495	2.838	3.068
2.514	1.517	1.768	1.649	1.370	2.731
3.769	4.142	4.281	4.185	4.407	2.830
2.446	2.578	2.323	3.055	2.154	2.863
6.336	6.288	6.649	6.438	6.361	6.025
6.177	6.052	6.005	6.088	5.846	6.474
4.124	3.121	3.464	4.364	2.767	4.952
6.117	5.613	6.113	5.730	5.735	6.117
4.326	2.410	3.317	2.273	3.394	2.988
2.663	2.345	3.045	2.488	2.101	2.117
2.782	3.745	2.901	1.878	2.018	2.582
4.320	2.469	4.133	4.160	3.031	4.944
3.892	2.799	3.434	3.738	2.939	4.746
8.285	9.966	9.078	9.241	9.058	8.935
5.810	8.180	6.853	7.203	7.721	6.034
5.476	4.899	5.587	5.621	5.264	5.678
2.453	4.838	4.249	3.107	3.548	3.436
6.152	5.710	6.348	5.976	6.021	5.708
6.282	6.732	6.879	6.676	6.988	6.730
4.007	3.929	3.811	3.821	3.897	4.325
5.466	5.367	5.575	5.837	5.648	6.072
3.923	4.099	3.951	3.909	4.409	3.921
3.742	3.683	4.025	4.357	4.158	4.097
2.519	1.945	1.710	1.722	2.056	3.116
4.049	4.635	3.899	4.561	4.377	3.898
4.820	4.645	4.828	4.931	5.055	5.018
3.203	3.593	3.263	3.704	4.316	3.601
4.473	5.885	4.638	4.119	4.644	4.144
2.126	1.781	1.659	1.718	2.236	1.977
3.658	2.617	3.275	3.623	2.701	4.396
2.216	2.120	2.179	2.244	2.334	2.958
2.717	2.468	2.543	3.298	2.861	2.984
3.466	1.014	5.869	2.305	4.866	1.869
2.250	2.781	3.808	2.702	3.872	1.736
3.033	3.016	2.995	2.934	2.634	2.825
2.412	2.397	2.415	2.628	3.275	3.132
2.900	1.998	1.636	2.984	2.579	3.953
3.494	2.889	2.485	3.106	3.529	3.819
4.069	3.724	4.075	4.599	3.758	4.575
3.106	3.051	2.999	3.066	3.041	3.214
3.863	3.755	3.545	4.131	4.108	3.333
6.276	6.944	6.369	6.386	6.999	6.303
6.610	6.749	7.483	6.497	6.983	6.094
6.505	7.229	7.197	7.353	7.205	6.624
3.761	2.985	3.713	3.565	3.730	3.642
2.723	2.931	2.527	2.660	2.572	4.085
6.958	7.052	7.127	6.661	7.079	6.605
4.071	3.605	3.554	3.719	3.934	3.531

4.743	5.656	5.204	5.389	5.317	4.557
2.524	2.556	2.355	2.701	2.330	2.430
4.229	5.380	3.839	4.659	4.851	3.943
4.234	4.363	3.928	4.655	4.226	3.817
5.225	5.858	5.892	6.051	6.399	4.984
3.505	3.783	3.855	4.595	3.940	3.805
3.170	2.585	2.680	2.961	3.400	2.959
8.380	8.484	7.802	8.672	8.144	8.867
6.150	6.433	6.075	6.565	6.298	6.657
3.808	3.936	3.676	4.134	3.323	3.654
2.980	2.894	3.461	4.022	3.284	2.685
4.781	4.264	4.472	4.681	4.140	4.838
2.925	3.097	2.649	3.352	2.971	3.131
4.775	5.814	5.080	4.875	5.397	4.493
4.449	4.497	4.373	4.549	4.149	4.783
2.881	2.708	2.380	2.365	2.561	3.314
5.339	4.166	4.741	5.108	3.915	6.016
5.210	5.001	5.242	4.914	5.042	4.832
4.249	3.979	4.556	4.049	4.695	5.066
3.445	4.395	3.920	3.083	3.631	3.580
5.690	5.306	5.537	5.722	5.524	5.973
7.083	6.738	7.175	7.291	6.785	7.301
5.545	5.346	5.739	6.372	5.822	5.822
5.658	5.611	5.705	5.472	5.943	5.736
3.236	3.774	3.013	3.405	3.518	3.251
2.222	2.102	2.333	2.725	2.044	2.592
3.261	3.724	2.255	2.453	2.826	2.784
3.666	3.228	3.159	3.334	3.364	3.726
2.485	2.031	2.025	2.787	2.555	2.313
8.656	8.531	8.905	8.956	8.627	8.750
3.377	3.563	2.960	3.247	4.047	2.321
4.889	6.271	4.594	5.409	6.172	4.425
3.506	3.276	4.247	4.045	3.335	3.611
5.995	4.572	4.951	5.811	4.338	6.129
2.130	3.536	2.403	3.004	3.581	2.947
3.562	4.262	3.625	3.577	5.574	4.680
5.644	6.102	6.199	6.582	6.545	5.279
2.701	2.305	2.403	2.678	2.725	2.312
3.181	2.223	3.270	3.214	1.804	3.481
6.978	7.982	7.022	7.460	7.269	7.029
6.988	7.968	7.008	7.446	7.300	7.040
6.994	7.960	7.003	7.446	7.269	7.003
6.100	6.862	6.055	6.221	6.177	6.041
6.089	6.850	6.054	6.219	6.131	6.022
4.442	5.698	5.603	5.142	5.872	3.419
2.545	2.297	2.660	1.775	1.967	2.355
8.879	9.126	9.026	9.284	9.356	9.074
2.715	2.644	2.257	2.935	3.473	2.827
5.013	5.756	4.975	5.639	5.080	5.048
5.536	6.180	5.603	6.076	6.209	5.432

3.485	4.231	3.004	3.847	3.631	4.078
4.532	4.902	5.068	5.055	4.720	4.626
6.473	6.528	6.979	6.813	6.986	7.028
8.491	7.996	8.242	8.163	7.965	8.432
4.895	3.895	4.348	5.069	3.559	5.728
4.817	3.972	4.436	4.824	4.384	5.718
6.410	6.177	5.935	6.062	5.829	6.438
4.454	4.129	4.650	4.040	4.398	4.411
1.715	2.039	2.846	3.902	2.382	2.617
3.356	3.346	3.133	3.158	2.472	3.783
2.488	2.630	2.330	3.100	2.630	3.879
5.519	5.573	5.703	6.110	5.814	6.167
5.043	6.203	5.014	5.638	6.023	5.131
5.314	5.900	5.517	5.710	5.911	5.363
6.775	6.661	6.882	6.939	6.989	6.755
4.221	5.201	4.312	4.819	5.358	2.999
4.756	4.578	5.326	4.712	5.343	5.099
9.316	9.038	8.972	10.164	8.430	10.114
5.796	6.366	6.435	6.446	6.406	6.344
3.350	3.198	3.066	3.281	3.352	4.171
2.246	2.309	1.810	2.448	1.804	2.566
4.214	3.298	4.206	4.059	4.138	4.393
2.767	2.672	2.635	2.867	2.685	3.169
5.969	4.964	5.893	5.683	6.091	6.347
4.990	4.903	5.078	4.854	4.573	4.481
5.304	6.784	5.964	5.800	5.252	5.781
8.264	8.656	8.499	9.048	9.039	8.366
4.750	4.825	4.848	5.359	4.869	5.055
9.934	9.814	10.009	10.128	9.824	10.127
4.552	5.020	5.908	5.005	6.108	5.015
4.677	4.933	5.898	5.170	5.937	4.941
4.970	4.689	5.032	5.127	5.078	4.983
4.938	4.782	5.180	5.065	5.067	5.049
7.171	5.673	7.085	6.686	6.662	6.550
7.970	7.934	8.159	8.145	7.806	8.082
4.998	3.230	4.461	3.916	4.069	4.621
3.356	3.694	3.988	4.014	3.031	4.770
2.186	1.467	2.001	2.318	1.770	2.161
2.090	1.904	1.621	2.469	1.403	3.050
6.966	7.146	6.993	7.511	7.479	7.427
6.684	6.095	7.065	6.952	6.292	7.358
4.275	4.919	5.427	4.837	5.611	4.941
7.923	7.863	8.179	7.706	7.679	7.863
5.125	3.965	5.542	5.407	4.788	5.775
3.498	4.116	3.298	3.775	3.540	3.582
3.692	3.750	3.385	3.795	3.972	3.766
3.112	3.468	2.663	3.182	2.840	4.320
6.488	6.504	6.391	6.775	6.567	6.692
5.531	5.751	5.941	6.011	6.089	6.066
1.694	2.365	1.929	1.696	1.861	1.536

4.413	5.019	4.152	4.250	4.117	4.742
4.784	5.775	5.599	6.868	6.130	5.297
2.557	3.286	2.169	2.610	2.488	2.654
5.116	5.053	5.161	4.562	4.652	4.769
5.485	5.964	5.612	5.845	5.525	5.000
2.194	2.484	2.563	2.200	2.181	2.335
3.687	4.563	3.588	4.789	4.134	3.342
4.223	4.774	4.000	5.277	4.632	3.842
2.334	1.965	1.902	1.740	2.308	2.441
2.875	2.834	3.084	3.089	2.713	3.463
3.605	3.667	3.596	3.655	3.259	3.872
2.871	3.785	3.188	2.621	3.687	3.226
3.208	3.365	3.422	2.745	2.960	3.168
2.651	3.560	2.303	3.754	3.372	2.345
4.220	5.645	4.970	5.512	5.051	4.603
4.245	5.330	4.758	5.396	4.471	4.203
2.725	1.884	2.985	2.652	2.103	2.378
6.843	7.356	7.012	6.920	7.269	7.345
5.281	5.115	5.670	5.618	5.353	5.503
3.782	4.231	4.030	3.797	3.742	3.674
5.009	4.779	3.915	5.407	4.612	4.116
3.452	3.974	3.126	3.477	3.190	2.801
2.705	2.815	2.831	3.200	2.155	2.777
3.257	3.398	2.979	3.870	3.456	2.727
3.145	2.892	3.775	2.793	2.920	3.289
4.648	4.747	4.755	4.443	3.897	4.823
4.664	4.615	4.437	4.344	4.278	4.619
5.466	6.416	6.044	6.148	7.080	5.845
6.334	7.285	6.655	6.520	6.996	6.243
3.567	2.647	3.126	2.737	3.275	2.988
3.927	4.118	3.678	4.351	4.250	4.245
4.084	4.449	3.962	3.437	3.777	4.035
4.698	4.563	4.784	5.023	3.868	5.100
5.615	5.504	5.604	5.899	5.703	5.735
4.887	5.222	5.489	5.410	5.328	5.198
3.068	2.369	3.006	3.022	3.202	3.989
5.667	6.901	5.133	6.489	6.356	5.854
2.745	2.949	2.181	2.441	1.417	2.730
4.338	4.195	4.054	4.440	3.896	4.533
7.788	8.193	7.770	8.139	7.658	7.518
4.896	5.391	5.079	5.032	5.198	5.316
5.337	5.200	5.459	5.785	5.123	5.735
4.724	4.912	4.665	5.230	4.963	5.026
2.458	1.770	2.295	3.062	2.014	3.056
5.539	5.962	5.172	5.183	5.215	5.678
7.032	8.000	7.930	7.687	8.785	6.745
2.987	2.755	3.152	3.068	1.948	1.878
8.049	7.427	7.945	7.504	8.229	6.001
4.384	4.460	5.243	4.787	4.189	4.353
6.849	6.696	7.118	6.715	6.892	6.974

4.776	4.057	5.020	5.007	4.200	5.065
4.530	5.338	4.416	5.302	4.866	4.475
5.108	4.086	4.110	4.755	3.469	4.952
2.505	1.946	2.013	2.631	2.118	2.842
4.721	4.891	5.544	4.782	5.622	4.010
3.284	1.997	3.529	2.819	3.712	2.255
4.767	6.160	6.439	5.477	6.392	4.991
7.797	7.799	7.766	7.887	7.571	7.736
3.457	3.611	3.261	3.911	3.664	5.122
3.416	3.484	3.597	4.005	3.367	4.704
2.913	2.947	3.065	3.273	2.530	2.451
4.565	4.670	4.604	4.875	5.301	4.954
3.980	3.702	3.333	2.859	3.128	3.354
1.628	1.377	1.882	1.726	2.298	1.668
2.061	2.008	3.222	2.446	1.955	1.632
6.065	5.837	6.041	6.389	5.946	6.026
6.664	7.225	6.699	7.111	6.783	6.883
4.112	3.653	4.417	4.260	4.279	4.090
4.789	5.008	5.821	4.777	5.340	4.372
5.877	5.196	5.331	5.740	4.418	5.835
4.638	5.024	5.003	5.065	5.215	5.563
2.984	3.595	4.595	3.424	4.262	2.579
2.724	4.052	2.405	2.517	2.639	2.405
5.228	5.578	5.655	5.213	4.995	5.087
2.897	5.127	3.149	3.488	3.884	2.288
2.923	2.714	2.470	2.788	2.825	2.698
3.899	3.245	4.084	3.677	3.712	3.883
5.539	6.017	5.446	5.388	5.793	5.814
4.862	5.080	4.912	5.249	5.459	4.465
2.963	3.038	3.057	2.708	2.725	2.889
2.712	3.088	2.735	2.839	3.643	2.448
5.189	6.038	6.076	6.127	6.192	5.760
3.541	2.804	2.227	3.701	2.606	4.076
4.657	4.635	5.053	4.260	5.032	4.428
1.646	1.716	1.656	1.866	1.688	1.361
4.120	3.551	4.548	4.455	4.409	4.568
4.183	4.221	4.606	4.651	4.360	4.086
3.137	3.466	2.544	3.075	2.226	3.406
4.202	4.658	5.568	4.643	4.552	4.472
5.609	5.445	6.658	6.416	6.575	5.990
5.727	6.557	6.371	6.093	5.254	5.555
3.984	3.859	4.004	3.076	3.558	3.986
4.139	4.073	4.295	4.488	3.915	4.404
4.497	3.419	5.059	4.838	5.066	4.727
3.139	2.786	2.635	2.728	2.943	2.858
4.364	3.962	4.471	4.626	4.548	4.326
4.319	3.787	4.174	4.291	3.598	4.351
7.524	8.008	7.885	7.825	7.877	7.280
5.621	5.397	5.189	5.702	5.619	5.162
4.956	4.512	5.715	5.180	5.049	5.545

2.549	4.596	1.983	2.290	2.921	2.428
6.528	6.115	6.673	6.586	6.632	6.083
2.693	3.155	2.670	3.149	3.006	3.219
6.939	6.652	7.132	7.376	6.880	6.856
2.178	2.231	1.988	1.997	2.395	3.420
5.664	5.765	5.845	5.863	5.678	5.408
2.513	3.126	2.318	2.450	1.994	1.922
4.078	4.199	4.507	4.565	4.431	4.180
8.137	8.961	8.088	8.872	8.773	7.786
5.214	5.817	6.280	5.523	5.672	4.472
4.868	5.326	5.645	4.731	5.091	4.382
3.559	3.529	2.680	3.799	2.831	3.479
3.117	5.202	3.722	4.445	4.454	2.981
4.457	3.879	5.178	4.332	4.118	5.009
5.268	4.279	5.388	4.809	4.496	5.836
3.753	4.195	4.330	4.931	3.475	3.963
3.539	3.545	4.445	4.042	3.534	3.351
3.933	3.823	4.660	4.396	4.084	4.549
3.058	3.341	2.826	3.066	2.825	3.183
5.116	4.409	5.099	4.921	4.358	5.469
5.508	6.022	5.887	5.379	5.589	5.175
5.024	4.940	4.628	5.293	5.346	5.318
5.078	4.642	5.659	5.334	5.236	6.115
4.251	4.121	4.320	4.094	4.319	3.914
2.649	2.401	2.372	2.381	2.681	2.259
4.937	7.213	4.951	4.955	4.459	4.268
4.856	4.538	5.328	4.868	5.111	5.074
1.897	1.522	2.158	2.270	3.880	1.770
4.075	3.370	4.482	3.858	3.892	3.839
2.959	3.886	2.413	2.065	2.392	2.587
4.087	4.115	3.860	3.720	4.141	4.110
2.605	3.028	2.785	1.712	2.435	1.640
2.521	1.933	3.044	2.642	2.642	1.727
7.781	7.386	7.410	7.528	6.985	7.623
1.303	1.122	1.103	1.242	1.358	1.003
5.710	6.510	6.465	6.679	6.666	5.681
1.274	1.316	1.230	1.733	2.050	2.337
3.898	4.369	4.084	4.039	4.018	3.903
3.032	2.899	3.562	2.349	2.537	2.281
2.823	2.334	2.608	2.380	2.351	3.153
1.830	1.376	1.349	1.199	1.482	1.632
3.718	3.299	2.649	3.644	3.157	4.362
1.946	1.732	1.832	1.995	1.985	2.206
2.400	1.902	2.747	1.973	2.505	2.372
2.548	2.542	2.277	2.420	2.649	2.661
2.016	1.725	1.613	1.656	1.749	2.392
2.375	2.902	2.474	2.783	2.336	3.149
1.127	0.727	0.804	0.726	1.177	1.283
1.893	1.442	2.284	1.088	1.747	1.555
2.594	1.670	2.249	3.130	1.625	2.173

3.629	3.015	3.061	3.629	3.675	3.590
3.241	2.786	3.671	3.976	4.571	3.806
2.953	3.269	2.801	2.863	3.157	3.480
3.388	1.743	3.573	2.475	2.157	3.065
2.302	3.180	3.199	2.529	1.853	2.595
3.781	3.825	3.468	2.303	2.366	3.123
2.657	3.434	3.913	3.493	3.357	4.239
3.418	2.948	4.146	3.905	3.119	3.323
2.447	2.817	2.293	2.041	2.051	2.410
2.529	2.317	3.630	3.252	3.790	3.308
1.852	2.422	2.077	2.263	2.745	2.543
3.818	3.321	3.983	3.688	3.371	3.657
3.330	2.188	3.345	2.702	3.068	3.585
2.531	3.083	2.664	1.956	3.077	2.708
2.720	2.001	2.475	2.748	2.155	2.494
3.144	2.955	2.166	2.573	2.521	2.872
3.083	3.226	3.199	2.934	2.856	3.717
1.469	1.090	1.318	0.858	0.847	1.070
3.164	2.214	3.382	3.431	3.132	4.164
1.929	1.586	2.222	1.943	2.193	2.317
4.626	5.122	4.974	4.815	4.911	4.550
3.143	3.248	3.091	2.765	2.839	3.188
3.038	3.162	2.847	4.058	2.835	2.702
2.216	2.787	2.339	2.821	1.810	1.671
4.730	4.423	4.346	4.639	5.221	4.610
2.627	2.005	3.324	2.412	2.835	2.646
1.952	2.502	1.824	1.940	1.537	1.733
2.665	1.818	2.369	2.416	2.240	2.135
1.938	2.218	1.936	2.174	1.839	2.184
4.361	4.916	4.705	3.721	4.467	3.774
2.435	3.100	2.024	1.859	3.078	3.252
2.304	1.677	2.254	2.391	1.298	2.856
4.040	4.271	3.461	3.753	3.684	4.063
4.565	4.516	4.860	5.154	4.731	4.370
5.853	5.947	6.659	6.059	4.768	5.586
3.849	3.378	3.786	4.088	4.046	3.460
6.532	5.986	6.358	6.214	6.190	6.931
6.371	6.731	6.402	6.774	6.799	6.511
2.176	1.761	2.167	2.178	1.853	2.279
7.705	6.970	8.146	7.591	7.590	7.514
4.399	5.596	4.119	4.062	4.542	4.447
4.093	4.284	3.872	4.251	4.341	4.048
5.271	5.624	5.658	6.035	5.927	5.840
4.971	6.906	5.624	6.793	6.321	5.483
4.140	4.792	4.567	4.230	5.108	3.788
3.359	3.354	3.517	4.197	3.402	3.363
4.131	4.239	4.920	3.951	4.012	3.920
5.977	6.403	6.004	6.842	6.508	6.023
6.405	8.213	7.413	7.286	7.873	6.576
2.720	2.032	2.710	3.033	1.658	2.472

3.015	4.142	2.745	2.871	2.439	2.822
6.424	7.368	6.404	7.036	7.136	6.862
4.941	4.508	4.422	4.414	4.399	4.514
1.710	1.103	1.640	1.997	1.821	2.150
2.129	1.895	1.920	1.934	1.780	2.367
5.836	5.566	5.882	5.450	5.360	6.430
6.729	6.795	6.864	7.118	6.887	7.222
2.596	1.910	2.492	2.246	2.047	2.498
5.581	5.868	6.106	5.802	6.060	5.924
4.594	4.592	4.824	4.688	5.299	5.091
6.320	6.822	6.086	7.164	6.334	7.032
4.614	5.860	3.258	6.293	4.216	4.817
2.852	3.280	2.348	1.871	2.604	4.000
3.186	2.510	1.894	2.853	2.131	3.526
4.142	4.906	3.587	4.292	5.346	4.958
5.685	5.554	4.802	5.772	4.307	6.444
6.673	7.973	7.948	7.255	8.011	6.984
2.542	3.863	4.205	3.512	2.425	2.240
3.816	3.422	4.794	3.726	3.838	3.331
2.312	1.367	1.977	2.741	3.736	2.957
2.449	5.378	2.700	2.999	2.442	2.014
8.874	9.788	9.922	8.421	9.693	8.240
2.029	3.241	3.325	3.355	3.699	2.249
5.108	3.180	4.229	3.630	4.360	3.353
2.534	1.214	4.277	1.395	3.485	3.246
3.439	1.988	2.665	2.502	2.591	2.318
4.062	3.079	3.858	3.659	3.499	4.124
4.034	3.839	4.341	4.087	3.732	3.607
2.835	3.140	2.873	2.840	2.925	2.419
2.289	2.239	2.199	2.043	2.136	1.873
7.439	7.852	7.714	7.598	7.718	7.400
3.934	3.679	3.331	4.368	4.401	3.893
7.381	7.600	7.642	7.619	7.394	7.606
4.919	6.473	5.127	5.407	5.310	4.579
7.669	9.433	7.581	8.978	8.825	8.166
7.971	8.125	8.024	7.437	8.355	7.714
6.504	5.772	6.541	6.467	6.326	6.628
4.871	5.413	4.480	4.910	4.213	5.414
5.091	6.744	5.194	5.423	5.869	5.554
4.738	5.447	5.070	4.495	5.489	4.798
4.051	3.569	3.425	3.484	3.341	3.721
7.500	7.992	8.010	7.978	8.128	7.243
2.535	2.027	1.748	4.075	1.823	1.710
2.683	2.749	1.946	2.535	2.273	2.532
3.057	2.558	2.493	3.096	3.121	3.243
4.537	4.539	4.871	4.203	4.364	4.295
3.472	4.159	4.135	3.639	4.410	3.551
4.961	4.511	4.665	4.959	4.576	4.947
5.063	4.023	4.918	5.762	4.811	4.945
1.899	1.476	2.462	2.877	2.756	1.893

2.720	2.539	2.246	2.383	3.606	3.707
5.839	6.132	6.241	4.894	5.818	6.203
4.777	5.872	5.908	5.063	4.497	4.439
2.479	2.355	1.904	2.991	2.346	2.243
5.576	5.662	5.875	5.827	5.604	5.848
2.529	2.538	2.469	2.473	2.737	2.676
3.954	5.444	3.967	4.884	4.714	4.468
2.949	4.400	2.782	3.445	3.527	2.170
4.933	5.344	5.140	6.343	5.765	4.733
4.375	4.868	2.630	4.506	4.122	3.636
3.944	4.450	3.573	3.679	3.454	3.016
8.358	7.474	8.360	7.865	7.752	8.205
3.634	3.224	2.934	4.059	3.293	3.553
4.539	5.721	5.086	5.603	5.568	4.452
4.110	3.889	3.941	4.136	3.999	4.450
1.194	1.047	1.013	0.950	1.034	1.539
3.181	3.746	3.812	3.795	3.217	3.917
5.019	4.436	5.148	5.180	4.724	5.568
6.000	6.821	6.669	6.816	6.617	5.673
6.947	5.398	7.610	6.817	6.892	7.342
2.038	3.338	2.117	2.613	3.692	2.261
2.780	1.695	1.953	1.849	1.757	2.288
3.538	4.234	3.714	3.758	3.918	3.353
2.378	2.425	2.434	2.349	2.067	2.389
5.896	6.144	6.017	6.163	5.818	6.084
4.922	4.337	4.425	5.076	4.738	5.114
5.762	5.226	5.657	5.982	5.793	6.091
5.118	4.887	5.080	4.665	5.158	5.081
2.165	1.747	2.052	2.080	1.753	2.730
2.290	2.245	2.662	1.989	2.144	2.034
4.989	5.205	4.881	5.227	5.282	5.165
4.289	3.971	4.443	4.343	3.682	4.926
3.151	2.461	2.363	2.427	2.600	2.551
3.251	2.209	2.272	3.070	1.829	3.014
4.382	4.151	4.983	4.319	4.749	4.368
4.450	4.804	4.662	4.592	4.993	4.425
3.410	3.743	3.068	3.648	3.701	3.536
4.841	5.251	5.373	5.134	4.968	4.524
2.877	3.220	3.438	3.244	3.272	2.986
3.043	2.798	2.733	3.737	3.474	3.698
4.164	4.099	4.690	4.333	4.227	4.650
2.424	2.104	2.833	1.881	1.889	2.425
3.326	2.865	3.936	3.649	3.475	3.109
5.948	6.316	5.753	5.957	5.759	5.835
5.952	6.365	5.786	6.000	5.765	5.856
4.600	5.621	5.441	5.840	5.373	4.650
8.641	9.109	7.877	8.284	8.764	6.992
1.661	1.982	1.926	1.400	2.030	1.630
4.010	3.687	4.538	3.862	4.619	4.167
5.534	5.792	5.622	5.885	5.593	5.410

7.783	7.995	7.820	8.144	7.807	7.663
7.885	8.087	7.922	8.252	7.908	7.760
7.748	7.870	7.720	8.130	7.790	7.655
7.110	7.597	7.641	6.880	7.679	6.628
7.200	6.836	7.164	7.126	6.962	7.328
4.582	4.553	4.494	4.680	4.931	4.686
2.193	2.598	2.377	2.063	2.540	1.288
3.350	3.697	2.452	3.406	2.923	2.923
3.212	3.017	2.177	3.076	3.244	3.135
2.677	2.626	2.110	2.812	3.352	1.772
3.900	4.819	3.694	4.156	3.969	4.122
2.393	2.880	2.545	2.889	2.442	2.338
3.774	2.947	3.652	3.875	3.739	3.877
5.301	5.764	5.394	5.566	5.094	5.291
3.597	5.071	5.799	1.468	5.480	0.978
4.301	4.404	3.654	4.446	4.590	4.628
4.183	5.083	3.751	4.450	4.695	4.438
3.503	4.085	3.623	4.054	4.102	3.628
4.609	5.765	5.230	5.004	5.897	4.587
3.475	5.063	3.244	4.028	4.411	3.933
3.837	5.121	2.185	3.370	2.463	2.134
3.798	5.136	2.333	3.289	2.471	2.012
4.047	3.294	4.050	4.262	4.434	4.051
2.613	4.244	2.941	4.512	3.935	3.724
5.297	4.551	4.692	5.272	5.086	5.687
3.361	4.478	4.240	4.365	4.747	3.942
5.367	6.334	5.564	6.570	5.639	5.202
8.355	8.246	8.747	8.398	8.345	8.352
6.123	5.379	6.235	6.314	6.159	6.206
5.033	5.113	4.479	4.782	5.111	5.053
5.364	5.313	5.135	4.922	5.001	4.807
2.867	2.846	3.411	2.737	2.989	2.547
5.239	5.572	5.651	5.352	5.551	5.631
2.566	3.782	2.388	4.647	2.109	2.168
3.092	2.939	3.307	3.639	3.109	3.296
5.851	6.035	6.195	6.078	5.783	5.742
5.744	6.041	5.699	5.810	6.077	5.705
2.323	2.845	2.532	2.576	2.370	2.266
1.825	1.780	2.079	2.163	1.325	1.654
2.529	2.096	2.221	2.097	1.486	1.362
4.420	4.741	4.264	4.035	4.799	4.430
2.046	1.374	1.649	2.414	1.582	2.787
4.041	4.416	4.760	4.583	4.572	4.325
4.654	3.429	5.245	4.281	5.220	4.867
5.137	5.098	5.041	5.034	4.649	5.000
5.745	5.610	5.995	5.737	5.705	5.931
3.996	3.751	4.275	3.724	3.702	4.755
4.465	3.567	4.415	4.361	2.869	4.785
2.555	2.384	2.617	2.498	2.300	3.821

5.072	4.940	4.911	4.891	4.902	4.805
10.334	9.781	10.287	10.455	9.392	10.194
7.018	6.725	7.035	7.858	6.166	6.829
2.314	3.325	2.042	2.891	3.238	2.205
3.583	3.679	3.914	3.544	3.744	3.402
6.538	7.712	6.558	6.817	7.623	6.244
2.606	2.893	2.974	3.666	3.002	2.887
5.156	5.859	4.833	5.497	5.331	5.169
4.804	4.338	3.946	5.009	4.325	4.547
4.782	5.122	4.795	4.774	4.753	5.060
1.728	1.976	1.980	1.736	2.051	1.580
5.746	5.385	5.748	5.526	5.728	5.642
2.386	2.298	1.839	2.733	2.832	2.309
8.226	7.942	8.608	8.434	8.264	8.345
5.085	4.836	4.199	5.374	4.810	5.195
4.649	5.010	5.078	5.088	4.770	5.476
2.946	4.007	3.134	3.984	2.827	3.877
3.800	3.560	3.169	4.053	3.356	4.234
3.876	2.763	4.066	3.874	4.076	3.843
3.297	2.789	3.349	3.538	3.121	2.967
2.605	3.049	1.379	1.990	2.138	1.377
2.593	2.321	3.805	2.713	3.285	2.974
5.621	5.038	6.619	5.980	6.367	5.967
5.256	5.231	5.798	3.981	5.599	5.161
3.637	4.075	3.302	3.872	3.711	4.129
5.843	5.706	5.700	6.173	5.986	6.019
3.588	1.834	2.582	3.134	2.765	1.991
2.136	2.718	2.749	1.881	2.356	1.698
2.998	2.417	3.493	0.960	2.640	2.897
2.795	2.849	2.615	3.149	2.624	2.318
5.994	5.188	6.243	5.673	5.741	6.348
4.182	3.758	4.041	4.429	4.004	4.985
5.618	5.117	5.043	5.686	5.416	6.132
3.496	3.195	4.038	3.257	3.755	3.903
4.526	4.338	4.905	4.051	5.284	4.171
2.714	2.436	4.005	2.099	3.321	2.770
1.163	1.641	1.283	1.013	0.991	1.204
2.307	2.080	2.941	2.160	3.133	2.137
2.695	1.970	2.878	2.126	2.630	3.540
2.588	2.217	3.173	2.803	1.633	3.037
5.975	6.276	5.855	6.002	6.248	5.694
4.106	4.424	3.666	4.602	3.709	4.033
2.454	2.142	2.222	1.906	1.566	2.527
1.543	1.050	1.278	1.771	1.403	2.031
2.826	2.302	2.176	2.596	2.398	2.105
2.336	2.502	2.692	2.108	1.835	1.977
1.446	1.637	1.170	1.258	1.610	1.697
2.854	3.582	4.362	3.223	2.393	2.275
5.971	5.532	5.886	5.568	5.894	5.913
2.576	2.370	2.072	2.649	2.022	2.249

2.130	2.391	2.444	2.226	2.567	2.944
3.349	3.169	3.203	3.361	3.177	3.525
2.639	2.802	2.446	2.652	2.241	2.826
6.232	5.935	5.903	5.932	5.529	6.361
6.809	6.634	6.765	6.819	6.977	6.939
3.192	3.516	3.551	3.456	3.639	3.018
4.386	2.833	3.774	2.479	2.196	3.046
3.450	2.714	2.919	2.362	3.028	3.300
4.277	4.136	5.002	4.200	4.397	4.390
3.463	1.691	1.870	1.578	1.800	1.511
3.202	1.937	1.931	2.008	1.935	1.731
3.106	2.484	2.336	2.264	2.673	2.451
3.435	2.495	2.725	2.488	2.541	3.400
4.080	2.992	3.731	3.004	2.850	3.608
4.640	2.752	3.716	3.048	3.148	3.219
3.387	2.499	2.062	1.943	2.258	2.036
8.032	7.947	8.713	8.084	8.485	7.990
7.064	7.147	7.172	7.517	7.063	7.361
5.114	5.190	5.984	4.973	5.824	5.521
7.579	8.355	8.861	7.201	9.112	7.286
4.752	4.272	5.182	5.064	4.886	5.052
5.217	5.210	5.905	5.299	5.424	5.378
5.806	5.675	5.741	5.926	5.827	5.494
4.680	4.261	4.929	4.869	4.651	4.788
4.753	4.086	4.253	5.015	4.568	4.407
3.289	3.092	3.408	3.340	2.981	3.110
8.803	8.342	8.754	9.183	8.447	8.937
3.721	3.139	3.296	3.490	3.320	3.614
2.739	3.098	3.036	3.302	3.554	2.616
3.065	3.064	3.758	3.623	3.851	2.765
2.914	2.165	3.019	2.821	2.323	2.084
1.783	2.934	4.114	1.644	5.009	1.859
3.259	2.778	3.198	3.142	3.073	2.387
8.929	8.744	9.196	9.247	8.861	8.912
7.123	5.835	6.246	7.343	5.684	7.905
5.419	5.318	5.384	5.679	5.379	5.905
3.410	2.570	3.151	3.024	3.413	4.197
2.967	2.273	3.270	2.857	3.339	2.578
3.227	2.652	4.187	3.390	3.049	1.659
7.794	7.098	7.638	7.561	7.293	8.074
3.210	2.808	3.415	2.681	3.123	3.096
4.313	3.737	4.105	4.085	4.358	4.920
5.313	5.203	5.730	5.494	5.352	6.102
8.249	7.975	8.194	7.926	8.005	8.013
8.592	8.735	8.923	8.819	8.675	8.888
5.508	6.476	6.213	6.338	6.650	5.681
5.567	6.950	5.986	6.157	6.328	5.475
6.536	6.341	6.832	6.685	6.202	6.851
6.969	6.812	7.281	6.947	6.889	7.064
4.974	6.111	4.701	5.935	5.559	5.037

2.892	3.267	2.816	2.963	2.815	2.766
1.999	3.640	2.320	2.475	3.051	2.050
3.039	4.342	2.344	3.606	3.246	3.059
3.499	3.155	3.294	3.738	3.606	4.201
5.099	5.411	5.624	5.411	5.835	5.462
4.783	5.376	4.999	4.992	4.881	5.180
2.897	3.018	2.943	4.266	4.317	2.062
3.593	3.690	3.361	3.529	3.300	3.666
4.819	4.277	4.831	4.543	4.849	4.810
3.732	4.299	4.286	4.567	4.194	4.527
2.975	3.243	3.791	2.337	3.014	2.331
5.742	4.413	5.995	5.533	6.064	5.994
3.340	3.570	3.323	3.087	2.690	3.524
2.628	2.215	3.162	1.944	3.376	2.309
2.395	2.331	2.800	2.394	2.839	2.718
6.402	5.627	6.451	6.228	6.084	6.772
5.584	5.502	5.953	6.110	5.810	5.843
2.307	2.814	3.822	3.278	1.753	2.646
2.926	3.495	3.277	2.961	3.936	2.478
2.084	1.814	2.795	1.895	1.930	2.628
2.225	2.124	1.849	1.817	1.964	1.788
2.114	2.418	1.961	2.255	2.442	2.819
1.751	1.733	1.555	2.007	1.434	2.738
7.004	7.345	7.476	7.218	7.686	6.785
3.760	4.284	4.896	3.745	4.257	2.473
7.952	7.630	8.208	7.976	8.124	7.724
6.473	6.467	6.790	6.813	6.355	6.456
2.510	1.748	2.042	1.349	2.930	1.903
2.717	2.898	2.779	2.607	3.152	4.373
4.709	6.703	3.422	3.949	4.293	4.813
5.060	5.893	5.904	6.253	6.480	5.132
5.400	4.987	5.442	3.944	5.024	5.547
6.860	6.111	7.548	6.829	6.870	6.697
3.481	3.719	3.331	3.722	3.363	3.307
4.877	5.861	4.360	3.939	4.532	4.094
5.600	5.506	6.180	6.282	5.808	6.295
3.821	1.345	1.476	1.343	1.632	1.121
9.654	9.557	9.936	9.861	10.054	9.671
4.678	4.337	4.613	4.199	4.220	4.686
3.150	3.667	2.737	3.722	3.908	3.688
4.252	4.240	4.283	4.201	4.027	4.842
5.623	6.477	5.649	6.082	6.153	5.985
6.777	5.885	6.388	6.032	6.241	6.767
2.103	2.609	1.738	2.298	2.441	2.080
9.014	9.754	9.180	9.012	9.306	8.304
5.421	5.858	5.843	5.852	6.556	5.498
5.260	5.523	5.169	5.535	6.079	5.426
3.947	4.278	3.780	4.006	3.326	4.073
5.427	5.423	6.223	5.409	5.730	5.510
3.452	3.228	3.680	3.145	3.773	4.058

4.227	5.201	4.378	4.045	5.263	3.725
6.122	6.111	6.143	6.215	5.834	6.161
5.785	6.250	5.559	6.055	5.841	5.496
2.836	2.861	3.045	2.965	2.658	2.500
4.325	4.501	4.369	4.375	4.243	4.601
4.439	5.051	5.640	4.760	5.056	4.452
3.341	2.608	4.559	3.579	2.697	3.436
7.148	6.055	6.814	7.064	6.459	7.106
5.163	4.839	4.963	5.451	4.894	5.392
4.563	4.105	4.253	4.020	4.645	3.754
3.013	2.036	2.628	3.027	3.054	2.762
5.639	5.519	5.901	5.507	5.554	4.542
6.420	6.278	6.400	6.458	6.280	6.797
6.315	6.052	6.391	6.528	6.057	6.743
6.934	6.367	6.818	6.885	6.841	7.025
3.275	3.502	4.235	3.393	3.190	3.980
4.197	4.402	4.694	4.359	4.190	5.437
2.705	4.222	2.727	3.094	2.638	3.817
3.624	3.655	3.291	4.371	3.329	6.231
2.863	4.631	3.539	3.802	4.222	2.766
3.948	3.954	4.378	3.963	3.939	4.077
6.674	6.793	6.908	7.165	6.813	7.076
6.492	6.389	6.691	7.103	6.790	7.226
6.529	6.932	6.181	6.896	6.750	6.243
3.749	3.043	2.709	2.722	2.775	3.115
3.373	4.733	3.596	3.463	3.811	2.863
4.506	5.074	4.907	4.589	5.474	4.778
4.958	7.415	4.180	7.409	7.583	4.726
5.218	6.595	5.496	5.393	5.132	5.241
3.653	4.217	3.983	4.100	4.457	3.952
4.238	4.744	6.014	4.822	6.390	4.164
5.693	6.369	7.190	6.935	5.805	7.130
3.641	2.847	3.258	3.220	3.213	3.758
3.567	4.941	4.141	4.767	5.218	4.441
8.123	7.213	8.935	8.864	8.476	8.666
6.981	6.134	6.222	6.421	5.603	7.241
3.646	4.271	3.607	3.795	3.423	4.337
3.378	2.642	3.296	3.181	2.828	3.006
8.841	9.479	9.009	9.252	9.148	8.649
5.306	5.576	5.229	5.882	5.441	5.192
3.291	2.191	2.469	2.436	1.745	2.469
3.046	2.139	1.100	1.622	1.931	2.134
3.918	2.952	3.485	3.359	3.111	3.744
3.779	3.349	4.021	3.206	3.945	3.760
8.878	9.176	8.587	8.657	9.330	8.056
5.301	5.377	5.176	5.939	5.390	5.642
3.006	2.440	2.849	1.474	3.266	2.288
3.561	2.623	3.362	2.725	3.645	3.469
5.160	5.067	5.040	5.157	5.081	4.943
5.298	5.711	4.735	5.604	5.228	5.278

4.864	5.516	5.446	5.605	5.227	5.322
3.582	3.064	3.244	2.644	2.684	3.348
3.697	4.005	3.533	4.322	3.909	4.377
3.178	2.871	3.337	2.847	3.324	4.190
3.680	4.391	3.353	4.959	3.615	4.414
6.898	6.561	7.140	6.948	7.183	6.970
6.854	6.466	6.913	6.799	6.263	6.951
5.666	5.332	5.746	5.624	5.540	5.775
2.984	3.473	4.186	3.618	3.929	3.237
3.033	3.427	2.948	2.874	2.712	3.328
5.712	5.166	5.367	4.976	5.323	5.478
5.332	4.772	4.561	4.550	4.004	5.286
3.420	3.201	2.496	2.770	1.908	3.251
2.135	2.041	1.890	2.205	2.076	1.683
6.065	6.177	6.221	5.776	6.030	6.373
3.935	4.630	3.829	4.139	3.913	3.645
5.930	6.389	5.765	6.172	6.203	5.848
5.064	4.551	5.706	5.598	5.473	5.635
5.283	5.302	5.416	5.983	5.110	5.535
4.523	3.827	5.168	4.678	4.198	3.853
3.911	3.790	3.465	4.187	3.816	3.931
2.569	2.763	2.939	3.140	2.117	3.304
5.029	5.641	5.382	5.482	5.627	5.182
3.240	4.489	4.432	1.931	4.212	1.898
7.225	7.366	7.569	7.341	7.294	7.377
5.576	5.851	6.253	5.908	5.721	5.771
2.730	2.774	2.623	2.661	2.310	2.944
4.603	6.076	4.831	5.493	5.383	4.248
5.017	3.698	3.958	5.061	4.085	5.223
5.336	3.054	4.629	5.102	3.488	5.893
3.665	3.248	3.254	3.412	2.856	4.084
8.307	8.824	8.561	8.627	8.700	8.337
2.524	4.361	3.404	3.536	3.416	2.904
3.733	4.196	3.805	4.326	3.942	4.945
2.776	2.760	3.045	3.058	3.184	3.352
4.040	4.609	4.182	4.525	4.027	4.421
4.761	5.061	4.957	4.983	5.485	5.042
8.371	8.297	9.163	8.389	8.890	8.040
7.519	7.695	8.170	7.710	7.822	7.594
3.009	3.249	4.223	3.509	3.451	3.664
6.086	5.650	5.851	6.382	6.212	6.752
3.331	3.454	3.916	3.779	3.061	2.809
7.604	7.551	7.706	7.839	7.513	7.537
7.118	7.443	7.522	7.878	7.499	6.994
5.425	4.381	4.673	5.728	4.271	6.049
6.927	7.056	7.247	7.445	6.942	6.876
5.212	5.878	5.543	5.772	5.306	5.615
3.309	3.131	2.836	2.384	3.675	2.144
7.074	7.211	7.110	7.236	7.409	6.979
3.393	3.859	3.875	3.434	2.665	2.673

6.355	7.048	6.346	6.993	7.068	6.622
3.533	3.581	3.781	4.043	4.143	3.191
3.974	4.725	3.978	3.736	3.549	3.853
6.513	7.065	6.476	6.517	6.662	6.170
3.457	3.313	3.244	3.521	3.461	4.376
5.741	6.079	5.476	5.906	5.561	5.552
4.039	3.716	3.852	4.083	3.520	3.814
4.178	4.119	4.448	4.074	3.905	4.025
6.218	7.174	7.478	7.029	7.322	6.743
4.802	5.472	4.819	4.699	5.309	5.175
8.149	9.083	8.396	8.842	8.872	8.238
1.907	3.688	2.214	3.961	2.815	1.956
2.120	4.334	3.256	1.615	3.065	2.853
2.356	2.211	1.968	2.402	2.149	2.125
3.176	3.476	2.457	3.891	2.961	4.104
2.654	1.820	2.623	2.868	2.018	2.887
2.565	2.959	2.676	3.027	2.317	3.832
7.206	7.105	7.364	7.555	7.176	7.213
5.960	7.167	6.104	7.054	6.465	6.659
5.353	3.752	5.091	4.692	4.458	6.068
5.236	3.959	5.316	5.525	4.914	5.240
5.586	5.970	5.365	5.876	6.134	5.313
2.427	3.179	2.768	2.723	3.038	2.361
4.216	4.046	3.267	4.059	3.836	4.132
3.627	3.167	3.204	3.483	3.015	3.100
6.203	6.169	5.482	6.074	5.740	6.392
4.116	4.953	4.072	4.080	3.442	2.894
10.069	9.868	10.323	10.221	10.043	10.336
6.309	5.512	6.875	6.037	5.942	6.061
7.460	7.542	7.921	7.786	7.528	7.611
5.457	5.565	5.944	6.005	5.780	5.589
6.704	6.915	7.007	7.103	6.669	6.629
3.840	4.583	3.959	4.323	4.132	3.600
5.022	4.157	4.832	4.347	5.383	4.716
3.708	2.744	2.744	4.058	2.882	4.406
3.123	2.995	3.393	2.620	2.600	2.886
3.630	4.186	4.079	3.879	3.366	3.754
3.891	4.037	4.383	3.842	3.911	3.910
3.740	3.931	3.839	4.179	3.808	4.025
5.085	5.197	4.680	5.308	5.074	6.446
2.488	2.427	2.182	1.594	1.141	1.737
3.498	3.985	3.590	3.701	3.031	3.526
6.804	6.181	6.302	6.960	6.240	7.065
1.937	1.716	1.293	1.498	1.846	2.125
3.368	3.590	3.652	3.837	3.767	3.834
4.112	4.522	3.288	3.902	4.101	4.117
4.804	5.172	4.554	5.316	4.162	4.772
5.271	4.927	5.736	5.110	5.679	5.857
1.574	0.920	1.257	1.312	1.054	1.968
4.080	4.984	4.575	3.402	3.867	4.517

3.332	3.990	3.584	3.651	4.011	4.084
4.181	3.566	4.549	4.397	3.893	4.062
6.829	6.634	7.008	6.979	7.056	6.986
4.026	3.913	4.497	4.252	4.291	4.302
2.944	2.344	2.260	3.221	2.307	3.128
3.736	3.493	3.885	3.099	2.852	3.879
3.234	3.045	2.767	3.051	3.309	2.851
6.299	6.608	6.638	6.808	6.899	6.408
4.115	4.330	3.452	3.162	3.936	4.089
3.446	4.161	3.387	3.586	4.241	3.774
6.273	5.834	6.498	6.816	6.321	6.588
5.571	5.476	5.597	5.890	6.194	5.767
2.509	3.785	3.438	2.982	2.711	1.886
5.663	5.878	5.590	5.216	5.671	5.315
8.539	8.351	8.851	8.903	8.917	8.466
5.947	5.601	5.714	5.659	5.166	6.166
6.615	5.973	6.845	6.575	6.944	6.851
4.080	3.989	3.690	4.425	3.329	4.512
2.123	1.978	2.369	2.480	1.726	2.232
4.015	4.169	3.804	3.968	4.442	4.319
4.030	4.255	4.240	4.540	4.280	4.210
2.944	2.549	2.551	2.778	2.444	4.123
6.754	6.570	6.820	7.169	6.716	7.260
3.985	4.172	3.846	4.061	3.807	3.954
5.608	5.468	5.982	5.660	5.885	5.700
5.238	5.974	5.450	5.671	5.658	5.356
3.248	3.486	2.418	2.784	2.357	2.062
3.644	2.366	3.901	3.392	3.610	3.512
3.356	3.167	3.705	3.280	3.429	3.518
3.346	3.519	3.743	4.051	3.745	3.862
7.523	7.933	8.069	7.964	7.069	7.194
6.367	7.238	7.090	7.343	7.396	6.784
4.195	3.894	4.545	4.151	4.587	4.515
4.094	5.466	5.023	4.887	5.197	4.440
4.623	5.195	4.732	4.730	4.313	4.793
5.198	5.161	5.656	5.408	5.564	5.457
5.368	5.183	5.625	5.400	6.025	6.160
5.805	6.075	5.074	5.821	6.441	6.024
5.957	6.055	5.875	6.049	5.698	6.187
4.105	3.883	3.950	4.586	3.897	4.622
3.913	4.055	3.591	4.275	4.003	3.078
5.547	5.895	5.610	5.201	6.067	5.312
4.862	4.313	4.872	4.318	4.578	4.202
4.913	6.034	5.649	5.582	5.441	4.688
5.712	8.186	6.153	6.051	7.457	5.942
4.072	4.244	3.980	5.008	4.644	4.222
3.153	2.346	2.507	3.168	2.859	2.908
7.747	8.525	8.269	9.472	9.054	8.931
5.040	5.138	6.000	5.474	5.495	4.236
2.253	2.253	1.800	1.982	2.106	2.137

7.372	8.576	7.719	7.364	8.060	8.149
7.396	7.561	7.366	7.449	7.381	7.309
7.808	7.685	8.139	8.233	7.790	8.066
5.379	4.901	5.288	5.385	5.322	5.238
6.307	5.556	5.462	7.249	5.580	6.468
4.267	3.263	4.331	4.606	3.538	4.132
5.075	4.774	4.939	6.042	5.353	5.595
4.810	4.376	4.651	5.417	4.967	4.968
2.329	1.600	2.301	2.122	1.186	2.076
4.318	4.859	4.304	4.798	4.939	4.174
7.102	7.068	6.925	7.580	7.113	7.434
6.904	6.475	7.121	6.814	6.414	7.066
5.749	5.318	4.654	5.238	4.958	5.029
5.490	5.999	6.049	6.242	6.031	5.956
5.196	5.986	4.639	5.146	5.071	4.524
2.594	2.087	3.168	2.296	2.896	2.103
5.992	5.861	6.629	5.838	5.884	6.111
4.514	4.302	4.525	4.449	4.747	4.480
3.323	2.453	3.708	3.003	4.075	2.589
3.891	4.165	4.138	4.139	4.588	3.744
5.759	6.148	5.908	6.095	6.334	5.779
5.725	5.502	5.771	5.964	5.508	6.028
6.864	6.586	6.842	6.940	6.909	7.057
3.145	3.272	2.864	2.995	2.864	3.805
4.028	4.320	4.360	4.305	3.920	4.324
4.901	5.101	4.835	5.168	4.966	5.446
4.724	5.028	5.173	5.425	4.571	4.913
5.470	6.513	5.314	6.758	6.121	5.428
6.692	6.658	6.734	6.681	6.786	6.387
4.154	4.215	4.208	4.162	3.746	4.102
3.922	2.456	3.690	3.957	3.789	3.818
4.909	3.784	4.143	4.456	4.088	4.890
5.195	5.403	5.132	4.941	5.643	5.053
3.759	4.272	4.193	4.343	4.217	3.263
3.136	2.237	3.051	2.677	2.525	3.188
2.581	2.219	2.048	2.245	2.069	2.122
4.779	4.107	4.756	4.960	4.528	5.967
4.651	3.850	5.055	4.050	3.706	5.528
5.655	5.865	5.679	5.965	6.092	5.769
3.596	3.438	3.560	3.227	3.382	3.541
2.279	2.325	1.613	1.663	1.897	2.502
2.370	2.828	3.217	3.059	3.059	2.485
4.031	3.985	3.604	4.059	3.939	5.070
6.613	6.324	7.126	7.042	6.706	6.902
4.793	5.048	4.820	5.335	5.127	5.408
8.474	7.916	8.987	8.433	8.559	8.750
2.979	3.195	3.273	4.314	4.221	2.161
5.714	5.583	6.064	5.884	5.922	5.573
3.081	3.718	3.320	3.250	3.318	2.569
3.536	3.664	3.335	2.794	2.597	2.289

5.858	5.429	6.046	6.032	6.482	6.090
3.111	2.119	2.260	2.132	3.696	3.286
3.784	3.025	3.441	3.448	3.688	3.477
4.745	4.461	4.209	4.527	3.876	5.090
3.689	3.624	3.937	3.987	3.535	4.117
7.999	7.138	8.505	8.242	8.544	8.228
2.835	2.447	2.723	3.261	2.591	3.690
5.102	4.935	5.634	5.082	6.009	5.073
4.638	4.250	5.031	4.920	4.788	4.948
4.903	5.101	5.775	5.283	5.440	5.177
1.786	1.557	1.499	1.426	1.515	1.854
1.883	1.545	1.712	1.733	1.767	1.874
3.392	3.451	3.803	3.988	3.692	3.501
3.859	3.936	4.093	4.374	4.416	4.165
4.636	4.803	4.811	5.121	4.738	4.818
3.372	4.891	4.031	4.246	3.929	3.479
6.318	6.460	6.345	6.355	6.654	6.191
5.969	6.931	5.790	6.025	6.440	6.189
3.017	2.507	3.396	3.467	2.358	2.593
4.344	4.422	4.378	4.660	4.839	4.668
4.273	3.850	4.103	4.206	3.495	4.545
3.778	2.053	1.531	1.410	1.052	1.253
5.474	5.218	5.789	5.593	5.693	5.928
4.469	4.293	4.207	4.578	4.645	4.643
5.484	5.344	5.178	5.973	5.594	5.732
3.919	4.003	4.409	4.009	4.258	4.328
2.393	2.524	2.404	2.014	2.276	2.210
4.418	4.838	5.140	4.142	5.435	5.049
2.562	2.120	3.292	2.464	3.140	3.392
2.726	2.777	3.517	2.563	2.837	3.096
4.192	2.861	3.674	3.769	3.624	2.648
4.198	3.647	3.860	4.538	4.112	4.161
2.559	2.050	2.191	2.175	1.441	2.958
2.927	4.602	2.606	3.475	2.416	2.976
3.205	4.079	2.835	3.524	4.509	2.754
2.687	2.523	2.887	3.142	3.021	3.288
5.130	4.795	5.167	5.135	4.866	5.610
4.273	4.810	4.293	4.274	2.953	3.432
3.602	3.905	3.447	3.810	4.419	3.260
2.211	2.633	2.457	3.352	2.211	2.211
5.264	5.062	5.479	4.906	5.712	3.942
6.205	6.447	6.920	6.465	6.886	6.409
4.850	3.979	4.483	4.405	4.432	4.529
6.110	6.017	6.253	6.406	5.989	5.899
4.836	5.475	4.720	5.082	4.618	5.028
3.789	3.052	2.592	3.729	3.153	4.662
4.033	4.070	4.145	4.031	3.452	4.017
3.173	3.057	3.796	3.351	2.605	4.323
5.188	5.253	5.012	6.024	5.008	6.194
3.644	3.253	3.440	4.440	3.648	4.638

4.021	4.330	4.865	4.664	3.902	4.634
3.149	3.134	3.773	3.174	2.911	3.579
3.735	3.367	3.382	3.489	3.601	3.828
2.694	1.751	2.504	2.572	2.322	2.114
4.350	3.942	4.175	4.515	4.186	4.369
4.918	4.465	5.240	4.502	4.746	4.579
3.986	4.651	4.223	4.357	4.722	4.851
2.370	2.689	2.237	3.047	3.185	3.496
2.647	3.347	1.690	3.017	3.036	3.072
4.454	3.905	3.841	4.516	4.595	4.613
3.951	3.028	3.809	3.111	3.957	2.964
2.861	3.181	2.176	2.495	1.582	2.529
5.090	5.469	5.052	5.623	5.292	5.085
6.518	7.000	6.565	6.783	6.814	6.404
2.207	3.500	2.160	2.446	2.697	3.058
4.215	4.549	3.769	4.281	3.811	3.940
3.087	3.210	2.436	3.404	2.829	3.224
3.368	3.725	3.195	2.775	2.504	2.934
2.396	3.598	2.206	2.337	2.836	3.553
5.476	5.085	5.730	5.380	5.534	5.560
2.495	1.933	2.044	2.602	1.543	2.845
3.296	2.653	2.214	2.527	3.067	2.926
2.701	2.704	3.343	2.978	3.601	2.532
3.721	3.814	3.554	3.999	3.516	3.948
3.715	4.533	3.681	4.438	4.035	3.767
4.728	4.047	4.388	4.469	4.944	4.366
4.258	4.750	4.174	4.162	4.260	4.808
6.655	6.650	6.603	6.782	6.742	7.212
4.607	5.535	6.262	6.043	6.019	5.041
4.727	4.478	4.883	4.586	4.904	4.939
4.336	4.590	4.239	4.690	4.724	5.209
5.605	4.609	6.272	5.526	5.674	6.110
5.287	5.907	6.376	6.067	5.994	6.100
1.489	1.511	1.797	1.793	1.601	1.978
4.352	2.990	4.292	4.515	3.714	5.084
5.185	4.859	5.230	5.530	5.425	5.383
4.697	3.813	3.411	4.112	4.804	4.132
5.524	5.982	5.530	5.204	5.508	5.215
4.328	5.149	3.716	4.932	4.706	5.223
5.857	6.168	5.383	5.412	5.597	5.528
2.019	1.693	2.355	2.613	1.979	2.234
3.448	4.931	4.070	4.822	4.972	3.732
5.806	5.484	5.793	5.751	6.068	5.584
2.738	2.883	2.515	2.774	2.509	2.675
5.606	4.421	5.321	5.329	4.849	6.171
5.969	5.702	6.131	6.371	6.161	6.408
3.059	3.415	3.178	3.046	3.118	2.054
3.579	4.048	4.112	3.601	3.598	3.376
4.757	4.130	4.585	4.607	4.355	4.843
6.175	6.094	6.527	6.281	6.347	6.107

6.730	6.621	6.975	6.624	6.648	6.606
3.316	1.835	3.647	3.125	3.892	4.026
4.371	4.709	4.225	4.459	3.857	4.610
6.179	5.896	6.306	6.061	6.035	6.204
4.224	4.122	4.710	3.187	3.651	4.284
5.603	5.735	5.833	5.844	5.772	5.716
5.312	4.872	5.633	5.496	5.592	5.725
5.953	5.835	5.583	5.759	5.901	5.974
8.220	8.557	8.297	7.794	8.526	7.929
4.575	4.998	4.748	5.180	4.712	4.776
4.069	3.834	4.443	4.296	3.917	4.111
1.710	1.938	1.705	1.830	2.090	1.752
3.943	3.530	3.411	3.152	3.148	4.441
4.558	4.458	4.359	4.376	4.429	4.655
4.117	4.105	4.784	4.987	4.735	5.006
3.413	3.593	3.532	4.326	4.202	4.269
7.971	7.983	8.016	8.187	8.126	7.910
4.691	4.644	4.599	5.458	4.488	5.015
5.849	5.731	6.076	6.070	5.664	6.167
2.395	2.808	2.538	2.546	1.966	3.438
3.306	5.146	4.170	3.940	3.497	2.764
7.412	9.222	8.802	8.729	8.869	7.621
3.726	6.444	3.692	5.435	4.353	4.701
4.234	6.183	4.609	7.177	6.452	6.125
3.639	4.761	3.733	5.192	3.841	4.387
4.063	5.500	5.053	4.429	5.757	4.143
1.978	4.119	3.969	1.511	5.065	3.349
4.897	5.396	5.074	5.212	5.474	4.752
2.828	4.809	3.114	2.889	2.950	2.858
6.811	6.831	7.110	7.263	7.194	7.020
4.665	4.510	4.467	3.879	4.226	4.971
4.210	5.812	4.594	5.456	5.215	4.686
2.291	2.572	1.614	2.284	2.074	2.879
2.611	2.963	2.802	1.730	1.806	2.801
2.091	3.206	2.298	2.559	1.730	2.921
3.695	3.979	4.203	4.327	4.337	4.074
2.831	4.266	3.188	3.945	3.849	3.054
3.293	3.518	3.029	3.780	2.936	2.372
5.301	5.133	5.244	5.192	5.431	5.157
4.537	4.126	4.754	4.059	4.638	4.875
3.330	3.730	3.083	3.292	3.220	3.190
5.460	5.565	5.047	5.410	5.871	3.976
3.353	2.301	3.157	3.262	2.340	4.033
2.944	2.574	2.682	3.326	3.132	3.505
4.364	4.425	4.749	4.483	4.996	5.798
3.590	3.352	4.296	3.872	3.958	3.759
4.209	2.730	3.047	3.566	3.906	4.615
2.911	2.948	2.204	2.321	2.990	3.546
2.530	2.757	2.052	2.506	2.096	2.910
2.328	2.007	2.259	2.337	2.300	1.962

2.308	3.501	1.537	3.125	3.419	1.887
3.435	2.149	2.713	3.337	2.188	3.783
2.559	3.801	1.501	2.784	2.046	3.448
4.482	4.220	4.383	4.154	4.334	3.974
5.031	5.638	6.447	5.150	5.802	4.667
7.508	7.747	7.522	7.595	7.449	7.528
7.090	6.323	6.484	6.102	6.455	7.141
6.797	8.114	7.762	7.543	8.061	6.972
3.547	4.749	4.490	3.895	4.110	3.725
5.656	5.798	5.832	6.061	5.830	5.755
2.356	1.926	2.596	2.777	2.592	2.020
2.824	4.038	3.221	3.130	2.988	3.168
4.631	5.336	4.974	5.596	5.185	3.992
4.568	4.435	4.401	5.454	4.027	3.833
5.869	4.970	5.448	5.615	4.996	6.333
6.045	6.407	6.334	6.384	6.312	6.000
3.187	3.936	3.557	3.120	3.547	3.628
5.625	5.885	5.914	6.255	5.916	6.010
3.431	3.479	3.213	3.457	3.903	3.894
3.699	4.306	3.104	3.614	3.844	3.422
2.514	2.886	3.444	2.689	2.832	2.669
5.371	5.256	5.605	6.235	4.946	5.742
5.239	6.029	5.555	5.559	5.737	4.774
2.133	2.300	2.766	2.819	1.717	2.496
6.483	6.602	6.933	6.036	7.118	6.160
5.158	4.935	5.834	5.299	5.209	4.443
4.018	4.005	4.432	4.300	3.942	3.589
9.612	9.331	9.624	9.195	9.338	9.257
4.861	5.163	4.942	4.729	5.247	5.321
3.438	3.605	4.068	3.586	3.844	3.041
3.841	5.104	3.936	4.437	4.705	4.087
7.247	7.788	7.292	7.388	7.339	6.803
2.003	2.487	3.601	3.812	2.994	2.995
4.307	4.394	4.093	4.098	4.283	4.205
3.088	2.855	4.096	3.606	4.009	2.543
3.570	3.654	2.725	3.244	3.308	3.648
3.804	3.344	3.710	4.505	3.806	3.986
2.729	2.586	2.708	2.506	1.835	2.214
1.746	2.016	2.185	2.117	2.311	2.116
2.353	2.547	2.159	2.137	1.625	2.479
2.101	2.111	1.702	1.797	1.279	1.641
3.131	2.599	3.258	2.249	2.522	2.573
1.841	2.555	1.736	1.476	2.051	2.446
2.621	1.987	2.008	2.403	3.022	2.728
3.149	2.808	2.586	2.614	3.459	3.291
3.430	3.729	3.453	3.342	3.242	4.097
2.880	2.802	2.974	2.122	2.619	3.482
3.738	3.431	3.064	3.212	3.694	3.375
4.255	3.851	4.518	3.421	3.761	4.278
3.108	2.564	4.278	3.003	2.485	3.178

2.593	2.003	2.295	3.231	2.302	2.063
2.531	3.335	2.454	2.796	2.820	2.217
6.894	7.410	7.110	7.223	7.042	6.343
6.950	6.874	7.166	6.739	6.572	6.835
8.046	8.063	8.089	8.050	7.931	7.473
4.323	3.847	3.881	3.787	3.959	4.430
3.079	3.609	3.860	3.378	3.040	4.014
6.312	6.745	6.426	6.196	6.678	6.508
3.452	3.382	2.719	2.593	2.782	3.751
4.396	5.335	4.314	4.722	4.969	4.378
4.769	4.738	5.164	4.622	4.925	4.753
9.962	9.847	10.314	9.935	9.906	9.946
6.034	6.531	6.035	6.093	5.722	5.462
3.384	3.979	3.124	3.394	3.975	3.075
3.283	3.273	3.273	3.812	4.029	3.866
5.238	6.750	4.683	5.170	4.973	5.624
7.820	8.759	7.583	8.888	8.535	7.732
8.326	8.160	8.555	8.291	8.327	8.418
7.381	6.879	7.758	7.578	7.772	7.663
6.730	7.513	7.136	7.186	7.292	6.834
6.159	6.855	5.844	6.507	6.200	6.558
3.660	3.236	3.390	3.777	3.486	4.611
3.937	4.373	3.508	4.216	3.800	4.859
5.558	6.270	6.188	5.624	6.077	6.032
6.734	6.688	7.061	6.912	6.993	6.691
6.151	5.695	5.687	6.201	5.950	6.211
6.613	6.490	6.499	6.499	6.844	6.741
2.725	2.630	2.664	2.665	2.548	3.125
2.489	3.283	2.330	2.160	2.699	2.519
3.170	3.801	3.353	3.369	4.106	3.572
8.033	8.484	8.758	7.942	8.870	7.291
3.498	4.914	4.232	3.088	3.885	3.399
2.624	2.171	2.676	2.589	2.604	2.693
5.941	7.133	7.196	5.850	6.747	5.466
2.307	2.373	2.126	3.122	2.319	3.038
5.937	6.916	5.853	5.939	5.509	5.851
3.120	4.530	2.428	2.117	3.062	2.983
3.941	3.220	3.787	2.296	4.834	4.094
3.040	2.947	2.526	2.404	2.757	3.589
2.047	1.983	1.759	1.997	1.924	2.887
3.401	3.553	3.389	3.613	3.134	4.008
4.634	5.030	3.567	4.674	4.195	4.655
4.277	4.464	3.936	4.423	4.430	5.242
1.499	0.929	0.941	1.373	1.186	1.526
4.512	3.120	3.198	2.534	3.195	3.259
6.650	6.827	6.792	6.778	7.113	6.981
5.858	5.550	5.656	5.425	5.432	6.109
3.616	3.060	3.686	2.927	3.258	2.478
3.150	3.086	2.255	2.758	2.800	1.785
2.996	3.905	3.246	2.698	3.368	3.988

5.332	6.763	6.862	7.124	6.620	5.643
3.446	5.898	5.249	4.984	6.695	4.293
2.681	2.768	2.602	2.362	2.566	3.036
4.231	4.765	4.508	4.371	4.326	3.744
5.229	4.775	6.030	4.869	5.433	5.268
2.301	2.689	2.319	2.724	2.234	2.592
1.883	1.293	1.773	1.612	1.272	2.401
3.581	2.931	2.799	3.368	3.723	3.671
3.122	2.349	3.167	2.934	3.036	3.114
5.123	5.214	5.314	5.147	5.090	5.435
2.920	3.380	2.956	3.483	3.210	2.777
2.733	3.795	2.457	2.566	1.624	1.898
2.250	2.080	1.954	2.666	1.255	2.765
5.019	3.468	4.994	3.859	4.655	4.169
4.387	2.875	4.284	2.658	4.361	3.308
5.000	5.035	5.386	5.188	6.312	5.330
2.799	2.270	3.320	3.544	2.114	4.484
5.815	5.290	5.450	6.210	5.708	6.650
3.553	3.332	3.186	3.704	3.544	3.837
2.777	2.744	3.102	3.222	3.164	2.981
6.433	5.990	6.613	6.800	6.626	6.821
2.519	2.763	3.173	2.783	3.514	1.853
7.729	7.265	7.950	7.430	7.931	7.842
5.814	6.804	5.974	5.589	6.830	5.885
5.020	4.600	4.754	4.643	4.385	5.385
5.294	5.458	5.581	5.812	5.888	5.353
4.966	4.981	4.431	5.032	4.985	4.700
3.158	2.116	2.871	3.010	2.765	3.133
5.202	4.752	4.398	4.776	4.771	5.732
11.248	11.480	11.418	11.417	11.618	11.316
5.060	6.149	5.282	5.920	5.648	5.408
5.372	5.810	5.939	5.965	5.477	5.386
2.715	4.026	2.611	3.412	3.140	2.766
1.963	1.973	1.470	2.112	2.475	2.070
5.169	4.648	5.173	4.904	5.387	5.241
3.635	4.282	4.020	3.886	3.640	3.786
7.507	7.092	7.318	7.435	7.504	7.155
3.087	1.626	3.420	3.551	3.149	2.935
5.456	6.620	5.887	5.978	6.473	5.500
4.616	3.923	4.226	4.436	3.646	5.294
5.910	7.151	6.657	7.267	6.803	5.726
2.552	3.053	2.428	3.103	3.126	2.369
6.282	5.804	6.333	6.361	6.160	6.329
4.209	4.328	4.541	4.185	4.210	3.799
3.695	3.673	3.798	3.792	3.623	3.831
4.483	4.736	4.200	4.265	4.341	3.958
4.990	7.642	5.373	5.161	5.719	4.826
4.789	4.809	5.104	5.282	4.592	5.147
6.180	5.929	6.122	6.515	6.130	6.380
8.024	8.348	8.059	8.730	8.447	8.227

6.301	6.812	6.726	6.839	6.696	6.635
5.336	5.255	5.692	4.983	5.551	5.254
3.694	4.561	4.338	4.634	4.721	4.158
6.178	6.456	6.309	6.028	6.362	5.635
8.125	7.519	7.892	8.025	7.536	8.453
7.664	7.187	7.425	7.592	7.057	7.984
7.707	7.203	7.479	7.618	7.149	8.074
7.537	7.045	7.340	7.430	6.983	7.875
7.689	7.186	7.466	7.607	7.148	8.041
7.104	6.617	6.916	7.051	6.532	7.453
5.889	5.111	5.476	6.391	4.830	7.371
5.768	5.805	5.704	5.106	5.898	5.633
2.890	2.068	2.602	2.863	1.738	3.454
2.782	2.565	2.860	3.636	3.160	3.804
4.970	4.334	5.100	5.522	4.792	5.932
2.823	1.881	3.403	3.207	2.441	3.856
2.740	2.107	2.451	2.991	2.486	3.561
5.187	4.894	5.279	5.054	5.058	5.363
2.463	2.463	2.167	2.198	2.494	2.876
2.973	2.896	3.505	3.072	2.730	3.870
2.535	1.977	2.120	2.449	1.894	3.140
3.453	3.473	3.687	3.972	3.565	2.823
7.269	6.536	8.132	7.021	7.711	7.419
5.609	3.534	5.241	5.836	4.372	6.499
1.842	1.750	2.022	1.959	1.939	2.836
4.592	3.164	4.002	5.038	3.733	5.100
3.363	3.266	2.827	3.255	2.923	4.517
3.963	2.719	3.655	5.068	2.848	5.196
7.556	8.632	9.162	8.802	8.914	7.512
8.633	9.251	9.415	8.765	8.924	8.740
5.148	4.548	5.796	4.160	4.556	3.235
2.892	2.012	2.315	2.392	2.275	2.698
3.220	3.444	2.949	2.557	2.579	4.278
3.690	3.571	3.339	3.240	2.899	3.485
2.787	3.121	2.484	3.523	3.503	1.892
3.489	4.292	3.587	3.488	4.170	3.488
5.371	5.383	5.293	5.486	5.369	5.730
1.708	1.876	1.903	1.473	1.415	2.262
8.461	8.867	8.839	9.170	8.982	8.567
4.438	4.906	3.888	4.739	4.612	5.283
4.649	5.309	4.308	4.854	4.864	4.525
3.995	4.283	5.111	4.721	5.008	4.514
4.726	4.259	4.459	5.683	5.101	3.863
5.266	5.279	5.398	5.277	5.552	5.335
4.466	3.378	3.602	4.344	2.745	4.727
1.780	1.448	1.688	1.395	1.891	2.458
2.494	1.369	2.668	2.305	1.844	2.229
3.525	3.104	4.393	2.943	4.019	3.548
6.573	7.014	6.146	6.394	6.118	6.981
3.683	4.879	3.889	4.199	5.310	3.930

5.404	3.998	5.695	4.745	4.894	5.235
5.025	4.840	5.062	4.895	4.976	5.495
5.917	6.108	5.619	6.379	6.279	6.225
2.998	4.583	2.825	2.888	3.676	3.525
4.557	5.062	5.579	5.380	4.770	4.349
2.321	1.704	2.627	2.448	1.799	3.185
2.345	2.880	2.127	2.299	3.397	2.299
2.407	1.926	2.814	2.140	2.336	2.138
7.395	7.204	7.477	7.249	7.303	7.225
5.650	5.408	5.368	5.804	5.654	5.959
5.209	5.330	5.487	5.364	5.660	4.869
4.554	5.243	5.510	5.481	5.406	5.012
4.433	6.332	6.113	5.308	5.610	4.132
5.900	7.763	6.908	6.992	6.616	5.531
4.577	5.645	6.178	5.677	4.628	5.382
3.202	2.791	2.755	3.886	2.915	2.997
5.345	6.077	6.090	6.140	5.885	5.506
7.015	6.864	7.292	6.979	7.086	6.916
4.903	5.100	5.442	6.039	5.501	4.820
2.564	3.256	2.687	2.751	2.625	2.397
1.961	1.732	1.907	1.873	1.759	1.719
2.852	2.977	2.608	2.814	2.793	2.921
2.873	2.282	2.150	3.124	2.027	3.174
6.019	5.518	5.945	5.887	5.700	6.145
5.644	5.519	6.507	6.216	6.375	6.135
7.636	7.630	7.690	8.116	7.590	7.568
3.943	4.114	4.160	3.925	3.866	3.811
6.326	5.564	6.620	5.652	6.157	6.012
3.418	4.849	3.616	3.626	4.363	2.957
2.372	3.183	3.032	1.967	2.324	2.356
5.621	5.017	5.099	5.481	5.135	6.047
4.950	5.100	5.834	5.780	5.982	5.070
3.165	2.570	2.751	2.041	3.178	3.021
3.658	4.258	4.490	4.352	4.226	3.791
3.485	3.177	3.460	3.655	3.661	3.367
5.279	4.974	4.721	5.395	5.215	5.304
2.066	1.563	2.501	2.156	2.017	2.341
3.217	2.553	3.295	3.089	2.659	3.608
5.049	4.611	5.221	5.009	5.575	5.242
3.263	2.567	3.178	3.201	2.076	3.590
2.107	2.599	2.499	2.391	2.032	2.005
3.635	3.225	3.776	3.013	3.380	3.797
5.979	6.491	6.196	5.832	6.852	5.654
4.956	4.769	4.372	4.211	4.406	5.305
2.659	2.790	2.563	3.576	3.633	3.735
4.492	4.488	4.639	4.939	4.480	4.147
2.787	2.845	2.529	3.244	2.720	2.889
2.521	3.257	1.918	3.122	2.904	2.387
4.908	6.559	4.604	6.154	5.538	4.778
2.249	2.025	2.441	3.812	2.555	2.469

8.045	8.021	7.838	8.110	7.907	8.569
2.720	2.762	3.138	3.213	2.988	2.731
2.687	2.204	3.016	2.967	2.755	2.432
4.955	5.843	4.706	5.852	5.620	4.635
3.699	4.117	3.455	4.542	3.679	3.586
4.673	5.812	3.758	5.394	4.978	5.288
4.197	4.830	3.843	4.951	4.706	3.490
4.333	4.365	4.691	4.417	4.306	4.113
3.114	3.524	3.080	4.535	3.884	3.221
6.416	6.361	6.507	6.339	6.183	6.148
9.170	8.337	10.171	8.931	8.995	9.535
2.563	3.334	3.307	3.471	2.597	2.765
4.378	4.384	4.575	4.679	3.929	4.537
3.994	5.317	2.828	3.045	3.599	2.843
4.767	4.988	3.131	3.490	2.163	4.126
2.684	3.021	2.972	3.452	2.868	2.815
5.624	5.868	5.704	5.803	5.328	5.452
2.908	4.775	3.184	2.141	2.686	2.075
3.140	2.577	2.288	2.967	3.320	2.793
5.642	5.490	5.806	5.751	5.301	5.710
3.619	4.817	4.955	4.635	4.424	4.184
4.848	6.665	5.577	5.790	6.085	4.877
3.646	3.543	3.181	3.828	4.020	3.012
1.702	1.854	1.281	1.928	1.481	2.093
2.290	2.249	2.475	1.875	2.255	2.522
4.398	5.384	3.721	5.046	4.593	4.951
7.101	7.338	7.055	7.932	6.889	7.274
3.939	3.901	3.631	3.279	3.892	3.307
3.703	3.566	3.669	3.223	3.804	3.293
4.853	4.662	5.522	5.055	4.834	5.052
3.773	3.477	4.265	3.237	3.939	4.054
6.713	7.985	7.186	6.996	7.901	6.564
4.991	4.648	4.540	5.241	5.083	3.681
5.794	6.717	6.070	6.490	6.523	6.308
5.396	5.518	5.931	5.435	6.683	5.027
3.046	3.225	2.987	3.105	3.228	3.122
2.083	2.387	1.914	2.416	2.594	2.942
2.453	2.569	2.032	1.933	2.211	2.541
5.737	3.521	5.153	6.138	3.895	7.090
3.668	3.904	3.794	3.816	3.129	5.190
3.555	3.366	3.223	4.161	3.125	5.017
3.793	3.108	3.404	3.868	2.634	4.325
4.621	5.305	4.792	5.088	5.788	4.407
4.384	4.271	4.040	3.915	4.620	3.943
5.717	5.652	5.935	5.996	5.704	5.834
3.747	2.847	3.352	3.971	3.549	4.796
4.163	4.247	3.614	4.205	3.100	4.834
8.928	8.833	9.167	8.986	8.575	9.020
7.568	7.736	7.779	7.644	7.647	6.993
6.178	5.529	6.528	5.987	5.738	5.906

2.650	3.160	2.623	2.961	2.872	2.306
9.078	9.685	9.941	9.566	9.765	9.215
3.759	2.683	4.136	4.558	4.159	3.482
6.527	6.513	6.759	6.930	6.869	6.353
6.674	8.472	6.690	7.964	7.473	6.409
3.914	5.184	4.014	4.949	4.713	3.072
6.187	5.475	5.952	5.751	6.310	6.090
6.196	6.529	6.171	6.624	6.346	6.707
5.387	5.358	5.672	5.602	5.774	5.542
6.707	7.317	7.450	6.751	7.206	6.554
5.475	5.510	5.834	6.207	5.776	5.256
3.899	5.729	4.024	4.837	4.642	3.680
4.724	5.175	4.833	5.418	4.990	5.054
3.042	2.809	3.404	3.281	3.305	2.561
3.382	3.384	3.190	3.250	3.610	3.980
9.356	8.733	9.208	9.420	9.113	9.600
3.079	3.222	3.216	2.124	2.139	3.019
4.745	5.533	5.704	5.428	5.211	4.999
2.783	2.212	2.254	3.004	2.297	2.809
3.243	3.015	3.095	3.182	2.798	3.120
6.020	6.112	6.346	6.216	6.565	6.015
3.291	3.318	2.610	2.752	2.903	2.973
2.431	2.653	2.535	2.332	2.696	2.952
7.246	5.643	6.726	7.240	5.875	7.799
4.509	4.400	3.628	4.455	3.966	5.256
3.580	3.907	4.593	3.600	3.882	3.236
6.232	6.984	6.871	6.669	6.464	5.934
5.484	5.725	5.607	5.978	5.633	6.045
2.463	3.656	2.441	2.315	3.465	1.978
4.396	4.091	4.844	4.769	5.067	4.945
3.941	4.354	4.394	4.479	4.368	4.192
5.078	5.308	5.481	5.521	5.522	5.484
2.498	3.138	2.345	3.138	2.932	2.727
2.795	2.589	2.784	1.896	2.136	2.759
3.900	2.402	3.646	3.503	3.094	3.701
2.488	1.197	1.535	1.292	1.570	2.343
3.533	3.994	3.858	2.412	3.317	3.845
6.186	5.720	5.706	5.584	5.817	6.177
5.908	5.420	5.467	5.359	5.542	6.013
4.079	3.102	3.072	3.359	3.860	4.028
1.571	1.828	1.767	2.311	1.503	1.442
7.842	7.629	7.381	7.331	7.513	8.059
7.844	7.729	7.431	7.327	7.554	8.114
3.896	3.972	3.762	4.062	3.531	3.702
3.298	3.810	4.041	2.930	3.201	2.091
3.290	3.809	3.880	3.004	2.751	2.608
5.962	5.497	5.782	5.910	6.107	5.733
7.014	6.739	6.964	6.706	6.923	6.951
6.347	6.096	6.211	6.144	6.420	6.154
6.430	6.088	6.353	6.310	6.530	6.324

3.465	4.141	4.077	2.691	2.465	3.519
8.838	8.487	8.987	8.761	8.937	9.015
5.844	6.240	6.183	6.419	6.219	6.306
6.638	6.539	6.856	6.542	7.121	7.029
4.285	4.040	4.313	4.494	4.184	4.564
7.556	6.889	6.976	7.884	7.073	7.930
7.340	7.371	7.370	7.762	7.391	7.577
4.638	4.253	5.180	4.363	4.996	4.477
5.470	5.653	5.505	5.600	5.941	5.931
4.779	4.798	4.802	4.981	4.632	5.223
4.558	4.246	5.166	4.606	5.002	4.475
3.538	4.094	2.569	3.675	3.336	2.903
2.893	2.102	2.772	2.957	2.361	2.486
3.001	4.699	2.496	3.496	2.863	4.156
5.643	5.366	6.304	5.248	5.595	5.836
5.332	3.877	4.755	5.417	4.772	5.493
5.607	6.171	5.514	6.081	5.484	5.543
6.434	6.728	6.126	5.923	6.437	6.370
5.059	5.012	4.873	5.232	4.858	4.742
6.699	7.102	6.932	7.117	7.065	6.882
8.170	8.196	7.745	7.817	8.011	8.312
5.741	5.289	6.137	5.894	5.650	6.074
3.867	3.359	3.668	3.834	2.629	4.371
5.364	7.314	7.413	5.016	7.867	6.601
3.579	3.338	3.376	3.290	3.226	4.448
4.219	4.676	4.737	4.408	4.271	4.118
5.394	5.438	5.131	5.877	5.493	5.516
3.402	2.503	3.227	3.495	2.961	3.869
3.290	2.648	2.934	3.379	2.197	3.374
6.643	6.214	7.193	6.738	6.863	6.422
3.504	4.554	3.416	2.951	4.470	3.042
2.965	2.962	3.048	2.636	3.887	1.969
3.252	3.943	2.686	2.599	3.092	3.067
4.787	4.090	5.189	5.272	4.626	5.373
2.942	3.731	2.715	3.548	3.867	3.169
2.867	3.468	2.691	2.693	1.454	1.535
2.463	2.705	2.485	2.563	1.259	2.763
1.725	2.210	1.615	1.620	1.431	1.175
5.532	5.726	6.214	6.398	6.379	6.079
6.810	6.416	6.940	7.316	6.437	7.258
5.066	4.920	4.782	5.055	4.402	4.863
3.095	2.747	3.951	2.960	3.335	2.986
2.549	1.395	2.618	2.524	2.315	3.008
3.161	2.775	3.568	3.062	3.485	3.684
5.115	4.754	5.707	6.041	5.473	5.395
7.259	9.013	7.916	8.582	8.624	7.009
2.540	3.427	1.784	2.905	2.165	2.605
4.222	3.905	4.033	4.357	4.523	4.616
2.661	2.039	1.773	2.183	1.524	2.951
2.333	2.510	2.473	2.906	2.392	3.360

4.050	4.062	4.809	4.219	3.540	4.200
6.303	6.761	5.103	6.803	6.313	5.733
8.990	8.294	8.979	9.045	8.613	9.297
4.410	5.260	4.759	5.178	3.584	5.144
2.160	1.776	2.370	2.126	2.227	2.660
3.134	3.148	3.253	3.135	3.252	3.668
8.057	8.434	7.885	8.871	8.094	8.184
3.550	3.480	3.420	3.439	2.760	3.666
3.915	3.815	3.986	4.350	3.882	4.131
1.672	1.018	1.266	2.891	2.015	1.695
4.558	6.246	5.827	5.094	6.616	5.381
3.106	3.161	3.127	2.946	3.359	3.800
3.418	3.289	2.335	4.381	3.463	4.424
3.875	2.755	4.423	3.013	4.601	3.510
3.017	2.455	2.450	2.978	2.198	2.984
5.173	4.961	5.110	5.500	4.976	5.611
3.881	3.972	4.058	3.847	3.838	3.803
2.616	3.652	2.298	2.020	2.380	1.740
10.013	9.772	10.231	9.897	10.008	10.344
3.524	3.451	3.628	4.042	3.422	4.505
6.836	6.407	6.695	6.971	6.583	7.071
6.289	5.796	6.177	6.009	6.528	6.210
5.252	5.720	4.916	5.595	5.383	4.708
3.889	4.687	3.625	3.973	4.605	3.424
2.679	2.487	2.473	3.682	3.971	2.614
5.849	5.554	5.747	5.669	5.691	5.903
5.723	4.817	6.180	5.699	5.662	5.682
2.164	2.081	2.257	3.598	1.236	3.633
3.623	3.259	3.834	3.585	3.185	3.130
2.446	2.774	2.620	2.702	2.066	2.471
3.870	3.200	3.819	3.613	2.794	3.844
5.821	5.315	6.118	5.182	6.551	6.431
4.087	3.477	3.820	3.617	3.138	3.890
3.952	3.543	5.316	3.572	5.198	3.705
4.036	4.015	4.206	3.937	3.183	4.922
5.690	5.249	6.104	5.573	5.781	6.141
6.122	5.564	6.187	6.025	5.790	6.255
6.516	6.329	7.000	6.283	6.257	6.994
4.220	3.567	4.656	4.708	4.316	4.878
3.984	2.637	4.561	3.970	3.678	4.898
5.262	5.076	5.078	4.981	4.956	6.145
5.113	5.083	4.761	4.679	5.126	5.471
5.807	5.665	5.715	5.837	5.780	6.092
6.014	4.761	5.139	5.930	4.244	6.926
3.725	3.998	4.270	3.494	3.673	4.598
3.960	3.955	3.446	4.554	3.337	5.284
4.088	3.936	4.231	3.693	3.215	3.980
4.154	3.659	4.182	3.668	4.388	4.273
2.658	2.861	2.580	2.929	2.791	3.045
6.169	6.119	5.955	5.809	5.774	6.421

3.215	3.227	3.303	3.663	2.757	4.172
1.791	1.559	1.439	1.733	1.968	1.216
6.840	6.969	6.654	6.564	7.457	7.452
5.054	5.230	5.110	5.331	4.816	6.074
2.856	2.842	2.914	2.540	2.779	3.584
2.143	1.925	2.044	1.998	1.827	2.436
7.087	8.175	7.490	7.660	8.152	6.588
4.913	4.717	4.717	5.801	5.273	5.310
3.296	2.974	3.329	3.746	3.457	4.492
2.593	2.787	2.454	2.118	2.651	2.097
3.757	2.848	3.604	2.550	3.419	2.984
2.886	1.978	2.543	3.743	2.821	2.647
2.098	2.302	2.770	1.885	2.605	2.559
1.918	1.816	1.605	1.186	1.273	2.492
3.268	3.622	3.610	3.535	3.205	3.000
4.006	4.250	3.714	4.277	4.281	4.552
7.043	7.197	6.970	7.094	6.850	6.872
3.310	3.431	3.495	2.935	3.600	3.344
6.200	5.880	6.538	6.097	6.193	6.534
5.559	5.186	5.540	5.955	5.400	5.418
2.679	3.248	2.199	1.602	1.451	2.358
5.294	4.396	5.910	5.945	5.740	5.710
3.770	3.844	4.532	4.266	4.228	4.035
6.127	6.139	7.064	6.446	6.128	5.667
4.556	4.738	4.738	4.927	5.636	4.826
4.619	3.461	3.996	5.890	5.752	4.583
4.826	2.779	3.645	5.298	5.362	4.302
5.197	5.387	5.332	5.268	5.539	5.256
6.598	5.856	5.928	6.272	6.411	7.347
5.108	3.894	4.860	4.822	4.797	5.034
5.190	5.296	5.680	6.089	6.284	5.591
5.123	5.343	4.984	5.344	5.059	5.705
3.012	3.362	3.925	3.359	3.497	3.392
1.917	2.711	2.067	2.517	2.430	1.608
5.801	5.303	6.228	6.406	5.459	5.618
7.057	6.921	7.351	7.724	6.938	7.535
5.554	5.616	5.817	5.839	6.023	5.973
3.076	3.155	2.325	3.069	2.605	3.142
4.953	5.887	5.321	5.397	4.882	5.197
5.400	5.713	5.711	4.537	6.111	5.356
2.529	2.896	2.289	1.832	1.505	1.889
4.601	4.631	4.126	4.608	4.775	4.244
4.984	5.066	6.092	4.967	5.146	4.188
7.945	7.743	8.258	7.814	8.164	7.830
4.174	3.599	4.782	3.979	4.162	4.083
3.308	3.839	3.499	3.526	3.265	3.506
2.518	3.292	2.877	2.281	3.379	2.644
5.192	5.653	5.992	5.786	5.103	5.924
6.612	6.165	6.355	7.109	7.061	6.961
3.053	3.705	3.464	3.275	3.882	2.717

5.359	5.514	4.861	5.439	5.721	5.098
2.879	4.626	2.767	2.643	2.994	2.538
4.783	4.712	4.679	5.518	4.996	4.632
6.125	5.794	5.905	5.887	5.801	5.365
4.483	4.765	4.457	4.787	4.659	4.272
9.133	9.740	9.096	9.251	9.198	9.267
3.959	3.735	3.726	4.216	3.888	3.634
5.637	5.379	6.147	5.917	5.687	5.943
7.542	7.542	7.968	7.792	7.924	7.666
3.075	3.212	2.817	3.016	2.614	2.817
7.790	9.922	8.923	9.892	8.903	8.032
4.406	5.298	4.548	4.993	4.569	4.344
8.403	8.213	8.681	8.433	7.830	8.486
4.221	2.743	2.564	4.042	1.881	4.855
2.635	1.599	1.903	2.705	1.816	2.749
2.189	1.561	1.425	1.987	1.924	2.165
3.430	3.688	3.474	3.739	4.009	3.805
3.497	4.242	4.422	3.724	4.326	3.592
4.333	4.953	4.307	5.196	5.161	4.017
5.939	6.615	5.963	6.989	6.810	5.683
3.320	3.101	2.305	2.883	2.899	2.575
5.074	6.006	5.726	5.489	5.091	5.204
2.305	2.252	3.269	2.377	2.562	3.054
3.372	4.610	2.967	3.947	4.222	3.082
4.122	3.860	4.361	3.623	3.652	3.487
5.067	5.558	5.244	5.209	5.218	5.244
3.619	3.393	3.028	3.145	3.512	3.609
3.295	3.263	3.320	2.971	3.102	3.214
2.554	1.887	1.801	2.335	1.453	2.563
3.381	2.522	3.425	3.811	3.081	2.579
3.232	2.449	3.214	3.526	3.020	2.398
2.989	2.338	3.399	2.821	2.743	2.388
3.647	3.794	2.751	2.961	3.201	3.520
3.243	3.442	3.615	3.836	4.366	3.387
3.197	3.445	3.274	4.029	4.388	3.521
5.378	7.471	5.661	6.857	6.081	5.926
6.353	7.093	7.063	6.840	7.217	5.690
3.271	3.290	3.411	3.409	3.610	3.450
3.508	3.722	3.430	3.054	3.360	4.151
4.642	5.826	5.054	5.815	6.069	5.048
3.976	5.112	4.525	5.081	5.267	4.253
3.816	4.817	4.479	4.578	4.877	3.878
3.139	3.129	2.958	3.592	2.618	2.988
4.595	6.442	4.558	5.681	5.661	4.395
5.833	5.754	5.977	5.930	5.926	5.508
4.421	4.614	4.242	4.334	4.555	4.104
4.071	4.429	4.293	4.300	4.366	4.746
7.405	7.023	7.232	7.385	7.142	7.621
6.467	6.612	6.765	6.920	6.851	6.303
5.017	5.277	5.189	5.406	5.011	5.131

4.111	4.360	3.971	3.738	4.365	4.537
4.025	3.691	3.829	3.971	3.328	4.966
2.306	2.044	1.829	2.071	1.925	3.024
5.805	5.178	5.676	5.408	5.075	6.178
7.961	8.059	8.372	8.156	8.006	8.455
2.780	2.812	3.410	2.657	2.739	2.637
2.587	2.144	2.795	2.079	2.891	2.609
2.523	3.108	2.719	3.094	2.329	2.570
3.494	3.721	4.124	3.960	3.571	3.984
7.769	6.959	7.624	7.351	7.551	7.541
4.515	4.277	4.904	4.730	4.780	4.924
6.878	6.751	6.953	6.838	6.814	7.062
4.751	4.477	4.491	3.996	3.857	4.641
3.920	4.115	4.289	3.625	4.124	3.982
5.106	5.389	5.454	5.280	5.381	5.805
7.496	7.932	7.551	7.295	7.871	6.847
5.905	6.615	4.926	5.629	6.004	5.725
3.453	4.278	2.797	3.967	3.414	3.351
5.632	5.521	5.287	4.819	5.283	5.412
4.185	4.646	4.670	5.727	4.732	4.494
6.286	5.994	6.327	6.912	6.445	6.415
3.026	3.356	3.098	3.159	3.780	2.841
3.536	4.213	4.426	3.821	5.155	4.279
7.610	8.016	8.236	8.064	8.192	7.311
5.185	4.237	5.204	4.998	4.739	5.902
2.912	2.779	2.793	2.235	2.521	3.198
3.533	3.955	3.911	4.913	3.606	3.911
4.904	4.591	5.365	4.530	5.150	4.539
4.878	4.101	5.191	5.344	4.871	5.162
8.813	9.501	9.555	9.791	9.510	8.856
3.373	4.372	3.336	3.430	4.850	3.172
3.580	4.703	2.669	3.273	4.292	1.928
5.115	4.007	4.869	4.705	4.509	4.937
4.061	3.768	3.568	3.727	3.923	3.936
8.000	8.519	8.215	8.009	9.113	7.314
7.039	6.944	7.184	6.830	7.098	7.208
6.443	5.543	6.447	5.755	5.515	6.325
2.324	2.932	2.613	2.394	3.140	2.109
10.258	9.918	10.261	9.732	9.885	10.303
5.790	5.687	5.570	4.724	5.837	6.210
4.910	5.890	4.817	5.719	5.048	4.994
8.268	9.489	7.942	8.833	8.584	8.457
6.704	7.081	6.527	6.736	6.601	6.187
4.280	4.964	3.993	4.838	4.376	3.567
2.347	2.948	3.063	1.996	2.101	1.366
5.102	5.122	5.592	4.801	5.404	3.651
2.613	3.614	1.635	2.733	3.203	1.882
3.657	3.547	4.128	4.563	3.991	4.152
3.770	4.021	3.432	4.896	4.736	3.151
3.196	3.120	3.101	3.656	3.008	3.941

4.895	6.600	3.769	6.166	4.516	4.466
3.284	4.057	1.926	3.175	4.166	2.182
5.702	6.474	6.051	6.347	6.062	5.324
5.510	6.253	4.187	5.202	6.173	4.619
2.911	2.577	3.577	4.408	3.184	3.331
2.428	3.428	2.352	2.139	2.911	1.962
3.145	4.563	3.325	4.088	3.025	2.978
2.820	2.530	3.412	2.442	3.723	3.085
5.301	7.476	5.456	6.509	7.221	4.332
4.554	4.342	4.202	3.685	4.400	3.575
6.023	5.904	4.841	4.732	5.825	6.353
4.315	4.273	4.598	3.689	4.344	4.544
8.875	9.599	8.646	8.954	9.381	8.905
3.757	4.293	4.313	4.748	5.821	2.830
8.200	9.844	7.874	8.800	8.233	7.745
10.080	10.257	10.096	9.782	10.197	9.907
3.566	4.780	3.965	4.814	4.141	4.180
4.635	5.715	5.193	5.710	5.276	4.597
4.821	5.221	4.629	4.942	5.096	4.526
5.284	6.218	5.469	5.468	5.805	5.341
4.857	5.765	3.190	4.471	3.950	3.551
4.562	4.357	4.953	4.709	4.741	4.713
7.477	6.575	7.891	7.543	7.561	7.924
6.395	6.269	6.000	6.451	6.246	6.793
5.863	7.009	5.202	6.149	6.654	4.525
4.006	5.022	4.329	4.588	5.674	3.942
6.518	7.491	6.538	9.138	8.282	6.392
4.874	5.652	5.048	5.642	5.488	4.793
5.757	6.438	6.057	6.436	5.985	5.853
7.687	8.639	7.988	8.393	7.811	7.998
7.487	8.290	8.021	7.780	8.203	7.388
9.138	10.129	9.384	9.867	9.546	9.396
8.780	10.115	9.149	9.508	9.836	8.953
7.707	9.237	8.005	8.467	8.766	7.866
8.966	9.951	9.223	9.549	9.888	8.939
7.362	8.037	7.923	7.999	8.107	7.264
7.433	7.993	8.020	7.938	8.229	7.409
8.243	9.127	8.094	8.913	8.757	8.156
7.909	8.704	7.781	8.535	8.370	7.908
7.634	8.364	7.436	8.208	8.089	7.654
5.947	7.200	5.741	6.296	6.079	6.034
6.081	7.171	6.662	7.076	7.662	5.995
5.979	7.186	6.147	7.054	7.502	5.706
5.881	6.965	6.293	6.945	7.454	5.715
6.240	7.360	6.728	7.267	7.683	6.198
6.245	7.304	6.599	7.181	7.689	6.174
6.159	7.412	6.873	7.283	7.699	6.276
6.163	7.502	6.816	7.308	7.717	6.255
5.048	6.417	5.714	6.186	6.544	5.164
5.869	7.237	6.535	6.978	7.317	6.058

5.861	7.218	6.461	6.978	7.317	6.058
5.170	6.360	5.611	5.904	6.274	5.237
5.224	6.371	5.650	5.974	6.280	5.255
3.812	5.047	4.232	4.426	4.671	3.861
3.905	5.312	4.089	4.622	4.924	3.913
5.653	6.608	5.899	6.272	6.344	5.427
5.656	6.577	6.218	6.591	6.665	5.294
5.663	6.609	6.105	6.601	6.732	5.269
5.890	6.805	6.432	6.854	7.006	5.489
4.770	5.720	5.197	5.671	5.862	4.394
4.733	5.632	5.372	5.532	5.887	4.281
6.095	7.050	6.634	7.375	7.230	6.153
7.313	8.296	7.886	8.535	8.354	7.364
7.284	8.250	7.776	8.480	8.271	7.387
8.147	9.088	8.745	9.327	9.122	8.178
3.850	4.880	4.425	4.701	4.777	4.367
2.647	4.093	3.706	3.048	4.093	4.503
5.576	7.222	4.937	7.033	7.713	5.380
2.987	3.171	3.081	3.830	4.099	2.924
2.733	2.931	2.723	3.505	3.742	2.547
5.941	5.647	7.804	5.402	5.714	4.836
6.542	6.251	8.348	6.379	6.525	5.583
5.621	6.830	6.517	6.853	7.437	6.560
4.441	5.565	5.615	5.714	6.320	5.216
5.310	6.429	6.139	6.532	6.958	6.165
5.255	6.355	6.300	6.315	6.920	5.907
5.109	6.205	5.973	6.250	6.726	5.864
5.993	7.140	6.817	7.036	7.440	6.649
5.070	6.239	5.965	6.200	6.799	5.827
5.244	6.118	6.134	6.375	6.579	4.813
3.617	3.579	3.693	3.181	3.635	3.442
3.048	3.970	2.989	4.160	4.151	3.417
2.958	4.008	2.957	3.888	3.617	3.208
2.972	4.096	3.217	3.888	3.435	3.070
3.325	4.157	2.971	4.028	4.093	3.238
3.091	3.866	2.750	3.700	3.765	3.096
8.634	10.090	9.966	10.216	9.718	9.497
9.755	10.610	10.253	10.677	10.830	9.708
9.996	10.722	10.630	10.824	10.933	10.097
10.130	10.904	10.581	11.009	11.078	10.143
10.147	10.911	10.669	10.999	11.062	10.133
2.438	9.392	8.995	9.151	9.452	8.309
4.430	5.291	4.123	5.262	5.181	4.053
4.445	5.200	4.064	4.925	4.967	4.004
7.759	8.370	8.415	7.710	8.288	8.060
6.850	7.597	7.574	7.589	7.654	7.000
4.826	3.092	6.217	3.241	2.893	5.753
8.815	9.592	9.639	9.541	9.538	9.055
6.814	8.734	7.139	8.335	8.997	6.244
5.187	5.650	4.036	5.691	6.858	3.762

3.030	9.868	9.060	9.513	9.934	8.160
3.034	3.386	2.627	3.176	3.277	3.107
9.048	9.716	9.497	9.555	9.490	8.859
9.084	9.736	9.395	9.610	9.519	8.891
5.227	6.279	5.758	5.989	5.933	5.269
5.697	6.532	6.121	6.294	6.235	5.838
5.320	6.155	5.828	6.056	5.950	5.356
5.403	6.199	5.849	6.006	5.917	5.392
3.700	4.105	3.772	4.309	4.456	3.843
4.116	4.640	4.159	4.760	4.630	4.247
3.998	4.471	3.951	4.485	4.741	4.142
5.450	6.545	5.559	5.443	5.577	4.959
6.086	5.468	5.995	5.753	5.444	6.502
2.363	3.046	3.042	2.948	3.441	3.020
2.525	2.244	3.331	2.013	3.007	2.697
5.556	5.702	5.215	5.825	5.383	5.627
4.398	4.564	4.196	4.655	4.234	4.089
6.270	6.842	6.039	6.691	6.542	6.271
3.491	3.895	3.886	3.227	3.825	2.794
4.540	4.831	4.346	5.259	4.423	4.555
6.327	5.403	6.226	7.624	5.635	6.755
3.789	4.421	4.252	3.677	4.051	3.808
3.072	3.310	3.835	2.941	3.687	3.197
4.892	4.382	5.266	4.968	4.955	5.163
4.731	5.582	4.659	5.606	5.206	4.610
5.237	7.095	5.450	5.785	6.566	5.237
3.421	3.146	3.443	3.259	3.176	3.854
4.024	4.054	4.234	4.498	4.498	3.912
2.476	3.083	2.316	2.120	2.799	2.475
4.840	5.497	4.939	5.519	5.237	4.993
6.816	6.762	7.276	7.604	6.761	6.455
4.632	4.811	5.381	5.107	5.416	4.762
2.951	1.729	2.754	1.437	2.650	3.710
3.929	4.143	4.197	4.410	4.596	3.791
6.025	5.905	7.317	5.814	6.292	5.896
2.880	3.191	2.749	2.822	3.060	2.442
4.976	5.002	4.966	4.694	4.643	5.020
4.892	5.531	5.478	5.407	5.337	4.636
1.794	0.922	2.432	1.421	2.077	2.691
4.710	4.398	4.845	4.355	4.225	4.129
2.924	3.000	3.603	3.681	3.252	3.222
3.513	2.787	1.929	2.729	2.374	4.006
3.520	3.274	2.443	3.527	3.261	4.476
5.094	3.684	5.575	4.930	5.385	5.231
4.432	4.317	4.864	4.726	4.489	4.391
6.037	6.144	6.131	5.442	5.891	6.101
4.612	4.545	4.161	3.842	3.954	4.308
5.002	4.678	3.993	4.893	4.227	5.120
4.788	4.632	5.237	4.524	4.576	5.315
3.517	3.956	3.751	3.244	3.768	4.336

3.910	3.979	4.063	3.795	4.384	4.175
4.375	4.335	4.476	4.390	3.954	5.101
4.671	4.650	4.352	4.510	4.392	5.704
3.388	2.878	2.887	2.948	3.159	4.072
4.246	4.067	4.294	4.594	4.285	5.881
4.723	3.599	4.699	5.398	4.046	6.098
2.908	3.054	3.251	2.930	2.668	2.791
4.695	3.735	4.656	5.003	4.919	5.601
3.166	2.942	2.849	3.140	3.099	3.461
5.572	4.879	5.455	5.180	5.141	5.243
3.733	3.539	3.160	3.671	3.488	3.654
3.392	3.311	3.333	3.738	3.680	2.455
3.505	3.710	3.611	3.795	3.408	4.110
3.750	3.816	3.604	3.817	4.238	4.046
2.748	2.570	2.156	2.082	2.319	3.685
3.792	3.705	3.749	4.268	3.666	4.499
4.071	3.476	3.531	4.152	3.993	4.282
3.774	3.322	4.161	4.543	4.301	3.838
5.082	8.226	3.656	5.730	5.914	2.754
3.179	3.134	2.963	3.366	2.834	3.366
5.217	4.694	4.615	5.290	5.171	5.930
2.435	2.942	2.517	2.444	3.097	2.513
3.610	5.100	3.103	5.203	4.341	3.713
3.656	4.007	3.713	3.171	4.008	3.389
2.719	3.167	2.995	3.357	3.151	2.908
5.467	5.018	5.337	5.539	5.159	5.718
7.179	7.678	7.661	8.061	7.997	7.209
5.732	6.304	6.292	5.982	6.295	6.168
1.914	1.534	1.737	2.621	2.114	2.428
3.014	4.652	3.339	3.766	3.542	4.578
5.954	4.243	6.029	6.050	6.103	6.416
3.462	2.948	3.286	2.995	2.737	3.962
2.473	1.496	3.179	2.592	1.995	3.035
5.374	5.581	5.561	6.005	6.194	5.762
3.517	4.195	4.010	4.237	3.814	3.458
3.525	2.785	3.237	3.333	3.059	4.078
2.301	1.707	1.825	1.807	2.231	1.871
5.655	5.898	6.053	6.347	6.164	6.010
2.620	2.501	1.866	2.648	2.049	3.172
2.751	1.859	3.583	2.529	2.841	3.518
1.702	2.782	1.396	2.137	1.742	1.366
5.110	5.267	4.918	5.262	5.032	6.196
3.405	3.806	3.324	3.994	3.649	4.124
2.660	2.338	2.542	2.862	2.846	3.206
3.615	3.637	3.363	3.806	3.064	4.750
4.713	4.510	4.222	4.927	4.222	4.628
2.249	2.479	2.171	2.225	2.295	2.531
3.404	3.325	3.729	3.694	3.993	2.771
4.611	4.758	5.216	4.994	5.752	4.993
5.492	5.508	5.260	5.391	5.111	5.019

1.837	3.054	2.722	2.526	2.037	1.826
5.000	5.229	5.165	5.514	5.869	4.854
3.423	2.849	3.468	3.559	3.943	3.215
3.647	3.819	3.461	3.925	3.693	3.738
2.959	2.401	2.786	2.805	3.205	3.176
5.679	5.500	6.069	6.544	5.323	5.845
4.095	4.596	3.683	3.746	4.293	3.537
3.728	4.312	3.882	4.320	4.199	3.625
6.361	6.197	6.739	6.890	6.648	6.750
4.421	4.461	4.998	5.170	4.829	4.567
3.805	4.687	4.433	4.868	5.236	4.157
4.366	4.446	4.167	4.836	4.528	4.658
3.104	3.044	2.822	2.555	3.198	2.941
5.097	5.147	5.857	4.291	5.113	4.524
2.471	2.429	3.120	2.366	3.303	1.983
6.890	6.099	6.175	6.704	5.339	7.163
1.957	2.212	1.580	1.952	1.290	1.903
2.885	3.923	2.966	2.848	3.175	3.161
4.114	3.758	3.890	4.192	4.190	4.350
11.004	11.427	11.651	10.958	11.941	10.662
11.092	11.499	11.723	11.037	12.020	10.737
10.614	11.026	11.283	10.522	11.649	10.187
10.333	10.690	10.980	10.211	11.336	9.793
10.351	10.876	11.071	10.342	11.444	9.952
10.391	10.862	11.103	10.368	11.458	9.963
10.782	11.261	11.439	10.673	11.686	10.384
10.480	10.833	11.168	10.395	11.378	10.108
4.716	5.009	5.348	4.813	5.513	4.684
4.511	4.553	5.067	4.724	5.260	4.253
9.277	9.022	9.505	9.696	9.193	9.427
6.265	5.204	6.521	6.457	6.960	6.827
9.581	10.480	9.763	9.286	10.153	9.403
3.379	4.226	3.918	1.889	4.934	2.435
2.944	3.927	3.547	2.185	3.117	3.013
9.539	10.091	10.254	9.366	10.855	9.360
1.284	1.049	1.518	1.123	1.196	1.368
6.287	6.186	7.045	6.264	6.885	5.935
2.910	2.848	2.859	2.715	2.986	2.540
5.558	5.676	5.897	5.731	6.357	5.524
4.109	5.367	4.065	4.680	4.144	4.259
7.982	8.422	8.778	7.583	9.238	7.900
4.990	5.247	5.940	5.682	5.977	5.316
2.042	2.240	2.014	2.798	1.689	2.743
2.640	1.547	2.702	2.680	3.413	3.153
2.006	1.883	1.612	1.810	1.742	2.131
1.698	2.086	1.537	1.587	1.852	2.788
4.852	3.978	4.763	4.288	4.871	5.482
3.878	2.524	3.309	4.064	3.113	5.216
1.718	1.520	1.640	1.502	2.005	1.604
2.464	2.031	2.402	2.648	2.481	2.695

3.379	2.561	3.423	3.884	3.284	3.881
2.694	2.277	2.443	2.684	2.301	2.344
2.508	2.863	2.588	1.910	3.799	2.664
2.517	2.135	2.921	2.444	2.661	2.649
4.086	3.662	4.189	4.685	4.827	3.745
3.054	3.275	1.874	3.426	2.628	3.396
3.760	3.158	3.380	2.452	2.801	3.198
5.147	4.712	4.773	4.973	4.729	4.662
5.000	5.329	4.982	5.054	5.145	5.306
3.597	2.965	3.408	2.790	3.135	3.122
2.766	2.623	2.563	2.654	2.762	3.332
5.203	6.403	5.682	5.946	6.485	5.928
5.512	6.908	5.502	5.297	5.683	6.204
3.344	4.127	3.578	3.626	3.811	3.345
2.435	2.150	1.893	2.519	2.054	2.486
5.081	5.088	5.473	5.647	5.474	5.497
5.004	5.321	4.670	5.297	4.919	5.731
6.557	6.936	6.685	7.009	6.362	6.613
3.988	3.661	4.330	4.827	4.408	4.386
4.968	5.836	5.215	6.431	5.597	4.644
6.386	6.024	7.254	6.199	5.834	6.164
3.446	2.837	3.883	3.752	3.851	3.716
4.889	5.410	4.831	4.672	4.435	4.726
4.898	7.132	5.022	5.851	5.540	5.067
4.964	7.323	5.168	5.843	5.713	5.013
5.442	7.596	5.483	6.450	6.063	5.535
5.048	5.264	4.874	5.262	4.678	5.297
4.466	5.945	5.482	5.027	6.465	3.802
3.798	5.316	4.413	4.668	5.028	4.027
7.619	8.384	8.585	8.329	8.285	8.038
3.822	3.733	3.505	3.665	3.765	4.382
5.965	8.435	7.011	7.533	7.797	6.681
5.103	5.936	4.072	5.410	5.222	5.216
4.280	4.456	4.008	4.094	4.131	4.591
4.845	5.443	4.532	4.928	5.482	5.369
7.774	8.554	7.479	8.014	8.918	8.417
3.492	3.995	3.796	3.518	4.456	2.984
4.298	4.177	4.451	3.867	4.696	4.914
4.423	4.904	5.310	4.411	4.461	4.618
3.676	3.891	3.928	3.438	3.832	4.364
7.738	8.899	8.449	8.747	8.562	7.294
8.558	9.060	8.625	9.702	9.046	8.385
5.154	5.802	5.320	5.328	5.613	5.262
3.963	5.114	3.979	4.394	4.362	4.258
4.722	5.069	5.231	5.069	5.132	4.783
4.203	4.446	4.674	4.530	4.663	4.233
4.130	4.479	4.398	4.376	4.520	4.115
3.392	3.553	3.600	3.533	3.596	3.182
3.031	3.093	2.776	3.275	2.115	2.732
5.817	6.130	6.028	6.208	6.174	5.612

3.493	4.095	3.367	3.408	3.973	3.355
5.582	5.238	5.529	5.807	5.535	5.981
0.850	1.041	1.139	0.701	1.183	0.967
5.757	5.166	5.633	5.399	5.879	6.147
3.447	3.450	3.913	3.901	3.686	2.754
4.834	4.783	5.145	5.272	5.178	4.345
3.108	3.082	3.206	3.259	3.168	3.023
3.456	3.301	3.170	2.909	3.245	4.011
3.311	3.031	3.605	2.885	2.435	2.902
3.018	3.738	2.405	2.896	2.731	3.452
4.692	6.128	3.465	4.943	3.791	3.965
5.020	7.447	5.011	6.165	5.947	4.411
7.085	8.268	7.532	7.117	7.672	6.585
4.927	5.615	4.615	4.021	4.505	3.930
8.443	8.940	8.887	9.422	9.006	8.757
1.635	2.275	1.674	2.031	2.276	2.766
2.285	3.058	2.617	3.016	2.486	2.759
4.122	5.067	4.009	5.072	4.618	3.791
3.926	4.455	3.870	4.132	4.230	3.302
4.264	4.024	2.883	4.515	4.995	4.526
2.989	3.073	2.713	1.681	2.359	2.446
3.187	3.374	3.464	3.626	2.417	2.896
2.811	2.899	2.025	3.154	2.520	3.313
4.038	3.339	2.817	3.239	3.175	2.477
3.030	3.322	2.983	2.543	2.358	2.302
2.181	1.835	1.674	2.723	2.438	1.899
5.984	5.380	6.136	5.613	5.747	6.332
3.819	3.894	3.785	2.989	2.936	2.072
2.692	3.761	2.347	2.321	2.251	2.620
2.788	2.157	3.194	1.938	2.445	2.246
3.440	3.392	3.888	3.045	3.652	3.293
2.193	2.949	3.251	2.725	1.612	2.627
3.123	3.052	3.069	3.026	2.407	2.860
4.827	5.214	4.689	4.587	4.913	5.105
3.193	3.447	2.460	3.045	2.866	3.045
2.842	2.362	2.750	1.827	3.097	2.471
4.803	3.037	4.116	4.722	3.567	5.101
3.035	3.201	3.535	2.108	3.201	1.887
3.058	2.178	3.168	2.037	3.591	2.448
3.825	3.853	3.999	2.605	2.681	2.983
4.714	5.052	3.995	4.000	3.625	4.553
2.899	2.424	2.589	2.677	2.401	2.300
2.630	1.575	2.414	3.183	4.437	3.473
1.920	2.177	2.452	2.882	2.701	1.422
3.753	4.044	4.649	4.329	4.580	3.648
5.295	5.679	5.330	5.665	5.222	4.977
4.881	4.389	4.452	4.872	4.436	4.323
4.601	6.429	4.481	6.368	5.310	5.358
4.228	4.064	4.582	3.686	4.304	4.193
2.341	2.839	2.380	2.550	3.085	2.539

6.592	6.446	7.193	6.654	6.625	6.857
5.918	6.477	6.217	5.976	6.232	5.690
3.005	4.061	2.982	4.091	4.006	3.800
3.074	4.090	3.391	4.674	4.028	3.565
3.823	3.315	4.389	4.438	4.506	3.838
5.114	6.264	6.065	5.123	6.394	4.688
4.776	5.413	5.015	5.199	5.380	4.715
1.861	3.630	2.175	2.630	3.451	1.796
4.435	4.290	4.808	4.483	4.523	4.036
2.513	2.310	2.388	1.773	2.373	1.814
1.330	1.226	1.251	1.303	2.048	1.553
6.987	7.587	7.766	7.918	7.946	7.019
3.537	4.262	3.468	3.742	4.228	3.193
4.577	3.814	4.829	3.926	4.148	5.113
7.155	8.019	6.728	8.263	7.635	6.931
5.251	4.920	5.642	5.390	5.489	5.058
5.035	5.460	6.246	5.994	6.162	4.197
4.428	5.575	4.499	4.459	5.067	4.664
3.518	2.591	3.092	3.111	2.988	2.527
1.920	3.043	2.088	2.782	2.798	2.607
2.572	3.460	2.707	3.228	2.909	4.274
5.274	6.806	5.637	5.481	6.290	5.142
2.587	3.961	2.963	2.449	1.807	1.896
3.081	3.387	3.570	2.988	3.395	2.732
5.679	5.002	6.189	5.278	5.901	6.149
1.009	0.696	1.015	0.711	0.882	0.929
3.749	4.188	4.532	4.513	4.089	4.207
2.612	2.267	2.173	2.148	2.107	2.527
2.259	3.176	2.614	3.202	2.342	2.526
3.127	2.828	2.553	3.785	3.563	4.558
2.795	2.653	2.817	3.823	1.967	3.694
3.229	5.073	3.642	2.862	4.389	3.245
2.616	2.811	2.084	2.268	2.687	3.067
3.952	5.219	4.900	5.194	5.486	5.117
3.347	4.315	2.954	3.595	3.203	2.689
3.606	4.601	4.256	3.275	3.939	3.261
4.228	5.381	4.710	4.069	4.217	4.009
5.691	7.176	6.690	7.036	6.926	5.700
5.596	6.241	5.797	5.699	5.817	5.441
3.192	3.005	4.085	2.636	3.657	2.697
4.663	4.660	4.281	3.773	4.057	3.970
6.017	5.912	6.388	6.822	6.353	6.163
2.779	2.464	2.155	2.062	2.725	2.517
6.005	6.178	6.216	5.732	6.028	5.790
3.030	3.437	3.202	3.015	3.519	3.141
1.656	1.485	1.478	1.769	1.188	1.881
4.455	4.393	4.376	4.699	4.322	4.241
5.723	5.810	5.372	5.895	5.952	6.047
3.475	3.707	3.833	3.736	3.537	3.724
6.618	6.558	6.987	6.793	6.939	7.033

3.239	3.155	3.310	2.922	3.360	3.218
6.555	6.113	6.424	6.292	6.170	6.531
6.626	7.342	6.631	7.172	7.207	6.116
4.342	4.708	3.646	4.437	4.358	4.878
7.121	6.958	6.918	7.529	7.066	7.671
2.315	2.557	1.841	2.676	1.980	2.542
6.203	6.557	6.477	6.548	6.602	6.220
2.850	2.445	2.512	2.267	2.281	2.487
7.371	7.802	7.500	8.496	6.737	7.460
5.121	5.332	5.264	5.528	5.484	5.263
2.996	2.462	2.369	2.867	2.924	2.812
1.873	1.052	0.815	1.805	2.805	1.222
3.957	4.079	4.405	4.238	4.889	4.005
4.389	5.085	4.768	4.810	4.844	4.507
6.125	6.134	6.096	6.098	5.827	6.228
5.020	4.647	6.364	5.689	5.125	4.483
4.000	4.343	3.472	5.141	3.770	4.639
4.779	4.879	5.184	4.922	5.130	4.889
6.519	6.412	6.777	6.995	6.661	6.772
3.466	3.554	3.593	3.617	3.267	3.687
6.555	7.017	6.839	6.807	6.456	6.879
3.508	3.341	3.296	3.955	3.851	4.142
4.806	3.951	5.476	3.801	3.818	5.162
6.020	5.603	5.972	5.735	5.511	6.255
5.605	5.184	5.542	5.070	5.597	6.134
3.423	4.276	3.691	3.172	4.704	3.890
8.644	7.824	8.470	8.186	8.162	8.890
2.545	2.623	2.732	3.119	2.392	2.723
8.379	7.247	8.254	7.741	7.977	8.663
8.648	7.931	8.498	8.188	8.190	8.802
2.908	2.301	2.067	3.363	2.165	2.529
3.608	3.486	3.974	3.867	3.218	4.004
2.420	1.919	1.540	2.278	2.278	1.866
5.467	5.069	5.095	5.018	4.924	5.808
8.031	7.329	8.094	7.722	7.494	8.289
9.176	8.400	8.974	8.841	8.667	9.559
7.484	6.836	7.539	6.994	6.987	7.744
7.052	5.775	7.245	6.802	6.704	7.528
5.665	4.887	5.644	5.291	5.496	6.015
3.176	3.110	3.084	3.562	3.023	3.103
7.204	6.380	7.391	6.752	6.738	7.702
6.293	6.017	6.429	5.787	5.821	6.393
6.593	5.980	6.830	6.421	6.474	7.133
6.121	5.573	6.062	5.883	5.732	6.634
3.474	3.701	2.999	3.623	2.429	3.492
6.882	6.126	7.021	6.592	6.521	7.336
6.029	5.802	6.193	5.260	5.636	6.029
5.044	3.886	4.336	4.242	4.697	4.564
3.075	3.044	3.791	3.174	3.554	3.215
7.572	8.302	8.194	7.790	8.055	7.716

3.555	4.319	4.882	3.225	5.242	3.067
6.727	6.422	6.736	6.724	6.389	6.794
4.147	4.171	5.043	4.204	4.759	4.300
3.897	3.366	3.098	3.693	2.668	4.452
3.863	2.996	3.497	3.436	3.229	3.503
5.201	5.832	5.131	5.027	5.310	5.364
3.131	3.500	2.981	3.161	3.500	3.209
5.462	6.120	5.966	5.951	5.847	4.558
3.724	3.596	3.939	4.052	4.560	3.693
6.011	6.004	6.400	6.442	6.454	6.480
3.451	4.174	3.561	3.032	3.544	3.654
6.207	6.708	6.905	6.059	6.827	6.253
3.388	4.244	3.382	3.268	3.930	3.887
4.619	6.136	4.544	6.222	5.536	4.720
5.845	5.871	5.805	5.891	6.307	5.929
2.856	2.820	2.488	3.005	2.021	2.429
4.617	4.961	4.987	4.726	4.521	5.199
3.069	2.569	2.658	3.353	3.055	3.091
7.274	7.405	8.133	7.749	8.034	7.039
3.455	3.044	3.602	2.948	3.528	3.773
2.504	2.528	2.589	2.087	2.021	2.374
4.327	3.768	4.328	4.179	4.653	4.123
3.624	3.598	3.005	2.997	2.991	3.887
3.565	3.922	3.457	3.637	3.341	3.548
4.917	4.617	5.594	5.479	4.968	5.353
2.579	3.330	3.145	2.320	2.271	2.859
3.271	3.436	3.354	3.604	3.443	3.045
4.063	4.315	3.477	3.280	3.872	4.052
3.668	3.135	3.402	3.019	3.525	3.366
7.074	6.910	7.147	7.290	7.197	6.990
5.381	5.530	5.342	5.753	5.320	5.192
2.480	2.693	1.797	1.961	1.891	1.598
5.455	5.540	5.770	5.188	5.569	5.485
5.062	5.737	5.230	5.702	5.352	5.289
2.677	4.176	3.357	2.840	3.529	2.640
4.097	5.086	4.855	4.716	4.362	4.312
5.588	6.068	5.827	6.132	5.747	5.338
3.781	3.681	3.811	3.692	3.447	3.888
4.411	4.608	5.501	4.462	4.178	4.488
3.926	5.400	3.528	4.817	5.197	3.951
4.727	6.299	5.782	5.594	6.309	4.844
9.137	9.084	9.506	9.523	9.244	9.345
5.531	6.844	7.001	6.270	7.288	5.747
4.502	4.724	4.934	4.740	4.542	4.476
3.824	4.494	4.068	4.811	4.544	3.679
2.401	2.644	2.113	2.800	2.417	3.215
3.279	5.442	4.161	4.441	3.627	3.594
3.716	3.711	3.734	3.597	3.934	3.396
7.858	8.131	7.911	7.661	8.078	7.844
6.136	6.210	6.208	5.862	5.981	5.846

8.417	7.918	8.424	8.066	8.170	8.431
5.387	6.012	6.780	6.066	6.774	6.047
4.233	4.078	4.214	4.428	4.851	6.025
6.535	6.739	6.069	6.534	6.241	6.647
2.097	2.295	1.956	3.136	1.968	1.647
8.832	8.753	8.855	8.855	9.006	8.937
4.804	4.570	5.064	4.030	4.603	4.798
5.375	5.443	6.281	5.799	6.198	5.508
1.693	1.039	1.434	1.920	3.115	1.448
7.665	7.688	7.811	7.684	8.078	7.732
4.031	5.275	4.714	4.269	4.286	4.396
2.625	3.471	3.349	3.155	3.004	2.089
2.731	2.890	2.666	3.349	3.085	2.664
4.440	5.162	4.859	4.828	5.043	4.288
7.419	7.131	7.555	7.397	7.629	7.635
4.562	3.947	5.099	4.582	4.472	4.743
3.597	3.741	3.516	4.318	3.248	3.770
3.788	4.992	4.740	5.300	4.704	3.658
4.568	3.510	4.396	4.510	4.109	4.790
7.096	7.278	7.497	7.027	7.045	6.956
3.346	3.453	3.634	4.570	3.516	3.494
1.612	1.299	1.674	1.726	1.458	1.592
4.193	4.402	4.714	4.704	3.924	4.357
2.643	3.322	2.990	4.190	3.335	2.492
4.999	4.222	4.657	4.887	4.092	5.812
3.349	2.908	3.252	3.591	3.216	4.271
5.634	5.560	5.471	5.883	5.508	5.712
8.791	9.527	8.769	8.344	8.558	8.226
5.120	5.054	4.881	5.341	4.916	4.980
7.999	8.235	8.524	8.961	8.901	8.658
4.534	4.181	4.974	4.740	4.357	5.062
5.569	5.166	5.772	5.094	5.196	5.676
6.089	6.417	5.903	6.347	5.864	5.859
3.662	3.409	2.418	3.532	3.923	3.576
3.402	3.896	4.312	4.006	3.330	3.527
5.095	5.228	4.826	5.069	4.960	5.280
1.248	1.268	1.402	0.744	1.400	1.505
4.044	4.479	3.766	4.628	4.272	4.149
1.333	1.355	0.990	1.197	1.572	1.284
4.219	3.811	4.597	4.353	4.538	4.473
5.184	4.906	5.016	5.013	5.209	5.617
3.905	4.058	4.020	4.634	4.545	3.544
2.397	2.228	2.460	2.514	3.184	3.138
4.208	2.075	2.323	4.028	2.840	4.609
5.252	4.262	5.013	5.576	4.461	5.951
4.071	4.318	4.246	4.078	4.339	4.722
2.712	2.442	2.985	3.454	3.245	4.437
4.002	4.670	4.817	3.932	4.458	2.532
5.674	4.362	5.625	4.806	4.735	5.592
3.703	3.556	3.862	3.849	3.792	4.334

2.652	2.641	2.575	2.616	2.474	2.835
3.709	3.588	3.720	3.761	3.329	4.293
4.497	4.023	4.575	4.852	4.347	4.860
2.815	2.207	2.933	2.598	3.106	4.008
3.116	2.315	2.602	3.399	1.907	4.055
3.469	2.443	2.810	4.237	2.446	4.933
4.226	3.467	3.885	3.736	4.016	4.425
4.067	3.946	5.161	5.423	4.137	4.488
6.561	5.736	6.511	6.344	6.334	6.952
5.700	5.922	5.950	5.998	5.658	6.169
2.661	2.761	2.707	1.829	2.269	2.147
3.050	3.149	2.512	2.138	2.336	2.537
4.856	5.673	5.673	5.117	6.899	3.805
4.021	3.994	4.374	4.222	3.776	3.543
2.640	2.096	2.291	2.293	2.300	2.205
2.423	1.631	2.007	2.155	2.330	2.972
5.382	6.289	4.820	5.104	6.918	5.206
3.049	3.469	2.688	3.011	3.993	3.002
3.496	2.515	3.565	3.250	3.365	4.023
2.565	2.142	2.724	2.261	1.878	2.990
1.953	1.595	1.897	1.740	1.856	2.008
3.055	2.613	2.996	3.678	2.911	4.037
3.022	2.533	2.983	3.377	2.486	4.466
5.603	4.961	5.894	5.916	5.801	6.597
4.568	4.276	4.234	5.514	4.628	5.431
3.808	3.724	3.604	3.502	3.265	3.388
5.045	4.614	5.414	4.802	5.428	4.771
3.230	2.860	3.097	2.983	2.763	4.493
3.409	3.484	3.347	1.967	3.882	4.594
8.564	8.024	8.950	8.294	8.257	8.773
3.460	2.239	3.157	3.587	3.140	3.196
1.930	2.117	2.149	1.764	1.814	2.391
5.744	4.013	5.853	5.117	5.042	6.211
6.296	6.452	6.235	6.175	6.686	6.221
4.435	3.954	4.309	4.163	4.568	4.592
4.744	3.723	4.859	4.667	4.795	5.203
3.632	3.381	3.054	3.078	3.137	3.236
3.183	3.593	3.145	3.544	4.159	3.810
3.219	3.297	3.108	3.803	2.896	4.991
3.081	3.665	3.060	3.346	3.956	3.377
5.661	5.441	5.433	5.938	5.291	5.640
2.782	2.351	2.502	2.212	2.544	2.874
2.818	2.716	1.951	2.374	2.713	2.601
2.710	2.452	2.492	3.065	3.036	2.219
2.863	3.815	2.860	2.254	2.759	2.926
4.591	4.634	4.900	4.196	4.966	4.677
4.921	3.804	4.470	4.554	4.231	5.274
3.004	2.437	2.831	2.484	2.385	3.008
3.493	4.526	3.958	4.028	3.647	3.427
3.077	1.990	2.581	3.230	2.492	3.793

5.679	5.281	6.531	5.380	5.530	5.549
4.334	4.526	4.054	4.705	5.030	4.281
3.081	3.151	3.377	3.185	3.121	3.397
6.330	6.227	6.338	6.044	6.495	6.825
4.223	4.339	3.583	3.729	3.819	4.690
6.896	6.156	7.215	7.496	7.264	7.508
3.448	4.515	3.442	3.821	3.784	3.297
3.566	4.040	3.396	3.842	4.085	3.208
3.666	3.606	2.709	3.772	3.132	2.710
4.165	3.663	4.297	3.738	2.977	4.240
2.784	3.052	2.366	2.952	2.442	2.968
3.808	3.980	3.442	3.684	3.634	4.232
5.247	5.113	5.521	5.578	5.246	6.111
2.302	2.027	2.216	2.965	2.455	3.542
1.880	2.092	1.523	1.935	1.753	1.943
2.821	1.984	2.881	2.480	2.131	2.306
3.075	2.898	3.422	3.003	2.649	3.426
4.180	3.644	3.400	3.793	3.137	4.399
4.006	3.839	4.468	4.148	4.980	4.610
2.307	2.002	1.726	1.443	2.018	2.123
4.601	4.572	4.194	4.756	4.571	4.812
4.664	5.431	4.457	4.587	5.046	4.571
7.398	7.838	7.371	7.849	7.746	7.151
3.405	3.280	3.646	3.679	3.023	4.093
7.746	7.526	7.829	8.140	7.763	7.580
3.235	3.721	4.078	3.371	4.111	3.523
3.584	4.509	4.073	4.155	4.086	3.827
3.391	5.580	6.011	1.588	5.456	1.403
5.404	5.398	5.871	6.028	5.490	5.895
5.168	5.604	5.004	4.944	4.203	6.076
4.908	4.759	5.325	4.521	4.365	4.612
4.302	0.911	3.843	4.904	4.472	4.147
4.185	3.757	3.013	4.461	3.322	5.022
3.635	2.975	4.049	3.936	3.026	4.338
5.234	4.862	5.764	5.138	5.637	5.065
4.831	4.954	4.971	5.392	5.139	4.892
4.729	4.738	5.357	5.434	5.075	4.766
3.994	4.948	4.249	4.665	4.499	4.472
5.192	5.671	6.011	5.443	5.850	5.130
4.208	2.922	2.820	4.826	3.840	4.850
6.739	6.889	6.925	6.893	6.884	6.755
7.212	7.062	7.503	7.065	7.366	7.226
6.879	6.206	6.527	6.889	6.288	7.250
5.682	5.052	5.324	5.457	5.629	5.709
4.929	4.596	5.106	4.575	4.897	5.040
4.828	4.339	4.414	3.809	4.537	4.615
3.422	2.892	3.557	3.619	3.334	3.719
3.544	4.417	3.629	3.881	3.968	3.271
3.928	3.554	3.787	4.177	3.201	4.165
5.240	3.838	4.105	5.354	3.961	5.913

6.939	6.521	7.342	6.833	7.073	7.220
5.843	5.461	6.193	5.334	5.760	5.546
2.406	2.641	2.390	2.871	2.014	2.603
4.622	4.661	4.057	4.271	4.071	3.616
7.376	7.341	7.532	7.920	7.744	7.641
3.996	3.503	3.821	3.912	4.137	4.966
7.019	6.468	7.103	7.034	6.612	6.967
6.405	5.205	6.508	5.705	6.104	5.657
5.025	4.468	4.615	4.725	4.374	4.998
3.936	3.583	4.147	4.012	3.880	3.746
4.874	4.868	4.451	4.598	4.714	5.111
5.338	4.845	5.175	5.707	5.247	5.726
6.158	6.073	6.178	6.431	6.290	6.441
2.493	2.154	2.313	1.991	1.750	1.831
2.670	2.822	2.719	3.043	2.987	3.846
5.501	4.976	5.414	4.912	5.222	5.583
5.100	4.348	5.364	5.117	4.624	5.580
5.606	5.480	5.862	6.060	5.653	5.999
8.275	7.847	8.327	8.112	8.017	8.502
2.780	2.769	2.749	2.770	3.383	3.718
2.850	3.150	3.050	3.117	2.135	3.342
4.685	4.343	4.466	3.568	4.386	4.084
2.165	1.385	1.353	1.524	1.844	1.841
4.684	4.209	4.972	4.504	4.726	5.010
3.288	3.219	3.407	3.349	3.192	2.798
6.035	5.434	6.366	5.979	5.998	5.851
4.017	3.239	3.866	4.292	4.109	4.949
7.603	7.400	7.582	7.525	7.477	7.668
2.550	2.672	2.687	2.625	2.609	2.668
4.266	4.314	4.231	4.096	4.738	3.150
8.681	8.249	8.479	7.977	8.043	8.453
7.549	6.653	7.502	7.734	7.022	7.688
4.824	4.500	5.200	5.224	4.539	4.788
7.608	6.722	7.220	7.823	6.870	7.864
6.521	5.880	6.040	6.606	5.903	6.715
3.269	3.774	3.624	2.851	2.794	2.121
2.842	3.094	2.603	2.458	2.868	3.217
8.760	7.680	8.407	8.508	7.825	9.228
9.554	9.109	9.445	9.293	9.305	9.351
8.602	8.042	8.523	8.275	8.104	8.676
4.401	3.950	4.488	4.149	4.799	3.870
7.941	7.422	7.916	8.080	7.495	8.137
3.017	2.829	3.251	3.077	2.991	3.899
2.487	2.325	2.194	1.648	2.830	1.938
4.895	4.125	4.937	5.087	4.602	5.579
5.843	5.235	6.367	6.046	5.684	5.974
4.875	4.569	5.247	4.399	4.797	4.420
2.114	2.821	2.401	2.347	2.030	2.937
3.348	3.202	2.929	2.130	2.844	2.817
5.377	5.048	5.117	5.576	4.944	5.623

5.564	6.231	5.448	5.190	5.385	5.667
6.321	6.408	6.747	6.525	6.470	6.281
5.049	4.533	4.577	4.540	4.756	4.679
6.005	6.950	5.746	5.074	5.650	4.191
5.900	6.076	5.961	6.311	6.035	5.885
3.454	3.590	2.988	3.451	3.044	3.683
6.991	8.048	7.472	7.727	7.649	7.284
5.868	5.703	6.462	5.841	5.787	5.674
2.701	2.581	3.197	3.132	3.301	2.630
6.919	6.977	7.177	6.992	6.743	6.731
6.960	6.791	6.699	7.004	6.976	7.090
3.618	3.052	3.926	3.478	3.114	3.760
4.459	4.092	4.157	4.894	4.795	4.486
3.742	2.456	4.102	3.961	3.087	5.106
5.322	5.538	5.658	5.475	5.530	5.678
2.367	2.300	2.537	2.644	2.553	2.593
4.540	4.450	4.881	5.215	4.766	4.620
4.666	5.160	4.653	5.227	5.093	4.821
6.810	6.866	7.051	6.781	6.693	6.839
3.732	3.985	3.696	3.689	4.037	3.569
5.603	5.468	5.842	5.806	5.933	5.999
4.394	4.250	4.431	4.565	4.439	4.545
2.821	2.521	3.117	2.768	2.667	3.607
4.277	5.584	6.222	4.805	5.800	3.864
2.866	2.626	2.872	3.967	2.897	3.635
3.349	2.817	3.677	3.354	3.313	3.375
9.826	9.940	9.908	9.701	9.701	9.787
3.800	2.912	2.627	3.242	4.284	4.068
2.925	2.851	2.542	2.440	2.414	2.914
2.532	2.785	3.159	2.945	2.377	3.551
4.851	3.842	4.285	4.401	4.304	5.154
1.440	0.981	1.171	1.226	1.369	1.890
3.761	4.065	3.551	3.803	3.581	3.604
6.172	6.970	6.654	6.816	6.640	6.738
5.558	5.535	5.968	5.710	5.731	5.901
4.613	4.783	4.601	4.424	4.827	4.908
5.495	5.715	5.614	5.374	5.661	5.676
2.502	3.807	2.209	1.638	2.203	3.146
3.863	4.482	4.128	4.246	3.988	3.947
2.300	2.371	3.779	2.713	3.197	1.836
3.396	4.203	4.687	4.502	3.429	2.878
3.687	2.993	2.951	3.795	2.715	4.176
2.196	1.910	2.144	2.674	2.362	2.231
4.112	3.716	3.298	3.278	3.783	3.597
3.494	3.330	3.666	4.093	2.722	2.684
2.601	2.195	2.746	3.019	2.475	2.347
3.085	3.392	2.495	2.537	2.369	2.288
4.217	4.342	3.952	3.931	4.265	4.137
5.561	4.755	5.691	5.406	4.440	5.559
2.735	3.439	3.775	3.925	4.842	2.624

4.922	3.804	4.254	4.846	3.878	5.627
2.805	3.059	2.676	3.346	2.824	3.310
4.569	4.541	4.610	4.549	4.365	3.914
2.225	2.637	2.728	1.975	2.357	3.050
2.890	2.706	4.115	3.486	3.001	2.104
5.451	5.733	5.581	5.623	6.499	5.820
4.397	5.282	4.725	5.830	5.432	4.651
4.388	4.242	5.282	5.023	4.915	4.544
4.458	5.301	4.288	4.454	4.327	4.217
5.831	5.846	5.820	6.006	6.058	5.625
2.451	2.750	2.444	3.074	2.417	2.043
5.637	6.475	5.853	6.202	6.107	5.262
8.226	8.300	8.247	8.119	8.298	8.231
8.634	8.463	9.038	8.916	8.773	8.897
5.954	5.777	6.392	5.924	5.910	6.021
2.586	2.597	2.785	3.220	2.456	2.245
6.042	6.718	6.266	6.916	6.668	6.499
9.289	10.646	10.190	10.569	10.606	9.167
4.865	4.403	4.583	4.450	4.721	5.062
4.562	3.969	4.769	4.719	4.187	4.581
5.250	5.846	5.153	5.780	5.538	4.846
5.107	5.507	5.506	5.259	4.938	4.954
2.489	2.444	2.727	1.916	2.342	2.952
5.759	5.889	6.467	6.414	6.558	6.136
2.691	1.976	2.686	2.388	2.390	2.205
3.777	3.495	3.308	4.414	2.123	3.986
4.343	4.349	4.240	4.978	4.376	4.261
4.079	4.141	4.429	3.687	5.123	4.206
6.681	8.039	6.678	7.373	7.164	6.620
6.209	7.426	6.988	7.171	7.079	6.713
3.699	4.265	3.673	4.428	4.288	2.817
3.825	3.771	4.048	3.770	3.564	4.049
5.064	4.894	4.810	5.900	4.710	4.760
5.110	5.625	4.869	5.399	6.205	4.752
6.210	5.648	6.263	7.164	5.814	6.527
2.555	2.526	2.022	1.994	2.009	2.053
2.782	2.238	2.431	2.494	2.087	1.674
5.162	5.298	5.292	5.077	5.115	5.061
6.310	5.777	6.042	6.173	5.766	6.581
7.390	7.523	7.485	6.850	7.352	7.093
4.298	4.524	5.135	4.214	5.347	4.326
4.374	3.675	4.438	3.953	4.366	3.978
3.844	3.412	3.254	4.070	3.947	3.790
6.509	6.871	6.575	6.798	6.384	6.239
3.121	4.622	3.552	3.220	4.067	3.244
7.373	7.752	7.386	7.715	7.263	7.015
4.810	6.297	4.599	4.998	5.460	5.233
3.418	3.917	3.152	3.133	3.313	3.149
5.741	5.914	5.922	6.677	6.213	6.181
6.859	5.926	7.199	6.991	6.635	7.572

4.533	5.015	4.118	4.993	4.481	5.092
6.062	6.888	6.182	6.265	6.966	6.420
6.409	6.044	6.758	6.869	6.867	6.636
6.812	6.214	7.362	7.137	6.696	7.070
3.486	2.460	3.166	2.587	3.040	3.523
3.171	2.573	3.077	2.800	3.041	2.628
4.107	4.907	3.580	5.068	4.547	3.693
3.736	2.999	2.993	3.395	3.329	4.254
2.723	2.205	2.479	2.542	2.922	2.499
6.430	6.292	6.728	6.207	6.600	6.097
2.381	3.083	1.781	3.193	1.497	3.587
3.063	2.309	2.141	2.435	2.172	2.435
3.181	2.630	2.910	2.124	4.103	2.797
2.734	3.041	2.423	2.194	2.649	2.782
4.492	4.344	5.260	4.587	4.807	5.016
7.587	7.020	8.157	7.708	7.721	7.707
3.033	3.769	2.700	4.613	3.428	2.551
2.877	3.235	2.956	3.607	3.471	2.688
3.547	3.565	3.666	3.198	4.857	3.835
2.239	4.950	2.153	2.271	4.012	3.781
3.080	5.099	4.256	4.119	4.663	2.792
3.609	4.988	3.826	4.106	3.956	3.736
4.870	5.376	5.139	5.193	4.886	4.946
2.728	2.127	2.063	3.034	1.579	3.801
5.425	4.914	4.860	5.403	4.712	5.777
6.421	6.827	6.861	7.004	7.125	6.644
3.048	3.630	2.115	2.889	2.662	3.552
4.531	5.328	4.937	5.170	5.057	4.453
3.030	3.115	3.375	3.048	3.005	3.733
3.703	3.588	3.885	3.095	3.801	3.241
3.800	4.235	4.519	4.148	3.288	4.819
4.929	3.348	3.674	5.082	4.214	5.495
5.141	4.941	4.837	4.535	5.399	5.425
3.228	3.518	3.407	3.130	2.611	2.789
3.999	3.648	3.504	4.028	4.954	4.251
3.706	3.621	3.012	3.572	2.995	3.091
4.379	4.018	3.840	4.204	4.417	4.446
2.905	4.833	3.987	2.630	4.650	2.861
2.624	2.326	1.829	2.026	1.825	2.637
3.449	3.134	3.276	3.492	3.380	3.284
5.844	5.564	6.174	6.267	5.540	5.822
2.416	1.732	2.481	2.223	2.624	2.558
2.981	2.731	2.419	2.134	2.961	2.339
2.568	2.314	2.293	3.154	2.830	2.504
4.121	3.871	4.346	3.976	4.922	4.202
2.757	1.892	3.982	1.669	3.465	2.409
1.247	1.808	0.705	1.048	1.308	1.567
3.423	1.476	4.659	3.330	1.576	1.646
2.041	1.935	2.792	1.580	2.462	2.557
3.473	2.330	3.522	2.750	3.603	3.979

1.659	1.246	1.528	1.910	1.689	1.541
2.250	2.026	2.306	2.066	2.315	2.141
3.573	3.413	3.079	3.719	3.329	3.358
1.342	0.929	0.833	0.671	0.752	1.179
2.483	1.743	2.393	2.870	2.098	2.694
1.395	1.163	1.422	1.376	1.191	1.875
3.643	4.196	3.572	4.202	4.270	3.883
3.740	4.125	3.493	3.696	3.233	3.565
3.492	3.835	3.927	4.594	4.058	3.445
2.151	2.333	1.860	1.589	2.333	1.604
3.474	2.742	2.274	2.572	2.237	4.232
3.155	2.624	2.878	2.077	2.988	2.835
2.730	2.027	2.390	2.725	2.585	2.781
2.904	2.504	4.037	1.747	1.879	1.612
2.472	2.425	2.902	1.899	2.359	2.837
1.141	0.781	1.370	0.600	0.984	1.139
2.661	3.146	3.146	3.010	2.911	2.541
2.894	2.382	2.553	2.351	2.553	2.221
3.524	2.783	3.112	3.393	2.738	3.625
2.403	1.720	2.596	2.193	1.462	2.849
3.786	3.364	3.427	4.004	2.862	4.219
3.872	3.527	3.366	3.722	3.363	3.596
2.378	1.110	1.963	1.734	1.903	3.095
4.843	4.727	5.548	4.785	5.193	5.423
4.801	4.101	5.385	4.300	4.511	3.799
2.753	3.052	1.983	2.754	1.655	2.451
2.151	2.208	2.482	2.746	2.379	1.433
3.501	3.419	3.171	3.130	2.245	3.337
5.305	5.176	5.450	5.026	4.985	5.238
5.444	4.297	5.477	4.544	4.807	5.590
2.822	1.656	2.269	1.790	1.397	2.625
4.545	3.552	4.758	4.515	4.437	5.054
6.680	5.793	7.264	6.683	6.524	6.754
2.163	1.954	1.878	1.835	1.899	1.928
3.047	2.581	3.110	2.869	2.987	3.168
4.215	3.981	4.318	4.425	4.301	4.245
1.553	2.002	2.346	3.158	2.976	2.804
2.584	3.501	3.468	2.396	2.581	2.704
4.810	4.611	5.110	4.823	4.802	5.061
4.293	3.737	5.796	4.546	5.413	3.673
3.752	3.655	2.930	3.700	2.869	3.917
3.746	2.998	4.189	3.163	4.224	3.184
2.092	2.643	2.789	1.757	2.707	2.032
2.721	2.005	2.588	2.181	3.466	2.776
3.856	3.282	3.836	3.742	4.041	3.683
4.417	4.357	4.872	4.329	4.689	4.703
3.355	2.833	3.304	3.277	3.628	3.555
2.780	2.974	2.988	2.329	3.092	2.640
8.137	7.853	7.824	7.393	7.370	7.872
3.729	3.099	4.134	3.888	4.001	3.716

2.185	2.027	2.577	2.870	1.610	3.691
3.062	3.468	2.591	2.802	3.692	2.916
3.472	2.637	3.552	2.964	3.291	3.440
3.118	2.590	2.140	1.734	2.383	3.392
2.994	2.983	2.921	3.001	3.175	2.752
3.959	3.974	4.357	4.931	4.750	4.883
1.930	2.096	2.580	1.847	2.051	1.932
4.618	4.706	4.872	4.404	4.683	4.621
4.241	5.321	4.603	4.460	4.395	4.948
4.353	4.937	3.847	4.654	4.658	3.858
5.776	5.410	5.082	4.525	6.540	4.493
3.189	3.189	3.371	2.914	2.999	3.604
5.467	6.537	6.745	5.968	5.598	4.856
4.911	4.439	5.576	5.357	4.500	5.113
4.624	3.946	5.229	3.851	4.220	3.735
5.078	4.851	5.147	4.276	5.306	4.495
7.278	7.506	7.254	7.585	7.343	6.551
7.078	7.225	7.027	7.469	7.079	6.418
7.106	7.323	7.111	7.487	7.143	6.416
7.310	7.558	7.350	7.586	7.299	6.720
5.101	6.348	4.956	6.116	4.561	5.065
1.727	1.801	1.605	1.985	1.559	2.171
2.320	2.041	2.340	2.278	2.354	2.199
2.613	2.257	2.174	3.021	2.717	2.637
2.687	3.066	3.488	3.576	3.676	3.259
5.188	5.006	5.042	4.565	5.314	4.104
3.918	3.928	4.164	3.685	3.937	3.043
5.480	4.219	6.092	5.337	5.338	4.800
7.342	7.482	7.960	7.674	8.161	7.337
3.564	3.500	3.941	3.766	3.538	4.158
6.376	6.434	7.026	6.428	6.366	6.615
4.169	4.006	3.775	4.382	2.715	5.309
2.832	2.588	3.014	2.736	2.271	3.153
4.167	4.347	4.147	4.105	4.088	4.726
3.180	2.870	3.213	3.052	3.140	3.978
3.412	3.773	3.451	3.977	3.071	3.398
3.410	3.414	3.685	4.270	2.604	3.072
2.475	2.330	2.403	2.336	2.666	2.191
3.452	2.660	3.382	2.958	3.436	3.452
2.445	3.529	3.353	3.000	2.455	3.207
4.205	3.956	4.494	3.813	3.764	3.503
2.877	2.631	3.397	2.688	2.755	2.640
1.671	1.786	1.693	1.769	1.475	2.175
2.815	2.569	2.929	2.701	2.760	2.637
3.231	3.190	3.328	2.875	3.073	3.081
1.440	1.716	1.341	1.446	1.308	1.482
3.528	2.925	2.778	3.851	3.346	4.197
2.390	4.073	2.585	2.625	3.182	2.435
1.917	2.160	1.956	2.131	1.491	1.895
3.886	3.681	3.768	3.541	3.422	4.222

5.025	3.229	5.753	3.573	4.937	4.568
2.304	2.895	2.389	2.444	2.360	2.672
5.070	4.964	5.457	4.554	5.596	5.312
2.798	2.444	2.432	2.609	2.384	3.441
4.293	1.936	4.717	1.170	5.741	2.658
3.881	3.710	4.496	3.932	4.281	3.349
5.769	5.199	6.182	5.137	5.128	5.675
5.200	5.405	5.230	3.962	4.274	5.112
1.154	1.350	1.169	0.998	1.451	1.487
1.091	0.859	1.596	0.908	1.403	1.070
3.294	3.298	3.109	3.226	3.291	3.371
3.683	3.637	3.658	3.429	2.717	4.852
3.856	2.534	2.066	2.829	2.534	3.889
7.166	6.811	7.710	7.401	7.347	7.126
6.383	6.665	7.007	6.670	6.294	6.059
3.457	4.893	3.375	3.971	2.177	3.268
6.925	7.764	7.591	7.430	7.408	6.871
7.599	6.671	8.152	7.881	8.038	7.971
1.788	1.889	1.732	1.866	1.728	1.916
7.351	7.247	7.505	7.319	7.511	7.251
3.484	3.979	2.872	3.412	3.863	3.018
2.964	2.488	4.279	3.505	3.761	2.925
6.672	6.122	6.768	6.551	6.400	6.872
6.679	6.489	7.032	7.061	7.063	7.061
4.820	4.834	4.765	5.268	4.657	4.916
4.990	5.438	4.854	5.379	4.866	4.770
3.737	4.998	4.497	4.443	4.239	4.239
4.152	3.253	4.481	3.934	3.823	4.313
5.316	5.543	5.632	6.144	5.823	5.431
3.377	3.926	3.089	3.223	3.615	3.551
3.717	4.292	3.514	4.429	3.680	2.810
2.934	3.195	3.497	3.408	2.690	3.000
5.814	5.251	5.876	5.690	5.197	5.955
2.960	2.960	3.211	3.115	2.277	4.349
4.630	3.405	4.995	5.100	4.931	5.075
3.208	2.844	3.166	2.910	2.900	3.500
3.615	3.437	4.040	4.369	3.769	3.792
2.539	2.102	2.133	2.135	2.586	2.147
3.700	3.714	4.441	3.641	4.291	3.694
1.360	1.456	1.510	1.050	1.752	1.737
3.813	3.429	4.236	4.128	4.034	3.478
2.587	2.202	2.390	2.736	2.893	1.745
2.656	1.685	2.391	1.685	1.838	1.796
4.549	4.298	5.282	4.549	4.103	4.565
1.969	1.969	2.059	2.376	2.028	2.252
3.925	3.756	3.404	3.587	3.334	3.854
3.802	3.218	3.793	3.534	3.365	3.374
2.398	2.926	2.538	2.523	2.627	2.903
1.942	1.755	1.823	1.596	1.658	2.192
2.078	2.346	2.132	2.067	2.186	2.761

5.071	4.717	5.270	4.248	5.058	5.332
4.595	4.637	4.599	4.238	5.716	4.553
2.845	2.839	2.313	2.661	2.486	3.111
2.917	2.642	3.620	3.186	2.177	3.186
2.650	1.734	2.139	4.719	2.476	4.496
3.172	2.969	2.873	3.310	3.336	3.024
3.368	4.376	3.265	3.721	3.974	2.863
4.408	3.844	3.953	5.083	4.315	4.680
2.376	2.952	2.856	2.659	2.389	2.617
2.944	3.216	2.916	2.484	2.630	2.092
2.816	3.029	2.783	2.628	2.913	3.510
2.294	1.935	1.577	2.859	2.321	2.740
3.012	3.342	2.772	3.408	2.712	2.790
2.668	3.021	2.946	2.649	2.870	3.453
2.035	2.263	2.088	2.232	1.853	2.616
2.905	3.424	2.763	2.911	2.965	2.465
4.398	4.473	4.477	4.344	4.393	3.797
2.510	2.792	1.828	2.880	2.377	2.455
1.936	1.681	2.040	2.323	2.469	1.968
3.296	3.352	3.292	3.076	3.190	4.112
4.350	4.781	4.342	4.074	5.091	4.319
2.079	1.709	2.370	2.178	2.436	2.457
2.591	3.022	2.554	2.183	2.141	2.606
3.130	2.507	2.652	3.269	2.557	3.197
3.197	2.771	3.482	2.882	3.248	3.393
3.442	3.378	2.682	3.511	3.724	3.300
2.511	2.219	1.893	2.420	2.560	2.558
5.236	5.304	5.353	5.087	5.308	4.935
1.922	1.597	2.384	1.928	2.675	1.983
2.615	3.250	3.264	2.631	2.817	2.298
2.323	2.053	2.461	1.846	2.353	2.753
8.136	8.553	7.997	8.649	8.004	8.171
2.504	2.363	2.644	2.657	2.414	2.800
1.743	1.673	2.147	1.450	1.766	2.436
4.385	3.699	4.967	4.071	4.353	4.408
2.753	2.256	2.581	2.369	2.522	2.934
3.814	4.285	3.934	3.844	3.868	4.115
1.548	1.902	1.602	1.441	1.432	1.431
3.310	3.898	4.623	3.143	3.839	3.380
6.107	6.159	6.070	6.328	5.424	5.648
2.408	3.371	2.670	2.225	2.509	2.923
3.159	3.434	2.831	2.938	3.772	2.789
5.086	5.271	4.928	4.526	5.051	3.852
8.567	8.338	8.450	8.234	7.956	8.332
8.543	8.334	8.454	8.256	7.939	8.330
4.788	4.348	5.344	4.161	5.872	5.258
4.103	3.700	3.777	4.002	4.022	3.884
6.394	6.527	7.272	6.601	6.913	6.207
5.439	5.525	6.227	5.634	6.025	4.991
5.680	5.559	6.291	6.003	6.048	5.440

5.724	5.665	6.271	6.054	5.865	5.332
5.259	5.081	5.681	5.532	4.954	4.567
4.082	5.221	5.631	2.055	5.423	5.287
2.296	2.632	2.432	2.806	2.046	3.023
3.890	3.569	4.007	4.232	4.223	3.850
4.568	4.195	4.668	4.530	4.439	3.982
4.288	4.479	3.977	4.434	4.036	4.191
9.498	10.192	9.185	8.947	7.988	8.810
3.625	3.609	4.262	4.055	3.893	4.469
3.724	3.357	4.108	3.159	3.947	3.509
3.431	3.451	3.380	3.057	2.097	3.045
4.597	3.753	5.503	5.061	4.919	4.461
1.649	1.812	1.983	1.850	1.788	2.232
3.064	3.277	3.019	3.750	3.863	3.167
2.881	2.770	3.071	3.527	2.356	2.771
3.344	3.733	3.275	4.133	3.954	4.154
3.077	3.355	3.123	3.317	3.348	3.008
4.087	3.397	4.096	3.515	3.309	4.173
3.809	3.205	3.554	3.021	3.537	3.846
4.185	4.471	4.151	4.160	4.348	4.253
2.266	2.328	2.588	2.549	2.248	2.365
2.813	2.309	2.584	2.694	1.454	2.410
2.854	3.243	2.100	2.690	3.533	2.438
2.340	2.871	2.848	2.864	2.817	2.738
2.506	1.657	3.589	3.233	1.640	3.239
4.855	4.775	6.391	5.245	5.414	5.723
2.882	2.872	2.223	1.940	1.947	3.341
4.286	3.571	4.059	3.912	3.920	3.520
2.898	2.981	3.112	2.663	3.091	3.395
3.605	3.524	3.052	3.954	3.954	3.494
2.171	2.583	2.189	1.645	2.272	2.495
2.915	2.647	2.538	2.372	2.292	2.752
2.533	2.573	2.553	1.895	2.272	2.878
3.075	2.505	2.763	3.042	2.838	3.630
2.444	1.738	2.608	1.868	2.798	2.504
3.849	3.074	4.159	4.106	4.145	3.779
2.619	3.050	4.310	1.918	2.650	3.236
3.607	4.023	3.997	3.671	4.722	3.403
3.387	3.237	3.863	2.604	3.296	2.601
3.929	3.160	3.525	3.502	3.672	4.400
4.194	3.038	3.835	3.090	4.709	3.909
2.248	2.429	1.830	2.476	1.943	2.156
2.872	2.359	2.573	2.882	2.572	2.867
2.400	1.085	2.395	2.303	2.426	3.095
2.956	2.371	2.271	2.514	2.767	2.941
2.750	2.448	2.595	2.236	2.769	3.074
3.482	3.276	3.820	3.168	4.067	3.529
1.684	2.301	2.222	1.900	1.222	2.088
2.855	1.093	1.370	2.684	1.509	1.113
4.299	3.623	3.943	4.220	4.530	4.351

5.481	5.222	5.392	5.612	5.159	5.418
2.919	2.264	2.860	3.216	3.392	3.406
1.661	1.252	1.979	1.510	1.823	2.225
5.538	5.822	5.979	5.643	6.039	4.556
2.846	2.669	3.135	2.467	4.232	1.912
3.416	3.958	3.447	3.212	3.198	3.475
4.596	4.607	4.856	5.029	4.477	4.202
2.377	2.761	3.138	2.427	1.778	3.177
2.594	2.551	3.319	1.959	2.963	2.835
2.884	3.346	3.417	2.630	4.209	2.486
3.077	3.256	3.893	4.428	4.790	3.862
3.380	2.706	3.563	3.481	3.823	3.474
5.219	5.180	4.906	5.225	4.546	5.145
2.461	2.895	2.718	2.947	2.560	2.749
3.232	3.160	3.128	2.955	2.416	3.749
2.679	3.208	3.291	3.305	3.364	2.769
2.711	3.027	2.958	3.153	1.880	2.669
3.954	5.090	5.294	4.313	4.913	3.777
3.260	3.740	3.315	2.785	4.159	3.862
3.790	3.337	3.708	3.532	3.191	3.581
4.062	3.943	4.848	4.299	3.572	3.558
1.676	1.408	1.357	1.458	1.328	1.423
3.378	4.346	3.589	3.966	4.024	3.276
3.385	2.984	2.923	3.027	3.406	3.764
2.952	3.364	2.777	2.539	2.927	3.468
5.582	5.784	5.281	5.098	5.742	6.125
4.183	2.840	3.638	4.345	4.168	4.030
2.974	3.119	2.287	2.295	1.904	2.275
4.786	4.094	5.685	5.718	5.734	4.733
3.649	3.358	3.880	3.344	2.982	3.899
3.286	3.639	3.590	3.639	3.192	3.469
2.969	2.261	2.035	2.998	2.826	2.509
3.416	3.652	4.364	2.989	3.526	3.374
3.398	2.266	2.526	2.583	2.255	3.023
2.219	2.831	2.261	2.541	1.205	2.312
4.454	4.540	5.307	4.836	4.501	4.473
2.578	3.167	2.771	2.727	2.445	3.119
3.379	4.148	3.861	3.348	3.359	3.421
3.589	3.693	4.613	3.564	4.088	3.162
2.577	2.019	2.369	3.229	2.693	2.817
3.709	3.349	3.507	4.045	3.295	3.512
3.077	2.862	3.066	3.652	2.783	3.079
4.333	4.451	3.841	4.700	2.934	3.462
4.148	4.589	5.005	5.196	4.516	4.575
5.030	4.428	4.085	4.220	5.149	4.261
8.200	8.390	8.221	8.249	7.912	8.054
2.116	1.469	3.158	2.324	2.292	2.977
4.921	3.371	5.107	4.313	4.532	4.263
2.273	2.414	2.260	2.447	2.260	2.052
1.888	1.751	2.077	2.154	2.054	3.485

4.278	3.436	5.014	4.477	4.386	4.673
2.933	2.419	2.409	2.618	2.697	2.695
1.494	1.508	1.286	1.818	2.122	1.283
3.243	3.144	3.906	3.814	3.205	2.494
3.092	3.166	3.166	3.887	3.095	3.294
2.986	3.363	3.441	3.679	3.706	3.075
2.755	2.125	3.756	2.516	2.165	2.440
3.352	2.909	3.481	2.910	3.408	3.063
4.450	4.598	4.302	4.351	4.837	4.573
3.573	3.238	4.229	3.693	3.639	3.007
2.340	2.759	2.372	2.204	2.344	2.485
3.626	2.457	3.680	3.035	3.694	3.410
6.566	6.843	6.434	6.909	6.190	6.521
1.822	1.490	1.482	1.821	1.510	1.452
1.727	1.889	2.019	1.737	1.826	1.848
3.187	2.780	3.861	3.053	3.293	3.238
3.149	4.346	3.760	3.121	3.924	3.736
4.047	3.425	4.503	3.845	3.835	4.714
3.455	3.212	3.607	3.646	3.350	3.708
1.132	1.273	1.013	0.912	1.024	0.672
5.904	6.077	6.519	5.944	5.295	6.341
2.681	1.955	2.411	2.328	2.373	2.948
2.475	3.571	2.186	3.257	3.466	2.205
1.465	1.115	1.206	1.526	1.050	1.489
4.326	5.225	4.015	6.024	5.278	2.852
3.151	2.258	3.094	2.723	3.564	3.093
4.166	3.984	4.467	4.452	3.967	4.352
2.298	2.522	2.549	2.225	1.782	2.251
3.695	4.120	5.751	5.457	6.077	3.456
3.120	3.518	2.881	2.988	2.359	2.988
5.503	6.163	7.195	6.306	7.823	6.730
1.471	1.137	1.205	1.535	0.804	1.602
2.883	3.456	2.744	2.923	3.058	2.822
3.110	2.631	3.122	2.726	3.625	2.642
1.532	1.086	1.181	1.048	1.228	1.698
1.619	1.557	1.478	1.157	1.050	1.810
3.651	4.115	4.138	3.951	4.391	4.007
2.760	3.106	3.715	2.816	2.890	3.673
3.333	3.727	2.491	2.721	3.201	3.223
3.950	3.282	3.167	3.727	3.956	3.773
3.166	3.413	3.967	4.507	4.534	4.185
5.163	4.531	5.466	5.631	6.032	4.407
2.302	1.278	2.058	2.651	2.098	1.878
1.257	0.865	1.643	0.898	1.195	3.105
2.190	2.087	1.801	2.467	3.533	2.209
2.530	2.120	1.787	1.789	3.057	3.072
2.513	2.695	3.338	1.851	3.492	2.364
3.316	2.936	3.479	3.006	3.314	3.133
2.365	2.907	2.931	2.756	3.405	2.239
1.199	0.623	0.648	0.557	0.762	1.476

2.678	2.394	3.425	2.886	3.325	1.845
2.586	2.515	3.304	2.561	2.994	2.651
1.938	2.019	0.943	1.870	2.205	1.803
2.165	1.459	1.861	1.653	1.730	2.002
3.010	3.246	3.148	2.959	3.705	2.641
2.007	1.333	2.095	1.909	1.316	1.782
3.345	3.470	4.348	3.106	3.711	3.332
1.206	1.238	1.234	0.760	1.432	1.110
3.601	3.585	2.817	3.577	4.175	3.085
2.513	2.491	1.933	1.949	2.533	2.513
3.588	3.089	3.847	3.125	3.429	3.396
3.338	2.943	3.429	3.573	3.422	3.107
2.506	2.487	1.790	2.470	1.219	2.209
3.309	3.237	2.981	3.274	2.780	3.075
2.758	2.312	2.287	2.726	2.978	2.778
3.306	3.279	2.665	3.330	3.879	3.200
4.451	3.887	4.869	4.482	4.818	4.441
3.242	1.317	2.497	2.237	2.310	1.163
2.857	3.839	3.271	3.313	4.273	3.430
2.434	2.427	2.657	2.488	2.918	2.395
2.170	1.639	2.920	1.211	3.018	0.687
4.400	5.585	4.920	5.764	4.233	4.942
6.124	6.530	5.998	6.592	5.973	6.158
6.188	6.405	6.025	6.560	5.925	6.275
7.441	7.776	7.274	7.824	7.279	7.445
4.033	5.070	4.706	5.223	4.638	3.347
2.050	2.149	2.079	2.076	1.959	1.986
3.333	3.070	2.965	3.914	3.336	2.444
3.382	3.247	3.546	3.258	4.000	3.840
4.377	2.736	4.873	4.084	3.032	4.107
2.561	2.536	2.167	1.951	2.194	2.406
2.523	1.940	1.908	1.994	1.441	1.877
3.904	3.699	3.441	3.086	3.452	4.346
4.904	4.197	5.152	4.921	4.416	5.171
2.823	3.197	2.306	2.270	2.624	2.581
5.336	5.280	5.915	5.740	5.783	5.429
2.637	3.052	3.538	3.305	3.473	2.000
3.990	2.516	3.885	2.924	3.421	2.483
7.490	8.427	7.516	8.603	7.585	7.809
7.740	7.957	7.886	8.367	7.740	7.655
3.308	3.557	3.171	2.352	3.074	3.598
3.045	3.352	3.416	2.860	4.038	2.250
2.363	1.692	1.849	2.218	2.341	2.744
2.154	2.903	4.578	2.911	3.711	3.167
2.534	2.117	2.805	3.157	1.959	3.306
1.594	1.086	0.891	1.913	1.687	1.917
1.768	1.190	1.441	1.700	1.630	1.528
3.345	1.509	3.317	3.694	3.311	3.380
5.136	4.794	5.400	5.592	5.313	5.239
2.146	2.994	2.619	2.764	2.021	2.927

3.285	4.454	3.984	3.587	3.866	2.970
1.548	1.166	1.658	1.516	1.224	1.232
1.806	1.764	1.873	2.059	2.167	1.718
1.752	1.973	1.419	1.338	1.627	1.761
2.893	2.832	3.701	3.073	2.957	2.727
2.602	2.325	3.354	2.958	2.547	2.443
1.789	1.513	1.460	1.262	1.107	2.024
4.695	4.315	4.469	4.131	4.181	4.335
3.329	2.900	3.171	3.374	4.512	3.420
3.003	2.665	2.645	2.995	2.131	1.976
2.759	2.476	2.507	2.203	1.726	2.427
4.209	4.324	4.914	4.875	4.605	4.811
4.834	3.809	5.479	4.578	5.770	5.206
4.702	3.201	4.564	3.765	4.318	4.837
1.838	1.632	2.516	1.897	1.938	2.300
2.790	3.340	3.434	3.020	3.093	2.763
3.141	2.591	3.361	3.180	3.068	3.473
6.921	7.126	7.444	6.230	6.924	6.872
4.246	3.810	4.590	4.627	4.741	3.825
3.955	3.696	4.444	4.701	4.135	4.274
3.868	4.026	4.238	4.320	4.040	3.937
3.695	3.089	4.094	2.407	3.376	3.672
7.772	7.374	7.512	7.658	7.110	8.211
5.203	4.626	5.186	4.854	5.282	4.856
4.365	4.132	3.936	4.855	3.941	3.830
3.146	3.197	2.642	3.284	2.731	2.607
4.122	4.221	4.396	4.362	4.269	4.276
2.388	2.138	3.675	3.245	2.347	2.619
4.191	3.938	3.982	3.131	4.184	4.308
4.751	4.514	4.592	4.434	4.588	5.264
1.577	1.693	1.654	1.317	1.189	2.044
3.675	2.863	3.582	3.498	3.608	4.128
1.572	1.840	2.172	1.973	1.977	2.028
1.657	1.773	1.378	1.355	2.182	1.918
2.073	2.643	2.651	2.272	1.884	2.257
3.632	3.406	4.162	3.752	3.247	3.625
2.480	2.564	2.853	3.800	3.141	2.582
4.204	3.611	3.901	4.776	3.862	4.825
3.125	3.097	2.686	1.603	1.888	2.192
2.998	2.376	2.714	3.307	3.316	3.503
4.716	3.825	4.073	5.434	7.371	6.494
2.536	2.304	2.715	2.702	3.111	2.644
3.866	3.637	4.031	3.270	3.668	4.087
3.433	2.796	3.818	4.293	3.988	3.080
2.445	2.323	2.489	3.378	1.488	3.556
4.126	4.082	3.306	3.679	3.929	4.174
2.035	1.730	1.537	1.336	1.467	0.987
2.530	1.803	2.695	3.288	2.105	2.262
2.569	2.904	2.348	2.761	1.994	2.496
2.728	2.811	2.292	3.029	2.815	3.582

0.905	1.216	0.672	0.414	0.815	0.944
3.617	4.001	3.676	3.911	3.354	3.276
2.465	2.334	2.585	1.606	1.996	2.994
2.336	2.673	1.435	1.989	2.597	1.852
2.560	3.629	3.293	2.859	3.218	2.580
2.533	2.475	2.639	2.397	2.737	2.842
3.717	3.114	2.829	2.826	2.960	3.178
2.302	2.641	2.810	2.236	2.344	2.540
4.277	4.671	4.691	2.835	4.381	4.309
1.881	2.069	2.343	2.527	2.279	1.633
2.599	2.440	2.882	2.854	3.507	3.208
2.265	1.707	2.276	2.431	2.137	1.732
4.760	5.084	4.318	4.556	4.527	5.467
2.897	1.948	3.967	2.084	3.273	1.238
2.448	2.241	2.369	2.927	2.130	1.997
2.776	2.590	3.199	2.409	2.666	2.212
2.784	3.412	3.294	3.340	4.538	2.202
4.025	4.591	4.564	3.897	5.357	3.673
2.388	2.367	2.318	1.882	3.056	3.278
3.087	3.536	3.899	2.400	3.298	3.927
3.925	3.552	3.626	3.278	3.667	3.820
2.775	3.923	3.157	2.737	3.087	3.483
0.886	0.862	1.068	1.286	1.415	1.329
2.306	2.258	1.959	3.108	1.481	1.959
3.541	4.174	3.806	3.949	4.450	4.592
2.599	2.797	2.235	2.699	2.468	2.748
2.032	2.185	1.939	2.246	1.317	2.461
4.585	3.957	4.019	4.671	4.802	4.987
2.827	2.887	2.729	3.602	3.879	2.819
2.517	2.679	2.630	4.254	3.401	2.090
4.658	4.924	4.751	4.309	4.890	5.536
5.249	4.885	5.196	4.585	4.080	4.611
2.759	2.569	3.521	2.653	2.935	3.132
5.032	4.699	5.089	3.961	4.845	5.463
2.739	3.222	3.470	3.190	3.159	3.190
5.214	5.692	5.342	6.026	5.333	5.757
3.450	2.918	3.163	3.564	3.165	3.137
2.577	2.940	3.729	2.845	3.323	2.703
3.904	3.745	4.293	4.003	6.045	3.629
3.390	4.390	3.654	3.635	3.871	3.939
6.656	6.189	7.233	6.658	7.219	6.917
3.355	3.195	3.960	3.483	4.006	3.939
2.425	2.218	1.829	3.126	3.032	2.926
4.913	4.844	5.041	5.021	4.994	4.875
3.680	3.820	3.966	3.755	3.264	3.995
2.999	3.469	3.346	2.793	3.213	3.122
2.380	2.315	2.315	2.547	2.532	2.640
2.086	2.047	1.585	1.856	1.779	1.944
3.607	3.628	3.080	3.845	3.952	3.356
2.402	2.456	2.444	2.797	2.211	2.643

4.096	2.658	4.169	3.608	4.056	4.136
2.539	2.283	2.825	2.453	2.496	3.299
2.081	2.300	3.268	2.987	2.837	2.085
2.592	2.585	1.755	2.867	1.899	2.389
1.659	1.768	1.476	1.723	1.573	1.671
2.104	2.318	2.639	2.595	2.488	1.922
2.231	2.976	2.124	2.480	2.404	3.152
2.609	2.505	1.843	1.880	2.150	2.142
3.417	2.749	3.384	3.602	2.846	3.198
4.529	4.723	5.117	4.001	4.451	4.102
3.984	4.944	4.561	4.977	4.344	4.713
2.025	2.295	2.263	2.436	2.543	2.242
5.109	5.752	5.204	6.378	4.891	5.101
3.020	3.795	3.073	3.430	2.472	4.001
3.109	3.097	3.625	3.530	3.116	3.083
3.205	4.053	3.321	3.012	3.737	3.834
4.920	4.473	5.155	5.174	4.912	4.052
2.907	2.644	3.763	1.429	4.182	2.879
3.272	3.490	2.806	3.008	2.006	3.874
2.586	2.773	1.859	3.228	2.704	1.658
3.460	4.501	3.506	1.755	3.609	4.285
4.124	4.341	4.492	3.776	4.734	4.176
3.184	3.548	2.815	3.324	3.054	3.202
4.601	5.159	4.714	5.021	5.253	5.100
2.579	2.425	2.252	2.380	2.304	3.151
3.071	2.796	2.883	2.417	2.324	2.370
1.908	1.287	1.396	1.877	1.918	2.075
2.886	3.505	3.484	2.975	3.866	3.765
2.671	2.677	2.525	1.337	2.544	2.426
6.107	4.766	5.800	5.480	5.808	6.260
3.520	2.621	3.139	3.042	2.676	2.989
3.690	3.486	4.565	4.251	4.715	2.893
5.136	4.788	6.049	4.459	5.160	5.849
2.884	2.710	3.335	2.143	1.886	2.031
2.658	1.858	2.233	3.454	1.509	2.080
3.008	2.817	2.643	3.278	2.373	2.517
2.176	2.425	2.156	2.135	1.643	2.019
1.459	1.427	1.124	1.450	1.334	2.705
4.318	4.307	2.769	4.109	4.389	4.636
1.798	2.349	1.976	2.118	1.662	2.011
2.258	1.944	2.220	2.584	2.062	2.090
7.382	8.292	7.346	8.431	7.419	6.935
6.860	7.659	6.858	7.926	6.803	5.842
4.326	4.444	4.193	4.805	4.581	3.938
3.675	2.976	3.859	3.120	3.653	3.171
7.742	6.687	7.519	7.165	7.464	7.475
9.973	9.738	9.799	9.705	9.448	9.857
2.779	2.984	3.348	2.630	2.324	2.557
2.966	2.928	3.136	3.325	2.749	4.125
3.561	3.162	3.935	3.425	3.091	3.569

2.425	2.014	2.726	2.544	3.220	2.387
4.114	4.240	3.755	3.452	3.708	3.046
4.159	4.633	2.204	6.032	4.249	2.679
1.881	1.655	1.935	2.340	2.022	2.221
5.108	4.453	6.056	6.405	6.022	6.014
4.826	5.312	5.391	4.191	5.094	5.189
1.833	2.315	1.916	1.496	1.239	2.559
4.722	4.346	5.163	4.751	4.443	4.782
5.688	4.926	5.798	5.345	5.148	5.563
5.176	4.133	6.316	5.695	5.615	5.543
4.717	4.407	5.344	5.281	4.927	4.890
2.734	3.340	4.402	3.546	2.473	3.174
2.608	1.751	2.187	1.900	1.833	1.875
1.682	2.099	1.991	1.340	1.340	2.414
5.079	4.435	4.764	4.628	4.621	5.376
1.950	1.873	2.376	2.111	1.910	2.565
4.091	3.727	4.335	4.035	4.673	4.657
5.755	3.578	2.661	3.841	3.479	6.511
4.141	2.858	4.099	2.515	2.888	3.667
2.882	3.970	2.538	3.045	3.373	2.920
4.621	4.894	4.428	4.523	5.275	4.304
6.035	4.834	5.209	6.567	6.368	6.235
5.697	5.955	5.955	5.154	5.710	4.713
8.276	8.294	8.778	7.958	8.928	7.888
0.925	0.941	0.886	1.047	1.266	1.132
9.932	9.758	10.039	10.184	10.067	9.860
2.986	3.527	3.128	3.255	2.503	2.952
5.638	4.865	5.902	4.647	4.507	6.357
3.858	4.083	4.028	2.686	4.555	4.462
2.828	2.367	2.694	2.596	2.534	2.789
1.806	2.272	1.931	1.881	1.636	2.876
3.519	3.753	3.362	3.971	3.235	3.945
2.047	2.410	2.148	1.985	1.763	1.821
3.392	3.603	4.227	3.054	3.162	3.264
6.001	6.761	6.103	6.459	5.818	5.709
2.728	2.064	2.777	1.859	2.108	2.887
2.083	1.976	1.725	2.328	1.493	2.718
2.215	1.702	1.459	2.503	1.613	2.056
2.066	2.022	1.843	1.952	1.902	2.925
4.301	4.281	4.309	5.979	4.834	5.267
2.526	2.212	2.522	2.388	2.737	3.376
3.396	2.620	3.179	2.799	3.332	2.983
2.542	2.425	2.731	3.105	2.611	2.914
4.220	3.810	3.417	3.833	4.020	4.432
2.297	3.761	2.460	2.136	2.716	2.162
3.581	3.558	3.458	3.477	3.520	3.163
2.499	2.660	1.952	3.414	1.403	1.303
3.662	3.739	3.303	4.081	3.500	3.633
5.142	4.960	6.073	5.574	5.583	5.306
2.943	3.279	2.497	2.977	2.903	2.554

3.271	3.694	3.768	3.574	3.568	3.156
4.927	5.585	5.138	5.407	5.789	5.429
4.199	4.768	4.577	4.237	4.569	4.212
9.162	9.086	9.180	8.914	9.137	8.866
1.772	1.091	1.568	2.139	2.053	1.601
3.403	2.883	4.830	3.509	4.302	3.936
3.640	4.735	3.886	4.651	4.690	3.583
2.765	3.728	2.962	3.889	3.644	3.170
7.529	8.210	7.590	8.296	8.155	7.505
5.842	6.384	6.152	6.447	6.396	6.242
5.406	5.076	5.337	5.524	5.091	6.042
3.137	2.772	2.842	2.421	3.532	3.780
6.599	6.161	6.761	6.634	6.497	6.838
6.858	6.397	6.579	6.756	6.194	6.819
4.420	3.865	4.323	4.197	3.769	4.804
6.771	5.990	6.886	6.509	6.626	6.719
3.355	4.401	3.630	3.533	3.496	2.760
3.849	4.290	4.239	4.512	4.306	3.526
4.447	4.007	5.052	4.309	4.942	4.785
4.770	4.123	5.072	4.700	4.496	5.032
4.744	4.822	4.298	5.203	4.499	5.334
2.619	2.218	3.006	2.978	2.923	2.602
5.157	5.749	4.673	5.730	5.517	4.635
3.330	3.382	4.491	4.197	4.273	3.191
3.331	3.726	3.672	3.653	3.380	3.854
2.887	2.587	2.928	3.257	2.413	3.512
2.880	2.106	2.758	2.360	2.076	2.663
3.696	5.336	3.392	4.288	3.441	3.230
4.035	4.228	4.082	4.197	4.716	4.017
5.399	5.949	5.541	5.717	5.387	5.165
4.299	4.296	4.954	4.804	4.200	4.267
4.773	4.506	4.776	5.069	4.936	4.800
6.631	5.718	6.612	6.387	6.547	6.822
6.955	6.450	7.675	7.016	6.784	6.904
5.584	3.912	4.833	4.432	5.961	4.782
4.088	4.702	4.125	4.480	4.081	4.956
4.890	5.275	5.477	4.125	4.406	4.693
4.986	4.490	4.657	5.195	4.871	4.826
2.568	2.748	2.815	2.979	1.841	2.566
6.211	6.007	6.416	6.320	6.127	6.600
2.798	2.382	2.187	2.495	2.527	2.982
3.159	2.257	3.284	3.412	2.710	2.730
2.788	3.701	3.276	3.827	3.774	2.746
3.910	5.227	4.831	5.348	4.081	3.246
6.298	6.158	6.615	6.378	6.168	6.445
4.969	3.346	5.246	4.346	4.499	3.870
3.549	4.020	3.886	4.840	3.914	3.612
4.564	3.671	4.825	4.444	4.753	4.313
2.237	2.300	1.447	1.919	2.940	2.555
3.075	3.010	3.734	3.366	3.322	3.380

3.028	2.858	3.493	3.206	3.197	4.210
3.946	4.107	3.669	3.459	2.805	4.073
4.962	4.831	5.007	5.352	5.243	5.479
2.865	1.556	2.044	1.369	1.805	2.062
4.025	2.652	2.606	2.827	2.852	3.073
3.615	3.256	3.153	3.583	3.388	3.951
5.905	5.905	6.137	5.956	6.149	6.405
1.228	1.756	1.430	1.259	1.142	2.158
4.872	6.214	5.045	4.758	6.627	6.110
4.713	4.160	4.532	3.949	4.426	4.571
4.849	4.422	5.117	4.777	4.835	4.696
1.232	1.460	1.585	1.352	1.064	1.487
4.343	3.447	3.801	4.088	3.692	5.508
3.054	2.038	2.333	2.063	1.882	2.010
3.453	3.221	3.211	2.785	3.156	3.091
5.620	5.903	5.849	6.384	5.864	5.813
2.820	1.281	2.014	1.144	1.651	1.443
5.191	4.777	5.808	5.448	5.207	5.158
4.997	4.711	4.987	5.267	5.138	5.173
3.630	4.451	3.735	4.533	3.910	3.489
5.763	6.305	6.623	5.908	7.126	5.407
4.514	4.004	5.202	3.873	4.203	5.020
3.923	3.943	4.333	3.889	4.301	4.550
3.506	2.976	3.969	3.751	3.430	4.186
3.593	4.139	4.188	3.905	3.989	3.671
6.120	5.011	6.746	5.911	5.764	6.981
5.208	4.721	5.681	5.356	5.252	5.569
4.420	4.139	4.760	4.258	4.508	4.126
2.778	2.034	3.089	2.451	2.283	3.705
3.132	3.464	3.624	3.632	3.377	4.138
4.645	5.343	4.510	4.703	4.336	4.279
3.284	2.294	3.314	3.173	2.781	3.906
3.304	3.618	2.854	2.970	3.688	2.954
4.519	4.813	3.813	3.941	4.233	3.848
7.333	6.671	6.965	7.987	6.761	7.553
3.427	5.010	3.767	4.722	4.757	3.458
3.654	5.100	3.959	4.752	4.834	3.617
3.544	5.041	3.819	4.765	4.696	3.387
3.672	5.017	3.968	4.761	4.834	3.664
3.929	5.313	4.196	4.834	4.883	4.173
3.618	5.009	3.876	4.676	4.885	3.489
2.278	3.596	2.379	2.959	3.189	1.811
5.897	6.288	5.928	6.093	6.161	5.758
4.324	5.068	4.583	4.704	5.305	4.622
6.512	6.483	6.557	6.178	6.340	6.110
4.886	5.455	4.910	6.522	4.517	4.696
6.456	6.829	6.465	7.017	7.371	6.622
5.617	3.221	6.149	6.986	6.879	4.746
5.835	5.725	6.039	6.369	6.105	6.216
2.775	5.224	2.588	3.462	3.596	2.821

4.360	5.067	5.298	5.438	4.841	6.013
7.719	7.101	8.180	8.045	7.711	7.841
3.533	3.725	2.937	3.062	3.020	3.711
3.101	3.203	3.075	2.965	2.496	3.488
4.655	5.300	5.014	5.803	5.671	4.903
7.026	8.378	8.008	8.166	8.581	7.523
1.468	1.182	1.382	1.626	1.360	1.423
3.016	3.511	2.756	3.492	3.839	2.965
3.773	4.759	3.790	4.386	4.481	4.125
5.963	7.601	6.465	7.175	6.562	6.178
3.819	3.374	3.472	3.651	3.556	3.949
4.843	3.605	4.176	3.849	3.911	4.720
4.791	5.019	4.873	5.471	4.793	5.293
0.999	1.612	0.909	1.102	1.336	1.288
4.252	4.467	4.534	4.510	4.594	4.804
3.886	3.859	3.424	3.686	3.100	3.563
3.382	3.180	3.254	3.561	2.828	3.996
3.053	3.103	3.652	3.829	3.732	2.739
7.081	8.597	7.687	7.360	8.256	7.192
5.091	7.999	6.068	4.881	7.094	4.887
3.212	2.825	2.951	2.799	3.261	3.109
2.798	3.672	3.769	3.625	3.311	3.635
4.886	4.850	4.828	5.029	4.915	4.951
4.335	3.261	3.656	3.524	3.462	3.788
2.694	2.340	2.538	3.002	1.902	4.185
4.538	3.491	4.050	4.951	3.687	5.888
2.323	2.491	2.430	2.432	2.360	2.689
2.037	1.952	2.090	1.776	1.760	1.486
6.032	5.783	5.895	5.986	5.748	6.097
1.655	1.623	1.374	1.854	1.608	1.723
4.550	6.090	5.782	5.884	6.158	4.382
3.951	4.163	3.934	3.641	3.772	3.217
2.548	3.167	1.981	1.918	1.647	3.140
4.582	5.255	4.461	3.678	4.922	4.573
8.913	8.839	8.882	9.103	8.635	8.796
7.087	6.965	7.047	7.317	6.907	7.204
4.605	4.373	4.134	4.862	4.089	5.114
2.884	2.647	2.847	2.683	2.723	3.122
5.775	5.582	5.814	6.222	5.911	6.169
2.225	2.128	1.845	2.223	2.092	2.508
3.632	3.152	3.223	3.583	3.256	3.802
5.872	5.748	6.287	5.913	6.149	5.672
3.185	2.980	4.015	2.451	2.893	3.128
5.815	5.302	5.990	6.318	6.080	6.527
2.523	2.972	2.883	2.470	2.625	3.460
6.273	6.497	6.642	6.448	6.437	6.133
2.514	2.567	1.975	1.585	1.875	1.671
9.005	8.916	8.779	9.231	9.000	8.916
2.058	2.177	1.824	2.073	1.644	2.682
9.896	9.798	10.415	9.857	10.012	9.862

3.541	4.661	3.524	3.936	3.524	3.618
3.941	4.340	3.692	3.304	3.782	3.910
2.755	3.196	3.130	2.491	3.118	2.902
3.226	3.305	3.838	2.847	3.695	3.234
6.150	5.960	6.547	6.160	6.742	5.752
5.694	6.344	6.593	5.784	6.730	4.881
7.485	7.248	7.518	7.251	7.333	7.826
3.096	2.992	2.651	3.437	2.728	3.547
6.709	7.594	7.036	6.961	7.669	5.870
7.426	7.233	8.092	7.449	7.414	7.489
5.124	6.142	5.337	6.110	5.884	4.848
3.884	3.093	3.632	3.633	3.091	3.845
4.856	5.227	4.712	4.879	5.227	4.908
4.811	5.042	4.571	5.051	4.918	5.026
7.703	8.077	8.044	8.200	8.685	7.592
8.389	7.629	7.865	8.552	7.783	8.414
3.958	4.145	4.218	4.773	5.000	5.004
7.732	6.601	7.351	7.605	6.734	8.384
7.238	7.097	7.339	7.532	7.004	7.471
3.624	3.490	3.614	3.662	3.153	3.798
5.477	4.973	5.407	5.578	5.407	4.753
4.928	5.206	5.350	5.149	4.956	5.384
4.303	4.848	4.571	4.676	4.384	4.092
3.984	4.327	3.982	4.125	4.894	4.094
4.934	5.391	5.162	5.360	5.369	4.959
5.048	5.223	6.633	5.112	5.074	4.501
5.296	5.365	6.204	5.501	5.128	4.476
3.910	2.795	3.630	3.639	3.291	3.973
7.721	7.310	7.638	7.530	7.407	7.755
2.479	2.987	2.052	3.471	2.486	2.565
4.529	4.666	4.632	4.558	4.334	4.052
8.862	8.517	8.889	8.387	8.752	8.850
7.790	7.379	8.013	7.498	7.817	7.899
6.299	5.662	5.908	6.089	5.679	6.468
5.641	6.037	6.052	5.847	5.635	5.969
8.589	8.312	8.812	8.585	8.562	8.603
6.340	3.740	4.989	6.241	4.360	7.000
5.606	5.979	5.382	5.370	5.544	5.090
4.979	4.660	4.607	5.079	4.904	5.691
2.526	2.832	3.221	1.954	1.205	3.180
2.967	3.280	2.909	3.246	3.603	3.449
4.200	4.070	4.276	4.295	4.485	4.511
2.839	3.221	2.259	2.754	3.085	2.341
3.528	2.929	4.062	3.441	3.231	3.794
4.105	4.467	4.056	4.205	3.988	4.698
5.494	5.018	4.585	5.194	4.268	5.470
5.430	5.008	5.734	5.884	5.328	5.976
6.917	6.888	7.067	6.899	7.020	6.526
5.074	5.815	5.190	5.717	5.719	5.118
4.394	3.835	4.826	4.708	4.255	4.790

4.069	4.250	3.517	2.189	3.815	3.639
5.044	5.124	5.279	5.445	4.749	5.154
2.801	2.880	1.887	2.116	2.321	2.097
2.436	1.965	1.958	2.364	2.336	2.984
6.103	6.314	6.739	6.581	7.369	5.963
3.375	3.089	3.219	3.508	3.223	4.499
8.564	8.444	8.438	8.505	8.588	8.238
6.984	7.111	7.225	7.162	6.979	6.892
2.613	3.484	2.475	2.733	3.575	3.425
5.298	4.847	5.424	5.369	5.090	5.697
5.551	5.279	5.404	5.333	5.588	5.809
1.643	1.261	1.839	1.053	1.341	1.327
5.953	5.820	6.262	6.610	6.206	6.185
5.287	3.809	3.937	4.864	4.371	5.520
6.457	6.305	6.739	6.729	6.142	6.919
4.067	3.848	3.978	3.878	3.905	4.294
3.501	4.435	3.948	3.979	3.396	4.444
7.805	7.672	8.519	7.863	7.741	7.648
3.462	3.800	3.146	3.669	3.471	3.956
2.802	2.965	3.069	3.735	2.931	2.806
3.693	3.854	4.266	3.893	3.959	3.607
3.667	4.354	3.465	3.959	3.081	2.487
6.410	6.766	6.092	6.513	6.457	6.232
2.164	3.512	2.292	2.668	3.640	2.292
4.262	4.990	5.111	4.217	4.904	3.983
3.968	4.511	4.306	4.076	3.564	3.246
3.917	3.861	4.508	3.886	3.044	3.580
4.652	4.341	4.090	4.974	4.731	4.778
3.922	4.884	4.081	4.566	4.692	3.492
5.930	6.619	6.313	6.468	6.233	5.864
4.775	5.105	4.839	5.151	5.010	5.026
7.106	7.054	7.260	7.199	7.025	7.120
5.460	5.681	5.982	5.495	5.657	5.523
6.312	6.786	6.760	7.020	6.780	6.549
1.879	1.841	1.795	2.151	1.821	2.291
3.868	4.326	3.962	4.037	2.993	4.286
4.477	4.639	3.751	4.886	3.720	4.104
5.810	5.444	5.901	6.009	5.799	5.919
2.193	4.647	3.261	2.307	2.449	1.377
4.008	2.801	3.915	4.683	3.361	4.397
3.133	2.258	2.732	2.730	3.073	2.913
3.724	3.419	3.364	3.743	2.862	3.259
1.077	0.756	0.911	0.901	0.909	0.978
7.577	8.033	8.397	7.448	7.825	7.656
5.138	5.639	5.170	5.488	4.700	5.406
4.654	5.955	4.917	5.570	5.205	4.324
4.468	5.206	4.796	5.458	5.051	5.201
3.823	4.045	4.536	4.503	4.424	4.215
4.208	4.416	4.595	4.211	4.085	4.752
2.532	4.457	2.412	2.942	2.394	2.550

4.670	4.232	4.186	4.647	4.470	4.629
3.689	3.306	3.946	3.696	3.973	4.068
5.207	4.671	5.701	4.967	5.175	5.345
3.294	2.259	3.139	3.172	2.943	3.084
4.841	5.847	5.450	4.711	5.359	4.795
3.792	3.054	3.930	4.472	4.270	3.936
6.510	6.608	6.667	6.400	6.526	6.566
6.112	5.873	6.034	6.335	6.129	6.570
5.166	4.855	4.860	5.212	4.518	5.364
3.174	2.910	2.376	3.084	2.255	2.494
5.001	4.618	5.153	5.450	5.316	5.109
3.215	4.446	3.843	4.125	3.419	2.764
3.491	3.875	3.522	4.137	3.914	3.492
6.239	6.445	6.296	6.170	6.513	6.379
4.146	3.203	4.084	3.455	3.656	4.760
7.817	7.339	8.285	7.587	7.749	7.616
4.114	3.629	4.344	3.858	3.635	4.538
4.161	4.454	3.749	4.875	3.932	3.925
3.083	3.878	2.306	2.157	2.544	3.118
0.914	0.572	1.201	0.970	0.945	1.097
2.824	3.596	3.193	3.181	2.334	3.762
3.634	2.980	3.524	3.976	3.432	4.175
4.598	5.413	4.089	3.842	4.536	3.881
4.369	4.778	4.820	4.776	4.929	4.489
7.217	7.081	7.310	7.348	7.056	8.121
4.459	5.575	5.190	4.492	4.772	4.271
2.927	3.949	2.620	2.888	3.268	3.122
4.797	4.226	5.042	4.875	5.355	5.138
3.607	3.534	4.146	4.326	4.060	3.154
3.151	2.311	2.657	3.355	2.996	3.050
4.377	4.858	4.914	4.898	4.970	5.073
2.752	2.678	2.907	2.374	3.637	2.981
5.403	5.977	5.589	5.832	5.700	5.841
8.404	8.182	8.975	8.322	8.442	8.403
4.336	5.610	5.244	5.451	5.741	5.858
1.900	2.097	2.151	2.198	1.949	2.015
4.818	5.082	5.005	5.308	5.125	4.871
3.872	4.024	4.553	3.175	4.376	4.149
3.572	3.614	3.346	2.795	3.338	4.103
4.898	5.434	4.902	4.913	5.491	5.372
2.950	3.283	3.365	2.867	3.645	2.505
3.674	4.619	3.770	4.360	4.219	3.380
4.865	5.094	4.286	5.659	4.726	4.302
3.501	3.985	3.140	3.470	3.690	3.551
4.780	4.843	4.293	4.941	4.682	5.813
3.680	4.630	4.345	3.952	4.337	3.306
3.296	2.248	3.264	3.357	3.269	3.152
4.953	5.713	4.708	4.971	5.428	4.605
5.698	6.655	6.375	6.711	6.322	6.279
4.000	3.693	4.671	3.833	3.928	4.469

3.377	2.832	3.039	3.165	2.752	3.399
2.556	2.680	2.422	3.058	1.956	3.737
2.923	2.490	3.303	2.584	2.646	2.907
2.256	2.655	2.435	2.094	2.252	2.789
4.941	4.757	5.009	4.387	5.057	5.092
2.190	2.383	2.704	2.662	3.593	3.344
4.499	4.340	4.573	3.716	4.395	4.604
1.668	1.482	1.849	1.486	1.990	2.206
4.419	4.389	4.797	4.849	4.506	4.742
4.968	5.325	4.415	4.743	5.001	5.714
6.400	6.302	5.591	6.263	6.286	5.882
3.591	3.335	3.688	3.569	3.219	3.747
4.760	5.130	4.931	4.931	4.895	4.690
5.787	7.232	6.135	6.315	5.797	4.824
5.215	5.132	5.253	5.542	5.866	5.408
3.416	3.651	4.433	3.870	4.138	4.020
4.734	5.504	5.100	5.513	5.031	4.691
4.762	5.493	4.895	5.331	5.032	4.727
3.361	2.958	3.017	3.264	2.834	3.595
3.739	4.204	3.362	3.866	3.467	3.737
5.653	5.496	5.777	5.892	5.786	5.835
4.338	3.219	4.241	4.057	4.266	4.525
7.365	7.154	7.152	7.564	7.030	7.457
6.721	6.982	6.928	6.855	6.973	6.706
4.778	5.467	4.963	5.932	5.646	5.087
2.986	1.849	3.335	2.370	2.662	3.012
2.251	2.003	3.246	2.229	2.686	2.327
2.587	2.530	2.764	2.450	2.840	2.313
2.698	3.049	3.317	2.479	2.573	2.801
4.429	5.155	4.680	5.037	5.023	4.528
4.253	4.148	4.954	3.547	4.322	2.181
2.853	2.840	2.315	2.217	2.276	1.944
2.854	3.629	1.476	1.558	1.740	2.290
3.464	4.348	3.975	2.791	4.137	3.142
3.819	3.213	3.211	3.940	3.943	3.807
5.669	5.272	5.553	6.002	5.943	5.642
3.401	3.027	3.176	3.404	3.084	4.123
2.736	1.819	1.945	3.016	3.882	3.592
3.313	2.877	3.567	2.904	4.220	2.910
3.878	3.832	3.224	3.741	3.623	3.467
2.397	2.629	2.579	1.861	1.547	2.794
2.358	2.429	3.194	3.303	2.412	2.452
5.676	6.131	5.952	5.917	5.631	5.691
3.345	2.613	4.861	2.935	3.752	3.183
6.522	6.271	7.191	6.202	6.141	6.587
2.871	3.189	3.223	3.271	2.737	2.559
2.382	1.948	2.451	2.217	2.034	2.765
2.180	2.611	2.272	2.127	2.240	2.328
4.022	3.201	4.026	3.352	4.089	4.316
2.226	2.173	1.727	2.910	2.542	2.542

2.673	2.081	1.579	2.363	3.122	2.479
3.218	2.810	4.445	2.600	2.138	2.208
3.271	3.264	3.033	3.066	3.384	2.396
4.195	3.201	4.255	2.850	3.196	3.762
1.736	2.288	2.288	2.964	3.869	1.652
4.341	4.496	4.662	4.375	4.902	4.443
1.641	1.698	3.282	1.643	3.209	1.591
2.958	2.455	2.857	2.963	3.165	2.635
4.276	3.952	4.146	4.514	3.916	4.789
2.285	2.388	1.807	2.057	1.065	2.385
3.984	4.602	3.813	4.607	3.936	4.172
3.869	4.154	4.144	4.128	3.829	3.022
3.041	2.924	2.225	3.555	2.762	2.648
4.382	5.084	6.368	5.184	6.157	4.494
3.349	4.328	2.533	2.732	3.826	1.670
4.649	4.824	5.486	4.915	5.297	5.442
2.859	2.502	2.955	2.515	2.596	2.902
2.384	2.467	1.337	1.901	2.484	2.107
2.957	3.257	2.610	3.026	2.994	2.869
2.504	2.978	2.484	2.671	3.019	2.541
1.221	1.615	1.520	1.283	1.353	0.965
1.841	2.446	2.132	2.306	3.078	2.156
1.801	1.596	1.986	1.127	1.794	2.279
1.337	1.330	2.354	2.628	1.858	1.343
2.558	2.147	3.426	2.854	3.264	1.760
2.771	2.948	1.943	2.745	3.192	2.666
3.542	3.173	3.602	3.706	3.615	3.934
3.099	3.480	3.386	3.356	3.256	2.420
2.376	2.218	4.016	1.750	2.126	2.061
3.841	4.437	3.161	4.147	4.528	4.203
2.290	2.063	2.730	2.197	2.778	1.856
3.967	4.663	3.809	4.912	3.191	4.162
2.427	1.690	3.050	2.751	2.969	3.488
3.890	3.722	3.698	2.995	2.780	3.173
2.668	3.288	2.089	3.201	2.614	3.946
2.791	2.338	2.949	2.765	3.503	3.631
2.268	2.921	3.043	2.985	3.224	2.935
4.228	4.617	4.167	4.981	4.959	4.566
3.778	3.721	3.383	3.790	2.826	3.673
2.690	3.192	1.729	2.817	2.436	2.761
3.335	2.421	3.763	3.059	3.661	3.478
2.996	2.141	4.155	3.213	3.039	3.387
2.181	2.068	1.975	1.081	1.070	1.488
2.403	1.954	3.090	2.386	2.974	2.940
3.431	3.253	3.292	3.130	3.837	2.887
4.024	4.638	4.527	3.393	3.485	3.554
3.055	4.321	3.624	3.862	3.178	3.720
4.421	4.050	4.389	3.102	4.369	3.451
3.305	3.667	4.294	2.907	3.131	2.130
4.923	4.832	4.588	3.848	4.932	3.815

2.824	2.705	2.538	2.298	1.867	2.954
3.147	3.919	1.441	2.773	3.903	2.887
3.625	3.129	2.728	4.247	3.659	4.233
3.600	3.933	3.395	3.927	3.946	3.750
2.622	2.377	3.292	2.746	2.758	2.611
3.201	2.744	3.049	2.928	3.837	3.748
1.414	2.138	2.042	2.394	2.140	2.155
4.049	3.030	4.307	4.112	3.001	3.045
2.523	1.344	2.187	2.005	2.224	2.417
6.413	6.657	5.808	5.204	6.166	6.254
2.689	2.763	1.844	2.858	2.751	2.879
1.443	1.303	1.498	1.564	0.903	1.429
4.296	4.427	4.005	4.031	4.178	3.861
2.909	2.079	2.632	2.627	2.979	2.596
4.014	3.082	4.065	3.648	2.673	3.107
4.914	5.245	5.648	5.598	5.794	4.474
4.265	2.676	4.154	4.150	3.257	3.613
2.454	3.108	2.152	3.024	3.839	2.767
2.127	1.719	2.203	2.137	2.443	2.238
3.488	3.194	3.353	3.654	2.680	3.781
2.159	3.040	2.211	3.274	3.084	2.121
3.299	4.839	4.130	3.762	4.015	2.610
2.198	2.163	1.874	3.123	2.627	2.245
4.814	4.707	5.313	5.088	5.172	5.244
2.744	3.244	3.646	2.788	3.728	2.569
2.491	1.985	1.658	3.224	2.988	1.407
2.090	3.509	3.117	3.579	2.526	2.642
2.764	2.442	3.036	1.986	3.416	2.884
2.645	1.913	2.904	1.776	2.781	0.975
3.429	2.998	3.829	3.965	3.445	2.384
2.518	1.888	2.399	2.687	2.470	2.903
4.102	4.357	3.596	4.269	3.593	3.119
4.075	3.648	2.457	3.178	3.526	3.336
2.512	3.416	2.512	2.111	2.291	1.784
4.630	4.777	5.794	4.906	4.843	4.120
5.594	4.683	4.282	4.790	3.740	5.295
4.026	4.420	4.499	3.917	5.112	3.945
5.837	6.134	4.883	5.672	6.078	6.358
7.002	6.585	7.013	6.099	4.247	6.621
2.907	2.949	3.077	3.230	2.952	2.767
4.435	4.493	4.375	4.478	4.181	4.181
2.765	2.848	3.466	3.004	3.029	2.625
2.490	2.385	3.233	1.985	2.044	2.838
4.215	3.924	3.212	4.551	2.575	3.332
3.411	3.553	2.526	2.573	2.891	2.652
1.925	2.433	2.008	2.420	2.479	2.047
1.973	1.757	1.988	1.679	2.236	1.979
2.579	1.219	2.576	2.988	2.410	3.345
4.069	3.552	4.475	4.484	3.971	4.138
3.375	3.707	3.162	3.892	3.201	2.205

3.684	3.467	3.831	3.706	1.891	2.641
2.521	2.889	2.344	2.392	1.318	2.534
6.535	6.049	6.995	6.570	6.256	6.443
2.936	1.993	2.385	3.853	2.531	3.931
5.116	5.044	4.654	4.639	4.249	4.060
7.025	5.885	6.158	5.759	6.386	6.960
2.777	3.214	3.214	4.133	3.318	2.935
3.354	1.663	3.918	3.008	3.917	3.866
2.801	2.885	2.814	1.950	3.813	3.197
2.285	2.744	2.597	2.871	2.406	2.246
2.427	2.065	3.166	2.838	2.266	1.723
2.056	3.564	3.899	2.118	3.049	3.305
5.251	4.798	4.685	4.867	4.326	4.528
3.790	3.463	4.458	4.120	4.670	4.855
2.887	2.678	3.449	2.722	3.571	4.177
3.011	2.888	2.503	3.738	3.561	1.189
3.665	3.554	4.107	3.884	3.708	4.010
0.975	1.070	0.747	0.970	1.294	1.105
3.263	3.649	1.928	3.251	1.912	2.473
3.023	4.105	4.669	2.541	3.419	3.133
2.959	3.376	4.215	4.009	4.132	2.998
2.772	1.849	3.643	2.320	2.992	3.820
4.229	4.297	3.369	4.096	4.689	4.516
3.671	3.274	4.516	3.728	3.564	1.402
2.424	2.215	2.276	2.232	1.942	2.232
2.669	2.251	3.171	2.842	2.876	1.549
2.731	2.867	3.009	2.590	2.122	2.114
3.314	3.185	3.128	2.899	3.955	2.846
3.165	3.991	2.603	2.972	3.723	2.017
2.239	1.746	2.016	1.693	3.294	1.894
4.438	3.682	4.044	3.544	4.268	4.223
2.452	2.297	2.547	2.911	2.379	3.166
2.284	2.955	3.337	2.954	2.815	3.102
3.286	3.329	4.071	3.222	3.908	4.174
3.277	2.694	3.324	2.515	2.654	3.208
3.624	2.503	3.891	3.475	3.312	2.998
3.681	2.327	3.969	3.299	3.792	3.113
2.428	1.782	3.164	2.404	2.094	2.268
5.167	5.070	5.679	4.953	6.095	5.457
2.451	2.370	2.247	2.625	3.274	2.782
2.862	2.488	2.789	2.673	3.847	3.542
2.295	1.811	2.402	2.661	1.051	2.501
7.031	5.774	5.861	5.757	6.894	5.816
3.453	3.627	4.543	3.816	4.454	3.418
2.070	1.703	2.702	2.628	2.859	3.009
5.387	4.961	4.885	5.593	5.337	5.785
5.884	5.355	5.393	4.853	5.298	6.047
2.276	1.833	2.567	2.264	2.981	2.156
3.473	4.235	5.094	5.145	3.951	3.625
1.959	2.148	2.393	1.919	1.874	2.137

2.893	2.834	2.470	3.149	2.443	2.880
3.840	4.048	4.422	3.426	4.239	3.846
3.864	3.447	3.892	3.830	3.829	4.222
3.029	2.948	3.128	1.347	2.443	2.316
3.889	4.402	5.140	3.454	4.718	3.528
3.644	2.062	4.462	3.201	2.793	3.425
1.546	1.356	1.439	1.936	1.674	2.140
3.562	4.612	4.666	4.661	4.034	2.939
2.866	2.238	1.754	2.795	3.051	2.927
4.327	4.310	4.931	4.281	4.849	4.575
3.579	2.074	3.947	3.077	3.072	4.332
2.891	3.630	3.592	2.630	3.866	3.045
5.159	5.360	5.622	5.185	4.895	5.006
2.564	2.758	1.119	2.471	1.287	1.524
1.447	0.704	1.015	1.482	1.020	2.757
2.886	2.701	2.542	2.197	2.327	2.345
4.655	4.932	4.124	4.212	3.676	3.721
2.572	3.460	2.734	2.620	2.279	2.760
2.721	2.265	3.045	3.049	3.158	2.611
2.220	2.452	2.585	2.308	3.258	1.516
3.112	3.180	3.262	2.574	3.493	3.286
2.676	2.324	1.932	3.527	2.581	2.400
2.460	2.598	3.075	2.332	2.043	2.845
2.957	2.817	3.348	3.070	2.995	4.557
3.683	3.147	1.727	2.696	3.029	1.995
3.789	3.752	2.909	3.147	3.199	2.188
4.780	5.011	4.304	4.858	3.604	4.760
1.670	0.985	1.778	1.565	2.060	2.769
5.942	6.218	5.827	5.094	6.035	5.062
4.803	5.175	4.227	4.328	4.695	3.804
2.538	2.938	1.437	2.051	1.678	3.095
2.009	2.721	3.329	1.683	2.285	1.778
3.055	3.051	2.612	2.951	3.096	3.000
5.555	5.616	5.669	5.791	5.763	5.662
7.128	7.900	7.313	7.787	7.915	7.566
5.612	5.448	6.169	5.165	6.356	4.998
5.908	5.905	6.142	6.414	6.446	6.135
3.374	3.890	3.929	3.423	3.434	3.379
3.901	3.793	3.982	4.077	4.258	4.022
9.112	8.823	9.392	9.173	9.326	9.151
2.672	4.307	2.013	1.987	2.392	2.246
4.437	5.795	5.955	4.634	5.671	4.401
7.622	6.933	7.347	8.026	6.947	7.894
5.470	4.949	5.353	5.516	5.526	5.275
3.565	3.833	3.978	4.086	3.723	3.747
6.055	5.941	5.626	6.360	5.688	5.771
6.228	6.945	6.778	7.402	6.548	7.018
5.750	5.744	6.042	5.730	5.156	5.183
2.971	5.490	1.918	2.485	2.531	2.579
3.610	3.206	3.421	3.523	3.802	4.035

5.596	6.891	5.302	6.501	6.133	4.548
5.446	5.447	5.807	5.357	5.611	5.948
3.441	3.366	3.317	2.867	2.835	3.565
5.115	6.085	5.864	4.759	5.719	4.881
3.453	3.819	2.721	3.452	2.684	3.749
2.082	2.874	2.418	2.408	2.923	1.362
2.757	3.548	2.798	2.874	2.562	2.425
2.726	3.034	2.513	1.517	1.906	1.958
3.006	4.640	2.220	3.433	2.371	2.164
2.550	2.589	3.211	3.168	2.899	2.497
4.335	3.502	4.206	4.493	3.921	4.105
4.076	5.888	4.033	4.959	5.170	3.876
5.500	4.446	4.912	4.731	4.855	4.733
5.743	5.174	5.301	5.726	5.563	6.127
7.720	8.140	7.613	7.802	8.151	7.706
3.275	2.587	3.088	2.584	2.708	2.587
8.114	7.902	8.502	7.946	7.923	7.982
9.113	8.615	8.984	8.745	8.643	9.008
7.738	7.780	8.103	8.216	8.221	7.881
2.770	2.054	2.082	3.447	2.767	2.834
8.479	8.514	8.776	8.960	8.493	8.680
8.558	8.624	8.839	9.084	8.493	8.707
3.799	4.350	3.290	4.554	4.248	3.283
3.097	2.512	2.899	2.068	2.728	2.852
4.025	4.496	4.493	3.770	4.362	4.111
4.681	5.191	4.976	4.477	5.539	4.916
3.522	3.967	3.099	2.734	3.326	3.776
2.639	3.219	2.370	2.997	2.734	2.792
3.686	3.778	3.622	3.631	4.307	4.259
3.566	4.054	4.571	4.004	4.243	4.142
5.960	5.995	6.137	5.952	6.177	6.074
2.922	3.073	2.840	2.661	3.278	2.795
6.746	7.224	6.723	7.298	6.380	6.651
4.822	5.993	4.269	5.294	5.462	5.167
4.252	3.771	3.914	3.946	4.150	4.424
7.264	6.906	7.197	6.853	6.718	7.016
2.954	2.978	2.500	2.764	2.330	3.280
3.378	3.985	3.566	3.951	2.880	3.658
3.317	2.783	3.215	3.445	2.969	3.490
2.777	2.956	3.173	1.800	3.142	2.175
5.632	7.885	3.116	4.434	4.700	4.410
3.257	5.079	4.492	3.475	3.260	3.601
4.049	4.694	4.235	4.733	4.849	3.637
3.940	4.606	4.277	3.729	4.110	3.778
4.216	5.019	4.532	3.970	4.366	3.825
4.908	4.923	5.093	5.155	4.940	4.419
3.570	3.840	3.318	3.881	4.449	3.732
6.485	6.172	6.677	6.701	6.723	6.933
3.772	3.626	4.187	4.091	3.282	4.187
3.521	3.098	3.911	3.853	3.681	4.528

5.032	5.436	5.677	5.653	5.418	4.499
6.888	6.627	6.928	6.651	7.000	6.742
3.460	3.820	3.430	3.425	3.254	3.240
5.747	5.505	6.085	6.100	6.054	6.127
5.696	5.446	5.714	5.631	5.974	5.874
4.949	4.278	5.229	5.358	5.117	5.319
5.694	5.169	6.231	5.967	5.758	5.968
4.153	4.108	4.552	4.287	4.413	4.552
5.713	6.234	6.285	6.315	6.083	5.951
5.332	5.636	5.246	5.794	5.672	5.185
5.607	5.908	6.201	6.147	6.110	6.045
3.307	3.767	2.900	3.922	3.403	3.043
4.528	4.566	4.379	4.449	4.670	4.605
9.266	8.885	9.101	9.297	9.185	9.471
5.936	6.057	6.293	6.246	6.162	6.050
3.927	4.057	3.865	5.075	3.834	4.175
2.292	2.207	2.686	2.544	1.884	2.367
4.226	4.220	3.925	4.784	3.806	4.102
5.008	5.307	5.593	5.351	5.124	5.319
7.338	7.212	7.381	7.940	7.781	7.190
3.329	3.971	5.502	3.654	5.319	3.115
3.425	4.183	4.106	3.734	4.366	2.592
3.929	5.484	4.841	5.196	5.787	4.230
5.522	6.414	7.187	5.758	7.109	5.415
5.101	4.962	5.425	5.432	5.406	5.237
5.361	5.557	5.559	5.437	5.530	5.693
3.717	3.488	3.141	4.358	4.071	3.890
7.784	7.251	7.936	7.479	7.714	7.764
2.537	3.625	2.775	4.250	2.661	2.212
7.036	6.501	7.091	8.464	6.502	7.453
9.599	10.014	9.721	9.809	9.783	9.455
2.604	3.828	3.601	3.455	4.456	2.573
3.489	2.819	4.225	3.568	3.144	3.173
2.649	2.451	2.437	3.111	3.132	2.764
2.151	1.909	3.419	2.771	2.131	2.326
3.590	2.941	2.884	2.874	3.736	3.050
4.084	4.937	5.333	3.485	3.985	3.666
7.128	7.040	7.594	6.907	7.047	6.700
5.013	4.875	5.848	4.679	5.366	3.820
7.234	6.505	6.913	7.245	7.535	6.782
5.543	3.790	3.609	4.458	4.536	4.983
2.960	2.318	3.938	1.914	2.401	1.466
3.902	4.666	3.900	3.940	4.435	3.399
8.163	8.135	8.157	7.895	7.600	7.121
9.842	9.814	10.003	9.938	9.977	9.137
3.369	3.398	3.587	3.173	3.199	3.783
4.141	3.691	4.593	3.528	4.128	4.324
3.577	3.222	4.029	3.933	4.545	4.059
4.012	3.887	4.087	4.320	4.132	4.119
3.557	4.945	3.089	3.395	3.611	2.192

3.763	3.335	3.648	3.492	4.105	3.109
5.130	5.429	6.015	5.818	5.826	5.071
3.724	3.817	3.473	4.176	4.150	4.557
9.257	9.031	9.417	9.473	9.007	9.396
4.519	5.014	4.959	5.071	5.731	5.040
6.035	5.971	6.060	6.164	5.981	6.245
6.773	6.732	6.741	6.770	6.550	6.973
5.583	5.055	5.477	5.754	5.341	5.820
4.867	4.932	5.123	4.947	4.870	5.349
3.427	2.551	2.912	3.928	3.983	3.788
3.934	4.178	4.573	3.959	4.361	4.112
5.568	5.514	5.669	6.092	5.878	5.817
3.911	4.737	4.397	3.714	4.027	4.022
4.391	3.175	4.112	3.338	3.945	3.531
7.227	6.824	7.094	6.603	6.565	7.016
6.534	6.572	6.400	6.837	6.463	6.407
8.576	8.207	8.615	7.963	8.524	8.406
10.467	10.550	10.680	10.253	9.832	10.423
2.984	1.488	2.636	2.097	1.577	3.088
7.252	5.958	6.457	5.655	6.409	6.759
8.369	8.069	7.838	7.166	8.138	8.227
5.260	4.551	4.709	5.124	4.275	5.367
6.792	6.253	5.696	5.338	6.280	6.140
9.588	9.291	9.077	8.280	8.785	9.746
8.429	8.366	8.530	8.271	7.777	8.570
4.963	3.388	3.552	3.266	2.688	3.804
9.075	9.434	9.598	9.272	8.107	8.307
6.469	5.808	5.660	4.713	4.748	6.814
8.422	8.207	7.933	7.023	7.314	8.322
10.072	9.940	10.065	9.387	9.689	9.392
4.140	4.367	1.778	3.008	3.229	2.817
4.943	3.839	4.922	4.367	4.415	4.495
5.803	5.400	5.723	5.389	5.709	5.702
3.212	3.138	3.235	2.970	2.477	4.287
5.709	5.211	5.927	5.641	5.295	5.604
4.095	4.319	3.758	4.535	4.450	4.015
3.553	3.302	2.104	2.672	3.268	3.746
2.336	1.198	1.981	2.787	1.919	3.272
3.372	3.020	4.070	3.798	3.461	3.559
7.258	6.874	7.437	7.238	6.849	7.221
5.692	5.879	5.039	6.642	4.974	5.414
4.398	3.778	4.318	5.515	3.746	4.148
6.086	7.379	6.018	6.851	6.783	6.328
3.205	4.965	4.076	3.125	4.429	3.807
5.927	7.906	6.546	6.326	6.726	5.345
6.136	6.232	6.390	6.382	6.202	6.295
5.506	6.354	5.120	5.674	5.665	5.163
2.454	2.637	2.599	2.606	2.523	2.259
2.426	3.581	2.140	2.139	2.471	2.464
3.304	3.685	5.072	3.499	4.428	3.682

7.326	7.008	7.383	7.360	7.252	7.597
4.057	3.799	3.751	4.235	3.453	3.996
3.346	4.221	3.632	4.065	3.733	3.255
6.189	5.177	5.840	5.914	5.653	6.496
5.986	6.453	5.569	6.729	4.693	4.979
5.453	4.878	5.318	5.076	4.867	5.431
3.951	4.974	4.564	4.918	4.314	3.863
3.700	3.630	3.802	5.216	3.733	3.981
3.360	2.532	3.238	3.602	3.679	3.596
4.677	5.788	4.972	4.924	5.260	5.443
5.818	5.700	6.061	6.137	5.898	5.320
3.944	2.638	3.048	3.530	3.220	2.943
2.807	1.942	3.297	2.241	1.806	3.056
4.809	4.829	4.953	4.316	4.852	3.953
4.252	4.036	4.725	4.546	4.808	4.869
5.709	6.099	6.389	3.792	4.310	2.448
4.679	5.401	4.359	4.791	4.188	4.899
5.940	6.714	5.796	6.381	6.138	6.118
7.231	7.220	7.226	7.351	6.326	7.070
7.070	6.811	7.430	7.076	7.304	7.017
5.686	6.760	5.509	6.206	6.253	4.833
3.113	5.177	3.769	4.486	4.390	3.010
7.579	7.032	7.425	7.549	7.141	7.811
5.736	5.321	6.194	5.433	5.987	5.449
6.756	6.510	6.878	6.923	6.965	6.963
7.674	8.097	7.888	7.797	7.906	7.538
6.128	7.477	7.001	6.746	7.535	5.906
3.300	3.473	3.227	3.179	3.531	2.602
2.848	2.810	3.603	3.068	3.536	2.483
7.011	7.037	6.856	6.827	6.774	6.873
4.635	4.482	4.577	4.721	4.673	4.925
6.431	6.161	6.366	6.486	6.266	6.483
4.634	4.084	4.525	5.027	3.837	5.697
3.291	3.547	3.062	3.793	3.701	4.291
4.313	4.848	5.538	5.095	5.182	5.051
6.263	5.394	6.912	5.563	6.648	6.215
2.794	2.454	2.296	2.085	2.518	1.175
5.565	5.110	5.774	5.991	5.689	5.856
3.204	2.141	3.510	3.090	2.857	3.695
3.482	4.753	4.701	4.289	4.894	3.730
3.979	4.702	3.983	4.214	4.621	3.591
6.503	6.389	6.411	6.544	5.767	6.004
3.639	3.668	4.066	4.092	3.500	3.749
7.597	7.140	7.581	7.187	6.879	7.636
4.678	4.472	4.936	4.500	5.116	4.055
2.759	2.373	2.638	2.728	1.885	2.163
5.640	6.819	5.645	6.718	6.272	5.793
4.817	5.549	5.041	5.399	5.326	5.312
4.405	5.006	4.081	4.525	5.086	4.885
6.594	7.056	6.334	7.766	7.443	6.994

8.583	9.725	9.739	8.743	9.859	8.482
6.062	5.022	6.138	6.097	5.444	6.548
6.348	5.667	6.340	6.510	6.105	6.857
3.942	4.788	4.038	3.916	4.156	4.454
6.499	5.182	6.541	6.864	5.754	7.340
5.613	5.567	5.648	5.718	5.745	6.065
7.483	7.267	7.640	7.858	7.248	7.717
4.053	5.352	3.723	6.560	3.707	4.239
6.024	5.965	6.215	5.830	5.849	5.941
3.976	4.584	3.630	3.866	4.142	3.967
7.169	6.444	6.264	6.669	6.375	7.190
7.329	6.907	7.446	7.241	7.253	7.480
3.546	3.190	4.211	3.848	3.985	4.081
3.331	3.117	3.742	3.480	2.292	3.317
3.442	3.535	4.060	3.349	3.530	3.609
2.876	1.925	2.798	2.102	1.948	2.849
6.215	5.390	6.296	6.302	6.034	7.056
6.467	6.313	6.289	6.162	6.534	5.798
8.287	8.062	8.342	8.101	8.204	8.157
7.887	7.826	8.056	7.848	7.883	7.825
5.840	5.560	6.134	5.856	5.721	6.036
7.207	6.835	7.417	7.122	7.443	6.743
2.621	2.849	3.367	1.654	3.704	3.210
8.117	8.363	8.877	7.887	8.656	7.935
6.732	6.062	7.347	6.173	6.387	6.415
8.734	8.458	8.680	8.505	8.659	8.533
5.422	5.224	6.307	5.413	6.453	4.698
3.953	4.324	3.644	4.028	4.195	3.736
2.327	2.169	2.288	2.822	2.450	2.763
8.054	7.440	8.147	7.868	8.041	8.519
6.080	5.705	6.365	6.591	6.299	6.909
5.496	5.471	5.590	5.747	5.486	5.666
5.061	5.050	5.213	4.946	5.060	5.025
5.729	5.552	5.413	6.226	5.260	6.412
5.041	4.700	5.113	5.343	4.529	5.700
3.518	4.881	3.335	3.090	3.603	2.870
5.019	7.255	5.549	6.344	6.610	4.942
4.606	6.525	4.663	5.594	5.417	4.400
3.601	3.584	3.766	4.230	3.510	3.217
6.179	6.442	6.019	6.994	6.760	6.713
8.709	8.526	9.102	8.788	8.941	8.962
5.830	7.056	6.171	7.024	6.572	5.514
3.241	2.740	3.397	2.369	2.630	2.309
5.048	5.012	5.081	4.975	5.315	5.174
4.037	6.271	2.493	3.386	3.705	3.784
5.955	6.442	5.555	5.766	6.006	6.081
6.955	6.082	7.178	7.135	6.989	6.975
1.634	1.858	2.248	1.010	2.177	0.807
3.330	4.445	4.734	1.813	4.865	2.425
6.855	6.200	6.199	6.695	5.696	7.182

9.100	9.105	8.806	8.251	8.143	8.798
7.938	7.838	8.023	8.168	7.811	7.690
7.382	7.157	7.441	7.413	7.268	7.641
6.770	6.538	6.712	6.882	6.766	6.957
5.587	5.333	5.659	5.852	5.668	5.884
3.692	3.570	3.489	3.684	3.238	4.334
3.812	3.212	4.564	3.267	4.080	3.573
4.567	4.542	5.415	4.963	5.132	5.570
6.169	6.038	6.317	6.019	5.925	5.715
6.674	6.067	6.905	6.242	6.433	6.370
6.645	6.183	6.648	6.990	6.756	7.122
7.374	6.354	6.820	7.372	6.587	7.545
7.120	7.206	6.981	7.306	6.970	7.044
2.606	2.072	2.331	2.675	2.083	3.019
2.119	1.921	1.928	2.326	1.923	2.602
3.905	3.961	3.587	4.036	3.691	4.165
2.665	2.429	2.673	3.260	2.510	2.683
6.725	6.903	6.816	6.972	6.748	6.987
3.253	3.072	3.674	4.097	3.791	3.322
3.132	3.129	3.223	3.635	3.006	3.139
4.813	4.864	4.965	4.886	4.893	4.976
4.180	4.137	4.290	4.544	4.037	4.546
4.616	3.895	3.285	4.398	4.968	3.864
10.546	10.191	10.921	10.985	10.744	10.083
3.073	1.965	2.588	3.230	2.431	2.996
6.955	6.571	7.061	6.816	6.772	6.861
4.986	4.203	4.639	5.451	4.949	4.507
4.362	4.063	3.570	4.632	4.491	4.414
6.540	6.112	6.304	6.594	6.233	7.102
5.791	5.976	6.037	6.097	6.086	5.811
5.635	5.857	5.688	6.352	6.184	5.831
5.535	5.007	5.232	5.193	5.648	5.833
9.079	5.557	6.667	8.832	6.383	9.731
10.046	6.783	8.858	10.187	7.579	10.727
9.930	6.942	8.437	9.944	7.598	10.449
6.228	5.933	6.438	6.229	6.083	6.738
1.935	1.298	1.990	1.731	1.757	1.749
2.544	2.066	2.329	1.594	1.967	3.124
4.608	4.942	4.321	5.111	4.560	4.953
3.597	2.378	2.348	2.819	2.846	3.130
6.844	6.220	7.060	6.485	6.912	6.964
3.049	3.072	3.506	3.003	1.967	3.301
4.295	2.868	3.704	3.916	3.850	4.164
5.942	6.026	5.979	5.926	5.663	6.005
5.684	5.986	6.145	5.678	5.670	6.345
6.591	6.423	6.478	6.016	6.419	6.338
3.556	2.706	3.072	3.946	2.648	4.318
5.068	5.177	5.456	5.921	5.486	5.628
2.671	3.506	2.630	2.632	2.894	2.874
4.742	4.794	4.224	4.668	4.075	4.275

1.559	1.328	1.125	1.209	1.473	2.041
2.373	1.447	2.339	2.496	1.333	2.720
5.732	5.881	5.851	5.966	5.466	6.231
3.515	4.089	4.054	4.076	4.145	3.492
4.519	4.485	4.536	5.053	4.817	4.153
4.820	5.444	4.443	5.866	5.097	4.343
3.584	3.446	3.303	3.630	4.457	3.584
6.836	7.166	7.699	7.334	7.467	6.944
2.354	3.148	2.271	2.512	2.939	1.698
3.287	2.689	3.584	4.109	3.981	3.327
5.851	6.997	7.396	6.121	6.863	5.221
3.386	3.712	3.544	3.753	4.248	3.804
7.647	8.119	7.697	7.556	7.791	7.111
2.846	3.900	2.428	3.794	3.573	2.982
4.837	5.038	5.776	5.299	4.535	5.004
2.278	2.287	2.292	2.694	2.149	2.637
3.706	4.062	4.329	4.249	4.119	3.829
4.468	4.921	4.605	4.131	3.779	3.801
8.494	8.221	8.747	8.624	8.493	8.735
4.084	4.164	4.756	4.313	4.032	3.576
2.066	2.193	2.087	2.678	2.188	2.245
5.261	5.796	5.382	5.956	5.942	5.495
3.686	3.398	3.558	3.227	3.164	3.605
4.548	4.227	4.463	4.308	4.644	4.227
3.236	4.098	3.104	3.127	2.873	3.667
5.894	6.019	5.828	6.276	6.199	6.311
5.086	5.605	5.445	5.720	5.176	4.806
4.141	4.721	4.326	4.450	4.671	4.573
4.312	4.409	4.243	3.954	4.623	4.290
5.596	6.282	5.805	6.390	5.949	5.673
4.301	4.222	4.040	4.609	4.502	4.455
4.651	4.449	4.783	4.662	3.884	4.543
4.682	4.957	5.239	5.086	5.108	4.727
3.708	4.202	3.672	4.093	3.944	4.067
6.766	6.372	6.731	6.953	6.315	7.085
4.358	3.985	4.264	4.437	4.442	4.830
4.955	5.130	5.212	5.339	5.227	4.977
3.180	2.722	3.789	3.800	2.169	3.262
3.218	3.386	2.972	3.357	3.063	3.685
8.490	8.092	8.695	8.490	8.485	8.453
2.904	3.424	3.946	3.845	3.551	2.884
6.054	5.371	5.534	6.084	5.185	6.073
3.482	4.234	2.892	3.695	3.840	3.345
4.084	3.937	4.312	4.050	4.129	4.108
3.331	3.107	3.187	3.728	3.674	3.579
6.327	5.884	6.393	5.836	5.933	6.502
2.955	2.560	3.649	2.662	3.330	4.134
3.370	2.742	2.985	2.469	2.858	2.731
7.542	7.509	7.635	7.386	7.156	7.407
4.162	4.337	3.347	4.086	4.159	4.244

4.708	4.319	4.978	4.997	4.395	5.566
2.612	1.893	2.814	2.998	2.546	1.854
7.687	7.314	7.848	7.781	7.940	7.896
3.255	3.711	3.682	3.713	3.643	3.383
3.668	4.285	3.887	4.091	3.960	2.772
2.479	2.197	1.773	2.530	1.624	2.425
4.555	5.167	4.707	5.077	4.823	3.609
5.264	4.781	5.443	5.944	4.963	4.912
3.151	2.079	2.688	3.109	3.278	3.572
3.299	2.355	3.350	3.350	2.572	2.618
3.081	4.002	3.672	3.432	3.939	3.230
5.716	4.039	4.802	5.659	4.761	6.179
3.282	3.786	4.511	3.888	3.393	3.824
4.104	4.009	4.350	4.691	4.451	3.934
2.628	2.037	2.796	3.751	2.237	3.101
5.067	4.310	4.699	3.522	4.127	4.348
3.459	4.626	3.898	3.572	4.478	3.657
6.925	6.859	7.263	7.373	7.128	7.081
5.199	5.624	5.645	5.966	5.895	6.069
4.657	5.367	4.805	4.855	5.239	4.395
2.377	3.054	2.527	3.148	2.453	1.582
4.421	5.632	4.438	5.064	5.413	4.437
4.692	4.485	4.689	4.955	4.641	5.458
4.044	4.320	3.479	4.787	3.981	4.254
6.840	6.890	7.116	7.313	6.497	6.925
8.782	8.958	9.096	8.801	8.720	9.166
8.839	8.994	9.074	8.794	8.829	9.207
4.124	3.947	3.340	4.246	3.645	3.781
4.076	3.716	3.994	3.564	3.880	3.637
1.739	1.446	1.665	2.074	2.115	2.713
3.442	4.288	4.570	3.681	3.536	4.256
3.169	3.847	2.499	2.885	2.642	3.629
7.016	6.716	6.861	6.657	6.498	6.866
7.537	7.224	7.691	7.361	7.910	6.778
6.219	5.847	6.723	6.089	6.640	5.847
3.764	4.104	4.034	5.283	4.547	4.090
4.331	5.777	3.279	3.338	3.550	3.943
4.447	4.156	3.867	4.435	4.611	4.609
3.342	3.976	2.947	3.872	3.027	3.635
2.890	3.028	2.827	2.540	2.075	3.874
2.207	1.884	1.782	2.016	1.880	2.293
5.982	5.596	6.566	5.814	5.770	6.087
2.871	3.224	2.316	2.337	3.340	2.357
7.733	7.652	7.945	8.120	7.702	7.964
9.248	9.825	9.438	9.831	9.702	8.951
4.052	4.268	3.787	3.606	3.766	3.884
2.920	2.647	3.186	4.210	3.064	3.668
9.403	9.096	9.185	9.049	8.805	9.218
7.895	8.130	7.903	7.372	7.349	7.652
8.938	8.760	8.748	8.541	8.463	8.741

9.415	9.192	9.136	9.159	8.982	9.358
9.222	9.008	8.940	8.972	8.765	9.008
5.843	5.920	5.732	5.920	4.970	5.947
7.877	7.592	7.650	7.669	7.072	7.590
5.918	6.129	6.184	5.806	6.399	5.448
3.906	3.079	3.035	4.103	3.964	4.523
2.833	4.051	3.670	2.945	3.350	3.334
3.299	3.332	2.959	2.567	3.077	2.596
2.706	2.894	1.918	2.613	2.015	2.679
5.509	5.126	6.261	5.568	4.978	6.922
2.660	2.785	2.618	2.265	1.812	2.353
7.792	7.241	7.556	7.595	7.229	8.071
4.193	3.848	3.798	4.013	3.780	5.058
5.136	5.407	5.359	5.325	3.766	3.587
4.302	3.620	4.803	4.012	3.187	4.227
3.450	2.692	2.646	2.766	3.126	2.801
3.490	2.874	3.122	3.992	3.145	4.446
3.726	4.028	4.164	4.333	3.021	5.619
2.183	2.917	2.214	2.379	3.229	2.987
2.532	2.267	2.800	2.073	2.604	2.712
5.730	6.709	6.418	6.463	6.821	5.915
3.962	3.526	4.199	4.178	3.909	4.018
5.972	6.329	5.606	5.972	5.929	5.445
5.551	5.356	6.047	5.334	5.487	5.700
1.694	1.419	1.945	1.461	1.858	1.697
4.061	4.253	4.584	4.248	4.469	4.590
2.738	2.892	2.522	2.686	2.557	2.896
4.860	4.984	4.880	5.077	5.265	5.600
5.288	4.844	5.313	5.185	5.293	5.680
4.888	5.070	3.986	3.804	4.021	3.442
5.238	6.761	5.237	4.733	5.465	4.436
4.311	3.025	4.866	3.984	4.418	2.229
5.365	6.018	5.976	5.886	5.937	5.881
6.998	6.782	7.042	7.126	6.998	6.765
7.964	7.564	8.409	8.024	8.320	8.216
2.116	2.326	2.213	1.902	1.991	2.219
4.011	2.464	3.384	3.833	2.731	4.096
2.703	2.092	2.298	2.653	2.339	2.924
1.776	1.749	1.916	1.649	1.173	2.187
2.048	1.900	2.359	1.487	1.680	2.038
4.844	4.538	5.324	5.133	5.217	4.874
3.327	3.021	3.109	2.206	2.505	2.251
4.093	4.772	3.455	4.017	3.225	4.792
4.226	5.107	4.151	5.080	3.975	4.858
6.420	6.831	5.481	7.004	6.030	6.926
5.680	5.999	4.544	5.691	5.465	5.840
4.558	5.361	5.252	4.839	4.961	4.685
3.701	3.308	3.811	3.805	3.334	4.143
4.807	5.218	3.847	5.503	5.187	5.213
5.758	5.373	6.089	5.631	5.869	6.586

6.508	5.945	6.624	6.283	6.861	6.594
3.778	4.720	3.755	3.078	3.673	4.182
7.431	6.424	6.991	7.394	6.384	8.059
7.457	6.734	7.069	7.491	6.767	8.156
2.818	2.818	2.535	2.942	2.197	3.125
7.172	7.122	7.155	7.160	6.776	7.530
4.647	4.955	5.066	5.047	5.385	4.343
4.857	5.261	4.318	4.951	4.768	4.783
3.418	2.614	3.787	3.878	2.821	3.887
6.575	6.262	7.005	6.727	6.751	6.860
5.203	4.577	4.883	5.209	4.982	5.285
4.422	4.070	4.600	4.188	4.813	4.839
3.370	2.850	3.774	2.884	3.703	3.651
5.260	5.232	5.258	5.578	5.026	5.051
2.552	3.013	2.299	2.156	2.863	2.273
4.574	5.373	4.505	4.924	4.790	5.076
4.441	4.255	4.736	4.438	4.782	4.760
4.827	4.981	5.390	5.426	5.052	4.463
6.002	5.358	6.961	6.069	6.560	6.362
5.812	6.109	6.059	6.240	5.830	5.837
3.378	4.681	4.181	3.710	3.726	2.966
3.465	3.847	4.367	4.277	3.909	3.671
3.543	3.543	3.442	3.411	2.912	3.109
3.461	2.449	3.241	4.079	3.441	3.777
2.990	2.633	2.391	3.311	2.450	2.480
3.890	4.552	4.505	3.924	4.295	4.239
3.960	4.713	4.288	4.397	4.242	4.165
7.849	7.671	7.673	8.092	7.795	7.854
8.968	8.490	9.044	8.895	8.819	9.133
2.070	2.515	3.960	2.749	2.071	2.483
2.141	3.085	2.872	2.789	2.744	2.249
2.493	1.853	2.792	2.908	2.109	2.814
4.531	4.813	4.420	4.718	5.127	4.948
4.753	5.188	4.591	4.892	5.123	5.132
5.545	6.020	5.773	5.980	6.032	5.872
8.333	8.575	8.359	8.099	9.169	8.295
2.212	2.312	2.584	2.888	1.444	1.299
2.498	2.276	2.762	2.709	3.064	1.505
4.571	4.320	3.993	3.464	4.939	3.123
1.886	2.419	1.748	2.383	1.649	2.420
2.648	2.255	2.297	3.397	2.386	2.177
6.489	6.658	6.763	7.034	6.540	7.114
4.203	4.304	4.207	4.119	4.189	4.549
2.819	2.013	2.129	3.072	1.462	3.005
3.807	4.433	3.672	4.944	4.209	4.325
3.626	4.409	3.878	3.786	4.047	3.560
4.018	3.377	3.891	3.967	2.665	4.089
3.358	3.223	3.224	3.431	2.420	4.858
3.538	3.704	3.297	3.737	3.805	4.130
3.648	4.237	3.762	3.644	3.396	3.010

5.096	4.897	5.497	4.904	5.266	4.873
3.438	4.805	3.401	3.595	3.475	3.918
3.832	5.481	3.805	4.406	4.601	4.462
2.961	3.940	2.833	3.008	3.321	3.064
2.150	2.866	2.846	2.233	1.852	2.709
5.076	5.788	5.259	6.506	5.834	5.585
3.017	2.218	3.431	2.653	2.616	3.560
2.123	1.904	1.999	2.115	1.649	2.206
2.618	2.427	2.554	3.325	2.232	3.456
2.022	2.559	2.356	2.580	2.142	2.426
3.170	3.061	2.031	2.820	2.480	3.100
6.005	5.828	6.046	6.247	6.250	6.222
5.252	4.055	5.479	5.351	4.931	5.406
3.572	3.905	3.158	3.373	3.571	2.981
5.962	3.827	5.348	5.790	4.470	6.286
9.499	9.244	9.915	10.024	9.631	9.754
5.501	6.276	5.292	4.979	5.563	5.267
4.413	4.292	4.516	4.533	4.178	4.712
5.398	5.480	5.330	5.632	5.525	5.378
3.929	3.129	3.630	3.723	3.331	3.770
6.378	5.989	6.343	6.694	5.966	6.721
2.504	1.013	2.326	2.147	2.065	3.287
1.995	2.674	2.225	2.116	1.693	2.350
2.864	2.756	2.329	3.379	2.535	2.361
3.429	2.689	1.791	1.960	2.146	1.933
3.940	4.519	4.990	4.346	4.978	3.947
3.390	4.850	3.024	4.523	3.557	3.627
6.696	6.309	6.707	5.956	5.833	6.895
4.745	4.477	4.709	4.537	4.291	4.673
6.117	7.193	7.180	6.995	7.484	6.318
4.301	4.852	3.891	2.300	3.586	4.162
6.068	5.067	6.111	6.042	5.434	6.794
6.444	6.507	6.632	6.709	6.640	6.717
6.475	5.464	5.543	6.176	6.243	7.445
3.504	3.668	3.649	3.439	3.109	4.173
4.556	4.230	4.507	4.852	3.919	5.628
3.224	2.801	3.102	2.126	2.986	3.759
4.181	3.301	3.876	4.655	3.799	4.314
3.209	3.315	3.257	3.704	4.244	2.881
2.434	1.898	2.581	3.419	2.167	1.987
2.912	3.628	3.271	2.899	2.809	2.845
2.586	2.149	1.991	2.730	2.260	3.107
3.511	3.603	3.502	3.666	3.712	4.236
2.741	2.319	2.807	2.799	2.694	3.417
2.506	3.114	1.543	2.032	1.530	1.733
4.107	4.790	4.365	4.198	4.705	3.916
2.726	2.512	3.542	2.829	2.074	2.940
4.138	4.172	4.491	4.523	3.326	4.438
6.003	5.223	5.931	5.757	4.052	6.250
2.600	1.739	2.618	2.436	2.072	1.859

2.889	2.495	3.082	2.671	3.778	3.813
2.949	3.285	3.429	1.698	2.580	2.233
4.701	3.892	4.373	4.393	3.502	4.557
2.619	1.248	1.855	2.558	2.414	2.465
3.660	3.955	3.399	3.024	3.313	3.743
3.716	3.366	4.588	3.377	3.720	3.995
2.563	2.065	2.420	2.316	2.738	1.994
2.662	4.083	2.832	2.442	2.068	2.279
3.842	3.664	2.809	3.673	3.439	3.522
3.745	3.422	4.657	2.956	3.012	4.064
3.592	4.869	3.006	4.141	3.474	3.849
3.873	4.275	3.300	4.076	3.834	3.317
4.785	4.659	4.377	4.851	5.157	4.964
5.446	5.384	5.276	5.387	5.403	5.866
2.419	2.538	3.089	2.621	2.266	2.399
4.901	4.916	4.651	5.150	4.874	4.513
2.402	2.697	2.365	2.793	2.468	2.235
6.990	7.441	8.250	7.839	7.667	7.223
5.007	5.306	4.861	4.793	5.100	4.499
5.079	4.737	4.661	5.119	4.483	5.194
2.743	3.349	2.315	3.151	3.458	2.314
3.894	3.501	3.319	3.716	3.347	4.135
3.047	2.949	2.992	2.922	3.167	3.285
5.117	4.890	5.604	5.848	5.293	5.471
2.212	3.318	2.799	2.623	2.445	2.405
5.858	6.774	6.616	6.876	7.299	6.271
6.973	7.770	7.486	7.715	7.656	7.165
2.239	2.470	2.150	1.381	2.118	1.664
1.857	1.527	2.080	2.021	1.871	1.809
3.526	3.405	4.015	3.876	4.654	3.588
2.862	3.207	2.222	2.957	3.081	3.009
4.453	4.657	4.611	4.279	4.631	4.735
5.830	5.021	5.610	5.813	5.416	5.476
7.737	7.582	7.504	7.777	7.667	7.545
3.729	3.290	3.546	4.675	3.707	3.953
2.247	2.191	3.025	1.596	2.746	3.066
6.814	6.256	6.753	6.771	6.711	7.035
2.537	2.873	3.637	2.521	2.158	2.346
1.703	1.504	2.029	1.611	1.283	1.507
5.816	4.915	5.239	5.676	5.153	5.620
6.484	5.841	6.675	7.186	6.564	7.134
2.957	3.587	2.954	1.864	2.703	3.035
3.764	3.764	3.839	3.551	4.269	4.085
3.105	3.786	3.084	3.233	3.465	2.968
2.709	2.284	1.983	2.367	2.108	3.105
2.832	4.191	3.544	3.634	3.425	3.246
3.917	3.921	3.605	3.654	2.789	4.127
1.456	1.247	1.045	1.263	1.484	1.537
6.436	6.077	6.752	6.747	6.733	6.406
5.017	4.533	5.725	4.587	5.093	5.040

5.142	6.308	3.283	6.511	5.579	4.755
3.716	3.198	3.023	3.523	3.639	3.302
7.308	7.896	7.668	6.934	8.500	6.130
2.667	3.440	3.033	2.803	2.859	3.249
1.027	0.948	1.024	0.807	0.705	1.111
2.075	1.350	1.648	2.142	1.551	3.082
3.547	3.350	3.892	3.621	3.711	3.931
4.027	3.602	3.786	3.499	3.704	3.716
2.742	2.943	2.276	2.026	2.204	2.859
6.717	6.793	6.628	6.791	6.451	6.369
5.121	3.155	4.631	4.889	3.889	5.779
5.833	6.168	5.671	6.009	6.033	5.521
9.386	9.568	9.655	9.866	9.865	9.436
8.308	7.771	8.357	8.369	8.062	8.420
4.471	5.002	4.329	4.862	5.017	4.562
5.419	5.270	5.578	5.062	5.548	5.574
2.131	1.449	1.896	1.267	1.533	2.545
5.445	6.014	5.437	5.701	4.691	5.721
8.849	8.201	8.613	7.731	7.960	8.564
6.214	6.510	5.758	6.791	6.073	6.406
6.331	6.211	6.847	6.494	6.533	6.244
4.754	3.486	4.540	4.747	3.554	5.759
3.036	3.497	2.959	3.022	2.996	4.295
4.740	4.759	5.022	4.808	4.281	4.281
4.341	4.334	4.260	4.214	3.481	4.537
5.186	5.935	4.635	5.322	5.322	5.201
6.592	6.703	6.949	6.678	6.899	6.747
6.556	6.375	6.619	6.873	6.896	6.460
2.402	2.387	2.576	2.376	2.168	2.441
4.219	4.547	4.305	4.622	4.652	4.510
4.801	2.702	3.841	4.227	3.833	5.214
5.249	4.845	5.330	4.869	5.261	5.109
3.555	3.805	3.709	2.948	3.303	3.429
3.591	2.745	2.709	3.466	3.235	3.505
4.651	5.443	4.719	4.937	4.915	4.588
6.721	6.603	6.975	7.070	6.369	6.494
4.668	5.405	4.237	4.473	4.185	4.480
9.565	8.929	9.153	9.006	8.526	9.116
5.137	4.981	5.873	5.297	5.186	5.130
6.136	5.747	5.889	5.759	5.735	5.958
5.962	5.521	5.958	5.535	5.568	5.933
4.898	4.345	4.582	4.321	4.403	4.799
2.480	2.563	2.401	2.193	2.073	1.639
3.363	3.301	3.355	3.244	3.980	3.073
4.905	4.443	4.280	4.810	5.095	5.158
5.090	4.868	5.538	5.743	5.533	5.515
6.035	5.533	5.375	6.694	5.335	7.182
5.014	5.901	4.647	5.282	5.588	5.468
6.088	6.489	6.434	7.005	6.849	6.547
4.063	3.939	4.075	4.080	4.606	4.395

7.917	8.205	8.237	8.171	8.254	7.679
4.939	5.552	5.343	5.552	5.536	5.326
1.561	2.390	1.404	1.826	1.961	1.140
3.685	4.883	4.580	4.583	5.178	3.440
2.609	2.855	2.320	2.700	2.251	2.965
4.473	4.780	5.041	4.602	4.820	4.531
2.127	2.041	2.573	2.086	2.041	3.362
2.219	3.029	3.263	2.200	2.352	2.426
3.063	3.471	2.763	3.356	3.225	3.607
5.326	5.060	5.154	5.469	5.316	5.521
4.341	4.120	3.788	4.209	4.077	4.210
6.023	5.392	6.302	5.826	5.669	5.551
3.894	3.218	3.865	3.105	3.957	3.521
3.526	2.934	2.976	2.935	3.138	3.623
6.873	6.860	6.455	7.004	6.678	7.095
4.875	4.689	5.049	4.923	5.172	5.334
6.289	5.357	6.287	5.421	5.845	6.564
2.544	3.115	3.768	2.253	2.452	2.091
4.471	3.860	5.124	3.881	4.466	5.261
5.614	5.710	6.458	6.564	6.862	6.397
3.398	3.256	3.080	3.391	2.562	3.706
5.315	5.062	5.507	5.342	5.317	5.724
3.404	3.774	3.162	3.943	3.691	4.807
8.198	7.360	8.542	7.961	8.332	8.424
5.650	5.403	5.848	6.337	6.143	6.042
5.460	5.383	5.850	6.238	5.648	6.089
3.232	4.414	4.376	3.981	4.851	3.897
4.572	4.614	5.102	5.484	5.189	5.079
4.671	4.837	4.826	5.436	5.008	5.424
3.876	4.418	4.060	4.646	4.803	4.679
4.524	4.088	4.607	4.997	5.066	4.986
3.721	4.089	4.010	4.320	3.973	3.946
3.580	3.314	3.253	3.561	3.888	4.047
5.547	6.251	6.595	6.941	6.633	5.791
4.618	4.019	5.363	5.366	5.375	4.566
4.581	4.612	5.138	5.160	4.321	4.543
2.174	2.288	2.577	2.445	2.443	2.470
3.925	2.961	3.990	3.990	4.466	4.551
4.557	3.819	4.911	4.595	4.843	4.444
4.977	3.730	4.256	4.263	4.390	4.693
3.033	4.613	2.603	4.503	3.891	2.720
2.839	3.312	2.752	3.823	3.779	3.044
4.473	3.931	4.332	4.679	3.878	5.315
5.718	5.646	5.790	5.821	5.349	5.806
3.907	4.991	3.920	4.319	4.009	4.145
2.545	2.790	2.105	2.518	2.345	2.598
2.566	2.605	2.402	2.241	2.552	2.597
2.969	1.283	2.398	3.583	2.091	3.288
4.843	4.406	4.449	5.630	4.207	6.482
3.573	3.168	3.651	4.268	3.703	4.243

5.771	5.386	5.314	5.639	5.705	6.136
5.369	3.727	4.939	5.541	3.208	6.457
6.078	6.363	6.317	6.352	5.977	5.837
1.783	2.290	1.624	2.602	2.202	2.097
6.140	6.009	6.440	6.601	6.229	6.352
4.470	4.342	4.404	5.663	4.353	4.647
3.375	4.676	4.703	5.438	4.842	3.196
3.678	3.312	2.776	4.036	4.133	3.468
5.108	5.265	4.966	4.563	4.407	5.228
6.094	6.055	5.701	6.075	5.633	6.206
4.400	3.235	3.988	4.173	4.852	4.968
2.045	1.953	1.945	1.856	2.350	1.804
5.349	5.604	3.930	6.042	4.884	6.100
4.719	4.360	4.527	4.849	4.350	5.071
4.767	4.646	5.479	4.837	5.443	5.512
2.867	2.863	2.466	2.455	2.921	2.765
4.894	4.406	5.184	5.205	4.975	4.695
4.403	4.881	5.314	4.772	4.912	3.724
4.352	4.420	4.654	4.383	4.103	4.413
5.539	5.541	6.269	5.623	6.075	5.569
3.439	3.312	3.657	3.172	3.673	3.633
3.315	3.731	2.710	3.830	3.007	3.588
5.250	6.360	5.726	5.217	4.878	6.198
6.461	6.284	5.975	5.858	6.119	6.556
6.610	6.484	6.919	6.680	6.813	6.735
6.235	5.886	6.035	6.341	6.302	6.611
5.222	5.080	5.515	4.900	5.387	4.845
4.848	4.574	3.975	4.786	4.888	4.625
3.892	2.738	3.005	2.615	2.185	4.116
6.357	6.016	6.446	5.727	5.744	6.655
4.409	4.600	4.860	4.578	4.644	3.235
5.170	6.016	5.789	5.280	3.221	4.446
6.409	6.428	6.458	5.813	5.745	5.566
6.358	6.671	6.564	5.733	6.606	5.931
3.451	3.400	3.110	3.937	3.011	3.908
6.236	6.458	5.754	6.082	6.897	5.719
5.027	4.734	4.932	5.318	5.081	4.851
7.703	7.424	8.204	7.676	7.702	7.261
5.558	6.631	5.258	5.487	5.400	5.010
4.996	4.859	4.730	5.170	5.416	4.964
3.856	4.342	3.835	4.693	3.907	4.476
5.108	4.864	5.642	5.326	5.369	4.950
2.850	3.100	3.080	3.080	3.393	3.135
4.171	4.058	4.855	4.838	3.954	4.361
6.972	6.858	6.777	6.769	7.007	7.367
4.886	5.938	4.585	5.225	5.900	5.048
6.457	6.148	5.832	6.510	6.695	5.918
6.891	6.753	7.161	6.766	7.098	7.173
2.330	2.490	2.336	2.426	2.997	2.319
5.740	5.700	5.786	5.723	5.157	6.341

2.236	2.265	2.089	3.336	2.011	3.475
5.083	4.942	5.275	4.876	5.090	5.131
6.039	5.737	5.796	5.507	5.189	6.037
3.235	2.924	2.958	3.589	3.086	3.572
4.357	4.530	4.253	4.125	3.917	3.954
3.325	3.674	3.693	2.902	4.102	3.669
7.131	6.225	7.133	7.662	7.175	7.851
5.821	5.190	5.360	6.011	5.230	6.646
3.372	2.488	3.215	2.745	2.923	3.835
6.957	6.981	7.938	7.472	7.096	6.953
4.379	4.611	4.264	4.897	4.462	4.833
2.740	2.337	2.875	2.498	2.164	4.029
6.615	6.846	7.000	6.360	6.532	5.317
4.142	4.204	3.803	4.178	3.370	4.820
5.507	5.663	5.814	5.934	5.668	5.781
2.890	3.212	2.980	3.104	2.595	3.454
5.788	5.986	5.631	6.169	6.005	6.106
4.155	3.811	3.186	3.407	3.761	4.028
4.746	5.112	5.117	5.395	5.305	5.116
7.183	6.866	7.601	7.224	7.043	7.433
3.032	2.881	3.841	3.274	3.235	3.367
4.419	3.876	4.394	4.858	4.438	4.878
5.890	6.580	5.831	7.206	6.398	5.828
8.266	8.805	8.808	8.897	8.696	7.945
4.252	3.905	3.872	4.277	3.815	4.496
7.638	7.164	7.387	7.319	7.448	7.469
9.027	9.584	8.993	9.230	8.830	8.934
6.412	7.562	6.354	6.893	8.053	5.842
3.477	2.757	3.684	3.330	2.869	2.550
4.266	4.240	3.969	4.313	3.957	4.410
6.136	5.935	6.516	5.843	6.303	6.516
3.348	4.056	3.826	3.839	4.120	3.171
3.197	3.454	2.969	3.524	3.701	3.527
4.552	4.485	4.620	4.630	4.534	4.701
5.108	5.191	5.080	4.810	5.605	5.267
5.878	7.145	6.299	6.589	5.951	5.831
4.980	4.899	4.907	5.450	5.103	5.231
5.275	5.117	5.085	5.656	4.641	5.900
6.548	6.336	6.796	6.474	6.515	6.303
6.884	6.869	6.858	6.965	6.893	6.948
7.175	6.923	7.096	6.160	7.012	7.003
5.876	5.992	6.194	6.079	5.795	5.612
2.548	2.642	2.765	2.337	3.131	2.488
5.700	5.350	5.656	5.378	5.717	5.848
6.135	5.997	6.257	6.416	6.302	6.183
5.468	5.761	5.992	5.812	6.267	5.778
5.503	5.678	6.160	6.148	6.022	5.460
4.240	4.196	3.735	4.081	4.117	4.599
4.760	3.980	4.666	4.896	4.168	4.555
4.532	4.911	4.442	5.102	4.336	4.603

2.684	3.075	2.840	2.433	2.474	2.437
8.173	7.822	8.434	8.082	8.191	8.506
5.288	5.103	5.077	5.638	5.361	5.648
3.681	3.409	3.547	3.427	4.350	3.899
5.366	5.623	5.223	5.325	5.362	4.929
4.451	3.908	4.238	3.912	4.022	3.963
4.599	4.676	4.838	5.056	4.919	5.072
5.651	5.310	5.731	5.330	5.822	5.434
5.526	4.841	5.131	5.364	5.387	5.550
7.520	6.656	8.262	7.377	7.682	7.639
5.005	5.821	5.091	5.074	5.416	4.774
3.063	4.019	3.475	5.780	4.944	1.896
4.824	5.028	4.057	3.994	4.669	3.538
6.698	6.819	6.811	7.381	7.049	6.797
4.508	4.499	4.749	4.196	3.208	3.509
4.085	4.111	4.291	4.836	3.932	4.235
7.284	7.321	7.263	6.311	7.191	7.488
6.187	6.383	5.881	5.508	5.945	6.461
7.344	7.663	6.988	6.473	7.064	7.590
4.518	3.718	4.204	4.524	3.788	5.110
3.392	2.594	2.862	3.111	2.697	3.153
5.556	6.235	5.440	5.755	6.156	5.401
5.811	5.795	6.733	6.200	5.744	5.965
4.434	3.634	3.998	3.776	4.689	4.553
4.031	4.080	4.208	4.641	3.972	3.953
3.999	4.073	4.300	4.489	4.640	4.090
6.376	6.201	6.451	6.373	6.151	6.510
5.596	5.463	5.848	5.900	5.866	5.851
7.617	7.848	7.603	7.746	7.681	7.670
4.367	4.138	3.934	4.466	3.392	4.545
2.607	2.192	2.211	2.096	2.292	2.387
5.490	7.184	5.711	6.895	6.536	5.859
6.521	6.566	6.847	6.681	6.873	6.685
5.602	5.392	5.958	6.042	5.818	5.905
5.243	6.566	5.396	6.472	6.257	5.418
8.082	7.906	8.251	8.025	8.183	8.424
5.043	5.050	5.228	4.958	4.966	4.907
4.796	4.759	4.876	5.132	4.921	5.293
4.169	5.210	4.072	4.243	4.819	3.870
2.543	3.474	2.640	2.975	3.381	1.919
3.124	3.787	3.852	4.006	4.548	3.125
5.808	6.927	5.753	6.810	6.469	5.938
3.889	3.995	4.456	4.326	4.152	4.151
2.203	2.248	1.871	1.569	3.047	2.535
8.871	8.727	9.394	8.814	8.754	9.068
8.070	7.940	8.108	7.842	7.851	8.365
5.554	5.602	5.501	5.788	5.778	5.576
3.650	3.272	3.334	3.806	3.409	3.544
4.230	3.594	4.937	4.923	4.271	4.684
3.644	3.640	4.107	4.494	3.695	3.502

6.614	7.290	7.824	7.014	7.649	7.215
6.255	6.207	6.480	6.430	6.205	6.714
5.221	5.189	4.816	5.355	5.404	5.273
4.267	4.431	4.229	4.072	4.341	4.601
3.594	3.203	2.963	3.400	3.366	4.258
2.720	2.236	2.299	2.210	2.299	2.965
4.005	4.036	4.591	4.069	4.388	4.010
7.869	7.731	7.949	8.048	7.780	8.250
3.152	3.770	2.970	3.209	3.228	2.703
5.719	4.893	5.684	6.121	5.310	6.315
4.878	4.508	5.614	5.289	5.431	5.226
4.167	3.961	4.287	4.495	4.045	4.557
4.350	5.596	3.706	3.692	3.799	3.621
9.210	8.731	9.120	8.772	8.718	8.823
3.809	4.932	3.896	4.322	3.839	3.992
4.882	5.675	4.294	5.468	5.602	5.495
8.483	8.278	8.739	8.554	8.459	8.653
4.994	5.839	5.109	5.458	5.243	4.535
5.387	5.798	5.323	6.190	5.237	5.515
3.793	3.857	3.151	4.840	3.910	3.709
4.558	6.741	6.560	5.161	7.402	5.382
3.542	4.074	3.296	3.353	3.556	4.062
4.373	5.075	3.291	3.588	4.424	3.785
4.395	5.144	4.800	4.965	5.046	4.770
6.016	6.590	5.444	6.014	6.243	6.223
2.615	3.417	3.233	2.633	2.900	3.476
5.203	6.804	5.996	5.286	6.526	4.812
4.330	6.145	5.348	4.249	5.754	4.151
6.619	6.000	6.505	7.028	6.706	7.108
1.813	1.813	1.432	1.823	2.119	1.874
5.343	6.435	5.192	5.890	5.831	5.107
5.535	5.755	5.615	5.727	5.834	5.244
5.550	4.774	5.214	5.408	5.135	6.006
5.104	5.486	4.776	5.431	5.556	5.068
7.726	7.962	7.635	7.940	7.720	8.061
4.907	6.366	4.954	5.913	5.644	4.727
3.424	2.193	3.399	3.622	3.660	4.163
7.037	6.711	7.241	6.841	6.816	6.865
3.814	3.939	4.570	3.625	3.993	4.154
5.796	5.375	6.005	5.835	6.146	6.217
6.441	6.470	6.405	6.340	6.216	6.565
6.173	6.196	6.441	6.555	6.422	6.189
5.943	5.794	6.009	6.132	5.809	6.018
4.281	4.899	4.410	5.036	4.654	4.539
5.177	4.382	5.372	5.181	4.134	5.383
6.640	6.367	6.451	6.476	6.651	6.413
4.547	6.263	3.503	5.760	5.090	4.951
5.586	5.333	5.940	5.912	5.622	6.239
4.620	5.800	4.099	4.130	4.981	3.627
3.341	3.910	3.252	3.328	3.496	3.803

6.952	6.507	6.967	6.329	6.680	6.690
3.874	3.419	4.024	3.338	4.527	4.433
6.295	6.132	6.505	6.166	6.069	6.116
4.581	4.968	4.376	4.049	4.007	4.526
2.925	3.694	3.217	3.733	2.836	2.553
4.829	5.104	5.053	4.828	5.020	4.681
3.115	2.895	2.515	3.130	3.271	3.433
5.046	4.660	4.906	5.301	4.733	5.041
5.032	5.052	5.168	5.000	5.078	5.293
5.873	6.585	6.470	6.204	6.129	6.440
4.892	3.999	4.880	3.802	4.724	3.865
3.562	5.096	3.350	3.960	4.930	3.450
6.527	7.096	7.042	7.109	6.889	6.392
3.790	3.685	3.829	3.648	3.821	3.110
2.695	3.330	2.879	2.925	2.600	3.759
4.920	6.217	5.533	6.360	5.858	4.937
2.786	2.611	2.897	3.177	2.748	2.044
7.819	7.557	7.631	8.471	7.766	8.647
2.646	2.561	2.229	2.250	2.401	1.534
6.896	7.059	7.076	6.197	7.177	6.404
7.167	7.042	7.473	7.264	8.076	6.926
5.777	6.030	6.049	5.489	6.274	5.880
6.142	7.043	6.265	6.429	6.543	5.822
2.688	3.256	2.464	3.681	3.256	2.783
5.497	6.560	7.246	6.288	6.833	5.589
6.792	6.552	7.565	7.110	7.526	6.941
8.723	8.972	9.039	8.172	9.356	8.228
2.881	3.047	2.113	4.506	3.015	2.177
5.451	6.227	6.209	6.513	6.056	5.791
9.267	9.869	9.550	9.555	9.878	9.083
5.423	5.412	5.246	5.102	5.346	5.120
5.004	5.007	5.909	5.330	4.719	5.933
5.052	4.817	4.983	5.005	5.400	4.972
4.522	4.187	3.959	4.672	3.550	4.241
6.667	6.370	7.018	6.843	6.624	6.765
6.932	7.015	7.031	6.825	7.108	6.717
5.670	5.555	5.837	5.531	5.506	5.786
6.103	6.580	6.169	6.234	6.265	5.489
10.788	10.818	10.910	11.071	11.005	10.749
6.983	7.242	6.775	7.047	6.931	6.997
4.881	4.348	4.682	5.557	5.200	5.636
4.964	5.395	5.361	5.664	5.518	4.951
4.907	4.186	4.477	4.954	4.368	4.332
3.297	3.638	3.343	2.829	3.026	3.445
3.158	3.089	2.742	3.040	3.023	3.626
4.273	4.489	4.402	4.334	4.487	4.320
1.789	2.180	2.281	1.669	1.651	1.933
7.499	7.229	7.795	7.420	7.760	7.784
6.466	5.710	6.139	6.196	6.369	6.740
4.081	2.930	3.686	3.991	2.897	4.348

3.117	3.254	3.005	3.674	3.581	3.754
3.312	2.914	3.557	3.786	2.944	3.969
3.278	2.772	3.490	3.727	2.883	3.938
3.331	2.916	3.562	3.784	2.991	3.925
3.212	2.887	3.357	3.540	2.957	3.564
5.350	5.423	5.275	5.527	4.947	6.221
3.912	3.624	3.355	3.795	2.646	4.800
2.787	2.719	2.884	3.219	2.136	3.222
4.574	4.984	5.566	4.818	4.547	4.677
4.771	4.785	5.236	5.267	5.574	4.864
4.158	5.611	3.955	4.327	4.585	3.891
3.519	3.871	3.019	3.675	4.006	3.162
6.652	6.058	6.448	6.028	5.794	6.974
3.398	3.758	3.775	3.872	3.552	3.316
3.820	4.399	4.395	4.767	4.558	3.901
6.171	6.200	6.533	6.676	6.421	6.657
5.669	6.026	5.826	5.884	5.762	5.907
5.463	5.473	5.134	5.219	5.803	4.914
5.963	5.731	5.925	6.164	5.917	5.886
5.160	5.355	4.616	5.022	4.994	5.468
4.511	4.714	4.488	4.720	4.821	4.925
2.666	2.717	1.736	2.860	2.230	1.992
2.860	2.754	2.197	3.018	2.504	3.168
2.968	3.047	2.886	2.803	2.907	3.033
3.716	3.376	3.209	4.974	3.996	2.464
6.624	7.195	6.994	6.976	6.585	6.782
5.895	6.078	6.175	6.337	6.339	6.185
3.442	2.365	3.464	2.917	2.103	3.145
3.920	3.375	3.864	4.832	4.158	4.061
4.896	5.143	5.000	5.252	5.015	5.201
2.847	4.594	3.099	5.321	3.142	2.300
3.025	2.773	2.684	3.883	2.831	3.419
2.996	3.299	2.906	3.000	2.716	3.664
6.512	6.300	6.009	5.376	5.918	6.273
2.968	3.402	2.918	2.596	2.230	3.744
3.806	4.104	3.875	3.071	2.570	2.329
2.819	2.805	2.109	2.093	2.001	2.633
5.488	5.287	5.561	6.104	5.424	5.777
2.486	2.536	2.406	2.680	2.476	2.573
1.300	1.482	1.204	0.976	0.864	1.211
3.120	2.903	3.211	2.594	2.770	2.287
2.988	3.677	3.144	1.353	4.168	3.258
3.259	2.654	2.783	2.727	2.655	3.549
6.853	6.653	6.963	6.779	6.901	6.919
2.923	3.198	2.943	3.193	3.332	2.893
3.103	2.260	2.988	2.907	2.337	2.778
2.548	2.494	2.123	2.898	2.377	2.664
3.968	4.167	4.304	4.587	3.937	4.209
2.598	7.016	2.790	1.403	2.325	1.654
7.745	8.016	7.520	7.582	7.492	6.830

5.129	5.598	5.319	5.799	4.754	4.283
3.235	2.717	2.853	3.563	2.745	3.158
3.695	3.616	3.186	3.643	3.318	3.399
6.491	6.375	6.171	6.272	6.387	6.106
4.815	4.340	5.091	4.823	5.135	4.829
2.453	2.068	2.817	1.780	2.093	3.025
3.065	3.084	2.352	2.902	2.773	2.232
2.782	2.094	1.661	2.438	0.954	1.759
3.067	3.555	3.694	2.680	3.370	2.758
5.728	5.809	6.351	5.544	6.441	6.056
3.803	3.201	4.351	3.529	3.790	3.729
3.987	4.413	4.193	3.578	3.805	3.695
7.224	7.452	7.352	7.266	7.387	7.629
5.974	6.096	5.953	6.081	6.113	6.324
5.968	6.001	5.951	6.051	5.929	6.392
7.080	7.137	7.101	7.310	7.028	7.429
6.691	6.657	6.587	6.656	6.541	7.163
5.299	5.289	5.148	5.745	5.192	5.311
4.825	4.817	4.719	5.004	4.845	5.262
2.981	2.878	2.831	3.123	2.382	2.877
6.497	6.249	6.327	6.298	6.284	6.753
5.414	5.584	5.352	5.636	5.095	5.407
7.299	7.105	7.122	7.236	6.956	7.543
7.926	7.920	7.743	7.812	7.844	8.083
6.023	5.533	5.944	6.217	6.008	6.129
3.747	3.871	3.560	3.768	2.502	4.181
5.702	5.439	5.115	6.007	5.597	5.789
2.640	3.002	2.229	2.960	2.726	3.183
3.568	2.298	2.968	3.574	3.398	3.720
5.452	5.063	5.352	5.576	5.027	5.648
2.888	2.757	3.063	3.554	3.416	2.348
6.602	6.251	6.793	7.157	6.540	6.914
4.519	4.208	4.695	4.458	4.722	4.774
5.849	5.945	5.897	6.004	6.027	5.866
6.080	6.359	6.119	6.094	5.975	6.387
4.816	4.569	4.810	4.634	4.130	4.734
3.131	2.294	2.762	3.683	2.389	3.425
4.587	6.187	5.279	4.972	6.150	4.393
6.018	5.975	6.023	6.366	6.238	6.657
4.859	5.811	5.035	5.700	5.367	4.795
5.981	6.689	6.214	6.593	6.516	5.993
5.007	5.872	5.090	5.684	5.503	5.020
5.077	5.890	5.172	5.746	5.363	5.126
5.060	5.792	5.001	5.797	5.338	4.848
6.021	6.156	6.157	6.563	6.356	5.519
5.433	5.171	5.885	5.070	5.531	5.536
3.202	3.228	3.684	3.269	3.155	3.555
4.069	3.786	3.860	4.106	3.756	3.440
3.135	4.130	3.928	3.056	3.729	3.101
7.383	7.090	7.084	6.645	7.862	6.888

6.300	5.886	6.156	6.084	6.078	6.588
6.745	6.800	6.516	6.647	6.728	6.854
5.896	4.320	5.636	5.707	5.404	6.947
5.001	3.804	5.164	3.807	4.754	5.011
5.130	4.201	5.184	5.476	5.282	5.353
3.847	3.692	4.406	3.555	3.935	3.572
5.593	5.183	5.537	5.641	5.785	5.803
6.126	6.221	6.369	6.632	6.027	6.058
7.331	6.843	7.352	7.427	7.395	7.962
5.909	5.596	6.139	6.190	5.689	6.139
3.976	4.259	3.719	3.948	3.525	3.525
7.352	6.868	7.112	7.449	6.886	7.659
2.868	2.461	3.310	2.865	3.284	2.396
4.622	4.083	4.762	4.715	4.280	4.578
2.769	3.371	2.518	3.365	1.983	2.289
5.110	4.540	5.331	5.435	5.160	4.946
5.473	5.523	5.758	6.078	5.923	5.870
3.979	5.286	3.750	5.627	4.187	3.629
3.656	4.239	3.559	3.083	3.711	3.580
4.202	4.159	4.245	4.721	3.780	4.340
4.129	4.685	4.501	4.353	4.765	3.400
4.815	5.372	5.107	5.242	5.166	4.756
3.584	3.426	3.239	3.924	3.399	3.815
5.605	5.400	5.644	5.653	5.234	5.675
4.804	4.940	4.623	5.179	5.167	5.482
5.162	4.636	5.238	5.061	4.755	4.575
3.164	3.418	2.765	2.705	2.521	2.301
3.685	3.256	3.776	3.755	3.270	3.692
5.602	6.532	5.322	5.184	5.632	5.406
5.492	5.737	5.163	5.680	5.429	5.363
8.219	7.674	8.418	8.420	8.176	8.695
7.834	8.070	8.040	7.871	7.806	7.815
2.773	2.734	2.138	2.781	2.062	1.773
2.514	4.080	1.388	1.544	1.425	1.280
4.740	4.535	4.407	4.858	4.274	5.225
6.613	6.598	6.628	6.778	6.683	6.315
3.163	2.971	2.588	3.353	2.819	2.567
5.760	5.722	5.894	5.743	5.247	5.544
4.626	4.591	5.077	5.415	4.696	5.425
7.104	6.955	7.446	7.025	7.239	7.420
2.106	2.240	2.404	2.146	1.803	2.165
5.068	4.071	4.276	4.925	4.276	5.242
2.929	2.723	3.020	2.726	2.547	2.823
5.355	5.202	5.346	5.671	5.445	5.647
4.346	4.413	4.660	4.160	4.502	4.596
3.699	3.579	4.054	3.348	3.308	3.899
3.005	3.274	3.062	3.141	2.573	4.064
5.541	5.655	5.644	5.895	5.510	5.798
4.075	3.480	4.288	4.412	3.954	4.465
2.315	2.389	2.349	2.360	2.237	3.011

8.542	8.722	8.407	8.483	8.421	8.481
5.119	4.561	4.658	4.827	4.855	4.990
5.135	4.604	5.689	5.896	5.884	4.688
2.588	2.315	2.687	2.306	2.411	3.516
4.939	3.872	6.116	4.524	4.959	4.825
2.623	3.543	3.202	3.183	1.847	3.158
2.778	4.555	3.481	1.741	4.107	2.275
1.735	1.573	2.229	2.093	1.877	3.311
3.636	3.600	3.888	3.436	3.106	4.020
3.235	2.984	2.396	3.339	2.557	3.452
4.739	6.292	4.219	4.691	5.007	4.761
5.170	4.136	4.170	4.544	5.014	5.387
2.736	5.738	3.399	2.726	2.219	2.250
2.211	2.161	1.525	2.378	2.138	2.628
2.162	1.895	2.103	1.997	1.506	1.748
4.983	5.635	5.152	5.390	5.426	4.658
6.297	6.011	6.098	6.252	6.250	6.892
2.864	2.610	2.327	3.055	2.415	3.023
5.952	6.429	6.335	6.784	6.613	5.933
3.407	3.960	3.214	3.660	3.785	2.522
2.698	2.797	3.211	2.664	2.576	2.829
5.225	5.331	5.226	5.672	4.771	5.431
5.012	5.944	4.927	5.515	5.490	4.645
4.570	4.091	4.584	3.967	4.539	4.725
4.300	4.878	4.379	4.646	4.370	4.529
6.790	6.297	6.807	6.473	6.447	6.866
2.487	2.312	2.546	2.858	2.502	2.494
3.933	5.360	3.395	4.294	4.298	2.940
3.777	3.365	3.545	3.628	3.668	4.021
3.338	2.926	3.159	2.994	3.620	3.620
3.601	3.152	3.367	3.578	3.547	3.959
3.824	3.294	3.529	3.849	3.420	4.324
4.025	3.581	3.576	4.350	3.093	4.210
4.634	5.234	4.830	4.216	5.054	4.369
4.618	5.386	5.619	5.151	5.921	5.122
2.285	2.506	2.169	2.068	2.142	2.275
2.646	4.050	3.601	3.750	3.331	3.564
2.399	2.221	2.834	2.900	3.017	2.117
2.718	2.430	1.740	3.015	2.075	2.171
6.707	6.297	7.181	6.642	6.793	6.988
4.681	4.803	4.994	5.002	4.829	4.393
3.982	3.560	3.672	4.008	3.879	4.453
7.368	6.570	7.445	7.279	7.162	8.086
3.135	4.091	2.940	3.466	2.946	3.457
5.136	5.322	5.309	5.247	5.538	5.291
5.327	6.092	5.079	5.449	5.519	5.183
5.483	6.607	5.652	6.387	6.200	5.560
5.490	6.428	5.642	6.391	6.203	5.474
3.606	4.378	4.099	4.279	3.918	3.769
3.065	3.887	3.556	3.903	3.596	3.456

2.726	3.558	3.144	3.626	3.274	2.930
3.548	4.390	4.020	4.505	4.203	4.003
3.698	4.534	4.210	4.468	4.281	4.039
3.467	4.349	3.970	4.178	4.220	3.883
6.838	6.541	6.791	7.153	6.668	6.947
3.414	3.894	3.865	3.938	4.347	2.931
4.729	4.995	5.023	5.350	5.325	5.158
4.849	3.755	4.939	4.109	5.060	6.224
5.505	6.136	5.153	5.508	4.867	5.263
5.066	5.125	5.575	5.260	4.778	4.285
3.982	4.955	4.934	5.315	4.497	4.148
3.676	4.909	3.652	4.897	4.654	3.619
7.557	8.363	7.569	7.894	7.950	7.517
5.139	4.434	4.980	4.186	4.856	5.125
3.781	3.346	3.661	2.716	2.896	3.199
4.826	4.393	5.405	5.562	6.025	5.343
2.845	2.947	3.409	3.089	3.061	3.172
3.094	2.874	3.765	3.473	3.916	3.910
3.095	2.748	2.395	3.383	2.640	2.737
6.337	6.502	6.210	6.821	6.404	6.702
3.392	3.786	2.274	2.133	2.603	1.318
8.638	8.659	8.671	8.488	8.526	8.441
2.634	4.073	2.149	2.407	2.869	2.834
2.467	3.218	1.838	3.153	1.861	2.883
3.381	3.780	3.063	3.539	3.471	3.721
5.271	5.014	5.507	5.672	5.738	5.605
3.470	4.241	3.321	3.606	3.277	3.317
6.003	6.434	6.344	6.304	6.402	6.037
4.049	5.420	4.089	5.311	4.747	3.284
4.180	5.155	4.213	4.300	4.343	3.684
4.063	4.064	3.873	4.468	4.474	4.616
1.809	1.703	1.259	2.016	1.667	1.847
4.899	4.910	5.247	5.132	5.346	4.583
6.181	6.340	6.682	6.213	5.551	5.920
5.706	5.834	5.305	5.413	5.286	5.060
4.447	4.694	4.846	4.834	4.876	4.864
7.985	7.755	8.015	7.791	7.712	8.319
5.379	6.417	5.139	5.973	6.260	6.105
3.427	4.326	3.751	4.060	4.770	3.132
4.759	5.637	5.652	5.375	4.842	4.904
2.502	2.724	2.721	3.202	2.486	1.983
3.160	3.326	3.053	3.945	3.306	3.851
5.121	4.959	5.340	4.837	4.820	5.295
4.722	3.365	4.441	4.375	3.810	5.546
4.880	5.929	4.716	5.626	5.618	5.202
6.153	7.562	6.800	7.289	6.894	5.770
3.570	3.997	3.522	3.424	3.997	3.627
7.263	6.894	7.582	7.338	7.291	7.696
4.463	4.072	3.992	3.907	3.978	4.339
1.413	1.860	1.406	1.692	1.661	2.149

3.652	4.672	3.943	3.795	2.480	3.029
5.973	6.513	6.030	6.359	6.265	6.277
6.127	6.000	6.483	5.950	6.175	6.466
7.018	6.669	6.923	7.027	6.873	7.063
5.010	4.193	4.581	4.971	4.189	5.289
4.889	4.119	4.482	4.591	4.104	5.624
3.541	4.011	3.485	4.128	3.493	3.947
2.571	2.888	2.669	2.215	2.378	3.158
4.407	3.697	4.373	4.386	3.678	5.429
6.365	6.603	5.700	6.281	6.125	6.009
7.571	7.909	7.816	7.827	7.998	7.589
3.026	3.339	3.325	3.024	2.818	2.714
5.382	5.563	5.945	5.685	5.653	5.595
4.881	3.938	5.190	4.967	4.754	4.836
3.941	4.290	4.570	3.290	4.674	3.633
3.927	4.153	3.935	4.012	4.020	4.324
4.770	5.192	5.588	5.193	5.303	5.290
3.317	1.703	1.962	3.375	2.926	3.379
5.019	5.750	5.429	5.907	5.808	5.192
3.923	4.733	4.377	4.612	4.333	4.237
4.347	3.905	4.816	4.152	4.353	5.720
1.972	1.755	2.200	1.832	1.901	2.704
8.408	7.431	8.342	8.105	7.776	8.896
7.763	6.730	7.796	7.319	6.920	8.111
4.506	5.668	4.501	5.084	4.536	4.593
6.275	6.793	6.569	6.726	6.462	6.413
4.293	4.664	4.944	4.524	5.170	4.221
8.150	8.079	8.431	8.420	8.195	8.120
4.559	4.975	4.897	4.860	4.463	4.627
3.829	5.641	3.876	4.854	4.648	3.799
3.796	3.811	3.861	4.004	4.171	3.655
4.948	4.672	4.893	4.593	4.455	4.840
4.965	6.655	5.312	6.334	6.412	5.168
5.633	4.815	6.125	5.806	5.855	5.932
4.241	4.408	4.665	4.225	4.629	4.173
2.680	3.538	3.038	1.825	1.854	3.101
8.234	7.834	8.473	8.167	7.681	8.589
9.307	8.867	9.647	9.238	9.153	9.327
3.450	3.377	3.367	3.553	3.015	3.831
5.413	5.562	6.034	5.796	5.979	5.404
3.610	3.857	3.847	4.568	3.840	3.601
5.319	6.018	5.667	5.891	5.761	5.757
5.723	5.927	5.686	5.983	5.479	5.190
5.412	5.372	5.349	5.379	5.667	5.312
6.599	6.508	6.418	6.424	6.315	6.650
3.726	3.422	4.343	4.228	4.067	4.157
2.067	3.380	2.814	2.650	2.177	2.174
6.655	6.791	7.165	7.198	7.325	7.330
7.329	6.682	6.823	6.790	6.786	7.501
4.027	3.431	3.913	3.961	3.455	4.088

4.809	5.319	5.038	4.573	4.950	4.118
4.007	3.388	4.193	4.602	4.010	4.300
4.855	5.935	5.537	4.873	5.705	4.412
4.274	4.836	4.796	4.852	4.465	4.478
4.004	3.654	4.510	3.967	3.661	4.324
4.554	5.342	5.175	4.771	5.790	4.019
3.731	4.256	3.707	4.783	3.928	5.297
6.144	6.525	6.638	7.265	7.257	6.231
3.558	3.840	3.390	4.344	4.578	3.987
4.384	4.457	4.106	4.096	4.569	4.110
2.159	2.372	1.823	2.379	1.798	2.038
3.572	4.365	4.123	3.757	5.297	3.800
5.147	5.218	5.034	5.677	5.529	5.494
5.270	4.219	4.404	5.067	4.275	5.351
4.160	3.504	2.670	4.849	2.502	4.924
3.971	2.921	3.522	3.883	2.888	4.848
5.897	5.787	4.727	5.877	5.399	5.473
6.980	7.002	7.328	7.150	6.967	6.971
3.736	4.508	4.074	5.066	4.368	3.659
2.654	2.340	2.730	2.825	2.606	3.208
4.544	4.785	4.168	4.856	4.488	4.610
2.461	5.418	2.346	2.769	3.926	2.424
6.168	5.964	6.550	6.639	6.007	6.428
8.261	8.225	8.344	8.788	8.305	8.877
7.735	6.765	7.397	8.046	7.433	8.110
4.129	4.108	4.616	5.899	4.980	5.284
4.040	3.147	3.084	4.437	3.148	4.652
4.143	4.391	3.757	3.366	3.934	4.263
3.689	3.779	3.478	4.059	3.613	4.143
7.207	7.334	7.282	6.607	7.475	7.369
4.228	4.286	4.726	3.979	4.336	4.234
4.235	4.619	4.532	4.455	4.661	4.520
4.867	4.622	4.701	5.673	5.318	5.603
3.973	3.742	3.478	4.674	4.120	3.966
3.966	4.008	3.710	4.370	4.262	4.109
2.914	2.218	2.468	1.885	2.274	2.311
4.686	4.486	4.531	4.485	4.272	4.487
3.348	3.051	3.200	3.227	3.229	3.531
6.286	6.466	6.217	6.402	6.064	6.322
7.939	7.944	8.237	8.369	8.474	8.310
4.083	3.181	3.590	3.953	3.370	4.487
6.018	6.098	6.108	5.574	5.933	6.046
5.216	6.470	5.011	5.613	5.684	5.972
4.759	4.955	5.140	4.096	5.258	4.825
5.355	5.889	5.614	5.545	5.476	5.480
7.028	6.688	8.416	7.443	7.535	6.725
5.472	5.397	5.448	5.864	5.524	5.816
4.044	4.389	4.917	4.637	4.417	3.633
4.710	4.608	4.165	4.659	4.511	5.441
4.537	4.233	4.247	4.785	3.620	4.893

7.157	7.118	7.349	7.235	7.069	7.133
4.588	5.400	4.850	5.093	4.240	5.026
6.162	6.090	6.189	6.321	6.311	6.786
5.417	5.101	5.548	5.567	4.824	6.076
1.566	1.587	1.348	2.129	1.576	2.512
3.378	3.157	2.683	3.167	2.366	3.637
5.853	5.950	5.865	6.162	6.006	6.018
3.848	4.276	3.827	4.028	4.134	4.028
3.334	4.181	3.114	3.341	3.708	3.366
3.336	3.491	3.180	3.696	3.059	3.345
5.752	5.727	5.183	5.422	5.809	5.356
4.984	5.798	5.036	5.449	5.709	5.788
6.513	6.347	6.566	6.696	6.365	6.678
4.556	4.599	5.485	4.741	5.457	4.579
4.092	3.726	4.043	4.325	3.726	3.204
5.079	5.555	5.471	5.299	5.348	4.618
5.450	5.780	5.546	5.286	5.991	4.524
8.171	8.151	7.964	7.928	7.752	7.231
5.973	5.963	5.676	5.715	6.031	5.335
6.569	6.584	6.402	6.315	6.221	5.478
6.369	6.524	6.778	6.105	6.555	5.713
8.540	8.703	8.417	7.976	8.527	7.457
6.637	6.871	6.830	6.456	6.679	6.209
6.288	6.163	6.532	5.963	6.053	5.811
7.041	7.078	6.596	6.392	6.590	5.509
3.705	3.928	4.860	4.804	4.001	4.140
4.523	4.730	4.940	4.764	5.049	4.350
7.081	7.270	7.158	6.982	7.097	6.270
3.126	3.201	3.829	3.181	3.225	3.055
6.520	6.730	6.596	6.525	6.302	6.581
4.251	5.132	3.987	5.055	4.510	4.144
7.346	7.441	8.405	7.403	8.298	7.174
4.160	3.810	4.623	4.358	3.600	4.473
5.071	5.323	5.224	6.081	5.650	5.714
4.207	4.173	4.191	4.286	4.950	4.368
6.672	6.240	6.997	6.791	6.527	6.964
2.438	2.405	2.978	2.308	2.326	2.261
3.755	3.492	3.628	3.590	4.156	3.501
2.153	2.612	2.136	2.527	1.951	2.298
6.011	5.937	6.600	5.801	6.139	6.080
3.564	5.449	3.641	4.498	4.307	2.737
3.133	3.604	3.274	3.346	2.750	3.561
3.155	3.995	3.278	3.286	2.862	2.471
5.785	5.782	5.450	5.823	5.112	6.098
2.982	2.800	3.193	2.694	2.407	2.987
5.293	5.342	5.238	5.927	4.778	5.709
3.044	3.977	3.429	4.207	3.534	3.060
10.057	9.972	10.192	10.098	9.826	9.833
4.700	5.348	5.377	4.820	5.404	4.738
6.676	6.690	6.854	7.106	6.868	6.517

6.189	5.877	5.908	6.238	6.056	6.383
3.958	2.801	4.041	3.175	3.861	3.816
6.402	6.210	6.575	6.407	6.405	6.628
5.764	6.200	5.915	6.118	6.331	6.242
5.591	5.548	5.887	6.283	6.096	5.874
5.864	6.155	5.939	5.746	6.117	5.860
4.318	3.940	4.251	4.478	3.303	5.506
4.623	4.190	4.491	4.233	4.311	5.048
3.320	3.319	3.321	3.556	3.221	4.218
4.695	4.481	5.138	4.780	4.445	4.607
2.011	1.700	1.511	1.529	1.364	2.199
5.385	5.512	4.688	5.856	4.880	5.842
4.357	4.206	3.832	3.167	4.294	3.889
2.966	4.124	3.454	3.789	4.180	3.239
4.353	4.224	4.956	4.561	4.790	4.262
2.788	3.484	3.485	3.127	2.825	1.872
5.245	5.083	4.735	4.879	4.780	5.271
4.083	4.357	3.666	4.152	3.475	4.367
3.683	3.595	4.567	4.537	3.455	4.277
3.962	4.910	4.242	4.781	4.502	3.492
7.122	6.473	6.912	7.447	6.723	7.269
4.610	4.751	4.646	5.058	4.584	4.667
4.341	4.355	4.737	4.614	4.380	4.029
3.717	4.259	3.300	3.707	3.456	2.711
2.667	2.009	2.228	2.053	2.136	2.306
3.117	3.551	3.281	4.155	4.241	1.519
2.032	2.055	2.136	1.974	1.770	2.054
4.850	6.152	5.147	6.297	6.271	5.604
6.415	6.293	6.577	6.332	6.382	7.034
4.209	4.407	4.345	4.519	3.950	3.664
5.447	6.190	5.838	6.282	6.301	5.840
3.278	2.685	2.474	2.876	2.125	3.374
5.759	5.113	5.441	5.740	5.225	5.742
7.718	7.531	8.021	7.822	7.963	7.617
4.077	4.050	4.855	3.895	4.225	3.830
7.450	7.338	7.597	7.654	7.506	7.722
6.174	6.515	7.299	6.222	6.518	5.631
5.963	6.362	6.088	6.498	6.537	5.826
7.471	7.926	7.822	7.770	7.606	7.543
4.950	4.273	4.297	4.760	4.403	5.386
5.145	5.629	4.898	5.080	4.545	5.705
4.059	4.717	4.088	4.213	4.788	4.332
3.980	4.043	4.085	4.460	4.415	4.629
6.834	6.095	6.694	6.534	6.657	6.919
3.644	3.150	3.463	3.221	3.477	3.792
4.488	2.928	3.983	5.387	4.301	4.601
7.293	7.393	7.420	7.756	7.170	7.411
4.726	4.842	4.049	4.673	4.538	4.913
4.136	4.095	3.928	4.540	4.725	4.902
5.229	5.267	5.294	5.227	5.420	5.235

3.459	3.035	3.405	3.719	3.130	3.671
5.145	5.163	4.702	5.091	5.432	5.115
3.370	2.377	3.806	4.016	3.572	4.629
5.178	5.915	5.578	5.533	5.607	5.634
3.163	3.140	3.588	3.614	4.756	3.213
8.082	7.754	8.863	8.233	8.419	8.397
3.956	3.599	3.400	3.782	3.609	3.511
5.668	5.588	5.228	5.019	5.739	4.900
7.269	6.866	7.324	7.711	7.194	6.989
4.120	4.366	4.028	4.009	4.175	4.543
7.452	7.104	8.124	7.168	7.515	7.237
4.365	3.829	4.356	3.765	5.084	3.325
4.816	4.785	4.382	5.376	4.288	4.723
2.883	2.637	3.361	2.767	3.370	3.072
6.750	6.574	6.827	6.821	6.906	6.873
7.075	6.818	7.188	7.141	7.204	7.214
8.066	7.808	8.145	8.130	8.238	8.220
2.116	2.724	1.899	1.601	1.961	1.648
8.834	9.283	9.032	8.649	9.062	7.852
4.123	5.188	4.608	5.197	5.080	3.830
5.444	5.741	5.472	5.557	5.622	5.287
3.423	3.812	3.343	3.285	3.420	2.830
4.913	6.220	6.304	6.455	6.071	5.456
3.930	3.738	3.766	3.713	3.959	4.288
5.100	6.744	4.705	6.119	5.320	5.499
7.281	8.264	8.320	6.765	8.257	6.126
2.672	3.063	2.806	2.870	3.561	3.090
2.707	1.760	1.742	1.547	1.881	1.873
3.478	2.576	3.125	3.705	2.891	3.256
4.696	3.008	4.274	4.163	3.434	3.918
4.462	3.051	4.400	4.293	3.202	5.266
5.172	3.355	4.286	4.978	3.987	5.509
3.768	2.798	4.490	3.831	3.891	4.087
5.281	4.974	5.636	5.122	5.223	5.452
6.158	6.401	6.126	6.327	6.168	6.435
3.631	4.133	3.318	4.040	3.516	3.093
5.750	7.262	5.596	6.949	6.467	6.000
2.755	1.762	3.104	2.637	2.875	3.120
4.632	4.322	3.857	4.816	4.128	5.234
3.968	3.596	3.907	3.153	4.015	3.909
2.044	2.100	1.665	1.995	2.126	2.086
9.847	9.705	9.894	9.723	9.608	9.507
4.982	5.050	4.182	4.742	4.835	4.840
3.278	3.351	2.973	3.413	2.915	3.711
2.884	3.990	2.430	3.740	3.344	2.823
4.350	5.549	4.289	4.427	4.189	4.368
7.001	6.863	7.543	7.155	7.492	7.277
5.099	4.882	5.327	5.316	5.140	5.428
5.691	5.111	4.754	6.764	5.921	5.965
2.585	2.157	2.137	1.802	2.137	2.137

2.274	2.467	1.714	2.003	1.862	2.488
3.542	3.556	3.630	3.754	3.308	3.622
5.125	5.119	5.109	4.552	5.435	4.880
2.588	2.138	3.320	2.999	3.229	2.339
4.459	3.343	3.716	4.255	3.201	5.082
4.792	3.969	4.162	4.272	3.533	5.030
4.205	3.883	4.080	4.171	3.893	5.296
4.914	4.470	4.561	5.168	4.580	5.622
2.736	2.733	2.623	2.668	2.342	3.045
2.652	2.649	2.063	2.243	2.866	2.599
3.254	4.397	2.829	3.508	4.358	1.942
4.384	5.002	4.172	4.827	4.620	4.292
5.617	5.968	6.525	5.953	6.333	5.426
3.057	2.404	2.942	2.378	2.272	3.260
4.350	3.853	4.199	4.813	4.589	4.645
2.340	2.881	2.210	2.361	3.830	2.469
6.430	6.675	6.527	6.761	6.986	6.402
6.983	6.857	7.173	7.489	6.960	7.141
3.144	2.682	3.043	3.463	3.149	2.746
4.988	5.208	5.391	5.436	5.247	5.380
4.743	4.601	4.605	5.117	5.015	4.842
5.912	5.891	6.469	5.813	6.613	6.314
11.429	11.284	11.282	11.096	11.116	11.247
5.517	4.567	5.223	4.938	4.772	5.309
11.552	11.269	11.336	10.910	11.185	11.354
3.521	2.669	3.849	2.976	3.936	3.938
1.333	0.996	1.593	1.131	1.596	1.017
6.533	6.275	6.521	5.753	6.391	5.463
2.113	2.227	2.195	1.541	2.462	2.139
2.936	2.131	3.647	2.001	2.413	2.865
3.322	2.664	3.254	3.978	2.648	2.782
3.025	2.965	2.909	2.868	3.650	2.566
3.243	2.703	2.476	2.923	3.445	2.923
6.053	5.697	5.906	5.819	5.830	5.290
2.884	2.188	2.759	2.165	1.869	1.807
3.562	3.810	1.280	3.513	3.446	3.853
4.256	4.395	3.673	3.713	4.210	3.053
2.691	1.998	2.000	2.544	2.437	2.791
2.876	2.556	2.130	3.005	3.725	3.076
4.740	4.767	5.478	5.436	5.224	4.727
2.853	2.139	2.448	2.815	2.019	2.279
6.503	5.911	6.257	5.945	6.047	6.077
4.501	3.396	5.944	4.837	5.228	5.397
2.987	2.764	3.471	2.445	4.135	2.602
2.595	2.845	2.379	3.651	2.624	2.701
4.577	4.360	4.598	4.423	4.658	4.415
2.262	2.586	2.640	3.031	2.045	2.367
3.945	3.750	3.776	4.474	3.652	4.204
2.820	2.212	3.179	2.978	1.818	1.632
3.791	3.381	4.383	3.312	4.056	2.358

2.976	3.162	2.976	2.343	3.419	3.326
3.660	3.514	3.838	3.233	3.950	4.025
2.412	3.162	3.577	2.725	2.354	4.391
3.932	3.872	3.872	5.558	4.165	4.872
3.399	3.627	3.571	3.035	3.084	2.041
6.311	6.246	6.363	6.066	6.138	6.240
3.073	2.327	3.035	2.262	2.272	2.127
3.129	3.265	3.654	2.629	3.388	2.395
6.802	6.817	6.444	5.848	6.299	6.313
3.525	2.257	4.408	3.119	3.196	4.514
3.684	3.710	4.109	2.993	4.483	4.238
3.649	3.140	3.967	3.046	2.789	2.865
3.747	3.301	3.737	3.537	3.272	3.768
2.294	2.383	1.960	3.155	1.337	2.724
2.079	1.121	2.588	1.663	1.688	1.939
2.800	2.976	3.099	4.002	2.885	2.576
5.677	5.429	6.363	5.728	5.463	5.106
2.686	1.765	2.315	2.656	2.480	3.405
2.221	1.887	1.344	1.727	1.718	2.545
4.743	4.715	5.119	3.867	4.764	4.310
9.823	9.375	9.783	9.296	9.400	9.555
10.425	10.061	10.475	9.903	10.072	10.042
2.500	2.728	2.017	1.978	3.152	2.090
2.827	2.475	3.088	2.974	2.718	2.930
2.886	1.903	1.467	3.287	3.215	3.103
4.489	3.958	4.923	4.056	4.738	4.321
3.344	3.279	2.903	3.075	2.831	2.648
2.484	3.300	2.299	2.846	1.793	2.603
2.741	1.732	3.185	2.183	2.080	2.499
3.198	3.761	2.442	2.351	3.797	2.966
2.401	2.757	2.585	1.874	1.466	3.052
4.009	3.744	4.378	3.489	3.695	4.697
2.891	3.042	2.723	2.679	2.984	2.756
4.121	2.995	3.498	3.418	3.854	3.805
2.462	2.280	1.786	1.701	4.083	1.912
1.845	2.599	2.546	1.232	2.241	1.160
4.014	4.188	4.419	3.896	4.286	4.147
2.480	3.286	3.102	2.043	2.053	2.804
2.111	2.635	1.366	2.670	1.333	1.340
2.187	3.159	2.997	2.365	2.808	3.860
3.628	3.649	3.108	3.126	2.652	3.695
3.922	3.426	3.042	3.946	3.168	3.097
2.552	2.553	1.854	1.429	2.401	3.308
3.133	3.196	3.570	3.265	3.585	2.405
2.060	2.175	2.196	1.803	2.464	2.316
3.195	3.445	3.700	2.767	3.656	2.440
3.849	3.836	4.646	3.877	4.229	3.524
5.095	4.887	5.192	4.608	4.539	4.828
5.046	5.135	4.907	4.645	4.540	5.000
3.092	2.541	2.895	3.118	2.630	3.428

2.710	3.026	3.510	2.506	2.675	2.663
3.189	3.215	2.022	3.691	3.261	3.615
3.334	3.248	3.498	3.716	2.309	2.889
2.680	2.800	2.107	2.268	2.341	3.554
3.360	2.431	3.797	3.041	3.958	2.887
4.821	5.360	5.100	5.065	4.987	5.228
3.683	3.744	3.963	3.913	4.438	3.705
3.466	3.511	4.544	3.535	4.342	4.709
3.236	3.171	3.192	2.445	2.704	2.666
2.823	3.380	3.546	2.255	2.652	3.140
3.044	3.277	2.939	2.546	3.093	1.986
4.792	4.864	4.127	4.450	3.619	4.709
3.475	3.861	3.252	3.312	3.265	3.890
2.684	3.276	2.260	3.123	3.031	2.585
2.928	2.024	3.351	3.295	2.889	3.183
3.357	2.925	3.795	2.827	2.335	2.997
1.757	1.541	1.222	1.434	1.482	1.818
2.167	2.336	3.073	1.918	2.258	2.258
3.654	3.266	3.581	2.858	3.344	3.090
5.118	4.258	4.392	4.892	4.623	5.140
3.064	1.636	3.072	4.028	1.874	2.494
4.141	3.537	3.974	3.705	3.439	4.677
2.403	1.924	3.192	3.174	2.333	3.170
2.522	2.469	1.869	1.993	1.919	2.479
2.556	3.801	1.580	3.211	2.945	2.336
6.742	6.260	7.100	6.268	5.999	6.056
3.506	3.708	2.730	4.216	3.884	3.321
5.257	5.205	5.186	4.737	4.449	5.059
5.707	5.350	5.609	5.654	5.506	5.617
6.343	7.982	5.302	8.278	7.957	5.520
3.724	4.496	2.287	6.094	5.664	2.621
6.311	6.995	6.919	6.853	8.038	5.539
3.799	4.184	4.495	4.471	4.958	3.610
5.581	5.820	5.937	6.512	6.719	5.402
4.458	4.323	5.827	3.986	4.814	4.613
8.815	8.338	8.809	8.596	8.618	8.166
3.457	3.784	3.611	3.402	4.283	3.592
7.914	7.872	8.029	8.076	8.104	8.235
5.797	6.002	6.266	6.107	6.238	6.011
3.522	3.966	3.653	3.616	2.940	3.959
4.386	5.010	6.151	4.132	5.297	4.419
4.455	4.448	4.237	4.406	4.653	4.272
5.047	5.274	4.379	4.920	5.238	4.859
2.757	2.669	2.863	3.014	2.311	3.055
8.367	8.291	8.701	8.439	8.310	8.339
3.854	4.122	3.801	4.270	3.713	4.333
2.804	2.094	2.659	2.841	3.346	3.204
1.787	1.441	1.414	2.706	1.656	1.519
5.449	6.592	6.287	5.537	6.604	4.699
5.509	5.952	5.762	5.697	5.918	5.719

5.204	5.855	6.100	5.932	5.688	4.827
4.269	4.934	5.421	4.619	5.270	4.417
6.979	6.085	6.602	7.003	6.184	7.075
3.231	3.057	2.979	3.108	2.590	4.512
3.354	2.341	2.307	2.760	2.807	3.326
4.026	3.915	4.304	4.461	3.788	5.096
2.410	3.234	3.145	2.828	2.417	3.402
5.723	6.035	6.181	6.321	6.267	6.482
4.648	4.741	4.831	4.890	5.113	4.810
7.078	7.350	7.313	7.141	7.362	7.083
3.230	2.919	3.496	3.252	3.422	4.215
5.223	4.817	5.099	4.974	4.847	5.290
5.117	5.081	6.338	6.202	6.134	5.900
5.797	5.180	6.774	6.022	6.352	5.626
5.716	5.344	5.829	5.597	5.739	5.759
3.079	2.880	2.960	3.253	3.968	2.962
6.084	6.090	6.290	6.243	5.902	6.160
2.853	2.865	3.202	2.586	3.133	3.499
3.280	3.327	3.438	2.856	2.873	3.530
6.206	6.422	6.194	6.356	6.473	6.333
4.358	4.193	3.966	4.430	4.121	4.533
5.344	5.049	5.217	5.743	4.885	5.918
5.026	5.177	5.416	5.147	5.373	5.443
6.488	7.220	6.587	6.851	6.353	6.376
5.287	5.311	5.404	5.490	5.518	5.504
4.038	4.284	3.954	4.736	4.145	4.312
6.894	5.942	6.901	6.450	6.538	6.942
4.677	5.209	4.728	4.820	4.779	4.501
3.716	4.855	3.957	3.371	5.053	3.603
3.745	3.391	4.567	3.436	2.082	4.334
10.507	10.514	10.136	8.513	10.425	10.584
3.548	3.218	3.903	3.262	3.980	2.777
2.144	2.845	4.128	1.991	2.114	4.127
2.633	2.717	2.577	2.849	1.832	2.809
3.001	2.297	2.879	3.032	2.172	2.690
6.505	6.206	6.394	5.907	6.178	5.932
4.247	3.417	4.440	4.361	4.912	4.092
5.288	5.587	4.850	5.021	5.222	4.836
3.415	2.371	2.524	2.574	3.245	3.381
5.333	4.630	5.501	4.426	5.380	5.010
4.571	3.261	3.514	3.998	4.228	6.068
2.848	2.868	2.634	3.463	2.831	2.604
4.357	4.162	4.524	4.695	5.018	4.712
4.512	3.386	4.177	3.553	4.159	3.599
5.443	4.477	5.550	5.017	5.193	5.098
7.146	7.207	6.303	6.317	6.493	6.694
2.681	3.029	2.915	2.647	3.092	3.037
2.418	2.763	3.475	2.813	2.853	3.897
7.943	7.779	7.776	7.298	7.394	7.711
3.602	3.808	4.681	2.357	4.121	2.992

5.049	4.293	3.928	4.927	4.488	4.224
3.374	3.489	3.321	3.330	4.048	4.281
2.621	2.910	2.817	2.527	1.923	3.038
2.545	2.188	3.684	1.191	3.311	2.273
3.723	3.575	3.837	3.645	3.363	3.975
2.560	2.599	2.661	2.714	2.383	2.735
5.180	6.166	4.298	5.649	6.199	5.215
3.035	3.358	2.592	2.093	2.343	2.985
2.913	3.612	3.189	3.396	2.399	2.609
9.278	8.886	8.767	8.558	8.699	8.555
5.215	4.944	5.401	4.831	4.954	4.756
4.633	5.019	5.336	4.221	4.739	4.734
4.401	4.014	4.464	3.241	3.993	3.294
2.657	2.758	3.261	2.404	3.301	3.296
2.597	2.229	3.095	1.520	2.516	2.579
3.376	2.957	4.165	3.148	2.669	2.803
4.544	4.818	4.378	5.784	4.613	4.594
3.001	3.400	4.014	2.912	3.439	3.412
3.377	3.248	3.245	1.912	2.541	3.094
4.612	5.227	5.784	5.422	5.128	3.823
5.817	5.652	6.465	5.344	5.533	5.984
2.551	2.659	2.817	1.571	2.256	1.334
2.925	3.329	4.226	4.436	3.549	3.163
10.605	10.596	10.383	10.129	10.092	10.331
6.493	5.577	6.726	5.438	6.391	5.663
2.430	2.680	3.684	2.252	3.746	1.920
3.092	3.936	3.281	3.343	2.571	3.257
3.952	4.602	4.881	5.562	4.006	5.502
3.548	3.908	2.864	5.716	2.471	1.848
7.866	7.469	8.258	8.259	7.926	6.858
5.846	5.774	5.642	5.753	5.039	5.671
2.371	3.890	2.170	2.940	1.913	3.521
1.192	1.613	2.412	1.245	2.683	1.661
5.843	6.073	5.862	5.685	6.235	5.376
2.962	3.459	3.611	2.026	1.196	1.417
3.121	3.936	2.770	2.989	2.906	1.968
7.165	6.582	8.147	6.738	7.944	6.972
5.897	5.367	5.680	4.875	5.269	5.563
5.204	5.107	5.319	5.263	5.069	4.890
2.726	2.682	4.431	2.498	1.718	3.024
2.450	1.558	3.515	2.835	3.573	2.285
3.416	4.673	4.045	3.640	3.282	4.107
3.303	3.056	3.056	3.396	2.900	2.851
4.861	4.272	4.643	3.877	4.907	4.474
2.333	2.526	2.912	1.875	2.874	2.346
3.277	2.733	3.219	3.055	2.748	3.592
2.829	3.280	2.775	3.262	3.603	2.642
3.097	2.710	2.928	3.046	3.870	3.070
4.878	4.514	3.538	4.993	4.209	4.959
2.731	2.930	1.777	1.940	1.976	1.595

3.469	3.224	4.116	1.811	1.922	3.000
2.536	1.548	3.312	2.073	1.779	2.567
4.986	4.779	6.056	5.007	4.937	5.236
1.570	1.119	3.580	1.955	1.480	1.596
1.917	3.291	2.437	2.825	1.724	2.074
4.400	3.159	4.447	3.893	4.370	4.809
4.277	3.691	5.032	3.211	4.024	3.155
7.387	7.099	7.372	6.680	7.612	6.821
4.289	3.069	3.328	3.134	3.396	3.444
4.313	3.790	4.436	4.058	4.442	4.585
2.534	1.626	0.985	2.343	1.486	2.761
2.686	1.781	2.374	1.709	3.002	2.315
3.392	3.060	3.602	3.857	2.269	2.822
3.981	3.136	4.238	3.222	3.732	4.285
2.342	1.433	3.187	3.052	3.229	3.102
3.015	3.838	3.053	3.016	3.115	2.273
2.368	3.290	2.881	1.855	1.770	2.567
4.276	3.668	4.678	3.338	4.274	3.954
4.309	4.559	4.451	4.153	4.480	4.199
3.645	3.727	4.167	3.597	3.831	3.949
4.051	4.016	4.511	4.042	4.119	4.299
3.771	3.718	4.389	3.583	3.958	3.853
2.565	0.535	3.449	0.891	2.276	2.588
3.082	3.425	3.356	2.664	2.409	1.983
3.390	3.157	4.038	3.353	2.528	4.095
3.637	2.539	4.232	3.128	3.659	2.292
2.994	1.726	2.351	3.259	2.855	2.367
3.136	3.535	2.851	2.713	2.503	3.317
3.705	4.204	2.890	3.132	2.909	3.223
3.492	3.107	3.371	3.308	3.490	3.637
2.813	3.713	3.333	3.045	2.963	2.803
3.970	3.520	4.408	3.839	3.278	3.653
3.939	4.775	4.673	4.806	3.318	4.016
2.847	1.641	2.382	3.144	2.612	1.987
3.901	4.558	4.940	4.145	5.135	3.784
2.258	1.784	3.125	2.258	1.662	1.983
2.570	2.226	2.093	0.855	1.311	2.696
2.839	2.489	2.325	1.947	2.366	2.586
2.817	3.349	2.561	2.878	2.709	2.482
3.078	3.024	4.590	4.284	3.332	2.575
3.302	2.878	2.133	3.146	3.498	2.597
2.478	1.909	2.514	1.166	1.369	2.224
3.506	3.696	3.536	3.260	4.302	3.492
4.277	3.659	4.418	2.916	4.532	4.811
2.404	0.955	4.275	3.312	3.133	1.940
3.074	3.050	4.558	2.592	2.742	3.418
3.989	2.648	4.890	2.263	4.259	4.235
2.941	2.298	3.050	3.321	2.364	2.944
2.546	1.722	2.347	3.064	2.716	2.176
4.404	4.367	5.216	4.396	4.509	4.640

6.347	5.738	5.835	5.318	6.171	6.042
2.882	1.521	2.590	2.228	2.522	3.223
2.694	2.566	3.131	2.255	1.732	1.411
4.171	3.471	3.096	3.526	1.938	3.205
3.418	3.008	4.683	3.326	3.547	3.965
4.338	4.020	4.512	3.938	4.454	4.680
2.787	2.569	3.498	3.091	2.748	3.050
3.919	4.063	3.664	3.693	3.784	3.313
2.720	3.156	3.239	2.941	3.672	2.360
6.775	5.484	7.361	4.435	6.733	7.107
1.652	1.674	1.132	1.594	1.776	1.633
2.803	3.078	2.972	2.782	2.987	3.380
3.680	2.749	3.683	3.195	3.653	3.932
3.616	3.783	4.466	3.976	3.321	3.748
4.378	4.948	4.199	4.293	5.196	4.176
3.131	3.550	4.700	3.815	3.039	3.723
5.066	5.660	5.010	5.116	5.303	5.041
2.648	2.547	1.049	2.146	2.787	2.432
5.652	5.130	5.914	5.332	5.295	5.369
1.980	2.224	4.012	1.896	3.906	2.177
3.768	3.796	4.051	4.112	4.552	3.402
3.012	2.742	3.405	2.380	2.903	1.851
5.019	3.608	4.714	4.639	4.874	4.647
1.729	1.487	1.462	2.071	1.891	1.867
3.529	2.416	3.628	3.781	3.380	3.045
4.873	4.389	4.564	4.027	3.296	2.842
2.466	2.101	3.273	2.813	2.693	2.578
3.100	2.156	3.186	2.218	3.631	1.906
6.644	6.283	6.005	5.013	6.113	5.965
3.320	1.702	3.040	2.412	2.955	2.516
2.688	2.315	2.751	2.465	2.135	3.949
3.917	3.905	4.438	3.426	3.309	3.667
4.590	4.278	3.626	4.090	4.060	4.748
2.789	3.742	2.643	2.725	2.606	2.003
2.727	3.941	3.367	2.126	1.809	1.995
3.521	4.586	3.646	4.130	4.144	4.029
2.186	1.987	2.690	2.622	2.382	2.419
3.169	2.237	3.705	2.135	2.751	2.262
1.953	0.355	2.725	1.638	3.403	1.852
3.449	2.817	3.776	3.155	3.644	3.189
2.492	1.890	2.260	1.841	1.765	2.766
2.798	3.588	2.197	3.029	1.615	1.604
2.428	1.980	2.707	2.346	2.842	2.161
6.510	6.096	6.280	5.197	6.024	6.207
2.682	1.992	2.799	2.167	1.752	3.583
4.426	3.441	3.445	4.409	4.355	3.758
4.972	5.441	5.564	3.370	4.910	5.248
2.522	2.546	2.820	2.320	2.839	3.273
4.129	4.123	4.389	3.724	4.039	4.974
2.170	1.793	2.241	3.095	3.173	2.454

6.592	6.802	6.851	6.312	5.813	6.432
3.344	2.060	3.449	1.961	1.501	2.206
5.782	4.417	5.035	5.312	5.185	6.086
2.735	2.223	2.537	2.193	3.561	4.262
4.696	3.732	5.172	4.341	3.988	4.626
3.176	2.729	2.652	3.080	1.490	3.118
4.297	4.248	4.099	3.016	3.914	4.429
4.778	4.740	4.343	4.115	4.794	5.078
4.077	3.668	3.569	3.429	4.599	4.091
2.789	2.368	3.673	2.406	2.921	3.243
1.630	1.635	1.872	0.994	1.232	1.362
2.116	2.091	3.777	1.663	2.417	3.010
2.744	1.748	2.921	1.787	1.755	2.835
4.078	3.453	3.605	2.968	3.680	4.352
2.596	3.448	2.674	2.365	2.851	2.574
5.385	4.764	5.597	5.863	4.519	5.311
4.181	3.507	3.988	4.252	3.591	3.197
3.277	2.547	2.413	3.217	3.250	2.942
3.404	2.537	3.658	3.028	2.028	3.105
4.347	3.630	3.995	3.049	2.879	3.107
2.652	1.400	1.560	1.832	2.494	2.189
3.898	3.849	4.861	3.722	3.549	4.494
2.612	2.828	3.438	3.665	4.142	2.465
1.918	1.173	1.295	0.878	1.783	1.324
5.171	4.244	5.784	4.808	5.199	4.524
1.261	1.228	1.347	1.319	1.103	1.306
2.935	1.808	3.076	2.069	2.076	4.223
4.430	4.624	4.114	3.791	3.561	3.881
3.158	2.232	3.307	2.692	2.918	2.844
2.692	1.951	2.275	2.680	2.683	2.108
2.185	2.342	2.656	2.202	1.295	1.835
7.871	7.443	7.646	6.569	7.364	7.632
4.867	4.696	4.323	3.394	4.236	4.766
2.975	2.838	2.638	2.074	2.604	2.879
3.136	4.329	3.599	4.099	3.520	2.263
3.108	3.389	3.367	2.434	2.729	3.761
7.054	6.605	6.844	5.654	6.431	6.693
3.861	3.944	4.270	3.675	3.905	4.029
3.172	2.849	2.914	2.298	2.476	2.182
2.361	2.799	2.743	2.158	3.327	2.742
2.576	2.528	1.910	1.600	1.678	1.242
1.392	1.312	2.795	2.364	1.278	0.699
2.802	1.261	2.358	2.377	2.105	2.509
3.181	3.597	3.247	3.661	3.090	1.406
2.872	2.486	3.692	2.929	3.766	4.312
3.410	2.098	3.531	2.401	3.500	3.567
2.762	2.009	2.789	2.275	3.271	2.648
4.592	4.637	4.884	4.460	4.507	5.284
2.542	1.545	2.350	2.455	2.233	3.100
2.602	2.752	3.524	2.267	2.829	3.488

2.090	2.665	3.275	2.528	1.946	2.458
2.219	3.295	2.571	2.288	2.990	3.167
3.545	4.067	2.797	4.479	3.293	3.260
6.739	7.098	6.334	6.952	6.055	5.493
6.216	6.674	5.888	6.471	5.811	5.087
2.900	2.514	3.064	2.978	3.199	2.627
2.789	3.968	2.743	2.395	2.277	2.804
2.756	2.402	1.971	1.995	3.128	2.956
2.053	2.912	1.884	1.746	3.119	1.708
2.566	2.783	2.639	3.294	3.407	3.158
2.492	2.995	1.651	3.201	2.005	2.637
5.694	5.830	5.940	4.824	4.947	5.539
5.476	5.436	5.843	6.062	5.761	6.357
6.178	6.071	6.167	6.024	6.043	6.363
4.742	4.902	4.749	5.007	4.520	4.381
4.366	2.726	3.252	2.280	2.695	2.808
2.939	2.480	3.235	2.781	1.350	2.967
5.047	4.342	4.970	5.127	5.149	5.827
1.749	1.389	1.504	1.219	1.497	1.225
3.173	3.202	3.009	3.369	2.943	3.055
2.863	2.329	2.952	2.124	1.927	3.118
3.952	3.778	4.201	3.893	3.750	4.227
2.226	2.344	2.058	2.771	2.841	2.795
3.600	3.880	3.901	3.824	4.338	2.901
3.943	4.554	4.180	3.375	3.969	3.213
3.487	3.412	3.432	3.462	3.244	4.043
3.232	2.033	3.054	3.089	3.130	1.794
2.315	2.514	2.260	2.578	2.287	2.819
2.310	3.193	2.678	2.785	2.739	2.444
4.196	3.711	4.767	4.357	4.725	4.233
3.704	3.468	4.547	4.182	4.501	2.948
3.722	2.887	3.589	3.876	3.948	3.933
3.029	2.740	2.603	3.600	3.135	2.963
3.235	2.996	4.364	3.130	3.086	3.485
3.286	3.766	4.116	2.883	2.228	1.901
2.110	5.776	3.120	1.809	3.329	1.711
6.540	5.932	6.905	6.266	6.762	6.382
5.642	5.242	5.459	6.107	5.144	4.834
2.272	2.597	3.305	2.498	3.367	2.909
4.163	3.542	4.792	4.626	5.010	3.655
3.832	2.676	4.204	3.206	3.250	3.764
3.301	3.537	3.772	2.743	4.019	2.797
3.983	3.800	4.759	3.723	5.583	4.922
4.372	4.710	4.906	4.727	5.040	4.793
3.468	2.642	3.517	2.323	2.961	3.240
3.254	3.929	2.982	2.461	2.670	3.146
4.991	6.403	6.151	5.294	5.383	4.878
2.417	2.960	2.291	2.594	2.154	1.216
2.520	2.437	2.715	2.370	2.762	2.176
2.571	2.746	2.947	2.102	3.003	2.076

3.665	3.727	3.510	3.115	3.501	3.170
2.900	2.474	3.104	2.932	2.541	2.915
3.271	2.665	3.337	4.206	3.447	3.652
3.084	2.646	2.973	3.843	3.412	3.339
3.748	4.038	3.692	1.668	3.556	2.033
3.890	3.460	4.290	4.082	3.646	3.956
5.210	4.923	5.273	5.135	4.768	5.920
3.995	2.855	4.003	4.605	3.369	2.627
1.254	1.509	1.023	1.047	1.404	1.023
2.203	2.365	2.639	2.556	2.873	2.313
3.520	3.943	3.193	3.172	4.165	3.776
2.232	1.981	2.013	1.931	2.277	2.473
4.234	4.223	4.382	4.071	3.109	4.541
1.681	2.227	1.708	2.535	1.931	1.614
3.948	3.412	3.719	4.016	3.476	3.655
3.264	2.976	4.657	3.408	2.583	2.350
3.459	3.496	3.471	3.423	3.845	3.991
1.930	1.583	1.062	1.448	1.316	2.128
1.554	1.628	1.400	1.580	1.316	2.048
1.254	1.040	1.054	1.245	0.752	1.506
2.853	2.880	3.014	2.762	2.705	3.179
2.929	2.726	2.862	2.532	3.586	2.759
2.695	2.682	2.659	2.278	2.448	3.013
3.520	3.671	3.765	2.704	4.122	3.296
2.112	2.374	2.320	1.918	2.222	2.005
1.483	1.140	1.487	1.169	0.871	1.715
4.244	5.130	4.448	4.408	4.172	4.090
3.138	2.754	2.307	3.303	3.064	2.742
3.154	2.707	3.064	3.754	2.921	3.110
2.353	1.830	2.281	2.033	2.434	3.387
3.716	3.264	3.999	3.741	3.327	3.647
2.894	2.850	2.680	2.638	2.230	2.788
2.859	3.732	3.927	2.626	3.496	3.647
2.829	1.222	2.365	1.859	2.429	2.540
2.134	2.034	1.501	2.210	2.520	1.502
1.843	1.452	2.209	2.035	1.841	2.002
4.255	3.735	4.532	5.620	4.192	4.305
1.962	1.503	2.406	1.596	2.042	2.132
3.505	3.291	3.629	3.424	4.012	3.454
3.568	3.003	4.253	3.843	3.770	4.310
4.341	4.774	3.526	3.563	4.655	4.753
4.513	4.843	4.200	4.366	4.539	4.856
5.237	4.863	4.653	5.179	5.155	5.123
3.184	3.052	2.985	2.808	2.283	3.393
2.585	2.125	3.151	1.980	2.071	4.809
2.396	1.408	2.305	2.054	2.462	2.427
2.526	2.321	3.254	1.744	3.937	1.587
4.198	4.169	5.649	4.163	5.390	3.807
1.814	1.582	2.376	2.449	1.141	3.064
2.423	3.089	2.463	2.333	2.141	2.128

2.372	2.154	2.088	2.198	2.280	2.050
2.076	1.828	2.729	2.027	1.718	2.115
2.873	3.050	2.695	2.368	2.285	3.020
4.966	4.638	4.579	5.044	6.001	4.622
8.287	7.534	8.699	8.838	7.975	8.152
4.448	4.938	4.644	5.368	4.679	4.633
4.640	4.695	4.916	4.897	4.728	4.465
2.848	1.685	3.293	3.007	2.860	2.796
6.543	5.804	7.283	6.160	5.922	5.901
3.181	2.512	2.922	2.664	2.984	2.383
2.990	2.921	2.937	2.824	3.538	2.806
3.016	2.970	2.843	2.459	2.480	2.692
2.360	2.914	2.764	2.231	2.655	2.769
2.968	2.307	3.009	2.454	3.144	2.633
3.154	3.171	4.093	3.491	4.363	2.207
1.794	1.471	1.794	1.370	1.595	1.327
2.437	2.121	2.921	1.678	2.597	1.824
2.170	3.200	2.631	2.214	2.241	1.884
3.838	3.448	3.491	4.045	4.714	3.901
2.446	3.413	2.017	2.449	2.459	2.874
3.996	4.136	4.700	4.039	4.623	3.791
2.314	1.933	1.788	1.780	2.692	1.535
4.021	4.559	4.536	4.833	4.417	4.120
2.516	2.634	3.215	2.862	1.636	1.862
5.193	4.963	5.756	5.112	4.904	5.415
2.363	1.677	2.371	2.630	2.995	3.071
2.689	3.298	2.071	1.212	2.794	2.506
6.392	4.090	7.598	7.273	7.142	7.173
2.058	2.502	2.347	2.034	1.698	2.189
2.461	2.389	1.517	3.898	2.389	2.389
4.654	5.080	5.013	5.246	4.806	5.246
3.494	2.955	3.330	2.868	2.784	3.199
1.816	1.256	2.253	1.333	2.001	1.319
3.556	4.731	3.849	3.827	3.629	3.425
1.676	1.681	1.898	1.996	1.665	1.677
4.355	3.921	4.900	4.585	4.868	4.090
2.654	3.417	2.838	3.201	2.695	3.228
2.779	2.248	3.594	3.126	3.587	2.430
2.390	2.134	1.890	2.218	2.350	2.171
3.912	3.222	3.872	4.015	4.310	4.234
2.310	4.886	3.260	2.590	3.402	1.838
2.051	1.876	1.825	2.214	1.344	2.596
2.879	3.064	4.035	2.894	3.552	2.734
3.573	3.436	3.655	3.287	3.663	3.721
4.531	3.223	4.232	3.348	3.685	4.374
3.632	2.875	4.028	2.996	3.386	3.146
2.943	2.817	4.006	3.093	3.002	2.980
2.417	3.550	2.648	2.956	2.232	2.128
4.732	4.125	4.744	4.473	4.487	4.898
2.547	2.262	2.900	2.631	3.676	2.564

2.871	2.160	3.114	3.418	2.910	3.354
2.370	2.249	2.023	2.213	2.546	3.238
1.263	1.228	0.993	1.252	1.306	0.997
2.671	3.047	2.550	2.240	2.682	2.525
3.551	3.102	3.564	3.366	3.087	3.801
3.882	3.085	3.295	3.732	3.748	3.289
3.518	2.965	4.980	3.536	3.414	3.458
2.293	1.552	2.738	2.417	2.562	2.012
3.383	3.504	3.716	3.058	3.794	3.121
4.321	4.219	4.085	4.306	3.782	4.045
3.045	2.955	3.133	3.082	3.126	3.178
3.367	3.146	2.526	2.101	2.910	3.171
2.439	2.445	3.036	4.650	3.304	2.790
4.058	3.971	4.551	6.233	4.876	4.273
1.947	1.654	2.389	3.954	2.544	2.044
3.732	3.409	4.141	3.695	3.528	4.086
2.612	2.212	1.972	1.833	1.862	1.612
2.697	2.098	2.880	2.134	1.981	2.390
1.716	2.127	1.923	1.401	1.939	2.540
3.916	4.388	3.824	4.034	3.571	4.156
2.561	2.079	2.655	2.399	2.752	2.815
2.978	3.078	3.372	2.852	3.762	3.057
2.887	3.237	3.236	2.994	3.682	2.326
2.928	3.164	3.401	2.819	3.811	2.421
4.475	3.964	4.831	4.077	4.681	4.057
5.568	5.643	5.442	5.106	5.106	5.520
2.228	2.100	2.331	2.818	1.648	2.355
2.599	2.182	2.545	2.553	2.048	2.486
3.258	2.432	3.869	2.496	2.350	2.689
3.483	3.171	2.601	3.233	2.720	2.925
2.901	1.375	2.470	2.833	1.273	2.652
3.312	3.035	4.056	3.204	4.085	3.748
2.876	2.783	2.600	2.783	2.565	2.600
1.947	2.595	2.305	2.305	2.031	2.305
1.905	1.611	1.917	2.139	2.338	2.297
2.843	3.753	3.927	3.158	3.720	3.339
4.287	3.810	5.298	2.747	4.789	4.150
6.346	5.903	6.120	6.837	6.334	6.506
4.217	2.915	4.940	3.924	4.184	3.564
2.141	2.254	1.849	2.092	1.985	1.793
2.919	2.506	3.796	2.910	3.820	2.474
2.271	1.587	2.715	2.642	2.131	2.682
3.072	2.514	2.378	2.603	2.038	2.360
3.062	2.109	2.819	2.766	3.425	3.245
3.520	3.362	2.685	3.517	3.245	3.299
4.062	4.024	3.512	4.374	3.565	3.700
3.973	3.486	3.845	4.074	3.653	4.079
2.683	2.239	1.814	2.025	2.254	2.506
2.538	2.414	2.589	2.372	2.079	2.365
2.807	2.264	3.010	3.269	2.606	2.455

2.192	2.038	1.980	2.105	2.442	2.499
3.329	3.252	3.153	3.366	3.372	2.547
3.272	2.483	3.706	3.169	3.073	3.137
4.092	4.508	4.890	4.461	4.553	4.575
4.803	5.251	5.139	5.174	4.936	5.061
2.156	2.689	2.224	1.884	2.667	3.132
2.384	2.951	2.056	2.488	2.630	2.521
3.103	2.791	2.833	3.013	2.526	3.287
2.644	3.109	2.436	2.773	2.687	2.680
2.876	2.994	3.069	1.936	2.568	3.274
4.852	3.101	4.895	4.611	4.312	4.657
2.046	2.399	2.388	1.929	2.369	1.820
4.781	4.797	4.882	5.001	4.038	4.924
3.313	2.608	3.267	3.006	2.672	3.211
1.750	1.353	1.677	1.872	1.333	2.185
1.955	1.831	2.673	1.915	2.289	1.661
2.853	2.674	2.718	2.763	2.591	2.569
2.101	1.472	1.664	2.339	2.270	1.447
4.016	3.594	4.548	4.936	4.611	3.781
2.837	3.339	3.311	3.824	3.539	2.769
4.091	4.065	4.084	4.213	4.656	4.012
5.591	5.034	4.601	4.936	5.191	5.151
2.297	2.020	2.680	2.725	1.788	2.678
2.836	4.145	3.589	3.684	4.455	2.746
2.275	1.820	2.620	2.133	2.126	2.560
3.719	3.461	4.258	4.059	3.029	3.067
3.899	2.575	3.511	4.586	2.802	4.190
0.942	0.858	0.507	0.798	1.069	1.416
2.919	3.668	2.514	3.614	3.878	2.860
3.489	3.567	3.246	3.587	3.774	3.549
3.598	2.923	3.670	3.402	3.634	3.694
1.897	1.791	1.817	1.800	1.695	1.611
2.587	2.973	2.685	1.817	2.287	2.021
4.669	4.624	4.888	5.145	5.331	5.377
2.667	3.001	2.025	2.374	2.498	2.876
4.563	3.026	5.293	2.959	5.336	4.392
2.423	2.644	3.010	2.910	2.097	2.050
1.749	2.036	1.801	2.062	1.813	1.482
5.556	5.568	6.147	5.048	5.863	5.369
2.573	3.296	2.747	2.511	2.416	1.831
3.267	3.182	2.495	2.373	2.339	3.448
1.703	1.527	1.158	1.283	1.136	1.014
2.466	2.008	1.760	1.715	2.092	2.451
3.752	4.428	4.053	3.738	3.973	3.621
5.525	5.232	5.855	6.191	5.538	5.932
1.680	1.346	1.975	1.748	1.461	1.521
2.928	3.028	2.464	2.445	2.770	2.642
2.839	3.248	2.287	2.129	2.366	3.155
2.245	2.311	3.022	1.924	2.215	1.012
2.824	1.921	3.729	1.630	2.587	2.373

4.321	4.254	4.006	3.713	4.362	3.618
3.768	3.194	3.909	3.552	3.201	3.703
2.801	3.069	3.327	2.907	3.086	3.229
1.120	1.012	1.537	1.565	1.146	0.991
3.429	2.470	3.828	3.253	2.957	3.964
4.296	4.768	4.116	3.620	4.952	3.740
3.756	2.880	3.487	2.553	4.326	4.473
1.923	2.766	1.170	1.727	2.028	2.195
2.382	1.751	1.885	1.925	2.076	2.137
4.076	3.571	4.944	4.535	4.202	4.058
4.092	3.126	4.352	3.479	3.310	3.898
1.994	2.427	2.146	1.491	2.007	1.806
4.202	4.622	5.017	4.084	3.978	4.060
2.811	2.841	1.890	3.650	2.753	2.867
4.881	4.497	4.172	4.133	3.877	5.492
2.567	2.355	2.668	2.816	3.024	2.104
3.689	3.480	3.451	3.045	3.688	3.536
3.177	3.019	2.502	3.059	3.497	3.044
2.720	1.885	2.444	2.608	2.175	1.829
3.479	2.463	2.548	2.800	1.578	3.761
2.306	1.547	1.918	1.202	1.706	2.100
2.596	2.351	1.827	2.476	2.090	2.236
5.030	5.655	5.339	5.867	5.016	5.139
1.959	1.890	1.976	2.035	1.809	2.696
2.940	3.205	3.260	2.317	2.809	2.306
3.126	3.306	2.950	2.545	2.592	2.217
4.392	4.312	5.399	4.813	6.661	4.570
1.894	1.657	1.627	1.781	1.732	1.737
1.791	1.951	2.164	1.543	1.804	2.005
3.073	2.946	2.620	2.456	3.370	2.723
2.587	2.220	3.030	3.626	3.143	2.784
2.330	2.363	2.272	2.895	2.499	2.567
2.406	2.075	2.999	2.236	2.443	2.270
3.412	2.576	3.610	3.065	2.901	3.563
4.325	4.530	4.162	4.959	4.158	5.307
1.986	3.017	2.030	3.127	2.558	1.962
6.002	5.601	6.425	6.073	5.977	5.810
2.649	2.198	3.296	2.228	3.306	1.387
2.921	2.589	3.807	3.212	3.675	3.050
2.896	3.588	3.737	2.964	3.826	3.574
4.827	4.416	5.050	5.021	4.849	5.406
1.963	1.462	1.919	1.861	2.145	2.008
4.712	3.405	4.703	4.648	4.511	4.214
3.078	2.340	3.659	2.149	3.483	3.542
2.792	1.779	1.973	3.171	3.552	1.914
2.849	3.081	2.664	3.172	2.321	2.725
2.713	3.042	2.558	1.817	0.897	2.277
4.743	3.753	5.159	4.374	4.390	4.932
1.594	1.923	2.064	1.655	1.716	2.013
2.554	2.362	2.734	2.405	3.264	2.074

3.134	4.058	3.668	2.908	2.765	2.734
3.001	2.917	3.499	3.686	3.202	3.283
1.545	1.915	1.718	1.309	1.252	1.740
2.949	2.429	3.587	3.702	3.646	2.632
1.350	1.250	1.626	1.672	0.897	1.796
4.011	4.614	3.916	4.853	4.577	4.367
2.003	1.513	2.044	2.001	2.062	2.005
2.139	1.533	2.131	2.432	2.754	2.025
3.056	2.622	3.629	3.110	3.576	2.422
3.584	3.741	4.250	4.033	2.903	3.766
8.809	8.945	8.930	8.586	8.492	8.455
2.486	2.305	2.227	1.989	1.783	3.218
2.819	1.726	2.204	2.235	1.772	2.511
2.796	2.738	3.468	2.803	2.259	3.056
3.341	3.957	3.255	4.072	3.503	3.911
2.746	2.584	3.144	3.418	3.634	1.744
2.801	3.174	3.264	3.753	3.009	2.475
4.215	3.893	3.907	4.146	3.998	3.779
2.010	1.252	2.682	3.244	2.024	1.896
7.150	6.770	7.413	6.627	6.608	6.702
3.033	3.719	3.403	2.634	3.818	3.337
2.568	2.839	3.935	3.301	3.976	2.060
1.025	1.345	1.175	1.819	1.071	1.303
2.489	2.391	3.379	4.208	2.869	3.136
3.844	3.536	4.070	4.327	3.844	4.056
2.244	3.183	3.260	2.748	2.778	2.030
2.986	2.307	2.444	2.487	2.883	3.092
3.241	3.706	3.268	2.988	2.798	3.587
2.378	1.775	2.751	2.500	2.505	2.502
1.426	1.339	1.317	1.224	1.158	1.519
1.640	1.191	1.646	1.668	0.820	1.534
1.676	1.538	1.555	1.379	1.527	1.955
1.347	1.062	1.020	0.553	0.827	0.985
2.132	1.428	1.771	1.954	1.969	2.266
2.848	2.475	2.599	2.788	3.091	3.420
5.050	3.587	4.797	5.265	4.104	5.155
3.792	2.990	3.519	3.172	3.656	4.055
2.948	3.252	2.450	3.158	2.554	3.581
1.017	0.874	0.747	0.457	0.742	0.417
2.500	2.218	2.511	2.218	2.555	2.984
3.153	2.435	2.955	2.680	3.225	2.693
1.401	1.213	1.361	1.744	0.697	2.523
4.074	3.918	5.045	4.239	4.938	3.338
2.078	1.591	2.498	1.848	2.745	2.235
2.529	2.218	2.998	2.705	3.212	2.889
3.787	3.318	4.106	3.380	4.110	2.827
1.152	0.557	0.914	0.976	1.229	0.953
2.446	1.697	3.319	2.602	2.720	2.653
5.335	6.305	5.426	6.282	5.726	5.579
2.921	3.021	3.064	2.279	3.233	2.141

6.768	6.294	6.994	6.796	7.069	7.058
2.747	2.608	2.828	2.505	2.837	2.532
2.158	2.237	1.837	1.819	2.021	2.422
2.179	1.107	2.501	2.105	1.882	2.259
4.658	4.133	5.092	4.398	4.260	3.722
3.027	2.882	2.866	3.601	2.495	3.758
3.534	3.424	3.516	3.408	3.334	3.369
2.146	1.606	2.345	1.951	1.656	1.650
3.219	3.487	3.425	2.684	3.317	3.197
4.624	4.524	4.474	4.373	4.353	4.049
3.268	3.468	2.965	3.225	3.576	3.029
7.014	5.960	7.737	7.200	7.241	7.113
2.798	2.389	1.986	3.142	2.871	2.486
1.379	0.874	0.976	1.087	1.608	1.507
2.200	2.747	1.933	2.031	2.527	2.042
7.554	7.003	7.512	7.476	7.568	7.405
3.231	2.744	3.218	3.815	3.655	3.199
4.797	3.837	4.712	4.852	4.062	4.483
5.250	4.774	5.697	4.347	4.631	4.764
2.939	2.164	2.794	3.049	2.356	3.403
3.043	1.683	1.595	1.369	1.671	0.535
3.730	3.761	3.186	3.231	3.723	3.578
2.511	2.496	2.769	2.912	2.717	2.793
2.971	2.070	3.035	2.831	2.392	3.233
2.709	3.035	2.565	2.461	2.938	2.905
7.557	6.196	7.783	7.450	7.639	7.437
2.042	2.198	2.283	1.723	2.240	2.214
1.360	1.270	1.537	0.948	0.852	0.816
2.356	2.389	1.651	2.719	2.006	2.389
2.795	2.565	2.996	2.150	3.159	3.063
2.628	2.341	2.594	2.747	2.697	2.606
3.123	3.137	2.699	3.045	2.735	2.866
3.505	3.951	3.821	3.361	3.386	3.293
2.755	3.478	2.879	2.815	3.224	2.269
3.231	2.439	2.131	1.844	2.236	3.352
2.323	2.430	2.752	2.554	3.076	2.327
2.682	2.271	1.251	2.453	2.891	2.897
2.757	2.824	2.928	2.599	2.730	2.971
2.702	2.329	2.465	2.677	2.931	3.045
3.377	4.022	3.094	3.342	2.829	2.854
3.003	3.421	3.755	3.497	4.191	3.383
2.605	1.496	2.746	2.332	2.766	2.430
2.538	2.836	3.150	2.379	2.936	2.790
3.526	3.672	3.173	3.542	3.254	3.375
3.816	3.150	4.215	6.652	3.499	4.597
4.773	4.598	4.552	4.676	4.506	4.312
4.297	4.440	4.038	4.416	4.058	5.149
4.895	3.734	4.509	4.838	4.080	5.842
2.568	3.275	2.912	2.848	2.788	2.394
10.298	10.473	10.163	10.294	10.483	10.020

4.003	4.861	3.723	4.275	3.786	3.565
3.205	3.709	3.353	3.919	3.155	3.161
7.504	7.843	7.575	7.520	7.408	7.216
8.809	9.030	8.873	8.792	8.874	8.451
3.157	3.300	3.136	3.303	2.301	3.045
5.475	5.407	5.704	5.464	5.565	6.115
4.021	4.179	4.100	3.838	4.782	4.516
5.146	4.856	5.601	5.881	5.563	5.513
2.629	1.525	2.456	2.937	1.907	3.230
4.334	5.022	4.809	4.807	4.099	4.039
3.430	4.037	3.519	3.671	3.528	3.191
3.647	4.088	3.915	4.483	4.217	4.175
3.989	4.474	4.065	4.262	4.208	4.188
2.707	2.606	2.739	2.866	3.115	2.494
3.284	3.343	3.042	3.139	3.073	3.357
1.918	2.329	1.996	2.874	2.372	1.879
4.629	5.567	5.454	5.290	5.457	4.995
3.154	3.566	4.077	3.834	2.952	3.544
8.678	9.147	8.612	8.408	8.667	8.100
3.630	4.003	4.329	4.385	3.872	2.697
9.714	10.466	9.752	10.177	10.067	9.506
10.993	11.617	11.004	11.379	11.279	10.827
4.752	5.073	5.384	5.804	5.035	5.302
2.851	2.574	3.416	3.026	3.165	2.698
10.707	10.944	10.830	10.677	11.040	10.682
9.013	9.359	8.895	9.094	8.959	8.846
4.408	4.611	4.711	4.259	4.655	3.664
3.825	3.368	3.427	3.712	4.301	3.908
5.551	5.340	5.736	5.832	5.843	5.530
4.012	3.556	3.847	4.447	3.552	3.775
1.898	1.720	3.665	2.142	1.739	3.626
2.528	2.094	1.935	2.025	1.934	2.015
3.588	3.839	3.217	4.013	3.239	4.135
3.202	2.626	3.492	3.086	2.344	2.570
4.574	4.433	5.196	5.356	5.233	5.179
6.532	7.497	6.434	7.090	7.166	6.924
7.175	7.723	7.159	7.158	7.467	7.491
5.533	6.247	5.898	5.495	5.917	5.416
2.226	2.116	2.393	2.309	2.373	2.332
5.494	5.925	6.337	6.638	6.256	5.618
5.001	5.428	5.263	5.741	5.988	4.907
3.193	4.383	3.156	3.276	3.939	3.663
3.961	4.242	4.057	4.385	4.365	4.115
3.956	3.930	3.945	4.501	4.318	3.793
4.811	4.850	4.435	3.957	4.143	4.849
2.691	2.637	4.839	3.818	1.517	1.310
2.350	2.795	2.097	2.657	2.463	2.769
5.158	5.680	5.487	5.506	5.901	5.574
4.142	3.250	3.156	3.101	3.115	3.584
5.479	5.147	4.964	4.867	5.395	5.141

3.949	1.849	2.328	2.421	2.109	2.758
2.667	2.374	2.584	2.830	2.785	3.248
1.839	2.439	1.692	2.022	1.930	1.363
4.971	4.866	4.699	5.240	4.930	5.312
6.756	6.419	6.414	7.193	6.591	7.364
6.247	6.217	6.425	6.354	6.098	6.201
1.312	1.357	1.567	1.085	1.364	1.792
7.024	6.009	6.522	6.830	6.138	7.162
3.892	3.216	3.039	3.297	3.354	4.231
3.537	3.637	3.524	4.255	3.340	3.987
3.657	3.539	3.775	3.644	3.220	3.840
2.135	2.211	2.272	2.341	2.376	1.620
1.357	1.312	1.408	2.202	1.403	1.955
3.079	3.536	3.091	3.045	2.882	3.073
2.838	2.719	3.505	3.226	2.658	3.813
6.069	5.994	6.073	6.501	6.263	6.275
5.025	4.804	5.312	5.089	5.310	5.166
7.029	6.374	6.707	7.080	6.089	7.232
3.770	3.972	3.195	3.711	2.912	4.027
2.781	3.147	3.602	3.152	3.588	1.772
4.117	3.928	4.300	4.052	3.859	4.317
2.787	3.830	3.369	3.327	2.886	2.579
3.826	3.711	3.482	3.999	3.531	3.693
6.066	5.451	6.101	6.132	6.004	6.285
4.805	4.672	4.555	4.945	4.721	5.293
2.805	2.956	2.362	3.515	2.206	3.711
1.166	0.950	1.060	1.201	1.089	0.955
4.741	4.633	4.637	4.722	4.966	4.141
6.698	6.008	6.747	7.071	6.951	7.029
4.651	4.329	4.740	4.368	4.424	4.089
3.951	4.035	4.539	4.167	4.615	4.226
4.316	5.486	4.638	4.891	5.202	4.143
4.219	7.461	5.450	4.384	3.268	2.553
4.887	5.865	4.618	5.515	5.149	4.938
2.634	3.208	2.150	2.775	2.189	3.046
5.306	5.693	5.569	5.492	5.180	5.005
5.066	7.275	5.181	6.050	4.362	3.678
5.469	8.587	5.973	6.248	6.164	4.167
10.920	10.581	11.104	10.904	10.590	10.675
3.813	3.267	4.328	3.833	4.157	4.108
5.814	5.783	6.097	5.811	5.180	5.513
3.267	2.765	3.376	3.406	3.376	3.356
6.410	5.900	6.013	5.927	5.829	6.478
4.351	3.908	4.110	4.046	4.215	4.644
5.711	5.481	5.680	4.984	5.417	5.576
4.363	5.115	4.199	4.814	3.941	4.709
5.091	4.669	5.372	4.998	4.122	5.045
2.994	2.625	2.624	2.195	3.033	2.528
2.582	2.591	2.155	2.257	2.464	2.742
3.879	4.541	4.154	4.271	4.993	4.497

4.928	5.836	5.294	5.606	5.902	5.735
7.737	8.592	8.033	7.671	8.138	7.414
3.240	3.921	3.874	3.158	3.958	3.158
3.333	3.664	2.617	3.144	3.100	3.655
2.009	1.820	1.823	1.600	1.979	1.619
8.360	7.972	8.584	8.245	8.266	8.063
4.350	6.052	4.594	4.980	5.303	4.374
8.070	8.577	9.011	8.073	8.347	7.560
5.825	5.717	6.095	5.600	5.865	5.216
2.301	3.193	1.983	2.539	1.726	2.694
3.690	2.907	3.292	3.851	3.259	3.609
7.103	6.800	7.090	6.978	7.060	7.183
4.959	6.094	5.804	5.263	5.787	4.806
7.846	8.477	8.256	9.079	8.994	8.114
4.609	4.590	4.553	4.946	4.859	4.631
7.280	6.892	7.432	7.490	7.261	7.544
6.610	6.937	6.637	7.078	6.614	6.387
6.926	6.491	6.520	7.183	6.458	7.361
7.877	8.204	7.765	8.296	8.326	8.156
2.600	2.085	2.726	2.044	2.742	2.327
9.281	9.037	8.901	8.133	8.586	9.106
8.123	7.443	6.894	6.182	8.411	7.708
4.075	4.513	4.716	3.483	4.265	3.684
6.282	6.287	5.776	5.368	5.667	5.681
5.260	5.046	4.341	4.391	4.577	4.708
5.379	6.373	5.859	5.341	5.786	5.082
6.211	6.386	5.911	5.348	5.790	5.958
6.173	5.852	5.679	4.812	5.545	6.023
7.841	7.408	7.140	6.432	7.386	7.250
8.837	8.683	8.946	7.957	8.997	8.599
8.522	6.321	9.479	8.222	8.650	8.640
7.942	7.762	7.759	7.266	7.280	7.655
8.625	8.343	8.411	7.574	7.761	7.889
5.753	5.665	5.303	4.362	5.338	5.124
2.898	3.095	2.774	2.677	2.713	2.364
7.849	7.585	8.088	7.956	7.837	7.668
10.914	10.585	11.088	11.259	10.839	11.057
6.396	6.738	6.159	6.760	6.151	6.742
4.957	4.314	5.289	5.484	5.219	5.473
7.357	6.738	7.517	7.786	7.074	7.429
4.321	4.025	4.265	4.518	4.175	4.889
4.127	5.835	5.337	5.224	5.672	4.165
6.525	7.906	6.158	8.063	7.522	6.111
3.238	3.388	3.622	4.027	3.380	3.680
1.990	1.902	2.230	1.979	1.850	2.092
2.272	1.964	2.715	2.226	2.549	3.370
5.677	4.742	4.966	5.915	6.155	6.592
6.360	6.175	5.992	6.586	5.929	6.677
4.398	3.654	4.367	4.511	4.233	5.459
5.224	4.710	5.354	5.446	4.854	6.273

4.172	4.102	4.434	5.363	4.344	5.321
4.631	3.405	3.680	4.454	3.145	4.709
3.919	4.646	3.613	4.350	3.794	4.538
4.361	2.916	3.361	4.597	3.270	5.174
5.454	4.380	4.587	5.853	4.650	6.427
4.166	3.707	4.414	3.749	3.995	4.429
3.437	3.492	2.912	2.929	3.653	3.113
2.898	2.951	3.114	2.224	2.673	2.478
2.589	2.950	1.694	2.435	1.649	2.661
2.902	2.428	2.597	3.080	2.907	2.960
3.175	3.457	3.088	2.131	2.724	2.492
4.240	3.904	5.020	4.802	4.641	4.612
2.668	3.155	1.887	3.387	2.842	4.254
6.040	5.900	6.144	5.910	5.836	6.522
8.376	7.916	8.995	8.717	8.455	8.077
5.262	6.066	6.266	5.336	6.224	4.868
3.572	3.500	3.418	4.065	3.214	3.449
3.193	3.727	4.109	3.251	3.110	3.020
4.271	5.791	4.521	4.514	5.154	4.258
4.948	4.533	3.818	3.904	5.925	4.302
5.643	4.858	1.113	4.476	5.412	5.868
4.680	4.581	3.673	3.611	5.569	4.225
4.296	3.930	2.611	3.867	4.733	3.719
4.485	4.040	4.698	3.830	3.353	4.656
1.800	2.144	1.726	1.646	1.498	2.071
5.692	5.973	4.001	4.588	5.163	5.064
2.338	1.720	1.326	2.388	1.563	1.861
3.378	3.062	3.731	2.676	3.076	3.235
4.898	5.176	5.494	5.207	5.479	4.694
4.833	4.964	5.251	5.299	5.204	5.102
7.383	7.255	7.214	7.174	7.678	7.187
2.237	1.642	3.305	3.607	1.958	2.235
5.851	4.750	6.045	6.142	5.628	6.368
2.872	2.887	2.680	1.125	3.499	2.379
2.339	2.162	2.470	1.951	2.694	2.931
2.972	2.932	3.034	3.250	2.929	2.889
5.995	5.928	6.232	6.469	6.088	6.353
4.865	5.954	4.765	5.576	5.825	5.062
4.376	5.753	5.869	4.969	5.815	3.146
7.133	7.801	7.321	7.677	7.475	7.547
2.673	3.443	1.598	4.453	1.421	4.506
4.964	5.258	5.399	5.065	5.866	4.436
5.439	6.344	5.202	5.744	5.182	4.715
5.575	6.353	6.421	5.332	6.823	5.489
4.166	4.071	4.729	3.577	4.489	4.259
7.598	7.590	7.170	7.426	6.987	7.621
3.336	3.676	3.816	3.840	3.324	3.221
5.703	5.269	5.772	5.662	5.480	5.568
5.484	4.915	6.220	5.549	5.818	5.189
5.112	5.006	5.301	5.095	5.095	5.811

2.954	2.872	3.411	2.850	2.872	2.854
2.982	3.999	3.021	4.609	3.282	3.584
4.575	3.889	4.880	4.290	4.411	4.200
6.525	6.614	6.787	6.780	6.214	6.341
3.985	3.264	3.762	3.622	3.777	4.467
3.469	3.144	3.755	3.869	3.459	3.461
6.682	7.141	7.002	6.879	6.828	6.620
3.658	3.015	3.870	3.585	3.584	3.890
6.338	6.113	6.616	6.697	6.201	6.609
4.640	4.863	4.835	4.924	4.714	5.032
2.328	2.373	2.228	1.802	2.409	2.405
6.394	5.892	6.837	6.536	6.525	6.879
6.396	5.881	6.380	6.812	6.550	6.470
4.892	5.103	4.936	5.223	5.246	5.314
7.567	7.951	7.403	7.614	7.465	6.914
5.416	5.811	5.941	4.662	5.631	5.138
5.923	5.865	5.998	6.248	6.025	5.773
6.924	6.676	7.166	7.246	6.634	6.303
5.861	5.818	6.238	5.654	5.657	5.593
2.777	2.564	2.322	3.678	2.385	3.113
8.552	7.940	8.752	8.254	8.561	9.033
7.238	6.874	7.131	7.184	7.106	7.412
4.855	4.779	4.755	4.290	5.249	5.237
4.627	5.152	4.868	5.127	4.980	5.079
7.322	7.538	7.063	7.349	7.597	7.245
4.604	4.713	4.907	5.109	4.918	5.410
4.100	4.409	4.195	3.945	3.496	3.785
2.233	2.683	3.044	2.204	3.663	2.576
3.430	3.980	3.798	3.608	3.923	3.306
6.926	7.125	7.475	7.215	7.579	6.554
4.184	3.627	3.998	4.131	4.022	4.448
2.659	4.682	3.467	4.416	4.359	2.767
5.626	6.637	6.340	6.541	7.170	5.747
2.725	3.609	2.815	3.665	3.122	2.175
5.636	6.854	5.864	6.423	6.235	5.510
5.750	6.199	5.653	6.139	6.181	5.688
3.295	4.439	3.021	4.115	3.748	4.173
6.075	6.442	6.438	6.915	5.804	6.047
3.548	4.108	4.153	3.532	2.672	3.103
3.244	4.366	2.194	2.379	2.765	2.365
5.202	6.320	7.040	5.626	5.874	3.728
5.353	7.347	5.069	6.911	6.164	5.253
7.008	7.426	6.830	7.200	7.522	6.632
5.769	7.089	6.950	5.987	7.846	5.692
7.646	7.598	7.896	7.756	7.699	7.905
3.587	3.010	4.124	3.560	4.037	3.903
3.142	2.611	3.161	2.697	2.922	3.951
4.022	3.762	4.108	3.844	4.411	3.914
3.233	3.077	2.862	3.386	3.389	3.954
6.283	6.101	6.745	6.425	6.437	6.212

4.554	4.416	4.848	5.060	5.036	4.670
4.301	4.994	4.647	4.260	3.653	4.517
4.622	5.067	4.596	4.576	4.921	4.495
3.256	3.468	3.865	3.860	3.039	3.851
5.963	5.428	5.632	5.699	5.064	6.277
6.072	5.190	5.782	6.237	5.057	6.468
6.973	6.540	6.941	6.522	7.343	6.411
4.202	5.651	5.475	5.257	5.477	4.668
3.568	3.778	3.992	3.855	4.193	3.159
4.194	3.559	2.994	4.099	3.516	4.354
6.488	5.982	6.416	6.182	5.843	5.518
3.486	4.350	3.476	3.465	3.916	3.081
4.098	5.642	3.227	2.810	3.570	3.056
6.778	7.393	6.772	7.502	7.287	6.787
3.278	3.655	3.632	3.709	3.796	3.678
6.796	6.707	7.649	6.542	7.271	6.337
4.960	5.078	5.213	5.638	4.834	4.764
3.687	4.255	3.990	4.408	4.263	3.548
4.000	3.681	4.160	3.963	4.376	3.854
3.769	3.770	4.051	3.793	3.212	3.481
4.747	3.837	3.352	4.440	3.414	5.213
4.759	4.577	5.094	4.450	4.791	4.950
6.401	6.718	6.255	6.094	6.293	6.685
7.022	7.036	7.093	7.626	6.980	7.475
3.283	3.719	3.024	3.912	3.300	3.451
2.707	2.467	2.418	3.130	2.138	2.468
5.049	4.875	5.486	5.162	5.698	4.872
8.320	8.082	8.278	8.005	8.054	8.348
5.509	5.869	5.392	5.507	5.371	5.179
2.722	2.899	2.066	2.857	2.978	2.522
3.822	4.575	3.295	4.308	4.170	4.149
4.646	4.724	4.749	4.489	4.377	4.415
4.008	3.346	3.756	4.075	3.372	3.585
3.820	4.379	3.399	3.460	4.779	3.483
3.207	3.782	3.149	3.098	3.382	2.991
6.297	6.006	6.468	6.356	6.198	6.785
5.691	5.627	5.993	5.883	5.983	6.111
1.821	1.613	1.531	1.923	1.912	1.876
3.378	3.439	4.064	3.443	3.682	3.213
3.465	2.842	3.263	3.368	3.344	4.443
2.642	2.830	1.634	2.630	2.494	2.543
4.665	4.852	5.091	4.502	4.085	5.402
4.947	5.176	4.986	4.850	4.775	5.350
5.931	6.737	6.118	5.859	6.209	5.927
5.533	5.077	5.504	5.253	5.275	6.447
5.289	5.456	5.969	5.020	5.968	5.376
2.933	2.832	2.808	2.219	2.076	3.248
3.739	3.947	4.118	3.683	4.096	3.618
2.950	2.995	3.450	2.772	2.820	2.554
3.285	3.824	3.567	3.666	3.688	4.126

3.923	3.346	4.635	3.762	3.768	4.725
4.177	3.986	3.976	4.095	4.008	4.855
5.439	6.437	5.326	6.064	5.749	5.491
4.143	4.785	4.945	4.601	4.584	4.200
5.247	5.218	5.321	4.694	4.721	5.281
2.692	2.667	2.254	2.748	2.585	2.482
3.815	3.324	3.707	3.839	3.510	3.903
5.232	5.077	5.737	5.739	4.701	5.522
2.801	2.294	2.610	2.207	1.653	3.366
4.056	3.834	5.253	4.574	4.837	4.887
6.459	6.530	6.634	6.935	6.509	6.216
5.406	6.553	5.395	6.642	6.560	4.910
2.186	2.499	2.339	2.148	2.606	2.782
2.977	4.213	3.560	4.933	3.753	3.652
3.681	5.856	4.970	4.407	4.976	3.875
4.576	5.512	5.758	5.591	5.977	4.564
3.802	5.329	4.587	5.624	5.575	3.939
5.879	6.660	5.867	5.144	5.675	4.309
3.670	3.566	4.404	3.941	3.693	4.014
5.985	6.197	5.978	5.535	5.815	5.965
6.268	5.987	6.834	6.100	6.472	6.282
7.452	7.529	7.795	7.131	7.677	7.578
3.702	4.845	5.286	3.829	5.331	3.540
6.034	6.005	6.398	6.218	5.790	5.839
3.788	3.683	3.436	3.503	3.946	3.475
1.710	1.580	1.737	1.389	1.506	1.929
4.547	4.288	4.652	4.627	4.759	4.838
2.513	2.498	2.496	2.716	2.380	2.517
2.197	2.945	2.184	2.659	3.080	2.169
6.816	7.016	6.953	6.738	6.824	6.809
5.145	6.310	6.049	5.658	6.046	4.537
8.094	7.453	8.390	8.028	8.048	7.947
4.165	6.107	6.316	5.779	6.750	4.164
5.503	5.599	5.452	5.888	4.983	5.974
4.038	1.298	2.425	3.230	3.271	4.766
2.184	1.779	1.938	1.747	2.019	2.228
5.085	7.086	6.507	6.544	7.044	4.963
6.575	6.619	6.917	7.287	7.159	6.802
5.271	3.847	4.453	5.435	3.313	6.752
2.998	2.435	3.084	3.401	3.171	3.037
3.773	3.774	3.615	4.344	3.533	3.604
5.292	5.672	5.870	5.350	5.448	5.053
3.912	4.072	3.505	2.376	3.070	3.343
3.514	2.948	2.476	3.220	2.513	3.219
9.734	8.237	10.804	9.317	9.583	9.528
5.151	4.586	5.902	5.266	5.822	5.611
4.215	5.198	4.471	4.800	4.669	4.597
3.522	3.388	4.507	3.864	4.393	3.504
2.715	2.684	1.930	1.781	2.081	1.595
3.305	4.042	3.510	3.578	3.584	3.565

5.561	6.909	5.755	5.988	6.487	5.158
5.296	5.731	5.676	6.264	5.946	5.158
3.964	3.722	2.907	3.130	4.283	3.950
4.150	4.672	4.345	4.607	4.470	3.925
5.628	5.838	6.240	5.264	5.734	5.324
5.483	5.849	5.931	6.337	5.867	5.633
4.341	2.866	3.778	5.484	3.154	6.231
3.356	2.285	2.589	2.706	3.073	2.200
2.107	2.230	1.754	2.513	2.177	2.417
4.204	2.932	3.042	3.658	3.236	3.943
5.324	5.261	5.781	5.479	5.432	5.003
5.333	5.728	5.170	5.334	5.133	5.734
8.872	7.982	8.221	8.697	8.043	9.356
4.799	6.515	5.278	6.067	5.849	5.155
2.123	1.589	2.093	2.092	2.135	2.852
1.446	1.017	1.327	1.069	1.819	1.060
5.285	4.847	5.899	5.663	5.456	5.504
3.939	3.827	3.336	3.823	4.608	4.178
3.356	2.982	3.643	3.430	3.073	3.885
2.526	2.608	2.093	3.027	2.018	2.291
3.484	3.964	3.524	3.122	3.186	3.075
8.439	7.977	8.551	8.474	8.220	8.825
4.510	4.535	4.236	4.303	3.918	5.352
4.716	3.744	4.962	4.028	4.584	4.616
6.552	6.492	6.374	6.024	6.422	6.771
5.596	5.115	4.895	5.597	4.757	5.817
5.765	5.610	5.577	5.748	5.582	5.926
4.791	4.617	5.003	4.937	4.702	4.807
3.390	3.271	2.977	2.693	2.590	2.952
6.543	6.096	6.995	7.019	6.737	7.079
4.228	6.108	4.472	5.068	5.395	4.299
3.758	3.639	3.709	3.639	3.970	3.994
7.422	7.019	7.463	7.501	7.280	7.444
6.751	6.196	7.137	6.426	6.721	6.427
6.740	6.780	7.154	6.485	6.799	6.462
4.215	2.569	4.345	4.286	2.499	3.639
5.123	5.115	5.398	5.731	5.066	5.627
6.356	6.925	6.786	6.683	6.473	5.682
7.512	7.315	7.454	7.893	7.445	8.012
4.798	4.555	5.029	5.636	4.817	5.322
5.571	5.397	5.689	5.876	5.620	6.067
5.680	5.821	5.845	6.045	5.533	5.824
2.007	2.343	2.111	2.283	2.177	2.334
2.283	2.080	2.224	2.854	2.864	2.756
2.331	2.226	2.380	2.954	1.857	3.185
5.849	6.219	5.981	6.313	5.712	6.128
4.777	4.138	3.866	4.658	4.594	4.260
4.446	5.852	4.963	5.419	5.215	4.807
5.006	4.730	5.103	5.656	4.613	4.995
4.798	5.625	5.264	4.937	5.356	4.594

5.282	4.035	4.159	4.198	4.792	4.271
4.040	3.380	3.946	4.774	2.618	3.609
4.609	4.532	4.782	4.873	4.643	5.290
5.202	7.476	5.320	5.832	5.337	5.139
4.716	4.859	4.510	4.352	4.024	4.698
4.420	5.220	4.760	5.404	5.363	3.606
2.609	2.153	2.486	2.097	1.978	2.882
4.274	4.114	4.512	4.615	4.695	4.001
6.792	6.283	7.075	7.236	6.756	7.147
5.779	6.352	5.937	6.800	6.477	6.176
7.071	6.961	7.920	7.390	7.303	7.111
3.669	2.814	3.404	3.512	2.564	3.965
4.955	5.523	5.258	5.067	5.213	5.757
5.312	5.401	5.502	5.695	5.172	5.465
5.822	5.474	6.106	6.365	5.924	5.911
4.398	4.477	5.344	4.702	5.035	4.898
4.413	4.570	4.310	4.297	4.818	4.418
6.187	6.443	6.777	6.894	6.894	6.515
4.343	3.896	4.632	4.526	3.970	4.753
4.425	4.183	4.292	4.714	4.429	4.646
5.121	5.004	5.555	5.849	5.400	5.469
3.139	4.548	3.607	3.600	5.012	2.887
4.641	4.746	4.827	5.254	4.986	4.986
5.127	5.729	4.942	4.766	4.939	4.736
2.323	2.384	1.933	2.359	2.075	2.326
7.048	6.638	7.033	6.804	7.044	6.892
4.043	3.639	4.375	4.041	4.378	4.375
2.358	3.044	2.466	2.911	1.701	2.350
4.637	4.612	4.277	4.243	4.493	4.977
5.987	5.853	6.531	6.315	5.986	6.055
4.781	3.921	4.915	4.803	4.638	4.594
5.797	6.179	6.510	5.771	6.860	5.368
3.284	3.348	2.874	3.755	3.145	2.852
5.059	5.151	4.714	5.640	5.592	4.835
4.403	4.500	5.243	4.488	5.112	4.294
5.178	4.706	5.119	5.497	5.267	5.782
3.947	5.223	4.421	4.851	5.035	4.280
8.683	8.732	8.979	8.791	8.503	8.327
3.877	5.032	4.401	4.726	5.297	4.882
3.349	3.006	3.708	3.315	3.546	4.036
5.509	5.984	5.570	5.360	5.503	5.070
4.267	4.332	3.564	4.176	3.546	4.215
5.148	5.484	5.234	5.188	5.119	4.833
3.496	4.422	3.523	3.670	2.713	3.295
3.815	3.292	3.437	2.183	4.716	3.091
4.114	2.934	3.932	4.535	3.150	5.590
3.590	2.855	3.586	3.090	3.662	3.880
4.989	4.407	4.545	5.137	4.252	5.254
8.870	8.371	9.513	9.072	9.181	9.248
4.210	4.323	4.301	5.026	4.317	4.734

5.331	5.254	5.449	5.492	5.649	5.419
3.522	2.583	3.175	3.831	3.020	3.903
4.196	5.182	4.442	4.510	4.975	3.945
3.128	3.546	2.651	3.165	2.710	2.907
7.105	7.521	7.615	7.727	7.219	7.005
2.294	2.977	1.750	1.938	1.939	2.054
3.476	3.679	3.189	3.611	4.078	3.286
5.076	1.183	3.984	4.886	3.993	5.364
8.258	7.539	8.168	8.168	7.795	8.715
6.179	5.190	5.440	5.984	5.488	7.007
3.613	3.567	3.231	2.500	2.717	2.614
4.228	2.706	2.974	4.101	2.813	5.459
4.521	3.761	4.357	5.065	4.498	4.969
3.140	3.548	2.532	2.152	2.414	2.443
4.778	1.731	3.650	5.979	2.693	5.545
5.542	6.384	5.509	6.883	5.915	6.011
2.572	2.783	2.542	3.265	2.236	3.871
7.078	6.780	6.999	7.257	7.012	7.217
4.062	2.977	2.850	4.570	2.990	4.827
8.110	7.597	8.034	7.852	8.496	8.269
6.166	4.979	5.435	6.557	4.755	6.192
6.115	6.692	6.983	6.550	7.193	5.761
1.526	1.092	1.214	1.594	1.191	1.266
8.187	8.567	8.126	7.937	8.486	7.103
4.330	5.358	3.950	5.071	4.741	4.682
2.611	3.788	2.827	3.400	2.765	2.496
4.007	4.006	3.602	4.088	4.190	2.600
4.079	3.902	4.287	4.197	4.058	4.513
4.588	4.798	4.583	4.472	3.955	5.832
3.007	2.713	2.603	2.369	2.508	3.465
4.727	5.341	4.445	5.296	4.514	4.865
4.934	5.424	4.739	5.936	5.424	5.463
2.204	2.253	1.922	1.906	1.263	2.563
7.713	7.717	8.110	8.406	8.149	7.885
2.675	2.810	2.499	3.242	2.259	3.128
8.151	7.724	8.136	8.724	8.146	8.592
6.021	6.069	5.763	6.153	5.743	6.283
4.980	4.015	5.055	5.244	4.660	5.550
3.778	3.889	3.006	4.681	4.414	4.550
7.437	7.811	7.972	7.462	8.289	7.427
5.611	5.881	5.537	5.619	5.584	5.621
6.091	5.712	6.105	6.077	5.974	6.357
4.644	5.471	6.324	6.040	5.948	5.124
3.384	5.066	3.661	4.540	3.420	3.756
5.679	6.694	6.048	6.283	6.499	5.666
6.473	6.496	6.903	6.625	6.544	6.465
3.975	4.543	4.388	4.083	4.072	3.839
1.765	2.956	1.339	1.994	1.254	1.206
3.258	4.389	4.382	3.683	4.470	2.264
4.305	5.070	4.078	4.788	4.838	4.584

2.373	3.404	2.564	3.264	3.110	2.804
6.055	6.635	6.056	7.099	7.064	6.397
4.421	4.815	4.083	4.793	4.590	5.377
4.909	4.251	4.124	4.413	3.650	4.838
2.851	2.627	2.361	2.596	2.208	2.761
5.161	4.418	4.614	5.187	4.460	4.742
5.392	4.763	5.011	4.866	5.244	5.841
4.514	3.801	4.084	4.493	3.983	5.125
2.168	1.815	2.160	2.408	1.711	2.452
4.414	4.819	5.893	4.858	5.490	5.149
4.568	4.287	4.979	4.306	4.546	4.581
5.180	7.576	4.681	4.587	5.500	2.231
5.267	5.285	5.366	5.617	5.401	5.687
7.968	7.757	8.148	7.733	8.079	7.795
2.396	1.642	2.149	1.705	2.301	2.076
7.542	7.195	7.743	7.611	7.357	7.488
3.393	4.359	3.146	3.877	2.471	2.962
6.191	6.158	6.364	6.274	6.304	6.100
3.685	3.734	4.438	3.657	3.410	3.652
5.552	5.481	5.107	4.985	5.184	5.554
6.514	6.445	6.899	6.601	6.456	7.034
4.562	3.437	4.093	4.941	3.896	5.518
2.018	1.148	1.895	1.290	1.923	2.142
2.302	2.655	2.510	2.521	2.947	2.175
4.442	4.372	5.137	4.408	4.493	3.686
2.890	3.487	3.291	3.091	2.899	3.231
3.744	4.131	3.551	3.938	3.647	4.081
4.302	4.165	4.166	4.163	4.207	3.903
3.195	2.725	2.205	2.632	2.572	2.927
6.114	5.508	6.772	5.852	6.203	5.843
6.811	6.540	7.078	7.246	7.017	6.433
2.809	3.020	3.093	2.860	2.842	3.516
5.666	5.322	5.175	5.657	5.075	5.911
4.124	4.361	4.172	4.178	4.016	4.276
4.798	4.067	4.421	3.926	4.274	4.866
3.271	3.045	3.238	3.015	4.188	3.002
4.191	5.278	5.067	5.694	4.615	4.197
7.531	8.028	7.663	7.791	8.072	7.877
9.047	7.027	8.293	9.569	7.663	9.940
1.828	1.635	1.976	2.022	1.806	2.145
3.525	3.512	3.328	3.504	3.382	3.439
5.857	4.484	4.329	3.700	5.498	4.033
5.456	3.668	3.888	3.405	5.371	3.207
6.240	4.406	4.195	3.469	6.316	4.269
4.938	4.902	4.478	3.771	4.916	3.315
3.802	2.284	3.372	3.279	4.182	2.541
3.774	2.066	3.399	1.434	3.589	3.239
4.495	4.356	4.559	4.106	4.496	4.487
3.224	2.508	3.105	2.834	2.735	3.158
2.694	1.376	1.540	1.436	2.026	1.373

6.199	5.824	5.908	5.952	5.425	5.780
3.669	3.073	3.127	3.319	3.007	3.534
5.651	4.457	4.865	5.858	4.960	6.492
6.521	5.335	5.680	6.205	5.293	6.387
5.243	5.604	4.589	4.510	5.359	4.129
5.102	5.317	4.902	4.329	4.417	4.440
4.992	6.194	4.830	5.419	5.086	4.301
3.948	5.575	4.131	3.924	3.914	4.208
3.141	3.516	3.689	3.952	3.772	3.690
4.943	3.474	5.086	4.508	3.439	5.603
4.821	5.665	5.209	4.995	4.930	4.694
7.861	7.866	8.175	7.736	8.264	8.252
7.667	7.847	8.133	7.446	7.873	7.039
4.405	4.309	4.241	3.672	4.199	4.391
6.360	6.323	6.105	5.631	5.859	7.829
6.192	6.248	6.061	5.198	5.986	5.913
7.936	7.463	7.109	6.354	7.284	7.376
4.361	4.079	4.131	3.894	4.408	3.881
3.587	3.833	4.220	3.724	3.355	3.120
7.425	7.561	7.313	6.868	7.206	7.348
9.491	9.344	9.403	9.011	8.732	9.275
9.788	9.747	9.681	9.330	9.480	9.464
6.105	6.787	6.428	6.827	6.217	5.497
5.191	4.923	5.085	4.428	4.647	4.539
4.153	4.912	4.874	4.613	4.208	4.052
1.807	2.697	3.825	2.718	3.456	3.044
10.228	10.151	10.218	9.075	9.778	10.171
9.567	9.486	9.597	9.376	9.485	9.255
5.276	4.236	4.839	4.118	4.977	5.880
4.042	3.381	3.187	2.972	3.427	2.720
2.624	3.024	2.092	2.660	1.908	2.544
9.280	9.470	8.485	8.612	8.918	9.032
5.357	4.261	5.341	4.427	4.746	5.097
3.918	2.347	4.177	3.491	3.183	4.587
10.172	10.281	10.243	9.941	10.404	10.322
9.764	9.285	9.168	7.947	9.108	8.997
9.036	8.553	8.799	7.121	8.514	8.691
11.133	10.926	10.880	10.814	10.794	10.883
11.524	11.649	11.505	11.526	11.465	11.281
10.251	9.943	9.724	8.848	9.697	9.975
5.539	5.345	5.591	5.040	5.809	5.717
9.934	9.746	9.929	9.485	9.926	9.776
7.239	7.441	7.014	7.217	7.224	6.805
5.933	6.517	6.113	5.919	5.627	5.594
4.646	4.606	4.599	4.004	4.704	4.016
9.729	9.556	8.876	8.485	9.027	8.686
7.852	8.234	7.987	7.807	7.784	7.595
7.852	8.095	7.386	7.023	7.791	7.470
3.604	3.850	4.449	3.211	3.734	2.936
10.465	10.547	10.221	9.868	10.139	10.125

2.838	2.986	3.056	2.808	3.034	3.226
4.134	4.229	4.366	2.975	2.460	2.774
3.740	4.318	4.745	4.046	4.065	2.887
5.887	6.318	6.177	6.140	6.129	5.767
8.925	9.403	8.897	8.309	8.874	8.372
6.113	6.612	6.326	6.215	6.376	5.845
7.363	7.228	7.101	6.641	7.500	7.175
8.857	8.509	8.192	6.846	8.746	8.811
3.869	3.429	3.874	3.258	3.124	3.603
7.719	7.893	7.659	6.035	7.164	7.103
10.779	9.967	10.535	8.329	10.238	10.635
9.444	9.678	9.219	8.985	9.095	8.974
7.726	8.009	7.104	6.517	7.156	7.032
4.463	4.422	4.187	4.125	5.072	3.919
3.127	3.489	3.296	2.265	2.813	3.176
7.922	8.117	7.944	7.683	7.630	7.737
7.276	6.993	6.976	7.051	6.230	6.631
4.392	4.655	3.282	3.916	4.345	3.885
8.067	7.686	7.497	6.078	6.912	7.175
5.721	6.582	5.706	5.891	5.874	5.446
6.510	7.068	6.913	6.555	6.802	6.263
8.911	9.658	9.107	9.174	9.091	8.587
10.462	10.287	9.710	9.356	10.187	11.215
3.702	3.991	3.798	3.456	3.350	3.197
7.448	7.711	7.813	7.408	7.838	7.160
6.320	5.586	6.260	5.832	5.666	6.715
5.350	6.700	6.015	6.101	5.637	5.545
3.148	1.702	3.929	2.928	3.191	4.451
3.000	2.334	3.024	3.013	2.201	3.900
5.324	4.366	4.882	4.613	3.642	5.748
1.811	0.896	2.433	1.446	1.445	2.376
2.744	0.800	2.200	3.147	3.394	4.724
3.517	3.062	3.595	3.218	3.092	3.251
8.650	7.892	8.382	7.527	7.030	9.044
1.187	1.797	2.214	0.513	1.163	1.045
2.393	1.284	2.888	2.254	1.965	3.103
9.297	8.947	8.762	8.400	8.749	9.716
2.738	1.761	2.735	1.904	2.254	3.128
5.384	4.492	4.959	4.695	4.963	5.789
4.646	3.865	4.473	3.629	3.537	5.179
2.775	1.571	3.035	3.741	2.014	3.347
3.368	1.762	3.105	2.750	3.584	4.392
2.604	1.051	2.701	2.983	0.966	3.816
2.604	1.094	3.066	1.972	0.518	3.406
5.317	4.204	5.346	5.141	4.672	6.272
4.242	3.866	4.153	3.246	3.646	4.425
1.499	2.268	1.878	1.440	1.403	3.048
4.079	4.247	3.262	4.035	4.189	4.776
6.426	5.283	6.294	6.395	5.288	7.054
8.747	8.213	8.452	8.152	7.975	9.227

2.635	2.069	3.118	2.474	2.018	3.438
2.444	1.346	2.938	1.184	1.242	3.201
1.195	0.895	1.260	2.437	0.688	2.172
1.975	2.624	0.423	3.190	1.378	1.625
2.102	1.490	3.315	0.846	1.049	1.649
4.385	3.479	4.747	3.768	3.547	4.893
4.890	4.384	5.669	4.852	5.415	5.925
3.586	2.623	3.437	3.092	2.898	4.078
9.868	9.125	9.878	9.391	9.336	10.372
10.459	9.834	10.482	9.957	9.825	10.897
2.197	0.463	2.056	1.716	2.066	2.362
7.929	7.231	8.212	7.305	7.349	8.493
7.311	6.177	7.250	6.844	6.665	7.700
2.150	0.946	2.291	2.267	1.959	3.857
7.209	5.889	7.972	7.042	6.919	8.372
6.875	6.021	6.960	6.016	5.722	7.555
4.659	3.659	4.889	3.459	4.146	5.375
4.526	3.020	4.467	4.280	4.195	5.186
11.009	10.506	11.097	10.774	10.612	11.539
3.777	3.601	3.846	3.571	2.936	4.826
2.018	2.315	1.988	1.693	1.653	2.578
10.451	9.940	10.496	10.047	9.960	11.013
2.408	2.774	2.851	3.068	3.149	3.684
10.934	10.451	10.981	10.614	10.454	11.409
10.922	10.414	10.971	10.605	10.440	11.399
10.881	10.403	10.903	10.581	10.431	11.303
1.795	2.827	2.164	1.572	1.891	3.129
8.047	7.682	7.621	7.219	7.664	8.036
9.873	9.329	9.749	9.088	9.277	9.775
2.133	2.037	2.561	1.552	1.581	2.079
7.828	6.952	7.591	7.092	7.245	7.625
6.115	6.160	6.940	6.527	6.322	6.855
5.366	4.157	5.432	4.345	4.833	5.270
3.730	3.302	3.668	3.322	3.373	3.832
6.206	5.598	6.096	5.111	5.371	6.387
5.341	4.711	5.198	3.999	5.138	5.476
7.087	5.975	6.852	6.247	6.312	6.982
6.157	5.591	6.241	5.796	5.173	6.576
8.753	8.304	8.785	8.663	8.694	8.578
8.603	8.291	8.679	8.520	8.534	8.366
6.717	6.992	6.800	6.420	6.702	6.247
4.693	6.138	5.344	5.354	5.320	4.808
2.165	1.686	2.777	2.493	2.995	2.607
3.867	3.528	3.248	3.151	4.036	3.126
3.268	3.612	2.534	3.674	3.189	2.720
5.347	6.064	5.646	5.654	5.412	4.850
5.038	5.556	4.795	4.921	4.655	4.636
5.550	5.914	5.689	4.952	5.522	5.306
7.225	7.583	7.699	7.284	7.274	7.194
4.912	4.911	5.076	4.524	4.120	5.054

5.318	6.047	6.846	5.650	7.014	5.549
9.634	9.953	9.625	9.410	9.419	9.346
8.809	9.211	8.411	7.693	8.170	8.029
3.024	3.011	3.342	3.072	2.813	2.222
3.610	5.057	4.526	5.226	5.216	4.569
6.481	6.656	7.028	6.001	6.274	6.224
2.609	1.821	1.773	1.897	1.963	1.825
3.053	3.503	1.697	2.821	3.954	1.851
3.461	3.423	3.877	4.138	3.865	3.646
5.139	5.973	5.485	5.273	5.020	5.038
4.017	4.667	3.889	5.308	4.123	3.802
8.468	8.917	8.234	7.767	8.173	7.995
6.354	6.709	5.744	5.343	5.794	6.043
9.660	9.321	9.419	8.857	9.284	9.294
5.759	5.947	6.124	5.914	6.157	5.880
5.140	5.860	6.041	5.514	5.314	5.510
2.294	3.024	4.699	1.708	2.548	2.913
4.945	5.421	5.608	4.939	5.067	4.986
6.430	6.530	6.892	6.485	6.412	5.998
3.638	4.018	4.001	4.766	3.027	3.654
7.255	7.378	7.263	6.775	6.919	6.904
9.159	8.916	9.126	8.866	8.809	8.808
6.421	6.288	6.680	5.682	6.852	6.006
6.958	7.057	7.029	6.334	6.921	6.198
5.626	5.491	5.097	4.906	5.209	4.747
2.284	2.598	2.955	3.071	4.025	2.704
3.185	3.478	3.406	3.440	3.081	2.546
11.497	11.457	11.569	11.396	11.392	11.389
2.870	3.520	3.584	3.870	2.329	2.610
9.472	9.492	9.612	9.150	9.236	8.859
3.099	3.840	2.903	2.763	3.454	2.254
2.569	2.741	3.016	2.405	2.998	1.833
5.475	6.152	5.176	5.445	5.932	5.169
3.982	3.926	4.330	3.840	4.191	4.329
2.729	2.579	3.096	3.411	2.931	3.238
8.529	8.374	8.428	8.026	7.923	8.029
6.428	6.865	6.526	6.104	6.081	5.948
3.734	3.644	3.700	3.001	3.324	1.816
7.933	7.968	7.277	7.311	6.988	7.295
5.049	5.278	5.704	5.082	5.592	4.599
8.727	8.721	8.459	8.192	8.134	8.122
2.236	2.085	3.138	2.388	1.803	2.442
2.180	2.920	2.817	2.471	2.122	3.150
5.503	4.941	5.223	5.205	4.973	5.692
4.327	4.272	4.498	4.635	3.772	5.205
9.136	8.333	9.441	9.347	8.467	9.104
6.335	5.575	6.454	6.342	5.634	6.350
5.813	6.300	6.524	6.725	6.786	6.054
4.390	4.555	4.418	3.560	4.555	4.614
5.670	4.383	6.116	5.430	5.549	6.439

6.489	6.240	6.250	6.416	6.558	6.342
4.198	4.347	3.781	4.361	4.124	4.634
3.555	3.271	4.283	3.714	3.573	3.234
4.683	4.765	5.247	5.020	5.010	4.817
2.748	3.828	2.434	3.488	2.992	2.301
7.154	7.354	7.674	7.411	7.378	7.346
2.230	2.311	2.713	3.121	2.924	2.548
5.247	5.275	5.599	5.687	5.525	5.240
6.147	6.651	6.029	6.929	6.284	6.934
5.958	6.854	6.557	6.512	6.950	6.603
6.393	6.054	5.876	5.840	5.680	6.508
6.386	8.159	8.460	7.407	7.301	6.933
5.292	5.632	5.266	6.040	6.054	5.382
5.883	5.751	5.887	5.930	5.731	6.200
4.388	4.999	4.764	4.526	5.033	4.090
4.421	4.954	4.606	5.179	4.619	4.034
5.873	5.239	5.668	5.723	4.838	6.159
2.245	1.986	1.905	1.990	1.742	1.968
4.854	4.370	4.478	4.609	4.111	4.986
4.057	4.923	4.891	4.203	4.632	4.468
4.751	4.397	4.906	4.854	4.480	5.571
2.643	2.409	3.888	2.431	3.109	2.613
1.878	1.983	2.084	2.425	1.403	2.022
4.130	3.814	4.262	4.217	3.700	3.963
2.540	2.836	3.009	2.598	2.268	2.908
3.004	3.579	3.285	3.544	3.407	3.122
2.714	2.546	2.633	2.794	2.634	2.028
4.734	4.825	4.548	4.596	4.543	4.366
2.573	2.899	2.329	3.194	2.119	3.999
3.944	3.261	4.245	3.648	4.030	4.076
4.810	4.629	5.048	4.640	4.903	4.965
5.983	6.012	6.298	5.268	5.936	5.964
2.947	2.295	3.167	3.141	3.664	2.719
7.013	7.198	7.287	7.233	7.425	7.222
5.008	6.155	4.707	5.447	5.653	5.019
5.213	6.169	5.287	6.071	6.117	5.245
3.016	3.397	2.512	2.717	3.268	3.117
3.042	1.561	1.590	1.899	1.988	1.332
2.941	2.599	2.156	2.973	2.996	3.425
3.283	2.296	1.873	1.844	1.862	1.836
3.368	2.668	2.904	2.262	2.292	2.160
7.933	7.669	7.860	7.662	7.470	7.560
6.389	6.776	6.630	6.919	6.522	6.077
2.604	1.877	2.145	1.426	2.200	2.214
3.948	3.899	3.923	3.059	2.731	3.453
2.814	3.386	3.719	3.742	3.045	3.358
2.159	2.387	1.713	2.545	2.401	2.680
1.936	1.341	1.940	1.532	1.603	2.143
3.918	4.263	4.507	4.626	3.827	4.082
5.081	5.266	4.998	5.429	5.076	5.170

3.441	2.180	2.721	2.923	3.496	3.061
4.489	4.326	3.959	4.560	4.088	4.548
3.582	3.146	3.443	3.510	3.189	3.343
4.459	4.402	5.622	4.173	4.880	3.809
2.821	3.481	2.839	1.796	2.850	2.124
3.679	4.147	3.667	4.538	4.081	3.408
3.571	2.706	3.121	4.581	3.368	5.011
5.499	6.874	5.677	6.282	6.049	5.312
8.187	7.848	8.262	8.181	8.194	8.332
3.802	3.301	3.736	3.954	4.137	3.502
2.632	3.440	2.264	2.537	2.924	2.836
5.313	5.819	5.095	5.444	5.366	5.666
4.057	3.833	3.415	3.694	2.542	2.962
3.637	5.007	4.123	4.566	5.006	3.080
2.454	3.080	1.919	2.426	3.290	2.325
6.394	6.392	6.947	6.509	6.413	7.081
7.090	6.756	7.131	7.503	7.215	7.258
6.598	5.843	6.346	6.822	6.153	6.284
11.344	11.792	11.499	11.669	11.568	11.344
2.949	3.686	3.476	3.768	2.706	3.197
4.115	4.003	4.418	4.308	4.166	3.953
3.084	3.549	3.067	3.247	3.323	2.612
2.563	2.270	2.509	2.443	1.451	1.813
4.261	4.966	4.846	4.488	4.571	3.972
4.422	3.973	4.317	3.839	4.382	4.146
3.913	3.787	3.818	3.370	3.818	3.666
4.347	3.518	3.633	4.280	3.686	4.644
4.507	3.931	4.458	4.320	3.199	5.051
4.767	4.215	4.083	4.452	3.934	5.019
6.988	6.753	7.305	7.361	7.004	6.989
5.245	6.113	6.525	5.057	6.662	4.626
5.847	6.075	5.941	6.411	6.330	6.032
2.602	2.321	1.708	2.054	2.498	3.280
3.437	2.806	3.733	3.620	3.928	3.373
6.858	5.937	6.828	8.041	5.704	7.380
4.673	6.205	4.622	5.806	5.698	4.673
8.901	8.914	8.602	8.415	8.064	8.535
2.313	2.875	1.927	2.444	2.143	2.586
5.362	5.685	5.216	5.842	5.941	5.973
6.584	6.146	7.190	6.809	7.042	6.392
7.562	7.689	7.639	7.700	7.692	7.389
7.864	7.788	7.402	8.162	7.844	7.115
9.548	10.727	10.384	9.490	10.058	9.112
2.095	2.024	2.662	1.942	1.942	1.478
3.806	3.721	3.830	3.536	4.055	3.749
6.711	6.932	6.886	7.197	7.229	7.042
4.821	5.330	5.037	4.917	5.080	5.128
7.107	6.516	7.141	7.104	7.071	7.460
6.395	6.634	7.219	6.702	6.832	5.757
4.285	5.116	4.158	4.321	4.494	4.129

2.800	2.841	2.812	3.282	3.325	3.075
4.512	4.887	4.459	5.444	4.933	4.876
4.649	4.090	3.977	4.354	3.949	4.392
5.008	5.173	4.530	4.748	5.293	4.972
3.017	3.253	3.137	3.181	3.291	2.669
7.250	7.379	7.449	7.537	7.521	7.241
5.109	5.505	4.926	5.598	5.348	5.252
5.369	6.068	5.153	5.502	5.035	5.373
6.592	5.719	6.536	7.656	6.823	7.520
4.118	4.092	3.823	4.087	3.412	4.539
1.654	1.482	1.870	1.181	1.142	1.938
1.865	2.317	1.999	1.687	2.047	1.943
3.250	3.086	3.327	3.734	3.223	4.060
6.606	6.540	6.970	6.027	6.600	6.501
3.936	4.661	4.705	3.797	4.083	3.991
3.587	4.041	3.597	3.027	3.270	4.475
2.812	2.113	3.103	2.901	2.910	2.034
4.581	4.950	5.088	5.298	4.919	4.211
2.829	1.788	2.860	2.371	2.636	2.386
5.858	6.893	5.730	5.626	6.372	5.441
6.056	6.307	6.423	6.219	6.341	6.153
2.255	1.998	2.141	2.520	2.292	2.487
6.484	6.351	6.753	6.601	6.743	7.078
4.547	3.830	3.897	4.683	3.159	5.707
4.686	6.069	4.665	5.419	5.916	4.878
5.129	5.596	4.686	5.332	5.156	5.471
3.010	3.038	2.463	3.385	3.788	4.313
5.340	7.060	5.492	5.385	6.679	4.967
4.054	3.493	3.404	3.338	3.286	4.025
3.157	2.965	3.540	2.766	3.005	3.211
4.839	4.885	5.189	5.280	5.467	5.015
2.744	2.770	2.354	3.043	3.336	2.518
4.071	3.961	4.260	4.195	4.209	4.527
4.267	3.981	4.119	3.584	4.436	3.807
6.305	6.454	6.585	5.993	6.479	6.014
5.698	4.630	5.370	6.387	5.059	6.312
3.465	4.168	3.175	2.960	3.508	3.809
5.590	5.254	5.532	5.555	5.375	6.005
7.099	7.327	7.051	7.556	7.360	7.951
6.380	6.685	6.510	6.622	6.636	6.208
8.752	9.240	8.865	9.171	9.098	8.930
2.587	2.795	2.950	3.010	2.663	3.306
4.546	5.756	4.754	4.854	5.422	4.375
5.510	5.023	5.753	5.484	5.604	5.549
3.866	5.578	3.759	4.569	5.716	4.336
3.297	2.914	3.663	3.989	4.514	2.364
1.992	1.811	2.300	1.710	2.268	2.268
4.808	5.030	5.564	5.200	5.570	5.352
5.986	7.077	6.673	6.732	7.425	6.051
2.813	4.713	3.329	3.118	3.664	4.113

4.439	4.162	3.958	3.712	4.500	4.848
3.073	3.451	2.988	3.304	2.875	3.476
5.505	6.846	5.420	5.309	5.795	5.358
4.215	4.459	4.224	4.355	4.605	4.189
6.466	5.835	7.145	6.276	6.805	6.661
4.906	4.224	5.044	4.858	4.980	4.488
8.702	6.361	7.484	8.709	7.053	8.868
6.421	5.661	6.147	5.982	6.287	6.743
6.289	6.165	6.884	7.158	5.997	6.114
9.184	9.619	9.512	9.379	9.712	9.116
4.367	3.776	3.635	4.428	3.713	4.350
5.440	5.488	5.680	5.525	6.142	5.474
3.713	3.399	3.949	3.858	3.594	4.037
5.832	5.146	5.587	5.918	5.573	6.094
2.080	2.121	2.185	2.398	2.198	2.061
4.544	4.247	4.656	4.370	4.499	4.625
3.022	2.972	3.337	3.387	3.066	3.257
3.811	3.533	3.943	4.127	3.604	4.393
3.179	3.794	3.619	3.775	3.954	2.913
7.509	6.127	6.663	7.423	6.057	8.192
6.207	6.019	6.209	6.152	6.065	5.869
5.865	6.481	5.967	6.384	6.078	5.747
3.804	5.293	3.853	4.546	4.696	3.790
7.171	6.872	7.526	7.465	7.569	7.423
5.426	5.129	5.170	5.470	5.081	5.861
2.010	2.083	2.159	2.376	1.893	2.247
6.097	5.155	6.027	6.012	6.089	6.354
4.375	3.995	4.359	4.212	3.828	4.356
1.993	1.674	2.150	2.352	1.914	2.618
5.473	5.760	5.694	6.111	5.543	5.646
6.420	5.892	6.379	6.152	6.285	6.405
5.887	6.334	6.520	6.189	6.568	5.794
5.178	4.835	4.871	5.110	4.811	4.957
4.525	4.810	4.431	4.761	5.200	4.449
2.401	2.263	1.587	2.752	2.847	2.408
2.138	2.523	2.502	2.617	2.899	2.222
5.542	5.123	5.591	6.547	5.700	5.173
4.976	3.476	4.020	5.384	4.040	5.538
5.622	5.883	6.204	6.265	6.596	5.836
4.051	3.870	3.222	3.895	4.049	2.553
6.715	6.427	6.654	6.198	6.102	6.365
5.454	5.739	5.523	5.769	5.736	5.630
4.641	4.723	5.011	4.559	4.117	4.789
5.795	5.574	5.164	6.274	5.860	5.624
3.872	3.534	4.072	3.781	3.449	4.528
4.086	4.148	3.890	3.887	3.508	4.496
2.936	2.634	2.679	2.668	3.784	3.175
6.550	6.416	5.978	7.357	5.940	7.353
4.941	4.380	3.589	5.716	3.165	5.832
6.066	3.501	4.770	5.995	4.287	6.429

3.334	3.434	3.011	3.514	3.098	3.318
5.309	5.382	5.379	5.349	5.961	5.266
3.477	3.200	3.360	3.756	3.099	4.431
2.670	2.573	2.485	2.180	1.817	3.040
3.743	3.151	3.483	4.188	3.521	3.887
3.411	3.474	4.707	4.395	4.073	4.005
4.357	5.330	4.106	5.247	5.265	4.134
5.806	4.876	5.118	6.030	4.344	6.739
6.053	4.462	4.983	5.761	4.112	6.555
3.449	2.832	2.748	3.768	3.181	4.300
3.257	3.130	2.744	3.615	2.254	3.313
6.433	5.843	6.023	6.028	5.954	6.321
4.496	5.324	4.642	4.468	4.328	4.575
5.245	5.006	5.050	4.661	4.788	4.757
5.223	5.221	5.145	4.629	4.795	5.035
4.589	4.530	4.211	4.756	4.547	4.437
7.249	7.969	7.553	7.723	7.470	7.134
4.647	3.780	4.580	4.846	4.184	5.270
8.049	7.935	8.222	8.115	7.725	7.914
2.263	2.954	2.932	1.634	2.301	2.158
4.385	4.744	4.210	3.963	4.354	4.181
1.158	0.839	1.248	1.381	1.566	1.748
5.059	4.138	4.515	5.008	3.898	5.153
4.811	4.460	4.915	4.827	4.545	4.964
7.555	7.489	7.825	7.875	7.593	7.389
3.244	2.757	3.026	3.600	2.214	4.092
3.923	3.379	3.692	3.777	3.417	5.119
2.709	1.863	2.533	2.935	2.575	2.943
3.612	3.646	3.321	3.829	2.527	4.507
4.983	5.159	5.035	5.110	4.572	5.111
3.177	2.805	3.070	3.659	2.496	3.618
5.457	4.929	5.521	5.887	5.727	5.575
6.087	2.538	4.787	5.933	3.720	6.662
3.810	3.388	3.126	4.280	2.949	4.373
6.635	4.758	6.160	6.823	5.378	7.474
4.814	4.238	4.252	4.944	4.121	5.632
3.374	3.472	3.066	3.278	3.314	3.260
2.859	3.469	2.820	2.412	2.722	3.198
4.830	5.235	4.996	4.958	5.010	4.896
4.482	5.521	6.631	5.065	6.517	4.446
5.278	4.861	6.038	4.855	4.661	5.037
2.844	2.101	2.717	3.148	2.585	4.153
3.477	3.247	3.281	2.763	3.104	3.228
3.381	3.691	3.666	3.514	3.123	4.227
3.021	2.527	2.839	3.511	3.002	4.135
3.646	3.681	3.956	3.246	3.970	3.827
3.921	3.760	4.102	3.428	3.328	4.161
3.535	2.840	3.008	3.664	2.751	3.358
3.038	2.811	3.023	2.792	3.023	3.041
2.493	2.394	2.685	2.494	2.725	2.704

4.283	4.347	4.049	4.666	4.599	4.455
5.650	5.789	6.735	6.004	6.367	6.057
4.802	4.745	4.336	4.571	4.915	4.728
3.954	4.110	4.109	4.262	4.044	3.889
3.425	5.150	3.829	4.790	4.838	4.218
5.458	6.574	5.063	6.073	5.017	5.196
3.135	4.063	3.624	3.715	3.767	3.249
5.453	4.270	4.961	5.653	4.194	6.155
2.856	2.777	2.399	2.895	3.043	2.558
5.374	5.545	5.287	5.629	5.328	5.425
6.872	6.774	7.052	7.073	6.976	6.872
4.618	5.561	5.183	5.228	5.320	5.149
4.855	5.803	5.393	5.426	5.523	5.343
4.149	4.568	4.107	3.897	4.396	4.361
2.120	1.388	2.131	2.343	2.535	1.844
3.361	2.872	3.350	3.073	3.104	3.340
4.072	3.535	4.194	2.380	4.491	4.630
2.821	2.492	2.925	1.916	2.454	2.729
3.077	2.136	3.802	3.090	3.391	3.392
4.336	4.426	4.758	4.230	4.893	5.074
3.931	3.356	4.323	2.359	4.444	3.931
3.803	3.295	4.821	4.132	4.333	4.090
6.696	6.817	7.003	6.851	6.770	6.602
5.845	6.803	6.047	6.874	5.869	5.691
5.221	5.442	5.333	5.758	5.476	5.327
5.054	4.944	4.507	5.121	5.251	5.657
4.608	5.170	5.770	4.418	5.175	3.997
4.363	4.687	4.656	5.079	3.922	5.326
4.786	4.666	4.275	5.268	4.882	4.919
5.517	5.687	6.246	5.803	5.881	6.194
4.516	3.573	4.271	4.216	4.371	4.467
4.208	4.169	4.755	4.068	4.231	4.442
1.505	2.048	1.577	1.721	1.555	2.236
4.824	5.023	5.079	5.082	5.052	5.291
2.632	2.102	2.327	2.636	3.207	2.331
2.899	3.064	2.433	2.936	2.600	2.409
3.861	3.777	3.488	3.714	3.420	3.548
3.873	4.766	4.442	4.350	4.817	3.320
5.236	4.759	6.702	6.307	6.385	5.142
4.348	4.441	4.660	4.389	4.779	4.248
5.023	4.060	4.372	4.743	4.394	5.065
3.805	4.290	3.604	4.786	4.641	4.151
3.072	2.800	2.963	2.897	2.773	2.273
3.085	2.404	3.284	3.298	2.520	3.629
6.720	6.752	6.998	6.653	6.927	6.870
3.035	4.110	3.381	3.088	2.967	2.967
6.959	6.516	6.879	6.315	6.249	6.460
4.798	5.214	4.813	5.361	5.230	4.579
4.221	4.829	3.883	4.449	4.494	4.075
3.304	4.190	3.763	3.033	3.536	3.124

4.449	4.369	4.477	4.859	4.338	4.503
5.825	5.561	6.066	6.064	6.195	5.635
2.857	2.116	2.809	2.165	1.943	2.691
3.272	1.704	2.100	1.707	2.561	2.429
3.185	3.801	3.215	3.028	3.723	3.642
1.207	1.169	1.501	1.315	1.580	0.962
2.391	2.882	2.903	2.671	2.163	3.214
5.146	4.242	5.153	5.279	5.154	5.568
4.637	3.936	4.849	3.965	4.050	4.602
3.560	3.810	3.690	4.487	3.470	2.815
3.223	3.300	2.954	4.185	2.952	2.671
3.738	3.379	3.441	3.762	3.329	3.853
4.370	5.490	5.696	4.972	4.414	5.819
3.616	2.108	1.624	1.189	1.165	2.288
2.818	1.898	2.381	2.082	2.550	1.859
2.789	2.931	2.928	2.441	3.133	2.584
4.684	5.201	4.889	3.444	5.247	4.899
6.364	6.405	6.297	5.846	6.154	6.507
5.176	4.981	5.301	5.621	4.854	6.271
3.694	3.278	4.014	4.214	3.573	4.441
4.871	3.776	5.164	4.808	4.361	5.012
2.716	2.295	2.440	2.185	2.319	2.748
4.880	5.519	5.314	5.364	5.276	4.239
5.972	6.232	5.781	5.256	5.501	5.938
3.076	3.430	2.608	3.721	2.549	3.934
2.668	2.909	2.994	2.664	2.717	2.731
2.959	3.135	2.431	3.372	2.363	3.690
5.623	5.190	5.596	6.251	5.951	5.658
4.884	5.279	5.196	5.320	5.258	5.523
2.867	1.565	2.865	2.611	2.732	1.792
2.244	3.065	1.567	2.175	2.443	2.788
3.514	3.397	3.570	3.697	3.364	3.731
3.767	3.101	3.581	3.053	3.321	3.453
10.535	10.130	10.519	11.172	10.194	10.760
4.400	4.864	4.592	4.406	5.148	5.359
2.164	3.104	2.311	2.845	2.963	1.983
2.980	2.830	2.350	2.713	2.433	2.488
6.434	6.873	6.294	6.728	6.948	6.541
2.113	1.730	2.106	2.545	2.103	2.037
6.009	6.458	6.710	6.521	5.803	6.103
6.209	6.945	6.457	6.432	6.437	5.750
4.850	6.307	4.803	5.406	5.256	4.540
7.405	7.851	7.866	8.402	8.783	7.696
2.023	1.994	3.068	2.643	4.078	2.537
5.255	4.547	5.464	4.790	4.838	5.264
4.238	5.567	5.490	4.521	6.167	3.977
6.501	7.424	6.687	7.637	6.596	6.761
6.626	7.842	6.419	7.142	7.378	6.721
3.660	3.567	3.395	4.472	3.227	3.714
3.328	3.767	4.075	3.340	3.684	3.049

6.031	7.372	5.653	5.433	6.325	5.899
3.553	3.703	3.762	3.626	3.071	2.988
2.943	2.607	3.809	3.150	3.846	2.441
4.000	4.300	4.824	4.890	4.486	4.409
3.553	5.032	5.435	4.414	4.124	3.448
5.647	7.109	7.692	5.654	6.063	5.484
7.395	8.026	7.780	8.381	8.243	7.802
5.919	5.550	6.124	5.750	5.768	5.369
3.861	4.737	4.148	4.028	3.909	4.662
2.138	2.341	1.961	2.458	2.504	2.310
5.549	6.281	7.114	6.097	6.789	5.264
4.558	4.300	3.787	5.120	3.013	5.243
2.749	2.689	3.097	3.256	2.608	2.887
4.215	3.935	4.644	4.123	4.784	4.226
1.997	1.794	2.195	1.610	2.785	1.073
3.335	3.729	3.253	3.254	3.469	3.088
4.193	4.256	3.931	4.131	3.620	4.722
3.520	3.532	4.279	4.136	4.216	3.701
3.271	2.544	2.507	3.244	2.324	3.644
4.910	4.792	4.762	5.128	4.818	5.395
5.541	5.470	5.862	5.873	5.743	5.463
2.639	2.712	3.322	2.987	2.714	3.155
2.948	3.308	3.221	3.864	3.221	2.287
2.521	2.632	2.788	2.104	2.258	2.653
3.326	4.772	3.642	3.298	3.615	3.563
5.595	6.033	5.134	5.922	6.026	6.145
4.181	4.122	4.441	4.284	4.194	4.077
7.241	8.191	7.564	7.778	7.885	7.441
8.208	8.319	8.406	7.950	8.274	8.114
7.883	7.785	7.834	8.229	7.602	8.030
6.255	5.211	5.038	6.723	5.731	5.104
7.413	7.702	8.462	7.545	8.699	7.014
5.148	5.421	5.208	5.787	5.577	5.329
5.360	5.177	5.369	5.460	5.537	5.495
7.948	7.602	8.497	8.283	8.199	8.006
2.617	3.086	2.792	1.995	3.226	3.164
2.978	3.615	2.554	3.686	3.369	3.119
4.914	4.533	4.622	4.942	4.252	4.800
5.467	5.313	5.178	5.093	5.158	5.152
3.312	3.207	2.630	3.391	2.986	3.348
3.378	3.863	2.989	2.980	3.205	3.075
7.349	7.948	7.400	7.456	7.518	7.127
5.871	5.837	6.131	5.979	5.805	6.074
4.019	5.468	4.746	5.781	4.660	4.421
3.108	3.759	3.335	4.789	4.003	3.410
5.250	6.592	6.443	6.787	6.494	5.092
4.485	5.899	5.241	5.537	5.921	4.689
6.098	6.443	6.851	6.863	6.567	5.906
4.797	5.337	5.757	6.397	6.315	4.472
3.214	3.844	3.743	3.538	4.311	2.676

4.160	5.798	4.261	5.783	5.209	4.778
1.835	4.248	2.412	2.260	3.516	1.983
3.855	4.223	4.203	4.465	4.704	4.045
6.365	7.588	7.199	6.894	6.144	7.397
3.338	3.763	4.046	3.519	2.965	4.571
5.450	6.037	6.287	6.649	6.363	5.751
4.244	4.715	3.962	4.704	4.826	4.497
4.906	5.170	4.924	4.933	5.194	4.556
2.590	2.324	3.437	3.080	2.439	2.780
5.750	6.376	6.237	6.410	6.264	5.931
6.993	7.157	6.987	7.366	7.000	7.315
3.670	4.559	3.316	4.748	3.932	3.614
3.961	3.154	3.511	3.376	4.147	3.517
5.892	6.188	6.405	6.128	6.093	5.967
2.735	3.588	2.502	3.609	3.413	2.980
3.748	4.476	3.870	4.658	4.400	3.744
5.476	5.564	5.702	6.462	5.969	5.826
5.107	4.277	4.713	4.812	4.713	5.322
5.898	6.097	5.813	5.921	5.917	5.577
4.869	5.226	4.336	4.943	5.093	4.629
2.393	2.360	2.608	2.491	2.550	2.686
4.043	4.142	4.061	5.743	4.625	4.438
2.893	2.828	2.843	2.527	2.632	2.185
3.662	3.497	3.684	4.117	3.541	4.511
3.007	2.862	3.099	2.846	2.632	3.092
5.702	6.911	5.748	6.684	6.691	5.350
4.251	4.665	4.673	5.022	4.453	4.227
4.803	4.355	4.571	4.551	4.199	3.907
5.734	6.849	6.632	6.518	6.641	5.497
6.906	6.541	7.901	7.338	7.245	7.193
4.509	4.337	4.321	4.741	3.665	5.135
6.826	6.468	7.016	7.051	6.552	6.841
6.373	5.967	6.771	7.022	7.189	6.641
6.368	6.189	6.495	6.172	6.210	6.214
1.949	1.943	1.213	1.754	1.522	1.929
1.650	1.627	1.481	1.203	1.457	1.788
3.913	4.860	4.209	4.317	5.766	3.640
3.738	3.764	3.594	4.104	5.140	4.201
4.536	5.065	4.070	4.318	4.043	5.278
5.977	6.119	6.219	6.253	6.351	5.581
7.719	7.187	8.649	7.691	7.537	7.772
5.278	5.040	5.514	5.727	5.907	5.357
2.593	2.808	1.900	3.201	3.376	2.085
2.468	2.211	2.407	2.318	2.782	2.919
4.963	5.002	5.075	5.436	4.819	5.669
3.067	4.043	2.761	4.110	3.376	3.007
4.694	5.416	5.136	5.578	5.257	4.928
6.217	6.885	6.462	6.978	6.971	6.142
4.286	4.639	3.795	4.950	4.360	4.580
5.329	4.121	5.234	5.094	4.569	5.680

3.455	3.005	3.033	3.216	3.148	3.161
4.352	3.007	3.941	4.052	3.858	4.641
5.048	5.333	5.082	5.421	5.627	5.080
6.293	6.218	6.259	5.891	6.331	6.613
5.164	4.880	5.333	5.112	5.430	5.032
3.931	4.476	4.043	4.689	4.392	4.121
3.568	2.452	3.081	3.893	2.512	4.537
3.365	3.460	2.997	3.257	3.250	2.901
5.446	5.984	5.659	5.707	5.700	5.664
5.015	5.851	5.606	5.044	5.341	5.108
3.931	4.804	3.972	5.096	3.473	3.908
5.483	5.697	5.602	5.721	5.398	5.702
4.082	4.274	4.187	3.966	4.006	4.557
3.444	3.082	3.635	3.448	3.282	3.771
3.038	2.439	2.543	2.983	2.923	2.841
3.888	2.714	3.476	3.331	3.522	3.572
3.982	3.551	3.270	3.499	3.518	4.697
3.116	2.219	2.680	3.742	3.059	3.546
3.644	4.000	3.749	3.563	3.189	3.271
5.224	5.054	5.149	4.943	5.085	5.404
4.190	4.113	4.126	4.296	4.401	4.198
2.392	1.324	2.789	2.662	2.150	2.273
7.125	6.898	7.459	7.655	7.843	7.823
3.624	4.345	3.552	4.161	3.790	3.566
4.393	3.514	5.189	4.333	4.836	4.450
2.819	3.409	2.019	2.100	1.761	1.898
3.996	3.041	3.411	4.705	3.404	5.217
4.989	4.774	5.233	5.039	5.275	4.578
5.242	5.048	5.885	5.903	5.525	5.335
3.294	2.271	3.144	2.616	2.922	2.818
3.512	3.789	3.643	3.574	3.248	3.735
2.664	2.779	2.952	3.659	3.176	2.944
2.956	4.094	2.814	3.693	4.151	2.611
4.833	4.746	4.910	4.764	5.007	4.844
5.636	4.221	5.389	5.523	4.704	6.274
4.192	4.831	4.192	4.510	4.903	4.351
2.639	2.970	2.448	2.492	2.144	2.654
5.571	5.283	5.518	5.180	5.264	5.803
6.510	6.766	6.742	7.073	6.744	6.531
4.257	3.236	3.846	3.923	3.762	3.497
2.683	3.215	3.466	3.225	3.215	3.167
2.221	4.003	3.442	3.375	3.654	1.957
2.620	1.925	2.429	3.012	2.826	2.474
2.481	3.317	2.925	2.565	1.795	3.270
2.736	2.689	2.953	2.738	2.293	2.715
7.265	7.459	7.592	7.396	7.357	7.537
3.443	4.361	2.800	2.521	2.889	4.085
6.877	6.830	7.073	6.900	6.426	7.043
6.523	6.925	6.917	6.311	6.911	7.125
5.330	4.602	5.920	5.061	5.219	5.051

4.103	3.171	3.909	4.503	3.912	3.970
8.152	8.243	7.823	8.127	8.130	7.958
8.084	7.491	8.304	8.346	8.018	8.351
2.336	2.758	2.334	2.304	1.591	2.419
3.896	3.650	5.094	4.601	3.723	3.570
5.409	5.740	5.747	5.858	5.719	5.337
4.601	5.046	5.043	5.180	4.310	3.874
2.398	3.028	2.727	2.097	2.147	1.388
5.919	5.880	6.018	5.982	6.107	6.011
6.147	6.267	6.554	6.616	6.726	6.442
6.216	6.623	6.120	6.705	6.342	6.646
5.577	5.647	5.784	6.126	5.635	5.991
6.773	7.682	7.806	7.834	6.682	6.870
4.469	5.105	5.095	4.908	5.049	4.099
5.624	6.655	6.459	5.662	6.521	5.239
3.058	4.295	3.275	3.555	3.679	3.543
2.317	3.168	2.394	3.153	3.002	1.919
2.344	2.833	2.139	2.369	3.123	2.502
3.883	3.757	4.229	4.427	4.639	3.478
3.261	4.266	3.870	4.073	2.987	2.578
2.991	3.771	3.334	3.870	2.763	3.232
3.346	3.619	3.166	2.940	3.430	2.936
4.097	5.354	4.471	4.689	4.760	3.521
5.501	6.995	6.083	6.696	5.511	6.252
2.676	1.757	2.692	2.975	2.756	1.970
3.328	4.834	3.846	3.873	4.613	2.543
5.927	6.289	6.440	6.436	6.825	5.874
7.264	9.156	8.301	8.019	8.754	6.720
7.966	8.256	8.031	8.283	7.987	7.301
3.403	3.275	3.149	2.941	3.043	3.731
5.333	6.458	4.653	6.528	5.390	5.324
3.560	5.084	5.349	4.413	4.659	3.702
2.949	3.551	2.654	2.615	3.328	3.173
2.516	3.300	3.735	3.273	4.470	3.066
6.486	6.039	6.932	6.712	6.730	5.902
2.805	3.578	2.009	1.750	2.586	3.045
1.978	2.268	1.723	2.180	1.417	2.301
2.053	1.795	1.712	1.773	2.140	2.248
5.510	5.428	5.677	5.138	5.419	4.763
5.693	5.696	5.612	5.356	5.907	5.347
4.778	4.895	3.476	4.445	4.170	4.659
4.313	4.665	3.595	3.817	4.143	4.540
4.652	5.259	4.079	5.104	4.895	4.272
4.975	5.393	4.235	4.980	5.181	4.603
4.793	5.284	4.141	4.739	4.851	4.669
5.291	5.218	5.975	5.610	5.336	5.283
3.019	2.736	2.034	2.986	2.410	2.546
5.448	5.763	5.244	5.213	5.331	5.522
6.415	5.647	6.850	6.516	6.622	6.239
6.212	6.076	5.965	6.486	6.069	6.383

5.872	6.132	5.866	6.054	5.964	6.368
3.852	3.870	4.171	4.125	3.657	3.773
5.993	5.879	5.942	6.280	6.156	6.382
6.008	6.416	6.119	6.701	6.567	6.480
5.517	5.388	5.557	5.891	5.691	5.264
3.091	3.598	2.486	3.536	3.169	3.095
5.090	5.568	5.162	5.471	5.620	5.386
3.657	4.149	3.679	3.787	4.360	2.817
4.255	4.229	4.339	5.223	4.400	4.284
8.836	8.377	8.273	8.241	7.827	8.572
3.966	4.521	3.882	4.327	4.707	4.769
4.166	4.553	4.259	5.371	4.815	4.626
2.485	2.838	2.168	2.396	2.066	2.456
2.812	2.657	2.415	3.047	1.927	3.019
2.909	3.464	2.618	2.902	2.519	3.579
4.304	5.467	4.139	4.341	4.283	3.914
4.218	4.529	4.076	4.326	3.817	3.854
5.866	6.737	5.738	6.790	5.881	5.853
8.512	8.460	8.344	8.321	8.586	8.788
8.462	7.525	8.493	8.213	8.189	8.219
6.992	6.710	6.795	6.650	6.783	7.619
2.343	2.702	2.795	3.370	2.467	3.003
2.550	2.169	1.833	2.078	2.089	2.085
5.003	5.052	5.581	5.634	6.248	5.598
8.713	8.942	8.606	8.111	7.661	8.134
3.772	2.002	3.461	3.757	3.650	4.391
2.787	2.827	3.412	2.831	2.792	2.763
2.236	2.424	2.331	2.477	2.728	2.291
4.003	4.930	3.734	4.404	4.539	3.976
3.974	4.992	4.731	4.443	4.308	3.907
3.100	3.766	2.453	3.328	3.667	2.297
3.023	2.965	2.730	3.003	2.813	2.772
5.205	5.285	5.399	5.729	5.314	5.112
3.299	2.602	2.694	2.943	2.870	3.260
2.120	2.666	2.916	1.621	3.057	1.925
3.235	4.028	2.022	2.316	0.649	2.424
8.417	7.772	8.256	7.727	7.607	7.980
2.505	3.951	3.414	2.587	3.023	2.403
2.207	1.883	1.634	2.280	1.832	2.112
4.747	5.048	5.082	4.863	5.038	5.655
4.322	4.176	4.453	4.849	4.372	4.572
2.300	1.998	2.514	1.978	1.452	1.598
6.263	6.206	6.397	6.667	6.143	6.781
3.297	2.649	2.956	2.274	3.154	2.865
3.817	3.701	4.229	3.203	4.360	3.701
3.028	4.709	2.859	3.890	2.970	2.490
4.997	5.997	4.990	5.140	5.408	5.375
2.604	3.742	2.764	2.712	2.781	3.335
2.615	2.863	2.702	1.711	3.178	2.788
3.932	5.152	3.900	4.255	3.307	3.988

3.116	2.785	2.626	4.599	2.876	3.313
3.634	3.418	4.141	2.868	4.241	1.687
3.048	3.162	2.946	2.648	3.099	3.191
4.839	4.631	5.814	4.307	5.338	4.524
3.331	5.106	4.675	3.715	4.837	3.744
5.940	7.764	6.115	6.990	6.987	6.127
2.773	3.659	2.277	3.796	2.316	2.352
3.834	4.438	3.576	4.391	3.914	4.510
2.988	3.711	2.428	3.207	2.455	3.442
2.673	3.537	3.031	3.049	1.569	4.290
2.305	2.805	2.210	2.102	2.210	3.765
2.857	3.182	2.810	2.879	2.962	3.256
2.266	2.609	2.243	2.654	2.352	2.893
2.253	2.754	2.237	2.828	2.411	2.920
3.819	5.057	5.090	4.877	4.762	4.202
6.759	5.822	7.310	7.167	6.585	6.952
2.610	3.037	2.429	2.431	2.643	3.394
3.462	4.155	3.364	3.925	4.222	3.790
6.199	6.953	6.970	6.808	6.797	5.808
5.395	5.095	5.581	5.836	5.648	5.749
3.662	3.490	3.107	3.926	4.260	3.846
3.701	3.324	4.075	3.844	3.506	3.836
7.240	6.696	7.010	7.713	6.927	7.689
5.202	6.315	5.871	5.905	6.214	5.384
5.054	5.489	4.569	5.409	5.733	5.553
3.235	4.156	3.421	4.136	4.066	3.161
4.788	4.568	5.237	4.469	5.173	4.843
5.383	5.854	5.283	5.456	5.854	5.168
3.397	2.947	3.714	3.843	3.740	3.966
2.519	2.915	2.041	3.598	3.261	2.940
4.596	4.764	5.029	4.723	4.365	4.354
5.070	5.476	5.103	5.724	5.315	4.972
4.016	2.699	3.203	3.720	3.282	3.395
4.615	3.905	4.100	3.608	4.412	4.273
6.285	5.804	6.266	6.217	6.430	6.590
5.914	5.934	6.113	5.611	6.084	6.109
3.340	3.871	3.509	3.635	3.520	3.826
4.994	5.011	5.419	5.219	5.530	4.690
3.068	4.314	3.119	3.116	3.246	3.135
3.921	3.882	4.166	4.587	3.826	3.600
3.837	3.328	3.813	3.817	3.722	3.824
4.091	3.809	2.900	4.145	2.963	3.740
3.031	2.901	3.216	2.723	2.872	3.273
1.723	2.168	1.864	1.643	1.714	2.581
3.037	2.775	2.699	3.727	3.332	3.272
3.449	2.680	3.519	4.133	1.684	4.589
2.136	1.948	1.405	2.338	2.417	1.579
4.216	4.382	4.155	4.883	4.767	4.449
4.227	4.330	4.481	4.435	4.484	4.072
6.705	6.386	7.179	6.506	6.762	6.712

4.012	4.000	4.022	3.742	3.748	3.197
4.385	4.296	4.532	4.678	4.407	4.662
5.880	6.296	5.808	5.967	5.804	6.354
4.610	4.026	4.891	4.653	4.387	4.607
4.700	4.675	5.827	5.386	4.947	4.998
5.542	5.324	5.506	5.468	5.074	5.443
4.080	4.067	3.884	3.152	3.954	3.754
3.356	2.861	3.578	2.925	2.843	3.418
5.718	5.950	6.299	5.910	6.168	5.960
4.383	4.078	4.577	4.047	4.242	4.627
3.997	3.709	3.458	4.173	3.586	3.502
3.050	3.374	2.305	3.436	3.504	3.586
5.670	4.220	5.233	5.099	5.226	6.550
2.828	2.854	2.868	2.921	3.747	3.235
5.980	5.970	5.843	6.479	6.037	6.151
4.382	4.709	4.458	4.979	4.710	5.040
4.161	3.924	3.504	3.437	3.644	4.388
6.135	6.712	7.045	6.924	6.927	6.891
4.639	4.415	4.790	4.793	4.463	5.273
5.545	5.680	5.746	5.952	6.132	5.596
6.192	5.642	6.291	6.335	5.721	6.546
3.593	3.519	4.152	3.150	4.162	3.184
3.777	3.992	3.723	4.206	3.774	3.889
4.105	5.056	3.866	4.919	4.519	4.027
3.638	3.017	3.441	3.508	3.540	3.459
6.534	6.049	6.342	6.562	6.039	6.712
6.984	6.423	6.974	6.753	6.629	7.125
7.118	6.580	7.225	7.074	7.205	7.283
4.637	3.988	4.436	4.066	3.918	4.616
2.476	2.911	2.023	2.722	2.768	3.038
6.186	5.547	6.268	6.211	6.040	5.753
3.066	3.409	2.992	3.269	3.598	2.367
5.059	4.904	5.163	4.745	4.719	5.354
6.928	6.533	6.913	7.154	6.846	7.328
4.955	3.718	4.785	3.808	4.618	4.519
5.637	5.522	5.595	5.966	5.727	5.922
3.285	2.768	3.344	2.229	2.756	3.488
5.633	5.110	5.732	5.559	5.751	5.949
3.580	2.971	2.820	2.823	3.102	3.062
3.822	3.533	2.937	3.331	2.971	3.677
2.352	1.949	1.658	1.837	2.536	2.104
5.007	5.037	5.107	5.531	5.114	5.815
3.009	2.694	3.491	2.781	2.712	3.115
5.039	5.713	5.386	5.487	6.315	5.034
2.810	2.705	2.538	2.770	2.874	2.705
4.376	3.461	4.500	4.665	4.622	4.724
4.663	4.565	4.205	4.638	4.533	4.675
4.696	4.366	4.672	4.850	4.221	5.185
2.950	2.040	2.418	2.169	2.186	2.178
3.126	2.733	3.104	3.303	3.429	3.497

5.405	6.357	6.137	6.288	5.835	5.084
5.563	5.356	5.848	5.757	5.238	5.804
4.991	4.651	5.428	5.463	3.969	5.709
2.152	1.620	2.536	3.000	2.525	2.719
4.492	4.995	5.020	4.872	5.167	4.506
3.608	3.453	3.395	4.095	3.824	3.503
6.228	6.420	6.313	6.553	6.828	6.131
7.138	6.644	7.524	7.091	6.999	7.812
4.502	3.965	4.585	4.042	3.534	4.539
4.848	4.015	5.445	4.967	4.302	4.306
4.376	2.983	6.217	3.548	4.540	3.137
4.490	4.734	4.860	4.839	4.856	4.310
3.138	2.058	4.309	3.916	3.913	4.025
4.414	4.477	3.618	4.436	4.271	4.081
3.589	3.792	3.716	4.805	3.319	3.117
2.567	2.716	3.494	1.851	3.436	2.177
3.649	5.668	5.909	1.881	6.292	1.894
6.050	5.801	6.077	5.915	5.802	5.941
8.202	8.015	8.343	8.497	7.970	8.232
6.719	6.280	6.852	5.959	6.124	6.890
2.483	2.531	3.098	1.993	2.140	2.795
2.366	1.802	2.546	1.608	2.075	2.425
3.114	2.628	2.400	2.255	1.569	2.199
2.652	2.770	2.337	2.596	2.627	3.559
2.633	5.394	2.871	2.251	2.572	2.234
8.175	8.397	8.071	8.475	7.880	8.101
5.118	5.540	5.373	5.944	5.359	5.221
4.876	5.791	5.531	6.014	4.605	5.125
5.953	6.346	6.414	6.351	6.745	6.309
3.187	3.133	3.358	3.093	2.707	4.355
4.360	4.167	3.799	4.264	4.254	4.612
5.237	4.888	4.867	5.107	4.898	5.854
3.310	3.172	3.337	3.291	4.365	3.269
4.428	4.451	5.312	4.678	6.119	4.557
3.110	5.200	5.600	1.134	5.138	1.488
3.133	5.175	5.704	1.065	5.291	1.245
4.920	4.280	5.252	5.408	4.907	5.297
8.943	8.993	9.461	9.016	9.451	8.484
4.831	5.736	5.145	5.024	4.731	4.747
4.504	5.254	4.742	5.187	5.052	4.600
3.661	4.456	3.243	3.419	4.099	3.735
8.762	10.414	9.516	9.793	10.062	8.742
2.631	3.815	2.127	1.769	2.381	1.942
3.973	3.981	4.106	4.197	4.350	4.350
2.204	2.044	1.845	1.813	2.507	2.110
5.731	6.066	6.143	5.911	6.111	5.689
4.885	5.511	4.669	5.266	4.898	4.625
4.588	5.926	4.460	5.776	5.017	5.176
5.245	5.221	4.704	4.461	5.067	4.599
6.606	7.198	6.746	6.806	7.012	5.935

7.356	7.688	7.824	7.794	8.025	6.986
5.328	5.104	5.513	5.265	4.592	5.349
5.871	6.266	5.864	5.963	5.233	5.894
5.974	5.360	5.402	6.019	5.674	5.921
2.804	3.104	2.203	3.000	2.812	1.947
4.565	3.211	3.947	4.302	3.499	5.307
3.687	3.821	4.249	4.248	4.172	4.018
2.526	2.293	2.837	2.752	2.504	2.264
6.369	6.706	6.391	6.844	6.499	6.738
3.249	3.103	2.914	2.846	3.209	3.438
7.199	7.218	7.409	7.302	7.561	7.263
6.993	6.658	7.219	7.264	6.950	7.257
4.699	4.478	4.179	4.511	4.731	4.932
4.691	4.049	4.064	3.713	4.073	4.314
3.002	2.530	2.773	2.959	3.499	2.816
4.662	4.913	5.882	5.434	5.766	4.523
6.851	6.020	6.664	6.866	6.264	7.058
4.617	4.520	4.666	5.064	5.082	5.068
3.235	3.753	3.364	2.926	3.353	3.634
4.060	5.456	3.921	4.452	4.360	4.012
5.954	5.152	6.579	6.249	6.013	6.334
5.452	6.463	6.910	6.634	7.074	5.781
3.550	3.909	3.497	3.252	4.338	2.673
3.501	4.103	4.056	3.613	4.044	3.545
9.889	9.656	10.165	10.100	9.788	10.175
5.817	5.912	6.303	6.367	5.896	6.428
4.781	4.400	4.535	4.474	4.563	4.967
4.062	3.317	3.893	3.700	3.357	3.426
5.755	5.876	5.776	6.368	6.225	6.241
3.081	3.869	3.905	3.977	3.650	4.120
5.929	6.216	5.876	6.555	6.748	6.454
4.907	4.750	4.492	4.588	4.074	5.559
4.813	4.415	4.788	4.953	4.418	6.679
4.420	5.151	4.416	4.597	4.798	3.737
2.972	2.187	2.641	2.594	2.301	3.708
6.680	6.779	6.661	6.514	6.396	6.516
2.626	2.601	3.019	3.220	2.308	3.191
3.764	2.455	3.309	3.472	3.853	4.596
5.012	4.258	5.060	4.562	4.691	5.797
5.847	5.257	5.622	5.627	4.785	6.413
4.165	5.010	4.259	4.522	4.916	4.035
4.320	4.912	4.626	5.148	4.726	4.568
5.509	5.485	5.796	5.432	5.737	6.023
2.926	2.678	3.379	3.201	3.125	3.210
3.327	3.250	3.775	3.483	3.840	3.687
3.440	3.727	4.087	3.760	3.559	3.498
8.183	8.257	8.340	8.366	8.177	8.429
4.466	4.096	3.892	4.634	4.337	4.644
5.821	5.503	6.047	5.842	5.764	5.278
4.269	3.609	4.641	4.047	3.764	3.636

7.179	6.920	7.215	7.163	7.205	7.343
4.555	4.130	4.870	3.560	4.752	4.608
4.371	4.274	4.670	3.467	4.407	4.604
4.528	4.463	4.987	3.543	4.711	4.615
3.980	4.345	4.530	3.859	4.632	4.107
6.190	6.371	6.478	6.393	6.638	6.397
2.952	2.398	2.902	3.098	3.154	3.220
4.284	4.748	4.179	4.541	4.805	4.689
7.106	7.252	7.411	7.890	7.305	7.445
3.221	3.422	4.482	3.613	3.722	2.610
5.762	5.515	5.898	5.904	5.603	5.934
2.792	2.163	2.255	2.838	2.743	2.606
6.916	6.824	6.620	7.105	7.161	6.574
8.122	7.638	8.319	8.195	7.950	8.140
3.968	5.802	6.525	1.260	6.244	2.261
2.906	3.690	2.721	2.996	3.753	3.746
4.884	5.057	5.194	4.997	5.355	5.139
7.375	7.367	7.743	7.363	7.509	7.378
3.191	0.959	2.252	3.143	2.553	3.426
2.634	4.671	5.490	1.263	4.899	0.786
4.438	3.855	4.319	4.147	4.250	4.531
7.937	6.011	6.888	7.761	6.556	8.159
7.948	8.214	8.248	8.346	8.199	7.926
6.128	5.445	6.579	6.773	6.248	6.534
5.778	6.329	5.534	6.021	5.587	5.918
4.995	6.885	5.050	5.895	6.175	5.030
5.360	5.033	5.256	5.251	4.835	5.268
5.746	5.968	5.938	5.955	5.527	5.666
5.920	7.297	6.032	5.792	5.894	5.886
7.073	7.787	7.406	7.295	7.421	6.892
8.438	7.624	7.572	8.369	7.275	8.927
5.236	6.666	5.508	6.046	5.751	4.779
2.203	2.677	1.660	1.999	2.279	1.891
2.050	2.129	1.881	2.158	2.753	1.937
5.505	5.864	6.160	5.145	5.556	5.871
7.400	7.491	8.197	7.490	7.569	7.315
2.435	2.884	3.301	2.623	3.060	1.746
5.753	7.181	6.508	6.801	6.374	6.258
6.006	6.127	6.210	6.709	6.225	6.578
3.474	3.186	4.209	3.191	3.607	3.356
3.297	3.568	3.536	4.367	3.884	3.631
8.360	8.010	8.173	8.434	8.053	8.588
6.160	7.050	5.886	5.308	6.989	4.276
3.225	4.166	2.422	4.006	3.572	3.692
6.379	6.195	6.245	6.252	6.603	6.569
4.176	5.257	3.763	4.710	4.243	3.431
3.367	3.032	3.599	3.486	2.967	2.560
6.536	7.395	6.776	6.950	7.061	6.756
5.690	6.850	5.932	6.063	6.064	5.623
2.634	2.708	2.467	2.873	2.500	2.910

3.006	2.800	2.959	3.090	3.103	2.804
5.033	4.923	4.902	5.203	4.648	5.350
2.772	3.557	3.531	2.762	3.765	2.905
3.718	3.678	4.204	3.812	3.762	3.593
2.550	4.551	2.912	2.367	2.955	2.280
2.395	5.890	2.950	2.392	2.723	2.190
5.553	5.664	5.889	5.811	6.049	5.664
5.571	5.335	5.901	5.766	5.441	5.909
3.929	4.281	3.771	3.948	4.229	4.249
4.981	5.151	5.188	5.395	4.948	5.309
8.318	8.492	8.701	8.594	8.793	8.665
5.700	5.677	5.735	5.847	5.889	5.696
3.064	2.609	2.902	3.357	3.397	3.225
4.314	4.561	4.433	4.542	4.412	4.361
2.104	2.534	2.140	2.558	1.901	2.391
6.197	7.877	6.229	6.768	7.404	5.697
1.540	0.336	0.949	1.443	0.389	2.122
4.434	3.022	3.574	4.691	3.141	5.767
1.733	1.378	1.922	2.232	1.866	2.239
5.647	5.406	5.818	5.320	5.677	5.311
3.618	3.641	3.741	3.945	3.361	5.078
2.391	2.075	1.756	2.147	1.817	2.650
3.202	3.014	3.442	2.711	3.491	2.289
6.517	6.669	6.517	6.956	6.787	6.832
6.915	6.605	7.218	5.030	7.115	8.513
2.717	2.365	1.748	1.893	2.542	1.422
4.350	4.856	4.387	3.363	4.307	5.012
5.691	5.502	6.131	4.457	5.987	6.218
5.387	5.652	5.505	5.908	5.701	5.356
4.916	3.232	3.962	2.733	4.106	3.346
5.438	5.818	5.643	5.985	6.134	5.597
2.549	1.513	2.251	1.560	2.332	1.319
3.189	2.170	2.696	2.875	2.869	3.515
3.096	2.490	2.946	2.963	2.552	3.271
2.699	2.218	2.581	2.761	2.431	2.849
2.916	2.655	2.572	2.061	2.370	2.617
6.191	7.034	5.582	6.565	6.571	5.959
4.579	4.312	4.229	4.706	4.622	4.779
5.154	5.980	5.391	6.184	6.218	5.055
5.683	5.566	5.932	5.663	5.441	5.667
8.854	8.975	8.851	9.481	9.095	8.608
3.557	2.316	2.467	3.089	3.271	3.756
6.090	5.901	5.338	6.357	4.725	4.547
5.584	5.945	5.428	5.799	6.302	5.797
7.043	6.820	7.346	7.557	7.367	7.079
2.174	2.339	1.504	2.523	1.952	2.061
7.788	8.030	8.055	7.615	8.197	7.499
2.811	2.585	3.584	3.318	3.803	2.425
6.719	6.404	6.604	6.675	6.721	6.491
4.020	4.201	3.978	4.046	3.682	4.679

6.874	6.867	7.278	6.922	7.186	6.860
4.545	5.503	5.419	5.379	5.479	4.666
3.461	4.421	3.925	4.013	3.918	3.335
3.155	3.673	3.281	3.577	3.238	3.727
6.923	6.712	7.467	7.204	6.915	6.769
3.919	3.824	4.670	4.281	3.949	3.366
4.918	2.203	3.060	2.757	3.240	3.892
5.568	5.372	5.900	5.885	5.451	6.427
5.801	5.535	5.877	5.745	5.511	5.754
5.300	5.961	5.295	5.434	5.808	5.650
5.908	6.058	6.084	6.288	6.775	6.292
5.850	5.396	5.625	5.811	5.562	6.182
3.777	2.215	3.060	2.512	3.240	2.172
6.577	6.097	6.605	7.303	6.538	7.340
7.799	6.610	7.747	7.194	7.836	7.459
4.944	3.993	4.342	3.902	3.953	4.192
3.559	4.076	3.347	3.701	3.615	3.715
4.198	4.799	4.515	4.643	5.066	4.730
3.877	1.458	3.485	2.263	2.832	2.518
2.629	1.769	1.939	1.980	1.731	2.075
7.013	6.858	7.121	7.279	6.972	7.379
3.163	3.809	2.644	3.276	3.275	2.615
4.449	4.187	4.110	4.683	4.513	4.726
4.859	2.544	3.982	3.158	3.549	3.215
3.293	3.254	3.496	3.573	3.378	3.620
3.648	3.695	3.689	3.784	3.620	3.877
4.704	4.417	5.038	5.057	5.166	3.965
3.597	2.089	2.985	2.223	2.850	3.031
5.071	5.093	5.517	5.618	5.617	5.041
5.800	5.603	5.437	5.951	5.576	5.797
3.605	4.412	4.037	4.481	4.325	4.127
6.684	6.977	7.310	7.079	7.014	6.408
2.853	3.009	2.666	2.436	2.548	2.301
8.746	8.474	8.943	8.801	8.571	8.645
5.216	4.373	5.492	5.453	4.844	5.502
3.976	3.337	3.943	3.987	3.499	3.925
2.869	2.867	2.866	2.548	2.566	3.249
3.476	3.564	3.539	3.884	4.028	4.396
3.830	3.894	3.752	3.682	3.220	3.345
3.888	2.600	3.887	4.060	3.207	3.844
3.062	2.970	2.948	2.556	2.572	3.737
8.874	8.876	9.229	8.945	9.019	8.939
5.738	5.767	5.662	5.306	5.583	5.877
2.241	2.323	2.161	2.432	2.249	2.334
6.386	5.721	6.038	5.935	6.237	6.111
5.113	5.045	5.685	4.802	5.415	5.000
6.269	6.904	5.846	6.240	6.883	6.850
6.311	6.633	6.393	6.921	6.790	6.225
2.621	2.784	2.842	3.885	3.590	2.405
3.125	2.875	2.880	3.699	3.986	2.935

5.533	1.503	1.594	9.056	1.443	9.441
2.878	2.739	3.611	3.436	3.244	3.564
4.226	4.088	4.251	4.208	4.607	5.052
3.425	3.053	3.571	3.094	3.513	3.861
3.011	2.457	2.673	2.412	3.559	3.423
2.103	2.357	2.198	1.896	1.489	2.410
3.163	3.719	2.947	3.167	3.223	2.420
2.581	2.323	2.723	2.345	2.320	2.706
6.024	6.061	5.858	6.277	6.203	6.270
7.112	7.118	7.555	7.309	7.341	7.192
4.223	4.646	4.605	4.643	4.671	4.399
2.101	2.683	2.444	2.510	2.807	2.761
4.802	5.264	4.390	4.205	4.238	5.340
3.958	3.933	3.849	3.667	3.489	4.196
2.433	2.541	2.100	2.120	2.327	2.373
4.315	3.764	4.962	5.153	4.889	3.592
4.075	5.109	3.821	4.602	4.696	3.889
3.597	3.201	2.776	3.383	3.344	4.121
2.106	2.344	3.044	2.237	2.181	2.817
5.678	5.719	5.766	5.782	6.092	5.518
4.463	4.660	4.690	3.840	4.816	4.240
8.778	9.246	8.808	8.655	8.705	8.604
5.082	5.662	6.361	5.487	5.395	5.323
2.602	2.823	3.826	2.523	2.781	2.374
4.549	5.073	4.644	5.328	4.590	5.144
5.377	5.558	5.239	5.610	5.460	4.727
6.198	6.309	6.567	6.078	6.621	6.679
6.984	7.252	6.930	7.392	7.340	7.494
3.502	3.773	3.990	3.302	3.547	3.960
3.982	4.458	3.743	3.590	4.609	4.261
3.723	4.083	3.455	3.190	3.510	3.739
4.070	4.743	4.219	4.892	4.533	3.346
3.386	3.430	3.610	3.241	2.535	3.061
7.318	6.953	7.397	7.062	7.197	7.396
3.126	3.604	3.296	3.447	3.816	2.817
6.704	6.809	7.302	7.538	7.570	7.207
6.416	6.234	6.292	6.241	6.358	6.835
9.117	8.615	8.583	9.116	8.590	9.475
4.023	3.808	3.857	4.529	4.243	4.547
3.244	3.104	3.713	2.639	3.313	2.782
5.421	5.173	5.661	5.579	5.379	5.419
5.432	6.016	5.386	5.329	6.330	5.276
3.158	1.174	1.448	1.812	1.439	1.250
3.518	3.406	3.592	3.384	3.806	3.713
2.235	2.182	1.748	2.178	1.339	1.977
3.290	2.996	2.407	2.970	3.220	3.076
3.869	3.565	4.261	3.283	4.099	2.578
6.089	5.822	6.494	6.098	6.423	5.226
5.453	5.296	5.342	5.498	5.346	5.323
6.855	6.203	7.070	6.296	6.809	6.612

3.243	2.663	4.648	4.052	4.478	3.228
5.121	5.640	4.963	5.422	5.661	5.584
4.452	4.458	5.422	5.098	5.272	4.740
5.932	5.621	6.262	6.098	6.435	6.063
4.453	3.238	4.155	4.174	4.598	4.762
3.636	3.742	3.957	4.158	3.745	4.037
6.535	6.371	6.781	6.664	6.505	7.266
4.652	4.407	4.958	5.302	5.384	5.088
5.093	5.418	5.116	5.286	5.168	5.355
4.247	4.049	4.153	4.424	4.917	3.936
3.587	3.765	3.394	3.070	3.379	3.157
6.082	5.674	6.476	6.449	6.098	6.243
4.288	4.555	4.566	4.188	4.105	3.529
4.564	4.688	4.672	4.691	4.479	5.049
4.665	4.038	4.347	3.845	4.566	4.226
6.816	7.796	7.325	6.611	7.480	6.620
3.542	2.604	3.060	4.252	2.625	5.013
7.383	7.475	7.106	7.130	7.416	7.485
4.747	4.389	4.581	4.320	4.485	4.807
5.690	5.289	5.862	5.551	5.937	6.247
3.739	4.111	3.383	3.064	3.920	4.359
3.943	4.631	4.077	4.033	3.882	4.010
3.941	3.268	3.956	4.492	3.964	4.483
7.041	6.758	7.111	7.066	6.907	6.814
7.417	7.317	7.901	7.052	7.127	7.002
4.191	3.287	4.901	3.674	3.892	3.444
6.021	6.151	6.351	6.342	5.948	6.190
2.560	2.614	2.328	2.916	2.923	2.411
2.805	1.440	2.350	1.725	2.170	3.176
8.373	8.143	8.354	8.553	8.487	8.644
2.741	3.006	3.430	2.878	2.518	3.068
6.783	6.428	6.536	6.811	6.545	7.042
4.663	4.290	5.200	5.326	5.126	4.783
7.298	7.594	7.720	7.415	7.267	7.337
5.233	5.102	5.969	5.332	6.136	5.932
3.851	3.305	3.650	3.082	4.360	4.227
3.834	3.584	3.594	3.848	3.799	4.244
8.558	9.204	9.715	8.908	8.463	8.066
5.282	5.322	5.168	5.652	5.176	4.594
7.175	7.480	8.091	7.277	7.708	6.692
3.595	3.364	2.797	4.255	3.682	3.665
4.622	4.465	3.808	4.301	4.457	4.971
3.222	2.897	3.503	3.498	3.163	2.796
2.613	2.525	2.781	3.020	2.277	3.076
2.298	3.461	2.740	1.858	2.766	1.929
7.289	6.805	7.538	6.927	7.430	7.332
2.915	3.124	3.870	2.344	3.171	2.712
3.268	4.038	3.137	3.524	3.606	2.931
6.228	5.847	6.004	6.038	5.931	6.556
5.833	5.355	5.960	5.408	6.009	5.820

5.255	5.633	5.760	5.355	5.617	6.329
3.654	4.456	3.512	3.458	3.747	3.550
4.712	5.400	4.585	4.610	4.614	4.709
3.534	3.829	3.140	3.913	3.161	3.855
4.375	4.558	4.660	4.636	4.475	5.024
3.784	4.302	3.877	4.814	4.496	3.092
5.739	5.833	6.276	5.592	6.699	5.666
6.788	6.981	7.449	7.332	7.486	6.930
3.616	3.694	3.871	4.065	2.935	4.110
6.023	7.073	6.200	6.675	6.810	6.159
2.805	2.048	1.868	1.926	2.217	2.566
3.110	2.994	3.572	3.452	3.301	2.938
6.219	5.596	5.939	5.490	6.265	6.101
4.836	4.454	5.810	5.411	5.146	4.850
4.115	4.744	3.690	4.801	5.387	3.529
4.488	4.111	4.940	4.889	4.813	4.244
2.829	3.333	4.103	3.415	3.608	3.283
5.039	4.921	5.640	4.897	5.272	5.177
5.792	5.099	5.586	6.109	5.852	6.008
3.762	3.064	3.767	3.462	3.682	4.294
2.926	3.796	3.493	2.199	3.174	2.979
4.098	3.890	4.495	4.243	4.153	3.811
4.098	3.588	4.220	3.773	4.598	4.638
4.380	5.034	5.000	5.385	4.500	4.618
3.668	4.079	3.392	3.344	4.059	3.550
5.225	4.961	5.636	5.600	5.619	4.995
3.589	3.533	3.583	3.868	4.540	3.662
2.844	2.835	3.747	3.076	3.549	3.225
1.857	1.194	1.345	2.297	1.542	2.321
2.243	2.316	2.721	2.373	2.340	2.582
1.428	1.323	1.343	1.711	1.750	1.796
4.234	4.855	3.759	4.409	4.545	3.966
3.557	4.183	3.750	3.886	3.773	2.914
3.271	3.664	4.077	4.224	3.699	4.287
2.836	3.195	3.085	2.959	3.513	3.870
4.812	4.630	5.256	5.334	5.409	5.382
4.165	4.510	4.186	4.479	4.296	4.470
3.741	3.432	3.647	3.832	4.229	4.239
2.367	3.964	3.301	2.744	3.664	2.565
2.797	3.282	2.315	4.238	3.956	3.743
3.733	4.193	3.978	4.623	4.518	3.489
2.704	2.464	2.276	2.924	2.276	2.817
5.189	4.589	5.689	4.817	5.421	4.724
6.768	6.686	7.139	7.208	7.198	7.409
4.344	4.300	4.115	4.907	4.632	4.794
3.065	2.807	2.937	3.475	3.660	3.747
3.698	4.494	4.506	5.326	4.560	3.358
3.087	4.115	3.831	3.791	3.587	3.459
4.867	4.900	4.844	4.511	4.788	5.243
4.340	4.087	3.839	5.279	4.618	4.474

5.196	5.032	5.188	5.693	5.614	5.753
1.864	2.067	2.263	2.022	2.365	2.360
2.588	2.142	2.538	2.275	2.281	3.235
4.719	4.486	5.438	4.639	4.622	4.961
3.144	3.851	4.045	4.367	4.253	4.192
2.930	2.689	3.052	3.254	4.064	3.851
3.750	3.820	3.547	4.297	3.772	3.190
2.852	3.406	3.063	3.207	3.891	3.020
5.466	5.371	6.528	5.733	5.818	5.898
4.021	3.878	3.910	4.190	4.392	4.443
2.358	2.413	1.869	3.244	3.141	2.250
4.066	3.893	4.786	4.234	4.252	3.803
4.138	3.730	4.965	4.864	4.351	4.310
3.267	3.230	2.883	3.769	4.223	3.016
7.216	6.766	7.141	6.786	6.945	6.975
3.448	3.903	3.448	3.807	3.997	3.758
2.899	3.135	2.808	2.465	3.279	2.950
3.565	3.400	4.214	3.274	2.637	3.385
2.136	1.225	2.215	1.922	1.164	1.652
3.671	3.558	3.830	4.288	4.418	4.442
3.272	3.045	2.983	3.179	3.086	3.174
2.099	1.796	2.194	2.226	1.884	1.970
5.180	5.080	5.167	5.415	5.726	5.229
3.454	3.070	4.196	3.916	3.871	3.940
3.845	4.093	3.996	4.549	4.863	4.003
7.392	7.251	6.797	7.367	6.955	7.154
2.363	1.978	1.531	2.183	2.212	2.639
1.650	1.674	1.716	1.644	1.474	2.064
4.734	4.622	3.953	3.772	4.498	3.443
4.549	4.530	5.518	4.490	5.199	4.433
2.473	2.121	2.609	2.248	2.550	2.436
4.865	4.588	4.926	4.821	4.632	4.097
3.574	3.800	4.146	4.190	3.790	4.111
3.250	3.636	2.878	3.570	3.361	3.636
4.868	4.852	5.214	4.764	5.107	5.181
3.991	4.500	3.931	4.532	4.340	4.613
5.248	5.668	6.337	5.958	6.832	6.244
4.236	4.539	4.072	5.411	5.045	4.423
3.672	3.712	3.866	4.143	4.557	3.175
2.367	1.708	3.104	2.853	2.827	1.835
4.129	3.717	4.096	5.534	4.110	5.025
5.645	4.996	6.139	5.360	5.754	5.830
4.182	4.028	3.913	4.677	4.491	4.595
2.787	3.206	3.008	3.389	3.302	3.144
6.551	5.741	6.504	6.224	6.355	6.519
4.633	4.824	4.494	5.212	5.128	5.087
2.537	1.397	2.303	1.557	1.731	2.188
4.617	4.446	4.467	4.853	5.288	5.277
4.881	4.448	5.543	5.230	4.907	5.654
3.095	3.104	3.751	3.319	3.766	3.274

4.061	4.190	3.638	3.365	3.810	4.099
4.472	4.765	5.159	4.578	4.497	4.262
3.142	2.887	3.083	3.262	3.487	3.621
3.702	3.902	3.561	3.369	4.127	3.668
4.694	4.583	4.515	4.743	4.746	4.831
5.885	6.144	6.240	6.041	6.280	6.173
4.983	5.194	5.443	5.322	5.749	5.204
4.070	4.308	4.054	4.339	4.281	4.729
3.562	3.775	3.663	3.286	3.535	3.909
5.600	5.442	6.000	5.301	5.804	6.129
2.340	2.701	2.191	2.434	2.087	3.399
6.669	6.424	6.874	6.489	6.735	7.010
3.268	3.198	3.455	3.420	3.535	3.759
4.204	3.892	4.331	4.339	4.372	4.634
3.293	3.617	3.765	3.435	4.116	3.578
3.764	3.146	3.559	4.110	4.287	4.112
3.343	3.223	3.934	4.429	3.803	3.302
3.768	4.293	4.486	4.907	4.420	4.460
2.464	1.420	3.461	2.980	2.542	2.688
3.167	2.879	2.658	3.587	3.211	3.560
3.422	3.507	3.315	3.837	4.253	3.982
3.131	2.015	3.341	2.819	3.670	2.506
2.409	1.880	2.192	1.666	2.572	2.417
4.087	4.621	4.426	4.095	5.325	3.932
3.525	3.099	3.926	3.212	3.620	3.479
2.894	2.521	3.038	3.069	2.926	2.993
3.982	4.862	3.524	4.516	3.339	3.701
3.495	3.841	3.265	3.006	3.979	3.851
4.534	3.888	5.025	4.554	4.976	5.312
1.871	1.602	2.058	1.631	1.595	2.321
4.648	5.100	4.955	5.118	5.324	5.194
4.394	4.073	4.882	5.064	4.813	4.644
3.678	3.498	3.673	4.310	4.256	3.908
3.451	2.475	3.822	3.822	3.785	4.175
3.710	3.932	4.021	4.150	4.049	4.236
2.312	1.400	2.535	2.916	2.175	1.782
3.553	3.934	4.291	4.529	3.351	4.499
3.393	3.688	3.563	3.757	3.485	3.804
4.458	4.428	5.505	4.737	4.916	4.831
4.484	4.255	4.571	4.538	4.637	4.637
7.044	6.905	7.677	7.112	7.120	7.067
4.204	4.049	4.459	5.072	4.210	5.030
3.967	3.386	4.204	3.985	4.254	3.997
7.093	7.010	7.161	7.188	7.252	6.941
3.269	3.815	3.418	4.051	3.416	3.527
2.897	3.351	3.561	3.453	3.595	3.130
1.779	1.727	2.508	2.493	2.302	2.408
2.760	2.198	2.970	3.464	3.244	3.000
2.680	3.162	2.421	3.309	2.135	3.157
3.022	2.596	3.301	2.865	3.722	3.321

2.909	3.281	2.939	3.094	3.492	3.705
3.257	3.083	3.037	2.987	2.599	3.364
3.433	3.129	3.913	4.503	4.193	3.418
2.189	3.343	2.942	2.613	2.910	2.397
4.685	4.727	5.182	4.826	5.107	4.300
4.115	4.115	4.237	4.797	4.198	5.154
2.085	2.589	2.292	2.989	1.686	2.597
3.462	3.653	4.354	3.931	3.827	4.272
4.199	4.295	3.870	4.562	4.300	5.035
2.408	3.091	4.074	2.677	2.979	2.131
2.764	1.820	2.626	2.988	1.901	2.734
4.194	3.913	4.434	3.648	4.357	4.576
2.959	3.053	3.634	3.144	2.732	2.743
3.023	3.357	2.820	2.739	3.349	3.871
3.334	2.323	2.994	3.723	3.560	3.400
5.789	6.003	6.016	6.064	6.263	5.665
2.693	2.127	2.545	2.121	1.957	2.288
3.886	3.630	3.216	4.215	3.705	3.220
4.744	4.542	4.512	5.104	4.970	4.878
3.571	3.674	4.118	4.170	4.310	2.497
5.299	5.948	5.814	5.467	5.197	5.800
3.645	3.260	3.741	3.554	4.135	3.220
6.106	6.326	6.163	6.443	6.348	5.995
4.408	4.525	4.321	4.337	4.545	4.206
4.163	4.429	3.368	5.042	3.678	4.531
3.504	2.982	2.987	3.034	2.817	2.975
3.074	3.009	1.947	2.648	3.186	2.953
5.993	5.980	6.252	6.547	6.303	6.038
3.282	2.794	2.900	3.172	2.383	3.997
3.239	3.178	2.639	3.002	2.592	3.030
3.402	3.669	4.353	3.573	4.387	3.245
3.328	3.706	2.658	2.937	2.730	3.276
4.231	4.512	4.413	4.646	3.999	4.548
2.697	2.573	3.476	4.032	2.767	3.537
2.092	2.543	2.092	2.918	2.369	2.137
5.371	5.409	5.940	5.815	5.713	5.570
2.260	1.897	2.083	1.829	2.691	2.475
3.468	3.842	2.979	3.405	3.690	3.501
3.968	4.445	3.176	4.233	4.454	4.817
2.583	2.497	2.729	2.688	2.926	1.886
4.556	4.836	4.959	4.216	4.916	4.634
4.018	2.934	3.620	4.268	3.579	3.897
2.718	2.637	3.044	3.001	3.042	2.203
5.040	4.202	4.977	4.997	4.703	5.358
2.328	1.782	2.351	2.097	1.387	2.828
3.640	3.214	3.860	4.738	3.382	4.152
3.697	3.248	3.776	4.052	3.686	3.938
3.362	3.013	3.923	3.208	3.620	2.637
3.199	3.268	3.330	2.963	3.284	3.623
2.530	3.344	2.758	2.758	2.337	3.097

2.991	3.314	3.988	3.196	3.036	3.641
2.533	2.334	2.819	2.713	2.905	3.154
3.509	4.501	3.975	4.022	4.087	3.499
3.535	3.258	3.505	3.819	4.052	3.301
3.912	3.304	4.086	4.791	3.422	4.043
3.210	3.306	3.450	3.908	3.800	3.532
3.954	3.767	3.189	3.656	3.115	2.906
3.918	4.376	4.425	4.126	3.795	4.295
2.575	3.106	2.499	3.278	2.669	3.451
5.360	5.076	5.644	5.515	6.020	4.972
7.316	7.121	7.230	7.524	7.568	7.842

6.778684388	6.583011092	6.775370013	6.540650507	6.316606382	6.229008921
10.18182093	9.821587955	10.50098526	9.934522235	10.73363618	9.93225179
3.30012106	3.332429611	3.985124684	3.008866224	3.358279086	2.998397593
1.759522326	1.81790987	1.99849857	1.614825304	2.017231369	1.383576404
3.101385979	3.712436819	2.319355314	3.577605332	3.546771744	1.967398072
4.564529292	4.910428707	3.358353062	4.462231521	3.271189974	5.604771636
7.865820846	7.295510332	8.163127541	7.682995486	7.945442985	8.304599353
6.341929298	5.978353955	6.321117958	6.331693312	5.829363716	6.731362492
2.879045797	3.155784994	2.773704523	2.725342002	2.752532377	2.695041938
5.191725795	5.220809763	4.603362037	5.177525596	5.125024131	5.684752142
3.37275136	4.023750325	2.984760793	3.755448523	2.887433607	3.39789916
2.436728936	2.529902447	2.697403687	2.321251489	2.391406808	2.321251489
3.20115402	2.765566688	3.241561607	3.392198439	3.24615055	3.254836204
7.567313692	6.2905637	8.426225863	7.806580699	7.693854238	7.575362931
6.508238881	5.725331468	6.362184812	6.584109061	5.942820402	7.103608339
8.868798249	8.394800463	8.371118442	9.159389304	8.441662988	9.436177959
3.095827585	2.160184761	2.863120941	3.481530439	2.966014682	3.060495037
2.392396245	2.34507845	1.954013344	2.442315743	2.014536623	2.909805378
6.563671638	5.821469094	6.516646411	6.679902553	6.357177893	7.042178387
6.866885801	6.001004482	7.148248567	6.463506777	7.051990563	7.117375292
7.31569346	6.828969448	7.81945852	7.501972706	7.38295438	7.176753643
2.73288876	3.247729662	2.552388605	2.847998018	3.010631896	2.010550405
1.987510002	1.71231675	2.159230285	1.873083145	1.49740081	2.416328539
3.50253886	3.832003472	2.918166898	3.571134994	3.140022956	4.153607798
6.63016417	6.422439753	6.7745064	6.721278669	6.728801465	6.802927556
4.838082601	4.754121605	5.24165841	5.05390277	4.730323034	4.295650084
1.721290154	1.147521179	1.605757923	1.966032639	1.54520326	1.787902664
4.461651281	4.580659133	3.917866296	4.255938977	4.935556719	4.731637847
3.464590617	3.489460029	3.071392137	3.602534282	3.842960716	3.45415061
5.357209719	5.513404297	5.439167442	5.073601468	5.554076389	4.903421015
2.301063584	1.943664922	2.714570769	2.465094515	1.920163155	2.19116955
3.997518617	3.944094361	4.021167688	3.976098825	3.929432037	3.879688642
3.635454074	2.635262646	4.095122484	3.865653089	3.784778223	3.76659469
5.383883447	5.233000434	5.147536831	5.396828453	5.896915266	5.230008789
5.689447214	5.263572507	5.825295532	6.499673186	5.010366887	5.817855656
2.603715151	2.970317718	3.285741164	1.870132537	2.259348901	3.128622954
5.250190158	4.470002705	5.785859394	5.329760586	5.281853439	5.13491743

1.420604297	1.102211312	1.832518049	1.400020188	1.412695644	1.3562418
2.911095685	2.929035812	2.749200293	2.971887916	3.318030841	3.508971779
3.93036935	4.764258603	3.861867747	4.264984597	3.365763196	3.302446978
7.20032291	7.032668772	7.534556862	6.980681878	7.164103118	7.250846357
6.936512118	7.114827108	7.062317435	7.289924512	6.975165396	6.323115731
9.097318264	9.124303981	9.146041916	9.401666594	8.985407358	9.084179604
2.445303044	2.064827547	2.731245248	2.299180589	2.755280909	2.697799273
5.900655609	6.181036203	5.908904321	5.886279797	5.600073465	5.951168362
6.414009801	6.495449949	6.04612724	6.574363503	6.293741151	6.400437946
6.939816385	6.810488755	6.721001829	7.066811088	7.186329045	6.977339524
5.010290622	5.070969186	4.885693506	5.289966752	5.099760503	5.008561673
1.930798104	2.378163175	1.612801512	2.173561926	2.302643721	1.854783971
8.238887638	8.344685544	8.410155355	8.613027679	8.05497581	8.228192799
9.448534333	9.226230381	9.437693828	9.65263133	9.231455588	9.648426324
6.77289274	6.062662067	6.687010214	7.954231109	6.055306234	6.891684599
2.950170709	3.05757372	3.673248921	2.571886835	2.759072771	2.512846614
3.595823262	3.34837827	3.128909032	3.501859001	3.488877055	3.743257122
8.357732409	7.832358267	8.483830879	8.35840843	8.097291688	8.698397565
6.25227224	5.798275314	6.429598815	6.305686561	6.169428281	6.285067167
3.616411719	1.827644203	3.716519964	4.442451727	3.329243612	4.536528135
3.204496106	1.942531563	3.399502832	2.948966721	3.610619529	3.627903663
2.950791313	2.441128248	2.613148461	3.004981484	3.138101188	3.440745009
4.014008972	3.481993592	4.789232467	2.555986058	4.95286759	4.709019667
3.845856107	3.89119509	4.394495316	2.908017558	4.124351245	3.840284213
2.896566853	2.770733192	3.011696683	2.242991604	3.04652516	2.781138906
1.932027926	1.389857731	2.18367234	1.907377084	2.560742457	1.826874583
3.495145161	3.08494012	3.268106072	3.452632173	3.626855793	3.195426843
2.134036345	2.065601305	1.676759417	2.119086122	2.017827021	2.139313703
3.588666745	3.420848585	3.069367341	3.30122162	3.142224498	4.128426841
3.137736872	2.796246417	2.939282519	3.197991616	3.424209843	3.395970557
7.548491456	7.30354697	7.745345015	7.385526019	7.769835923	7.400030803
3.032832029	3.399742598	3.094114931	3.118538776	3.012320245	2.493547946
5.996505595	5.849866911	5.855015857	6.276655633	6.070052406	5.945182606
4.655691314	4.717625069	4.670357018	4.316597055	4.291963955	5.000880028
8.528547731	8.456294902	8.663356957	8.372202243	8.209715696	8.451286083
3.211169894	3.831262189	2.613065681	4.139382121	2.706142411	3.422796172
4.497510679	5.607981031	4.520035943	4.33806879	4.083761588	4.028319305
8.634814344	8.132899805	8.719942811	8.728635297	8.39005254	8.844388146
5.216868829	5.405133416	5.235774849	5.621301897	5.176904919	5.397777677
3.152860348	3.646665001	3.049024828	2.558890647	3.322160976	3.017837869
7.328136956	7.200507268	7.164541616	7.351795206	7.213998628	7.477275034
2.844028629	3.367142021	2.292482395	2.802839238	3.40444149	2.665114223
3.975610624	4.095504147	3.967159306	4.217972874	3.509202034	3.991794068
3.067021054	3.065856412	3.528857893	3.876475906	3.215741116	2.539741703
4.788031547	3.879482163	4.075382642	5.044685945	3.982750717	5.884354758
5.971874235	6.036497371	6.432889556	5.812410174	5.836701868	6.060136154
5.837052522	5.714792907	5.733430464	6.103293498	5.765780164	5.926276257
6.880890836	8.104924833	6.043744599	7.223466971	6.042326215	6.342153536
5.359414374	4.884826051	5.039075699	5.364839992	5.312781086	5.786533156
1.781547633	1.811147599	2.042374319	1.426429503	1.798073777	1.942594396

7.258363494	6.768188094	7.415281093	7.397156353	7.338203696	7.308660735
2.789591805	2.724333574	2.838755581	2.648401499	3.217273519	2.343268901
2.785623574	2.90081038	1.911278056	3.353064917	2.341970568	3.423912884
5.043089407	4.832732745	4.946428502	5.286661378	4.89206158	5.231963977
3.449473954	3.731839722	3.726031927	3.579140082	3.592558219	2.744985678
6.370722264	6.470344852	6.302256292	6.101899351	6.47031947	6.372481985
7.662419575	7.510682807	7.779972569	7.813276694	7.35293781	7.775522553
1.705750617	2.020546561	1.289877122	1.861753642	1.442582837	2.066930756
4.559828613	6.009792338	4.060806075	5.389365128	4.087102363	3.54212458
7.062142527	6.347409985	6.74970076	7.239332812	6.557805136	7.531626695
4.529486808	4.272194229	4.712633178	4.752996335	4.219884088	4.994227981
2.987170876	2.81587821	3.154752545	2.540382771	3.583225156	2.665659701
7.042394202	6.837180863	7.286713133	6.863020341	7.15456015	7.044847549
3.701557824	5.809932518	2.465254301	2.799742692	2.871769437	2.617163287
5.313499578	5.44206559	5.745334204	5.282540232	5.823139356	4.604620383
4.856941613	4.550197822	4.726990239	4.849154715	4.903992563	5.034308565
5.087923909	5.780149234	5.019911715	5.189225682	5.117797882	4.971566514
8.915941246	8.520398164	9.035079632	8.874675754	8.80690261	9.090886624
2.61240736	2.74539512	2.804922322	2.655559185	2.204536626	2.290770608
5.660749927	5.547566196	6.189851499	6.286415409	5.377774858	5.233032893
2.778433769	3.08880244	2.117950405	3.780587749	2.607147933	2.595608488
5.809304623	5.662163276	6.102182096	5.879319516	5.95582258	5.510090877
3.591811914	3.741534193	3.440916239	4.147034563	3.317552872	3.212196858
3.375664932	4.60975826	3.547086864	4.122536782	3.042238039	2.375932729
3.238013425	2.86286938	3.270738254	3.311279607	4.041360228	3.247046028
2.026414681	1.441450448	1.836269294	2.368746434	1.966045891	2.106462231
3.994202459	3.708654518	3.640057241	4.398288362	4.031327181	4.15732798
2.662710906	3.135360599	2.219726174	3.248415623	2.483451207	1.902283331
5.842635411	5.409000788	6.083019586	5.713305207	5.853866252	6.040321614
4.732344467	4.795253585	4.920197353	4.891176549	4.501054407	4.47776678
6.106572296	5.960177439	5.848242164	6.464111779	6.01636353	6.558775114
3.720945156	4.53415254	3.357406481	3.782214102	3.625547394	3.255464818
3.458686946	3.264193914	3.328102944	3.607622929	3.124591804	3.725874095
5.692197793	5.410949101	5.784757108	5.599350789	6.03345324	5.731101427
6.545597817	4.652660668	6.110964189	7.442299184	5.128226294	7.731056526
4.649515629	4.005586011	4.250100997	5.069483954	4.015147286	5.414221857
3.898780213	2.270919273	4.101676342	3.877671205	3.713824217	4.819787766
7.623743951	7.930000408	7.623040275	7.657269825	7.558892159	7.510732193
4.573935457	5.033513396	4.806745631	4.635692745	4.586480493	4.035450307
3.680445439	3.311461233	3.46983866	4.094268483	3.006326715	3.801571251
6.259085976	6.429656418	6.43822505	6.339025382	6.058104936	6.031818275
3.405175065	3.763772994	3.132917336	3.761337221	3.089364348	3.546759303
5.465780168	5.295405799	6.291194611	5.270570976	5.440643545	5.543413789
1.777024222	1.893000007	1.570509621	1.730792417	2.213313796	1.596990049
0.987005621	0.859883306	1.040013068	0.949419021	0.426450999	1.087484279
6.920463677	7.019230822	6.97629147	6.75008582	6.936513128	7.149888473
5.242312528	5.083445363	4.879269014	5.642600301	5.325343092	5.255821474
5.212146175	5.01783241	5.301504468	5.22798429	5.529109443	5.094677936
3.497132592	3.668483982	4.106251578	3.4730788	3.219347017	3.029379776
2.942083399	2.433913446	3.318525654	3.267009162	2.46878427	2.542713349

4.790825945	5.157260116	5.112121214	4.898929648	4.494762245	4.290412087
3.422500641	3.627694933	2.970836388	3.26921384	3.045895283	3.685304156
2.061666878	1.730599544	2.160060317	1.910045035	2.278673449	2.33565998
5.850052584	5.527090213	6.56123321	5.847686813	5.542759015	5.767837169
4.849752558	4.168010666	5.4259016	4.794375248	5.192894392	4.678928386
2.561957041	2.109098185	2.844570881	2.394396857	1.475877079	3.303707253
8.617175899	8.477803214	9.094972752	8.568523781	8.857505124	8.438287648
3.700550233	3.799470824	3.752708328	3.647883743	3.457541177	3.651098176
4.008898273	3.662946228	3.714557012	4.055108668	3.547184513	4.597000259
5.912103317	6.009404742	5.988532462	6.094090026	6.164603912	5.423930957
2.686870422	3.000455187	2.624526169	2.680271866	2.568597602	2.652546923
3.970439991	2.650304721	3.778435115	4.34339261	3.154807618	4.711662778
2.277976649	2.075489227	2.318191746	2.185115288	2.25601447	2.053942644
7.138895792	6.784426443	6.82266656	7.111920972	7.145220967	7.567546497
4.242090168	4.256750442	3.936382964	4.096750959	4.243275195	4.868533012
2.558364331	2.371117834	2.651274515	1.968386872	2.455199835	2.408422127
3.308528727	3.256555419	2.970120293	3.233070203	3.234464431	3.98280367
4.175263216	4.337462582	4.622294788	3.900201847	4.041529439	3.972270648
2.828551867	2.279029801	2.372477274	2.859324597	2.659787461	3.489013267
2.958744606	3.167108308	2.351337376	3.878046637	2.243852428	3.029997791
7.624956051	7.864955023	7.433110468	7.660907814	7.296582627	7.755760655
6.337384785	5.634607265	6.523101825	6.139350296	6.679845639	6.672701347
2.878905941	2.89324127	2.846852389	3.007212986	2.611097834	3.117175755
4.40357919	4.160729505	4.827349986	3.936178383	4.418720442	4.662525902
1.967093351	2.224481156	1.818693872	1.999668454	1.324148646	2.258462664
4.201235905	4.337292873	4.785317505	5.295699418	3.653730942	3.908603262
7.105014026	6.701341679	7.515627391	7.048843585	6.904108469	7.218834126
5.083633981	5.065415821	5.239858274	4.882794028	4.953798131	5.471450766
4.348347435	4.326429549	5.101785046	4.11590661	4.431709556	3.755752086
6.942110575	6.59884147	6.971556172	6.672458484	7.087001295	7.225817674
9.279385709	9.017992927	9.510207156	9.245479397	9.326208464	9.35039371
4.412875598	4.580998628	5.136963498	4.363125966	4.053463189	3.985262115
5.361297866	5.271908259	5.5888155	5.395307974	5.344495897	5.285550538
6.923887945	6.82930443	7.044533672	7.086904778	6.714339146	6.980425046
6.323365119	6.268508503	6.064504485	6.270670548	6.071497883	6.637692625
4.064172703	3.62144417	3.939000645	4.343021546	3.635715355	4.380696934
1.615679549	1.785844217	1.868166636	1.315432678	1.255999686	1.27267159
4.972411019	4.684951633	5.236942995	5.09153966	5.007605138	4.793236081
4.522540963	3.384996259	4.961034652	4.883581248	4.705872212	4.37500838
1.25085546	1.496416564	1.480532302	0.896000102	1.023368288	1.415167129
3.315722611	3.148528579	3.515380479	3.255098012	3.40601847	3.204285845
3.031078108	3.004776975	3.492333773	2.490525085	2.530406847	3.031584937
5.046376386	4.486392537	5.179463509	5.110767083	5.422643574	5.429055542
6.485792721	6.089252304	6.450057238	6.792278817	6.617792436	6.56587309
5.353969734	4.849469099	5.48564007	5.439597967	5.188851443	5.603595243
3.461374866	2.656171127	2.749550831	3.035740459	3.620579392	4.339573192
3.350115475	4.197341947	3.134149642	3.007303443	2.515195813	3.312530533
5.537525954	4.78000107	5.226386943	5.906073698	4.959570012	6.061321131
3.390759017	4.03716028	3.424564429	2.6913144	3.380879488	4.072485937
3.092256478	2.888391521	3.297239292	3.389282743	3.491611342	2.28553787

2.554512128	3.404606101	2.481534148	1.614598509	3.948181649	2.448784232
4.673119929	4.23070219	4.577947107	4.830151629	4.209538961	4.998363469
4.519503531	4.267253198	4.690478194	4.237116686	4.354892774	4.899237027
4.875013444	4.693168599	5.100835099	5.705524551	4.26058118	4.659149484
3.674956093	3.596789623	3.576140747	3.245037439	3.738853011	3.844155156
4.204056053	4.576401955	4.082398067	4.205760861	3.839394363	4.593975599
5.982245518	5.584746231	5.916561477	6.219057557	5.865578756	6.275117886
1.689856404	1.409993249	1.935211464	1.543534255	1.707163936	1.893461708
2.956442812	2.945194484	3.199420623	2.738226894	2.174489213	3.088955979
4.10594971	4.53230661	4.224053493	3.871935577	3.738482106	4.281614732
4.442492708	4.09233263	3.933331799	4.353793821	3.784705243	5.405365432
4.884400744	5.254012492	3.873446033	5.960135396	5.076341943	4.858763253
6.069765291	5.316324747	7.097424326	5.787830256	6.342439766	5.885691284
5.105284947	4.077676513	4.949835286	5.015873289	5.070314414	5.662922993
7.182693215	6.396991058	7.21506734	7.213849032	7.042996813	7.474565339
4.211082401	3.786295188	4.291034223	4.226568249	4.110811696	4.716981605
6.831691499	6.718642182	6.979771598	6.815475766	7.107629448	6.712079791
4.830144776	4.842027037	4.756654092	4.760907901	4.501236067	4.949328126
4.969820009	5.064673167	5.85889787	3.549993513	5.675893383	4.355871441
2.039187918	2.135300221	1.853392044	2.04592932	1.58858724	2.23974812
6.450467348	6.089001984	6.397025964	6.708316728	5.965411503	6.744370265
3.525518655	3.625822134	3.426815128	3.939569323	3.557293093	3.548120472
2.781572731	3.32682635	2.88898116	2.991640645	2.222815851	2.519839915
3.451349566	3.514491874	3.774723009	3.874728455	3.489195732	2.828686662
4.824612682	4.127428775	4.865219249	5.341296228	4.62465499	5.029785233
4.124783292	5.03521843	3.199544311	4.69705058	4.74926077	3.360900178
4.609617626	2.883461701	4.813424516	5.186509124	4.586522991	5.161160669
5.549689687	5.30191398	5.736041489	5.085479453	6.109550722	5.153163934
5.366413263	3.448425595	6.36809892	5.095805656	5.034712523	4.692641897
3.365403569	3.946824848	3.07119189	3.353518867	2.862753847	3.375018219
3.566673535	4.12554137	3.443316065	3.732964601	3.756866803	3.260877439
3.191741055	3.194848795	2.92251514	3.066501576	2.821403664	3.860760981
3.374299288	3.322813217	3.435478899	3.32679005	2.816630406	3.686622943
3.653190152	3.41702863	3.862152057	3.368600351	3.314585072	4.302943676
6.039098257	5.967711109	6.04818149	5.675676084	6.266696564	6.122936299
4.053351209	3.876557441	4.207094975	4.285004779	3.776997586	4.270411894
4.812246674	4.597997879	4.125594111	4.926136479	4.321393551	5.213792271
2.965071395	3.118179143	2.382949032	2.962703113	3.567270941	2.621117126
5.506982471	4.672709956	5.573337784	5.676660236	5.358005228	5.378982626
10.4717827	9.981414443	10.5682862	10.20596969	10.04487262	10.95620293
5.355876088	5.06592958	5.731143238	5.312487175	5.269804411	5.498000295
5.421092749	4.862653252	5.603805752	5.232474452	5.367576547	5.529051415
6.761590105	6.528810104	6.954932236	6.719732356	6.407363316	7.016230411
3.408230047	3.764432541	3.465506106	4.108850641	2.78553559	2.886546232
2.774484467	3.616336378	2.92095955	2.032045822	2.955890732	2.806642212
3.475183536	3.033459433	2.705668607	3.413343915	4.064857529	3.549990231
2.897438916	2.20460233	2.528781294	3.58929934	3.351403746	2.723004826
2.556572646	2.812505642	2.861179721	4.301128096	2.094557869	1.82374106
5.276204764	5.3320218	5.241437893	5.410672978	5.232274113	5.293711059
3.672773713	4.752653992	3.960249342	3.429870736	3.692297549	3.754788486

5.031556706	4.093911944	5.061775776	4.722459793	4.708802419	5.546575329
5.751945222	5.814550333	5.801464108	5.909480277	5.470964193	5.757162069
2.206306592	2.136266997	2.26542559	2.056347162	1.654292619	2.316773091
3.94575768	3.968211998	3.351290826	4.304502995	3.516274661	4.412991892
7.548413567	7.696072598	7.720381377	7.780686602	7.522718712	7.430041988
3.906509901	3.533074325	4.386949271	4.411077284	3.537530975	3.979526893
6.716142507	6.512251046	6.685319107	6.441122638	6.581228205	7.082380046
9.611147018	9.551596606	9.719495953	9.774847191	9.468719449	9.669012298
3.595848543	3.945106494	3.459445437	3.437734306	3.570522635	3.403476305
4.269646043	4.488005107	4.00351289	4.035146871	4.210988397	4.418241755
4.311065182	4.614392772	3.911180188	3.603230811	4.580997302	4.611231522
4.017450741	3.603809382	3.741598213	3.324892349	4.521854955	4.80728311
1.532661618	1.18671382	1.4339514	1.843519986	1.27350258	1.902049338
5.073597016	4.94958609	5.460617911	5.571464574	4.944236831	5.088334848
5.381578755	4.732031034	5.855719844	5.751489313	5.228953818	5.33591226
5.567842763	6.224059285	4.993913206	6.831229252	5.304614548	5.366615777
5.272431834	5.640614162	5.259230714	5.153290995	5.270674593	5.039426841
2.881729923	3.365016745	2.51382256	3.544076632	2.314804087	3.143894399
5.179189566	5.357187596	5.42748854	5.047743705	5.030176594	4.72991048
3.236402167	2.539740914	3.477313221	3.287613341	3.401929999	3.431008726
2.912113749	2.683666318	2.868477299	2.807147941	2.296595333	3.496618772
2.833305318	2.706676059	3.058163068	2.614651966	2.303377448	3.097817611
8.977544693	8.738728321	9.3382746	8.831023428	8.966481456	8.82974906
6.380917629	5.720135712	6.085733044	6.631071634	5.884134226	7.267107401
3.657855979	3.474288182	4.43656426	3.814638398	2.620328108	3.770549414
4.437276049	4.332217353	4.636854626	4.526355179	4.342397411	4.778605466
7.950138743	7.650997254	8.238992123	8.059229063	7.929913397	8.032832921
5.004599805	5.264639559	4.593912156	5.097917587	4.985703086	5.009884408
7.770261183	6.998308886	8.14853689	7.549114243	7.640300761	8.142350234
3.532099865	3.974509632	3.237226474	3.741198081	3.061738342	3.835700272
5.419891892	5.433607695	5.192555705	5.459778407	5.225090949	5.588551769
3.737630946	4.206209245	3.007902839	3.571346953	2.838647988	4.485468547
5.500846995	4.948048274	5.585776631	5.573732282	6.079961553	5.41661062
3.754092664	3.714614202	3.646773711	3.646353615	2.94101508	3.95958608
5.464545194	5.325019864	5.954390207	5.200732497	5.326205966	5.574700539
4.691115321	4.718735658	4.867306032	4.478702314	4.554749909	4.801894487
3.951718716	4.361907483	3.377347837	3.898400706	4.643150756	3.786621695
3.045384772	2.722735338	3.482200988	2.135875444	3.729526171	3.254304003
6.030995609	5.971165352	6.547382981	5.755074708	7.421038631	4.984371881
2.572383785	3.312234858	2.955740704	2.237116722	3.054616453	1.661757845
5.352868349	3.379131536	5.290488909	5.591562225	4.88526778	6.449410343
3.49342626	3.746062826	3.23607577	3.375535276	2.886765487	4.234538436
2.99391794	2.560921015	3.289073977	2.455200449	3.096147122	3.250901829
4.06384759	3.760619789	3.899484606	3.931156767	3.916089846	4.300605074
1.902697992	2.191903525	1.147821553	1.842975948	1.5785851	2.544800341
2.677266115	2.543957557	2.597024317	3.546627883	2.806606426	2.831805889
3.853798694	4.161789146	4.195154113	3.567718172	3.895139771	3.715985295
4.523056388	3.940592435	4.689979962	4.186950095	4.542569236	4.997589558
3.708874089	3.755845485	3.54891777	3.132714554	2.710904831	4.513505026
8.541193713	8.290538435	8.685481151	8.877246644	8.442352848	8.49313937

3.488441992	4.002265951	3.13314205	3.637787784	3.848201364	3.152944438
5.855763805	5.989689961	5.908431928	5.992380915	6.004037463	5.449581805
6.320652204	6.062977006	6.312472393	6.383263028	6.049082358	6.631189029
3.415276597	3.284604386	3.798530217	2.840312775	4.347687284	3.143615343
3.334925143	4.340620848	3.31147819	3.629466871	3.480331604	2.442819907
2.200169049	1.498773005	2.013009924	1.808093874	1.751098409	2.341709597
3.960261911	4.481279436	3.781661742	3.877084861	3.370571182	3.953127471
2.784972608	2.574637069	2.506668637	2.981850776	2.512743142	3.159669307
2.034961271	1.771362518	1.884909084	2.278959133	2.007617557	2.231163809
5.322925354	5.010040935	5.227604188	5.689451353	5.429321298	5.444401356
7.09733614	7.124817106	7.308284588	6.60491374	7.302216073	6.826381585
6.148043203	5.460660218	6.170219705	6.27341157	6.237148233	6.234979708
3.403409718	3.821205605	2.449284458	3.614325492	3.088932052	3.858623672
3.650529719	3.559420258	3.546337259	3.844859471	3.418416727	3.744767594
3.951418026	3.899963879	3.806040127	3.499950521	3.419796789	4.288589488
9.473278893	9.03016802	9.774656397	9.454237316	9.406193595	9.851612309
2.771684709	2.524641434	2.315245203	2.356281652	2.865778621	3.484747078
4.05752038	4.717463575	3.704840508	3.760619789	4.032277006	4.068306825
4.804764816	4.528281908	5.0602243	4.526355179	4.33963224	4.686914868
6.11490544	5.930360576	5.860914127	6.365394343	5.930779852	6.474540817
2.47754369	2.226203968	1.784633471	2.212485906	2.017827021	2.761258147
3.407561421	2.886212754	4.084552375	3.094590753	3.923000879	3.311239081
3.086073547	3.116634108	2.931493662	3.861640217	2.850029415	2.929463934
3.145280958	3.901189989	3.952458578	2.890647208	3.901707188	2.010943595
3.663927708	2.532633388	4.361655446	2.792207519	3.014876317	4.299058543
6.342416568	6.307546471	6.233894837	6.377322681	6.104652247	6.590507389
5.686264112	4.761701793	5.950169388	6.077693576	5.601838208	5.869658527
5.785738159	6.279830831	5.665701754	5.933244164	5.463605403	5.597178623
4.687938345	4.186565086	4.82614076	4.034323988	4.767316475	5.30053855
4.29616762	4.454340429	4.717109092	3.602600378	4.796226467	3.955897733
3.983206165	3.593216957	4.519261579	3.95226969	3.827196526	3.558556735
4.21186965	4.116490975	4.265732861	4.292966285	4.664322825	3.936898935
6.611025376	6.229761571	6.903486254	6.833111659	6.820952671	6.362749979
4.277184444	4.069595038	4.254622984	4.641608789	3.901722603	3.982802161
5.065840922	5.920937464	4.46776493	4.733422636	4.30387048	5.389664405
4.02386043	4.386660573	3.850613633	3.880202372	4.12204935	3.910390627
6.922226223	7.468754096	6.706363384	6.84730617	7.033265587	6.738910439
3.374082549	3.170508228	3.041993836	3.65283159	3.41871201	3.138737803
0.978904609	1.202627248	0.963294038	0.921199504	0.764673585	0.933862907
3.281637917	3.83829668	3.284341919	3.226061511	1.507281967	4.64352343
5.579902828	4.255113926	6.158790381	5.550902909	5.831300961	5.951591244
6.167741892	5.941279197	6.166103356	6.353829259	6.246643177	6.226644082
6.755613571	4.922145498	6.143208658	7.954829775	5.232180177	8.080532344
3.072700935	3.142483975	2.419941854	3.270457908	2.647057214	3.81103019
2.562610004	2.050955454	2.925499618	2.602979285	3.410085982	2.204591676
4.177345442	4.769511766	3.979177797	4.559704169	3.804516753	3.780700918
3.913766498	4.616744583	3.456758102	4.899307372	3.995543247	3.696890528
3.426628519	2.846517903	4.25410279	3.978070585	3.543833873	2.859885741
4.601497179	4.478678973	4.509136765	5.090441463	4.175170045	4.749539416
2.233661622	2.1863415	1.771769434	2.113696374	2.590269398	2.308446512

2.104404433	2.881147119	1.848396019	1.54742599	1.696647863	2.454918138
3.66990962	3.373093217	3.132122358	2.868285453	3.654862653	4.478881228
4.613516677	4.757376539	3.53686828	5.502449598	3.05177273	6.893552087
3.620109112	2.974904982	3.129329129	3.774032335	3.10095447	4.249803101
5.238389756	5.196766702	5.378793563	5.560329678	4.688068696	5.144027662
5.251930671	4.628130372	5.217161758	5.242209004	5.762525802	4.887028403
2.727341543	2.338089952	3.1014272	2.483089832	2.479755638	2.47932483
5.833381194	5.507131431	6.241903682	5.761373833	5.97744854	5.506260943
1.363066351	1.167479957	1.282334974	1.230762308	1.61908329	1.509056855
3.541522186	2.825633944	3.547975776	3.946537884	3.530082286	3.849528583
6.493085737	6.36586362	7.290429742	6.663065109	7.004955412	5.857251064
3.393954024	3.525334318	3.18089412	3.302148403	3.747472184	3.371631262
2.600894533	2.770869694	2.228471208	2.542927365	2.812232946	2.235571374
8.166757701	8.000948679	8.194302411	8.251731561	7.734778943	8.505089774
5.218205785	4.932654678	4.907108101	5.15223341	5.176945422	5.643537867
6.485055081	6.411110791	6.34453377	6.263907695	6.909594919	6.762165989
5.092676613	4.353963654	5.180413257	5.368783953	4.886834669	5.535830461
8.490724565	8.201897419	8.959464861	8.193489701	8.39067272	8.436754978
11.62932366	11.57881928	11.76088485	11.69580552	11.5820758	11.64833222
3.340559135	3.797538881	3.098205499	3.569636881	3.347327311	2.998446672
1.746766211	1.457456473	1.634816923	1.400909821	2.044001361	1.802617685
4.761982649	4.671497701	4.600692333	4.815087925	4.795211695	5.098667016
4.592339876	4.835450092	4.295397119	4.939687771	4.571546012	4.461749373
3.276094332	2.775602219	3.248229104	3.553294669	3.62647952	2.932810957
3.3306241	3.17254823	3.644869048	3.353239181	3.513589641	3.67587763
3.775506669	3.765312003	3.846987828	3.857289632	3.200047884	3.966775088
4.955034266	4.223846355	5.174602718	4.529074688	5.161812186	5.10680331
4.953908735	3.798770061	4.622391257	5.19331024	4.003798511	5.917635715
7.050134264	6.674258674	6.888820033	7.230392151	6.742817343	7.427183158
2.073052854	1.925768318	1.871160348	2.275727233	2.290587541	2.044009847
5.785407302	4.753965217	5.254433667	6.187270316	4.725999346	6.862429635
4.545368975	4.03466652	4.851607927	4.036265058	5.206816811	4.609633432
4.348556212	3.200510207	4.735955793	3.885695566	4.385260718	4.861548272
6.415391507	6.232927213	6.097000495	6.484125558	6.267056931	6.562084293
4.663866711	4.828980677	4.795663069	4.591454278	5.410071215	4.094735314
1.923777638	2.259423731	1.462008596	1.774644933	1.54484331	1.849219663
6.936584129	7.140618434	6.61011213	7.287510846	6.655319913	6.936278298
4.954378242	4.669241064	4.583107792	5.268691616	4.796371485	5.313209368
3.428340856	3.387613683	3.155701324	3.267421797	3.979449905	2.890794801
6.451120373	6.568974658	6.363843302	6.505300765	6.857955554	6.213269681
4.533195158	4.296816276	4.1124997	4.596647072	3.710355317	5.558918127
5.754941526	4.959368464	6.375100521	5.561718462	6.050307299	5.869736299
5.500805724	5.16759646	5.828398929	5.632605116	5.247030562	5.460973034
1.134599472	0.781434587	1.179575877	1.182012288	0.997754162	1.367108251
4.128413304	4.377722799	3.926466425	4.772208355	3.660232516	4.151008287
2.574684868	3.297775539	3.222663075	2.128467476	2.407444623	2.354130088
6.118487126	6.12575165	6.594607009	5.873829402	6.310985126	5.825157699
5.405354379	4.955283633	5.657243649	5.10210236	5.12923401	5.509080062
6.052115241	6.216698191	6.495995601	6.327741104	6.238466988	5.850517452
2.430508905	2.742056154	2.430948775	2.503295092	2.020735507	2.539962494

4.916950837	4.609046537	4.889092374	5.117548893	5.234262958	4.952082242
3.27791642	3.100911972	3.337438357	2.659599648	3.213972457	3.477801558
3.915902303	3.510383555	4.511994803	3.154563329	3.977614506	3.801248141
5.273998163	5.574588706	5.179654635	5.01028614	4.926002329	5.43855059
5.483933755	5.028409896	5.362532486	5.483894355	4.746901524	5.832177326
2.018549833	1.320084112	1.961482003	2.324299577	2.357813235	2.005552925
2.693767145	2.648262102	1.809643981	1.312870772	3.7233366	2.636583867
3.980633104	3.652937651	3.777167366	4.453695961	3.537843194	4.055500549
3.545392027	2.312063998	3.584850953	4.342910875	3.985433277	3.561581909
6.002500212	6.325616047	6.236790056	6.367184813	5.840292465	5.747208552
4.132623052	4.164161819	4.399868511	4.276171512	4.264226554	3.761959787
6.342907635	6.094509858	6.324583545	6.400711738	6.290809852	6.482721594
4.778577254	4.598374621	4.701639391	4.902827127	4.193535152	4.897122371
3.486461654	3.619201174	3.769385576	3.257032064	3.442462351	3.452677255
1.555032758	1.802572349	1.854579937	1.299586006	1.095945404	1.297625019
7.1720547	7.000278084	7.225294218	7.276932228	7.046871499	7.35153628

12.ALS.SP.F	01.CTL.SP.M	02.CTL.SP.F	03.CTL.SP.M	04.CTL.SP.F	05.CTL.SP.F
4.108	3.194	3.483	2.341	3.412	3.978
8.003	7.016	8.343	7.490	7.963	8.403
4.224	4.499	3.806	4.021	4.167	4.001
3.134	2.501	3.124	3.007	3.565	2.050
5.529	5.321	5.055	4.862	5.248	5.238
4.368	4.269	4.039	3.746	4.436	4.929
4.307	4.283	4.311	4.178	3.895	4.574
6.867	6.753	6.802	6.865	6.606	6.873
5.434	5.134	5.184	4.691	5.251	5.398
6.567	5.869	6.711	6.256	6.806	6.485
6.883	5.916	6.117	6.345	6.928	5.535
2.762	2.264	2.029	2.357	1.765	2.378
2.221	2.388	1.939	3.080	2.037	1.590
6.622	6.187	6.035	5.315	6.270	6.909
6.463	7.736	5.914	3.859	6.139	7.057
7.485	7.418	6.941	7.142	7.343	7.150
5.687	5.863	5.331	4.154	5.344	6.122
5.591	6.274	5.888	5.304	5.911	6.213
2.194	2.154	2.307	3.070	2.147	2.417
3.734	6.595	4.708	4.222	4.178	5.475
5.197	4.887	5.264	4.239	5.691	5.755
5.794	5.876	5.857	6.376	5.896	6.148
4.039	4.333	5.384	3.944	5.352	4.414
5.677	7.201	5.637	5.487	6.217	5.962
5.213	4.140	4.602	4.569	4.389	4.232
4.632	5.211	4.951	4.843	5.146	5.006
5.320	4.138	4.897	5.064	5.353	4.881
5.118	5.321	6.875	6.485	5.799	6.078
3.878	3.224	3.951	4.408	3.061	3.986
2.414	2.206	2.257	2.456	2.724	3.627
5.729	5.039	5.032	5.181	5.020	5.083
3.487	3.164	3.338	2.876	4.324	3.864
2.852	2.315	3.117	2.962	2.562	2.476
2.672	2.430	3.512	3.795	3.220	2.922
6.516	6.213	5.358	5.365	6.262	5.894
4.518	4.732	4.680	4.279	3.808	4.336
5.451	6.355	5.319	5.158	6.229	5.467
4.973	5.489	5.509	4.584	5.523	5.507
5.415	4.848	5.399	6.122	4.932	4.703
5.046	5.309	4.587	5.085	4.422	6.079
6.220	6.417	6.310	5.957	6.713	5.988
7.355	7.715	7.418	7.360	7.623	7.200
5.581	4.323	5.305	4.712	4.678	4.950
6.553	6.377	6.966	7.339	6.906	6.750
2.509	2.679	2.432	3.381	3.041	3.036
3.432	3.604	3.548	3.849	2.659	3.939
6.073	5.871	6.815	6.002	6.886	6.419
4.945	4.568	4.512	4.168	5.185	4.857
3.936	4.184	3.961	2.914	3.782	3.145

2.357	2.724	3.030	3.925	2.502	2.353
3.214	2.910	3.959	3.318	3.859	2.338
2.708	2.876	2.887	3.502	2.724	2.105
5.505	5.227	5.694	5.993	5.885	5.555
2.104	1.970	2.882	3.389	2.181	1.962
2.278	2.493	1.678	1.304	3.053	2.802
4.822	4.430	4.694	2.917	5.372	6.079
4.220	3.273	3.597	3.155	2.812	2.639
5.176	5.160	4.352	3.538	4.309	4.341
3.652	3.598	3.976	4.617	4.044	3.579
3.846	3.607	2.125	2.909	3.648	3.036
4.387	4.769	3.612	5.011	4.802	4.577
1.881	2.243	1.608	2.243	1.969	1.710
2.345	3.597	3.753	3.588	4.510	5.201
2.341	3.020	3.300	3.937	2.554	3.554
4.840	3.715	4.051	3.557	3.688	5.009
3.673	3.544	3.489	2.241	3.801	3.656
1.666	2.455	2.858	2.776	2.877	3.053
2.525	3.665	2.552	2.365	2.973	3.409
2.473	1.963	2.809	3.470	2.912	1.812
3.498	1.669	1.179	0.948	3.961	2.715
2.100	2.708	2.395	3.867	3.101	2.412
2.832	2.305	3.322	2.546	3.405	2.023
2.870	3.167	2.723	3.148	3.257	4.094
2.149	1.596	1.981	2.833	1.573	3.028
2.496	2.250	2.490	2.718	2.525	2.237
3.044	2.260	2.890	2.486	3.127	3.200
1.717	2.357	2.351	2.341	2.142	4.113
2.552	2.818	2.947	3.679	3.103	2.559
3.107	2.750	2.136	2.720	2.742	3.451
2.531	3.142	2.196	3.664	3.352	2.803
1.743	1.860	1.739	2.274	2.453	2.655
3.038	2.131	1.609	1.299	3.107	3.048
3.348	3.584	3.877	3.976	3.130	3.392
2.387	2.009	2.123	2.763	2.266	1.972
2.802	3.090	3.167	2.888	2.295	2.455
2.521	3.484	1.534	2.324	2.521	2.484
2.569	2.315	1.849	2.722	2.152	2.096
4.824	4.125	4.343	3.492	5.280	4.802
2.332	2.184	3.313	2.155	3.011	1.803
2.425	2.570	2.294	1.792	3.011	2.983
3.760	4.434	0.879	1.002	2.085	1.761
3.151	2.693	2.630	2.398	3.410	2.001
1.718	1.398	1.147	1.597	1.103	1.418
1.817	1.722	1.890	2.024	2.026	1.680
1.353	2.007	1.034	1.700	1.774	1.178
3.983	4.280	4.054	3.469	3.394	4.362
1.856	3.442	2.461	2.183	2.484	2.567
3.394	3.658	3.555	4.026	3.814	3.658
2.425	2.419	3.027	2.350	2.162	2.134

2.055	2.197	1.966	1.952	2.823	1.180
3.252	3.745	2.416	3.387	2.880	3.898
3.183	2.944	2.597	2.307	2.935	3.104
2.718	3.099	2.904	2.843	2.874	3.084
2.779	2.696	2.945	3.637	3.076	2.223
2.298	2.385	2.770	2.649	2.209	2.668
2.318	2.188	2.341	2.014	1.690	2.080
2.911	2.157	2.278	1.551	1.841	2.354
0.636	0.917	1.145	1.007	1.076	0.515
5.067	4.870	5.684	5.138	5.562	5.611
3.097	2.254	2.738	2.819	2.616	3.223
4.844	4.666	5.270	4.723	5.369	6.221
3.204	3.984	3.828	5.100	3.066	3.922
3.290	4.086	3.009	3.556	3.178	2.608
2.071	1.699	2.170	2.381	2.332	2.198
2.156	1.799	0.891	0.763	1.459	1.351
2.426	2.323	3.371	3.546	2.840	2.389
4.700	4.699	4.912	5.240	4.835	5.055
3.603	3.438	3.156	3.166	3.476	3.749
1.437	1.094	1.301	1.489	0.923	1.464
2.085	2.621	2.844	1.922	3.180	2.584
1.988	2.696	1.984	3.332	1.989	2.025
2.621	2.088	4.813	5.343	4.618	3.108
4.247	3.373	3.146	2.752	2.766	3.691
1.289	1.902	1.297	1.325	1.454	0.975
3.109	3.161	3.332	2.148	4.263	2.948
1.614	2.859	3.089	3.851	1.598	2.156
4.095	2.963	3.261	3.785	4.005	3.486
2.738	2.391	3.033	3.349	3.417	1.960
1.853	1.535	1.793	2.671	3.181	2.198
4.419	3.715	3.443	3.241	4.534	4.562
2.443	1.497	2.760	2.353	4.183	2.464
6.312	5.530	5.905	6.621	5.838	6.026
2.192	2.746	4.428	3.654	3.458	2.781
5.426	5.847	5.466	5.542	5.922	5.316
2.185	2.890	2.460	2.285	2.322	2.685
4.109	3.467	2.440	3.724	4.180	3.673
5.746	6.157	6.094	5.158	5.412	5.605
6.784	5.980	7.142	7.221	7.313	6.269
4.033	3.529	4.316	3.595	4.259	3.971
4.139	3.713	4.151	4.096	3.320	3.840
5.665	4.899	5.753	5.452	4.918	5.503
4.509	5.423	4.914	3.681	4.232	4.744
3.522	4.843	3.747	3.904	5.130	3.159
8.544	8.900	8.610	8.344	7.875	8.612
4.631	4.749	4.757	4.178	4.901	4.188
4.063	4.175	4.818	4.345	4.521	4.197
2.870	2.108	3.299	2.477	2.727	3.135
2.391	3.271	2.527	2.237	2.894	4.871
6.499	6.057	6.725	6.469	6.847	6.317

3.921	3.804	3.959	4.230	4.952	3.829
7.933	8.171	6.548	6.339	7.607	7.241
7.420	7.237	6.712	6.453	7.031	7.413
1.756	2.229	2.383	1.540	2.502	2.022
5.646	5.334	4.472	4.388	4.447	5.451
3.995	3.306	4.056	3.604	3.364	3.321
5.715	4.588	5.634	4.682	5.897	5.987
5.479	4.445	5.307	5.616	5.688	4.783
6.535	7.037	5.626	5.874	6.547	6.086
2.464	2.007	2.586	2.797	1.991	2.699
3.181	4.736	2.987	3.829	3.564	2.904
2.964	2.921	2.909	2.136	2.391	3.342
4.062	4.269	3.602	3.475	4.404	4.266
4.308	3.615	3.449	4.010	3.678	4.091
4.142	4.937	2.909	4.035	3.643	4.776
5.405	6.577	6.108	3.891	6.499	6.611
6.037	5.699	5.642	5.498	5.834	6.377
6.222	5.746	6.558	5.788	5.854	6.314
5.065	5.070	5.778	4.645	4.669	5.585
5.408	5.134	4.903	4.816	5.289	5.620
2.862	2.842	2.636	3.210	2.649	2.803
4.914	5.059	4.586	4.444	4.339	4.813
4.050	3.669	3.554	3.004	3.758	3.192
4.464	4.171	4.363	3.789	4.406	4.486
3.894	4.196	3.632	2.877	2.680	3.699
4.156	2.747	5.609	4.497	5.992	4.189
4.742	4.799	5.679	5.947	5.126	5.277
4.583	2.936	4.387	3.720	3.452	3.166
3.835	3.724	3.330	3.307	3.850	4.184
4.194	3.422	3.664	3.718	4.490	3.959
4.456	4.384	3.084	3.308	3.825	4.157
4.978	4.693	3.516	3.827	3.793	4.864
6.901	6.466	6.483	6.933	6.903	6.715
7.250	7.238	7.153	7.061	5.834	7.249
6.891	6.971	6.634	4.990	6.950	7.032
2.567	2.946	3.151	4.169	4.068	2.474
3.036	2.610	2.952	3.524	3.341	2.824
5.879	6.755	5.191	5.661	5.037	4.892
2.226	2.295	2.538	2.478	3.743	2.990
2.884	2.889	3.351	2.595	2.693	3.225
1.876	4.630	2.470	2.212	2.962	2.907
3.290	3.914	4.094	3.666	3.563	2.866
4.883	1.981	3.336	3.715	3.727	3.301
2.406	1.457	2.676	2.551	2.466	2.665
3.084	3.876	4.204	3.903	3.254	2.941
2.723	2.193	1.572	1.668	1.419	1.386
6.333	6.076	6.623	4.822	6.904	6.292
3.514	3.964	3.392	3.511	4.208	3.231
1.720	2.946	2.708	3.183	2.234	3.340
3.950	3.808	3.869	2.946	3.918	3.950

7.645	8.210	6.024	6.444	6.553	6.637
4.719	5.946	3.797	4.299	5.021	4.674
3.348	3.740	3.762	4.370	3.026	2.865
4.549	5.314	5.122	4.563	6.166	4.628
2.785	3.214	2.424	2.899	2.649	3.110
2.555	3.434	3.158	2.825	3.191	2.080
5.006	5.651	5.608	4.573	5.422	6.220
5.297	5.410	4.968	4.625	4.742	5.018
1.580	2.205	2.809	1.837	2.261	2.163
4.173	3.656	3.399	2.528	2.904	4.163
6.430	5.392	6.589	6.088	6.881	6.023
4.360	4.694	4.079	4.372	4.113	4.405
4.719	5.025	4.682	3.924	4.627	5.311
7.695	6.834	7.384	7.372	7.483	6.410
4.703	5.018	4.613	4.992	4.240	4.606
3.808	3.298	3.665	2.017	2.576	3.843
7.073	5.685	7.072	7.139	6.857	6.148
2.083	2.115	3.616	2.580	2.852	1.814
2.832	4.479	2.077	1.864	2.323	1.634
5.950	5.749	5.399	5.631	6.134	5.618
5.363	5.359	5.714	6.422	6.104	5.756
5.177	7.182	5.608	6.586	5.825	5.279
4.276	4.228	2.945	2.774	4.411	3.709
3.784	3.873	4.778	4.866	4.208	4.377
4.815	4.979	4.713	4.089	5.246	5.848
4.257	3.717	4.029	3.237	4.362	3.714
3.690	3.290	3.552	3.593	3.298	3.141
3.343	3.428	3.147	3.381	2.871	2.630
3.605	2.534	2.254	2.786	2.281	2.543
2.741	4.075	2.900	2.963	2.805	2.972
4.017	3.253	3.312	4.060	3.865	3.622
6.274	5.898	6.603	5.806	6.099	6.370
7.317	6.608	6.742	7.827	6.734	7.123
4.976	5.776	5.273	5.013	4.987	5.134
3.442	4.471	3.107	3.481	3.100	3.463
9.020	9.083	9.259	9.335	8.831	8.869
3.665	3.762	3.196	2.994	3.187	4.118
3.716	3.615	2.995	3.389	4.104	4.302
2.704	2.707	1.938	2.111	2.266	3.219
4.325	4.046	4.301	4.941	3.945	3.681
6.022	5.025	5.832	5.224	5.459	6.186
5.515	4.747	5.046	4.829	5.029	5.248
4.294	3.350	4.135	4.165	4.150	4.147
6.792	6.951	6.529	6.603	6.353	6.839
5.573	5.190	5.255	3.664	5.389	5.720
2.662	2.926	3.379	3.605	2.926	3.029
5.690	4.243	4.460	3.874	4.373	4.893
2.601	2.976	2.547	2.556	1.934	2.536
6.203	6.223	5.849	6.323	6.477	6.650
3.326	2.843	2.121	3.421	2.713	2.558

3.623	3.314	3.297	3.897	3.527	3.320
1.885	1.817	2.063	1.410	1.628	1.640
5.595	5.735	6.614	5.851	6.958	5.833
6.623	6.478	6.216	6.546	6.586	6.695
5.357	5.193	4.864	4.448	5.298	5.338
6.092	5.356	5.632	4.918	6.269	5.882
2.703	2.827	3.162	2.262	2.384	3.080
2.705	2.191	1.471	1.686	1.935	1.661
7.464	7.190	7.280	5.876	7.873	7.595
6.029	5.166	6.228	5.198	6.504	6.495
7.751	7.093	7.642	7.457	8.066	6.927
4.379	4.689	5.234	3.772	4.987	4.889
7.742	7.673	7.714	8.181	8.304	7.049
5.782	7.161	7.020	7.384	5.912	7.252
8.057	7.754	6.248	6.105	6.974	7.285
4.903	4.382	4.111	4.660	4.428	4.627
6.459	6.907	6.028	4.488	6.586	8.107
6.135	6.589	5.947	4.339	6.116	7.600
6.519	6.945	6.315	4.525	6.537	8.071
4.205	4.165	4.578	4.244	3.891	5.002
5.110	4.816	5.773	6.062	5.132	4.638
1.199	0.923	1.077	1.251	0.681	1.129
1.964	1.397	0.966	1.643	1.228	1.049
6.256	4.521	5.062	5.421	5.107	5.934
7.487	6.018	7.344	6.645	6.790	7.299
7.539	7.438	7.551	7.228	7.897	7.578
2.094	2.356	2.444	2.503	2.466	2.279
1.708	1.842	1.533	2.186	1.890	1.695
4.689	5.098	4.171	3.811	4.515	4.399
2.820	2.583	3.197	3.205	3.362	2.712
3.735	2.590	4.448	4.246	3.678	3.498
2.835	2.379	3.345	3.478	2.291	2.205
7.423	7.232	7.391	7.885	7.323	7.323
3.310	2.809	3.043	2.648	3.267	3.877
3.282	1.109	1.522	1.817	1.209	2.530
5.093	5.129	5.668	5.458	5.513	5.079
6.226	5.952	5.641	5.378	5.522	6.186
2.622	4.885	2.613	1.981	5.404	4.707
6.548	6.286	6.208	5.373	6.524	6.443
7.632	7.372	7.550	7.866	8.052	7.436
3.966	4.183	4.837	3.531	3.903	4.699
3.134	3.620	4.494	3.827	3.863	4.384
2.358	2.320	2.442	2.352	2.285	2.463
6.701	6.661	7.057	6.441	7.401	7.205
4.428	4.380	5.038	3.238	4.640	4.740
3.402	3.920	3.211	3.215	3.202	3.989
3.194	3.668	1.771	3.029	3.671	2.618
2.317	2.090	3.526	2.596	2.965	3.612
4.501	4.320	3.678	3.417	4.316	4.158
3.590	3.288	2.226	2.257	3.041	2.907

2.596	2.625	3.099	2.272	2.983	2.893
3.288	4.814	1.711	3.458	3.797	2.538
5.459	6.070	4.414	3.850	5.176	6.228
6.960	7.457	6.310	5.352	6.868	8.250
4.895	5.194	3.937	3.022	3.900	5.037
5.419	5.704	4.500	5.069	5.593	5.514
5.595	5.469	5.104	5.234	5.295	5.526
3.103	3.662	4.012	4.060	3.679	4.062
2.468	2.468	3.004	3.195	1.710	2.548
3.481	2.644	1.954	2.798	1.713	2.136
6.701	6.292	5.972	6.416	6.153	6.044
2.864	2.321	2.685	2.177	1.669	2.638
2.114	1.950	2.446	2.267	2.611	2.267
5.634	4.542	5.654	5.009	4.197	4.888
2.134	1.916	2.142	1.703	1.917	2.560
5.460	5.724	5.932	5.959	5.550	5.771
4.627	5.172	4.611	4.395	4.730	5.389
7.008	4.949	6.391	5.573	6.044	6.439
3.921	3.116	3.318	3.280	3.825	3.029
5.178	4.675	4.261	4.544	4.871	5.245
4.817	5.322	3.785	3.973	4.711	4.199
5.270	5.177	4.484	4.434	5.274	4.923
4.275	4.727	3.507	3.925	4.817	3.959
6.447	7.625	4.532	7.905	5.507	5.906
6.202	5.854	5.878	4.554	5.771	6.379
6.549	7.310	5.902	6.531	6.475	5.534
3.424	3.420	3.033	2.743	3.217	3.935
5.930	4.006	5.435	5.102	4.615	5.385
8.989	8.477	8.443	8.457	8.533	8.737
7.767	7.232	7.305	6.994	7.020	7.499
1.799	2.495	2.734	2.438	2.042	2.761
6.435	5.380	5.656	5.932	5.192	5.764
7.438	7.107	7.526	7.201	7.667	7.176
2.632	2.456	3.165	4.113	1.936	2.658
5.932	4.970	5.104	4.943	5.629	5.239
6.678	6.143	5.407	5.213	6.558	6.496
6.515	6.754	4.623	4.648	6.249	6.417
6.454	6.172	6.198	5.398	6.358	6.571
6.577	6.474	5.690	5.812	6.316	6.655
6.002	5.595	5.864	5.205	5.900	5.606
4.850	3.744	4.216	4.648	3.814	4.557
4.365	2.259	3.646	3.352	3.715	3.665
3.268	3.497	3.173	3.344	3.264	2.426
6.598	6.337	5.737	6.089	5.560	5.955
6.932	6.588	5.941	6.643	5.859	6.211
4.120	2.720	3.551	3.811	4.186	2.477
2.691	1.711	1.018	1.124	1.374	0.775
2.423	3.649	1.193	1.785	1.810	1.825
3.299	3.018	3.176	4.242	3.656	3.699
5.469	5.324	4.755	5.205	5.217	5.391

3.943	1.673	2.639	2.711	2.783	2.055
5.385	5.134	5.309	4.454	4.080	5.179
3.529	3.014	2.371	2.623	1.389	2.851
3.996	3.916	3.669	3.738	3.906	3.914
1.992	2.671	3.012	2.712	2.223	1.683
3.084	3.080	3.167	2.853	2.559	2.901
2.299	2.619	3.489	2.300	2.294	2.468
2.706	2.825	2.983	2.315	2.767	2.316
7.004	7.243	6.548	7.117	6.942	7.176
4.735	6.155	4.561	6.520	4.552	4.919
8.404	8.672	8.993	9.098	9.015	8.651
4.391	4.252	3.635	3.765	4.017	4.471
5.938	5.796	6.182	5.619	6.358	6.420
3.643	3.469	3.120	3.581	4.159	3.618
4.311	3.783	4.031	4.215	3.816	3.763
5.751	5.495	4.942	4.353	4.367	5.304
4.175	3.008	3.340	3.201	3.234	3.418
6.031	4.944	5.686	5.429	5.667	5.662
4.397	5.584	4.105	4.314	4.612	4.524
8.839	8.827	8.936	8.696	8.998	9.015
3.479	2.861	3.289	2.799	2.177	3.111
5.933	5.493	4.972	5.321	4.914	5.481
5.750	5.980	5.645	5.921	6.097	6.276
8.656	9.170	8.107	7.919	8.689	8.732
4.318	4.167	4.609	4.472	4.399	3.560
4.694	4.919	5.129	5.113	4.954	5.224
1.958	3.042	2.417	2.297	4.321	2.283
7.073	6.989	7.099	7.551	7.518	7.107
5.411	5.662	5.276	5.833	5.467	5.276
5.842	7.039	5.209	5.413	5.438	6.932
4.355	5.163	4.850	4.753	4.779	5.322
2.698	3.300	4.300	2.361	4.650	4.583
8.281	8.780	7.848	8.306	7.961	8.444
2.036	1.830	1.770	1.308	2.394	1.686
1.965	2.671	2.693	2.394	2.295	2.240
4.867	4.515	3.799	4.023	4.679	4.164
6.791	6.687	6.729	6.438	7.059	6.843
8.457	8.934	8.591	7.994	8.724	9.082
8.589	8.894	8.633	8.327	8.280	8.442
5.667	5.046	5.113	4.465	5.224	5.594
2.913	3.916	2.472	2.323	2.908	2.650
2.321	1.468	2.044	1.687	2.266	2.927
1.511	1.845	1.890	2.342	1.951	1.914
2.741	3.394	1.851	3.445	2.838	3.344
6.125	5.234	4.549	4.429	4.846	4.473
4.052	4.241	1.957	3.602	3.633	3.710
2.531	2.098	2.120	3.889	3.235	1.983
2.998	3.298	3.237	3.921	3.426	3.180
4.086	4.478	4.098	5.801	3.647	4.064
4.597	3.568	3.759	3.277	4.175	4.944

4.388	2.583	3.926	4.120	3.998	3.896
5.150	4.860	5.135	4.469	5.721	5.768
3.809	4.899	4.906	4.765	4.224	4.113
5.067	5.249	5.025	5.419	4.795	5.396
5.226	5.226	4.615	4.518	4.202	5.369
5.236	5.145	5.313	4.868	5.010	5.282
5.850	5.453	5.503	5.228	5.526	5.521
3.169	3.025	2.421	2.771	3.226	2.944
5.486	5.670	5.287	5.434	5.369	5.703
6.426	5.261	6.065	6.071	5.801	6.240
4.369	5.334	5.145	3.853	5.033	5.802
6.244	5.489	6.888	6.635	7.318	5.400
6.519	6.330	6.033	5.642	6.616	6.639
6.636	5.074	6.902	6.711	6.897	6.199
8.532	9.165	8.497	7.677	8.741	9.090
9.134	9.265	9.526	8.360	9.480	9.423
4.503	5.421	4.224	3.740	5.314	5.615
3.518	4.324	4.814	4.789	3.968	3.702
3.406	3.291	3.588	3.212	4.106	4.278
3.687	3.456	3.109	2.943	2.650	3.400
5.481	5.755	3.551	3.389	5.246	5.536
3.913	3.926	3.583	2.787	2.825	4.261
9.135	9.473	9.373	9.145	9.060	9.177
4.942	4.420	4.492	4.540	4.576	4.533
4.390	4.258	3.824	2.994	3.483	4.013
3.490	2.169	1.787	1.784	2.052	1.982
6.733	6.318	5.155	5.421	5.576	6.307
4.921	5.226	5.451	5.038	5.272	5.839
7.481	7.192	7.178	6.650	7.393	7.165
5.898	5.610	5.699	5.599	6.354	5.882
5.063	5.420	5.056	5.130	5.631	5.565
8.166	8.047	7.850	7.532	8.483	8.140
4.041	4.356	4.408	4.975	4.147	4.340
5.114	5.279	5.850	4.758	5.139	4.147
2.311	4.036	2.132	2.015	2.948	2.779
3.927	3.628	4.318	4.080	4.066	3.821
5.343	5.348	5.979	6.057	5.512	5.661
6.224	5.661	5.665	5.556	5.894	6.130
3.938	4.023	3.256	3.804	3.827	4.103
5.690	5.646	5.868	5.508	5.126	5.826
6.651	6.637	6.710	6.032	6.539	6.761
4.207	5.094	3.627	4.466	3.981	4.482
7.388	7.593	7.708	6.822	8.220	8.202
2.521	2.336	2.478	2.436	2.908	2.978
5.889	6.053	5.853	5.648	6.257	6.084
5.231	5.535	4.429	4.635	4.744	4.984
4.076	3.566	3.413	3.399	3.733	3.962
3.996	3.511	3.882	3.971	4.591	4.147
3.998	3.413	3.244	3.395	3.416	3.592
6.911	6.481	7.046	6.615	7.208	6.904

5.741	5.144	5.133	4.036	5.090	5.044
3.410	3.129	3.657	2.848	3.061	3.804
6.980	6.439	6.198	6.565	6.666	6.487
5.561	4.468	3.502	5.138	4.661	4.331
3.204	3.598	3.911	3.512	3.190	3.512
2.363	2.993	2.391	2.433	2.809	3.742
3.550	3.584	3.704	2.401	3.152	3.663
7.162	6.825	6.955	7.401	7.210	6.598
7.948	7.749	7.949	8.195	7.679	8.004
6.513	6.730	6.632	6.553	4.323	6.187
4.361	4.476	4.726	4.327	4.010	4.784
4.640	4.051	4.628	4.341	4.256	4.484
4.366	4.883	4.539	4.371	4.664	4.547
3.609	4.123	3.609	3.524	3.483	4.404
3.024	3.439	2.007	4.018	3.044	3.308
5.275	5.069	4.973	5.202	5.172	5.473
5.437	4.972	6.022	5.469	5.497	5.303
6.083	6.526	6.248	6.430	6.578	6.170
6.354	5.696	5.827	5.981	5.424	5.724
7.958	7.580	7.473	7.884	7.494	7.281
3.206	2.886	3.620	4.083	3.113	2.683
2.321	3.143	3.148	1.784	2.872	1.932
5.868	5.368	5.686	5.700	4.933	5.524
4.083	4.363	3.977	3.813	3.922	4.788
3.167	3.750	3.752	4.589	3.360	3.470
6.046	6.331	6.289	6.309	6.786	6.666
5.248	4.841	5.501	5.567	5.375	4.727
1.592	2.849	2.825	2.740	3.202	2.331
8.089	7.538	7.872	8.004	8.151	7.952
2.612	2.537	2.307	3.231	2.294	2.338
7.376	6.611	7.021	6.629	7.060	6.974
6.589	5.614	4.684	5.086	4.845	5.403
6.596	6.927	5.227	5.387	5.896	5.972
6.326	6.025	5.508	5.326	5.730	6.109
4.945	4.504	4.623	4.844	4.478	4.980
3.241	3.217	3.950	4.265	3.959	3.270
6.593	6.957	6.449	6.181	4.821	6.366
2.630	2.819	2.062	2.154	3.394	2.929
4.160	3.425	3.132	3.641	3.917	4.110
9.891	9.925	9.815	9.699	9.955	9.856
2.824	4.587	3.266	4.675	1.500	2.514
2.283	2.125	2.599	3.854	2.790	2.241
4.145	4.736	4.098	4.595	3.692	4.159
5.921	5.567	5.595	5.084	5.347	5.556
7.564	7.157	7.714	8.020	7.901	7.279
4.996	5.513	4.954	4.738	5.374	5.049
3.698	3.412	2.941	2.653	3.670	3.611
4.974	5.556	5.073	4.412	4.750	5.760
4.127	2.844	3.791	3.596	3.417	3.752
4.638	4.125	5.335	5.014	4.896	4.292

6.055	5.800	4.989	6.895	6.239	5.414
7.945	9.167	7.593	7.486	8.518	8.396
7.879	8.581	8.852	8.938	9.022	9.135
4.542	4.305	4.801	3.596	4.930	4.865
2.066	1.970	3.266	2.868	2.513	2.142
5.517	5.042	4.166	3.639	4.775	5.118
2.260	3.031	2.241	2.195	2.399	2.134
3.582	4.406	3.924	3.759	3.608	3.533
7.679	7.172	7.350	7.086	8.023	7.351
4.288	2.670	4.008	3.708	3.496	3.027
5.853	6.339	5.752	5.754	5.815	6.010
5.168	5.439	5.017	4.511	5.395	5.586
6.318	5.540	7.146	6.857	6.778	5.671
3.012	2.806	2.902	2.715	2.693	2.940
6.417	6.053	6.008	5.170	6.126	6.127
4.341	2.616	4.491	4.079	3.939	3.378
4.645	3.686	3.891	3.823	4.260	3.440
5.103	6.221	4.973	5.334	4.595	5.376
7.944	6.625	7.491	7.132	7.330	7.226
3.537	3.791	3.275	2.798	3.867	3.394
4.150	4.225	3.455	3.673	3.319	3.961
5.875	6.154	5.756	5.526	6.787	6.368
7.320	6.074	7.042	7.106	7.226	6.735
5.499	5.754	5.936	4.630	5.670	5.913
4.457	4.274	4.009	4.428	4.341	4.535
3.653	3.745	3.625	3.707	3.248	4.067
7.128	5.728	6.861	6.952	6.047	6.805
3.661	4.524	4.365	4.696	5.263	3.810
4.378	5.104	3.832	4.082	4.271	4.586
4.085	4.844	3.646	4.232	4.367	4.129
6.310	6.563	6.407	6.775	6.601	6.306
3.207	2.863	2.304	2.343	3.021	3.348
5.747	6.007	4.929	5.551	5.501	5.688
5.160	5.234	4.634	3.707	5.172	5.743
6.458	6.395	6.064	5.663	6.237	6.445
6.208	5.936	5.248	6.211	5.948	6.165
5.450	6.035	5.076	5.250	5.774	5.819
4.088	3.375	3.511	3.866	3.853	4.253
5.765	5.307	5.410	4.873	5.520	5.771
3.736	3.425	3.898	3.714	4.015	3.738
6.799	7.404	6.945	6.362	7.245	7.144
6.310	6.614	6.298	5.867	6.593	6.754
7.997	7.472	7.812	7.208	7.611	7.444
2.437	4.752	2.449	3.447	3.419	2.628
6.680	6.217	6.754	4.654	7.187	6.748
6.988	6.997	6.355	6.483	6.664	7.225
5.714	5.629	4.661	5.155	5.062	5.451
7.530	7.467	7.629	7.938	6.971	7.518
5.887	5.387	5.815	6.244	6.710	5.303
1.763	1.888	1.709	1.985	1.387	1.545

5.079	4.941	4.584	4.594	4.884	4.848
3.829	4.068	3.023	4.003	4.097	3.637
4.896	4.163	4.822	4.476	3.892	4.710
9.457	9.468	9.557	9.194	9.311	9.727
8.219	8.207	7.896	8.124	7.688	8.097
2.453	2.173	3.088	2.317	2.663	1.941
6.588	6.707	6.666	6.378	6.830	6.882
5.919	5.005	6.078	5.680	4.883	5.591
4.279	3.344	4.174	3.260	2.230	3.787
7.855	7.739	7.261	7.354	7.330	7.584
1.852	1.558	2.820	2.465	1.973	2.184
2.273	2.208	2.713	3.801	1.622	2.267
8.418	8.329	8.170	8.093	8.039	8.460
9.893	10.032	9.671	9.455	9.640	9.722
3.601	3.422	3.542	4.859	3.832	3.179
4.723	5.226	4.773	4.354	4.292	4.874
8.307	8.499	8.158	7.576	8.478	8.497
9.221	9.346	9.183	8.610	9.402	9.215
9.835	10.092	9.342	9.269	9.800	9.597
7.394	6.933	7.175	6.578	6.556	7.231
8.159	8.087	7.806	7.846	7.680	8.444
7.687	7.470	7.118	7.103	7.171	7.559
5.152	5.481	5.224	4.915	5.142	5.248
6.567	6.161	6.088	5.990	6.380	6.516
3.433	3.531	3.587	2.725	3.279	3.719
8.995	8.444	8.552	7.942	8.960	9.178
6.765	6.084	6.651	6.392	5.362	6.739
3.352	4.300	4.004	2.598	4.298	2.906
4.490	4.228	4.341	3.871	4.692	5.483
6.216	6.149	6.099	6.011	6.154	6.235
5.401	5.197	4.398	4.308	4.494	5.418
6.739	6.778	6.593	5.774	6.861	6.641
6.272	6.973	6.719	7.263	6.195	6.762
3.103	3.869	2.482	3.688	4.349	3.412
6.804	6.349	6.243	6.639	6.451	6.411
7.762	7.832	7.570	7.699	7.401	7.439
3.149	2.847	2.732	4.171	2.073	2.354
3.172	2.947	2.962	4.284	2.642	3.158
6.589	6.105	6.104	6.068	6.276	6.144
5.149	5.743	5.585	4.092	6.071	5.605
3.632	4.527	2.766	3.106	2.713	2.268
8.749	8.394	7.927	7.633	8.301	8.550
4.458	4.297	4.202	2.960	3.862	4.086
4.413	4.223	4.496	3.844	3.917	3.966
6.176	5.608	5.992	5.583	6.168	6.384
7.177	6.576	7.626	7.186	7.639	6.788
2.211	1.903	0.953	1.476	2.019	1.445
5.077	4.589	4.477	4.152	5.125	5.325
4.848	4.690	5.036	4.901	4.134	4.322
2.464	1.908	2.116	2.986	2.010	2.033

4.697	4.924	4.641	4.335	5.484	4.573
7.661	7.045	6.364	6.791	6.577	6.699
6.085	5.062	5.116	4.656	4.471	5.793
3.895	4.317	3.656	4.431	3.109	3.457
3.359	2.851	2.218	2.112	3.029	3.700
4.042	3.362	2.890	1.986	2.615	2.922
2.211	2.080	1.954	2.091	2.483	1.589
7.932	8.187	8.058	7.188	8.398	8.374
4.385	5.696	3.745	4.245	4.314	4.801
3.244	4.451	2.809	3.244	3.244	3.763
1.948	2.564	2.215	2.115	2.295	3.305
4.141	4.269	4.031	4.808	4.325	4.354
6.413	8.131	6.110	7.420	6.249	6.950
4.431	4.433	4.324	3.991	4.176	4.177
4.859	3.860	5.412	5.744	4.421	3.970
4.834	4.543	5.215	5.087	4.770	4.348
7.451	6.416	6.637	6.778	6.535	6.511
4.683	5.285	3.999	5.014	4.381	4.406
4.219	3.470	4.437	4.261	3.807	3.823
3.458	3.357	3.175	4.508	2.969	3.205
6.628	4.985	5.681	5.850	5.727	5.589
5.716	6.628	6.720	6.560	6.973	5.892
5.701	6.129	5.798	5.605	5.804	6.060
5.591	4.705	4.502	4.684	5.037	4.502
3.024	3.177	2.081	2.368	1.961	1.626
8.286	8.196	8.303	8.628	6.918	8.158
4.024	3.493	3.112	2.529	2.251	3.439
4.144	4.610	4.226	3.888	3.989	4.424
5.258	5.727	4.624	4.394	4.667	5.185
8.003	7.891	7.580	7.774	8.175	7.905
4.877	3.655	4.629	5.195	4.569	4.740
4.641	5.458	4.190	4.191	5.437	4.563
6.527	6.668	5.975	6.353	6.134	6.543
6.165	5.997	5.628	5.399	5.960	5.826
6.731	6.055	6.814	7.399	7.178	6.101
4.244	4.180	4.549	5.601	4.858	4.031
7.056	7.148	7.530	7.422	7.416	7.408
3.943	3.601	3.400	3.328	4.383	3.832
5.471	6.075	5.291	4.595	5.569	5.859
2.929	2.057	2.577	2.023	2.230	1.952
2.850	2.640	3.379	3.753	2.754	3.023
4.133	2.618	3.818	3.323	3.581	3.173
3.077	1.946	3.076	3.422	2.625	2.453
7.242	6.170	6.417	7.270	6.834	6.427
3.726	3.979	2.120	2.641	3.511	4.421
4.700	5.177	4.427	4.345	3.896	3.994
8.634	9.276	7.315	8.076	8.100	8.103
2.802	2.676	2.992	3.470	2.054	2.400
5.311	5.036	4.468	5.110	5.058	4.958
4.210	4.502	4.666	4.285	4.573	5.175

2.892	2.097	2.120	2.237	2.199	2.337
5.040	5.449	5.081	5.748	4.977	5.157
4.372	4.310	4.343	2.644	4.060	4.265
4.075	3.615	4.030	3.922	4.274	4.027
4.465	4.410	4.628	4.314	4.961	4.583
3.813	3.562	3.687	3.441	4.055	4.383
3.000	3.078	3.283	3.833	2.737	3.128
2.386	2.078	1.893	2.852	2.205	2.035
2.511	2.133	1.864	2.005	1.931	2.447
4.174	4.089	3.900	3.741	3.428	3.486
5.528	5.391	5.821	5.369	4.624	6.123
7.823	8.233	7.857	6.091	8.087	7.806
3.244	4.479	2.581	3.262	3.160	4.753
8.487	7.693	8.140	7.945	6.829	8.022
4.493	3.886	3.898	5.082	4.441	4.636
4.781	4.113	4.656	4.416	3.669	4.656
6.504	7.433	6.952	6.980	6.303	6.930
3.138	4.999	4.116	3.625	3.525	2.867
4.864	4.367	5.146	4.857	5.446	5.508
3.382	1.787	2.321	2.406	2.201	2.586
3.858	3.918	4.461	4.369	3.614	3.344
7.707	8.402	7.432	7.346	7.391	7.199
8.227	8.558	8.825	8.372	8.716	8.833
3.817	3.798	3.967	3.283	3.383	3.448
4.622	4.661	4.131	4.068	5.310	3.945
4.722	5.362	5.393	4.379	5.255	5.856
3.886	4.542	3.371	2.459	4.083	3.407
3.588	2.786	2.779	3.116	3.062	2.229
3.420	4.221	3.251	2.721	4.178	3.735
3.098	4.167	5.207	3.811	4.866	5.702
5.237	5.063	4.133	4.564	4.181	4.142
7.796	7.594	7.454	6.770	7.940	8.091
3.376	3.397	2.807	3.963	2.925	2.803
2.645	3.881	3.560	3.524	4.384	2.808
2.998	3.576	1.565	3.070	3.154	1.672
4.220	4.353	3.483	4.642	4.712	4.086
5.187	4.167	5.175	5.424	5.371	4.121
5.308	5.539	4.974	5.291	5.607	5.680
3.578	3.888	3.351	3.801	2.635	3.551
4.237	4.110	4.259	3.916	4.077	4.060
8.627	8.303	8.199	8.139	8.291	8.272
5.897	5.741	5.154	5.507	5.421	5.955
4.095	3.390	2.360	3.654	4.276	3.512
3.125	2.726	3.013	3.525	3.727	3.955
4.157	4.461	3.514	2.926	4.626	4.061
4.638	5.084	4.566	5.894	5.181	4.844
6.998	7.498	7.352	7.274	7.466	7.182
3.523	3.827	3.058	3.681	3.584	3.892
6.960	6.561	6.571	6.788	6.706	6.885
6.160	5.636	5.770	6.038	6.063	6.207

2.570	2.873	2.346	2.342	2.416	2.553
3.035	3.173	2.332	2.837	3.284	3.317
7.562	6.237	7.432	6.954	7.726	6.722
5.771	4.339	4.867	5.146	5.001	4.776
6.569	5.370	6.048	6.368	6.055	5.428
4.027	4.453	4.059	3.505	3.878	4.076
4.514	4.774	4.469	4.916	4.389	4.514
6.494	6.252	6.400	6.054	6.051	6.138
5.636	5.149	5.818	5.344	5.054	5.195
4.926	3.834	5.362	4.915	4.358	4.272
2.961	2.590	2.887	3.017	2.351	2.820
2.970	3.047	3.659	3.789	3.031	3.220
6.383	6.426	5.630	5.807	5.597	5.879
5.182	4.990	5.325	5.013	5.073	5.801
5.069	4.779	4.360	3.304	3.808	4.773
2.395	2.405	2.330	2.684	2.685	2.495
2.925	2.514	4.103	3.360	3.336	2.347
2.836	4.318	3.473	2.760	2.890	4.202
6.457	5.648	6.004	5.437	6.010	6.189
4.486	4.552	5.198	4.985	4.014	4.609
5.570	5.188	5.528	5.668	5.405	5.454
8.745	8.995	8.167	7.804	8.719	8.174
4.514	4.826	4.590	2.953	4.451	5.529
7.092	6.768	6.963	6.906	6.937	7.054
6.654	7.410	6.644	5.701	7.267	6.536
2.723	2.925	1.868	2.341	3.358	2.992
4.515	3.860	4.427	4.684	4.736	4.152
3.431	3.770	3.323	3.126	2.843	3.875
7.404	7.836	5.004	6.055	7.360	7.542
3.282	3.440	3.360	4.290	2.987	2.986
5.941	5.702	5.402	5.377	5.681	5.401
3.500	2.897	3.043	2.408	3.184	3.833
3.666	4.301	4.617	3.320	5.030	4.484
3.210	3.556	2.468	3.857	3.422	3.590
1.827	2.045	2.121	1.808	1.470	1.627
3.475	4.802	3.721	4.782	4.443	4.145
4.398	4.498	4.235	4.943	4.304	4.569
5.425	5.039	5.415	4.200	5.407	4.601
5.725	5.332	5.449	4.172	5.657	5.149
4.169	3.292	2.877	3.908	3.644	3.077
3.279	3.173	3.274	2.534	3.695	3.448
4.437	3.184	3.438	4.847	2.843	3.061
3.780	3.426	3.670	3.733	2.657	3.738
4.225	4.253	3.870	3.433	3.533	3.480
5.024	2.473	4.063	1.758	3.253	3.234
2.084	3.731	3.701	4.535	0.977	2.347
2.230	2.769	2.703	3.928	2.546	3.089
1.669	2.736	2.234	3.164	1.182	1.592
5.931	5.575	5.708	5.691	5.944	5.611
5.720	5.862	4.915	4.820	5.672	6.190

4.655	4.582	4.843	4.227	4.718	4.782
4.345	3.783	3.788	4.133	3.584	4.358
3.585	4.959	3.640	4.912	2.624	3.834
4.201	3.790	3.998	4.106	3.959	3.775
6.125	6.102	6.270	6.051	6.622	6.217
2.800	2.949	3.226	3.008	2.435	2.748
3.681	3.908	4.257	3.497	3.303	3.932
1.245	0.878	1.264	1.305	1.877	0.693
5.586	4.747	5.191	5.487	4.682	5.440
2.143	2.332	2.285	2.239	1.848	2.542
3.951	4.154	4.209	4.869	3.924	3.713
3.843	3.807	4.284	3.831	3.779	4.281
6.070	5.450	6.073	6.325	6.333	5.524
3.974	4.011	4.485	4.000	4.443	4.366
4.493	4.754	5.157	5.232	4.296	4.564
5.419	5.703	5.570	5.303	5.756	5.414
6.793	6.412	6.452	6.323	6.436	6.233
4.790	4.436	4.140	4.825	4.153	4.340
6.693	6.733	6.653	5.515	7.417	7.392
5.050	5.250	4.620	4.884	5.482	4.979
4.910	5.042	4.803	4.275	5.443	4.553
2.870	3.362	3.049	3.216	2.666	2.964
4.082	4.105	4.193	4.137	4.245	4.185
3.311	2.509	3.810	2.945	2.937	3.247
2.690	3.227	3.538	3.739	2.691	2.399
4.775	4.150	3.659	4.215	3.722	3.908
6.436	6.786	5.994	5.611	6.093	6.666
5.128	4.766	5.055	4.881	5.070	4.942
2.076	2.495	2.556	2.337	1.889	1.838
4.702	5.231	5.209	4.569	4.271	3.938
3.198	3.564	2.704	2.647	2.403	2.976
6.000	5.819	5.324	5.453	5.405	5.552
3.058	2.859	3.260	2.705	1.790	2.852
4.297	4.222	3.617	3.451	4.370	4.549
3.852	3.253	3.818	3.423	3.761	3.844
3.333	3.538	3.350	3.089	4.611	3.847
3.201	3.743	3.694	4.265	3.170	3.296
1.976	2.933	1.962	2.832	4.172	1.785
2.806	3.468	3.505	4.192	2.902	2.577
4.338	5.010	4.187	3.170	4.290	4.737
3.655	4.277	3.675	3.477	5.323	3.959
3.080	3.471	3.160	2.183	3.490	2.871
2.168	2.851	1.637	1.871	2.066	2.326
2.901	4.343	4.525	4.564	4.025	4.169
2.546	3.225	2.271	3.176	3.088	3.524
6.481	6.447	6.495	4.908	6.657	6.453
3.051	2.524	3.003	2.502	3.284	3.052
3.809	3.819	3.873	3.890	3.587	3.856
2.829	3.615	3.165	4.208	2.018	3.152
6.163	6.944	4.621	3.950	6.295	6.744

5.924	6.266	6.073	6.673	4.404	5.196
3.721	3.804	3.924	4.534	2.807	3.446
6.525	6.195	6.522	5.847	6.613	6.007
5.649	5.597	5.744	6.473	5.658	5.184
3.424	3.095	3.434	3.046	3.120	3.460
2.718	1.312	2.217	1.931	2.569	1.873
2.590	3.684	4.035	3.031	3.721	3.256
3.908	3.776	3.776	3.776	2.469	3.066
4.337	3.676	4.628	4.297	4.138	4.809
4.451	4.945	4.651	4.585	5.057	4.634
5.481	4.835	6.583	6.258	6.563	5.682
4.041	3.865	3.876	4.040	3.849	3.109
3.952	3.682	3.437	3.029	3.909	3.796
3.264	5.369	3.458	4.626	1.410	3.919
2.660	2.348	2.710	3.157	2.660	2.443
3.979	3.551	3.610	3.860	3.340	3.034
3.641	4.517	3.266	2.627	3.310	4.590
7.200	7.935	5.941	5.092	6.296	7.819
7.603	8.257	6.529	5.653	6.925	8.213
3.354	4.779	4.488	4.927	3.887	4.717
3.796	3.434	4.218	4.048	3.854	3.471
5.178	3.014	3.458	1.906	3.880	4.631
2.096	2.181	1.765	2.053	2.238	3.129
5.214	4.589	5.646	5.293	4.320	4.786
3.435	3.512	4.545	3.906	3.458	3.383
1.850	2.446	3.597	2.043	2.711	2.199
4.920	6.192	4.126	4.779	4.452	5.271
4.527	5.225	3.778	3.581	4.357	4.256
6.517	7.416	5.581	5.643	6.256	6.228
3.753	4.587	3.589	3.264	4.247	3.887
3.746	4.831	3.554	3.279	4.093	3.725
3.754	4.808	3.682	3.296	4.200	3.822
3.776	4.937	3.691	3.334	4.220	3.881
3.682	4.296	3.718	2.591	4.232	3.747
3.524	3.658	3.609	2.496	3.487	3.886
2.786	4.090	3.365	3.463	2.184	2.806
3.073	2.750	2.529	3.211	2.491	2.423
1.953	1.968	1.694	2.366	1.499	1.499
4.177	4.124	4.343	3.545	3.792	2.991
3.299	2.707	2.775	3.115	3.272	2.868
4.588	4.539	3.638	4.981	4.081	5.048
3.976	3.239	4.057	3.959	4.435	3.993
4.882	4.682	4.921	3.944	5.139	5.146
1.768	1.350	1.724	1.571	1.807	1.308
4.893	4.961	4.958	5.181	4.153	4.413
3.693	3.745	4.859	5.091	4.512	3.915
6.271	7.338	6.962	7.365	7.371	7.245
3.178	3.977	3.284	2.428	3.102	3.211
3.644	3.177	3.537	3.213	3.292	2.742
6.100	6.250	6.278	6.173	6.264	6.445

2.399	1.914	2.604	2.783	2.528	2.209
3.867	3.262	3.765	4.554	3.548	3.858
3.415	3.277	2.408	3.286	2.984	3.626
6.179	6.039	6.006	5.709	6.540	5.986
4.859	4.626	4.798	4.118	4.917	4.733
5.841	4.874	5.592	5.522	4.846	4.995
3.737	3.041	2.880	2.734	3.041	3.523
3.862	3.825	4.194	3.529	5.342	4.043
3.539	2.834	3.314	3.284	3.687	2.792
9.337	9.235	8.737	7.752	8.799	10.090
3.511	4.499	3.707	3.096	4.543	4.328
4.675	4.641	4.202	4.303	4.805	5.146
5.491	5.426	5.357	3.810	5.329	5.360
2.127	1.693	2.233	1.978	1.614	1.692
4.138	4.170	2.857	3.477	3.161	3.342
3.955	4.003	4.010	3.237	4.353	4.354
2.361	2.140	1.728	1.671	2.298	1.536
1.956	2.929	1.940	1.842	2.432	2.346
8.336	8.688	8.460	8.115	8.349	8.048
8.615	8.973	8.778	8.398	8.692	8.347
8.507	8.881	8.644	8.264	8.593	8.241
8.625	9.001	8.762	8.391	8.709	8.353
8.667	9.072	8.763	8.460	8.728	8.370
8.661	9.052	8.811	8.458	8.719	8.355
8.648	9.034	8.811	8.458	8.717	8.355
7.658	8.076	7.781	7.443	7.833	7.405
7.782	8.213	7.972	7.624	7.938	7.538
3.061	2.021	2.810	2.325	2.401	1.690
3.709	3.730	3.365	3.677	4.082	3.726
2.634	4.196	3.226	4.474	2.734	3.062
3.048	1.595	3.668	3.048	2.545	2.422
4.304	4.052	3.763	3.225	4.684	3.990
5.615	6.821	3.641	3.587	5.898	4.244
3.005	3.779	2.490	2.712	3.917	2.836
5.783	5.335	5.135	4.405	5.377	5.451
3.811	6.612	4.964	2.993	6.819	6.824
4.675	4.121	4.091	3.064	3.922	4.597
2.081	1.858	1.551	1.733	1.631	2.248
3.740	3.489	4.184	3.737	4.238	3.614
4.625	5.724	4.693	5.758	3.113	4.750
3.602	2.617	3.693	2.943	3.903	3.503
2.734	2.862	3.438	3.431	3.721	3.355
3.973	2.696	3.529	4.370	4.668	3.565
6.335	6.454	6.100	6.088	6.191	6.210
5.228	5.511	5.040	4.837	5.666	5.339
2.630	3.137	3.762	4.314	2.011	3.454
5.443	3.410	5.180	3.607	5.136	5.232
4.424	4.667	3.909	3.469	4.078	4.615
3.429	2.360	3.046	3.892	2.820	3.038
7.506	7.931	7.581	7.260	7.716	8.013

7.731	7.959	7.760	7.564	8.120	8.092
7.416	7.667	7.430	7.424	7.674	7.662
5.288	4.177	3.807	4.816	4.507	4.666
6.199	6.574	5.070	4.564	5.552	4.943
2.103	2.696	3.518	2.240	2.987	3.383
1.955	3.386	3.106	3.516	1.195	2.689
5.021	5.150	4.669	5.102	5.040	4.870
3.528	3.505	3.477	4.709	3.121	3.613
3.551	3.852	3.233	3.577	2.675	3.316
2.694	3.420	3.484	4.330	2.673	2.963
2.538	5.721	1.844	1.794	2.348	3.137
2.929	2.433	3.302	2.522	2.519	2.544
2.875	3.721	2.508	2.267	2.199	2.463
5.578	5.966	5.604	5.312	6.088	6.011
5.006	5.134	4.946	4.904	5.161	4.983
3.562	3.908	2.501	2.270	3.017	3.201
2.097	2.209	2.379	2.167	2.860	1.961
4.226	4.994	3.516	3.372	4.387	4.410
3.315	4.944	3.548	5.647	1.469	2.522
2.917	1.946	2.093	2.137	2.065	2.540
4.901	5.390	5.735	5.613	5.829	5.434
2.989	2.204	2.767	3.557	2.009	2.786
2.993	3.720	2.761	2.954	3.355	2.953
3.285	3.291	3.458	3.113	3.381	3.116
3.882	4.368	4.053	4.539	4.389	3.979
3.955	3.875	4.376	4.876	3.369	3.724
5.110	5.380	4.441	4.354	4.886	4.784
4.831	5.145	3.862	4.600	4.715	4.943
3.824	3.363	2.724	2.843	4.071	3.107
4.601	4.627	4.955	4.609	4.636	4.530
5.415	4.795	5.454	4.549	5.294	5.432
6.274	5.452	6.002	5.402	5.448	5.984
6.625	6.252	7.023	7.769	6.274	6.199
3.647	3.391	2.934	3.382	3.240	3.104
6.105	6.169	6.858	6.799	5.694	6.117
2.061	1.957	3.346	2.726	2.101	2.302
5.092	4.763	4.429	2.373	5.143	4.999
4.310	4.720	4.888	5.389	4.601	5.052
0.874	1.125	1.988	2.109	2.316	1.530
4.861	5.348	4.752	5.807	5.138	4.427
4.975	5.075	4.946	3.301	5.166	4.828
5.747	4.655	5.532	6.854	5.669	4.681
4.344	4.555	4.355	4.656	3.981	4.830
2.370	2.135	2.667	2.513	2.532	2.410
4.910	5.353	5.356	5.746	3.901	5.208
5.006	5.330	5.131	4.750	4.536	5.433
5.908	5.125	5.822	5.509	5.123	5.890
5.260	4.511	5.276	4.493	5.559	5.598
4.949	4.901	3.988	4.033	4.563	4.963
1.954	2.720	2.012	1.889	2.766	2.588

5.293	3.936	5.332	4.726	5.397	5.107
5.863	2.832	4.470	3.416	4.197	4.632
3.999	2.778	2.515	2.603	2.551	2.914
4.113	3.501	3.525	3.701	3.366	3.448
6.985	6.335	6.517	6.012	6.630	6.688
5.959	4.373	5.220	4.416	4.612	5.244
5.507	4.796	5.262	4.991	4.758	5.013
5.869	5.498	5.778	5.582	5.590	5.609
4.754	4.695	4.887	4.542	4.337	4.758
3.280	3.312	3.269	3.469	2.975	3.289
6.468	5.439	6.201	5.856	6.026	5.920
5.847	5.059	4.816	5.104	4.307	5.449
6.322	4.691	5.364	5.218	4.528	5.677
6.153	4.854	6.293	5.436	6.265	4.792
3.624	1.754	3.115	1.273	2.171	1.782
3.696	2.597	3.573	2.897	2.875	2.207
7.142	6.076	7.320	6.654	6.636	7.295
8.205	8.146	7.998	8.210	7.509	8.305
5.464	5.495	4.954	5.471	4.820	5.451
7.348	7.694	7.165	6.264	6.414	7.667
5.668	4.938	4.929	5.304	5.156	5.088
5.431	4.958	5.714	5.280	5.576	5.003
7.733	5.892	6.518	5.090	6.032	6.583
7.885	6.493	7.289	6.059	5.607	7.249
5.818	6.291	5.717	5.326	5.850	5.716
1.902	2.354	2.857	2.787	2.778	2.433
4.355	4.050	4.504	3.650	4.212	4.057
3.967	3.732	4.109	4.115	3.684	4.043
5.144	3.965	4.738	5.607	3.714	4.356
4.692	3.678	3.539	4.480	4.131	3.652
4.230	3.706	3.865	3.715	2.788	4.304
7.364	4.475	6.635	5.450	6.511	6.524
7.613	5.971	6.567	6.396	6.656	6.329
6.650	6.275	5.625	5.560	6.243	6.155
7.410	6.485	7.946	7.727	7.640	6.808
7.636	6.571	7.205	7.001	7.000	7.116
6.547	5.478	6.099	6.710	4.999	5.917
4.181	2.960	3.865	4.467	3.817	3.480
4.317	3.569	4.596	4.479	4.400	4.125
4.767	4.413	5.277	4.341	4.795	4.889
6.042	3.902	4.331	4.245	4.574	4.960
3.086	2.822	3.277	3.314	2.972	3.006
7.367	6.834	7.222	7.329	7.009	7.345
6.463	6.277	6.348	6.482	6.106	6.366
6.537	6.485	5.656	5.897	6.227	6.122
6.914	6.807	7.141	7.309	6.496	7.204
3.210	3.344	3.276	3.234	3.567	3.885
2.887	2.156	2.733	2.198	2.138	2.056
7.586	7.477	7.859	6.472	8.098	7.724
6.670	6.610	5.916	6.073	5.875	6.075

6.605	6.708	7.416	7.241	6.959	6.378
4.278	4.278	4.348	4.551	3.863	4.137
2.032	2.486	2.780	3.552	3.471	2.556
4.574	5.040	5.036	4.805	5.183	5.044
7.989	6.622	8.337	6.787	8.525	8.025
6.340	5.581	5.599	4.877	4.301	6.390
3.108	2.698	3.168	3.831	1.764	2.216
3.816	3.506	4.666	5.297	3.883	3.944
3.026	2.472	3.617	3.087	2.913	2.326
7.214	6.761	6.643	6.142	6.944	7.200
6.026	5.138	5.889	6.412	5.184	5.321
2.594	2.573	1.736	1.666	1.663	3.024
3.550	2.810	2.982	3.303	2.587	3.502
3.791	3.412	2.643	2.678	2.223	2.850
2.357	1.826	1.734	1.976	2.048	2.803
2.716	3.251	3.549	3.793	2.745	2.020
6.581	7.224	7.269	6.546	7.328	7.074
3.096	3.254	2.884	3.685	3.432	3.412
3.842	3.165	4.119	3.951	4.084	4.308
4.685	5.498	4.930	4.372	5.615	5.213
7.835	8.280	7.611	7.717	7.929	8.003
3.503	2.527	3.093	2.581	2.661	3.278
4.708	2.571	3.433	3.410	3.898	3.615
3.199	3.241	3.700	3.831	3.749	3.579
2.684	2.009	2.954	2.542	2.057	1.896
6.001	5.734	5.384	5.010	5.839	5.791
2.413	3.088	3.163	2.106	2.504	3.183
5.533	5.744	5.865	5.749	5.891	5.972
3.842	4.147	4.367	3.843	4.379	4.548
2.016	2.235	1.990	2.719	1.632	2.279
4.901	4.232	4.666	3.649	4.970	5.139
2.908	2.342	2.568	2.751	1.963	2.518
5.627	5.654	5.446	5.513	5.380	5.683
4.901	5.329	4.054	3.653	5.178	5.485
5.127	6.022	5.595	7.172	4.839	5.278
5.524	5.731	5.874	5.887	5.188	5.670
3.862	4.140	3.522	4.107	2.534	3.399
4.547	3.511	4.181	3.948	3.872	4.287
5.196	4.938	4.945	4.704	4.827	5.278
2.725	2.680	3.748	3.848	2.115	2.826
2.316	2.716	3.208	2.931	3.411	3.762
6.391	7.007	6.774	5.687	6.844	6.872
5.593	5.337	5.167	5.641	5.416	5.811
4.374	2.780	2.582	3.250	4.660	3.958
3.492	4.090	3.029	1.992	2.893	3.642
6.127	5.693	5.622	5.895	5.678	6.043
4.878	5.008	5.211	5.519	4.295	4.847
2.315	1.575	2.276	2.161	2.469	2.161
4.891	4.716	4.689	4.725	4.433	4.676
2.883	4.301	4.047	5.141	4.122	3.233

4.043	4.167	3.920	3.962	3.841	5.011
3.447	3.714	3.032	3.727	4.175	4.055
2.613	3.246	2.398	4.034	2.987	3.343
3.731	3.892	4.095	3.186	4.284	3.654
4.670	4.670	4.431	4.138	3.781	4.562
2.986	3.359	3.051	4.800	2.281	2.570
2.983	2.071	3.057	2.295	2.467	2.460
2.003	3.296	2.260	2.493	3.154	3.323
3.473	4.233	4.145	4.423	3.216	4.180
3.424	4.072	3.625	4.452	2.225	3.431
3.724	3.746	3.288	3.937	3.753	3.398
3.384	3.347	2.931	4.217	3.749	3.890
3.485	3.791	3.329	4.148	2.491	3.122
4.340	4.369	3.894	4.766	3.811	3.905
2.811	2.768	2.797	3.345	2.026	2.297
2.640	3.340	1.532	4.572	1.722	1.738
2.496	3.812	3.202	3.566	3.016	3.394
3.370	4.118	3.765	4.632	2.884	3.112
7.665	7.912	7.566	7.670	7.639	7.815
2.382	3.611	2.761	3.529	1.356	2.482
2.821	2.708	3.768	2.727	2.651	2.743
2.197	2.049	2.275	1.987	1.680	2.049
1.863	2.121	1.981	2.019	2.771	1.619
3.531	4.263	4.256	4.789	2.724	4.289
4.887	4.283	4.154	2.882	4.582	3.669
4.063	6.248	4.000	4.150	4.442	4.424
3.603	4.573	4.786	4.375	3.855	3.733
5.378	4.775	5.647	4.112	5.541	5.516
1.986	3.504	2.766	3.512	1.998	2.788
5.978	6.330	5.744	5.319	6.244	6.088
3.093	3.575	4.040	4.198	3.171	3.301
3.180	3.478	3.085	2.179	3.190	3.797
2.714	1.555	1.639	1.425	2.119	1.591
3.106	2.949	2.758	3.986	2.622	3.006
9.962	7.465	6.948	5.746	7.524	8.554
2.024	3.761	1.817	2.160	2.260	2.525
2.346	3.601	3.330	2.846	2.986	3.469
3.257	3.467	4.938	3.630	4.074	3.970
3.690	2.825	3.885	3.290	2.556	3.740
3.698	5.038	2.771	4.597	3.364	4.259
2.422	2.430	2.657	2.491	2.266	2.701
2.085	2.384	2.218	1.887	2.686	3.241
4.993	4.805	6.020	5.233	5.473	6.273
4.842	4.088	3.317	4.067	3.246	4.609
5.491	5.688	5.487	4.970	5.650	5.462
3.859	2.936	2.679	2.825	3.142	3.207
4.866	4.059	5.088	3.461	5.514	5.251
3.003	1.930	2.347	3.153	1.755	2.533
3.024	3.344	2.892	3.571	3.301	3.012
6.939	7.023	5.827	5.696	6.689	6.661

1.638	2.233	2.585	3.060	1.538	1.618
4.236	3.191	4.272	3.697	4.058	4.431
7.410	7.200	6.601	7.718	6.669	6.894
4.478	4.060	3.345	2.163	3.223	4.853
2.169	2.506	3.227	2.204	3.356	2.724
3.892	3.691	3.496	3.723	3.414	3.823
5.346	4.993	4.829	4.880	5.196	5.136
2.492	2.617	2.421	2.159	2.914	3.073
3.877	4.012	4.419	3.519	4.195	3.997
5.371	5.276	5.243	4.967	5.826	5.580
6.792	7.038	5.766	4.014	6.264	7.288
4.481	5.737	5.585	5.887	5.622	5.539
6.978	8.861	3.570	5.022	6.240	6.076
7.502	7.749	7.060	6.596	7.766	7.795
2.513	2.469	2.770	3.208	5.504	2.831
2.853	3.517	2.208	2.733	5.652	1.852
3.906	4.955	4.215	3.046	4.454	5.028
7.507	6.693	6.552	6.237	6.476	7.028
2.400	1.879	1.802	2.991	2.206	1.703
7.318	7.070	6.644	6.195	6.990	7.310
4.051	3.728	3.170	2.528	3.319	4.621
5.361	5.346	4.674	5.638	4.467	4.876
3.676	2.634	3.279	2.794	3.202	3.338
4.415	5.454	3.938	2.247	4.275	4.643
3.024	3.802	3.494	3.260	3.415	3.713
3.714	3.085	2.673	2.199	2.792	3.817
3.090	3.252	2.970	2.413	4.100	3.224
4.971	5.095	5.772	5.546	4.757	5.459
6.413	6.633	5.774	5.261	6.203	6.638
5.099	4.114	4.295	3.549	5.152	4.878
3.643	4.120	3.656	3.522	3.798	4.129
3.890	4.623	4.312	3.723	3.960	4.730
7.205	8.119	6.628	6.757	7.283	7.479
5.214	4.980	3.422	3.946	4.147	4.282
2.546	2.099	1.958	0.945	1.573	1.603
2.692	2.917	3.960	2.399	3.074	2.879
6.365	7.100	6.290	5.623	6.371	6.935
3.960	4.614	4.297	3.965	4.052	4.374
5.855	6.270	5.810	6.012	5.939	6.358
9.295	9.515	8.831	7.405	9.066	9.670
2.882	3.655	2.771	1.540	3.377	4.808
6.689	6.745	6.728	5.892	6.711	6.971
3.673	3.864	3.950	3.477	4.038	3.892
4.218	4.423	4.836	4.226	3.703	4.404
5.990	5.467	6.096	5.493	5.497	5.858
4.388	3.285	2.991	1.868	4.185	4.441
5.577	5.123	4.521	3.482	5.524	5.875
4.124	4.285	4.161	3.395	4.463	4.110
6.102	5.660	5.473	4.889	4.761	5.554
5.241	5.124	5.302	3.285	4.790	4.677

9.837	10.514	9.732	10.289	9.664	10.106
4.371	4.639	3.606	3.450	3.807	3.807
4.867	4.558	4.261	4.200	3.935	4.659
4.718	4.186	2.976	3.607	5.301	4.716
4.924	5.033	4.718	4.335	5.197	5.173
5.164	5.436	4.737	5.042	5.019	4.937
6.602	6.582	6.685	7.062	7.106	6.020
3.036	2.936	2.827	3.046	2.849	2.868
5.798	4.908	3.635	3.732	4.635	5.060
3.446	2.769	3.077	3.095	3.862	2.395
4.022	3.631	2.721	2.740	2.785	3.588
4.824	3.985	3.380	3.033	5.091	3.033
5.732	5.108	5.538	5.511	5.840	5.281
6.552	5.335	5.882	5.279	5.575	5.676
4.590	3.387	4.046	3.587	3.216	3.808
6.562	6.099	3.186	4.594	3.931	4.136
6.692	6.759	6.586	7.645	7.181	6.507
4.609	4.896	5.459	4.976	5.381	4.920
3.688	3.490	2.735	3.161	3.104	3.263
4.264	3.162	3.611	2.200	3.153	2.997
3.844	3.548	3.310	3.487	3.250	4.028
6.993	5.498	5.381	6.072	5.968	5.361
3.741	3.598	3.206	2.962	2.545	2.871
6.748	4.851	5.251	4.650	6.171	6.006
2.603	1.154	1.417	1.742	1.288	1.679
3.522	3.453	4.257	4.747	4.079	4.307
7.586	6.595	7.032	7.362	7.171	7.156
6.397	6.519	6.668	5.769	7.177	7.112
4.889	5.545	4.152	4.841	4.541	4.840
4.527	4.376	3.853	3.762	4.138	4.392
2.783	4.860	4.463	4.583	4.052	4.130
5.774	6.053	5.888	5.412	5.336	5.919
4.285	3.876	4.946	4.357	3.722	4.173
5.074	4.617	4.708	5.069	4.868	4.753
4.322	4.750	4.544	4.442	4.027	4.190
3.374	3.633	3.217	3.184	3.227	2.971
5.004	5.053	4.879	4.143	4.744	5.094
6.540	5.939	5.965	6.084	6.649	6.582
4.870	5.544	3.114	4.151	3.522	4.684
5.057	5.384	5.501	4.196	5.865	4.966
4.416	4.394	5.031	4.597	4.811	5.069
2.232	3.406	2.183	2.271	2.216	2.591
1.810	2.773	2.416	2.208	2.214	2.879
4.932	4.940	5.247	5.251	4.151	4.796
1.902	2.287	1.841	1.989	1.658	2.455
3.643	2.992	4.249	3.614	3.670	3.427
6.489	6.154	6.387	5.597	6.361	5.887
3.015	3.627	3.121	3.106	3.831	3.441
4.583	4.439	4.264	3.915	3.924	4.015
5.666	5.394	6.408	6.067	5.748	5.761

2.868	3.298	3.617	3.532	2.871	3.706
4.874	4.824	4.853	4.939	3.669	5.062
3.767	3.220	2.470	3.376	3.191	2.764
5.118	4.894	4.970	5.957	5.110	4.553
4.350	4.230	5.047	4.349	4.524	5.312
4.911	5.714	3.965	4.658	4.760	4.912
7.487	8.048	5.730	7.305	7.672	6.989
4.456	4.471	4.552	3.560	5.069	4.738
5.331	4.423	4.390	5.575	5.072	5.124
2.966	2.703	2.666	2.316	3.107	3.533
3.485	3.308	3.305	3.242	3.017	3.438
7.555	6.918	6.778	6.929	7.562	6.976
3.146	2.760	4.114	3.867	3.284	4.026
6.540	4.219	5.569	5.151	5.164	5.453
4.912	2.799	3.707	3.903	4.326	4.231
3.089	2.846	3.043	3.508	3.616	3.488
5.222	7.290	6.130	5.776	7.847	4.416
5.123	4.973	5.739	5.484	5.031	5.427
5.518	5.912	6.895	6.344	5.971	5.815
4.697	4.828	4.379	3.789	4.216	4.471
3.773	3.651	3.648	3.568	3.042	3.942
3.289	3.833	3.834	3.105	3.671	3.923
4.802	4.843	5.086	4.621	4.929	4.835
5.191	4.908	5.577	5.621	4.715	5.090
4.275	4.339	4.394	4.344	3.701	3.694
5.982	5.808	5.187	5.548	6.348	6.594
2.664	2.672	2.336	2.244	3.210	3.347
4.049	3.382	3.782	2.560	3.532	3.733
6.641	6.904	6.468	5.781	6.794	6.375
7.853	7.050	7.285	6.960	6.734	7.228
2.600	3.030	2.373	2.389	1.849	2.754
4.693	5.001	4.972	3.956	5.032	4.715
7.052	6.502	6.539	6.366	6.785	6.517
3.162	3.754	3.803	3.335	2.532	3.162
3.371	3.395	4.471	4.607	4.199	3.338
5.220	4.746	3.595	4.760	5.235	4.529
3.542	5.056	3.134	3.431	4.093	3.827
3.968	4.262	4.312	4.217	4.371	4.056
4.966	6.458	3.998	4.134	5.137	3.937
3.972	4.835	3.904	2.787	3.697	4.836
2.251	2.320	2.773	2.532	2.858	2.403
5.813	5.694	5.212	5.235	5.304	5.744
4.952	5.216	4.502	4.303	4.920	4.742
2.163	2.205	2.730	3.587	3.020	2.205
3.185	3.582	3.664	4.099	2.964	2.906
2.242	2.493	2.158	2.168	1.749	2.487
3.834	3.409	3.465	3.847	3.239	3.482
6.729	6.681	5.076	4.015	5.053	6.907
4.516	5.371	3.685	3.116	3.339	4.870
4.040	2.552	5.055	4.560	4.607	2.962

4.938	4.970	6.109	4.328	6.512	5.982
3.607	3.945	4.364	3.839	4.218	3.806
3.557	3.078	2.725	3.107	3.114	3.234
2.289	3.468	2.901	3.125	1.399	2.338
3.579	4.019	3.725	3.458	3.418	4.351
2.351	2.212	2.254	1.733	2.387	3.383
6.158	6.131	6.187	5.965	6.456	6.473
6.906	6.247	6.460	6.674	5.885	6.344
5.322	3.834	4.409	4.700	3.949	4.510
6.176	5.911	6.088	6.109	6.415	6.243
3.272	4.974	4.301	5.496	3.722	4.120
2.359	3.767	2.632	2.573	2.000	3.301
3.311	2.350	1.587	3.297	3.016	3.229
4.996	4.625	5.114	4.559	3.564	4.885
5.337	3.531	4.178	3.888	3.788	4.621
7.984	10.716	6.778	7.953	6.740	10.151
6.132	8.493	4.122	3.447	4.643	7.669
5.693	5.390	5.489	5.604	5.618	5.584
2.493	2.562	2.131	2.286	1.943	2.738
6.170	6.454	5.757	6.194	6.450	5.967
6.999	6.294	6.226	6.240	6.343	6.487
4.011	3.835	4.015	4.680	4.387	3.674
5.842	5.685	5.103	5.086	5.747	5.526
4.739	4.009	4.108	3.927	4.065	4.119
4.302	3.908	3.498	3.820	3.803	3.495
1.956	2.774	2.200	2.665	2.003	3.294
3.728	4.665	4.151	3.868	3.820	4.015
4.593	4.959	4.583	5.001	4.780	4.971
2.928	3.306	3.520	2.730	3.331	2.751
4.841	5.712	4.792	4.253	4.621	3.754
1.448	2.033	2.827	1.366	1.871	1.706
3.832	4.021	4.199	3.960	3.083	3.700
2.980	2.376	1.916	2.109	2.023	2.774
3.222	2.557	3.142	2.528	2.517	3.134
2.075	1.852	1.981	2.766	6.164	1.622
1.825	2.275	1.720	2.585	2.416	2.265
3.613	3.146	3.407	2.782	3.181	3.159
3.190	2.263	2.972	2.186	2.100	2.716
2.758	2.758	2.516	3.969	2.741	3.219
4.101	3.205	3.803	4.251	3.472	3.590
4.881	4.187	4.145	4.389	3.953	3.827
2.921	2.778	3.339	3.131	3.451	3.262
3.646	3.633	3.444	4.078	4.269	3.476
6.284	6.710	5.734	6.164	6.336	6.002
6.901	7.217	6.329	6.082	6.787	5.878
6.789	6.518	6.471	5.869	6.654	6.524
3.088	3.218	4.198	4.865	3.184	3.273
3.061	2.613	2.688	2.603	3.165	2.984
6.472	7.160	7.435	5.707	7.435	7.220
4.049	3.998	3.534	4.576	4.311	3.762

4.889	5.067	4.894	4.099	4.812	4.956
2.128	2.270	2.237	2.727	2.427	2.461
3.971	4.303	3.834	4.679	3.943	4.120
3.541	4.149	4.869	4.328	4.444	3.770
5.179	5.112	5.300	4.295	5.416	5.771
3.579	3.728	3.729	2.232	4.712	3.129
3.098	3.128	3.617	2.945	2.956	3.196
8.842	7.933	8.516	7.452	8.947	8.718
6.378	6.343	5.955	6.203	6.095	6.219
4.233	4.070	4.366	3.784	3.078	4.186
3.649	3.009	3.140	2.341	2.185	2.916
5.060	4.860	5.061	5.060	4.458	4.978
2.715	3.247	2.671	3.184	2.719	2.500
4.668	5.301	3.616	4.378	5.205	4.771
4.470	3.872	4.551	5.211	4.314	4.822
1.995	2.740	2.576	2.953	2.119	2.645
6.236	5.387	6.163	6.007	4.455	5.846
5.109	4.467	5.821	4.948	5.737	5.086
4.712	3.718	4.337	3.334	4.223	4.852
3.168	4.071	3.363	3.468	3.675	3.060
5.758	5.505	6.163	4.282	6.487	5.908
7.083	7.131	7.170	6.788	7.325	7.074
6.104	5.040	5.907	6.054	5.846	5.088
5.708	6.380	5.481	4.634	5.844	5.693
3.398	3.466	3.659	3.373	2.896	3.161
2.486	2.051	2.287	1.751	2.114	2.546
3.786	4.283	2.931	3.464	3.192	2.460
3.084	3.054	3.755	4.711	3.312	3.213
1.928	2.175	2.872	2.033	3.013	2.343
8.747	8.964	8.475	7.780	8.793	8.665
3.149	3.432	3.378	4.086	3.503	3.757
4.894	6.018	4.105	3.943	4.535	5.108
2.950	3.443	3.511	3.108	3.691	4.061
5.945	6.029	6.244	6.292	5.547	6.684
2.581	2.600	1.614	1.975	2.571	1.682
3.590	4.145	3.130	2.424	3.643	4.250
5.787	5.619	5.371	3.952	6.375	6.334
2.427	1.810	3.898	2.204	3.729	2.329
3.620	3.404	3.840	3.096	2.885	3.144
6.772	7.467	6.727	6.472	6.720	7.275
6.772	7.459	6.759	6.490	6.720	7.274
6.785	7.526	6.462	6.651	6.746	7.300
5.922	6.293	6.146	5.931	5.822	6.196
5.865	6.166	6.173	5.913	5.897	6.143
4.393	4.765	3.874	4.131	4.489	4.681
1.728	2.475	2.967	2.216	2.925	2.087
9.183	9.618	8.330	8.410	8.568	9.287
2.377	2.891	1.415	2.782	3.173	2.302
4.699	5.531	5.234	3.365	5.393	5.121
5.413	6.052	5.637	4.910	5.506	5.424

3.144	2.722	3.670	2.791	4.140	3.747
4.514	4.552	3.961	4.217	4.796	4.509
6.933	6.908	6.120	6.388	6.612	6.835
8.364	8.540	8.641	8.654	8.288	8.530
5.744	5.189	5.722	5.290	4.618	5.386
5.867	4.989	5.345	4.882	4.435	5.629
6.635	6.133	6.596	6.621	6.402	6.593
4.272	4.507	4.654	4.168	4.831	4.045
3.545	1.852	1.564	1.677	1.278	1.791
3.782	3.068	3.992	3.065	3.342	3.800
2.241	2.482	1.937	2.970	2.354	3.042
5.903	5.912	5.577	5.083	5.135	5.868
4.900	5.171	4.524	4.478	5.081	5.038
5.331	5.363	5.320	4.548	5.426	5.582
6.879	6.451	7.211	5.250	7.437	7.008
3.899	4.801	3.869	3.136	4.669	4.202
5.000	4.587	5.024	4.332	5.452	4.587
10.391	9.456	9.914	8.263	9.328	9.238
5.952	6.299	5.392	5.119	5.830	5.940
4.200	2.763	4.017	2.366	4.606	3.174
2.411	1.874	2.054	2.395	2.834	2.365
4.592	3.741	4.502	4.302	4.294	3.902
3.107	2.664	2.425	2.553	2.812	2.886
6.664	5.848	6.260	5.740	6.094	6.042
4.658	4.795	4.973	5.829	4.790	4.634
5.644	6.007	5.413	5.261	5.276	4.437
8.271	8.982	7.554	7.718	7.824	8.736
4.881	3.357	5.089	4.359	5.506	5.079
10.311	9.538	10.050	9.427	10.195	10.019
5.333	5.296	4.331	4.469	3.951	5.315
5.370	5.323	4.801	4.810	3.862	5.299
5.135	4.858	4.864	4.922	4.864	5.090
5.269	5.080	4.690	5.408	4.668	4.786
6.317	6.832	7.305	8.076	7.498	6.345
8.268	7.351	8.197	7.540	8.279	7.998
5.197	4.716	5.109	5.297	5.342	4.820
5.128	2.763	3.674	3.305	3.901	3.883
2.313	2.185	2.162	1.712	2.586	2.429
3.036	2.290	2.222	1.904	2.013	2.559
7.196	7.910	6.863	6.775	6.354	7.067
7.203	6.499	7.391	5.874	6.837	7.427
4.022	3.977	4.491	4.116	4.368	4.264
7.982	7.618	8.195	6.831	8.641	8.458
6.561	5.138	5.315	4.826	5.110	5.536
3.418	4.060	2.976	2.947	3.672	3.975
3.143	3.532	4.029	3.432	3.375	4.189
4.155	2.618	3.195	3.173	4.173	3.216
6.730	6.297	6.608	5.377	6.993	6.993
6.044	5.735	5.718	4.648	5.712	5.670
1.531	1.619	1.713	1.743	1.826	1.700

4.855	5.120	4.161	4.885	4.416	4.019
6.341	6.178	3.942	3.551	3.876	6.001
2.669	2.058	3.110	3.093	2.169	2.562
5.351	4.714	5.585	5.138	5.636	4.652
4.746	5.643	5.324	5.241	5.418	5.733
2.403	2.164	2.281	2.388	2.167	2.181
3.472	4.026	3.680	3.919	3.248	3.547
4.242	4.427	4.039	4.083	5.298	3.790
1.951	2.220	2.726	2.551	1.838	2.112
3.646	2.763	2.830	3.760	3.241	2.701
3.697	3.539	4.008	3.517	3.474	3.320
3.144	3.084	2.970	2.752	2.581	2.942
4.350	3.639	2.244	4.075	3.959	3.268
2.567	3.074	2.055	2.752	3.413	2.482
5.249	5.950	3.330	3.715	3.387	4.609
4.772	5.364	3.300	4.113	3.841	4.563
2.441	2.997	2.731	2.427	2.746	2.250
7.257	7.211	6.831	6.211	7.086	6.940
5.547	4.639	5.721	4.782	5.792	5.566
3.935	3.664	3.747	3.606	3.986	3.635
4.277	5.474	4.695	5.380	5.548	4.827
3.685	4.462	2.954	3.165	3.243	3.426
3.067	3.324	1.428	3.241	2.177	2.859
3.859	3.497	2.849	3.110	3.048	3.706
3.279	2.219	3.850	4.192	3.016	2.813
4.342	4.245	5.247	4.511	5.038	4.361
4.049	4.355	4.993	4.803	4.974	4.614
6.449	4.848	5.249	4.779	5.012	7.014
6.364	6.714	5.992	6.103	6.387	6.375
3.811	2.795	4.035	4.799	2.678	3.597
4.210	3.933	3.753	3.544	4.118	4.301
3.961	3.902	4.301	4.028	4.053	4.037
5.227	4.976	4.371	4.604	4.771	5.336
5.791	5.857	5.715	5.004	5.609	5.849
5.506	5.232	4.647	3.786	5.037	5.125
3.468	2.871	3.413	3.204	2.968	3.092
5.186	5.884	5.392	3.776	5.568	6.785
2.867	3.419	3.098	2.646	1.616	3.507
5.064	4.120	4.921	4.177	4.188	4.457
7.361	7.786	7.563	7.272	7.794	8.238
5.391	5.106	4.524	4.992	4.751	5.150
5.578	5.558	5.450	5.472	5.181	5.201
5.084	4.734	4.918	3.449	5.357	4.776
2.682	2.025	2.143	3.501	2.348	2.506
5.654	5.538	6.022	6.600	4.620	5.277
6.954	8.067	6.229	5.828	6.703	7.460
2.163	2.330	2.618	1.947	3.278	4.566
6.462	8.092	8.450	8.324	8.842	6.036
4.823	4.763	4.050	3.341	4.871	4.573
6.900	7.245	6.719	5.913	7.324	7.086

5.702	4.694	5.261	4.612	4.847	5.295
4.528	4.161	4.428	4.339	5.273	4.679
4.777	4.945	5.562	5.789	4.531	5.451
3.150	2.361	3.212	2.760	1.762	3.187
4.465	4.557	5.073	4.161	5.572	4.547
3.333	2.966	3.819	3.661	2.721	3.383
4.626	4.817	3.896	4.557	5.150	4.779
7.942	7.794	8.086	7.555	7.812	7.622
5.160	3.698	3.572	3.141	3.711	4.254
4.538	3.089	3.374	3.065	4.205	3.293
3.631	2.833	3.463	2.590	2.278	3.460
4.830	5.142	4.400	4.464	3.991	4.830
3.978	4.371	4.897	4.865	3.335	3.544
1.806	1.847	1.259	1.677	0.904	2.559
2.317	2.643	0.989	1.733	1.778	2.644
6.476	6.353	5.422	5.688	5.992	6.530
6.728	6.772	6.716	5.074	6.880	7.365
4.092	3.617	4.541	3.906	4.558	4.034
4.706	4.355	4.514	3.514	4.902	4.874
5.963	5.709	6.250	5.467	6.123	6.211
4.388	4.673	4.505	3.705	5.588	4.991
2.821	3.334	2.043	3.055	3.035	4.019
2.011	3.812	2.911	1.971	1.957	2.887
5.578	5.480	5.112	5.248	5.218	4.967
2.995	2.992	2.957	3.893	2.164	2.553
2.529	2.898	2.721	2.612	4.203	2.460
3.760	3.727	3.864	3.486	4.195	3.966
5.349	5.196	5.961	5.311	5.598	5.441
4.598	4.941	4.960	4.511	5.130	4.913
3.258	2.848	2.985	3.262	2.622	2.499
3.142	2.600	2.143	2.396	3.296	2.560
5.075	5.317	5.042	4.755	5.268	5.381
4.349	3.789	3.770	4.142	3.092	4.114
5.030	4.938	4.333	4.986	4.366	4.750
1.878	1.492	1.459	1.609	1.549	1.741
4.956	3.598	4.231	4.212	4.385	3.961
4.601	3.841	4.192	4.501	4.131	4.377
3.467	2.915	3.835	2.726	3.210	3.079
4.531	4.133	4.169	3.668	4.304	4.259
6.832	5.919	5.424	4.995	5.297	5.544
6.250	6.403	4.637	6.261	5.572	5.231
4.165	3.836	4.301	4.716	3.734	3.783
4.623	4.336	4.396	3.776	4.069	4.440
4.385	4.919	4.212	3.833	4.974	4.646
3.272	2.979	3.109	4.151	3.476	2.888
4.021	4.631	3.860	3.968	4.901	4.631
3.816	3.970	4.957	4.198	4.557	4.268
7.241	7.421	7.595	7.690	7.703	7.053
6.032	5.879	5.639	5.058	5.789	5.630
6.081	4.749	4.855	4.782	5.139	4.739

2.685	2.465	2.936	1.800	1.887	3.610
6.477	6.763	6.113	6.352	6.687	6.642
3.435	3.091	2.275	2.445	2.650	3.456
6.843	6.477	7.231	6.323	7.322	7.290
2.052	1.851	2.495	2.184	1.993	1.795
5.537	5.442	5.603	5.115	6.257	5.785
1.625	1.718	2.115	3.429	2.770	2.087
4.320	3.996	3.951	3.814	4.483	3.892
8.121	8.152	8.137	7.082	8.382	8.605
5.177	4.933	5.158	4.430	5.704	5.080
4.525	4.431	5.165	4.620	5.090	4.447
2.865	5.718	3.228	3.301	2.707	3.166
2.881	3.514	2.559	2.230	3.372	3.817
5.137	4.113	4.630	4.431	4.766	4.462
6.284	5.106	5.502	5.886	5.153	5.497
3.329	4.081	3.967	3.244	3.304	3.834
5.738	3.850	3.240	3.977	2.875	3.740
3.902	4.115	3.650	4.589	3.850	3.499
3.463	2.585	3.474	3.389	3.060	3.125
5.682	4.801	5.231	5.095	5.390	4.908
5.479	5.793	5.184	5.335	5.898	4.585
5.124	4.976	4.858	5.109	4.985	5.098
6.170	4.368	5.449	4.776	5.359	5.307
4.605	4.181	4.514	4.174	4.372	4.493
2.468	2.932	2.681	2.909	1.732	2.783
4.068	4.668	4.730	4.740	4.984	4.795
5.480	4.976	4.451	4.377	5.287	4.951
1.604	2.084	2.186	2.026	1.552	1.468
4.701	4.222	3.977	3.690	4.367	3.840
2.192	2.588	2.842	3.347	2.950	3.002
4.053	4.661	3.831	3.786	4.006	3.890
2.633	2.842	2.350	2.033	2.169	3.371
2.934	3.158	2.755	3.150	2.151	1.878
7.698	7.714	7.801	7.989	7.886	7.867
1.016	1.319	1.640	1.525	1.173	1.197
5.604	6.723	5.477	3.893	5.960	5.850
1.346	1.491	1.235	1.479	0.930	1.304
3.997	4.034	4.254	3.540	3.871	3.809
3.035	3.848	2.686	3.909	2.673	2.753
3.213	2.555	2.999	3.267	2.993	2.568
2.514	1.729	2.326	1.800	1.683	1.871
4.048	3.786	3.968	4.370	3.045	3.459
1.488	1.881	1.699	2.412	1.733	2.081
2.659	3.733	2.219	1.187	3.080	2.306
2.295	2.503	2.633	2.957	2.459	2.190
1.688	1.333	2.166	2.490	1.826	2.316
2.718	2.331	2.753	2.419	2.484	1.756
0.905	1.167	0.875	1.299	1.746	0.735
1.699	2.282	1.859	1.442	1.948	2.232
2.607	2.643	2.835	2.813	2.526	2.293

3.828	4.024	3.663	3.661	3.666	4.118
3.562	2.973	2.778	2.739	4.316	3.027
3.173	2.751	3.241	3.880	2.354	3.174
2.963	4.393	3.367	2.113	3.087	4.022
2.026	2.848	2.055	2.473	1.984	2.389
4.130	3.089	3.072	4.943	3.368	3.362
2.166	3.445	2.257	3.759	1.581	2.290
3.215	2.573	3.676	3.563	3.874	3.496
2.947	2.527	2.400	3.299	1.236	2.533
2.127	2.261	1.830	0.987	3.102	3.131
1.816	1.584	2.035	1.110	1.614	2.701
3.530	3.469	4.062	3.728	3.921	3.926
3.486	3.620	2.888	2.838	3.187	2.888
2.595	2.761	1.874	1.939	2.664	3.294
2.120	2.270	3.152	1.916	2.672	3.019
2.933	2.488	3.129	2.339	3.397	3.972
3.870	2.833	3.045	3.350	3.000	3.276
1.876	1.346	1.121	1.188	1.179	1.647
4.169	3.540	3.547	2.650	4.019	3.249
2.197	1.772	1.712	2.322	1.810	1.635
4.897	5.062	4.485	4.382	5.001	4.892
3.072	3.024	2.583	3.866	2.793	3.253
3.201	3.227	2.987	3.181	2.906	3.026
2.496	2.506	1.757	2.061	2.164	2.128
4.885	4.944	4.041	4.287	5.120	4.679
2.878	2.638	2.669	2.458	2.347	2.444
2.421	1.998	1.602	2.459	2.127	1.763
1.934	2.504	2.979	2.152	3.119	2.539
2.236	1.735	1.585	2.719	1.674	1.842
5.665	4.111	4.863	4.593	4.431	4.023
2.928	2.309	2.678	2.148	2.555	2.481
2.519	1.688	2.070	2.484	2.797	2.126
3.666	4.093	4.019	3.832	3.939	3.969
4.184	4.583	4.693	4.036	4.770	3.932
5.229	5.526	5.296	7.094	6.422	5.233
3.701	4.719	3.750	3.335	4.046	3.674
6.663	6.377	6.938	7.045	6.349	6.615
6.696	6.632	6.210	6.009	6.533	6.332
2.711	1.916	2.419	2.416	2.276	2.229
7.602	7.335	7.890	7.158	8.194	7.819
6.010	5.425	3.933	4.153	4.133	4.422
4.245	4.353	3.982	4.001	3.991	4.327
5.586	5.369	4.925	4.257	6.016	5.610
5.408	5.498	4.285	3.758	4.534	6.205
3.477	3.715	4.431	3.741	4.201	4.025
3.667	3.569	3.489	3.808	4.029	2.787
4.663	4.257	4.640	3.971	3.438	4.195
6.465	5.725	5.662	5.155	6.405	6.238
6.821	6.566	6.071	5.567	6.501	7.235
2.191	2.306	2.987	2.201	2.726	2.484

2.120	2.482	3.310	2.724	2.556	3.457
6.067	6.351	6.262	6.429	6.157	7.094
5.068	5.217	4.604	5.576	4.297	4.741
2.400	1.882	1.342	1.920	1.745	2.151
1.944	2.072	2.446	2.263	1.961	2.041
5.749	5.376	6.206	5.417	6.236	6.054
7.123	7.413	6.287	6.471	6.661	6.995
2.379	2.070	2.638	3.021	2.382	2.767
6.024	5.827	5.628	5.103	5.739	5.813
5.434	4.326	4.043	3.691	5.289	5.215
6.818	6.177	6.873	5.685	6.482	6.335
3.912	3.903	5.633	3.135	5.041	5.650
4.237	2.492	1.705	1.426	4.436	4.726
1.906	3.706	2.933	2.107	3.814	3.754
4.048	4.019	4.809	4.587	3.642	3.842
6.479	5.046	6.479	7.267	4.553	4.943
6.823	6.659	6.698	5.471	7.140	6.820
2.856	3.524	2.043	1.909	2.225	2.825
4.867	4.418	4.037	3.254	3.580	3.552
2.472	2.972	1.691	1.221	2.394	2.097
2.636	1.941	2.229	2.263	3.266	2.030
8.385	8.642	8.609	8.433	9.642	7.982
2.591	1.881	1.851	1.550	1.683	2.932
4.044	6.113	5.526	5.635	4.603	5.349
2.832	1.934	1.684	4.223	3.065	1.881
2.524	4.624	3.460	4.029	2.693	3.562
4.435	3.571	3.610	5.381	4.255	3.922
4.327	4.090	4.198	4.020	4.025	4.579
2.109	2.385	3.347	2.756	2.758	2.872
1.875	2.782	3.101	1.691	1.832	2.422
7.195	7.745	7.206	6.960	7.411	7.725
4.962	3.243	4.048	4.337	4.139	3.795
7.093	7.254	7.586	6.866	7.501	7.445
4.638	5.150	4.805	3.649	4.768	5.400
7.677	8.081	7.379	5.215	8.055	9.122
8.066	7.800	7.797	8.036	8.478	7.528
6.715	6.236	6.753	6.521	6.644	6.560
5.529	5.210	5.314	4.728	4.393	5.027
6.118	7.037	4.081	4.353	5.045	5.308
4.447	5.091	4.751	4.257	4.961	5.120
3.363	3.900	4.177	4.498	4.043	3.278
7.622	7.758	7.548	6.625	7.899	7.490
3.304	2.843	1.930	1.697	3.132	2.906
3.507	2.468	3.253	2.628	2.978	3.359
3.151	2.904	3.046	3.188	3.252	2.999
4.432	4.356	4.804	4.721	4.330	4.837
3.084	3.522	3.239	3.374	3.097	3.606
5.140	4.587	5.267	5.108	4.905	4.929
5.708	4.635	5.157	4.250	5.819	5.181
1.980	1.587	1.403	1.302	2.952	2.644

2.778	2.447	1.208	3.141	2.530	3.335
6.214	5.614	5.620	4.982	6.366	6.341
4.492	5.036	4.510	4.837	5.246	4.214
1.955	2.679	2.388	3.191	2.123	2.432
6.033	5.069	5.670	5.285	6.107	5.621
2.834	2.713	2.475	2.512	2.446	2.910
4.231	4.484	4.335	2.506	3.840	4.340
2.226	3.886	2.624	2.236	2.511	3.065
4.672	4.791	4.621	4.321	5.017	5.747
4.001	4.113	4.965	4.513	4.309	4.146
3.573	4.095	4.180	4.065	3.959	4.194
8.232	7.540	9.002	8.520	8.837	7.866
4.336	3.317	3.706	4.015	3.559	3.651
4.757	5.326	4.382	3.825	4.508	4.895
4.823	3.829	4.607	4.489	4.290	4.329
1.462	0.960	1.191	1.490	1.382	1.092
4.215	3.344	3.132	2.386	3.297	3.435
5.601	4.589	5.498	5.259	5.168	4.906
6.214	6.353	5.951	5.124	6.389	5.986
6.998	5.173	7.621	6.989	7.893	6.163
2.114	2.096	2.051	1.750	1.850	2.350
2.745	3.716	3.061	4.451	1.471	2.505
3.205	3.958	3.153	2.659	3.862	3.570
2.124	2.786	2.353	2.378	2.525	2.355
5.578	5.947	5.893	5.634	5.952	5.651
4.860	4.475	5.184	5.368	4.680	4.601
6.156	4.996	6.408	5.660	5.702	5.669
4.985	4.595	5.172	5.594	5.427	5.223
2.399	1.940	2.421	2.421	2.415	1.779
3.149	2.541	1.969	1.283	2.255	3.019
4.956	5.312	4.954	4.913	4.924	4.953
5.197	3.595	4.728	5.078	4.145	4.701
3.094	4.071	3.554	3.580	2.421	2.629
2.897	3.255	2.973	3.835	2.804	2.757
4.192	3.894	4.646	4.205	5.430	3.506
4.382	4.901	4.309	4.269	4.461	4.717
3.585	4.202	3.472	3.305	3.685	2.544
5.138	5.176	4.504	5.106	5.027	5.040
3.383	3.148	3.521	2.278	2.914	2.413
3.894	3.271	3.104	2.871	2.789	3.386
4.416	4.842	4.136	4.399	4.148	3.947
2.704	2.208	2.704	2.549	2.147	2.304
3.518	2.944	3.634	3.458	3.824	3.269
5.592	5.764	6.212	6.057	5.900	5.629
5.560	5.825	6.162	6.071	5.897	5.659
4.682	5.051	4.429	4.331	4.456	4.638
7.225	9.219	7.558	9.419	8.717	7.814
1.415	1.645	1.383	1.759	1.645	1.563
3.663	3.390	4.554	2.831	4.245	5.022
5.385	6.198	5.644	5.009	5.517	5.174

7.524	8.286	7.802	7.274	7.784	7.559
7.604	8.376	7.920	7.369	7.900	7.646
7.436	8.238	7.809	7.202	7.800	7.459
5.956	7.736	6.881	7.182	6.891	7.232
7.191	7.336	7.104	7.406	6.998	7.403
4.831	4.871	4.631	4.116	4.597	4.592
2.875	1.415	1.696	2.296	3.406	2.296
2.829	2.771	3.208	4.010	2.923	2.441
3.636	2.619	3.372	3.318	3.905	3.190
2.991	2.796	1.826	2.341	3.291	3.035
3.600	3.213	4.598	3.913	3.802	3.876
2.882	2.179	2.358	2.315	2.730	2.274
4.001	3.354	3.802	2.950	4.079	3.988
5.450	5.644	5.141	5.101	5.495	5.663
5.609	5.837	1.384	5.399	1.693	1.930
4.699	4.923	4.308	3.059	3.808	4.958
5.105	4.189	4.450	2.953	4.516	4.658
4.266	3.700	3.808	2.726	3.397	3.986
5.044	4.937	4.801	5.230	4.239	3.869
3.453	3.505	2.965	2.568	3.523	3.791
2.764	3.366	3.149	3.326	4.973	3.048
2.988	3.382	2.992	3.320	4.980	3.060
3.619	4.332	4.066	4.034	3.932	4.289
2.477	2.337	2.824	2.242	2.824	3.018
5.096	4.961	5.665	5.866	5.570	4.494
3.659	3.397	2.590	2.947	3.336	3.974
5.748	5.982	4.498	4.883	4.778	5.995
8.399	8.256	8.449	8.258	8.483	8.348
6.815	6.178	6.442	6.145	5.986	6.115
5.100	4.921	5.584	5.312	4.516	5.035
5.527	5.440	5.573	5.419	4.799	5.804
2.417	3.173	2.824	2.374	2.647	2.974
5.118	5.063	4.965	5.152	5.343	5.475
2.191	4.525	2.106	1.569	2.015	2.557
3.079	2.919	3.153	2.862	3.397	3.568
5.421	5.421	5.593	5.341	6.116	6.082
5.840	6.256	5.954	5.474	5.601	5.717
2.390	2.264	2.883	2.243	2.220	2.314
2.420	1.453	1.693	1.935	1.685	1.921
1.279	2.911	3.281	2.723	1.113	2.210
4.123	4.652	4.229	3.597	4.695	4.441
3.085	1.623	1.963	3.473	2.311	1.733
3.770	4.400	3.624	3.717	3.912	4.168
5.272	4.271	4.725	4.685	4.817	4.159
5.660	5.400	5.239	5.081	4.644	5.179
6.233	5.674	5.445	6.195	5.840	5.519
4.792	4.207	4.137	4.665	3.451	4.000
5.009	4.148	5.008	4.032	4.740	4.807
3.399	3.168	2.244	2.504	1.953	3.337

4.897	4.968	5.396	5.055	4.905	5.133
9.497	10.347	10.647	9.629	10.574	10.437
6.662	7.450	7.038	6.552	6.943	6.658
2.495	3.117	1.978	1.892	2.931	1.703
4.106	3.519	3.988	3.144	3.458	3.804
6.037	7.382	5.899	6.135	6.382	6.646
2.042	3.148	2.222	2.500	2.962	2.077
4.988	4.979	5.355	5.091	5.405	5.166
4.861	4.930	4.817	4.465	4.890	5.403
5.095	5.211	4.453	3.988	4.846	5.335
1.774	1.540	1.820	1.921	1.644	1.583
5.966	5.655	6.215	5.861	5.684	5.802
2.891	2.239	1.830	2.010	3.140	2.417
8.544	8.495	8.130	7.897	8.296	8.215
5.413	5.419	5.266	4.792	4.615	5.965
5.013	4.761	5.043	3.423	5.008	4.913
4.093	3.640	2.596	2.640	3.026	3.391
4.149	3.626	3.809	3.920	3.710	3.734
4.034	3.510	3.587	3.661	4.603	4.541
3.445	3.999	2.976	2.310	3.868	3.314
2.187	1.708	3.173	2.643	2.276	3.270
2.276	2.204	2.184	3.029	3.073	2.021
6.380	6.019	5.276	4.874	6.032	6.071
6.247	4.817	5.485	3.641	5.917	4.953
3.912	3.682	3.361	3.627	4.099	3.440
5.915	5.845	5.845	5.546	5.979	5.814
3.272	4.032	3.894	4.590	3.293	3.074
2.450	2.096	2.048	2.021	2.218	2.629
3.202	2.541	2.681	3.863	3.755	2.406
1.868	2.505	2.951	2.088	3.032	2.968
6.580	5.394	5.958	5.825	6.452	6.415
5.069	3.609	4.761	4.414	4.352	4.332
6.038	5.868	5.733	5.845	5.370	5.825
3.987	3.133	3.515	3.691	3.972	3.545
5.031	4.819	4.333	4.357	4.653	4.812
3.255	3.559	1.885	3.118	2.888	2.321
1.710	1.252	0.931	1.119	1.192	0.966
2.855	2.055	2.328	2.282	2.219	2.085
3.648	2.586	2.468	2.903	2.598	2.625
2.912	2.422	2.515	2.264	2.657	3.019
6.444	6.439	5.780	6.190	6.186	5.845
4.110	4.382	4.110	5.146	3.280	3.617
1.837	2.619	2.287	2.663	2.841	1.853
1.829	1.448	1.609	1.742	1.108	1.523
2.052	2.818	2.633	2.843	3.122	2.745
2.065	2.218	2.546	2.321	2.041	2.143
0.848	1.170	1.272	1.344	1.697	1.627
2.346	2.738	1.786	1.767	3.931	3.269
6.243	6.242	5.944	6.092	5.870	6.140
2.653	3.376	2.468	2.389	2.649	2.781

2.963	2.239	2.199	2.424	2.088	1.979
3.238	3.086	3.473	3.378	3.474	3.354
2.420	2.698	2.881	2.237	2.562	2.729
6.838	5.665	6.845	6.156	6.266	6.474
7.099	6.912	6.808	6.666	6.800	7.294
3.460	3.368	2.956	3.535	2.749	3.493
4.554	5.790	4.644	6.107	2.898	4.528
3.031	3.768	3.635	4.347	2.960	3.657
4.805	4.306	4.224	5.012	3.999	3.857
3.270	4.751	3.945	5.596	1.525	3.573
2.980	3.511	3.949	4.513	2.142	2.690
3.121	3.928	3.054	3.750	2.275	3.295
3.703	3.946	3.420	4.280	2.855	3.414
4.233	4.669	4.508	4.744	3.787	3.923
4.507	5.494	4.999	6.177	3.326	4.152
3.209	3.758	3.862	4.410	2.991	3.154
8.035	8.784	7.913	8.588	7.317	7.883
7.252	7.128	7.074	6.757	6.962	7.288
5.222	5.414	4.995	5.973	4.359	5.375
7.555	7.473	7.315	9.038	7.007	6.947
5.195	4.925	5.066	4.005	4.751	4.946
5.266	5.310	5.200	5.335	5.277	5.139
5.798	6.534	5.604	5.150	5.596	6.097
4.984	4.879	4.105	4.618	4.956	4.899
4.650	4.856	4.195	4.974	4.336	5.300
3.427	3.317	3.615	3.081	3.931	3.008
9.091	9.037	8.670	8.297	8.713	9.254
3.386	4.140	3.916	4.182	3.158	3.697
2.967	3.088	2.859	2.358	2.980	3.440
3.381	2.785	3.333	3.345	3.381	3.282
1.737	3.170	3.208	2.821	3.331	2.464
1.733	1.534	1.047	2.431	2.071	1.420
3.241	2.949	2.930	4.071	3.311	3.138
8.835	9.364	8.930	8.426	8.858	8.976
8.043	7.468	8.107	7.545	6.504	7.793
5.995	5.675	5.243	4.661	5.506	5.890
3.759	3.513	3.447	3.020	3.654	3.745
2.643	2.407	3.512	4.276	2.570	2.940
2.601	3.466	3.469	3.776	2.954	2.717
8.238	7.782	7.867	7.749	7.759	8.196
3.532	2.977	2.947	2.612	3.587	4.514
5.177	4.572	3.632	5.724	4.127	4.546
6.597	5.022	5.822	5.222	5.595	5.580
8.276	8.179	8.192	8.349	8.253	8.395
8.845	8.671	8.533	7.771	8.954	8.999
6.010	6.219	4.867	4.636	5.273	6.192
5.279	5.276	5.296	5.046	5.648	6.151
6.855	6.638	6.591	6.220	6.728	6.930
7.199	6.853	6.610	7.052	7.169	7.140
4.733	5.251	4.755	4.168	5.405	5.518

2.108	2.633	2.722	2.883	2.961	3.069
1.857	1.577	2.063	2.065	2.136	1.889
3.666	3.359	2.498	3.473	2.464	2.972
3.933	3.585	3.479	3.690	3.455	3.472
4.875	5.675	5.261	4.893	5.173	5.304
5.014	4.469	4.953	4.367	4.930	5.227
2.541	2.206	3.572	3.020	2.969	2.474
3.360	3.106	4.350	3.979	3.119	3.017
4.613	4.452	5.072	4.705	4.698	4.758
3.948	3.344	3.777	2.478	4.135	4.390
3.381	2.919	2.911	3.301	3.369	2.211
6.329	5.473	6.052	5.444	6.184	5.518
3.283	3.307	3.332	3.166	3.315	3.457
2.639	2.343	2.455	2.381	2.529	2.781
1.809	2.580	2.452	1.680	2.437	2.357
6.667	6.279	6.708	6.486	6.706	6.291
5.854	5.577	5.487	5.306	5.989	5.644
2.227	1.932	2.440	2.617	1.713	2.445
2.260	3.855	3.149	2.980	2.538	2.783
2.223	1.354	1.361	1.900	3.142	2.338
3.252	2.031	1.935	2.163	2.351	2.979
2.133	1.636	2.454	2.200	1.840	2.115
2.294	2.232	2.023	1.743	1.590	1.974
7.225	7.332	6.569	5.959	7.228	7.533
4.100	4.156	2.363	2.569	4.871	4.234
7.609	8.156	7.957	7.785	8.074	8.006
6.191	5.916	6.814	5.546	7.054	6.854
1.877	2.106	3.195	3.427	1.818	1.745
3.694	1.579	3.604	2.681	2.778	3.206
7.017	5.883	4.598	5.060	4.672	4.361
5.604	5.899	4.520	3.872	5.180	5.300
5.466	4.444	5.573	6.333	5.502	4.890
6.358	6.472	6.485	7.194	6.936	6.987
3.843	3.408	3.797	2.819	3.214	3.817
4.367	5.348	4.105	4.446	7.481	3.646
6.141	6.202	5.764	4.971	5.708	5.579
2.832	5.330	4.121	6.046	1.947	3.570
9.450	9.941	9.263	9.427	9.520	9.799
5.359	4.668	4.953	4.883	4.698	4.064
3.728	2.989	2.992	2.934	3.509	3.351
4.475	3.603	4.334	4.954	3.818	4.128
6.418	5.937	5.112	5.341	5.336	5.663
6.351	5.780	7.247	6.324	7.420	6.986
2.201	1.815	2.201	1.899	2.081	2.077
8.643	9.106	8.885	8.656	9.815	8.307
5.644	4.870	5.412	5.382	6.071	5.293
4.884	6.006	4.328	5.077	5.033	5.217
3.872	3.757	4.600	3.372	4.230	3.720
5.815	5.737	5.143	5.351	5.634	5.133
3.514	3.529	3.960	2.912	3.542	3.600

4.491	3.817	4.256	4.474	4.798	3.693
6.177	6.444	6.308	5.712	6.287	6.085
5.856	6.198	5.455	5.798	5.389	5.959
2.479	3.160	3.132	2.166	4.017	2.292
4.314	4.814	4.622	4.231	4.231	3.776
5.258	2.798	3.563	5.276	4.698	4.823
4.111	2.807	2.441	3.316	4.162	4.405
7.178	7.194	7.305	6.536	7.098	7.655
5.341	5.368	4.981	4.933	4.878	5.425
4.050	4.777	4.544	5.710	3.856	4.507
2.324	3.507	3.531	2.999	2.674	2.935
5.512	5.891	5.318	5.658	5.471	5.659
6.407	6.424	6.400	6.643	6.289	6.448
6.930	6.133	6.175	7.045	6.337	6.102
7.207	6.805	7.179	6.898	6.988	7.136
3.511	2.870	3.595	3.253	3.612	3.357
5.882	4.948	3.615	3.720	4.644	4.752
4.789	3.445	2.550	2.607	2.546	3.143
6.607	1.908	3.977	2.287	4.929	5.356
3.446	2.920	1.812	2.462	3.071	3.503
4.213	3.991	3.319	3.240	4.428	4.443
6.881	6.988	6.597	5.585	6.747	6.967
7.172	6.430	6.508	5.508	7.027	6.471
6.009	7.071	6.033	6.314	6.530	6.761
4.032	4.416	4.233	4.039	3.218	4.019
3.106	3.021	3.031	4.258	3.458	3.249
3.929	4.082	4.503	4.058	5.330	4.171
4.100	6.552	3.082	1.762	4.642	7.069
5.263	5.799	5.216	4.523	4.825	6.026
4.331	4.170	3.577	3.020	3.683	3.598
4.268	4.700	4.194	3.994	4.272	4.257
6.836	5.325	3.444	6.362	6.556	5.996
3.745	3.719	5.066	3.954	3.114	3.338
3.866	4.269	2.725	3.307	3.738	4.388
9.297	7.437	8.685	8.792	8.563	7.066
7.357	7.137	7.384	8.002	6.461	7.225
3.167	3.903	4.036	3.345	3.686	2.748
2.668	2.885	3.485	3.566	3.562	3.748
8.697	9.077	8.569	8.849	8.678	8.908
5.368	6.030	5.620	4.012	5.552	5.630
2.833	4.283	2.714	5.211	2.015	3.076
2.215	3.578	4.148	4.925	1.534	2.569
3.478	3.806	3.840	3.971	4.127	4.158
4.018	4.245	3.737	3.675	3.303	4.118
9.095	9.042	8.584	8.558	9.123	9.457
5.014	5.173	5.329	5.340	5.505	4.996
2.801	3.093	3.186	4.049	1.999	3.035
4.044	3.629	3.573	3.837	3.480	3.817
4.951	5.400	5.033	5.212	5.195	4.796
5.370	5.428	5.774	4.660	4.947	5.812

5.541	6.106	4.595	3.684	3.748	5.193
2.693	4.368	3.064	3.829	3.762	2.850
4.784	4.192	3.893	3.075	3.090	4.194
4.338	3.233	3.557	3.353	3.161	3.585
4.242	4.447	2.645	3.693	4.424	3.447
7.305	6.956	6.842	6.947	6.905	7.070
6.939	6.661	7.360	6.523	6.932	7.266
5.332	5.327	6.039	5.394	6.115	4.887
3.258	2.907	2.804	2.732	3.514	3.107
4.240	3.631	2.640	3.066	2.712	3.748
5.581	5.518	5.745	6.940	6.360	4.445
5.038	5.408	6.203	4.609	5.585	5.552
2.190	3.562	3.750	3.944	3.252	2.693
2.140	1.695	2.368	1.780	2.357	2.446
6.487	6.351	5.840	6.295	5.925	6.290
3.147	4.190	3.970	3.807	2.967	4.314
5.600	6.513	5.756	6.288	5.898	5.707
5.868	5.690	5.505	4.581	4.653	4.694
4.980	4.766	5.731	4.640	5.638	5.649
2.916	5.276	4.509	3.636	4.613	4.318
4.348	4.120	4.348	4.242	3.386	3.786
3.417	2.659	2.553	1.710	2.464	3.223
5.474	5.363	5.067	5.138	4.761	5.176
4.113	4.758	1.887	3.999	2.429	2.007
7.429	7.278	7.040	7.150	7.186	7.622
6.235	5.399	5.105	4.854	5.804	5.637
2.624	2.885	2.956	3.541	2.141	2.624
4.563	5.802	3.714	4.414	3.447	5.066
4.881	4.920	5.185	4.949	4.660	5.590
6.424	4.791	6.164	5.898	4.819	6.291
3.933	3.831	4.559	3.639	2.844	4.217
8.317	8.503	7.866	7.888	8.572	8.471
2.332	2.832	2.291	1.799	2.193	2.969
4.800	3.533	3.554	3.363	4.305	4.127
3.115	2.402	2.923	2.997	2.903	3.057
4.474	4.495	4.102	3.134	3.974	4.337
4.842	5.128	4.763	4.143	4.841	5.081
8.604	9.445	7.456	7.870	8.161	8.434
7.604	7.832	7.477	7.069	7.458	7.624
4.248	3.242	2.776	3.228	2.937	3.588
7.158	6.371	5.808	6.502	5.468	6.869
3.071	3.231	3.763	3.383	4.020	2.980
7.350	7.447	7.731	6.355	8.127	7.720
6.933	7.008	7.243	5.881	7.399	7.763
6.074	5.877	5.898	5.872	4.692	5.698
7.220	7.192	6.640	5.990	7.230	7.292
5.324	5.766	4.801	4.670	4.955	5.377
3.148	3.284	3.578	3.688	2.743	3.041
6.879	7.117	7.339	6.456	7.371	6.913
3.425	3.073	3.504	2.559	3.771	4.004

6.016	6.938	5.967	5.482	6.488	6.435
3.401	3.461	3.949	3.420	2.895	3.707
3.813	4.839	3.586	3.194	3.879	4.223
6.369	6.672	6.927	4.189	7.090	7.131
3.329	2.832	3.600	3.577	3.619	3.135
5.547	6.138	5.516	5.323	5.637	5.684
4.211	4.243	3.874	4.016	4.204	4.117
4.827	4.717	3.679	4.380	4.308	4.268
6.886	7.206	5.662	5.262	6.189	6.235
5.546	5.335	4.621	5.088	4.497	4.274
7.958	8.959	7.758	7.653	7.781	8.552
1.507	2.310	1.174	1.519	1.165	3.418
2.446	1.622	2.860	1.850	1.412	2.027
2.313	2.086	2.368	2.402	2.103	2.160
4.579	2.818	3.309	3.837	3.411	2.524
2.791	2.293	2.569	2.546	3.419	2.700
3.656	2.179	3.101	1.965	2.963	3.289
7.348	7.302	7.201	6.543	7.267	7.273
6.088	6.167	6.199	5.844	5.552	6.108
4.733	3.887	6.142	6.620	5.763	3.874
5.019	4.710	5.297	5.998	5.125	5.309
5.411	6.472	5.186	5.394	5.379	5.081
1.972	3.290	1.988	2.821	2.112	1.881
4.270	4.667	4.319	4.285	3.368	4.462
3.763	3.141	3.764	3.052	4.676	3.281
6.712	6.268	5.825	5.802	6.404	6.875
3.829	3.758	4.254	4.395	4.920	4.173
10.288	9.997	10.149	9.404	10.377	10.150
6.948	5.311	6.281	5.894	6.925	7.032
7.605	7.891	7.397	6.121	7.904	7.664
5.897	5.778	5.466	4.493	6.051	5.528
6.447	6.619	6.967	4.825	7.527	6.722
3.906	4.160	3.745	3.308	3.978	4.417
4.668	4.968	5.571	4.319	5.340	4.602
4.028	4.136	4.460	3.670	3.033	4.128
3.270	3.032	3.213	3.301	3.227	3.022
4.502	3.396	2.973	2.810	4.344	4.102
4.740	3.859	3.721	3.139	4.212	4.351
4.438	3.961	3.407	3.414	3.726	4.239
6.790	5.083	5.243	5.227	5.137	5.456
2.774	1.879	2.499	2.011	2.742	3.454
2.976	3.565	3.182	2.783	3.777	4.019
7.269	6.542	6.702	6.989	6.885	7.379
2.324	2.004	2.197	1.929	1.744	2.090
3.906	3.749	2.714	3.441	3.080	3.171
3.890	4.305	3.954	4.122	4.112	4.256
4.680	4.539	4.997	5.491	4.825	4.473
5.811	5.616	5.230	5.686	4.824	5.205
1.441	1.514	1.480	2.367	1.070	1.710
4.253	3.797	4.279	4.141	4.216	4.202

3.933	3.492	3.026	3.611	3.420	3.472
4.044	4.094	4.668	4.192	3.705	4.080
6.616	5.977	7.264	7.366	7.388	6.312
4.122	3.893	4.487	3.202	4.506	4.200
2.881	3.505	2.501	3.117	2.506	3.110
3.623	3.295	3.908	3.818	4.511	3.692
2.828	3.010	3.093	3.665	2.971	3.581
6.414	6.363	6.123	6.155	6.613	6.185
3.314	4.418	4.382	3.683	3.741	3.952
4.069	2.504	3.509	3.014	3.648	3.652
6.526	6.516	6.195	5.191	6.782	6.642
5.559	6.404	5.281	3.979	6.151	5.782
3.596	2.157	1.944	2.046	2.006	3.620
5.738	5.202	5.842	5.601	5.509	5.733
8.637	8.806	8.133	8.380	8.439	8.656
6.311	5.939	5.737	5.376	6.447	5.653
7.162	6.388	6.734	6.888	6.900	5.946
4.768	3.714	4.234	4.466	3.669	4.195
2.272	2.237	2.341	2.056	2.287	2.254
4.379	4.251	4.135	3.692	3.670	3.590
4.219	3.708	4.016	2.730	4.766	4.238
4.062	3.210	3.314	3.199	2.294	3.402
6.975	6.944	6.615	5.995	6.873	7.095
3.871	3.735	3.718	4.111	4.242	4.487
5.818	5.642	5.179	4.581	6.545	5.997
5.626	6.161	4.789	4.245	5.008	5.449
1.997	3.619	3.233	3.491	2.862	2.510
4.246	4.102	3.869	4.184	3.055	3.513
3.949	3.952	3.050	3.205	3.956	3.129
4.004	3.350	3.207	3.236	3.193	3.839
7.240	8.036	7.421	7.076	7.575	7.257
7.409	6.684	5.511	6.131	6.103	6.524
4.398	3.610	4.343	4.049	4.370	4.428
3.928	4.208	4.102	3.173	4.394	4.216
4.921	4.593	4.474	4.402	4.451	5.237
5.501	5.387	4.516	4.503	5.788	5.227
5.806	5.614	5.378	5.166	5.392	5.495
6.004	5.916	5.723	4.537	6.301	5.788
6.186	5.525	6.300	6.099	6.265	6.019
4.643	4.322	3.959	3.681	4.501	4.399
3.391	4.115	4.029	3.659	4.862	3.353
5.103	5.510	5.495	5.389	6.694	4.747
4.312	4.651	5.340	4.569	4.931	4.913
5.178	5.469	4.437	4.071	4.872	4.736
5.537	7.311	4.587	4.092	5.827	5.899
4.582	5.045	4.546	3.766	4.113	3.754
3.524	2.422	3.268	5.040	2.835	2.886
8.790	8.328	6.773	6.414	7.505	8.442
4.234	4.104	4.750	5.012	7.014	3.597
1.778	2.758	2.094	2.106	2.318	2.531

8.051	6.529	7.826	6.477	8.299	7.542
7.633	6.993	7.436	7.241	7.421	7.989
7.900	7.410	8.251	7.161	8.201	7.912
4.850	5.180	6.123	4.197	5.865	5.364
6.546	6.486	6.707	6.717	6.199	6.504
4.216	4.255	5.246	4.803	3.987	4.126
4.768	4.387	5.363	5.208	4.974	5.320
4.910	4.398	5.190	5.114	4.729	4.758
2.570	1.820	3.161	2.621	2.179	1.774
3.936	3.988	4.517	4.113	4.735	3.959
7.016	6.403	7.628	6.431	7.359	7.265
6.963	6.962	6.954	6.799	7.148	6.985
4.688	5.595	6.173	6.388	5.267	5.290
6.320	5.685	5.792	4.494	5.537	6.167
4.226	4.462	5.504	4.456	5.401	6.025
2.171	2.811	2.006	2.650	2.944	2.792
6.658	6.290	6.478	6.384	6.140	6.761
3.988	4.431	4.282	4.642	4.756	4.431
3.452	3.614	2.774	3.300	3.617	2.531
3.827	3.719	3.699	3.655	4.090	4.395
5.830	5.930	5.468	5.673	5.843	5.932
6.193	5.523	6.174	5.794	5.599	5.590
7.030	6.794	6.832	7.216	6.850	7.016
2.758	3.839	3.043	2.950	2.881	3.395
4.666	3.487	3.931	3.612	4.563	4.026
5.191	4.876	4.879	4.812	5.055	4.973
4.599	5.389	4.386	3.613	4.823	4.632
5.534	6.330	4.917	4.673	5.093	6.122
6.338	6.908	6.891	6.504	6.569	6.407
3.575	4.616	4.110	3.994	4.099	3.777
4.308	3.710	4.323	3.627	4.257	3.913
5.201	4.970	4.858	5.048	5.069	5.154
4.800	5.488	4.828	5.444	5.136	5.047
4.754	3.881	2.767	4.358	3.745	3.992
4.112	2.710	3.937	3.288	3.187	3.036
2.344	2.440	3.521	2.900	1.855	2.700
5.868	4.364	5.470	5.151	4.964	4.635
5.101	4.016	4.618	4.752	5.118	4.795
5.673	5.837	5.525	5.223	5.770	5.796
3.650	3.890	3.886	3.483	3.791	3.745
2.216	1.965	3.074	2.410	1.981	2.393
3.373	2.593	1.826	3.007	1.976	2.397
4.867	3.468	4.395	4.285	4.214	4.353
7.054	6.876	6.175	6.172	6.805	6.669
5.337	4.772	5.257	4.909	4.797	4.680
8.929	8.139	8.302	8.971	8.787	8.149
2.900	2.468	2.675	2.979	3.116	3.498
6.029	5.861	5.495	5.142	6.256	5.911
3.501	3.174	2.787	2.846	3.039	3.173
3.182	2.820	3.135	4.637	3.136	3.620

5.957	6.179	5.224	5.505	6.053	6.098
2.667	2.869	3.334	3.265	2.915	2.305
3.365	3.507	4.419	4.237	3.965	2.954
5.013	5.039	4.770	4.540	4.363	5.099
4.142	3.805	3.680	3.407	3.774	3.625
8.767	7.862	7.932	7.272	8.604	7.470
4.056	2.676	3.314	2.757	2.702	2.759
5.757	5.110	5.021	4.898	5.640	4.894
5.236	4.865	4.171	4.315	4.766	4.543
5.234	4.600	4.747	4.729	5.521	4.662
1.687	1.642	1.922	1.961	1.796	1.901
2.097	1.715	2.022	2.182	1.858	2.132
3.173	3.499	3.048	2.429	3.837	3.729
4.309	4.309	3.430	3.873	4.042	3.585
4.416	4.410	4.171	4.628	4.434	4.912
3.839	3.912	3.260	2.835	3.963	2.938
6.266	6.623	6.482	6.026	6.081	6.437
5.951	7.135	5.398	4.942	5.374	6.832
2.933	3.028	3.746	3.096	2.199	2.481
4.232	4.475	4.662	4.115	4.275	4.366
4.295	4.124	4.197	4.624	4.064	4.407
3.561	4.553	4.930	5.483	2.109	3.804
6.037	5.180	5.373	5.599	5.719	5.534
4.327	4.681	3.965	3.643	4.653	4.828
5.411	6.088	5.167	5.465	5.738	5.386
4.640	4.198	3.357	3.180	4.259	4.594
2.191	2.123	2.245	3.032	2.221	2.386
4.178	4.368	4.469	4.143	4.642	4.642
2.905	2.264	1.327	2.444	2.890	2.699
3.273	2.859	2.251	3.096	3.104	2.605
4.668	4.701	4.284	5.497	4.367	3.297
5.019	4.462	3.484	4.399	3.983	4.367
3.016	3.333	1.803	4.343	2.968	1.662
2.503	2.644	2.545	2.529	4.559	2.630
3.287	3.431	2.519	2.324	3.113	3.765
3.442	2.960	2.625	2.718	2.696	2.528
4.696	5.350	5.801	6.420	4.298	5.126
3.902	4.601	4.899	4.049	4.392	4.218
3.881	3.864	3.757	3.638	3.381	3.051
2.018	2.094	3.021	1.916	2.132	2.395
5.212	4.699	3.956	5.320	5.951	5.310
6.800	5.841	6.174	6.403	6.126	6.230
4.501	5.108	5.866	5.121	3.675	4.647
5.990	6.305	5.944	5.396	6.422	6.124
5.060	4.779	4.800	4.881	5.378	5.067
2.315	1.975	4.636	4.445	5.303	3.350
4.145	3.625	3.355	3.152	4.625	4.488
4.177	3.006	3.169	2.580	3.298	3.746
5.448	4.916	5.317	4.788	5.358	5.414
4.356	2.745	4.254	3.682	3.500	3.800

4.462	4.765	3.925	2.869	4.221	4.411
3.604	3.035	3.231	3.901	2.819	3.329
3.984	3.700	3.401	4.103	3.418	4.322
2.264	2.171	3.195	3.448	1.883	2.798
4.549	4.616	4.837	4.590	4.053	4.221
4.373	4.824	5.006	4.822	5.613	4.153
3.575	3.555	4.099	4.006	4.327	3.927
2.798	1.995	3.079	1.908	2.504	2.788
3.315	2.386	3.740	2.464	2.114	2.746
4.996	4.092	4.788	4.382	4.446	5.298
2.840	4.206	3.768	3.163	4.608	4.182
2.805	3.786	3.343	1.649	3.779	2.336
5.048	4.962	5.162	5.669	4.770	4.683
6.263	6.932	6.498	6.669	6.263	6.111
2.335	2.488	2.622	1.727	2.154	2.335
3.908	3.617	4.763	4.043	3.654	4.601
2.919	2.411	3.180	3.302	1.971	3.656
2.950	2.683	3.586	3.266	3.751	3.926
2.285	2.113	2.516	1.917	2.287	3.567
5.473	5.703	5.177	4.748	5.564	5.897
2.546	2.078	2.127	2.469	2.884	2.783
3.404	4.077	3.109	3.950	2.653	3.121
2.178	2.420	2.324	3.261	2.978	2.655
4.488	3.559	3.522	2.821	4.068	4.284
4.184	3.719	3.420	3.475	3.429	4.393
4.482	5.109	4.089	4.296	5.275	5.122
4.022	4.547	3.561	3.588	4.940	5.022
7.119	6.957	6.767	5.635	6.682	7.015
4.602	6.021	4.079	3.107	4.396	4.859
4.807	4.866	4.518	4.557	4.580	5.178
5.021	4.396	4.482	3.505	4.575	4.851
5.928	5.128	6.392	5.640	6.105	4.854
6.244	5.250	5.096	4.223	5.921	5.593
1.311	1.125	1.559	1.720	1.329	1.526
4.968	4.825	4.605	4.238	3.783	4.796
5.442	4.983	5.474	4.438	5.477	5.335
4.902	4.818	5.093	5.167	4.258	4.678
4.851	5.508	5.797	5.818	6.040	4.545
4.920	4.754	4.825	4.411	3.730	3.893
5.707	6.249	5.892	5.768	5.839	5.777
2.760	2.353	2.093	1.633	2.173	1.996
3.714	3.461	2.709	2.664	3.707	4.325
5.830	5.213	5.668	6.406	5.855	5.834
2.734	2.879	2.953	2.749	2.558	2.217
6.481	5.180	5.957	5.882	5.558	5.701
6.179	5.873	5.756	6.060	6.104	6.009
3.841	2.651	3.855	3.412	2.312	3.127
4.045	4.033	4.045	3.723	2.993	3.265
5.017	4.876	4.559	4.819	4.626	5.059
6.515	5.895	6.394	6.253	6.384	6.050

6.727	6.465	6.812	7.124	7.000	6.254
3.634	3.206	2.816	3.229	4.303	3.493
4.993	3.972	4.594	4.652	4.386	4.823
5.752	5.692	6.570	6.659	6.098	5.968
3.937	4.244	4.055	4.163	4.040	4.777
6.032	5.987	5.487	5.025	5.743	5.866
5.686	5.197	5.300	4.759	5.612	5.643
5.918	6.357	6.064	5.814	5.757	5.981
7.589	8.481	8.239	7.316	8.785	8.459
4.897	4.939	3.985	4.369	4.735	4.700
4.266	4.235	3.884	4.444	3.962	4.111
1.584	1.508	1.482	1.825	1.588	1.961
4.035	4.129	4.126	4.085	3.554	3.860
4.590	4.354	4.727	4.675	4.787	4.762
4.778	4.018	4.133	4.366	4.122	4.205
3.535	3.548	3.003	2.855	3.514	3.909
8.111	7.963	7.936	7.365	8.268	8.088
5.207	4.556	4.611	4.255	4.866	5.351
6.429	5.986	5.883	5.600	5.634	6.263
2.337	2.707	3.005	2.264	2.546	2.454
2.900	3.844	2.656	2.163	3.904	3.605
8.311	8.491	6.432	4.760	7.419	8.804
2.992	4.325	2.951	2.116	2.518	5.846
5.501	4.842	3.815	1.569	4.156	6.605
3.561	4.041	3.305	2.961	3.396	4.477
4.694	5.218	3.254	3.159	3.101	4.585
3.003	2.085	1.840	1.999	1.801	2.011
4.600	5.156	4.940	4.611	5.059	4.704
2.639	2.453	2.686	2.916	3.204	2.812
7.000	7.157	6.712	6.659	6.853	6.443
3.571	3.733	5.792	4.902	5.146	4.369
4.064	4.840	3.283	3.317	4.055	4.967
1.812	1.961	2.720	2.210	1.932	2.837
1.460	2.128	2.609	1.849	2.503	3.536
2.280	1.351	2.355	2.504	1.995	2.708
4.122	3.628	3.913	3.664	3.619	3.886
2.682	2.805	2.572	3.457	2.515	2.278
2.666	3.439	3.384	3.271	3.474	3.010
4.752	5.116	5.894	5.894	5.097	4.436
4.592	4.084	4.567	4.260	4.601	5.063
3.878	3.239	3.374	2.774	4.851	3.144
4.906	6.036	5.292	6.136	4.829	4.896
4.334	3.049	3.817	4.398	2.993	3.725
3.938	3.099	3.419	2.575	2.602	3.312
5.701	4.568	4.386	4.168	4.478	5.129
4.285	2.906	3.999	3.700	4.659	2.970
4.082	4.306	4.541	4.633	4.016	3.507
3.272	2.454	2.673	3.344	3.392	3.191
2.953	2.677	2.367	2.645	2.997	2.364
2.185	2.206	2.439	2.334	2.249	2.028

4.171	3.792	1.911	2.026	2.294	2.065
3.677	3.558	3.981	3.396	3.249	3.948
3.961	3.456	1.472	3.050	2.602	2.331
4.320	4.752	4.537	4.452	4.459	4.451
5.321	5.717	3.938	4.977	5.139	5.457
7.371	7.553	7.597	6.253	8.015	7.872
7.305	6.365	7.561	7.927	7.250	6.120
7.045	6.338	6.889	5.679	7.398	7.192
3.570	3.905	3.358	3.226	3.573	3.701
5.678	5.539	5.836	4.742	5.950	5.917
2.607	2.579	2.166	2.625	1.325	2.450
2.983	3.155	2.636	2.683	3.223	2.682
4.622	4.741	4.787	4.206	4.484	4.824
4.695	5.796	3.884	5.319	4.692	3.547
6.429	5.585	6.207	5.819	6.200	6.143
6.312	6.037	5.789	5.982	6.920	5.547
3.477	3.304	2.958	3.542	3.236	2.791
5.762	5.799	5.762	5.064	5.655	6.102
3.196	3.302	4.178	3.191	3.191	3.980
4.257	4.228	3.967	3.397	3.943	3.302
2.629	2.074	2.290	2.304	3.136	2.486
5.390	4.733	6.264	5.215	5.552	5.277
5.093	5.497	5.388	4.857	5.627	4.864
2.817	2.322	2.217	2.446	1.769	2.598
6.332	6.926	6.248	7.714	5.636	6.181
5.650	6.009	3.489	5.571	5.244	4.845
4.572	4.020	3.750	3.932	4.586	4.302
9.721	9.443	9.595	10.095	9.552	9.549
5.112	5.054	4.977	3.399	5.206	5.599
3.452	2.945	3.752	3.475	3.750	4.003
3.545	3.912	3.771	3.541	3.743	4.193
7.246	7.553	7.330	7.265	6.976	7.312
2.625	2.420	1.818	1.746	1.307	2.206
4.887	4.243	4.475	4.578	4.038	4.343
3.504	2.579	2.678	2.836	3.623	3.219
3.611	3.009	4.675	4.059	3.402	3.278
3.840	3.838	3.669	2.893	4.213	4.173
2.304	2.304	2.943	2.594	2.465	3.067
2.029	1.856	1.866	1.486	1.661	1.827
2.157	2.379	2.153	2.440	2.828	2.894
2.185	2.010	2.205	2.821	1.730	1.947
3.299	2.720	3.530	3.539	3.453	2.735
1.416	1.697	1.994	1.586	2.064	1.958
1.993	2.892	2.922	2.246	2.674	2.553
3.047	2.966	3.574	3.722	2.831	3.337
4.105	3.650	3.498	3.400	3.165	3.635
3.770	2.521	3.758	2.989	2.866	3.195
3.182	3.286	3.291	4.865	4.120	3.111
4.120	4.330	4.284	4.086	3.321	5.451
2.491	3.395	3.258	2.604	3.186	3.060

3.017	2.743	2.353	2.320	2.725	2.583
2.785	2.786	2.497	2.681	2.445	2.753
6.358	7.657	6.665	6.925	6.640	6.628
6.646	6.956	7.102	6.693	7.319	7.000
7.723	8.071	8.326	7.787	8.040	7.647
4.096	4.226	4.303	5.693	4.244	4.220
3.845	2.232	3.687	2.950	3.971	3.413
6.611	6.884	6.406	7.272	5.824	5.898
3.072	3.143	3.767	3.301	3.510	4.019
4.806	4.715	3.780	4.239	4.957	4.282
4.598	5.164	4.247	4.146	4.988	4.904
9.993	9.680	9.985	9.574	10.322	10.147
5.819	5.428	6.318	6.325	6.429	5.694
3.310	4.667	3.412	2.363	3.453	3.326
3.236	3.529	3.306	1.578	3.839	3.466
5.145	5.974	5.357	5.362	5.757	4.676
8.265	8.776	7.616	7.912	7.639	7.995
8.417	8.250	8.206	7.737	8.843	8.596
7.713	7.239	7.462	7.603	7.464	7.054
7.011	7.040	6.034	6.783	6.941	6.707
5.805	7.288	5.855	6.195	4.844	6.709
4.199	3.788	3.660	3.757	3.736	3.843
5.227	3.773	3.852	3.940	4.111	4.397
6.109	5.676	4.959	5.215	5.907	5.724
6.496	6.714	6.921	6.224	6.863	6.954
6.480	6.287	6.386	5.764	6.116	6.642
7.338	6.060	7.019	5.038	7.025	6.952
3.406	2.446	2.719	2.630	2.709	2.503
2.382	2.515	3.306	2.969	2.103	1.936
2.527	2.597	3.190	2.584	3.287	3.650
7.764	8.030	7.888	8.076	8.081	7.554
3.303	2.798	4.022	3.673	3.264	3.789
2.811	2.904	1.983	2.946	2.036	2.570
6.419	6.747	5.325	5.449	5.890	5.507
3.018	1.840	1.804	2.562	2.308	2.645
5.738	6.250	5.611	6.059	7.016	5.436
2.256	3.890	3.159	2.400	3.741	2.677
4.724	3.777	3.583	4.514	4.449	3.846
3.329	2.307	3.654	3.065	2.561	3.812
2.813	1.936	1.804	2.513	1.849	2.060
3.917	3.253	3.573	4.012	3.320	2.964
4.136	4.737	5.390	4.576	3.981	4.441
4.226	4.571	4.899	4.963	3.595	3.690
0.973	1.372	1.728	1.954	0.831	1.116
3.441	4.928	4.487	6.193	3.678	4.401
6.616	6.290	6.829	6.153	6.966	6.755
5.758	5.502	6.506	6.868	5.334	5.338
3.827	4.061	3.239	4.038	3.147	3.496
2.163	3.424	2.942	3.626	3.387	3.313
3.162	2.801	3.611	3.162	2.431	2.667

5.962	6.262	4.004	3.238	5.317	6.270
5.920	2.582	2.709	2.213	2.973	4.955
3.648	2.608	2.762	2.594	2.793	3.024
4.052	3.935	4.260	4.711	4.477	4.767
5.251	4.600	5.741	5.353	6.201	5.008
2.318	2.576	1.933	2.164	2.651	2.325
1.519	2.813	1.728	1.555	1.263	2.193
3.060	3.061	3.916	3.447	3.890	3.470
3.242	3.149	3.036	2.943	3.708	3.186
5.344	4.885	4.901	4.567	5.487	5.718
2.925	2.777	2.328	3.612	2.823	3.173
3.367	3.357	2.137	2.413	2.340	2.612
2.603	1.787	2.136	2.619	2.349	2.613
5.170	5.308	5.330	5.449	4.518	5.005
4.652	5.033	4.630	5.481	3.659	4.136
5.371	5.457	4.950	5.109	4.510	4.921
4.546	2.921	2.590	3.065	2.676	3.864
6.618	5.794	5.843	5.755	6.034	6.531
3.615	3.561	3.851	3.537	3.416	3.437
3.146	3.250	2.842	3.217	2.873	2.649
7.085	6.216	6.700	6.411	6.524	6.429
3.255	3.138	2.260	2.888	3.045	1.665
7.804	7.740	7.638	7.208	7.897	8.098
5.572	5.905	5.883	5.812	7.349	4.904
5.288	5.150	5.347	5.466	5.024	5.306
5.691	5.690	5.125	5.137	5.363	5.056
4.022	6.203	4.516	4.252	4.668	4.909
2.672	3.341	3.437	3.454	2.942	3.115
6.073	5.633	5.227	4.343	5.253	5.732
11.043	11.269	11.249	11.016	11.142	11.451
5.513	5.030	5.255	3.444	5.585	5.838
5.210	5.713	5.208	4.532	5.707	5.348
3.505	2.737	2.624	2.456	2.766	3.013
2.329	1.832	1.996	2.539	1.687	1.914
4.994	5.493	5.567	5.518	5.423	4.437
3.864	3.828	3.785	2.795	3.740	3.962
7.178	7.118	7.983	7.739	7.727	6.940
3.253	3.231	3.744	2.978	2.575	3.148
4.889	5.288	5.910	4.914	5.416	5.536
4.469	5.350	5.303	5.050	3.863	4.592
6.376	6.317	5.297	4.924	5.551	6.955
2.216	2.425	2.907	1.772	3.488	2.695
6.554	6.618	6.146	6.434	6.342	6.000
4.574	4.782	4.037	5.130	4.697	3.300
3.818	4.087	3.791	3.138	3.852	3.426
4.582	4.560	5.502	4.956	5.189	3.706
4.050	5.021	5.101	5.168	5.000	5.191
4.834	5.095	4.713	3.700	5.003	5.162
6.442	6.185	6.392	5.773	6.292	6.215
7.834	7.991	8.304	7.338	8.314	7.879

6.360	6.157	6.727	6.200	6.197	6.338
5.573	5.308	5.440	5.433	5.497	5.285
4.012	4.019	3.355	2.644	3.468	4.624
6.189	6.632	5.707	5.622	6.645	5.890
8.518	7.761	8.371	8.650	8.191	7.730
8.044	7.307	7.882	8.192	7.708	7.295
8.099	7.343	7.942	8.217	7.754	7.343
7.930	7.190	7.776	8.062	7.576	7.159
8.097	7.326	7.902	8.196	7.770	7.326
7.520	6.754	7.308	7.647	7.134	6.750
7.412	5.298	6.614	5.946	5.957	6.578
5.353	5.681	5.619	5.936	5.718	5.746
3.439	2.832	3.329	3.223	2.254	3.302
3.568	2.723	2.831	2.946	2.730	2.970
6.189	5.348	5.599	4.661	4.995	5.535
4.528	1.966	3.552	2.600	2.907	3.787
3.197	2.474	3.128	3.095	2.462	3.280
5.966	4.777	5.196	5.051	6.023	5.064
3.308	2.333	2.802	2.035	2.751	2.700
3.866	3.186	2.914	2.744	2.780	3.500
2.653	2.671	1.809	2.959	2.613	2.620
4.588	3.853	3.541	3.430	4.151	2.767
7.809	6.725	7.252	7.317	8.141	6.908
6.739	6.054	6.552	5.924	5.216	6.487
2.891	1.376	2.169	2.147	1.806	2.013
6.014	4.983	5.504	4.845	3.809	5.165
4.397	2.540	3.270	3.917	3.454	3.851
5.196	3.463	4.661	3.705	3.844	4.903
8.596	9.950	5.978	6.324	6.400	8.317
9.489	9.496	7.727	8.025	8.640	8.166
4.275	5.537	3.613	7.369	6.641	2.997
3.340	3.080	3.170	3.489	2.673	1.933
1.835	3.262	3.028	3.668	2.960	2.633
3.222	3.671	3.672	4.315	3.422	3.366
2.565	2.630	3.255	1.910	2.992	3.248
3.049	3.488	3.483	3.488	3.488	3.261
5.773	5.093	5.116	5.131	5.580	5.834
2.142	1.442	1.560	2.004	1.962	1.667
8.313	8.950	7.979	7.311	8.748	8.804
5.234	4.686	4.568	4.246	4.143	5.080
4.894	5.301	3.759	4.789	4.538	4.336
5.013	3.892	3.592	3.777	4.092	3.817
5.137	4.679	4.699	4.431	4.612	5.227
5.215	5.212	5.011	4.683	5.810	5.628
4.409	4.817	5.136	4.944	3.904	4.098
2.712	1.959	1.952	1.385	2.116	1.662
1.333	2.123	2.766	2.351	2.633	2.053
3.879	3.652	3.678	4.085	3.802	4.024
7.218	7.304	6.655	6.708	5.656	7.526
3.894	2.874	4.783	2.456	4.529	4.457

4.598	4.952	6.243	6.267	5.647	4.297
5.272	4.670	4.957	4.889	5.346	5.387
6.133	6.952	5.315	5.310	5.819	6.333
5.194	4.048	2.313	2.817	3.411	2.915
4.585	4.942	4.280	4.377	4.819	4.588
2.710	1.758	1.973	1.686	3.171	2.552
2.066	2.324	1.981	2.299	2.483	2.569
3.374	3.091	2.915	2.111	2.274	2.149
7.422	7.430	7.230	6.558	7.808	7.754
6.295	5.928	5.719	5.454	5.306	6.059
5.083	6.552	5.112	4.683	4.892	5.267
5.129	5.773	3.538	3.197	4.953	4.920
4.509	6.689	3.671	2.531	3.815	5.466
6.123	7.632	4.792	4.617	5.191	6.784
6.476	5.944	4.594	4.195	4.348	4.114
3.727	2.625	3.016	3.494	3.590	3.409
5.922	5.587	4.761	3.968	5.947	5.862
7.438	7.060	6.773	6.823	7.026	7.385
5.007	5.637	4.349	4.232	4.906	5.396
2.204	2.896	2.593	2.736	2.244	2.473
1.703	2.597	1.520	1.905	2.035	2.071
2.715	2.658	2.821	3.427	2.646	2.717
3.447	2.743	2.945	2.346	3.800	2.884
6.119	5.925	6.133	5.056	6.401	6.519
5.992	6.090	5.184	4.684	6.028	5.928
7.129	7.308	7.904	7.574	7.808	7.508
4.056	3.954	3.872	4.595	3.936	3.367
5.775	5.644	6.570	7.351	6.264	5.924
2.841	4.467	3.681	2.363	3.041	3.703
1.973	2.493	1.957	2.627	2.933	1.501
5.915	5.817	5.788	5.967	5.171	5.881
5.441	4.731	5.159	5.053	5.001	4.688
2.519	3.382	3.647	4.049	2.518	2.562
3.488	4.270	2.980	3.652	3.522	3.735
2.685	3.607	3.556	3.186	3.535	3.782
5.290	4.864	5.106	5.364	5.630	5.577
2.228	2.543	1.935	1.941	1.808	2.249
2.996	2.713	2.991	2.848	3.639	2.755
5.372	5.128	4.980	4.713	5.123	5.389
3.802	3.260	3.737	3.257	3.188	3.101
2.334	1.766	2.122	2.631	2.227	1.826
3.874	3.774	3.730	4.043	2.994	3.829
5.189	7.355	5.253	5.593	5.848	5.461
6.060	5.204	4.970	5.456	4.714	5.286
3.777	3.024	1.848	2.700	2.578	3.291
4.760	4.875	4.081	4.253	4.353	4.741
3.071	2.915	2.726	2.814	2.961	2.994
2.976	2.261	2.540	2.501	2.151	2.758
4.236	5.824	4.577	4.018	4.079	5.436
2.391	1.946	2.227	2.454	2.579	2.239

8.148	8.484	7.887	7.915	7.775	8.227
3.095	2.454	3.819	2.604	2.885	1.938
2.548	3.271	2.509	2.943	2.498	2.756
5.446	4.736	4.814	4.273	4.751	5.702
3.986	3.638	2.710	3.523	3.814	4.481
4.624	4.674	4.354	3.973	4.446	5.162
3.841	3.362	3.968	4.868	3.816	4.666
4.494	3.364	4.642	4.829	4.620	4.229
2.032	3.220	3.234	3.554	2.831	2.993
6.142	6.326	6.612	7.141	6.291	6.029
9.231	8.484	9.650	9.859	9.928	8.190
3.346	2.656	1.635	3.067	2.442	2.848
3.760	3.931	5.184	3.000	4.888	4.912
3.034	4.522	3.319	3.660	5.752	3.226
4.413	4.572	4.986	6.000	5.249	3.210
2.901	3.198	2.847	3.712	2.206	2.408
5.279	5.733	5.846	4.524	6.198	5.653
5.198	3.528	2.613	3.707	2.891	1.757
2.287	3.026	3.608	3.347	3.034	3.087
5.337	5.901	5.417	4.659	6.439	5.809
4.721	3.551	3.738	2.359	4.241	3.707
5.383	5.258	4.498	3.529	5.019	5.498
3.541	2.840	3.785	3.481	3.617	3.985
1.967	1.273	2.201	2.064	1.499	1.802
2.434	2.306	2.359	2.317	2.212	2.245
5.055	4.969	4.184	3.493	4.688	4.521
7.835	7.119	7.227	6.347	7.166	7.060
4.650	3.356	4.570	4.571	3.498	3.618
3.617	4.051	3.593	3.837	4.155	3.536
4.774	4.228	4.914	4.864	5.223	5.559
3.866	3.241	3.945	4.134	3.660	4.410
6.649	7.074	6.605	5.471	7.202	6.871
3.070	5.555	3.858	5.351	5.318	4.181
6.159	5.869	5.731	4.692	5.745	5.976
5.192	6.406	5.207	5.100	4.773	5.615
3.079	3.097	2.624	2.756	3.133	3.842
2.689	2.178	2.036	2.262	2.318	2.165
2.316	2.377	2.427	2.652	2.341	1.879
6.990	6.083	6.839	6.108	4.755	7.255
5.203	3.087	3.498	3.468	3.694	4.349
5.523	2.552	3.777	3.828	3.761	4.488
4.247	3.850	4.151	4.515	3.330	4.473
4.478	6.334	3.672	3.577	4.081	5.141
3.777	4.649	4.620	4.390	4.025	4.555
6.037	6.171	5.843	5.444	5.407	6.165
5.058	3.667	3.787	3.816	3.495	4.777
4.847	4.017	4.428	4.167	3.937	4.580
8.637	8.566	9.150	8.456	9.261	9.165
7.364	7.513	7.619	7.976	7.417	7.462
6.284	5.555	6.402	6.674	6.498	6.048

3.125	2.263	2.900	2.294	2.928	2.911
9.729	10.058	8.060	8.350	9.064	9.560
2.761	3.919	3.613	2.930	5.033	2.903
6.153	6.488	6.635	6.239	6.707	6.580
6.077	7.171	6.708	5.040	6.558	7.176
3.659	4.069	3.795	3.178	3.819	4.511
5.673	5.493	6.758	6.529	6.462	5.397
6.381	6.330	6.322	5.714	6.251	6.381
5.143	5.557	5.031	5.484	5.639	5.138
6.591	6.933	6.611	6.638	6.524	6.492
5.821	5.674	4.894	4.040	5.940	5.787
3.802	4.519	3.228	3.500	3.262	4.440
4.703	4.703	4.651	4.256	4.912	4.663
3.333	2.244	3.199	3.534	3.290	2.945
3.784	3.360	3.812	3.603	3.209	3.946
9.612	9.222	9.422	9.545	9.310	9.366
3.218	2.466	3.223	2.862	3.361	3.178
5.639	4.661	4.437	3.849	4.906	5.324
3.137	2.662	3.146	3.005	2.787	2.734
3.739	3.183	3.149	3.268	2.915	4.480
6.053	6.309	5.575	6.386	5.743	5.946
2.717	2.607	3.384	4.307	2.567	3.375
2.817	2.435	2.492	2.186	2.343	2.766
7.775	7.601	7.980	7.327	6.764	7.864
5.126	4.527	4.480	4.671	4.351	4.808
3.316	4.339	3.157	3.702	3.497	3.722
5.268	6.430	5.647	7.108	6.330	6.114
6.072	5.949	4.689	5.198	5.164	6.120
1.856	2.218	2.922	2.308	2.323	2.647
5.035	4.798	3.928	4.359	4.355	4.846
4.031	4.278	3.950	3.636	3.704	4.522
4.766	4.801	4.917	5.017	5.214	5.408
2.354	2.603	2.811	1.971	2.452	2.575
3.453	2.815	2.713	2.808	2.472	2.551
3.423	3.897	3.928	4.667	3.464	4.412
2.928	2.610	2.263	2.903	2.852	1.518
4.295	2.724	3.643	4.610	3.606	3.979
6.337	5.954	6.327	6.058	6.701	6.278
6.077	5.650	5.875	5.662	6.459	6.174
4.028	4.178	4.075	4.716	3.530	3.795
2.769	1.268	2.679	1.604	1.245	1.506
8.141	7.694	8.262	7.920	8.256	7.230
8.234	7.596	8.266	7.968	8.316	7.172
4.157	4.087	4.116	4.367	3.840	3.528
3.126	2.976	3.133	3.883	3.126	3.424
3.287	2.975	3.258	3.937	3.190	3.321
5.780	5.868	5.784	6.042	6.259	5.946
6.965	6.695	7.069	6.805	7.288	7.512
6.328	6.165	6.578	6.131	6.620	6.381
6.452	6.274	6.523	6.126	6.783	6.570

3.104	4.400	2.692	2.895	3.414	3.254
8.654	8.719	8.983	9.110	8.912	8.581
6.308	6.112	5.424	5.073	6.116	6.128
6.792	6.926	6.439	5.898	6.790	6.941
3.560	3.605	5.082	4.326	5.083	3.762
7.957	7.988	7.896	7.692	7.067	7.880
7.620	7.201	7.424	7.228	7.323	7.622
4.790	4.127	4.489	5.090	5.277	4.398
5.864	5.620	5.740	4.733	5.726	5.828
5.075	4.876	4.982	4.428	5.134	4.681
4.957	4.797	4.947	3.806	4.891	4.421
3.524	3.703	3.319	3.637	3.247	3.664
2.944	3.368	3.056	3.068	3.094	2.265
3.441	3.404	2.651	2.269	3.960	2.998
5.747	5.301	5.802	5.690	6.114	5.298
5.844	5.315	5.554	5.606	5.034	5.277
5.775	6.158	5.425	5.294	6.766	4.960
6.375	6.821	7.042	5.334	6.154	6.867
5.182	4.336	5.759	3.821	5.550	5.698
6.527	6.344	7.099	6.672	6.876	6.222
8.365	8.439	8.068	8.493	7.785	8.314
5.687	5.551	6.285	4.639	5.455	5.486
4.251	3.867	4.033	4.288	3.887	3.774
5.367	5.046	4.930	4.832	6.321	5.281
3.955	3.226	3.908	3.323	3.680	3.830
4.108	4.146	4.395	4.602	4.083	4.114
5.641	4.967	5.439	5.671	5.309	5.468
3.667	3.016	4.013	3.980	2.488	3.361
3.136	2.929	3.484	3.839	3.427	3.132
6.191	6.498	6.356	6.998	7.033	6.225
3.850	4.361	3.158	3.136	3.750	3.268
2.242	2.578	2.785	2.460	3.549	3.166
3.432	3.906	3.530	2.943	3.128	2.703
5.398	4.756	4.688	4.493	5.489	4.793
3.677	2.739	2.696	2.981	2.911	3.228
2.378	3.335	2.723	3.518	2.241	2.466
3.536	3.345	2.076	2.147	2.169	2.915
1.806	2.029	1.966	1.829	1.889	1.166
6.334	5.948	4.971	4.868	5.269	6.128
7.239	7.212	6.518	5.668	7.232	7.457
5.638	5.314	4.839	5.924	5.077	4.719
3.133	2.335	3.627	4.426	3.713	2.006
2.628	2.511	2.846	2.580	2.062	2.820
3.605	3.062	2.871	3.156	3.349	3.407
5.692	5.297	4.443	4.749	5.420	5.685
6.886	7.911	7.090	5.230	7.427	7.832
2.523	2.147	2.183	2.681	2.828	1.855
4.685	3.969	4.439	3.600	4.714	4.827
2.039	2.487	3.362	3.216	2.299	2.335
3.282	2.687	2.683	2.360	1.869	2.357

4.334	4.764	3.495	4.021	3.728	3.733
5.340	5.808	6.385	5.581	6.580	7.302
8.999	8.633	9.240	8.323	9.357	8.966
4.943	4.526	4.235	5.214	4.755	3.385
2.408	2.229	1.817	1.802	2.473	2.357
2.932	3.438	2.658	3.411	3.042	3.098
8.230	8.396	7.651	7.709	7.895	8.264
3.752	3.097	3.299	4.347	3.747	3.773
3.891	3.623	4.267	3.670	3.328	4.152
1.905	1.490	1.784	1.620	1.788	1.775
5.347	4.952	4.175	3.344	4.222	5.374
4.222	2.343	3.627	3.093	3.238	3.433
3.392	3.081	4.193	3.453	3.189	3.698
3.433	3.761	3.423	3.619	4.834	3.528
3.658	3.128	2.690	3.622	2.772	3.489
6.044	5.819	5.202	5.074	4.778	5.312
4.461	5.084	3.821	3.103	3.801	3.956
2.910	4.158	2.156	2.621	2.910	1.694
10.229	9.560	10.216	10.078	10.358	9.898
4.324	3.571	4.209	2.718	3.751	3.607
7.060	6.452	6.960	6.841	6.956	6.971
6.400	5.974	6.285	6.586	6.526	5.670
4.525	5.238	5.134	4.699	5.615	5.395
4.770	5.668	3.374	3.837	3.949	3.425
2.084	3.699	2.194	2.725	2.793	1.626
6.034	6.226	6.215	6.245	5.586	5.836
5.729	5.133	5.802	6.289	6.262	5.010
2.148	2.848	1.743	1.804	1.797	1.517
3.883	3.493	3.742	3.827	4.077	2.553
3.188	3.199	2.627	2.493	2.017	2.373
4.562	4.421	3.939	3.987	3.567	3.456
6.172	5.773	5.197	5.905	5.739	5.887
4.409	3.958	5.065	4.376	3.415	3.815
5.898	5.797	2.881	2.680	3.545	3.079
5.348	3.575	4.708	4.231	3.782	4.314
6.570	5.323	5.812	5.588	5.878	5.904
5.900	5.965	6.347	5.603	6.399	6.313
6.666	6.192	6.958	6.815	6.202	6.428
4.558	3.680	4.259	4.225	4.430	4.253
4.625	3.611	4.421	3.588	4.283	4.101
6.469	4.784	5.737	4.915	5.845	5.653
5.870	4.820	5.343	5.546	4.915	4.955
5.940	5.469	5.794	5.760	6.033	5.950
7.249	5.832	7.028	6.892	5.444	6.488
4.608	4.145	4.133	3.225	3.750	4.218
5.700	4.284	3.208	3.550	4.242	4.868
4.218	3.913	4.433	4.430	4.069	4.101
4.615	3.671	4.423	4.250	4.233	4.338
2.771	2.396	2.888	2.261	3.186	2.369
5.876	6.086	6.122	6.524	6.178	6.390

4.430	2.852	3.099	3.559	3.196	3.509
1.944	1.915	1.437	2.499	1.573	1.442
7.650	6.514	7.011	6.406	6.979	7.146
6.630	4.802	4.847	5.322	5.496	5.423
3.692	3.054	3.084	2.880	2.471	3.151
2.451	2.261	2.626	2.361	1.677	2.158
7.020	7.289	7.041	6.222	7.540	7.269
4.675	5.310	4.428	5.146	4.948	4.895
4.575	2.828	3.346	3.211	3.701	3.861
2.498	2.331	2.606	2.505	2.687	2.285
2.856	3.477	3.319	4.925	3.872	2.950
3.041	3.370	2.717	2.304	2.087	2.881
2.343	2.134	1.849	2.298	2.219	1.797
2.000	1.460	1.874	1.720	2.237	2.204
2.746	3.710	3.508	3.224	3.057	3.278
4.145	4.011	4.057	3.654	3.890	4.353
6.821	6.869	7.078	6.589	7.461	7.106
3.348	3.208	3.717	3.438	3.369	3.233
6.605	6.150	6.366	5.448	6.480	6.581
5.691	4.853	6.283	6.294	6.043	4.807
1.932	2.055	2.765	2.935	2.462	2.451
5.795	5.110	5.667	5.522	5.336	5.272
4.508	3.433	4.165	3.829	3.585	3.148
6.101	4.125	6.893	5.705	6.582	6.084
4.148	2.713	4.927	5.379	4.199	5.322
5.212	4.982	6.397	1.927	5.012	3.260
4.965	4.774	5.756	3.694	4.749	4.034
5.533	5.122	5.201	5.084	5.168	5.294
6.138	6.690	6.082	6.631	6.545	7.281
4.859	5.496	5.282	4.859	5.086	5.163
5.234	5.399	4.798	4.910	5.570	5.492
5.226	5.313	5.388	5.006	5.064	4.832
3.192	3.105	2.878	1.866	3.272	4.031
2.383	2.844	1.379	1.298	1.861	1.577
5.621	5.282	6.269	5.870	6.464	5.944
7.318	7.291	7.168	6.360	7.258	7.796
5.717	6.099	5.368	4.125	6.076	6.030
3.857	3.325	3.311	3.056	2.941	3.137
4.916	5.316	4.244	4.982	4.998	5.121
5.232	5.495	5.134	5.018	5.404	5.981
1.782	2.568	2.917	2.454	2.169	2.958
4.353	5.152	4.611	3.625	4.082	5.732
5.564	5.009	4.445	5.339	4.509	5.835
8.060	8.013	7.497	8.086	7.734	8.406
4.856	4.326	3.736	3.242	4.492	4.727
3.399	3.104	3.527	2.948	3.374	3.755
3.194	2.672	3.045	2.088	2.345	2.500
5.151	4.678	5.023	5.026	6.133	5.133
6.231	6.252	6.758	6.187	6.803	6.955
2.546	3.399	3.083	2.546	2.953	2.928

4.888	6.178	5.294	4.939	5.439	5.093
3.325	4.337	2.752	2.992	2.324	2.415
4.435	4.666	4.868	4.507	5.004	4.344
5.320	5.978	5.963	6.653	5.977	6.059
4.381	4.708	4.485	4.738	4.046	4.679
9.210	9.535	8.826	8.248	9.261	9.155
3.620	4.616	4.025	3.396	3.961	3.328
5.995	6.134	5.472	5.269	5.704	5.521
7.610	7.420	7.415	7.325	8.013	7.572
2.850	2.492	3.572	2.291	3.118	3.315
8.548	9.727	6.176	6.060	6.322	9.216
5.155	5.452	4.493	4.047	3.437	5.389
7.631	8.435	8.534	7.356	8.686	8.210
4.286	4.425	4.906	4.497	3.605	4.676
2.450	2.585	2.929	2.196	2.744	2.703
2.667	1.830	2.324	2.619	2.145	2.624
3.803	3.840	2.758	2.961	3.600	3.439
4.158	3.718	3.138	3.882	4.054	3.017
4.307	4.520	4.370	3.954	4.404	4.647
6.271	5.622	5.891	4.882	6.121	6.718
2.109	3.404	3.137	2.610	3.715	2.923
5.173	5.537	4.973	4.540	5.188	4.959
2.647	2.343	2.417	1.535	2.121	2.763
3.272	3.096	2.872	3.650	4.046	3.314
2.637	4.350	4.110	3.699	4.577	4.076
4.902	5.245	5.026	5.241	4.585	5.029
3.877	3.486	3.330	3.811	3.844	3.549
5.028	4.310	3.535	2.752	3.225	3.152
2.200	2.586	2.621	2.533	3.112	2.340
2.792	2.792	3.984	3.333	3.719	3.665
2.731	2.708	3.673	3.319	3.798	3.161
2.580	2.560	3.352	3.168	3.797	2.580
3.239	3.118	3.893	3.702	4.192	3.309
3.502	3.165	2.682	3.168	3.568	3.893
2.629	3.350	2.704	2.881	3.444	3.866
5.896	6.072	4.641	4.302	5.006	6.264
6.796	7.187	5.513	5.073	6.268	6.802
3.810	3.823	3.143	3.165	3.831	3.292
4.067	3.228	3.169	4.137	2.632	3.622
5.486	4.770	4.852	3.839	4.392	4.657
4.380	4.117	3.717	3.148	4.128	4.029
4.244	3.896	3.681	3.393	3.914	3.830
3.701	2.493	3.745	2.501	3.300	3.668
4.021	5.010	4.532	3.002	4.319	5.225
5.647	6.288	6.217	5.795	5.575	5.810
3.928	5.060	4.062	3.982	4.453	4.484
4.404	4.065	4.543	3.684	4.337	4.323
7.735	7.285	7.250	7.570	7.435	7.553
6.632	6.900	6.507	6.430	6.562	5.857
4.470	5.429	4.679	5.080	4.585	5.317

4.631	4.225	4.008	4.026	4.338	4.339
4.653	4.073	4.505	4.010	3.514	4.507
2.426	2.182	1.867	3.063	2.468	2.569
6.275	5.691	6.088	5.716	5.893	5.943
8.066	7.597	8.420	7.559	8.369	7.899
3.665	2.641	2.346	2.230	3.041	2.894
2.919	2.266	2.718	3.166	2.502	2.851
2.347	2.678	2.677	2.448	2.273	2.693
3.636	3.506	3.584	2.665	3.897	4.253
7.358	7.114	8.078	7.841	8.119	7.495
3.980	4.451	4.050	4.959	4.532	3.964
7.061	6.768	7.006	6.812	7.196	6.978
4.914	5.380	5.129	4.952	5.567	4.215
4.738	3.920	3.873	4.028	3.905	3.665
6.699	4.438	5.049	4.699	5.612	5.834
7.055	7.757	6.796	7.524	8.265	7.036
5.294	5.694	6.248	5.370	5.945	5.447
3.608	3.986	3.261	3.976	2.682	3.566
5.334	5.610	5.535	6.626	5.239	5.541
4.625	4.593	4.116	3.267	4.110	4.973
6.099	5.467	7.256	6.208	6.964	5.891
2.567	2.990	2.733	2.484	3.404	3.219
4.254	3.870	3.495	3.056	3.588	3.744
7.368	7.311	7.551	7.117	8.293	7.195
6.436	4.930	4.933	5.227	5.053	5.116
3.697	3.207	3.190	2.898	2.716	2.692
3.550	3.055	3.728	3.743	3.590	3.782
4.592	5.007	4.443	5.390	5.078	5.143
5.333	4.582	4.846	5.183	4.626	4.832
9.265	9.295	8.166	8.196	8.955	8.966
3.322	4.996	1.671	3.475	2.755	3.221
3.070	4.897	3.115	3.312	2.899	2.817
4.780	5.353	4.366	6.454	5.553	3.825
3.517	3.596	4.218	5.148	3.724	3.629
7.548	9.515	6.243	8.439	7.129	7.336
7.071	6.799	7.156	6.720	7.239	7.211
5.730	5.757	6.677	7.159	6.722	5.866
3.146	2.149	2.888	2.056	2.613	2.192
10.175	9.924	10.434	10.682	10.274	10.171
5.540	5.627	5.530	5.965	6.194	5.584
4.871	5.527	4.379	4.680	4.932	5.121
8.190	8.448	8.250	7.357	8.854	8.054
6.509	6.996	6.682	6.121	6.789	6.850
4.190	5.276	3.707	2.654	4.332	5.316
1.567	3.019	2.224	2.506	2.765	2.196
4.029	5.765	4.825	3.503	6.035	5.271
1.644	2.309	2.972	1.701	2.104	3.233
3.776	4.944	3.041	2.996	3.127	3.948
4.128	4.782	4.385	1.994	3.886	3.977
3.791	3.086	2.626	2.808	3.562	3.289

5.350	6.756	4.168	2.534	5.164	5.979
3.271	4.387	3.045	1.784	3.385	4.474
5.936	5.696	5.690	4.563	5.768	6.281
5.106	6.512	4.260	4.390	5.369	6.741
3.026	3.408	2.272	1.386	3.895	3.608
2.167	3.660	2.253	1.659	2.176	2.740
2.345	3.665	3.527	2.935	2.460	4.007
1.493	3.450	3.367	2.303	2.131	3.695
3.877	6.385	4.907	3.197	5.428	6.346
3.709	5.006	4.660	5.097	3.887	4.627
5.319	6.133	6.523	6.435	4.954	6.535
3.569	4.629	4.494	3.747	4.537	3.871
8.656	8.723	8.758	8.749	8.955	9.166
3.460	4.213	3.653	3.691	3.063	4.329
8.082	9.062	7.729	7.006	8.069	9.016
9.819	10.022	10.040	10.021	10.426	10.131
3.558	4.204	3.380	2.380	3.435	4.625
4.803	5.331	4.589	3.944	4.180	5.201
3.750	4.922	4.861	4.619	4.732	5.005
5.504	5.717	4.604	6.222	4.752	5.558
4.571	5.846	5.098	3.932	4.241	4.635
5.016	4.845	4.050	4.742	4.401	4.822
8.196	7.631	7.101	7.839	7.567	7.205
6.711	6.773	6.424	6.335	6.033	6.540
4.975	6.930	5.050	5.258	5.854	5.371
4.031	4.190	4.141	3.928	4.198	3.406
7.458	6.793	7.957	5.056	6.080	6.805
5.689	4.784	5.313	4.542	4.821	4.548
6.295	5.811	5.955	5.210	5.761	5.626
8.124	7.706	7.514	6.864	7.934	7.619
7.644	7.738	7.202	6.925	7.789	7.526
9.661	9.200	8.993	8.458	9.305	9.146
9.257	8.971	8.568	8.230	8.972	8.832
8.185	7.819	7.387	7.256	7.882	7.795
9.259	8.998	8.690	8.598	9.173	9.011
7.926	7.885	6.990	6.788	7.658	7.292
8.100	8.067	7.120	6.791	7.709	7.274
8.494	8.265	8.123	7.816	8.295	8.411
8.171	7.936	7.765	7.463	7.969	8.071
7.875	7.764	7.490	7.259	7.646	7.696
6.145	5.969	5.479	6.280	5.936	6.089
6.069	6.037	5.990	4.799	6.593	6.372
5.930	5.980	5.808	5.078	6.250	6.227
5.911	5.886	5.731	4.566	6.417	6.176
5.941	6.020	6.151	5.177	6.665	6.640
5.795	6.093	6.258	5.056	6.645	6.624
6.282	5.894	6.114	4.750	6.605	6.802
6.290	5.889	6.161	4.736	6.645	6.787
5.119	4.713	5.057	3.500	5.295	6.075
5.774	5.523	5.916	4.310	6.264	6.743

5.733	5.567	5.916	4.310	6.154	6.748
5.416	4.865	5.024	4.010	5.681	5.547
5.509	4.967	5.183	4.041	5.674	5.614
4.024	3.480	4.182	2.687	4.074	4.065
4.008	3.649	3.701	2.755	4.351	4.358
5.879	5.387	6.064	4.499	5.706	6.134
5.527	5.404	5.995	4.538	6.281	5.398
5.522	5.391	6.011	4.535	6.302	5.440
5.690	5.631	6.229	4.783	6.553	5.625
4.511	4.600	5.066	3.800	5.307	4.521
4.366	4.575	5.125	3.519	5.296	4.520
6.291	6.040	6.605	5.313	6.524	5.213
7.458	7.234	7.806	6.528	7.688	6.575
7.390	7.216	7.826	6.558	7.699	6.389
8.182	8.067	8.584	7.340	8.509	7.468
4.168	3.757	4.186	2.857	4.416	3.776
3.245	3.310	3.944	1.996	1.338	2.940
6.237	5.247	5.784	4.263	6.675	5.074
3.754	2.907	2.981	1.970	3.721	2.601
3.418	2.520	2.827	1.753	3.471	2.380
4.816	4.672	6.664	4.799	7.111	6.957
5.540	5.347	7.221	5.229	7.796	7.475
5.704	5.818	5.548	3.895	5.850	6.231
4.177	4.391	4.372	2.850	4.733	5.148
5.670	5.424	5.444	3.571	5.120	6.111
5.268	5.363	5.278	3.687	5.187	6.034
5.096	5.163	5.168	3.544	5.061	5.841
6.038	5.850	6.169	4.532	5.962	6.648
4.934	5.174	5.126	3.474	5.101	5.790
5.748	4.884	5.037	4.085	6.032	5.580
3.302	3.002	3.880	5.165	3.333	3.067
3.617	2.930	3.400	2.022	2.939	3.408
3.748	2.666	3.158	2.247	2.889	3.238
3.695	2.670	3.192	2.287	2.893	3.238
3.139	2.854	3.538	2.887	3.467	3.378
2.859	2.570	3.312	2.676	3.246	3.050
8.595	9.615	8.293	7.161	8.679	9.065
10.098	9.839	9.711	8.483	10.108	10.061
10.319	10.122	9.954	8.634	10.404	10.313
10.419	10.237	10.095	8.872	10.472	10.441
10.436	10.273	10.092	8.870	10.489	10.461
0.711	8.372	1.384	0.742	0.966	1.570
4.762	5.213	3.799	3.390	4.767	4.363
4.763	5.108	3.997	3.338	4.742	4.407
7.954	7.052	8.003	6.780	8.218	8.379
6.890	6.537	7.228	5.556	7.000	7.558
6.576	2.961	5.865	2.657	5.316	4.955
9.015	8.610	9.105	7.477	8.964	9.510
7.723	7.898	5.976	4.585	8.076	6.430
7.118	7.080	3.935	3.992	5.162	3.457

1.226	8.550	1.866	1.329	2.401	1.818
2.746	3.069	2.825	2.888	3.696	2.575
8.979	9.368	8.849	8.440	9.096	9.115
9.043	9.417	8.907	8.497	9.105	9.132
5.712	5.454	5.445	4.584	5.296	5.126
6.088	6.015	5.723	5.204	5.812	5.632
5.765	5.520	5.302	4.681	5.355	5.255
5.801	5.798	5.464	4.792	5.415	5.285
4.197	4.122	3.536	3.350	3.419	3.845
4.560	4.616	3.921	3.784	3.902	4.235
4.445	4.406	3.816	3.916	3.691	3.950
5.321	6.348	4.687	5.496	5.190	5.456
6.531	5.328	6.491	5.575	6.551	5.870
2.659	2.511	2.058	2.314	1.983	2.680
2.648	2.603	1.703	1.902	3.224	2.856
5.771	5.456	5.855	5.326	5.418	5.682
3.979	4.778	4.561	4.485	4.054	4.376
6.023	7.199	5.773	6.515	5.188	6.694
3.509	4.190	3.351	2.649	3.570	3.410
4.735	4.395	4.315	4.074	5.039	5.045
6.511	6.800	6.695	6.146	5.987	6.405
4.521	4.274	3.107	3.369	4.171	4.388
3.082	3.474	2.013	2.924	3.436	3.288
4.823	4.803	5.156	5.415	4.904	4.065
3.825	5.183	4.363	4.641	5.012	4.879
5.490	5.994	5.057	4.483	4.979	5.701
4.037	2.683	3.897	3.739	3.484	3.236
3.610	3.816	3.643	3.352	4.312	4.635
1.871	2.126	2.770	2.376	2.280	2.071
5.160	4.764	4.948	5.036	4.629	5.444
6.295	7.041	7.200	5.218	7.461	6.736
4.660	4.137	4.625	4.053	5.134	4.733
3.376	3.075	3.444	2.884	1.772	4.082
4.139	4.085	3.372	3.611	3.806	4.276
6.773	6.291	5.437	6.359	5.729	5.855
3.384	2.437	3.178	2.103	3.744	2.454
4.920	5.092	4.633	5.466	4.568	5.094
5.014	4.947	4.575	4.236	5.224	5.072
3.446	2.335	1.590	1.036	2.037	2.682
5.401	4.607	5.093	4.782	5.048	4.046
3.637	3.100	3.342	2.324	2.794	2.852
3.276	3.017	4.553	4.196	2.937	3.122
3.392	2.800	3.476	4.395	3.576	3.639
5.109	4.361	5.219	5.228	5.367	5.539
4.190	3.835	4.708	4.453	4.905	4.064
6.123	6.075	6.058	6.648	6.378	6.161
4.693	4.120	4.671	5.241	4.844	4.709
5.171	4.806	5.528	5.141	5.146	5.150
4.984	4.741	4.096	5.507	5.090	4.448
4.341	3.121	4.033	3.834	3.867	3.043

3.292	3.595	3.881	4.857	4.426	3.306
5.615	4.170	4.146	4.430	4.422	5.033
5.059	4.622	4.704	5.861	4.582	4.446
3.078	2.748	3.498	4.258	3.392	3.177
5.546	3.238	4.177	4.834	5.114	4.716
5.801	3.943	5.168	5.307	4.393	5.319
2.450	2.696	2.954	2.984	2.648	2.694
5.778	4.685	4.374	5.789	4.411	4.091
3.033	3.256	3.398	3.281	3.308	2.938
4.551	5.330	5.757	6.364	5.612	5.132
4.036	4.033	3.410	3.776	3.468	4.300
2.364	2.354	3.114	4.191	4.146	3.120
3.162	3.158	3.977	3.741	4.052	2.807
3.307	3.530	3.441	3.064	4.156	4.180
5.674	2.163	3.176	3.001	2.666	2.805
4.709	3.838	4.402	3.999	3.804	3.059
4.786	3.804	4.043	3.798	4.707	3.835
3.527	4.138	3.989	3.242	4.043	3.350
5.095	8.619	2.754	2.190	3.450	6.852
2.930	3.309	3.366	3.137	3.260	3.380
5.815	4.543	5.271	5.310	5.954	4.965
1.639	3.394	2.046	2.748	2.182	2.114
2.963	2.672	4.764	2.190	3.560	4.936
3.782	3.929	3.405	3.111	3.846	3.548
3.280	2.601	2.690	2.908	2.609	2.809
5.745	5.257	5.805	5.641	5.133	6.007
7.208	7.431	6.783	6.262	7.487	7.468
6.185	5.986	5.256	5.162	6.000	5.807
1.712	2.054	1.634	2.346	1.894	2.146
2.903	2.563	2.957	2.050	2.999	4.289
6.735	5.368	5.874	6.373	6.754	5.117
3.580	3.640	2.973	3.719	3.755	3.055
2.378	2.164	2.510	2.412	2.662	2.669
5.505	4.697	5.540	4.370	5.492	5.467
3.897	3.861	3.102	3.782	2.825	3.834
4.341	3.665	4.431	3.744	3.145	3.686
2.000	2.187	2.412	2.268	2.389	2.280
5.735	5.926	5.190	5.912	5.758	5.267
3.091	3.140	2.645	2.353	1.954	3.171
4.761	2.479	2.680	2.963	2.326	2.817
1.326	1.145	2.141	1.952	1.738	2.256
5.846	4.771	5.762	3.633	5.876	5.555
3.696	2.972	3.622	3.185	3.546	2.867
3.461	2.462	2.592	3.052	2.550	2.590
3.330	3.067	3.718	3.342	4.079	3.383
4.862	4.857	4.950	4.996	4.784	4.288
2.945	1.967	2.568	2.363	2.440	2.009
3.206	3.902	3.459	3.813	3.382	3.669
5.043	4.976	4.433	4.098	5.193	4.819
5.598	5.471	5.058	5.387	5.703	5.696

2.330	1.851	2.061	1.649	1.569	1.996
5.141	5.542	5.158	4.746	5.197	4.664
3.721	3.577	4.360	3.503	3.573	3.191
3.546	4.530	3.268	2.283	3.435	4.636
3.862	2.559	3.245	3.661	2.606	2.800
6.104	6.287	5.509	5.460	5.640	5.717
3.888	3.858	4.469	3.681	4.000	3.891
3.431	4.001	3.822	3.949	3.643	3.562
6.778	6.315	6.990	6.060	6.490	6.575
5.022	4.614	4.250	3.474	4.831	4.547
3.894	3.909	3.641	3.314	4.599	3.488
5.076	3.932	4.141	5.385	4.490	4.163
3.495	3.522	2.156	3.767	2.536	3.681
4.458	5.410	4.713	5.681	4.019	6.140
2.007	2.424	1.664	2.617	1.799	2.494
7.171	7.538	7.188	7.143	6.084	7.243
1.955	1.992	2.371	1.762	1.517	2.063
3.061	2.552	3.045	3.762	2.686	2.819
3.773	4.423	3.982	4.016	3.927	4.725
10.876	11.497	10.876	11.695	10.469	10.572
10.961	11.588	11.001	11.752	10.544	10.642
10.416	11.068	10.545	11.366	10.027	10.177
10.078	10.734	10.313	11.126	9.731	9.880
10.330	10.801	10.208	11.170	9.667	9.913
10.319	10.818	10.308	11.210	9.703	9.909
10.708	11.222	10.697	11.493	10.158	10.316
10.374	10.915	10.419	11.228	9.829	10.004
4.764	5.175	4.202	5.477	4.203	4.407
4.387	4.843	4.042	5.249	4.181	4.296
9.259	8.704	9.616	8.780	9.600	9.282
6.832	6.807	6.056	6.549	5.705	6.437
8.986	10.487	9.455	9.592	9.120	9.627
3.424	3.123	2.681	3.502	3.477	3.650
3.858	1.510	2.590	2.763	2.633	3.529
8.937	10.411	8.990	10.378	8.895	9.327
1.182	1.287	1.607	1.244	1.120	0.990
5.730	6.552	6.342	6.310	6.039	5.947
2.491	3.402	2.760	2.491	3.021	3.037
5.404	5.259	5.593	4.970	6.032	5.296
4.491	4.610	4.090	3.981	3.971	4.247
8.085	8.697	7.633	9.126	7.200	7.838
5.091	6.276	4.750	4.044	4.649	4.814
2.636	1.766	2.506	2.088	1.898	2.332
2.131	2.627	2.409	3.373	2.435	2.589
2.398	1.520	2.207	1.863	2.470	2.133
2.446	1.623	1.831	1.864	1.863	1.722
4.874	4.766	5.390	4.881	4.884	4.894
5.821	3.679	4.418	4.027	3.358	4.933
1.413	2.048	1.867	2.173	1.415	1.553
2.074	2.536	2.533	2.515	2.117	2.693

4.582	3.165	3.531	3.459	3.590	3.759
2.410	2.715	2.648	2.946	2.570	2.806
2.559	2.347	2.461	2.952	2.017	2.866
2.944	2.375	2.620	2.869	2.489	2.730
3.575	4.697	4.149	3.713	4.175	3.752
2.183	3.493	2.914	2.739	3.736	2.745
3.429	4.368	4.357	3.973	3.545	3.176
4.614	5.290	5.391	5.607	5.589	4.946
4.709	4.501	5.400	4.395	5.439	4.788
3.459	3.669	3.584	4.368	2.693	3.338
3.355	2.363	3.055	3.114	2.350	3.043
5.884	6.050	4.650	3.924	5.104	5.524
6.354	5.375	5.701	6.222	5.452	4.875
3.106	3.611	2.672	4.277	2.959	2.962
2.987	2.486	2.913	2.741	1.771	2.387
5.665	5.441	4.869	4.225	5.333	5.418
5.191	5.126	4.552	4.900	5.041	5.439
5.860	7.304	6.743	5.317	7.399	6.321
3.809	4.215	2.910	3.804	4.758	4.269
4.491	4.491	4.920	5.480	4.324	5.840
6.209	6.114	6.610	6.563	7.044	5.864
3.787	3.125	3.736	3.242	3.547	3.680
4.545	4.988	5.465	4.657	4.688	4.726
4.637	5.845	4.847	3.920	4.318	4.968
4.443	5.771	4.729	3.918	4.561	5.182
4.972	6.192	5.330	4.475	5.022	5.619
5.325	5.186	5.140	4.384	5.343	5.687
4.628	4.326	4.378	4.996	4.214	4.010
3.748	5.263	3.184	3.180	2.342	4.551
8.754	7.802	7.080	7.077	7.461	8.183
4.423	3.121	4.020	4.613	3.473	4.156
7.021	6.963	5.047	3.572	5.539	7.248
5.341	5.261	5.159	4.639	4.634	5.733
5.519	3.814	3.972	3.595	4.786	4.678
7.010	4.173	4.325	4.314	5.330	4.930
10.122	7.359	8.060	7.167	7.846	7.497
4.010	3.718	3.317	3.130	3.415	4.004
5.332	3.679	4.856	3.184	4.667	4.873
5.140	4.186	4.570	4.202	5.023	4.603
5.294	2.732	3.750	2.954	4.721	4.578
7.983	8.622	7.429	7.160	7.286	7.565
8.124	9.433	7.710	7.750	8.762	8.728
5.401	5.601	4.733	4.797	4.931	5.301
3.765	5.355	3.585	3.975	3.483	3.753
4.509	6.043	4.123	3.618	4.220	5.012
3.798	5.499	3.464	3.200	3.709	4.508
3.682	5.302	3.493	3.283	3.732	4.672
3.011	4.513	2.765	3.099	2.598	3.496
2.266	2.405	2.798	3.484	2.822	3.319
5.588	6.054	5.521	5.126	6.074	6.164

3.139	4.252	3.400	3.344	3.320	3.311
5.681	5.749	5.311	5.772	5.428	5.771
1.216	0.827	0.864	0.644	0.854	0.897
6.215	5.622	5.528	5.772	5.925	6.261
4.115	3.691	3.095	2.543	3.955	4.127
4.441	4.831	5.227	4.606	4.583	5.184
3.122	2.675	2.992	3.039	3.393	3.435
3.714	3.786	2.864	3.345	3.861	3.870
2.544	3.354	3.549	3.657	3.334	2.315
2.286	2.945	2.732	3.471	3.628	2.392
4.167	5.610	3.774	5.168	4.072	4.941
5.399	7.046	4.305	4.377	4.360	5.065
6.610	7.592	6.624	8.158	6.843	6.545
4.770	4.930	4.822	5.987	5.940	3.335
8.834	8.899	8.179	8.420	8.120	8.260
2.462	1.365	1.949	1.273	1.573	1.963
2.344	2.803	2.459	2.479	2.325	1.736
4.271	4.708	3.848	3.692	3.970	4.608
3.914	4.138	3.824	3.405	4.656	3.717
4.039	5.214	4.722	3.578	3.974	4.449
3.071	3.427	3.339	3.033	2.869	2.421
2.298	2.438	4.112	4.048	3.228	2.977
3.497	3.171	3.613	2.829	2.235	2.587
2.920	6.534	3.852	4.594	2.416	2.788
2.314	2.543	2.495	2.828	3.190	4.045
1.952	2.040	2.375	2.106	1.863	2.046
6.013	5.692	5.800	6.461	6.192	6.050
2.921	3.406	3.404	4.111	3.201	4.434
2.415	3.004	3.475	2.839	1.968	2.608
3.047	3.476	3.718	2.349	2.166	1.849
2.858	3.297	3.573	2.654	3.584	3.829
2.612	1.660	1.085	2.666	2.888	2.820
2.281	2.956	3.477	3.694	2.777	2.630
5.257	4.574	4.461	4.665	5.378	5.436
3.306	3.045	2.510	4.452	2.188	3.266
2.343	2.993	2.180	3.510	2.251	2.567
4.935	4.712	4.935	6.119	4.444	5.490
3.723	3.129	3.144	1.739	3.611	2.970
3.600	2.689	2.873	3.499	2.732	3.007
3.605	3.951	3.226	4.084	3.835	3.791
4.078	4.407	4.990	5.446	4.222	4.090
2.683	2.650	3.981	3.166	2.568	2.366
2.712	2.312	2.825	1.884	2.825	2.842
3.582	1.835	2.774	2.076	1.753	1.058
4.460	3.629	4.474	3.423	3.870	3.312
5.162	5.439	5.232	5.695	4.959	5.188
4.785	4.329	5.083	5.021	4.939	5.022
4.155	4.235	4.954	3.734	4.628	5.704
4.417	4.310	4.290	4.697	3.779	4.310
1.760	1.882	1.588	2.408	2.526	2.864

7.047	6.876	6.453	6.188	6.793	6.897
5.699	6.133	5.804	6.057	5.837	5.841
2.781	3.509	2.783	2.982	2.640	3.218
3.028	3.402	2.716	2.497	2.810	3.531
4.155	4.032	3.099	3.634	3.826	4.092
5.250	5.346	5.246	4.351	5.192	5.190
4.514	5.404	4.674	3.954	4.611	5.235
1.613	1.613	2.013	2.161	1.533	2.202
4.388	4.344	4.831	4.178	4.608	3.738
1.587	2.114	2.243	2.415	2.128	3.000
0.821	1.049	1.528	1.067	1.382	1.565
7.283	7.381	6.399	6.431	6.909	7.248
3.412	3.715	3.416	3.384	3.529	3.409
3.975	4.031	4.505	5.365	4.778	4.107
6.932	7.372	7.138	6.075	7.201	7.846
4.794	4.901	5.834	5.730	5.299	4.792
5.194	5.420	4.414	4.171	5.267	5.925
4.772	4.507	4.766	4.196	4.302	4.831
3.536	3.809	2.678	3.545	3.812	3.426
1.810	1.458	2.504	1.264	1.908	2.376
2.858	3.007	2.497	1.140	2.462	3.881
5.100	6.193	4.330	4.931	5.231	5.277
1.827	3.644	1.857	2.320	4.001	1.469
3.546	3.626	2.527	2.124	3.352	3.529
6.183	6.108	5.049	5.049	5.938	5.779
1.611	0.462	1.547	0.980	1.141	0.676
3.668	5.032	1.817	4.543	3.019	3.063
2.512	2.251	2.267	2.549	3.097	1.971
1.968	2.790	1.773	2.002	1.770	2.694
3.653	3.739	4.226	1.641	2.706	2.336
3.615	4.511	2.114	2.389	2.389	2.603
2.813	2.927	3.562	2.757	3.136	3.186
2.284	3.032	2.567	2.914	2.678	2.513
4.262	4.447	3.813	2.618	4.506	4.638
4.984	3.941	3.587	3.349	3.372	2.971
3.435	4.328	3.832	3.647	3.280	3.250
3.989	5.120	4.316	4.501	3.904	3.654
6.119	6.408	4.906	4.627	5.472	6.412
5.548	5.733	5.499	5.379	5.519	5.786
2.413	3.480	4.031	2.870	3.010	2.493
4.481	5.080	3.957	4.671	5.290	4.495
6.091	6.086	5.606	5.410	6.504	6.073
2.772	3.125	2.731	2.727	2.550	2.879
5.926	6.082	5.938	5.236	6.385	6.330
2.788	2.785	2.843	2.721	2.823	3.488
1.682	1.199	1.858	1.814	2.273	1.403
4.187	5.057	4.287	4.114	4.240	4.579
6.027	5.999	6.074	5.138	6.163	5.760
3.978	4.223	2.997	3.366	3.444	4.077
7.149	6.687	6.633	6.261	6.995	6.615

3.329	3.749	3.394	2.746	3.313	3.197
6.177	6.352	6.615	6.169	6.737	6.649
6.073	6.415	6.577	6.408	6.378	7.150
4.683	4.100	4.978	4.538	3.872	4.585
7.539	7.565	7.149	6.716	6.893	7.691
2.480	2.225	2.435	2.060	2.240	2.553
6.330	6.228	6.391	6.797	5.916	5.642
2.106	2.716	3.104	3.441	3.413	2.046
7.446	7.476	7.320	7.185	7.607	7.217
5.282	5.861	4.516	5.143	4.479	5.241
2.628	2.527	3.475	3.411	2.312	2.999
2.710	2.087	2.803	2.382	1.320	1.376
3.822	4.458	4.123	3.741	4.066	3.805
4.613	4.714	4.247	4.103	4.429	4.760
6.533	6.221	6.290	6.063	6.108	6.297
5.310	5.150	5.137	4.621	5.316	4.763
3.964	4.128	4.113	3.439	4.295	4.813
4.971	5.153	4.387	4.201	4.866	5.352
6.678	6.444	6.783	6.060	6.881	6.420
3.777	3.787	3.530	3.465	2.980	4.041
6.830	6.649	6.627	5.569	7.036	6.744
4.055	3.050	4.663	4.546	3.643	3.906
6.094	4.780	5.225	4.780	5.286	4.849
6.524	6.260	5.900	6.085	5.944	6.125
6.398	5.394	5.979	6.018	5.289	5.784
3.750	2.522	3.864	3.381	3.373	3.719
8.959	8.969	8.552	8.926	8.383	8.871
3.654	2.272	3.567	1.931	2.160	2.603
8.974	8.671	8.390	8.606	8.204	8.510
8.949	8.834	8.833	9.049	8.444	8.731
3.557	3.181	3.308	2.303	2.898	2.618
4.334	3.276	3.974	4.021	3.692	3.977
2.967	2.091	3.042	1.739	2.722	2.659
5.731	5.766	6.022	6.090	4.976	5.172
8.319	8.180	7.974	8.400	7.902	8.404
9.674	9.328	9.188	9.431	9.053	9.412
7.906	7.803	7.393	7.687	7.408	7.681
7.389	7.392	7.092	7.334	6.833	7.332
6.032	5.955	5.742	6.065	5.333	5.497
3.883	3.254	3.573	2.023	4.068	3.445
7.715	7.305	7.158	7.553	7.061	7.564
6.743	6.366	6.585	7.086	6.277	6.398
7.168	6.768	6.397	6.617	6.402	6.911
6.436	6.382	6.232	6.230	6.047	6.410
4.139	4.049	3.050	3.946	3.164	3.465
7.471	7.159	6.638	6.809	6.755	7.494
6.273	5.967	6.476	6.746	6.023	6.065
5.417	5.286	5.122	5.526	5.032	4.560
3.551	3.465	2.952	2.568	3.435	3.196
7.699	7.911	7.220	7.253	7.554	7.778

3.741	3.858	3.624	3.545	3.626	3.466
6.939	6.365	7.177	6.946	6.917	6.739
4.888	4.179	4.204	3.844	4.570	4.341
4.739	3.297	4.344	4.888	3.515	3.918
3.743	4.024	3.296	4.813	3.021	3.909
4.937	5.211	4.945	5.543	4.956	5.396
3.411	3.559	3.297	2.206	3.596	3.477
5.903	6.116	4.303	5.308	5.607	5.196
3.725	4.314	3.265	3.173	3.637	4.394
6.461	6.117	6.009	5.366	6.390	6.340
4.619	3.435	2.912	2.859	3.718	3.529
6.430	6.983	5.395	5.995	6.323	6.182
3.716	3.808	2.857	3.604	3.163	3.214
4.732	5.199	4.255	3.982	4.347	5.380
6.485	6.207	6.033	5.847	5.378	5.980
2.665	2.992	3.028	2.676	2.149	2.403
5.173	4.203	4.613	5.185	4.046	4.706
3.436	2.829	3.177	3.597	2.978	3.393
7.167	8.198	6.644	7.009	7.007	7.017
3.129	3.031	3.758	4.136	3.816	2.794
2.488	2.459	2.435	2.179	2.914	2.110
3.760	4.647	4.249	4.757	4.437	3.687
3.446	3.377	3.751	3.434	4.209	3.278
3.829	3.712	3.665	3.463	3.352	3.416
5.583	5.156	4.846	4.789	4.620	5.423
2.507	3.878	1.428	2.546	3.240	2.330
3.655	3.209	2.731	3.303	3.632	3.547
4.333	4.670	4.797	4.261	4.093	3.640
3.669	3.294	3.473	4.022	4.149	3.794
7.263	7.254	7.018	6.413	7.323	7.381
5.327	5.313	5.691	4.260	5.797	5.852
2.695	2.431	1.917	3.118	3.265	1.729
5.821	5.122	5.060	4.149	6.407	6.281
5.352	5.267	5.084	5.124	4.846	5.497
2.559	2.631	2.599	2.023	2.906	3.117
4.605	5.129	3.811	3.882	4.238	3.721
5.290	5.574	5.647	5.470	5.571	5.664
3.389	3.890	3.464	4.023	4.063	2.653
5.146	5.014	4.827	3.773	4.476	4.200
3.602	3.715	3.723	3.070	4.065	5.200
4.737	4.573	4.860	3.724	4.930	5.136
9.071	9.251	8.982	8.900	9.184	9.141
6.160	5.090	6.150	4.396	5.928	5.931
4.624	5.109	4.680	4.120	4.412	4.157
2.988	4.282	3.407	2.861	3.711	4.428
1.985	2.542	2.183	3.136	2.034	2.533
3.940	3.954	3.194	2.552	4.079	3.204
4.098	4.507	3.413	3.021	3.752	4.252
7.293	8.031	7.771	7.243	8.158	7.750
5.453	5.688	6.342	6.454	6.502	5.445

8.299	8.467	7.993	8.691	8.769	7.801
5.446	4.889	4.807	4.845	6.385	5.131
4.972	4.044	5.861	2.842	4.863	4.148
6.376	6.008	7.166	7.237	5.971	6.674
2.096	2.007	2.594	1.860	1.831	2.158
8.876	8.762	8.650	8.949	8.802	8.577
3.946	4.627	4.311	4.846	4.913	5.083
6.423	6.189	4.359	4.959	5.272	5.258
1.450	1.646	1.994	2.128	1.165	1.545
7.807	7.839	7.624	7.641	7.457	7.961
4.737	4.720	3.932	3.414	4.358	3.805
3.135	3.477	2.251	2.683	2.344	2.530
1.834	3.323	2.091	2.248	3.276	2.688
5.143	4.722	4.011	4.066	4.542	4.594
7.712	7.580	7.476	7.993	7.221	7.416
4.779	4.256	4.835	4.382	4.631	4.592
3.762	3.413	4.795	3.335	3.689	3.546
4.094	4.750	2.576	3.243	3.334	4.436
4.790	4.296	5.109	5.294	4.042	4.034
7.196	7.517	6.799	6.495	7.428	7.233
3.585	3.122	3.577	2.976	3.173	4.078
1.655	1.782	1.599	1.008	1.588	2.002
3.239	4.437	4.276	2.848	4.576	4.278
2.446	2.999	2.358	2.762	2.205	2.956
6.132	4.643	5.217	5.696	4.266	5.480
4.038	2.885	3.514	3.388	3.450	3.393
5.246	5.088	5.868	5.401	6.531	5.981
8.174	9.017	8.863	8.294	9.186	8.350
4.734	5.300	5.458	4.683	5.805	4.841
8.559	8.396	8.064	7.576	7.998	8.093
5.135	4.493	4.430	4.801	5.073	4.413
5.400	5.081	5.844	5.826	5.879	4.863
5.947	6.513	6.376	5.583	6.416	5.349
3.527	3.699	3.747	4.510	3.325	3.344
4.201	3.128	3.199	2.020	4.232	3.913
5.433	5.290	5.262	4.730	5.316	5.264
1.380	1.103	1.133	1.379	1.179	1.562
4.962	3.331	4.598	3.220	4.665	4.554
1.043	1.547	1.234	1.198	1.294	1.446
4.921	4.128	4.292	4.409	4.382	4.345
5.410	5.300	5.739	5.213	5.020	5.516
3.744	4.234	3.961	3.590	4.541	3.563
2.907	2.373	2.187	1.779	3.003	2.762
4.038	4.797	4.731	4.805	3.406	4.594
5.913	4.917	5.845	5.939	5.140	5.586
4.165	4.365	4.036	3.776	4.044	4.397
4.386	2.245	3.223	2.159	3.311	3.763
4.248	3.505	3.766	3.882	4.671	3.803
5.455	5.886	5.974	6.157	5.425	5.342
4.389	3.602	3.874	3.662	3.578	3.924

3.047	2.730	3.128	2.646	2.120	2.904
4.028	3.632	3.547	4.574	3.654	3.511
5.057	4.362	5.118	4.434	4.531	4.119
4.315	2.518	3.265	3.064	2.114	3.688
4.099	3.309	4.076	2.866	2.893	3.382
5.138	3.095	3.557	3.149	3.547	4.904
5.023	4.324	4.886	4.425	3.972	3.965
3.495	4.671	3.352	3.533	4.716	3.540
7.059	6.157	7.133	7.252	6.685	6.208
6.237	6.630	5.351	5.248	5.051	5.910
2.440	2.082	3.054	3.776	2.449	2.492
2.612	3.515	3.402	3.466	2.420	2.739
4.002	6.326	4.418	4.761	4.561	4.000
3.447	4.108	4.388	4.245	3.613	4.266
2.126	3.437	2.641	3.274	2.303	1.842
2.887	2.513	2.840	2.536	2.033	2.205
5.609	6.084	5.266	4.167	5.396	5.562
2.902	3.860	2.965	2.763	2.252	3.370
3.882	3.823	3.735	3.816	2.842	3.625
3.104	2.399	2.594	2.926	2.501	2.712
1.941	1.615	2.053	1.500	2.073	1.867
4.720	2.593	3.261	3.232	3.231	3.186
4.493	2.776	3.341	2.616	3.209	3.899
7.046	5.046	5.511	4.607	6.523	5.635
5.563	4.345	4.167	4.727	4.539	4.864
3.337	3.326	3.698	4.067	4.172	3.367
5.370	4.294	5.363	5.230	5.751	4.203
3.940	2.756	4.078	3.218	2.696	3.538
4.445	3.291	4.182	3.539	3.742	3.756
8.470	7.581	8.959	8.724	9.032	8.383
3.063	3.802	3.550	3.560	3.331	3.116
1.746	2.218	2.159	2.457	1.395	1.322
5.485	4.660	6.199	7.554	5.744	4.793
6.132	6.126	6.027	5.791	6.709	6.702
4.645	3.609	4.655	3.289	5.339	4.704
5.497	4.651	5.254	4.728	5.239	4.742
3.201	4.016	3.390	4.332	3.497	3.717
2.899	2.804	2.793	2.549	3.951	3.560
5.059	2.434	4.193	2.266	3.364	3.921
2.647	4.269	3.073	2.646	3.073	2.850
5.976	5.396	6.249	5.573	6.210	5.085
2.633	2.670	3.401	2.367	2.951	3.042
3.794	2.972	2.479	2.557	2.653	2.985
2.524	2.796	1.946	2.106	2.928	3.259
3.493	2.573	2.912	2.702	2.626	3.111
4.876	4.279	5.055	5.024	5.040	3.638
5.380	5.249	5.359	5.322	4.166	5.154
2.884	3.161	3.267	3.465	2.723	3.002
3.465	3.258	3.742	3.508	2.960	3.498
3.129	3.353	3.305	3.528	2.703	3.097

5.972	6.105	5.505	5.310	6.122	5.590
3.786	4.374	4.291	3.743	4.123	4.659
3.471	3.154	3.068	2.864	2.907	3.644
7.118	6.813	5.882	5.962	5.710	6.694
3.629	3.961	4.348	5.468	3.991	4.043
7.044	5.799	7.318	7.701	7.329	6.160
3.161	3.722	3.459	3.300	3.254	3.317
2.692	3.813	4.202	3.340	3.276	3.784
2.442	3.274	3.883	2.465	4.351	3.753
3.637	3.975	4.656	3.435	4.566	3.959
4.347	2.451	3.393	4.401	2.471	2.553
4.128	3.455	4.338	4.475	3.617	3.724
6.115	5.011	5.730	5.129	5.151	5.463
2.794	2.228	2.049	2.408	2.319	2.641
2.445	1.874	1.804	1.130	2.210	2.157
2.593	2.809	3.352	2.808	2.612	2.878
4.765	2.755	2.470	3.109	4.039	3.070
4.638	3.960	4.253	4.600	4.187	4.285
4.734	4.618	3.759	3.808	4.284	4.221
2.062	2.259	2.137	2.446	3.082	1.834
4.863	4.792	4.476	4.872	4.792	4.365
5.010	5.352	4.524	4.493	4.222	4.806
7.346	7.642	6.790	7.385	7.277	7.772
5.117	3.567	3.609	3.418	3.491	3.501
7.790	7.926	7.402	7.224	7.983	7.846
4.404	3.589	2.737	2.760	3.555	3.312
3.792	4.264	3.912	3.501	3.022	4.169
5.602	5.441	1.251	4.988	1.481	1.677
5.503	5.594	5.374	4.685	6.095	5.951
5.011	5.291	6.044	4.382	6.431	4.235
4.519	4.889	4.552	4.459	5.418	5.610
4.304	4.422	4.888	5.388	3.865	4.501
4.300	4.089	3.279	4.163	4.668	4.887
4.489	3.512	3.761	3.566	3.591	3.733
4.763	5.734	5.246	4.730	5.261	5.182
4.814	5.037	4.504	4.295	5.100	4.960
5.852	6.139	3.708	4.404	3.930	4.600
4.693	4.271	3.939	3.560	3.689	4.582
5.659	5.602	4.469	4.939	4.924	5.456
5.401	3.964	4.274	4.477	4.068	4.505
6.835	6.949	6.715	6.073	6.927	6.814
7.306	7.346	7.175	7.345	7.207	7.211
7.430	6.872	7.219	7.194	6.440	7.289
5.741	5.785	5.502	5.663	5.908	5.947
5.664	4.720	4.744	4.879	5.115	5.187
4.768	5.232	5.177	5.268	4.903	4.950
3.684	3.396	2.901	3.260	3.582	3.734
3.610	3.816	3.675	2.409	3.652	4.161
4.422	3.606	4.075	3.595	4.058	4.264
5.667	5.283	5.630	5.358	4.807	5.335

7.281	7.137	7.115	6.424	7.127	7.174
5.287	5.469	6.144	6.928	6.118	5.756
2.521	3.104	2.711	1.787	2.440	2.318
4.268	4.777	4.281	4.933	5.515	4.332
7.724	7.351	6.891	6.800	7.912	7.535
5.291	3.343	4.321	3.615	4.499	4.499
6.898	6.975	6.778	6.432	7.505	6.933
6.347	6.736	5.806	7.200	6.034	6.474
5.055	4.980	5.295	5.014	5.013	4.978
3.643	4.049	4.082	4.580	3.783	3.791
4.686	5.310	4.694	5.582	5.158	4.532
5.859	5.577	5.333	5.651	5.195	5.343
6.724	6.158	5.994	5.843	6.434	6.443
2.257	2.356	2.992	3.140	2.420	2.126
2.718	2.363	2.297	1.894	2.812	3.648
5.472	5.184	6.230	6.467	5.434	5.017
5.785	5.403	5.356	5.163	5.118	5.408
6.227	5.816	5.949	4.794	5.713	6.054
8.496	7.834	8.282	8.404	8.429	8.409
3.676	2.969	2.827	2.585	2.607	2.749
3.829	2.263	2.778	3.011	3.272	2.967
4.660	4.731	4.540	4.733	4.857	4.678
1.528	2.134	2.752	2.768	1.853	1.772
4.999	4.210	5.058	4.836	5.349	4.389
3.510	3.438	3.070	3.220	3.179	3.340
6.115	6.064	5.785	6.506	5.970	5.927
5.145	3.812	4.094	4.170	4.121	4.329
7.667	7.518	7.658	7.479	7.596	7.746
2.672	2.752	2.832	2.668	2.219	2.790
4.789	4.558	3.432	4.971	4.115	3.911
8.076	8.351	8.833	9.484	8.344	8.521
7.649	7.771	7.868	7.580	7.264	7.722
4.981	3.914	5.357	5.227	5.456	4.606
7.735	7.840	7.763	7.386	7.482	7.978
6.757	6.591	6.996	7.087	6.114	6.579
3.077	4.151	2.655	3.130	2.912	3.419
3.374	2.933	3.189	3.503	2.355	2.397
9.196	8.852	9.227	9.392	8.290	8.893
9.210	9.600	9.357	9.734	9.500	9.597
8.616	8.714	8.564	8.823	8.665	8.597
4.276	3.945	5.062	4.357	4.408	4.220
8.133	8.134	8.127	7.814	7.455	8.374
3.393	2.811	3.659	3.361	2.735	3.436
1.208	2.390	2.871	2.825	1.753	2.296
5.852	3.963	5.337	5.164	4.859	4.988
6.276	5.296	6.248	6.194	6.581	5.386
4.561	5.152	4.955	5.051	5.270	4.668
2.720	1.833	1.879	2.890	2.327	2.257
3.236	3.954	2.792	4.052	3.046	2.543
5.254	5.110	6.137	5.689	5.144	5.452

5.719	5.267	5.577	5.282	5.701	5.357
6.358	6.354	6.064	6.532	6.712	6.286
5.119	5.481	4.604	5.937	5.281	3.997
5.722	6.487	6.100	5.719	7.230	4.741
6.236	5.973	5.824	5.559	6.295	5.949
2.635	3.530	3.875	3.401	4.282	2.269
7.117	7.087	6.661	6.595	7.279	7.139
5.995	5.711	6.193	5.656	6.671	5.818
3.600	2.927	2.352	2.393	3.609	2.688
7.156	7.556	5.832	7.035	7.306	6.367
6.932	7.028	7.255	6.875	7.020	7.046
3.758	3.755	3.530	4.610	3.285	3.701
4.503	4.338	4.333	4.563	4.668	4.633
5.284	2.887	4.762	4.009	3.689	4.257
5.662	5.111	5.756	5.227	5.038	5.402
2.654	2.229	2.256	2.160	3.051	2.371
5.121	4.605	4.204	4.138	4.624	4.535
4.966	4.603	5.028	4.429	4.776	4.817
6.921	7.093	6.454	6.946	6.736	6.904
4.678	3.769	3.744	4.490	3.807	3.507
5.992	5.288	5.569	4.943	5.581	5.964
4.428	4.287	4.858	3.857	4.726	4.240
3.229	2.381	2.110	3.449	3.088	3.686
4.744	4.452	3.371	3.665	4.447	4.307
2.977	2.607	2.824	2.602	2.701	3.368
3.366	4.001	4.053	3.567	3.005	2.729
9.674	9.673	10.261	8.626	10.300	9.938
3.381	4.415	3.670	4.559	3.308	3.718
2.966	3.221	3.062	3.107	3.608	2.271
2.914	2.433	2.428	3.484	2.369	2.539
5.263	4.208	5.378	5.893	4.627	4.686
1.147	1.155	1.524	1.864	1.430	1.111
4.437	4.045	4.024	3.757	3.474	3.974
6.449	6.415	5.961	5.580	5.954	6.782
5.821	5.766	5.114	5.617	5.867	5.897
4.843	4.462	4.574	4.713	4.762	4.799
5.497	5.542	5.241	5.283	5.586	5.706
3.696	2.610	2.676	2.561	2.879	1.817
3.829	3.961	2.723	3.150	4.316	4.257
2.030	2.476	2.527	1.373	1.760	2.676
3.357	3.869	3.069	2.473	4.275	3.091
4.659	4.129	4.548	3.870	2.680	4.251
3.625	2.576	1.318	2.075	2.699	2.538
3.454	3.422	4.031	3.851	4.863	4.568
2.673	3.464	3.582	3.500	3.111	3.241
2.654	2.624	2.122	2.773	2.853	3.033
2.922	3.327	3.927	3.502	2.590	2.590
3.931	4.232	4.495	3.931	3.976	4.480
6.179	4.973	6.153	6.137	5.961	4.943
3.496	3.264	2.348	2.066	2.073	3.285

5.736	5.077	5.758	4.857	4.582	5.445
3.290	2.900	3.514	2.680	2.976	2.467
4.819	4.946	4.493	4.799	4.502	4.452
2.672	2.219	1.921	2.801	2.223	1.606
2.828	2.590	2.598	2.435	3.006	3.481
5.955	6.144	4.884	5.044	5.438	5.346
4.674	4.904	3.192	3.415	4.636	4.782
5.178	4.138	4.466	4.843	4.593	4.049
5.361	5.830	3.825	4.645	4.145	4.105
5.507	5.884	5.963	6.127	5.919	5.380
3.081	2.535	2.223	2.867	2.076	2.227
5.443	6.329	5.441	5.706	5.799	5.278
8.074	8.223	8.322	7.860	8.444	8.296
8.685	8.744	8.505	8.186	8.759	8.689
6.022	5.850	6.075	5.160	6.370	6.243
2.546	2.504	2.198	3.068	2.586	2.918
6.878	5.969	5.875	5.260	5.985	6.612
9.580	10.088	8.782	7.551	9.342	9.750
4.806	4.768	5.304	4.810	4.758	4.984
4.649	4.318	4.475	4.253	5.307	4.427
5.251	5.474	5.516	4.591	4.737	5.623
5.190	5.393	4.734	5.439	5.152	4.918
2.867	2.406	3.268	2.634	2.904	1.806
6.135	5.510	6.221	5.433	6.124	5.622
2.868	3.061	3.306	3.064	2.616	2.180
2.485	4.027	3.779	2.714	4.598	3.361
4.704	4.661	4.570	4.211	4.374	4.184
4.012	3.979	4.449	3.727	4.134	4.113
5.951	7.364	6.174	5.990	6.460	7.164
6.622	6.374	5.732	5.442	6.201	6.811
3.476	4.431	3.715	2.723	4.567	3.944
4.299	3.986	3.147	3.948	4.285	3.913
4.975	4.887	4.487	3.707	5.817	5.705
5.053	5.526	4.726	5.185	4.539	5.245
6.998	6.525	6.279	6.536	5.564	5.711
1.996	1.987	2.943	2.733	1.878	2.486
2.418	2.177	2.750	2.639	3.394	2.271
4.850	4.568	5.416	4.850	5.911	5.081
6.515	6.264	6.619	6.006	6.514	6.648
7.239	7.249	7.352	7.706	7.343	7.420
4.287	4.498	3.717	4.640	4.209	4.477
4.105	4.484	3.372	4.484	4.644	4.698
3.338	3.491	3.764	4.798	4.147	3.374
5.998	6.553	6.832	6.434	6.195	6.688
3.280	3.034	2.979	2.852	3.532	3.310
7.537	7.359	7.635	6.275	7.670	7.519
4.735	5.281	4.008	3.971	4.615	5.991
3.011	3.755	2.368	3.247	2.867	3.985
6.594	6.051	5.794	5.055	5.660	6.053
7.216	6.102	6.864	7.302	7.419	6.445

5.139	5.021	4.618	4.559	4.041	5.240
6.070	5.811	6.017	5.109	6.125	6.787
6.050	7.072	5.873	5.961	6.684	6.304
6.699	7.321	6.501	5.256	7.148	6.790
4.051	3.346	4.310	4.128	2.749	2.592
3.382	3.358	3.591	3.358	2.827	3.124
4.156	3.765	3.983	4.133	4.051	4.500
3.887	3.421	4.103	3.674	3.962	3.574
3.149	2.502	2.712	3.130	2.929	2.645
6.469	6.502	6.523	6.207	6.729	6.128
2.641	1.920	3.129	2.770	2.020	2.730
2.501	2.975	3.092	3.756	2.071	2.621
3.156	3.551	3.072	3.547	2.965	2.870
2.413	2.819	3.010	2.865	2.796	2.406
4.263	4.708	4.556	4.121	4.457	4.533
7.325	7.655	7.172	7.807	8.031	7.034
2.678	2.968	2.995	2.204	2.362	4.076
2.293	3.026	2.602	3.073	2.313	3.150
3.068	2.571	3.977	2.880	4.917	3.460
2.149	2.659	1.671	2.447	2.360	2.090
3.342	3.672	2.982	2.621	2.544	3.048
3.958	3.765	3.052	3.084	3.829	3.675
5.010	4.525	5.260	4.435	5.012	5.394
2.898	2.325	2.166	2.661	3.653	3.146
5.967	5.329	6.107	5.286	5.263	5.792
6.721	7.103	5.937	5.609	6.342	6.782
2.683	2.794	3.420	3.541	3.326	2.560
4.744	5.068	3.702	4.173	4.607	5.072
3.764	3.431	2.943	3.096	2.875	3.013
4.035	3.309	2.907	3.604	4.761	3.099
5.112	3.362	3.841	3.440	4.277	4.087
5.621	4.793	5.336	5.810	4.638	5.300
5.023	4.809	5.415	5.161	5.005	5.199
2.965	2.945	2.899	4.252	2.261	3.569
4.194	4.113	3.736	3.206	4.698	3.630
3.845	3.668	3.915	4.005	3.638	3.556
4.702	4.455	3.862	4.476	4.743	4.375
3.036	3.999	1.777	3.036	1.839	2.649
2.214	2.204	3.082	3.879	2.830	1.944
3.760	2.921	3.615	3.760	3.874	3.607
5.989	6.177	5.864	5.093	6.119	6.267
2.081	2.330	2.745	2.233	3.160	1.472
3.179	2.314	4.180	2.950	3.035	2.646
2.138	2.504	3.403	2.017	2.504	2.267
4.886	3.572	4.338	4.092	4.343	4.051
1.220	2.865	2.275	1.892	2.638	3.306
1.312	1.333	1.655	0.914	1.608	0.910
2.926	3.618	3.735	2.237	3.301	4.696
1.885	2.413	1.425	2.034	2.010	2.462
3.751	2.583	3.587	3.587	3.703	3.650

2.190	1.714	1.785	1.566	1.580	1.521
2.058	1.839	2.896	2.533	2.853	1.819
3.454	3.187	3.859	4.505	3.436	3.570
0.809	0.894	1.291	1.795	1.383	1.340
2.602	2.386	2.825	2.167	2.945	2.726
1.531	1.412	1.136	1.428	1.469	1.384
3.536	3.270	4.184	3.109	3.767	3.760
3.857	3.979	4.071	3.027	4.301	3.377
3.330	3.575	4.002	4.179	3.387	3.119
2.333	2.823	2.262	2.130	2.031	1.851
3.794	2.532	4.215	1.932	4.557	4.498
3.572	3.043	3.058	3.406	3.255	3.599
2.113	2.763	3.044	2.365	2.058	2.533
1.474	3.408	1.817	1.345	3.792	2.087
1.991	2.024	3.538	2.276	2.156	2.474
1.076	1.174	1.576	1.145	1.075	0.864
3.146	2.653	2.302	1.997	3.129	2.982
1.983	2.932	3.495	2.558	2.428	2.904
3.458	4.074	3.267	4.438	2.584	3.602
3.196	2.813	2.251	2.325	2.000	2.533
3.801	3.436	4.529	4.101	3.569	4.209
3.890	3.469	4.311	5.289	3.152	3.470
3.272	1.489	2.740	3.517	2.187	2.530
6.113	4.132	4.109	3.901	5.625	5.748
5.280	4.620	4.064	4.248	5.554	5.821
2.805	3.523	2.511	3.989	1.820	2.740
2.182	2.047	1.899	2.111	2.793	2.237
4.484	3.462	3.898	4.274	2.921	4.122
5.692	5.324	5.339	5.396	5.258	5.591
6.232	4.896	5.888	5.822	5.563	6.041
2.204	3.075	3.691	3.232	1.695	1.824
4.565	3.838	4.714	4.479	5.231	5.122
6.815	6.752	6.387	6.629	6.645	6.867
2.022	2.230	2.719	2.048	2.291	1.954
3.136	3.469	3.363	3.066	3.346	3.310
4.925	4.272	4.483	4.040	3.967	4.426
3.362	1.659	0.964	1.206	2.091	1.970
3.262	3.137	2.254	2.665	2.594	2.777
5.031	4.746	4.518	4.597	4.673	5.233
3.567	3.750	4.741	4.082	5.075	3.450
3.917	3.847	4.160	4.215	2.742	3.978
3.434	3.504	4.676	4.031	4.440	3.503
2.229	2.295	1.763	2.214	2.421	2.253
2.924	2.704	2.174	3.256	2.631	2.794
4.271	4.060	3.770	3.718	3.768	3.946
5.366	4.548	4.428	4.646	4.640	4.834
3.626	3.074	3.692	3.355	4.027	3.489
2.539	3.497	3.217	3.356	2.231	2.275
8.251	8.164	8.193	8.874	7.880	7.744
4.270	3.468	4.003	4.257	3.595	3.290

2.210	1.506	3.178	2.078	1.981	1.905
3.667	2.731	2.397	2.454	3.854	3.556
2.906	3.023	3.519	3.497	4.075	3.310
1.898	2.468	3.274	2.634	3.779	3.629
3.258	2.595	3.554	2.801	2.997	3.060
4.463	3.661	3.494	4.111	4.540	4.055
1.998	1.646	1.685	2.177	2.089	1.749
5.129	4.895	5.040	4.336	4.536	4.617
4.503	3.679	3.510	5.474	4.331	4.485
4.309	5.491	3.811	3.755	4.144	4.094
4.845	5.603	5.254	5.918	6.240	5.717
3.423	3.109	3.209	3.405	3.272	2.937
6.151	5.941	4.533	3.997	5.470	6.278
5.105	5.069	4.332	4.297	4.899	4.878
5.384	4.486	4.097	4.591	4.979	4.873
4.810	5.094	4.945	6.253	5.006	4.913
7.096	7.526	7.468	7.255	7.304	7.213
6.942	7.317	7.308	6.993	7.075	6.964
6.931	7.272	7.204	6.977	7.017	6.990
7.191	7.554	7.519	7.267	7.304	7.268
5.288	5.477	4.456	4.636	4.648	5.938
1.506	1.666	1.847	1.905	2.070	1.361
2.967	2.314	2.457	2.335	2.133	2.731
2.347	2.451	2.744	3.432	2.629	2.199
3.829	2.845	2.550	2.534	1.800	3.324
4.944	5.485	5.105	4.816	5.323	5.464
4.120	3.421	4.603	4.691	3.878	3.647
4.818	5.365	5.398	5.760	5.824	4.703
7.148	7.304	7.652	6.599	7.689	7.387
4.407	3.328	3.320	2.205	4.447	3.838
6.736	6.169	6.436	5.405	7.109	6.637
4.306	4.186	4.806	4.436	3.848	4.437
3.405	2.357	2.611	2.695	3.110	3.417
5.020	4.041	4.804	5.324	2.767	4.335
3.555	3.231	3.249	2.780	3.325	4.059
3.237	3.655	3.409	3.299	3.126	3.555
2.808	3.893	3.511	2.765	3.407	3.290
2.565	2.356	2.597	2.551	2.679	1.841
3.065	3.076	3.397	2.725	4.385	3.417
3.221	2.044	2.771	2.146	2.194	1.949
4.055	3.837	4.487	4.330	4.012	4.062
2.435	2.453	2.841	3.628	2.556	2.830
2.596	2.128	1.826	1.742	1.210	1.633
4.093	2.643	2.482	2.317	2.945	2.761
2.262	2.882	3.475	2.954	2.897	3.486
1.477	1.166	1.779	1.440	1.651	1.325
3.478	3.840	3.764	4.029	3.372	3.254
2.708	2.879	2.973	1.770	2.168	2.247
2.850	1.558	1.792	2.333	2.024	1.838
4.216	3.735	4.307	2.057	4.539	4.493

4.024	3.699	5.539	5.429	6.186	4.873
2.924	1.463	2.308	2.961	2.586	1.860
5.474	5.186	4.942	3.986	5.413	5.561
2.686	2.746	2.583	1.865	3.630	2.487
3.029	2.308	5.329	4.952	5.592	1.481
3.781	4.286	3.857	2.523	4.563	4.036
6.087	6.463	5.672	5.981	5.110	5.812
5.238	4.746	4.996	5.687	5.472	5.044
0.969	1.473	1.054	1.266	0.962	1.130
1.153	1.384	1.132	1.216	1.014	0.882
3.223	3.334	3.623	3.303	3.146	3.211
4.923	3.006	4.127	3.475	4.253	4.211
3.604	4.698	3.870	5.146	2.416	4.656
7.043	7.063	7.221	6.316	7.695	7.287
6.470	6.193	6.438	4.839	6.895	6.712
3.227	5.112	3.661	1.886	2.937	3.251
7.552	7.198	6.938	6.109	7.030	7.021
8.230	7.050	7.644	7.960	8.006	6.783
2.693	1.535	1.918	1.682	1.990	2.269
7.089	6.799	7.656	7.040	7.637	7.285
2.950	3.412	2.673	3.387	4.019	3.711
2.510	2.565	3.229	3.321	4.014	2.182
6.770	6.811	6.508	6.407	6.629	6.826
7.083	6.934	6.629	6.037	6.891	6.908
5.212	5.336	4.627	4.387	4.833	4.757
5.108	5.549	4.709	4.734	5.038	4.868
5.175	4.239	3.066	2.985	2.966	4.671
4.451	3.533	4.297	4.615	4.780	3.660
5.299	5.646	5.112	5.275	4.873	5.560
3.116	3.686	3.639	3.943	2.887	3.374
3.465	5.098	2.748	3.486	3.882	3.253
4.213	3.441	2.702	2.146	3.544	2.660
6.155	6.227	5.800	5.957	5.769	5.995
4.062	2.818	2.839	3.366	2.491	3.577
5.053	4.498	4.255	4.597	4.915	4.563
3.614	3.026	2.612	3.682	2.994	3.481
3.426	4.273	3.276	2.908	3.883	3.131
1.643	2.938	2.050	2.808	2.069	2.655
3.073	3.738	4.145	4.016	4.066	3.847
1.547	1.177	1.141	1.665	1.254	1.519
4.281	3.762	3.562	3.739	4.385	3.882
2.492	2.404	2.771	3.272	2.472	2.049
2.400	1.834	3.487	3.020	2.406	3.211
4.363	4.708	3.765	5.097	5.000	4.610
1.868	1.674	2.254	2.689	1.265	1.772
3.450	3.513	4.024	4.518	3.187	3.773
3.661	3.770	4.272	4.369	3.085	3.453
1.575	2.040	2.695	2.612	3.217	2.554
1.340	2.000	1.999	2.107	1.495	1.884
2.777	2.037	1.849	2.275	2.013	2.190

5.229	4.888	5.519	5.662	5.374	4.459
4.707	4.202	4.454	3.780	5.745	4.601
2.702	2.613	2.748	2.826	3.176	2.934
4.001	3.474	2.561	3.592	2.926	3.218
3.497	2.388	3.523	2.790	1.552	3.229
2.462	3.058	2.992	3.312	3.450	3.476
3.494	4.152	3.202	2.755	3.121	3.462
4.976	4.594	4.262	4.339	4.813	4.156
2.431	2.297	2.559	2.484	2.442	2.481
2.479	2.547	3.196	2.944	2.277	3.253
3.257	3.111	2.698	2.325	3.166	3.138
3.188	2.050	2.632	2.487	2.297	2.233
2.661	3.467	3.225	2.632	3.204	2.247
3.429	2.131	2.765	2.051	3.090	3.561
2.326	1.517	1.787	2.194	2.080	2.105
2.500	3.221	3.350	2.662	3.231	2.792
3.541	4.413	4.029	5.023	4.412	4.303
2.294	2.037	2.876	2.353	2.915	2.352
2.474	1.984	1.408	2.995	1.686	1.838
3.950	2.823	3.481	3.858	2.915	3.244
4.442	4.125	4.025	3.567	5.147	4.936
2.741	1.590	2.341	2.787	2.281	2.060
2.320	3.893	2.605	2.823	2.807	1.806
2.900	3.465	2.883	3.248	3.164	2.975
3.508	3.086	3.113	2.468	3.285	3.468
3.534	4.349	2.455	3.805	3.514	3.893
2.468	2.560	2.643	2.233	2.079	2.520
4.670	5.443	4.571	4.929	5.560	5.900
2.882	1.764	1.365	2.089	1.849	1.970
3.199	3.124	2.575	2.051	2.160	3.385
3.226	2.182	2.295	1.236	2.772	3.120
8.048	8.595	8.062	7.836	7.989	8.298
2.545	1.795	2.774	2.700	2.962	2.568
1.739	1.656	1.600	2.530	1.468	1.662
4.897	4.038	4.605	4.486	5.096	4.539
3.111	2.850	3.268	3.389	1.821	2.468
4.058	3.349	4.645	3.868	3.910	3.723
1.377	1.862	1.542	1.687	1.364	1.525
4.608	3.692	2.737	2.736	3.385	3.476
5.761	6.481	5.913	5.957	5.967	6.481
2.868	2.184	3.114	2.181	2.426	1.673
3.337	2.802	3.447	3.463	3.321	3.207
4.805	5.188	5.266	5.663	4.991	5.310
8.795	8.281	8.586	8.811	8.639	8.521
8.808	8.163	8.602	8.812	8.622	8.499
4.167	3.460	5.311	4.951	4.932	5.257
4.559	4.395	3.581	3.661	4.050	4.521
7.056	6.295	6.303	6.516	6.208	6.906
6.054	5.043	5.643	5.708	5.412	5.539
5.870	5.144	6.058	6.173	5.225	5.623

6.062	5.177	5.991	6.206	5.366	5.656
4.999	4.633	5.357	5.251	5.093	5.546
4.125	3.782	3.000	3.844	5.188	4.366
2.413	2.047	2.241	2.074	3.126	2.139
4.600	3.460	3.779	3.802	3.867	4.136
5.479	4.721	4.306	3.378	5.203	5.175
4.004	4.337	4.642	4.989	3.886	4.382
9.280	8.964	9.816	10.459	9.075	8.807
4.491	3.615	3.470	3.218	4.069	4.235
3.457	3.961	3.712	4.077	3.712	3.633
3.297	3.278	3.823	3.656	3.244	3.239
4.440	4.305	4.965	4.837	4.858	3.984
3.123	2.067	1.620	1.511	1.658	2.055
3.387	3.263	2.488	3.051	3.527	3.318
3.520	3.050	2.876	2.820	2.307	3.202
2.822	3.527	2.821	3.241	3.812	3.820
3.108	3.164	3.340	2.538	2.687	3.376
3.634	3.896	4.172	4.459	4.559	4.210
3.189	3.692	4.352	4.281	3.305	4.105
4.674	4.205	4.521	4.138	3.830	4.412
2.476	2.509	2.140	2.163	2.032	2.242
2.246	2.700	3.489	3.003	2.759	2.725
2.930	3.539	2.858	2.695	2.304	2.966
2.386	2.300	1.800	3.003	2.784	2.413
3.134	4.125	3.307	1.372	1.515	2.183
5.353	3.325	5.565	5.851	6.278	3.399
2.999	3.043	2.279	2.773	2.729	3.418
4.310	4.321	5.250	5.096	4.121	3.611
2.599	2.602	3.286	2.804	2.954	3.089
3.889	3.604	3.996	3.987	3.734	3.969
2.756	1.743	2.328	1.699	2.833	2.286
2.106	2.434	2.818	3.057	3.480	2.573
3.242	2.658	2.570	2.788	2.698	2.433
3.183	3.071	2.984	2.741	3.241	3.370
1.563	2.817	2.440	3.343	1.646	2.328
4.412	3.516	4.066	3.042	4.274	3.642
2.369	1.966	2.345	3.080	2.532	2.502
3.813	4.000	3.678	3.732	3.844	3.278
3.723	3.078	3.187	3.609	3.765	4.138
3.139	3.883	4.472	3.864	4.034	3.613
3.710	4.119	4.281	4.190	3.910	4.133
2.956	2.183	3.241	2.208	1.505	2.092
3.183	3.290	2.081	2.723	2.917	3.090
2.935	3.300	2.188	1.994	3.311	1.571
3.127	2.691	3.338	2.394	2.875	2.535
2.482	2.267	2.861	2.324	3.345	2.878
4.188	3.201	3.657	3.856	3.401	3.296
1.972	1.879	1.594	1.408	1.800	1.843
1.962	1.935	3.791	3.311	2.772	2.112
4.408	4.110	4.176	3.416	4.825	4.996

5.338	5.308	5.910	4.808	5.904	5.359
2.921	2.533	3.222	2.900	2.971	2.627
2.737	1.479	1.575	1.764	1.548	1.997
5.096	5.710	6.093	3.957	5.839	5.972
1.948	3.139	2.170	3.725	2.209	2.917
3.575	2.423	3.867	3.429	3.482	4.039
4.626	3.745	4.540	4.036	5.103	4.464
2.214	3.090	2.442	2.013	2.010	2.482
3.958	2.203	2.643	2.229	3.611	1.789
2.592	4.281	2.254	1.955	2.942	2.863
3.418	3.452	3.095	2.136	3.196	3.676
3.313	3.505	3.022	3.252	3.881	2.849
4.897	4.616	4.819	4.442	5.959	5.713
2.638	2.382	2.859	2.543	2.492	2.148
2.570	3.790	4.668	2.136	2.946	2.946
2.608	2.758	1.794	2.581	3.074	3.483
2.184	2.338	2.694	2.751	2.425	3.819
3.869	5.014	3.776	3.464	3.696	3.899
3.630	2.912	3.481	3.529	4.421	2.767
3.907	3.548	3.263	3.648	4.090	4.636
3.805	4.062	3.679	3.861	4.571	4.358
1.926	1.281	1.856	1.569	1.826	1.752
3.361	3.488	3.293	3.602	2.801	3.587
2.917	3.252	4.045	3.116	3.133	3.968
2.946	2.246	3.886	2.890	3.003	3.242
5.641	5.230	5.882	5.206	6.058	5.930
4.782	3.946	4.434	4.096	4.324	3.891
1.854	3.225	2.522	3.222	3.185	2.326
4.549	4.563	4.678	4.846	4.959	5.075
4.034	3.816	3.559	3.542	3.585	4.052
3.791	3.364	3.401	2.797	3.668	2.811
2.190	2.181	3.506	2.446	3.002	3.464
2.873	3.616	3.011	3.433	3.105	3.862
3.487	3.851	3.845	5.021	2.291	3.080
3.479	1.713	3.642	1.396	3.029	1.941
4.233	4.789	4.040	4.301	4.605	4.489
1.858	2.288	3.383	3.550	3.325	1.271
3.378	3.664	3.103	2.838	4.087	3.382
3.802	3.799	3.615	3.573	3.790	3.652
2.845	2.384	2.779	2.732	2.609	2.871
3.695	4.072	4.111	4.256	3.271	3.057
3.496	3.304	3.465	3.619	2.588	2.792
4.344	3.464	4.912	4.501	4.168	4.608
4.358	4.248	3.581	4.602	4.282	4.934
3.997	4.643	5.357	4.727	5.194	4.442
8.079	8.345	8.509	7.897	8.088	8.426
2.215	1.681	2.433	2.195	1.481	2.615
4.863	4.902	4.382	4.521	5.275	5.608
2.391	1.987	2.127	1.850	2.941	1.827
3.337	1.556	2.155	1.438	2.427	1.908

4.870	3.971	4.353	4.007	5.161	3.945
2.647	2.821	2.894	2.946	2.479	2.695
1.398	1.293	1.485	1.682	1.759	1.526
3.777	3.838	2.781	3.015	3.463	3.443
3.190	3.519	2.877	2.078	3.506	3.404
3.686	4.042	2.720	2.409	3.287	2.946
2.135	3.020	2.199	3.524	2.704	2.810
2.303	3.148	3.111	3.096	3.290	4.354
3.893	4.083	4.875	4.274	4.421	4.741
3.795	3.530	3.298	3.851	3.939	3.685
3.187	2.204	1.984	2.918	2.204	2.194
3.264	2.981	4.318	3.625	4.286	3.247
6.660	6.875	6.782	5.867	6.189	7.207
1.567	1.843	1.654	2.053	1.982	1.792
1.277	1.402	2.244	1.722	1.209	1.577
3.018	2.667	2.679	3.027	3.822	3.705
3.066	3.783	3.378	3.589	3.547	2.539
4.682	4.137	2.937	4.868	4.297	4.148
3.933	2.531	3.389	1.830	4.270	4.753
1.126	0.860	0.960	1.257	1.122	1.555
6.157	6.368	5.512	4.589	6.724	6.522
2.079	2.513	2.699	2.698	2.975	2.703
3.197	2.879	2.277	2.016	2.264	2.894
0.960	1.248	1.426	1.932	1.382	1.382
4.095	4.581	3.715	3.968	4.137	4.939
2.657	2.390	3.984	3.877	2.608	2.682
4.317	4.079	4.368	3.574	4.142	4.786
2.645	1.934	2.416	2.288	2.454	2.524
3.780	4.656	2.543	2.757	4.151	3.275
2.626	2.413	2.988	3.376	3.459	3.493
6.118	5.051	4.969	4.728	6.008	5.375
1.395	1.917	1.577	1.642	1.110	1.277
3.144	2.787	2.776	1.518	3.202	2.960
2.150	3.808	2.770	3.445	2.652	2.690
1.428	1.535	1.522	1.867	1.519	1.176
1.782	1.214	1.270	1.993	1.785	1.777
3.612	3.994	3.779	3.546	3.702	3.192
2.947	3.442	2.615	1.993	2.613	2.592
3.575	2.383	3.550	4.456	3.036	2.956
3.754	3.123	4.238	3.483	4.655	4.453
3.707	3.334	2.949	2.594	3.144	4.193
4.095	5.984	5.252	3.637	6.731	4.062
3.336	2.590	1.740	1.901	2.934	2.712
2.385	0.753	0.954	1.382	1.197	2.036
2.188	2.410	2.415	1.817	1.901	2.028
3.862	1.688	1.901	4.865	3.129	2.578
1.821	2.172	2.897	3.201	2.037	1.940
2.382	2.912	3.111	3.965	3.992	2.953
1.722	2.232	2.848	1.721	1.786	3.071
1.296	1.101	1.226	0.967	0.879	1.291

2.368	3.037	2.351	1.897	2.856	3.229
2.822	1.992	2.765	3.026	2.483	2.728
1.583	1.619	1.878	1.907	2.242	2.244
2.428	2.428	2.160	2.247	2.177	2.063
3.078	2.531	2.870	2.429	3.113	3.135
1.911	1.580	1.310	2.462	1.951	2.698
3.874	2.964	3.262	3.502	3.356	2.858
1.850	0.847	1.069	1.107	1.878	1.164
3.623	2.693	3.725	4.256	3.406	3.419
2.928	2.502	3.043	2.853	2.641	1.971
3.630	3.191	4.214	3.418	3.485	3.836
3.276	3.143	3.284	3.282	3.709	3.484
1.721	2.265	3.829	2.549	2.542	2.701
3.135	3.471	3.081	3.137	3.168	3.685
3.816	2.391	3.411	2.738	2.414	1.907
3.861	2.175	3.344	3.846	4.268	3.353
3.818	4.054	4.435	4.580	4.825	4.186
3.071	4.414	3.394	4.108	2.237	2.868
3.145	3.059	3.140	1.920	3.002	3.260
2.423	2.452	2.386	3.123	2.142	2.399
2.477	3.368	1.441	2.219	1.638	1.614
4.625	4.840	4.382	2.780	4.123	5.750
6.386	6.674	6.083	5.341	6.010	6.447
6.304	6.672	6.288	5.478	5.998	6.440
7.492	7.860	7.433	7.191	7.147	7.620
5.020	5.023	3.878	2.559	4.191	4.098
1.846	2.003	2.211	2.185	1.822	2.107
3.346	3.273	3.611	2.787	3.685	3.516
3.689	4.359	4.138	2.948	3.207	3.020
4.615	4.485	4.014	4.609	3.727	4.084
2.547	3.043	2.438	2.459	2.326	2.140
2.858	2.611	3.534	3.619	1.455	2.650
4.527	3.773	3.731	3.086	4.377	4.148
5.240	4.352	5.236	3.198	5.740	5.701
1.785	2.068	3.467	2.445	4.077	2.511
5.187	4.659	5.130	5.048	6.209	5.494
1.594	3.022	1.506	3.185	2.361	3.675
4.974	4.868	3.726	3.058	5.359	3.855
8.046	8.459	7.158	6.501	7.312	7.799
7.939	7.991	7.749	7.202	7.939	7.907
2.960	3.006	3.472	3.558	3.190	3.310
3.294	2.343	2.651	3.300	3.530	2.988
2.463	2.137	2.629	2.408	2.386	2.532
1.906	1.513	1.923	2.127	1.987	2.263
3.081	2.883	2.185	1.995	3.545	2.638
1.913	1.877	1.996	1.143	1.634	0.998
2.075	2.086	1.910	2.175	1.244	1.142
2.167	3.261	2.861	3.041	4.124	3.782
5.435	5.353	4.941	3.674	5.733	5.424
1.921	2.235	1.724	2.345	2.223	2.504

2.945	3.280	3.308	3.233	3.167	3.164
1.841	1.607	1.023	1.587	1.610	1.783
2.136	1.817	1.977	1.421	2.104	2.137
1.597	1.163	1.497	2.372	1.584	1.908
2.965	2.679	2.625	3.539	2.732	3.210
3.110	2.697	2.743	2.108	2.673	2.880
1.787	1.719	1.714	2.103	1.731	1.772
4.303	4.056	4.947	4.664	5.124	4.666
3.128	2.849	3.613	3.189	4.245	3.095
3.156	2.930	3.517	2.690	3.274	2.989
2.204	2.362	3.054	3.724	2.840	2.585
4.540	4.906	3.795	4.587	3.924	3.823
3.248	5.594	4.059	4.476	4.343	4.876
4.233	3.987	5.082	6.131	4.830	4.065
2.200	1.790	2.058	1.501	1.929	2.094
3.128	2.469	2.409	2.624	2.931	3.870
4.018	3.615	2.895	1.980	3.471	3.856
6.765	6.706	7.229	5.738	7.242	7.985
4.428	3.554	3.991	4.640	4.738	4.460
4.460	3.156	4.125	2.840	4.700	4.072
4.213	3.971	4.580	3.826	3.234	4.705
3.066	3.961	3.559	3.509	4.518	3.249
8.040	7.432	7.878	8.053	7.626	7.744
5.581	5.884	4.953	4.503	5.072	4.964
3.181	4.460	4.620	3.822	4.567	4.437
2.351	3.110	2.815	3.894	3.449	2.941
4.225	4.173	4.096	3.544	4.281	3.848
2.576	2.605	1.736	2.285	2.492	2.241
4.253	3.946	4.546	4.878	3.960	3.956
4.974	4.967	5.039	3.643	5.077	4.681
1.912	1.175	1.466	1.913	1.933	1.712
4.022	3.368	4.289	3.845	3.620	4.027
1.858	1.493	1.505	1.838	0.955	2.127
1.023	1.652	1.905	2.585	1.158	1.525
1.872	1.789	1.781	2.293	2.082	1.782
3.402	3.406	3.485	4.258	3.386	4.157
3.452	2.405	2.451	2.420	2.141	3.093
4.013	3.811	4.335	4.563	3.892	4.364
1.801	3.321	3.223	3.572	3.612	2.337
2.638	2.025	3.543	2.281	3.330	3.736
7.316	4.966	5.429	3.549	4.986	5.838
2.969	2.460	2.742	2.306	2.319	2.753
4.341	3.164	4.552	4.388	3.724	3.473
2.233	2.780	4.258	2.536	2.825	3.080
2.675	2.136	2.454	2.512	2.425	2.591
3.884	3.756	3.989	3.396	4.096	5.394
1.853	2.005	1.248	2.324	2.298	1.770
2.259	2.363	3.035	1.863	2.007	1.958
2.644	1.892	3.353	2.366	2.644	2.254
2.835	2.560	2.795	2.856	2.626	2.976

1.009	0.524	0.956	1.245	0.745	0.779
3.893	3.452	3.849	2.620	4.528	4.194
2.559	1.767	2.802	2.134	3.018	2.671
2.493	2.485	2.533	2.777	1.921	1.740
2.157	4.117	1.921	1.447	2.470	2.873
2.836	1.867	2.787	2.628	2.836	2.547
3.544	3.676	4.173	3.599	3.626	3.637
2.328	2.961	2.067	1.944	2.064	1.945
4.227	4.616	4.546	4.734	3.775	4.260
1.547	2.136	1.532	1.752	1.538	1.433
3.334	2.475	2.793	2.324	2.785	3.213
1.421	2.529	2.239	2.306	2.240	1.784
4.402	4.374	5.404	4.041	5.109	5.200
2.717	3.338	2.639	2.963	2.481	2.657
1.580	2.535	2.428	2.269	2.274	2.928
2.802	2.810	2.999	3.873	1.854	2.532
1.996	5.177	1.446	2.251	2.835	1.898
3.803	4.254	4.396	5.265	3.700	2.530
2.773	1.992	2.999	2.291	2.622	2.240
3.769	3.377	2.939	3.469	2.941	2.474
4.115	3.269	3.591	4.499	4.289	3.240
2.278	3.024	2.682	3.492	2.264	2.823
1.038	0.848	1.251	0.772	0.926	0.790
1.450	3.454	2.734	1.392	2.086	2.259
4.362	3.826	3.183	2.546	4.345	3.885
2.544	2.543	2.454	2.381	3.110	2.754
1.790	2.447	2.121	1.386	2.328	2.333
5.301	4.632	4.487	3.699	4.482	5.225
3.003	2.477	3.121	1.944	3.114	3.832
2.810	2.886	2.194	1.968	2.296	2.727
5.815	4.543	5.471	5.079	5.677	5.014
4.860	4.940	5.264	5.619	4.788	5.286
3.491	2.492	2.732	2.050	3.482	2.935
5.319	4.996	4.669	5.580	5.256	4.836
3.868	3.788	2.068	2.053	2.911	2.741
4.889	5.655	4.522	4.849	6.109	5.475
3.585	2.774	3.121	3.499	3.509	3.499
2.513	2.978	2.140	2.581	2.414	3.088
4.956	6.601	2.639	3.900	3.084	3.729
4.020	2.288	3.722	2.304	3.639	3.893
7.049	6.667	6.587	6.954	6.675	6.748
2.665	3.245	3.238	2.324	3.857	3.611
2.223	2.275	2.140	1.959	2.498	2.917
4.875	4.733	4.407	4.859	4.689	4.631
4.027	4.339	3.022	3.647	3.905	4.052
2.785	2.691	2.921	2.873	3.543	3.175
1.814	2.042	2.388	2.033	3.316	2.511
1.786	1.722	2.371	3.452	2.031	1.368
3.883	3.737	3.825	3.090	4.371	3.630
3.203	2.933	2.579	1.651	2.653	2.205

5.281	3.946	4.921	4.950	2.657	4.247
3.272	2.464	2.867	2.172	2.671	2.814
3.159	2.028	1.510	1.721	2.118	2.526
2.393	2.192	2.623	2.020	2.500	3.124
1.280	1.264	2.066	1.653	1.403	1.942
1.550	2.194	2.141	2.281	2.066	2.083
2.458	2.004	2.870	2.862	1.896	2.227
1.971	2.144	2.240	3.502	2.332	2.173
3.673	3.723	3.251	2.970	3.903	4.111
4.311	4.403	5.025	4.392	4.308	4.827
3.855	3.629	3.630	3.584	4.070	5.015
1.545	2.126	3.206	1.625	1.659	1.922
5.230	5.756	5.002	4.544	4.899	5.385
3.656	3.545	2.436	3.232	3.015	2.962
3.224	3.254	3.655	2.165	3.068	3.393
2.675	4.005	2.467	4.262	1.865	2.663
4.767	4.504	5.249	4.930	4.480	5.637
1.944	2.114	2.926	2.926	3.330	2.832
2.208	3.221	3.113	3.750	3.322	3.411
1.787	2.919	2.043	2.907	2.536	2.971
2.326	5.268	2.386	4.784	1.050	3.398
4.633	4.253	4.491	2.632	4.559	4.281
2.986	4.032	2.635	3.497	3.284	2.429
4.304	4.767	4.428	4.690	4.378	4.529
2.436	2.341	2.355	2.517	2.323	2.820
2.583	2.672	2.942	3.455	3.396	2.557
1.479	2.058	1.998	2.157	1.865	1.385
3.814	3.173	2.850	2.688	2.417	3.810
2.260	3.136	1.764	2.840	2.989	2.995
4.993	5.686	6.336	6.827	6.154	5.232
3.861	3.185	3.063	2.506	4.286	4.709
3.304	3.582	3.604	2.900	4.361	3.643
5.259	5.631	5.000	4.366	5.860	5.195
2.906	2.972	3.280	2.288	2.739	3.309
3.068	2.541	3.359	2.392	3.031	1.740
2.817	3.180	3.351	4.130	2.343	2.472
2.537	2.259	1.874	2.575	2.509	1.747
2.174	1.619	1.254	2.300	1.447	1.123
4.719	3.517	5.296	3.130	5.133	4.538
2.821	2.060	1.600	0.644	2.862	1.677
2.271	2.328	2.427	2.042	2.152	2.210
6.962	7.721	6.702	6.667	6.759	8.369
6.502	7.166	6.299	6.121	6.242	7.841
3.580	4.714	3.202	4.434	3.705	5.312
3.757	3.062	4.194	3.639	3.863	3.719
8.064	7.115	8.200	8.131	8.220	6.828
10.061	9.948	9.999	10.205	10.011	10.044
1.842	3.213	3.804	1.473	3.451	2.350
2.373	2.472	3.359	1.283	3.377	3.533
3.978	4.035	3.372	3.110	3.976	3.544

2.415	3.500	2.319	1.933	2.132	2.568
3.893	3.867	4.507	4.002	4.206	4.164
3.919	6.045	2.227	1.230	4.905	5.202
2.315	2.040	1.765	2.521	1.619	1.171
4.976	4.510	4.705	5.487	4.828	6.331
5.038	4.807	5.146	3.351	5.560	6.098
2.776	1.641	1.902	1.516	1.842	1.565
3.912	4.966	4.777	4.250	4.866	5.002
5.247	5.260	5.757	5.897	6.122	5.152
6.344	5.771	4.768	3.863	5.544	5.564
5.039	5.463	4.536	3.240	4.905	5.295
3.962	2.430	3.217	2.543	1.156	3.006
2.568	2.764	1.916	3.014	3.370	2.411
1.761	1.501	1.621	1.299	2.503	1.570
5.396	4.957	5.489	4.804	5.130	4.951
2.185	1.906	1.858	2.504	1.878	2.223
4.285	4.218	3.958	3.859	4.218	4.076
2.282	5.614	3.995	6.209	6.583	6.496
3.986	4.643	4.740	2.220	5.017	5.772
3.211	2.699	3.195	2.634	3.365	2.699
4.971	4.508	4.290	4.318	4.363	5.363
5.738	4.915	6.072	5.328	6.824	6.271
6.042	5.819	6.757	5.346	6.024	6.022
8.279	8.941	7.802	9.194	7.737	8.070
0.709	0.985	0.857	0.863	0.944	0.618
10.169	9.685	10.126	9.554	10.022	10.105
2.382	2.693	3.331	3.192	2.621	3.156
5.145	6.022	5.690	5.598	5.004	5.859
4.353	3.238	4.485	3.726	3.221	4.312
2.940	2.332	2.348	3.045	3.094	3.105
1.636	1.576	1.852	2.721	0.769	2.419
3.356	4.207	3.895	3.064	2.790	3.498
1.750	1.936	2.215	2.237	2.174	1.912
2.042	3.804	3.750	2.386	3.401	3.347
6.344	6.589	6.123	5.129	5.783	6.750
2.617	3.386	3.079	3.720	1.448	2.540
1.722	2.730	2.131	1.959	2.527	1.444
2.422	2.109	2.496	2.562	1.898	2.316
3.106	1.834	2.083	2.020	2.158	2.197
4.234	5.507	4.269	3.566	3.888	4.446
3.112	2.518	2.413	2.105	2.517	2.991
3.557	2.584	4.308	4.065	3.051	3.160
2.573	2.763	2.337	2.006	2.660	2.997
3.547	3.796	5.031	4.168	4.169	4.400
2.672	2.671	1.991	2.271	2.402	2.094
3.381	3.916	3.458	3.833	3.393	3.668
3.147	2.726	2.224	3.942	2.043	1.775
4.048	3.489	3.340	3.633	3.753	3.762
5.608	6.064	4.325	5.034	5.220	5.010
2.827	3.915	2.830	2.512	3.085	2.659

3.368	2.705	3.413	3.574	2.902	3.323
6.412	5.705	4.392	4.096	4.371	5.435
3.566	4.327	3.963	4.069	4.082	4.356
8.790	9.494	9.119	9.028	8.900	9.362
1.272	1.222	1.514	1.556	3.337	1.409
2.707	3.305	3.565	3.718	4.042	3.023
3.345	3.205	3.575	2.888	4.223	4.202
2.678	2.194	2.748	1.734	3.253	3.755
7.270	7.747	7.429	6.157	7.794	8.040
6.047	5.755	5.764	5.146	6.197	5.922
5.636	5.865	5.733	5.678	4.784	5.809
3.279	2.179	3.295	3.507	3.222	2.787
7.027	6.344	6.397	6.400	6.738	6.875
7.033	6.519	7.156	7.112	6.990	6.658
4.970	4.780	4.479	4.861	4.143	4.625
7.049	6.592	7.026	6.842	6.827	6.653
5.050	3.978	3.371	3.593	3.441	2.108
3.630	4.686	3.650	3.422	3.918	3.702
4.732	3.628	4.542	4.130	5.490	4.304
5.704	4.337	5.068	4.763	4.879	4.364
5.719	4.081	5.321	4.641	4.880	5.356
3.799	2.369	2.471	2.673	3.033	2.977
4.903	4.758	4.992	4.588	5.508	5.700
2.781	4.291	3.500	2.583	3.865	2.306
3.465	3.024	3.333	4.268	3.016	3.316
3.498	3.072	2.577	3.524	2.769	3.177
3.459	2.425	3.341	3.194	2.791	2.809
3.523	4.355	2.994	4.204	3.289	3.208
4.466	4.494	3.590	4.543	4.260	3.475
5.095	5.504	5.360	5.173	5.357	5.463
4.726	4.448	4.535	4.313	4.646	4.314
4.872	5.004	4.385	5.132	4.536	5.077
7.002	6.207	6.840	6.802	6.967	6.584
6.566	7.005	6.824	6.103	7.468	6.946
5.194	6.355	5.634	6.273	5.365	5.029
4.252	4.335	4.718	3.919	4.029	3.928
4.468	4.831	5.243	5.185	5.368	4.613
5.173	4.838	5.202	5.117	4.546	5.252
3.312	3.105	2.902	2.870	2.372	1.958
6.949	5.620	6.191	6.659	6.478	5.989
2.483	2.509	2.852	3.192	2.630	3.132
3.087	3.223	2.050	3.552	2.998	3.328
2.209	3.356	2.169	2.145	2.815	3.600
4.520	4.723	3.276	3.661	3.782	2.725
5.166	6.172	6.673	7.340	6.063	6.194
5.217	4.822	5.478	5.629	4.746	3.776
3.812	3.782	4.000	3.635	3.750	2.577
4.958	3.908	4.927	3.533	4.869	5.083
2.511	1.955	2.195	2.421	2.483	2.275
3.496	3.784	2.625	2.508	2.729	3.610

3.230	2.892	3.071	3.611	2.359	3.218
4.211	3.155	4.616	4.518	3.813	4.295
5.243	5.138	4.866	3.650	5.509	5.197
2.534	3.731	3.508	3.741	1.735	2.789
4.304	4.209	4.722	5.040	3.315	3.949
3.628	3.589	3.664	3.238	3.817	4.135
6.660	5.386	6.278	6.233	5.808	6.312
1.505	0.972	1.265	1.762	1.053	0.890
5.703	5.131	4.523	4.290	4.230	6.145
4.529	5.033	4.829	5.152	4.401	4.656
4.693	4.579	4.563	3.938	4.959	5.396
2.112	1.251	1.139	1.111	1.369	1.056
6.079	4.975	4.598	4.677	4.350	4.504
2.519	3.396	2.786	3.885	2.546	3.015
2.994	3.280	3.359	4.366	3.121	3.614
5.921	6.028	5.262	4.114	5.923	6.237
2.411	3.987	3.427	3.672	1.515	2.746
5.449	4.953	5.182	4.748	5.488	5.315
5.269	5.162	4.719	5.117	4.840	5.348
3.167	3.809	4.164	3.287	3.510	3.676
5.855	6.036	5.261	4.747	5.957	6.223
4.889	4.239	4.970	5.465	4.772	3.794
4.628	3.472	4.759	4.173	3.812	3.729
3.975	3.158	4.136	3.887	3.820	3.034
3.966	3.918	4.098	2.742	3.522	3.972
7.263	6.252	5.884	6.392	5.899	6.207
5.782	5.286	5.101	5.227	5.285	5.593
5.143	4.385	4.394	4.312	4.507	4.524
2.776	2.801	3.277	2.190	2.931	3.137
3.584	3.371	3.213	2.844	3.266	2.956
4.279	4.203	4.824	4.956	4.280	4.509
3.818	2.985	2.948	3.235	3.573	3.729
2.748	3.136	2.970	3.352	2.773	3.239
4.064	4.350	5.100	5.129	4.256	4.325
7.159	7.620	7.596	7.365	7.084	7.320
3.782	3.194	3.751	2.833	3.411	3.930
3.987	3.407	4.038	3.001	3.495	4.334
3.935	3.306	3.977	2.863	3.373	4.246
3.997	3.488	3.965	3.005	3.463	4.390
4.214	3.794	4.260	3.223	3.780	4.548
3.920	3.251	4.021	2.919	3.423	4.428
3.042	2.447	2.560	2.408	2.190	2.179
5.751	6.560	5.741	5.745	5.915	6.067
4.297	4.471	3.893	4.215	4.933	4.171
6.002	6.514	6.926	6.720	6.421	6.131
4.501	4.904	5.257	3.965	5.920	4.905
6.435	7.085	5.958	6.388	6.143	6.699
2.978	3.739	6.161	3.516	6.038	7.914
5.852	6.216	5.844	4.441	6.179	6.144
4.632	3.383	2.795	2.306	2.831	2.965

5.641	4.271	4.577	3.138	5.224	4.905
7.816	7.220	8.134	8.006	8.208	7.578
3.997	3.398	3.330	3.696	4.214	3.202
3.149	3.160	3.203	3.288	2.576	3.394
4.887	4.596	4.546	3.809	5.217	5.064
7.263	7.111	6.463	5.350	7.219	8.304
1.561	1.486	1.710	1.472	1.542	1.240
3.121	3.785	2.104	2.701	2.611	3.824
3.599	3.758	3.545	3.474	3.710	4.186
5.988	6.287	5.169	4.925	6.275	6.673
3.842	3.224	4.120	4.261	3.236	4.246
4.925	4.758	5.739	5.648	4.810	4.116
4.910	5.403	4.405	4.087	5.155	4.859
1.410	1.016	0.286	0.865	1.446	1.311
4.257	4.678	4.184	3.523	4.040	4.333
3.685	4.006	3.993	4.401	3.700	3.912
3.382	2.918	2.945	4.536	3.630	2.966
4.552	2.946	2.933	3.292	3.245	3.551
6.266	8.216	6.616	6.569	7.591	6.261
4.702	6.714	4.087	4.220	5.080	5.780
3.172	3.106	3.358	3.101	3.266	3.215
3.477	3.013	2.791	2.235	2.407	3.724
4.635	4.742	5.070	4.246	4.900	4.853
4.023	4.969	4.519	4.523	3.884	4.397
4.542	2.106	2.411	2.619	3.431	3.268
6.097	3.616	4.525	4.535	4.622	5.830
2.647	2.456	2.301	1.973	2.105	2.664
2.206	1.621	1.900	2.260	1.985	1.797
6.278	6.183	6.192	6.069	6.119	6.281
2.334	1.931	2.112	1.293	1.382	1.712
4.690	4.535	3.738	4.190	4.835	5.210
3.569	4.058	3.879	4.235	3.542	3.350
1.409	3.070	2.222	1.961	2.562	2.563
4.382	5.317	3.741	4.098	4.724	5.062
8.751	8.705	9.298	7.956	9.391	8.984
7.249	6.736	7.269	6.532	7.470	7.169
5.187	4.495	5.116	4.849	4.302	4.818
2.718	3.150	2.421	3.026	3.227	3.031
6.307	5.608	5.765	6.118	5.282	5.590
2.316	1.838	2.477	2.698	1.880	2.360
4.261	3.447	3.679	3.926	3.598	3.912
6.068	5.852	5.838	5.807	5.828	5.962
3.242	2.915	2.825	3.031	3.554	3.657
6.548	5.979	5.555	5.078	6.340	6.160
2.860	2.903	1.390	2.346	2.825	2.776
6.051	6.354	6.037	5.537	6.503	6.571
2.264	2.837	2.473	3.277	1.813	2.577
8.950	8.814	9.203	8.369	9.126	8.980
2.446	2.138	2.228	2.263	2.326	2.151
9.951	10.215	9.765	9.662	9.843	10.046

4.705	4.446	3.282	3.363	3.817	3.451
3.276	4.697	3.843	3.930	4.197	3.300
2.890	2.343	2.914	2.813	2.640	2.734
2.659	2.778	2.948	3.549	3.335	3.494
6.168	6.040	6.109	6.367	6.410	5.812
5.815	6.254	5.407	5.122	5.670	5.706
7.534	7.623	7.193	7.737	7.407	7.623
3.507	1.978	3.311	2.532	3.581	3.537
6.547	7.717	6.143	5.848	6.419	7.163
7.583	7.613	7.353	6.814	7.666	7.836
4.923	5.270	5.003	4.719	4.986	5.535
4.205	4.222	3.869	3.841	3.868	4.092
3.928	5.521	4.498	5.240	4.196	4.884
4.969	4.784	4.931	4.806	4.730	4.739
7.786	8.182	7.271	7.058	7.157	8.321
8.287	8.492	8.704	8.556	8.062	8.642
4.158	3.579	3.329	4.075	4.478	3.575
8.577	7.641	7.900	8.015	7.552	8.392
7.505	7.543	7.058	6.981	7.094	7.631
4.188	4.213	3.594	3.783	3.418	3.576
5.551	5.676	6.215	4.712	6.003	5.137
5.280	4.944	4.968	5.337	4.783	4.696
4.322	4.060	4.780	4.311	3.799	4.172
4.526	4.376	3.662	3.067	3.998	4.274
4.842	5.383	4.286	4.671	4.847	4.962
4.123	5.893	3.417	4.525	6.094	4.814
4.734	6.455	3.231	4.682	7.056	4.661
4.162	3.793	4.306	4.353	3.624	4.391
7.482	7.506	7.727	7.730	7.806	7.798
1.636	2.845	2.201	1.963	2.394	3.090
4.736	4.109	4.831	5.235	4.998	4.422
8.744	8.586	9.032	9.021	8.837	9.072
7.733	7.748	7.522	7.631	8.154	7.849
6.351	6.069	6.684	6.468	6.242	6.351
6.255	5.161	5.937	5.073	5.735	5.810
8.432	8.434	8.552	8.329	8.755	8.678
6.839	6.810	7.010	7.289	5.060	7.087
5.152	5.814	5.552	6.296	5.426	5.272
5.416	5.424	4.832	4.967	4.730	5.189
2.343	3.031	2.609	2.897	3.331	1.702
3.655	3.286	3.088	2.991	2.583	3.013
4.569	4.085	4.063	3.777	4.499	4.433
3.201	3.203	1.812	3.538	2.524	2.646
3.591	3.162	4.029	3.803	3.894	2.949
4.888	4.815	4.321	3.231	4.513	4.271
5.605	5.320	6.003	5.996	4.884	5.832
5.885	5.364	5.166	5.442	5.308	5.849
6.597	7.339	6.607	6.832	6.834	6.850
5.055	5.219	5.197	4.689	4.812	4.847
4.836	4.393	4.866	4.536	4.089	4.616

3.383	3.272	4.397	3.924	4.527	3.873
5.549	4.891	5.028	5.140	4.957	5.213
2.515	2.850	2.470	4.334	2.156	2.708
2.686	2.620	2.248	2.456	2.757	2.650
6.462	6.665	5.579	5.524	6.202	5.727
4.355	2.929	4.161	2.615	3.952	2.936
8.433	8.057	8.998	8.843	8.717	8.551
7.048	6.698	6.961	6.654	7.448	6.833
2.515	2.221	2.876	2.261	3.098	2.224
5.842	5.327	5.351	5.130	5.337	5.645
5.670	5.713	5.584	5.427	5.495	5.459
1.880	1.773	1.729	1.646	1.366	1.787
6.025	5.605	5.959	5.323	6.562	5.633
5.580	5.536	5.896	5.964	4.605	5.413
6.914	6.545	6.574	6.128	6.720	6.726
4.045	4.349	3.683	3.842	4.080	4.469
3.761	3.603	3.609	2.591	3.670	4.335
7.772	8.534	7.599	7.234	8.085	7.775
3.745	3.389	3.417	3.731	3.604	3.106
2.576	2.757	3.014	1.833	2.915	2.859
4.060	4.177	3.625	3.702	3.405	3.795
3.712	4.219	3.327	3.081	3.153	3.327
6.195	6.681	6.072	6.325	6.597	6.300
2.826	2.136	2.199	1.924	1.740	3.109
4.575	4.242	4.197	3.875	4.594	3.647
3.701	3.858	4.091	3.664	3.755	4.201
3.681	4.043	3.703	3.633	3.898	4.362
4.506	4.791	4.500	4.230	4.947	5.057
3.834	4.325	3.540	2.789	4.697	4.317
5.504	6.199	6.250	3.954	6.766	6.616
4.987	4.216	5.155	4.531	4.812	5.184
7.343	6.949	7.265	6.673	7.345	7.136
5.804	5.573	5.941	5.316	5.358	5.424
6.805	6.722	6.294	4.861	6.760	6.478
3.381	1.572	1.628	1.889	2.297	1.597
4.319	4.440	4.253	3.804	3.330	3.908
4.582	4.518	4.918	4.668	4.144	4.721
6.196	5.210	6.219	6.172	5.880	5.288
1.355	2.305	1.760	2.935	1.181	2.071
4.284	3.694	4.217	3.556	4.334	4.645
3.175	2.944	3.102	3.628	2.357	3.752
3.540	4.085	4.396	3.955	3.122	3.783
1.165	1.089	1.150	1.031	1.226	1.088
7.805	7.885	7.067	7.346	7.847	7.586
5.514	5.130	4.546	4.839	4.999	5.740
4.408	4.728	4.232	3.111	4.875	5.779
5.370	4.659	4.393	3.560	4.518	5.233
4.444	4.024	3.436	3.166	3.965	4.355
4.605	4.359	3.855	4.285	3.748	4.548
2.776	2.377	2.836	2.130	3.255	2.460

4.347	5.075	4.796	4.739	4.374	4.743
3.064	3.963	3.583	3.831	4.101	3.264
5.709	5.244	5.181	5.617	5.459	5.117
4.016	3.034	3.425	3.530	3.442	3.239
5.664	5.170	4.827	4.433	5.040	4.866
4.584	3.852	3.574	2.907	4.536	4.036
6.594	6.633	6.510	6.079	6.939	6.574
6.424	6.192	6.272	5.774	6.228	6.251
5.417	5.173	4.968	5.068	5.344	5.395
2.232	2.285	3.581	3.318	2.878	3.259
5.231	5.426	4.837	4.931	5.090	4.764
3.286	5.281	1.900	1.920	4.794	2.989
3.231	3.250	3.600	2.992	3.716	3.847
6.370	6.540	6.192	6.235	6.338	5.955
4.726	3.895	4.243	4.396	4.228	4.598
7.730	7.750	8.112	7.530	8.220	7.574
4.364	3.871	4.446	3.729	4.579	4.083
4.271	4.418	3.463	4.685	3.957	4.354
3.816	2.789	2.953	3.601	3.683	2.929
1.189	0.774	1.207	0.821	0.873	0.860
2.238	2.944	2.956	2.845	3.244	2.473
4.114	3.817	4.023	3.480	3.359	3.700
4.696	5.143	3.953	3.686	5.567	5.094
4.662	4.430	4.288	4.098	4.427	4.622
6.652	6.425	7.639	7.796	7.198	6.816
4.949	5.258	4.044	3.766	4.334	4.210
2.993	3.567	2.207	2.303	2.848	3.416
5.185	4.775	4.575	4.444	5.008	5.106
3.775	4.041	2.765	3.349	3.772	3.755
3.008	3.468	3.817	2.944	2.863	3.383
4.614	4.726	4.059	3.720	4.568	4.604
2.514	2.908	2.872	2.809	2.417	2.989
6.029	5.706	5.426	4.648	5.473	5.336
8.233	8.011	8.334	8.553	9.144	7.842
4.789	4.680	4.070	3.741	5.053	3.992
1.772	1.968	1.868	1.813	1.868	1.988
5.491	5.454	4.545	4.004	4.898	5.052
3.072	4.542	4.141	3.576	3.380	4.695
4.354	2.953	3.456	2.759	3.752	4.167
5.205	4.518	5.459	5.136	5.693	4.238
2.881	2.774	2.712	2.414	4.743	2.177
2.946	3.557	3.261	3.681	3.433	4.093
4.068	5.396	4.216	4.963	4.613	4.729
3.263	3.619	3.731	3.839	3.573	3.629
5.221	5.125	5.445	4.174	4.647	4.610
3.877	3.665	3.803	3.171	3.291	3.726
3.217	3.204	2.888	3.294	3.185	3.805
4.650	5.298	4.895	4.820	4.820	4.976
6.350	5.733	5.738	5.270	5.230	6.426
4.525	2.900	4.337	3.905	4.891	3.655

3.587	2.981	3.400	3.491	3.824	3.371
2.933	2.199	2.517	2.912	2.016	2.480
3.242	2.833	3.507	3.176	3.516	2.092
1.896	3.005	2.187	2.335	2.389	1.661
5.154	4.165	5.270	5.101	5.587	4.773
2.085	2.769	1.271	1.269	2.939	3.200
4.715	4.489	4.435	3.556	5.135	4.840
1.394	1.584	1.353	1.943	1.433	2.251
4.839	4.337	4.540	4.479	4.380	4.910
5.258	4.642	5.060	3.616	5.992	5.672
6.298	5.843	6.431	6.002	6.959	6.627
3.295	3.414	3.494	3.922	3.613	3.302
4.759	4.794	4.688	3.991	5.077	4.842
5.824	6.275	4.939	5.875	6.642	5.483
5.088	5.759	4.750	5.105	4.803	5.857
3.989	3.413	3.520	2.735	3.609	3.244
4.917	5.084	3.627	4.696	4.903	5.124
4.943	5.257	3.645	4.711	4.876	5.040
3.747	3.214	3.061	3.039	3.712	3.453
3.684	4.140	3.659	4.833	3.362	3.302
5.950	5.575	5.685	5.563	5.995	5.694
4.441	4.282	3.948	4.026	4.478	4.942
7.162	7.706	7.411	7.885	7.063	6.842
7.097	7.131	6.263	6.750	6.581	6.274
5.048	4.639	4.819	3.942	5.114	4.980
2.683	3.695	2.225	3.780	3.245	2.928
2.366	2.343	2.393	1.649	2.766	2.658
2.761	2.095	2.477	4.041	2.253	2.449
2.838	3.679	3.017	2.113	2.254	3.267
4.453	4.669	4.165	4.042	4.370	4.696
4.660	3.316	3.694	4.510	5.256	4.720
2.554	2.118	2.399	3.856	2.775	2.476
2.826	2.757	2.588	2.959	3.437	2.246
3.736	3.503	2.712	3.924	3.190	3.658
3.786	3.259	2.096	4.832	4.264	4.169
5.478	5.102	6.156	6.265	4.438	5.823
4.614	3.329	3.752	3.416	3.215	3.930
3.829	2.304	4.675	3.313	2.566	2.340
3.593	2.600	3.474	3.197	4.142	3.496
3.667	3.734	4.323	3.893	3.415	4.461
2.109	2.098	2.177	3.443	1.647	2.631
2.266	2.473	1.642	1.991	2.688	2.761
4.824	5.928	5.388	6.065	5.365	5.398
4.344	2.723	4.265	2.609	3.383	4.016
6.615	6.377	6.787	6.380	6.364	7.248
2.688	3.029	2.919	3.703	1.611	2.841
3.024	2.375	2.672	2.256	2.714	2.664
2.024	1.922	2.120	2.905	1.788	2.187
3.362	4.203	4.588	3.345	3.440	3.864
2.060	2.419	1.055	3.222	1.057	2.754

2.504	3.060	2.719	3.036	1.336	2.519
3.279	2.855	2.400	3.344	3.933	4.154
2.518	3.285	2.971	3.609	2.583	3.160
3.552	4.384	4.293	3.756	4.219	4.470
2.327	0.802	2.184	2.702	3.146	0.798
4.894	4.320	3.886	4.202	4.888	4.655
2.236	1.558	1.431	1.753	1.844	1.031
2.726	2.125	3.844	3.362	2.618	2.275
4.054	4.691	4.395	4.792	3.502	3.683
2.941	2.259	2.570	1.707	2.344	2.735
4.773	4.460	4.020	3.538	3.880	4.328
3.455	4.132	4.085	3.530	3.468	3.826
2.677	3.706	2.490	4.257	2.453	1.977
4.956	5.623	4.357	4.107	4.107	4.882
2.787	3.626	2.287	3.788	3.171	2.856
5.564	2.871	5.275	4.257	5.606	4.548
2.850	2.125	3.483	2.713	2.842	2.891
2.444	2.274	2.368	2.000	1.798	2.557
3.196	3.200	2.542	2.656	3.231	2.924
2.410	2.260	2.410	3.416	2.409	2.171
1.410	1.079	1.107	1.183	1.440	1.274
1.103	2.019	1.486	1.822	2.184	1.847
2.270	1.755	1.602	1.895	2.246	1.504
2.674	1.935	1.094	1.013	1.116	1.048
2.841	2.859	2.058	4.148	2.615	2.420
2.578	2.625	2.440	3.050	3.076	2.429
4.241	3.987	2.451	2.367	4.976	3.706
3.267	3.412	2.635	3.924	2.874	2.700
3.128	2.938	2.437	2.980	1.918	2.329
3.575	3.439	3.531	5.226	4.415	3.349
2.438	2.353	2.110	2.324	2.179	2.456
4.342	5.430	3.509	3.602	3.557	3.472
1.529	0.873	3.121	2.093	2.661	2.892
3.503	3.769	3.642	4.645	3.402	4.148
2.679	2.774	3.387	3.229	2.085	2.220
2.517	2.701	2.596	2.713	3.930	2.992
4.199	2.935	2.319	1.723	2.630	1.891
5.009	4.545	4.013	3.576	4.331	3.832
3.694	3.518	4.215	4.162	3.526	3.457
2.613	1.612	2.538	4.103	3.070	2.265
3.408	3.520	3.032	4.329	3.692	3.260
2.573	2.412	3.514	2.699	3.499	3.031
2.194	2.698	2.255	2.860	1.538	2.461
3.129	2.177	2.035	2.358	2.966	2.963
3.593	2.851	4.172	4.444	3.583	2.754
4.038	4.458	3.699	3.622	4.761	3.262
4.049	3.276	3.050	3.681	2.636	2.930
4.541	3.513	5.234	5.059	4.359	3.946
3.815	3.017	4.475	2.202	4.072	3.183
4.848	5.093	4.722	5.302	4.098	4.867

2.017	2.739	2.386	3.811	2.538	2.504
3.461	2.620	3.979	3.722	2.145	2.699
3.314	3.964	2.928	3.701	4.381	2.610
3.521	3.914	3.617	3.800	3.763	3.464
2.244	2.719	1.858	1.871	2.796	2.919
3.628	3.692	3.285	3.237	3.141	3.248
1.739	1.388	2.335	0.137	1.817	1.618
2.335	4.241	3.498	4.032	4.040	3.969
3.217	1.965	2.165	2.883	2.167	3.281
5.919	5.774	6.745	7.217	6.514	6.143
1.868	2.069	2.709	3.830	2.291	2.265
2.181	0.908	1.345	1.866	2.151	1.207
4.049	3.765	5.050	4.428	3.687	4.635
3.108	2.286	3.702	3.343	2.638	2.364
3.490	4.095	4.426	3.441	4.125	3.948
5.312	5.785	2.904	4.189	4.930	5.875
4.426	4.379	4.688	4.553	4.677	4.030
3.864	1.928	3.464	2.661	2.888	1.798
2.923	2.214	2.342	1.103	2.635	2.230
3.480	3.367	3.066	4.293	2.968	3.770
1.799	2.361	2.540	2.073	1.658	1.701
4.200	3.132	3.072	3.453	2.785	3.045
3.216	2.173	2.977	2.594	1.713	1.465
5.138	5.400	3.536	5.645	3.959	5.377
3.711	3.804	2.328	2.461	3.244	2.488
2.582	2.366	4.172	3.084	1.671	1.621
1.632	3.412	1.187	2.923	1.881	2.164
3.069	3.430	2.703	2.041	2.871	3.375
3.683	3.377	2.533	1.080	3.517	2.814
2.529	4.126	3.132	3.662	3.475	3.058
2.103	2.536	2.007	2.320	2.032	2.660
4.439	3.739	4.887	4.397	4.238	3.497
3.275	4.067	3.648	5.138	3.510	3.864
1.968	2.749	1.630	1.767	2.944	2.852
5.441	5.013	4.480	4.479	5.307	3.874
4.641	4.285	5.837	6.300	7.080	4.947
4.434	4.079	4.313	3.865	2.920	4.348
6.552	5.340	6.569	6.176	6.439	5.519
6.473	6.342	7.088	8.093	6.352	7.126
2.706	2.924	3.050	3.617	3.344	3.332
4.705	4.537	4.401	4.831	3.695	4.627
3.101	2.741	2.431	2.718	2.444	2.801
3.456	2.705	2.590	2.588	3.110	2.002
3.603	3.826	4.392	5.018	4.048	3.497
3.110	3.076	2.053	4.886	3.542	2.933
1.706	1.596	0.943	1.626	3.193	2.581
1.922	1.954	2.327	2.567	1.794	1.531
2.176	2.801	2.164	3.127	2.014	1.776
3.129	4.044	5.027	3.970	3.714	4.505
3.162	4.527	2.365	3.958	2.354	1.990

3.748	3.279	2.776	4.617	4.308	3.379
0.348	1.593	2.524	3.321	2.382	2.031
7.058	6.690	6.738	7.137	6.162	6.323
1.860	3.570	4.714	3.386	2.210	1.737
4.806	5.004	4.700	5.372	5.143	5.672
7.685	7.232	7.628	7.298	6.409	6.965
3.214	3.192	2.509	2.787	2.296	3.009
3.445	3.616	3.080	3.855	3.365	3.554
3.536	2.944	2.288	3.877	3.215	1.870
2.285	2.186	3.324	1.822	1.514	2.107
3.005	1.371	1.844	2.751	2.101	3.556
3.167	1.586	0.568	2.443	1.957	3.759
4.402	5.449	5.730	5.362	4.680	4.834
4.644	3.997	3.425	3.280	3.417	4.084
3.157	2.211	3.565	3.425	2.352	3.383
2.576	2.878	2.265	3.387	3.057	3.165
4.182	3.606	3.768	3.751	3.362	3.488
1.322	0.806	0.778	0.982	1.276	1.037
3.605	3.883	3.189	2.826	3.349	2.810
3.671	3.643	3.005	3.432	4.182	1.868
1.766	4.227	1.389	1.826	4.185	2.728
1.766	3.060	2.881	4.059	1.902	2.032
3.170	4.258	4.446	4.504	4.186	3.976
3.534	3.501	3.513	3.353	3.444	4.195
1.397	2.526	2.899	2.475	1.873	2.394
3.134	3.603	2.289	2.383	2.113	2.803
3.355	2.729	3.418	2.964	1.928	2.458
3.488	3.786	2.429	3.306	3.771	3.509
2.252	2.474	3.575	3.330	2.260	4.161
1.996	2.174	2.156	1.944	2.769	2.120
4.470	4.223	5.247	3.509	4.810	4.741
2.693	2.504	2.526	2.026	1.945	2.692
2.378	2.823	1.443	2.114	2.798	2.472
3.644	3.358	2.522	3.438	3.654	3.902
3.768	3.079	4.336	4.338	2.975	2.516
3.431	4.151	4.437	4.149	2.526	3.721
3.631	4.134	4.519	4.206	2.524	3.796
2.529	1.922	2.593	2.233	1.869	3.475
5.502	5.973	4.128	3.979	6.512	5.288
2.907	2.084	2.592	2.582	2.410	2.746
3.914	3.013	2.938	2.331	3.119	2.127
2.110	1.817	2.152	2.475	1.979	2.153
6.556	6.970	6.270	7.862	7.211	7.306
3.852	4.080	3.016	3.611	3.510	2.858
3.002	1.369	1.822	2.071	2.371	1.831
6.073	4.867	5.643	5.637	5.187	5.187
6.060	5.604	5.991	6.187	6.324	6.188
2.791	2.147	2.644	1.426	2.302	2.206
4.598	4.856	2.132	3.175	3.814	2.502
1.885	2.076	2.939	1.601	1.958	1.592

2.664	2.698	3.807	3.181	2.380	3.168
4.564	4.311	3.211	3.437	4.136	4.233
3.804	3.793	3.725	4.521	3.546	4.110
3.031	2.708	2.729	2.897	2.837	3.985
4.390	4.720	3.025	3.015	4.103	4.472
3.410	4.205	4.073	3.964	3.348	3.348
2.222	1.510	1.238	1.123	1.733	1.814
4.404	3.448	3.854	3.433	2.548	4.741
2.684	2.972	3.341	3.412	3.211	2.449
5.067	4.564	3.428	4.303	4.652	4.450
4.011	3.501	3.529	2.343	4.225	4.019
2.021	3.281	1.990	3.401	3.491	2.261
4.660	5.580	4.614	4.483	5.505	5.598
3.038	2.728	2.143	2.864	3.006	2.709
1.865	1.834	1.297	1.076	1.382	2.038
2.505	3.002	3.078	3.329	3.613	2.400
4.615	4.819	4.371	5.213	4.733	3.776
2.974	2.708	3.899	2.088	2.184	2.697
3.475	2.828	3.611	2.406	2.821	2.443
2.260	1.932	2.305	2.685	2.353	1.955
3.389	2.697	2.881	3.580	3.237	3.323
2.973	2.302	2.139	2.055	2.959	2.765
3.244	3.584	2.167	2.134	1.999	2.562
4.674	1.576	3.010	5.039	2.922	3.646
4.098	3.885	4.716	3.234	3.421	3.488
3.768	3.258	4.829	3.863	3.798	3.622
4.384	4.521	5.081	5.522	4.256	4.364
2.195	1.849	1.018	1.592	2.113	2.060
5.846	5.817	5.896	6.556	5.560	5.980
4.768	4.519	4.908	5.148	5.004	4.458
1.390	2.379	2.108	2.768	1.659	2.663
2.072	2.454	2.050	2.107	1.929	1.476
3.100	2.734	3.268	2.485	3.478	3.135
6.087	5.399	5.722	5.564	5.453	5.656
7.547	7.369	6.407	6.522	6.918	7.777
5.111	6.226	4.957	6.909	5.134	4.812
6.213	6.123	5.978	5.504	5.786	6.024
3.900	3.564	3.354	2.835	3.039	3.726
3.732	4.565	3.774	4.416	3.385	3.597
8.929	8.913	9.391	9.112	9.394	8.803
4.144	3.592	2.717	2.612	2.600	2.083
6.138	4.295	4.172	4.950	4.058	4.452
7.895	7.842	8.047	7.854	7.014	7.854
5.264	5.230	5.578	5.795	5.559	5.329
4.484	3.906	3.354	3.257	3.527	3.630
5.888	6.350	6.011	6.014	6.223	5.636
7.040	6.195	5.702	5.621	6.421	7.125
5.007	5.045	6.426	5.654	6.928	4.838
4.906	4.342	2.445	3.314	3.022	2.237
3.809	3.507	3.636	3.946	3.474	3.543

5.089	7.005	4.788	4.597	5.268	6.452
5.686	5.285	5.843	5.386	5.817	5.434
3.502	3.158	3.718	3.725	3.718	2.879
4.726	5.955	5.329	4.490	5.393	4.563
4.954	2.879	4.129	3.335	3.055	4.084
2.998	2.996	2.052	1.821	2.110	1.951
3.177	2.602	3.150	3.764	2.930	2.020
2.185	4.085	2.043	1.970	3.048	2.091
2.193	4.306	3.192	2.311	2.819	2.915
2.499	2.934	2.232	1.628	2.637	3.083
4.737	4.764	5.308	4.541	3.600	4.133
4.600	3.964	3.779	3.192	4.292	5.265
5.514	5.693	5.638	6.640	4.896	5.531
6.252	5.778	5.701	5.991	5.866	5.923
7.114	8.133	7.853	7.893	7.448	7.251
3.112	3.053	3.541	2.356	3.399	4.223
7.913	7.608	8.282	7.560	8.752	8.111
8.734	8.429	9.355	8.781	9.586	9.335
7.908	7.510	7.730	7.736	8.137	7.890
2.941	3.077	2.052	2.156	3.223	3.332
8.844	8.549	8.760	7.485	8.706	8.895
8.845	8.523	8.761	7.593	8.888	8.969
3.584	4.019	3.216	3.243	3.987	4.516
2.942	3.284	3.022	4.145	2.822	3.182
4.733	3.579	3.344	3.855	4.593	4.677
4.946	4.745	4.624	4.439	4.958	4.821
3.920	3.789	3.543	4.276	3.138	3.284
3.466	2.554	3.064	2.343	2.361	2.640
4.011	4.036	4.282	3.417	3.313	3.706
4.325	4.459	2.640	2.865	3.743	4.079
6.375	5.981	6.487	4.910	6.329	6.139
3.242	2.751	2.828	2.493	3.450	2.502
6.537	6.610	6.510	6.626	6.790	7.130
4.478	4.343	5.755	3.542	5.733	4.700
4.566	5.239	3.788	4.411	3.820	4.083
7.123	7.094	7.096	7.711	7.646	6.876
3.459	3.348	2.948	2.635	2.838	3.201
4.941	3.337	3.406	2.275	4.484	3.613
3.512	3.203	3.613	3.124	3.573	3.590
3.525	2.880	2.794	3.023	2.649	3.153
8.099	7.215	4.732	6.963	5.261	4.291
3.818	3.496	2.490	2.982	3.955	2.796
4.207	4.278	3.575	3.480	4.448	4.141
3.517	4.526	3.689	3.653	4.392	3.075
3.915	4.907	3.996	3.950	4.424	3.508
4.089	4.893	5.083	4.647	5.210	4.640
3.535	4.431	3.140	2.944	3.324	4.039
6.937	6.219	6.425	6.151	6.823	6.651
4.187	4.561	3.961	3.616	3.311	3.307
3.230	3.808	3.460	3.468	3.794	3.693

4.056	5.186	5.418	4.251	5.169	5.250
6.490	6.412	7.017	7.213	7.336	5.753
3.970	2.997	3.588	3.464	3.044	4.270
6.209	5.757	5.898	5.589	5.880	6.090
5.964	5.527	5.802	5.192	5.883	6.106
4.978	5.013	5.257	5.176	4.894	4.789
6.045	6.136	4.951	5.946	5.591	5.683
4.504	4.348	3.811	3.443	4.297	4.506
6.306	6.230	5.589	5.427	5.652	5.627
5.520	5.726	5.734	5.235	5.160	5.397
6.101	5.813	4.902	5.329	5.737	5.717
3.697	3.844	2.937	3.606	2.841	3.314
4.750	4.023	4.723	4.640	4.337	4.936
9.391	9.255	9.043	9.260	9.222	9.387
6.242	6.092	5.834	5.711	5.883	6.122
4.071	4.027	3.144	3.675	4.509	4.376
2.195	2.682	2.325	1.851	1.820	2.466
4.970	4.407	4.282	2.728	4.430	4.536
5.245	5.185	4.986	4.656	5.091	5.725
7.195	7.559	6.744	6.919	7.361	7.535
4.452	3.828	2.995	3.526	2.652	3.471
4.107	3.635	2.874	2.730	3.063	4.396
4.199	3.961	3.479	3.282	3.429	4.869
6.126	6.136	4.373	4.643	5.637	6.352
5.571	5.386	4.590	5.112	5.339	5.115
5.561	5.880	5.823	4.719	5.455	5.577
3.436	3.519	3.772	3.577	3.957	3.658
7.766	7.688	7.775	8.278	7.951	7.109
2.493	2.441	2.209	2.963	2.218	2.559
7.057	7.849	7.098	6.360	7.044	6.898
9.557	9.808	9.404	9.152	9.578	9.902
3.009	2.647	2.220	2.080	2.590	2.897
4.779	3.796	3.017	2.968	3.766	3.700
2.451	2.114	2.882	3.223	2.546	2.617
2.430	3.444	1.985	1.465	2.615	1.661
3.135	3.061	4.135	4.704	3.395	3.229
6.890	6.514	1.934	2.964	4.485	3.801
7.328	7.210	6.422	7.452	6.772	6.997
6.362	3.215	3.642	4.532	7.042	4.516
7.465	4.698	7.984	8.286	7.395	7.152
5.901	3.581	5.241	7.293	5.155	6.374
3.559	3.578	1.214	2.441	4.122	2.499
4.819	4.578	3.199	4.687	3.672	2.908
8.533	9.024	7.227	8.345	7.810	7.539
9.882	10.106	8.815	9.451	9.992	10.079
3.694	3.409	3.464	3.483	3.353	3.564
4.342	4.093	4.464	3.828	4.536	3.975
3.853	3.817	3.072	3.079	4.366	4.182
4.264	4.161	3.704	3.619	3.947	4.174
2.605	4.276	3.150	4.222	2.623	3.593

3.599	3.594	3.784	4.136	3.751	3.500
5.765	6.245	4.276	5.107	4.197	5.370
3.830	3.954	4.424	3.515	3.950	3.961
9.051	9.064	9.197	8.729	9.675	9.500
4.823	5.580	3.671	4.623	4.478	5.070
6.354	6.144	5.808	5.557	6.305	6.352
6.723	7.082	6.659	6.965	6.702	6.932
5.752	5.132	5.966	4.347	5.984	6.197
4.906	5.152	5.374	4.231	5.092	5.056
3.246	3.473	3.784	3.145	3.768	3.298
3.981	4.250	3.424	3.318	4.266	4.160
5.493	5.175	5.446	5.548	5.661	5.914
3.679	4.105	3.852	3.937	3.822	4.379
3.936	4.093	3.882	5.698	4.093	5.058
6.803	6.880	7.559	8.160	6.574	6.892
6.374	6.126	6.758	6.185	7.192	6.479
7.932	8.046	8.817	9.266	8.597	8.316
9.921	10.256	10.840	11.361	10.098	9.868
3.209	2.027	5.561	4.107	0.576	2.840
6.932	5.672	7.884	9.453	6.215	7.248
8.156	7.838	9.193	9.941	7.473	7.578
5.425	5.223	5.879	7.905	3.622	4.648
6.682	5.437	7.779	8.835	5.575	6.562
9.606	8.793	10.372	10.877	8.851	9.224
7.862	7.930	9.131	10.080	7.486	7.851
3.535	4.154	5.384	6.626	4.510	4.558
8.317	8.800	9.607	10.622	8.622	7.578
6.388	5.725	7.536	8.762	5.363	6.187
7.892	7.398	9.283	10.026	7.836	8.030
10.015	9.193	10.532	11.215	9.408	9.937
4.265	4.112	4.491	4.533	3.674	3.970
4.636	4.457	5.245	6.878	3.896	4.606
5.695	5.745	5.321	5.427	6.275	6.181
4.330	2.860	3.622	3.183	3.576	3.737
6.095	5.959	6.006	5.434	5.976	5.963
3.647	4.635	3.760	3.938	4.036	3.809
2.533	3.815	3.713	3.825	3.710	3.581
2.132	2.929	1.937	3.090	1.591	2.453
3.962	3.554	3.731	2.813	3.247	3.565
6.990	6.906	7.602	6.906	7.469	7.408
4.991	5.719	6.271	5.202	5.633	5.740
4.751	4.202	4.440	4.547	4.456	4.539
6.003	6.651	6.254	4.736	5.727	6.738
5.169	2.906	2.560	2.846	3.255	4.113
6.052	6.529	5.388	5.776	5.995	6.049
6.165	5.914	6.106	6.097	6.224	6.211
5.292	5.555	5.607	4.698	6.245	5.425
2.675	2.042	2.659	2.966	2.431	1.793
2.264	2.924	2.478	1.899	1.927	3.244
3.510	3.136	3.125	2.867	3.944	3.180

7.663	7.502	7.283	7.264	7.344	7.697
4.121	4.037	4.192	4.002	4.420	3.582
3.597	3.673	2.888	3.137	3.360	3.360
6.255	6.128	6.599	6.462	5.957	6.510
6.049	7.237	5.781	6.206	5.799	5.515
5.349	4.960	5.294	5.326	5.706	5.758
3.890	4.427	3.803	3.394	3.645	3.911
3.453	3.408	3.326	3.093	3.871	3.820
3.614	3.380	3.478	2.790	3.626	3.262
5.345	4.486	4.786	4.866	5.145	4.534
5.496	6.307	5.360	5.174	6.152	5.699
3.871	4.110	3.874	5.455	3.261	3.959
2.711	2.364	2.603	2.762	3.505	2.237
4.853	4.946	5.014	4.756	5.265	4.515
4.808	4.286	4.394	4.436	4.138	4.310
3.108	5.265	6.492	3.421	6.201	6.022
5.421	5.302	4.988	4.862	4.457	4.468
5.734	6.507	5.946	5.628	5.896	5.667
7.197	7.201	7.250	6.281	7.847	7.499
7.242	7.154	6.962	6.895	7.111	7.224
5.406	5.497	5.970	4.716	5.604	6.469
4.125	4.670	1.807	1.064	2.520	3.881
7.977	7.756	7.582	7.567	7.495	8.053
5.044	5.971	5.383	5.713	6.085	5.300
7.032	6.954	6.605	6.730	6.836	6.634
7.722	7.649	7.539	7.185	8.099	7.792
6.410	7.347	5.717	5.215	5.812	6.203
3.285	2.997	3.167	4.071	3.880	2.878
3.215	2.454	2.832	3.216	3.071	2.499
6.754	6.950	7.274	6.362	7.258	7.175
4.947	4.527	4.743	4.647	4.948	4.622
6.490	6.444	6.314	5.727	6.739	6.957
5.828	4.015	5.167	4.934	4.690	5.325
3.844	3.274	2.908	3.543	3.673	3.544
5.476	4.511	3.305	4.065	4.392	4.555
6.537	6.683	5.762	5.950	6.443	6.536
2.544	1.758	1.749	3.990	2.513	3.515
5.725	5.718	5.408	5.554	5.753	5.491
4.421	3.215	3.093	3.470	2.848	3.412
4.161	3.868	3.045	3.162	2.908	4.178
3.981	4.329	3.249	4.396	4.351	3.570
6.327	6.526	6.620	6.193	6.726	6.229
3.749	3.749	4.459	3.749	3.378	3.896
7.274	7.294	7.724	8.095	7.644	7.377
5.113	4.947	4.321	4.576	4.783	4.907
3.127	2.938	2.452	2.306	3.826	3.247
5.694	6.025	5.581	5.165	5.497	5.963
4.776	5.022	4.656	4.523	5.232	4.724
4.043	3.975	4.333	4.754	4.314	4.378
7.081	6.917	5.711	6.401	6.229	7.432

9.340	10.503	7.113	7.579	8.018	9.066
6.986	6.043	6.404	5.891	6.065	6.536
6.791	5.951	6.880	6.253	6.453	6.719
3.790	3.790	4.175	3.776	3.659	3.673
7.566	6.516	6.802	6.872	6.154	7.340
6.195	5.455	5.319	5.410	6.179	5.920
7.520	7.506	7.642	7.032	7.444	7.901
3.395	5.124	4.025	2.862	4.402	4.782
6.208	5.881	5.793	5.883	6.152	6.515
4.142	3.959	3.877	4.561	3.764	4.101
7.187	6.724	7.688	7.724	7.048	6.959
7.267	7.379	7.508	6.933	7.701	6.960
4.238	3.250	3.492	3.889	4.358	3.081
2.962	3.135	2.763	3.421	3.562	3.470
3.058	2.899	3.810	2.938	3.902	3.350
3.715	3.176	2.649	2.755	2.899	2.918
7.151	6.119	6.340	6.239	6.335	6.721
6.768	6.046	6.948	6.177	6.861	6.827
8.277	8.237	8.276	8.285	8.457	8.370
8.019	7.780	8.000	7.895	7.886	8.009
6.119	6.125	6.263	4.916	6.050	6.228
7.168	7.243	7.298	7.419	7.286	7.169
1.729	3.074	2.149	3.256	2.283	2.208
8.768	7.970	8.294	8.118	8.269	7.777
6.866	6.631	6.417	6.389	7.412	7.129
8.719	8.555	8.728	8.803	8.808	8.798
5.997	5.627	4.782	6.323	5.155	5.842
3.972	3.736	3.993	2.783	4.727	4.256
2.420	2.042	2.418	2.020	2.645	2.345
8.481	8.006	7.804	8.052	8.149	8.116
6.860	5.606	6.289	7.228	6.547	5.378
5.380	5.791	5.344	5.058	5.312	5.607
5.290	4.917	5.181	4.995	5.202	5.047
6.708	5.340	6.074	5.721	5.970	5.866
5.160	5.043	5.172	4.818	5.140	5.543
2.984	4.023	3.901	2.575	3.645	3.909
5.401	5.567	4.232	3.927	5.349	5.584
4.307	5.390	4.507	3.641	4.243	4.882
3.434	3.646	3.969	2.494	4.267	3.852
6.465	6.115	5.967	6.453	6.022	6.315
8.952	9.042	8.412	8.698	8.844	8.708
5.482	6.142	5.629	4.210	5.719	7.078
4.004	2.965	3.049	2.699	4.122	3.901
5.184	5.173	4.942	4.995	5.414	4.956
6.003	5.836	3.037	4.897	4.007	2.416
5.413	6.299	6.280	6.512	5.871	5.591
6.938	7.557	7.057	6.609	6.678	7.193
2.736	2.214	1.128	2.194	1.418	1.089
4.823	5.130	1.647	4.857	1.956	2.055
7.214	6.515	7.397	7.410	6.606	7.238

8.486	8.615	9.284	10.181	8.465	8.908
7.677	7.984	7.805	7.763	8.046	7.989
7.644	7.526	7.376	7.316	7.263	7.486
7.013	6.921	6.718	6.314	6.901	7.078
5.576	5.682	5.532	5.084	5.796	5.785
4.017	3.396	3.547	3.661	3.763	4.129
3.900	3.881	3.911	3.766	4.310	3.102
5.774	3.562	4.870	5.184	4.958	3.942
6.005	6.366	5.938	5.914	6.505	5.985
6.330	6.194	6.977	7.528	6.880	5.952
6.942	6.669	7.028	6.497	6.573	6.819
7.559	7.205	7.935	7.658	7.176	7.751
7.078	7.081	7.293	6.599	7.407	7.227
3.242	2.402	3.053	2.863	2.644	2.391
3.752	1.902	2.240	2.088	2.177	2.629
4.219	3.904	4.254	4.148	3.886	3.735
2.787	1.596	3.438	3.613	2.685	1.510
6.782	6.789	6.922	6.373	6.629	6.853
3.392	3.568	2.509	3.114	3.423	3.280
3.547	3.302	2.719	2.524	3.305	3.557
5.194	5.118	4.659	4.465	4.673	5.250
4.516	4.185	4.326	3.600	4.357	4.492
3.035	6.260	4.198	4.283	3.912	4.134
10.605	11.465	9.724	10.643	10.199	10.619
3.106	2.456	3.926	3.064	3.361	2.590
6.765	6.820	6.948	5.587	7.634	7.158
5.411	5.478	5.316	5.108	4.678	5.123
3.910	4.122	4.448	4.502	4.140	4.817
7.133	6.831	6.875	6.297	6.325	6.943
5.732	5.930	5.950	5.641	5.558	5.807
5.627	5.868	5.466	5.104	5.699	5.805
6.083	5.948	5.689	6.043	5.156	5.533
8.649	10.309	9.824	10.108	7.256	9.783
10.348	10.770	10.804	10.836	8.740	10.815
9.963	10.849	10.696	10.597	8.318	10.637
6.892	5.924	6.645	5.583	6.518	6.417
2.324	1.839	1.529	2.136	2.354	1.762
3.322	2.894	2.663	2.862	1.956	2.983
5.222	5.048	4.361	3.435	4.605	5.486
3.249	3.937	3.474	5.546	2.892	3.262
6.668	6.331	6.838	6.956	6.976	6.910
3.875	3.935	3.631	3.328	1.952	3.207
4.615	4.575	4.224	4.796	3.662	4.699
5.723	5.858	6.030	5.684	5.986	6.131
5.882	5.007	6.269	5.883	5.446	5.775
6.174	6.386	6.197	7.123	7.357	6.168
3.903	3.651	3.725	3.667	2.651	3.879
6.339	4.943	4.664	5.672	4.473	5.519
2.889	2.584	2.936	3.199	2.393	2.706
4.901	4.680	4.619	5.169	4.701	4.645

1.611	1.666	1.666	1.655	1.503	1.688
2.576	2.179	2.935	2.098	1.941	3.113
5.926	5.952	5.372	5.747	6.115	5.807
3.776	3.033	3.488	3.410	3.690	4.072
4.312	4.655	4.268	4.151	5.017	4.420
4.796	4.488	4.785	4.767	4.492	5.610
3.462	4.198	3.327	3.572	3.459	3.215
6.915	7.067	6.470	6.528	7.045	6.871
2.942	2.328	2.057	2.230	2.445	1.933
3.545	2.840	3.890	2.578	3.540	3.776
6.494	7.373	4.067	5.883	5.910	5.190
3.453	3.451	2.829	3.626	3.733	3.269
7.510	8.454	6.882	7.993	7.690	6.782
3.331	3.117	3.058	3.173	2.307	2.886
5.322	5.376	4.530	4.431	4.803	4.859
2.888	2.040	2.578	2.065	2.564	2.484
3.399	4.551	3.723	2.998	3.262	4.169
3.690	5.164	4.416	3.429	4.467	4.497
8.685	8.508	8.608	8.478	8.428	8.542
3.858	4.153	5.019	4.118	3.585	3.671
2.445	1.754	2.019	1.905	2.328	2.039
5.625	5.198	5.156	3.916	5.728	5.946
4.092	3.939	3.329	3.730	3.692	3.722
4.468	4.040	4.673	4.967	5.070	4.255
3.145	3.153	3.607	2.894	3.486	3.287
5.722	5.945	6.235	5.639	5.909	5.858
5.049	6.190	4.589	5.324	4.976	4.217
4.270	3.981	4.377	4.278	4.483	4.181
4.472	4.834	4.562	4.261	4.086	3.970
4.989	5.286	6.171	4.942	5.893	5.779
4.647	4.124	4.444	4.023	4.461	4.452
4.793	4.573	4.761	4.836	5.011	4.525
4.393	5.115	4.102	4.235	4.657	5.240
4.603	3.865	4.191	3.609	3.711	3.893
6.950	6.517	6.778	6.758	6.740	6.768
3.678	3.922	4.761	2.321	5.173	4.874
5.424	5.153	5.082	4.666	5.578	5.082
4.159	2.940	4.216	3.210	2.940	3.285
3.813	3.547	3.604	2.863	3.526	3.230
8.531	8.401	8.648	7.683	8.659	8.600
3.064	2.145	3.510	2.884	3.227	2.432
5.736	5.534	5.990	6.433	6.177	5.955
2.994	4.109	3.270	2.928	2.955	3.672
4.217	3.932	4.421	4.088	3.978	3.997
3.777	3.480	3.393	3.279	3.057	3.616
5.604	5.352	7.216	5.518	7.101	6.528
2.768	3.149	2.910	2.623	2.774	2.926
2.746	3.695	3.681	3.999	2.795	3.052
7.067	7.515	7.709	7.281	7.835	7.415
4.137	3.933	4.632	4.063	4.347	4.059

5.529	4.778	4.917	4.671	4.793	4.886
2.546	2.357	2.546	3.048	2.714	2.113
8.213	7.930	7.601	7.142	7.831	7.782
3.663	3.158	2.969	2.899	3.494	3.452
3.172	3.271	4.462	3.741	3.626	3.338
2.185	2.287	2.520	2.886	2.527	2.536
4.329	5.042	4.069	3.922	4.908	4.477
5.170	5.247	4.795	4.827	5.215	5.818
3.711	2.847	3.840	3.054	3.593	3.057
4.137	3.676	3.058	4.105	2.514	3.350
3.371	2.967	2.764	2.461	3.361	4.095
6.432	5.775	6.294	5.762	5.874	6.009
3.783	3.267	3.343	3.139	3.142	3.448
4.404	3.681	3.868	4.669	4.203	4.131
3.222	2.051	2.652	2.274	2.925	3.520
4.393	5.030	5.746	5.557	5.779	4.048
3.311	4.884	2.075	2.360	3.175	3.823
7.248	6.993	6.658	6.639	7.200	7.171
5.476	5.689	4.673	4.446	5.217	5.549
5.521	5.236	4.587	3.847	3.963	4.902
2.271	3.042	2.077	1.923	1.926	2.651
4.803	4.937	4.288	4.136	4.314	3.932
5.603	4.900	4.618	4.666	4.830	4.796
4.717	4.203	3.886	3.130	4.098	4.315
7.211	6.633	7.127	6.764	6.832	7.211
9.196	8.486	8.975	8.907	8.674	9.113
9.207	8.565	8.999	8.967	8.707	9.169
4.420	3.888	4.666	3.888	5.077	3.609
3.837	3.724	4.041	4.306	4.217	4.024
3.150	1.374	1.750	1.664	2.250	1.491
4.383	3.159	3.892	3.413	3.180	3.421
3.293	2.729	3.344	3.045	2.988	3.787
6.714	6.499	7.574	7.334	7.402	6.447
7.642	7.018	7.698	7.806	7.636	7.612
6.310	6.621	6.352	6.321	5.854	5.774
4.265	3.854	4.599	4.240	3.592	2.671
4.376	3.969	4.086	3.025	7.976	3.787
4.806	4.115	4.866	4.728	4.437	4.581
4.133	3.291	3.599	3.204	3.768	3.088
2.154	2.563	3.010	2.551	2.679	2.808
2.293	1.758	2.647	2.454	2.026	2.066
6.230	5.108	5.981	6.117	6.527	5.991
2.393	2.966	2.974	2.387	3.350	3.203
7.951	7.880	7.627	7.327	7.752	7.650
8.909	9.381	8.986	8.731	9.507	9.460
4.152	3.805	4.103	4.386	3.998	4.097
3.449	3.522	3.016	2.119	3.381	2.438
9.260	9.355	9.382	9.742	9.383	9.484
7.756	7.565	8.004	8.511	7.398	8.089
9.077	8.865	8.821	9.197	9.023	8.969

9.539	9.431	9.333	9.619	9.506	9.488
9.310	9.214	9.097	9.437	9.306	9.275
5.747	6.086	5.462	5.531	5.887	6.321
7.880	7.754	7.991	8.094	7.871	7.855
5.667	5.694	5.463	6.000	6.510	6.126
4.231	3.696	4.570	3.949	3.516	3.902
3.519	3.285	2.733	2.279	2.641	2.994
2.892	2.893	3.209	3.132	4.858	2.911
2.191	3.627	2.656	2.689	1.880	2.866
7.588	6.645	5.468	3.119	6.164	6.221
2.595	2.875	3.271	2.906	1.596	2.529
8.239	7.569	8.314	8.105	7.655	7.981
5.369	4.127	4.395	4.100	4.684	4.619
5.357	8.055	3.350	5.020	4.163	5.047
5.269	4.635	4.356	5.030	4.243	4.002
3.097	3.342	3.484	4.078	3.429	3.057
4.300	2.659	3.986	3.022	3.916	3.451
5.463	2.734	4.079	3.790	3.912	4.418
2.366	1.996	2.192	2.153	1.981	2.403
2.868	2.795	2.421	1.783	2.985	3.085
5.640	5.794	5.617	4.637	6.136	6.007
4.864	3.624	4.849	3.782	3.849	4.902
6.224	6.070	5.930	6.240	5.814	5.658
5.944	5.228	5.508	5.886	5.886	5.342
1.212	1.491	1.818	1.795	1.542	1.773
4.002	3.896	4.109	4.314	3.960	3.713
3.078	2.985	2.454	2.860	3.071	2.529
5.265	4.985	4.662	5.714	4.468	4.639
5.705	5.201	5.703	5.383	5.416	5.205
3.958	5.278	5.172	5.322	5.194	3.691
4.251	6.007	5.733	4.142	5.295	4.510
3.308	5.338	4.686	4.724	3.652	3.530
5.966	5.959	5.642	3.921	5.051	5.793
6.601	6.486	7.240	6.739	7.525	6.753
8.034	8.253	7.748	8.094	7.725	8.101
2.649	2.172	2.266	2.118	2.245	1.808
4.491	5.239	4.375	4.015	2.596	4.671
2.848	2.140	3.097	3.143	2.255	3.043
3.341	1.868	2.134	1.526	1.443	2.244
2.080	2.046	2.445	2.083	1.931	1.912
5.126	4.641	4.814	4.950	5.329	4.517
3.401	3.358	3.132	4.161	3.169	3.241
5.169	3.893	4.283	4.586	4.484	3.965
5.260	4.520	4.311	4.617	3.443	4.558
7.245	6.672	6.876	6.460	5.920	6.926
6.284	5.647	5.711	5.568	5.679	6.009
4.940	4.719	4.540	4.622	4.552	4.775
4.839	3.628	3.851	4.078	3.795	4.057
5.312	5.328	4.427	4.784	4.474	4.991
7.130	5.373	6.053	6.212	6.104	5.833

6.995	6.688	6.308	5.942	6.534	6.853
2.951	3.458	4.147	3.702	3.697	3.725
8.194	7.558	7.780	7.465	6.956	8.221
8.127	7.763	7.956	7.830	6.746	8.276
3.082	3.039	3.256	3.164	2.406	2.855
6.808	7.113	7.336	7.524	6.951	7.093
5.017	4.887	5.062	3.558	4.615	4.981
4.664	4.899	5.296	4.472	4.248	5.343
3.944	2.632	3.834	2.737	4.099	3.398
6.674	6.548	6.697	5.795	6.942	6.809
5.396	4.970	5.226	5.106	5.179	5.395
5.277	3.774	4.183	5.078	5.090	3.706
2.926	3.515	4.164	2.553	3.748	3.269
5.021	5.342	5.612	5.963	4.576	4.922
2.556	1.636	3.419	3.211	2.382	2.203
4.819	4.674	4.705	4.441	4.906	4.607
4.997	4.459	4.172	4.538	4.300	4.657
4.904	4.418	5.144	3.551	5.337	5.246
6.264	4.918	6.324	6.355	6.397	5.715
6.206	6.153	5.815	6.199	5.576	5.886
3.279	3.812	3.441	2.730	2.838	3.363
3.642	3.814	3.481	3.174	3.976	3.044
4.084	3.598	4.086	3.240	3.463	3.794
4.306	3.080	3.224	3.725	3.359	4.203
3.019	2.946	2.557	3.101	2.279	3.647
3.637	4.224	3.711	3.910	4.272	3.684
4.073	4.675	3.594	2.797	4.048	4.644
8.213	7.751	7.941	7.612	7.896	7.959
9.216	8.470	9.117	9.296	8.922	9.145
2.898	2.486	2.052	1.187	2.567	1.890
2.625	2.009	2.042	2.393	1.954	1.940
2.357	2.619	1.773	2.058	3.575	2.663
4.943	4.855	3.735	4.168	4.733	5.034
5.223	5.172	4.067	4.824	4.745	4.671
5.885	5.887	5.665	4.186	5.848	5.642
7.915	8.709	8.297	8.483	8.039	8.316
2.294	2.361	2.997	3.313	1.688	1.573
2.260	1.919	2.464	2.260	2.277	3.485
4.097	4.225	5.074	5.309	4.600	3.318
2.589	1.745	1.640	2.291	2.135	1.958
2.516	2.684	2.218	2.496	3.683	2.391
6.833	6.857	6.322	5.515	6.646	6.724
4.665	4.616	3.917	4.433	4.086	4.473
3.273	2.353	3.297	2.226	2.671	3.626
3.987	3.572	3.558	4.652	3.477	3.902
3.935	4.076	3.234	3.678	3.247	3.560
3.733	4.183	4.269	3.676	3.801	4.187
4.642	3.103	3.814	2.869	3.372	3.918
4.093	3.510	3.846	4.004	4.015	3.636
3.311	3.712	4.193	3.426	3.553	3.058

5.442	5.481	4.561	4.172	5.331	5.342
4.598	3.131	3.625	2.892	3.654	3.792
5.081	3.831	4.113	3.520	3.541	4.553
3.997	2.598	3.411	2.353	3.042	3.457
2.756	2.514	2.557	1.403	1.684	1.927
5.334	6.162	4.779	4.256	4.739	5.449
2.551	3.895	2.679	3.496	2.473	2.459
2.022	2.467	1.776	3.045	1.615	1.774
3.331	2.661	2.471	2.682	2.251	2.882
2.816	1.746	1.851	1.972	2.506	2.175
2.290	3.891	2.795	3.433	3.679	2.850
6.586	5.988	5.785	5.716	6.249	6.060
5.305	5.745	4.939	4.788	5.094	5.470
3.671	4.292	3.143	4.096	2.272	3.785
6.657	6.089	6.718	5.819	5.502	6.631
9.564	9.164	9.640	9.599	9.759	9.305
4.998	5.361	6.076	5.195	6.987	4.468
4.781	4.227	4.620	4.291	4.460	4.593
5.695	5.731	5.240	5.025	5.499	5.667
4.031	4.330	4.181	4.149	3.517	3.690
6.769	5.459	7.275	7.469	6.714	5.634
3.508	2.448	2.881	2.429	2.226	3.142
1.709	2.002	1.530	1.927	2.386	1.285
2.067	2.332	2.895	2.866	4.436	2.290
2.938	3.047	3.458	4.035	4.690	2.326
3.776	4.237	3.902	3.140	3.897	4.259
3.109	2.793	3.547	3.214	3.700	3.837
6.302	5.548	7.183	7.269	7.017	6.447
4.273	4.272	5.435	5.018	4.969	4.301
6.049	6.270	6.049	4.236	6.438	6.631
3.174	4.015	3.948	5.172	5.795	2.772
6.695	4.979	6.657	5.929	6.433	5.884
6.807	6.653	6.183	6.235	6.370	6.766
7.566	5.417	6.498	6.455	5.992	7.657
3.940	3.380	3.869	3.321	3.286	4.074
5.422	4.731	4.817	4.567	4.474	4.579
3.860	2.972	3.417	2.849	3.405	4.190
3.383	3.895	4.530	3.567	4.359	4.348
3.635	3.204	3.209	3.394	3.081	3.103
2.346	2.586	2.580	2.919	1.977	2.198
3.460	2.627	3.230	3.776	2.512	2.619
1.977	2.901	2.723	2.384	2.477	2.558
3.406	3.308	3.559	4.294	2.688	3.809
3.997	2.660	2.704	2.858	2.900	2.894
2.320	1.543	2.842	2.523	2.612	2.244
3.605	4.411	4.044	3.784	4.719	3.409
3.249	2.591	2.778	2.661	2.727	3.053
4.423	4.246	5.064	3.323	3.780	4.577
6.954	5.864	6.283	5.775	5.950	6.693
3.942	2.431	2.035	1.739	3.208	3.497

2.650	3.243	3.111	3.473	3.275	1.896
3.943	2.461	2.549	2.664	3.194	3.632
5.573	4.912	4.706	4.584	4.932	4.758
3.114	2.801	2.980	3.111	2.402	2.509
3.813	2.897	3.449	4.065	3.575	3.539
3.451	4.326	3.274	2.451	4.584	4.160
1.519	3.637	1.570	2.633	2.211	2.389
2.943	3.094	2.950	3.000	2.670	1.907
3.882	3.385	3.949	3.473	4.253	4.813
3.449	3.585	4.028	2.030	4.432	4.964
3.672	3.843	3.432	3.969	2.970	3.825
3.108	4.369	3.778	3.872	4.010	3.413
4.929	4.834	5.079	4.646	4.588	5.061
5.623	5.227	5.537	5.350	5.429	5.694
2.990	2.686	2.293	2.309	1.946	2.532
4.370	5.086	5.082	4.423	4.833	5.135
2.391	3.221	1.955	2.346	2.026	2.908
7.007	6.577	6.549	6.641	7.926	6.831
4.491	4.508	4.950	4.568	5.901	4.754
5.277	5.168	5.533	4.853	4.835	5.530
2.866	2.810	2.231	2.772	2.104	3.149
4.149	4.410	3.594	3.911	3.890	3.992
3.691	3.183	3.611	3.599	2.643	2.603
4.894	4.850	5.425	4.346	5.677	4.827
1.733	1.900	1.875	2.005	2.182	3.110
6.563	7.462	3.951	5.029	5.324	6.506
7.483	7.241	6.714	6.041	6.898	7.563
2.609	1.667	2.694	2.316	2.772	1.750
1.728	1.684	1.788	1.977	1.803	1.903
2.853	3.654	3.585	3.719	4.180	2.517
1.780	2.809	2.915	2.957	2.651	3.001
5.055	4.281	4.608	4.104	4.840	4.853
5.871	5.788	5.667	5.568	5.825	6.056
7.910	7.974	7.379	7.428	7.894	8.026
4.677	3.500	3.620	3.386	4.009	3.743
2.287	2.100	2.595	2.309	2.485	2.141
6.852	6.622	7.033	7.009	6.898	6.643
2.463	2.479	1.641	2.458	2.860	3.669
1.272	2.144	1.405	1.492	1.622	1.824
5.924	5.990	5.975	5.315	5.549	6.281
6.764	6.841	6.608	5.703	6.606	7.117
2.917	3.289	2.903	3.849	1.410	3.119
5.016	3.689	3.313	4.181	3.821	3.919
3.355	2.905	2.811	3.031	3.340	3.060
2.548	2.251	3.128	2.645	2.502	2.377
3.129	3.010	3.177	2.463	3.240	3.087
3.522	3.431	4.083	4.286	3.828	4.174
1.473	1.739	1.652	1.017	1.072	1.831
6.726	6.343	6.609	7.109	6.482	6.455
4.340	5.372	5.367	5.237	5.554	4.173

4.683	4.942	5.139	3.975	5.814	5.974
3.367	2.901	3.826	3.930	4.468	3.452
6.869	8.366	6.588	7.695	6.808	6.936
3.366	3.119	2.796	2.258	3.078	2.522
1.207	0.725	1.232	0.938	1.407	0.942
2.089	2.113	2.347	2.715	1.748	2.715
3.670	4.354	3.313	2.546	3.797	3.384
3.901	4.036	4.544	4.777	3.807	3.856
3.304	2.307	3.206	2.699	2.405	3.060
6.261	6.717	6.795	6.197	6.996	6.970
5.819	5.001	5.446	5.670	4.466	5.487
5.744	6.101	6.045	4.774	6.105	5.786
9.549	8.824	9.606	8.622	9.726	9.531
8.446	8.342	8.465	8.179	8.242	8.503
4.537	4.328	4.548	4.399	3.944	5.182
5.381	5.400	5.282	5.941	5.497	4.869
2.339	2.022	1.958	2.079	2.197	2.050
5.214	5.447	5.069	5.586	5.575	5.358
8.578	8.431	8.929	9.344	8.781	8.755
6.364	6.363	6.563	5.995	6.186	6.686
6.203	6.811	5.848	5.881	6.558	6.305
6.157	4.644	5.390	5.021	4.300	5.356
4.209	2.503	2.882	3.385	3.087	3.462
4.296	4.605	4.719	4.578	4.436	5.007
4.672	4.812	4.632	4.241	3.764	4.494
5.196	5.522	4.777	5.466	5.169	5.820
7.043	6.820	6.323	6.388	6.498	6.933
6.566	6.130	6.514	6.616	6.931	6.313
2.273	2.761	2.593	2.198	2.101	2.368
4.180	4.270	4.371	4.256	4.010	4.137
4.635	4.653	5.352	6.034	4.063	3.994
5.352	5.371	4.822	5.193	5.404	5.226
3.129	2.747	3.113	4.027	4.643	3.439
3.721	4.797	3.233	3.797	2.821	4.200
4.467	5.547	4.003	4.827	4.202	4.266
6.308	6.763	7.009	5.679	7.098	6.861
4.443	4.874	4.742	4.539	5.762	3.791
8.863	8.896	9.981	10.090	9.477	9.300
4.918	5.022	5.311	5.302	5.263	4.903
5.994	6.101	5.782	6.571	6.384	5.866
6.024	5.814	6.178	6.425	5.983	5.771
4.980	4.929	5.531	5.833	4.427	4.476
2.325	1.900	2.479	2.877	3.001	2.379
3.022	3.452	3.251	4.610	3.035	3.269
5.257	5.081	4.405	4.650	5.257	4.802
5.625	5.258	4.886	5.099	5.430	4.916
7.357	6.354	6.325	6.254	5.530	6.394
5.770	4.708	5.229	4.801	5.346	5.008
7.066	5.978	5.796	5.145	6.493	6.581
4.560	4.160	3.937	3.351	4.089	4.532

7.749	8.162	7.856	7.187	7.852	8.296
5.982	5.475	4.536	4.553	4.632	5.635
1.172	1.301	1.377	1.401	1.830	1.687
4.171	4.175	3.366	3.252	3.532	3.945
2.763	2.278	3.173	2.900	2.445	2.485
4.166	4.280	4.205	4.779	5.381	3.920
3.193	1.483	2.104	2.776	2.359	1.794
1.726	1.525	2.124	2.953	1.861	2.056
3.903	2.904	2.498	2.185	4.254	3.790
5.701	4.460	5.303	4.789	5.906	5.525
3.231	4.293	4.285	3.252	5.720	3.939
6.148	5.449	6.256	6.359	6.023	5.726
4.178	4.174	4.260	3.381	4.190	3.846
3.454	3.610	3.436	4.355	3.407	3.367
6.765	6.535	7.138	6.591	7.012	6.876
4.912	4.878	4.996	5.037	4.802	4.739
6.410	5.971	6.588	6.899	6.233	5.833
4.099	2.539	1.691	3.198	1.927	2.900
5.244	3.808	5.244	4.862	4.525	4.510
6.018	6.124	5.694	5.644	5.645	4.998
3.830	2.898	3.795	3.587	3.566	4.016
5.966	5.017	5.929	5.503	5.387	5.471
4.541	3.178	3.326	2.833	4.070	3.874
8.643	8.141	7.960	8.318	8.446	7.970
6.004	5.974	5.110	4.527	6.098	6.237
5.846	5.817	4.840	4.448	6.214	6.088
4.669	3.771	2.498	2.526	3.690	3.983
5.457	5.475	4.419	3.683	5.135	4.551
5.319	5.399	4.621	3.336	5.054	5.170
3.818	4.635	3.699	2.441	4.277	4.189
5.000	4.721	3.788	3.924	5.317	4.606
4.597	4.102	3.422	2.874	3.662	4.150
3.699	4.070	3.224	2.901	3.753	3.712
6.053	6.713	5.174	3.535	6.297	5.343
5.288	5.649	3.668	3.256	4.946	4.573
4.926	5.025	4.330	3.244	5.163	4.414
2.748	2.769	1.774	1.836	2.443	2.169
3.836	4.187	3.338	3.585	3.953	4.236
4.818	4.712	4.100	4.606	4.889	4.545
4.806	5.101	5.037	5.554	4.579	5.207
3.480	3.381	2.947	2.204	2.949	3.510
3.500	2.859	3.281	2.401	2.633	2.553
5.553	4.610	5.009	4.131	4.369	5.127
5.710	5.789	5.739	5.620	5.585	5.959
4.018	4.027	3.904	4.843	4.172	3.560
1.817	3.114	1.769	2.368	2.576	2.310
1.941	2.619	2.786	2.234	2.207	2.216
3.939	2.342	3.573	2.560	2.523	4.233
6.010	5.028	5.320	5.221	4.701	5.710
5.287	4.293	3.804	2.504	3.031	4.693

6.157	5.516	6.174	5.903	5.484	6.075
6.178	5.274	5.889	5.896	4.856	6.124
5.849	6.149	6.238	4.772	6.727	6.287
2.810	1.588	1.738	1.638	1.823	1.977
6.410	6.392	6.357	5.735	5.937	6.273
4.351	4.449	4.448	4.281	4.948	4.461
4.265	3.148	3.045	2.891	3.049	3.467
3.752	3.162	4.399	3.204	3.845	4.130
4.295	5.451	5.226	6.080	5.677	5.466
5.611	5.479	6.361	5.788	6.396	6.606
5.635	4.665	3.995	4.402	4.628	4.558
2.209	1.890	2.170	1.906	2.218	2.292
5.241	6.079	5.745	3.390	5.738	6.006
5.639	4.576	4.782	4.786	4.397	5.059
5.519	4.988	4.486	3.936	5.021	4.840
2.623	2.465	3.430	3.511	2.930	2.869
5.459	5.045	4.727	4.976	5.321	4.531
4.675	4.346	3.869	4.440	4.228	4.465
4.542	4.384	5.160	4.143	3.557	4.182
5.626	5.805	5.284	5.031	5.753	5.489
3.594	3.544	2.789	3.068	4.076	3.723
3.586	3.852	3.561	2.091	4.308	3.107
5.765	5.451	5.326	4.665	5.505	5.746
6.801	6.380	6.464	6.971	6.571	6.266
7.001	6.555	6.388	6.526	6.780	6.708
6.591	6.778	5.690	6.134	5.641	6.332
5.189	5.247	5.403	5.576	5.535	4.676
4.078	4.444	5.019	5.032	5.402	4.044
3.659	4.685	3.512	4.504	4.207	4.057
6.137	6.392	6.844	7.321	6.448	5.262
3.776	5.055	4.571	4.240	5.019	3.001
5.100	4.787	6.196	5.211	6.164	5.385
5.624	5.471	6.375	6.410	7.054	6.908
5.571	6.886	5.961	6.404	6.237	6.537
3.783	3.159	3.435	3.111	3.755	3.180
6.328	7.395	5.261	5.954	5.785	6.194
4.947	5.666	5.161	4.618	4.576	5.350
7.801	7.975	6.642	7.685	8.103	7.701
5.916	6.525	4.842	5.199	5.272	5.521
5.082	4.945	4.629	5.156	5.351	4.723
4.521	4.054	3.853	2.550	3.748	4.026
4.875	5.211	5.181	4.867	5.300	4.823
2.882	3.234	2.590	2.572	3.282	2.584
4.646	4.352	4.076	4.383	4.314	4.595
6.879	7.046	7.361	6.850	7.146	6.768
5.126	5.567	4.328	4.325	4.770	5.137
6.747	6.732	6.981	6.520	6.009	6.043
7.251	7.181	6.696	6.951	6.775	6.943
2.660	2.596	1.438	1.881	2.677	3.162
6.206	5.561	5.986	5.874	5.690	5.688

3.996	1.612	1.948	1.552	3.505	2.538
5.044	5.292	5.027	5.561	5.121	4.441
6.125	6.086	6.550	6.637	5.708	5.550
3.396	3.339	3.702	2.717	3.317	3.233
3.757	4.032	4.783	4.109	5.209	3.995
4.221	3.249	3.540	3.337	3.408	3.115
8.165	6.920	7.681	6.960	7.293	7.314
6.850	5.628	6.278	5.796	5.880	5.942
3.821	3.119	3.109	3.061	3.472	3.853
7.558	7.019	6.400	6.787	7.350	6.835
4.519	4.319	4.533	4.655	4.321	4.607
3.162	2.386	3.871	2.210	2.399	3.608
6.245	7.016	5.916	7.095	6.916	5.656
4.002	4.821	4.362	3.646	4.321	4.061
6.121	5.341	5.739	4.047	5.930	6.071
2.686	2.643	3.451	2.643	3.471	2.476
6.057	6.021	5.786	5.517	5.825	6.116
3.646	3.463	4.997	3.811	4.423	4.123
4.565	5.092	5.048	3.880	5.096	4.568
7.517	6.898	7.136	6.700	7.296	7.667
2.642	2.538	2.667	2.741	3.198	3.371
4.292	4.043	4.900	4.667	4.309	4.632
5.717	6.741	6.153	4.839	5.897	5.631
8.306	8.783	8.350	7.452	8.089	8.208
4.252	3.992	4.485	4.438	4.050	4.353
7.254	8.137	7.439	8.192	7.039	7.497
8.813	9.189	8.714	8.835	9.060	9.332
6.093	7.312	5.658	5.840	6.326	6.365
2.314	3.469	4.184	3.475	2.971	3.575
4.589	4.301	4.500	4.606	3.800	4.131
6.663	6.493	5.424	5.754	6.133	6.702
4.158	3.304	3.789	2.110	3.367	3.726
3.833	3.249	3.419	2.550	2.870	3.768
4.460	4.900	4.510	4.675	4.416	4.695
5.600	4.661	5.618	5.259	5.137	5.618
5.389	6.162	5.629	5.016	5.871	6.524
5.774	5.419	5.031	4.857	4.127	5.583
5.481	5.088	5.668	5.299	5.211	5.557
6.001	6.293	6.760	6.961	6.528	6.506
7.128	6.971	6.735	6.507	6.739	7.201
6.760	7.154	7.062	7.721	7.011	6.992
5.954	6.431	5.824	5.306	5.824	5.711
1.694	2.854	2.509	2.562	2.208	2.389
6.003	5.857	5.901	5.026	5.850	6.004
5.940	5.830	6.661	6.159	6.325	5.690
5.736	5.638	5.147	5.090	5.969	5.343
5.536	5.433	5.557	4.815	5.738	5.949
3.581	4.079	4.677	3.784	4.661	4.471
4.721	5.022	4.698	4.980	4.590	4.865
4.753	4.129	4.532	4.425	4.375	4.866

2.314	3.469	2.279	2.503	4.071	2.219
8.422	7.978	8.125	8.544	8.273	7.910
5.769	5.223	5.066	5.051	5.802	5.381
3.862	4.124	3.920	3.439	3.884	3.403
4.867	5.615	5.411	5.588	5.345	4.961
4.471	4.436	4.091	4.542	4.692	4.818
5.146	5.108	4.234	3.972	4.813	4.849
4.949	5.010	5.952	6.162	6.115	4.961
5.763	6.461	5.440	5.850	4.876	5.433
7.718	6.690	7.841	7.743	8.153	7.162
4.876	5.007	4.323	4.837	4.867	5.601
4.009	5.956	1.867	1.912	1.699	4.110
4.183	4.244	5.141	4.341	4.855	4.573
7.188	6.832	6.727	6.516	6.399	6.852
4.256	4.330	4.670	4.664	4.466	5.063
3.886	3.735	4.244	3.460	4.503	4.298
7.693	7.318	7.317	7.546	7.255	7.383
6.437	6.786	6.241	6.300	5.995	6.076
7.715	8.045	7.392	7.229	7.212	7.457
5.073	4.362	4.591	4.544	4.318	4.549
3.314	3.648	3.745	3.332	2.874	3.017
4.669	5.993	5.271	5.294	5.685	5.829
6.024	5.900	6.121	3.903	6.677	6.221
4.907	5.522	4.349	5.018	3.759	4.202
3.715	5.060	4.197	2.904	4.419	3.748
4.090	4.695	3.464	3.157	3.986	4.263
6.335	6.224	6.054	5.548	7.238	6.514
5.817	5.921	5.692	4.085	5.848	6.156
7.433	7.473	7.799	6.946	7.770	7.765
4.464	4.734	4.761	2.858	4.328	4.934
2.466	2.393	2.322	3.045	2.577	3.091
6.009	6.191	4.902	4.497	4.992	6.374
6.922	6.923	5.875	6.400	6.623	6.504
6.027	5.773	5.575	5.569	5.749	5.630
5.076	5.353	5.299	3.423	5.368	6.206
8.163	7.947	7.866	8.180	8.642	7.844
4.786	4.924	5.042	5.209	4.888	5.017
5.189	5.010	4.475	5.175	4.742	4.718
4.267	4.320	4.074	3.833	4.183	4.094
2.237	2.556	2.511	2.234	2.264	2.898
3.025	2.817	3.302	2.448	3.121	3.443
5.842	6.693	4.686	5.271	5.012	6.551
4.466	4.083	3.695	3.889	3.962	3.740
2.225	2.159	1.815	2.739	1.970	2.437
9.059	8.783	8.727	8.538	8.913	9.026
8.200	7.870	8.272	8.209	8.066	8.175
5.525	5.878	5.581	4.977	5.851	5.460
4.009	3.591	3.385	3.940	3.492	4.409
4.496	2.987	4.073	4.783	4.797	3.859
3.762	2.877	3.100	2.856	4.235	4.399

8.135	5.935	6.762	7.087	6.609	5.691
6.443	6.281	6.198	6.420	6.237	6.732
5.150	5.474	5.532	5.637	4.502	5.212
4.253	4.621	3.964	4.408	4.180	4.168
4.607	3.190	3.966	3.655	3.766	3.908
2.171	2.545	2.414	3.328	2.693	2.108
4.010	4.302	4.004	3.548	4.010	4.010
7.957	8.102	7.736	7.209	8.277	8.107
3.190	3.246	3.007	3.573	3.238	2.854
6.353	5.368	5.728	4.380	6.653	6.168
5.446	4.268	4.831	5.049	5.524	4.787
4.721	4.476	4.017	4.257	3.721	4.286
5.198	5.349	3.611	3.482	6.874	3.581
8.725	8.617	9.410	9.257	9.422	9.520
4.027	4.001	4.204	3.036	4.014	3.903
5.022	5.304	4.934	4.015	4.914	5.417
8.321	8.349	8.447	8.378	8.702	8.333
4.775	5.255	4.678	4.299	4.877	5.827
5.589	5.860	5.421	4.054	6.061	5.807
3.579	4.352	3.478	3.840	3.776	3.734
5.142	5.396	4.104	2.974	4.272	5.581
3.877	3.653	3.328	3.698	4.359	3.711
4.290	5.511	3.241	3.303	3.608	5.163
5.377	4.349	4.145	3.434	4.788	4.788
6.752	6.043	6.833	5.519	5.868	6.082
2.873	2.800	2.961	2.470	2.355	2.637
5.476	5.725	4.939	4.903	4.825	5.059
4.870	4.187	4.333	3.567	4.898	4.582
7.220	6.382	6.850	7.188	6.810	6.362
1.700	1.824	1.783	2.001	2.001	1.667
4.920	5.031	5.642	4.682	5.304	6.056
4.950	5.508	6.003	5.706	5.413	5.321
6.156	5.766	5.695	5.793	5.386	5.880
4.164	5.438	4.997	4.477	4.989	5.343
6.937	7.720	7.906	7.755	7.791	7.670
4.658	5.067	4.708	3.742	5.256	5.506
4.339	3.452	3.253	3.408	3.923	3.759
6.922	7.301	6.916	6.111	7.194	7.270
5.716	3.759	3.427	3.967	4.043	3.726
5.908	5.607	5.818	5.759	6.282	6.114
6.466	6.636	6.346	5.674	6.875	6.576
6.417	5.993	6.542	5.225	6.676	6.114
6.290	5.897	6.333	5.865	5.491	6.228
4.257	2.942	4.888	3.230	4.980	5.132
5.508	5.056	5.193	4.745	5.302	5.541
6.304	6.667	7.036	6.871	6.491	6.337
4.057	4.498	4.789	3.284	4.570	5.414
6.033	6.014	5.569	4.907	5.641	5.536
5.713	5.575	4.299	4.240	4.660	4.441
3.969	3.114	3.573	3.765	4.132	2.602

6.876	6.240	7.387	7.175	7.660	6.737
4.567	3.234	4.146	4.074	3.510	4.032
5.991	5.909	6.511	5.995	6.544	6.253
4.493	4.081	4.525	4.760	4.808	4.478
2.812	3.159	2.811	2.733	2.948	2.441
5.158	4.207	5.051	4.623	5.045	4.881
3.346	2.921	3.583	3.295	2.784	2.910
5.449	4.510	4.923	5.444	5.038	5.311
4.815	5.260	4.595	4.812	5.096	5.338
6.349	6.419	5.945	5.197	5.590	5.965
4.127	4.155	4.925	4.155	6.225	3.829
3.338	4.080	3.262	3.190	3.615	3.533
6.179	6.822	6.488	5.850	6.353	6.295
3.314	3.039	4.721	3.934	4.095	3.464
2.773	2.824	2.748	2.847	2.399	2.884
4.848	6.155	4.337	3.293	4.629	5.790
2.933	2.568	2.527	3.264	2.499	2.968
8.741	7.859	7.897	7.410	7.910	7.323
2.736	4.670	2.263	2.647	2.639	1.723
6.228	7.346	6.945	7.732	6.341	6.408
6.982	8.132	6.698	7.555	6.825	6.439
5.435	6.449	5.387	5.776	5.808	5.655
5.633	6.966	5.713	6.037	5.832	5.993
2.501	2.573	3.040	2.734	2.758	3.005
6.612	6.304	4.677	4.135	5.161	6.311
7.571	6.682	6.251	7.070	7.103	6.421
8.448	9.441	8.724	8.714	8.605	8.145
3.047	2.433	2.770	2.538	3.736	2.620
5.732	5.896	5.118	4.313	5.609	6.024
9.423	9.404	9.173	8.851	9.401	9.358
4.908	5.436	5.737	5.670	5.443	5.572
5.951	4.874	4.918	5.420	5.469	4.916
4.608	4.869	5.606	4.907	4.988	5.026
4.921	4.405	4.710	5.268	4.031	4.744
6.674	6.324	6.763	6.894	6.909	6.277
6.481	7.113	6.939	6.838	6.725	6.929
5.910	5.063	5.881	5.572	6.403	5.792
5.325	6.232	5.892	5.930	6.370	6.189
10.809	10.934	10.639	10.520	10.660	11.171
7.613	7.331	7.023	6.755	6.976	6.842
5.018	5.504	4.514	3.960	5.376	5.409
5.159	5.552	4.892	3.681	5.498	5.083
4.318	4.172	5.621	4.732	4.949	4.854
3.852	3.567	2.708	3.296	3.369	3.930
3.930	3.256	3.180	3.197	3.363	3.048
4.201	4.323	4.968	4.311	3.725	4.160
1.563	1.508	2.198	1.785	1.529	1.724
7.654	7.692	7.522	7.267	7.453	7.752
6.813	6.130	6.790	7.168	6.036	6.521
4.945	4.499	4.987	3.493	3.771	4.589

3.741	2.292	3.379	3.326	3.127	3.504
3.922	3.585	3.429	3.875	2.940	3.162
4.210	3.203	3.241	3.841	2.904	3.620
3.920	3.673	3.437	3.882	2.937	3.156
3.864	3.232	3.214	3.420	2.795	3.769
6.077	5.065	6.051	5.314	5.382	5.449
4.024	3.445	4.731	3.701	3.692	4.455
3.955	2.325	3.180	2.205	2.999	3.580
5.134	4.868	4.092	4.556	4.225	4.514
4.668	4.842	4.176	4.766	4.985	4.763
4.092	4.828	4.084	4.067	4.528	3.460
2.870	3.467	3.166	4.019	4.053	3.075
6.832	6.433	7.081	6.711	6.768	6.804
3.675	3.316	3.453	3.316	3.267	3.445
4.126	4.333	3.380	3.454	3.990	3.896
6.519	6.606	5.849	5.444	6.349	6.477
5.235	5.412	5.832	4.964	5.901	6.142
4.659	5.044	5.708	5.503	5.574	5.555
6.646	6.089	5.812	5.555	6.035	6.184
5.216	5.326	5.285	4.781	5.254	5.643
4.848	4.730	4.586	3.963	4.512	4.892
2.333	1.681	3.639	2.936	2.815	2.129
2.754	2.708	2.700	3.048	3.273	3.179
3.336	2.734	3.071	3.096	2.680	2.983
3.520	4.422	2.896	2.316	3.801	4.435
6.368	6.414	6.827	6.088	7.163	6.766
6.160	5.912	5.941	5.625	6.257	6.298
3.249	3.118	3.007	3.542	3.959	3.426
4.174	4.204	3.724	4.050	3.686	3.894
5.367	4.998	4.804	4.095	5.365	4.861
2.369	3.711	2.564	3.254	2.913	2.466
2.828	2.216	2.891	4.094	1.967	3.484
3.252	2.390	3.581	2.792	3.535	2.733
5.474	6.158	7.073	6.465	6.804	6.204
3.181	4.127	2.847	2.426	3.104	3.004
3.134	3.610	3.870	4.502	3.323	3.858
2.191	2.684	3.169	2.499	3.597	2.681
5.860	5.266	5.869	5.425	5.580	5.494
2.866	2.493	2.531	2.614	2.329	2.328
1.092	1.643	1.145	1.491	1.186	1.122
2.513	3.707	3.053	3.801	2.944	2.416
2.463	3.677	2.717	3.311	3.408	2.854
3.036	3.304	2.868	2.758	3.760	3.881
6.797	6.759	6.723	7.157	6.832	6.973
3.187	2.848	2.972	2.349	3.035	3.429
2.931	2.635	3.478	3.600	3.418	3.016
2.938	2.541	2.566	2.478	2.966	2.687
3.974	4.300	3.360	3.927	4.074	3.874
2.060	1.929	1.881	2.388	4.624	3.062
6.954	7.453	8.092	6.934	8.041	7.965

2.782	4.624	4.331	3.304	6.049	6.347
3.405	3.158	3.017	3.264	3.375	3.686
4.086	3.942	4.086	4.419	2.897	3.595
6.295	6.498	6.592	6.900	6.921	5.887
4.954	4.493	4.652	5.196	4.811	4.738
1.916	1.936	3.726	2.453	2.676	2.160
2.370	3.348	3.793	2.906	3.008	2.601
2.143	2.908	2.354	3.637	3.328	2.988
3.336	3.022	3.476	2.250	3.059	2.984
5.640	6.181	5.424	6.664	5.011	5.788
3.387	4.033	3.547	4.127	3.541	3.782
3.185	3.524	4.424	4.196	4.552	3.805
7.700	7.212	7.445	6.118	7.776	7.934
6.360	5.368	6.383	5.764	6.574	6.335
6.449	5.882	6.185	5.646	6.125	6.413
7.551	6.934	7.537	6.485	7.445	7.526
7.128	6.251	6.861	6.497	6.867	7.464
5.412	5.275	5.665	4.829	5.511	5.530
4.589	4.789	4.857	4.454	5.288	5.245
3.460	3.407	2.473	2.457	3.013	3.578
6.779	6.135	6.547	6.833	6.478	6.998
5.638	5.155	5.508	5.474	5.417	5.935
7.600	7.132	7.416	7.292	7.490	7.705
8.368	7.898	7.820	7.552	8.038	8.634
6.507	5.738	6.153	5.879	5.915	6.625
4.756	3.667	5.029	2.909	4.053	3.994
6.136	6.005	5.557	4.884	5.954	6.273
2.895	2.980	2.487	2.738	2.963	2.456
3.382	4.236	3.436	3.439	2.825	3.997
5.548	5.648	5.873	5.136	5.541	5.610
3.366	2.857	2.458	3.394	2.570	3.146
6.850	6.575	6.558	6.223	6.671	6.567
4.519	4.523	4.741	4.784	4.507	4.419
5.874	6.318	5.749	5.270	5.834	5.817
6.072	6.197	5.908	6.190	6.084	5.961
4.723	4.573	5.212	5.320	4.872	4.543
3.262	3.250	2.999	3.461	3.070	3.398
4.642	4.846	3.816	4.939	4.331	4.393
6.615	5.601	6.202	5.288	6.334	6.052
4.856	5.243	4.296	4.881	4.239	5.011
5.913	6.263	5.541	6.174	5.288	6.099
4.893	5.318	4.790	4.905	4.296	5.171
5.022	5.293	5.091	4.944	4.314	5.249
4.758	5.311	4.986	4.899	4.383	5.176
6.035	6.967	6.114	6.246	5.380	4.940
5.000	4.626	6.111	5.979	5.813	4.344
3.972	3.422	3.480	3.154	2.935	3.237
4.323	4.560	4.649	5.031	2.926	3.738
3.173	3.741	2.759	2.884	3.261	2.863
6.672	8.208	7.226	7.831	7.041	6.496

6.590	6.302	6.521	6.288	6.598	5.868
6.598	6.845	6.787	7.124	6.512	6.639
6.547	6.073	6.150	5.126	6.418	6.484
4.836	4.989	4.779	3.901	5.912	5.526
5.668	5.093	4.548	4.851	5.584	5.180
3.359	4.067	4.091	3.581	3.567	3.940
5.750	5.744	5.561	5.587	5.691	5.760
6.487	6.285	5.933	5.970	5.917	6.363
8.050	7.393	7.215	7.431	7.373	7.579
6.361	6.032	5.935	5.615	5.980	6.319
3.575	3.894	3.709	4.381	4.072	3.936
7.895	7.502	7.402	7.624	6.914	7.505
3.280	3.308	2.462	2.186	3.364	3.547
4.659	4.703	4.728	4.523	4.335	4.889
2.538	2.931	3.411	3.103	2.698	2.245
5.410	5.227	5.599	5.118	4.597	4.897
6.257	5.626	5.312	4.097	6.191	6.135
3.348	3.533	3.916	3.633	3.829	4.641
4.213	3.853	4.074	3.432	3.595	3.273
4.529	3.758	4.376	3.969	4.122	5.038
3.780	4.284	3.648	3.734	4.310	3.465
5.142	5.155	4.823	4.389	5.044	4.874
3.177	3.804	4.012	3.609	3.207	3.388
5.710	5.418	5.633	5.860	5.699	5.473
5.211	4.810	4.887	4.946	4.698	5.129
4.642	4.923	5.239	5.898	5.022	4.916
2.952	3.177	2.765	2.716	3.907	2.735
3.486	3.927	3.829	3.687	3.428	3.689
5.234	5.670	5.669	6.640	6.650	3.547
5.293	5.383	5.597	5.162	5.549	5.712
8.839	8.445	8.198	7.870	8.177	8.502
8.011	7.845	7.874	8.051	7.638	7.471
1.888	1.561	3.914	2.157	3.428	2.367
0.931	2.531	1.808	1.354	6.626	1.324
5.372	4.805	5.190	4.391	4.814	4.505
6.768	6.337	6.530	6.976	6.880	6.565
2.710	3.308	3.110	3.992	2.627	2.626
5.711	5.709	5.630	4.742	6.060	6.083
5.398	5.267	3.843	3.898	5.322	5.198
7.457	6.847	7.182	6.966	7.199	7.190
2.657	2.439	1.913	2.812	1.872	1.719
5.361	4.696	5.314	5.383	4.850	5.558
2.567	2.498	3.463	3.608	2.741	3.121
5.644	5.563	5.138	5.719	5.504	4.993
4.733	4.271	4.225	4.126	4.215	4.814
4.203	3.410	4.069	3.890	3.635	3.480
4.407	1.963	3.409	3.214	3.216	3.275
5.720	5.842	5.682	5.322	5.426	5.374
5.185	3.843	3.946	3.953	4.250	3.854
3.170	2.125	2.298	2.079	2.663	2.272

8.165	8.264	8.595	8.480	8.660	8.689
4.717	5.040	5.276	5.404	5.245	4.597
5.615	5.132	4.864	5.274	5.518	4.875
3.306	2.528	2.544	2.749	2.445	2.643
4.947	4.736	4.480	5.751	5.540	4.378
3.022	3.063	2.474	1.893	3.827	3.399
3.068	3.206	2.006	3.359	2.215	2.235
3.923	1.524	1.628	1.230	2.263	2.222
4.020	2.874	3.906	3.498	4.087	3.832
3.442	3.674	3.112	3.696	2.810	3.727
5.495	4.976	4.576	4.219	4.700	5.262
5.178	5.036	5.653	4.702	5.950	4.659
2.571	2.580	2.416	2.945	2.777	2.970
1.632	1.926	2.225	2.755	2.174	2.390
2.178	1.625	2.178	2.102	2.452	2.437
5.241	5.985	4.462	4.490	4.908	5.549
6.594	6.189	6.328	6.447	6.268	6.635
2.992	2.894	3.458	2.888	3.075	2.718
6.475	5.961	5.754	5.536	5.910	6.108
3.482	3.074	3.540	2.924	3.654	3.622
2.442	2.841	3.102	2.391	2.797	3.052
5.572	5.235	5.313	4.524	5.203	4.944
4.878	5.949	5.098	4.989	4.418	5.133
4.312	4.387	4.416	5.617	4.794	4.433
3.726	4.157	4.555	4.189	4.395	4.565
6.401	6.477	6.663	6.616	7.051	6.862
3.254	2.544	2.666	3.281	2.731	2.265
5.390	5.193	3.626	4.452	3.887	3.423
3.911	3.693	3.908	4.171	3.957	3.480
3.176	3.194	3.528	3.620	3.899	3.313
3.756	3.535	3.803	3.992	3.791	3.358
4.396	3.273	4.566	3.885	3.738	3.883
4.400	3.978	3.816	4.416	4.288	4.604
4.497	4.676	4.579	4.386	4.832	4.701
4.714	5.083	4.212	4.033	4.900	4.578
2.421	2.308	2.122	3.213	1.631	2.323
3.403	2.556	2.125	2.718	2.685	3.094
2.011	2.636	2.691	1.450	2.896	3.020
1.992	2.353	2.740	2.516	3.211	2.316
6.707	6.095	6.751	6.552	6.866	6.903
4.218	4.912	4.933	2.935	5.910	5.240
4.731	3.972	4.320	4.114	3.533	3.927
7.880	6.305	7.966	8.191	7.761	6.315
3.365	2.908	3.184	3.360	2.995	3.713
5.254	4.989	5.086	4.629	5.599	5.446
4.940	5.136	5.490	5.181	5.037	5.566
6.042	5.968	5.290	5.255	5.151	5.678
6.023	5.929	5.262	5.246	5.131	5.730
3.933	3.487	3.715	3.462	3.611	3.480
3.895	3.214	2.824	2.563	3.211	3.255

3.480	2.896	2.641	2.242	2.784	2.806
4.382	3.798	3.324	3.070	3.539	3.769
4.322	3.657	3.612	3.390	3.696	3.877
4.033	3.250	3.383	3.209	3.442	3.837
6.808	7.134	6.828	6.613	6.526	7.164
3.254	3.293	2.788	3.309	3.230	3.977
5.287	5.340	4.450	4.185	4.975	4.768
3.969	4.416	4.843	4.352	5.148	5.227
5.412	5.627	4.978	5.423	5.509	5.974
4.968	4.792	4.812	4.913	5.045	5.404
5.368	4.054	2.921	3.415	4.369	4.379
3.695	3.534	4.030	3.159	3.522	4.764
7.488	8.083	7.212	7.185	7.618	7.531
5.021	4.725	5.227	6.207	5.340	4.403
3.367	4.148	3.681	4.526	3.240	3.560
5.773	5.326	4.913	4.262	4.850	4.676
3.453	2.417	3.735	3.012	3.327	2.540
3.266	3.096	3.208	2.068	3.300	4.022
2.349	2.863	2.328	3.482	3.518	3.239
6.467	6.722	6.361	6.009	6.104	6.537
2.187	3.540	3.043	3.948	5.400	2.371
8.463	8.680	8.849	8.479	8.929	8.442
2.381	3.282	2.274	1.760	3.069	2.682
3.056	2.489	2.753	2.291	2.561	2.504
3.745	3.861	3.154	2.940	3.619	3.559
5.668	5.477	4.697	5.462	4.820	5.644
3.317	4.037	3.281	2.559	3.712	3.459
5.951	6.447	5.965	6.023	5.375	6.672
3.602	3.770	3.935	3.198	4.132	4.504
3.792	4.761	4.220	3.736	4.457	4.345
3.539	4.103	4.605	3.811	4.003	3.790
1.480	1.754	1.624	1.372	2.186	1.682
4.680	4.722	4.597	4.673	4.954	5.051
5.347	6.520	5.671	6.755	5.389	6.582
5.384	6.211	5.961	6.371	5.155	5.358
4.806	4.392	4.846	4.654	4.557	4.452
8.527	7.474	8.431	8.280	8.476	7.657
5.959	5.933	4.992	4.610	5.260	5.640
3.200	3.660	3.115	3.122	3.623	3.938
5.447	5.288	4.327	3.980	4.412	5.446
2.131	2.954	1.832	2.878	2.652	2.108
3.622	3.719	3.220	3.433	3.338	3.184
4.919	5.004	5.364	5.440	4.861	5.159
5.741	4.597	5.326	5.027	4.520	5.163
4.474	4.783	4.497	4.216	4.839	5.537
6.437	6.657	5.864	4.798	6.050	6.860
2.563	3.205	3.991	3.688	3.056	3.801
7.871	7.215	7.081	6.452	7.768	7.547
3.800	4.474	3.973	4.299	4.847	4.886
1.621	1.244	1.663	1.405	1.260	1.364

3.478	4.177	3.380	2.625	4.312	3.896
6.322	6.328	5.929	5.462	5.842	6.365
6.422	6.222	6.056	5.472	6.003	6.499
7.474	7.713	7.135	6.922	6.894	7.055
6.051	4.927	5.485	5.340	4.509	5.455
5.742	4.409	5.484	5.016	5.095	5.093
4.201	3.507	3.602	3.076	3.542	4.372
3.882	1.943	2.896	2.522	3.304	2.810
5.904	3.347	5.044	5.340	4.625	4.657
5.892	6.378	6.583	5.864	6.637	6.263
7.564	7.823	7.257	7.335	7.708	7.420
2.845	2.580	2.751	3.668	3.483	2.762
5.763	5.949	5.184	5.396	5.181	5.365
5.003	5.100	5.041	4.872	5.158	4.629
3.489	4.472	4.081	3.807	3.915	3.619
4.120	4.353	3.498	3.663	3.974	4.170
5.660	5.510	4.415	3.593	4.834	5.442
2.487	4.159	4.166	3.485	1.846	4.013
5.575	5.490	4.910	3.985	5.611	5.093
3.725	4.180	3.338	3.893	3.865	3.992
6.168	4.340	4.877	3.953	4.290	4.897
2.380	1.542	2.465	2.337	2.016	1.735
8.710	8.279	8.563	8.679	8.346	8.755
8.107	7.624	8.005	8.204	7.510	8.064
4.295	4.635	4.705	4.390	3.970	4.949
5.990	6.120	5.980	6.734	6.146	6.538
3.803	4.971	3.748	3.779	3.628	4.886
8.399	8.286	7.920	7.744	8.347	8.393
4.646	4.664	4.198	4.424	4.821	4.396
2.870	4.018	4.095	3.294	2.958	4.371
4.186	3.905	4.009	4.098	3.597	3.793
4.654	4.653	4.918	4.988	5.011	5.059
5.279	5.989	4.412	3.503	4.819	5.763
6.004	4.043	7.166	6.429	6.379	4.085
4.019	4.065	4.316	4.079	4.835	4.059
2.587	3.155	2.551	3.311	2.485	2.472
8.393	8.246	8.237	8.709	8.024	8.277
9.323	9.165	9.210	9.042	9.560	9.427
4.019	3.522	3.341	3.794	3.180	3.753
5.386	4.812	6.012	5.562	5.775	5.106
3.372	4.416	3.240	4.338	3.324	3.992
5.710	5.866	4.910	4.247	6.075	5.362
5.458	5.912	5.864	4.919	5.862	6.004
4.914	5.597	5.340	5.180	5.478	5.180
7.018	6.377	6.763	6.492	6.717	6.947
3.905	3.141	4.270	2.893	4.875	3.852
2.140	2.543	1.715	2.132	2.306	1.906
7.281	6.931	6.503	6.170	6.694	6.861
7.115	6.770	7.563	7.640	7.294	7.502
4.257	4.517	4.145	4.176	3.439	4.114

4.203	5.382	4.737	4.787	4.181	4.607
4.215	4.096	4.208	4.219	3.459	4.728
4.812	5.083	4.431	5.079	4.482	4.566
4.830	4.422	4.388	3.967	4.477	4.140
4.396	3.958	4.694	4.336	4.055	4.235
4.845	5.268	4.400	3.986	4.022	4.934
5.314	3.706	3.514	3.525	3.442	4.928
6.256	6.347	5.714	6.177	6.025	6.351
3.949	3.467	3.297	3.473	3.559	4.077
3.829	4.461	4.661	4.606	4.429	3.847
1.940	2.074	2.553	2.412	1.911	1.997
3.837	4.075	3.609	3.374	3.240	3.686
5.295	5.943	4.847	5.069	5.010	4.514
5.533	5.284	5.741	5.837	4.856	5.480
5.449	4.175	4.674	3.644	4.431	4.981
4.681	3.923	4.253	4.035	4.260	4.496
5.471	6.256	6.128	6.940	5.276	5.818
7.087	7.092	6.831	6.870	6.989	7.239
4.213	4.226	3.407	3.480	4.022	3.518
3.036	2.989	2.580	2.809	2.314	2.617
4.529	4.856	4.352	4.356	4.576	4.693
2.478	2.308	1.481	1.474	1.790	3.769
5.964	6.135	6.176	6.257	6.224	6.084
8.555	8.423	8.258	8.166	8.240	8.261
7.893	7.925	7.954	7.959	7.494	7.845
5.596	4.301	4.054	1.834	4.861	4.650
4.227	4.431	4.443	3.594	3.791	4.495
4.277	4.204	4.816	4.111	3.545	4.634
3.848	3.860	3.372	3.477	4.322	3.380
7.494	7.471	7.190	6.244	7.539	7.543
4.442	4.057	4.904	3.785	5.136	4.286
4.349	4.604	4.329	3.907	4.102	4.173
5.155	4.981	5.132	4.151	5.230	5.752
4.327	3.959	4.066	4.119	4.041	4.245
4.370	4.152	3.849	3.651	4.012	4.139
3.172	2.588	2.919	3.097	3.302	3.183
4.295	4.503	4.993	4.698	4.944	4.705
4.031	3.286	3.369	3.504	3.578	3.499
6.033	6.396	6.083	6.463	6.145	6.497
8.127	8.122	7.615	7.713	7.764	7.947
4.772	4.464	4.140	4.077	3.750	4.503
5.404	6.033	5.985	6.652	6.007	5.426
4.819	4.828	6.042	4.417	5.292	5.715
4.635	4.738	4.887	4.941	4.875	4.534
5.075	4.831	5.866	3.249	6.284	5.805
7.192	5.832	7.002	8.246	7.754	6.394
5.573	6.031	5.167	4.972	5.306	5.951
4.406	4.323	2.978	3.806	4.146	4.816
5.351	4.500	5.014	4.732	4.729	5.032
4.728	4.081	5.129	5.269	3.675	5.194

7.209	7.365	7.180	6.916	7.362	7.226
4.261	4.374	4.406	4.513	5.203	4.787
6.593	6.092	5.961	6.340	6.444	6.124
6.319	5.235	5.906	5.709	5.044	5.733
1.777	1.826	2.166	0.895	1.406	1.767
3.672	3.082	3.390	3.509	3.266	3.352
5.808	5.907	5.746	5.847	5.945	6.046
3.425	3.074	4.028	4.018	3.947	3.856
3.298	3.395	3.210	3.123	3.614	3.451
2.569	2.592	4.101	3.929	2.837	3.052
5.635	5.808	5.724	5.723	5.788	5.676
5.414	5.172	5.053	3.978	4.885	5.647
6.832	6.602	6.350	6.477	6.678	6.567
5.293	4.738	3.997	4.081	4.793	5.008
3.726	4.976	4.839	3.926	4.114	2.970
4.935	5.230	5.997	5.336	4.890	4.494
5.025	5.720	5.866	6.022	5.403	5.124
7.677	8.459	8.966	8.600	7.815	7.176
5.438	5.902	6.882	6.272	5.604	5.622
6.315	7.031	7.504	6.582	6.395	5.824
6.353	6.611	7.044	6.921	6.105	5.624
8.264	8.723	9.171	9.097	8.274	7.756
6.202	6.944	7.143	6.996	6.361	5.952
5.811	6.434	7.181	6.256	6.123	5.790
6.200	7.405	7.945	7.003	6.905	6.088
3.429	4.169	4.265	4.311	3.135	4.027
4.586	4.418	5.756	5.115	4.023	3.695
6.343	7.425	8.214	7.192	6.788	6.121
3.957	3.317	2.964	3.162	2.716	3.238
6.510	6.616	6.333	6.161	6.625	6.632
3.870	4.139	4.527	3.938	4.360	4.250
7.529	8.593	6.035	7.045	6.974	8.081
4.800	4.232	4.257	3.577	4.232	4.503
5.222	5.013	4.830	5.079	4.246	5.449
4.105	5.038	4.659	4.001	3.439	4.804
7.136	6.292	6.954	5.777	7.165	7.147
2.047	2.006	3.208	1.992	2.092	3.019
3.980	3.871	4.043	3.397	3.664	3.368
2.146	1.957	2.393	2.527	1.835	2.177
6.135	5.084	6.533	5.255	6.432	6.438
3.334	3.729	3.914	3.261	3.761	3.865
2.970	2.881	3.615	3.387	3.149	3.060
2.504	2.747	2.914	3.278	3.224	3.278
5.330	6.081	5.697	6.037	5.636	5.897
2.545	3.117	2.947	2.735	3.450	2.630
5.170	5.420	5.540	5.235	5.088	4.608
2.615	2.813	3.232	2.539	3.101	3.135
9.628	10.005	10.181	9.379	10.420	10.196
4.364	4.714	4.906	4.301	5.079	4.672
6.738	6.233	6.336	6.941	6.892	6.595

6.087	5.956	6.103	6.313	6.485	6.240
4.174	4.542	3.768	4.717	3.545	3.822
6.705	6.669	6.447	6.589	6.112	6.425
6.093	6.182	5.249	5.909	5.642	6.208
5.788	5.366	5.690	4.110	6.233	5.887
5.730	6.082	5.786	5.468	6.140	5.745
5.627	4.163	4.740	4.357	4.119	5.303
5.024	4.488	4.564	4.515	4.575	4.947
4.351	2.895	3.870	3.474	3.236	3.588
4.640	5.101	3.943	3.607	5.560	4.889
1.616	2.779	1.858	2.098	1.475	1.543
5.367	5.107	5.369	4.411	5.567	5.682
3.825	4.129	4.100	5.022	4.406	3.971
3.131	3.469	2.804	2.277	2.753	3.113
4.433	4.705	4.107	4.578	4.088	4.732
2.380	2.810	2.259	2.397	2.606	3.089
5.860	5.310	5.405	5.400	5.231	5.456
5.097	4.202	4.574	3.841	3.947	4.207
4.250	3.886	4.168	1.904	3.150	4.160
3.953	4.510	3.387	4.024	4.689	3.288
7.383	7.134	7.467	6.843	6.967	7.419
5.220	4.707	4.641	3.594	4.904	5.118
4.334	4.191	4.080	4.371	4.331	4.732
3.374	3.999	3.592	3.758	3.470	3.940
2.536	1.918	2.988	3.340	2.840	2.094
3.090	2.678	3.112	2.866	3.533	3.282
2.676	1.780	2.260	2.314	2.036	1.962
5.517	5.823	4.284	3.672	4.751	5.400
6.769	6.188	6.397	5.933	6.908	6.704
4.654	4.591	4.093	3.854	4.634	4.007
5.935	5.824	4.941	4.519	5.518	5.774
3.404	3.378	3.675	3.908	2.742	3.568
5.974	5.549	6.416	5.877	5.269	5.832
7.650	8.020	7.538	6.821	8.073	7.984
4.179	4.076	4.467	3.958	3.762	4.375
7.718	7.328	7.529	7.209	7.494	7.513
6.372	6.441	5.752	5.336	6.354	6.166
5.807	6.695	5.830	5.267	6.056	5.810
7.043	7.370	7.306	6.640	7.865	7.660
5.211	4.987	5.428	5.233	4.621	5.264
6.839	5.997	5.076	5.189	5.305	5.666
4.113	3.705	4.257	3.800	4.195	4.425
4.116	3.351	4.230	5.018	3.694	3.803
6.735	6.672	6.871	6.565	7.138	6.913
3.513	3.660	3.933	3.780	3.347	3.701
5.349	4.648	4.669	4.723	4.098	4.754
7.170	7.354	7.349	6.666	7.583	7.378
4.809	5.120	4.673	5.062	4.777	4.571
4.484	3.578	3.857	3.720	4.549	4.985
5.373	5.235	4.977	4.802	5.424	5.368

3.490	3.520	3.478	3.397	3.194	3.726
4.532	5.413	5.428	6.012	4.466	4.441
3.921	2.867	3.803	2.221	4.036	4.241
5.112	5.305	5.536	3.787	5.512	5.533
3.207	3.321	2.631	2.993	2.424	4.022
8.251	7.949	8.051	8.428	8.384	7.560
3.511	4.099	4.284	4.854	3.304	3.511
5.052	6.035	6.079	5.752	5.959	4.741
7.180	7.654	6.892	6.890	7.269	7.197
4.217	4.170	4.609	4.497	3.522	4.305
7.742	7.274	7.016	8.389	7.965	6.695
4.436	4.695	3.778	4.588	4.535	4.172
4.507	4.387	4.878	5.264	4.843	4.340
3.362	2.798	2.957	2.183	3.191	3.097
7.272	7.080	6.718	6.351	6.950	6.792
7.593	7.420	7.035	6.749	7.221	7.102
8.495	8.416	8.051	7.760	8.198	8.040
1.372	1.992	2.761	2.094	2.599	1.164
8.609	9.302	8.263	7.570	9.519	8.936
3.792	4.227	4.059	3.001	3.873	4.944
5.328	4.969	5.715	4.888	6.207	6.056
3.277	3.555	3.134	4.198	3.502	3.105
5.454	5.542	3.891	3.024	4.341	6.323
4.659	4.189	3.952	4.128	3.074	4.207
5.288	5.522	4.870	4.033	4.990	5.806
7.287	8.458	6.589	6.725	7.182	7.091
2.857	2.375	2.735	2.816	2.536	2.946
2.700	2.581	2.887	2.780	2.601	2.714
3.808	3.697	4.271	3.859	2.443	3.861
4.926	4.191	4.647	5.131	4.974	4.793
5.510	4.468	4.360	5.119	4.510	4.474
5.654	5.793	5.845	5.592	4.030	5.937
4.113	3.262	3.424	2.730	4.935	4.044
5.481	4.401	6.086	5.664	5.675	4.808
6.629	6.152	6.322	6.112	6.371	6.087
3.549	3.734	3.271	4.029	3.154	3.492
5.881	6.065	5.057	5.198	5.555	6.086
3.361	2.963	3.510	2.366	2.399	2.710
4.294	4.515	4.984	3.619	4.939	4.160
3.893	3.676	4.185	5.067	3.834	3.903
2.032	2.133	1.984	2.407	1.889	2.206
9.478	10.164	9.316	9.640	10.179	9.510
4.896	4.422	5.435	4.957	5.372	4.666
3.696	3.428	3.659	3.297	3.047	3.479
2.306	2.538	3.409	2.263	2.687	2.950
4.559	4.609	4.596	3.951	4.212	4.044
7.117	7.154	6.893	6.720	7.032	7.021
5.552	4.862	5.145	5.355	4.879	4.899
5.300	5.651	5.251	5.000	6.012	5.971
2.226	3.476	3.182	3.154	1.595	2.107

2.700	2.547	2.062	2.718	2.463	2.125
3.190	3.491	3.638	4.524	2.863	3.014
5.028	5.441	5.204	4.274	5.225	5.776
3.204	2.913	2.038	2.129	3.050	2.691
4.121	4.444	5.302	4.449	4.153	5.047
4.945	4.648	4.896	5.071	4.510	5.419
5.484	3.993	4.186	4.503	4.079	4.553
5.981	4.321	4.821	5.474	5.074	5.100
3.582	2.298	3.087	3.154	1.775	2.875
1.826	2.842	2.876	2.460	2.649	2.304
3.157	3.495	3.153	2.958	3.249	3.499
4.578	4.312	4.374	4.216	4.361	4.571
5.618	5.489	5.480	5.357	6.003	5.571
2.824	3.451	3.404	3.480	2.657	2.370
4.616	4.375	4.813	4.985	3.419	4.730
2.218	1.823	2.418	1.705	2.560	2.847
6.590	6.760	5.705	6.305	6.372	6.392
7.520	7.102	6.586	6.666	7.212	7.335
3.086	2.749	3.233	4.047	2.479	2.739
5.259	5.046	5.013	4.422	5.369	4.894
5.003	4.867	4.853	4.529	4.894	4.791
6.068	6.204	5.564	6.003	5.866	6.007
11.278	11.336	11.594	11.647	11.168	11.272
5.640	5.610	5.212	6.279	5.149	5.604
11.348	11.308	11.993	12.273	11.121	11.153
4.953	3.527	3.095	2.973	3.982	4.203
0.747	1.213	1.650	1.469	0.670	1.235
6.349	6.238	6.923	7.479	6.018	6.251
2.214	1.858	2.210	2.067	2.034	1.886
3.152	2.851	2.363	2.306	3.983	3.532
2.459	3.585	1.718	3.987	4.144	3.316
2.156	3.562	2.402	3.789	2.822	2.347
3.706	3.064	3.418	3.071	3.023	2.923
5.954	5.391	6.470	6.919	5.399	5.985
2.137	1.967	3.207	3.572	2.712	2.763
3.206	3.592	3.528	1.863	4.765	4.718
3.151	4.862	4.303	4.156	4.233	4.044
2.336	2.432	2.302	2.687	2.483	2.916
3.061	2.295	2.478	2.644	3.273	3.603
4.844	5.232	4.518	3.019	4.979	5.281
3.288	2.474	4.213	2.937	2.067	2.692
6.173	6.386	6.609	7.574	5.993	6.094
5.759	5.138	4.689	3.723	4.857	5.010
2.764	2.578	3.010	3.273	3.146	2.764
2.389	2.710	2.596	1.884	2.860	3.559
4.903	4.512	3.951	4.073	4.286	5.682
3.202	1.690	2.585	3.467	1.356	2.877
3.044	4.579	3.249	4.263	3.338	4.402
2.232	2.430	3.625	1.787	3.097	2.858
3.720	2.535	3.174	6.031	4.031	3.660

2.135	2.942	2.893	4.415	2.824	2.473
3.980	1.972	4.186	5.138	3.586	3.167
3.028	2.370	3.147	1.858	2.023	2.370
2.547	3.500	4.822	3.872	3.629	3.607
3.062	3.846	3.659	4.447	2.490	3.530
6.460	6.157	6.197	7.185	6.033	5.846
1.352	2.511	3.000	3.932	3.065	2.381
3.218	3.412	4.692	3.727	2.458	2.824
6.059	6.378	7.167	8.502	5.992	5.974
3.129	4.125	2.940	3.250	4.013	3.483
3.668	2.777	3.651	4.275	3.855	3.832
3.172	3.387	3.597	4.172	3.781	3.640
3.431	3.805	3.863	4.110	3.237	3.692
3.657	2.393	3.580	2.044	1.107	1.811
2.663	1.601	1.133	3.044	2.177	1.716
3.458	2.869	2.516	2.726	2.812	3.710
5.486	5.183	6.069	7.440	4.766	4.643
2.331	3.329	2.656	2.931	2.420	2.233
3.523	2.361	3.494	1.645	1.354	2.383
5.174	4.460	5.167	5.253	4.137	4.519
9.591	9.455	10.174	10.672	9.468	9.249
10.281	10.054	10.638	11.312	10.160	9.918
2.559	1.488	2.751	3.185	3.247	1.968
2.994	2.155	3.224	3.956	2.433	2.308
2.929	3.120	2.216	3.595	2.274	2.886
5.094	4.479	4.110	3.172	4.938	5.052
2.152	2.856	3.386	4.168	2.900	2.922
2.990	2.614	2.712	3.167	1.806	2.283
1.518	3.288	2.993	1.452	2.184	3.089
2.478	3.364	2.420	3.848	3.152	3.202
2.061	2.313	1.923	2.530	2.224	2.680
4.876	4.545	4.542	5.522	3.337	3.920
2.640	3.160	2.737	2.814	2.598	2.596
3.607	3.609	4.416	5.656	3.275	3.466
3.673	1.480	2.752	2.560	3.452	2.956
2.367	1.434	1.751	2.346	1.492	2.149
4.701	3.575	3.831	3.879	4.542	4.561
2.108	1.195	2.071	3.194	2.592	3.436
2.434	2.759	2.273	1.855	1.335	1.027
2.649	2.094	1.968	1.289	3.089	3.314
3.556	3.072	3.535	3.394	4.567	3.690
3.829	4.194	3.815	4.292	3.786	3.629
2.256	2.675	1.446	2.549	3.145	2.009
3.031	2.904	3.187	3.087	3.865	2.864
2.065	1.747	1.977	1.199	3.221	2.001
4.053	2.955	3.346	2.714	3.902	3.485
5.043	3.963	3.861	4.024	3.201	3.831
4.370	5.042	5.089	6.124	4.081	4.804
4.591	4.793	4.504	6.933	4.480	4.821
4.084	2.671	2.858	3.730	4.279	1.802

2.394	3.802	2.348	2.402	2.834	3.179
2.321	3.511	3.802	3.707	1.799	3.012
2.689	2.549	3.498	3.940	3.911	3.079
2.286	2.367	3.614	3.357	2.033	2.196
3.929	3.055	3.011	3.908	3.822	3.508
4.258	4.832	5.039	5.344	4.747	4.407
3.742	3.735	3.929	2.491	4.336	3.815
4.290	4.433	2.587	1.670	4.812	3.799
3.037	3.464	3.155	3.293	2.689	2.584
4.125	2.976	2.561	3.062	3.273	3.521
2.455	2.654	3.539	3.318	2.766	2.505
4.429	4.983	4.644	5.594	4.071	4.609
4.047	3.292	3.525	2.518	4.803	2.599
2.699	2.549	2.500	4.220	2.453	2.372
2.715	2.396	2.400	3.129	3.130	2.850
3.129	3.186	3.533	3.062	2.804	3.484
2.388	1.926	1.926	1.395	1.854	2.125
2.142	2.127	2.258	3.115	1.821	1.928
3.090	3.092	4.095	4.142	3.732	3.776
5.145	5.197	5.528	5.355	4.749	5.187
3.346	3.119	3.549	3.459	2.933	1.582
4.352	3.255	4.373	4.427	4.513	4.049
2.366	2.236	2.279	2.555	2.685	2.150
1.952	2.586	2.294	4.566	1.656	1.901
3.277	2.940	2.476	2.049	2.643	3.680
6.135	7.011	6.997	8.332	4.869	6.188
3.753	3.053	3.486	3.639	3.831	3.487
4.745	5.262	4.777	5.883	4.691	5.133
6.136	5.557	6.063	5.304	5.569	5.428
5.143	7.390	4.481	3.604	6.381	8.406
2.484	4.707	3.590	1.658	3.431	5.179
6.265	7.179	5.506	4.791	6.350	6.897
4.087	4.210	3.298	3.956	3.764	4.182
5.786	5.717	4.993	4.948	5.657	5.962
6.497	4.210	4.456	4.286	4.298	4.415
8.434	8.446	9.031	9.300	9.044	8.084
4.292	4.358	2.539	3.936	3.358	3.179
8.241	7.680	7.886	8.030	8.053	7.943
6.001	5.876	5.986	5.022	6.065	5.836
3.652	3.586	3.987	3.511	3.291	3.137
6.054	5.060	3.760	3.409	4.588	4.254
3.638	3.890	4.475	3.616	5.208	5.387
5.259	4.867	4.851	5.168	5.113	4.865
3.004	3.126	3.014	2.997	1.804	3.046
8.292	8.167	8.291	7.859	8.602	8.611
3.476	4.109	3.452	3.524	3.691	4.372
3.174	2.753	2.030	2.435	3.149	3.103
1.525	1.558	2.646	1.355	1.868	1.833
5.027	6.052	4.853	5.559	4.878	5.335
5.599	5.270	5.634	5.541	5.688	5.494

5.660	6.108	4.559	3.896	5.033	5.715
4.510	4.316	4.450	4.158	4.283	3.898
7.137	6.971	7.225	7.173	6.581	7.376
3.794	2.795	2.501	3.540	3.761	3.477
3.709	4.023	3.800	3.965	2.436	3.617
4.570	4.031	4.023	4.603	4.237	3.501
1.999	2.164	2.249	1.241	2.417	3.110
5.746	6.017	5.855	5.112	6.010	5.646
4.842	4.706	4.818	4.101	4.417	5.096
6.938	6.859	7.118	6.717	7.392	7.311
3.359	3.460	3.337	3.116	2.883	2.556
5.172	5.271	4.505	5.392	5.322	5.611
6.081	3.919	5.877	4.996	5.526	4.679
6.211	5.185	6.018	6.500	6.046	6.157
5.549	5.646	5.648	5.484	6.124	5.699
3.026	3.067	3.553	2.822	3.291	3.101
6.442	6.145	6.095	6.157	5.836	6.209
2.798	2.988	3.085	2.994	2.967	2.650
3.245	3.443	4.242	3.680	2.852	2.503
6.185	6.275	6.203	6.419	6.076	6.036
4.489	4.794	4.074	4.055	4.453	4.275
5.819	4.928	5.663	5.169	5.634	5.425
5.383	5.112	5.220	4.570	4.727	5.343
6.479	6.570	6.374	6.419	6.330	6.432
5.815	5.513	4.860	4.569	5.774	5.579
4.457	4.478	4.148	3.812	4.080	4.067
7.316	6.408	6.782	7.168	7.159	7.092
4.156	4.710	4.126	4.851	4.746	4.905
4.161	4.340	4.609	2.088	3.146	3.329
4.128	3.912	4.437	3.607	4.566	3.902
10.770	9.804	10.766	11.007	10.423	10.284
4.787	3.856	3.804	2.065	2.899	4.239
1.745	1.139	1.997	2.547	3.320	1.868
2.129	2.802	2.157	4.059	1.910	2.172
2.768	3.092	3.240	3.866	2.735	2.720
6.130	6.172	6.633	7.333	5.907	6.375
3.963	4.408	4.328	3.648	4.263	4.313
5.054	4.934	5.912	5.879	4.995	4.859
2.931	4.147	2.524	4.380	1.837	3.922
5.253	5.057	5.280	6.010	4.680	5.075
4.039	4.625	4.191	5.532	4.369	4.502
2.552	3.063	3.063	2.983	2.364	2.471
5.379	4.382	3.288	4.551	4.131	5.015
3.407	3.970	3.975	5.353	4.714	5.143
5.651	4.817	5.305	6.459	4.929	5.569
6.447	6.891	7.561	8.223	6.652	6.560
2.812	2.383	3.127	2.315	3.259	2.610
3.805	1.420	2.232	2.044	3.495	3.475
7.658	7.386	7.928	9.004	7.284	7.839
5.422	4.736	2.832	4.833	2.340	3.520

3.797	4.915	5.476	5.970	4.041	4.507
3.916	4.208	3.135	3.354	4.031	2.616
2.618	1.969	3.194	2.485	2.752	3.175
2.743	2.715	3.290	1.317	2.041	3.531
4.043	3.484	3.784	3.645	3.645	3.970
1.718	2.045	2.594	2.551	2.627	2.626
5.530	6.264	4.512	4.906	4.692	4.759
3.714	3.031	2.693	3.129	2.591	3.355
1.577	3.085	2.913	3.310	3.481	1.622
8.555	9.211	9.306	10.375	8.617	8.980
5.537	5.350	5.286	5.180	5.570	4.836
4.872	4.357	4.045	5.459	4.603	4.733
3.566	3.325	5.041	5.707	4.008	4.252
3.014	3.214	1.942	2.349	3.286	2.400
3.016	2.289	3.430	2.409	2.329	2.772
3.187	3.296	2.806	3.543	3.803	3.492
5.187	4.129	4.457	4.441	5.418	4.259
3.471	2.736	2.389	2.431	3.551	3.658
3.042	2.718	3.757	4.437	3.576	3.269
5.265	4.713	4.643	4.885	5.062	4.813
5.416	5.554	6.175	6.012	6.166	6.217
2.619	2.688	2.497	2.920	2.719	2.238
3.977	3.946	2.089	2.236	2.321	3.176
10.300	10.183	10.889	11.129	10.433	10.351
7.340	6.157	6.405	6.348	6.941	6.781
3.813	2.384	1.887	1.466	3.135	3.391
4.157	3.292	2.530	3.822	3.279	3.692
4.871	3.744	4.610	4.697	4.271	3.469
0.711	4.394	2.145	2.293	3.743	4.218
6.278	9.104	7.331	8.084	6.970	7.150
5.468	5.930	5.569	5.693	5.249	5.761
1.500	3.058	1.904	2.485	1.523	3.720
1.994	1.801	0.561	0.857	1.644	1.263
5.507	5.575	5.657	6.520	6.557	5.924
1.704	2.876	2.244	4.173	3.024	3.631
2.585	4.040	2.660	3.513	2.199	2.717
7.298	6.461	7.406	6.687	8.151	6.524
6.180	6.283	5.574	5.368	5.796	6.723
5.046	4.871	5.627	5.590	4.520	5.058
2.606	2.707	2.031	2.912	2.498	3.784
3.952	2.314	1.853	3.173	3.144	2.566
4.640	4.043	2.478	2.570	3.674	4.400
2.804	1.874	3.669	4.145	3.275	2.807
4.601	5.091	4.364	5.873	3.995	4.989
2.789	2.710	2.281	1.992	1.405	2.526
2.125	3.226	2.907	3.726	3.197	2.954
2.561	2.956	2.568	3.784	1.868	2.840
3.860	3.464	3.189	2.326	4.848	2.489
5.662	4.429	5.954	4.662	5.356	5.114
1.608	2.897	2.738	3.784	2.526	2.447

3.017	3.056	3.311	4.247	3.757	3.429
2.791	2.643	2.719	3.081	2.251	2.451
4.403	4.164	4.382	6.193	4.732	5.904
2.863	1.649	0.926	2.682	1.122	1.642
2.939	1.983	1.745	2.043	2.448	1.059
3.609	3.475	4.589	5.368	5.040	4.228
3.886	3.886	3.855	4.242	5.211	5.339
7.252	7.424	7.568	7.460	7.617	7.437
3.243	3.872	4.484	5.351	4.079	4.216
4.716	4.380	3.927	3.677	4.577	4.923
3.011	3.688	3.048	2.571	2.225	2.638
1.657	2.033	2.749	3.050	2.299	2.970
3.112	2.895	3.827	2.588	4.206	3.964
4.146	3.303	4.254	3.386	4.993	5.058
2.329	2.632	1.633	2.700	2.424	3.187
3.084	2.705	2.708	2.666	3.088	2.567
2.657	2.515	1.529	3.524	3.410	1.523
4.528	3.559	4.632	3.934	4.960	4.821
4.816	4.694	3.470	4.080	4.190	4.973
4.485	3.440	3.709	3.966	3.199	3.658
4.694	4.236	4.119	4.310	3.268	3.980
4.497	3.803	3.930	3.975	3.152	3.808
3.064	3.071	2.409	3.702	1.310	1.819
3.320	2.590	3.020	2.854	3.308	4.007
2.860	4.134	2.902	1.996	3.629	4.070
3.937	3.647	3.611	3.333	3.655	3.659
2.922	3.957	2.827	4.199	2.283	2.123
2.756	3.155	3.001	4.190	2.771	2.508
3.003	3.781	3.386	3.874	4.272	3.211
3.027	2.689	3.262	3.249	3.933	4.098
3.762	3.097	1.815	2.395	3.641	3.113
3.208	3.816	4.358	4.858	3.620	3.550
3.938	4.132	4.272	4.368	2.838	4.535
3.575	2.868	2.827	1.879	3.266	3.039
2.528	3.598	4.424	4.334	3.525	4.781
2.059	1.510	1.971	3.700	3.133	1.791
1.181	3.638	2.305	2.591	0.966	3.107
2.366	3.604	2.894	3.953	2.231	2.138
3.206	2.743	2.600	3.058	2.542	2.608
4.197	4.262	2.172	2.539	3.071	3.676
2.803	3.117	3.481	3.293	3.287	3.154
1.727	2.095	2.530	3.400	1.414	3.004
2.618	3.576	3.387	3.798	3.698	3.127
3.645	4.102	5.234	4.235	3.844	4.325
2.825	2.052	1.442	2.847	2.607	3.493
4.285	3.719	2.161	2.015	2.562	3.693
5.284	3.867	3.802	3.898	3.616	4.317
3.556	2.732	3.361	2.281	2.373	3.942
2.347	3.385	2.394	2.037	2.584	2.283
4.605	4.650	4.640	4.376	4.482	5.405

5.931	5.934	6.674	7.140	5.839	6.085
2.368	3.466	3.006	1.840	3.223	3.204
2.865	2.034	1.953	2.688	2.638	3.520
3.670	4.408	5.047	3.729	4.708	3.248
4.180	3.270	2.722	3.620	3.513	3.606
4.957	4.535	3.866	4.672	4.514	4.838
2.584	2.653	2.194	2.712	2.631	2.866
2.829	3.636	3.704	4.604	3.594	3.757
2.205	3.480	2.562	2.624	2.355	2.778
5.986	5.158	6.707	8.077	6.773	7.222
1.433	1.461	1.722	1.692	1.876	1.388
4.000	2.738	3.001	2.586	2.944	2.783
3.134	3.499	3.157	5.316	2.995	3.911
3.509	3.809	3.105	2.779	3.210	4.770
3.835	4.163	4.760	4.883	4.460	4.355
2.808	2.718	3.494	2.700	3.897	3.348
5.738	5.872	3.776	4.735	4.842	5.913
2.813	0.887	2.932	3.080	3.789	3.293
5.675	5.321	5.177	5.177	6.496	6.533
3.225	3.016	0.996	2.176	1.539	2.360
4.899	3.628	3.329	3.640	4.213	3.743
2.471	2.551	2.708	3.218	3.257	2.599
4.892	5.026	5.027	4.409	5.877	5.879
1.423	1.497	1.490	1.772	1.905	1.918
4.004	3.663	3.518	3.038	3.202	4.774
4.732	4.258	3.697	5.564	5.441	5.760
3.191	2.589	2.120	3.801	2.716	2.671
3.013	2.568	4.073	3.485	2.513	2.536
5.974	6.475	6.585	7.830	6.070	6.491
3.489	3.379	3.428	3.120	3.317	3.860
3.315	3.918	2.030	2.406	1.943	2.977
4.454	2.994	3.569	3.593	3.184	5.136
4.769	4.090	5.080	4.333	4.081	5.117
1.586	3.332	2.658	3.527	2.592	2.155
1.838	1.781	2.343	2.757	2.742	3.776
2.405	3.462	4.040	3.245	2.950	3.734
2.063	2.299	2.989	1.833	1.594	2.704
3.390	3.281	1.787	2.734	2.453	4.704
3.185	2.835	0.433	2.418	2.579	1.763
3.430	2.957	3.571	3.736	3.341	4.158
2.690	2.691	3.284	1.089	2.717	2.336
1.701	2.519	3.246	3.553	3.689	1.933
3.721	2.931	2.345	1.620	2.963	3.047
6.063	6.346	6.749	7.766	5.454	6.755
2.397	2.025	3.203	2.073	1.646	3.965
4.269	4.357	4.358	4.019	4.593	4.422
5.840	4.987	4.988	5.132	4.713	5.002
2.333	1.767	2.411	2.967	2.907	2.796
3.714	4.351	4.304	3.696	4.271	4.000
2.846	1.851	3.125	1.892	2.666	1.664

5.503	6.442	6.791	6.620	6.285	6.704
3.830	2.983	2.782	3.637	4.124	4.673
5.785	4.783	6.846	6.848	4.271	5.648
4.871	1.868	2.673	3.531	2.879	2.697
4.448	4.526	4.530	4.663	4.977	5.579
3.409	2.808	2.935	4.726	2.060	2.808
4.250	3.763	3.916	5.215	3.929	5.201
4.130	4.422	4.753	5.119	5.087	5.126
4.022	3.555	5.737	4.549	3.547	3.523
2.889	2.893	1.671	3.302	2.608	3.256
1.027	1.076	1.191	1.689	2.239	1.576
3.150	2.122	2.518	1.486	1.615	3.003
2.824	2.663	2.730	3.042	2.718	2.938
3.637	4.058	4.965	4.683	3.774	3.101
2.067	2.489	3.344	2.592	2.489	2.726
5.245	4.513	5.527	5.936	6.194	5.450
3.988	3.227	4.549	5.145	3.715	4.483
3.030	2.446	4.113	3.936	2.684	2.719
3.417	3.529	3.276	2.920	3.694	3.805
4.133	3.751	4.601	4.696	4.146	4.364
2.674	1.812	2.069	2.309	3.746	3.436
3.997	3.614	3.189	4.606	4.164	4.012
2.868	1.625	0.802	3.937	3.143	2.926
1.046	2.011	0.874	0.885	2.057	2.412
5.285	4.917	4.570	5.185	5.580	5.773
2.363	0.974	1.384	1.703	1.018	1.191
3.259	2.821	2.243	2.906	3.405	4.457
3.763	2.022	4.293	5.432	5.706	4.565
3.784	3.000	3.023	3.444	2.773	3.525
2.793	2.549	2.588	2.416	3.142	2.294
2.638	1.260	2.835	2.433	2.177	1.626
7.779	7.290	8.036	8.614	7.818	7.960
5.016	4.451	5.483	5.636	4.027	4.974
3.487	2.725	3.125	3.798	2.206	3.378
4.114	3.116	3.416	3.008	3.738	3.246
3.188	2.155	2.903	3.123	3.748	3.435
6.912	6.366	7.197	7.892	7.000	7.059
3.567	3.585	3.429	4.251	3.795	3.281
3.290	3.027	2.842	3.175	2.937	2.959
2.020	1.891	1.767	1.817	3.821	1.928
1.072	1.122	2.490	3.909	2.773	2.193
0.955	2.649	0.906	0.332	2.053	0.550
3.330	2.730	2.878	3.072	2.344	3.183
3.937	3.622	3.788	2.866	2.885	3.980
3.351	2.569	2.444	2.854	3.630	2.423
2.674	3.577	3.371	2.930	3.745	4.045
3.368	2.011	3.316	2.898	3.869	1.631
4.801	4.402	4.755	4.794	5.320	4.584
2.872	1.674	2.360	3.842	2.592	2.788
3.910	2.733	1.657	2.453	3.384	2.829

2.514	2.873	1.245	1.036	2.058	2.719
1.932	1.770	1.080	2.176	2.288	3.058
3.064	4.626	3.051	3.250	3.299	2.729
6.239	7.587	6.812	6.841	5.882	6.069
5.793	6.892	6.471	6.006	5.147	6.153
2.772	3.513	3.057	2.693	2.680	2.684
1.908	2.946	2.596	3.344	2.344	2.329
2.160	2.897	3.080	3.310	2.712	2.342
3.584	2.431	2.403	3.403	1.266	1.427
3.341	2.434	3.372	2.316	2.208	2.208
1.155	2.365	2.446	2.423	1.824	2.274
4.726	4.346	6.220	6.755	5.861	5.480
6.627	5.317	5.436	5.090	5.939	5.743
6.500	6.499	5.663	6.467	6.167	6.182
4.517	5.643	4.517	4.687	4.297	4.412
3.619	4.999	5.319	6.373	2.637	4.206
3.047	3.336	2.338	4.142	2.236	2.628
6.062	4.995	4.601	5.920	5.285	4.845
1.430	1.382	1.950	1.770	1.828	1.443
2.999	3.690	3.201	2.697	3.616	3.613
2.975	2.499	2.486	2.886	2.999	3.477
4.136	4.073	3.342	3.795	4.453	4.041
2.330	2.878	2.091	2.268	1.504	2.580
3.050	3.591	2.556	4.273	3.925	3.641
3.573	4.000	3.545	3.386	3.961	4.355
4.118	3.297	3.167	3.356	3.251	4.167
3.130	3.449	3.751	3.161	3.488	2.185
2.433	2.279	2.383	2.151	2.774	2.098
2.949	1.998	2.384	3.204	2.535	2.023
4.277	4.344	4.583	3.611	3.804	4.750
3.766	3.822	3.275	4.267	3.859	3.478
3.741	3.930	3.949	3.214	3.824	4.199
4.390	2.805	2.509	2.546	3.278	3.528
4.559	2.895	2.431	2.978	3.744	3.727
3.327	4.019	2.629	3.058	2.522	3.703
1.467	3.262	1.960	1.501	2.299	1.829
6.640	6.368	6.526	5.498	7.167	6.925
5.166	6.153	5.972	3.962	5.153	5.803
1.836	2.503	2.017	2.800	2.084	2.060
4.625	4.274	4.079	2.938	4.582	5.152
3.369	2.811	4.541	5.527	3.803	3.191
3.518	3.473	3.045	3.843	3.133	3.309
3.170	4.503	3.988	4.260	4.477	2.338
4.458	4.820	4.133	3.739	4.914	4.509
3.522	3.124	4.136	4.288	2.895	3.566
2.641	2.611	3.303	4.085	3.111	3.023
4.505	5.712	4.144	5.113	4.923	4.918
2.101	2.293	1.989	3.098	3.016	1.969
3.470	2.348	2.050	3.313	2.406	2.236
2.271	3.028	2.116	2.810	2.476	2.564

3.405	3.709	3.457	4.669	3.683	3.159
3.889	3.147	3.276	1.800	3.452	2.946
2.944	3.007	3.193	2.728	3.540	3.843
2.550	2.979	3.097	2.048	3.416	3.696
3.052	3.475	3.382	6.096	2.878	3.271
3.460	3.634	3.837	4.601	4.143	3.655
5.664	4.987	5.100	4.871	5.406	5.920
3.636	4.672	3.570	4.371	4.571	3.926
1.914	0.878	1.730	1.318	1.378	1.405
2.619	2.155	2.813	2.822	1.847	2.348
3.266	3.248	3.354	2.774	3.907	4.228
2.093	1.903	1.876	2.977	2.266	1.792
2.936	4.739	3.687	4.065	5.295	3.883
2.051	1.196	1.669	1.658	1.472	1.879
3.439	3.130	4.808	4.046	3.457	3.875
2.475	3.675	2.928	2.036	4.023	3.427
3.783	3.859	2.822	2.669	3.155	4.035
2.321	1.222	2.022	2.874	1.556	2.172
1.535	1.437	1.524	1.343	1.792	1.368
1.812	0.829	1.356	1.597	0.974	1.263
2.746	2.708	2.970	3.159	2.553	3.040
2.637	3.890	2.327	2.193	2.889	3.734
2.192	2.888	2.546	2.944	2.384	2.102
3.676	3.212	3.413	3.409	4.036	4.100
2.571	1.604	2.569	2.445	1.900	2.656
1.393	1.212	1.164	1.774	1.211	2.249
2.884	4.221	4.259	2.871	5.009	4.706
2.620	3.546	3.975	3.008	2.700	2.447
3.193	3.216	3.228	2.735	3.504	3.240
3.107	1.522	2.311	2.519	2.431	2.840
4.088	3.633	3.872	3.212	3.724	4.319
2.787	2.631	3.517	2.819	2.309	2.873
3.647	2.971	3.732	2.227	2.420	2.757
1.732	2.476	2.842	1.980	2.767	3.641
2.326	1.503	2.772	2.400	2.285	1.980
1.631	1.602	1.999	1.902	2.011	1.777
4.866	4.625	3.946	3.480	5.365	4.137
1.869	1.852	1.121	2.143	2.125	2.602
3.876	3.811	3.009	3.336	4.292	3.012
3.810	3.023	4.077	3.053	3.872	3.800
3.111	4.697	4.592	3.585	4.171	4.359
3.232	4.904	4.756	4.369	4.490	4.525
5.332	5.700	4.941	5.184	5.181	5.406
3.851	3.555	3.238	2.682	3.176	3.223
3.929	1.924	1.876	2.354	3.607	3.939
2.983	2.860	2.382	1.825	2.856	2.357
2.552	3.321	1.594	3.198	1.909	2.037
5.171	4.021	4.238	3.744	4.256	4.712
1.954	1.683	1.493	1.083	2.992	2.291
2.292	1.761	2.577	1.772	2.774	3.393

2.183	2.400	2.187	2.350	2.248	2.650
1.629	2.100	1.762	1.778	3.154	1.353
2.955	3.029	3.041	2.821	2.367	2.977
4.156	6.225	5.098	4.284	4.928	4.607
8.390	8.295	8.089	7.940	8.319	8.196
4.331	4.827	4.805	3.074	4.675	4.778
4.885	4.824	4.094	4.620	5.135	4.955
3.179	2.515	2.083	2.209	3.644	3.816
6.095	6.888	6.046	6.504	6.905	6.266
2.568	3.057	2.753	4.394	2.879	3.123
3.685	3.438	3.359	2.834	3.959	2.443
2.675	2.783	3.374	2.600	2.970	3.589
3.686	3.185	1.642	2.495	2.477	2.570
2.541	3.165	2.419	2.295	3.244	3.541
2.739	2.956	2.834	2.821	3.766	2.680
1.356	2.084	1.508	2.304	2.117	1.727
2.184	3.406	2.284	1.691	3.181	2.408
1.698	2.083	1.728	3.178	2.114	1.803
4.200	3.656	3.924	3.268	4.322	4.093
2.719	1.687	2.657	3.107	2.270	2.203
4.139	3.392	3.856	4.627	4.387	4.350
1.664	2.238	2.947	2.546	1.793	2.060
3.783	4.455	3.427	3.224	4.101	4.338
2.734	2.441	2.203	2.340	2.516	2.866
5.100	4.943	4.805	6.074	5.137	4.916
3.035	1.704	2.026	1.752	3.387	2.702
2.235	2.626	3.219	2.738	3.366	1.927
6.370	8.547	7.034	2.322	7.309	6.030
1.886	1.934	1.597	2.523	2.337	2.300
3.052	2.534	2.214	1.940	3.291	1.917
5.166	4.416	5.042	3.822	4.729	4.995
3.785	2.624	4.047	3.433	4.259	3.415
1.893	2.176	1.718	1.578	2.868	1.194
3.464	3.201	4.501	3.513	4.195	2.793
1.289	1.957	1.634	1.845	1.545	1.243
4.843	4.588	3.450	4.015	4.277	5.253
2.763	2.949	2.578	2.375	2.109	3.170
2.881	2.860	2.265	2.648	2.875	2.864
3.176	2.576	2.152	2.213	2.997	2.341
4.517	4.174	3.742	3.193	3.551	4.531
2.665	3.191	1.716	2.468	2.278	2.505
2.186	2.089	1.987	1.993	2.145	2.035
3.123	2.888	2.718	2.461	3.280	3.724
4.020	3.457	3.655	2.466	4.400	4.320
3.852	4.507	5.375	4.404	4.370	4.789
3.624	3.441	3.451	2.167	4.497	4.717
3.129	3.302	3.064	2.688	2.956	3.045
2.950	3.014	2.057	1.325	3.591	2.596
4.868	4.979	4.224	4.967	4.753	4.825
2.549	2.876	2.631	1.947	3.329	2.753

2.430	2.901	2.194	3.423	2.862	3.289
2.323	1.834	2.998	2.849	2.870	2.223
1.022	1.125	1.346	1.226	0.971	1.496
3.415	2.452	2.979	2.576	2.853	2.998
3.513	3.591	3.811	3.914	3.146	3.952
3.282	3.306	3.776	4.828	3.933	4.200
4.003	3.135	3.684	3.132	4.925	3.519
2.955	2.061	1.940	3.283	2.019	1.868
2.576	2.980	3.313	3.538	2.822	3.392
3.724	4.897	4.490	4.604	3.750	3.742
3.397	2.647	2.978	3.811	3.483	2.931
2.757	2.680	3.662	3.024	3.915	3.925
3.115	2.549	2.409	2.138	2.636	2.311
4.740	4.315	4.049	3.205	4.245	4.018
2.215	1.980	2.149	1.268	2.226	1.780
4.496	3.736	3.472	3.915	3.707	3.851
1.990	2.703	3.041	2.414	2.401	1.621
2.389	2.140	2.402	2.389	3.228	2.389
1.668	1.597	2.043	1.187	1.822	1.890
3.719	3.556	4.116	4.252	4.133	4.296
2.537	2.691	2.865	2.949	2.483	1.864
3.643	2.982	3.904	3.130	2.916	2.396
2.318	2.973	3.994	3.202	2.780	2.303
2.187	2.999	4.063	3.303	2.512	2.386
5.078	4.615	4.216	4.035	4.850	5.045
5.338	5.119	6.244	7.011	4.876	4.615
2.351	2.358	2.134	1.829	2.132	2.714
2.127	5.791	2.145	1.628	2.189	2.379
3.296	3.688	3.280	4.025	2.863	2.827
3.013	2.948	3.335	4.907	3.347	2.920
2.434	3.365	1.528	2.064	4.108	2.432
3.077	3.404	2.920	3.283	4.101	2.486
2.918	3.124	2.191	3.386	3.212	2.791
2.807	1.200	2.258	2.011	2.528	1.768
2.437	2.739	1.710	2.158	1.822	1.248
2.883	2.407	2.985	2.921	2.511	3.173
4.386	4.612	3.415	3.792	4.805	4.836
6.956	6.262	5.756	5.948	6.764	6.608
4.266	3.614	4.339	3.723	5.654	2.977
2.099	1.829	2.238	2.559	2.109	2.109
3.969	3.166	2.363	2.340	3.008	3.580
2.376	2.095	1.822	2.546	2.876	1.821
3.259	3.635	3.494	3.446	2.678	2.994
4.201	4.014	2.108	2.553	3.057	3.797
3.363	3.151	3.334	4.077	3.151	3.732
3.768	3.194	5.304	3.700	4.648	3.785
4.004	3.628	4.016	4.074	3.509	4.285
2.205	1.721	3.207	2.719	3.393	2.353
1.857	2.773	2.589	3.313	2.184	2.342
2.615	2.418	2.541	2.450	2.481	3.314

1.688	2.299	1.808	2.299	2.299	1.792
2.828	2.854	3.390	2.842	3.914	3.123
3.551	3.974	2.986	2.613	3.408	3.499
5.412	4.207	4.320	3.617	4.388	4.703
5.241	5.608	4.603	4.020	4.696	5.003
1.612	1.810	2.725	2.356	1.591	1.756
2.109	3.030	2.376	2.273	2.765	2.065
2.789	3.351	3.714	2.762	2.723	3.453
2.671	2.849	2.230	2.813	2.917	2.675
3.577	3.442	2.328	1.739	2.649	4.090
4.680	4.857	5.243	3.624	5.517	4.884
2.185	2.506	2.094	1.836	1.890	2.473
4.241	4.102	4.582	5.603	4.589	4.992
3.096	3.304	2.930	2.203	3.924	4.481
2.734	2.105	1.993	1.540	1.362	1.714
2.250	1.275	2.055	2.163	2.450	1.893
2.854	2.478	2.620	2.371	3.389	2.735
2.394	1.671	2.549	1.925	1.929	2.440
4.615	4.143	3.046	4.301	4.678	4.232
2.464	2.719	2.913	2.814	2.907	2.850
4.318	3.776	4.246	3.915	4.272	4.231
5.557	5.386	5.892	6.125	5.632	5.789
2.437	1.940	1.947	2.921	2.284	2.386
2.454	3.363	2.663	2.148	2.731	3.181
1.813	2.217	2.305	1.993	2.320	2.192
3.379	3.543	3.393	4.696	3.210	3.301
4.457	3.099	4.637	2.338	4.451	4.486
0.668	0.821	1.028	1.212	0.775	0.938
2.807	3.187	3.066	1.792	3.603	3.288
3.469	3.740	2.818	3.707	3.778	3.488
4.107	3.408	3.850	4.405	3.684	3.166
1.567	1.609	1.833	2.206	1.977	1.663
2.004	1.988	2.844	3.677	2.652	2.190
5.182	5.059	4.632	3.300	4.747	4.699
2.620	2.735	2.547	3.345	2.210	2.936
5.485	4.167	4.137	4.747	4.795	5.212
2.395	2.811	2.078	2.751	2.165	3.013
2.012	1.794	1.693	1.905	2.082	1.402
5.813	5.400	4.916	5.149	5.525	6.354
2.150	1.913	1.662	2.530	3.397	2.720
2.888	3.011	3.470	3.470	3.082	3.178
1.414	1.269	1.297	2.237	2.228	1.791
2.000	2.456	2.206	2.892	3.399	2.073
3.675	4.113	3.891	2.926	3.822	3.781
5.647	5.267	4.979	4.701	6.057	5.967
1.923	1.446	1.983	1.716	1.577	1.476
2.595	2.832	3.671	3.604	2.421	2.507
3.318	1.154	3.765	1.250	4.111	3.816
2.832	2.692	2.794	2.188	1.655	2.173
2.794	2.781	2.578	2.771	3.122	3.224

3.824	4.289	4.677	4.871	4.116	3.829
3.013	3.175	3.944	3.406	4.447	3.846
2.708	3.188	2.657	2.951	3.133	2.404
1.254	1.062	1.354	1.113	1.298	1.019
3.773	3.079	3.281	3.397	3.912	2.934
3.828	3.960	4.683	4.788	3.732	3.935
3.596	3.767	3.299	3.319	3.849	4.346
2.078	2.081	1.868	2.487	1.524	1.972
2.096	2.301	2.799	2.429	2.381	2.082
2.829	4.114	4.357	3.826	3.862	3.603
4.038	3.475	4.092	4.672	4.224	4.195
2.261	1.807	2.438	2.282	1.406	2.427
4.965	4.831	3.946	3.296	4.949	4.306
3.813	2.742	2.789	1.874	2.892	3.380
5.342	4.193	4.407	5.150	5.839	5.577
3.310	2.683	2.573	2.139	1.818	3.528
3.765	3.768	3.917	3.095	3.765	4.091
2.143	2.769	3.366	3.573	3.523	2.108
3.263	2.579	3.329	4.032	1.710	2.370
3.678	2.474	3.831	4.830	2.910	3.745
1.986	3.276	2.694	1.202	2.029	2.670
2.375	2.474	3.925	1.916	2.224	1.904
4.725	5.391	5.143	4.404	4.612	5.021
1.860	2.421	2.874	1.258	2.113	1.726
3.030	3.578	2.801	2.594	3.047	2.748
2.939	3.100	3.209	4.275	2.526	2.481
4.333	5.543	4.208	3.225	4.018	4.818
2.145	1.775	1.810	2.110	1.902	2.018
1.657	2.500	1.702	1.276	1.566	1.762
3.202	2.955	3.185	3.030	4.034	2.464
3.402	2.919	1.744	2.522	3.085	2.201
2.556	2.272	1.959	2.627	2.241	2.561
2.522	2.407	2.738	1.977	2.228	2.679
4.257	3.144	3.799	4.029	3.695	3.238
4.754	4.618	4.780	3.205	5.051	4.611
2.173	2.072	1.522	2.110	2.267	1.876
6.815	5.992	6.029	6.150	5.951	6.483
3.348	2.393	2.283	2.699	3.220	4.041
3.248	2.804	2.668	3.273	1.953	3.539
3.479	3.179	2.578	2.912	2.696	3.134
4.977	5.240	4.311	5.153	4.671	4.725
2.227	1.934	1.995	2.270	1.186	2.260
5.300	4.362	4.869	4.699	5.171	4.609
3.422	2.441	3.356	2.745	4.135	3.135
2.840	2.155	2.843	2.807	2.888	2.690
2.793	3.088	2.848	2.602	3.288	2.565
2.670	3.227	3.096	2.457	1.463	2.189
4.896	4.474	5.031	4.162	5.255	5.011
1.705	1.600	1.608	1.871	1.401	1.590
3.347	3.346	1.487	2.455	3.482	2.026

3.109	3.170	2.364	3.186	3.636	2.586
3.189	3.175	2.828	2.903	3.111	2.970
1.447	1.747	2.011	1.082	1.287	1.513
3.032	2.939	3.506	2.671	2.883	2.421
1.784	1.101	1.390	1.637	1.317	1.418
4.753	4.718	3.967	3.696	3.252	4.917
1.944	2.026	2.335	2.542	1.687	1.729
2.581	2.417	1.601	2.322	2.453	2.314
3.537	2.681	2.631	2.876	3.472	2.932
3.848	3.395	3.365	4.008	3.131	4.071
8.319	8.547	8.834	9.165	8.514	8.914
2.099	2.296	2.971	2.157	2.685	2.483
2.293	2.710	2.639	3.474	2.024	2.695
2.737	2.692	3.348	2.202	3.237	2.819
3.341	4.332	2.664	3.331	2.497	4.192
1.747	3.413	2.298	1.762	2.890	3.103
2.460	3.592	2.631	2.817	2.259	2.499
4.091	4.294	4.651	4.773	3.762	3.597
1.779	1.183	1.842	1.977	2.129	2.373
7.265	6.762	6.971	7.784	7.106	7.355
2.960	3.808	3.149	2.622	3.129	3.350
2.776	2.235	2.131	1.925	3.184	3.497
0.709	1.014	0.710	1.041	1.568	1.086
3.040	1.786	2.666	2.134	2.513	2.388
4.585	4.633	3.510	3.126	3.671	4.479
1.880	2.288	2.288	2.268	2.654	2.023
3.156	2.458	3.994	2.612	2.949	2.906
3.420	3.468	2.072	4.480	3.029	2.928
3.478	2.589	2.042	2.053	2.819	2.722
1.523	1.652	1.445	1.449	1.049	1.385
2.253	0.729	1.984	2.026	1.562	1.946
1.475	1.895	1.246	1.646	1.706	1.664
1.445	0.892	1.252	1.705	1.498	1.461
2.900	2.301	2.453	2.346	1.468	2.635
3.452	3.169	3.166	2.254	3.007	2.685
5.031	4.235	5.240	4.459	5.974	5.770
3.635	3.724	3.647	4.728	2.823	4.084
3.651	2.385	2.342	4.180	4.223	2.728
0.830	1.166	0.832	0.975	0.991	1.364
2.349	2.815	1.878	1.646	2.546	3.253
3.273	2.633	3.247	2.950	3.572	2.919
2.589	1.759	1.473	1.600	1.196	1.813
4.479	4.824	3.952	4.118	3.813	3.487
2.390	2.078	1.817	2.114	2.007	2.006
2.847	2.743	2.143	2.260	2.415	2.676
4.553	3.230	4.443	4.376	4.026	3.499
1.037	0.909	0.860	1.696	1.095	0.948
2.747	2.350	2.942	1.957	2.280	2.448
5.193	5.225	5.341	4.386	5.528	5.877
2.008	2.356	2.304	3.711	3.066	3.138

7.113	7.131	6.620	6.698	6.940	7.005
3.450	2.486	3.335	3.844	2.458	2.911
2.063	1.754	1.940	2.577	2.716	2.558
2.950	1.756	1.714	2.230	3.079	2.244
3.704	4.654	4.652	4.737	4.760	4.997
3.075	3.412	3.073	2.110	3.417	3.003
3.137	2.835	4.005	3.395	4.308	3.764
1.795	2.750	1.380	2.381	2.325	2.262
2.769	3.648	2.486	3.950	3.472	3.191
4.291	5.061	4.538	4.361	4.434	4.118
3.171	3.552	3.227	4.422	2.467	3.286
7.496	6.800	6.998	6.415	7.781	6.939
2.433	2.815	2.542	3.008	2.829	2.357
1.448	1.303	1.447	1.627	1.098	1.222
2.241	1.927	2.357	2.345	1.878	2.320
7.651	7.834	7.633	6.920	8.194	7.591
2.649	3.109	3.170	3.364	3.724	2.983
4.996	4.017	4.993	4.252	5.600	5.049
4.665	5.164	5.470	4.364	5.748	5.750
2.352	3.037	2.160	2.980	2.994	2.399
2.661	4.331	2.854	4.305	1.571	3.059
4.141	3.641	3.977	3.372	3.807	4.082
2.443	2.760	1.909	2.460	3.096	2.145
3.025	3.099	2.966	3.751	3.203	2.627
3.047	2.822	3.406	3.411	2.468	2.966
7.030	7.597	7.172	7.665	7.910	7.808
2.472	1.832	1.979	1.804	2.197	2.428
1.298	1.308	1.253	1.467	1.381	1.141
2.134	2.074	3.353	1.828	2.720	2.523
2.958	2.519	2.913	3.421	1.973	3.202
3.031	2.730	2.438	2.586	2.747	2.647
3.169	2.450	3.782	2.942	3.199	2.660
2.759	3.621	3.658	3.839	3.199	2.888
2.230	3.010	3.353	3.575	1.961	2.033
2.631	3.118	3.683	3.412	3.606	2.629
2.350	2.081	2.769	2.258	2.507	1.843
1.739	2.278	3.172	2.619	2.122	2.851
3.436	2.376	3.180	2.842	2.914	1.356
2.601	2.740	3.281	2.779	2.459	2.433
2.979	3.287	4.070	3.779	2.631	2.937
2.794	3.893	2.611	2.647	3.417	1.812
2.315	1.765	2.540	3.407	2.604	2.765
3.203	2.983	2.485	2.758	2.476	2.396
3.971	3.426	3.942	3.836	3.598	3.386
4.263	3.499	4.053	3.745	3.653	3.499
4.994	5.981	3.682	5.224	4.229	4.671
4.938	4.431	4.646	3.788	4.887	4.724
6.002	4.947	5.354	5.177	4.326	5.364
2.820	2.758	2.288	1.972	2.797	2.826
10.017	10.429	10.423	10.237	10.141	10.190

3.584	4.332	4.404	3.735	3.404	4.171
2.599	3.622	2.735	3.005	3.235	3.397
7.214	7.286	7.481	7.711	7.141	7.638
8.562	8.822	8.806	9.155	8.760	8.892
3.328	2.806	3.672	2.921	3.069	3.325
6.299	5.823	5.396	5.029	6.484	5.812
4.432	4.388	4.225	2.509	4.910	4.725
5.453	5.668	4.753	3.837	4.898	5.639
3.321	3.103	2.425	1.587	2.235	2.581
4.257	4.758	4.432	4.294	3.970	4.378
3.797	3.718	3.062	3.508	3.024	3.599
4.559	4.059	3.153	3.048	4.063	4.083
3.951	3.948	4.339	3.898	3.981	4.131
2.061	2.658	2.741	2.574	2.025	3.516
3.293	3.078	3.132	3.529	3.345	3.215
2.108	2.377	1.663	2.534	1.113	2.328
4.890	4.345	4.145	4.962	5.620	4.629
2.958	3.149	2.455	2.814	3.425	3.468
8.361	8.764	8.794	8.936	8.287	8.776
4.300	3.679	3.038	3.779	3.833	3.902
9.555	10.069	9.440	9.443	9.498	9.977
10.899	11.435	10.747	10.508	10.967	11.176
4.882	5.327	4.011	3.811	5.085	5.094
3.165	3.335	2.424	3.409	2.641	2.843
10.687	10.558	10.608	10.832	10.646	10.724
8.552	9.087	9.015	9.021	8.769	9.094
4.848	4.867	3.522	4.685	4.506	4.713
4.594	4.159	3.780	4.530	3.140	4.864
5.908	5.666	5.381	5.248	5.729	5.749
4.547	3.135	4.425	3.716	4.418	3.942
2.908	1.352	1.940	3.014	2.207	1.255
1.814	1.716	2.804	2.878	2.428	2.089
3.643	3.103	3.398	3.899	4.089	3.341
3.613	2.266	4.009	3.217	3.325	2.605
5.489	4.322	4.457	4.030	4.703	4.617
6.819	7.109	6.476	6.270	6.770	5.808
7.373	7.324	6.890	6.632	7.365	7.566
5.946	5.897	4.933	5.636	5.601	5.608
2.594	2.034	2.408	1.943	2.786	2.465
5.958	6.179	5.126	4.599	5.497	5.955
5.741	5.742	5.223	3.845	5.429	4.848
3.341	2.473	1.591	4.121	3.484	4.613
2.929	4.333	4.538	3.884	3.900	3.483
4.250	4.406	3.969	3.750	3.744	3.838
4.971	4.864	5.430	5.186	4.158	5.347
3.788	3.186	2.260	1.697	3.150	3.393
4.344	1.833	2.847	1.703	2.625	2.709
5.574	5.298	4.842	4.010	5.556	5.739
4.088	4.509	4.190	5.348	3.365	4.255
5.317	5.532	5.385	5.632	5.808	5.340

3.658	4.575	4.451	5.257	3.074	4.307
3.187	2.171	2.763	2.958	2.757	2.776
2.423	1.554	1.894	2.069	2.300	1.704
5.248	5.264	4.922	4.175	4.858	5.639
7.184	6.567	6.720	6.507	6.841	7.064
6.346	6.140	6.135	5.821	6.585	6.103
1.461	1.497	1.276	1.157	1.280	1.063
7.143	7.168	6.939	7.251	6.857	7.029
3.922	3.993	4.103	4.820	3.205	3.735
3.657	3.442	3.487	3.896	3.659	3.499
3.289	3.497	3.466	3.720	3.871	4.079
1.729	1.520	2.376	2.088	2.544	2.089
0.893	1.023	1.109	1.422	1.493	0.998
3.599	2.804	3.211	2.352	2.868	3.758
3.389	2.815	3.378	2.605	2.312	2.629
6.091	5.873	6.215	5.051	6.566	6.467
4.948	5.258	4.732	5.558	4.885	5.012
7.168	7.040	7.320	7.233	6.902	7.264
3.848	3.810	3.248	4.129	3.896	4.026
3.281	2.812	3.087	2.399	2.252	2.437
4.626	3.820	3.943	4.304	4.508	4.204
3.309	2.879	2.535	2.571	2.840	3.137
3.722	3.670	3.833	4.083	3.744	3.898
6.359	6.011	6.603	5.743	5.901	6.114
5.254	4.574	5.067	4.323	4.823	5.204
3.114	2.800	2.570	2.088	3.104	3.126
1.094	0.837	1.211	1.481	1.541	0.854
4.360	4.755	4.621	4.630	5.040	5.003
6.592	6.525	6.456	6.785	7.369	6.414
3.716	4.569	4.840	4.046	5.231	4.713
4.025	4.334	3.332	4.148	3.597	4.148
4.109	5.253	3.527	3.876	3.876	5.102
3.004	4.797	3.250	2.691	4.668	3.991
4.485	5.435	4.689	4.469	5.165	4.924
3.149	3.677	2.440	2.275	1.802	3.620
5.142	5.298	5.287	5.365	5.028	5.698
4.048	6.185	3.845	3.690	4.612	6.031
4.434	6.014	3.947	4.352	5.038	6.931
10.532	10.725	10.765	10.855	11.261	10.898
4.024	3.889	3.575	3.170	4.011	4.368
5.601	5.853	6.290	5.402	5.635	5.433
2.788	3.340	3.758	3.361	2.934	3.208
6.677	5.770	6.938	6.878	6.831	6.117
4.088	4.227	3.985	5.235	4.420	4.413
4.983	5.538	5.960	6.197	5.396	5.571
4.340	5.513	3.910	4.416	4.201	4.034
5.072	4.865	5.308	4.596	5.562	5.231
2.690	3.398	3.202	3.532	2.628	2.977
2.741	2.302	2.739	3.030	2.702	1.669
4.312	3.760	4.020	3.249	4.423	3.850

5.757	4.045	5.191	4.224	5.849	5.281
7.831	8.120	7.396	7.595	7.696	7.962
3.201	2.718	3.763	3.029	3.670	2.697
3.144	3.144	3.099	2.135	4.118	3.892
2.619	2.231	2.162	2.213	1.991	2.416
8.162	8.053	8.338	8.468	8.603	8.283
4.080	4.350	3.732	4.162	4.461	4.592
8.370	8.714	7.358	7.338	8.224	8.246
5.075	5.969	5.771	5.142	6.215	6.011
2.206	2.463	2.439	1.928	2.199	2.526
4.126	3.134	3.641	4.504	3.898	3.737
6.875	7.469	7.005	7.080	7.015	7.152
5.561	6.118	4.132	4.676	4.469	5.088
7.838	7.815	7.520	6.933	8.298	8.409
4.821	4.763	4.673	4.478	4.617	4.639
7.679	7.168	7.240	7.030	7.342	7.644
5.874	6.456	6.875	6.389	7.048	6.488
6.906	6.909	7.198	6.937	6.829	7.231
8.154	8.187	7.661	7.280	7.420	8.686
2.525	2.703	2.406	3.076	2.599	2.121
9.144	9.139	9.577	9.916	8.922	8.949
7.555	8.087	8.484	9.027	7.610	6.826
3.624	3.009	4.336	5.973	3.741	3.839
5.833	6.085	6.783	6.995	6.048	6.010
4.842	4.443	4.761	6.466	5.821	5.466
5.257	5.133	5.781	5.790	5.060	5.585
6.168	6.025	6.405	7.447	5.716	5.359
6.034	5.374	7.423	6.885	5.505	5.207
7.625	7.219	8.352	8.966	7.727	7.113
8.555	8.907	8.540	10.131	8.827	7.531
8.949	9.635	9.391	9.546	6.808	6.753
7.452	7.554	8.042	8.607	8.066	7.649
8.037	7.987	9.019	9.613	8.361	8.469
5.080	5.340	5.634	7.133	5.649	5.027
3.363	2.477	3.144	2.104	3.488	2.544
7.483	7.667	8.174	7.240	8.111	8.031
10.982	10.785	11.003	10.584	11.055	10.956
6.730	6.281	6.593	4.912	6.774	7.109
5.827	5.462	5.067	4.183	4.077	6.001
7.348	7.600	7.999	7.049	6.806	7.467
4.734	4.057	4.570	3.594	4.568	4.919
4.497	4.566	3.594	3.163	3.651	5.413
5.649	6.166	6.183	5.535	6.294	8.002
3.347	3.863	2.879	2.650	3.231	3.000
2.077	2.313	1.725	1.914	2.338	1.658
2.777	2.829	1.549	1.301	3.307	2.631
6.617	6.093	5.914	6.128	5.381	6.086
6.329	6.533	6.776	6.983	5.596	6.547
5.950	4.191	4.309	4.041	4.108	5.432
6.032	5.433	5.199	5.056	5.327	5.811

5.836	4.410	3.751	4.330	4.362	4.528
4.192	4.833	5.207	5.617	3.631	4.812
6.302	4.809	3.453	3.821	3.752	4.574
5.258	5.184	5.160	5.548	2.785	5.315
6.188	6.073	6.260	5.741	4.788	6.263
4.424	4.202	4.552	4.483	3.483	4.654
2.920	3.672	3.782	2.817	4.200	3.017
2.726	2.752	3.592	2.382	2.572	3.210
2.789	2.152	3.604	3.886	1.508	2.680
2.522	2.694	3.122	3.501	2.830	2.503
3.081	2.526	4.304	3.506	3.483	3.052
3.929	3.774	4.108	4.333	4.707	3.792
2.767	1.613	3.890	2.987	2.417	3.059
6.020	6.294	6.199	5.702	6.184	6.113
8.628	8.727	7.815	8.495	8.965	7.127
5.482	5.684	4.896	4.527	5.475	5.305
3.464	3.683	3.571	3.926	3.396	3.376
3.594	2.828	3.531	2.533	3.752	3.560
3.695	5.176	3.633	4.584	3.620	4.293
4.329	5.323	4.094	5.603	5.314	4.537
6.250	4.994	6.928	6.162	4.702	5.139
4.553	5.194	4.334	4.798	5.010	4.327
3.938	4.461	3.875	4.834	4.239	4.026
4.117	4.261	4.318	4.676	4.770	4.596
2.372	2.210	1.413	1.601	1.897	2.035
5.123	5.462	5.392	7.289	6.150	4.743
1.978	2.959	2.101	2.550	2.079	2.635
3.225	4.067	3.134	2.647	3.044	3.150
4.577	5.031	4.707	4.292	5.114	5.285
4.781	4.902	4.532	4.261	4.833	5.289
7.088	7.257	7.281	6.828	7.548	7.807
3.539	2.352	2.133	1.179	3.094	2.613
6.366	5.048	6.696	4.714	6.335	6.012
1.883	2.565	4.068	2.579	2.510	3.349
2.395	2.223	2.591	2.127	2.493	2.624
3.162	2.705	3.056	3.142	3.129	2.874
6.443	6.277	5.855	5.882	5.954	6.241
4.800	5.516	4.354	4.003	4.878	5.090
5.373	5.826	3.292	3.243	4.288	4.288
7.531	7.484	6.859	6.204	7.268	7.579
1.746	6.083	2.142	1.250	1.316	3.518
5.258	4.404	5.403	5.231	5.773	4.204
5.051	5.293	5.040	4.924	6.307	5.638
5.378	5.041	5.948	5.025	6.193	5.255
4.602	4.309	4.310	3.616	4.403	4.107
7.491	7.630	7.680	6.923	8.001	7.382
3.985	3.930	4.165	2.335	3.288	2.947
6.297	6.079	5.393	5.213	6.523	6.229
5.675	5.342	5.646	5.798	6.107	4.403
5.501	5.112	5.413	5.015	5.201	5.323

3.167	3.846	2.933	3.130	2.511	2.758
3.184	2.962	2.822	3.138	2.798	3.094
4.545	4.863	4.028	4.876	4.449	4.293
6.254	6.389	6.460	5.660	7.107	6.785
4.090	3.761	4.555	3.526	3.887	4.238
4.219	2.931	4.430	3.657	3.225	3.012
6.921	7.113	6.752	6.015	6.923	7.065
3.674	3.507	3.535	4.305	3.674	3.375
6.491	5.979	6.136	5.530	6.744	6.777
5.411	4.949	4.689	4.737	4.218	4.875
2.392	2.640	2.770	1.999	2.425	2.188
7.066	5.868	6.490	6.491	6.876	6.205
6.682	6.479	6.191	6.128	6.612	6.643
4.955	4.991	4.928	4.681	4.773	5.155
7.115	7.950	7.491	7.238	7.285	7.740
5.030	5.681	5.237	4.744	5.723	5.474
5.823	6.042	5.800	5.268	6.100	6.012
6.796	7.030	7.005	6.114	7.180	7.112
5.657	5.855	5.790	6.017	6.027	5.764
2.449	2.286	2.904	3.314	2.429	2.484
9.017	8.495	8.929	8.986	8.622	8.642
7.442	6.962	7.404	7.359	7.135	7.079
5.138	4.747	5.562	5.397	4.572	4.904
4.918	4.514	4.556	4.508	4.431	5.293
7.057	7.793	7.019	6.964	7.302	7.325
4.547	5.012	4.378	4.026	4.804	4.904
4.373	4.099	4.212	4.181	3.861	4.073
1.610	3.387	2.916	1.678	2.205	1.704
3.186	3.215	3.805	2.824	3.656	3.428
7.102	7.225	6.939	6.505	7.153	6.743
4.396	4.268	3.734	4.628	4.416	4.275
2.926	3.843	1.413	1.837	2.300	3.350
5.776	7.226	4.371	4.192	4.778	6.489
2.285	4.317	2.550	1.943	2.641	2.559
6.238	6.125	5.226	4.413	5.493	6.415
5.738	6.055	5.659	5.583	5.754	5.815
3.496	4.278	2.570	1.853	3.664	3.997
6.211	5.977	5.817	5.048	6.588	6.629
4.079	3.461	3.730	2.831	3.011	4.326
1.781	3.053	3.406	3.106	4.521	2.239
3.703	6.762	3.214	6.238	7.144	3.687
5.744	6.260	5.005	3.255	6.293	5.345
7.109	7.931	6.584	6.608	6.772	7.039
5.711	6.594	5.501	7.000	4.562	5.433
7.422	7.990	7.626	6.182	8.256	8.165
3.042	3.539	3.123	4.383	4.302	2.823
3.830	2.706	3.455	3.415	3.286	3.097
3.572	3.238	4.279	3.797	4.203	3.695
3.924	3.133	3.434	3.519	3.133	3.880
6.241	6.321	5.668	6.592	6.694	6.331

4.458	5.901	4.452	4.237	3.886	4.496
4.550	4.492	4.404	4.460	4.418	4.411
4.921	4.907	4.754	3.950	4.447	4.828
3.553	2.810	4.014	2.596	3.682	3.719
6.297	6.113	6.177	6.295	5.559	6.016
6.651	5.973	6.555	6.422	5.442	6.397
6.660	7.188	7.096	6.789	7.093	6.888
4.096	6.475	3.487	3.043	2.933	5.007
3.220	3.723	2.935	2.951	3.981	4.141
4.128	3.974	4.412	4.117	4.338	4.503
5.475	6.000	7.304	6.223	7.244	5.707
3.524	3.035	4.164	3.273	3.984	3.214
5.713	5.974	3.594	4.373	3.817	2.880
6.992	6.930	6.545	6.300	7.011	7.028
4.343	3.015	3.587	3.226	3.009	3.843
7.263	7.426	5.871	6.508	7.405	6.482
5.458	4.937	4.870	3.089	5.860	5.564
4.004	3.694	3.743	3.410	3.613	4.084
4.158	4.204	3.632	4.086	3.889	4.406
3.420	3.162	3.744	3.394	4.548	4.016
4.874	4.841	5.501	5.170	4.260	5.117
5.165	4.821	5.084	4.758	5.049	5.000
6.512	6.050	6.628	7.202	6.445	6.233
7.279	6.958	7.369	6.018	7.185	7.724
4.054	3.215	2.904	3.569	3.126	3.285
3.272	2.741	2.495	2.770	2.552	2.863
5.363	5.099	4.345	5.821	4.847	4.895
8.141	8.141	8.463	8.786	8.070	8.455
4.810	5.553	5.470	5.473	5.330	5.682
3.149	2.857	3.084	3.021	1.977	2.486
3.808	4.114	3.730	3.709	3.114	4.400
4.455	4.518	4.539	4.584	4.840	4.428
4.363	3.882	3.367	3.695	4.551	4.004
4.038	4.227	4.204	3.812	3.938	4.501
2.557	3.610	2.800	3.961	2.985	2.649
6.926	6.107	6.384	6.449	6.296	6.398
5.559	6.181	5.500	5.481	5.737	5.429
1.841	1.752	1.631	2.126	1.899	1.778
3.243	3.157	3.294	3.047	3.919	3.118
5.007	3.149	3.996	3.882	3.039	3.529
1.930	3.002	2.262	2.860	2.659	2.596
5.308	4.452	4.662	5.871	4.614	4.239
5.528	5.267	4.680	4.731	5.281	4.928
6.054	6.780	5.523	5.499	5.673	6.125
5.963	5.130	6.052	6.430	4.913	5.877
5.857	5.874	4.725	4.627	5.649	5.351
2.642	2.377	3.494	3.776	2.875	2.454
4.040	3.644	4.077	2.292	4.382	4.045
2.997	3.609	3.144	2.681	2.770	3.094
3.849	3.902	2.339	2.817	3.295	3.922

4.606	3.667	4.129	4.117	4.171	3.761
4.527	4.203	3.935	4.539	3.748	4.858
5.079	5.535	5.642	4.957	5.187	5.928
4.334	4.561	4.007	3.819	4.146	4.181
4.859	5.209	5.553	4.975	5.208	5.366
2.856	2.758	3.040	2.892	2.606	2.852
3.995	3.329	4.098	4.671	4.061	3.340
5.357	5.075	5.229	4.605	5.642	5.014
3.827	2.603	3.454	3.233	2.527	2.824
4.923	3.199	4.572	3.983	5.328	3.368
6.307	6.601	6.615	5.771	6.886	6.428
6.060	6.842	4.347	4.231	5.008	5.656
2.357	1.774	2.213	3.259	2.341	1.591
4.045	2.462	1.542	1.661	3.529	4.680
2.749	4.659	2.724	3.565	2.818	4.524
4.859	4.934	4.514	3.410	4.840	4.638
4.884	4.797	2.313	2.752	3.101	5.193
5.332	7.053	5.318	5.393	6.140	4.971
4.847	3.904	2.639	3.169	4.595	4.268
6.194	6.150	6.343	5.828	6.021	6.143
6.760	6.101	6.213	6.403	6.503	5.932
7.485	7.276	7.606	7.394	7.806	7.461
4.559	3.722	4.067	3.097	3.383	3.926
5.826	6.053	6.186	5.712	6.378	5.832
3.681	3.682	3.950	3.780	3.998	3.854
1.649	1.400	2.067	2.158	1.545	1.585
4.656	4.779	4.487	4.686	4.219	4.498
2.206	2.885	2.702	2.702	2.367	2.229
2.170	2.480	2.210	2.765	1.760	1.416
6.377	7.033	6.570	6.652	6.937	6.727
5.829	5.635	4.384	4.773	5.271	5.347
8.323	8.404	7.580	7.698	8.248	8.366
5.298	5.470	2.433	2.952	3.627	4.700
5.351	5.892	5.934	5.279	5.147	5.331
3.919	5.284	5.088	5.363	2.246	4.180
2.767	1.940	2.552	2.015	2.467	2.270
6.076	6.571	2.806	3.266	5.307	5.880
6.660	6.281	6.875	6.655	6.916	6.189
6.689	5.308	6.197	5.433	4.683	6.240
3.689	2.947	2.983	2.956	2.744	3.223
3.239	3.864	3.745	3.316	3.824	3.817
5.247	5.943	5.500	3.488	6.082	5.294
2.445	4.698	5.130	2.444	5.834	2.791
4.247	2.598	3.730	4.147	3.590	3.155
8.908	8.818	9.114	10.224	10.998	8.753
5.723	4.489	5.205	4.807	5.972	4.737
4.184	3.790	4.632	3.918	4.224	4.530
4.251	3.602	3.708	3.409	3.461	3.175
2.143	3.545	1.992	2.777	3.745	2.562
3.072	3.275	3.667	3.067	3.509	3.558

5.656	6.848	5.034	4.556	5.087	5.510
5.159	5.366	4.955	5.089	5.936	5.040
4.089	4.843	2.938	3.126	3.654	4.753
3.878	5.102	3.795	3.060	4.192	4.543
5.466	6.003	5.405	5.032	6.831	5.072
5.656	5.576	5.745	4.298	5.727	5.722
5.741	3.894	5.347	4.213	4.076	5.416
2.695	3.175	3.415	2.673	3.093	3.922
3.080	1.874	2.656	2.204	1.739	1.939
3.762	5.153	5.122	4.678	2.612	4.777
5.284	6.295	4.702	4.522	5.820	4.758
5.511	5.567	5.579	5.168	4.492	5.590
8.920	8.816	9.222	9.499	8.662	8.499
5.503	5.898	3.887	4.099	4.308	5.007
2.092	2.047	2.507	1.508	2.575	2.408
1.526	1.333	1.667	1.284	1.268	1.492
5.896	5.339	4.886	4.788	5.828	5.358
4.483	4.024	3.452	3.332	4.355	3.820
4.201	3.595	3.224	3.509	4.066	2.737
2.079	1.893	2.544	2.975	2.130	2.623
2.910	3.951	2.822	2.945	4.173	2.649
8.519	8.321	8.596	8.045	8.580	8.640
5.849	4.043	4.779	4.689	4.920	4.765
4.131	3.833	5.112	4.689	5.782	3.667
5.768	6.789	6.750	6.374	6.471	6.632
6.038	6.040	5.559	5.351	5.475	6.019
5.522	5.706	6.035	4.451	6.382	6.188
5.047	5.116	4.941	4.489	4.867	4.557
3.888	3.191	3.851	3.300	3.171	3.508
6.482	5.544	6.475	7.087	7.538	5.460
4.583	5.054	3.891	3.492	3.205	4.808
4.408	3.963	4.096	3.558	3.653	3.983
7.261	7.360	7.781	7.825	7.429	6.871
6.862	6.672	6.579	6.376	7.019	6.919
6.397	6.866	6.880	5.812	7.218	6.543
2.020	3.518	4.843	4.558	4.434	5.063
5.595	5.781	4.857	4.221	5.480	5.547
6.483	7.011	5.406	5.677	6.428	6.609
7.682	7.665	7.553	7.386	7.540	7.529
5.309	5.283	4.617	3.957	5.457	5.356
5.946	5.317	5.673	5.146	5.885	5.319
5.707	5.631	5.943	5.512	5.948	5.747
1.740	1.698	1.795	2.069	2.147	2.097
2.339	2.104	2.414	2.438	2.473	2.060
3.273	2.094	2.637	2.217	2.182	2.779
5.732	5.700	5.745	5.880	6.060	5.935
4.858	5.242	4.256	5.566	5.154	4.312
4.558	4.345	4.473	4.558	4.061	4.872
4.135	5.227	4.746	5.430	5.186	4.247
4.439	4.777	5.145	4.110	4.983	5.229

4.685	4.640	6.208	6.447	5.215	5.072
4.048	4.327	3.015	4.607	4.350	3.821
5.878	4.619	4.904	4.067	4.409	5.001
5.628	6.300	4.015	4.201	3.987	5.923
4.766	4.847	4.360	5.021	5.083	4.361
4.701	5.385	4.092	3.150	4.116	4.803
2.525	3.011	2.643	2.948	2.446	2.418
4.347	4.516	4.362	4.272	3.959	4.471
7.157	6.903	6.830	6.192	6.970	7.082
5.842	6.057	5.552	4.693	5.935	6.144
7.180	6.806	7.009	6.519	7.530	7.034
4.403	3.320	4.361	4.480	3.310	3.976
5.594	5.352	5.022	4.052	5.493	5.235
5.429	4.991	5.878	4.249	5.845	5.288
6.037	5.745	5.682	5.563	6.194	5.882
4.702	4.916	3.773	3.787	4.838	4.702
3.738	5.160	3.596	4.558	4.800	4.297
6.416	6.264	6.385	5.753	6.331	6.345
4.471	4.770	4.929	3.896	4.175	4.402
4.208	4.741	4.388	3.854	4.907	4.681
5.204	5.592	4.455	4.794	5.487	5.331
2.959	2.959	3.276	2.637	3.310	3.556
4.908	5.070	4.363	3.529	5.055	4.645
4.856	5.265	4.865	5.547	5.446	5.086
2.373	2.049	2.587	2.378	2.119	2.712
7.205	6.614	7.730	5.940	7.487	7.314
4.076	4.440	4.027	3.896	3.827	4.488
2.463	2.806	1.909	2.010	1.757	2.757
4.749	4.681	5.024	5.041	4.328	4.753
5.939	5.826	5.999	5.989	6.580	5.553
4.532	4.705	4.840	5.319	4.772	4.841
6.197	7.782	4.046	5.337	4.802	6.456
3.159	3.643	3.018	3.037	3.416	3.712
4.860	5.237	4.976	5.120	4.833	4.884
4.176	3.964	4.787	5.074	4.615	4.162
5.502	5.157	5.127	5.013	5.140	5.235
4.281	4.512	3.829	3.427	3.885	4.151
8.455	8.527	8.703	8.204	9.206	8.562
4.249	4.475	3.382	3.340	3.516	4.514
3.991	3.575	3.766	3.240	3.714	3.440
5.328	5.403	6.044	3.874	6.019	5.690
4.527	4.112	4.304	4.558	4.342	4.108
4.690	5.273	5.537	4.646	5.521	5.080
1.888	2.932	2.590	3.074	5.741	3.448
4.215	3.768	4.718	4.168	2.952	3.178
5.771	3.699	4.464	4.704	4.081	4.907
3.551	3.547	3.714	3.514	3.390	3.532
5.204	4.921	5.357	5.249	4.432	5.146
9.301	8.189	9.102	9.137	9.524	7.892
5.163	4.837	4.114	2.678	4.483	4.272

5.475	5.319	5.070	4.894	5.541	5.774
3.963	3.433	3.658	3.360	3.613	3.634
4.117	4.777	4.164	3.995	3.648	4.205
3.438	3.273	3.536	3.459	2.875	2.360
7.102	7.100	7.282	6.261	7.245	7.348
1.854	2.119	2.065	2.155	3.360	1.750
3.490	4.018	3.356	3.452	3.005	3.390
4.979	6.710	5.706	6.036	2.792	6.116
8.603	7.416	8.757	8.392	8.800	7.656
7.378	5.879	6.848	6.940	5.708	5.712
2.679	3.936	3.654	3.402	4.928	2.762
5.340	4.988	4.326	4.720	3.394	5.042
5.558	4.458	4.798	4.344	4.507	5.434
2.144	3.400	3.149	2.349	4.309	2.436
6.020	5.384	6.081	5.118	3.862	6.159
6.122	6.643	4.741	4.713	5.255	5.985
3.629	2.343	2.406	2.798	2.692	2.981
6.759	7.034	7.138	6.619	7.429	6.761
4.479	3.786	4.613	5.377	3.541	3.707
8.222	8.068	8.337	8.051	8.595	7.561
5.560	6.606	6.419	5.624	5.959	6.663
5.104	7.889	5.037	5.801	6.961	4.548
1.232	1.538	1.634	1.153	1.571	1.497
7.811	8.998	8.234	8.218	7.289	7.871
4.408	4.697	3.793	3.952	4.394	4.982
2.076	2.341	2.645	3.053	2.279	3.098
3.638	4.704	4.142	3.889	3.491	3.321
4.563	3.778	4.049	4.216	4.218	4.586
5.538	4.248	4.789	3.841	5.124	4.756
3.161	3.259	3.151	3.191	3.122	3.168
4.834	4.918	4.452	4.416	4.642	5.064
5.527	5.252	5.080	4.149	4.811	5.269
1.502	1.984	2.158	2.064	3.213	1.952
7.728	7.627	7.680	7.192	7.987	7.426
3.419	2.961	2.836	2.031	2.649	2.646
8.761	8.300	8.009	8.267	8.135	8.171
6.572	5.823	6.315	6.220	5.893	6.572
5.671	5.229	4.987	5.225	4.567	5.528
4.585	3.738	4.194	3.387	3.476	4.324
7.264	7.634	7.263	6.631	7.664	7.769
6.029	6.010	5.090	5.254	6.156	5.751
6.472	5.795	6.189	6.056	5.950	5.988
5.185	5.201	4.469	3.495	5.124	4.584
3.704	3.713	3.660	3.137	3.114	3.411
5.744	5.748	5.736	4.835	5.551	6.106
6.420	6.239	6.590	6.347	6.807	6.440
4.464	4.931	3.352	4.840	3.806	3.020
1.369	1.011	1.402	2.005	2.223	1.665
3.465	3.502	2.243	2.913	4.162	3.093
4.294	4.281	4.219	4.235	4.789	4.265

2.220	2.639	2.220	1.739	2.504	2.449
6.502	6.935	6.216	4.783	6.127	5.774
5.699	3.922	4.576	3.992	4.671	4.706
5.540	5.477	5.182	5.325	4.722	5.254
3.291	3.205	2.469	3.080	2.282	3.149
5.230	5.827	5.581	5.624	4.927	5.186
6.156	4.618	5.411	6.444	5.427	5.511
5.022	4.696	4.907	4.192	4.418	4.634
2.989	2.456	2.542	2.752	1.569	2.153
5.560	4.661	4.059	4.031	5.035	4.302
4.892	4.053	5.303	5.391	4.802	3.613
3.496	6.884	3.899	3.357	8.357	3.855
5.359	5.307	4.916	5.110	5.408	5.542
7.944	8.005	7.682	8.077	7.747	8.465
1.988	1.228	2.677	3.341	1.785	2.360
7.110	7.797	7.644	7.047	7.689	7.217
2.832	2.806	3.603	3.881	3.544	3.414
6.065	6.176	6.144	6.208	6.197	6.029
3.520	3.982	2.960	4.307	3.832	3.980
5.533	5.365	6.078	5.684	5.221	5.719
6.844	6.786	6.637	5.925	6.668	6.994
5.889	4.345	5.366	4.612	3.793	5.178
1.981	2.158	2.333	2.046	1.654	2.854
1.980	1.899	2.221	1.335	2.576	1.770
4.748	5.024	3.681	5.000	3.963	4.216
2.902	3.316	2.884	1.769	3.757	2.480
3.921	3.489	4.145	2.871	3.775	3.881
4.486	4.467	4.131	3.901	4.630	4.335
3.092	2.938	3.345	4.109	2.786	3.396
5.865	5.544	6.427	6.527	6.510	5.070
6.886	6.804	6.829	7.049	7.247	5.774
3.415	2.714	2.782	3.164	2.926	2.808
5.783	5.669	5.846	5.741	5.471	6.010
4.597	4.370	4.374	3.918	3.963	4.265
4.749	4.801	4.596	4.438	5.265	5.265
3.006	3.513	2.864	3.625	3.692	2.887
4.380	4.401	3.900	4.152	4.772	3.825
7.481	7.719	7.448	7.198	7.547	7.468
9.950	9.238	9.676	9.540	8.405	9.930
2.160	1.509	2.018	2.179	1.415	1.806
3.556	3.578	3.792	3.425	3.500	3.722
5.797	4.470	5.987	6.965	6.840	6.062
4.405	4.083	6.196	6.502	6.559	5.351
5.789	4.814	6.738	7.670	7.532	6.840
4.264	4.077	5.435	5.602	6.075	5.210
2.625	3.244	2.992	4.608	4.874	3.808
2.535	2.775	3.251	6.090	2.371	4.847
4.333	4.890	4.128	4.689	4.333	4.240
3.119	2.888	3.439	3.230	2.502	3.510
1.853	0.893	2.454	4.229	4.430	2.585

6.236	6.378	6.295	5.982	6.116	6.370
3.935	3.263	3.605	4.505	3.420	3.752
6.674	5.588	6.195	6.272	5.021	6.082
6.387	6.528	7.245	7.418	5.754	6.899
4.294	6.449	4.630	4.711	5.670	4.759
4.682	4.359	5.238	5.960	4.948	4.906
4.756	5.595	4.113	4.211	4.591	5.421
4.459	4.388	3.640	3.370	3.762	4.140
3.746	3.416	2.997	2.583	3.223	3.254
6.081	6.061	5.100	4.657	4.052	5.344
4.892	4.534	4.789	5.435	4.593	4.476
7.775	7.066	8.089	8.208	8.467	7.416
7.006	7.514	7.586	9.011	7.538	6.760
4.075	4.030	4.635	5.812	4.394	3.633
5.507	5.654	5.709	7.374	7.922	4.881
6.811	6.289	6.341	7.679	5.044	5.571
8.295	7.121	8.515	8.932	7.602	6.942
3.898	3.937	4.541	5.382	4.125	3.708
4.285	3.559	3.010	2.947	5.067	3.133
7.611	7.357	7.712	7.987	7.117	6.993
9.360	9.553	9.624	10.134	8.739	9.584
9.856	9.620	9.767	10.293	9.979	9.237
6.167	6.510	5.303	5.573	6.122	6.544
4.680	4.937	5.091	6.382	5.131	4.448
3.762	4.002	4.202	5.508	3.894	4.022
2.925	2.208	1.257	1.602	2.011	2.034
10.230	9.649	10.539	11.027	10.236	9.691
9.467	9.670	9.717	9.967	9.297	9.231
6.702	4.522	6.073	7.015	4.947	3.980
3.367	4.447	4.577	4.938	3.195	3.268
2.267	2.400	3.116	4.163	2.113	1.959
9.219	9.118	9.269	10.038	9.174	8.825
5.616	5.216	4.605	6.207	5.410	5.013
4.451	4.327	2.629	4.799	4.313	2.930
10.396	10.023	10.064	11.046	10.318	9.167
9.645	8.772	10.319	10.508	9.704	9.352
9.000	8.264	9.422	10.052	8.805	8.557
10.760	11.134	11.385	11.355	11.034	10.886
11.250	11.512	11.621	11.548	11.383	11.494
10.210	9.483	10.855	10.843	10.117	9.610
5.364	5.197	5.599	5.681	6.060	5.491
9.756	9.693	9.955	10.655	9.979	9.297
6.966	7.169	7.412	7.779	7.084	7.230
5.695	6.179	4.916	7.007	5.539	6.032
4.472	4.353	5.195	5.476	4.203	4.193
9.468	9.518	9.691	10.798	9.514	9.211
7.116	7.483	8.132	8.562	7.571	7.664
7.826	7.471	7.902	8.189	7.831	7.792
3.761	3.507	3.737	4.219	3.666	3.422
10.181	10.195	10.656	10.844	10.259	10.476

2.478	2.643	2.486	3.327	2.517	2.735
2.699	3.907	4.348	3.758	4.446	4.628
4.443	4.078	3.448	3.748	3.845	4.468
6.292	5.601	6.157	6.309	5.601	6.054
9.321	8.963	8.717	9.877	8.308	8.646
5.928	6.315	6.391	6.346	5.624	6.202
7.471	7.032	7.063	8.652	6.941	6.952
8.456	8.377	9.260	10.237	8.019	7.943
3.722	3.479	4.214	4.394	3.081	4.354
7.664	6.394	8.245	9.390	7.573	7.079
10.959	10.228	11.107	11.339	10.288	10.880
9.038	9.447	9.718	9.937	9.081	9.279
6.961	7.009	8.303	8.597	7.359	7.405
4.169	4.287	4.147	5.328	4.105	4.011
2.916	3.384	3.521	3.595	3.342	2.966
7.438	7.914	8.388	8.663	7.498	8.011
6.554	7.599	7.324	8.076	6.496	6.854
4.363	4.255	4.710	5.359	4.503	3.607
7.762	7.350	8.284	9.354	7.899	7.550
5.783	5.869	5.644	5.867	5.565	5.894
6.808	6.271	6.501	6.713	6.616	6.446
8.637	9.018	8.832	8.760	8.803	8.956
11.230	9.829	10.255	11.270	10.598	10.631
3.594	3.683	3.865	4.582	2.878	3.411
7.236	7.257	7.475	8.463	6.958	6.870
7.250	5.955	6.415	7.751	5.935	5.677
5.676	5.724	5.249	5.160	5.222	5.427
5.497	2.820	3.402	3.628	2.007	4.623
4.632	3.267	3.393	3.238	2.766	3.576
6.063	5.475	5.550	5.950	4.875	5.736
2.678	1.898	1.843	1.535	1.883	2.176
4.503	0.929	2.942	3.283	3.330	3.480
4.346	2.583	4.244	3.389	3.328	3.360
9.476	8.178	9.517	9.829	7.718	8.402
2.756	1.354	1.212	1.661	0.651	1.346
4.252	2.308	3.243	3.033	0.902	3.161
9.939	8.340	9.701	10.047	9.055	9.341
3.033	2.811	3.968	3.756	2.260	2.413
6.121	5.525	5.792	5.830	5.121	5.532
5.724	4.177	4.864	5.723	4.257	4.774
4.566	2.033	2.023	3.901	3.621	3.338
5.017	3.307	3.945	3.669	2.384	3.188
3.930	1.639	2.653	2.724	2.867	4.061
4.117	2.183	0.949	3.309	3.446	3.722
6.495	4.813	5.560	6.131	5.009	5.514
5.291	3.436	4.539	4.366	3.888	5.364
3.126	1.833	1.134	1.905	1.529	1.418
5.262	2.444	4.831	5.058	4.445	3.776
7.190	5.952	7.229	7.392	5.833	6.871
9.767	8.074	9.268	9.821	8.279	8.752

4.051	2.578	2.897	3.291	3.079	2.458
4.370	2.135	1.864	2.966	2.181	3.752
3.317	1.653	1.182	1.260	0.602	1.259
2.309	0.514	2.278	1.293	1.774	3.060
3.220	0.681	2.230	2.557	3.083	3.193
5.613	3.240	4.725	5.312	4.442	5.076
6.572	4.358	5.567	5.147	4.436	5.782
3.952	2.814	4.222	4.247	3.201	4.061
10.532	9.378	10.264	10.491	9.523	10.279
11.046	9.969	10.838	10.998	10.164	10.977
3.403	2.024	1.940	2.548	1.875	2.919
8.802	7.265	8.334	9.256	7.242	8.310
8.089	6.826	8.011	8.658	6.461	7.857
3.648	2.378	2.660	2.301	2.133	2.411
8.766	7.003	8.034	8.120	6.872	8.304
7.494	6.471	7.268	7.891	6.151	7.494
6.140	3.621	4.991	4.558	4.765	4.961
5.662	3.862	5.059	5.777	3.395	4.931
11.646	10.654	11.441	11.427	10.848	11.220
4.645	3.533	4.159	4.528	3.203	4.520
2.831	1.489	2.811	3.283	1.462	1.754
11.119	10.109	10.823	10.986	10.195	10.751
4.994	2.345	4.087	1.445	1.694	3.819
11.516	10.611	11.255	11.376	10.737	11.254
11.515	10.556	11.280	11.372	10.724	11.250
11.413	10.544	11.136	11.399	10.727	11.116
3.000	1.482	2.671	1.591	1.712	2.728
8.391	7.455	8.200	9.068	7.729	8.151
9.856	9.416	10.114	10.725	9.520	9.826
2.858	2.188	2.613	2.207	1.614	2.027
7.676	7.780	8.022	8.737	7.115	7.817
7.154	2.553	7.351	7.875	6.221	6.935
5.468	5.075	5.589	6.073	5.097	5.578
4.693	3.273	3.316	4.902	3.309	4.384
6.722	5.798	6.209	6.711	5.906	6.610
5.450	4.660	5.388	6.205	5.109	6.014
7.420	6.652	7.665	8.114	6.608	7.079
6.419	5.753	5.635	7.222	5.881	6.817
8.509	8.500	9.031	8.800	8.848	8.749
8.449	8.374	8.850	8.646	8.713	8.661
6.259	5.760	7.171	7.047	6.862	6.693
4.610	4.856	4.932	4.076	5.144	4.134
2.885	2.345	2.026	2.699	1.639	2.596
4.280	3.621	4.681	4.100	3.831	2.463
3.120	2.815	3.405	3.392	3.518	2.578
4.882	5.860	5.181	4.874	5.579	5.711
4.774	4.856	5.352	5.048	5.331	4.828
5.083	5.330	4.536	6.133	6.140	6.086
7.390	7.111	7.496	6.668	7.111	7.219
4.506	5.638	3.478	4.593	4.816	5.326

5.665	4.128	5.429	6.649	5.167	5.026
9.256	9.499	9.733	9.858	9.507	9.555
8.229	8.275	9.149	9.470	8.810	8.500
2.956	3.161	3.312	1.494	2.817	3.517
4.249	4.347	2.985	2.166	3.486	4.133
6.112	6.548	6.323	7.063	5.505	6.799
2.185	2.093	2.545	3.880	1.773	2.552
2.856	3.347	3.372	3.573	2.967	2.162
4.378	3.897	2.754	2.566	3.586	4.251
4.910	5.071	5.165	4.701	5.527	5.076
3.983	3.873	4.910	3.643	3.719	3.907
8.616	8.853	8.633	9.365	7.725	8.133
6.094	6.133	6.218	6.896	5.924	6.343
9.328	9.293	10.108	9.706	9.570	9.628
5.414	5.652	5.834	5.950	5.510	5.795
5.161	4.881	4.923	5.650	5.099	5.120
2.016	2.749	1.960	2.313	2.853	1.834
4.924	4.691	4.370	4.880	4.787	5.710
6.039	6.461	6.261	6.736	6.203	6.586
4.136	3.480	4.473	3.568	3.320	3.845
6.868	7.029	7.710	7.552	6.804	7.094
8.873	8.854	9.142	9.733	8.934	9.099
6.395	7.063	6.355	7.411	6.105	5.513
6.378	6.515	7.306	8.394	6.699	5.941
5.181	5.077	5.914	6.382	5.435	4.944
2.705	2.759	2.475	1.886	2.429	1.834
2.744	3.026	3.037	3.771	2.672	3.319
11.570	11.342	11.563	11.605	11.602	11.263
2.026	2.871	2.579	2.490	2.641	3.182
9.340	9.306	9.822	9.902	9.016	9.259
3.281	3.076	3.485	4.449	2.200	2.717
2.623	2.657	2.274	3.765	1.490	2.270
5.176	4.579	6.002	6.002	5.212	5.884
3.417	3.801	3.674	3.909	3.364	3.670
3.364	2.713	2.650	2.817	2.661	2.661
8.377	8.342	8.677	9.000	8.294	8.612
6.041	6.081	6.468	7.436	5.991	6.201
3.958	3.097	3.615	3.455	3.952	3.802
7.272	7.695	8.057	7.960	7.592	8.392
5.258	5.189	5.167	5.580	5.179	4.405
8.407	8.847	8.635	9.254	8.448	8.205
3.213	1.965	2.342	2.645	1.804	2.342
2.124	2.452	0.879	2.047	2.108	2.741
5.874	5.756	6.144	6.183	4.652	5.601
5.260	4.155	4.484	4.888	4.297	4.915
9.531	8.656	9.493	9.441	8.955	9.106
6.347	5.719	6.668	6.912	6.935	5.225
6.316	6.740	5.213	5.748	5.911	5.541
5.167	3.881	4.561	4.507	4.065	5.037
6.437	5.697	5.500	5.468	5.686	6.516

6.346	6.123	6.273	6.261	6.810	6.731
4.175	4.717	4.121	4.314	3.831	4.676
3.740	3.977	3.700	3.458	3.120	4.060
4.224	4.621	4.799	3.991	4.660	4.918
2.539	2.518	2.900	2.300	1.816	3.583
7.296	6.937	7.307	6.009	7.808	7.633
3.169	1.929	1.626	2.681	2.489	2.976
5.118	5.668	4.926	5.693	5.007	5.187
6.384	6.232	5.744	4.819	6.720	6.552
6.489	6.297	5.094	5.226	6.262	6.616
6.097	6.116	6.306	7.017	6.422	6.342
8.008	7.469	5.072	5.236	5.213	7.213
5.288	5.332	5.204	4.735	5.667	5.803
6.290	5.806	5.751	6.227	6.046	5.732
4.400	5.790	3.721	3.596	3.710	4.790
4.672	4.446	4.521	3.947	4.472	4.218
5.922	5.813	5.747	5.635	5.813	6.254
2.617	1.753	2.712	2.132	2.651	2.316
5.523	4.769	4.763	5.082	4.857	5.207
5.022	4.912	3.633	3.907	3.545	4.782
5.488	4.141	4.974	4.527	5.082	4.668
2.338	2.933	2.791	2.961	2.918	2.880
2.850	1.786	1.928	1.739	1.977	1.918
4.128	3.641	4.889	4.922	4.381	3.335
2.724	2.847	3.272	1.914	2.083	2.990
3.893	3.710	2.834	2.329	3.498	2.819
2.414	2.205	2.454	3.536	2.818	2.304
4.486	4.339	4.439	5.333	4.895	4.775
2.451	2.780	3.596	2.492	1.681	2.352
3.985	3.447	4.327	3.755	4.100	3.897
4.992	4.663	4.632	5.277	5.325	4.598
6.031	5.039	6.449	5.899	6.631	5.903
3.343	2.946	3.043	3.670	2.445	2.757
7.508	7.384	6.878	6.733	7.078	6.998
5.321	5.326	4.346	4.576	4.750	5.448
5.572	5.285	4.796	4.525	5.347	5.919
3.260	2.701	3.283	3.343	2.735	2.754
2.844	4.002	2.972	4.388	1.965	3.243
3.714	3.077	2.368	3.181	3.163	3.306
2.706	4.563	3.769	4.065	1.897	3.317
3.453	3.660	3.655	4.139	2.981	2.804
7.713	7.681	7.896	8.050	8.266	7.688
6.789	6.531	6.065	5.599	6.666	6.954
2.681	2.951	2.969	3.733	2.171	2.296
3.973	4.904	4.399	5.202	2.365	4.270
2.773	2.102	2.894	3.411	3.045	3.045
1.971	1.773	2.033	2.395	2.154	1.982
2.194	1.391	2.508	2.155	1.719	1.868
4.270	4.624	2.957	3.351	4.291	3.990
5.090	5.466	4.589	4.411	5.235	5.580

3.788	3.999	3.235	3.824	2.911	3.648
4.410	4.863	4.507	4.710	4.418	4.708
3.782	3.916	3.501	3.694	3.272	3.831
4.950	4.729	4.173	4.000	4.828	4.173
2.780	2.786	2.694	2.243	2.661	4.231
3.669	4.772	3.660	3.075	3.102	3.814
4.878	3.486	4.206	3.128	3.695	4.775
5.409	5.314	5.485	4.688	5.573	6.156
8.451	8.137	8.344	8.068	8.371	8.219
4.289	4.012	3.281	3.242	4.419	4.219
2.537	3.028	3.039	2.172	2.177	2.624
5.840	5.008	5.328	5.064	5.922	5.919
4.277	4.412	4.736	4.439	3.725	3.409
3.314	2.606	4.058	3.313	4.364	3.574
2.531	3.179	2.265	2.431	1.997	2.456
6.472	6.721	6.080	6.079	6.413	6.507
7.236	6.757	7.381	7.266	7.262	7.158
6.478	7.099	7.030	7.285	6.562	5.950
11.166	11.848	10.892	10.419	11.249	11.972
2.284	2.859	2.600	3.183	3.140	3.356
4.354	4.447	3.674	4.490	4.020	3.854
3.651	2.525	3.912	3.084	4.015	3.135
3.119	1.915	3.204	2.438	2.568	2.400
4.248	4.363	3.579	4.215	4.696	3.954
4.496	5.111	4.341	4.701	3.792	4.488
4.068	4.929	3.846	3.471	3.798	4.258
5.151	4.148	4.583	4.072	4.415	4.907
4.561	3.815	5.076	4.709	4.080	4.855
4.792	4.501	5.106	5.118	4.732	4.657
6.748	6.897	6.857	6.582	7.269	7.192
6.107	5.269	4.954	4.207	6.720	4.908
5.605	6.421	5.383	5.306	5.810	6.080
2.848	2.361	2.623	3.161	2.685	2.706
3.064	2.311	3.419	3.774	3.843	3.551
6.999	7.290	7.053	6.435	6.747	6.774
5.229	5.743	3.451	3.709	4.245	5.719
8.083	8.279	8.782	9.406	8.689	8.933
3.145	2.045	2.410	3.780	1.704	2.468
5.586	4.949	5.450	5.219	5.795	5.055
7.245	6.623	6.375	6.517	6.779	6.602
7.670	7.598	7.413	7.635	7.790	7.703
7.530	7.861	8.622	7.707	8.282	7.351
9.769	10.450	8.544	8.737	9.498	9.889
3.138	2.230	2.093	2.076	1.740	1.607
3.453	3.387	3.542	4.243	4.000	4.042
6.949	7.297	6.461	6.172	6.645	6.755
4.745	4.867	4.889	4.264	4.868	4.914
7.223	7.241	6.978	7.022	7.104	7.253
6.682	6.712	6.225	6.333	6.845	5.474
4.228	4.731	3.845	4.121	4.531	4.446

3.270	2.957	2.361	2.990	2.961	2.516
4.625	5.220	4.264	3.858	4.129	4.608
4.360	4.620	4.815	4.724	4.615	4.626
4.885	5.082	5.547	5.644	4.622	5.055
2.770	2.316	3.604	2.953	3.802	2.275
7.418	7.464	6.919	7.531	7.136	7.138
5.100	4.776	5.509	4.447	5.697	4.952
5.328	5.507	5.441	5.259	5.148	5.382
8.601	6.603	7.732	6.226	6.532	7.678
5.315	4.459	3.523	4.593	4.636	3.754
2.052	1.131	1.885	2.242	1.546	1.952
2.124	1.714	2.380	1.382	2.204	1.920
3.479	3.456	3.253	3.489	3.074	3.550
6.483	6.699	6.396	5.565	7.091	7.148
4.597	4.263	3.625	4.277	3.349	3.849
3.558	3.645	3.864	3.295	3.777	3.523
3.092	2.527	2.853	2.383	3.192	2.755
4.421	4.725	4.184	4.650	4.612	4.818
2.900	2.189	3.370	3.946	2.988	2.430
6.447	6.154	5.816	5.704	5.970	5.190
6.102	6.219	6.202	5.140	6.374	5.929
2.862	1.923	1.898	2.560	2.426	2.525
6.448	6.498	6.371	6.973	6.420	6.214
5.782	4.771	5.147	5.143	4.038	5.144
4.752	5.088	4.151	4.046	4.235	5.074
4.728	5.526	5.339	4.628	5.213	5.810
3.773	2.947	3.744	3.117	2.557	3.510
4.689	6.542	4.664	4.209	4.424	6.246
4.181	3.692	4.700	4.657	3.936	3.804
3.643	3.003	3.003	3.960	2.868	2.937
4.730	5.219	4.395	4.360	4.912	5.139
3.017	2.958	2.985	2.518	2.519	3.361
3.996	4.255	4.318	3.849	4.189	4.352
3.875	4.249	4.192	5.149	4.588	3.787
5.629	6.240	5.980	6.470	6.727	6.128
5.918	5.970	5.193	5.761	5.894	5.691
3.695	3.548	2.734	4.493	3.146	3.142
6.045	5.044	5.502	5.502	6.148	5.696
8.457	7.083	6.632	6.450	7.283	7.616
6.570	6.578	6.008	5.483	6.792	6.940
9.065	9.298	8.342	8.445	8.565	8.852
3.811	1.924	2.854	2.334	2.197	3.583
4.734	5.012	4.346	3.941	4.639	4.568
5.740	5.564	5.331	5.439	5.403	5.676
5.005	6.235	2.465	2.880	4.268	3.462
2.633	4.930	2.643	3.157	2.553	3.246
2.259	2.174	2.063	2.076	1.820	2.302
5.169	4.481	4.903	4.165	5.456	4.801
5.495	6.231	5.295	4.968	6.136	6.829
4.874	3.919	1.916	2.346	2.802	2.894

4.686	5.229	4.602	5.415	3.647	5.350
3.096	3.209	2.830	2.861	3.017	3.183
5.279	5.182	5.268	5.278	5.984	5.790
4.651	4.717	4.190	3.591	4.285	4.193
6.348	6.247	6.612	7.178	7.047	5.922
4.831	4.510	5.004	4.783	5.376	5.156
9.208	9.248	9.396	9.291	7.581	9.310
6.762	6.054	6.580	6.700	6.508	6.506
6.437	5.939	6.766	5.755	5.173	6.516
9.229	9.615	8.660	8.758	9.234	9.245
4.912	4.308	4.412	3.881	4.562	4.714
5.321	5.626	5.045	4.965	5.652	5.383
4.058	4.139	3.780	4.448	2.907	3.943
5.887	5.683	5.989	5.512	6.116	6.080
2.132	2.106	1.971	1.977	1.981	2.653
4.531	4.703	4.338	5.078	4.545	4.688
3.663	3.153	2.687	2.953	2.920	3.182
4.703	3.749	3.961	3.384	4.143	3.994
3.994	2.758	3.227	2.903	3.297	2.993
8.483	7.543	8.355	8.270	6.767	8.222
6.110	6.274	6.283	5.579	6.427	6.340
5.975	6.108	5.800	5.707	5.719	6.018
3.488	4.003	3.547	2.981	3.742	4.414
7.269	7.071	7.096	6.544	7.330	7.474
6.261	5.274	5.247	4.750	5.750	6.019
2.856	1.855	1.776	1.987	2.199	2.221
6.587	5.929	6.643	6.152	6.446	5.943
4.457	3.891	4.594	4.576	4.783	4.493
2.224	1.566	1.996	2.002	1.861	2.396
5.413	5.964	5.554	4.059	5.753	5.601
6.562	6.488	6.590	6.230	6.345	6.651
5.845	5.923	5.970	5.646	6.106	6.109
5.498	5.533	4.929	5.571	5.107	5.275
4.987	5.560	3.919	3.786	5.030	4.207
2.182	2.749	2.172	3.106	2.576	1.603
2.436	2.958	1.767	1.956	1.809	2.200
5.318	5.862	5.386	5.435	5.719	5.247
6.055	5.428	4.824	5.265	4.503	5.658
6.097	5.542	5.073	5.026	5.815	6.131
4.054	3.742	3.745	4.733	3.968	3.854
6.484	6.897	6.391	6.904	6.679	6.773
5.880	5.728	5.319	4.728	5.712	5.575
5.388	4.627	4.535	4.947	4.795	4.984
5.201	6.181	6.048	6.414	4.713	5.946
4.650	3.930	4.186	4.242	3.156	4.690
4.483	4.041	4.000	3.810	3.972	3.962
2.956	3.173	2.899	2.533	2.915	3.327
7.359	6.721	6.787	6.228	6.269	7.106
5.833	5.429	4.950	4.997	4.625	5.545
6.690	6.681	6.688	6.917	4.725	6.744

3.913	4.335	3.172	2.922	2.882	3.484
5.457	5.534	5.198	5.245	5.181	5.441
4.494	3.624	3.425	3.311	3.440	3.812
2.590	1.541	2.936	2.587	2.293	3.831
3.555	3.610	3.852	4.121	3.934	3.341
3.817	4.073	3.200	3.266	3.734	3.710
4.126	4.659	3.921	3.419	4.383	4.932
6.908	6.134	6.274	6.042	5.188	6.497
6.819	5.757	6.706	6.096	5.730	6.729
4.367	3.308	3.910	4.055	3.001	3.555
2.910	2.817	3.081	4.390	2.781	3.545
6.440	6.272	6.699	6.981	6.255	6.523
4.328	4.303	4.316	4.354	4.602	5.096
5.029	5.466	5.510	5.370	5.254	5.125
5.044	5.466	5.441	5.334	5.233	5.176
4.867	4.545	4.986	4.496	4.630	4.589
7.036	7.564	7.202	6.248	7.299	7.644
5.550	5.001	4.804	5.145	4.438	4.798
7.736	7.563	8.242	7.029	8.733	8.190
2.444	2.238	1.701	2.855	2.363	2.236
4.327	4.376	4.361	4.758	4.070	4.290
2.397	0.216	1.475	1.000	1.309	1.208
5.444	4.799	5.585	4.862	5.425	5.177
5.035	4.830	5.014	4.524	4.836	4.999
7.339	7.880	7.523	6.582	7.662	7.626
4.292	2.982	3.459	3.490	3.216	3.661
5.821	3.937	3.975	4.372	4.098	4.089
2.935	2.589	2.962	2.796	2.336	2.634
4.299	3.782	3.649	3.950	3.245	3.757
5.825	4.851	5.556	4.542	5.450	5.372
4.079	2.633	3.336	3.171	3.409	3.988
5.935	5.226	5.655	5.715	5.500	5.340
6.418	6.646	7.256	7.348	4.314	6.818
3.924	4.059	4.107	3.526	3.322	3.944
7.968	6.879	7.234	6.549	6.400	7.421
5.913	4.506	5.074	4.942	4.673	5.321
4.510	3.256	3.536	2.562	4.044	3.686
3.667	2.855	2.758	2.929	2.862	3.128
5.061	5.123	4.826	4.177	4.720	5.525
4.949	5.198	3.948	3.360	4.504	4.346
4.583	5.045	4.802	5.722	5.777	4.371
5.060	2.024	3.073	3.018	2.535	3.528
3.279	3.485	2.728	4.297	3.561	2.922
3.453	3.826	2.630	3.776	2.539	4.019
5.328	2.491	2.956	2.807	3.205	3.884
5.661	2.466	3.867	3.283	4.022	4.115
4.441	3.786	4.211	4.192	3.825	3.769
4.160	3.840	3.520	3.491	3.202	3.626
2.442	2.972	4.052	2.603	2.327	2.775
3.994	2.472	2.251	2.122	2.452	2.964

4.406	4.767	4.163	4.081	4.531	4.396
6.359	5.934	4.870	5.211	5.676	6.027
4.754	5.162	4.668	4.411	4.807	5.292
4.398	4.194	3.586	3.957	3.724	4.032
3.433	2.634	3.676	2.293	3.498	4.296
5.180	6.343	4.471	6.065	5.515	4.925
3.449	4.374	2.788	3.104	2.650	3.140
5.884	5.475	5.892	5.988	5.027	5.622
2.735	2.905	2.794	3.323	2.684	3.114
5.274	5.440	5.236	4.904	5.446	5.664
7.029	6.907	6.473	6.814	6.961	7.117
5.352	5.170	4.063	4.220	4.487	4.635
5.652	5.398	4.379	4.424	4.685	4.842
4.036	3.770	4.576	3.961	4.473	4.048
2.385	1.811	1.793	2.073	2.187	2.850
4.223	3.736	2.654	2.994	3.736	4.361
4.537	3.676	3.611	3.168	4.936	4.707
3.162	2.880	2.543	2.546	2.748	3.231
3.980	3.859	3.571	3.315	2.463	2.302
5.028	4.862	3.169	4.382	4.659	4.575
4.249	3.892	3.858	3.506	4.678	4.241
4.352	4.178	2.977	3.671	4.392	3.775
6.535	7.013	6.663	5.543	6.857	7.128
6.036	6.226	5.703	4.904	5.843	6.384
5.532	5.629	5.039	4.107	5.207	5.660
5.707	4.420	5.725	5.256	5.361	5.197
4.890	4.847	4.398	3.887	4.644	5.030
5.832	4.517	4.756	3.606	4.203	5.052
4.485	4.678	5.008	5.632	4.522	4.965
6.117	5.583	5.835	5.560	5.229	5.930
4.578	4.982	4.675	4.953	4.019	4.357
3.907	4.209	4.125	4.260	4.189	3.956
1.529	1.528	1.610	2.035	1.210	1.330
5.190	5.115	4.717	4.392	4.855	4.804
2.552	2.573	3.035	3.572	2.349	1.817
3.040	2.740	2.956	2.601	3.522	2.683
3.410	3.902	3.874	5.174	3.560	2.969
3.908	4.177	3.339	3.485	3.872	3.898
5.737	5.970	3.872	6.507	4.271	6.598
4.174	3.857	4.602	4.073	4.277	4.764
5.309	4.856	5.385	4.810	5.002	4.654
4.536	3.790	3.573	3.277	4.073	4.082
2.840	2.726	2.909	3.469	3.028	2.981
3.508	2.588	3.248	3.151	3.382	3.054
6.843	6.816	6.578	6.671	6.742	6.848
2.967	3.272	3.026	2.385	2.967	3.661
6.559	6.528	6.640	7.280	7.084	7.234
4.630	5.121	4.874	4.689	4.521	4.869
4.376	4.044	4.433	4.078	4.602	4.310
3.279	3.229	3.606	3.816	2.620	3.581

4.536	4.618	4.655	4.230	4.232	4.764
6.154	6.082	5.633	5.410	5.921	5.692
2.047	2.995	3.432	3.234	1.918	3.051
3.126	3.870	3.819	3.530	2.292	4.262
3.168	3.660	2.928	2.726	3.528	3.250
1.007	1.076	1.179	1.505	1.353	1.127
1.427	1.872	2.396	1.898	3.339	3.086
5.205	4.816	5.297	4.728	5.448	5.565
4.509	4.772	4.853	5.203	4.496	4.335
3.842	3.757	2.834	3.642	3.037	3.392
4.034	3.963	3.187	2.884	3.288	2.925
4.110	3.537	4.104	3.735	3.939	3.681
4.972	4.549	5.072	4.092	3.927	4.260
3.953	5.065	4.437	4.451	2.194	3.337
2.501	2.864	3.512	3.372	2.318	2.618
3.264	3.026	3.468	3.391	2.069	2.743
3.303	5.214	3.443	4.366	4.849	5.747
6.291	6.144	6.519	6.541	6.504	6.147
6.462	4.812	5.850	5.129	5.215	5.655
5.113	2.567	4.743	4.332	4.048	3.451
5.201	4.695	4.915	5.238	4.942	4.974
3.647	2.087	3.232	3.432	2.319	2.907
4.580	5.330	4.843	4.390	4.998	5.080
5.805	6.144	6.157	6.409	5.653	6.043
4.023	2.998	2.351	2.905	3.778	2.667
2.723	3.891	2.371	2.301	1.753	3.135
2.296	3.258	2.141	3.325	3.183	2.943
5.772	5.638	5.403	4.969	5.765	6.093
5.110	5.187	4.300	4.535	5.269	5.042
2.853	3.025	2.876	3.590	2.392	2.810
1.802	1.669	2.048	2.722	2.753	1.627
3.213	3.334	3.189	3.678	3.810	3.635
3.107	3.988	3.617	4.275	3.466	3.654
10.643	10.376	10.803	9.935	10.848	10.494
4.618	4.254	4.340	3.771	5.079	4.767
2.295	2.042	1.894	1.717	2.433	2.540
3.359	4.102	2.319	3.085	3.186	2.671
6.726	6.824	6.166	6.377	6.129	6.472
2.455	2.992	1.680	2.323	1.695	2.027
6.041	6.552	5.691	5.597	5.851	5.884
5.652	6.527	6.237	5.843	5.703	6.341
4.898	5.611	4.227	4.427	4.903	4.653
7.583	8.754	6.628	6.973	6.988	7.591
2.006	1.565	2.063	3.023	1.828	1.985
4.602	4.576	4.813	6.596	5.230	4.505
5.214	4.986	3.840	3.509	3.916	4.507
6.872	6.391	6.156	5.305	6.700	7.524
6.861	7.485	6.314	6.201	6.432	6.748
3.758	4.128	2.974	3.812	4.055	4.094
3.213	4.016	3.088	3.150	3.130	3.019

6.165	6.925	6.032	5.635	5.676	5.732
3.573	3.817	4.181	2.865	3.424	3.627
3.346	3.517	2.515	2.273	3.400	3.157
4.966	4.238	3.513	3.065	4.684	4.484
4.256	4.967	2.660	2.957	3.067	3.727
6.598	6.995	4.904	5.111	5.772	5.026
8.022	7.539	7.628	6.916	7.749	7.019
6.339	5.432	5.864	6.212	6.274	5.814
3.851	3.770	4.371	3.000	4.479	4.184
2.303	1.931	2.131	1.796	1.796	2.027
6.043	6.045	5.236	4.385	6.080	5.302
4.871	3.982	4.890	4.233	4.233	5.149
2.915	2.370	3.506	2.766	3.171	2.223
4.150	4.491	3.933	4.457	4.108	4.006
2.295	1.829	2.710	1.543	1.496	2.136
2.899	3.927	3.241	2.883	3.573	3.499
3.788	3.869	4.682	3.549	5.129	3.858
3.992	3.524	3.571	2.805	3.530	3.805
3.721	3.228	3.516	3.912	2.766	3.562
4.869	5.075	4.589	4.555	5.142	5.439
5.999	5.651	5.569	5.088	5.728	6.495
2.793	2.455	2.380	3.098	2.314	3.026
3.055	3.243	2.934	3.221	2.341	3.187
2.922	2.667	2.344	2.639	2.661	2.250
3.115	3.842	3.390	3.007	2.859	3.801
5.227	5.918	5.364	5.059	6.344	5.864
4.103	4.047	4.328	3.983	4.086	4.405
7.101	8.263	6.904	6.688	6.969	7.547
8.053	8.109	8.377	7.754	8.459	8.549
8.119	8.102	7.840	8.337	7.780	7.664
5.500	6.941	6.359	5.477	6.539	4.479
8.078	8.297	6.672	7.249	7.182	7.064
5.923	5.401	5.127	4.452	5.207	5.612
5.393	5.118	5.145	4.836	6.027	5.800
7.890	7.587	8.015	7.755	8.434	7.734
2.735	2.190	2.190	2.991	2.519	2.760
3.003	3.128	2.733	2.478	2.733	3.768
4.833	4.658	4.626	5.191	4.946	4.901
4.952	5.555	5.642	4.723	5.803	5.591
2.946	3.637	3.233	3.587	3.061	3.340
3.955	4.177	2.527	3.320	3.421	4.223
6.935	7.525	7.347	7.421	7.252	7.347
6.028	5.948	5.955	5.372	6.058	6.190
4.635	4.811	3.470	2.496	3.450	5.284
3.170	3.581	3.076	1.793	3.067	4.516
6.406	6.148	3.850	3.543	5.324	6.014
5.296	4.176	3.736	3.282	4.977	5.606
6.050	5.020	6.378	6.235	7.325	5.790
5.174	5.845	4.035	4.016	4.740	4.964
3.076	3.633	2.446	2.600	3.172	3.546

4.753	3.891	4.306	2.871	4.510	5.140
2.138	2.760	1.968	0.912	1.753	1.968
4.219	4.216	4.009	2.846	3.968	4.066
7.197	6.572	5.856	6.454	5.766	6.824
3.507	3.177	3.329	3.524	3.506	3.347
5.782	5.343	5.607	4.591	5.598	6.526
3.708	4.437	4.041	3.267	4.308	4.869
4.776	5.080	4.611	4.367	5.153	5.043
2.503	2.387	2.983	2.324	2.521	2.419
5.436	5.860	5.456	4.799	6.097	5.896
7.802	6.585	7.195	5.652	7.498	7.674
3.350	3.468	3.541	3.699	3.293	4.389
4.067	3.391	4.306	4.074	4.141	3.753
6.206	5.735	5.995	5.343	6.373	5.804
3.391	3.312	3.096	2.129	2.701	2.569
3.582	4.028	4.032	3.297	3.788	3.720
6.085	6.118	5.401	4.523	5.737	5.714
5.329	5.349	5.071	5.102	4.714	5.564
5.421	6.045	5.817	5.362	6.110	5.993
4.518	5.194	5.056	4.058	4.961	5.331
2.143	2.727	2.295	2.643	2.237	2.334
3.691	3.671	4.585	3.048	4.038	5.153
2.584	2.168	3.024	2.486	2.840	3.184
4.261	3.835	3.782	3.851	3.518	3.909
2.901	2.663	2.738	3.090	2.863	2.933
5.028	6.153	5.537	5.058	5.150	6.054
3.645	4.238	4.132	3.433	5.007	4.062
3.929	4.463	4.824	3.219	5.541	5.406
6.201	7.080	4.779	4.381	6.032	6.001
7.541	6.636	6.705	5.954	7.255	7.213
5.183	4.052	5.111	4.303	4.833	4.962
6.863	6.693	7.112	6.657	6.835	7.251
6.286	6.627	5.945	5.812	6.596	6.380
6.158	6.278	6.432	5.396	6.817	6.567
1.497	2.152	2.041	2.539	2.075	1.594
1.727	1.340	1.412	2.315	1.534	2.117
4.015	3.529	3.246	3.423	4.428	4.684
5.480	4.186	4.094	3.788	3.459	4.083
4.343	4.648	4.780	4.613	4.019	4.446
6.279	5.950	6.231	4.989	6.383	6.241
8.283	7.667	7.182	7.294	8.051	8.079
5.636	5.462	4.858	4.074	5.852	5.888
3.161	2.507	2.931	2.374	2.712	3.060
2.793	2.136	2.693	2.539	2.617	2.541
5.130	4.751	4.817	4.875	5.174	5.077
3.405	3.073	2.958	2.402	3.732	3.706
4.437	5.072	4.846	4.613	4.257	4.514
6.510	6.355	6.128	6.180	6.209	6.004
4.582	4.066	4.419	4.289	4.287	4.396
5.695	4.981	5.862	5.176	5.653	5.691

3.236	3.314	3.070	3.908	3.573	3.468
4.159	3.820	4.533	4.354	4.488	4.429
4.905	5.480	5.013	4.305	5.210	4.936
6.601	6.495	6.381	5.957	6.349	6.627
5.483	5.696	4.786	5.326	4.910	5.587
4.384	3.954	3.935	3.461	4.252	4.395
4.691	2.992	3.844	3.319	2.832	4.418
3.013	3.641	2.946	3.178	3.567	3.139
5.821	5.385	5.967	4.741	5.770	5.358
5.769	5.517	4.764	4.994	4.831	4.847
4.118	3.401	3.855	4.401	4.362	3.325
5.100	5.172	5.890	4.164	6.070	5.873
4.229	3.310	4.414	3.750	4.141	4.198
3.301	3.034	3.887	3.436	3.395	3.662
2.983	2.549	3.914	2.977	2.012	3.211
3.452	4.011	4.085	4.909	3.373	3.900
3.923	4.057	4.408	3.625	3.746	3.965
2.559	3.054	3.454	4.390	2.811	3.061
3.302	3.663	4.153	3.323	3.956	3.675
4.939	5.627	5.151	4.843	5.180	5.193
4.273	4.237	4.833	3.409	4.589	4.239
2.255	1.952	2.735	2.088	2.932	1.959
7.921	6.933	7.429	6.903	7.237	7.467
3.319	3.849	3.716	3.912	3.297	4.053
5.249	4.811	4.300	4.046	4.550	4.446
1.990	2.636	3.434	2.238	4.757	1.567
5.023	3.889	4.943	4.183	3.703	4.510
4.542	4.732	5.532	5.153	5.640	4.196
5.768	5.595	4.904	4.870	5.216	5.464
3.034	3.386	3.292	3.187	3.250	3.460
3.383	3.936	3.405	2.493	4.247	3.603
2.501	2.858	2.270	2.530	3.118	2.728
2.935	3.457	3.039	3.143	2.483	2.727
5.106	5.068	4.555	5.041	4.975	4.771
5.973	5.193	6.243	5.976	5.465	6.151
4.619	4.721	3.878	4.275	3.905	4.115
3.324	3.056	2.367	2.624	2.123	3.371
6.100	5.222	5.437	5.610	5.929	5.755
6.632	6.474	6.504	6.638	6.809	6.568
4.470	4.914	4.135	4.337	3.995	4.548
3.156	2.689	2.439	2.534	2.489	3.449
2.203	2.437	2.203	1.545	2.348	2.339
3.165	1.997	3.079	2.037	2.818	3.148
3.301	2.391	2.965	3.213	1.981	2.369
2.839	2.942	2.241	2.684	2.781	2.753
7.217	7.688	7.301	6.418	7.437	7.219
2.815	2.622	4.670	3.686	3.804	3.372
6.633	6.392	7.376	5.908	7.302	7.140
7.416	6.931	6.599	5.506	6.557	6.597
4.498	4.878	5.577	5.424	5.554	4.951

3.957	4.150	4.357	3.996	4.769	4.175
7.832	9.013	7.772	7.491	8.373	7.852
8.229	7.971	8.171	8.318	7.935	8.296
2.912	2.287	2.254	2.432	2.375	2.495
5.162	3.880	3.242	5.657	3.592	3.282
5.516	5.686	5.518	5.166	5.369	5.775
4.392	4.635	4.383	4.831	4.823	4.738
3.369	3.775	1.799	2.765	1.948	1.300
6.156	6.446	5.879	5.856	5.766	5.917
6.476	6.595	6.033	5.820	6.068	6.178
6.572	6.406	6.455	4.501	6.652	6.533
6.041	5.999	5.536	4.985	5.193	6.062
7.267	6.840	6.966	6.191	7.113	6.830
5.142	3.850	4.441	3.857	4.985	4.700
5.505	6.662	4.708	5.024	6.385	4.739
4.383	3.442	2.930	3.389	2.755	2.331
2.225	2.311	2.245	2.167	2.006	2.388
1.884	2.797	2.480	2.155	2.223	2.273
3.678	4.229	4.101	4.097	3.669	4.098
3.051	2.954	3.107	3.179	3.491	3.602
3.097	3.874	2.860	3.241	2.790	2.521
2.631	2.804	3.979	3.308	3.212	3.587
3.930	4.646	3.829	3.459	4.715	4.109
5.725	6.552	4.894	4.791	5.159	5.871
2.544	2.124	2.989	1.957	3.867	2.338
3.411	3.097	3.839	2.601	3.825	3.021
6.020	6.362	5.591	5.593	5.810	5.921
7.959	8.317	6.446	6.139	7.115	7.529
7.490	7.853	8.209	6.353	8.508	8.606
2.955	3.150	4.067	3.772	2.963	3.144
5.476	5.317	6.567	4.280	5.183	5.952
4.444	3.781	3.531	2.750	3.392	4.685
3.342	3.580	2.497	2.736	2.811	2.737
2.983	3.656	1.838	1.760	2.488	2.069
6.272	6.663	6.381	5.955	6.914	6.606
2.799	2.840	3.365	2.116	3.077	2.519
1.673	1.848	1.799	2.186	1.825	2.080
2.391	1.806	1.762	2.337	2.104	2.171
4.623	5.587	5.610	5.356	5.792	5.457
5.816	5.241	5.779	5.973	5.614	5.734
4.588	4.813	5.083	4.933	5.080	4.206
4.289	4.488	4.542	3.952	4.697	4.255
4.859	5.067	4.801	4.357	5.099	4.371
5.053	5.159	4.943	5.457	5.430	4.438
4.814	5.017	4.544	5.209	5.194	4.256
4.744	5.053	5.356	5.567	5.349	4.942
2.610	2.585	3.252	3.687	2.407	2.714
5.302	5.306	5.779	5.735	5.325	5.352
6.940	6.316	6.399	6.245	6.788	6.184
6.526	6.479	6.053	5.704	6.177	6.578

6.251	6.474	5.747	6.030	5.503	5.788
4.210	4.039	3.635	3.771	3.737	4.107
6.367	6.147	6.022	5.549	6.234	6.136
6.165	6.365	5.899	5.203	5.944	6.325
5.858	5.422	5.978	5.722	5.400	5.345
3.142	2.711	3.487	3.392	2.787	3.622
5.350	5.626	4.323	4.474	4.794	5.176
3.035	3.985	4.551	3.412	3.252	3.712
4.358	4.735	3.817	4.082	3.882	4.368
8.040	8.400	9.153	9.390	8.579	8.817
4.289	4.041	3.958	3.710	4.464	4.569
4.658	4.378	4.026	3.013	4.566	4.412
3.547	2.646	2.712	2.027	2.253	2.226
3.212	2.959	3.026	3.185	2.475	2.537
2.842	3.414	3.108	2.358	3.405	2.194
4.171	4.877	4.398	4.430	3.748	3.702
4.137	5.142	3.933	4.310	4.453	3.941
5.743	5.765	5.753	5.508	5.861	6.455
9.394	8.763	8.703	8.376	8.542	8.335
8.600	8.166	8.932	8.805	8.184	8.055
7.105	7.187	7.072	7.212	6.560	7.225
2.405	1.873	2.911	3.863	1.945	1.596
1.802	2.566	3.045	2.239	1.925	2.452
5.497	5.910	4.023	4.431	4.379	5.483
8.390	8.545	8.654	9.496	8.405	8.411
4.732	3.979	3.783	3.468	4.394	3.868
2.537	3.251	2.561	2.644	2.959	3.054
2.499	1.955	2.507	1.874	1.756	3.061
3.836	3.767	3.872	3.290	4.669	4.346
3.716	4.536	3.571	3.883	3.854	3.855
2.597	3.012	3.725	2.304	2.826	3.487
3.231	3.372	2.903	3.103	3.325	2.658
5.388	5.478	5.158	4.580	5.161	5.176
2.518	3.067	3.373	3.191	3.099	3.174
3.097	1.456	2.069	1.623	3.347	2.341
1.848	1.833	2.492	4.096	3.330	3.154
7.561	7.874	8.639	8.782	8.518	8.448
2.877	2.608	2.239	2.737	2.110	2.732
2.080	2.160	2.176	2.046	2.367	2.196
5.834	4.328	4.936	4.180	4.893	5.690
4.427	4.517	3.427	3.423	4.959	4.775
2.487	2.361	3.152	2.084	2.546	2.091
6.503	6.237	6.048	5.445	6.843	6.405
0.832	3.532	3.660	2.969	3.158	3.060
3.978	2.797	4.629	4.216	4.306	3.183
3.205	3.276	3.189	1.442	3.476	2.884
4.073	4.275	5.789	4.496	4.872	5.113
1.977	2.236	2.832	1.969	2.920	3.143
2.025	1.881	3.542	3.211	2.159	2.954
2.514	3.836	4.306	3.402	3.928	4.192

3.492	2.523	3.258	3.077	3.199	3.113
4.116	4.203	3.597	2.362	4.152	3.555
2.718	2.946	3.852	3.205	3.233	3.102
4.029	4.897	4.761	5.013	5.265	3.853
3.108	3.923	3.108	2.713	3.150	3.062
5.665	5.955	5.339	5.151	6.032	6.648
2.784	3.125	2.099	2.081	3.407	2.815
4.266	3.607	4.436	4.064	3.862	3.944
1.457	2.367	2.301	1.617	4.092	3.918
2.384	1.428	3.043	1.191	3.098	4.922
2.021	1.870	2.750	1.857	2.283	2.646
3.451	2.672	2.971	2.664	2.762	3.223
3.166	2.473	1.971	2.176	2.061	2.359
2.415	1.939	2.545	2.164	2.309	2.322
4.365	3.732	4.337	3.343	3.878	3.258
6.950	6.027	7.256	7.309	6.913	6.050
2.478	2.121	2.709	3.285	2.788	2.352
4.147	3.576	3.087	3.665	2.959	4.218
6.790	6.320	5.888	5.688	6.275	6.521
5.481	5.427	5.243	5.030	5.533	5.640
3.928	3.731	3.679	3.778	3.893	3.544
3.746	3.746	4.063	3.094	3.853	4.171
7.570	7.415	7.217	7.648	6.901	7.340
5.774	5.195	5.104	3.804	5.829	5.563
5.224	5.252	4.952	4.702	4.203	5.508
3.578	3.335	2.800	3.139	3.239	3.221
4.973	4.572	4.674	5.036	5.001	5.125
5.697	5.670	5.402	5.460	5.106	5.465
3.949	2.961	3.133	3.522	3.732	3.727
2.670	1.753	2.414	2.633	2.861	2.803
4.668	4.479	4.857	4.163	5.105	4.303
4.691	4.901	5.611	4.082	4.793	5.239
4.761	3.780	3.879	4.377	4.571	3.596
4.562	4.489	4.641	4.055	5.426	4.744
6.781	6.489	6.290	6.598	6.247	6.214
6.251	6.007	5.852	6.008	5.721	6.142
2.844	3.345	3.666	3.121	3.534	3.291
5.347	5.664	4.388	5.132	4.523	4.675
2.683	3.259	3.430	2.237	2.873	2.805
4.474	4.318	3.692	3.613	3.920	3.919
4.168	3.984	4.342	3.172	3.914	4.162
3.923	4.491	3.771	4.924	3.827	3.785
3.626	3.318	3.094	3.134	3.029	2.775
3.058	1.457	1.979	2.163	1.435	1.810
3.431	3.026	2.518	3.260	2.925	3.271
4.796	3.343	3.372	3.908	3.312	4.069
2.141	2.023	2.421	2.250	2.151	2.112
3.590	4.804	4.111	3.739	3.682	4.338
4.331	4.314	4.194	2.649	4.667	4.931
6.503	6.464	6.483	7.178	7.062	6.432

3.457	4.132	3.763	3.132	4.612	4.906
4.627	4.532	3.982	3.856	4.505	4.794
6.005	5.980	6.064	5.685	5.847	6.075
4.925	4.910	4.312	4.535	4.830	4.633
5.601	5.569	4.605	3.756	4.238	5.168
5.148	5.414	5.700	5.268	5.773	5.295
3.694	3.950	4.057	4.413	4.342	4.113
3.798	2.270	3.985	2.706	4.914	2.442
5.971	5.562	5.901	5.635	6.209	5.413
4.492	4.693	4.206	4.173	4.371	4.723
2.677	4.351	4.238	4.499	4.149	3.037
3.483	2.847	3.595	2.798	3.146	3.318
6.272	5.701	5.874	6.396	5.360	6.377
3.047	3.198	2.271	3.109	2.783	2.279
6.122	5.727	6.153	4.955	6.679	6.011
4.749	5.049	4.003	4.162	4.028	4.777
4.246	3.978	4.553	4.113	5.293	3.712
5.873	6.179	5.964	6.216	6.481	5.950
4.790	4.694	4.572	4.350	5.014	4.691
5.382	5.718	5.304	4.710	6.258	5.929
6.491	5.998	6.365	6.393	5.958	6.367
3.159	3.692	3.548	2.698	3.797	3.671
4.306	3.610	3.772	4.193	3.857	3.669
3.526	4.271	4.001	3.948	3.850	4.713
3.242	3.679	4.157	3.969	3.201	3.317
7.000	6.397	6.726	6.801	6.397	6.594
7.340	6.986	6.659	7.359	7.191	6.537
7.580	7.200	7.245	7.270	7.164	7.200
4.676	4.630	4.952	4.639	4.773	4.456
2.822	2.598	2.730	2.388	3.380	2.196
6.198	5.932	6.922	5.722	6.763	5.217
2.516	2.620	2.816	2.705	2.741	3.809
4.974	4.714	5.494	5.210	4.981	5.432
7.254	7.086	6.978	7.182	6.699	7.018
5.204	5.302	5.196	5.084	4.819	4.864
6.066	5.494	5.241	5.433	6.014	5.804
3.344	3.309	3.435	2.998	3.362	3.906
6.151	6.084	5.329	5.451	5.681	5.654
3.331	3.905	3.318	4.940	2.815	3.065
3.641	4.143	3.927	4.269	3.817	4.070
3.422	2.308	2.548	2.277	2.504	2.081
5.455	5.155	4.834	4.895	5.032	5.164
2.953	2.831	2.906	3.249	3.078	2.848
5.246	5.346	4.676	4.595	4.831	5.171
2.704	2.497	3.045	3.214	2.582	3.268
4.893	4.600	4.588	4.191	4.173	4.704
4.735	4.956	4.531	4.914	4.538	4.788
4.747	4.305	4.953	5.199	5.232	4.394
2.890	3.380	2.989	3.864	2.241	2.564
3.552	3.191	2.850	2.795	3.165	3.738

5.836	5.231	5.287	5.413	5.499	5.816
5.975	5.350	5.594	5.639	5.717	5.902
5.526	4.656	5.474	4.920	4.638	5.719
2.858	1.877	1.983	2.697	2.386	1.860
4.683	4.940	3.804	4.819	3.829	4.961
3.896	3.473	3.280	2.839	3.907	4.103
5.903	6.497	6.043	5.578	6.713	6.175
7.808	6.926	6.995	7.099	7.247	7.376
4.866	4.318	4.539	4.043	5.181	5.241
5.021	4.813	4.855	5.052	5.453	4.470
4.138	3.848	4.566	4.931	4.834	4.039
4.449	4.715	4.380	3.457	5.301	5.275
3.591	3.036	3.150	4.415	4.015	1.989
4.553	4.607	4.448	4.013	4.634	4.636
2.609	2.242	3.898	4.397	3.732	3.140
4.256	3.343	1.648	2.900	1.790	2.186
6.163	5.748	1.291	5.397	1.738	1.872
6.056	5.382	6.504	6.042	6.414	5.693
8.299	7.994	8.334	7.933	8.397	8.083
6.843	6.239	7.190	7.261	6.861	6.587
2.499	2.584	2.800	2.330	2.348	2.643
2.071	2.351	3.110	3.025	1.642	2.383
1.769	2.961	3.796	4.553	2.888	2.651
3.309	2.659	3.082	2.861	2.416	2.691
1.604	2.040	2.311	2.351	4.257	2.313
7.409	8.494	7.994	7.961	7.729	8.469
5.115	5.487	4.922	4.458	5.044	5.210
5.104	5.742	4.352	4.009	5.224	5.739
5.918	6.455	6.115	5.560	5.827	6.031
3.784	2.659	3.378	3.858	2.851	3.536
4.564	4.410	4.146	4.305	4.503	4.453
6.005	5.428	5.377	5.473	5.070	5.498
3.723	2.971	3.937	3.868	3.436	3.166
4.419	4.049	4.558	4.603	5.244	3.934
5.259	5.490	1.604	4.183	1.754	1.102
5.449	5.394	1.512	4.382	1.629	1.013
4.910	5.296	4.441	4.716	5.045	5.244
8.878	8.950	8.488	8.729	9.648	8.529
4.259	4.466	4.537	5.161	4.984	4.787
4.775	4.679	4.219	4.410	4.043	4.967
4.082	3.234	3.749	2.836	3.558	4.707
9.051	9.204	8.577	7.159	8.908	9.264
2.059	3.226	3.088	2.640	2.464	2.643
4.015	4.274	3.498	3.792	4.013	4.015
2.253	1.989	2.144	2.520	2.417	2.237
5.870	7.137	5.159	5.027	5.476	5.793
4.252	4.799	5.274	4.621	5.058	4.835
5.040	3.652	5.090	4.004	5.071	5.303
5.734	5.177	5.512	5.025	5.259	5.275
6.787	7.496	5.881	6.306	6.415	6.561

6.923	7.590	7.258	7.484	7.434	7.056
5.694	5.315	5.329	4.882	5.510	5.661
5.843	6.074	5.281	5.625	5.830	6.270
5.936	6.070	6.134	6.351	5.597	6.108
2.802	3.787	2.795	1.978	3.295	2.689
5.209	4.596	5.064	4.806	3.680	5.085
4.013	4.116	3.143	3.437	3.572	4.069
2.853	2.302	2.783	1.739	2.646	2.536
6.813	6.598	6.605	5.371	6.605	6.416
3.618	2.919	3.242	3.481	3.198	3.518
7.692	7.308	7.160	6.543	7.363	7.409
7.431	7.243	6.725	6.320	7.183	7.285
3.924	4.267	4.031	5.406	5.071	4.739
4.682	4.983	4.619	5.467	4.506	4.599
3.146	3.287	3.050	3.766	2.643	2.419
5.540	5.309	3.701	4.046	4.680	5.198
6.991	6.946	7.163	6.629	6.646	7.227
4.847	4.690	4.215	4.387	4.727	5.068
3.327	3.807	2.722	2.869	3.575	3.793
3.880	3.718	4.273	2.613	4.046	4.967
6.443	4.871	5.823	5.927	6.655	6.064
6.284	5.888	4.882	4.979	5.101	6.006
3.767	3.584	3.065	3.720	3.775	3.386
3.848	3.674	3.125	3.675	3.462	3.792
9.805	9.937	10.169	8.972	10.276	9.999
6.170	6.169	5.374	5.417	5.655	6.382
4.935	5.056	4.853	5.137	4.275	4.623
3.798	5.164	3.822	4.367	3.683	4.267
5.859	5.384	5.992	5.384	6.493	5.837
3.812	3.202	2.451	2.728	3.300	3.629
6.235	5.325	6.282	5.669	6.658	6.050
5.516	4.991	5.341	4.525	4.973	5.406
6.837	4.587	5.357	4.997	4.416	5.365
3.603	4.210	4.284	4.773	3.896	4.367
2.674	3.360	3.600	2.988	2.561	2.590
6.396	6.748	6.964	6.371	6.492	6.635
2.570	2.822	2.297	2.160	2.434	2.424
4.601	3.909	3.875	4.002	3.597	3.898
5.946	4.725	5.057	4.714	5.400	5.289
6.685	5.697	6.485	6.071	5.660	6.200
4.261	4.128	3.668	3.788	4.207	4.222
4.454	4.556	4.028	3.781	4.269	4.448
5.609	5.700	5.360	5.917	5.574	5.602
2.822	2.688	2.875	3.031	3.045	2.869
3.899	3.342	3.220	2.560	3.352	3.971
3.834	3.923	2.810	2.713	3.727	3.739
8.475	8.244	7.921	8.204	8.163	8.430
4.430	4.378	4.529	4.481	4.400	4.363
5.984	6.183	6.073	6.187	5.264	6.425
3.212	3.738	4.377	3.029	5.408	3.899

7.607	7.149	7.044	7.181	7.202	7.371
4.935	4.335	4.453	4.806	3.860	4.991
4.508	4.375	3.747	4.650	3.680	4.850
4.979	4.569	3.935	4.573	3.967	4.977
4.216	4.305	4.003	3.176	4.388	4.556
6.315	6.472	5.825	5.970	6.277	6.554
4.284	3.287	2.832	2.935	2.612	3.244
4.570	4.902	4.035	3.520	4.748	4.146
7.401	7.390	6.867	6.443	7.141	7.438
4.270	3.822	3.058	1.905	3.921	3.857
5.914	5.704	5.982	5.615	5.772	5.981
2.041	3.043	2.478	3.546	2.513	2.869
7.558	7.360	6.416	6.780	7.399	6.484
8.235	8.239	7.912	8.150	8.221	7.843
6.471	6.351	1.543	5.692	2.622	1.526
4.079	2.992	2.824	2.848	2.686	3.504
5.148	5.370	4.893	4.426	5.257	4.959
7.538	7.330	7.456	7.459	7.883	7.060
2.657	4.558	2.682	3.674	1.755	4.457
5.031	4.654	0.817	4.200	0.527	0.570
4.121	3.464	5.174	5.208	4.611	4.105
7.777	8.308	8.155	8.216	7.453	8.343
7.801	7.933	7.848	7.300	8.175	8.087
6.257	6.238	5.587	5.475	6.816	6.077
5.158	5.778	5.516	5.710	5.621	5.858
5.106	5.361	4.312	3.817	5.309	6.158
4.960	4.795	5.811	5.303	5.818	5.185
5.654	5.048	6.245	5.633	6.090	5.765
5.757	5.936	5.862	5.849	5.682	6.066
7.076	7.123	7.222	6.724	7.365	7.014
8.946	8.891	8.801	8.441	7.929	8.769
5.143	5.133	5.484	4.221	5.167	5.792
2.099	2.373	2.055	2.554	2.235	2.099
1.709	2.379	2.444	2.067	1.613	2.172
6.039	5.440	5.447	4.540	6.332	5.644
6.978	7.593	7.578	7.629	7.716	6.418
1.596	2.102	2.168	3.380	2.322	1.894
6.098	6.795	5.271	4.811	6.436	5.808
6.330	6.293	5.881	5.549	5.953	6.430
3.938	3.997	3.274	3.438	3.471	3.669
3.922	2.994	4.115	3.755	2.561	2.362
8.622	8.495	8.490	8.187	8.120	8.647
5.512	7.601	5.015	6.571	5.437	5.050
3.260	3.518	3.184	2.948	3.227	3.199
6.538	6.478	6.172	6.809	6.236	6.277
5.152	4.233	3.980	3.400	4.039	5.209
3.902	3.520	3.260	3.385	3.877	3.091
6.339	7.201	6.140	6.133	6.732	6.640
4.884	6.589	4.739	6.020	5.233	5.888
2.715	2.733	2.102	3.404	2.010	2.861

2.362	2.736	3.673	3.249	2.783	2.719
4.287	4.934	4.792	5.368	4.765	5.247
2.969	3.302	2.579	2.884	2.679	2.138
3.947	2.969	3.822	3.854	3.979	3.917
1.168	2.593	1.833	1.649	4.528	2.748
2.492	2.641	1.924	1.624	3.014	2.449
6.005	5.681	5.668	4.235	5.570	6.100
5.634	5.646	5.419	5.274	5.740	5.985
3.904	4.212	3.877	2.855	4.195	4.514
5.395	5.395	4.809	4.081	5.346	5.162
8.652	8.798	8.271	7.874	8.169	8.490
6.338	5.909	5.719	5.000	5.797	6.308
3.257	2.691	3.916	2.536	3.125	3.088
4.676	4.121	4.097	4.547	4.075	4.607
2.534	2.501	2.331	1.616	2.002	2.203
4.868	7.476	4.401	4.913	6.282	6.875
2.188	1.709	2.156	1.284	0.999	2.244
5.235	4.669	5.045	4.950	3.675	5.283
2.044	1.741	2.815	2.031	1.698	1.112
5.887	4.966	6.077	5.627	6.079	5.822
4.982	2.891	4.074	3.408	3.952	3.784
2.366	2.406	2.020	2.859	2.192	2.570
3.221	3.468	3.803	2.107	3.465	3.242
6.565	6.920	6.587	5.687	6.556	6.757
5.657	6.378	6.930	8.319	6.237	7.054
1.840	1.951	2.118	3.412	3.190	3.619
4.810	4.385	3.894	5.859	3.714	4.873
5.163	5.507	7.041	6.764	4.686	6.562
5.354	5.204	5.397	4.712	5.760	5.729
4.599	5.386	5.253	6.150	4.530	4.337
6.190	6.244	5.327	4.586	5.171	5.978
2.367	3.065	2.721	3.096	1.982	2.133
3.516	2.980	3.535	3.515	2.888	3.216
3.022	3.605	3.283	3.715	2.230	3.197
2.644	3.254	3.021	3.135	1.838	2.761
2.822	3.138	3.025	2.723	2.340	3.666
6.050	6.322	5.627	6.760	6.214	5.834
5.099	4.453	4.119	4.595	4.632	5.067
4.716	5.325	4.556	4.823	5.171	5.597
5.264	5.881	5.649	5.029	5.917	6.107
8.773	9.112	8.588	8.707	8.877	8.839
2.802	4.018	3.499	3.914	3.245	3.033
5.886	6.755	5.338	6.773	6.122	5.786
5.666	5.185	5.549	5.198	6.083	5.944
7.416	7.576	7.023	6.450	7.201	7.026
1.782	1.639	1.610	2.615	2.352	2.445
7.927	7.957	7.411	7.876	7.899	7.652
3.225	2.039	2.884	2.534	2.915	3.452
6.614	7.007	6.603	7.091	6.645	6.438
5.508	4.692	4.461	4.273	3.844	4.498

7.109	6.744	6.645	6.575	7.564	6.632
4.893	4.571	3.630	4.848	4.530	4.729
3.310	3.614	3.352	2.990	3.219	4.116
3.378	3.150	2.423	2.541	3.332	3.946
7.031	6.591	6.908	6.570	7.804	6.627
3.788	3.693	3.862	3.379	5.324	3.242
4.782	5.770	5.391	6.618	3.216	4.791
6.624	5.315	5.755	4.581	5.929	5.868
6.088	6.758	5.706	5.562	5.674	5.819
5.945	5.384	5.012	4.665	5.729	5.841
6.340	6.532	5.365	5.329	5.932	6.154
6.157	5.955	6.052	5.610	5.812	6.013
3.084	4.113	5.300	3.765	2.833	4.317
7.249	6.602	6.583	6.271	6.702	6.873
7.334	7.565	7.746	8.556	7.893	7.221
4.726	5.421	5.166	5.614	4.744	4.754
4.076	4.100	3.385	3.321	3.684	3.643
4.367	4.354	4.418	3.085	4.642	4.364
3.598	5.214	4.445	5.215	2.046	3.673
2.596	2.408	2.770	3.798	2.404	2.198
7.345	7.238	6.825	6.670	6.936	7.254
2.888	2.898	3.397	2.986	4.274	2.546
4.737	4.808	4.490	4.741	4.454	4.808
4.865	6.200	5.390	6.696	3.286	4.761
3.186	3.609	2.925	3.399	3.402	3.531
3.859	3.963	3.765	3.776	3.440	3.181
5.004	4.507	4.813	4.297	5.966	4.289
3.629	3.239	5.210	3.523	4.059	2.349
5.236	4.975	5.068	4.014	5.398	5.526
5.932	5.733	5.899	6.012	5.768	5.834
3.893	3.383	3.761	3.219	3.682	3.839
6.844	6.988	6.407	6.199	6.898	6.511
2.617	3.899	2.769	2.184	3.297	2.117
8.593	8.633	8.492	8.949	8.888	8.768
5.818	4.330	5.824	5.162	5.900	4.639
4.590	4.571	3.224	3.877	3.780	4.349
3.256	2.844	2.643	2.604	3.113	2.623
4.331	4.755	3.150	2.359	3.623	3.047
3.755	3.743	4.109	3.964	4.009	3.423
3.912	3.680	4.238	5.322	3.425	2.814
3.796	2.549	2.778	4.065	2.678	3.057
9.064	9.329	8.604	7.918	9.193	9.171
5.346	5.338	6.141	6.242	5.649	5.689
2.280	2.155	2.260	2.229	2.720	2.062
6.163	6.428	6.307	6.845	6.180	6.125
5.103	5.281	5.090	5.031	4.843	5.087
7.835	5.818	6.047	6.476	6.784	6.388
6.451	7.377	5.573	6.051	6.062	6.310
2.563	2.478	2.370	2.255	2.981	2.466
2.158	2.471	3.260	2.828	3.842	2.837

2.114	1.824	9.366	1.709	9.416	9.612
3.855	2.916	3.168	3.045	2.855	3.003
4.976	3.699	4.659	4.570	4.303	4.555
4.242	2.870	3.503	3.508	3.613	3.847
3.148	2.455	2.320	3.053	3.606	3.876
1.949	1.782	2.218	1.529	2.240	2.266
2.541	3.361	3.020	3.063	3.653	2.611
2.480	2.303	2.239	2.401	3.284	3.323
5.946	6.405	5.803	5.425	6.180	6.100
7.237	7.208	7.084	6.960	7.181	7.080
4.831	5.109	3.806	3.200	4.388	4.321
2.453	2.111	1.807	1.649	2.465	2.497
4.524	5.516	4.288	4.325	4.790	5.042
4.227	3.332	4.645	3.678	4.043	4.418
1.985	2.260	2.157	2.645	2.460	3.027
5.050	4.826	4.306	3.741	4.253	4.299
3.898	5.026	3.721	3.423	4.308	4.065
2.955	2.642	3.877	4.544	3.541	3.344
2.735	2.311	2.237	1.990	2.469	1.967
5.649	6.130	5.438	5.892	5.893	5.360
4.174	4.221	4.483	4.230	4.383	4.807
8.401	9.053	8.561	8.829	8.618	8.938
6.419	5.611	3.828	4.593	5.199	5.010
3.036	3.745	1.814	2.475	2.205	2.393
4.301	5.238	4.752	4.104	4.285	4.865
5.221	5.640	5.481	5.116	5.250	5.281
6.719	6.574	6.210	5.359	6.605	6.406
7.026	7.105	6.765	6.259	7.183	7.442
3.282	3.899	3.404	4.447	3.544	3.081
3.763	3.613	5.164	2.941	3.661	4.006
2.922	3.800	4.004	3.638	2.976	3.597
3.430	5.829	3.381	3.377	3.706	3.981
3.085	3.258	3.145	3.663	3.515	2.961
7.226	7.425	7.377	7.670	7.269	7.317
4.155	3.445	3.282	2.665	2.644	3.692
7.623	6.957	6.343	6.099	6.495	6.954
6.801	6.225	6.521	6.282	6.303	6.926
9.260	9.305	9.573	9.542	8.469	9.289
4.740	4.133	4.605	4.165	3.541	4.303
3.211	2.618	4.780	2.533	3.215	3.504
5.708	5.428	5.470	5.802	5.492	4.901
5.710	6.289	4.547	4.924	5.592	5.654
3.390	4.156	4.164	4.094	1.385	4.004
3.560	3.668	3.425	3.005	3.610	3.794
1.945	2.076	2.816	2.314	2.351	2.114
2.714	3.065	3.773	3.723	3.537	2.911
3.777	4.932	2.440	4.956	4.048	3.531
6.061	7.141	4.520	6.835	6.444	5.681
5.078	5.386	5.669	5.752	5.634	5.124
6.947	6.868	6.769	6.916	7.221	6.669

3.868	4.559	2.843	2.945	3.785	2.074
5.882	5.481	5.266	4.433	5.545	5.341
5.035	4.690	4.177	4.370	4.605	4.552
6.027	5.858	5.985	5.634	6.341	6.036
4.543	4.596	5.069	3.636	4.258	4.658
4.216	3.496	3.443	3.476	4.014	4.317
7.041	6.509	6.140	6.778	6.640	6.560
5.014	4.789	4.287	4.097	4.840	4.966
4.952	4.989	5.344	4.974	4.804	4.893
4.661	4.535	3.919	4.314	4.039	3.997
4.122	3.625	3.224	3.448	4.081	3.536
6.230	6.136	6.260	5.431	6.467	6.374
4.203	4.768	4.293	4.091	4.106	3.872
5.030	4.638	4.702	4.855	4.569	4.300
5.200	4.960	4.681	4.607	4.738	4.767
6.931	6.837	6.436	7.041	6.726	6.780
5.007	2.586	4.369	3.467	3.376	3.802
7.328	7.200	7.518	7.400	7.397	7.213
4.272	4.891	5.248	4.704	4.700	4.744
6.239	5.553	5.395	5.964	5.955	5.892
3.225	3.745	2.692	4.708	3.203	4.236
3.774	3.345	4.144	3.783	4.010	4.306
4.144	4.146	3.418	3.038	4.535	4.285
6.766	6.495	7.020	6.663	7.697	7.114
7.146	7.848	7.019	6.778	7.468	7.198
4.836	4.274	3.304	3.795	4.357	4.738
6.595	5.911	5.806	5.698	6.422	6.475
2.930	2.556	2.813	2.763	2.296	2.672
3.055	3.018	2.739	2.565	2.910	2.540
8.855	8.466	8.360	7.850	8.425	8.450
2.815	2.700	2.918	2.715	3.091	2.586
7.092	6.875	6.726	6.783	6.827	6.866
4.754	4.839	4.493	4.371	4.742	4.904
7.020	7.608	7.012	6.965	7.478	7.347
5.888	5.629	5.021	4.890	5.817	4.676
4.349	3.655	4.412	3.144	3.837	4.104
4.393	3.713	4.250	3.273	3.924	4.040
9.092	9.041	7.739	7.583	8.960	8.496
4.697	5.786	5.182	5.195	5.324	5.232
7.664	7.673	6.646	7.012	7.195	6.880
4.539	3.799	3.835	2.971	2.585	4.169
4.796	4.861	4.308	4.192	5.037	4.691
3.890	3.414	3.593	2.297	3.289	3.579
2.971	2.580	1.848	2.888	2.622	3.270
2.988	2.972	1.837	2.834	2.118	0.983
7.424	7.028	7.240	6.413	7.871	7.485
3.305	3.600	2.588	2.176	2.765	3.241
2.944	4.475	3.206	2.690	2.359	3.706
6.437	6.305	6.173	6.525	6.183	6.498
6.300	5.698	5.900	5.948	6.209	5.129

6.560	4.953	5.314	5.041	5.813	4.760
3.543	3.696	3.521	3.516	4.476	3.402
3.651	4.973	4.948	4.399	4.709	4.709
3.445	3.188	3.531	3.632	3.684	3.614
4.681	4.353	3.975	3.692	5.002	4.725
3.422	4.311	3.602	2.953	3.483	3.831
5.960	6.010	5.666	4.222	6.302	6.320
6.972	6.969	6.665	6.719	6.711	6.761
4.034	3.628	4.283	3.378	3.438	3.685
6.346	6.418	5.582	5.961	5.860	5.864
2.217	3.599	3.194	3.154	1.804	2.995
2.927	2.881	2.908	2.936	3.755	3.082
6.338	6.351	6.042	6.205	6.158	6.233
6.006	4.701	4.095	4.832	4.681	5.491
4.821	4.843	3.743	3.796	4.286	4.203
4.248	4.949	4.821	3.824	4.546	4.444
3.818	3.338	2.672	1.995	2.826	2.818
5.620	4.820	4.631	5.159	4.878	5.406
5.893	5.696	5.429	5.243	6.251	6.623
3.694	3.761	3.282	4.129	4.197	3.538
3.244	2.561	2.151	3.776	3.616	3.201
3.554	4.055	3.978	4.578	4.346	3.340
4.039	3.966	3.144	3.996	4.343	4.880
5.109	4.963	4.481	3.596	4.885	4.041
3.949	4.445	3.136	3.126	3.695	3.594
5.451	5.487	5.077	4.753	5.873	5.413
4.047	4.007	2.499	3.345	4.589	3.503
3.287	3.189	2.964	2.950	2.573	2.610
1.838	2.329	1.706	2.054	1.728	1.965
3.034	2.918	2.390	2.275	2.058	2.862
1.284	1.672	1.615	1.464	1.124	1.368
4.840	4.368	3.810	3.995	4.075	4.567
3.101	4.390	3.292	3.071	3.171	4.086
4.290	4.246	3.552	2.110	3.082	4.110
3.903	2.643	2.527	3.009	3.217	3.119
5.485	5.366	4.307	3.908	4.742	5.093
4.478	4.368	3.329	3.319	4.859	4.421
3.582	4.079	3.479	3.733	3.642	3.679
3.516	2.225	2.616	2.526	1.473	2.471
2.805	3.437	2.207	2.482	3.198	2.677
3.694	4.277	3.290	3.233	4.284	4.082
2.946	2.790	3.240	1.997	2.548	2.018
5.409	5.150	4.813	5.567	5.678	5.027
7.691	6.831	6.863	5.460	7.149	6.966
4.241	4.799	4.412	4.027	4.074	4.967
3.132	3.525	2.643	2.031	3.720	3.819
3.967	3.736	2.577	3.136	4.186	4.332
3.971	2.765	2.669	2.627	3.579	3.472
4.689	4.510	4.660	5.235	5.216	4.536
4.792	4.617	4.219	3.568	4.379	4.339

5.730	5.434	5.380	3.686	5.406	5.765
2.123	2.145	1.622	1.814	2.567	1.744
2.723	2.436	1.979	2.300	3.091	2.937
4.284	4.306	4.590	4.275	5.604	5.346
3.331	3.694	2.766	1.290	3.865	4.209
3.534	3.129	1.953	1.770	3.241	3.897
3.686	3.618	3.737	3.599	3.893	3.745
3.563	2.659	2.592	2.555	3.193	2.977
5.545	5.857	4.749	5.504	5.558	6.082
3.652	4.190	3.341	3.883	4.192	4.173
2.115	2.082	2.974	2.412	2.038	2.310
4.520	4.427	4.264	3.699	3.872	4.153
4.604	4.405	4.051	4.119	4.396	3.901
3.407	3.420	4.147	3.121	3.241	2.271
6.747	7.162	7.394	7.701	6.735	7.123
3.880	4.307	2.911	3.259	3.268	3.904
2.030	2.520	3.418	2.926	3.135	3.135
3.559	3.595	4.057	2.996	3.881	3.417
2.541	1.879	1.897	2.471	1.996	2.282
4.362	3.866	3.541	2.903	3.767	3.643
3.555	3.246	3.887	3.754	3.045	3.233
2.099	1.963	1.939	2.101	1.992	2.286
5.546	5.372	5.688	4.761	5.001	5.144
4.237	3.664	2.905	3.470	3.598	3.367
4.755	4.133	3.650	3.845	3.748	4.236
6.960	7.353	7.251	7.537	7.281	7.165
2.479	2.368	3.024	2.427	2.434	2.389
2.128	1.579	1.608	1.621	1.515	1.926
4.333	5.387	4.564	5.457	4.207	4.224
4.955	4.660	4.665	4.102	4.303	5.067
3.178	2.317	2.883	1.723	2.501	2.619
5.031	5.197	4.758	4.875	4.690	5.067
3.782	3.879	3.208	3.253	3.517	4.106
3.747	3.163	3.144	2.512	3.786	3.069
5.114	4.875	5.081	4.500	4.774	4.920
4.690	4.421	4.538	2.122	4.527	3.938
6.376	5.145	4.977	4.889	5.626	5.168
5.014	4.527	3.860	3.875	3.797	5.014
4.060	3.992	3.873	2.850	4.138	3.432
3.276	1.948	3.217	1.806	1.620	2.832
4.745	3.971	4.079	2.690	4.686	4.451
6.009	6.064	5.515	5.249	5.256	6.059
4.387	4.001	4.583	4.006	3.909	4.615
3.362	2.665	2.810	2.240	2.914	2.803
6.407	6.607	6.502	6.946	6.687	6.175
4.707	5.255	4.449	3.876	5.153	4.641
2.192	2.007	3.032	2.868	2.664	2.160
5.341	5.208	4.345	3.914	4.915	4.312
5.737	5.167	4.825	4.416	4.909	5.304
2.942	3.379	2.822	2.739	2.026	3.780

4.009	4.558	3.372	5.228	4.155	3.751
4.708	4.346	4.518	3.745	4.833	4.227
3.545	3.565	2.554	3.479	3.639	3.225
4.564	3.463	4.034	3.846	3.345	3.809
5.117	4.628	4.718	4.770	4.639	4.956
6.442	6.075	5.748	4.921	6.094	6.446
5.561	5.171	4.708	3.870	5.426	5.726
4.349	4.073	4.082	4.726	4.060	4.335
3.906	3.750	3.374	3.593	3.555	3.750
5.885	5.624	5.724	5.522	5.620	5.715
3.170	2.496	2.104	2.132	2.719	2.616
7.207	6.256	6.681	5.728	7.284	7.119
3.522	3.631	3.235	3.003	3.225	3.181
4.861	4.286	4.318	4.442	4.183	4.584
2.603	3.930	3.302	3.754	2.761	3.684
3.818	3.671	4.124	3.073	4.136	4.155
4.290	3.495	3.370	2.213	3.678	3.333
4.329	4.382	3.258	3.758	3.994	3.925
3.274	1.860	2.777	3.087	2.359	3.365
3.513	3.398	2.725	2.968	3.253	3.266
3.956	3.615	2.302	3.604	3.144	4.018
3.217	3.373	2.687	2.839	2.983	3.330
2.999	2.470	2.470	2.443	2.203	2.792
3.763	4.588	3.980	4.768	2.548	4.200
4.036	3.848	3.639	3.269	3.779	3.776
2.932	3.334	2.945	3.093	2.321	2.586
4.816	4.121	4.366	3.313	3.724	4.003
3.170	3.792	3.124	3.045	3.708	4.228
5.016	4.993	4.909	3.712	4.734	4.488
1.801	1.659	1.643	1.654	2.234	2.302
5.356	4.758	4.891	3.997	4.442	4.916
4.862	4.545	4.015	3.692	4.598	4.817
3.707	4.082	3.613	3.018	3.367	4.147
3.475	3.664	2.446	3.292	3.335	3.683
4.564	3.988	3.235	3.207	3.690	3.473
2.391	1.820	2.307	2.898	2.210	3.101
4.113	3.740	3.779	2.973	3.780	2.986
3.281	2.727	3.767	3.719	2.780	3.965
5.455	4.974	3.831	3.378	4.797	5.502
5.248	4.183	4.227	4.111	4.375	4.933
7.183	7.289	6.740	6.860	7.048	7.010
5.024	4.673	4.688	3.242	4.674	3.883
4.577	3.810	4.014	4.044	3.780	4.157
7.444	6.657	7.152	6.982	7.358	7.350
3.622	3.936	3.505	2.441	3.466	3.133
3.675	2.913	2.479	1.986	3.448	3.557
2.205	1.771	1.250	2.085	1.251	2.793
3.496	2.512	2.659	2.332	3.327	3.159
2.725	2.870	2.363	2.177	2.434	2.835
2.897	2.732	3.221	3.763	2.859	2.832

2.773	3.366	2.034	2.607	3.260	3.501
3.144	3.618	3.466	2.670	3.201	3.336
3.585	4.003	3.192	2.940	3.624	3.851
2.550	2.713	1.859	2.407	2.233	1.961
5.492	4.984	4.183	3.189	4.487	5.892
4.789	4.027	3.780	3.994	4.415	4.446
3.507	1.276	2.529	1.465	2.368	2.747
4.509	3.767	3.083	2.994	3.936	4.080
4.862	4.071	4.464	3.167	4.468	4.526
3.815	2.769	1.165	2.621	2.304	3.280
3.574	2.788	3.196	1.546	2.988	3.500
4.446	4.465	4.016	3.705	4.440	4.845
3.184	2.884	2.993	2.502	3.236	3.185
3.221	3.356	3.063	2.403	3.386	3.346
3.798	3.349	3.573	3.535	3.169	3.551
6.016	5.895	5.132	6.242	5.627	5.585
2.957	2.617	3.084	3.329	2.217	2.294
4.159	3.587	4.364	3.924	3.565	3.806
5.166	4.512	5.169	4.453	4.726	4.811
3.665	4.472	3.994	2.983	3.512	3.303
5.948	5.540	4.757	4.724	5.387	6.007
3.321	4.099	3.455	3.872	3.702	3.429
6.198	5.988	6.046	5.672	6.549	6.054
4.666	4.583	4.071	5.215	4.171	4.808
3.753	4.430	4.164	4.602	3.679	4.254
3.380	2.694	3.245	3.524	4.190	4.018
4.010	2.905	2.460	2.916	3.870	3.534
6.618	6.422	5.993	5.225	5.930	6.161
3.849	2.833	3.364	3.867	2.936	3.911
3.450	2.607	3.823	3.465	3.104	3.653
3.968	3.071	2.848	5.200	3.254	3.005
3.905	3.360	3.681	2.991	3.222	3.601
4.314	3.781	4.551	4.107	4.104	4.493
2.006	2.817	2.392	1.885	2.742	3.113
2.420	2.575	1.728	1.210	2.251	1.816
5.704	5.411	5.046	5.541	6.028	5.239
2.142	2.279	2.352	2.370	2.162	1.974
3.564	3.865	3.751	3.290	3.086	3.629
4.913	3.973	4.139	3.562	2.816	5.140
2.402	2.104	2.794	2.342	2.660	1.776
4.136	5.517	4.479	4.591	4.375	4.687
3.819	4.308	4.575	4.072	3.856	3.411
2.703	3.126	2.698	2.360	2.934	2.569
5.361	4.827	4.797	5.351	5.040	4.920
2.987	1.801	2.259	1.876	2.699	3.127
3.750	4.156	3.298	3.708	3.561	4.107
4.245	3.151	3.361	4.026	3.980	4.188
3.154	3.085	3.535	2.585	4.299	3.630
3.934	3.257	2.685	2.782	3.726	3.351
2.396	2.989	2.597	2.052	2.751	1.915

3.453	3.039	2.811	2.556	3.476	3.500
2.709	1.514	3.022	2.203	3.279	2.879
3.541	3.843	2.970	3.450	4.068	3.450
4.290	3.848	3.325	3.133	3.897	4.296
3.710	3.901	3.340	3.814	3.979	4.055
3.399	3.481	3.599	3.180	2.922	3.140
3.478	3.810	4.342	3.367	3.964	3.849
4.185	4.046	3.962	2.841	4.126	4.401
3.190	2.724	1.972	1.738	3.045	3.634
5.788	5.798	4.816	4.703	5.847	5.484
7.827	7.443	7.131	6.988	7.366	7.468

6.898880963	6.627637406	6.16900505	6.566978491	6.088530116	6.784253155
10.07795389	10.08697378	9.786424372	10.66927892	10.5714719	9.664785174
3.01735724	2.656357835	3.694123066	3.067279809	3.91027313	3.316833603
1.634921583	1.882782287	1.837374064	1.854056551	1.28965443	1.66305134
3.388084053	2.360723914	4.27598503	3.153300662	3.634172571	2.309035555
5.542964693	5.489512529	5.095864399	4.733104485	3.946988398	5.850402433
7.945806821	7.011548513	7.984142147	8.725672164	8.35717591	7.025472334
6.789013166	6.106398838	6.477460256	6.074234782	6.489587121	6.378971667
3.06046233	3.031194444	2.799869679	3.75584711	2.554424459	2.490484911
5.487794776	5.304819585	5.02419486	5.68312819	4.77879019	5.469044236
3.113274714	3.142758595	3.791832263	4.130975358	3.19060028	2.867649426
2.290499318	2.488460279	2.007264394	2.546071259	3.036425759	2.518133413
3.371234546	3.435404358	3.082332819	3.088268384	2.682395806	3.706147874
7.723928463	6.3385345	7.837517637	8.3063533	8.823001785	6.159935808
7.215594909	6.637224272	6.930784311	6.252524626	6.529404084	7.044173099
9.307489257	9.097371765	9.264679725	8.830566811	8.536728825	9.36963013
3.948954947	2.824337194	2.63273612	2.958196309	3.770526891	2.948595276
2.62188314	2.14596981	2.737353549	2.578187971	2.619908499	2.239848907
7.042178387	6.197908422	6.294670478	7.021067848	6.761471247	6.732386863
7.33005805	6.389738155	7.103982492	7.118974767	7.418529062	6.307929535
7.117127297	7.499944872	7.263286079	7.210115463	7.683234637	6.956211762
2.790521344	2.79302007	2.673037057	2.652288409	2.553818327	3.238953066
2.338549326	2.278936569	1.946262064	1.642904115	1.776989975	2.073483692
3.535271547	4.36720967	3.661427196	4.764814388	1.370125842	4.154947659
6.264164374	6.611106099	6.709775787	6.539672904	6.74387855	6.517112847
4.883546865	4.894281641	5.379540232	4.898603519	4.402320489	5.32269082
2.159302876	1.40634614	1.979655984	1.426003525	1.771621537	1.729359156
4.406008782	4.903931678	4.208521497	4.005985466	4.601716927	4.430898631
3.394571092	3.861922921	3.303157506	4.314216456	3.582008368	2.751417699
5.739718094	5.507811531	4.661102067	5.176955312	5.112776857	6.238880232
2.458040997	2.420565383	2.618772328	1.755086849	2.19468487	2.687228629
4.294268288	3.946631653	4.27644933	3.749670634	3.644748393	3.843248152
3.734989102	3.739349126	3.157498654	3.270914719	4.583800518	3.191803363
5.462237127	5.355549219	5.071299899	5.486401364	5.77403926	5.454452549
5.790952621	5.529596267	5.918138791	5.394561558	5.759990287	5.41507565
2.043430902	2.416994251	2.913045731	3.031552457	2.510159796	2.326367593
5.425863878	5.611284333	4.442796506	4.884920005	5.989955796	5.312001962

1.34011892	1.386311225	1.862128696	1.455367821	1.420636167	1.090895137
2.066930756	2.096609916	3.16688677	3.658421994	2.579787266	2.764129931
4.083187608	4.094639064	4.036128821	3.397767481	3.755987904	4.331881436
7.180140948	7.467251712	7.105538781	6.898070944	7.205386369	7.328694653
6.789608843	6.875484244	7.358431345	6.920683236	6.969175904	6.877249689
8.785951523	9.247413747	8.965761009	8.75016975	9.133092284	9.087010206
2.183263959	2.80280624	2.301285339	2.747435694	2.520346758	1.807354992
5.955934815	6.197377407	6.023708193	5.309582938	5.689231302	6.163089869
6.622818483	6.572329728	6.131975316	6.434474666	6.416257191	6.81806756
6.932276017	6.963670772	6.848800745	6.68244452	7.199721828	6.776246828
4.760692476	5.280169643	4.680205326	5.184341035	4.819807452	5.38753537
1.336926991	2.207603384	1.857118519	2.063799177	1.222415655	2.207603384
7.846610947	8.323842316	8.125263998	7.935438141	8.032009905	8.467248028
9.426108555	9.475638458	9.475175036	8.711946799	9.592500415	9.676130833
6.917252664	6.997998984	6.885303868	6.986114886	6.504418526	7.001488802
3.052680262	2.078043739	3.446569205	3.989371488	2.925938266	2.161516751
4.270610903	3.506323887	3.586497668	3.526729498	3.482661674	4.234875314
8.626703855	8.33852287	8.193985205	8.551897747	8.46406677	8.344930756
6.612450731	6.61241455	5.769257126	5.725834316	6.427789023	6.829129568
3.941302189	2.963114097	4.603679401	4.276438138	3.809963621	3.534799918
3.633923289	3.237832354	3.519788507	3.217820851	2.855564048	3.483975465
3.12854755	2.833396646	2.65589888	3.207862971	2.731387003	3.138101188
3.690329481	4.256800906	3.585895482	4.994669451	3.654292724	3.537164372
3.981685658	4.688888385	3.495971786	3.855944572	3.917279898	3.530446364
3.608130029	3.323738563	2.105789357	3.568419912	2.828728216	2.417922526
1.652202436	2.021452615	1.579767366	1.568577968	2.685147145	2.321733502
4.270499349	3.190328285	2.844044818	3.111733964	4.019142783	4.253657026
2.697087071	2.120094017	2.383634523	1.82120511	2.492725149	1.748197355
4.366244252	3.483927247	4.051170088	3.851668243	3.499957733	3.930027128
3.161056541	3.89703408	3.147204083	2.978992753	3.07389605	3.715190518
7.616996152	7.638025068	7.087213471	7.49949973	7.40595141	8.159000713
3.025016508	3.13771389	3.360939354	2.463999719	2.891989348	2.95028495
6.03493256	6.166301668	6.087881205	5.87609328	5.818132166	6.216901087
4.992900833	4.54384492	4.641144374	4.815284557	4.646923363	5.162437889
9.090651272	8.471008059	8.393507895	8.45788755	8.658064	8.527137781
2.468456848	2.749175067	3.246932839	2.945135665	3.339600599	3.148777596
4.311578215	4.940282483	4.094521144	3.540137382	3.865591576	6.073847043
8.948474248	8.619879928	8.631933167	8.474620416	8.586124771	9.086973563
4.528042386	5.473465424	4.907884906	4.478334402	5.698784772	4.946056437
3.255023841	3.041091605	2.850784484	3.337946386	3.547695329	2.693671788
7.606077733	7.452348213	7.183664512	7.349000069	7.296945252	7.331666757
2.594490645	2.662809553	3.4493752	3.386165486	2.518626911	2.384385071
3.99899126	3.972997799	3.845943601	3.531703633	4.42958375	3.873787021
2.258812764	2.720387309	3.216270991	2.568921986	3.241229923	3.075355735
5.973056402	5.013116196	5.169271832	5.159283524	4.068891134	5.222441336
5.710800627	5.44607679	5.750144503	6.482255727	6.564955236	5.736200319
5.681551486	6.464424896	5.316309377	5.2986652	5.965612579	6.21997553
7.435378783	7.607128832	6.953526235	7.861736603	6.912942996	6.145703151
5.824723778	4.859511393	5.837545014	5.744692163	5.882212743	4.858240762
1.611068061	1.251555064	2.204279327	2.063411869	1.569019379	1.74840752

7.277557208	7.222944731	7.362944045	7.207810221	7.286192692	7.178507921
2.904633326	2.855842891	2.952982058	3.082249892	2.886606172	2.499252927
2.716056557	2.598968937	3.004153573	3.41773989	2.247076544	2.301432343
4.959713156	4.490869522	5.218980725	3.138875754	6.095131334	5.535917455
3.386411985	3.438487658	2.757941598	3.726031927	4.066546261	3.564868208
6.435531541	6.385975094	6.347984385	7.012636526	6.30833757	6.426259363
7.857650317	7.297131839	7.912249958	6.62637184	8.224165667	8.109223271
1.608654306	2.066930756	1.047918119	1.761432092	1.667328024	1.631398637
4.355031343	5.658405546	4.258561946	4.790935359	4.63314981	3.970800558
7.866230607	7.262692081	7.546945066	7.008955741	6.626516137	7.427687897
4.288138871	4.091216451	4.613701054	4.095341938	5.487927559	3.739110539
3.08865899	3.191824828	2.615352978	2.717420832	3.506820719	3.471420344
7.013358379	7.107202186	6.978579894	6.864249275	7.229489626	6.969375815
5.533010923	5.205861413	3.240282735	4.300248438	3.461417867	2.743599336
4.927358756	5.317578272	4.772302691	5.383493387	5.390705476	5.277755313
5.031966977	4.568729696	5.040982928	4.751515926	5.241100465	4.833359883
4.394856986	5.445524018	5.308650164	4.059100484	4.935477646	5.512200636
9.081558273	8.584171678	9.149114344	8.343482357	9.16756539	8.958658257
3.03641823	2.153589253	2.728406897	3.893825506	2.696140992	1.959008466
5.177695025	5.282575222	6.552330834	3.13373679	6.345357203	5.56659827
2.407580604	2.665252205	2.747523932	3.73682919	3.215333066	2.533501504
5.802662902	5.667315347	5.982277876	5.353322634	6.197895067	5.967018175
3.750152313	3.543051234	3.741534193	2.784346544	2.969137577	4.168817886
2.467288894	4.734755134	2.340922425	2.596521655	4.231668174	3.102132456
2.748672684	3.089091826	3.661137843	3.172222774	3.768303236	2.866191812
2.36661248	1.820367853	2.02794257	2.290204162	1.517474552	2.495349386
4.087315079	4.09795578	4.559452442	3.899858364	3.951423736	3.768048806
3.044050401	3.46846908	2.655904153	1.9235561	2.389500983	3.189426582
6.01791164	5.768105159	5.894135089	5.290479927	6.004291788	6.018527003
4.859680705	4.879703337	4.868573777	3.950535914	4.898800862	4.972602648
5.748428293	6.634644128	6.156011218	5.603304959	5.95494472	6.294001539
3.719609751	4.108132476	3.447504467	3.656770432	4.291143892	3.500176445
3.636633365	3.105013992	3.510884339	3.679759949	3.557662276	3.677135234
5.646065402	5.734443448	5.551674117	5.083806343	6.015357462	5.882416643
8.062380449	6.440038801	7.550521655	6.783204843	6.224727984	7.802318979
5.054943285	4.638055545	5.096490815	5.458034891	4.082083321	5.008768945
4.497473846	4.670764478	4.122452376	3.751955957	3.749276817	4.778993646
7.423151209	7.624585617	7.463399663	7.450284156	7.645408968	7.702960937
4.288058747	4.672655859	4.635734533	4.489492804	4.489359494	4.639366779
4.484734809	3.322560012	3.635702532	3.874913849	3.996385058	4.052870551
6.206006981	6.287128719	6.593007137	5.735639917	6.282606651	6.197668099
3.087333321	4.038455685	3.17800243	2.426372904	3.458137118	3.487234556
5.013251287	5.202818642	5.662041282	5.215630544	6.24745484	4.944976536
1.590783023	1.441916521	1.513695785	2.193572859	1.62330108	1.948530249
1.452667316	0.416461364	1.28651583	1.058658375	1.543268174	0.675831247
6.619049863	6.801780623	7.183646048	6.389461596	7.118761753	6.880998074
5.307626634	5.417241327	5.157473469	5.035125099	5.254075463	5.308365736
5.053893265	5.077019672	5.035021081	6.104067341	4.540166646	5.215518483
3.421363604	4.244577497	2.960035442	3.239346827	3.327143916	4.116917285
3.57054457	3.128456509	2.771572865	3.334915603	3.079408111	2.697856589

4.837513209	4.389548355	5.107389748	4.206331294	4.662299251	5.353074658
3.887386649	3.228666337	3.600554551	3.761749739	3.435626071	3.089254444
2.0215816	1.901101553	2.499744233	1.948769553	1.915587763	2.198918964
5.770515274	5.294912954	6.120880794	5.470020154	6.419231509	5.566071001
4.891778211	4.949485421	4.328559787	4.369959181	5.367558543	5.011391454
3.174243317	3.700637979	2.186729937	2.358913866	2.761256622	2.378949083
8.215892335	8.341171495	8.846777388	8.586099326	8.775360129	8.562491188
3.855761352	3.809791878	3.573277329	4.279082658	3.181386422	3.631241543
4.52708858	3.701254957	4.275337966	4.640523478	3.828429226	4.082318279
5.744222006	5.750528019	6.100854712	5.496166296	6.012052935	5.87545353
2.633895265	2.415172916	2.501053528	2.178574589	2.783989095	2.849789918
5.089077167	3.767493128	4.472046326	3.320483703	3.753450369	4.595220928
2.722627085	1.754454266	2.19096163	1.997925966	2.747664085	2.804127553
7.355597016	7.128417534	7.227516784	7.360553737	7.083453287	7.134888692
4.121110012	4.367317779	4.410070709	4.299012838	4.270610903	4.300849104
3.444784899	3.476392594	2.746549435	2.385519975	2.283981245	2.289887554
3.237276943	3.082732859	3.435434406	2.823436684	4.26527728	3.410837626
4.243162032	3.687477619	4.261927217	3.867254574	4.543309957	4.149964022
3.384222787	2.937258269	2.385740443	3.239560117	2.388661804	2.963829211
3.028060864	1.991911908	3.05865238	3.569835316	3.331606574	3.597674835
7.697710354	7.683595964	7.761114421	7.817007055	7.455056613	7.847139788
6.439700103	6.11674994	6.579213175	6.172558153	6.719172955	6.275228626
2.762061697	2.952676951	2.964977879	3.008302073	2.736460533	3.14070493
4.451543766	4.470023551	4.406645522	4.427974923	4.412808377	4.023193847
2.130412778	1.360713278	2.298374502	1.350952048	2.17285707	2.225609022
3.284417617	4.291940725	3.858693909	3.978737156	4.638371475	3.665261038
7.207020002	6.767654127	7.247896525	7.149996305	7.289538285	7.322680961
4.833778228	5.007960059	5.353801072	5.570689708	5.094479853	5.07546176
4.398673072	4.01823126	4.064453023	4.554224641	4.691636571	4.353117605
7.137204473	6.941558633	6.970803475	7.273785216	7.212925201	6.817732518
9.281668063	9.181498263	9.312692751	9.00941895	9.473267826	9.392574327
4.308825132	3.985262115	4.281294109	4.095703156	5.032759957	4.271585529
5.362078484	5.470158783	5.294872365	4.370043751	5.677926485	5.646220975
6.8318581	6.4738034	6.642480736	5.790722925	8.00440765	7.391836792
6.669423771	6.43205357	6.537486328	6.286230715	6.098665521	6.417212149
4.52088158	3.895144295	4.02785257	4.453503272	4.117788354	4.003340677
2.243785125	1.348508216	1.66599421	2.019994136	1.498057729	1.872668426
5.064306747	5.254481834	4.978432995	3.857245573	5.352067067	5.331178944
4.760664804	4.816972599	4.191629161	3.51115329	5.360379663	4.411346693
1.252692912	0.923882819	1.684660969	1.0908862	1.440532346	0.987915616
3.316986996	2.941183189	3.846305292	3.179340831	3.637985977	3.271527304
3.583413627	2.773445579	3.200081435	2.617978757	3.749857752	3.287903606
4.687228213	4.850131833	5.225625196	5.652893659	5.016577177	4.632333924
6.367873526	6.421118289	6.214871146	6.083723582	6.847479849	6.452644251
5.518849649	5.378454309	5.163257905	5.228086322	5.728833353	5.084594625
4.443297036	2.746793164	3.739023137	3.376681328	3.750299111	3.627521791
3.87018258	2.672384775	4.442683255	2.579490298	3.332510381	3.284178001
6.221552729	5.629568201	5.473735794	5.806876175	5.150962357	5.605409958
2.691524348	3.233269591	3.503925051	3.721521153	2.988244926	3.595537237
3.139563371	3.057037092	1.620513868	2.636017302	3.552266888	3.741599537

1.497517315	2.796466496	2.317964114	2.61788952	2.708732194	1.859358836
5.245364984	4.975724184	5.270446356	4.700876445	4.250472373	4.503162827
4.717241454	3.428410865	5.074787761	4.273049301	4.843235625	4.417635567
4.894103139	4.426455117	5.101551854	5.841198567	4.844112509	4.486576609
4.007794346	3.750498658	4.002271367	3.745672814	3.431149091	3.93863297
3.878458557	2.951546784	4.869740517	4.114577917	4.275995238	4.427593773
6.062178739	5.78532556	5.80833944	5.455092417	6.36428713	6.116141431
1.609134471	1.541848552	1.77146243	1.535383917	1.889436396	2.045887852
3.639055129	3.145779745	2.194913634	2.643420995	3.004261093	3.874112238
4.029245358	4.000652046	4.619048656	4.300744998	2.740448933	4.224839635
5.158139159	4.041123106	5.149274811	3.977090753	4.55524624	5.007226535
4.354319169	4.09828301	5.301312878	4.529642821	6.022775451	4.56120593
5.886787648	4.679125421	5.778007787	6.960878704	6.926855124	5.463832332
5.778799342	4.83099659	5.023074899	4.908122411	5.742213159	5.254755322
7.704698805	7.130987331	7.408276313	7.11119727	7.262094386	7.444198102
4.087473888	4.089994943	4.425278482	4.239213968	4.148504635	4.395201075
6.687723639	7.453033107	6.530800251	6.784162301	6.672176173	6.770326421
5.127158609	4.428382981	5.136783363	5.180683032	4.652604083	4.49071549
5.36736213	5.669852386	3.788863191	4.793085479	5.00145573	5.402750014
2.416439457	1.651926472	2.462839649	2.62146567	1.892579264	1.946008454
6.837740418	6.591275949	6.618023409	6.354113983	6.051577145	7.038575415
3.025579766	2.833510121	3.76544564	3.466198137	3.419151488	3.937119137
2.784127366	2.623324325	2.693218547	2.956964134	2.036151353	3.329313938
3.187785821	3.747532188	3.0629607	3.881696708	3.311470134	3.625522436
4.920660968	5.130572681	4.629109158	4.678957908	4.470475598	5.185609978
3.746969805	4.032277006	4.207214926	4.003823152	4.151779015	4.33185889
4.953813465	5.311894204	4.602174887	4.458258502	4.30923562	5.498926171
5.957664229	5.484233151	5.407728519	5.082096895	5.792989119	6.130732327
7.664644972	4.810844677	6.831272026	7.217965836	4.439148786	5.023978026
3.54902764	3.570232629	3.288024108	3.185780249	3.626501952	3.274186295
3.114260622	3.426358089	4.133135672	2.771588789	3.500636782	3.496936688
3.325729245	3.688522328	3.313186449	3.577096357	1.885960434	3.172562068
3.695896535	3.311259059	3.161796401	3.393245806	4.237019602	3.208819495
3.685984786	3.702396453	3.946640848	3.584374098	3.768357835	3.512516383
6.187168619	6.183564778	5.483636121	6.467414143	5.940015419	6.149703953
3.86564166	3.839474737	4.371383696	4.172110663	3.428674985	4.277601228
5.729816684	3.728331518	5.095280311	4.451034049	5.612556846	5.525839788
3.175550315	3.277353148	3.169829254	2.736774222	3.28862514	2.898340057
6.334283194	5.574089747	4.940112299	5.494402869	5.525541496	5.767367617
11.03590254	10.09909459	10.80161396	10.85250158	10.30118927	10.81850175
5.288929338	5.394641482	5.547633437	5.742573884	5.303055221	5.088491321
5.887023772	5.210188234	5.316775068	4.959706098	5.986354779	5.421392065
6.967784504	6.800055444	6.774233569	6.43779123	6.909546898	7.180537782
3.472478187	3.925102481	2.525665817	3.515765395	3.809740363	3.513900428
2.368360427	2.655691393	3.543123936	3.372652663	1.794565222	2.893983178
4.122781964	4.32542849	3.405878798	3.241710476	2.532865844	3.64044653
2.954562143	2.962564002	2.593539038	2.711675967	3.271286499	3.129663375
1.400083966	2.817015292	1.598683873	2.213260425	3.358216463	3.177195346
5.121961764	5.506769643	5.27540943	5.432986567	5.439644976	5.404789168
2.393064661	2.294880716	4.232248874	5.148744897	3.495930178	2.839311474

6.111725416	4.095812605	5.841737335	4.860156315	5.659497958	5.085493685
5.789389375	5.971018896	5.84415133	6.00778214	5.603595204	5.74372126
2.774209393	2.15181577	2.388038075	2.161214762	2.356624904	1.882909451
4.165139644	3.728872936	3.981474143	4.230258753	3.937113622	4.04130309
7.167787591	7.737730214	7.280598567	7.018291881	7.668500796	7.68604181
3.623572342	3.867979124	4.713109711	3.48325288	3.88764247	3.51611527
7.022148456	6.285758481	6.706561053	6.480189337	6.996061917	7.593584986
9.44512457	9.532942214	9.702914597	9.193202624	9.901189686	9.58136469
3.728361744	3.072061715	3.974618786	3.929688904	3.742129169	3.625068283
4.504658465	3.925292967	4.61577533	4.660324331	4.724604986	4.712527799
4.584766987	3.911180188	4.263021465	4.295538429	4.519924998	4.965545825
4.067495026	3.022645022	4.700340667	4.706800215	4.674400993	4.2085873
1.594205094	1.569171501	1.395987284	1.740757625	1.404354361	1.481616045
4.45808197	5.134020175	5.534068094	4.548068275	5.30914066	4.647675196
5.360035976	5.865328456	5.189886515	5.133745124	5.261811106	5.56898658
4.739967546	4.518728095	6.898095414	3.990644578	6.425496883	6.488854322
5.309164326	5.866220966	5.45623717	4.698823159	4.952896852	5.187047669
2.442331753	2.635805718	2.504135267	3.546038799	2.196797318	3.155978276
5.454898197	5.500134015	4.851663569	5.116484738	4.918982729	5.606854045
3.24087955	3.217076033	2.770084073	3.424685655	3.328164579	3.788745662
3.276987031	2.659196156	3.002448386	3.11531536	2.807178697	2.922503028
3.167456657	3.29338991	2.804864596	3.186433828	2.520150768	2.256973809
9.129447798	8.850279913	9.107590324	9.051351993	8.596230727	9.39629036
6.738970434	6.586590487	7.063630134	5.908641598	6.309049256	6.944531557
3.879459842	3.341472328	2.851062066	1.964475692	4.495385672	4.892069748
4.040961769	4.260824762	4.508353594	3.765288663	5.038087594	4.612459262
7.816153944	7.828803907	8.037161256	7.282489056	8.140933532	8.227051817
5.103499818	5.072127382	5.179337313	4.748449036	5.190466651	5.140946418
8.178666398	6.658587329	7.923546487	8.502606201	8.439681797	6.957485909
3.366952613	3.41731389	2.902622409	4.382117014	3.99907918	3.350067788
5.593437338	5.037367398	5.480718336	4.601499729	6.061571852	5.715751543
4.264345956	2.90040374	3.88170992	4.029200378	5.115996322	4.274269089
5.426085602	5.9824397	5.335141878	5.655056495	5.065144797	5.515483598
4.583415718	3.650877169	3.709026381	3.65944428	3.909125122	3.692685241
5.440242065	5.355137628	5.385138498	5.391632257	5.629740439	5.536355104
4.750292368	4.200328843	4.907479527	4.963213224	5.103346938	4.734229454
3.612572047	4.522791177	4.741381846	3.105108207	4.14653163	3.555046337
2.922858906	2.792543139	2.953803369	3.347396009	3.292711768	2.619149065
5.547752243	6.217420598	5.361243417	6.283167462	6.35873982	5.69357815
2.240254196	2.774994319	2.001270175	1.936599821	3.200998127	2.61302522
6.583131512	5.233133019	5.47250735	5.874757782	4.669902119	6.101241402
3.438826408	3.460757038	3.717898208	3.774196633	2.945527638	4.018633368
3.287503384	2.783784924	3.117412375	4.041656238	2.891122188	2.482663925
4.556063273	3.667476467	3.598585254	4.21428867	4.104650779	4.268738167
2.074455655	2.625450571	1.452663295	1.7436854	1.840561555	1.685286169
1.768291034	1.89733385	2.278055883	3.009143365	2.366726843	2.875735326
3.614693082	4.023792396	4.453131317	3.989296534	4.479636437	3.857233427
4.804939759	4.184046649	4.54496198	3.943800763	5.268324076	4.850023245
4.546658073	3.280649195	3.769173397	4.036476713	3.743900969	4.065527299
8.418443531	8.24519474	8.777235747	7.805867146	9.035390316	8.586941064

3.181622595	4.47830109	3.350856264	3.817104975	2.590406812	3.329746828
5.76955324	6.130621866	6.035472469	5.783877191	5.955984548	5.946758861
6.503548426	6.216797677	6.427453897	6.079844265	6.365395464	6.722972427
3.10481104	3.480800653	3.219232386	4.807903908	4.058792847	2.801875742
2.784213863	3.327734844	3.064638507	3.505460188	3.110701749	3.279878999
3.755561116	1.221577295	2.390145056	2.3843093	2.227334991	2.809050097
4.27230305	4.75200975	4.064813267	4.610253722	3.128300095	3.870149947
2.953314446	2.677694473	3.005209337	3.583046097	2.901751623	2.48165403
2.055131722	1.953110221	2.747694872	2.117482572	2.155139291	1.248053224
5.15201576	5.426995475	5.586464797	5.222077767	5.043647948	5.670506615
7.436806065	7.873205559	6.121420836	7.10892777	7.055759699	7.091268045
6.526382886	6.369310726	5.86672754	5.935552733	6.208886287	6.461458075
3.568946633	3.184703669	4.105705525	3.527431381	3.290028178	3.2290209
3.809159295	3.923442165	3.881619549	3.309481522	3.35404566	3.639968076
4.772110747	3.566736758	4.267397023	4.873992677	3.606961596	3.850638868
9.305469492	9.445170154	9.398521319	9.578130778	9.8893775	9.095108653
3.062093653	2.044753903	2.619693574	3.798284809	2.952005619	2.994263025
4.044491987	3.617369859	4.614944491	3.616007491	4.069179435	3.777364349
5.665167048	4.827091289	5.211591219	5.231198867	4.740351294	4.935082986
6.145579906	5.952445405	6.256178009	6.581779531	5.969800771	5.98733037
3.882487225	2.588556126	2.748071838	2.360079254	2.814942482	2.572357988
3.122363193	2.202711548	3.633081378	3.56851436	4.505848028	3.103007106
2.843113095	3.388565899	3.647626361	3.052353499	2.792676371	2.930161244
2.237046628	3.103454183	3.581948608	3.005976513	2.715577447	3.456106668
5.029511465	3.261407548	3.448271403	5.132045611	4.461139776	4.042848856
6.470358887	5.793049203	6.643700349	5.631813117	6.570968061	6.800082015
5.840532756	4.720730779	5.602839653	5.90728228	6.014798461	5.923971669
5.756890949	5.791858355	5.579515813	5.289070221	6.536013358	5.767910345
5.048906448	4.107810029	5.015722524	3.01629749	5.848744403	5.096490801
4.269515709	4.956717951	3.991282179	3.776949273	4.699300115	4.117008011
4.433943541	3.594879097	4.1980462	4.095866104	4.365469899	3.787180692
3.978538771	4.518258309	4.287128925	3.509837165	4.688015966	3.268377435
6.530727926	6.478437728	6.620126172	6.403048807	6.821201783	6.453360265
4.79433709	3.821854851	4.797207575	5.0514472	3.994173699	4.137813746
5.604771644	4.518648041	5.656634454	5.595597824	6.065953425	4.613064796
4.006407956	4.34533974	3.910211133	4.551870123	3.728356908	3.424159489
6.751655394	6.865184766	6.79792794	6.242351447	6.899064935	7.567492832
3.807019775	3.465927142	3.084084423	3.394408896	2.928604108	3.993523142
1.123151965	0.882946886	1.210419886	0.887232578	0.758405608	0.968983504
3.168871563	2.664198455	3.291362194	3.226061511	2.509046809	4.699902752
5.716121162	5.420871976	5.493090285	5.145697942	6.127203479	6.151011296
6.084367608	6.520961684	6.031366362	6.538674402	5.700570405	6.700589426
8.16096148	7.355396816	7.365514766	6.632419548	6.467307744	8.105976005
3.132773883	3.502606143	2.944456678	2.867623565	2.852843021	2.938523254
2.165037687	3.335430371	1.963586394	3.446156481	2.687914586	1.942948619
4.187836978	3.940637173	4.860196753	3.399105206	4.602174887	4.267205086
2.80188363	3.359941535	3.975819286	3.652100354	4.221181671	4.657994936
3.0627323	3.950221748	2.760170477	3.320955869	3.777628584	2.622718335
4.594265064	4.476175643	4.644122424	5.190960916	4.332055063	4.666361259
2.443619254	2.811079451	1.969180036	1.393247912	2.54609354	2.963612422

2.216242521	2.247218718	1.979224605	2.513581493	1.561400439	2.077188128
4.487320925	3.986286944	3.989934827	4.400855173	3.122350308	4.214599866
3.97384485	3.861202698	4.507442864	3.6996923	2.907442612	6.512113705
4.513392383	3.924517168	3.59077371	3.915588358	3.585172917	3.954761782
5.474251687	5.283402629	4.662365154	5.05676377	5.425568845	5.044209556
5.762150303	5.738210042	4.784464792	4.721795248	5.651010454	4.791859001
3.470315526	2.499630813	2.829998733	1.954033903	3.162961521	2.941449636
6.022106998	5.972810813	5.087970817	5.625009811	6.208303176	5.832955406
1.382324357	1.46368439	1.014004813	1.467022923	1.172656505	1.340784376
3.560493362	2.895672446	3.708514658	2.92213761	3.721335864	4.391023362
5.766611464	6.253450338	6.779186317	6.690591673	6.671577652	6.060422919
3.22329074	2.939176505	2.996414661	3.468745197	3.838169754	2.995522207
3.030132336	2.678855124	2.717592379	2.807145475	2.566655824	2.215120158
8.30362122	8.298046396	8.269647028	8.015870855	8.261554435	8.20635201
5.505940037	5.565018099	5.100183253	5.250007153	5.206540676	5.146319927
6.23198506	6.471459018	5.901313689	6.691294538	6.442647814	6.256757877
5.217414716	5.446317546	5.032976163	5.269086856	4.977036396	5.195563185
8.770665988	8.187354881	8.624799547	8.948239823	8.80285151	7.976317178
11.52283644	11.72908409	11.65860668	11.53819566	11.58427187	11.60864506
3.240923778	3.572334149	2.993448825	2.943209941	3.757559598	3.336058116
2.127800907	1.678748904	1.17471162	2.156587185	1.56519618	1.922881312
4.578636496	4.51316975	5.177840906	4.36673562	4.906911508	4.479645296
4.441063159	4.046169502	4.830639904	4.126417318	4.844267274	4.921375483
3.529965708	2.935275718	3.058297885	3.357349565	3.455386408	2.784217776
2.612182479	2.649929088	3.457831398	4.482962665	3.785578634	2.790669437
4.023339936	3.404900947	4.009895615	3.933724374	3.497732273	4.060242477
5.521649616	4.818601074	4.346854492	4.654919802	5.536437186	5.621820084
6.203881394	4.929429278	5.42188222	4.695001695	4.926971367	5.533299319
7.346607246	6.130380311	7.718078701	7.407361588	7.697117018	6.0131406
2.02064562	1.981001944	2.115662841	1.876539488	2.199298446	2.177697479
6.947980553	5.60018947	6.492431901	6.575167165	5.490838738	6.546216192
4.542408796	5.00911473	4.922058328	4.646800972	4.289727377	4.573655655
5.034855905	4.089186089	4.010372301	4.8519072	4.545776831	4.655396793
6.841180565	6.374444092	6.638167388	6.349127333	6.234280629	6.736801917
4.25353329	4.771439769	4.199514094	5.572570744	4.330552229	4.866473103
2.659650231	1.509363452	2.441866863	2.116302972	1.929588399	1.494419952
6.997082016	7.529559719	6.740863878	6.567251372	6.431602285	7.449869336
5.08969477	4.963115196	5.169125956	5.475192767	4.422913478	4.792615963
3.895594004	4.191566244	3.945417827	3.005576146	3.089807042	3.063634841
6.192200589	6.340107429	6.569950084	6.645742468	6.545080621	6.321898201
4.934140182	4.463378761	4.991895214	5.331931286	4.17612853	4.83351577
5.708161813	5.470816882	5.975084026	5.987355912	6.038537855	5.870443007
5.673562845	5.283011346	5.359419806	4.775279391	6.160644303	5.490832055
1.306340671	1.007860365	1.253771605	1.23018465	1.114022479	0.951390979
3.888077764	4.175906446	3.578316666	3.603219092	4.620003963	4.433805411
2.04452379	2.87014687	1.876385439	2.257080512	2.532808213	2.041239251
5.975626299	5.674867058	6.129476278	7.168794531	6.324780638	6.076072271
6.074627905	5.097168458	5.466128011	5.797372409	5.390092526	5.686948116
5.179082373	6.263895009	5.361528103	6.605680132	5.927347556	6.190813079
2.333005326	2.459781041	2.168549322	2.336941629	2.281757914	2.375362464

4.694800969	4.358361038	4.352458926	5.304572516	5.266734188	5.563497714
3.880654163	3.211273422	3.177183799	2.766806314	3.585787725	3.604029151
4.546150387	3.491411501	4.230492622	3.136878266	4.613969667	4.229460875
5.512739284	5.21804136	5.572331337	4.911963301	5.412914134	5.034956869
6.446051846	6.087632373	5.564274478	5.760273928	4.684189619	5.894619381
2.135639271	1.895280187	1.642034092	2.062255879	1.828597634	3.273136693
4.035575142	2.590305617	2.367050344	2.885922329	3.556951127	2.881941541
4.40425765	3.477298263	4.555762916	4.825360136	4.547611736	2.414148219
3.486855716	3.970376703	4.389768312	2.514219459	3.754064604	3.191106223
5.498835751	6.2332949	6.197778114	5.693604879	6.26006656	5.190531486
3.929074532	4.095486553	4.170455695	4.707886258	3.787498947	3.948532695
6.463851756	6.2115517	6.354223219	5.852054192	6.572910563	6.517260165
5.377795549	4.956009658	4.666742533	4.65944504	4.994793792	5.028649335
3.378193308	3.021465002	3.845994767	2.117344021	4.001843263	3.974905063
1.980012942	1.283811634	1.55700683	1.465376105	1.762007318	1.690071081
7.131476704	7.34753597	6.82145954	6.735291492	7.414641378	7.304023707

06.CTL.SP.M	s.post	s.unscaled	t.ord	p.ord	df.res
3.918	0.506	0.577	0.992	0.334	19.000
7.953	0.503	0.577	1.574	0.132	19.000
4.430	0.458	0.577	1.273	0.218	19.000
2.818	0.459	0.577	-0.188	0.853	19.000
4.182	0.447	0.577	0.657	0.519	19.000
4.720	0.420	0.577	0.773	0.449	19.000
3.933	0.357	0.577	-0.718	0.481	19.000
6.407	0.406	0.577	0.056	0.956	19.000
5.227	0.370	0.577	0.546	0.591	19.000
6.592	0.444	0.577	-0.197	0.846	19.000
6.518	0.612	0.577	1.621	0.122	19.000
1.822	0.447	0.577	-0.203	0.842	19.000
2.410	0.495	0.577	0.800	0.434	19.000
6.009	0.404	0.577	0.148	0.884	19.000
7.904	1.083	0.577	-0.217	0.831	19.000
7.445	0.747	0.577	0.160	0.874	19.000
5.679	0.611	0.577	-0.461	0.650	19.000
5.771	0.353	0.577	-0.651	0.523	19.000
2.918	0.404	0.577	-1.323	0.202	19.000
5.986	0.862	0.577	0.397	0.695	19.000
5.647	0.470	0.577	1.003	0.328	19.000
5.026	0.362	0.577	-1.236	0.232	19.000
3.520	0.466	0.577	-0.910	0.374	19.000
5.946	0.552	0.577	0.710	0.486	19.000
3.723	0.691	0.577	0.141	0.889	19.000
4.877	0.377	0.577	-0.287	0.777	19.000
5.139	0.391	0.577	2.150	0.045	19.000
6.053	0.553	0.577	-1.315	0.204	19.000
2.917	0.618	0.577	0.677	0.507	19.000
2.127	0.517	0.577	-0.501	0.622	19.000
5.008	0.320	0.577	3.162	0.005	19.000
4.549	0.466	0.577	1.510	0.148	19.000
3.227	0.354	0.577	-1.163	0.259	19.000
2.892	0.425	0.577	-0.939	0.359	19.000
6.445	0.475	0.577	1.026	0.318	19.000
3.761	0.457	0.577	1.763	0.094	19.000
5.530	0.516	0.577	1.483	0.154	19.000
5.164	0.450	0.577	0.164	0.871	19.000
5.080	0.602	0.577	0.202	0.842	19.000
5.421	0.581	0.577	1.229	0.234	19.000
6.304	0.667	0.577	0.320	0.752	19.000
7.644	0.336	0.577	0.245	0.809	19.000
4.183	0.635	0.577	0.350	0.730	19.000
5.898	0.426	0.577	-0.408	0.688	19.000
3.498	0.492	0.577	0.094	0.926	19.000
2.765	0.393	0.577	0.150	0.883	19.000
6.538	0.657	0.577	-0.506	0.618	19.000
5.005	0.471	0.577	-0.350	0.730	19.000
3.901	0.479	0.577	0.937	0.361	19.000

2.606	0.430	0.577	-2.161	0.044	19.000
3.551	0.514	0.577	0.432	0.671	19.000
2.876	0.538	0.577	-0.523	0.607	19.000
4.725	0.421	0.577	-0.845	0.409	19.000
2.622	0.538	0.577	-0.543	0.593	19.000
3.597	0.577	0.577	-0.168	0.868	19.000
4.105	0.818	0.577	-0.393	0.698	19.000
3.144	0.470	0.577	0.546	0.591	19.000
4.796	0.443	0.577	0.915	0.372	19.000
4.003	0.424	0.577	-0.848	0.407	19.000
3.522	0.432	0.577	-1.011	0.325	19.000
3.765	0.569	0.577	-0.872	0.394	19.000
2.163	0.470	0.577	-0.262	0.796	19.000
4.569	0.506	0.577	-3.887	0.001	19.000
3.106	0.451	0.577	-1.428	0.169	19.000
3.183	0.583	0.577	0.275	0.786	19.000
3.882	0.469	0.577	0.737	0.470	19.000
2.589	0.482	0.577	0.178	0.861	19.000
2.626	0.460	0.577	0.914	0.372	19.000
3.153	0.450	0.577	0.574	0.572	19.000
4.209	1.176	0.577	-0.063	0.951	19.000
2.241	0.464	0.577	-0.931	0.364	19.000
2.483	0.399	0.577	-0.161	0.874	19.000
2.726	0.425	0.577	-1.949	0.066	19.000
2.200	0.517	0.577	-0.474	0.641	19.000
2.850	0.390	0.577	-0.290	0.775	19.000
2.892	0.451	0.577	0.809	0.429	19.000
1.694	0.797	0.577	0.169	0.867	19.000
3.054	0.593	0.577	-0.499	0.623	19.000
2.519	0.440	0.577	0.671	0.510	19.000
3.827	0.465	0.577	-1.559	0.135	19.000
2.048	0.479	0.577	-0.398	0.695	19.000
2.214	0.718	0.577	0.512	0.614	19.000
4.128	0.440	0.577	-1.487	0.154	19.000
1.888	0.456	0.577	-0.184	0.856	19.000
2.423	0.429	0.577	0.188	0.853	19.000
3.066	0.498	0.577	0.414	0.684	19.000
2.314	0.456	0.577	0.513	0.614	19.000
4.127	0.872	0.577	-0.157	0.877	19.000
2.661	0.411	0.577	-1.874	0.076	19.000
2.711	0.541	0.577	0.568	0.577	19.000
3.186	0.956	0.577	1.485	0.154	19.000
2.992	0.428	0.577	1.123	0.275	19.000
1.469	0.610	0.577	0.525	0.606	19.000
2.005	0.476	0.577	0.076	0.940	19.000
1.386	0.845	0.577	-0.262	0.796	19.000
5.071	0.436	0.577	0.067	0.948	19.000
3.672	0.448	0.577	-0.860	0.400	19.000
4.254	0.413	0.577	0.546	0.592	19.000
3.685	0.490	0.577	-0.632	0.535	19.000

2.323	0.506	0.577	0.501	0.622	19.000
2.945	0.501	0.577	0.071	0.944	19.000
2.851	0.473	0.577	-0.206	0.839	19.000
3.534	0.423	0.577	-1.661	0.113	19.000
3.515	0.392	0.577	-0.439	0.665	19.000
2.486	0.522	0.577	-0.405	0.690	19.000
2.315	0.393	0.577	-1.052	0.306	19.000
2.566	0.395	0.577	0.506	0.618	19.000
1.093	0.475	0.577	0.091	0.928	19.000
3.846	0.543	0.577	-0.253	0.803	19.000
2.320	0.535	0.577	0.543	0.593	19.000
3.710	0.586	0.577	-0.368	0.717	19.000
4.480	0.433	0.577	-2.917	0.009	19.000
3.210	0.488	0.577	1.030	0.316	19.000
2.867	0.418	0.577	-0.637	0.532	19.000
1.613	0.765	0.577	0.433	0.670	19.000
2.587	0.479	0.577	-0.700	0.493	19.000
4.572	0.335	0.577	0.506	0.618	19.000
2.831	0.508	0.577	1.105	0.283	19.000
1.471	0.562	0.577	-0.451	0.657	19.000
3.481	0.416	0.577	-2.093	0.050	19.000
2.124	0.485	0.577	-1.243	0.229	19.000
3.645	0.886	0.577	-1.961	0.065	19.000
3.469	0.454	0.577	3.632	0.002	19.000
1.193	0.566	0.577	-0.397	0.696	19.000
4.032	0.503	0.577	0.957	0.351	19.000
3.039	0.503	0.577	-2.615	0.017	19.000
2.193	0.555	0.577	1.416	0.173	19.000
2.627	0.525	0.577	-0.910	0.374	19.000
2.797	0.670	0.577	0.413	0.684	19.000
3.813	0.464	0.577	0.886	0.387	19.000
2.060	1.012	0.577	0.311	0.759	19.000
4.962	0.611	0.577	-1.333	0.198	19.000
2.338	0.545	0.577	-2.240	0.037	19.000
5.428	0.393	0.577	-0.509	0.617	19.000
2.887	0.464	0.577	1.375	0.185	19.000
3.330	0.523	0.577	0.414	0.684	19.000
5.923	0.489	0.577	0.065	0.949	19.000
6.940	0.467	0.577	-1.141	0.268	19.000
3.724	0.368	0.577	0.470	0.644	19.000
3.969	0.439	0.577	0.653	0.522	19.000
4.799	0.577	0.577	-1.111	0.280	19.000
5.346	0.623	0.577	0.896	0.381	19.000
4.104	0.470	0.577	-0.811	0.428	19.000
8.213	0.465	0.577	-0.158	0.876	19.000
4.994	0.437	0.577	2.163	0.044	19.000
3.588	0.412	0.577	-1.099	0.286	19.000
3.855	0.434	0.577	1.345	0.194	19.000
2.561	0.757	0.577	0.756	0.459	19.000
6.973	0.462	0.577	0.505	0.619	19.000

3.817	0.346	0.577	0.337	0.740	19.000
8.018	0.633	0.577	1.494	0.152	19.000
6.710	0.346	0.577	1.900	0.073	19.000
2.359	0.431	0.577	-0.251	0.805	19.000
4.888	0.426	0.577	3.228	0.004	19.000
3.343	0.501	0.577	-0.392	0.699	19.000
6.119	0.460	0.577	1.351	0.193	19.000
5.337	0.401	0.577	-0.983	0.338	19.000
6.433	0.415	0.577	1.358	0.190	19.000
2.699	0.481	0.577	0.382	0.707	19.000
2.822	0.655	0.577	1.123	0.275	19.000
3.410	0.458	0.577	-0.687	0.500	19.000
4.007	0.420	0.577	2.129	0.047	19.000
3.177	0.661	0.577	-0.200	0.843	19.000
4.170	0.646	0.577	1.913	0.071	19.000
5.556	0.552	0.577	-0.666	0.513	19.000
5.303	0.340	0.577	0.631	0.536	19.000
6.105	0.400	0.577	-0.673	0.509	19.000
3.848	0.573	0.577	-1.087	0.291	19.000
5.179	0.330	0.577	1.308	0.206	19.000
2.797	0.412	0.577	0.634	0.534	19.000
4.591	0.440	0.577	-0.124	0.902	19.000
2.969	0.497	0.577	1.197	0.246	19.000
4.145	0.347	0.577	1.197	0.246	19.000
3.453	0.421	0.577	1.889	0.074	19.000
4.591	0.749	0.577	-0.951	0.353	19.000
5.115	0.427	0.577	-1.401	0.177	19.000
3.357	0.624	0.577	0.684	0.502	19.000
4.681	0.428	0.577	1.366	0.188	19.000
3.579	0.363	0.577	0.987	0.336	19.000
3.383	0.451	0.577	1.174	0.255	19.000
3.551	0.518	0.577	0.522	0.608	19.000
6.355	0.412	0.577	0.727	0.476	19.000
5.726	0.799	0.577	-0.509	0.617	19.000
7.284	0.734	0.577	0.620	0.542	19.000
3.137	0.507	0.577	-1.818	0.085	19.000
3.625	0.374	0.577	-0.124	0.903	19.000
5.521	0.486	0.577	0.558	0.583	19.000
2.774	0.638	0.577	0.911	0.374	19.000
3.848	0.507	0.577	-0.359	0.724	19.000
3.362	0.672	0.577	0.526	0.605	19.000
2.857	0.483	0.577	0.122	0.904	19.000
1.565	1.209	0.577	1.854	0.079	19.000
2.884	0.428	0.577	-1.802	0.087	19.000
3.810	0.548	0.577	-1.251	0.226	19.000
2.369	0.591	0.577	1.528	0.143	19.000
6.928	0.744	0.577	0.357	0.725	19.000
3.548	0.385	0.577	1.651	0.115	19.000
3.127	0.544	0.577	-0.655	0.520	19.000
3.231	0.494	0.577	-0.605	0.552	19.000

7.146	0.591	0.577	0.966	0.346	19.000
4.430	0.544	0.577	-0.492	0.628	19.000
3.404	0.411	0.577	-0.632	0.535	19.000
5.239	0.534	0.577	-1.331	0.199	19.000
1.609	0.562	0.577	-0.372	0.714	19.000
3.005	0.437	0.577	-0.451	0.657	19.000
5.715	0.553	0.577	0.836	0.413	19.000
4.131	0.427	0.577	0.526	0.605	19.000
3.682	0.477	0.577	-0.710	0.486	19.000
3.412	0.404	0.577	2.595	0.018	19.000
5.310	0.875	0.577	-0.553	0.587	19.000
4.255	0.440	0.577	0.805	0.431	19.000
4.466	0.461	0.577	1.117	0.278	19.000
7.022	0.426	0.577	0.809	0.429	19.000
3.957	0.485	0.577	0.899	0.380	19.000
2.717	0.685	0.577	0.810	0.428	19.000
6.413	0.483	0.577	-0.372	0.714	19.000
2.904	0.484	0.577	0.662	0.516	19.000
3.160	0.750	0.577	0.880	0.390	19.000
6.151	0.477	0.577	-0.444	0.662	19.000
5.875	0.380	0.577	0.508	0.617	19.000
7.367	0.701	0.577	-0.891	0.384	19.000
3.772	0.433	0.577	1.430	0.169	19.000
4.534	0.481	0.577	-0.532	0.601	19.000
5.173	0.564	0.577	1.236	0.231	19.000
3.601	0.534	0.577	-0.337	0.740	19.000
4.056	0.574	0.577	-1.139	0.269	19.000
3.368	0.484	0.577	0.811	0.428	19.000
2.078	0.527	0.577	0.560	0.582	19.000
2.766	0.542	0.577	0.170	0.867	19.000
3.550	0.674	0.577	0.781	0.444	19.000
6.220	0.386	0.577	1.221	0.237	19.000
6.268	0.548	0.577	-0.306	0.763	19.000
4.894	0.475	0.577	0.197	0.846	19.000
3.566	0.416	0.577	1.017	0.322	19.000
8.933	0.413	0.577	-1.437	0.167	19.000
2.807	0.527	0.577	1.441	0.166	19.000
2.940	0.523	0.577	-1.014	0.323	19.000
2.187	0.483	0.577	0.952	0.353	19.000
3.961	0.481	0.577	1.128	0.273	19.000
3.177	0.843	0.577	0.113	0.912	19.000
5.115	0.406	0.577	0.703	0.490	19.000
4.239	0.416	0.577	0.519	0.610	19.000
6.634	0.327	0.577	1.810	0.086	19.000
5.324	0.489	0.577	1.076	0.296	19.000
2.256	0.471	0.577	-1.315	0.204	19.000
4.235	0.944	0.577	0.692	0.497	19.000
2.733	0.414	0.577	1.297	0.210	19.000
5.687	0.480	0.577	-0.608	0.550	19.000
3.390	0.411	0.577	-0.403	0.692	19.000

2.977	0.605	0.577	-0.851	0.405	19.000
1.257	0.454	0.577	-0.164	0.871	19.000
7.074	0.434	0.577	-0.901	0.379	19.000
6.496	0.292	0.577	1.188	0.250	19.000
4.900	0.351	0.577	0.887	0.386	19.000
5.688	0.406	0.577	1.456	0.162	19.000
2.647	0.411	0.577	-0.382	0.707	19.000
2.474	0.621	0.577	0.546	0.591	19.000
7.395	0.589	0.577	0.207	0.838	19.000
5.679	0.629	0.577	-0.475	0.641	19.000
7.813	0.462	0.577	0.912	0.373	19.000
5.142	0.545	0.577	0.314	0.757	19.000
7.705	0.432	0.577	-0.391	0.700	19.000
4.926	0.815	0.577	-1.387	0.182	19.000
7.204	0.467	0.577	3.007	0.007	19.000
4.116	0.418	0.577	2.489	0.022	19.000
7.133	0.900	0.577	1.014	0.324	19.000
6.681	0.802	0.577	0.994	0.333	19.000
7.133	0.879	0.577	0.940	0.359	19.000
3.983	0.469	0.577	-0.546	0.591	19.000
4.726	0.461	0.577	-1.550	0.138	19.000
0.954	0.696	0.577	0.049	0.961	19.000
1.627	0.551	0.577	0.320	0.752	19.000
4.672	0.516	0.577	0.771	0.450	19.000
6.222	0.519	0.577	0.124	0.903	19.000
7.603	0.320	0.577	1.121	0.276	19.000
1.784	0.429	0.577	1.012	0.324	19.000
1.480	0.922	0.577	-0.103	0.919	19.000
5.044	0.427	0.577	0.864	0.398	19.000
2.961	0.487	0.577	0.084	0.934	19.000
3.910	0.466	0.577	-0.810	0.428	19.000
2.529	0.433	0.577	-1.779	0.091	19.000
7.003	0.358	0.577	-1.498	0.151	19.000
2.346	0.510	0.577	-0.798	0.435	19.000
1.400	0.578	0.577	1.380	0.184	19.000
5.149	0.350	0.577	-0.907	0.376	19.000
5.590	0.370	0.577	1.343	0.195	19.000
6.302	1.021	0.577	-1.610	0.124	19.000
6.472	0.467	0.577	0.778	0.446	19.000
8.071	0.529	0.577	0.239	0.814	19.000
3.637	0.460	0.577	0.229	0.822	19.000
4.028	0.480	0.577	-0.356	0.726	19.000
2.295	0.456	0.577	0.078	0.939	19.000
7.108	0.390	0.577	0.435	0.668	19.000
4.512	0.484	0.577	0.733	0.472	19.000
3.853	0.407	0.577	2.026	0.057	19.000
2.531	0.503	0.577	0.065	0.949	19.000
1.127	0.609	0.577	-0.135	0.894	19.000
4.071	0.367	0.577	2.486	0.022	19.000
3.436	0.535	0.577	0.209	0.837	19.000

2.141	0.403	0.577	1.381	0.183	19.000
4.504	0.824	0.577	0.643	0.528	19.000
6.230	0.698	0.577	1.396	0.179	19.000
7.616	0.778	0.577	1.621	0.122	19.000
4.252	0.649	0.577	1.323	0.202	19.000
5.612	0.467	0.577	1.937	0.068	19.000
4.821	0.317	0.577	1.093	0.288	19.000
3.922	0.510	0.577	-1.232	0.233	19.000
1.998	0.457	0.577	-0.059	0.954	19.000
1.459	0.445	0.577	3.060	0.006	19.000
6.430	0.463	0.577	0.943	0.358	19.000
2.644	0.441	0.577	1.315	0.204	19.000
2.657	0.471	0.577	0.745	0.466	19.000
4.115	0.719	0.577	-0.168	0.868	19.000
1.678	0.449	0.577	1.038	0.312	19.000
6.088	0.319	0.577	-1.261	0.222	19.000
4.549	0.461	0.577	-0.077	0.939	19.000
4.202	0.964	0.577	0.218	0.830	19.000
3.398	0.473	0.577	1.456	0.162	19.000
4.381	0.335	0.577	2.632	0.016	19.000
5.033	0.466	0.577	1.819	0.085	19.000
5.644	0.346	0.577	0.555	0.585	19.000
3.809	0.564	0.577	0.290	0.775	19.000
6.932	0.710	0.577	-0.566	0.578	19.000
5.747	0.562	0.577	0.320	0.752	19.000
8.428	0.731	0.577	0.399	0.694	19.000
3.993	0.505	0.577	1.981	0.062	19.000
3.929	0.787	0.577	0.061	0.952	19.000
8.284	0.297	0.577	0.893	0.383	19.000
6.891	0.356	0.577	0.311	0.759	19.000
2.249	0.463	0.577	-0.271	0.789	19.000
4.980	0.504	0.577	0.090	0.929	19.000
7.418	0.320	0.577	1.423	0.171	19.000
2.963	0.439	0.577	-0.464	0.648	19.000
4.635	0.377	0.577	1.722	0.101	19.000
6.046	0.469	0.577	2.076	0.052	19.000
5.852	0.609	0.577	1.473	0.157	19.000
6.591	0.435	0.577	0.342	0.736	19.000
6.292	0.325	0.577	1.314	0.204	19.000
6.201	0.334	0.577	-0.362	0.722	19.000
4.242	0.650	0.577	-0.587	0.564	19.000
3.158	0.505	0.577	1.388	0.181	19.000
2.999	0.489	0.577	0.599	0.556	19.000
5.812	0.503	0.577	0.580	0.569	19.000
5.930	0.532	0.577	0.496	0.625	19.000
2.144	0.692	0.577	0.780	0.445	19.000
0.896	1.111	0.577	1.524	0.144	19.000
1.836	0.615	0.577	3.205	0.005	19.000
3.901	0.517	0.577	0.157	0.877	19.000
5.124	0.359	0.577	-0.938	0.360	19.000

2.836	0.689	0.577	0.716	0.483	19.000
3.742	0.752	0.577	-0.489	0.630	19.000
2.205	0.796	0.577	0.224	0.825	19.000
3.283	0.489	0.577	0.461	0.650	19.000
3.193	0.583	0.577	0.263	0.796	19.000
2.933	0.529	0.577	0.192	0.849	19.000
2.547	0.540	0.577	-1.734	0.099	19.000
2.900	0.638	0.577	-0.554	0.586	19.000
6.495	0.481	0.577	-1.361	0.189	19.000
5.677	0.636	0.577	-0.150	0.882	19.000
8.812	0.373	0.577	-1.170	0.256	19.000
3.614	0.533	0.577	-0.114	0.910	19.000
5.895	0.432	0.577	0.542	0.594	19.000
2.645	0.598	0.577	-0.464	0.648	19.000
4.209	0.397	0.577	1.154	0.263	19.000
4.596	0.424	0.577	1.452	0.163	19.000
2.830	0.444	0.577	1.153	0.263	19.000
5.140	0.482	0.577	0.212	0.835	19.000
4.894	0.444	0.577	-0.588	0.563	19.000
9.174	0.697	0.577	-0.207	0.838	19.000
2.609	0.943	0.577	0.559	0.582	19.000
4.334	0.494	0.577	0.167	0.869	19.000
6.019	0.477	0.577	-0.518	0.610	19.000
8.754	0.419	0.577	2.456	0.024	19.000
4.156	0.480	0.577	1.208	0.242	19.000
5.203	0.375	0.577	-1.305	0.208	19.000
2.312	0.620	0.577	-1.086	0.291	19.000
7.387	0.428	0.577	0.113	0.912	19.000
4.835	0.326	0.577	0.170	0.867	19.000
6.173	0.543	0.577	1.508	0.148	19.000
5.241	0.457	0.577	0.339	0.738	19.000
4.404	0.654	0.577	-2.052	0.054	19.000
8.350	0.451	0.577	1.282	0.215	19.000
1.578	0.384	0.577	-0.441	0.664	19.000
2.372	0.432	0.577	0.178	0.861	19.000
2.801	0.521	0.577	2.585	0.018	19.000
7.068	0.432	0.577	1.178	0.253	19.000
8.717	0.437	0.577	0.258	0.799	19.000
7.946	0.524	0.577	0.181	0.858	19.000
5.709	0.443	0.577	3.457	0.003	19.000
1.994	0.486	0.577	0.196	0.847	19.000
3.421	0.643	0.577	0.050	0.960	19.000
2.116	0.382	0.577	-0.086	0.932	19.000
2.817	0.528	0.577	-0.389	0.701	19.000
5.502	0.666	0.577	1.177	0.254	19.000
3.979	0.578	0.577	0.990	0.335	19.000
2.049	0.540	0.577	-1.625	0.121	19.000
3.127	0.480	0.577	-0.380	0.708	19.000
4.279	0.439	0.577	-0.152	0.881	19.000
4.512	0.757	0.577	0.103	0.919	19.000

3.807	0.509	0.577	0.902	0.378	19.000
5.095	0.451	0.577	-0.944	0.357	19.000
3.728	0.521	0.577	-0.278	0.784	19.000
4.868	0.455	0.577	-0.556	0.585	19.000
4.845	0.453	0.577	0.982	0.338	19.000
4.278	0.595	0.577	-0.473	0.641	19.000
5.299	0.378	0.577	1.039	0.312	19.000
2.628	0.349	0.577	0.452	0.657	19.000
5.611	0.313	0.577	-0.193	0.849	19.000
5.192	0.506	0.577	0.308	0.761	19.000
5.265	0.687	0.577	0.573	0.573	19.000
6.508	0.512	0.577	-0.166	0.870	19.000
6.488	0.319	0.577	1.457	0.161	19.000
6.772	0.523	0.577	1.902	0.072	19.000
9.316	0.800	0.577	0.624	0.540	19.000
9.532	0.426	0.577	-0.402	0.692	19.000
5.324	0.741	0.577	1.866	0.078	19.000
4.331	0.527	0.577	0.367	0.718	19.000
3.475	0.379	0.577	-0.097	0.923	19.000
3.714	0.393	0.577	1.869	0.077	19.000
6.218	0.854	0.577	2.172	0.043	19.000
3.529	0.491	0.577	3.478	0.003	19.000
9.197	0.401	0.577	-0.202	0.842	19.000
4.719	0.294	0.577	3.247	0.004	19.000
3.504	0.424	0.577	2.746	0.013	19.000
2.056	0.511	0.577	1.863	0.078	19.000
6.207	0.483	0.577	1.583	0.130	19.000
5.640	0.495	0.577	-1.846	0.081	19.000
7.468	0.329	0.577	1.523	0.144	19.000
6.055	0.401	0.577	0.656	0.520	19.000
5.425	0.411	0.577	0.772	0.450	19.000
8.306	1.072	0.577	0.725	0.477	19.000
4.625	0.397	0.577	-1.545	0.139	19.000
4.564	0.580	0.577	1.578	0.131	19.000
2.963	0.948	0.577	1.617	0.122	19.000
3.579	0.405	0.577	0.533	0.600	19.000
5.402	0.350	0.577	-0.125	0.902	19.000
6.039	0.405	0.577	0.625	0.539	19.000
3.945	0.471	0.577	-0.625	0.539	19.000
5.704	0.388	0.577	-0.341	0.737	19.000
6.201	0.314	0.577	1.086	0.291	19.000
4.059	0.575	0.577	0.468	0.645	19.000
7.628	0.422	0.577	-0.401	0.693	19.000
2.548	0.503	0.577	2.165	0.043	19.000
5.750	0.429	0.577	0.561	0.582	19.000
5.053	0.404	0.577	2.497	0.022	19.000
3.451	0.685	0.577	0.816	0.425	19.000
3.894	0.504	0.577	0.737	0.470	19.000
3.616	0.509	0.577	0.233	0.818	19.000
6.966	0.498	0.577	-0.101	0.920	19.000

4.929	0.460	0.577	1.852	0.080	19.000
3.461	0.505	0.577	2.447	0.024	19.000
6.550	0.344	0.577	2.046	0.055	19.000
3.525	0.633	0.577	1.588	0.129	19.000
2.547	0.410	0.577	-0.484	0.634	19.000
2.192	0.501	0.577	-0.206	0.839	19.000
3.969	0.501	0.577	2.108	0.049	19.000
6.605	0.513	0.577	-0.479	0.637	19.000
7.576	0.328	0.577	-1.024	0.319	19.000
4.489	1.025	0.577	-1.178	0.253	19.000
4.953	0.367	0.577	0.726	0.477	19.000
2.786	0.670	0.577	0.293	0.773	19.000
4.188	0.386	0.577	1.433	0.168	19.000
4.187	0.417	0.577	0.728	0.476	19.000
2.918	0.469	0.577	-0.619	0.543	19.000
2.970	0.665	0.577	-0.093	0.927	19.000
5.285	0.460	0.577	0.131	0.897	19.000
6.695	0.381	0.577	-0.616	0.545	19.000
5.419	0.430	0.577	0.314	0.757	19.000
7.501	0.351	0.577	0.530	0.602	19.000
3.401	0.433	0.577	-0.324	0.749	19.000
3.106	0.444	0.577	-0.387	0.703	19.000
5.313	0.631	0.577	-0.399	0.694	19.000
4.203	0.429	0.577	0.801	0.433	19.000
2.833	0.429	0.577	-1.022	0.319	19.000
6.423	0.584	0.577	-0.640	0.530	19.000
4.836	0.391	0.577	0.800	0.433	19.000
2.331	0.440	0.577	-1.507	0.148	19.000
8.115	0.396	0.577	1.021	0.320	19.000
2.231	0.476	0.577	-0.491	0.629	19.000
6.681	0.526	0.577	0.325	0.748	19.000
6.272	0.444	0.577	2.628	0.017	19.000
6.266	0.457	0.577	1.238	0.231	19.000
5.899	0.341	0.577	1.750	0.096	19.000
4.835	0.402	0.577	-0.133	0.896	19.000
3.959	0.636	0.577	-1.234	0.232	19.000
6.094	0.681	0.577	0.717	0.482	19.000
2.095	0.672	0.577	-1.151	0.264	19.000
2.970	0.574	0.577	0.533	0.600	19.000
9.949	0.319	0.577	0.414	0.684	19.000
2.819	0.666	0.577	-3.066	0.006	19.000
1.803	0.420	0.577	-2.916	0.009	19.000
3.603	0.475	0.577	-1.039	0.312	19.000
5.236	0.324	0.577	2.225	0.038	19.000
7.716	0.393	0.577	-0.846	0.408	19.000
5.205	0.437	0.577	0.989	0.335	19.000
2.795	0.671	0.577	0.652	0.522	19.000
5.392	0.502	0.577	1.410	0.175	19.000
2.327	0.723	0.577	0.283	0.780	19.000
4.465	0.402	0.577	-0.986	0.337	19.000

6.342	0.617	0.577	-0.345	0.734	19.000
9.014	0.576	0.577	0.054	0.957	19.000
9.143	0.613	0.577	0.320	0.752	19.000
4.596	0.517	0.577	0.238	0.815	19.000
2.133	0.522	0.577	-1.776	0.092	19.000
4.654	0.454	0.577	0.499	0.623	19.000
2.536	0.396	0.577	0.945	0.356	19.000
3.630	0.377	0.577	0.734	0.472	19.000
7.808	0.383	0.577	1.744	0.097	19.000
2.375	0.552	0.577	0.381	0.708	19.000
6.263	0.323	0.577	1.813	0.086	19.000
5.785	0.383	0.577	0.443	0.663	19.000
7.156	0.462	0.577	-0.608	0.551	19.000
2.598	0.412	0.577	-0.896	0.381	19.000
5.911	0.321	0.577	2.147	0.045	19.000
3.724	0.440	0.577	0.592	0.561	19.000
2.682	0.795	0.577	0.351	0.730	19.000
4.672	0.414	0.577	-0.882	0.389	19.000
7.278	0.761	0.577	0.317	0.754	19.000
4.062	0.473	0.577	0.584	0.566	19.000
3.957	0.600	0.577	0.133	0.895	19.000
6.193	0.542	0.577	1.018	0.322	19.000
6.593	0.452	0.577	0.783	0.443	19.000
5.700	0.436	0.577	0.637	0.532	19.000
4.032	0.374	0.577	-0.313	0.757	19.000
3.767	0.367	0.577	0.193	0.849	19.000
5.317	0.727	0.577	-0.215	0.832	19.000
3.616	0.693	0.577	-0.182	0.858	19.000
4.815	0.631	0.577	1.684	0.109	19.000
4.300	0.424	0.577	0.702	0.491	19.000
6.325	0.423	0.577	-0.538	0.597	19.000
2.005	0.439	0.577	-0.873	0.394	19.000
5.271	0.350	0.577	0.878	0.391	19.000
4.212	0.449	0.577	1.924	0.069	19.000
6.078	0.322	0.577	0.579	0.570	19.000
6.448	0.427	0.577	0.630	0.536	19.000
5.723	0.352	0.577	-1.288	0.213	19.000
3.887	0.411	0.577	1.286	0.214	19.000
5.165	0.403	0.577	0.393	0.699	19.000
3.807	0.436	0.577	0.159	0.875	19.000
7.363	0.407	0.577	1.076	0.295	19.000
6.351	0.423	0.577	0.469	0.644	19.000
7.330	0.320	0.577	0.968	0.345	19.000
2.846	0.563	0.577	-1.732	0.099	19.000
7.236	0.819	0.577	-0.052	0.959	19.000
6.790	0.475	0.577	0.491	0.629	19.000
5.243	0.385	0.577	1.884	0.075	19.000
6.939	0.428	0.577	-1.101	0.285	19.000
6.350	0.511	0.577	0.127	0.901	19.000
1.816	0.371	0.577	-0.128	0.899	19.000

4.582	0.332	0.577	1.256	0.224	19.000
3.686	0.432	0.577	0.926	0.366	19.000
2.515	0.796	0.577	-0.704	0.490	19.000
9.182	0.321	0.577	-1.212	0.240	19.000
7.719	0.333	0.577	1.544	0.139	19.000
2.538	0.441	0.577	1.950	0.066	19.000
6.385	0.709	0.577	0.152	0.881	19.000
4.819	0.857	0.577	-0.459	0.652	19.000
1.736	0.989	0.577	-0.683	0.503	19.000
7.195	0.381	0.577	1.599	0.126	19.000
1.961	0.457	0.577	-1.493	0.152	19.000
2.717	0.509	0.577	-1.946	0.067	19.000
8.206	0.329	0.577	0.818	0.423	19.000
9.725	0.344	0.577	0.849	0.407	19.000
5.597	0.521	0.577	-1.979	0.062	19.000
4.773	0.349	0.577	1.054	0.305	19.000
8.151	0.346	0.577	0.747	0.464	19.000
9.061	0.333	0.577	1.271	0.219	19.000
9.750	0.371	0.577	1.681	0.109	19.000
6.589	0.520	0.577	0.577	0.571	19.000
7.784	0.422	0.577	0.503	0.621	19.000
7.254	0.459	0.577	1.066	0.300	19.000
5.204	0.321	0.577	-1.255	0.225	19.000
6.031	0.461	0.577	1.176	0.254	19.000
2.764	0.480	0.577	2.254	0.036	19.000
8.830	0.372	0.577	-0.136	0.893	19.000
4.735	0.924	0.577	-0.778	0.446	19.000
3.719	0.571	0.577	0.799	0.434	19.000
4.427	0.497	0.577	1.036	0.313	19.000
5.947	0.306	0.577	0.480	0.636	19.000
4.750	0.340	0.577	1.972	0.063	19.000
6.799	0.323	0.577	0.700	0.492	19.000
6.205	0.852	0.577	-0.417	0.681	19.000
3.600	0.505	0.577	-0.599	0.556	19.000
6.162	0.512	0.577	-0.181	0.858	19.000
7.233	0.372	0.577	0.209	0.837	19.000
2.561	0.481	0.577	0.077	0.939	19.000
2.661	0.489	0.577	-0.335	0.741	19.000
6.386	0.342	0.577	1.996	0.060	19.000
6.060	0.487	0.577	0.826	0.419	19.000
3.273	0.608	0.577	-0.481	0.636	19.000
8.409	0.380	0.577	2.176	0.042	19.000
3.787	0.405	0.577	1.649	0.116	19.000
2.778	0.751	0.577	-0.639	0.530	19.000
5.565	0.504	0.577	0.262	0.796	19.000
6.806	0.546	0.577	0.524	0.606	19.000
1.329	0.539	0.577	0.972	0.343	19.000
5.533	0.430	0.577	1.590	0.128	19.000
3.948	0.790	0.577	-0.312	0.759	19.000
2.407	0.436	0.577	0.783	0.443	19.000

5.210	0.415	0.577	0.798	0.435	19.000
7.158	0.347	0.577	4.655	0.000	19.000
4.252	0.876	0.577	0.417	0.681	19.000
3.262	0.451	0.577	-0.587	0.564	19.000
3.966	0.573	0.577	0.549	0.589	19.000
3.201	0.459	0.577	4.184	0.001	19.000
2.368	0.403	0.577	-0.373	0.713	19.000
8.772	0.495	0.577	0.140	0.891	19.000
4.878	0.465	0.577	0.584	0.566	19.000
4.036	0.485	0.577	0.177	0.861	19.000
2.313	0.449	0.577	-0.951	0.353	19.000
3.592	0.434	0.577	1.089	0.290	19.000
7.447	0.575	0.577	-0.159	0.875	19.000
4.464	0.405	0.577	0.774	0.449	19.000
4.873	0.616	0.577	-0.874	0.393	19.000
4.323	0.488	0.577	-0.178	0.861	19.000
6.205	0.494	0.577	0.349	0.731	19.000
4.642	0.580	0.577	-0.174	0.864	19.000
3.729	0.448	0.577	0.793	0.438	19.000
2.643	0.449	0.577	-0.914	0.372	19.000
4.794	0.634	0.577	0.057	0.955	19.000
6.364	0.514	0.577	-0.581	0.568	19.000
5.474	0.446	0.577	0.552	0.587	19.000
5.218	0.415	0.577	0.630	0.536	19.000
2.414	0.404	0.577	1.766	0.093	19.000
7.505	0.776	0.577	0.336	0.740	19.000
3.246	0.464	0.577	1.712	0.103	19.000
4.372	0.424	0.577	1.592	0.128	19.000
5.165	0.438	0.577	2.149	0.045	19.000
7.907	0.387	0.577	-0.474	0.641	19.000
4.434	0.426	0.577	0.797	0.436	19.000
4.892	0.456	0.577	0.815	0.425	19.000
6.308	0.376	0.577	-0.083	0.935	19.000
6.230	0.386	0.577	0.934	0.362	19.000
6.348	0.441	0.577	0.279	0.784	19.000
4.527	0.389	0.577	-1.576	0.131	19.000
7.567	0.482	0.577	-1.155	0.262	19.000
3.496	0.416	0.577	0.799	0.434	19.000
5.028	0.456	0.577	-0.188	0.853	19.000
2.423	0.373	0.577	1.494	0.152	19.000
2.423	0.450	0.577	-0.269	0.791	19.000
2.284	0.813	0.577	0.488	0.631	19.000
3.126	0.739	0.577	0.376	0.711	19.000
6.743	0.375	0.577	1.257	0.224	19.000
3.215	0.716	0.577	3.037	0.007	19.000
4.991	0.409	0.577	0.378	0.710	19.000
8.582	0.473	0.577	2.085	0.051	19.000
2.479	0.527	0.577	-0.548	0.590	19.000
5.024	0.419	0.577	0.470	0.644	19.000
5.108	0.396	0.577	-2.430	0.025	19.000

1.677	0.560	0.577	1.410	0.175	19.000
4.336	0.452	0.577	-1.284	0.215	19.000
3.443	0.429	0.577	2.438	0.025	19.000
3.958	0.355	0.577	-0.508	0.618	19.000
4.773	0.419	0.577	0.080	0.937	19.000
4.034	0.449	0.577	-0.929	0.364	19.000
2.908	0.418	0.577	-0.924	0.367	19.000
2.388	0.560	0.577	-1.041	0.311	19.000
2.812	0.464	0.577	-0.142	0.888	19.000
3.475	0.465	0.577	-0.530	0.603	19.000
3.867	0.559	0.577	0.710	0.486	19.000
8.316	0.805	0.577	0.318	0.754	19.000
3.751	0.674	0.577	0.506	0.618	19.000
7.098	0.699	0.577	-0.444	0.662	19.000
4.032	0.470	0.577	-1.017	0.322	19.000
3.605	0.565	0.577	-0.314	0.757	19.000
6.334	0.509	0.577	0.950	0.354	19.000
3.340	0.508	0.577	-1.139	0.269	19.000
5.063	0.456	0.577	-0.847	0.408	19.000
2.199	0.431	0.577	0.703	0.490	19.000
3.994	0.352	0.577	-3.130	0.006	19.000
8.602	0.411	0.577	0.906	0.376	19.000
8.658	0.387	0.577	0.079	0.938	19.000
3.367	0.379	0.577	1.585	0.130	19.000
4.038	0.529	0.577	0.203	0.841	19.000
5.628	0.515	0.577	0.755	0.459	19.000
2.979	0.565	0.577	1.215	0.239	19.000
3.145	0.430	0.577	1.398	0.178	19.000
2.968	0.707	0.577	-0.562	0.581	19.000
4.253	0.790	0.577	0.209	0.837	19.000
4.775	0.399	0.577	2.273	0.035	19.000
7.971	0.391	0.577	2.036	0.056	19.000
3.146	0.523	0.577	-0.842	0.410	19.000
2.967	0.655	0.577	-0.552	0.587	19.000
1.362	1.053	0.577	-0.427	0.674	19.000
3.341	0.639	0.577	0.589	0.563	19.000
4.864	0.510	0.577	0.069	0.946	19.000
5.957	0.378	0.577	-2.050	0.054	19.000
2.149	0.547	0.577	-0.365	0.719	19.000
3.742	0.468	0.577	0.195	0.847	19.000
7.964	0.328	0.577	2.000	0.060	19.000
5.775	0.349	0.577	1.363	0.189	19.000
3.460	0.545	0.577	-0.653	0.522	19.000
2.468	0.622	0.577	-1.121	0.276	19.000
3.297	0.499	0.577	1.427	0.170	19.000
4.946	0.407	0.577	-0.971	0.344	19.000
7.345	0.667	0.577	-0.150	0.882	19.000
2.796	0.454	0.577	0.738	0.470	19.000
6.408	0.320	0.577	-0.543	0.593	19.000
6.261	0.325	0.577	1.850	0.080	19.000

2.530	0.325	0.577	2.351	0.030	19.000
3.668	0.451	0.577	-0.608	0.550	19.000
7.070	0.547	0.577	0.603	0.554	19.000
4.898	0.641	0.577	0.065	0.949	19.000
5.322	0.474	0.577	0.969	0.345	19.000
3.964	0.366	0.577	0.086	0.933	19.000
4.298	0.379	0.577	-0.517	0.611	19.000
5.833	0.416	0.577	-0.311	0.759	19.000
5.418	0.367	0.577	-0.762	0.456	19.000
4.204	0.861	0.577	-0.218	0.830	19.000
2.082	0.474	0.577	1.572	0.133	19.000
2.648	0.445	0.577	0.156	0.878	19.000
5.740	0.461	0.577	1.831	0.083	19.000
4.999	0.380	0.577	-0.461	0.650	19.000
3.657	0.534	0.577	0.902	0.378	19.000
2.833	0.433	0.577	-0.080	0.937	19.000
2.208	0.461	0.577	-0.724	0.478	19.000
3.929	0.562	0.577	0.091	0.929	19.000
6.328	0.372	0.577	0.552	0.587	19.000
4.895	0.411	0.577	-1.593	0.128	19.000
4.846	0.421	0.577	-0.270	0.790	19.000
8.841	0.462	0.577	1.066	0.300	19.000
4.552	0.592	0.577	2.473	0.023	19.000
6.758	0.322	0.577	-0.232	0.819	19.000
7.108	0.611	0.577	0.807	0.430	19.000
2.113	0.574	0.577	0.842	0.410	19.000
4.178	0.605	0.577	-0.424	0.676	19.000
3.380	0.487	0.577	0.813	0.426	19.000
7.549	0.788	0.577	1.538	0.140	19.000
3.436	0.828	0.577	-0.515	0.612	19.000
6.062	0.475	0.577	0.585	0.566	19.000
3.080	0.464	0.577	1.354	0.192	19.000
4.570	0.731	0.577	0.049	0.961	19.000
2.549	0.439	0.577	0.829	0.417	19.000
1.649	0.487	0.577	-0.796	0.436	19.000
4.628	0.540	0.577	-0.077	0.940	19.000
3.784	0.480	0.577	-0.463	0.648	19.000
4.830	0.428	0.577	0.315	0.756	19.000
5.493	0.413	0.577	0.499	0.623	19.000
3.115	0.708	0.577	-0.254	0.803	19.000
3.288	0.531	0.577	-0.196	0.847	19.000
3.650	0.540	0.577	-0.454	0.655	19.000
2.970	0.400	0.577	0.382	0.707	19.000
3.370	0.402	0.577	1.861	0.078	19.000
1.653	1.378	0.577	0.766	0.453	19.000
1.915	0.748	0.577	-3.105	0.006	19.000
2.497	0.495	0.577	-0.853	0.404	19.000
1.601	0.637	0.577	-2.090	0.050	19.000
5.850	0.302	0.577	2.244	0.037	19.000
5.384	0.394	0.577	0.752	0.461	19.000

4.505	0.337	0.577	-0.160	0.874	19.000
2.762	0.385	0.577	0.317	0.755	19.000
2.897	0.605	0.577	-2.858	0.010	19.000
4.020	0.402	0.577	0.356	0.726	19.000
6.161	0.479	0.577	-0.791	0.439	19.000
3.254	0.426	0.577	-0.739	0.469	19.000
3.208	0.378	0.577	-0.386	0.704	19.000
1.480	0.749	0.577	0.293	0.772	19.000
4.241	0.517	0.577	-0.216	0.831	19.000
1.759	0.647	0.577	0.358	0.724	19.000
3.689	0.382	0.577	1.396	0.179	19.000
3.732	0.370	0.577	0.738	0.470	19.000
5.860	0.487	0.577	-0.718	0.481	19.000
3.934	0.478	0.577	-1.529	0.143	19.000
3.252	0.467	0.577	-0.345	0.734	19.000
5.279	0.346	0.577	-0.254	0.802	19.000
6.385	0.360	0.577	0.296	0.771	19.000
4.055	0.369	0.577	0.529	0.603	19.000
7.518	0.444	0.577	1.021	0.320	19.000
5.343	0.471	0.577	0.085	0.933	19.000
5.095	0.427	0.577	-0.437	0.667	19.000
2.868	0.393	0.577	-0.777	0.447	19.000
4.814	0.406	0.577	-0.905	0.377	19.000
3.403	0.509	0.577	-1.162	0.260	19.000
2.821	0.394	0.577	-1.086	0.291	19.000
3.161	0.512	0.577	0.175	0.863	19.000
6.409	0.431	0.577	2.083	0.051	19.000
4.464	0.404	0.577	-0.660	0.517	19.000
1.658	0.557	0.577	0.148	0.884	19.000
4.876	0.452	0.577	-2.120	0.047	19.000
2.925	0.380	0.577	1.278	0.217	19.000
5.543	0.349	0.577	1.396	0.179	19.000
1.971	0.585	0.577	-2.082	0.051	19.000
4.451	0.506	0.577	1.607	0.125	19.000
4.099	0.350	0.577	0.774	0.448	19.000
4.779	0.509	0.577	-0.946	0.356	19.000
4.108	0.441	0.577	-1.506	0.149	19.000
2.215	0.545	0.577	-0.276	0.786	19.000
3.060	0.406	0.577	0.082	0.935	19.000
5.062	0.675	0.577	1.752	0.096	19.000
4.276	0.521	0.577	-0.052	0.959	19.000
3.341	0.448	0.577	-0.634	0.534	19.000
2.352	0.488	0.577	0.967	0.345	19.000
4.339	0.432	0.577	-2.346	0.030	19.000
2.691	0.431	0.577	-0.086	0.933	19.000
6.906	0.470	0.577	0.842	0.410	19.000
4.081	0.561	0.577	0.529	0.603	19.000
3.623	1.020	0.577	0.094	0.926	19.000
1.369	0.720	0.577	-3.128	0.006	19.000
7.324	0.794	0.577	0.942	0.358	19.000

5.123	0.855	0.577	-2.886	0.009	19.000
2.445	0.492	0.577	-1.709	0.104	19.000
6.798	0.493	0.577	0.329	0.746	19.000
5.811	0.440	0.577	-1.440	0.166	19.000
2.538	0.444	0.577	-0.335	0.741	19.000
2.301	0.457	0.577	1.552	0.137	19.000
3.103	0.405	0.577	-1.235	0.232	19.000
3.321	0.464	0.577	0.205	0.840	19.000
3.329	0.541	0.577	-1.186	0.250	19.000
4.609	0.372	0.577	0.701	0.492	19.000
5.466	0.505	0.577	-1.361	0.189	19.000
3.941	0.475	0.577	0.375	0.712	19.000
2.848	0.360	0.577	0.998	0.331	19.000
2.047	0.843	0.577	-2.192	0.041	19.000
3.188	0.442	0.577	1.300	0.209	19.000
3.040	0.430	0.577	1.410	0.175	19.000
4.229	0.706	0.577	1.393	0.180	19.000
7.006	0.831	0.577	2.101	0.049	19.000
8.090	0.764	0.577	1.986	0.062	19.000
3.056	0.576	0.577	-1.165	0.258	19.000
3.914	0.576	0.577	-0.056	0.956	19.000
2.029	1.343	0.577	0.743	0.467	19.000
1.367	0.532	0.577	1.904	0.072	19.000
4.965	0.748	0.577	-0.619	0.543	19.000
3.492	0.490	0.577	-0.168	0.868	19.000
3.527	0.425	0.577	-1.237	0.231	19.000
4.970	0.532	0.577	0.910	0.374	19.000
4.990	0.468	0.577	0.733	0.473	19.000
6.831	0.495	0.577	1.274	0.218	19.000
3.862	0.456	0.577	1.352	0.192	19.000
3.681	0.468	0.577	0.969	0.345	19.000
3.795	0.461	0.577	0.971	0.344	19.000
3.817	0.477	0.577	0.933	0.363	19.000
3.701	0.544	0.577	1.839	0.082	19.000
4.025	0.416	0.577	0.748	0.463	19.000
2.593	0.428	0.577	-2.571	0.019	19.000
1.702	0.662	0.577	0.520	0.609	19.000
1.582	0.394	0.577	1.812	0.086	19.000
3.150	0.548	0.577	-0.661	0.516	19.000
3.231	0.471	0.577	0.057	0.955	19.000
5.131	0.535	0.577	-0.890	0.385	19.000
4.124	0.367	0.577	0.275	0.786	19.000
5.405	0.667	0.577	0.276	0.785	19.000
2.018	0.683	0.577	0.149	0.883	19.000
4.190	0.448	0.577	0.125	0.902	19.000
4.315	0.433	0.577	-1.410	0.175	19.000
7.179	0.324	0.577	-1.635	0.119	19.000
3.102	0.545	0.577	0.709	0.487	19.000
2.812	0.408	0.577	0.106	0.916	19.000
5.744	0.333	0.577	-0.381	0.707	19.000

3.063	0.495	0.577	0.999	0.331	19.000
4.010	0.410	0.577	0.220	0.828	19.000
2.940	0.520	0.577	-1.152	0.264	19.000
5.813	0.490	0.577	0.085	0.933	19.000
4.797	0.340	0.577	-0.131	0.897	19.000
5.211	0.406	0.577	0.582	0.568	19.000
2.463	0.941	0.577	0.097	0.924	19.000
5.037	0.535	0.577	-0.374	0.712	19.000
3.751	0.411	0.577	1.805	0.087	19.000
9.445	0.727	0.577	1.574	0.132	19.000
4.065	0.541	0.577	1.321	0.202	19.000
4.280	0.400	0.577	-0.312	0.758	19.000
5.094	0.400	0.577	0.728	0.476	19.000
1.832	0.342	0.577	0.856	0.403	19.000
3.799	0.436	0.577	-0.160	0.874	19.000
4.331	0.394	0.577	0.441	0.664	19.000
1.496	0.428	0.577	1.890	0.074	19.000
2.134	1.178	0.577	-0.617	0.545	19.000
8.360	0.706	0.577	0.302	0.766	19.000
8.649	0.722	0.577	0.288	0.777	19.000
8.548	0.712	0.577	0.282	0.781	19.000
8.650	0.723	0.577	0.272	0.789	19.000
8.687	0.719	0.577	0.267	0.792	19.000
8.694	0.740	0.577	0.254	0.802	19.000
8.682	0.733	0.577	0.243	0.811	19.000
7.782	0.645	0.577	0.397	0.696	19.000
7.876	0.663	0.577	0.291	0.774	19.000
1.944	0.449	0.577	1.632	0.119	19.000
4.068	0.422	0.577	0.683	0.503	19.000
2.635	0.497	0.577	-2.277	0.035	19.000
2.336	0.654	0.577	-0.867	0.397	19.000
4.318	0.499	0.577	0.635	0.533	19.000
6.792	1.064	0.577	1.377	0.184	19.000
3.479	0.655	0.577	0.588	0.564	19.000
5.460	0.366	0.577	1.496	0.151	19.000
7.007	0.967	0.577	-0.834	0.415	19.000
4.678	0.456	0.577	0.710	0.486	19.000
1.903	0.376	0.577	-0.202	0.842	19.000
3.892	0.394	0.577	-1.422	0.171	19.000
3.051	0.682	0.577	-0.821	0.422	19.000
3.460	0.457	0.577	0.317	0.754	19.000
3.361	0.431	0.577	0.600	0.556	19.000
4.924	0.827	0.577	-0.114	0.910	19.000
6.229	0.353	0.577	0.740	0.469	19.000
5.431	0.357	0.577	1.526	0.143	19.000
1.968	0.623	0.577	-1.600	0.126	19.000
4.164	0.644	0.577	0.465	0.647	19.000
4.111	0.417	0.577	2.159	0.044	19.000
2.301	0.530	0.577	-0.239	0.813	19.000
8.171	0.404	0.577	0.344	0.735	19.000

8.325	0.386	0.577	0.212	0.834	19.000
7.948	0.326	0.577	-0.142	0.889	19.000
3.724	0.526	0.577	1.501	0.150	19.000
5.054	0.626	0.577	2.673	0.015	19.000
1.891	0.463	0.577	-0.803	0.432	19.000
1.861	0.541	0.577	-3.006	0.007	19.000
5.222	0.435	0.577	0.687	0.501	19.000
4.272	0.506	0.577	-1.196	0.247	19.000
3.009	0.439	0.577	-1.556	0.136	19.000
3.321	0.410	0.577	-1.597	0.127	19.000
4.627	0.934	0.577	-0.661	0.516	19.000
3.122	0.450	0.577	0.081	0.936	19.000
2.571	0.493	0.577	1.662	0.113	19.000
6.138	0.400	0.577	-0.601	0.555	19.000
5.366	0.318	0.577	-0.304	0.765	19.000
3.717	0.601	0.577	-0.221	0.827	19.000
2.252	0.397	0.577	1.288	0.213	19.000
4.092	0.457	0.577	-0.733	0.472	19.000
1.765	0.895	0.577	-2.169	0.043	19.000
1.348	0.616	0.577	0.520	0.609	19.000
5.671	0.354	0.577	-1.019	0.321	19.000
2.654	0.479	0.577	-0.816	0.425	19.000
2.858	0.498	0.577	0.387	0.703	19.000
3.113	0.464	0.577	-0.764	0.454	19.000
4.191	0.404	0.577	-0.180	0.859	19.000
3.954	0.434	0.577	-2.906	0.009	19.000
4.441	0.423	0.577	0.257	0.800	19.000
3.901	0.442	0.577	0.822	0.422	19.000
3.834	0.399	0.577	2.963	0.008	19.000
5.296	0.352	0.577	1.624	0.121	19.000
5.607	0.580	0.577	0.157	0.877	19.000
4.946	0.588	0.577	1.113	0.280	19.000
6.117	0.765	0.577	-0.149	0.883	19.000
3.121	0.365	0.577	1.470	0.158	19.000
5.702	0.550	0.577	-0.631	0.536	19.000
4.094	0.643	0.577	-1.107	0.282	19.000
5.390	0.596	0.577	1.328	0.200	19.000
3.960	0.484	0.577	-0.874	0.393	19.000
1.909	0.868	0.577	-0.804	0.431	19.000
5.187	0.438	0.577	-0.933	0.362	19.000
4.505	0.420	0.577	1.961	0.065	19.000
5.821	0.654	0.577	-0.470	0.644	19.000
3.882	0.415	0.577	0.940	0.359	19.000
1.471	0.819	0.577	-0.053	0.958	19.000
4.179	0.772	0.577	-1.040	0.311	19.000
4.268	0.383	0.577	-0.088	0.930	19.000
4.458	0.635	0.577	0.052	0.959	19.000
5.046	0.749	0.577	-0.220	0.828	19.000
4.362	0.575	0.577	0.549	0.590	19.000
3.020	0.445	0.577	-0.079	0.938	19.000

4.337	0.456	0.577	0.228	0.822	19.000
2.926	0.958	0.577	1.023	0.319	19.000
2.386	0.517	0.577	1.456	0.162	19.000
2.054	0.495	0.577	1.076	0.296	19.000
6.074	0.419	0.577	0.198	0.845	19.000
3.815	0.724	0.577	0.887	0.386	19.000
4.312	0.623	0.577	-0.756	0.459	19.000
5.317	0.672	0.577	-0.238	0.815	19.000
4.049	0.821	0.577	-0.770	0.451	19.000
2.774	0.552	0.577	0.156	0.878	19.000
5.700	0.636	0.577	0.348	0.731	19.000
3.508	0.979	0.577	-0.262	0.796	19.000
3.606	1.178	0.577	0.579	0.570	19.000
5.686	0.456	0.577	1.157	0.262	19.000
2.347	0.523	0.577	1.013	0.324	19.000
2.831	0.898	0.577	0.521	0.608	19.000
6.693	0.436	0.577	-0.624	0.540	19.000
7.346	0.875	0.577	-0.235	0.817	19.000
4.965	0.498	0.577	-0.247	0.808	19.000
5.797	0.732	0.577	0.358	0.725	19.000
4.692	0.770	0.577	0.306	0.763	19.000
5.339	0.473	0.577	0.162	0.873	19.000
5.256	0.967	0.577	0.831	0.417	19.000
4.768	1.411	0.577	-0.096	0.925	19.000
5.841	0.505	0.577	2.328	0.031	19.000
2.764	0.489	0.577	-0.985	0.337	19.000
3.882	0.391	0.577	1.726	0.101	19.000
3.427	0.398	0.577	-0.159	0.875	19.000
3.894	0.747	0.577	-0.237	0.815	19.000
3.495	0.448	0.577	2.273	0.035	19.000
2.043	1.295	0.577	-0.466	0.647	19.000
5.584	1.133	0.577	0.687	0.500	19.000
5.788	0.677	0.577	0.236	0.816	19.000
5.777	0.336	0.577	3.223	0.004	19.000
7.080	0.529	0.577	-0.992	0.334	19.000
6.775	0.462	0.577	-0.107	0.916	19.000
5.103	0.758	0.577	-0.793	0.438	19.000
3.165	0.820	0.577	-0.144	0.887	19.000
3.650	0.687	0.577	-0.318	0.754	19.000
4.379	0.719	0.577	0.280	0.782	19.000
3.747	1.133	0.577	0.941	0.359	19.000
1.862	0.543	0.577	-0.406	0.689	19.000
6.950	0.387	0.577	-0.715	0.483	19.000
5.752	0.461	0.577	0.489	0.631	19.000
5.913	0.347	0.577	1.695	0.106	19.000
6.152	0.800	0.577	-0.555	0.585	19.000
4.161	0.420	0.577	1.584	0.130	19.000
3.413	0.517	0.577	-0.568	0.577	19.000
7.126	0.738	0.577	0.270	0.790	19.000
5.525	0.371	0.577	0.324	0.749	19.000

7.483	0.601	0.577	-1.234	0.232	19.000
3.234	0.412	0.577	-1.004	0.328	19.000
3.482	0.487	0.577	-0.621	0.542	19.000
4.782	0.351	0.577	0.560	0.582	19.000
8.378	0.810	0.577	0.142	0.889	19.000
5.496	1.055	0.577	-0.106	0.917	19.000
2.301	0.549	0.577	-1.162	0.260	19.000
3.373	0.475	0.577	-0.188	0.853	19.000
2.507	0.543	0.577	-0.853	0.404	19.000
6.660	0.353	0.577	0.966	0.346	19.000
5.150	0.621	0.577	-0.767	0.453	19.000
2.957	0.450	0.577	2.845	0.010	19.000
3.529	0.411	0.577	1.738	0.098	19.000
3.443	0.473	0.577	3.231	0.004	19.000
2.108	0.421	0.577	1.767	0.093	19.000
3.288	0.680	0.577	0.067	0.947	19.000
7.141	0.444	0.577	-0.333	0.742	19.000
2.945	0.399	0.577	0.258	0.799	19.000
3.304	0.446	0.577	-1.278	0.217	19.000
4.695	0.419	0.577	0.508	0.617	19.000
8.176	0.395	0.577	-0.200	0.844	19.000
2.121	0.556	0.577	1.294	0.211	19.000
2.900	1.145	0.577	0.841	0.411	19.000
4.593	0.440	0.577	-0.083	0.935	19.000
2.557	0.893	0.577	-0.776	0.448	19.000
6.172	0.428	0.577	0.725	0.478	19.000
3.088	0.460	0.577	-0.138	0.891	19.000
5.550	0.382	0.577	-0.623	0.541	19.000
4.258	0.374	0.577	-0.490	0.630	19.000
2.206	0.731	0.577	-0.313	0.758	19.000
5.230	0.444	0.577	0.786	0.442	19.000
2.112	0.455	0.577	-0.468	0.645	19.000
5.178	0.372	0.577	-0.985	0.337	19.000
5.059	0.485	0.577	2.167	0.043	19.000
4.253	0.613	0.577	-2.081	0.051	19.000
5.235	0.456	0.577	-2.116	0.048	19.000
2.345	0.535	0.577	-2.045	0.055	19.000
3.056	0.540	0.577	0.330	0.745	19.000
4.450	0.333	0.577	1.743	0.097	19.000
2.881	0.446	0.577	-2.210	0.040	19.000
4.897	0.617	0.577	-1.830	0.083	19.000
7.023	0.420	0.577	-0.347	0.732	19.000
4.683	0.487	0.577	0.296	0.770	19.000
1.790	0.799	0.577	2.063	0.053	19.000
2.129	0.711	0.577	1.529	0.143	19.000
4.979	0.476	0.577	0.340	0.738	19.000
4.362	0.408	0.577	-2.457	0.024	19.000
2.047	0.538	0.577	-0.204	0.841	19.000
4.420	0.314	0.577	1.922	0.070	19.000
3.085	0.513	0.577	-2.603	0.017	19.000

3.652	0.463	0.577	-0.419	0.680	19.000
2.961	0.421	0.577	0.474	0.641	19.000
2.659	0.489	0.577	-1.778	0.091	19.000
5.015	0.531	0.577	0.565	0.578	19.000
3.648	0.465	0.577	-1.588	0.129	19.000
2.030	0.569	0.577	-1.761	0.094	19.000
2.018	0.768	0.577	0.445	0.662	19.000
3.385	0.500	0.577	-0.374	0.713	19.000
3.573	0.553	0.577	-0.132	0.897	19.000
2.210	0.551	0.577	-2.271	0.035	19.000
3.424	0.614	0.577	0.093	0.927	19.000
2.287	0.545	0.577	-1.623	0.121	19.000
2.667	0.487	0.577	-1.593	0.128	19.000
3.891	0.416	0.577	-1.518	0.145	19.000
2.589	0.478	0.577	-1.094	0.288	19.000
2.194	0.637	0.577	-1.808	0.086	19.000
2.180	0.425	0.577	-3.254	0.004	19.000
3.135	0.454	0.577	-1.787	0.090	19.000
7.224	0.369	0.577	-0.104	0.918	19.000
1.639	0.521	0.577	-2.493	0.022	19.000
2.583	0.529	0.577	-0.914	0.372	19.000
1.910	0.380	0.577	-1.869	0.077	19.000
1.740	0.444	0.577	-0.644	0.528	19.000
3.560	0.496	0.577	0.457	0.653	19.000
4.207	0.447	0.577	2.668	0.015	19.000
4.079	0.492	0.577	-0.606	0.552	19.000
3.472	0.393	0.577	-1.320	0.203	19.000
5.567	0.541	0.577	1.901	0.073	19.000
2.243	0.498	0.577	-2.506	0.021	19.000
6.013	0.482	0.577	1.132	0.272	19.000
3.698	0.413	0.577	-0.157	0.877	19.000
3.265	0.516	0.577	0.647	0.526	19.000
1.708	0.836	0.577	0.367	0.718	19.000
3.195	0.531	0.577	2.864	0.010	19.000
9.778	1.030	0.577	2.597	0.018	19.000
3.172	0.619	0.577	1.027	0.317	19.000
2.303	0.561	0.577	0.193	0.849	19.000
4.612	0.586	0.577	0.818	0.423	19.000
2.376	0.710	0.577	0.233	0.818	19.000
3.829	0.603	0.577	-0.940	0.359	19.000
2.487	0.419	0.577	-1.253	0.225	19.000
2.262	0.472	0.577	0.237	0.815	19.000
5.126	0.475	0.577	-0.249	0.806	19.000
3.961	0.476	0.577	1.625	0.121	19.000
5.334	0.306	0.577	-0.676	0.507	19.000
2.868	0.420	0.577	1.307	0.207	19.000
4.918	0.694	0.577	0.103	0.919	19.000
2.927	0.440	0.577	0.659	0.518	19.000
3.568	0.401	0.577	0.412	0.685	19.000
6.737	0.408	0.577	2.188	0.041	19.000

1.543	0.483	0.577	-1.417	0.173	19.000
3.906	0.423	0.577	0.535	0.599	19.000
6.804	0.439	0.577	-0.194	0.848	19.000
3.821	0.705	0.577	2.802	0.011	19.000
2.748	0.420	0.577	1.221	0.237	19.000
4.085	0.433	0.577	1.691	0.107	19.000
4.824	0.336	0.577	0.441	0.664	19.000
2.345	0.605	0.577	-0.503	0.621	19.000
3.195	0.455	0.577	-2.182	0.042	19.000
5.643	0.492	0.577	1.110	0.281	19.000
7.040	0.818	0.577	1.776	0.092	19.000
5.078	0.416	0.577	-0.541	0.595	19.000
8.243	1.160	0.577	1.229	0.234	19.000
7.659	0.415	0.577	0.607	0.551	19.000
3.992	0.865	0.577	-0.641	0.529	19.000
4.677	1.108	0.577	-0.096	0.924	19.000
4.340	0.775	0.577	2.298	0.033	19.000
6.309	0.486	0.577	1.920	0.070	19.000
2.123	0.554	0.577	1.394	0.179	19.000
6.668	0.784	0.577	0.735	0.472	19.000
4.288	0.626	0.577	2.354	0.030	19.000
4.131	0.706	0.577	-0.941	0.358	19.000
2.737	0.450	0.577	0.897	0.381	19.000
5.119	0.705	0.577	1.902	0.072	19.000
3.269	0.515	0.577	1.965	0.064	19.000
2.727	0.514	0.577	3.472	0.003	19.000
3.551	0.536	0.577	2.271	0.035	19.000
5.834	0.563	0.577	1.180	0.252	19.000
6.747	0.676	0.577	2.352	0.030	19.000
4.972	0.655	0.577	0.539	0.596	19.000
4.447	0.463	0.577	1.672	0.111	19.000
3.868	0.419	0.577	-0.517	0.611	19.000
7.579	0.966	0.577	0.649	0.524	19.000
5.425	0.821	0.577	0.876	0.392	19.000
1.534	0.460	0.577	3.667	0.002	19.000
3.288	0.443	0.577	-0.815	0.425	19.000
6.742	0.504	0.577	1.534	0.142	19.000
4.096	0.350	0.577	1.306	0.207	19.000
6.173	0.309	0.577	1.008	0.326	19.000
9.414	0.660	0.577	1.816	0.085	19.000
4.647	0.751	0.577	-0.129	0.899	19.000
7.053	0.651	0.577	0.902	0.379	19.000
4.049	0.296	0.577	-0.359	0.724	19.000
4.085	0.458	0.577	0.396	0.697	19.000
5.156	0.468	0.577	0.803	0.432	19.000
4.433	0.727	0.577	1.860	0.078	19.000
6.135	0.737	0.577	1.892	0.074	19.000
4.411	0.465	0.577	0.690	0.499	19.000
6.369	0.536	0.577	2.465	0.023	19.000
5.261	0.565	0.577	2.154	0.044	19.000

10.708	0.562	0.577	0.136	0.894	19.000
4.734	0.407	0.577	2.388	0.027	19.000
4.444	0.332	0.577	-0.078	0.938	19.000
4.608	0.784	0.577	0.151	0.881	19.000
5.132	0.452	0.577	0.522	0.608	19.000
5.621	0.530	0.577	0.549	0.589	19.000
7.042	0.379	0.577	0.909	0.375	19.000
3.111	0.323	0.577	0.459	0.651	19.000
5.585	0.593	0.577	1.277	0.217	19.000
2.769	0.541	0.577	1.138	0.269	19.000
3.915	0.482	0.577	3.591	0.002	19.000
2.926	0.613	0.577	0.615	0.546	19.000
4.723	0.689	0.577	0.558	0.584	19.000
4.447	0.790	0.577	0.530	0.602	19.000
3.071	0.804	0.577	0.180	0.859	19.000
3.863	0.884	0.577	1.307	0.207	19.000
6.707	0.432	0.577	-0.651	0.523	19.000
4.764	0.749	0.577	-0.183	0.857	19.000
4.334	0.430	0.577	1.486	0.154	19.000
3.133	0.587	0.577	1.514	0.146	19.000
3.132	0.419	0.577	1.973	0.063	19.000
5.800	0.431	0.577	4.412	0.000	19.000
2.628	0.680	0.577	0.094	0.926	19.000
5.118	0.582	0.577	1.347	0.194	19.000
1.850	1.110	0.577	0.351	0.730	19.000
3.614	0.495	0.577	-1.474	0.157	19.000
6.357	0.697	0.577	0.282	0.781	19.000
6.692	0.707	0.577	-0.162	0.873	19.000
4.514	0.427	0.577	1.477	0.156	19.000
4.187	0.471	0.577	0.830	0.417	19.000
4.471	0.511	0.577	-0.884	0.388	19.000
5.455	0.426	0.577	0.147	0.884	19.000
4.268	0.571	0.577	-1.080	0.294	19.000
4.140	0.443	0.577	0.591	0.562	19.000
4.011	0.347	0.577	1.473	0.157	19.000
3.305	0.453	0.577	0.354	0.728	19.000
3.950	0.397	0.577	0.898	0.380	19.000
6.302	0.473	0.577	0.255	0.802	19.000
5.430	0.593	0.577	2.670	0.015	19.000
6.103	0.628	0.577	0.438	0.666	19.000
4.773	0.327	0.577	-1.357	0.191	19.000
2.726	0.420	0.577	1.449	0.164	19.000
2.382	0.467	0.577	0.910	0.374	19.000
4.574	0.452	0.577	-0.345	0.734	19.000
1.553	0.394	0.577	0.115	0.910	19.000
4.028	0.473	0.577	-0.317	0.755	19.000
6.363	0.499	0.577	0.231	0.820	19.000
3.028	0.392	0.577	-0.595	0.559	19.000
4.148	0.507	0.577	0.775	0.448	19.000
5.450	0.492	0.577	-1.160	0.260	19.000

3.695	0.437	0.577	1.309	0.206	19.000
3.998	0.862	0.577	-0.743	0.467	19.000
3.689	0.718	0.577	1.338	0.197	19.000
5.242	0.584	0.577	-0.055	0.957	19.000
4.397	0.476	0.577	1.772	0.092	19.000
5.019	0.424	0.577	0.967	0.346	19.000
7.816	0.599	0.577	0.664	0.515	19.000
4.710	0.504	0.577	1.451	0.163	19.000
4.881	0.578	0.577	-0.449	0.659	19.000
3.288	0.485	0.577	1.041	0.311	19.000
3.001	0.473	0.577	1.178	0.253	19.000
7.175	0.469	0.577	1.636	0.118	19.000
3.588	0.723	0.577	-0.235	0.817	19.000
4.436	0.902	0.577	0.583	0.567	19.000
3.894	0.796	0.577	0.846	0.408	19.000
2.959	0.504	0.577	-0.104	0.919	19.000
4.877	0.965	0.577	-0.711	0.486	19.000
4.520	0.564	0.577	-1.363	0.189	19.000
6.618	0.387	0.577	-2.248	0.037	19.000
3.660	0.392	0.577	1.902	0.072	19.000
2.825	0.360	0.577	0.410	0.686	19.000
3.546	0.457	0.577	-1.110	0.281	19.000
4.748	0.381	0.577	-0.767	0.453	19.000
4.589	0.520	0.577	-0.984	0.337	19.000
2.699	0.584	0.577	1.051	0.306	19.000
6.148	0.456	0.577	0.365	0.719	19.000
2.664	0.412	0.577	0.453	0.656	19.000
3.358	0.360	0.577	1.488	0.153	19.000
6.669	0.368	0.577	0.179	0.860	19.000
7.200	0.715	0.577	0.183	0.856	19.000
1.604	0.508	0.577	-1.289	0.213	19.000
4.852	0.520	0.577	0.327	0.747	19.000
6.400	0.470	0.577	1.434	0.168	19.000
3.213	0.526	0.577	1.047	0.308	19.000
4.361	0.462	0.577	-0.305	0.764	19.000
5.810	0.505	0.577	1.194	0.247	19.000
3.575	0.515	0.577	0.161	0.874	19.000
4.682	0.608	0.577	1.854	0.079	19.000
5.019	0.778	0.577	0.171	0.866	19.000
4.307	0.608	0.577	2.108	0.049	19.000
2.427	0.357	0.577	-0.143	0.888	19.000
5.793	0.410	0.577	0.207	0.838	19.000
4.861	0.397	0.577	1.828	0.083	19.000
3.293	0.429	0.577	-2.294	0.033	19.000
2.891	0.533	0.577	0.541	0.595	19.000
1.663	0.428	0.577	0.166	0.870	19.000
3.568	0.480	0.577	0.455	0.654	19.000
6.206	0.745	0.577	3.553	0.002	19.000
4.001	0.625	0.577	0.137	0.893	19.000
3.097	0.625	0.577	0.764	0.454	19.000

4.986	0.770	0.577	-0.442	0.664	19.000
3.763	0.437	0.577	-1.038	0.312	19.000
3.026	0.489	0.577	0.184	0.856	19.000
1.853	0.537	0.577	-1.953	0.066	19.000
3.641	0.445	0.577	0.540	0.596	19.000
2.708	0.405	0.577	0.491	0.629	19.000
6.802	0.417	0.577	-0.070	0.945	19.000
5.452	0.591	0.577	0.144	0.887	19.000
3.341	1.080	0.577	-0.179	0.860	19.000
5.937	0.531	0.577	-0.644	0.528	19.000
3.345	0.521	0.577	-4.484	0.000	19.000
1.708	0.519	0.577	-0.828	0.418	19.000
3.214	0.526	0.577	-0.138	0.891	19.000
3.172	0.976	0.577	-0.580	0.569	19.000
3.347	0.835	0.577	-0.113	0.912	19.000
7.373	1.265	0.577	0.920	0.369	19.000
6.487	1.159	0.577	1.607	0.125	19.000
5.169	0.350	0.577	-0.109	0.915	19.000
3.060	0.543	0.577	3.564	0.002	19.000
6.092	0.444	0.577	-0.655	0.521	19.000
6.105	0.432	0.577	2.293	0.033	19.000
3.450	0.400	0.577	-0.192	0.850	19.000
5.651	0.374	0.577	1.333	0.198	19.000
3.307	0.497	0.577	0.857	0.402	19.000
3.926	0.670	0.577	0.870	0.395	19.000
2.174	0.557	0.577	-1.296	0.211	19.000
3.775	0.390	0.577	0.648	0.525	19.000
4.627	0.453	0.577	0.095	0.925	19.000
3.583	0.528	0.577	1.161	0.260	19.000
3.704	0.830	0.577	0.453	0.656	19.000
2.953	0.758	0.577	-0.675	0.508	19.000
2.983	0.657	0.577	-0.614	0.546	19.000
2.100	0.744	0.577	0.540	0.595	19.000
2.423	0.578	0.577	0.511	0.615	19.000
6.409	1.370	0.577	-0.520	0.609	19.000
2.241	0.537	0.577	1.676	0.110	19.000
2.524	0.453	0.577	-0.118	0.908	19.000
2.236	0.407	0.577	1.938	0.068	19.000
2.196	0.639	0.577	-0.631	0.536	19.000
2.642	0.613	0.577	-0.458	0.652	19.000
3.912	0.602	0.577	0.542	0.594	19.000
2.672	0.371	0.577	-0.299	0.769	19.000
4.278	0.400	0.577	-0.512	0.615	19.000
6.708	0.417	0.577	1.189	0.249	19.000
7.368	0.626	0.577	0.453	0.656	19.000
6.996	0.645	0.577	1.406	0.176	19.000
3.829	0.586	0.577	-0.862	0.399	19.000
2.284	0.649	0.577	0.622	0.541	19.000
6.792	0.546	0.577	-0.384	0.705	19.000
4.249	0.463	0.577	-1.283	0.215	19.000

4.627	0.386	0.577	2.092	0.050	19.000
3.025	0.397	0.577	-0.507	0.618	19.000
4.496	0.435	0.577	0.869	0.396	19.000
3.842	0.460	0.577	-0.555	0.586	19.000
5.457	0.593	0.577	1.387	0.181	19.000
3.499	0.525	0.577	1.351	0.193	19.000
3.181	0.462	0.577	-0.846	0.408	19.000
8.716	0.472	0.577	0.324	0.749	19.000
6.087	0.343	0.577	1.511	0.147	19.000
3.364	0.418	0.577	0.079	0.938	19.000
4.287	0.532	0.577	1.113	0.280	19.000
4.269	0.614	0.577	-0.545	0.592	19.000
3.229	0.450	0.577	0.239	0.814	19.000
5.381	0.671	0.577	0.669	0.512	19.000
3.924	0.454	0.577	0.081	0.936	19.000
4.252	0.504	0.577	-1.106	0.283	19.000
4.178	1.082	0.577	-0.441	0.664	19.000
5.200	0.467	0.577	-0.697	0.494	19.000
5.029	0.873	0.577	0.467	0.646	19.000
3.033	0.400	0.577	0.859	0.401	19.000
5.796	0.543	0.577	-0.165	0.871	19.000
7.008	0.308	0.577	-0.153	0.880	19.000
5.336	0.485	0.577	1.144	0.267	19.000
5.914	0.730	0.577	0.082	0.935	19.000
2.862	0.408	0.577	0.707	0.488	19.000
2.580	0.381	0.577	0.798	0.435	19.000
3.236	0.473	0.577	-1.062	0.302	19.000
3.951	0.398	0.577	-1.640	0.117	19.000
2.473	0.429	0.577	-0.887	0.386	19.000
9.256	0.558	0.577	0.289	0.776	19.000
2.104	0.549	0.577	-0.493	0.627	19.000
5.623	0.610	0.577	1.085	0.291	19.000
3.222	0.409	0.577	0.322	0.751	19.000
5.173	0.713	0.577	-1.576	0.132	19.000
2.335	0.420	0.577	3.799	0.001	19.000
3.780	0.874	0.577	1.173	0.255	19.000
6.215	0.745	0.577	0.933	0.363	19.000
2.237	0.548	0.577	-0.689	0.499	19.000
2.720	0.573	0.577	-0.710	0.486	19.000
7.210	0.408	0.577	1.253	0.225	19.000
7.227	0.403	0.577	1.229	0.234	19.000
7.279	0.417	0.577	1.093	0.288	19.000
6.211	0.341	0.577	0.689	0.499	19.000
6.239	0.348	0.577	0.594	0.559	19.000
4.712	0.672	0.577	1.385	0.182	19.000
2.602	0.370	0.577	-2.187	0.041	19.000
9.062	0.533	0.577	0.931	0.363	19.000
3.728	0.578	0.577	0.106	0.917	19.000
5.436	0.584	0.577	0.525	0.606	19.000
5.689	0.391	0.577	1.363	0.189	19.000

3.836	0.448	0.577	0.677	0.507	19.000
5.161	0.408	0.577	1.271	0.219	19.000
5.979	0.365	0.577	2.180	0.042	19.000
8.292	0.387	0.577	-1.454	0.162	19.000
3.166	0.938	0.577	-0.284	0.779	19.000
3.625	0.799	0.577	0.097	0.923	19.000
6.117	0.393	0.577	-1.103	0.284	19.000
4.517	0.446	0.577	-0.543	0.593	19.000
2.125	0.603	0.577	3.183	0.005	19.000
2.869	0.676	0.577	-0.183	0.857	19.000
2.143	0.441	0.577	1.265	0.221	19.000
5.541	0.380	0.577	1.809	0.086	19.000
5.966	0.511	0.577	1.467	0.159	19.000
5.644	0.450	0.577	1.209	0.242	19.000
7.291	0.860	0.577	0.139	0.891	19.000
4.648	0.598	0.577	0.576	0.571	19.000
4.556	0.370	0.577	1.334	0.198	19.000
9.695	0.791	0.577	0.404	0.691	19.000
6.195	0.382	0.577	2.643	0.016	19.000
3.174	0.702	0.577	0.443	0.662	19.000
1.954	0.590	0.577	-0.060	0.953	19.000
4.543	0.569	0.577	-0.290	0.775	19.000
3.265	0.384	0.577	0.440	0.665	19.000
5.830	0.743	0.577	-0.062	0.951	19.000
4.919	0.549	0.577	-0.706	0.489	19.000
5.433	0.583	0.577	1.598	0.127	19.000
8.772	0.467	0.577	1.427	0.170	19.000
5.110	0.691	0.577	0.516	0.612	19.000
10.376	0.610	0.577	0.271	0.789	19.000
3.950	0.927	0.577	1.422	0.171	19.000
3.969	0.972	0.577	1.114	0.279	19.000
5.220	0.379	0.577	0.189	0.852	19.000
4.998	0.484	0.577	0.465	0.647	19.000
6.972	0.465	0.577	-2.540	0.020	19.000
8.455	0.640	0.577	0.242	0.811	19.000
4.702	0.592	0.577	-2.076	0.052	19.000
2.611	0.767	0.577	1.545	0.139	19.000
2.040	0.685	0.577	-0.423	0.677	19.000
1.556	0.549	0.577	0.476	0.640	19.000
6.829	0.555	0.577	0.976	0.341	19.000
6.075	0.682	0.577	0.338	0.739	19.000
4.434	0.368	0.577	3.636	0.002	19.000
7.796	0.592	0.577	-0.123	0.903	19.000
4.827	0.707	0.577	0.484	0.634	19.000
3.359	0.488	0.577	0.434	0.669	19.000
3.593	0.375	0.577	-0.291	0.774	19.000
2.298	0.549	0.577	0.988	0.335	19.000
6.661	0.412	0.577	0.542	0.594	19.000
5.705	0.350	0.577	2.627	0.017	19.000
1.566	0.413	0.577	0.554	0.586	19.000

3.874	0.550	0.577	0.333	0.743	19.000
5.155	0.704	0.577	2.767	0.012	19.000
2.353	0.401	0.577	0.409	0.687	19.000
4.972	0.483	0.577	-0.683	0.503	19.000
5.551	0.508	0.577	-0.121	0.905	19.000
1.985	0.404	0.577	0.764	0.454	19.000
3.699	0.402	0.577	1.363	0.189	19.000
3.703	0.536	0.577	0.744	0.466	19.000
2.556	0.471	0.577	-1.045	0.309	19.000
1.954	0.554	0.577	0.792	0.438	19.000
3.770	0.500	0.577	0.068	0.947	19.000
2.896	0.354	0.577	2.300	0.033	19.000
2.065	0.548	0.577	0.385	0.705	19.000
2.127	0.514	0.577	1.096	0.287	19.000
4.326	0.613	0.577	2.532	0.020	19.000
4.288	0.508	0.577	1.927	0.069	19.000
3.201	0.448	0.577	-1.255	0.225	19.000
6.780	0.321	0.577	2.410	0.026	19.000
5.189	0.768	0.577	0.384	0.705	19.000
4.054	0.689	0.577	0.277	0.784	19.000
4.129	0.453	0.577	-1.908	0.072	19.000
3.460	0.425	0.577	-0.324	0.750	19.000
3.199	0.460	0.577	0.391	0.700	19.000
3.331	0.475	0.577	0.454	0.655	19.000
2.778	0.458	0.577	0.052	0.959	19.000
4.488	0.447	0.577	-0.583	0.567	19.000
4.245	0.434	0.577	-1.131	0.272	19.000
5.894	0.562	0.577	2.550	0.020	19.000
6.431	0.402	0.577	1.586	0.129	19.000
3.499	0.498	0.577	-1.613	0.123	19.000
3.912	0.331	0.577	1.391	0.180	19.000
4.184	0.350	0.577	-0.855	0.403	19.000
4.132	0.425	0.577	0.266	0.793	19.000
5.659	0.331	0.577	0.585	0.565	19.000
5.496	0.402	0.577	2.182	0.042	19.000
2.863	0.402	0.577	0.497	0.625	19.000
6.596	0.667	0.577	0.771	0.450	19.000
2.182	0.750	0.577	-0.663	0.515	19.000
4.167	0.537	0.577	0.079	0.938	19.000
8.073	0.414	0.577	-0.065	0.949	19.000
4.857	0.305	0.577	2.606	0.017	19.000
5.158	0.368	0.577	0.761	0.456	19.000
5.112	0.403	0.577	1.177	0.254	19.000
2.226	0.436	0.577	0.090	0.929	19.000
5.179	0.584	0.577	-0.175	0.863	19.000
7.904	0.855	0.577	1.194	0.247	19.000
3.186	0.660	0.577	-1.205	0.243	19.000
8.546	1.019	0.577	-1.196	0.246	19.000
4.707	0.731	0.577	0.562	0.581	19.000
6.808	0.410	0.577	0.149	0.883	19.000

3.949	0.600	0.577	0.178	0.861	19.000
4.303	0.435	0.577	1.195	0.247	19.000
4.371	0.633	0.577	-1.923	0.070	19.000
1.746	0.720	0.577	-0.121	0.905	19.000
4.416	0.521	0.577	0.533	0.601	19.000
3.157	0.505	0.577	-1.157	0.262	19.000
5.403	0.656	0.577	2.246	0.037	19.000
7.913	0.449	0.577	-0.052	0.959	19.000
2.364	1.043	0.577	0.986	0.336	19.000
3.473	0.744	0.577	1.137	0.270	19.000
2.851	0.480	0.577	0.253	0.803	19.000
4.564	0.454	0.577	1.189	0.249	19.000
2.868	0.667	0.577	-1.418	0.172	19.000
1.523	0.432	0.577	0.686	0.501	19.000
2.577	0.581	0.577	0.575	0.572	19.000
6.403	0.546	0.577	0.167	0.869	19.000
7.175	0.562	0.577	0.712	0.485	19.000
4.014	0.410	0.577	0.089	0.930	19.000
6.575	0.756	0.577	0.451	0.657	19.000
5.500	0.403	0.577	-2.128	0.047	19.000
4.367	0.499	0.577	1.383	0.183	19.000
2.417	0.601	0.577	1.532	0.142	19.000
2.808	0.547	0.577	-0.161	0.874	19.000
5.345	0.629	0.577	0.316	0.755	19.000
2.825	0.640	0.577	1.496	0.151	19.000
2.645	0.483	0.577	-0.902	0.378	19.000
4.157	0.401	0.577	-0.797	0.435	19.000
5.729	0.328	0.577	0.627	0.538	19.000
4.717	0.362	0.577	0.538	0.597	19.000
3.564	0.462	0.577	-0.066	0.948	19.000
3.276	0.410	0.577	1.213	0.240	19.000
5.373	0.416	0.577	3.016	0.007	19.000
2.342	1.216	0.577	-0.313	0.758	19.000
4.570	0.351	0.577	0.475	0.640	19.000
2.024	0.699	0.577	0.111	0.913	19.000
4.333	0.542	0.577	0.908	0.375	19.000
4.053	0.398	0.577	1.114	0.279	19.000
3.060	0.482	0.577	-0.382	0.706	19.000
4.677	0.413	0.577	2.373	0.028	19.000
6.472	0.683	0.577	1.669	0.111	19.000
6.261	0.537	0.577	0.891	0.384	19.000
3.534	0.659	0.577	-0.512	0.615	19.000
3.815	0.447	0.577	0.637	0.531	19.000
4.399	0.835	0.577	0.160	0.874	19.000
2.228	0.465	0.577	-1.007	0.327	19.000
4.190	0.368	0.577	-0.201	0.843	19.000
3.963	0.372	0.577	-1.648	0.116	19.000
7.685	0.326	0.577	1.074	0.296	19.000
5.733	0.421	0.577	-0.451	0.657	19.000
5.472	0.422	0.577	1.679	0.109	19.000

2.594	0.691	0.577	0.623	0.541	19.000
6.614	0.506	0.577	-0.339	0.738	19.000
2.240	0.403	0.577	1.897	0.073	19.000
6.987	0.481	0.577	0.065	0.949	19.000
2.750	1.180	0.577	0.220	0.828	19.000
5.784	0.409	0.577	0.082	0.935	19.000
2.958	0.499	0.577	-0.935	0.361	19.000
4.333	0.425	0.577	1.227	0.235	19.000
8.465	0.583	0.577	0.837	0.413	19.000
5.981	0.596	0.577	0.760	0.457	19.000
5.457	0.545	0.577	0.251	0.805	19.000
3.232	0.887	0.577	-0.637	0.532	19.000
3.209	0.613	0.577	2.211	0.039	19.000
4.338	0.625	0.577	0.396	0.697	19.000
4.461	0.620	0.577	-0.225	0.825	19.000
4.085	0.512	0.577	0.942	0.358	19.000
3.551	0.565	0.577	1.672	0.111	19.000
3.893	0.411	0.577	1.355	0.191	19.000
2.714	0.400	0.577	0.277	0.785	19.000
5.271	0.429	0.577	-0.531	0.602	19.000
6.253	0.465	0.577	0.302	0.766	19.000
5.118	0.319	0.577	0.586	0.565	19.000
5.212	0.659	0.577	1.095	0.287	19.000
3.774	0.342	0.577	-0.135	0.894	19.000
2.854	0.461	0.577	-0.841	0.411	19.000
5.706	0.614	0.577	0.128	0.899	19.000
5.094	0.352	0.577	1.211	0.241	19.000
2.065	0.592	0.577	0.842	0.410	19.000
4.352	0.421	0.577	-0.219	0.829	19.000
3.025	0.464	0.577	-1.393	0.180	19.000
4.350	0.339	0.577	-0.537	0.597	19.000
2.863	0.556	0.577	-0.695	0.495	19.000
2.034	0.499	0.577	-0.116	0.909	19.000
7.432	0.387	0.577	-1.683	0.109	19.000
0.963	0.730	0.577	-0.354	0.727	19.000
6.357	0.649	0.577	1.386	0.182	19.000
1.204	0.586	0.577	1.107	0.282	19.000
3.884	0.380	0.577	0.857	0.402	19.000
2.320	0.496	0.577	-0.876	0.392	19.000
2.559	0.528	0.577	-0.479	0.638	19.000
1.568	0.399	0.577	-1.109	0.281	19.000
3.680	0.448	0.577	-0.756	0.459	19.000
1.869	0.508	0.577	-0.243	0.810	19.000
1.875	0.573	0.577	-0.116	0.909	19.000
2.548	0.481	0.577	-0.267	0.792	19.000
1.965	0.542	0.577	-0.653	0.521	19.000
2.506	0.526	0.577	1.125	0.275	19.000
0.942	0.742	0.577	-0.407	0.688	19.000
1.595	0.518	0.577	-0.838	0.412	19.000
2.452	0.435	0.577	-1.444	0.165	19.000

2.644	0.462	0.577	-0.618	0.544	19.000
3.615	0.565	0.577	1.431	0.169	19.000
2.320	0.667	0.577	0.411	0.686	19.000
3.346	0.624	0.577	-1.889	0.074	19.000
2.066	0.520	0.577	0.848	0.407	19.000
4.853	0.606	0.577	-1.560	0.135	19.000
2.612	0.560	0.577	2.301	0.033	19.000
3.328	0.580	0.577	0.070	0.945	19.000
2.688	0.486	0.577	-0.073	0.942	19.000
3.866	0.940	0.577	0.896	0.381	19.000
2.071	0.454	0.577	1.780	0.091	19.000
3.804	0.361	0.577	-1.244	0.229	19.000
4.555	0.495	0.577	-0.924	0.367	19.000
2.653	0.469	0.577	0.555	0.585	19.000
3.291	0.439	0.577	-1.575	0.132	19.000
3.536	0.442	0.577	-1.903	0.072	19.000
2.997	0.539	0.577	0.673	0.509	19.000
2.334	0.482	0.577	-1.048	0.308	19.000
1.979	0.743	0.577	0.537	0.597	19.000
2.323	0.375	0.577	0.761	0.456	19.000
3.933	0.392	0.577	1.208	0.242	19.000
3.339	0.463	0.577	-0.413	0.684	19.000
2.901	0.410	0.577	0.431	0.671	19.000
2.682	0.531	0.577	0.329	0.746	19.000
5.309	0.485	0.577	-0.152	0.880	19.000
3.207	0.391	0.577	0.270	0.790	19.000
1.763	0.394	0.577	0.195	0.847	19.000
2.695	0.420	0.577	-2.216	0.039	19.000
2.072	0.400	0.577	0.744	0.466	19.000
4.143	0.506	0.577	0.608	0.550	19.000
2.441	0.450	0.577	1.066	0.300	19.000
2.661	0.455	0.577	-0.534	0.599	19.000
4.387	0.376	0.577	-1.146	0.266	19.000
5.378	0.445	0.577	0.282	0.781	19.000
5.548	0.641	0.577	-0.366	0.718	19.000
3.571	0.386	0.577	-0.520	0.609	19.000
5.867	0.409	0.577	-0.634	0.534	19.000
6.512	0.376	0.577	1.442	0.166	19.000
1.801	0.441	0.577	-0.073	0.942	19.000
7.832	0.867	0.577	-0.245	0.809	19.000
4.330	0.552	0.577	1.195	0.247	19.000
3.904	0.397	0.577	0.378	0.710	19.000
5.452	0.421	0.577	2.183	0.042	19.000
5.545	0.654	0.577	2.760	0.012	19.000
4.730	0.452	0.577	0.728	0.476	19.000
2.471	0.447	0.577	0.888	0.386	19.000
4.285	0.386	0.577	0.753	0.461	19.000
6.680	0.459	0.577	1.517	0.146	19.000
6.492	0.593	0.577	2.652	0.016	19.000
3.617	0.464	0.577	-1.398	0.178	19.000

3.561	0.492	0.577	-0.552	0.587	19.000
6.251	0.442	0.577	1.563	0.135	19.000
5.212	0.736	0.577	-0.836	0.414	19.000
1.218	0.717	0.577	0.316	0.755	19.000
1.991	0.979	0.577	-0.247	0.807	19.000
5.726	0.503	0.577	-0.327	0.747	19.000
6.548	0.350	0.577	1.579	0.131	19.000
2.699	0.794	0.577	-0.663	0.515	19.000
5.375	0.329	0.577	2.506	0.021	19.000
5.002	0.435	0.577	1.618	0.122	19.000
6.367	0.598	0.577	1.067	0.299	19.000
4.320	0.927	0.577	0.189	0.852	19.000
2.328	0.907	0.577	0.352	0.729	19.000
2.803	0.612	0.577	-1.910	0.071	19.000
3.954	0.457	0.577	1.463	0.160	19.000
5.820	1.191	0.577	-0.161	0.874	19.000
7.254	0.566	0.577	2.414	0.026	19.000
2.728	0.617	0.577	1.694	0.107	19.000
4.056	0.471	0.577	0.664	0.515	19.000
3.496	0.647	0.577	0.574	0.573	19.000
2.965	0.680	0.577	1.367	0.188	19.000
9.938	0.635	0.577	0.513	0.614	19.000
2.275	0.545	0.577	3.209	0.005	19.000
3.421	0.574	0.577	-3.764	0.001	19.000
2.415	0.893	0.577	0.363	0.721	19.000
2.263	0.498	0.577	-3.457	0.003	19.000
3.631	0.492	0.577	-0.998	0.331	19.000
3.295	0.545	0.577	-0.140	0.890	19.000
2.889	0.367	0.577	-0.624	0.540	19.000
1.907	0.456	0.577	-0.879	0.390	19.000
7.589	0.409	0.577	0.629	0.537	19.000
4.044	0.454	0.577	0.665	0.514	19.000
7.633	0.355	0.577	0.631	0.535	19.000
5.744	0.595	0.577	0.927	0.365	19.000
8.162	0.882	0.577	1.371	0.186	19.000
8.184	0.464	0.577	-0.064	0.949	19.000
6.309	0.437	0.577	-0.391	0.700	19.000
4.555	0.593	0.577	0.338	0.739	19.000
4.723	0.726	0.577	1.592	0.128	19.000
4.249	0.436	0.577	0.900	0.379	19.000
4.410	0.396	0.577	-2.681	0.015	19.000
7.681	0.634	0.577	0.841	0.411	19.000
2.702	0.721	0.577	-0.193	0.849	19.000
1.411	0.573	0.577	-0.267	0.793	19.000
2.957	0.389	0.577	-0.553	0.587	19.000
4.175	0.496	0.577	-0.299	0.768	19.000
3.992	0.442	0.577	1.442	0.166	19.000
4.969	0.529	0.577	-0.512	0.615	19.000
5.334	0.526	0.577	-0.113	0.911	19.000
1.504	0.826	0.577	0.651	0.523	19.000

3.660	0.885	0.577	0.276	0.786	19.000
6.109	0.690	0.577	0.182	0.858	19.000
4.822	0.623	0.577	0.699	0.493	19.000
2.060	0.500	0.577	-0.613	0.547	19.000
5.702	0.423	0.577	0.995	0.332	19.000
2.121	0.359	0.577	0.509	0.616	19.000
4.221	0.569	0.577	1.930	0.069	19.000
3.370	0.529	0.577	0.454	0.655	19.000
5.101	0.685	0.577	0.937	0.361	19.000
4.203	0.501	0.577	-1.412	0.174	19.000
3.172	0.401	0.577	-1.480	0.155	19.000
8.381	0.746	0.577	-0.800	0.433	19.000
3.553	0.529	0.577	-0.213	0.833	19.000
4.299	0.442	0.577	2.654	0.016	19.000
3.116	0.491	0.577	0.337	0.740	19.000
1.047	0.464	0.577	-0.073	0.943	19.000
3.491	0.413	0.577	2.672	0.015	19.000
4.692	0.585	0.577	0.256	0.801	19.000
6.197	0.465	0.577	1.758	0.095	19.000
7.845	1.306	0.577	-0.123	0.903	19.000
2.129	0.448	0.577	2.570	0.019	19.000
1.477	0.643	0.577	-1.842	0.081	19.000
4.023	0.416	0.577	0.699	0.493	19.000
1.871	0.527	0.577	-0.256	0.801	19.000
6.296	0.350	0.577	0.416	0.682	19.000
5.224	0.447	0.577	-0.648	0.525	19.000
6.137	0.416	0.577	0.243	0.810	19.000
4.697	0.474	0.577	-0.520	0.609	19.000
2.015	0.422	0.577	-0.165	0.870	19.000
2.673	0.539	0.577	0.250	0.805	19.000
4.878	0.497	0.577	0.448	0.659	19.000
3.487	0.546	0.577	0.422	0.678	19.000
2.650	0.433	0.577	-2.357	0.029	19.000
3.881	0.509	0.577	-2.342	0.030	19.000
4.609	0.476	0.577	0.285	0.778	19.000
4.042	0.357	0.577	1.080	0.294	19.000
3.251	0.417	0.577	0.600	0.556	19.000
4.195	0.375	0.577	1.154	0.263	19.000
2.988	0.425	0.577	1.619	0.122	19.000
2.836	0.428	0.577	1.454	0.162	19.000
3.514	0.370	0.577	1.255	0.225	19.000
2.632	0.474	0.577	-0.432	0.670	19.000
2.829	0.406	0.577	0.451	0.657	19.000
6.126	0.371	0.577	-0.417	0.681	19.000
6.098	0.367	0.577	-0.338	0.739	19.000
4.693	0.476	0.577	2.427	0.025	19.000
9.122	0.657	0.577	-1.472	0.157	19.000
1.968	1.341	0.577	0.080	0.937	19.000
4.015	0.603	0.577	0.216	0.831	19.000
5.662	0.452	0.577	0.313	0.757	19.000

7.990	0.462	0.577	0.163	0.872	19.000
8.101	0.464	0.577	0.139	0.891	19.000
7.980	0.468	0.577	0.070	0.945	19.000
6.740	0.489	0.577	-0.164	0.871	19.000
6.949	0.307	0.577	-0.749	0.463	19.000
4.683	0.338	0.577	0.706	0.488	19.000
2.051	0.485	0.577	0.343	0.735	19.000
4.750	0.627	0.577	-0.809	0.428	19.000
2.865	0.424	0.577	-0.701	0.492	19.000
2.775	0.489	0.577	-0.234	0.817	19.000
3.996	0.521	0.577	0.519	0.610	19.000
2.501	0.390	0.577	1.305	0.208	19.000
4.469	0.569	0.577	-0.267	0.792	19.000
4.760	0.367	0.577	0.672	0.510	19.000
5.339	1.779	0.577	0.402	0.692	19.000
4.751	0.470	0.577	0.379	0.709	19.000
4.334	0.498	0.577	1.383	0.183	19.000
3.401	0.370	0.577	2.405	0.027	19.000
4.578	0.504	0.577	2.179	0.042	19.000
4.496	0.626	0.577	1.421	0.172	19.000
5.159	0.910	0.577	-1.423	0.171	19.000
5.052	0.900	0.577	-1.316	0.204	19.000
3.626	0.383	0.577	-0.473	0.642	19.000
2.432	0.527	0.577	3.278	0.004	19.000
5.223	0.458	0.577	-0.891	0.384	19.000
3.922	0.413	0.577	3.894	0.001	19.000
6.067	0.493	0.577	1.651	0.115	19.000
8.337	0.386	0.577	0.290	0.775	19.000
5.874	0.406	0.577	0.279	0.783	19.000
4.828	0.433	0.577	-0.386	0.704	19.000
5.151	0.384	0.577	-1.222	0.237	19.000
3.212	0.572	0.577	-0.124	0.903	19.000
5.436	0.379	0.577	1.218	0.238	19.000
2.627	0.845	0.577	0.584	0.566	19.000
2.653	0.369	0.577	0.720	0.481	19.000
6.553	0.404	0.577	0.112	0.912	19.000
5.464	0.546	0.577	0.359	0.723	19.000
2.015	0.378	0.577	0.882	0.389	19.000
2.261	0.675	0.577	0.188	0.853	19.000
2.936	0.480	0.577	-2.781	0.012	19.000
4.906	0.424	0.577	-0.091	0.928	19.000
1.177	0.911	0.577	0.174	0.863	19.000
4.428	0.471	0.577	1.338	0.197	19.000
5.268	0.721	0.577	0.143	0.888	19.000
5.279	0.435	0.577	-0.234	0.818	19.000
5.799	0.330	0.577	0.798	0.435	19.000
3.515	0.536	0.577	0.535	0.599	19.000
4.055	0.602	0.577	-0.809	0.429	19.000
2.124	0.616	0.577	0.746	0.465	19.000

4.976	0.566	0.577	-0.531	0.602	19.000
10.372	0.482	0.577	-1.432	0.168	19.000
7.466	0.506	0.577	-0.466	0.646	19.000
2.263	0.466	0.577	1.441	0.166	19.000
3.584	0.493	0.577	0.515	0.612	19.000
6.781	0.520	0.577	0.954	0.352	19.000
2.725	0.479	0.577	1.099	0.286	19.000
4.941	0.434	0.577	0.509	0.616	19.000
4.318	0.395	0.577	-1.419	0.172	19.000
4.858	0.366	0.577	0.811	0.427	19.000
1.859	0.432	0.577	0.505	0.619	19.000
5.257	0.421	0.577	-0.345	0.734	19.000
2.678	0.583	0.577	0.277	0.785	19.000
8.321	0.416	0.577	0.573	0.573	19.000
4.456	0.731	0.577	-0.249	0.806	19.000
4.747	0.851	0.577	0.779	0.446	19.000
2.381	0.689	0.577	1.646	0.116	19.000
4.000	0.511	0.577	-0.154	0.880	19.000
3.353	0.440	0.577	-0.404	0.691	19.000
3.318	0.518	0.577	-0.313	0.758	19.000
2.560	0.869	0.577	-1.054	0.305	19.000
3.047	0.522	0.577	0.979	0.340	19.000
5.456	0.458	0.577	1.673	0.111	19.000
6.724	0.861	0.577	0.146	0.885	19.000
3.614	0.476	0.577	0.714	0.484	19.000
6.026	0.327	0.577	0.490	0.630	19.000
2.645	0.498	0.577	-3.404	0.003	19.000
1.803	0.513	0.577	0.572	0.574	19.000
2.746	0.599	0.577	-1.085	0.291	19.000
3.228	0.500	0.577	-0.767	0.453	19.000
5.920	0.404	0.577	-0.145	0.886	19.000
3.622	0.661	0.577	0.486	0.633	19.000
5.066	0.681	0.577	-0.109	0.915	19.000
3.119	0.749	0.577	0.410	0.686	19.000
4.181	0.431	0.577	0.433	0.670	19.000
2.514	0.518	0.577	0.870	0.395	19.000
1.516	0.592	0.577	0.399	0.694	19.000
2.876	0.430	0.577	1.020	0.321	19.000
2.992	0.840	0.577	0.193	0.849	19.000
2.654	0.889	0.577	0.072	0.944	19.000
5.412	0.469	0.577	0.414	0.683	19.000
4.104	0.475	0.577	-0.057	0.955	19.000
2.463	0.766	0.577	-0.869	0.396	19.000
1.826	0.410	0.577	0.080	0.937	19.000
2.798	0.515	0.577	-1.822	0.084	19.000
2.749	0.437	0.577	-0.571	0.574	19.000
1.568	0.456	0.577	-0.293	0.772	19.000
3.632	0.726	0.577	0.387	0.703	19.000
5.535	0.325	0.577	-0.879	0.390	19.000
1.790	0.428	0.577	-1.011	0.325	19.000

1.850	0.502	0.577	1.562	0.135	19.000
3.328	0.346	0.577	-0.416	0.682	19.000
2.725	0.353	0.577	-0.424	0.676	19.000
5.985	0.734	0.577	-0.322	0.751	19.000
6.373	0.435	0.577	0.260	0.797	19.000
3.052	0.429	0.577	1.040	0.311	19.000
2.348	0.927	0.577	-2.081	0.051	19.000
2.330	0.478	0.577	-2.017	0.058	19.000
4.262	0.487	0.577	0.745	0.465	19.000
1.387	0.904	0.577	-2.606	0.017	19.000
2.410	0.571	0.577	-3.232	0.004	19.000
2.331	0.454	0.577	-2.138	0.046	19.000
2.696	0.548	0.577	-1.653	0.115	19.000
2.851	0.567	0.577	-1.979	0.063	19.000
3.691	0.673	0.577	-2.966	0.008	19.000
2.144	0.505	0.577	-3.540	0.002	19.000
7.705	0.437	0.577	0.724	0.478	19.000
7.172	0.315	0.577	1.353	0.192	19.000
4.571	0.457	0.577	1.298	0.210	19.000
7.693	0.649	0.577	1.203	0.244	19.000
4.820	0.364	0.577	1.025	0.318	19.000
5.040	0.385	0.577	0.971	0.344	19.000
5.854	0.379	0.577	-0.315	0.756	19.000
4.621	0.320	0.577	0.466	0.647	19.000
4.857	0.356	0.577	-1.441	0.166	19.000
2.781	0.368	0.577	-0.331	0.745	19.000
8.845	0.327	0.577	-0.069	0.945	19.000
3.232	0.402	0.577	-1.603	0.125	19.000
1.710	0.437	0.577	1.456	0.162	19.000
2.262	0.442	0.577	1.376	0.185	19.000
2.489	0.418	0.577	-2.422	0.026	19.000
2.196	0.737	0.577	2.371	0.028	19.000
3.157	0.424	0.577	-1.236	0.232	19.000
9.018	0.391	0.577	0.179	0.860	19.000
5.320	1.165	0.577	-0.370	0.715	19.000
5.539	0.403	0.577	0.880	0.390	19.000
3.080	0.466	0.577	-0.215	0.832	19.000
2.097	0.553	0.577	-0.422	0.678	19.000
2.977	0.548	0.577	-0.924	0.367	19.000
7.411	0.348	0.577	-0.847	0.408	19.000
2.624	0.546	0.577	-0.310	0.760	19.000
3.275	0.931	0.577	0.141	0.889	19.000
4.635	0.619	0.577	1.140	0.269	19.000
8.124	0.415	0.577	-0.809	0.429	19.000
8.622	0.574	0.577	0.640	0.530	19.000
5.863	0.431	0.577	2.998	0.007	19.000
5.985	0.518	0.577	1.508	0.148	19.000
6.112	0.384	0.577	0.453	0.656	19.000
6.992	0.654	0.577	0.155	0.878	19.000
4.748	0.614	0.577	0.986	0.336	19.000

3.083	0.336	0.577	-0.644	0.528	19.000
2.263	0.426	0.577	2.401	0.027	19.000
3.468	0.486	0.577	1.197	0.246	19.000
3.315	0.541	0.577	0.480	0.637	19.000
4.287	0.415	0.577	1.488	0.153	19.000
4.754	0.411	0.577	1.296	0.210	19.000
3.144	0.619	0.577	0.772	0.449	19.000
3.986	0.530	0.577	-0.344	0.735	19.000
5.230	0.383	0.577	-0.820	0.422	19.000
4.270	0.499	0.577	1.953	0.066	19.000
3.142	0.510	0.577	0.135	0.894	19.000
5.778	0.493	0.577	-0.070	0.945	19.000
3.464	0.575	0.577	-0.270	0.790	19.000
3.280	0.503	0.577	-0.070	0.945	19.000
2.864	0.503	0.577	0.294	0.772	19.000
5.943	0.459	0.577	-0.372	0.714	19.000
5.502	0.331	0.577	1.687	0.108	19.000
2.695	0.572	0.577	1.299	0.209	19.000
2.250	0.465	0.577	0.533	0.600	19.000
2.408	0.531	0.577	0.412	0.685	19.000
1.892	0.766	0.577	-0.192	0.850	19.000
2.442	0.346	0.577	1.328	0.200	19.000
0.944	0.613	0.577	0.556	0.585	19.000
7.405	0.508	0.577	0.952	0.353	19.000
4.365	0.901	0.577	0.346	0.733	19.000
7.736	0.431	0.577	-0.309	0.761	19.000
6.657	0.579	0.577	0.109	0.914	19.000
2.770	0.879	0.577	-0.953	0.353	19.000
2.454	0.571	0.577	1.543	0.139	19.000
3.682	1.030	0.577	0.486	0.633	19.000
5.587	0.553	0.577	2.459	0.024	19.000
5.657	0.504	0.577	-1.120	0.277	19.000
7.086	0.468	0.577	-0.463	0.648	19.000
3.828	0.465	0.577	0.250	0.805	19.000
4.238	0.978	0.577	-0.558	0.583	19.000
5.373	0.351	0.577	2.511	0.021	19.000
1.914	0.931	0.577	-3.672	0.002	19.000
9.970	0.344	0.577	0.608	0.551	19.000
4.801	0.395	0.577	-0.517	0.611	19.000
3.123	0.386	0.577	2.092	0.050	19.000
4.677	0.465	0.577	0.346	0.733	19.000
6.349	0.407	0.577	2.282	0.034	19.000
6.906	0.518	0.577	-1.633	0.119	19.000
2.547	0.498	0.577	0.428	0.674	19.000
9.317	0.513	0.577	0.061	0.952	19.000
5.494	0.470	0.577	1.684	0.109	19.000
5.896	0.510	0.577	0.587	0.564	19.000
4.003	0.434	0.577	-0.239	0.814	19.000
5.565	0.412	0.577	1.145	0.266	19.000
3.171	0.625	0.577	0.298	0.769	19.000

4.321	0.478	0.577	1.052	0.306	19.000
5.900	0.358	0.577	-0.087	0.932	19.000
5.913	0.383	0.577	0.287	0.778	19.000
2.250	0.478	0.577	-0.308	0.762	19.000
4.278	0.340	0.577	0.461	0.650	19.000
5.478	0.594	0.577	1.648	0.116	19.000
2.915	0.664	0.577	0.382	0.707	19.000
7.098	0.416	0.577	-1.617	0.122	19.000
5.396	0.339	0.577	-0.102	0.920	19.000
3.985	0.446	0.577	-1.690	0.107	19.000
2.430	0.377	0.577	-1.913	0.071	19.000
5.835	0.393	0.577	-1.035	0.314	19.000
6.317	0.415	0.577	0.073	0.943	19.000
6.098	0.410	0.577	0.605	0.552	19.000
6.597	0.348	0.577	-0.451	0.657	19.000
2.966	0.401	0.577	1.668	0.112	19.000
3.502	0.604	0.577	1.709	0.104	19.000
1.936	0.528	0.577	2.687	0.015	19.000
3.285	1.035	0.577	1.430	0.169	19.000
3.410	0.593	0.577	2.411	0.026	19.000
4.265	0.536	0.577	0.435	0.668	19.000
7.158	0.385	0.577	1.312	0.205	19.000
7.010	0.376	0.577	2.065	0.053	19.000
6.466	0.362	0.577	-0.148	0.884	19.000
2.572	0.797	0.577	-1.351	0.192	19.000
3.221	0.501	0.577	0.757	0.459	19.000
4.892	0.644	0.577	0.717	0.482	19.000
6.641	1.480	0.577	0.973	0.343	19.000
4.920	0.578	0.577	0.862	0.400	19.000
3.872	0.400	0.577	2.422	0.026	19.000
4.011	0.730	0.577	1.806	0.087	19.000
6.472	0.772	0.577	2.086	0.051	19.000
2.653	0.494	0.577	-1.040	0.311	19.000
2.972	0.532	0.577	3.142	0.005	19.000
8.197	0.711	0.577	1.014	0.323	19.000
5.678	0.789	0.577	-0.970	0.344	19.000
4.156	0.478	0.577	0.439	0.666	19.000
3.019	0.408	0.577	-1.988	0.061	19.000
8.966	0.327	0.577	1.307	0.207	19.000
4.991	0.456	0.577	0.548	0.590	19.000
2.449	0.650	0.577	-2.320	0.032	19.000
1.522	0.762	0.577	-2.471	0.023	19.000
3.603	0.308	0.577	-4.253	0.000	19.000
3.594	0.436	0.577	-0.256	0.800	19.000
8.502	0.523	0.577	-0.196	0.847	19.000
5.461	0.352	0.577	0.702	0.491	19.000
2.672	0.561	0.577	-1.436	0.167	19.000
3.029	0.484	0.577	-0.888	0.385	19.000
5.323	0.508	0.577	-0.402	0.692	19.000
5.167	0.425	0.577	0.097	0.924	19.000

5.858	0.607	0.577	1.558	0.136	19.000
3.617	0.457	0.577	-2.446	0.024	19.000
3.740	0.717	0.577	1.018	0.322	19.000
2.179	0.723	0.577	0.676	0.507	19.000
3.423	0.728	0.577	1.054	0.305	19.000
6.664	0.468	0.577	0.448	0.659	19.000
6.381	0.391	0.577	-0.635	0.533	19.000
6.234	0.602	0.577	-0.293	0.773	19.000
2.841	0.367	0.577	3.373	0.003	19.000
2.401	0.515	0.577	0.730	0.474	19.000
5.262	0.525	0.577	-1.272	0.219	19.000
4.638	0.545	0.577	-1.930	0.069	19.000
3.317	0.654	0.577	-1.935	0.068	19.000
2.165	0.451	0.577	-0.506	0.619	19.000
5.687	0.393	0.577	0.539	0.596	19.000
4.364	0.451	0.577	-0.201	0.843	19.000
5.421	0.383	0.577	0.326	0.748	19.000
5.260	0.613	0.577	1.086	0.291	19.000
5.273	0.443	0.577	0.421	0.679	19.000
4.785	0.576	0.577	-1.192	0.248	19.000
3.583	0.397	0.577	0.056	0.956	19.000
2.805	0.458	0.577	1.445	0.165	19.000
4.668	0.313	0.577	3.182	0.005	19.000
4.358	1.031	0.577	0.409	0.687	19.000
7.075	0.314	0.577	1.238	0.231	19.000
6.659	0.606	0.577	1.025	0.318	19.000
2.232	0.382	0.577	-0.371	0.715	19.000
5.175	0.593	0.577	1.373	0.186	19.000
4.797	0.516	0.577	-1.744	0.097	19.000
4.053	0.984	0.577	-0.901	0.379	19.000
2.900	0.835	0.577	-0.377	0.710	19.000
8.543	0.395	0.577	1.201	0.244	19.000
3.062	0.511	0.577	2.657	0.016	19.000
3.516	0.485	0.577	2.137	0.046	19.000
2.377	0.353	0.577	1.771	0.093	19.000
4.200	0.636	0.577	0.848	0.407	19.000
4.610	0.436	0.577	1.233	0.233	19.000
8.862	0.459	0.577	0.736	0.471	19.000
7.654	0.566	0.577	0.722	0.479	19.000
2.285	0.464	0.577	2.693	0.014	19.000
5.496	0.849	0.577	0.459	0.652	19.000
2.606	0.402	0.577	0.083	0.935	19.000
8.246	0.723	0.577	-0.048	0.963	19.000
7.417	0.450	0.577	1.021	0.320	19.000
4.512	0.796	0.577	-0.453	0.656	19.000
7.219	0.470	0.577	0.756	0.459	19.000
5.700	0.357	0.577	2.021	0.058	19.000
3.521	0.468	0.577	-1.572	0.132	19.000
7.249	0.367	0.577	0.337	0.739	19.000
3.444	0.553	0.577	-0.213	0.834	19.000

6.818	0.441	0.577	1.322	0.202	19.000
3.766	0.428	0.577	0.660	0.517	19.000
4.125	0.454	0.577	-0.125	0.902	19.000
7.072	0.789	0.577	0.060	0.953	19.000
3.976	0.476	0.577	0.307	0.762	19.000
6.150	0.350	0.577	-0.316	0.756	19.000
3.782	0.412	0.577	-0.773	0.449	19.000
3.716	0.483	0.577	0.196	0.847	19.000
6.755	0.682	0.577	2.088	0.050	19.000
5.000	0.380	0.577	1.856	0.079	19.000
8.193	0.491	0.577	1.450	0.163	19.000
1.853	0.673	0.577	1.873	0.077	19.000
2.950	0.715	0.577	1.802	0.087	19.000
3.015	0.497	0.577	-0.555	0.586	19.000
3.159	0.881	0.577	0.713	0.485	19.000
2.398	0.672	0.577	-0.367	0.718	19.000
1.893	1.152	0.577	0.685	0.502	19.000
7.649	0.454	0.577	0.339	0.738	19.000
5.888	0.449	0.577	2.476	0.023	19.000
5.835	0.699	0.577	-1.268	0.220	19.000
4.976	0.486	0.577	-0.850	0.406	19.000
6.005	0.410	0.577	0.413	0.684	19.000
2.470	0.414	0.577	1.087	0.291	19.000
4.194	0.449	0.577	-1.106	0.283	19.000
3.851	0.469	0.577	-1.255	0.225	19.000
6.041	0.626	0.577	-0.280	0.782	19.000
3.194	0.554	0.577	-0.713	0.484	19.000
10.340	0.652	0.577	0.273	0.788	19.000
6.411	0.973	0.577	-0.127	0.900	19.000
7.785	0.457	0.577	0.788	0.440	19.000
5.426	0.478	0.577	1.228	0.234	19.000
7.564	0.866	0.577	0.164	0.871	19.000
3.432	0.374	0.577	1.264	0.221	19.000
5.330	0.461	0.577	-1.282	0.215	19.000
2.822	0.874	0.577	-0.414	0.684	19.000
2.945	0.426	0.577	-0.691	0.498	19.000
4.156	0.478	0.577	1.198	0.246	19.000
4.064	0.421	0.577	1.062	0.302	19.000
3.695	0.401	0.577	1.373	0.186	19.000
4.366	0.701	0.577	1.134	0.271	19.000
2.345	0.589	0.577	-1.430	0.169	19.000
3.660	0.516	0.577	-0.096	0.924	19.000
6.326	0.507	0.577	-0.450	0.657	19.000
[truncated: 403,258 more chars]
