# Supplementary material for: Amyotrophic lateral sclerosis, gene deregulation in the anterior horn of the spinal cord and frontal cortex area 8: implications in frontotemporal lobar degeneration
Source: Aging (Albany NY). 2017 Mar 9;9(3):823–51. doi: 10.18632/aging.101195 (PMC5391234; doi:10.18632/aging.101195)
Supplement: Supplementary file 3 [file aging-09-823-s003.pdf]

Gene symbol	logFC	Fold Change	AveExpr	t
A1BG-AS1	0.053	1.037	3.668	0.173
A2M//HDAC1	-0.100	0.933	7.771	-0.327
A2M-AS1	-0.433	0.741	4.273	-1.560
A2ML1	-0.258	0.836	2.737	-0.927
AACS	0.497	1.412	5.535	1.840
AAED1	0.359	1.283	4.270	1.413
AAGAB	0.445	1.362	4.495	2.058
AAK1	0.592	1.508	7.244	2.410
AASDHPPT	0.392	1.312	5.760	1.747
AASS	-0.543	0.686	6.842	-1.927
AATK	-0.449	0.733	6.203	-1.669
ABCA1	-1.034	0.488	5.808	-2.789
ABCA10	0.527	1.441	2.297	1.943
ABCA17P	0.162	1.119	2.505	0.541
ABCA2	-0.923	0.527	9.158	-2.783
ABCA5	0.240	1.181	6.465	0.980
ABCA6	-1.673	0.314	5.813	-2.552
ABCA8	-1.552	0.341	6.900	-3.431
ABCA9	-0.372	0.772	5.280	-1.006
ABCC1	-0.200	0.870	5.556	-0.936
ABCC11	0.109	1.078	2.134	0.445
ABCC3	-0.471	0.721	3.902	-0.902
ABCC4	-0.454	0.730	5.072	-1.594
ABCC5	0.193	1.143	6.195	0.882
ABCC8	0.231	1.174	4.582	0.819
ABCC9	0.561	1.475	6.267	1.678
ABCD2	1.043	2.061	5.017	2.493
ABCD4	-0.270	0.829	4.718	-1.183
ABCG1	-0.102	0.932	5.472	-0.431
ABCG2	-0.297	0.814	5.791	-0.888
ABCG4	0.853	1.806	4.414	2.278
ABHD12B//MIR4454	0.981	1.974	3.146	3.135
ABHD13	0.375	1.297	5.667	1.934
ABHD15	-0.871	0.547	3.657	-3.084
ABHD16B	0.171	1.126	2.402	0.798
ABHD17A	0.341	1.266	2.873	1.323
ABHD17B	-0.301	0.812	6.296	-1.045
ABHD3	-0.557	0.680	4.498	-2.015
ABHD4	-0.374	0.771	5.625	-1.199
ABHD5	-0.495	0.709	4.976	-1.820
ABHD8	0.747	1.678	5.807	2.048
ABI3	-0.532	0.691	4.734	-1.513
ABI3BP	-0.740	0.599	5.402	-1.832
ABL1	-0.564	0.676	7.261	-2.773
ABLIM2	0.768	1.703	5.351	1.998
ABLIM3	-0.106	0.929	6.305	-0.411
ABRACL	0.587	1.503	3.256	1.972
ABT1	0.040	1.028	4.191	0.170
ABTB1	0.403	1.322	3.754	1.693

ABTB2	-0.876	0.545	5.749	-2.203
AC002117.1	-0.506	0.704	4.514	-1.773
AC002310.12	-0.440	0.737	3.805	-1.515
AC002310.7	0.004	1.003	2.511	0.017
AC002429.5	0.152	1.111	3.586	0.487
AC004540.4	0.151	1.110	2.952	0.464
AC004951.6	-0.469	0.723	5.354	-1.837
AC005220.3	-0.428	0.743	2.299	-1.315
AC005237.4	-0.112	0.925	2.093	-0.321
AC005518.2	-0.362	0.778	4.455	-0.731
AC006003.3	0.285	1.219	3.375	1.003
AC006070.12	0.512	1.426	4.592	1.911
AC006160.5	-0.402	0.757	3.770	-1.566
AC006947.1	-0.273	0.827	2.863	-1.044
AC007036.4	-0.499	0.708	4.274	-1.450
AC007098.1	0.803	1.745	2.363	2.824
AC007106.1	-0.083	0.944	2.755	-0.270
AC007383.3	-0.036	0.975	3.021	-0.132
AC008269.2	0.580	1.495	4.640	1.642
AC008277.1	-0.342	0.789	3.666	-1.206
AC008440.10	-0.899	0.536	2.785	-3.080
AC008440.5	0.054	1.038	3.147	0.195
AC008746.3	-0.241	0.846	2.486	-0.885
AC009110.1	-1.410	0.376	2.183	-1.979
AC009262.2	0.393	1.313	2.875	1.400
AC009495.2	-0.214	0.862	2.371	-0.885
AC009495.3	0.237	1.179	3.163	0.922
AC011196.3	0.257	1.195	2.444	0.821
AC011247.3	0.224	1.168	2.200	0.949
AC011330.5//PPIP5K1	0.673	1.595	3.255	2.463
AC011891.5	-0.689	0.620	2.313	-1.429
AC011995.1	0.699	1.623	3.483	1.947
AC012074.2//LOC100131510	0.350	1.274	3.103	1.312
AC012442.6	-0.376	0.770	2.917	-1.337
AC012507.4	0.272	1.207	2.363	0.937
AC012593.1	-0.811	0.570	2.322	-1.865
AC015936.3	-0.458	0.728	5.909	-1.411
AC016745.3	0.275	1.210	3.716	1.032
AC017002.2	0.605	1.521	2.197	2.188
AC018685.1	0.313	1.242	2.896	1.205
AC018816.3	0.022	1.015	2.564	0.072
AC020594.5	-0.740	0.599	2.258	-2.683
AC023481.1	-1.460	0.364	3.740	-2.765
AC025165.8	0.611	1.527	2.459	2.457
AC026202.3	-0.600	0.660	2.534	-1.832
AC053503.6	0.495	1.410	2.617	0.856
AC061961.2	0.411	1.329	3.113	1.584
AC067956.1	0.940	1.918	2.470	2.546
AC067961.1	0.439	1.356	2.323	1.523
AC068490.1	1.479	2.788	2.391	2.891

AC068580.5	0.076	1.054	3.923	0.288
AC068580.6	-0.005	0.996	2.394	-0.020
AC073283.7	-0.413	0.751	3.713	-1.651
AC073834.3	-0.147	0.903	2.601	-0.494
AC073842.19	0.467	1.382	2.455	1.523
AC074183.4	-0.257	0.837	3.293	-0.846
AC074363.1//B3GALT1	0.917	1.889	3.368	3.200
AC078852.2	-0.358	0.780	2.802	-1.398
AC078883.4	0.344	1.269	2.737	1.450
AC079154.1	0.923	1.897	2.959	2.922
AC079354.5	0.036	1.026	2.240	0.153
AC079779.4	0.057	1.040	2.400	0.238
AC079790.2	1.132	2.192	1.519	3.938
AC083899.3	0.244	1.184	5.425	0.742
AC084149.2	-0.305	0.810	2.577	-0.940
AC090044.2	-0.482	0.716	4.578	-1.358
AC091729.8	-0.517	0.699	3.424	-1.969
AC092168.2	0.124	1.090	3.192	0.419
AC092171.4	0.211	1.158	2.362	0.835
AC092661.1	1.861	3.632	2.372	4.017
AC092675.3	-0.272	0.828	2.548	-0.937
AC092835.2	-0.063	0.957	5.260	-0.309
AC093110.3	-0.560	0.678	3.305	-1.822
AC093388.3	-0.514	0.700	5.549	-2.112
AC093843.1	0.560	1.474	1.829	1.646
AC102953.6	-0.435	0.740	2.340	-1.728
AC103564.7	0.293	1.225	2.259	0.999
AC107072.2	-0.454	0.730	3.060	-0.846
AC109826.1//LOC101927070	-0.147	0.903	3.377	-0.536
AC113617.1	1.154	2.225	1.834	3.364
AC114730.2	0.140	1.102	3.125	0.459
AC114730.7	-0.051	0.965	2.324	-0.168
AC114877.3	0.233	1.175	4.062	0.693
AC123886.2	0.281	1.215	3.154	0.884
AC140481.4	-0.586	0.666	2.300	-1.443
AC144449.1//LOC101929231	-0.472	0.721	3.858	-1.677
AC144835.1	-1.209	0.433	2.649	-1.974
AC159540.1//LOC100506123	0.152	1.111	5.848	0.410
AC159540.2	-0.038	0.974	2.940	-0.114
ACACB	-0.336	0.792	5.301	-1.413
ACAP1	0.025	1.018	2.364	0.090
ACAP2-IT1	0.748	1.680	4.178	2.361
ACAT2	0.203	1.151	5.767	0.687
ACBD7	-0.063	0.957	6.707	-0.223
ACCS	-0.412	0.752	3.703	-1.850
ACER2	0.194	1.144	4.122	0.731
ACHE	-0.330	0.795	4.853	-0.945
ACKR1//DARC	0.657	1.576	4.827	1.740
ACKR3	0.008	1.006	3.786	0.028
ACLY	0.109	1.078	8.268	0.387

ACOT2	0.131	1.095	4.868	0.497
ACOX2	-0.521	0.697	3.930	-2.088
ACP5	-0.059	0.960	2.705	-0.225
ACPP	-0.233	0.851	3.097	-0.508
ACSBG1	-0.215	0.862	5.859	-0.769
ACSF2	-0.138	0.908	3.818	-0.661
ACSL1	-0.674	0.627	7.092	-1.757
ACSL4	0.295	1.227	7.376	1.410
ACSL4	0.082	1.058	2.346	0.313
ACSL5	0.543	1.457	5.348	2.107
ACSL6//LOC728637//AC034228.7	0.668	1.589	3.736	2.205
ACSM5	0.005	1.004	4.847	0.018
ACSS1	-0.146	0.904	5.329	-0.600
ACSS3	0.235	1.177	6.228	0.935
ACTA1	0.329	1.256	2.688	1.129
ACTA2	0.217	1.162	3.404	0.547
ACTA2-AS1	0.535	1.449	3.341	1.930
ACTL6A	-0.463	0.725	4.024	-1.823
ACTL6B	0.970	1.959	4.456	2.424
ACTN1	0.786	1.724	4.334	2.010
ACTN2	-0.202	0.869	6.026	-0.605
ACTR10	0.294	1.226	6.153	1.431
ACTR1B	0.483	1.398	6.252	1.996
ACTR3B	0.323	1.251	5.503	0.931
ACTR6	0.315	1.244	5.569	1.576
ACTRT3	-0.291	0.817	3.075	-1.165
ACVR1B	0.664	1.584	5.096	2.494
ACVR1C	0.322	1.250	4.310	1.071
ACVR2A	0.369	1.291	4.720	1.753
ACVRL1	0.304	1.234	3.491	1.191
ACY3	-1.047	0.484	4.157	-2.310
ACYP2//LOC101927144	-0.774	0.585	5.007	-2.993
ADAM11	1.181	2.268	4.601	3.128
ADAM12	-0.057	0.961	3.774	-0.221
ADAM15	-0.103	0.931	4.414	-0.415
ADAM1A	0.065	1.046	4.317	0.296
ADAM20	0.325	1.253	3.972	1.191
ADAM20P1	0.336	1.262	4.607	1.071
ADAM22	0.493	1.407	7.233	1.974
ADAM23	0.909	1.878	7.424	1.880
ADAM28	-0.908	0.533	6.105	-2.044
ADAM33	0.017	1.012	2.990	0.055
ADAM8	0.027	1.019	2.849	0.120
ADAMTS1	-0.257	0.837	5.375	-0.873
ADAMTS12	-0.950	0.518	2.602	-2.458
ADAMTS14	0.075	1.053	2.921	0.243
ADAMTS15	-0.012	0.992	2.679	-0.030
ADAMTS16	0.691	1.614	2.365	2.230
ADAMTS17	0.157	1.115	3.513	0.538
ADAMTS18	-0.545	0.685	3.296	-0.745

ADAMTS19	0.299	1.230	2.726	1.154
ADAMTS2	0.234	1.176	3.432	0.706
ADAMTS3	0.955	1.938	2.648	2.668
ADAMTS4	-0.683	0.623	5.767	-1.516
ADAMTS5	-0.215	0.862	3.436	-0.921
ADAMTS7P1	-0.132	0.913	2.741	-0.400
ADAMTS8	0.089	1.064	3.976	0.298
ADAMTS9	0.227	1.170	6.614	0.634
ADAMTS9-AS2	0.326	1.253	4.541	0.988
ADAMTSL2	-0.352	0.784	3.657	-1.414
ADAMTSL3	-0.477	0.719	4.734	-1.475
ADAMTSL4	-0.302	0.811	2.276	-0.886
ADAMTSL5	0.445	1.361	2.894	1.684
ADAP2	-0.801	0.574	5.306	-2.392
ADARB1	0.392	1.312	5.324	1.515
ADARB2-AS1	-0.149	0.902	2.074	-0.515
ADAT2	0.307	1.237	3.677	1.255
ADCY1	1.341	2.533	7.038	2.530
ADCY10P1	-1.013	0.495	4.277	-3.806
ADCY7	-0.567	0.675	4.582	-2.031
ADCY8	-0.260	0.835	6.761	-1.006
ADCY9	0.511	1.425	5.023	1.742
ADCYAP1	0.324	1.252	3.357	0.782
ADCYAP1R1	0.074	1.052	6.506	0.252
ADD2	1.060	2.084	6.151	2.360
ADD3-AS1//RP11-451M19.3	-0.260	0.835	2.689	-0.886
ADH1C	0.419	1.337	2.516	0.922
ADIPOR2	-0.866	0.549	5.496	-3.002
ADIRF	0.024	1.017	5.323	0.105
ADM	-0.453	0.730	5.195	-1.068
ADNP2	0.088	1.063	4.140	0.335
ADORA2B	1.014	2.020	4.531	3.480
ADORA3	-0.718	0.608	4.862	-2.101
ADPRHL1	0.762	1.696	4.183	2.357
ADRA1B	0.726	1.654	3.647	2.090
ADRA1D	0.154	1.113	3.429	0.527
ADRA2A	-0.062	0.958	2.901	-0.193
ADRA2C	-0.001	1.000	2.986	-0.002
ADRB1	1.013	2.018	4.636	2.484
ADRBK1	0.391	1.311	6.521	1.673
ADRBK2	0.877	1.836	7.454	2.644
ADSSL1	-0.497	0.708	4.219	-2.282
AEBP1//MIR4649	-0.244	0.844	4.316	-0.848
AEN	-0.256	0.837	3.450	-1.017
AES	0.365	1.288	9.069	1.457
AF127936.5	-0.435	0.740	3.613	-1.364
AF131215.3	-0.256	0.837	3.465	-0.808
AF131215.4	-0.153	0.899	2.746	-0.523
AFAP1L2	0.339	1.265	4.278	1.163
AFF2	0.520	1.434	5.749	1.019

AFF3	0.534	1.448	5.483	2.171
AFMID	-0.599	0.660	4.129	-2.375
AFTPH	0.348	1.273	7.175	1.758
AGA	-0.191	0.876	5.038	-0.645
AGAP1-IT1	-0.777	0.584	2.936	-2.726
AGAP2	1.826	3.546	5.769	3.194
AGAP2-AS1	0.673	1.595	3.032	2.684
AGAP5//AGAP4//AGAP11//AGAP10//LC	-0.244	0.845	6.087	-0.840
AGBL3	0.497	1.412	2.898	1.997
AGBL4	1.075	2.107	3.982	2.938
AGBL4-IT1	0.528	1.442	1.908	1.921
AGFG2	-0.171	0.888	5.941	-0.651
AGGF1	0.210	1.156	6.930	1.187
AGK	0.473	1.388	5.435	2.226
AGL	0.508	1.422	5.974	2.066
AGMAT	0.452	1.368	3.122	1.813
AGMO	-0.820	0.567	2.121	-2.178
AGPAT4	-0.838	0.560	6.837	-2.350
AGPAT4-IT1	-0.781	0.582	5.273	-2.052
AGT	-0.397	0.760	7.225	-1.419
AGTRAP//BRAF	-0.694	0.618	4.310	-2.104
AHNAK	-0.364	0.777	7.055	-1.392
AHNAK2	0.565	1.479	6.240	1.144
AHR	-0.223	0.857	7.045	-0.788
AHSA2	-0.405	0.755	4.675	-1.601
AIF1	-0.418	0.748	5.891	-0.861
AIF1	-0.386	0.765	6.229	-0.708
AIF1	-0.353	0.783	6.254	-0.662
AIF1L	-0.390	0.763	3.813	-1.374
AIFM3	0.257	1.195	4.974	0.922
AIM1	0.552	1.466	2.344	2.002
AJ006998.2	2.165	4.484	2.176	5.135
AJ009632.3	0.963	1.949	1.839	2.887
AJAP1	0.232	1.175	6.057	0.744
AK5	0.535	1.448	7.739	1.701
AKAP11	0.325	1.253	8.000	1.678
AKAP12	0.899	1.864	5.890	2.295
AKAP3	-0.522	0.696	2.506	-2.009
AKAP5	1.319	2.496	2.883	2.362
AKNA	-0.432	0.741	4.214	-1.670
AKR1C1	-0.029	0.980	3.029	-0.099
AKR7A3	-0.082	0.945	3.669	-0.291
AKR7L	0.313	1.243	2.477	1.196
AKT3	0.476	1.391	7.672	2.196
AKT3-IT1	-0.323	0.799	3.034	-1.047
AL121578.2	0.429	1.346	2.257	1.224
ALAD	-0.474	0.720	4.924	-2.239
ALAS1	0.368	1.291	6.079	1.644
ALAS2	-0.425	0.745	3.480	-0.688
ALCAM	-0.644	0.640	6.368	-2.275

ALDH1A1	-0.631	0.646	7.244	-1.969
ALDH1B1	0.384	1.305	4.408	1.379
ALDH3B1	-0.250	0.841	3.607	-0.860
ALDH8A1	0.010	1.007	2.524	0.036
ALDH9A1	-0.351	0.784	6.813	-1.485
ALG1//NAGPA-AS1	0.040	1.028	4.703	0.136
ALG10	-0.218	0.860	3.917	-0.884
ALG10B//ALG10	0.182	1.135	3.009	0.598
ALG1L2	-0.015	0.989	2.467	-0.041
ALKBH1	0.185	1.137	4.578	0.832
ALMS1-IT1	-0.241	0.846	2.650	-0.744
ALOX12B	0.775	1.712	1.994	2.275
ALOX12P2	0.482	1.397	3.150	1.979
ALOX15B	-0.145	0.904	3.150	-0.291
ALOX5	-0.309	0.807	5.041	-0.732
ALOX5AP	-0.276	0.826	6.867	-0.586
ALPK1	-0.201	0.870	4.035	-0.511
ALPL	0.745	1.675	5.542	2.630
ALS2	0.339	1.265	5.716	1.767
ALS2CR11	-0.793	0.577	3.157	-2.566
ALX4	-0.060	0.959	2.522	-0.218
AMER1	0.246	1.186	2.794	0.914
AMER2	-0.793	0.577	6.424	-2.833
AMER3	1.036	2.050	3.197	3.877
AMICA1	-0.019	0.987	2.259	-0.065
AMIGO1	0.611	1.527	5.352	1.403
AMMECR1	0.125	1.090	2.432	0.460
AMN1	0.271	1.207	6.142	1.405
AMPD3//CAND1.11	-0.519	0.698	4.586	-1.860
AMPH	1.111	2.159	6.617	1.902
AMZ2P1	0.111	1.080	3.764	0.388
ANAPC1//LOC730268//AC083899.3	0.294	1.226	5.203	1.446
ANAPC1P1	0.254	1.192	2.794	0.829
ANG//RNASE4	-0.119	0.921	4.262	-0.423
ANGEL2	0.110	1.079	5.366	0.524
ANGPT1	-0.180	0.883	3.739	-0.526
ANGPT2	-0.599	0.660	5.994	-1.393
ANGPTL1	0.172	1.126	3.059	0.582
ANGPTL2	-1.004	0.499	5.161	-2.953
ANGPTL4	0.127	1.092	6.286	0.288
ANGPTL6	-0.065	0.956	3.520	-0.213
ANK1	0.289	1.222	5.017	0.607
ANK2	0.208	1.155	8.932	1.157
ANK3	0.213	1.159	7.720	0.991
ANKEF1	-0.482	0.716	2.598	-1.719
ANKFN1	-0.451	0.732	5.117	-1.478
ANKFY1	-0.533	0.691	7.185	-2.752
ANKLE1	0.204	1.152	2.710	0.768
ANKMY2	0.795	1.735	5.684	3.480
ANKRD10-IT1	-0.228	0.854	6.178	-0.804

ANKRD10-IT1	-0.002	0.999	6.002	-0.006
ANKRD13A	-0.429	0.743	6.146	-1.630
ANKRD13C	0.330	1.257	6.641	1.677
ANKRD16	0.383	1.304	5.988	1.896
ANKRD18CP//RP11-498P14.5	0.635	1.553	4.256	1.613
ANKRD19P	1.025	2.035	3.840	3.353
ANKRD2	0.297	1.228	3.651	1.002
ANKRD20A3//ANKRD20A2//ANKRD20A4	0.553	1.467	6.323	1.814
ANKRD20A3//ANKRD20A2//ANKRD20A4	0.516	1.430	6.553	1.602
ANKRD20A5P	1.435	2.704	3.881	3.426
ANKRD20A7P	0.342	1.267	2.484	0.508
ANKRD22	0.260	1.198	2.265	0.700
ANKRD24	0.488	1.402	4.327	1.557
ANKRD26	0.246	1.186	5.397	1.132
ANKRD26P3	0.009	1.006	2.669	0.021
ANKRD29	0.561	1.475	5.289	1.233
ANKRD30B	1.105	2.152	2.850	2.293
ANKRD32	0.653	1.572	4.404	2.205
ANKRD33B	0.661	1.581	3.105	1.874
ANKRD34A	0.556	1.470	3.568	1.736
ANKRD34B	-0.405	0.755	2.378	-1.238
ANKRD34C	0.658	1.577	3.527	1.701
ANKRD36B	0.007	1.005	6.793	0.025
ANKRD37	-0.266	0.832	5.210	-0.690
ANKRD40	-0.715	0.609	8.442	-3.163
ANKRD42	0.485	1.400	4.462	1.504
ANKRD44	-0.714	0.610	5.837	-2.728
ANKRD44-IT1	-0.382	0.767	3.134	-1.057
ANKRD49	0.293	1.225	4.405	1.220
ANKRD50	0.052	1.037	5.504	0.202
ANKRD55	-0.172	0.888	3.433	-0.640
ANKS1B	0.715	1.642	6.124	2.451
ANKZF1	-0.364	0.777	4.504	-1.353
ANLN	-1.277	0.413	8.442	-3.025
ANO3	1.530	2.889	4.259	2.680
ANO4	-0.003	0.998	5.640	-0.009
ANO5	0.755	1.687	6.503	2.614
ANO6	0.184	1.136	8.305	0.724
ANP32A-IT1	-1.064	0.478	4.199	-3.664
ANP32B	-0.743	0.597	4.529	-3.278
ANPEP	0.064	1.045	2.316	0.169
ANTXR1	-0.276	0.826	6.587	-1.064
ANTXR2	-0.280	0.824	5.055	-1.418
ANXA1	0.308	1.238	5.307	0.935
ANXA11	0.436	1.353	5.057	1.576
ANXA13	-0.395	0.760	2.866	-0.998
ANXA2P2	0.162	1.119	7.708	0.594
ANXA2P3	0.469	1.384	2.088	2.019
ANXA2R	-0.332	0.795	2.369	-1.270
ANXA3	0.384	1.305	4.647	1.219

ANXA4	-0.261	0.834	6.693	-0.999
ANXA5	-0.668	0.629	8.208	-2.528
ANXA6	0.736	1.666	8.863	2.322
AOAH	-0.202	0.870	5.110	-0.751
AOC3	-0.040	0.972	2.560	-0.137
AP000282.2	-0.386	0.765	2.396	-0.993
AP000438.2	0.221	1.165	2.309	0.954
AP000473.5	-0.488	0.713	3.946	-1.529
AP000640.10	0.237	1.179	2.879	0.742
AP000688.29	-0.564	0.676	5.158	-1.398
AP001056.1	-0.218	0.860	3.046	-0.623
AP001347.6	0.552	1.466	2.280	1.687
AP001469.7	-0.191	0.876	3.250	-0.659
AP001476.4	0.088	1.063	4.224	0.330
AP001631.9	-0.333	0.794	3.083	-1.286
AP002954.3	-0.567	0.675	3.715	-1.237
AP006216.11	0.479	1.394	4.326	1.555
AP006222.2	0.688	1.611	5.236	2.522
AP006621.6	-0.107	0.928	3.993	-0.340
AP1S1	0.689	1.612	5.469	2.499
AP1S2	-0.271	0.829	5.020	-0.987
AP1S3	0.473	1.388	2.260	1.766
AP3B2	0.744	1.674	5.495	2.065
AP3M2	0.455	1.371	5.931	1.987
AP4S1	0.222	1.166	3.202	1.050
AP5M1	0.158	1.115	5.865	0.830
APBA1	0.522	1.436	6.511	1.704
APBB1IP	-0.992	0.503	4.668	-2.386
APCDD1	0.035	1.025	6.207	0.114
API5	0.177	1.130	6.711	0.916
APLN	-0.493	0.711	5.898	-1.554
APLNR	-0.601	0.659	7.340	-1.239
APLP1	-0.745	0.597	8.997	-2.886
APOBEC3C	-0.115	0.923	4.571	-0.257
APOBEC3D	-0.049	0.967	3.718	-0.154
APOBEC3G	-0.216	0.861	3.373	-0.941
APOBR	-0.727	0.604	2.866	-3.050
APOC1	-0.247	0.843	4.538	-0.477
APOC4-APOC2//APOC2//APOC4	-0.555	0.681	3.621	-1.866
APOD	-0.599	0.660	8.827	-2.463
APOE	-0.123	0.919	4.391	-0.688
APOL2	0.397	1.317	4.232	1.547
APOL4	0.395	1.315	2.110	1.275
APOL6	-0.273	0.827	5.540	-0.934
APOO	0.260	1.198	5.535	0.869
APPBP2	0.118	1.085	7.672	0.594
APPL2	-0.564	0.677	5.772	-2.319
APRT	-0.126	0.917	5.233	-0.505
AQP1//RP5-877J2.1	-0.904	0.534	6.920	-1.393
AQP11	-0.008	0.994	4.699	-0.035

AQP4-AS1	-0.214	0.862	4.866	-0.609
AQP5	-0.392	0.762	2.682	-1.287
AQP9	0.131	1.095	3.361	0.229
AR	-0.556	0.680	3.621	-2.268
ARAP1	-0.573	0.672	5.332	-2.707
ARAP2//LOC101928667	-0.279	0.824	6.081	-1.137
ARC	0.568	1.482	3.797	1.990
ARF3	0.398	1.318	5.854	1.695
ARFGEF2	0.481	1.396	6.998	2.527
ARG2	0.373	1.295	4.734	1.071
ARHGAP12	-0.111	0.926	7.404	-0.435
ARHGAP15//LOC101928361	-0.079	0.947	2.409	-0.261
ARHGAP17	-0.598	0.660	5.772	-2.305
ARHGAP18	0.248	1.187	4.715	1.013
ARHGAP20	0.818	1.763	4.427	1.973
ARHGAP22	-0.826	0.564	3.828	-2.707
ARHGAP22-IT1	-0.626	0.648	3.227	-2.031
ARHGAP23	-0.762	0.590	6.547	-2.525
ARHGAP24	-0.257	0.837	4.769	-0.922
ARHGAP25	0.107	1.077	3.543	0.349
ARHGAP26	0.521	1.435	6.903	2.497
ARHGAP26-IT1	0.324	1.251	4.575	0.844
ARHGAP27	-0.102	0.932	3.096	-0.411
ARHGAP28	0.317	1.246	2.666	1.045
ARHGAP30	-0.124	0.917	3.204	-0.409
ARHGAP32	0.939	1.918	7.579	3.024
ARHGAP35	0.189	1.140	8.140	0.952
ARHGAP36	-0.752	0.594	4.599	-1.210
ARHGAP4	-0.152	0.900	4.177	-0.686
ARHGAP44	0.763	1.697	5.133	1.881
ARHGAP6	-0.069	0.953	4.249	-0.294
ARHGDIB	-0.224	0.856	3.622	-0.889
ARHGDIG	0.308	1.238	5.318	0.765
ARHGDIG	-0.094	0.937	3.201	-0.331
ARHGEF1//LOC100505585	-0.459	0.727	4.991	-1.647
ARHGEF10	-0.702	0.615	6.013	-3.043
ARHGEF11	0.344	1.269	6.020	1.322
ARHGEF12	0.494	1.408	7.885	2.323
ARHGEF15	-0.238	0.848	3.091	-0.907
ARHGEF19	0.067	1.048	2.411	0.249
ARHGEF25	0.874	1.832	6.050	2.286
ARHGEF28	0.856	1.810	4.969	2.807
ARHGEF3	0.497	1.411	4.428	1.914
ARHGEF33	0.317	1.245	3.605	1.220
ARHGEF37	-0.748	0.596	5.851	-2.115
ARHGEF40	-0.489	0.713	4.704	-2.065
ARHGEF5	-0.752	0.594	2.332	-2.822
ARHGEF6	-0.619	0.651	7.664	-2.584
ARHGEF7-IT1	0.357	1.281	2.659	1.238
ARHGEF9	0.649	1.569	7.535	2.040

ARID5A	0.210	1.156	5.304	0.779
ARID5B	0.427	1.345	6.475	1.543
ARL14EP	0.202	1.150	6.285	0.978
ARL15	0.731	1.660	5.088	3.005
ARL4A	0.898	1.864	4.079	2.333
ARL4C	0.833	1.781	6.470	2.019
ARL4D	0.518	1.432	2.935	1.273
ARL6	0.603	1.519	4.428	1.734
ARL6IP1//RP11-1035H13.3	0.327	1.254	10.236	1.692
ARMC3	0.146	1.106	2.364	0.362
ARMC4	-0.128	0.915	1.956	-0.505
ARMC9	0.609	1.526	4.566	2.117
ARMCX1	0.155	1.114	5.901	0.791
ARNT2	0.366	1.289	7.892	1.538
ARNTL	0.606	1.522	5.752	2.291
ARNTL2	1.052	2.073	4.095	2.587
ARPC1B	-0.106	0.929	5.000	-0.348
ARPC5L	0.475	1.390	4.872	1.900
ARPP21	1.152	2.222	4.560	2.631
ARRB1	0.149	1.109	4.934	0.614
ARRDC2	-0.753	0.593	5.593	-2.014
ARRDC3	-0.913	0.531	8.026	-2.615
ARRDC4	-0.795	0.576	8.005	-2.142
ARSD	-0.191	0.876	4.357	-0.610
ARSF	-0.677	0.625	2.300	-2.141
ARSG	0.013	1.009	4.979	0.048
ARSJ	0.374	1.296	2.732	1.561
ARSK	0.494	1.409	4.186	2.164
ARX	0.943	1.923	3.535	2.367
ASAH1	0.057	1.040	7.151	0.245
ASAP1-IT2	-0.047	0.968	3.049	-0.140
ASAP3	0.021	1.015	5.618	0.107
ASB2	0.529	1.443	2.115	2.031
ASCC2	-0.466	0.724	5.105	-2.009
ASCL1	-0.251	0.840	6.345	-0.896
ASGR1	0.257	1.195	2.924	1.030
ASH2L	0.390	1.310	6.358	2.002
ASIC1	-0.002	0.998	4.004	-0.009
ASIC2	0.891	1.854	4.540	1.850
ASNS	0.498	1.413	5.361	1.986
ASPA	-0.670	0.628	7.176	-1.456
ASPDH	0.384	1.305	4.188	1.342
ASPHD2	0.552	1.466	4.480	1.518
ASS1	0.642	1.561	6.266	1.958
ASTN1	0.491	1.405	7.270	1.795
ASUN	-0.463	0.726	5.538	-1.753
ASXL3	0.249	1.188	4.707	1.098
ATAD2	0.574	1.489	4.077	2.585
ATCAY	0.872	1.831	7.165	1.982
ATE1-AS1//RP11-500G22.2	-0.907	0.533	4.337	-2.160

ATF3	-0.478	0.718	4.158	-1.252
ATF5	-0.071	0.952	4.074	-0.278
ATF7//NPFF//RP11-793H13.10	-0.662	0.632	6.106	-2.587
ATF7IP2	0.313	1.242	2.849	1.179
ATG13	-0.237	0.848	5.856	-1.119
ATG16L1	0.225	1.169	5.412	0.826
ATG2B	0.143	1.104	6.561	0.734
ATG4C	-0.583	0.668	6.307	-2.255
ATG5	0.542	1.456	5.901	2.541
ATHL1	0.165	1.121	3.507	0.664
ATL1	0.399	1.319	5.985	1.636
ATL2	0.619	1.536	4.354	2.347
ATL3	-0.324	0.799	6.874	-1.312
ATM//NPAT	-0.539	0.688	6.375	-2.108
ATMIN	0.340	1.266	7.928	1.754
ATOH8	-0.372	0.773	2.616	-1.093
ATP10B	-1.054	0.482	6.061	-2.126
ATP11A	-0.797	0.576	6.583	-2.770
ATP11C	-0.475	0.719	5.165	-2.038
ATP13A2	0.486	1.401	7.485	1.877
ATP13A4	-0.443	0.736	5.997	-1.430
ATP13A5-AS1	0.417	1.335	2.007	1.853
ATP1A1	0.606	1.522	5.125	3.016
ATP1A1-AS1	-0.396	0.760	4.015	-1.512
ATP1A3//LOC101927137	0.590	1.505	4.602	1.224
ATP1B1	0.163	1.120	9.698	0.839
ATP2A2	0.227	1.171	8.517	1.125
ATP2A3	-0.101	0.932	2.600	-0.378
ATP2B1	1.368	2.581	7.614	3.189
ATP2B2	1.325	2.505	6.237	2.551
ATP2B3	1.203	2.302	4.108	2.008
ATP2B4	0.471	1.386	7.781	2.042
ATP2C2	0.269	1.205	2.218	0.973
ATP4B	-0.254	0.839	2.060	-0.824
ATP5A1	0.434	1.351	8.595	2.181
ATP5B	0.401	1.320	10.183	1.927
ATP5SL	0.073	1.052	5.123	0.343
ATP5SL	0.093	1.067	3.648	0.295
ATP6V0A1	0.270	1.206	8.753	1.290
ATP6V0C	0.258	1.196	9.554	1.279
ATP6V0E1	-0.259	0.836	9.537	-1.152
ATP6V1A	0.727	1.655	7.578	2.308
ATP6V1B2	0.550	1.464	8.519	2.152
ATP6V1C1	0.771	1.707	8.078	2.777
ATP6V1D	0.507	1.421	5.437	2.605
ATP6V1H	0.777	1.714	6.969	2.784
ATP7B	0.259	1.196	3.880	0.890
ATP8A1	-0.120	0.920	8.915	-0.532
ATP8A2	0.854	1.807	6.622	1.525
ATP8B1	0.649	1.568	4.107	1.877

ATP8B4	-0.397	0.760	4.403	-1.317
ATPAF1	0.167	1.123	6.522	0.905
ATR	0.190	1.141	5.225	0.925
ATRN	0.141	1.103	6.979	0.722
ATRNL1	1.478	2.785	7.799	2.865
ATXN3//RP11-529H20.6	-0.130	0.914	3.301	-0.426
ATXN7L3	0.668	1.589	6.897	2.158
ATXN7L3B	0.363	1.286	8.034	1.614
AURKB	-0.177	0.885	2.474	-0.608
AVIL//PP12719	-0.370	0.774	3.073	-1.248
AVL9	0.448	1.364	6.726	2.162
AVPI1	0.219	1.164	5.751	0.744
AZGP1	-0.345	0.787	2.905	-0.938
B2M	0.201	1.149	8.007	0.871
B3GALNT1	0.057	1.040	4.270	0.232
B3GALT1	1.148	2.216	4.751	2.525
B3GALT2	0.846	1.798	6.864	2.775
B3GAT2	-0.554	0.681	6.700	-1.675
B3GNT1	0.683	1.605	6.556	2.569
B3GNT4	0.561	1.475	2.366	1.717
B3GNT9	-0.482	0.716	4.700	-1.852
B4GALNT1	1.175	2.258	5.123	2.455
B4GALNT3	0.387	1.308	2.462	1.466
B4GALT1	-0.123	0.919	4.596	-0.488
B4GALT5	0.469	1.384	7.421	2.228
B4GALT6	1.103	2.148	5.965	2.078
B9D2	-0.132	0.912	3.724	-0.484
BAALC-AS1//RP11-318M2.2	0.849	1.801	3.012	2.448
BAALCOS//C8orf56	0.331	1.258	3.186	1.193
BAAT	0.447	1.363	2.298	1.833
BACE1	-0.730	0.603	7.894	-2.437
BACE2	-0.385	0.766	4.239	-1.368
BACE2-IT1	-0.279	0.824	3.281	-0.950
BACH1-IT3	-0.421	0.747	2.358	-1.550
BAG2	-0.158	0.896	4.615	-0.600
BAG3	-0.176	0.885	6.218	-0.505
BAG4	0.655	1.574	4.957	2.667
BAI1	0.723	1.650	4.987	1.938
BAI2	0.552	1.466	5.225	1.865
BAI3	0.688	1.611	7.389	2.299
BAIAP2	0.767	1.701	5.026	2.183
BAIAP2-AS1	0.411	1.329	4.227	1.513
BAIAP2L2	0.049	1.034	3.296	0.178
BAIAP3	0.630	1.548	5.710	1.641
BAMBI	-0.874	0.546	5.988	-2.808
BAP1	0.715	1.641	6.205	2.650
BARD1	-0.211	0.864	4.529	-0.840
BARX2	0.206	1.153	1.937	0.841
BASP1	1.245	2.370	8.665	2.649
BATF3	0.070	1.050	3.318	0.249

BAX	-0.243	0.845	4.188	-0.947
BAZ1A	-0.421	0.747	4.916	-1.587
BAZ2B	-0.563	0.677	7.629	-2.399
BBOX1	-0.358	0.780	4.146	-1.387
BBS10	-0.049	0.966	5.264	-0.178
BBS7	0.599	1.515	6.834	2.631
BBX	-0.676	0.626	5.819	-2.894
BCAN	-0.403	0.756	6.658	-1.510
BCAR3	0.055	1.039	4.225	0.233
BCAS1	-0.960	0.514	6.748	-3.288
BCAS4	0.371	1.293	4.202	1.474
BCAT1	0.235	1.177	5.739	0.853
BCDIN3D-AS1	0.589	1.504	2.731	2.607
BCHE	-0.371	0.773	3.069	-1.364
BCL11A	1.405	2.648	4.255	2.853
BCL11B	1.469	2.769	3.522	3.281
BCL2	-0.467	0.724	6.521	-2.055
BCL2A1	0.352	1.276	3.559	0.812
BCL3//MIR8085	0.121	1.088	4.179	0.489
BCL6	0.028	1.020	8.153	0.098
BCL6B	-0.534	0.691	2.660	-1.673
BCL7A	0.389	1.310	5.532	1.533
BCL7C	-0.431	0.742	4.179	-1.798
BCMO1	0.746	1.677	2.451	2.201
BCO2	-0.249	0.841	5.061	-0.911
BCS1L	0.066	1.047	4.242	0.253
BDH1	0.349	1.274	4.398	1.624
BDH2	-0.458	0.728	4.338	-1.807
BDNF-AS//BDNF-AS1	-0.399	0.758	3.652	-1.468
BEAN1	0.365	1.288	3.345	1.442
BEGAIN	0.534	1.448	2.487	1.576
BEND4	0.580	1.495	2.549	2.061
BEND5	0.316	1.245	4.256	1.123
BEND6	0.602	1.518	5.969	1.779
BEST1	-0.601	0.659	7.087	-1.234
BEST3	0.238	1.179	3.337	0.583
BEX1	0.850	1.802	8.278	2.009
BEX2	0.260	1.198	4.609	0.913
BEX5	0.782	1.719	4.728	2.287
BGN	0.511	1.426	6.883	1.661
BHLHA15	-0.098	0.934	3.335	-0.318
BHLHB9	0.325	1.253	5.592	1.177
BHLHE22	0.240	1.181	2.614	0.917
BHLHE23	-0.148	0.903	3.424	-0.693
BHLHE40	0.505	1.419	7.525	2.027
BHLHE41	-0.574	0.672	8.257	-2.452
BHMT2	0.266	1.203	3.378	1.159
BICC1	0.616	1.532	4.500	1.923
BIN2	-0.541	0.687	5.061	-1.736
BIRC3	-0.188	0.878	3.684	-0.550

BIRC7	0.098	1.071	2.907	0.378
BLID	-0.743	0.597	3.195	-1.735
BLNK	-0.848	0.556	4.295	-1.772
BMP2	0.267	1.203	4.550	1.105
BMP2K	-0.083	0.944	7.423	-0.352
BMP3	0.347	1.271	3.259	1.094
BMP4	0.228	1.171	3.695	0.574
BMP5	0.808	1.750	2.391	1.266
BMP6	0.545	1.459	4.246	1.409
BMP7	-0.520	0.697	4.572	-1.683
BMP8A	-0.386	0.765	5.005	-1.687
BMP8B	-0.934	0.523	3.039	-2.822
BMPER	0.767	1.702	4.588	2.705
BMPR2	0.466	1.381	8.766	2.349
BMS1//LOC101929959	0.219	1.164	5.999	1.037
BMS1P20//LL22NC03-80A10.12	0.420	1.338	3.620	1.273
BMS1P4//BMS1//RP11-574K11.28	-0.183	0.881	3.015	-0.484
BNC2	-0.376	0.771	3.455	-1.244
BOC	-0.512	0.701	4.358	-2.076
BOK	-1.468	0.361	6.769	-3.636
BOLA3-AS1	0.244	1.184	4.014	0.887
BRAF//KIAA1549	0.352	1.276	7.012	1.816
BRCC3	0.099	1.071	6.446	0.505
BRI3	0.431	1.348	2.880	1.578
BRI3	-0.089	0.940	2.387	-0.453
BRINP1//DBC1	0.535	1.449	7.358	1.616
BRINP2//FAM5B	0.998	1.997	5.627	2.571
BRINP3//FAM5C	0.253	1.192	6.373	0.881
BRMS1L	0.302	1.233	4.297	1.360
BRPF3	0.002	1.002	4.857	0.011
BRSK1	0.247	1.187	6.616	0.982
BRSK2	0.297	1.229	5.625	1.338
BSN	1.220	2.330	5.422	2.342
BSPRY	0.515	1.429	2.918	1.793
BST1	-0.161	0.894	2.977	-0.598
BST2	0.204	1.152	5.725	0.730
BTBD10	0.493	1.407	5.701	2.145
BTBD11	0.288	1.221	4.476	0.890
BTBD16	-0.272	0.828	2.431	-1.038
BTBD17	-0.134	0.911	2.766	-0.480
BTBD19//PLK3	-0.331	0.795	3.155	-0.973
BTBD3	-0.387	0.765	6.467	-1.718
BTBD6	0.663	1.583	4.763	2.662
BTBD8	-0.344	0.788	4.901	-1.173
BTBD9	0.527	1.440	5.913	2.064
BTG2	-0.030	0.979	7.920	-0.108
BTG3	-0.259	0.836	3.726	-0.930
BTK	-0.557	0.680	4.408	-1.552
BTRC	0.334	1.260	7.369	1.708
BVES	-1.156	0.449	6.506	-3.124

BVES-AS1	-0.890	0.540	2.735	-2.559
BZRAP1	0.866	1.822	4.576	2.364
BZW2	0.950	1.932	3.927	3.224
C10orf10	0.832	1.781	6.564	1.745
C10orf105	-1.492	0.356	2.943	-2.975
C10orf107	-0.089	0.940	5.145	-0.308
C10orf11	-0.591	0.664	2.939	-2.106
C10orf128	-1.001	0.500	4.017	-2.261
C10orf25	-0.083	0.944	3.233	-0.313
C10orf35	0.928	1.903	2.054	3.149
C10orf54	-0.540	0.688	4.029	-1.652
C10orf85	-0.667	0.630	4.139	-2.292
C10orf88	0.200	1.149	5.328	0.772
C10orf90	-0.931	0.524	4.686	-2.273
C11orf24	-0.609	0.656	5.106	-2.436
C11orf39	-0.621	0.650	3.344	-1.929
C11orf39	-0.708	0.612	3.364	-1.650
C11orf70	-0.001	0.999	3.245	-0.004
C11orf74	0.647	1.566	3.763	2.676
C11orf80	0.382	1.303	4.172	1.568
C11orf87	2.178	4.526	4.632	2.611
C11orf88	-0.266	0.832	1.741	-0.587
C11orf91	0.701	1.626	2.728	2.337
C11orf97//RP11-867G2.2	0.613	1.529	2.031	1.589
C12orf29	0.413	1.331	6.209	2.258
C12orf4	0.171	1.126	6.003	0.714
C12orf43	0.495	1.409	4.883	2.426
C12orf45	0.189	1.140	4.062	0.810
C12orf55	-0.148	0.903	2.977	-0.404
C12orf76	-0.779	0.583	4.246	-3.203
C14orf132	0.282	1.216	6.222	0.972
C14orf142	0.404	1.323	2.980	1.566
C14orf2	0.488	1.402	4.005	2.130
C14orf23	1.492	2.812	2.568	3.288
C14orf37	0.351	1.275	5.326	1.120
C15orf27	0.626	1.544	2.810	1.598
C15orf39	0.219	1.164	3.916	0.948
C15orf52	-0.312	0.806	3.751	-1.392
C15orf59	0.107	1.077	6.031	0.362
C15orf61	0.454	1.370	4.577	1.567
C15orf65	-0.033	0.977	4.722	-0.117
C16orf13	0.219	1.164	5.806	1.046
C16orf45	0.355	1.279	6.956	1.627
C16orf52//VWA3A//LOC101930115	0.532	1.446	4.961	2.380
C16orf70	-0.003	0.998	7.285	-0.012
C16orf87	0.548	1.462	5.483	1.921
C16orf89	-0.066	0.955	4.565	-0.257
C16orf93	0.386	1.306	3.127	1.620
C17orf100	0.232	1.175	4.534	0.945
C17orf107	0.185	1.137	3.170	0.600

C17orf50	0.194	1.144	2.751	0.813
C17orf51	0.622	1.539	4.460	2.006
C17orf58	0.358	1.281	3.340	1.367
C17orf62	-0.135	0.911	6.266	-0.517
C17orf75	0.378	1.299	5.349	1.545
C17orf96	0.640	1.558	2.722	1.896
C17orf97	-0.150	0.901	4.718	-0.548
C18orf21	0.252	1.191	3.411	1.094
C18orf25	0.415	1.333	5.983	1.965
C18orf42	-0.119	0.921	2.703	-0.336
C18orf54	-0.420	0.747	4.303	-1.372
C19orf12	0.439	1.356	4.055	2.070
C19orf18	0.027	1.019	3.450	0.086
C19orf26	0.184	1.136	3.706	0.689
C19orf33//CTB-102L5.4	0.123	1.089	2.379	0.374
C19orf35	-0.204	0.868	2.961	-0.830
C19orf38	-0.771	0.586	4.418	-1.885
C19orf54	-0.407	0.754	3.776	-1.292
C19orf57	-0.318	0.802	2.847	-1.172
C19orf81	0.302	1.233	2.351	1.023
C19orf82	-0.405	0.755	4.103	-1.546
C19orf83//AC006132.1	-0.067	0.955	2.722	-0.256
C1GALT1	-0.345	0.787	6.293	-1.213
C1orf110	0.005	1.004	2.879	0.016
C1orf115	2.059	4.168	5.240	3.334
C1orf158	-0.430	0.742	1.816	-0.987
C1orf162	-0.279	0.824	5.579	-0.581
C1orf192	-0.005	0.996	4.436	-0.010
C1orf194	0.103	1.074	3.180	0.345
C1orf198	-0.628	0.647	5.994	-2.103
C1orf21	0.498	1.412	5.816	1.870
C1orf216	0.483	1.397	3.563	1.794
C1orf220	0.129	1.094	2.522	0.467
C1orf226//NOS1AP	-0.553	0.682	2.904	-2.252
C1orf228	-0.037	0.974	3.285	-0.133
C1orf233	0.305	1.235	4.361	0.931
C1orf54	-0.409	0.753	4.495	-1.813
C1orf61	-0.552	0.682	5.606	-1.805
C1orf64	0.434	1.351	3.861	1.508
C1orf74	0.005	1.003	3.876	0.022
C1orf87	-0.354	0.783	2.383	-0.693
C1orf94	-0.112	0.925	2.575	-0.417
C1orf95	0.213	1.159	3.945	0.818
C1QA	-0.558	0.679	3.526	-1.306
C1QB	-0.320	0.801	6.710	-0.636
C1QC	-0.288	0.819	7.174	-0.623
C1QL1	-0.415	0.750	3.998	-1.191
C1QL2	0.146	1.107	4.540	0.419
C1QL3	2.132	4.382	5.352	2.621
C1QL4	-0.235	0.850	2.194	-0.728

C1QTNF4	0.880	1.840	5.101	1.942
C1QTNF6	-0.155	0.898	3.533	-0.522
C1QTNF8	0.065	1.046	2.445	0.252
C1R//C1RL	0.438	1.355	4.399	1.359
C1RL	0.214	1.160	4.122	0.756
C1S	0.393	1.313	5.773	1.312
C2//CFB//XXbac-BPG116M5.17	-0.109	0.927	3.719	-0.385
C2//CFB//XXbac-BPG116M5.17	-0.103	0.931	3.825	-0.357
C2//CFB//XXbac-BPG116M5.17	-0.096	0.935	3.789	-0.345
C2//CFB//XXbac-BPG116M5.17	-0.086	0.942	3.828	-0.312
C2//XXbac-BPG116M5.17	0.075	1.053	3.770	0.228
C20orf197	-0.287	0.820	3.436	-1.139
C20orf26	0.052	1.037	2.758	0.200
C21orf128	0.832	1.780	3.163	2.073
C21orf140//AP000322.54	-0.153	0.900	2.187	-0.640
C21orf15//LINC00417//CYP4F29P	-0.095	0.936	3.433	-0.288
C21orf37	-1.275	0.413	4.392	-1.769
C21orf49	-0.197	0.872	2.924	-0.691
C21orf62	-0.654	0.635	3.993	-2.019
C21orf88	-0.660	0.633	3.836	-2.972
C21orf91	-1.035	0.488	4.614	-2.560
C22orf24	0.734	1.663	2.390	1.775
C22orf39//HIRA	-0.236	0.849	4.821	-0.869
C22orf46	-0.488	0.713	3.820	-1.864
C2CD2	-0.522	0.696	3.194	-1.581
C2CD2	-0.297	0.814	6.825	-1.511
C2CD4C	0.116	1.084	3.343	0.470
C2CD5	0.305	1.236	6.657	1.515
C2orf15//MRPL30//LOC101927110//RP1	0.650	1.569	3.037	2.167
C2orf40	-0.106	0.929	3.342	-0.427
C2orf50	0.436	1.353	3.203	1.384
C2orf68	-0.672	0.628	5.684	-2.265
C2orf69	0.253	1.192	4.975	1.230
C2orf72	0.109	1.079	5.520	0.445
C2orf80	1.311	2.481	4.203	2.300
C2orf82	-0.428	0.743	3.901	-1.321
C2orf88	-0.472	0.721	3.117	-1.895
C3	-0.972	0.510	8.694	-2.210
C3AR1	-0.263	0.834	3.750	-0.802
C3orf14	0.413	1.331	5.037	1.706
C3orf17	0.032	1.023	5.372	0.133
C3orf33	0.666	1.587	2.255	3.216
C3orf35	-0.060	0.959	3.694	-0.227
C3orf58	-0.320	0.801	4.328	-1.340
C3orf67-AS1//RP11-147N17.1	0.428	1.345	2.456	1.651
C3orf80//RP11-431I8.1	2.268	4.816	3.489	3.178
C4A//C4B//C4B_2	-0.409	0.753	7.186	-0.957
C4B//C4A//C4B_2	-0.414	0.751	7.480	-0.946
C4B//C4B_2//C4A	-0.507	0.704	7.356	-1.177
C4B//C4B_2//C4A	-0.493	0.711	7.464	-1.126

C4B//C4B_2//C4A	-0.490	0.712	7.482	-1.104
C4B//C4B_2//C4A	-0.483	0.716	7.483	-1.077
C4B//C4B_2//C4A	-0.411	0.752	7.513	-0.945
C4B//C4B_2//C4A//STK19//DAQB-331I1	-0.367	0.775	6.703	-0.940
C4B//C4B_2//C4A//STK19//DAQB-331I1	-0.340	0.790	6.810	-0.847
C4orf19	0.662	1.582	2.325	2.434
C4orf32	-0.632	0.645	3.891	-2.473
C4orf47	-0.477	0.719	2.827	-1.584
C4orf50	0.632	1.550	3.004	1.596
C5	-0.407	0.754	3.986	-1.346
C5AR1	0.260	1.197	4.639	0.403
C5AR2//GPR77	0.130	1.095	2.983	0.329
C5orf22	0.360	1.283	5.808	1.622
C5orf27	0.069	1.049	4.425	0.118
C5orf30	0.076	1.054	4.711	0.276
C5orf34	1.027	2.037	2.123	4.512
C5orf38	-0.799	0.575	3.137	-3.345
C5orf49	0.402	1.321	4.456	0.973
C5orf55	0.055	1.039	3.453	0.198
C5orf63	-0.261	0.835	3.182	-0.999
C5orf64	-0.713	0.610	3.812	-1.424
C6orf106	0.314	1.243	6.704	1.470
C6orf120	0.333	1.260	5.692	1.541
C6orf165//RP3-382I10.7	0.927	1.901	5.110	2.379
C6orf165//RP3-382I10.7	0.494	1.408	3.064	1.310
C6orf211	0.210	1.157	4.895	0.831
C6orf47	0.393	1.313	2.573	1.224
C6orf48	-0.380	0.768	8.647	-1.707
C6orf48	-0.392	0.762	7.128	-1.698
C6orf48	-0.387	0.765	7.564	-1.653
C6orf48	-0.327	0.797	7.250	-1.653
C6orf48	-0.390	0.763	7.378	-1.595
C6orf48	-0.276	0.826	7.238	-1.406
C6orf57	0.353	1.277	3.919	1.379
C6orf62	0.173	1.128	4.644	0.545
C7	0.918	1.890	5.134	2.422
C7orf34	-0.308	0.808	2.613	-1.100
C7orf57	-0.169	0.890	1.764	-0.515
C7orf60	0.477	1.392	5.462	1.814
C7orf61//TSC22D4	-0.379	0.769	3.479	-1.237
C7orf63	0.534	1.448	3.254	2.009
C7orf65	0.104	1.074	2.969	0.417
C8orf22	-0.093	0.937	2.436	-0.165
C8orf31	-0.375	0.771	2.692	-1.376
C8orf4	0.147	1.107	2.894	0.492
C8orf44-SGK3//SGK3//C8orf44	-0.546	0.685	5.675	-2.255
C8orf46	-0.468	0.723	3.198	-1.286
C8orf46	-0.025	0.983	5.435	-0.130
C8orf58	-0.648	0.638	2.475	-2.698
C8orf88//RP11-122A3.2	-0.396	0.760	3.846	-1.430

C9orf117	-0.329	0.796	2.395	-0.607
C9orf129	0.837	1.786	2.841	2.243
C9orf142	-0.498	0.708	5.243	-2.327
C9orf153	-0.152	0.900	2.411	-0.524
C9orf16	0.485	1.399	3.489	1.608
C9orf170	-0.595	0.662	3.128	-2.119
C9orf172	0.229	1.172	4.560	0.937
C9orf24	-0.076	0.949	3.818	-0.290
C9orf3	-0.541	0.687	4.550	-2.112
C9orf41	0.316	1.245	5.031	1.181
C9orf47//S1PR3	0.199	1.148	3.086	0.825
C9orf89	0.231	1.173	4.648	1.082
C9orf91	0.856	1.809	5.884	2.437
CA10	0.745	1.676	6.510	2.093
CA11	1.113	2.163	7.457	2.405
CA13//LOC100507258	0.433	1.350	3.581	1.960
CA14	-0.856	0.552	4.550	-1.747
CA2	-0.993	0.503	6.047	-2.981
CA4	0.312	1.241	3.011	0.800
CA5B	0.145	1.106	4.905	0.403
CA5BP1	-0.192	0.876	4.624	-0.653
CA7	1.584	2.998	2.647	3.013
CA8	-0.198	0.872	5.157	-0.745
CAAP1	0.044	1.031	5.162	0.172
CABLES1	0.441	1.358	5.953	1.115
CABLES2	0.235	1.177	4.911	0.935
CABP1	1.726	3.309	3.593	3.480
CABP7	-0.453	0.731	4.209	-0.969
CABYR	-0.038	0.974	5.247	-0.164
CACNA1A//LOC100507353	0.876	1.836	6.071	2.279
CACNA1B	1.008	2.012	5.409	2.222
CACNA1C	0.831	1.779	4.954	2.389
CACNA1C-AS2	0.381	1.302	2.453	1.413
CACNA1D	0.295	1.227	5.248	1.068
CACNA1E	1.009	2.013	5.147	1.740
CACNA1G	0.508	1.422	3.137	1.624
CACNA1H	0.263	1.200	3.630	0.876
CACNA1I	1.308	2.476	4.275	3.012
CACNA2D1	0.408	1.327	7.062	1.606
CACNA2D2	0.269	1.205	5.088	0.613
CACNA2D3	0.958	1.943	5.671	2.541
CACNB1	0.857	1.811	6.210	2.104
CACNB2	1.295	2.454	5.348	2.604
CACNB3	0.570	1.484	3.769	1.705
CACNB4	1.092	2.131	6.722	2.836
CACNG2	1.135	2.196	5.740	1.916
CACNG3	1.808	3.501	6.867	2.533
CACNG4	-0.274	0.827	5.726	-0.994
CACNG5	0.156	1.114	2.314	0.491
CACNG8	1.549	2.926	3.831	2.850

CADM2	0.533	1.447	7.285	2.018
CADM3	0.932	1.908	8.080	1.759
CADM3-AS1//CTA-134P22.2	0.362	1.285	5.382	1.200
CADPS	0.942	1.922	7.471	2.125
CADPS2	1.465	2.761	6.078	3.144
CAHM	-0.662	0.632	5.175	-2.309
CALB1	0.349	1.274	6.451	0.597
CALB2	-0.647	0.639	6.456	-0.757
CALCRL	-0.447	0.733	5.731	-1.462
CALHM1	0.670	1.591	2.962	2.260
CALHM2	-0.092	0.938	3.979	-0.388
CALM1//CALM2//CALM3	0.474	1.389	4.317	1.969
CALY	0.662	1.582	4.939	1.464
CAMK1	0.432	1.349	4.374	1.593
CAMK1D	1.024	2.033	6.976	2.756
CAMK1G	1.777	3.426	4.743	2.265
CAMK2A	1.859	3.628	7.727	2.711
CAMK2B	1.077	2.109	7.122	2.624
CAMK2D	0.698	1.623	6.579	3.434
CAMK2G	0.390	1.310	7.369	1.217
CAMK2N1	0.294	1.226	7.530	1.053
CAMK2N2	0.140	1.102	5.591	0.304
CAMK4	1.202	2.300	4.898	2.420
CAMKK1	1.151	2.221	4.970	2.769
CAMKK2	1.208	2.310	5.837	2.773
CAMKV	1.958	3.885	5.720	2.853
CAMSAP2	0.500	1.414	8.705	2.421
CAMSAP3	0.772	1.708	3.348	2.349
CAMTA1	0.243	1.184	7.376	1.038
CAMTA2	0.574	1.489	6.843	2.058
CAND1	0.278	1.213	6.554	1.324
CAP2	1.310	2.480	7.795	2.703
CAPG	-0.063	0.957	3.239	-0.248
CAPN13	-0.468	0.723	2.372	-1.497
CAPN3//RP11-164J13.1	-1.057	0.480	7.006	-2.365
CAPRIN2	0.250	1.189	6.333	1.114
CAPS	-0.866	0.549	5.653	-2.380
CAPS2	0.950	1.932	4.210	3.807
CARD11	-0.190	0.877	2.756	-0.643
CARD8	-0.627	0.648	4.698	-2.946
CARNS1	-1.176	0.442	7.175	-2.398
CARTPT	0.267	1.203	5.209	0.418
CASC1	0.457	1.372	2.549	1.375
CASC10	-0.334	0.793	4.118	-1.162
CASC14//RP1-67M12.2//CASC15	-0.282	0.822	3.053	-0.858
CASD1	0.338	1.264	7.303	1.582
CASKIN1	0.787	1.726	5.843	2.093
CASP1	0.015	1.011	2.267	0.056
CASP10	-0.401	0.757	3.090	-1.611
CASP4	0.357	1.280	2.880	1.244

CASP6	0.122	1.088	2.103	0.477
CASQ1	1.188	2.278	4.097	2.885
CAT	-0.616	0.653	6.851	-2.288
CATIP-AS1//AC021016.6	-0.110	0.926	2.993	-0.457
CATSPER2//LOC101930343	-0.100	0.933	3.810	-0.372
CAV1	-0.468	0.723	4.744	-1.846
CBFB	-0.535	0.690	7.628	-2.236
CBLN1	-0.062	0.958	2.982	-0.184
CBLN2	2.023	4.065	4.872	2.918
CBLN3	-0.089	0.940	3.569	-0.334
CBLN4	1.392	2.625	3.479	2.575
CBR1	-0.551	0.683	5.697	-2.125
CBR3	0.111	1.080	2.984	0.397
CBR3-AS1	0.410	1.329	2.145	1.511
CBX6	0.391	1.311	6.194	1.689
CC2D1B	-0.546	0.685	3.930	-2.413
CCBE1	1.203	2.303	2.776	2.717
CCDC102B	0.049	1.034	4.842	0.181
CCDC103//FAM187A	0.682	1.604	2.584	2.475
CCDC104	0.520	1.433	5.676	2.308
CCDC109B	0.141	1.103	4.650	0.481
CCDC11	-0.680	0.624	4.883	-1.833
CCDC110	0.455	1.371	2.469	1.299
CCDC113	0.825	1.772	5.656	2.986
CCDC114	0.051	1.036	2.622	0.157
CCDC122	-0.096	0.936	3.710	-0.292
CCDC132	0.306	1.236	5.414	1.513
CCDC135	0.073	1.052	2.592	0.269
CCDC13-AS1	-0.676	0.626	2.975	-1.808
CCDC14	-0.247	0.843	6.463	-0.969
CCDC144B//CCDC144CP	0.094	1.067	5.551	0.317
CCDC144B//RP11-219A15.1	-0.454	0.730	3.693	-1.054
CCDC144B//RP11-219A15.1	-0.509	0.703	4.080	-1.052
CCDC144CP	-0.501	0.707	5.903	-1.736
CCDC146	-0.110	0.927	4.500	-0.446
CCDC147-AS1	0.917	1.888	2.556	2.816
CCDC149	0.391	1.312	5.053	2.058
CCDC151	-0.348	0.786	3.576	-1.120
CCDC152	-0.351	0.784	3.985	-1.250
CCDC159	-0.483	0.715	3.516	-1.896
CCDC160	-0.861	0.550	2.706	-2.911
CCDC163P	-0.230	0.853	3.689	-0.715
CCDC170	0.324	1.252	3.671	1.153
CCDC173	0.257	1.195	2.681	0.747
CCDC177	1.229	2.344	3.608	2.644
CCDC18	0.017	1.012	2.811	0.057
CCDC184//C12orf68	0.286	1.219	4.200	0.853
CCDC19	-0.062	0.958	2.584	-0.186
CCDC3	1.010	2.014	4.243	2.718
CCDC30	0.112	1.081	4.200	0.445

CCDC30	-0.093	0.938	3.094	-0.314
CCDC30	-0.056	0.962	3.379	-0.171
CCDC34	0.248	1.188	2.811	0.857
CCDC37	-0.358	0.780	1.824	-0.930
CCDC39//LOC101928882	0.316	1.245	2.922	1.228
CCDC40	-0.354	0.782	3.190	-1.288
CCDC6	0.324	1.252	7.942	1.450
CCDC60	0.157	1.115	1.892	0.498
CCDC64	0.528	1.442	3.201	1.648
CCDC65	0.675	1.597	2.075	2.934
CCDC68	0.938	1.916	2.491	3.489
CCDC69	0.371	1.294	3.836	1.237
CCDC71	-0.003	0.998	4.398	-0.010
CCDC71L	0.468	1.383	4.358	1.570
CCDC8	-0.250	0.841	3.674	-1.050
CCDC80	0.072	1.051	4.794	0.219
CCDC81	-0.564	0.676	2.104	-1.872
CCDC85A	0.707	1.632	6.082	2.421
CCDC85B	0.013	1.009	3.951	0.053
CCER2//CTC-360G5.1	-0.075	0.949	3.283	-0.241
CCK	1.573	2.974	3.019	3.107
CCKBR	0.981	1.974	3.074	2.821
CCL19	0.683	1.606	3.308	2.126
CCL2	1.776	3.424	7.375	2.846
CCL20	0.071	1.051	2.474	0.190
CCL3L3//CCL3L1	-0.291	0.817	2.932	-0.855
CCL4L2//CCL4L1//CCL4	0.117	1.084	2.189	0.304
CCL5	-0.181	0.882	3.518	-0.512
CCM2L	0.206	1.154	3.525	0.769
CCNA1	0.880	1.841	3.741	2.048
CCNA2	-0.764	0.589	3.797	-2.092
CCNB1	0.413	1.331	2.684	1.630
CCNB3//BCOR	-0.545	0.685	2.293	-1.907
CCND1	-0.376	0.771	5.011	-1.306
CCND2-AS1//RP11-264F23.3	0.794	1.733	4.049	2.751
CCNDBP1	0.069	1.049	5.792	0.373
CCNE1	0.208	1.155	3.673	0.817
CCNE2	-1.173	0.443	4.465	-2.790
CCNF	0.468	1.383	2.933	1.756
CCNG2	0.299	1.230	3.604	1.232
CCNL1	-0.098	0.934	6.530	-0.397
CCNO	0.286	1.219	2.170	0.977
CCNT2-AS1//AC016725.4	0.166	1.122	4.308	0.646
CCP110	-0.400	0.758	7.312	-1.506
CCR1	-0.095	0.936	3.774	-0.223
CCRL2	-0.058	0.960	2.783	-0.229
CCRN4L	0.403	1.322	4.214	1.537
CCSAP	0.145	1.106	5.560	0.712
CCSER1	1.033	2.047	3.099	2.821
CCT6P1	0.182	1.135	3.576	0.663

CD109	0.037	1.026	4.848	0.126
CD14	-0.290	0.818	6.125	-0.585
CD151	-0.001	0.999	5.107	-0.005
CD163	0.461	1.377	6.080	0.657
CD164	-0.480	0.717	7.282	-1.910
CD177	0.123	1.089	3.349	0.236
CD177//CD177P1//CTC-490G23.5	-0.142	0.906	3.334	-0.212
CD180	-0.168	0.890	4.577	-0.359
CD200	0.472	1.387	7.505	1.604
CD209	0.277	1.212	2.157	0.827
CD22	-1.384	0.383	6.646	-2.917
CD226	-0.355	0.782	3.580	-0.938
CD24	0.551	1.465	4.319	1.288
CD274	0.215	1.161	3.262	0.789
CD300A	-0.277	0.826	4.219	-0.648
CD300C	-0.010	0.993	3.435	-0.032
CD300LF	-0.292	0.817	2.825	-0.939
CD33	-0.340	0.790	3.206	-1.049
CD34	0.369	1.292	5.680	1.082
CD37	-0.766	0.588	5.702	-1.872
CD38	-0.109	0.927	4.382	-0.275
CD4	-0.195	0.874	3.716	-0.694
CD40	-0.324	0.799	3.848	-1.278
CD44	-1.008	0.497	6.118	-1.722
CD44//RP1-68D18.2	-0.550	0.683	3.664	-1.106
CD48	-0.034	0.977	1.680	-0.122
CD5	0.068	1.048	2.771	0.253
CD53	-0.577	0.670	6.268	-1.889
CD58	-0.342	0.789	4.045	-1.612
CD63	-0.165	0.892	5.834	-0.880
CD68//SNORA67//LOC101928634//RP1	-0.443	0.735	8.608	-1.109
CD69	0.241	1.182	2.501	0.529
CD74	-0.511	0.702	6.003	-1.297
CD81	-0.293	0.816	3.481	-1.630
CD82	-0.311	0.806	4.090	-1.123
CD83	0.497	1.411	5.804	1.753
CD84	-0.476	0.719	3.418	-1.081
CD86	-0.744	0.597	4.878	-1.667
CD9	-0.427	0.744	3.975	-1.517
CD93	0.361	1.285	5.419	1.113
CD97	-0.424	0.745	4.850	-1.239
CD99	-0.857	0.552	9.192	-2.522
CD99P1	-0.197	0.872	3.883	-0.799
CDADC1	0.258	1.195	4.672	1.282
CDC14B	-0.848	0.556	4.203	-1.786
CDC40	0.558	1.473	5.563	2.041
CDC42EP1	-0.738	0.599	4.821	-2.302
CDC42EP2	-0.523	0.696	3.884	-1.626
CDC42EP4	-0.144	0.905	6.264	-0.629
CDC42SE2	0.237	1.179	3.304	1.214

CDC7	0.915	1.885	5.253	2.546
CDCA7L	-0.347	0.786	3.282	-1.059
CDCP1	0.135	1.098	3.380	0.463
CDH1	-0.353	0.783	3.586	-0.951
CDH10	1.059	2.084	6.352	2.540
CDH12	0.881	1.841	3.076	2.035
CDH13	1.261	2.396	6.425	2.634
CDH18	1.098	2.140	4.615	2.255
CDH19	-0.893	0.538	4.632	-1.670
CDH20	-0.712	0.610	6.721	-2.723
CDH22	1.146	2.213	5.185	2.526
CDH26	-0.093	0.937	3.255	-0.359
CDH3	0.751	1.683	3.387	2.113
CDH4	0.451	1.367	4.095	1.777
CDH5	0.559	1.473	6.304	2.144
CDH7	0.687	1.610	3.882	1.669
CDH8	0.596	1.512	6.497	1.693
CDH9	1.986	3.962	3.412	2.956
CDHR1	0.451	1.367	4.338	1.505
CDK14	1.140	2.203	7.809	2.700
CDK18	-1.031	0.489	5.930	-2.410
CDK2	0.479	1.394	4.652	1.854
CDK20	0.642	1.561	4.696	2.254
CDK2AP2	-0.622	0.650	4.065	-2.009
CDK5	0.454	1.370	6.231	1.760
CDK5R1	1.101	2.145	5.455	2.395
CDK5R2	0.655	1.574	4.684	1.892
CDK6	-0.481	0.716	4.891	-1.794
CDK7	0.298	1.229	4.739	1.417
CDKL1	0.705	1.630	3.739	2.571
CDKL2	0.518	1.432	5.315	2.158
CDKL5	0.763	1.697	7.043	2.663
CDKN1A	0.097	1.070	4.377	0.271
CDKN1C	-0.962	0.513	4.994	-2.529
CDKN2AIPNL	0.332	1.259	5.093	1.681
CDKN2B	-0.345	0.787	2.581	-1.357
CDKN2C	-0.032	0.978	2.648	-0.112
CDKN2D	0.751	1.683	5.186	2.748
CDKN3	0.700	1.624	2.289	2.931
CDNF	-0.119	0.921	3.388	-0.417
CDO1	0.545	1.459	6.640	1.805
CDON	0.333	1.260	3.728	1.403
CDR2//LOC101060399	0.559	1.473	4.733	1.821
CDR2L	-0.792	0.578	5.295	-2.656
CDRT15	-0.187	0.878	3.592	-0.709
CDS1	1.139	2.202	5.287	2.182
CDYL2	0.870	1.828	5.667	2.462
CDYL2	0.889	1.852	4.247	2.047
CEBPA	-0.523	0.696	4.468	-1.815
CEBPB	0.252	1.191	4.646	0.980

CEBPD	0.185	1.137	6.707	0.511
CECR1	-0.369	0.774	4.247	-1.210
CECR2	-0.887	0.541	4.449	-2.537
CECR6	0.386	1.307	3.570	1.315
CECR7	-0.203	0.868	3.488	-0.710
CELF2	0.715	1.641	7.659	2.516
CELF3	0.962	1.948	4.307	2.196
CELF4	1.261	2.396	6.275	2.309
CELF5	1.040	2.057	5.031	2.157
CELSR3	0.518	1.432	3.645	1.699
CEMIP//KIAA1199	1.361	2.569	5.461	2.329
CEND1	0.725	1.653	5.733	2.124
CENPB	-0.524	0.695	5.531	-2.237
CENPBD1	-0.032	0.978	4.752	-0.136
CENPBD1P1//RPL23AP79	0.386	1.307	4.281	1.649
CENPJ	0.055	1.039	3.793	0.251
CENPP	0.320	1.249	3.727	1.159
CENPV	-0.109	0.927	5.257	-0.471
CEP170B	0.643	1.562	5.353	2.041
CEP19	0.555	1.469	4.727	1.570
CEP192	-0.445	0.734	6.167	-1.612
CEP57L1	0.158	1.115	3.111	0.633
CEP76	0.069	1.049	3.854	0.318
CEP85	-0.484	0.715	5.356	-1.847
CEP97	-0.240	0.847	6.774	-1.079
CERCAM	-0.654	0.635	6.699	-1.512
CERKL	0.561	1.475	2.381	1.823
CERS2	-1.024	0.492	4.437	-3.251
CERS6	0.502	1.416	7.244	1.765
CES1//LOC100653057	0.280	1.215	3.132	0.881
CES1//LOC100653057//LOC100653086/	0.365	1.288	3.625	1.307
CES4A//CES8	-0.215	0.862	5.301	-0.703
CFB//XXbac-BPG116M5.17	-0.113	0.924	3.561	-0.363
CFD	-0.111	0.926	4.213	-0.302
CFH	0.626	1.544	4.671	1.330
CFI	0.255	1.193	3.717	0.693
CFL1P1	0.217	1.162	2.874	1.005
CFL2	-0.597	0.661	5.660	-2.409
CFLAR	-0.383	0.767	4.705	-1.596
CFP	-0.082	0.945	2.363	-0.315
CGB//CGB5//CGB8//CGB1	0.110	1.079	3.291	0.342
CGN	-0.603	0.658	2.181	-2.328
CGREF1	0.491	1.406	4.173	1.692
CH25H	0.743	1.674	5.486	1.648
CHAC1	0.261	1.198	4.317	0.689
CHAD	-0.086	0.942	3.924	-0.228
CHADL	-0.510	0.702	4.777	-1.095
CHAF1A	0.420	1.338	3.998	1.588
CHAF1B	0.717	1.643	3.619	2.421
CHAT	-0.328	0.797	1.965	-1.008

CHCHD1	0.003	1.002	4.178	0.012
CHCHD7	0.248	1.187	2.704	1.010
CHD1L	-0.340	0.790	6.109	-1.348
CHD3	0.921	1.893	6.672	2.575
CHD5	1.545	2.918	5.307	2.362
CHD7	-0.792	0.577	5.703	-2.464
CHDC2	-0.618	0.651	3.029	-1.960
CHEK2	0.028	1.019	2.343	0.089
CHFR//RNU6-327P	-0.027	0.981	2.484	-0.086
CHGA	1.287	2.440	5.141	2.178
CHGB	0.964	1.951	4.481	1.907
CHI3L1	1.194	2.288	8.000	1.559
CHI3L2	-0.813	0.569	4.845	-1.158
CHIC1	0.161	1.118	5.882	0.761
CHIT1	0.340	1.266	2.675	1.035
CHKA	-0.512	0.701	6.140	-1.903
CHL1	0.719	1.646	6.977	2.747
CHL1-AS2	-0.052	0.964	4.252	-0.216
CHM	0.406	1.325	6.132	1.794
CHML	0.753	1.685	4.833	2.504
CHN1	1.435	2.703	5.007	3.538
CHODL	-0.598	0.661	2.149	-1.772
CHPF	0.573	1.488	3.890	2.426
CHRAC1	-0.274	0.827	4.648	-0.999
CHRD	0.690	1.614	3.532	2.160
CHRDL1	1.284	2.434	5.299	2.553
CHRM1	1.173	2.255	2.809	2.557
CHRM2	-0.139	0.908	3.814	-0.350
CHRM3	1.349	2.548	3.331	2.997
CHRM4	0.573	1.487	3.355	1.636
CHRM5	0.167	1.123	2.655	0.201
CHRNA1	0.330	1.257	2.518	1.016
CHRNA4//LOC102723788	0.452	1.368	3.170	1.649
CHRNA6	0.063	1.045	2.850	0.255
CHRNA7//CHRFAM7A	0.071	1.051	2.941	0.184
CHRNB2	0.650	1.570	4.168	1.751
CHST1	0.756	1.689	4.806	2.074
CHST10	0.142	1.104	3.434	0.633
CHST14	-0.409	0.753	3.480	-1.688
CHST15	0.614	1.530	6.630	2.430
CHST3	-0.681	0.624	6.234	-1.798
CHST6	-0.188	0.878	6.084	-0.481
CHST9	-0.496	0.709	3.426	-1.395
CHSY3	0.996	1.994	3.404	2.534
CHURC1-FNTB//FNTB	-0.681	0.624	6.384	-2.059
CIART//C1orf51	0.299	1.230	4.270	1.067
CIB1	-0.129	0.914	4.692	-0.553
CIDEA	0.540	1.454	2.767	2.247
CIDEB	-0.329	0.796	3.986	-1.248
CIDECP	0.370	1.293	4.317	1.332

CIITA	-0.814	0.569	4.825	-2.265
CINP	-0.625	0.649	3.819	-1.964
CIRBP	0.605	1.521	3.400	2.164
CIRBP	0.111	1.080	8.323	0.388
CIRH1A	0.241	1.182	6.611	1.159
CISD1	0.529	1.443	4.133	2.088
CISH	0.149	1.109	2.804	0.464
CIT//MIR1178	0.833	1.781	5.328	2.239
CITED1	0.276	1.210	3.175	1.012
CITED2	0.809	1.752	5.289	1.990
CKAP2	0.521	1.435	4.761	1.897
CKM	0.534	1.448	2.595	1.750
CKMT1A//CKMT1B	1.132	2.191	5.884	1.727
CKMT2	-0.588	0.665	4.964	-2.079
CLCA4	-1.076	0.474	4.115	-2.036
CLCF1	0.245	1.185	3.345	1.010
CLCN2	-0.744	0.597	5.316	-2.262
CLCN4	-0.013	0.991	7.469	-0.071
CLDN10	0.683	1.605	6.256	2.323
CLDN11	-1.063	0.479	5.205	-2.403
CLDN15	-0.523	0.696	3.124	-2.117
CLDN16	0.910	1.879	2.564	3.950
CLDN19	0.160	1.117	2.823	0.558
CLDN3	-0.041	0.972	3.273	-0.169
CLDN9	-0.573	0.672	2.677	-2.204
CLDND1	-0.983	0.506	8.345	-2.907
CLEC18B	-0.231	0.852	3.267	-0.638
CLEC18C//CLEC18B	-0.291	0.817	3.106	-0.876
CLEC2B	0.065	1.046	4.463	0.177
CLEC2D	0.094	1.067	3.246	0.378
CLEC2L	0.990	1.987	6.236	2.295
CLEC4A	0.097	1.070	2.457	0.381
CLEC5A	-0.760	0.590	3.134	-1.436
CLEC7A	-0.338	0.791	5.035	-0.750
CLEC9A	-1.022	0.492	2.414	-3.079
CLGN	0.588	1.503	3.394	1.696
CLIC1	0.027	1.019	6.483	0.109
CLIC1	0.014	1.009	6.498	0.054
CLIC1	0.005	1.003	6.481	0.020
CLIC1//DDAH2	-0.147	0.903	5.558	-0.713
CLIC1//DDAH2	-0.148	0.903	5.539	-0.702
CLIC2	0.928	1.903	4.643	2.280
CLIC3	0.205	1.153	2.084	0.916
CLIC4	-0.223	0.857	8.652	-0.691
CLK2P	-0.581	0.669	2.660	-1.660
CLMN	-0.633	0.645	5.470	-1.685
CLMP//ASAM	0.034	1.024	4.840	0.097
CLN3//RP11-435I10.4	-0.588	0.665	5.476	-2.487
CLN6	-0.236	0.849	3.684	-0.871
CLN8	-0.209	0.865	4.469	-0.846

CLOCK	0.337	1.263	6.982	1.526
CLSTN1	0.610	1.526	8.836	2.600
CLSTN2	1.404	2.645	5.925	2.471
CLSTN3	0.959	1.944	5.534	1.982
CLTB	0.427	1.344	6.469	1.792
CLTCL1	-0.369	0.774	3.983	-1.366
CLUL1	0.527	1.441	2.678	1.443
CLVS1	0.764	1.698	4.025	1.866
CLVS2	1.315	2.488	5.826	2.505
CLVS2	1.355	2.558	5.238	2.445
CLYBL	0.455	1.371	2.693	1.703
CMAS	0.394	1.314	6.145	1.713
CMKLR1	-0.470	0.722	4.765	-1.518
CMTM3	-0.268	0.831	4.933	-0.982
CMTM5	-1.408	0.377	6.206	-2.704
CMTM7	-0.245	0.844	3.829	-0.677
CMYA5	-0.262	0.834	4.631	-1.170
CNDP1	-0.814	0.569	9.308	-1.700
CNEP1R1//TMEM188	0.233	1.175	6.268	1.006
CNGA3	-0.632	0.645	3.183	-1.487
CNGB1	0.562	1.476	2.587	1.572
CNIH2	0.886	1.848	4.570	2.572
CNIH3	0.381	1.302	3.217	1.639
CNKSR2	1.267	2.406	7.076	2.815
CNKSR3	-0.455	0.730	4.627	-1.367
CNN1	0.670	1.591	4.600	1.715
CNN2	-0.597	0.661	5.254	-1.690
CNN3	0.265	1.201	8.002	0.936
CNNM1	0.985	1.980	4.436	2.227
CNP	-0.946	0.519	4.582	-2.261
CNP	-0.738	0.599	9.602	-1.999
CNR1	0.688	1.611	6.099	1.226
CNR1	0.714	1.640	6.063	1.213
CNRIP1	0.536	1.450	5.364	2.338
CNST	0.697	1.621	5.676	2.379
CNTFR	-0.175	0.886	6.965	-0.621
CNTN2	-1.047	0.484	7.640	-2.703
CNTN3	0.640	1.558	5.526	1.784
CNTN4	1.237	2.357	4.555	2.665
CNTN5	1.029	2.041	3.204	2.480
CNTN6	0.955	1.939	2.540	2.874
CNTNAP1	0.778	1.715	7.341	2.316
CNTNAP2	0.822	1.767	7.428	1.989
CNTNAP3B//CNTNAP3//CNTNAP3P2	0.505	1.420	4.654	2.267
CNTNAP4	-0.950	0.517	7.517	-2.651
CNTNAP5	0.973	1.963	6.245	2.271
COA6//SEC61A1	0.590	1.506	3.986	1.997
COA7//SELRC1	0.327	1.254	4.156	1.441
COCH	0.468	1.383	3.360	1.406
COG1	0.466	1.382	6.910	1.869

COG6	0.205	1.152	6.107	0.966
COL10A1	0.327	1.254	2.061	1.307
COL12A1	0.871	1.828	4.414	2.616
COL14A1	-0.225	0.856	4.102	-0.528
COL15A1	0.056	1.039	2.224	0.229
COL16A1	-0.666	0.630	4.634	-2.277
COL18A1	-0.321	0.801	4.820	-1.042
COL19A1	0.999	1.999	2.615	4.084
COL1A1	-0.114	0.924	3.478	-0.470
COL1A2	0.562	1.477	4.012	1.734
COL24A1	0.739	1.669	2.705	2.592
COL25A1	-0.140	0.907	3.151	-0.418
COL26A1	0.625	1.543	4.275	2.067
COL27A1	0.030	1.021	2.778	0.138
COL28A1	0.125	1.091	3.047	0.378
COL3A1	0.526	1.440	2.547	1.690
COL4A1	0.457	1.372	4.338	1.230
COL4A2	0.513	1.427	4.211	1.665
COL4A3	0.101	1.072	2.290	0.372
COL4A3BP	0.185	1.137	7.414	0.954
COL4A5	-0.841	0.558	5.054	-1.807
COL5A2	1.531	2.891	4.526	3.669
COL6A2	0.356	1.280	4.260	1.298
COL6A3	0.176	1.130	3.060	0.683
COL8A1	-0.219	0.859	2.194	-0.787
COL8A2	-0.429	0.743	2.929	-1.492
COL9A1	-0.215	0.862	2.973	-0.774
COL9A2	-0.289	0.818	4.202	-1.068
COL9A3	-0.297	0.814	4.194	-1.131
COLEC12	0.608	1.525	5.659	1.787
COLGALT1//GLT25D1	-0.048	0.967	6.224	-0.197
COLQ	0.305	1.236	3.535	1.013
COMMD8	0.229	1.172	4.350	1.142
COMT	-0.542	0.687	3.718	-2.557
COPG2	0.256	1.195	5.045	0.997
COPS4	0.386	1.307	6.025	1.930
COPZ2	-0.201	0.870	4.577	-0.721
COQ10B	0.344	1.269	5.422	1.416
COQ3	0.464	1.379	3.543	1.905
CORO1A	-0.072	0.951	5.807	-0.178
CORO2A	0.400	1.319	3.036	0.879
CORO6	0.744	1.674	4.745	2.286
COTL1	-0.453	0.731	7.210	-1.806
COX10	0.207	1.154	5.378	1.118
COX11	0.381	1.302	5.698	1.710
COX18	0.382	1.303	5.026	1.564
COX6A2	-0.043	0.970	2.305	-0.164
COX7A1	0.595	1.511	6.020	1.683
CP	-0.285	0.821	6.592	-0.550
CPA2	-0.774	0.585	2.254	-1.938

CPAMD8	-1.315	0.402	5.984	-2.131
CPB2-AS1	-0.534	0.691	4.648	-1.205
CPEB2	-0.576	0.671	6.922	-2.321
CPEB3	0.803	1.745	5.684	2.211
CPED1	-0.029	0.980	4.408	-0.110
CPLX1	0.792	1.731	5.218	2.067
CPLX2	0.612	1.528	3.320	1.403
CPLX3//RP11-414J4.2	0.336	1.262	4.546	1.064
CPLX3//RP11-414J4.2//LMAN1L	0.125	1.090	2.746	0.408
CPM	-0.712	0.610	5.087	-1.792
CPNE2	-1.044	0.485	7.451	-3.842
CPNE4	1.769	3.407	4.775	2.801
CPNE5	1.076	2.108	4.592	2.388
CPNE7	0.732	1.661	3.422	2.518
CPNE8	0.892	1.856	4.959	3.241
CPNE9	0.543	1.457	4.143	1.344
CPO	0.336	1.263	2.006	1.286
CPOX	-0.948	0.518	5.953	-2.869
CPOX	0.218	1.163	2.320	0.619
CPPED1	-0.658	0.634	6.639	-1.934
CPQ	-0.692	0.619	3.845	-2.790
CPS1	-0.782	0.582	4.710	-1.707
CPT1C	0.214	1.160	6.103	0.877
CPT2	-0.483	0.715	4.781	-1.599
CPVL	-0.179	0.883	2.820	-0.493
CPXM1	0.082	1.058	2.507	0.246
CPXM2	-1.237	0.424	4.287	-3.248
CR1	-0.066	0.955	2.871	-0.171
CRABP1	-0.144	0.905	4.844	-0.330
CRABP2	0.731	1.660	2.831	2.501
CRADD	-0.790	0.578	3.772	-3.249
CRB2	-0.137	0.909	5.223	-0.691
CREB3L2	-0.626	0.648	4.557	-2.855
CREB3L3	0.524	1.438	3.227	1.873
CREB3L4	0.039	1.027	2.642	0.155
CREB5//LOC401317	-0.509	0.703	3.714	-1.503
CREG1	0.185	1.137	5.421	0.732
CREG2	2.058	4.165	4.962	2.797
CRELD1	-0.053	0.964	5.034	-0.251
CRH	-0.644	0.640	2.178	-1.521
CRHBP	0.033	1.023	5.205	0.101
CRHR1-IT1//MGC57346//AC126544.2	0.162	1.119	4.660	0.672
CRHR2	0.426	1.344	3.560	1.461
CRIPAK	0.121	1.088	4.291	0.485
CRISPLD1	-0.286	0.820	5.422	-0.692
CRISPLD2	0.017	1.012	5.608	0.054
CRLF1	0.445	1.362	4.247	1.116
CRMP1	0.570	1.484	4.785	2.105
CRNDE	-1.177	0.442	3.545	-2.328
CROCCP2	-0.252	0.840	2.985	-0.894

CROT	0.483	1.397	4.582	2.164
CRTAC1	0.317	1.245	4.558	1.404
CRTAP	-0.148	0.902	7.276	-0.751
CRTC1	0.544	1.458	5.573	1.701
CRTC3	-0.703	0.614	5.409	-2.760
CRY2	0.409	1.328	5.337	1.603
CRYAB	-0.990	0.503	7.989	-3.569
CRYBB1	0.426	1.343	2.680	1.019
CRYBG3	-0.802	0.574	6.125	-2.618
CRYGS	-0.277	0.826	3.159	-1.133
CRYL1	-0.298	0.813	6.525	-1.024
CRYM	2.267	4.814	3.933	3.172
CSAD	-0.335	0.793	5.433	-1.354
CSAG1//CSAG3	0.126	1.091	2.296	0.416
CSF1	-0.650	0.637	3.714	-2.522
CSF1R	-0.723	0.606	7.734	-2.046
CSF2RA	-1.012	0.496	4.682	-2.805
CSF2RA	-0.651	0.637	4.322	-1.972
CSF3	-0.175	0.886	3.395	-0.326
CSF3R	-0.428	0.744	3.039	-1.152
CSMD1	0.972	1.962	5.365	2.569
CSMD3	0.686	1.609	5.840	1.830
CSNK1E//RP3-449O17.1	-0.056	0.962	4.017	-0.180
CSNK2A3	0.176	1.130	4.160	0.515
CSPG4	-0.160	0.895	3.817	-0.645
CSPG4P1Y//CSPG4P2Y//CSPG4P3Y//DNM	0.393	1.313	3.348	1.620
CSPG5	0.580	1.495	5.480	2.234
CSRNP1	0.248	1.187	5.107	0.880
CSRNP2	0.132	1.096	5.420	0.685
CSRNP3	1.067	2.095	6.284	2.674
CSRP2BP//PET117	0.442	1.359	4.689	2.133
CST4	0.468	1.383	2.416	1.676
CST7	-0.078	0.947	4.350	-0.209
CSTF3	0.289	1.222	5.249	1.357
CTA-212D2.2	-0.097	0.935	2.237	-0.271
CTA-797E19.1	-0.511	0.702	3.875	-2.003
CTAG2	0.086	1.061	2.608	0.305
CTAGE6	-0.231	0.852	3.332	-0.758
CTAGE8//CTAGE4//CTAGE9//CTAGE6//C	-0.140	0.908	3.731	-0.681
CTB-118N6.3	-0.620	0.651	2.326	-1.841
CTB-131B5.2	-0.397	0.760	2.240	-1.313
CTB-134H23.2//LOC613037//NPIPB9//N	-0.470	0.722	7.339	-2.005
CTB-25B13.12	-0.567	0.675	2.343	-2.275
CTB-78F1.1	1.325	2.505	1.981	2.997
CTBS	-0.133	0.912	5.795	-0.338
CTC-218H9.1	1.115	2.166	2.017	3.141
CTC-255N20.1	-0.323	0.800	3.488	-1.402
CTC-301O7.4	-0.195	0.874	2.761	-0.648
CTC-304I17.5	0.315	1.244	3.145	0.985
CTC-321K16.1	0.660	1.580	1.996	2.733

CTC-329D1.3	-0.485	0.714	3.177	-1.618
CTC-338M12.9	-0.040	0.973	3.501	-0.146
CTC-340D7.1	0.597	1.513	2.384	1.940
CTC-367F4.1	0.160	1.118	2.658	0.462
CTC-458G6.2	-0.919	0.529	3.155	-2.570
CTC-467M3.1	0.980	1.972	2.987	3.366
CTC-467M3.3	0.273	1.209	2.164	0.832
CTC-498M16.4	0.281	1.215	2.861	0.882
CTC-552D5.1	0.871	1.830	1.688	1.939
CTC-806A22.1	0.596	1.511	2.079	1.897
CTD-2014B16.3	0.458	1.374	2.333	1.738
CTD-2015H6.3	0.487	1.401	4.039	1.740
CTD-2017C7.2	-0.660	0.633	3.368	-1.931
CTD-2020K17.3	0.241	1.181	3.441	0.595
CTD-2026D20.2	-1.067	0.477	3.064	-2.824
CTD-2033D15.1	0.218	1.163	2.416	0.692
CTD-2054N24.1	0.298	1.230	3.454	0.812
CTD-2054N24.2	-0.327	0.797	3.004	-0.965
CTD-2058B24.3	-0.535	0.690	3.532	-1.523
CTD-2152M20.2	-0.168	0.890	2.576	-0.572
CTD-2175A23.1	-1.029	0.490	2.498	-1.808
CTD-2184D3.6	1.059	2.084	2.691	3.856
CTD-2193P3.2	0.425	1.343	3.980	1.946
CTD-2201G16.1	-0.060	0.959	2.961	-0.199
CTD-2215E18.1	0.071	1.051	2.584	0.251
CTD-2223O18.1	-0.189	0.877	2.352	-0.711
CTD-2269F5.1	0.079	1.056	3.330	0.295
CTD-2277K2.1	0.733	1.662	3.686	2.244
CTD-2281E23.2	1.037	2.052	1.618	3.555
CTD-2298J14.2	1.159	2.233	4.124	2.577
CTD-2320G14.2	0.648	1.567	2.253	2.856
CTD-2369P2.10//FDX1L	0.346	1.271	5.073	1.457
CTD-2501E16.2//LOC101928784	-0.336	0.792	2.942	-1.200
CTD-2531D15.5	0.645	1.564	3.327	2.598
CTD-2538C1.2	0.301	1.232	3.037	1.121
CTD-2555A7.2	0.396	1.315	2.685	1.229
CTD-2555K7.2	-0.368	0.775	2.556	-1.061
CTD-2555O16.2	-0.236	0.849	4.427	-0.804
CTD-2562J17.4	0.383	1.304	2.987	1.618
CTD-2562J17.9	0.479	1.394	2.207	2.012
CTD-2574D22.2	-0.076	0.948	2.392	-0.300
CTD-2647L4.5	-0.528	0.694	4.173	-1.807
CTD-3064C13.1	0.115	1.083	2.275	0.475
CTD-3092A11.1	-0.234	0.851	4.498	-0.763
CTD-3138B18.5	0.082	1.058	2.752	0.299
CTD-3195I5.5//C17orf85	0.128	1.092	2.499	0.462
CTDSP1	-0.407	0.754	3.626	-1.785
CTF1	-0.004	0.998	4.389	-0.013
CTGF	-0.031	0.979	5.520	-0.081
CTH	0.387	1.308	4.203	1.655

CTIF	0.256	1.194	6.680	1.032
CTNNA1	-0.462	0.726	6.326	-2.031
CTNNA2	0.858	1.813	2.914	3.211
CTNNA3	-1.416	0.375	6.741	-2.697
CTNNAL1	0.417	1.335	4.579	1.246
CTNS	-0.513	0.701	3.954	-2.134
CTPS2	-0.323	0.799	5.410	-1.268
CTSC	0.093	1.066	5.139	0.234
CTSH	0.359	1.282	4.066	1.309
CTSK	0.225	1.169	3.598	0.829
CTSLP2//CTSLP7//CTSLP4//CTSLP1//CTS	-0.106	0.929	3.948	-0.453
CTSO	0.180	1.133	5.951	0.648
CTSS	-0.505	0.705	6.175	-1.407
CTSV	-0.373	0.772	2.449	-1.328
CTSW	-0.252	0.840	2.535	-0.846
CTSZ	-0.161	0.894	6.093	-0.602
CTXN1	1.090	2.129	5.402	2.446
CTXN2	0.657	1.577	2.642	1.514
CTXN3	1.616	3.065	3.183	2.727
CUEDC1	-0.612	0.654	3.810	-1.921
CUEDC1	-0.363	0.778	5.371	-1.192
CUL1	0.213	1.159	7.211	1.003
CUX2	0.940	1.918	3.258	1.953
CWC22	0.392	1.312	6.066	1.968
CWF19L1	0.117	1.084	5.109	0.443
CX3CL1	0.705	1.630	6.765	1.946
CX3CR1	-1.113	0.462	4.346	-1.983
CXCL10	-0.577	0.670	2.558	-1.050
CXCL11	-0.257	0.837	2.549	-0.695
CXCL12	0.067	1.047	3.787	0.240
CXCL14	1.843	3.588	6.880	2.556
CXCL16	-0.215	0.862	6.342	-0.627
CXCL2	0.031	1.022	2.810	0.084
CXCL5	0.069	1.049	3.916	0.243
CXCL9	-0.096	0.936	1.966	-0.245
CXCR1	0.323	1.251	2.642	0.783
CXCR4	-0.764	0.589	7.503	-1.989
CXorf21	0.092	1.066	2.346	0.279
CXorf22	-0.492	0.711	3.814	-1.414
CXorf28	-1.061	0.479	2.453	-1.962
CXorf30	-0.531	0.692	2.443	-1.763
CXXC4	0.017	1.012	4.450	0.057
CYB561	0.784	1.722	4.244	2.376
CYB561A3//CYBASC3	-0.175	0.886	2.496	-0.789
CYB561D2//XXcos-LUCA11.5//XXcos-LU(	-0.054	0.963	2.453	-0.195
CYB5A	-0.248	0.842	7.284	-1.002
CYB5R2	0.094	1.067	3.832	0.343
CYB5R3	-0.215	0.862	6.998	-1.000
CYBA	-0.376	0.771	4.584	-1.043
CYBB	-0.325	0.798	7.387	-0.608

CYBRD1	-0.256	0.837	7.699	-0.912
CYFIP2	0.627	1.544	7.131	2.368
CYGB	0.563	1.477	5.220	1.569
CYP1B1	0.908	1.876	5.227	2.066
CYP20A1	-0.546	0.685	4.814	-2.066
CYP21A1P	-0.142	0.906	3.662	-0.593
CYP26B1	0.526	1.440	2.292	1.762
CYP27A1	-1.188	0.439	6.988	-3.094
CYP2B7P//CYP2B7P1	0.743	1.673	3.062	1.701
CYP2D7P//CYP2D6//CYP2D7P1	0.155	1.113	2.707	0.446
CYP2E1	0.420	1.337	3.222	1.782
CYP2J2	-0.922	0.528	4.288	-3.069
CYP2S1	-0.151	0.901	3.549	-0.565
CYP46A1	0.748	1.679	5.487	2.333
CYP4F11	0.236	1.177	4.823	0.740
CYP4F26P//RP11-255A11.21	0.265	1.201	2.549	0.529
CYP4F3	-1.661	0.316	2.314	-3.098
CYP4X1	1.010	2.013	4.598	1.909
CYP7B1	-0.957	0.515	5.797	-2.292
CYR61	0.176	1.130	4.713	0.466
CYSLTR2	0.490	1.405	2.593	1.619
CYTH1	-0.559	0.679	5.582	-2.185
CYTH3	0.437	1.354	2.906	2.013
CYTH4	-0.193	0.875	3.962	-0.561
CYTIP	-0.250	0.841	2.472	-0.781
CYTL1	-0.198	0.872	4.711	-0.477
CYYR1	0.267	1.203	4.017	0.881
D2HGDH	-0.049	0.966	3.594	-0.203
DAAM2	-1.193	0.437	7.492	-2.641
DAB1	0.353	1.277	4.225	1.103
DAB2//LOC101926921	0.038	1.027	4.354	0.143
DACH1	0.628	1.546	4.405	2.115
DACH2	0.361	1.285	1.743	1.287
DACT1	-0.003	0.998	3.521	-0.012
DAGLA	0.872	1.830	5.623	2.461
DAGLB	0.284	1.218	6.483	1.009
DAO	-1.643	0.320	5.270	-2.078
DAPK1	0.655	1.575	7.127	1.983
DAPK2	-0.615	0.653	3.542	-2.318
DAPP1	-0.222	0.857	2.120	-0.818
DAW1	0.310	1.240	2.402	0.795
DBF4B	-0.556	0.680	3.587	-2.207
DBH	-0.435	0.740	2.637	-1.362
DBI	-0.213	0.863	5.400	-1.006
DBNDD1	0.435	1.352	5.053	1.606
DBP	0.371	1.293	6.050	1.471
DBX2	-0.739	0.599	4.453	-2.577
DCAF12L2	0.261	1.198	2.317	1.020
DCAF13P3	-0.122	0.919	2.437	-0.373
DCAF6	0.840	1.790	5.595	2.789

DCC	0.593	1.509	5.233	1.794
DCDC1//DCDC5	-0.116	0.923	2.722	-0.441
DCDC2	0.763	1.698	2.938	2.476
DCHS2	0.201	1.149	4.339	0.696
DCLRE1A	0.404	1.323	5.007	1.867
DCN	0.436	1.353	3.045	1.729
DCP2	0.346	1.271	5.373	1.526
DCT	0.175	1.129	3.247	0.682
DCTN1-AS1	0.170	1.125	3.796	0.655
DCUN1D3	0.350	1.274	4.547	1.561
DCX	0.475	1.390	2.775	1.658
DDAH1	0.287	1.220	3.783	1.170
DDAH2	-0.266	0.832	5.284	-1.184
DDAH2	-0.255	0.838	5.299	-1.145
DDB2	0.033	1.023	4.365	0.113
DDIT4	-1.243	0.422	7.493	-3.125
DDN	2.522	5.745	3.560	3.107
DDO	-0.290	0.818	3.773	-0.795
DDR1//MIR4640	-0.868	0.548	7.217	-3.092
DDR1//MIR4640	-0.854	0.553	7.114	-3.051
DDR1//MIR4640	-0.816	0.568	7.068	-2.881
DDR1//MIR4640	-0.712	0.610	4.977	-2.599
DDR1//MIR4640//Error in gsub("^[[:space:]]+|[[:space:]		1.000		
DDR2	-0.249	0.842	6.330	-0.839
DDX1	0.294	1.226	7.490	1.581
DDX10	0.393	1.313	4.951	1.918
DDX11L10	0.395	1.315	2.300	1.345
DDX11L2	0.052	1.037	3.052	0.137
DDX11L2//DDX11L9//DDX11L10//DDX1	0.313	1.242	3.009	1.221
DDX11L5	0.007	1.005	2.634	0.023
DDX12P	-0.231	0.852	3.783	-0.730
DDX20	0.227	1.170	2.849	0.959
DDX25	0.792	1.732	4.463	2.299
DDX39A	-0.505	0.704	5.058	-2.272
DDX3Y	-0.011	0.992	4.317	-0.010
DDX55	-0.012	0.991	4.448	-0.044
DDX60	-0.146	0.904	4.050	-0.482
DDX60L	-0.501	0.707	3.429	-2.234
DEDD2	0.243	1.183	5.119	0.795
DEF6	-0.603	0.658	3.395	-1.592
DEFA1B//DEFA1//DEFA3	-0.737	0.600	3.061	-1.353
DEFA1B//DEFA1//DEFA3	-0.704	0.614	3.054	-1.276
DENND1B	0.262	1.199	4.261	1.131
DENND1C	-0.164	0.892	2.921	-0.515
DENND2A	-0.404	0.756	4.734	-1.455
DENND2D	0.110	1.079	3.260	0.441
DENND3	0.078	1.056	5.322	0.262
DENND5A	-0.420	0.748	8.167	-1.795
DENND5B	0.293	1.225	6.649	1.194
DENND6B	0.516	1.430	5.178	1.970

DENR	0.372	1.294	5.518	1.601
DEPDC1B	-0.519	0.698	2.779	-1.496
DEPTOR	-0.729	0.603	5.372	-3.181
DES	0.944	1.924	2.678	1.847
DFFB	-0.054	0.963	3.468	-0.243
DFNA5	-0.196	0.873	5.553	-0.800
DFNB31	-0.927	0.526	5.512	-2.805
DGAT2	0.587	1.502	2.809	2.567
DGCR10	0.583	1.498	2.403	1.427
DGCR11	0.036	1.025	1.799	0.123
DGCR14//TSSK2	-0.194	0.874	4.638	-0.756
DGCR9//AC000095.10	0.974	1.965	3.143	1.766
DGKA	0.368	1.291	4.453	1.291
DGKB	1.024	2.033	5.618	2.346
DGKE	0.674	1.596	5.774	2.562
DGKG	0.368	1.291	6.141	1.841
DGKH	0.635	1.553	4.725	1.958
DGKI	0.812	1.756	5.271	2.230
DGKK	-0.669	0.629	2.689	-1.795
DGKZ//DGKZP1	1.011	2.015	5.549	2.947
DHCR24	-0.666	0.630	9.759	-2.282
DHCR7	-0.507	0.704	6.661	-1.655
DHH	-0.198	0.871	2.291	-0.703
DHRS11	0.840	1.790	4.349	2.810
DHRS3	-0.423	0.746	6.141	-1.342
DHRS4	-0.109	0.927	2.721	-0.375
DHRS4L1//DHRS4L2	0.276	1.211	5.397	1.052
DHX57	0.328	1.255	4.873	1.369
DIAPH1	0.260	1.198	5.399	1.029
DIEXF	0.125	1.090	5.146	0.562
DIO2	0.865	1.822	2.101	3.307
DIP2A	-0.631	0.646	5.573	-2.477
DIP2A-IT1	-0.662	0.632	2.360	-1.874
DIP2B	-0.636	0.644	8.267	-2.524
DIRAS1	1.208	2.311	5.748	2.731
DIRAS2	1.642	3.122	6.071	3.186
DIRAS3	-0.396	0.760	3.383	-0.950
DISP2	0.589	1.504	4.369	1.901
DKFZP434I0714//MIR4453//RP11-555K1	-0.169	0.890	4.047	-0.633
DKFZp434J0226//AC006262.6	-0.433	0.741	3.326	-1.381
DKFZP434L187	-1.281	0.412	2.139	-2.435
DKFZp451B082	-0.427	0.744	2.635	-1.352
DKFZP586I1420	-0.012	0.991	5.907	-0.044
DKFZp667F0711	-0.174	0.886	4.629	-0.333
DKK2	0.057	1.040	3.923	0.199
DLAT	0.252	1.191	6.716	1.236
DLD	0.376	1.298	6.273	1.900
DLEC1	-0.216	0.861	2.683	-0.717
DLEU2	-0.796	0.576	2.312	-2.563
DLEU2L	1.078	2.111	2.962	2.971

DLEU7	0.458	1.373	3.034	1.511
DLG2	0.121	1.087	6.537	0.495
DLG3	0.905	1.872	5.155	2.262
DLG4	1.049	2.070	6.250	2.544
DLG5	-0.270	0.829	6.513	-1.252
DLGAP1	1.463	2.756	4.612	3.227
DLGAP1-AS1	-0.361	0.778	4.385	-1.384
DLGAP1-AS2	-0.004	0.997	2.784	-0.013
DLGAP1-AS3	1.006	2.008	1.809	2.804
DLGAP1-AS5	0.054	1.038	2.255	0.206
DLGAP2	1.370	2.585	3.627	2.695
DLGAP3	1.178	2.262	3.580	2.187
DLGAP4	0.532	1.446	6.497	1.876
DLL1	-0.609	0.656	4.837	-2.013
DLL4	-0.185	0.880	4.115	-0.642
DLX1	0.579	1.494	2.875	1.249
DLX2	0.378	1.300	1.934	1.523
DLX5	0.575	1.490	2.948	1.843
DLX6	0.229	1.172	2.508	0.863
DLX6-AS1	0.867	1.824	1.860	3.139
DMBT1	-0.922	0.528	2.480	-2.097
DMD	0.159	1.116	6.258	0.806
DMKN	0.309	1.239	2.613	1.192
DMRT2	-0.679	0.625	2.486	-2.235
DMRTC1//DMRTC1B	0.585	1.500	3.587	2.788
DMRTC1B//DMRTC1	0.718	1.645	2.891	3.361
DMTN	0.960	1.946	6.960	2.161
DMXL2	0.705	1.630	7.349	2.674
DNA2	-0.319	0.802	3.523	-1.229
DNAAF1	0.347	1.272	3.751	0.618
DNAH10	-0.511	0.702	2.975	-1.766
DNAH10OS	0.663	1.583	4.775	2.248
DNAH11	-0.396	0.760	2.265	-0.723
DNAH17	-0.377	0.770	2.638	-1.636
DNAH17-AS1//RP11-559N14.5	-0.865	0.549	2.457	-2.356
DNAH2	0.202	1.151	2.473	0.586
DNAH5	-0.168	0.890	2.708	-0.610
DNAH6	0.569	1.484	3.371	1.716
DNAH7	0.553	1.467	4.215	1.612
DNAH9	-0.490	0.712	3.567	-1.203
DNAI1	0.115	1.083	2.735	0.374
DNAJA1	-0.076	0.949	8.364	-0.286
DNAJA2	0.302	1.233	7.471	1.585
DNAJA4	-0.002	0.999	5.626	-0.007
DNAJB1	-0.345	0.787	7.592	-0.878
DNAJB14	0.199	1.148	5.174	0.901
DNAJB2	-0.672	0.628	5.161	-2.879
DNAJC12	0.029	1.020	6.062	0.125
DNAJC19	0.145	1.106	5.046	0.748
DNAJC27	0.024	1.017	5.034	0.110

DNAJC28	0.349	1.274	3.701	1.566
DNAJC6	0.061	1.043	9.207	0.309
DNAL4	0.235	1.177	3.740	0.966
DNAPTP3	0.239	1.181	2.873	0.904
DNASE1	-0.289	0.819	3.274	-1.077
DNASE1L2	-0.414	0.751	2.761	-1.635
DNASE2B	-0.044	0.970	2.181	-0.098
DND1	-0.764	0.589	2.861	-2.976
DNER	-0.664	0.631	8.651	-2.805
DNM1	1.341	2.534	8.136	1.902
DNM1L	0.563	1.477	5.914	2.308
DNM1L	0.263	1.200	3.807	0.934
DNM1P35	0.250	1.189	3.129	0.745
DNM1P46	-0.285	0.821	3.009	-0.859
DNM2	-0.503	0.706	5.493	-1.897
DNM3	-0.029	0.980	8.080	-0.138
DNM3-IT1	-0.573	0.672	3.144	-1.733
DOC2A	1.576	2.982	5.559	2.797
DOC2B	0.500	1.414	5.776	1.332
DOCK1	-0.630	0.646	7.872	-2.507
DOCK10	-1.133	0.456	8.371	-3.260
DOCK11	-0.218	0.860	5.558	-0.835
DOCK2	-0.568	0.674	5.119	-1.812
DOCK3	0.509	1.423	7.275	2.191
DOCK5	-1.158	0.448	6.762	-2.924
DOCK8	-1.098	0.467	4.567	-2.952
DOK1	-0.243	0.845	2.542	-1.195
DOK2	0.126	1.091	2.127	0.488
DOK3	-0.148	0.903	2.849	-0.503
DOK4	0.678	1.600	4.009	2.071
DOK5	0.260	1.197	5.552	1.035
DOK6	1.276	2.421	6.190	1.915
DOPEY2	0.679	1.601	5.133	2.729
DPCR1	0.237	1.179	3.012	0.633
DPF1	0.413	1.332	4.038	1.289
DPF3	-0.476	0.719	4.496	-2.050
DPH6	-0.027	0.981	3.847	-0.091
DPH6-AS1	0.072	1.051	3.122	0.233
DPP10	0.658	1.578	6.171	2.207
DPP10-AS1//AC066593.1	0.804	1.745	4.069	2.309
DPP10-AS2//AC068542.2	0.083	1.059	2.581	0.271
DPP4	0.505	1.419	2.607	1.656
DPP6	0.392	1.312	6.984	1.409
DPP8	0.324	1.252	6.069	1.619
DPRXP4	-0.312	0.806	2.356	-0.900
DPT	0.373	1.295	2.786	1.323
DPY19L1P1//AC018633.4	0.538	1.452	2.240	1.671
DPY19L2P1	1.311	2.481	3.164	2.828
DPY19L2P3//AC007276.5	0.572	1.487	2.448	2.734
DPY19L2P4	0.801	1.742	2.587	2.157

DPYD	-0.575	0.671	6.872	-1.868
DPYD-IT1	-0.995	0.502	3.652	-1.823
DPYSL3	-0.553	0.682	7.216	-2.121
DPYSL5	-1.074	0.475	5.900	-3.062
DRD1	1.476	2.781	3.100	2.773
DRGX	0.537	1.451	2.754	1.551
DRP2	1.272	2.416	5.375	2.040
DSC2	0.049	1.034	4.959	0.146
DSCAM	0.048	1.034	5.492	0.156
DSEL	-0.444	0.735	6.694	-1.567
DSN1	0.643	1.561	3.767	2.283
DSP	0.659	1.579	4.514	1.114
DSTYK	0.265	1.202	6.228	1.246
DTHD1	-0.524	0.695	2.077	-0.930
DTNA	0.001	1.001	9.309	0.004
DTX1	0.164	1.120	4.871	0.684
DTX2	-0.550	0.683	3.280	-2.353
DTX2P1-UPK3BP1-PMS2P11	-0.878	0.544	4.129	-3.121
DTX3L	0.046	1.033	5.519	0.188
DTX4	-0.823	0.565	6.406	-2.625
DUOX1	-0.468	0.723	2.271	-1.552
DUSP1	0.299	1.230	8.171	0.962
DUSP10	-0.390	0.763	5.467	-1.371
DUSP14	0.043	1.030	5.378	0.140
DUSP15//C20orf57	-0.280	0.823	3.665	-1.067
DUSP16	-0.634	0.645	5.587	-2.536
DUSP19	0.825	1.772	4.388	2.181
DUSP2	0.408	1.327	4.238	1.410
DUSP26	0.214	1.160	6.497	0.988
DUSP3	0.330	1.257	6.088	1.421
DUSP5	0.199	1.148	2.950	0.687
DUSP7	-0.366	0.776	4.676	-1.777
DUSP8//LOC101927562	0.706	1.631	4.654	2.103
DUXAP10	0.366	1.289	4.910	1.019
DUXAP10//LOC100507460//CTD-2314B2	0.438	1.355	3.916	1.090
DYNC1I1	0.307	1.237	7.408	1.218
DYNC1LI1	0.430	1.348	5.449	2.095
DYNLL1-AS1	-0.051	0.965	4.324	-0.188
DYNLRB2	-0.609	0.655	2.638	-2.671
DYNLT1	-0.203	0.868	5.165	-0.856
DYNLT3	0.467	1.382	6.913	1.857
DZIP1	0.477	1.392	6.645	1.924
DZIP3	0.419	1.337	7.301	1.990
E2F3	0.375	1.297	3.671	1.546
EBF1	-0.184	0.880	3.800	-0.503
EBF2	-0.010	0.993	2.604	-0.031
EBF3	-0.818	0.567	3.085	-1.305
EBI3	-0.400	0.758	2.855	-1.113
EBLN2	-0.519	0.698	3.915	-1.600
EBLN3//RP11-220I1.1	0.335	1.261	7.065	1.435

ECD//RP11-152N13.3	0.048	1.034	6.929	0.211
ECE1	-0.152	0.900	6.121	-0.691
ECEL1	-0.941	0.521	3.221	-1.950
ECHDC3	0.170	1.125	3.108	0.561
ECM2	-0.323	0.799	3.931	-0.828
ECRP	0.044	1.031	4.994	0.049
ECSCR	-0.130	0.914	4.935	-0.373
ECT2	0.690	1.613	4.277	2.849
EDA2R	-0.901	0.536	4.269	-2.037
EDIL3	-0.771	0.586	9.316	-2.770
EDN1	0.362	1.285	6.437	0.775
EDN3	-0.403	0.756	3.624	-1.346
EDNRA	0.308	1.238	3.996	0.957
EDNRB	-0.194	0.874	7.814	-0.449
EEF1A2	0.911	1.880	7.312	1.907
EEF1D//RP11-661A12.7	0.006	1.004	3.568	0.020
EEF1DP3	0.106	1.076	3.290	0.429
EEF1G//MIR3654//EEF1GP1//RP11-864I	-0.154	0.899	8.629	-0.777
EEPD1	-0.437	0.739	5.276	-1.581
EFCAB1	0.063	1.044	2.608	0.159
EFCAB12	0.454	1.370	2.433	0.983
EFCAB6	0.444	1.360	3.884	2.381
EFCAB7	0.361	1.285	4.317	1.369
EFEMP1	-0.351	0.784	8.494	-1.108
EFEMP2	-0.090	0.940	4.920	-0.420
EFHB	-0.175	0.886	2.525	-0.514
EFHC2	0.608	1.524	3.543	2.076
EFHD1	-1.037	0.487	4.847	-3.375
EFHD2	0.607	1.523	5.699	2.360
EFNA1	-0.394	0.761	4.837	-1.073
EFNA2	0.379	1.300	3.090	1.371
EFNA3//RP11-540D14.8	0.805	1.747	4.749	1.854
EFNA5	1.097	2.140	4.388	2.507
EFNB2	0.886	1.848	4.676	2.008
EFNB3	1.068	2.097	4.474	1.965
EFR3A	0.862	1.818	7.567	3.040
EFR3B	0.071	1.050	7.036	0.298
EFS	-0.877	0.544	5.187	-2.407
EGF	0.079	1.057	3.030	0.357
EGFEM1P	0.464	1.380	3.097	1.489
EGFR	-0.041	0.972	5.759	-0.130
EGR1	0.167	1.123	5.072	0.507
EGR3	0.851	1.804	3.614	2.149
EGR4	-0.104	0.930	2.317	-0.382
EHBP1	0.560	1.474	6.461	2.353
EHBP1L1	-0.446	0.734	3.598	-1.633
EHD2	0.141	1.103	5.673	0.608
EHD3	0.833	1.782	6.120	2.245
EHD4	-0.080	0.946	4.978	-0.299
EHF	-0.385	0.766	3.794	-1.105

EID2	0.618	1.534	4.379	2.567
EID2B	0.699	1.623	3.346	2.518
EIF1AD	0.132	1.096	5.588	0.697
EIF1AY	-0.001	0.999	3.558	-0.002
EIF4A2//SNORA63//SNORD2//SNORA4/,	0.174	1.128	7.665	0.918
EIF4B	-0.103	0.931	5.487	-0.281
EIF4E1B	0.464	1.379	2.895	2.006
EIF4EBP1	-0.192	0.875	4.449	-0.535
EIF5A2	0.539	1.453	5.429	1.725
ELAVL2	0.934	1.911	6.245	1.568
ELAVL4	1.007	2.009	4.566	1.991
ELF1	-0.595	0.662	7.982	-2.485
ELF4	-0.281	0.823	2.555	-0.906
ELFN1	0.373	1.295	4.211	1.270
ELFN2//RP1-63G5.5	0.586	1.501	3.290	2.744
ELK1	0.647	1.566	4.738	1.680
ELK3	-0.195	0.874	4.434	-0.738
ELL2	-0.043	0.971	8.446	-0.153
ELMO1	-0.987	0.505	7.368	-2.879
ELMOD1//LOC643923	1.009	2.013	6.772	1.963
ELMOD1//LOC643923	0.346	1.271	3.605	1.233
ELN	-0.011	0.992	3.046	-0.046
ELOVL1//MIR6734	-1.294	0.408	7.016	-2.954
ELOVL2	-0.043	0.970	7.062	-0.159
ELOVL4	0.903	1.869	6.143	1.871
ELOVL5	-0.231	0.852	6.838	-0.812
ELP5	0.054	1.038	5.648	0.248
EMBP1	-0.018	0.988	3.068	-0.063
EMC10	-0.437	0.739	6.873	-1.964
EMC3-AS1//AC034193.5	0.118	1.085	3.586	0.353
EMD	-0.042	0.971	6.304	-0.158
EMID1	-0.068	0.954	3.835	-0.263
EMILIN1	-0.205	0.868	3.438	-0.745
EMILIN2	-0.762	0.590	5.541	-1.595
EMILIN3	0.008	1.005	3.455	0.027
EML1	-0.399	0.758	5.550	-1.687
EML3	-0.243	0.845	5.231	-1.147
EML5	0.549	1.463	4.429	2.203
EML6	0.649	1.568	4.747	2.217
EMP1	0.453	1.369	5.950	1.097
EMP2	-0.248	0.842	4.731	-1.078
EMP3	-0.094	0.937	7.612	-0.315
EMR1	0.182	1.134	2.000	0.447
EMR4P	-0.692	0.619	2.155	-1.597
EMX1	0.387	1.307	2.445	1.286
EMX2	1.189	2.280	4.314	2.229
EMX2OS	0.756	1.688	3.143	1.856
ENC1	1.929	3.808	4.411	2.765
ENDOD1	-0.349	0.785	7.143	-1.268
ENG	0.470	1.385	6.038	1.729

ENHO	-0.119	0.921	4.908	-0.281
ENKUR	-0.186	0.879	4.700	-0.632
ENO1	0.097	1.069	5.422	0.389
ENO1-IT1	-0.259	0.835	2.343	-1.034
ENO2	0.632	1.550	4.513	2.325
ENO4	0.064	1.046	3.227	0.227
ENOSF1	-1.291	0.409	5.737	-3.404
ENPP1	-0.236	0.849	3.695	-0.705
ENPP2	-1.046	0.484	9.718	-2.648
ENPP2	-0.907	0.533	5.609	-1.539
ENPP4	-0.177	0.885	7.604	-0.638
ENPP5	0.360	1.283	6.269	1.242
ENPP6	-1.322	0.400	6.213	-2.522
ENTPD1	-0.498	0.708	3.650	-2.199
ENTPD2	-0.026	0.982	4.306	-0.093
ENTPD3	0.824	1.770	4.532	1.557
ENTPD3-AS1	-0.696	0.617	2.964	-2.699
ENTPD4	-0.422	0.746	3.330	-1.460
ENTPD5	-0.271	0.828	3.804	-1.065
ENTPD6	0.570	1.485	6.102	2.181
ENTPD7	0.411	1.329	4.060	1.689
EPB41L4B	0.904	1.872	6.009	2.130
EPC1//IGHV3-33	-0.527	0.694	2.236	-1.479
EPCAM	-0.280	0.823	3.570	-0.897
EPDR1	0.499	1.413	6.929	1.624
EPHA10	0.435	1.352	2.264	1.768
EPHA2	-0.032	0.978	3.381	-0.132
EPHA3	0.562	1.476	4.300	2.031
EPHA4	1.445	2.723	5.834	2.626
EPHA5	0.464	1.380	5.755	1.368
EPHA6	0.596	1.511	2.182	1.815
EPHA7	0.967	1.955	4.903	2.314
EPHB1	0.405	1.324	3.979	1.652
EPHB6	1.966	3.907	5.178	3.684
EPHX1	-0.382	0.767	6.585	-1.518
EPHX2	-0.301	0.812	3.879	-1.157
EPHX4	1.345	2.541	4.051	2.702
EPN2-IT1//EPN2	-0.657	0.634	3.569	-2.172
EPN3	0.521	1.435	3.485	1.875
EPS8	-0.414	0.751	6.173	-1.689
EPS8L2	0.087	1.062	3.525	0.359
EPSTI1	-0.194	0.874	3.419	-0.748
EPT1	0.295	1.227	6.793	1.152
ERAP1	-0.199	0.871	5.564	-0.676
ERAP2	-0.167	0.890	2.522	-0.484
ERBB2	-0.431	0.742	5.157	-1.870
ERBB2IP	-0.362	0.778	8.312	-1.413
ERBB3	-1.328	0.398	5.572	-2.906
ERBB4	0.062	1.044	7.111	0.261
ERC2	1.063	2.089	5.035	2.644

ERC2-IT1	0.519	1.433	2.352	1.913
ERF	-0.532	0.691	3.907	-2.113
ERI2	-0.200	0.870	4.143	-0.664
ERICH3//C1orf173	1.274	2.419	4.078	2.365
ERLEC1	0.370	1.293	7.188	1.765
ERMAP	-0.172	0.888	3.651	-0.866
ERMARD//C6orf70	-0.089	0.940	5.524	-0.315
ERMN	-1.062	0.479	8.472	-2.621
ERO1L	0.369	1.291	5.659	1.279
ESPNP	0.144	1.105	2.675	0.442
ESRRG	0.156	1.114	4.106	0.463
ESYT3	0.690	1.613	3.808	2.079
ETAA1	0.322	1.250	3.745	1.404
ETNPPL	-0.503	0.706	7.691	-1.217
ETS2	0.337	1.263	7.769	1.310
ETV1	0.611	1.528	4.323	2.631
ETV6//INO80D	0.156	1.115	6.444	0.663
EVI2A	-1.005	0.498	4.069	-2.254
EVI2B//CTD-2370N5.3	-0.684	0.622	3.972	-2.044
EXOC6	0.578	1.493	5.144	2.139
EXOC8	0.339	1.264	5.721	1.409
EXOSC3	-0.162	0.894	5.896	-0.806
EXOSC5	-0.017	0.988	5.998	-0.059
EXOSC6	0.388	1.309	6.264	1.662
EXTL1	0.901	1.868	3.162	2.531
EXTL2	0.402	1.321	4.777	1.641
EYA1	0.211	1.157	4.356	0.702
EYA2	-0.360	0.779	5.225	-1.269
EYA4	-0.983	0.506	4.244	-3.510
F11R//RP11-544M22.13	-0.129	0.915	4.891	-0.425
F13A1	0.751	1.684	5.968	1.366
F2R	0.067	1.047	4.018	0.250
F2RL2	-0.698	0.616	2.673	-1.522
F3	1.121	2.175	7.890	2.949
F5	-0.432	0.741	4.374	-0.939
F7	0.286	1.219	2.350	1.016
F8A1//F8A2//F8A3	0.468	1.383	8.091	1.540
F8A1//F8A2//F8A3	0.144	1.105	7.733	0.720
FA2H	-1.552	0.341	7.133	-3.132
FAAH	-0.476	0.719	5.271	-1.797
FABP3	0.659	1.579	6.589	1.456
FABP6	0.200	1.149	4.474	0.631
FABP7	0.227	1.170	5.562	0.780
FADS2	-0.344	0.788	4.477	-1.409
FADS6	0.235	1.177	2.618	0.751
FAH	-0.084	0.943	4.061	-0.367
FAHD2CP	-0.607	0.657	6.766	-1.705
FAIM2	0.240	1.181	7.240	1.195
FAM101B	0.354	1.278	5.261	1.288
FAM102B	0.968	1.956	6.427	2.974

FAM105A	-0.265	0.832	4.975	-0.888
FAM106A//FAM106CP	-0.430	0.742	2.803	-1.661
FAM106CP	-0.337	0.792	6.211	-0.869
FAM107A	0.064	1.045	4.417	0.227
FAM110C	0.338	1.264	3.336	1.051
FAM111A	-0.431	0.742	3.549	-1.790
FAM114A1	-0.309	0.807	5.426	-1.382
FAM115A//LOC100294033	0.529	1.443	6.219	2.233
FAM115C//LOC101928422	0.261	1.198	3.340	0.951
FAM117B	0.287	1.221	7.380	1.116
FAM118A	-0.464	0.725	3.047	-1.734
FAM124A	-0.628	0.647	4.057	-1.928
FAM126B	0.763	1.697	5.747	2.879
FAM127B	-0.073	0.951	4.800	-0.236
FAM129A	0.130	1.095	5.227	0.378
FAM129B	-0.092	0.938	5.993	-0.392
FAM131A	0.465	1.380	4.416	1.944
FAM133A	0.249	1.188	4.086	0.836
FAM135B	0.545	1.459	5.215	1.898
FAM13A	0.396	1.315	5.441	1.707
FAM13A-AS1	-0.351	0.784	4.062	-1.275
FAM153A//FAM153B//LOC100507387	0.900	1.866	3.954	1.974
FAM154B	0.015	1.011	2.651	0.055
FAM155A	0.648	1.567	5.721	1.711
FAM155A-IT1	-0.028	0.981	5.056	-0.074
FAM160B1	0.388	1.309	6.071	1.829
FAM162B	1.270	2.411	4.538	3.239
FAM163A	0.174	1.128	2.432	0.580
FAM163B	1.646	3.129	5.323	2.908
FAM163B	0.478	1.392	3.140	1.392
FAM169A	0.226	1.169	7.175	1.083
FAM171A2	0.916	1.887	5.685	2.593
FAM171B	0.445	1.361	9.006	1.847
FAM177B	-0.118	0.922	2.933	-0.380
FAM178A	-0.694	0.618	5.746	-2.790
FAM178B	-0.099	0.934	2.905	-0.431
FAM179A	0.074	1.052	2.957	0.243
FAM179B	0.111	1.080	6.405	0.489
FAM181A-AS1	-0.113	0.925	2.790	-0.385
FAM181B	-0.272	0.828	3.224	-1.009
FAM184A	0.588	1.503	5.031	2.619
FAM189A1	0.772	1.707	4.370	2.735
FAM189A2	-0.330	0.796	7.379	-0.822
FAM196A	-0.214	0.862	3.099	-0.749
FAM196B	-0.693	0.619	5.408	-2.714
FAM198A//KRBOX1	-0.194	0.874	4.507	-0.718
FAM198B	-0.037	0.975	4.883	-0.147
FAM19A1	1.025	2.035	2.407	2.982
FAM19A2	0.752	1.684	2.466	2.402
FAM19A4	-0.774	0.585	3.623	-2.487

FAM200A	-0.430	0.742	4.312	-2.190
FAM200B	0.053	1.037	5.052	0.237
FAM20A	0.073	1.052	3.325	0.256
FAM20B	0.411	1.329	6.762	1.840
FAM20C	-0.058	0.960	5.645	-0.181
FAM212B//LOC101928718	0.699	1.623	3.566	1.742
FAM213B	0.495	1.409	4.680	1.979
FAM215A	-0.298	0.813	3.102	-0.845
FAM216A	0.406	1.325	4.596	1.745
FAM216B	-0.247	0.842	2.366	-0.411
FAM217B	0.517	1.431	6.199	2.305
FAM218A	-0.461	0.726	4.612	-1.813
FAM220A//FAM220BP//FAM220CP	0.272	1.208	5.945	1.103
FAM221A	0.083	1.059	4.132	0.292
FAM222A	-0.965	0.512	3.861	-2.623
FAM224A//FAM224B	0.209	1.156	2.561	0.782
FAM225A//FAM225B	-0.898	0.536	4.109	-2.704
FAM225B	-0.229	0.853	2.502	-0.615
FAM226B//FAM226A//RP1-172N19.5	0.183	1.136	3.085	0.682
FAM227A	-0.158	0.896	3.750	-0.437
FAM228A//RP11-507M3.1	0.753	1.685	4.479	2.495
FAM231D//FAM231B//LOC100996721//	0.783	1.720	2.555	1.797
FAM26E	-0.171	0.888	2.921	-0.450
FAM26F	0.029	1.021	3.011	0.095
FAM27C//FAM27B//FAM182B	0.801	1.743	3.050	3.052
FAM27E2//FAM27E3//FAM27E4P	-0.510	0.702	4.939	-1.313
FAM43A	-0.193	0.875	4.136	-0.620
FAM46A	-0.215	0.862	3.381	-0.970
FAM46B	-0.062	0.958	2.252	-0.236
FAM46C	-0.132	0.913	4.290	-0.331
FAM49A	0.942	1.921	7.030	3.164
FAM57B//LOC100996332	0.504	1.418	4.739	1.446
FAM63A	-1.029	0.490	5.634	-3.018
FAM65B	0.838	1.788	5.385	2.325
FAM66A	-0.078	0.947	3.931	-0.135
FAM66B	0.249	1.189	4.247	0.675
FAM66E	0.143	1.104	3.351	0.388
FAM69B	0.686	1.609	5.793	2.358
FAM69C	-0.278	0.825	4.026	-0.932
FAM76B	0.013	1.009	4.263	0.045
FAM78B	0.354	1.278	4.055	0.954
FAM81A	1.283	2.433	4.439	3.651
FAM83D	0.687	1.610	2.302	1.686
FAM83H	0.303	1.234	2.502	0.997
FAM84A	1.066	2.094	4.905	2.555
FAM84B	-0.338	0.791	4.607	-1.240
FAM86A//FAM86B2//FAM86KP	-0.217	0.861	3.975	-0.838
FAM86B3P	0.127	1.092	2.435	0.482
FAM86C2P	-0.013	0.991	2.557	-0.046
FAM86DP	0.236	1.178	4.674	0.869

FAM86EP	0.146	1.106	3.691	0.500
FAM86HP	0.578	1.493	3.051	1.353
FAM89A//MIR1182	0.052	1.037	4.781	0.253
FAM89B//SSSCA1	0.247	1.187	6.915	0.985
FAM90A1	-0.425	0.745	2.341	-1.245
FAM90A10P	-0.012	0.992	3.933	-0.045
FAM99A	0.135	1.098	2.930	0.484
FAM99B	-0.768	0.587	3.075	-2.647
FANCA	0.084	1.060	2.509	0.288
FANCL	-0.493	0.710	5.341	-1.742
FANCM	-0.056	0.962	2.615	-0.211
FANK1	0.227	1.170	2.879	0.781
FAP	-0.112	0.925	2.978	-0.419
FAR1	-0.495	0.710	7.840	-1.894
FAR1-IT1	-0.511	0.702	3.678	-1.424
FAR2	1.012	2.017	4.619	2.976
FAR2P2//FAR2P3//AC140481.1	-0.259	0.835	5.069	-0.944
FAR2P2//FAR2P3//AC140481.1	-0.165	0.892	4.664	-0.609
FARSB	0.346	1.271	7.150	1.443
FAS	-0.014	0.990	4.831	-0.036
FASTKD1	0.107	1.077	5.175	0.569
FASTKD2	0.368	1.291	4.982	1.622
FAT1	-0.557	0.680	6.981	-2.243
FAT3	0.653	1.573	6.218	1.849
FAT4	-0.565	0.676	5.675	-2.084
FATE1	0.534	1.448	1.870	1.740
FAXC	0.959	1.945	5.316	2.044
FAXDC2	-0.763	0.589	5.001	-2.096
FBLL1	0.801	1.743	4.843	1.968
FBLN1	-0.046	0.969	4.907	-0.188
FBLN2	0.150	1.109	4.077	0.574
FBLN5	0.092	1.066	5.328	0.393
FBLN7	0.650	1.569	2.673	2.164
FBP1	-0.179	0.883	3.292	-0.576
FBXL14	0.314	1.243	6.063	1.470
FBXL15	0.518	1.432	3.092	2.160
FBXL16	1.504	2.837	6.649	2.585
FBXL17	0.472	1.387	6.443	2.012
FBXL18	0.277	1.212	3.362	0.907
FBXL19	0.589	1.504	4.151	1.852
FBXL2	0.541	1.455	5.231	1.968
FBXL20	0.333	1.260	6.684	1.641
FBXL7	-0.586	0.666	6.444	-2.589
FBXO15	-0.540	0.688	3.400	-1.703
FBXO16//ZNF395	0.536	1.450	5.035	1.702
FBXO2	0.026	1.018	5.683	0.111
FBXO27	0.286	1.219	4.530	1.117
FBXO27	0.069	1.049	5.198	0.265
FBXO28	0.137	1.100	6.160	0.695
FBXO3	0.274	1.209	5.832	1.312

FBXO31	0.318	1.246	6.202	1.303
FBXO32	-0.592	0.663	7.725	-2.115
FBXO33	0.548	1.462	5.509	1.879
FBXO34	0.820	1.765	4.828	2.428
FBXO40	0.849	1.801	2.485	2.249
FBXO41//LOC101927826	1.167	2.246	4.657	2.899
FBXO44	0.383	1.304	4.743	1.522
FBXO45	0.196	1.145	4.897	0.863
FBXO48	0.033	1.023	4.015	0.133
FBXO7	-0.508	0.703	7.841	-2.095
FBXO9	0.203	1.151	5.250	0.845
FBXW7	1.071	2.100	7.079	2.674
FBXW9	0.352	1.277	2.539	1.137
FCAR	0.087	1.062	3.177	0.233
FCER1G	-0.075	0.949	7.262	-0.128
FCGBP	0.182	1.135	3.697	0.292
FCGR1A//FCGR1B	-0.243	0.845	5.193	-0.443
FCGR1B	-0.422	0.746	3.905	-1.361
FCGR2A	-0.256	0.838	3.953	-0.660
FCGR2B	-0.087	0.941	2.285	-0.210
FCGRT	-0.441	0.737	4.487	-1.986
FCN1	-0.044	0.970	2.848	-0.155
FDFT1	-0.389	0.764	5.673	-1.422
FDX1L	0.401	1.320	2.953	1.722
FEM1B	0.221	1.166	7.289	1.076
FERMT1	-0.633	0.645	4.397	-1.843
FERMT3	-0.311	0.806	4.254	-0.942
FEZF2	1.655	3.150	3.553	3.039
FFAR3	0.374	1.296	2.460	1.020
FFAR4	0.487	1.401	2.761	1.079
FGD1	0.526	1.440	4.352	2.062
FGD2	-0.799	0.575	3.832	-2.762
FGD3	-0.425	0.745	2.647	-1.420
FGD5	0.422	1.340	3.321	1.735
FGD6	0.414	1.332	5.385	1.542
FGF1	-0.789	0.579	4.146	-2.562
FGF10	0.269	1.205	3.309	0.814
FGF11//RP11-104H15.7//RP11-104H15.	-0.419	0.748	4.889	-1.392
FGF12	0.916	1.887	4.285	1.815
FGF13	0.873	1.832	3.866	2.220
FGF14	1.294	2.452	5.815	2.652
FGF14-AS2//RP11-811P12.4	-0.346	0.787	3.397	-1.059
FGF14-IT1	0.449	1.365	4.271	1.224
FGF17	-0.008	0.995	3.110	-0.027
FGF18	0.470	1.385	2.817	1.956
FGF5	0.534	1.448	2.730	2.010
FGF7	-0.127	0.916	2.125	-0.334
FGF9	1.057	2.081	4.162	2.137
FGFBP2	-0.011	0.993	2.275	-0.029
FGFR1	-0.369	0.775	4.013	-2.048

FGFR1OP2	-0.297	0.814	5.261	-0.900
FGFR2	-0.693	0.619	7.082	-2.114
FGFR3	-0.123	0.918	6.562	-0.374
FGL2	0.021	1.014	6.548	0.056
FGR	-0.129	0.915	3.546	-0.572
FH	0.334	1.260	5.983	1.487
FHDC1	0.077	1.055	2.586	0.264
FHL2	0.698	1.622	3.244	2.022
FHL3	0.001	1.000	4.352	0.002
FHL5	0.059	1.042	4.667	0.150
FHOD3	0.480	1.394	6.157	1.372
FIBIN	0.237	1.179	5.804	1.051
FICD	-0.321	0.800	3.089	-1.641
FIG4	0.198	1.147	6.186	0.902
FIGNL1	0.121	1.087	3.825	0.485
FILIP1	0.679	1.601	3.933	2.478
FIRRE//RP11-453F18__B.1	-0.240	0.847	2.450	-1.055
FJX1	0.413	1.331	5.448	1.412
FKBP10	-0.052	0.965	4.973	-0.239
FKBP11	0.721	1.649	4.144	2.676
FKBP1B	1.133	2.193	3.072	3.327
FKBP4	-0.252	0.840	6.621	-0.871
FKBP5//LOC285847	-0.193	0.875	5.133	-0.574
FKSG29	-0.199	0.871	3.919	-0.765
FKSG49	-0.164	0.892	9.278	-0.699
FKTN	-0.079	0.947	5.117	-0.295
FLG-AS1	-0.313	0.805	3.586	-1.097
FLI1	0.062	1.044	3.763	0.238
FLII	-0.200	0.871	6.975	-0.982
FLJ16779	0.106	1.076	2.232	0.397
FLJ20021//AP001816.1	0.489	1.404	4.932	1.534
FLJ21369	-0.325	0.798	3.255	-1.009
FLJ22184//CTD-3193O13.9	0.698	1.622	4.076	1.792
FLJ25917//RP5-1158E12.3	0.513	1.427	1.777	1.419
FLJ27354//RP5-1007M22.2	-0.163	0.893	3.882	-0.521
FLJ31104//CTD-2031P19.3	-0.245	0.844	2.491	-0.933
FLJ31183	-0.221	0.858	2.193	-1.015
FLJ33360//CTD-2324F15.2	0.250	1.189	2.379	0.894
FLJ33534//AC062028.1	0.720	1.647	2.347	2.471
FLJ35934//RP1-37N7.3	-0.104	0.930	2.657	-0.441
FLJ37035//RP11-383C5.5	0.309	1.239	2.126	1.272
FLJ37201	-0.642	0.641	2.632	-2.504
FLJ38717	-0.590	0.665	3.181	-2.258
FLJ41130	0.792	1.732	4.200	1.992
FLJ41278//RP11-766N7.3	0.915	1.885	3.466	2.114
FLJ41733//AP001046.6	-0.300	0.812	3.327	-1.086
FLJ42627//RP11-20I23.13	0.300	1.231	4.578	1.115
FLJ43315	-0.079	0.947	2.882	-0.271
FLJ43681//LOC101930151//AC139099.3	0.429	1.347	2.745	1.725
FLJ44635//NHSL2	0.066	1.047	2.878	0.258

FLNA	-0.067	0.955	6.235	-0.269
FLNB	-0.660	0.633	6.351	-2.634
FLNC	-0.852	0.554	6.718	-2.320
FLRT2//LOC100506718	0.705	1.631	4.943	1.755
FLRT3	1.107	2.154	4.211	2.896
FLT1	0.322	1.250	6.560	1.179
FLT3	0.424	1.342	3.223	1.334
FLT3LG//CTD-3148I10.15	-0.184	0.880	4.253	-0.821
FLVCR2	-0.143	0.906	2.944	-0.599
FMN1	0.812	1.755	5.219	2.581
FMNL2	-0.765	0.589	9.652	-2.659
FMNL3	-0.390	0.763	5.679	-1.438
FMO2	0.421	1.338	3.128	1.397
FMO4	-0.672	0.628	3.064	-1.810
FMO5	-0.690	0.620	3.184	-2.219
FMOD	0.909	1.878	4.729	2.257
FN1	0.404	1.323	7.682	1.363
FNBP1	-0.657	0.634	8.013	-2.288
FNBP1L	-0.221	0.858	7.107	-0.989
FNDC3B//LOC101928615	-0.111	0.926	6.673	-0.524
FNDC4	0.721	1.648	6.196	1.875
FNDC5	0.455	1.371	4.255	1.437
FNDC9	0.307	1.237	4.696	0.707
FNIP2	0.281	1.215	6.086	1.318
FNTA//RP11-598P20.5	-0.463	0.725	6.509	-1.976
FOCAD	0.653	1.572	6.589	2.560
FOLH1//FOLH1B	-1.317	0.401	6.356	-2.285
FOLH1B	-0.310	0.806	2.631	-1.102
FOLR1	-0.357	0.781	2.119	-1.188
FOLR2	0.359	1.282	3.203	1.135
FOS	0.334	1.260	7.068	0.844
FOSB	0.427	1.345	3.716	1.228
FOSL1	0.251	1.190	2.696	0.784
FOSL2	0.436	1.353	6.033	1.199
FOXB1	-0.426	0.744	2.051	-1.705
FOXC1	0.398	1.318	5.794	1.259
FOXC2	-0.448	0.733	2.786	-1.244
FOXD4	0.421	1.339	4.160	0.950
FOXD4L1	0.129	1.093	3.078	0.384
FOXD4L5//FOXD4L4//FOXD4L6//FOXD4l	0.366	1.289	2.490	1.116
FOXD4L6	0.238	1.179	3.417	0.745
FOXD4L6//FOXD4L5//FOXD4L3//FOXD4l	0.319	1.247	3.752	1.226
FOXF1	0.141	1.103	4.412	0.485
FOXF2	0.401	1.320	4.622	1.401
FOXG1	1.982	3.950	3.405	2.438
FOXJ1	-0.331	0.795	3.471	-0.996
FOXN2	-0.644	0.640	6.710	-2.369
FOXO4	-0.586	0.666	5.668	-2.093
FOXP1-IT1	-0.304	0.810	3.287	-1.012
FOXP2	0.362	1.285	3.330	1.207

FOXQ1	0.257	1.195	3.227	0.973
FPR1	0.399	1.318	5.205	0.708
FPR3	0.614	1.531	3.338	1.239
FRAS1	0.855	1.808	3.547	2.170
FRAT2	0.360	1.283	4.386	1.315
FREM1	-0.313	0.805	4.608	-0.993
FREM2	0.010	1.007	2.856	0.033
FREM3	1.443	2.720	3.043	2.381
FRG1//LOC100289097//LOC101930278/	0.048	1.034	3.401	0.173
FRK	-0.430	0.742	3.076	-1.462
FRMD4B	-1.011	0.496	4.936	-2.608
FRMD8	0.006	1.004	2.887	0.022
FRMD8P1	-0.071	0.952	2.689	-0.195
FRMPD1	-0.123	0.918	2.480	-0.447
FRMPD2//FRMPD2P1//LOC101930006/	0.820	1.766	5.308	1.964
FRMPD2//LOC101930006	-0.891	0.539	3.536	-2.087
FRMPD3	-0.408	0.754	4.669	-1.406
FRMPD4	2.199	4.593	4.889	2.789
FRRS1L	1.001	2.002	6.782	2.314
FRS3	0.428	1.345	3.885	2.251
FRY	0.435	1.352	6.932	1.569
FRY	-0.041	0.972	3.482	-0.153
FRY-AS1	0.586	1.501	2.591	2.037
FRYL	-0.710	0.611	7.654	-2.438
FRZB	0.859	1.814	5.999	1.743
FSD1	0.324	1.252	5.396	1.067
FSTL1	0.442	1.358	5.147	2.183
FSTL3	0.056	1.039	4.502	0.207
FSTL4	1.093	2.133	3.827	2.540
FSTL5	-0.144	0.905	5.618	-0.512
FTL//SRGAP1//GBP4	-0.370	0.774	10.983	-1.818
FUCA1	0.200	1.149	5.262	0.679
FUCA2	-0.444	0.735	5.152	-1.724
FUOM	-0.001	0.999	2.649	-0.006
FUT1	0.373	1.295	2.369	1.500
FUT10	-0.345	0.787	5.355	-1.435
FUT4	-0.589	0.665	3.682	-2.304
FXYD1//CTD-2527I21.4	-0.243	0.845	6.939	-1.158
FXYD4	-0.410	0.753	3.165	-1.355
FXYD5	-0.295	0.815	4.974	-1.097
FXYD7//CTD-2527I21.4	0.622	1.539	5.252	1.665
FYB	-0.460	0.727	5.504	-1.146
FYCO1	-0.551	0.683	5.975	-2.516
FZD2	0.110	1.079	2.570	0.405
FZD3	0.987	1.981	6.859	3.069
FZD4	0.489	1.403	4.599	1.672
FZD5	-0.551	0.682	3.629	-2.213
FZD7	-0.282	0.822	3.927	-0.750
G0S2	-0.307	0.809	4.627	-0.772
G2E3	0.098	1.070	5.089	0.399

G3BP2	0.393	1.313	6.647	1.923
G6PC3	-0.243	0.845	7.914	-1.049
GAA	-0.236	0.849	6.175	-1.311
GAB1	-0.647	0.638	5.830	-2.165
GAB2	-0.566	0.675	5.018	-1.935
GAB3	-0.130	0.914	3.601	-0.458
GABARAPL1	0.329	1.256	6.351	1.289
GABBR1	0.337	1.263	8.011	1.356
GABBR1	0.345	1.270	8.488	1.347
GABBR1	0.326	1.254	8.064	1.311
GABBR1	0.321	1.250	7.895	1.284
GABBR1	0.307	1.237	8.050	1.246
GABBR1	0.312	1.241	7.470	1.230
GABBR2	1.690	3.227	7.282	2.329
GABPB2	-0.736	0.600	5.594	-3.228
GABRA1	2.073	4.207	5.002	2.649
GABRA2	1.121	2.175	4.294	2.852
GABRA3	1.163	2.239	6.160	2.329
GABRA4	1.908	3.753	4.954	2.666
GABRA5	1.982	3.950	4.329	3.186
GABRB1	0.578	1.493	5.974	1.871
GABRB2	2.429	5.387	4.684	3.188
GABRB3	0.700	1.624	3.965	2.221
GABRD	2.170	4.499	4.010	3.347
GABRE//MIR224//MIR452	0.477	1.391	3.283	1.518
GABRG1	0.088	1.063	7.434	0.295
GABRG2	1.515	2.857	6.796	1.897
GABRG3	1.278	2.426	2.736	3.770
GABRQ	0.255	1.194	4.212	0.432
GAD1	1.189	2.280	4.319	2.570
GAD2	1.331	2.515	5.282	2.042
GADD45A	0.834	1.782	7.831	1.447
GADD45B	1.083	2.118	8.276	2.795
GADD45G	-0.486	0.714	4.504	-0.858
GAL	-0.341	0.790	2.652	-0.994
GAL3ST2	-0.588	0.665	2.995	-1.821
GAL3ST3	0.329	1.256	3.725	1.436
GAL3ST4	-0.218	0.860	2.579	-0.781
GALM	0.213	1.159	3.491	0.783
GALNT11	0.522	1.436	5.773	2.100
GALNT14	0.450	1.366	2.080	1.657
GALNT15	-0.862	0.550	7.548	-1.927
GALNT16	0.690	1.613	4.889	2.079
GALNT18	0.342	1.267	4.886	1.212
GALNT3	-0.100	0.933	3.824	-0.379
GALNT6	-0.456	0.729	4.583	-1.274
GALNT7	-0.645	0.639	5.197	-2.478
GALNT9	1.013	2.018	5.021	2.214
GALNTL6	0.985	1.980	2.423	2.505
GALR3	-0.422	0.746	2.269	-1.632

GANC	-0.563	0.677	5.516	-2.101
GANC//RP11-164J13.1//CAPN3	-0.658	0.634	3.356	-1.710
GAP43	1.070	2.099	7.213	2.292
GAPT	-0.764	0.589	3.356	-1.736
GAREML	-0.307	0.808	4.898	-0.861
GARNL3	0.073	1.052	5.409	0.387
GARS	0.595	1.511	6.300	2.184
GAS2L3	0.538	1.452	3.119	1.512
GAS5//SNORD81//SNORD79//SNORD80	-0.090	0.939	4.552	-0.368
GAS5-AS1//GAS5	0.231	1.174	2.606	0.734
GAS6-AS1	-0.155	0.898	2.257	-0.602
GAS6-AS2	0.426	1.344	3.135	1.279
GAS7	0.436	1.353	7.734	1.864
GBA2	0.151	1.111	6.075	0.721
GBE1	-0.141	0.907	5.193	-0.519
GBP1	0.382	1.303	4.083	1.024
GBP2	0.308	1.238	5.477	0.631
GBP2	0.161	1.118	4.176	0.282
GBP3	-0.926	0.526	4.596	-2.661
GBP4	-0.063	0.957	4.390	-0.131
GBX2	-0.120	0.920	2.870	-0.485
GCA	-0.089	0.940	5.696	-0.287
GCC2	0.457	1.372	7.406	2.334
GCLM	0.517	1.431	5.140	2.007
GCNT1	0.585	1.500	2.980	2.360
GCNT4	1.988	3.968	2.925	4.322
GCSHP3//AC007383.4	0.132	1.096	3.109	0.613
GCSHP3//NDUFS1	0.527	1.441	6.291	2.055
GDA	2.849	7.204	4.772	3.142
GDAP1	0.506	1.420	6.515	1.929
GDAP1L1	0.853	1.806	3.587	2.082
GDAP2	0.190	1.141	6.190	0.793
GDF1//CERS1	-0.492	0.711	7.142	-2.335
GDF10	-0.024	0.983	4.235	-0.103
GDF11	-0.358	0.780	6.017	-1.374
GDF15	0.033	1.023	3.047	0.095
GDF7	0.348	1.273	2.446	1.053
GDPD1	0.345	1.270	6.013	1.306
GDPD2	-0.260	0.835	5.021	-1.103
GDPD3	-0.389	0.763	2.518	-1.348
GEM	0.495	1.409	3.704	1.619
GEMIN7	-0.385	0.766	3.647	-1.441
GEMIN8	-0.008	0.994	4.937	-0.033
GEMIN8P4	0.649	1.568	2.319	2.577
GEN1	0.315	1.244	3.332	1.127
GFAP	-0.283	0.822	10.929	-0.811
GFM2	0.170	1.125	5.481	0.827
GFOD1	0.615	1.531	3.894	1.584
GFOD1-AS1	0.710	1.636	2.575	2.309
GFOD2	0.660	1.580	3.989	1.998

GFPT2	0.084	1.060	5.403	0.248
GFRA1	0.050	1.035	5.070	0.170
GFRA2	1.472	2.774	3.782	2.891
GGA3	0.214	1.160	5.565	1.026
GGCT	0.780	1.717	4.737	3.018
GGH	0.494	1.409	3.199	2.403
GGT2	-0.236	0.849	2.551	-0.787
GGT5	0.376	1.298	4.431	0.926
GGTA1P	0.397	1.317	2.592	1.320
GHITM	0.430	1.347	8.504	1.923
GHR	0.238	1.179	2.703	0.910
GHRLOS	-0.589	0.665	2.895	-2.585
GIMAP4	-0.277	0.825	4.877	-0.979
GIMAP6	-0.118	0.921	3.652	-0.423
GIMAP7	0.015	1.011	4.666	0.046
GIMAP8	-0.082	0.945	3.971	-0.251
GINS3	-0.329	0.796	4.192	-1.295
GIPC3	-0.044	0.970	3.117	-0.126
GIT1	0.329	1.257	6.745	1.346
GJA1//IFT140	-0.380	0.769	8.955	-1.131
GJA4	0.028	1.019	2.742	0.092
GJB1	-1.288	0.409	3.786	-2.779
GJB2	0.520	1.434	3.580	1.076
GJB6	0.929	1.904	4.566	1.553
GJC1	0.434	1.351	3.280	1.381
GJC2	-1.305	0.405	5.015	-3.753
GJC3	-0.182	0.881	2.696	-0.409
GJD2	0.219	1.164	3.391	0.712
GK5	-0.285	0.821	5.406	-1.014
GK-IT1	0.225	1.169	3.845	0.726
GLA	-0.034	0.977	5.104	-0.110
GLB1L	0.156	1.114	3.409	0.557
GLB1L3	0.882	1.844	2.403	2.943
GLCCI1	0.307	1.237	2.773	1.485
GLDC	0.830	1.778	4.868	2.640
GLDN	-0.938	0.522	6.917	-2.232
GLI2	0.089	1.063	3.566	0.330
GLI3	0.241	1.182	3.927	1.116
GLIDR//LOC102724238//LOC554249//RI	-0.411	0.752	4.951	-1.566
GLIDR//RP11-211N8.2	-0.027	0.982	3.664	-0.095
GLIPR1	0.385	1.306	7.029	1.170
GLIPR1L2	-0.665	0.631	4.709	-1.799
GLIPR2	-0.309	0.807	5.667	-0.886
GLIS3	-0.533	0.691	4.919	-1.818
GLMN	0.719	1.646	3.692	2.773
GLP1R	-0.390	0.763	2.357	-1.493
GLP2R	1.323	2.502	3.008	2.724
GLRA1	-1.427	0.372	4.668	-1.544
GLRA2	0.520	1.434	4.233	1.019
GLRA3	1.622	3.079	4.914	2.205

GLRB	1.185	2.274	4.709	2.315
GLRX	0.237	1.179	4.697	0.625
GLRX2	0.412	1.331	4.564	1.851
GLS	0.879	1.839	6.556	2.799
GLS2	0.846	1.797	4.756	1.726
GLT1D1	0.925	1.899	4.943	2.424
GLTP	-1.243	0.423	8.323	-3.299
GLTSCR2//SNORD23	-0.404	0.756	7.087	-2.118
GLUD1	-0.371	0.773	5.880	-1.295
GLUD1P7	0.445	1.361	2.865	1.693
GLUL	-0.455	0.729	8.667	-1.435
GLYATL2	0.167	1.123	3.401	0.375
GM2A	-0.400	0.758	6.202	-1.808
GMFG	-0.283	0.822	6.432	-0.650
GMIP	-0.229	0.853	3.980	-0.740
GMPR	-0.907	0.533	5.157	-2.399
GMPS	0.319	1.247	6.728	1.627
GNA12	-0.166	0.891	5.142	-0.772
GNA13	-0.356	0.782	6.407	-1.460
GNA14	0.086	1.061	5.424	0.257
GNA15	0.363	1.286	3.908	0.988
GNAI2	-0.191	0.876	4.631	-0.839
GNAI3//RP5-1160K1.3	0.547	1.461	3.253	2.301
GNAL	0.687	1.610	4.039	1.657
GNAO1	0.164	1.120	9.735	0.820
GNB1L//C22orf29	0.190	1.140	3.194	0.689
GNB4	0.138	1.100	4.918	0.579
GNB5	0.987	1.983	4.188	2.203
GNB5	0.504	1.418	3.235	1.902
GNG12	-0.467	0.723	5.459	-1.790
GNG13	0.714	1.640	3.669	1.842
GNG2	0.196	1.145	2.753	0.747
GNG3	0.928	1.903	8.009	1.771
GNG4	0.155	1.113	4.500	0.524
GNG5	-0.244	0.844	3.501	-0.892
GNG5P2	0.075	1.054	5.872	0.254
GNL3//SNORD19B	0.261	1.199	5.928	1.131
GNLY	0.009	1.006	2.492	0.030
GNPDA2	0.753	1.685	5.150	2.921
GNPNAT1	0.311	1.240	4.481	1.431
GNPTAB	0.375	1.296	5.533	1.768
GNRHR2	0.164	1.120	3.010	0.677
GOLGA2P1	0.560	1.474	2.689	1.680
GOLGA2P5//GOLGA2B	-0.095	0.936	3.391	-0.327
GOLGA2P6//MTPAP	-0.007	0.995	2.045	-0.023
GOLGA2P7//LOC642423//LOC10192947	0.791	1.730	3.591	1.793
GOLGA6L10//GOLGA6L4//GOLGA6L9//G	0.005	1.004	5.770	0.022
GOLGA6L4//GOLGA6L5P//GOLGA6L17P/	0.002	1.001	5.523	0.006
GOLGA7B	0.724	1.651	4.176	2.216
GOLGA7B	0.620	1.537	2.233	1.733

GOLGA8A//GOLGA8B//LOC101930583	0.120	1.087	7.451	0.526
GOLGA8A//GOLGA8B//LOC101930583	0.097	1.070	7.490	0.418
GOLGA8EP	-0.318	0.802	3.684	-1.181
GOLGA8J	-0.471	0.721	3.073	-1.526
GOLGA8J	-0.358	0.780	3.070	-1.032
GOLGA8M//GOLGA8J//GOLGA8K//GOL(	-0.296	0.814	5.556	-1.250
GOLGA8O//GOLGA8N//GOLGA8Q//GOL	-0.487	0.714	6.517	-1.845
GOLGA8O//GOLGA8R//GOLGA8N//GOL(	-0.552	0.682	5.946	-2.319
GOLGA8O//GOLGA8R//GOLGA8N//GOL(	-0.520	0.697	6.092	-2.121
GOLGA8S	0.081	1.058	3.455	0.228
GOLIM4	-0.072	0.951	8.350	-0.345
GOLT1B	0.257	1.195	6.272	1.127
GOPC	0.420	1.338	7.188	2.004
GORASP1	0.044	1.031	4.040	0.171
GOT1	0.567	1.481	8.249	1.787
GOT2	0.439	1.355	7.789	2.324
GPAM	-0.202	0.869	5.018	-0.849
GPATCH1	-0.028	0.981	5.970	-0.133
GPATCH2	0.534	1.448	5.179	2.679
GPATCH2L	0.054	1.038	5.122	0.244
GPATCH4	0.254	1.193	3.682	0.974
GPC2	0.007	1.005	2.434	0.032
GPC3	0.196	1.145	2.806	0.598
GPC4	-0.164	0.892	5.379	-0.763
GPC5	0.124	1.090	5.463	0.479
GPC6	-0.252	0.840	5.599	-0.895
GPCPD1	0.103	1.074	6.558	0.390
GPD1	-0.461	0.727	4.266	-1.107
GPD1L	0.346	1.271	7.013	1.394
GPI	0.545	1.459	8.480	2.018
GPIHBP1	-1.048	0.484	5.359	-1.993
GPLD1	0.338	1.264	4.308	1.075
GPNMB	-0.261	0.834	5.228	-0.594
GPR101	-0.177	0.884	3.264	-0.658
GPR108//MIR6791	-0.183	0.881	4.008	-0.858
GPR116	0.421	1.339	5.759	2.141
GPR12	0.817	1.762	4.202	1.920
GPR123	0.220	1.165	3.264	0.886
GPR125	-0.365	0.776	6.387	-1.420
GPR126	0.052	1.037	3.297	0.175
GPR132	-0.246	0.843	2.582	-0.884
GPR133	0.007	1.005	2.972	0.027
GPR137C	0.183	1.135	5.443	0.783
GPR141	-0.279	0.824	2.737	-1.263
GPR146//RP11-449P15.1	0.291	1.223	2.575	0.913
GPR149	0.088	1.063	2.566	0.245
GPR150	1.215	2.321	2.331	4.301
GPR153	0.330	1.257	3.523	0.901
GPR155	0.653	1.573	6.330	2.114
GPR158	0.674	1.595	7.471	1.694

GPR162	0.922	1.894	5.615	2.317
GPR17	-0.823	0.565	3.136	-2.189
GPR176	0.524	1.438	2.832	1.829
GPR179	0.675	1.597	3.424	3.018
GPR180	0.219	1.164	5.696	0.878
GPR183	0.243	1.183	6.349	0.469
GPR20	-0.070	0.953	2.798	-0.229
GPR21//RABGAP1	0.800	1.741	5.205	2.007
GPR22	2.278	4.851	3.876	3.197
GPR26	0.690	1.613	3.796	1.292
GPR3	0.433	1.350	4.006	1.408
GPR34	-1.165	0.446	5.673	-2.885
GPR37//SEL1L2	-1.288	0.409	8.623	-3.033
GPR37L1	-0.372	0.773	6.898	-1.203
GPR4	0.519	1.433	4.550	1.539
GPR45	0.609	1.525	2.542	2.287
GPR52	-0.023	0.984	3.594	-0.086
GPR56	0.168	1.124	7.922	0.749
GPR6	0.185	1.137	3.610	0.641
GPR61	0.806	1.749	2.918	2.559
GPR62	-0.747	0.596	3.573	-2.098
GPR63	1.509	2.846	3.068	3.109
GPR65	-0.405	0.755	4.512	-1.104
GPR83	0.894	1.858	4.085	1.597
GPR85	0.605	1.520	4.013	1.582
GPR88	-0.416	0.750	3.999	-1.345
GPR98	0.851	1.804	4.365	1.661
GPRASP1	0.417	1.335	5.853	1.556
GPRASP2//ARMCX5//ARMCX5-GPRASP2	0.273	1.209	4.522	0.934
GPRC5A	0.139	1.101	2.334	0.391
GPRC5B	-0.559	0.679	9.856	-2.240
GPRIN1	0.423	1.341	4.314	1.083
GPSM2	-0.518	0.698	6.659	-2.125
GPSM3	-0.120	0.920	3.071	-0.442
GPT2	-0.291	0.817	5.960	-1.310
GPX1	-0.325	0.798	4.873	-1.433
GPX3	0.732	1.661	3.856	1.774
GPX8	0.061	1.043	2.363	0.184
GRAMD1B	0.605	1.521	6.377	1.769
GRAMD3	-0.435	0.739	5.501	-1.494
GRAPL	-0.696	0.617	2.392	-1.690
GRASP	0.587	1.502	3.731	2.267
GRB14	0.732	1.661	3.283	1.885
GREB1L	-0.617	0.652	3.774	-1.925
GREM1	-0.995	0.502	5.995	-2.749
GREM2	0.657	1.577	4.149	1.784
GRHL1	0.518	1.432	1.979	1.682
GRHL3	-0.173	0.887	3.206	-0.328
GRIA1	1.018	2.024	5.069	2.189
GRIA2	0.821	1.767	6.772	2.621

GRIA3	0.717	1.644	6.672	2.513
GRIA4	0.174	1.128	7.020	0.777
GRID2	-0.130	0.914	4.554	-0.537
GRIK1	0.743	1.674	4.811	1.813
GRIK2	0.721	1.649	6.015	1.931
GRIK3	0.261	1.198	5.391	1.084
GRIK5	0.587	1.503	6.032	2.252
GRIN1	1.555	2.939	6.872	1.903
GRIN2A	1.678	3.200	5.381	2.852
GRIN2B	1.709	3.269	5.809	2.488
GRIN3A	0.739	1.669	4.568	2.491
GRIP1	0.453	1.369	4.497	1.641
GRIP2	0.124	1.090	3.110	0.448
GRIPAP1	0.201	1.150	6.392	1.030
GRM1	0.918	1.890	4.438	2.162
GRM2	0.583	1.498	2.430	1.336
GRM3	-0.244	0.844	7.632	-0.966
GRM5	1.380	2.602	6.382	2.582
GRM7//LOC100996542	0.646	1.565	3.396	1.886
GRM8	0.634	1.552	2.485	2.757
GRN	-0.295	0.815	6.920	-1.072
GRPEL2	0.611	1.527	5.391	2.073
GRPR	0.486	1.400	3.847	1.201
GRTP1-AS1//LOC101930262//LOC10193	-0.191	0.876	2.158	-0.920
GS1-115G20.1	0.458	1.374	3.297	1.617
GS1-259H13.2//ZNF655	0.362	1.285	2.935	1.077
GS1-421I3.4	0.659	1.579	2.293	2.556
GS52	0.479	1.393	2.139	1.604
GSDMD	-0.097	0.935	2.976	-0.425
GSK3A	0.423	1.341	6.365	1.869
GSKIP	0.208	1.155	4.453	1.027
GSN	-0.941	0.521	6.339	-2.911
GSPT2	0.427	1.345	3.597	1.890
GSTA4	0.353	1.277	6.542	1.605
GSTM2	-0.279	0.824	5.233	-0.904
GSTM2P1	-0.373	0.772	2.300	-1.267
GSTM3	0.752	1.685	5.424	2.598
GSTM4	0.304	1.235	4.110	1.201
GSTM5	0.455	1.370	5.932	0.838
GSTT1	0.108	1.078	4.612	0.173
GSTT2	1.245	2.370	4.910	1.884
GSTT2//GSTT2B	0.761	1.695	4.769	1.517
GTDC1	0.215	1.161	5.623	1.183
GTF2H2C_2//GTF2H2C//GTF2H2//GTF2I	-0.588	0.665	6.335	-2.339
GTF2IRD2B//GTF2IRD2//GTF2IRD2P1	-0.649	0.638	4.843	-2.588
GTF3C3	0.012	1.008	5.771	0.053
GTF3C4	0.315	1.244	5.560	1.584
GTPBP8	0.020	1.014	3.434	0.073
GUCA1B	0.734	1.663	2.185	2.496
GUCY1A2	1.106	2.152	5.448	2.539

GUCY1A3	0.231	1.173	6.304	0.866
GUCY1B3	0.892	1.856	8.037	2.487
GUF1	0.189	1.140	6.020	0.714
GULP1	1.006	2.009	3.738	2.930
GUSB	-0.435	0.740	4.864	-1.751
GUSBP1//LOC100996497//GUSBP4//LIN	-0.427	0.744	5.458	-1.572
GUSBP10	-0.688	0.621	2.078	-2.377
GUSBP2	-0.776	0.584	4.362	-2.124
GUSBP3	-0.220	0.859	4.946	-0.579
GUSBP3//SMA4//LOC653080//SMA5//G	-0.568	0.675	7.713	-1.942
GUSBP3//SMA4//LOC653080//SMA5//G	-0.624	0.649	7.302	-2.090
GUSBP4//XXbac-BPGBPG55C20.2	0.244	1.184	4.387	0.758
GUSBP5	0.524	1.438	3.802	2.188
GUSBP9//GUSBP2//GUSBP3//SMA4//SM	-0.476	0.719	8.676	-1.611
GVINP1	0.086	1.061	2.729	0.321
GXYLT2	-0.211	0.864	5.202	-0.770
GYG2	-0.972	0.510	5.502	-2.574
GYPC	-0.272	0.828	2.598	-1.096
GYS1	-0.288	0.819	5.030	-1.182
H19//MIR675//RPS12	0.246	1.186	2.982	0.735
H2AFJ	-0.478	0.718	4.475	-1.592
H2AFX	-0.376	0.770	5.695	-1.705
H2AFZ	0.473	1.388	4.786	2.178
H3F3B//H3F3A//MIR4738	-0.399	0.758	8.938	-1.376
H6PD	-0.207	0.866	3.495	-0.799
HAAO	-0.270	0.829	2.658	-0.985
HACE1	0.339	1.265	6.047	1.767
HADHB	-0.402	0.757	7.335	-1.864
HAGLR//HOXD-AS1	-0.916	0.530	2.712	-2.957
HAMP	0.001	1.001	7.735	0.002
HAP1	0.654	1.573	4.359	1.547
HAPLN2	-1.690	0.310	7.392	-3.063
HAPLN4//AC138430.4	1.440	2.712	4.695	2.134
HAR1A	0.292	1.225	2.592	1.176
HAR1B	0.411	1.330	2.654	1.129
HARBI1	-0.394	0.761	3.803	-1.682
HAS2	-0.100	0.933	3.408	-0.419
HAUS4//MIR4707//RP11-298I3.5	-0.167	0.891	4.153	-0.714
HAVCR2	-0.502	0.706	5.548	-1.478
HBB	-0.306	0.809	2.471	-1.074
HBEGF	-0.505	0.705	4.895	-1.708
HBG1	-0.558	0.679	2.374	-2.187
HCAR3//GPR109B	0.314	1.243	3.140	1.077
HCCAT5	-0.288	0.819	3.389	-1.147
HCCS	0.619	1.536	5.662	2.228
HCG17	0.240	1.181	3.813	1.063
HCG18	0.165	1.122	3.827	0.496
HCG2040054//AC138655.4	-0.407	0.754	2.236	-1.587
HCG22	-0.978	0.508	2.597	-2.756
HCG22	-1.012	0.496	2.809	-2.707

HCG22	-0.927	0.526	2.924	-2.506
HCG27//MICB	-0.349	0.785	3.380	-1.169
HCG8	-0.615	0.653	3.373	-1.934
HCG8	-0.422	0.746	3.415	-1.511
HCK	-0.482	0.716	5.318	-1.227
HCLS1	-0.219	0.859	5.898	-0.542
HCN1	2.045	4.128	6.266	2.433
HCN3	0.396	1.316	3.575	1.653
HCN4	0.659	1.579	3.616	1.941
HCP5	0.284	1.218	4.038	1.019
HCP5	0.201	1.149	3.775	0.654
HCP5	-0.011	0.992	4.647	-0.039
HCRTR2	-0.027	0.981	3.255	-0.095
HCST	-0.292	0.817	4.384	-0.701
HDAC1	-0.733	0.602	5.529	-3.169
HDAC7	-0.035	0.976	4.050	-0.168
HDAC9	0.101	1.073	4.627	0.462
HDGFRP3//RP11-382A20.3	0.429	1.347	7.721	2.175
HEATR5A//RP11-176H8.1	-0.474	0.720	6.423	-1.986
HEBP2	-0.245	0.844	4.762	-1.054
HECTD2	0.528	1.442	4.667	1.840
HECW1	1.340	2.531	5.025	2.845
HECW1-IT1	0.811	1.754	2.694	2.153
HECW2	0.076	1.054	6.389	0.338
HEG1	-0.301	0.812	7.778	-0.878
HELB	0.072	1.051	2.703	0.277
HELB	-0.015	0.990	2.299	-0.066
HELZ2	0.015	1.011	2.304	0.074
HENMT1	0.115	1.083	4.329	0.439
HEPACAM//HEPN1	-0.880	0.543	7.256	-3.160
HEPN1	-0.542	0.687	4.212	-1.763
HERC1	0.441	1.357	7.389	2.347
HERC2P7	-0.830	0.562	4.604	-2.210
HERC5	-0.539	0.688	3.737	-2.512
HERC6	0.700	1.625	5.868	2.283
HERPUD1	0.032	1.023	7.209	0.123
HES1	0.547	1.461	5.283	2.055
HES4	-0.008	0.995	3.448	-0.027
HEXIM1	-0.052	0.965	5.735	-0.201
HEY1	0.184	1.136	4.428	0.655
HEY2	-0.763	0.589	5.828	-2.291
HEYL	-0.474	0.720	2.951	-1.274
HFE	-0.222	0.857	2.788	-1.021
HGF	1.445	2.723	4.126	4.136
HHATL	-0.894	0.538	7.081	-2.080
HHIP	-0.830	0.563	5.376	-1.978
HHIP-AS1	-0.293	0.816	3.168	-1.102
HHIPL1	0.374	1.296	4.082	1.476
HIBCH	-0.834	0.561	4.770	-3.138
HID1	-0.178	0.884	5.288	-0.720

HIF1A	0.155	1.114	8.861	0.667
HIF1A-AS2	0.205	1.152	3.139	0.484
HIF1A-AS2	-0.006	0.996	2.962	-0.013
HIF3A	-0.499	0.708	4.685	-1.503
HIGD1B//EFTUD2	-0.539	0.688	4.093	-2.064
HILPDA	-0.031	0.979	7.211	-0.059
HIP1	-0.927	0.526	6.756	-2.813
HIP1R	-0.849	0.555	5.898	-2.759
HIPK1-AS1//RP5-1073O3.7	-0.593	0.663	2.675	-1.959
HIPK2	-0.615	0.653	9.837	-2.260
HIRA//C22orf39	-0.263	0.833	5.910	-1.122
HIST1H1C	-0.102	0.932	5.068	-0.381
HIST1H1E	-0.014	0.990	8.269	-0.053
HIST1H2AC	-0.971	0.510	6.433	-3.823
HIST1H2AE	0.217	1.162	4.448	0.527
HIST1H2AI//HIST1H2AK//HIST1H2AL//H	-0.609	0.656	2.245	-1.617
HIST1H2AK	-0.319	0.801	4.778	-0.669
HIST1H2BB	-0.306	0.809	2.399	-0.715
HIST1H2BD	-0.025	0.983	3.829	-0.069
HIST1H2BF	-0.267	0.831	3.695	-0.579
HIST1H2BG//HIST1H2BF//HIST1H2BE//H	-0.114	0.924	3.321	-0.329
HIST1H2BG//HIST1H2BJ	-0.146	0.904	4.682	-0.237
HIST1H2BH	-0.839	0.559	2.964	-1.793
HIST1H2BK	-0.213	0.863	5.871	-0.668
HIST1H2BM	-0.217	0.860	4.987	-0.395
HIST1H2BN	-0.114	0.924	2.983	-0.285
HIST1H2BO	0.081	1.057	2.226	0.247
HIST1H3A	0.393	1.313	3.672	0.953
HIST1H3E	-1.240	0.423	2.701	-2.361
HIST1H3F	-0.467	0.723	4.158	-1.128
HIST1H3I	-0.525	0.695	4.535	-0.767
HIST1H4B//HIST1H4I//HIST1H4A//HIST1	-0.791	0.578	3.994	-2.670
HIST1H4D	0.127	1.092	5.671	0.355
HIST1H4H	-0.838	0.560	4.127	-2.234
HIST1H4K//HIST1H4J//HIST1H4I//HIST1I	0.503	1.417	9.098	2.047
HIST1H4L	-0.551	0.683	3.451	-1.329
HIST2H2AA4//HIST2H2AA3	-0.393	0.761	7.750	-1.008
HIST2H2AC	-0.259	0.835	9.673	-1.299
HIST2H2BA	-0.301	0.811	6.633	-1.287
HIST2H2BC	-0.695	0.618	5.059	-1.981
HIST2H2BE	-0.870	0.547	5.040	-2.542
HIST2H2BF	-0.076	0.948	3.891	-0.236
HIST2H3D	-0.063	0.958	4.950	-0.173
HIST2H3PS2//RP5-998N21.10	-0.694	0.618	4.450	-2.242
HIST2H4B//HIST2H4A//HIST1H4I//HIST1	-0.776	0.584	5.631	-2.072
HIST3H2BB	0.140	1.102	4.301	0.335
HIVEP1	0.387	1.307	4.990	1.730
HIVEP2	0.700	1.624	8.110	2.167
HK1	0.637	1.555	6.770	2.278
HK2	-0.913	0.531	5.059	-2.064

HLA-A	0.272	1.207	4.671	1.055
HLA-A	-0.127	0.916	5.499	-0.533
HLA-A	-0.085	0.943	7.420	-0.304
HLA-A	0.028	1.019	7.322	0.112
HLA-A	0.019	1.013	4.245	0.067
HLA-A	-0.019	0.987	6.575	-0.038
HLA-A//HLA-B//HLA-H	-0.007	0.995	8.872	-0.027
HLA-B	0.140	1.102	7.492	0.503
HLA-B	0.086	1.061	8.606	0.324
HLA-B	0.075	1.053	8.657	0.288
HLA-C	-0.520	0.697	5.570	-2.101
HLA-C	-0.243	0.845	7.932	-0.968
HLA-C	-0.196	0.873	7.577	-0.793
HLA-C	0.169	1.125	7.253	0.654
HLA-C	-0.123	0.918	7.337	-0.532
HLA-C	0.130	1.094	7.299	0.481
HLA-DMA	-0.407	0.754	5.630	-1.323
HLA-DMA	-0.327	0.797	5.476	-0.940
HLA-DMA	-0.262	0.834	5.666	-0.778
HLA-DMB//HLA-DMA	-0.309	0.807	5.708	-0.840
HLA-DMB//HLA-DMA	-0.287	0.819	5.724	-0.816
HLA-DMB//HLA-DMA//XXbac-BPG181M	-0.334	0.793	5.743	-0.864
HLA-DMB//HLA-DMA//XXbac-BPG181M	-0.278	0.825	5.745	-0.728
HLA-DMB//XXbac-BPG181M17.5	-0.451	0.731	5.454	-1.104
HLA-DMB//XXbac-BPG181M17.5	-0.410	0.753	5.467	-1.008
HLA-DMB//XXbac-BPG181M17.5	-0.376	0.771	4.686	-0.955
HLA-DOA	-0.522	0.696	5.127	-1.730
HLA-DOA	-0.422	0.746	4.733	-1.299
HLA-DOA	-0.403	0.756	3.331	-1.281
HLA-DOA	-0.423	0.746	4.700	-1.270
HLA-DOA	-0.408	0.754	3.424	-1.242
HLA-DPA1	-0.353	0.783	5.153	-0.983
HLA-DPA1	-0.345	0.787	5.142	-0.952
HLA-DPA1	-0.326	0.798	4.232	-0.929
HLA-DPA1	-0.339	0.791	5.358	-0.904
HLA-DPA1	-0.309	0.807	4.139	-0.808
HLA-DPB1	-0.352	0.783	7.614	-0.954
HLA-DPB1	-0.325	0.798	5.631	-0.891
HLA-DPB1	-0.233	0.851	6.806	-0.666
HLA-DPB1	-0.229	0.853	6.816	-0.635
HLA-DPB2	0.360	1.284	3.709	1.093
HLA-DQA1//LOC100509457	-0.372	0.773	2.585	-1.041
HLA-DQA1//LOC100509457	-0.263	0.833	2.438	-0.941
HLA-DQA1//LOC100509457	-0.279	0.824	2.661	-0.928
HLA-DQA1//LOC100509457	-0.125	0.917	5.233	-0.277
HLA-DQA1//LOC100509457	0.085	1.061	5.723	0.175
HLA-DQA1//LOC100509457	-0.085	0.943	5.045	-0.168
HLA-DQA2//HLA-DQA1//LOC100509457	0.145	1.105	4.080	0.391
HLA-DQA2//HLA-DQA1//LOC100509457	0.124	1.090	4.691	0.337
HLA-DQA2//HLA-DQA1//LOC100509457	0.126	1.091	5.293	0.332

HLA-DQA2//HLA-DQA1//LOC100509457	0.109	1.078	4.700	0.302
HLA-DQA2//HLA-DQA1//LOC100509457	0.105	1.076	4.879	0.294
HLA-DQA2//HLA-DQA1//LOC100509457	0.109	1.079	4.914	0.292
HLA-DQA2//HLA-DQA1//LOC100509457	0.075	1.054	5.572	0.221
HLA-DQB1//LOC101060835	-0.098	0.934	4.931	-0.290
HLA-DQB1-AS1//XXbac-BPG254F23.5	-0.008	0.994	3.357	-0.026
HLA-DQB2	0.197	1.146	3.048	0.756
HLA-DQB2	0.167	1.123	2.824	0.657
HLA-DQB2	0.101	1.073	2.789	0.403
HLA-DQB2	0.091	1.065	2.778	0.355
HLA-DQB2	0.046	1.033	2.819	0.170
HLA-DRA	-0.100	0.933	8.107	-0.171
HLA-DRA//HLA-DQA1	-0.233	0.851	9.471	-0.537
HLA-DRA//HLA-DQA1	-0.221	0.858	9.494	-0.505
HLA-DRA//HLA-DQA1	-0.173	0.887	9.130	-0.401
HLA-DRA//HLA-DQA1	-0.171	0.888	9.349	-0.389
HLA-DRB1//LOC100996809	2.404	5.291	4.266	1.494
HLA-DRB1//LOC100996809//HLA-DRB3/	0.028	1.020	4.042	0.089
HLA-DRB1//LOC100996809//HLA-DRB3/	-0.003	0.998	4.118	-0.010
HLA-DRB1//LOC100996809//HLA-DRB4/	0.132	1.096	7.156	0.404
HLA-DRB3	0.793	1.733	3.835	1.106
HLA-DRB3	0.051	1.036	6.330	0.153
HLA-DRB3//HLA-DRB1//LOC100996809/	0.030	1.021	8.158	0.080
HLA-DRB4	0.104	1.075	6.699	0.172
HLA-DRB4	0.010	1.007	5.181	0.014
HLA-DRB5	2.204	4.609	4.521	1.384
HLA-DRB6	0.139	1.101	2.598	0.632
HLA-E	0.106	1.076	8.630	0.424
HLA-E	0.098	1.071	8.663	0.399
HLA-F	0.089	1.064	5.482	0.445
HLA-F	-0.060	0.959	5.114	-0.256
HLA-F	-0.026	0.982	5.196	-0.123
HLA-F	-0.023	0.984	5.111	-0.099
HLA-G	-0.242	0.846	3.998	-1.283
HLA-G	-0.124	0.918	3.846	-0.640
HLA-G	-0.108	0.928	3.581	-0.559
HLA-L	-0.088	0.941	5.280	-0.304
HLF	1.520	2.867	6.970	2.791
HLX	-0.091	0.939	2.521	-0.428
HMBOX1-IT1	-0.134	0.911	2.444	-0.429
HMCES//C3orf37	0.427	1.345	5.874	1.962
HMG20B	-0.548	0.684	4.025	-2.450
HMGA1//HMGA1P8	0.261	1.198	6.237	0.859
HMGB2	-0.360	0.779	3.238	-1.224
HMGCL	-0.033	0.978	4.377	-0.131
HMGCR	0.369	1.291	7.102	1.310
HMGCS1	0.345	1.270	6.481	1.072
HMGN1//LOC101927733	-0.485	0.715	3.959	-1.729
HMGN2	-0.383	0.767	3.141	-1.221
HMGN5	0.025	1.017	4.527	0.102

HMHA1	-0.549	0.683	4.486	-1.854
HMOX1	-0.424	0.745	5.164	-1.298
HMP19	0.757	1.690	3.888	2.539
HMSD	-0.736	0.600	3.598	-2.246
HMX3	-0.170	0.889	2.479	-0.620
HN1	0.457	1.373	5.298	1.742
HN1L	-1.513	0.350	6.028	-2.928
HNMT	-0.462	0.726	4.523	-1.804
HNRNPA1L2//MRPS31P4	0.070	1.049	3.166	0.172
HNRNPLL	0.395	1.315	4.341	1.868
HNRNPU-AS1	-0.124	0.918	6.208	-0.447
HOGA1	-0.491	0.711	2.566	-1.584
HOMER1	1.024	2.033	5.935	2.835
HOMER3	-0.502	0.706	4.629	-1.886
HOOK1	1.801	3.484	3.404	2.660
HOTAIRM1	-1.042	0.486	3.595	-2.579
HOXA1	-0.553	0.682	2.580	-2.087
HOXA10//HOXA9//MIR196B	-0.104	0.930	2.586	-0.333
HOXA10-AS//MIR196B//HOXA-AS4	-0.216	0.861	2.916	-0.759
HOXA2	-1.647	0.319	3.690	-2.515
HOXA4	-1.122	0.459	3.457	-1.756
HOXA5	-1.942	0.260	4.606	-2.768
HOXA6	-0.397	0.760	3.705	-1.165
HOXA7	-0.412	0.752	3.783	-1.447
HOXB2	-0.908	0.533	3.826	-2.103
HOXB3	-0.418	0.749	2.826	-1.096
HOXB4	-0.329	0.796	3.201	-0.917
HOXB5	-1.327	0.399	3.386	-2.262
HOXB6	-0.407	0.754	3.614	-1.257
HOXB7	-0.231	0.852	2.499	-0.862
HOXB8	-0.047	0.968	3.407	-0.131
HOXB9	0.094	1.068	3.790	0.258
HOXB-AS3	-0.672	0.628	2.494	-2.139
HOXC10	0.529	1.443	3.553	1.711
HOXC6//HOXC4//HOXC5//RP11-834C11	-0.377	0.770	2.419	-1.867
HOXC8	0.081	1.057	3.914	0.340
HOXC9	0.147	1.107	2.560	0.710
HOXC-AS1	-0.273	0.828	2.322	-0.921
HOXC-AS2	-0.348	0.786	3.193	-1.269
HOXD1	-1.204	0.434	3.356	-2.864
HOXD10	0.134	1.098	2.578	0.318
HOXD3//HOXD4//LOC401021	-1.103	0.465	3.166	-2.570
HOXD4	-0.624	0.649	3.294	-1.612
HOXD4//HOXD3//LOC401021	-0.645	0.640	3.615	-1.638
HOXD8	-0.291	0.817	2.992	-1.096
HP	-0.137	0.910	3.528	-0.151
HPCAL1	0.078	1.055	3.413	0.330
HPCAL4	1.103	2.148	5.978	2.747
HPD	-0.293	0.816	2.127	-1.162
HPGDS	-0.554	0.681	3.603	-1.185

HPN	-0.495	0.710	3.535	-1.708
HPN-AS1	-0.344	0.788	3.016	-1.056
HPRT1	0.782	1.720	6.058	2.744
HPS3	-0.381	0.768	7.223	-1.596
HPS5	-0.320	0.801	5.754	-1.229
HPS6	-0.183	0.881	2.173	-0.736
HPSE	-0.057	0.961	3.189	-0.167
HPSE2	-0.484	0.715	5.326	-1.122
HR	0.108	1.077	3.478	0.409
HRASLS	-0.038	0.974	3.089	-0.140
HRASLS5	-0.103	0.931	5.311	-0.327
HRH2	0.828	1.776	3.967	2.983
HRH3	1.064	2.091	4.317	2.247
HRK	0.604	1.520	2.328	2.339
HS2ST1//RP5-1052I5.2	0.580	1.495	6.049	2.597
HS3ST2	1.221	2.331	3.220	3.231
HS3ST3B1	0.020	1.014	2.854	0.047
HS3ST4	0.338	1.264	2.259	1.071
HS3ST5	-0.499	0.708	5.327	-1.452
HS6ST1	0.140	1.102	3.755	0.512
HS6ST2	0.288	1.221	3.114	0.974
HS6ST3	1.071	2.101	6.228	2.057
HSD11B1	0.149	1.109	4.161	0.501
HSD11B1L	0.751	1.683	5.029	2.677
HSD11B2	0.161	1.118	2.605	0.519
HSD17B1	-0.462	0.726	3.486	-1.543
HSD17B1//RP11-400F19.6	0.325	1.253	5.105	1.347
HSD17B10	-0.633	0.645	5.120	-2.911
HSD17B13	0.490	1.404	2.266	2.008
HSD17B14	0.241	1.182	4.760	1.111
HSD17B1P1	-0.115	0.923	3.568	-0.392
HSD17B3	-0.773	0.585	3.327	-2.016
HSD17B6	0.397	1.317	3.193	1.538
HSD17B7P2	0.065	1.046	5.471	0.264
HSD17B8	-0.186	0.879	3.598	-0.681
HSD3B7	-0.008	0.995	3.635	-0.032
HSDL1	0.496	1.411	7.020	1.803
HSF2	0.470	1.385	5.092	1.810
HSF4	0.054	1.038	4.169	0.187
HSFX2//HSFX1	0.648	1.567	3.613	1.863
HSFX2//HSFX1	0.259	1.197	4.793	0.952
HSP90AB3P	0.403	1.322	5.316	1.006
HSP90AB4P	-0.606	0.657	2.601	-1.646
HSPA12A	0.365	1.288	7.258	1.112
HSPA12A//RP11-498B4.5	0.569	1.484	2.205	2.427
HSPA12B	0.231	1.174	3.080	0.829
HSPA14	0.215	1.161	4.410	0.996
HSPA1A//HSPA1B//HSPA1L	-0.674	0.627	10.751	-2.149
HSPA1A//HSPA1B//HSPA1L	-0.664	0.631	10.836	-2.132
HSPA1A//HSPA1B//HSPA1L	-0.673	0.627	10.322	-2.031

HSPA1A//HSPA1B//HSPA1L	-0.631	0.646	10.031	-1.937
HSPA1B//HSPA1A//HSPA1L	-0.629	0.647	10.505	-2.013
HSPA1B//HSPA1A//HSPA1L	-0.605	0.657	10.198	-1.956
HSPA1B//HSPA1A//HSPA1L	-0.614	0.653	10.116	-1.853
HSPA1B//HSPA1A//HSPA1L	-0.614	0.653	10.094	-1.799
HSPA1L	-0.246	0.843	4.528	-0.923
HSPA1L	-0.175	0.886	4.806	-0.657
HSPA2	-1.259	0.418	8.820	-3.128
HSPA4L	0.282	1.216	6.997	0.958
HSPA5	-0.304	0.810	9.479	-1.112
HSPA6	-0.476	0.719	3.039	-1.186
HSPA7	0.095	1.068	2.580	0.222
HSPB1	-0.367	0.775	9.423	-0.975
HSPB3	1.078	2.111	2.145	2.332
HSPB6	-0.497	0.709	5.515	-1.556
HSPB7	-0.427	0.744	2.581	-1.790
HSPB8	-0.131	0.913	5.241	-0.518
HSPG2	-0.170	0.889	4.030	-0.768
HSPH1	0.239	1.180	8.690	0.662
HTATIP2	-0.452	0.731	5.338	-1.366
HTR1A	0.687	1.610	3.286	1.265
HTR1B	0.952	1.935	3.328	2.414
HTR1E	1.683	3.212	3.327	3.031
HTR1F	0.965	1.951	2.658	2.591
HTR2A	1.779	3.432	6.275	3.030
HTR2C	-0.501	0.706	3.909	-0.773
HTR3B	0.890	1.853	2.856	1.743
HTR4	0.310	1.239	3.115	1.169
HTR5A	0.990	1.987	4.418	2.056
HTR5AOS//AC093726.4	1.201	2.299	3.634	2.528
HTRA4	-0.417	0.749	2.396	-1.655
HUNK	1.221	2.331	3.601	2.829
HVCN1	-0.447	0.734	3.664	-1.678
HYAL1	-0.188	0.878	2.845	-0.648
HYDIN//HYDIN2//LOC101930373	-0.550	0.683	3.281	-2.017
HYDIN//HYDIN2//LOC101930373	-0.141	0.907	4.650	-0.461
HYI	-0.366	0.776	4.727	-1.525
IBA57-AS1//C1orf148	-0.288	0.819	3.276	-1.143
ICA1	1.029	2.041	3.588	2.949
ICA1L	0.549	1.463	5.107	2.613
ICAM1	0.347	1.272	5.086	1.024
ICAM2	0.288	1.221	5.649	1.009
ICAM3	0.413	1.331	3.133	1.514
ICAM5	1.593	3.018	3.362	2.773
ICE1//CTC-501N16.1	0.114	1.082	5.644	0.534
ICOSLG	-0.647	0.639	4.831	-2.139
ID1	0.484	1.399	6.316	1.377
ID2	0.271	1.207	4.062	0.982
ID3	-0.350	0.785	4.554	-1.049
ID4	-0.031	0.979	6.174	-0.117

IDH3A	0.400	1.319	3.296	1.947
IDI2-AS1	0.137	1.100	3.861	0.641
IDS	0.486	1.401	8.599	1.960
IER2	0.119	1.086	4.546	0.537
IER3	-0.022	0.985	4.695	-0.062
IER3	-0.011	0.992	4.722	-0.030
IER3	0.010	1.007	5.144	0.029
IER3IP1//RP11-49K24.6	0.445	1.362	5.263	1.870
IER5	0.494	1.408	4.913	1.251
IER5L//MGAT2	0.345	1.270	3.946	0.868
IFI16	-0.309	0.807	7.478	-1.070
IFI27	-0.082	0.945	3.648	-0.337
IFI30//PIK3R2//AC007192.4	-0.106	0.929	5.916	-0.177
IFI35	0.307	1.237	4.789	1.011
IFI44	-0.473	0.721	3.920	-1.412
IFI44L	-0.147	0.903	4.527	-0.376
IFI6	1.164	2.241	8.223	2.430
IFIH1	0.134	1.097	3.479	0.527
IFIT1	0.581	1.496	4.893	1.897
IFIT2	-0.716	0.609	4.444	-2.592
IFIT3	0.428	1.345	3.999	1.150
IFITM1	0.481	1.395	7.494	1.410
IFITM2	0.308	1.238	8.026	0.726
IFITM3	0.258	1.196	4.924	0.825
IFITM4P	0.303	1.233	3.030	0.977
IFITM4P	0.342	1.268	3.755	0.975
IFITM4P	0.319	1.247	3.942	0.817
IFITM4P	0.307	1.237	4.468	0.781
IFITM4P	-0.125	0.917	3.777	-0.373
IFNA14	0.106	1.076	2.986	0.399
IFNGR1	-0.544	0.686	5.590	-2.222
IFRD2	-0.370	0.774	3.330	-1.472
IFT57	0.509	1.423	5.907	2.410
IFT80//RP11-432B6.3	1.107	2.154	1.841	2.870
IFT80//RP11-432B6.3	-0.110	0.927	6.223	-0.453
IGANRP	-0.377	0.770	3.321	-0.953
IGDCC4	-0.265	0.832	4.283	-1.035
IGF1	0.561	1.475	3.405	2.329
IGF2BP2	-0.269	0.830	2.986	-1.096
IGFALS	0.261	1.199	2.901	1.048
IGFBP2	0.482	1.396	3.092	1.632
IGFBP3	0.104	1.075	4.460	0.244
IGFBP4	0.603	1.519	5.114	1.144
IGFBP5	-0.531	0.692	6.747	-1.495
IGFBP6	-0.029	0.980	4.340	-0.080
IGFBP7	0.262	1.199	8.443	1.081
IGFBPL1	0.586	1.501	2.349	1.889
IGFL4	0.350	1.275	3.089	1.216
IGFLR1//AD000671.6	-0.439	0.738	3.952	-1.432
IGHA2	0.139	1.101	3.813	0.421

IGHD2-15	0.013	1.009	4.422	0.042
IGHG4//IGHG1	-0.049	0.966	2.603	-0.201
IGHJ1//SKAP2//IGHG1	-0.243	0.845	2.902	-0.721
IGHJ2//SKAP2//IGHG1//IGHD	-0.108	0.928	2.815	-0.361
IGHJ3//SKAP2//IGHG1//IGHA1//IGH//IG	-0.003	0.998	3.245	-0.006
IGHM//IGHG1	-0.461	0.727	2.692	-1.129
IGHV1-24	0.716	1.642	2.283	2.311
IGHV4-34//SKAP2//IGHG1	-0.620	0.651	2.572	-1.946
IGIP	0.320	1.248	6.259	1.578
IGKJ5//IGKC	-0.932	0.524	3.249	-2.609
IGKV1-16//IGKC	0.245	1.185	2.546	0.814
IGKV1-17	0.219	1.164	2.533	0.715
IGKV1D-16//IGKC	-0.137	0.909	3.001	-0.421
IGKV1OR2-3//IGKV1-12//IGKC	-0.777	0.584	2.416	-2.340
IGKV3-15//IGKC	-0.750	0.594	2.662	-2.868
IGLJ2	0.126	1.091	4.768	0.455
IGLJ4	0.108	1.077	3.037	0.336
IGLL3P	-0.566	0.675	2.650	-1.469
IGLON5	0.247	1.187	4.730	0.521
IGLV@//IGHG1//IGLC1//IGLJ3//IGLC2	0.511	1.425	3.067	1.210
IGLV2-11	-0.278	0.825	3.012	-0.801
IGLV2-23//IGLC1	-0.278	0.825	3.328	-1.018
IGLV2-8	-0.091	0.939	4.182	-0.357
IGLV3-10//IGLC1	-0.399	0.758	2.653	-1.440
IGLV3-25	0.144	1.105	2.760	0.389
IGLV3-27	0.342	1.267	2.013	0.936
IGSF1	-0.506	0.704	3.661	-1.664
IGSF11	-0.535	0.690	5.410	-2.052
IGSF21	0.676	1.597	5.708	2.387
IGSF3	0.448	1.364	4.801	1.672
IGSF6	-0.063	0.957	4.493	-0.167
IGSF9B	0.434	1.351	4.433	1.661
IKBIP	-0.046	0.968	2.330	-0.169
IKBKAP	-0.075	0.949	7.021	-0.370
IKBKB	-0.363	0.778	5.650	-1.565
IKBKE	-0.350	0.785	3.057	-1.205
IKZF1	-0.437	0.739	3.140	-1.640
IKZF2	-0.606	0.657	3.815	-2.116
IL10RA	-0.415	0.750	4.658	-1.226
IL10RB//AP000295.9	-0.556	0.680	4.641	-2.226
IL10RB-AS1	-0.071	0.952	1.890	-0.228
IL11RA	-0.436	0.739	4.206	-1.636
IL12RB1	-0.050	0.966	2.042	-0.224
IL12RB2	1.354	2.555	2.381	2.903
IL13RA1	-0.204	0.868	6.730	-0.646
IL16	-0.223	0.857	3.250	-1.079
IL17D	-0.356	0.782	4.314	-1.302
IL17RA	-0.609	0.656	6.943	-1.907
IL17RD	-0.214	0.862	4.944	-0.856
IL18	-0.413	0.751	4.610	-1.132

IL18BP	-0.133	0.912	4.462	-0.488
IL18R1	0.070	1.049	3.165	0.253
IL1A	-0.261	0.835	1.985	-0.888
IL1B	0.279	1.213	2.843	0.770
IL1R1	0.576	1.491	5.034	1.804
IL1R2	-0.348	0.785	2.450	-0.762
IL1RAP	0.305	1.236	3.250	1.157
IL1RAPL1	-0.228	0.854	6.241	-0.840
IL1RAPL2	1.187	2.276	1.533	3.784
IL1RL1	0.822	1.768	4.158	1.761
IL20RA	-0.018	0.987	2.189	-0.076
IL21R	-0.027	0.982	2.237	-0.100
IL23A//TRBJ2-6	-0.337	0.792	3.097	-0.705
IL2RA	-0.071	0.952	2.496	-0.200
IL2RG	0.000	1.000	3.018	0.000
IL32	0.030	1.021	2.252	0.136
IL33	0.421	1.339	4.369	1.279
IL34	0.409	1.327	3.814	1.066
IL3RA	-0.395	0.761	3.616	-1.462
IL3RA	-0.258	0.836	4.152	-0.935
IL4R	0.106	1.076	5.724	0.305
IL6R	-0.344	0.788	5.379	-1.712
IL7R	-0.491	0.711	2.804	-1.610
ILDR2	0.686	1.609	5.110	2.004
IMPA1	0.440	1.356	6.513	1.884
IMPACT	-0.238	0.848	6.062	-1.074
IMPG2	-0.178	0.884	2.856	-0.773
INA	1.150	2.218	7.951	1.387
INF2	-0.512	0.701	5.724	-2.004
INHBA	-0.020	0.986	2.777	-0.088
INHBA-AS1	0.592	1.507	1.967	1.775
INHBB	-0.319	0.802	4.337	-1.164
INIP	-0.029	0.980	6.085	-0.132
INO80C	-0.268	0.831	3.875	-1.114
INPP4A	0.474	1.389	7.167	1.867
INPP4B	0.577	1.492	3.743	2.406
INPP5A	0.215	1.161	6.725	1.106
INPP5D	-0.660	0.633	6.104	-2.235
INPP5E	0.244	1.184	4.630	0.923
INPP5F	0.155	1.114	7.444	0.588
INPP5J	0.044	1.031	2.636	0.188
INPPL1	-0.235	0.850	5.987	-1.160
INS//IGF2//INS-IGF2	0.126	1.091	2.340	0.528
INSIG1	0.100	1.072	7.295	0.365
INSIG2	0.363	1.286	5.679	1.415
INSM1	0.061	1.043	2.952	0.216
INTS4L1	-0.053	0.964	2.173	-0.126
INTS5	0.251	1.190	4.348	1.010
INTS7	-0.338	0.791	4.889	-1.656
IP6K1	0.274	1.209	6.472	1.424

IP6K3	-1.086	0.471	4.731	-2.501
IPCEF1//CNKSR3	1.397	2.634	5.180	2.641
IPO11	0.084	1.060	5.283	0.415
IPO13	-0.594	0.662	6.487	-2.544
IPO5P1//RP11-15H20.6	-0.141	0.907	3.833	-0.634
IPO9	0.277	1.212	7.044	1.251
IPW//SNORD107//PWARSN//SNORD116	0.207	1.154	3.793	0.502
IPW//SNORD107//PWARSN//SNORD116	-0.014	0.991	4.917	-0.034
IPW//SNORD107//PWARSN//SNORD116	0.087	1.062	6.241	0.360
IPW//SNORD107//PWARSN//SNORD116	0.006	1.004	5.867	0.017
IPW//SNORD107//PWARSN//SNORD116	0.831	1.779	4.076	2.616
IPW//SNORD107//PWARSN//SNORD116	0.845	1.797	3.475	2.613
IPW//SNORD107//PWARSN//SNORD116	0.581	1.496	3.876	2.164
IPW//SNORD107//PWARSN//SNORD116	0.515	1.429	2.425	1.387
IPW//SNORD107//PWARSN//SNORD116	-0.290	0.818	5.150	-1.043
IPW//SNORD107//PWARSN//SNORD116	0.285	1.218	2.821	0.730
IPW//SNORD107//PWARSN//SNORD116	-0.145	0.904	8.670	-0.535
IPW//SNORD107//PWARSN//SNORD116	0.151	1.111	3.353	0.526
IPW//SNORD107//PWARSN//SNORD116	-0.124	0.918	8.109	-0.462
IPW//SNORD107//PWARSN//SNORD116	0.141	1.103	3.149	0.457
IPW//SNORD107//PWARSN//SNORD116	0.130	1.094	7.137	0.450
IPW//SNORD107//PWARSN//SNORD116	-0.120	0.920	9.250	-0.442
IPW//SNORD107//PWARSN//SNORD116	-0.090	0.939	5.798	-0.342
IPW//SNORD107//PWARSN//SNORD116	-0.087	0.941	8.709	-0.330
IPW//SNORD107//PWARSN//SNORD116	-0.085	0.942	7.336	-0.313
IPW//SNORD107//PWARSN//SNORD116	-0.083	0.944	6.014	-0.260
IPW//SNORD107//PWARSN//SNORD116	0.061	1.043	7.233	0.215
IPW//SNORD107//PWARSN//SNORD116	0.053	1.037	8.409	0.189
IPW//SNORD107//PWARSN//SNORD116	0.039	1.028	6.953	0.150
IPW//SNORD107//PWARSN//SNORD116	-0.030	0.979	6.324	-0.124
IPW//SNORD107//PWARSN//SNORD116	-0.004	0.997	7.559	-0.014
IPW//SNORD107//PWARSN//SNORD116	-0.363	0.778	5.782	-1.133
IQCA1	0.255	1.193	5.119	1.017
IQCJ//IQCJ-SCHIP1//SCHIP1	0.202	1.150	3.685	0.846
IQGAP1	-0.627	0.647	7.515	-2.168
IQGAP2	-0.032	0.978	3.198	-0.109
IQSEC1	0.491	1.405	7.182	1.925
IQSEC2	0.993	1.990	4.958	2.774
IQSEC3	1.030	2.042	4.400	2.164
IQUB	0.608	1.524	3.907	2.058
IRAK1	0.027	1.019	4.836	0.131
IRAK1BP1//RP11-173D14.1	-0.176	0.885	3.471	-0.737
IRAK3	-0.062	0.958	5.310	-0.197
IRAK4	-0.490	0.712	3.594	-1.765
IREB2	0.388	1.309	6.534	1.738
IRF1	0.223	1.167	3.451	0.747
IRF2BP2	-0.444	0.735	6.206	-1.522
IRF7	-0.333	0.794	3.247	-1.450
IRF8	-0.335	0.793	4.753	-0.946
IRF9//RP11-468E2.4	-0.373	0.772	5.599	-1.532

IRF9//RP11-468E2.4//RNF31	-0.198	0.872	2.676	-0.687
IRGQ	0.330	1.257	5.026	1.437
IRS1	0.401	1.321	3.368	1.488
IRS2	-0.111	0.926	7.337	-0.401
IRX1	-0.478	0.718	2.668	-1.440
IRX2	-0.254	0.838	2.067	-1.015
IRX3	-0.444	0.735	3.720	-1.505
IRX5	-0.079	0.947	3.071	-0.287
ISCA1	0.141	1.103	3.933	0.772
ISCU	0.268	1.204	5.419	1.236
ISG20	-0.059	0.960	2.511	-0.157
ISLR	0.583	1.498	3.229	2.401
ISLR2	1.071	2.101	2.371	3.104
ISM1//C20orf82	0.020	1.014	3.652	0.057
ISOC1	-0.636	0.643	7.034	-2.755
ISPD	0.124	1.089	3.968	0.550
ITFG1	0.454	1.370	7.564	1.958
ITFG2//LOC100507424	-0.581	0.668	3.067	-2.254
ITFG3	-0.717	0.609	4.738	-1.852
ITGA10	0.068	1.049	2.076	0.251
ITGA2	-1.383	0.383	5.372	-2.768
ITGA3	0.456	1.372	5.205	1.646
ITGA4	0.266	1.203	2.782	0.889
ITGA5	-0.034	0.977	4.048	-0.136
ITGA7	-0.164	0.892	5.114	-0.804
ITGA8	0.829	1.777	3.926	2.380
ITGA9	0.444	1.360	4.953	1.373
ITGAL	-0.633	0.645	3.688	-1.541
ITGAM	-0.432	0.741	4.800	-1.335
ITGAV	-0.442	0.736	8.925	-2.311
ITGAX	-0.414	0.751	5.152	-0.901
ITGB1	-0.206	0.867	4.326	-0.899
ITGB2	-0.343	0.789	3.499	-1.053
ITGB2-AS1	-0.361	0.778	2.286	-1.405
ITGB3//RP11-290H9.2	0.064	1.045	3.493	0.179
ITGB3BP	0.171	1.126	3.845	0.596
ITGB4	-0.307	0.808	7.000	-0.910
ITGB5	-0.448	0.733	5.476	-1.797
ITGB8	-0.693	0.619	8.023	-2.468
ITGBL1	-0.324	0.799	5.073	-0.794
ITIH2	0.916	1.887	3.819	2.071
ITM2A	0.327	1.254	6.409	1.226
ITPKA	1.517	2.862	3.092	2.895
ITPKB	-0.615	0.653	8.172	-2.217
ITPKB-IT1	-0.520	0.697	3.889	-1.406
ITPKC	0.602	1.518	5.435	2.000
ITPR1	1.631	3.098	6.218	3.023
ITPR1-AS1	0.840	1.790	2.171	2.122
ITPR2	0.233	1.176	7.405	1.150
ITPR3	0.177	1.131	3.995	0.633

ITPRIP	-0.349	0.785	2.549	-1.354
ITPRIPL1	0.038	1.027	2.622	0.122
ITPRIPL2	0.019	1.013	4.452	0.081
ITSN1	0.295	1.227	7.715	1.280
JADE3//PHF16	-0.906	0.534	4.266	-2.798
JAG2	0.010	1.007	3.765	0.037
JAK3	0.156	1.114	3.864	0.418
JAKMIP1	0.993	1.991	5.237	2.489
JAM3	-0.848	0.556	7.032	-3.013
JAZF1	0.429	1.346	3.775	1.830
JAZF1-AS1	0.583	1.498	2.283	1.920
JPH1	0.804	1.746	4.744	2.246
JPH2	-0.069	0.954	2.413	-0.265
JPH3	1.256	2.389	5.704	2.284
JPH4	0.942	1.921	4.305	2.234
JPX	-0.280	0.824	5.444	-0.997
JUNB	0.048	1.034	7.887	0.162
JUP	-0.686	0.622	4.885	-2.372
KAL1	-0.181	0.882	7.716	-0.748
KALRN	1.232	2.349	5.381	3.146
KANK1//RP11-31F19.1	-0.639	0.642	5.198	-2.270
KANK2	0.376	1.297	5.586	1.575
KANK3	0.019	1.013	3.399	0.072
KANK4	-0.962	0.513	3.384	-2.067
KATNB1	0.653	1.573	5.460	2.863
KB-1107E3.1	0.526	1.440	1.838	1.477
KB-1507C5.3	-0.189	0.877	4.163	-0.590
KBTBD12	0.771	1.706	1.965	2.626
KBTBD3	-0.016	0.989	4.723	-0.080
KBTBD6	0.132	1.096	5.594	0.571
KBTBD7	0.340	1.266	4.398	1.261
KBTBD8	0.530	1.444	3.057	1.826
KCNA1	1.166	2.244	4.718	2.024
KCNA2	0.854	1.807	6.412	1.895
KCNA3	0.378	1.299	4.812	1.012
KCNA4	1.018	2.025	4.238	1.839
KCNA5	0.352	1.276	3.524	0.930
KCNA6//GALNT8	0.368	1.290	5.870	1.274
KCNAB1	0.919	1.890	4.524	3.143
KCNAB2	0.942	1.921	3.478	2.495
KCNAB3//RP11-1099M24.7	0.825	1.771	4.232	2.341
KCNB1	1.376	2.595	5.482	2.792
KCNB2	0.826	1.772	3.849	1.522
KCNC1	1.128	2.186	4.040	2.175
KCNC2	1.843	3.589	5.182	2.435
KCNC4	0.312	1.242	4.319	0.879
KCND1	-0.288	0.819	3.827	-0.869
KCND2	0.245	1.185	6.977	0.725
KCND3	0.818	1.764	6.611	2.400
KCND3-IT1	0.216	1.162	2.626	0.671

KCNE1	-0.248	0.842	2.615	-1.007
KCNE4	-0.181	0.882	4.446	-0.426
KCNF1	0.924	1.897	4.324	2.230
KCNG1	0.040	1.028	2.652	0.126
KCNG3	1.267	2.406	3.179	2.689
KCNH1	1.092	2.132	6.521	2.144
KCNH1-IT1	0.747	1.679	3.536	2.315
KCNH2	0.227	1.171	3.501	0.753
KCNH3	1.915	3.771	4.003	3.078
KCNH4	1.085	2.121	2.456	3.235
KCNH5	1.356	2.559	4.370	2.644
KCNH7	1.319	2.495	4.738	2.363
KCNH8	-0.716	0.609	5.883	-1.553
KCNIP1	0.307	1.238	4.866	1.060
KCNIP2	0.903	1.870	4.378	2.187
KCNIP3	0.480	1.395	5.630	1.360
KCNIP4	1.139	2.203	4.316	2.151
KCNIP4-IT1	1.417	2.670	4.637	2.480
KCNJ10	-0.858	0.552	8.237	-2.672
KCNJ11	0.193	1.143	3.512	0.759
KCNJ12//KCNJ18//LOC100996843	0.117	1.084	2.287	0.493
KCNJ16	-0.321	0.801	5.184	-0.622
KCNJ2	-0.959	0.514	6.282	-3.273
KCNJ2-AS1	-0.832	0.562	4.394	-2.243
KCNJ3	1.124	2.179	5.933	2.483
KCNJ4	1.602	3.036	4.334	2.798
KCNJ5	0.197	1.146	3.064	0.725
KCNJ6	1.460	2.751	4.689	2.097
KCNJ8	0.522	1.436	3.138	1.858
KCNJ9	0.701	1.625	6.229	2.370
KCNK1	0.259	1.196	3.293	0.983
KCNK10	0.039	1.028	2.987	0.134
KCNK13	-0.305	0.810	2.287	-1.184
KCNK15	0.657	1.576	2.872	2.699
KCNK2	0.653	1.573	4.341	2.249
KCNK3	0.153	1.112	4.952	0.376
KCNK4//RP11-783K16.10	0.290	1.223	3.072	1.140
KCNK6	-0.505	0.705	3.474	-2.058
KCNK9	0.569	1.483	3.525	1.583
KCNMA1	1.023	2.032	6.237	2.797
KCNMB1	-0.319	0.802	3.742	-1.209
KCNMB2	0.220	1.165	3.573	0.928
KCNMB4	-0.120	0.920	6.624	-0.335
KCNN1	1.228	2.342	4.862	2.718
KCNN3	-0.606	0.657	6.730	-1.848
KCNN4	0.374	1.296	3.391	1.450
KCNQ1	0.099	1.071	3.275	0.386
KCNQ1DN	-0.140	0.907	2.979	-0.433
KCNQ1OT1//LOC101927338	-0.826	0.564	3.777	-1.897
KCNQ2	1.002	2.003	4.625	2.320

KCNQ2	1.344	2.538	3.943	2.235
KCNQ3	1.178	2.263	6.285	2.868
KCNQ5	1.886	3.695	3.908	3.207
KCNQ5-IT1	1.083	2.119	2.794	2.371
KCNS1	1.705	3.261	3.739	2.882
KCNS2	1.484	2.797	4.103	2.665
KCNT1	1.299	2.461	4.789	2.468
KCNT2	1.034	2.048	4.646	2.767
KCNV1	2.217	4.649	3.474	3.038
KCTD1	0.799	1.740	5.195	2.463
KCTD11	-0.488	0.713	4.391	-2.147
KCTD12	-0.402	0.757	7.126	-1.777
KCTD16	1.479	2.787	4.819	2.615
KCTD3	-0.237	0.848	7.502	-1.044
KCTD6	0.044	1.031	3.919	0.168
KDELC1	-0.107	0.929	4.072	-0.373
KDM5D	-0.386	0.766	4.341	-0.348
KDM6A	-0.500	0.707	5.574	-1.789
KDR	0.352	1.277	5.029	0.851
KEL	-1.130	0.457	4.479	-2.980
KGFLP1	1.196	2.291	4.460	1.823
KGFLP2	0.555	1.469	4.751	1.516
KHDRBS2	1.063	2.090	5.053	2.604
KHDRBS3	0.716	1.643	5.771	2.193
KHNYN	-0.472	0.721	4.734	-2.060
KIAA0040	0.358	1.282	4.499	1.035
KIAA0226L	-0.600	0.660	4.201	-2.751
KIAA0247	-0.086	0.942	5.082	-0.378
KIAA0319	1.102	2.147	4.835	2.454
KIAA0355	-0.513	0.701	6.692	-2.065
KIAA0430	0.102	1.073	7.672	0.539
KIAA0513	0.884	1.845	7.451	2.221
KIAA0753	0.236	1.178	5.899	0.952
KIAA0754//RP4-562N20.1	-0.764	0.589	4.569	-2.861
KIAA0825	0.545	1.459	5.092	1.776
KIAA0895	0.398	1.318	3.777	1.562
KIAA0922	-0.276	0.826	3.906	-1.033
KIAA1024	-0.231	0.852	4.188	-0.721
KIAA1045	1.293	2.450	6.027	2.302
KIAA1107	0.716	1.642	7.794	2.716
KIAA1161	-0.204	0.868	5.850	-0.687
KIAA1211L	1.763	3.395	3.892	2.830
KIAA1217	0.594	1.509	4.834	1.673
KIAA1279	0.128	1.093	7.841	0.597
KIAA1324	0.754	1.687	5.023	1.988
KIAA1324L	-0.817	0.568	6.991	-2.807
KIAA1377	0.500	1.414	6.604	1.590
KIAA1407	-0.037	0.975	4.589	-0.144
KIAA1456//LOC101927137	0.550	1.464	4.119	2.206
KIAA1462	0.534	1.448	5.189	1.866

KIAA1467	0.755	1.687	6.129	2.288
KIAA1468	0.096	1.069	6.618	0.532
KIAA1522	0.422	1.340	2.645	1.532
KIAA1524	0.702	1.627	3.032	2.319
KIAA1549	0.401	1.320	5.677	1.191
KIAA1549L	1.527	2.881	6.210	2.506
KIAA1586	0.436	1.353	6.262	1.811
KIAA1598	-0.593	0.663	8.123	-2.138
KIAA1614-AS1//RP11-46A10.4	-0.348	0.786	3.132	-1.282
KIAA1644	0.948	1.929	4.102	2.111
KIAA1671	0.459	1.375	4.566	1.903
KIAA1751//C1orf222	0.555	1.469	2.017	2.205
KIAA1755	-1.016	0.494	4.499	-2.639
KIAA1804//RP5-862P8.2	0.740	1.671	3.709	2.911
KIAA1958	-0.962	0.513	5.910	-3.666
KIAA2022	1.095	2.136	5.108	2.250
KIF13B	-1.044	0.485	7.174	-3.107
KIF17	0.520	1.434	2.982	1.675
KIF19	-0.497	0.709	3.978	-1.425
KIF1C	-0.775	0.584	7.907	-2.529
KIF21A	0.284	1.218	7.703	1.160
KIF21B	0.320	1.248	5.306	1.057
KIF3A	0.491	1.405	8.005	1.882
KIF3B	0.427	1.344	7.225	1.811
KIF3C	0.734	1.664	6.963	2.056
KIF4A	-0.166	0.892	3.182	-0.578
KIF4B	0.740	1.670	3.009	2.911
KIF5A	1.085	2.122	9.486	2.556
KIF5B	-0.391	0.763	9.156	-1.896
KIF5C	0.207	1.154	8.877	1.117
KIF6	-0.446	0.734	4.076	-1.354
KIFAP3	0.523	1.437	8.457	2.124
KIFC2	0.657	1.576	3.758	1.923
KIR2DL1	0.002	1.002	2.132	0.009
KIRREL3	-0.192	0.875	5.388	-0.695
KIT	0.409	1.328	6.274	1.371
KITLG	1.135	2.196	5.558	2.778
KL	0.055	1.039	2.443	0.202
KLC1//XRCC3	-0.499	0.707	3.115	-1.719
KLC2	0.465	1.380	5.818	1.592
KLF10	0.274	1.209	5.343	1.136
KLF12	0.507	1.421	6.672	2.322
KLF15	-0.759	0.591	4.588	-2.403
KLF2	-0.046	0.969	5.442	-0.109
KLF3	-0.550	0.683	5.820	-2.413
KLF4	-0.091	0.939	3.068	-0.285
KLF6	-0.112	0.925	6.911	-0.546
KLF7-IT1	-0.500	0.707	5.382	-1.435
KLF8	0.820	1.765	3.229	2.764
KLF9	0.212	1.159	6.939	0.776

KLHDC3	0.660	1.580	7.564	2.572
KLHDC8A//LOC102724743	0.044	1.031	3.479	0.142
KLHDC9	0.736	1.666	4.667	2.520
KLHL1	0.086	1.062	3.721	0.164
KLHL11	0.359	1.283	5.983	1.692
KLHL14	-0.246	0.843	2.702	-0.958
KLHL15	0.317	1.246	5.030	1.411
KLHL18	0.310	1.240	5.224	1.345
KLHL2	0.070	1.050	7.226	0.330
KLHL29	0.950	1.932	4.269	2.662
KLHL4	-0.605	0.658	5.996	-1.972
KLHL42	-0.023	0.984	4.775	-0.125
KLHL42//RP11-860B13.3	0.846	1.798	3.486	2.411
KLHL6	-0.163	0.893	4.006	-0.411
KLHL7-AS1	0.541	1.455	2.975	2.116
KLK5	0.063	1.045	3.413	0.220
KLK6	-1.539	0.344	8.883	-2.707
KLK7	1.506	2.841	4.615	3.245
KLKB1	-0.535	0.690	2.877	-1.963
KLRC3//RP11-277P12.6	-0.137	0.910	2.696	-0.369
KNDC1	0.462	1.378	4.961	1.374
KNG1	0.558	1.472	1.771	1.468
KNSTRN	0.121	1.087	4.091	0.532
KPNA2	0.533	1.447	6.769	2.402
KPNA5	0.371	1.293	6.065	1.778
KRAS	0.587	1.502	5.064	2.740
KRR1	0.272	1.207	5.924	1.415
KRT14	-0.037	0.975	2.389	-0.119
KRT16P2	0.460	1.376	3.980	1.682
KRT16P3//KRT16P1//KRT16P2	0.260	1.197	2.354	0.769
KRT18P54	0.107	1.077	3.446	0.308
KRT222//RP5-1028K7.3	1.235	2.354	4.896	2.255
KRT81	0.341	1.267	2.072	1.089
KRT81//RP11-845M18.6	-0.314	0.805	3.703	-1.306
KRTAP10-9	-0.222	0.857	3.275	-0.744
KRTAP5-2	0.869	1.826	3.243	2.935
KRTAP6-2	0.181	1.134	2.640	0.700
KRTCAP2//RP11-201K10.3	-0.593	0.663	3.861	-3.148
KSR2	0.551	1.466	5.881	1.552
KYNU	-0.115	0.924	2.767	-0.352
L1CAM	1.170	2.250	5.580	2.018
L2HGDH	0.336	1.262	3.303	1.399
L3MBTL4//CIAO1	-0.185	0.880	4.876	-0.871
LA16c-23H5.4	-0.526	0.695	2.220	-1.838
LA16c-431H6.6	-0.851	0.554	2.792	-2.145
LACC1	-0.678	0.625	5.722	-1.752
LAIR1	-0.437	0.739	4.158	-1.135
LAMA1	-0.227	0.855	4.264	-0.915
LAMB1	1.012	2.016	4.415	2.354
LAMB2	-0.177	0.885	5.429	-0.883

LAMB2P1//RP11-694I15.7	0.331	1.258	2.793	1.034
LAMC1	0.368	1.290	5.538	1.617
LAMC3	0.130	1.094	2.880	0.524
LAMP1	-0.569	0.674	7.884	-2.506
LAMP2	-0.471	0.722	8.590	-1.851
LAMP5	0.423	1.341	7.097	0.585
LAMTOR3	0.462	1.378	5.836	2.030
LANCL2	0.051	1.036	6.409	0.241
LANCL3	0.374	1.296	2.988	1.500
LAP3	-0.051	0.965	6.008	-0.202
LAPTM5	-0.324	0.799	9.023	-0.667
LARGE	0.566	1.480	5.351	2.110
LARP6	-0.524	0.696	4.498	-1.985
LAT2	-0.350	0.784	5.038	-1.499
LATS2	-0.393	0.761	4.901	-1.828
LAYN	-0.582	0.668	3.690	-2.093
LCA10//U52112.12	0.021	1.015	2.341	0.078
LCAT	-0.546	0.685	5.464	-2.403
LCE3A	0.077	1.055	2.490	0.279
LCE3B	0.051	1.036	3.219	0.133
LCLAT1	0.371	1.294	4.764	1.808
LCN12	0.234	1.176	3.791	0.952
LCP1	-0.565	0.676	6.305	-1.590
LCP2	0.234	1.176	6.426	1.010
LCTL	-0.648	0.638	3.040	-1.849
LDB2	1.434	2.702	4.738	3.268
LDB3	-1.035	0.488	4.483	-2.073
LDHA	0.286	1.219	5.112	1.046
LDLR	0.138	1.100	6.014	0.429
LDLRAD2	0.155	1.113	2.135	0.640
LDLRAD3	-0.399	0.759	5.838	-1.518
LDLRAD4-AS1	-0.326	0.798	2.255	-1.188
LDLRAP1	-1.203	0.434	4.629	-3.037
LDOC1	-0.037	0.975	6.605	-0.176
LENG8	-0.632	0.645	6.995	-2.412
LEPREL1	-0.619	0.651	4.004	-2.100
LETM2	0.088	1.063	4.456	0.362
LFNG	-0.376	0.770	3.440	-1.552
LGALS1	-0.420	0.748	6.000	-1.793
LGALS3	0.878	1.838	3.244	3.094
LGALS3BP	-0.924	0.527	6.671	-2.409
LGALS9B	-0.226	0.855	4.604	-0.720
LGALS9C//LGALS9B	0.129	1.093	3.078	0.422
LGALSL	0.618	1.535	6.576	2.153
LGI1	0.423	1.340	7.448	1.273
LGI2	0.149	1.109	5.049	0.416
LGMN	0.296	1.228	6.319	1.102
LGR4	-0.041	0.972	6.248	-0.146
LGR5	-1.677	0.313	6.212	-3.066
LGR6	-0.296	0.814	3.110	-0.691

LHFPL1	0.564	1.478	3.303	2.394
LHFPL2	-0.324	0.799	3.918	-1.297
LHFPL4	0.790	1.729	4.243	2.274
LHFPL5//RP3-422H11.8	0.479	1.394	3.282	1.523
LHPP	-0.456	0.729	6.146	-2.063
LHX2	1.720	3.294	4.208	2.552
LHX3	-0.335	0.793	2.650	-1.130
LHX4-AS1//RP5-1180C10.2	-0.669	0.629	3.101	-2.553
LHX6	0.531	1.445	3.026	1.591
LIAS	0.048	1.034	4.899	0.225
LIFR	-0.513	0.701	7.413	-1.869
LILRA1	-0.414	0.750	2.849	-1.166
LILRA2	-0.501	0.706	2.828	-1.599
LILRA4	-0.627	0.648	3.369	-1.357
LILRA6	-0.312	0.806	2.414	-0.781
LILRB3	-0.446	0.734	2.949	-1.318
LILRB4	-0.409	0.753	3.636	-1.431
LIMA1	-0.393	0.762	4.773	-1.663
LIME1//ZGPAT//RP4-583P15.15	-0.705	0.613	2.631	-2.086
LIMK1	0.616	1.532	5.690	1.624
LIMK2	0.079	1.056	5.862	0.266
LIMS2	-0.225	0.856	2.793	-0.886
LIMS3-LOC440895//LIMS3//LIMS3L//AC	-0.010	0.993	4.376	-0.038
LIN7A	0.887	1.849	3.878	2.589
LINC00032	-0.722	0.606	3.436	-2.155
LINC00086	0.853	1.807	4.576	2.355
LINC00087	0.875	1.834	5.440	1.712
LINC00094	0.401	1.321	5.369	1.716
LINC00173	-0.398	0.759	3.792	-1.394
LINC00173	-0.233	0.851	3.065	-0.623
LINC00202-1	0.579	1.494	3.863	2.068
LINC00271	0.165	1.121	4.118	0.652
LINC00278	-0.022	0.985	3.044	-0.047
LINC00280	0.163	1.120	2.244	0.517
LINC00282	-0.283	0.822	3.693	-1.290
LINC00294	0.321	1.250	6.360	1.396
LINC00298//AC007464.1	0.110	1.080	2.510	0.392
LINC00310	-0.311	0.806	2.737	-1.202
LINC00315	-0.405	0.755	2.503	-1.481
LINC00320	-0.575	0.671	4.420	-1.666
LINC00323	-0.861	0.550	2.065	-1.676
LINC00326	1.013	2.018	1.603	3.413
LINC00328	0.236	1.178	2.840	0.364
LINC00339	-0.269	0.830	2.441	-1.010
LINC00341//SYNE3	-0.625	0.648	3.834	-1.810
LINC00342	-0.279	0.824	3.209	-0.786
LINC00390//RP11-478K15.1	0.105	1.075	2.182	0.243
LINC00391	-0.279	0.824	2.349	-0.908
LINC00403	1.037	2.052	4.416	2.389
LINC00440	0.891	1.855	1.594	3.255

LINC00441	0.015	1.011	2.236	0.052
LINC00443	0.475	1.390	1.857	1.338
LINC00461//MIR9-2	0.126	1.091	5.579	0.505
LINC00467	-0.446	0.734	3.562	-1.880
LINC00471	-0.141	0.907	3.894	-0.528
LINC00472//RP3-331H24.5	-0.141	0.907	3.854	-0.504
LINC00473	0.694	1.618	2.407	2.615
LINC00476	0.060	1.042	3.463	0.139
LINC00476//LOC101930476	-0.108	0.928	3.048	-0.383
LINC00484	-0.246	0.843	2.479	-0.920
LINC00499	0.050	1.035	2.993	0.075
LINC00506	-0.001	0.999	2.819	-0.004
LINC00507	1.661	3.163	2.589	2.245
LINC00515//AP000223.42	0.242	1.183	3.148	0.919
LINC00539//LINC00422	-0.204	0.868	2.571	-0.791
LINC00566	0.136	1.099	3.229	0.492
LINC00577	0.688	1.611	2.787	2.149
LINC00594	0.799	1.740	4.491	1.884
LINC00599//MIR124-1	1.082	2.117	3.237	2.077
LINC00599//MIR124-1	0.421	1.339	4.204	1.092
LINC00601	-0.864	0.550	4.729	-1.742
LINC00609	-1.357	0.391	4.089	-2.272
LINC00614	0.453	1.369	2.790	1.131
LINC00621	0.929	1.904	2.698	2.549
LINC00622	1.224	2.336	4.495	2.117
LINC00630//LOC102725166//LL0XNC01-	0.337	1.263	5.620	1.787
LINC00632	0.109	1.078	5.780	0.316
LINC00633//LOC101928677	-0.346	0.787	2.253	-0.921
LINC00639	-0.997	0.501	4.021	-1.720
LINC00641	0.300	1.231	7.123	1.387
LINC00643//RP11-355I22.6	0.287	1.220	2.478	1.082
LINC00662//CTC-459F4.2	-0.001	1.000	3.611	-0.003
LINC00665	0.012	1.008	3.214	0.044
LINC00672	0.247	1.187	4.821	1.120
LINC00675	0.186	1.138	2.006	0.693
LINC00685	-0.757	0.592	2.609	-3.087
LINC00693	-0.367	0.776	4.601	-1.442
LINC00836	-0.897	0.537	3.778	-1.870
LINC00839	0.396	1.316	3.837	1.074
LINC00842	-0.316	0.803	3.546	-0.924
LINC00862	-0.541	0.687	2.365	-1.542
LINC00877	-0.310	0.807	2.480	-1.198
LINC00882	0.182	1.135	4.146	0.888
LINC00883	-0.311	0.806	4.810	-1.189
LINC00884	0.456	1.372	3.634	1.703
LINC00886	-0.185	0.879	2.389	-0.685
LINC00888	-0.124	0.918	7.559	-0.581
LINC00888	0.123	1.089	4.108	0.499
LINC00889	1.499	2.826	3.597	2.800
LINC00893	0.345	1.271	3.161	1.191

LINC00899	-0.623	0.649	3.012	-1.808
LINC00920	-1.227	0.427	2.696	-3.174
LINC00936	1.000	2.000	3.725	2.787
LINC00950	0.502	1.416	4.773	1.998
LINC00951	0.119	1.086	2.303	0.414
LINC00957	-0.213	0.863	5.094	-1.100
LINC00961	0.185	1.137	4.546	0.693
LINC00963	-0.214	0.862	4.058	-0.774
LINC00969//SDHAP2	-0.416	0.749	5.271	-1.269
LINC00982	0.803	1.745	3.719	3.216
LINC00984	0.305	1.235	5.626	0.797
LINC00987	-0.477	0.718	4.799	-1.232
LINC00997	-0.193	0.875	4.568	-0.654
LINC01000	-0.688	0.621	4.884	-2.332
LINC01000//LINC01061//LOC101060341	-0.549	0.683	7.091	-1.986
LINC01000//LINC01061//LOC101060341	-0.503	0.706	6.888	-1.967
LINC01000//LINC01061//LOC101060341	-0.502	0.706	6.906	-1.923
LINC01000//LINC01061//LOC729737//L	-0.563	0.677	7.111	-2.273
LINC01002	0.080	1.057	4.986	0.244
LINC01007//RP5-1059M17.1	1.205	2.306	2.848	1.970
LINC01018//CTD-2195M18.1	0.594	1.509	2.809	2.104
LINC01030//AB015752.4	-0.422	0.746	2.541	-1.488
LINC01057//RP11-86H7.1	0.130	1.094	2.816	0.436
LINC01061	-0.678	0.625	4.680	-1.734
LINC01061//LOC729218//LOC10192691	-0.569	0.674	6.786	-2.259
LINC01063//AC023797.1	0.030	1.021	3.742	0.104
LINC01088//RP11-438E5.1	-1.872	0.273	4.189	-3.726
LINC01094//RP11-792D21.2	-0.208	0.866	6.487	-0.648
LINC01102//AC013402.2	-0.069	0.953	4.218	-0.228
LINC01105//LOC400940//AC073479.1	-0.966	0.512	6.290	-2.616
LINC01106//LINC01123	1.082	2.118	4.706	2.104
LINC01122//AC007092.1	0.615	1.532	3.298	1.946
LINC01123//AC013268.5	1.271	2.414	5.304	2.285
LINC01125//AC017099.3	0.123	1.089	3.186	0.441
LINC01128//RP11-206L10.11	0.741	1.671	3.818	2.544
LINC01137//RP11-422J8.1	-0.883	0.542	2.964	-2.702
LINC01140//RP5-1052I5.1	0.255	1.193	2.712	0.869
LINC01144	-0.449	0.733	3.309	-1.618
LINC01149//XXbac-BPG181B23.4	-0.272	0.828	2.798	-0.783
LINC01158//AC018730.1	-0.270	0.829	4.450	-1.314
LINC01166//RP13-137A17.4	0.076	1.054	3.780	0.225
LINC01170//RP11-93O7.5	-0.447	0.734	2.588	-1.444
LINC01197//CTD-2536I1.1	1.011	2.016	2.502	2.532
LINC01212//RP11-460N16.1	-0.595	0.662	2.677	-1.959
LINC01234//RP11-438N16.1	0.568	1.482	3.413	1.754
LINC01250//AC019118.2	0.825	1.772	2.001	2.272
LINC01252//RP11-434C1.1	0.573	1.488	4.040	1.650
LINC01272//RP11-290F20.3	-0.028	0.981	2.425	-0.099
LINC01277//RP11-439L18.3	0.611	1.528	2.097	2.422
LINC01285//RP4-562J12.2	-0.389	0.763	3.742	-0.929

LINC01289//RP11-579E24.1	0.728	1.656	1.594	2.116
LINC01315//Z83851.1	-0.470	0.722	3.983	-1.638
LINC01338	-1.449	0.366	4.363	-2.738
LINC01351//RP11-547I7.2	0.303	1.234	2.939	1.098
LINC01354//RP5-855F14.1	0.095	1.068	5.399	0.217
LINC01355//RP5-1057J7.6	-0.288	0.819	5.204	-1.074
LINC01359//RP11-182I10.3	-0.006	0.996	2.546	-0.017
LINC01377//CTD-2029E14.1	-1.026	0.491	2.729	-1.251
LINC01422//RP1-90L6.2	-0.281	0.823	3.823	-0.736
LINC-PINT//MIR29A//MIR29B1//AC0587	0.062	1.044	5.821	0.213
LINCR-0001//RP11-981G7.2	0.499	1.414	5.203	1.600
LINCR-0002//RP11-197K6.1	0.822	1.768	1.785	2.207
LINCR-0003//CTD-2023N9.3	1.771	3.414	2.430	2.745
LINGO1	0.473	1.388	3.826	1.565
LINGO2	1.008	2.011	4.702	1.918
LINGO4	-0.869	0.547	2.789	-1.636
LIPA//LOC101926887	-0.646	0.639	7.073	-2.037
LIPE	-1.048	0.484	5.907	-2.240
LIPG	-0.488	0.713	3.052	-1.008
LITAF	-0.770	0.586	6.708	-2.104
LIX1	-0.307	0.808	7.348	-0.880
LL0XNC01-16G2.1	0.605	1.521	2.181	2.245
LLGL1	-0.627	0.647	6.823	-1.849
LLGL2	-0.384	0.766	3.371	-1.380
LMAN1L//CPLX3//RP11-414J4.2	-0.276	0.826	2.471	-0.812
LMBR1	0.292	1.224	6.953	1.445
LMBRD2	0.591	1.507	7.331	2.468
LMCD1	-0.351	0.784	4.613	-1.270
LMLN	0.051	1.036	5.249	0.246
LMNA	-0.354	0.782	4.603	-1.498
LMNB1	0.345	1.270	4.148	1.145
LMO3	0.823	1.769	4.901	2.401
LMO4	1.189	2.280	6.051	3.048
LMO7//RP11-29G8.3	1.109	2.157	4.315	3.274
LMOD1	0.658	1.578	3.595	1.881
LMOD1//RP11-307B6.3	-0.247	0.843	3.212	-0.806
LMTK2	1.018	2.025	6.585	2.776
LMTK3	0.831	1.779	5.178	2.388
LMX1B	0.162	1.119	2.772	0.544
LNP1	0.644	1.562	5.237	2.080
LNX1	1.045	2.064	4.326	2.089
LOC100126784//RP11-359E10.1	0.701	1.625	3.740	2.489
LOC100127909	-0.027	0.982	2.147	-0.112
LOC100127910//RP1-79C4.4	-0.303	0.811	3.580	-1.037
LOC100128253//RP5-1063M23.1	1.300	2.462	1.934	3.535
LOC100128288	-0.453	0.730	3.901	-1.742
LOC100128340	0.022	1.015	2.917	0.049
LOC100128343	-0.076	0.949	2.302	-0.294
LOC100128364	-0.240	0.847	2.342	-0.661
LOC100128508	-0.449	0.732	4.562	-1.394

LOC100128554//RP5-944M2.3	0.635	1.553	2.324	2.055
LOC100128563	-0.160	0.895	3.616	-0.605
LOC100128751	-0.491	0.711	2.340	-1.311
LOC100129027//AL592528.1	-0.096	0.936	3.315	-0.451
LOC100129129//AF131216.5	-0.269	0.830	4.102	-0.923
LOC100129138//RP11-364B6.1	-0.482	0.716	2.410	-1.722
LOC100129223	0.120	1.087	2.166	0.547
LOC100129233	1.132	2.191	2.425	3.674
LOC100129361	-0.076	0.949	5.229	-0.309
LOC100129434//AC007743.1	-0.183	0.881	4.330	-0.570
LOC100129455	-0.381	0.768	2.195	-1.986
LOC100129473	-0.894	0.538	3.083	-2.000
LOC100129518//SOD2	-0.269	0.830	3.153	-0.544
LOC100129534//RP4-740C4.6	0.421	1.339	3.348	1.740
LOC100129540//RP11-16K12.1//RASGRI	-0.711	0.611	3.356	-2.334
LOC100129550//RP11-67L2.2	0.297	1.228	4.706	1.210
LOC100129697//RP11-830F9.6	-0.011	0.993	2.666	-0.041
LOC100129831	-0.454	0.730	2.697	-1.744
LOC100129936//CTA-373H7.7	0.945	1.925	3.362	2.993
LOC100129973//RP11-430B1.2	0.908	1.877	2.671	3.002
LOC100130027	0.400	1.320	3.253	1.451
LOC100130093//SNAP47//JMJD4	0.719	1.646	3.103	2.742
LOC100130169	0.554	1.468	3.155	1.712
LOC100130172//RP11-325L7.1	0.702	1.627	2.460	2.462
LOC100130193	-0.301	0.811	2.652	-1.240
LOC100130285	0.337	1.263	4.085	1.378
LOC100130298//AC022182.3	-0.136	0.910	2.098	-0.591
LOC100130357//RP1-257A7.4	-0.074	0.950	2.258	-0.241
LOC100130370//RP11-1055B8.3	-0.367	0.775	3.331	-1.318
LOC100130691//AC074286.1//NFE2L2	-0.151	0.901	4.311	-0.523
LOC100130705//RP11-309L24.4	0.604	1.520	2.861	1.647
LOC100130876	-0.508	0.703	2.601	-1.588
LOC100130950//RP11-333E1.1	-0.016	0.989	3.665	-0.055
LOC100130987	0.203	1.151	3.218	0.862
LOC100130992	-0.338	0.791	3.474	-1.040
LOC100131262	-0.195	0.874	3.501	-0.634
LOC100131510	0.111	1.080	2.624	0.346
LOC100131541	-0.547	0.685	3.862	-1.468
LOC100131564//RP4-717I23.3	-0.632	0.645	4.916	-1.944
LOC100131802	0.277	1.212	2.328	0.932
LOC100131826	0.503	1.417	3.363	1.832
LOC100132004//AC125634.1//ANKRD3(	0.260	1.197	2.816	0.818
LOC100132062//LOC100133331//LINC0	-0.467	0.724	7.928	-1.973
LOC100132111//AL450992.2	0.407	1.326	2.895	1.403
LOC100132215//AC009501.4	0.162	1.119	2.187	0.597
LOC100132352	0.282	1.216	4.608	1.019
LOC100132731//RP11-723O4.6//KIAA12	0.069	1.049	2.872	0.232
LOC100132741//LINC00891	-0.134	0.911	3.896	-0.486
LOC100132891//RP11-383H13.1	0.952	1.935	1.932	3.452
LOC100133050	-0.426	0.744	2.414	-1.440

LOC100133106	-0.914	0.531	3.624	-3.698
LOC100133299	0.089	1.063	3.698	0.296
LOC100133331//LOC101929819//LINC0	-0.391	0.763	5.744	-1.587
LOC100133445//RP3-395M20.8	0.142	1.103	2.300	0.561
LOC100133669//RP11-273G15.2	0.775	1.711	3.590	2.301
LOC100134868	-0.364	0.777	4.939	-1.343
LOC100190986//LOC102725125	-0.363	0.777	8.167	-1.258
LOC100190986//LOC102725125	-0.361	0.778	8.129	-1.214
LOC100233156	-0.406	0.755	4.757	-0.796
LOC100270746	-0.714	0.609	4.037	-2.359
LOC100272216	-0.448	0.733	5.405	-1.466
LOC100272216	-0.350	0.785	5.388	-1.214
LOC100272216	-0.412	0.752	5.201	-1.191
LOC100272216	-0.364	0.777	6.269	-1.126
LOC100272216	-0.350	0.785	4.758	-0.969
LOC100286906//AC008060.7	-0.117	0.922	4.336	-0.196
LOC100286925//LL22NC03-86G7.1	0.519	1.433	2.590	2.020
LOC100287225//RP11-267C16.1	-0.488	0.713	3.950	-1.433
LOC100287497//LOC100287934//LOC1(	0.304	1.234	4.954	1.100
LOC100287704	-0.112	0.925	4.055	-0.392
LOC100287834	-0.499	0.708	9.111	-1.275
LOC100287846//LOC286063//RP11-113	-0.030	0.979	4.302	-0.111
LOC100287934//LOC101930657//RP11-	-0.106	0.929	3.963	-0.470
LOC100288160	-0.135	0.911	3.305	-0.517
LOC100288181//CTD-2292P10.4	0.527	1.441	4.561	1.653
LOC100288310//RP11-531A24.3	0.662	1.582	2.765	1.775
LOC100288842//RP11-27I1.4	-0.391	0.763	3.292	-1.378
LOC100288911//RP11-490M8.1	0.644	1.562	3.607	2.093
LOC100289455	-0.545	0.685	3.880	-1.784
LOC100289473//RP4-673D20.3	0.022	1.016	4.212	0.090
LOC100289495	0.554	1.468	3.332	2.424
LOC100294341//LOC101929774//RP11-	-0.517	0.699	3.587	-1.984
LOC100294362//CTD-2047H16.4	0.323	1.251	3.489	1.227
LOC100379224//AC084219.4	-0.179	0.883	4.751	-0.827
LOC100505501//RP11-25K19.1	0.570	1.485	2.610	2.763
LOC100505502	-0.032	0.978	2.426	-0.118
LOC100505549//ATP8B1	-0.738	0.600	2.626	-2.772
LOC100505564//RP11-876N24.4	0.185	1.137	2.244	0.828
LOC100505570//AP006285.6	-0.579	0.669	2.399	-1.277
LOC100505835//CTD-2081K17.2	-0.513	0.701	4.179	-0.824
LOC100505912	-0.295	0.815	2.682	-1.044
LOC100505942	-0.165	0.892	4.020	-0.558
LOC100506022	-0.782	0.582	2.761	-3.108
LOC100506023	0.039	1.028	3.881	0.150
LOC100506036//RP11-353K11.1	-0.624	0.649	2.283	-2.151
LOC100506083//RP11-529K1.2	-0.328	0.797	2.614	-1.067
LOC100506114//CTD-2545M3.8	-0.559	0.679	2.605	-1.842
LOC100506124//TTC21B	0.209	1.156	2.771	0.847
LOC100506127//RP11-111M22.2	0.649	1.569	3.502	2.918
LOC100506136//RP11-682N22.1	-0.430	0.742	2.540	-1.428

LOC100506207//RP11-314C16.1	-0.191	0.876	3.785	-0.685
LOC100506217//RP11-823P9.3	0.112	1.081	2.851	0.312
LOC100506258//CTD-2516F10.2	-0.445	0.735	3.670	-1.454
LOC100506276	-0.084	0.943	3.627	-0.292
LOC100506299//AC011747.7	-0.249	0.842	3.622	-0.934
LOC100506384//RP4-719C8.1	-0.954	0.516	4.036	-2.447
LOC100506411//RP6-65G23.3	0.941	1.920	3.034	2.108
LOC100506458	-0.461	0.726	2.996	-1.725
LOC100506497//CTB-113D17.1	0.336	1.262	2.536	0.878
LOC100506504//RP11-181G12.2	0.018	1.013	2.590	0.073
LOC100506514	0.108	1.078	2.807	0.377
LOC100506544//RP4-756G23.5	-0.345	0.787	3.511	-1.090
LOC100506563	0.495	1.409	2.189	1.661
LOC100506596	-0.362	0.778	2.289	-1.042
LOC100506674//RP11-53O19.1	0.376	1.297	4.746	1.381
LOC100506713//AC027763.2	-0.462	0.726	5.286	-1.811
LOC100506713//AC027763.2	-0.034	0.977	5.507	-0.108
LOC100506725//AC018647.3	-0.644	0.640	3.000	-2.292
LOC100506731//RP11-497E19.1	1.684	3.212	2.932	3.367
LOC100506851//RP1-232L24.3	-0.334	0.794	5.681	-1.069
LOC100506885//RP4-625H18.2	0.255	1.193	2.590	0.682
LOC100507002	0.088	1.063	3.260	0.352
LOC100507006//AC074289.1	-0.431	0.742	4.309	-1.384
LOC100507054//RP11-1398P2.1	-0.146	0.903	2.246	-0.596
LOC100507140//AC007163.3	-0.481	0.717	3.071	-1.128
LOC100507144//RP4-607I7.1	-0.309	0.807	2.335	-0.785
LOC100507194//RP1-40E16.9	-0.417	0.749	3.303	-1.175
LOC100507316//RP13-582O9.5	-0.473	0.720	3.402	-1.790
LOC100507336//RP1-223B1.1	-1.832	0.281	4.245	-2.823
LOC100507351//RP11-13K12.1	1.297	2.456	3.542	2.672
LOC100507395	-0.007	0.995	3.672	-0.021
LOC100507460	0.173	1.127	3.089	0.671
LOC100507494	-0.160	0.895	2.639	-0.520
LOC100507507//AC093673.5	0.385	1.306	4.264	1.222
LOC100507516//RP11-400K9.4	-0.706	0.613	3.352	-1.472
LOC100507520//RP11-1055B8.4	-0.323	0.799	3.669	-1.227
LOC100507530//CTD-2541M15.3	-0.437	0.739	4.074	-1.596
LOC100507534//AC140542.2	0.225	1.169	1.956	0.909
LOC100507535//CTD-2521M24.9//MVB	-0.303	0.811	3.370	-1.309
LOC100507554//AC098820.3	-0.685	0.622	3.012	-2.021
LOC100507564//RP5-1024G6.5	-0.435	0.739	2.857	-1.494
LOC100507577//LONP2	-0.291	0.817	5.749	-1.143
LOC100507599//CTA-445C9.14	0.807	1.750	4.503	2.542
LOC100508046//POTEH-AS1//LOC10192	-0.194	0.874	2.280	-0.738
LOC100508227	-0.463	0.725	4.425	-1.465
LOC100508782//PPIP5K1	0.967	1.955	4.238	2.549
LOC100630918	0.313	1.243	3.639	1.276
LOC100652758	-0.253	0.839	2.576	-0.875
LOC100652768	-0.207	0.866	3.187	-0.686
LOC100652824//AC079354.1	0.308	1.238	3.064	0.841

LOC100652901	-0.504	0.705	2.433	-1.186
LOC100652999//RP11-793H13.3	-0.744	0.597	4.320	-2.648
LOC100653225//OVOS2//LOC10050944	0.562	1.476	2.662	1.276
LOC100653515//CTD-2373H9.6	-0.667	0.630	3.425	-2.392
LOC100653515//CTD-2373H9.6	-0.553	0.681	3.564	-1.513
LOC100996348//RP11-317J10.2	-0.302	0.811	2.791	-1.167
LOC100996385//RP11-826N14.2//RP11-	1.081	2.116	4.782	2.241
LOC100996385//RP11-826N14.2//RP11-	1.083	2.118	4.260	2.186
LOC100996437//RP4-756H11.3	-0.736	0.600	4.038	-1.884
LOC100996557//RP11-535M15.1	0.268	1.204	4.861	0.957
LOC100996664//CTD-2325P2.4	-0.252	0.840	4.622	-0.869
LOC101060341//LINC01347//LOC10106	-0.604	0.658	7.974	-2.371
LOC101060602//AC025627.7	-0.147	0.903	2.375	-0.549
LOC101060691	-0.428	0.743	4.607	-1.228
LOC101926898//RP11-795J1.1	-0.781	0.582	2.415	-2.119
LOC101926908//RP11-313C15.1	0.359	1.283	2.589	0.941
LOC101926964//RP11-436K8.1	-0.646	0.639	4.134	-1.663
LOC101926977//RP11-267M23.4	-0.404	0.756	2.707	-1.470
LOC101926996//AC003102.3	0.460	1.376	2.035	1.723
LOC101927040//RP11-26J3.1	-0.158	0.896	2.738	-0.525
LOC101927043	-0.524	0.695	3.108	-1.712
LOC101927055//RP11-88L24.4	0.500	1.415	3.774	1.534
LOC101927057//OMG	-0.245	0.844	3.146	-0.796
LOC101927063//RP11-310E22.4	-0.141	0.907	3.553	-0.552
LOC101927085//RP11-363E6.3	-0.298	0.813	4.146	-1.275
LOC101927184//LOC100507487//RP11-	-0.913	0.531	3.462	-2.944
LOC101927226//AP000442.1	-0.234	0.851	2.173	-1.019
LOC101927248	-0.268	0.831	3.655	-0.756
LOC101927270//LOC100996442//LOC1(	-0.218	0.860	6.450	-0.837
LOC101927286//CTD-2147F2.2	1.615	3.062	2.517	4.229
LOC101927286//CTD-2147F2.2	0.606	1.522	1.949	2.381
LOC101927300//RP11-441O15.3	-0.108	0.928	3.085	-0.399
LOC101927314//RP1-104O17.1//RP1-1C	-0.285	0.821	3.462	-0.970
LOC101927326//LOC100130157//RP3-4	-1.287	0.410	3.529	-4.043
LOC101927359//RP11-9G1.3	-0.185	0.880	4.585	-0.573
LOC101927397	-0.767	0.588	3.102	-1.738
LOC101927406//AC018799.1	1.528	2.884	1.594	6.082
LOC101927468	-0.787	0.579	5.922	-2.254
LOC101927473	0.722	1.649	2.809	2.940
LOC101927507//ANO7P1	-0.004	0.997	2.457	-0.014
LOC101927531//RP4-809F18.1	1.024	2.033	1.920	2.477
LOC101927532//RP11-552D4.1	-0.617	0.652	4.019	-1.494
LOC101927533//FLJ16124//AC074391.1	0.025	1.018	2.951	0.085
LOC101927621//AC074138.3	0.295	1.227	4.623	1.312
LOC101927636//RP11-673E1.1	0.511	1.425	2.987	1.402
LOC101927646	0.084	1.060	3.882	0.171
LOC101927667//AC012309.5	-0.818	0.567	3.021	-3.076
LOC101927692//RP11-106M7.1	0.207	1.155	5.327	0.515
LOC101927699//RP11-38L15.3	0.980	1.972	5.446	2.356
LOC101927728//RP11-567L7.6	1.565	2.958	2.441	3.358

LOC101927773	-0.063	0.957	2.976	-0.212
LOC101927789	-0.745	0.597	2.788	-2.693
LOC101927871	0.790	1.729	1.796	2.233
LOC101927885//CTB-78F1.2	0.673	1.594	1.928	2.259
LOC101927895//RP1-43E13.2//LOC2845	-0.572	0.673	3.614	-2.220
LOC101927902//RP11-325F22.2	0.194	1.144	2.656	0.729
LOC101927993//RP11-274J16.5	-0.309	0.807	3.098	-0.920
LOC101928020//AC007038.7	0.076	1.054	3.803	0.286
LOC101928054//RP11-705C15.3	-0.455	0.729	3.138	-1.484
LOC101928058//RP11-439C15.4	-0.346	0.787	4.952	-0.757
LOC101928062//RP11-320P7.1	-0.543	0.686	2.262	-1.753
LOC101928067//RP1-156L9.1	1.135	2.196	1.798	2.810
LOC101928100//RP11-277P12.20	-0.543	0.686	2.299	-1.787
LOC101928102//LOC102723678//LOC10	-0.088	0.941	2.503	-0.157
LOC101928102//RP4-610C12.3	0.290	1.223	2.375	0.707
LOC101928111//AC062017.1	-0.169	0.890	3.006	-0.661
LOC101928113	-0.678	0.625	4.407	-1.144
LOC101928161//AC010890.1	-0.159	0.895	2.525	-0.509
LOC101928174//RP11-814P5.1	0.911	1.881	1.716	2.639
LOC101928188//RP11-426C22.4	-0.671	0.628	2.333	-1.709
LOC101928246	0.236	1.177	2.968	0.944
LOC101928258	0.522	1.436	2.061	2.220
LOC101928279	0.172	1.127	2.471	0.718
LOC101928290//RP11-695J4.2	-0.143	0.906	2.713	-0.537
LOC101928300//RP11-728G15.1	1.134	2.194	2.727	2.269
LOC101928304//RP11-557H15.4	-0.488	0.713	3.348	-1.376
LOC101928335//RP6-191P20.4	0.777	1.714	2.048	1.706
LOC101928344//LINC01347//LOC72921	-0.389	0.763	6.627	-1.451
LOC101928370//RP4-575N6.4	0.303	1.233	3.447	1.094
LOC101928387	0.513	1.427	2.935	1.726
LOC101928424//RP11-315O6.1	-0.273	0.828	3.224	-1.079
LOC101928433//RP1-151F17.1	-0.054	0.963	3.765	-0.245
LOC101928436//RP11-153F1.1	0.520	1.434	2.665	1.301
LOC101928470//RP11-490D19.6	-0.480	0.717	3.226	-1.744
LOC101928483//RP11-611D20.2//LOC28	-0.064	0.957	2.646	-0.208
LOC101928489//RP11-553A21.3	-0.062	0.958	3.321	-0.210
LOC101928545//AC007551.3	-0.452	0.731	4.224	-1.932
LOC101928564	-0.380	0.769	2.325	-1.067
LOC101928576//AP001055.6	-0.177	0.885	3.051	-0.718
LOC101928583//RP11-373E16.3	-0.319	0.802	2.245	-1.046
LOC101928590//RP11-806K15.1	0.687	1.610	2.027	2.383
LOC101928633	0.170	1.125	2.245	0.391
LOC101928670//FAM157A	-0.256	0.837	4.598	-0.685
LOC101928670//RP11-206L10.3//RP11-3	-0.321	0.800	5.953	-1.255
LOC101928670//RP11-206L10.3//RP11-3	-0.335	0.793	5.961	-1.249
LOC101928706//LOC101929823//LINC0	-0.447	0.734	7.219	-1.928
LOC101928706//LOC101929823//RP5-8	0.341	1.267	4.500	0.820
LOC101928737//RP11-482M8.1	0.423	1.340	2.302	1.896
LOC101928738//RP11-353N14.4	-0.519	0.698	3.032	-1.597
LOC101928762//STX4	-0.714	0.610	3.231	-2.204

LOC101928792	-1.844	0.279	3.907	-4.399
LOC101928794//MAST4-AS1	-0.167	0.890	2.217	-0.707
LOC101928817//RP11-356K23.1	-0.072	0.951	2.031	-0.195
LOC101928829//RP11-452L6.7	-0.481	0.716	4.028	-1.615
LOC101928832	-0.948	0.518	4.174	-1.737
LOC101928913//RP11-222A11.1	-0.793	0.577	4.564	-1.715
LOC101928913//RP11-222A11.1	-0.131	0.913	2.346	-0.420
LOC101928979	-0.124	0.917	3.304	-0.322
LOC101928989//RP11-179A16.1	0.214	1.160	3.182	0.502
LOC101929008//LOC101929038//LOC10	-0.323	0.799	7.479	-0.959
LOC101929038//LOC101926894//LINC0	-0.264	0.833	7.667	-0.991
LOC101929054	-0.029	0.980	2.943	-0.123
LOC101929066//CTD-2547L16.1	0.014	1.010	3.305	0.045
LOC101929108//RP11-259O2.1	0.467	1.383	2.464	1.965
LOC101929118//RP11-10L7.1	0.213	1.159	2.652	0.510
LOC101929132	0.100	1.072	2.887	0.284
LOC101929179//RP11-143M1.3	1.375	2.594	2.078	5.299
LOC101929237//RP11-87E22.2	0.408	1.326	2.074	1.144
LOC101929289	-0.246	0.843	3.150	-0.722
LOC101929302//RP11-509J21.1	-0.291	0.817	4.090	-0.745
LOC101929302//RP11-509J21.3	-0.494	0.710	4.751	-1.327
LOC101929307	0.052	1.037	2.269	0.187
LOC101929331//RP11-344B5.2	-0.455	0.729	2.987	-1.827
LOC101929514	0.494	1.409	2.106	2.160
LOC101929515//LINC00951//RP11-552E	1.063	2.089	2.337	3.098
LOC101929544	1.407	2.651	2.164	4.547
LOC101929587//LOC101927777//LOC10	0.835	1.784	3.532	2.937
LOC101929595//RP11-286E11.1	-0.685	0.622	2.644	-2.138
LOC101929625	1.059	2.084	2.424	2.750
LOC101929634//RP11-982M15.8	-0.272	0.828	4.287	-1.180
LOC101929643	0.166	1.122	3.236	0.574
LOC101929668//LOC100507419//RP11-	-0.003	0.998	2.674	-0.010
LOC101929679	-0.313	0.805	2.463	-1.211
LOC101929709//RP11-37B2.1	-0.147	0.903	5.137	-0.522
LOC101929761//RP11-195F19.9//LOC73	-0.117	0.922	4.479	-0.444
LOC101929777//WNT3	0.382	1.303	4.874	0.624
LOC101929787	-1.213	0.431	3.942	-2.780
LOC101929920//LOC101930237//AB015	-0.592	0.664	2.147	-2.121
LOC101929949//RP11-982M15.6	-0.350	0.784	3.031	-1.331
LOC101929964//LINC01184//CTC-228N2	0.094	1.067	3.320	0.305
LOC101930100//LOC101928669//LOC10	-0.042	0.971	6.777	-0.105
LOC101930275//RP11-806O11.1	-0.883	0.542	3.918	-2.693
LOC101930276	-0.655	0.635	3.788	-1.192
LOC101930277//RP11-166O4.1	-0.018	0.988	4.016	-0.057
LOC101930305//RP11-341G23.4	-0.225	0.856	3.381	-0.683
LOC101930415//RP11-732M18.3//TULP	0.110	1.079	8.127	0.528
LOC101930421//RP11-809C18.3	-0.522	0.696	5.280	-1.216
LOC101930532//LOC101930156//RP11-	-0.279	0.824	4.213	-1.051
LOC101930595//RP11-326C3.2//ATHL1	-0.412	0.752	2.748	-1.654
LOC101930599//LENG8-AS1	-0.552	0.682	4.116	-2.084

LOC101930660//RP11-380O24.1	-0.489	0.712	3.933	-1.887
LOC101930660//RP11-380O24.1	-0.506	0.704	2.461	-1.516
LOC101930660//RP11-380O24.1	0.130	1.094	4.606	0.490
LOC102546299//CTC-340A15.2	1.156	2.229	2.003	4.265
LOC102577424//RP11-375B1.3	0.401	1.320	4.305	1.184
LOC102723350//RP11-262I2.2	0.426	1.344	2.294	1.600
LOC102723392	0.597	1.513	2.001	1.720
LOC102723403	0.863	1.819	2.429	2.999
LOC102723466//RP11-441F2.2	-0.568	0.675	3.520	-2.028
LOC102723493//RP11-798K3.2	-0.118	0.921	2.515	-0.500
LOC102723521//RP11-513I15.6	0.568	1.483	4.403	1.992
LOC102723544//RP11-283I3.4	-0.659	0.633	2.562	-2.018
LOC102723619//RP11-686G8.2	-0.019	0.987	3.213	-0.060
LOC102723630//LOC100505874	0.742	1.672	5.201	1.073
LOC102723676	0.949	1.931	3.108	2.557
LOC102723678//LOC102723709//RP11-	-0.616	0.652	3.428	-1.913
LOC102723724//RP3-460G2.2	-0.162	0.894	3.029	-0.414
LOC102723736//RP1-153P14.5	-0.064	0.957	2.654	-0.223
LOC102723779//RP11-326I11.3	-0.526	0.695	3.911	-1.539
LOC102723817//RP11-100E13.1	1.057	2.081	2.410	3.502
LOC102723828//RP11-734I18.1	0.058	1.041	2.182	0.187
LOC102723854//AC016735.2	-0.331	0.795	2.362	-1.348
LOC102723894//AP003774.6	-0.203	0.869	2.880	-0.725
LOC102723895//RP11-886D15.1	0.973	1.963	1.502	3.377
LOC102723899//CDK14//CLDN12//AC0(	1.108	2.156	4.468	2.763
LOC102723927//AC093642.3	-0.104	0.930	2.525	-0.375
LOC102724094//RP11-395B7.4	0.392	1.312	2.507	1.174
LOC102724129	0.386	1.307	2.508	1.050
LOC102724155	0.114	1.082	2.805	0.443
LOC102724156	0.584	1.499	3.729	2.315
LOC102724312	-0.448	0.733	2.242	-1.616
LOC102724314//FAM95B1//LOC101928	-0.073	0.951	4.194	-0.241
LOC102724351	0.466	1.381	2.254	1.610
LOC102724425	0.901	1.868	3.349	2.838
LOC102724530	0.016	1.011	2.500	0.061
LOC102724532//LOC100506325//RP11-	0.040	1.028	4.484	0.147
LOC102724596//RP11-1079K10.2	-1.423	0.373	2.784	-2.651
LOC102724596//RP11-1079K10.3	-1.053	0.482	2.465	-2.400
LOC102724640	-0.054	0.963	2.824	-0.182
LOC102724646	0.154	1.113	2.430	0.319
LOC102724679//AP000343.2	-0.470	0.722	4.454	-1.195
LOC102724687	0.016	1.011	2.628	0.057
LOC102724731	-0.479	0.718	3.273	-1.268
LOC102724768	-0.594	0.663	3.743	-1.898
LOC102724814//RP11-66N24.3	-0.342	0.789	2.810	-1.294
LOC102724834	1.961	3.895	2.773	2.928
LOC102724842//RP11-597D13.8	-0.836	0.560	2.500	-2.020
LOC102724851	-0.308	0.808	5.558	-0.929
LOC102724883	-0.164	0.892	4.148	-0.634
LOC102724970//LOC100133616	0.249	1.189	2.759	0.901

LOC102725086//AC090952.5	-0.450	0.732	2.270	-1.607
LOC102725236//BAGE2//RP11-763B22.9	-0.203	0.869	4.596	-0.724
LOC102725258	-0.298	0.813	2.856	-0.873
LOC102725378	-0.036	0.975	2.370	-0.118
LOC102725394	-0.273	0.828	5.251	-0.624
LOC102725417	0.168	1.123	4.704	0.759
LOC102725458	-0.109	0.928	2.970	-0.353
LOC103344931//RP11-57H14.4	0.174	1.128	5.268	0.720
LOC145474	-0.016	0.989	3.139	-0.062
LOC150776//AC093838.4	-0.291	0.818	5.316	-1.091
LOC151760//RP11-553A10.1	0.440	1.356	3.484	1.552
LOC153684//AC025171.1	-0.707	0.613	2.677	-2.355
LOC154761//RP11-61L23.2	0.088	1.063	4.012	0.170
LOC166994	-0.117	0.922	3.319	-0.306
LOC220729//AC024560.3	0.249	1.188	7.262	1.200
LOC257396//CTD-2366F13.1	-0.358	0.780	3.716	-1.273
LOC283045	0.404	1.323	2.839	1.287
LOC283335//RP11-983P16.4	-0.103	0.931	4.620	-0.323
LOC283588//RPS6KA5	0.691	1.615	4.278	2.893
LOC283692//RP11-752G15.3	-0.269	0.830	2.861	-0.964
LOC283693//RP11-752G15.9	-0.261	0.835	2.395	-0.886
LOC283731//RP11-247C2.2	0.480	1.394	2.138	1.506
LOC284023//AC025335.1	0.303	1.234	3.824	1.182
LOC284395//CTC-525D6.1	0.039	1.027	2.727	0.149
LOC284412	-0.559	0.679	4.086	-1.469
LOC284578	0.423	1.341	3.631	0.924
LOC284648//RP11-317P15.4	-0.026	0.982	2.089	-0.109
LOC284926	-0.109	0.927	2.606	-0.365
LOC284930//RP11-191L9.4	0.433	1.350	2.238	1.117
LOC285484//RP11-586D19.1	0.512	1.426	2.840	1.650
LOC285696//AC091878.1	1.375	2.593	3.592	2.823
LOC285857	-0.346	0.787	2.260	-1.274
LOC286367	0.461	1.377	3.743	1.788
LOC286437	-0.487	0.713	4.559	-1.882
LOC374443//RP11-705C15.2//RP11-705	-0.240	0.847	3.705	-0.751
LOC374890//AC011516.2	0.658	1.578	2.324	2.025
LOC388572//LOC101060494//LOC1019	-0.349	0.785	5.018	-1.234
LOC388849//AC006547.14	0.319	1.248	3.218	1.215
LOC389607	-0.303	0.810	3.015	-1.213
LOC389705	-0.190	0.877	3.569	-0.499
LOC389831//LOC102724951	-0.835	0.560	4.708	-1.988
LOC389834	-0.311	0.806	2.874	-0.571
LOC390705//RP11-17M15.2	-0.159	0.896	2.886	-0.543
LOC390937//CTD-2575K13.6	0.063	1.045	2.119	0.210
LOC399491//RP11-958N24.2	-1.361	0.389	3.705	-2.255
LOC399715//RP11-563J2.2	-0.583	0.667	3.719	-1.585
LOC399900	-0.589	0.665	3.185	-1.855
LOC400043//RP11-834C11.4	-1.145	0.452	3.741	-3.036
LOC400553//RP11-863P13.4	0.115	1.083	2.714	0.425
LOC400558//RP11-46C24.3	-0.270	0.829	2.598	-1.211

LOC400622//RP11-13K12.2	0.046	1.032	2.131	0.178
LOC400706	0.511	1.425	4.019	1.239
LOC400743//RP11-380J14.1	-0.820	0.567	2.370	-2.682
LOC400927//RP3-449O17.1	-0.078	0.948	5.539	-0.273
LOC400940//AC073479.1	-0.815	0.568	3.125	-2.051
LOC401134//RP11-707A18.1	0.116	1.083	3.770	0.356
LOC401320//LOC101928623//AC005154	-0.430	0.742	5.157	-1.456
LOC401357	0.318	1.247	2.436	1.180
LOC401357	-0.043	0.970	2.405	-0.136
LOC407835//RP11-286H14.4	-0.044	0.970	2.834	-0.151
LOC414300//RP11-61E11.2	-0.193	0.875	2.465	-0.787
LOC440040//RP11-707M1.1	1.280	2.428	2.477	2.309
LOC440149	-0.765	0.589	4.085	-1.891
LOC440300	0.598	1.513	2.177	2.004
LOC440896	0.570	1.485	2.404	2.088
LOC441081//LOC101929573//LOC10106	-0.549	0.683	6.943	-1.446
LOC441081//LOC101929573//LOC10106	-0.471	0.721	6.405	-1.241
LOC441081//LOC102725466//LOC16699	-0.515	0.700	3.858	-1.458
LOC441204	0.272	1.207	3.710	1.098
LOC441268//RP11-481A20.11	-0.155	0.898	7.343	-0.497
LOC613037//LOC101929910//RP11-347	-0.402	0.757	9.656	-1.697
LOC642236//RP11-764K9.4	-0.327	0.797	2.647	-0.902
LOC642361//LOC439994//RP11-182L21.	0.089	1.063	3.213	0.230
LOC642776//RP11-761E20.1	0.072	1.051	3.706	0.276
LOC642846//RP11-22B23.1	0.264	1.201	2.318	1.041
LOC642852	0.527	1.441	4.149	2.311
LOC642934	-0.326	0.798	3.193	-0.450
LOC643355//RP11-88H9.2	0.020	1.014	2.376	0.063
LOC644172//RP11-259G18.2//RP11-707	0.112	1.081	5.208	0.296
LOC644450	-0.377	0.770	4.773	-0.696
LOC644669//AP005901.1	0.347	1.272	2.174	1.239
LOC644794//RP11-458F8.4	-0.880	0.543	4.335	-2.387
LOC645321//RP11-849I19.1	-0.506	0.704	5.155	-2.100
LOC645513//RP11-33B1.1	-0.099	0.934	5.152	-0.335
LOC645513//RP11-33B1.1	-0.059	0.960	5.661	-0.168
LOC645752//RP11-114H24.2	0.007	1.005	2.711	0.014
LOC646214	-0.017	0.988	2.413	-0.060
LOC646241//RP11-454P21.1	0.152	1.111	2.069	0.530
LOC646719	0.427	1.344	5.216	1.948
LOC646778//RP11-549B18.1	0.490	1.405	2.647	1.353
LOC647264//LOC101927016	0.370	1.293	4.157	1.330
LOC647859//RP11-195E2.1	-1.223	0.428	4.286	-2.186
LOC650226//RP13-492C18.2	-0.778	0.583	3.343	-1.420
LOC651337	-0.134	0.911	2.735	-0.522
LOC652276//AC141586.5	0.063	1.044	4.982	0.254
LOC653513	-0.038	0.974	5.918	-0.093
LOC654342	-0.054	0.963	5.818	-0.161
LOC727896	-0.318	0.802	8.585	-1.107
LOC728084//RP11-13A1.1	1.183	2.271	1.814	2.710
LOC728392//NLRP1	0.184	1.136	9.756	0.719

LOC728485//CTD-2162K18.4	0.703	1.628	3.200	2.412
LOC728554//RP11-423H2.1	0.160	1.117	5.776	0.531
LOC728613//RP11-43F13.1	-0.050	0.966	4.124	-0.145
LOC728730//AC007246.3	0.513	1.427	3.032	2.239
LOC728739//RP11-554D14.2	0.014	1.010	2.287	0.044
LOC728752//CTD-2630F21.1	-0.178	0.884	3.773	-0.666
LOC729305//RP11-469N6.1	-0.014	0.991	2.282	-0.042
LOC729658	-0.783	0.581	3.209	-1.772
LOC729737	-0.493	0.711	5.825	-1.796
LOC729739//MIR7975//CTD-2311M21.2	0.128	1.093	2.484	0.405
LOC729770//CTD-2308L22.1	0.372	1.294	2.372	1.023
LOC729870	0.477	1.392	2.188	1.875
LOC729911//RP11-17L5.4	0.651	1.570	2.935	1.719
LOC729970//RP4-639F20.1	0.365	1.288	4.255	1.127
LOC730081//RP5-884G6.2	0.244	1.184	2.925	1.087
LOC730183//RP11-146F11.1	-0.307	0.808	3.158	-1.167
LOC81691//AC004381.6	0.469	1.384	2.903	2.162
LOC90784//POLR1A	0.249	1.188	4.347	1.040
LOC93432//RP11-1220K2.2	-0.713	0.610	3.389	-3.006
LOC93432//RP11-1220K2.2	0.059	1.042	2.168	0.157
LOC93432//RP11-1220K2.2	-0.024	0.983	2.528	-0.082
LOC93622//AC093323.3	0.285	1.218	4.028	1.121
LONRF1	0.426	1.344	5.461	1.719
LONRF2	0.647	1.566	7.862	1.976
LOX	0.364	1.287	2.609	1.589
LOXL1	0.086	1.061	3.014	0.375
LOXL2	-0.280	0.824	4.520	-0.914
LOXL3	-0.527	0.694	3.901	-2.209
LPAR1	-1.217	0.430	8.463	-3.514
LPAR3	0.758	1.691	2.292	1.739
LPAR4	-0.472	0.721	3.623	-1.197
LPAR5	-0.630	0.646	3.554	-1.721
LPAR6	-0.446	0.734	2.723	-1.374
LPCAT2	-0.842	0.558	7.050	-2.362
LPCAT3	-0.251	0.840	5.758	-1.162
LPCAT4	0.647	1.566	6.008	1.955
LPEQ6126	0.131	1.095	3.066	0.408
LPGAT1	-0.108	0.928	6.975	-0.558
LPHN1	0.551	1.465	7.190	2.166
LPHN2//LOC101927458	1.252	2.381	5.328	2.605
LPHN3	0.287	1.220	7.098	1.384
LPL	0.098	1.071	3.490	0.281
LPP	0.142	1.103	3.569	0.598
LPP-AS2	-0.268	0.830	4.275	-0.853
LPPR1	-0.790	0.578	4.981	-2.216
LPPR3	1.154	2.225	4.515	2.181
LPPR4//RP4-788L13.1	1.443	2.719	5.972	2.459
LPPR5	0.921	1.893	4.004	1.855
LPXN	0.131	1.095	5.112	0.440
LRAT//LOC101927965	-0.381	0.768	2.665	-1.012

LRCH4	-0.514	0.701	3.254	-1.623
LRFN1	0.553	1.467	3.481	1.696
LRFN2	1.007	2.009	3.530	2.373
LRFN5	1.382	2.607	5.492	2.800
LRG1	0.134	1.097	3.555	0.382
LRIG3	-0.607	0.657	3.932	-2.189
LRP10	-0.280	0.824	4.895	-1.251
LRP11	0.041	1.029	4.778	0.194
LRP12	0.433	1.350	5.433	1.696
LRP1B	0.424	1.342	6.929	1.937
LRP2	-1.784	0.290	6.489	-2.817
LRP2BP	-0.657	0.634	5.169	-1.906
LRP3	0.588	1.503	4.638	2.014
LRP5	-0.518	0.699	4.536	-1.878
LRP8	0.420	1.338	5.168	1.665
LRRC1	-0.891	0.539	5.580	-2.506
LRRC10B	0.039	1.027	3.100	0.102
LRRC16A	0.486	1.401	6.552	1.993
LRRC2	1.043	2.060	3.435	2.592
LRRC24	-0.043	0.971	3.190	-0.161
LRRC25	-0.163	0.893	2.599	-0.511
LRRC32	0.159	1.117	4.315	0.411
LRRC37A2	-0.600	0.660	6.276	-1.982
LRRC37A3	0.208	1.155	4.775	0.542
LRRC37A6P	0.047	1.033	3.611	0.168
LRRC37B//LOC101929841	0.027	1.019	4.725	0.097
LRRC38	0.481	1.396	2.390	1.907
LRRC39	0.490	1.405	3.870	1.729
LRRC3B	0.247	1.187	3.594	0.930
LRRC4	0.795	1.735	4.420	2.058
LRRC40	0.341	1.267	5.444	1.312
LRRC46	-0.161	0.894	2.088	-0.449
LRRC48	-0.324	0.799	3.259	-0.916
LRRC49	0.477	1.392	3.997	1.893
LRRC4C	0.315	1.244	6.515	1.303
LRRC53	0.627	1.545	1.899	1.417
LRRC55	0.684	1.607	4.774	1.854
LRRC57	0.341	1.266	5.329	1.580
LRRC6	0.620	1.536	4.843	2.444
LRRC63	-0.942	0.521	4.263	-1.979
LRRC7	1.012	2.017	1.758	3.281
LRRC7	1.032	2.045	5.759	2.079
LRRC71	-0.112	0.925	2.409	-0.496
LRRC73	0.939	1.917	3.700	2.376
LRRC8B	0.412	1.330	6.388	1.223
LRRC9	0.159	1.117	2.082	0.400
LRRCC1	-0.209	0.865	4.855	-0.676
LRRFIP1	0.618	1.535	5.274	2.546
LRRK1	0.555	1.469	4.174	1.909
LRRK2	0.438	1.354	5.969	1.463

LRRN1	-0.026	0.982	4.897	-0.091
LRRN2	0.122	1.088	4.390	0.468
LRRN3	0.882	1.843	4.482	2.291
LRRTM1	0.955	1.938	4.679	2.586
LRRTM2	0.461	1.376	7.272	1.273
LRRTM3//RP11-68P3.2	0.748	1.680	6.297	2.167
LRRTM4	1.017	2.023	5.519	2.336
LRTM2	0.371	1.293	3.419	1.065
LSM11	0.877	1.837	4.017	2.576
LSM7	-0.125	0.917	4.340	-0.540
LSMEM2	-0.428	0.743	3.117	-1.547
LSP1	0.121	1.088	2.931	0.473
LSR	-0.214	0.862	4.138	-0.838
LSS	-0.941	0.521	6.877	-3.012
LST1	-0.423	0.746	3.855	-1.516
LST1	-0.421	0.747	3.633	-1.479
LST1	-0.407	0.754	3.637	-1.431
LST1	-0.411	0.752	3.564	-1.371
LST1	-0.375	0.771	3.478	-1.328
LST1	-0.384	0.766	3.527	-1.306
LTB4R	-0.036	0.975	2.412	-0.117
LTBP1	-0.088	0.941	5.644	-0.353
LTBP2	0.166	1.122	3.990	0.706
LTBP3	-0.307	0.808	5.913	-1.452
LTBP4	0.389	1.309	4.724	1.110
LTBR	-0.238	0.848	6.085	-0.961
LTF	-0.199	0.871	4.217	-0.270
LTN1	0.066	1.047	6.147	0.251
LUCAT1	-0.286	0.820	2.744	-0.671
LUM	0.772	1.707	3.045	2.234
LURAP1L	0.163	1.119	5.127	0.551
LUZP1	0.616	1.533	5.638	1.898
LUZP2	-0.528	0.693	7.175	-1.661
LY6E	0.328	1.255	3.666	1.161
LY6H	0.735	1.665	3.479	2.319
LY75-CD302//LY75//CD302	-0.013	0.991	4.657	-0.051
LY86	-0.468	0.723	6.828	-1.044
LY86-AS1	1.003	2.004	2.368	2.100
LYAR	-0.284	0.821	3.085	-0.981
LYL1	-0.360	0.779	3.739	-1.591
LYN	-0.025	0.983	5.840	-0.073
LYNX1	0.411	1.330	4.015	1.620
LYPD1	-0.564	0.676	4.312	-1.757
LYPD5	0.229	1.172	5.475	0.974
LYPD8//XX-CR54.3	1.095	2.136	1.792	2.578
LYSMD1	0.019	1.013	4.686	0.085
LYSMD2	0.655	1.575	4.160	2.471
LYSMD4	0.069	1.049	3.697	0.257
LYST	-0.157	0.897	3.261	-0.492
LYVE1	0.820	1.765	6.937	1.624

LYZ	-0.433	0.741	4.831	-0.843
LZTFL1	0.446	1.362	3.530	2.049
LZTS1	1.068	2.096	4.032	2.186
LZTS2	-0.675	0.626	4.566	-2.645
LZTS3//RP5-1187M17.10	0.761	1.695	4.833	2.407
MAATS1	0.035	1.025	4.015	0.154
MAB21L1//MIR548F5	-0.359	0.780	2.582	-0.912
MAB21L2	-1.486	0.357	3.728	-1.909
MACROD2	0.578	1.493	2.989	2.658
MAD2L1	0.235	1.177	2.767	0.962
MADCAM1	-0.559	0.679	2.254	-2.042
MADD	0.453	1.369	6.467	1.927
MAEL	0.759	1.693	2.151	3.146
MAFB	0.170	1.125	4.694	0.543
MAFF	0.333	1.260	3.591	1.408
MAFG-AS1	-0.053	0.964	2.482	-0.162
MAFIP	-0.448	0.733	4.370	-1.287
MAG	-1.163	0.447	8.093	-2.134
MAGED1	0.381	1.302	7.210	1.461
MAGEE1	0.488	1.403	5.154	1.539
MAGEE2	0.649	1.568	3.504	1.535
MAGEH1	0.036	1.025	6.314	0.170
MAGEL2	-0.137	0.909	2.578	-0.558
MAGI1//MAGI1-IT1	0.190	1.141	3.953	0.887
MAGI2-AS3	-0.551	0.683	5.978	-2.155
MAGI2-IT1	-0.589	0.665	3.168	-1.652
MAGI3	0.617	1.533	6.335	2.129
MAGOH//MAGOHB	0.175	1.129	2.743	0.658
MAGT1	-0.541	0.687	6.022	-2.168
MAK	0.182	1.134	1.998	0.764
MAL	-1.123	0.459	8.484	-2.797
MAL2	1.754	3.374	3.710	2.912
MALAT1	-0.309	0.807	9.692	-1.334
MALL	0.246	1.186	3.534	0.765
MAMDC2	1.008	2.011	3.877	3.158
MAMDC2-AS1//RP11-195E11.2	-0.229	0.853	2.551	-1.038
MAMDC4	-0.427	0.744	3.012	-1.665
MAML2	-0.430	0.742	5.737	-1.988
MAN1A1	0.427	1.344	5.924	1.200
MAN1A2	0.251	1.190	7.963	1.153
MAN1B1-AS1	-0.186	0.879	3.120	-0.626
MAN1C1	0.135	1.098	6.306	0.413
MAN2A1	-1.010	0.497	7.123	-2.671
MAN2B1	-0.418	0.749	4.842	-1.503
MANEAL	0.106	1.076	4.100	0.481
MANF	-0.037	0.975	4.560	-0.142
MANSC1	0.443	1.360	5.317	2.019
MAOB	0.049	1.034	7.345	0.174
MAP1A	0.377	1.299	8.590	1.502
MAP1LC3B2	-0.484	0.715	3.914	-1.338

MAP2	0.867	1.823	8.295	2.060
MAP2K1	0.781	1.718	7.753	2.763
MAP2K4	0.656	1.575	4.312	2.347
MAP2K6	0.042	1.029	5.618	0.172
MAP3K10	0.656	1.575	5.550	2.366
MAP3K11	-0.716	0.609	4.132	-2.608
MAP3K14	-0.159	0.895	3.923	-0.663
MAP3K6	0.527	1.441	4.602	2.283
MAP3K8	-0.590	0.664	4.665	-1.100
MAP3K8	-0.130	0.914	4.799	-0.313
MAP3K9	1.039	2.055	4.495	2.515
MAP4	-0.446	0.734	7.361	-2.166
MAP4K1	-0.359	0.780	2.341	-1.370
MAP4K2	0.432	1.349	4.840	1.671
MAP4K4	-0.704	0.614	8.473	-2.547
MAP4K5	-0.636	0.643	7.647	-2.591
MAP6	0.441	1.358	6.469	1.593
MAP6D1	-0.712	0.611	5.625	-2.657
MAP7	-0.798	0.575	8.246	-2.909
MAP7D2	1.084	2.120	6.614	1.446
MAP7D3	-0.154	0.899	5.011	-0.703
MAP9	0.724	1.652	5.534	2.571
MAPK12	0.149	1.109	2.489	0.405
MAPK13	0.857	1.811	3.765	2.710
MAPK6	0.308	1.238	4.672	1.570
MAPK7	-0.305	0.810	2.705	-1.165
MAPK8	0.199	1.148	3.880	0.828
MAPK8IP1	0.061	1.043	4.182	0.204
MAPK8IP2	0.772	1.708	5.822	2.118
MAPK9	0.562	1.476	6.000	2.320
MAPKAPK2	-0.536	0.690	6.424	-2.460
MAPKAPK3	-0.047	0.968	4.912	-0.208
MAPRE3	0.580	1.495	4.850	2.196
MAPT-IT1	-0.296	0.815	3.730	-1.044
MARC2	0.739	1.669	5.526	3.261
MARCH10	-0.049	0.967	2.485	-0.170
MARCH11	0.548	1.462	2.998	1.722
MARCH3	-0.961	0.514	5.555	-2.486
MARCH4	0.726	1.654	4.003	1.663
MARCKS	-0.408	0.754	8.048	-1.992
MARCKSL1	-0.331	0.795	6.776	-1.351
MARCO	-0.141	0.907	2.585	-0.516
MARK1	0.551	1.466	5.831	2.097
MARK4	0.499	1.413	5.738	2.180
MAS1	1.899	3.730	2.850	2.925
MASP1	-0.316	0.803	5.154	-0.893
MAST1	0.727	1.655	5.544	1.633
MAST3	0.644	1.563	7.377	2.198
MAST4	0.678	1.600	4.551	2.539
MASTL	0.429	1.346	4.000	1.461

MAT2A	-0.494	0.710	7.689	-1.938
MATK	0.804	1.746	4.032	2.699
MATN1-AS1	-0.189	0.877	2.750	-0.660
MATN2//LOC100506558	-0.221	0.858	3.418	-1.141
MATN3	0.345	1.271	3.362	0.960
MAVS	-0.538	0.689	6.062	-2.321
MB21D1	-0.213	0.863	2.335	-0.756
MB21D2	0.531	1.445	4.863	1.797
MBD6	-0.457	0.729	3.720	-1.998
MBLAC1	0.556	1.470	4.074	2.236
MBLAC2	0.143	1.104	5.020	0.644
MBNL3	-0.681	0.624	3.753	-1.834
MBOAT1	-1.718	0.304	5.089	-3.110
MBOAT7	0.353	1.277	5.169	1.657
MBP	-0.352	0.783	6.846	-1.526
MBTPS1	0.084	1.060	5.914	0.431
MBTPS2	0.302	1.233	6.865	1.101
MC4R	0.957	1.941	2.780	2.238
MCAM//MIR6756	-0.049	0.967	3.767	-0.141
MCAT	0.099	1.071	4.790	0.317
MCC	-0.221	0.858	5.576	-0.920
MCEMP1//C19orf59	0.000	1.000	2.344	-0.001
MCF2	0.446	1.362	4.552	1.171
MCF2L2	0.728	1.656	6.130	2.598
MCF2L-AS1	0.040	1.028	3.126	0.114
MCHR1	0.731	1.659	3.948	2.223
MCHR2	2.462	5.511	2.676	3.292
MCL1	-0.181	0.882	7.345	-0.826
MCM4	0.397	1.317	5.516	1.586
MCM5	-0.499	0.708	4.476	-1.245
MCM6	0.503	1.418	4.963	1.951
MCM7	-0.738	0.599	4.472	-2.849
MCM8	-0.079	0.947	4.311	-0.360
MCTP1	1.160	2.235	5.141	3.135
MCTP2	0.541	1.455	2.891	1.827
MCU	0.362	1.286	4.909	1.675
MDFIC	-0.406	0.755	3.093	-1.526
MDGA2	0.508	1.422	5.269	1.797
MDH1	0.677	1.599	7.026	2.466
MDH1B	-0.244	0.845	3.315	-0.820
MDK	-0.404	0.756	4.695	-1.610
MDM1	-0.218	0.860	3.989	-0.743
MDM4	-0.462	0.726	6.325	-2.020
ME3	0.588	1.503	5.300	2.186
MEAF6	0.331	1.258	6.489	1.748
MED12L	0.924	1.898	5.725	3.365
MED15P9//MED15P2	0.101	1.072	2.732	0.377
MED31	0.411	1.329	5.499	2.179
MEDAG	0.158	1.116	3.128	0.305
MEF2C	1.403	2.644	4.465	3.130

MEF2D	0.411	1.330	6.691	1.798
MEG3	1.888	3.700	6.140	2.441
MEG3	0.677	1.598	5.176	2.128
MEG8//SNORD112	0.427	1.344	4.375	1.264
MEGF10	-0.867	0.548	7.556	-2.789
MEIS1	-1.144	0.453	3.932	-3.801
MEIS3P1	0.267	1.203	4.301	0.807
MEOX2	-0.400	0.758	2.936	-1.254
MEPE	1.433	2.701	2.116	2.467
MESP2	0.068	1.049	2.946	0.241
MEST	0.644	1.562	4.238	2.104
MET	-0.479	0.718	4.879	-1.183
METAP1	0.286	1.219	4.927	1.418
METRN	-0.492	0.711	6.660	-1.664
METRNL	0.405	1.324	4.238	1.354
METTL1	0.063	1.045	2.952	0.232
METTL10//RP11-12J10.3	0.137	1.099	5.188	0.616
METTL15	0.007	1.005	3.958	0.027
METTL16//LOC284009	-0.458	0.728	3.134	-1.804
METTL23	-0.276	0.826	4.634	-1.092
METTL25	0.010	1.007	3.171	0.049
METTL2A	0.147	1.107	5.875	0.693
METTL7A	-0.390	0.763	8.258	-1.495
METTL7B	0.585	1.500	4.493	1.728
MEX3B	0.151	1.110	2.221	0.671
MFAP3	0.253	1.191	5.266	1.126
MFAP3	-0.304	0.810	3.506	-0.769
MFAP3//PCNXL2//LOC101927169	1.041	2.057	4.101	3.091
MFAP4	0.527	1.441	4.561	1.738
MFAP5	-0.162	0.894	2.609	-0.472
MFNG	0.046	1.033	3.414	0.169
MFSD2A	-0.185	0.880	4.262	-0.684
MFSD3	0.237	1.178	3.761	0.980
MFSD4	1.073	2.104	5.998	2.193
MFSD7	0.119	1.086	3.630	0.476
MFSD9	0.054	1.038	3.494	0.214
MGAT1//LOC102724833	-0.139	0.908	4.671	-0.648
MGAT4A	0.473	1.388	6.242	2.156
MGAT4C	0.261	1.199	4.486	0.903
MGAT5B	1.502	2.833	4.388	2.872
MGC12916//RP11-214O1.2	0.081	1.058	2.496	0.256
MGC16025	-0.381	0.768	2.867	-1.082
MGC16142	-0.583	0.667	2.730	-2.042
MGC16275//CTD-2514K5.2	-0.691	0.619	4.706	-1.724
MGC24103	-0.460	0.727	2.037	-1.286
MGC27345//RBM28	-0.545	0.685	4.657	-2.148
MGC2889	0.190	1.141	2.365	0.650
MGC57346//CRHR1-IT1//AC126544.2//(	0.278	1.212	5.033	1.271
MGC70870	-0.958	0.515	4.620	-2.010
MGC72080	-0.349	0.785	5.932	-1.288

MGLL	0.545	1.459	3.855	2.247
MGME1	-0.308	0.808	4.641	-1.237
MGP	0.273	1.208	5.401	0.675
MGST1	0.020	1.014	5.003	0.087
MGST3	0.042	1.030	3.776	0.162
MICA	0.189	1.140	4.665	0.754
MICA	0.186	1.138	4.671	0.741
MICAL2	1.123	2.179	4.100	2.887
MICALL1	-0.626	0.648	4.138	-1.940
MICALL2	0.006	1.004	3.454	0.023
MICU1	0.164	1.121	6.104	0.851
MICU3	0.645	1.563	4.857	2.358
MID1IP1	-0.717	0.609	6.898	-2.743
MIDN	0.092	1.066	6.472	0.384
MIF4GD	-0.408	0.753	4.721	-1.522
MINOS1P1	-0.266	0.832	2.926	-0.733
MIPEPP3	-0.158	0.896	2.480	-0.577
MIR106B	-0.273	0.828	2.550	-1.013
MIR1180	-0.883	0.542	2.675	-2.547
MIR1184-1//MIR1184-2//MIR1184-3	0.372	1.294	4.995	1.704
MIR1200	-0.472	0.721	4.117	-1.074
MIR1208	-0.225	0.856	2.518	-0.806
MIR1226	0.067	1.048	2.398	0.205
MIR1227	0.289	1.222	3.591	1.018
MIR1229	-0.366	0.776	3.279	-0.820
MIR1231	-0.118	0.922	3.584	-0.301
MIR1238	-0.074	0.950	5.043	-0.221
MIR124-2HG//LINC00966	1.530	2.888	4.664	2.947
MIR1243	-0.500	0.707	3.253	-1.012
MIR1250	-0.542	0.687	3.340	-1.679
MIR1254-1	-0.209	0.865	3.623	-0.573
MIR1260A	0.348	1.273	2.433	1.039
MIR127	0.231	1.173	3.259	0.728
MIR1270	0.100	1.072	2.756	0.378
MIR1282	-0.008	0.994	5.229	-0.030
MIR1290	0.224	1.168	3.748	0.534
MIR1299	-0.207	0.866	6.526	-0.654
MIR134	-0.032	0.978	3.054	-0.103
MIR137//MIR137HG//MIR2682	0.832	1.780	2.855	2.834
MIR138-1	0.003	1.002	2.350	0.010
MIR139	0.447	1.363	3.550	1.688
MIR147A	0.566	1.480	2.351	1.584
MIR149	0.142	1.103	2.458	0.390
MIR151A	-0.654	0.636	2.996	-1.552
MIR151B	0.051	1.036	3.046	0.179
MIR1538	-0.361	0.779	3.808	-1.533
MIR1587	-0.187	0.879	2.323	-0.357
MIR181A1HG	-0.587	0.666	4.644	-1.942
MIR181B1	-0.300	0.812	2.538	-0.679
MIR181C	0.162	1.119	2.893	0.564

MIR185	-0.014	0.990	4.107	-0.053
MIR191	-0.253	0.839	2.312	-0.831
MIR1910	-0.028	0.981	4.004	-0.109
MIR1913	0.157	1.115	3.760	0.539
MIR197//GNAI3	0.277	1.212	2.862	0.809
MIR21//VMP1	0.128	1.093	5.018	0.360
MIR210HG	-0.287	0.819	2.935	-0.694
MIR219A2//MIR219B//RP11-339B21.12	-0.546	0.685	4.903	-1.045
MIR219B	-0.559	0.679	2.945	-1.667
MIR2278	-0.233	0.851	2.320	-0.862
MIR22HG//MIR22	0.624	1.541	3.173	2.627
MIR23B//C9orf3//MIR24-1	-0.270	0.830	2.936	-0.770
MIR23C	0.718	1.645	1.682	2.811
MIR25	0.535	1.449	2.117	1.689
MIR2682//MIR137HG//MIR137	0.364	1.287	2.256	1.234
MIR27B//C9orf3//MIR24-1	0.146	1.106	2.103	0.355
MIR29C//MIR29B2//C1orf132	-0.103	0.931	3.336	-0.267
MIR3065	0.243	1.183	2.700	0.865
MIR3120	-0.575	0.671	3.931	-1.410
MIR3132	-0.430	0.742	2.960	-1.656
MIR3135B	-0.315	0.804	2.376	-0.834
MIR3137	0.391	1.311	3.666	1.021
MIR3139	-0.753	0.593	2.424	-2.565
MIR3143	0.437	1.354	4.160	1.201
MIR3147	0.229	1.172	1.910	0.635
MIR3151	0.152	1.111	3.437	0.583
MIR3155A//MIR3155B	-0.146	0.904	2.764	-0.427
MIR3157	-0.208	0.866	2.932	-0.585
MIR3161	-0.191	0.876	2.874	-0.697
MIR3175	-0.279	0.824	4.487	-1.088
MIR3180-4	0.143	1.104	3.509	0.532
MIR3180-4//MIR3180-5	-0.119	0.921	2.617	-0.335
MIR3192	0.060	1.043	3.357	0.196
MIR3198-1	0.030	1.021	3.208	0.110
MIR31HG	0.876	1.835	2.609	2.391
MIR32	0.560	1.475	2.766	1.935
MIR324	-0.124	0.917	3.473	-0.430
MIR326	-0.677	0.626	3.994	-2.343
MIR338//MIR3065	-0.388	0.764	3.488	-1.270
MIR339	-0.206	0.867	4.006	-0.653
MIR33B	0.401	1.320	3.367	1.049
MIR3619	0.050	1.036	4.682	0.157
MIR3622A	0.641	1.559	2.454	2.101
MIR3622B//MIR3622A	-0.565	0.676	3.004	-1.503
MIR3650	0.039	1.028	3.636	0.123
MIR3661	0.445	1.362	3.984	1.892
MIR3662	-0.069	0.953	2.781	-0.195
MIR3667	-0.472	0.721	3.215	-1.761
MIR3671	-0.405	0.755	2.147	-1.300
MIR3689A	0.133	1.096	3.485	0.419

MIR3689B	-0.345	0.787	2.442	-1.217
MIR3689F	0.047	1.033	5.855	0.174
MIR3691	-0.243	0.845	2.323	-0.800
MIR370	0.156	1.114	1.906	0.607
MIR375	-0.004	0.997	3.901	-0.015
MIR378E	-0.296	0.815	2.671	-0.965
MIR378I	-0.349	0.785	3.631	-1.042
MIR3916//RP11-488L18.4	-0.599	0.660	5.051	-1.419
MIR3936	-0.147	0.903	4.078	-0.475
MIR3939	0.958	1.943	2.879	2.133
MIR3942	-0.495	0.710	2.312	-1.505
MIR3944	0.036	1.025	3.184	0.143
MIR3972	0.109	1.078	2.412	0.384
MIR3978	0.441	1.357	3.776	1.370
MIR412	-0.028	0.980	2.116	-0.102
MIR421	-0.110	0.927	5.320	-0.338
MIR4288	0.191	1.142	3.262	0.626
MIR4292	-0.029	0.980	2.390	-0.072
MIR4294	-0.426	0.744	2.584	-0.942
MIR4295	-0.428	0.743	2.864	-1.346
MIR4300	-0.407	0.754	2.219	-0.904
MIR4312	-0.458	0.728	2.911	-1.491
MIR4314	-0.297	0.814	2.503	-1.108
MIR4316	-0.048	0.967	3.722	-0.144
MIR4324	0.324	1.252	3.689	0.867
MIR4420	-0.176	0.885	2.038	-0.598
MIR4441	-0.216	0.861	4.812	-0.662
MIR4450	-1.017	0.494	4.678	-1.949
MIR4451	-1.118	0.461	3.167	-2.401
MIR4454	0.239	1.180	6.165	0.796
MIR4461	-0.437	0.739	6.289	-1.059
MIR4476	-0.464	0.725	2.866	-1.404
MIR4481	-0.328	0.797	4.105	-1.192
MIR4482	-0.337	0.791	3.083	-1.289
MIR4486	-0.207	0.866	2.542	-0.660
MIR4492	0.187	1.138	3.884	0.656
MIR4497	-0.106	0.929	2.867	-0.295
MIR4500	1.138	2.201	2.562	2.842
MIR4500HG	0.760	1.694	2.581	2.183
MIR4502	0.417	1.335	2.613	1.235
MIR4516//LOC102724992//LOC101927[illegible]	0.127	1.092	3.937	0.326
MIR4518	-0.105	0.930	3.207	-0.235
MIR4519	0.691	1.615	3.351	1.607
MIR4522	0.072	1.051	2.324	0.186
MIR4525	-0.354	0.783	6.348	-1.340
MIR4530	0.365	1.288	2.930	0.680
MIR4533	-0.062	0.958	4.825	-0.247
MIR4534	-0.604	0.658	6.730	-1.749
MIR4638	-0.589	0.665	3.154	-2.220
MIR4644	-0.498	0.708	3.463	-1.322

MIR4646	-0.003	0.998	2.752	-0.007
MIR4655	0.020	1.014	2.235	0.064
MIR4663	-0.219	0.859	2.679	-0.638
MIR4668	-0.240	0.847	1.924	-0.652
MIR4673	-0.156	0.898	4.829	-0.643
MIR4677	-0.457	0.729	4.228	-1.363
MIR4686	-0.145	0.905	3.053	-0.485
MIR4689	-0.210	0.865	2.797	-0.619
MIR4697HG//MIR4697	0.575	1.490	4.215	1.921
MIR4705	1.106	2.152	1.506	2.914
MIR4710	-0.651	0.637	2.711	-1.874
MIR4730	-0.015	0.989	3.502	-0.037
MIR4742	-0.221	0.858	3.332	-0.451
MIR4746	-0.270	0.829	2.623	-0.632
MIR4748	0.168	1.124	3.998	0.643
MIR4756	-0.072	0.951	3.492	-0.202
MIR4757	-0.661	0.633	2.273	-2.742
MIR4780	-0.178	0.884	2.391	-0.545
MIR486-1	0.164	1.120	2.687	0.578
MIR502	0.305	1.235	3.115	1.105
MIR513A2	0.035	1.025	3.052	0.085
MIR516B1	0.524	1.438	2.245	1.581
MIR539	0.005	1.004	4.292	0.021
MIR548AL	0.335	1.262	2.713	1.328
MIR548F2	0.025	1.018	3.029	0.089
MIR548H2	0.330	1.257	3.657	1.079
MIR548I2	-0.627	0.648	2.955	-1.294
MIR548I2//MIR548I1//MIR548I3	-0.457	0.729	3.379	-1.074
MIR548I2//MIR548I1//MIR548I3	-0.284	0.822	3.336	-0.703
MIR548L	-0.004	0.997	2.473	-0.012
MIR548V	-1.328	0.398	5.193	-2.695
MIR550A1//MIR550B1	-0.041	0.972	2.669	-0.143
MIR550A3	0.011	1.008	3.110	0.030
MIR552	-0.345	0.787	2.265	-1.226
MIR570	-0.473	0.721	6.440	-1.377
MIR571	-0.330	0.795	3.848	-1.246
MIR573	-0.246	0.843	2.532	-0.884
MIR574	-0.183	0.881	5.026	-0.725
MIR575	-1.056	0.481	5.244	-2.828
MIR577	-0.948	0.518	2.431	-2.199
MIR579	-0.261	0.835	4.046	-0.634
MIR585	0.396	1.316	2.183	1.594
MIR587	0.142	1.103	2.868	0.471
MIR597	-0.159	0.895	3.972	-0.586
MIR600HG//MIR600	0.745	1.676	4.799	2.078
MIR601	-0.786	0.580	2.784	-2.331
MIR604	-0.110	0.927	3.453	-0.286
MIR606	0.181	1.134	3.354	0.532
MIR628	0.750	1.682	2.440	2.115
MIR637//DAPK3	-0.286	0.820	3.651	-0.636

MIR642A//MIR642B	0.325	1.252	2.639	0.952
MIR644A	-0.517	0.699	4.301	-1.201
MIR645	0.102	1.073	3.723	0.279
MIR646	-0.654	0.635	3.056	-2.092
MIR647	-0.336	0.792	4.935	-1.382
MIR648	-0.144	0.905	2.248	-0.405
MIR654	0.716	1.643	2.020	1.795
MIR657	-0.310	0.807	3.076	-0.850
MIR662	0.348	1.273	2.637	1.225
MIR663B	-0.010	0.993	4.296	-0.033
MIR6735//SZT2-AS1	-0.131	0.913	2.623	-0.458
MIR7-3HG	0.967	1.954	4.109	2.054
MIR744	-0.412	0.752	2.824	-1.422
MIR760	0.404	1.323	3.429	1.494
MIR7641-2	-0.198	0.872	2.171	-0.587
MIR765	-0.460	0.727	2.369	-1.799
MIR770//MEG3	1.165	2.242	4.197	1.977
MIR877	-0.301	0.812	2.924	-0.726
MIR877	-0.227	0.854	3.230	-0.623
MIR92B	-0.153	0.899	4.261	-0.535
MIR9-3	0.624	1.541	2.129	1.613
MIR941-1//DNAJC5//MIR941-4	-0.393	0.762	5.444	-1.576
MIR941-1//MIR941-2//MIR941-3//MIR9	-0.282	0.823	4.188	-1.074
MIRLET7A1	0.327	1.255	2.284	0.785
MIRLET7D	-0.189	0.877	2.352	-0.704
MITF	-0.514	0.700	2.940	-1.998
MKKS	0.424	1.342	6.194	2.153
MKL2	1.388	2.618	8.113	2.948
MKNK2	-0.370	0.774	5.496	-1.034
MKRN2	0.274	1.209	6.354	1.412
MKRN3	-0.541	0.687	3.419	-1.747
MKX	0.397	1.317	4.186	1.393
MLC1	-0.195	0.874	8.797	-0.950
MLIP	1.179	2.264	3.438	2.025
MLKL	0.000	1.000	4.635	0.001
MLLT11	0.937	1.915	8.193	2.288
MLLT3	0.360	1.284	5.838	1.752
MLXIPL	0.156	1.114	3.732	0.558
MMAA	0.344	1.269	4.035	1.658
MMAB	-0.269	0.830	5.751	-1.123
MMD	0.612	1.528	7.006	2.115
MMD2	-0.290	0.818	5.289	-0.894
MME	0.093	1.067	2.683	0.195
MMEL1	0.174	1.129	3.344	0.796
MMP14	-0.523	0.696	4.859	-1.194
MMP16	0.608	1.524	5.975	2.309
MMP17	0.420	1.338	3.748	1.521
MMP2	-0.600	0.660	4.464	-1.992
MMP24	0.951	1.933	4.156	2.137
MMP24-AS1	-0.562	0.677	2.197	-1.863

MMP24-AS1//EDEM2//RP4-614O4.11	-0.921	0.528	5.211	-3.567
MMP28	-0.076	0.949	3.027	-0.261
MMP8	-0.935	0.523	2.580	-1.692
MMRN1	-0.151	0.901	2.707	-0.395
MMS22L	-0.327	0.797	2.607	-1.250
MN1	0.169	1.124	4.439	0.548
MNDA	-0.095	0.936	4.080	-0.281
MNS1	0.345	1.270	5.072	1.288
MOAP1	0.609	1.526	6.370	1.965
MOB3A	-0.512	0.701	7.466	-2.409
MOB3B	-1.199	0.436	7.565	-3.371
MOB3B	-0.579	0.669	2.726	-1.812
MOBP	-1.355	0.391	8.161	-2.673
MOCS2	0.184	1.136	8.190	0.961
MOCS3	0.478	1.393	3.490	1.523
MOG	-1.082	0.472	8.090	-2.092
MOG	-1.092	0.469	8.141	-2.091
MOGS	-0.046	0.969	3.911	-0.168
MOK	0.186	1.137	2.927	0.650
MORC3	0.191	1.141	4.495	0.778
MORC4	-0.500	0.707	4.567	-1.804
MORN1	0.717	1.643	3.534	2.935
MORN3	0.810	1.753	2.939	2.992
MORN4	0.651	1.570	4.227	2.069
MOSPD1	0.175	1.129	4.301	0.585
MOSPD2	-0.800	0.574	5.905	-2.443
MOXD1	1.384	2.609	4.200	2.516
MPC2	0.451	1.367	7.186	1.851
MPEG1	-0.401	0.757	4.572	-1.103
MPLKIP	0.035	1.025	4.721	0.171
MPP3	-0.034	0.977	4.286	-0.125
MPP6	0.394	1.314	7.310	1.617
MPP7	0.909	1.878	3.754	2.507
MPPED1	1.553	2.934	4.697	2.657
MPPED2	-0.111	0.926	3.749	-0.364
MPRIP-AS1	-0.143	0.906	2.643	-0.545
MPZ	-0.273	0.828	4.068	-0.400
MPZL2	0.459	1.374	3.267	1.306
MR1	0.157	1.115	3.938	0.683
MRAP2	0.945	1.925	4.422	1.975
MRC1	0.669	1.590	4.473	1.557
MRC1	0.658	1.577	4.205	1.505
MRC2	-0.684	0.623	4.262	-2.343
MREG	0.314	1.243	3.780	1.165
MRFAP1L1	0.293	1.225	6.983	1.405
MRGBP	-0.106	0.929	4.069	-0.418
MRGPRE	-0.037	0.975	3.484	-0.140
MRI1	0.012	1.008	4.991	0.041
MRO	-0.346	0.787	6.608	-1.239
MRPL1	0.225	1.169	3.866	0.925

MRPL15	0.395	1.315	6.263	1.765
MRPL30//LOC101927110//RP11-111H13	0.268	1.204	6.064	1.416
MRPL30//LOC101927110//RP11-111H13	0.001	1.001	5.297	0.004
MRPL35	0.347	1.272	6.210	1.451
MRPL42	0.434	1.351	4.573	1.900
MRPL45	0.452	1.368	6.234	2.335
MRPL46//RP11-97O12.7	0.066	1.047	5.725	0.321
MRPL50	0.237	1.178	6.363	0.944
MRPS12	-0.071	0.952	3.663	-0.307
MRPS17//RP11-15K19.2	0.005	1.003	4.894	0.023
MRPS21	0.358	1.281	9.556	1.781
MRPS30	0.590	1.505	6.551	2.756
MRPS31P5	-0.533	0.691	4.104	-1.992
MRPS36	0.320	1.248	2.647	1.351
MRRF	-0.053	0.964	4.367	-0.173
MRS2	0.401	1.320	5.533	1.848
MRTO4	0.353	1.277	4.937	1.455
MRVI1	0.061	1.043	6.954	0.250
MS4A14	-0.328	0.796	3.430	-0.769
MS4A4A	0.275	1.210	3.089	0.705
MS4A6A	-0.030	0.979	3.790	-0.082
MS4A7	0.048	1.034	5.267	0.110
MSANTD4	0.155	1.113	5.601	0.824
MSH2	0.373	1.295	5.891	1.675
MSH5//MSH5-SAPCD1//SAPCD1	-0.298	0.814	3.274	-1.207
MSH5//MSH5-SAPCD1//SAPCD1	-0.247	0.843	4.086	-0.910
MSI2	-0.395	0.761	7.352	-1.596
MSLN	-0.080	0.946	2.695	-0.244
MSMO1	0.203	1.151	7.149	0.641
MSN	-0.378	0.769	9.050	-1.288
MSR1	0.122	1.088	2.492	0.454
MSRA	0.498	1.412	3.763	1.450
MST1L	0.436	1.353	2.540	1.765
MSTN	0.391	1.311	2.531	1.012
MSX1	-0.142	0.907	3.136	-0.537
MT1A	1.021	2.029	4.342	1.529
MT1CP	-0.064	0.956	6.753	-0.238
MT1E	-0.313	0.805	4.804	-0.504
MT1F	-0.333	0.794	6.892	-0.626
MT1G	-0.001	0.999	5.649	-0.002
MT1JP	0.037	1.026	2.633	0.081
MT1L//NUTF2//MT1M	0.691	1.614	3.777	1.804
MT1M	0.439	1.355	7.883	1.265
MT1X	0.094	1.067	9.610	0.307
MT2A	0.058	1.041	6.779	0.217
MTA3	0.520	1.434	3.841	2.193
MTCL1//SOGA2	0.209	1.156	4.421	0.999
MTERF1//MTERF	-0.024	0.983	3.979	-0.090
MTFMT	0.313	1.243	4.428	1.365
MTFP1//RP4-539M6.19	0.139	1.101	3.313	0.344

MTHFD1//CTD-2555O16.4	-0.224	0.856	3.320	-0.796
MTHFD2	-0.471	0.721	4.689	-1.437
MTHFSD	-0.241	0.846	3.984	-0.893
MTMR10	-0.727	0.604	8.709	-2.103
MTMR11	-0.251	0.840	4.592	-0.845
MTMR12	0.308	1.238	6.442	1.580
MTMR4	0.149	1.109	7.135	0.813
MTMR6	-0.064	0.957	6.483	-0.303
MTMR7	0.029	1.020	5.930	0.107
MTMR9	0.391	1.311	5.306	1.843
MTMR9LP	-0.621	0.650	3.252	-2.083
MTO1	-0.140	0.908	4.387	-0.661
MTPAP	0.438	1.355	6.112	2.275
MTPN	0.628	1.546	4.111	2.668
MTRNR2L2	-0.255	0.838	3.826	-0.739
MTRNR2L8//MIR4485	-0.160	0.895	6.693	-0.612
MTSS1	-0.555	0.681	6.509	-2.183
MTSS1L	-0.240	0.847	7.979	-1.124
MT-TA	0.003	1.002	10.177	0.010
MT-TD	-0.674	0.627	3.118	-1.075
MT-TG	0.602	1.518	6.800	1.127
MT-TH	-0.011	0.992	7.912	-0.033
MT-TI	0.024	1.017	5.115	0.037
MT-TL2	-0.063	0.957	6.515	-0.147
MT-TM	-0.176	0.885	9.248	-0.510
MT-TN	0.162	1.119	8.010	0.419
MT-TP	-0.014	0.990	3.842	-0.037
MT-TQ	0.153	1.112	8.604	0.385
MT-TR	0.162	1.119	6.044	0.310
MT-TS1//UIMC1	-0.040	0.972	7.823	-0.118
MT-TS2	0.095	1.068	9.637	0.316
MT-TT	0.641	1.559	3.760	1.533
MT-TW	-0.070	0.952	4.821	-0.210
MTUS1	-0.872	0.546	5.525	-2.968
MTUS2	0.879	1.839	3.929	1.966
MTX3	0.450	1.366	6.037	1.788
MUC1	0.245	1.185	3.607	1.020
MUC20	0.095	1.068	3.389	0.274
MUM1L1	1.050	2.071	3.662	1.882
MURC	-0.526	0.694	3.831	-1.919
MVB12B	-1.003	0.499	6.693	-3.027
MVD	-0.114	0.924	5.388	-0.406
MVK	-0.510	0.702	4.233	-1.604
MVP	-0.232	0.852	5.722	-0.586
MX2	-0.096	0.935	3.273	-0.304
MXD1	0.040	1.028	6.176	0.124
MXRA7	0.571	1.485	6.017	1.887
MXRA8	-0.132	0.913	4.810	-0.461
MYBPC1	0.445	1.362	2.657	1.756
MYBPH	0.362	1.285	2.348	1.199

MYC	0.221	1.165	3.297	0.728
MYCBP2	0.585	1.500	7.914	2.401
MYCN	-0.546	0.685	3.947	-1.948
MYD88	0.294	1.226	3.317	1.148
MYH10	0.731	1.659	6.663	2.093
MYH11	0.467	1.383	5.811	1.108
MYH14	-0.588	0.665	5.096	-2.383
MYL12A	0.022	1.015	3.852	0.084
MYL3	-0.169	0.890	3.265	-0.477
MYL5	0.032	1.022	3.103	0.115
MYL9	0.294	1.226	6.421	0.714
MYLIP	-0.165	0.892	5.375	-0.753
MYLK	-0.620	0.651	5.173	-1.977
MYLK3	0.181	1.134	3.176	0.535
MYLK4	-0.053	0.964	3.080	-0.149
MYO15B	-0.623	0.649	4.379	-2.146
MYO16	0.662	1.582	4.765	2.263
MYO1A	-0.419	0.748	4.032	-0.701
MYO1B	0.547	1.462	5.252	1.484
MYO1C	0.071	1.050	5.678	0.309
MYO1D	-1.091	0.469	6.730	-2.522
MYO1E	-0.864	0.549	6.643	-2.612
MYO1F	-0.591	0.664	5.236	-1.703
MYO1G	-0.083	0.944	3.129	-0.157
MYO5A	0.731	1.660	8.324	2.729
MYO5B	0.518	1.432	3.737	1.672
MYO5C	-0.360	0.779	4.747	-1.042
MYO9A	0.363	1.286	7.145	1.978
MYO9B	-0.893	0.539	7.282	-2.917
MYOF	-0.287	0.820	5.798	-0.810
MYOM1	-0.945	0.519	4.940	-3.091
MYOM2	0.792	1.731	3.626	2.269
MYOZ1	-0.470	0.722	2.734	-1.768
MYPOP	0.535	1.448	3.615	1.849
MYRF	-1.212	0.432	6.428	-3.271
MYRIP	0.839	1.789	5.215	2.926
MYSM1	0.288	1.221	6.714	1.470
MYT1L	1.526	2.879	6.029	2.617
MZT1	0.687	1.610	3.964	2.567
N4BP2L1	0.556	1.470	5.140	2.173
N4BP2L2-IT2	-0.474	0.720	6.105	-1.448
N4BP3//RP11-889L3.3	0.366	1.289	2.570	0.861
NAA30	0.363	1.286	6.080	1.706
NAALAD2	0.347	1.272	3.720	1.180
NABP1	-0.173	0.887	3.740	-0.672
NACA	-0.288	0.819	3.889	-1.188
NACAD	-0.701	0.615	5.983	-2.322
NACC1	-0.151	0.901	3.740	-0.445
NACC2	-0.335	0.793	7.157	-1.435
NAF1	0.429	1.346	5.036	2.083

NAG20	0.330	1.257	3.033	0.952
NAGA	-0.037	0.975	5.487	-0.146
NAGLU//RP11-400F19.8	-0.351	0.784	4.700	-1.436
NAGPA	0.867	1.823	4.966	3.098
NAIP//RP11-1319K7.1	-0.427	0.744	6.497	-1.159
NAMPT//NAMPTL	0.686	1.609	8.312	1.596
NAP1L2	1.059	2.084	7.003	2.568
NAP1L3	0.514	1.428	7.160	1.671
NAP1L5	0.795	1.735	7.618	2.142
NAPA-AS1	-0.319	0.801	3.916	-1.265
NAPB	1.137	2.199	7.547	2.127
NAPEPLD	0.255	1.193	6.461	1.199
NAPG	0.495	1.410	7.864	2.313
NAPSB	-0.354	0.782	4.046	-0.603
NASP	-0.530	0.692	5.823	-2.053
NAT14	0.462	1.378	4.124	1.742
NAT8L	0.411	1.330	7.254	1.459
NAV2	-0.531	0.692	6.815	-1.974
NAV2//RP1-34H18.1	0.357	1.280	4.350	1.156
NAV2-AS1	-0.100	0.933	2.984	-0.369
NAV2-AS2	-0.279	0.824	3.245	-1.095
NAV2-IT1	-0.723	0.606	2.470	-2.393
NBEA	0.820	1.765	7.125	2.290
NBPF1//NBPF11//NBPF15	-0.574	0.672	5.881	-1.981
NBPF11//NBPF12//NBPF10//NBPF9//NE	-0.401	0.758	7.889	-1.951
NBPF11//NBPF12//NBPF15//NBPF8//NE	-0.401	0.757	7.592	-1.992
NBPF12	-0.267	0.831	5.718	-1.000
NBPF14//NBPF10//NBPF26	-0.254	0.838	6.810	-1.177
NBPF19//AC023137.2	-1.032	0.489	2.622	-2.312
NBPF20	-0.455	0.729	7.869	-1.381
NBPF20	-0.454	0.730	6.368	-1.368
NBPF20//NBPF14//NBPF8//NBPF23//NE	-0.453	0.731	8.348	-2.060
NBPF25P//NOTCH2NL//LOC100996763	-0.386	0.765	5.426	-1.091
NBR2	-0.229	0.853	3.829	-0.790
NCALD	0.519	1.433	3.012	2.046
NCAM2	-0.078	0.947	8.416	-0.408
NCAN	0.537	1.451	6.677	1.504
NCAPD2	-0.369	0.774	5.329	-1.434
NCBP2-AS2	0.349	1.274	5.541	1.877
NCDN	0.554	1.468	6.455	1.819
NCEH1	1.163	2.240	5.827	2.519
NCF1C	-1.105	0.465	3.402	-2.885
NCF2	-0.147	0.903	4.979	-0.369
NCF4	-0.187	0.878	4.483	-0.549
NCK1-AS1//RP11-85F14.5	-0.295	0.815	3.510	-0.968
NCK2	0.411	1.329	6.697	1.821
NCKAP1	0.570	1.484	9.218	2.619
NCKAP1L	-0.463	0.725	5.501	-1.072
NCKAP5	-0.791	0.578	5.262	-2.219
NCKAP5-IT1	-0.756	0.592	2.983	-1.645

NCKIPSD	0.501	1.416	5.620	2.213
NCMAP	-0.381	0.768	3.419	-0.668
NCOA5	-0.604	0.658	6.060	-2.270
NCOA7	0.055	1.039	7.321	0.240
NCR3LG1	1.206	2.307	4.191	2.538
NCRNA00185//TTTY14	0.166	1.122	2.041	0.436
NCRNA00185//TTTY14//LOC102723889	-0.069	0.953	3.963	-0.091
NCS1	1.063	2.089	7.348	2.276
ND6	-0.140	0.907	8.506	-0.525
NDE1	-1.190	0.438	7.450	-3.103
NDEL1	0.491	1.406	7.734	2.225
NDFIP1	0.333	1.260	7.192	1.662
NDFIP2	0.741	1.671	6.328	2.709
NDN	-0.089	0.940	4.023	-0.397
NDNF	-0.864	0.549	3.216	-2.419
NDP	0.064	1.045	5.194	0.234
NDRG1	-0.790	0.578	5.802	-2.716
NDRG2	-0.581	0.669	6.408	-2.281
NDRG3	0.438	1.354	7.248	1.755
NDRG4	0.723	1.651	7.610	1.999
NDST1	-0.345	0.787	6.750	-1.688
NDST3	1.201	2.300	3.819	2.432
NDST4	-0.743	0.598	2.309	-2.172
NDUFA5	0.524	1.438	4.343	2.674
NDUFA6-AS1	0.614	1.531	2.954	1.435
NDUFA9//RP11-500M8.7	0.200	1.148	7.133	1.073
NDUFAF1	0.637	1.555	3.799	2.638
NDUFAF5	0.618	1.534	3.684	2.499
NDUFAF7	0.302	1.233	5.250	1.616
NDUFB5	0.565	1.480	4.771	2.245
NEAT1//MIR612	-0.593	0.663	9.809	-1.398
NEAT1//MIR612	-0.374	0.772	3.756	-0.964
NEBL-AS1	0.147	1.107	2.950	0.539
NECAB1	0.591	1.506	7.615	1.853
NECAB2	0.728	1.656	5.470	1.689
NECAB3	0.054	1.038	4.472	0.209
NECAP1	0.554	1.468	7.131	1.817
NECAP2	-0.618	0.652	5.476	-3.000
NEDD1	-0.055	0.963	5.501	-0.250
NEDD4L	0.854	1.808	6.402	2.145
NEFH	0.699	1.624	8.367	0.822
NEFL	1.068	2.097	10.027	1.256
NEFM	0.746	1.677	9.736	1.100
NEGR1	0.901	1.867	7.098	2.830
NEGR1-IT1	0.438	1.355	2.165	1.546
NEK10	0.749	1.680	3.163	2.033
NEK3	-0.711	0.611	4.519	-1.795
NEK5//LOC100506859	-0.465	0.725	2.967	-1.307
NEK7	-0.357	0.781	6.538	-1.523
NELL1	0.987	1.982	4.078	2.139

NELL2	1.374	2.592	5.369	2.699
NENF	-0.313	0.805	5.597	-1.417
NES	-0.526	0.695	5.297	-2.102
NET1	-0.199	0.871	6.177	-0.875
NETO1	1.631	3.097	5.139	2.858
NETO2	0.584	1.499	6.126	1.408
NEURL1//NEURL	0.980	1.973	4.576	2.641
NEURL1B	0.241	1.182	2.943	0.913
NEURL4	0.187	1.138	4.950	0.845
NEUROD1	0.586	1.501	1.823	1.999
NEUROD2	1.545	2.917	3.574	2.692
NEUROD6	2.540	5.814	3.535	3.457
NF2	0.268	1.204	6.236	1.254
NFAM1	-0.244	0.844	3.524	-1.053
NFATC1	-0.440	0.737	4.131	-1.833
NFATC2	-0.276	0.826	4.452	-1.047
NFATC4	-0.198	0.872	3.322	-0.880
NFE2L2	-0.389	0.764	6.800	-1.726
NFE2L3	-0.611	0.655	2.136	-1.988
NFIA-AS2//RP4-668G5.1	-0.404	0.756	2.896	-1.157
NFIL3	0.445	1.362	5.813	0.946
NFKB2	-0.446	0.734	3.336	-1.872
NFKBIA	-0.115	0.923	7.231	-0.426
NFKBIE	-0.080	0.946	3.257	-0.338
NFKBIZ	-0.032	0.978	4.709	-0.135
NGB	-0.117	0.922	2.893	-0.411
NGEF	0.880	1.841	4.580	2.125
NGFR	-0.810	0.570	3.681	-2.386
NGRN	0.425	1.343	8.900	1.876
NGRN	0.292	1.224	4.256	1.121
NHLRC1	0.084	1.060	2.630	0.383
NHLRC3	-0.044	0.970	5.475	-0.158
NHS	0.595	1.510	4.132	2.088
NHSL1	-0.572	0.673	4.236	-2.434
NHSL2	0.333	1.260	3.444	1.112
NICN1	0.312	1.241	6.297	1.335
NID1	0.423	1.341	4.891	1.509
NID2	-0.213	0.863	3.942	-0.897
NIM1K//NIM1	0.490	1.405	4.672	1.721
NINJ1	-0.230	0.852	5.347	-0.901
NINJ2	-0.895	0.538	4.055	-2.926
NINL	-0.291	0.817	4.295	-1.121
NIPA2	0.241	1.182	5.156	1.069
NIPAL2	1.165	2.242	4.769	2.680
NIPAL3	-0.612	0.654	6.974	-2.371
NIPAL4	-1.437	0.369	3.833	-2.677
NIPSNAP1	0.326	1.254	5.548	1.384
NIPSNAP3B	1.145	2.211	4.007	2.624
NKAIN1	-0.769	0.587	3.697	-3.378
NKAIN2	-0.570	0.674	8.380	-2.040

NKAIN3	0.166	1.122	5.906	0.646
NKAIN4	-0.068	0.954	2.953	-0.271
NKD1	-0.799	0.575	5.448	-2.683
NKG7	-0.237	0.849	3.108	-0.894
NKIRAS1	0.241	1.182	4.446	1.287
NKRF	0.393	1.313	3.963	1.625
NKX2-2	-0.986	0.505	5.745	-2.602
NKX3-1	0.135	1.098	3.111	0.483
NKX6-1	0.004	1.003	2.693	0.021
NKX6-2	-1.177	0.442	6.861	-2.686
NLE1	0.230	1.173	4.463	1.064
NLGN1	0.413	1.332	5.443	1.563
NLGN4Y-AS1	-0.011	0.992	2.537	-0.041
NLK	0.602	1.518	8.637	2.097
NLRC5	-0.288	0.819	3.097	-1.303
NLRP1	-0.917	0.530	3.283	-3.213
NLRP2	0.418	1.336	2.531	1.626
NLRP3	0.047	1.033	4.240	0.149
NMB	-0.993	0.502	4.593	-3.145
NME5	0.649	1.568	3.559	1.821
NME9	0.212	1.158	3.438	0.652
NMI	0.248	1.188	3.217	0.936
NMNAT2	1.299	2.461	6.681	2.247
NMRK1	0.458	1.374	3.850	1.921
NMT2	0.188	1.140	4.497	0.887
NMUR2	-0.282	0.823	2.513	-0.939
NNAT	1.210	2.314	5.312	2.371
NNMT//LOC101928916	0.287	1.220	3.063	0.761
NNT	0.510	1.424	7.511	2.278
NOA1	0.029	1.020	5.758	0.127
NOD1	-0.152	0.900	4.401	-0.569
NOD2	-0.100	0.933	2.104	-0.387
NOL3	0.019	1.013	4.459	0.075
NOL4	0.942	1.921	5.731	2.441
NOL6	0.392	1.312	4.426	1.577
NOMO1	0.282	1.216	7.257	1.361
NOMO2//NOMO3//NOMO1	0.330	1.257	9.309	1.642
NOMO2//NOMO3//NOMO1	0.325	1.253	9.272	1.583
NOP14-AS1	-0.541	0.687	3.890	-1.943
NOS1AP	0.488	1.403	4.237	1.938
NOS2	1.080	2.114	2.429	3.300
NOS3	0.429	1.346	3.793	1.475
NOSTRIN	-0.031	0.978	3.066	-0.115
NOTCH1	-0.740	0.599	6.517	-2.949
NOTCH2NL	-0.479	0.717	5.764	-1.494
NOTCH2NL	0.119	1.086	7.181	0.512
NOTCH3	0.228	1.171	4.127	0.789
NOV	1.308	2.475	4.722	1.755
NOVA2	0.385	1.306	4.894	1.623
NOXA1	0.501	1.415	3.700	1.632

NOXO1	0.192	1.143	2.652	0.728
NPAP1	-0.804	0.573	2.779	-2.571
NPAS1	0.118	1.085	2.313	0.470
NPAS3	-0.444	0.735	5.750	-1.738
NPB	-0.012	0.992	2.442	-0.041
NPC1	-0.772	0.586	7.543	-2.325
NPC2//MIR4709	-0.629	0.646	8.661	-1.936
NPFF//RP11-793H13.10	-0.802	0.573	3.725	-3.704
NPFFR1	0.074	1.053	3.005	0.247
NPIPB11//NPIPB5//NPIPB3//NPIPB4//NI	-0.421	0.747	9.019	-1.758
NPIPB3	-0.391	0.763	8.293	-1.251
NPIPB3//NPIPB5//NPIPB4	-0.219	0.859	7.464	-0.863
NPIPB4//LOC613037//NPIPB5//NPIPA5/	-0.436	0.739	8.616	-1.741
NPIPB5//NPIPB3//NPIPB4//LOC613037/	-0.442	0.736	9.092	-1.857
NPIPB5//NPIPB4//LOC613037//NPIPA5/	-0.365	0.777	8.918	-1.542
NPIPB6	-0.580	0.669	5.592	-1.682
NPIPB8//NPIPB9//NPIPB6//NPIPB15//NI	-0.479	0.718	7.508	-2.090
NPL	-0.778	0.583	5.531	-2.586
NPM2	0.929	1.904	4.550	2.560
NPM3	0.311	1.240	3.310	1.274
NPNT	-0.068	0.954	3.266	-0.223
NPPA	-0.328	0.796	2.661	-0.915
NPPC	-0.005	0.996	5.628	-0.011
NPR3	-0.217	0.860	2.661	-0.738
NPTN	0.770	1.706	8.338	2.363
NPTX1	1.081	2.115	5.611	1.921
NPTX2	-0.465	0.725	5.365	-0.736
NPTXR	0.852	1.805	5.147	2.065
NPW	0.028	1.019	2.890	0.143
NPY	0.649	1.568	3.934	1.720
NPY1R	1.214	2.320	4.752	2.379
NPY2R	0.342	1.267	3.220	1.023
NPY5R	0.741	1.671	2.830	2.547
NQO1	0.101	1.072	5.950	0.347
NQO2//RP1-90J20.11	0.477	1.392	4.668	1.266
NR1D1	0.580	1.495	6.338	1.996
NR1D2	0.303	1.234	6.067	1.426
NR2E1	1.543	2.914	3.091	2.510
NR2F1-AS1	0.610	1.526	4.516	2.753
NR2F2	-0.156	0.898	2.612	-0.575
NR2F6	0.114	1.083	5.236	0.470
NR3C2	0.394	1.314	5.703	1.716
NR4A1	0.020	1.014	4.106	0.067
NR4A2	0.496	1.411	4.763	1.260
NR4A3	-0.070	0.952	3.400	-0.160
NRBF2	0.132	1.096	5.779	0.490
NRBP2//MIR6845	-0.781	0.582	6.422	-2.968
NRCAM	0.575	1.490	8.430	2.643
NREP	0.485	1.399	2.733	1.898
NRG1	0.269	1.205	4.108	0.590

NRG1-IT1	0.191	1.142	2.926	0.610
NRG1-IT2	0.329	1.256	2.225	0.981
NRG1-IT3	0.093	1.066	2.250	0.331
NRG3	0.262	1.199	5.228	1.183
NRG4	0.011	1.008	3.302	0.036
NRGN	2.340	5.063	6.031	3.065
NRIP2	-0.043	0.971	4.749	-0.159
NRIP3	0.865	1.822	5.399	1.600
NRN1	1.266	2.405	7.810	2.410
NRP1	0.781	1.719	5.816	2.374
NRP2	-0.232	0.852	4.552	-0.995
NRSN1	0.918	1.889	4.821	2.090
NRSN2	0.835	1.784	5.332	2.388
NRXN1	0.921	1.894	6.909	2.440
NRXN3	0.681	1.603	6.897	2.052
NS3BP	-0.461	0.726	3.831	-1.172
NSF//LOC101930324	0.749	1.681	8.140	1.665
NSF//NSFP1//LOC101930324	0.785	1.723	8.276	1.806
NSG1	0.384	1.305	3.073	1.178
NSMF//MIR7114	0.722	1.650	7.221	2.330
NSUN5P1	-0.499	0.708	4.826	-1.527
NT5C	-0.538	0.689	4.490	-2.346
NT5C1A	0.395	1.315	3.677	1.043
NT5C3B	0.470	1.385	6.518	1.831
NT5DC1	-0.322	0.800	6.590	-1.162
NT5DC3	0.816	1.760	5.706	2.424
NT5E	-0.127	0.916	4.481	-0.441
NTM-IT	-0.515	0.700	3.020	-1.775
NTN1	-0.494	0.710	4.458	-1.702
NTN3	0.425	1.343	2.350	1.497
NTN4	0.805	1.748	5.350	2.672
NTNG1	0.744	1.675	4.977	2.391
NTNG2	0.220	1.164	4.879	0.817
NTSR2	-0.249	0.841	6.119	-0.825
NUAK1	0.659	1.579	6.655	2.239
NUAK2	0.262	1.199	3.316	0.770
NUDT1	-0.435	0.739	3.448	-1.849
NUDT10	0.525	1.439	4.018	1.802
NUDT11	1.276	2.421	4.594	2.322
NUDT13	-0.413	0.751	2.753	-1.575
NUDT16P1//NUDT16P	-0.222	0.857	3.720	-0.994
NUDT19	-0.160	0.895	4.295	-0.628
NUDT21	0.283	1.216	8.210	1.543
NUDT3	0.348	1.273	9.258	1.528
NUDT4	0.618	1.534	2.790	1.957
NUDT4P1//NUDT4P2	0.931	1.907	2.737	3.554
NUDT9P1	0.743	1.674	2.742	2.516
NUFIP1	0.344	1.269	4.924	1.469
NUMBL	0.636	1.554	5.093	2.338
NUP155	0.170	1.125	6.014	0.700

NUPR1	-0.573	0.672	7.437	-1.587
NUPR1L	0.359	1.282	2.565	0.975
NUSAP1	-0.215	0.861	2.318	-0.763
NUTM2D	-0.024	0.983	3.937	-0.087
NWD1	-0.436	0.739	4.263	-1.348
NWD2//KIAA1239	1.333	2.520	3.044	2.653
NXNL2	-0.006	0.996	2.419	-0.021
NXPE3	-0.420	0.748	6.832	-1.628
NXPH1	0.575	1.489	4.981	1.955
NXPH2	0.482	1.397	3.064	1.366
NXPH3	0.373	1.295	4.241	1.135
NXT1	0.134	1.098	3.551	0.585
NYAP1	0.540	1.454	4.129	1.642
NYAP2	1.226	2.339	4.100	2.450
NYNRIN	-0.140	0.908	3.664	-0.502
OAF	0.583	1.498	2.040	2.269
OAF	0.581	1.496	3.843	2.184
OARD1	-0.710	0.611	4.837	-2.618
OAS1	-0.366	0.776	3.342	-1.328
OAS2	-0.110	0.926	3.940	-0.389
OAS3	0.159	1.116	3.023	0.595
OASL	0.297	1.229	2.237	1.167
OBFC1	-0.088	0.941	5.324	-0.311
OBP2A	-0.230	0.853	2.741	-0.794
OCA2	0.680	1.602	2.707	1.788
OCEL1	0.538	1.452	3.940	1.707
OCIAD2	0.023	1.016	3.007	0.078
OCLM	-0.343	0.788	2.307	-1.324
OCLN	-0.359	0.780	2.900	-1.381
OCRL	0.553	1.468	6.606	2.387
ODF2L	0.375	1.296	5.392	1.507
ODF3B	-0.452	0.731	3.083	-1.432
OGDHL	0.527	1.441	6.297	1.032
OGFRL1	-0.179	0.883	9.038	-0.795
OGN	1.566	2.962	5.037	2.816
OIP5-AS1	0.566	1.481	4.982	2.771
OLA1	0.308	1.238	5.818	1.587
OLFM1	0.863	1.819	4.594	2.505
OLFM2	0.555	1.470	6.587	1.618
OLFM3	1.299	2.461	3.841	2.376
OLFM4	0.010	1.007	2.355	0.031
OLFML1	0.148	1.108	2.659	0.473
OLFML2A	0.066	1.047	2.815	0.183
OLFML2B	0.410	1.328	4.028	1.489
OLFML3	-0.230	0.853	3.470	-0.741
OLIG1	-0.980	0.507	6.322	-2.790
OLIG2	-1.223	0.428	4.611	-3.823
OLR1	-1.064	0.478	5.591	-2.351
OMD	0.827	1.775	3.845	1.626
OMG//LOC101927057	0.126	1.092	6.592	0.428

OPA1	0.296	1.228	6.877	1.612
OPALIN	-0.329	0.796	6.749	-0.741
OPCML	1.346	2.543	5.959	2.814
OPCML	0.462	1.377	4.236	1.306
OPCML-IT2	0.849	1.802	4.059	1.947
OPLAH	-0.426	0.745	3.667	-1.477
OPN3	1.181	2.267	4.227	3.011
OPN4	0.285	1.219	2.689	1.074
OPRD1	0.723	1.651	3.515	1.958
OPRK1	0.867	1.824	3.190	2.450
OPRL1	0.175	1.129	4.043	0.617
OPRM1	0.997	1.996	3.409	3.242
OR14I1	1.792	3.463	2.508	2.551
OR2A1-AS1//LOC101928605//RP4-545C	-0.154	0.899	1.958	-0.552
OR2A9P//OR2A20P//OR2A1-AS1//LOC1(	-0.157	0.897	3.704	-0.586
OR2AK2	-0.156	0.897	2.980	-0.526
OR2L13	-0.354	0.782	4.548	-1.138
OR2L1P	-0.266	0.831	5.958	-0.640
OR2L2	-0.418	0.749	2.915	-0.975
OR2L3	0.046	1.032	2.833	0.146
OR2L5	-0.322	0.800	3.220	-0.728
OR2L8	-0.473	0.720	4.498	-1.250
OR2M4	0.168	1.123	2.642	0.470
OR2T12	-0.233	0.851	3.376	-0.825
OR6W1P	-0.742	0.598	3.361	-2.026
OR7A5	-0.988	0.504	2.562	-1.649
OR7C1	-0.838	0.559	2.719	-1.890
OR7E47P	0.398	1.318	2.531	1.172
OR7E91P	0.341	1.267	3.536	1.289
OR9A2	-1.166	0.446	2.894	-2.115
ORAI1	-0.082	0.945	3.459	-0.291
ORAI3	-0.387	0.765	3.375	-1.577
ORC4	0.494	1.409	5.284	2.232
ORC5	0.324	1.252	5.812	1.821
ORC6	0.480	1.395	2.910	1.628
ORMDL2	-0.614	0.654	4.568	-2.527
OSBPL10	0.491	1.406	2.823	1.833
OSBPL11	-0.143	0.905	6.941	-0.495
OSBPL3	0.733	1.662	5.332	2.420
OSBPL6	0.604	1.520	5.667	2.217
OSCAR	-0.073	0.951	2.683	-0.236
OSCP1	0.582	1.497	4.235	2.448
OSER1-AS1	-0.439	0.738	3.270	-1.703
OSGIN1	-0.259	0.836	3.142	-1.025
OSGIN2	0.008	1.005	4.851	0.028
OSM	-0.017	0.988	2.029	-0.065
OSMR	0.690	1.614	5.789	1.574
OSTF1	0.234	1.176	7.661	0.968
OSTM1-AS1	0.391	1.311	2.463	1.295
OTOGL	1.254	2.385	2.580	3.498

OTOS	0.173	1.127	3.569	0.560
OTUB2	0.524	1.438	2.931	1.987
OTUD3	0.658	1.578	4.987	2.722
OTUD7A	-0.229	0.853	5.999	-1.145
OTUD7B	-0.789	0.579	7.194	-2.772
OVGP1	0.600	1.516	3.964	2.284
OVOL3	-0.447	0.734	2.499	-1.788
OXCT1	0.477	1.392	7.328	2.008
OXCT2	-0.391	0.762	2.546	-1.241
OXGR1	0.177	1.130	2.013	0.548
OXR1	1.133	2.193	6.363	3.345
OXR1//RP11-227D13.2	0.674	1.596	7.349	1.957
OXT	0.217	1.162	3.024	0.566
OXTR	0.104	1.075	3.898	0.301
P2RX1	-0.372	0.773	2.888	-1.453
P2RX3	-0.143	0.906	2.318	-0.618
P2RX4	-0.102	0.932	3.051	-0.392
P2RX6	0.374	1.296	3.845	1.376
P2RX6P	0.763	1.697	1.982	1.647
P2RX7	-0.856	0.552	6.426	-2.458
P2RY1	-0.523	0.696	5.034	-1.655
P2RY12	-0.495	0.709	5.572	-1.032
P2RY13	-0.578	0.670	4.963	-1.218
P2RY14	-0.119	0.921	3.947	-0.476
P4HA1	-0.915	0.530	6.985	-2.303
PABPC1L	-0.084	0.943	2.699	-0.286
PABPC1L2A//PABPC1L2B//RP11-493K23	1.064	2.091	1.598	3.172
PABPC1L2B//PABPC1L2A	1.049	2.070	2.766	2.161
PABPC5	-0.574	0.672	3.721	-1.988
PACRG	0.253	1.192	3.990	0.988
PACRG-AS1	-0.715	0.609	2.836	-2.290
PACS2	-0.702	0.615	6.154	-2.827
PACSIN1	1.262	2.399	5.998	2.084
PACSIN3	-0.400	0.758	5.458	-1.271
PADI2	-0.755	0.593	8.633	-1.810
PAFAH1B1	0.205	1.152	8.606	1.112
PAFAH1B3	0.291	1.223	4.523	1.072
PAGR1//CTD-2574D22.6	-0.182	0.881	5.093	-0.881
PAH//LOC101929036	0.435	1.352	2.123	1.767
PAIP2//CTB-43P18.1	0.069	1.049	5.622	0.257
PAIP2B//VPS36	-0.974	0.509	8.307	-2.608
PAK1	1.187	2.277	7.128	3.180
PAK2	-0.350	0.785	6.200	-1.404
PAK3	1.033	2.046	5.693	2.150
PAK7	1.521	2.870	4.566	2.634
PALD1	-0.551	0.683	4.276	-2.293
PALM2-AKAP2//AKAP2//PALM2	0.265	1.202	4.798	1.215
PALMD	-0.151	0.900	5.052	-0.578
PAM	0.424	1.342	6.914	1.609
PAMR1	-0.109	0.927	5.936	-0.375

PANK1	0.532	1.446	2.684	2.289
PANX1	0.600	1.516	4.948	1.968
PANX2	0.741	1.672	4.790	1.577
PAPD7	0.178	1.131	5.520	0.948
PAPLN	-0.031	0.979	3.495	-0.112
PAPPA	-0.427	0.744	2.880	-1.160
PAPSS2	0.237	1.179	4.708	0.855
PAQR4	-0.768	0.587	6.284	-2.346
PAQR5	0.154	1.113	4.660	0.504
PAQR6	-1.283	0.411	8.355	-3.423
PAQR8	-0.727	0.604	4.954	-2.338
PARD3	-0.493	0.710	5.664	-2.316
PARD3B	-0.668	0.630	5.319	-2.874
PARD6A	0.497	1.411	4.930	1.621
PARD6G	-0.532	0.692	2.146	-2.101
PARD6G-AS1//AC139100.2	-0.432	0.741	3.565	-1.404
PARG	0.226	1.170	5.166	0.995
PARK2	0.570	1.484	5.857	2.090
PARM1	1.306	2.473	7.229	2.102
PARP12	-0.246	0.843	4.807	-0.941
PARP14	0.228	1.172	5.989	0.799
PARP2	0.468	1.384	4.595	1.998
PARP4	-0.796	0.576	7.510	-2.465
PARP9	-0.145	0.904	4.865	-0.616
PARS2	0.127	1.092	3.105	0.465
PART1	1.736	3.330	2.763	3.423
PARVG	-0.401	0.757	3.471	-1.397
PAX2	-0.418	0.748	2.374	-1.387
PAX3	-0.372	0.773	3.839	-1.205
PAX8	-0.243	0.845	2.055	-0.700
PAX8-AS1//AC016683.6	-0.245	0.844	2.294	-0.772
PAXBP1-AS1	-0.351	0.784	3.204	-1.082
PBLD	0.082	1.059	5.069	0.331
PBX1	-0.479	0.718	4.231	-1.526
PBX3	-0.917	0.530	4.801	-2.601
PBXIP1	-0.190	0.877	5.439	-0.778
PCBD2	-0.518	0.698	3.551	-1.985
PCBP3	-0.266	0.831	3.438	-0.970
PCBP4	-0.644	0.640	6.528	-2.203
PCDH1	0.248	1.188	5.224	1.165
PCDH10	0.444	1.360	6.956	1.627
PCDH11Y//PCDH11X	0.477	1.392	3.810	0.787
PCDH15	-0.302	0.811	4.540	-0.996
PCDH18	0.051	1.036	5.650	0.214
PCDH19	0.772	1.708	4.385	1.762
PCDH20//LOC101926951	0.434	1.351	2.132	1.594
PCDH7	0.831	1.778	5.994	2.301
PCDH8	1.318	2.492	4.683	2.051
PCDH9	-0.223	0.857	8.556	-0.888
PCDHB10//PCDHB9	0.287	1.220	5.965	1.072

PCDHB12	0.567	1.481	5.763	1.868
PCDHB13	0.685	1.608	4.035	2.487
PCDHB14	0.318	1.247	5.244	1.139
PCDHB15	0.167	1.123	4.900	0.622
PCDHB16	-0.099	0.934	4.026	-0.314
PCDHB18	0.549	1.463	4.884	2.128
PCDHB19P//AC005752.8	0.569	1.484	4.046	2.121
PCDHB2	0.089	1.064	3.895	0.295
PCDHB4	0.190	1.141	5.757	0.478
PCDHB6	0.200	1.149	4.899	0.510
PCDHB7	0.054	1.038	4.494	0.167
PCDHB8	0.268	1.204	2.469	1.062
PCDHB9	0.372	1.294	3.954	1.335
PCDHGB8P	-0.430	0.742	4.445	-1.529
PCDP1	0.600	1.516	5.094	2.220
PCED1B-AS1	-0.313	0.805	3.061	-0.956
PCK2	-0.143	0.905	2.665	-0.553
PCLO	1.440	2.714	5.550	2.741
PCMT1	0.317	1.246	6.088	1.575
PCNXL2	0.548	1.463	6.861	2.368
PCOLCE	0.305	1.236	3.768	1.169
PCOLCE2	0.120	1.087	2.163	0.495
PCP2	-0.007	0.995	2.198	-0.025
PCP4	0.825	1.772	3.320	1.803
PCP4L1	0.580	1.494	5.586	0.998
PCSK1	1.560	2.949	4.771	2.622
PCSK1N	0.276	1.211	6.031	1.001
PCSK2	0.971	1.961	6.295	1.556
PCSK6//LOC100507472	-1.191	0.438	5.366	-2.732
PCTP	0.753	1.686	2.114	2.870
PCYOX1	0.222	1.166	6.597	1.112
PCYOX1L	0.406	1.325	4.805	1.749
PCYT1B	0.107	1.077	6.348	0.542
PDCD1LG2	-0.591	0.664	3.519	-1.570
PDCD6IPP2	0.529	1.443	3.951	1.972
PDCL3P4	-0.635	0.644	4.974	-1.534
PDDC1	-0.300	0.812	5.715	-1.406
PDE10A	0.511	1.426	5.195	1.521
PDE10A	0.319	1.247	2.309	1.238
PDE1A	0.928	1.903	6.150	2.002
PDE1B	1.045	2.064	5.742	2.510
PDE1C	-0.572	0.672	5.185	-1.777
PDE2A	1.150	2.220	4.025	2.483
PDE3A	-0.245	0.844	4.638	-1.118
PDE3B	0.313	1.242	4.734	1.139
PDE4A	0.602	1.518	4.857	2.196
PDE4B	-0.420	0.748	5.436	-1.859
PDE4D	0.470	1.385	3.808	1.941
PDE5A	0.425	1.343	3.565	1.265
PDE6B	-0.461	0.727	5.029	-1.267

PDE7A	-0.059	0.960	4.594	-0.252
PDE7B	0.009	1.006	6.531	0.034
PDE8A	-0.769	0.587	6.457	-2.359
PDE8B	0.354	1.278	6.706	1.496
PDE9A	-0.735	0.601	4.952	-3.118
PDGFB	-0.438	0.738	4.511	-1.677
PDGFD	0.060	1.043	3.514	0.116
PDGFRA//FIP1L1	-0.586	0.666	6.188	-1.964
PDGFRL	0.172	1.127	4.380	0.543
PDIA2	-0.822	0.566	4.901	-1.604
PDIA3P1	-0.385	0.766	5.860	-1.170
PDIA4	-0.750	0.595	5.913	-2.626
PDIK1L	0.360	1.283	3.896	1.598
PDK1	0.155	1.113	6.183	0.497
PDK3	0.683	1.606	5.677	2.057
PDK4	-0.938	0.522	7.349	-2.637
PDLIM1	0.196	1.146	5.234	0.508
PDLIM3	-0.593	0.663	4.492	-2.545
PDLIM4	-0.028	0.981	3.509	-0.090
PDLIM5	-0.244	0.844	4.853	-1.088
PDP1	1.136	2.198	3.902	3.060
PDP2	0.129	1.093	4.663	0.556
PDPK1//LOC101060747//PDPK2	0.274	1.209	7.401	1.322
PDPN	-0.066	0.955	4.430	-0.213
PDPR	-0.715	0.609	6.211	-2.825
PDS5B	0.657	1.577	7.555	2.598
PDSS2	-0.213	0.863	2.575	-0.773
PDXP//SH3BP1	0.637	1.555	6.356	2.006
PDYN	0.189	1.140	2.232	0.548
PDZD2	-0.045	0.969	5.387	-0.186
PDZD4	0.962	1.948	6.296	2.588
PDZD7	-0.116	0.923	3.307	-0.411
PDZRN3	0.809	1.753	4.594	2.413
PEBP4	-0.228	0.854	3.535	-0.853
PEG10	0.076	1.054	7.631	0.238
PEG3//ZIM2	0.331	1.258	6.394	1.028
PEG3-AS1	0.289	1.222	3.791	0.961
PELI1	-0.187	0.878	6.911	-0.809
PELI3	0.442	1.359	4.937	2.057
PENK	-0.735	0.601	2.500	-1.716
PER1//MIR6883	-0.111	0.926	6.198	-0.375
PERP	0.256	1.195	4.066	0.930
PEX1	-0.105	0.930	5.884	-0.391
PEX10	0.145	1.106	2.677	0.621
PEX11B	0.232	1.174	6.360	1.159
PEX11G	-0.450	0.732	3.809	-1.839
PEX12	-0.431	0.742	5.098	-1.873
PEX5L	-0.046	0.969	7.577	-0.213
PEX5L-AS1	-0.020	0.986	3.351	-0.072
PFDN4	0.300	1.231	4.745	1.341

PFKFB2	0.332	1.258	5.900	1.167
PFKFB3	0.007	1.005	7.743	0.027
PFKM	0.516	1.430	4.389	2.126
PFKP	0.290	1.223	7.553	1.077
PFN1	-0.333	0.794	8.636	-1.547
PFN2	0.477	1.392	7.579	2.359
PGAM2	-0.973	0.509	5.665	-2.399
PGAM4	-0.001	0.999	3.427	-0.003
PGAM5	0.231	1.174	4.563	0.964
PGAP1	-0.062	0.958	6.517	-0.265
PGAP3	-0.012	0.992	3.874	-0.043
PGBD2	-0.614	0.654	3.454	-2.622
PGBD4	0.125	1.091	5.039	0.696
PGBD5	0.606	1.522	5.903	1.871
PGLS	-0.388	0.764	4.735	-1.534
PGM2	-0.007	0.995	5.997	-0.024
PGM2L1	1.547	2.923	6.009	2.975
PGM5	-0.268	0.831	2.838	-1.015
PGP	0.483	1.398	5.622	1.929
PGPEP1//FKBP8	-0.285	0.821	6.079	-1.471
PHACTR1	1.318	2.492	3.781	2.743
PHACTR4	-0.587	0.666	6.690	-2.634
PHC1	-0.512	0.701	6.698	-2.397
PHC2	-0.267	0.831	5.607	-1.152
PHEX	-0.018	0.987	2.192	-0.084
PHF14	0.195	1.144	5.993	0.979
PHGDH	-0.568	0.674	5.797	-2.304
PHKA1	-0.200	0.871	5.378	-0.826
PHKA2	-0.537	0.689	5.451	-2.187
PHKG1	-0.184	0.880	3.822	-0.610
PHLDA3	-0.414	0.751	4.310	-1.510
PHLDB1//MIR6716	-0.810	0.570	4.335	-2.494
PHLDB2	0.670	1.591	2.736	1.990
PHLPP1	-0.736	0.600	8.135	-2.854
PHLPP2	0.395	1.315	6.015	1.586
PHOSPHO2-KLHL23//PHOSPHO2//KLHL2	0.015	1.010	3.960	0.061
PHPT1	-0.376	0.770	4.991	-1.814
PHTF1	0.446	1.363	4.752	1.936
PHTF2	0.128	1.093	5.028	0.572
PHYHD1	-0.639	0.642	4.740	-1.977
PHYHIP	1.482	2.793	6.339	2.637
PHYHIPL	-0.404	0.756	7.626	-1.454
PHYKPL	-0.560	0.679	4.782	-2.148
PI15	1.240	2.361	3.496	1.748
PI16	-1.211	0.432	4.042	-2.892
PI4K2A//RP11-548K23.11	0.275	1.210	7.120	1.290
PI4K2B//SEPSECS-AS1	-0.984	0.506	4.245	-3.193
PI4K2B//SEPSECS-AS1	-0.039	0.973	3.868	-0.152
PI4KA	0.730	1.659	7.887	2.123
PI4KA//PI4KAP2//PI4KAP1	0.614	1.530	6.586	2.011

PI4KA//PI4KAP2//PI4KAP1	0.552	1.466	7.818	1.598
PIANP	0.899	1.865	5.131	2.371
PID1	0.309	1.239	3.347	1.221
PIEZO1	-0.568	0.674	5.055	-2.026
PIEZO2	-1.172	0.444	5.575	-2.164
PIFO	-0.124	0.917	3.851	-0.405
PIGBOS1//RP11-139H15.1	0.013	1.009	4.015	0.042
PIGF	-0.478	0.718	4.116	-1.809
PIGM	-0.715	0.609	6.235	-2.477
PIGN	-0.258	0.836	5.822	-0.937
PIGT	-0.633	0.645	7.360	-2.747
PIGZ	0.176	1.130	4.724	0.558
PIH1D2	0.253	1.191	2.286	1.007
PIK3AP1	-0.131	0.913	5.456	-0.374
PIK3CA	0.452	1.368	6.923	2.251
PIK3CB	0.261	1.198	6.144	1.359
PIK3CG	0.116	1.084	5.266	0.294
PIK3R1	0.334	1.260	8.515	1.616
PIK3R2//AC007192.4	0.427	1.344	5.379	1.947
PIK3R3//LOC102724021//RP11-322N21.	0.629	1.547	5.198	2.923
PIK3R5	-0.808	0.571	3.804	-3.182
PIK3R6	-0.530	0.692	2.297	-2.100
PILRA	-0.412	0.752	2.913	-1.544
PIM1	0.128	1.093	5.723	0.369
PIM2	0.352	1.277	4.277	1.325
PIN4P1	-0.308	0.808	2.320	-1.099
PIP4K2A	-0.727	0.604	8.654	-2.176
PIP4K2B	0.410	1.328	8.427	1.988
PIP4K2C	0.564	1.478	6.180	2.210
PIP5K1B	0.547	1.461	4.386	1.552
PIP5K1C	0.619	1.536	6.849	1.923
PIPOX	0.028	1.020	4.398	0.096
PIR-FIGF//PIR//FIGF	-0.286	0.820	3.658	-0.963
PIRT	-0.569	0.674	5.963	-1.103
PITPNB	0.368	1.290	6.640	1.785
PITPNM1	0.748	1.679	5.408	2.411
PITPNM2	0.685	1.608	4.503	2.571
PITPNM3	1.695	3.238	4.607	2.894
PITRM1-AS1	-0.066	0.955	2.376	-0.264
PIWIL4	-0.187	0.878	4.005	-0.694
PJA1	0.183	1.135	5.579	0.801
PJA2	0.427	1.345	8.300	2.005
PKDCC	0.241	1.182	3.434	0.945
PKI55//RP11-566E18.3	0.767	1.702	6.403	2.164
PKIA	0.458	1.374	5.417	1.850
PKNOX2	1.073	2.103	4.610	3.016
PKP2	0.674	1.596	4.346	1.136
PKP4	-0.693	0.619	8.625	-2.616
PLA1A	0.206	1.153	3.836	0.798
PLA2G15	0.199	1.148	5.215	0.686

PLA2G16	-0.899	0.536	8.115	-3.068
PLA2G4A	0.240	1.181	4.853	0.814
PLA2G4C	-0.584	0.667	5.388	-1.945
PLA2G4C	-0.307	0.808	3.619	-1.014
PLA2G7	0.280	1.214	5.337	0.669
PLA2R1	0.304	1.235	3.553	0.793
PLAC8	-0.333	0.794	4.058	-0.750
PLAGL1	0.541	1.455	4.833	2.192
PLAT	0.374	1.296	5.926	1.337
PLAU	-0.096	0.936	2.478	-0.488
PLAUR	0.332	1.259	4.887	1.085
PLBD1	0.042	1.029	4.158	0.132
PLCB1	1.275	2.421	7.656	2.921
PLCB1-IT1//LOC100128668	0.516	1.430	2.279	1.620
PLCB2	-0.606	0.657	4.836	-1.910
PLCB3	-0.286	0.820	5.197	-1.475
PLCB4	-0.055	0.963	5.978	-0.169
PLCD1	-0.445	0.735	4.552	-1.661
PLCD3	-0.507	0.704	6.819	-1.742
PLCE1	0.097	1.069	6.108	0.491
PLCG2	-0.292	0.817	4.703	-0.815
PLCH1	1.663	3.166	4.495	3.124
PLCL1	-0.676	0.626	6.892	-2.353
PLCXD1	-0.073	0.951	4.545	-0.218
PLCXD3	0.225	1.168	5.875	0.686
PLD1	-1.081	0.473	6.126	-3.047
PLD2	-0.138	0.909	5.748	-0.471
PLD3	0.279	1.213	6.278	1.288
PLD4	-0.624	0.649	3.868	-1.570
PLD5	-0.260	0.835	5.293	-0.991
PLEC	-0.165	0.892	6.186	-0.894
PLEK	-0.398	0.759	4.310	-0.972
PLEKHA1	0.471	1.386	6.403	2.011
PLEKHA4	0.125	1.090	3.902	0.384
PLEKHA6	0.306	1.236	6.913	1.143
PLEKHA6	-0.062	0.958	3.356	-0.232
PLEKHA8P1//RP11-139E19.1	0.199	1.148	3.764	0.681
PLEKHB1	-0.615	0.653	5.773	-2.076
PLEKHF1	-0.489	0.713	4.251	-2.070
PLEKHG1	-0.601	0.659	6.546	-1.799
PLEKHG2	0.123	1.089	2.837	0.488
PLEKHG3	-0.713	0.610	4.644	-2.386
PLEKHG5	0.851	1.803	3.560	3.185
PLEKHH1	-1.178	0.442	7.103	-2.256
PLEKHH2	-0.110	0.926	4.788	-0.392
PLEKHM2	0.488	1.403	7.917	2.099
PLEKHM3	0.267	1.204	5.617	1.311
PLEKHO2//AC069368.3	-0.017	0.988	5.830	-0.078
PLIN1	-0.307	0.808	4.277	-0.781
PLIN2	-0.124	0.917	3.507	-0.431

PLIN3	-0.809	0.571	6.112	-2.206
PLIN4	-0.738	0.600	3.413	-2.925
PLK2	1.574	2.977	4.106	3.033
PLK3	-0.711	0.611	4.580	-1.875
PLK5	0.229	1.172	2.928	0.801
PLLP	-0.767	0.588	7.947	-1.851
PLN	0.690	1.614	2.597	1.668
PLOD1	-0.615	0.653	6.696	-2.186
PLOD2	0.001	1.001	6.951	0.003
PLOD3	-0.960	0.514	5.372	-3.225
PLP2	-0.293	0.816	5.579	-1.002
PLS1	0.471	1.386	3.250	1.650
PLSCR1	0.385	1.306	5.563	1.006
PLSCR4	0.276	1.210	6.783	1.134
PLTP	-0.177	0.884	8.217	-0.633
PLVAP	-0.010	0.993	3.041	-0.026
PLXDC1	0.321	1.249	5.386	1.354
PLXDC2	-0.585	0.667	9.067	-2.367
PLXDC2	-0.302	0.811	5.581	-1.139
PLXNA1	0.339	1.265	5.629	1.282
PLXNA2	0.770	1.705	5.904	2.226
PLXNA3	-0.214	0.862	4.653	-0.938
PLXNA4	0.614	1.530	4.998	1.645
PLXNB1	-0.632	0.645	6.289	-2.347
PLXNB2	-0.288	0.819	6.457	-1.513
PLXNB3	-1.082	0.472	5.231	-3.124
PLXND1	-0.387	0.765	5.652	-1.651
PMP2	-0.047	0.968	10.309	-0.169
PMP22	-0.714	0.610	6.682	-2.364
PMS2	0.235	1.177	5.473	0.844
PMS2CL	0.067	1.047	5.408	0.256
PMS2P1	-0.128	0.915	4.464	-0.522
PMS2P9	-0.392	0.762	3.262	-1.188
PNCK	0.658	1.577	3.654	2.093
PNKD	0.149	1.109	4.545	0.605
PNLDC1	0.040	1.028	2.323	0.137
PNMA1	0.383	1.304	7.936	1.900
PNMA2	0.677	1.599	6.853	1.915
PNMA3	0.638	1.556	4.524	1.521
PNMA5	0.207	1.154	3.501	0.767
PNMA6A//PNMA6C//PNMA6D	0.761	1.695	3.878	2.816
PNMA6A//PNMA6C//PNMA6D	0.713	1.639	3.887	2.660
PNMA6A//PNMA6C//PNMA6D	0.622	1.539	3.711	2.363
PNMA6A//PNMA6C//PNMA6D	0.674	1.596	3.703	2.124
PNMAL1	0.669	1.589	6.083	2.018
PNMAL2	0.512	1.426	4.571	1.356
PNOC	0.145	1.106	3.003	0.418
PNP	-0.005	0.997	4.669	-0.017
POC1B//POC1B-GALNT4//GALNT4	-0.237	0.848	4.805	-0.917
PODN	0.186	1.137	4.026	0.574

PODNL1	-0.328	0.796	3.080	-1.294
PODXL2	0.318	1.246	6.705	1.297
POLD1	-0.087	0.942	3.264	-0.364
POLM	-0.093	0.938	3.713	-0.438
POLR2B	0.158	1.116	6.651	0.763
POLR2G	-0.376	0.770	5.567	-1.475
POLR2L	-0.433	0.741	5.061	-2.104
POLR3A	0.567	1.481	6.517	2.341
POLR3B	0.028	1.019	5.491	0.127
POLR3F	0.140	1.102	5.027	0.642
POM121L10P	0.282	1.216	2.287	0.979
POM121L1P	0.295	1.227	2.797	1.021
POM121L8P	0.503	1.417	2.745	1.988
POM121L9P	-0.142	0.906	2.664	-0.387
POMGNT1	-0.324	0.799	6.458	-1.476
POMK//SGK196	0.370	1.293	6.589	1.559
POMZP3	-0.027	0.982	3.550	-0.096
PON3	-0.228	0.854	2.880	-0.771
POPDC3	0.229	1.172	4.515	0.955
PORCN	0.333	1.259	5.479	1.459
POSTN	0.475	1.390	2.993	1.088
POTEF	0.313	1.242	3.033	1.019
POU3F1	0.276	1.211	3.115	1.000
POU3F2	-0.489	0.713	6.224	-2.180
POU4F1	-0.618	0.652	2.841	-1.325
POU5F1P4	0.251	1.190	3.612	0.892
POU5F2	-0.146	0.904	2.546	-0.513
POU6F1	0.304	1.235	5.864	1.298
POU6F2	0.624	1.542	3.291	2.064
PP12613	0.794	1.734	1.668	2.276
PP13	-0.116	0.923	3.031	-0.253
PP13	0.031	1.021	3.036	0.112
PP13439	-1.150	0.451	2.973	-3.154
PPAP2C	-1.341	0.395	6.575	-3.586
PPAPDC1A	-1.154	0.449	6.322	-3.754
PPAPDC2	0.309	1.239	3.394	1.608
PPARGC1A	0.517	1.431	3.367	2.149
PPARGC1B	0.303	1.234	2.893	1.293
PPAT	0.413	1.332	4.436	1.790
PPBP	-0.011	0.993	2.319	-0.016
PPDPF	-0.848	0.556	7.126	-2.751
PPEF1	0.049	1.034	4.565	0.091
PPFIA2	0.656	1.575	7.134	2.293
PPFIA3	0.818	1.763	3.806	2.504
PPFIA4	0.904	1.871	4.317	2.140
PPFIBP1	0.569	1.483	6.751	2.494
PPFIBP2	-0.741	0.598	4.726	-2.823
PPIAL4B	0.460	1.376	2.352	1.396
PPIAL4B//PPIAL4C//PPIAL4D//PPIAL4F//	0.158	1.116	3.043	0.640
PPIAL4B//PPIAL4C//PPIAL4E	-0.034	0.977	2.333	-0.090

PPIAP30	0.234	1.176	3.162	0.799
PPID	0.287	1.220	6.272	1.034
PPIL6	0.580	1.494	3.928	2.611
PPIP5K1	-0.157	0.897	3.775	-0.585
PPIP5K1//AC011330.5	0.404	1.323	6.998	1.688
PPIP5K1//LOC100508782//AC011330.5	0.459	1.374	5.215	2.089
PPIP5K1//LOC100508782//AC011330.5	0.422	1.340	6.794	1.846
PPIP5K1//LOC100508782//AC011330.5	0.399	1.319	6.517	1.755
PPIP5K1//LOC100508782//AC011330.5	0.335	1.262	5.831	1.751
PPIP5K1//LOC100508782//AC011330.5	0.358	1.282	7.149	1.512
PPIP5K1//LOC100508782//AC011330.5	0.300	1.231	5.874	1.501
PPIP5K1//LOC100508782//AC011330.5	0.621	1.538	4.568	1.452
PPIP5K1//LOC100508782//AC011330.5	0.377	1.298	3.485	1.443
PPIP5K1//LOC100508782//AC011330.5	0.293	1.225	6.429	1.348
PPIP5K1//LOC100508782//AC011330.5	0.278	1.213	6.490	1.338
PPIP5K1//LOC100508782//AC011330.5	0.292	1.225	8.357	1.303
PPIP5K1//LOC100508782//AC011330.5	0.296	1.228	7.854	1.141
PPIP5K1//LOC100508782//AC011330.5	0.247	1.187	7.629	1.034
PPIP5K1//LOC100508782//AC011330.5	0.225	1.169	7.578	0.955
PPIP5K2	0.149	1.109	5.986	0.630
PPL	1.415	2.667	3.431	3.685
PPM1E	0.705	1.630	4.195	1.906
PPM1H	0.014	1.009	6.067	0.066
PPM1J	0.994	1.991	3.071	3.555
PPM1K	0.228	1.171	7.058	0.929
PPM1L	0.489	1.404	5.025	2.300
PPME1	0.451	1.367	6.340	2.014
PPP1R12A	0.557	1.471	6.518	2.745
PPP1R13B	0.351	1.276	5.191	1.427
PPP1R13L	-0.357	0.781	3.794	-1.307
PPP1R14A	-0.894	0.538	5.766	-1.879
PPP1R14C	0.691	1.615	3.662	1.600
PPP1R15A	0.074	1.052	4.514	0.237
PPP1R16B	0.221	1.166	6.901	0.793
PPP1R18	-0.298	0.814	4.659	-1.110
PPP1R18	-0.261	0.834	4.731	-1.067
PPP1R18	-0.250	0.841	4.674	-0.962
PPP1R18	-0.186	0.879	5.700	-0.796
PPP1R18	-0.111	0.926	4.626	-0.412
PPP1R1A	0.432	1.349	5.837	1.438
PPP1R1B	0.137	1.100	5.467	0.462
PPP1R1C	-0.220	0.858	3.303	-0.821
PPP1R32	-0.217	0.860	3.755	-0.771
PPP1R3B	-0.123	0.919	2.939	-0.425
PPP1R3C	-0.248	0.842	7.013	-0.939
PPP1R9A	0.352	1.276	6.638	1.870
PPP1R9B	0.693	1.617	7.080	2.689
PPP2R2B//RNA5SP196	0.510	1.424	6.441	1.294
PPP2R2B-IT1	-1.120	0.460	4.537	-2.158
PPP2R3A	-0.296	0.814	5.188	-1.082

PPP2R3B//LOC102725016	-0.459	0.728	3.544	-1.995
PPP2R5D	0.479	1.393	6.099	2.250
PPP2R5E	0.378	1.299	6.618	1.915
PPP3CA	1.095	2.135	8.269	2.842
PPP3CB	0.665	1.586	6.532	2.538
PPP3CB-AS1//RP11-137L10.6	-0.512	0.701	3.522	-2.538
PPP3R1//RP11-474G23.1	0.836	1.786	7.987	2.650
PPP4R1L	-0.413	0.751	2.651	-1.360
PPP4R4	0.822	1.767	5.132	2.302
PPP5D1	-0.090	0.940	2.663	-0.305
PPTC7	0.375	1.297	5.526	1.445
PPWD1	-0.196	0.873	5.865	-0.698
PQLC3	-0.396	0.760	4.725	-1.472
PRAM1	-1.014	0.495	3.549	-2.865
PRB1	0.902	1.869	3.993	3.306
PRB2	0.306	1.236	2.939	0.792
PRC1	0.321	1.249	4.384	1.188
PRDM1	-0.005	0.997	3.686	-0.015
PRDM10	0.187	1.138	5.314	0.924
PRDM11//LOC101060179	0.082	1.059	3.833	0.298
PRDM2	0.212	1.159	5.909	1.088
PRDM4	0.169	1.124	5.305	0.773
PRDM5	-0.337	0.792	4.674	-1.252
PRDM6	0.342	1.268	2.802	1.403
PRDM8	0.804	1.746	4.195	2.865
PRELP	0.252	1.191	5.301	0.587
PREP	0.615	1.531	5.925	2.609
PREPL	0.459	1.375	8.870	1.951
PREX1	-0.677	0.625	7.450	-2.458
PRF1	-0.396	0.760	2.171	-1.222
PRG4	0.169	1.125	1.728	0.253
PRICKLE1	0.842	1.793	5.480	2.280
PRICKLE2	0.770	1.706	6.921	2.621
PRICKLE4//TOMM6	-0.410	0.753	2.844	-1.363
PRIMA1	-1.750	0.297	4.914	-3.830
PRKAA2	0.650	1.569	5.653	1.887
PRKACB	0.193	1.143	7.585	1.063
PRKAG2-AS1	0.515	1.429	2.667	1.734
PRKAR1B	0.943	1.923	5.512	2.307
PRKAR1B	0.372	1.294	3.052	1.302
PRKAR2B	1.173	2.255	6.204	3.105
PRKCB	1.125	2.181	5.200	3.180
PRKCDBP	-0.203	0.869	3.461	-0.805
PRKCE	0.834	1.783	4.428	2.521
PRKCG	2.406	5.302	4.438	3.183
PRKCI	0.400	1.320	6.028	1.704
PRKCQ	-0.092	0.938	4.462	-0.307
PRKCQ-AS1	-0.495	0.710	2.816	-1.522
PRKCSH	-0.307	0.808	8.186	-1.526
PRKD1	-0.232	0.851	4.640	-1.221

PRKD3	-0.356	0.782	4.874	-1.283
PRKG2	0.543	1.457	3.376	1.798
PRKX	-1.018	0.494	4.381	-2.241
PRKXP1//RNU6-322P	-0.579	0.669	2.902	-1.145
PRKY	-0.052	0.965	3.151	-0.121
PRLR	0.144	1.105	1.924	0.414
PRMT6	-0.435	0.740	3.811	-1.687
PRMT8	1.263	2.400	4.280	2.923
PROCR	0.015	1.010	4.467	0.053
PRODH//LOC102724788	0.063	1.045	4.718	0.217
PROK2	0.411	1.330	2.749	0.909
PROKR2	1.028	2.039	2.847	2.600
PRORSD1P	0.247	1.187	2.254	1.071
PROS1	0.533	1.447	4.992	1.902
PRPF19	0.341	1.267	6.646	1.580
PRPH	-0.492	0.711	2.647	-0.974
PRPH2	0.015	1.010	3.298	0.039
PRPS2	0.841	1.791	6.775	2.680
PRR11	-0.197	0.872	3.262	-0.737
PRR16	-0.115	0.923	3.028	-0.470
PRR18	-0.549	0.684	5.256	-1.406
PRR24	0.234	1.176	5.053	0.783
PRR34-AS1//RP6-109B7.3	-0.618	0.651	4.399	-2.333
PRR5L	-1.166	0.446	3.761	-3.338
PRRG1	-0.577	0.670	6.442	-1.978
PRRG3	1.054	2.076	3.256	2.404
PRRG4	-0.525	0.695	3.595	-1.338
PRRT1//LOC100507547	0.285	1.219	3.736	1.069
PRRT1//LOC100507547	0.096	1.069	3.862	0.391
PRRT1//LOC100507547	0.076	1.054	3.995	0.307
PRRT2	0.895	1.859	4.365	2.313
PRRT3	0.595	1.510	4.499	1.860
PRRT3-AS1	-0.641	0.641	4.230	-2.382
PRRX1	-0.015	0.990	4.782	-0.054
PRSS12	0.821	1.766	2.768	2.879
PRSS23	0.448	1.364	3.071	1.832
PRSS23//CTD-2005H7.1	-0.571	0.673	2.187	-2.050
PRSS35	0.425	1.343	2.682	1.765
PRTFDC1	-0.457	0.728	6.574	-1.803
PRTG	-0.991	0.503	4.316	-2.296
PRX	-0.103	0.931	3.248	-0.198
PSD	1.007	2.009	4.907	2.413
PSD2	-0.112	0.925	7.183	-0.320
PSD4	-0.402	0.757	2.883	-1.404
PSEN1	-0.709	0.612	5.136	-2.478
PSMB10//CTC-479C5.12	-0.248	0.842	5.007	-1.207
PSMB8	0.308	1.238	5.435	1.213
PSMB8	0.309	1.239	5.437	1.192
PSMB8-AS1//XXbac-BPG246D15.8	-0.076	0.949	3.556	-0.345
PSMB9	-0.318	0.802	2.690	-1.717

PSMB9	-0.238	0.848	3.032	-1.206
PSMB9	-0.147	0.903	3.488	-0.681
PSMB9//PSMB8-AS1//XXbac-BPG246D1	-0.202	0.870	3.669	-1.021
PSMB9//PSMB8-AS1//XXbac-BPG246D1	-0.116	0.922	3.442	-0.557
PSMD12	0.494	1.408	7.240	2.279
PSMD3	-0.008	0.994	3.542	-0.028
PSMD5	0.096	1.069	5.263	0.459
PSMD5-AS1	-0.206	0.867	4.894	-0.556
PSMG3	0.216	1.162	5.727	0.905
PSRC1	-0.431	0.742	4.851	-1.543
PSTPIP2	0.118	1.085	3.903	0.403
PTAFR	-0.730	0.603	3.651	-2.044
PTBP1//MIR4745	-0.361	0.779	7.309	-1.656
PTCH1	-0.457	0.728	4.978	-1.752
PTCH2	-0.137	0.909	3.455	-0.475
PTCHD1	0.474	1.389	5.300	1.730
PTCHD2	-0.241	0.846	3.269	-0.934
PTCHD4	0.769	1.704	3.799	2.122
PTCRA	-0.154	0.899	2.835	-0.582
PTDSS1	0.433	1.350	6.781	1.758
PTGDR	-0.460	0.727	2.661	-0.920
PTGDS	-0.399	0.758	8.315	-1.819
PTGER4	-0.672	0.628	2.435	-2.002
PTGES	-0.276	0.826	2.703	-0.966
PTGES3L-AARSD1//PTGES3L//AARSD1	0.211	1.157	3.755	0.941
PTGFRN	0.406	1.325	5.548	1.493
PTGIS	0.319	1.248	3.346	1.235
PTGR1	0.442	1.359	6.451	1.608
PTGS1	-0.879	0.544	3.752	-2.434
PTGS2	0.898	1.863	4.117	2.427
PTH1R	-0.245	0.844	3.848	-1.132
PTH2R	1.269	2.409	2.483	3.268
PTK2B	1.484	2.797	5.771	3.091
PTMA//MIR1244-1//MIR1244-2//MIR12	-0.385	0.766	5.709	-1.182
PTMS	0.612	1.528	5.606	1.879
PTN	0.725	1.653	4.868	3.001
PTN	0.425	1.342	8.215	1.818
PTP4A3	0.646	1.565	5.690	2.247
PTPLAD2	-0.261	0.834	3.380	-0.999
PTPN2	0.235	1.177	4.893	0.947
PTPN20B//PTPN20A	0.335	1.262	2.836	0.889
PTPN3	1.032	2.045	3.842	2.180
PTPN4	0.627	1.545	5.781	2.590
PTPN5	1.268	2.408	5.503	2.190
PTPN6	-0.468	0.723	4.696	-1.579
PTPRC	-0.539	0.688	5.689	-1.363
PTPRCAP	-0.405	0.755	3.262	-1.688
PTPRD	-0.242	0.845	7.632	-1.007
PTPRD-AS1//RP11-75C9.1	-0.606	0.657	4.391	-1.972
PTPRD-AS2//RP11-87N24.2	0.846	1.797	2.156	3.226

PTPRH	-0.755	0.593	3.295	-1.953
PTPRJ	-0.127	0.915	6.406	-0.607
PTPRK	0.188	1.139	6.761	0.904
PTPRM	0.371	1.293	7.264	1.580
PTPRN	1.018	2.024	5.566	2.201
PTPRN2	1.335	2.523	5.717	2.820
PTPRO	0.668	1.589	4.514	1.838
PTPRR	1.054	2.076	3.514	2.347
PTPRT	1.469	2.769	5.690	2.629
PTRF	-0.511	0.702	5.538	-1.830
PTTG1IP	-0.043	0.971	7.292	-0.179
PTTG2	-0.241	0.846	3.064	-0.846
PURB//MIR4657	0.349	1.274	5.926	1.628
PURG	0.414	1.332	5.100	1.845
PUS10	-0.211	0.864	4.149	-0.846
PUS7	0.386	1.306	4.302	1.973
PUS7L	0.141	1.102	5.374	0.541
PVALB	0.532	1.446	3.301	1.226
PVR	0.176	1.130	5.585	0.744
PVRL2	0.406	1.325	3.901	1.875
PWAR1	0.745	1.675	5.322	1.627
PWAR4//SNORD115-23	0.713	1.639	2.247	2.614
PWAR5	0.505	1.419	8.716	1.716
PWARSN//IPW//SNORD107//SNORD116	0.375	1.297	8.053	1.187
PWP2	-0.385	0.766	4.694	-1.410
PXDC1	0.163	1.119	5.981	0.731
PXDN	-0.211	0.864	3.917	-0.761
PXK	-0.916	0.530	8.100	-2.811
PXN	-0.402	0.757	4.234	-1.975
PYCARD	-0.466	0.724	3.423	-1.010
PYCR1	-0.478	0.718	3.594	-2.034
PYCR2//MIR6741//RP4-559A3.7	-0.493	0.710	4.469	-2.186
PYGL	-0.127	0.915	4.940	-0.343
PYGM	-0.359	0.780	5.251	-0.779
PYROXD2//MIR1287	-0.754	0.593	3.892	-2.794
PYY2	0.050	1.035	2.527	0.160
QDPR	-0.455	0.729	8.226	-1.723
QKI	-0.819	0.567	8.957	-2.883
QPCT	0.305	1.235	3.852	1.214
QPRT//LOC101929880	0.264	1.201	5.334	0.893
QRICH2	0.020	1.014	3.896	0.067
QRSL1	-0.086	0.942	5.665	-0.339
QSOX1//FLJ23867	-0.018	0.988	5.413	-0.078
R3HCC1	-0.648	0.638	5.193	-2.535
R3HDM1	0.952	1.935	7.477	2.623
RAB11B-AS1//RP11-886P16.3	-0.693	0.619	3.736	-1.872
RAB11FIP1	0.204	1.152	2.419	0.821
RAB11FIP2	0.435	1.352	7.299	2.026
RAB11FIP4	0.142	1.104	7.574	0.726
RAB11FIP5	0.453	1.369	4.112	1.733

RAB13	-0.092	0.938	4.147	-0.369
RAB15	0.861	1.816	4.674	2.126
RAB20	0.191	1.141	4.510	0.701
RAB24//MXD3	0.372	1.294	4.768	1.953
RAB26	1.143	2.209	4.764	2.963
RAB27A	0.212	1.158	4.424	0.766
RAB27B	1.440	2.713	5.397	2.452
RAB29//RAB7L1	-0.051	0.965	6.018	-0.241
RAB32	-0.258	0.836	3.510	-0.936
RAB34//NARR	-0.167	0.891	3.940	-0.771
RAB37	0.488	1.402	2.426	1.990
RAB39A	0.233	1.175	4.287	0.690
RAB39B	0.279	1.213	5.793	1.130
RAB3A	0.851	1.804	5.719	2.014
RAB3B	0.498	1.412	4.672	0.889
RAB3C	0.786	1.725	4.747	1.511
RAB3D	0.066	1.047	5.540	0.228
RAB3GAP1	0.370	1.292	7.380	2.135
RAB3IL1	-0.009	0.994	3.873	-0.037
RAB40A	0.122	1.088	2.978	0.514
RAB40C	0.452	1.368	4.864	1.999
RAB42	-0.127	0.916	2.094	-0.264
RAB4A//SPHAR	0.199	1.148	6.575	1.045
RAB6A//WTH3DI	0.268	1.204	8.834	1.410
RAB6B	0.826	1.772	8.299	2.127
RAB6C-AS1//AC079776.7	-0.090	0.940	4.967	-0.197
RAB9B	0.305	1.235	4.520	0.858
RAC3	0.233	1.175	4.240	0.797
RAD1	0.392	1.312	4.093	1.598
RAD50	0.171	1.126	7.479	0.735
RAD51-AS1	-0.569	0.674	4.236	-1.968
RAD51C	0.367	1.289	4.848	1.650
RAD51D	0.113	1.081	5.487	0.402
RAD54B//FSBP	0.355	1.279	4.355	1.613
RAE1	0.234	1.176	4.345	1.124
RAET1E-AS1//RP11-244K5.1//RP11-244I	-0.148	0.902	2.742	-0.593
RAI14	-0.512	0.701	4.144	-2.139
RAI2	0.449	1.365	3.544	1.843
RALB	0.605	1.521	6.562	2.610
RALGDS	-0.370	0.774	7.680	-1.344
RALYL	0.910	1.879	4.775	2.349
RAMP1	-0.066	0.955	5.427	-0.299
RAMP2	-0.349	0.785	5.062	-0.931
RAMP2-AS1//CTD-3193K9.7	-0.367	0.776	4.457	-1.676
RAMP3	0.053	1.037	4.898	0.131
RANBP3L	0.627	1.544	7.651	1.692
RANBP6	0.270	1.206	6.146	1.153
RAP1B	0.223	1.167	4.045	0.780
RAP1GAP	1.111	2.161	5.508	2.809
RAP1GAP2	1.074	2.105	5.226	2.215

RAP2A	0.326	1.253	7.551	1.460
RAP2B	0.357	1.281	4.612	1.327
RAPGEF2	0.493	1.407	6.894	2.122
RAPGEF4	1.143	2.208	6.815	2.700
RAPGEF4-AS1	0.505	1.419	2.328	1.642
RAPGEFL1	0.902	1.869	3.855	2.120
RAPH1	0.400	1.320	6.285	1.662
RARA//BCOR	0.608	1.525	3.954	2.374
RARB	0.610	1.526	4.246	1.643
RARG	-0.051	0.965	3.036	-0.192
RARRES2	-0.327	0.797	5.083	-1.722
RARRES3	-0.424	0.745	4.875	-1.330
RASA1	0.384	1.305	6.932	2.041
RASA2	0.329	1.256	4.989	1.455
RASA4//RASA4B	-0.231	0.852	3.672	-0.741
RASA4//RASA4B//AC004985.12//RP11-5	-0.109	0.927	4.878	-0.430
RASA4//RASA4B//LOC102725198//RASA	0.010	1.007	5.163	0.039
RASA4//RASA4B//POLR2J4//AC004985.1	-0.261	0.834	7.582	-0.928
RASA4//RASA4B//POLR2J4//AC004985.1	-0.146	0.904	5.453	-0.589
RASA4//RASA4B//RASA4CP//LOC102725	-0.071	0.952	6.278	-0.267
RASA4//RASA4B//RASA4CP//LOC102725	0.159	1.117	6.159	0.607
RASA4//RASA4B//RP11-514P8.6//RP11-	-0.221	0.858	7.907	-0.767
RASA4//RASA4B//RP11-514P8.6//RP11-	-0.084	0.944	6.173	-0.322
RASA4//RASA4B//RP11-514P8.6//RP11-	-0.260	0.835	5.841	-1.125
RASA4//RASA4CP//LOC102725198//RAS	-0.070	0.952	6.574	-0.237
RASA4B//RASA4	-0.132	0.913	3.954	-0.357
RASA4B//RASA4	-0.081	0.945	4.296	-0.274
RASA4B//RASA4//RP11-514P8.8//RASA4	-0.150	0.901	6.560	-0.521
RASAL1	0.105	1.075	3.843	0.381
RASAL2	0.735	1.664	6.895	2.907
RASAL3	-0.551	0.683	3.985	-1.898
RASD1	0.731	1.660	7.383	1.649
RASD2	1.074	2.106	5.024	2.530
RASGEF1A	0.286	1.219	5.344	1.093
RASGEF1B	-0.685	0.622	4.164	-2.292
RASGRF2	0.535	1.449	7.384	1.520
RASGRF2-AS1//CTC-459I6.1	0.640	1.558	2.420	2.365
RASGRP1	1.442	2.717	4.790	3.256
RASGRP2	0.376	1.297	2.545	1.394
RASGRP3	-1.367	0.388	5.535	-2.975
RASGRP4	-0.253	0.839	3.401	-0.777
RASIP1	0.268	1.204	3.420	1.152
RASL10A	0.454	1.369	3.271	1.481
RASL10B	0.516	1.430	5.868	1.813
RASL11B	1.426	2.687	3.729	3.849
RASL12	-0.786	0.580	4.558	-2.785
RASSF1	-0.230	0.853	3.064	-0.917
RASSF2	-1.213	0.432	9.236	-2.925
RASSF3	0.069	1.049	4.440	0.273
RASSF4	-0.373	0.772	6.472	-1.358

RASSF8	-0.322	0.800	5.851	-1.471
RASSF9	-0.943	0.520	3.014	-2.479
RB1CC1	0.499	1.414	6.888	2.392
RBBP5	0.306	1.236	6.434	1.331
RBBP9	0.081	1.058	5.832	0.277
RBCK1	-0.361	0.779	5.673	-1.548
RBFOX1	1.256	2.388	5.493	2.290
RBFOX2	0.615	1.531	5.351	2.094
RBFOX3	1.803	3.488	4.648	3.003
RBM11	0.030	1.021	4.616	0.109
RBM24	0.504	1.418	2.242	1.567
RBM3	-0.050	0.966	5.045	-0.143
RBM38	0.160	1.118	3.900	0.659
RBM47	0.031	1.022	2.898	0.132
RBM48	-0.161	0.895	4.812	-0.723
RBM5-AS1	-0.032	0.978	2.699	-0.102
RBMS3	0.021	1.015	5.058	0.076
RBP1	-0.344	0.788	3.301	-1.600
RBP4	1.092	2.132	5.313	2.410
RBP7	-0.492	0.711	3.513	-1.035
RBPMS	0.044	1.031	3.813	0.153
RCAN2	0.326	1.253	7.878	1.360
RCAN3	-0.785	0.580	4.634	-2.542
RCHY1	0.315	1.244	4.733	1.569
RCN3	-0.056	0.962	3.354	-0.243
RCOR2	0.454	1.370	2.989	1.794
RCSD1	-0.284	0.821	3.017	-0.841
RCVRN	0.513	1.427	2.353	1.550
RDH10	0.126	1.091	4.698	0.415
RDX	-0.711	0.611	6.169	-2.270
REC8	-0.246	0.843	3.970	-1.027
RECQL	0.043	1.030	5.777	0.174
REEP1	0.486	1.400	3.288	1.606
REEP2	0.397	1.317	5.961	1.329
REEP3	-0.833	0.561	7.366	-2.489
REEP4	-0.346	0.787	3.743	-1.597
REEP5	0.233	1.176	7.909	1.210
REL	-0.047	0.968	6.012	-0.174
RELA	-0.305	0.810	5.809	-1.268
RELL1	-0.211	0.864	7.082	-0.721
RELL2	0.644	1.562	5.144	1.842
RELN	0.390	1.311	5.684	0.785
RENBP	-0.587	0.666	3.698	-2.212
REPIN1	0.313	1.242	4.311	1.257
REPS2	0.401	1.320	7.377	1.853
RERG	0.191	1.142	3.836	0.698
RET//KIF5B	0.558	1.472	4.188	1.277
RETSAT	-0.237	0.849	7.032	-1.200
REXO1//MIR1909	0.458	1.374	4.915	1.834
RFC3	0.246	1.186	4.389	0.916

RFFL//RAD51D//RAD51L3-RFFL	-0.676	0.626	5.090	-2.467
RFK	0.170	1.125	3.798	0.818
RFNG	-0.126	0.917	4.891	-0.450
RFPL1S	1.504	2.837	4.651	2.657
RFT1	0.012	1.008	5.475	0.047
RFTN1	0.554	1.468	3.544	1.631
RFTN2	-0.803	0.573	8.079	-2.549
RFX1	-0.084	0.943	3.566	-0.358
RFX2	-0.391	0.763	4.937	-1.689
RFX4	-0.254	0.839	6.555	-1.003
RGAG4	0.175	1.129	4.499	0.661
RGCC	-0.624	0.649	7.568	-1.929
RGL3	-0.152	0.900	3.856	-0.563
RGMA	-0.148	0.903	4.362	-0.616
RGPD4	0.193	1.143	3.329	0.919
RGPD6//RGPD8//RGPD5//RGPD4//RGPI	0.225	1.168	8.470	1.207
RGPD6//RGPD8//RGPD5//RGPD4//RGPI	0.184	1.136	7.471	0.975
RGPD6//RGPD8//RGPD5//RGPD4//RGPI	0.181	1.134	7.143	0.964
RGR	0.787	1.725	2.887	1.734
RGS1	-0.714	0.610	7.146	-1.321
RGS10	-0.527	0.694	3.925	-1.438
RGS11	-0.400	0.758	5.390	-1.534
RGS14	0.443	1.360	3.540	1.631
RGS16	0.392	1.312	4.917	0.830
RGS17	0.438	1.355	4.288	1.680
RGS19	-0.239	0.848	5.066	-0.754
RGS2	-0.031	0.979	6.838	-0.088
RGS20	0.470	1.385	3.106	2.100
RGS22	-0.077	0.948	2.010	-0.320
RGS4	1.987	3.963	5.053	2.871
RGS6	0.552	1.467	4.762	1.535
RGS7	1.229	2.344	5.510	2.509
RGS7BP	1.384	2.611	6.231	2.200
RGS8	-0.229	0.853	4.082	-0.563
RGS9	-0.643	0.640	4.908	-2.038
RHBDD2	0.067	1.048	6.595	0.299
RHBDF1	-0.078	0.948	3.333	-0.316
RHBDF2	-0.782	0.582	5.545	-2.407
RHBDL1	0.468	1.383	3.305	1.565
RHBDL2	-0.719	0.607	4.268	-1.627
RHBDL3	0.424	1.342	4.197	1.529
RHCG	0.832	1.781	2.388	3.847
RHEBL1	0.908	1.877	2.216	2.879
RHOB	-0.400	0.758	9.291	-1.720
RHOBTB2	0.669	1.589	5.372	2.031
RHOF	0.193	1.143	3.588	0.744
RHOG	-0.696	0.617	2.717	-1.912
RHOJ	0.657	1.577	4.905	2.353
RHOT1	0.311	1.241	7.535	1.699
RHOU//DUSP5P1	-0.414	0.750	5.147	-1.589

RHPN2	0.530	1.443	5.297	1.517
RIBC2	0.054	1.038	2.243	0.181
RIIAD1	0.750	1.682	2.576	2.274
RILP	0.291	1.224	3.676	1.061
RIMBP2	0.692	1.615	5.400	1.827
RIMBP2//RP11-117L5.4	0.575	1.490	2.870	1.927
RIMBP3	-0.193	0.875	2.922	-0.661
RIMKLA	0.752	1.684	5.098	1.705
RIMS1	0.991	1.987	5.540	2.309
RIMS2	1.300	2.462	5.488	2.845
RIMS3	1.677	3.197	6.109	2.868
RIMS4	0.695	1.619	3.743	1.684
RIN1	0.314	1.243	2.580	1.171
RIN3	-0.216	0.861	2.982	-0.645
RINT1	0.498	1.412	4.762	2.465
RIPK2	-0.097	0.935	5.534	-0.442
RIPPLY2	0.417	1.335	3.142	1.681
RIT2	0.823	1.769	4.323	2.142
RITA1//C12orf52	0.161	1.118	4.784	0.549
RLBP1	1.596	3.022	2.836	4.475
RLF	0.200	1.149	6.848	0.932
RLIM	0.420	1.338	7.353	2.038
RLN3	-0.482	0.716	2.966	-1.832
RMDN1	-0.228	0.854	4.898	-0.993
RMND1	0.196	1.146	5.125	0.929
RMND5A	0.093	1.067	6.477	0.444
RMRP//RP11-331F9.10	-0.249	0.842	10.953	-1.445
RMST	-0.127	0.916	4.984	-0.402
RN7SK	-0.451	0.731	11.098	-1.946
RN7SKP105	-0.146	0.904	3.803	-0.403
RN7SKP106	1.467	2.765	1.873	3.625
RN7SKP111	-0.351	0.784	6.019	-1.300
RN7SKP134	0.225	1.169	2.341	0.935
RN7SKP154	-1.224	0.428	2.655	-2.494
RN7SKP16	-0.637	0.643	3.925	-2.080
RN7SKP173	0.140	1.102	3.104	0.326
RN7SKP174	0.121	1.088	2.816	0.447
RN7SKP207	-0.137	0.909	2.913	-0.549
RN7SKP231	-0.017	0.988	5.644	-0.061
RN7SKP232	-0.262	0.834	2.492	-1.072
RN7SKP233	-0.405	0.755	3.136	-0.713
RN7SKP25	-0.757	0.592	3.883	-2.549
RN7SKP262	-0.080	0.946	2.309	-0.324
RN7SKP283	-0.016	0.989	2.674	-0.055
RN7SKP40	-0.644	0.640	2.839	-1.888
RN7SKP5	-1.306	0.405	4.208	-2.211
RN7SKP53	0.520	1.433	3.262	1.260
RN7SKP55	-0.284	0.821	6.015	-1.184
RN7SKP56	-0.627	0.647	4.569	-1.226
RN7SKP62	-0.137	0.910	2.901	-0.511

RN7SKP65	-0.183	0.881	2.645	-0.469
RN7SKP7	-0.056	0.962	4.456	-0.205
RN7SKP75	-0.018	0.988	2.654	-0.051
RN7SKP80	-0.217	0.860	4.011	-0.737
RN7SKP86	0.350	1.275	2.544	0.751
RNA5-8SP2	0.103	1.074	3.375	0.231
RNA5-8SP4	-0.062	0.958	2.772	-0.186
RNA5-8SP6	-0.080	0.946	3.436	-0.226
RNA5SP111	0.002	1.001	3.324	0.005
RNA5SP113	0.268	1.204	3.838	0.633
RNA5SP122	0.454	1.370	3.101	1.314
RNA5SP125	0.652	1.571	2.970	2.117
RNA5SP129	0.072	1.051	3.287	0.208
RNA5SP132	-0.094	0.937	5.958	-0.447
RNA5SP134	0.396	1.316	2.463	1.060
RNA5SP145	0.478	1.392	2.809	1.199
RNA5SP150	-0.339	0.790	5.774	-0.976
RNA5SP153	-0.178	0.884	3.218	-0.378
RNA5SP156	-0.036	0.976	3.752	-0.100
RNA5SP161	-0.056	0.962	3.599	-0.230
RNA5SP174	-0.241	0.846	3.268	-1.168
RNA5SP176	0.564	1.478	2.223	1.539
RNA5SP180	0.583	1.498	2.254	1.585
RNA5SP182	0.173	1.127	3.039	0.588
RNA5SP19	0.356	1.280	5.250	0.878
RNA5SP190	-0.005	0.996	2.450	-0.020
RNA5SP191	0.980	1.972	3.175	1.669
RNA5SP195	-0.326	0.798	4.510	-1.016
RNA5SP199	0.334	1.261	9.939	1.303
RNA5SP199	0.222	1.166	9.273	0.894
RNA5SP20	-0.199	0.871	2.265	-0.589
RNA5SP207	-0.271	0.829	2.482	-1.009
RNA5SP217	-0.373	0.772	3.373	-0.927
RNA5SP218	0.162	1.119	2.427	0.459
RNA5SP237	-0.334	0.793	4.277	-0.913
RNA5SP242	0.054	1.038	2.831	0.203
RNA5SP248	-0.296	0.814	2.580	-1.047
RNA5SP25	-0.546	0.685	2.550	-1.475
RNA5SP256	-0.238	0.848	3.064	-0.769
RNA5SP259	0.266	1.203	2.636	0.821
RNA5SP260	-0.454	0.730	4.065	-0.943
RNA5SP289	0.170	1.125	2.674	0.604
RNA5SP290	-0.047	0.968	3.352	-0.142
RNA5SP294	0.006	1.004	2.737	0.013
RNA5SP300	0.052	1.037	2.288	0.114
RNA5SP33	-0.556	0.680	4.226	-1.982
RNA5SP341	0.035	1.025	2.380	0.103
RNA5SP343	0.560	1.474	2.267	1.438
RNA5SP344	-0.261	0.834	2.549	-0.721
RNA5SP345	-0.059	0.960	3.366	-0.189

RNA5SP361	-0.407	0.754	3.247	-1.437
RNA5SP366	0.704	1.629	2.802	1.871
RNA5SP370	-0.618	0.652	2.971	-2.362
RNA5SP372	-0.608	0.656	2.191	-1.559
RNA5SP383	-0.331	0.795	3.233	-0.981
RNA5SP395	0.023	1.016	3.633	0.060
RNA5SP399	-0.162	0.894	4.599	-0.520
RNA5SP403	-0.288	0.819	4.663	-0.897
RNA5SP405	-0.696	0.617	3.014	-1.963
RNA5SP406	0.215	1.161	2.719	0.666
RNA5SP408	0.890	1.853	2.647	2.330
RNA5SP409	0.611	1.527	3.467	1.618
RNA5SP415	-0.278	0.824	2.648	-0.695
RNA5SP425	-0.228	0.854	3.538	-0.678
RNA5SP427	-0.502	0.706	2.485	-1.424
RNA5SP428	-0.371	0.773	4.838	-1.324
RNA5SP434	-0.348	0.786	3.729	-1.173
RNA5SP435	0.568	1.483	4.000	1.315
RNA5SP441	0.330	1.257	2.739	1.091
RNA5SP450	-0.288	0.819	2.706	-0.741
RNA5SP455	-0.066	0.955	2.591	-0.250
RNA5SP473	-0.470	0.722	4.220	-1.898
RNA5SP474	-0.844	0.557	3.395	-2.272
RNA5SP478	-0.083	0.944	2.609	-0.282
RNA5SP486	-0.368	0.775	2.866	-1.325
RNA5SP488	-0.459	0.727	3.114	-1.538
RNA5SP497	0.374	1.296	1.986	1.190
RNA5SP513	-0.022	0.985	2.419	-0.066
RNA5SP55	-0.152	0.900	3.113	-0.499
RNA5SP60	0.306	1.236	4.978	1.146
RNA5SP64	0.395	1.315	2.872	0.840
RNA5SP65	-0.215	0.861	3.948	-0.691
RNA5SP68	-0.189	0.877	2.386	-0.647
RNA5SP69	0.111	1.080	2.457	0.320
RNA5SP82	0.196	1.145	2.727	0.523
RNA5SP85	0.318	1.247	6.222	0.765
RNA5SP93	0.349	1.274	3.372	1.378
RNA5SP99	0.120	1.087	4.633	0.331
RNASE1	-0.508	0.703	5.614	-1.568
RNASE2	-0.163	0.893	5.822	-0.203
RNASE3	0.065	1.046	3.402	0.096
RNASE6	-0.571	0.673	5.815	-1.230
RNASEL	0.298	1.229	4.165	1.180
RNASET2	-0.678	0.625	5.408	-2.402
RND1	1.208	2.309	5.108	2.789
RND2	-0.547	0.684	7.959	-1.695
RND3	0.523	1.437	3.581	1.798
RNF11	0.398	1.318	8.372	2.154
RNF111	0.041	1.029	6.246	0.205
RNF112	0.397	1.317	3.930	1.442

RNF113B	0.001	1.001	2.370	0.005
RNF122	0.110	1.079	4.228	0.296
RNF125//RP11-53I6.2	-1.124	0.459	4.091	-2.379
RNF126	0.222	1.166	4.900	0.772
RNF128	0.659	1.579	3.027	2.460
RNF13	-0.429	0.743	8.090	-1.758
RNF130	-0.724	0.605	6.863	-2.868
RNF135	-0.511	0.702	3.479	-1.514
RNF139-AS1	-0.007	0.995	3.109	-0.026
RNF141	-0.292	0.817	6.386	-1.176
RNF144A-AS1	0.931	1.907	2.831	2.154
RNF144B	0.069	1.049	5.233	0.214
RNF145	0.351	1.276	5.957	1.783
RNF149	0.154	1.112	5.380	0.496
RNF150	0.243	1.184	4.676	1.015
RNF157	0.582	1.497	7.294	2.095
RNF165	1.087	2.124	4.100	2.413
RNF175	0.937	1.914	4.143	1.978
RNF182	0.194	1.144	4.364	0.770
RNF185-AS1	0.266	1.203	2.644	0.760
RNF19B	0.074	1.053	6.226	0.363
RNF2	0.069	1.049	5.182	0.311
RNF208	0.549	1.463	2.745	2.233
RNF213	-0.713	0.610	6.601	-2.622
RNF216P1	-0.527	0.694	3.336	-1.781
RNF219	0.354	1.279	5.542	1.446
RNF219-AS1	-0.355	0.782	5.319	-1.020
RNF219-AS1	-0.013	0.991	5.161	-0.039
RNF220	-0.714	0.609	5.538	-2.311
RNF32	-0.223	0.857	2.988	-0.827
RNF41	0.173	1.127	6.568	0.818
RNF43	0.547	1.461	3.208	1.811
RNF5P1	-0.221	0.858	3.365	-0.783
RNF6	0.436	1.353	6.623	1.935
RNF8	0.186	1.138	4.660	0.903
RNFT2	0.554	1.468	5.888	2.211
RNGTT	0.262	1.199	5.585	1.356
RNH1	-0.530	0.692	6.242	-2.246
RNMT	0.450	1.366	5.814	1.840
RNMTL1	0.419	1.337	4.465	1.954
RNPC3	0.167	1.123	7.070	0.701
RNPEPL1	-0.444	0.735	4.391	-2.032
RNPS1//MIR572	-0.043	0.971	3.903	-0.100
RNU105B	0.683	1.605	4.358	1.458
RNU11	-0.485	0.715	9.485	-1.228
RNU1-103P	-0.333	0.794	3.296	-0.720
RNU1-106P	-0.614	0.654	2.135	-1.431
RNU1-112P	0.118	1.085	2.402	0.361
RNU1-119P	-0.172	0.888	2.520	-0.714
RNU1-119P//RNU1-78P	-0.210	0.865	5.830	-0.779

RNU1-122P	0.534	1.448	4.253	1.591
RNU1-132P	0.239	1.180	3.903	0.841
RNU1-136P	0.044	1.031	4.799	0.172
RNU1-138P	0.059	1.041	2.686	0.152
RNU1-13P	0.163	1.120	4.864	0.510
RNU1-14P	-0.332	0.795	3.943	-0.927
RNU1-150P	0.080	1.057	2.578	0.334
RNU1-16P	-0.047	0.968	4.423	-0.154
RNU1-18P	0.093	1.066	3.674	0.328
RNU1-19P	-0.158	0.896	4.932	-0.616
RNU12	-0.321	0.800	6.189	-1.081
RNU1-23P	0.456	1.372	3.128	1.416
RNU1-24P	0.438	1.355	3.320	1.012
RNU1-38P	-0.311	0.806	7.243	-0.985
RNU1-41P//RNU1-48P	0.334	1.260	4.197	0.559
RNU1-42P	-0.476	0.719	3.881	-1.654
RNU1-44P	0.034	1.024	3.127	0.094
RNU1-47P	-0.477	0.718	2.585	-1.718
RNU1-51P	0.008	1.006	1.986	0.020
RNU1-56P	-0.445	0.734	3.587	-1.751
RNU1-58P	0.113	1.081	2.519	0.385
RNU1-59P	0.185	1.137	4.391	0.464
RNU1-74P	-0.308	0.808	2.734	-1.076
RNU1-82P	0.207	1.154	2.471	0.539
RNU1-83P	-0.464	0.725	8.129	-1.535
RNU1-87P	-0.397	0.759	4.866	-1.399
RNU1-91P	-0.349	0.785	4.498	-1.381
RNU1-94P	-0.746	0.596	3.906	-2.213
RNU2-58P	-0.160	0.895	2.875	-0.568
RNU2-7P	-0.321	0.800	2.779	-0.927
RNU4-24P	-0.356	0.781	3.142	-1.128
RNU4-32P//MMAB	-0.364	0.777	4.667	-1.137
RNU4-33P//RNU4-87P	0.118	1.085	3.182	0.415
RNU4-61P	0.152	1.111	2.790	0.463
RNU4-62P	-0.261	0.834	4.265	-0.635
RNU4-78P	-0.527	0.694	5.598	-1.815
RNU4-82P	-0.011	0.992	2.286	-0.038
RNU4-87P	0.437	1.354	3.626	1.253
RNU4ATAC	-0.185	0.880	10.048	-0.898
RNU4ATAC11P	-0.303	0.811	6.015	-0.715
RNU4ATAC16P	-0.418	0.748	2.795	-0.814
RNU4ATAC18P	-0.926	0.526	2.958	-1.960
RNU5B-1	0.045	1.032	4.253	0.120
RNU5D-1	-0.309	0.807	3.074	-0.469
RNU5E-1	-0.392	0.762	7.511	-0.826
RNU5E-9P	-0.210	0.865	5.344	-0.782
RNU5F-1	0.364	1.287	2.310	0.828
RNU6-1003P	1.127	2.184	1.764	3.755
RNU6-1005P	-0.213	0.863	5.646	-0.699
RNU6-1010P	0.183	1.135	2.330	0.436

RNU6-1013P	0.025	1.017	2.659	0.057
RNU6-1014P	-1.768	0.294	5.915	-2.399
RNU6-1016P	-0.214	0.862	5.408	-0.571
RNU6-1017P	-0.404	0.756	4.872	-1.832
RNU6-1052P	-0.422	0.746	2.552	-0.892
RNU6-1053P	-0.067	0.955	2.650	-0.143
RNU6-1054P	0.318	1.247	3.555	0.777
RNU6-1057P	-0.450	0.732	4.232	-1.226
RNU6-1064P	-0.382	0.767	2.945	-1.223
RNU6-1067P	-0.017	0.988	4.397	-0.067
RNU6-1073P	-0.167	0.891	2.501	-0.584
RNU6-1080P	-0.208	0.866	3.043	-0.775
RNU6-1087P	0.206	1.154	2.765	0.693
RNU6-108P	-0.596	0.661	2.930	-1.571
RNU6-1099P	0.156	1.114	4.815	0.418
RNU6-1105P	-0.257	0.837	2.102	-0.966
RNU6-1112P	-0.880	0.543	2.986	-2.468
RNU6-1122P	-0.320	0.801	2.427	-1.054
RNU6-1123P	0.602	1.518	5.057	1.739
RNU6-1141P	-0.920	0.529	2.120	-2.037
RNU6-1162P	0.291	1.223	2.082	0.999
RNU6-1181P	-0.049	0.967	3.842	-0.114
RNU6-1184P	0.139	1.101	3.893	0.296
RNU6-118P	-0.312	0.805	7.024	-1.270
RNU6-119P	-0.263	0.833	3.546	-0.992
RNU6-1226P	-0.449	0.732	4.210	-1.502
RNU6-1262P	-0.230	0.852	3.222	-0.538
RNU6-126P	-0.978	0.508	2.183	-2.258
RNU6-1274P	-0.316	0.803	2.033	-1.091
RNU6-1279P	-0.305	0.810	3.270	-0.721
RNU6-1283P	0.034	1.024	2.945	0.100
RNU6-1284P	-0.322	0.800	3.759	-0.752
RNU6-1295P	-0.558	0.679	2.455	-1.427
RNU6-1298P	-0.151	0.901	2.878	-0.538
RNU6-1305P	0.840	1.790	2.646	2.313
RNU6-130P	0.096	1.069	4.069	0.317
RNU6-1319P//RNU6-747P//RNU6-241P/	-0.251	0.840	4.080	-0.837
RNU6-1320P//RNU6-1269P//RNU6-599I	0.917	1.888	4.366	2.646
RNU6-1320P//RNU6-1269P//RNU6-599I	0.822	1.767	4.003	2.573
RNU6-1320P//RNU6-1269P//RNU6-599I	0.863	1.819	4.112	2.672
RNU6-1327P	-1.035	0.488	2.527	-2.069
RNU6-1340P	-0.660	0.633	2.754	-1.952
RNU6-135P	0.174	1.128	3.314	0.498
RNU6-137P	0.050	1.035	3.592	0.153
RNU6-143P	1.076	2.109	3.121	2.418
RNU6-165P	0.042	1.029	2.874	0.160
RNU6-171P	-0.300	0.812	3.614	-1.180
RNU6-176P	0.205	1.153	2.980	0.579
RNU6-177P	-0.564	0.677	2.901	-1.577
RNU6-178P	-0.112	0.925	3.522	-0.444

RNU6-188P	0.066	1.047	4.036	0.180
RNU6-195P	0.076	1.054	2.536	0.210
RNU6-196P	0.265	1.202	4.020	0.570
RNU6-202P	-0.846	0.556	2.236	-2.373
RNU6-204P	-0.209	0.865	1.643	-0.559
RNU6-208P	-0.184	0.880	2.442	-0.668
RNU6-216P	-0.057	0.961	3.973	-0.169
RNU6-220P	-0.136	0.910	2.632	-0.481
RNU6-226P	0.759	1.692	4.054	1.732
RNU6-252P	0.001	1.001	2.985	0.003
RNU6-271P	0.607	1.523	1.780	1.751
RNU6-283P//RNU6-683P	0.189	1.140	3.423	0.684
RNU6-28P//RNU6-738P//APBA3	0.010	1.007	3.787	0.028
RNU6-294P	-0.263	0.833	2.790	-0.621
RNU6-313P	0.434	1.351	2.774	1.144
RNU6-316P	1.083	2.119	2.891	2.484
RNU6-321P	1.056	2.080	3.943	2.300
RNU6-326P	-0.129	0.914	2.506	-0.433
RNU6-331P	0.194	1.144	2.636	0.712
RNU6-341P	0.137	1.100	4.601	0.372
RNU6-353P	-0.026	0.982	5.686	-0.114
RNU6-359P	-0.031	0.979	2.548	-0.104
RNU6-360P	0.160	1.118	2.607	0.494
RNU6-367P	-0.005	0.996	3.551	-0.017
RNU6-371P	-0.276	0.826	3.264	-0.779
RNU6-377P	-0.101	0.932	4.223	-0.297
RNU6-386P	-0.564	0.677	2.725	-2.022
RNU6-395P	-0.060	0.960	3.455	-0.238
RNU6-398P	0.152	1.111	2.662	0.504
RNU6-411P	-2.260	0.209	5.458	-3.174
RNU6-418P	0.886	1.848	1.908	3.470
RNU6-429P	-0.186	0.879	2.907	-0.473
RNU6-431P	-0.153	0.899	3.515	-0.393
RNU6-433P	-0.669	0.629	3.635	-1.505
RNU6-439P	-0.084	0.944	4.326	-0.308
RNU6-441P	-0.182	0.882	3.130	-0.570
RNU6-447P//RNU6-1118P//RNU6-1076I	-0.324	0.799	4.910	-0.935
RNU6-447P//RNU6-1118P//RNU6-1076I	-0.226	0.855	5.059	-0.743
RNU6-457P	-0.527	0.694	2.835	-0.912
RNU6-466P	-1.516	0.350	5.075	-3.199
RNU6-469P	0.141	1.103	2.576	0.314
RNU6-482P	0.193	1.143	3.968	0.597
RNU6-522P	-0.299	0.813	2.654	-0.648
RNU6-529P	-0.393	0.761	5.862	-1.365
RNU6-530P	-0.578	0.670	4.869	-1.423
RNU6-548P	0.452	1.368	2.006	1.897
RNU6-552P	0.079	1.056	3.551	0.272
RNU6-560P	-0.243	0.845	4.390	-0.655
RNU6-564P	-0.723	0.606	2.651	-2.080
RNU6-572P	-0.106	0.929	2.645	-0.318

RNU6-580P	-0.172	0.888	5.715	-0.556
RNU6-59P	-0.255	0.838	2.755	-0.868
RNU6-605P	-0.264	0.833	2.760	-0.747
RNU6-606P	-0.555	0.681	3.675	-1.562
RNU6-611P	-0.831	0.562	4.163	-2.722
RNU6-618P	0.058	1.041	2.744	0.172
RNU6-621P	0.269	1.205	2.330	0.720
RNU6-622P	-0.224	0.856	3.504	-0.736
RNU6-623P	-0.489	0.713	2.496	-1.595
RNU6-652P	-0.696	0.617	2.822	-1.544
RNU6-653P	0.046	1.032	2.469	0.097
RNU6-654P	-0.280	0.824	3.218	-1.038
RNU6-680P	-0.505	0.705	2.332	-1.460
RNU6-700P	-0.189	0.877	2.538	-0.478
RNU6-701P	-0.021	0.986	2.652	-0.054
RNU6-702P	-0.026	0.982	5.919	-0.080
RNU6-703P	-0.103	0.931	2.381	-0.289
RNU6-711P	0.123	1.089	4.087	0.484
RNU6-729P	0.982	1.975	5.280	2.268
RNU6-731P	-0.094	0.937	2.481	-0.287
RNU6-742P	-0.127	0.916	3.811	-0.518
RNU6-748P	0.195	1.145	2.321	0.564
RNU6-758P	-0.719	0.608	5.493	-2.152
RNU6-759P	-0.031	0.979	2.839	-0.065
RNU6-760P	0.547	1.461	5.378	1.341
RNU6-767P	-0.562	0.677	2.989	-1.211
RNU6-787P	0.104	1.075	4.439	0.365
RNU6-798P	-0.090	0.940	2.762	-0.230
RNU6-801P	-0.447	0.733	3.959	-1.275
RNU6-817P	-0.812	0.569	4.689	-2.646
RNU6-818P	-0.400	0.758	3.638	-1.095
RNU6-823P	-0.334	0.794	2.651	-0.892
RNU6-831P	0.726	1.654	2.003	1.505
RNU6-856P	0.027	1.019	2.392	0.077
RNU6-876P	0.584	1.499	2.804	1.159
RNU6-878P	-0.443	0.736	3.569	-1.404
RNU6-882P	0.234	1.176	2.509	0.806
RNU6-883P	-0.596	0.661	4.811	-1.241
RNU6-892P	0.325	1.253	4.024	1.060
RNU6-909P	0.205	1.152	2.879	0.734
RNU6-912P//RNU6-1319P//RNU6-747P/	-0.197	0.872	3.085	-0.565
RNU6-925P	-0.120	0.920	3.614	-0.445
RNU6-928P	-0.312	0.806	2.218	-0.990
RNU6-936P	-0.050	0.966	3.694	-0.147
RNU6-942P	-1.254	0.419	2.914	-2.478
RNU6-945P	-0.106	0.929	2.062	-0.224
RNU6-946P	-0.248	0.842	4.808	-0.740
RNU6-954P	0.757	1.690	1.731	2.556
RNU6-957P	-0.927	0.526	2.387	-1.753
RNU6-979P	-0.251	0.840	3.941	-0.474

RNU6-984P	0.336	1.262	3.120	1.261
RNU6-992P	-0.319	0.802	2.550	-1.118
RNU6-999P	0.426	1.344	2.306	1.454
RNU6ATAC//RNU6ATAC2P	0.203	1.151	7.236	0.662
RNU6ATAC10P	0.713	1.639	4.442	1.737
RNU6ATAC11P	-0.316	0.803	2.668	-1.219
RNU6ATAC24P//PPP1R2	0.083	1.059	3.683	0.274
RNU6ATAC27P	-0.166	0.891	2.859	-0.535
RNU6ATAC2P//RNU6ATAC	0.203	1.151	6.460	0.728
RNU6ATAC36P	0.176	1.130	4.054	0.597
RNU6ATAC5P	0.312	1.242	2.943	1.004
RNU6ATAC6P	0.390	1.311	2.011	1.524
RNU6ATAC9P	-0.179	0.883	3.112	-0.668
RNU7-13P//RNU7-41P	-0.231	0.852	2.248	-0.543
RNU7-151P	-0.023	0.984	1.946	-0.061
RNU7-156P	1.990	3.972	2.915	2.626
RNU7-170P	0.342	1.267	2.475	1.153
RNU7-181P	-0.530	0.693	2.830	-1.239
RNU7-187P	-0.905	0.534	3.356	-1.905
RNU7-193P	-0.483	0.716	2.981	-1.618
RNU7-194P	-0.361	0.778	2.579	-1.066
RNU7-35P	0.163	1.119	4.548	0.532
RNU7-40P	-0.378	0.769	3.289	-0.820
RNU7-57P//RNU7-189P	-0.011	0.992	2.604	-0.034
RNU7-77P	-0.592	0.664	2.951	-1.658
RNU7-80P	-0.499	0.708	2.262	-1.533
RNU7-96P	-0.592	0.663	2.291	-1.538
RNVU1-14	-0.241	0.846	2.716	-0.711
RNVU1-15	-0.217	0.860	6.023	-0.633
RNVU1-15	-0.086	0.942	5.616	-0.274
RNVU1-3	-0.130	0.914	2.800	-0.455
RNVU1-6	0.236	1.178	2.429	0.802
RNVU1-8	0.107	1.077	2.317	0.390
RNY3P2	0.229	1.172	1.971	0.584
RNY3P9	-0.346	0.787	2.557	-1.353
RNY4P13	-0.511	0.702	1.996	-1.517
RNY4P18	-0.220	0.858	4.974	-0.514
RNY4P19	0.748	1.680	4.391	1.847
ROBO2	0.746	1.677	6.263	2.480
ROCK1P1	1.277	2.423	1.411	2.314
ROCK2	0.725	1.653	6.663	2.818
ROM1	-0.036	0.976	4.318	-0.155
ROPN1L	0.156	1.114	3.668	0.296
ROPN1L//RP11-1C1.7	0.092	1.066	2.781	0.282
RORB	1.524	2.876	6.389	2.938
ROS1//SLC34A2	1.625	3.084	2.584	3.422
RP1-102E24.8	-0.708	0.612	2.859	-2.178
RP11-1008C21.2	-0.254	0.838	2.813	-0.986
RP11-102L12.2	0.355	1.279	4.232	1.619
RP11-1049A21.2	-0.385	0.766	2.154	-1.645

RP11-1049H7.2	0.419	1.337	3.727	1.290
RP11-1079K10.4	-0.477	0.719	3.843	-1.752
RP11-108K3.4	-0.634	0.644	3.485	-1.912
RP11-108M9.2//RP5-875O13.6	-0.549	0.684	3.083	-1.579
RP11-10A14.4//LOC102724880	-0.312	0.805	2.372	-1.174
RP11-10J21.4	0.131	1.095	2.638	0.431
RP11-10K16.1	-0.508	0.703	4.209	-1.842
RP11-10L12.4	-0.662	0.632	3.988	-2.095
RP11-110I1.12	-0.442	0.736	3.840	-1.722
RP11-110I1.13	-0.047	0.968	3.147	-0.149
RP11-1114A5.4	-0.258	0.836	3.516	-0.713
RP11-111F5.1//RP11-204M4.1	-1.198	0.436	3.237	-2.914
RP11-1143G9.4	0.075	1.054	2.025	0.149
RP11-116N8.1	-1.598	0.330	5.871	-2.587
RP11-118B22.4	-0.705	0.613	5.185	-1.553
RP1-111C20.3	-0.102	0.932	2.325	-0.429
RP11-11N9.4	-0.434	0.740	3.908	-0.890
RP11-120J1.1	-0.168	0.890	3.592	-0.456
RP11-121A14.3//NEK6	-0.076	0.949	3.081	-0.282
RP11-122C5.3	-0.544	0.686	3.168	-1.308
RP11-122K13.12	0.088	1.063	4.315	0.317
RP11-123O10.4	0.325	1.253	3.574	0.877
RP11-124D2.3	-0.455	0.730	2.843	-1.433
RP11-124N14.3	0.060	1.042	4.640	0.182
RP11-124N2.1	0.182	1.134	2.474	0.636
RP11-134G8.7	-0.636	0.643	2.647	-2.306
RP11-134G8.8	-0.514	0.700	2.625	-1.790
RP11-13E1.5	-0.195	0.874	3.347	-0.767
RP11-143A22.1	0.243	1.184	2.844	0.909
RP11-144G6.4//RP11-96C23.10//RP11-4	-0.048	0.967	3.002	-0.144
RP11-144G6.4//RP11-96C23.10//RP11-4	0.037	1.026	2.799	0.112
RP11-14I17.2	-0.666	0.630	3.388	-1.388
RP11-154D6.1//LINC00472//RP3-331H2	-0.019	0.987	3.541	-0.077
RP11-156E6.1	0.368	1.291	5.843	1.538
RP11-158A14.1	-0.434	0.740	3.091	-1.125
RP11-158J3.2	0.945	1.925	2.281	1.881
RP11-159F24.6	-1.123	0.459	2.341	-3.085
RP11-161H23.5//RP11-386G11.10	-0.151	0.901	7.898	-0.580
RP11-164J13.1	-0.487	0.714	3.313	-1.482
RP11-164P12.4	0.647	1.566	1.952	2.258
RP11-165J3.6	0.246	1.186	2.522	0.917
RP11-166B2.1	-0.980	0.507	3.924	-2.374
RP11-169E6.1	0.260	1.197	2.143	1.120
RP11-169K16.4	-0.243	0.845	3.621	-0.977
RP11-16D22.2	-0.618	0.652	2.443	-1.450
RP11-16P20.3	-0.163	0.893	3.253	-0.592
RP11-171A24.3	1.466	2.763	2.608	3.473
RP11-174M13.2	0.540	1.454	1.914	1.832
RP11-181B11.1	0.628	1.545	1.715	1.948
RP11-184A2.2	1.338	2.528	3.501	3.398

RP11-190P13.2	-0.291	0.817	2.821	-0.913
RP11-196D18.1	0.131	1.095	2.474	0.504
RP11-196G18.22	-0.442	0.736	3.954	-1.202
RP11-1C8.4	0.634	1.552	2.596	1.987
RP11-203E8.1	0.952	1.935	2.137	3.382
RP11-203F10.5	-0.369	0.774	3.577	-0.945
RP11-206L10.4	0.069	1.049	2.719	0.234
RP11-207C16.4	-0.382	0.767	3.049	-1.369
RP11-212D3.4	-0.141	0.907	2.352	-0.522
RP11-216M21.7	-0.394	0.761	3.895	-1.513
RP11-229C3.2	1.559	2.947	2.878	3.944
RP11-229P13.15	0.012	1.008	2.761	0.044
RP1-122P22.2	-0.394	0.761	3.259	-1.439
RP11-22P4.1	0.189	1.140	2.829	0.681
RP11-236B18.5	-0.596	0.662	2.384	-1.762
RP11-238K6.2	0.283	1.216	2.214	0.932
RP11-244F12.2	0.866	1.823	2.238	3.407
RP11-244N9.4//XPA	-0.259	0.836	4.182	-0.701
RP11-247L20.4	0.860	1.815	2.538	2.405
RP11-250B2.3	-0.490	0.712	3.486	-1.793
RP11-253I19.3	0.168	1.124	4.082	0.634
RP11-257K9.8	0.185	1.137	4.210	0.625
RP11-259K15.2	-0.940	0.521	3.447	-1.687
RP11-261C10.7	-0.229	0.853	4.278	-0.702
RP11-263K19.4	0.059	1.042	4.852	0.260
RP11-264A11.1	-0.018	0.987	2.998	-0.065
RP11-270M14.5	0.076	1.054	2.569	0.256
RP11-272L13.3	1.126	2.182	3.549	1.871
RP11-272P10.2	-0.185	0.879	2.503	-0.643
RP11-274H2.3	-0.201	0.870	2.478	-0.520
RP11-274J7.2	-0.714	0.610	4.113	-2.030
RP11-275H4.1	-0.023	0.984	2.462	-0.042
RP11-276M12.1	-0.400	0.758	2.366	-1.434
RP1-127H14.3	0.452	1.368	2.559	1.826
RP11-281A20.2	-0.861	0.550	2.077	-2.293
RP11-281O15.4	-0.027	0.982	3.174	-0.110
RP11-282K24.3	-0.009	0.994	4.588	-0.025
RP11-283C24.1	-0.969	0.511	7.689	-2.593
RP11-283G6.3	-0.195	0.874	4.719	-0.726
RP11-284M14.1	-0.292	0.817	4.524	-1.138
RP11-286E11.2	-0.653	0.636	2.672	-1.672
RP11-288G11.3	-1.707	0.306	5.981	-3.858
RP11-288I21.1	0.116	1.084	2.872	0.438
RP11-290D2.3	0.202	1.151	2.967	0.653
RP11-293M10.6	0.446	1.362	3.469	1.399
RP11-295K2.3	0.684	1.607	2.870	2.419
RP11-305F18.1	1.004	2.006	3.104	2.689
RP11-305L7.3	-0.295	0.815	3.010	-0.870
RP11-30J20.1	0.732	1.661	2.080	2.011
RP11-30K9.4	-0.333	0.794	2.421	-1.103

RP11-313F23.4	0.446	1.363	2.382	1.458
RP11-314N13.3	-0.381	0.768	3.773	-1.505
RP11-314P15.2	1.416	2.669	3.297	3.105
RP11-315E17.1	-0.807	0.572	3.990	-1.752
RP11-315I14.2//LOC101929563	0.828	1.775	2.898	2.089
RP11-316M1.12	-0.568	0.675	3.838	-1.858
RP11-317G6.1	0.618	1.535	3.059	1.956
RP11-318M2.3	0.609	1.526	4.897	2.172
RP11-319G6.3	-0.296	0.815	2.527	-0.822
RP11-320M2.1	0.025	1.018	2.504	0.080
RP11-320P7.2	-1.427	0.372	5.920	-1.512
RP11-324O2.6	0.329	1.256	2.421	1.111
RP11-326C3.11	0.081	1.058	4.086	0.285
RP11-326C3.7	0.557	1.471	2.362	1.446
RP11-326I11.4	0.419	1.337	4.785	1.661
RP11-32B5.7	-0.121	0.920	3.495	-0.337
RP11-334E6.3	0.809	1.752	2.159	2.264
RP11-334J6.6	-0.113	0.925	3.469	-0.351
RP11-337L12.1	0.520	1.434	2.049	1.733
RP11-33B1.3	-0.012	0.992	4.783	-0.042
RP11-342M1.3	-0.272	0.828	2.594	-1.210
RP11-344N10.5	0.120	1.087	3.178	0.408
RP11-348F1.3	0.248	1.188	2.745	0.766
RP11-350F4.2	0.308	1.238	4.216	1.229
RP11-353N4.5	-0.391	0.762	2.604	-0.933
RP11-355I22.2	0.944	1.924	2.559	2.915
RP11-356M20.3	-0.945	0.519	2.922	-2.489
RP11-359J14.2	0.391	1.311	4.011	1.378
RP11-360F5.3	-0.507	0.704	2.909	-1.455
RP11-370I10.6//LOC102723866	0.056	1.039	4.218	0.175
RP11-374A22.1	-0.817	0.568	3.748	-1.978
RP11-379B18.5//LOC101927056	-0.486	0.714	3.124	-1.548
RP11-380G5.2	0.230	1.173	2.563	0.730
RP11-381K20.2//LOC101928005	-0.506	0.704	4.712	-1.785
RP11-383B4.4	-0.535	0.690	2.737	-1.678
RP11-385D13.1	-0.231	0.852	2.673	-0.837
RP11-386G11.3	1.120	2.174	3.312	2.455
RP11-386G11.5	0.669	1.590	1.679	2.067
RP11-386G21.2	0.459	1.374	3.030	1.580
RP11-388P9.2	0.315	1.244	3.975	1.325
RP11-389O22.1	-0.621	0.650	3.665	-2.159
RP11-38P22.2	-0.413	0.751	3.718	-0.972
RP11-395I14.2	-0.134	0.911	2.432	-0.434
RP11-396B14.2	0.298	1.229	3.169	1.133
RP11-397D12.4	-0.190	0.877	3.802	-0.827
RP11-398K22.12	0.227	1.171	3.399	0.892
RP11-3D4.2	-0.220	0.858	3.175	-0.773
RP11-402N8.1//RP11-449H15.2	0.599	1.515	2.419	1.317
RP11-402N8.1//RP11-449H15.2	0.562	1.476	3.228	1.276
RP11-402N8.1//RP11-449H15.2	0.592	1.507	4.712	1.199

RP11-405A12.2	0.687	1.610	2.767	2.310
RP11-407G23.3	-0.366	0.776	4.111	-1.367
RP11-417L19.2	0.026	1.018	2.337	0.094
RP11-418I22.2//LOC101927026	-0.176	0.885	2.509	-0.671
RP11-430H10.4	0.531	1.444	2.058	1.710
RP11-432J22.2//LOC102724037	0.121	1.088	4.110	0.468
RP11-432M8.12	0.883	1.844	2.459	3.813
RP11-432M8.3//RP11-432M8.4//RP11-4	0.930	1.905	3.139	3.036
RP11-432M8.3//RP11-432M8.4//RP11-4	0.985	1.979	2.995	2.462
RP11-432M8.3//RP11-432M8.4//RP11-4	0.688	1.611	2.979	1.656
RP11-436D23.1	0.520	1.434	2.510	1.710
RP11-436H11.5	-0.072	0.951	4.309	-0.249
RP11-436I9.5	0.162	1.118	5.141	0.531
RP11-445H22.4	0.009	1.006	2.635	0.036
RP11-445N18.5	-0.398	0.759	2.790	-0.598
RP11-44N11.1	-0.740	0.599	3.055	-2.285
RP11-458D21.1//RP4-791M13.3	0.010	1.007	4.154	0.025
RP11-463O12.3	-0.334	0.793	3.257	-1.266
RP11-464F9.21	-0.050	0.966	2.222	-0.102
RP11-466L17.1	-0.421	0.747	3.059	-1.438
RP11-467H10.2	-0.046	0.969	2.589	-0.186
RP11-467K18.2	-0.043	0.970	2.332	-0.153
RP11-468H14.2	0.075	1.053	2.216	0.302
RP11-469A15.2	-0.709	0.612	2.615	-2.948
RP1-146A15.1	-0.697	0.617	4.492	-1.660
RP11-473I1.9	0.384	1.305	6.801	1.624
RP11-47I22.1	-0.870	0.547	3.656	-1.657
RP11-480I12.5	0.417	1.336	2.204	1.498
RP11-484O2.1	0.011	1.007	3.098	0.034
RP11-486G15.2	0.350	1.274	2.669	1.182
RP11-486O12.2	-0.619	0.651	2.862	-2.120
RP11-486O13.4	0.310	1.239	3.349	0.898
RP11-488C13.6	-0.368	0.775	3.120	-1.649
RP11-489O18.1	-0.917	0.530	3.778	-2.820
RP11-490H24.5	0.336	1.262	4.184	1.468
RP11-495K9.7	0.132	1.096	2.277	0.524
RP11-495O11.1	0.653	1.572	2.626	2.434
RP11-496D24.2	-0.860	0.551	2.649	-2.252
RP11-497E19.2	0.114	1.082	2.320	0.401
RP11-498J9.2	0.025	1.018	3.017	0.094
RP11-49O14.2	-0.436	0.739	3.078	-1.096
RP11-503E24.2	-0.161	0.894	4.577	-0.591
RP11-504P24.2	-0.629	0.647	4.842	-2.257
RP11-505E24.3	0.224	1.168	2.458	0.684
RP11-506M12.1	-0.412	0.751	2.552	-1.531
RP11-507B12.2//LOC101928828	0.108	1.078	2.605	0.447
RP11-507K2.3	0.199	1.148	2.965	0.813
RP11-509E10.1	-0.048	0.967	3.400	-0.127
RP11-509J21.2	-0.463	0.726	4.006	-1.035
RP11-513D5.5	-0.371	0.773	2.266	-1.479

RP11-513I15.6	0.641	1.559	4.913	2.065
RP11-514F3.5	-0.576	0.671	2.788	-1.728
RP11-51G5.1	0.592	1.508	2.348	1.490
RP11-521D12.1	-0.553	0.682	2.173	-1.197
RP11-527D7.1	0.481	1.396	2.996	1.988
RP11-527F13.1	0.383	1.304	2.211	1.334
RP11-531A24.5	0.048	1.034	4.271	0.176
RP11-536K7.3	-0.087	0.942	2.805	-0.348
RP11-539G18.2	-0.405	0.755	3.919	-1.856
RP11-539I5.1	0.235	1.177	5.818	0.904
RP11-543C4.1	0.528	1.442	2.429	1.963
RP11-555M1.3	0.167	1.122	2.147	0.464
RP11-556E13.1	-0.281	0.823	2.715	-0.867
RP11-556G22.3	0.643	1.562	2.700	2.372
RP11-561I11.3	-0.219	0.859	3.686	-0.541
RP11-561O23.8	0.888	1.851	4.416	2.062
RP11-562L8.1	1.166	2.244	1.488	3.396
RP11-563N6.6	-0.784	0.581	2.731	-1.986
RP11-56G10.2	0.491	1.406	3.953	1.719
RP11-574K11.5	0.586	1.501	3.916	2.148
RP11-579E24.2	0.767	1.702	2.496	2.308
RP11-57H14.2	-0.042	0.971	2.391	-0.147
RP11-582J16.4	0.662	1.582	4.814	2.322
RP11-586K2.1	0.758	1.691	3.100	2.386
RP11-588P7.1	0.339	1.265	4.903	0.665
RP11-589M4.1	-0.031	0.979	2.512	-0.102
RP11-594C13.1	0.654	1.573	2.239	1.528
RP11-597D13.7	-1.213	0.431	5.309	-2.217
RP11-5L12.1	-0.533	0.691	2.253	-1.432
RP11-5N11.2	0.049	1.034	3.121	0.169
RP11-5N11.3	0.454	1.369	1.886	1.353
RP11-5N11.7	0.323	1.251	2.563	1.033
RP11-603J24.17	-0.164	0.892	3.767	-0.605
RP11-605F22.2	1.003	2.004	1.992	3.243
RP11-617F23.1	0.221	1.166	5.582	0.775
RP11-624C23.1	0.794	1.733	2.126	2.760
RP11-624G17.3	-0.237	0.849	2.596	-0.933
RP11-627D16.1	-1.716	0.304	4.712	-2.467
RP11-631M21.1//AP005530.2	-0.982	0.506	3.227	-1.569
RP11-638I2.2	-0.156	0.897	2.475	-0.476
RP11-642D21.1	0.192	1.142	3.107	0.503
RP11-644F5.11	-0.349	0.785	3.898	-1.471
RP11-64B16.3	-0.648	0.638	3.285	-2.701
RP11-64K12.4	0.388	1.309	3.159	1.421
RP11-653B10.1	0.930	1.906	1.812	3.133
RP11-660L16.2	-0.263	0.833	3.409	-0.947
RP11-661A12.5	-0.270	0.829	3.897	-1.039
RP11-666A20.4	-0.001	0.999	3.638	-0.002
RP11-66B24.4	1.274	2.418	2.810	3.336
RP11-66N11.8	-0.294	0.816	2.289	-1.166

RP11-672F9.1	0.255	1.194	4.211	0.806
RP11-673E1.4	1.047	2.067	4.880	2.846
RP11-675F6.3	0.724	1.651	2.816	1.814
RP11-678G14.3	0.247	1.187	3.025	0.965
RP1-167F1.2//LOC102724609	-0.573	0.672	2.530	-2.035
RP11-680F8.4	-0.755	0.593	4.163	-1.815
RP11-685N10.1	-0.405	0.755	2.865	-0.905
RP11-688I9.4	-0.705	0.614	4.998	-1.725
RP11-691N7.6	-0.164	0.893	2.680	-0.550
RP11-692D12.1	-0.268	0.830	3.741	-1.015
RP11-696P8.2	0.452	1.368	3.299	1.382
RP11-69A21.2	0.050	1.035	2.502	0.147
RP11-701H24.3	0.634	1.551	3.421	1.495
RP11-706O15.1	0.289	1.221	2.524	0.819
RP11-706O15.7	0.504	1.418	2.460	1.257
RP11-707G14.1	0.361	1.285	5.520	1.405
RP11-707G14.7	-0.334	0.794	2.177	-1.294
RP11-707P17.2	-0.728	0.604	2.929	-2.064
RP11-70C1.3	-0.025	0.983	2.835	-0.096
RP11-70J12.1	-0.562	0.678	4.097	-1.195
RP11-711K1.7	0.484	1.399	2.178	1.745
RP11-712L6.7	0.122	1.088	2.106	0.445
RP11-716H6.1	-0.408	0.754	2.862	-1.507
RP11-71N10.1	-0.752	0.594	3.119	-2.147
RP11-723D22.2	-0.142	0.906	3.026	-0.527
RP11-728F11.4	0.237	1.179	2.795	0.928
RP11-736K20.5	0.560	1.474	2.675	2.631
RP11-737F9.2	-0.323	0.799	3.233	-1.083
RP11-739P1.2	-0.469	0.722	4.587	-1.439
RP11-73E17.2	-0.298	0.813	2.209	-1.155
RP11-745L13.2	0.239	1.180	6.193	0.872
RP11-745L13.2	0.355	1.279	2.756	0.825
RP11-760H22.2	-0.688	0.621	3.071	-1.998
RP11-762H8.4	0.293	1.225	3.639	1.305
RP11-768G7.2	-0.103	0.931	4.778	-0.373
RP11-769N19.2	0.389	1.309	2.250	1.268
RP11-774O3.3	-0.194	0.874	4.953	-0.737
RP11-776H12.1	-0.679	0.625	3.094	-2.063
RP11-77P16.4	-0.038	0.974	2.818	-0.102
RP11-77P6.2	-0.090	0.940	3.123	-0.356
RP11-783K16.10//KCNK4	0.012	1.009	2.542	0.034
RP11-785H5.2	-0.492	0.711	4.412	-1.388
RP11-798M19.3	0.370	1.292	2.150	1.373
RP11-815J21.1	-0.140	0.908	2.407	-0.411
RP11-815J21.2	-0.084	0.943	2.673	-0.289
RP11-819C21.1	0.429	1.346	3.533	2.176
RP11-81K13.1	0.886	1.847	2.197	3.025
RP11-82L18.2	0.389	1.309	3.227	1.527
RP11-839G9.1	0.971	1.961	2.005	3.204
RP11-841O20.2	0.695	1.619	4.592	1.972

RP11-85M11.2	0.527	1.440	2.420	2.175
RP11-85O21.5	0.556	1.470	2.632	1.916
RP11-861E21.2	-0.623	0.649	2.920	-1.630
RP11-862L9.3	-0.619	0.651	3.437	-1.642
RP11-864N7.2	-0.386	0.765	8.365	-1.668
RP11-867G2.8	0.472	1.387	2.854	1.492
RP11-867G23.12	-0.120	0.920	2.176	-0.515
RP11-867G23.8//LOC102724064	-0.122	0.919	3.129	-0.474
RP11-86H7.7//LOC102723542	-0.088	0.941	3.436	-0.259
RP1-187B23.1	-0.360	0.779	2.369	-1.102
RP11-883G14.1	-0.132	0.912	2.714	-0.515
RP11-897M7.1	-0.277	0.825	3.664	-1.231
RP11-90D4.3	0.289	1.222	2.409	0.815
RP11-90J19.1	0.093	1.066	6.744	0.339
RP11-946L20.2//LOC101927459	-0.883	0.542	3.704	-1.831
RP11-950C14.3	0.076	1.054	3.342	0.271
RP11-956J14.1	-0.020	0.986	2.173	-0.064
RP11-958J22.1	0.320	1.248	2.703	0.847
RP11-966I7.1	0.833	1.781	2.097	1.876
RP11-977B10.2	-0.362	0.778	2.771	-1.189
RP11-989F5.3	-0.018	0.987	4.155	-0.072
RP11-99H8.1	0.160	1.117	2.327	0.603
RP11-9L18.3	0.087	1.062	2.970	0.300
RP11-9M16.2	-0.360	0.779	3.009	-1.239
RP1-20N2.6//FUCA2	0.404	1.323	2.736	1.277
RP1-212P9.2	0.853	1.807	1.915	2.413
RP1-239B22.5	0.952	1.935	2.175	2.581
RP1-249F5.3	0.667	1.588	2.276	1.854
RP1-251M9.2	1.028	2.040	1.718	2.840
RP1-269M15.3	1.603	3.038	3.720	2.622
RP1-293L6.1	0.340	1.266	3.302	1.205
RP13-216E22.4	-0.263	0.834	4.766	-0.703
RP13-317D12.3	0.260	1.197	3.563	0.882
RP13-401N8.1	-0.520	0.697	3.301	-1.345
RP13-578N3.3	1.329	2.512	1.870	2.542
RP13-635I23.3	-0.194	0.874	2.315	-0.719
RP1-45C12.1	-0.120	0.920	2.826	-0.484
RP1-46F2.3	-0.251	0.840	2.192	-0.627
RP1-5O6.4	-0.432	0.741	4.266	-1.331
RP1-69D17.4	0.171	1.126	2.337	0.598
RP1-8B1.4//LOC102725160	0.453	1.368	3.231	1.625
RP1-90G24.10	0.226	1.169	4.293	0.540
RP1-90G24.6	0.856	1.810	1.732	2.171
RP1-97G4.1	0.358	1.282	2.746	1.078
RP2	-0.326	0.798	5.244	-1.211
RP3-323P24.3	-0.097	0.935	3.509	-0.315
RP3-325F22.5	-0.378	0.769	2.719	-1.352
RP3-368A4.5	-0.451	0.732	6.971	-1.841
RP3-377H17.2	0.656	1.576	3.006	2.149
RP3-380B8.4	0.360	1.284	2.333	1.308

RP3-395P12.2	0.158	1.116	2.314	0.536
RP3-404F18.5	-0.399	0.759	3.911	-1.496
RP3-405J10.3	-0.295	0.815	3.055	-1.038
RP3-418C23.2//LOC101927314	-0.417	0.749	3.413	-1.604
RP3-428L16.1	0.598	1.513	2.240	2.126
RP3-462E2.3	0.084	1.060	3.388	0.297
RP3-508I15.18//RP3-508I15.19	-0.601	0.659	4.282	-2.208
RP3-510D11.1	0.108	1.078	3.051	0.403
RP3-525N10.2	0.232	1.174	7.264	0.853
RP4-537K23.4	-0.127	0.916	2.769	-0.397
RP4-541C22.5	1.139	2.203	1.918	2.834
RP4-564M11.2	1.207	2.309	3.143	2.873
RP4-565E6.1	-0.266	0.832	7.305	-0.943
RP4-580N22.2	-0.348	0.786	2.797	-1.570
RP4-613B23.1//LOC101928323	-0.575	0.671	4.395	-1.901
RP4-630C24.3	-0.983	0.506	4.729	-1.848
RP4-647C14.3	0.072	1.051	2.939	0.235
RP4-666F24.3	-0.139	0.908	1.918	-0.349
RP4-758J18.7	-0.468	0.723	3.597	-1.800
RP4-771M4.3	-0.467	0.723	2.809	-1.266
RP4-782G3.1	1.229	2.344	3.983	2.770
RP4-784A16.5	0.114	1.082	3.408	0.294
RP4-791M13.3	-0.487	0.713	7.501	-1.739
RP4-803J11.2	-0.191	0.876	2.348	-0.809
RP4-809F18.2	0.682	1.604	1.660	2.341
RP5-1031D4.2	-0.201	0.870	2.396	-0.698
RP5-1050D4.2	0.520	1.434	3.094	1.909
RP5-1056L3.1	-0.352	0.784	2.966	-1.303
RP5-1104E15.6	-0.359	0.780	2.887	-1.400
RP5-1157M23.2//LOC101929054	-0.010	0.993	3.264	-0.032
RP5-1160K1.8	-0.106	0.929	2.674	-0.370
RP5-1172N10.2	0.210	1.157	2.797	0.823
RP5-1177I5.3	-0.577	0.670	2.381	-1.996
RP5-827C21.2	0.770	1.706	2.757	2.302
RP5-857K21.1	0.302	1.233	2.937	0.910
RP5-884C9.2	0.177	1.130	3.093	0.671
RP5-940J5.3	-0.767	0.588	3.090	-2.841
RP5-965F6.2	-0.227	0.854	3.256	-0.722
RP5-968J1.1	0.185	1.137	2.774	0.514
RP6-109B7.4	-0.498	0.708	2.850	-1.831
RPARP-AS1//RP11-18I14.10	0.591	1.507	3.882	1.972
RPE65	0.331	1.258	3.571	0.654
RPGR	-0.036	0.976	4.550	-0.131
RPGRIP1L	0.620	1.537	4.873	1.914
RPH3A	0.919	1.891	5.867	1.918
RPL12	-0.053	0.964	2.582	-0.202
RPL12//RPL12P4//NBEAL1//RPL12P8//A	-0.158	0.896	9.898	-0.831
RPL13//SNORD68	-0.114	0.924	3.725	-0.455
RPL13A//SNORD35A//SNORD34//SNORI	-0.033	0.977	2.974	-0.135
RPL13AP20//RP11-392P7.2	-0.134	0.912	7.186	-0.593

RPL19	-0.274	0.827	8.464	-1.415
RPL19P12	-0.090	0.939	3.346	-0.288
RPL23AP32	-0.267	0.831	5.765	-0.840
RPL23AP53	0.407	1.326	4.469	1.216
RPL23AP60//XX-C2158C6.2//AL672183.:	0.629	1.546	5.582	2.056
RPL23AP7//RPL23AP82//AC002055.4	1.200	2.298	3.415	2.986
RPL29P2//RP11-199F11.1	-0.370	0.774	3.757	-1.707
RPL39	-0.344	0.788	4.088	-1.594
RPLP0	-0.461	0.726	3.139	-2.345
RPP14	0.552	1.466	4.440	2.067
RPP30	0.352	1.276	4.411	1.747
RPP40	0.305	1.236	3.041	1.180
RPRM	0.666	1.587	3.546	2.903
RPRML	0.970	1.959	2.703	2.458
RPS15A	-0.179	0.883	4.939	-0.558
RPS15AP10	-0.111	0.926	3.208	-0.403
RPS19	-0.478	0.718	8.146	-2.035
RPS2//SNORA64//AC005363.9	-0.442	0.736	3.371	-1.365
RPS2//SNORA64//RPS2P55//RPS2P46//I	-0.229	0.853	9.475	-0.930
RPS2//SNORA64//RPS2P7//RPS2P46//RI	-0.095	0.936	10.794	-0.436
RPS25	-0.214	0.862	5.080	-0.797
RPS26//LOC100996747//LOC10192987€	0.320	1.248	3.171	1.304
RPS27	-0.263	0.833	10.423	-1.473
RPS5	-0.574	0.672	8.619	-2.913
RPS6KA2-IT1	-0.563	0.677	4.401	-2.094
RPS6KA6	0.447	1.363	4.596	1.247
RPS6KC1	0.440	1.357	6.056	2.368
RPS6KL1	0.179	1.132	4.290	0.696
RPSAP52	-0.190	0.877	2.185	-0.488
RPSAP58	-0.269	0.830	2.158	-0.976
RPUSD1	0.070	1.050	3.996	0.283
RRAD	-0.433	0.741	2.720	-1.262
RRAGB	0.226	1.170	5.179	1.002
RRAS	-0.106	0.929	6.426	-0.442
RRBP1	-0.639	0.642	6.778	-2.406
RREB1	-0.349	0.785	5.178	-1.584
RRH	0.623	1.541	2.448	2.472
RRM2B	0.229	1.172	5.949	0.922
RRN3	0.464	1.380	5.696	1.653
RRN3P2	-0.270	0.830	3.239	-0.596
RRN3P3//LOC101060405//RP11-21M24	0.242	1.183	4.424	0.703
RRNAD1	-0.494	0.710	3.852	-2.139
RRP12	0.089	1.064	4.848	0.320
RRP7B	-0.786	0.580	3.069	-1.107
RSAD2	0.427	1.344	2.659	1.385
RSC1A1//DDI2	0.143	1.105	5.579	0.614
RSPH1	0.200	1.149	3.721	0.587
RSPH3	0.325	1.253	5.519	1.416
RSPH4A	-0.099	0.934	2.936	-0.225
RSPO2	1.161	2.236	3.821	2.547

RSPO3	1.550	2.928	2.748	4.173
RTCA	0.271	1.207	5.284	1.208
RTF1	0.529	1.443	7.159	1.982
RTKN	-1.114	0.462	5.907	-3.138
RTKN2	0.550	1.464	1.920	1.516
RTN1	0.636	1.554	7.437	2.310
RTN2	0.609	1.525	4.283	1.620
RTN3	0.327	1.254	9.054	1.689
RTN4IP1	0.052	1.037	4.000	0.234
RTN4R	0.353	1.277	3.171	1.294
RTN4RL1	1.258	2.392	4.439	2.480
RTN4RL2	0.449	1.365	2.474	1.180
RTP1	0.529	1.443	1.938	1.500
RTP4	-0.242	0.846	3.009	-0.853
RTP5//CXXC11	0.325	1.253	3.057	1.170
RTTN	-0.649	0.638	5.847	-1.991
RUNDC1	0.383	1.304	5.489	1.543
RUNDC3A	0.914	1.884	7.695	2.369
RUNDC3B	0.531	1.445	4.293	1.557
RUNX1-IT1	-0.356	0.781	2.608	-1.049
RUNX1T1	0.608	1.524	4.538	2.209
RUNX2	-0.145	0.904	2.741	-0.611
RUSC1	0.430	1.347	3.950	1.748
RUSC2	0.357	1.281	6.591	1.651
RWDD2A	0.557	1.471	5.391	2.192
RWDD2B	0.516	1.430	3.281	1.450
RXFP1	2.521	5.741	2.785	3.948
RXRG	-0.738	0.600	4.425	-3.638
RYR2	1.285	2.436	5.987	2.612
RYR3	-0.363	0.777	6.445	-1.431
S100A1	-0.161	0.894	4.044	-0.694
S100A10	-0.360	0.779	3.344	-1.399
S100A11	0.039	1.027	4.012	0.119
S100A12	-0.581	0.668	4.126	-0.696
S100A16	-0.102	0.932	4.555	-0.378
S100A3	0.554	1.468	2.429	1.590
S100A6	-0.248	0.842	4.850	-1.257
S100A8	0.229	1.172	4.901	0.380
S100A9	0.066	1.047	5.300	0.101
S100B	-0.967	0.512	10.047	-2.507
S100PBP	-0.396	0.760	3.812	-1.461
S1PR5	-0.935	0.523	5.321	-2.533
SACS	0.395	1.315	7.228	1.915
SAE1	0.245	1.185	3.460	1.054
SALL1	-1.015	0.495	6.244	-4.053
SALL2	-0.548	0.684	6.064	-2.030
SALL3	-0.411	0.752	3.980	-1.340
SAMD1	-0.419	0.748	5.210	-2.081
SAMD11	-0.340	0.790	3.820	-1.196
SAMD12	0.355	1.279	5.598	1.547

SAMD15	0.081	1.058	3.025	0.321
SAMD4A	-0.586	0.666	5.368	-2.230
SAMD5	0.394	1.314	2.933	1.370
SAMD9	-0.100	0.933	3.854	-0.449
SAMD9L	-0.259	0.835	4.919	-0.857
SAMHD1	-0.481	0.716	7.676	-2.005
SAP25//LRCH4	-0.562	0.677	3.201	-1.840
SAPCD1//MSH5-SAPCD1	-0.215	0.861	3.174	-0.767
SAPCD1-AS1//XXbac-BPG32J3.18	0.028	1.020	2.377	0.099
SASH1	-0.478	0.718	8.037	-1.926
SASH3	-0.559	0.679	4.113	-1.735
SAT1	0.305	1.236	7.847	1.080
SATB1	0.579	1.494	5.850	2.356
SATB2	1.123	2.178	6.541	2.547
SATL1	0.282	1.215	2.750	0.846
SBK1	0.608	1.524	4.124	1.639
SBNO1	0.286	1.220	7.469	1.457
SBNO2	-0.077	0.948	4.782	-0.262
SBSPON	-1.281	0.412	6.021	-2.020
SC22CB-1E7.1	-0.390	0.763	5.696	-1.457
SCAI	0.953	1.937	5.646	3.042
SCAMP1	0.384	1.305	7.740	1.774
SCAMP2	-0.706	0.613	6.101	-2.759
SCAMP4	-0.590	0.664	4.356	-2.362
SCAMP5	0.659	1.579	7.671	2.117
SCARA3	0.500	1.414	7.427	1.595
SCARNA1	-0.152	0.900	2.169	-0.649
SCARNA10	-0.606	0.657	8.615	-2.183
SCARNA11	-0.062	0.958	6.856	-0.171
SCARNA14	0.097	1.070	3.203	0.250
SCARNA2	-0.390	0.763	5.708	-1.552
SCARNA20	-0.148	0.902	4.436	-0.359
SCARNA21	0.252	1.191	5.237	0.800
SCARNA22	0.314	1.243	5.650	1.108
SCARNA27	0.113	1.081	5.216	0.342
SCARNA3	-0.249	0.842	6.747	-0.810
SCARNA4	0.208	1.155	7.685	0.543
SCARNA5	0.259	1.197	8.099	0.423
SCARNA6	-0.172	0.888	7.408	-0.798
SCARNA7	-0.255	0.838	7.772	-0.962
SCARNA8	0.185	1.137	4.759	0.587
SCART1//RP11-108K14.4	-0.023	0.984	2.695	-0.082
SCCPDH	-0.617	0.652	7.533	-2.463
SCD	-0.950	0.518	10.462	-3.063
SCFD2	0.195	1.144	6.677	0.706
SCG2	0.293	1.225	5.299	0.718
SCG3	0.582	1.497	7.889	1.812
SCG5	0.389	1.310	4.919	1.488
SCIMP	-0.509	0.703	4.037	-1.199
SCIN	-0.619	0.651	5.881	-1.169

SCML1	0.585	1.500	3.498	2.462
SCML2	0.224	1.168	2.302	1.086
SCN11A	-0.452	0.731	2.374	-1.254
SCN1A	1.288	2.442	6.693	2.353
SCN1B	0.747	1.678	6.831	2.158
SCN2A	1.718	3.289	5.957	2.743
SCN2B	1.078	2.111	6.114	2.314
SCN3A	0.258	1.196	6.065	0.661
SCN3B	1.435	2.703	5.589	2.486
SCN4B	0.798	1.739	4.726	1.907
SCN7A	-0.198	0.872	3.828	-0.396
SCN8A	1.474	2.777	6.601	2.306
SCN8A	1.393	2.627	5.392	2.013
SCN9A	-0.250	0.841	3.898	-0.688
SCNN1D	-0.125	0.917	3.476	-0.458
SCOC-AS1//RP11-425I13.3	-0.460	0.727	2.775	-1.536
SCRT1	1.456	2.744	3.359	2.563
SCRT2	0.244	1.185	2.961	0.945
SCUBE1	-0.152	0.900	2.886	-0.491
SCUBE2	-0.426	0.744	3.819	-1.501
SCX//SCXB	-0.086	0.942	2.771	-0.211
SDAD1	0.398	1.318	6.412	1.945
SDC4//ROS1	-0.409	0.753	8.065	-0.980
SDCBP	0.271	1.206	5.143	1.057
SDCBP2//FKBP1A-SDCBP2	0.743	1.674	3.802	2.833
SDCBP2-AS1	0.097	1.070	3.443	0.360
SDF2L1	-0.529	0.693	4.246	-1.502
SDHAP1//SDHAP2	-0.034	0.977	5.069	-0.091
SDHAP2//LINC00969	-0.445	0.735	4.665	-1.268
SDHAP2//LINC00969	-0.742	0.598	4.890	-0.959
SDHAP2//LINC00969	-0.230	0.853	4.410	-0.712
SDHAP2//LINC00969//LOC102725383	-0.498	0.708	3.917	-1.459
SDHC//RP11-122G18.5	0.149	1.109	4.655	0.610
SDIM1	0.960	1.945	2.019	3.594
SDPR	0.018	1.013	5.365	0.051
SDR16C5	0.994	1.991	2.618	2.754
SDS	0.347	1.271	3.240	1.294
SDSL	-0.393	0.762	4.855	-1.503
SEC11A	-0.331	0.795	4.661	-1.519
SEC11C	-0.521	0.697	6.898	-2.240
SEC14L1P1	0.187	1.138	2.925	0.446
SEC14L5	-0.441	0.736	6.002	-1.121
SEC14L6	-0.180	0.883	2.790	-0.518
SEC16B//RP4-798P15.3	0.613	1.529	3.211	2.828
SEC16B//RP4-798P15.3	-0.565	0.676	2.527	-2.119
SEC16B//RP4-798P15.3	-0.270	0.829	2.552	-0.673
SEC23A	0.435	1.352	6.459	2.042
SEC24D	0.693	1.616	5.004	2.308
SECTM1	0.404	1.323	4.280	0.859
SEH1L	0.452	1.368	7.851	1.736

SEL1L3	0.558	1.472	4.848	1.762
SELE	-0.049	0.966	2.840	-0.057
SELENBP1	-0.202	0.869	4.908	-0.727
SELL	-0.804	0.573	4.967	-2.212
SELPLG	-1.131	0.457	5.167	-3.290
SEMA3A	0.685	1.607	4.739	2.599
SEMA3B//MIR6872	-0.948	0.518	6.835	-1.985
SEMA3B-AS1//U73167.7	-0.412	0.752	3.210	-1.119
SEMA3C	-0.301	0.812	5.955	-1.096
SEMA3D	-0.173	0.887	4.942	-0.564
SEMA3E	0.480	1.395	5.108	1.920
SEMA3F	0.097	1.069	3.025	0.331
SEMA3G	0.354	1.278	3.293	1.275
SEMA4B	-0.395	0.761	4.236	-1.685
SEMA4C	-0.449	0.733	5.029	-1.933
SEMA4D	-0.905	0.534	5.951	-2.636
SEMA4F	0.253	1.191	4.548	0.887
SEMA4G	0.236	1.177	3.499	0.868
SEMA5A	-0.449	0.732	6.526	-1.930
SEMA5B	0.254	1.192	4.002	0.911
SEMA6A	-0.895	0.538	6.096	-2.886
SEMA6B	1.039	2.055	5.379	2.770
SEMA6C	0.527	1.441	2.544	2.154
SEMA6D	-0.200	0.870	6.870	-0.943
SENP5	0.197	1.146	6.856	1.051
SEPHS2	0.516	1.430	5.290	2.690
SEPN1	-0.247	0.843	7.040	-1.149
SEPP1	-1.263	0.417	4.850	-3.522
SEPSECS	-0.372	0.773	5.822	-1.401
SEPT10	-0.703	0.614	6.167	-1.999
SEPT11	0.468	1.383	6.045	2.076
SEPT12	-0.046	0.968	2.612	-0.176
SEPT3	0.934	1.910	9.240	2.413
SEPT4	-0.500	0.707	7.136	-1.692
SEPT5-GP1BB//GP1BB//SEPT5	0.671	1.592	5.393	1.639
SEPT6	0.278	1.213	5.135	1.278
SEPT9	0.057	1.041	6.935	0.303
SERAC1	0.011	1.008	5.098	0.055
SERGEF	0.270	1.206	4.367	1.230
SERHL	-0.618	0.652	2.408	-1.512
SERINC2	-0.205	0.867	3.230	-0.999
SERINC5	-0.741	0.598	6.890	-2.733
SERP2	0.256	1.194	4.568	1.147
SERPINA1	-0.126	0.917	2.710	-0.291
SERPINA3//RP11-986E7.7	0.775	1.711	5.019	1.148
SERPINA5	0.236	1.178	2.445	0.712
SERPINB1	0.085	1.061	5.535	0.235
SERPINB6	-0.559	0.679	5.453	-2.468
SERPINB8	-0.233	0.851	3.458	-0.694
SERPINB9	0.021	1.015	6.070	0.080

SERPINB9P1//RP11-420G6.4	-0.136	0.910	3.427	-0.421
SERPIND1	0.549	1.463	2.846	1.224
SERPINE1	-0.994	0.502	4.291	-1.426
SERPINF1	1.135	2.196	6.405	1.883
SERPING1	0.297	1.229	6.374	1.103
SERPINH1	-0.313	0.805	5.473	-0.754
SERPINI1	0.635	1.553	8.433	2.074
SERPINI2	-0.836	0.560	2.971	-2.087
SERTAD4	0.360	1.283	3.462	1.388
SERTAD4-AS1	-0.876	0.545	3.823	-2.924
SERTM1	1.439	2.711	4.553	2.533
SESN1	0.205	1.153	6.723	0.882
SESN2	0.364	1.287	4.575	1.165
SETBP1	0.667	1.588	4.613	2.452
SETDB2	0.077	1.055	4.969	0.348
SEZ6	0.358	1.282	3.939	1.110
SEZ6L	0.405	1.324	6.413	1.188
SEZ6L2	0.602	1.518	6.633	1.713
SFMBT2	-0.591	0.664	6.534	-2.848
SFN	0.817	1.762	4.245	1.544
SFR1	-0.527	0.694	3.669	-1.556
SFRP1	-0.467	0.723	4.223	-1.976
SFRP2	-0.326	0.797	5.544	-0.659
SFRP4	-0.144	0.905	2.931	-0.577
SFRP5	-0.279	0.824	3.379	-0.557
SFT2D2	-0.367	0.775	6.747	-1.563
SGCZ	1.021	2.029	3.802	2.853
SGIP1	0.334	1.261	6.555	1.650
SGK1	-1.012	0.496	6.468	-2.721
SGK2	-0.896	0.537	4.459	-1.774
SGK223//PRAGMIN	0.544	1.458	4.267	1.815
SGK494	0.189	1.140	4.444	0.763
SGMS2	-0.148	0.902	3.431	-0.609
SGPP2	0.320	1.248	4.886	0.741
SGSM1	0.432	1.349	5.091	1.214
SGSM2	0.482	1.396	6.680	1.798
SGTB	0.722	1.649	7.721	2.416
SH2B2	0.159	1.116	3.185	0.673
SH2D5	0.348	1.273	3.047	1.193
SH3BGR	-0.364	0.777	4.925	-1.174
SH3BGRL2	0.632	1.550	8.616	2.058
SH3BGRL3	-0.369	0.775	5.093	-1.846
SH3BP1	-0.110	0.927	2.605	-0.404
SH3BP2	-0.138	0.909	3.438	-0.540
SH3BP4	-0.689	0.620	4.450	-2.834
SH3BP5-AS1	-0.697	0.617	3.826	-2.279
SH3D21	0.144	1.105	3.844	0.414
SH3GL1	0.029	1.020	3.133	0.103
SH3GL2	1.395	2.630	7.047	2.593
SH3PXD2B	-0.401	0.757	5.465	-1.505

SH3RF2	0.646	1.565	2.118	2.424
SH3TC1	-0.482	0.716	3.085	-2.014
SH3TC2	-1.193	0.437	4.977	-2.506
SHANK1	1.194	2.288	4.431	2.254
SHANK2	0.360	1.284	2.546	1.244
SHANK2	-0.011	0.992	2.275	-0.048
SHANK3	0.919	1.890	5.457	2.095
SHB//RP11-613M10.9	-0.330	0.795	4.909	-1.246
SHC1	0.114	1.082	5.744	0.487
SHC3	1.360	2.567	6.587	2.726
SHC4	-1.042	0.486	5.350	-2.651
SHD	0.165	1.121	3.120	0.555
SHISA2	-0.735	0.601	3.631	-2.193
SHISA3	-0.036	0.976	2.754	-0.136
SHISA5	0.360	1.283	3.446	1.467
SHISA6	-0.882	0.543	6.950	-1.736
SHISA7	0.220	1.165	4.415	0.801
SHISA9	0.723	1.650	4.770	1.845
SHKBP1	-0.574	0.672	5.219	-2.134
SHMT1//MIR6778	-0.148	0.902	4.167	-0.609
SHROOM1	-1.154	0.449	4.872	-4.059
SHROOM2	0.552	1.466	3.075	2.219
SHROOM3	-0.121	0.920	3.631	-0.417
SHROOM4	-0.806	0.572	4.958	-2.205
SIAH2	0.198	1.147	5.515	0.761
SIAH3	0.905	1.873	3.268	2.084
SIDT1	0.324	1.252	4.666	1.017
SIDT2	-0.577	0.670	6.211	-2.500
SIGLEC10	-0.485	0.714	5.213	-1.068
SIGLEC11	0.404	1.323	2.317	1.567
SIGLEC14	0.022	1.015	3.153	0.047
SIGLEC7	-0.253	0.839	3.626	-0.578
SIGLEC8	-0.806	0.572	4.363	-2.355
SIGLEC9	0.283	1.216	4.027	0.582
SIK1	0.400	1.320	5.212	0.988
SIK3-IT1	-0.462	0.726	3.837	-1.170
SIN3B	0.447	1.363	6.214	2.034
SIPA1L1	0.443	1.360	6.683	1.754
SIRPA	0.495	1.409	7.787	2.360
SIRPB2	-0.393	0.762	3.788	-1.347
SIRT2	-0.827	0.564	5.615	-3.053
SIRT3	0.244	1.185	4.022	1.212
SIRT4	0.347	1.272	1.992	1.378
SIRT5	0.582	1.497	5.006	2.649
SIX4	0.519	1.433	2.766	1.794
SIX5	-0.118	0.921	2.226	-0.446
SKAP2	-0.310	0.807	6.412	-1.498
SLA	-0.189	0.877	4.911	-0.537
SLAIN1	-0.808	0.571	7.776	-2.450
SLAMF8	0.481	1.396	4.386	0.882

SLBP	0.252	1.191	5.776	1.046
SLC10A4	-0.352	0.784	3.653	-0.558
SLC10A5	0.262	1.199	2.763	0.949
SLC11A1	-0.350	0.785	4.777	-0.557
SLC12A4	-0.071	0.952	6.218	-0.282
SLC12A5	1.370	2.585	6.183	1.800
SLC12A8	-0.045	0.970	3.258	-0.156
SLC12A9	-0.420	0.748	3.480	-1.937
SLC13A3	-0.636	0.643	5.129	-1.555
SLC13A4	1.369	2.583	3.605	2.092
SLC13A5	0.870	1.827	3.932	2.212
SLC14A1	-1.031	0.489	9.047	-1.933
SLC15A2	0.175	1.129	5.644	0.627
SLC15A3	-0.336	0.792	3.918	-1.425
SLC16A10	0.450	1.366	4.225	1.906
SLC16A12	0.688	1.611	2.373	1.718
SLC16A14	0.618	1.535	4.077	1.942
SLC16A3//MIR6787	-0.340	0.790	5.227	-0.882
SLC16A4	-0.267	0.831	4.842	-0.999
SLC16A6	0.041	1.028	4.195	0.116
SLC16A7	0.352	1.276	4.531	1.250
SLC16A9	-0.698	0.617	5.381	-2.054
SLC17A5	0.284	1.218	5.835	1.121
SLC17A6	-0.123	0.918	5.175	-0.170
SLC17A7	3.036	8.199	5.603	3.410
SLC17A8	0.444	1.360	2.429	1.837
SLC18A3	-0.272	0.828	3.371	-0.608
SLC19A2	0.407	1.326	5.431	1.497
SLC1A1	0.729	1.658	5.933	2.151
SLC1A2	0.749	1.681	9.287	2.475
SLC1A5	-0.442	0.736	4.644	-1.185
SLC1A6	0.632	1.549	2.687	1.942
SLC22A10	0.671	1.592	1.773	2.186
SLC22A15	-0.590	0.664	5.450	-2.190
SLC22A3	-0.535	0.690	3.410	-1.813
SLC22A4	0.101	1.072	3.547	0.367
SLC22A6	0.074	1.052	3.422	0.227
SLC22A9	0.472	1.387	2.736	1.585
SLC24A1	-0.096	0.936	3.122	-0.325
SLC24A2	0.097	1.069	9.042	0.474
SLC24A3	0.017	1.012	5.111	0.052
SLC24A4	-0.716	0.609	4.168	-2.063
SLC25A1	-0.857	0.552	5.905	-3.497
SLC25A12	0.885	1.846	6.238	2.440
SLC25A13	-0.851	0.554	5.431	-2.227
SLC25A14	0.435	1.352	5.344	1.714
SLC25A16//LINC00849	0.517	1.431	3.413	2.217
SLC25A18	0.288	1.221	6.631	0.957
SLC25A19	0.182	1.134	4.559	0.510
SLC25A22	1.054	2.076	4.421	3.392

SLC25A23	0.462	1.378	7.742	2.084
SLC25A27	0.143	1.104	7.077	0.676
SLC25A29	-0.692	0.619	6.243	-2.384
SLC25A3	0.274	1.209	4.121	0.676
SLC25A32	0.279	1.213	5.670	1.181
SLC25A37	-0.075	0.949	6.174	-0.265
SLC25A4	0.407	1.326	8.151	1.806
SLC25A40	0.570	1.484	5.440	2.006
SLC25A44	0.209	1.156	6.021	1.001
SLC25A46	0.414	1.332	6.117	2.210
SLC25A48	-0.756	0.592	4.020	-1.892
SLC26A4	1.110	2.158	2.817	3.127
SLC26A4-AS1	1.975	3.931	3.706	3.310
SLC26A8	0.791	1.731	3.070	2.383
SLC27A1	-0.480	0.717	5.719	-2.137
SLC27A2	1.271	2.414	2.599	2.915
SLC27A6	-0.704	0.614	3.679	-1.779
SLC29A1	0.263	1.200	4.925	1.138
SLC29A2	-0.325	0.798	4.606	-1.166
SLC29A3	-0.838	0.559	4.822	-3.040
SLC29A4	0.341	1.267	4.754	0.826
SLC2A12	0.579	1.494	4.855	1.584
SLC2A13	1.245	2.370	5.826	2.632
SLC2A3	0.811	1.754	5.126	1.628
SLC2A4RG	-0.506	0.704	4.357	-2.359
SLC2A5	-0.269	0.830	4.204	-0.789
SLC30A3	1.039	2.055	3.226	3.117
SLC30A4	0.199	1.148	4.956	0.844
SLC30A9	0.350	1.275	7.228	1.605
SLC31A1	0.266	1.203	6.105	1.080
SLC31A2	-1.149	0.451	6.861	-2.782
SLC32A1	0.366	1.289	4.180	0.853
SLC35A1//C6orf165//RP3-382I10.7	0.509	1.423	5.625	2.104
SLC35B2//MIR4647	-0.534	0.691	5.146	-1.945
SLC35B4	0.171	1.126	6.315	0.904
SLC35D1	-0.422	0.747	4.698	-1.556
SLC35D2	-0.859	0.551	5.449	-2.001
SLC35E2	0.335	1.261	4.744	1.079
SLC35F1	1.073	2.104	7.097	4.020
SLC35F3	0.673	1.595	4.939	2.290
SLC35G5//SLC35G4P//SLC35G6	-0.002	0.999	2.988	-0.005
SLC36A1	0.299	1.230	4.773	1.183
SLC36A4	0.429	1.347	5.465	1.801
SLC37A2	-0.220	0.858	2.964	-0.680
SLC37A3	0.314	1.244	5.011	1.324
SLC38A1	0.084	1.060	9.188	0.345
SLC38A10	-0.317	0.803	4.834	-1.319
SLC38A11	0.682	1.604	3.073	2.211
SLC38A3	-0.245	0.844	6.746	-0.780
SLC38A5	0.384	1.305	3.214	0.950

SLC38A6	0.246	1.186	4.389	1.135
SLC38A8	0.297	1.229	2.613	1.054
SLC39A10	0.869	1.827	5.294	2.758
SLC39A11	-0.826	0.564	5.888	-3.137
SLC39A12	0.393	1.313	5.272	1.659
SLC39A14	0.382	1.303	5.709	0.850
SLC39A3	0.182	1.134	3.477	0.717
SLC39A8	-0.065	0.956	4.803	-0.298
SLC40A1	-0.679	0.625	4.136	-2.164
SLC41A2	0.481	1.396	5.702	1.996
SLC43A3	-0.320	0.801	3.903	-1.320
SLC44A1	-1.082	0.472	8.913	-3.298
SLC44A2	-0.592	0.664	8.210	-2.179
SLC44A3	-0.409	0.753	4.021	-1.409
SLC44A5	1.241	2.363	3.898	3.321
SLC45A3	-0.962	0.514	4.977	-1.864
SLC45A4	0.656	1.576	4.815	1.935
SLC46A1	-0.468	0.723	4.869	-1.842
SLC47A1	0.864	1.820	3.420	1.782
SLC47A2	-0.657	0.634	2.891	-1.600
SLC4A10	1.590	3.011	5.719	2.314
SLC4A1AP	0.351	1.275	5.651	1.665
SLC4A2	-0.723	0.606	3.252	-3.493
SLC4A3	0.352	1.276	5.133	1.278
SLC4A4	0.199	1.148	8.761	0.599
SLC4A5//RP11-287D1.3	0.023	1.016	4.222	0.068
SLC4A7	0.604	1.520	5.797	2.762
SLC4A8	0.330	1.257	3.980	1.166
SLC50A1	-0.015	0.990	4.082	-0.059
SLC51B	0.033	1.023	2.767	0.137
SLC52A2//GPR172A	0.175	1.129	2.702	0.581
SLC5A11	-1.578	0.335	6.596	-3.198
SLC5A5	0.564	1.478	2.209	1.722
SLC5A6	0.117	1.085	3.769	0.493
SLC5A7	-0.863	0.550	3.494	-0.967
SLC6A1	0.149	1.109	8.567	0.570
SLC6A11	0.195	1.144	6.164	0.446
SLC6A13	0.084	1.060	3.348	0.257
SLC6A15	0.975	1.966	5.218	1.873
SLC6A17	1.477	2.783	6.072	2.920
SLC6A20	0.588	1.503	3.103	1.411
SLC6A5	-1.713	0.305	3.553	-1.697
SLC6A6	0.245	1.185	5.695	0.638
SLC6A7	1.556	2.941	3.918	2.662
SLC6A9	-1.356	0.391	6.319	-3.649
SLC7A10	0.401	1.320	4.177	0.894
SLC7A11	-0.018	0.988	7.824	-0.073
SLC7A14	0.278	1.212	6.085	0.804
SLC7A2	0.626	1.543	5.847	1.259
SLC7A3	-0.205	0.868	1.890	-0.687

SLC7A5	-0.031	0.979	7.849	-0.105
SLC7A6	0.324	1.252	4.640	1.233
SLC7A7	-0.337	0.792	2.366	-1.227
SLC7A8	0.758	1.691	4.148	2.020
SLC8A1	0.791	1.731	4.831	2.696
SLC8A2	1.421	2.677	5.910	2.793
SLC8A3	-0.346	0.787	3.317	-1.298
SLC8B1	-0.190	0.876	4.481	-0.851
SLC9A1	0.383	1.304	5.313	1.342
SLC9A2	0.131	1.095	2.270	0.436
SLC9A3R1	0.242	1.183	7.578	0.976
SLC9A5	0.729	1.657	3.096	2.335
SLC9A6	0.679	1.601	8.944	2.273
SLC9A7	0.634	1.552	5.616	1.505
SLC9A7	0.379	1.300	6.525	1.154
SLC9A7P1	0.595	1.511	4.563	1.541
SLC9A9	-0.631	0.646	7.077	-2.023
SLC9B2	0.510	1.424	6.169	1.803
SLCO1A2	-0.722	0.606	6.031	-2.166
SLCO1C1	1.076	2.108	5.629	3.169
SLCO2A1	0.180	1.133	3.726	0.620
SLCO2B1	-0.402	0.757	5.378	-1.373
SLCO3A1	-0.724	0.605	6.280	-2.889
SLCO4A1	0.312	1.241	4.266	0.908
SLCO4C1	1.596	3.023	2.560	3.788
SLED1//RP11-701P16.1	-0.466	0.724	2.882	-1.158
SLFN11	-0.447	0.734	4.128	-1.933
SLFN12	-0.281	0.823	2.327	-1.121
SLFN5	0.020	1.014	6.107	0.061
SLIT1//ARHGAP19-SLIT1//ARHGAP19	0.990	1.986	5.338	2.992
SLIT2	0.687	1.610	5.477	1.503
SLIT2-IT1	0.133	1.097	3.195	0.364
SLIT3	0.484	1.399	5.332	1.305
SLITRK1	0.164	1.120	6.369	0.561
SLITRK3	0.845	1.796	5.324	2.106
SLITRK4	1.245	2.370	3.440	2.436
SLITRK5	0.724	1.652	5.441	2.451
SLITRK5	0.595	1.511	4.917	1.769
SLN	1.394	2.627	2.555	3.825
SLPI	0.516	1.430	4.156	0.628
SLU7	0.391	1.311	5.759	2.023
SMA5//SMA4//GUSBP3//LOC653080//G	-0.611	0.655	7.674	-2.151
SMAD5	-0.903	0.535	2.142	-2.177
SMAD9	-0.788	0.579	6.802	-2.563
SMAGP	0.073	1.052	3.317	0.223
SMAP1	0.617	1.534	6.489	3.337
SMG1P7//RP11-296I10.6	-0.274	0.827	3.699	-1.008
SMIM10	-0.532	0.692	4.836	-2.255
SMIM13	0.143	1.104	7.121	0.674
SMIM17	0.837	1.786	5.133	1.620

SMIM18	0.221	1.165	2.197	0.664
SMIM24//C19orf77	0.757	1.690	2.713	2.022
SMIM3	-0.150	0.901	4.238	-0.531
SMIM5	-1.012	0.496	2.759	-2.525
SMIM6	-0.787	0.580	3.576	-1.955
SMIM8	0.223	1.167	4.716	0.991
SMKR1	0.452	1.368	3.394	1.355
SMO	-0.294	0.816	4.679	-1.276
SMOC1	-1.043	0.485	5.590	-2.950
SMOX	-0.572	0.672	6.355	-2.091
SMPD3	0.565	1.480	3.520	1.960
SMYD2	0.988	1.984	6.382	2.854
SMYD3	0.381	1.303	4.517	1.699
SMYD3-IT1	0.057	1.040	4.781	0.223
SNAI1	0.334	1.260	2.995	1.262
SNAI2	0.368	1.290	4.470	1.107
SNAI3-AS1	0.429	1.346	4.496	1.540
SNAP23	-0.527	0.694	7.427	-2.172
SNAP25	1.032	2.045	9.944	1.697
SNAP25-AS1	0.703	1.628	2.241	2.883
SNAP47//LOC100130093	0.470	1.385	3.870	2.315
SNAP91	0.946	1.927	7.349	2.227
SNAR-B2//SNAR-B1	-0.230	0.853	5.001	-0.571
SNAR-C2//SNAR-C4//SNAR-C5//SNAR-C1	-0.254	0.838	4.502	-0.586
SNAR-C2//SNAR-C4//SNAR-C5//SNAR-C1	-0.413	0.751	5.329	-0.731
SNAR-C2//SNAR-C5//SNAR-C1	-0.315	0.804	4.369	-0.805
SNAR-D	-0.033	0.977	3.357	-0.094
SNAR-F	-1.173	0.444	3.089	-1.930
SNAR-G1	-0.105	0.930	4.176	-0.460
SNAR-G2	-0.311	0.806	2.860	-1.266
SNAR-H	-0.331	0.795	1.894	-0.728
SNCA	0.636	1.554	6.617	3.191
SNCAIP	0.373	1.295	3.531	1.396
SNCB	1.294	2.452	6.651	2.341
SNCG	0.271	1.207	6.206	0.629
SNED1	-0.252	0.840	4.619	-0.827
SNHG10//SCARNA13	-0.098	0.935	4.483	-0.411
SNHG12//SNORA44//SNORA61//SNORA	-0.163	0.893	4.482	-0.496
SNHG15//SNORA9//LOC101928927	-0.345	0.787	3.958	-1.332
SNHG18//CTD-2001E22.2	-0.806	0.572	3.227	-2.177
SNHG24//RP11-909M7.3	0.307	1.237	5.274	0.596
SNHG7//SNORA17//SNORA43	-0.052	0.964	4.527	-0.241
SNN	0.697	1.621	8.153	2.566
SNORA10//RPS2//SNORA64	-0.332	0.795	6.654	-1.043
SNORA11//MAGED2	0.032	1.023	9.550	0.102
SNORA11B	-0.590	0.664	3.791	-1.769
SNORA13	0.127	1.092	5.429	0.288
SNORA14A	-0.412	0.752	5.874	-1.044
SNORA14B	-0.013	0.991	6.899	-0.040
SNORA15	-0.054	0.963	3.963	-0.230

SNORA16B	-0.578	0.670	3.752	-1.716
SNORA21//RPL23	0.136	1.099	6.775	0.678
SNORA22	-0.167	0.891	8.990	-0.670
SNORA23	-0.177	0.885	8.947	-0.610
SNORA24//SNHG8	0.026	1.018	5.642	0.097
SNORA26	-0.280	0.824	4.420	-1.096
SNORA28	-0.208	0.866	3.892	-0.710
SNORA2A	0.431	1.348	2.472	0.931
SNORA31	-0.302	0.811	2.244	-0.981
SNORA33	-0.390	0.763	8.874	-1.013
SNORA34//MIR1291	-0.203	0.869	9.183	-1.039
SNORA35	0.128	1.093	4.515	0.256
SNORA36B//MIR664A	-0.085	0.942	3.298	-0.263
SNORA36C	-0.205	0.868	2.412	-0.618
SNORA37	-0.540	0.688	8.356	-2.077
SNORA38	-0.476	0.719	4.638	-1.407
SNORA38	-0.465	0.725	3.216	-0.992
SNORA38B	-0.137	0.910	9.049	-0.269
SNORA45B	-0.681	0.624	8.224	-1.791
SNORA46	-0.186	0.879	7.665	-0.482
SNORA47	-0.599	0.660	10.675	-3.020
SNORA48//EIF4A1//SNORA67//SNORD1	-0.241	0.846	11.099	-1.347
SNORA54	-0.834	0.561	9.137	-2.314
SNORA55	-0.232	0.851	4.927	-0.946
SNORA57//METTL12	-0.039	0.973	9.140	-0.166
SNORA59B//SNORA59A	0.231	1.173	6.744	1.155
SNORA5A//TBRG4	-0.501	0.707	5.507	-1.753
SNORA5C//TBRG4	-0.776	0.584	4.158	-2.800
SNORA60	-0.551	0.683	8.740	-1.698
SNORA64//RPS2	-0.391	0.763	6.986	-1.643
SNORA65	0.119	1.086	7.104	0.616
SNORA66//RPL5	-0.191	0.876	3.442	-0.680
SNORA68//RPL18A	-0.184	0.880	9.855	-0.950
SNORA70B	-0.419	0.748	2.900	-1.275
SNORA70D	-0.201	0.870	2.815	-0.719
SNORA70E	-0.335	0.793	3.284	-0.699
SNORA70G	0.109	1.079	3.750	0.338
SNORA71A	-0.352	0.784	5.564	-1.659
SNORA71B//SNHG17	-0.031	0.979	8.138	-0.106
SNORA71C	-0.531	0.692	5.699	-2.276
SNORA71D	-0.022	0.985	6.503	-0.080
SNORA72//RPL30	0.072	1.051	7.426	0.186
SNORA74A	-0.021	0.985	3.397	-0.079
SNORA74B	-0.470	0.722	6.468	-0.988
SNORA75	-0.677	0.626	9.566	-1.374
SNORA76A	-0.316	0.804	8.677	-1.437
SNORA76C//RP11-329L6.1	-0.591	0.664	6.309	-1.751
SNORA77	-0.126	0.917	4.105	-0.537
SNORA79	-0.505	0.705	2.971	-1.667
SNORA7B	-0.489	0.712	7.154	-1.695

SNORA80A	0.129	1.094	6.314	0.492
SNORA80B	-0.017	0.988	3.895	-0.057
SNORA80E	-0.401	0.757	6.747	-1.155
SNORA84//MIR3651	-0.026	0.982	5.361	-0.133
SNORA9//SNHG15//LOC101928927	0.070	1.050	6.048	0.347
SNORD10//EIF4A1//SNORA67//SNORA4	-0.348	0.786	8.373	-1.585
SNORD100//SNORA33	-0.811	0.570	9.728	-2.127
SNORD103A//SNORD103B	-0.015	0.990	3.366	-0.056
SNORD104	-0.454	0.730	6.621	-1.518
SNORD109B//SNORD109A//RP11-701H2	0.663	1.584	6.434	1.643
SNORD111	-0.291	0.817	5.441	-1.110
SNORD113-1	0.849	1.801	4.211	1.359
SNORD113-3	0.864	1.820	3.753	1.854
SNORD113-4//SNHG23//AL132709.5	0.515	1.429	5.577	1.204
SNORD113-5	0.743	1.674	2.194	2.264
SNORD113-8	1.089	2.128	3.510	1.873
SNORD113-9	0.754	1.686	3.897	1.768
SNORD114-1	0.563	1.477	8.583	1.060
SNORD114-10	0.738	1.668	1.814	1.692
SNORD114-11	1.097	2.139	3.108	1.990
SNORD114-12	0.739	1.669	9.262	1.790
SNORD114-13	1.004	2.005	3.010	1.889
SNORD114-14	0.428	1.345	5.376	1.073
SNORD114-15	0.781	1.718	4.988	1.638
SNORD114-16	0.960	1.945	3.702	1.636
SNORD114-17	0.846	1.798	3.997	1.624
SNORD114-2	0.960	1.946	3.395	1.656
SNORD114-20	-0.259	0.836	3.131	-0.326
SNORD114-21	0.794	1.733	5.691	1.588
SNORD114-22	0.994	1.991	4.686	1.928
SNORD114-23	0.967	1.955	2.337	1.956
SNORD114-25	1.231	2.347	4.152	1.678
SNORD114-26//SNHG24//RP11-909M7.:	1.059	2.083	4.605	1.843
SNORD114-27	0.157	1.115	4.492	0.561
SNORD114-28	1.115	2.165	6.652	1.856
SNORD114-3	0.731	1.659	8.943	1.603
SNORD114-30	-0.106	0.929	3.039	-0.214
SNORD114-31	0.474	1.389	2.942	0.896
SNORD114-4	0.843	1.794	1.991	2.162
SNORD114-6	0.632	1.550	2.335	1.047
SNORD114-7	0.003	1.002	2.711	0.005
SNORD114-9	0.945	1.925	4.802	1.486
SNORD115-1//SNORD115-13//SNORD11	0.518	1.432	10.603	1.732
SNORD115-10//SNORD115-21	0.954	1.937	5.423	1.800
SNORD115-15//SNORD115-21	0.528	1.442	3.764	1.326
SNORD115-17//SNORD115-18//SNORD1	0.660	1.580	9.964	1.747
SNORD115-20//IPW//SNORD107//PWAI	0.508	1.422	10.523	1.443
SNORD115-21	0.479	1.394	2.392	1.345
SNORD115-25	0.459	1.375	7.977	1.087
SNORD115-27	0.547	1.461	7.359	1.226

SNORD115-3	1.326	2.508	2.935	2.446
SNORD115-30	0.804	1.746	7.912	1.355
SNORD115-31	0.652	1.572	6.843	1.411
SNORD115-32	1.199	2.296	4.979	2.243
SNORD115-38	1.140	2.203	4.928	2.023
SNORD115-4//SNORD115-6//SNORD115	0.525	1.438	11.172	1.687
SNORD115-40	0.841	1.791	4.167	1.911
SNORD115-41	0.605	1.521	2.298	1.584
SNORD115-44//SNORD115-1//SNORD11	0.666	1.586	10.604	1.850
SNORD115-45	0.534	1.448	3.669	0.941
SNORD115-5//SNORD115-9//SNORD115	0.544	1.458	11.040	1.730
SNORD115-5//SNORD115-9//SNORD115	0.527	1.441	11.025	1.652
SNORD115-5//SNORD115-9//SNORD115	0.485	1.400	10.981	1.658
SNORD115-8	1.177	2.260	2.541	2.941
SNORD116-1	0.199	1.148	7.871	0.694
SNORD116-14//SNRPN	0.194	1.144	9.539	0.771
SNORD116-16	0.719	1.646	2.215	2.233
SNORD116-20//SNORD116-19//SNORD1	-0.041	0.972	7.432	-0.163
SNORD116-21//SNORD116-19//SNORD1	0.929	1.904	6.446	1.323
SNORD116-23	0.348	1.273	5.106	1.111
SNORD116-25	0.621	1.537	4.066	1.684
SNORD116-26	0.470	1.385	6.203	1.368
SNORD116-27	0.786	1.724	5.203	2.095
SNORD116-29	0.369	1.292	6.987	1.082
SNORD116-30	0.663	1.583	6.211	1.801
SNORD117	-0.449	0.733	8.189	-2.431
SNORD117	-0.416	0.750	8.351	-2.260
SNORD12	-0.165	0.892	6.133	-0.690
SNORD123	-0.734	0.601	4.488	-1.650
SNORD124	-0.591	0.664	2.396	-2.078
SNORD125//MIR3653	-0.335	0.793	3.250	-0.917
SNORD126	0.104	1.075	2.976	0.355
SNORD12B	-0.105	0.930	5.062	-0.416
SNORD12C	-0.410	0.752	4.299	-1.617
SNORD13P2	-0.503	0.705	5.201	-1.459
SNORD13P3	0.202	1.150	7.005	0.770
SNORD14A	-0.424	0.746	4.475	-1.264
SNORD14E	-0.131	0.913	5.153	-0.347
SNORD15B	-0.295	0.815	9.129	-1.466
SNORD17	-0.619	0.651	7.683	-2.083
SNORD18A//RPL4//SNORD16//SNORD18	-0.006	0.996	2.779	-0.017
SNORD18C//RPL4//SNORD16//SNORD18	0.237	1.178	4.214	0.596
SNORD2//EIF4A2//SNORA63//SNORA4/	-0.128	0.915	5.976	-0.524
SNORD20	-0.278	0.825	1.890	-1.029
SNORD23//GLTSCR2	0.831	1.779	2.765	2.186
SNORD29//SNORD31//SNORD30//SNOR	0.138	1.101	3.856	0.536
SNORD35B//RPS11	-0.430	0.742	4.860	-1.897
SNORD37	-0.064	0.956	4.016	-0.198
SNORD3C	-0.477	0.718	8.050	-1.607
SNORD3D//RP11-160E2.6	0.041	1.029	5.839	0.184

SNORD42A//RPL23A	-0.425	0.745	9.067	-2.360
SNORD4B//RPL23A//SNORD42A	-0.479	0.718	5.548	-2.252
SNORD51	-0.108	0.928	4.977	-0.375
SNORD56B	0.130	1.094	2.296	0.332
SNORD58A//RPL17//SNORD58B//SNORI	-0.320	0.801	4.603	-1.337
SNORD58C//RPL17//SNORD58B//SNORI	-0.035	0.976	5.924	-0.140
SNORD59B	0.226	1.170	3.780	0.814
SNORD60	-0.410	0.753	6.697	-1.961
SNORD61//RBMX//LOC101928747	-0.383	0.767	8.668	-1.709
SNORD62A//SNORD62B	0.116	1.084	5.802	0.341
SNORD66	-0.443	0.735	6.034	-1.431
SNORD67	-0.090	0.939	5.078	-0.369
SNORD7	-0.492	0.711	2.711	-1.765
SNORD70	0.069	1.049	2.939	0.283
SNORD71	0.166	1.122	11.202	0.638
SNORD75	-0.146	0.904	2.644	-0.395
SNORD82	-0.224	0.856	8.829	-0.901
SNORD83A	0.364	1.287	2.617	1.110
SNORD85	-0.082	0.945	2.383	-0.275
SNORD88A	-0.436	0.739	4.974	-1.391
SNORD88B//C19orf48//SNORD88C	-0.126	0.916	3.784	-0.446
SNORD88C//C19orf48	-0.168	0.890	2.598	-0.668
SNORD9	-0.037	0.974	8.082	-0.170
SNORD90	-0.317	0.803	6.119	-1.276
SNORD91A	0.432	1.349	3.477	1.300
SNORD91B	-0.516	0.700	7.377	-2.234
SNORD92	0.065	1.046	4.766	0.234
SNORD94	-0.544	0.686	8.106	-2.523
SNORD96B	0.415	1.333	2.527	1.373
SNORD99	-0.559	0.679	2.048	-1.784
SNPH	0.911	1.881	5.991	1.873
SNRPN//IPW//SNORD107//PWARSN//SI	0.484	1.399	4.959	1.258
SNRPN//SNURF	0.261	1.198	9.522	1.000
SNTA1	-0.263	0.833	6.186	-0.963
SNTB2	0.470	1.385	6.383	1.648
SNTG1	1.014	2.019	5.512	2.544
SNURF//IPW//SNORD107//PWARSN//SI	0.577	1.492	6.420	1.613
SNX1	-0.573	0.672	6.133	-2.421
SNX10	0.830	1.778	4.741	2.063
SNX16	0.164	1.120	3.951	0.673
SNX18	-0.535	0.690	4.592	-1.949
SNX20	-0.289	0.818	2.445	-0.890
SNX22	-0.505	0.705	4.300	-1.571
SNX29//LOC101929304//AC007601.2	-0.700	0.615	7.125	-2.394
SNX32	1.063	2.089	3.233	2.557
SNX33	-0.120	0.920	5.028	-0.586
SNX4	0.075	1.053	6.512	0.259
SOAT1	0.024	1.017	6.124	0.097
SOBP	0.208	1.155	6.509	1.022
SOCS3	1.317	2.492	6.432	2.513

SOCS6	-0.467	0.723	5.546	-1.882
SOCS7	0.327	1.254	6.300	1.443
SOD2//LOC100129518	0.252	1.191	4.162	0.803
SOD3	0.574	1.488	4.245	2.016
SOGA3//KIAA0408//RP3-403A15.5	0.396	1.316	6.121	1.579
SOHLH1	0.669	1.590	2.652	2.298
SORBS2	0.749	1.681	5.287	2.000
SORCS1	1.183	2.271	5.011	2.772
SORCS3	0.491	1.405	5.364	1.891
SOS1-IT1	-0.439	0.738	2.866	-1.143
SOSTDC1	1.185	2.274	2.884	3.014
SOWAHA	0.346	1.271	4.705	0.946
SOWAHB	1.277	2.423	3.499	2.998
SOWAHC	-0.371	0.774	2.912	-1.414
SOX1	0.567	1.481	2.886	1.830
SOX10	-1.363	0.389	5.406	-4.319
SOX13	-0.350	0.784	4.343	-1.596
SOX18	-0.117	0.922	2.563	-0.357
SOX2-OT	-0.685	0.622	3.948	-2.574
SOX5	0.536	1.449	5.125	2.295
SOX8	-1.018	0.494	5.787	-3.141
SOX9	0.033	1.023	8.204	0.147
SOX9-AS1//AC005152.2	-0.365	0.776	2.813	-1.274
SP1	-0.398	0.759	6.846	-1.929
SP100	-0.068	0.954	4.771	-0.259
SP110	-0.287	0.819	5.076	-0.902
SP9	0.025	1.018	3.419	0.085
SPAG17	-0.305	0.809	1.964	-0.727
SPAG5	0.486	1.401	3.344	1.571
SPAG6	-0.259	0.836	2.477	-0.690
SPAG8	-0.158	0.896	2.797	-0.522
SPARC	-1.033	0.489	6.921	-3.246
SPATA13//RP11-307N16.6	-0.782	0.582	6.134	-2.543
SPATA13//RP11-309I15.1//RP11-307N1(	-0.758	0.591	3.558	-2.748
SPATA17	0.184	1.136	2.231	0.600
SPATA18	0.240	1.181	3.542	0.545
SPATA2L	0.063	1.044	3.011	0.244
SPATA31C1	0.088	1.063	2.420	0.307
SPATA41	-0.208	0.866	2.146	-0.743
SPATA6	-0.504	0.705	4.941	-1.663
SPATA6	-0.292	0.817	3.967	-0.902
SPATA6L	0.096	1.069	3.528	0.320
SPATA7	0.308	1.238	4.741	1.510
SPATS2	0.676	1.598	4.069	2.512
SPDYA	0.103	1.074	4.125	0.305
SPDYE6//SPDYE2B//SPDYE2	-0.318	0.802	2.730	-0.982
SPEG//LOC100996693	0.147	1.107	3.670	0.558
SPHKAP	1.125	2.182	4.769	2.128
SPI1	-0.418	0.748	5.303	-1.531
SPIN1	0.275	1.210	8.535	1.540

SPIN3	0.309	1.239	4.403	1.198
SPINT1	-0.524	0.695	2.514	-2.106
SPINT2	0.727	1.655	5.793	2.413
SPIRE2	0.564	1.478	4.072	1.420
SPN//LOC101929889	-0.963	0.513	3.356	-2.400
SPOCD1	-0.199	0.871	2.243	-0.801
SPOCK1	0.064	1.045	6.833	0.286
SPOCK2	0.411	1.330	6.969	1.431
SPOCK3	-0.659	0.634	6.363	-2.615
SPON1	-0.466	0.724	6.611	-1.471
SPP1	-0.487	0.714	10.916	-1.265
SPPL2C//IMP5	-0.302	0.811	2.967	-1.125
SPR	-0.002	0.999	4.710	-0.005
SPRED2	0.229	1.172	4.477	0.934
SPRN	1.116	2.167	3.922	2.342
SPRR2F	0.068	1.048	2.107	0.241
SPRY1	0.409	1.328	4.472	1.292
SPRY3	0.264	1.201	4.137	0.783
SPRY3	0.066	1.047	4.649	0.248
SPRYD3	0.601	1.516	6.741	2.385
SPRYD7	0.243	1.183	5.691	1.169
SPTB	0.378	1.299	4.636	1.029
SPTBN2	1.014	2.020	5.175	2.434
SPTBN4	0.655	1.575	5.042	2.026
SPTLC2	-0.651	0.637	6.869	-2.169
SPTLC3	0.065	1.046	4.957	0.160
SPTSSA	-0.015	0.990	5.713	-0.063
SPTSSB	1.430	2.694	3.539	2.834
SPX//C12orf39	0.203	1.151	3.257	0.585
SQLE	-0.484	0.715	6.750	-1.409
SQRDL//RP11-96O20.4	-0.080	0.946	4.602	-0.204
SQSTM1	-0.155	0.898	8.059	-0.647
SRD5A1	0.161	1.118	2.488	0.486
SREBF1	-0.666	0.630	4.993	-2.379
SRGAP1	-0.350	0.785	6.219	-1.319
SRGAP2	-0.575	0.671	7.319	-2.809
SRGAP2//SRGAP2B//SRGAP2C	-0.840	0.559	7.162	-2.652
SRGN	0.031	1.021	9.004	0.079
SRL	-0.584	0.667	2.178	-2.020
SRP14-AS1	-0.640	0.642	3.549	-2.398
SRP68	0.199	1.148	7.240	0.980
SRPK1	0.455	1.371	5.338	1.850
SRPK2	-0.007	0.995	7.507	-0.038
SRPX	-0.963	0.513	5.748	-2.396
SRPX2	-0.029	0.980	3.602	-0.135
SRRM3	0.696	1.620	4.271	2.238
SRRM4	1.526	2.881	4.606	2.958
SRSF12	0.460	1.376	3.412	1.897
SS18L2	0.021	1.014	4.998	0.083
SSBP3	0.317	1.246	4.663	1.559

SSBP4	0.628	1.546	5.124	1.846
SSC5D	-0.041	0.972	2.849	-0.160
SSFA2	-0.397	0.759	7.046	-1.901
SSH3	-0.383	0.767	4.907	-1.732
SSSCA1	0.227	1.170	5.658	0.968
SSSCA1-AS1	-0.132	0.913	3.997	-0.466
SST	0.230	1.173	6.793	0.344
SSTR1	0.735	1.665	3.554	1.835
SSTR2	1.141	2.205	4.880	2.484
SSTR3	0.542	1.456	2.144	1.637
SSTR4	0.701	1.626	2.209	2.108
SSX2IP	1.121	2.176	4.340	2.822
ST18	-1.280	0.412	6.029	-2.554
ST3GAL1	0.464	1.380	4.249	1.952
ST3GAL2	0.194	1.144	3.882	0.780
ST3GAL6-AS1	-0.400	0.758	2.784	-1.352
ST6GAL1	-0.479	0.717	4.273	-1.869
ST6GAL2	0.605	1.521	3.535	1.948
ST6GALNAC2	-0.452	0.731	4.757	-1.136
ST6GALNAC3	-0.563	0.677	5.932	-2.057
ST6GALNAC5	1.732	3.321	3.629	2.859
ST6GALNAC6	0.397	1.317	6.853	1.777
ST8SIA3	1.260	2.395	6.004	2.084
ST8SIA4	-0.057	0.961	4.788	-0.199
ST8SIA5	1.060	2.085	5.802	2.307
ST8SIA6	-0.197	0.873	3.296	-0.630
STAB1	-0.137	0.909	5.072	-0.303
STAC2	1.347	2.545	4.510	3.067
STAG3	-0.578	0.670	3.025	-2.172
STAMBPL1	0.599	1.514	5.261	2.260
STAR	0.910	1.879	3.109	3.019
STARD10	0.157	1.115	4.456	0.722
STARD13	-0.100	0.933	3.921	-0.412
STARD3	-0.514	0.700	6.052	-2.187
STARD4	0.423	1.340	5.982	1.445
STARD8	0.326	1.254	3.494	1.203
STARD9	-0.424	0.745	5.500	-1.561
STAT1	0.433	1.350	7.361	1.663
STAT2	-0.595	0.662	6.118	-2.167
STAT3	-0.174	0.887	8.600	-0.836
STAT4	1.375	2.595	4.037	2.652
STAT6	0.332	1.258	4.617	1.309
STAU2	0.568	1.482	6.038	2.567
STC1	0.180	1.133	3.931	0.292
STC2	-0.135	0.911	3.032	-0.375
STEAP1B	0.053	1.038	2.272	0.215
STEAP2	1.004	2.006	5.739	2.592
STEAP3	-0.664	0.631	5.279	-1.694
STEAP4	0.926	1.901	2.991	2.702
STH	-0.308	0.808	4.543	-0.746

STIL	0.362	1.285	3.494	1.657
STK10	-0.278	0.825	5.074	-0.999
STK24	0.428	1.345	4.578	1.945
STK32A	-1.447	0.367	5.702	-3.497
STK33	-0.407	0.754	4.488	-1.434
STMN2	1.236	2.356	8.996	1.975
STMN3	0.685	1.608	6.579	2.376
STMN4	0.099	1.071	6.380	0.275
STOM	0.067	1.048	8.829	0.326
STOML1	0.177	1.130	4.569	0.731
STON1-GTF2A1L//STON1//GTF2A1L	-1.248	0.421	4.166	-3.019
STOX1	0.733	1.662	4.661	2.138
STOX2	0.182	1.134	6.123	0.924
STPG1	0.321	1.249	2.646	1.780
STRBP	0.903	1.870	5.317	3.026
STRC	0.515	1.429	3.458	2.346
STRIP2	0.535	1.449	4.418	1.652
STS	0.795	1.735	6.937	2.023
STX11	0.058	1.041	3.099	0.240
STX1A	1.075	2.107	4.267	3.343
STX1B	1.065	2.092	8.049	1.761
STX2	-0.289	0.818	5.837	-1.402
STX4	-0.244	0.844	5.692	-1.130
STXBP1	0.923	1.896	9.600	2.455
STXBP2	-0.324	0.799	3.720	-1.001
STXBP3	-0.526	0.694	7.085	-2.157
STXBP5	0.827	1.774	6.345	2.628
STXBP5-AS1	0.568	1.482	2.486	2.352
STXBP5L	1.249	2.377	6.955	3.158
STXBP6	-0.022	0.985	4.742	-0.108
STYK1	1.155	2.227	3.179	2.191
STYX	-0.270	0.829	5.650	-0.981
SUCLA2	0.336	1.263	6.792	1.693
SUCLG2	0.063	1.045	5.738	0.273
SUDS3	0.324	1.252	5.459	1.476
SULF1	0.446	1.362	4.457	1.650
SULT1A2	-0.034	0.977	2.435	-0.117
SULT1B1	0.533	1.447	2.258	2.068
SULT1C4	-0.085	0.943	5.261	-0.311
SULT4A1	1.299	2.461	5.892	2.082
SUMO4	-0.157	0.897	5.816	-0.490
SUN2	-0.663	0.632	6.355	-2.240
SUN2	-0.193	0.875	3.494	-0.706
SUPV3L1	0.236	1.178	5.928	1.221
SUSD1	0.447	1.364	5.105	1.398
SUSD2	-0.830	0.562	4.849	-1.812
SUSD3	-0.481	0.716	3.571	-1.471
SUSD4	0.853	1.806	4.732	1.873
SUSD5	0.632	1.550	4.466	2.529
SUV39H2	0.290	1.223	3.193	1.183

SV2A	0.848	1.799	7.502	1.921
SV2B	1.668	3.178	6.627	2.702
SV2C	0.366	1.289	5.849	0.516
SVEP1	-0.156	0.897	3.597	-0.526
SVIL	-0.296	0.814	4.821	-1.177
SVOP	1.252	2.382	4.940	2.513
SYBU	0.660	1.580	4.825	1.504
SYCE1	0.679	1.601	3.006	2.055
SYDE1	-0.316	0.804	3.439	-1.399
SYDE2	0.511	1.425	4.084	1.780
SYK	-0.898	0.537	4.020	-2.755
SYN1	1.326	2.507	7.087	2.148
SYN2	1.443	2.719	6.939	2.086
SYN3	0.773	1.708	4.304	1.784
SYNDIG1L//TMEM90A	-0.337	0.791	2.929	-1.179
SYNE1	0.632	1.550	6.685	2.155
SYNE3	-0.805	0.572	4.191	-2.688
SYNGAP1	0.721	1.648	5.584	2.414
SYNGAP1	0.690	1.613	5.560	2.322
SYNGR1	0.683	1.605	4.952	3.001
SYNGR2	-0.840	0.559	6.853	-2.430
SYNGR3	0.923	1.896	5.264	1.934
SYNJ1	0.774	1.710	7.210	2.759
SYNJ2	-0.948	0.518	7.467	-2.473
SYNJ2-IT1	-0.747	0.596	2.355	-2.206
SYNPO	0.346	1.271	4.606	1.032
SYNPO2	-0.028	0.981	6.259	-0.117
SYNPR	1.285	2.437	2.245	3.224
SYP	0.526	1.440	5.512	1.596
SYP-AS1	0.878	1.838	5.279	2.913
SYPL1	-0.886	0.541	7.031	-2.727
SYT1	1.562	2.953	4.700	2.862
SYT10	0.489	1.403	4.350	1.300
SYT12	0.562	1.476	2.887	2.182
SYT13	1.188	2.279	4.542	2.455
SYT14	0.461	1.377	5.530	1.803
SYT16	1.392	2.625	4.374	2.762
SYT17	0.456	1.372	6.014	1.638
SYT2	0.900	1.866	5.961	1.098
SYT3	0.573	1.488	4.235	1.465
SYT4	1.518	2.864	7.506	1.948
SYT5	1.002	2.003	5.664	2.368
SYT6	0.191	1.142	3.287	0.635
SYT7	1.730	3.318	4.035	3.263
SYTL2	0.529	1.443	5.245	2.316
SYTL3	-0.090	0.939	4.419	-0.224
SYTL4	-0.730	0.603	4.543	-2.168
SYTL5	0.496	1.410	3.672	0.917
TAB1//LOC100506472	-0.094	0.937	3.228	-0.322
TAB3-AS2	0.188	1.139	3.736	0.667

TAC1	0.317	1.246	3.284	0.594
TAC3	0.664	1.585	3.700	1.397
TACC2	0.364	1.287	4.153	1.479
TACR1	0.098	1.071	3.653	0.376
TACR2	-0.197	0.873	3.054	-0.668
TACR3	-0.868	0.548	2.833	-2.635
TADA1	0.302	1.233	4.715	1.411
TAF1D//SNORA8//SNORA1//SNORA18//	0.476	1.390	6.145	2.035
TAF4B	0.615	1.531	5.178	2.500
TAF5	0.171	1.126	4.437	0.789
TAGAP	-0.348	0.786	3.423	-0.914
TAGLN	0.008	1.006	5.023	0.024
TAGLN2	-0.322	0.800	2.879	-1.221
TAGLN3	0.913	1.883	6.386	2.172
TAL1	-0.822	0.566	2.694	-3.476
TALDO1	-0.539	0.688	5.122	-2.333
TANC1	-0.878	0.544	6.402	-2.680
TANC2	0.205	1.153	7.452	1.026
TAP1//PSMB8-AS1	0.134	1.098	4.608	0.574
TAP1//PSMB8-AS1	0.097	1.069	4.822	0.422
TAPBPL	-0.397	0.760	3.795	-1.810
TARBP1	0.465	1.380	4.951	1.447
TAS2R10	0.329	1.256	2.561	1.128
TAS2R13	-0.338	0.791	3.442	-0.988
TAS2R14	-0.388	0.764	4.239	-0.907
TAS2R19	-0.158	0.896	2.727	-0.538
TAS2R20	-0.257	0.837	3.073	-0.688
TAS2R3	-0.185	0.880	4.775	-0.687
TAS2R31//TAS2R45	-0.281	0.823	3.809	-0.795
TAS2R4	-0.286	0.820	4.193	-0.918
TBC1D12	-0.920	0.528	6.320	-2.576
TBC1D2	-0.403	0.756	5.653	-1.305
TBC1D22B	0.032	1.023	5.595	0.139
TBC1D24	0.840	1.790	5.603	2.375
TBC1D2B	0.038	1.026	4.550	0.147
TBC1D30	0.748	1.680	5.302	1.926
TBC1D4	0.148	1.108	5.122	0.499
TBC1D9	0.766	1.701	6.046	2.670
TBKBP1	0.456	1.371	4.613	1.709
TBPL1	0.390	1.310	4.735	1.514
TBR1	1.967	3.908	3.143	2.970
TBRG1	0.565	1.479	5.326	2.968
TBX10	-0.344	0.788	2.605	-1.263
TBX15	0.884	1.845	3.033	3.089
TBX3	0.250	1.189	3.646	0.917
TBXAS1	-0.452	0.731	3.544	-1.461
TC2N	0.801	1.742	5.326	1.574
TCEA3	0.077	1.055	3.907	0.323
TCEAL5	0.597	1.513	5.209	2.269
TCEB3-AS1//RP5-886K2.3	-0.283	0.822	4.039	-1.156

TCEB3C//LOC101929870//LOC1010604€	-0.177	0.885	3.440	-0.588
TCEB3CL2//TCEB3C//LOC101060464//L(	-0.427	0.744	2.660	-2.014
TCERG1L	0.379	1.300	3.766	1.009
TCF12	-0.691	0.619	6.651	-3.084
TCF19	-0.254	0.839	2.894	-1.001
TCFL5	-0.685	0.622	6.518	-2.903
TCIRG1	-0.209	0.865	4.539	-0.958
TCN2	0.149	1.109	3.989	0.634
TCP10L//LINC00846	-0.212	0.863	3.633	-0.650
TCP11L1	0.276	1.211	4.855	1.429
TCP11L2	-0.804	0.573	5.698	-2.745
TCTE1	-0.070	0.952	2.555	-0.268
TCTEX1D1	-0.077	0.948	2.843	-0.182
TDH	-0.073	0.951	2.887	-0.242
TDO2	1.043	2.061	1.702	3.566
TDRD9	0.170	1.125	2.490	0.542
TDRKH	0.325	1.252	5.436	1.385
TDRP	0.179	1.132	4.680	0.704
TEAD2	0.529	1.443	3.429	1.713
TEAD3	0.466	1.382	3.002	1.722
TECTA	-0.061	0.958	3.552	-0.276
TEF	0.338	1.264	4.188	1.303
TEK	0.189	1.140	5.152	0.711
TEKT1	-0.194	0.874	2.458	-0.391
TEKT2	-0.051	0.965	2.291	-0.206
TEKT4	-0.193	0.875	2.682	-0.728
TEKT4P2//MAFIP//LOC100233156	-1.000	0.500	4.451	-1.928
TENC1	-0.364	0.777	5.940	-1.522
TENM1	0.601	1.517	5.751	1.734
TENM2	1.032	2.046	4.743	2.097
TENM3	0.450	1.366	5.265	1.228
TENM4//ODZ4	-0.037	0.974	2.449	-0.125
TEP1	-0.464	0.725	4.686	-1.736
TERC	-0.136	0.910	5.601	-0.558
TERF2IP	0.496	1.410	3.220	1.374
TES	-0.193	0.875	2.560	-0.621
TESC	0.791	1.730	3.363	2.320
TESK2	-0.921	0.528	5.406	-3.068
TESPA1	2.945	7.701	4.465	3.190
TEX10	0.318	1.246	5.375	1.665
TEX26	0.161	1.118	2.263	0.584
TEX29	1.133	2.193	3.149	3.423
TEX30	0.095	1.068	3.892	0.520
TEX9	-0.184	0.880	3.241	-0.872
TF	-1.231	0.426	9.937	-2.734
TFB2M	0.169	1.124	4.975	0.687
TFEC	-0.167	0.891	2.130	-0.741
TFPI	-0.102	0.932	2.597	-0.394
TFPT	0.394	1.314	7.010	1.629
TFR2	0.634	1.552	2.607	1.891

TFRC	0.652	1.571	6.156	2.452
TGFA	-0.886	0.541	4.530	-2.643
TGFB1	-0.503	0.706	5.654	-1.902
TGFB1I1	-0.256	0.837	4.587	-0.809
TGFB2	0.454	1.369	7.469	1.471
TGFB2-AS1//RP11-224O19.2	-0.330	0.796	2.177	-1.068
TGFB3	-0.647	0.639	4.927	-1.666
TGFBI	0.395	1.315	3.997	1.166
TGFBR1	-0.220	0.859	6.431	-0.706
TGFBR2	-0.227	0.854	6.441	-0.878
TGFBR3L	0.410	1.328	4.017	1.189
TGIF1	-0.083	0.944	3.046	-0.374
TGM2	0.535	1.449	5.901	1.562
THAP10	0.227	1.170	4.059	0.821
THAP9	-0.577	0.670	3.240	-1.763
THAP9-AS1	0.414	1.332	4.397	1.575
THBD	0.264	1.201	3.609	0.701
THBS1	0.400	1.319	5.555	0.991
THBS2	-0.678	0.625	7.091	-2.252
THBS4	-0.465	0.725	5.278	-1.559
THEM6	0.008	1.006	4.153	0.032
THEMIS	0.291	1.224	2.395	1.168
THEMIS2	-0.354	0.782	5.230	-0.837
THNSL2	-0.392	0.762	4.226	-1.003
THPO	0.118	1.085	2.814	0.400
THRB	1.173	2.255	5.246	2.579
THRB-IT1	0.711	1.636	2.512	1.996
THSD1	0.633	1.550	3.586	2.408
THSD4	0.594	1.509	3.873	2.117
THUMPD2	0.321	1.249	4.021	1.562
THY1	0.484	1.399	3.631	1.193
THYN1	0.445	1.361	5.199	1.970
TIA1	-0.335	0.793	5.822	-1.328
TIAM2	1.090	2.129	3.511	2.826
TIFA	0.513	1.427	3.228	1.774
TIGD2	-0.391	0.763	2.900	-1.662
TIMELESS	-0.170	0.889	3.314	-0.594
TIMM22	0.023	1.016	6.086	0.084
TIMM23B	-0.101	0.933	4.777	-0.365
TIMM8A	0.093	1.066	4.594	0.446
TIMP1	0.180	1.133	6.705	0.583
TIMP2	-0.340	0.790	7.751	-1.481
TIMP3	-0.006	0.996	7.601	-0.025
TIMP4	-0.737	0.600	5.236	-1.711
TIPARP	0.193	1.143	7.210	0.592
TIPRL	0.432	1.349	5.869	1.703
TJAP1	-0.861	0.550	5.206	-2.382
TJP2	-0.952	0.517	7.171	-2.566
TK2	0.060	1.042	2.898	0.230
TLCD2	0.102	1.073	2.926	0.380

TLE1	0.190	1.141	5.098	0.843
TLE2	-0.055	0.963	5.016	-0.258
TLL1	0.561	1.475	3.663	2.378
TLL2	-0.760	0.591	3.310	-2.120
TLN1	-0.672	0.628	6.876	-2.848
TLN2	0.327	1.254	6.314	1.694
TLR1	-0.138	0.909	4.220	-0.356
TLR10	-0.401	0.758	3.516	-1.035
TLR2	0.069	1.049	5.295	0.151
TLR3	-0.415	0.750	4.425	-1.067
TLR4	-0.687	0.621	6.039	-1.980
TLR5	-0.003	0.998	4.873	-0.008
TLR6	-0.434	0.740	3.145	-1.441
TLR7	-0.604	0.658	4.147	-1.473
TLR8	-0.282	0.823	2.090	-0.745
TM2D2	0.202	1.150	4.348	0.867
TM4SF1	0.431	1.348	6.690	1.509
TM4SF18	0.324	1.252	3.647	1.217
TM6SF1	0.501	1.415	5.910	1.861
TMBIM1//MIR6513	-0.269	0.830	3.764	-0.792
TMBIM4//RP11-745O10.4	-0.445	0.735	4.682	-1.817
TMC1	0.040	1.028	2.380	0.184
TMC6	-0.882	0.543	5.321	-2.203
TMC7	-1.050	0.483	6.849	-2.342
TMC8	-0.110	0.927	3.449	-0.368
TMCC1-AS1//AL449209.1	0.129	1.094	4.170	0.504
TMCC2	-0.351	0.784	3.392	-1.360
TMCC3	-0.899	0.536	5.658	-2.823
TMCO4	-0.124	0.918	2.746	-0.452
TMCO6	-0.344	0.788	3.759	-1.425
TMED8	0.376	1.298	6.114	1.505
TMEFF2	0.248	1.187	5.590	1.036
TMEM104	-0.628	0.647	5.347	-2.320
TMEM106A	-0.736	0.600	4.369	-2.824
TMEM108	0.630	1.548	2.842	2.024
TMEM119	-0.666	0.630	3.965	-1.927
TMEM125	-0.286	0.820	2.612	-1.075
TMEM130	1.213	2.318	6.640	1.833
TMEM132B	0.985	1.979	4.854	2.628
TMEM132D	1.184	2.273	3.848	2.792
TMEM132E	-0.284	0.821	5.117	-0.918
TMEM139	-1.540	0.344	3.468	-3.390
TMEM139//AC073342.12	-1.041	0.486	4.204	-2.832
TMEM140	-1.427	0.372	5.589	-2.612
TMEM144	-0.804	0.573	6.780	-1.933
TMEM145	0.449	1.365	4.900	1.438
TMEM14A	0.297	1.228	7.129	1.410
TMEM150C	0.726	1.654	6.778	2.277
TMEM151A	-0.776	0.584	6.001	-2.693
TMEM151B//RP11-444E17.6	0.452	1.368	2.154	1.386

TMEM155	1.940	3.836	2.984	3.199
TMEM156	-0.449	0.732	3.825	-1.292
TMEM158	0.415	1.333	4.245	1.142
TMEM160	0.290	1.223	4.763	1.091
TMEM163	0.184	1.136	5.920	0.713
TMEM165	-0.906	0.534	7.445	-2.103
TMEM168	-0.162	0.894	5.417	-0.582
TMEM169	-0.050	0.966	3.084	-0.159
TMEM17	0.296	1.228	2.810	1.372
TMEM170B	0.303	1.234	5.598	1.354
TMEM173	-0.128	0.915	3.001	-0.449
TMEM176A	-0.634	0.644	4.256	-2.407
TMEM176B	-0.729	0.603	5.808	-2.569
TMEM177	0.523	1.437	4.725	2.336
TMEM178B	0.646	1.565	5.781	1.698
TMEM179	0.189	1.140	4.389	0.663
TMEM179	0.058	1.041	3.556	0.260
TMEM179B//MIR6748	-0.449	0.733	4.957	-1.619
TMEM181	0.235	1.177	6.666	1.107
TMEM183B//RP11-651P23.4	0.393	1.313	5.776	1.543
TMEM185A	0.424	1.342	4.532	1.627
TMEM196	0.616	1.533	4.624	1.245
TMEM198	0.135	1.098	3.437	0.522
TMEM198B	-0.626	0.648	5.405	-2.128
TMEM2	0.628	1.545	5.443	2.477
TMEM200A	0.221	1.165	2.736	0.931
TMEM204	0.193	1.143	4.303	0.615
TMEM206	-0.852	0.554	5.148	-2.107
TMEM220	-0.168	0.890	3.948	-0.681
TMEM221	0.420	1.338	2.972	1.215
TMEM229A	-0.488	0.713	3.751	-2.093
TMEM229B	0.602	1.518	2.988	2.142
TMEM232	0.308	1.238	3.949	1.136
TMEM235	-0.622	0.650	3.745	-1.824
TMEM240	0.766	1.701	3.746	1.877
TMEM241	0.706	1.631	4.068	2.408
TMEM242	0.286	1.219	5.584	1.380
TMEM25	0.391	1.311	4.552	1.597
TMEM254-AS1	0.164	1.120	2.409	0.555
TMEM255A	-1.117	0.461	6.551	-2.504
TMEM255B	-0.096	0.936	3.527	-0.384
TMEM257	0.044	1.031	4.915	0.151
TMEM30B	0.198	1.147	2.428	0.472
TMEM35	0.701	1.626	5.040	1.394
TMEM38A	0.409	1.328	5.258	1.410
TMEM38B	-0.434	0.740	5.276	-1.717
TMEM44	0.386	1.307	3.302	1.420
TMEM44-AS1	-0.940	0.521	3.400	-3.223
TMEM45A	-0.504	0.705	2.775	-1.887
TMEM51	-0.112	0.925	2.933	-0.400

TMEM56//TMEM56-RWDD3//RWDD3//	0.079	1.056	5.266	0.447
TMEM59L	0.697	1.621	6.330	1.654
TMEM60	0.264	1.201	4.654	1.234
TMEM61	0.180	1.133	2.292	0.759
TMEM63A	-1.303	0.405	7.885	-2.756
TMEM63C	0.390	1.310	5.792	1.293
TMEM65	0.571	1.486	6.905	2.471
TMEM67	0.327	1.254	4.277	1.277
TMEM70	0.074	1.053	3.186	0.346
TMEM71	0.052	1.037	2.326	0.175
TMEM72-AS1	0.249	1.188	2.768	0.874
TMEM74B	0.713	1.639	3.162	2.056
TMEM81	0.149	1.108	3.076	0.542
TMEM87A	-0.687	0.621	7.126	-2.473
TMEM88	-0.385	0.766	3.331	-1.035
TMEM88B	-0.764	0.589	6.518	-2.073
TMEM98	-0.855	0.553	6.465	-2.055
TMEM9B-AS1	-0.836	0.560	4.828	-2.155
TMIE	-0.361	0.779	3.946	-1.296
TMOD1	0.210	1.157	7.913	0.798
TMOD2	0.665	1.586	8.610	2.604
TMPRSS3	0.430	1.347	2.536	1.339
TMPRSS5	-0.384	0.766	3.926	-0.977
TMSB4X	-0.043	0.970	5.326	-0.173
TMSB4XP4	-0.003	0.998	4.348	-0.010
TMSB4Y	-0.534	0.691	2.674	-1.178
TMTC1	0.505	1.419	6.189	2.202
TMTC2	-0.790	0.578	6.151	-2.172
TMTC3	0.300	1.231	6.648	1.536
TMTC4	-0.531	0.692	6.227	-1.493
TMX3	0.395	1.315	6.055	1.735
TMX4	0.606	1.522	7.453	2.351
TNC	-1.191	0.438	5.863	-2.277
TNFAIP2	-0.086	0.942	4.348	-0.332
TNFAIP3	-0.162	0.894	5.136	-0.409
TNFAIP6	0.028	1.020	3.513	0.100
TNFAIP8	-0.280	0.824	2.198	-1.229
TNFAIP8L1	0.797	1.738	3.211	2.032
TNFAIP8L3	0.319	1.248	4.226	1.175
TNFRSF10C//LOC254896	0.252	1.191	3.218	0.938
TNFRSF10D	-0.451	0.731	3.242	-1.643
TNFRSF11A	0.298	1.230	3.343	1.149
TNFRSF11B	0.642	1.560	4.103	2.056
TNFRSF12A	0.052	1.037	5.414	0.148
TNFRSF13C	-0.152	0.900	2.456	-0.429
TNFRSF14	0.071	1.051	3.469	0.223
TNFRSF1A	-0.342	0.789	5.557	-1.527
TNFRSF1B	-0.251	0.840	6.924	-0.552
TNFRSF21	-0.492	0.711	7.681	-1.505
TNFRSF25	0.576	1.490	3.438	2.056

TNFSF10	-0.227	0.854	4.997	-0.567
TNFSF13B	0.208	1.155	3.813	0.700
TNFSF14	-0.146	0.903	2.769	-0.638
TNFSF8	-0.150	0.901	2.601	-0.436
TNKS	-0.198	0.872	6.799	-0.943
TNNC1	-0.036	0.975	2.348	-0.121
TNNT1	0.623	1.540	2.379	2.432
TNNT2	0.902	1.868	2.399	3.121
TNRC6C-AS1	-1.164	0.446	5.361	-2.556
TNS1	-0.587	0.666	4.777	-2.069
TNS3	-0.518	0.698	5.303	-2.756
TNXB	0.432	1.349	4.100	1.759
TNXB	0.287	1.220	4.426	1.078
TNXB//TNXA	0.221	1.165	4.513	0.922
TNXB//TNXA	0.122	1.089	4.641	0.474
TNXB//TNXA	0.042	1.030	4.335	0.166
TOB2	-0.502	0.706	5.096	-1.903
TOB2P1	-0.146	0.904	2.635	-0.511
TOLLIP	0.468	1.383	5.808	2.033
TOM1L1	-0.010	0.993	6.078	-0.030
TOMM20	0.431	1.348	6.539	1.913
TOMM34	0.697	1.621	6.618	2.308
TOMM40L	0.429	1.347	4.274	1.949
TOMM70A	0.406	1.325	6.493	2.170
TOR1A	0.328	1.256	6.492	1.515
TOX	1.112	2.161	6.481	3.645
TOX2//C20orf100	0.418	1.336	3.610	1.349
TP53	-0.072	0.952	4.964	-0.252
TP53I3	-0.163	0.893	3.567	-0.581
TP53INP1	-0.484	0.715	4.184	-1.734
TP53INP2	-1.177	0.442	7.811	-2.894
TP53RK	-0.281	0.823	4.339	-1.128
TP53TG5	-0.653	0.636	4.318	-1.676
TPBG	0.239	1.180	2.735	0.807
TPBGL	-0.414	0.750	3.117	-1.227
TPCN1	-0.555	0.681	5.850	-2.162
TPD52L1	0.006	1.004	5.626	0.023
TPGS1	0.208	1.155	3.053	0.724
TPI1	0.410	1.329	4.367	1.500
TPM2	-0.150	0.901	3.965	-0.568
TPMT	0.619	1.536	6.387	2.089
TPPP	-0.041	0.972	8.980	-0.195
TPPP3	-0.713	0.610	7.821	-1.744
TPRG1L	0.624	1.542	7.471	2.210
TPRN	-0.444	0.735	2.976	-1.620
TPSB2//TPSAB1	0.367	1.290	2.542	0.996
TPSD1	0.010	1.007	2.161	0.039
TPST1	0.232	1.175	5.041	0.771
TPT1//LOC101928826	-0.667	0.630	7.722	-2.434
TPTE2P1	0.300	1.231	4.739	0.767

TPTE2P5	-0.068	0.954	3.209	-0.235
TPX2	0.936	1.913	2.743	2.709
TRADD	-0.112	0.925	4.069	-0.402
TRAF1	-0.810	0.570	4.081	-2.368
TRAF3IP3	-0.359	0.780	2.685	-1.240
TRAF5	0.470	1.385	3.327	2.106
TRAFD1	-0.683	0.623	5.084	-2.502
TRAJ11	-0.059	0.960	2.800	-0.281
TRAJ32//YME1L1	-0.350	0.785	2.308	-1.111
TRAJ59	-0.786	0.580	2.737	-1.462
TRAK2	-0.670	0.629	7.677	-2.626
TRAM1	-0.142	0.906	2.492	-0.525
TRAM1L1	-0.163	0.893	2.410	-0.808
TRANK1	0.821	1.766	5.375	2.167
TRAPPC13//CTC-534A2.2	0.289	1.222	4.765	1.098
TRAPPC2	0.136	1.099	2.437	0.472
TRAPPC6B	0.651	1.570	6.912	2.540
TRAV1-1	0.062	1.044	2.982	0.209
TRAV38-1//YME1L1	0.161	1.118	3.670	0.527
TRAV38-2DV8//YME1L1	-0.404	0.756	3.313	-0.992
TRBC2//IL23A	0.759	1.692	5.188	1.962
TRBJ2-1//IL23A	0.387	1.308	2.681	1.332
TRBJ2-5//EPC1	-0.566	0.675	2.263	-1.709
TRBJ2-7//IL23A	0.081	1.058	3.456	0.215
TRBV20OR9-2	0.088	1.063	3.037	0.311
TRBV23-1	-0.140	0.908	3.521	-0.369
TRBV7-1//TRBV7-2	-0.441	0.737	2.945	-1.626
TRDN	0.205	1.153	3.697	0.668
TREM1	0.082	1.058	3.254	0.261
TREM2	-0.426	0.744	5.456	-1.086
TREML1	-0.530	0.693	2.450	-1.682
TRERF1	0.563	1.477	4.454	1.548
TRGJ2//TRGJ1//TARP	0.020	1.014	2.280	0.052
TRGJP1//TARP	0.175	1.129	2.730	0.273
TRGJP2//TARP//TRGC2	0.708	1.633	4.754	0.928
TRGV9//TARP	-0.116	0.923	2.302	-0.414
TRH	-0.434	0.740	3.001	-1.567
TRHDE	1.773	3.418	4.042	3.237
TRHDE-AS1	0.938	1.915	3.132	2.358
TRIB1	0.109	1.079	4.300	0.428
TRIL	-0.532	0.691	6.247	-1.453
TRIM17	0.392	1.312	3.003	1.309
TRIM21	-0.182	0.881	3.546	-0.615
TRIM22	-0.014	0.991	5.857	-0.049
TRIM23	0.262	1.200	6.071	1.241
TRIM32	0.323	1.251	4.286	1.230
TRIM36	0.150	1.110	4.060	0.629
TRIM37	0.928	1.902	7.967	2.943
TRIM38	-0.387	0.765	5.065	-1.148
TRIM47	0.079	1.056	4.922	0.269

TRIM5	0.044	1.031	3.213	0.203
TRIM52	-0.186	0.879	5.138	-0.861
TRIM56	-0.441	0.737	5.136	-1.896
TRIM59	-0.710	0.611	3.706	-1.672
TRIM61	-0.482	0.716	2.824	-1.583
TRIM62	-0.985	0.505	4.589	-3.411
TRIM65	-0.152	0.900	4.947	-0.585
TRIM66	-0.661	0.632	4.043	-1.886
TRIM66	0.121	1.088	3.784	0.380
TRIM74	-0.711	0.611	3.450	-2.092
TRIM9	0.291	1.223	6.619	1.370
TRIO	0.432	1.350	6.167	1.834
TRIOBP//NOL12	-0.286	0.820	3.161	-1.299
TRIP10	0.281	1.215	4.797	1.193
TRIP6//MIR6875	-0.294	0.816	5.443	-0.976
TRIQK	0.393	1.313	3.477	1.386
TRMT12	0.055	1.039	4.550	0.234
TRMT61B	-0.226	0.855	4.131	-0.971
TRNP1	0.528	1.442	4.485	1.405
TRO	0.319	1.248	3.392	1.438
TRPC3	-0.065	0.956	2.347	-0.189
TRPC4	0.932	1.908	3.601	2.671
TRPC5	0.830	1.778	4.338	1.409
TRPC6	0.931	1.907	2.333	3.514
TRPM3	-0.466	0.724	7.404	-1.326
TRPM4	0.087	1.062	4.080	0.337
TRPM6	-0.708	0.612	4.031	-1.929
TRPS1	-0.525	0.695	6.464	-2.354
TRPV5	-1.113	0.462	3.391	-2.505
TRUB1	0.672	1.594	5.020	2.977
TRUB2	0.123	1.089	6.312	0.587
TSC22D1	0.393	1.313	4.952	1.810
TSC22D1-AS1	0.417	1.335	4.251	1.832
TSC22D2	0.622	1.539	5.713	2.025
TSC22D4	-0.660	0.633	4.887	-2.282
TSEN54	-0.492	0.711	3.631	-2.019
TSHR	-0.080	0.946	2.763	-0.225
TSHZ2	0.079	1.056	5.580	0.329
TSHZ3	0.400	1.319	4.671	1.622
TSKU	-0.122	0.919	3.432	-0.452
TSPAN11	-0.434	0.740	3.090	-1.687
TSPAN13	1.216	2.323	6.738	2.171
TSPAN14	0.546	1.460	3.467	1.839
TSPAN15	-0.851	0.555	5.590	-2.062
TSPAN17	0.266	1.202	4.952	1.104
TSPAN18	-0.280	0.824	3.825	-0.937
TSPAN2	0.504	1.418	4.014	1.476
TSPAN33	-0.183	0.881	6.137	-0.875
TSPAN5	0.292	1.224	5.187	1.330
TSPAN6	-0.082	0.945	5.306	-0.358

TSPAN7//RP5-972B16.2	0.634	1.552	6.882	2.088
TSPAN8	-0.980	0.507	3.008	-2.537
TSPAN9	0.589	1.505	4.154	2.955
TSPO	-0.351	0.784	3.841	-1.299
TSPY26P	0.054	1.038	3.762	0.218
TSPYL1	0.825	1.771	7.123	2.477
TSPYL2	0.622	1.539	7.385	2.409
TSPYL4	0.273	1.209	7.694	1.144
TSPYL5	0.290	1.223	4.842	1.295
TSSC1-IT1	0.037	1.026	2.621	0.132
TSSK3	-0.441	0.737	3.212	-1.243
TST	-0.703	0.614	5.957	-2.167
TSTD1	0.639	1.557	3.495	1.876
TTBK1	0.279	1.213	5.341	1.216
TTBK2	0.410	1.328	7.421	1.833
TTC18	-0.346	0.787	4.502	-1.146
TTC19	0.491	1.405	6.035	2.345
TTC21B//LOC100506124	0.127	1.092	6.001	0.646
TTC21B//LOC100506124	0.067	1.048	3.316	0.270
TTC25	0.005	1.003	3.387	0.017
TTC26	0.101	1.073	3.685	0.392
TTC30B	-0.090	0.939	2.429	-0.327
TTC33	0.702	1.627	5.784	2.983
TTC34	0.127	1.092	2.653	0.515
TTC38	-0.230	0.853	5.009	-0.883
TTC39A	0.337	1.263	2.952	1.159
TTC39B	0.399	1.319	4.748	1.230
TTC39C	0.829	1.776	4.971	3.002
TTC3P1	-0.019	0.987	5.066	-0.070
TTC40	-0.023	0.984	2.616	-0.093
TTC5	0.428	1.346	3.510	1.912
TTC7A	-0.378	0.769	5.278	-1.374
TTC7B	0.453	1.369	6.044	2.114
TTC9	1.080	2.115	5.444	1.862
TTC9B	1.251	2.379	3.141	3.017
TTF1	0.138	1.100	4.949	0.589
TTF2	-0.295	0.815	3.457	-0.923
TTLL4	-0.670	0.629	5.910	-2.485
TTLL7	-0.236	0.849	7.527	-0.943
TTLL7-IT1	-0.898	0.537	4.598	-2.341
TTLL9	-1.138	0.455	4.437	-3.677
TTN	-0.936	0.523	4.153	-1.672
TTN-AS1	0.041	1.029	4.620	0.136
TTPA	-0.557	0.680	3.569	-1.384
TTPAL	0.189	1.140	4.630	0.839
TTR	0.709	1.635	3.506	1.487
TTTY10	-0.536	0.690	2.997	-1.160
TTTY15	-0.052	0.965	4.857	-0.045
TTYH1	-0.296	0.815	5.683	-1.298
TTYH2	-1.059	0.480	7.825	-2.714

TUB	0.213	1.159	6.769	0.800
TUBA3C//TUBA3D	0.395	1.315	2.381	1.425
TUBA3FP	-0.430	0.742	2.419	-1.346
TUBA4B	-0.151	0.901	2.461	-0.466
TUBA8	0.854	1.807	3.371	2.256
TUBB1	-0.294	0.816	2.466	-0.637
TUBB2A	0.119	1.086	7.602	0.463
TUBB2B	-0.116	0.922	4.925	-0.402
TUBB6	0.105	1.075	4.733	0.323
TUBGCP4	0.329	1.257	6.621	1.344
TUNAR//LINC00617	0.818	1.763	3.731	2.310
TUSC2	0.262	1.199	4.624	1.193
TUSC3	0.946	1.927	5.790	2.618
TVP23A	0.293	1.225	3.794	1.065
TXLNB	-0.308	0.808	4.199	-0.974
TXLNGY//TXLNG2P	0.108	1.078	4.067	0.109
TXLNGY//TXLNG2P	0.077	1.055	3.987	0.083
TXNDC16	0.225	1.168	5.371	1.016
TXNIP	-0.996	0.501	8.580	-2.601
TYK2	-0.256	0.837	4.675	-1.074
TYMP//SCO2	-0.045	0.969	4.439	-0.216
TYMS	-0.621	0.650	3.644	-1.351
TYROBP	-0.417	0.749	8.375	-0.978
TYRP1	0.181	1.134	2.630	0.599
TYW3	0.278	1.212	4.378	1.397
U91328.19	0.155	1.113	2.438	0.702
UACA	-0.505	0.705	5.261	-1.726
UAP1	0.471	1.386	5.823	1.584
UAP1L1	-0.433	0.741	4.568	-1.637
UBA7//MIR5193	-0.395	0.760	4.516	-1.118
UBALD1	0.319	1.248	5.369	1.311
UBALD2	-0.188	0.878	6.088	-0.684
UBC	-0.357	0.781	6.758	-1.173
UBE2E2	0.165	1.121	5.648	0.796
UBE2MP1	0.359	1.282	6.058	1.509
UBE2O	0.437	1.354	6.329	1.727
UBE2Q2L//RP11-182J1.16	-0.077	0.948	2.845	-0.220
UBE2QL1	1.114	2.164	5.480	2.035
UBE2T	0.748	1.679	4.035	1.983
UBE2W//RP11-463D19.2	0.484	1.399	4.285	2.206
UBL7-AS1	-0.492	0.711	2.497	-1.541
UBLCP1	0.198	1.147	6.870	0.890
UBP1	0.276	1.211	3.496	1.289
UBQLN2	0.203	1.151	7.618	1.003
UBR3	0.321	1.249	7.444	1.608
UBTD1	-0.242	0.845	4.246	-1.048
UBXN10	0.167	1.123	4.637	0.536
UBXN10-AS1//RP3-340N1.5	-0.049	0.966	3.081	-0.177
UBXN2B	0.432	1.349	5.152	1.488
UCHL1	0.434	1.351	7.102	1.675

UCHL5	0.263	1.200	5.069	1.296
UCN	-0.195	0.873	3.490	-0.752
UCP2	-0.687	0.621	3.662	-1.765
UG0898H09	-0.070	0.953	6.257	-0.241
UGCG	0.509	1.423	6.060	1.884
UGDH-AS1	-0.787	0.579	3.416	-2.440
UGP2	0.438	1.355	4.159	2.075
UGT8	-1.225	0.428	9.349	-2.717
UHRF1BP1L	0.483	1.397	6.404	1.974
ULK1	0.383	1.304	5.047	1.663
ULK4	0.038	1.027	3.782	0.140
ULK4P1//RP13-395E19.3//ULK4P3//ULK	-0.454	0.730	5.372	-1.538
ULK4P3	0.062	1.044	3.037	0.179
ULK4P3//ULK4P1//RP13-395E19.3//CHR	-0.450	0.732	5.583	-1.468
UNC13A	1.466	2.763	6.083	2.446
UNC13C	1.654	3.146	6.023	2.683
UNC13D	-0.698	0.617	3.982	-2.262
UNC5A	1.363	2.571	3.924	2.669
UNC5B	-0.903	0.535	6.311	-3.125
UNC5C	-0.594	0.663	5.563	-2.231
UNC5CL//RP1-34B21.5	-0.083	0.944	2.589	-0.301
UNC5D	1.095	2.136	4.758	2.130
UNC79	0.616	1.532	5.864	1.873
UNC80	0.956	1.940	6.503	2.155
UNC93B1	-0.294	0.816	4.053	-1.166
UNC93B3//UNC93B1//UNC93B7//UNC9	-0.073	0.950	4.223	-0.311
UPF2	0.425	1.343	5.921	1.757
UPP2	0.476	1.391	3.478	1.639
UPRT	0.222	1.166	3.775	0.957
UQCRB	0.343	1.269	3.832	1.499
UQCRC2	0.363	1.286	8.615	2.013
UQCRFS1	0.385	1.306	4.660	1.517
UQCRH//UQCRHL	0.333	1.259	4.050	1.542
UQCRHL//RP11-169K16.7	0.322	1.250	6.254	1.202
USH1C	-1.054	0.482	3.477	-2.870
USP11	0.496	1.410	7.602	2.325
USP17L15//USP17L6P//USP17L9P//USP1	0.181	1.133	4.568	0.650
USP17L15//USP17L6P//USP17L9P//USP1	0.225	1.169	4.602	0.800
USP17L15//USP17L6P//USP17L9P//USP1	0.122	1.088	4.435	0.434
USP17L5	0.442	1.359	4.290	1.438
USP25	0.596	1.512	6.672	2.815
USP27X	0.489	1.403	3.713	1.428
USP28	0.123	1.089	4.674	0.496
USP32	-0.045	0.969	7.579	-0.229
USP32P2	0.736	1.666	3.661	1.942
USP33	0.269	1.205	6.136	1.471
USP40	-0.508	0.703	5.400	-1.958
USP43	0.497	1.411	3.126	1.602
USP53	-0.191	0.876	6.652	-0.787
USP54	-0.970	0.510	7.779	-3.228

USP9Y	0.158	1.116	5.172	0.135
UTP14A	-0.105	0.930	3.235	-0.406
UTP23	0.057	1.040	5.378	0.255
UTRN	-0.355	0.782	6.937	-1.474
UTS2	-0.381	0.768	2.285	-0.818
UTY	-0.146	0.904	3.510	-0.141
VAC14-AS1	-0.190	0.876	4.115	-0.620
VAMP1	0.124	1.090	7.972	0.335
VAMP2//RP11-599B13.6	0.332	1.259	7.771	1.668
VAMP3	-0.534	0.691	7.659	-1.907
VAMP4	0.400	1.319	6.450	1.552
VAMP5	-0.262	0.834	5.275	-1.153
VAMP8	-0.547	0.684	4.699	-1.531
VANGL2	-0.578	0.670	4.877	-2.327
VASH1	-0.676	0.626	5.492	-2.689
VASP	-0.545	0.686	5.666	-2.257
VAT1	-0.527	0.694	6.576	-2.154
VAT1L	-0.058	0.961	7.969	-0.145
VAV1	-0.608	0.656	4.883	-1.816
VAV3	0.353	1.277	2.509	1.493
VAX1	-0.281	0.823	2.393	-0.950
VCAM1	-0.182	0.881	5.033	-0.471
VCAN	-0.918	0.529	6.898	-2.740
VCAN-AS1	-0.250	0.841	1.877	-0.869
VCL	0.197	1.146	6.110	0.681
VCPIP1	0.302	1.233	6.558	1.362
VCPKMT//METTL21D	0.218	1.163	4.022	0.954
VCX2//VCX	0.216	1.162	3.470	0.695
VDAC3	0.274	1.209	8.659	1.354
VEGFA	-0.473	0.721	5.518	-0.883
VEGFC	0.016	1.011	3.252	0.062
VEZF1	-0.738	0.600	6.217	-3.208
VGF	0.260	1.198	4.633	0.667
VGLL3	-0.415	0.750	2.993	-1.576
VIM	0.206	1.154	6.136	0.888
VIM-AS1	-0.030	0.980	5.032	-0.094
VIP	0.750	1.682	3.672	1.703
VIPR1	1.100	2.143	3.709	2.594
VMAC	-0.655	0.635	4.601	-2.259
VMO1	-0.024	0.983	2.623	-0.103
VN1R1	0.076	1.054	4.111	0.392
VNN1	-0.332	0.795	2.409	-0.701
VNN2	0.104	1.074	2.516	0.233
VPS11	0.230	1.173	5.916	0.946
VPS13A	0.776	1.712	6.139	2.950
VPS33A//RP11-512M8.5	0.228	1.172	4.260	0.987
VPS33B	0.446	1.363	5.364	1.973
VPS35	0.299	1.230	8.852	1.488
VPS36	0.365	1.288	6.224	1.594
VPS37D	-0.098	0.934	2.753	-0.329

VRK1	0.258	1.196	3.446	1.156
VRK2	-0.980	0.507	4.186	-2.616
VSIG1	0.509	1.423	2.578	1.669
VSIG4	-0.451	0.731	6.044	-0.782
VSNL1	1.045	2.064	2.481	2.116
VSTM2A	1.607	3.047	5.823	2.165
VSTM2A-OT1//GS1-18A18.1	1.699	3.247	3.198	2.877
VSTM2B	0.175	1.129	6.069	0.801
VSTM2L	1.038	2.054	4.765	2.184
VSTM5	0.291	1.223	2.711	1.099
VSX1	-0.335	0.793	2.851	-1.296
VTA1	0.291	1.224	6.956	1.379
VTRNA1-1	-0.384	0.766	5.430	-0.725
VTRNA1-2	0.035	1.024	2.010	0.117
VTRNA1-3	-0.256	0.838	3.889	-0.607
VTRNA2-1	-0.763	0.589	4.591	-1.527
VWA1	-0.492	0.711	5.171	-1.415
VWA3A	0.589	1.504	4.222	1.574
VWA5A	-0.162	0.894	5.507	-0.560
VWA5B1	-0.338	0.791	1.943	-1.168
VWA5B2	0.555	1.469	3.393	1.713
VWA7	0.324	1.252	3.055	1.104
VWA7	0.263	1.200	3.355	0.884
VWC2	0.810	1.753	4.732	2.481
VWC2L	0.852	1.805	3.635	1.913
VWF	-0.007	0.995	5.970	-0.025
WAC-AS1	0.155	1.113	4.917	0.746
WAS	-0.464	0.725	4.799	-1.573
WASF1	0.889	1.852	6.429	2.933
WASF2	-0.625	0.648	8.447	-2.488
WASH3P	-0.084	0.943	3.081	-0.337
WASH3P//ABC7-42389800N19.1//WASH	-0.157	0.897	5.681	-0.428
WASH5P	-0.381	0.768	5.572	-1.522
WASL	0.381	1.302	7.659	1.645
WBP11P1	0.650	1.569	2.119	2.699
WBP1L	-0.123	0.918	7.468	-0.675
WBP2NL	0.105	1.076	2.855	0.346
WBP5	-0.318	0.802	6.357	-1.546
WBSCR17	1.610	3.053	5.236	2.718
WDFY2	-0.734	0.601	6.696	-2.921
WDFY3-AS1	-0.349	0.785	5.013	-1.488
WDFY4	-0.647	0.639	3.307	-2.267
WDHD1	0.315	1.244	3.657	1.073
WDR11	-0.295	0.815	6.681	-1.084
WDR11-AS1	-0.291	0.817	3.731	-0.894
WDR16	0.200	1.149	3.971	0.411
WDR17	0.901	1.867	6.436	2.612
WDR19	0.284	1.217	6.075	1.286
WDR3	0.360	1.283	5.776	1.629
WDR36	0.158	1.116	6.460	0.712

WDR37	0.505	1.419	6.253	2.201
WDR38	-0.877	0.545	2.822	-2.247
WDR47	0.483	1.398	7.450	1.752
WDR49	-1.046	0.484	6.582	-2.758
WDR52	-0.144	0.905	4.419	-0.587
WDR54	0.605	1.521	4.049	2.676
WDR5B	-0.626	0.648	4.481	-2.168
WDR63	0.217	1.162	3.021	0.450
WDR65	-0.253	0.839	2.291	-0.877
WDR7	0.389	1.310	7.554	2.004
WDR86-AS1	0.061	1.043	2.936	0.234
WDR92//RP11-474G23.1	0.075	1.053	4.742	0.326
WDR96	-0.265	0.832	3.877	-0.496
WEE1	-0.164	0.893	3.012	-0.818
WFDC1	0.748	1.680	4.368	2.393
WHAMMP1	-0.577	0.670	4.366	-1.641
WIF1	1.726	3.308	4.038	2.826
WIPF1	-0.752	0.594	4.684	-2.000
WIPF2	0.185	1.137	6.072	0.903
WIPF3	1.185	2.274	4.693	2.746
WIPI1	-0.250	0.841	6.531	-1.075
WISP1	-0.251	0.840	2.432	-0.992
WLS	-0.029	0.980	6.558	-0.133
WNK1	-0.461	0.726	8.467	-2.151
WNK2	0.529	1.443	5.425	1.585
WNK3	-0.038	0.974	4.407	-0.155
WNT10B	0.843	1.794	3.501	2.584
WNT3//LOC101929777	0.476	1.391	3.763	1.394
WNT5A-AS1	-0.037	0.975	3.317	-0.192
WNT7A	-0.217	0.860	3.811	-0.677
WNT9A	0.045	1.031	3.057	0.152
WSB1	-0.682	0.623	8.719	-2.190
WSCD1//LOC339166	-0.704	0.614	5.455	-3.061
WSCD2	0.640	1.558	2.893	2.092
WWC1	-0.038	0.974	5.839	-0.200
WWTR1	-0.202	0.869	4.472	-0.860
XAF1	0.189	1.140	6.405	0.596
XBP1	0.073	1.052	6.243	0.296
XG//XGY2	-0.232	0.851	2.394	-0.859
XGY2//XG//SRY//RPS4Y1	-0.512	0.701	2.583	-1.587
XIST	0.142	1.103	3.852	0.070
XK	0.938	1.915	4.460	3.138
XKR4	1.202	2.300	4.083	2.796
XKR4	0.709	1.635	5.149	1.785
XKR6	0.320	1.249	3.873	1.284
XKR6//AF131215.2	0.215	1.161	3.295	0.692
XKR7	0.441	1.357	2.291	1.530
XKR8	-0.219	0.859	2.838	-0.967
XKR9	0.558	1.473	2.894	2.059
XPC	-0.431	0.742	5.895	-1.638

XPR1	0.261	1.198	7.494	1.286
XRCC4	-0.022	0.985	4.457	-0.080
XRCC6BP1	0.654	1.573	2.837	2.401
XRRA1	-0.426	0.744	4.838	-1.590
XXbac-B476C20.13	-0.168	0.890	4.147	-0.664
XXbac-B476C20.14	-0.464	0.725	2.503	-1.883
XXbac-BPG116M5.15	0.141	1.103	4.202	0.337
XXbac-BPG116M5.17//C2//CFB	-0.069	0.953	3.907	-0.232
XXbac-BPG154L12.4	0.289	1.222	3.434	1.006
XXbac-BPG55C20.7	-0.974	0.509	2.353	-3.678
YAP1	-0.012	0.992	5.306	-0.059
YARS2	0.191	1.141	4.766	0.916
YBX1	-0.512	0.701	8.285	-2.322
YBX3	0.535	1.449	4.962	1.543
YBX3P1//CSDAP1	0.404	1.324	2.479	1.323
YEATS4	0.132	1.096	4.993	0.501
YIF1A	-0.197	0.872	4.914	-0.905
YIPF4	-0.067	0.955	6.677	-0.305
YIPF5	0.134	1.097	7.430	0.639
YJEFN3//CTC-260F20.3	0.379	1.301	4.113	1.294
YME1L1	0.210	1.157	3.827	0.852
YME1L1//TRAJ17//TRAV20//TRAC//TRD	-0.526	0.695	3.614	-1.354
YME1L1//TRAV12-2	-0.258	0.836	3.253	-0.974
YOD1	0.035	1.024	4.039	0.093
YPEL1	0.459	1.375	3.708	1.543
YPEL3	0.075	1.054	7.615	0.337
YPEL4	0.470	1.385	3.892	1.803
YTHDF1	0.217	1.163	6.893	0.961
YWHAB	0.321	1.250	6.829	1.589
YWHAG	0.582	1.497	9.525	1.862
YWHAH	0.474	1.389	4.644	1.827
YY2	-0.182	0.881	3.143	-0.601
ZADH2	0.094	1.068	5.793	0.423
ZAK//AC013461.1	-0.034	0.977	5.264	-0.130
ZBBX	1.724	3.304	3.249	2.632
ZBED8//C5orf54	-0.492	0.711	3.918	-2.314
ZBED9//SCAND3	1.022	2.031	2.975	2.246
ZBTB12	-0.609	0.656	3.156	-2.502
ZBTB16	-0.557	0.680	3.406	-1.281
ZBTB16	0.111	1.080	5.897	0.323
ZBTB18	0.651	1.570	6.038	2.616
ZBTB20	-0.800	0.574	6.414	-2.750
ZBTB20//MIR568	0.198	1.147	3.820	0.448
ZBTB24	0.036	1.025	5.650	0.160
ZBTB33	0.480	1.395	4.921	2.341
ZBTB37	-0.618	0.652	5.823	-2.353
ZBTB39	-0.176	0.885	4.521	-0.684
ZBTB40-IT1	-0.251	0.840	3.675	-0.776
ZBTB44	0.023	1.016	6.969	0.119
ZBTB6	0.306	1.237	5.132	1.406

ZBTB7B	-0.586	0.666	4.765	-2.354
ZBTB7C	0.129	1.094	3.983	0.500
ZBTB8A//RP1-27O5.3	0.318	1.246	3.740	1.302
ZC2HC1A	0.411	1.330	6.509	1.733
ZC3H12A//MIR6732	-0.208	0.866	3.934	-0.909
ZC3H12B	0.550	1.465	5.248	2.076
ZC3H8	0.058	1.041	4.956	0.199
ZC3HAV1	-0.404	0.756	6.638	-1.451
ZCCHC12	0.229	1.172	4.470	0.389
ZCCHC24	-0.633	0.645	6.897	-2.376
ZCWPW1	-0.216	0.861	4.354	-0.920
ZDBF2	0.640	1.558	6.157	2.185
ZDHHC11	-0.773	0.585	3.772	-2.309
ZDHHC12	-0.246	0.843	3.718	-0.929
ZDHHC13	0.188	1.139	4.491	0.685
ZDHHC14	-0.771	0.586	6.637	-3.392
ZDHHC20	-1.087	0.471	7.162	-3.280
ZDHHC20-IT1	-0.762	0.590	4.022	-1.844
ZDHHC21	0.517	1.431	6.600	2.062
ZDHHC22	0.580	1.495	3.962	2.196
ZDHHC23	0.989	1.985	3.406	2.211
ZDHHC8P1	0.402	1.322	2.951	1.319
ZDHHC9	-0.694	0.618	8.342	-2.456
ZEB2-AS1	-0.228	0.854	2.680	-0.891
ZER1	0.403	1.322	7.289	1.978
ZFAND1	0.394	1.314	5.138	1.826
ZFAS1	-0.271	0.829	7.020	-1.420
ZFHX3	-0.409	0.753	5.541	-1.617
ZFP1	0.236	1.178	6.019	0.939
ZFP14	-0.256	0.838	4.207	-0.918
ZFP28	0.018	1.012	4.165	0.078
ZFP36	0.811	1.754	7.914	1.801
ZFP36L1	-0.405	0.755	4.540	-1.717
ZFP36L2	0.103	1.074	6.473	0.286
ZFP69B	1.057	2.080	4.288	2.699
ZFP82	0.168	1.123	4.940	0.707
ZFP90	0.516	1.430	3.576	1.953
ZFP92	0.731	1.660	3.087	2.404
ZFY	0.261	1.198	2.779	0.583
ZFYVE16	-0.530	0.692	7.030	-1.564
ZFYVE28	0.333	1.259	2.889	1.178
ZFYVE28	0.323	1.251	3.184	1.076
ZFYVE9	0.529	1.443	6.738	2.240
ZHX2	-0.547	0.684	5.691	-1.868
ZIC1	0.503	1.417	5.310	1.648
ZIC2	0.140	1.102	3.888	0.523
ZIC5	0.418	1.336	4.724	1.531
ZIK1	0.273	1.208	3.822	1.165
ZKSCAN2	0.019	1.013	4.897	0.091
ZKSCAN3	0.155	1.113	4.132	0.493

ZMAT1	-0.575	0.671	5.760	-1.482
ZMAT3	0.257	1.195	7.308	1.322
ZMAT4	1.074	2.105	4.781	2.699
ZMYM6NB//ZMYM6//RP11-244H3.4	-0.094	0.937	6.006	-0.351
ZMYND10	0.049	1.035	2.332	0.163
ZMYND15	-0.238	0.848	2.885	-1.079
ZMYND8	-0.135	0.911	6.423	-0.680
ZNF100	0.220	1.164	5.212	0.777
ZNF114	0.347	1.272	4.115	1.192
ZNF117	-0.463	0.725	4.634	-1.716
ZNF132	-0.169	0.889	3.636	-0.719
ZNF134	0.249	1.188	5.502	1.177
ZNF138	-0.356	0.781	6.107	-1.528
ZNF14	-0.025	0.983	4.109	-0.096
ZNF140	0.599	1.514	3.220	1.881
ZNF154	0.343	1.269	4.332	1.395
ZNF17	0.124	1.090	4.481	0.477
ZNF184	0.514	1.428	5.379	1.710
ZNF185	0.219	1.164	3.386	0.867
ZNF189	-0.120	0.921	5.645	-0.521
ZNF192P1	-0.098	0.935	4.067	-0.294
ZNF200	0.115	1.083	3.336	0.502
ZNF204P	1.059	2.083	2.807	2.407
ZNF208	-0.474	0.720	2.626	-1.011
ZNF215	0.560	1.474	1.921	1.930
ZNF217	-0.038	0.974	3.962	-0.160
ZNF22	-0.210	0.865	3.405	-0.785
ZNF223	0.361	1.284	3.776	1.346
ZNF224	-0.194	0.874	4.027	-0.652
ZNF225	0.080	1.057	3.429	0.340
ZNF227	-0.060	0.959	5.154	-0.240
ZNF229//CTC-512J12.4//ZFP28	-0.120	0.920	4.351	-0.407
ZNF23//AC010547.9	0.167	1.123	4.005	0.637
ZNF230	0.566	1.480	3.082	1.675
ZNF232	0.202	1.150	3.146	0.639
ZNF233	0.326	1.254	2.038	1.278
ZNF234	-0.012	0.992	4.141	-0.045
ZNF239	0.049	1.034	2.946	0.168
ZNF248	0.126	1.091	5.664	0.541
ZNF25	0.702	1.627	7.603	2.428
ZNF253	-0.067	0.955	4.593	-0.263
ZNF256	0.282	1.216	3.697	1.018
ZNF267	0.138	1.100	4.200	0.431
ZNF274	-0.095	0.936	3.335	-0.417
ZNF276	-0.368	0.775	4.557	-1.592
ZNF280B	0.358	1.282	5.011	1.157
ZNF280C	0.186	1.137	5.633	0.666
ZNF280D//LOC145783	-0.638	0.643	4.920	-2.250
ZNF280D//LOC145783	-0.126	0.917	2.303	-0.512
ZNF280D//LOC145783	-0.009	0.994	2.813	-0.031

ZNF284//ZNF223//AC084219.2	0.061	1.043	4.028	0.171
ZNF285	0.105	1.076	3.141	0.319
ZNF3	-0.751	0.594	3.826	-3.021
ZNF300	0.005	1.003	3.013	0.018
ZNF302	-0.159	0.895	5.790	-0.635
ZNF304	0.369	1.291	4.364	1.500
ZNF311	-0.187	0.878	2.232	-0.682
ZNF320	0.019	1.013	4.274	0.072
ZNF322	0.018	1.013	4.552	0.083
ZNF324B	-0.023	0.984	3.333	-0.076
ZNF326	-0.538	0.689	6.753	-2.378
ZNF329	0.289	1.222	3.874	1.291
ZNF33B	0.372	1.294	3.092	1.146
ZNF34	-0.342	0.789	3.728	-1.399
ZNF346-IT1	0.456	1.371	2.206	1.574
ZNF350	0.142	1.103	4.051	0.573
ZNF350-AS1//HCCAT3	-0.173	0.887	3.061	-0.672
ZNF365	0.310	1.240	2.640	1.471
ZNF37BP	0.355	1.279	5.663	1.616
ZNF382	0.452	1.368	4.155	1.973
ZNF383	0.297	1.228	4.322	1.220
ZNF385A	0.114	1.082	6.743	0.514
ZNF385B	1.531	2.889	3.479	2.809
ZNF385D	0.147	1.107	2.202	0.514
ZNF391	0.196	1.145	3.594	0.665
ZNF395//FBXO16	-0.063	0.957	4.250	-0.237
ZNF404	-0.431	0.741	4.905	-1.621
ZNF416	0.038	1.027	2.965	0.138
ZNF417	-0.167	0.891	5.032	-0.701
ZNF418	-0.434	0.740	4.080	-1.919
ZNF419	0.418	1.336	3.556	1.684
ZNF420	-0.146	0.904	5.280	-0.736
ZNF429	0.247	1.187	5.705	0.901
ZNF43	-0.132	0.913	4.488	-0.408
ZNF436	0.266	1.203	5.518	0.933
ZNF440	0.405	1.324	4.882	1.480
ZNF441	0.180	1.133	4.037	0.683
ZNF443	0.216	1.161	2.573	0.624
ZNF449	0.381	1.302	4.535	1.120
ZNF451	-0.248	0.842	5.987	-1.018
ZNF454	0.341	1.267	4.762	1.386
ZNF461	0.396	1.316	3.398	1.788
ZNF462	-0.819	0.567	6.223	-3.159
ZNF468	0.169	1.124	5.111	0.575
ZNF48	0.142	1.103	2.373	0.553
ZNF480	-0.413	0.751	4.792	-1.278
ZNF483	0.438	1.355	5.707	1.694
ZNF486	-0.391	0.762	3.349	-1.163
ZNF487	0.546	1.460	4.274	1.751
ZNF488	-0.634	0.644	4.244	-1.815

ZNF502	-0.535	0.690	3.353	-1.620
ZNF503//RP11-399K21.11	0.091	1.065	3.529	0.380
ZNF510	0.175	1.129	5.120	0.978
ZNF512//RP11-158I13.2	0.060	1.043	6.380	0.290
ZNF518A	-0.391	0.763	5.200	-1.310
ZNF518B	0.068	1.048	4.713	0.288
ZNF519	0.762	1.695	4.177	2.554
ZNF521	-0.614	0.653	5.548	-2.539
ZNF527	0.089	1.064	2.613	0.341
ZNF534	0.122	1.089	2.538	0.434
ZNF536	-0.927	0.526	6.515	-2.402
ZNF542P//ZNF542//LOC283788	0.092	1.066	3.787	0.454
ZNF547	0.015	1.010	4.653	0.062
ZNF549	-0.063	0.957	3.702	-0.202
ZNF554	0.843	1.794	4.135	3.250
ZNF557	-0.098	0.934	4.032	-0.333
ZNF558	0.315	1.244	4.270	1.306
ZNF563	0.250	1.189	2.583	0.641
ZNF567	0.401	1.321	3.754	1.588
ZNF569	-0.251	0.840	3.911	-0.977
ZNF571	-0.084	0.944	3.233	-0.277
ZNF572	-0.388	0.764	2.527	-1.250
ZNF581	-0.213	0.863	4.161	-0.654
ZNF582	0.226	1.170	3.779	0.919
ZNF582-AS1//AC006116.25	0.134	1.097	3.235	0.640
ZNF584	0.317	1.245	4.277	1.151
ZNF587B	-0.163	0.893	3.728	-0.625
ZNF594	-0.234	0.850	4.904	-0.867
ZNF596	-0.149	0.902	2.160	-0.579
ZNF597	-0.037	0.974	5.270	-0.168
ZNF607//CTD-2528L19.4	-0.131	0.913	4.948	-0.576
ZNF611	0.178	1.131	4.079	0.762
ZNF613	0.343	1.269	3.744	1.131
ZNF615	0.251	1.190	4.267	1.033
ZNF616	0.180	1.133	2.603	0.621
ZNF624	0.013	1.009	3.879	0.052
ZNF626	0.322	1.250	3.852	1.383
ZNF638//ZNF638-IT1	-0.111	0.926	4.671	-0.287
ZNF649	-0.368	0.775	4.599	-1.397
ZNF652	-0.818	0.567	6.978	-2.816
ZNF658//ZNF658B//CTD-2173L22.4	0.496	1.410	4.950	1.860
ZNF667	0.214	1.160	4.506	1.013
ZNF667-AS1//LOC100288114	0.075	1.053	7.459	0.347
ZNF669	0.393	1.313	3.786	1.463
ZNF674-AS1	0.070	1.050	3.455	0.270
ZNF675	1.013	2.019	2.534	3.775
ZNF681	0.722	1.650	3.631	2.682
ZNF689	0.264	1.201	3.029	1.015
ZNF69	-0.697	0.617	2.893	-2.735
ZNF691	-0.462	0.726	3.075	-1.696

ZNF697	0.414	1.333	3.464	1.646
ZNF699	0.124	1.090	3.851	0.475
ZNF702P	0.610	1.527	2.981	1.684
ZNF708	-0.361	0.779	4.744	-0.892
ZNF710	-0.437	0.739	3.237	-2.067
ZNF711	0.449	1.365	4.867	1.770
ZNF714	0.652	1.571	2.644	1.680
ZNF718//ZNF595	-0.035	0.976	4.052	-0.146
ZNF721//ABCA11P	0.281	1.215	4.590	1.097
ZNF724P	0.554	1.469	2.795	1.613
ZNF727P//RP11-3N2.13	-0.276	0.826	4.465	-1.082
ZNF727P//RP11-3N2.13	0.017	1.012	2.920	0.043
ZNF749	-0.008	0.995	3.310	-0.032
ZNF75D	-0.117	0.922	3.398	-0.498
ZNF764	-0.196	0.873	3.474	-0.687
ZNF770	0.455	1.371	6.360	1.916
ZNF771	0.192	1.142	2.799	0.803
ZNF774	0.195	1.145	4.119	0.800
ZNF776	0.268	1.204	5.235	1.246
ZNF780A	0.438	1.354	4.065	1.478
ZNF780B	0.142	1.103	5.807	0.653
ZNF781//ZFP30	-0.308	0.808	3.898	-1.425
ZNF783	-0.378	0.769	5.837	-1.371
ZNF786	0.356	1.280	4.862	1.147
ZNF787	-0.102	0.932	4.128	-0.326
ZNF789	-0.282	0.823	3.367	-1.142
ZNF790-AS1	0.127	1.092	3.115	0.418
ZNF791	0.197	1.146	6.429	0.955
ZNF804A	0.871	1.829	3.891	1.869
ZNF804B	1.213	2.318	3.919	2.950
ZNF812//ZNF562	-0.804	0.573	3.179	-2.190
ZNF814	-0.104	0.931	3.780	-0.422
ZNF815P	0.284	1.217	3.522	1.196
ZNF821	0.410	1.329	4.635	1.605
ZNF826P	-0.453	0.731	3.348	-1.313
ZNF829	0.315	1.244	2.346	1.230
ZNF83	-0.624	0.649	5.389	-2.493
ZNF831	0.814	1.758	2.810	2.319
ZNF835	-0.074	0.950	3.815	-0.359
ZNF836	-0.037	0.975	4.668	-0.117
ZNF843	-0.110	0.927	2.378	-0.401
ZNF844	-0.675	0.627	4.660	-2.220
ZNF850	-0.417	0.749	3.826	-1.607
ZNF852	-0.038	0.974	3.042	-0.158
ZNF91	0.556	1.470	5.433	1.979
ZNF91	0.578	1.493	2.930	1.831
ZNF93	0.122	1.088	3.909	0.452
ZNRF2P1	-0.058	0.960	4.007	-0.240
ZRANB2-AS2	0.113	1.081	3.492	0.402
ZRANB3	0.170	1.125	3.652	0.777

ZSCAN12	0.263	1.200	3.026	1.099
ZSCAN12P1	-0.858	0.552	3.359	-2.615
ZSCAN16	-0.109	0.927	2.841	-0.374
ZSCAN22	0.028	1.019	3.670	0.104
ZSCAN23	-0.107	0.929	3.850	-0.399
ZSCAN31	-0.263	0.833	3.964	-0.904
ZSWIM1	-0.179	0.883	3.712	-0.819
ZSWIM7	-0.560	0.678	3.682	-2.452
ZUFSP	0.164	1.121	4.366	0.732
ZWILCH	0.745	1.675	3.347	2.046
ZXDA	-0.023	0.984	3.892	-0.088
ZXDB	-0.172	0.887	5.899	-0.686
ZYG11B	0.590	1.506	7.983	2.546
		1.000		
		1.000		

P.Value	adj.P.Val	B	logFC validation	ALS.FC	CTL.FC
0.864	0.927	-5.701	0.053	3.843	3.790
0.746	0.853	-5.665	-0.100	7.365	7.464
0.131	0.313	-4.641	-0.433	3.986	4.419
0.363	0.554	-5.323	-0.258	2.506	2.764
0.078	0.247	-4.250	0.497	6.316	5.819
0.170	0.360	-4.827	0.359	4.274	3.914
0.050	0.208	-3.912	0.445	5.135	4.689
0.024	0.173	-3.315	0.592	8.134	7.541
0.093	0.268	-4.385	0.392	6.585	6.193
0.065	0.229	-4.118	-0.543	6.186	6.729
0.108	0.286	-4.495	-0.449	5.713	6.162
0.010	0.157	-2.616	-1.034	4.456	5.489
0.063	0.227	-4.093	0.527	2.824	2.298
0.593	0.745	-5.579	0.162	2.757	2.594
0.010	0.157	-2.628	-0.923	8.163	9.086
0.336	0.532	-5.278	0.240	6.954	6.714
0.017	0.162	-3.059	-1.673	4.305	5.979
0.002	0.157	-1.332	-1.552	5.640	7.193
0.324	0.520	-5.255	-0.372	5.023	5.396
0.358	0.552	-5.316	-0.200	5.141	5.341
0.660	0.793	-5.623	0.109	1.949	1.840
0.375	0.565	-5.343	-0.471	2.115	2.587
0.123	0.304	-4.596	-0.454	4.486	4.939
0.386	0.574	-5.359	0.193	6.785	6.592
0.421	0.607	-5.408	0.231	4.941	4.710
0.106	0.284	-4.482	0.561	6.654	6.093
0.020	0.166	-3.168	1.043	6.361	5.318
0.248	0.446	-5.084	-0.270	4.288	4.559
0.670	0.801	-5.629	-0.102	5.785	5.887
0.383	0.572	-5.355	-0.297	5.528	5.825
0.031	0.182	-3.547	0.853	5.637	4.784
0.004	0.157	-1.935	0.981	4.397	3.415
0.064	0.229	-4.107	0.375	6.284	5.909
0.005	0.157	-2.038	-0.871	2.930	3.801
0.433	0.617	-5.423	0.171	2.199	2.028
0.198	0.392	-4.932	0.341	2.900	2.559
0.306	0.503	-5.219	-0.301	6.386	6.687
0.055	0.216	-3.981	-0.557	4.197	4.754
0.242	0.439	-5.068	-0.374	5.119	5.493
0.081	0.252	-4.280	-0.495	4.334	4.830
0.051	0.209	-3.929	0.747	6.857	6.111
0.143	0.327	-4.702	-0.532	3.752	4.285
0.079	0.249	-4.262	-0.740	3.967	4.707
0.010	0.157	-2.646	-0.564	6.678	7.242
0.057	0.218	-4.008	0.768	6.414	5.645
0.685	0.811	-5.637	-0.106	5.862	5.968
0.060	0.221	-4.048	0.587	3.828	3.241
0.866	0.928	-5.701	0.040	4.491	4.451
0.103	0.280	-4.461	0.403	4.349	3.946

0.037	0.190	-3.674	-0.876	4.649	5.525
0.088	0.262	-4.348	-0.506	4.069	4.575
0.142	0.326	-4.699	-0.440	3.595	4.034
0.987	0.993	-5.715	0.004	2.418	2.413
0.630	0.771	-5.605	0.152	3.885	3.733
0.647	0.784	-5.615	0.151	3.262	3.110
0.078	0.248	-4.254	-0.469	5.031	5.500
0.200	0.396	-4.941	-0.428	1.940	2.368
0.751	0.856	-5.667	-0.112	1.633	1.745
0.471	0.650	-5.469	-0.362	4.212	4.574
0.326	0.522	-5.258	0.285	3.747	3.462
0.067	0.233	-4.142	0.512	4.940	4.427
0.130	0.311	-4.634	-0.402	3.442	3.844
0.306	0.504	-5.220	-0.273	2.542	2.816
0.160	0.348	-4.781	-0.499	4.002	4.501
0.009	0.157	-2.548	0.803	3.247	2.444
0.789	0.880	-5.681	-0.083	1.760	1.843
0.896	0.943	-5.707	-0.036	2.955	2.991
0.113	0.292	-4.532	0.580	5.746	5.166
0.239	0.437	-5.060	-0.342	3.622	3.964
0.005	0.157	-2.046	-0.899	2.287	3.186
0.847	0.915	-5.697	0.054	3.280	3.226
0.384	0.573	-5.357	-0.241	2.055	2.296
0.059	0.221	-4.037	-1.410	1.152	2.562
0.174	0.363	-4.841	0.393	3.320	2.927
0.385	0.573	-5.357	-0.214	1.935	2.150
0.365	0.557	-5.327	0.237	3.518	3.280
0.419	0.606	-5.406	0.257	2.927	2.670
0.352	0.546	-5.305	0.224	2.015	1.791
0.021	0.168	-3.222	0.673	3.996	3.323
0.165	0.354	-4.806	-0.689	1.686	2.376
0.063	0.226	-4.088	0.699	4.460	3.761
0.201	0.397	-4.944	0.350	3.622	3.272
0.193	0.387	-4.916	-0.376	2.669	3.046
0.357	0.551	-5.314	0.272	2.781	2.509
0.074	0.242	-4.212	-0.811	1.850	2.661
0.171	0.361	-4.828	-0.458	5.029	5.487
0.312	0.509	-5.231	0.275	4.104	3.829
0.038	0.192	-3.699	0.605	2.583	1.978
0.240	0.437	-5.061	0.313	3.235	2.922
0.943	0.970	-5.712	0.022	2.505	2.483
0.013	0.160	-2.816	-0.740	1.799	2.540
0.011	0.157	-2.662	-1.460	2.313	3.773
0.021	0.168	-3.232	0.611	2.953	2.341
0.079	0.249	-4.261	-0.600	2.078	2.677
0.400	0.588	-5.380	0.495	2.816	2.321
0.126	0.306	-4.609	0.411	3.655	3.245
0.017	0.162	-3.070	0.940	4.093	3.153
0.140	0.324	-4.690	0.439	3.023	2.584
0.008	0.157	-2.419	1.479	4.232	2.752

0.776	0.871	-5.676	0.076	3.757	3.681
0.984	0.992	-5.715	-0.005	2.066	2.071
0.111	0.290	-4.519	-0.413	3.297	3.709
0.625	0.767	-5.602	-0.147	2.588	2.735
0.140	0.324	-4.689	0.467	3.044	2.577
0.405	0.593	-5.387	-0.257	3.231	3.488
0.004	0.157	-1.804	0.917	4.531	3.613
0.174	0.364	-4.844	-0.358	2.540	2.898
0.159	0.348	-4.781	0.344	2.670	2.326
0.007	0.157	-2.358	0.923	4.002	3.078
0.880	0.935	-5.704	0.036	2.528	2.492
0.814	0.895	-5.689	0.057	2.673	2.616
0.001	0.157	-0.267	1.132	2.735	1.603
0.465	0.644	-5.462	0.244	5.938	5.694
0.356	0.550	-5.312	-0.305	2.224	2.529
0.187	0.379	-4.892	-0.482	3.934	4.416
0.060	0.222	-4.054	-0.517	2.830	3.346
0.679	0.808	-5.634	0.124	3.010	2.886
0.412	0.599	-5.396	0.211	2.651	2.440
0.000	0.157	-0.100	1.861	4.429	2.568
0.358	0.551	-5.315	-0.272	2.183	2.455
0.760	0.862	-5.671	-0.063	5.591	5.653
0.080	0.252	-4.277	-0.560	2.822	3.383
0.045	0.201	-3.824	-0.514	5.443	5.957
0.112	0.291	-4.526	0.560	2.805	2.245
0.096	0.273	-4.413	-0.435	1.892	2.327
0.327	0.523	-5.261	0.293	2.502	2.209
0.406	0.593	-5.388	-0.454	2.463	2.917
0.596	0.747	-5.582	-0.147	2.980	3.127
0.002	0.157	-1.470	1.154	3.057	1.903
0.650	0.786	-5.617	0.140	2.841	2.701
0.868	0.928	-5.702	-0.051	2.236	2.287
0.494	0.667	-5.494	0.233	4.782	4.549
0.385	0.574	-5.358	0.281	3.853	3.572
0.161	0.350	-4.789	-0.586	1.819	2.405
0.106	0.284	-4.483	-0.472	3.443	3.915
0.060	0.221	-4.046	-1.209	1.984	3.193
0.685	0.811	-5.637	0.152	6.241	6.089
0.910	0.951	-5.709	-0.038	2.996	3.034
0.170	0.360	-4.827	-0.336	4.863	5.199
0.929	0.961	-5.711	0.025	1.935	1.909
0.026	0.176	-3.403	0.748	5.290	4.542
0.498	0.670	-5.497	0.203	5.914	5.711
0.825	0.901	-5.692	-0.063	6.727	6.790
0.076	0.245	-4.234	-0.412	3.217	3.629
0.471	0.650	-5.469	0.194	4.438	4.244
0.353	0.548	-5.308	-0.330	4.483	4.814
0.094	0.270	-4.395	0.657	5.112	4.455
0.978	0.989	-5.714	0.008	3.514	3.506
0.702	0.822	-5.646	0.109	8.178	8.069

0.623	0.766	-5.601	0.131	4.914	4.783
0.047	0.204	-3.864	-0.521	3.405	3.925
0.824	0.900	-5.691	-0.059	2.246	2.305
0.616	0.760	-5.595	-0.233	2.813	3.046
0.449	0.631	-5.444	-0.215	4.900	5.115
0.515	0.682	-5.514	-0.138	3.406	3.544
0.091	0.267	-4.371	-0.674	6.156	6.830
0.171	0.361	-4.829	0.295	7.854	7.558
0.757	0.860	-5.669	0.082	2.612	2.531
0.045	0.203	-3.833	0.543	5.794	5.251
0.037	0.190	-3.671	0.668	4.422	3.753
0.986	0.992	-5.715	0.005	3.961	3.956
0.554	0.715	-5.549	-0.146	5.509	5.655
0.359	0.552	-5.317	0.235	6.144	5.910
0.270	0.468	-5.139	0.329	3.055	2.726
0.589	0.742	-5.577	0.217	3.197	2.980
0.065	0.229	-4.114	0.535	4.265	3.730
0.080	0.251	-4.276	-0.463	3.525	3.988
0.023	0.173	-3.292	0.970	5.888	4.918
0.055	0.216	-3.989	0.786	4.624	3.838
0.550	0.711	-5.546	-0.202	6.201	6.404
0.165	0.354	-4.804	0.294	6.723	6.429
0.057	0.218	-4.010	0.483	6.803	6.320
0.360	0.553	-5.319	0.323	6.507	6.184
0.127	0.308	-4.620	0.315	6.080	5.765
0.255	0.452	-5.103	-0.291	3.114	3.405
0.020	0.166	-3.166	0.664	5.973	5.309
0.294	0.491	-5.195	0.322	5.336	5.013
0.092	0.267	-4.376	0.369	5.348	4.979
0.245	0.442	-5.076	0.304	3.491	3.188
0.029	0.178	-3.491	-1.047	3.344	4.391
0.006	0.157	-2.220	-0.774	4.420	5.194
0.004	0.157	-1.950	1.181	6.300	5.119
0.827	0.902	-5.692	-0.057	3.487	3.544
0.681	0.809	-5.635	-0.103	4.006	4.109
0.769	0.868	-5.674	0.065	4.812	4.746
0.245	0.442	-5.076	0.325	4.293	3.968
0.294	0.491	-5.195	0.336	5.321	4.985
0.059	0.221	-4.045	0.493	8.057	7.564
0.072	0.239	-4.189	0.909	8.840	7.931
0.052	0.210	-3.935	-0.908	4.867	5.775
0.957	0.978	-5.713	0.017	2.931	2.914
0.906	0.949	-5.708	0.027	2.552	2.525
0.391	0.578	-5.366	-0.257	5.003	5.260
0.021	0.168	-3.231	-0.950	1.670	2.620
0.810	0.893	-5.687	0.075	2.825	2.751
0.976	0.988	-5.714	-0.012	2.104	2.116
0.035	0.186	-3.629	0.691	3.093	2.403
0.595	0.746	-5.581	0.157	3.603	3.445
0.463	0.643	-5.460	-0.545	2.593	3.138

0.259	0.457	-5.114	0.299	3.424	3.125
0.487	0.662	-5.486	0.234	3.530	3.296
0.013	0.160	-2.845	0.955	3.828	2.874
0.142	0.326	-4.698	-0.683	4.750	5.432
0.366	0.557	-5.328	-0.215	2.912	3.127
0.692	0.816	-5.641	-0.132	2.588	2.720
0.768	0.867	-5.674	0.089	4.508	4.419
0.532	0.696	-5.530	0.227	6.307	6.080
0.332	0.528	-5.271	0.326	4.638	4.312
0.170	0.360	-4.825	-0.352	3.684	4.035
0.153	0.340	-4.750	-0.477	4.227	4.704
0.384	0.572	-5.356	-0.302	1.772	2.074
0.105	0.282	-4.474	0.445	3.137	2.692
0.025	0.174	-3.348	-0.801	4.472	5.273
0.142	0.326	-4.699	0.392	6.025	5.633
0.611	0.757	-5.592	-0.149	1.642	1.791
0.221	0.417	-5.007	0.307	3.900	3.593
0.018	0.163	-3.101	1.341	9.018	7.677
0.001	0.157	-0.547	-1.013	3.569	4.583
0.053	0.213	-3.956	-0.567	4.012	4.580
0.324	0.520	-5.254	-0.260	6.164	6.423
0.094	0.270	-4.393	0.511	5.658	5.147
0.441	0.625	-5.434	0.324	3.713	3.389
0.803	0.889	-5.685	0.074	6.552	6.478
0.026	0.176	-3.405	1.060	7.405	6.346
0.384	0.573	-5.356	-0.260	2.493	2.752
0.365	0.557	-5.327	0.419	2.436	2.017
0.006	0.157	-2.201	-0.866	4.781	5.647
0.918	0.956	-5.710	0.024	4.682	4.658
0.296	0.493	-5.198	-0.453	3.948	4.401
0.741	0.849	-5.663	0.088	4.546	4.458
0.002	0.157	-1.230	1.014	5.264	4.250
0.046	0.203	-3.842	-0.718	4.082	4.800
0.027	0.176	-3.410	0.762	5.100	4.338
0.047	0.204	-3.861	0.726	4.432	3.706
0.603	0.752	-5.586	0.154	3.706	3.552
0.849	0.917	-5.698	-0.062	3.304	3.365
0.998	0.999	-5.715	-0.001	2.852	2.853
0.020	0.167	-3.184	1.013	6.129	5.116
0.107	0.285	-4.489	0.391	6.986	6.595
0.014	0.160	-2.891	0.877	8.610	7.733
0.031	0.181	-3.539	-0.497	3.678	4.176
0.404	0.592	-5.386	-0.244	3.212	3.456
0.319	0.517	-5.245	-0.256	3.095	3.351
0.157	0.345	-4.772	0.365	9.465	9.100
0.185	0.376	-4.884	-0.435	3.422	3.858
0.427	0.612	-5.416	-0.256	3.396	3.652
0.606	0.753	-5.589	-0.153	2.890	3.043
0.256	0.453	-5.104	0.339	4.435	4.096
0.318	0.516	-5.243	0.520	6.652	6.132

0.040	0.194	-3.727	0.534	6.215	5.681
0.026	0.175	-3.379	-0.599	3.845	4.444
0.091	0.266	-4.370	0.348	7.786	7.438
0.525	0.690	-5.523	-0.191	4.710	4.901
0.012	0.159	-2.735	-0.777	2.615	3.392
0.004	0.157	-1.817	1.826	8.088	6.262
0.013	0.160	-2.816	0.673	3.767	3.094
0.409	0.596	-5.392	-0.244	5.922	6.166
0.057	0.218	-4.009	0.497	3.281	2.784
0.007	0.157	-2.327	1.075	5.388	4.313
0.066	0.231	-4.128	0.528	2.517	1.989
0.521	0.687	-5.519	-0.171	5.527	5.698
0.246	0.444	-5.080	0.210	7.436	7.226
0.035	0.187	-3.636	0.473	6.076	5.604
0.049	0.207	-3.899	0.508	6.459	5.951
0.082	0.253	-4.289	0.452	3.845	3.394
0.039	0.193	-3.715	-0.820	1.799	2.618
0.027	0.176	-3.422	-0.838	5.941	6.779
0.051	0.208	-3.922	-0.781	4.286	5.067
0.168	0.358	-4.819	-0.397	6.577	6.973
0.046	0.203	-3.839	-0.694	3.357	4.051
0.176	0.366	-4.852	-0.364	6.127	6.491
0.264	0.461	-5.124	0.565	6.537	5.972
0.438	0.622	-5.430	-0.223	6.612	6.834
0.122	0.303	-4.587	-0.405	4.456	4.861
0.398	0.585	-5.376	-0.418	5.038	5.456
0.485	0.661	-5.484	-0.386	5.359	5.745
0.514	0.681	-5.513	-0.353	5.409	5.762
0.181	0.373	-4.872	-0.390	3.137	3.528
0.365	0.557	-5.327	0.257	5.100	4.843
0.056	0.218	-4.002	0.552	2.293	1.740
0.000	0.083	2.274	2.165	4.625	2.460
0.008	0.157	-2.426	0.963	2.874	1.911
0.464	0.644	-5.461	0.232	7.078	6.846
0.101	0.278	-4.450	0.535	9.122	8.588
0.106	0.284	-4.482	0.325	8.581	8.256
0.030	0.180	-3.518	0.899	6.823	5.925
0.055	0.216	-3.991	-0.522	2.307	2.830
0.026	0.176	-3.400	1.319	4.850	3.530
0.107	0.286	-4.494	-0.432	3.548	3.980
0.922	0.958	-5.710	-0.029	3.028	3.057
0.773	0.870	-5.675	-0.082	3.677	3.759
0.243	0.440	-5.070	0.313	2.634	2.321
0.038	0.191	-3.685	0.476	8.419	7.943
0.305	0.503	-5.218	-0.323	3.027	3.351
0.232	0.429	-5.041	0.429	2.945	2.516
0.034	0.185	-3.614	-0.474	4.305	4.779
0.113	0.291	-4.528	0.368	6.541	6.173
0.498	0.669	-5.497	-0.425	2.916	3.341
0.032	0.182	-3.552	-0.644	6.067	6.711

0.060	0.222	-4.054	-0.631	6.334	6.965
0.180	0.371	-4.866	0.384	4.886	4.502
0.398	0.586	-5.377	-0.250	3.058	3.308
0.972	0.986	-5.714	0.010	2.697	2.687
0.150	0.336	-4.738	-0.351	6.392	6.743
0.893	0.941	-5.706	0.040	4.916	4.877
0.385	0.574	-5.358	-0.218	3.938	4.155
0.555	0.715	-5.550	0.182	3.238	3.056
0.967	0.984	-5.714	-0.015	2.284	2.299
0.413	0.601	-5.398	0.185	5.061	4.876
0.464	0.644	-5.461	-0.241	2.254	2.496
0.032	0.182	-3.551	0.775	2.917	2.141
0.059	0.221	-4.038	0.482	3.774	3.291
0.773	0.870	-5.675	-0.145	2.537	2.682
0.471	0.650	-5.469	-0.309	4.226	4.535
0.563	0.721	-5.556	-0.276	6.163	6.439
0.614	0.758	-5.594	-0.201	3.430	3.631
0.014	0.160	-2.915	0.745	5.894	5.149
0.089	0.264	-4.356	0.339	6.334	5.995
0.017	0.162	-3.034	-0.793	2.117	2.910
0.829	0.904	-5.693	-0.060	2.535	2.595
0.370	0.560	-5.334	0.246	3.248	3.002
0.009	0.157	-2.531	-0.793	6.081	6.874
0.001	0.157	-0.398	1.036	4.501	3.465
0.949	0.974	-5.713	-0.019	2.022	2.040
0.173	0.362	-4.839	0.611	6.401	5.790
0.650	0.786	-5.617	0.125	2.843	2.718
0.172	0.362	-4.835	0.271	6.720	6.449
0.075	0.243	-4.220	-0.519	4.071	4.591
0.069	0.235	-4.156	1.111	8.258	7.147
0.702	0.822	-5.645	0.111	4.084	3.973
0.161	0.349	-4.786	0.294	5.725	5.431
0.415	0.602	-5.400	0.254	2.833	2.579
0.676	0.805	-5.632	-0.119	3.668	3.787
0.605	0.753	-5.588	0.110	5.785	5.675
0.603	0.752	-5.587	-0.180	3.167	3.346
0.176	0.365	-4.850	-0.599	5.361	5.959
0.566	0.722	-5.559	0.172	3.056	2.884
0.007	0.157	-2.298	-1.004	3.969	4.973
0.776	0.871	-5.676	0.127	5.808	5.681
0.833	0.907	-5.694	-0.065	3.305	3.370
0.550	0.710	-5.545	0.289	5.455	5.166
0.258	0.456	-5.111	0.208	9.471	9.263
0.331	0.527	-5.268	0.213	8.421	8.207
0.098	0.275	-4.426	-0.482	2.533	3.015
0.152	0.339	-4.746	-0.451	4.457	4.908
0.011	0.157	-2.686	-0.533	6.603	7.136
0.450	0.631	-5.444	0.204	2.696	2.492
0.002	0.157	-1.231	0.795	6.585	5.791
0.429	0.615	-5.419	-0.228	5.968	6.196

0.996	0.998	-5.715	-0.002	5.967	5.970
0.116	0.296	-4.548	-0.429	5.791	6.220
0.106	0.284	-4.484	0.330	7.188	6.857
0.070	0.236	-4.166	0.383	6.511	6.129
0.119	0.299	-4.571	0.635	4.789	4.154
0.003	0.157	-1.492	1.025	4.765	3.740
0.326	0.522	-5.258	0.297	4.303	4.006
0.082	0.253	-4.288	0.553	6.977	6.425
0.122	0.302	-4.585	0.516	7.140	6.623
0.002	0.157	-1.343	1.435	5.280	3.845
0.616	0.760	-5.596	0.342	3.527	3.185
0.491	0.665	-5.490	0.260	2.019	1.758
0.132	0.315	-4.645	0.488	5.342	4.855
0.268	0.467	-5.136	0.246	5.872	5.627
0.983	0.991	-5.715	0.009	2.745	2.736
0.229	0.426	-5.031	0.561	6.418	5.857
0.031	0.180	-3.522	1.105	3.872	2.766
0.037	0.190	-3.671	0.653	5.415	4.762
0.073	0.241	-4.199	0.661	3.985	3.324
0.095	0.271	-4.401	0.556	4.551	3.995
0.227	0.424	-5.026	-0.405	2.199	2.604
0.101	0.278	-4.450	0.658	4.924	4.266
0.980	0.989	-5.714	0.007	6.865	6.857
0.497	0.669	-5.496	-0.266	4.892	5.158
0.004	0.157	-1.879	-0.715	7.705	8.420
0.145	0.329	-4.713	0.485	5.296	4.810
0.011	0.159	-2.731	-0.714	5.149	5.862
0.301	0.498	-5.209	-0.382	2.693	3.075
0.234	0.430	-5.045	0.293	4.906	4.613
0.841	0.912	-5.696	0.052	6.067	6.016
0.528	0.693	-5.526	-0.172	3.467	3.639
0.022	0.169	-3.242	0.715	7.249	6.534
0.188	0.380	-4.897	-0.364	4.202	4.566
0.006	0.157	-2.156	-1.277	7.251	8.528
0.013	0.160	-2.822	1.530	6.492	4.961
0.993	0.996	-5.715	-0.003	6.218	6.221
0.015	0.160	-2.945	0.755	7.551	6.797
0.476	0.653	-5.474	0.184	7.817	7.633
0.001	0.157	-0.846	-1.064	3.406	4.470
0.003	0.157	-1.646	-0.743	3.650	4.393
0.867	0.928	-5.702	0.064	2.073	2.009
0.298	0.494	-5.202	-0.276	5.671	5.947
0.169	0.359	-4.820	-0.280	4.521	4.801
0.359	0.552	-5.316	0.308	4.414	4.106
0.128	0.309	-4.621	0.436	5.289	4.853
0.328	0.523	-5.262	-0.395	1.940	2.335
0.558	0.717	-5.552	0.162	6.991	6.829
0.054	0.215	-3.975	0.469	2.750	2.281
0.216	0.411	-4.992	-0.332	2.083	2.414
0.234	0.431	-5.046	0.384	5.131	4.746

0.327	0.523	-5.261	-0.261	6.275	6.536
0.018	0.163	-3.103	-0.668	7.301	7.970
0.029	0.178	-3.471	0.736	9.718	8.981
0.459	0.640	-5.456	-0.202	4.441	4.642
0.892	0.941	-5.706	-0.040	2.343	2.383
0.330	0.526	-5.267	-0.386	2.277	2.664
0.349	0.544	-5.300	0.221	2.765	2.545
0.139	0.323	-4.682	-0.488	3.778	4.267
0.465	0.645	-5.462	0.237	2.999	2.762
0.174	0.364	-4.844	-0.564	4.932	5.496
0.539	0.701	-5.536	-0.218	2.220	2.438
0.104	0.281	-4.470	0.552	2.559	2.007
0.516	0.683	-5.515	-0.191	3.078	3.270
0.744	0.851	-5.664	0.088	4.106	4.018
0.210	0.405	-4.973	-0.333	2.727	3.060
0.228	0.424	-5.027	-0.567	3.026	3.592
0.132	0.315	-4.648	0.479	5.098	4.619
0.018	0.163	-3.115	0.688	5.809	5.121
0.737	0.847	-5.661	-0.107	3.496	3.603
0.019	0.165	-3.156	0.689	6.298	5.609
0.333	0.529	-5.271	-0.271	4.978	5.249
0.090	0.264	-4.358	0.473	2.808	2.335
0.049	0.207	-3.901	0.744	6.553	5.809
0.058	0.219	-4.026	0.455	6.624	6.169
0.304	0.501	-5.215	0.222	3.681	3.459
0.414	0.602	-5.399	0.158	6.351	6.193
0.101	0.278	-4.447	0.522	7.551	7.029
0.025	0.175	-3.359	-0.992	3.551	4.543
0.910	0.952	-5.709	0.035	6.073	6.038
0.368	0.559	-5.332	0.177	7.152	6.975
0.133	0.315	-4.649	-0.493	4.732	5.225
0.227	0.424	-5.025	-0.601	5.304	5.904
0.008	0.157	-2.428	-0.745	8.351	9.096
0.800	0.888	-5.684	-0.115	3.630	3.745
0.879	0.934	-5.704	-0.049	2.970	3.019
0.356	0.550	-5.311	-0.216	2.954	3.170
0.005	0.157	-2.105	-0.727	1.879	2.606
0.637	0.777	-5.610	-0.247	3.309	3.556
0.074	0.241	-4.211	-0.555	2.923	3.477
0.021	0.168	-3.222	-0.599	8.077	8.676
0.497	0.669	-5.497	-0.123	3.946	4.068
0.134	0.318	-4.659	0.397	4.702	4.305
0.214	0.409	-4.985	0.395	2.171	1.776
0.359	0.552	-5.317	-0.273	4.833	5.106
0.393	0.581	-5.370	0.260	6.103	5.842
0.558	0.717	-5.552	0.118	8.152	8.034
0.029	0.178	-3.476	-0.564	5.284	5.848
0.618	0.762	-5.597	-0.126	4.919	5.044
0.176	0.365	-4.850	-0.904	4.908	5.813
0.972	0.986	-5.714	-0.008	5.117	5.125

0.548	0.709	-5.544	-0.214	4.349	4.563
0.210	0.404	-4.972	-0.392	2.371	2.763
0.821	0.899	-5.691	0.131	3.495	3.363
0.032	0.183	-3.564	-0.556	2.933	3.489
0.012	0.160	-2.772	-0.573	4.674	5.247
0.266	0.465	-5.131	-0.279	6.135	6.414
0.058	0.219	-4.020	0.568	4.168	3.600
0.102	0.280	-4.458	0.398	6.372	5.974
0.018	0.163	-3.105	0.481	7.743	7.262
0.294	0.491	-5.195	0.373	5.339	4.965
0.667	0.799	-5.627	-0.111	7.077	7.188
0.797	0.886	-5.683	-0.079	1.795	1.874
0.030	0.179	-3.500	-0.598	5.189	5.787
0.321	0.518	-5.249	0.248	4.355	4.107
0.060	0.221	-4.047	0.818	5.598	4.780
0.012	0.160	-2.772	-0.826	3.072	3.897
0.053	0.213	-3.956	-0.626	2.608	3.234
0.018	0.163	-3.109	-0.762	5.795	6.556
0.365	0.557	-5.327	-0.257	4.224	4.481
0.730	0.843	-5.659	0.107	3.441	3.334
0.019	0.166	-3.160	0.521	7.458	6.938
0.407	0.594	-5.389	0.324	4.745	4.422
0.685	0.811	-5.637	-0.102	2.790	2.892
0.306	0.503	-5.219	0.317	2.770	2.453
0.686	0.812	-5.637	-0.124	2.572	2.696
0.006	0.157	-2.157	0.939	8.875	7.936
0.350	0.545	-5.302	0.189	8.634	8.445
0.237	0.434	-5.055	-0.752	3.284	4.035
0.499	0.670	-5.498	-0.152	3.617	3.769
0.072	0.239	-4.188	0.763	6.620	5.857
0.771	0.868	-5.675	-0.069	3.743	3.812
0.382	0.571	-5.354	-0.224	3.118	3.342
0.451	0.632	-5.446	0.308	6.062	5.753
0.744	0.851	-5.664	-0.094	3.326	3.420
0.112	0.291	-4.525	-0.459	4.249	4.708
0.005	0.157	-2.120	-0.702	5.229	5.932
0.198	0.392	-4.933	0.344	6.539	6.195
0.029	0.178	-3.469	0.494	8.486	7.992
0.373	0.564	-5.340	-0.238	2.779	3.017
0.805	0.890	-5.686	0.067	2.224	2.157
0.031	0.181	-3.533	0.874	7.298	6.424
0.010	0.157	-2.581	0.856	5.644	4.788
0.067	0.232	-4.138	0.497	4.868	4.371
0.234	0.431	-5.046	0.317	3.887	3.570
0.045	0.201	-3.820	-0.748	4.895	5.643
0.049	0.207	-3.901	-0.489	3.870	4.359
0.009	0.157	-2.552	-0.752	1.709	2.461
0.016	0.162	-3.002	-0.619	6.909	7.528
0.227	0.424	-5.026	0.357	3.112	2.755
0.052	0.211	-3.942	0.649	8.604	7.955

0.443	0.626	-5.437	0.210	5.045	4.835
0.135	0.319	-4.664	0.427	7.109	6.682
0.337	0.533	-5.279	0.202	6.806	6.604
0.006	0.157	-2.194	0.731	5.914	5.182
0.028	0.178	-3.451	0.898	5.169	4.271
0.054	0.215	-3.974	0.833	7.112	6.279
0.215	0.409	-4.988	0.518	3.872	3.354
0.095	0.271	-4.404	0.603	5.633	5.031
0.103	0.280	-4.462	0.327	10.785	10.458
0.720	0.835	-5.654	0.146	2.194	2.048
0.618	0.762	-5.597	-0.128	1.586	1.715
0.044	0.201	-3.817	0.609	5.511	4.902
0.436	0.620	-5.428	0.155	6.354	6.199
0.137	0.320	-4.670	0.366	8.473	8.106
0.031	0.180	-3.525	0.606	6.619	6.013
0.016	0.162	-2.995	1.052	5.519	4.467
0.731	0.843	-5.659	-0.106	4.543	4.649
0.069	0.235	-4.160	0.475	5.427	4.952
0.014	0.160	-2.913	1.152	6.505	5.353
0.545	0.706	-5.541	0.149	5.399	5.250
0.055	0.216	-3.982	-0.753	4.867	5.620
0.015	0.160	-2.943	-0.913	7.151	8.064
0.042	0.197	-3.776	-0.795	6.480	7.276
0.547	0.709	-5.543	-0.191	4.040	4.231
0.042	0.197	-3.776	-0.677	2.032	2.709
0.962	0.981	-5.714	0.013	5.368	5.354
0.131	0.313	-4.640	0.374	3.164	2.790
0.040	0.194	-3.738	0.494	4.787	4.293
0.026	0.176	-3.392	0.943	4.774	3.831
0.809	0.893	-5.687	0.057	6.647	6.591
0.890	0.940	-5.706	-0.047	2.776	2.822
0.916	0.954	-5.709	0.021	5.079	5.058
0.053	0.213	-3.956	0.529	2.489	1.961
0.055	0.216	-3.991	-0.466	4.602	5.068
0.379	0.569	-5.349	-0.251	6.100	6.350
0.313	0.510	-5.233	0.257	3.336	3.079
0.056	0.218	-4.002	0.390	6.900	6.510
0.993	0.996	-5.715	-0.002	4.249	4.251
0.076	0.245	-4.234	0.891	5.921	5.031
0.058	0.219	-4.026	0.498	5.922	5.424
0.158	0.346	-4.774	-0.670	6.723	7.394
0.191	0.384	-4.909	0.384	5.027	4.643
0.141	0.326	-4.695	0.552	5.535	4.983
0.061	0.224	-4.071	0.642	6.585	5.942
0.085	0.257	-4.316	0.491	7.946	7.455
0.092	0.267	-4.376	-0.463	5.134	5.596
0.283	0.481	-5.169	0.249	5.352	5.103
0.016	0.162	-3.000	0.574	4.788	4.214
0.059	0.220	-4.034	0.872	8.655	7.783
0.040	0.194	-3.745	-0.907	3.839	4.746

0.222	0.419	-5.011	-0.478	3.223	3.702
0.783	0.876	-5.679	-0.071	3.751	3.822
0.016	0.162	-2.996	-0.662	5.387	6.049
0.250	0.447	-5.088	0.313	3.358	3.045
0.274	0.472	-5.149	-0.237	6.042	6.279
0.416	0.603	-5.402	0.225	5.952	5.727
0.470	0.649	-5.468	0.143	7.045	6.902
0.033	0.184	-3.587	-0.583	6.250	6.833
0.018	0.162	-3.080	0.542	6.634	6.092
0.513	0.680	-5.512	0.165	3.130	2.965
0.114	0.293	-4.539	0.399	6.859	6.460
0.027	0.176	-3.428	0.619	5.313	4.693
0.201	0.397	-4.944	-0.324	6.346	6.670
0.045	0.203	-3.832	-0.539	5.981	6.520
0.092	0.267	-4.375	0.340	8.527	8.188
0.285	0.482	-5.174	-0.372	2.034	2.406
0.043	0.200	-3.801	-1.054	5.060	6.114
0.010	0.157	-2.651	-0.797	5.891	6.688
0.052	0.212	-3.945	-0.475	4.659	5.134
0.072	0.240	-4.194	0.486	7.975	7.489
0.165	0.354	-4.805	-0.443	5.767	6.209
0.076	0.245	-4.230	0.417	2.559	2.142
0.006	0.157	-2.174	0.606	5.770	5.164
0.143	0.327	-4.703	-0.396	3.965	4.360
0.232	0.429	-5.041	0.590	5.669	5.079
0.409	0.597	-5.393	0.163	10.202	10.038
0.271	0.469	-5.142	0.227	9.122	8.895
0.709	0.827	-5.649	-0.101	2.442	2.543
0.004	0.157	-1.828	1.368	9.385	8.016
0.017	0.162	-3.062	1.325	8.072	6.748
0.056	0.216	-3.992	1.203	6.114	4.912
0.052	0.211	-3.938	0.471	8.269	7.798
0.340	0.535	-5.284	0.269	2.639	2.370
0.418	0.605	-5.404	-0.254	1.700	1.954
0.039	0.192	-3.711	0.434	9.177	8.742
0.065	0.229	-4.118	0.401	10.843	10.442
0.734	0.845	-5.660	0.073	5.508	5.435
0.770	0.868	-5.674	0.093	3.570	3.476
0.209	0.404	-4.969	0.270	9.406	9.136
0.212	0.408	-4.981	0.258	10.041	9.784
0.260	0.458	-5.116	-0.259	9.107	9.366
0.029	0.178	-3.494	0.727	8.679	7.952
0.041	0.195	-3.759	0.550	9.386	8.836
0.010	0.157	-2.639	0.771	9.217	8.446
0.015	0.161	-2.962	0.507	6.070	5.563
0.010	0.157	-2.625	0.777	8.069	7.292
0.382	0.571	-5.353	0.259	4.347	4.088
0.599	0.749	-5.584	-0.120	9.153	9.273
0.140	0.324	-4.686	0.854	8.025	7.171
0.072	0.240	-4.194	0.649	4.823	4.174

0.200	0.395	-4.939	-0.397	3.908	4.305
0.374	0.565	-5.341	0.167	7.041	6.873
0.364	0.555	-5.324	0.190	5.660	5.469
0.477	0.654	-5.475	0.141	7.441	7.299
0.008	0.157	-2.469	1.478	9.943	8.465
0.674	0.804	-5.631	-0.130	3.038	3.168
0.041	0.195	-3.750	0.668	7.860	7.191
0.119	0.299	-4.570	0.363	8.762	8.398
0.549	0.710	-5.545	-0.177	2.021	2.198
0.224	0.420	-5.015	-0.370	2.883	3.253
0.040	0.194	-3.743	0.448	7.386	6.938
0.464	0.644	-5.461	0.219	5.990	5.770
0.357	0.551	-5.314	-0.345	2.593	2.938
0.392	0.580	-5.368	0.201	7.667	7.466
0.819	0.897	-5.690	0.057	4.544	4.488
0.018	0.163	-3.109	1.148	6.503	5.355
0.010	0.157	-2.643	0.846	8.352	7.506
0.106	0.284	-4.486	-0.554	5.865	6.418
0.017	0.162	-3.029	0.683	7.408	6.725
0.098	0.275	-4.428	0.561	3.422	2.861
0.076	0.245	-4.232	-0.482	4.047	4.530
0.021	0.169	-3.236	1.175	6.523	5.348
0.155	0.343	-4.762	0.387	2.808	2.421
0.630	0.771	-5.605	-0.123	4.141	4.264
0.035	0.186	-3.631	0.469	7.952	7.484
0.048	0.205	-3.879	1.103	7.609	6.507
0.632	0.772	-5.606	-0.132	3.755	3.887
0.022	0.169	-3.249	0.849	3.408	2.559
0.244	0.442	-5.074	0.331	3.158	2.827
0.079	0.249	-4.259	0.447	2.808	2.361
0.022	0.171	-3.269	-0.730	7.180	7.910
0.183	0.374	-4.879	-0.385	3.535	3.921
0.351	0.546	-5.304	-0.279	2.764	3.043
0.134	0.317	-4.655	-0.421	2.141	2.562
0.554	0.714	-5.549	-0.158	4.804	4.962
0.618	0.762	-5.597	-0.176	5.244	5.420
0.013	0.160	-2.848	0.655	5.981	5.327
0.064	0.228	-4.102	0.723	5.860	5.137
0.074	0.242	-4.212	0.552	6.111	5.559
0.030	0.179	-3.510	0.688	8.665	7.976
0.039	0.192	-3.708	0.767	5.919	5.152
0.143	0.327	-4.702	0.411	4.675	4.264
0.860	0.925	-5.700	0.049	3.434	3.386
0.113	0.292	-4.533	0.630	6.339	5.709
0.009	0.157	-2.579	-0.874	5.024	5.897
0.014	0.160	-2.879	0.715	6.947	6.233
0.409	0.596	-5.392	-0.211	4.068	4.279
0.408	0.596	-5.391	0.206	1.475	1.269
0.014	0.160	-2.881	1.245	10.034	8.789
0.805	0.890	-5.686	0.070	3.443	3.373

0.352	0.547	-5.306	-0.243	3.783	4.026
0.125	0.306	-4.606	-0.421	4.350	4.771
0.024	0.173	-3.335	-0.563	7.109	7.672
0.178	0.368	-4.857	-0.358	3.421	3.779
0.860	0.925	-5.700	-0.049	5.640	5.689
0.014	0.160	-2.915	0.599	7.720	7.121
0.008	0.157	-2.413	-0.676	5.325	6.001
0.144	0.327	-4.706	-0.403	6.409	6.812
0.817	0.897	-5.690	0.055	3.996	3.941
0.003	0.157	-1.626	-0.960	5.673	6.633
0.153	0.340	-4.751	0.371	4.883	4.511
0.402	0.589	-5.382	0.235	6.262	6.026
0.015	0.160	-2.958	0.589	3.463	2.873
0.185	0.376	-4.884	-0.371	2.985	3.357
0.009	0.157	-2.492	1.405	6.108	4.703
0.003	0.157	-1.640	1.469	5.043	3.574
0.050	0.208	-3.917	-0.467	5.999	6.466
0.425	0.610	-5.413	0.352	3.274	2.921
0.629	0.770	-5.604	0.121	3.881	3.760
0.923	0.959	-5.710	0.028	7.762	7.734
0.107	0.285	-4.489	-0.534	2.443	2.977
0.138	0.321	-4.677	0.389	6.328	5.939
0.084	0.256	-4.312	-0.431	3.635	4.066
0.037	0.190	-3.678	0.746	2.970	2.224
0.371	0.562	-5.337	-0.249	5.036	5.285
0.802	0.889	-5.685	0.066	4.394	4.329
0.117	0.297	-4.556	0.349	5.102	4.753
0.083	0.255	-4.299	-0.458	3.780	4.238
0.154	0.341	-4.758	-0.399	3.334	3.734
0.162	0.351	-4.791	0.365	3.860	3.495
0.128	0.308	-4.620	0.534	3.237	2.703
0.050	0.207	-3.907	0.580	3.267	2.688
0.272	0.471	-5.145	0.316	5.126	4.810
0.087	0.261	-4.339	0.602	7.007	6.404
0.229	0.426	-5.030	-0.601	5.967	6.568
0.565	0.722	-5.558	0.238	2.994	2.756
0.055	0.216	-3.990	0.850	9.563	8.714
0.370	0.560	-5.334	0.260	5.206	4.946
0.031	0.181	-3.532	0.782	5.805	5.023
0.109	0.288	-4.506	0.511	7.074	6.563
0.753	0.857	-5.668	-0.098	3.019	3.117
0.250	0.448	-5.090	0.325	6.465	6.140
0.368	0.559	-5.331	0.240	3.050	2.810
0.495	0.667	-5.494	-0.148	3.067	3.215
0.053	0.214	-3.962	0.505	7.467	6.962
0.022	0.169	-3.242	-0.574	7.495	8.069
0.257	0.455	-5.109	0.266	3.174	2.907
0.066	0.230	-4.125	0.616	4.955	4.339
0.095	0.271	-4.401	-0.541	4.366	4.907
0.587	0.740	-5.575	-0.188	3.585	3.774

0.709	0.827	-5.649	0.098	2.838	2.740
0.095	0.271	-4.402	-0.743	2.580	3.323
0.088	0.263	-4.349	-0.848	3.368	4.216
0.280	0.478	-5.163	0.267	4.511	4.243
0.728	0.840	-5.657	-0.083	6.914	6.997
0.284	0.482	-5.173	0.347	3.685	3.338
0.571	0.726	-5.563	0.228	4.133	3.905
0.217	0.412	-4.995	0.808	2.981	2.173
0.171	0.361	-4.831	0.545	4.574	4.029
0.105	0.283	-4.475	-0.520	3.970	4.489
0.104	0.281	-4.469	-0.386	4.465	4.851
0.009	0.157	-2.552	-0.934	2.388	3.322
0.012	0.160	-2.775	0.767	5.589	4.822
0.027	0.176	-3.423	0.466	9.477	9.011
0.310	0.507	-5.227	0.219	6.435	6.215
0.215	0.409	-4.988	0.420	4.159	3.739
0.632	0.772	-5.606	-0.183	2.908	3.091
0.225	0.422	-5.020	-0.376	2.631	3.007
0.048	0.205	-3.884	-0.512	3.402	3.914
0.001	0.157	-0.904	-1.468	5.365	6.833
0.384	0.572	-5.356	0.244	4.633	4.389
0.081	0.253	-4.285	0.352	7.638	7.286
0.618	0.762	-5.597	0.099	6.840	6.741
0.127	0.308	-4.617	0.431	2.958	2.527
0.654	0.790	-5.620	-0.089	2.013	2.103
0.119	0.298	-4.567	0.535	7.907	7.372
0.016	0.162	-3.025	0.998	7.018	6.020
0.387	0.575	-5.360	0.253	7.029	6.776
0.186	0.378	-4.889	0.302	4.789	4.487
0.992	0.996	-5.715	0.002	5.238	5.235
0.336	0.532	-5.276	0.247	7.329	7.082
0.193	0.386	-4.915	0.297	6.187	5.890
0.027	0.177	-3.435	1.220	7.169	5.949
0.085	0.257	-4.319	0.515	3.284	2.770
0.555	0.715	-5.550	-0.161	2.589	2.751
0.472	0.650	-5.470	0.204	5.441	5.237
0.042	0.197	-3.771	0.493	6.565	6.072
0.382	0.571	-5.353	0.288	4.872	4.585
0.309	0.507	-5.226	-0.272	2.138	2.410
0.635	0.774	-5.608	-0.134	2.566	2.700
0.340	0.535	-5.284	-0.331	2.477	2.808
0.098	0.275	-4.426	-0.387	6.778	7.165
0.013	0.160	-2.856	0.663	5.379	4.716
0.252	0.450	-5.094	-0.344	4.763	5.107
0.049	0.207	-3.902	0.527	6.851	6.325
0.915	0.954	-5.709	-0.030	7.172	7.202
0.361	0.553	-5.320	-0.259	3.146	3.405
0.133	0.316	-4.652	-0.557	3.534	4.091
0.100	0.277	-4.440	0.334	8.084	7.751
0.004	0.157	-1.959	-1.156	5.414	6.570

0.017	0.162	-3.046	-0.890	2.238	3.128
0.026	0.176	-3.397	0.866	5.392	4.526
0.003	0.157	-1.756	0.950	4.911	3.961
0.093	0.269	-4.387	0.832	6.242	5.410
0.006	0.157	-2.253	-1.492	1.762	3.253
0.760	0.862	-5.671	-0.089	4.509	4.598
0.045	0.203	-3.835	-0.591	2.274	2.865
0.033	0.184	-3.576	-1.001	3.055	4.056
0.757	0.860	-5.670	-0.083	3.067	3.150
0.004	0.157	-1.907	0.928	2.975	2.046
0.111	0.290	-4.518	-0.540	3.321	3.861
0.031	0.180	-3.523	-0.667	3.574	4.240
0.447	0.629	-5.441	0.200	5.847	5.646
0.032	0.182	-3.556	-0.931	3.885	4.816
0.022	0.171	-3.270	-0.609	4.600	5.209
0.065	0.229	-4.114	-0.621	3.159	3.779
0.111	0.290	-4.521	-0.708	3.089	3.796
0.997	0.999	-5.715	-0.001	3.043	3.044
0.013	0.160	-2.830	0.647	4.504	3.857
0.129	0.311	-4.630	0.382	4.724	4.342
0.015	0.160	-2.952	2.178	7.509	5.331
0.563	0.721	-5.556	-0.266	1.167	1.432
0.028	0.177	-3.444	0.701	3.035	2.333
0.125	0.306	-4.603	0.613	2.756	2.143
0.033	0.184	-3.580	0.413	6.804	6.391
0.482	0.658	-5.480	0.171	6.588	6.418
0.023	0.173	-3.289	0.495	5.464	4.970
0.426	0.612	-5.415	0.189	4.487	4.299
0.690	0.815	-5.639	-0.148	2.564	2.712
0.004	0.157	-1.799	-0.779	4.112	4.891
0.340	0.535	-5.285	0.282	6.479	6.197
0.130	0.311	-4.633	0.404	3.343	2.939
0.043	0.200	-3.796	0.488	4.663	4.175
0.003	0.157	-1.626	1.492	4.744	3.252
0.273	0.471	-5.148	0.351	5.947	5.596
0.123	0.304	-4.591	0.626	3.775	3.149
0.352	0.547	-5.305	0.219	3.690	3.471
0.176	0.366	-4.851	-0.312	3.284	3.596
0.720	0.835	-5.654	0.107	6.313	6.206
0.130	0.311	-4.632	0.454	5.459	5.005
0.908	0.950	-5.708	-0.033	4.951	4.984
0.306	0.503	-5.219	0.219	6.278	6.059
0.116	0.296	-4.552	0.355	7.756	7.401
0.025	0.175	-3.369	0.532	5.890	5.359
0.991	0.995	-5.715	-0.003	7.595	7.598
0.066	0.231	-4.127	0.548	6.195	5.647
0.799	0.887	-5.684	-0.066	4.253	4.320
0.118	0.298	-4.562	0.386	3.541	3.156
0.354	0.548	-5.308	0.232	5.046	4.813
0.554	0.714	-5.549	0.185	3.491	3.306

0.424	0.610	-5.413	0.194	2.635	2.441
0.056	0.217	-3.995	0.622	5.487	4.866
0.184	0.375	-4.881	0.358	3.829	3.471
0.610	0.756	-5.591	-0.135	5.928	6.063
0.135	0.318	-4.661	0.378	6.163	5.785
0.070	0.236	-4.166	0.640	3.691	3.051
0.589	0.741	-5.576	-0.150	4.972	5.121
0.284	0.482	-5.173	0.252	4.000	3.748
0.061	0.223	-4.059	0.415	6.588	6.173
0.740	0.849	-5.663	-0.119	3.185	3.304
0.182	0.374	-4.875	-0.420	4.023	4.443
0.049	0.207	-3.893	0.439	4.610	4.171
0.932	0.963	-5.711	0.027	3.162	3.135
0.497	0.669	-5.496	0.184	4.001	3.817
0.712	0.828	-5.650	0.123	2.201	2.078
0.414	0.602	-5.399	-0.204	2.488	2.692
0.071	0.238	-4.182	-0.771	3.606	4.377
0.208	0.403	-4.967	-0.407	3.139	3.546
0.252	0.450	-5.096	-0.318	2.502	2.820
0.316	0.514	-5.239	0.302	2.547	2.245
0.135	0.318	-4.660	-0.405	3.945	4.349
0.800	0.888	-5.684	-0.067	2.397	2.464
0.237	0.434	-5.053	-0.345	5.961	6.307
0.987	0.993	-5.715	0.005	2.571	2.566
0.003	0.157	-1.532	2.059	7.929	5.869
0.333	0.529	-5.271	-0.430	1.147	1.578
0.566	0.723	-5.559	-0.279	4.732	5.011
0.992	0.996	-5.715	-0.005	3.997	4.003
0.733	0.844	-5.660	0.103	3.162	3.060
0.046	0.203	-3.839	-0.628	5.232	5.860
0.073	0.241	-4.206	0.498	6.360	5.862
0.085	0.257	-4.317	0.483	4.360	3.877
0.644	0.782	-5.614	0.129	2.905	2.776
0.033	0.184	-3.592	-0.553	2.131	2.684
0.896	0.943	-5.707	-0.037	3.148	3.185
0.361	0.553	-5.320	0.305	4.970	4.665
0.082	0.253	-4.290	-0.409	3.923	4.332
0.083	0.255	-4.302	-0.552	5.207	5.759
0.144	0.328	-4.708	0.434	4.131	3.697
0.983	0.991	-5.715	0.005	4.244	4.240
0.495	0.667	-5.494	-0.354	1.644	1.998
0.680	0.808	-5.634	-0.112	2.146	2.257
0.421	0.607	-5.408	0.213	4.465	4.252
0.203	0.398	-4.951	-0.558	2.633	3.191
0.530	0.695	-5.528	-0.320	5.960	6.280
0.539	0.701	-5.536	-0.288	6.380	6.668
0.245	0.442	-5.076	-0.415	3.654	4.069
0.679	0.808	-5.634	0.146	5.412	5.266
0.015	0.160	-2.932	2.132	8.558	6.426
0.473	0.652	-5.471	-0.235	1.814	2.049

0.063	0.227	-4.095	0.880	5.923	5.043
0.606	0.754	-5.589	-0.155	3.276	3.431
0.803	0.890	-5.685	0.065	2.303	2.238
0.186	0.378	-4.890	0.438	3.862	3.425
0.457	0.637	-5.452	0.214	3.866	3.652
0.201	0.397	-4.944	0.393	5.183	4.790
0.703	0.823	-5.646	-0.109	3.363	3.472
0.724	0.838	-5.656	-0.103	3.460	3.563
0.733	0.844	-5.660	-0.096	3.440	3.536
0.758	0.860	-5.670	-0.086	3.506	3.592
0.821	0.899	-5.691	0.075	3.546	3.470
0.265	0.464	-5.129	-0.287	3.088	3.375
0.843	0.913	-5.696	0.052	2.764	2.712
0.049	0.206	-3.888	0.832	4.203	3.372
0.528	0.693	-5.526	-0.153	2.354	2.507
0.776	0.871	-5.676	-0.095	3.254	3.349
0.089	0.264	-4.354	-1.275	2.327	3.601
0.496	0.668	-5.495	-0.197	2.732	2.929
0.054	0.215	-3.975	-0.654	3.163	3.817
0.006	0.157	-2.260	-0.660	3.304	3.964
0.017	0.162	-3.045	-1.035	3.705	4.739
0.088	0.262	-4.345	0.734	3.585	2.851
0.393	0.581	-5.370	-0.236	4.870	5.106
0.074	0.242	-4.214	-0.488	3.100	3.588
0.126	0.307	-4.613	-0.522	2.794	3.316
0.143	0.327	-4.704	-0.297	6.339	6.636
0.642	0.780	-5.613	0.116	3.627	3.511
0.142	0.326	-4.700	0.305	7.363	7.057
0.040	0.194	-3.734	0.650	3.802	3.152
0.673	0.803	-5.630	-0.106	2.722	2.828
0.179	0.369	-4.861	0.436	3.761	3.325
0.032	0.183	-3.570	-0.672	4.941	5.613
0.230	0.427	-5.034	0.253	5.463	5.210
0.660	0.793	-5.623	0.109	5.888	5.778
0.030	0.179	-3.509	1.311	6.255	4.945
0.198	0.393	-4.934	-0.428	3.265	3.694
0.070	0.236	-4.167	-0.472	2.464	2.936
0.036	0.189	-3.662	-0.972	7.427	8.400
0.430	0.616	-5.420	-0.263	3.057	3.320
0.100	0.277	-4.443	0.413	5.816	5.404
0.895	0.943	-5.707	0.032	5.642	5.610
0.004	0.157	-1.771	0.666	2.994	2.328
0.822	0.899	-5.691	-0.060	3.929	3.989
0.192	0.385	-4.912	-0.320	4.408	4.728
0.111	0.290	-4.519	0.428	3.147	2.719
0.004	0.157	-1.850	2.268	6.313	4.045
0.348	0.543	-5.297	-0.409	5.635	6.044
0.353	0.548	-5.307	-0.414	5.917	6.331
0.250	0.448	-5.091	-0.507	5.731	6.238
0.271	0.469	-5.142	-0.493	5.841	6.334

0.280	0.478	-5.163	-0.490	5.851	6.341
0.292	0.489	-5.189	-0.483	5.847	6.330
0.354	0.548	-5.308	-0.411	5.950	6.361
0.356	0.550	-5.313	-0.367	5.306	5.673
0.405	0.593	-5.387	-0.340	5.413	5.753
0.022	0.171	-3.273	0.662	2.597	1.935
0.020	0.167	-3.203	-0.632	3.587	4.219
0.126	0.306	-4.609	-0.477	2.317	2.794
0.123	0.304	-4.593	0.632	3.978	3.346
0.190	0.383	-4.905	-0.407	3.642	4.049
0.690	0.815	-5.640	0.260	3.690	3.431
0.745	0.852	-5.665	0.130	2.684	2.554
0.117	0.297	-4.558	0.360	6.521	6.161
0.907	0.950	-5.708	0.069	3.170	3.102
0.785	0.878	-5.680	0.076	5.341	5.265
0.000	0.157	0.955	1.027	3.034	2.007
0.003	0.157	-1.510	-0.799	2.078	2.877
0.340	0.535	-5.284	0.402	4.802	4.400
0.844	0.914	-5.697	0.055	3.547	3.492
0.327	0.523	-5.261	-0.261	2.765	3.025
0.167	0.356	-4.813	-0.713	3.296	4.009
0.154	0.341	-4.756	0.314	7.341	7.026
0.136	0.319	-4.665	0.333	6.149	5.816
0.025	0.175	-3.371	0.927	6.230	5.303
0.202	0.397	-4.946	0.494	3.619	3.125
0.414	0.602	-5.399	0.210	5.561	5.351
0.232	0.429	-5.041	0.393	2.461	2.068
0.100	0.277	-4.441	-0.380	7.953	8.333
0.102	0.279	-4.454	-0.392	6.453	6.845
0.111	0.290	-4.517	-0.387	6.887	7.274
0.111	0.290	-4.517	-0.327	6.664	6.991
0.123	0.304	-4.595	-0.390	6.686	7.076
0.172	0.362	-4.835	-0.276	6.670	6.946
0.180	0.371	-4.866	0.353	4.522	4.169
0.591	0.743	-5.578	0.173	4.877	4.704
0.023	0.173	-3.294	0.918	4.900	3.982
0.282	0.480	-5.167	-0.308	2.373	2.681
0.611	0.757	-5.592	-0.169	1.264	1.433
0.082	0.253	-4.289	0.477	6.125	5.648
0.228	0.424	-5.027	-0.379	3.134	3.513
0.055	0.216	-3.991	0.534	3.728	3.194
0.681	0.808	-5.634	0.104	2.788	2.684
0.870	0.929	-5.702	-0.093	1.719	1.812
0.181	0.372	-4.870	-0.375	2.423	2.798
0.627	0.768	-5.603	0.147	3.010	2.864
0.033	0.184	-3.586	-0.546	5.254	5.800
0.210	0.405	-4.974	-0.468	3.091	3.560
0.898	0.944	-5.707	-0.025	5.830	5.855
0.012	0.160	-2.789	-0.648	2.158	2.806
0.165	0.354	-4.806	-0.396	3.425	3.821

0.550	0.710	-5.545	-0.329	1.888	2.217
0.034	0.185	-3.606	0.837	4.084	3.247
0.028	0.178	-3.463	-0.498	4.667	5.165
0.605	0.753	-5.588	-0.152	2.178	2.330
0.120	0.301	-4.578	0.485	4.116	3.631
0.044	0.201	-3.813	-0.595	2.786	3.381
0.358	0.551	-5.315	0.229	5.041	4.812
0.774	0.870	-5.676	-0.076	3.930	4.006
0.045	0.201	-3.824	-0.541	4.044	4.585
0.249	0.446	-5.086	0.316	5.642	5.326
0.417	0.604	-5.404	0.199	2.589	2.389
0.289	0.488	-5.184	0.231	4.481	4.250
0.022	0.171	-3.267	0.856	7.079	6.223
0.047	0.203	-3.856	0.745	7.759	7.014
0.024	0.173	-3.326	1.113	9.032	7.919
0.061	0.224	-4.067	0.433	4.089	3.657
0.093	0.268	-4.385	-0.856	3.556	4.412
0.006	0.157	-2.242	-0.993	5.427	6.419
0.431	0.616	-5.421	0.312	3.700	3.389
0.690	0.815	-5.640	0.145	5.146	5.001
0.520	0.686	-5.519	-0.192	4.497	4.688
0.006	0.157	-2.179	1.584	4.646	3.062
0.463	0.643	-5.460	-0.198	5.226	5.424
0.865	0.928	-5.701	0.044	5.511	5.467
0.276	0.474	-5.153	0.441	6.753	6.312
0.359	0.552	-5.317	0.235	5.506	5.271
0.002	0.157	-1.231	1.726	5.974	4.247
0.342	0.537	-5.288	-0.453	3.423	3.875
0.871	0.930	-5.702	-0.038	5.605	5.643
0.031	0.182	-3.544	0.876	7.356	6.480
0.035	0.188	-3.641	1.008	6.374	5.366
0.025	0.175	-3.354	0.831	5.834	5.002
0.170	0.360	-4.826	0.381	2.620	2.239
0.296	0.493	-5.198	0.295	5.859	5.565
0.094	0.270	-4.395	1.009	6.882	5.872
0.117	0.297	-4.556	0.508	3.731	3.222
0.390	0.577	-5.365	0.263	4.001	3.739
0.006	0.157	-2.182	1.308	5.630	4.322
0.121	0.301	-4.580	0.408	8.008	7.600
0.546	0.707	-5.542	0.269	5.533	5.264
0.018	0.162	-3.081	0.958	7.242	6.284
0.046	0.203	-3.838	0.857	7.438	6.582
0.015	0.161	-2.964	1.295	7.151	5.856
0.101	0.278	-4.445	0.570	4.692	4.122
0.009	0.157	-2.524	1.092	8.187	7.095
0.067	0.232	-4.136	1.135	7.576	6.441
0.018	0.163	-3.094	1.808	9.815	8.007
0.330	0.525	-5.265	-0.274	5.599	5.874
0.628	0.769	-5.603	0.156	2.490	2.335
0.009	0.157	-2.499	1.549	5.604	4.055

0.054	0.215	-3.977	0.533	8.211	7.679
0.091	0.266	-4.368	0.932	8.837	7.905
0.241	0.439	-5.066	0.362	5.826	5.464
0.044	0.200	-3.804	0.942	8.591	7.649
0.004	0.157	-1.918	1.465	7.952	6.487
0.029	0.178	-3.494	-0.662	4.640	5.302
0.556	0.716	-5.551	0.349	6.965	6.615
0.456	0.637	-5.452	-0.647	6.383	7.030
0.156	0.344	-4.766	-0.447	5.045	5.492
0.033	0.184	-3.577	0.670	3.807	3.137
0.701	0.822	-5.645	-0.092	3.647	3.739
0.060	0.222	-4.053	0.474	5.102	4.627
0.156	0.343	-4.764	0.662	5.972	5.310
0.124	0.305	-4.598	0.432	4.891	4.459
0.011	0.157	-2.678	1.024	8.226	7.202
0.032	0.183	-3.568	1.777	7.389	5.612
0.012	0.160	-2.764	1.859	10.499	8.640
0.015	0.160	-2.927	1.077	8.569	7.492
0.002	0.157	-1.325	0.698	7.377	6.679
0.235	0.432	-5.049	0.390	7.848	7.458
0.302	0.500	-5.212	0.294	8.344	8.049
0.764	0.864	-5.672	0.140	5.737	5.597
0.023	0.173	-3.299	1.202	6.982	5.781
0.010	0.157	-2.654	1.151	6.582	5.431
0.010	0.157	-2.645	1.208	7.687	6.479
0.009	0.157	-2.493	1.958	7.967	6.010
0.023	0.173	-3.297	0.500	9.361	8.861
0.027	0.176	-3.423	0.772	4.356	3.584
0.309	0.507	-5.226	0.243	7.873	7.629
0.050	0.208	-3.912	0.574	7.763	7.189
0.197	0.392	-4.931	0.278	7.100	6.822
0.012	0.160	-2.778	1.310	9.692	8.382
0.806	0.891	-5.686	-0.063	2.635	2.698
0.147	0.331	-4.722	-0.468	2.126	2.595
0.026	0.176	-3.395	-1.057	5.867	6.924
0.276	0.474	-5.154	0.250	6.785	6.535
0.025	0.175	-3.370	-0.866	3.926	4.792
0.001	0.157	-0.545	0.950	5.006	4.056
0.526	0.691	-5.524	-0.190	2.422	2.612
0.007	0.157	-2.311	-0.627	3.995	4.621
0.024	0.173	-3.337	-1.176	5.835	7.012
0.680	0.808	-5.634	0.267	5.214	4.947
0.181	0.373	-4.872	0.457	2.883	2.427
0.256	0.454	-5.106	-0.334	3.970	4.305
0.399	0.586	-5.378	-0.282	3.304	3.586
0.126	0.307	-4.612	0.338	8.027	7.689
0.047	0.203	-3.855	0.787	6.789	6.001
0.955	0.977	-5.713	0.015	1.877	1.861
0.120	0.300	-4.574	-0.401	2.628	3.029
0.225	0.422	-5.020	0.357	2.599	2.243

0.638	0.777	-5.610	0.122	1.968	1.847
0.008	0.157	-2.430	1.188	5.807	4.620
0.031	0.181	-3.530	-0.616	6.292	6.907
0.652	0.788	-5.618	-0.110	2.603	2.713
0.713	0.829	-5.651	-0.100	3.918	4.019
0.077	0.246	-4.241	-0.468	4.087	4.555
0.035	0.186	-3.619	-0.535	7.004	7.539
0.855	0.922	-5.699	-0.062	3.010	3.072
0.007	0.157	-2.365	2.023	7.344	5.321
0.741	0.850	-5.663	-0.089	3.299	3.388
0.016	0.162	-3.018	1.392	5.728	4.336
0.044	0.200	-3.804	-0.551	5.347	5.899
0.694	0.817	-5.642	0.111	3.205	3.095
0.143	0.327	-4.705	0.410	2.566	2.156
0.104	0.281	-4.467	0.391	6.909	6.519
0.023	0.173	-3.312	-0.546	3.327	3.873
0.012	0.160	-2.753	1.203	4.165	2.962
0.858	0.924	-5.700	0.049	4.974	4.926
0.020	0.167	-3.199	0.682	3.249	2.567
0.029	0.178	-3.494	0.520	6.281	5.762
0.634	0.774	-5.608	0.141	4.239	4.098
0.079	0.249	-4.260	-0.680	4.235	4.916
0.206	0.401	-4.959	0.455	3.206	2.751
0.006	0.157	-2.232	0.825	6.469	5.644
0.876	0.933	-5.703	0.051	2.221	2.170
0.773	0.869	-5.675	-0.096	3.491	3.587
0.143	0.327	-4.702	0.306	6.038	5.733
0.790	0.881	-5.681	0.073	2.472	2.399
0.083	0.254	-4.298	-0.676	2.425	3.100
0.342	0.537	-5.288	-0.247	6.112	6.359
0.754	0.858	-5.668	0.094	5.784	5.691
0.302	0.500	-5.211	-0.454	3.871	4.325
0.303	0.500	-5.212	-0.509	4.226	4.735
0.095	0.271	-4.400	-0.501	5.854	6.354
0.660	0.793	-5.623	-0.110	4.330	4.440
0.009	0.157	-2.565	0.917	3.573	2.657
0.050	0.208	-3.913	0.391	5.604	5.213
0.273	0.471	-5.148	-0.348	3.367	3.715
0.223	0.419	-5.013	-0.351	3.737	4.088
0.069	0.236	-4.165	-0.483	3.090	3.573
0.007	0.157	-2.379	-0.861	2.071	2.932
0.481	0.658	-5.480	-0.230	3.103	3.333
0.260	0.457	-5.115	0.324	3.495	3.171
0.462	0.643	-5.459	0.257	2.788	2.530
0.014	0.160	-2.889	1.229	5.485	4.256
0.955	0.977	-5.713	0.017	2.689	2.672
0.402	0.589	-5.382	0.286	4.640	4.354
0.854	0.921	-5.699	-0.062	2.141	2.203
0.012	0.160	-2.751	1.010	5.486	4.477
0.660	0.793	-5.623	0.112	4.552	4.440

0.756	0.859	-5.669	-0.093	3.110	3.203
0.866	0.928	-5.701	-0.056	3.610	3.666
0.399	0.587	-5.379	0.248	3.301	3.052
0.361	0.553	-5.321	-0.358	1.260	1.619
0.231	0.428	-5.037	0.316	3.214	2.898
0.210	0.404	-4.971	-0.354	2.792	3.147
0.159	0.348	-4.781	0.324	8.462	8.138
0.623	0.766	-5.600	0.157	1.664	1.507
0.112	0.290	-4.523	0.528	4.014	3.486
0.007	0.157	-2.335	0.675	2.736	2.061
0.002	0.157	-1.212	0.938	3.580	2.642
0.227	0.424	-5.027	0.371	3.809	3.438
0.992	0.996	-5.715	-0.003	4.504	4.506
0.129	0.310	-4.629	0.468	4.490	4.022
0.304	0.501	-5.215	-0.250	3.189	3.439
0.828	0.903	-5.693	0.072	4.062	3.990
0.073	0.241	-4.203	-0.564	1.433	1.998
0.023	0.173	-3.297	0.707	6.431	5.724
0.958	0.978	-5.713	0.013	4.290	4.277
0.812	0.894	-5.688	-0.075	3.263	3.338
0.005	0.157	-1.993	1.573	5.238	3.665
0.009	0.157	-2.554	0.981	4.702	3.720
0.043	0.200	-3.802	0.683	3.430	2.746
0.009	0.157	-2.505	1.776	7.080	5.304
0.850	0.919	-5.698	0.071	2.146	2.074
0.400	0.588	-5.380	-0.291	2.567	2.857
0.763	0.864	-5.672	0.117	1.856	1.740
0.613	0.758	-5.594	-0.181	2.609	2.790
0.449	0.631	-5.444	0.206	3.772	3.565
0.051	0.209	-3.928	0.880	4.851	3.971
0.047	0.203	-3.857	-0.764	3.374	4.138
0.116	0.296	-4.548	0.413	3.261	2.848
0.068	0.234	-4.149	-0.545	1.794	2.339
0.203	0.398	-4.951	-0.376	4.323	4.699
0.011	0.157	-2.688	0.794	4.420	3.627
0.712	0.828	-5.650	0.069	6.270	6.201
0.421	0.607	-5.409	0.208	4.418	4.210
0.010	0.157	-2.613	-1.173	3.528	4.701
0.091	0.267	-4.372	0.468	3.637	3.169
0.229	0.427	-5.032	0.299	4.070	3.771
0.695	0.818	-5.642	-0.098	6.313	6.411
0.338	0.533	-5.280	0.286	2.624	2.338
0.524	0.689	-5.523	0.166	4.747	4.582
0.145	0.329	-4.711	-0.400	7.480	7.880
0.826	0.901	-5.692	-0.095	3.263	3.358
0.821	0.899	-5.690	-0.058	2.585	2.643
0.137	0.320	-4.671	0.403	4.765	4.362
0.483	0.659	-5.482	0.145	6.238	6.093
0.009	0.157	-2.555	1.033	4.322	3.288
0.514	0.681	-5.513	0.182	3.640	3.458

0.901	0.946	-5.707	0.037	4.068	4.031
0.564	0.721	-5.557	-0.290	5.342	5.632
0.996	0.998	-5.715	-0.001	4.662	4.664
0.517	0.684	-5.516	0.461	5.548	5.086
0.068	0.233	-4.145	-0.480	6.776	7.256
0.816	0.896	-5.689	0.123	3.486	3.363
0.834	0.907	-5.694	-0.142	3.179	3.321
0.723	0.837	-5.655	-0.168	4.129	4.298
0.121	0.302	-4.583	0.472	8.510	8.038
0.416	0.603	-5.402	0.277	2.098	1.821
0.007	0.157	-2.368	-1.384	5.509	6.893
0.357	0.551	-5.314	-0.355	2.860	3.216
0.209	0.404	-4.971	0.551	4.262	3.711
0.437	0.621	-5.429	0.215	3.543	3.328
0.523	0.689	-5.522	-0.277	3.544	3.821
0.974	0.987	-5.714	-0.010	3.040	3.050
0.357	0.551	-5.313	-0.292	2.029	2.321
0.304	0.502	-5.216	-0.340	2.575	2.915
0.289	0.488	-5.184	0.369	5.957	5.588
0.073	0.241	-4.202	-0.766	4.192	4.957
0.786	0.878	-5.680	-0.109	4.081	4.190
0.494	0.667	-5.493	-0.195	3.118	3.312
0.213	0.408	-4.982	-0.324	3.350	3.674
0.097	0.274	-4.420	-1.008	4.051	5.059
0.279	0.477	-5.161	-0.550	2.348	2.898
0.904	0.948	-5.708	-0.034	1.202	1.236
0.802	0.889	-5.685	0.068	2.576	2.508
0.071	0.238	-4.177	-0.577	5.440	6.017
0.120	0.299	-4.573	-0.342	3.530	3.872
0.387	0.575	-5.361	-0.165	5.387	5.552
0.278	0.477	-5.159	-0.443	7.551	7.994
0.601	0.751	-5.586	0.241	1.613	1.372
0.206	0.401	-4.961	-0.511	4.783	5.294
0.116	0.295	-4.548	-0.293	2.911	3.204
0.272	0.470	-5.144	-0.311	3.657	3.969
0.092	0.267	-4.376	0.497	6.186	5.689
0.290	0.488	-5.185	-0.476	2.568	3.044
0.108	0.287	-4.497	-0.744	3.740	4.484
0.142	0.326	-4.697	-0.427	3.464	3.892
0.276	0.475	-5.154	0.361	5.152	4.791
0.227	0.424	-5.025	-0.424	4.339	4.763
0.018	0.163	-3.116	-0.857	7.633	8.491
0.432	0.616	-5.422	-0.197	3.353	3.550
0.212	0.407	-4.978	0.258	5.177	4.920
0.086	0.258	-4.329	-0.848	3.667	4.514
0.052	0.211	-3.939	0.558	6.483	5.925
0.030	0.179	-3.505	-0.738	3.984	4.722
0.117	0.296	-4.554	-0.523	3.505	4.028
0.535	0.698	-5.533	-0.144	5.557	5.701
0.236	0.433	-5.051	0.237	3.794	3.557

0.017	0.162	-3.071	0.915	6.220	5.305
0.300	0.497	-5.206	-0.347	3.206	3.553
0.647	0.784	-5.616	0.135	3.069	2.934
0.351	0.545	-5.303	-0.353	3.280	3.634
0.018	0.162	-3.082	1.059	7.910	6.851
0.053	0.212	-3.950	0.881	4.413	3.532
0.014	0.160	-2.909	1.261	8.123	6.862
0.033	0.184	-3.586	1.098	6.358	5.260
0.107	0.286	-4.494	-0.893	4.102	4.996
0.012	0.159	-2.742	-0.712	6.226	6.938
0.018	0.163	-3.108	1.146	5.987	4.841
0.723	0.837	-5.655	-0.093	2.908	3.001
0.045	0.201	-3.823	0.751	3.877	3.126
0.088	0.261	-4.343	0.451	4.787	4.336
0.042	0.197	-3.773	0.559	6.713	6.154
0.108	0.286	-4.495	0.687	5.232	4.545
0.103	0.280	-4.461	0.596	7.824	7.227
0.007	0.157	-2.292	1.986	6.420	4.434
0.145	0.329	-4.713	0.451	5.101	4.650
0.012	0.160	-2.785	1.140	9.306	8.166
0.024	0.173	-3.317	-1.031	4.614	5.645
0.076	0.245	-4.229	0.479	4.628	4.149
0.033	0.184	-3.587	0.642	5.553	4.911
0.055	0.216	-3.990	-0.622	3.478	4.100
0.091	0.266	-4.367	0.454	7.064	6.610
0.024	0.174	-3.343	1.101	7.059	5.958
0.070	0.237	-4.172	0.655	5.748	5.094
0.085	0.257	-4.318	-0.481	4.765	5.247
0.169	0.359	-4.822	0.298	5.214	4.916
0.016	0.162	-3.026	0.705	4.601	3.896
0.041	0.195	-3.750	0.518	6.226	5.707
0.013	0.160	-2.855	0.763	8.283	7.520
0.789	0.880	-5.681	0.097	3.870	3.772
0.018	0.163	-3.102	-0.962	4.004	4.966
0.105	0.283	-4.478	0.332	5.728	5.395
0.187	0.379	-4.893	-0.345	2.227	2.573
0.911	0.952	-5.709	-0.032	2.681	2.713
0.011	0.157	-2.693	0.751	6.036	5.284
0.007	0.157	-2.340	0.700	3.012	2.312
0.680	0.808	-5.634	-0.119	3.069	3.189
0.083	0.255	-4.301	0.545	7.463	6.918
0.173	0.362	-4.838	0.333	4.380	4.047
0.080	0.252	-4.277	0.559	5.583	5.024
0.014	0.160	-2.868	-0.792	4.489	5.281
0.485	0.661	-5.484	-0.187	3.468	3.655
0.039	0.192	-3.709	1.139	6.928	5.789
0.021	0.168	-3.223	0.870	6.748	5.878
0.051	0.210	-3.931	0.889	5.635	4.745
0.082	0.253	-4.287	-0.523	3.727	4.250
0.336	0.532	-5.278	0.252	4.449	4.198

0.614	0.758	-5.594	0.185	6.072	5.887
0.238	0.435	-5.056	-0.369	3.518	3.887
0.018	0.162	-3.088	-0.887	3.546	4.434
0.200	0.396	-4.941	0.386	4.313	3.926
0.484	0.660	-5.483	-0.203	3.496	3.699
0.019	0.164	-3.126	0.715	8.466	7.751
0.038	0.191	-3.686	0.962	5.653	4.691
0.029	0.178	-3.493	1.261	8.171	6.910
0.041	0.195	-3.751	1.040	6.696	5.656
0.102	0.279	-4.453	0.518	4.383	3.865
0.028	0.178	-3.458	1.361	5.795	4.434
0.044	0.200	-3.805	0.725	6.967	6.242
0.034	0.185	-3.616	-0.524	4.700	5.224
0.893	0.941	-5.706	-0.032	5.094	5.127
0.112	0.290	-4.523	0.386	4.772	4.385
0.804	0.890	-5.686	0.055	4.160	4.105
0.258	0.455	-5.109	0.320	4.141	3.821
0.641	0.779	-5.612	-0.109	5.733	5.842
0.052	0.211	-3.940	0.643	6.163	5.520
0.129	0.310	-4.628	0.555	5.769	5.214
0.119	0.299	-4.572	-0.445	6.121	6.567
0.533	0.696	-5.531	0.158	3.541	3.384
0.753	0.857	-5.668	0.069	4.249	4.179
0.077	0.246	-4.239	-0.484	5.219	5.703
0.291	0.489	-5.187	-0.240	6.925	7.165
0.143	0.327	-4.704	-0.654	5.885	6.539
0.080	0.251	-4.276	0.561	2.949	2.388
0.003	0.157	-1.702	-1.024	3.477	4.502
0.090	0.264	-4.360	0.502	8.098	7.596
0.387	0.575	-5.361	0.280	2.924	2.643
0.203	0.398	-4.950	0.365	3.392	3.026
0.488	0.664	-5.487	-0.215	5.559	5.774
0.720	0.834	-5.654	-0.113	3.154	3.268
0.765	0.865	-5.672	-0.111	3.662	3.773
0.196	0.390	-4.924	0.626	4.847	4.220
0.495	0.667	-5.494	0.255	3.054	2.799
0.325	0.521	-5.256	0.217	3.357	3.139
0.024	0.173	-3.318	-0.597	5.486	6.084
0.123	0.304	-4.594	-0.383	4.228	4.612
0.756	0.859	-5.669	-0.082	2.097	2.179
0.735	0.846	-5.661	0.110	3.191	3.081
0.028	0.178	-3.459	-0.603	1.898	2.501
0.103	0.280	-4.463	0.491	5.105	4.613
0.112	0.290	-4.524	0.743	4.795	4.052
0.497	0.669	-5.497	0.261	4.707	4.447
0.821	0.899	-5.691	-0.086	3.845	3.932
0.284	0.482	-5.172	-0.510	3.848	4.359
0.125	0.306	-4.604	0.420	4.376	3.956
0.023	0.173	-3.296	0.717	4.605	3.888
0.323	0.520	-5.253	-0.328	1.528	1.856

0.991	0.995	-5.715	0.003	4.552	4.549
0.322	0.519	-5.251	0.248	3.062	2.814
0.190	0.382	-4.903	-0.340	5.684	6.024
0.016	0.162	-3.018	0.921	7.686	6.765
0.026	0.176	-3.401	1.545	7.509	5.964
0.021	0.168	-3.219	-0.792	4.930	5.722
0.061	0.224	-4.067	-0.618	2.032	2.650
0.930	0.962	-5.711	0.028	2.147	2.119
0.932	0.963	-5.711	-0.027	2.168	2.195
0.039	0.193	-3.716	1.287	6.938	5.651
0.068	0.234	-4.149	0.964	5.682	4.718
0.131	0.314	-4.642	1.194	7.927	6.732
0.258	0.455	-5.109	-0.813	2.689	3.501
0.454	0.634	-5.449	0.161	6.423	6.262
0.310	0.508	-5.228	0.340	2.470	2.129
0.069	0.235	-4.155	-0.512	5.937	6.449
0.011	0.157	-2.695	0.719	7.825	7.106
0.831	0.905	-5.693	-0.052	4.514	4.567
0.085	0.257	-4.317	0.406	6.939	6.533
0.019	0.164	-3.147	0.753	6.102	5.349
0.002	0.157	-1.110	1.435	6.973	5.539
0.089	0.263	-4.350	-0.598	1.658	2.255
0.023	0.173	-3.288	0.573	3.955	3.382
0.328	0.523	-5.262	-0.274	4.297	4.571
0.040	0.194	-3.745	0.690	4.068	3.378
0.017	0.162	-3.059	1.284	6.770	5.487
0.017	0.162	-3.050	1.173	4.370	3.197
0.729	0.841	-5.658	-0.139	4.046	4.185
0.006	0.157	-2.212	1.349	5.144	3.795
0.114	0.293	-4.541	0.573	4.266	3.693
0.842	0.912	-5.696	0.167	2.116	1.949
0.319	0.517	-5.246	0.330	2.697	2.367
0.112	0.290	-4.522	0.452	3.582	3.129
0.801	0.888	-5.685	0.063	3.129	3.067
0.855	0.922	-5.699	0.071	3.161	3.090
0.092	0.267	-4.379	0.650	5.353	4.702
0.049	0.206	-3.887	0.756	5.913	5.156
0.532	0.696	-5.530	0.142	3.901	3.759
0.104	0.281	-4.468	-0.409	2.898	3.307
0.023	0.172	-3.280	0.614	7.203	6.590
0.084	0.256	-4.311	-0.681	5.357	6.038
0.634	0.774	-5.608	-0.188	5.216	5.404
0.175	0.365	-4.847	-0.496	2.957	3.453
0.018	0.163	-3.093	0.996	4.555	3.559
0.050	0.208	-3.910	-0.681	5.454	6.135
0.296	0.493	-5.199	0.299	4.834	4.535
0.585	0.739	-5.574	-0.129	4.334	4.463
0.034	0.184	-3.600	0.540	3.385	2.845
0.223	0.420	-5.015	-0.329	3.427	3.755
0.195	0.389	-4.922	0.370	4.689	4.319

0.032	0.183	-3.569	-0.814	3.670	4.484
0.061	0.223	-4.061	-0.625	3.591	4.215
0.040	0.194	-3.739	0.605	4.102	3.497
0.702	0.822	-5.645	0.111	8.273	8.162
0.257	0.455	-5.108	0.241	7.108	6.867
0.047	0.204	-3.864	0.529	4.765	4.237
0.647	0.784	-5.615	0.149	2.500	2.351
0.034	0.185	-3.614	0.833	6.491	5.659
0.321	0.518	-5.249	0.276	3.565	3.290
0.058	0.219	-4.021	0.809	6.138	5.329
0.069	0.236	-4.165	0.521	5.375	4.854
0.092	0.268	-4.381	0.534	2.753	2.219
0.096	0.273	-4.413	1.132	7.264	6.133
0.048	0.205	-3.878	-0.588	4.478	5.065
0.052	0.212	-3.948	-1.076	3.240	4.316
0.322	0.519	-5.252	0.245	3.268	3.023
0.033	0.184	-3.574	-0.744	4.531	5.275
0.944	0.970	-5.712	-0.013	7.895	7.908
0.029	0.178	-3.469	0.683	7.229	6.546
0.024	0.173	-3.328	-1.063	4.111	5.174
0.044	0.201	-3.816	-0.523	2.631	3.154
0.001	0.157	-0.243	0.910	3.348	2.438
0.582	0.736	-5.571	0.160	2.590	2.430
0.867	0.928	-5.702	-0.041	3.013	3.054
0.037	0.190	-3.672	-0.573	2.689	3.262
0.008	0.157	-2.387	-0.983	7.418	8.401
0.529	0.694	-5.527	-0.231	2.977	3.208
0.389	0.577	-5.364	-0.291	2.741	3.033
0.861	0.926	-5.700	0.065	3.813	3.748
0.709	0.827	-5.649	0.094	2.974	2.880
0.030	0.180	-3.517	0.990	7.424	6.433
0.706	0.826	-5.648	0.097	2.387	2.290
0.163	0.352	-4.798	-0.760	1.895	2.655
0.460	0.641	-5.457	-0.338	3.946	4.284
0.005	0.157	-2.048	-1.022	1.667	2.688
0.102	0.280	-4.457	0.588	4.082	3.493
0.914	0.954	-5.709	0.027	5.800	5.773
0.958	0.978	-5.713	0.014	5.829	5.815
0.984	0.991	-5.715	0.005	5.789	5.785
0.483	0.659	-5.482	-0.147	4.825	4.972
0.489	0.664	-5.489	-0.148	4.804	4.952
0.031	0.182	-3.544	0.928	5.052	4.124
0.368	0.559	-5.332	0.205	1.920	1.715
0.496	0.668	-5.496	-0.223	8.122	8.345
0.109	0.288	-4.507	-0.581	2.262	2.843
0.104	0.282	-4.472	-0.633	5.088	5.721
0.924	0.959	-5.710	0.034	4.568	4.534
0.020	0.167	-3.179	-0.588	4.936	5.524
0.392	0.580	-5.368	-0.236	3.680	3.916
0.405	0.593	-5.387	-0.209	4.131	4.341

0.140	0.324	-4.686	0.337	7.501	7.164
0.015	0.161	-2.972	0.610	9.706	9.097
0.021	0.168	-3.206	1.404	7.908	6.504
0.059	0.220	-4.034	0.959	6.812	5.852
0.085	0.257	-4.320	0.427	6.892	6.465
0.184	0.375	-4.882	-0.369	3.344	3.713
0.161	0.350	-4.789	0.527	3.376	2.848
0.074	0.241	-4.211	0.764	5.214	4.450
0.019	0.164	-3.146	1.315	7.877	6.562
0.022	0.170	-3.254	1.355	7.448	6.092
0.101	0.278	-4.448	0.455	2.993	2.538
0.099	0.276	-4.433	0.394	6.848	6.454
0.142	0.326	-4.696	-0.470	3.965	4.435
0.335	0.531	-5.276	-0.268	4.203	4.471
0.012	0.160	-2.776	-1.408	4.775	6.183
0.504	0.674	-5.504	-0.245	3.154	3.399
0.253	0.451	-5.097	-0.262	4.213	4.475
0.102	0.279	-4.452	-0.814	8.746	9.559
0.324	0.520	-5.255	0.233	6.622	6.389
0.150	0.335	-4.735	-0.632	2.550	3.181
0.129	0.310	-4.626	0.562	3.277	2.715
0.016	0.162	-3.023	0.886	5.496	4.610
0.114	0.292	-4.535	0.381	3.868	3.487
0.009	0.157	-2.565	1.267	8.990	7.723
0.184	0.375	-4.880	-0.455	4.110	4.564
0.099	0.275	-4.431	0.670	5.002	4.331
0.103	0.281	-4.466	-0.597	4.563	5.160
0.358	0.551	-5.315	0.265	7.652	7.388
0.035	0.186	-3.633	0.985	5.907	4.921
0.033	0.184	-3.575	-0.946	3.760	4.707
0.057	0.218	-4.006	-0.738	8.782	9.520
0.231	0.428	-5.038	0.688	7.701	7.013
0.236	0.433	-5.053	0.714	7.583	6.869
0.028	0.177	-3.443	0.536	6.065	5.529
0.025	0.175	-3.370	0.697	6.789	6.092
0.540	0.702	-5.537	-0.175	7.013	7.187
0.012	0.160	-2.779	-1.047	6.657	7.704
0.086	0.259	-4.332	0.640	6.860	6.221
0.013	0.160	-2.850	1.237	6.129	4.892
0.020	0.167	-3.190	1.029	4.975	3.945
0.008	0.157	-2.451	0.955	3.466	2.511
0.029	0.178	-3.481	0.778	7.996	7.218
0.058	0.219	-4.023	0.822	8.610	7.788
0.032	0.183	-3.566	0.505	4.969	4.464
0.014	0.160	-2.877	-0.950	6.579	7.530
0.032	0.182	-3.558	0.973	7.880	6.907
0.057	0.218	-4.010	0.590	4.773	4.182
0.162	0.351	-4.792	0.327	4.872	4.545
0.172	0.362	-4.834	0.468	3.708	3.240
0.073	0.241	-4.206	0.466	7.558	7.092

0.343	0.539	-5.290	0.205	6.600	6.396
0.203	0.398	-4.950	0.327	2.571	2.244
0.015	0.160	-2.942	0.871	4.831	3.960
0.602	0.751	-5.586	-0.225	2.572	2.797
0.821	0.899	-5.690	0.056	1.843	1.788
0.032	0.182	-3.549	-0.666	3.850	4.516
0.307	0.505	-5.222	-0.321	3.940	4.261
0.000	0.157	0.043	0.999	3.529	2.530
0.643	0.780	-5.613	-0.114	3.027	3.142
0.095	0.271	-4.404	0.562	3.958	3.396
0.016	0.162	-2.987	0.739	3.667	2.928
0.679	0.808	-5.634	-0.140	3.233	3.373
0.049	0.207	-3.898	0.625	5.338	4.712
0.891	0.941	-5.706	0.030	2.471	2.442
0.709	0.827	-5.649	0.125	2.870	2.745
0.103	0.281	-4.465	0.526	2.540	2.014
0.230	0.428	-5.035	0.457	4.197	3.741
0.108	0.287	-4.500	0.513	4.138	3.626
0.713	0.829	-5.651	0.101	2.044	1.943
0.349	0.544	-5.300	0.185	7.948	7.763
0.083	0.254	-4.298	-0.841	4.247	5.088
0.001	0.157	-0.836	1.531	6.107	4.576
0.206	0.401	-4.960	0.356	3.907	3.550
0.501	0.671	-5.500	0.176	2.770	2.594
0.438	0.622	-5.431	-0.219	1.460	1.680
0.148	0.333	-4.728	-0.429	2.270	2.699
0.446	0.629	-5.440	-0.215	2.660	2.875
0.296	0.493	-5.198	-0.289	3.638	3.927
0.269	0.467	-5.137	-0.297	3.668	3.965
0.086	0.258	-4.327	0.608	5.729	5.121
0.845	0.914	-5.697	-0.048	5.892	5.940
0.321	0.518	-5.249	0.305	3.923	3.618
0.264	0.462	-5.126	0.229	4.819	4.590
0.017	0.162	-3.051	-0.542	3.104	3.646
0.328	0.524	-5.263	0.256	5.529	5.272
0.065	0.229	-4.114	0.386	6.633	6.246
0.477	0.655	-5.476	-0.201	3.763	3.963
0.169	0.359	-4.823	0.344	5.935	5.591
0.068	0.234	-4.152	0.464	4.256	3.792
0.860	0.925	-5.700	-0.072	5.746	5.818
0.388	0.575	-5.362	0.400	3.743	3.344
0.031	0.181	-3.533	0.744	5.581	4.837
0.083	0.255	-4.299	-0.453	6.340	6.793
0.274	0.472	-5.150	0.207	5.833	5.626
0.100	0.277	-4.437	0.381	6.223	5.842
0.130	0.312	-4.636	0.382	5.423	5.042
0.871	0.930	-5.702	-0.043	2.101	2.145
0.105	0.283	-4.476	0.595	6.904	6.309
0.587	0.740	-5.575	-0.285	5.601	5.886
0.064	0.228	-4.102	-0.774	1.300	2.074

0.043	0.199	-3.794	-1.315	3.445	4.760
0.240	0.437	-5.061	-0.534	4.504	5.037
0.029	0.178	-3.472	-0.576	6.670	7.246
0.036	0.189	-3.661	0.803	7.077	6.274
0.914	0.954	-5.709	-0.029	4.101	4.130
0.049	0.207	-3.899	0.792	6.178	5.386
0.173	0.362	-4.838	0.612	4.573	3.962
0.297	0.494	-5.201	0.336	4.448	4.112
0.687	0.812	-5.638	0.125	2.415	2.290
0.085	0.257	-4.320	-0.712	4.550	5.262
0.001	0.157	-0.471	-1.044	6.511	7.555
0.010	0.157	-2.592	1.769	6.814	5.046
0.025	0.175	-3.355	1.076	6.170	5.094
0.019	0.164	-3.122	0.732	4.339	3.606
0.003	0.157	-1.721	0.892	5.708	4.816
0.191	0.383	-4.907	0.543	4.938	4.395
0.210	0.405	-4.973	0.336	2.512	2.176
0.008	0.157	-2.461	-0.948	5.285	6.233
0.542	0.703	-5.538	0.218	2.611	2.393
0.064	0.229	-4.107	-0.658	6.122	6.780
0.010	0.157	-2.614	-0.692	3.167	3.859
0.100	0.277	-4.442	-0.782	4.080	4.862
0.389	0.576	-5.364	0.214	6.720	6.506
0.122	0.304	-4.590	-0.483	4.451	4.935
0.626	0.768	-5.602	-0.179	2.237	2.417
0.807	0.892	-5.687	0.082	2.343	2.261
0.003	0.157	-1.706	-1.237	2.518	3.755
0.865	0.928	-5.701	-0.066	2.483	2.549
0.744	0.851	-5.664	-0.144	4.520	4.664
0.019	0.165	-3.153	0.731	3.267	2.536
0.003	0.157	-1.706	-0.790	3.297	4.086
0.496	0.668	-5.495	-0.137	4.751	4.888
0.009	0.157	-2.488	-0.626	3.830	4.456
0.073	0.241	-4.201	0.524	3.810	3.286
0.878	0.934	-5.704	0.039	2.440	2.402
0.145	0.329	-4.715	-0.509	3.396	3.904
0.471	0.650	-5.469	0.185	5.398	5.214
0.010	0.157	-2.601	2.058	7.769	5.711
0.804	0.890	-5.686	-0.053	5.371	5.424
0.141	0.325	-4.691	-0.644	2.382	3.025
0.921	0.957	-5.710	0.033	6.246	6.213
0.508	0.677	-5.507	0.162	5.140	4.978
0.156	0.344	-4.767	0.426	4.312	3.885
0.632	0.772	-5.606	0.121	4.162	4.041
0.495	0.668	-5.495	-0.286	4.674	4.961
0.958	0.978	-5.713	0.017	5.332	5.315
0.275	0.473	-5.151	0.445	4.890	4.445
0.045	0.203	-3.836	0.570	5.713	5.143
0.028	0.178	-3.460	-1.177	1.818	2.995
0.380	0.569	-5.350	-0.252	2.826	3.078

0.040	0.194	-3.739	0.483	5.104	4.621
0.173	0.362	-4.837	0.317	5.164	4.848
0.460	0.640	-5.456	-0.148	6.836	6.984
0.101	0.278	-4.451	0.544	6.409	5.866
0.011	0.157	-2.672	-0.703	4.850	5.553
0.121	0.302	-4.585	0.409	5.763	5.354
0.001	0.157	-1.045	-0.990	6.767	7.757
0.318	0.516	-5.244	0.426	2.910	2.484
0.015	0.160	-2.938	-0.802	5.304	6.105
0.268	0.466	-5.135	-0.277	3.291	3.567
0.315	0.513	-5.238	-0.298	5.901	6.199
0.004	0.157	-1.861	2.267	6.992	4.725
0.188	0.380	-4.896	-0.335	4.988	5.323
0.681	0.809	-5.635	0.126	2.278	2.152
0.018	0.163	-3.115	-0.650	2.804	3.454
0.051	0.210	-3.931	-0.723	6.739	7.461
0.010	0.157	-2.585	-1.012	3.399	4.410
0.060	0.221	-4.049	-0.651	3.326	3.977
0.747	0.853	-5.666	-0.175	3.318	3.493
0.260	0.457	-5.115	-0.428	2.268	2.696
0.017	0.162	-3.029	0.972	6.804	5.832
0.079	0.249	-4.265	0.686	6.885	6.199
0.859	0.924	-5.700	-0.056	4.121	4.176
0.611	0.757	-5.593	0.176	4.623	4.447
0.525	0.690	-5.524	-0.160	3.437	3.598
0.118	0.298	-4.561	0.393	3.846	3.453
0.035	0.186	-3.621	0.580	6.263	5.683
0.387	0.575	-5.361	0.248	4.760	4.513
0.500	0.670	-5.499	0.132	5.877	5.745
0.013	0.160	-2.834	1.067	7.937	6.870
0.043	0.199	-3.790	0.442	5.419	4.977
0.106	0.284	-4.485	0.468	2.539	2.071
0.836	0.908	-5.694	-0.078	3.641	3.719
0.187	0.379	-4.892	0.289	5.721	5.433
0.789	0.880	-5.681	-0.097	2.389	2.486
0.056	0.217	-4.000	-0.511	3.407	3.918
0.763	0.864	-5.672	0.086	2.473	2.387
0.456	0.636	-5.451	-0.231	3.033	3.265
0.502	0.672	-5.501	-0.140	3.314	3.454
0.077	0.247	-4.248	-0.620	1.812	2.431
0.201	0.397	-4.943	-0.397	1.735	2.132
0.056	0.217	-3.996	-0.470	6.788	7.258
0.032	0.182	-3.552	-0.567	1.938	2.504
0.006	0.157	-2.210	1.325	3.532	2.207
0.738	0.848	-5.662	-0.133	5.510	5.643
0.004	0.157	-1.924	1.115	3.220	2.105
0.173	0.363	-4.839	-0.323	2.772	3.094
0.523	0.689	-5.521	-0.195	2.498	2.693
0.334	0.530	-5.273	0.315	3.750	3.435
0.011	0.159	-2.723	0.660	2.667	2.007

0.118	0.298	-4.564	-0.485	2.803	3.289
0.885	0.937	-5.705	-0.040	3.347	3.387
0.064	0.227	-4.098	0.597	3.228	2.631
0.648	0.784	-5.616	0.160	3.049	2.889
0.016	0.162	-3.027	-0.919	2.381	3.300
0.002	0.157	-1.466	0.980	4.040	3.061
0.413	0.601	-5.398	0.273	2.590	2.317
0.386	0.574	-5.360	0.281	3.352	3.071
0.064	0.228	-4.100	0.871	2.878	2.006
0.069	0.236	-4.163	0.596	2.747	2.151
0.094	0.271	-4.398	0.458	2.491	2.033
0.094	0.270	-4.396	0.487	4.841	4.354
0.065	0.229	-4.112	-0.660	2.880	3.540
0.557	0.716	-5.551	0.241	4.011	3.771
0.009	0.157	-2.549	-1.067	2.524	3.592
0.495	0.668	-5.494	0.218	2.517	2.299
0.424	0.610	-5.413	0.298	3.576	3.277
0.344	0.539	-5.291	-0.327	2.781	3.108
0.140	0.324	-4.689	-0.535	3.350	3.885
0.573	0.728	-5.564	-0.168	2.637	2.805
0.083	0.254	-4.298	-1.029	1.606	2.635
0.001	0.157	-0.441	1.059	3.933	2.873
0.063	0.226	-4.089	0.425	4.512	4.087
0.844	0.914	-5.696	-0.060	2.672	2.732
0.804	0.890	-5.686	0.071	2.599	2.527
0.484	0.659	-5.483	-0.189	2.059	2.248
0.770	0.868	-5.674	0.079	3.834	3.755
0.034	0.185	-3.604	0.733	4.625	3.892
0.002	0.157	-1.075	1.037	2.507	1.470
0.016	0.162	-3.014	1.159	5.666	4.508
0.008	0.157	-2.487	0.648	2.879	2.231
0.157	0.345	-4.772	0.346	5.611	5.265
0.242	0.439	-5.067	-0.336	2.600	2.936
0.015	0.161	-2.975	0.645	3.941	3.296
0.273	0.471	-5.146	0.301	3.539	3.238
0.230	0.428	-5.035	0.396	3.356	2.960
0.299	0.496	-5.205	-0.368	2.349	2.717
0.429	0.615	-5.419	-0.236	3.992	4.228
0.118	0.298	-4.564	0.383	3.558	3.175
0.055	0.216	-3.986	0.479	2.725	2.246
0.766	0.866	-5.673	-0.076	2.333	2.409
0.083	0.254	-4.298	-0.528	3.453	3.981
0.639	0.778	-5.611	0.115	2.619	2.504
0.453	0.634	-5.448	-0.234	4.422	4.655
0.767	0.866	-5.673	0.082	2.995	2.913
0.648	0.784	-5.616	0.128	2.857	2.730
0.086	0.259	-4.330	-0.407	3.075	3.481
0.990	0.995	-5.715	-0.004	4.156	4.160
0.936	0.966	-5.712	-0.031	5.218	5.250
0.110	0.289	-4.514	0.387	4.858	4.471

0.312	0.509	-5.231	0.256	7.058	6.802
0.053	0.213	-3.955	-0.462	5.871	6.334
0.004	0.157	-1.782	0.858	4.197	3.339
0.012	0.160	-2.791	-1.416	4.991	6.407
0.224	0.421	-5.017	0.417	4.786	4.369
0.043	0.199	-3.789	-0.513	3.479	3.993
0.217	0.411	-4.994	-0.323	5.109	5.432
0.817	0.896	-5.689	0.093	4.763	4.671
0.202	0.398	-4.948	0.359	4.078	3.719
0.415	0.602	-5.400	0.225	3.860	3.635
0.654	0.790	-5.620	-0.106	3.607	3.713
0.523	0.689	-5.521	0.180	5.805	5.625
0.172	0.362	-4.833	-0.505	5.125	5.630
0.196	0.390	-4.926	-0.373	2.152	2.525
0.405	0.593	-5.387	-0.252	1.961	2.213
0.553	0.713	-5.548	-0.161	5.431	5.593
0.022	0.170	-3.252	1.090	6.710	5.620
0.143	0.327	-4.701	0.657	3.939	3.282
0.011	0.159	-2.734	1.616	5.300	3.684
0.066	0.231	-4.127	-0.612	3.050	3.663
0.245	0.442	-5.075	-0.363	4.718	5.081
0.326	0.522	-5.258	0.213	7.704	7.491
0.062	0.225	-4.078	0.940	4.675	3.735
0.060	0.222	-4.056	0.392	6.601	6.209
0.661	0.794	-5.624	0.117	5.520	5.403
0.063	0.226	-4.089	0.705	7.422	6.717
0.058	0.220	-4.031	-1.113	3.386	4.499
0.304	0.501	-5.215	-0.577	1.811	2.388
0.494	0.667	-5.493	-0.257	2.104	2.361
0.812	0.894	-5.688	0.067	3.228	3.162
0.017	0.162	-3.053	1.843	9.257	7.413
0.536	0.699	-5.534	-0.215	5.414	5.629
0.934	0.964	-5.712	0.031	2.769	2.738
0.810	0.893	-5.687	0.069	3.964	3.894
0.808	0.892	-5.687	-0.096	1.411	1.508
0.441	0.625	-5.434	0.323	2.713	2.390
0.058	0.219	-4.023	-0.764	5.480	6.244
0.782	0.876	-5.679	0.092	2.170	2.078
0.170	0.360	-4.824	-0.492	2.849	3.341
0.061	0.223	-4.064	-1.061	1.672	2.734
0.090	0.265	-4.362	-0.531	1.616	2.147
0.955	0.977	-5.713	0.017	5.040	5.023
0.025	0.175	-3.376	0.784	4.925	4.141
0.438	0.622	-5.430	-0.175	2.094	2.269
0.847	0.916	-5.697	-0.054	2.727	2.781
0.326	0.522	-5.259	-0.248	6.903	7.151
0.735	0.846	-5.660	0.094	3.678	3.584
0.327	0.523	-5.260	-0.215	6.403	6.618
0.307	0.504	-5.221	-0.376	3.830	4.205
0.549	0.710	-5.545	-0.325	6.480	6.805

0.370	0.561	-5.335	-0.256	7.272	7.528
0.026	0.176	-3.391	0.627	8.209	7.582
0.129	0.310	-4.629	0.563	5.842	5.278
0.049	0.207	-3.900	0.908	5.475	4.568
0.049	0.207	-3.899	-0.546	4.479	5.025
0.558	0.717	-5.553	-0.142	3.469	3.611
0.090	0.265	-4.363	0.526	2.965	2.439
0.005	0.157	-2.019	-1.188	5.602	6.790
0.101	0.278	-4.450	0.743	4.090	3.348
0.659	0.793	-5.623	0.155	2.869	2.714
0.087	0.260	-4.335	0.420	3.693	3.273
0.005	0.157	-2.069	-0.922	3.560	4.481
0.577	0.732	-5.568	-0.151	3.356	3.507
0.028	0.178	-3.452	0.748	6.557	5.809
0.466	0.645	-5.463	0.236	4.709	4.473
0.601	0.750	-5.585	0.265	3.217	2.953
0.005	0.157	-2.010	-1.661	0.880	2.541
0.068	0.234	-4.146	1.010	6.091	5.082
0.031	0.180	-3.523	-0.957	5.186	6.143
0.645	0.782	-5.614	0.176	4.593	4.418
0.118	0.298	-4.563	0.490	3.082	2.592
0.038	0.192	-3.705	-0.559	5.156	5.716
0.055	0.216	-3.984	0.437	3.506	3.069
0.580	0.734	-5.570	-0.193	3.503	3.696
0.442	0.625	-5.435	-0.250	1.736	1.987
0.638	0.777	-5.610	-0.198	4.143	4.341
0.387	0.575	-5.361	0.267	3.998	3.731
0.841	0.912	-5.696	-0.049	3.360	3.409
0.014	0.160	-2.896	-1.193	6.103	7.296
0.280	0.478	-5.164	0.353	5.100	4.747
0.887	0.938	-5.705	0.038	3.815	3.776
0.045	0.201	-3.821	0.628	5.017	4.389
0.210	0.405	-4.973	0.361	2.551	2.190
0.991	0.995	-5.715	-0.003	3.561	3.564
0.021	0.168	-3.224	0.872	6.708	5.836
0.322	0.519	-5.252	0.284	6.910	6.626
0.048	0.205	-3.881	-1.643	2.602	4.245
0.058	0.220	-4.031	0.655	7.775	7.120
0.029	0.178	-3.477	-0.615	3.151	3.766
0.421	0.607	-5.408	-0.222	1.734	1.956
0.434	0.618	-5.425	0.310	2.557	2.247
0.037	0.189	-3.667	-0.556	3.250	3.806
0.185	0.377	-4.887	-0.435	2.726	3.161
0.324	0.520	-5.255	-0.213	4.705	4.918
0.121	0.301	-4.580	0.435	5.523	5.088
0.154	0.341	-4.755	0.371	6.536	6.165
0.016	0.162	-3.013	-0.739	3.401	4.140
0.317	0.516	-5.242	0.261	2.647	2.386
0.712	0.828	-5.650	-0.122	2.486	2.608
0.010	0.157	-2.615	0.840	6.642	5.802

0.085	0.257	-4.317	0.593	6.512	5.919
0.663	0.795	-5.625	-0.116	2.501	2.617
0.020	0.167	-3.199	0.763	3.396	2.633
0.493	0.667	-5.492	0.201	4.359	4.158
0.074	0.241	-4.209	0.404	5.730	5.326
0.096	0.272	-4.411	0.436	2.809	2.373
0.140	0.324	-4.686	0.346	6.020	5.674
0.501	0.671	-5.501	0.175	3.539	3.364
0.519	0.686	-5.518	0.170	4.522	4.352
0.131	0.313	-4.640	0.350	5.024	4.674
0.110	0.289	-4.510	0.475	3.481	3.006
0.253	0.451	-5.098	0.287	4.383	4.096
0.247	0.445	-5.083	-0.266	4.457	4.723
0.263	0.461	-5.123	-0.255	4.481	4.736
0.911	0.952	-5.709	0.033	3.761	3.729
0.004	0.157	-1.956	-1.243	5.889	7.132
0.005	0.157	-1.991	2.522	6.970	4.447
0.434	0.618	-5.425	-0.290	3.314	3.604
0.005	0.157	-2.022	-0.868	5.994	6.863
0.005	0.157	-2.103	-0.854	5.896	6.750
0.008	0.157	-2.439	-0.816	5.872	6.687
0.015	0.161	-2.973	-0.712	3.938	4.650
0.409	0.596	-5.393	-0.249	5.369	5.618
0.126	0.307	-4.614	0.294	8.021	7.727
0.067	0.231	-4.132	0.393	5.506	5.113
0.191	0.383	-4.906	0.395	2.579	2.184
0.892	0.941	-5.706	0.052	2.925	2.873
0.233	0.430	-5.044	0.313	3.049	2.736
0.982	0.991	-5.715	0.007	2.626	2.619
0.472	0.650	-5.470	-0.231	3.443	3.673
0.347	0.542	-5.296	0.227	3.324	3.097
0.030	0.179	-3.511	0.792	5.698	4.906
0.032	0.182	-3.557	-0.505	4.448	4.953
0.992	0.996	-5.715	-0.011	4.839	4.850
0.965	0.982	-5.714	-0.012	4.546	4.558
0.634	0.774	-5.607	-0.146	3.604	3.750
0.035	0.186	-3.621	-0.501	2.826	3.326
0.434	0.618	-5.425	0.243	5.456	5.214
0.124	0.305	-4.599	-0.603	2.681	3.284
0.188	0.380	-4.898	-0.737	2.269	3.006
0.214	0.409	-4.984	-0.704	2.270	2.974
0.269	0.467	-5.136	0.262	4.689	4.427
0.611	0.757	-5.592	-0.164	2.607	2.772
0.158	0.346	-4.774	-0.404	4.026	4.430
0.663	0.795	-5.625	0.110	2.731	2.621
0.795	0.885	-5.683	0.078	5.057	4.978
0.085	0.257	-4.316	-0.420	7.701	8.121
0.244	0.442	-5.073	0.293	7.348	7.055
0.060	0.222	-4.052	0.516	5.669	5.153

0.122	0.303	-4.587	0.372	6.022	5.650
0.147	0.331	-4.723	-0.519	2.424	2.943
0.004	0.157	-1.844	-0.729	4.989	5.718
0.077	0.246	-4.240	0.944	3.143	2.199
0.810	0.893	-5.687	-0.054	3.774	3.828
0.431	0.616	-5.422	-0.196	5.108	5.304
0.010	0.157	-2.584	-0.927	4.688	5.615
0.017	0.162	-3.033	0.587	3.565	2.978
0.166	0.355	-4.809	0.583	3.310	2.727
0.903	0.947	-5.708	0.036	1.443	1.407
0.457	0.637	-5.453	-0.194	4.782	4.976
0.090	0.264	-4.358	0.974	4.815	3.840
0.208	0.403	-4.968	0.368	4.905	4.537
0.027	0.176	-3.430	1.024	7.192	6.169
0.017	0.162	-3.042	0.674	6.867	6.193
0.078	0.247	-4.249	0.368	6.706	6.337
0.061	0.224	-4.071	0.635	5.774	5.139
0.035	0.186	-3.629	0.812	6.754	5.942
0.085	0.257	-4.316	-0.669	2.316	2.985
0.007	0.157	-2.308	1.011	6.718	5.708
0.031	0.181	-3.539	-0.666	8.987	9.653
0.110	0.289	-4.514	-0.507	6.070	6.577
0.489	0.664	-5.488	-0.198	1.948	2.146
0.009	0.157	-2.575	0.840	5.562	4.723
0.192	0.384	-4.910	-0.423	5.316	5.739
0.711	0.828	-5.650	-0.109	2.621	2.730
0.303	0.501	-5.213	0.276	5.742	5.466
0.183	0.374	-4.878	0.328	5.291	4.963
0.313	0.511	-5.234	0.260	5.783	5.522
0.579	0.733	-5.569	0.125	5.528	5.403
0.003	0.157	-1.587	0.865	2.915	2.049
0.020	0.167	-3.197	-0.631	5.083	5.714
0.073	0.241	-4.199	-0.662	1.916	2.578
0.018	0.163	-3.111	-0.636	7.894	8.530
0.011	0.159	-2.726	1.208	7.192	5.983
0.004	0.157	-1.833	1.642	8.288	6.645
0.351	0.546	-5.304	-0.396	3.258	3.655
0.069	0.235	-4.158	0.589	5.335	4.747
0.532	0.696	-5.530	-0.169	4.195	4.364
0.179	0.370	-4.864	-0.433	3.174	3.607
0.022	0.171	-3.272	-1.281	1.251	2.532
0.188	0.380	-4.898	-0.427	2.282	2.709
0.965	0.982	-5.714	-0.012	5.974	5.986
0.742	0.850	-5.663	-0.174	3.806	3.980
0.844	0.914	-5.696	0.057	4.159	4.101
0.228	0.425	-5.029	0.252	7.188	6.936
0.069	0.235	-4.160	0.376	6.908	6.531
0.480	0.656	-5.479	-0.216	2.118	2.334
0.017	0.162	-3.040	-0.796	1.976	2.772
0.006	0.157	-2.263	1.078	3.728	2.650

0.143	0.327	-4.705	0.458	3.666	3.209
0.625	0.767	-5.602	0.121	7.212	7.092
0.033	0.184	-3.575	0.905	6.602	5.697
0.017	0.162	-3.074	1.049	7.537	6.487
0.222	0.419	-5.011	-0.270	6.015	6.285
0.003	0.157	-1.750	1.463	6.522	5.059
0.178	0.369	-4.860	-0.361	3.977	4.338
0.989	0.995	-5.715	-0.004	2.713	2.717
0.010	0.157	-2.587	1.006	2.984	1.978
0.839	0.911	-5.695	0.054	2.095	2.041
0.012	0.160	-2.794	1.370	5.334	3.964
0.038	0.192	-3.701	1.178	5.282	4.104
0.072	0.240	-4.196	0.532	7.296	6.764
0.055	0.216	-3.985	-0.609	4.517	5.126
0.527	0.692	-5.525	-0.185	4.032	4.216
0.223	0.419	-5.014	0.579	3.879	3.300
0.140	0.324	-4.689	0.378	2.557	2.179
0.077	0.246	-4.245	0.575	3.697	3.121
0.396	0.584	-5.375	0.229	2.895	2.667
0.004	0.157	-1.928	0.867	2.826	1.959
0.046	0.203	-3.850	-0.922	1.472	2.394
0.428	0.613	-5.417	0.159	6.730	6.571
0.244	0.442	-5.074	0.309	2.954	2.645
0.035	0.186	-3.620	-0.679	2.254	2.932
0.010	0.157	-2.618	0.585	4.204	3.620
0.002	0.157	-1.477	0.718	3.599	2.881
0.040	0.194	-3.744	0.960	8.359	7.399
0.013	0.160	-2.834	0.705	8.288	7.583
0.230	0.428	-5.036	-0.319	3.575	3.894
0.542	0.704	-5.539	0.347	3.923	3.576
0.090	0.264	-4.358	-0.511	2.482	2.993
0.034	0.184	-3.599	0.663	5.566	4.903
0.476	0.654	-5.474	-0.396	1.565	1.961
0.114	0.293	-4.540	-0.377	2.006	2.383
0.027	0.176	-3.410	-0.865	1.313	2.178
0.563	0.721	-5.557	0.202	2.396	2.193
0.547	0.709	-5.543	-0.168	2.483	2.650
0.099	0.275	-4.430	0.569	3.907	3.338
0.119	0.299	-4.572	0.553	5.033	4.480
0.240	0.438	-5.063	-0.490	2.807	3.297
0.711	0.828	-5.650	0.115	2.660	2.545
0.777	0.872	-5.677	-0.076	8.588	8.663
0.125	0.306	-4.608	0.302	7.977	7.675
0.994	0.997	-5.715	-0.002	5.998	6.000
0.388	0.576	-5.363	-0.345	7.155	7.500
0.376	0.566	-5.344	0.199	5.639	5.440
0.008	0.157	-2.441	-0.672	4.627	5.299
0.901	0.946	-5.708	0.029	6.390	6.362
0.462	0.642	-5.458	0.145	5.488	5.343
0.914	0.954	-5.709	0.024	5.494	5.470

0.130	0.311	-4.633	0.349	4.374	4.025
0.760	0.862	-5.671	0.061	9.687	9.626
0.343	0.539	-5.290	0.235	4.079	3.844
0.374	0.565	-5.342	0.239	2.954	2.715
0.292	0.490	-5.189	-0.289	3.158	3.447
0.114	0.293	-4.541	-0.414	2.672	3.086
0.923	0.958	-5.710	-0.044	1.991	2.035
0.006	0.157	-2.252	-0.764	2.168	2.931
0.010	0.157	-2.585	-0.664	7.965	8.628
0.069	0.235	-4.157	1.341	10.126	8.784
0.030	0.178	-3.495	0.563	6.657	6.094
0.359	0.552	-5.317	0.263	4.416	4.153
0.463	0.643	-5.460	0.250	3.519	3.270
0.398	0.586	-5.377	-0.285	2.781	3.066
0.069	0.236	-4.165	-0.503	4.766	5.269
0.892	0.941	-5.706	-0.029	8.455	8.484
0.095	0.271	-4.405	-0.573	2.815	3.388
0.010	0.157	-2.600	1.576	7.732	6.156
0.195	0.389	-4.921	0.500	6.316	5.817
0.019	0.164	-3.142	-0.630	7.218	7.848
0.003	0.157	-1.683	-1.133	7.390	8.524
0.411	0.598	-5.395	-0.218	5.101	5.319
0.082	0.253	-4.291	-0.568	4.068	4.636
0.038	0.192	-3.695	0.509	8.309	7.800
0.007	0.157	-2.354	-1.158	5.871	7.029
0.007	0.157	-2.299	-1.098	3.321	4.419
0.243	0.441	-5.071	-0.243	2.083	2.326
0.630	0.771	-5.605	0.126	2.026	1.900
0.619	0.763	-5.598	-0.148	2.376	2.524
0.049	0.207	-3.892	0.678	4.849	4.171
0.311	0.508	-5.229	0.260	6.004	5.744
0.067	0.232	-4.137	1.276	7.794	6.518
0.011	0.159	-2.731	0.679	5.726	5.047
0.533	0.696	-5.531	0.237	3.106	2.869
0.209	0.404	-4.970	0.413	4.808	4.395
0.051	0.209	-3.925	-0.476	3.974	4.450
0.928	0.961	-5.711	-0.027	3.645	3.672
0.817	0.897	-5.690	0.072	3.300	3.228
0.037	0.189	-3.667	0.658	7.009	6.351
0.029	0.178	-3.494	0.804	5.354	4.550
0.789	0.880	-5.681	0.083	2.585	2.503
0.110	0.289	-4.513	0.505	3.066	2.561
0.171	0.361	-4.831	0.392	7.885	7.493
0.118	0.298	-4.563	0.324	6.633	6.309
0.376	0.566	-5.345	-0.312	1.994	2.305
0.198	0.392	-4.932	0.373	2.760	2.387
0.107	0.286	-4.492	0.538	2.632	2.094
0.009	0.157	-2.541	1.311	4.953	3.642
0.011	0.159	-2.721	0.572	3.002	2.430
0.041	0.195	-3.751	0.801	3.823	3.022

0.073	0.241	-4.208	-0.575	6.258	6.834
0.080	0.251	-4.274	-0.995	2.883	3.877
0.044	0.201	-3.811	-0.553	6.151	6.704
0.005	0.157	-2.081	-1.074	4.667	5.741
0.010	0.157	-2.646	1.476	4.840	3.365
0.133	0.316	-4.653	0.537	2.796	2.259
0.052	0.211	-3.941	1.272	6.619	5.346
0.885	0.937	-5.705	0.049	4.429	4.380
0.877	0.933	-5.703	0.048	5.799	5.751
0.130	0.311	-4.632	-0.444	6.339	6.783
0.031	0.181	-3.538	0.643	4.394	3.752
0.276	0.474	-5.154	0.659	4.669	4.010
0.224	0.421	-5.018	0.265	6.821	6.556
0.361	0.553	-5.321	-0.524	1.086	1.610
0.997	0.998	-5.715	0.001	8.878	8.878
0.500	0.671	-5.500	0.164	5.231	5.068
0.027	0.176	-3.416	-0.550	2.889	3.439
0.004	0.157	-1.965	-0.878	3.465	4.343
0.852	0.920	-5.698	0.046	5.156	5.110
0.015	0.160	-2.925	-0.823	5.825	6.648
0.133	0.316	-4.651	-0.468	2.131	2.599
0.345	0.540	-5.294	0.299	7.403	7.104
0.182	0.374	-4.876	-0.390	5.057	5.447
0.890	0.940	-5.706	0.043	5.434	5.391
0.296	0.493	-5.199	-0.280	3.236	3.516
0.018	0.162	-3.088	-0.634	5.275	5.909
0.039	0.192	-3.710	0.825	5.796	4.971
0.171	0.361	-4.830	0.408	4.315	3.907
0.333	0.528	-5.271	0.214	7.032	6.818
0.168	0.358	-4.817	0.330	6.568	6.238
0.498	0.670	-5.498	0.199	3.229	3.030
0.088	0.261	-4.342	-0.366	4.819	5.185
0.046	0.203	-3.840	0.706	5.676	4.970
0.318	0.516	-5.243	0.366	5.298	4.932
0.286	0.484	-5.177	0.438	4.697	4.258
0.234	0.431	-5.047	0.307	8.060	7.754
0.046	0.203	-3.852	0.430	6.004	5.574
0.852	0.920	-5.698	-0.051	4.266	4.317
0.013	0.160	-2.838	-0.609	2.102	2.711
0.400	0.588	-5.380	-0.203	4.654	4.858
0.075	0.244	-4.224	0.467	7.695	7.229
0.066	0.230	-4.124	0.477	7.192	6.715
0.058	0.219	-4.020	0.419	7.971	7.552
0.135	0.318	-4.660	0.375	4.111	3.736
0.619	0.763	-5.598	-0.184	2.923	3.107
0.975	0.988	-5.714	-0.010	2.028	2.038
0.204	0.399	-4.952	-0.818	1.530	2.348
0.276	0.475	-5.155	-0.400	2.153	2.552
0.122	0.303	-4.589	-0.519	3.520	4.040
0.164	0.353	-4.799	0.335	7.530	7.195

0.834	0.907	-5.694	0.048	7.213	7.165
0.496	0.668	-5.496	-0.152	5.607	5.759
0.062	0.226	-4.083	-0.941	2.504	3.446
0.580	0.734	-5.569	0.170	2.790	2.619
0.416	0.603	-5.401	-0.323	2.972	3.295
0.961	0.980	-5.714	0.044	4.543	4.499
0.712	0.828	-5.650	-0.130	4.389	4.520
0.009	0.157	-2.500	0.690	5.050	4.360
0.052	0.212	-3.946	-0.901	3.358	4.259
0.010	0.157	-2.653	-0.771	8.757	9.528
0.446	0.628	-5.439	0.362	6.891	6.529
0.190	0.383	-4.905	-0.403	3.550	3.953
0.348	0.543	-5.298	0.308	4.090	3.782
0.657	0.792	-5.621	-0.194	7.124	7.318
0.068	0.234	-4.149	0.911	8.434	7.523
0.984	0.991	-5.715	0.006	3.421	3.415
0.671	0.802	-5.629	0.106	3.492	3.386
0.445	0.627	-5.438	-0.154	8.207	8.360
0.126	0.307	-4.613	-0.437	4.927	5.363
0.875	0.932	-5.703	0.063	2.407	2.344
0.335	0.531	-5.275	0.454	2.659	2.206
0.025	0.175	-3.368	0.444	4.396	3.952
0.183	0.374	-4.878	0.361	5.136	4.775
0.279	0.477	-5.160	-0.351	7.916	8.267
0.678	0.808	-5.633	-0.090	4.389	4.479
0.612	0.757	-5.593	-0.175	2.170	2.344
0.048	0.205	-3.884	0.608	3.992	3.384
0.002	0.157	-1.448	-1.037	4.005	5.042
0.026	0.176	-3.405	0.607	6.455	5.847
0.293	0.491	-5.193	-0.394	4.277	4.671
0.182	0.374	-4.876	0.379	3.106	2.727
0.075	0.245	-4.228	0.805	6.086	5.281
0.019	0.164	-3.141	1.097	6.138	5.041
0.055	0.216	-3.992	0.886	5.982	5.097
0.061	0.223	-4.060	1.068	5.935	4.867
0.005	0.157	-2.126	0.862	8.701	7.839
0.768	0.867	-5.674	0.071	7.345	7.275
0.024	0.173	-3.322	-0.877	4.245	5.122
0.724	0.838	-5.656	0.079	2.778	2.698
0.149	0.334	-4.733	0.464	3.298	2.834
0.897	0.943	-5.707	-0.041	6.004	6.046
0.617	0.761	-5.596	0.167	5.223	5.055
0.041	0.196	-3.765	0.851	4.719	3.868
0.706	0.825	-5.647	-0.104	2.530	2.634
0.027	0.176	-3.417	0.560	7.138	6.578
0.115	0.294	-4.544	-0.446	3.016	3.462
0.548	0.709	-5.544	0.141	5.434	5.292
0.034	0.184	-3.602	0.833	7.503	6.670
0.767	0.867	-5.673	-0.080	4.546	4.626
0.280	0.478	-5.163	-0.385	3.016	3.401

0.017	0.162	-3.033	0.618	5.219	4.602
0.019	0.164	-3.123	0.699	4.369	3.671
0.492	0.666	-5.492	0.132	6.033	5.901
0.998	0.999	-5.715	-0.001	3.756	3.757
0.368	0.559	-5.331	0.174	8.146	7.972
0.781	0.874	-5.678	-0.103	5.126	5.229
0.056	0.217	-3.995	0.464	3.368	2.904
0.597	0.747	-5.582	-0.192	3.906	4.099
0.097	0.273	-4.417	0.539	6.463	5.924
0.129	0.311	-4.631	0.934	8.058	7.124
0.057	0.219	-4.019	1.007	6.207	5.200
0.020	0.167	-3.182	-0.595	7.165	7.760
0.374	0.564	-5.340	-0.281	1.999	2.280
0.216	0.411	-4.991	0.373	4.608	4.235
0.011	0.157	-2.702	0.586	4.001	3.415
0.105	0.283	-4.479	0.647	5.670	5.023
0.468	0.647	-5.465	-0.195	3.808	4.003
0.879	0.934	-5.704	-0.043	8.400	8.442
0.008	0.157	-2.442	-0.987	6.531	7.518
0.061	0.223	-4.063	1.009	7.936	6.926
0.229	0.426	-5.031	0.346	4.055	3.708
0.964	0.982	-5.714	-0.011	2.720	2.731
0.007	0.157	-2.296	-1.294	5.680	6.974
0.875	0.932	-5.703	-0.043	6.834	6.878
0.073	0.241	-4.203	0.903	7.544	6.642
0.424	0.610	-5.413	-0.231	6.504	6.734
0.806	0.891	-5.686	0.054	5.957	5.904
0.950	0.974	-5.713	-0.018	3.025	3.043
0.061	0.223	-4.061	-0.437	6.381	6.817
0.727	0.840	-5.657	0.118	3.900	3.781
0.876	0.933	-5.703	-0.042	6.046	6.088
0.794	0.884	-5.683	-0.068	4.041	4.110
0.463	0.643	-5.460	-0.205	2.759	2.963
0.123	0.304	-4.596	-0.762	4.048	4.810
0.978	0.989	-5.714	0.008	3.412	3.404
0.104	0.281	-4.470	-0.399	5.549	5.948
0.262	0.460	-5.121	-0.243	4.571	4.814
0.037	0.190	-3.674	0.549	5.247	4.698
0.036	0.188	-3.650	0.649	5.691	5.042
0.283	0.481	-5.171	0.453	5.420	4.967
0.291	0.489	-5.188	-0.248	4.318	4.566
0.755	0.858	-5.669	-0.094	6.747	6.841
0.659	0.793	-5.622	0.182	1.773	1.591
0.123	0.304	-4.592	-0.692	1.374	2.066
0.210	0.405	-4.974	0.387	2.841	2.455
0.035	0.186	-3.630	1.189	5.984	4.795
0.075	0.244	-4.226	0.756	4.171	3.415
0.011	0.157	-2.661	1.929	7.197	5.268
0.216	0.411	-4.993	-0.349	6.837	7.185
0.096	0.272	-4.411	0.470	6.038	5.567

0.781	0.874	-5.678	-0.119	4.659	4.778
0.533	0.696	-5.531	-0.186	4.146	4.332
0.700	0.821	-5.645	0.097	5.245	5.148
0.311	0.508	-5.230	-0.259	1.975	2.234
0.028	0.178	-3.465	0.632	5.335	4.702
0.822	0.899	-5.691	0.064	3.011	2.946
0.002	0.157	-1.387	-1.291	4.585	5.875
0.488	0.663	-5.487	-0.236	3.237	3.474
0.014	0.160	-2.883	-1.046	8.704	9.750
0.136	0.320	-4.669	-0.907	4.394	5.300
0.529	0.694	-5.527	-0.177	7.552	7.729
0.226	0.423	-5.022	0.360	7.165	6.806
0.018	0.163	-3.114	-1.322	4.906	6.229
0.037	0.190	-3.680	-0.498	3.037	3.535
0.926	0.960	-5.711	-0.026	3.695	3.721
0.132	0.315	-4.645	0.824	6.006	5.182
0.012	0.160	-2.786	-0.696	2.500	3.196
0.157	0.345	-4.769	-0.422	2.591	3.013
0.297	0.493	-5.200	-0.271	3.428	3.699
0.039	0.192	-3.710	0.570	6.793	6.223
0.104	0.281	-4.466	0.411	4.471	4.061
0.043	0.200	-3.796	0.904	7.238	6.334
0.152	0.338	-4.745	-0.527	1.953	2.480
0.378	0.568	-5.348	-0.280	3.512	3.793
0.117	0.297	-4.556	0.499	7.410	6.912
0.089	0.264	-4.355	0.435	2.932	2.497
0.896	0.943	-5.707	-0.032	3.161	3.192
0.053	0.213	-3.956	0.562	4.888	4.326
0.014	0.160	-2.923	1.445	7.761	6.316
0.183	0.374	-4.879	0.464	6.402	5.937
0.082	0.253	-4.287	0.596	3.305	2.710
0.029	0.178	-3.485	0.967	6.227	5.260
0.111	0.290	-4.518	0.405	4.609	4.205
0.001	0.157	-0.804	1.966	7.391	5.424
0.142	0.326	-4.696	-0.382	6.083	6.465
0.258	0.456	-5.111	-0.301	3.442	3.744
0.012	0.160	-2.781	1.345	6.131	4.786
0.040	0.194	-3.726	-0.657	3.173	3.830
0.073	0.240	-4.198	0.521	4.184	3.662
0.104	0.281	-4.466	-0.414	5.629	6.043
0.723	0.837	-5.655	0.087	3.148	3.061
0.461	0.642	-5.458	-0.194	3.053	3.247
0.260	0.457	-5.115	0.295	7.436	7.141
0.505	0.674	-5.504	-0.199	5.351	5.550
0.632	0.772	-5.606	-0.167	2.142	2.309
0.073	0.241	-4.204	-0.431	4.422	4.853
0.170	0.360	-4.826	-0.362	7.785	8.147
0.008	0.157	-2.390	-1.328	4.536	5.864
0.796	0.885	-5.683	0.062	7.626	7.564
0.014	0.160	-2.890	1.063	6.634	5.572

0.067	0.232	-4.140	0.519	2.856	2.338
0.045	0.201	-3.823	-0.532	3.408	3.940
0.512	0.680	-5.512	-0.200	4.014	4.214
0.026	0.176	-3.396	1.274	5.934	4.660
0.090	0.264	-4.360	0.370	7.774	7.404
0.395	0.582	-5.372	-0.172	3.211	3.383
0.756	0.859	-5.669	-0.089	5.305	5.394
0.015	0.160	-2.933	-1.062	7.416	8.478
0.213	0.408	-4.981	0.369	6.110	5.741
0.663	0.795	-5.625	0.144	2.527	2.383
0.647	0.784	-5.616	0.156	4.770	4.614
0.048	0.205	-3.879	0.690	4.594	3.905
0.173	0.362	-4.837	0.322	4.097	3.774
0.235	0.432	-5.048	-0.503	7.541	8.044
0.202	0.397	-4.946	0.337	8.190	7.853
0.014	0.160	-2.915	0.611	4.864	4.253
0.513	0.681	-5.512	0.156	6.153	5.997
0.033	0.184	-3.588	-1.005	3.301	4.305
0.052	0.210	-3.935	-0.684	3.068	3.753
0.042	0.198	-3.781	0.578	5.941	5.363
0.171	0.361	-4.831	0.339	6.334	5.996
0.428	0.614	-5.418	-0.162	6.202	6.364
0.953	0.976	-5.713	-0.017	6.144	6.161
0.109	0.288	-4.504	0.388	6.783	6.395
0.018	0.163	-3.099	0.901	4.265	3.364
0.113	0.292	-4.533	0.402	5.642	5.240
0.489	0.664	-5.488	0.211	5.053	4.842
0.216	0.411	-4.993	-0.360	4.687	5.047
0.002	0.157	-1.167	-0.983	3.269	4.251
0.674	0.804	-5.631	-0.129	4.515	4.644
0.184	0.375	-4.882	0.751	6.197	5.446
0.805	0.890	-5.686	0.067	3.791	3.725
0.141	0.324	-4.691	-0.698	1.915	2.613
0.007	0.157	-2.305	1.121	8.061	6.940
0.357	0.551	-5.313	-0.432	3.382	3.813
0.319	0.517	-5.246	0.286	2.744	2.459
0.136	0.319	-4.668	0.468	8.795	8.327
0.478	0.655	-5.476	0.144	8.150	8.006
0.004	0.157	-1.942	-1.552	5.463	7.015
0.084	0.256	-4.314	-0.476	5.010	5.486
0.158	0.346	-4.774	0.659	7.346	6.687
0.534	0.697	-5.532	0.200	4.947	4.747
0.443	0.626	-5.436	0.227	6.124	5.897
0.171	0.361	-4.831	-0.344	3.887	4.231
0.460	0.640	-5.456	0.235	3.253	3.018
0.717	0.832	-5.652	-0.084	3.635	3.719
0.101	0.278	-4.444	-0.607	6.022	6.628
0.243	0.441	-5.071	0.240	7.790	7.550
0.209	0.404	-4.971	0.354	5.340	4.986
0.006	0.157	-2.256	0.968	7.645	6.677

0.383	0.572	-5.354	-0.265	4.734	4.999
0.109	0.288	-4.506	-0.430	2.873	3.304
0.393	0.581	-5.370	-0.337	6.172	6.509
0.822	0.899	-5.691	0.064	4.399	4.335
0.303	0.501	-5.213	0.338	3.595	3.257
0.085	0.258	-4.323	-0.431	2.826	3.257
0.179	0.370	-4.864	-0.309	4.754	5.062
0.035	0.186	-3.623	0.529	6.991	6.462
0.351	0.546	-5.303	0.261	3.386	3.125
0.275	0.473	-5.151	0.287	8.083	7.796
0.095	0.271	-4.404	-0.464	2.715	3.179
0.065	0.229	-4.117	-0.628	3.590	4.218
0.008	0.157	-2.441	0.763	6.968	6.205
0.815	0.896	-5.689	-0.073	4.718	4.791
0.709	0.827	-5.649	0.130	4.773	4.643
0.698	0.820	-5.644	-0.092	5.231	5.323
0.063	0.227	-4.092	0.465	5.038	4.574
0.411	0.598	-5.395	0.249	4.538	4.289
0.069	0.236	-4.162	0.545	6.109	5.564
0.100	0.277	-4.442	0.396	5.943	5.548
0.214	0.409	-4.985	-0.351	3.976	4.327
0.059	0.221	-4.046	0.900	5.430	4.530
0.956	0.978	-5.713	0.015	2.957	2.942
0.099	0.276	-4.436	0.648	6.964	6.316
0.942	0.969	-5.712	-0.028	5.539	5.567
0.079	0.249	-4.266	0.388	6.636	6.248
0.003	0.157	-1.726	1.270	6.329	5.059
0.567	0.723	-5.560	0.174	2.826	2.652
0.008	0.157	-2.386	1.646	7.508	5.863
0.176	0.366	-4.851	0.478	3.896	3.419
0.289	0.488	-5.184	0.226	7.777	7.552
0.016	0.162	-2.985	0.916	6.947	6.031
0.077	0.246	-4.239	0.445	9.763	9.319
0.707	0.826	-5.648	-0.118	2.620	2.737
0.010	0.157	-2.614	-0.694	5.331	6.025
0.670	0.801	-5.629	-0.099	2.552	2.651
0.810	0.893	-5.687	0.074	2.670	2.596
0.629	0.770	-5.604	0.111	6.982	6.871
0.704	0.824	-5.646	-0.113	2.606	2.719
0.322	0.519	-5.252	-0.272	2.671	2.943
0.015	0.160	-2.936	0.588	5.782	5.194
0.011	0.159	-2.719	0.772	5.423	4.651
0.419	0.606	-5.405	-0.330	6.392	6.722
0.461	0.642	-5.458	-0.214	3.110	3.325
0.012	0.160	-2.758	-0.693	5.193	5.886
0.480	0.656	-5.478	-0.194	4.137	4.330
0.884	0.937	-5.705	-0.037	4.606	4.643
0.006	0.157	-2.241	1.025	3.752	2.726
0.024	0.173	-3.330	0.752	3.565	2.813
0.020	0.167	-3.178	-0.774	3.338	4.112

0.038	0.192	-3.695	-0.430	4.376	4.806
0.814	0.895	-5.689	0.053	5.453	5.400
0.800	0.888	-5.684	0.073	2.934	2.861
0.078	0.247	-4.249	0.411	7.438	7.027
0.858	0.924	-5.700	-0.058	5.121	5.179
0.094	0.270	-4.392	0.699	4.623	3.924
0.059	0.221	-4.038	0.495	5.214	4.719
0.406	0.594	-5.388	-0.298	2.889	3.187
0.093	0.269	-4.389	0.406	5.275	4.869
0.685	0.811	-5.637	-0.247	1.765	2.012
0.030	0.179	-3.500	0.517	7.144	6.627
0.082	0.253	-4.290	-0.461	4.527	4.988
0.280	0.478	-5.164	0.272	6.565	6.293
0.773	0.869	-5.675	0.083	4.216	4.133
0.015	0.160	-2.928	-0.965	2.985	3.951
0.441	0.625	-5.434	0.209	2.925	2.716
0.012	0.160	-2.777	-0.898	3.074	3.973
0.544	0.706	-5.541	-0.229	2.136	2.364
0.501	0.671	-5.501	0.183	3.421	3.237
0.666	0.798	-5.626	-0.158	3.536	3.695
0.020	0.166	-3.164	0.753	5.182	4.429
0.084	0.256	-4.313	0.783	3.117	2.335
0.656	0.791	-5.621	-0.171	2.729	2.900
0.925	0.959	-5.711	0.029	2.656	2.626
0.005	0.157	-2.103	0.801	3.684	2.883
0.201	0.396	-4.943	-0.510	4.499	5.009
0.541	0.703	-5.538	-0.193	4.060	4.253
0.341	0.537	-5.287	-0.215	2.923	3.137
0.816	0.896	-5.689	-0.062	2.115	2.177
0.744	0.851	-5.664	-0.132	3.270	3.402
0.004	0.157	-1.877	0.942	8.198	7.257
0.161	0.349	-4.786	0.504	5.145	4.641
0.006	0.157	-2.169	-1.029	4.563	5.592
0.028	0.178	-3.465	0.838	6.353	5.515
0.893	0.942	-5.706	-0.078	4.338	4.416
0.506	0.675	-5.506	0.249	4.648	4.399
0.701	0.822	-5.645	0.143	3.417	3.273
0.026	0.176	-3.408	0.686	6.663	5.977
0.360	0.553	-5.319	-0.278	4.117	4.395
0.964	0.982	-5.714	0.013	4.285	4.273
0.349	0.544	-5.300	0.354	5.121	4.767
0.001	0.157	-0.873	1.283	5.976	4.693
0.104	0.281	-4.471	0.687	3.116	2.430
0.328	0.524	-5.263	0.303	2.698	2.395
0.017	0.162	-3.055	1.066	6.126	5.059
0.227	0.424	-5.024	-0.338	4.230	4.568
0.410	0.597	-5.394	-0.217	3.621	3.838
0.634	0.774	-5.608	0.127	2.282	2.155
0.964	0.982	-5.714	-0.013	2.306	2.319
0.393	0.581	-5.370	0.236	5.066	4.830

0.621	0.765	-5.599	0.146	3.850	3.704
0.188	0.380	-4.897	0.578	3.792	3.214
0.803	0.889	-5.685	0.052	4.380	4.327
0.334	0.530	-5.273	0.247	7.418	7.171
0.224	0.421	-5.018	-0.425	1.987	2.412
0.964	0.982	-5.714	-0.012	3.713	3.725
0.633	0.773	-5.607	0.135	2.878	2.743
0.014	0.160	-2.884	-0.768	2.529	3.297
0.776	0.871	-5.676	0.084	2.456	2.372
0.094	0.270	-4.392	-0.493	4.933	5.427
0.834	0.907	-5.694	-0.056	2.847	2.903
0.442	0.625	-5.435	0.227	2.820	2.593
0.679	0.808	-5.634	-0.112	3.116	3.229
0.070	0.236	-4.168	-0.495	7.447	7.942
0.167	0.356	-4.813	-0.511	3.262	3.773
0.006	0.157	-2.253	1.012	5.944	4.932
0.354	0.548	-5.308	-0.259	5.451	5.710
0.548	0.709	-5.544	-0.165	4.960	5.125
0.162	0.350	-4.790	0.346	7.772	7.426
0.972	0.986	-5.714	-0.014	4.534	4.548
0.574	0.729	-5.566	0.107	5.700	5.593
0.117	0.297	-4.559	0.368	5.663	5.295
0.034	0.185	-3.606	-0.557	6.319	6.876
0.076	0.245	-4.236	0.653	7.199	6.545
0.047	0.204	-3.870	-0.565	5.339	5.903
0.094	0.270	-4.395	0.534	2.481	1.947
0.052	0.210	-3.935	0.959	6.942	5.982
0.046	0.203	-3.850	-0.763	4.314	5.077
0.060	0.222	-4.055	0.801	5.712	4.911
0.852	0.920	-5.698	-0.046	4.287	4.333
0.571	0.726	-5.563	0.150	3.642	3.492
0.698	0.820	-5.643	0.092	4.925	4.832
0.040	0.194	-3.739	0.650	3.604	2.954
0.570	0.725	-5.562	-0.179	2.519	2.697
0.154	0.341	-4.756	0.314	6.543	6.229
0.041	0.195	-3.746	0.518	3.791	3.274
0.016	0.162	-2.999	1.504	8.704	7.200
0.055	0.216	-3.985	0.472	7.114	6.642
0.373	0.563	-5.339	0.277	3.815	3.539
0.076	0.245	-4.232	0.589	4.976	4.388
0.060	0.222	-4.054	0.541	6.135	5.594
0.113	0.292	-4.533	0.333	7.346	7.013
0.016	0.162	-2.992	-0.586	5.829	6.415
0.101	0.278	-4.447	-0.540	3.172	3.712
0.101	0.278	-4.449	0.536	5.995	5.459
0.913	0.953	-5.709	0.026	5.231	5.205
0.274	0.473	-5.150	0.286	5.152	4.866
0.793	0.883	-5.682	0.069	5.635	5.566
0.494	0.667	-5.493	0.137	6.724	6.587
0.202	0.397	-4.945	0.274	6.445	6.171

0.204	0.399	-4.954	0.318	6.715	6.398
0.045	0.201	-3.820	-0.592	6.919	7.511
0.072	0.240	-4.192	0.548	6.657	6.109
0.023	0.172	-3.284	0.820	6.063	5.243
0.034	0.184	-3.597	0.849	3.736	2.887
0.008	0.157	-2.404	1.167	6.249	5.082
0.140	0.324	-4.690	0.383	5.197	4.814
0.396	0.584	-5.374	0.196	5.519	5.323
0.895	0.943	-5.707	0.033	4.419	4.386
0.046	0.203	-3.852	-0.508	7.376	7.884
0.406	0.594	-5.388	0.203	5.856	5.653
0.013	0.160	-2.834	1.071	8.911	7.840
0.266	0.464	-5.130	0.352	2.773	2.421
0.818	0.897	-5.690	0.087	2.850	2.763
0.899	0.945	-5.707	-0.075	6.413	6.488
0.773	0.869	-5.675	0.182	3.286	3.104
0.662	0.794	-5.624	-0.243	5.139	5.382
0.186	0.378	-4.888	-0.422	3.634	4.056
0.515	0.682	-5.514	-0.256	3.221	3.476
0.835	0.908	-5.694	-0.087	1.741	1.829
0.058	0.219	-4.027	-0.441	3.697	4.138
0.878	0.934	-5.704	-0.044	2.637	2.681
0.167	0.357	-4.815	-0.389	5.437	5.826
0.097	0.274	-4.420	0.401	3.405	3.004
0.292	0.490	-5.191	0.221	7.790	7.569
0.077	0.246	-4.245	-0.633	3.975	4.608
0.355	0.549	-5.310	-0.311	3.717	4.028
0.005	0.157	-2.128	1.655	5.880	4.225
0.317	0.516	-5.242	0.374	2.691	2.317
0.291	0.489	-5.188	0.487	3.535	3.048
0.050	0.207	-3.906	0.526	5.094	4.569
0.011	0.157	-2.668	-0.799	3.073	3.873
0.168	0.358	-4.817	-0.425	1.851	2.276
0.095	0.271	-4.402	0.422	3.714	3.292
0.136	0.319	-4.665	0.414	5.784	5.370
0.017	0.162	-3.042	-0.789	3.305	4.094
0.424	0.609	-5.412	0.269	3.396	3.127
0.176	0.366	-4.851	-0.419	4.218	4.637
0.081	0.253	-4.287	0.916	5.865	4.949
0.036	0.188	-3.645	0.873	5.213	4.340
0.014	0.160	-2.876	1.294	7.743	6.449
0.300	0.497	-5.206	-0.346	2.817	3.163
0.232	0.429	-5.041	0.449	4.884	4.435
0.979	0.989	-5.714	-0.008	3.340	3.348
0.062	0.224	-4.074	0.470	3.379	2.909
0.055	0.216	-3.989	0.534	3.544	3.010
0.742	0.850	-5.663	-0.127	1.512	1.639
0.043	0.198	-3.784	1.057	5.784	4.727
0.977	0.988	-5.714	-0.011	1.761	1.772
0.051	0.209	-3.929	-0.369	3.438	3.807

0.377	0.566	-5.345	-0.297	5.092	5.389
0.045	0.201	-3.821	-0.693	6.226	6.918
0.711	0.828	-5.650	-0.123	6.167	6.290
0.956	0.977	-5.713	0.021	5.859	5.839
0.572	0.728	-5.564	-0.129	3.179	3.308
0.149	0.335	-4.735	0.334	6.430	6.096
0.794	0.884	-5.683	0.077	2.844	2.767
0.054	0.215	-3.971	0.698	3.847	3.150
0.998	0.999	-5.715	0.001	3.873	3.873
0.882	0.936	-5.704	0.059	4.857	4.798
0.182	0.373	-4.875	0.480	6.862	6.382
0.303	0.501	-5.214	0.237	5.536	5.299
0.113	0.292	-4.533	-0.321	2.613	2.934
0.376	0.566	-5.344	0.198	6.726	6.528
0.632	0.772	-5.606	0.121	4.271	4.150
0.020	0.167	-3.195	0.679	4.527	3.848
0.301	0.499	-5.210	-0.240	2.061	2.301
0.170	0.361	-4.827	0.413	5.670	5.257
0.813	0.895	-5.688	-0.052	4.535	4.587
0.013	0.160	-2.830	0.721	5.053	4.332
0.003	0.157	-1.546	1.133	4.523	3.389
0.392	0.580	-5.368	-0.252	6.608	6.859
0.571	0.726	-5.563	-0.193	4.963	5.156
0.451	0.632	-5.446	-0.199	3.635	3.834
0.491	0.665	-5.490	-0.164	8.933	9.097
0.770	0.868	-5.674	-0.079	5.230	5.309
0.283	0.481	-5.170	-0.313	3.487	3.801
0.814	0.895	-5.688	0.062	3.461	3.399
0.335	0.531	-5.276	-0.200	6.535	6.735
0.695	0.818	-5.642	0.106	1.949	1.843
0.138	0.321	-4.675	0.489	5.869	5.380
0.323	0.519	-5.252	-0.325	3.070	3.395
0.085	0.257	-4.321	0.698	5.120	4.422
0.168	0.358	-4.819	0.513	2.624	2.112
0.607	0.754	-5.590	-0.163	3.845	4.008
0.360	0.552	-5.318	-0.245	2.299	2.544
0.320	0.517	-5.247	-0.221	2.351	2.572
0.380	0.569	-5.350	0.250	2.635	2.385
0.021	0.168	-3.207	0.720	3.179	2.460
0.663	0.795	-5.625	-0.104	2.291	2.395
0.215	0.410	-4.989	0.309	2.547	2.238
0.019	0.164	-3.147	-0.642	2.438	3.079
0.033	0.184	-3.581	-0.590	2.993	3.582
0.057	0.219	-4.018	0.792	5.347	4.554
0.045	0.201	-3.822	0.915	4.559	3.644
0.288	0.486	-5.181	-0.300	2.940	3.240
0.275	0.474	-5.152	0.300	5.237	4.937
0.789	0.880	-5.681	-0.079	2.651	2.730
0.097	0.273	-4.417	0.429	3.204	2.775
0.798	0.887	-5.684	0.066	3.182	3.116

0.790	0.881	-5.681	-0.067	5.469	5.535
0.014	0.160	-2.909	-0.660	5.417	6.076
0.029	0.178	-3.475	-0.852	4.890	5.742
0.091	0.267	-4.374	0.705	6.182	5.476
0.008	0.157	-2.409	1.107	5.753	4.646
0.249	0.447	-5.088	0.322	6.888	6.566
0.194	0.388	-4.920	0.424	3.425	3.001
0.419	0.606	-5.406	-0.184	3.800	3.985
0.554	0.715	-5.549	-0.143	2.433	2.576
0.016	0.162	-3.007	0.812	6.136	5.324
0.013	0.160	-2.862	-0.765	8.882	9.647
0.163	0.352	-4.795	-0.390	5.129	5.518
0.175	0.364	-4.845	0.421	3.028	2.608
0.082	0.254	-4.295	-0.672	2.296	2.968
0.036	0.188	-3.647	-0.690	2.545	3.235
0.033	0.184	-3.583	0.909	4.586	3.676
0.185	0.377	-4.886	0.404	7.493	7.090
0.031	0.181	-3.530	-0.657	7.293	7.951
0.332	0.528	-5.270	-0.221	6.590	6.812
0.605	0.753	-5.588	-0.111	6.316	6.426
0.072	0.240	-4.197	0.721	6.555	5.834
0.163	0.352	-4.797	0.455	5.086	4.631
0.486	0.662	-5.485	0.307	5.476	5.170
0.199	0.394	-4.938	0.281	6.546	6.265
0.059	0.221	-4.043	-0.463	5.972	6.435
0.017	0.162	-3.045	0.653	7.484	6.831
0.031	0.181	-3.534	-1.317	5.291	6.608
0.281	0.479	-5.166	-0.310	2.297	2.607
0.246	0.443	-5.079	-0.357	1.480	1.837
0.267	0.465	-5.133	0.359	3.279	2.921
0.407	0.594	-5.389	0.334	6.116	5.782
0.231	0.428	-5.037	0.427	4.020	3.593
0.441	0.625	-5.433	0.251	2.929	2.678
0.242	0.439	-5.068	0.436	6.083	5.648
0.101	0.278	-4.445	-0.426	1.343	1.769
0.219	0.415	-5.003	0.398	5.838	5.440
0.225	0.422	-5.020	-0.448	2.301	2.749
0.351	0.546	-5.304	0.421	4.692	4.270
0.704	0.824	-5.646	0.129	3.253	3.124
0.275	0.473	-5.151	0.366	3.076	2.709
0.463	0.643	-5.460	0.238	3.479	3.241
0.232	0.428	-5.039	0.319	4.200	3.881
0.632	0.772	-5.606	0.141	4.388	4.247
0.174	0.363	-4.841	0.401	5.120	4.719
0.022	0.171	-3.267	1.982	6.754	4.772
0.329	0.524	-5.263	-0.331	2.912	3.243
0.026	0.176	-3.389	-0.644	6.315	6.958
0.047	0.203	-3.855	-0.586	5.251	5.838
0.321	0.518	-5.250	-0.304	2.985	3.289
0.239	0.436	-5.059	0.362	4.093	3.731

0.340	0.535	-5.284	0.257	3.402	3.145
0.485	0.661	-5.484	0.399	4.650	4.251
0.227	0.424	-5.025	0.614	2.431	1.816
0.040	0.194	-3.730	0.855	4.877	4.023
0.200	0.396	-4.941	0.360	4.716	4.356
0.330	0.526	-5.266	-0.313	3.738	4.051
0.974	0.987	-5.714	0.010	3.370	3.360
0.025	0.175	-3.368	1.443	5.225	3.781
0.864	0.927	-5.701	0.048	3.408	3.360
0.156	0.344	-4.766	-0.430	2.871	3.302
0.015	0.160	-2.957	-1.011	4.107	5.118
0.983	0.991	-5.715	0.006	2.743	2.737
0.847	0.915	-5.697	-0.071	2.665	2.736
0.658	0.793	-5.622	-0.123	2.678	2.802
0.061	0.223	-4.061	0.820	6.416	5.596
0.047	0.204	-3.866	-0.891	2.501	3.392
0.172	0.362	-4.835	-0.408	3.846	4.254
0.010	0.157	-2.615	2.199	8.051	5.852
0.029	0.178	-3.484	1.001	8.288	7.286
0.033	0.184	-3.592	0.428	4.491	4.063
0.129	0.310	-4.630	0.435	7.593	7.158
0.879	0.934	-5.704	-0.041	4.074	4.115
0.052	0.212	-3.946	0.586	2.787	2.201
0.022	0.171	-3.266	-0.710	7.200	7.909
0.094	0.270	-4.392	0.859	6.508	5.649
0.296	0.493	-5.199	0.324	6.079	5.755
0.039	0.192	-3.707	0.442	5.044	4.602
0.838	0.910	-5.695	0.056	4.177	4.121
0.018	0.162	-3.082	1.093	5.369	4.277
0.613	0.758	-5.594	-0.144	6.059	6.203
0.081	0.252	-4.282	-0.370	10.419	10.789
0.504	0.673	-5.503	0.200	5.268	5.068
0.097	0.273	-4.418	-0.444	4.530	4.973
0.995	0.998	-5.715	-0.001	2.289	2.290
0.146	0.330	-4.718	0.373	2.958	2.584
0.164	0.353	-4.800	-0.345	5.430	5.775
0.030	0.179	-3.502	-0.589	3.261	3.850
0.258	0.455	-5.109	-0.243	6.311	6.553
0.187	0.380	-4.894	-0.410	3.080	3.490
0.283	0.481	-5.170	-0.295	4.051	4.346
0.108	0.287	-4.500	0.622	6.439	5.817
0.263	0.461	-5.122	-0.460	4.399	4.860
0.019	0.164	-3.126	-0.551	5.156	5.706
0.689	0.814	-5.639	0.110	2.559	2.450
0.005	0.157	-2.068	0.987	8.040	7.053
0.107	0.286	-4.491	0.489	5.256	4.768
0.036	0.189	-3.657	-0.551	3.224	3.775
0.460	0.641	-5.456	-0.282	3.241	3.523
0.447	0.629	-5.442	-0.307	3.807	4.113
0.693	0.816	-5.641	0.098	5.376	5.278

0.066	0.230	-4.124	0.393	7.332	6.938
0.304	0.502	-5.216	-0.243	7.526	7.769
0.202	0.397	-4.945	-0.236	5.703	5.939
0.040	0.194	-3.737	-0.647	5.457	6.105
0.064	0.228	-4.106	-0.566	4.335	4.902
0.651	0.787	-5.618	-0.130	3.041	3.171
0.209	0.404	-4.970	0.329	6.727	6.398
0.187	0.379	-4.894	0.337	8.636	8.299
0.190	0.383	-4.905	0.345	9.145	8.801
0.202	0.397	-4.945	0.326	8.687	8.361
0.211	0.406	-4.976	0.321	8.517	8.196
0.224	0.421	-5.017	0.307	8.665	8.358
0.230	0.427	-5.034	0.312	8.088	7.777
0.028	0.178	-3.459	1.690	9.609	7.919
0.003	0.157	-1.747	-0.736	5.104	5.840
0.014	0.160	-2.881	2.073	8.543	6.470
0.009	0.157	-2.494	1.121	6.291	5.170
0.028	0.178	-3.459	1.163	7.907	6.744
0.013	0.160	-2.848	1.908	8.103	6.195
0.004	0.157	-1.834	1.982	7.112	5.130
0.073	0.241	-4.203	0.578	7.104	6.526
0.004	0.157	-1.829	2.429	8.365	5.935
0.036	0.188	-3.643	0.700	5.237	4.538
0.003	0.157	-1.504	2.170	6.891	4.721
0.141	0.326	-4.695	0.477	3.230	2.754
0.770	0.868	-5.674	0.088	7.569	7.481
0.069	0.236	-4.165	1.515	9.047	7.532
0.001	0.157	-0.623	1.278	4.196	2.918
0.669	0.801	-5.628	0.255	3.982	3.727
0.016	0.162	-3.027	1.189	5.918	4.729
0.052	0.211	-3.937	1.331	7.364	6.033
0.160	0.349	-4.785	0.834	8.015	7.181
0.010	0.157	-2.604	1.083	8.224	7.141
0.399	0.587	-5.378	-0.486	3.929	4.414
0.330	0.525	-5.265	-0.341	2.416	2.756
0.081	0.252	-4.278	-0.588	2.582	3.170
0.163	0.352	-4.798	0.329	4.160	3.831
0.442	0.625	-5.435	-0.218	2.200	2.418
0.441	0.625	-5.434	0.213	3.505	3.292
0.046	0.203	-3.845	0.522	6.422	5.900
0.110	0.289	-4.511	0.450	2.657	2.207
0.065	0.229	-4.118	-0.862	5.904	6.765
0.048	0.205	-3.879	0.690	5.573	4.883
0.237	0.434	-5.054	0.342	5.252	4.910
0.708	0.826	-5.648	-0.100	3.158	3.258
0.214	0.409	-4.987	-0.456	4.164	4.621
0.020	0.167	-3.195	-0.645	4.727	5.372
0.036	0.189	-3.656	1.013	6.505	5.492
0.019	0.164	-3.145	0.985	3.578	2.593
0.115	0.295	-4.545	-0.422	2.086	2.509

0.046	0.203	-3.842	-0.563	4.883	5.446
0.100	0.277	-4.438	-0.658	2.746	3.404
0.031	0.180	-3.522	1.070	8.455	7.386
0.095	0.271	-4.401	-0.764	2.219	2.984
0.397	0.585	-5.376	-0.307	4.474	4.781
0.702	0.823	-5.646	0.073	5.833	5.760
0.038	0.192	-3.705	0.595	6.929	6.334
0.143	0.327	-4.703	0.538	3.117	2.579
0.716	0.831	-5.652	-0.090	4.281	4.371
0.470	0.649	-5.467	0.231	2.992	2.761
0.552	0.713	-5.548	-0.155	1.987	2.141
0.213	0.408	-4.981	0.426	4.136	3.710
0.074	0.242	-4.215	0.436	8.386	7.950
0.478	0.655	-5.476	0.151	6.568	6.416
0.608	0.755	-5.590	-0.141	5.050	5.191
0.316	0.513	-5.238	0.382	3.371	2.989
0.534	0.697	-5.531	0.308	4.772	4.464
0.780	0.874	-5.678	0.161	3.493	3.332
0.013	0.160	-2.858	-0.926	3.518	4.444
0.897	0.943	-5.707	-0.063	3.811	3.874
0.632	0.772	-5.606	-0.120	2.455	2.575
0.776	0.872	-5.677	-0.089	5.708	5.797
0.028	0.178	-3.450	0.457	8.038	7.581
0.056	0.217	-3.994	0.517	5.454	4.938
0.026	0.176	-3.405	0.585	3.704	3.119
0.000	0.157	0.549	1.988	5.158	3.170
0.545	0.707	-5.542	0.132	3.487	3.354
0.050	0.208	-3.918	0.527	7.164	6.636
0.004	0.157	-1.922	2.849	8.491	5.643
0.065	0.229	-4.115	0.506	7.405	6.899
0.048	0.204	-3.873	0.853	4.872	4.019
0.435	0.619	-5.426	0.190	6.627	6.437
0.028	0.178	-3.448	-0.492	6.343	6.835
0.919	0.957	-5.710	-0.024	4.515	4.539
0.182	0.373	-4.873	-0.358	5.682	6.040
0.925	0.959	-5.711	0.033	2.502	2.470
0.302	0.500	-5.212	0.348	2.663	2.314
0.203	0.398	-4.951	0.345	6.722	6.377
0.281	0.478	-5.164	-0.260	4.638	4.899
0.190	0.382	-4.903	-0.389	1.864	2.253
0.118	0.298	-4.563	0.495	3.782	3.287
0.162	0.351	-4.792	-0.385	3.695	4.080
0.974	0.987	-5.714	-0.008	4.629	4.638
0.016	0.162	-3.014	0.649	2.912	2.263
0.270	0.468	-5.141	0.315	3.730	3.415
0.425	0.610	-5.413	-0.283	10.010	10.294
0.416	0.603	-5.402	0.170	5.979	5.809
0.126	0.306	-4.610	0.615	5.024	4.410
0.029	0.178	-3.493	0.710	3.362	2.652
0.057	0.218	-4.008	0.660	4.803	4.143

0.807	0.891	-5.686	0.084	4.789	4.705
0.866	0.928	-5.701	0.050	5.278	5.228
0.008	0.157	-2.418	1.472	5.437	3.965
0.315	0.513	-5.237	0.214	6.000	5.786
0.006	0.157	-2.170	0.780	5.385	4.605
0.024	0.173	-3.328	0.494	3.866	3.372
0.439	0.622	-5.431	-0.236	2.324	2.560
0.363	0.555	-5.324	0.376	3.896	3.519
0.199	0.393	-4.935	0.397	2.983	2.587
0.066	0.230	-4.125	0.430	9.210	8.780
0.371	0.562	-5.337	0.238	2.667	2.430
0.016	0.162	-2.999	-0.589	2.819	3.407
0.337	0.533	-5.279	-0.277	4.425	4.702
0.676	0.805	-5.632	-0.118	3.429	3.547
0.964	0.982	-5.714	0.015	4.501	4.486
0.804	0.890	-5.686	-0.082	3.635	3.717
0.207	0.402	-4.963	-0.329	3.824	4.153
0.901	0.946	-5.707	-0.044	2.951	2.995
0.190	0.383	-4.905	0.329	7.357	7.027
0.269	0.467	-5.137	-0.380	8.498	8.878
0.927	0.961	-5.711	0.028	2.639	2.611
0.010	0.157	-2.635	-1.288	2.474	3.762
0.292	0.490	-5.190	0.520	3.712	3.192
0.133	0.316	-4.650	0.929	5.425	4.496
0.179	0.370	-4.864	0.434	3.992	3.558
0.001	0.157	-0.660	-1.305	3.668	4.973
0.686	0.812	-5.637	-0.182	2.129	2.311
0.483	0.659	-5.482	0.219	4.020	3.802
0.320	0.518	-5.248	-0.285	5.035	5.320
0.475	0.653	-5.473	0.225	3.667	3.442
0.913	0.954	-5.709	-0.034	4.883	4.918
0.583	0.736	-5.572	0.156	3.303	3.148
0.007	0.157	-2.317	0.882	3.613	2.730
0.150	0.336	-4.738	0.307	3.460	3.153
0.014	0.160	-2.897	0.830	5.618	4.788
0.035	0.186	-3.625	-0.938	6.048	6.986
0.744	0.851	-5.664	0.089	3.296	3.207
0.275	0.473	-5.151	0.241	4.399	4.158
0.130	0.311	-4.634	-0.411	4.762	5.172
0.925	0.959	-5.711	-0.027	3.512	3.539
0.253	0.451	-5.098	0.385	7.311	6.926
0.084	0.256	-4.310	-0.665	4.373	5.038
0.384	0.573	-5.357	-0.309	5.042	5.351
0.081	0.252	-4.282	-0.533	3.979	4.512
0.010	0.157	-2.646	0.719	4.747	4.028
0.148	0.332	-4.727	-0.390	2.222	2.611
0.012	0.159	-2.739	1.323	4.437	3.114
0.135	0.319	-4.662	-1.427	2.873	4.299
0.318	0.516	-5.244	0.520	4.858	4.338
0.037	0.190	-3.671	1.622	7.002	5.380

0.029	0.178	-3.482	1.185	6.461	5.276
0.538	0.700	-5.535	0.237	4.714	4.477
0.076	0.245	-4.233	0.412	5.143	4.731
0.010	0.157	-2.597	0.879	7.872	6.993
0.097	0.273	-4.415	0.846	6.218	5.372
0.023	0.173	-3.291	0.925	6.310	5.385
0.003	0.157	-1.603	-1.243	7.018	8.261
0.044	0.201	-3.815	-0.404	6.365	6.769
0.207	0.402	-4.964	-0.371	5.453	5.823
0.103	0.280	-4.461	0.445	3.237	2.793
0.164	0.353	-4.800	-0.455	7.659	8.115
0.711	0.828	-5.650	0.167	3.172	3.005
0.083	0.254	-4.297	-0.400	5.593	5.993
0.521	0.687	-5.520	-0.283	5.738	6.020
0.466	0.645	-5.463	-0.229	3.735	3.964
0.024	0.173	-3.336	-0.907	3.710	4.617
0.116	0.296	-4.552	0.319	7.339	7.020
0.447	0.629	-5.441	-0.166	4.714	4.880
0.157	0.345	-4.768	-0.356	5.737	6.093
0.800	0.888	-5.684	0.086	5.273	5.188
0.333	0.528	-5.271	0.363	3.813	3.450
0.410	0.597	-5.393	-0.191	4.259	4.450
0.030	0.179	-3.507	0.547	3.742	3.195
0.110	0.289	-4.511	0.687	5.047	4.361
0.420	0.607	-5.407	0.164	10.279	10.115
0.497	0.669	-5.496	0.190	3.563	3.374
0.568	0.724	-5.560	0.138	4.815	4.677
0.037	0.190	-3.675	0.987	5.804	4.817
0.069	0.235	-4.156	0.504	4.092	3.589
0.086	0.258	-4.323	-0.467	4.485	4.952
0.077	0.247	-4.247	0.714	4.695	3.981
0.462	0.643	-5.459	0.196	3.084	2.889
0.089	0.263	-4.351	0.928	9.567	8.639
0.605	0.753	-5.588	0.155	4.595	4.440
0.381	0.570	-5.351	-0.244	3.186	3.430
0.802	0.889	-5.685	0.075	5.447	5.372
0.269	0.467	-5.137	0.261	6.359	6.098
0.976	0.988	-5.714	0.009	2.444	2.435
0.007	0.157	-2.361	0.753	6.167	5.415
0.165	0.354	-4.805	0.311	5.042	4.731
0.089	0.264	-4.355	0.375	6.088	5.713
0.504	0.674	-5.504	0.164	3.509	3.346
0.105	0.284	-4.480	0.560	2.982	2.423
0.746	0.853	-5.665	-0.095	3.114	3.209
0.982	0.990	-5.715	-0.007	1.928	1.934
0.085	0.257	-4.319	0.791	4.039	3.248
0.983	0.991	-5.715	0.005	5.481	5.476
0.995	0.998	-5.715	0.002	5.246	5.245
0.036	0.188	-3.651	0.724	4.951	4.227
0.095	0.271	-4.406	0.620	3.095	2.475

0.603	0.752	-5.587	0.120	7.181	7.061
0.679	0.808	-5.634	0.097	7.218	7.120
0.249	0.446	-5.086	-0.318	3.297	3.616
0.140	0.324	-4.686	-0.471	2.615	3.086
0.312	0.509	-5.232	-0.358	2.680	3.039
0.223	0.419	-5.012	-0.296	5.040	5.336
0.077	0.246	-4.243	-0.487	5.786	6.273
0.029	0.178	-3.476	-0.552	5.273	5.825
0.044	0.201	-3.811	-0.520	5.487	6.007
0.822	0.899	-5.691	0.081	3.559	3.478
0.733	0.844	-5.660	-0.072	7.795	7.867
0.270	0.469	-5.141	0.257	6.642	6.385
0.056	0.217	-3.998	0.420	7.921	7.501
0.865	0.928	-5.701	0.044	3.847	3.804
0.086	0.258	-4.328	0.567	9.371	8.804
0.029	0.178	-3.468	0.439	8.423	7.984
0.404	0.592	-5.385	-0.202	5.301	5.503
0.896	0.943	-5.707	-0.028	6.349	6.377
0.013	0.160	-2.824	0.534	5.830	5.296
0.809	0.893	-5.687	0.054	5.667	5.613
0.340	0.535	-5.283	0.254	4.080	3.826
0.975	0.987	-5.714	0.007	2.095	2.088
0.555	0.715	-5.550	0.196	2.414	2.219
0.452	0.633	-5.447	-0.164	4.979	5.143
0.636	0.775	-5.609	0.124	5.840	5.716
0.379	0.569	-5.349	-0.252	5.368	5.620
0.699	0.821	-5.644	0.103	6.809	6.706
0.279	0.477	-5.160	-0.461	3.191	3.652
0.175	0.365	-4.849	0.346	7.414	7.068
0.054	0.215	-3.976	0.545	9.167	8.622
0.057	0.219	-4.016	-1.048	4.344	5.392
0.293	0.490	-5.191	0.338	5.072	4.735
0.558	0.717	-5.552	-0.261	4.264	4.526
0.517	0.684	-5.516	-0.177	2.810	2.987
0.399	0.587	-5.379	-0.183	3.586	3.768
0.042	0.197	-3.778	0.421	6.321	5.900
0.066	0.231	-4.130	0.817	5.584	4.767
0.384	0.572	-5.356	0.220	3.544	3.324
0.168	0.358	-4.818	-0.365	5.930	6.295
0.862	0.926	-5.701	0.052	2.985	2.933
0.385	0.574	-5.358	-0.246	2.125	2.371
0.979	0.989	-5.714	0.007	2.732	2.726
0.441	0.625	-5.434	0.183	6.146	5.964
0.218	0.413	-4.999	-0.279	2.085	2.363
0.370	0.560	-5.335	0.291	2.687	2.396
0.808	0.892	-5.687	0.088	2.678	2.590
0.000	0.157	0.505	1.215	3.699	2.484
0.376	0.566	-5.344	0.330	4.019	3.689
0.045	0.201	-3.822	0.653	7.199	6.546
0.103	0.280	-4.460	0.674	8.492	7.818

0.029	0.178	-3.480	0.922	6.784	5.862
0.038	0.192	-3.698	-0.823	2.683	3.506
0.079	0.249	-4.266	0.524	3.499	2.975
0.006	0.157	-2.170	0.675	3.968	3.293
0.388	0.576	-5.363	0.219	6.234	6.015
0.643	0.780	-5.613	0.243	5.081	4.838
0.821	0.899	-5.691	-0.070	3.027	3.097
0.056	0.217	-3.993	0.800	6.642	5.842
0.004	0.157	-1.812	2.278	6.758	4.480
0.208	0.403	-4.967	0.690	5.502	4.812
0.171	0.361	-4.832	0.433	4.116	3.683
0.008	0.157	-2.430	-1.165	4.511	5.676
0.006	0.157	-2.139	-1.288	7.585	8.873
0.240	0.438	-5.063	-0.372	6.529	6.900
0.136	0.320	-4.669	0.519	4.767	4.248
0.031	0.181	-3.531	0.609	3.237	2.627
0.932	0.963	-5.711	-0.023	4.031	4.054
0.461	0.641	-5.457	0.168	7.743	7.575
0.527	0.692	-5.525	0.185	3.849	3.663
0.017	0.162	-3.048	0.806	4.062	3.255
0.046	0.203	-3.848	-0.747	2.740	3.488
0.005	0.157	-1.988	1.509	5.345	3.836
0.280	0.478	-5.164	-0.405	3.589	3.994
0.123	0.304	-4.593	0.894	5.462	4.568
0.126	0.307	-4.612	0.605	4.921	4.317
0.191	0.383	-4.906	-0.416	4.045	4.461
0.109	0.288	-4.506	0.851	6.424	5.573
0.132	0.315	-4.646	0.417	6.717	6.300
0.359	0.552	-5.317	0.273	5.307	5.034
0.699	0.820	-5.644	0.139	2.165	2.026
0.034	0.185	-3.612	-0.559	9.344	9.903
0.289	0.488	-5.184	0.423	5.204	4.780
0.044	0.200	-3.804	-0.518	6.205	6.723
0.662	0.795	-5.624	-0.120	2.719	2.839
0.202	0.397	-4.947	-0.291	5.498	5.789
0.164	0.353	-4.801	-0.325	4.342	4.667
0.088	0.262	-4.346	0.732	4.059	3.328
0.856	0.922	-5.699	0.061	1.939	1.877
0.089	0.264	-4.354	0.605	7.327	6.722
0.148	0.332	-4.726	-0.435	5.073	5.509
0.103	0.281	-4.466	-0.696	2.083	2.779
0.032	0.183	-3.566	0.587	4.247	3.660
0.071	0.238	-4.182	0.732	4.492	3.760
0.066	0.230	-4.122	-0.617	3.459	4.076
0.011	0.157	-2.692	-0.995	5.565	6.560
0.086	0.259	-4.332	0.657	4.772	4.115
0.105	0.283	-4.477	0.518	2.724	2.206
0.746	0.852	-5.665	-0.173	1.979	2.152
0.038	0.192	-3.697	1.018	6.623	5.606
0.015	0.160	-2.932	0.821	8.284	7.463

0.019	0.164	-3.132	0.717	7.756	7.039
0.444	0.627	-5.438	0.174	7.626	7.452
0.596	0.747	-5.582	-0.130	4.724	4.854
0.082	0.253	-4.289	0.743	6.076	5.333
0.065	0.229	-4.111	0.721	7.126	6.405
0.289	0.487	-5.182	0.261	5.809	5.548
0.033	0.184	-3.591	0.587	6.581	5.994
0.069	0.235	-4.155	1.555	8.823	7.268
0.009	0.157	-2.494	1.678	7.894	6.216
0.020	0.167	-3.177	1.709	8.529	6.821
0.020	0.167	-3.172	0.739	5.609	4.870
0.113	0.292	-4.533	0.453	5.132	4.678
0.658	0.792	-5.622	0.124	3.576	3.452
0.313	0.510	-5.233	0.201	6.840	6.639
0.040	0.194	-3.742	0.918	6.068	5.149
0.193	0.387	-4.916	0.583	3.526	2.943
0.343	0.538	-5.290	-0.244	8.207	8.451
0.016	0.162	-3.005	1.380	8.322	6.942
0.071	0.238	-4.180	0.646	4.227	3.581
0.011	0.157	-2.676	0.634	3.221	2.587
0.294	0.491	-5.194	-0.295	6.343	6.638
0.049	0.206	-3.888	0.611	6.155	5.544
0.241	0.438	-5.065	0.486	4.459	3.973
0.367	0.558	-5.329	-0.191	1.667	1.857
0.118	0.298	-4.566	0.458	3.433	2.975
0.292	0.490	-5.190	0.362	3.235	2.873
0.017	0.162	-3.052	0.659	2.694	2.035
0.121	0.302	-4.583	0.479	2.744	2.265
0.674	0.804	-5.631	-0.097	2.596	2.693
0.073	0.241	-4.206	0.423	6.993	6.569
0.314	0.512	-5.236	0.208	4.948	4.739
0.007	0.157	-2.380	-0.941	5.098	6.039
0.070	0.237	-4.175	0.427	4.116	3.688
0.121	0.302	-4.582	0.353	7.049	6.697
0.375	0.565	-5.342	-0.279	4.741	5.020
0.217	0.412	-4.994	-0.373	1.982	2.355
0.015	0.161	-2.974	0.752	5.820	5.068
0.241	0.438	-5.065	0.304	4.393	4.089
0.410	0.597	-5.394	0.455	5.895	5.441
0.864	0.927	-5.701	0.108	4.580	4.471
0.071	0.239	-4.184	1.245	5.799	4.554
0.142	0.326	-4.697	0.761	5.360	4.599
0.248	0.446	-5.084	0.215	6.103	5.888
0.028	0.177	-3.441	-0.588	5.876	6.464
0.016	0.162	-2.993	-0.649	4.417	5.066
0.958	0.978	-5.713	0.012	6.135	6.124
0.126	0.306	-4.610	0.315	6.125	5.810
0.943	0.969	-5.712	0.020	3.663	3.643
0.019	0.166	-3.162	0.734	2.676	1.943
0.018	0.162	-3.084	1.106	6.930	5.824

0.395	0.582	-5.372	0.231	6.994	6.763
0.020	0.167	-3.179	0.892	9.462	8.570
0.482	0.658	-5.480	0.189	6.480	6.291
0.007	0.157	-2.342	1.006	5.037	4.031
0.092	0.267	-4.379	-0.435	4.358	4.793
0.128	0.310	-4.625	-0.427	5.266	5.692
0.025	0.175	-3.374	-0.688	1.482	2.171
0.044	0.200	-3.806	-0.776	3.756	4.532
0.568	0.724	-5.560	-0.220	4.682	4.902
0.063	0.227	-4.095	-0.568	7.132	7.699
0.047	0.204	-3.861	-0.624	6.699	7.323
0.456	0.637	-5.452	0.244	4.715	4.470
0.038	0.192	-3.699	0.524	4.519	3.995
0.120	0.300	-4.573	-0.476	8.090	8.566
0.751	0.856	-5.667	0.086	2.773	2.687
0.449	0.630	-5.443	-0.211	4.875	5.087
0.016	0.162	-3.019	-0.972	3.741	4.713
0.284	0.481	-5.172	-0.272	1.838	2.110
0.248	0.446	-5.085	-0.288	4.572	4.860
0.469	0.648	-5.467	0.246	3.081	2.835
0.124	0.305	-4.599	-0.478	3.854	4.332
0.100	0.278	-4.444	-0.376	5.263	5.640
0.039	0.193	-3.716	0.473	5.335	4.862
0.181	0.372	-4.870	-0.399	8.420	8.819
0.432	0.617	-5.422	-0.207	2.960	3.167
0.334	0.530	-5.274	-0.270	2.302	2.572
0.089	0.264	-4.357	0.339	6.566	6.227
0.074	0.242	-4.214	-0.402	6.777	7.179
0.007	0.157	-2.289	-0.916	1.919	2.835
0.998	0.999	-5.715	0.001	6.995	6.994
0.134	0.318	-4.658	0.654	4.440	3.786
0.005	0.157	-2.079	-1.690	5.471	7.161
0.043	0.199	-3.789	1.440	6.447	5.007
0.251	0.448	-5.091	0.292	2.940	2.648
0.270	0.468	-5.139	0.411	3.513	3.101
0.105	0.283	-4.477	-0.394	3.819	4.212
0.679	0.808	-5.634	-0.100	2.940	3.040
0.482	0.658	-5.481	-0.167	3.690	3.857
0.152	0.339	-4.746	-0.502	4.602	5.104
0.293	0.491	-5.192	-0.306	1.746	2.052
0.100	0.277	-4.440	-0.505	4.221	4.726
0.038	0.192	-3.701	-0.558	1.933	2.491
0.292	0.489	-5.189	0.314	2.887	2.574
0.262	0.460	-5.121	-0.288	2.716	3.004
0.035	0.186	-3.632	0.619	6.560	5.941
0.298	0.495	-5.202	0.240	4.260	4.019
0.624	0.767	-5.601	0.165	4.394	4.229
0.125	0.306	-4.606	-0.407	1.947	2.354
0.011	0.157	-2.680	-0.978	1.788	2.766
0.012	0.160	-2.771	-1.012	1.982	2.995

0.019	0.164	-3.144	-0.927	2.109	3.036
0.253	0.451	-5.098	-0.349	3.117	3.467
0.064	0.229	-4.108	-0.615	3.038	3.654
0.143	0.327	-4.705	-0.422	3.128	3.550
0.231	0.428	-5.037	-0.482	4.479	4.961
0.592	0.744	-5.579	-0.219	5.047	5.267
0.022	0.171	-3.276	2.045	9.259	7.213
0.111	0.290	-4.517	0.396	4.000	3.604
0.064	0.227	-4.097	0.659	4.027	3.368
0.318	0.516	-5.243	0.284	3.827	3.543
0.519	0.686	-5.518	0.201	3.479	3.278
0.969	0.985	-5.714	-0.011	4.151	4.162
0.925	0.959	-5.711	-0.027	3.354	3.381
0.490	0.664	-5.489	-0.292	3.704	3.996
0.004	0.157	-1.867	-0.733	4.822	5.555
0.868	0.928	-5.702	-0.035	3.695	3.730
0.648	0.784	-5.616	0.101	5.097	4.995
0.039	0.193	-3.721	0.429	8.326	7.896
0.058	0.219	-4.028	-0.474	6.000	6.474
0.302	0.500	-5.211	-0.245	4.316	4.561
0.078	0.247	-4.250	0.528	5.477	4.949
0.009	0.157	-2.508	1.340	6.822	5.482
0.041	0.195	-3.757	0.811	3.606	2.796
0.738	0.848	-5.662	0.076	7.162	7.086
0.388	0.576	-5.363	-0.301	7.291	7.592
0.784	0.877	-5.679	0.072	2.545	2.473
0.948	0.973	-5.713	-0.015	1.984	1.999
0.941	0.969	-5.712	0.015	1.979	1.964
0.664	0.796	-5.626	0.115	5.118	5.003
0.004	0.157	-1.886	-0.880	6.418	7.298
0.090	0.265	-4.363	-0.542	3.539	4.081
0.027	0.176	-3.427	0.441	8.168	7.727
0.036	0.189	-3.662	-0.830	4.184	5.014
0.019	0.164	-3.133	-0.539	3.121	3.660
0.031	0.181	-3.539	0.700	6.775	6.075
0.903	0.947	-5.708	0.032	6.953	6.921
0.050	0.208	-3.917	0.547	5.016	4.469
0.979	0.989	-5.714	-0.008	3.375	3.383
0.842	0.912	-5.696	-0.052	6.013	6.065
0.518	0.685	-5.517	0.184	4.449	4.266
0.031	0.180	-3.524	-0.763	4.868	5.631
0.214	0.409	-4.986	-0.474	2.416	2.890
0.317	0.515	-5.242	-0.222	2.349	2.571
0.000	0.157	0.153	1.445	5.109	3.663
0.048	0.205	-3.878	-0.894	5.878	6.772
0.059	0.221	-4.040	-0.830	5.095	5.924
0.281	0.479	-5.166	-0.293	3.310	3.603
0.152	0.339	-4.749	0.374	4.645	4.271
0.004	0.157	-1.929	-0.834	4.229	5.063
0.478	0.655	-5.476	-0.178	5.572	5.749

0.511	0.679	-5.510	0.155	8.670	8.515
0.633	0.773	-5.607	0.205	2.791	2.586
0.990	0.995	-5.715	-0.006	2.425	2.431
0.145	0.329	-4.714	-0.499	4.205	4.704
0.049	0.207	-3.902	-0.539	4.009	4.547
0.953	0.976	-5.713	-0.031	6.269	6.299
0.009	0.157	-2.569	-0.927	5.933	6.859
0.011	0.157	-2.674	-0.849	5.145	5.993
0.061	0.224	-4.068	-0.593	2.518	3.111
0.033	0.184	-3.577	-0.615	9.158	9.773
0.272	0.471	-5.145	-0.263	6.004	6.267
0.707	0.826	-5.648	-0.102	5.014	5.116
0.958	0.978	-5.713	-0.014	8.091	8.105
0.001	0.157	-0.512	-0.971	5.660	6.631
0.603	0.752	-5.587	0.217	4.727	4.510
0.118	0.298	-4.566	-0.609	1.897	2.506
0.510	0.679	-5.509	-0.319	4.434	4.753
0.481	0.658	-5.480	-0.306	2.087	2.393
0.945	0.971	-5.713	-0.025	3.802	3.827
0.568	0.724	-5.560	-0.267	3.309	3.576
0.745	0.851	-5.664	-0.114	3.263	3.377
0.815	0.896	-5.689	-0.146	4.234	4.380
0.085	0.257	-4.318	-0.839	2.242	3.081
0.510	0.679	-5.509	-0.213	5.758	5.971
0.696	0.819	-5.643	-0.217	4.437	4.654
0.778	0.872	-5.677	-0.114	2.759	2.873
0.807	0.892	-5.687	0.081	2.014	1.933
0.350	0.545	-5.302	0.393	4.329	3.937
0.026	0.176	-3.402	-1.240	1.917	3.157
0.270	0.468	-5.140	-0.467	3.540	4.008
0.450	0.631	-5.445	-0.525	3.134	3.659
0.013	0.160	-2.842	-0.791	3.261	4.052
0.726	0.838	-5.656	0.127	5.643	5.516
0.035	0.186	-3.621	-0.838	3.546	4.383
0.051	0.210	-3.931	0.503	9.535	9.033
0.196	0.390	-4.925	-0.551	2.552	3.103
0.323	0.519	-5.253	-0.393	6.919	7.312
0.206	0.401	-4.959	-0.259	9.125	9.385
0.210	0.404	-4.972	-0.301	6.152	6.453
0.059	0.220	-4.034	-0.695	4.835	5.530
0.018	0.162	-3.077	-0.870	4.308	5.178
0.815	0.896	-5.689	-0.076	3.837	3.913
0.864	0.927	-5.701	-0.063	4.942	5.005
0.034	0.185	-3.608	-0.694	3.736	4.430
0.049	0.206	-3.890	-0.776	5.395	6.171
0.740	0.849	-5.663	0.140	4.102	3.962
0.096	0.272	-4.409	0.387	5.567	5.181
0.040	0.194	-3.734	0.700	9.107	8.407
0.031	0.182	-3.546	0.637	7.518	6.881
0.050	0.207	-3.903	-0.913	3.743	4.657

0.302	0.499	-5.210	0.272	4.319	4.047
0.599	0.748	-5.584	-0.127	4.918	5.045
0.763	0.864	-5.672	-0.085	6.826	6.910
0.912	0.952	-5.709	0.028	6.940	6.912
0.947	0.973	-5.713	0.019	4.193	4.174
0.970	0.985	-5.714	-0.019	6.063	6.083
0.978	0.989	-5.714	-0.007	8.290	8.297
0.619	0.763	-5.598	0.140	6.944	6.804
0.749	0.854	-5.666	0.086	8.087	8.001
0.776	0.871	-5.676	0.075	8.087	8.012
0.046	0.203	-3.844	-0.520	4.711	5.231
0.342	0.538	-5.288	-0.243	7.271	7.514
0.435	0.619	-5.427	-0.196	6.924	7.121
0.519	0.686	-5.518	0.169	6.946	6.777
0.599	0.749	-5.584	-0.123	6.780	6.903
0.635	0.774	-5.608	0.130	6.944	6.814
0.198	0.392	-4.932	-0.407	4.696	5.103
0.356	0.550	-5.312	-0.327	4.493	4.819
0.444	0.627	-5.437	-0.262	4.693	4.955
0.409	0.596	-5.392	-0.309	4.657	4.965
0.422	0.608	-5.410	-0.287	4.664	4.952
0.396	0.583	-5.374	-0.334	4.657	4.991
0.474	0.652	-5.472	-0.278	4.699	4.977
0.280	0.478	-5.164	-0.451	4.345	4.796
0.323	0.520	-5.253	-0.410	4.378	4.788
0.348	0.544	-5.299	-0.376	3.645	4.021
0.096	0.272	-4.409	-0.522	4.041	4.563
0.206	0.401	-4.959	-0.422	3.630	4.052
0.212	0.407	-4.979	-0.403	2.279	2.682
0.216	0.411	-4.991	-0.423	3.614	4.036
0.226	0.423	-5.021	-0.408	2.364	2.771
0.335	0.531	-5.275	-0.353	4.145	4.499
0.350	0.545	-5.302	-0.345	4.127	4.471
0.362	0.554	-5.322	-0.326	3.218	3.544
0.375	0.565	-5.342	-0.339	4.327	4.665
0.427	0.612	-5.416	-0.309	3.080	3.389
0.349	0.544	-5.300	-0.352	6.499	6.851
0.381	0.571	-5.352	-0.325	4.570	4.895
0.512	0.680	-5.511	-0.233	5.809	6.041
0.531	0.695	-5.529	-0.229	5.785	6.013
0.285	0.482	-5.174	0.360	3.368	3.008
0.308	0.505	-5.223	-0.372	1.697	2.069
0.356	0.550	-5.311	-0.263	1.748	2.012
0.362	0.554	-5.323	-0.279	1.886	2.165
0.784	0.877	-5.679	-0.125	4.331	4.456
0.863	0.927	-5.701	0.085	4.934	4.848
0.868	0.928	-5.702	-0.085	4.151	4.236
0.699	0.820	-5.644	0.145	3.224	3.079
0.739	0.848	-5.662	0.124	3.841	3.717
0.743	0.851	-5.664	0.126	4.442	4.316

0.765	0.865	-5.672	0.109	3.817	3.708
0.771	0.869	-5.675	0.105	4.023	3.917
0.773	0.869	-5.675	0.109	3.992	3.883
0.827	0.902	-5.692	0.075	4.685	4.609
0.774	0.871	-5.676	-0.098	4.145	4.244
0.979	0.989	-5.714	-0.008	3.148	3.156
0.457	0.637	-5.453	0.197	2.702	2.505
0.517	0.684	-5.516	0.167	2.489	2.321
0.691	0.815	-5.640	0.101	2.320	2.219
0.725	0.838	-5.656	0.091	2.296	2.206
0.867	0.928	-5.701	0.046	2.229	2.183
0.866	0.928	-5.701	-0.100	6.907	7.007
0.596	0.747	-5.582	-0.233	8.321	8.554
0.618	0.762	-5.597	-0.221	8.358	8.579
0.692	0.816	-5.640	-0.173	8.023	8.196
0.701	0.822	-5.645	-0.171	8.227	8.398
0.148	0.332	-4.726	2.404	4.719	2.316
0.930	0.962	-5.711	0.028	3.504	3.476
0.992	0.996	-5.715	-0.003	3.587	3.590
0.689	0.814	-5.639	0.132	6.366	6.234
0.279	0.477	-5.161	0.793	3.295	2.502
0.880	0.935	-5.704	0.051	5.499	5.448
0.937	0.966	-5.712	0.030	7.141	7.111
0.865	0.928	-5.701	0.104	6.060	5.956
0.989	0.994	-5.715	0.010	4.998	4.988
0.179	0.369	-4.861	2.204	4.660	2.456
0.533	0.696	-5.531	0.139	2.188	2.049
0.675	0.805	-5.631	0.106	8.068	7.962
0.694	0.816	-5.641	0.098	8.104	8.005
0.660	0.793	-5.623	0.089	5.030	4.941
0.800	0.888	-5.684	-0.060	4.567	4.627
0.903	0.947	-5.708	-0.026	4.707	4.733
0.922	0.958	-5.710	-0.023	4.647	4.670
0.211	0.406	-4.977	-0.242	3.528	3.769
0.528	0.693	-5.527	-0.124	3.409	3.533
0.581	0.735	-5.570	-0.108	3.166	3.274
0.764	0.864	-5.672	-0.088	4.983	5.071
0.010	0.157	-2.612	1.520	8.838	7.319
0.673	0.803	-5.630	-0.091	2.281	2.372
0.671	0.802	-5.630	-0.134	2.211	2.345
0.061	0.223	-4.064	0.427	6.437	6.010
0.022	0.169	-3.245	-0.548	3.380	3.928
0.399	0.586	-5.378	0.261	6.272	6.011
0.232	0.429	-5.041	-0.360	2.748	3.108
0.897	0.943	-5.707	-0.033	4.098	4.131
0.202	0.397	-4.946	0.369	7.669	7.300
0.294	0.491	-5.194	0.345	6.819	6.474
0.096	0.272	-4.411	-0.485	3.699	4.184
0.233	0.430	-5.044	-0.383	2.859	3.242
0.919	0.957	-5.710	0.025	4.124	4.099

0.076	0.245	-4.229	-0.549	3.818	4.367
0.206	0.401	-4.960	-0.424	4.470	4.895
0.018	0.162	-3.084	0.757	4.775	4.017
0.034	0.184	-3.601	-0.736	2.673	3.409
0.541	0.703	-5.538	-0.170	2.406	2.576
0.094	0.270	-4.393	0.457	5.837	5.380
0.007	0.157	-2.346	-1.513	4.320	5.833
0.083	0.255	-4.303	-0.462	3.939	4.401
0.865	0.928	-5.701	0.070	3.625	3.555
0.073	0.241	-4.207	0.395	4.851	4.456
0.659	0.793	-5.622	-0.124	6.171	6.295
0.126	0.306	-4.609	-0.491	1.922	2.414
0.009	0.157	-2.527	1.024	7.595	6.571
0.071	0.238	-4.180	-0.502	3.883	4.385
0.013	0.160	-2.861	1.801	5.941	4.141
0.016	0.162	-3.011	-1.042	1.892	2.934
0.047	0.204	-3.866	-0.553	1.645	2.198
0.742	0.850	-5.663	-0.104	1.878	1.983
0.455	0.636	-5.450	-0.216	2.208	2.424
0.019	0.164	-3.127	-1.647	1.319	2.966
0.091	0.267	-4.372	-1.122	1.746	2.867
0.010	0.157	-2.655	-1.942	2.021	3.963
0.255	0.452	-5.103	-0.397	2.696	3.093
0.160	0.349	-4.784	-0.412	2.404	2.816
0.046	0.203	-3.840	-0.908	2.232	3.141
0.283	0.481	-5.171	-0.418	1.635	2.053
0.368	0.559	-5.331	-0.329	2.227	2.555
0.033	0.184	-3.574	-1.327	1.437	2.764
0.220	0.416	-5.005	-0.407	2.180	2.587
0.397	0.585	-5.375	-0.231	1.522	1.753
0.897	0.943	-5.707	-0.047	2.176	2.223
0.798	0.887	-5.684	0.094	2.705	2.611
0.042	0.198	-3.781	-0.672	1.704	2.376
0.099	0.276	-4.436	0.529	2.456	1.927
0.074	0.241	-4.209	-0.377	1.442	1.819
0.737	0.847	-5.661	0.081	2.420	2.340
0.485	0.660	-5.484	0.147	1.439	1.292
0.366	0.557	-5.328	-0.273	1.139	1.412
0.216	0.411	-4.992	-0.348	2.592	2.940
0.008	0.157	-2.472	-1.204	2.240	3.443
0.753	0.857	-5.668	0.134	2.283	2.149
0.016	0.162	-3.026	-1.103	1.713	2.817
0.119	0.299	-4.572	-0.624	2.125	2.750
0.114	0.293	-4.538	-0.645	2.572	3.217
0.284	0.481	-5.171	-0.291	1.884	2.175
0.881	0.935	-5.704	-0.137	1.679	1.815
0.744	0.851	-5.664	0.078	3.755	3.677
0.011	0.157	-2.697	1.103	7.391	6.288
0.256	0.454	-5.106	-0.293	1.582	1.875
0.247	0.445	-5.082	-0.554	3.042	3.596

0.100	0.277	-4.440	-0.495	3.120	3.615
0.301	0.499	-5.209	-0.344	2.921	3.265
0.011	0.157	-2.701	0.782	7.155	6.373
0.123	0.304	-4.594	-0.381	6.814	7.196
0.231	0.428	-5.036	-0.320	5.346	5.666
0.469	0.648	-5.466	-0.183	2.296	2.479
0.868	0.928	-5.702	-0.057	2.922	2.979
0.272	0.471	-5.146	-0.484	4.390	4.874
0.686	0.812	-5.638	0.108	3.668	3.561
0.890	0.940	-5.706	-0.038	3.755	3.793
0.746	0.853	-5.665	-0.103	4.970	5.073
0.006	0.157	-2.239	0.828	4.681	3.853
0.034	0.184	-3.600	1.064	5.791	4.727
0.028	0.177	-3.441	0.604	2.902	2.298
0.015	0.161	-2.977	0.580	6.589	6.009
0.003	0.157	-1.742	1.221	4.577	3.356
0.963	0.981	-5.714	0.020	2.742	2.722
0.294	0.491	-5.195	0.338	3.002	2.664
0.159	0.347	-4.778	-0.499	5.123	5.622
0.613	0.758	-5.594	0.140	4.016	3.876
0.339	0.535	-5.283	0.288	3.648	3.360
0.050	0.208	-3.913	1.071	8.045	6.974
0.621	0.764	-5.599	0.149	4.814	4.665
0.013	0.160	-2.828	0.751	5.866	5.115
0.608	0.755	-5.590	0.161	2.976	2.815
0.135	0.319	-4.663	-0.462	3.296	3.758
0.190	0.383	-4.904	0.325	5.540	5.215
0.007	0.157	-2.379	-0.633	4.465	5.098
0.056	0.216	-3.992	0.490	2.685	2.195
0.277	0.475	-5.156	0.241	4.469	4.229
0.698	0.820	-5.644	-0.115	3.643	3.758
0.055	0.215	-3.980	-0.773	2.528	3.301
0.137	0.320	-4.670	0.397	3.621	3.224
0.794	0.884	-5.682	0.065	5.160	5.095
0.502	0.672	-5.502	-0.186	3.031	3.216
0.975	0.987	-5.714	-0.008	3.401	3.409
0.083	0.255	-4.304	0.496	7.844	7.347
0.082	0.254	-4.294	0.470	5.852	5.382
0.853	0.921	-5.699	0.054	4.192	4.138
0.074	0.242	-4.215	0.648	4.574	3.927
0.350	0.545	-5.302	0.259	5.262	5.002
0.324	0.520	-5.255	0.403	5.882	5.479
0.112	0.291	-4.526	-0.606	2.378	2.984
0.277	0.475	-5.156	0.365	8.102	7.738
0.023	0.172	-3.286	0.569	2.863	2.294
0.415	0.602	-5.400	0.231	3.252	3.021
0.329	0.524	-5.263	0.215	4.899	4.684
0.041	0.196	-3.764	-0.674	9.971	10.645
0.043	0.199	-3.792	-0.664	10.064	10.729
0.053	0.213	-3.956	-0.673	9.508	10.181

0.064	0.228	-4.103	-0.631	9.258	9.888
0.055	0.216	-3.983	-0.629	9.728	10.356
0.062	0.224	-4.074	-0.605	9.437	10.042
0.076	0.245	-4.231	-0.614	9.323	9.937
0.084	0.256	-4.310	-0.614	9.304	9.918
0.365	0.557	-5.327	-0.246	4.306	4.552
0.517	0.684	-5.516	-0.175	4.642	4.817
0.004	0.157	-1.949	-1.259	7.594	8.853
0.347	0.543	-5.297	0.282	7.840	7.558
0.277	0.475	-5.156	-0.304	9.149	9.453
0.247	0.444	-5.081	-0.476	2.359	2.835
0.826	0.902	-5.692	0.095	2.054	1.959
0.339	0.534	-5.282	-0.367	8.948	9.315
0.028	0.178	-3.453	1.078	3.696	2.618
0.132	0.315	-4.646	-0.497	4.373	4.870
0.086	0.258	-4.323	-0.427	2.081	2.509
0.609	0.756	-5.591	-0.131	4.710	4.842
0.449	0.631	-5.444	-0.170	3.637	3.807
0.514	0.681	-5.513	0.239	9.400	9.161
0.184	0.375	-4.882	-0.452	5.171	5.623
0.217	0.412	-4.996	0.687	4.848	4.161
0.023	0.173	-3.309	0.952	4.616	3.664
0.006	0.157	-2.143	1.683	5.728	4.045
0.016	0.162	-2.989	0.965	4.041	3.076
0.006	0.157	-2.145	1.779	8.875	7.096
0.447	0.629	-5.441	-0.501	3.598	4.100
0.094	0.270	-4.391	0.890	4.640	3.751
0.253	0.451	-5.098	0.310	4.035	3.725
0.050	0.208	-3.916	0.990	5.971	4.980
0.018	0.163	-3.103	1.201	5.469	4.268
0.110	0.289	-4.514	-0.417	1.925	2.342
0.009	0.157	-2.538	1.221	5.390	4.169
0.106	0.284	-4.482	-0.447	2.943	3.390
0.523	0.689	-5.522	-0.188	2.655	2.843
0.054	0.215	-3.978	-0.550	2.836	3.385
0.649	0.785	-5.617	-0.141	4.251	4.392
0.140	0.324	-4.687	-0.366	4.182	4.549
0.264	0.461	-5.124	-0.288	3.013	3.302
0.007	0.157	-2.305	1.029	4.983	3.954
0.015	0.160	-2.947	0.549	5.683	5.134
0.315	0.513	-5.238	0.347	4.683	4.336
0.323	0.519	-5.252	0.288	5.694	5.406
0.143	0.327	-4.701	0.413	2.989	2.577
0.010	0.157	-2.647	1.593	5.290	3.697
0.598	0.748	-5.583	0.114	6.106	5.992
0.042	0.198	-3.781	-0.647	4.190	4.837
0.181	0.372	-4.869	0.484	6.306	5.821
0.335	0.531	-5.276	0.271	4.155	3.884
0.304	0.502	-5.215	-0.350	3.693	4.043
0.907	0.950	-5.708	-0.031	5.984	6.014

0.063	0.226	-4.088	0.400	3.971	3.572
0.528	0.693	-5.526	0.137	4.284	4.147
0.061	0.224	-4.068	0.486	9.337	8.850
0.596	0.747	-5.582	0.119	4.302	4.183
0.951	0.975	-5.713	-0.022	4.111	4.133
0.976	0.988	-5.714	-0.011	4.113	4.124
0.977	0.989	-5.714	0.010	4.511	4.501
0.073	0.241	-4.205	0.445	5.703	5.258
0.222	0.419	-5.012	0.494	5.255	4.762
0.393	0.581	-5.370	0.345	3.894	3.549
0.295	0.492	-5.196	-0.309	6.733	7.041
0.739	0.848	-5.662	-0.082	3.363	3.445
0.861	0.926	-5.700	-0.106	5.015	5.121
0.322	0.519	-5.251	0.307	4.562	4.255
0.170	0.361	-4.828	-0.473	3.167	3.639
0.710	0.828	-5.649	-0.147	3.764	3.912
0.023	0.172	-3.280	1.164	8.907	7.743
0.603	0.752	-5.587	0.134	3.374	3.240
0.069	0.236	-4.164	0.581	5.708	5.127
0.016	0.162	-2.986	-0.716	3.866	4.582
0.261	0.459	-5.118	0.428	4.340	3.912
0.171	0.361	-4.830	0.481	7.171	6.690
0.475	0.653	-5.473	0.308	7.469	7.161
0.417	0.604	-5.403	0.258	4.650	4.392
0.338	0.533	-5.280	0.303	2.788	2.485
0.339	0.534	-5.283	0.342	3.460	3.118
0.422	0.608	-5.410	0.319	3.729	3.411
0.442	0.625	-5.435	0.307	4.226	3.919
0.712	0.829	-5.650	-0.125	3.315	3.440
0.693	0.816	-5.641	0.106	3.169	3.063
0.035	0.188	-3.641	-0.544	4.970	5.514
0.153	0.341	-4.754	-0.370	2.915	3.285
0.024	0.173	-3.317	0.509	6.514	6.006
0.008	0.157	-2.460	1.107	3.422	2.315
0.654	0.790	-5.620	-0.110	6.681	6.791
0.350	0.545	-5.301	-0.377	2.874	3.252
0.310	0.508	-5.228	-0.265	3.523	3.788
0.028	0.178	-3.458	0.561	4.015	3.454
0.283	0.481	-5.171	-0.269	2.362	2.631
0.304	0.502	-5.216	0.261	2.820	2.558
0.115	0.295	-4.546	0.482	3.490	3.009
0.809	0.893	-5.687	0.104	4.419	4.315
0.264	0.461	-5.124	0.603	5.157	4.554
0.147	0.332	-4.725	-0.531	5.973	6.503
0.937	0.966	-5.712	-0.029	3.874	3.903
0.290	0.488	-5.185	0.262	8.296	8.034
0.071	0.238	-4.177	0.586	3.115	2.529
0.235	0.432	-5.050	0.350	3.924	3.574
0.165	0.353	-4.803	-0.439	3.337	3.776
0.678	0.807	-5.633	0.139	3.734	3.595

0.966	0.983	-5.714	0.013	4.657	4.643
0.843	0.913	-5.696	-0.049	2.389	2.439
0.478	0.655	-5.476	-0.243	2.553	2.796
0.721	0.835	-5.654	-0.108	2.712	2.820
0.995	0.998	-5.715	-0.003	2.946	2.950
0.270	0.468	-5.139	-0.461	2.287	2.748
0.029	0.178	-3.490	0.716	2.835	2.120
0.063	0.226	-4.089	-0.620	2.001	2.621
0.127	0.308	-4.617	0.320	6.795	6.475
0.015	0.160	-2.955	-0.932	2.512	3.444
0.423	0.609	-5.411	0.245	2.559	2.314
0.481	0.658	-5.480	0.219	2.529	2.310
0.677	0.806	-5.633	-0.137	2.495	2.632
0.028	0.177	-3.440	-0.777	1.998	2.775
0.008	0.157	-2.464	-0.750	1.936	2.686
0.653	0.789	-5.619	0.126	4.698	4.573
0.740	0.849	-5.662	0.108	3.015	2.908
0.154	0.341	-4.757	-0.566	2.332	2.898
0.607	0.754	-5.589	0.247	5.090	4.842
0.238	0.434	-5.056	0.511	3.430	2.919
0.430	0.616	-5.421	-0.278	2.930	3.208
0.318	0.516	-5.244	-0.278	2.926	3.204
0.724	0.838	-5.656	-0.091	3.824	3.915
0.162	0.351	-4.793	-0.399	2.377	2.776
0.700	0.821	-5.645	0.144	2.798	2.654
0.358	0.552	-5.316	0.342	1.964	1.622
0.109	0.288	-4.502	-0.506	2.995	3.502
0.051	0.208	-3.923	-0.535	5.020	5.555
0.025	0.175	-3.356	0.676	6.503	5.827
0.107	0.285	-4.490	0.448	5.143	4.696
0.868	0.928	-5.702	-0.063	3.933	3.996
0.109	0.288	-4.506	0.434	4.904	4.470
0.867	0.928	-5.701	-0.046	2.193	2.239
0.715	0.831	-5.652	-0.075	7.333	7.408
0.130	0.312	-4.635	-0.363	5.090	5.452
0.240	0.437	-5.061	-0.350	2.587	2.937
0.113	0.292	-4.534	-0.437	2.580	3.017
0.044	0.201	-3.819	-0.606	3.321	3.927
0.231	0.428	-5.038	-0.415	3.653	4.068
0.035	0.187	-3.635	-0.556	4.046	4.602
0.822	0.899	-5.691	-0.071	1.507	1.578
0.114	0.293	-4.540	-0.436	3.726	4.162
0.825	0.901	-5.692	-0.050	1.758	1.808
0.008	0.157	-2.396	1.354	4.246	2.893
0.524	0.689	-5.523	-0.204	6.011	6.216
0.291	0.489	-5.187	-0.223	2.717	2.940
0.205	0.400	-4.955	-0.356	3.983	4.339
0.068	0.234	-4.149	-0.609	6.236	6.844
0.400	0.587	-5.379	-0.214	4.511	4.725
0.268	0.467	-5.136	-0.413	3.645	4.058

0.630	0.771	-5.605	-0.133	4.202	4.335
0.802	0.889	-5.685	0.070	3.115	3.045
0.383	0.572	-5.355	-0.261	1.585	1.846
0.449	0.630	-5.443	0.279	2.926	2.647
0.083	0.255	-4.302	0.576	4.832	4.255
0.453	0.634	-5.448	-0.348	2.167	2.515
0.258	0.456	-5.111	0.305	3.499	3.193
0.409	0.596	-5.392	-0.228	6.673	6.901
0.001	0.157	-0.593	1.187	2.771	1.584
0.090	0.265	-4.365	0.822	4.808	3.986
0.940	0.967	-5.712	-0.018	1.893	1.912
0.921	0.958	-5.710	-0.027	1.992	2.019
0.488	0.663	-5.487	-0.337	2.683	3.020
0.843	0.913	-5.696	-0.071	1.967	2.038
1.000	1.000	-5.715	0.000	2.548	2.547
0.893	0.941	-5.706	0.030	1.917	1.887
0.212	0.408	-4.981	0.421	4.465	4.044
0.297	0.493	-5.200	0.409	4.395	3.987
0.156	0.344	-4.766	-0.395	3.309	3.703
0.359	0.552	-5.316	-0.258	3.837	4.095
0.763	0.864	-5.672	0.106	5.318	5.212
0.099	0.276	-4.435	-0.344	4.866	5.210
0.120	0.300	-4.576	-0.491	2.172	2.664
0.056	0.217	-3.998	0.686	6.222	5.536
0.071	0.239	-4.184	0.440	7.145	6.705
0.293	0.491	-5.192	-0.238	5.625	5.863
0.447	0.629	-5.441	-0.178	3.024	3.202
0.178	0.368	-4.857	1.150	9.886	8.737
0.056	0.217	-3.998	-0.512	5.152	5.664
0.930	0.962	-5.711	-0.020	2.406	2.426
0.088	0.262	-4.346	0.592	2.670	2.078
0.255	0.453	-5.104	-0.319	4.080	4.399
0.896	0.943	-5.707	-0.029	6.395	6.424
0.276	0.474	-5.154	-0.268	4.014	4.282
0.074	0.241	-4.210	0.474	7.866	7.392
0.024	0.173	-3.324	0.577	4.621	4.044
0.279	0.477	-5.162	0.215	7.174	6.959
0.035	0.186	-3.620	-0.660	5.103	5.763
0.365	0.556	-5.326	0.244	5.017	4.774
0.562	0.721	-5.556	0.155	7.795	7.640
0.852	0.920	-5.698	0.044	2.992	2.948
0.257	0.455	-5.107	-0.235	5.486	5.721
0.602	0.751	-5.586	0.126	2.125	2.000
0.718	0.834	-5.653	0.100	7.157	7.057
0.169	0.360	-4.824	0.363	6.357	5.994
0.831	0.905	-5.693	0.061	3.112	3.051
0.901	0.946	-5.707	-0.053	2.547	2.599
0.322	0.519	-5.251	0.251	4.757	4.506
0.110	0.289	-4.513	-0.338	5.042	5.380
0.167	0.356	-4.812	0.274	6.985	6.711

0.019	0.165	-3.152	-1.086	3.688	4.774
0.014	0.160	-2.896	1.397	7.109	5.712
0.682	0.809	-5.635	0.084	5.761	5.677
0.017	0.162	-3.074	-0.594	6.020	6.614
0.532	0.695	-5.530	-0.141	4.094	4.235
0.223	0.419	-5.012	0.277	7.591	7.314
0.620	0.764	-5.599	0.207	4.072	3.865
0.973	0.987	-5.714	-0.014	5.079	5.093
0.722	0.836	-5.655	0.087	6.578	6.491
0.986	0.993	-5.715	0.006	6.128	6.123
0.015	0.160	-2.942	0.831	4.973	4.142
0.015	0.160	-2.947	0.845	4.198	3.353
0.040	0.194	-3.740	0.581	4.373	3.792
0.178	0.368	-4.858	0.515	2.863	2.347
0.307	0.504	-5.222	-0.290	4.751	5.041
0.472	0.650	-5.470	0.285	3.099	2.814
0.598	0.747	-5.583	-0.145	8.744	8.889
0.604	0.752	-5.587	0.151	3.346	3.195
0.648	0.784	-5.616	-0.124	8.212	8.336
0.651	0.787	-5.618	0.141	3.621	3.480
0.656	0.791	-5.621	0.130	7.507	7.377
0.662	0.795	-5.624	-0.120	9.351	9.471
0.735	0.846	-5.661	-0.090	5.950	6.040
0.744	0.851	-5.664	-0.087	8.848	8.935
0.757	0.859	-5.669	-0.085	7.482	7.568
0.797	0.886	-5.683	-0.083	5.715	5.798
0.832	0.906	-5.693	0.061	7.542	7.481
0.852	0.920	-5.698	0.053	8.600	8.547
0.882	0.936	-5.704	0.039	7.327	7.287
0.902	0.947	-5.708	-0.030	6.567	6.597
0.989	0.994	-5.715	-0.004	7.720	7.723
0.268	0.466	-5.135	-0.363	5.404	5.767
0.319	0.517	-5.245	0.255	5.623	5.368
0.405	0.593	-5.387	0.202	4.291	4.089
0.040	0.194	-3.733	-0.627	6.898	7.526
0.914	0.954	-5.709	-0.032	2.504	2.537
0.066	0.230	-4.122	0.491	7.980	7.489
0.010	0.157	-2.644	0.993	6.158	5.165
0.040	0.194	-3.739	1.030	5.636	4.606
0.050	0.208	-3.913	0.608	4.538	3.930
0.897	0.943	-5.707	0.027	4.418	4.390
0.468	0.647	-5.465	-0.176	3.657	3.833
0.845	0.915	-5.697	-0.062	4.967	5.029
0.090	0.264	-4.360	-0.490	3.073	3.563
0.094	0.271	-4.398	0.388	7.119	6.731
0.462	0.643	-5.459	0.223	3.402	3.179
0.141	0.324	-4.691	-0.444	5.786	6.230
0.159	0.348	-4.781	-0.333	2.721	3.055
0.353	0.548	-5.307	-0.335	4.336	4.672
0.138	0.322	-4.678	-0.373	5.017	5.390

0.498	0.670	-5.498	-0.198	2.528	2.726
0.163	0.352	-4.797	0.330	5.482	5.152
0.149	0.335	-4.734	0.401	3.936	3.535
0.692	0.816	-5.640	-0.111	7.174	7.285
0.162	0.351	-4.794	-0.478	1.613	2.091
0.320	0.517	-5.246	-0.254	1.537	1.792
0.145	0.329	-4.713	-0.444	2.921	3.365
0.776	0.871	-5.676	-0.079	2.590	2.669
0.447	0.629	-5.442	0.141	4.380	4.239
0.228	0.425	-5.028	0.268	5.923	5.655
0.877	0.933	-5.703	-0.059	2.318	2.377
0.024	0.173	-3.332	0.583	3.418	2.835
0.005	0.157	-1.998	1.071	3.608	2.537
0.955	0.977	-5.713	0.020	3.310	3.290
0.011	0.157	-2.681	-0.636	6.511	7.148
0.587	0.740	-5.575	0.124	4.535	4.412
0.061	0.224	-4.070	0.454	8.314	7.859
0.033	0.184	-3.587	-0.581	2.427	3.008
0.076	0.245	-4.231	-0.717	3.630	4.347
0.804	0.890	-5.686	0.068	1.876	1.808
0.010	0.157	-2.656	-1.383	4.469	5.852
0.112	0.291	-4.526	0.456	5.401	4.945
0.382	0.571	-5.354	0.266	2.765	2.499
0.893	0.941	-5.706	-0.034	3.672	3.706
0.429	0.615	-5.419	-0.164	4.427	4.591
0.025	0.175	-3.369	0.829	4.608	3.778
0.182	0.373	-4.873	0.444	5.612	5.169
0.136	0.319	-4.665	-0.633	2.815	3.448
0.194	0.387	-4.917	-0.432	4.170	4.602
0.029	0.178	-3.489	-0.442	8.365	8.807
0.376	0.566	-5.344	-0.414	3.956	4.370
0.377	0.567	-5.346	-0.206	3.928	4.134
0.302	0.500	-5.212	-0.343	2.808	3.151
0.172	0.362	-4.835	-0.361	1.894	2.255
0.859	0.925	-5.700	0.064	3.257	3.193
0.557	0.716	-5.551	0.171	4.062	3.891
0.371	0.562	-5.337	-0.307	5.918	6.224
0.084	0.256	-4.313	-0.448	4.626	5.074
0.021	0.168	-3.212	-0.693	7.324	8.016
0.435	0.618	-5.426	-0.324	4.116	4.440
0.049	0.207	-3.892	0.916	3.579	2.663
0.232	0.428	-5.039	0.327	6.504	6.177
0.008	0.157	-2.412	1.517	4.959	3.442
0.036	0.188	-3.650	-0.615	7.090	7.705
0.172	0.362	-4.835	-0.520	2.771	3.291
0.056	0.218	-4.005	0.602	5.519	4.917
0.006	0.157	-2.159	1.631	8.214	6.582
0.044	0.200	-3.809	0.840	3.128	2.288
0.261	0.459	-5.118	0.233	7.175	6.941
0.532	0.696	-5.530	0.177	3.735	3.558

0.188	0.380	-4.896	-0.349	2.053	2.402
0.904	0.948	-5.708	0.038	2.515	2.477
0.936	0.966	-5.712	0.019	4.231	4.212
0.212	0.408	-4.980	0.295	8.149	7.855
0.010	0.157	-2.599	-0.906	3.426	4.331
0.971	0.985	-5.714	0.010	3.884	3.874
0.679	0.808	-5.634	0.156	3.599	3.444
0.020	0.167	-3.175	0.993	6.627	5.634
0.006	0.157	-2.180	-0.848	6.462	7.310
0.079	0.249	-4.265	0.429	4.278	3.849
0.066	0.231	-4.129	0.583	3.357	2.774
0.034	0.184	-3.602	0.804	5.767	4.963
0.793	0.883	-5.682	-0.069	1.861	1.929
0.031	0.181	-3.537	1.256	7.177	5.920
0.035	0.186	-3.622	0.942	5.754	4.812
0.328	0.524	-5.263	-0.280	5.123	5.402
0.873	0.931	-5.703	0.048	7.030	6.983
0.026	0.175	-3.383	-0.686	4.329	5.015
0.461	0.642	-5.458	-0.181	6.959	7.140
0.004	0.157	-1.914	1.232	6.864	5.632
0.032	0.182	-3.560	-0.639	4.545	5.184
0.128	0.309	-4.621	0.376	5.260	4.884
0.943	0.970	-5.712	0.019	3.267	3.247
0.049	0.207	-3.898	-0.962	2.580	3.543
0.008	0.157	-2.472	0.653	6.195	5.542
0.152	0.339	-4.747	0.526	2.750	2.223
0.560	0.719	-5.554	-0.189	4.009	4.197
0.015	0.160	-2.924	0.771	3.125	2.354
0.937	0.966	-5.712	-0.016	5.140	5.156
0.573	0.728	-5.564	0.132	6.107	5.975
0.219	0.414	-5.001	0.340	5.021	4.681
0.080	0.250	-4.271	0.530	3.879	3.349
0.054	0.214	-3.966	1.166	6.395	5.229
0.070	0.236	-4.167	0.854	8.174	7.320
0.321	0.518	-5.250	0.378	5.696	5.318
0.078	0.247	-4.251	1.018	6.032	5.014
0.361	0.553	-5.320	0.352	3.136	2.784
0.214	0.409	-4.987	0.368	6.599	6.231
0.004	0.157	-1.920	0.919	5.777	4.859
0.020	0.166	-3.163	0.942	4.868	3.926
0.027	0.177	-3.438	0.825	5.210	4.385
0.010	0.157	-2.610	1.376	7.240	5.864
0.141	0.324	-4.691	0.826	5.221	4.395
0.039	0.193	-3.721	1.128	5.692	4.563
0.022	0.171	-3.271	1.843	7.948	6.105
0.387	0.575	-5.361	0.312	4.664	4.352
0.393	0.581	-5.370	-0.288	3.203	3.492
0.475	0.653	-5.473	0.245	7.604	7.359
0.024	0.173	-3.335	0.818	7.893	7.075
0.509	0.677	-5.508	0.216	2.870	2.653

0.323	0.520	-5.254	-0.248	2.280	2.528
0.674	0.804	-5.631	-0.181	3.715	3.896
0.035	0.186	-3.629	0.924	5.228	4.304
0.901	0.946	-5.707	0.040	2.916	2.876
0.013	0.160	-2.805	1.267	4.745	3.479
0.042	0.197	-3.773	1.092	8.209	7.117
0.029	0.178	-3.482	0.747	4.405	3.658
0.459	0.639	-5.455	0.227	3.665	3.437
0.005	0.157	-2.051	1.915	6.644	4.729
0.003	0.157	-1.734	1.085	3.658	2.573
0.014	0.160	-2.889	1.356	6.305	4.950
0.026	0.176	-3.399	1.319	7.129	5.810
0.133	0.316	-4.650	-0.716	5.562	6.278
0.299	0.496	-5.205	0.307	5.154	4.847
0.038	0.192	-3.700	0.903	5.676	4.773
0.186	0.378	-4.889	0.480	6.518	6.038
0.041	0.195	-3.761	1.139	6.054	4.915
0.020	0.167	-3.191	1.417	6.633	5.217
0.013	0.160	-2.837	-0.858	7.469	8.327
0.455	0.636	-5.450	0.193	3.889	3.696
0.626	0.768	-5.602	0.117	2.704	2.587
0.540	0.702	-5.537	-0.321	4.646	4.967
0.003	0.157	-1.656	-0.959	5.733	6.692
0.034	0.185	-3.606	-0.832	3.930	4.762
0.020	0.167	-3.185	1.124	7.817	6.693
0.010	0.157	-2.599	1.602	6.220	4.618
0.475	0.653	-5.474	0.197	2.855	2.658
0.046	0.203	-3.850	1.460	6.741	5.281
0.075	0.244	-4.223	0.522	3.343	2.821
0.026	0.175	-3.386	0.701	7.253	6.553
0.335	0.531	-5.275	0.259	4.134	3.875
0.894	0.942	-5.706	0.039	3.261	3.222
0.247	0.445	-5.083	-0.305	1.702	2.006
0.012	0.160	-2.786	0.657	3.156	2.500
0.034	0.184	-3.596	0.653	4.360	3.707
0.710	0.828	-5.649	0.153	5.234	5.081
0.265	0.463	-5.127	0.290	3.485	3.195
0.050	0.208	-3.913	-0.505	2.988	3.492
0.126	0.307	-4.611	0.569	4.437	3.868
0.010	0.157	-2.600	1.023	7.388	6.365
0.238	0.435	-5.056	-0.319	2.888	3.207
0.362	0.554	-5.322	0.220	4.120	3.900
0.740	0.849	-5.663	-0.120	6.783	6.903
0.012	0.160	-2.751	1.228	6.369	5.142
0.076	0.245	-4.238	-0.606	6.100	6.706
0.159	0.348	-4.781	0.374	3.412	3.038
0.703	0.823	-5.646	0.099	3.024	2.925
0.668	0.800	-5.628	-0.140	2.484	2.624
0.069	0.236	-4.164	-0.826	3.125	3.951
0.029	0.178	-3.475	1.002	6.041	5.038

0.035	0.186	-3.620	1.344	5.810	4.467
0.008	0.157	-2.463	1.178	7.860	6.682
0.004	0.157	-1.791	1.886	6.489	4.603
0.026	0.175	-3.385	1.083	4.345	3.262
0.008	0.157	-2.436	1.705	5.902	4.197
0.013	0.160	-2.850	1.484	5.878	4.395
0.021	0.168	-3.212	1.299	6.349	5.050
0.010	0.157	-2.658	1.034	5.659	4.624
0.005	0.157	-2.130	2.217	6.180	3.963
0.021	0.168	-3.221	0.799	6.264	5.466
0.042	0.196	-3.767	-0.488	3.725	4.213
0.088	0.261	-4.342	-0.402	6.526	6.928
0.015	0.160	-2.943	1.479	6.949	5.470
0.306	0.504	-5.220	-0.237	7.092	7.329
0.868	0.928	-5.702	0.044	4.344	4.300
0.712	0.828	-5.650	-0.107	4.278	4.385
0.731	0.843	-5.659	-0.386	4.687	5.072
0.086	0.258	-4.324	-0.500	5.326	5.826
0.403	0.590	-5.383	0.352	5.084	4.731
0.006	0.157	-2.245	-1.130	3.517	4.647
0.080	0.251	-4.275	1.196	5.578	4.382
0.142	0.326	-4.699	0.555	5.784	5.229
0.015	0.161	-2.965	1.063	7.088	6.025
0.038	0.192	-3.691	0.716	6.764	6.048
0.050	0.208	-3.909	-0.472	4.264	4.736
0.311	0.508	-5.229	0.358	4.185	3.827
0.011	0.157	-2.688	-0.600	3.775	4.375
0.709	0.827	-5.649	-0.086	4.678	4.765
0.021	0.169	-3.238	1.102	6.174	5.072
0.049	0.207	-3.902	-0.513	6.294	6.807
0.595	0.746	-5.581	0.102	8.207	8.105
0.036	0.188	-3.644	0.884	8.618	7.735
0.350	0.545	-5.302	0.236	6.373	6.136
0.008	0.157	-2.477	-0.764	3.733	4.497
0.088	0.262	-4.344	0.545	5.903	5.358
0.131	0.313	-4.639	0.398	4.357	3.959
0.311	0.508	-5.230	-0.276	4.012	4.288
0.478	0.655	-5.476	-0.231	4.370	4.601
0.030	0.179	-3.505	1.293	7.827	6.534
0.012	0.160	-2.754	0.716	9.059	8.343
0.499	0.670	-5.498	-0.204	5.880	6.084
0.009	0.157	-2.538	1.763	6.420	4.657
0.107	0.285	-4.490	0.594	5.643	5.049
0.556	0.716	-5.550	0.128	8.268	8.140
0.058	0.219	-4.023	0.754	6.404	5.649
0.010	0.157	-2.581	-0.817	6.608	7.425
0.124	0.305	-4.602	0.500	7.358	6.858
0.887	0.938	-5.705	-0.037	4.266	4.303
0.037	0.190	-3.669	0.550	4.673	4.123
0.074	0.241	-4.210	0.534	5.897	5.363

0.031	0.181	-3.529	0.755	7.356	6.601
0.599	0.749	-5.584	0.096	7.064	6.968
0.138	0.322	-4.677	0.422	3.283	2.861
0.029	0.178	-3.476	0.702	3.618	2.916
0.245	0.442	-5.075	0.401	6.216	5.815
0.019	0.164	-3.144	1.527	8.233	6.706
0.082	0.254	-4.293	0.436	7.067	6.631
0.042	0.198	-3.782	-0.593	7.666	8.259
0.212	0.407	-4.978	-0.348	3.110	3.458
0.045	0.201	-3.826	0.948	5.846	4.899
0.068	0.235	-4.154	0.459	4.924	4.465
0.037	0.190	-3.671	0.555	2.478	1.923
0.014	0.160	-2.900	-1.016	3.715	4.731
0.007	0.157	-2.379	0.740	4.595	3.855
0.001	0.157	-0.841	-0.962	5.292	6.254
0.033	0.184	-3.594	1.095	6.758	5.664
0.005	0.157	-1.993	-1.044	6.163	7.207
0.106	0.285	-4.487	0.520	3.678	3.159
0.166	0.356	-4.811	-0.497	3.418	3.915
0.018	0.163	-3.102	-0.775	6.895	7.670
0.257	0.455	-5.108	0.284	8.122	7.837
0.300	0.498	-5.208	0.320	5.981	5.661
0.072	0.239	-4.187	0.491	8.834	8.343
0.082	0.254	-4.292	0.427	7.762	7.335
0.050	0.208	-3.915	0.734	7.958	7.223
0.568	0.724	-5.561	-0.166	3.122	3.287
0.007	0.157	-2.379	0.740	3.545	2.804
0.017	0.162	-3.052	1.085	11.027	9.942
0.070	0.236	-4.166	-0.391	8.655	9.046
0.275	0.473	-5.151	0.207	9.416	9.209
0.188	0.380	-4.895	-0.446	3.558	4.004
0.044	0.200	-3.805	0.523	9.346	8.823
0.066	0.230	-4.124	0.657	4.802	4.146
0.993	0.996	-5.715	0.002	1.999	1.996
0.493	0.667	-5.493	-0.192	5.747	5.939
0.182	0.374	-4.876	0.409	6.920	6.511
0.010	0.157	-2.636	1.135	7.036	5.901
0.842	0.912	-5.696	0.055	2.598	2.543
0.098	0.274	-4.425	-0.499	2.854	3.353
0.124	0.305	-4.600	0.465	6.615	6.151
0.267	0.465	-5.132	0.274	5.229	4.955
0.029	0.178	-3.470	0.507	7.254	6.747
0.024	0.173	-3.328	-0.759	3.860	4.619
0.914	0.954	-5.709	-0.046	5.048	5.094
0.023	0.173	-3.311	-0.550	5.332	5.882
0.778	0.872	-5.677	-0.091	2.718	2.810
0.590	0.743	-5.577	-0.112	6.457	6.569
0.164	0.353	-4.799	-0.500	4.556	5.056
0.011	0.157	-2.663	0.820	4.049	3.229
0.445	0.627	-5.438	0.212	7.053	6.840

0.016	0.162	-3.023	0.660	8.607	7.947
0.889	0.939	-5.705	0.044	3.407	3.364
0.018	0.164	-3.119	0.736	5.279	4.543
0.871	0.930	-5.702	0.086	3.604	3.517
0.103	0.281	-4.463	0.359	6.753	6.394
0.347	0.542	-5.297	-0.246	2.837	3.082
0.171	0.361	-4.829	0.317	5.574	5.257
0.191	0.383	-4.907	0.310	5.826	5.516
0.744	0.851	-5.664	0.070	7.692	7.622
0.013	0.160	-2.856	0.950	5.259	4.309
0.060	0.221	-4.049	-0.605	5.966	6.571
0.902	0.947	-5.708	-0.023	5.153	5.175
0.024	0.173	-3.314	0.846	4.584	3.738
0.685	0.811	-5.637	-0.163	3.135	3.298
0.044	0.201	-3.818	0.541	3.228	2.687
0.828	0.903	-5.692	0.063	3.536	3.473
0.012	0.160	-2.772	-1.539	7.037	8.576
0.003	0.157	-1.713	1.506	6.535	5.028
0.061	0.223	-4.063	-0.535	2.663	3.198
0.715	0.831	-5.652	-0.137	2.568	2.705
0.181	0.373	-4.872	0.462	5.503	5.040
0.155	0.342	-4.759	0.558	2.514	1.956
0.600	0.749	-5.584	0.121	4.473	4.352
0.024	0.173	-3.331	0.533	7.418	6.884
0.088	0.261	-4.341	0.371	6.701	6.330
0.011	0.158	-2.708	0.587	5.812	5.225
0.169	0.360	-4.824	0.272	6.488	6.217
0.906	0.949	-5.708	-0.037	2.092	2.128
0.105	0.283	-4.476	0.460	4.227	3.767
0.449	0.631	-5.444	0.260	2.361	2.101
0.761	0.862	-5.671	0.107	3.316	3.209
0.033	0.184	-3.587	1.235	6.964	5.729
0.286	0.484	-5.178	0.341	1.961	1.620
0.203	0.398	-4.951	-0.314	3.408	3.721
0.464	0.644	-5.461	-0.222	3.078	3.301
0.007	0.157	-2.332	0.869	4.433	3.564
0.490	0.664	-5.489	0.181	2.482	2.301
0.004	0.157	-1.909	-0.593	3.217	3.809
0.133	0.316	-4.652	0.551	6.652	6.100
0.728	0.840	-5.657	-0.115	2.221	2.336
0.054	0.215	-3.976	1.170	7.062	5.892
0.174	0.364	-4.843	0.336	3.877	3.541
0.392	0.580	-5.368	-0.185	5.165	5.350
0.078	0.247	-4.252	-0.526	1.739	2.265
0.042	0.197	-3.771	-0.851	2.139	2.990
0.092	0.267	-4.378	-0.678	5.420	6.099
0.267	0.465	-5.133	-0.437	3.277	3.714
0.369	0.559	-5.333	-0.227	3.744	3.971
0.027	0.176	-3.415	1.012	4.810	3.798
0.385	0.574	-5.358	-0.177	4.906	5.083

0.311	0.508	-5.230	0.331	3.246	2.915
0.118	0.298	-4.566	0.368	5.493	5.125
0.605	0.753	-5.588	0.130	2.724	2.594
0.019	0.164	-3.143	-0.569	7.208	7.777
0.076	0.245	-4.234	-0.471	8.196	8.667
0.564	0.721	-5.557	0.423	7.942	7.519
0.053	0.213	-3.958	0.462	6.587	6.124
0.811	0.894	-5.688	0.051	6.903	6.852
0.146	0.330	-4.718	0.374	3.600	3.226
0.842	0.912	-5.696	-0.051	5.608	5.659
0.511	0.679	-5.510	-0.324	8.099	8.423
0.045	0.202	-3.829	0.566	6.297	5.731
0.058	0.219	-4.028	-0.524	4.189	4.712
0.146	0.330	-4.719	-0.350	4.534	4.884
0.080	0.250	-4.268	-0.393	4.397	4.790
0.047	0.203	-3.855	-0.582	3.414	3.997
0.939	0.967	-5.712	0.021	2.163	2.142
0.024	0.173	-3.328	-0.546	4.567	5.114
0.783	0.876	-5.679	0.077	2.461	2.384
0.896	0.943	-5.707	0.051	2.898	2.846
0.083	0.254	-4.297	0.371	5.348	4.976
0.350	0.545	-5.302	0.234	3.501	3.267
0.124	0.305	-4.602	-0.565	5.428	5.993
0.322	0.519	-5.251	0.234	6.358	6.124
0.076	0.245	-4.237	-0.648	1.900	2.547
0.003	0.157	-1.667	1.434	6.467	5.033
0.049	0.206	-3.889	-1.035	3.316	4.351
0.306	0.503	-5.219	0.286	5.158	4.872
0.672	0.802	-5.630	0.138	5.770	5.633
0.528	0.693	-5.526	0.155	2.009	1.855
0.142	0.326	-4.696	-0.399	5.165	5.564
0.246	0.443	-5.079	-0.326	1.807	2.133
0.006	0.157	-2.132	-1.203	3.417	4.620
0.862	0.926	-5.700	-0.037	6.999	7.036
0.023	0.173	-3.312	-0.632	6.291	6.924
0.046	0.203	-3.844	-0.619	3.160	3.779
0.721	0.835	-5.654	0.088	4.750	4.662
0.133	0.316	-4.652	-0.376	2.908	3.285
0.085	0.257	-4.319	-0.420	5.233	5.653
0.005	0.157	-2.019	0.878	3.561	2.683
0.024	0.173	-3.317	-0.924	5.362	6.287
0.478	0.655	-5.477	-0.226	4.032	4.258
0.676	0.806	-5.632	0.129	2.853	2.724
0.041	0.195	-3.758	0.618	7.547	6.929
0.215	0.409	-4.988	0.423	8.287	7.864
0.681	0.809	-5.635	0.149	5.543	5.394
0.281	0.479	-5.165	0.296	6.540	6.244
0.885	0.937	-5.705	-0.041	5.981	6.022
0.005	0.157	-2.074	-1.677	4.615	6.292
0.496	0.668	-5.495	-0.296	2.728	3.024

0.024	0.174	-3.344	0.564	3.893	3.329
0.207	0.402	-4.961	-0.324	3.416	3.740
0.032	0.182	-3.553	0.790	5.369	4.579
0.140	0.324	-4.689	0.479	4.202	3.723
0.050	0.207	-3.905	-0.456	5.604	6.060
0.017	0.162	-3.060	1.720	7.002	5.283
0.269	0.468	-5.138	-0.335	2.416	2.751
0.017	0.162	-3.058	-0.669	2.772	3.441
0.124	0.305	-4.600	0.531	3.716	3.185
0.824	0.900	-5.691	0.048	5.249	5.200
0.073	0.241	-4.206	-0.513	6.933	7.446
0.255	0.452	-5.102	-0.414	2.283	2.697
0.122	0.304	-4.590	-0.501	2.410	2.912
0.187	0.379	-4.892	-0.627	2.750	3.377
0.442	0.625	-5.435	-0.312	1.901	2.213
0.199	0.394	-4.938	-0.446	2.035	2.482
0.165	0.354	-4.804	-0.409	3.177	3.586
0.109	0.288	-4.503	-0.393	4.333	4.725
0.047	0.204	-3.868	-0.705	2.220	2.925
0.117	0.297	-4.557	0.616	6.398	5.783
0.792	0.882	-5.682	0.079	5.054	4.975
0.384	0.572	-5.356	-0.225	2.462	2.687
0.970	0.985	-5.714	-0.010	3.973	3.983
0.016	0.162	-2.992	0.887	5.118	4.232
0.041	0.195	-3.755	-0.722	2.802	3.524
0.027	0.176	-3.413	0.853	5.587	4.734
0.099	0.276	-4.434	0.875	6.670	5.795
0.098	0.275	-4.429	0.401	5.898	5.497
0.175	0.365	-4.848	-0.398	3.296	3.694
0.539	0.701	-5.536	-0.233	2.847	3.080
0.049	0.207	-3.896	0.579	4.541	3.962
0.520	0.687	-5.519	0.165	3.982	3.817
0.963	0.982	-5.714	-0.022	2.769	2.791
0.610	0.756	-5.592	0.163	2.181	2.018
0.209	0.404	-4.968	-0.283	3.839	4.122
0.175	0.365	-4.847	0.321	7.072	6.750
0.698	0.820	-5.644	0.110	2.744	2.634
0.241	0.438	-5.064	-0.311	2.495	2.806
0.151	0.338	-4.742	-0.405	2.227	2.631
0.108	0.287	-4.499	-0.575	4.299	4.874
0.106	0.284	-4.485	-0.861	1.015	1.876
0.002	0.157	-1.370	1.013	2.521	1.508
0.719	0.834	-5.653	0.236	2.780	2.543
0.322	0.519	-5.252	-0.269	2.643	2.912
0.082	0.254	-4.293	-0.625	2.969	3.594
0.439	0.623	-5.431	-0.279	2.850	3.129
0.810	0.893	-5.687	0.105	2.796	2.691
0.373	0.563	-5.339	-0.279	2.358	2.636
0.025	0.175	-3.353	1.037	5.999	4.962
0.003	0.157	-1.694	0.891	2.617	1.726

0.959	0.979	-5.714	0.015	2.019	2.004
0.193	0.386	-4.915	0.475	2.603	2.128
0.618	0.762	-5.597	0.126	5.881	5.755
0.072	0.239	-4.190	-0.446	3.067	3.513
0.602	0.751	-5.586	-0.141	4.028	4.169
0.619	0.763	-5.598	-0.141	3.969	4.110
0.015	0.160	-2.943	0.694	3.142	2.447
0.891	0.941	-5.706	0.060	3.755	3.695
0.705	0.825	-5.647	-0.108	3.051	3.158
0.366	0.558	-5.329	-0.246	2.230	2.476
0.941	0.968	-5.712	0.050	3.496	3.446
0.997	0.999	-5.715	-0.001	3.235	3.236
0.034	0.184	-3.602	1.661	5.156	3.495
0.367	0.558	-5.330	0.242	3.637	3.395
0.436	0.620	-5.428	-0.204	2.409	2.612
0.627	0.769	-5.603	0.136	3.167	3.031
0.041	0.196	-3.764	0.688	3.618	2.931
0.071	0.239	-4.183	0.799	5.791	4.992
0.048	0.205	-3.881	1.082	4.872	3.790
0.285	0.483	-5.175	0.421	4.957	4.536
0.094	0.270	-4.392	-0.864	3.885	4.748
0.032	0.182	-3.557	-1.357	2.702	4.058
0.269	0.467	-5.136	0.453	3.242	2.789
0.017	0.162	-3.066	0.929	3.755	2.826
0.044	0.201	-3.816	1.224	6.358	5.133
0.086	0.258	-4.327	0.337	6.170	5.834
0.755	0.858	-5.669	0.109	6.362	6.253
0.366	0.557	-5.328	-0.346	1.898	2.244
0.098	0.274	-4.423	-0.997	2.941	3.938
0.178	0.368	-4.857	0.300	7.792	7.492
0.290	0.488	-5.184	0.287	3.110	2.823
0.998	0.999	-5.715	-0.001	4.053	4.053
0.965	0.982	-5.714	0.012	3.441	3.429
0.273	0.471	-5.147	0.247	5.535	5.288
0.495	0.667	-5.494	0.186	1.936	1.750
0.005	0.157	-2.031	-0.757	2.005	2.762
0.162	0.351	-4.791	-0.367	4.120	4.486
0.073	0.241	-4.205	-0.897	2.639	3.536
0.293	0.491	-5.192	0.396	4.285	3.889
0.364	0.556	-5.325	-0.316	3.272	3.588
0.136	0.319	-4.664	-0.541	2.221	2.762
0.242	0.440	-5.069	-0.310	2.084	2.394
0.383	0.572	-5.355	0.182	4.588	4.405
0.246	0.443	-5.078	-0.311	4.903	5.215
0.101	0.278	-4.447	0.456	4.179	3.723
0.500	0.671	-5.499	-0.185	1.872	2.057
0.567	0.723	-5.559	-0.124	7.067	7.191
0.622	0.765	-5.600	0.123	4.523	4.399
0.010	0.157	-2.595	1.499	5.785	4.286
0.245	0.442	-5.076	0.345	3.388	3.042

0.083	0.254	-4.297	-0.623	2.355	2.978
0.004	0.157	-1.857	-1.227	1.982	3.208
0.010	0.157	-2.620	1.000	5.057	4.058
0.057	0.218	-4.008	0.502	5.608	5.106
0.682	0.809	-5.635	0.119	2.691	2.572
0.282	0.480	-5.167	-0.213	5.432	5.646
0.495	0.667	-5.494	0.185	4.726	4.540
0.446	0.629	-5.440	-0.214	3.606	3.820
0.216	0.411	-4.992	-0.416	4.835	5.251
0.004	0.157	-1.772	0.803	4.701	3.898
0.433	0.618	-5.424	0.305	5.688	5.383
0.229	0.427	-5.033	-0.477	4.359	4.837
0.519	0.686	-5.518	-0.193	4.527	4.720
0.028	0.178	-3.454	-0.688	4.441	5.129
0.058	0.219	-4.027	-0.549	6.618	7.167
0.060	0.222	-4.056	-0.503	6.396	6.899
0.066	0.230	-4.124	-0.502	6.472	6.974
0.032	0.182	-3.556	-0.563	6.602	7.165
0.809	0.893	-5.687	0.080	4.747	4.667
0.060	0.222	-4.052	1.205	4.705	3.500
0.046	0.203	-3.837	0.594	3.643	3.049
0.149	0.335	-4.734	-0.422	2.280	2.702
0.667	0.798	-5.627	0.130	2.594	2.464
0.095	0.271	-4.404	-0.678	3.952	4.629
0.033	0.184	-3.579	-0.569	6.316	6.885
0.918	0.956	-5.710	0.030	3.623	3.593
0.001	0.157	-0.717	-1.872	1.966	3.838
0.523	0.689	-5.522	-0.208	5.286	5.494
0.821	0.899	-5.691	-0.069	4.720	4.789
0.015	0.160	-2.941	-0.966	5.546	6.512
0.046	0.203	-3.838	1.082	5.929	4.846
0.063	0.226	-4.089	0.615	4.125	3.510
0.031	0.181	-3.535	1.271	7.109	5.837
0.663	0.795	-5.625	0.123	3.195	3.072
0.017	0.162	-3.075	0.741	4.626	3.886
0.012	0.160	-2.781	-0.883	2.051	2.934
0.393	0.581	-5.370	0.255	3.141	2.887
0.118	0.298	-4.565	-0.449	3.068	3.517
0.441	0.625	-5.434	-0.272	2.663	2.935
0.201	0.396	-4.942	-0.270	4.720	4.990
0.824	0.900	-5.691	0.076	4.141	4.065
0.161	0.350	-4.788	-0.447	2.093	2.540
0.018	0.163	-3.097	1.011	3.826	2.815
0.061	0.224	-4.068	-0.595	2.129	2.724
0.092	0.267	-4.375	0.568	4.065	3.497
0.032	0.182	-3.556	0.825	3.051	2.226
0.111	0.290	-4.521	0.573	4.964	4.391
0.922	0.958	-5.710	-0.028	2.151	2.179
0.023	0.173	-3.294	0.611	2.538	1.927
0.362	0.554	-5.322	-0.389	3.414	3.804

0.044	0.201	-3.819	0.728	2.492	1.765
0.114	0.293	-4.537	-0.470	3.261	3.731
0.011	0.158	-2.713	-1.449	3.220	4.670
0.283	0.481	-5.170	0.303	3.627	3.324
0.830	0.904	-5.693	0.095	4.758	4.663
0.293	0.491	-5.193	-0.288	5.108	5.396
0.987	0.993	-5.715	-0.006	2.340	2.345
0.222	0.419	-5.011	-1.026	1.056	2.082
0.468	0.647	-5.466	-0.281	3.582	3.862
0.833	0.907	-5.694	0.062	6.022	5.960
0.122	0.303	-4.588	0.499	5.659	5.160
0.037	0.189	-3.667	0.822	2.875	2.053
0.011	0.157	-2.699	1.771	4.817	3.045
0.130	0.311	-4.634	0.473	4.686	4.213
0.067	0.231	-4.132	1.008	6.263	5.255
0.114	0.293	-4.539	-0.869	1.666	2.535
0.052	0.212	-3.945	-0.646	6.579	7.225
0.034	0.185	-3.611	-1.048	4.736	5.784
0.323	0.519	-5.253	-0.488	2.329	2.817
0.046	0.203	-3.838	-0.770	5.772	6.543
0.387	0.575	-5.361	-0.307	6.784	7.091
0.034	0.184	-3.602	0.605	2.840	2.235
0.076	0.245	-4.237	-0.627	5.922	6.550
0.180	0.371	-4.865	-0.384	3.111	3.495
0.425	0.610	-5.413	-0.276	1.630	1.906
0.161	0.349	-4.787	0.292	7.468	7.176
0.021	0.168	-3.213	0.591	8.209	7.618
0.216	0.411	-4.992	-0.351	3.917	4.268
0.808	0.892	-5.687	0.051	5.679	5.628
0.147	0.331	-4.721	-0.354	3.945	4.299
0.263	0.461	-5.123	0.345	4.315	3.970
0.024	0.173	-3.332	0.823	6.226	5.403
0.005	0.157	-2.110	1.189	7.321	6.132
0.003	0.157	-1.653	1.109	5.920	4.811
0.072	0.239	-4.189	0.658	3.832	3.173
0.428	0.614	-5.417	-0.247	3.163	3.410
0.010	0.157	-2.641	1.018	8.051	7.033
0.025	0.175	-3.356	0.831	6.176	5.345
0.591	0.743	-5.578	0.162	2.454	2.292
0.048	0.205	-3.877	0.644	6.179	5.536
0.047	0.204	-3.863	1.045	6.144	5.098
0.020	0.167	-3.175	0.701	4.155	3.455
0.912	0.952	-5.709	-0.027	1.931	1.958
0.310	0.507	-5.227	-0.303	3.225	3.528
0.002	0.157	-1.117	1.300	3.187	1.887
0.094	0.270	-4.393	-0.453	3.686	4.139
0.961	0.980	-5.714	0.022	3.079	3.057
0.771	0.868	-5.675	-0.076	2.045	2.121
0.514	0.682	-5.513	-0.240	2.245	2.485
0.175	0.365	-4.848	-0.449	4.343	4.792

0.050	0.208	-3.918	0.635	2.991	2.356
0.550	0.711	-5.546	-0.160	3.388	3.548
0.202	0.397	-4.945	-0.491	1.816	2.307
0.656	0.791	-5.621	-0.096	2.901	2.997
0.365	0.557	-5.326	-0.269	3.483	3.752
0.097	0.274	-4.420	-0.482	2.096	2.578
0.589	0.742	-5.577	0.120	2.592	2.472
0.001	0.157	-0.824	1.132	3.257	2.125
0.760	0.862	-5.671	-0.076	5.412	5.488
0.574	0.728	-5.565	-0.183	3.861	4.044
0.058	0.219	-4.027	-0.381	1.366	1.747
0.056	0.218	-4.004	-0.894	2.657	3.551
0.591	0.744	-5.578	-0.269	3.267	3.536
0.094	0.270	-4.396	0.421	3.865	3.445
0.028	0.178	-3.450	-0.711	2.820	3.531
0.238	0.434	-5.056	0.297	5.157	4.860
0.968	0.984	-5.714	-0.011	2.826	2.837
0.093	0.270	-4.390	-0.454	2.347	2.801
0.006	0.157	-2.218	0.945	4.362	3.417
0.006	0.157	-2.202	0.908	3.499	2.591
0.159	0.348	-4.780	0.400	3.768	3.368
0.011	0.157	-2.704	0.719	3.657	2.937
0.099	0.276	-4.434	0.554	3.773	3.219
0.021	0.168	-3.223	0.702	3.200	2.498
0.227	0.424	-5.024	-0.301	2.248	2.550
0.180	0.371	-4.868	0.337	4.051	3.715
0.560	0.718	-5.554	-0.136	1.615	1.751
0.812	0.894	-5.688	-0.074	2.447	2.521
0.200	0.394	-4.938	-0.367	3.060	3.428
0.605	0.753	-5.588	-0.151	4.100	4.251
0.112	0.291	-4.526	0.604	3.914	3.310
0.125	0.306	-4.604	-0.508	2.400	2.908
0.956	0.978	-5.713	-0.016	3.885	3.901
0.397	0.585	-5.376	0.203	3.579	3.376
0.308	0.505	-5.224	-0.338	3.583	3.921
0.532	0.696	-5.530	-0.195	3.507	3.702
0.732	0.843	-5.659	0.111	2.895	2.784
0.155	0.342	-4.759	-0.547	3.114	3.661
0.063	0.227	-4.093	-0.632	4.291	4.923
0.360	0.553	-5.319	0.277	2.748	2.471
0.079	0.249	-4.261	0.503	4.293	3.790
0.421	0.607	-5.409	0.260	3.426	3.167
0.060	0.221	-4.047	-0.467	7.392	7.859
0.173	0.362	-4.838	0.407	3.508	3.101
0.556	0.715	-5.550	0.162	2.691	2.529
0.318	0.516	-5.243	0.282	4.996	4.714
0.819	0.897	-5.690	0.069	3.108	3.039
0.631	0.772	-5.606	-0.134	3.801	3.935
0.002	0.157	-1.289	0.952	2.880	1.928
0.162	0.351	-4.793	-0.426	2.006	2.432

0.001	0.157	-0.776	-0.914	3.090	4.005
0.770	0.868	-5.674	0.089	3.840	3.752
0.125	0.306	-4.606	-0.391	5.249	5.639
0.580	0.735	-5.570	0.142	2.066	1.924
0.030	0.179	-3.507	0.775	4.470	3.695
0.191	0.384	-4.909	-0.364	4.770	5.134
0.220	0.416	-5.004	-0.363	7.650	8.013
0.236	0.433	-5.052	-0.361	7.583	7.945
0.433	0.618	-5.424	-0.406	4.464	4.870
0.026	0.176	-3.407	-0.714	3.637	4.351
0.155	0.342	-4.761	-0.448	4.731	5.179
0.236	0.433	-5.051	-0.350	4.776	5.126
0.245	0.442	-5.076	-0.412	4.551	4.963
0.271	0.469	-5.142	-0.364	5.733	6.097
0.342	0.537	-5.287	-0.350	4.088	4.438
0.846	0.915	-5.697	-0.117	4.255	4.371
0.054	0.215	-3.973	0.519	3.050	2.531
0.164	0.353	-4.802	-0.488	3.646	4.134
0.282	0.480	-5.167	0.304	5.552	5.248
0.698	0.820	-5.644	-0.112	3.795	3.907
0.214	0.409	-4.986	-0.499	8.652	9.151
0.912	0.953	-5.709	-0.030	4.748	4.778
0.642	0.780	-5.613	-0.106	4.239	4.344
0.610	0.756	-5.592	-0.135	3.300	3.435
0.111	0.290	-4.517	0.527	4.759	4.232
0.088	0.262	-4.345	0.662	4.079	3.417
0.180	0.371	-4.867	-0.391	3.139	3.530
0.047	0.203	-3.855	0.644	4.684	4.041
0.087	0.259	-4.333	-0.545	3.985	4.530
0.929	0.961	-5.711	0.022	4.475	4.453
0.023	0.173	-3.291	0.554	3.840	3.286
0.058	0.220	-4.029	-0.517	2.978	3.495
0.231	0.428	-5.038	0.323	3.543	3.220
0.416	0.603	-5.402	-0.179	5.184	5.364
0.011	0.157	-2.665	0.570	3.210	2.639
0.907	0.950	-5.708	-0.032	2.275	2.307
0.010	0.157	-2.648	-0.738	1.991	2.729
0.415	0.603	-5.401	0.185	2.014	1.829
0.213	0.409	-4.984	-0.579	1.835	2.414
0.418	0.604	-5.404	-0.513	2.819	3.332
0.306	0.504	-5.220	-0.295	2.482	2.777
0.582	0.736	-5.571	-0.165	3.860	4.025
0.005	0.157	-1.989	-0.782	2.145	2.926
0.882	0.936	-5.704	0.039	4.181	4.142
0.041	0.195	-3.760	-0.624	1.982	2.606
0.296	0.493	-5.199	-0.328	2.360	2.688
0.077	0.247	-4.247	-0.559	1.930	2.489
0.405	0.593	-5.386	0.209	3.124	2.915
0.007	0.157	-2.366	0.649	4.367	3.717
0.166	0.355	-4.808	-0.430	2.554	2.984

0.500	0.671	-5.499	-0.191	3.558	3.749
0.758	0.860	-5.670	0.112	3.000	2.887
0.158	0.347	-4.776	-0.445	3.316	3.761
0.772	0.869	-5.675	-0.084	3.934	4.018
0.359	0.552	-5.317	-0.249	3.350	3.599
0.022	0.170	-3.251	-0.954	3.604	4.559
0.045	0.202	-3.831	0.941	4.376	3.435
0.097	0.273	-4.416	-0.461	2.952	3.413
0.388	0.576	-5.362	0.336	2.883	2.547
0.942	0.969	-5.712	0.018	2.358	2.340
0.709	0.827	-5.649	0.108	3.010	2.902
0.286	0.484	-5.177	-0.345	3.248	3.594
0.109	0.288	-4.506	0.495	2.865	2.370
0.308	0.505	-5.222	-0.362	2.147	2.509
0.180	0.370	-4.865	0.376	5.503	5.128
0.082	0.254	-4.292	-0.462	4.888	5.351
0.915	0.954	-5.709	-0.034	5.411	5.445
0.031	0.180	-3.522	-0.644	2.688	3.332
0.002	0.157	-1.464	1.684	5.095	3.412
0.295	0.492	-5.197	-0.334	5.664	5.997
0.502	0.672	-5.501	0.255	2.514	2.260
0.728	0.841	-5.658	0.088	3.104	3.016
0.179	0.369	-4.861	-0.431	3.741	4.171
0.557	0.716	-5.551	-0.146	1.912	2.058
0.270	0.468	-5.140	-0.481	3.145	3.626
0.440	0.624	-5.433	-0.309	1.443	1.752
0.251	0.449	-5.092	-0.417	2.851	3.268
0.086	0.258	-4.323	-0.473	3.159	3.633
0.009	0.157	-2.550	-1.832	2.312	4.144
0.013	0.160	-2.838	1.297	5.270	3.974
0.984	0.991	-5.715	-0.007	4.276	4.284
0.508	0.677	-5.508	0.173	3.406	3.233
0.608	0.754	-5.590	-0.160	2.509	2.669
0.233	0.430	-5.043	0.385	4.492	4.107
0.153	0.341	-4.754	-0.706	2.892	3.598
0.231	0.428	-5.038	-0.323	3.495	3.818
0.123	0.304	-4.594	-0.437	3.880	4.317
0.372	0.562	-5.338	0.225	2.490	2.264
0.203	0.398	-4.948	-0.303	2.988	3.290
0.054	0.215	-3.972	-0.685	2.348	3.033
0.148	0.332	-4.727	-0.435	2.487	2.922
0.264	0.462	-5.125	-0.291	5.755	6.046
0.018	0.162	-3.078	0.807	5.411	4.604
0.467	0.646	-5.464	-0.194	1.791	1.985
0.155	0.343	-4.763	-0.463	3.616	4.079
0.017	0.162	-3.065	0.967	5.444	4.477
0.214	0.409	-4.985	0.313	4.050	3.736
0.390	0.577	-5.365	-0.253	2.282	2.535
0.499	0.670	-5.498	-0.207	2.798	3.006
0.409	0.596	-5.392	0.308	3.254	2.945

0.247	0.444	-5.081	-0.504	2.272	2.776
0.014	0.160	-2.883	-0.744	3.661	4.405
0.214	0.409	-4.985	0.562	3.004	2.442
0.025	0.174	-3.348	-0.667	2.999	3.667
0.143	0.327	-4.702	-0.553	3.109	3.663
0.254	0.452	-5.101	-0.302	2.814	3.116
0.034	0.185	-3.610	1.081	6.620	5.539
0.038	0.192	-3.702	1.083	6.099	5.016
0.071	0.239	-4.184	-0.736	3.521	4.257
0.348	0.543	-5.298	0.268	5.481	5.213
0.393	0.581	-5.370	-0.252	4.408	4.660
0.026	0.175	-3.385	-0.604	7.426	8.029
0.588	0.741	-5.576	-0.147	2.419	2.566
0.231	0.428	-5.036	-0.428	4.311	4.739
0.044	0.201	-3.813	-0.781	2.126	2.907
0.356	0.550	-5.311	0.359	3.229	2.870
0.109	0.288	-4.503	-0.646	3.516	4.162
0.154	0.341	-4.756	-0.404	2.432	2.835
0.097	0.274	-4.420	0.460	2.835	2.375
0.604	0.753	-5.587	-0.158	2.022	2.180
0.099	0.276	-4.434	-0.524	2.726	3.250
0.138	0.321	-4.675	0.500	4.631	4.130
0.434	0.618	-5.425	-0.245	3.566	3.811
0.586	0.739	-5.574	-0.141	3.880	4.021
0.214	0.409	-4.985	-0.298	3.666	3.964
0.007	0.157	-2.316	-0.913	2.828	3.741
0.318	0.516	-5.243	-0.234	1.745	1.979
0.456	0.637	-5.452	-0.268	3.742	4.010
0.410	0.597	-5.394	-0.218	6.189	6.408
0.000	0.157	0.352	1.615	4.301	2.687
0.025	0.175	-3.367	0.606	2.501	1.895
0.693	0.816	-5.641	-0.108	3.128	3.236
0.341	0.537	-5.287	-0.285	3.596	3.881
0.000	0.157	-0.044	-1.287	2.964	4.251
0.572	0.727	-5.564	-0.185	5.006	5.191
0.094	0.271	-4.398	-0.767	2.232	2.999
0.000	0.022	4.203	1.528	3.002	1.474
0.033	0.184	-3.588	-0.787	5.430	6.217
0.007	0.157	-2.322	0.722	3.523	2.801
0.989	0.994	-5.715	-0.004	2.161	2.164
0.020	0.167	-3.196	1.024	3.107	2.083
0.148	0.332	-4.726	-0.617	3.208	3.825
0.933	0.964	-5.711	0.025	2.885	2.860
0.201	0.397	-4.944	0.295	5.232	4.937
0.173	0.363	-4.840	0.511	4.001	3.489
0.865	0.928	-5.701	0.084	3.543	3.459
0.005	0.157	-2.053	-0.818	2.591	3.409
0.611	0.757	-5.592	0.207	4.582	4.375
0.027	0.176	-3.412	0.980	6.597	5.618
0.003	0.157	-1.481	1.565	4.457	2.892

0.834	0.907	-5.694	-0.063	2.965	3.028
0.012	0.160	-2.798	-0.745	2.192	2.936
0.035	0.186	-3.624	0.790	2.654	1.864
0.033	0.184	-3.579	0.673	2.712	2.039
0.036	0.188	-3.645	-0.572	3.052	3.623
0.473	0.652	-5.471	0.194	2.413	2.219
0.366	0.557	-5.329	-0.309	2.770	3.080
0.777	0.872	-5.677	0.076	3.870	3.794
0.150	0.336	-4.739	-0.455	2.376	2.831
0.456	0.637	-5.452	-0.346	4.607	4.953
0.092	0.267	-4.377	-0.543	1.969	2.512
0.009	0.157	-2.575	1.135	2.775	1.640
0.086	0.258	-4.328	-0.543	2.017	2.561
0.876	0.933	-5.703	-0.088	2.380	2.468
0.486	0.662	-5.485	0.290	2.340	2.050
0.515	0.682	-5.514	-0.169	2.726	2.894
0.263	0.461	-5.124	-0.678	3.727	4.405
0.615	0.760	-5.595	-0.159	2.452	2.611
0.014	0.160	-2.898	0.911	2.944	2.033
0.100	0.277	-4.439	-0.671	1.574	2.245
0.354	0.548	-5.309	0.236	3.392	3.156
0.036	0.188	-3.645	0.522	2.591	2.069
0.479	0.656	-5.478	0.172	3.067	2.895
0.596	0.747	-5.582	-0.143	2.246	2.389
0.032	0.182	-3.562	1.134	4.286	3.152
0.181	0.372	-4.870	-0.488	2.925	3.413
0.100	0.277	-4.443	0.777	3.354	2.577
0.159	0.348	-4.779	-0.389	6.077	6.466
0.284	0.482	-5.173	0.303	3.512	3.209
0.097	0.273	-4.415	0.513	3.742	3.229
0.291	0.489	-5.188	-0.273	2.769	3.042
0.808	0.892	-5.687	-0.054	4.226	4.280
0.205	0.400	-4.956	0.520	3.408	2.888
0.093	0.270	-4.390	-0.480	2.998	3.478
0.837	0.909	-5.695	-0.064	2.458	2.521
0.835	0.908	-5.694	-0.062	3.269	3.331
0.065	0.229	-4.110	-0.452	3.766	4.218
0.296	0.493	-5.199	-0.380	1.740	2.120
0.479	0.656	-5.478	-0.177	2.790	2.967
0.306	0.503	-5.219	-0.319	1.800	2.119
0.025	0.175	-3.364	0.687	2.845	2.158
0.699	0.821	-5.644	0.170	2.518	2.348
0.499	0.670	-5.499	-0.256	4.340	4.596
0.221	0.417	-5.008	-0.321	5.488	5.810
0.223	0.420	-5.014	-0.335	5.520	5.855
0.065	0.229	-4.116	-0.447	6.694	7.141
0.420	0.606	-5.407	0.341	4.849	4.508
0.070	0.236	-4.165	0.423	2.826	2.404
0.123	0.304	-4.593	-0.519	2.505	3.023
0.037	0.190	-3.673	-0.714	2.560	3.273

0.000	0.157	0.715	-1.844	2.644	4.488
0.486	0.662	-5.485	-0.167	1.893	2.060
0.847	0.915	-5.697	-0.072	1.739	1.811
0.119	0.298	-4.568	-0.481	3.981	4.462
0.095	0.271	-4.400	-0.948	2.889	3.838
0.099	0.275	-4.431	-0.793	3.168	3.962
0.678	0.808	-5.633	-0.131	1.953	2.084
0.750	0.855	-5.667	-0.124	3.859	3.984
0.620	0.763	-5.598	0.214	2.757	2.543
0.347	0.542	-5.296	-0.323	7.014	7.337
0.331	0.527	-5.268	-0.264	7.342	7.606
0.903	0.947	-5.708	-0.029	2.633	2.662
0.964	0.982	-5.714	0.014	3.511	3.496
0.061	0.223	-4.060	0.467	2.907	2.440
0.615	0.759	-5.595	0.213	2.743	2.529
0.779	0.873	-5.677	0.100	3.215	3.115
0.000	0.081	2.615	1.375	3.371	1.996
0.264	0.461	-5.124	0.408	2.725	2.318
0.477	0.655	-5.476	-0.246	3.049	3.295
0.463	0.643	-5.460	-0.291	3.466	3.757
0.196	0.390	-4.927	-0.494	3.975	4.469
0.853	0.921	-5.699	0.052	2.216	2.163
0.080	0.250	-4.270	-0.455	2.224	2.679
0.040	0.194	-3.745	0.494	3.043	2.548
0.005	0.157	-2.011	1.063	3.415	2.353
0.000	0.157	1.030	1.407	3.454	2.047
0.007	0.157	-2.328	0.835	4.604	3.769
0.042	0.198	-3.782	-0.685	2.214	2.900
0.011	0.157	-2.691	1.059	3.838	2.779
0.249	0.447	-5.088	-0.272	3.914	4.186
0.571	0.726	-5.563	0.166	3.178	3.011
0.992	0.996	-5.715	-0.003	2.536	2.539
0.237	0.434	-5.054	-0.313	2.243	2.555
0.606	0.754	-5.589	-0.147	4.940	5.087
0.661	0.794	-5.623	-0.117	4.453	4.570
0.539	0.701	-5.536	0.382	5.210	4.828
0.010	0.157	-2.634	-1.213	2.751	3.963
0.044	0.201	-3.811	-0.592	2.029	2.621
0.195	0.389	-4.923	-0.350	2.917	3.267
0.763	0.864	-5.672	0.094	3.489	3.395
0.917	0.956	-5.710	-0.042	6.611	6.653
0.012	0.160	-2.797	-0.883	3.029	3.912
0.244	0.442	-5.074	-0.655	3.068	3.723
0.955	0.977	-5.713	-0.018	4.025	4.043
0.501	0.671	-5.500	-0.225	3.140	3.365
0.602	0.752	-5.586	0.110	8.641	8.531
0.235	0.432	-5.049	-0.522	5.155	5.677
0.303	0.501	-5.214	-0.279	4.106	4.385
0.111	0.290	-4.516	-0.412	2.278	2.690
0.048	0.204	-3.871	-0.552	3.717	4.269

0.071	0.238	-4.179	-0.489	3.507	3.996
0.142	0.326	-4.698	-0.506	2.058	2.563
0.629	0.770	-5.604	0.130	4.531	4.401
0.000	0.157	0.429	1.156	3.068	1.911
0.247	0.445	-5.083	0.401	5.243	4.842
0.122	0.303	-4.589	0.426	3.095	2.669
0.098	0.274	-4.423	0.597	2.731	2.133
0.006	0.157	-2.208	0.863	3.184	2.321
0.053	0.213	-3.960	-0.568	3.106	3.674
0.622	0.765	-5.599	-0.118	2.169	2.287
0.057	0.219	-4.017	0.568	4.951	4.383
0.054	0.215	-3.976	-0.659	2.087	2.746
0.953	0.976	-5.713	-0.019	3.449	3.468
0.293	0.491	-5.193	0.742	5.569	4.827
0.017	0.162	-3.051	0.949	4.139	3.189
0.067	0.232	-4.139	-0.616	2.629	3.245
0.682	0.809	-5.635	-0.162	2.536	2.698
0.826	0.901	-5.692	-0.064	2.735	2.799
0.136	0.320	-4.668	-0.526	3.544	4.070
0.002	0.157	-1.184	1.057	3.697	2.639
0.853	0.921	-5.699	0.058	1.905	1.846
0.190	0.382	-4.903	-0.331	1.980	2.311
0.475	0.653	-5.473	-0.203	2.844	3.047
0.002	0.157	-1.443	0.973	2.716	1.743
0.011	0.157	-2.665	1.108	5.963	4.855
0.711	0.828	-5.650	-0.104	2.598	2.702
0.251	0.449	-5.093	0.392	2.996	2.603
0.304	0.501	-5.214	0.386	2.446	2.060
0.662	0.794	-5.624	0.114	3.098	2.985
0.029	0.178	-3.482	0.584	4.411	3.826
0.119	0.298	-4.567	-0.448	1.833	2.282
0.812	0.894	-5.688	-0.073	4.113	4.186
0.120	0.300	-4.575	0.466	2.814	2.348
0.009	0.157	-2.522	0.901	4.419	3.517
0.951	0.975	-5.713	0.016	2.937	2.921
0.884	0.937	-5.705	0.040	4.223	4.183
0.014	0.160	-2.876	-1.423	2.082	3.504
0.024	0.173	-3.335	-1.053	2.041	3.093
0.857	0.923	-5.699	-0.054	2.917	2.972
0.752	0.856	-5.667	0.154	1.940	1.786
0.243	0.441	-5.071	-0.470	4.508	4.978
0.955	0.977	-5.713	0.016	2.775	2.760
0.216	0.411	-4.993	-0.479	3.005	3.484
0.069	0.236	-4.163	-0.594	3.356	3.950
0.207	0.402	-4.964	-0.342	2.479	2.821
0.007	0.157	-2.347	1.961	5.748	3.786
0.054	0.215	-3.973	-0.836	2.403	3.238
0.362	0.554	-5.322	-0.308	5.396	5.704
0.532	0.696	-5.530	-0.164	4.349	4.513
0.376	0.566	-5.344	0.249	3.079	2.830

0.121	0.301	-4.579	-0.450	2.308	2.758
0.476	0.653	-5.474	-0.203	4.476	4.678
0.391	0.579	-5.366	-0.298	2.547	2.846
0.907	0.950	-5.708	-0.036	1.939	1.975
0.539	0.701	-5.536	-0.273	5.542	5.815
0.455	0.636	-5.451	0.168	4.499	4.331
0.727	0.840	-5.657	-0.109	2.982	3.091
0.478	0.655	-5.477	0.174	5.695	5.521
0.951	0.975	-5.713	-0.016	3.234	3.250
0.286	0.483	-5.176	-0.291	5.109	5.399
0.133	0.316	-4.651	0.440	3.866	3.427
0.027	0.176	-3.412	-0.707	2.166	2.873
0.866	0.928	-5.701	0.088	3.882	3.795
0.762	0.864	-5.672	-0.117	2.890	3.007
0.241	0.439	-5.066	0.249	7.939	7.690
0.215	0.409	-4.988	-0.358	3.813	4.171
0.210	0.404	-4.972	0.404	3.437	3.034
0.749	0.855	-5.666	-0.103	4.228	4.331
0.008	0.157	-2.416	0.691	5.240	4.549
0.344	0.539	-5.291	-0.269	2.498	2.767
0.384	0.572	-5.356	-0.261	2.280	2.541
0.145	0.329	-4.711	0.480	2.595	2.115
0.248	0.446	-5.085	0.303	4.216	3.913
0.883	0.936	-5.704	0.039	2.981	2.942
0.154	0.341	-4.757	-0.559	3.831	4.391
0.364	0.556	-5.326	0.423	4.926	4.503
0.914	0.954	-5.709	-0.026	1.705	1.732
0.719	0.834	-5.653	-0.109	2.714	2.823
0.275	0.473	-5.151	0.433	3.148	2.715
0.111	0.290	-4.521	0.512	3.841	3.329
0.009	0.157	-2.551	1.375	5.626	4.251
0.214	0.409	-4.987	-0.346	1.979	2.325
0.086	0.258	-4.326	0.461	4.445	3.984
0.071	0.239	-4.186	-0.487	4.370	4.857
0.459	0.640	-5.456	-0.240	2.952	3.192
0.054	0.214	-3.965	0.658	2.902	2.244
0.229	0.426	-5.030	-0.349	4.556	4.906
0.236	0.433	-5.050	0.319	3.400	3.080
0.236	0.433	-5.053	-0.303	2.654	2.957
0.622	0.765	-5.600	-0.190	3.735	3.924
0.058	0.219	-4.024	-0.835	4.110	4.945
0.573	0.728	-5.565	-0.311	2.724	3.035
0.592	0.744	-5.579	-0.159	2.582	2.741
0.836	0.908	-5.694	0.063	1.780	1.717
0.033	0.184	-3.586	-1.361	3.302	4.662
0.126	0.306	-4.609	-0.583	2.831	3.415
0.075	0.245	-4.227	-0.589	2.880	3.469
0.006	0.157	-2.133	-1.145	2.002	3.146
0.674	0.804	-5.631	0.115	2.972	2.856
0.237	0.434	-5.055	-0.270	2.213	2.483

0.860	0.925	-5.700	0.046	2.528	2.483
0.227	0.424	-5.025	0.511	5.162	4.651
0.013	0.160	-2.820	-0.820	1.800	2.619
0.787	0.879	-5.680	-0.078	5.199	5.276
0.051	0.208	-3.923	-0.815	2.502	3.317
0.725	0.838	-5.656	0.116	3.823	3.708
0.158	0.346	-4.774	-0.430	4.877	5.307
0.249	0.447	-5.087	0.318	2.328	2.010
0.893	0.941	-5.706	-0.043	2.265	2.308
0.881	0.935	-5.704	-0.044	2.765	2.809
0.439	0.623	-5.431	-0.193	2.687	2.880
0.029	0.178	-3.493	1.280	4.153	2.873
0.070	0.237	-4.174	-0.765	3.503	4.267
0.056	0.217	-3.999	0.598	2.721	2.123
0.047	0.204	-3.864	0.570	2.909	2.339
0.161	0.349	-4.786	-0.549	6.066	6.615
0.226	0.423	-5.022	-0.471	5.612	6.083
0.157	0.345	-4.771	-0.515	3.105	3.620
0.283	0.481	-5.169	0.272	4.035	3.763
0.624	0.766	-5.601	-0.155	7.012	7.167
0.102	0.280	-4.456	-0.402	9.203	9.605
0.376	0.565	-5.343	-0.327	2.414	2.741
0.820	0.898	-5.690	0.089	3.455	3.367
0.785	0.878	-5.679	0.072	3.922	3.851
0.308	0.505	-5.223	0.264	2.276	2.012
0.029	0.178	-3.489	0.527	4.710	4.183
0.657	0.792	-5.621	-0.326	2.075	2.401
0.950	0.974	-5.713	0.020	2.818	2.798
0.770	0.868	-5.674	0.112	5.081	4.968
0.493	0.667	-5.492	-0.377	4.395	4.772
0.227	0.424	-5.024	0.347	2.617	2.271
0.025	0.175	-3.356	-0.880	3.518	4.397
0.046	0.203	-3.844	-0.506	4.489	4.995
0.741	0.849	-5.663	-0.099	5.429	5.527
0.868	0.928	-5.702	-0.059	5.923	5.982
0.989	0.994	-5.715	0.007	2.280	2.274
0.953	0.976	-5.713	-0.017	2.513	2.530
0.600	0.750	-5.585	0.152	2.498	2.345
0.063	0.226	-4.086	0.427	5.713	5.286
0.188	0.380	-4.897	0.490	3.555	3.065
0.195	0.389	-4.923	0.370	4.326	3.956
0.038	0.192	-3.703	-1.223	3.123	4.346
0.168	0.358	-4.817	-0.778	2.486	3.265
0.606	0.753	-5.589	-0.134	2.341	2.475
0.802	0.889	-5.685	0.063	5.350	5.287
0.927	0.960	-5.711	-0.038	5.884	5.922
0.873	0.931	-5.703	-0.054	5.981	6.035
0.279	0.477	-5.161	-0.318	8.706	9.023
0.012	0.160	-2.766	1.183	3.390	2.206
0.479	0.655	-5.477	0.184	9.621	9.437

0.024	0.173	-3.313	0.703	3.833	3.129
0.600	0.749	-5.585	0.160	6.232	6.073
0.886	0.938	-5.705	-0.050	4.294	4.344
0.034	0.185	-3.613	0.513	3.630	3.118
0.965	0.982	-5.714	0.014	2.692	2.678
0.512	0.680	-5.511	-0.178	3.906	4.084
0.966	0.983	-5.714	-0.014	2.568	2.582
0.089	0.263	-4.350	-0.783	2.673	3.455
0.085	0.257	-4.314	-0.493	5.256	5.749
0.689	0.814	-5.639	0.128	2.474	2.346
0.316	0.514	-5.240	0.372	2.940	2.567
0.072	0.240	-4.197	0.477	2.558	2.082
0.098	0.274	-4.424	0.651	4.114	3.463
0.270	0.469	-5.141	0.365	4.122	3.757
0.287	0.485	-5.179	0.244	3.385	3.141
0.254	0.452	-5.101	-0.307	2.903	3.211
0.040	0.194	-3.743	0.469	3.452	2.983
0.308	0.506	-5.224	0.249	4.826	4.577
0.006	0.157	-2.192	-0.713	2.875	3.587
0.876	0.933	-5.703	0.059	1.941	1.883
0.935	0.965	-5.712	-0.024	2.574	2.598
0.273	0.471	-5.146	0.285	4.553	4.268
0.098	0.274	-4.425	0.426	5.836	5.410
0.059	0.221	-4.042	0.647	8.960	8.313
0.125	0.306	-4.603	0.364	2.499	2.135
0.711	0.828	-5.650	0.086	2.644	2.559
0.369	0.560	-5.333	-0.280	3.541	3.821
0.037	0.189	-3.665	-0.527	3.223	3.749
0.002	0.157	-1.160	-1.217	7.128	8.345
0.094	0.270	-4.396	0.758	3.355	2.597
0.243	0.440	-5.070	-0.472	3.448	3.919
0.098	0.274	-4.423	-0.630	2.840	3.470
0.181	0.373	-4.872	-0.446	2.118	2.565
0.026	0.176	-3.401	-0.842	5.899	6.741
0.256	0.454	-5.105	-0.251	5.292	5.543
0.062	0.224	-4.075	0.647	6.993	6.345
0.686	0.812	-5.638	0.131	3.078	2.948
0.582	0.736	-5.571	-0.108	7.297	7.405
0.040	0.194	-3.736	0.551	7.978	7.426
0.015	0.161	-2.962	1.252	7.054	5.802
0.179	0.369	-4.861	0.287	7.687	7.400
0.781	0.874	-5.678	0.098	3.442	3.344
0.555	0.715	-5.550	0.142	3.212	3.071
0.402	0.589	-5.382	-0.268	3.837	4.105
0.036	0.188	-3.653	-0.790	4.734	5.524
0.039	0.192	-3.711	1.154	5.747	4.593
0.021	0.168	-3.228	1.443	7.971	6.528
0.075	0.245	-4.227	0.921	5.853	4.932
0.664	0.796	-5.625	0.131	5.110	4.979
0.321	0.518	-5.249	-0.381	1.519	1.900

0.117	0.297	-4.557	-0.514	2.730	3.243
0.102	0.280	-4.457	0.553	4.351	3.798
0.026	0.175	-3.382	1.007	4.958	3.951
0.010	0.157	-2.595	1.382	7.384	6.001
0.706	0.825	-5.647	0.134	3.380	3.246
0.038	0.192	-3.697	-0.607	3.353	3.960
0.222	0.419	-5.012	-0.280	4.151	4.431
0.848	0.916	-5.697	0.041	5.191	5.150
0.102	0.280	-4.457	0.433	6.361	5.928
0.064	0.228	-4.103	0.424	7.550	7.125
0.009	0.157	-2.561	-1.784	5.038	6.822
0.068	0.234	-4.151	-0.657	4.756	5.414
0.055	0.216	-3.982	0.588	5.370	4.782
0.072	0.240	-4.193	-0.518	3.948	4.466
0.108	0.287	-4.500	0.420	5.660	5.239
0.019	0.164	-3.145	-0.891	4.817	5.708
0.920	0.957	-5.710	0.039	3.623	3.585
0.057	0.219	-4.015	0.486	7.084	6.598
0.016	0.162	-2.986	1.043	4.855	3.813
0.873	0.931	-5.703	-0.043	3.334	3.376
0.614	0.759	-5.594	-0.163	2.049	2.212
0.684	0.811	-5.636	0.159	4.499	4.340
0.059	0.220	-4.033	-0.600	6.003	6.603
0.593	0.745	-5.579	0.208	4.975	4.767
0.868	0.928	-5.702	0.047	3.451	3.403
0.923	0.959	-5.710	0.027	4.953	4.926
0.068	0.234	-4.149	0.481	2.796	2.315
0.096	0.272	-4.411	0.490	4.835	4.345
0.361	0.553	-5.321	0.247	4.162	3.915
0.050	0.208	-3.913	0.795	5.494	4.699
0.201	0.397	-4.945	0.341	6.031	5.690
0.657	0.792	-5.621	-0.161	1.682	1.843
0.368	0.559	-5.332	-0.324	2.778	3.102
0.070	0.236	-4.170	0.477	4.740	4.263
0.204	0.399	-4.954	0.315	7.189	6.874
0.169	0.359	-4.821	0.627	2.801	2.174
0.075	0.245	-4.228	0.684	4.566	3.882
0.127	0.307	-4.615	0.341	5.859	5.518
0.022	0.170	-3.255	0.620	5.514	4.894
0.059	0.221	-4.037	-0.942	3.160	4.101
0.003	0.157	-1.641	1.012	2.732	1.720
0.048	0.205	-3.878	1.032	7.816	6.784
0.624	0.767	-5.601	-0.112	2.143	2.255
0.025	0.175	-3.376	0.939	4.688	3.749
0.233	0.430	-5.043	0.412	7.267	6.856
0.693	0.816	-5.641	0.159	1.997	1.838
0.505	0.674	-5.505	-0.209	4.310	4.519
0.017	0.162	-3.072	0.618	5.862	5.243
0.068	0.234	-4.146	0.555	4.933	4.378
0.156	0.344	-4.765	0.438	6.193	5.755

0.928	0.961	-5.711	-0.026	5.290	5.317
0.644	0.781	-5.613	0.122	4.774	4.653
0.031	0.180	-3.524	0.882	5.913	5.031
0.016	0.162	-2.998	0.955	6.176	5.221
0.215	0.409	-4.987	0.461	8.694	8.233
0.040	0.194	-3.734	0.748	7.791	7.043
0.028	0.178	-3.446	1.017	7.234	6.217
0.297	0.494	-5.201	0.371	4.350	3.979
0.016	0.162	-3.016	0.877	5.186	4.309
0.594	0.745	-5.580	-0.125	3.978	4.102
0.134	0.318	-4.658	-0.428	2.740	3.168
0.640	0.778	-5.611	0.121	2.682	2.560
0.410	0.597	-5.394	-0.214	3.824	4.038
0.006	0.157	-2.181	-0.941	5.948	6.889
0.142	0.326	-4.699	-0.423	3.177	3.600
0.152	0.338	-4.745	-0.421	2.978	3.399
0.165	0.354	-4.805	-0.407	3.003	3.410
0.183	0.374	-4.877	-0.411	2.904	3.316
0.196	0.390	-4.927	-0.375	2.880	3.255
0.203	0.398	-4.951	-0.384	2.902	3.286
0.907	0.950	-5.708	-0.036	2.237	2.274
0.727	0.839	-5.657	-0.088	5.265	5.353
0.487	0.662	-5.486	0.166	3.476	3.310
0.159	0.347	-4.779	-0.307	5.220	5.527
0.277	0.475	-5.157	0.389	4.642	4.253
0.346	0.541	-5.295	-0.238	5.369	5.607
0.789	0.880	-5.681	-0.199	2.831	3.030
0.804	0.890	-5.686	0.066	6.406	6.340
0.509	0.677	-5.508	-0.286	1.907	2.194
0.035	0.186	-3.621	0.772	3.244	2.472
0.587	0.740	-5.575	0.163	5.495	5.332
0.069	0.236	-4.162	0.616	6.570	5.954
0.109	0.288	-4.506	-0.528	6.259	6.787
0.257	0.454	-5.107	0.328	4.057	3.730
0.029	0.178	-3.476	0.735	4.314	3.579
0.960	0.979	-5.714	-0.013	4.320	4.333
0.306	0.504	-5.220	-0.468	5.832	6.301
0.046	0.203	-3.844	1.003	3.922	2.919
0.336	0.532	-5.277	-0.284	2.861	3.145
0.124	0.305	-4.600	-0.360	3.279	3.638
0.943	0.969	-5.712	-0.025	5.311	5.336
0.118	0.298	-4.562	0.411	4.549	4.138
0.091	0.267	-4.371	-0.564	3.776	4.341
0.340	0.535	-5.283	0.229	6.200	5.971
0.016	0.162	-3.012	1.095	3.103	2.007
0.933	0.964	-5.711	0.019	5.020	5.002
0.021	0.168	-3.208	0.655	4.998	4.343
0.799	0.887	-5.684	0.069	4.086	4.017
0.627	0.769	-5.603	-0.157	3.104	3.261
0.117	0.297	-4.556	0.820	6.965	6.145

0.407	0.594	-5.389	-0.433	3.848	4.281
0.051	0.209	-3.926	0.446	4.225	3.779
0.038	0.192	-3.703	1.068	5.534	4.466
0.014	0.160	-2.888	-0.675	3.858	4.533
0.024	0.173	-3.321	0.761	5.856	5.094
0.879	0.934	-5.704	0.035	4.075	4.039
0.371	0.561	-5.335	-0.359	2.050	2.409
0.068	0.234	-4.146	-1.486	1.949	3.435
0.013	0.160	-2.864	0.578	3.631	3.053
0.345	0.540	-5.294	0.235	3.278	3.043
0.052	0.211	-3.938	-0.559	2.273	2.832
0.065	0.229	-4.119	0.453	7.224	6.772
0.004	0.157	-1.914	0.759	3.052	2.293
0.592	0.744	-5.579	0.170	4.427	4.257
0.171	0.361	-4.832	0.333	3.507	3.174
0.873	0.931	-5.703	-0.053	2.566	2.619
0.210	0.404	-4.972	-0.448	3.915	4.363
0.043	0.199	-3.788	-1.163	6.608	7.770
0.157	0.344	-4.768	0.381	7.537	7.156
0.136	0.320	-4.669	0.488	6.008	5.520
0.137	0.321	-4.674	0.649	4.585	3.936
0.866	0.928	-5.701	0.036	6.798	6.762
0.582	0.736	-5.571	-0.137	2.911	3.048
0.383	0.572	-5.355	0.190	4.453	4.263
0.041	0.195	-3.753	-0.551	5.738	6.289
0.111	0.290	-4.518	-0.589	2.864	3.453
0.043	0.200	-3.797	0.617	7.068	6.451
0.516	0.683	-5.515	0.175	2.885	2.710
0.040	0.194	-3.732	-0.541	5.400	5.941
0.452	0.633	-5.447	0.182	1.821	1.639
0.010	0.157	-2.600	-1.123	7.325	8.448
0.007	0.157	-2.378	1.754	6.424	4.670
0.194	0.388	-4.920	-0.309	9.255	9.564
0.452	0.632	-5.447	0.246	3.414	3.168
0.004	0.157	-1.889	1.008	4.479	3.472
0.309	0.506	-5.226	-0.229	2.096	2.325
0.108	0.287	-4.501	-0.427	2.526	2.953
0.058	0.219	-4.024	-0.430	5.013	5.443
0.241	0.439	-5.066	0.427	6.228	5.802
0.260	0.457	-5.115	0.251	8.640	8.389
0.537	0.700	-5.534	-0.186	3.019	3.205
0.683	0.810	-5.636	0.135	5.810	5.675
0.013	0.160	-2.840	-1.010	6.177	7.188
0.145	0.329	-4.714	-0.418	4.080	4.497
0.635	0.774	-5.608	0.106	4.559	4.453
0.888	0.939	-5.705	-0.037	4.239	4.276
0.054	0.215	-3.975	0.443	6.046	5.603
0.863	0.927	-5.701	0.049	6.784	6.735
0.145	0.329	-4.716	0.377	9.180	8.802
0.193	0.386	-4.914	-0.484	3.279	3.763

0.050	0.208	-3.909	0.867	9.485	8.618
0.011	0.157	-2.665	0.781	8.694	7.914
0.027	0.176	-3.427	0.656	5.405	4.749
0.865	0.928	-5.701	0.042	5.902	5.861
0.026	0.176	-3.394	0.656	6.426	5.770
0.015	0.160	-2.957	-0.716	3.457	4.173
0.513	0.681	-5.513	-0.159	3.584	3.744
0.031	0.181	-3.538	0.527	4.394	3.867
0.282	0.480	-5.167	-0.590	3.688	4.278
0.756	0.859	-5.669	-0.130	4.422	4.551
0.019	0.164	-3.128	1.039	5.880	4.840
0.040	0.194	-3.736	-0.446	6.835	7.281
0.183	0.374	-4.877	-0.359	1.967	2.325
0.107	0.286	-4.492	0.432	5.432	5.000
0.017	0.162	-3.068	-0.704	7.759	8.463
0.016	0.162	-2.987	-0.636	7.181	7.817
0.124	0.305	-4.598	0.441	7.045	6.603
0.013	0.160	-2.865	-0.712	5.042	5.754
0.007	0.157	-2.384	-0.798	7.456	8.255
0.160	0.349	-4.786	1.084	7.948	6.864
0.489	0.664	-5.488	-0.154	4.380	4.534
0.016	0.162	-3.025	0.724	6.491	5.767
0.689	0.814	-5.639	0.149	2.568	2.419
0.012	0.160	-2.767	0.857	4.890	4.033
0.129	0.310	-4.628	0.308	5.263	4.955
0.255	0.452	-5.103	-0.305	2.408	2.712
0.416	0.603	-5.401	0.199	4.382	4.183
0.840	0.912	-5.696	0.061	4.148	4.086
0.044	0.201	-3.814	0.772	6.858	6.086
0.029	0.178	-3.475	0.562	6.815	6.254
0.021	0.168	-3.228	-0.536	5.639	6.174
0.837	0.909	-5.695	-0.047	4.512	4.558
0.038	0.191	-3.686	0.580	5.584	5.004
0.306	0.504	-5.220	-0.296	3.527	3.823
0.003	0.157	-1.680	0.739	6.361	5.622
0.866	0.928	-5.701	-0.049	2.385	2.434
0.097	0.274	-4.421	0.548	3.938	3.391
0.020	0.167	-3.179	-0.961	4.178	5.139
0.109	0.288	-4.504	0.726	4.935	4.209
0.057	0.219	-4.018	-0.408	7.329	7.737
0.189	0.381	-4.899	-0.331	6.323	6.654
0.610	0.757	-5.592	-0.141	2.248	2.389
0.046	0.203	-3.849	0.551	6.669	6.118
0.039	0.192	-3.712	0.499	6.235	5.736
0.007	0.157	-2.353	1.899	5.307	3.408
0.380	0.569	-5.350	-0.316	3.983	4.299
0.115	0.295	-4.544	0.727	6.552	5.824
0.037	0.191	-3.682	0.644	8.640	7.996
0.018	0.162	-3.083	0.678	5.490	4.813
0.156	0.344	-4.767	0.429	4.509	4.080

0.064	0.228	-4.101	-0.494	7.259	7.753
0.012	0.160	-2.786	0.804	4.999	4.195
0.515	0.682	-5.515	-0.189	2.474	2.663
0.265	0.462	-5.127	-0.221	2.864	3.085
0.346	0.541	-5.295	0.345	3.304	2.958
0.029	0.178	-3.472	-0.538	5.408	5.945
0.457	0.637	-5.453	-0.213	2.020	2.233
0.084	0.256	-4.313	0.531	5.609	5.078
0.057	0.218	-4.008	-0.457	3.236	3.693
0.034	0.186	-3.618	0.556	4.633	4.077
0.525	0.691	-5.524	0.143	5.543	5.400
0.079	0.249	-4.259	-0.681	3.013	3.695
0.005	0.157	-1.987	-1.718	3.104	4.822
0.110	0.289	-4.511	0.353	5.658	5.305
0.139	0.324	-4.685	-0.352	6.337	6.689
0.670	0.802	-5.629	0.084	6.330	6.246
0.281	0.479	-5.166	0.302	7.376	7.074
0.034	0.185	-3.615	0.957	4.101	3.144
0.889	0.940	-5.706	-0.049	3.566	3.614
0.754	0.858	-5.668	0.099	5.293	5.195
0.367	0.558	-5.329	-0.221	5.164	5.385
0.999	0.999	-5.715	0.000	2.306	2.306
0.252	0.450	-5.096	0.446	5.444	4.998
0.015	0.161	-2.975	0.728	7.317	6.589
0.910	0.952	-5.709	0.040	3.314	3.274
0.035	0.188	-3.641	0.731	4.787	4.056
0.003	0.157	-1.617	2.462	5.841	3.378
0.417	0.604	-5.403	-0.181	6.838	7.019
0.125	0.306	-4.607	0.397	6.047	5.650
0.225	0.421	-5.019	-0.499	3.787	4.286
0.062	0.225	-4.081	0.503	5.390	4.886
0.009	0.157	-2.500	-0.738	3.829	4.567
0.722	0.836	-5.655	-0.079	4.505	4.585
0.004	0.157	-1.935	1.160	6.663	5.502
0.080	0.250	-4.269	0.541	3.365	2.824
0.106	0.284	-4.486	0.362	5.505	5.143
0.140	0.324	-4.686	-0.406	2.227	2.634
0.084	0.256	-4.313	0.508	5.585	5.077
0.021	0.168	-3.217	0.677	8.019	7.342
0.420	0.606	-5.407	-0.244	3.310	3.554
0.120	0.300	-4.575	-0.404	4.062	4.465
0.465	0.644	-5.462	-0.218	3.948	4.166
0.054	0.215	-3.972	-0.462	5.844	6.306
0.038	0.192	-3.703	0.588	5.940	5.352
0.093	0.268	-4.384	0.331	7.017	6.686
0.002	0.157	-1.468	0.924	6.909	5.985
0.709	0.827	-5.649	0.101	2.641	2.540
0.039	0.193	-3.715	0.411	6.187	5.777
0.763	0.864	-5.672	0.158	2.885	2.726
0.004	0.157	-1.947	1.403	6.181	4.779

0.084	0.256	-4.312	0.411	7.340	6.929
0.022	0.171	-3.261	1.888	8.004	6.116
0.043	0.200	-3.799	0.677	5.952	5.275
0.218	0.412	-4.997	0.427	4.947	4.520
0.010	0.157	-2.615	-0.867	6.853	7.720
0.001	0.157	-0.558	-1.144	3.138	4.282
0.427	0.613	-5.417	0.267	4.578	4.311
0.221	0.417	-5.008	-0.400	2.619	3.018
0.021	0.168	-3.215	1.433	4.165	2.732
0.812	0.894	-5.688	0.068	2.992	2.924
0.046	0.203	-3.837	0.644	5.211	4.568
0.248	0.446	-5.084	-0.479	5.027	5.506
0.169	0.359	-4.820	0.286	5.488	5.202
0.109	0.288	-4.502	-0.492	5.761	6.252
0.188	0.380	-4.896	0.405	3.990	3.586
0.818	0.897	-5.690	0.063	2.897	2.833
0.543	0.705	-5.540	0.137	5.595	5.459
0.979	0.989	-5.714	0.007	4.221	4.215
0.083	0.255	-4.302	-0.458	3.004	3.462
0.285	0.483	-5.175	-0.276	4.484	4.761
0.961	0.980	-5.714	0.010	3.581	3.571
0.495	0.667	-5.494	0.147	6.239	6.092
0.147	0.332	-4.725	-0.390	7.873	8.263
0.096	0.272	-4.412	0.585	4.377	3.792
0.508	0.677	-5.508	0.151	2.582	2.432
0.271	0.469	-5.142	0.253	5.713	5.460
0.449	0.631	-5.444	-0.304	2.931	3.235
0.005	0.157	-2.024	1.041	5.235	4.195
0.094	0.271	-4.398	0.527	4.306	3.778
0.641	0.779	-5.612	-0.162	2.073	2.235
0.867	0.928	-5.702	0.046	3.042	2.995
0.500	0.671	-5.500	-0.185	3.600	3.784
0.336	0.532	-5.278	0.237	4.165	3.928
0.038	0.192	-3.690	1.073	7.811	6.738
0.639	0.777	-5.610	0.119	3.425	3.306
0.832	0.906	-5.693	0.054	3.854	3.800
0.523	0.689	-5.522	-0.139	4.254	4.393
0.041	0.195	-3.753	0.473	6.685	6.212
0.375	0.565	-5.343	0.261	5.057	4.796
0.008	0.157	-2.457	1.502	6.446	4.943
0.800	0.888	-5.684	0.081	2.344	2.263
0.289	0.488	-5.184	-0.381	2.631	3.012
0.052	0.211	-3.938	-0.583	2.876	3.459
0.097	0.274	-4.418	-0.691	4.090	4.781
0.210	0.405	-4.974	-0.460	1.290	1.750
0.042	0.196	-3.766	-0.545	4.591	5.136
0.522	0.688	-5.520	0.190	3.192	3.002
0.215	0.410	-4.990	0.278	5.708	5.431
0.055	0.216	-3.989	-0.958	3.658	4.616
0.209	0.404	-4.971	-0.349	5.392	5.741

0.034	0.184	-3.600	0.545	4.503	3.958
0.228	0.424	-5.027	-0.308	4.272	4.580
0.506	0.675	-5.505	0.273	5.001	4.728
0.932	0.963	-5.711	0.020	4.693	4.672
0.872	0.931	-5.703	0.042	3.864	3.821
0.458	0.638	-5.454	0.189	4.479	4.290
0.466	0.645	-5.463	0.186	4.506	4.320
0.008	0.157	-2.426	1.123	5.586	4.463
0.064	0.227	-4.098	-0.626	3.466	4.092
0.982	0.990	-5.715	0.006	3.156	3.150
0.403	0.591	-5.384	0.164	6.611	6.446
0.026	0.176	-3.408	0.645	5.892	5.247
0.011	0.157	-2.704	-0.717	6.059	6.775
0.704	0.824	-5.646	0.092	6.139	6.048
0.140	0.324	-4.690	-0.408	4.119	4.528
0.470	0.649	-5.468	-0.266	2.897	3.163
0.569	0.725	-5.561	-0.158	2.520	2.678
0.321	0.518	-5.249	-0.273	2.348	2.620
0.017	0.162	-3.070	-0.883	2.089	2.972
0.101	0.278	-4.445	0.372	5.606	5.234
0.293	0.491	-5.193	-0.472	3.866	4.338
0.428	0.614	-5.417	-0.225	2.299	2.524
0.840	0.911	-5.695	0.067	2.266	2.199
0.318	0.516	-5.244	0.289	3.765	3.476
0.420	0.606	-5.407	-0.366	2.655	3.021
0.766	0.865	-5.673	-0.118	3.355	3.473
0.827	0.902	-5.692	-0.074	4.331	4.405
0.007	0.157	-2.310	1.530	6.783	5.253
0.321	0.518	-5.250	-0.500	3.393	3.893
0.106	0.284	-4.482	-0.542	3.057	3.599
0.572	0.727	-5.564	-0.209	3.387	3.596
0.309	0.506	-5.225	0.348	2.742	2.394
0.473	0.652	-5.471	0.231	3.455	3.224
0.709	0.827	-5.649	0.100	3.071	2.971
0.976	0.988	-5.714	-0.008	4.729	4.737
0.598	0.748	-5.583	0.224	4.163	3.939
0.519	0.686	-5.518	-0.207	6.428	6.636
0.919	0.957	-5.710	-0.032	3.195	3.227
0.009	0.157	-2.529	0.832	3.812	2.980
0.992	0.996	-5.715	0.003	2.489	2.486
0.104	0.281	-4.469	0.447	3.441	2.994
0.126	0.306	-4.609	0.566	2.742	2.177
0.700	0.821	-5.644	0.142	2.474	2.332
0.133	0.316	-4.652	-0.654	2.563	3.217
0.859	0.924	-5.700	0.051	3.008	2.957
0.138	0.321	-4.676	-0.361	3.585	3.946
0.724	0.837	-5.656	-0.187	2.410	2.597
0.063	0.227	-4.095	-0.587	4.498	5.085
0.503	0.673	-5.503	-0.300	3.006	3.306
0.578	0.732	-5.568	0.162	2.996	2.834

0.958	0.978	-5.713	-0.014	3.942	3.956
0.414	0.601	-5.399	-0.253	2.300	2.553
0.914	0.954	-5.709	-0.028	3.828	3.856
0.595	0.746	-5.581	0.157	3.771	3.614
0.426	0.612	-5.415	0.277	2.950	2.673
0.722	0.836	-5.655	0.128	5.241	5.113
0.494	0.667	-5.493	-0.287	2.530	2.817
0.306	0.503	-5.219	-0.546	4.552	5.098
0.108	0.287	-4.497	-0.559	2.809	3.368
0.397	0.585	-5.375	-0.233	2.266	2.499
0.014	0.160	-2.922	0.624	3.729	3.105
0.448	0.630	-5.443	-0.270	3.163	3.432
0.009	0.157	-2.574	0.718	2.502	1.783
0.104	0.281	-4.467	0.535	2.512	1.977
0.229	0.426	-5.031	0.364	2.928	2.565
0.726	0.839	-5.657	0.146	2.638	2.493
0.792	0.882	-5.682	-0.103	4.043	4.146
0.395	0.582	-5.373	0.243	2.805	2.562
0.171	0.361	-4.829	-0.575	3.949	4.524
0.110	0.289	-4.512	-0.430	2.522	2.952
0.412	0.599	-5.396	-0.315	2.110	2.425
0.317	0.515	-5.242	0.391	3.598	3.207
0.017	0.162	-3.035	-0.753	2.131	2.884
0.241	0.438	-5.065	0.437	4.493	4.056
0.531	0.695	-5.529	0.229	1.387	1.159
0.565	0.722	-5.558	0.152	3.341	3.189
0.673	0.803	-5.630	-0.146	2.624	2.770
0.563	0.721	-5.557	-0.208	2.885	3.093
0.492	0.666	-5.492	-0.191	2.913	3.104
0.287	0.485	-5.179	-0.279	4.350	4.629
0.600	0.749	-5.584	0.143	3.437	3.295
0.740	0.849	-5.663	-0.119	2.526	2.645
0.846	0.915	-5.697	0.060	3.433	3.373
0.913	0.953	-5.709	0.030	3.408	3.378
0.025	0.174	-3.349	0.876	3.845	2.969
0.064	0.228	-4.106	0.560	3.281	2.721
0.671	0.802	-5.629	-0.124	3.505	3.630
0.027	0.177	-3.435	-0.677	3.639	4.315
0.216	0.411	-4.992	-0.388	3.346	3.734
0.520	0.686	-5.518	-0.206	3.680	3.886
0.304	0.502	-5.215	0.401	3.643	3.242
0.876	0.933	-5.703	0.050	4.690	4.639
0.046	0.203	-3.842	0.641	2.632	1.991
0.145	0.329	-4.715	-0.565	2.585	3.150
0.903	0.947	-5.708	0.039	3.709	3.670
0.070	0.237	-4.172	0.445	4.566	4.121
0.847	0.915	-5.697	-0.069	2.890	2.959
0.090	0.265	-4.366	-0.472	2.907	3.379
0.205	0.401	-4.957	-0.405	2.350	2.755
0.679	0.808	-5.633	0.133	3.348	3.215

0.235	0.432	-5.048	-0.345	2.323	2.669
0.863	0.927	-5.701	0.047	5.497	5.450
0.431	0.616	-5.422	-0.243	1.898	2.140
0.550	0.710	-5.545	0.156	2.475	2.320
0.988	0.994	-5.715	-0.004	3.580	3.584
0.344	0.539	-5.291	-0.296	2.380	2.676
0.308	0.505	-5.223	-0.349	3.389	3.738
0.168	0.358	-4.819	-0.599	4.638	5.237
0.639	0.778	-5.610	-0.147	4.095	4.242
0.043	0.199	-3.791	0.958	3.499	2.541
0.145	0.329	-4.712	-0.495	2.162	2.657
0.887	0.938	-5.705	0.036	2.943	2.908
0.704	0.824	-5.646	0.109	2.513	2.405
0.183	0.374	-4.877	0.441	4.194	3.754
0.920	0.957	-5.710	-0.028	1.825	1.853
0.738	0.848	-5.662	-0.110	5.652	5.761
0.537	0.700	-5.534	0.191	3.640	3.448
0.943	0.970	-5.712	-0.029	2.364	2.393
0.355	0.550	-5.311	-0.426	2.484	2.910
0.190	0.383	-4.906	-0.428	2.716	3.144
0.375	0.565	-5.342	-0.407	1.699	2.106
0.148	0.333	-4.729	-0.458	2.227	2.684
0.278	0.477	-5.159	-0.297	2.383	2.680
0.887	0.938	-5.705	-0.048	3.394	3.442
0.394	0.581	-5.371	0.324	3.903	3.578
0.555	0.715	-5.550	-0.176	1.516	1.692
0.514	0.682	-5.513	-0.216	4.703	4.918
0.063	0.226	-4.085	-1.017	3.681	4.699
0.024	0.173	-3.332	-1.118	1.422	2.540
0.434	0.618	-5.425	0.239	6.594	6.355
0.300	0.497	-5.206	-0.437	5.725	6.162
0.173	0.362	-4.837	-0.464	2.547	3.012
0.245	0.442	-5.075	-0.328	3.584	3.912
0.209	0.404	-4.970	-0.337	3.111	3.448
0.515	0.682	-5.514	-0.207	2.395	2.602
0.518	0.685	-5.517	0.187	3.996	3.810
0.771	0.868	-5.675	-0.106	2.503	2.608
0.009	0.157	-2.514	1.138	3.737	2.599
0.039	0.192	-3.708	0.760	3.684	2.923
0.228	0.425	-5.029	0.417	2.946	2.529
0.747	0.853	-5.666	0.127	3.923	3.796
0.816	0.896	-5.689	-0.105	3.050	3.155
0.120	0.301	-4.579	0.691	3.620	2.929
0.854	0.921	-5.699	0.072	2.447	2.376
0.192	0.385	-4.913	-0.354	5.937	6.290
0.503	0.672	-5.502	0.365	3.220	2.855
0.807	0.891	-5.686	-0.062	4.767	4.830
0.092	0.268	-4.382	-0.604	6.379	6.983
0.036	0.188	-3.645	-0.589	2.939	3.527
0.198	0.392	-4.933	-0.498	3.338	3.835

0.994	0.997	-5.715	-0.003	2.570	2.572
0.950	0.974	-5.713	0.020	2.060	2.040
0.529	0.694	-5.527	-0.219	2.788	3.007
0.521	0.687	-5.519	-0.240	1.033	1.273
0.526	0.691	-5.524	-0.156	4.638	4.794
0.185	0.377	-4.886	-0.457	4.140	4.597
0.632	0.772	-5.606	-0.145	2.934	3.079
0.542	0.703	-5.538	-0.210	2.595	2.805
0.066	0.231	-4.128	0.575	4.998	4.422
0.007	0.157	-2.374	1.106	2.629	1.524
0.073	0.241	-4.199	-0.651	2.004	2.655
0.971	0.985	-5.714	-0.015	3.707	3.722
0.656	0.791	-5.621	-0.221	3.368	3.590
0.533	0.696	-5.531	-0.270	2.335	2.605
0.526	0.692	-5.525	0.168	3.950	3.782
0.842	0.912	-5.696	-0.072	3.441	3.513
0.011	0.158	-2.706	-0.661	1.952	2.613
0.590	0.743	-5.578	-0.178	2.008	2.186
0.569	0.725	-5.561	0.164	2.758	2.594
0.280	0.478	-5.163	0.305	3.099	2.794
0.933	0.964	-5.711	0.035	3.077	3.042
0.126	0.307	-4.614	0.524	2.610	2.085
0.984	0.991	-5.715	0.005	4.353	4.348
0.196	0.390	-4.926	0.335	3.064	2.729
0.930	0.962	-5.711	0.025	3.507	3.482
0.291	0.489	-5.188	0.330	4.001	3.672
0.208	0.402	-4.965	-0.627	2.398	3.025
0.293	0.491	-5.192	-0.457	2.978	3.434
0.489	0.664	-5.488	-0.284	3.060	3.344
0.991	0.995	-5.715	-0.004	2.550	2.554
0.012	0.160	-2.795	-1.328	4.338	5.667
0.888	0.939	-5.705	-0.041	2.748	2.790
0.977	0.988	-5.714	0.011	3.197	3.186
0.232	0.428	-5.039	-0.345	2.156	2.501
0.181	0.372	-4.869	-0.473	6.040	6.513
0.224	0.421	-5.017	-0.330	3.827	4.158
0.385	0.574	-5.358	-0.246	2.586	2.832
0.475	0.653	-5.474	-0.183	4.504	4.687
0.009	0.157	-2.541	-1.056	4.178	5.234
0.037	0.190	-3.680	-0.948	1.999	2.947
0.532	0.695	-5.529	-0.261	4.001	4.262
0.123	0.304	-4.596	0.396	2.589	2.192
0.641	0.779	-5.612	0.142	3.001	2.860
0.563	0.721	-5.556	-0.159	3.892	4.051
0.048	0.205	-3.881	0.745	6.239	5.494
0.028	0.178	-3.454	-0.786	2.357	3.144
0.777	0.872	-5.677	-0.110	2.709	2.819
0.599	0.749	-5.584	0.181	3.366	3.185
0.045	0.201	-3.820	0.750	3.689	2.938
0.531	0.695	-5.529	-0.286	3.235	3.520

0.350	0.545	-5.302	0.325	2.727	2.403
0.241	0.438	-5.065	-0.517	3.804	4.321
0.783	0.876	-5.679	0.102	4.022	3.921
0.047	0.203	-3.858	-0.654	2.749	3.403
0.179	0.370	-4.863	-0.336	4.527	4.863
0.689	0.814	-5.639	-0.144	2.114	2.259
0.085	0.257	-4.316	0.716	3.022	2.306
0.403	0.591	-5.384	-0.310	2.631	2.941
0.232	0.429	-5.040	0.348	2.800	2.453
0.974	0.987	-5.714	-0.010	4.139	4.149
0.651	0.787	-5.618	-0.131	2.480	2.611
0.051	0.208	-3.919	0.967	6.034	5.067
0.167	0.357	-4.815	-0.412	3.162	3.574
0.148	0.332	-4.726	0.404	3.948	3.543
0.562	0.721	-5.556	-0.198	1.540	1.738
0.084	0.256	-4.310	-0.460	1.895	2.355
0.059	0.221	-4.041	1.165	5.847	4.682
0.474	0.652	-5.472	-0.301	2.422	2.723
0.539	0.701	-5.536	-0.227	2.839	3.066
0.597	0.747	-5.583	-0.153	3.771	3.924
0.119	0.299	-4.571	0.624	2.849	2.225
0.127	0.308	-4.620	-0.393	4.829	5.222
0.293	0.491	-5.193	-0.282	3.529	3.810
0.440	0.624	-5.432	0.327	2.426	2.099
0.488	0.663	-5.487	-0.189	2.459	2.647
0.057	0.218	-4.007	-0.514	2.582	3.096
0.041	0.195	-3.757	0.424	7.009	6.585
0.007	0.157	-2.307	1.388	9.649	8.261
0.311	0.508	-5.229	-0.370	5.206	5.576
0.170	0.361	-4.827	0.274	6.826	6.552
0.093	0.268	-4.385	-0.541	3.018	3.559
0.176	0.365	-4.850	0.397	4.673	4.276
0.351	0.546	-5.303	-0.195	8.336	8.531
0.054	0.214	-3.964	1.179	4.820	3.642
0.999	0.999	-5.715	0.000	4.309	4.308
0.031	0.181	-3.530	0.937	9.390	8.453
0.092	0.267	-4.379	0.360	6.522	6.162
0.581	0.736	-5.571	0.156	3.966	3.810
0.110	0.289	-4.510	0.344	4.511	4.167
0.272	0.470	-5.145	-0.269	5.370	5.639
0.045	0.201	-3.820	0.612	7.793	7.181
0.380	0.569	-5.350	-0.290	4.777	5.067
0.847	0.916	-5.697	0.093	2.230	2.137
0.433	0.618	-5.424	0.174	3.137	2.963
0.243	0.441	-5.072	-0.523	3.688	4.211
0.029	0.178	-3.492	0.608	6.776	6.168
0.141	0.325	-4.692	0.420	4.420	4.000
0.057	0.219	-4.017	-0.600	3.303	3.903
0.042	0.198	-3.783	0.951	5.382	4.430
0.074	0.242	-4.215	-0.562	1.789	2.351

0.001	0.157	-1.050	-0.921	4.391	5.312
0.796	0.885	-5.683	-0.076	3.204	3.280
0.103	0.280	-4.462	-0.935	2.205	3.140
0.696	0.819	-5.642	-0.151	2.392	2.542
0.223	0.419	-5.013	-0.327	2.349	2.675
0.589	0.741	-5.576	0.169	4.740	4.570
0.781	0.874	-5.678	-0.095	3.664	3.758
0.210	0.404	-4.972	0.345	5.140	4.795
0.061	0.223	-4.060	0.609	7.415	6.805
0.024	0.173	-3.317	-0.512	6.890	7.402
0.002	0.157	-1.456	-1.199	6.359	7.559
0.082	0.253	-4.290	-0.579	2.082	2.662
0.013	0.160	-2.835	-1.355	6.562	7.917
0.346	0.541	-5.294	0.184	8.630	8.446
0.140	0.324	-4.689	0.478	4.582	4.104
0.047	0.203	-3.857	-1.082	6.949	8.030
0.047	0.203	-3.859	-1.092	6.978	8.070
0.868	0.928	-5.702	-0.046	3.962	4.008
0.522	0.688	-5.520	0.186	3.078	2.892
0.444	0.627	-5.437	0.191	4.947	4.756
0.083	0.255	-4.303	-0.500	3.991	4.491
0.007	0.157	-2.333	0.717	3.967	3.250
0.006	0.157	-2.221	0.810	3.550	2.740
0.049	0.207	-3.895	0.651	5.037	4.387
0.564	0.721	-5.557	0.175	4.840	4.665
0.022	0.170	-3.258	-0.800	5.323	6.124
0.019	0.164	-3.127	1.384	6.317	4.933
0.076	0.245	-4.234	0.451	7.882	7.431
0.281	0.478	-5.164	-0.401	3.923	4.325
0.866	0.928	-5.701	0.035	5.083	5.048
0.901	0.946	-5.708	-0.034	4.371	4.405
0.118	0.298	-4.566	0.394	7.736	7.342
0.019	0.164	-3.143	0.909	5.160	4.251
0.014	0.160	-2.866	1.553	6.736	5.184
0.719	0.834	-5.653	-0.111	4.203	4.314
0.590	0.743	-5.578	-0.143	2.409	2.552
0.692	0.816	-5.641	-0.273	2.317	2.590
0.203	0.398	-4.951	0.459	3.147	2.689
0.501	0.671	-5.500	0.157	3.715	3.558
0.059	0.221	-4.043	0.945	5.810	4.865
0.132	0.315	-4.646	0.669	5.089	4.420
0.145	0.329	-4.712	0.658	4.818	4.160
0.027	0.177	-3.434	-0.684	3.259	3.943
0.255	0.452	-5.102	0.314	4.060	3.746
0.172	0.362	-4.835	0.293	7.575	7.281
0.680	0.808	-5.634	-0.106	4.252	4.358
0.890	0.940	-5.706	-0.037	3.316	3.353
0.967	0.984	-5.714	0.012	4.903	4.891
0.227	0.424	-5.025	-0.346	6.193	6.540
0.364	0.555	-5.324	0.225	4.398	4.173

0.090	0.264	-4.360	0.395	6.896	6.501
0.169	0.359	-4.822	0.268	6.572	6.304
0.997	0.998	-5.715	0.001	5.624	5.623
0.159	0.348	-4.780	0.347	6.874	6.527
0.069	0.235	-4.159	0.434	5.132	4.698
0.028	0.178	-3.449	0.452	6.786	6.333
0.751	0.856	-5.667	0.066	6.094	6.028
0.354	0.548	-5.309	0.237	7.055	6.818
0.761	0.863	-5.671	-0.071	3.931	4.002
0.982	0.990	-5.715	0.005	5.274	5.269
0.087	0.260	-4.337	0.358	10.091	9.733
0.011	0.157	-2.679	0.590	7.414	6.825
0.057	0.219	-4.017	-0.533	3.868	4.401
0.189	0.381	-4.899	0.320	3.197	2.877
0.864	0.927	-5.701	-0.053	4.452	4.505
0.076	0.245	-4.237	0.401	6.152	5.751
0.158	0.346	-4.775	0.353	5.431	5.078
0.805	0.890	-5.686	0.061	6.507	6.446
0.449	0.631	-5.444	-0.328	2.812	3.140
0.487	0.662	-5.486	0.275	2.642	2.367
0.935	0.965	-5.712	-0.030	3.060	3.091
0.913	0.954	-5.709	0.048	4.572	4.524
0.418	0.604	-5.404	0.155	6.102	5.947
0.106	0.284	-4.486	0.373	6.566	6.193
0.239	0.436	-5.059	-0.298	2.621	2.918
0.371	0.562	-5.337	-0.247	3.399	3.646
0.123	0.304	-4.594	-0.395	6.739	7.133
0.809	0.893	-5.687	-0.080	2.570	2.651
0.527	0.692	-5.525	0.203	7.305	7.102
0.209	0.404	-4.971	-0.378	8.176	8.554
0.654	0.789	-5.620	0.122	1.955	1.833
0.159	0.348	-4.780	0.498	4.263	3.765
0.090	0.264	-4.359	0.436	2.618	2.182
0.321	0.518	-5.249	0.391	2.970	2.579
0.596	0.747	-5.582	-0.142	2.830	2.971
0.139	0.323	-4.682	1.021	4.835	3.814
0.814	0.895	-5.689	-0.064	6.324	6.388
0.619	0.763	-5.597	-0.313	4.325	4.638
0.537	0.699	-5.534	-0.333	6.424	6.757
0.999	0.999	-5.715	-0.001	6.307	6.308
0.936	0.966	-5.712	0.037	2.494	2.457
0.083	0.255	-4.302	0.691	3.860	3.169
0.218	0.412	-4.997	0.439	7.956	7.518
0.761	0.863	-5.671	0.094	9.434	9.341
0.830	0.904	-5.693	0.058	6.461	6.404
0.038	0.192	-3.692	0.520	4.583	4.062
0.327	0.523	-5.261	0.209	4.863	4.654
0.929	0.961	-5.711	-0.024	4.206	4.230
0.185	0.376	-4.884	0.313	4.986	4.673
0.733	0.844	-5.660	0.139	3.260	3.121

0.434	0.618	-5.425	-0.224	2.831	3.055
0.163	0.352	-4.797	-0.471	3.665	4.136
0.380	0.569	-5.350	-0.241	3.985	4.226
0.046	0.203	-3.839	-0.727	7.730	8.457
0.406	0.594	-5.388	-0.251	4.214	4.466
0.127	0.307	-4.615	0.308	7.003	6.695
0.424	0.609	-5.412	0.149	7.624	7.475
0.764	0.864	-5.672	-0.064	6.863	6.927
0.916	0.954	-5.709	0.029	6.351	6.322
0.077	0.246	-4.245	0.391	5.915	5.524
0.048	0.204	-3.872	-0.621	2.736	3.358
0.514	0.682	-5.513	-0.140	4.662	4.802
0.032	0.182	-3.552	0.438	6.849	6.410
0.013	0.160	-2.845	0.628	4.570	3.942
0.467	0.646	-5.464	-0.255	3.462	3.717
0.546	0.708	-5.542	-0.160	6.246	6.405
0.039	0.192	-3.707	-0.555	6.192	6.747
0.272	0.470	-5.144	-0.240	7.362	7.602
0.992	0.996	-5.715	0.003	9.965	9.962
0.293	0.490	-5.191	-0.674	3.243	3.917
0.271	0.469	-5.141	0.602	7.122	6.520
0.974	0.987	-5.714	-0.011	7.645	7.656
0.970	0.985	-5.714	0.024	5.161	5.137
0.885	0.937	-5.705	-0.063	6.575	6.639
0.615	0.759	-5.595	-0.176	9.031	9.207
0.679	0.808	-5.634	0.162	7.749	7.587
0.970	0.985	-5.714	-0.014	3.480	3.494
0.704	0.824	-5.646	0.153	8.302	8.150
0.759	0.861	-5.670	0.162	6.095	5.933
0.907	0.950	-5.708	-0.040	7.496	7.536
0.754	0.858	-5.668	0.095	9.391	9.295
0.138	0.321	-4.676	0.641	4.183	3.542
0.836	0.908	-5.694	-0.070	4.921	4.991
0.006	0.157	-2.268	-0.872	4.856	5.728
0.061	0.223	-4.059	0.879	5.084	4.206
0.086	0.258	-4.327	0.450	6.684	6.234
0.317	0.516	-5.242	0.245	3.194	2.949
0.786	0.878	-5.680	0.095	3.597	3.503
0.071	0.239	-4.186	1.050	5.749	4.698
0.066	0.231	-4.130	-0.526	3.897	4.423
0.006	0.157	-2.151	-1.003	5.615	6.618
0.689	0.814	-5.639	-0.114	5.105	5.218
0.121	0.302	-4.583	-0.510	3.788	4.297
0.563	0.721	-5.557	-0.232	4.940	5.172
0.764	0.864	-5.672	-0.096	2.718	2.814
0.902	0.947	-5.708	0.040	6.166	6.126
0.071	0.238	-4.179	0.571	6.122	5.552
0.649	0.785	-5.617	-0.132	3.957	4.089
0.091	0.267	-4.373	0.445	3.069	2.624
0.242	0.439	-5.067	0.362	2.416	2.053

0.473	0.652	-5.471	0.221	3.041	2.820
0.024	0.173	-3.332	0.585	8.847	8.262
0.063	0.226	-4.086	-0.546	3.619	4.165
0.262	0.460	-5.120	0.294	3.224	2.930
0.047	0.203	-3.856	0.731	7.743	7.012
0.278	0.477	-5.160	0.467	6.007	5.539
0.025	0.175	-3.364	-0.588	4.549	5.137
0.934	0.964	-5.711	0.022	3.499	3.477
0.637	0.777	-5.610	-0.169	2.552	2.721
0.909	0.951	-5.709	0.032	2.830	2.798
0.482	0.658	-5.481	0.294	6.088	5.794
0.459	0.639	-5.455	-0.165	5.713	5.878
0.059	0.221	-4.040	-0.620	4.157	4.777
0.597	0.747	-5.583	0.181	2.838	2.656
0.883	0.936	-5.705	-0.053	3.511	3.564
0.042	0.196	-3.769	-0.623	3.669	4.292
0.033	0.184	-3.573	0.662	5.478	4.817
0.489	0.664	-5.489	-0.419	2.711	3.131
0.150	0.336	-4.739	0.547	6.086	5.538
0.760	0.862	-5.671	0.071	5.317	5.247
0.018	0.163	-3.115	-1.091	5.678	6.769
0.015	0.160	-2.949	-0.864	5.663	6.528
0.101	0.278	-4.448	-0.591	4.346	4.937
0.876	0.933	-5.703	-0.083	2.531	2.613
0.011	0.159	-2.730	0.731	9.585	8.854
0.107	0.285	-4.491	0.518	4.396	3.878
0.307	0.505	-5.222	-0.360	3.562	3.922
0.059	0.221	-4.039	0.363	7.700	7.336
0.007	0.157	-2.368	-0.893	6.285	7.178
0.425	0.611	-5.414	-0.287	4.890	5.177
0.005	0.157	-2.025	-0.945	3.846	4.791
0.032	0.182	-3.561	0.792	4.459	3.667
0.089	0.264	-4.355	-0.470	2.205	2.674
0.076	0.245	-4.236	0.535	4.172	3.637
0.003	0.157	-1.660	-1.212	5.217	6.429
0.007	0.157	-2.350	0.839	6.259	5.420
0.154	0.341	-4.756	0.288	7.209	6.922
0.015	0.160	-2.940	1.526	8.273	6.748
0.017	0.162	-3.032	0.687	4.846	4.159
0.039	0.194	-3.724	0.556	5.863	5.307
0.160	0.349	-4.783	-0.474	5.701	6.176
0.398	0.585	-5.376	0.366	2.861	2.495
0.100	0.277	-4.443	0.363	6.770	6.407
0.249	0.447	-5.088	0.347	4.427	4.080
0.507	0.677	-5.507	-0.173	3.415	3.588
0.246	0.443	-5.079	-0.288	3.523	3.811
0.029	0.178	-3.471	-0.701	5.178	5.879
0.660	0.793	-5.623	-0.151	3.678	3.829
0.164	0.353	-4.800	-0.335	6.667	7.001
0.048	0.204	-3.871	0.429	5.649	5.220

0.350	0.545	-5.302	0.330	3.660	3.330
0.885	0.937	-5.705	-0.037	5.018	5.054
0.163	0.352	-4.798	-0.351	4.154	4.505
0.005	0.157	-2.010	0.867	5.942	5.075
0.257	0.455	-5.108	-0.427	5.875	6.303
0.123	0.304	-4.594	0.686	7.991	7.305
0.017	0.162	-3.031	1.059	8.620	7.561
0.107	0.286	-4.492	0.514	8.310	7.796
0.042	0.197	-3.776	0.795	8.665	7.870
0.217	0.412	-4.996	-0.319	3.578	3.897
0.043	0.200	-3.801	1.137	9.287	8.150
0.242	0.439	-5.068	0.255	7.410	7.156
0.029	0.178	-3.486	0.495	8.518	8.022
0.552	0.713	-5.547	-0.354	3.604	3.958
0.051	0.208	-3.921	-0.530	5.327	5.857
0.094	0.270	-4.393	0.462	4.495	4.033
0.157	0.345	-4.770	0.411	7.833	7.422
0.059	0.221	-4.045	-0.531	5.999	6.530
0.258	0.456	-5.111	0.357	5.214	4.857
0.715	0.831	-5.652	-0.100	2.649	2.749
0.284	0.482	-5.172	-0.279	2.833	3.112
0.025	0.174	-3.347	-0.723	1.808	2.531
0.031	0.180	-3.526	0.820	8.479	7.659
0.059	0.220	-4.034	-0.574	5.014	5.588
0.062	0.225	-4.082	-0.401	7.288	7.689
0.057	0.219	-4.017	-0.401	7.040	7.441
0.327	0.523	-5.260	-0.267	5.403	5.671
0.250	0.448	-5.090	-0.254	6.286	6.540
0.029	0.178	-3.489	-1.032	1.988	3.019
0.179	0.370	-4.864	-0.455	7.189	7.644
0.183	0.374	-4.879	-0.454	5.827	6.281
0.050	0.208	-3.909	-0.453	7.757	8.209
0.285	0.483	-5.176	-0.386	5.078	5.464
0.437	0.621	-5.429	-0.229	3.552	3.781
0.051	0.210	-3.932	0.519	3.957	3.437
0.687	0.812	-5.638	-0.078	8.752	8.830
0.145	0.329	-4.713	0.537	7.416	6.880
0.164	0.353	-4.800	-0.369	4.912	5.281
0.072	0.240	-4.194	0.349	6.236	5.887
0.081	0.252	-4.281	0.554	7.441	6.888
0.018	0.164	-3.120	1.163	7.292	6.128
0.008	0.157	-2.431	-1.105	2.703	3.809
0.715	0.831	-5.652	-0.147	4.310	4.457
0.588	0.741	-5.576	-0.187	3.949	4.137
0.342	0.538	-5.289	-0.295	3.238	3.533
0.081	0.252	-4.278	0.411	7.275	6.865
0.015	0.160	-2.937	0.570	9.991	9.421
0.294	0.491	-5.194	-0.463	4.631	5.094
0.036	0.188	-3.648	-0.791	4.661	5.452
0.113	0.291	-4.528	-0.756	2.470	3.226

0.036	0.189	-3.657	0.501	6.476	5.974
0.510	0.679	-5.510	-0.381	2.408	2.789
0.032	0.182	-3.560	-0.604	5.888	6.492
0.813	0.894	-5.688	0.055	7.790	7.735
0.018	0.162	-3.085	1.206	5.614	4.408
0.667	0.798	-5.627	0.166	2.482	2.316
0.929	0.961	-5.711	-0.069	4.331	4.400
0.032	0.182	-3.551	1.063	8.643	7.580
0.604	0.753	-5.588	-0.140	8.055	8.195
0.005	0.157	-1.999	-1.190	6.307	7.497
0.035	0.187	-3.637	0.491	8.362	7.870
0.109	0.288	-4.504	0.333	7.811	7.478
0.012	0.160	-2.768	0.741	7.501	6.760
0.694	0.817	-5.642	-0.089	4.319	4.408
0.023	0.173	-3.299	-0.864	2.119	2.983
0.817	0.896	-5.689	0.064	5.551	5.487
0.012	0.160	-2.755	-0.790	5.061	5.851
0.031	0.182	-3.542	-0.581	5.954	6.535
0.091	0.267	-4.374	0.438	8.074	7.636
0.057	0.218	-4.006	0.723	8.445	7.722
0.104	0.281	-4.468	-0.345	6.170	6.515
0.022	0.171	-3.277	1.201	5.818	4.617
0.039	0.194	-3.726	-0.743	1.952	2.695
0.013	0.160	-2.835	0.524	5.086	4.562
0.164	0.353	-4.799	0.614	3.573	2.959
0.294	0.491	-5.193	0.200	7.609	7.409
0.014	0.160	-2.900	0.637	4.576	3.939
0.019	0.165	-3.156	0.618	4.542	3.924
0.119	0.298	-4.567	0.302	5.810	5.508
0.034	0.184	-3.603	0.565	5.634	5.069
0.174	0.364	-4.844	-0.593	8.659	9.252
0.344	0.539	-5.292	-0.374	3.001	3.375
0.594	0.746	-5.581	0.147	3.089	2.942
0.076	0.245	-4.231	0.591	8.737	8.146
0.104	0.281	-4.467	0.728	6.465	5.737
0.836	0.908	-5.694	0.054	4.720	4.666
0.081	0.253	-4.283	0.554	8.056	7.502
0.006	0.157	-2.204	-0.618	4.706	5.324
0.805	0.890	-5.686	-0.055	5.168	5.223
0.042	0.196	-3.770	0.854	7.832	6.978
0.419	0.606	-5.406	0.699	8.760	8.060
0.221	0.417	-5.006	1.068	11.115	10.047
0.282	0.480	-5.167	0.746	10.500	9.754
0.009	0.157	-2.536	0.901	8.452	7.551
0.135	0.318	-4.660	0.438	2.724	2.286
0.053	0.212	-3.952	0.749	4.325	3.576
0.085	0.257	-4.316	-0.711	3.900	4.611
0.203	0.398	-4.950	-0.465	2.463	2.928
0.140	0.324	-4.689	-0.357	6.079	6.436
0.042	0.198	-3.780	0.987	5.700	4.713

0.012	0.160	-2.787	1.374	7.532	6.158
0.169	0.359	-4.821	-0.313	5.080	5.394
0.046	0.203	-3.841	-0.526	4.443	4.969
0.390	0.577	-5.365	-0.199	5.772	5.971
0.008	0.157	-2.483	1.631	7.822	6.191
0.171	0.361	-4.832	0.584	7.271	6.687
0.014	0.160	-2.895	0.980	5.794	4.813
0.370	0.560	-5.335	0.241	3.244	3.003
0.406	0.594	-5.388	0.187	5.417	5.230
0.057	0.218	-4.006	0.586	2.481	1.895
0.012	0.160	-2.800	1.545	5.848	4.303
0.002	0.157	-1.278	2.540	6.576	4.036
0.221	0.418	-5.009	0.268	6.849	6.581
0.302	0.500	-5.212	-0.244	3.169	3.413
0.079	0.249	-4.260	-0.440	3.446	3.886
0.305	0.503	-5.218	-0.276	3.803	4.078
0.387	0.575	-5.361	-0.198	2.895	3.093
0.097	0.273	-4.416	-0.389	6.320	6.709
0.058	0.219	-4.024	-0.611	1.439	2.051
0.258	0.456	-5.111	-0.404	2.112	2.516
0.353	0.548	-5.307	0.445	5.652	5.206
0.073	0.241	-4.201	-0.446	2.838	3.284
0.673	0.804	-5.631	-0.115	6.722	6.837
0.738	0.848	-5.662	-0.080	3.395	3.475
0.894	0.942	-5.706	-0.032	4.375	4.407
0.685	0.811	-5.637	-0.117	3.384	3.501
0.044	0.200	-3.804	0.880	5.854	4.974
0.025	0.175	-3.359	-0.810	2.551	3.361
0.072	0.240	-4.196	0.425	9.525	9.100
0.273	0.471	-5.147	0.292	4.585	4.293
0.705	0.824	-5.647	0.084	3.161	3.077
0.876	0.932	-5.703	-0.044	5.445	5.489
0.047	0.204	-3.864	0.595	5.041	4.446
0.022	0.171	-3.274	-0.572	3.671	4.242
0.277	0.475	-5.155	0.333	3.799	3.465
0.194	0.387	-4.918	0.312	6.817	6.505
0.144	0.328	-4.707	0.423	4.791	4.369
0.378	0.568	-5.347	-0.213	3.412	3.625
0.098	0.274	-4.422	0.490	5.321	4.831
0.376	0.566	-5.344	-0.230	4.811	5.042
0.007	0.157	-2.351	-0.895	3.238	4.133
0.273	0.471	-5.147	-0.291	3.820	4.111
0.295	0.492	-5.197	0.241	5.675	5.434
0.013	0.160	-2.822	1.165	6.349	5.185
0.026	0.175	-3.386	-0.612	6.884	7.496
0.013	0.160	-2.828	-1.437	2.522	3.959
0.179	0.369	-4.861	0.326	6.228	5.902
0.015	0.160	-2.928	1.145	5.459	4.314
0.002	0.157	-1.442	-0.769	3.711	4.480
0.052	0.211	-3.941	-0.570	7.967	8.536

0.524	0.689	-5.523	0.166	6.238	6.072
0.789	0.880	-5.681	-0.068	2.670	2.738
0.013	0.160	-2.816	-0.799	4.564	5.363
0.380	0.569	-5.350	-0.237	2.561	2.798
0.210	0.404	-4.972	0.241	4.949	4.708
0.117	0.297	-4.555	0.393	4.770	4.377
0.015	0.161	-2.968	-0.986	4.737	5.724
0.633	0.773	-5.607	0.135	3.230	3.095
0.984	0.991	-5.715	0.004	2.293	2.289
0.013	0.160	-2.811	-1.177	5.575	6.752
0.297	0.494	-5.202	0.230	4.976	4.746
0.131	0.312	-4.638	0.413	6.330	5.917
0.968	0.984	-5.714	-0.011	2.541	2.553
0.046	0.203	-3.849	0.602	9.922	9.320
0.204	0.399	-4.954	-0.288	2.561	2.849
0.004	0.157	-1.778	-0.917	2.349	3.266
0.116	0.296	-4.553	0.418	3.011	2.593
0.883	0.936	-5.704	0.047	3.886	3.839
0.004	0.157	-1.916	-0.993	3.353	4.346
0.081	0.252	-4.278	0.649	4.401	3.752
0.520	0.687	-5.519	0.212	3.834	3.622
0.358	0.552	-5.316	0.248	3.213	2.965
0.034	0.184	-3.600	1.299	8.664	7.365
0.066	0.231	-4.127	0.458	4.402	3.944
0.383	0.572	-5.355	0.188	4.917	4.729
0.357	0.551	-5.313	-0.282	2.110	2.392
0.026	0.175	-3.386	1.210	6.695	5.485
0.454	0.634	-5.449	0.287	2.535	2.247
0.031	0.182	-3.546	0.510	8.302	7.792
0.900	0.945	-5.707	0.029	6.066	6.037
0.575	0.729	-5.566	-0.152	3.836	3.988
0.702	0.822	-5.645	-0.100	1.681	1.781
0.941	0.968	-5.712	0.019	4.214	4.196
0.022	0.171	-3.260	0.942	7.225	6.283
0.127	0.308	-4.619	0.392	4.940	4.549
0.186	0.378	-4.888	0.282	7.783	7.501
0.113	0.292	-4.531	0.330	9.904	9.574
0.126	0.307	-4.611	0.325	9.871	9.545
0.063	0.227	-4.094	-0.541	3.464	4.005
0.064	0.228	-4.102	0.488	4.845	4.357
0.003	0.157	-1.600	1.080	3.523	2.444
0.153	0.340	-4.750	0.429	4.040	3.611
0.909	0.951	-5.709	-0.031	2.956	2.987
0.007	0.157	-2.305	-0.740	5.731	6.471
0.148	0.332	-4.725	-0.479	4.995	5.474
0.613	0.758	-5.594	0.119	6.915	6.796
0.437	0.621	-5.429	0.228	4.307	4.079
0.091	0.267	-4.373	1.308	6.018	4.711
0.117	0.297	-4.557	0.385	5.610	5.226
0.115	0.295	-4.546	0.501	4.224	3.723

0.473	0.652	-5.471	0.192	2.573	2.381
0.016	0.162	-3.025	-0.804	2.142	2.947
0.642	0.780	-5.612	0.118	2.593	2.475
0.094	0.271	-4.398	-0.444	5.238	5.682
0.968	0.984	-5.714	-0.012	2.083	2.094
0.028	0.178	-3.465	-0.772	6.830	7.602
0.064	0.228	-4.104	-0.629	7.570	8.200
0.001	0.157	-0.762	-0.802	3.008	3.810
0.807	0.891	-5.686	0.074	2.892	2.818
0.091	0.266	-4.369	-0.421	8.536	8.957
0.222	0.419	-5.012	-0.391	7.817	8.208
0.396	0.584	-5.375	-0.219	6.986	7.204
0.094	0.270	-4.394	-0.436	8.144	8.580
0.075	0.244	-4.225	-0.442	8.601	9.044
0.136	0.319	-4.665	-0.365	8.509	8.874
0.105	0.283	-4.477	-0.580	5.076	5.657
0.047	0.204	-3.861	-0.479	7.010	7.488
0.016	0.162	-2.998	-0.778	4.674	5.452
0.017	0.162	-3.046	0.929	5.805	4.876
0.214	0.409	-4.987	0.311	3.648	3.338
0.825	0.901	-5.692	-0.068	3.409	3.476
0.369	0.559	-5.333	-0.328	2.608	2.936
0.991	0.996	-5.715	-0.005	5.447	5.452
0.468	0.647	-5.465	-0.217	2.683	2.900
0.026	0.176	-3.399	0.770	9.428	8.657
0.066	0.231	-4.128	1.081	7.683	6.603
0.469	0.648	-5.466	-0.465	5.544	6.009
0.049	0.207	-3.901	0.852	6.596	5.743
0.887	0.938	-5.705	0.028	2.618	2.590
0.098	0.274	-4.424	0.649	4.688	4.039
0.025	0.175	-3.371	1.214	6.145	4.930
0.316	0.514	-5.240	0.342	4.266	3.924
0.017	0.162	-3.069	0.741	3.547	2.806
0.732	0.843	-5.659	0.101	5.920	5.819
0.217	0.412	-4.995	0.477	5.616	5.139
0.057	0.218	-4.011	0.580	7.082	6.502
0.166	0.356	-4.810	0.303	6.744	6.441
0.019	0.164	-3.136	1.543	5.508	3.965
0.011	0.157	-2.684	0.610	5.184	4.574
0.570	0.726	-5.562	-0.156	2.372	2.528
0.643	0.780	-5.613	0.114	5.536	5.421
0.099	0.275	-4.430	0.394	6.345	5.951
0.947	0.973	-5.713	0.020	3.723	3.703
0.219	0.415	-5.002	0.496	4.566	4.070
0.874	0.932	-5.703	-0.070	2.734	2.804
0.629	0.770	-5.604	0.132	5.986	5.854
0.007	0.157	-2.269	-0.781	5.430	6.211
0.014	0.160	-2.893	0.575	9.182	8.606
0.069	0.236	-4.162	0.485	3.615	3.130
0.561	0.719	-5.554	0.269	4.640	4.372

0.548	0.709	-5.544	0.191	3.369	3.178
0.336	0.532	-5.277	0.329	2.770	2.441
0.744	0.851	-5.664	0.093	2.589	2.496
0.248	0.446	-5.084	0.262	5.685	5.424
0.971	0.986	-5.714	0.011	3.595	3.584
0.005	0.157	-2.077	2.340	9.342	7.002
0.875	0.932	-5.703	-0.043	5.016	5.059
0.122	0.303	-4.588	0.865	6.354	5.489
0.024	0.173	-3.316	1.266	9.926	8.660
0.026	0.175	-3.380	0.781	6.414	5.633
0.329	0.525	-5.265	-0.232	4.174	4.406
0.047	0.204	-3.861	0.918	6.450	5.532
0.025	0.175	-3.355	0.835	6.229	5.394
0.022	0.171	-3.263	0.921	8.474	7.553
0.051	0.208	-3.922	0.681	7.669	6.988
0.252	0.450	-5.096	-0.461	3.664	4.126
0.108	0.287	-4.500	0.749	9.426	8.677
0.083	0.255	-4.300	0.785	9.634	8.849
0.250	0.447	-5.089	0.384	3.579	3.195
0.028	0.178	-3.457	0.722	7.711	6.989
0.139	0.324	-4.684	-0.499	4.574	5.073
0.027	0.176	-3.429	-0.538	3.833	4.371
0.307	0.504	-5.221	0.395	4.136	3.741
0.079	0.249	-4.263	0.470	7.156	6.686
0.256	0.454	-5.106	-0.322	6.356	6.678
0.023	0.173	-3.290	0.816	6.779	5.964
0.663	0.795	-5.625	-0.127	4.362	4.488
0.088	0.262	-4.344	-0.515	2.396	2.911
0.101	0.278	-4.448	-0.494	3.349	3.843
0.147	0.331	-4.723	0.425	2.375	1.950
0.013	0.160	-2.837	0.805	6.451	5.645
0.025	0.174	-3.350	0.744	5.847	5.103
0.422	0.608	-5.409	0.220	4.942	4.722
0.417	0.604	-5.403	-0.249	5.968	6.217
0.034	0.185	-3.613	0.659	7.807	7.148
0.448	0.630	-5.443	0.262	3.184	2.922
0.076	0.245	-4.236	-0.435	2.926	3.361
0.084	0.256	-4.306	0.525	4.891	4.366
0.029	0.178	-3.471	1.276	6.477	5.201
0.128	0.309	-4.622	-0.413	2.419	2.833
0.330	0.526	-5.266	-0.222	3.249	3.471
0.536	0.698	-5.533	-0.160	4.381	4.541
0.135	0.319	-4.664	0.283	8.740	8.457
0.139	0.323	-4.683	0.348	9.783	9.435
0.062	0.224	-4.071	0.618	3.526	2.908
0.002	0.157	-1.076	0.931	3.574	2.643
0.019	0.164	-3.125	0.743	3.431	2.687
0.154	0.341	-4.757	0.344	5.379	5.035
0.028	0.177	-3.442	0.636	5.663	5.027
0.490	0.664	-5.490	0.170	6.410	6.240

0.125	0.306	-4.606	-0.573	6.110	6.683
0.339	0.534	-5.283	0.359	3.187	2.828
0.452	0.633	-5.448	-0.215	2.041	2.256
0.931	0.963	-5.711	-0.024	3.842	3.866
0.190	0.382	-4.902	-0.436	4.006	4.442
0.014	0.160	-2.874	1.333	4.862	3.528
0.984	0.991	-5.715	-0.006	2.245	2.251
0.116	0.296	-4.551	-0.420	6.794	7.214
0.062	0.224	-4.075	0.575	6.069	5.494
0.184	0.375	-4.882	0.482	3.772	3.290
0.267	0.465	-5.133	0.373	4.671	4.298
0.564	0.721	-5.557	0.134	3.374	3.240
0.113	0.292	-4.532	0.540	4.761	4.221
0.022	0.169	-3.244	1.226	5.429	4.203
0.620	0.763	-5.598	-0.140	3.586	3.726
0.032	0.183	-3.562	0.583	2.483	1.900
0.039	0.192	-3.706	0.581	4.422	3.841
0.015	0.160	-2.938	-0.710	4.140	4.851
0.196	0.390	-4.926	-0.366	2.750	3.115
0.700	0.821	-5.645	-0.110	3.557	3.667
0.557	0.716	-5.552	0.159	2.959	2.800
0.254	0.452	-5.101	0.297	2.279	1.982
0.758	0.860	-5.670	-0.088	5.197	5.285
0.435	0.619	-5.426	-0.230	2.412	2.642
0.086	0.258	-4.326	0.680	3.791	3.111
0.100	0.277	-4.442	0.538	4.222	3.685
0.938	0.967	-5.712	0.023	3.297	3.274
0.197	0.392	-4.930	-0.343	2.180	2.523
0.179	0.370	-4.864	-0.359	2.703	3.062
0.025	0.175	-3.356	0.553	7.460	6.906
0.144	0.329	-4.710	0.375	5.838	5.463
0.165	0.353	-4.803	-0.452	2.374	2.826
0.312	0.509	-5.231	0.527	7.258	6.731
0.434	0.618	-5.425	-0.179	8.335	8.514
0.009	0.157	-2.564	1.566	5.443	3.876
0.010	0.157	-2.651	0.566	5.864	5.298
0.125	0.306	-4.605	0.308	6.386	6.078
0.019	0.164	-3.146	0.863	5.969	5.105
0.118	0.298	-4.564	0.555	7.098	6.542
0.025	0.175	-3.376	1.299	6.069	4.770
0.976	0.988	-5.714	0.010	2.680	2.670
0.640	0.779	-5.611	0.148	2.676	2.528
0.856	0.923	-5.699	0.066	2.827	2.761
0.149	0.334	-4.732	0.410	4.083	3.673
0.466	0.645	-5.463	-0.230	3.218	3.448
0.010	0.157	-2.613	-0.980	5.577	6.557
0.001	0.157	-0.510	-1.223	3.936	5.158
0.027	0.176	-3.420	-1.064	4.027	5.090
0.116	0.296	-4.553	0.827	4.148	3.320
0.672	0.803	-5.630	0.126	7.258	7.131

0.119	0.299	-4.572	0.296	7.389	7.093
0.465	0.645	-5.462	-0.329	6.907	7.236
0.009	0.157	-2.567	1.346	8.112	6.766
0.204	0.398	-4.951	0.462	5.200	4.739
0.063	0.226	-4.088	0.849	5.498	4.648
0.152	0.339	-4.748	-0.426	3.034	3.460
0.006	0.157	-2.183	1.181	5.820	4.639
0.293	0.491	-5.192	0.285	3.142	2.856
0.061	0.224	-4.071	0.723	4.437	3.714
0.022	0.169	-3.246	0.867	4.441	3.574
0.543	0.705	-5.539	0.175	4.622	4.447
0.003	0.157	-1.720	0.997	4.537	3.541
0.017	0.162	-3.062	1.792	5.151	3.359
0.586	0.739	-5.574	-0.154	1.528	1.682
0.563	0.721	-5.557	-0.157	3.094	3.251
0.603	0.752	-5.587	-0.156	3.100	3.256
0.266	0.464	-5.129	-0.354	4.754	5.108
0.528	0.693	-5.526	-0.266	5.933	6.199
0.339	0.534	-5.282	-0.418	3.115	3.532
0.885	0.937	-5.705	0.046	2.693	2.647
0.473	0.652	-5.472	-0.322	3.388	3.710
0.223	0.419	-5.013	-0.473	4.192	4.665
0.642	0.780	-5.612	0.168	2.946	2.778
0.417	0.604	-5.403	-0.233	3.005	3.237
0.053	0.214	-3.963	-0.742	2.550	3.292
0.112	0.290	-4.522	-0.988	2.233	3.222
0.070	0.237	-4.175	-0.838	2.434	3.272
0.252	0.450	-5.095	0.398	2.544	2.146
0.209	0.404	-4.970	0.341	3.575	3.234
0.044	0.201	-3.819	-1.166	1.412	2.578
0.773	0.870	-5.675	-0.082	3.137	3.219
0.127	0.308	-4.619	-0.387	2.741	3.127
0.035	0.186	-3.625	0.494	6.078	5.584
0.081	0.252	-4.278	0.324	6.364	6.040
0.116	0.296	-4.551	0.480	3.580	3.100
0.018	0.163	-3.105	-0.614	3.954	4.568
0.079	0.249	-4.260	0.491	3.502	3.010
0.625	0.767	-5.602	-0.143	6.493	6.636
0.023	0.173	-3.299	0.733	6.177	5.444
0.036	0.188	-3.650	0.604	6.729	6.125
0.816	0.896	-5.689	-0.073	2.487	2.559
0.022	0.169	-3.248	0.582	5.034	4.452
0.101	0.278	-4.447	-0.439	3.208	3.647
0.315	0.513	-5.238	-0.259	2.718	2.976
0.978	0.989	-5.714	0.008	4.447	4.439
0.949	0.974	-5.713	-0.017	1.634	1.651
0.128	0.309	-4.622	0.690	5.613	4.923
0.342	0.537	-5.288	0.234	8.226	7.992
0.207	0.402	-4.963	0.391	3.016	2.625
0.002	0.157	-1.194	1.254	4.061	2.808

0.580	0.735	-5.570	0.173	3.598	3.425
0.058	0.219	-4.026	0.524	3.375	2.850
0.012	0.159	-2.743	0.658	5.816	5.158
0.263	0.461	-5.123	-0.229	6.220	6.449
0.010	0.157	-2.648	-0.789	6.210	6.999
0.031	0.181	-3.536	0.600	4.413	3.813
0.086	0.258	-4.327	-0.447	2.401	2.847
0.055	0.216	-3.992	0.477	8.196	7.719
0.226	0.423	-5.022	-0.391	2.272	2.663
0.588	0.741	-5.576	0.177	2.540	2.363
0.003	0.157	-1.508	1.133	7.793	6.660
0.061	0.224	-4.071	0.674	8.546	7.872
0.576	0.731	-5.567	0.217	3.277	3.060
0.766	0.866	-5.673	0.104	3.926	3.822
0.159	0.347	-4.777	-0.372	2.333	2.705
0.542	0.704	-5.539	-0.143	1.984	2.126
0.699	0.820	-5.644	-0.102	2.855	2.956
0.181	0.372	-4.870	0.374	4.142	3.768
0.112	0.291	-4.525	0.763	3.035	2.272
0.021	0.168	-3.230	-0.856	5.872	6.728
0.110	0.289	-4.514	-0.523	4.840	5.362
0.312	0.509	-5.232	-0.495	5.302	5.797
0.235	0.431	-5.047	-0.578	4.428	5.006
0.638	0.777	-5.610	-0.119	4.338	4.457
0.030	0.179	-3.503	-0.915	6.119	7.035
0.777	0.872	-5.677	-0.084	2.438	2.522
0.004	0.157	-1.861	1.064	2.834	1.769
0.040	0.194	-3.744	1.049	4.145	3.096
0.058	0.219	-4.023	-0.574	3.516	4.090
0.333	0.529	-5.271	0.253	4.265	4.012
0.031	0.180	-3.526	-0.715	2.625	3.340
0.009	0.157	-2.542	-0.702	5.248	5.950
0.047	0.204	-3.870	1.262	7.877	6.615
0.215	0.410	-4.990	-0.400	4.818	5.218
0.082	0.254	-4.294	-0.755	7.252	8.007
0.276	0.475	-5.155	0.205	9.080	8.876
0.294	0.491	-5.194	0.291	4.603	4.313
0.387	0.575	-5.360	-0.182	4.648	4.830
0.089	0.264	-4.357	0.435	2.512	2.077
0.799	0.887	-5.684	0.069	5.842	5.773
0.015	0.160	-2.957	-0.974	7.499	8.473
0.004	0.157	-1.846	1.187	8.716	7.528
0.173	0.362	-4.837	-0.350	5.818	6.168
0.041	0.196	-3.763	1.033	7.306	6.273
0.014	0.160	-2.908	1.521	6.814	5.293
0.030	0.180	-3.520	-0.551	3.560	4.111
0.236	0.433	-5.051	0.265	5.491	5.226
0.568	0.724	-5.561	-0.151	4.779	4.930
0.120	0.300	-4.576	0.424	7.363	6.939
0.711	0.828	-5.650	-0.109	5.159	5.269

0.031	0.180	-3.527	0.532	3.299	2.767
0.060	0.222	-4.055	0.600	5.934	5.334
0.127	0.308	-4.619	0.741	5.578	4.836
0.352	0.547	-5.305	0.178	5.980	5.802
0.911	0.952	-5.709	-0.031	3.503	3.534
0.257	0.455	-5.108	-0.427	2.068	2.495
0.400	0.588	-5.380	0.237	4.794	4.557
0.027	0.176	-3.429	-0.768	5.435	6.203
0.619	0.763	-5.598	0.154	4.807	4.654
0.002	0.157	-1.347	-1.283	6.681	7.964
0.028	0.177	-3.443	-0.727	4.306	5.034
0.029	0.178	-3.481	-0.493	5.038	5.531
0.008	0.157	-2.453	-0.668	4.365	5.033
0.117	0.298	-4.560	0.497	5.414	4.917
0.046	0.203	-3.843	-0.532	1.644	2.176
0.173	0.362	-4.837	-0.432	3.569	4.001
0.329	0.525	-5.265	0.226	5.609	5.382
0.047	0.204	-3.861	0.570	6.796	6.226
0.046	0.203	-3.841	1.306	9.130	7.824
0.356	0.550	-5.311	-0.246	4.212	4.458
0.432	0.617	-5.423	0.228	5.654	5.425
0.057	0.218	-4.008	0.468	5.316	4.847
0.021	0.168	-3.219	-0.796	6.559	7.355
0.543	0.705	-5.540	-0.145	4.372	4.517
0.646	0.783	-5.615	0.127	3.403	3.277
0.002	0.157	-1.348	1.736	4.973	3.237
0.175	0.364	-4.846	-0.401	2.588	2.989
0.178	0.368	-4.857	-0.418	1.871	2.289
0.240	0.437	-5.061	-0.372	2.845	3.216
0.490	0.664	-5.489	-0.243	1.613	1.856
0.447	0.629	-5.442	-0.245	2.089	2.334
0.289	0.488	-5.184	-0.351	2.990	3.341
0.744	0.851	-5.664	0.082	4.809	4.727
0.140	0.324	-4.686	-0.479	4.070	4.548
0.015	0.161	-2.970	-0.917	3.573	4.490
0.444	0.627	-5.438	-0.190	4.812	5.001
0.058	0.220	-4.029	-0.518	3.032	3.550
0.341	0.537	-5.287	-0.266	3.387	3.653
0.037	0.190	-3.675	-0.644	5.836	6.479
0.255	0.452	-5.103	0.248	5.665	5.416
0.116	0.296	-4.552	0.444	8.093	7.649
0.438	0.622	-5.431	0.477	5.222	4.745
0.329	0.524	-5.263	-0.302	4.360	4.662
0.832	0.906	-5.694	0.051	5.324	5.272
0.090	0.265	-4.364	0.772	5.933	5.161
0.123	0.304	-4.597	0.434	2.855	2.421
0.030	0.179	-3.507	0.831	7.094	6.263
0.051	0.208	-3.923	1.318	6.480	5.162
0.383	0.572	-5.355	-0.223	8.818	9.041
0.294	0.491	-5.194	0.287	6.294	6.007

0.073	0.241	-4.208	0.567	6.195	5.628
0.020	0.167	-3.178	0.685	4.666	3.981
0.265	0.463	-5.129	0.318	5.833	5.515
0.540	0.702	-5.537	0.167	4.983	4.816
0.756	0.859	-5.669	-0.099	3.847	3.946
0.043	0.200	-3.799	0.549	5.431	4.882
0.044	0.201	-3.811	0.569	4.474	3.904
0.770	0.868	-5.674	0.089	4.263	4.174
0.637	0.776	-5.609	0.190	5.636	5.446
0.615	0.759	-5.595	0.200	5.108	4.908
0.868	0.928	-5.702	0.054	4.318	4.264
0.298	0.495	-5.204	0.268	2.763	2.495
0.194	0.387	-4.918	0.372	4.166	3.793
0.139	0.323	-4.681	-0.430	4.080	4.510
0.036	0.188	-3.645	0.600	5.887	5.287
0.348	0.543	-5.298	-0.313	2.591	2.904
0.585	0.738	-5.574	-0.143	2.109	2.252
0.011	0.158	-2.708	1.440	7.434	5.994
0.128	0.309	-4.621	0.317	6.681	6.364
0.026	0.176	-3.391	0.548	7.748	7.200
0.254	0.451	-5.099	0.305	3.605	3.300
0.625	0.767	-5.601	0.120	1.911	1.791
0.981	0.990	-5.715	-0.007	1.888	1.895
0.083	0.255	-4.305	0.825	4.266	3.440
0.328	0.524	-5.262	0.580	6.520	5.940
0.015	0.160	-2.930	1.560	6.668	5.108
0.327	0.523	-5.260	0.276	6.492	6.216
0.132	0.315	-4.647	0.971	8.033	7.061
0.011	0.159	-2.725	-1.191	3.919	5.110
0.008	0.157	-2.459	0.753	2.620	1.867
0.277	0.475	-5.155	0.222	7.107	6.885
0.092	0.268	-4.382	0.406	5.307	4.901
0.593	0.745	-5.579	0.107	6.803	6.696
0.129	0.310	-4.628	-0.591	2.729	3.320
0.060	0.221	-4.048	0.529	4.591	4.062
0.137	0.321	-4.675	-0.635	4.657	5.292
0.172	0.362	-4.834	-0.300	5.255	5.554
0.141	0.325	-4.692	0.511	6.301	5.789
0.227	0.424	-5.026	0.319	2.784	2.465
0.056	0.218	-4.002	0.928	7.557	6.629
0.019	0.164	-3.136	1.045	7.385	6.340
0.088	0.261	-4.342	-0.572	5.070	5.643
0.020	0.167	-3.186	1.150	6.017	4.866
0.274	0.472	-5.150	-0.245	4.174	4.419
0.266	0.464	-5.129	0.313	5.096	4.783
0.038	0.191	-3.686	0.602	5.699	5.097
0.075	0.243	-4.221	-0.420	4.960	5.380
0.064	0.227	-4.097	0.470	4.429	3.959
0.218	0.412	-4.997	0.425	4.018	3.593
0.217	0.412	-4.995	-0.461	4.296	4.757

0.803	0.889	-5.685	-0.059	4.826	4.885
0.973	0.986	-5.714	0.009	6.719	6.710
0.026	0.176	-3.405	-0.769	5.781	6.550
0.147	0.332	-4.724	0.354	7.381	7.027
0.005	0.157	-1.969	-0.735	4.295	5.030
0.106	0.284	-4.483	-0.438	4.100	4.538
0.909	0.951	-5.709	0.060	3.591	3.531
0.061	0.223	-4.061	-0.586	5.813	6.399
0.592	0.744	-5.579	0.172	4.512	4.340
0.121	0.302	-4.583	-0.822	4.266	5.088
0.253	0.451	-5.098	-0.385	5.308	5.694
0.014	0.160	-2.922	-0.750	5.116	5.866
0.123	0.304	-4.592	0.360	4.537	4.178
0.624	0.766	-5.601	0.155	6.226	6.071
0.050	0.208	-3.914	0.683	6.787	6.103
0.014	0.160	-2.904	-0.938	6.464	7.402
0.616	0.760	-5.595	0.196	4.956	4.760
0.017	0.162	-3.072	-0.593	3.597	4.190
0.929	0.961	-5.711	-0.028	2.876	2.904
0.287	0.485	-5.179	-0.244	4.407	4.652
0.005	0.157	-2.087	1.136	5.526	4.390
0.583	0.737	-5.572	0.129	5.116	4.987
0.198	0.392	-4.933	0.274	8.035	7.761
0.833	0.907	-5.694	-0.066	3.670	3.736
0.009	0.157	-2.546	-0.715	5.633	6.349
0.015	0.161	-2.975	0.657	8.559	7.902
0.447	0.629	-5.441	-0.213	2.613	2.826
0.056	0.217	-3.995	0.637	7.332	6.695
0.589	0.741	-5.576	0.189	1.990	1.801
0.854	0.921	-5.699	-0.045	5.707	5.753
0.016	0.162	-2.995	0.962	7.268	6.306
0.685	0.811	-5.637	-0.116	3.311	3.427
0.023	0.173	-3.310	0.809	5.440	4.631
0.402	0.589	-5.382	-0.228	3.429	3.657
0.814	0.895	-5.689	0.076	8.088	8.012
0.314	0.511	-5.235	0.331	7.166	6.835
0.345	0.541	-5.294	0.289	4.514	4.226
0.426	0.612	-5.415	-0.187	6.554	6.741
0.050	0.208	-3.914	0.442	5.669	5.226
0.099	0.275	-4.430	-0.735	1.781	2.516
0.711	0.828	-5.650	-0.111	5.810	5.921
0.361	0.553	-5.321	0.256	4.167	3.910
0.699	0.820	-5.644	-0.105	6.061	6.166
0.540	0.702	-5.537	0.145	2.462	2.317
0.257	0.455	-5.109	0.232	6.999	6.768
0.078	0.247	-4.251	-0.450	3.467	3.917
0.073	0.241	-4.200	-0.431	5.053	5.484
0.833	0.907	-5.694	-0.046	7.928	7.974
0.943	0.970	-5.712	-0.020	3.592	3.612
0.192	0.385	-4.911	0.300	5.243	4.943

0.254	0.452	-5.100	0.332	5.891	5.559
0.979	0.989	-5.714	0.007	7.007	7.000
0.044	0.200	-3.803	0.516	4.901	4.385
0.292	0.489	-5.189	0.290	7.780	7.490
0.134	0.318	-4.659	-0.333	8.007	8.340
0.026	0.176	-3.406	0.477	8.194	7.717
0.024	0.173	-3.335	-0.973	4.107	5.080
0.998	0.999	-5.715	-0.001	3.677	3.678
0.344	0.539	-5.292	0.231	5.025	4.793
0.793	0.883	-5.682	-0.062	6.812	6.874
0.966	0.983	-5.714	-0.012	4.161	4.173
0.015	0.160	-2.930	-0.614	3.233	3.847
0.493	0.666	-5.492	0.125	5.628	5.503
0.073	0.241	-4.204	0.606	7.018	6.412
0.138	0.321	-4.675	-0.388	4.142	4.530
0.981	0.990	-5.715	-0.007	5.947	5.954
0.006	0.157	-2.254	1.547	7.841	6.294
0.320	0.517	-5.247	-0.268	2.467	2.735
0.065	0.229	-4.115	0.483	6.243	5.760
0.154	0.341	-4.754	-0.285	5.491	5.775
0.011	0.157	-2.702	1.318	5.538	4.221
0.014	0.160	-2.909	-0.587	6.126	6.714
0.024	0.173	-3.340	-0.512	6.089	6.601
0.260	0.458	-5.116	-0.267	5.135	5.402
0.934	0.964	-5.712	-0.018	1.815	1.833
0.337	0.532	-5.278	0.195	6.440	6.246
0.030	0.179	-3.502	-0.568	5.092	5.660
0.417	0.604	-5.403	-0.200	4.939	5.139
0.038	0.192	-3.701	-0.537	4.920	5.457
0.548	0.709	-5.543	-0.184	3.369	3.553
0.144	0.327	-4.706	-0.414	3.736	4.150
0.020	0.166	-3.166	-0.810	3.590	4.401
0.058	0.219	-4.020	0.670	3.207	2.537
0.009	0.157	-2.491	-0.736	7.654	8.390
0.125	0.306	-4.606	0.395	6.945	6.550
0.952	0.975	-5.713	0.015	4.234	4.220
0.082	0.253	-4.288	-0.376	4.455	4.832
0.064	0.228	-4.105	0.446	5.523	5.076
0.573	0.728	-5.564	0.128	5.373	5.245
0.059	0.221	-4.040	-0.639	3.519	4.158
0.014	0.160	-2.903	1.482	8.135	6.653
0.158	0.346	-4.776	-0.404	7.503	7.907
0.042	0.196	-3.766	-0.560	4.140	4.700
0.093	0.268	-4.383	1.240	4.123	2.883
0.008	0.157	-2.418	-1.211	2.845	4.056
0.209	0.404	-4.969	0.275	7.561	7.286
0.004	0.157	-1.820	-0.984	3.660	4.644
0.881	0.935	-5.704	-0.039	3.540	3.579
0.044	0.200	-3.807	0.730	8.983	8.252
0.055	0.216	-3.987	0.614	7.402	6.788

0.122	0.304	-4.590	0.552	8.687	8.135
0.026	0.175	-3.386	0.899	6.353	5.453
0.233	0.430	-5.044	0.309	3.704	3.395
0.054	0.214	-3.964	-0.568	4.170	4.739
0.040	0.194	-3.739	-1.172	4.534	5.706
0.689	0.814	-5.639	-0.124	3.243	3.368
0.967	0.983	-5.714	0.013	3.970	3.958
0.082	0.254	-4.296	-0.478	3.829	4.307
0.020	0.167	-3.196	-0.715	5.713	6.429
0.358	0.551	-5.315	-0.258	5.821	6.080
0.011	0.157	-2.697	-0.633	6.709	7.341
0.582	0.736	-5.571	0.176	5.323	5.147
0.324	0.520	-5.254	0.253	2.256	2.003
0.712	0.828	-5.650	-0.131	4.869	5.000
0.033	0.184	-3.593	0.452	7.477	7.024
0.186	0.378	-4.890	0.261	6.739	6.479
0.771	0.868	-5.675	0.116	5.016	4.900
0.119	0.298	-4.568	0.334	9.127	8.793
0.063	0.226	-4.088	0.427	6.011	5.585
0.007	0.157	-2.356	0.629	5.854	5.225
0.004	0.157	-1.840	-0.808	2.832	3.639
0.046	0.203	-3.845	-0.530	1.615	2.145
0.135	0.319	-4.662	-0.412	2.132	2.544
0.715	0.831	-5.652	0.128	5.437	5.310
0.197	0.392	-4.930	0.352	4.691	4.339
0.282	0.480	-5.168	-0.308	2.255	2.563
0.039	0.193	-3.720	-0.727	7.967	8.693
0.058	0.219	-4.024	0.410	9.052	8.642
0.036	0.189	-3.662	0.564	7.130	6.566
0.133	0.316	-4.651	0.547	5.535	4.988
0.066	0.230	-4.124	0.619	7.715	7.096
0.924	0.959	-5.710	0.028	4.459	4.431
0.345	0.540	-5.293	-0.286	3.395	3.681
0.281	0.478	-5.165	-0.569	4.448	5.017
0.086	0.259	-4.331	0.368	7.175	6.807
0.024	0.173	-3.314	0.748	6.033	5.285
0.016	0.162	-3.024	0.685	5.104	4.419
0.008	0.157	-2.413	1.695	6.615	4.920
0.794	0.884	-5.682	-0.066	2.159	2.225
0.494	0.667	-5.493	-0.187	3.806	3.993
0.430	0.616	-5.421	0.183	6.071	5.888
0.056	0.217	-3.996	0.427	8.957	8.530
0.354	0.548	-5.308	0.241	3.857	3.617
0.040	0.194	-3.740	0.767	7.534	6.766
0.076	0.245	-4.235	0.458	6.050	5.591
0.006	0.157	-2.173	1.073	5.581	4.508
0.267	0.465	-5.132	0.674	4.755	4.080
0.015	0.160	-2.942	-0.693	7.825	8.518
0.432	0.617	-5.423	0.206	3.782	3.576
0.499	0.670	-5.499	0.199	5.481	5.282

0.005	0.157	-2.070	-0.899	7.214	8.113
0.423	0.609	-5.412	0.240	4.740	4.499
0.063	0.226	-4.091	-0.584	4.949	5.533
0.320	0.518	-5.248	-0.307	3.236	3.543
0.510	0.679	-5.509	0.280	5.518	5.238
0.435	0.619	-5.427	0.304	3.643	3.339
0.460	0.641	-5.457	-0.333	3.695	4.028
0.038	0.192	-3.693	0.541	5.266	4.725
0.193	0.386	-4.915	0.374	5.925	5.551
0.629	0.771	-5.605	-0.096	2.018	2.114
0.288	0.487	-5.182	0.332	4.389	4.057
0.896	0.943	-5.707	0.042	3.581	3.539
0.007	0.157	-2.360	1.275	9.405	8.130
0.118	0.298	-4.561	0.516	3.080	2.563
0.068	0.233	-4.145	-0.606	3.833	4.438
0.153	0.340	-4.749	-0.286	4.679	4.966
0.867	0.928	-5.702	-0.055	6.470	6.525
0.109	0.288	-4.506	-0.445	3.720	4.165
0.094	0.270	-4.392	-0.507	5.562	6.069
0.628	0.769	-5.603	0.097	5.752	5.655
0.423	0.609	-5.411	-0.292	4.065	4.357
0.004	0.157	-1.959	1.663	6.494	4.832
0.027	0.176	-3.416	-0.676	6.441	7.116
0.829	0.904	-5.693	-0.073	5.020	5.093
0.499	0.670	-5.498	0.225	6.019	5.794
0.005	0.157	-2.113	-1.081	5.209	6.290
0.642	0.780	-5.612	-0.138	5.101	5.239
0.209	0.404	-4.971	0.279	6.760	6.482
0.129	0.310	-4.628	-0.624	2.885	3.509
0.331	0.527	-5.268	-0.260	5.377	5.637
0.380	0.569	-5.350	-0.165	5.707	5.872
0.340	0.535	-5.285	-0.398	3.621	4.019
0.055	0.216	-3.987	0.471	7.410	6.939
0.704	0.824	-5.646	0.125	3.129	3.004
0.264	0.462	-5.125	0.306	7.189	6.883
0.818	0.897	-5.690	-0.062	3.184	3.246
0.502	0.672	-5.501	0.199	3.656	3.457
0.048	0.205	-3.883	-0.615	4.940	5.555
0.049	0.207	-3.893	-0.489	3.720	4.209
0.084	0.256	-4.310	-0.601	5.810	6.411
0.630	0.771	-5.605	0.123	2.690	2.568
0.025	0.175	-3.358	-0.713	3.975	4.688
0.004	0.157	-1.835	0.851	4.518	3.667
0.033	0.184	-3.584	-1.178	5.840	7.018
0.698	0.820	-5.644	-0.110	4.463	4.573
0.046	0.203	-3.847	0.488	8.506	8.018
0.202	0.397	-4.946	0.267	6.382	6.115
0.938	0.967	-5.712	-0.017	5.506	5.523
0.442	0.625	-5.435	-0.307	3.798	4.105
0.670	0.802	-5.629	-0.124	3.128	3.253

0.037	0.190	-3.669	-0.809	5.078	5.887
0.007	0.157	-2.352	-0.738	2.721	3.459
0.006	0.157	-2.139	1.574	6.313	4.739
0.072	0.240	-4.197	-0.711	3.437	4.147
0.431	0.616	-5.421	0.229	3.236	3.007
0.076	0.245	-4.233	-0.767	7.496	8.262
0.108	0.287	-4.496	0.690	3.117	2.427
0.038	0.192	-3.703	-0.615	6.254	6.869
0.998	0.999	-5.715	0.001	6.646	6.645
0.003	0.157	-1.754	-0.960	4.362	5.321
0.326	0.522	-5.258	-0.293	4.724	5.017
0.111	0.290	-4.520	0.471	3.955	3.484
0.324	0.520	-5.254	0.385	5.286	4.900
0.267	0.466	-5.134	0.276	6.695	6.420
0.532	0.696	-5.530	-0.177	7.580	7.757
0.980	0.989	-5.714	-0.010	3.154	3.164
0.188	0.380	-4.896	0.321	6.033	5.712
0.026	0.176	-3.393	-0.585	8.371	8.956
0.266	0.464	-5.129	-0.302	5.210	5.512
0.212	0.407	-4.978	0.339	6.166	5.827
0.035	0.187	-3.636	0.770	7.048	6.278
0.357	0.551	-5.314	-0.214	4.149	4.363
0.112	0.291	-4.528	0.614	5.998	5.384
0.027	0.176	-3.426	-0.632	5.504	6.136
0.143	0.327	-4.702	-0.288	5.820	6.109
0.004	0.157	-1.959	-1.082	4.153	5.235
0.111	0.290	-4.519	-0.387	5.000	5.387
0.867	0.928	-5.702	-0.047	9.702	9.749
0.026	0.176	-3.398	-0.714	5.901	6.614
0.407	0.594	-5.389	0.235	6.141	5.906
0.800	0.888	-5.684	0.067	5.723	5.656
0.606	0.753	-5.589	-0.128	4.165	4.293
0.246	0.443	-5.078	-0.392	2.978	3.370
0.047	0.203	-3.856	0.658	4.509	3.851
0.550	0.711	-5.546	0.149	4.869	4.720
0.892	0.941	-5.706	0.040	2.879	2.839
0.069	0.235	-4.159	0.383	8.563	8.180
0.067	0.232	-4.136	0.677	7.720	7.043
0.141	0.325	-4.692	0.638	5.509	4.871
0.450	0.631	-5.445	0.207	3.823	3.616
0.009	0.157	-2.564	0.761	4.799	4.038
0.013	0.160	-2.860	0.713	4.782	4.069
0.026	0.176	-3.399	0.622	4.513	3.891
0.044	0.200	-3.804	0.674	4.411	3.737
0.054	0.215	-3.976	0.669	7.122	6.453
0.187	0.379	-4.894	0.512	5.681	5.169
0.680	0.808	-5.634	0.145	3.190	3.045
0.986	0.993	-5.715	-0.005	4.563	4.568
0.368	0.559	-5.331	-0.237	4.553	4.790
0.571	0.726	-5.563	0.186	3.847	3.661

0.207	0.402	-4.964	-0.328	2.469	2.797
0.206	0.401	-4.961	0.318	7.096	6.779
0.719	0.834	-5.653	-0.087	2.928	3.015
0.665	0.797	-5.626	-0.093	3.252	3.344
0.453	0.633	-5.448	0.158	7.081	6.923
0.153	0.340	-4.749	-0.376	5.193	5.569
0.046	0.203	-3.839	-0.433	4.529	4.963
0.027	0.177	-3.438	0.567	7.385	6.819
0.900	0.945	-5.707	0.028	5.893	5.866
0.527	0.692	-5.525	0.140	5.534	5.394
0.337	0.533	-5.279	0.282	2.220	1.938
0.317	0.515	-5.241	0.295	2.960	2.665
0.058	0.219	-4.023	0.503	2.758	2.255
0.702	0.822	-5.645	-0.142	1.507	1.650
0.152	0.339	-4.749	-0.324	5.996	6.320
0.132	0.314	-4.643	0.370	7.375	7.005
0.924	0.959	-5.710	-0.027	3.687	3.714
0.448	0.630	-5.442	-0.228	2.452	2.681
0.349	0.544	-5.300	0.229	5.203	4.975
0.157	0.345	-4.770	0.333	6.152	5.819
0.287	0.485	-5.179	0.475	3.070	2.595
0.318	0.516	-5.244	0.313	3.189	2.876
0.327	0.523	-5.260	0.276	3.316	3.040
0.039	0.192	-3.712	-0.489	5.982	6.471
0.197	0.392	-4.930	-0.618	2.343	2.960
0.381	0.570	-5.352	0.251	3.879	3.628
0.613	0.758	-5.593	-0.146	2.448	2.594
0.206	0.401	-4.960	0.304	6.342	6.038
0.050	0.207	-3.903	0.624	4.453	3.828
0.032	0.182	-3.549	0.794	2.590	1.796
0.802	0.889	-5.685	-0.116	3.001	3.118
0.911	0.952	-5.709	0.031	3.183	3.152
0.004	0.157	-1.898	-1.150	2.229	3.378
0.001	0.157	-1.009	-1.341	5.474	6.815
0.001	0.157	-0.657	-1.154	5.122	6.276
0.120	0.301	-4.578	0.309	3.966	3.657
0.041	0.196	-3.764	0.517	4.157	3.640
0.208	0.403	-4.965	0.303	3.417	3.113
0.085	0.258	-4.323	0.413	5.047	4.634
0.987	0.993	-5.715	-0.011	1.859	1.870
0.011	0.157	-2.688	-0.848	6.176	7.024
0.928	0.961	-5.711	0.049	4.179	4.130
0.030	0.180	-3.521	0.656	8.107	7.452
0.019	0.164	-3.147	0.818	4.964	4.146
0.042	0.198	-3.779	0.904	5.571	4.667
0.020	0.166	-3.166	0.569	7.466	6.897
0.009	0.157	-2.550	-0.741	4.199	4.941
0.175	0.365	-4.847	0.460	2.585	2.125
0.528	0.693	-5.526	0.158	3.348	3.190
0.929	0.961	-5.711	-0.034	2.339	2.372

0.432	0.617	-5.423	0.234	3.303	3.068
0.311	0.508	-5.229	0.287	6.888	6.600
0.015	0.160	-2.952	0.580	4.457	3.877
0.564	0.721	-5.557	-0.157	3.553	3.710
0.104	0.281	-4.468	0.404	7.524	7.120
0.047	0.204	-3.862	0.459	5.864	5.406
0.077	0.246	-4.241	0.422	7.376	6.954
0.091	0.267	-4.374	0.399	7.228	6.829
0.092	0.267	-4.379	0.335	6.566	6.231
0.143	0.327	-4.703	0.358	7.790	7.432
0.146	0.330	-4.718	0.300	6.519	6.219
0.159	0.347	-4.778	0.621	5.788	5.168
0.161	0.350	-4.789	0.377	4.271	3.895
0.190	0.382	-4.903	0.293	7.014	6.721
0.193	0.386	-4.914	0.278	7.124	6.845
0.205	0.399	-4.955	0.292	8.968	8.675
0.265	0.462	-5.126	0.296	8.572	8.276
0.311	0.508	-5.229	0.247	8.144	7.897
0.349	0.544	-5.299	0.225	8.146	7.921
0.534	0.697	-5.532	0.149	6.390	6.240
0.001	0.157	-0.802	1.415	4.961	3.546
0.068	0.234	-4.151	0.705	5.450	4.746
0.948	0.973	-5.713	0.014	6.791	6.778
0.002	0.157	-1.075	0.994	3.705	2.711
0.361	0.553	-5.321	0.228	7.599	7.372
0.030	0.179	-3.510	0.489	5.821	5.331
0.055	0.216	-3.983	0.451	7.074	6.623
0.011	0.157	-2.699	0.557	7.251	6.695
0.166	0.355	-4.809	0.351	5.911	5.559
0.203	0.398	-4.950	-0.357	3.144	3.501
0.072	0.240	-4.191	-0.894	4.647	5.540
0.122	0.303	-4.589	0.691	4.707	4.015
0.815	0.896	-5.689	0.074	4.102	4.029
0.435	0.619	-5.427	0.221	7.824	7.603
0.277	0.475	-5.157	-0.298	3.953	4.251
0.296	0.493	-5.199	-0.261	4.041	4.302
0.345	0.540	-5.294	-0.250	3.990	4.241
0.434	0.618	-5.425	-0.186	5.107	5.293
0.684	0.811	-5.636	-0.111	4.093	4.204
0.163	0.352	-4.796	0.432	5.811	5.379
0.648	0.784	-5.616	0.137	5.615	5.478
0.419	0.606	-5.406	-0.220	3.142	3.362
0.448	0.630	-5.442	-0.217	3.398	3.615
0.674	0.804	-5.631	-0.123	2.449	2.572
0.357	0.551	-5.313	-0.248	6.662	6.909
0.073	0.241	-4.205	0.352	7.236	6.884
0.013	0.160	-2.805	0.693	7.853	7.159
0.208	0.402	-4.965	0.510	7.387	6.877
0.041	0.195	-3.748	-1.120	3.659	4.779
0.290	0.488	-5.185	-0.296	5.054	5.350

0.057	0.218	-4.013	-0.459	3.015	3.474
0.033	0.184	-3.594	0.479	6.899	6.420
0.067	0.232	-4.136	0.378	7.267	6.890
0.009	0.157	-2.514	1.095	9.795	8.700
0.018	0.162	-3.086	0.665	7.516	6.851
0.018	0.162	-3.085	-0.512	2.866	3.378
0.014	0.160	-2.879	0.836	9.157	8.321
0.186	0.378	-4.888	-0.413	2.153	2.566
0.030	0.179	-3.504	0.822	6.196	5.374
0.763	0.864	-5.672	-0.090	2.548	2.637
0.161	0.350	-4.788	0.375	6.170	5.795
0.492	0.666	-5.491	-0.196	5.952	6.148
0.154	0.341	-4.754	-0.396	4.236	4.632
0.008	0.157	-2.468	-1.014	2.350	3.363
0.003	0.157	-1.590	0.902	4.812	3.910
0.436	0.619	-5.427	0.306	3.523	3.217
0.246	0.443	-5.079	0.321	4.707	4.386
0.988	0.994	-5.715	-0.005	3.136	3.140
0.364	0.556	-5.326	0.187	5.788	5.602
0.768	0.867	-5.674	0.082	4.198	4.116
0.287	0.485	-5.179	0.212	6.385	6.173
0.447	0.629	-5.441	0.169	5.783	5.614
0.222	0.419	-5.011	-0.337	4.146	4.483
0.173	0.362	-4.838	0.342	2.806	2.463
0.008	0.157	-2.470	0.804	5.270	4.466
0.563	0.721	-5.556	0.252	5.139	4.886
0.015	0.160	-2.955	0.615	6.760	6.145
0.062	0.225	-4.081	0.459	9.748	9.289
0.021	0.168	-3.231	-0.677	6.606	7.283
0.233	0.430	-5.043	-0.396	1.594	1.990
0.802	0.889	-5.685	0.169	1.365	1.196
0.031	0.182	-3.544	0.842	6.725	5.883
0.015	0.160	-2.933	0.770	7.669	6.899
0.185	0.377	-4.886	-0.410	2.451	2.861
0.001	0.157	-0.498	-1.750	3.099	4.849
0.071	0.238	-4.179	0.650	6.869	6.219
0.298	0.495	-5.203	0.193	8.132	7.939
0.095	0.271	-4.404	0.515	3.490	2.975
0.030	0.178	-3.497	0.943	6.717	5.774
0.205	0.400	-4.955	0.372	3.496	3.124
0.005	0.157	-1.996	1.173	7.695	6.522
0.004	0.157	-1.845	1.125	6.656	5.532
0.429	0.614	-5.418	-0.203	3.071	3.274
0.018	0.164	-3.117	0.834	5.659	4.825
0.004	0.157	-1.838	2.406	7.087	4.681
0.101	0.278	-4.446	0.400	6.688	6.288
0.761	0.863	-5.671	-0.092	4.713	4.805
0.140	0.324	-4.690	-0.495	2.946	3.440
0.140	0.324	-4.685	-0.307	7.683	7.990
0.233	0.430	-5.044	-0.232	4.182	4.414

0.211	0.406	-4.977	-0.356	4.253	4.609
0.084	0.256	-4.312	0.543	4.440	3.897
0.034	0.185	-3.609	-1.018	3.252	4.270
0.263	0.461	-5.122	-0.579	2.689	3.268
0.905	0.949	-5.708	-0.052	3.297	3.349
0.682	0.809	-5.635	0.144	1.790	1.646
0.104	0.281	-4.471	-0.435	3.742	4.177
0.007	0.157	-2.356	1.263	6.221	4.958
0.958	0.978	-5.713	0.015	3.994	3.979
0.830	0.904	-5.693	0.063	4.495	4.432
0.372	0.562	-5.338	0.411	2.761	2.350
0.015	0.161	-2.972	1.028	4.174	3.146
0.294	0.491	-5.195	0.247	2.630	2.383
0.069	0.235	-4.157	0.533	5.147	4.614
0.127	0.307	-4.615	0.341	7.183	6.842
0.339	0.534	-5.283	-0.492	1.433	1.925
0.970	0.985	-5.714	0.015	3.849	3.834
0.013	0.160	-2.823	0.841	7.871	7.030
0.468	0.647	-5.465	-0.197	2.979	3.176
0.643	0.780	-5.613	-0.115	3.295	3.410
0.172	0.362	-4.834	-0.549	4.931	5.480
0.441	0.625	-5.434	0.234	5.105	4.872
0.028	0.178	-3.452	-0.618	3.984	4.602
0.003	0.157	-1.523	-1.166	2.471	3.638
0.059	0.221	-4.039	-0.577	5.879	6.456
0.024	0.173	-3.327	1.054	4.576	3.522
0.193	0.386	-4.915	-0.525	2.752	3.276
0.295	0.492	-5.197	0.285	4.374	4.088
0.699	0.820	-5.644	0.096	4.222	4.126
0.761	0.863	-5.671	0.076	4.323	4.247
0.029	0.178	-3.486	0.895	5.455	4.560
0.075	0.243	-4.219	0.595	5.425	4.830
0.025	0.175	-3.366	-0.641	3.403	4.044
0.957	0.978	-5.713	-0.015	4.570	4.585
0.008	0.157	-2.442	0.821	3.754	2.933
0.079	0.249	-4.262	0.448	3.250	2.802
0.051	0.209	-3.925	-0.571	1.580	2.150
0.090	0.264	-4.360	0.425	3.137	2.712
0.083	0.255	-4.305	-0.457	6.147	6.604
0.030	0.180	-3.516	-0.991	3.364	4.355
0.845	0.914	-5.697	-0.103	2.707	2.810
0.023	0.173	-3.310	1.007	6.427	5.421
0.752	0.856	-5.667	-0.112	6.900	7.012
0.172	0.362	-4.836	-0.402	2.256	2.658
0.020	0.167	-3.194	-0.709	4.645	5.355
0.239	0.436	-5.059	-0.248	4.495	4.743
0.236	0.433	-5.053	0.308	5.250	4.942
0.244	0.442	-5.074	0.309	5.232	4.923
0.733	0.844	-5.660	-0.076	3.212	3.288
0.098	0.275	-4.428	-0.318	2.145	2.464

0.239	0.437	-5.060	-0.238	2.512	2.750
0.502	0.672	-5.501	-0.147	2.959	3.106
0.317	0.515	-5.242	-0.202	3.194	3.396
0.583	0.736	-5.572	-0.116	3.004	3.120
0.031	0.182	-3.544	0.494	7.960	7.466
0.978	0.989	-5.714	-0.008	3.519	3.527
0.650	0.786	-5.617	0.096	5.647	5.551
0.583	0.737	-5.572	-0.206	4.926	5.132
0.374	0.565	-5.341	0.216	6.149	5.933
0.135	0.319	-4.663	-0.431	4.419	4.850
0.691	0.815	-5.640	0.118	3.398	3.280
0.052	0.210	-3.935	-0.730	2.920	3.650
0.110	0.289	-4.513	-0.361	6.711	7.072
0.092	0.267	-4.378	-0.457	4.756	5.213
0.639	0.778	-5.611	-0.137	3.342	3.479
0.096	0.272	-4.410	0.474	5.755	5.280
0.359	0.552	-5.317	-0.241	3.413	3.654
0.044	0.200	-3.808	0.769	4.747	3.978
0.566	0.722	-5.559	-0.154	2.679	2.833
0.091	0.266	-4.370	0.433	7.403	6.971
0.366	0.558	-5.329	-0.460	2.162	2.622
0.081	0.252	-4.281	-0.399	7.797	8.196
0.056	0.218	-4.002	-0.672	1.769	2.442
0.343	0.538	-5.290	-0.276	2.699	2.975
0.355	0.550	-5.311	0.211	4.183	3.972
0.148	0.332	-4.727	0.406	5.929	5.523
0.228	0.425	-5.029	0.319	3.360	3.041
0.120	0.301	-4.578	0.442	7.048	6.606
0.022	0.171	-3.273	-0.879	2.751	3.630
0.023	0.172	-3.286	0.898	4.501	3.603
0.268	0.467	-5.136	-0.245	3.420	3.666
0.003	0.157	-1.667	1.269	3.990	2.721
0.005	0.157	-2.025	1.484	7.485	6.002
0.248	0.446	-5.085	-0.385	5.078	5.464
0.072	0.240	-4.191	0.612	6.008	5.396
0.006	0.157	-2.202	0.725	5.520	4.795
0.081	0.252	-4.282	0.425	8.679	8.254
0.034	0.184	-3.600	0.646	6.056	5.410
0.327	0.523	-5.261	-0.261	2.943	3.205
0.353	0.548	-5.307	0.235	4.868	4.633
0.383	0.572	-5.354	0.335	3.363	3.027
0.039	0.192	-3.712	1.032	4.954	3.922
0.016	0.162	-2.990	0.627	6.893	6.266
0.038	0.192	-3.695	1.268	7.142	5.874
0.127	0.308	-4.616	-0.468	4.033	4.501
0.185	0.377	-4.885	-0.539	4.539	5.078
0.104	0.281	-4.469	-0.405	2.731	3.136
0.323	0.520	-5.254	-0.242	7.804	8.046
0.060	0.221	-4.048	-0.606	4.226	4.832
0.003	0.157	-1.752	0.846	3.253	2.407

0.062	0.225	-4.078	-0.755	2.540	3.295
0.549	0.710	-5.545	-0.127	6.634	6.761
0.374	0.565	-5.342	0.188	7.488	7.299
0.127	0.307	-4.615	0.371	7.742	7.371
0.037	0.190	-3.678	1.018	6.798	5.781
0.009	0.157	-2.556	1.335	7.409	6.074
0.078	0.247	-4.252	0.668	5.757	5.089
0.027	0.176	-3.427	1.054	4.958	3.904
0.014	0.160	-2.917	1.469	7.797	6.328
0.079	0.249	-4.264	-0.511	4.502	5.013
0.859	0.925	-5.700	-0.043	6.848	6.891
0.406	0.593	-5.388	-0.241	2.983	3.224
0.116	0.296	-4.551	0.349	6.535	6.186
0.077	0.246	-4.242	0.414	5.619	5.205
0.406	0.593	-5.388	-0.211	4.234	4.445
0.060	0.221	-4.047	0.386	4.831	4.445
0.593	0.745	-5.580	0.141	5.782	5.641
0.232	0.428	-5.039	0.532	3.945	3.412
0.464	0.644	-5.461	0.176	5.976	5.800
0.072	0.240	-4.197	0.406	3.873	3.468
0.116	0.296	-4.552	0.745	6.517	5.772
0.015	0.160	-2.946	0.713	2.851	2.137
0.098	0.275	-4.429	0.505	9.436	8.931
0.246	0.444	-5.080	0.375	8.720	8.344
0.171	0.361	-4.830	-0.385	4.540	4.925
0.471	0.650	-5.469	0.163	5.630	5.467
0.454	0.634	-5.449	-0.211	3.248	3.459
0.009	0.157	-2.574	-0.916	7.479	8.395
0.059	0.221	-4.043	-0.402	3.560	3.962
0.322	0.519	-5.252	-0.466	2.478	2.944
0.053	0.212	-3.952	-0.478	3.031	3.509
0.038	0.192	-3.702	-0.493	3.821	4.314
0.735	0.845	-5.660	-0.127	4.356	4.483
0.443	0.626	-5.436	-0.359	4.571	4.930
0.010	0.157	-2.607	-0.754	3.038	3.792
0.874	0.932	-5.703	0.050	2.400	2.350
0.097	0.274	-4.419	-0.455	7.993	8.448
0.008	0.157	-2.435	-0.819	8.155	8.975
0.236	0.433	-5.051	0.305	4.415	4.110
0.380	0.569	-5.350	0.264	5.240	4.976
0.947	0.972	-5.713	0.020	4.089	4.069
0.738	0.848	-5.662	-0.086	5.733	5.819
0.938	0.967	-5.712	-0.018	5.120	5.138
0.018	0.163	-3.091	-0.648	4.644	5.292
0.015	0.160	-2.930	0.952	8.977	8.025
0.073	0.241	-4.202	-0.693	3.209	3.902
0.419	0.606	-5.406	0.204	2.647	2.443
0.053	0.214	-3.963	0.435	7.952	7.517
0.475	0.653	-5.473	0.142	8.126	7.984
0.095	0.271	-4.406	0.453	4.549	4.096

0.715	0.831	-5.652	-0.092	3.434	3.526
0.043	0.200	-3.801	0.861	5.811	4.950
0.490	0.664	-5.489	0.191	4.043	3.852
0.062	0.225	-4.078	0.372	5.272	4.901
0.007	0.157	-2.278	1.143	6.172	5.028
0.451	0.632	-5.446	0.212	4.159	3.947
0.021	0.169	-3.241	1.440	7.555	6.115
0.812	0.894	-5.688	-0.051	5.551	5.602
0.358	0.551	-5.315	-0.258	3.068	3.326
0.448	0.630	-5.442	-0.167	3.467	3.634
0.058	0.219	-4.021	0.488	3.039	2.552
0.496	0.668	-5.496	0.233	4.854	4.621
0.269	0.467	-5.137	0.279	6.525	6.246
0.055	0.216	-3.982	0.851	6.926	6.074
0.382	0.571	-5.354	0.498	5.599	5.101
0.143	0.327	-4.705	0.786	6.121	5.334
0.821	0.899	-5.691	0.066	5.426	5.360
0.043	0.198	-3.787	0.370	7.961	7.592
0.970	0.985	-5.714	-0.009	3.702	3.710
0.612	0.757	-5.593	0.122	3.323	3.202
0.057	0.218	-4.007	0.452	5.445	4.992
0.794	0.884	-5.683	-0.127	1.207	1.334
0.306	0.503	-5.219	0.199	7.077	6.878
0.171	0.361	-4.830	0.268	9.467	9.199
0.043	0.200	-3.801	0.826	9.434	8.608
0.845	0.915	-5.697	-0.090	5.313	5.403
0.399	0.587	-5.378	0.305	5.350	5.045
0.433	0.617	-5.424	0.233	4.551	4.318
0.123	0.304	-4.592	0.392	4.676	4.284
0.469	0.648	-5.467	0.171	7.841	7.670
0.060	0.222	-4.054	-0.569	3.877	4.446
0.111	0.290	-4.521	0.367	5.559	5.192
0.691	0.815	-5.640	0.113	6.054	5.941
0.119	0.299	-4.572	0.355	4.923	4.568
0.271	0.470	-5.143	0.234	4.793	4.559
0.558	0.717	-5.553	-0.148	2.761	2.909
0.042	0.198	-3.780	-0.512	3.415	3.927
0.077	0.246	-4.245	0.449	3.987	3.538
0.015	0.160	-2.952	0.605	7.213	6.608
0.191	0.384	-4.908	-0.370	7.030	7.400
0.027	0.176	-3.423	0.910	6.130	5.220
0.767	0.866	-5.673	-0.066	4.833	4.898
0.361	0.553	-5.320	-0.349	4.497	4.847
0.106	0.284	-4.484	-0.367	3.896	4.263
0.897	0.943	-5.707	0.053	4.342	4.289
0.103	0.280	-4.462	0.627	8.507	7.880
0.260	0.457	-5.115	0.270	6.957	6.687
0.443	0.626	-5.436	0.223	3.973	3.750
0.009	0.157	-2.577	1.111	6.942	5.831
0.036	0.189	-3.654	1.074	6.628	5.554

0.157	0.345	-4.769	0.326	8.142	7.816
0.196	0.390	-4.927	0.357	4.711	4.354
0.044	0.200	-3.808	0.493	7.842	7.349
0.012	0.160	-2.785	1.143	8.878	7.735
0.113	0.292	-4.531	0.505	3.296	2.791
0.044	0.201	-3.812	0.902	5.012	4.110
0.109	0.288	-4.504	0.400	6.912	6.512
0.026	0.175	-3.380	0.608	4.343	3.735
0.113	0.292	-4.531	0.610	5.481	4.872
0.850	0.918	-5.698	-0.051	2.743	2.794
0.097	0.274	-4.420	-0.327	4.300	4.627
0.195	0.389	-4.923	-0.424	4.226	4.650
0.052	0.211	-3.940	0.384	7.560	7.177
0.158	0.346	-4.774	0.329	5.384	5.055
0.465	0.645	-5.462	-0.231	3.252	3.483
0.671	0.802	-5.629	-0.109	4.531	4.640
0.969	0.985	-5.714	0.010	4.905	4.894
0.362	0.554	-5.322	-0.261	7.008	7.269
0.561	0.720	-5.555	-0.146	4.958	5.104
0.792	0.882	-5.682	-0.071	6.113	6.184
0.549	0.710	-5.545	0.159	6.034	5.875
0.450	0.631	-5.445	-0.221	7.269	7.490
0.750	0.856	-5.667	-0.084	5.673	5.757
0.271	0.469	-5.143	-0.260	5.338	5.599
0.815	0.896	-5.689	-0.070	6.379	6.450
0.724	0.838	-5.656	-0.132	3.887	4.019
0.787	0.879	-5.680	-0.081	3.889	3.970
0.607	0.754	-5.589	-0.150	6.034	6.184
0.706	0.826	-5.648	0.105	4.528	4.424
0.008	0.157	-2.388	0.735	7.694	6.960
0.069	0.236	-4.162	-0.551	3.285	3.836
0.112	0.290	-4.522	0.731	7.625	6.894
0.018	0.163	-3.100	1.074	6.488	5.414
0.285	0.482	-5.174	0.286	5.545	5.259
0.031	0.180	-3.523	-0.685	3.667	4.353
0.141	0.325	-4.693	0.535	8.396	7.861
0.026	0.176	-3.395	0.640	2.777	2.137
0.003	0.157	-1.692	1.442	6.723	5.281
0.176	0.365	-4.849	0.376	3.109	2.733
0.006	0.157	-2.255	-1.367	4.213	5.580
0.445	0.627	-5.438	-0.253	2.850	3.104
0.260	0.458	-5.116	0.268	3.816	3.548
0.151	0.338	-4.743	0.454	3.693	3.239
0.082	0.253	-4.290	0.516	6.341	5.825
0.001	0.157	-0.456	1.426	5.437	4.011
0.010	0.157	-2.623	-0.786	3.289	4.076
0.368	0.559	-5.331	-0.230	2.728	2.958
0.007	0.157	-2.353	-1.213	7.752	8.964
0.787	0.879	-5.680	0.069	4.026	3.957
0.186	0.379	-4.891	-0.373	5.981	6.354

0.154	0.341	-4.755	-0.322	5.361	5.683
0.020	0.167	-3.193	-0.943	1.640	2.583
0.025	0.174	-3.348	0.499	7.644	7.145
0.195	0.389	-4.922	0.306	7.120	6.814
0.784	0.877	-5.679	0.081	5.963	5.882
0.134	0.317	-4.656	-0.361	5.235	5.596
0.031	0.180	-3.526	1.256	7.351	6.095
0.047	0.203	-3.854	0.615	6.521	5.906
0.006	0.157	-2.200	1.803	6.892	5.090
0.914	0.954	-5.709	0.030	4.553	4.523
0.130	0.311	-4.632	0.504	2.965	2.461
0.887	0.938	-5.705	-0.050	4.662	4.712
0.516	0.683	-5.515	0.160	3.755	3.594
0.896	0.943	-5.707	0.031	2.466	2.434
0.476	0.654	-5.475	-0.161	5.124	5.285
0.920	0.957	-5.710	-0.032	2.544	2.576
0.940	0.967	-5.712	0.021	4.945	4.924
0.122	0.303	-4.588	-0.344	2.680	3.024
0.024	0.173	-3.317	1.092	7.195	6.102
0.311	0.508	-5.229	-0.492	2.823	3.316
0.880	0.934	-5.704	0.044	3.483	3.438
0.186	0.378	-4.889	0.326	8.928	8.602
0.018	0.162	-3.079	-0.785	4.117	4.902
0.129	0.310	-4.629	0.315	5.296	4.981
0.810	0.893	-5.687	-0.056	3.063	3.119
0.085	0.257	-4.317	0.454	3.836	3.382
0.408	0.596	-5.391	-0.284	2.651	2.934
0.134	0.317	-4.654	0.513	2.940	2.427
0.681	0.809	-5.635	0.126	4.066	3.940
0.032	0.182	-3.560	-0.711	5.430	6.142
0.314	0.512	-5.236	-0.246	3.548	3.794
0.863	0.927	-5.701	0.043	5.825	5.782
0.121	0.301	-4.580	0.486	3.812	3.326
0.196	0.390	-4.925	0.397	6.518	6.121
0.020	0.167	-3.174	-0.833	6.500	7.333
0.123	0.304	-4.593	-0.346	3.138	3.484
0.237	0.434	-5.055	0.233	8.462	8.229
0.863	0.927	-5.701	-0.047	5.672	5.719
0.216	0.411	-4.993	-0.305	5.353	5.658
0.478	0.655	-5.476	-0.211	6.462	6.674
0.077	0.247	-4.247	0.644	5.830	5.187
0.440	0.624	-5.432	0.390	6.318	5.928
0.036	0.189	-3.658	-0.587	2.811	3.398
0.220	0.416	-5.006	0.313	4.673	4.360
0.076	0.245	-4.231	0.401	8.312	7.912
0.491	0.665	-5.491	0.191	4.262	4.071
0.213	0.408	-4.983	0.558	4.200	3.642
0.241	0.439	-5.067	-0.237	6.566	6.803
0.078	0.249	-4.258	0.458	5.419	4.961
0.369	0.559	-5.332	0.246	4.631	4.384

0.021	0.168	-3.214	-0.676	4.530	5.206
0.421	0.607	-5.409	0.170	4.289	4.119
0.656	0.792	-5.621	-0.126	4.622	4.748
0.014	0.160	-2.866	1.504	6.678	5.173
0.963	0.982	-5.714	0.012	5.596	5.583
0.115	0.295	-4.547	0.554	4.031	3.477
0.017	0.162	-3.066	-0.803	7.509	8.312
0.724	0.837	-5.656	-0.084	3.305	3.389
0.104	0.281	-4.467	-0.391	4.153	4.544
0.326	0.522	-5.258	-0.254	5.671	5.924
0.515	0.682	-5.514	0.175	4.951	4.776
0.065	0.229	-4.116	-0.624	7.338	7.962
0.578	0.733	-5.569	-0.152	3.344	3.496
0.543	0.705	-5.540	-0.148	3.862	4.010
0.367	0.558	-5.330	0.193	3.807	3.613
0.239	0.436	-5.059	0.225	8.976	8.751
0.339	0.534	-5.282	0.184	7.939	7.755
0.344	0.539	-5.292	0.181	7.602	7.420
0.095	0.271	-4.404	0.787	4.292	3.505
0.198	0.393	-4.934	-0.714	4.963	5.677
0.163	0.352	-4.795	-0.527	3.153	3.680
0.137	0.321	-4.675	-0.400	5.081	5.480
0.115	0.295	-4.546	0.443	3.962	3.519
0.414	0.602	-5.399	0.392	4.546	4.154
0.105	0.284	-4.480	0.438	4.867	4.429
0.458	0.638	-5.454	-0.239	4.619	4.858
0.931	0.962	-5.711	-0.031	6.217	6.248
0.046	0.203	-3.844	0.470	3.634	3.164
0.751	0.856	-5.667	-0.077	1.638	1.715
0.008	0.157	-2.457	1.987	7.991	6.005
0.137	0.321	-4.673	0.552	5.541	4.988
0.019	0.164	-3.138	1.229	7.419	6.190
0.037	0.190	-3.679	1.384	8.438	7.054
0.578	0.733	-5.568	-0.229	4.246	4.475
0.052	0.212	-3.944	-0.643	4.132	4.775
0.767	0.867	-5.673	0.067	7.006	6.939
0.755	0.858	-5.668	-0.078	2.978	3.055
0.024	0.173	-3.321	-0.782	4.563	5.344
0.130	0.311	-4.634	0.468	4.131	3.663
0.116	0.296	-4.552	-0.719	3.582	4.301
0.139	0.323	-4.682	0.424	4.799	4.375
0.001	0.157	-0.460	0.832	3.240	2.407
0.008	0.157	-2.441	0.908	3.218	2.310
0.098	0.274	-4.423	-0.400	8.571	8.971
0.053	0.213	-3.956	0.669	6.284	5.616
0.464	0.644	-5.461	0.193	3.996	3.803
0.067	0.233	-4.141	-0.696	2.034	2.730
0.027	0.176	-3.417	0.657	5.752	5.094
0.102	0.279	-4.453	0.311	8.157	7.846
0.124	0.305	-4.603	-0.414	4.877	5.292

0.142	0.326	-4.697	0.530	5.186	4.657
0.858	0.924	-5.700	0.054	2.163	2.109
0.032	0.182	-3.554	0.750	3.351	2.601
0.299	0.496	-5.204	0.291	3.997	3.706
0.080	0.250	-4.269	0.692	6.134	5.442
0.065	0.229	-4.119	0.575	3.337	2.762
0.514	0.682	-5.514	-0.193	2.767	2.959
0.101	0.278	-4.445	0.752	6.481	5.729
0.029	0.178	-3.493	0.991	7.154	6.163
0.009	0.157	-2.508	1.300	7.454	6.154
0.008	0.157	-2.464	1.677	8.245	6.568
0.105	0.282	-4.475	0.695	5.168	4.473
0.252	0.450	-5.096	0.314	2.823	2.510
0.525	0.690	-5.523	-0.216	2.506	2.722
0.021	0.168	-3.218	0.498	5.338	4.840
0.662	0.795	-5.624	-0.097	5.197	5.294
0.105	0.283	-4.479	0.417	3.668	3.251
0.042	0.197	-3.776	0.823	5.574	4.751
0.588	0.741	-5.576	0.161	5.290	5.129
0.000	0.157	0.875	1.596	4.072	2.477
0.360	0.553	-5.319	0.200	7.290	7.090
0.052	0.212	-3.945	0.420	7.872	7.452
0.079	0.249	-4.261	-0.482	2.573	3.054
0.330	0.526	-5.266	-0.228	4.494	4.722
0.362	0.554	-5.321	0.196	5.584	5.387
0.661	0.794	-5.624	0.093	6.990	6.897
0.161	0.350	-4.788	-0.249	10.414	10.663
0.691	0.815	-5.640	-0.127	4.575	4.702
0.063	0.226	-4.089	-0.451	10.530	10.981
0.690	0.815	-5.639	-0.146	3.923	4.069
0.001	0.157	-0.928	1.467	3.345	1.878
0.205	0.401	-4.958	-0.351	5.488	5.839
0.358	0.552	-5.316	0.225	2.705	2.480
0.020	0.166	-3.165	-1.224	1.809	3.033
0.048	0.205	-3.877	-0.637	3.172	3.809
0.747	0.853	-5.666	0.140	3.138	2.999
0.658	0.793	-5.622	0.121	2.747	2.626
0.588	0.741	-5.576	-0.137	2.594	2.731
0.952	0.975	-5.713	-0.017	5.354	5.371
0.294	0.491	-5.194	-0.262	2.319	2.581
0.482	0.658	-5.481	-0.405	2.655	3.060
0.017	0.162	-3.065	-0.757	3.367	4.124
0.749	0.854	-5.666	-0.080	2.035	2.115
0.957	0.978	-5.713	-0.016	2.795	2.811
0.071	0.238	-4.178	-0.644	2.420	3.064
0.036	0.189	-3.661	-1.306	2.730	4.036
0.219	0.415	-5.002	0.520	4.185	3.665
0.248	0.445	-5.083	-0.284	5.564	5.848
0.232	0.428	-5.039	-0.627	3.979	4.606
0.614	0.759	-5.594	-0.137	2.709	2.846

0.643	0.780	-5.613	-0.183	2.508	2.691
0.839	0.911	-5.695	-0.056	4.304	4.360
0.960	0.979	-5.714	-0.018	2.863	2.880
0.468	0.647	-5.465	-0.217	4.035	4.252
0.460	0.640	-5.456	0.350	2.694	2.344
0.819	0.898	-5.690	0.103	3.116	3.014
0.854	0.921	-5.699	-0.062	2.560	2.622
0.823	0.900	-5.691	-0.080	3.095	3.175
0.996	0.998	-5.715	0.002	3.887	3.885
0.533	0.696	-5.530	0.268	3.763	3.495
0.201	0.396	-4.942	0.454	3.204	2.750
0.044	0.201	-3.817	0.652	3.343	2.691
0.837	0.909	-5.695	0.072	3.102	3.031
0.659	0.793	-5.622	-0.094	5.553	5.648
0.299	0.496	-5.205	0.396	2.473	2.077
0.242	0.439	-5.068	0.478	2.741	2.264
0.338	0.534	-5.281	-0.339	4.743	5.082
0.709	0.827	-5.649	-0.178	2.835	3.012
0.921	0.958	-5.710	-0.036	3.706	3.742
0.820	0.898	-5.690	-0.056	3.779	3.834
0.254	0.452	-5.100	-0.241	2.745	2.986
0.136	0.320	-4.668	0.564	2.321	1.757
0.126	0.306	-4.609	0.583	2.838	2.254
0.562	0.721	-5.556	0.173	3.195	3.022
0.388	0.576	-5.363	0.356	5.022	4.666
0.984	0.991	-5.715	-0.005	2.295	2.300
0.107	0.286	-4.494	0.980	4.803	3.823
0.319	0.517	-5.246	-0.326	4.125	4.451
0.204	0.399	-4.954	0.334	9.747	9.413
0.380	0.569	-5.350	0.222	8.970	8.748
0.561	0.720	-5.555	-0.199	1.944	2.143
0.323	0.519	-5.252	-0.271	1.938	2.209
0.363	0.554	-5.323	-0.373	3.482	3.855
0.650	0.786	-5.617	0.162	2.144	1.981
0.370	0.560	-5.334	-0.334	3.850	4.184
0.841	0.912	-5.696	0.054	2.590	2.536
0.305	0.503	-5.217	-0.296	2.438	2.734
0.153	0.340	-4.750	-0.546	2.340	2.886
0.449	0.631	-5.444	-0.238	2.915	3.154
0.420	0.606	-5.407	0.266	3.093	2.827
0.355	0.549	-5.309	-0.454	3.805	4.258
0.552	0.712	-5.547	0.170	2.578	2.408
0.888	0.939	-5.705	-0.047	2.811	2.858
0.990	0.995	-5.715	0.006	2.980	2.974
0.910	0.951	-5.709	0.052	2.703	2.650
0.059	0.220	-4.034	-0.556	4.013	4.569
0.919	0.957	-5.710	0.035	2.244	2.209
0.163	0.352	-4.796	0.560	2.824	2.264
0.477	0.655	-5.476	-0.261	2.373	2.634
0.852	0.920	-5.698	-0.059	3.228	3.287

0.163	0.352	-4.797	-0.407	2.563	2.971
0.073	0.241	-4.203	0.704	3.597	2.893
0.026	0.176	-3.401	-0.618	2.434	3.052
0.132	0.314	-4.643	-0.608	1.942	2.549
0.336	0.532	-5.277	-0.331	3.013	3.343
0.953	0.976	-5.713	0.023	3.224	3.201
0.607	0.754	-5.590	-0.162	4.165	4.327
0.378	0.568	-5.348	-0.288	4.220	4.508
0.061	0.223	-4.063	-0.696	2.536	3.232
0.512	0.680	-5.511	0.215	2.798	2.584
0.028	0.178	-3.456	0.890	2.632	1.742
0.118	0.298	-4.565	0.611	4.096	3.485
0.493	0.667	-5.493	-0.278	2.527	2.806
0.504	0.673	-5.503	-0.228	3.618	3.846
0.167	0.356	-4.812	-0.502	1.998	2.501
0.197	0.392	-4.931	-0.371	4.557	4.928
0.252	0.450	-5.094	-0.348	3.461	3.809
0.200	0.396	-4.941	0.568	4.517	3.949
0.286	0.483	-5.176	0.330	2.577	2.247
0.466	0.645	-5.463	-0.288	2.224	2.512
0.805	0.890	-5.686	-0.066	2.239	2.304
0.069	0.236	-4.162	-0.470	3.572	4.042
0.032	0.182	-3.556	-0.844	2.777	3.621
0.780	0.874	-5.678	-0.083	2.403	2.486
0.197	0.391	-4.929	-0.368	2.608	2.976
0.136	0.320	-4.669	-0.459	2.791	3.251
0.245	0.443	-5.077	0.374	2.500	2.127
0.948	0.973	-5.713	-0.022	2.592	2.614
0.622	0.765	-5.600	-0.152	2.685	2.837
0.263	0.461	-5.122	0.306	5.196	4.890
0.409	0.596	-5.392	0.395	3.055	2.659
0.496	0.668	-5.495	-0.215	3.725	3.940
0.524	0.689	-5.522	-0.189	2.107	2.296
0.751	0.856	-5.667	0.111	2.670	2.559
0.605	0.753	-5.588	0.196	2.855	2.659
0.451	0.632	-5.446	0.318	6.067	5.749
0.180	0.372	-4.868	0.349	3.365	3.016
0.743	0.851	-5.664	0.120	4.214	4.094
0.129	0.311	-4.630	-0.508	5.269	5.777
0.841	0.912	-5.696	-0.163	4.972	5.135
0.924	0.959	-5.711	0.065	2.969	2.903
0.230	0.427	-5.034	-0.571	4.713	5.284
0.249	0.447	-5.087	0.298	4.452	4.154
0.024	0.173	-3.330	-0.678	4.605	5.283
0.010	0.157	-2.616	1.208	6.175	4.967
0.102	0.280	-4.459	-0.547	6.903	7.450
0.084	0.256	-4.312	0.523	3.799	3.276
0.041	0.195	-3.756	0.398	8.991	8.593
0.839	0.911	-5.695	0.041	6.591	6.550
0.162	0.350	-4.790	0.397	4.532	4.135

0.996	0.998	-5.715	0.001	2.121	2.120
0.769	0.868	-5.674	0.110	3.684	3.574
0.025	0.175	-3.371	-1.124	3.179	4.302
0.447	0.629	-5.442	0.222	4.893	4.671
0.021	0.168	-3.226	0.659	3.648	2.989
0.091	0.266	-4.369	-0.429	7.539	7.968
0.008	0.157	-2.463	-0.724	6.278	7.003
0.142	0.327	-4.700	-0.511	2.738	3.249
0.980	0.989	-5.714	-0.007	3.393	3.400
0.251	0.448	-5.091	-0.292	5.979	6.271
0.041	0.195	-3.756	0.931	4.521	3.589
0.832	0.906	-5.694	0.069	4.849	4.780
0.087	0.259	-4.334	0.351	6.492	6.141
0.624	0.767	-5.601	0.154	5.398	5.244
0.320	0.517	-5.247	0.243	4.929	4.686
0.046	0.203	-3.852	0.582	8.118	7.535
0.023	0.173	-3.310	1.087	5.582	4.495
0.059	0.221	-4.040	0.937	5.776	4.840
0.448	0.630	-5.443	0.194	4.659	4.465
0.454	0.635	-5.450	0.266	2.811	2.544
0.719	0.834	-5.654	0.074	6.571	6.497
0.758	0.860	-5.670	0.069	5.681	5.612
0.035	0.186	-3.623	0.549	3.434	2.885
0.015	0.160	-2.931	-0.713	5.626	6.339
0.087	0.260	-4.336	-0.527	3.047	3.574
0.160	0.349	-4.785	0.354	6.186	5.832
0.318	0.516	-5.243	-0.355	4.478	4.833
0.969	0.985	-5.714	-0.013	4.745	4.758
0.029	0.178	-3.490	-0.714	4.997	5.712
0.416	0.603	-5.401	-0.223	2.714	2.937
0.421	0.607	-5.408	0.173	7.133	6.961
0.082	0.254	-4.292	0.547	3.805	3.258
0.441	0.625	-5.433	-0.221	3.375	3.596
0.064	0.228	-4.107	0.436	7.251	6.814
0.375	0.565	-5.342	0.186	5.129	4.943
0.036	0.189	-3.661	0.554	6.733	6.178
0.187	0.379	-4.894	0.262	6.175	5.914
0.034	0.184	-3.601	-0.530	5.599	6.129
0.078	0.247	-4.250	0.450	6.514	6.064
0.062	0.225	-4.077	0.419	5.009	4.590
0.490	0.664	-5.489	0.167	7.470	7.303
0.053	0.213	-3.954	-0.444	3.826	4.270
0.921	0.958	-5.710	-0.043	3.838	3.880
0.157	0.345	-4.771	0.683	5.448	4.765
0.231	0.428	-5.036	-0.485	8.297	8.781
0.478	0.655	-5.477	-0.333	2.781	3.114
0.165	0.354	-4.804	-0.614	1.419	2.032
0.721	0.835	-5.654	0.118	2.294	2.176
0.482	0.658	-5.481	-0.172	2.099	2.270
0.443	0.626	-5.437	-0.210	5.187	5.397

0.124	0.305	-4.600	0.534	4.595	4.060
0.408	0.596	-5.391	0.239	3.686	3.447
0.865	0.928	-5.701	0.044	4.393	4.350
0.880	0.935	-5.704	0.059	2.238	2.179
0.614	0.759	-5.595	0.163	4.604	4.441
0.363	0.554	-5.323	-0.332	3.287	3.619
0.741	0.850	-5.663	0.080	2.339	2.260
0.879	0.934	-5.704	-0.047	4.256	4.303
0.745	0.852	-5.665	0.093	3.246	3.154
0.543	0.705	-5.540	-0.158	4.441	4.599
0.290	0.488	-5.185	-0.321	5.281	5.603
0.169	0.359	-4.823	0.456	3.732	3.276
0.321	0.518	-5.249	0.438	4.080	3.642
0.334	0.530	-5.274	-0.311	6.495	6.806
0.581	0.736	-5.571	0.334	4.868	4.535
0.111	0.290	-4.516	-0.476	2.769	3.244
0.926	0.960	-5.711	0.034	2.682	2.648
0.098	0.275	-4.427	-0.477	2.276	2.753
0.984	0.991	-5.715	0.008	1.370	1.362
0.092	0.267	-4.379	-0.445	3.187	3.632
0.704	0.824	-5.646	0.113	2.587	2.474
0.647	0.784	-5.615	0.185	3.452	3.267
0.292	0.490	-5.190	-0.308	2.340	2.648
0.595	0.746	-5.581	0.207	2.165	1.958
0.137	0.321	-4.673	-0.464	6.953	7.418
0.174	0.364	-4.843	-0.397	4.347	4.744
0.179	0.370	-4.864	-0.349	4.058	4.407
0.036	0.189	-3.657	-0.746	3.309	4.054
0.575	0.730	-5.566	-0.160	2.835	2.995
0.363	0.554	-5.323	-0.321	2.859	3.180
0.270	0.468	-5.140	-0.356	2.812	3.169
0.266	0.465	-5.131	-0.364	4.410	4.774
0.682	0.809	-5.635	0.118	3.204	3.086
0.647	0.784	-5.616	0.152	2.522	2.370
0.531	0.695	-5.529	-0.261	3.473	3.734
0.081	0.253	-4.286	-0.527	5.118	5.645
0.970	0.985	-5.714	-0.011	2.177	2.188
0.222	0.418	-5.009	0.437	4.163	3.726
0.378	0.567	-5.346	-0.185	9.502	9.687
0.481	0.658	-5.480	-0.303	5.495	5.797
0.423	0.609	-5.411	-0.418	2.646	3.064
0.061	0.224	-4.068	-0.926	2.125	3.051
0.905	0.949	-5.708	0.045	4.087	4.042
0.643	0.781	-5.613	-0.309	2.731	3.040
0.417	0.604	-5.402	-0.392	7.107	7.498
0.441	0.625	-5.434	-0.210	4.840	5.049
0.415	0.603	-5.401	0.364	2.286	1.922
0.001	0.157	-0.656	1.127	2.598	1.471
0.491	0.665	-5.490	-0.213	5.345	5.557
0.666	0.798	-5.627	0.183	2.136	1.953

0.955	0.977	-5.713	0.025	2.312	2.288
0.024	0.173	-3.336	-1.768	3.523	5.291
0.573	0.728	-5.564	-0.214	4.981	5.195
0.079	0.249	-4.262	-0.404	4.337	4.741
0.381	0.570	-5.352	-0.422	2.076	2.498
0.887	0.938	-5.705	-0.067	2.556	2.623
0.444	0.627	-5.438	0.318	3.527	3.209
0.231	0.428	-5.038	-0.450	4.044	4.494
0.233	0.429	-5.042	-0.382	2.505	2.887
0.947	0.973	-5.713	-0.017	4.099	4.117
0.564	0.721	-5.558	-0.167	2.473	2.640
0.446	0.628	-5.440	-0.208	2.873	3.081
0.495	0.667	-5.494	0.206	2.703	2.497
0.129	0.310	-4.626	-0.596	2.340	2.936
0.679	0.808	-5.634	0.156	4.958	4.802
0.343	0.539	-5.290	-0.257	1.691	1.948
0.021	0.168	-3.212	-0.880	2.318	3.198
0.302	0.500	-5.211	-0.320	2.238	2.559
0.094	0.270	-4.396	0.602	5.417	4.815
0.052	0.212	-3.947	-0.920	1.929	2.849
0.328	0.523	-5.262	0.291	2.075	1.785
0.911	0.952	-5.709	-0.049	3.398	3.447
0.770	0.868	-5.674	0.139	3.793	3.654
0.216	0.411	-4.991	-0.312	6.593	6.905
0.331	0.526	-5.267	-0.263	3.148	3.411
0.146	0.330	-4.716	-0.449	3.839	4.288
0.595	0.746	-5.581	-0.230	3.034	3.264
0.033	0.184	-3.581	-0.978	1.538	2.516
0.286	0.483	-5.176	-0.316	1.447	1.763
0.478	0.655	-5.476	-0.305	3.119	3.424
0.921	0.958	-5.710	0.034	2.924	2.890
0.459	0.640	-5.456	-0.322	3.443	3.765
0.166	0.355	-4.810	-0.558	2.067	2.625
0.595	0.746	-5.581	-0.151	2.620	2.771
0.029	0.178	-3.486	0.840	3.334	2.493
0.754	0.858	-5.668	0.096	4.007	3.911
0.411	0.598	-5.395	-0.251	3.621	3.872
0.014	0.160	-2.886	0.917	5.085	4.168
0.016	0.162	-3.022	0.822	4.669	3.848
0.013	0.160	-2.838	0.863	4.829	3.966
0.049	0.207	-3.894	-1.035	2.156	3.190
0.062	0.225	-4.080	-0.660	2.159	2.819
0.623	0.766	-5.600	0.174	3.355	3.181
0.879	0.934	-5.704	0.050	3.755	3.705
0.023	0.173	-3.302	1.076	4.073	2.996
0.874	0.932	-5.703	0.042	2.714	2.672
0.249	0.447	-5.087	-0.300	3.612	3.912
0.568	0.724	-5.560	0.205	2.626	2.421
0.127	0.308	-4.619	-0.564	2.440	3.003
0.661	0.794	-5.624	-0.112	3.147	3.259

0.859	0.924	-5.700	0.066	4.005	3.940
0.835	0.908	-5.694	0.076	2.397	2.321
0.573	0.728	-5.565	0.265	4.141	3.876
0.026	0.175	-3.382	-0.846	1.813	2.659
0.581	0.735	-5.570	-0.209	0.978	1.186
0.510	0.679	-5.509	-0.184	2.177	2.362
0.867	0.928	-5.702	-0.057	3.897	3.954
0.635	0.774	-5.608	-0.136	2.330	2.467
0.096	0.271	-4.407	0.759	5.212	4.453
0.998	0.999	-5.715	0.001	2.891	2.890
0.092	0.267	-4.380	0.607	1.709	1.102
0.501	0.671	-5.500	0.189	3.449	3.259
0.978	0.989	-5.714	0.010	3.409	3.400
0.540	0.702	-5.537	-0.263	2.884	3.147
0.263	0.461	-5.124	0.434	2.484	2.050
0.020	0.167	-3.184	1.083	4.238	3.154
0.030	0.179	-3.509	1.056	4.501	3.445
0.669	0.800	-5.628	-0.129	1.971	2.100
0.483	0.659	-5.482	0.194	2.922	2.728
0.713	0.829	-5.651	0.137	4.772	4.635
0.910	0.951	-5.709	-0.026	5.228	5.253
0.918	0.956	-5.710	-0.031	2.424	2.456
0.626	0.767	-5.602	0.160	2.801	2.640
0.987	0.993	-5.715	-0.005	3.428	3.433
0.443	0.626	-5.437	-0.276	2.745	3.021
0.769	0.868	-5.674	-0.101	3.994	4.095
0.054	0.215	-3.971	-0.564	2.276	2.840
0.814	0.895	-5.689	-0.060	3.114	3.173
0.619	0.763	-5.598	0.152	2.569	2.416
0.004	0.157	-1.857	-2.260	3.110	5.370
0.002	0.157	-1.251	0.886	2.731	1.845
0.640	0.779	-5.611	-0.186	2.702	2.888
0.697	0.820	-5.643	-0.153	3.408	3.562
0.145	0.329	-4.713	-0.669	3.190	3.860
0.761	0.862	-5.671	-0.084	4.188	4.272
0.574	0.728	-5.565	-0.182	2.770	2.951
0.359	0.552	-5.317	-0.324	4.430	4.754
0.464	0.644	-5.461	-0.226	4.604	4.830
0.370	0.561	-5.335	-0.527	2.943	3.470
0.004	0.157	-1.806	-1.516	3.728	5.244
0.756	0.859	-5.669	0.141	2.797	2.656
0.556	0.715	-5.550	0.193	4.091	3.898
0.523	0.689	-5.521	-0.299	2.311	2.609
0.184	0.376	-4.884	-0.393	5.368	5.761
0.167	0.357	-4.814	-0.578	4.639	5.217
0.069	0.236	-4.164	0.452	2.571	2.119
0.788	0.879	-5.680	0.079	3.701	3.622
0.518	0.685	-5.517	-0.243	4.221	4.464
0.048	0.205	-3.877	-0.723	2.289	3.013
0.753	0.857	-5.668	-0.106	2.316	2.422

0.583	0.736	-5.572	-0.172	5.018	5.190
0.394	0.581	-5.371	-0.255	2.345	2.600
0.462	0.643	-5.459	-0.264	2.621	2.885
0.131	0.312	-4.638	-0.555	3.136	3.691
0.012	0.159	-2.743	-0.831	3.423	4.254
0.865	0.928	-5.701	0.058	2.857	2.800
0.478	0.655	-5.477	0.269	2.159	1.891
0.469	0.648	-5.466	-0.224	3.199	3.423
0.123	0.304	-4.595	-0.489	2.472	2.960
0.135	0.319	-4.663	-0.696	2.295	2.991
0.924	0.959	-5.710	0.046	2.926	2.880
0.309	0.507	-5.226	-0.280	2.875	3.154
0.157	0.345	-4.768	-0.505	2.040	2.545
0.637	0.776	-5.609	-0.189	2.428	2.618
0.957	0.978	-5.713	-0.021	2.777	2.798
0.937	0.966	-5.712	-0.026	5.551	5.577
0.775	0.871	-5.676	-0.103	2.123	2.226
0.633	0.773	-5.607	0.123	4.046	3.922
0.032	0.183	-3.564	0.982	6.102	5.120
0.777	0.872	-5.677	-0.094	2.294	2.388
0.609	0.756	-5.591	-0.127	3.377	3.504
0.578	0.733	-5.568	0.195	2.357	2.162
0.041	0.195	-3.759	-0.719	4.070	4.789
0.949	0.974	-5.713	-0.031	2.731	2.762
0.192	0.384	-4.911	0.547	5.496	4.949
0.237	0.434	-5.055	-0.562	2.666	3.228
0.718	0.833	-5.653	0.104	4.385	4.280
0.820	0.898	-5.690	-0.090	2.495	2.585
0.214	0.409	-4.986	-0.447	3.512	3.959
0.014	0.160	-2.886	-0.812	4.283	5.096
0.284	0.482	-5.172	-0.400	3.039	3.439
0.381	0.570	-5.352	-0.334	2.249	2.582
0.145	0.329	-4.712	0.726	2.957	2.231
0.940	0.967	-5.712	0.027	2.398	2.370
0.257	0.455	-5.109	0.584	3.424	2.841
0.173	0.362	-4.837	-0.443	3.024	3.467
0.428	0.614	-5.418	0.234	2.504	2.271
0.226	0.423	-5.023	-0.596	3.952	4.548
0.299	0.496	-5.205	0.325	4.262	3.937
0.470	0.648	-5.467	0.205	2.762	2.558
0.577	0.732	-5.568	-0.197	2.870	3.068
0.660	0.793	-5.623	-0.120	3.230	3.350
0.332	0.527	-5.269	-0.312	1.916	2.228
0.885	0.937	-5.705	-0.050	3.346	3.396
0.020	0.167	-3.195	-1.254	2.220	3.474
0.824	0.900	-5.691	-0.106	2.525	2.631
0.466	0.645	-5.463	-0.248	4.384	4.632
0.017	0.162	-3.053	0.757	2.556	1.799
0.092	0.267	-4.377	-0.927	1.384	2.311
0.640	0.778	-5.611	-0.251	3.530	3.781

0.219	0.414	-5.001	0.336	3.367	3.031
0.274	0.472	-5.150	-0.319	2.372	2.691
0.158	0.347	-4.776	0.426	2.683	2.257
0.514	0.681	-5.513	0.203	6.907	6.704
0.095	0.271	-4.400	0.713	4.620	3.907
0.234	0.431	-5.046	-0.316	2.282	2.598
0.786	0.878	-5.680	0.083	4.042	3.959
0.597	0.747	-5.582	-0.166	2.477	2.643
0.473	0.652	-5.471	0.203	6.212	6.009
0.556	0.716	-5.551	0.176	4.341	4.165
0.325	0.521	-5.256	0.312	3.139	2.827
0.140	0.324	-4.688	0.390	2.479	2.089
0.510	0.679	-5.510	-0.179	2.736	2.915
0.592	0.744	-5.579	-0.231	1.850	2.081
0.952	0.975	-5.713	-0.023	1.740	1.763
0.015	0.160	-2.923	1.990	5.567	3.577
0.260	0.457	-5.114	0.342	2.565	2.223
0.227	0.424	-5.024	-0.530	2.171	2.701
0.068	0.234	-4.152	-0.905	3.091	3.996
0.118	0.298	-4.565	-0.483	2.494	2.977
0.296	0.493	-5.199	-0.361	2.201	2.562
0.599	0.749	-5.584	0.163	4.495	4.333
0.420	0.606	-5.407	-0.378	2.676	3.054
0.973	0.987	-5.714	-0.011	2.729	2.741
0.110	0.289	-4.510	-0.592	2.721	3.313
0.138	0.321	-4.676	-0.499	1.919	2.418
0.137	0.320	-4.670	-0.592	1.781	2.373
0.483	0.659	-5.482	-0.241	1.708	1.949
0.532	0.696	-5.530	-0.217	5.329	5.547
0.786	0.878	-5.680	-0.086	5.057	5.143
0.653	0.789	-5.619	-0.130	2.642	2.772
0.430	0.615	-5.420	0.236	2.224	1.988
0.699	0.821	-5.644	0.107	2.072	1.965
0.565	0.722	-5.558	0.229	1.766	1.537
0.188	0.380	-4.898	-0.346	2.066	2.412
0.142	0.326	-4.697	-0.511	1.296	1.807
0.612	0.757	-5.593	-0.220	4.283	4.503
0.077	0.246	-4.240	0.748	5.201	4.453
0.020	0.167	-3.190	0.746	7.234	6.488
0.029	0.178	-3.485	1.277	2.579	1.302
0.009	0.157	-2.560	0.725	7.579	6.854
0.878	0.934	-5.704	-0.036	3.870	3.905
0.770	0.868	-5.674	0.156	3.794	3.638
0.780	0.874	-5.678	0.092	2.820	2.729
0.007	0.157	-2.327	1.524	8.576	7.052
0.002	0.157	-1.351	1.625	4.583	2.958
0.039	0.193	-3.715	-0.708	2.172	2.880
0.334	0.530	-5.273	-0.254	2.779	3.034
0.118	0.298	-4.562	0.355	4.706	4.351
0.112	0.291	-4.527	-0.385	1.703	2.088

0.209	0.404	-4.969	0.419	4.068	3.649
0.092	0.267	-4.378	-0.477	3.547	4.024
0.067	0.233	-4.141	-0.634	3.066	3.700
0.127	0.308	-4.616	-0.549	2.909	3.458
0.251	0.449	-5.093	-0.312	2.173	2.485
0.670	0.801	-5.629	0.131	2.801	2.670
0.077	0.247	-4.247	-0.508	3.865	4.373
0.046	0.203	-3.853	-0.662	3.829	4.491
0.097	0.274	-4.421	-0.442	3.761	4.203
0.883	0.936	-5.704	-0.047	3.136	3.183
0.483	0.659	-5.481	-0.258	3.480	3.738
0.007	0.157	-2.374	-1.198	2.666	3.863
0.883	0.936	-5.704	0.075	1.560	1.485
0.016	0.162	-2.995	-1.598	4.301	5.899
0.133	0.316	-4.650	-0.705	4.474	5.179
0.671	0.802	-5.629	-0.102	2.066	2.168
0.382	0.571	-5.353	-0.434	3.277	3.711
0.652	0.788	-5.618	-0.168	3.469	3.637
0.780	0.874	-5.678	-0.076	2.746	2.822
0.203	0.398	-4.949	-0.544	1.795	2.339
0.754	0.858	-5.668	0.088	4.082	3.993
0.389	0.576	-5.363	0.325	4.104	3.779
0.164	0.353	-4.802	-0.455	2.301	2.755
0.857	0.923	-5.699	0.060	4.048	3.989
0.531	0.695	-5.529	0.182	2.743	2.561
0.030	0.178	-3.498	-0.636	2.365	3.002
0.086	0.258	-4.324	-0.514	2.430	2.944
0.450	0.631	-5.445	-0.195	3.035	3.230
0.372	0.562	-5.338	0.243	2.874	2.631
0.887	0.938	-5.705	-0.048	2.625	2.673
0.911	0.952	-5.709	0.037	2.484	2.447
0.177	0.368	-4.856	-0.666	3.037	3.702
0.940	0.967	-5.712	-0.019	3.191	3.210
0.137	0.320	-4.670	0.368	6.696	6.327
0.271	0.469	-5.142	-0.434	2.129	2.563
0.072	0.239	-4.189	0.945	3.881	2.937
0.005	0.157	-2.036	-1.123	1.684	2.807
0.567	0.723	-5.560	-0.151	7.437	7.588
0.151	0.337	-4.741	-0.487	2.786	3.273
0.033	0.184	-3.581	0.647	2.947	2.300
0.368	0.559	-5.331	0.246	3.029	2.783
0.026	0.175	-3.379	-0.980	3.246	4.226
0.273	0.471	-5.147	0.260	2.608	2.349
0.338	0.533	-5.280	-0.243	3.311	3.554
0.159	0.348	-4.781	-0.618	1.313	1.930
0.559	0.718	-5.553	-0.163	2.826	2.989
0.002	0.157	-1.245	1.466	4.304	2.838
0.079	0.249	-4.262	0.540	2.585	2.045
0.063	0.226	-4.086	0.628	2.570	1.942
0.002	0.157	-1.400	1.338	4.923	3.585

0.370	0.560	-5.334	-0.291	2.586	2.878
0.619	0.763	-5.597	0.131	2.385	2.254
0.241	0.438	-5.064	-0.442	4.176	4.618
0.058	0.219	-4.025	0.634	3.403	2.769
0.002	0.157	-1.433	0.952	3.471	2.519
0.354	0.548	-5.308	-0.369	2.677	3.046
0.817	0.896	-5.689	0.069	2.484	2.415
0.183	0.374	-4.878	-0.382	2.741	3.123
0.606	0.754	-5.589	-0.141	2.187	2.328
0.143	0.327	-4.702	-0.394	3.896	4.290
0.001	0.157	-0.255	1.559	4.571	3.012
0.965	0.982	-5.714	0.012	2.750	2.738
0.162	0.351	-4.794	-0.394	2.837	3.232
0.502	0.672	-5.502	0.189	2.544	2.355
0.090	0.265	-4.364	-0.596	2.013	2.608
0.360	0.553	-5.319	0.283	2.520	2.237
0.002	0.157	-1.382	0.866	3.131	2.265
0.490	0.664	-5.489	-0.259	3.805	4.063
0.024	0.173	-3.324	0.860	3.656	2.796
0.085	0.257	-4.319	-0.490	3.138	3.628
0.532	0.695	-5.530	0.168	4.590	4.422
0.537	0.700	-5.535	0.185	4.320	4.135
0.104	0.281	-4.470	-0.940	2.421	3.361
0.489	0.664	-5.489	-0.229	3.993	4.221
0.797	0.886	-5.683	0.059	4.565	4.506
0.949	0.974	-5.713	-0.018	2.851	2.870
0.800	0.888	-5.684	0.076	2.558	2.482
0.073	0.241	-4.203	1.126	4.982	3.856
0.526	0.691	-5.524	-0.185	2.585	2.770
0.607	0.754	-5.590	-0.201	2.294	2.495
0.053	0.213	-3.957	-0.714	3.344	4.058
0.967	0.983	-5.714	-0.023	3.004	3.027
0.164	0.353	-4.801	-0.400	2.095	2.495
0.080	0.250	-4.270	0.452	3.126	2.674
0.030	0.180	-3.520	-0.861	1.646	2.507
0.913	0.953	-5.709	-0.027	3.566	3.592
0.980	0.989	-5.714	-0.009	4.240	4.249
0.016	0.162	-2.985	-0.969	6.519	7.488
0.475	0.653	-5.473	-0.195	4.735	4.930
0.266	0.464	-5.130	-0.292	4.171	4.463
0.107	0.286	-4.491	-0.653	2.148	2.801
0.001	0.157	-0.436	-1.707	4.627	6.334
0.665	0.797	-5.626	0.116	2.876	2.760
0.520	0.686	-5.518	0.202	2.983	2.780
0.174	0.364	-4.843	0.446	4.386	3.940
0.023	0.173	-3.300	0.684	3.541	2.857
0.013	0.160	-2.805	1.004	3.957	2.953
0.393	0.581	-5.369	-0.295	2.589	2.883
0.055	0.216	-3.988	0.732	2.960	2.227
0.280	0.478	-5.164	-0.333	2.340	2.673

0.157	0.345	-4.771	0.446	2.779	2.333
0.145	0.329	-4.712	-0.381	3.424	3.805
0.005	0.157	-1.995	1.416	4.885	3.468
0.092	0.267	-4.377	-0.807	3.411	4.218
0.047	0.204	-3.862	0.828	4.214	3.386
0.075	0.244	-4.224	-0.568	3.135	3.703
0.062	0.224	-4.074	0.618	4.003	3.384
0.040	0.194	-3.726	0.609	4.898	4.288
0.419	0.606	-5.405	-0.296	2.398	2.694
0.937	0.966	-5.712	0.025	2.499	2.474
0.143	0.327	-4.703	-1.427	4.509	5.936
0.277	0.475	-5.157	0.329	2.969	2.640
0.778	0.872	-5.677	0.081	4.104	4.023
0.161	0.349	-4.786	0.557	2.478	1.922
0.109	0.288	-4.506	0.419	4.917	4.499
0.739	0.848	-5.662	-0.121	3.616	3.736
0.032	0.183	-3.571	0.809	3.051	2.241
0.728	0.841	-5.658	-0.113	3.167	3.280
0.095	0.271	-4.405	0.520	2.727	2.206
0.967	0.983	-5.714	-0.012	5.146	5.157
0.237	0.434	-5.055	-0.272	2.178	2.450
0.687	0.812	-5.638	0.120	3.571	3.450
0.451	0.632	-5.446	0.248	3.304	3.055
0.230	0.428	-5.035	0.308	4.652	4.343
0.360	0.552	-5.318	-0.391	2.276	2.667
0.007	0.157	-2.371	0.944	3.651	2.707
0.020	0.167	-3.175	-0.945	2.261	3.206
0.180	0.371	-4.868	0.391	4.670	4.279
0.158	0.346	-4.775	-0.507	2.437	2.944
0.862	0.926	-5.701	0.056	4.313	4.257
0.059	0.221	-4.039	-0.817	3.587	4.404
0.134	0.317	-4.657	-0.486	2.932	3.418
0.472	0.650	-5.470	0.230	2.670	2.440
0.086	0.259	-4.330	-0.506	4.487	4.993
0.106	0.284	-4.482	-0.535	2.534	3.070
0.410	0.597	-5.394	-0.231	2.316	2.547
0.021	0.169	-3.237	1.120	4.918	3.797
0.049	0.207	-3.897	0.669	2.568	1.899
0.127	0.307	-4.615	0.459	3.633	3.175
0.197	0.392	-4.930	0.315	4.688	4.373
0.041	0.195	-3.748	-0.621	3.350	3.970
0.340	0.535	-5.285	-0.413	3.598	4.011
0.668	0.800	-5.628	-0.134	2.468	2.602
0.268	0.466	-5.135	0.298	3.147	2.849
0.416	0.603	-5.402	-0.190	3.292	3.482
0.381	0.570	-5.352	0.227	3.855	3.628
0.446	0.629	-5.440	-0.220	3.171	3.392
0.200	0.395	-4.938	0.599	2.978	2.379
0.214	0.409	-4.984	0.562	3.968	3.406
0.242	0.439	-5.068	0.592	5.358	4.766

0.029	0.178	-3.492	0.687	3.456	2.768
0.184	0.375	-4.881	-0.366	4.238	4.604
0.926	0.960	-5.711	0.026	2.431	2.405
0.508	0.677	-5.508	-0.176	2.418	2.594
0.100	0.277	-4.438	0.531	2.603	2.072
0.644	0.781	-5.613	0.121	4.370	4.249
0.001	0.157	-0.533	0.883	2.840	1.957
0.006	0.157	-2.133	0.930	3.650	2.720
0.021	0.168	-3.223	0.985	3.583	2.599
0.110	0.289	-4.513	0.688	3.411	2.723
0.100	0.277	-4.438	0.520	3.431	2.911
0.805	0.890	-5.686	-0.072	4.104	4.176
0.600	0.749	-5.584	0.162	4.876	4.714
0.972	0.986	-5.714	0.009	3.063	3.054
0.555	0.715	-5.550	-0.398	2.931	3.329
0.031	0.181	-3.534	-0.740	2.651	3.391
0.980	0.989	-5.714	0.010	4.738	4.728
0.217	0.412	-4.996	-0.334	3.122	3.456
0.919	0.957	-5.710	-0.050	1.852	1.902
0.163	0.352	-4.796	-0.421	2.433	2.855
0.854	0.921	-5.699	-0.046	2.343	2.389
0.879	0.934	-5.704	-0.043	2.486	2.529
0.765	0.865	-5.672	0.075	2.476	2.402
0.007	0.157	-2.307	-0.709	1.642	2.351
0.109	0.288	-4.507	-0.697	4.385	5.082
0.117	0.297	-4.556	0.384	7.454	7.071
0.110	0.289	-4.512	-0.870	2.707	3.576
0.146	0.331	-4.720	0.417	2.571	2.153
0.973	0.987	-5.714	0.011	3.121	3.111
0.248	0.446	-5.085	0.350	3.248	2.898
0.044	0.201	-3.812	-0.619	2.593	3.212
0.378	0.567	-5.347	0.310	3.812	3.503
0.112	0.290	-4.522	-0.368	2.633	3.001
0.009	0.157	-2.557	-0.917	3.098	4.014
0.154	0.341	-4.758	0.336	4.661	4.326
0.605	0.753	-5.588	0.132	2.185	2.053
0.022	0.171	-3.274	0.653	3.220	2.567
0.033	0.184	-3.591	-0.860	2.064	2.925
0.692	0.816	-5.640	0.114	2.558	2.444
0.925	0.960	-5.711	0.025	2.822	2.796
0.284	0.481	-5.171	-0.436	2.675	3.110
0.560	0.718	-5.554	-0.161	4.668	4.829
0.033	0.184	-3.582	-0.629	4.362	4.990
0.500	0.671	-5.500	0.224	2.793	2.569
0.138	0.322	-4.679	-0.412	2.469	2.881
0.659	0.793	-5.623	0.108	2.246	2.137
0.424	0.610	-5.412	0.199	3.380	3.180
0.900	0.945	-5.707	-0.048	3.930	3.978
0.311	0.508	-5.229	-0.463	3.091	3.554
0.152	0.338	-4.745	-0.371	2.229	2.600

0.049	0.207	-3.901	0.641	5.480	4.839
0.096	0.273	-4.412	-0.576	2.085	2.661
0.149	0.334	-4.731	0.592	3.263	2.671
0.243	0.440	-5.070	-0.553	2.029	2.582
0.058	0.219	-4.024	0.481	3.501	3.019
0.194	0.388	-4.919	0.383	2.632	2.249
0.862	0.926	-5.700	0.048	4.394	4.345
0.730	0.843	-5.659	-0.087	2.520	2.607
0.075	0.244	-4.226	-0.405	3.437	3.843
0.375	0.565	-5.342	0.235	6.473	6.238
0.061	0.223	-4.062	0.528	2.812	2.284
0.647	0.784	-5.615	0.167	2.676	2.509
0.394	0.582	-5.371	-0.281	2.062	2.342
0.026	0.175	-3.384	0.643	3.568	2.925
0.593	0.745	-5.579	-0.219	3.626	3.845
0.050	0.207	-3.906	0.888	5.581	4.693
0.002	0.157	-1.404	1.166	2.751	1.584
0.058	0.219	-4.027	-0.784	1.933	2.716
0.098	0.274	-4.424	0.491	4.703	4.212
0.042	0.196	-3.766	0.586	4.598	4.012
0.030	0.178	-3.495	0.767	3.669	2.902
0.885	0.937	-5.705	-0.042	2.313	2.355
0.029	0.178	-3.470	0.662	5.103	4.442
0.025	0.175	-3.359	0.758	4.041	3.283
0.512	0.680	-5.511	0.339	5.540	5.201
0.920	0.957	-5.710	-0.031	2.539	2.570
0.139	0.323	-4.683	0.654	3.065	2.411
0.036	0.188	-3.651	-1.213	4.334	5.547
0.164	0.353	-4.803	-0.533	1.658	2.191
0.867	0.928	-5.702	0.049	3.250	3.201
0.188	0.380	-4.898	0.454	2.577	2.123
0.311	0.508	-5.230	0.323	3.097	2.774
0.551	0.711	-5.546	-0.164	3.605	3.769
0.003	0.157	-1.717	1.003	3.170	2.167
0.446	0.628	-5.440	0.221	5.652	5.430
0.011	0.157	-2.672	0.794	3.054	2.261
0.360	0.552	-5.318	-0.237	2.253	2.489
0.021	0.168	-3.214	-1.716	2.303	4.019
0.129	0.310	-4.629	-0.982	3.162	4.144
0.638	0.777	-5.610	-0.156	2.654	2.810
0.619	0.763	-5.598	0.192	3.694	3.502
0.154	0.341	-4.755	-0.349	3.442	3.791
0.012	0.160	-2.783	-0.648	2.590	3.238
0.168	0.357	-4.816	0.388	3.623	3.235
0.004	0.157	-1.939	0.930	3.002	2.072
0.352	0.547	-5.306	-0.263	3.274	3.537
0.309	0.506	-5.225	-0.270	3.382	3.652
0.998	0.999	-5.715	-0.001	3.620	3.621
0.003	0.157	-1.528	1.274	4.435	3.161
0.255	0.452	-5.102	-0.294	2.246	2.540

0.428	0.613	-5.417	0.255	4.527	4.271
0.009	0.157	-2.507	1.047	6.525	5.478
0.082	0.253	-4.288	0.724	4.091	3.367
0.344	0.539	-5.291	0.247	2.674	2.427
0.053	0.212	-3.950	-0.573	1.941	2.514
0.082	0.253	-4.287	-0.755	3.572	4.327
0.374	0.565	-5.341	-0.405	2.616	3.021
0.097	0.273	-4.416	-0.705	4.902	5.607
0.587	0.740	-5.575	-0.164	2.634	2.798
0.320	0.517	-5.247	-0.268	3.757	4.026
0.179	0.370	-4.864	0.452	3.841	3.390
0.884	0.937	-5.705	0.050	2.483	2.433
0.147	0.332	-4.725	0.634	4.071	3.437
0.420	0.607	-5.408	0.289	3.227	2.938
0.220	0.416	-5.006	0.504	2.789	2.285
0.172	0.362	-4.836	0.361	6.109	5.747
0.207	0.402	-4.964	-0.334	2.185	2.519
0.050	0.207	-3.903	-0.728	2.584	3.313
0.924	0.959	-5.711	-0.025	2.705	2.730
0.243	0.441	-5.072	-0.562	3.130	3.692
0.093	0.269	-4.388	0.484	2.814	2.330
0.660	0.793	-5.623	0.122	2.483	2.360
0.144	0.328	-4.709	-0.408	2.511	2.919
0.042	0.196	-3.767	-0.752	2.520	3.271
0.603	0.752	-5.587	-0.142	3.184	3.326
0.362	0.554	-5.322	0.237	3.324	3.087
0.014	0.160	-2.914	0.560	3.264	2.704
0.289	0.487	-5.183	-0.323	2.908	3.231
0.163	0.352	-4.795	-0.469	4.443	4.912
0.259	0.456	-5.113	-0.298	2.023	2.321
0.391	0.579	-5.367	0.239	6.469	6.231
0.417	0.604	-5.403	0.355	3.078	2.724
0.057	0.218	-4.009	-0.688	2.672	3.360
0.204	0.398	-4.952	0.293	4.264	3.971
0.713	0.829	-5.650	-0.103	4.599	4.702
0.216	0.411	-4.993	0.389	2.790	2.401
0.468	0.647	-5.466	-0.194	5.245	5.439
0.050	0.207	-3.904	-0.679	2.730	3.409
0.920	0.957	-5.710	-0.038	2.964	3.001
0.725	0.838	-5.656	-0.090	3.403	3.493
0.973	0.987	-5.714	0.012	2.637	2.624
0.177	0.368	-4.856	-0.492	3.868	4.361
0.182	0.373	-4.873	0.370	2.822	2.452
0.685	0.811	-5.637	-0.140	2.091	2.231
0.775	0.871	-5.676	-0.084	2.085	2.169
0.039	0.193	-3.719	0.429	4.187	3.758
0.006	0.157	-2.155	0.886	3.382	2.497
0.139	0.323	-4.683	0.389	3.621	3.232
0.004	0.157	-1.796	0.971	3.165	2.194
0.060	0.221	-4.048	0.695	5.332	4.636

0.039	0.193	-3.720	0.527	3.203	2.676
0.067	0.232	-4.135	0.556	3.417	2.861
0.116	0.296	-4.548	-0.623	2.381	3.004
0.113	0.292	-4.532	-0.619	2.844	3.463
0.108	0.286	-4.496	-0.386	7.772	8.158
0.148	0.333	-4.729	0.472	3.630	3.158
0.611	0.757	-5.592	-0.120	1.789	1.909
0.640	0.778	-5.611	-0.122	3.400	3.523
0.798	0.886	-5.684	-0.088	3.310	3.398
0.281	0.479	-5.165	-0.360	1.780	2.140
0.611	0.757	-5.592	-0.132	2.427	2.559
0.230	0.427	-5.034	-0.277	3.046	3.323
0.423	0.609	-5.410	0.289	2.928	2.639
0.738	0.848	-5.662	0.093	6.489	6.396
0.079	0.249	-4.263	-0.883	3.480	4.363
0.788	0.880	-5.681	0.076	3.569	3.493
0.950	0.974	-5.713	-0.020	1.415	1.435
0.405	0.593	-5.387	0.320	3.507	3.187
0.072	0.240	-4.196	0.833	3.604	2.772
0.246	0.443	-5.078	-0.362	2.509	2.871
0.944	0.970	-5.712	-0.018	4.362	4.380
0.552	0.712	-5.547	0.160	2.286	2.125
0.766	0.866	-5.673	0.087	3.140	3.053
0.227	0.424	-5.024	-0.360	2.530	2.889
0.213	0.409	-4.984	0.404	3.234	2.830
0.023	0.173	-3.311	0.853	2.958	2.105
0.016	0.162	-3.007	0.952	3.346	2.393
0.076	0.245	-4.229	0.667	3.351	2.683
0.009	0.157	-2.517	1.028	2.882	1.854
0.015	0.160	-2.931	1.603	6.373	4.770
0.239	0.437	-5.061	0.340	3.916	3.576
0.489	0.664	-5.488	-0.263	4.528	4.791
0.386	0.574	-5.360	0.260	3.612	3.352
0.191	0.383	-4.906	-0.520	3.316	3.836
0.018	0.162	-3.079	1.329	3.711	2.382
0.479	0.656	-5.478	-0.194	2.024	2.218
0.632	0.772	-5.606	-0.120	2.554	2.675
0.536	0.699	-5.534	-0.251	2.762	3.013
0.195	0.389	-4.923	-0.432	4.102	4.534
0.555	0.715	-5.550	0.171	2.637	2.465
0.117	0.297	-4.555	0.453	4.091	3.638
0.594	0.745	-5.580	0.226	5.007	4.782
0.040	0.194	-3.728	0.856	2.945	2.089
0.291	0.489	-5.188	0.358	3.172	2.813
0.237	0.434	-5.054	-0.326	4.739	5.065
0.755	0.858	-5.669	-0.097	3.478	3.575
0.189	0.380	-4.899	-0.378	2.453	2.831
0.077	0.247	-4.249	-0.451	6.881	7.332
0.041	0.196	-3.764	0.656	3.622	2.965
0.203	0.398	-4.949	0.360	2.771	2.411

0.597	0.747	-5.582	0.158	2.573	2.415
0.147	0.331	-4.723	-0.399	3.119	3.518
0.309	0.506	-5.226	-0.295	2.877	3.172
0.121	0.302	-4.583	-0.417	3.145	3.561
0.044	0.200	-3.803	0.598	2.839	2.241
0.769	0.868	-5.674	0.084	3.659	3.574
0.037	0.189	-3.665	-0.601	3.734	4.335
0.690	0.815	-5.640	0.108	2.889	2.781
0.402	0.589	-5.382	0.232	7.602	7.371
0.695	0.818	-5.642	-0.127	2.803	2.930
0.009	0.157	-2.530	1.139	3.197	2.058
0.008	0.157	-2.453	1.207	4.801	3.594
0.355	0.549	-5.310	-0.266	6.953	7.219
0.129	0.310	-4.628	-0.348	2.144	2.492
0.069	0.235	-4.158	-0.575	3.810	4.385
0.076	0.246	-4.238	-0.983	3.862	4.845
0.816	0.896	-5.689	0.072	3.116	3.044
0.730	0.842	-5.658	-0.139	1.410	1.548
0.084	0.256	-4.309	-0.468	3.266	3.734
0.217	0.412	-4.995	-0.467	2.782	3.249
0.010	0.157	-2.652	1.229	5.870	4.641
0.771	0.868	-5.675	0.114	4.170	4.057
0.094	0.271	-4.397	-0.487	7.339	7.826
0.426	0.612	-5.415	-0.191	2.498	2.689
0.027	0.177	-3.437	0.682	2.491	1.809
0.492	0.666	-5.491	-0.201	2.406	2.607
0.068	0.234	-4.146	0.520	3.693	3.174
0.204	0.399	-4.954	-0.352	3.121	3.473
0.174	0.363	-4.841	-0.359	2.532	2.891
0.975	0.987	-5.714	-0.010	3.037	3.047
0.715	0.831	-5.652	-0.106	2.494	2.601
0.418	0.605	-5.404	0.210	2.870	2.660
0.057	0.218	-4.010	-0.577	1.990	2.567
0.030	0.179	-3.506	0.770	3.495	2.725
0.371	0.562	-5.337	0.302	3.213	2.911
0.509	0.677	-5.508	0.177	3.631	3.454
0.009	0.157	-2.515	-0.767	2.463	3.230
0.477	0.655	-5.476	-0.227	3.132	3.360
0.612	0.757	-5.593	0.185	3.200	3.015
0.079	0.249	-4.262	-0.498	2.732	3.230
0.060	0.221	-4.049	0.591	4.608	4.016
0.519	0.686	-5.518	0.331	3.167	2.836
0.897	0.943	-5.707	-0.036	4.378	4.413
0.067	0.232	-4.138	0.620	5.726	5.106
0.067	0.231	-4.132	0.919	7.462	6.543
0.841	0.912	-5.696	-0.053	2.421	2.474
0.414	0.601	-5.399	-0.158	9.406	9.564
0.653	0.789	-5.619	-0.114	3.379	3.493
0.893	0.942	-5.706	-0.033	2.643	2.676
0.558	0.717	-5.553	-0.134	6.790	6.923

0.169	0.359	-4.823	-0.274	7.962	8.236
0.776	0.871	-5.676	-0.090	3.551	3.641
0.409	0.596	-5.392	-0.267	5.782	6.048
0.235	0.432	-5.049	0.407	5.024	4.617
0.050	0.208	-3.915	0.629	6.220	5.591
0.006	0.157	-2.232	1.200	4.963	3.762
0.100	0.277	-4.442	-0.370	3.222	3.592
0.123	0.304	-4.596	-0.344	3.538	3.882
0.027	0.176	-3.430	-0.461	2.465	2.926
0.049	0.207	-3.899	0.552	5.282	4.730
0.093	0.269	-4.386	0.352	4.953	4.601
0.249	0.447	-5.087	0.305	3.605	3.300
0.008	0.157	-2.395	0.666	4.237	3.571
0.021	0.168	-3.230	0.970	3.903	2.933
0.582	0.736	-5.571	-0.179	4.828	5.007
0.690	0.815	-5.640	-0.111	3.025	3.136
0.053	0.212	-3.949	-0.478	7.375	7.853
0.185	0.376	-4.884	-0.442	2.683	3.125
0.361	0.553	-5.320	-0.229	8.977	9.206
0.667	0.798	-5.627	-0.095	10.430	10.525
0.433	0.617	-5.424	-0.214	5.051	5.265
0.204	0.399	-4.954	0.320	3.609	3.289
0.153	0.341	-4.753	-0.263	9.914	10.177
0.007	0.157	-2.375	-0.574	7.910	8.484
0.047	0.203	-3.855	-0.563	4.060	4.622
0.224	0.421	-5.017	0.447	5.647	5.200
0.026	0.176	-3.391	0.440	6.766	6.326
0.493	0.667	-5.492	0.179	4.722	4.543
0.630	0.771	-5.605	-0.190	1.994	2.183
0.339	0.534	-5.282	-0.269	1.911	2.181
0.780	0.874	-5.678	0.070	4.428	4.358
0.218	0.413	-4.999	-0.433	2.093	2.526
0.326	0.522	-5.259	0.226	5.649	5.423
0.662	0.795	-5.624	-0.106	6.004	6.110
0.024	0.173	-3.323	-0.639	5.877	6.516
0.126	0.306	-4.609	-0.349	4.443	4.792
0.021	0.168	-3.205	0.623	2.961	2.337
0.365	0.557	-5.327	0.229	6.227	5.998
0.111	0.290	-4.516	0.464	6.429	5.965
0.557	0.716	-5.551	-0.270	2.906	3.176
0.488	0.664	-5.487	0.242	5.031	4.789
0.042	0.198	-3.781	-0.494	3.377	3.871
0.752	0.856	-5.667	0.089	5.089	5.000
0.279	0.477	-5.161	-0.786	2.892	3.678
0.178	0.369	-4.860	0.427	2.954	2.527
0.545	0.706	-5.541	0.143	5.863	5.720
0.562	0.721	-5.556	0.200	3.786	3.585
0.169	0.359	-4.822	0.325	5.927	5.602
0.823	0.900	-5.691	-0.099	2.557	2.655
0.017	0.162	-3.069	1.161	5.622	4.461

0.000	0.157	0.233	1.550	4.509	2.959
0.238	0.436	-5.058	0.271	5.730	5.459
0.059	0.220	-4.034	0.529	7.830	7.301
0.004	0.157	-1.930	-1.114	4.914	6.027
0.142	0.326	-4.698	0.550	2.815	2.265
0.029	0.178	-3.491	0.636	8.447	7.811
0.118	0.298	-4.562	0.609	5.251	4.642
0.104	0.281	-4.468	0.327	9.695	9.368
0.817	0.896	-5.689	0.052	4.426	4.374
0.207	0.402	-4.964	0.353	3.785	3.432
0.020	0.167	-3.190	1.258	6.036	4.778
0.249	0.447	-5.088	0.449	3.113	2.664
0.146	0.330	-4.718	0.529	2.765	2.237
0.402	0.589	-5.382	-0.242	2.733	2.975
0.253	0.451	-5.097	0.325	3.265	2.940
0.057	0.219	-4.019	-0.649	5.180	5.829
0.135	0.319	-4.664	0.383	6.161	5.778
0.026	0.176	-3.389	0.914	9.058	8.145
0.132	0.315	-4.646	0.531	5.239	4.708
0.304	0.502	-5.215	-0.356	2.056	2.412
0.037	0.189	-3.664	0.608	5.294	4.686
0.547	0.708	-5.543	-0.145	2.376	2.522
0.093	0.268	-4.384	0.430	4.393	3.963
0.111	0.290	-4.519	0.357	7.363	7.006
0.038	0.192	-3.692	0.557	6.278	5.722
0.160	0.348	-4.781	0.516	3.987	3.472
0.001	0.157	-0.246	2.521	5.985	3.463
0.001	0.157	-0.901	-0.738	3.767	4.505
0.015	0.160	-2.949	1.285	7.985	6.701
0.165	0.354	-4.804	-0.363	5.953	6.316
0.494	0.667	-5.494	-0.161	3.498	3.659
0.174	0.364	-4.843	-0.360	2.313	2.673
0.906	0.950	-5.708	0.039	3.469	3.430
0.493	0.666	-5.492	-0.581	3.634	4.215
0.709	0.827	-5.649	-0.102	4.024	4.126
0.124	0.305	-4.602	0.554	2.442	1.887
0.220	0.416	-5.006	-0.248	4.196	4.444
0.707	0.826	-5.648	0.229	4.828	4.599
0.920	0.957	-5.710	0.066	4.900	4.835
0.019	0.164	-3.142	-0.967	8.669	9.636
0.156	0.344	-4.768	-0.396	3.517	3.914
0.018	0.163	-3.095	-0.935	4.354	5.290
0.067	0.232	-4.137	0.395	7.949	7.554
0.302	0.500	-5.211	0.245	3.854	3.608
0.000	0.157	-0.024	-1.015	5.276	6.291
0.053	0.213	-3.957	-0.548	5.537	6.085
0.192	0.385	-4.912	-0.411	3.451	3.862
0.048	0.205	-3.875	-0.419	4.630	5.049
0.243	0.441	-5.071	-0.340	2.957	3.297
0.134	0.318	-4.658	0.355	6.460	6.105

0.751	0.856	-5.667	0.081	3.310	3.229
0.035	0.186	-3.628	-0.586	4.313	4.899
0.183	0.374	-4.877	0.394	3.580	3.185
0.657	0.792	-5.621	-0.100	3.453	3.553
0.399	0.587	-5.379	-0.259	4.354	4.613
0.056	0.217	-3.997	-0.481	7.233	7.714
0.078	0.247	-4.249	-0.562	2.684	3.247
0.450	0.631	-5.445	-0.215	2.944	3.159
0.922	0.958	-5.710	0.028	2.848	2.820
0.066	0.230	-4.120	-0.478	7.503	7.981
0.095	0.271	-4.402	-0.559	3.250	3.809
0.291	0.489	-5.187	0.305	7.633	7.328
0.027	0.176	-3.411	0.579	6.703	6.123
0.017	0.162	-3.068	1.123	8.059	6.936
0.406	0.593	-5.388	0.282	3.345	3.063
0.114	0.292	-4.535	0.608	5.028	4.420
0.157	0.345	-4.772	0.286	8.082	7.796
0.795	0.885	-5.683	-0.077	4.224	4.301
0.054	0.215	-3.973	-1.281	2.998	4.278
0.158	0.346	-4.773	-0.390	5.095	5.485
0.005	0.157	-2.121	0.953	7.229	6.275
0.088	0.262	-4.346	0.384	8.394	8.010
0.011	0.157	-2.673	-0.706	5.173	5.880
0.026	0.176	-3.400	-0.590	3.556	4.146
0.044	0.201	-3.817	0.659	8.902	8.243
0.123	0.304	-4.595	0.500	7.061	6.561
0.522	0.688	-5.521	-0.152	1.721	1.873
0.039	0.192	-3.708	-0.606	7.810	8.417
0.866	0.928	-5.701	-0.062	5.854	5.916
0.805	0.890	-5.686	0.097	2.320	2.223
0.133	0.316	-4.651	-0.390	5.148	5.539
0.722	0.836	-5.655	-0.148	3.789	3.937
0.431	0.616	-5.421	0.252	5.090	4.838
0.279	0.477	-5.160	0.314	5.365	5.051
0.735	0.846	-5.661	0.113	4.514	4.401
0.426	0.611	-5.414	-0.249	5.763	6.012
0.592	0.744	-5.579	0.208	6.659	6.451
0.676	0.805	-5.632	0.259	7.858	7.599
0.432	0.617	-5.423	-0.172	6.957	7.129
0.345	0.540	-5.293	-0.255	7.041	7.296
0.563	0.721	-5.556	0.185	4.108	3.923
0.936	0.965	-5.712	-0.023	2.499	2.522
0.021	0.168	-3.221	-0.617	6.895	7.512
0.005	0.157	-2.080	-0.950	9.422	10.372
0.487	0.662	-5.486	0.195	7.007	6.813
0.479	0.656	-5.478	0.293	5.663	5.369
0.082	0.253	-4.291	0.582	8.810	8.228
0.149	0.335	-4.734	0.389	5.722	5.332
0.242	0.439	-5.068	-0.509	3.119	3.628
0.254	0.451	-5.099	-0.619	4.636	5.255

0.021	0.168	-3.224	0.585	3.919	3.333
0.288	0.486	-5.181	0.224	2.749	2.524
0.221	0.418	-5.009	-0.452	2.060	2.513
0.027	0.176	-3.416	1.288	8.420	7.132
0.041	0.195	-3.749	0.747	7.795	7.048
0.011	0.157	-2.703	1.718	8.433	6.715
0.029	0.178	-3.484	1.078	7.877	6.800
0.515	0.682	-5.514	0.258	7.003	6.745
0.020	0.167	-3.181	1.435	7.520	6.086
0.068	0.234	-4.149	0.798	5.647	4.849
0.695	0.818	-5.642	-0.198	3.282	3.479
0.030	0.179	-3.499	1.474	8.715	7.241
0.055	0.216	-3.984	1.393	7.421	6.027
0.498	0.670	-5.497	-0.250	3.494	3.744
0.651	0.787	-5.618	-0.125	3.595	3.720
0.137	0.320	-4.672	-0.460	2.501	2.961
0.017	0.162	-3.040	1.456	5.117	3.660
0.354	0.548	-5.308	0.244	3.243	2.998
0.627	0.769	-5.603	-0.152	2.676	2.828
0.146	0.330	-4.717	-0.426	2.995	3.421
0.835	0.908	-5.694	-0.086	2.629	2.714
0.063	0.226	-4.091	0.398	7.028	6.629
0.336	0.532	-5.278	-0.409	7.453	7.862
0.300	0.498	-5.208	0.271	4.919	4.648
0.009	0.157	-2.531	0.743	4.487	3.744
0.722	0.836	-5.655	0.097	3.619	3.522
0.145	0.329	-4.715	-0.529	3.720	4.249
0.928	0.961	-5.711	-0.034	5.154	5.188
0.216	0.411	-4.993	-0.445	4.375	4.820
0.347	0.542	-5.296	-0.742	4.199	4.941
0.483	0.659	-5.482	-0.230	4.159	4.389
0.157	0.345	-4.769	-0.498	3.503	4.002
0.547	0.709	-5.543	0.149	5.122	4.973
0.001	0.157	-0.993	0.960	2.722	1.762
0.960	0.979	-5.714	0.018	5.404	5.386
0.011	0.157	-2.682	0.994	3.756	2.763
0.207	0.402	-4.964	0.347	3.393	3.046
0.145	0.329	-4.715	-0.393	4.481	4.874
0.141	0.326	-4.695	-0.331	4.147	4.478
0.034	0.185	-3.611	-0.521	6.148	6.668
0.659	0.793	-5.623	0.187	3.516	3.329
0.273	0.471	-5.147	-0.441	5.909	6.350
0.609	0.756	-5.591	-0.180	2.569	2.749
0.009	0.157	-2.541	0.613	3.773	3.160
0.044	0.201	-3.814	-0.565	2.372	2.937
0.507	0.676	-5.507	-0.270	2.309	2.579
0.052	0.211	-3.938	0.435	7.062	6.627
0.030	0.178	-3.495	0.693	5.279	4.586
0.399	0.586	-5.378	0.404	3.970	3.567
0.095	0.271	-4.401	0.452	8.650	8.198

0.090	0.265	-4.363	0.558	5.482	4.924
0.955	0.977	-5.713	-0.049	2.893	2.942
0.474	0.652	-5.472	-0.202	4.600	4.802
0.036	0.189	-3.659	-0.804	4.049	4.853
0.003	0.157	-1.621	-1.131	3.891	5.023
0.015	0.161	-2.973	0.685	5.671	4.986
0.058	0.220	-4.029	-0.948	5.605	6.553
0.274	0.472	-5.149	-0.412	2.679	3.091
0.283	0.481	-5.171	-0.301	6.082	6.382
0.578	0.733	-5.568	-0.173	4.214	4.388
0.066	0.231	-4.129	0.480	5.263	4.782
0.743	0.851	-5.664	0.097	3.127	3.030
0.214	0.409	-4.986	0.354	3.481	3.127
0.104	0.282	-4.473	-0.395	3.764	4.158
0.065	0.229	-4.108	-0.449	4.517	4.966
0.014	0.160	-2.905	-0.905	4.847	5.752
0.383	0.572	-5.355	0.253	5.385	5.132
0.393	0.581	-5.370	0.236	3.561	3.325
0.065	0.229	-4.114	-0.449	5.993	6.442
0.371	0.561	-5.336	0.254	4.547	4.293
0.008	0.157	-2.429	-0.895	5.280	6.175
0.010	0.157	-2.651	1.039	6.575	5.536
0.041	0.195	-3.756	0.527	3.100	2.573
0.355	0.549	-5.310	-0.200	7.156	7.356
0.303	0.501	-5.214	0.197	7.429	7.232
0.013	0.160	-2.804	0.516	5.877	5.360
0.261	0.459	-5.118	-0.247	6.417	6.664
0.002	0.157	-1.143	-1.263	3.569	4.833
0.174	0.363	-4.841	-0.372	5.492	5.864
0.057	0.218	-4.006	-0.703	5.023	5.726
0.048	0.205	-3.883	0.468	6.551	6.083
0.862	0.926	-5.700	-0.046	2.421	2.468
0.023	0.173	-3.311	0.934	10.479	9.545
0.103	0.280	-4.463	-0.500	6.777	7.278
0.114	0.292	-4.535	0.671	6.318	5.647
0.213	0.408	-4.983	0.278	5.627	5.349
0.764	0.864	-5.672	0.057	6.551	6.494
0.957	0.978	-5.713	0.011	5.462	5.451
0.230	0.427	-5.034	0.270	4.842	4.572
0.143	0.327	-4.703	-0.618	2.045	2.663
0.327	0.523	-5.261	-0.205	2.788	2.994
0.011	0.159	-2.722	-0.741	6.309	7.051
0.262	0.460	-5.121	0.256	5.170	4.913
0.774	0.870	-5.676	-0.126	2.091	2.216
0.262	0.460	-5.120	0.775	4.375	3.600
0.483	0.659	-5.482	0.236	2.148	1.911
0.816	0.896	-5.689	0.085	5.171	5.086
0.021	0.168	-3.213	-0.559	4.725	5.284
0.494	0.667	-5.494	-0.233	3.215	3.449
0.937	0.966	-5.712	0.021	5.949	5.928

0.678	0.807	-5.633	-0.136	3.187	3.323
0.232	0.429	-5.041	0.549	3.039	2.491
0.166	0.356	-4.811	-0.994	2.657	3.651
0.071	0.239	-4.186	1.135	7.775	6.640
0.281	0.478	-5.165	0.297	5.784	5.487
0.458	0.638	-5.454	-0.313	4.556	4.869
0.048	0.206	-3.887	0.635	9.602	8.967
0.047	0.204	-3.865	-0.836	1.830	2.665
0.177	0.368	-4.856	0.360	3.978	3.619
0.007	0.157	-2.354	-0.876	3.176	4.052
0.018	0.163	-3.094	1.439	6.668	5.230
0.386	0.574	-5.359	0.205	7.273	7.068
0.255	0.452	-5.102	0.364	4.690	4.326
0.021	0.169	-3.240	0.667	5.242	4.575
0.731	0.843	-5.659	0.077	5.312	5.236
0.277	0.475	-5.157	0.358	4.688	4.330
0.246	0.443	-5.078	0.405	7.250	6.845
0.099	0.276	-4.433	0.602	7.667	7.066
0.009	0.157	-2.502	-0.591	5.861	6.452
0.135	0.319	-4.662	0.817	4.247	3.429
0.132	0.315	-4.646	-0.527	3.419	3.946
0.059	0.221	-4.042	-0.467	4.228	4.695
0.516	0.683	-5.515	-0.326	4.656	4.983
0.569	0.725	-5.561	-0.144	2.167	2.310
0.583	0.736	-5.572	-0.279	2.494	2.773
0.131	0.312	-4.637	-0.367	6.325	6.692
0.009	0.157	-2.493	1.021	4.643	3.623
0.111	0.290	-4.521	0.334	7.181	6.847
0.012	0.160	-2.746	-1.012	5.468	6.480
0.088	0.262	-4.347	-0.896	3.313	4.209
0.082	0.253	-4.287	0.544	4.984	4.440
0.452	0.633	-5.448	0.189	5.014	4.825
0.548	0.709	-5.544	-0.148	3.062	3.210
0.465	0.645	-5.463	0.320	5.517	5.197
0.236	0.433	-5.052	0.432	5.645	5.213
0.084	0.256	-4.312	0.482	7.233	6.751
0.023	0.173	-3.306	0.722	8.754	8.032
0.507	0.676	-5.506	0.159	3.005	2.846
0.244	0.442	-5.074	0.348	3.639	3.291
0.251	0.449	-5.093	-0.364	4.485	4.849
0.050	0.208	-3.912	0.632	9.376	8.743
0.077	0.246	-4.241	-0.369	4.522	4.891
0.689	0.814	-5.639	-0.110	2.405	2.515
0.594	0.746	-5.580	-0.138	2.819	2.957
0.009	0.157	-2.530	-0.689	3.921	4.610
0.031	0.182	-3.544	-0.697	3.388	4.085
0.682	0.809	-5.635	0.144	3.894	3.749
0.919	0.957	-5.710	0.029	3.094	3.065
0.016	0.162	-2.985	1.395	8.538	7.143
0.145	0.329	-4.712	-0.401	4.909	5.310

0.023	0.173	-3.291	0.646	2.816	2.170
0.055	0.216	-3.983	-0.482	2.429	2.910
0.019	0.164	-3.144	-1.193	3.900	5.093
0.033	0.184	-3.587	1.194	6.002	4.808
0.225	0.422	-5.019	0.360	3.116	2.756
0.962	0.981	-5.714	-0.011	2.031	2.043
0.046	0.203	-3.852	0.919	6.707	5.789
0.224	0.421	-5.017	-0.330	4.599	4.929
0.630	0.771	-5.605	0.114	5.481	5.367
0.012	0.159	-2.735	1.360	8.450	7.090
0.014	0.160	-2.876	-1.042	4.674	5.716
0.584	0.737	-5.573	0.165	3.575	3.411
0.038	0.192	-3.691	-0.735	3.016	3.751
0.893	0.941	-5.706	-0.036	2.532	2.567
0.155	0.342	-4.759	0.360	3.545	3.185
0.095	0.271	-4.401	-0.882	5.457	6.339
0.430	0.616	-5.421	0.220	4.953	4.733
0.077	0.246	-4.242	0.723	5.777	5.055
0.043	0.199	-3.789	-0.574	4.529	5.103
0.548	0.709	-5.544	-0.148	3.876	4.024
0.000	0.157	-0.011	-1.154	3.959	5.113
0.036	0.188	-3.647	0.552	3.846	3.293
0.681	0.808	-5.634	-0.121	3.421	3.541
0.037	0.190	-3.671	-0.806	4.151	4.957
0.454	0.634	-5.449	0.198	5.849	5.651
0.048	0.204	-3.871	0.905	4.350	3.444
0.319	0.517	-5.245	0.324	5.147	4.823
0.019	0.165	-3.155	-0.577	5.591	6.168
0.296	0.493	-5.198	-0.485	4.402	4.887
0.130	0.311	-4.632	0.404	2.533	2.129
0.963	0.981	-5.714	0.022	2.784	2.762
0.568	0.724	-5.561	-0.253	2.996	3.249
0.027	0.176	-3.413	-0.806	3.255	4.061
0.566	0.722	-5.559	0.283	3.778	3.495
0.333	0.528	-5.271	0.400	4.910	4.509
0.253	0.451	-5.097	-0.462	3.545	4.007
0.053	0.212	-3.951	0.447	6.728	6.281
0.092	0.267	-4.375	0.443	7.303	6.860
0.026	0.176	-3.404	0.495	8.378	7.883
0.190	0.383	-4.904	-0.393	3.197	3.590
0.005	0.157	-2.101	-0.827	4.719	5.546
0.237	0.434	-5.054	0.244	4.503	4.258
0.180	0.371	-4.868	0.347	2.532	2.185
0.014	0.160	-2.880	0.582	5.776	5.195
0.085	0.257	-4.317	0.519	3.348	2.830
0.659	0.793	-5.623	-0.118	2.009	2.127
0.147	0.331	-4.721	-0.310	5.818	6.128
0.596	0.747	-5.582	-0.189	4.229	4.418
0.022	0.169	-3.245	-0.808	7.022	7.830
0.386	0.574	-5.359	0.481	4.033	3.551

0.305	0.503	-5.218	0.252	6.191	5.939
0.582	0.736	-5.571	-0.352	3.525	3.876
0.352	0.546	-5.304	0.262	3.595	3.333
0.582	0.736	-5.572	-0.350	3.549	3.899
0.780	0.874	-5.678	-0.071	5.609	5.680
0.084	0.256	-4.309	1.370	8.028	6.658
0.877	0.933	-5.704	-0.045	3.443	3.488
0.064	0.228	-4.103	-0.420	2.981	3.400
0.132	0.315	-4.648	-0.636	4.523	5.159
0.047	0.203	-3.858	1.369	4.441	3.072
0.036	0.189	-3.660	0.870	5.087	4.217
0.065	0.229	-4.110	-1.031	7.920	8.951
0.536	0.699	-5.534	0.175	6.094	5.919
0.167	0.356	-4.812	-0.336	3.171	3.507
0.068	0.234	-4.151	0.450	4.984	4.534
0.098	0.275	-4.426	0.688	2.745	2.058
0.063	0.227	-4.095	0.618	5.119	4.500
0.386	0.574	-5.360	-0.340	4.441	4.781
0.327	0.523	-5.261	-0.267	4.005	4.272
0.908	0.951	-5.709	0.041	4.623	4.583
0.223	0.419	-5.013	0.352	5.049	4.697
0.051	0.208	-3.919	-0.698	4.722	5.419
0.273	0.471	-5.146	0.284	6.159	5.875
0.867	0.928	-5.701	-0.123	5.908	6.031
0.002	0.157	-1.374	3.036	10.127	7.092
0.078	0.248	-4.254	0.444	2.883	2.440
0.549	0.710	-5.545	-0.272	2.736	3.008
0.147	0.331	-4.723	0.407	5.742	5.335
0.041	0.195	-3.760	0.729	6.929	6.200
0.020	0.167	-3.199	0.749	10.331	9.582
0.247	0.445	-5.082	-0.442	3.728	4.170
0.063	0.227	-4.095	0.632	3.636	3.004
0.038	0.192	-3.703	0.671	2.574	1.903
0.038	0.192	-3.695	-0.590	5.168	5.758
0.082	0.253	-4.290	-0.535	3.154	3.689
0.716	0.832	-5.652	0.101	3.117	3.016
0.822	0.899	-5.691	0.074	3.436	3.363
0.126	0.306	-4.609	0.472	3.318	2.846
0.748	0.853	-5.666	-0.096	2.778	2.874
0.640	0.778	-5.611	0.097	9.759	9.662
0.959	0.979	-5.714	0.017	5.674	5.657
0.050	0.207	-3.904	-0.716	3.382	4.098
0.002	0.157	-1.196	-0.857	4.866	5.722
0.022	0.171	-3.262	0.885	7.544	6.660
0.035	0.186	-3.633	-0.851	4.660	5.511
0.099	0.276	-4.432	0.435	6.153	5.717
0.036	0.188	-3.650	0.517	3.899	3.382
0.348	0.543	-5.298	0.288	6.779	6.491
0.614	0.759	-5.595	0.182	4.606	4.424
0.002	0.157	-1.413	1.054	5.646	4.592

0.047	0.204	-3.870	0.462	8.393	7.931
0.505	0.674	-5.505	0.143	7.578	7.435
0.025	0.175	-3.363	-0.692	5.356	6.048
0.505	0.674	-5.505	0.274	4.708	4.434
0.249	0.447	-5.087	0.279	6.257	5.978
0.793	0.884	-5.682	-0.075	5.854	5.929
0.083	0.255	-4.300	0.407	9.004	8.597
0.056	0.217	-3.995	0.570	6.281	5.711
0.326	0.523	-5.259	0.209	6.519	6.311
0.036	0.189	-3.663	0.414	6.757	6.343
0.070	0.237	-4.172	-0.756	3.651	4.407
0.004	0.157	-1.952	1.110	4.219	3.109
0.003	0.157	-1.581	1.975	6.207	4.232
0.025	0.175	-3.363	0.791	4.136	3.345
0.043	0.198	-3.784	-0.480	5.226	5.706
0.007	0.157	-2.371	1.271	4.261	2.990
0.087	0.260	-4.339	-0.704	2.305	3.009
0.266	0.464	-5.130	0.263	5.216	4.953
0.254	0.452	-5.101	-0.325	4.066	4.391
0.005	0.157	-2.126	-0.838	4.260	5.099
0.416	0.603	-5.402	0.341	4.869	4.528
0.126	0.306	-4.610	0.579	6.229	5.650
0.014	0.160	-2.913	1.245	7.621	6.376
0.116	0.296	-4.551	0.811	5.166	4.355
0.026	0.176	-3.406	-0.506	3.789	4.295
0.438	0.622	-5.430	-0.269	3.592	3.860
0.005	0.157	-1.971	1.039	4.605	3.566
0.406	0.594	-5.389	0.199	5.750	5.551
0.121	0.302	-4.582	0.350	7.813	7.463
0.290	0.488	-5.186	0.266	6.341	6.074
0.010	0.157	-2.630	-1.149	5.875	7.024
0.402	0.589	-5.382	0.366	5.061	4.695
0.046	0.203	-3.838	0.509	6.389	5.881
0.063	0.226	-4.090	-0.534	4.602	5.136
0.375	0.565	-5.342	0.171	6.867	6.695
0.132	0.315	-4.646	-0.422	4.544	4.966
0.056	0.218	-4.004	-0.859	4.567	5.425
0.291	0.489	-5.187	0.335	5.318	4.983
0.000	0.157	-0.095	1.073	8.418	7.345
0.031	0.180	-3.527	0.673	5.939	5.265
0.996	0.998	-5.715	-0.002	2.872	2.874
0.248	0.446	-5.084	0.299	5.323	5.024
0.084	0.256	-4.307	0.429	5.915	5.486
0.503	0.672	-5.502	-0.220	2.308	2.529
0.198	0.392	-4.931	0.314	5.417	5.103
0.733	0.844	-5.660	0.084	8.923	8.839
0.199	0.394	-4.936	-0.317	4.414	4.731
0.036	0.189	-3.661	0.682	4.306	3.625
0.443	0.626	-5.436	-0.245	6.343	6.589
0.351	0.546	-5.304	0.384	3.281	2.896

0.267	0.465	-5.133	0.246	4.844	4.598
0.302	0.500	-5.211	0.297	2.982	2.685
0.011	0.157	-2.675	0.869	6.527	5.658
0.004	0.157	-1.932	-0.826	5.260	6.086
0.110	0.288	-4.508	0.393	6.139	5.746
0.403	0.591	-5.384	0.382	5.629	5.248
0.480	0.656	-5.479	0.182	3.839	3.657
0.768	0.867	-5.674	-0.065	4.449	4.514
0.040	0.194	-3.739	-0.679	3.348	4.027
0.057	0.218	-4.011	0.481	6.463	5.983
0.199	0.393	-4.935	-0.320	3.280	3.599
0.003	0.157	-1.605	-1.082	7.917	9.000
0.039	0.193	-3.714	-0.592	7.400	7.992
0.171	0.361	-4.831	-0.409	3.470	3.880
0.003	0.157	-1.559	1.241	5.037	3.796
0.074	0.242	-4.215	-0.962	3.894	4.855
0.064	0.228	-4.106	0.656	5.883	5.227
0.077	0.247	-4.247	-0.468	4.339	4.808
0.087	0.260	-4.335	0.864	3.942	3.078
0.122	0.303	-4.588	-0.657	1.704	2.361
0.029	0.178	-3.485	1.590	8.226	6.635
0.108	0.287	-4.501	0.351	6.284	5.933
0.002	0.157	-1.203	-0.723	2.572	3.296
0.213	0.408	-4.982	0.352	5.586	5.234
0.554	0.715	-5.549	0.199	8.617	8.418
0.946	0.972	-5.713	0.023	4.010	3.988
0.011	0.157	-2.666	0.604	6.566	5.962
0.254	0.452	-5.101	0.330	4.719	4.389
0.953	0.976	-5.713	-0.015	3.768	3.783
0.892	0.941	-5.706	0.033	2.423	2.390
0.567	0.723	-5.559	0.175	2.562	2.387
0.004	0.157	-1.808	-1.578	5.037	6.616
0.097	0.274	-4.421	0.564	2.537	1.974
0.627	0.768	-5.603	0.117	4.110	3.993
0.343	0.538	-5.289	-0.863	1.849	2.712
0.574	0.728	-5.565	0.149	9.036	8.887
0.659	0.793	-5.623	0.195	6.307	6.112
0.799	0.887	-5.684	0.084	3.501	3.417
0.073	0.241	-4.201	0.975	7.009	6.033
0.007	0.157	-2.362	1.477	8.470	6.994
0.170	0.361	-4.828	0.588	3.712	3.124
0.102	0.279	-4.455	-1.713	1.559	3.272
0.529	0.694	-5.527	0.245	5.671	5.426
0.013	0.160	-2.856	1.556	5.973	4.416
0.001	0.157	-0.878	-1.356	4.792	6.148
0.380	0.569	-5.350	0.401	4.673	4.272
0.942	0.969	-5.712	-0.018	7.520	7.538
0.429	0.615	-5.419	0.278	6.469	6.191
0.220	0.415	-5.004	0.626	5.751	5.125
0.499	0.670	-5.498	-0.205	2.318	2.522

0.917	0.956	-5.710	-0.031	7.563	7.594
0.229	0.426	-5.031	0.324	4.952	4.628
0.231	0.428	-5.038	-0.337	1.762	2.099
0.054	0.215	-3.973	0.758	4.889	4.131
0.012	0.160	-2.792	0.791	5.987	5.195
0.010	0.157	-2.608	1.421	7.976	6.556
0.206	0.401	-4.960	-0.346	3.578	3.924
0.403	0.591	-5.384	-0.190	4.025	4.215
0.191	0.384	-4.909	0.383	5.671	5.288
0.666	0.798	-5.627	0.131	2.578	2.447
0.338	0.534	-5.281	0.242	7.407	7.165
0.028	0.178	-3.448	0.729	3.833	3.104
0.032	0.182	-3.556	0.679	10.072	9.393
0.145	0.329	-4.712	0.634	6.626	5.992
0.260	0.457	-5.114	0.379	7.240	6.861
0.136	0.319	-4.666	0.595	5.521	4.926
0.054	0.214	-3.968	-0.631	6.194	6.825
0.083	0.255	-4.304	0.510	7.000	6.490
0.040	0.194	-3.735	-0.722	5.557	6.279
0.004	0.157	-1.867	1.076	6.724	5.649
0.541	0.703	-5.538	0.180	3.848	3.668
0.182	0.373	-4.874	-0.402	4.571	4.973
0.008	0.157	-2.423	-0.724	5.619	6.343
0.372	0.562	-5.338	0.312	4.614	4.302
0.001	0.157	-0.585	1.596	4.340	2.744
0.258	0.455	-5.109	-0.466	1.936	2.402
0.065	0.229	-4.110	-0.447	3.526	3.973
0.273	0.471	-5.146	-0.281	1.840	2.121
0.952	0.975	-5.713	0.020	5.863	5.843
0.006	0.157	-2.221	0.990	6.622	5.631
0.145	0.329	-4.715	0.687	6.711	6.024
0.719	0.834	-5.654	0.133	3.758	3.625
0.204	0.399	-4.952	0.484	5.992	5.508
0.580	0.735	-5.570	0.164	7.567	7.403
0.045	0.203	-3.835	0.845	6.720	5.875
0.022	0.171	-3.270	1.245	5.459	4.214
0.022	0.169	-3.243	0.724	6.475	5.751
0.089	0.264	-4.353	0.595	5.606	5.011
0.001	0.157	-0.507	1.394	3.847	2.454
0.536	0.698	-5.533	0.516	3.595	3.079
0.054	0.215	-3.968	0.391	6.408	6.018
0.041	0.195	-3.761	-0.611	7.051	7.662
0.039	0.193	-3.717	-0.903	1.601	2.504
0.017	0.162	-3.040	-0.788	5.633	6.421
0.825	0.901	-5.692	0.073	3.339	3.266
0.003	0.157	-1.525	0.617	7.139	6.522
0.323	0.520	-5.253	-0.274	3.537	3.811
0.033	0.184	-3.586	-0.532	3.899	4.431
0.506	0.676	-5.506	0.143	7.752	7.609
0.118	0.298	-4.561	0.837	6.176	5.340

0.513	0.680	-5.512	0.221	2.669	2.449
0.054	0.215	-3.969	0.757	3.486	2.729
0.600	0.749	-5.585	-0.150	3.915	4.065
0.018	0.163	-3.109	-1.012	1.922	2.935
0.062	0.225	-4.076	-0.787	2.891	3.678
0.331	0.527	-5.268	0.223	5.355	5.132
0.188	0.380	-4.895	0.452	4.131	3.680
0.214	0.409	-4.985	-0.294	4.115	4.409
0.007	0.157	-2.303	-1.043	4.507	5.550
0.047	0.203	-3.858	-0.572	5.514	6.087
0.061	0.224	-4.067	0.565	4.431	3.866
0.009	0.157	-2.490	0.988	7.799	6.811
0.102	0.279	-4.453	0.381	5.076	4.694
0.825	0.901	-5.692	0.057	5.019	4.962
0.218	0.413	-5.000	0.334	2.889	2.555
0.279	0.477	-5.160	0.368	4.603	4.235
0.136	0.319	-4.668	0.429	5.273	4.844
0.039	0.194	-3.725	-0.527	6.870	7.397
0.102	0.280	-4.456	1.032	11.652	10.620
0.008	0.157	-2.433	0.703	3.005	2.302
0.029	0.178	-3.484	0.470	4.542	4.072
0.035	0.187	-3.634	0.946	8.792	7.846
0.573	0.728	-5.564	-0.230	4.605	4.835
0.563	0.721	-5.557	-0.254	4.121	4.375
0.471	0.650	-5.469	-0.413	4.928	5.341
0.428	0.614	-5.418	-0.315	3.940	4.255
0.926	0.960	-5.711	-0.033	3.266	3.299
0.065	0.229	-4.114	-1.173	2.281	3.454
0.649	0.785	-5.617	-0.105	3.828	3.933
0.217	0.412	-4.995	-0.311	2.464	2.775
0.473	0.652	-5.472	-0.331	1.437	1.768
0.004	0.157	-1.824	0.636	7.608	6.972
0.175	0.364	-4.846	0.373	3.767	3.395
0.027	0.177	-3.437	1.294	8.503	7.209
0.535	0.698	-5.532	0.271	6.358	6.087
0.416	0.603	-5.401	-0.252	4.072	4.324
0.684	0.811	-5.636	-0.098	3.985	4.082
0.624	0.767	-5.601	-0.163	3.780	3.943
0.195	0.389	-4.921	-0.345	3.552	3.897
0.039	0.193	-3.717	-0.806	2.562	3.368
0.556	0.716	-5.551	0.307	5.935	5.629
0.812	0.894	-5.688	-0.052	4.044	4.097
0.017	0.162	-3.034	0.697	8.769	8.072
0.307	0.504	-5.221	-0.332	5.428	5.759
0.919	0.957	-5.710	0.032	8.887	8.855
0.089	0.264	-4.354	-0.590	2.940	3.529
0.775	0.871	-5.676	0.127	4.564	4.437
0.307	0.504	-5.220	-0.412	5.388	5.800
0.969	0.984	-5.714	-0.013	6.099	6.113
0.820	0.898	-5.690	-0.054	3.669	3.723

0.098	0.275	-4.429	-0.578	3.524	4.103
0.504	0.673	-5.503	0.136	6.199	6.063
0.509	0.678	-5.509	-0.167	8.518	8.685
0.547	0.709	-5.543	-0.177	8.040	8.217
0.923	0.959	-5.710	0.026	5.034	5.009
0.283	0.481	-5.171	-0.280	3.684	3.964
0.484	0.660	-5.483	-0.208	3.357	3.564
0.360	0.553	-5.319	0.431	2.464	2.033
0.336	0.532	-5.277	-0.302	1.844	2.146
0.321	0.518	-5.248	-0.390	7.343	7.733
0.309	0.506	-5.225	-0.203	8.720	8.923
0.800	0.888	-5.684	0.128	3.838	3.709
0.795	0.884	-5.683	-0.085	2.911	2.997
0.542	0.704	-5.539	-0.205	2.182	2.387
0.048	0.205	-3.882	-0.540	7.231	7.770
0.172	0.361	-4.833	-0.476	3.834	4.310
0.331	0.526	-5.267	-0.465	2.313	2.778
0.790	0.881	-5.681	-0.137	7.699	7.836
0.085	0.258	-4.322	-0.681	6.575	7.257
0.634	0.774	-5.607	-0.186	6.389	6.575
0.006	0.157	-2.166	-0.599	10.007	10.606
0.190	0.383	-4.905	-0.241	10.545	10.787
0.029	0.178	-3.485	-0.834	7.749	8.583
0.353	0.548	-5.307	-0.232	4.166	4.398
0.870	0.929	-5.702	-0.039	8.341	8.381
0.259	0.456	-5.112	0.231	6.401	6.170
0.092	0.267	-4.377	-0.501	4.782	5.282
0.010	0.157	-2.595	-0.776	3.337	4.113
0.102	0.279	-4.454	-0.551	7.748	8.299
0.113	0.292	-4.530	-0.391	5.882	6.272
0.543	0.705	-5.540	0.119	6.492	6.372
0.502	0.672	-5.502	-0.191	3.133	3.324
0.351	0.546	-5.304	-0.184	9.245	9.429
0.214	0.409	-4.985	-0.419	2.534	2.953
0.479	0.655	-5.477	-0.201	2.630	2.831
0.491	0.665	-5.490	-0.335	2.656	2.991
0.739	0.848	-5.662	0.109	3.632	3.523
0.110	0.288	-4.509	-0.352	4.884	5.236
0.917	0.955	-5.710	-0.031	7.297	7.327
0.032	0.182	-3.550	-0.531	4.900	5.432
0.937	0.966	-5.712	-0.022	5.650	5.672
0.854	0.921	-5.699	0.072	6.230	6.158
0.937	0.966	-5.712	-0.021	3.072	3.093
0.333	0.529	-5.271	-0.470	5.102	5.572
0.182	0.373	-4.873	-0.677	8.238	8.914
0.163	0.352	-4.796	-0.316	7.821	8.136
0.092	0.267	-4.379	-0.591	4.766	5.357
0.596	0.747	-5.582	-0.126	3.727	3.852
0.108	0.287	-4.497	-0.505	2.598	3.103
0.103	0.280	-4.459	-0.489	6.139	6.629

0.627	0.769	-5.603	0.129	5.628	5.499
0.955	0.977	-5.713	-0.017	3.516	3.533
0.259	0.457	-5.113	-0.401	5.570	5.971
0.895	0.943	-5.707	-0.026	4.867	4.893
0.732	0.843	-5.659	0.070	5.459	5.389
0.125	0.306	-4.608	-0.348	7.524	7.873
0.043	0.200	-3.801	-0.811	8.556	9.367
0.956	0.977	-5.713	-0.015	3.066	3.081
0.142	0.326	-4.696	-0.454	5.429	5.883
0.113	0.292	-4.530	0.663	6.976	6.313
0.277	0.475	-5.157	-0.291	5.057	5.348
0.186	0.378	-4.890	0.849	5.579	4.730
0.075	0.245	-4.228	0.864	4.945	4.081
0.240	0.437	-5.062	0.515	6.374	5.859
0.032	0.183	-3.571	0.743	2.981	2.237
0.073	0.241	-4.200	1.089	4.731	3.641
0.089	0.264	-4.356	0.754	4.772	4.018
0.299	0.496	-5.205	0.563	9.048	8.485
0.103	0.280	-4.463	0.738	2.686	1.948
0.058	0.219	-4.021	1.097	4.359	3.262
0.086	0.258	-4.324	0.739	9.742	9.003
0.071	0.238	-4.176	1.004	4.001	2.997
0.294	0.491	-5.194	0.428	5.718	5.289
0.114	0.293	-4.537	0.781	5.921	5.140
0.114	0.293	-4.540	0.960	5.089	4.129
0.117	0.297	-4.556	0.846	5.108	4.262
0.110	0.289	-4.513	0.960	4.799	3.838
0.747	0.853	-5.666	-0.259	3.697	3.955
0.125	0.306	-4.604	0.794	6.511	5.717
0.065	0.229	-4.117	0.994	5.787	4.794
0.062	0.224	-4.073	0.967	3.399	2.432
0.106	0.284	-4.483	1.231	5.352	4.121
0.077	0.246	-4.245	1.059	5.637	4.579
0.580	0.734	-5.570	0.157	4.541	4.384
0.075	0.244	-4.226	1.115	7.603	6.488
0.121	0.302	-4.584	0.731	9.618	8.887
0.832	0.906	-5.694	-0.106	3.300	3.405
0.379	0.569	-5.348	0.474	3.778	3.304
0.040	0.194	-3.742	0.843	2.992	2.149
0.305	0.503	-5.217	0.632	3.099	2.467
0.996	0.998	-5.715	0.003	3.472	3.469
0.150	0.335	-4.736	0.945	5.796	4.851
0.096	0.271	-4.407	0.518	11.031	10.513
0.084	0.256	-4.308	0.954	6.208	5.255
0.197	0.391	-4.929	0.528	4.378	3.849
0.093	0.268	-4.385	0.660	10.481	9.821
0.161	0.350	-4.789	0.508	10.935	10.427
0.191	0.383	-4.906	0.479	2.967	2.488
0.287	0.485	-5.180	0.459	8.297	7.837
0.232	0.428	-5.039	0.547	7.813	7.266

0.022	0.170	-3.252	1.326	4.327	3.001
0.187	0.380	-4.895	0.804	8.963	8.159
0.170	0.361	-4.828	0.652	7.298	6.646
0.034	0.185	-3.606	1.199	6.008	4.809
0.054	0.214	-3.968	1.140	6.019	4.879
0.104	0.281	-4.469	0.525	11.624	11.099
0.068	0.233	-4.144	0.841	4.981	4.140
0.126	0.306	-4.610	0.605	2.847	2.242
0.076	0.245	-4.235	0.666	11.147	10.481
0.356	0.550	-5.311	0.534	4.785	4.251
0.096	0.272	-4.410	0.544	11.468	10.924
0.111	0.290	-4.518	0.527	11.442	10.915
0.110	0.289	-4.510	0.485	11.378	10.893
0.007	0.157	-2.321	1.177	3.651	2.474
0.494	0.667	-5.493	0.199	7.940	7.741
0.448	0.630	-5.442	0.194	9.478	9.285
0.035	0.186	-3.623	0.719	2.708	1.990
0.872	0.930	-5.702	-0.041	7.232	7.273
0.198	0.392	-4.931	0.929	7.016	6.087
0.277	0.475	-5.157	0.348	5.258	4.909
0.105	0.282	-4.474	0.621	4.787	4.167
0.183	0.374	-4.879	0.470	6.633	6.163
0.046	0.203	-3.852	0.786	5.671	4.884
0.290	0.488	-5.185	0.369	7.328	6.959
0.084	0.256	-4.307	0.663	6.736	6.073
0.023	0.172	-3.279	-0.449	7.564	8.012
0.033	0.184	-3.577	-0.416	7.775	8.190
0.496	0.668	-5.496	-0.165	5.486	5.652
0.111	0.290	-4.520	-0.734	3.555	4.288
0.048	0.205	-3.880	-0.591	2.102	2.693
0.368	0.559	-5.331	-0.335	2.560	2.895
0.725	0.838	-5.656	0.104	2.782	2.678
0.681	0.809	-5.635	-0.105	4.655	4.759
0.118	0.298	-4.566	-0.410	3.351	3.761
0.157	0.345	-4.770	-0.503	4.621	5.124
0.448	0.630	-5.443	0.202	6.779	6.577
0.218	0.412	-4.997	-0.424	3.884	4.308
0.731	0.843	-5.659	-0.131	4.461	4.591
0.155	0.342	-4.761	-0.295	8.490	8.785
0.048	0.204	-3.873	-0.619	6.399	7.018
0.986	0.993	-5.715	-0.006	2.574	2.581
0.556	0.716	-5.551	0.237	4.348	4.111
0.605	0.753	-5.588	-0.128	5.408	5.537
0.313	0.511	-5.234	-0.278	1.335	1.613
0.038	0.192	-3.703	0.831	3.054	2.223
0.597	0.747	-5.582	0.138	4.130	3.992
0.069	0.236	-4.165	-0.430	4.243	4.673
0.845	0.914	-5.697	-0.064	3.829	3.893
0.121	0.301	-4.579	-0.477	7.435	7.912
0.855	0.922	-5.699	0.041	5.517	5.476

0.026	0.176	-3.405	-0.425	8.412	8.837
0.033	0.184	-3.591	-0.479	4.977	5.456
0.711	0.828	-5.650	-0.108	4.508	4.616
0.742	0.851	-5.664	0.130	2.083	1.953
0.193	0.386	-4.916	-0.320	3.939	4.259
0.890	0.940	-5.706	-0.035	5.373	5.407
0.423	0.609	-5.411	0.226	3.897	3.671
0.061	0.223	-4.066	-0.410	5.994	6.403
0.100	0.277	-4.439	-0.383	8.064	8.447
0.736	0.846	-5.661	0.116	5.247	5.131
0.165	0.354	-4.805	-0.443	4.950	5.394
0.716	0.831	-5.652	-0.090	4.716	4.806
0.090	0.264	-4.360	-0.492	2.513	3.004
0.779	0.873	-5.678	0.069	2.745	2.676
0.529	0.694	-5.527	0.166	10.989	10.823
0.696	0.819	-5.643	-0.146	2.253	2.399
0.376	0.566	-5.344	-0.224	8.108	8.332
0.277	0.475	-5.157	0.364	2.299	1.935
0.785	0.878	-5.680	-0.082	2.117	2.199
0.176	0.366	-4.852	-0.436	4.172	4.608
0.659	0.793	-5.623	-0.126	3.486	3.612
0.510	0.679	-5.509	-0.168	2.159	2.327
0.867	0.928	-5.701	-0.037	7.757	7.794
0.214	0.409	-4.985	-0.317	5.701	6.018
0.205	0.401	-4.958	0.432	3.700	3.269
0.035	0.186	-3.621	-0.516	6.807	7.322
0.817	0.896	-5.689	0.065	4.383	4.318
0.018	0.163	-3.113	-0.544	7.343	7.888
0.182	0.373	-4.874	0.415	2.920	2.505
0.087	0.259	-4.332	-0.559	1.370	1.929
0.073	0.241	-4.200	0.911	7.120	6.209
0.220	0.416	-5.004	0.484	5.760	5.275
0.327	0.523	-5.260	0.261	10.140	9.879
0.345	0.539	-5.292	-0.263	5.999	6.262
0.112	0.290	-4.524	0.470	6.913	6.443
0.017	0.162	-3.075	1.014	7.240	6.226
0.119	0.299	-4.570	0.577	7.524	6.946
0.023	0.173	-3.297	-0.573	5.502	6.076
0.050	0.207	-3.905	0.830	5.766	4.936
0.507	0.676	-5.506	0.164	4.428	4.264
0.063	0.226	-4.085	-0.535	4.111	4.646
0.382	0.571	-5.353	-0.289	1.857	2.146
0.129	0.310	-4.626	-0.505	3.780	4.285
0.024	0.174	-3.344	-0.700	6.573	7.273
0.017	0.162	-3.051	1.063	4.597	3.534
0.563	0.721	-5.557	-0.120	4.637	4.757
0.798	0.886	-5.684	0.075	6.739	6.664
0.924	0.959	-5.710	0.024	5.935	5.911
0.317	0.515	-5.241	0.208	6.959	6.751
0.019	0.164	-3.131	1.317	6.478	5.160

0.071	0.239	-4.187	-0.467	5.395	5.862
0.161	0.350	-4.789	0.327	6.889	6.562
0.429	0.615	-5.419	0.252	3.917	3.665
0.055	0.215	-3.979	0.574	4.226	3.653
0.127	0.307	-4.616	0.396	6.762	6.366
0.030	0.180	-3.512	0.669	3.575	2.906
0.056	0.218	-4.004	0.749	6.263	5.514
0.010	0.157	-2.647	1.183	6.351	5.168
0.070	0.237	-4.173	0.491	6.194	5.703
0.264	0.462	-5.125	-0.439	2.786	3.225
0.006	0.157	-2.178	1.185	4.493	3.307
0.353	0.548	-5.307	0.346	5.584	5.239
0.006	0.157	-2.209	1.277	5.141	3.864
0.170	0.360	-4.825	-0.371	2.354	2.724
0.079	0.249	-4.265	0.567	3.495	2.929
0.000	0.157	0.544	-1.363	4.115	5.478
0.123	0.304	-4.593	-0.350	3.820	4.170
0.724	0.838	-5.656	-0.117	2.346	2.463
0.016	0.162	-3.020	-0.685	3.617	4.302
0.030	0.180	-3.518	0.536	5.732	5.196
0.004	0.157	-1.924	-1.018	5.054	6.072
0.884	0.937	-5.705	0.033	7.910	7.876
0.214	0.409	-4.987	-0.365	2.409	2.774
0.065	0.229	-4.116	-0.398	6.283	6.681
0.798	0.886	-5.684	-0.068	4.270	4.338
0.376	0.566	-5.344	-0.287	4.481	4.768
0.933	0.964	-5.711	0.025	3.855	3.830
0.474	0.652	-5.472	-0.305	1.261	1.566
0.129	0.310	-4.627	0.486	4.030	3.544
0.496	0.668	-5.496	-0.259	2.118	2.376
0.606	0.753	-5.589	-0.158	2.493	2.651
0.003	0.157	-1.710	-1.033	5.404	6.437
0.018	0.162	-3.077	-0.782	5.303	6.085
0.011	0.157	-2.694	-0.758	2.825	3.582
0.554	0.715	-5.549	0.184	2.205	2.021
0.591	0.743	-5.578	0.240	3.472	3.232
0.809	0.893	-5.687	0.063	2.975	2.912
0.761	0.863	-5.671	0.088	2.686	2.598
0.464	0.644	-5.461	-0.208	2.340	2.548
0.109	0.288	-4.503	-0.504	4.464	4.967
0.376	0.565	-5.343	-0.292	3.674	3.966
0.751	0.856	-5.667	0.096	3.901	3.805
0.144	0.327	-4.706	0.308	5.278	4.970
0.019	0.164	-3.132	0.676	5.067	4.391
0.763	0.864	-5.672	0.103	3.719	3.616
0.335	0.531	-5.276	-0.318	2.555	2.872
0.582	0.736	-5.571	0.147	3.611	3.463
0.043	0.200	-3.799	1.125	6.487	5.362
0.138	0.322	-4.679	-0.418	4.624	5.043
0.136	0.319	-4.667	0.275	9.051	8.776

0.242	0.439	-5.068	0.309	5.218	4.908
0.045	0.203	-3.834	-0.524	2.032	2.556
0.023	0.173	-3.310	0.727	6.589	5.863
0.168	0.358	-4.817	0.564	4.725	4.161
0.024	0.173	-3.334	-0.963	2.228	3.191
0.431	0.616	-5.421	-0.199	1.826	2.025
0.777	0.872	-5.677	0.064	7.216	7.152
0.165	0.354	-4.805	0.411	7.011	6.599
0.015	0.160	-2.944	-0.659	6.033	6.692
0.154	0.341	-4.755	-0.466	6.523	6.989
0.217	0.412	-4.996	-0.487	10.094	10.581
0.271	0.469	-5.143	-0.302	2.693	2.994
0.996	0.998	-5.715	-0.002	4.761	4.762
0.359	0.552	-5.317	0.229	4.951	4.722
0.027	0.177	-3.436	1.116	5.359	4.244
0.812	0.894	-5.688	0.068	1.807	1.739
0.208	0.403	-4.967	0.409	4.786	4.377
0.441	0.625	-5.433	0.264	4.612	4.349
0.806	0.891	-5.686	0.066	5.058	4.992
0.025	0.175	-3.360	0.601	7.520	6.920
0.253	0.451	-5.099	0.243	6.224	5.981
0.314	0.511	-5.235	0.378	5.264	4.886
0.022	0.171	-3.274	1.014	6.595	5.581
0.054	0.214	-3.964	0.655	5.892	5.237
0.040	0.194	-3.731	-0.651	6.363	7.013
0.874	0.932	-5.703	0.065	4.350	4.285
0.950	0.974	-5.713	-0.015	5.439	5.454
0.009	0.157	-2.530	1.430	5.423	3.994
0.564	0.721	-5.557	0.203	3.180	2.977
0.171	0.361	-4.830	-0.484	6.392	6.876
0.840	0.911	-5.695	-0.080	4.099	4.179
0.524	0.689	-5.522	-0.155	7.311	7.466
0.631	0.772	-5.606	0.161	2.655	2.493
0.025	0.175	-3.371	-0.666	4.038	4.705
0.199	0.394	-4.937	-0.350	5.510	5.860
0.009	0.157	-2.578	-0.575	6.704	7.279
0.014	0.160	-2.875	-0.840	6.060	6.900
0.938	0.967	-5.712	0.031	8.222	8.192
0.054	0.215	-3.972	-0.584	1.895	2.479
0.024	0.173	-3.337	-0.640	2.963	3.604
0.336	0.532	-5.278	0.199	7.747	7.548
0.076	0.245	-4.235	0.455	6.032	5.577
0.970	0.985	-5.714	-0.007	7.951	7.958
0.024	0.174	-3.341	-0.963	4.386	5.348
0.894	0.942	-5.706	-0.029	2.773	2.802
0.034	0.185	-3.615	0.696	5.001	4.305
0.007	0.157	-2.288	1.526	6.422	4.896
0.069	0.236	-4.164	0.460	4.167	3.707
0.934	0.964	-5.712	0.021	5.343	5.322
0.132	0.314	-4.643	0.317	5.103	4.786

0.077	0.246	-4.240	0.628	5.633	5.005
0.874	0.932	-5.703	-0.041	2.624	2.665
0.069	0.235	-4.158	-0.397	6.512	6.909
0.096	0.271	-4.407	-0.383	4.380	4.763
0.342	0.538	-5.288	0.227	6.075	5.848
0.645	0.782	-5.614	-0.132	4.022	4.153
0.734	0.845	-5.660	0.230	6.838	6.608
0.078	0.249	-4.258	0.735	4.659	3.924
0.020	0.167	-3.184	1.141	6.283	5.142
0.114	0.293	-4.538	0.542	2.998	2.456
0.045	0.203	-3.832	0.701	2.883	2.182
0.009	0.157	-2.551	1.121	6.013	4.892
0.017	0.162	-3.057	-1.280	4.748	6.028
0.062	0.225	-4.080	0.464	4.642	4.178
0.443	0.626	-5.436	0.194	4.270	4.076
0.188	0.380	-4.898	-0.400	2.600	3.000
0.073	0.241	-4.207	-0.479	3.548	4.027
0.063	0.226	-4.086	0.605	4.819	4.214
0.267	0.465	-5.132	-0.452	3.186	3.638
0.050	0.208	-3.913	-0.563	5.380	5.943
0.008	0.157	-2.480	1.732	6.003	4.271
0.088	0.261	-4.342	0.397	7.375	6.978
0.047	0.204	-3.870	1.260	8.302	7.042
0.844	0.914	-5.696	-0.057	4.543	4.599
0.030	0.178	-3.497	1.060	7.097	6.037
0.534	0.697	-5.532	-0.197	3.255	3.452
0.765	0.865	-5.672	-0.137	4.503	4.640
0.005	0.157	-2.072	1.347	5.979	4.631
0.040	0.194	-3.726	-0.578	2.548	3.126
0.033	0.184	-3.577	0.599	5.909	5.310
0.006	0.157	-2.167	0.910	3.951	3.041
0.477	0.654	-5.475	0.157	4.895	4.738
0.684	0.811	-5.636	-0.100	3.653	3.754
0.038	0.192	-3.700	-0.514	5.558	6.072
0.161	0.350	-4.788	0.423	6.570	6.147
0.240	0.438	-5.063	0.326	3.656	3.330
0.131	0.313	-4.640	-0.424	5.151	5.575
0.109	0.288	-4.502	0.433	7.600	7.168
0.040	0.194	-3.734	-0.595	5.421	6.016
0.411	0.598	-5.395	-0.174	8.171	8.345
0.014	0.160	-2.875	1.375	6.095	4.719
0.202	0.398	-4.948	0.332	4.637	4.305
0.017	0.162	-3.032	0.568	6.916	6.348
0.773	0.869	-5.675	0.180	3.575	3.395
0.711	0.828	-5.650	-0.135	2.642	2.776
0.832	0.906	-5.693	0.053	2.547	2.494
0.016	0.162	-2.986	1.004	7.026	6.022
0.103	0.280	-4.460	-0.664	3.825	4.488
0.012	0.160	-2.781	0.926	3.061	2.134
0.463	0.643	-5.460	-0.308	4.558	4.865

0.110	0.289	-4.512	0.362	4.083	3.721
0.327	0.523	-5.261	-0.278	4.363	4.641
0.063	0.226	-4.090	0.428	5.098	4.671
0.002	0.157	-1.195	-1.447	4.054	5.500
0.164	0.353	-4.801	-0.407	3.902	4.309
0.059	0.221	-4.044	1.236	10.403	9.167
0.025	0.175	-3.376	0.685	7.212	6.527
0.786	0.878	-5.680	0.099	6.440	6.341
0.747	0.853	-5.665	0.067	8.346	8.278
0.472	0.650	-5.469	0.177	5.013	4.836
0.006	0.157	-2.168	-1.248	2.006	3.253
0.042	0.198	-3.782	0.733	6.038	5.305
0.364	0.556	-5.326	0.182	6.611	6.429
0.087	0.260	-4.338	0.321	3.383	3.063
0.006	0.157	-2.154	0.903	6.684	5.781
0.027	0.176	-3.429	0.515	4.074	3.560
0.111	0.290	-4.518	0.535	5.241	4.707
0.054	0.215	-3.969	0.795	7.974	7.179
0.812	0.894	-5.688	0.058	2.773	2.715
0.003	0.157	-1.512	1.075	5.741	4.666
0.090	0.265	-4.365	1.065	9.410	8.345
0.173	0.363	-4.839	-0.289	5.349	5.639
0.269	0.468	-5.138	-0.244	5.239	5.483
0.021	0.169	-3.235	0.923	10.910	9.987
0.327	0.523	-5.260	-0.324	3.193	3.518
0.041	0.195	-3.752	-0.526	6.604	7.131
0.014	0.160	-2.920	0.827	7.760	6.933
0.027	0.176	-3.418	0.568	3.173	2.605
0.004	0.157	-1.889	1.249	8.604	7.355
0.915	0.954	-5.709	-0.022	5.225	5.247
0.038	0.192	-3.694	1.155	5.013	3.858
0.336	0.532	-5.277	-0.270	5.574	5.844
0.103	0.280	-4.461	0.336	7.457	7.121
0.787	0.879	-5.680	0.063	5.434	5.371
0.152	0.339	-4.749	0.324	6.032	5.708
0.111	0.290	-4.521	0.446	4.491	4.045
0.908	0.950	-5.708	-0.034	2.488	2.522
0.049	0.207	-3.897	0.533	2.464	1.930
0.758	0.860	-5.670	-0.085	4.890	4.975
0.048	0.205	-3.874	1.299	7.722	6.423
0.628	0.770	-5.604	-0.157	5.655	5.812
0.034	0.185	-3.611	-0.663	5.788	6.450
0.487	0.662	-5.486	-0.193	3.023	3.216
0.234	0.430	-5.045	0.236	6.432	6.196
0.174	0.364	-4.844	0.447	5.788	5.341
0.082	0.253	-4.291	-0.830	3.462	4.292
0.154	0.341	-4.755	-0.481	2.936	3.417
0.073	0.241	-4.200	0.853	6.063	5.210
0.018	0.163	-3.102	0.632	5.235	4.603
0.248	0.446	-5.084	0.290	3.605	3.315

0.066	0.231	-4.127	0.848	8.903	8.055
0.012	0.160	-2.781	1.668	9.480	7.811
0.610	0.757	-5.592	0.366	6.241	5.876
0.603	0.752	-5.587	-0.156	3.773	3.929
0.250	0.448	-5.090	-0.296	4.042	4.339
0.019	0.164	-3.131	1.252	7.084	5.832
0.145	0.329	-4.713	0.660	5.704	5.044
0.050	0.208	-3.918	0.679	3.865	3.185
0.174	0.364	-4.843	-0.316	2.997	3.313
0.087	0.260	-4.337	0.511	4.732	4.221
0.011	0.157	-2.681	-0.898	2.975	3.873
0.042	0.196	-3.766	1.326	9.283	7.957
0.047	0.204	-3.867	1.443	9.022	7.579
0.087	0.259	-4.333	0.773	5.612	4.839
0.250	0.447	-5.088	-0.337	2.531	2.869
0.041	0.195	-3.754	0.632	7.486	6.854
0.013	0.160	-2.807	-0.805	3.356	4.160
0.023	0.173	-3.309	0.721	6.505	5.784
0.029	0.178	-3.470	0.690	6.478	5.788
0.006	0.157	-2.204	0.683	5.737	5.054
0.023	0.172	-3.281	-0.840	5.836	6.676
0.064	0.229	-4.108	0.923	6.411	5.488
0.011	0.157	-2.673	0.774	8.500	7.726
0.021	0.167	-3.203	-0.948	6.375	7.323
0.037	0.190	-3.670	-0.747	1.970	2.717
0.312	0.509	-5.231	0.346	5.084	4.738
0.907	0.950	-5.708	-0.028	5.989	6.017
0.003	0.157	-1.756	1.285	3.928	2.643
0.123	0.304	-4.594	0.526	6.494	5.968
0.007	0.157	-2.375	0.878	6.278	5.401
0.011	0.159	-2.733	-0.886	5.959	6.845
0.008	0.157	-2.474	1.562	7.092	5.530
0.205	0.400	-4.957	0.489	4.930	4.441
0.039	0.192	-3.709	0.562	3.431	2.869
0.021	0.169	-3.236	1.188	6.164	4.976
0.083	0.255	-4.304	0.461	6.294	5.833
0.011	0.157	-2.667	1.392	6.379	4.987
0.114	0.293	-4.537	0.456	6.796	6.339
0.283	0.481	-5.170	0.900	6.903	6.003
0.155	0.343	-4.762	0.573	5.085	4.512
0.063	0.226	-4.085	1.518	9.398	7.880
0.026	0.176	-3.391	1.002	7.118	6.116
0.531	0.695	-5.529	0.191	3.237	3.045
0.003	0.157	-1.677	1.730	6.159	4.428
0.029	0.178	-3.480	0.529	5.879	5.350
0.824	0.900	-5.691	-0.090	3.733	3.823
0.040	0.194	-3.733	-0.730	3.491	4.221
0.368	0.559	-5.331	0.496	4.600	4.104
0.750	0.855	-5.667	-0.094	3.084	3.178
0.511	0.679	-5.510	0.188	4.100	3.913

0.558	0.717	-5.552	0.317	3.451	3.134
0.175	0.364	-4.846	0.664	3.898	3.233
0.152	0.338	-4.745	0.364	4.632	4.268
0.710	0.828	-5.649	0.098	3.966	3.867
0.510	0.679	-5.509	-0.197	3.064	3.261
0.014	0.160	-2.906	-0.868	2.545	3.413
0.170	0.361	-4.828	0.302	5.276	4.973
0.053	0.212	-3.949	0.476	6.634	6.158
0.019	0.165	-3.155	0.615	5.993	5.378
0.437	0.621	-5.429	0.171	4.958	4.787
0.370	0.560	-5.334	-0.348	2.712	3.060
0.981	0.990	-5.715	0.008	4.521	4.513
0.234	0.430	-5.045	-0.322	2.146	2.468
0.040	0.194	-3.726	0.913	8.048	7.135
0.002	0.157	-1.239	-0.822	2.135	2.957
0.028	0.178	-3.452	-0.539	4.530	5.069
0.013	0.160	-2.823	-0.878	5.360	6.239
0.315	0.513	-5.237	0.205	7.945	7.740
0.571	0.726	-5.563	0.134	4.298	4.164
0.677	0.806	-5.632	0.097	4.474	4.377
0.082	0.254	-4.294	-0.397	3.149	3.545
0.160	0.349	-4.785	0.465	5.352	4.888
0.270	0.468	-5.140	0.329	3.229	2.900
0.333	0.528	-5.271	-0.338	3.364	3.703
0.373	0.564	-5.340	-0.388	4.269	4.657
0.595	0.746	-5.581	-0.158	2.651	2.809
0.498	0.669	-5.497	-0.257	2.808	3.065
0.498	0.670	-5.498	-0.185	4.935	5.120
0.434	0.618	-5.425	-0.281	3.607	3.888
0.367	0.558	-5.331	-0.286	4.261	4.548
0.016	0.162	-3.016	-0.920	5.371	6.291
0.204	0.399	-4.952	-0.403	5.020	5.423
0.890	0.940	-5.706	0.032	5.881	5.849
0.026	0.175	-3.378	0.840	6.581	5.741
0.885	0.937	-5.705	0.038	4.345	4.308
0.066	0.230	-4.120	0.748	6.432	5.684
0.622	0.765	-5.600	0.148	5.589	5.441
0.013	0.160	-2.841	0.766	6.783	6.016
0.100	0.277	-4.439	0.456	5.115	4.660
0.142	0.327	-4.700	0.390	5.492	5.103
0.006	0.157	-2.264	1.967	5.854	3.887
0.007	0.157	-2.268	0.565	6.022	5.457
0.218	0.413	-4.998	-0.344	2.446	2.789
0.005	0.157	-2.027	0.884	3.745	2.861
0.368	0.559	-5.331	0.250	3.713	3.463
0.156	0.344	-4.768	-0.452	2.725	3.177
0.128	0.309	-4.623	0.801	5.533	4.732
0.749	0.855	-5.666	0.077	3.414	3.337
0.032	0.182	-3.561	0.597	5.938	5.341
0.259	0.456	-5.112	-0.283	3.851	4.135

0.562	0.721	-5.556	-0.177	3.401	3.577
0.055	0.216	-3.982	-0.427	2.149	2.577
0.322	0.519	-5.252	0.379	4.704	4.325
0.005	0.157	-2.037	-0.691	6.133	6.824
0.327	0.523	-5.260	-0.254	2.487	2.741
0.008	0.157	-2.395	-0.685	5.915	6.600
0.347	0.542	-5.296	-0.209	4.049	4.258
0.532	0.696	-5.530	0.149	3.747	3.597
0.521	0.687	-5.520	-0.212	3.777	3.989
0.165	0.355	-4.807	0.276	5.412	5.136
0.011	0.157	-2.699	-0.804	5.055	5.858
0.791	0.882	-5.682	-0.070	2.493	2.563
0.857	0.923	-5.699	-0.077	2.880	2.957
0.811	0.893	-5.688	-0.073	2.388	2.461
0.001	0.157	-1.052	1.043	2.785	1.742
0.593	0.745	-5.579	0.170	2.608	2.437
0.178	0.369	-4.860	0.325	5.955	5.630
0.488	0.663	-5.487	0.179	4.999	4.819
0.099	0.276	-4.434	0.529	3.505	2.976
0.097	0.274	-4.421	0.466	2.947	2.481
0.785	0.878	-5.680	-0.061	3.817	3.878
0.204	0.399	-4.955	0.338	4.913	4.575
0.483	0.659	-5.482	0.189	5.641	5.452
0.699	0.820	-5.644	-0.194	1.940	2.135
0.838	0.910	-5.695	-0.051	2.020	2.071
0.473	0.652	-5.471	-0.193	2.410	2.603
0.065	0.229	-4.117	-1.000	3.753	4.753
0.141	0.324	-4.691	-0.364	5.341	5.704
0.095	0.271	-4.404	0.601	6.485	5.884
0.046	0.203	-3.850	1.032	6.226	5.193
0.231	0.428	-5.037	0.450	6.022	5.572
0.902	0.947	-5.708	-0.037	2.197	2.235
0.095	0.271	-4.401	-0.464	4.130	4.593
0.582	0.736	-5.571	-0.136	5.242	5.378
0.182	0.373	-4.872	0.496	3.483	2.987
0.540	0.702	-5.537	-0.193	2.271	2.464
0.029	0.178	-3.473	0.791	4.277	3.486
0.005	0.157	-2.069	-0.921	4.606	5.527
0.004	0.157	-1.826	2.945	8.293	5.348
0.108	0.287	-4.500	0.318	5.867	5.549
0.565	0.722	-5.558	0.161	1.946	1.784
0.002	0.157	-1.348	1.133	4.720	3.587
0.608	0.754	-5.590	0.095	4.367	4.272
0.392	0.580	-5.368	-0.184	2.839	3.023
0.011	0.159	-2.721	-1.231	8.594	9.825
0.499	0.670	-5.498	0.169	5.459	5.290
0.466	0.645	-5.463	-0.167	1.773	1.940
0.697	0.820	-5.643	-0.102	2.278	2.380
0.116	0.296	-4.549	0.394	7.716	7.322
0.070	0.237	-4.173	0.634	3.465	2.831

0.021	0.169	-3.240	0.652	6.529	5.877
0.014	0.160	-2.891	-0.886	3.794	4.680
0.069	0.235	-4.156	-0.503	4.737	5.240
0.426	0.612	-5.415	-0.256	3.969	4.225
0.154	0.341	-4.755	0.454	7.446	6.992
0.296	0.493	-5.198	-0.330	1.783	2.113
0.108	0.287	-4.498	-0.647	4.400	5.046
0.254	0.452	-5.101	0.395	3.448	3.053
0.486	0.662	-5.486	-0.220	5.965	6.185
0.388	0.576	-5.363	-0.227	5.878	6.105
0.246	0.443	-5.078	0.410	4.614	4.204
0.711	0.828	-5.650	-0.083	2.587	2.670
0.131	0.313	-4.639	0.535	5.991	5.456
0.420	0.606	-5.407	0.227	4.790	4.563
0.090	0.265	-4.363	-0.577	3.109	3.687
0.128	0.309	-4.622	0.414	4.704	4.291
0.490	0.664	-5.489	0.264	3.324	3.059
0.331	0.527	-5.268	0.400	5.243	4.843
0.033	0.184	-3.592	-0.678	6.036	6.714
0.131	0.314	-4.642	-0.465	4.380	4.845
0.974	0.987	-5.714	0.008	4.279	4.270
0.254	0.452	-5.100	0.291	2.740	2.448
0.411	0.598	-5.394	-0.354	4.299	4.653
0.325	0.522	-5.258	-0.392	3.742	4.134
0.692	0.816	-5.641	0.118	2.862	2.744
0.016	0.162	-3.010	1.173	6.957	5.784
0.057	0.218	-4.011	0.711	3.481	2.771
0.024	0.173	-3.320	0.633	4.233	3.600
0.044	0.201	-3.817	0.594	3.912	3.318
0.131	0.312	-4.638	0.321	4.494	4.173
0.244	0.442	-5.074	0.484	4.438	3.953
0.060	0.222	-4.052	0.445	5.730	5.285
0.196	0.390	-4.926	-0.335	5.841	6.175
0.009	0.157	-2.545	1.090	4.888	3.798
0.088	0.262	-4.346	0.513	3.698	3.184
0.109	0.288	-4.504	-0.391	3.080	3.471
0.558	0.717	-5.552	-0.170	3.022	3.191
0.934	0.964	-5.712	0.023	6.551	6.528
0.719	0.834	-5.653	-0.101	4.674	4.775
0.659	0.793	-5.623	0.093	5.084	4.991
0.565	0.722	-5.558	0.180	5.991	5.810
0.151	0.338	-4.743	-0.340	7.078	7.419
0.981	0.990	-5.715	-0.006	7.263	7.269
0.099	0.276	-4.436	-0.737	4.106	4.843
0.559	0.718	-5.553	0.193	6.820	6.626
0.101	0.278	-4.447	0.432	6.677	6.246
0.025	0.175	-3.365	-0.861	4.543	5.405
0.017	0.162	-3.033	-0.952	5.732	6.683
0.820	0.898	-5.690	0.060	3.120	3.060
0.707	0.826	-5.648	0.102	2.791	2.689

0.407	0.595	-5.390	0.190	5.539	5.349
0.798	0.887	-5.684	-0.055	4.673	4.727
0.025	0.175	-3.372	0.561	4.475	3.915
0.044	0.201	-3.811	-0.760	2.840	3.600
0.009	0.157	-2.501	-0.672	5.968	6.640
0.103	0.280	-4.460	0.327	6.918	6.591
0.725	0.838	-5.656	-0.138	3.856	3.994
0.310	0.508	-5.228	-0.401	3.396	3.796
0.881	0.935	-5.704	0.069	4.807	4.738
0.296	0.493	-5.199	-0.415	3.618	4.033
0.059	0.221	-4.037	-0.687	5.411	6.097
0.994	0.997	-5.715	-0.003	4.529	4.532
0.162	0.351	-4.791	-0.434	2.659	3.094
0.153	0.341	-4.753	-0.604	3.292	3.897
0.463	0.643	-5.460	-0.282	1.711	1.992
0.394	0.582	-5.371	0.202	4.748	4.546
0.144	0.328	-4.707	0.431	6.895	6.464
0.235	0.432	-5.048	0.324	3.949	3.625
0.074	0.243	-4.218	0.501	6.307	5.806
0.436	0.619	-5.427	-0.269	3.007	3.276
0.081	0.253	-4.283	-0.445	4.184	4.629
0.856	0.922	-5.699	0.040	2.079	2.039
0.037	0.190	-3.674	-0.882	4.176	5.058
0.027	0.177	-3.436	-1.050	5.968	7.018
0.716	0.831	-5.652	-0.110	3.011	3.121
0.618	0.763	-5.597	0.129	4.650	4.521
0.186	0.378	-4.889	-0.351	3.008	3.358
0.009	0.157	-2.551	-0.899	4.764	5.663
0.655	0.790	-5.620	-0.124	2.411	2.535
0.166	0.356	-4.811	-0.344	3.381	3.725
0.145	0.329	-4.713	0.376	6.766	6.390
0.310	0.508	-5.228	0.248	6.386	6.138
0.029	0.178	-3.474	-0.628	4.461	5.089
0.009	0.157	-2.548	-0.736	3.464	4.201
0.054	0.214	-3.966	0.630	3.632	3.002
0.065	0.229	-4.118	-0.666	3.295	3.962
0.292	0.490	-5.191	-0.286	2.310	2.596
0.079	0.249	-4.260	1.213	8.233	7.020
0.014	0.160	-2.920	0.985	6.543	5.558
0.010	0.157	-2.610	1.184	5.467	4.283
0.367	0.558	-5.330	-0.284	4.123	4.407
0.002	0.157	-1.418	-1.540	1.666	3.206
0.009	0.157	-2.534	-1.041	3.284	4.325
0.015	0.160	-2.949	-1.427	4.295	5.723
0.065	0.229	-4.109	-0.804	5.994	6.797
0.163	0.352	-4.796	0.449	5.542	5.093
0.171	0.361	-4.830	0.297	7.637	7.341
0.032	0.182	-3.548	0.726	7.466	6.740
0.012	0.160	-2.798	-0.776	5.248	6.024
0.178	0.369	-4.858	0.452	2.792	2.339

0.004	0.157	-1.806	1.940	5.554	3.615
0.208	0.403	-4.967	-0.449	3.182	3.631
0.264	0.462	-5.126	0.415	4.673	4.259
0.286	0.483	-5.176	0.290	5.178	4.888
0.482	0.658	-5.481	0.184	5.873	5.689
0.046	0.203	-3.839	-0.906	6.579	7.485
0.565	0.722	-5.558	-0.162	5.342	5.504
0.875	0.932	-5.703	-0.050	3.505	3.555
0.182	0.374	-4.875	0.296	3.289	2.993
0.188	0.380	-4.896	0.303	6.332	6.029
0.658	0.792	-5.622	-0.128	2.651	2.778
0.024	0.173	-3.321	-0.634	3.196	3.830
0.017	0.162	-3.029	-0.729	4.722	5.451
0.028	0.178	-3.447	0.523	5.382	4.859
0.102	0.279	-4.455	0.646	6.770	6.124
0.513	0.681	-5.512	0.189	4.756	4.567
0.797	0.886	-5.684	0.058	3.873	3.815
0.118	0.298	-4.563	-0.449	4.507	4.956
0.279	0.477	-5.160	0.235	7.186	6.951
0.135	0.319	-4.663	0.393	6.631	6.238
0.116	0.296	-4.553	0.424	5.190	4.766
0.225	0.421	-5.018	0.616	6.135	5.518
0.606	0.753	-5.589	0.135	3.709	3.574
0.043	0.200	-3.799	-0.626	4.849	5.475
0.020	0.167	-3.197	0.628	6.014	5.386
0.361	0.553	-5.319	0.221	3.169	2.948
0.544	0.705	-5.540	0.193	4.652	4.459
0.045	0.203	-3.833	-0.852	4.288	5.139
0.502	0.672	-5.502	-0.168	3.643	3.812
0.236	0.433	-5.051	0.420	3.418	2.998
0.047	0.203	-3.856	-0.488	3.832	4.320
0.042	0.197	-3.776	0.602	3.400	2.798
0.267	0.465	-5.132	0.308	4.480	4.172
0.080	0.251	-4.273	-0.622	3.278	3.901
0.072	0.240	-4.195	0.766	4.932	4.166
0.024	0.173	-3.320	0.706	4.987	4.281
0.180	0.371	-4.865	0.286	6.203	5.917
0.123	0.304	-4.592	0.391	5.137	4.746
0.584	0.737	-5.573	0.164	2.599	2.436
0.019	0.164	-3.147	-1.117	5.056	6.173
0.704	0.824	-5.647	-0.096	3.278	3.374
0.881	0.935	-5.704	0.044	5.398	5.355
0.641	0.779	-5.612	0.198	2.450	2.252
0.175	0.365	-4.849	0.701	6.486	5.785
0.171	0.361	-4.830	0.409	5.820	5.411
0.098	0.275	-4.428	-0.434	4.895	5.328
0.168	0.358	-4.818	0.386	3.790	3.404
0.004	0.157	-1.759	-0.940	2.738	3.678
0.071	0.238	-4.179	-0.504	2.437	2.941
0.693	0.816	-5.641	-0.112	2.613	2.725

0.659	0.793	-5.622	0.079	5.747	5.668
0.111	0.290	-4.516	0.697	7.624	6.927
0.229	0.426	-5.030	0.264	5.097	4.833
0.455	0.636	-5.451	0.180	1.993	1.813
0.011	0.157	-2.679	-1.303	6.463	7.766
0.208	0.402	-4.965	0.390	6.271	5.881
0.021	0.168	-3.207	0.571	7.517	6.946
0.213	0.409	-4.984	0.327	4.733	4.407
0.732	0.844	-5.659	0.074	3.471	3.397
0.862	0.926	-5.701	0.052	1.986	1.934
0.390	0.578	-5.365	0.249	3.055	2.806
0.050	0.208	-3.916	0.713	4.087	3.374
0.593	0.745	-5.579	0.149	3.545	3.397
0.021	0.167	-3.203	-0.687	6.512	7.199
0.311	0.508	-5.229	-0.385	3.107	3.492
0.049	0.206	-3.888	-0.764	5.741	6.506
0.050	0.208	-3.917	-0.855	5.710	6.565
0.041	0.195	-3.753	-0.836	3.973	4.809
0.207	0.402	-4.962	-0.361	3.685	4.047
0.433	0.617	-5.423	0.210	7.840	7.630
0.015	0.161	-2.963	0.665	9.525	8.860
0.193	0.385	-4.913	0.430	2.962	2.532
0.338	0.533	-5.280	-0.384	3.528	3.912
0.864	0.927	-5.701	-0.043	5.078	5.121
0.992	0.996	-5.715	-0.003	4.050	4.053
0.250	0.447	-5.089	-0.534	2.651	3.184
0.037	0.190	-3.676	0.505	6.702	6.197
0.039	0.194	-3.725	-0.790	5.603	6.393
0.137	0.320	-4.672	0.300	7.159	6.859
0.148	0.332	-4.727	-0.531	5.793	6.324
0.095	0.271	-4.402	0.395	6.632	6.237
0.027	0.176	-3.421	0.606	8.130	7.524
0.032	0.182	-3.549	-1.191	3.902	5.093
0.743	0.851	-5.664	-0.086	3.935	4.021
0.686	0.812	-5.637	-0.162	4.307	4.469
0.921	0.958	-5.710	0.028	3.627	3.599
0.231	0.428	-5.036	-0.280	1.737	2.017
0.053	0.213	-3.954	0.797	4.520	3.723
0.251	0.448	-5.092	0.319	4.692	4.373
0.357	0.551	-5.313	0.252	3.195	2.943
0.113	0.292	-4.531	-0.451	3.076	3.527
0.261	0.459	-5.118	0.298	3.626	3.327
0.050	0.208	-3.915	0.642	4.265	3.623
0.884	0.937	-5.705	0.052	4.960	4.908
0.672	0.802	-5.630	-0.152	2.257	2.409
0.825	0.901	-5.692	0.071	3.374	3.303
0.139	0.324	-4.684	-0.342	4.755	5.097
0.586	0.739	-5.574	-0.251	5.932	6.183
0.145	0.329	-4.712	-0.492	7.142	7.634
0.050	0.208	-3.915	0.576	3.910	3.334

0.576	0.730	-5.566	-0.227	4.339	4.566
0.490	0.664	-5.489	0.208	3.631	3.423
0.529	0.694	-5.527	-0.146	2.405	2.552
0.666	0.798	-5.627	-0.150	2.092	2.242
0.355	0.549	-5.310	-0.198	7.062	7.259
0.905	0.949	-5.708	-0.036	1.926	1.962
0.022	0.171	-3.277	0.623	3.184	2.561
0.004	0.157	-1.964	0.902	3.292	2.391
0.017	0.162	-3.052	-1.164	4.201	5.364
0.049	0.207	-3.894	-0.587	3.839	4.426
0.011	0.157	-2.679	-0.518	4.634	5.152
0.091	0.266	-4.368	0.432	4.180	3.747
0.291	0.489	-5.189	0.287	4.420	4.133
0.365	0.557	-5.327	0.221	4.351	4.130
0.640	0.778	-5.611	0.122	4.380	4.258
0.869	0.928	-5.702	0.042	3.963	3.921
0.069	0.235	-4.155	-0.502	4.572	5.074
0.614	0.759	-5.594	-0.146	2.391	2.537
0.053	0.213	-3.953	0.468	6.485	6.017
0.976	0.988	-5.714	-0.010	5.675	5.685
0.067	0.233	-4.140	0.431	7.109	6.678
0.029	0.178	-3.494	0.697	7.684	6.987
0.063	0.226	-4.084	0.429	4.905	4.476
0.040	0.194	-3.729	0.406	7.165	6.758
0.142	0.326	-4.699	0.328	6.981	6.653
0.001	0.157	-0.887	1.112	8.089	6.977
0.189	0.382	-4.901	0.418	4.360	3.942
0.803	0.889	-5.685	-0.072	4.603	4.675
0.566	0.723	-5.559	-0.163	3.391	3.553
0.095	0.271	-4.404	-0.484	3.724	4.208
0.008	0.157	-2.413	-1.177	6.386	7.563
0.270	0.468	-5.140	-0.281	4.348	4.629
0.106	0.284	-4.486	-0.653	3.830	4.483
0.427	0.613	-5.416	0.239	3.088	2.849
0.231	0.428	-5.037	-0.414	3.278	3.692
0.040	0.194	-3.742	-0.555	5.409	5.964
0.982	0.990	-5.715	0.006	6.085	6.079
0.476	0.653	-5.474	0.208	3.280	3.072
0.146	0.330	-4.719	0.410	4.615	4.205
0.575	0.730	-5.566	-0.150	3.658	3.809
0.047	0.204	-3.863	0.619	7.152	6.533
0.847	0.915	-5.697	-0.041	9.393	9.434
0.093	0.270	-4.390	-0.713	6.873	7.586
0.036	0.189	-3.663	0.624	8.361	7.736
0.118	0.298	-4.562	-0.444	2.776	3.219
0.329	0.524	-5.264	0.367	2.715	2.348
0.970	0.985	-5.714	0.010	2.037	2.027
0.448	0.630	-5.442	0.232	4.883	4.651
0.022	0.171	-3.274	-0.667	6.499	7.166
0.450	0.631	-5.445	0.300	6.016	5.716

0.816	0.896	-5.689	-0.068	3.593	3.661
0.012	0.160	-2.768	0.936	3.685	2.749
0.691	0.815	-5.640	-0.112	3.952	4.065
0.026	0.176	-3.391	-0.810	3.551	4.361
0.227	0.424	-5.024	-0.359	2.082	2.441
0.045	0.203	-3.835	0.470	3.972	3.501
0.019	0.165	-3.152	-0.683	4.488	5.171
0.781	0.874	-5.678	-0.059	2.488	2.547
0.277	0.475	-5.156	-0.350	2.089	2.439
0.156	0.344	-4.766	-0.786	2.321	3.107
0.015	0.160	-2.924	-0.670	6.831	7.501
0.604	0.752	-5.587	-0.142	2.107	2.249
0.427	0.612	-5.416	-0.163	2.673	2.836
0.040	0.194	-3.733	0.821	6.232	5.411
0.283	0.481	-5.170	0.289	5.323	5.034
0.641	0.779	-5.612	0.136	2.821	2.684
0.018	0.162	-3.081	0.651	7.872	7.222
0.836	0.908	-5.695	0.062	3.131	3.069
0.603	0.752	-5.587	0.161	3.572	3.412
0.330	0.526	-5.267	-0.404	3.225	3.629
0.061	0.223	-4.064	0.759	5.718	4.959
0.195	0.389	-4.922	0.387	2.823	2.436
0.100	0.277	-4.439	-0.566	1.609	2.176
0.832	0.906	-5.693	0.081	3.025	2.944
0.759	0.861	-5.670	0.088	2.909	2.821
0.715	0.831	-5.652	-0.140	3.443	3.582
0.116	0.296	-4.553	-0.441	2.641	3.081
0.510	0.679	-5.509	0.205	2.599	2.394
0.796	0.885	-5.683	0.082	2.743	2.661
0.288	0.486	-5.181	-0.426	4.332	4.758
0.105	0.283	-4.476	-0.530	1.760	2.289
0.134	0.317	-4.656	0.563	5.247	4.684
0.959	0.979	-5.714	0.020	1.630	1.610
0.787	0.879	-5.680	0.175	2.723	2.548
0.362	0.554	-5.322	0.708	5.290	4.582
0.682	0.809	-5.635	-0.116	2.119	2.235
0.130	0.311	-4.632	-0.434	2.795	3.229
0.003	0.157	-1.729	1.773	6.735	4.962
0.026	0.176	-3.408	0.938	4.417	3.480
0.672	0.803	-5.630	0.109	4.390	4.281
0.159	0.347	-4.778	-0.532	5.377	5.909
0.202	0.397	-4.947	0.392	3.578	3.186
0.544	0.706	-5.541	-0.182	3.282	3.464
0.961	0.980	-5.714	-0.014	5.209	5.223
0.226	0.423	-5.023	0.262	6.858	6.595
0.230	0.428	-5.035	0.323	5.090	4.767
0.535	0.698	-5.532	0.150	4.522	4.372
0.007	0.157	-2.316	0.928	9.252	8.324
0.262	0.460	-5.119	-0.387	4.318	4.705
0.790	0.881	-5.681	0.079	4.670	4.591

0.841	0.912	-5.696	0.044	2.929	2.885
0.397	0.585	-5.376	-0.186	5.370	5.556
0.069	0.236	-4.165	-0.441	4.534	4.975
0.107	0.285	-4.490	-0.710	3.495	4.205
0.126	0.306	-4.610	-0.482	2.683	3.165
0.002	0.157	-1.374	-0.985	4.014	4.999
0.564	0.721	-5.557	-0.152	4.651	4.804
0.071	0.238	-4.181	-0.661	3.316	3.977
0.707	0.826	-5.648	0.121	3.874	3.753
0.047	0.203	-3.858	-0.711	2.958	3.669
0.183	0.374	-4.877	0.291	7.107	6.816
0.078	0.249	-4.258	0.432	6.622	6.190
0.206	0.401	-4.959	-0.286	2.703	2.989
0.244	0.442	-5.073	0.281	4.622	4.341
0.338	0.534	-5.281	-0.294	4.673	4.966
0.178	0.369	-4.858	0.393	4.027	3.635
0.817	0.896	-5.689	0.055	5.174	5.119
0.341	0.536	-5.285	-0.226	4.362	4.588
0.172	0.362	-4.835	0.528	5.481	4.954
0.163	0.352	-4.795	0.319	3.922	3.603
0.852	0.920	-5.698	-0.065	2.748	2.812
0.013	0.160	-2.839	0.932	4.632	3.700
0.171	0.361	-4.831	0.830	5.697	4.867
0.002	0.157	-1.159	0.931	3.146	2.215
0.197	0.391	-4.928	-0.466	6.747	7.213
0.739	0.848	-5.662	0.087	3.895	3.808
0.065	0.229	-4.116	-0.708	3.361	4.070
0.027	0.176	-3.415	-0.525	5.930	6.455
0.019	0.164	-3.145	-1.113	2.279	3.391
0.006	0.157	-2.251	0.672	5.997	5.325
0.563	0.721	-5.556	0.123	6.764	6.641
0.082	0.254	-4.294	0.393	5.555	5.162
0.079	0.249	-4.262	0.417	4.750	4.334
0.054	0.214	-3.964	0.622	6.864	6.243
0.031	0.181	-3.540	-0.660	3.930	4.590
0.054	0.215	-3.974	-0.492	3.053	3.545
0.824	0.900	-5.691	-0.080	2.138	2.218
0.745	0.852	-5.665	0.079	5.296	5.217
0.117	0.297	-4.558	0.400	5.225	4.825
0.655	0.790	-5.620	-0.122	3.010	3.133
0.104	0.281	-4.470	-0.434	2.770	3.205
0.040	0.194	-3.728	1.216	8.695	7.479
0.078	0.247	-4.251	0.546	4.306	3.760
0.050	0.207	-3.907	-0.851	4.636	5.487
0.280	0.478	-5.163	0.266	5.505	5.240
0.358	0.551	-5.315	-0.280	3.471	3.751
0.152	0.339	-4.748	0.504	4.349	3.846
0.390	0.578	-5.365	-0.183	5.715	5.898
0.195	0.389	-4.924	0.292	5.882	5.590
0.723	0.837	-5.655	-0.082	4.889	4.971

0.047	0.204	-3.864	0.634	7.991	7.357
0.018	0.162	-3.088	-0.980	1.856	2.837
0.007	0.157	-2.294	0.589	4.774	4.185
0.206	0.401	-4.959	-0.351	3.244	3.595
0.829	0.904	-5.693	0.054	4.074	4.019
0.020	0.167	-3.195	0.825	8.261	7.436
0.024	0.173	-3.318	0.622	8.223	7.600
0.263	0.461	-5.124	0.273	8.450	8.176
0.207	0.402	-4.964	0.290	5.417	5.127
0.896	0.943	-5.707	0.037	2.669	2.632
0.225	0.422	-5.020	-0.441	2.861	3.302
0.040	0.194	-3.734	-0.703	5.423	6.126
0.072	0.240	-4.196	0.639	4.333	3.694
0.235	0.432	-5.050	0.279	5.847	5.568
0.079	0.249	-4.259	0.410	8.140	7.730
0.263	0.461	-5.122	-0.346	4.098	4.444
0.027	0.176	-3.430	0.491	6.649	6.158
0.524	0.689	-5.523	0.127	6.430	6.303
0.790	0.880	-5.681	0.067	3.549	3.482
0.987	0.993	-5.715	0.005	3.486	3.481
0.698	0.820	-5.643	0.101	3.850	3.748
0.746	0.853	-5.665	-0.090	2.518	2.608
0.006	0.157	-2.238	0.702	6.831	6.129
0.611	0.757	-5.592	0.127	2.361	2.234
0.386	0.574	-5.359	-0.230	4.584	4.814
0.257	0.455	-5.109	0.337	3.341	3.004
0.230	0.427	-5.034	0.399	5.315	4.916
0.006	0.157	-2.200	0.829	5.816	4.987
0.945	0.970	-5.713	-0.019	5.472	5.491
0.927	0.960	-5.711	-0.023	2.586	2.609
0.067	0.233	-4.142	0.428	4.083	3.655
0.182	0.373	-4.872	-0.378	4.650	5.028
0.045	0.201	-3.822	0.453	6.761	6.308
0.074	0.242	-4.217	1.080	6.455	5.375
0.006	0.157	-2.170	1.251	4.688	3.438
0.561	0.720	-5.555	0.138	5.315	5.177
0.365	0.556	-5.326	-0.295	3.084	3.379
0.020	0.167	-3.181	-0.670	5.127	5.796
0.355	0.549	-5.310	-0.236	7.727	7.963
0.027	0.177	-3.438	-0.898	4.348	5.246
0.001	0.157	-0.819	-1.138	3.462	4.600
0.107	0.285	-4.490	-0.936	3.552	4.488
0.893	0.941	-5.706	0.041	4.684	4.643
0.179	0.369	-4.861	-0.557	3.465	4.022
0.410	0.597	-5.393	0.189	5.064	4.875
0.149	0.335	-4.735	0.709	3.819	3.110
0.257	0.455	-5.107	-0.536	2.944	3.479
0.965	0.982	-5.714	-0.052	5.610	5.661
0.206	0.401	-4.960	-0.296	5.185	5.481
0.012	0.160	-2.759	-1.059	6.824	7.883

0.431	0.616	-5.421	0.213	7.064	6.851
0.166	0.356	-4.811	0.395	2.471	2.076
0.190	0.383	-4.905	-0.430	2.393	2.823
0.645	0.782	-5.615	-0.151	2.200	2.351
0.033	0.184	-3.584	0.854	4.504	3.650
0.530	0.694	-5.528	-0.294	2.020	2.314
0.647	0.784	-5.616	0.119	7.107	6.988
0.691	0.815	-5.640	-0.116	4.484	4.600
0.749	0.855	-5.666	0.105	4.370	4.265
0.191	0.384	-4.908	0.329	7.314	6.985
0.029	0.178	-3.492	0.818	4.651	3.833
0.244	0.442	-5.073	0.262	5.100	4.838
0.015	0.160	-2.938	0.946	6.891	5.945
0.297	0.494	-5.201	0.293	4.363	4.070
0.339	0.535	-5.283	-0.308	3.512	3.820
0.914	0.954	-5.709	0.108	4.679	4.571
0.934	0.965	-5.712	0.077	4.517	4.440
0.319	0.517	-5.246	0.225	5.937	5.712
0.015	0.161	-2.970	-0.996	7.584	8.580
0.293	0.491	-5.192	-0.256	4.306	4.562
0.831	0.905	-5.693	-0.045	4.109	4.154
0.189	0.381	-4.899	-0.621	3.190	3.811
0.338	0.533	-5.280	-0.417	7.271	7.687
0.554	0.715	-5.549	0.181	2.881	2.700
0.175	0.364	-4.846	0.278	4.865	4.587
0.489	0.664	-5.489	0.155	2.837	2.683
0.097	0.273	-4.415	-0.505	4.415	4.920
0.126	0.306	-4.609	0.471	6.054	5.583
0.114	0.293	-4.538	-0.433	3.993	4.426
0.274	0.472	-5.149	-0.395	3.870	4.265
0.202	0.397	-4.945	0.319	5.832	5.513
0.500	0.671	-5.500	-0.188	5.343	5.531
0.252	0.450	-5.095	-0.357	5.809	6.167
0.434	0.618	-5.425	0.165	6.128	5.963
0.144	0.328	-4.707	0.359	6.472	6.113
0.097	0.273	-4.414	0.437	7.094	6.656
0.828	0.903	-5.692	-0.077	2.935	3.013
0.053	0.212	-3.949	1.114	7.260	6.146
0.058	0.220	-4.031	0.748	5.415	4.667
0.037	0.190	-3.669	0.484	4.983	4.498
0.136	0.319	-4.666	-0.492	2.166	2.658
0.382	0.571	-5.353	0.198	7.364	7.166
0.209	0.404	-4.970	0.276	3.949	3.673
0.326	0.522	-5.258	0.203	8.071	7.869
0.120	0.301	-4.577	0.321	8.037	7.716
0.305	0.502	-5.216	-0.242	3.750	3.992
0.597	0.747	-5.582	0.167	4.964	4.797
0.861	0.926	-5.700	-0.049	3.167	3.217
0.149	0.335	-4.734	0.432	5.552	5.120
0.106	0.284	-4.486	0.434	7.726	7.292

0.207	0.402	-4.963	0.263	5.565	5.302
0.459	0.640	-5.455	-0.195	3.575	3.771
0.090	0.264	-4.359	-0.687	2.696	3.383
0.812	0.894	-5.688	-0.070	6.455	6.525
0.071	0.239	-4.183	0.509	6.415	5.907
0.022	0.171	-3.262	-0.787	2.829	3.616
0.048	0.205	-3.885	0.438	4.915	4.476
0.012	0.160	-2.753	-1.225	8.060	9.286
0.060	0.221	-4.046	0.483	7.110	6.627
0.109	0.288	-4.503	0.383	5.619	5.236
0.890	0.940	-5.706	0.038	3.759	3.721
0.137	0.320	-4.670	-0.454	4.541	4.995
0.859	0.924	-5.700	0.062	2.581	2.519
0.154	0.341	-4.758	-0.450	4.731	5.181
0.022	0.170	-3.252	1.466	8.208	6.742
0.013	0.160	-2.816	1.654	7.952	6.299
0.033	0.184	-3.575	-0.698	3.143	3.841
0.013	0.160	-2.843	1.363	5.863	4.500
0.004	0.157	-1.955	-0.903	5.490	6.393
0.035	0.186	-3.627	-0.594	5.398	5.992
0.766	0.865	-5.673	-0.083	2.397	2.481
0.043	0.200	-3.795	1.095	6.466	5.371
0.073	0.241	-4.200	0.616	7.106	6.491
0.041	0.195	-3.753	0.956	7.803	6.847
0.254	0.452	-5.101	-0.294	3.586	3.880
0.758	0.860	-5.670	-0.073	3.848	3.921
0.091	0.267	-4.372	0.425	6.510	6.084
0.114	0.293	-4.536	0.476	4.262	3.786
0.348	0.543	-5.298	0.222	4.206	3.984
0.146	0.331	-4.720	0.343	4.280	3.937
0.055	0.216	-3.985	0.363	9.198	8.835
0.142	0.326	-4.697	0.385	5.151	4.766
0.136	0.319	-4.665	0.333	4.531	4.198
0.241	0.438	-5.065	0.322	6.948	6.626
0.008	0.157	-2.460	-1.054	2.283	3.337
0.028	0.178	-3.465	0.496	8.300	7.804
0.522	0.688	-5.521	0.181	4.723	4.543
0.431	0.616	-5.422	0.225	4.793	4.568
0.668	0.800	-5.628	0.122	4.597	4.476
0.163	0.352	-4.795	0.442	4.704	4.262
0.009	0.157	-2.566	0.596	7.391	6.794
0.166	0.355	-4.809	0.489	4.688	4.199
0.624	0.767	-5.601	0.123	5.001	4.878
0.821	0.899	-5.690	-0.045	7.882	7.928
0.063	0.227	-4.096	0.736	4.289	3.553
0.154	0.341	-4.754	0.269	6.674	6.405
0.061	0.224	-4.071	-0.508	4.799	5.308
0.122	0.303	-4.586	0.497	3.954	3.457
0.438	0.622	-5.431	-0.191	6.228	6.419
0.003	0.157	-1.749	-0.970	6.898	7.868

0.893	0.942	-5.706	0.158	6.139	5.981
0.688	0.814	-5.639	-0.105	3.213	3.318
0.801	0.888	-5.685	0.057	5.804	5.748
0.153	0.340	-4.752	-0.355	6.207	6.562
0.421	0.607	-5.409	-0.381	1.466	1.848
0.889	0.940	-5.706	-0.146	3.810	3.956
0.541	0.703	-5.538	-0.190	3.786	3.976
0.741	0.849	-5.663	0.124	8.485	8.361
0.108	0.287	-4.496	0.332	8.431	8.099
0.068	0.234	-4.149	-0.534	6.957	7.491
0.133	0.316	-4.652	0.400	6.922	6.522
0.260	0.457	-5.115	-0.262	4.595	4.857
0.138	0.322	-4.679	-0.547	3.698	4.245
0.028	0.178	-3.463	-0.578	4.177	4.755
0.013	0.160	-2.806	-0.676	4.825	5.501
0.033	0.184	-3.583	-0.545	4.988	5.533
0.041	0.195	-3.756	-0.527	5.616	6.143
0.886	0.937	-5.705	-0.058	7.600	7.658
0.081	0.253	-4.285	-0.608	3.941	4.549
0.148	0.332	-4.728	0.353	3.092	2.739
0.351	0.546	-5.304	-0.281	2.589	2.871
0.642	0.780	-5.612	-0.182	4.274	4.456
0.011	0.158	-2.710	-0.918	5.792	6.711
0.393	0.581	-5.370	-0.250	1.078	1.328
0.502	0.672	-5.502	0.197	6.180	5.983
0.185	0.377	-4.887	0.302	7.129	6.827
0.349	0.544	-5.301	0.218	4.680	4.462
0.493	0.667	-5.493	0.216	3.493	3.277
0.188	0.380	-4.896	0.274	9.161	8.887
0.385	0.574	-5.358	-0.473	4.735	5.208
0.951	0.975	-5.713	0.016	3.130	3.113
0.004	0.157	-1.788	-0.738	5.626	6.364
0.511	0.679	-5.510	0.260	5.138	4.877
0.128	0.308	-4.620	-0.415	2.419	2.834
0.383	0.572	-5.355	0.206	5.624	5.418
0.926	0.960	-5.711	-0.030	4.182	4.212
0.101	0.278	-4.448	0.750	5.181	4.431
0.016	0.162	-2.983	1.100	5.160	4.060
0.033	0.184	-3.579	-0.655	3.853	4.508
0.919	0.957	-5.710	-0.024	2.187	2.211
0.698	0.820	-5.644	0.076	4.518	4.442
0.490	0.664	-5.489	-0.332	1.989	2.320
0.818	0.897	-5.690	0.104	2.316	2.213
0.353	0.548	-5.307	0.230	6.278	6.048
0.007	0.157	-2.303	0.776	7.132	6.356
0.333	0.529	-5.272	0.228	4.673	4.445
0.060	0.221	-4.047	0.446	5.888	5.442
0.149	0.334	-4.733	0.299	9.416	9.117
0.123	0.304	-4.596	0.365	6.905	6.541
0.745	0.852	-5.665	-0.098	2.671	2.769

0.259	0.456	-5.112	0.258	3.970	3.712
0.015	0.160	-2.942	-0.980	3.411	4.391
0.108	0.286	-4.495	0.509	3.242	2.733
0.441	0.625	-5.434	-0.451	5.479	5.931
0.044	0.201	-3.819	1.045	4.243	3.198
0.040	0.194	-3.737	1.607	8.321	6.713
0.008	0.157	-2.445	1.699	5.605	3.907
0.431	0.616	-5.421	0.175	6.667	6.492
0.038	0.192	-3.705	1.038	6.306	5.267
0.282	0.480	-5.168	0.291	3.358	3.067
0.207	0.402	-4.963	-0.335	2.380	2.715
0.180	0.371	-4.867	0.291	7.482	7.191
0.475	0.653	-5.473	-0.384	3.723	4.107
0.908	0.950	-5.708	0.035	1.667	1.632
0.549	0.710	-5.545	-0.256	3.189	3.445
0.139	0.323	-4.683	-0.763	3.037	3.800
0.169	0.360	-4.824	-0.492	4.563	5.055
0.128	0.309	-4.623	0.589	4.470	3.880
0.580	0.735	-5.570	-0.162	5.244	5.406
0.254	0.451	-5.099	-0.338	1.458	1.796
0.099	0.276	-4.433	0.555	4.053	3.498
0.280	0.478	-5.163	0.324	3.685	3.360
0.385	0.574	-5.358	0.263	3.902	3.639
0.020	0.167	-3.189	0.810	5.747	4.937
0.067	0.232	-4.139	0.852	5.103	4.251
0.980	0.989	-5.714	-0.007	5.670	5.677
0.462	0.643	-5.459	0.155	5.345	5.190
0.128	0.309	-4.624	-0.464	3.920	4.384
0.007	0.157	-2.337	0.889	7.778	6.889
0.020	0.167	-3.176	-0.625	7.601	8.226
0.739	0.848	-5.662	-0.084	2.846	2.931
0.672	0.803	-5.630	-0.157	5.493	5.651
0.140	0.324	-4.690	-0.381	5.222	5.603
0.112	0.291	-4.528	0.381	8.418	8.037
0.012	0.160	-2.786	0.650	2.494	1.844
0.506	0.675	-5.505	-0.123	6.998	7.121
0.732	0.844	-5.659	0.105	2.771	2.666
0.135	0.318	-4.659	-0.318	5.862	6.180
0.012	0.160	-2.752	1.610	7.262	5.651
0.007	0.157	-2.360	-0.734	6.012	6.746
0.149	0.335	-4.734	-0.349	4.964	5.312
0.032	0.183	-3.566	-0.647	2.591	3.238
0.294	0.491	-5.193	0.315	4.200	3.884
0.289	0.487	-5.183	-0.295	6.204	6.500
0.380	0.569	-5.350	-0.291	3.334	3.624
0.685	0.811	-5.637	0.200	3.918	3.718
0.015	0.160	-2.949	0.901	7.670	6.769
0.210	0.405	-4.973	0.284	6.556	6.272
0.116	0.296	-4.550	0.360	6.284	5.924
0.483	0.659	-5.482	0.158	6.932	6.774

0.037	0.190	-3.677	0.505	7.002	6.497
0.034	0.184	-3.599	-0.877	1.882	2.759
0.092	0.267	-4.378	0.483	8.515	8.032
0.011	0.157	-2.675	-1.046	4.921	5.967
0.562	0.721	-5.556	-0.144	4.182	4.327
0.013	0.160	-2.830	0.605	4.807	4.201
0.040	0.194	-3.732	-0.626	4.182	4.807
0.657	0.792	-5.621	0.217	2.851	2.635
0.389	0.576	-5.363	-0.253	2.119	2.371
0.056	0.217	-3.998	0.389	8.276	7.887
0.817	0.896	-5.689	0.061	2.765	2.704
0.747	0.853	-5.666	0.075	5.078	5.004
0.624	0.767	-5.601	-0.265	3.374	3.639
0.421	0.607	-5.408	-0.164	2.549	2.713
0.024	0.174	-3.346	0.748	5.503	4.754
0.113	0.292	-4.534	-0.577	3.643	4.220
0.009	0.157	-2.545	1.726	5.895	4.168
0.056	0.218	-4.005	-0.752	3.698	4.450
0.375	0.565	-5.343	0.185	6.515	6.330
0.011	0.157	-2.697	1.185	6.196	5.011
0.292	0.490	-5.191	-0.250	6.088	6.338
0.330	0.526	-5.267	-0.251	1.977	2.228
0.896	0.943	-5.707	-0.029	6.119	6.149
0.041	0.195	-3.760	-0.461	7.952	8.413
0.125	0.306	-4.608	0.529	5.925	5.396
0.878	0.934	-5.704	-0.038	4.909	4.947
0.016	0.162	-3.002	0.843	4.637	3.794
0.175	0.365	-4.848	0.476	4.074	3.598
0.849	0.918	-5.698	-0.037	2.860	2.896
0.505	0.674	-5.504	-0.217	3.774	3.991
0.880	0.935	-5.704	0.045	3.058	3.013
0.038	0.192	-3.696	-0.682	8.103	8.785
0.005	0.157	-2.084	-0.704	4.843	5.547
0.047	0.203	-3.857	0.640	3.924	3.284
0.843	0.913	-5.696	-0.038	5.413	5.451
0.398	0.586	-5.377	-0.202	3.628	3.830
0.556	0.716	-5.551	0.189	6.389	6.200
0.769	0.868	-5.674	0.073	6.069	5.995
0.398	0.586	-5.377	-0.232	1.807	2.039
0.125	0.306	-4.605	-0.512	1.732	2.244
0.944	0.970	-5.712	0.142	2.826	2.684
0.004	0.157	-1.929	0.938	5.418	4.481
0.010	0.157	-2.602	1.202	5.764	4.563
0.086	0.259	-4.330	0.709	6.378	5.669
0.211	0.406	-4.976	0.320	4.465	4.145
0.496	0.668	-5.495	0.215	3.758	3.543
0.139	0.323	-4.681	0.441	2.766	2.326
0.343	0.538	-5.289	-0.219	2.451	2.670
0.050	0.208	-3.910	0.558	3.593	3.034
0.114	0.293	-4.538	-0.431	5.476	5.907

0.210	0.405	-4.974	0.261	7.956	7.695
0.937	0.966	-5.712	-0.022	4.477	4.499
0.024	0.173	-3.333	0.654	3.719	3.065
0.124	0.305	-4.602	-0.426	4.723	5.150
0.513	0.680	-5.512	-0.168	4.298	4.465
0.071	0.239	-4.185	-0.464	2.430	2.894
0.739	0.848	-5.662	0.141	4.016	3.875
0.819	0.897	-5.690	-0.069	3.543	3.612
0.324	0.520	-5.255	0.289	3.579	3.289
0.001	0.157	-0.818	-0.974	1.843	2.817
0.953	0.976	-5.713	-0.012	4.853	4.865
0.369	0.559	-5.332	0.191	5.234	5.043
0.029	0.178	-3.470	-0.512	7.490	8.003
0.135	0.319	-4.664	0.535	4.746	4.211
0.198	0.392	-4.932	0.404	2.407	2.002
0.621	0.764	-5.599	0.132	5.387	5.255
0.374	0.564	-5.341	-0.197	4.342	4.539
0.763	0.864	-5.672	-0.067	7.002	7.069
0.529	0.693	-5.527	0.134	7.850	7.716
0.207	0.402	-4.964	0.379	4.910	4.531
0.402	0.590	-5.383	0.210	3.693	3.482
0.188	0.380	-4.896	-0.526	2.877	3.403
0.339	0.534	-5.283	-0.258	2.730	2.988
0.927	0.960	-5.711	0.035	3.957	3.922
0.135	0.319	-4.663	0.459	4.433	3.974
0.739	0.848	-5.662	0.075	8.041	7.966
0.083	0.255	-4.304	0.470	4.769	4.299
0.346	0.541	-5.294	0.217	6.869	6.652
0.124	0.305	-4.603	0.321	7.431	7.109
0.074	0.242	-4.216	0.582	10.385	9.803
0.080	0.250	-4.269	0.474	5.437	4.962
0.553	0.713	-5.548	-0.182	2.997	3.180
0.676	0.805	-5.632	0.094	6.214	6.120
0.897	0.943	-5.707	-0.034	4.930	4.965
0.014	0.160	-2.913	1.724	5.056	3.332
0.029	0.178	-3.485	-0.492	4.055	4.546
0.034	0.184	-3.600	1.022	4.526	3.504
0.019	0.165	-3.151	-0.609	2.893	3.501
0.212	0.407	-4.979	-0.557	2.747	3.303
0.750	0.855	-5.667	0.111	5.786	5.675
0.015	0.160	-2.942	0.651	7.107	6.456
0.011	0.157	-2.691	-0.800	5.605	6.405
0.658	0.793	-5.622	0.198	4.242	4.044
0.874	0.932	-5.703	0.036	6.027	5.991
0.027	0.177	-3.438	0.480	5.394	4.914
0.027	0.176	-3.417	-0.618	5.285	5.902
0.500	0.671	-5.500	-0.176	4.612	4.788
0.445	0.628	-5.439	-0.251	3.395	3.646
0.907	0.950	-5.708	0.023	7.327	7.304
0.172	0.362	-4.834	0.306	5.619	5.313

0.027	0.176	-3.415	-0.586	4.021	4.607
0.621	0.765	-5.599	0.129	3.707	3.577
0.205	0.400	-4.955	0.318	4.138	3.821
0.095	0.271	-4.405	0.411	7.139	6.728
0.372	0.562	-5.338	-0.208	3.487	3.695
0.048	0.205	-3.883	0.550	6.183	5.632
0.844	0.914	-5.696	0.058	5.465	5.408
0.159	0.348	-4.780	-0.404	6.054	6.458
0.700	0.821	-5.645	0.229	5.488	5.259
0.025	0.175	-3.376	-0.633	6.054	6.686
0.366	0.557	-5.328	-0.216	3.956	4.172
0.038	0.192	-3.704	0.640	6.926	6.286
0.029	0.178	-3.494	-0.773	3.421	4.194
0.362	0.554	-5.321	-0.246	3.269	3.515
0.500	0.671	-5.499	0.188	5.134	4.946
0.002	0.157	-1.412	-0.771	5.851	6.622
0.003	0.157	-1.642	-1.087	6.379	7.465
0.077	0.246	-4.243	-0.762	3.523	4.285
0.050	0.207	-3.906	0.517	7.381	6.864
0.038	0.191	-3.686	0.580	4.872	4.292
0.036	0.189	-3.661	0.989	4.800	3.811
0.199	0.394	-4.936	0.402	3.595	3.192
0.021	0.169	-3.235	-0.694	7.858	8.551
0.381	0.570	-5.352	-0.228	2.374	2.602
0.059	0.221	-4.040	0.403	8.084	7.681
0.080	0.250	-4.271	0.394	5.734	5.340
0.168	0.358	-4.817	-0.271	6.517	6.788
0.118	0.298	-4.566	-0.409	4.977	5.386
0.357	0.551	-5.313	0.236	6.736	6.501
0.367	0.558	-5.331	-0.256	4.469	4.725
0.938	0.967	-5.712	0.018	4.494	4.476
0.084	0.256	-4.306	0.811	7.463	6.653
0.098	0.275	-4.427	-0.405	3.607	4.012
0.778	0.872	-5.677	0.103	5.594	5.491
0.012	0.160	-2.786	1.057	5.553	4.497
0.486	0.662	-5.485	0.168	5.463	5.296
0.062	0.225	-4.079	0.516	4.207	3.690
0.024	0.173	-3.327	0.731	3.913	3.182
0.565	0.722	-5.558	0.261	3.270	3.009
0.130	0.312	-4.636	-0.530	6.483	7.013
0.250	0.447	-5.089	0.333	2.949	2.616
0.292	0.490	-5.190	0.323	3.217	2.894
0.034	0.185	-3.612	0.529	7.633	7.104
0.073	0.241	-4.208	-0.547	5.252	5.799
0.112	0.290	-4.524	0.503	5.305	4.802
0.606	0.753	-5.589	0.140	4.188	4.048
0.138	0.322	-4.679	0.418	5.028	4.611
0.255	0.452	-5.102	0.273	4.273	4.000
0.928	0.961	-5.711	0.019	5.314	5.295
0.627	0.768	-5.603	0.155	4.478	4.324

0.151	0.337	-4.741	-0.575	5.324	5.899
0.198	0.392	-4.932	0.257	7.795	7.537
0.012	0.160	-2.786	1.074	6.545	5.471
0.729	0.841	-5.658	-0.094	5.652	5.747
0.872	0.930	-5.702	0.049	2.207	2.157
0.291	0.489	-5.187	-0.238	2.463	2.700
0.502	0.672	-5.502	-0.135	6.717	6.851
0.444	0.627	-5.438	0.220	5.501	5.281
0.244	0.442	-5.075	0.347	4.097	3.750
0.098	0.275	-4.429	-0.463	4.511	4.974
0.479	0.655	-5.477	-0.169	4.007	4.176
0.250	0.448	-5.090	0.249	6.024	5.775
0.139	0.323	-4.683	-0.356	6.274	6.630
0.924	0.959	-5.711	-0.025	4.529	4.555
0.072	0.239	-4.188	0.599	3.747	3.148
0.175	0.365	-4.848	0.343	4.816	4.472
0.637	0.777	-5.610	0.124	4.943	4.818
0.100	0.277	-4.438	0.514	6.444	5.930
0.394	0.582	-5.372	0.219	3.164	2.945
0.607	0.754	-5.589	-0.120	5.955	6.075
0.771	0.868	-5.675	-0.098	4.380	4.478
0.620	0.763	-5.598	0.115	3.693	3.579
0.024	0.173	-3.321	1.059	4.475	3.416
0.322	0.519	-5.251	-0.474	2.612	3.086
0.065	0.229	-4.113	0.560	2.705	2.146
0.874	0.932	-5.703	-0.038	3.557	3.595
0.440	0.624	-5.433	-0.210	3.101	3.311
0.190	0.383	-4.906	0.361	4.603	4.242
0.520	0.687	-5.519	-0.194	4.327	4.521
0.737	0.847	-5.661	0.080	3.800	3.720
0.812	0.894	-5.688	-0.060	5.256	5.316
0.688	0.813	-5.638	-0.120	4.359	4.479
0.530	0.694	-5.528	0.167	4.336	4.169
0.106	0.285	-4.487	0.566	3.665	3.099
0.528	0.693	-5.527	0.202	3.314	3.112
0.213	0.408	-4.982	0.326	2.574	2.247
0.964	0.982	-5.714	-0.012	4.389	4.400
0.868	0.928	-5.702	0.049	3.284	3.236
0.594	0.745	-5.580	0.126	6.294	6.169
0.023	0.172	-3.284	0.702	8.648	7.946
0.795	0.884	-5.683	-0.067	4.745	4.811
0.318	0.516	-5.244	0.282	4.417	4.135
0.670	0.802	-5.629	0.138	4.457	4.319
0.680	0.808	-5.634	-0.095	3.170	3.264
0.124	0.305	-4.600	-0.368	4.039	4.406
0.258	0.456	-5.111	0.358	5.843	5.484
0.511	0.680	-5.511	0.186	6.043	5.858
0.033	0.184	-3.594	-0.638	4.781	5.419
0.613	0.758	-5.594	-0.126	2.531	2.656
0.975	0.987	-5.714	-0.009	3.085	3.094

0.866	0.928	-5.701	0.061	4.554	4.493
0.752	0.857	-5.668	0.105	3.168	3.063
0.006	0.157	-2.163	-0.751	3.516	4.266
0.985	0.992	-5.715	0.005	2.915	2.911
0.531	0.695	-5.529	-0.159	5.865	6.024
0.146	0.330	-4.718	0.369	4.908	4.539
0.502	0.672	-5.501	-0.187	1.913	2.100
0.943	0.970	-5.712	0.019	4.412	4.393
0.935	0.965	-5.712	0.018	4.831	4.813
0.940	0.967	-5.712	-0.023	3.308	3.331
0.025	0.175	-3.374	-0.538	6.130	6.668
0.208	0.403	-4.968	0.289	4.305	4.016
0.263	0.461	-5.122	0.372	3.572	3.200
0.174	0.364	-4.843	-0.342	3.804	4.146
0.128	0.309	-4.623	0.456	2.721	2.265
0.572	0.727	-5.563	0.142	4.281	4.139
0.508	0.677	-5.507	-0.173	2.792	2.965
0.154	0.341	-4.755	0.310	3.438	3.128
0.119	0.298	-4.567	0.355	6.284	5.929
0.060	0.221	-4.048	0.452	4.938	4.487
0.234	0.430	-5.045	0.297	4.715	4.418
0.612	0.757	-5.593	0.114	6.267	6.153
0.009	0.157	-2.577	1.531	5.636	4.105
0.611	0.757	-5.593	0.147	2.812	2.665
0.512	0.680	-5.511	0.196	4.009	3.813
0.814	0.895	-5.689	-0.063	4.031	4.095
0.117	0.298	-4.560	-0.431	4.893	5.324
0.892	0.941	-5.706	0.038	3.495	3.456
0.490	0.664	-5.489	-0.167	5.223	5.391
0.066	0.231	-4.130	-0.434	4.179	4.613
0.105	0.282	-4.475	0.418	3.997	3.580
0.469	0.648	-5.466	-0.146	5.556	5.702
0.376	0.566	-5.345	0.247	6.335	6.087
0.687	0.812	-5.638	-0.132	4.716	4.848
0.360	0.552	-5.318	0.266	5.419	5.153
0.151	0.338	-4.743	0.405	5.528	5.122
0.501	0.671	-5.500	0.180	4.399	4.219
0.538	0.701	-5.536	0.216	2.789	2.573
0.273	0.471	-5.148	0.381	4.963	4.581
0.318	0.516	-5.244	-0.248	6.205	6.453
0.178	0.369	-4.859	0.341	5.490	5.149
0.086	0.258	-4.326	0.396	4.036	3.640
0.004	0.157	-1.888	-0.819	5.560	6.379
0.570	0.726	-5.562	0.169	5.575	5.406
0.585	0.739	-5.574	0.142	2.622	2.480
0.213	0.408	-4.982	-0.413	4.578	4.992
0.103	0.280	-4.460	0.438	6.645	6.207
0.256	0.453	-5.105	-0.391	3.268	3.659
0.092	0.267	-4.380	0.546	4.919	4.373
0.081	0.253	-4.286	-0.634	3.547	4.181

0.118	0.298	-4.561	-0.535	3.198	3.733
0.707	0.826	-5.648	0.091	3.301	3.210
0.337	0.533	-5.279	0.175	5.647	5.472
0.774	0.870	-5.676	0.060	6.769	6.709
0.202	0.397	-4.946	-0.391	4.989	5.380
0.775	0.871	-5.676	0.068	5.302	5.234
0.017	0.162	-3.056	0.762	5.200	4.439
0.018	0.162	-3.084	-0.614	5.068	5.682
0.736	0.846	-5.661	0.089	3.074	2.984
0.668	0.799	-5.627	0.122	2.643	2.521
0.024	0.173	-3.331	-0.927	5.776	6.703
0.654	0.789	-5.619	0.092	4.287	4.195
0.951	0.975	-5.713	0.015	5.041	5.026
0.841	0.912	-5.696	-0.063	3.990	4.053
0.003	0.157	-1.703	0.843	4.960	4.116
0.742	0.850	-5.663	-0.098	4.463	4.562
0.204	0.398	-4.951	0.315	4.599	4.285
0.527	0.693	-5.526	0.250	2.706	2.456
0.125	0.306	-4.604	0.401	4.576	4.175
0.338	0.534	-5.281	-0.251	4.098	4.348
0.784	0.877	-5.679	-0.084	3.409	3.493
0.223	0.419	-5.013	-0.388	2.510	2.898
0.519	0.686	-5.518	-0.213	3.978	4.190
0.367	0.558	-5.330	0.226	4.159	3.933
0.528	0.693	-5.527	0.134	3.670	3.537
0.261	0.458	-5.117	0.317	4.693	4.376
0.537	0.700	-5.535	-0.163	3.879	4.043
0.394	0.581	-5.371	-0.234	5.038	5.272
0.567	0.724	-5.560	-0.149	2.414	2.563
0.868	0.928	-5.702	-0.037	5.641	5.679
0.570	0.725	-5.562	-0.131	5.301	5.432
0.453	0.634	-5.449	0.178	4.496	4.319
0.269	0.467	-5.137	0.343	4.173	3.830
0.312	0.509	-5.231	0.251	4.766	4.515
0.540	0.702	-5.537	0.180	3.079	2.900
0.959	0.979	-5.714	0.013	3.932	3.919
0.179	0.369	-4.861	0.322	4.417	4.096
0.776	0.871	-5.676	-0.111	4.559	4.670
0.175	0.364	-4.845	-0.368	4.435	4.803
0.009	0.157	-2.564	-0.818	6.388	7.205
0.075	0.243	-4.220	0.496	5.796	5.300
0.321	0.518	-5.248	0.214	5.156	4.942
0.732	0.843	-5.659	0.075	7.859	7.784
0.156	0.344	-4.765	0.393	4.360	3.968
0.789	0.880	-5.681	0.070	3.794	3.724
0.001	0.157	-0.613	1.013	3.640	2.627
0.013	0.160	-2.818	0.722	4.811	4.089
0.320	0.517	-5.246	0.264	3.479	3.215
0.011	0.159	-2.719	-0.697	2.321	3.018
0.102	0.280	-4.458	-0.462	2.838	3.300

0.112	0.291	-4.526	0.414	4.038	3.623
0.639	0.778	-5.610	0.124	4.180	4.055
0.105	0.282	-4.474	0.610	3.848	3.237
0.381	0.570	-5.351	-0.361	4.474	4.835
0.049	0.207	-3.899	-0.437	2.687	3.124
0.089	0.263	-4.352	0.449	5.695	5.246
0.105	0.284	-4.480	0.652	3.323	2.671
0.885	0.937	-5.705	-0.035	4.334	4.369
0.283	0.481	-5.170	0.281	5.013	4.732
0.119	0.299	-4.571	0.554	3.127	2.572
0.289	0.488	-5.184	-0.276	4.592	4.868
0.966	0.983	-5.714	0.017	3.185	3.168
0.975	0.987	-5.714	-0.008	3.622	3.630
0.623	0.766	-5.600	-0.117	3.633	3.750
0.498	0.670	-5.498	-0.196	3.537	3.734
0.067	0.232	-4.135	0.455	7.113	6.659
0.429	0.615	-5.419	0.192	3.216	3.024
0.431	0.616	-5.422	0.195	4.587	4.391
0.224	0.421	-5.017	0.268	5.852	5.585
0.152	0.339	-4.747	0.438	4.751	4.314
0.520	0.686	-5.519	0.142	6.223	6.081
0.166	0.356	-4.811	-0.308	4.064	4.371
0.182	0.374	-4.876	-0.378	5.261	5.639
0.262	0.460	-5.121	0.356	5.538	5.182
0.747	0.853	-5.665	-0.102	4.051	4.153
0.264	0.462	-5.125	-0.282	3.323	3.605
0.679	0.808	-5.634	0.127	3.292	3.165
0.349	0.544	-5.300	0.197	6.850	6.653
0.073	0.241	-4.207	0.871	5.084	4.213
0.007	0.157	-2.303	1.213	5.462	4.250
0.038	0.192	-3.695	-0.804	2.244	3.048
0.676	0.806	-5.632	-0.104	3.991	4.095
0.243	0.441	-5.071	0.284	3.957	3.673
0.121	0.302	-4.582	0.410	5.204	4.794
0.201	0.397	-4.944	-0.453	3.610	4.063
0.230	0.428	-5.035	0.315	2.620	2.305
0.020	0.166	-3.168	-0.624	4.893	5.517
0.029	0.178	-3.476	0.814	3.894	3.080
0.722	0.836	-5.655	-0.074	4.138	4.212
0.908	0.950	-5.708	-0.037	5.209	5.246
0.692	0.816	-5.640	-0.110	2.127	2.237
0.036	0.188	-3.645	-0.675	4.372	5.047
0.120	0.301	-4.579	-0.417	3.571	3.987
0.876	0.933	-5.703	-0.038	3.346	3.385
0.059	0.221	-4.037	0.556	6.223	5.667
0.079	0.249	-4.263	0.578	3.950	3.372
0.655	0.791	-5.620	0.122	4.155	4.033
0.812	0.894	-5.688	-0.058	4.243	4.301
0.691	0.815	-5.640	0.113	3.750	3.638
0.445	0.627	-5.438	0.170	4.129	3.959

0.282	0.480	-5.168	0.263	3.574	3.311
0.015	0.160	-2.943	-0.858	3.049	3.907
0.712	0.828	-5.650	-0.109	2.988	3.097
0.918	0.956	-5.710	0.028	3.627	3.599
0.693	0.816	-5.641	-0.107	4.042	4.149
0.375	0.565	-5.342	-0.263	3.887	4.150
0.421	0.607	-5.408	-0.179	3.968	4.147
0.022	0.169	-3.242	-0.560	3.409	3.969
0.471	0.650	-5.469	0.164	4.791	4.626
0.051	0.210	-3.931	0.745	4.345	3.601
0.930	0.962	-5.711	-0.023	4.154	4.177
0.499	0.670	-5.499	-0.172	6.316	6.488
0.017	0.162	-3.070	0.590	8.922	8.331

19.ALS.FC.M	20.ALS.FC.F	21.ALS.FC.M	23.ALS.FC.M	24.ALS.FC.F	13.CTL.FC.F
3.833	3.324	4.189	3.802	4.066	4.149
7.304	7.382	7.634	7.271	7.231	8.253
4.420	3.203	4.035	4.224	4.047	4.737
3.194	1.998	2.359	2.668	2.310	3.170
6.542	6.145	6.377	6.310	6.207	5.063
3.758	4.805	4.293	4.307	4.203	3.449
5.082	5.395	4.974	5.181	5.040	4.539
8.278	8.043	8.138	8.061	8.148	6.900
6.511	6.487	6.573	6.762	6.593	5.427
6.204	6.382	5.705	6.322	6.314	7.850
5.362	5.868	5.570	5.785	5.979	6.451
4.640	3.965	4.365	4.720	4.587	6.894
2.698	2.514	2.678	3.390	2.841	2.226
3.108	2.270	2.661	2.901	2.844	2.063
7.968	8.144	7.916	8.301	8.483	9.797
7.014	6.771	7.161	7.177	6.644	6.680
4.094	3.848	4.037	5.000	4.549	7.651
5.420	5.037	5.543	6.359	5.843	8.218
5.518	4.465	4.442	5.566	5.124	6.466
4.787	5.140	5.338	5.256	5.183	5.643
1.278	2.585	2.147	1.918	1.818	1.554
1.670	1.944	2.412	2.262	2.289	4.407
4.615	4.516	4.161	4.522	4.614	5.553
6.965	6.772	6.743	6.733	6.712	6.028
4.952	5.029	4.965	4.829	4.927	4.947
6.423	7.311	6.981	6.311	6.242	6.358
6.123	6.364	6.304	6.411	6.603	4.821
4.666	4.511	3.937	4.104	4.224	4.693
5.889	5.589	5.779	5.838	5.828	5.767
4.858	5.980	5.817	5.583	5.403	6.188
5.537	5.778	5.707	5.397	5.767	4.067
4.208	4.556	4.331	4.217	4.671	2.584
6.273	6.214	6.456	6.200	6.275	5.220
3.009	2.779	2.756	3.383	2.721	4.306
2.369	2.095	2.045	2.166	2.317	2.337
2.925	3.110	2.858	2.545	3.061	2.514
5.911	6.707	6.206	6.492	6.614	6.625
4.169	4.532	4.351	3.678	4.254	5.200
5.087	5.599	4.962	5.005	4.941	6.081
4.259	4.175	4.245	4.369	4.624	5.291
6.871	6.883	6.972	6.617	6.944	5.067
3.203	3.938	3.857	3.917	3.847	4.550
4.887	3.236	3.365	3.910	4.438	6.213
6.746	6.676	6.550	6.717	6.702	7.360
6.590	6.395	6.408	6.145	6.531	4.855
5.862	5.671	5.729	6.074	5.972	6.586
3.108	4.064	4.270	3.998	3.700	2.420
4.358	4.375	4.672	4.385	4.667	4.270
4.426	4.298	4.033	4.527	4.460	3.627

4.743	4.342	4.750	4.268	5.142	6.838
3.292	4.194	3.670	4.613	4.575	5.003
3.732	3.511	3.162	4.026	3.542	3.932
2.630	2.820	2.573	2.034	2.032	2.519
4.207	3.506	3.981	4.075	3.656	3.140
3.008	3.453	3.559	3.475	2.813	2.975
5.078	4.885	4.504	5.348	5.341	5.765
1.681	2.149	2.235	1.914	1.722	3.052
1.479	1.830	1.637	1.707	1.513	2.163
5.028	3.647	3.970	4.060	4.353	5.809
4.454	3.767	3.113	4.007	3.394	4.177
4.904	4.813	5.003	4.862	5.116	4.091
3.370	3.297	3.483	3.375	3.685	4.486
2.438	2.436	2.602	2.355	2.882	2.752
4.113	3.648	3.219	4.319	4.712	4.910
2.850	4.023	3.409	2.382	3.573	1.793
1.907	1.698	1.639	2.013	1.545	2.645
2.765	3.421	2.692	2.980	2.917	3.183
5.982	5.528	5.738	6.197	5.285	4.475
3.609	3.684	3.304	3.369	4.143	4.475
2.261	1.826	2.568	2.444	2.337	3.382
3.356	3.727	3.425	2.365	3.529	2.926
2.336	1.894	2.093	2.065	1.886	2.673
1.357	1.034	1.626	0.966	0.777	4.004
3.137	3.138	2.706	3.974	3.644	2.680
1.652	1.711	2.183	1.889	2.242	2.305
3.594	3.161	3.646	4.149	3.037	2.912
3.213	2.429	2.912	3.323	2.761	2.060
2.683	1.927	1.504	2.011	1.949	1.619
4.303	3.201	4.414	3.968	4.097	2.847
1.454	2.051	1.752	1.665	1.510	3.453
4.587	3.989	4.763	4.778	4.180	2.372
3.701	3.862	2.974	3.804	3.770	3.088
2.766	2.339	2.420	3.028	2.794	2.871
3.286	2.893	2.713	2.384	2.628	2.274
1.010	1.879	1.375	2.038	2.951	3.570
4.801	5.802	5.487	4.911	4.145	5.916
3.701	4.285	4.039	5.070	3.425	4.168
2.781	2.561	2.248	2.947	2.377	1.733
3.348	3.252	2.972	3.616	2.986	2.238
2.483	2.451	1.922	2.521	3.147	2.699
1.529	1.228	1.912	1.973	2.355	2.804
2.226	2.106	1.696	2.636	2.904	5.822
2.965	2.706	3.213	3.451	2.428	2.301
1.902	1.777	3.128	1.734	1.847	2.497
2.205	3.839	2.150	3.133	2.753	1.782
4.379	3.729	3.231	3.415	3.524	2.977
4.398	3.334	4.278	4.429	4.026	1.766
3.215	2.542	3.013	3.139	3.207	2.212
4.139	4.850	5.081	2.520	4.568	1.585

3.986	3.680	3.479	3.684	3.955	3.460
1.878	2.343	2.528	1.738	1.842	1.953
3.617	3.370	3.375	2.759	3.363	4.123
2.828	2.618	2.299	2.622	2.573	2.573
3.374	2.708	3.409	3.292	2.437	1.529
2.712	3.356	4.002	2.623	3.462	3.339
4.119	4.183	4.645	4.610	5.096	2.731
3.281	2.308	2.533	2.002	2.579	3.289
2.333	3.079	2.829	2.439	2.670	2.246
4.401	3.715	4.082	4.220	3.592	2.801
2.235	2.023	2.822	2.754	2.807	2.849
2.819	2.950	2.295	2.699	2.602	2.369
2.046	2.945	2.850	2.803	3.031	1.634
6.087	6.345	5.481	5.841	5.935	5.419
1.463	2.460	2.226	2.661	2.310	3.371
4.955	3.555	3.445	3.578	4.137	4.617
3.172	2.791	2.556	2.744	2.884	3.516
3.228	2.909	3.009	3.767	2.137	3.271
2.253	2.826	2.587	2.827	2.766	2.133
4.195	4.843	4.738	4.286	4.084	1.587
1.865	2.269	2.386	2.173	2.224	3.409
5.588	5.539	5.679	5.523	5.626	5.642
2.409	3.225	2.308	2.945	3.225	4.078
5.349	5.170	5.462	5.629	5.603	6.210
3.015	2.644	2.937	2.871	2.559	0.944
1.767	2.436	1.570	1.763	1.925	2.148
3.665	2.192	2.543	2.142	1.969	2.047
2.767	2.346	2.258	3.628	1.318	4.626
2.940	3.579	3.024	2.857	2.498	3.071
2.679	2.895	3.314	3.889	2.507	1.112
2.729	2.198	2.978	3.048	3.252	2.712
2.529	2.043	1.942	2.406	2.260	2.312
4.621	5.409	4.855	4.782	4.242	4.233
4.427	4.468	3.509	3.569	3.293	3.477
2.801	1.667	1.567	1.096	1.966	2.501
3.189	3.382	3.395	3.562	3.689	4.796
1.308	1.654	2.201	2.225	2.530	4.009
6.180	5.912	6.014	6.917	6.184	6.566
3.703	2.962	2.472	2.932	2.912	2.317
4.938	4.506	4.308	5.420	5.145	5.601
2.668	1.814	1.951	1.340	1.901	2.043
5.116	5.054	5.746	5.341	5.193	3.574
5.391	6.807	5.721	5.617	6.034	5.767
7.160	6.772	6.100	7.039	6.564	7.014
3.023	2.984	3.208	3.703	3.167	3.836
4.405	4.218	4.714	4.338	4.516	3.463
4.338	4.215	4.726	4.501	4.637	4.928
5.502	4.842	5.118	4.903	5.194	4.790
3.618	2.950	3.732	3.623	3.648	3.961
7.914	8.594	8.130	7.969	8.283	8.014

4.636	4.998	4.959	4.688	5.292	4.334
3.708	2.972	3.374	3.664	3.304	4.420
2.067	2.588	2.269	1.997	2.309	2.881
2.760	3.763	2.974	2.230	2.339	2.736
5.133	4.678	4.639	4.946	5.104	6.326
3.599	3.336	3.573	3.285	3.238	3.781
6.389	6.145	5.716	6.175	6.357	7.383
7.804	7.966	7.969	7.835	7.695	7.372
2.468	2.575	2.458	2.279	3.283	2.193
5.952	5.683	6.118	5.704	5.514	4.905
4.524	4.529	4.134	4.418	4.503	3.683
3.835	4.089	3.774	4.302	3.808	4.837
5.487	5.095	5.264	5.953	5.747	5.520
6.554	5.796	6.096	6.186	6.090	6.276
2.264	3.607	3.717	3.118	2.569	2.882
3.968	2.904	2.159	3.040	3.916	3.077
4.319	4.051	4.122	4.416	4.417	3.440
3.450	3.508	3.429	3.517	3.720	4.168
6.167	5.393	6.233	5.884	5.762	3.736
5.056	4.642	4.641	4.399	4.381	2.810
6.249	6.047	6.243	6.261	6.207	6.501
6.508	6.929	6.720	6.653	6.807	6.009
6.860	7.007	6.727	6.749	6.670	6.313
6.532	6.149	6.477	6.663	6.714	6.212
5.775	6.389	6.151	6.108	5.977	5.427
2.768	2.876	3.578	2.966	3.384	2.590
5.966	6.275	5.920	5.869	5.834	4.388
5.410	5.503	5.378	5.403	4.984	3.745
5.236	5.555	5.239	5.429	5.281	4.576
3.458	3.673	3.461	3.603	3.262	2.920
3.725	3.305	2.717	3.197	3.773	5.922
4.452	4.142	4.453	4.542	4.511	5.737
6.132	6.464	6.260	6.293	6.352	4.717
3.667	3.751	3.736	2.871	3.409	3.672
3.767	3.868	4.130	4.077	4.190	4.728
4.665	4.613	4.661	5.102	5.017	4.778
4.441	3.624	4.350	4.997	4.052	3.724
5.779	4.939	4.910	5.688	5.289	5.673
8.005	8.180	8.173	7.929	7.998	7.371
8.856	8.926	8.765	8.834	8.820	7.772
5.208	4.953	4.683	4.796	4.693	7.094
3.480	2.570	2.709	2.875	3.021	2.671
2.715	2.625	2.218	2.772	2.429	2.544
5.337	5.508	5.001	4.388	4.781	4.770
2.159	1.643	1.068	1.288	2.190	3.400
2.142	2.665	2.811	2.835	3.674	3.137
2.175	2.441	2.181	1.695	2.026	2.209
2.881	3.049	3.877	2.750	2.909	1.710
3.639	3.363	2.892	4.153	3.966	3.090
2.167	1.371	3.168	2.855	3.404	1.936

3.906	3.066	3.496	3.450	3.202	2.464
4.382	3.587	3.552	3.004	3.124	3.716
3.953	4.343	3.935	3.679	3.232	1.758
4.770	4.843	4.506	4.383	5.247	6.466
3.053	2.705	2.800	3.089	2.914	3.646
2.277	3.129	3.366	2.693	1.475	2.153
4.395	4.646	4.897	4.455	4.150	3.912
6.697	6.693	6.325	5.664	6.156	6.682
4.870	4.099	4.911	4.677	4.631	3.705
3.512	3.438	3.781	3.733	3.954	3.966
4.515	3.920	3.866	4.444	4.392	5.358
2.243	1.988	1.396	0.997	2.237	2.094
3.174	2.936	3.472	2.516	3.585	2.509
4.227	4.974	4.871	4.164	4.125	5.665
6.006	6.227	5.834	5.853	6.206	5.676
1.610	1.820	1.552	1.437	1.790	2.219
3.374	4.170	3.671	4.102	4.182	3.625
9.195	8.955	8.835	9.141	8.965	6.819
3.606	3.412	3.421	3.881	3.526	5.008
3.589	4.386	4.192	3.596	4.298	5.002
5.832	6.207	6.112	6.302	6.366	7.212
5.885	5.811	5.486	5.513	5.593	3.855
2.455	4.557	3.311	3.962	4.280	3.271
6.842	6.359	6.650	6.372	6.536	6.845
7.401	7.516	7.453	7.385	7.270	5.790
2.504	1.901	2.585	2.862	2.610	3.046
2.126	3.082	2.326	2.376	2.270	1.773
4.652	4.939	4.587	4.902	4.826	6.056
4.768	4.467	4.765	4.445	4.963	5.057
4.384	3.699	3.816	3.772	4.069	4.573
4.502	4.485	4.582	4.578	4.580	4.754
5.651	5.119	5.583	4.678	5.291	3.849
4.044	4.536	4.520	3.588	3.720	5.271
5.061	5.047	5.020	5.178	5.196	3.762
4.662	4.336	4.660	3.948	4.555	2.854
3.649	3.701	4.434	2.865	3.883	4.194
2.878	3.345	3.536	3.254	3.504	3.205
2.900	3.090	2.600	2.597	3.074	2.117
6.296	6.486	6.155	5.894	5.812	3.809
7.223	7.064	6.841	6.850	6.952	5.920
8.442	8.737	8.728	8.552	8.592	7.375
3.525	3.488	3.692	3.716	3.971	4.650
3.846	3.065	2.965	3.041	3.143	4.521
3.415	3.236	2.924	2.596	3.305	3.531
9.464	9.288	9.744	9.435	9.393	8.219
2.826	4.253	3.380	3.620	3.033	4.624
4.385	2.729	3.139	3.870	2.858	4.251
3.239	2.575	2.886	3.996	1.753	3.499
4.295	4.701	4.527	4.325	4.325	3.222
6.764	6.727	6.522	6.552	6.696	4.990

6.348	6.359	6.021	6.094	6.253	4.922
3.757	3.753	3.742	3.995	3.981	4.819
7.671	7.960	7.803	7.810	7.686	6.783
4.420	4.431	4.690	4.717	5.290	5.404
2.776	2.664	2.748	2.368	2.519	3.670
8.140	8.185	8.113	7.942	8.061	4.526
3.896	3.997	3.865	3.536	3.542	2.452
6.045	5.481	5.379	6.551	6.153	6.829
3.597	3.047	3.446	3.188	3.127	2.493
5.482	5.085	5.268	5.714	5.393	3.601
3.112	1.451	2.474	3.027	2.521	1.918
5.716	5.677	5.421	5.344	5.476	6.224
7.214	7.448	7.515	7.501	7.501	6.850
5.936	6.147	5.954	6.018	6.327	5.318
6.060	6.605	6.351	6.662	6.615	6.554
3.072	4.070	3.905	3.950	4.231	3.174
1.612	1.525	1.701	2.078	2.076	3.322
5.809	5.603	5.778	6.108	6.407	7.681
4.174	3.960	3.884	4.595	4.818	6.111
6.673	6.191	6.692	6.625	6.701	8.131
3.767	3.289	3.387	3.019	3.324	4.183
6.418	5.668	5.845	6.306	6.397	7.346
6.472	6.840	6.570	6.341	6.461	6.186
6.435	6.851	6.646	6.501	6.625	6.893
4.479	4.207	4.466	4.859	4.270	5.837
4.769	5.904	5.764	4.163	4.588	5.840
5.035	6.275	6.137	4.390	4.958	6.124
5.090	6.325	6.128	4.519	4.983	6.248
3.001	3.132	3.001	3.006	3.548	3.915
5.342	4.594	5.184	5.185	5.196	4.888
2.630	2.287	2.184	2.201	2.160	1.527
4.831	4.435	4.622	4.826	4.409	1.118
4.026	1.661	2.693	2.808	3.181	1.248
7.133	7.263	7.032	6.891	7.073	6.478
9.118	9.118	9.100	9.204	9.072	7.485
8.386	8.742	8.510	8.563	8.704	8.005
7.016	7.063	6.571	6.680	6.787	5.579
2.016	1.982	2.895	2.100	2.543	2.997
4.984	5.024	4.990	4.461	4.791	1.717
3.803	3.422	3.573	3.569	3.373	4.108
3.472	3.206	2.717	3.146	2.601	2.679
4.083	3.882	3.282	3.957	3.183	3.813
2.179	2.654	2.364	3.014	2.961	2.247
8.515	8.392	8.326	8.446	8.417	7.373
3.099	3.119	2.634	3.417	2.867	3.980
2.782	3.364	2.358	3.080	3.142	1.999
4.225	4.179	4.222	4.368	4.530	5.137
6.417	6.744	6.568	6.320	6.655	5.977
2.693	3.388	3.622	2.666	2.211	2.647
5.999	5.978	5.952	6.248	6.159	7.041

5.913	6.658	6.112	6.372	6.617	8.194
4.615	4.866	4.852	4.999	5.099	3.876
3.006	3.413	2.377	3.596	2.897	2.842
3.375	2.448	2.203	3.209	2.251	2.377
6.467	6.534	6.280	6.363	6.318	7.132
4.109	5.204	5.052	5.270	4.947	4.928
3.650	3.904	4.233	3.908	3.993	4.164
3.249	3.033	3.459	3.388	3.062	2.946
2.554	2.284	1.731	3.250	1.603	2.339
5.167	5.245	4.963	4.714	5.219	4.508
1.737	2.495	2.035	2.782	2.224	3.078
1.896	3.555	3.202	2.659	3.273	1.172
3.800	3.942	3.444	3.810	3.872	3.117
2.372	2.658	2.790	2.840	2.026	2.356
3.932	4.675	4.338	4.147	4.038	4.843
6.555	6.281	6.626	5.481	5.869	6.243
3.638	3.660	3.254	3.251	3.348	3.631
5.672	6.181	5.748	6.128	5.742	4.556
6.385	6.394	6.234	6.359	6.297	5.702
2.380	1.931	2.083	2.121	2.069	3.784
2.894	2.447	2.347	2.333	2.656	2.999
3.329	3.396	3.314	2.697	3.503	2.550
5.484	6.040	6.282	6.229	6.370	7.123
4.803	4.464	4.587	4.259	4.393	2.876
2.070	1.791	1.935	2.024	2.289	2.007
6.579	6.469	6.570	6.406	5.980	5.151
2.945	2.640	2.553	3.092	2.984	2.537
6.476	6.680	6.915	6.667	6.860	6.069
3.886	3.906	4.117	3.948	4.501	4.836
8.508	8.455	8.255	8.121	7.951	6.259
3.629	3.899	4.288	4.505	4.097	5.063
5.544	5.991	5.912	5.744	5.435	5.184
2.500	2.682	3.800	2.755	2.425	3.191
3.531	3.766	3.842	3.652	3.547	4.348
5.692	6.083	5.649	5.764	5.737	5.755
3.406	2.436	2.837	3.277	3.877	4.298
5.689	5.115	5.459	5.293	5.247	5.149
3.295	2.337	2.869	3.191	3.589	2.944
3.712	3.641	4.180	3.971	4.340	5.376
6.328	5.296	5.763	5.800	5.852	4.607
3.070	3.671	3.371	2.894	3.517	3.693
5.238	5.722	5.300	5.386	5.629	5.739
9.529	9.438	9.404	9.535	9.450	9.095
8.308	8.350	8.404	8.605	8.437	7.840
2.041	2.358	2.622	2.536	3.110	2.810
4.360	4.184	4.422	4.780	4.540	5.558
6.541	6.521	6.433	6.759	6.760	7.532
2.899	2.568	2.629	2.514	2.871	2.524
6.142	6.832	6.561	6.685	6.708	5.438
6.102	5.819	5.824	6.064	6.033	6.631

5.864	6.042	5.788	5.987	6.156	6.616
5.762	5.812	5.525	6.069	5.788	6.604
6.992	7.482	7.236	7.001	7.227	6.750
6.575	6.601	6.229	6.542	6.611	5.977
5.170	4.743	4.330	5.491	4.211	4.156
5.585	4.299	4.552	4.786	4.603	3.076
3.135	4.849	4.856	4.241	4.432	3.522
7.592	6.550	6.961	7.039	6.743	5.654
7.800	6.643	7.192	7.241	6.823	5.764
6.306	4.814	4.754	4.742	5.785	3.154
5.022	3.634	0.980	3.951	4.046	0.535
1.835	1.766	1.976	2.322	2.194	1.507
5.229	5.046	5.241	5.538	5.659	3.913
5.890	5.850	5.621	6.284	5.716	5.372
1.919	2.376	3.271	3.615	2.544	1.782
6.385	6.730	6.266	6.248	6.463	5.816
4.368	2.935	4.347	4.157	3.551	1.308
5.576	5.230	5.409	5.719	5.140	4.420
3.816	4.233	4.109	3.982	3.785	2.013
4.645	4.088	4.690	4.827	4.504	3.157
1.982	2.469	2.100	2.145	2.300	3.814
4.647	4.943	5.311	4.642	5.077	3.693
6.946	6.873	6.246	7.410	6.848	6.970
4.705	4.421	5.691	4.772	4.872	4.945
7.690	7.720	7.587	7.796	7.733	8.823
5.493	5.339	5.379	5.158	5.108	4.429
4.841	5.084	5.098	5.571	5.149	6.585
2.222	2.939	2.401	3.044	2.858	3.573
4.546	5.310	4.942	4.921	4.813	3.921
5.866	6.300	6.329	5.955	5.887	5.778
3.297	3.566	4.075	3.425	2.972	3.654
7.435	7.191	7.064	7.534	7.022	5.676
4.056	4.264	4.068	4.389	4.233	4.595
6.754	6.759	7.321	7.773	7.647	9.366
6.448	6.331	6.891	6.221	6.567	3.046
6.042	6.036	6.349	6.287	6.375	5.356
7.462	7.546	7.649	7.552	7.549	6.392
7.922	8.130	7.927	7.534	7.570	8.287
3.695	3.401	3.241	3.481	3.211	4.761
3.474	3.932	3.470	3.655	3.718	4.563
2.208	2.208	1.781	1.824	2.342	1.864
5.646	5.714	5.359	5.922	5.716	7.068
4.475	4.344	4.350	4.589	4.848	5.150
5.179	4.197	4.120	4.063	4.511	4.540
5.325	5.269	5.523	5.148	5.179	4.790
1.795	1.956	2.109	1.722	2.119	3.658
7.694	6.887	6.743	6.846	6.787	7.750
3.093	2.833	2.142	3.012	2.669	2.321
2.078	2.358	1.719	2.580	1.679	2.630
5.894	4.798	4.595	5.264	5.104	4.974

6.154	6.562	5.970	6.250	6.437	7.208
7.422	7.244	7.375	7.305	7.162	8.779
9.413	9.957	9.650	9.744	9.823	8.611
4.521	4.700	4.456	4.113	4.412	5.079
2.553	2.075	2.189	1.705	3.192	2.303
1.985	1.971	2.663	2.158	2.610	2.426
2.888	3.010	2.654	2.589	2.684	2.164
3.734	3.793	3.525	4.102	3.737	4.351
2.637	3.130	3.375	2.416	3.439	2.643
5.467	4.737	4.509	4.756	5.191	5.404
2.275	2.509	2.073	2.153	2.087	2.985
2.976	2.614	2.347	2.174	2.681	1.582
2.824	2.964	3.754	3.055	2.795	2.568
4.461	3.872	4.062	4.117	4.017	4.531
3.111	2.849	3.015	2.492	2.170	3.666
2.714	2.912	2.470	2.743	4.289	4.480
4.654	5.632	5.062	4.915	5.230	4.301
6.445	5.376	5.949	5.692	5.583	5.461
3.249	3.745	3.382	4.013	3.089	3.747
6.407	6.491	6.427	6.136	6.028	4.757
5.312	5.240	4.456	5.160	4.723	5.763
2.418	2.360	3.285	3.281	2.697	2.055
6.575	6.436	6.720	6.573	6.460	5.586
6.460	6.746	6.599	6.687	6.626	5.856
3.667	3.717	3.642	3.977	3.403	3.078
6.262	6.458	6.257	6.561	6.217	5.914
7.751	7.468	7.773	7.477	7.289	6.248
3.159	4.505	3.807	3.445	2.840	5.275
6.595	5.401	5.746	6.057	6.568	6.126
7.078	7.162	7.202	7.108	7.208	6.734
4.624	4.385	4.996	4.744	4.912	6.294
5.747	5.994	5.451	4.579	4.746	7.928
8.386	8.003	8.360	8.394	8.612	9.430
3.127	3.865	3.904	3.351	3.904	4.279
2.665	3.106	3.207	2.928	2.942	3.544
2.602	2.994	3.311	2.720	3.143	3.366
1.650	1.962	1.933	1.507	2.345	2.376
3.635	2.921	3.347	3.422	3.222	3.255
2.672	2.859	3.421	3.024	2.637	3.467
8.710	7.498	8.023	8.336	7.818	8.948
3.982	4.054	3.871	3.869	3.954	4.239
5.184	4.265	4.505	4.686	4.868	3.951
1.913	2.285	3.210	1.516	1.929	1.689
4.551	4.644	5.563	4.093	5.313	5.558
6.108	6.329	5.993	5.917	6.168	5.537
7.718	8.448	8.257	8.069	8.267	7.703
5.441	5.050	5.028	5.611	5.292	6.653
4.681	5.103	5.218	4.566	5.025	5.126
3.967	5.671	4.897	4.676	5.329	8.107
5.154	4.923	5.130	4.989	5.388	4.648

3.780	4.326	4.844	4.598	4.199	4.566
2.545	2.502	2.248	3.016	1.546	2.952
3.996	2.970	3.499	3.609	3.399	2.363
2.421	2.754	2.982	3.133	3.376	4.029
4.609	4.547	4.609	4.650	4.956	5.503
6.027	5.739	6.137	6.508	6.263	6.730
5.319	3.913	3.565	3.868	4.176	2.838
6.532	6.275	6.467	6.166	6.419	5.473
7.601	8.126	7.648	7.683	7.657	7.080
5.098	5.716	5.567	5.115	5.197	3.912
7.073	7.233	6.961	7.152	6.965	7.837
1.849	1.920	1.801	1.768	1.636	2.414
5.132	5.328	4.919	5.350	5.214	6.508
4.946	4.555	4.129	4.212	3.934	4.072
5.269	5.568	5.861	5.589	5.705	3.400
3.142	3.052	3.200	2.824	3.141	4.635
2.031	2.490	2.571	3.125	2.825	3.869
5.383	5.680	5.668	6.053	6.189	7.255
4.435	4.245	4.012	4.062	4.368	5.107
3.498	3.061	4.037	3.288	3.320	3.975
7.730	7.456	7.399	7.528	7.178	6.750
5.461	4.206	4.405	5.343	4.311	5.204
2.527	2.848	2.762	2.796	3.019	3.045
3.717	2.372	2.508	2.626	2.626	2.074
2.733	2.973	2.183	2.627	2.342	3.150
9.064	8.752	8.810	9.009	8.741	7.044
8.709	8.651	8.733	8.533	8.547	8.005
3.118	3.182	3.366	3.206	3.548	4.323
3.728	4.007	3.651	3.289	3.409	4.083
6.521	6.538	6.806	6.510	6.726	5.311
4.119	3.648	3.259	3.890	3.800	4.349
3.315	3.044	3.647	2.682	2.903	3.649
6.085	5.858	6.149	6.022	6.194	5.118
3.502	2.841	3.145	3.805	3.336	3.477
4.212	4.208	4.493	4.196	4.134	4.956
5.073	5.241	5.286	5.365	5.183	6.494
6.521	6.522	6.579	6.311	6.761	5.723
8.436	8.507	8.536	8.515	8.435	7.717
2.873	2.852	2.529	2.748	2.892	3.022
2.461	1.968	2.043	2.602	2.047	2.359
7.705	7.374	7.086	6.942	7.383	5.225
5.463	5.913	6.004	5.312	5.529	4.217
4.612	5.170	4.883	4.841	4.833	3.683
3.869	3.685	3.805	4.189	3.887	3.351
4.603	4.843	4.910	5.028	5.092	6.624
3.934	3.698	4.190	3.720	3.807	4.338
1.732	1.627	2.062	1.203	1.922	2.466
6.811	7.006	6.799	7.088	6.842	8.388
4.060	2.683	3.015	2.940	2.864	2.420
8.614	8.464	8.690	8.547	8.706	7.293

5.583	4.717	4.971	4.742	5.210	4.742
7.056	7.370	7.010	7.061	7.048	6.043
6.841	6.784	6.825	6.808	6.769	6.341
5.748	6.012	6.074	5.604	6.131	4.454
5.371	5.552	5.409	4.922	4.593	2.920
7.069	7.252	7.021	6.919	7.300	5.233
3.166	4.036	4.423	3.635	4.098	2.291
5.466	5.822	5.838	5.483	5.558	3.599
10.639	10.888	10.784	10.834	10.781	10.098
2.096	2.599	2.156	2.061	2.058	1.972
1.481	1.475	1.682	1.814	1.480	1.844
5.851	5.332	5.736	5.592	5.045	4.103
6.338	6.365	6.522	6.306	6.242	5.675
8.630	8.565	8.506	8.376	8.287	7.506
6.658	6.733	6.558	6.738	6.409	5.515
5.530	5.475	5.644	5.524	5.421	3.460
4.617	4.838	4.787	4.287	4.188	4.958
5.120	5.578	5.371	5.465	5.602	4.404
6.508	6.253	6.666	6.615	6.484	4.390
5.479	5.514	5.098	5.460	5.447	5.428
4.664	4.889	4.674	4.747	5.361	5.615
7.249	6.737	7.640	7.124	7.006	8.497
6.637	6.690	6.324	6.380	6.371	8.209
3.888	4.221	3.682	4.209	4.202	4.864
2.284	1.515	2.120	1.783	2.458	2.919
5.253	5.068	5.615	5.282	5.620	5.660
3.437	2.992	2.786	3.505	3.101	2.596
4.678	5.156	5.069	4.547	4.488	3.968
4.364	4.817	4.840	4.953	4.899	2.530
6.654	6.640	6.574	6.804	6.565	7.433
3.126	3.031	2.209	3.058	2.454	2.605
5.357	5.149	5.108	4.795	4.987	5.433
2.179	2.113	2.973	2.539	2.643	1.781
4.758	4.563	4.619	4.605	4.466	5.459
6.329	5.578	6.025	6.562	6.006	6.236
3.561	3.423	3.478	2.804	3.417	2.474
6.859	7.053	6.852	6.819	6.916	6.451
4.301	4.126	3.993	4.210	4.614	4.488
5.740	6.104	5.856	5.671	6.236	5.238
5.906	5.986	5.830	6.010	5.878	5.051
6.120	6.374	6.440	7.225	7.457	7.752
5.260	4.825	4.683	5.156	5.211	3.551
5.545	5.723	5.459	5.359	5.586	3.743
6.741	6.642	6.664	6.216	6.659	5.447
8.201	7.747	7.889	8.015	7.876	7.318
4.924	5.051	5.483	5.183	5.027	5.800
5.502	5.113	5.515	5.580	5.050	4.546
4.801	5.205	4.967	4.400	4.569	3.844
8.683	8.640	8.756	8.564	8.634	6.546
4.258	3.820	3.484	3.955	3.680	4.624

3.750	3.354	2.988	2.881	3.144	3.493
3.350	3.829	3.811	3.812	3.951	3.648
5.269	5.345	5.328	5.553	5.440	6.522
3.013	3.062	3.645	3.672	3.399	3.067
6.018	6.119	5.902	5.968	6.201	6.193
5.818	5.970	5.839	5.957	6.177	5.432
6.948	6.962	7.059	7.145	7.111	6.625
6.147	6.138	6.172	6.648	6.146	7.102
6.382	6.690	6.611	6.705	6.781	5.703
2.464	3.685	3.220	3.394	2.889	2.558
6.605	7.049	6.998	6.707	6.936	5.789
5.153	5.249	5.213	5.326	5.622	3.913
5.979	6.706	6.658	6.127	6.262	6.982
5.833	5.991	6.045	6.174	5.861	6.927
8.280	8.885	8.445	8.478	8.549	7.892
2.306	2.317	1.907	1.484	2.156	2.756
5.071	4.611	4.761	5.133	5.723	7.311
5.586	6.077	6.043	5.620	6.129	6.961
4.463	4.893	4.700	4.566	4.673	5.535
7.957	8.153	7.935	7.790	8.040	7.346
6.000	5.457	5.256	6.301	5.819	7.359
3.075	2.206	2.316	2.771	2.427	1.688
5.903	5.768	5.601	5.875	5.706	4.745
3.839	4.496	3.899	3.804	3.785	4.817
5.642	5.423	5.765	5.706	5.807	4.832
10.054	10.313	10.136	10.175	10.329	9.850
9.257	9.169	9.115	9.012	9.057	8.405
2.705	2.497	2.577	2.163	2.267	3.082
9.352	9.300	9.405	9.442	9.424	6.520
8.021	8.271	7.915	7.887	8.267	6.080
6.264	6.033	5.991	5.901	6.382	4.321
8.375	8.340	8.402	8.162	8.066	7.425
2.165	3.197	2.014	2.981	2.838	2.184
1.561	1.433	1.666	2.100	1.741	1.513
9.011	9.345	9.203	9.164	9.159	8.543
10.707	10.984	10.850	10.710	10.963	10.006
5.203	5.201	5.861	5.516	5.758	5.037
3.545	3.558	3.626	3.486	3.634	4.189
9.279	9.582	9.503	9.272	9.395	8.673
10.003	9.961	10.182	9.797	10.265	9.423
9.151	9.222	9.057	9.025	9.080	10.013
8.426	8.805	8.693	8.596	8.873	7.132
9.305	9.451	9.502	9.273	9.401	8.188
8.951	9.292	9.377	9.297	9.170	7.583
5.769	6.232	6.117	6.208	6.026	5.269
7.965	8.343	8.271	7.849	7.919	6.590
4.312	4.398	4.240	4.625	4.160	3.941
9.185	9.058	9.210	9.155	9.155	9.380
8.121	8.030	7.919	8.007	8.048	6.834
4.687	5.074	4.724	5.007	4.623	3.592

4.162	4.029	4.225	3.637	3.487	5.380
6.981	7.053	7.092	7.031	7.046	6.936
5.563	5.619	5.659	5.829	5.628	5.408
7.406	7.590	7.381	7.330	7.497	7.187
10.032	10.027	9.970	9.922	9.764	6.427
3.550	2.989	2.055	3.083	3.512	3.314
7.951	7.960	7.806	7.975	7.606	6.178
8.481	8.953	8.854	8.743	8.777	7.867
2.045	2.195	1.843	2.045	1.978	2.085
2.756	2.548	3.087	2.700	3.327	2.647
7.259	7.755	7.455	7.242	7.218	6.821
5.835	5.923	6.109	5.849	6.230	5.615
2.188	2.526	2.440	3.627	2.182	2.524
7.427	7.767	7.871	7.377	7.891	7.841
4.728	4.375	4.502	4.574	4.543	4.393
6.506	6.417	6.334	6.556	6.701	3.952
7.951	7.899	8.582	8.777	8.554	6.346
6.262	5.752	5.721	5.714	5.873	7.277
7.269	7.564	7.587	7.295	7.324	6.235
3.993	3.237	3.320	3.083	3.478	2.066
3.853	4.185	3.718	4.215	4.265	4.578
6.509	6.620	6.622	6.343	6.521	4.960
2.628	2.886	2.432	3.637	2.456	2.430
4.545	3.925	4.101	3.788	4.348	4.413
7.840	8.072	7.955	7.854	8.040	7.155
7.529	7.642	7.674	7.482	7.720	6.048
4.092	4.263	3.688	3.374	3.357	3.956
3.126	3.580	4.326	2.784	3.223	2.438
2.937	3.336	3.283	3.283	2.952	3.186
3.019	3.376	2.065	2.591	2.992	1.842
7.034	7.281	7.133	7.167	7.283	8.593
3.472	3.284	3.669	3.712	3.541	4.211
2.095	3.037	2.675	2.937	3.077	3.417
1.951	2.240	2.199	2.220	2.095	3.423
4.866	4.509	5.221	4.797	4.629	4.404
5.673	4.633	5.797	4.580	5.537	5.338
6.028	6.060	6.146	5.764	5.909	4.773
5.833	5.811	5.894	5.670	6.091	4.388
6.014	6.151	6.190	6.057	6.143	4.720
8.874	8.404	8.691	8.770	8.585	7.023
6.435	5.514	6.051	5.688	5.907	4.083
4.337	5.193	4.439	4.466	4.939	3.744
3.369	3.289	3.506	3.636	3.370	3.384
6.741	6.023	6.402	6.640	5.887	4.957
4.943	4.683	4.839	5.265	5.388	6.174
6.957	6.990	7.003	6.893	6.894	5.436
4.109	3.928	4.004	3.798	4.502	4.736
1.737	1.179	1.578	1.323	1.559	1.528
9.969	9.887	10.130	10.067	10.114	7.448
3.827	2.755	3.409	3.376	3.849	2.927

3.970	3.769	3.908	3.610	3.656	3.997
4.265	4.264	4.497	4.241	4.483	4.914
7.270	6.894	6.858	7.286	7.238	8.084
3.408	3.361	3.760	3.382	3.197	4.544
5.588	5.375	5.947	5.742	5.547	5.412
7.201	8.103	7.748	7.821	7.728	6.701
5.418	5.388	5.079	5.321	5.417	6.268
6.523	6.253	6.127	6.637	6.506	6.852
3.977	3.720	4.020	4.383	3.883	4.448
5.360	5.605	5.246	6.085	6.071	7.338
5.163	4.959	5.072	4.597	4.622	4.042
5.680	6.603	6.495	6.165	6.366	5.690
3.259	3.787	3.055	3.453	3.759	2.625
2.818	2.559	3.053	3.032	3.464	3.367
6.077	5.805	6.240	6.215	6.201	3.095
4.949	5.047	5.014	4.812	5.391	2.495
5.950	5.926	5.966	6.151	6.005	6.859
3.484	3.314	3.371	3.090	3.108	2.849
3.861	3.836	3.813	4.033	3.861	3.644
8.055	7.850	7.647	7.595	7.665	7.614
2.686	2.436	2.531	2.239	2.323	2.279
6.406	5.824	6.623	6.389	6.399	5.361
3.151	3.455	3.793	4.021	3.755	4.191
2.520	3.366	4.441	2.142	2.379	2.050
5.462	4.729	4.820	5.077	5.092	5.149
4.256	4.470	4.190	4.328	4.729	4.196
5.252	4.962	4.880	5.049	5.367	4.569
3.716	4.153	3.368	3.882	3.780	4.738
3.132	3.147	3.059	3.371	3.963	4.535
4.039	3.415	4.328	3.664	3.855	3.161
3.930	3.479	3.457	2.195	3.125	1.940
3.359	2.988	3.819	2.671	3.500	2.131
4.881	5.349	4.565	5.314	5.520	4.249
6.564	7.048	7.134	7.173	7.115	5.843
5.908	5.661	5.436	6.167	6.664	7.366
2.618	4.038	3.397	2.518	2.400	2.409
9.514	9.616	9.690	9.405	9.591	8.130
5.441	5.164	5.301	5.261	4.864	4.506
5.782	5.823	5.979	5.735	5.705	4.387
7.149	7.001	7.362	6.761	7.097	5.787
3.098	3.320	3.655	2.667	2.355	3.206
6.700	6.554	6.584	5.987	6.498	5.497
3.345	3.194	2.627	2.917	3.165	2.454
2.977	2.873	3.231	3.344	2.910	3.264
7.466	7.583	7.321	7.303	7.661	6.873
7.262	7.649	7.436	7.613	7.515	8.807
3.007	3.355	3.107	3.654	2.744	3.134
5.483	4.660	5.053	4.717	4.862	3.506
4.263	4.660	4.682	4.450	3.773	4.999
3.702	3.738	3.155	3.524	3.807	3.059

3.105	2.229	2.879	2.551	3.426	3.336
2.547	2.417	1.728	3.312	2.896	4.546
3.008	3.776	3.964	3.149	2.942	4.839
4.246	4.747	4.273	4.182	5.104	3.724
6.860	7.036	6.822	6.876	6.975	7.572
2.956	3.530	3.845	4.001	4.092	3.049
4.358	3.759	4.095	4.156	4.295	2.538
4.494	1.773	2.174	2.843	3.620	0.924
5.689	4.284	3.720	4.530	4.647	3.955
4.545	3.468	3.629	4.131	4.075	4.811
4.867	3.957	4.658	4.094	4.747	5.136
2.171	1.688	2.245	2.927	2.908	3.017
5.652	5.414	5.528	5.878	5.473	3.843
9.463	9.687	9.428	9.421	9.386	8.679
6.240	6.100	6.871	6.738	6.225	5.697
3.560	4.927	4.451	4.124	3.733	2.945
3.219	1.798	2.678	4.072	2.774	3.788
2.993	2.696	2.513	2.501	2.451	3.919
3.612	3.167	3.366	3.833	3.032	4.353
5.136	4.900	5.349	5.625	5.816	7.370
4.470	4.679	4.919	4.397	4.700	4.107
7.595	7.677	7.570	7.708	7.639	7.008
6.677	6.951	6.858	6.949	6.765	6.505
2.551	3.678	2.715	3.100	2.744	2.656
1.854	1.841	2.107	2.284	1.980	2.702
7.673	8.130	7.873	7.966	7.893	7.383
7.286	6.760	6.810	7.093	7.140	5.158
7.214	6.616	6.901	7.196	7.217	6.100
4.688	5.077	4.729	4.764	4.685	4.055
5.312	5.069	5.370	5.277	5.162	4.301
7.215	7.216	7.343	7.487	7.383	6.134
6.151	6.234	6.330	5.998	6.221	5.625
7.268	7.176	7.096	7.157	7.151	4.446
3.208	3.983	3.402	2.942	2.887	2.908
2.386	2.680	2.482	2.428	2.970	2.301
5.500	5.287	5.791	4.990	5.636	5.125
6.821	6.367	6.695	6.617	6.327	5.670
4.596	5.308	4.798	4.732	4.928	4.723
1.889	2.238	2.043	1.749	2.771	2.848
2.649	2.434	2.354	2.708	2.686	2.745
2.871	2.150	2.512	2.045	2.808	3.439
6.896	6.556	6.800	6.853	6.787	7.262
5.565	5.727	5.217	5.020	5.367	3.981
4.676	4.703	4.650	5.149	4.639	5.274
7.218	6.716	6.847	6.785	6.690	5.942
7.825	6.925	6.869	6.635	7.607	7.389
2.726	3.594	3.164	3.159	3.088	3.646
3.515	3.624	3.617	3.360	3.556	4.448
8.059	8.126	8.183	8.092	7.962	7.404
5.369	5.367	5.399	5.308	5.628	7.176

2.456	2.128	2.219	2.355	2.033	3.768
5.585	5.594	5.336	5.448	4.995	4.228
4.937	5.122	4.596	4.973	4.926	3.438
6.193	6.449	7.204	5.363	6.002	6.219
1.806	1.515	1.702	2.429	1.356	4.612
4.458	4.639	4.304	3.925	5.218	5.325
2.249	2.510	2.184	2.359	2.068	3.931
3.261	2.957	3.057	3.180	2.820	4.802
2.940	2.776	3.199	3.217	3.205	2.833
2.565	3.605	2.980	2.470	3.255	1.352
4.016	3.627	3.709	2.800	2.452	3.793
3.063	4.215	3.729	3.449	3.413	4.375
5.437	6.086	6.187	5.613	5.911	5.518
3.073	3.892	3.879	4.277	4.304	5.735
4.326	4.906	4.557	4.413	4.796	5.322
2.986	2.632	3.298	3.050	3.828	3.868
2.374	3.266	2.796	3.498	3.511	3.845
3.290	2.642	3.309	3.158	2.814	2.927
4.746	4.165	4.292	4.638	4.681	3.921
4.549	4.657	4.858	4.844	4.709	3.822
7.636	7.470	7.849	7.166	7.425	3.223
1.142	1.171	1.382	0.975	1.164	1.502
3.645	3.174	2.769	2.655	2.930	2.769
3.217	2.590	3.340	1.836	2.796	1.517
6.719	6.767	6.935	6.679	6.917	5.936
6.557	6.357	6.926	6.783	6.318	6.062
5.305	5.339	5.790	5.287	5.603	4.765
4.601	4.583	4.469	4.478	4.304	4.239
2.664	2.133	2.803	2.803	2.416	2.965
3.718	4.201	4.124	4.352	4.164	4.901
6.696	6.323	6.662	6.444	6.270	6.461
2.828	3.555	3.419	3.245	3.667	2.316
4.536	4.705	4.756	4.651	4.667	3.703
4.984	4.528	5.304	4.414	4.488	1.554
5.603	6.472	5.763	6.006	5.891	5.187
3.291	4.299	3.559	3.976	3.751	1.688
3.659	3.341	3.495	4.117	3.837	3.469
3.566	3.307	2.910	3.289	3.348	3.680
6.398	6.358	6.123	6.241	6.444	6.047
5.539	5.589	5.129	5.669	5.369	4.380
4.581	4.412	5.432	5.194	5.133	4.650
6.135	6.084	6.528	6.246	6.398	5.564
7.675	7.748	7.890	7.641	7.828	7.015
5.828	6.039	5.789	6.074	5.722	5.123
7.638	7.717	7.594	7.408	7.618	7.405
6.143	6.302	6.398	6.024	6.108	4.823
4.827	3.704	3.944	4.379	4.413	4.873
3.418	4.049	3.613	3.262	3.366	3.097
5.226	4.566	5.401	4.938	5.097	4.494
3.127	3.512	3.628	3.742	3.445	2.731

2.738	2.661	2.708	2.761	2.306	1.695
5.807	5.387	5.360	5.448	5.435	3.896
3.459	3.475	4.084	3.982	4.145	3.198
5.593	6.187	6.168	5.593	6.098	6.346
5.837	6.284	6.329	6.147	6.219	5.149
3.226	3.867	4.145	3.404	3.812	1.949
4.573	4.807	5.012	4.814	5.651	4.537
3.512	4.197	4.012	4.278	4.000	3.403
6.571	6.611	6.532	6.631	6.594	5.543
2.866	3.513	2.734	3.223	3.588	3.691
3.962	4.173	3.673	4.013	4.293	4.914
4.601	4.641	4.564	4.420	4.827	3.762
2.822	3.788	2.893	3.079	3.226	3.726
4.315	3.948	3.699	3.832	4.214	3.255
2.478	1.469	2.540	2.256	2.261	2.471
2.585	2.636	2.528	2.290	2.399	2.813
3.730	3.923	3.836	2.833	3.711	4.539
2.595	3.661	3.356	2.712	3.371	4.040
2.871	2.064	2.390	2.834	2.349	3.029
2.246	2.863	3.085	2.443	2.100	1.374
3.659	4.512	3.825	3.539	4.188	4.651
3.071	2.185	2.422	1.914	2.396	2.607
5.731	5.935	6.008	6.128	6.005	6.523
2.546	2.120	2.580	2.812	2.800	3.812
8.086	8.219	8.292	7.357	7.689	3.672
1.273	1.241	1.291	0.920	1.012	1.561
5.391	4.383	4.932	4.394	4.559	4.984
4.408	3.917	3.303	4.361	3.997	4.320
3.135	2.977	3.520	3.183	2.997	3.048
5.128	5.235	5.134	5.317	5.345	6.695
6.422	6.492	6.298	6.420	6.171	5.959
4.651	4.468	4.235	4.221	4.224	3.188
2.846	2.870	2.739	3.297	2.772	2.763
2.197	1.956	1.927	2.292	2.283	3.100
3.263	3.263	2.977	3.263	2.971	3.556
4.927	4.501	5.186	4.982	5.255	4.668
4.484	4.120	3.828	3.281	3.902	4.801
5.044	5.007	5.307	5.457	5.219	6.600
3.690	4.235	4.442	3.865	4.422	2.791
4.223	4.391	4.201	4.522	3.885	4.175
1.624	2.189	1.626	1.127	1.654	2.045
1.988	2.104	2.099	1.999	2.538	3.082
4.128	4.692	4.328	4.439	4.737	4.199
2.586	2.788	3.585	2.173	2.035	3.235
5.982	6.425	6.741	5.182	5.469	6.236
6.471	6.772	6.913	5.843	5.903	6.570
3.766	3.502	3.793	3.526	3.681	4.942
6.260	4.520	5.643	5.316	5.324	4.225
8.438	8.730	8.947	8.289	8.385	5.091
1.755	1.895	1.751	1.683	1.987	2.322

5.837	5.552	5.967	6.156	6.102	4.436
3.477	3.185	3.103	3.653	2.962	3.610
2.419	2.293	2.497	2.332	1.972	2.114
3.993	4.203	4.314	3.175	3.626	3.422
3.885	4.272	3.832	3.449	3.892	3.400
5.294	5.389	5.612	4.685	4.937	4.792
3.225	3.702	3.776	3.033	3.079	3.300
3.340	3.782	3.884	3.153	3.141	3.388
3.364	3.779	3.858	3.117	3.082	3.390
3.275	3.839	4.040	3.151	3.225	3.349
3.431	4.183	4.049	2.933	3.132	3.559
2.890	3.162	2.982	3.064	3.344	3.972
3.002	2.754	2.794	2.379	2.890	2.472
4.918	3.820	4.059	4.471	3.749	2.610
2.478	2.925	2.232	2.044	2.092	2.681
3.626	2.284	3.682	3.599	3.080	3.347
2.461	2.959	1.732	2.481	2.000	5.976
2.588	2.967	3.145	2.499	2.459	3.188
3.324	3.530	3.306	2.525	3.131	4.194
2.546	3.361	3.655	3.213	3.746	4.132
3.190	3.895	3.536	4.157	3.747	5.619
4.365	3.512	3.888	3.064	3.093	1.670
5.181	3.971	5.354	5.220	4.623	5.330
2.928	2.832	2.810	3.522	3.406	4.058
3.047	2.704	2.572	2.942	2.706	4.288
6.450	6.369	6.529	6.149	6.198	6.759
3.147	3.742	4.104	3.881	3.260	2.773
7.452	7.215	7.512	7.671	6.963	6.797
3.180	4.315	4.024	3.889	3.601	2.110
2.956	2.454	2.919	2.564	2.717	3.288
3.683	3.867	4.196	3.617	3.443	2.627
4.514	4.967	5.021	5.014	5.192	6.118
5.196	5.767	5.555	5.293	5.505	5.099
6.106	5.699	5.705	5.984	5.945	5.379
6.271	6.389	6.462	6.281	5.874	2.871
3.080	3.007	2.964	3.436	3.840	4.750
2.607	2.342	2.363	2.566	2.442	3.678
6.967	8.035	7.877	7.023	7.235	9.190
2.777	3.225	3.635	2.607	3.042	3.230
5.617	5.648	5.977	6.052	5.788	4.708
5.469	5.606	5.499	5.800	5.838	5.570
2.905	2.842	3.527	2.625	3.072	1.880
4.190	3.564	3.685	4.281	3.925	3.866
4.358	4.080	4.348	4.938	4.316	4.762
3.212	2.579	3.425	3.359	3.161	1.971
5.953	6.507	6.747	6.237	6.120	1.517
5.005	6.204	5.942	5.554	5.469	7.457
5.221	6.504	6.237	5.860	5.765	7.784
4.990	6.397	6.070	5.565	5.631	7.653
5.103	6.504	6.198	5.670	5.731	7.759

5.184	6.512	6.233	5.572	5.752	7.772
5.184	6.514	6.201	5.572	5.764	7.774
5.248	6.526	6.267	5.903	5.803	7.794
4.861	5.797	5.556	5.235	5.081	7.000
4.992	5.953	5.686	5.256	5.178	7.082
2.409	2.268	2.943	2.149	3.217	2.361
3.416	3.416	3.602	3.725	3.777	4.208
2.439	2.247	2.282	2.670	1.947	2.680
3.337	4.043	4.240	4.177	4.092	2.319
3.364	3.660	3.239	4.047	3.899	4.674
5.068	3.090	3.847	2.596	3.852	2.513
3.261	2.517	3.055	2.476	2.112	2.359
6.061	6.741	6.594	6.496	6.714	5.846
2.617	3.679	3.299	3.249	3.008	4.562
5.258	5.429	5.392	5.583	5.044	4.804
2.850	3.435	3.141	2.958	2.786	1.548
2.090	2.498	1.627	2.319	1.856	3.628
4.857	4.963	5.267	4.246	4.675	3.761
2.325	3.946	3.558	3.591	4.316	3.443
2.928	2.951	2.278	2.976	2.692	3.851
3.207	2.984	3.419	3.524	3.347	5.510
7.243	7.487	7.313	7.293	7.368	6.436
5.620	6.580	6.120	6.076	6.352	5.415
6.538	6.420	5.421	6.357	6.416	5.366
3.547	3.534	3.657	3.549	3.810	2.409
5.368	5.533	5.690	5.451	5.762	4.473
2.971	3.069	2.214	2.294	1.758	2.202
7.713	8.233	7.960	7.885	7.974	8.916
6.178	6.756	6.478	6.291	6.563	7.323
6.713	7.273	6.852	6.670	6.927	7.955
6.553	6.870	6.661	6.447	6.789	7.324
6.411	6.963	6.671	6.472	6.915	7.739
6.545	6.904	6.636	6.474	6.791	7.281
4.624	4.203	4.662	4.526	4.597	4.224
4.474	4.914	4.890	5.216	4.892	5.398
5.553	4.744	5.070	4.874	4.261	3.455
2.075	2.551	2.544	2.186	2.510	3.045
1.133	1.475	1.370	1.002	1.340	1.368
5.961	6.085	6.390	6.005	6.184	5.120
2.842	2.938	3.436	3.014	3.439	3.521
3.818	3.457	3.427	4.194	3.747	3.239
2.576	2.903	2.445	3.249	2.766	3.066
2.072	1.417	2.090	1.415	1.599	3.010
1.998	3.007	2.431	2.177	2.500	2.545
2.799	3.021	3.423	2.684	3.125	2.519
5.256	5.082	5.074	5.665	5.192	6.307
2.810	2.715	3.807	3.537	2.587	4.712
6.019	5.837	5.853	5.896	5.546	5.598
2.606	1.707	1.993	2.242	2.243	3.250
3.883	3.219	3.200	3.608	3.216	3.972

1.870	1.902	2.008	1.643	2.020	1.792
3.774	4.052	4.649	3.913	4.032	2.348
4.422	4.342	4.848	4.780	4.942	5.172
2.918	2.003	1.983	2.050	1.934	1.686
4.217	4.483	3.735	4.019	4.125	3.066
2.503	2.541	2.472	3.497	2.918	4.033
5.324	4.676	5.320	4.896	4.987	4.210
3.751	3.751	4.152	4.102	3.891	3.331
3.840	4.009	3.945	4.302	4.121	5.014
5.813	5.451	6.014	5.324	5.609	4.931
3.008	2.545	2.590	2.074	2.725	2.672
4.565	4.578	4.272	4.324	4.664	4.538
7.038	7.209	6.971	6.876	7.298	5.379
7.750	7.853	7.803	7.587	7.802	6.413
9.183	8.882	9.078	9.054	8.966	5.989
4.661	3.736	3.814	3.886	4.350	3.582
2.675	3.572	3.211	4.069	4.252	5.535
5.484	5.173	5.613	5.408	5.456	6.324
3.972	3.465	3.916	3.737	3.411	2.712
5.479	5.288	4.497	5.294	5.174	5.454
4.702	3.781	4.702	4.702	4.596	3.870
4.876	4.637	4.951	4.161	4.606	1.104
5.218	5.289	5.287	5.261	5.078	4.885
5.399	5.279	5.695	5.608	5.574	5.315
6.969	6.233	6.604	7.203	6.758	5.775
5.304	5.737	5.658	5.116	5.713	4.905
6.090	5.891	5.623	6.167	6.098	3.197
3.031	3.466	3.611	3.836	3.168	4.464
5.698	5.569	5.666	5.586	5.505	5.069
7.398	7.335	7.281	7.408	7.361	5.902
6.473	6.469	6.460	6.320	6.149	5.190
5.979	5.767	5.857	5.661	5.903	4.802
3.026	2.496	2.816	2.026	2.737	1.833
6.054	5.631	5.707	6.239	5.664	5.313
7.106	6.691	7.060	6.731	6.822	6.052
3.919	3.781	3.858	3.505	3.590	3.058
4.085	4.013	4.243	3.487	4.180	3.412
5.775	5.690	5.390	5.402	5.890	3.034
8.128	7.993	8.161	8.037	7.722	7.162
5.392	5.654	5.369	5.412	5.838	5.232
7.435	7.258	7.175	7.287	7.056	5.568
7.411	7.494	7.408	7.389	7.489	5.393
7.290	7.184	7.327	6.917	7.035	4.892
4.878	4.786	4.314	4.761	4.722	3.208
8.011	8.288	8.137	8.216	8.282	6.431
7.530	7.753	7.652	7.287	7.660	4.998
9.807	9.590	10.137	9.556	9.984	6.707
5.395	5.164	5.788	5.649	6.001	5.512
2.116	2.755	2.864	2.352	2.365	1.686
5.633	5.582	5.518	5.844	5.440	2.246

8.131	8.266	8.203	8.293	8.164	7.265
8.830	8.846	8.958	8.697	8.853	7.509
5.856	6.184	6.006	5.549	5.535	4.737
8.586	8.597	8.529	8.674	8.570	7.532
7.743	7.930	7.877	8.247	7.962	4.692
4.439	4.388	4.920	4.501	4.955	5.804
6.915	7.342	7.216	6.426	6.925	7.465
6.422	6.502	6.564	6.159	6.267	9.350
5.369	4.992	4.932	5.283	4.648	6.703
3.809	3.994	3.853	3.761	3.616	2.545
3.368	3.820	3.916	3.725	3.408	3.367
4.937	5.040	5.282	5.117	5.133	4.305
5.921	6.190	6.084	5.836	5.830	4.867
4.919	4.945	4.830	5.032	4.729	4.018
8.314	8.317	8.026	8.171	8.300	6.438
7.583	7.490	7.462	7.122	7.287	4.423
10.768	10.008	10.643	10.734	10.343	7.429
8.764	8.140	8.576	8.704	8.661	6.885
7.208	7.310	7.413	7.546	7.410	6.277
7.627	8.068	7.658	7.958	7.929	7.537
8.409	8.425	8.396	8.173	8.315	7.069
5.453	6.067	5.744	5.701	5.718	5.527
6.865	6.751	6.990	7.134	7.172	4.545
6.584	6.532	6.763	6.379	6.652	4.235
7.547	7.658	7.711	7.671	7.849	4.830
7.991	7.965	8.030	7.913	7.938	3.715
9.369	9.482	9.320	9.321	9.314	8.540
4.335	4.323	4.325	4.353	4.445	2.704
7.861	7.730	7.742	8.122	7.910	7.446
7.909	7.591	7.861	7.674	7.780	6.210
6.955	7.215	7.176	7.093	7.060	6.326
9.560	9.710	9.693	9.714	9.784	7.234
2.784	2.851	2.811	2.565	2.162	3.007
1.820	2.032	2.091	2.096	2.591	2.580
5.422	5.730	5.647	6.034	6.503	8.096
6.773	6.713	6.709	7.101	6.631	6.689
3.517	3.766	3.645	4.620	4.082	6.020
5.122	4.814	5.063	4.878	5.155	3.424
2.199	2.468	2.639	2.460	2.345	3.248
4.024	4.065	4.172	3.931	3.781	5.023
5.281	5.639	5.558	6.009	6.691	8.393
5.005	4.937	5.160	6.110	4.859	2.969
2.827	2.609	2.890	3.156	2.934	1.914
4.288	3.695	4.087	3.836	3.945	3.572
3.380	2.226	3.936	3.706	3.272	3.289
7.754	8.244	8.083	7.932	8.121	7.368
6.859	6.765	6.671	6.746	6.901	5.224
2.389	1.588	1.844	1.868	1.695	1.821
2.349	2.911	2.179	3.130	2.573	3.138
2.977	2.495	2.621	2.006	2.897	1.974

1.993	2.048	1.727	2.230	1.846	2.372
6.052	5.147	5.704	5.984	6.149	3.859
6.733	5.672	6.482	6.370	6.204	6.882
3.176	2.869	2.367	2.216	2.387	2.715
3.940	3.665	3.485	4.835	3.666	4.226
4.292	3.756	4.142	4.060	4.186	4.499
6.846	7.094	6.837	7.178	7.065	7.905
3.129	3.791	3.149	2.548	2.432	3.431
7.647	7.662	7.091	7.012	7.310	2.308
3.221	3.766	3.589	2.576	3.344	3.730
5.917	5.955	5.975	5.223	5.572	3.004
5.207	5.317	5.096	5.519	5.598	6.178
3.151	2.781	3.443	3.329	3.322	3.436
2.289	2.554	2.500	2.489	2.998	1.964
7.145	6.768	7.080	6.832	6.721	5.666
3.228	3.407	3.209	3.501	3.290	4.454
4.430	4.939	4.298	2.918	4.241	2.791
4.521	5.426	4.788	5.028	5.109	5.514
2.707	3.740	3.455	3.129	3.214	2.085
5.965	6.240	6.254	6.560	6.388	5.495
4.105	4.596	4.484	3.751	4.260	4.048
4.506	3.875	4.478	4.543	3.775	5.021
2.977	3.390	3.738	2.456	3.470	1.990
6.415	6.437	6.530	6.388	6.577	5.571
2.218	1.966	2.353	2.254	2.312	1.827
2.515	3.172	3.801	3.862	4.106	4.421
5.917	6.042	6.119	6.075	6.039	5.515
2.532	2.810	2.026	2.488	2.503	2.666
2.081	2.363	2.469	2.809	2.403	3.882
5.790	5.867	6.166	6.291	6.448	7.088
6.700	5.031	5.631	5.880	5.678	5.742
4.616	3.312	2.778	4.602	4.047	3.871
5.005	3.931	3.556	4.349	4.288	5.679
6.830	5.185	5.534	6.083	5.636	6.190
4.278	4.365	4.380	4.205	4.420	4.197
3.459	3.931	3.758	3.100	3.617	1.630
5.506	5.723	5.702	5.423	5.667	5.019
3.656	2.714	4.106	3.157	3.204	3.134
3.940	3.940	3.680	3.753	3.374	4.328
3.113	2.650	3.194	3.145	3.348	3.760
1.799	1.799	2.218	2.417	2.121	3.322
3.258	2.356	3.760	2.626	3.513	3.909
3.582	3.017	3.268	3.821	3.789	3.637
2.769	2.309	3.310	2.876	2.673	2.797
5.656	5.718	5.797	4.947	5.305	2.698
2.483	2.594	2.718	3.165	2.485	2.910
4.434	5.061	4.503	4.478	4.723	3.446
2.156	2.012	2.039	2.476	2.019	1.806
5.592	5.654	5.807	5.534	4.845	3.365
4.611	4.214	5.053	4.842	4.042	4.078

3.051	3.090	3.061	3.504	2.845	3.185
4.094	3.345	3.592	3.704	3.315	3.350
3.556	3.310	3.573	2.880	3.185	2.465
1.401	1.456	0.975	1.326	1.145	1.789
3.190	3.115	3.178	3.250	3.336	2.451
2.929	2.726	2.454	3.067	2.785	3.190
8.743	8.336	8.494	8.381	8.355	7.762
1.282	1.767	1.827	1.882	1.564	1.412
3.860	4.393	3.635	4.230	3.951	2.935
3.026	2.855	2.790	2.328	2.680	1.551
3.779	3.700	3.619	3.339	3.465	2.256
3.445	4.375	4.004	3.598	3.625	3.289
3.892	4.664	4.432	4.622	4.908	4.788
4.526	4.324	4.445	4.345	4.810	3.975
3.198	3.310	3.322	3.025	3.089	3.801
4.007	4.294	3.924	4.043	4.040	5.197
1.520	1.578	1.433	1.555	1.080	2.127
6.339	6.497	6.279	6.440	6.601	6.117
4.138	4.013	4.866	4.266	4.166	3.949
3.534	2.944	3.212	3.011	3.612	4.431
5.174	5.004	5.304	5.508	5.201	2.219
4.778	4.492	4.729	4.560	4.949	3.136
3.771	4.033	2.895	2.699	3.752	2.237
8.324	6.481	7.258	5.002	8.333	6.828
2.320	2.057	1.971	2.202	2.179	2.064
2.623	2.883	2.094	2.469	2.765	3.056
1.918	2.320	2.165	1.470	1.409	1.704
2.733	2.416	2.368	2.867	2.658	2.994
3.364	4.201	3.790	3.756	3.747	3.276
4.632	5.282	4.963	4.717	4.662	3.714
3.508	2.869	3.976	3.983	2.532	4.011
2.970	3.085	3.615	3.149	3.485	2.471
1.494	1.385	2.218	1.802	2.069	2.915
4.019	4.220	4.777	3.986	4.613	4.964
4.078	4.999	4.800	3.765	4.460	3.347
6.187	6.173	6.287	6.319	6.384	5.754
3.841	4.907	4.437	4.232	4.672	3.793
3.093	3.269	3.393	3.648	4.236	5.251
3.586	3.939	3.872	3.484	3.303	2.804
3.926	3.872	4.307	4.186	4.062	3.279
6.621	6.130	6.097	6.424	6.291	6.700
2.420	2.559	2.810	2.655	2.675	1.703
4.483	4.693	4.743	5.247	4.570	4.282
7.141	7.507	7.573	7.743	7.438	7.781
3.178	3.585	3.228	2.774	3.548	3.808
2.620	2.442	2.870	2.748	2.244	2.188
5.126	4.611	4.355	4.375	5.358	3.782
6.343	6.416	6.238	6.073	6.118	5.827
4.952	3.882	4.091	4.469	4.214	2.397
3.621	3.579	3.584	3.584	3.833	2.606

3.594	4.195	3.906	4.244	4.403	5.512
5.142	6.073	5.634	4.646	5.217	5.478
4.929	4.889	4.630	4.337	4.525	5.248
6.194	5.374	6.103	4.856	5.210	4.144
6.895	6.516	6.711	6.952	6.804	7.372
4.028	2.944	2.770	3.914	3.774	2.059
3.518	2.713	1.928	3.734	4.001	2.586
3.639	4.217	5.140	3.802	3.849	4.738
8.486	8.371	8.797	8.324	8.572	7.280
2.658	1.898	2.875	1.469	1.589	0.971
4.772	5.008	5.492	6.040	6.233	7.749
2.362	3.286	2.831	3.001	2.821	3.541
4.384	4.205	4.596	4.803	3.321	2.308
3.120	3.646	3.583	3.542	3.825	2.608
3.303	3.973	3.891	2.926	3.627	3.721
2.746	3.365	3.551	2.377	3.162	2.948
1.780	2.221	2.500	1.742	1.904	2.890
2.271	2.830	2.822	2.346	2.605	3.228
5.596	6.413	5.760	6.485	5.531	5.415
4.671	4.031	4.581	3.415	4.261	5.444
2.859	4.476	3.800	4.583	4.689	4.861
3.193	3.423	3.358	2.813	2.803	3.546
3.874	3.059	3.520	3.027	3.271	4.204
3.848	4.434	4.669	3.312	3.992	7.253
2.907	1.198	2.274	2.352	3.009	3.886
1.316	1.458	1.304	1.161	0.770	1.125
2.247	2.116	2.693	2.664	3.158	2.247
5.476	5.502	5.744	5.283	5.197	6.159
3.692	3.879	3.196	3.514	3.369	4.399
5.450	5.472	5.530	5.211	5.272	5.712
7.594	7.826	8.117	6.746	7.473	8.528
1.358	2.726	1.219	1.327	1.436	1.839
4.682	5.386	4.929	4.651	4.265	6.334
2.957	2.873	3.110	2.866	2.749	3.627
3.327	3.730	3.824	3.736	3.670	3.936
6.108	6.296	6.311	6.123	6.091	5.602
2.890	2.606	3.037	1.563	2.745	3.886
3.617	4.144	4.506	2.715	3.718	4.638
3.096	3.713	3.272	3.451	3.789	4.343
5.625	5.475	4.904	4.406	5.349	4.452
4.500	4.198	4.305	4.036	4.657	5.500
8.076	6.849	7.861	7.673	7.708	9.673
3.705	2.759	3.512	3.297	3.490	3.716
4.745	5.447	5.170	5.092	5.434	4.709
3.507	3.637	3.615	3.353	4.222	4.596
6.242	6.665	6.444	6.512	6.552	5.223
3.990	3.715	3.759	4.032	4.424	5.350
3.263	3.159	3.709	3.746	3.650	4.426
5.670	5.300	5.469	5.263	6.083	6.291
3.765	3.844	3.764	3.946	3.652	3.216

5.658	6.667	6.570	5.693	6.513	4.709
2.686	2.721	3.313	3.833	3.479	3.101
3.172	2.805	3.810	2.614	2.944	2.915
3.389	3.073	2.849	3.500	3.590	3.476
7.827	7.911	8.060	7.969	7.784	6.484
4.712	4.145	4.449	4.311	4.447	1.949
8.042	8.417	8.287	7.700	8.167	5.752
6.420	6.522	6.297	6.325	6.227	4.183
3.223	4.249	4.003	4.703	4.333	5.227
6.529	5.928	5.999	6.466	6.208	7.504
6.034	6.159	6.109	5.756	5.876	4.167
3.024	2.914	2.524	2.990	3.089	2.921
3.413	4.921	3.791	3.717	3.543	1.952
4.998	4.283	4.958	4.744	4.953	3.480
7.406	6.501	6.058	6.508	7.093	6.287
4.954	5.303	5.256	5.330	5.317	3.779
7.851	7.818	8.043	7.639	7.767	6.289
6.737	6.010	6.748	6.308	6.296	2.145
5.147	5.317	5.334	4.903	4.804	4.312
9.234	9.417	9.330	9.332	9.217	7.340
4.160	4.552	4.461	4.931	4.968	6.621
4.964	4.770	4.693	4.123	4.593	4.009
5.575	5.550	5.811	5.523	5.309	4.473
3.724	3.136	4.071	3.261	3.197	3.787
7.114	7.044	6.979	7.069	7.114	6.057
7.191	6.981	6.994	7.085	7.045	4.764
5.496	5.646	6.098	5.476	6.024	4.509
4.518	4.513	4.785	4.861	5.150	5.212
5.013	5.207	5.538	4.999	5.313	4.707
5.015	4.143	4.269	5.098	4.480	3.270
5.925	6.479	6.255	6.227	6.243	5.169
8.017	8.358	8.546	8.219	8.274	6.956
4.441	3.730	3.829	3.602	3.747	4.134
3.491	4.241	4.340	3.848	4.101	5.381
5.623	5.725	5.977	5.679	5.634	5.131
2.109	2.444	2.348	2.093	2.144	2.411
2.995	2.442	2.496	2.786	2.688	2.490
5.888	6.331	6.273	5.681	6.006	4.473
2.231	2.763	3.579	3.355	3.135	2.009
2.826	3.534	2.274	3.351	3.361	4.195
7.364	7.505	7.448	7.504	7.494	6.394
4.819	4.315	4.189	4.685	3.894	3.447
5.167	6.045	5.600	5.412	5.691	4.417
4.386	4.308	4.236	4.664	4.851	5.579
3.177	3.886	2.935	3.664	3.677	3.940
6.516	7.277	6.859	6.868	7.119	5.345
6.854	6.845	6.813	6.554	6.675	4.939
5.926	6.101	6.004	4.983	5.160	3.346
3.828	4.169	3.957	2.912	3.767	4.614
5.050	4.418	4.362	4.020	4.396	3.973

6.745	5.847	5.981	5.668	6.120	5.601
3.563	3.463	3.821	3.135	3.606	4.669
3.401	3.209	3.463	3.851	3.807	5.114
4.596	4.302	4.220	4.279	4.166	3.157
3.607	3.142	3.790	3.487	3.453	4.979
8.534	8.244	8.473	8.539	8.541	7.424
5.749	5.551	6.083	5.439	5.445	3.851
8.022	8.227	8.309	8.088	8.210	5.850
6.923	6.560	6.877	6.498	6.624	4.833
4.121	4.649	4.541	4.269	4.337	2.907
6.815	5.405	5.512	5.419	5.826	3.494
6.795	6.860	7.009	7.162	7.008	5.508
4.636	4.719	4.747	4.750	4.650	5.361
4.866	5.095	5.224	4.942	5.344	4.567
4.702	5.074	4.674	4.789	4.619	3.709
4.460	4.061	3.929	4.307	4.043	4.051
3.676	3.732	4.770	4.339	4.189	3.928
5.593	5.967	5.882	5.422	5.800	5.061
6.198	5.991	6.280	6.162	6.184	4.643
5.784	5.707	6.009	5.778	5.569	4.842
6.290	5.942	6.067	6.265	6.043	7.057
3.699	3.707	3.445	3.387	3.468	3.103
4.009	4.165	4.375	4.405	4.290	3.860
5.094	5.289	5.101	5.298	5.311	6.241
6.692	7.112	6.792	7.111	6.918	6.973
5.155	5.651	6.019	5.892	6.708	6.880
2.836	3.226	2.509	3.332	2.841	1.853
3.551	3.526	3.265	3.455	3.590	5.026
8.051	8.093	8.122	8.187	8.036	6.911
3.930	1.714	3.262	2.947	2.765	2.791
3.808	3.502	3.100	3.417	3.133	3.122
5.716	5.358	5.406	5.634	5.683	5.166
3.082	3.435	3.483	2.991	2.780	3.072
3.876	3.441	3.813	3.774	3.405	3.197
5.705	4.604	5.082	4.582	4.261	3.501
2.628	3.345	3.464	3.380	2.452	3.102
3.466	3.392	3.275	3.291	3.359	2.963
5.050	5.493	5.493	5.687	5.708	5.966
4.417	4.444	3.717	4.304	4.259	4.940
2.315	2.205	2.027	1.701	2.239	2.761
4.194	2.761	3.672	2.373	2.956	2.321
1.537	1.856	1.993	2.127	1.975	2.255
4.784	4.981	5.380	5.116	5.263	3.774
4.823	5.365	4.648	4.267	4.874	5.008
4.622	4.517	5.054	4.376	4.968	3.932
3.429	3.179	4.134	4.390	4.095	4.153
3.291	3.665	3.833	4.136	4.316	5.198
4.443	3.894	4.317	4.637	4.589	3.084
4.710	4.652	4.574	4.670	4.418	3.155
1.609	1.812	1.414	1.412	1.394	2.348

4.083	5.083	4.509	4.372	4.715	4.200
2.823	3.376	2.988	2.962	3.159	2.897
5.672	5.486	5.580	5.909	5.772	6.552
7.587	7.799	7.732	7.691	7.622	5.778
7.696	7.549	7.370	7.442	7.488	5.064
4.604	4.779	4.713	5.305	5.250	6.462
1.934	1.924	2.168	2.278	1.855	3.165
2.009	2.438	2.793	1.845	1.651	2.629
1.907	2.394	2.275	1.825	2.437	1.722
6.666	7.201	6.677	7.255	6.892	5.504
5.327	5.864	5.619	5.909	5.693	4.334
7.487	9.754	8.887	6.614	6.891	6.563
2.363	3.253	3.196	2.624	2.008	4.305
6.343	6.488	6.492	6.271	6.521	5.961
2.448	2.407	2.921	1.960	2.613	1.550
5.832	5.732	5.979	6.106	6.037	6.670
7.882	7.652	7.855	7.863	7.872	6.562
4.210	4.188	4.966	4.735	4.472	4.547
6.784	7.086	7.015	6.918	6.895	6.257
5.994	5.736	6.114	6.430	6.234	4.723
7.004	7.009	6.995	7.127	6.731	4.232
1.792	1.467	1.540	1.799	1.691	2.545
3.734	4.429	3.857	3.830	3.927	3.076
4.325	4.113	4.336	4.345	4.367	4.786
4.207	4.164	4.041	4.065	3.863	2.991
7.192	6.706	6.587	6.882	6.485	3.549
4.484	4.198	4.659	4.013	4.496	1.786
3.362	3.853	4.477	4.266	4.271	3.950
5.408	4.878	5.346	5.363	4.726	2.412
3.894	4.700	4.508	3.766	4.464	3.288
2.309	1.616	2.805	1.995	1.855	1.853
2.599	2.732	2.539	2.477	3.139	1.959
2.922	3.731	3.829	3.382	4.045	3.273
2.977	3.449	3.030	2.891	3.300	3.077
2.559	2.881	3.651	3.522	3.195	2.750
5.357	5.298	5.126	5.544	5.438	4.051
6.266	5.847	5.948	5.852	5.648	3.693
3.863	3.860	4.117	3.925	3.740	3.058
3.021	3.195	2.924	3.087	2.264	3.993
7.289	7.330	7.208	7.071	7.120	6.312
5.850	4.966	5.068	5.419	5.481	6.534
4.572	5.881	5.556	4.505	5.568	5.936
2.926	3.380	2.868	2.977	2.635	4.260
4.436	4.338	4.639	4.891	4.468	2.308
5.193	5.552	5.344	5.444	5.740	6.789
4.624	5.245	4.891	4.623	4.786	4.083
4.362	4.618	4.222	4.105	4.362	4.566
3.592	3.169	3.535	3.271	3.356	2.418
3.086	3.423	4.012	3.201	3.413	3.787
4.763	4.723	4.983	4.309	4.669	3.713

3.424	3.953	4.141	3.538	3.296	5.292
3.354	3.274	3.790	3.651	3.885	4.290
3.519	4.528	3.628	4.255	4.578	2.673
7.867	8.389	7.995	8.415	8.698	8.932
7.012	7.103	7.018	7.077	7.328	6.471
4.937	4.832	4.681	4.639	4.738	3.837
2.771	2.539	2.347	1.679	3.166	2.503
6.862	6.227	6.396	6.661	6.310	4.838
3.412	4.075	3.723	3.431	3.183	2.502
6.216	6.019	6.105	6.180	6.170	4.212
5.262	5.564	5.260	5.298	5.491	4.063
2.459	3.478	2.799	2.716	2.312	2.075
7.403	7.101	7.100	7.481	7.237	5.738
4.296	3.784	4.463	4.912	4.933	5.657
1.968	2.398	3.724	3.934	4.175	4.963
3.507	3.318	2.840	3.337	3.337	3.082
4.103	4.537	4.454	4.885	4.678	5.816
7.731	7.917	7.971	7.755	8.102	7.321
7.823	6.810	7.405	6.902	7.204	6.148
3.672	4.150	4.085	4.234	4.414	5.784
2.670	2.436	2.577	3.055	2.417	2.855
4.008	2.874	2.881	3.555	3.422	2.408
2.736	2.444	2.892	2.299	2.578	2.388
3.084	2.894	3.092	2.921	3.075	2.737
3.137	3.127	2.693	2.695	1.794	3.258
7.066	7.600	7.208	7.439	7.778	9.000
3.319	3.313	2.378	3.482	2.392	3.637
2.908	2.704	2.444	2.487	3.164	2.259
3.925	3.955	4.217	3.454	3.514	3.280
3.227	2.603	2.730	3.125	3.185	3.452
7.141	7.607	7.369	7.311	7.691	6.044
2.874	1.940	2.815	2.220	2.083	2.247
1.553	2.122	2.380	1.236	2.184	2.519
4.464	4.200	3.924	3.407	3.734	5.555
1.854	1.673	1.700	1.376	1.730	3.609
3.629	4.515	4.008	4.433	3.824	3.414
6.120	5.978	5.680	5.331	5.890	6.023
6.246	5.970	5.692	5.379	5.856	6.016
6.143	5.932	5.635	5.354	5.883	6.037
4.973	4.816	4.876	4.576	4.883	5.618
4.961	4.816	4.803	4.603	4.838	5.611
5.095	5.278	4.860	4.876	5.149	3.250
2.594	1.756	1.810	1.552	1.889	1.594
8.078	8.458	8.027	7.974	8.075	8.981
2.514	2.635	1.727	1.352	3.083	2.561
4.554	5.229	4.808	5.444	5.406	6.242
4.474	4.637	4.311	4.516	4.903	5.742
4.667	5.208	4.970	5.023	4.812	5.683
3.351	3.553	4.098	3.581	3.816	4.327
4.074	3.980	3.768	4.210	4.624	4.666

7.523	7.443	7.484	7.452	7.602	6.890
9.726	9.577	9.650	9.789	9.790	8.428
7.926	7.999	7.825	7.942	7.846	6.003
6.975	6.392	7.025	6.700	6.967	5.264
7.193	6.851	6.814	6.832	6.772	5.925
3.164	3.016	3.520	3.229	3.789	4.295
3.617	3.871	2.688	2.767	3.935	1.920
5.252	4.987	5.402	5.215	5.212	3.354
7.841	8.134	7.942	7.849	7.619	5.399
7.433	7.542	7.523	7.271	7.470	4.880
2.850	3.193	2.630	3.292	3.001	2.550
6.623	7.255	6.911	6.629	6.821	6.156
4.282	4.065	4.285	3.476	3.717	4.700
3.944	4.115	4.635	3.743	4.578	5.059
4.289	4.165	4.878	4.938	5.606	7.738
3.340	2.827	3.610	2.668	3.323	3.595
4.347	3.862	4.129	4.565	4.161	4.829
8.168	8.925	7.968	9.138	9.530	10.352
6.344	7.105	6.718	6.475	6.466	6.224
3.174	2.888	1.662	2.352	2.672	4.746
3.777	3.281	3.679	3.430	2.218	2.008
5.491	5.124	5.642	5.475	5.747	3.981
3.692	4.146	4.194	3.664	3.644	4.306
9.132	8.847	9.042	9.032	8.897	6.632
3.823	4.177	4.013	4.060	4.476	5.196
5.232	5.082	4.448	4.834	5.412	3.838
5.573	4.715	4.096	4.300	4.134	5.180
7.645	8.001	7.863	7.288	7.465	7.809
6.041	5.739	5.631	6.078	6.044	4.044
2.587	4.430	3.763	3.599	4.424	5.224
8.013	9.009	8.450	8.977	9.460	10.358
8.060	7.642	7.985	7.535	7.283	7.337
7.923	7.374	7.882	7.473	7.264	7.203
6.141	5.919	6.337	5.941	5.988	4.952
6.651	7.194	6.755	6.610	6.734	5.121
7.492	6.298	6.841	7.254	7.178	7.022
6.308	6.546	6.579	6.632	7.220	8.584
7.374	6.770	6.915	6.802	6.441	4.628
6.243	6.049	6.101	6.079	6.174	3.672
5.151	5.061	4.987	4.823	4.851	3.261
3.828	3.212	3.918	3.026	3.347	1.780
7.931	8.218	8.133	7.778	7.921	6.773
8.483	8.850	8.589	8.637	8.492	7.653
4.965	4.996	4.979	5.142	4.763	4.231
6.082	6.664	6.532	6.642	6.977	8.310
7.966	7.597	7.862	7.995	7.980	5.915
4.518	5.002	4.957	4.987	4.400	3.232
4.412	5.166	4.807	5.071	4.903	3.945
3.825	3.570	4.158	3.296	3.690	2.531
7.618	7.588	7.603	7.446	7.537	6.784

6.646	6.548	6.679	6.637	6.492	6.073
2.722	3.140	2.372	2.225	2.398	2.453
4.533	5.001	4.788	5.069	4.762	3.301
2.718	2.787	2.573	2.061	2.719	3.918
1.692	1.883	1.890	1.542	2.209	2.237
4.084	3.666	3.057	4.239	4.204	5.386
4.607	4.252	3.527	3.600	3.714	4.586
3.184	4.123	3.401	3.515	3.423	1.823
2.996	3.035	3.280	2.834	2.992	2.994
4.908	3.395	3.775	3.526	4.188	3.536
3.593	3.302	4.050	3.633	3.759	2.031
3.052	3.684	2.908	2.786	3.732	4.624
6.036	5.434	5.219	4.972	5.028	3.614
2.532	2.498	2.479	2.356	2.492	2.328
2.596	2.970	2.975	3.036	2.774	2.927
3.384	2.463	2.482	2.023	2.348	1.405
4.736	4.641	4.122	3.373	4.115	3.835
4.334	4.447	3.917	3.916	4.077	3.409
1.799	2.267	2.481	1.570	2.102	3.025
7.710	8.011	8.103	7.840	8.076	7.585
3.946	4.395	4.062	4.172	4.658	6.601
6.457	6.194	5.831	5.996	6.059	3.425
4.061	4.285	3.862	3.638	3.688	3.919
3.216	2.273	2.894	2.582	2.883	2.617
1.745	1.347	1.297	1.487	1.426	1.935
1.863	2.922	2.129	2.388	2.047	3.359
2.829	2.406	2.647	2.500	2.920	3.212
3.697	3.793	3.559	3.291	3.849	4.373
3.359	3.760	3.656	3.503	4.063	4.259
5.622	6.114	6.345	4.912	5.652	5.072
6.284	6.108	6.048	5.426	5.593	5.575
4.485	3.629	4.186	3.572	3.743	3.852
4.406	5.172	5.325	4.671	4.521	4.569
3.470	2.886	3.218	3.116	2.827	3.903
5.450	5.764	5.370	5.500	5.558	5.361
6.256	6.848	6.750	6.618	6.692	6.022
3.639	4.228	3.802	3.242	3.902	4.820
5.797	6.158	5.877	5.725	6.118	5.309
3.943	4.550	4.131	4.367	4.289	3.223
5.562	5.995	5.971	5.543	5.657	5.134
2.952	4.121	3.938	3.779	3.926	2.677
5.505	5.774	5.351	5.524	5.752	4.593
6.137	6.714	6.262	6.111	6.476	7.295
5.714	5.758	5.705	5.901	6.086	5.598
6.182	6.581	6.204	6.063	6.083	5.305
5.324	5.557	5.381	5.357	5.498	5.145
2.067	1.948	2.568	2.105	1.820	2.040
6.804	6.458	7.149	7.278	6.830	5.174
5.213	5.309	6.705	5.556	5.222	6.649
1.502	1.079	1.045	1.109	1.764	3.496

4.132	2.956	3.056	3.490	3.591	7.148
4.061	4.242	4.469	4.855	4.893	5.856
6.224	6.746	6.847	6.784	6.748	7.390
6.788	7.309	6.899	7.141	7.251	5.072
4.399	3.711	3.824	4.259	4.312	4.944
5.890	6.442	6.280	6.282	5.996	5.450
4.467	4.118	4.554	5.090	4.638	3.956
4.264	4.453	4.066	4.489	4.970	4.571
2.595	1.967	2.454	2.337	2.721	2.618
4.598	4.535	4.331	4.642	4.644	5.258
6.374	6.443	6.437	6.339	6.960	8.104
6.629	6.649	7.131	6.558	7.105	3.407
6.349	6.022	6.299	6.144	6.035	4.130
3.643	4.260	4.623	4.471	4.698	3.197
5.878	5.528	6.227	5.372	5.534	4.268
4.381	5.079	5.178	5.088	4.963	3.952
2.539	2.385	2.432	3.113	2.091	1.791
4.790	5.059	5.349	5.490	5.739	6.724
2.565	2.587	2.115	2.364	3.422	3.029
5.824	6.156	6.060	6.256	6.312	7.348
3.262	2.772	3.240	3.280	3.279	4.219
3.777	4.013	4.312	4.201	4.097	5.722
6.639	6.710	6.584	6.626	7.041	6.254
3.805	4.501	4.897	4.293	4.760	5.164
2.692	2.066	2.474	1.551	2.404	1.738
2.405	2.198	1.942	2.405	2.765	1.902
2.483	2.848	2.169	1.953	3.135	5.391
3.134	2.471	1.868	2.873	2.068	2.239
5.502	3.901	4.259	4.216	4.723	4.512
3.714	2.999	3.196	2.898	3.529	1.851
3.140	3.144	3.319	3.556	3.325	4.629
4.647	4.315	4.691	5.023	5.079	4.925
3.822	3.799	3.872	3.989	3.667	4.614
3.766	3.896	4.251	3.207	3.928	2.755
2.512	2.077	2.482	2.331	2.798	2.892
3.050	3.381	3.018	3.393	4.135	4.541
5.432	5.713	5.428	5.097	5.321	4.812
7.970	7.826	7.843	7.406	7.802	3.124
5.769	5.084	5.628	5.152	5.223	5.016
1.765	2.440	1.902	2.909	2.892	4.914
5.055	7.033	6.436	6.030	6.677	5.346
5.701	4.524	5.128	5.168	5.180	4.704
4.258	4.692	3.828	4.236	4.546	3.249
4.501	3.997	4.107	4.110	4.095	4.887
4.264	4.454	4.853	4.338	5.463	6.203
5.700	5.476	4.817	5.057	5.611	5.235
5.643	4.167	4.902	5.231	4.507	3.155
5.527	5.884	5.843	5.575	5.737	4.366
2.016	1.705	1.645	1.887	1.838	5.212
2.347	3.373	2.798	2.516	3.095	2.213

4.903	5.191	5.394	5.142	4.889	4.510
4.988	5.034	5.314	5.067	5.418	4.321
7.007	6.867	6.598	6.911	6.796	7.104
6.777	6.204	6.485	6.264	6.316	4.911
4.686	4.896	4.977	4.755	4.938	5.880
5.996	5.892	5.600	5.416	5.910	5.305
7.080	6.312	6.735	6.799	6.910	8.593
2.475	2.928	2.795	2.685	3.665	2.236
5.369	4.933	5.146	5.437	5.634	6.952
3.681	3.274	3.202	2.951	3.346	4.034
5.490	6.261	5.929	5.963	5.861	7.201
6.741	7.280	7.177	6.705	7.059	2.120
5.017	5.055	4.607	5.094	5.166	5.955
2.233	2.632	2.233	1.918	2.374	2.006
3.097	2.573	2.970	2.469	2.913	4.109
6.918	7.228	7.058	6.123	6.368	8.311
3.440	3.300	3.733	3.299	3.222	5.041
3.774	3.417	3.323	3.240	2.876	4.835
3.318	3.908	2.840	3.240	3.286	2.827
2.568	2.526	2.311	2.135	1.800	3.192
7.024	6.568	6.818	7.070	6.541	5.025
7.116	6.518	6.977	7.043	6.771	5.428
3.788	4.879	3.725	3.514	4.698	5.125
4.624	4.502	4.820	4.778	4.388	4.100
3.584	2.543	3.651	3.800	3.609	3.611
3.886	3.571	3.869	4.175	3.730	3.230
6.411	5.924	6.371	6.158	6.452	5.103
5.258	4.572	4.400	4.330	5.241	4.302
5.681	6.191	5.699	5.876	5.939	5.423
7.902	7.776	8.135	7.898	7.974	5.678
5.206	5.472	5.496	5.581	5.342	4.384
2.999	2.684	2.541	2.927	1.544	2.982
3.740	3.579	3.233	4.177	3.475	3.704
5.678	5.681	5.578	5.809	5.862	5.409
2.469	2.249	3.186	2.400	1.639	1.930
3.323	3.106	3.290	3.506	3.808	4.810
3.146	2.338	2.043	2.884	1.951	2.302
2.817	2.701	2.770	3.095	3.783	2.420
3.346	3.001	3.266	3.714	3.242	4.034
1.877	1.841	1.591	1.898	1.851	3.767
1.996	1.552	1.399	1.981	1.748	2.415
6.986	6.547	6.428	7.187	6.792	7.742
2.035	2.039	1.994	1.749	1.871	2.205
4.266	3.397	4.162	2.754	3.080	1.276
5.654	5.826	5.777	4.903	5.392	5.982
4.019	2.335	3.191	3.927	2.627	1.443
2.644	2.936	2.673	3.026	2.579	3.202
2.680	2.532	2.703	2.376	2.198	2.331
4.267	3.702	3.638	3.260	3.883	2.396
2.612	2.072	2.729	2.646	3.277	2.126

2.750	2.437	3.051	2.421	3.358	4.256
3.624	3.916	2.996	3.105	3.093	3.510
3.141	3.024	3.302	3.805	2.869	1.666
3.262	2.917	3.458	2.977	2.631	2.760
2.553	2.493	1.768	2.817	2.273	4.379
3.693	4.539	4.082	4.183	3.703	2.506
2.758	2.513	2.716	2.835	2.128	1.941
3.756	3.164	2.399	3.982	3.457	4.552
4.018	2.522	3.279	1.637	2.932	0.910
3.363	2.491	2.428	2.617	2.837	1.542
2.077	2.662	1.963	2.602	3.150	1.780
4.966	5.231	4.743	4.588	4.675	3.827
2.612	2.547	3.040	3.049	3.150	4.136
3.888	3.919	4.867	3.588	3.794	2.612
2.773	1.765	2.328	3.046	2.709	4.548
3.178	2.901	2.074	1.918	2.513	1.476
4.006	2.970	3.319	4.026	3.558	2.814
2.846	3.098	2.856	2.695	2.408	3.221
3.399	3.353	3.131	3.847	3.021	4.566
2.889	1.887	2.708	3.171	2.527	2.275
0.818	1.922	1.530	1.889	1.873	2.875
3.568	4.764	3.504	3.951	3.877	2.475
5.010	4.357	4.530	4.115	4.549	3.462
2.532	2.231	3.214	2.903	2.482	3.017
2.370	2.280	3.271	2.388	2.684	3.012
2.102	1.315	2.682	1.923	2.270	2.075
3.945	3.550	4.302	3.444	3.927	4.369
4.956	4.140	4.347	5.195	4.487	3.122
3.054	1.563	2.290	3.425	2.202	1.575
5.402	5.848	5.828	5.720	5.534	3.777
3.331	3.237	2.933	2.457	2.440	1.903
5.188	5.739	5.786	5.372	5.969	5.022
2.492	2.918	2.428	2.451	2.711	3.284
4.091	3.962	4.267	3.632	3.753	2.663
3.666	3.496	3.806	3.422	3.307	2.701
3.470	3.027	3.573	3.043	3.664	2.103
2.483	2.370	2.399	2.608	1.885	3.411
3.756	4.166	3.507	4.270	4.260	5.182
3.426	3.753	3.617	3.832	3.162	3.052
2.814	3.494	2.184	2.286	2.849	2.296
1.757	2.431	2.359	2.994	2.124	1.842
3.496	3.145	3.644	3.550	3.429	4.756
2.350	2.952	2.605	2.293	2.895	2.454
4.277	3.886	4.756	4.267	4.922	5.302
3.443	2.536	3.073	3.073	2.848	2.629
2.906	2.539	2.217	3.591	3.032	2.596
3.225	3.388	2.751	3.106	2.903	3.907
4.254	3.696	4.069	4.379	4.382	4.596
6.048	5.298	4.282	5.267	5.197	5.207
4.660	5.149	4.746	4.910	4.824	4.197

7.098	7.095	7.026	6.996	7.075	6.340
5.746	6.063	6.036	5.563	5.949	6.595
4.206	4.294	4.059	4.417	4.009	2.742
4.309	4.904	4.801	5.126	5.817	7.929
5.057	4.788	4.644	4.814	4.626	3.737
3.438	3.510	3.809	2.918	3.721	3.853
5.015	5.090	4.967	5.234	5.239	5.768
4.926	5.477	5.074	4.067	4.274	4.545
4.382	3.934	3.611	4.487	3.974	3.578
3.936	3.871	3.694	3.912	3.886	3.684
3.751	3.316	3.446	3.740	3.782	3.971
5.535	6.095	5.836	5.758	5.801	6.004
4.946	5.658	5.542	4.497	4.982	6.069
2.056	2.406	2.199	1.915	2.183	3.017
1.834	1.585	2.355	2.095	1.935	2.330
5.402	5.529	5.814	5.225	5.188	6.055
7.052	5.980	7.266	6.450	6.804	4.395
3.556	4.252	4.245	3.617	4.026	1.997
5.580	5.549	5.520	4.786	5.066	1.567
2.954	2.997	2.808	2.767	3.725	3.875
4.361	4.754	4.285	5.112	5.079	6.003
7.401	7.950	7.788	7.770	7.610	7.067
5.116	4.780	4.693	4.388	4.397	2.181
6.396	6.750	6.577	6.652	6.630	5.728
5.432	5.687	5.376	5.423	5.680	5.006
7.633	7.513	7.406	7.195	7.364	6.095
2.521	4.080	4.144	2.923	3.261	5.836
1.522	1.624	1.771	2.369	1.771	1.945
1.658	2.752	2.294	2.202	1.613	1.973
2.788	3.709	3.293	2.973	3.378	3.311
9.344	9.314	9.101	8.794	9.730	6.341
5.646	5.567	5.852	4.758	5.245	6.215
2.333	3.611	3.140	2.564	2.199	2.901
4.460	3.995	4.234	3.539	3.590	3.554
1.713	1.616	1.457	1.083	1.189	2.159
3.546	2.521	2.234	2.290	2.974	2.269
6.226	5.080	5.244	5.627	5.223	6.043
1.600	3.342	1.641	1.817	2.452	2.648
2.920	3.118	2.795	2.928	2.485	3.235
2.199	0.794	1.612	2.094	1.664	2.422
1.827	1.418	1.389	1.579	1.866	1.992
4.926	4.862	5.142	5.242	5.027	4.332
4.906	5.184	5.039	4.341	5.155	4.253
2.066	1.958	2.028	2.334	2.084	2.685
3.178	3.158	3.011	2.196	2.092	2.685
6.886	7.064	7.243	6.536	6.787	7.279
3.589	3.831	3.669	3.390	3.913	3.792
6.589	6.272	6.426	6.365	6.362	7.195
4.024	4.533	4.077	3.137	3.378	4.094
6.174	7.177	7.260	5.744	6.044	7.186

7.692	6.511	7.159	7.506	7.490	8.288
8.368	8.067	8.156	8.148	8.304	6.990
6.110	5.963	5.696	5.359	6.081	5.066
5.999	4.934	5.443	5.461	5.539	4.014
4.659	4.223	4.501	4.736	4.279	5.236
3.068	3.219	3.893	3.656	3.508	3.643
2.841	2.236	3.548	3.071	3.131	2.208
5.327	5.550	5.637	5.519	5.978	7.714
2.981	4.501	3.798	4.617	4.554	3.162
2.530	2.576	3.934	2.876	2.430	3.188
3.596	3.759	4.203	3.474	3.432	2.731
3.469	3.555	2.993	4.072	3.711	4.920
3.369	3.635	3.812	2.491	3.472	3.337
6.797	6.361	6.588	6.323	6.716	4.891
4.578	5.333	4.635	4.439	4.558	4.921
3.747	1.608	4.407	3.961	2.363	1.120
0.946	0.287	0.548	1.296	1.323	5.132
6.341	5.873	6.131	6.089	6.022	4.216
4.899	4.560	5.127	5.567	5.775	6.615
5.904	4.599	3.995	3.846	4.623	3.703
2.865	2.828	3.573	3.156	2.988	1.983
5.306	5.050	5.096	5.055	5.275	6.220
3.405	3.785	3.376	3.505	3.458	2.838
3.482	3.565	4.012	3.016	3.442	4.417
1.773	1.753	1.713	1.843	1.599	1.892
4.542	4.134	4.723	3.482	3.836	4.733
4.091	4.210	3.595	4.289	3.804	3.286
3.389	3.303	2.807	3.869	3.429	3.573
5.789	5.768	5.882	6.331	6.743	8.614
5.479	4.425	5.151	5.117	5.329	3.935
4.151	3.548	3.732	3.601	4.040	3.924
4.443	4.726	5.115	5.319	5.481	4.194
2.377	2.802	2.446	2.797	2.332	1.495
3.702	3.370	3.460	3.084	4.191	3.776
6.873	6.511	6.561	6.693	6.900	5.200
6.918	7.157	7.103	6.557	6.816	6.113
2.697	2.842	3.052	2.025	2.396	7.720
8.204	7.620	7.728	7.714	7.610	6.880
2.984	2.929	2.739	3.285	3.818	3.924
1.258	1.915	2.049	1.844	1.602	2.042
2.627	2.266	2.833	2.558	2.502	2.078
3.546	3.363	3.212	2.927	3.202	4.134
2.704	2.257	3.333	2.817	2.522	2.144
4.764	4.847	4.792	4.698	4.422	5.668
5.563	5.465	5.668	5.233	5.687	4.574
6.510	6.436	6.732	6.130	6.871	5.725
3.267	2.998	3.562	3.410	3.767	5.100
1.821	2.802	3.232	2.805	2.572	1.883
2.724	2.515	2.267	2.824	2.101	3.126
6.838	6.439	6.819	6.577	6.538	4.914

6.582	6.597	6.357	6.504	6.519	5.565
2.535	2.578	2.525	2.611	2.256	2.272
3.173	3.909	3.129	3.113	3.657	2.168
4.501	4.210	4.458	4.211	4.414	5.185
5.328	5.833	6.007	5.724	5.757	4.866
3.158	2.621	2.624	2.727	2.918	1.879
5.896	6.000	6.349	6.025	5.829	5.126
3.684	3.762	3.527	3.799	2.923	3.950
4.691	4.441	4.233	4.421	4.824	3.693
5.070	4.812	4.960	5.203	5.077	4.235
2.990	3.240	3.644	3.922	3.612	2.126
4.578	4.277	4.400	4.458	4.203	4.000
4.416	4.373	4.555	4.402	4.539	5.668
4.429	4.410	4.569	4.419	4.579	5.620
3.401	4.394	3.459	3.627	3.926	4.428
5.756	5.775	6.089	6.146	5.679	7.418
7.133	6.805	7.300	6.670	6.940	1.801
3.212	3.252	3.297	3.322	3.486	4.867
5.986	5.938	6.013	5.920	6.115	7.579
5.898	5.869	5.872	5.836	6.007	7.473
5.791	5.915	5.917	5.754	5.983	7.399
3.857	3.667	4.086	4.133	3.947	5.361

5.780	5.015	5.164	5.386	5.499	6.371
7.904	8.242	8.036	7.943	7.979	7.389
5.480	5.167	5.938	5.464	5.479	4.646
2.546	2.296	2.780	2.499	2.775	1.773
1.914	3.044	3.420	2.996	3.252	3.019
2.408	3.010	3.557	3.118	3.152	2.475
2.592	3.256	2.178	2.606	2.498	2.913
3.614	4.072	2.978	3.492	3.058	4.069
3.040	3.603	3.349	3.178	3.449	2.524
5.598	5.960	5.681	5.852	5.399	4.296
4.210	4.794	4.115	4.330	4.792	5.154
6.004	1.611	5.427	5.602	5.552	1.049
4.334	4.594	4.468	4.411	4.922	5.063
3.050	3.778	3.393	3.775	4.025	4.467
2.429	2.787	2.992	2.778	3.142	3.512
5.230	5.499	5.918	5.256	5.378	4.018
3.242	2.450	2.684	2.189	2.838	4.046
2.364	2.167	2.416	2.056	2.340	2.129
2.385	2.155	2.406	2.106	2.297	1.853
4.769	4.408	4.867	4.838	4.565	4.294
2.735	2.859	2.897	2.591	1.955	2.419
3.496	4.016	4.323	4.167	4.129	4.732
2.677	3.047	3.166	2.232	2.532	2.554
5.020	5.238	4.915	5.164	4.946	4.681
7.616	7.664	7.643	7.800	7.782	8.663
7.446	7.452	7.388	7.219	7.236	6.667
5.776	5.744	5.565	5.731	5.528	4.898

5.772	6.110	6.214	5.956	6.061	5.316
2.438	2.257	2.868	2.275	2.281	2.459
4.609	4.809	5.045	5.305	5.177	5.969
3.388	2.904	1.923	3.523	3.978	1.378
3.619	4.085	3.456	4.066	3.644	3.518
4.836	5.199	5.211	4.873	5.421	6.106
4.287	4.678	4.801	4.720	4.953	6.042
3.332	3.878	3.557	3.525	3.533	2.797
3.518	2.475	3.672	3.081	3.802	1.866
1.559	1.229	1.319	1.538	1.568	1.341
4.811	4.166	4.982	4.772	5.179	4.310
5.399	3.961	4.443	5.355	4.915	2.207
5.481	4.886	4.851	4.582	4.725	3.761
7.425	7.289	7.279	7.071	6.898	5.336
6.642	6.852	7.052	6.829	6.961	5.730
6.716	6.759	6.592	6.789	6.672	5.812
5.547	5.599	5.815	5.969	5.940	4.529
6.885	6.384	6.864	6.882	6.758	5.061
2.331	1.958	2.662	2.594	2.035	2.661
6.882	6.489	6.793	6.580	6.847	4.643
8.750	9.121	9.092	8.938	9.033	10.200
6.025	5.994	5.866	6.194	6.270	6.576
2.096	2.148	1.380	1.911	2.205	2.427
5.357	5.560	5.502	5.516	5.877	4.119
5.713	5.341	5.519	5.025	4.985	6.135
2.235	3.313	2.082	2.924	2.550	2.467
5.849	5.726	6.034	5.673	5.429	4.645
5.273	5.611	5.104	5.260	5.206	4.514
6.116	5.638	5.821	5.741	5.596	5.153
5.209	5.476	5.573	5.688	5.694	5.013
2.428	3.081	3.670	2.190	3.204	1.746
5.057	5.073	4.945	5.293	5.048	6.374
1.136	1.747	2.243	2.179	2.276	3.684
7.655	7.977	7.641	7.972	8.227	8.864
6.980	7.204	7.149	7.233	7.393	5.061
8.014	8.220	8.358	8.398	8.448	5.679
3.157	3.887	3.261	3.118	2.869	3.624
5.738	5.211	5.471	5.125	5.132	4.427
4.331	3.727	4.367	4.581	3.969	4.338
3.232	3.691	2.632	3.417	2.897	3.976
1.258	1.611	1.125	1.039	1.223	5.334
1.940	2.544	2.156	2.646	2.125	3.191
5.818	6.055	5.851	6.015	6.130	6.713
3.397	3.872	3.412	4.255	4.096	5.716
3.889	4.231	4.064	4.515	4.094	3.602
6.940	7.345	7.124	7.216	7.314	6.837
6.608	6.899	6.930	7.056	7.047	6.115
2.022	2.021	2.472	2.088	1.989	2.518
1.570	2.138	1.828	2.127	2.218	3.073
4.116	3.243	3.126	4.605	3.548	2.557

3.081	3.797	3.814	3.551	4.088	2.237
7.410	7.093	7.171	7.310	7.078	6.755
6.630	6.729	6.468	6.635	6.545	4.786
7.724	7.325	7.697	7.546	7.392	5.347
5.980	6.135	5.892	5.915	6.153	6.409
6.630	6.487	6.315	6.564	6.614	4.020
4.418	3.384	4.308	3.929	3.846	4.862
3.373	2.744	2.613	2.387	2.446	2.668
1.784	3.326	3.382	2.923	3.505	1.023
2.327	1.787	2.176	2.382	1.801	2.878
5.556	5.289	4.925	5.441	5.460	2.136
5.441	4.962	5.369	5.286	5.352	2.951
7.461	7.228	7.423	7.031	7.338	6.004
4.843	4.443	3.708	4.550	5.041	5.004
3.937	4.332	4.211	3.723	3.956	3.713
3.672	4.030	3.863	3.508	4.323	1.544
2.410	2.162	2.716	2.495	3.001	1.596
3.242	3.811	3.798	3.463	4.170	2.117
3.205	3.190	3.010	2.509	2.563	1.843
2.577	2.745	2.837	2.840	3.131	1.558
1.693	1.162	1.104	1.327	2.077	3.317
6.780	6.538	6.792	6.885	6.655	6.186
2.695	3.034	2.988	3.212	2.840	2.480
2.449	1.974	2.053	2.289	2.503	3.431
3.990	4.453	4.122	4.261	4.195	3.650
3.224	3.656	3.427	3.875	3.812	2.792
8.466	8.032	8.665	8.176	8.457	6.656
8.171	8.128	8.387	8.413	8.342	7.166
3.494	3.151	3.580	3.986	3.662	3.295
4.323	3.699	4.180	4.015	3.395	2.647
2.460	2.401	2.678	2.396	2.476	2.957
5.904	5.809	5.151	5.254	5.710	4.573
1.675	1.827	1.612	1.407	1.306	1.433
1.710	1.903	2.303	1.991	2.124	2.831
1.670	1.095	1.232	1.305	1.261	3.630
2.397	2.411	2.477	2.335	2.360	1.597
2.574	2.213	2.446	2.683	2.498	2.622
4.061	3.666	4.039	4.219	3.551	2.503
5.074	4.756	5.248	5.312	4.773	4.303
3.206	2.529	2.630	2.833	2.837	3.514
2.974	2.172	2.586	2.920	2.647	2.457
9.001	8.355	8.792	8.539	8.251	7.984
7.839	8.063	8.036	7.965	7.983	7.327
5.958	6.013	6.343	6.013	5.664	5.316
7.680	6.827	7.642	6.775	6.850	6.957
5.354	5.693	5.542	5.849	5.758	5.195
4.947	4.451	4.641	4.449	4.649	5.352
6.297	6.550	6.561	6.374	6.170	5.809
5.571	5.557	5.392	5.524	5.393	5.391
5.365	5.752	5.617	5.136	5.598	5.129

4.060	4.548	4.461	4.695	4.107	3.505
9.623	9.689	9.673	9.738	9.712	9.350
4.302	3.728	4.101	4.200	4.064	4.054
2.987	2.876	2.752	3.343	2.813	3.010
2.950	2.631	2.992	3.595	3.624	3.430
2.821	2.371	2.795	2.366	3.006	3.418
2.059	2.037	2.048	1.817	1.995	1.884
2.625	2.263	2.030	1.764	2.156	2.505
7.710	8.034	7.934	8.046	8.100	9.365
10.112	10.236	9.988	10.080	10.213	7.982
6.297	6.918	6.653	6.710	6.707	5.666
4.821	4.248	4.471	4.550	3.992	3.712
3.543	3.967	3.173	3.366	3.549	2.899
3.023	2.960	2.466	3.411	2.044	3.308
4.453	4.880	4.825	4.618	5.051	5.924
8.301	8.409	8.352	8.562	8.649	8.428
2.650	2.916	2.316	3.129	3.063	4.095
7.904	7.677	7.775	7.560	7.743	4.062
6.428	6.385	6.272	6.076	6.422	5.599
7.092	7.137	6.978	7.378	7.506	8.558
6.998	7.251	7.238	7.781	7.683	9.318
4.838	5.115	5.292	5.059	5.202	5.274
4.240	4.228	4.336	3.864	3.670	5.263
8.224	8.463	8.258	8.282	8.317	7.128
5.452	5.605	5.560	6.372	6.365	7.898
2.667	4.000	3.722	2.943	3.275	5.307
2.155	1.857	2.416	2.018	1.970	2.661
2.329	2.063	2.187	1.489	2.063	1.876
2.578	2.452	2.184	1.954	2.711	2.126
4.880	4.952	4.934	4.609	4.871	3.204
5.908	6.120	6.239	5.785	5.970	5.443
7.830	7.847	7.973	7.678	7.641	5.298
5.457	6.355	5.541	5.563	5.714	5.151
3.177	2.021	3.435	3.020	3.879	3.305
4.610	4.913	4.933	4.991	4.593	3.679
4.098	3.567	3.567	4.304	4.332	5.057
3.458	3.756	3.554	3.706	3.750	4.577
3.525	2.762	3.975	3.076	3.161	2.877
7.076	7.014	7.062	7.051	6.843	6.374
5.664	4.627	5.593	5.735	5.150	3.969
2.720	2.466	2.644	2.647	2.448	3.942
2.676	3.247	2.846	3.020	3.540	1.715
8.039	7.936	7.849	7.742	7.859	7.178
6.298	6.923	6.716	6.510	6.719	6.113
2.113	1.987	1.842	2.163	1.863	3.082
2.939	2.634	2.686	2.496	3.043	2.088
2.918	2.270	1.943	2.662	3.366	2.801
5.384	4.373	4.928	5.289	4.793	2.053
3.114	3.302	2.812	2.442	3.341	2.432
3.458	3.584	4.559	3.953	3.561	1.835

6.311	6.194	6.382	6.256	6.150	7.316
3.618	2.827	2.238	3.217	2.512	5.041
6.243	6.398	6.282	5.843	5.991	7.867
4.575	4.588	4.830	4.591	4.750	6.341
4.976	4.952	5.266	4.624	4.382	1.958
2.948	2.803	2.436	3.153	2.637	2.223
6.813	6.450	6.725	6.663	6.443	4.227
4.306	4.843	4.920	3.899	4.175	4.915
6.044	5.478	5.586	6.068	5.818	5.464
6.078	6.304	6.554	6.432	6.326	6.803
3.849	4.738	4.597	4.290	4.499	3.162
6.308	3.482	4.094	4.445	5.017	2.828
6.713	7.095	6.697	6.691	6.911	6.326
0.603	0.947	1.097	1.443	1.338	1.356
8.647	9.126	9.268	8.714	8.637	9.321
5.107	5.234	5.289	5.202	5.326	4.871
2.491	3.167	2.821	2.816	3.151	3.679
3.191	3.727	4.124	3.546	2.739	4.931
4.958	5.199	5.738	4.872	5.014	4.935
5.616	5.455	5.942	5.973	6.137	7.015
2.049	2.201	2.201	2.042	2.161	3.709
7.667	7.862	6.763	7.298	7.427	7.024
4.675	4.883	5.016	5.274	5.439	5.118
5.516	5.403	5.851	4.978	5.423	4.887
3.441	3.314	3.181	3.068	3.175	4.569
5.444	5.096	5.195	5.325	5.316	6.098
5.929	5.151	6.187	5.823	5.889	3.773
3.952	4.909	4.434	4.178	4.102	3.693
6.971	6.972	7.331	6.912	6.973	6.415
6.484	6.525	6.649	6.319	6.860	5.486
3.830	3.259	2.780	2.965	3.313	3.013
4.627	4.719	4.973	4.885	4.891	4.971
5.720	5.594	5.846	5.609	5.613	3.422
5.418	4.751	5.455	5.206	5.661	4.660
5.862	3.722	5.117	4.528	4.254	4.267
8.161	8.116	7.838	8.050	8.136	7.672
5.864	6.067	5.974	6.204	5.912	5.221
4.761	3.920	4.281	3.996	4.373	4.670
2.035	2.064	1.856	2.474	2.080	2.983
4.703	4.759	4.742	4.558	4.510	5.273
7.215	8.100	7.665	7.731	7.766	6.795
7.361	7.186	7.096	7.224	7.093	6.323
7.943	7.774	8.058	8.073	8.007	7.047
4.219	4.191	4.038	4.148	3.959	3.306
3.054	3.473	2.950	2.347	2.788	2.834
2.154	1.961	2.181	1.891	1.954	1.744
1.432	1.960	1.438	1.112	1.707	2.410
2.229	2.708	2.317	2.125	1.384	2.383
3.704	3.076	3.064	3.870	3.887	4.216
7.365	7.971	7.387	7.359	7.566	6.915

7.015	7.227	7.162	7.258	7.400	7.283
5.657	5.849	5.688	5.439	5.402	5.757
2.330	2.660	2.074	2.799	2.658	4.801
2.389	2.725	3.297	2.889	2.647	3.639
3.921	1.710	2.297	3.499	3.434	4.527
4.920	5.012	6.019	3.676	3.089	3.870
4.920	4.777	3.760	4.909	3.580	4.907
4.781	5.429	5.292	4.778	4.969	3.779
3.764	2.750	3.649	3.147	3.482	5.680
8.331	8.853	8.380	9.146	9.077	9.951
8.044	6.617	5.304	7.145	7.345	6.366
3.994	3.493	3.542	3.097	3.626	4.201
4.127	4.172	4.369	3.716	4.067	2.836
7.064	7.177	6.625	7.403	7.352	8.785
8.390	8.655	8.214	8.528	8.381	6.918
3.090	3.880	3.334	3.369	3.434	3.469
3.410	3.945	3.321	3.661	3.122	3.922
8.208	8.359	8.261	8.106	8.099	8.635
5.082	4.943	4.857	4.782	4.970	5.317
2.445	2.902	1.917	2.279	2.493	1.950
3.097	2.888	2.745	2.160	2.408	1.612
4.350	4.340	4.504	4.371	4.416	3.901
5.393	4.844	5.223	5.244	4.979	3.830
7.508	7.752	7.849	8.364	8.109	9.428
4.066	4.602	4.484	4.447	4.347	5.189
2.415	1.645	3.054	1.512	2.222	2.521
3.923	3.812	4.277	3.957	3.991	2.985
3.927	3.808	3.937	3.986	4.366	4.991
6.457	6.634	6.367	6.493	6.322	6.110
3.851	4.321	4.597	4.242	4.373	4.916
2.491	2.864	3.693	3.399	3.085	2.163
6.112	6.239	6.315	5.679	6.085	4.004
6.428	6.162	5.906	6.330	5.866	3.762
5.885	6.277	5.948	5.992	5.811	3.285
6.229	5.882	6.202	5.679	5.685	3.190
8.536	8.707	8.660	8.834	8.768	7.272
7.262	7.422	7.176	7.492	7.375	7.107
3.942	4.106	3.941	4.413	4.821	5.977
2.459	2.970	2.760	2.470	3.229	3.070
3.421	3.077	3.143	3.186	3.663	2.070
6.492	5.672	5.357	6.169	6.332	5.953
6.169	5.233	4.846	4.818	5.047	4.389
5.355	4.203	4.746	4.330	4.959	2.431
2.777	2.888	2.487	2.129	2.367	1.941
7.093	7.349	7.088	7.059	7.103	5.988
3.059	3.163	3.330	2.548	2.979	4.030
5.682	5.014	5.428	5.467	5.577	5.556
7.286	7.714	7.431	7.266	7.821	5.347
5.353	4.711	4.189	4.047	4.428	4.846
2.924	2.928	3.254	2.852	3.123	3.878

5.118	5.306	5.317	5.170	5.185	4.078
3.965	4.407	4.691	4.396	4.388	3.271
5.757	6.093	6.261	6.034	6.020	5.570
4.119	2.063	4.108	4.355	4.134	1.617
8.099	8.041	8.085	8.307	8.198	7.535
5.554	5.333	4.473	4.827	5.441	5.914
2.902	3.559	3.445	3.304	3.629	2.719
3.878	3.525	4.521	3.946	3.661	4.233
5.967	6.817	6.486	6.355	6.689	5.656
8.016	7.984	8.042	8.219	8.030	5.713
6.152	6.071	6.505	6.363	5.943	4.655
7.220	7.264	6.990	7.024	7.327	8.109
1.965	2.258	1.887	1.645	2.242	1.894
4.304	4.855	4.683	4.406	4.793	3.267
4.226	3.951	3.929	3.883	4.015	3.028
6.303	5.839	5.768	5.371	5.068	4.258
3.470	4.236	4.363	3.509	3.463	4.769
8.565	8.449	8.484	8.199	8.301	8.302
6.344	6.248	6.122	6.865	7.075	8.228
7.874	8.047	7.936	7.890	7.930	6.480
4.362	3.453	4.044	3.938	4.476	3.532
2.996	2.779	2.688	2.486	2.651	3.025
5.386	5.296	5.582	6.066	6.070	7.941
6.654	7.085	7.011	6.659	6.762	7.342
7.448	7.486	7.715	7.480	7.591	5.746
6.732	6.818	6.347	6.137	6.484	7.111
5.800	6.247	5.872	5.833	6.033	5.504
2.780	3.712	2.924	2.750	2.959	3.955
6.224	6.560	6.487	6.119	6.512	7.099
4.386	3.263	3.945	3.836	4.066	3.471
5.897	6.203	6.381	5.622	6.129	6.634
4.375	3.994	4.110	4.032	3.695	3.824
2.671	3.157	2.671	2.451	2.843	3.217
3.864	3.579	4.194	4.139	4.463	5.982
3.856	2.879	3.352	4.114	2.858	3.702
5.389	5.521	5.647	5.518	5.669	5.904
4.377	4.856	4.723	4.546	4.351	5.261
5.368	5.140	5.189	5.392	5.148	4.255
5.652	5.558	5.866	5.991	5.388	4.773
7.273	5.293	4.996	4.026	5.513	5.613
4.481	4.265	4.768	3.854	4.222	4.543
7.132	6.905	6.853	6.308	6.536	7.544
1.840	2.290	1.805	1.724	1.206	1.790
1.313	1.004	1.925	1.417	1.212	3.205
2.951	2.899	2.535	2.375	3.448	2.153
6.349	5.691	5.802	6.073	6.006	2.309
4.361	4.021	3.844	4.559	4.069	1.860
7.443	7.257	7.213	7.229	6.841	3.316
6.777	7.096	6.583	6.732	6.995	7.680
6.052	6.160	5.671	6.312	5.993	5.657

4.651	4.560	4.356	4.676	5.050	5.078
3.913	3.494	4.417	4.298	4.607	5.151
5.443	5.343	5.178	4.989	5.273	5.323
2.048	2.177	2.058	1.874	1.717	2.099
5.391	5.486	5.154	5.310	5.331	4.220
2.815	2.832	3.167	3.071	3.167	3.728
4.041	4.381	4.265	5.276	4.960	6.805
3.367	3.132	2.824	3.504	3.360	4.066
8.065	8.598	8.218	9.293	9.345	10.681
3.775	4.212	3.790	4.771	5.420	6.743
7.241	7.748	7.471	7.571	7.729	7.876
6.899	7.075	7.382	7.315	7.154	6.012
5.418	4.771	4.301	4.755	5.287	7.195
2.948	2.919	3.137	2.942	3.241	3.897
4.030	3.437	3.606	3.441	3.959	4.743
5.538	6.531	5.889	5.973	6.098	4.517
2.500	2.178	2.707	2.896	2.221	3.930
2.502	3.083	2.387	2.023	2.961	3.240
3.028	3.584	3.257	3.685	3.584	4.374
6.575	7.074	6.737	6.831	6.748	5.661
4.202	4.557	4.268	4.782	4.548	3.224
7.315	7.385	7.266	7.320	6.906	4.743
2.617	2.191	0.974	2.115	1.868	2.985
3.110	3.821	4.883	2.745	3.003	3.282
7.334	7.528	7.583	7.266	7.341	6.690
3.037	2.687	2.734	3.071	3.131	2.039
3.634	2.641	2.954	2.831	3.743	3.221
4.879	4.447	5.142	5.089	4.885	3.610
8.012	7.990	7.849	7.495	7.458	5.352
6.145	6.078	6.781	6.439	6.564	5.280
3.727	2.462	3.642	3.494	3.201	2.299
6.291	6.156	6.143	6.069	6.476	4.777
4.695	4.032	4.583	4.925	4.810	4.202
7.204	7.557	7.465	7.389	7.339	3.925
6.305	5.805	5.998	6.261	6.044	6.226
3.652	3.202	3.516	3.219	3.623	4.404
6.316	5.902	6.222	6.232	5.982	3.251
3.048	2.941	2.818	3.604	3.453	3.966
4.364	4.286	4.046	4.078	4.145	2.978
5.757	5.918	5.463	5.367	5.640	6.454
2.861	3.224	3.285	3.494	2.875	3.071
2.752	3.440	2.901	2.724	3.448	3.458
7.207	7.618	7.634	7.474	7.248	6.816
5.134	5.306	5.519	5.465	5.332	5.779
2.183	1.704	1.728	2.465	2.628	2.852
4.262	4.464	4.430	4.478	4.476	5.787
7.548	7.944	7.644	7.876	7.914	8.686
3.584	4.324	4.213	5.272	5.286	6.835
7.589	7.580	7.429	7.750	7.781	7.415
6.504	6.612	6.473	6.863	6.720	5.102

3.734	2.181	2.371	3.393	2.602	2.408
3.562	3.699	3.482	3.352	2.943	3.749
3.580	4.108	3.884	4.210	4.288	4.761
5.778	5.957	5.969	5.903	6.065	2.946
7.647	8.016	7.769	7.616	7.825	7.053
3.431	3.148	2.769	3.411	3.298	3.727
4.681	5.335	5.354	5.515	5.640	6.031
6.731	7.221	7.089	7.966	8.072	9.434
5.681	6.331	6.310	6.170	6.058	5.300
3.011	2.448	2.609	2.073	2.494	2.300
4.563	4.570	4.743	4.991	4.985	4.796
5.122	4.415	4.711	4.442	4.282	3.811
3.755	4.009	3.994	4.075	4.651	3.382
7.843	7.068	6.936	7.990	7.868	8.430
8.223	8.403	8.282	8.244	7.799	7.222
4.548	4.693	4.940	4.917	5.224	3.840
6.194	6.273	6.196	5.772	6.333	5.961
2.443	3.057	3.033	4.227	3.744	5.053
3.179	2.977	3.314	2.875	2.997	4.477
5.951	6.011	5.897	6.111	5.734	4.592
6.142	6.619	6.322	6.207	6.380	5.692
6.272	6.041	6.380	6.233	6.085	6.593
6.004	5.659	6.451	6.049	6.555	5.970
6.800	6.885	6.543	6.834	6.853	5.936
4.349	4.325	4.159	4.144	4.348	2.200
5.604	5.649	5.616	5.511	5.831	4.831
5.622	4.881	4.711	5.305	4.746	4.442
5.112	4.237	4.640	4.322	5.122	5.674
3.193	3.278	2.939	3.645	3.289	5.307
4.425	4.857	4.660	4.147	4.484	3.975
7.013	5.881	6.938	5.682	5.474	4.515
3.614	3.463	3.913	4.187	3.780	3.968
2.089	2.071	1.634	1.695	2.086	4.542
8.198	8.597	8.103	7.530	7.877	7.302
3.574	3.633	3.491	3.215	2.994	4.965
1.997	3.167	3.011	3.111	2.435	2.007
8.963	8.313	8.766	9.176	8.757	8.787
8.338	7.966	8.273	7.941	8.229	7.633
5.056	5.224	5.184	5.673	6.177	8.104
4.975	4.961	4.772	5.069	5.273	5.756
7.252	7.198	7.546	7.381	7.354	6.449
4.590	5.172	4.967	4.798	5.210	5.001
6.619	6.037	6.068	6.147	5.747	5.758
3.557	4.017	3.907	4.112	3.843	4.734
2.743	3.460	3.656	3.155	3.251	2.580
3.582	3.631	3.782	3.445	3.737	4.081
5.782	6.450	5.962	5.707	6.206	7.627
7.767	7.685	7.793	7.839	7.867	7.098
5.851	5.248	5.346	5.504	4.752	4.188
7.418	7.951	7.676	7.387	7.791	5.970

4.765	5.344	4.812	4.683	4.065	4.969
3.801	2.675	2.782	2.811	2.298	3.697
7.005	6.033	5.552	6.289	5.983	7.009
4.526	4.063	4.549	4.473	4.383	4.365
3.253	3.799	3.952	3.227	3.745	2.847
2.461	2.933	2.786	3.053	2.897	4.044
4.731	4.871	4.702	4.662	4.802	5.544
6.939	7.199	7.082	6.780	6.954	6.070
3.380	2.800	3.335	3.596	3.818	2.652
8.126	8.041	8.282	7.965	8.003	6.802
2.384	3.013	2.775	2.721	2.683	2.264
3.607	3.360	3.498	3.701	3.783	4.736
6.866	7.066	7.059	6.835	7.012	5.588
4.777	4.329	5.172	4.369	4.941	4.393
4.828	4.823	4.717	4.452	5.047	4.264
5.794	4.850	5.166	5.304	5.041	6.193
5.286	4.938	5.003	5.095	4.870	3.875
4.021	4.787	4.716	4.661	4.505	3.908
6.217	6.046	6.149	5.989	6.143	5.296
6.016	5.682	6.119	6.237	5.661	5.153
4.014	3.571	3.871	4.549	3.875	4.833
6.061	5.583	4.993	5.905	4.610	3.702
3.035	3.136	3.154	3.219	2.242	2.549
7.059	6.950	6.763	6.927	7.120	6.097
5.917	4.553	5.652	6.348	5.228	6.099
6.483	6.899	6.774	6.565	6.459	5.932
6.396	6.184	6.495	6.115	6.457	3.710
3.591	2.982	2.920	2.024	2.611	2.970
7.338	7.616	7.618	7.639	7.330	3.830
4.279	3.793	3.399	4.541	3.469	2.935
7.674	7.833	7.848	7.749	7.783	7.405
7.087	6.990	7.160	6.616	6.883	4.845
9.886	9.855	9.656	9.790	9.630	8.911
2.676	2.969	2.720	2.214	2.518	2.948
5.467	4.905	5.167	5.560	5.556	6.366
2.292	2.883	2.611	2.601	2.373	3.423
2.699	2.351	2.616	2.888	2.794	2.887
7.005	6.844	7.011	7.021	7.028	6.351
2.471	2.429	2.894	2.189	3.046	2.724
2.893	2.856	2.720	2.595	2.292	3.854
5.726	5.737	5.817	5.981	5.648	4.997
5.267	5.540	5.809	5.314	5.185	4.198
6.840	5.844	6.555	5.854	6.869	8.197
2.551	3.434	3.217	2.774	3.575	2.939
4.920	4.924	5.417	5.320	5.384	5.791
4.536	4.233	3.872	4.024	4.019	5.270
4.746	4.194	4.351	4.921	4.820	5.515
4.017	3.536	4.117	3.930	3.158	2.066
3.533	2.931	3.844	3.886	3.631	2.167
3.424	3.345	2.939	3.609	3.373	4.636

4.309	4.580	4.418	4.264	4.309	4.607
5.348	5.485	5.663	5.495	5.272	4.827
3.598	2.303	2.583	2.769	3.420	2.271
7.544	7.419	7.520	7.260	7.447	6.513
4.819	5.474	5.306	4.837	5.169	5.310
4.480	4.874	4.649	4.564	4.546	2.430
5.179	5.433	5.589	4.777	5.091	4.112
2.037	2.267	3.069	3.429	3.645	3.032
4.966	5.101	5.133	5.690	5.483	4.280
1.877	1.850	1.866	1.539	1.693	1.116
7.260	7.046	7.148	7.170	7.095	6.163
3.964	4.619	4.634	4.437	4.979	4.920
6.313	6.736	6.626	6.417	6.735	5.578
3.621	4.241	4.419	4.120	4.681	3.640
2.867	2.817	2.881	3.097	3.265	4.399
3.653	1.914	3.144	3.359	2.553	2.329
2.977	2.537	2.901	3.546	3.410	4.944
2.033	2.475	2.058	2.322	1.791	3.708
3.603	2.664	3.863	3.136	3.838	3.169
3.866	3.501	3.525	3.699	3.091	3.681
5.318	5.440	4.885	5.101	5.164	3.672
3.497	3.943	2.634	2.634	2.878	2.236
3.075	3.210	2.847	2.209	2.304	2.999
2.566	2.835	2.835	2.393	2.650	2.679
3.344	4.092	2.985	4.271	3.728	2.107
4.972	4.798	3.078	4.778	4.870	4.677
3.573	4.261	4.347	4.317	3.803	4.261
3.105	2.939	2.711	2.718	3.140	3.140
1.973	1.750	1.963	2.252	2.638	2.205
3.528	2.824	3.341	3.704	2.955	2.501
7.976	8.389	8.333	8.073	8.220	6.695
5.392	5.255	5.166	4.847	5.064	4.149
4.171	4.678	4.360	4.827	4.779	6.221
6.687	6.457	6.481	6.386	5.755	5.129
4.247	5.008	4.603	4.908	2.926	4.647
5.263	5.284	3.436	3.970	5.287	3.663
3.330	3.593	2.792	3.938	3.430	2.944
6.209	7.208	6.474	6.624	6.799	5.616
3.679	3.937	4.114	4.409	4.444	5.065
4.048	4.720	3.788	4.454	4.417	4.334
4.916	5.436	5.455	4.863	4.933	4.289
5.898	6.137	5.832	6.075	5.937	4.106
2.896	3.543	3.434	2.733	2.975	1.075
2.658	2.783	3.155	2.704	2.190	1.713
6.175	6.199	6.054	6.048	6.153	3.584
4.133	4.305	3.825	4.473	4.414	5.299
3.693	3.471	3.910	2.949	4.083	3.785
2.328	2.788	2.178	1.776	2.338	2.124
2.002	2.196	2.300	2.525	2.508	2.530
4.729	4.977	5.938	4.877	4.807	4.747

3.219	4.049	4.061	3.716	4.205	3.403
2.697	4.899	2.868	3.906	4.590	3.105
4.688	4.663	4.335	4.025	4.189	4.508
7.645	7.173	7.829	7.181	7.264	6.621
2.335	1.893	1.178	1.273	3.257	2.079
3.413	3.737	3.354	4.152	3.910	4.415
2.848	3.179	2.570	2.895	2.897	2.897
2.468	2.866	3.039	2.290	1.980	2.299
2.084	2.774	2.371	2.465	2.587	2.247
4.762	4.668	5.089	5.427	4.722	5.868
1.974	3.248	2.625	3.389	3.001	3.263
2.639	2.623	3.318	3.016	2.503	2.359
2.871	3.199	3.728	3.098	2.685	3.592
7.363	7.350	7.299	7.717	7.509	8.401
3.201	3.493	3.139	3.844	2.632	4.058
5.771	5.992	6.113	6.034	5.811	3.981
5.618	5.640	5.905	5.242	4.848	4.866
4.766	5.277	4.934	4.805	5.016	5.314
7.454	7.945	7.960	7.796	7.707	7.137
4.433	4.469	4.386	4.569	4.814	4.384
5.747	5.577	5.801	5.946	5.430	5.319
5.678	5.521	5.681	5.843	5.595	5.263
6.314	6.095	6.057	6.603	6.525	7.621
7.422	7.035	7.145	7.291	7.100	6.273
5.279	5.229	5.305	5.347	5.533	5.745
2.731	2.639	3.386	2.302	1.348	1.314
6.807	6.994	7.184	6.855	6.869	4.641
3.886	4.370	3.894	4.636	4.785	5.971
5.460	5.429	5.859	5.933	5.881	4.630
4.692	4.405	4.185	4.204	3.949	4.631
3.880	3.829	3.796	3.360	3.342	3.500
5.654	4.620	4.991	4.802	4.556	4.921
3.231	3.735	3.829	3.122	4.101	2.133
2.514	3.032	2.705	2.211	2.131	3.331
6.626	6.962	6.457	6.493	6.178	6.044
3.687	3.844	4.015	3.460	3.952	2.722
8.859	8.513	8.908	8.580	8.660	5.918
6.943	7.285	7.035	7.142	7.162	6.314
3.644	3.557	3.915	4.025	3.937	2.665
5.055	5.413	4.892	4.683	4.839	3.393
6.157	5.983	6.476	5.987	6.073	5.013
7.394	7.561	7.131	7.294	7.352	6.691
5.996	5.645	5.673	5.756	6.074	6.812
2.947	3.186	3.347	3.637	2.744	3.683
5.780	5.584	6.174	6.142	6.293	4.909
5.278	5.050	5.466	5.082	5.279	5.496
5.499	4.494	5.633	4.960	5.175	4.588
5.667	5.162	6.070	5.486	5.788	5.145
6.669	6.689	6.938	6.729	6.597	6.292
6.367	6.239	6.497	6.606	6.515	5.880

6.931	6.421	6.816	6.794	6.615	5.555
6.809	6.958	6.620	7.147	7.061	7.807
6.539	7.051	6.723	6.425	6.549	5.280
6.339	6.289	6.095	5.808	5.785	4.131
3.430	3.049	4.097	4.166	3.935	1.511
6.373	6.152	6.351	6.099	6.270	4.242
5.500	5.253	5.311	4.826	5.096	4.017
5.462	5.581	5.714	5.050	5.786	5.240
4.187	4.597	4.917	4.082	4.314	4.390
7.232	7.301	7.295	7.424	7.627	8.504
5.418	5.717	5.700	6.131	6.312	5.671
8.814	8.793	8.894	9.013	9.039	7.049
2.546	2.546	3.444	2.455	2.874	1.624
3.496	2.800	2.294	2.603	3.056	2.410
6.707	6.513	7.028	5.429	6.387	6.503
3.026	4.143	4.635	1.873	2.753	3.067
5.015	5.569	5.242	4.436	5.434	6.152
3.328	3.619	3.914	3.407	3.900	5.019
3.224	3.655	3.474	2.707	3.043	3.819
2.243	1.048	2.506	1.400	1.509	2.730
3.817	3.555	3.785	3.729	3.598	4.639
2.240	2.439	2.783	2.753	2.970	3.025
5.578	5.788	4.945	5.327	5.547	6.257
2.961	3.288	3.375	3.667	3.734	2.496
7.575	8.126	7.745	7.602	7.901	7.186
3.664	4.047	3.861	4.312	3.990	4.779
3.652	3.645	3.810	3.666	3.813	3.977
5.065	6.166	6.101	5.846	6.222	2.292
2.320	2.931	3.674	2.103	2.427	2.675
3.777	3.487	3.520	3.663	3.228	2.008
5.282	5.388	5.382	4.839	4.581	3.884
3.108	3.072	2.998	2.995	3.195	4.720
1.821	1.887	2.380	1.951	1.216	2.459
3.962	3.774	3.518	3.677	3.641	2.561
5.848	5.644	5.568	5.991	5.867	4.944
3.224	3.073	3.423	3.226	3.578	4.622
3.193	2.958	3.908	3.634	3.286	2.649
4.317	4.326	4.628	3.915	3.905	4.368
5.597	5.749	5.892	6.222	5.862	4.039
5.060	5.169	5.210	5.307	5.321	3.818
7.706	7.938	7.738	7.639	7.693	5.097
2.576	3.185	2.323	3.176	2.824	3.250
5.501	3.750	4.691	5.834	4.646	4.135
4.071	2.938	3.047	3.455	3.191	2.603
3.637	3.016	3.020	3.316	3.903	2.563
3.449	3.593	3.690	3.492	3.497	2.352
1.981	1.491	1.178	1.370	1.540	1.933
5.700	6.090	5.840	5.591	5.697	4.667
2.271	1.646	1.650	1.757	1.482	1.520
3.569	3.462	3.629	3.272	3.260	3.787

4.963	4.348	5.052	5.646	5.449	5.501
5.834	6.297	6.126	6.246	6.626	7.622
6.306	5.907	5.561	6.406	6.656	7.129
6.388	5.749	5.849	5.413	5.898	6.310
3.306	3.253	3.206	3.261	2.872	3.590
6.038	6.895	6.434	6.343	6.438	5.785
2.669	2.723	3.232	2.349	3.248	1.708
3.512	3.954	4.340	3.783	3.648	2.019
4.097	4.379	3.835	3.189	3.867	3.790
5.616	4.524	3.527	5.084	5.536	4.445
6.996	6.879	6.655	6.887	6.890	5.863
5.669	5.559	5.526	5.419	5.509	5.982
2.313	2.769	2.594	2.840	2.548	3.001
6.725	6.803	6.957	6.424	6.722	6.233
3.880	4.390	4.750	4.340	3.995	4.389
3.901	4.226	4.457	4.775	5.274	3.937
2.036	1.775	2.168	2.040	2.285	2.571
5.760	5.717	5.961	5.234	5.678	5.487
4.672	4.688	4.423	4.521	4.372	4.899
5.008	5.099	5.094	4.914	5.152	3.402
4.534	4.811	4.452	4.472	4.343	2.351
6.735	6.296	6.809	6.690	6.508	6.344
5.228	5.114	4.709	4.861	4.902	5.122
3.890	3.245	3.368	3.782	3.889	4.815
9.021	8.761	8.513	9.566	8.803	9.448
4.785	5.558	5.088	5.390	5.328	5.426
3.660	3.494	3.985	2.748	3.551	3.601
3.474	3.819	3.800	3.099	3.112	3.585
6.383	6.647	6.517	6.606	6.523	6.916
2.128	1.379	2.116	2.195	1.926	1.875
5.933	5.604	6.348	5.714	5.748	4.249
3.235	2.957	3.060	2.710	3.387	4.140
5.434	5.112	5.226	4.582	5.246	3.681
3.695	1.614	2.689	2.493	2.630	1.116
3.443	4.116	3.511	4.318	3.838	4.719
1.573	2.609	2.030	2.506	2.779	2.438
2.480	2.161	1.877	2.788	2.447	2.329
3.171	2.437	2.219	2.102	3.247	2.259
3.193	2.508	3.699	3.283	3.213	2.270
2.359	2.566	2.440	2.292	1.796	2.374
2.030	2.639	2.432	2.937	2.695	1.650
1.964	1.913	3.291	2.293	2.727	3.125
2.351	3.038	2.762	3.323	3.489	3.674
5.752	4.905	5.503	5.341	5.232	3.844
4.001	4.583	4.829	4.427	4.954	3.179
3.173	3.158	3.089	2.223	3.058	3.793
5.232	5.055	5.348	5.408	5.140	4.796
3.668	2.343	2.191	2.399	2.654	2.864
3.399	2.995	2.815	3.367	3.444	2.914
2.869	3.504	3.590	2.991	2.956	3.263

5.877	5.553	5.069	5.412	5.432	6.049
6.041	5.160	5.130	5.161	5.591	6.566
4.949	4.957	4.844	4.850	4.852	7.263
6.808	5.875	6.108	6.427	5.690	4.687
5.281	5.972	5.706	6.010	5.795	3.294
7.018	6.714	7.463	6.632	6.611	5.909
3.461	3.488	3.488	3.189	3.499	2.985
3.873	3.632	4.043	3.374	4.080	3.810
2.582	2.399	2.432	2.203	2.548	2.943
6.240	6.101	5.796	6.463	6.080	5.710
8.795	8.512	8.616	9.334	9.154	10.345
5.318	5.198	4.741	5.175	5.211	6.269
3.196	2.604	3.685	2.582	3.075	2.858
1.783	2.502	2.618	1.986	2.589	3.641
2.487	2.628	2.589	2.524	2.494	3.212
5.184	4.003	4.140	4.492	5.110	3.575
7.715	7.655	7.639	6.809	7.649	7.138
7.041	7.155	7.134	7.513	7.623	8.700
6.512	6.489	6.758	6.637	6.556	7.643
6.887	6.177	6.156	6.220	6.139	6.670
6.193	6.829	6.769	6.326	6.657	5.000
5.249	5.041	4.646	5.157	5.338	4.756
5.003	5.480	5.764	5.599	5.536	3.842
6.634	6.718	6.701	6.134	6.543	6.109
5.592	6.093	6.176	5.988	6.009	6.823
7.530	7.474	7.480	7.707	7.229	6.520
4.022	5.176	5.474	5.693	6.091	7.569
1.578	2.041	2.528	2.456	2.882	2.552
1.920	1.611	1.627	0.996	1.246	2.531
3.563	2.851	3.703	2.798	3.481	2.451
7.308	6.156	5.481	5.367	6.267	6.296
4.022	4.575	3.922	3.964	3.616	2.259
3.650	3.192	2.518	2.536	2.749	2.178
6.736	5.862	5.854	5.715	6.249	4.439
1.482	1.450	1.158	1.408	1.215	2.532
6.263	5.673	5.539	5.909	5.808	5.178
2.622	2.425	1.831	2.601	2.025	1.984
5.117	3.554	5.574	4.644	4.570	3.117
3.367	2.452	4.067	2.663	3.717	2.079
3.210	2.215	3.073	3.660	3.220	2.083
3.866	3.679	2.922	3.608	3.320	2.095
4.297	3.406	4.493	4.692	4.110	3.487
4.366	4.288	4.096	4.592	4.598	3.616
5.006	5.577	5.138	4.870	5.008	4.397
6.871	6.539	6.728	6.744	6.886	1.229
2.885	2.605	2.879	2.733	3.459	3.467
5.957	6.504	6.316	6.509	6.287	7.379
5.470	5.036	5.384	5.333	5.034	5.446
2.787	2.410	2.998	3.550	3.177	4.399
3.643	3.997	4.236	4.400	4.190	4.820

3.077	4.085	3.742	2.946	3.162	2.978
5.345	4.856	4.727	3.468	4.856	3.838
2.185	2.290	2.777	1.607	3.295	2.044
4.937	4.893	4.887	5.065	4.603	3.861
4.664	4.657	4.852	4.575	4.833	3.402
4.049	3.427	3.582	3.871	3.760	5.129
3.040	3.522	4.303	3.470	2.515	2.205
5.669	4.892	6.043	4.050	5.470	2.149
3.336	3.258	3.707	3.514	3.223	3.615
2.657	2.939	2.851	3.036	2.874	3.795
3.956	3.795	3.731	4.456	4.600	5.999
2.686	3.468	2.670	2.496	2.395	2.234
2.530	3.136	2.785	1.825	3.052	2.973
2.280	2.952	2.604	2.990	2.567	2.486
5.386	6.651	6.371	6.617	7.055	5.386
2.689	2.468	2.404	2.588	2.357	4.504
3.853	3.990	3.876	3.616	3.895	5.181
8.001	8.167	8.089	7.941	8.058	3.776
8.376	8.379	8.266	8.298	8.120	6.439
4.439	4.275	4.765	4.603	4.372	3.839
7.715	7.619	7.612	7.486	7.532	7.010
4.339	4.144	4.075	4.226	3.586	4.068
2.568	3.217	2.491	2.583	3.078	2.079
6.969	7.084	7.100	7.564	7.282	8.569
8.049	5.655	5.766	6.165	6.904	4.954
5.834	6.066	6.089	6.097	6.310	4.991
5.467	4.832	5.082	4.691	5.145	4.520
4.244	3.976	4.321	4.229	4.114	4.159
5.541	5.347	5.315	5.419	5.225	3.316
5.732	6.270	6.057	6.199	6.035	5.732
10.396	10.312	10.850	10.234	10.303	10.936
5.271	5.759	5.090	5.035	5.184	5.134
4.488	4.577	4.089	4.817	4.676	5.607
1.451	2.591	2.535	2.298	2.570	2.455
2.838	3.136	3.137	2.666	3.012	2.240
5.287	5.307	5.189	5.614	5.756	6.000
3.432	2.897	2.810	3.396	3.768	3.493
6.627	6.239	6.300	6.225	6.164	7.066
3.047	2.870	2.988	2.954	3.541	3.317
4.308	4.405	3.858	3.875	3.810	4.279
6.662	6.154	6.751	6.328	6.297	4.879
4.525	4.897	4.376	3.851	4.348	5.561
4.922	5.296	5.398	4.916	5.246	6.236
2.005	2.887	2.811	2.557	2.537	2.923
7.983	8.145	8.148	7.963	7.959	6.566
5.442	5.440	5.148	4.827	5.424	4.167
3.125	3.205	3.279	3.023	3.488	3.981
3.150	2.600	3.447	3.296	3.711	4.580
4.307	3.448	3.984	3.886	3.410	3.877
5.494	5.162	5.562	5.412	5.248	5.048

7.219	7.511	7.352	7.209	7.367	6.423
7.145	7.631	7.460	7.463	7.928	8.221
5.851	5.679	5.622	5.600	5.761	5.908
5.504	5.146	5.366	5.947	5.323	6.571
3.891	4.271	4.287	4.776	4.453	5.572
2.998	3.155	3.248	2.510	3.296	3.627
7.007	6.588	6.362	6.642	7.035	5.787
8.549	8.610	8.568	8.573	8.878	7.869
9.025	9.134	9.037	9.133	9.396	8.379
8.590	8.681	8.603	8.626	8.935	7.914
8.422	8.503	8.462	8.414	8.786	7.836
8.568	8.633	8.585	8.611	8.929	7.950
7.982	8.100	8.021	7.983	8.355	7.322
9.586	9.601	9.657	9.499	9.702	6.409
5.254	4.551	5.311	5.304	5.099	6.041
8.019	8.857	8.797	8.411	8.633	3.489
6.441	6.224	6.282	6.239	6.267	3.816
8.040	8.110	8.184	7.583	7.617	5.607
7.871	8.181	8.350	7.947	8.164	5.128
6.707	7.181	7.384	6.976	7.313	3.328
7.259	6.979	7.267	7.188	6.826	5.811
8.203	8.409	8.500	8.348	8.365	3.405
5.605	4.951	5.024	5.283	5.324	3.655
6.345	6.856	7.164	6.913	7.175	2.423
3.239	3.671	3.376	2.934	2.932	2.538
7.797	7.225	7.148	8.051	7.625	7.513
8.910	8.922	9.270	9.022	9.110	6.233
4.295	4.189	4.082	4.154	4.260	1.636
3.626	4.606	4.132	3.523	4.023	3.852
5.351	6.069	5.929	5.955	6.288	3.071
6.513	7.984	7.272	7.333	7.717	4.325
8.691	8.219	8.011	6.865	8.289	6.484
9.391	7.701	8.091	7.146	8.791	6.931
4.042	3.491	3.976	4.218	3.916	4.707
2.373	2.389	2.950	1.797	2.570	3.259
2.282	2.637	2.923	2.124	2.946	3.042
4.100	4.263	4.344	3.837	4.256	3.431
2.054	2.326	2.285	2.147	2.190	2.926
4.400	3.268	3.470	2.886	3.503	3.964
6.517	6.508	6.124	6.583	6.379	5.406
2.526	2.357	2.793	2.711	2.897	1.865
5.812	6.277	5.703	5.583	6.144	8.139
5.297	5.816	5.647	5.437	5.670	4.342
5.340	5.199	5.680	4.974	5.068	4.457
3.298	3.017	3.194	3.268	3.014	3.797
3.679	4.299	3.644	4.396	4.804	4.814
4.702	4.475	4.870	4.963	4.626	5.970
6.760	6.341	6.481	6.072	6.872	4.581
3.947	3.266	3.724	3.269	3.683	1.249
2.280	2.222	2.563	1.820	1.547	2.320

5.097	4.771	4.753	4.850	4.942	6.315
2.888	1.858	2.454	3.318	3.211	4.065
8.259	8.405	8.646	8.513	8.454	6.259
2.329	2.634	2.513	1.597	2.024	4.129
4.830	4.254	3.735	4.879	4.675	5.246
6.125	5.488	5.794	5.987	5.771	5.592
6.848	7.068	6.794	6.891	7.045	5.842
2.736	3.517	3.387	3.110	2.836	2.249
4.645	4.463	3.704	4.436	4.159	4.257
3.753	3.283	3.223	2.445	2.257	2.511
2.265	2.011	2.243	1.571	1.844	2.765
4.216	3.667	4.418	4.038	4.341	2.776
8.311	8.335	8.266	8.555	8.461	7.486
6.587	6.641	6.663	6.475	6.472	6.258
5.157	5.007	5.255	5.034	4.800	5.454
3.403	3.755	3.404	2.612	3.683	3.734
4.783	5.870	4.807	4.506	3.894	4.417
3.309	4.324	4.281	3.029	2.523	3.247
3.978	3.637	3.439	2.291	4.243	4.217
3.898	5.177	3.999	2.638	3.343	3.317
2.541	2.552	2.387	2.104	2.690	2.846
5.684	5.774	5.409	5.662	6.008	6.111
7.795	8.260	7.907	8.158	8.068	7.195
4.927	5.769	5.535	5.333	5.708	4.715
3.380	3.791	4.327	3.640	3.381	2.734
4.814	5.208	5.317	4.966	5.486	1.452
3.150	3.716	3.629	3.654	3.285	2.896
6.921	7.222	7.402	7.437	6.836	6.241
8.332	8.885	8.613	8.142	8.485	1.583
7.413	7.544	7.338	7.374	7.354	6.500
4.663	4.877	4.840	4.957	5.025	3.095
6.577	6.634	6.608	6.810	6.508	6.476
6.262	6.422	6.436	6.131	6.464	7.249
4.598	4.199	4.261	4.495	5.020	4.432
5.971	5.452	5.762	6.009	5.217	6.130
2.888	2.481	2.743	2.097	2.303	2.621
2.153	2.883	2.823	2.538	2.916	1.601
6.595	6.514	6.945	6.980	6.575	5.573
4.666	4.336	4.889	4.704	4.597	5.418
1.656	1.986	2.064	1.405	2.207	3.176
4.530	3.372	3.214	3.856	3.939	3.427
2.896	3.940	4.370	3.948	3.322	3.215
4.376	4.785	4.765	4.643	4.577	4.328
2.546	2.692	3.337	2.762	3.223	2.167
3.620	3.216	3.725	4.243	3.850	3.695
10.052	10.574	9.956	9.935	9.534	11.536
5.880	5.600	6.384	5.952	6.077	5.316
5.271	4.981	4.819	5.113	4.937	3.376
3.348	3.841	3.916	2.736	2.969	1.942
4.734	4.975	5.103	4.383	4.821	3.340

4.853	4.956	4.658	4.533	4.946	5.237
5.225	5.631	4.878	4.940	5.714	4.945
5.489	5.728	5.492	5.104	5.370	2.524
6.004	5.864	6.271	5.984	5.875	5.288
5.252	5.479	5.707	5.129	5.356	4.455
3.935	4.048	4.007	3.565	3.774	3.013
2.657	2.122	1.616	2.697	2.530	2.960
3.761	4.362	3.527	4.214	3.614	3.497
3.308	3.136	2.719	2.387	3.365	2.719
9.008	9.356	9.235	9.189	9.261	8.378
2.816	2.708	2.714	2.687	2.412	3.076
2.794	2.911	2.596	3.015	2.778	3.192
4.326	4.814	4.745	3.996	4.246	5.058
3.175	3.407	3.905	3.179	3.480	3.734
4.530	5.276	4.432	4.254	4.015	4.674
3.955	4.070	3.495	3.087	3.569	4.091
3.599	4.012	3.864	4.078	3.569	4.776
2.957	3.597	2.796	2.938	2.468	2.229
7.414	7.461	7.463	7.273	7.172	6.083
8.798	8.036	8.189	9.056	8.410	9.578
3.020	2.061	2.055	3.384	2.673	2.221
1.730	2.796	2.240	2.822	2.780	4.943
4.342	3.122	3.218	3.832	4.047	3.301
5.794	4.689	4.922	5.859	5.862	4.418
3.819	4.108	4.097	4.185	3.752	2.997
3.648	3.848	2.945	3.774	4.125	5.574
2.184	1.935	1.976	2.213	2.335	2.549
4.234	3.687	4.285	3.503	4.392	4.067
5.187	5.129	4.619	5.131	5.109	5.912
3.783	3.847	2.679	4.126	3.900	3.628
4.632	5.206	5.119	4.543	4.916	4.719
2.701	3.168	3.265	3.855	3.527	4.082
3.751	4.027	3.113	3.355	3.818	1.742
3.614	3.324	3.373	3.581	3.409	2.635
5.268	5.786	5.921	5.375	5.740	4.725
5.077	6.120	5.662	6.297	7.085	7.724
3.441	3.373	2.679	3.670	3.315	3.211
4.352	4.162	4.171	4.689	4.623	3.714
5.144	4.568	4.578	4.836	4.683	5.398
3.853	2.971	3.212	3.695	3.832	3.318
7.074	7.412	7.381	7.366	7.324	6.423
4.032	4.633	4.268	4.823	4.106	5.723
4.651	5.393	5.319	4.626	5.222	6.177
4.234	3.862	3.860	4.229	3.709	5.193
4.668	4.806	4.636	4.914	4.711	3.600
2.698	1.459	2.429	2.180	2.342	2.766
5.338	4.200	5.321	3.236	4.091	1.929
2.920	3.282	3.313	2.247	2.601	6.742
4.158	4.806	5.248	4.659	5.420	3.327
7.173	6.877	7.625	6.734	6.602	3.539

6.198	6.530	6.599	6.489	6.487	3.734
4.433	4.914	4.979	4.577	4.668	3.878
5.039	5.348	4.989	5.022	5.315	4.439
7.594	8.099	7.843	7.947	7.879	6.261
6.215	6.136	6.120	6.416	6.203	4.000
6.256	6.143	6.446	6.484	6.220	4.360
6.461	7.086	6.873	7.202	7.467	9.124
6.479	6.478	6.248	6.202	6.418	7.165
5.642	5.081	5.019	5.971	5.551	6.342
3.233	3.649	2.979	2.912	3.413	3.225
8.165	7.672	6.990	7.538	7.931	8.352
2.694	2.614	3.197	4.255	3.100	4.690
5.468	5.792	5.320	5.622	5.764	6.527
5.382	6.174	6.176	5.193	5.762	5.787
3.669	4.200	3.623	3.488	3.696	3.864
3.529	3.796	3.865	3.799	3.560	6.308
7.234	7.553	7.265	7.265	7.376	6.584
4.673	5.141	4.603	4.610	4.541	5.414
5.928	5.399	5.529	6.105	5.725	6.396
5.060	5.124	5.594	5.270	5.319	6.101
3.163	4.138	4.449	3.174	4.140	2.826
4.417	4.604	3.807	3.927	4.541	4.881
3.449	3.655	4.050	3.714	3.840	2.828
5.034	4.941	5.366	4.834	5.063	3.576
10.263	10.316	10.351	10.215	10.249	9.593
4.063	3.254	3.774	3.337	3.388	2.632
4.513	4.904	5.087	4.533	5.038	4.935
5.741	6.231	5.638	5.611	5.800	3.468
4.005	3.859	4.245	4.034	4.319	2.918
4.925	4.428	4.378	4.214	4.479	5.407
4.721	4.598	5.038	4.575	4.544	2.520
3.456	2.650	3.485	3.050	2.781	2.157
9.504	9.495	9.788	9.373	9.676	8.161
4.316	4.822	4.771	4.480	4.584	3.362
3.509	3.318	2.257	3.469	3.377	3.303
5.687	5.206	5.717	5.202	5.425	5.502
6.104	6.546	6.303	6.373	6.471	6.067
2.367	2.860	2.744	1.651	2.599	1.843
6.050	6.381	6.367	5.949	6.090	4.992
4.839	5.088	5.256	5.131	4.896	4.385
5.959	6.061	6.172	6.143	6.106	5.484
3.205	3.192	4.065	3.219	3.865	3.049
3.174	3.346	1.732	3.239	3.421	1.853
2.632	2.986	3.244	3.480	3.229	3.367
2.349	2.099	1.747	1.516	1.928	2.216
4.611	2.549	4.572	4.136	4.326	2.135
5.684	5.110	4.892	5.881	5.839	5.858
5.400	4.926	4.638	5.724	5.543	5.616
5.191	4.990	5.024	4.915	4.634	2.653
3.585	3.138	3.138	3.373	2.239	1.500

7.252	7.021	6.601	7.612	7.419	6.857
7.329	7.139	6.663	7.661	7.298	7.101
2.989	3.370	3.506	3.345	3.278	3.944
2.383	2.680	1.676	3.269	3.066	3.630
2.469	2.779	1.567	3.411	3.175	3.505
4.737	5.093	4.665	5.597	5.107	5.805
5.690	5.916	5.387	6.183	5.754	6.896
5.320	5.137	4.966	5.536	5.408	6.402
5.579	5.337	5.164	5.806	5.547	6.488
3.628	4.081	3.469	3.113	3.505	4.150
7.973	7.461	7.766	7.959	7.817	8.515
6.597	6.588	6.781	6.830	6.414	6.195
7.930	7.995	7.935	7.889	7.855	7.155
3.939	3.719	3.956	3.740	3.883	3.869
9.160	9.408	9.291	9.434	9.561	8.150
8.223	8.664	8.497	8.426	8.306	7.564
5.542	5.146	5.111	5.520	5.183	5.425
6.242	6.353	6.346	6.458	6.346	6.156
5.976	5.856	5.671	5.780	5.868	4.997
5.548	5.837	5.615	5.640	5.694	5.788
3.989	4.108	4.458	3.679	4.168	3.128
2.310	2.363	1.853	2.084	1.866	2.361
2.427	2.273	2.585	1.900	2.886	2.371
5.223	4.771	5.075	4.905	4.919	5.461
5.596	5.780	5.984	6.050	5.789	5.691
5.890	5.113	4.980	5.329	5.526	6.126
6.424	6.925	6.565	6.963	7.168	7.080
2.597	3.249	3.200	3.184	3.726	5.484
7.312	7.612	7.493	7.316	7.337	7.073
9.194	9.049	9.100	9.271	9.220	7.961
3.895	4.471	3.958	4.455	4.941	6.586
4.615	5.209	4.714	5.401	5.423	4.138
4.439	4.281	5.407	3.513	3.683	4.335
2.278	2.692	2.632	3.198	3.250	3.424
3.687	3.522	3.428	3.641	3.650	4.354
6.076	6.304	6.501	6.414	6.310	5.432
5.260	5.216	5.980	5.594	5.870	3.881
3.338	3.625	3.343	3.626	3.789	2.930
6.144	5.998	5.572	6.462	5.473	6.957
3.555	2.781	3.242	2.869	2.479	2.740
2.012	1.934	2.148	2.434	2.097	2.177
2.093	2.947	3.028	2.964	2.630	2.534
5.785	6.339	6.315	6.039	6.252	5.825
1.647	1.972	2.307	2.236	2.262	2.516
2.974	3.247	2.079	2.532	2.603	2.049
2.667	2.354	2.676	2.823	2.869	2.294
3.371	4.152	3.823	3.460	3.688	2.066
4.366	4.113	4.013	4.173	3.431	2.070
7.080	7.429	7.315	7.073	7.097	5.916
8.451	8.637	8.440	8.240	8.690	8.168

7.137	6.574	6.857	6.707	6.645	4.515
3.084	1.974	2.331	3.412	2.614	3.653
3.512	3.888	3.595	3.412	3.089	1.849
3.887	3.363	4.396	4.129	4.065	3.216
5.967	6.443	6.402	6.166	6.192	5.283
5.771	5.805	5.037	4.167	4.625	4.744
2.814	2.987	3.229	2.754	3.351	3.134
7.161	6.345	6.666	6.572	6.467	4.719
6.706	6.597	7.174	6.728	6.586	1.237
5.951	5.733	5.920	4.420	5.486	4.779
4.982	4.008	3.603	3.400	4.587	3.035
3.559	4.804	5.369	4.206	4.617	6.540
6.810	7.619	7.047	8.154	8.297	9.702
6.551	6.047	6.289	6.634	7.123	7.675
5.550	4.097	4.170	4.961	5.056	4.515
3.113	3.660	3.379	3.368	2.663	1.988
4.144	3.635	4.661	3.699	4.018	3.424
7.858	7.969	7.748	7.472	7.667	7.751
3.994	3.960	4.136	3.609	3.543	3.107
4.346	3.749	4.054	4.123	4.037	2.354
2.379	2.859	2.175	2.991	3.298	4.123
5.436	5.456	5.306	5.029	5.497	2.800
3.611	3.289	3.899	2.955	4.191	4.201
6.247	4.971	5.121	5.363	5.606	3.028
4.416	5.027	5.418	4.933	4.812	3.464
4.170	3.971	4.077	4.008	4.000	5.095
6.674	6.247	6.214	6.867	6.119	3.116
6.723	6.650	6.653	6.639	6.920	5.854
5.205	5.072	5.400	5.419	5.441	4.161
2.448	1.746	1.652	2.169	2.812	1.862
9.231	9.093	9.181	9.629	9.586	10.439
5.390	5.191	5.146	5.041	5.251	3.764
5.890	6.301	6.249	6.247	6.341	7.119
3.103	2.871	3.111	2.128	2.381	2.850
5.150	5.469	6.002	5.511	5.360	5.735
4.420	4.313	4.478	4.307	4.192	4.727
3.949	3.949	4.015	4.300	4.084	2.103
1.817	2.028	2.488	1.487	1.873	1.723
7.279	7.377	7.351	7.333	7.297	5.974
5.237	4.866	4.555	5.603	5.105	6.216
2.484	2.377	2.497	1.214	1.844	1.719
4.097	4.609	4.314	4.164	4.051	3.168
5.085	4.419	4.175	4.759	4.022	3.184
3.052	3.872	2.827	3.905	3.641	3.928
5.452	4.710	5.711	5.741	6.210	6.501
4.366	5.013	5.154	4.533	4.794	3.619
2.453	2.088	3.537	3.004	2.540	1.946
2.187	1.362	2.117	1.715	2.511	2.267
6.735	6.557	6.703	6.628	6.492	5.313
8.416	7.995	8.275	8.317	8.417	6.557

7.733	7.842	7.702	7.750	7.754	6.150
7.663	7.431	7.699	7.519	7.817	7.270
5.114	4.281	4.976	4.466	4.783	5.120
6.216	5.771	6.158	6.133	6.102	4.670
7.472	6.722	7.149	7.261	7.026	5.747
5.375	6.054	5.934	5.599	6.084	5.698
6.526	6.582	6.704	6.533	6.562	5.495
9.115	8.641	8.864	8.770	8.725	6.702
7.894	8.124	7.724	7.793	7.935	5.768
8.555	8.729	8.625	8.204	8.535	4.985
5.477	5.824	5.838	5.260	5.643	4.944
5.180	4.944	5.253	5.232	5.050	4.529
3.403	3.861	3.066	3.201	4.351	3.001
6.734	6.778	6.902	6.928	6.859	6.336
6.402	5.944	6.322	5.689	5.982	4.837
3.546	3.308	4.236	3.262	3.278	1.563
8.075	8.018	8.236	8.246	8.461	8.739
8.484	8.044	8.510	8.314	8.257	5.152
4.646	3.985	4.175	4.278	4.053	3.339
2.850	3.365	3.596	2.762	3.530	2.126
6.685	6.221	6.412	6.137	6.263	7.039
5.872	6.510	6.244	5.803	6.344	4.421
4.270	4.017	4.676	4.365	4.965	3.501
1.370	1.563	1.790	1.489	2.120	2.019
3.483	3.307	3.335	3.950	3.091	2.955
2.645	4.057	2.760	3.423	3.290	3.598
3.261	2.124	2.310	3.433	2.340	2.042
2.206	2.970	2.986	2.343	3.214	1.650
2.360	2.638	2.635	2.355	2.990	2.781
6.955	7.038	7.122	6.967	6.881	5.491
4.811	4.811	5.312	4.790	5.014	4.487
5.238	4.869	4.883	5.053	5.449	7.127
3.766	4.201	4.500	4.175	3.935	2.867
7.039	7.021	7.122	7.272	6.794	6.228
5.143	4.391	4.437	5.014	4.721	5.989
1.799	1.575	2.552	1.911	2.073	2.979
5.904	6.053	6.115	5.240	5.787	5.160
4.326	4.979	4.017	4.242	4.401	4.494
6.778	5.181	5.906	6.112	5.500	7.183
4.719	5.138	5.819	2.295	4.927	4.501
3.541	6.180	6.207	6.993	6.076	4.934
3.493	5.209	5.409	6.722	5.965	4.794
6.352	5.725	6.135	6.007	6.293	5.514
5.500	5.939	5.976	6.110	5.855	6.634
4.153	4.163	4.341	4.752	4.674	5.348
5.859	6.361	6.045	6.204	6.206	6.086
5.877	6.325	6.325	5.969	6.129	5.432
3.311	3.801	3.824	3.677	3.704	3.622
2.008	3.180	2.862	2.730	2.602	2.678
6.754	7.207	6.823	6.773	7.092	4.776

7.193	6.942	7.071	6.861	6.903	6.275
9.349	9.454	9.572	9.389	9.549	7.633
6.257	6.461	6.601	6.571	6.512	5.966
4.812	4.811	5.311	5.153	5.099	3.093
4.622	4.312	4.177	4.598	4.081	4.914
4.937	5.355	5.253	6.090	4.693	5.815
1.658	0.896	1.088	2.320	1.451	2.571
3.923	3.378	3.117	4.560	3.799	5.282
4.592	5.049	3.952	5.580	4.237	5.557
6.963	7.084	6.780	7.806	7.025	8.492
6.610	6.627	6.401	7.269	6.590	8.138
5.527	4.303	3.840	5.002	4.901	4.823
4.756	4.121	4.560	4.474	4.684	3.403
8.118	8.001	7.598	8.839	7.893	9.456
3.298	2.540	3.293	2.218	2.515	2.682
5.120	5.192	4.747	4.880	4.438	5.935
3.244	3.832	4.287	3.661	3.683	6.590
2.079	1.700	2.070	1.902	1.441	2.167
4.721	4.675	4.848	3.992	4.625	5.269
2.802	3.362	3.286	3.030	2.925	2.672
3.671	4.676	3.882	3.740	3.302	4.358
5.441	5.260	5.311	5.113	5.192	5.389
5.155	5.664	5.450	5.134	5.273	4.247
8.331	8.487	8.215	8.482	8.585	9.517
2.197	3.050	3.017	3.356	3.181	3.422
2.090	1.701	2.669	2.161	2.890	2.981
6.421	6.712	6.635	6.561	6.503	6.068
6.921	6.795	6.580	6.748	6.840	7.752
2.002	1.966	1.877	1.861	1.890	3.323
7.655	7.504	7.135	5.975	6.707	6.333
3.991	5.079	4.793	3.838	4.498	3.266
5.095	5.282	5.281	5.736	5.962	8.639
6.646	6.430	6.403	6.143	6.611	3.840
2.653	3.375	2.653	3.013	3.007	2.472
3.605	2.768	3.993	3.548	3.650	2.591
3.616	3.991	3.725	4.028	3.733	3.805
3.097	2.779	3.265	2.383	3.176	3.168
3.483	4.185	3.704	3.390	3.687	3.884
3.921	5.108	5.102	4.365	4.514	5.511
1.852	2.238	1.915	2.079	0.646	1.879
4.629	4.397	4.149	3.745	4.183	5.001
2.842	1.673	1.793	1.843	1.511	2.345
3.356	3.088	3.094	2.589	2.310	2.064
2.910	2.827	2.682	2.659	2.500	3.284
6.410	6.757	6.804	6.467	6.364	4.771
4.250	4.372	4.263	4.261	4.154	3.419
4.032	4.245	3.919	4.939	4.835	4.711
2.181	1.512	2.078	1.881	2.082	2.510
1.659	2.124	1.826	1.924	1.405	3.734
2.006	1.849	2.062	2.163	1.831	3.940

2.067	2.037	2.272	2.251	1.919	3.966
2.145	3.479	3.592	3.312	3.059	4.030
3.120	2.639	2.396	3.440	3.597	3.434
3.184	2.647	2.590	3.714	3.506	3.607
4.519	4.715	4.715	3.990	4.455	5.309
5.327	5.111	5.143	4.658	4.997	5.781
9.170	9.354	9.284	8.971	9.515	5.715
3.934	4.203	4.388	3.743	3.731	3.673
3.792	3.331	4.923	4.321	3.767	2.768
3.280	3.988	3.969	3.652	4.248	3.434
2.376	3.659	3.854	3.507	4.000	3.099
3.467	4.364	4.658	3.802	4.463	3.725
3.508	3.093	3.631	2.982	3.555	2.922
3.387	4.014	4.292	3.284	3.546	4.432
4.775	4.810	4.780	5.091	4.651	5.773
3.814	3.779	3.833	3.503	3.544	4.003
4.919	5.032	5.066	5.319	5.147	4.540
8.149	8.337	8.266	8.356	8.520	7.356
5.960	5.958	5.849	6.253	5.982	6.912
4.170	4.572	4.473	4.142	4.225	4.291
5.474	5.420	5.580	5.504	5.407	4.158
6.782	6.749	6.752	7.023	6.801	4.191
3.827	3.144	3.109	4.790	3.161	2.136
7.086	6.957	7.224	7.243	7.298	6.526
7.699	7.025	6.648	7.494	7.589	8.587
2.617	2.087	2.967	2.637	2.419	2.491
2.037	1.755	2.201	2.141	1.788	2.051
2.386	2.364	1.700	1.837	1.609	1.684
4.725	5.364	5.344	5.198	4.961	4.319
6.714	5.961	6.195	6.647	6.575	8.096
3.707	3.859	3.446	3.423	3.262	5.015
8.153	8.243	8.123	8.244	8.076	7.352
3.250	4.160	4.187	4.834	4.490	5.459
3.115	2.747	3.048	3.273	3.423	3.938
6.419	6.776	6.682	6.685	7.312	5.542
7.099	7.158	7.173	6.603	6.731	6.670
5.085	4.962	4.827	4.960	5.246	4.771
3.078	3.594	3.271	3.115	3.817	3.083
6.124	5.771	6.158	6.242	5.772	5.477
4.401	4.903	4.568	3.939	4.436	4.073
4.989	4.910	4.706	4.983	4.753	6.867
2.342	2.770	2.267	2.276	2.425	3.662
2.329	2.346	2.756	2.153	2.162	2.728
4.930	5.749	5.822	4.792	4.250	3.007
5.623	5.206	5.982	6.055	6.525	7.838
4.369	4.993	4.654	5.730	5.727	6.339
3.096	3.639	3.312	3.592	2.909	4.149
4.675	4.834	4.578	4.733	4.402	3.738
4.582	3.823	4.067	4.530	4.142	5.528
5.685	5.118	5.538	5.833	5.683	5.486

8.781	8.989	8.475	8.467	8.640	8.851
2.568	3.235	2.891	2.503	2.758	2.287
1.813	1.734	2.801	2.690	3.088	1.930
4.766	3.825	3.767	4.796	3.872	4.723
3.609	3.715	4.580	4.282	3.858	4.192
6.849	5.775	6.775	5.576	6.369	6.244
6.001	5.730	5.519	6.343	6.071	7.497
5.383	4.725	4.727	5.563	5.326	6.561
1.886	2.275	2.933	2.575	2.923	2.926
8.960	8.867	9.058	9.378	9.529	10.293
6.049	5.530	6.126	6.058	6.254	5.940
4.977	5.169	5.513	5.030	4.381	4.579
7.852	8.268	8.277	7.853	8.204	7.915
5.595	5.459	5.949	5.996	5.299	6.526
4.243	5.011	4.907	4.616	4.857	3.694
1.659	1.431	2.015	2.224	2.156	3.025
4.266	4.759	4.835	4.232	4.076	3.777
1.828	1.825	1.844	2.177	2.761	1.455
3.858	3.519	4.403	3.636	3.592	3.448
2.596	3.239	4.209	2.744	3.758	3.588
2.738	3.509	3.131	2.956	3.981	3.392
4.097	4.624	4.525	3.676	4.246	4.352
1.984	1.797	2.879	2.267	2.283	2.397
6.027	5.647	6.161	5.373	5.582	5.904
4.177	3.349	5.142	4.927	4.592	3.919
2.716	3.173	2.809	2.846	2.249	2.149
1.914	1.730	2.522	2.004	1.900	2.132
4.709	4.393	4.245	4.725	3.574	3.570
1.241	1.962	2.485	1.653	2.244	1.357
2.899	4.523	2.831	3.617	3.833	3.678
3.342	3.781	4.351	2.771	1.425	3.210
3.961	2.804	3.499	3.090	2.952	3.606
5.241	6.123	5.991	5.750	5.112	5.393
3.349	3.450	3.946	3.365	3.618	3.605
9.483	9.498	9.800	9.715	9.180	8.575
2.121	2.707	2.805	2.229	2.897	3.002
7.134	6.913	7.132	6.603	6.815	7.663
9.074	8.935	9.334	9.094	9.189	9.938
6.156	6.037	6.140	6.082	6.343	6.556
5.035	4.688	4.885	4.798	4.771	5.266
4.312	3.880	4.395	4.471	4.481	5.186
3.794	4.075	3.953	3.471	3.892	3.853
4.503	6.128	5.335	4.342	4.405	4.543
3.664	3.843	4.253	3.753	3.167	4.151
5.197	5.300	6.013	5.124	5.339	5.282
3.515	4.134	4.699	4.031	4.130	4.210
5.532	5.281	5.494	5.851	5.678	4.985
9.013	9.256	9.084	9.111	9.072	7.513
7.476	7.645	7.502	7.486	7.478	6.247
3.606	3.722	4.206	3.666	3.517	5.050

3.633	4.632	4.264	4.076	4.989	4.101
4.854	5.027	5.097	4.383	5.229	5.095
6.944	7.341	6.759	5.937	7.146	6.862
7.147	7.110	7.199	6.370	6.872	6.963
3.947	4.326	4.726	3.826	4.140	4.539
4.424	7.237	6.489	5.894	6.274	6.746
8.236	8.665	8.336	7.633	8.582	8.171
6.800	7.186	7.258	6.469	7.007	6.663
7.952	8.334	8.352	7.616	8.179	7.928
7.914	8.408	8.363	7.625	8.125	7.898
4.269	5.084	5.121	4.278	4.803	5.152
6.687	7.533	7.642	6.969	7.524	7.626
6.331	7.180	7.287	6.635	7.188	7.178
6.977	6.717	7.082	6.699	7.257	6.646
6.260	7.020	7.103	6.517	7.002	7.035
7.054	6.739	7.199	6.577	7.150	6.722
4.901	4.530	4.869	4.597	4.582	5.705
4.704	4.400	4.696	4.466	4.197	5.403
4.886	4.582	4.854	4.577	4.564	5.599
4.363	4.658	5.114	4.815	4.332	5.735
4.341	4.724	5.159	4.705	4.392	5.663
4.360	4.923	5.122	4.503	4.376	5.784
4.366	4.972	5.157	4.500	4.501	5.792
3.956	4.987	4.889	3.937	3.955	5.535
3.996	4.996	4.878	3.912	4.107	5.524
3.263	4.254	4.160	3.211	3.336	4.661
3.796	4.108	4.576	3.887	3.836	5.401
3.445	3.668	4.154	3.463	3.421	4.900
2.180	2.260	2.770	2.167	2.020	3.502
3.404	3.738	4.108	3.475	3.345	4.908
2.271	2.382	2.862	2.298	2.006	3.500
4.083	4.536	4.687	3.723	3.696	4.980
4.141	4.402	4.696	3.656	3.737	4.987
3.240	3.482	3.631	2.687	3.050	4.124
4.360	4.678	4.856	3.904	3.835	5.219
3.119	3.374	3.633	2.642	2.632	3.997
6.585	7.166	6.666	6.032	6.046	7.566
4.548	5.294	4.738	4.152	4.119	5.491
5.855	6.498	5.824	5.385	5.481	6.693
5.800	6.514	5.883	5.348	5.379	6.661
3.124	3.670	4.458	2.994	2.594	3.670
1.291	1.927	2.018	1.482	1.765	2.601
1.552	1.733	2.201	1.393	1.863	2.212
1.634	1.983	2.491	1.478	1.842	2.543
4.211	4.701	4.909	3.878	3.957	5.194
5.012	5.002	5.069	4.950	4.636	4.584
4.430	4.085	4.204	4.232	3.805	4.027
3.180	3.600	3.539	2.677	3.122	3.364
3.822	4.076	4.175	3.310	3.820	3.894
4.439	4.708	4.814	3.928	4.320	4.535

3.914	4.030	4.209	3.208	3.722	3.931
4.013	4.337	4.349	3.558	3.857	4.030
4.024	4.197	4.393	3.402	3.944	4.200
4.668	4.847	5.209	4.152	4.547	4.894
3.918	4.690	4.574	3.631	3.913	4.226
3.371	2.903	3.020	2.939	3.508	3.938
2.834	2.945	2.399	2.716	2.616	2.328
2.709	2.606	2.206	2.580	2.342	2.406
2.345	2.940	1.903	2.218	2.194	1.707
2.341	2.940	1.863	2.184	2.154	1.702
2.358	2.587	2.356	2.094	1.751	1.776
7.541	8.271	7.356	5.868	5.497	7.255
8.202	8.960	8.912	7.790	7.740	9.305
8.348	8.997	8.840	7.822	7.783	9.328
7.990	8.628	8.551	7.399	7.545	8.900
8.261	8.842	8.690	7.691	7.651	9.116
7.046	7.210	7.034	1.055	1.252	1.639
3.313	3.937	3.622	3.096	3.550	3.549
3.337	3.963	3.592	3.345	3.696	3.516
6.774	6.635	6.618	5.691	6.112	6.377
4.455	2.558	2.764	2.839	3.861	1.909
5.863	5.733	5.682	4.863	5.354	5.572
7.789	7.511	7.311	6.213	6.882	7.343
6.135	6.718	6.833	5.235	5.377	6.774
4.722	4.593	5.104	5.315	5.256	5.628
7.131	7.311	6.944	0.970	0.946	1.565
2.191	1.791	2.376	2.219	2.362	2.508
8.232	8.286	8.103	7.709	8.008	8.094
8.254	8.339	8.133	7.744	8.049	8.225
4.958	5.131	5.238	4.690	5.134	4.971
4.363	4.934	4.612	4.198	4.729	4.517
4.685	4.839	4.776	4.467	4.767	4.706
4.346	4.902	4.866	4.370	4.751	4.575
3.648	3.460	3.529	3.386	3.616	3.921
3.318	3.373	3.400	3.400	3.554	3.500
3.085	3.088	3.271	3.112	3.274	3.215
4.095	5.053	4.852	5.418	5.499	5.070
9.212	8.797	8.779	8.740	8.665	5.997
2.912	2.048	2.237	2.181	2.028	2.769
1.846	2.650	2.295	2.135	2.129	3.322
6.464	6.545	6.102	6.376	6.699	5.545
3.334	3.691	3.681	2.950	3.243	4.062
6.324	6.385	6.127	6.286	6.237	5.613
3.030	2.637	2.876	2.474	2.721	3.374
3.690	4.405	4.198	3.898	4.302	4.862
7.598	8.020	7.667	7.603	7.456	7.096
6.486	7.404	6.806	6.585	6.815	6.524
3.451	3.036	3.944	4.334	3.729	4.733
3.182	2.479	2.892	2.952	2.789	3.740
3.783	4.605	3.894	4.185	4.152	4.754

4.184	4.142	3.686	3.444	3.635	4.587
4.134	4.262	4.962	4.178	4.816	4.657
4.950	4.660	4.889	4.770	4.605	3.575
2.471	2.802	2.735	2.456	2.899	4.211
2.585	2.309	1.963	2.070	3.105	3.088
5.472	5.805	6.029	6.059	5.823	5.078
4.049	4.474	4.262	4.100	4.712	7.008
3.734	3.749	4.098	3.951	4.163	4.854
2.859	3.719	3.468	3.905	4.172	3.570
4.613	5.020	4.799	4.983	4.841	4.032
6.447	5.952	5.852	6.358	6.246	6.625
1.892	1.913	1.924	1.558	2.325	2.529
7.584	7.668	7.849	7.333	7.542	5.293
4.036	3.891	3.351	3.914	4.221	5.021
6.004	6.067	6.125	5.808	5.702	1.569
1.760	2.175	2.086	1.411	2.028	4.507
1.551	1.863	1.614	1.597	1.598	3.192
1.675	2.135	2.118	1.698	1.767	2.137
2.283	2.562	2.073	1.779	2.342	2.364
1.143	1.247	1.751	1.127	1.325	5.846
1.836	1.762	1.703	1.521	1.904	5.143
1.947	2.418	1.589	1.957	2.196	6.748
2.495	2.734	2.516	2.968	2.767	4.177
2.386	2.466	2.789	2.151	2.229	3.368
2.093	1.873	2.866	2.144	2.185	4.976
1.963	1.378	1.790	1.593	1.450	3.419
1.799	2.100	2.250	2.181	2.803	3.758
1.487	1.589	1.699	1.125	1.286	4.331
2.471	1.984	2.513	1.957	1.978	3.148
1.631	1.792	1.433	1.467	1.287	2.437
2.379	2.242	1.476	2.224	2.558	2.707
2.056	2.879	2.674	2.886	3.032	2.366
1.700	1.736	2.142	1.450	1.491	3.796
2.188	2.383	2.220	2.558	2.933	2.255
1.410	1.531	1.513	1.344	1.412	2.349
2.544	2.274	2.644	2.022	2.618	1.916
1.566	1.315	1.334	1.551	1.426	0.967
1.445	1.249	0.886	1.046	1.071	1.252
1.980	2.827	2.492	2.895	2.764	3.162
1.741	2.274	1.898	2.283	3.002	4.503
2.470	1.937	2.515	2.083	2.410	1.905
1.680	1.897	1.819	1.560	1.611	4.550
1.748	2.482	1.989	1.995	2.413	4.618
2.577	2.447	2.895	2.812	2.127	4.867
2.107	1.758	2.059	1.429	2.069	3.097
2.224	1.222	1.703	1.457	1.787	1.213
3.880	3.366	3.772	4.052	3.705	3.366
7.416	7.231	7.530	7.529	7.251	5.830
1.768	1.654	1.592	1.375	1.520	2.118
2.827	3.544	3.595	2.392	2.852	4.707

3.020	2.869	3.313	2.856	3.543	3.904
2.473	3.448	2.839	3.054	2.794	2.676
7.082	7.184	7.163	7.263	7.085	5.369
6.622	6.852	7.094	6.699	6.805	7.294
5.289	5.093	5.173	5.776	5.401	5.920
2.438	1.732	2.913	2.284	2.111	2.478
2.706	2.861	3.051	2.704	3.287	3.273
4.092	4.034	4.164	4.742	4.921	6.130
3.655	3.302	3.656	4.064	3.664	4.097
3.798	4.073	3.810	3.491	3.603	3.623
4.938	4.797	5.480	4.338	5.296	5.923
4.699	4.888	4.701	4.663	4.454	3.048
6.091	5.557	5.849	5.403	6.054	4.494
3.482	3.008	2.873	2.725	2.424	2.268
6.423	6.615	6.852	6.519	6.535	6.084
4.177	4.523	4.653	4.770	4.761	2.765
3.377	2.300	3.173	1.374	3.486	2.433
2.991	3.404	2.363	3.004	3.246	2.436
5.132	4.680	5.165	5.189	5.451	6.203
4.289	3.995	3.940	3.977	3.879	3.725
3.606	3.711	4.044	3.050	3.828	2.619
8.267	8.272	8.099	7.704	7.882	5.752
4.744	5.032	4.093	5.226	4.973	4.228
5.979	5.779	5.903	5.647	6.023	4.611
3.876	2.936	2.955	2.863	2.252	2.752
3.448	3.111	3.200	4.099	2.621	3.571
5.724	5.337	5.857	5.502	5.279	4.650
4.090	4.717	4.621	4.475	4.421	5.558
2.966	3.025	2.527	2.959	1.948	2.883
4.470	4.197	4.558	4.430	4.692	4.692
3.750	3.328	3.435	3.422	4.280	3.502
1.850	2.842	2.494	2.597	2.857	3.802
3.531	3.103	3.525	3.915	4.029	3.324
5.504	5.191	5.021	4.639	5.446	5.393
2.687	3.094	3.443	2.726	3.204	4.244
2.969	3.734	3.520	2.973	3.812	3.552
7.722	8.000	7.931	7.685	7.881	6.909
5.529	6.192	5.652	6.008	5.878	4.861
4.769	3.926	4.295	4.292	3.680	3.527
4.936	4.685	4.392	4.322	4.538	3.064
5.565	5.483	4.881	4.800	5.578	4.866
5.991	6.326	6.297	5.564	5.233	5.658
2.302	2.278	2.893	2.267	2.149	4.194
8.153	8.022	7.994	8.106	8.237	6.925
2.639	3.260	2.424	2.738	3.252	1.816
3.221	3.483	3.430	3.097	3.030	2.288
4.510	5.018	5.098	4.999	4.873	4.420
10.623	9.735	10.333	9.439	9.726	10.691
10.706	9.808	10.417	9.576	9.815	10.731
10.262	9.199	9.789	9.020	9.269	10.111

9.974	9.058	9.506	8.796	8.955	9.865
10.397	9.473	10.056	9.280	9.432	10.315
10.067	9.104	9.738	9.075	9.199	9.964
10.074	8.978	9.655	8.849	9.058	9.883
10.098	9.132	9.644	8.774	8.872	9.859
4.148	4.581	4.847	4.121	3.835	4.171
4.213	4.508	5.309	4.600	4.579	4.678
7.133	7.278	7.303	8.039	8.218	9.904
7.762	7.800	8.101	7.916	7.621	6.896
9.604	9.044	9.640	8.760	8.698	9.292
3.152	2.013	1.707	2.050	2.871	2.640
2.769	1.635	2.845	1.935	1.087	2.007
9.626	9.035	9.618	8.403	8.060	8.934
2.891	4.379	4.371	3.031	3.808	1.264
4.866	3.937	4.546	3.968	4.546	6.192
2.408	2.041	2.162	2.019	1.778	3.156
4.700	4.651	4.936	4.436	4.830	5.687
4.039	3.720	3.447	3.582	3.396	3.867
9.635	9.205	9.713	9.371	9.075	7.967
4.948	5.120	5.452	5.197	5.137	5.334
5.492	4.535	5.225	4.532	4.455	2.351
5.474	4.366	4.602	4.194	4.446	2.787
5.891	5.647	5.899	5.545	5.661	2.123
3.846	4.228	4.115	3.736	4.278	2.019
8.802	8.988	9.118	8.599	8.866	5.624
3.181	3.658	3.977	3.747	3.428	4.978
4.663	4.698	4.878	4.298	4.663	1.857
4.097	3.928	4.309	3.987	3.853	2.799
5.792	6.227	6.240	5.650	5.944	3.020
5.364	5.860	5.923	5.121	5.077	2.796
1.715	2.375	1.962	1.725	1.848	2.293
5.087	5.322	5.480	5.156	5.904	3.396
3.006	3.245	3.131	2.622	2.713	3.632
1.893	2.813	2.802	3.057	2.712	2.427
2.805	2.425	3.120	2.907	2.921	3.712
4.713	4.065	4.286	4.344	3.848	5.428
4.203	4.143	3.917	4.314	4.335	4.998
2.932	2.794	3.252	3.116	2.973	2.679
4.879	5.023	4.774	5.225	5.014	3.505
5.724	5.837	5.406	5.918	5.532	5.073
5.084	4.690	4.879	4.095	4.665	4.605
6.078	5.559	5.481	5.437	5.917	4.662
3.252	2.727	3.470	2.886	2.610	2.367
5.330	5.489	5.572	5.066	4.996	2.093
5.920	6.281	5.998	6.259	6.068	5.730
3.806	4.284	3.648	4.388	4.825	4.806
6.720	7.037	5.633	6.124	6.015	6.149
4.427	4.033	4.249	4.029	4.037	3.942
3.442	4.165	3.526	4.035	3.296	4.708
5.615	6.175	6.161	6.361	5.607	6.321

3.807	4.228	3.772	3.917	4.133	3.137
4.383	3.889	4.314	4.434	4.399	4.268
9.434	9.404	9.329	9.277	9.238	8.494
4.545	4.045	4.459	4.106	4.355	4.571
4.366	4.306	3.874	3.901	4.111	4.011
4.283	4.246	4.228	3.836	3.972	3.912
4.821	4.760	4.343	4.278	4.354	4.300
5.453	5.920	5.711	5.487	5.943	4.996
5.377	5.164	5.386	5.099	5.250	4.347
4.112	4.050	4.057	3.057	4.193	3.244
6.966	6.752	6.731	6.285	6.929	7.683
3.401	3.335	3.335	3.567	3.178	3.594
4.931	5.873	5.806	3.667	4.800	5.071
4.213	4.814	5.087	4.291	4.405	3.958
2.606	3.305	3.096	3.249	3.579	4.508
3.162	3.603	3.477	3.861	4.718	4.185
7.825	8.126	9.971	8.104	10.510	6.457
2.953	3.546	3.715	2.984	3.672	3.220
5.475	5.693	6.090	5.502	5.783	4.370
3.486	3.695	3.870	4.015	4.264	4.920
3.545	3.850	4.752	4.232	5.323	3.524
7.353	7.424	7.375	6.966	6.736	6.179
7.674	8.560	7.713	7.195	6.203	6.589
4.919	5.009	4.770	4.408	4.145	4.307
2.983	3.020	3.374	2.330	2.233	2.149
3.893	3.523	3.880	3.157	2.849	2.668
3.997	4.050	4.457	3.275	2.868	3.089
4.493	4.589	4.943	3.828	3.279	3.544
3.408	3.605	3.612	2.891	3.057	3.033
3.293	2.961	3.324	2.627	3.641	2.780
4.960	4.985	4.900	4.868	5.135	6.089
3.103	2.820	3.035	2.613	3.002	3.483
6.639	6.665	6.431	6.519	6.318	5.592
2.666	4.040	3.211	3.192	4.002	0.891
6.524	6.730	6.683	6.798	6.669	6.663
3.272	2.579	2.566	3.272	2.681	4.618
3.438	3.518	3.777	3.435	3.446	4.330
3.360	4.385	4.246	3.996	4.085	3.385
2.826	2.493	1.668	2.339	2.482	2.603
2.822	2.516	2.734	3.007	3.019	2.251
3.600	3.439	3.329	3.380	3.703	2.298
4.754	4.554	4.181	4.474	4.129	3.418
5.620	5.537	5.451	4.612	4.568	3.160
6.856	5.034	5.533	6.324	6.116	6.035
3.999	3.646	3.997	3.807	3.924	3.308
8.132	8.556	8.235	8.102	8.455	8.745
2.872	2.901	3.753	2.440	3.608	1.379
4.070	4.207	3.617	3.885	3.839	2.934
3.143	3.495	4.062	2.613	3.374	3.987
4.422	3.328	4.154	3.590	3.176	3.251

4.690	4.752	4.767	4.295	4.781	4.705
2.490	2.198	2.642	2.106	2.512	2.456
2.391	3.151	2.256	2.845	2.120	3.315
2.264	2.509	3.521	2.581	2.686	2.562
3.584	2.435	3.485	1.984	3.242	3.242
2.434	2.365	2.330	2.369	1.935	2.167
3.935	1.981	2.184	3.542	2.534	1.815
1.128	2.211	2.238	2.168	2.259	2.079
6.621	6.844	6.903	6.837	6.769	6.437
2.908	2.124	2.603	2.604	2.320	3.425
3.101	2.025	2.496	2.538	2.633	1.821
2.090	2.852	2.189	2.920	2.594	2.398
2.715	1.869	2.595	2.531	2.765	3.497
1.501	1.934	2.168	1.538	2.849	2.747
2.414	2.135	1.731	1.831	1.569	2.756
4.412	5.239	4.689	3.756	5.396	4.731
3.028	3.060	3.173	3.154	2.662	1.954
2.863	1.946	2.471	1.938	2.440	4.854
4.718	5.322	5.046	4.938	5.423	4.707
2.245	3.350	3.646	3.322	4.588	2.845
3.250	2.929	3.835	2.189	2.446	2.165
3.035	3.187	2.776	2.703	2.929	2.563
3.875	3.857	3.809	3.573	4.005	4.204
2.390	2.036	2.640	1.987	2.834	2.709
3.455	2.533	2.083	2.596	3.320	2.617
2.105	1.440	2.418	2.768	1.088	2.728
3.222	3.262	2.787	2.693	3.013	4.514
5.103	4.721	4.907	5.326	5.042	6.195
6.883	6.376	6.573	6.243	6.438	4.988
5.278	5.208	5.002	5.106	5.123	5.130
3.737	4.444	3.961	3.902	3.621	4.315
5.103	4.608	5.233	4.727	4.847	3.944
2.528	2.485	2.361	1.925	1.666	1.444
7.740	6.929	7.365	7.555	7.075	7.467
5.170	5.117	5.016	5.024	5.122	6.009
2.748	2.963	2.480	2.217	2.528	3.215
2.661	2.748	2.443	2.258	2.790	3.553
3.418	3.077	2.957	3.582	3.569	4.227
3.924	3.697	3.869	2.966	3.810	3.798
3.824	4.535	4.026	3.984	3.863	4.559
1.593	1.429	1.636	1.074	1.801	1.578
3.819	3.680	2.900	4.079	4.151	4.381
1.586	1.672	1.994	1.597	1.944	1.922
4.047	4.035	4.836	4.396	3.918	1.568
5.920	6.302	5.874	5.932	6.027	6.956
2.658	2.797	2.847	2.608	2.677	2.593
3.836	4.147	4.052	4.268	3.614	4.917
6.006	6.569	6.269	6.072	6.262	7.379
4.672	4.307	4.594	4.547	4.436	5.440
4.121	3.877	3.714	2.978	3.535	4.387

4.053	4.658	4.716	3.718	3.864	4.661
3.099	3.896	3.098	3.118	2.362	3.037
1.043	2.070	1.907	1.119	1.789	2.773
3.446	2.848	2.518	2.325	3.493	3.146
4.764	5.372	5.186	4.329	4.508	4.525
2.872	2.244	1.689	2.189	1.841	1.660
3.749	3.416	3.306	3.546	3.477	2.568
6.464	6.807	6.457	6.843	6.796	6.507
2.263	3.063	2.535	3.023	2.970	0.870
5.419	5.958	4.020	5.032	3.610	3.803
2.192	1.825	1.702	1.727	2.021	2.315
1.708	2.627	1.983	1.729	1.915	1.870
1.802	1.910	3.304	3.958	2.440	2.906
2.297	2.244	1.700	1.551	2.042	1.753
2.887	2.609	2.529	1.987	2.726	2.010
2.114	1.902	1.737	2.074	1.755	1.620
4.211	4.809	4.952	3.910	4.442	4.216
4.076	4.255	4.972	4.261	4.411	3.498
3.345	2.888	3.180	4.003	3.128	3.479
3.783	3.347	3.834	4.494	3.728	3.797
5.378	5.449	5.431	5.337	4.994	5.246
5.107	4.749	4.540	4.767	5.168	5.739
2.525	1.852	2.087	2.097	2.302	2.102
6.287	6.196	6.031	6.501	6.096	3.949
6.937	7.336	7.040	7.184	7.228	6.515
5.381	5.450	5.595	5.955	5.745	6.563
2.732	2.879	3.464	3.113	2.932	2.879
9.665	9.947	9.855	10.022	9.942	8.397
4.935	4.982	5.228	5.139	5.477	5.878
2.922	2.721	2.173	1.913	2.302	2.932
2.884	1.870	3.087	2.530	2.981	1.014
3.284	4.850	3.994	4.488	3.784	4.690
6.330	6.440	6.605	6.295	6.306	6.004
3.506	4.187	4.062	3.965	4.353	3.870
7.872	7.932	7.893	7.756	7.875	7.204
4.911	4.404	4.451	4.824	4.516	3.826
7.250	7.307	7.108	6.971	7.237	6.681
5.048	5.561	5.322	4.810	4.775	6.432
5.162	5.042	5.075	5.089	4.718	4.473
7.909	7.622	7.785	7.902	7.758	7.599
2.812	3.254	2.548	2.811	3.535	2.540
5.627	5.509	5.342	5.591	5.361	6.197
2.772	2.086	1.894	1.700	2.174	1.901
7.270	7.687	6.850	7.084	6.895	7.608
6.061	6.656	6.637	6.092	6.336	5.257
2.415	3.116	4.002	2.966	3.062	2.432
2.653	2.095	3.376	2.181	2.427	1.558
4.951	4.901	5.138	4.373	4.424	4.080
4.945	4.904	5.464	4.952	4.945	5.102
6.953	6.994	7.023	6.895	7.062	6.155

3.647	2.697	3.568	3.963	4.566	5.126
7.066	7.101	6.934	7.160	7.285	4.634
5.712	5.568	6.028	6.013	5.484	5.569
5.793	6.077	5.808	6.256	6.168	7.005
4.500	3.674	3.845	4.283	4.167	3.928
7.534	7.642	7.577	7.542	7.661	7.331
4.359	3.377	4.195	4.218	4.210	3.859
6.024	4.191	4.746	5.208	5.226	5.584
7.150	6.234	6.419	6.879	6.208	6.708
6.900	5.133	5.897	6.652	6.059	6.251
5.125	4.785	4.857	5.738	4.360	4.191
4.628	4.231	3.552	4.504	4.075	3.951
4.737	4.363	4.146	4.450	4.168	3.790
3.248	2.501	2.459	3.656	2.449	2.798
4.940	4.250	4.437	5.368	4.760	4.951
4.075	3.064	2.601	3.286	2.467	3.026
9.012	8.282	8.650	9.088	8.689	9.061
3.316	3.562	3.798	2.820	3.236	2.997
8.554	7.926	7.804	8.775	7.998	8.348
4.472	3.483	3.397	3.281	3.472	3.291
7.708	7.425	7.158	7.986	7.256	7.160
9.715	8.982	9.118	9.768	9.171	9.634
6.090	5.794	5.633	6.522	5.711	5.845
8.968	8.573	8.534	9.339	8.826	9.029
7.795	7.107	7.167	7.884	7.458	7.572
6.157	5.158	5.645	6.203	5.410	6.312
7.771	7.276	7.352	7.833	7.480	7.494
8.798	8.222	8.481	8.970	8.529	8.541
7.626	7.112	7.019	7.705	7.171	7.150
6.861	6.524	6.134	6.900	6.416	6.577
8.054	7.435	7.414	8.111	7.584	7.897
6.010	4.372	5.366	5.804	5.468	5.753
5.723	5.118	5.685	5.898	5.689	5.176
4.623	4.086	4.027	4.857	3.860	4.006
7.063	6.878	6.755	6.874	6.922	7.718
2.816	2.286	2.640	2.479	2.300	2.201
8.014	7.994	7.936	7.872	8.081	7.004
6.272	6.103	6.091	6.015	6.306	4.485
5.551	5.415	5.700	5.689	5.824	3.780
5.113	4.145	4.539	4.656	4.237	3.304
3.881	4.483	4.769	4.571	4.385	4.295
3.629	3.411	3.899	3.954	3.392	3.449
5.428	4.800	4.815	5.118	4.674	5.163
2.526	3.125	3.068	3.049	3.599	3.596
6.913	7.211	7.288	7.022	7.162	6.217
3.655	3.177	3.102	2.746	4.328	3.435
6.160	5.221	5.619	5.958	5.974	6.379
2.718	3.112	2.216	2.505	3.055	3.157
3.865	4.650	5.111	4.308	3.750	4.934
4.809	4.850	5.233	4.698	5.494	5.395

3.047	1.803	2.814	2.580	2.395	2.676
5.104	5.541	5.464	5.694	5.607	4.824
4.157	3.932	3.773	3.729	4.088	2.794
7.480	7.145	7.256	6.939	7.049	6.765
1.768	1.239	1.925	1.244	1.891	2.239
1.279	1.987	1.584	1.082	1.754	1.640
2.932	3.119	2.969	2.302	3.284	4.228
2.602	2.511	2.830	2.270	2.735	3.423
4.241	4.298	4.194	4.416	4.749	4.078
6.011	5.981	5.918	5.847	5.856	5.195
2.307	2.979	1.974	1.850	2.477	2.487
3.673	3.303	2.876	3.597	3.640	2.519
4.048	3.843	3.649	3.199	3.304	1.689
3.253	3.248	3.325	3.350	3.376	3.569
6.129	6.415	6.614	6.935	6.464	7.439
4.431	4.343	4.429	5.018	4.456	4.036
8.210	8.412	8.365	8.196	8.385	7.400
1.991	2.924	1.708	2.685	2.826	3.369
3.488	3.842	3.557	3.741	3.523	5.048
1.913	1.693	1.461	1.931	2.384	1.837
4.563	4.405	4.051	4.573	4.752	6.982
5.281	5.718	5.516	5.288	5.204	4.647
3.265	2.761	2.970	2.240	2.590	1.766
4.341	3.859	3.579	3.128	3.453	3.840
4.675	4.580	4.009	4.414	4.456	5.136
5.027	4.141	4.582	4.449	4.841	2.485
5.791	5.703	5.378	5.378	5.812	4.667
2.532	2.967	3.451	2.285	2.839	3.986
4.564	4.179	4.540	3.793	3.774	5.110
8.484	8.559	8.040	8.343	8.399	9.292
3.989	3.591	4.407	3.679	4.114	5.920
4.442	3.736	3.752	3.947	3.762	4.220
2.753	2.748	3.361	2.626	2.552	3.514
1.953	2.445	1.745	1.600	1.726	2.617
3.658	3.908	3.392	1.909	3.420	3.107
3.853	3.624	3.939	4.670	4.224	3.404
5.668	6.295	6.140	5.820	5.665	7.305
4.984	4.494	4.773	4.147	4.733	5.698
7.501	7.232	6.995	7.771	7.119	8.787
4.838	4.007	3.522	4.046	4.168	6.350
5.107	3.260	2.733	2.878	3.918	2.401
6.359	6.436	6.258	6.935	6.534	5.914
5.413	4.959	4.958	4.642	4.823	1.988
7.002	7.030	7.281	6.940	7.199	8.417
3.407	2.610	2.816	3.050	1.973	4.133
6.062	4.963	5.547	5.141	5.880	4.690
8.475	7.978	8.181	8.207	8.227	5.002
3.587	2.377	3.643	3.329	2.702	1.394
7.391	7.321	7.112	6.972	7.077	7.563
3.973	3.698	3.311	3.973	3.721	3.973

2.198	2.186	2.037	1.790	2.054	2.639
3.025	2.726	2.335	2.358	2.131	1.565
4.340	4.166	4.517	3.677	4.455	4.604
8.165	7.996	8.079	8.324	8.184	7.396
3.525	3.234	3.658	3.301	3.409	4.935
3.510	4.279	4.365	3.828	3.439	3.714
3.523	3.980	3.510	3.092	3.891	3.112
6.669	6.537	6.537	6.606	6.788	5.258
6.485	6.064	6.423	6.685	6.655	7.558
4.440	4.559	3.976	4.283	4.132	3.747
3.947	3.562	3.597	2.891	2.789	1.793
6.000	5.935	5.880	5.398	5.623	4.766
1.535	2.042	1.874	1.520	2.332	1.858
7.255	7.311	6.940	7.245	7.134	4.622
6.010	5.485	5.713	5.797	5.762	3.672
4.950	5.110	5.125	5.401	5.027	6.241
7.815	7.046	6.623	6.530	7.138	7.351
4.124	4.259	4.470	4.182	4.610	5.486
6.958	6.867	7.077	6.936	6.956	8.345
6.935	6.731	6.772	7.109	6.772	5.190
4.537	4.265	4.301	4.963	4.657	5.975
5.757	5.412	4.588	5.132	5.411	4.907
3.455	3.166	3.565	3.378	2.768	3.545
2.342	2.629	3.199	1.863	2.868	4.591
5.978	6.370	6.215	6.121	6.290	5.134
2.256	3.042	3.140	3.271	2.041	1.470
3.752	3.885	3.752	4.369	4.286	5.115
3.172	3.135	3.524	3.025	2.766	1.398
5.290	5.030	5.018	5.172	5.188	5.154
6.001	5.907	6.240	6.377	6.010	5.457
5.014	5.078	4.873	5.141	4.997	3.774
3.483	4.289	3.906	4.114	3.603	2.693
6.141	6.357	6.415	6.351	6.711	4.123
8.190	8.170	8.220	8.087	8.201	6.680
5.711	5.800	5.785	5.679	5.504	4.669
6.192	5.703	6.419	5.956	5.891	3.385
3.893	2.818	3.597	2.249	3.120	3.553
6.611	6.455	6.700	6.647	6.579	5.715
5.540	5.822	5.883	5.753	5.890	3.585
4.958	4.966	4.682	4.709	5.024	3.270
5.082	5.383	5.146	5.407	5.029	3.323
7.296	7.328	7.301	6.959	7.315	4.030
5.060	5.313	5.477	5.093	5.162	3.865
5.712	5.826	5.652	5.580	5.689	3.496
7.714	7.936	8.104	7.927	8.060	4.545
4.863	4.918	4.402	4.662	4.477	3.863
2.878	3.244	3.373	3.347	3.175	4.099
7.973	7.236	7.631	7.573	7.607	7.502
8.237	7.792	7.914	7.751	7.771	5.457
2.868	2.760	2.372	3.706	2.644	2.539

1.844	2.305	2.285	2.613	2.351	2.656
3.678	3.988	3.904	3.393	3.612	3.419
4.771	5.557	5.344	5.212	5.255	3.070
3.274	2.671	3.093	2.643	2.899	3.822
4.773	4.973	4.895	4.661	4.425	2.186
8.337	8.123	8.156	8.095	8.336	5.364
4.467	4.454	4.406	4.583	4.115	2.958
3.778	3.851	3.559	3.292	3.843	3.810
6.677	6.482	6.765	6.510	6.786	3.140
3.404	3.319	3.746	4.046	3.776	2.207
6.534	6.464	6.218	5.919	6.391	3.209
7.564	6.830	7.181	6.916	7.155	5.567
5.313	5.481	4.448	6.157	6.409	7.154
4.744	5.557	5.419	4.833	5.220	4.624
5.994	5.280	5.833	5.433	5.839	3.546
6.444	6.287	6.636	6.551	6.671	5.225
6.557	5.188	6.194	6.735	5.596	3.005
7.608	5.399	6.577	7.621	5.962	3.825
7.703	7.094	7.247	7.621	7.680	9.120
3.791	4.423	3.749	3.506	3.977	3.692
2.956	2.373	2.868	2.725	2.598	2.083
4.893	4.294	4.712	4.912	4.419	5.832
5.553	5.529	5.924	5.817	5.840	7.221
3.679	3.698	3.928	4.052	4.294	5.297
7.895	7.640	7.968	7.749	7.833	5.703
5.544	6.732	6.737	6.551	5.533	3.892
2.892	2.628	2.847	2.994	2.914	3.494
7.067	6.704	6.840	6.493	6.599	4.416
3.068	3.797	3.681	2.962	3.205	2.654
7.290	7.334	7.358	6.834	7.451	5.831
4.061	3.887	4.524	4.281	3.918	3.377
3.058	3.177	3.166	3.461	3.444	2.402
1.598	1.909	1.761	1.706	1.534	1.602
2.795	3.558	3.408	2.992	3.028	2.464
4.693	4.164	4.174	4.062	4.708	3.515
5.087	5.097	5.225	5.325	5.436	4.265
3.449	3.437	3.734	2.983	3.824	2.921
2.906	2.851	3.307	3.017	2.858	3.251
4.391	4.890	4.554	4.174	4.176	2.880
7.609	7.438	7.094	7.420	7.378	5.327
3.130	2.508	2.929	2.657	3.219	4.074
4.053	4.016	4.296	4.356	3.879	3.261
6.556	6.983	6.697	6.924	6.757	7.073
6.285	6.396	6.692	6.526	5.949	3.723
6.204	5.949	6.086	6.420	5.839	7.639
3.045	4.017	3.568	2.815	3.617	3.104
2.872	2.845	3.384	2.764	3.253	2.950
2.203	2.496	2.706	2.048	2.965	3.016
2.523	3.828	2.988	3.245	3.041	4.653
6.084	6.163	5.983	5.755	6.220	3.769

6.085	5.865	5.886	5.398	5.818	2.879
7.799	8.099	7.837	7.718	7.849	6.060
6.600	6.529	6.601	6.569	6.146	3.011
5.299	3.403	4.145	4.844	4.036	2.655
5.969	5.543	5.842	5.936	6.223	2.313
6.193	5.980	5.505	6.036	5.676	2.720
6.497	5.976	6.221	6.470	6.583	3.518
5.700	5.676	5.826	5.392	5.699	2.822
5.619	6.751	6.630	5.977	5.925	1.845
6.381	6.245	6.393	6.144	6.158	4.492
3.577	3.574	3.707	3.983	3.784	4.732
6.489	6.757	6.566	6.522	6.297	7.079
7.091	6.991	6.965	6.814	6.884	3.299
6.861	7.269	7.049	7.119	7.162	7.696
4.581	4.325	4.374	4.054	4.385	3.597
4.155	4.138	4.402	4.631	4.066	3.719
5.798	0.505	5.587	5.978	5.566	1.581
5.283	5.421	5.140	5.399	5.391	6.556
5.932	4.936	4.773	4.601	5.177	4.210
3.196	3.781	3.852	3.264	3.493	5.657
4.413	6.298	5.731	5.837	5.612	4.484
5.626	6.319	5.901	5.468	5.605	4.263
7.555	6.626	7.026	7.494	6.739	4.321
6.911	6.509	6.866	6.935	6.598	5.218
4.190	4.627	3.775	4.304	4.423	5.066
4.457	4.493	3.987	3.731	4.259	3.829
3.948	3.804	4.014	3.425	3.684	4.843
4.678	4.664	4.689	4.679	4.681	5.052
6.029	6.177	6.206	6.393	6.067	4.364
6.451	6.197	6.050	6.301	6.470	7.179
8.113	8.033	8.262	8.439	8.186	7.728
8.705	8.681	8.606	8.503	8.596	7.056
6.546	6.000	6.520	6.644	6.153	5.947
3.594	3.792	3.174	4.022	4.082	5.226
5.920	5.690	5.957	6.087	5.862	5.141
4.341	4.489	4.582	4.084	4.288	3.435
4.205	3.501	4.248	4.328	3.780	3.891
3.850	4.786	4.345	4.448	4.422	4.537
7.742	7.933	7.883	7.635	7.943	5.375
8.931	9.105	9.010	9.197	9.053	7.481
6.468	5.448	5.525	6.088	5.873	5.946
6.411	6.177	6.821	6.619	6.074	2.623
5.677	5.299	5.664	5.750	5.827	4.508
7.892	8.286	8.414	8.286	8.462	7.885
6.419	6.339	6.639	6.301	6.319	4.720
6.522	6.415	6.525	6.702	6.877	7.702
7.721	7.361	7.352	7.316	7.041	6.635
4.088	4.264	3.982	4.573	4.425	5.439
4.570	4.634	4.545	4.806	4.808	4.196
5.605	6.041	6.199	5.657	5.983	4.725

7.493	7.426	7.359	7.206	7.295	5.925
6.939	6.850	7.241	7.243	7.049	6.841
2.836	3.067	3.500	3.623	3.387	2.589
3.420	3.858	3.429	3.711	3.674	2.845
6.562	6.148	6.275	6.037	6.061	5.945
8.337	8.203	8.323	8.157	8.143	5.784
6.753	7.293	7.028	7.034	7.228	6.339
7.343	7.618	7.567	7.879	7.925	8.728
2.718	2.491	3.230	3.540	3.572	3.855
5.750	5.831	6.131	5.757	5.763	5.465
5.138	4.869	4.965	5.004	4.644	4.658
2.776	2.190	2.072	2.585	2.764	1.479
3.676	3.440	3.319	3.956	4.183	5.191
4.512	4.680	4.373	5.037	4.375	3.088
5.308	5.073	5.155	5.760	5.161	6.712
6.303	6.894	6.912	6.698	6.986	4.470
5.998	6.114	5.950	6.517	6.236	7.962
3.728	3.563	3.822	3.399	3.880	2.454
3.400	3.526	3.125	3.724	3.315	3.587
6.683	6.718	6.765	7.145	7.163	8.165
7.899	8.281	8.112	8.174	8.142	7.872
5.995	5.951	5.904	5.930	6.123	5.019
8.555	9.028	8.820	8.958	8.807	7.793
7.651	7.841	7.670	7.925	7.725	6.967
7.907	7.953	8.232	7.741	7.954	6.328
3.130	3.016	3.244	3.082	3.136	3.130
3.807	2.966	3.663	3.961	3.327	2.934
11.060	10.987	10.896	11.227	10.965	8.823
8.685	8.635	8.537	8.801	8.618	9.188
9.377	9.480	9.308	9.496	9.420	8.720
3.008	3.431	3.829	3.647	3.875	4.597
8.979	9.563	9.475	9.355	9.360	8.295
4.801	4.786	4.905	4.632	4.887	3.356
2.095	1.492	2.306	2.234	1.867	2.177
5.744	5.532	5.821	5.900	5.735	5.656
6.480	7.180	6.868	6.861	7.212	6.617
7.134	7.132	7.037	6.998	6.879	4.776
2.580	2.401	2.554	3.101	2.357	2.108
2.751	2.210	2.952	2.903	3.453	3.633
6.519	6.575	6.621	6.625	6.737	5.384
5.618	4.878	5.120	4.937	5.593	4.442
7.083	7.306	7.221	7.362	7.297	6.349
3.690	3.759	3.725	4.386	3.741	4.307
5.535	4.304	5.022	5.288	5.093	4.926
5.252	5.214	5.300	5.418	5.477	6.157
3.027	2.710	1.947	3.248	2.658	2.509
6.699	6.429	6.600	6.164	6.394	6.632
4.455	4.228	4.518	4.880	4.700	6.167
4.381	3.724	4.209	3.973	3.957	3.301
7.170	7.244	6.750	6.807	7.292	6.586

8.411	8.763	8.686	8.421	8.750	7.180
2.879	3.528	3.730	3.440	3.460	3.510
4.880	5.564	5.732	5.185	5.036	4.094
3.359	3.548	3.954	3.551	3.607	3.761
6.417	6.919	6.829	6.767	6.833	5.908
2.530	2.992	2.654	3.138	2.869	2.832
5.612	5.773	5.373	5.152	5.959	5.012
5.784	6.087	5.967	5.658	5.636	4.892
7.763	7.650	7.710	7.682	7.654	6.978
5.179	5.341	5.405	5.045	5.325	2.992
5.824	5.866	6.221	6.021	5.896	6.622
5.032	5.211	4.971	5.256	5.294	5.171
3.741	5.234	4.605	4.333	5.008	2.410
3.535	3.048	2.982	2.365	3.746	3.438
2.666	3.777	3.246	3.271	3.180	2.846
3.875	3.187	3.026	3.230	4.361	3.499
6.404	6.370	6.828	7.521	8.062	9.909
6.303	7.061	6.515	6.199	6.596	3.209
3.260	2.260	1.973	3.029	2.790	3.916
2.203	2.811	2.406	3.313	2.109	3.348
5.841	5.263	5.254	5.451	5.703	4.803
2.112	2.914	3.054	1.358	3.133	1.292
4.272	4.639	4.385	4.255	4.813	4.252
7.373	7.560	7.520	7.246	7.388	6.437
6.626	6.662	6.809	6.757	6.651	5.758
5.654	6.009	5.947	5.626	5.823	4.920
6.294	6.478	6.340	6.884	6.446	5.932
1.908	2.016	2.256	1.962	2.317	2.445
4.026	4.311	4.472	4.170	4.157	4.117
1.852	2.289	3.027	2.163	2.475	1.462
3.431	3.000	3.907	3.814	2.428	3.697
6.692	6.834	7.028	7.006	7.260	4.563
2.428	1.570	2.834	1.602	1.374	1.731
3.314	3.052	3.774	3.393	3.505	4.267
2.644	3.319	3.590	3.412	2.426	3.755
4.486	4.803	4.330	4.789	3.758	2.436
2.230	2.970	2.642	2.379	2.189	2.266
3.219	2.853	3.564	3.256	3.192	3.734
6.876	6.439	6.728	6.670	6.546	5.972
2.387	2.823	1.886	2.099	1.912	2.680
7.327	7.013	7.000	6.922	7.047	5.066
3.711	4.310	3.426	4.036	3.903	3.138
5.191	4.874	5.211	5.420	5.128	4.780
1.652	2.558	1.352	0.915	2.219	2.753
2.068	2.310	1.756	2.005	2.558	3.860
4.454	5.437	5.718	5.481	6.013	6.082
3.300	3.888	3.189	2.817	3.190	3.587
3.544	3.706	3.542	3.831	4.097	4.546
5.156	4.821	4.585	4.808	4.679	2.941
5.070	5.077	4.732	4.747	4.903	5.620

2.212	4.094	2.859	3.575	3.488	2.723
5.540	5.532	5.393	5.507	5.492	5.294
2.793	3.008	2.581	2.783	2.453	2.843
7.181	7.206	7.105	7.227	7.319	8.226
8.053	8.269	7.889	8.471	8.299	9.093
8.643	7.772	7.877	7.655	7.763	6.954
6.449	6.762	6.623	6.485	6.614	5.689
6.959	6.938	6.887	6.767	6.966	6.520
3.591	4.127	3.714	3.482	3.083	2.969
5.369	5.769	5.807	5.336	5.757	6.243
8.382	8.616	8.706	7.084	7.705	8.793
6.495	6.209	6.032	6.260	6.489	5.188
3.419	4.383	4.263	4.166	4.713	4.674
4.666	4.260	4.808	4.521	4.413	5.036
4.868	4.265	4.161	4.227	4.463	5.181
3.120	3.642	3.218	3.437	3.654	4.087
2.094	2.411	2.204	2.280	1.829	2.064
5.042	4.384	4.413	4.469	4.530	5.967
2.914	2.560	3.152	1.955	1.722	2.148
3.491	2.788	2.548	3.131	2.529	2.814
5.038	5.399	5.484	5.294	5.525	4.684
3.744	3.068	3.713	3.349	3.631	4.135
5.444	5.936	6.096	5.029	4.636	5.864
6.382	6.301	6.696	5.983	6.431	6.246
1.846	1.913	2.132	1.372	2.234	3.462
6.502	6.107	6.720	6.678	6.329	3.985
2.338	3.410	3.100	3.572	4.159	5.791
5.487	5.358	4.891	4.955	5.100	4.188
6.197	5.971	5.462	5.223	5.999	5.845
2.101	2.174	1.782	2.101	1.888	1.504
5.316	5.199	5.136	5.017	5.160	6.027
1.963	1.826	2.408	1.460	1.380	2.160
3.044	3.835	3.255	3.157	3.795	5.542
6.961	6.697	7.163	7.063	7.112	6.726
6.618	5.883	5.871	6.622	6.463	7.646
3.543	3.094	3.098	3.074	2.991	4.725
4.700	4.468	5.212	4.686	4.686	4.343
3.319	2.785	2.746	3.017	2.674	3.404
5.356	5.083	5.362	5.094	5.271	6.191
3.389	3.853	3.995	3.322	3.248	2.407
4.749	5.400	5.770	5.096	5.797	6.990
4.067	3.850	4.187	4.124	3.929	4.223
3.119	2.800	3.105	2.465	2.774	3.291
7.422	7.498	7.626	7.447	7.745	6.137
8.125	8.284	8.491	8.408	8.127	7.309
4.921	5.876	5.801	5.249	5.868	6.277
6.511	6.635	6.780	5.995	6.782	5.978
6.129	6.331	5.620	5.985	5.838	6.673
4.410	4.693	4.303	4.589	5.082	7.487
2.877	2.609	2.927	2.554	2.673	3.660

3.452	3.636	4.090	4.045	4.241	2.649
3.260	3.534	3.623	3.143	3.523	4.178
5.596	5.266	5.091	5.271	5.623	4.081
4.727	4.223	4.171	4.011	3.878	2.847
5.888	5.148	5.641	5.464	5.879	6.582
7.455	6.383	6.862	7.001	7.310	2.173
2.002	3.267	2.448	1.817	2.546	3.227
2.949	2.466	2.467	3.192	2.788	3.385
3.297	4.094	3.679	3.862	3.648	2.764
4.992	5.079	5.578	5.327	5.267	4.946
6.828	6.925	6.694	7.210	7.008	7.967
2.147	2.534	2.400	2.190	2.145	2.496
1.974	2.855	2.613	2.277	2.334	3.261
2.442	2.454	3.208	2.765	2.882	4.248
2.178	1.936	1.166	2.263	1.964	2.146
1.941	2.088	2.332	1.775	2.041	1.626
2.969	3.864	3.264	2.702	3.085	3.847
4.238	4.370	4.357	4.508	4.189	5.292
2.295	1.992	1.929	2.410	2.474	3.605
6.183	6.450	6.354	6.265	6.739	5.402
5.095	5.372	5.113	4.579	5.109	5.595
2.320	2.602	2.308	2.721	2.358	2.718
4.096	3.926	3.849	3.588	4.408	4.305
5.117	5.453	5.150	4.920	4.953	3.782
2.639	2.929	2.825	2.609	3.008	4.380
5.768	5.843	5.528	5.559	5.237	3.418
6.496	6.592	6.945	6.572	6.744	5.438
5.789	5.914	5.910	5.828	6.050	4.910
2.935	3.048	3.551	3.040	3.904	4.249
2.744	2.661	2.965	1.837	4.028	3.234
5.266	4.185	4.676	4.486	4.092	4.091
3.807	3.878	3.431	4.451	4.342	4.576
2.834	1.987	3.438	3.113	2.471	2.077
1.545	2.311	2.094	2.692	2.264	2.077
4.084	3.655	3.686	4.008	3.760	4.293
7.074	7.068	7.303	7.012	6.901	6.213
2.977	2.451	2.063	2.888	3.342	2.832
2.612	2.144	2.637	2.651	2.429	2.822
2.487	2.076	1.905	1.684	2.981	2.043
3.878	4.013	4.704	4.387	4.513	5.002
1.328	1.283	0.915	0.778	0.769	3.734
2.736	1.960	1.668	3.026	3.213	1.082
3.353	2.779	1.489	4.686	1.593	4.821
2.346	2.414	3.034	2.626	2.794	2.583
2.992	3.056	2.327	3.327	3.145	4.218
3.109	2.222	2.150	3.638	3.132	3.566
2.463	2.691	3.411	2.677	2.739	1.405
1.932	2.166	2.591	2.534	2.565	3.491
5.966	6.227	5.613	5.991	6.199	3.910
2.674	2.442	3.055	2.045	2.870	1.146

2.201	1.955	1.530	2.047	2.364	3.121
2.925	1.567	3.209	2.748	2.564	1.300
5.561	5.911	5.918	6.073	5.943	5.864
3.207	2.882	3.106	3.156	2.981	3.987
3.455	3.491	3.988	4.511	4.696	4.351
3.957	4.211	3.670	3.865	4.142	4.620
3.495	2.949	3.095	3.579	2.590	1.995
3.594	3.468	4.361	4.100	3.251	4.250
2.490	2.656	3.127	3.887	3.094	3.588
2.293	1.867	2.936	1.950	2.101	3.099
4.923	2.486	2.645	4.727	2.698	1.833
3.114	2.812	3.165	3.387	3.695	3.119
5.021	4.819	5.658	5.006	5.277	1.242
3.782	3.111	3.736	4.051	3.503	3.798
2.248	2.398	2.888	2.697	1.811	2.562
2.974	3.010	2.825	3.475	3.548	2.915
3.531	3.395	3.494	3.775	3.896	2.518
5.804	6.178	6.110	5.458	5.406	4.097
5.083	5.280	4.499	5.281	4.217	2.522
5.007	4.697	5.044	5.304	4.734	3.516
2.939	3.580	3.946	4.548	4.410	6.109
2.253	2.334	2.487	3.102	3.330	5.083
3.938	3.443	2.754	3.115	2.961	1.898
4.061	3.688	3.170	4.033	3.822	2.070
6.485	6.293	6.190	6.756	6.065	2.799
5.869	6.316	6.224	6.321	6.122	5.702
6.987	5.739	6.254	6.832	5.995	6.272
1.726	1.436	1.334	2.591	2.401	2.299
2.224	2.903	3.042	2.834	3.703	5.393
7.875	7.882	7.775	7.819	7.611	7.034
2.477	3.292	3.260	3.155	3.367	2.588
3.717	3.852	4.142	4.352	4.199	4.036
3.427	3.656	3.373	3.505	3.244	3.481
5.275	5.524	5.497	5.589	5.789	4.552
2.516	2.063	1.744	1.348	2.008	2.304
1.845	1.620	1.799	2.498	2.262	3.097
4.251	4.317	3.368	4.141	4.521	5.334
2.880	2.206	2.321	3.311	2.477	5.081
4.245	4.548	4.863	3.740	4.028	2.661
3.826	3.059	3.355	3.339	2.780	4.278
2.303	2.169	1.886	2.340	2.407	2.778
1.634	2.057	2.043	2.470	2.213	2.818
4.704	4.563	4.395	4.952	4.325	4.700
5.116	4.500	4.923	5.143	4.834	5.439
4.125	4.145	4.405	4.112	4.110	3.210
1.949	1.848	1.965	1.790	1.805	2.464
7.296	6.883	7.164	7.134	6.855	6.933
4.188	4.850	4.444	4.924	4.208	3.929
5.505	5.854	6.123	5.518	5.924	4.036
3.311	3.175	3.474	3.799	3.179	3.717

2.039	2.246	2.635	2.704	2.153	3.555
2.315	2.040	2.190	1.477	1.886	4.425
4.779	4.557	5.516	5.002	5.432	2.792
5.798	5.925	5.516	5.344	5.457	4.891
2.581	2.756	3.142	2.771	2.207	1.684
5.549	5.084	5.595	5.502	5.432	5.488
4.748	4.930	4.485	4.938	4.528	4.861
3.354	3.851	3.673	3.502	3.650	3.992
4.684	4.236	5.237	5.739	4.278	5.897
4.610	4.415	4.788	4.579	5.115	3.124
5.753	5.650	5.825	5.630	5.579	4.990
3.761	4.414	4.190	4.972	4.460	4.864
4.591	4.383	4.755	4.316	4.590	4.611
4.303	4.351	4.364	4.713	4.476	5.500
6.732	6.527	6.219	6.809	6.803	7.206
6.357	6.273	6.309	6.554	6.486	6.519
6.553	6.369	6.083	6.680	6.675	6.849
6.679	6.608	6.181	6.800	6.740	7.257
5.046	4.871	4.532	4.613	4.673	4.070
4.572	4.820	5.683	3.615	4.835	1.469
3.328	3.640	3.852	3.284	4.110	2.383
1.998	2.293	2.657	1.942	2.508	3.487
2.522	3.054	3.454	1.600	2.338	2.740
3.866	3.399	4.357	4.009	4.128	5.767
6.415	6.230	5.916	6.495	6.524	6.946
3.360	3.680	4.026	3.477	3.572	4.245
2.817	1.339	1.512	2.186	1.977	6.180
6.102	4.999	5.274	5.035	5.019	6.721
4.629	4.586	4.888	4.600	4.896	4.669
5.071	5.435	5.439	5.792	5.992	7.329
6.100	5.920	5.888	5.787	5.948	3.949
4.235	3.498	4.537	4.393	3.964	2.275
7.417	6.490	7.203	7.379	7.056	4.606
3.242	3.356	2.826	3.146	3.404	3.405
4.507	4.312	4.591	4.758	4.963	3.415
2.000	2.524	1.800	1.743	2.190	3.536
2.922	2.997	3.362	2.707	3.719	3.011
2.911	2.660	3.130	3.767	2.871	3.638
2.865	2.647	1.537	3.581	2.685	2.126
4.790	4.365	4.819	4.916	4.709	4.690
3.756	4.270	4.113	4.609	3.957	4.431
1.776	2.047	2.453	2.177	2.015	3.489
4.333	3.742	3.667	3.966	3.423	1.370
1.932	1.892	2.129	1.857	2.835	2.562
4.097	3.971	3.827	4.277	4.151	2.583
3.567	1.632	3.006	3.885	3.165	1.787
4.979	4.841	5.198	5.094	4.710	3.907
1.844	2.588	2.276	2.285	1.762	1.994
2.024	2.349	2.577	3.147	2.594	2.712
2.639	3.076	3.772	3.803	3.780	4.745

3.788	1.707	2.435	1.710	2.821	1.251
3.248	3.522	3.108	3.026	3.400	3.728
4.181	1.595	3.769	3.912	2.644	5.427
3.671	3.135	3.831	3.823	3.674	3.048
6.192	3.576	4.835	4.656	4.532	5.328
5.166	4.704	5.180	5.406	5.082	5.918
1.865	2.323	1.673	3.265	2.572	3.037
1.015	0.757	1.351	1.017	1.139	5.118
3.212	3.978	2.979	3.886	3.854	4.284
6.879	5.639	5.527	6.175	5.890	5.790
6.187	4.957	5.282	6.316	5.553	4.876
2.521	2.563	3.578	3.858	1.855	1.075
4.803	3.769	5.460	5.044	5.008	0.857
4.620	4.241	5.156	4.290	5.122	3.505
6.790	5.821	6.331	6.219	6.151	3.862
1.542	1.700	1.303	1.773	2.013	3.093
6.340	6.560	6.608	6.602	6.783	7.600
4.545	4.397	4.701	4.743	5.296	6.636
2.178	2.261	2.174	2.867	2.166	3.196
5.980	5.814	5.591	5.490	5.987	7.346
6.595	6.792	6.750	7.120	6.664	8.227
2.617	3.184	3.651	2.290	2.455	2.193
5.908	5.817	5.984	5.774	6.128	7.575
3.208	2.999	2.689	3.201	3.461	3.473
1.701	1.379	1.523	1.975	1.570	2.604
7.452	7.566	7.380	7.369	7.572	6.939
8.237	8.282	8.241	8.140	8.145	6.795
3.825	4.134	3.811	3.550	4.265	5.475
5.843	5.399	5.814	5.671	5.668	5.614
3.980	3.972	3.738	3.845	4.189	4.598
4.610	3.960	4.214	4.345	4.445	3.522
6.371	6.027	6.349	6.189	6.194	4.754
7.252	7.528	7.331	7.206	7.289	4.828
6.079	5.659	5.909	6.046	5.905	4.154
3.638	3.710	4.292	3.197	4.322	2.891
2.767	3.023	3.339	3.009	3.676	3.218
7.953	8.303	8.108	7.899	7.992	5.866
6.328	6.210	6.356	5.895	6.092	4.251
2.248	2.305	2.270	3.094	2.354	2.267
5.825	6.415	6.202	6.209	6.247	4.673
6.020	6.040	6.408	6.009	6.241	3.502
4.086	3.978	4.547	3.852	4.315	2.858
1.498	2.081	1.435	2.321	2.319	2.360
3.561	3.434	2.804	3.344	2.983	3.716
3.693	3.644	3.353	1.584	3.662	1.508
3.509	3.578	3.355	3.944	4.042	4.482
3.156	3.878	3.053	3.242	2.064	3.041
2.082	1.681	2.371	1.697	2.395	2.374
2.748	1.895	1.479	2.846	2.256	2.709
5.139	4.199	3.569	4.728	4.080	5.440

2.263	3.387	2.826	3.057	3.423	1.445
3.018	4.151	3.240	2.805	3.727	4.097
1.406	2.237	1.169	1.730	2.539	2.962
2.739	2.848	2.950	2.893	3.076	3.176
3.283	3.445	3.212	3.665	3.808	4.503
1.684	2.567	2.149	1.825	2.254	2.486
2.409	2.729	2.790	2.630	2.402	2.484
4.432	2.905	2.289	3.154	3.505	1.705
5.272	5.032	5.797	5.381	5.577	5.300
2.935	4.329	4.374	3.864	3.805	4.853
1.767	1.477	1.092	1.290	1.203	1.984
2.192	2.890	2.119	2.958	3.126	4.082
2.749	3.092	3.493	3.396	3.608	3.791
4.095	3.316	4.301	4.048	3.567	3.378
2.043	3.296	3.296	2.957	2.509	3.928
4.696	5.567	5.274	5.105	5.142	5.130
2.677	2.814	2.725	3.069	2.846	2.437
2.498	2.461	2.301	1.884	2.590	2.484
4.702	4.728	3.650	4.076	4.653	3.276
2.879	3.862	3.811	3.143	3.801	1.972
3.572	4.047	3.873	3.443	3.906	3.042
3.360	4.293	3.780	3.669	3.181	2.096
4.334	3.638	2.739	4.391	3.766	3.118
3.064	2.409	3.161	3.627	3.741	1.804
2.307	1.970	2.430	2.544	1.990	2.476
3.905	4.196	4.369	4.042	3.744	3.464
1.276	1.557	1.777	1.794	1.670	2.383
1.948	2.285	2.070	2.951	2.983	3.091
3.003	2.749	3.087	3.269	3.193	4.345
3.841	3.623	4.258	4.147	4.633	4.649
4.126	3.680	4.367	3.694	3.704	2.350
2.755	2.235	2.136	1.943	2.931	3.098
3.925	4.186	2.777	4.497	4.041	3.835
3.831	3.321	3.490	3.367	3.887	3.092
3.437	2.547	4.263	3.586	4.082	4.199
3.572	3.225	4.191	2.898	3.649	3.883
2.850	2.893	2.827	2.560	3.348	1.756
2.958	3.261	2.211	3.802	3.339	4.794
4.125	4.479	3.706	4.890	4.256	6.133
2.475	2.536	2.642	2.894	3.193	2.605
4.649	4.156	4.058	4.506	4.097	3.696
3.688	2.825	3.096	4.062	3.461	2.693
7.461	7.501	6.729	7.748	7.523	8.001
2.547	3.522	3.737	3.590	4.142	2.606
2.887	2.064	2.573	2.748	3.184	2.122
4.964	5.325	5.105	4.696	4.894	4.674
2.805	3.388	3.218	3.221	2.906	3.081
3.513	3.638	3.504	3.715	4.635	4.084
3.042	2.767	2.986	2.270	3.335	1.445
2.123	2.335	2.004	2.016	1.551	2.181

3.276	3.220	2.868	3.510	2.578	4.413
3.999	3.192	3.751	4.359	3.901	3.201
5.292	5.484	4.706	5.612	5.149	5.902
1.754	1.929	2.853	1.967	1.828	1.962
4.630	4.230	4.644	4.679	4.168	2.757
5.233	4.589	5.121	4.426	4.482	5.374
7.797	7.530	6.941	8.201	7.779	8.899
7.796	7.409	6.861	8.120	7.732	8.869
4.760	3.067	5.312	5.566	3.615	6.046
3.813	3.371	3.317	4.018	3.663	4.557
4.653	4.723	4.056	5.405	4.818	5.735
4.703	4.651	4.140	5.372	5.015	5.361
4.431	4.659	4.054	5.310	4.300	5.576
5.785	5.625	5.161	6.511	5.584	7.081
4.023	4.023	3.472	4.793	4.131	5.162
3.998	4.118	4.723	4.487	3.947	4.673
2.931	2.915	3.363	2.918	3.120	1.645
2.887	3.660	3.653	3.826	4.204	5.051
5.697	5.477	5.281	5.618	5.686	5.328
3.047	4.205	4.043	3.742	3.938	3.371
9.023	8.147	8.179	9.530	8.383	9.122
4.510	5.121	4.643	4.615	4.851	4.824
4.757	3.834	3.979	4.474	4.149	3.929
3.594	3.236	2.800	3.541	3.330	3.897
4.921	4.457	4.822	4.936	4.660	4.615
4.026	3.993	3.913	4.225	4.239	3.332
2.895	2.713	3.586	2.711	3.791	3.397
4.484	4.713	5.022	4.505	4.696	2.893
4.223	3.738	4.314	3.573	4.076	3.954
4.948	4.507	3.660	4.739	4.523	4.539
3.194	4.570	3.590	3.605	4.240	3.535
3.007	3.641	2.541	3.060	2.640	3.683
3.493	3.674	3.298	3.816	3.437	3.128
4.757	5.257	5.646	4.871	5.391	4.852
3.130	3.551	3.275	2.928	3.164	2.245
1.839	2.458	2.195	1.870	3.016	2.230
2.275	2.274	2.040	1.847	1.519	3.208
1.948	2.644	1.814	1.784	1.881	1.953
0.990	2.202	2.581	1.629	1.771	2.200
3.133	2.874	2.291	2.319	3.478	5.981
2.637	1.710	2.260	3.273	2.530	2.722
4.309	3.096	4.175	3.806	3.912	3.751
1.876	1.803	2.173	2.456	2.415	3.002
4.007	4.174	3.954	4.459	4.312	4.542
1.604	1.900	1.529	2.196	2.680	3.415
1.552	2.783	1.994	3.183	2.288	2.861
1.985	1.772	1.986	1.669	2.239	2.302
3.516	2.636	2.788	3.040	3.641	3.297
4.556	4.130	4.360	4.547	4.240	3.127
2.977	2.126	2.353	2.890	2.423	2.641

3.652	3.447	3.313	3.313	4.062	4.416
4.189	2.822	2.989	1.949	3.049	2.983
2.728	3.316	3.459	3.388	3.689	4.610
4.318	3.896	3.341	4.041	4.073	4.511
3.370	3.310	2.821	3.665	3.583	4.499
3.490	3.444	3.870	3.618	3.599	5.243
3.811	4.397	4.892	4.363	4.419	2.420
2.592	2.873	2.700	2.972	3.620	3.792
3.455	2.235	2.707	3.549	2.468	3.602
2.656	2.048	2.182	2.226	2.680	2.161
2.825	2.706	3.027	3.394	3.097	3.347
3.351	2.608	3.890	2.982	3.411	3.717
1.959	3.185	2.545	3.434	3.201	2.405
1.415	2.265	2.577	2.070	2.406	1.860
5.365	5.242	5.494	5.901	5.514	4.925
4.982	4.640	4.754	5.076	4.990	5.732
5.465	5.525	5.318	5.538	5.208	6.299
2.606	2.449	2.502	2.992	2.893	3.937
5.221	4.727	5.271	5.097	5.159	1.860
5.740	5.271	6.011	5.756	5.542	5.743
2.922	1.987	3.485	2.001	2.177	2.417
3.362	3.317	2.693	3.001	3.146	3.314
4.153	3.515	3.653	3.699	3.684	4.878
2.137	1.797	2.018	1.851	1.757	1.696
2.632	3.168	2.878	3.664	3.385	4.011
1.372	1.309	2.277	1.002	1.255	2.571
2.774	3.107	3.104	2.315	2.954	3.806
2.937	3.125	3.106	3.047	3.582	3.949
1.986	2.909	2.251	2.640	1.773	6.258
4.906	5.187	5.538	5.475	5.247	2.904
4.100	4.723	4.405	3.816	4.338	3.891
3.596	2.928	3.501	3.272	3.730	3.062
1.887	2.430	2.042	3.507	2.677	2.670
5.082	4.538	4.498	3.776	4.569	3.370
3.274	1.726	2.491	3.425	3.546	3.509
3.413	3.648	3.361	3.692	3.361	4.575
4.445	3.493	3.412	4.681	3.367	4.422
1.789	3.170	2.432	2.365	2.692	1.547
2.801	2.361	3.257	3.293	3.225	3.222
2.162	1.870	2.855	2.666	2.188	3.156
1.984	2.386	2.628	2.882	2.553	2.935
5.774	5.529	5.861	6.078	5.535	6.568
4.803	5.556	5.688	5.203	5.803	4.033
1.506	1.365	2.373	1.938	1.772	1.584
3.890	3.445	3.569	3.651	3.527	5.179
5.641	5.039	5.571	5.632	5.337	3.767
3.211	4.156	4.147	4.127	4.606	3.634
1.462	3.197	1.942	2.552	2.256	2.361
2.804	2.759	3.091	2.665	2.672	3.713
3.656	3.049	3.461	3.049	3.052	2.473

2.462	2.467	0.896	2.658	2.875	2.643
3.619	3.845	4.202	3.251	3.391	4.888
3.186	3.578	3.218	3.330	1.708	3.282
3.050	3.023	2.835	2.890	3.199	4.164
3.365	3.175	2.492	3.020	3.495	4.044
2.601	3.273	2.937	2.475	2.786	2.871
6.431	6.737	6.905	6.695	6.332	3.945
6.023	6.326	6.086	6.172	5.887	3.481
3.446	3.817	3.025	3.300	4.018	4.646
5.691	5.575	5.432	4.987	5.718	4.643
4.396	4.014	4.845	4.591	4.194	4.905
7.568	7.485	6.723	7.943	7.410	8.538
2.138	2.843	2.379	2.096	2.640	2.795
4.731	4.657	3.227	4.606	4.332	5.317
1.516	2.462	2.199	2.260	2.192	3.491
3.835	2.687	3.119	4.379	2.126	2.846
3.569	2.552	3.338	4.381	3.742	5.869
2.147	2.330	2.869	2.066	2.747	2.794
3.034	2.719	2.728	2.848	2.848	1.922
1.507	2.759	1.919	1.755	2.169	2.781
2.396	3.166	2.539	2.951	2.580	3.625
5.233	4.501	4.627	4.724	4.069	4.179
3.850	3.097	3.812	3.875	3.195	3.358
4.334	3.541	3.541	4.064	3.918	3.960
3.538	3.381	3.950	3.639	3.821	4.441
3.414	2.497	3.062	2.591	2.574	4.731
1.835	1.577	1.440	1.765	2.108	2.204
3.477	3.315	3.518	4.147	4.256	4.587
6.258	6.110	5.483	6.937	6.157	6.580
4.181	4.004	4.914	4.451	3.956	2.135
2.977	2.044	2.875	2.236	2.372	1.690
3.709	2.506	3.344	2.953	3.130	3.471
3.345	3.342	3.510	3.900	3.883	4.772
3.167	2.280	3.105	3.117	3.150	3.997
4.722	4.805	4.901	5.369	5.233	4.946
1.517	2.129	2.016	2.744	2.753	4.356
2.739	2.818	3.362	3.486	2.605	0.752
6.082	5.159	4.986	5.776	5.148	6.835
2.880	4.209	3.719	3.425	3.382	2.526
2.358	1.903	2.264	1.924	2.354	1.998
4.502	1.478	3.531	3.758	2.264	1.275
3.006	3.471	3.863	2.993	2.709	4.043
2.641	3.468	2.396	2.856	3.062	2.810
5.403	5.802	4.893	4.789	5.275	4.846
4.905	3.307	3.919	4.177	3.697	1.926
3.703	3.668	3.438	3.555	3.351	2.823
1.743	2.988	2.884	2.583	2.756	3.720
4.811	4.572	4.092	4.732	4.703	5.822
6.683	6.421	6.650	6.537	6.696	5.032
4.139	4.605	5.022	3.916	4.601	1.548

2.609	3.551	3.102	2.638	2.925	3.313
2.093	1.945	2.736	1.991	2.192	2.657
2.490	1.810	2.960	2.497	3.513	0.949
3.519	1.810	2.713	3.490	2.030	2.296
3.122	3.369	2.601	3.244	2.922	3.785
2.794	2.548	2.156	2.260	2.306	2.011
2.503	2.676	3.036	2.622	3.014	3.036
3.877	3.694	3.487	4.173	4.120	4.195
2.113	2.238	2.414	2.740	2.373	3.846
4.661	4.992	4.168	4.838	4.375	5.250
1.727	1.807	1.612	2.214	2.487	3.033
3.146	2.379	2.554	2.340	3.458	1.831
2.036	1.496	2.239	2.466	1.848	3.067
3.048	2.847	2.162	1.535	2.311	1.711
2.124	1.808	3.102	3.165	1.501	1.467
2.523	2.789	3.007	2.511	2.797	3.086
3.843	2.720	3.300	4.281	4.493	6.239
1.979	2.814	3.042	2.067	2.358	2.275
3.203	3.446	2.816	2.522	2.733	1.752
1.477	0.942	1.734	2.277	1.439	2.045
3.788	3.643	3.111	3.463	2.953	4.067
2.359	2.566	2.436	3.054	2.538	1.813
3.263	2.180	3.069	2.993	3.831	2.959
2.054	2.434	2.279	1.989	2.476	2.760
3.319	4.408	4.312	4.521	4.869	1.972
2.340	3.082	2.987	2.873	3.343	3.863
2.635	3.460	3.540	3.444	3.694	1.224
5.838	6.409	5.498	6.684	5.953	6.776
3.151	3.842	3.741	3.645	3.179	3.590
3.275	3.245	4.220	4.594	3.377	2.593
2.839	2.989	2.830	2.716	2.471	4.155
3.809	4.307	4.262	4.365	4.387	4.154
3.922	3.597	3.233	3.676	2.610	2.730
2.623	3.008	3.194	3.278	2.889	4.186
2.240	3.126	2.266	2.113	2.544	2.626
2.969	3.143	3.454	3.329	3.450	3.990
3.715	3.668	3.748	4.052	3.646	4.392
2.013	1.342	1.403	2.197	1.747	2.237
2.506	3.111	3.031	2.333	2.969	3.107
1.593	1.920	1.582	1.771	2.133	2.180
3.082	3.449	2.409	2.859	2.428	1.601
3.417	1.277	2.128	2.752	3.015	1.503
3.667	4.404	4.800	4.637	4.193	4.857
5.414	5.601	4.919	6.044	5.464	5.878
5.462	5.730	4.954	6.051	5.403	6.043
6.651	6.690	6.230	7.125	6.775	7.445
4.312	4.964	5.502	4.697	4.772	5.075
2.272	2.959	2.573	3.096	3.232	1.683
2.787	2.254	2.038	2.740	2.704	2.686
2.865	2.086	2.679	2.777	2.391	3.339

3.233	1.605	1.554	2.801	4.028	4.661
1.760	1.779	2.617	1.967	1.339	2.592
1.954	1.991	1.571	1.652	1.525	1.486
3.848	3.639	4.081	4.253	4.086	5.100
2.184	3.009	2.771	3.455	3.028	4.984
3.214	2.522	2.779	3.601	3.725	5.978
1.891	2.237	1.683	1.986	1.966	2.794
3.580	3.604	4.615	3.819	3.679	3.608
2.094	2.533	2.530	3.545	3.083	3.192
6.933	7.471	6.023	7.514	7.130	7.715
7.428	7.388	6.700	7.725	7.470	8.286
2.583	2.545	2.575	2.645	2.817	2.951
3.800	3.052	3.888	3.005	3.810	2.841
3.243	3.031	2.397	2.973	2.893	2.407
3.780	2.154	2.960	2.694	2.124	2.338
2.102	3.210	3.865	3.569	3.328	3.391
2.978	3.235	3.623	3.825	3.193	1.722
3.030	1.872	2.868	2.857	2.998	1.479
2.591	3.570	3.119	2.807	3.158	3.158
3.312	3.608	2.677	3.619	4.115	5.010
3.338	4.146	3.629	4.414	4.348	5.979
2.570	2.380	1.724	1.843	2.561	2.791
2.190	2.792	1.912	2.025	2.202	2.709
3.177	3.167	2.362	3.492	3.016	1.983
3.718	3.332	4.000	3.540	2.485	1.736
3.695	2.096	3.695	4.024	3.758	1.383
4.502	4.475	4.460	4.968	4.615	2.830
1.850	2.139	2.123	2.244	2.717	3.192
4.150	2.994	3.153	4.232	4.660	2.066
4.231	3.710	3.254	4.321	4.055	4.515
3.171	2.828	3.154	2.943	3.794	3.646
2.586	2.847	2.339	2.564	2.345	2.454
2.148	2.445	1.987	1.783	2.850	2.343
5.102	4.496	5.192	5.102	4.807	5.991
5.091	4.403	4.137	4.434	4.199	4.708
6.062	5.254	6.444	5.213	3.076	4.450
2.305	3.556	3.155	1.879	2.859	4.798
1.501	1.397	2.039	3.086	2.121	2.615
2.155	3.209	3.076	2.783	3.361	3.143
3.361	3.222	3.151	4.143	3.569	3.948
6.888	5.606	6.420	7.902	6.239	7.767
2.745	3.210	3.061	2.889	3.240	4.729
1.980	2.430	2.920	4.054	3.958	4.741
4.344	3.829	3.974	4.457	3.521	4.705
3.888	3.116	3.281	3.479	1.935	4.002
8.464	8.788	8.723	8.548	8.680	8.171
4.647	5.224	5.588	4.648	5.667	5.799
3.882	4.047	4.300	4.373	3.929	4.806
2.282	2.646	2.211	2.060	2.191	2.495
4.109	3.557	3.345	3.797	3.776	4.703

3.533	3.206	3.354	3.642	3.797	4.566
2.557	1.588	1.553	2.580	2.010	2.326
4.818	5.053	4.369	4.211	4.203	4.700
2.896	3.008	2.969	3.457	3.008	1.257
5.414	4.576	5.546	5.457	5.222	4.100
3.373	2.870	2.952	3.690	2.590	1.922
2.731	2.161	3.116	2.521	3.123	1.646
3.088	3.441	2.161	3.492	3.740	2.568
3.491	3.534	2.812	3.110	2.582	3.685
2.156	1.867	2.352	2.330	2.139	2.578
5.037	5.561	4.900	4.653	4.606	3.688
2.573	1.285	2.072	2.999	1.504	2.221
3.620	2.768	3.616	3.743	3.496	3.220
3.832	4.784	6.577	6.016	6.633	5.070
4.777	3.614	3.333	5.296	3.674	2.659
3.151	2.273	2.464	2.751	2.508	3.252
2.545	2.431	2.655	2.707	2.342	3.130
2.328	2.690	2.530	3.316	2.810	3.029
3.514	3.404	3.582	3.417	3.803	5.070
3.031	4.150	4.195	3.383	3.724	2.119
2.125	1.926	2.105	1.449	1.918	1.586
2.427	1.761	2.097	1.846	1.769	2.814
2.241	2.928	2.910	2.361	3.780	3.272
2.626	2.476	3.384	2.377	2.718	1.631
5.636	6.716	5.953	5.638	5.872	4.363
2.722	1.762	2.465	2.606	3.436	2.885
3.320	1.849	2.882	3.204	3.722	2.268
2.462	3.343	2.349	2.305	1.770	1.420
3.639	3.290	2.872	3.031	2.659	2.366
4.701	3.876	4.602	4.685	4.190	3.657
1.542	1.868	1.724	2.015	2.017	3.090
4.941	3.575	3.909	4.288	3.850	3.827
3.044	2.334	2.968	2.972	2.754	2.076
4.761	3.856	4.471	4.578	4.428	2.843
2.552	2.584	3.285	3.475	2.791	2.709
3.909	4.032	4.425	4.253	4.495	4.519
1.985	1.197	2.597	2.330	2.299	3.179
2.204	2.003	2.284	1.887	1.825	2.839
2.953	2.731	2.491	3.597	2.816	2.738
1.714	1.861	2.540	1.976	1.606	2.282
4.535	4.203	4.957	4.593	4.250	4.995
2.589	3.115	2.066	3.129	2.979	2.562
2.616	3.105	2.930	3.819	2.558	4.547
3.071	3.121	2.967	3.910	3.711	4.414
2.416	2.799	2.289	2.584	2.308	2.458
6.486	5.897	6.107	5.141	5.108	1.671
2.259	2.663	2.573	2.253	2.264	4.681
5.439	5.179	5.195	5.914	5.252	6.313
3.782	4.433	4.469	4.517	4.541	4.754
2.926	4.252	3.383	2.459	2.374	2.748

2.264	2.066	1.942	2.888	2.380	3.478
4.592	4.271	4.493	4.840	4.183	5.042
2.092	3.218	2.638	2.648	2.140	2.812
2.042	2.179	1.181	2.263	2.029	2.296
6.018	4.884	6.068	5.335	5.406	6.054
4.605	4.597	4.469	4.690	4.133	3.952
3.121	2.329	2.451	3.623	3.388	3.791
5.818	5.388	5.582	5.961	5.725	5.538
3.696	2.680	3.072	3.661	3.061	3.386
5.365	5.063	5.304	5.236	4.574	5.517
2.872	4.529	3.791	3.901	4.239	3.499
2.188	2.475	1.822	1.865	2.480	3.648
3.506	3.810	4.399	3.678	4.018	3.029
2.585	3.886	2.027	2.414	3.536	2.329
8.154	7.824	7.975	7.912	7.828	7.211
4.052	3.814	4.131	3.350	3.718	3.838
3.272	3.268	3.888	3.449	3.310	2.917
3.980	4.220	4.224	4.266	4.451	4.631
5.205	5.161	5.114	5.433	5.288	4.036
2.620	2.203	2.467	2.450	2.750	2.965
2.265	2.281	2.014	2.799	2.039	3.676
2.731	2.925	2.432	2.426	2.460	1.554
4.225	4.305	4.443	3.770	4.337	3.483
2.994	2.843	3.226	2.777	3.066	2.416
4.037	3.889	3.252	3.974	4.005	4.624
4.792	4.851	5.427	4.776	4.784	3.464
1.875	1.575	1.429	1.997	1.651	1.982
2.298	2.618	2.602	2.846	3.208	3.407
3.556	2.571	3.282	3.773	2.558	1.720
4.100	3.368	4.004	3.660	4.074	2.735
5.654	5.543	5.591	5.870	5.470	2.539
1.929	2.108	2.210	1.684	1.964	2.154
4.410	4.561	4.424	4.398	4.431	3.770
4.131	3.980	4.764	4.638	4.336	4.774
2.990	3.419	3.431	2.594	2.327	3.747
3.847	3.282	2.430	1.966	2.985	1.892
4.928	4.566	4.325	4.808	4.155	4.491
3.203	3.854	3.341	2.799	3.801	2.748
3.071	2.965	2.484	2.647	2.102	3.325
3.679	2.976	4.004	4.283	3.732	3.468
4.386	5.075	3.728	3.551	3.809	5.946
2.564	1.781	2.695	4.013	2.565	4.342
2.312	1.851	2.986	2.704	3.056	3.127
1.567	2.826	1.669	1.267	1.571	2.089
2.472	2.610	4.206	4.032	3.189	4.308
2.251	3.262	2.988	2.931	2.725	4.516
3.023	2.760	2.781	3.167	2.670	2.727
1.767	2.073	2.192	2.032	1.945	4.807
2.973	3.711	3.043	2.995	2.137	2.168
1.819	2.678	2.028	2.034	2.505	2.146

2.251	2.278	2.608	2.598	2.906	2.038
5.614	5.233	5.035	5.089	4.841	2.633
1.917	1.905	1.800	1.455	1.920	3.115
5.425	5.101	4.749	5.231	5.488	5.837
2.345	2.109	2.432	2.918	2.707	4.251
3.480	4.038	3.917	3.685	3.996	4.641
5.318	4.922	4.657	4.803	4.683	5.303
2.681	2.164	2.019	1.746	3.030	2.185
2.409	2.258	2.150	2.676	1.831	2.714
2.258	2.641	2.770	2.923	3.233	2.981
2.383	3.268	2.664	2.789	2.333	2.190
5.144	4.193	4.170	3.505	3.753	0.930
2.658	3.612	3.704	3.688	3.849	5.028
2.929	2.155	2.518	2.829	3.174	1.792
3.106	1.994	3.324	3.399	2.723	2.877
6.107	6.025	5.832	6.306	6.060	7.239
5.759	5.569	5.340	5.892	5.500	6.483
2.808	3.537	2.544	3.919	2.716	3.438
4.887	3.906	3.718	3.652	4.010	3.718
7.258	6.905	7.024	6.882	6.992	8.488
9.288	8.979	8.740	9.641	9.370	10.231
2.712	2.111	2.630	2.772	1.843	2.926
3.028	3.862	2.794	3.386	4.206	3.295
4.474	3.210	3.631	4.534	3.762	3.909
2.227	2.420	2.189	1.878	2.666	1.803
4.483	4.234	5.158	4.942	4.731	4.463
1.905	2.401	2.104	1.992	1.972	1.075
3.034	2.658	3.320	2.914	2.164	1.607
5.353	5.498	5.138	5.440	3.974	5.768
4.519	3.621	3.896	6.019	3.920	6.891
2.500	1.879	3.026	3.021	2.660	1.864
3.687	3.029	3.265	4.015	3.593	4.949
4.188	4.334	4.420	4.861	4.645	5.573
5.732	5.768	4.993	5.562	5.087	5.514
6.362	5.547	5.686	6.249	5.771	6.312
2.129	3.280	1.960	1.373	2.658	1.311
3.030	1.744	2.207	3.241	2.342	2.531
2.662	2.164	2.722	2.722	2.218	1.537
5.741	5.456	6.006	6.036	5.324	4.915
3.302	3.679	4.060	3.249	3.483	1.982
4.703	4.518	4.499	3.709	4.200	3.520
2.850	4.058	2.838	2.372	3.498	3.569
2.947	1.642	2.179	3.155	2.509	4.912
2.577	2.699	2.349	1.970	2.109	2.129
5.033	4.971	5.588	5.471	5.687	4.840
5.192	6.842	5.873	5.184	6.327	6.833
6.206	5.342	6.274	6.246	5.836	5.806
8.938	8.405	9.075	8.684	8.428	8.638
2.733	3.574	4.131	2.781	3.730	1.154
9.808	9.672	9.624	9.477	9.522	9.978

3.465	4.084	3.991	3.910	3.713	3.182
6.416	5.638	6.154	6.683	6.270	5.905
4.038	4.226	5.066	4.361	3.779	4.228
3.655	3.636	3.297	3.823	3.742	3.045
2.959	2.605	2.927	2.272	2.696	2.272
3.480	4.103	3.886	3.563	4.497	3.415
1.951	2.237	2.762	2.652	3.240	1.762
2.283	2.600	2.704	2.869	2.908	4.724
5.445	5.484	4.563	5.636	5.154	5.729
2.493	2.258	2.244	2.567	2.806	2.595
2.349	2.535	3.723	2.737	3.354	1.853
1.867	3.022	2.363	2.885	2.656	2.168
4.326	3.167	4.139	4.792	4.147	2.597
3.960	4.739	4.297	3.892	3.721	3.642
3.132	3.470	3.390	3.194	3.739	3.154
2.602	2.546	3.217	3.199	2.952	3.266
3.161	3.400	3.394	3.579	3.727	2.648
4.986	5.205	4.595	4.756	4.590	4.887
3.458	2.365	3.586	2.530	2.433	3.281
2.043	2.029	1.550	2.043	2.043	2.279
2.470	2.524	2.656	2.817	2.401	1.445
4.109	4.568	5.006	4.403	4.677	3.692
5.812	6.013	5.870	5.718	5.767	4.912
9.077	9.010	8.891	8.950	8.871	7.726
2.901	2.300	2.589	2.518	2.186	1.880
2.841	2.222	2.567	2.780	2.812	3.162
3.715	3.369	3.558	3.095	3.970	3.643
3.237	3.760	2.690	3.699	2.727	4.084
6.848	6.615	6.970	7.404	7.803	9.119
3.807	2.742	3.891	2.895	3.439	1.709
3.833	2.665	3.794	3.413	3.533	4.393
2.422	3.267	3.729	2.404	2.378	4.286
2.010	2.084	2.693	1.724	2.081	3.161
5.417	6.353	6.379	5.690	5.658	7.032
5.241	5.387	5.535	5.245	5.051	6.048
7.176	6.842	6.908	6.980	7.058	5.763
2.944	3.104	3.228	3.129	2.988	2.671
7.027	7.377	7.278	7.421	7.380	7.285
7.973	7.959	8.065	7.918	7.974	6.900
7.119	7.020	7.147	6.936	7.049	3.985
7.715	7.588	7.662	7.878	7.593	7.015
3.042	3.553	3.635	3.783	3.197	2.608
3.510	3.328	2.913	3.129	3.183	3.527
4.256	3.169	4.025	3.765	3.970	4.935
4.152	4.807	4.612	5.135	4.965	6.183
5.673	5.722	5.992	5.647	5.700	3.014
8.176	8.118	8.031	7.719	7.811	4.955
5.515	5.882	6.091	5.697	6.080	2.451
4.928	5.119	5.067	5.282	5.156	5.219
1.222	1.751	1.104	1.966	1.552	3.184

2.968	2.529	3.370	2.379	2.403	4.115
4.128	4.387	4.537	3.854	4.849	2.859
5.449	4.771	4.789	4.820	4.960	2.877
7.570	7.692	7.354	7.309	6.993	4.929
3.485	3.593	3.479	2.861	3.484	2.589
3.522	2.903	3.377	3.575	3.390	4.349
4.439	4.236	4.161	3.805	4.114	4.811
4.991	5.196	5.115	5.093	5.562	4.821
6.034	6.384	6.326	6.592	6.468	5.258
7.400	7.449	7.587	7.613	7.698	7.416
4.678	4.613	4.917	5.571	5.412	8.167
4.162	4.753	5.218	5.182	4.467	5.610
5.208	5.582	5.614	5.101	5.344	4.326
4.085	3.862	4.054	3.878	3.861	4.626
5.451	5.731	5.652	5.561	5.903	5.202
4.786	4.606	4.434	5.106	5.154	6.249
4.522	3.261	4.153	3.242	2.938	3.072
7.225	6.778	7.073	7.151	7.191	6.559
4.607	4.943	5.412	4.816	4.497	2.315
3.277	3.426	3.378	3.299	3.289	2.922
2.240	2.389	2.135	1.469	2.012	1.999
5.073	4.389	4.314	4.389	4.331	4.342
6.310	6.105	5.951	5.977	5.671	6.826
5.240	4.579	4.942	5.616	4.499	3.622
3.615	4.167	3.009	3.013	3.450	3.942
4.818	4.818	4.987	4.969	5.174	4.767
2.638	3.043	2.841	2.775	2.682	2.043
4.089	5.054	4.767	5.091	5.176	4.504
3.917	4.404	4.754	3.890	3.847	3.942
5.423	5.859	5.525	5.240	5.422	3.735
5.586	6.326	6.118	6.038	6.087	5.253
1.436	1.704	1.828	2.117	1.323	1.585
2.956	2.590	2.554	2.739	3.049	3.176
4.343	4.818	4.789	4.965	4.784	4.000
7.352	7.180	7.179	6.939	7.298	6.606
3.211	1.897	4.170	1.931	2.798	1.498
3.735	4.897	5.293	4.366	4.537	3.958
5.886	6.061	6.044	5.815	5.488	5.111
5.536	5.210	5.505	5.697	5.620	4.234
2.748	3.088	2.822	3.115	4.025	5.312
2.796	2.009	1.741	3.503	3.612	1.424
8.017	7.619	7.845	7.953	7.643	5.553
2.029	2.454	2.196	1.826	2.212	1.978
4.920	4.362	5.063	4.575	4.521	2.160
7.210	7.386	7.488	6.940	7.313	6.216
1.587	1.847	1.789	2.954	1.808	1.596
3.915	4.143	4.415	4.270	4.806	5.491
6.141	5.707	5.522	6.091	5.848	4.973
4.748	4.994	4.993	4.913	5.019	3.942
6.302	6.168	6.145	6.207	6.143	5.318

5.534	5.151	5.120	5.426	5.219	5.147
5.022	4.468	4.843	4.774	4.763	4.917
5.591	5.891	6.008	6.041	6.035	4.178
6.150	6.349	6.226	5.942	6.211	4.003
8.464	8.893	8.866	8.504	8.744	6.937
7.875	7.539	7.958	7.873	7.710	5.921
7.543	7.035	7.390	7.185	7.018	5.087
4.540	4.440	4.575	4.048	4.149	2.952
5.106	5.667	5.237	4.840	5.081	3.214
3.937	4.170	3.981	3.699	4.101	3.676
2.933	2.286	2.811	2.940	2.731	3.215
2.509	2.911	2.802	2.945	2.239	2.602
3.653	3.969	3.871	3.915	3.712	4.426
5.802	5.862	5.906	6.136	6.032	7.630
2.947	3.137	3.585	3.312	2.903	4.260
3.008	2.636	3.310	3.109	2.826	3.765
3.010	2.626	3.307	3.254	2.816	3.774
2.978	2.720	3.349	3.013	2.462	3.679
2.786	2.692	3.306	2.994	2.625	3.594
2.702	2.658	3.219	3.115	2.815	3.512
1.619	2.279	1.821	2.379	3.090	3.194
5.616	4.937	5.429	5.146	5.197	5.693
4.129	3.468	3.447	2.911	3.426	3.652
5.440	5.404	5.045	5.046	5.163	6.182
4.870	4.433	4.323	4.659	4.924	5.091
5.402	5.490	5.690	5.187	5.075	5.980
2.448	3.007	3.502	2.322	2.876	1.901
6.117	6.751	6.391	6.436	6.334	6.438
2.018	1.708	1.944	1.936	1.930	2.698
3.281	3.116	3.512	3.059	3.253	2.406
5.213	5.689	5.338	5.353	5.881	5.432
6.667	6.450	6.520	6.709	6.506	4.993
6.056	6.266	6.433	6.096	6.444	7.977
3.888	3.596	4.314	4.180	4.310	3.163
4.331	4.394	4.622	4.247	3.975	2.420
4.483	4.574	4.272	4.145	4.126	4.707
6.017	6.029	6.708	4.952	5.456	7.162
3.900	3.915	4.338	3.520	3.937	1.277
2.588	3.027	2.957	2.736	2.997	3.136
3.136	3.741	3.545	2.835	3.136	3.624
5.342	5.830	5.594	4.552	5.238	5.163
4.635	4.212	4.881	4.360	4.660	3.889
4.065	3.583	3.922	3.469	3.842	5.259
6.225	6.190	6.512	6.012	6.058	5.512
3.820	2.031	3.005	3.245	3.411	0.884
5.228	5.020	5.158	5.003	4.692	4.573
4.904	4.941	5.191	4.995	4.960	3.895
4.313	4.095	4.121	3.749	4.154	3.819
2.649	2.714	2.652	3.788	3.715	3.592
8.041	6.607	7.800	6.182	6.196	4.716

4.598	3.412	4.242	3.484	3.505	3.806
4.234	3.924	4.369	4.504	4.094	3.388
5.932	5.305	5.508	5.254	5.669	3.408
3.938	3.901	3.695	3.954	3.800	4.600
5.816	5.750	5.902	5.801	6.008	3.859
3.855	4.059	4.013	4.471	3.976	4.501
2.505	2.043	1.739	1.695	2.269	1.956
1.815	1.786	2.120	1.534	2.492	4.677
3.756	3.748	3.547	3.648	3.458	3.444
2.714	3.827	3.241	3.053	3.554	3.178
1.566	2.260	2.439	3.210	1.891	2.260
7.331	7.288	7.237	7.186	7.079	6.176
3.379	3.405	2.828	2.412	3.238	2.303
4.278	4.549	4.649	4.306	4.354	3.402
3.951	3.184	3.459	3.542	3.399	2.751
2.124	2.494	3.090	2.340	2.781	2.237
4.390	3.036	3.835	4.757	3.556	4.628
5.609	6.533	6.449	6.904	7.543	9.206
7.820	7.511	7.411	7.378	7.562	7.276
5.778	6.064	5.970	6.116	6.112	5.098
4.901	4.309	5.394	4.817	3.503	2.642
6.825	6.909	6.936	6.764	6.559	6.159
2.838	2.819	2.857	2.887	3.155	3.143
4.505	4.370	4.311	4.456	4.621	3.955
6.148	5.484	5.668	5.868	5.524	6.818
3.301	1.993	2.231	3.394	3.402	4.906
7.017	6.980	6.960	7.075	7.308	6.178
2.976	2.283	3.116	3.039	3.010	2.817
5.314	5.517	5.484	5.332	5.352	6.429
1.761	2.237	1.454	1.732	1.920	1.572
6.744	7.356	7.063	7.640	7.819	9.338
6.378	6.687	6.822	5.897	6.334	2.783
9.899	9.241	8.943	9.179	9.012	10.243
4.277	4.007	3.162	2.774	2.849	3.524
4.900	4.202	4.569	4.561	4.166	2.484
2.476	1.734	1.887	2.156	2.226	2.824
2.958	2.566	2.385	2.467	2.252	2.620
5.523	4.748	4.725	5.010	5.059	5.917
6.751	6.137	6.628	5.868	5.758	4.914
8.640	8.734	8.718	8.654	8.453	7.618
2.577	2.856	3.478	2.841	3.344	3.547
6.010	5.900	5.830	5.623	5.685	5.782
5.938	6.108	5.980	6.458	6.404	7.836
3.577	4.468	4.056	4.267	4.030	4.992
4.508	4.685	4.708	4.535	4.356	4.212
4.727	4.114	4.691	3.931	3.731	4.385
5.952	6.352	5.875	6.033	6.017	4.933
6.595	7.060	7.021	6.732	6.510	7.531
9.226	9.066	9.183	9.175	9.248	8.447
3.712	4.801	3.041	2.122	2.718	4.567

9.613	9.288	9.489	9.649	9.387	8.033
8.552	8.773	8.741	8.689	8.717	6.835
5.292	5.571	5.504	5.222	5.433	3.772
5.949	5.922	6.135	5.821	5.684	6.072
6.335	6.425	6.510	6.526	6.331	4.926
3.365	2.741	3.730	3.709	3.738	4.367
3.791	3.297	3.962	3.245	3.627	3.661
4.602	4.804	4.354	3.959	4.248	4.211
4.499	4.361	2.974	4.006	2.599	4.792
4.772	4.454	4.133	4.713	4.038	4.093
5.735	5.996	5.813	5.905	5.950	4.017
6.784	6.678	6.744	7.023	6.947	7.606
1.869	2.291	2.015	1.968	1.690	2.562
5.557	5.235	5.184	5.690	5.494	4.450
7.716	7.576	7.753	7.863	7.884	9.193
7.142	6.897	7.150	7.510	7.205	8.240
6.882	7.126	7.040	7.122	7.053	6.240
4.622	4.883	4.954	5.154	5.597	6.189
7.542	7.279	7.142	7.719	7.600	8.913
7.870	8.090	8.019	8.027	7.733	6.847
4.542	4.046	4.418	4.178	4.716	4.658
6.366	6.443	6.469	6.606	6.572	5.506
2.758	2.159	2.397	2.597	2.929	1.923
4.938	4.399	5.038	4.963	5.109	3.011
5.263	5.543	5.285	4.956	5.266	4.513
2.549	2.332	2.497	2.252	2.409	2.903
4.330	4.533	4.033	4.569	4.447	4.052
3.682	4.325	4.281	4.419	4.030	4.579
6.816	6.838	6.907	6.758	6.969	5.407
6.743	6.935	6.849	6.868	6.682	5.719
5.711	5.748	5.695	5.572	5.468	6.669
4.540	4.642	4.404	4.289	4.683	5.197
5.461	5.562	5.625	5.688	5.582	4.740
3.346	3.371	3.292	3.831	3.798	3.812
6.274	6.238	6.183	6.683	6.425	5.439
2.288	2.310	2.546	2.402	2.382	2.048
3.760	4.334	4.501	3.398	3.700	2.972
4.086	4.113	4.318	3.913	4.462	6.655
4.537	5.164	5.086	5.157	4.731	3.418
7.414	7.222	7.262	7.519	7.227	8.309
6.089	6.708	6.063	6.185	6.569	6.986
2.620	2.007	2.251	2.309	2.055	1.769
6.486	6.681	6.592	6.696	6.890	5.747
6.053	6.540	6.118	6.237	6.226	5.205
5.399	5.333	5.242	5.040	5.520	1.421
4.055	3.896	3.918	3.477	4.567	6.084
6.385	6.751	6.436	6.637	6.550	5.177
8.972	8.384	8.658	8.606	8.578	6.865
5.709	5.535	5.343	5.431	5.435	3.977
4.070	5.045	4.550	4.501	4.380	3.319

7.495	6.790	6.965	7.672	7.371	8.093
4.706	5.058	4.955	5.063	5.214	3.498
2.315	2.565	2.616	2.488	2.385	3.221
2.669	3.051	2.938	2.574	3.087	3.470
3.327	3.658	3.547	3.149	2.839	3.296
5.210	5.430	5.673	5.459	5.266	6.354
1.866	2.292	2.104	2.295	1.540	2.292
5.738	5.536	5.965	5.390	5.414	4.471
2.775	3.164	3.573	3.095	3.575	3.707
4.014	4.994	4.805	4.834	4.520	3.717
5.264	5.552	5.545	5.592	5.761	4.997
3.034	2.838	3.314	3.437	2.442	3.793
3.714	2.900	2.799	2.593	3.514	6.476
5.789	5.489	5.926	5.598	5.486	4.870
6.122	6.234	6.245	6.562	6.522	6.986
6.601	6.017	6.214	6.426	6.391	5.974
6.931	7.836	7.250	7.200	7.664	7.022
3.834	4.711	4.550	3.191	4.221	1.629
3.593	3.856	2.682	3.842	3.856	4.159
4.827	5.364	5.213	5.514	5.548	4.871
4.968	5.103	5.110	5.395	5.243	6.230
3.091	2.184	1.650	2.774	1.830	1.719
5.014	5.592	5.537	5.430	5.648	4.848
7.468	7.419	7.092	7.667	6.941	5.996
2.963	3.089	3.703	3.600	3.214	2.474
4.655	4.874	4.813	4.590	5.001	3.157
5.593	6.021	6.160	5.789	5.641	0.855
7.173	6.616	6.649	6.671	7.081	7.196
5.916	6.108	5.979	6.016	6.216	5.514
3.608	3.872	3.815	3.637	4.000	4.982
4.859	5.841	5.381	5.265	5.603	4.594
3.135	3.968	4.061	3.916	4.065	5.004
4.144	4.781	4.483	4.419	4.700	4.384
6.678	6.670	6.607	6.945	6.413	3.966
3.347	2.967	3.518	3.504	3.491	2.804
5.445	5.857	5.794	5.207	5.224	4.654
2.068	2.122	1.920	2.543	2.484	3.581
5.620	5.487	5.592	5.547	5.679	5.040
8.014	8.171	7.946	8.020	7.943	6.886
2.715	3.466	3.703	3.237	3.432	3.440
4.147	3.913	4.147	3.837	4.265	5.264
3.518	4.097	3.893	4.152	4.082	5.042
5.832	5.787	5.669	6.009	5.923	6.871
6.206	5.685	6.006	5.961	5.844	5.591
6.925	7.115	6.984	6.938	7.124	6.633
6.926	6.968	6.884	6.947	6.820	5.395
3.424	2.209	2.068	3.024	2.478	2.332
6.214	6.347	6.245	6.028	6.102	5.796
3.832	2.348	2.705	2.757	2.781	2.237
6.306	6.338	6.034	6.109	6.120	4.082

7.523	7.224	7.380	7.213	7.363	6.172
9.008	7.320	7.954	8.390	7.348	5.196
6.520	5.646	5.504	6.172	5.917	4.948
5.363	4.683	4.758	5.210	4.723	4.454
7.109	6.367	6.294	7.299	7.194	8.372
2.915	3.002	2.880	3.748	3.143	5.013
3.418	4.805	4.930	4.652	5.085	4.192
2.820	2.547	2.556	2.538	2.633	3.813
4.299	4.033	4.650	3.918	3.927	0.596
2.944	2.871	3.350	3.435	2.363	2.614
5.639	4.875	5.386	5.064	5.091	3.491
4.998	4.347	4.922	6.168	4.697	4.348
4.939	5.749	5.508	5.552	5.690	4.807
5.678	5.738	5.973	5.572	5.843	7.129
4.628	3.987	3.249	3.847	4.239	3.080
2.887	2.972	3.235	2.571	2.819	3.079
5.537	5.796	5.680	5.431	5.532	5.640
4.091	4.264	4.071	4.558	4.122	4.028
3.072	2.822	2.976	3.097	3.052	3.479
3.778	4.826	4.381	4.484	4.953	4.675
3.497	3.262	3.698	3.743	3.705	3.645
5.736	6.104	6.238	6.684	6.431	5.729
8.141	7.443	7.632	8.282	7.868	8.888
4.791	4.234	4.316	4.344	4.200	3.185
2.395	2.998	2.407	2.391	2.719	1.818
5.621	5.897	5.879	5.626	5.542	5.016
2.570	3.108	2.952	2.529	3.498	4.016
4.969	4.845	5.564	5.188	5.611	4.144
5.072	3.931	3.699	4.009	4.817	3.960
2.207	1.546	2.729	2.108	1.774	2.584
2.499	3.492	3.440	3.056	2.723	2.975
3.465	3.498	3.663	3.868	3.506	4.045
4.429	3.859	4.466	3.943	4.128	3.419
7.998	7.813	7.959	7.460	7.825	5.696
3.560	3.248	3.418	3.473	3.427	3.606
3.776	3.864	4.002	3.223	4.408	3.399
4.220	4.047	4.441	4.391	4.172	4.448
6.480	6.819	6.831	6.508	6.786	5.895
5.289	4.770	4.889	5.149	5.190	5.243
6.678	6.347	6.671	6.363	6.169	4.034
2.495	2.496	2.105	2.410	2.214	2.742
2.957	2.788	2.833	2.252	2.325	3.870
2.416	2.647	3.179	3.459	2.680	2.495
3.521	4.072	4.335	3.860	4.663	5.829
0.966	1.778	1.627	0.880	1.198	2.144
4.901	3.878	4.462	4.981	4.733	5.346
3.975	2.250	3.274	3.701	2.760	2.568
5.955	5.495	5.701	5.741	5.651	4.919
2.590	3.249	3.695	4.488	4.266	5.580
5.423	5.070	5.290	5.572	5.606	6.264

4.374	4.430	4.562	4.466	4.683	3.434
4.257	4.420	4.204	4.338	4.142	4.909
6.493	4.175	4.336	5.091	4.909	4.728
4.420	4.993	4.893	4.626	4.531	4.923
4.089	3.993	2.921	4.022	4.292	4.012
4.856	4.605	4.237	4.409	4.289	4.336
4.854	4.616	4.192	4.537	4.332	4.546
5.688	5.555	5.599	5.459	5.630	3.164
3.342	3.302	3.159	3.723	3.805	4.359
2.997	3.241	3.129	2.901	3.513	3.362
6.821	6.447	6.597	6.673	6.515	5.822
5.798	6.017	5.922	5.987	5.735	4.469
6.021	5.894	6.104	6.019	6.257	7.284
6.717	6.146	5.626	5.757	6.450	6.267
4.027	4.419	4.032	3.732	4.387	4.866
3.159	2.632	2.390	3.432	2.874	3.479
2.516	2.118	2.809	2.588	2.568	3.083
2.091	2.948	2.238	2.086	2.375	2.573
2.646	3.055	2.268	1.345	1.133	2.615
5.599	5.706	5.555	5.162	6.007	4.913
4.238	2.581	3.841	4.706	3.964	5.191
2.690	2.306	2.613	2.000	1.886	2.013
2.405	1.973	2.312	2.246	2.392	2.305
3.862	3.771	4.093	3.282	3.816	3.612
2.827	2.789	3.287	2.176	2.195	3.370
3.080	3.990	2.520	3.387	3.800	3.709
3.376	4.682	4.973	4.493	4.131	5.253
6.972	6.538	6.986	6.811	6.607	3.697
3.205	3.140	3.205	4.394	3.020	4.339
3.589	2.438	3.250	3.159	2.850	2.967
4.173	2.884	3.020	3.615	3.241	4.424
3.047	2.013	3.641	1.991	3.017	2.134
3.921	3.531	3.510	2.882	3.430	2.012
3.123	2.928	2.627	3.473	3.205	3.117
4.969	4.725	4.989	4.192	4.769	5.322
4.698	4.296	4.014	4.634	3.174	4.112
6.862	5.985	5.957	7.574	5.764	7.215
2.377	3.599	3.009	3.517	3.475	3.015
3.260	3.884	3.968	4.083	3.864	2.300
1.986	3.064	2.445	2.704	2.244	2.559
3.342	3.813	3.461	3.128	3.461	3.196
3.403	2.829	2.542	2.215	2.723	2.653
2.665	2.207	3.477	1.789	2.230	1.166
3.176	1.566	2.668	2.517	2.888	2.795
2.328	3.196	2.869	3.016	3.630	3.320
3.948	3.634	3.568	3.594	3.182	3.441
2.837	1.203	2.790	3.389	1.833	4.198
3.982	4.345	4.588	4.972	4.602	5.834
3.081	2.097	3.086	3.691	3.074	2.260
3.232	3.024	2.579	2.813	3.334	2.304

4.312	3.389	4.241	3.627	4.141	3.734
1.608	1.975	2.352	2.865	2.701	2.913
3.955	3.834	3.951	4.091	3.309	3.511
4.355	3.188	4.051	4.297	2.963	3.741
3.128	2.842	3.466	2.626	2.687	3.579
5.185	5.552	5.108	4.647	5.713	5.446
2.460	2.479	2.596	2.574	2.539	2.450
4.423	4.083	4.710	4.572	4.975	5.573
3.057	2.101	2.656	3.087	3.141	3.565
2.407	2.602	1.848	2.259	2.212	2.237
3.384	4.576	3.393	3.837	3.456	2.820
2.895	3.087	2.919	2.805	4.109	2.500
2.744	1.828	1.714	3.197	3.026	1.943
2.961	1.718	2.011	2.846	3.025	2.040
3.601	2.181	3.343	3.259	2.257	2.049
2.138	2.007	3.095	2.879	3.073	1.750
3.544	3.781	4.495	4.569	3.826	4.384
3.222	2.453	3.147	2.448	2.754	2.403
4.325	3.855	3.874	3.870	3.823	5.700
2.911	2.587	2.519	2.458	2.134	2.182
1.438	2.984	2.054	1.535	2.541	2.841
3.736	4.061	3.274	3.013	3.906	1.773
2.241	2.102	1.637	2.197	2.478	3.610
4.302	4.825	4.359	4.508	4.470	3.804
1.498	1.340	1.404	1.726	0.970	1.360
3.291	3.365	3.093	3.957	2.999	2.565
2.839	2.574	2.111	2.247	3.349	2.422
2.832	2.788	2.616	3.504	2.685	4.032
3.076	2.635	3.601	2.282	2.970	2.935
4.897	3.632	4.188	4.654	4.377	4.938
3.871	3.971	3.093	2.885	3.367	3.237
2.732	3.005	2.214	1.768	2.911	2.357
3.458	3.486	3.909	3.089	3.224	3.268
3.456	3.411	3.153	3.474	3.546	3.114
4.193	4.348	3.755	3.842	3.088	2.039
3.290	2.722	2.761	4.102	3.531	3.483
3.718	3.746	2.827	3.615	3.623	3.583
4.359	3.072	3.557	3.421	3.783	4.246
2.896	3.326	2.967	3.972	3.567	4.287
4.001	2.991	4.383	3.528	3.495	4.283
3.327	4.688	3.315	3.706	3.179	3.537
4.781	5.169	4.489	4.393	4.615	4.993
2.358	2.662	2.650	2.853	2.637	2.034
2.888	2.387	3.020	1.871	2.759	3.271
4.073	3.399	3.516	3.993	3.564	3.904
4.014	5.081	4.838	4.393	4.503	3.998
2.871	3.128	2.546	2.552	3.352	3.017
2.585	3.671	3.248	2.293	2.739	3.035
2.200	2.455	2.198	1.887	3.012	2.590
2.935	3.541	3.267	3.128	3.869	2.725

2.558	2.417	2.616	2.487	1.540	2.417
5.813	5.397	5.523	5.473	5.278	5.454
1.977	1.514	1.357	2.412	2.228	2.654
2.793	2.610	1.943	2.783	2.247	2.783
3.580	3.206	3.887	3.653	3.573	3.337
2.245	2.682	1.991	2.332	2.649	3.811
3.648	3.426	3.466	4.008	2.396	3.935
5.213	4.275	4.457	4.536	4.709	6.225
4.498	4.091	3.541	4.498	3.847	4.897
3.476	4.195	3.779	1.857	4.190	3.369
2.124	2.032	1.994	2.247	2.410	4.198
3.099	3.355	2.599	2.382	3.280	3.204
2.344	2.913	2.332	1.886	3.093	2.547
4.483	3.912	4.031	4.104	4.442	4.428
1.918	1.607	1.586	1.865	2.148	2.401
6.210	4.983	5.493	6.035	5.538	6.208
3.548	2.975	3.403	3.958	4.315	3.175
1.985	2.954	2.380	2.033	2.467	1.894
2.936	1.888	2.816	3.090	1.690	2.833
2.704	2.750	2.247	3.457	2.423	3.953
1.178	1.378	1.438	2.546	1.953	2.901
3.058	1.981	1.728	2.082	2.285	2.755
2.510	2.554	2.142	2.696	2.014	3.095
3.045	2.908	4.098	2.816	4.102	3.992
3.506	4.130	4.348	3.787	3.743	3.908
1.183	1.581	1.653	1.829	1.334	1.508
5.480	4.525	3.606	5.038	4.864	5.169
3.689	3.663	2.955	4.879	3.221	6.305
0.596	2.909	0.696	0.952	1.960	2.824
7.049	6.826	6.263	6.412	6.422	6.734
6.134	6.421	4.247	6.238	5.587	6.193
2.646	2.726	3.144	2.449	1.772	2.653
2.907	2.782	4.561	4.130	3.539	4.068
2.772	3.630	2.951	2.645	3.555	3.314
2.633	1.986	2.177	2.588	2.591	3.232
4.079	3.786	3.788	4.006	4.323	3.844
2.156	1.849	2.769	3.050	2.688	2.660
4.313	2.782	3.866	4.364	3.359	2.758
3.770	3.505	3.650	3.434	4.059	2.226
2.644	3.120	3.373	2.839	2.753	2.569
4.059	3.785	4.381	3.600	3.790	4.338
2.473	3.120	3.502	3.475	2.680	2.961
3.879	3.369	3.992	3.037	3.825	3.221
1.894	2.628	2.833	2.117	2.765	2.001
6.032	5.772	5.696	6.491	5.693	6.677
3.454	3.535	2.970	4.000	2.139	2.324
5.079	4.806	4.494	4.768	4.689	5.444
6.123	6.196	6.262	6.719	6.597	7.830
3.604	2.658	2.919	3.475	2.037	3.956
3.965	2.216	3.759	2.947	3.800	4.296

2.027	2.389	2.854	3.316	2.262	3.162
1.930	1.731	2.857	1.940	1.842	1.945
3.385	2.765	2.552	3.114	2.125	2.675
0.945	1.184	0.792	1.201	1.042	1.566
4.361	4.910	4.405	5.017	4.496	5.198
3.842	3.756	4.070	4.737	4.296	5.624
3.032	2.712	3.232	2.645	3.049	3.078
2.409	3.170	2.833	1.837	2.726	2.531
5.208	5.107	4.869	5.162	4.643	3.988
3.414	1.357	3.284	3.974	1.118	0.722
3.020	2.283	1.793	1.487	1.437	1.793
3.380	3.272	4.969	3.139	3.773	3.704
3.298	3.435	3.188	4.588	2.332	3.540
3.178	2.494	1.732	2.151	2.120	3.209
4.272	3.968	3.528	3.830	4.150	3.893
3.141	3.243	3.474	3.503	3.844	4.007
1.696	1.366	2.232	2.106	2.360	3.102
2.236	2.163	2.152	1.082	2.406	2.831
3.287	2.810	2.763	2.661	2.268	3.416
3.441	3.152	2.817	2.956	3.128	3.393
3.451	3.765	2.283	3.720	2.167	3.537
3.369	1.813	2.494	3.299	2.074	3.203
4.604	3.728	4.286	4.521	4.627	4.640
2.460	3.504	2.754	2.824	3.778	2.289
2.886	3.462	3.009	3.833	4.346	3.320
4.038	3.526	3.146	4.917	4.380	3.838
3.261	2.020	1.785	3.210	1.713	3.877
4.115	2.585	2.185	3.389	2.613	4.162
4.064	2.534	2.484	3.283	2.936	4.030
2.053	2.382	3.028	2.382	2.905	2.756
4.000	3.905	4.133	4.781	4.874	6.799
1.736	2.502	3.243	3.494	2.765	2.225
3.082	3.399	2.690	3.616	3.200	2.922
2.782	2.073	2.080	1.879	1.965	2.315
5.312	5.423	6.541	7.115	5.810	7.002
3.992	4.109	3.210	3.693	4.134	4.167
2.412	2.811	2.215	2.389	3.104	2.756
4.155	4.506	5.309	4.568	3.983	4.746
3.760	3.990	4.191	4.388	4.563	6.821
1.567	1.893	2.026	2.143	2.364	4.341
4.288	3.729	3.706	4.521	3.759	4.084
2.210	3.026	2.945	2.582	2.179	1.782
2.669	3.487	3.238	2.401	3.212	2.624
4.602	3.483	3.455	4.246	3.671	4.477
6.550	6.196	6.101	6.455	5.895	4.078
2.035	1.445	2.703	2.984	2.621	2.837
3.390	2.568	2.175	2.504	2.908	4.160
2.977	3.634	3.619	3.348	3.252	4.266
3.631	3.701	3.358	3.971	3.783	2.302
3.830	3.030	3.865	3.822	1.625	2.344

3.170	2.174	2.250	3.312	2.730	2.963
4.118	3.820	2.764	4.353	3.964	5.266
4.109	3.768	3.950	4.559	3.726	3.115
2.759	2.802	2.899	2.696	2.589	3.838
4.998	4.243	4.459	4.594	4.342	5.161
1.785	1.954	2.513	1.920	2.400	3.675
1.977	3.676	2.611	3.814	3.033	2.303
2.956	2.825	2.442	1.811	3.124	2.634
2.163	3.150	2.893	2.469	3.328	2.809
4.329	4.270	4.371	4.321	3.404	4.020
2.437	2.620	2.179	2.544	2.620	2.458
5.600	5.001	6.889	5.959	6.721	3.750
2.763	2.533	3.778	3.800	2.938	3.734
3.484	3.920	4.145	4.053	4.137	3.009
2.086	2.080	0.865	1.207	1.465	2.235
2.161	1.897	2.091	1.745	1.579	2.295
6.500	5.265	5.585	6.342	5.540	3.808
1.948	2.637	2.043	2.642	2.841	4.015
2.631	3.061	2.498	2.876	3.130	4.262
3.184	3.465	4.279	3.687	4.239	3.960
1.918	4.042	2.835	2.067	3.383	1.059
4.980	4.556	4.702	4.673	5.233	5.328
3.978	3.044	3.593	3.403	3.624	3.977
2.836	1.805	2.635	2.021	2.835	1.912
2.224	2.158	3.189	2.482	2.241	2.441
2.383	2.665	2.723	2.578	2.562	3.600
6.832	7.141	7.122	6.807	7.141	6.323
9.874	9.763	9.588	9.586	9.435	6.859
5.587	4.744	5.739	4.908	5.051	4.954
6.881	7.019	6.819	6.516	6.896	6.298
2.664	3.166	2.957	3.346	2.955	2.554
4.685	4.810	4.998	4.232	4.639	4.108
8.572	8.230	8.172	8.537	8.168	9.228
5.072	4.295	5.384	5.159	4.192	1.720
5.064	3.850	3.654	4.335	4.639	4.380
9.038	9.520	9.520	9.314	9.560	7.762
6.766	6.502	6.500	6.555	6.287	5.513
3.742	4.834	3.488	4.024	3.742	3.616
4.372	4.387	4.634	4.169	4.993	4.061
5.248	5.352	5.549	5.089	5.610	6.041
7.645	7.981	8.117	7.539	7.683	6.488
4.984	4.332	4.419	4.945	5.206	5.815
2.232	2.346	2.130	2.255	2.188	2.217
2.851	3.213	3.603	3.007	3.012	2.927
3.521	3.799	4.269	2.994	3.859	4.674
7.029	6.580	6.857	6.852	6.564	5.244
4.536	4.095	4.807	4.407	4.255	3.361
3.738	3.021	3.642	3.167	2.948	4.366
5.384	5.596	5.447	5.301	5.181	3.632
1.668	2.160	1.712	1.577	1.830	3.266

4.411	4.286	4.542	4.360	4.356	5.740
3.188	2.962	3.180	3.437	3.252	3.398
2.624	1.789	1.620	2.974	2.021	2.070
2.906	2.513	2.376	2.244	1.919	2.737
2.509	2.651	1.975	2.125	2.482	2.783
4.641	4.617	4.739	4.464	5.237	3.928
3.847	3.949	3.869	3.037	3.616	3.972
5.151	5.119	5.315	5.123	4.994	4.913
7.246	7.368	7.570	7.397	7.491	6.522
6.934	6.713	7.246	6.764	6.795	7.461
6.250	6.401	6.330	6.002	6.813	8.517
2.208	1.964	1.821	2.186	2.233	3.779
5.836	6.171	6.536	6.967	7.299	9.671
8.594	8.711	8.666	8.436	8.745	8.111
4.051	4.884	4.785	4.624	4.566	3.621
5.713	7.274	6.822	7.315	7.620	9.241
5.788	7.358	6.840	7.355	7.552	9.264
3.731	4.165	3.915	3.987	4.012	3.793
3.378	2.994	2.937	3.267	2.812	2.923
4.737	4.699	4.841	5.134	5.325	5.049
3.817	3.851	4.119	4.192	3.975	5.240
3.739	3.720	4.244	4.154	3.978	3.215
3.529	3.776	3.347	3.429	3.668	2.307
4.778	5.331	5.200	5.060	4.818	3.819
4.337	5.073	4.997	4.677	5.113	3.870
4.846	5.203	5.394	5.732	5.440	6.432
6.350	6.065	6.448	6.226	6.496	2.678
7.942	8.108	7.888	7.577	7.895	6.963
4.118	4.167	3.855	3.508	3.967	4.950
4.837	5.201	5.299	4.993	5.085	4.735
3.903	4.417	4.673	4.338	4.527	4.501
7.956	7.923	7.672	7.539	7.592	7.132
5.374	5.298	5.053	5.387	4.689	3.413
6.718	6.706	6.863	6.514	6.882	3.187
4.139	4.076	4.422	4.444	3.935	5.333
2.750	2.051	2.328	2.352	2.565	2.742
2.921	1.772	2.018	2.641	2.233	3.453
3.939	3.161	2.536	3.635	2.464	2.152
3.517	3.542	3.920	3.730	3.865	3.675
5.504	6.122	5.683	5.883	5.858	3.716
6.371	4.672	5.255	4.570	4.577	3.722
6.079	4.475	4.947	4.291	4.297	3.258
3.071	3.708	3.101	2.723	3.690	4.948
3.819	4.048	4.019	4.071	4.341	3.406
7.533	7.422	7.762	7.658	7.496	6.879
3.760	4.856	4.234	4.566	3.841	4.255
3.238	3.464	3.179	3.335	3.366	3.539
4.904	5.304	4.749	5.216	4.341	5.164
6.607	5.884	6.084	6.129	6.265	6.944
4.364	4.345	4.727	4.454	4.099	3.854

6.979	6.849	6.873	6.781	6.999	6.243
6.607	6.548	6.707	6.512	6.486	6.121
5.269	5.392	6.061	5.378	6.022	4.667
6.894	6.856	6.891	7.044	6.686	6.188
4.572	5.676	5.158	4.990	5.263	4.216
6.537	7.012	6.791	6.858	6.730	5.768
5.765	6.364	6.076	6.062	6.203	5.960
7.019	7.005	7.141	6.999	7.110	6.278
3.815	3.905	4.219	4.022	3.695	3.819
5.005	5.259	5.542	5.323	5.240	4.917
9.984	10.103	10.236	10.110	10.021	9.498
7.439	7.356	7.473	7.536	7.269	6.221
3.744	3.951	4.079	3.791	3.775	4.361
2.968	3.696	3.203	3.071	3.048	2.394
4.334	4.741	3.799	4.657	4.728	4.645
6.251	6.060	5.904	6.127	6.418	5.403
5.029	5.659	5.544	5.299	5.624	4.874
6.251	6.798	6.700	6.356	6.429	6.975
3.733	2.665	2.893	2.171	2.598	3.678
3.120	2.434	3.005	1.669	2.981	2.511
3.381	3.387	3.782	2.213	2.538	3.228
5.230	4.367	4.822	4.151	4.291	4.341
6.072	6.102	6.105	6.174	6.056	5.634
6.371	6.609	6.755	6.566	6.529	5.803
2.505	2.716	2.296	2.594	2.991	3.724
2.658	3.706	3.153	3.634	3.846	4.399
6.857	6.485	6.526	6.914	6.911	8.036
1.748	3.044	3.044	2.792	2.223	2.525
7.136	7.967	7.447	6.839	7.136	7.128
8.907	8.214	7.984	7.737	8.039	9.503
1.707	1.963	2.258	1.921	1.927	1.564
5.041	3.722	3.962	4.662	3.929	3.564
2.327	2.374	3.169	2.684	2.537	2.127
3.656	2.615	2.894	3.585	2.102	1.831
2.544	2.887	3.048	3.067	2.602	2.876
5.930	4.300	4.516	4.438	4.989	3.363
6.681	5.697	6.671	6.411	6.158	6.090
5.041	2.952	4.146	4.662	4.826	5.971
6.208	5.638	7.175	6.819	6.278	7.381
5.856	5.518	6.830	6.757	6.573	5.674
2.429	2.789	2.120	2.972	2.161	2.077
3.791	3.113	4.389	3.725	4.280	2.691
7.949	7.911	8.325	7.519	8.077	7.117
9.931	8.810	9.752	9.207	9.471	9.554
6.517	6.101	6.430	6.596	6.663	6.042
4.583	4.767	4.344	4.754	4.466	3.602
4.673	4.666	4.744	5.186	5.045	4.860
3.823	4.349	4.471	4.209	4.177	4.722
4.392	5.331	5.085	5.054	5.068	3.957
3.605	3.258	2.755	3.292	3.391	1.995

2.745	3.119	2.518	2.946	2.825	3.810
3.727	3.903	3.438	3.484	3.771	4.547
3.864	3.795	4.024	3.992	4.249	4.363
7.511	7.757	7.467	7.910	8.003	9.475
4.222	4.517	4.314	3.645	4.374	4.487
7.021	7.134	6.921	7.081	6.859	6.473
7.519	7.789	7.908	7.466	7.437	7.233
6.688	7.144	7.129	6.942	6.411	6.742
6.604	6.428	6.492	6.286	5.945	6.570
5.819	6.099	5.786	5.911	5.957	5.188
2.383	2.132	2.782	2.801	3.583	3.848
4.559	4.860	4.622	4.496	4.774	4.828
6.695	7.080	6.873	6.872	6.723	5.972
4.482	5.301	4.238	4.487	4.343	3.961
3.570	2.686	3.476	3.855	3.725	3.892
6.574	6.532	6.464	6.199	5.459	6.348
6.038	6.172	6.139	6.261	6.352	7.263
7.652	7.226	7.075	7.367	7.489	7.686
10.297	9.853	10.493	9.728	9.453	10.146
3.821	2.354	4.574	2.961	2.506	2.269
7.320	6.347	7.323	7.940	6.680	5.712
7.924	7.184	8.035	7.688	7.393	7.350
5.821	4.583	5.596	5.246	4.558	1.903
6.792	5.984	6.907	6.911	6.283	5.945
9.386	8.412	9.332	9.033	8.993	8.353
8.058	7.317	8.574	7.599	7.199	7.834
3.835	3.423	4.044	3.358	2.739	2.795
8.650	8.483	8.544	8.106	7.728	8.222
6.638	5.805	6.320	5.828	5.883	4.283
7.638	7.511	7.542	7.359	7.429	7.329
9.536	9.346	9.775	9.701	8.596	8.614
2.866	4.450	4.770	4.592	4.238	2.965
5.143	4.976	4.870	4.746	4.870	4.854
4.697	4.817	4.796	4.877	5.093	6.346
5.690	4.782	4.982	5.290	4.679	3.262
6.352	6.773	6.679	6.919	6.695	5.998
3.211	3.875	3.340	2.872	2.673	3.090
2.845	3.851	3.665	4.501	3.124	4.090
5.355	5.606	6.500	5.826	5.457	2.168
3.508	4.003	3.870	4.213	3.890	4.222
5.525	5.280	5.371	5.862	6.038	7.403
4.661	5.604	5.273	4.862	5.124	5.433
3.457	4.458	3.490	3.921	3.611	5.026
4.344	5.392	5.087	4.585	5.292	5.664
2.909	2.727	2.620	2.389	2.942	2.897
6.454	6.145	6.214	5.904	6.111	5.506
6.113	6.135	6.101	6.195	6.066	6.024
3.750	3.984	3.893	3.848	4.313	5.083
2.834	2.662	2.897	3.797	3.156	2.086
2.502	3.110	2.339	2.204	1.924	1.985

3.958	2.787	2.356	2.689	3.416	2.967
8.882	8.922	8.902	8.754	8.773	7.669
2.825	3.377	4.153	3.831	3.908	3.860
3.194	3.262	3.387	2.858	3.419	3.189
7.746	7.703	7.779	7.783	7.704	6.361
6.899	5.329	4.442	6.371	6.993	5.383
4.373	4.739	4.239	4.548	4.845	5.632
3.917	3.468	3.236	3.237	3.639	3.816
2.722	3.145	3.174	2.315	1.404	2.555
2.320	2.879	2.323	2.741	3.887	3.131
7.032	5.711	4.863	6.416	6.419	5.290
5.406	6.079	5.480	5.752	5.848	5.840
3.814	4.321	4.184	4.188	4.279	5.711
3.070	2.894	2.753	2.711	2.762	2.333
4.133	3.134	3.405	3.694	3.186	2.826
3.810	3.414	3.233	3.693	4.196	5.106
5.376	5.516	5.672	5.328	5.500	4.216
2.526	2.853	2.647	2.464	3.067	4.113
6.321	6.284	5.807	6.066	5.952	4.868
5.558	5.151	5.156	5.486	5.235	5.329
5.448	5.501	5.305	5.865	6.272	7.715
5.088	5.715	5.703	5.888	5.922	7.311
4.211	4.772	4.698	4.062	3.988	5.245
2.644	2.812	2.515	1.925	2.757	2.413
9.492	9.581	9.565	9.676	9.613	8.153
4.068	4.372	4.206	4.420	4.914	2.760
4.158	3.149	3.223	3.736	3.542	5.448
7.634	7.855	7.859	7.570	7.580	6.906
6.407	6.170	5.811	6.392	6.644	7.763
5.208	4.633	5.010	4.612	4.988	5.994
4.176	3.338	3.892	4.135	3.691	5.565
4.007	4.029	4.586	5.061	4.611	3.298
2.174	1.505	2.262	2.392	2.690	3.386
4.173	4.270	4.606	3.601	4.207	3.092
4.573	5.093	4.952	5.549	5.920	7.233
6.042	6.690	6.098	6.068	6.399	4.617
7.059	7.186	7.043	7.459	7.301	6.902
8.387	8.317	8.177	8.199	8.288	5.622
4.578	4.863	4.907	4.846	5.039	3.842
5.640	6.099	5.721	5.993	5.862	4.830
6.218	5.025	5.204	6.303	5.757	6.856
2.001	2.424	3.191	3.410	3.279	1.995
6.670	6.989	6.855	6.525	6.812	5.845
4.574	4.122	4.215	4.871	4.352	4.166
3.654	3.809	3.425	3.294	2.894	3.316
3.186	3.244	3.555	3.676	3.955	3.875
4.971	5.101	4.736	5.572	5.509	6.385
3.956	4.431	3.749	2.874	3.380	3.169
6.756	6.801	6.502	6.546	6.729	7.408
5.629	5.506	5.782	5.670	5.657	5.108

4.281	2.787	3.399	4.461	3.373	3.286
4.985	5.071	5.313	5.044	4.675	5.376
3.681	4.432	4.030	4.114	4.511	4.530
5.701	6.047	6.044	6.080	5.838	4.934
5.288	6.277	5.677	6.110	6.025	7.674
8.411	8.013	7.956	7.742	7.834	6.816
8.547	8.866	8.605	8.612	8.471	6.715
8.148	8.356	8.288	8.444	8.313	6.996
8.232	8.688	8.778	8.764	8.862	7.532
3.790	3.678	3.664	3.387	3.370	3.499
9.084	9.463	9.142	9.316	9.429	7.254
7.368	7.581	7.383	7.459	7.261	6.972
8.307	8.721	8.518	8.414	8.627	7.606
2.625	4.500	4.399	3.617	2.878	4.550
5.208	5.207	4.992	5.672	5.556	6.117
4.484	4.492	4.814	4.543	4.142	3.740
7.693	7.799	7.994	7.798	7.881	6.932
6.309	5.660	5.552	6.167	6.305	7.677
5.184	5.005	5.093	5.452	5.334	4.587
2.604	2.700	2.151	2.818	2.970	3.260
2.912	2.791	2.614	3.079	2.766	3.817
2.311	1.369	1.426	1.724	2.211	3.160
8.364	8.498	8.451	8.583	8.498	6.747
4.570	4.373	4.911	5.950	5.265	6.293
7.412	7.273	7.045	7.593	7.117	8.287
7.247	6.976	6.766	7.356	6.855	8.062
5.967	4.867	5.304	5.730	5.149	6.265
6.529	6.105	6.213	6.504	6.078	7.061
2.157	1.554	2.036	2.417	1.776	3.330
8.226	6.543	6.486	7.577	7.111	8.587
6.435	5.232	5.462	6.357	5.648	7.039
8.298	7.436	7.488	8.057	7.505	8.833
5.374	5.643	4.746	5.548	4.077	5.382
3.578	3.401	3.517	3.559	3.704	3.350
4.095	3.948	3.503	4.160	4.077	3.105
8.769	8.574	8.861	8.750	8.808	8.694
7.617	7.132	7.124	7.607	7.602	5.296
4.612	4.961	4.713	5.127	5.147	5.786
6.206	6.321	6.197	6.059	6.399	5.644
7.792	7.166	7.510	7.327	7.411	6.073
7.339	7.270	7.390	7.149	7.310	5.223
2.789	3.418	3.418	2.289	1.603	4.280
4.138	4.605	4.799	3.701	4.307	4.707
3.749	4.040	4.374	3.599	3.984	3.964
3.004	3.011	3.179	3.219	3.775	4.181
7.091	7.364	7.173	7.221	7.528	6.210
9.982	10.012	9.925	9.983	10.054	9.013
4.365	5.407	4.875	4.234	4.273	5.803
4.294	4.435	4.354	5.049	5.173	6.099
2.308	1.807	2.798	2.795	2.644	4.745

6.665	6.450	6.532	6.512	6.220	5.281
2.407	2.681	2.046	2.314	2.592	3.064
6.356	5.238	6.272	5.991	5.582	6.271
7.593	8.118	7.828	7.541	7.870	7.109
5.569	5.847	5.717	5.748	5.189	3.921
2.988	1.159	2.781	2.958	2.527	1.076
4.894	1.876	4.793	5.350	4.741	1.704
8.666	8.588	8.780	8.610	8.569	6.760
8.313	8.287	7.985	8.049	7.643	8.212
6.277	5.965	5.874	6.789	6.632	8.287
8.254	8.473	8.295	8.417	8.369	7.247
7.693	7.818	7.775	7.830	7.937	7.103
7.534	7.812	7.448	7.291	7.419	6.033
3.676	4.554	4.172	4.673	4.521	4.096
2.101	2.337	2.211	1.537	2.410	4.072
5.259	5.253	5.994	5.557	5.692	5.592
5.224	4.682	4.747	5.164	5.486	6.331
6.042	5.874	5.699	6.263	5.894	6.808
7.898	8.462	8.210	7.937	7.865	6.913
8.360	8.223	8.318	8.582	8.744	7.342
5.905	6.210	6.196	6.249	6.292	6.976
5.982	5.684	6.171	5.866	5.387	2.998
1.833	2.279	1.750	2.151	1.749	2.996
5.095	4.919	5.030	5.157	5.227	4.132
3.874	3.473	4.042	3.565	2.911	2.031
7.444	7.627	7.759	7.463	7.751	7.093
4.106	4.988	4.903	4.185	4.698	3.910
4.365	4.761	4.605	4.507	4.472	3.554
5.711	5.936	5.926	5.765	5.710	5.225
5.251	5.720	5.700	5.604	5.897	4.524
9.198	9.466	7.532	8.486	8.615	10.423
3.278	2.874	2.989	2.770	3.093	3.507
2.768	3.263	2.937	3.223	3.254	3.350
8.776	8.697	8.641	8.904	8.666	7.411
6.428	6.165	6.419	6.861	6.452	4.349
5.434	4.533	4.811	4.323	4.502	4.288
7.941	8.010	8.278	8.154	7.897	6.914
4.607	4.715	4.887	4.664	4.655	5.732
4.909	5.041	5.431	5.301	5.160	5.345
7.850	7.717	7.960	7.903	7.733	5.851
8.304	9.236	8.561	8.739	8.958	8.798
11.013	11.364	10.959	11.087	11.152	10.538
10.443	10.608	10.506	10.410	10.532	10.007
8.598	8.386	8.300	8.348	8.626	6.515
3.553	2.459	2.165	3.037	2.406	2.046
4.458	4.118	4.313	4.457	4.279	2.909
3.281	4.207	4.070	3.632	4.312	5.517
2.426	2.409	2.547	2.376	2.558	2.815
5.848	6.036	5.976	6.276	6.258	6.892
5.553	5.592	5.898	6.051	5.407	3.110

7.622	7.522	7.594	7.593	7.330	5.586
4.985	4.979	5.036	5.181	5.222	5.778
4.910	4.471	4.100	4.341	4.394	5.470
5.667	5.887	5.711	5.725	5.870	6.421
7.602	8.005	7.756	7.879	7.869	4.750
7.226	7.214	7.315	7.263	7.335	4.854
5.822	5.384	5.910	5.750	6.101	3.803
3.302	3.042	4.079	2.943	2.856	2.635
5.431	5.201	5.678	5.447	5.328	4.934
2.716	2.843	1.705	3.154	1.990	1.356
5.796	5.667	5.734	5.947	6.095	2.673
6.172	6.608	6.722	6.764	6.613	1.527
6.870	7.021	6.824	6.702	6.828	6.157
3.551	2.809	3.377	2.698	3.412	3.270
4.028	3.211	3.234	3.233	3.527	4.146
3.750	3.878	3.655	3.539	4.192	4.624
3.062	3.200	2.681	2.669	2.862	3.211
6.264	6.168	6.523	6.263	6.382	6.866
1.690	1.497	1.370	1.032	1.607	2.647
2.089	2.136	2.441	2.030	1.863	4.142
6.552	5.520	5.325	5.200	5.662	4.312
3.147	2.886	3.038	2.413	2.705	3.500
7.090	6.628	6.505	6.637	6.750	6.764
3.462	3.185	3.517	3.396	3.416	3.442
4.832	4.566	4.019	4.169	4.288	5.004
3.832	2.779	3.489	3.066	3.755	3.097
6.400	5.951	5.595	5.053	6.272	4.089
2.817	2.872	1.989	2.520	2.556	3.927
9.732	9.439	9.529	9.449	9.475	8.664
4.464	5.257	4.260	4.536	4.407	4.322
3.843	3.344	3.073	2.522	3.023	2.761
5.001	5.486	5.516	5.760	5.459	6.148
5.203	4.905	5.468	4.861	4.770	3.863
3.994	3.572	3.486	3.704	3.598	4.928
4.282	3.514	3.780	3.836	3.582	3.046
6.840	6.780	6.976	6.677	6.813	5.905
5.406	4.888	4.695	4.253	4.715	4.618
3.397	3.644	3.339	3.371	3.307	4.102
5.428	5.146	5.351	5.592	5.089	4.083
4.800	5.132	5.067	4.325	4.733	5.425
2.915	3.159	3.716	2.908	3.491	4.603
3.447	3.633	3.630	4.221	4.169	3.811
5.651	5.893	5.772	5.437	5.621	5.180
6.386	5.610	6.452	6.904	6.394	3.931
6.648	7.122	6.895	6.798	6.955	7.759
2.329	2.450	2.145	2.803	2.884	4.730
5.961	6.355	6.218	6.128	6.480	5.647
4.591	6.112	5.573	5.698	5.319	3.545
3.615	3.526	3.604	4.054	3.756	4.448
7.724	7.948	8.151	7.878	8.132	9.264

6.364	6.196	5.849	6.503	6.279	6.079
2.658	2.349	2.832	3.020	2.492	3.084
4.633	4.300	4.504	4.722	4.659	5.624
2.699	2.791	2.714	2.108	2.494	2.837
4.707	5.016	4.859	5.057	5.109	4.498
4.799	5.068	4.650	4.617	4.713	3.609
4.584	4.548	4.666	4.900	4.988	6.572
3.216	3.400	2.614	3.072	3.848	2.888
2.259	2.418	2.403	2.151	2.235	2.208
5.173	5.506	5.260	5.598	6.338	7.563
4.717	5.118	5.300	4.728	5.017	4.689
6.528	6.104	6.302	6.465	6.249	5.581
2.216	2.467	2.794	3.066	2.164	2.442
9.862	9.924	10.094	9.759	9.970	8.483
2.378	2.316	3.170	2.196	2.745	2.702
2.454	2.618	2.138	2.587	1.950	4.073
3.103	3.518	1.907	3.035	3.490	3.008
4.177	3.998	3.607	3.748	3.902	4.840
3.308	3.884	3.285	2.716	3.570	4.820
3.852	4.512	4.319	4.667	4.656	3.292
4.593	2.952	3.825	3.837	3.963	3.111
3.049	3.348	3.399	2.786	3.482	2.390
8.653	8.645	8.628	8.582	8.810	6.300
3.888	4.500	4.241	4.458	4.924	3.524
4.851	5.047	5.042	5.010	4.636	4.677
2.389	2.107	2.679	1.685	1.688	2.290
6.931	6.822	6.672	6.145	6.904	3.624
2.737	2.847	2.043	2.386	2.660	2.307
8.414	8.279	8.366	8.278	8.172	7.609
5.780	6.372	6.185	5.950	6.045	5.780
3.719	3.734	3.911	3.889	3.926	4.561
1.626	1.660	1.938	1.593	1.590	1.819
3.953	4.513	4.429	4.010	4.167	4.265
7.200	7.302	7.378	7.218	7.024	5.494
4.802	4.834	4.994	4.904	5.168	4.556
7.859	7.758	7.875	7.814	7.610	7.173
9.935	9.921	9.850	9.826	9.988	9.113
9.976	9.880	9.776	9.750	9.970	9.119
2.974	3.823	3.399	3.748	3.374	4.459
4.991	4.937	4.410	4.755	5.132	3.715
3.403	3.589	3.399	3.612	3.613	1.783
4.921	3.774	3.461	4.025	4.017	4.308
2.372	3.508	2.568	3.129	3.204	2.946
5.714	5.661	5.458	6.036	5.788	7.004
5.816	4.973	4.441	5.389	4.354	6.073
6.997	6.628	7.096	7.099	6.756	7.355
4.104	4.808	3.936	4.322	4.365	3.812
6.583	5.661	5.938	5.626	6.284	2.987
5.633	5.601	5.835	5.506	5.477	4.745
3.972	4.319	3.816	4.157	4.853	2.995

2.711	2.386	2.388	2.652	2.729	2.246
2.187	2.504	2.016	2.102	1.903	4.045
2.052	2.733	2.752	2.358	3.069	2.005
5.337	5.124	5.175	5.337	5.218	6.371
2.144	2.236	1.975	1.709	2.350	2.387
6.771	6.929	6.451	6.887	7.113	8.319
7.508	7.490	7.956	7.291	7.605	9.281
2.913	2.453	2.878	3.563	3.234	4.329
3.254	2.215	3.006	2.422	3.565	3.064
8.628	8.309	8.086	8.938	8.717	9.524
7.962	7.616	7.019	8.400	8.089	9.203
7.027	7.060	6.689	7.188	6.965	7.692
8.315	7.865	7.604	8.647	8.288	9.269
8.770	8.386	8.205	9.045	8.600	9.681
8.669	8.263	8.142	8.876	8.594	9.502
5.640	4.427	4.692	5.702	4.921	6.083
7.175	6.894	6.649	7.309	7.020	8.084
4.545	4.548	4.731	4.859	4.686	6.714
5.624	5.880	5.527	6.059	5.935	3.584
3.869	3.705	4.028	3.166	3.474	3.087
3.680	2.920	3.649	3.037	3.757	2.954
2.156	2.945	3.085	2.448	2.407	1.731
4.848	5.693	5.686	5.306	5.702	5.178
2.218	2.407	2.814	3.174	2.802	2.737
9.479	9.477	9.447	9.429	9.306	7.939
7.810	7.397	7.839	7.698	7.672	7.003
5.384	5.445	5.750	5.561	5.582	4.531
6.801	6.614	6.687	6.441	6.435	5.236
2.836	2.685	2.573	2.784	2.211	2.463
4.215	4.689	4.344	5.255	4.937	2.519
6.435	5.858	6.251	6.128	6.051	2.923
4.152	3.941	4.987	3.992	4.258	2.792
3.378	3.655	3.871	3.519	3.313	2.056
5.941	6.043	6.316	5.430	5.868	5.949
5.813	5.482	5.343	5.977	5.464	5.433
7.390	7.202	7.314	6.633	6.872	5.440
6.733	6.722	6.825	6.747	6.692	6.125
6.021	4.832	5.506	5.352	5.831	1.194
5.303	4.932	5.181	5.282	5.221	4.317
2.726	2.539	2.518	1.674	2.402	2.455
5.798	5.637	5.659	5.485	5.100	4.985
6.529	6.316	6.424	6.303	6.154	5.571
3.974	4.116	3.498	3.355	3.670	3.628
3.895	4.555	4.762	4.581	5.038	3.077
3.113	2.601	3.035	2.156	2.765	1.451
5.631	6.189	6.139	5.891	6.081	5.654
5.492	5.338	5.267	5.572	5.480	6.975
9.087	9.319	9.142	9.193	9.167	8.592
3.639	3.156	3.624	3.784	3.870	2.897
4.344	4.612	4.490	4.920	4.836	4.611

3.620	2.848	3.247	4.090	3.042	3.341
2.849	2.335	2.641	3.293	2.733	2.607
2.492	2.477	2.730	2.832	2.414	2.103
5.519	5.852	5.660	5.866	5.530	5.509
3.098	3.231	3.752	4.022	3.874	3.178
9.453	9.205	9.409	9.288	9.356	4.263
4.642	5.388	4.981	5.152	4.916	4.453
6.310	6.333	6.466	6.022	6.639	4.844
9.494	10.121	10.029	9.979	10.008	7.791
6.528	6.095	6.637	6.011	6.799	4.603
4.467	4.169	4.170	4.262	3.804	4.675
6.455	6.557	6.029	6.557	6.653	4.256
5.801	6.449	6.336	6.041	6.519	4.582
8.755	8.344	8.336	8.561	8.373	6.733
7.806	7.638	7.657	7.633	7.608	6.702
3.531	2.675	4.720	4.638	2.758	4.205
9.504	9.527	9.408	9.456	9.236	8.572
9.757	9.740	9.567	9.636	9.468	8.464
3.681	3.447	3.718	3.442	3.605	3.358
7.998	7.628	7.677	7.647	7.606	6.398
4.532	4.594	4.467	5.085	4.194	5.107
3.978	3.755	3.893	3.825	3.714	4.899
3.709	4.064	4.119	4.470	4.318	2.590
7.040	7.486	7.259	6.988	7.008	6.188
6.103	6.421	6.216	6.594	6.444	7.315
6.817	7.113	6.719	6.745	6.503	5.702
4.561	3.744	4.475	4.455	4.574	4.944
2.502	2.383	2.115	2.460	2.520	3.915
3.719	3.365	3.137	3.379	3.146	4.688
1.974	2.040	3.005	2.400	2.457	2.125
5.979	6.848	6.713	6.248	6.464	4.729
5.633	5.778	6.101	5.647	6.077	4.356
4.964	5.147	4.916	4.620	5.061	4.902
6.037	5.619	6.004	6.048	6.131	6.920
7.676	7.788	7.818	7.740	8.014	6.046
3.617	3.279	3.331	2.538	3.156	3.503
2.859	3.032	3.081	2.812	2.846	3.532
4.155	5.100	5.279	4.888	5.033	3.954
6.587	6.619	6.648	6.450	6.083	2.964
2.011	2.350	2.553	2.679	2.504	2.735
3.404	3.194	3.525	2.962	3.159	3.578
3.929	4.498	4.659	4.440	4.378	4.624
8.552	9.044	8.706	8.684	8.715	8.316
9.754	9.656	9.985	9.786	9.734	9.095
3.257	3.957	3.701	3.206	3.506	2.132
3.107	4.055	3.873	3.829	3.005	2.095
3.875	2.952	3.654	3.813	2.860	3.151
5.293	5.033	5.396	5.664	5.508	4.381
5.631	5.667	5.839	5.553	5.626	4.265
6.260	6.462	6.529	6.319	6.479	6.164

6.166	5.726	6.752	5.191	6.715	7.648
3.224	3.069	3.244	3.431	2.967	2.126
2.081	2.125	2.203	2.035	1.760	1.568
3.907	3.334	3.437	3.698	4.832	4.542
3.894	3.717	3.919	4.294	4.204	4.469
4.833	4.436	5.342	5.233	4.464	2.043
1.817	2.414	2.877	2.087	2.030	2.511
6.712	6.979	6.541	6.566	7.172	7.273
5.589	6.472	6.145	5.885	6.251	4.653
3.117	4.108	3.645	4.135	3.854	3.063
4.661	4.187	4.828	4.608	5.070	3.152
2.937	3.435	3.503	3.333	3.663	2.924
4.271	5.053	4.880	4.334	5.267	3.607
5.265	5.507	5.668	5.328	5.377	2.704
3.270	3.405	3.969	3.548	3.736	3.743
2.435	2.283	2.201	2.806	2.693	1.244
4.581	4.341	4.374	4.491	4.322	3.422
3.885	4.204	3.974	4.476	4.163	5.312
2.625	2.501	2.842	2.544	3.237	3.451
2.929	3.930	3.549	3.676	3.703	3.448
2.201	2.975	3.196	3.131	3.292	2.499
2.575	1.914	2.343	1.939	2.622	1.872
4.827	5.446	5.218	5.342	5.151	5.615
2.070	2.469	3.095	2.290	2.137	2.947
4.066	3.679	3.755	3.763	3.690	1.122
3.958	4.556	4.107	4.173	4.319	4.012
3.476	3.721	2.931	3.225	3.131	2.515
2.204	1.468	2.134	2.515	2.581	2.939
2.739	2.732	2.817	2.838	2.391	2.838
7.474	7.762	7.524	7.221	7.318	6.643
5.618	5.878	5.793	6.148	5.752	5.568
2.492	2.996	2.556	1.971	1.855	2.892
7.282	7.033	7.372	7.231	7.371	7.009
8.142	8.487	8.180	8.484	8.383	9.366
6.727	4.369	4.638	5.206	6.273	2.326
6.034	5.952	5.575	5.805	5.954	4.759
6.222	6.371	6.417	6.440	6.478	5.760
6.126	6.127	5.864	5.842	5.884	4.367
7.236	7.170	6.969	7.117	6.997	6.179
5.872	6.292	6.161	5.957	6.063	3.457
2.856	2.904	2.626	2.789	2.226	1.771
3.281	2.619	2.432	2.457	2.594	2.241
3.250	2.963	2.318	2.904	2.703	2.365
4.120	3.908	4.321	4.058	4.007	3.598
3.448	3.350	3.197	2.902	3.195	3.465
5.471	4.923	5.403	6.099	5.991	6.868
3.809	3.566	3.894	4.072	4.336	5.200
3.382	4.415	4.311	3.776	4.248	5.916
5.164	3.311	3.607	4.380	4.277	2.925
7.078	7.256	7.354	7.376	7.224	6.866

7.137	7.466	7.307	7.604	7.430	6.821
5.488	7.091	7.147	7.127	7.681	7.637
8.170	8.281	8.104	8.109	7.898	5.410
5.841	4.248	5.065	5.909	4.939	4.755
6.280	4.844	5.101	6.335	4.928	4.842
3.044	3.664	2.554	2.369	3.538	4.088
5.533	5.779	6.094	5.747	5.947	3.834
3.237	3.680	2.990	3.285	2.517	2.670
4.282	5.023	4.655	4.050	4.176	2.525
4.376	4.702	4.561	4.204	4.361	3.331
4.415	4.520	5.188	4.603	4.381	3.868
4.702	4.476	4.892	4.274	4.342	2.961
5.304	4.366	6.024	4.859	5.203	1.051
1.609	1.196	1.566	1.700	1.568	1.892
3.203	2.749	2.900	3.335	3.285	3.843
3.941	2.691	2.463	3.439	2.967	3.944
5.053	4.622	3.951	5.417	4.726	5.386
6.476	5.442	5.293	6.629	5.825	7.626
3.460	3.177	2.132	4.068	2.737	4.868
2.646	2.618	2.608	2.902	2.691	1.620
4.719	1.731	3.013	4.115	3.362	4.464
4.624	4.034	2.637	5.003	4.662	5.520
3.148	2.828	2.432	3.685	2.634	3.105
2.868	3.372	3.166	2.995	2.622	4.065
2.858	2.172	2.275	2.970	2.477	4.274
1.299	1.437	3.401	2.952	2.079	2.532
2.692	1.856	2.414	3.007	2.202	2.104
3.082	2.121	2.346	2.194	2.979	1.178
3.887	3.662	3.048	3.582	3.695	3.304
1.409	1.075	1.382	1.894	1.301	3.625
3.205	3.564	3.201	3.119	2.598	2.952
3.075	2.695	2.889	2.262	2.782	3.047
5.917	6.203	5.886	6.237	6.147	4.941
6.425	6.338	6.483	6.349	6.227	5.782
3.732	3.725	3.519	3.575	3.351	2.665
4.299	3.733	4.257	3.945	3.535	4.898
3.727	3.312	3.026	3.734	3.709	2.713
6.516	6.573	6.308	6.731	6.337	6.808
5.946	6.106	6.113	6.275	6.447	5.209
6.887	6.605	6.639	6.792	6.724	5.630
2.583	2.605	2.392	2.229	2.624	2.426
4.784	5.179	5.323	4.909	4.975	4.137
2.947	3.220	3.591	3.234	3.049	3.693
2.636	2.435	2.757	2.642	3.118	3.221
4.253	4.688	4.099	4.750	4.443	5.254
2.089	1.401	1.301	1.574	1.806	1.690
5.657	6.308	5.717	4.856	5.528	4.918
8.084	8.383	8.394	8.007	8.261	7.435
3.752	2.624	2.997	2.896	2.811	2.470
3.847	3.398	4.286	4.355	4.422	2.016

3.582	4.078	3.129	3.834	3.368	3.477
3.542	3.420	3.610	2.890	3.410	2.369
5.528	6.245	5.819	5.499	5.990	4.636
6.304	6.462	5.870	6.220	6.246	6.268
5.941	6.256	6.237	6.151	6.465	7.796
4.315	4.728	3.964	4.860	4.200	3.980
2.550	2.138	2.325	2.328	2.662	3.332
8.167	8.110	8.042	8.290	8.371	7.057
2.128	2.460	2.007	2.678	2.086	3.149
3.652	2.229	2.446	1.931	2.440	1.721
7.737	7.790	7.785	8.038	7.616	5.614
8.539	8.315	8.751	8.560	8.566	6.696
3.724	2.717	3.501	3.347	3.095	2.708
3.212	3.242	5.541	3.260	4.375	3.714
1.742	2.140	2.822	2.645	2.315	3.734
1.971	2.101	1.853	1.843	2.150	2.076
2.976	3.083	2.395	2.743	3.077	3.196
4.450	3.600	4.227	4.037	4.395	3.602
4.331	1.135	3.855	2.264	3.590	1.078
6.082	5.395	5.653	6.240	5.991	6.756
5.142	4.283	4.301	5.387	5.086	5.955
4.760	6.080	5.613	5.022	5.034	6.754
4.463	5.021	4.977	3.830	3.849	6.110
4.324	4.647	4.425	4.028	4.266	4.005
6.373	5.238	6.910	5.999	6.077	6.709
3.124	2.755	1.676	2.753	1.882	3.220
2.800	2.926	2.802	2.967	2.675	1.582
3.700	4.822	4.152	4.103	3.950	2.176
2.801	3.135	3.887	3.900	3.856	4.076
4.525	3.897	4.586	4.275	4.044	3.922
2.504	2.103	2.859	2.800	2.859	3.654
4.980	5.332	5.203	5.342	5.383	6.346
7.835	7.772	8.188	7.775	7.816	5.975
4.611	5.089	4.744	4.657	4.988	5.936
7.063	7.240	6.949	7.159	7.849	9.457
8.900	9.281	9.044	9.064	9.113	8.682
4.614	4.614	4.755	4.368	4.667	4.091
4.521	4.751	4.551	4.702	4.712	5.289
2.881	2.799	2.206	2.493	2.181	2.000
4.934	6.742	5.807	5.798	5.930	5.158
6.832	7.371	7.516	7.713	8.061	8.901
8.821	8.830	8.673	8.812	8.442	6.910
5.642	5.948	6.023	5.815	5.663	6.462
7.232	7.455	7.371	7.132	7.340	5.032
6.768	6.846	6.872	6.806	6.779	2.593
3.350	3.846	3.536	3.577	3.491	4.744
5.445	5.161	5.524	5.537	5.790	5.089
4.787	4.881	4.807	4.904	4.516	5.308
7.275	7.471	7.456	7.309	7.305	6.573
4.803	5.373	5.147	4.900	5.573	6.743

3.455	3.578	3.483	3.007	2.971	2.329
5.934	5.930	6.069	5.516	6.221	4.622
5.321	6.056	5.325	5.668	5.519	4.288
5.978	6.017	5.918	6.152	5.834	5.333
3.698	2.818	3.382	3.916	3.703	3.639
2.053	2.449	1.686	2.098	2.055	2.912
4.744	5.103	4.964	4.643	4.518	3.831
5.135	5.322	5.502	5.612	5.605	6.590
5.141	4.433	4.329	4.941	5.192	4.867
6.416	6.696	6.472	6.630	7.191	9.102
4.247	4.047	4.085	4.372	4.781	5.413
5.272	4.981	4.906	5.016	5.013	6.168
4.489	4.324	4.115	4.372	4.527	5.756
5.453	5.576	5.561	5.036	5.443	4.030
1.616	1.622	1.971	1.396	1.615	2.672
3.865	3.193	3.747	3.686	3.353	3.261
5.484	5.971	5.592	5.637	5.358	4.888
6.618	7.030	6.979	6.320	7.033	5.987
8.983	9.546	9.156	8.970	8.995	7.420
3.628	4.531	4.598	3.984	4.321	5.005
5.254	6.297	5.765	5.237	5.715	5.705
5.104	5.406	5.422	5.327	5.318	4.381
6.323	6.603	6.701	6.584	6.587	8.235
3.784	4.464	4.731	4.391	4.491	4.481
2.583	3.185	4.143	3.408	3.698	3.167
5.123	4.871	5.057	5.224	4.589	1.755
2.781	2.623	2.902	2.045	2.592	2.911
1.866	2.451	1.937	1.444	1.657	2.084
2.772	2.962	2.619	2.810	3.061	4.603
1.757	1.508	1.767	1.378	1.657	1.870
3.066	2.200	1.805	1.717	1.658	1.849
2.810	2.522	2.885	3.469	3.261	4.026
4.476	4.913	4.749	4.934	4.974	5.393
4.557	4.114	3.602	4.158	3.918	4.931
3.926	3.601	3.412	3.347	3.578	5.780
4.829	4.556	4.988	4.656	5.030	5.610
2.637	3.053	2.808	3.266	3.395	3.583
3.648	3.703	3.201	3.075	3.305	4.341
5.686	5.734	5.470	5.895	6.393	7.241
6.003	5.346	5.750	5.552	5.672	4.777
8.069	8.057	8.086	8.012	8.240	7.044
5.237	3.303	5.279	6.252	6.039	2.320
4.613	4.060	4.310	4.301	4.514	4.728
5.906	5.612	5.227	4.771	5.103	5.015
6.500	5.832	5.990	5.729	5.616	4.005
2.850	3.057	3.259	1.989	3.121	1.523
7.242	6.933	7.276	6.944	7.076	5.776
6.817	6.656	6.492	6.260	6.174	2.470
8.769	8.645	8.862	8.958	8.858	9.331
6.098	6.556	6.351	5.994	6.472	6.095

6.283	6.159	6.090	6.199	6.243	5.676
4.944	4.611	4.687	4.766	4.321	3.585
5.999	5.653	6.149	5.631	5.733	5.351
4.676	5.079	4.983	5.114	5.064	5.155
3.360	4.196	3.634	3.995	4.048	4.260
5.735	5.377	5.179	5.511	5.352	5.054
4.495	4.408	4.452	4.622	4.390	4.460
4.028	4.117	4.550	4.255	4.364	4.319
5.290	6.449	5.525	5.416	5.502	6.009
5.132	5.572	4.349	5.388	5.101	5.434
4.231	4.692	3.339	4.844	4.484	4.814
3.054	2.278	2.001	2.965	3.515	2.731
3.870	4.092	4.563	3.913	4.390	3.489
4.147	3.629	4.110	4.609	3.904	5.271
6.009	6.026	5.664	5.926	5.807	4.961
2.432	2.643	2.940	2.584	2.357	3.038
1.707	2.995	1.805	2.040	1.996	2.420
7.235	7.355	7.604	7.478	7.498	4.772
6.510	6.847	6.702	6.586	6.760	5.816
7.847	7.640	7.793	7.797	7.665	6.554
3.813	3.435	3.350	3.668	3.761	3.213
1.977	1.777	1.921	1.446	2.436	1.574
2.220	2.189	1.678	2.128	1.227	1.920
3.954	4.417	4.103	4.025	4.830	2.336
5.895	6.748	6.780	6.321	6.854	6.327
6.224	6.832	6.595	7.021	6.666	3.941
6.721	6.398	6.676	6.038	6.625	5.785
8.063	7.931	8.027	8.059	8.083	7.053
3.473	3.980	3.793	4.023	4.326	5.969
2.142	3.697	2.184	2.225	2.854	1.768
6.838	7.342	7.189	7.012	7.153	6.690
5.103	5.306	5.251	5.471	5.406	4.834
6.658	6.821	6.927	6.754	6.857	6.464
2.335	3.200	3.235	2.244	2.632	4.293
4.567	4.610	3.969	4.666	5.141	4.251
3.692	4.766	4.360	5.263	5.207	5.409
5.397	5.113	5.344	5.107	5.313	5.826
6.558	5.990	6.582	6.127	6.248	4.844
3.524	2.254	2.351	3.149	2.642	1.932
7.648	7.396	7.832	7.362	7.548	5.606
7.479	7.315	7.359	7.448	7.325	5.093
4.693	4.854	5.021	5.386	5.400	6.111
6.325	6.102	6.193	5.503	5.961	3.262
4.533	4.119	3.728	4.425	4.064	4.765
5.116	4.884	5.156	5.349	4.974	4.094
5.438	5.893	5.601	5.786	5.779	4.591
5.089	5.007	4.798	4.934	4.974	5.647
4.761	4.274	4.491	4.802	3.816	3.757
4.430	4.385	3.643	3.520	4.114	3.114
3.850	4.041	4.151	4.740	4.699	4.806

4.624	4.760	4.801	4.928	5.016	4.302
6.840	6.516	6.548	6.979	6.714	6.411
5.463	5.543	5.468	6.172	6.259	7.286
7.429	7.570	7.384	7.253	7.271	6.679
4.394	4.000	4.222	4.599	4.259	5.344
3.824	3.967	4.246	4.462	4.001	4.732
3.934	2.985	2.824	4.083	4.130	3.924
6.059	5.379	5.669	6.215	5.745	6.300
5.111	4.312	4.657	4.342	4.139	4.122
4.012	4.040	4.189	4.357	4.733	6.045
5.922	4.750	5.224	5.692	4.953	6.521
5.486	5.065	5.590	4.785	4.655	5.752
4.359	4.824	4.520	4.464	4.519	3.982
5.861	6.386	6.260	6.332	6.291	5.585
6.655	6.819	6.963	6.796	6.700	5.344
6.736	6.513	5.924	6.770	6.378	7.639
5.822	4.447	4.663	4.761	5.088	5.813
3.650	3.587	3.573	3.756	3.418	4.946
2.904	3.204	2.856	2.315	3.103	3.544
4.944	4.157	4.058	4.405	4.472	5.120
5.537	5.365	5.714	5.522	5.494	3.680
5.025	5.201	5.228	5.097	5.028	4.628
8.074	8.081	8.035	8.076	7.908	7.244
3.296	4.185	4.108	3.135	3.626	4.299
5.644	6.008	5.394	5.031	6.091	6.757
8.405	8.650	8.538	8.791	8.414	6.978
2.503	2.614	2.196	2.779	2.974	2.651
7.471	7.034	7.557	7.224	7.376	5.606
2.130	2.167	1.935	2.081	1.634	1.637
6.163	5.412	5.732	5.769	5.461	5.104
7.465	7.153	7.177	7.249	7.294	5.483
3.697	3.396	3.026	2.754	3.681	3.172
5.735	5.444	5.557	5.143	5.321	4.111
3.571	3.211	3.449	3.308	3.606	4.231
8.367	8.204	8.171	7.681	8.015	7.477
7.153	7.196	6.904	7.297	7.280	6.291
4.130	4.915	4.762	4.118	4.647	3.333
6.719	6.499	6.366	6.500	6.684	6.779
5.548	5.776	5.685	5.588	5.746	4.796
1.629	1.901	1.868	1.667	1.839	3.286
6.144	5.729	5.651	5.691	5.832	5.866
4.225	4.620	3.761	4.168	4.060	3.892
6.095	6.199	5.738	6.255	6.016	6.491
2.570	2.274	2.218	2.421	2.828	2.708
6.795	7.190	7.249	6.773	6.991	6.289
3.533	3.606	3.399	3.535	3.263	4.528
5.432	4.677	5.156	4.836	5.165	5.622
7.886	7.854	7.796	8.111	7.995	7.920
3.702	4.380	3.443	3.546	2.889	3.227
4.985	5.236	5.381	5.245	5.369	4.599

5.897	6.043	5.671	5.802	6.042	5.657
7.462	7.209	6.774	6.513	7.078	7.254
4.973	4.879	4.816	4.852	4.985	4.128
7.605	7.691	7.982	7.959	7.663	7.021
7.971	8.149	8.042	7.912	7.962	8.793
8.207	8.203	8.265	8.271	8.021	7.425
3.703	4.134	4.225	4.033	4.439	4.983
3.404	4.173	3.295	4.009	3.503	3.407
5.114	4.913	5.096	5.148	4.851	3.841
6.922	6.687	7.134	6.720	6.595	6.455
4.021	4.223	4.373	3.958	4.228	4.121
2.785	2.941	3.518	3.378	3.542	3.437
5.352	5.504	5.760	5.838	5.688	5.094
7.171	6.881	7.230	6.988	6.818	5.769
4.011	4.463	4.051	4.107	4.079	4.713
6.447	5.964	5.863	5.871	5.591	6.263
7.620	8.068	7.894	7.696	7.930	4.683
2.602	2.681	2.105	2.557	2.390	2.430
6.199	6.487	6.130	6.148	6.253	5.177
5.366	5.757	5.392	5.443	5.495	6.170
5.391	5.154	5.708	5.722	5.716	2.506
5.880	5.972	6.078	6.559	6.141	7.021
6.458	5.976	5.905	6.039	6.064	6.597
5.236	4.846	4.889	5.105	5.599	5.317
1.797	1.945	1.745	1.729	1.856	2.065
6.485	6.350	6.438	6.666	6.261	6.061
5.068	4.962	4.994	5.094	5.340	6.381
4.775	4.928	4.865	5.186	4.942	6.192
5.014	4.887	4.721	5.164	4.814	5.944
3.254	3.616	3.534	3.682	2.758	4.786
3.736	3.870	3.587	3.754	3.734	4.242
3.203	3.543	3.518	3.630	4.059	4.906
3.722	2.768	2.615	3.409	3.521	2.666
7.529	7.475	7.310	8.030	7.923	8.759
7.097	6.883	6.910	6.983	6.850	5.673
4.390	3.994	4.302	4.592	3.893	4.204
4.404	4.483	4.956	3.950	4.483	4.624
5.386	5.525	5.719	5.623	5.360	4.670
4.992	5.957	5.154	5.602	5.161	5.079
4.021	2.888	3.733	3.555	3.396	5.105
8.328	8.071	8.095	8.069	8.114	5.753
7.289	7.278	7.443	7.867	7.636	8.360
4.197	4.131	4.083	4.053	4.237	5.422
3.803	5.487	4.680	3.088	3.555	1.564
3.054	2.327	2.871	2.781	3.190	4.179
7.307	7.863	7.634	7.407	7.593	6.802
4.337	3.530	2.989	3.833	3.609	5.390
2.823	3.850	3.684	3.715	3.626	4.239
9.252	8.198	9.239	9.014	9.211	7.347
7.505	6.730	7.496	7.718	7.561	6.201

8.882	7.877	8.863	9.017	8.798	7.609
6.284	6.551	6.452	6.182	6.294	4.125
3.687	3.455	4.068	3.590	3.721	3.326
4.573	4.111	4.161	3.789	4.218	5.064
4.236	3.977	4.318	4.886	5.252	6.503
2.954	3.219	3.723	3.051	3.268	3.815
3.171	4.460	4.355	3.946	3.919	4.030
4.106	3.819	3.727	3.883	3.610	4.891
5.596	5.660	5.479	5.668	6.164	7.196
5.693	5.638	6.064	5.838	5.874	6.303
6.906	6.702	6.479	6.728	6.728	7.914
5.110	5.338	5.071	5.516	5.581	5.197
1.940	2.217	3.027	1.868	2.228	1.642
4.613	5.450	5.196	4.214	4.872	4.757
7.468	7.487	7.586	7.272	7.570	6.698
6.307	7.052	6.818	6.678	6.840	6.128
5.023	5.632	5.545	4.205	4.677	5.172
9.223	8.972	9.087	9.228	9.126	8.602
5.923	6.251	6.142	5.866	5.873	4.866
5.773	6.338	5.633	5.807	5.720	4.753
2.924	3.304	2.963	2.589	2.378	3.652
1.521	1.854	1.873	1.281	1.546	2.354
1.647	2.316	2.274	2.485	1.940	2.817
6.004	5.197	5.350	4.987	5.649	5.164
4.775	4.920	4.882	4.398	4.480	3.668
2.174	2.217	2.242	2.278	2.362	2.483
7.590	8.021	7.626	8.369	8.228	9.483
9.134	9.013	9.088	8.992	9.030	8.094
7.180	7.215	7.292	6.945	7.018	5.762
5.472	5.743	5.537	5.330	5.593	4.780
7.898	7.555	7.698	7.566	7.857	6.139
4.931	4.340	4.217	4.243	4.565	4.800
3.593	3.573	3.272	3.491	3.047	4.022
4.746	3.921	5.160	3.404	5.007	7.075
7.093	7.267	7.277	7.218	7.022	6.484
5.856	6.295	6.211	5.648	6.156	4.757
5.503	5.190	4.972	4.880	4.976	3.565
6.596	6.626	6.650	6.515	6.689	3.394
2.299	1.492	2.426	2.299	2.279	2.461
3.441	3.434	3.657	4.448	4.049	4.667
6.121	6.107	6.333	6.049	5.745	5.055
8.743	9.136	8.953	9.007	8.948	8.035
3.906	3.785	3.769	4.052	3.776	2.958
7.527	7.460	7.463	7.745	7.474	6.375
6.012	6.203	6.232	5.874	5.926	5.793
5.480	5.489	5.695	5.642	5.599	3.471
5.285	4.327	4.543	4.654	4.964	3.106
7.818	7.711	7.781	7.841	7.974	9.129
3.982	3.740	3.372	3.947	3.870	3.613
5.308	5.818	5.792	4.985	5.503	4.797

7.049	7.252	7.051	7.287	7.430	8.716
4.738	4.662	5.370	4.458	4.471	3.753
4.619	5.133	4.753	5.128	5.114	5.703
3.519	3.337	2.451	3.294	3.579	3.179
5.245	5.429	5.908	5.281	5.726	5.132
4.322	3.314	3.069	3.735	3.775	3.603
3.467	3.288	3.644	4.064	4.013	4.250
5.465	5.308	5.160	5.187	5.211	5.061
6.562	5.330	6.252	5.672	5.811	5.569
2.095	1.739	2.419	2.066	1.773	2.331
4.673	4.223	4.197	4.034	4.818	3.794
3.546	3.718	3.886	3.156	3.598	3.868
9.057	9.514	9.456	9.363	9.636	7.203
3.849	2.214	3.213	3.532	2.591	2.343
3.341	4.136	4.092	4.040	3.555	5.071
4.904	4.693	4.654	4.676	4.469	5.121
6.521	6.330	6.306	6.598	6.593	7.037
3.729	4.092	3.558	3.402	3.821	5.068
5.413	5.917	5.499	5.395	5.587	7.320
5.686	5.776	6.118	5.486	5.693	6.054
3.976	4.520	4.297	3.891	3.639	5.149
6.430	7.047	6.772	5.682	6.541	2.796
6.112	6.497	6.522	6.660	6.414	7.294
5.371	3.978	5.209	4.888	5.655	5.643
5.916	6.154	6.050	6.035	5.940	5.942
4.684	5.117	5.103	5.505	5.638	6.897
4.712	5.305	5.366	4.869	5.251	5.979
6.984	6.629	6.773	6.890	6.526	6.154
2.642	3.014	2.779	2.899	3.090	5.111
4.956	5.362	5.386	5.487	5.695	5.319
5.959	5.839	5.608	5.526	5.601	6.281
3.624	4.065	3.861	3.334	3.220	4.267
7.471	7.294	7.335	7.590	7.362	6.430
2.701	3.226	3.282	3.281	3.154	3.339
7.453	7.100	7.072	7.152	7.169	6.822
3.598	2.835	2.859	3.622	3.007	3.121
3.774	2.946	4.028	3.672	3.858	3.254
5.107	4.413	4.808	5.263	5.110	6.303
3.846	3.789	3.657	3.806	3.504	4.218
6.125	5.821	5.468	5.898	5.738	7.171
2.818	3.307	2.811	2.347	2.168	2.674
3.475	3.960	3.956	3.926	4.555	5.179
5.035	4.160	4.508	4.491	4.395	3.314
5.310	5.487	5.369	6.408	6.625	8.351
4.661	4.614	4.271	4.143	4.625	5.204
8.664	8.536	8.391	8.565	8.375	7.509
6.460	6.402	6.397	6.276	6.376	5.598
5.696	5.209	5.427	5.323	5.872	6.037
4.119	3.682	2.994	4.290	3.906	5.090
3.201	3.160	3.101	3.048	3.130	3.310

5.066	5.107	5.233	4.635	5.348	6.560
3.065	3.138	2.579	2.540	2.285	3.702
6.458	6.478	6.300	6.148	6.180	3.399
2.995	3.532	3.584	3.566	3.507	4.250
3.959	3.055	3.221	3.169	2.776	3.029
6.772	7.581	7.113	7.731	8.282	9.143
3.689	2.561	1.759	3.620	3.955	2.554
6.525	6.092	6.410	6.353	5.888	6.622
6.865	6.600	6.717	6.459	6.590	6.771
4.391	4.117	4.693	4.303	4.305	5.327
5.014	4.877	4.961	4.222	4.545	5.271
3.741	3.621	3.740	4.404	4.268	2.800
5.678	5.325	5.603	4.512	5.311	4.596
6.926	6.466	6.819	6.496	6.769	6.726
8.184	6.862	7.700	7.526	7.628	8.104
3.203	3.774	2.811	2.936	3.047	3.462
5.882	5.988	6.203	6.034	6.056	5.034
8.401	8.311	8.541	8.322	8.283	9.313
4.920	5.313	5.560	5.292	4.965	5.693
6.392	5.913	6.194	6.260	6.070	4.937
7.227	7.131	7.020	6.971	6.890	5.519
3.769	4.103	4.265	4.129	4.478	4.721
6.036	5.819	6.101	6.037	5.996	5.314
5.399	5.438	5.384	5.575	5.725	6.688
5.893	6.123	5.545	5.935	5.606	6.655
3.837	3.937	3.991	4.352	4.646	6.139
4.926	5.124	5.110	5.017	4.822	5.653
9.232	10.141	9.967	9.698	9.473	10.653
5.765	5.701	6.070	5.749	6.218	7.373
5.816	6.350	6.014	6.111	6.415	6.026
5.342	5.949	5.792	5.702	5.830	5.876
4.370	4.213	4.539	3.646	4.059	4.289
3.248	2.541	2.142	4.035	2.924	3.852
4.875	4.640	4.679	4.283	4.066	3.027
5.223	4.782	4.934	4.469	4.936	4.188
2.882	2.933	2.493	3.272	2.816	1.782
8.540	8.624	8.733	8.556	8.361	7.951
7.711	7.706	7.565	7.709	7.907	6.755
5.522	5.397	5.562	5.671	5.393	4.401
3.961	4.006	4.004	3.249	3.895	3.354
4.670	4.993	4.978	4.648	4.707	3.620
4.680	4.945	4.954	4.644	4.688	3.735
4.400	4.577	4.905	4.200	4.485	3.690
4.471	4.375	4.868	4.283	4.060	2.941
7.179	7.204	7.165	7.127	6.933	6.004
5.706	5.551	5.632	5.814	5.702	4.814
2.975	3.605	3.422	3.106	2.843	2.077
5.558	4.047	4.302	4.442	4.469	4.447
4.466	4.878	4.535	4.409	4.476	5.226
3.761	3.319	3.893	4.163	4.097	3.263

2.383	2.209	2.362	2.635	2.754	2.804
6.910	7.217	7.058	7.106	7.191	6.492
2.890	2.718	3.569	2.730	2.734	2.573
3.189	3.479	2.965	3.256	3.369	3.644
6.883	7.323	7.187	6.977	7.034	6.610
5.041	5.231	5.180	5.101	5.413	5.999
4.623	4.296	4.860	4.671	4.197	5.246
7.323	7.281	7.631	7.370	7.322	6.214
5.851	5.346	6.012	6.119	6.139	5.529
5.150	5.768	5.239	5.611	5.903	4.975
2.017	2.603	1.894	2.488	2.097	1.566
2.204	2.738	3.749	3.090	3.017	2.770
2.113	3.356	2.541	3.129	2.652	1.707
1.344	1.707	1.626	1.563	1.297	1.763
5.946	6.279	5.907	5.867	5.982	6.589
7.436	7.645	7.155	7.240	7.402	6.380
3.341	4.206	3.557	3.930	3.401	4.180
2.487	2.150	2.332	2.733	2.559	3.671
4.850	5.488	5.103	5.235	5.341	4.525
6.112	6.306	6.080	5.926	6.335	5.555
2.688	3.326	2.828	3.049	3.461	1.921
2.816	3.418	3.010	3.612	3.089	2.598
2.972	3.142	3.518	3.456	3.494	2.623
5.871	5.719	6.034	6.366	5.921	6.755
2.198	2.163	2.708	2.027	2.616	5.155
3.571	4.194	4.129	3.985	3.519	3.597
2.672	2.305	1.805	2.527	2.929	3.546
6.200	6.318	6.425	6.386	6.380	5.431
4.531	3.597	4.609	4.886	4.641	3.094
3.385	2.196	1.900	2.814	2.654	1.528
3.144	1.492	4.106	2.947	3.319	2.088
3.074	2.877	3.277	3.283	3.404	2.402
2.005	2.148	2.128	2.245	2.619	4.159
5.204	5.284	5.307	5.762	5.813	7.289
4.898	5.199	5.641	5.104	4.769	6.847
3.673	4.181	3.938	3.964	4.074	3.588
4.081	4.374	4.157	4.015	4.160	3.401
3.407	3.375	3.427	3.392	3.481	2.842
4.835	5.386	5.145	5.006	4.862	3.970
2.666	1.408	1.729	2.163	1.328	1.645
6.360	5.853	6.534	5.891	6.244	7.617
3.361	3.915	5.084	3.712	4.824	4.946
8.207	8.023	8.153	8.214	7.939	6.759
4.688	5.101	5.070	4.935	5.024	3.181
5.395	5.548	5.721	5.862	5.330	3.250
7.728	7.337	7.588	7.415	7.264	6.444
3.994	4.121	4.262	4.275	4.346	5.227
2.502	2.093	3.085	2.957	2.288	1.984
3.540	3.118	3.463	3.507	3.112	2.523
2.476	2.209	1.749	3.420	1.839	1.799

3.321	3.555	3.172	3.069	3.398	3.122
7.193	6.633	7.097	6.883	6.634	6.004
4.708	4.531	4.562	4.367	4.114	3.636
4.327	3.640	2.803	3.461	3.534	3.780
7.646	7.368	7.435	7.670	7.501	6.943
5.850	5.749	5.900	5.950	5.872	4.968
7.279	7.193	7.471	7.548	7.389	6.546
7.224	7.290	7.224	7.246	7.155	6.545
6.459	6.504	6.575	6.642	6.652	6.050
8.073	7.594	7.823	7.891	7.570	6.990
6.593	6.517	6.429	6.545	6.512	5.760
6.225	5.112	5.866	6.021	5.719	4.470
4.104	4.345	4.258	4.411	4.239	3.168
7.033	6.899	7.088	7.205	6.843	6.511
7.178	7.117	7.040	7.275	7.007	6.398
9.237	8.731	9.008	9.016	8.847	8.385
8.775	8.496	8.590	8.554	8.446	7.929
8.418	7.714	8.159	8.275	8.154	7.426
8.342	7.951	8.227	8.357	7.854	7.604
6.079	6.516	6.251	6.635	6.467	6.067
4.727	5.307	5.058	4.846	4.867	2.475
5.272	5.522	5.584	5.292	5.582	4.236
6.589	6.891	6.775	6.823	6.878	6.458
3.082	4.198	3.887	3.623	3.734	2.776
7.551	7.798	7.578	7.556	7.513	6.759
5.715	6.001	6.043	5.506	5.837	4.889
6.836	7.177	7.135	7.039	7.182	6.078
7.366	7.065	7.347	7.405	7.075	6.216
6.143	5.799	5.826	5.951	5.834	5.109
3.816	2.517	3.070	3.114	3.204	3.646
4.066	4.397	4.563	4.798	5.408	6.702
4.342	5.239	5.004	4.359	4.590	1.888
4.434	4.287	3.497	3.922	4.370	3.634
7.875	7.876	7.849	7.531	7.992	6.847
4.125	4.247	4.403	3.288	3.703	4.689
4.290	4.143	4.441	3.592	3.741	4.687
4.167	4.270	4.410	3.437	3.669	4.695
5.343	5.204	5.299	4.672	5.019	5.655
4.398	4.288	4.366	3.404	4.009	4.593
5.850	5.688	5.989	5.877	5.654	4.977
5.932	5.219	5.426	5.879	5.618	5.008
3.250	2.906	3.223	3.276	3.056	3.209
3.258	3.400	3.504	3.434	3.396	3.563
2.516	2.340	2.637	2.318	2.435	2.815
6.988	6.415	6.257	6.745	6.902	6.927
7.121	7.201	7.413	7.154	7.290	6.592
7.851	7.786	7.777	7.906	7.943	6.307
7.375	7.355	7.225	7.427	7.554	6.928
3.299	3.089	3.297	4.454	4.154	6.552
4.883	5.164	4.868	5.141	5.214	5.706

2.634	2.926	3.309	2.888	3.317	3.557
6.734	7.043	7.103	6.896	6.719	6.174
7.064	7.265	7.349	7.448	7.210	6.515
9.781	9.939	9.791	9.800	9.662	7.486
7.525	7.603	7.469	7.494	7.487	6.239
3.065	2.808	2.583	2.789	3.084	3.457
9.126	9.079	9.398	9.233	8.950	7.270
2.085	2.237	1.922	2.302	2.219	3.683
6.193	6.437	6.333	5.861	6.154	4.646
2.618	2.429	2.834	2.426	2.432	2.566
5.791	6.533	6.089	6.272	6.164	5.269
5.929	5.842	5.852	6.149	5.988	6.393
3.994	4.086	4.338	4.629	4.134	5.301
1.806	2.892	1.959	2.871	2.220	3.947
5.487	4.719	4.347	4.539	4.968	3.274
3.668	3.416	3.187	3.758	3.584	2.183
4.464	4.861	4.489	5.220	4.502	4.406
3.489	3.157	3.074	2.695	3.264	2.482
6.088	5.608	5.890	5.607	5.748	5.224
4.503	4.348	4.826	3.893	3.419	3.813
6.506	6.221	6.447	6.466	6.287	5.745
5.523	6.113	5.731	5.770	5.778	5.109
4.315	4.215	4.204	4.206	3.791	5.554
3.273	2.507	2.737	2.899	2.613	2.159
5.487	5.238	5.539	5.027	5.060	3.866
6.222	4.531	4.776	5.228	4.936	4.626
6.617	6.817	6.727	6.876	6.763	5.685
9.705	9.686	9.709	9.840	9.802	8.799
6.435	6.634	6.356	6.552	7.052	7.675
2.035	1.566	1.474	1.399	1.495	1.521
2.121	0.979	1.239	1.224	1.264	1.049
6.886	6.799	6.722	6.401	6.817	4.903
7.558	7.874	7.694	7.540	7.679	6.527
2.099	2.170	2.162	2.829	2.994	2.829
1.692	3.507	3.205	3.476	3.617	5.880
6.721	6.903	6.969	7.008	6.741	5.640
7.998	8.003	8.180	8.162	8.318	7.539
3.237	3.329	3.752	3.482	3.650	1.900
6.521	6.827	6.745	6.646	6.846	5.146
4.171	3.580	3.496	2.879	3.354	2.956
7.437	7.876	7.795	7.776	7.593	5.609
7.003	6.796	6.034	6.806	6.644	4.691
2.940	2.650	3.488	2.690	3.587	3.387
5.553	5.718	5.683	5.861	5.482	3.820
7.219	6.974	7.283	6.836	7.125	2.187
6.531	7.010	6.601	6.612	6.687	5.552
4.166	4.689	4.601	5.038	5.072	5.406
2.566	3.053	2.676	3.022	3.410	3.813
7.599	7.858	7.518	7.629	7.810	8.486
4.140	4.223	4.189	4.386	3.971	4.797

4.561	4.247	4.048	4.283	4.128	5.153
4.597	4.299	4.847	4.073	4.386	3.080
3.028	3.462	3.128	3.623	3.018	5.203
3.382	2.711	1.457	3.140	2.754	5.095
3.564	2.147	3.210	3.671	3.893	2.469
1.641	1.789	1.471	1.715	2.334	1.327
3.493	3.308	3.460	4.158	4.290	4.162
5.779	6.415	6.281	6.100	6.528	4.605
4.094	4.186	3.758	3.781	4.151	3.989
4.351	3.816	4.363	5.088	4.858	4.464
3.382	3.367	2.680	2.350	2.024	1.429
3.604	4.081	5.336	4.540	3.310	1.963
2.178	2.754	2.635	2.640	2.945	2.635
5.252	5.521	5.011	4.940	5.012	4.535
7.154	7.343	7.189	7.157	7.073	6.473
1.536	1.814	1.315	1.321	1.178	1.913
4.353	3.380	3.687	4.590	3.237	2.500
7.674	8.125	7.870	7.805	7.882	5.798
3.018	3.280	2.686	2.724	3.186	3.868
3.335	3.092	2.730	3.730	3.586	3.034
4.555	4.846	4.330	5.209	5.716	6.006
5.391	4.960	5.053	4.851	5.272	4.102
4.133	3.497	3.925	4.399	3.966	4.899
2.293	2.314	1.987	2.905	2.857	4.282
5.851	5.762	5.629	6.271	5.881	7.122
5.081	4.469	4.962	4.248	4.119	2.872
2.355	3.101	2.808	2.562	2.932	3.386
4.708	4.130	4.124	4.233	4.673	3.542
4.568	3.635	4.084	4.579	4.245	3.505
4.724	3.783	4.046	4.673	4.389	3.628
5.225	5.542	5.566	5.409	5.535	3.664
5.254	5.406	5.470	5.579	5.417	3.886
3.375	2.354	4.175	3.744	3.368	4.247
4.559	4.558	4.634	4.143	4.955	4.641
3.763	3.862	3.628	4.026	3.490	2.201
3.380	3.660	3.156	3.126	2.931	3.277
1.323	1.739	1.504	1.684	1.649	2.562
3.534	3.012	2.609	3.620	2.912	2.829
6.190	5.999	6.049	6.254	6.245	6.989
2.768	3.556	3.074	3.592	3.829	5.746
2.386	2.755	2.898	2.355	3.142	2.717
6.612	6.427	6.500	6.230	6.367	4.359
7.141	6.535	6.225	7.299	7.298	7.677
2.048	2.349	1.913	2.301	2.671	2.774
4.320	4.594	4.644	4.867	4.802	5.902
4.230	4.473	4.639	4.426	4.707	4.902
4.672	5.561	5.699	4.855	5.463	4.857
4.724	5.590	5.701	4.685	5.458	4.859
2.649	3.409	3.364	2.994	3.644	3.437
2.117	2.231	2.418	2.029	1.932	2.629

2.503	2.561	2.892	2.300	2.306	3.053
2.970	2.896	3.313	2.858	2.757	3.461
3.126	3.214	3.593	3.029	3.007	3.636
2.741	3.213	3.406	2.889	2.771	3.304
7.621	8.231	7.992	7.944	8.011	7.170
3.598	3.749	3.503	3.286	3.461	3.313
5.340	5.804	5.612	5.811	5.666	5.088
4.697	4.585	5.013	5.822	4.513	5.809
6.009	6.277	6.027	6.233	6.200	5.193
4.511	4.067	4.785	4.261	4.469	5.038
3.762	3.552	2.966	2.884	3.825	3.201
2.748	3.291	3.386	2.538	2.637	4.187
7.026	6.640	6.377	6.781	6.730	7.053
4.931	4.547	4.556	4.972	4.775	5.264
3.692	3.389	3.261	3.763	2.606	3.403
5.884	5.847	5.725	5.356	5.961	4.755
3.919	3.332	3.467	2.916	3.431	3.129
4.396	4.690	4.731	4.729	5.191	3.711
2.784	2.270	2.772	3.176	2.391	2.082
7.273	7.729	7.417	7.289	7.308	6.313
2.492	1.900	1.434	2.671	2.313	1.873
8.232	7.376	7.329	7.870	8.178	8.523
1.667	1.772	2.062	1.883	1.463	2.847
2.982	2.708	2.996	2.976	1.836	2.994
3.852	4.337	3.871	4.464	4.391	3.727
5.815	6.088	6.100	5.904	5.736	5.130
3.734	3.317	3.018	3.415	3.317	2.723
7.111	7.106	7.210	6.902	6.913	6.002
2.737	2.740	2.916	2.538	2.826	4.287
4.862	4.707	4.233	4.431	4.270	2.851
3.082	3.799	3.379	3.596	3.246	3.771
4.079	3.803	4.102	4.655	3.312	1.535
7.561	7.553	7.381	7.624	7.308	4.634
5.006	5.369	5.272	4.914	4.831	5.650
5.963	5.872	6.079	5.829	6.296	4.582
5.792	6.054	5.357	4.933	5.465	4.301
8.972	8.384	8.664	8.553	8.822	8.020
5.768	6.402	6.338	5.957	5.816	5.276
3.318	2.868	3.175	2.890	2.466	3.245
4.847	4.892	4.905	4.974	4.719	4.396
4.019	3.345	3.395	2.062	3.993	3.761
4.706	4.831	5.197	5.064	4.974	3.233
7.208	6.770	6.857	6.949	6.682	5.420
7.188	7.409	7.115	7.066	6.932	4.453
3.820	4.700	4.014	3.704	3.926	4.954
4.819	5.044	4.492	4.070	4.271	5.712
2.925	2.707	2.691	2.787	2.543	3.291
7.733	7.824	7.586	7.988	7.890	8.266
3.944	3.831	4.151	4.720	4.485	4.909
3.343	2.995	3.456	2.958	3.512	1.792

2.070	2.978	2.141	2.698	2.813	3.036
6.781	6.428	6.771	6.559	6.631	6.109
7.632	7.535	7.410	7.501	7.360	6.878
7.562	7.794	7.778	7.559	8.017	7.158
6.942	6.880	6.748	6.623	6.798	5.179
7.450	7.372	7.479	7.305	7.439	5.587
5.943	5.787	5.784	5.822	5.452	4.171
4.490	4.644	5.256	5.463	4.935	2.862
7.926	7.898	7.653	7.695	7.814	5.163
4.942	4.175	4.165	4.612	4.615	5.654
7.168	6.851	6.781	6.782	6.659	7.393
3.213	2.802	2.776	3.119	3.005	4.276
6.292	6.760	6.760	6.516	6.348	5.951
5.479	5.841	5.546	5.698	5.533	5.045
4.288	4.149	4.071	4.547	4.114	4.650
4.462	4.912	4.848	5.034	4.897	4.239
5.848	5.762	5.974	5.734	5.590	5.752
3.490	4.270	3.755	3.870	4.339	3.538
6.249	6.041	5.761	5.779	6.050	5.495
4.092	3.799	3.686	3.887	3.903	3.587
7.228	5.624	6.430	7.130	6.172	5.377
3.287	2.754	2.727	2.759	2.726	1.938
9.666	9.314	9.310	9.729	9.161	8.980
8.902	8.489	8.505	9.072	8.630	8.226
4.448	4.662	4.346	4.703	4.544	4.733
5.759	5.945	5.805	4.950	5.689	5.935
3.120	3.384	3.285	3.447	3.004	3.237
7.292	7.167	7.379	7.672	7.883	9.151
3.808	3.527	3.394	3.396	3.672	4.285
2.265	2.774	3.475	2.273	1.604	3.073
3.050	2.610	3.976	2.489	3.030	3.597
3.932	3.507	3.853	3.934	3.879	5.186
4.324	4.912	4.720	3.953	3.871	4.111
4.937	3.971	4.422	4.592	4.934	5.849
2.896	2.823	2.903	3.479	3.089	4.467
2.716	2.029	3.020	2.032	2.202	2.402
7.927	8.074	7.656	8.302	8.007	8.551
8.067	7.955	7.996	8.501	8.258	9.861
4.336	4.568	4.855	3.980	4.338	3.847
4.741	5.682	4.982	5.026	5.768	5.386
4.258	4.625	3.974	3.740	3.850	3.640
5.662	5.790	5.592	5.729	5.894	5.857
5.123	5.029	5.218	4.982	5.249	5.620
4.344	5.009	4.926	4.204	4.735	5.589
9.345	8.708	9.010	9.045	8.779	6.852
2.826	3.345	3.810	2.326	3.738	4.842
3.094	2.307	2.848	2.597	2.388	2.080
7.986	7.951	7.871	7.920	8.031	7.227
8.056	8.134	8.047	8.111	8.283	7.659
4.291	4.759	4.890	4.487	4.319	3.272

3.339	3.593	3.796	3.341	3.101	3.759
6.057	5.827	5.687	5.769	5.715	4.105
4.172	3.912	4.149	4.016	3.965	3.982
5.154	5.366	5.313	5.248	5.281	4.405
6.418	6.343	6.117	5.839	6.141	4.009
4.385	3.899	4.168	4.179	4.166	4.374
7.467	7.605	7.935	7.296	7.471	4.988
5.333	5.794	5.723	5.295	5.611	5.626
2.986	3.052	3.012	3.238	3.052	4.441
3.339	3.607	3.914	2.966	3.511	4.036
3.017	3.487	2.766	3.128	2.799	2.353
4.446	4.996	5.484	4.369	4.975	3.631
6.376	6.697	6.560	6.300	6.691	5.853
7.042	6.714	7.251	7.099	6.521	5.557
4.688	5.946	5.719	5.811	5.832	5.072
6.079	6.326	6.290	5.867	6.040	4.614
5.429	5.339	5.462	5.435	5.465	5.329
7.896	8.047	7.999	8.049	7.816	7.451
3.587	3.847	3.532	3.635	3.907	3.986
3.830	3.270	2.971	3.322	3.221	3.290
5.346	5.520	5.545	5.415	5.398	4.238
0.985	1.495	1.235	1.394	0.924	1.431
6.790	7.191	7.002	7.165	7.237	6.600
9.380	9.627	9.571	9.259	9.498	8.805
9.377	9.623	9.423	9.355	9.390	7.908
5.013	5.567	5.078	4.873	6.032	5.678
5.661	5.228	5.188	5.461	5.210	4.696
4.878	4.854	4.747	3.847	4.432	4.766
4.553	4.339	5.053	4.554	4.878	3.572
7.826	7.312	8.084	8.062	7.919	7.577
4.199	3.454	3.616	3.720	4.395	4.412
5.226	5.591	5.908	5.500	5.571	4.614
5.833	6.003	5.935	5.941	6.558	5.514
4.827	5.039	4.683	5.213	4.854	4.396
4.719	4.833	4.829	4.796	4.785	3.946
2.994	2.156	2.623	2.765	3.266	2.746
3.410	3.409	3.209	3.605	3.444	4.614
3.712	4.073	4.368	3.748	4.036	3.254
6.954	7.452	7.026	7.194	7.439	6.044
7.047	7.116	7.156	6.575	7.258	7.482
6.324	5.981	6.187	6.112	6.047	4.261
4.965	5.160	5.184	4.419	4.434	5.095
4.942	4.180	4.815	4.647	3.903	5.847
4.179	3.755	3.399	4.154	3.993	4.714
4.233	4.652	4.259	3.890	4.675	5.234
8.759	8.242	8.204	8.626	8.703	7.823
6.675	7.136	6.954	7.008	7.014	6.177
3.963	4.089	3.817	4.074	3.923	4.017
6.791	7.159	6.848	6.884	7.029	5.055
6.477	6.456	6.750	6.668	6.790	4.593

8.132	8.177	8.185	8.304	7.911	7.214
4.654	4.751	4.661	4.727	4.764	4.575
7.935	7.844	7.823	7.931	7.679	6.627
8.845	8.749	8.983	8.929	8.884	5.789
3.101	3.654	3.131	3.103	3.493	2.337
5.365	4.870	4.876	5.089	4.861	3.160
7.054	7.092	6.875	6.557	6.980	5.896
4.588	4.435	4.496	4.170	4.028	3.388
5.459	5.073	5.769	5.659	5.445	3.400
2.931	2.383	2.474	2.585	3.340	2.707
4.269	3.917	4.467	4.293	4.555	4.914
4.082	4.252	5.162	3.594	4.038	4.822
7.549	7.475	7.567	7.653	7.557	6.936
5.519	5.238	5.208	5.683	5.271	4.903
3.022	3.489	3.102	3.677	2.970	3.162
4.495	4.620	4.332	4.651	4.558	4.639
4.833	4.979	4.749	4.983	4.977	4.821
7.085	6.909	6.708	7.377	6.960	7.468
4.940	4.924	4.578	5.399	4.951	5.434
6.102	5.950	5.943	6.363	6.206	5.933
6.145	5.773	5.892	6.343	6.017	5.517
7.318	7.180	6.912	7.632	7.300	7.809
5.601	5.820	5.241	5.749	5.954	5.719
5.361	5.460	5.080	5.646	5.145	5.782
6.516	6.120	6.104	6.878	6.279	6.111
4.464	4.668	3.773	3.663	2.869	3.627
4.246	3.906	3.270	4.043	3.978	4.340
5.992	6.000	5.769	6.385	6.024	6.074
4.874	3.991	4.586	4.173	5.018	3.791
7.760	7.741	7.593	7.685	7.693	6.494
3.702	2.900	3.620	3.086	3.119	4.493
8.214	7.710	7.660	7.196	7.345	6.444
7.123	6.462	6.403	5.989	6.463	4.117
5.645	5.960	5.589	5.285	5.245	4.956
3.254	3.647	3.930	3.865	3.642	4.950
8.891	8.169	8.375	8.089	8.453	7.009
3.396	2.326	2.809	2.182	3.172	2.202
6.532	6.773	6.759	6.503	7.049	3.974
2.704	3.384	3.218	3.010	3.227	2.118
3.536	4.087	4.198	4.556	4.687	6.572
2.809	2.883	3.188	2.645	2.726	2.917
4.117	3.472	3.672	4.041	3.776	2.821
3.614	3.512	4.117	3.278	3.943	2.232
6.295	6.356	6.254	6.126	6.673	5.452
5.133	5.470	5.563	5.731	5.287	3.187
3.055	3.639	3.401	2.798	3.554	4.796
2.557	2.872	2.511	2.601	3.101	3.101
7.552	7.603	7.563	7.811	8.230	9.943
4.260	3.892	4.122	3.763	4.092	4.567
5.943	6.163	5.515	6.140	6.146	6.923

5.229	5.480	5.096	5.501	5.496	6.429
1.490	1.238	1.861	1.508	2.103	3.758
7.402	7.711	7.647	7.736	7.725	6.573
6.983	7.185	7.275	7.117	7.042	6.253
5.433	6.465	6.059	5.971	5.887	5.922
5.024	5.429	5.259	5.149	5.314	5.730
7.516	7.227	7.520	7.351	7.141	5.312
6.518	6.448	6.615	6.501	6.523	5.009
6.882	6.848	7.061	6.806	6.864	2.846
4.182	4.883	4.415	4.767	4.520	4.917
2.696	2.655	3.067	3.091	3.314	1.922
4.249	5.091	4.417	4.389	5.165	5.908
3.360	3.894	4.066	3.265	4.189	3.258
2.612	2.570	2.659	2.126	2.362	2.230
5.225	4.774	5.054	5.464	5.102	4.785
2.238	3.649	1.946	2.678	2.210	2.843
5.176	5.099	4.722	4.656	5.070	5.204
2.396	2.803	2.581	2.494	3.125	3.286
7.635	6.694	7.506	7.098	7.042	5.072
1.956	2.997	2.306	3.226	3.631	4.475
3.790	3.638	3.076	3.753	3.157	3.902
8.704	8.937	8.769	8.974	9.256	7.973
3.641	4.074	4.203	4.193	4.474	5.333
5.075	5.345	5.313	5.255	5.494	4.977
2.749	3.158	2.780	3.290	3.339	2.958
3.822	3.980	4.023	3.581	3.774	2.988
2.731	2.689	2.820	2.495	2.518	3.425
2.397	3.523	3.212	2.516	3.052	1.446
3.986	4.217	4.181	4.281	3.663	4.284
5.253	5.118	5.248	5.691	5.841	6.591
4.037	3.669	3.298	3.152	3.585	4.105
5.374	6.265	5.793	5.853	5.840	6.142
3.715	3.714	3.751	3.909	3.971	2.760
6.340	6.705	6.643	6.318	6.585	5.780
6.262	6.820	6.237	6.353	6.828	7.736
3.094	3.236	3.248	3.129	2.985	3.763
8.405	8.549	8.309	8.419	8.629	7.772
5.593	5.803	5.687	5.602	5.676	5.578
5.558	5.498	5.289	5.076	5.342	5.726
6.673	6.501	6.735	6.183	6.220	7.485
5.312	6.015	5.590	5.949	6.285	4.646
6.183	6.787	6.324	5.858	6.439	4.665
2.888	2.606	2.732	2.973	2.856	4.460
4.430	5.024	5.002	4.606	4.301	3.951
8.479	8.211	8.309	8.315	8.246	7.164
4.405	4.077	4.388	4.128	4.312	3.470
4.035	4.387	4.038	4.134	4.404	3.426
6.521	6.625	6.620	6.385	6.680	7.127
5.585	5.323	5.289	5.429	5.471	4.243
3.750	5.155	4.651	4.782	4.814	4.132

4.076	4.498	4.491	4.997	4.588	6.008
4.066	4.617	4.383	4.450	3.928	3.619
4.471	4.658	4.679	4.565	4.738	4.307
6.354	7.065	6.511	6.687	6.771	4.552
5.550	5.895	5.312	5.469	5.752	5.559
4.215	3.982	4.217	3.672	4.068	2.829
8.064	6.819	7.125	7.950	7.584	8.764
3.264	3.072	3.410	3.171	3.607	3.230
4.517	4.096	3.918	4.092	4.141	4.900
5.826	5.497	5.245	5.828	5.957	6.665
4.996	4.801	5.007	5.046	4.904	3.843
7.974	6.893	6.908	7.574	7.339	8.088
3.763	3.377	2.744	3.712	3.125	3.706
3.632	4.301	3.777	3.672	3.926	4.458
4.000	3.978	3.431	3.850	3.775	3.366
8.799	8.998	9.016	9.122	8.944	8.464
7.828	7.903	8.013	8.135	7.814	7.521
7.481	7.567	7.670	7.802	7.489	7.195
4.636	4.271	4.361	4.424	3.766	1.853
5.268	5.440	5.247	4.217	4.643	6.176
3.084	3.281	3.552	2.899	2.948	4.890
5.205	4.893	5.021	5.280	5.004	5.866
4.099	4.085	3.836	3.739	4.050	2.904
5.678	4.473	4.059	3.655	4.862	4.315
4.310	4.979	5.083	4.972	4.994	4.381
4.289	4.583	4.886	4.522	4.817	4.563
6.495	6.223	6.445	6.075	5.847	6.026
3.660	3.326	4.162	3.164	3.858	2.900
1.732	1.569	1.822	1.674	1.394	1.977
7.581	7.989	8.137	8.103	8.146	3.862
5.585	5.635	5.742	5.505	5.237	5.044
7.228	7.534	7.391	7.528	7.414	4.806
8.372	8.379	8.372	8.408	8.658	6.337
3.917	4.415	4.438	4.218	4.241	5.219
3.995	4.131	3.886	4.214	4.434	5.472
7.094	6.686	7.092	7.063	7.095	6.441
2.977	2.982	2.647	3.172	3.110	3.664
4.641	4.618	4.677	4.068	4.810	5.790
4.003	4.123	4.414	3.846	4.269	3.187
3.142	3.468	3.418	3.811	4.069	5.200
4.400	4.880	4.661	5.103	4.951	3.940
3.101	3.253	3.512	2.902	3.431	2.305
3.109	4.023	3.465	2.535	2.957	1.696
8.605	8.308	8.663	8.706	8.574	9.639
6.307	6.302	6.256	6.183	6.374	4.803
4.219	4.112	4.091	3.640	3.915	3.041
2.138	2.346	1.781	1.526	2.381	2.694
5.702	5.687	5.884	5.650	5.835	4.153
8.045	8.319	8.249	8.172	8.003	7.477
4.523	5.099	4.524	4.902	5.339	5.614

4.050	5.821	5.792	5.088	5.181	5.114
2.219	2.378	2.613	1.734	1.871	1.408
3.696	2.143	4.379	3.030	3.507	1.937
3.951	3.889	3.675	3.904	4.568	3.324
6.230	6.285	6.008	6.132	6.016	4.570
4.169	2.929	2.658	3.870	3.057	2.913
3.735	2.690	1.923	2.737	2.748	2.406
6.185	6.826	6.522	6.307	6.564	4.945
7.103	6.579	7.404	7.429	7.252	5.103
7.465	7.310	7.477	7.608	7.411	5.242
8.303	8.195	8.127	8.287	8.310	5.147
5.233	5.440	5.145	5.062	4.961	3.775
2.791	2.969	3.013	2.649	2.696	1.403
2.417	2.626	2.595	2.625	2.270	2.523
4.827	5.477	5.191	5.411	5.782	4.451
5.240	5.525	5.169	4.908	5.142	5.720
3.447	3.833	3.874	3.599	3.589	2.548
5.864	5.717	5.634	5.409	5.245	4.871
5.490	5.431	5.162	4.876	5.491	4.655
4.341	3.488	4.595	3.442	4.494	1.268
7.065	7.432	7.236	7.373	7.346	6.736
7.685	8.026	7.738	8.109	7.803	6.983
2.266	2.480	2.963	2.778	2.376	3.672
4.029	4.676	4.263	4.739	4.766	5.516
5.199	5.709	5.730	5.492	5.788	4.708
6.898	7.096	7.244	6.934	6.779	6.625
10.445	10.224	10.463	10.703	10.235	10.979
4.552	4.316	4.805	4.605	4.598	5.427
10.447	10.267	10.867	10.432	10.636	10.648
3.792	3.597	3.448	4.902	3.874	4.503
3.870	3.564	2.707	3.612	2.972	0.975
5.960	5.630	5.530	5.202	5.118	5.338
2.313	2.244	2.540	3.097	3.329	2.743
1.751	1.302	1.180	2.578	2.237	4.515
3.340	2.930	3.022	3.170	3.397	3.954
3.568	2.374	2.599	3.377	3.773	3.181
2.850	3.130	2.397	2.656	2.704	2.296
2.647	2.934	2.156	3.012	2.218	3.299
5.887	5.885	5.591	4.693	4.714	5.285
2.596	1.756	2.404	2.176	2.665	2.643
3.178	3.240	2.241	2.254	2.364	5.270
3.911	2.802	3.645	2.406	4.072	4.014
2.080	1.558	2.037	2.273	2.226	2.458
2.937	2.374	2.675	2.848	3.143	3.622
2.159	2.409	2.434	2.431	2.665	4.208
2.880	1.978	2.104	3.475	3.216	5.491
4.965	3.334	3.511	4.875	4.240	2.899
5.906	5.899	5.457	5.498	5.059	5.607
4.560	3.919	3.261	3.861	4.293	5.496
2.395	2.764	2.690	2.664	3.033	2.576

2.347	3.947	2.281	1.834	2.129	2.179
4.655	4.546	3.729	4.065	4.524	4.797
3.847	2.366	2.305	3.306	2.490	2.706
3.867	4.396	3.851	4.425	3.638	3.759
1.894	3.237	2.658	3.333	2.348	3.266
3.251	3.063	3.344	3.648	2.275	2.551
2.571	2.056	2.756	2.328	3.089	2.637
2.421	3.078	3.303	3.475	3.199	3.471
4.058	3.435	3.274	4.939	3.730	3.356
3.216	3.680	4.071	4.099	3.750	3.277
3.345	2.666	3.834	3.121	3.054	2.624
3.803	3.467	3.002	3.185	3.256	2.136
3.360	3.327	1.938	3.938	2.949	4.090
5.472	5.881	5.306	5.768	5.340	5.555
2.655	2.629	2.185	2.464	2.430	2.740
3.939	2.704	2.437	2.704	1.923	1.757
4.421	5.147	4.169	5.312	4.665	4.896
3.491	3.052	1.179	4.425	2.027	4.464
3.962	3.880	3.237	4.423	3.030	4.601
4.241	4.234	3.142	3.637	3.640	3.640
3.010	2.799	2.678	2.558	2.678	3.182
2.570	1.930	2.316	2.512	2.277	1.659
3.275	2.789	2.902	2.746	2.475	1.459
2.914	2.568	3.499	4.208	2.788	3.168
5.123	5.432	5.352	5.159	4.046	3.747
2.287	2.531	2.050	1.735	2.871	2.420
5.661	3.306	5.209	5.928	3.913	2.686
4.872	3.611	3.678	4.113	4.351	5.202
9.725	9.900	10.135	9.927	9.049	9.165
8.841	9.132	9.378	9.142	8.357	8.544
2.228	1.858	1.245	2.033	2.356	2.544
1.586	2.256	1.631	2.006	2.211	2.596
3.118	4.076	3.250	4.044	2.920	4.769
2.030	2.856	2.147	2.178	1.507	1.428
4.068	3.464	3.514	4.242	3.962	4.931
2.793	2.267	2.975	2.650	2.267	2.110
2.156	2.791	2.810	2.406	2.028	3.258
1.566	2.658	2.405	2.063	3.010	1.949
2.792	2.743	2.561	3.312	3.168	2.496
3.358	1.962	3.514	3.662	2.968	3.116
3.686	3.348	4.465	3.599	3.925	4.524
3.018	2.590	2.246	2.316	2.719	3.517
2.168	2.773	3.769	2.532	2.813	2.898
3.379	2.318	1.616	4.393	3.196	3.683
4.445	1.717	2.328	2.944	2.079	1.665
3.872	3.647	3.161	4.808	4.577	5.193
2.438	1.633	2.451	2.671	2.028	2.247
2.893	3.053	2.788	2.519	2.866	1.868
2.707	2.256	2.009	2.766	2.127	3.848
3.690	2.475	2.815	3.608	3.551	2.856

2.723	2.636	3.013	2.427	2.017	2.558
3.914	3.571	3.743	3.041	3.716	2.251
2.337	2.477	2.731	2.877	1.747	3.350
2.809	1.315	1.579	1.933	2.071	3.778
2.426	3.041	2.794	3.380	3.422	3.447
4.178	2.924	2.324	3.715	2.976	3.751
4.581	4.653	4.331	3.908	3.354	3.741
4.178	4.672	4.327	4.014	3.910	4.657
2.005	3.105	3.247	2.223	2.100	2.997
3.196	3.257	2.787	2.051	2.700	2.999
2.702	2.609	1.482	3.816	2.553	1.333
2.581	4.910	4.124	4.482	4.382	3.788
1.789	2.206	3.324	2.724	2.593	2.609
3.106	3.638	3.448	4.844	3.055	4.429
1.533	2.372	1.638	1.925	2.523	2.804
5.132	4.556	4.365	4.820	3.912	6.068
3.960	3.433	3.518	3.659	2.733	4.605
5.081	4.033	4.014	4.912	4.546	4.891
2.587	3.179	3.098	2.471	1.549	2.149
2.248	2.370	1.699	2.976	1.825	2.573
2.225	2.076	1.949	2.678	2.264	2.298
3.879	3.231	3.506	3.760	3.484	3.682
2.761	2.611	2.369	2.530	3.612	2.618
2.389	2.228	2.548	2.378	2.471	3.177
2.977	2.679	2.618	2.546	2.221	3.616
2.279	3.129	2.849	2.788	2.911	3.734
3.681	1.551	2.234	2.796	2.240	2.247
3.260	1.936	2.361	3.586	1.816	3.288
1.509	3.354	3.245	2.020	3.300	2.793
5.496	4.657	5.362	5.425	5.041	5.039
3.540	1.526	3.990	3.707	2.509	3.117
3.830	3.476	3.279	4.320	3.721	4.948
2.986	1.362	1.818	2.607	1.763	2.442
2.635	2.998	2.212	2.866	2.637	3.050
3.242	2.751	1.776	3.248	3.257	2.971
6.466	6.013	6.426	6.144	5.285	5.465
3.161	3.241	3.396	3.908	3.117	2.542
4.665	4.723	4.433	3.611	3.640	3.176
5.102	5.124	5.141	5.268	5.712	6.359
4.211	5.859	6.236	4.539	4.016	4.599
2.230	4.765	3.646	2.433	1.768	1.982
5.175	4.887	5.092	3.903	4.509	5.706
4.566	4.364	4.949	4.207	4.175	3.510
4.548	4.692	4.756	4.592	4.436	5.679
6.938	5.700	5.957	5.917	6.362	3.781
6.500	7.025	6.672	7.201	7.117	8.338
4.242	3.453	3.661	3.661	3.979	2.655
8.995	8.931	9.047	8.979	9.001	8.270
6.556	6.409	6.719	6.620	6.650	6.420
4.729	4.718	4.136	4.414	4.663	3.589

1.674	2.467	2.258	2.226	1.980	2.174
4.342	3.389	3.318	3.030	4.343	3.892
2.410	3.101	3.445	3.449	3.488	5.531
3.827	5.444	4.918	5.064	5.214	3.695
3.504	3.960	4.024	3.264	3.488	2.481
7.442	7.680	7.447	7.610	7.519	8.569
6.427	6.125	6.188	6.291	6.359	7.314
1.808	3.626	2.907	3.376	1.974	3.732
3.238	3.805	3.179	3.310	3.434	3.501
5.566	6.152	5.908	6.168	6.102	6.873
4.454	4.248	4.885	4.743	4.274	2.546
4.919	4.815	4.748	5.001	4.761	4.287
6.463	6.511	6.718	6.311	6.457	5.791
5.686	5.339	5.324	5.374	5.266	4.940
4.795	4.966	5.042	4.896	4.949	4.220
8.039	8.293	8.059	8.098	8.098	7.301
5.599	5.701	5.600	5.410	5.599	4.192
5.338	5.771	6.182	5.729	5.862	3.837
4.939	4.578	4.503	4.607	4.668	4.547
2.107	3.201	3.459	2.468	2.817	2.955
6.266	6.739	6.692	6.426	6.732	6.185
5.417	5.760	5.827	5.749	5.654	4.941
3.439	3.112	3.541	3.445	3.634	2.105
5.281	5.830	5.744	5.558	5.715	7.114
2.488	3.439	3.255	2.924	3.130	3.302
6.142	6.308	6.344	6.244	5.893	5.248
4.482	3.903	4.415	4.727	4.864	6.045
5.020	4.547	4.214	4.968	4.976	5.920
4.424	4.400	5.167	5.549	5.448	5.919
2.425	2.639	2.886	2.798	2.825	3.225
7.121	7.328	7.150	6.901	7.167	6.366
4.470	3.928	3.442	2.975	4.212	2.945
3.215	3.896	3.551	3.757	2.457	3.524
7.160	7.451	7.281	7.205	7.157	6.435
4.835	5.193	5.189	5.383	5.044	4.695
6.554	6.872	6.776	6.550	6.910	5.822
5.981	6.208	6.444	6.176	6.070	5.443
5.630	5.675	5.533	5.399	5.757	6.287
6.026	6.834	6.596	6.452	6.663	5.855
4.886	5.081	5.162	4.930	4.987	3.990
7.877	6.906	7.424	7.752	7.391	7.297
3.717	4.038	3.860	3.753	3.761	4.121
4.347	3.957	3.957	3.769	3.159	3.662
6.102	4.671	4.549	6.258	5.659	4.252
8.961	7.635	8.289	8.952	7.647	8.621
3.263	2.733	2.117	1.919	3.874	3.507
1.347	0.959	1.682	1.584	1.522	1.864
3.137	2.285	2.041	1.609	2.399	2.117
2.067	2.231	1.895	1.941	2.360	2.041
5.628	5.588	5.130	5.203	4.385	5.382

4.399	5.012	4.462	4.941	4.159	3.708
3.983	4.147	3.936	2.492	3.870	2.927
4.766	4.378	3.835	4.566	4.423	4.460
1.751	2.455	2.208	2.399	2.377	2.524
4.702	4.083	4.492	5.027	4.718	4.279
3.327	3.296	3.040	3.107	3.665	3.630
2.376	2.394	2.414	2.214	2.298	2.516
4.861	4.332	3.554	4.682	3.849	4.780
3.286	3.052	3.270	3.416	3.208	3.181
4.375	4.719	4.444	4.295	4.370	4.958
5.797	5.279	5.457	5.384	4.490	5.624
4.073	4.173	3.659	3.869	2.888	2.940
4.894	3.978	3.475	4.504	3.548	3.730
7.171	7.067	6.732	6.338	5.167	6.521
5.296	2.516	5.501	5.777	5.252	3.056
2.488	3.163	2.903	2.809	2.482	3.647
2.602	3.489	1.717	3.024	2.580	2.524
2.367	1.892	2.476	2.150	2.494	3.664
1.334	0.985	1.675	1.410	1.446	1.506
3.064	3.742	3.416	3.278	2.434	3.750
2.599	2.788	2.538	1.740	3.267	2.927
3.252	4.017	4.364	2.771	2.855	4.463
2.273	2.465	2.013	2.439	2.511	2.688
1.694	2.885	2.922	1.894	1.428	2.211
7.278	7.363	6.943	7.286	5.896	7.779
4.663	4.201	3.742	4.404	4.722	5.920
4.226	4.142	4.295	3.722	3.904	4.942
3.029	3.464	3.325	3.401	3.325	4.691
2.463	2.349	2.477	3.337	3.551	3.474
2.355	2.927	3.481	3.151	2.380	4.268
3.266	2.578	2.336	3.421	2.460	3.813
4.336	4.822	3.678	4.271	4.945	5.619
3.367	3.365	3.176	3.456	2.657	3.199
2.782	3.064	2.666	2.642	1.459	2.333
4.124	2.830	3.010	3.738	3.662	3.984
5.482	4.939	4.671	5.494	5.004	5.930
2.210	1.987	1.843	2.415	2.427	2.718
4.094	4.095	4.449	4.338	3.837	4.093
9.640	9.611	9.509	9.784	8.966	9.837
5.312	4.604	5.091	5.995	6.471	6.793
2.754	2.066	2.195	3.416	2.801	4.806
1.619	1.816	2.354	3.186	1.650	4.245
3.744	4.693	3.742	4.080	4.177	3.350
2.544	3.613	3.344	2.973	1.180	3.818
8.025	7.477	7.817	6.806	5.409	7.715
5.175	4.936	4.735	4.841	4.511	4.552
2.017	3.179	3.047	1.336	1.851	1.367
2.156	3.272	2.034	2.765	2.762	1.111
6.204	4.872	5.010	5.310	5.326	6.004
2.648	2.117	2.332	2.003	1.582	1.539

2.446	2.119	1.489	2.861	2.647	3.634
2.797	2.847	3.213	4.368	4.391	8.445
5.279	4.793	4.262	5.601	4.967	6.337
4.324	4.733	4.324	3.895	4.410	4.897
1.491	1.784	1.382	3.100	2.625	3.410
3.439	2.206	3.230	1.677	2.231	1.526
3.064	3.132	3.746	3.496	4.198	3.640
3.676	4.674	3.322	4.344	4.206	4.892
2.340	2.860	2.652	2.902	1.773	3.259
4.530	3.891	4.271	3.873	3.932	4.110
1.985	2.185	3.050	2.664	2.481	2.428
2.219	3.351	3.241	2.745	2.807	3.134
3.182	2.092	2.271	2.677	3.292	2.047
2.394	1.774	2.893	2.348	2.288	2.278
4.185	5.419	4.222	5.261	5.703	4.939
1.738	1.306	1.893	1.795	1.723	1.783
2.992	2.528	2.500	1.720	1.849	2.592
2.361	2.077	3.045	1.783	1.926	2.141
5.602	5.597	5.020	5.282	5.585	3.906
1.816	1.883	2.308	2.145	1.492	2.788
2.209	2.235	1.306	2.737	1.891	1.820
4.395	3.571	2.439	3.715	2.873	2.899
3.684	3.640	4.323	4.571	2.746	4.677
6.913	6.519	6.362	6.506	6.663	7.319
3.401	2.645	3.447	3.504	2.742	3.333
4.020	3.542	4.218	4.452	2.962	5.132
2.292	3.978	3.106	2.571	3.225	3.646
1.407	1.051	1.209	2.988	1.037	3.366
1.287	1.414	2.184	1.383	0.967	1.914
3.297	2.886	2.149	4.070	3.194	3.770
3.983	2.940	2.536	3.283	1.877	4.137
4.210	2.210	3.311	4.287	3.196	4.261
1.605	1.923	2.458	2.363	1.984	2.514
2.583	2.360	2.784	2.523	2.848	2.929
3.680	3.259	3.145	3.413	3.173	2.659
4.291	4.012	3.166	4.626	3.939	4.285
3.775	3.416	2.730	4.572	3.611	4.545
6.354	4.382	4.582	5.203	4.902	3.832
5.876	3.966	4.238	4.754	4.511	3.873
5.919	4.051	4.557	4.962	4.658	3.966
1.818	2.063	2.647	2.028	2.222	4.338
1.872	1.831	1.971	2.446	2.675	2.730
2.727	3.262	3.189	4.359	3.238	2.940
4.399	2.938	3.459	4.583	3.398	4.361
4.714	3.656	3.355	4.343	4.295	2.209
2.250	2.926	2.532	2.786	3.076	2.372
3.764	3.609	3.290	3.247	4.148	4.237
3.481	1.537	2.458	2.625	3.029	1.243
2.569	1.880	1.862	2.892	2.995	3.734
3.146	3.432	2.740	2.889	3.528	3.408

3.554	4.080	3.269	5.270	3.853	4.809
2.547	1.633	2.136	3.488	2.182	2.250
4.177	3.549	4.080	4.976	3.922	4.930
1.764	1.983	1.204	2.582	1.531	2.895
1.662	0.546	0.812	1.015	0.854	1.139
2.238	2.271	2.047	2.393	1.939	2.082
4.270	3.625	3.932	4.045	3.613	3.932
2.465	2.629	1.726	2.572	2.259	2.049
5.316	5.629	5.102	5.190	4.823	3.270
3.264	2.463	3.147	2.784	2.797	3.425
1.756	1.515	1.619	2.620	1.035	0.871
4.374	2.977	3.488	3.611	2.793	3.634
3.735	3.200	2.828	4.316	2.969	4.371
2.543	2.766	2.643	3.715	2.755	3.616
2.473	2.497	2.442	2.302	2.707	1.606
5.462	3.402	3.538	4.247	4.539	3.101
4.987	4.315	4.269	5.045	3.891	2.593
2.019	1.801	1.927	2.097	2.009	1.813
2.566	2.862	3.209	3.178	2.794	2.361
5.145	4.929	3.877	5.060	4.849	5.562
5.521	5.066	5.372	5.110	5.069	5.387
2.767	2.586	1.877	2.177	2.715	2.280
2.743	2.679	3.287	3.174	2.120	3.318
3.411	3.526	3.282	3.712	3.207	3.209
2.810	2.430	2.549	3.578	2.361	3.710
4.312	3.393	3.298	4.701	4.266	4.710
2.940	2.204	2.598	2.010	1.628	2.712
2.960	3.561	3.280	2.853	2.913	3.235
2.141	3.216	2.143	3.296	2.047	2.478
3.576	2.888	1.744	3.889	3.450	8.163
3.255	1.721	2.538	3.634	2.506	1.947
2.255	2.037	2.480	2.894	3.843	2.480
3.836	3.144	3.543	3.617	2.902	3.892
3.694	1.989	3.046	3.533	3.690	4.999
4.310	4.221	4.504	3.673	4.232	4.515
3.168	2.573	2.321	3.613	2.174	3.227
4.651	4.169	3.491	5.207	4.634	5.363
5.016	4.383	3.625	5.375	4.619	5.501
2.810	1.518	1.455	5.200	3.731	2.594
2.571	3.866	3.135	4.552	4.518	6.471
2.479	3.289	2.409	3.708	2.103	4.068
4.550	3.427	4.650	4.001	3.826	4.591
2.707	1.707	1.602	2.984	2.553	4.291
5.756	5.134	5.243	5.535	5.170	5.787
4.663	3.786	4.709	5.573	4.466	6.105
2.891	2.685	2.510	2.860	1.908	1.722
3.787	4.023	3.336	3.565	3.793	3.692
4.951	3.826	3.909	4.389	4.028	4.146
1.735	2.219	2.171	2.520	2.801	2.839
1.912	2.467	2.520	2.437	2.247	2.622

5.718	4.819	5.088	5.197	4.268	5.416
1.486	2.441	2.670	2.815	2.314	2.693
2.601	2.206	2.386	2.668	3.245	2.507
2.953	3.192	2.644	3.426	3.463	4.332
3.007	3.408	3.631	3.489	3.580	4.015
3.030	2.265	3.411	3.407	2.173	2.982
2.305	1.318	1.752	2.915	2.507	1.656
2.866	3.211	3.155	3.089	3.672	3.481
2.658	2.444	2.401	2.705	2.150	2.115
3.290	2.178	1.528	2.377	2.103	3.463
3.306	2.857	2.512	3.239	2.715	3.458
3.145	2.182	2.779	3.589	2.680	3.424
2.018	1.889	1.426	2.725	2.141	3.123
2.519	1.518	3.025	2.232	2.846	2.614
3.290	2.699	1.627	3.219	3.050	3.648
5.732	5.221	6.008	5.741	5.053	5.478
1.903	2.077	2.567	1.580	2.488	2.601
3.256	4.222	3.725	4.543	4.481	4.248
6.674	5.351	5.483	6.726	6.276	4.701
2.939	3.031	1.681	1.886	1.929	3.717
3.762	3.426	2.959	3.298	3.440	3.596
1.593	2.260	3.031	3.103	1.799	1.613
4.212	4.266	3.615	4.442	3.812	5.828
3.489	2.441	1.968	3.801	1.954	3.207
5.970	5.144	5.749	5.237	5.381	4.665
1.868	2.404	2.101	4.012	2.946	2.946
4.476	4.349	4.221	4.482	4.396	3.760
2.929	2.390	2.864	3.030	1.265	2.059
4.272	3.563	3.065	3.484	3.175	3.916
3.415	4.374	4.316	4.572	4.740	5.629
2.787	2.823	4.060	2.870	2.653	2.494
2.590	2.246	1.303	2.941	2.163	2.053
4.354	1.456	1.895	4.138	2.942	3.400
2.331	2.114	1.728	2.894	2.921	1.668
5.443	2.055	1.604	4.623	3.398	2.549
3.303	3.216	3.512	2.373	2.718	4.207
2.641	2.178	2.943	2.489	2.270	2.489
4.054	4.316	3.480	3.809	4.100	5.959
4.302	3.851	4.052	4.654	4.451	3.524
2.856	2.507	2.675	2.636	3.136	2.716
2.753	2.451	3.210	3.692	2.247	4.132
3.718	2.618	3.158	3.138	3.516	3.507
1.732	2.259	1.427	2.210	1.951	2.395
3.083	2.090	3.619	4.191	3.750	4.065
1.464	1.378	1.916	3.056	3.287	3.360
2.452	3.308	0.952	3.695	2.218	2.021
4.995	4.497	4.197	4.740	3.491	5.160
3.528	1.809	1.997	2.874	2.571	1.703
1.151	1.129	1.151	1.846	1.642	2.955
4.298	4.077	2.525	3.983	2.767	5.182

3.850	2.989	3.251	3.707	3.040	2.546
2.549	1.932	2.679	2.178	2.522	3.538
3.115	2.738	2.760	2.431	2.373	2.117
7.650	5.904	6.968	7.646	6.368	6.636
5.217	2.714	4.619	6.031	4.519	4.029
2.346	2.158	2.355	2.203	2.347	2.642
3.841	3.907	3.929	4.059	4.474	3.886
2.935	1.664	2.987	2.595	2.204	3.458
6.691	5.476	6.293	6.771	5.828	5.794
3.797	4.796	4.274	4.211	4.625	3.728
3.499	2.797	3.360	3.955	2.086	2.321
2.697	1.795	2.188	3.233	2.485	1.886
2.043	2.825	3.361	2.448	3.005	3.073
1.491	2.347	2.787	1.664	0.961	3.303
2.397	0.879	1.243	1.787	2.394	2.317
6.761	4.295	5.824	6.290	4.667	2.424
2.303	2.942	2.492	2.492	2.595	2.138
1.606	2.526	1.502	2.486	2.736	2.881
2.363	3.537	2.936	3.670	2.948	5.256
1.769	2.610	2.655	2.858	2.578	3.022
2.443	1.865	2.119	2.696	1.882	3.297
4.812	3.732	4.170	5.369	4.394	5.200
2.761	1.932	2.531	3.364	2.790	3.839
2.419	2.354	2.871	3.390	2.613	2.144
3.462	2.573	2.000	2.934	2.637	2.805
2.746	1.646	1.812	1.728	1.664	3.139
2.319	0.469	1.478	2.202	2.437	2.623
1.191	2.279	1.805	1.929	1.337	2.229
5.517	6.136	5.517	5.090	4.386	5.660
4.990	5.745	5.156	4.809	4.585	5.269
2.678	2.863	2.490	2.637	2.541	2.533
2.057	2.328	2.431	2.252	2.053	1.969
2.273	1.532	2.543	1.881	2.134	1.544
1.652	1.910	1.533	2.634	1.100	1.206
2.546	2.195	1.923	1.551	2.115	2.894
1.572	1.402	1.436	0.779	1.290	1.901
3.605	4.227	5.109	4.793	3.679	4.910
5.159	5.196	5.036	5.214	5.402	3.188
7.173	7.294	7.382	6.834	7.487	6.243
0.638	3.251	4.261	0.748	3.997	0.895
7.453	7.848	7.545	7.628	7.421	6.076
3.846	3.794	3.948	3.917	3.842	4.282
3.521	3.358	3.945	3.832	4.312	2.903
2.923	3.380	2.392	2.578	2.829	3.012
8.408	8.611	8.268	8.801	8.793	5.723
4.883	4.167	4.489	5.129	4.246	1.664
1.878	2.164	1.881	2.229	2.707	3.271
2.937	2.845	2.543	2.833	2.739	3.475
4.796	4.393	4.703	4.731	4.907	3.870
1.541	1.558	2.297	1.558	1.563	2.036

3.678	3.946	3.970	4.534	4.211	2.779
3.692	3.895	3.906	2.837	3.405	3.655
3.102	2.754	3.100	3.303	3.070	4.514
2.850	1.825	3.718	2.912	3.240	3.014
2.471	2.060	2.240	1.931	2.163	2.287
3.011	2.829	1.807	2.931	3.426	2.301
4.006	3.503	3.903	4.359	3.553	4.302
3.488	3.734	3.729	4.362	3.832	3.750
3.400	3.546	4.002	4.029	3.827	4.524
2.699	3.485	3.153	2.751	3.594	3.135
3.802	3.704	2.756	3.541	3.596	4.227
3.067	2.981	2.025	2.787	2.467	4.189
1.442	1.717	1.810	1.292	1.541	1.196
3.604	3.915	3.889	4.780	5.316	7.405
3.417	5.073	4.274	5.193	4.414	4.939
1.771	2.156	2.119	2.268	2.018	2.268
2.966	3.235	3.341	3.480	3.364	5.478
3.191	3.149	3.801	3.405	3.798	3.810
2.771	2.483	2.807	2.780	2.890	3.144
2.015	1.721	1.388	1.835	2.017	3.520
3.953	4.545	3.554	4.200	4.156	4.336
4.245	3.364	4.443	4.719	3.751	3.928
2.349	2.313	1.985	2.218	2.637	3.164
4.482	4.159	4.078	3.480	4.041	4.390
2.498	3.182	2.897	2.320	2.816	1.988
2.287	2.622	2.134	2.542	2.241	3.514
2.543	2.582	2.307	2.516	2.201	2.265
2.104	3.414	3.076	3.347	3.234	3.432
2.984	3.010	2.859	2.660	2.859	2.808
2.477	2.964	1.939	3.324	2.422	3.853
2.176	2.728	1.855	3.110	2.551	3.450
2.980	2.525	2.538	3.835	3.306	4.041
2.865	3.483	3.252	3.486	2.868	3.959
6.659	6.770	6.696	6.803	6.550	5.966
2.137	2.178	1.822	2.181	2.330	4.214
4.457	3.839	4.326	3.660	3.124	0.862
1.780	2.051	2.119	1.254	1.214	3.688
7.245	8.063	7.532	7.173	7.174	8.080
2.389	2.766	2.877	2.796	3.104	4.343
3.261	2.532	2.560	3.439	2.945	1.933
2.707	3.676	2.762	3.028	2.972	2.325
3.225	2.533	2.860	4.632	2.979	4.330
2.536	2.456	3.014	2.830	2.206	2.754
3.529	3.433	3.128	2.910	3.553	3.835
1.196	1.613	1.418	1.136	1.200	3.506
2.795	3.355	2.617	2.682	2.683	3.575
4.373	4.331	4.335	4.392	4.089	1.920
3.149	1.900	2.423	3.661	1.794	1.611
2.328	2.469	2.772	3.255	2.027	1.229
5.031	4.634	4.595	4.861	5.495	2.419

2.285	2.382	2.812	2.864	2.589	3.610
2.282	2.240	2.448	1.872	3.083	1.684
4.584	4.306	4.318	3.690	3.982	4.597
3.713	3.432	2.941	3.499	3.433	2.386
3.489	3.739	3.143	3.333	3.653	1.750
2.232	2.954	2.375	2.930	2.896	4.169
1.522	2.732	2.562	2.844	2.758	3.154
2.449	2.661	2.257	3.202	3.136	3.033
2.022	2.206	2.421	2.118	2.167	2.436
4.072	3.117	3.854	4.719	3.718	4.507
4.258	4.480	4.909	4.645	4.565	1.878
2.844	3.284	2.883	2.365	2.374	2.094
2.686	2.618	2.789	2.944	3.148	4.017
2.291	2.949	2.339	2.635	2.504	2.988
1.490	2.305	2.841	1.525	1.902	2.868
2.134	2.443	2.748	2.800	2.472	1.556
3.144	3.451	2.821	2.860	3.381	1.051
3.585	4.219	3.496	3.799	3.924	5.117
4.101	3.804	3.268	3.510	3.595	1.878
3.226	2.797	3.280	3.299	3.087	4.585
4.366	4.693	4.711	4.358	4.822	4.060
4.439	4.426	4.106	4.139	4.490	3.646
1.652	1.799	2.370	3.084	3.202	4.907
3.910	4.018	3.783	4.721	3.530	4.448
4.729	4.979	4.641	4.059	4.415	5.036
3.386	2.211	2.510	2.470	3.680	3.079
2.782	2.187	1.875	3.199	2.747	1.900
4.958	4.820	5.233	5.895	4.003	2.512
2.660	2.325	2.505	3.148	2.286	2.636
2.424	2.078	2.671	2.114	2.182	2.289
3.786	3.289	2.426	3.797	3.422	5.035
3.859	3.258	3.605	1.371	2.926	2.454
2.797	1.752	1.582	2.089	2.254	2.575
3.605	3.478	2.803	3.170	2.577	2.360
1.506	1.375	1.684	2.171	1.494	4.063
3.234	3.491	3.496	3.680	3.927	3.076
4.832	3.573	4.081	4.700	4.016	5.442
5.969	7.248	6.461	6.305	6.611	8.240
4.461	4.775	5.053	4.680	4.706	5.289
4.072	4.048	4.370	4.219	4.145	5.431
1.902	2.227	2.691	1.628	2.291	3.140
4.530	3.940	4.389	5.076	5.200	6.632
2.942	3.112	2.452	2.879	2.995	2.505
3.480	2.725	2.587	2.555	3.567	3.024
4.588	3.759	4.342	4.705	4.537	3.371
3.735	3.184	2.919	4.034	3.830	2.592
3.967	3.144	4.297	4.461	3.915	2.420
2.458	2.627	2.207	2.901	2.749	3.038
3.445	2.851	2.438	2.470	3.595	1.214
2.649	2.413	1.823	2.408	2.408	3.283

3.087	2.143	3.133	2.922	2.609	2.041
3.907	3.179	3.634	2.730	3.671	4.120
5.257	4.895	5.301	4.437	4.533	1.921
2.996	2.891	3.540	3.863	3.768	5.253
4.848	3.461	4.345	4.097	4.319	2.616
3.245	3.007	3.244	3.071	3.109	4.185
3.732	4.834	4.060	3.570	3.818	2.988
4.852	5.730	4.675	4.751	4.478	4.299
2.048	2.959	1.714	2.515	2.756	3.942
2.337	2.484	2.346	2.279	3.049	2.916
4.024	5.443	5.358	3.728	3.993	6.126
3.501	2.525	3.085	2.541	3.193	1.701
4.784	4.158	3.523	3.954	4.102	4.044
2.076	3.536	2.389	1.547	2.843	1.838
4.753	5.059	4.675	4.941	5.159	5.453
3.442	2.652	3.279	4.842	3.864	3.488
3.051	2.509	2.735	3.223	3.736	2.043
3.432	3.570	2.934	3.085	2.817	4.191
3.321	2.327	2.394	3.307	2.283	1.351
5.272	4.816	5.018	5.637	4.985	5.297
2.143	2.475	2.112	1.928	2.231	2.695
2.847	4.127	3.471	3.514	3.895	3.440
3.155	3.343	3.356	3.342	3.323	2.654
4.460	4.570	4.417	4.859	4.952	4.580
2.680	1.662	2.515	2.064	2.457	2.541
3.429	4.674	3.835	3.362	2.956	1.989
2.245	1.986	1.895	2.326	2.851	4.019
4.796	4.393	4.313	5.202	4.645	4.114
2.055	2.533	2.358	2.373	2.868	3.959
4.386	4.024	3.633	4.989	4.534	5.115
3.530	2.986	3.872	4.370	3.177	5.687
3.156	2.762	2.676	3.745	2.322	4.350
3.132	2.525	2.490	2.709	2.495	2.791
4.882	3.730	4.480	4.846	4.500	5.255
2.502	2.080	2.387	2.945	2.756	4.191
1.889	2.355	2.620	2.336	2.379	2.633
5.089	4.853	5.304	4.424	4.917	2.489
2.633	2.438	3.025	2.462	2.283	1.205
4.188	3.170	3.305	3.973	3.532	3.149
4.463	5.261	4.670	4.694	4.352	3.830
3.714	2.614	3.684	3.709	3.028	4.648
3.803	3.107	2.867	4.200	4.014	5.035
2.993	2.191	2.180	2.704	2.270	2.808
2.998	3.400	3.045	2.925	3.365	2.486
3.607	2.777	3.625	3.178	3.274	3.754
3.821	4.428	4.101	3.420	3.504	3.397
3.178	2.479	3.098	3.797	3.306	3.549
2.085	4.574	3.306	1.725	3.201	1.621
3.036	5.670	4.013	2.859	4.261	2.723
4.267	7.090	5.650	4.196	5.586	3.726

3.373	3.529	3.312	3.692	3.373	2.576
3.850	4.231	3.914	4.662	4.534	4.823
3.080	2.350	2.509	2.438	1.778	1.993
2.179	2.265	2.794	2.164	2.687	2.571
2.973	2.012	2.804	3.132	2.094	2.111
4.976	3.794	4.632	4.184	4.265	4.540
3.332	2.185	2.853	3.034	2.797	1.740
4.576	3.287	3.310	3.730	3.350	2.202
4.820	2.962	2.711	3.964	3.459	1.937
4.772	2.666	2.319	4.006	3.294	2.122
3.508	3.339	3.373	3.877	3.060	2.432
3.993	4.082	4.004	4.032	4.408	4.405
4.936	4.687	5.002	4.968	4.787	5.213
2.752	2.973	3.545	2.631	3.412	2.462
3.195	3.160	3.702	2.479	2.120	2.727
2.707	2.473	2.882	2.910	2.280	4.053
4.713	4.894	4.877	4.772	4.434	4.667
2.839	3.258	3.255	2.999	3.258	3.452
0.739	1.819	2.076	1.896	2.730	3.881
2.477	2.588	2.651	2.525	1.925	3.609
2.540	2.245	1.725	2.600	2.606	2.496
2.790	2.748	2.332	2.134	2.426	2.291
2.269	2.788	2.079	2.855	2.391	2.095
1.696	1.763	1.720	1.417	1.615	3.176
4.411	3.771	4.386	5.010	4.349	6.110
7.209	7.586	7.364	7.417	7.695	6.883
2.935	3.005	2.183	2.853	2.557	5.527
2.737	3.136	2.088	2.064	2.829	1.511
3.402	3.056	2.850	3.348	2.951	3.254
2.536	2.848	3.518	3.952	3.387	2.870
2.728	2.333	2.613	2.632	2.660	2.915
4.064	3.281	3.607	4.423	3.688	3.776
2.428	2.782	2.802	2.249	2.903	2.897
2.862	3.242	2.915	3.116	3.353	4.708
4.499	4.759	4.819	4.695	4.536	4.122
2.395	1.963	2.572	1.989	2.005	2.292
3.724	3.259	3.499	3.277	2.341	2.703
1.780	1.749	2.003	2.178	2.612	3.994
2.400	2.175	2.953	2.859	2.404	1.966
2.925	2.579	3.407	2.759	2.438	2.611
2.476	2.457	2.258	3.195	2.987	4.646
5.076	4.061	4.681	4.787	4.735	5.457
4.385	4.497	3.752	4.874	4.300	5.650
2.813	2.392	3.520	3.230	2.011	2.406
2.492	2.823	2.492	2.241	2.296	2.344
2.481	2.306	2.256	2.314	1.873	2.546
3.265	3.477	4.148	3.129	2.878	2.493
4.686	2.902	3.749	4.665	3.649	4.011
2.486	3.267	2.804	3.489	3.412	5.351
1.903	2.733	1.696	2.551	2.262	2.570

5.207	6.247	5.443	5.224	5.278	4.643
2.047	2.005	1.892	2.531	1.952	3.416
2.354	3.259	3.863	2.529	4.311	1.283
2.055	1.789	1.903	1.994	2.403	2.124
3.632	3.375	3.729	3.180	3.586	2.999
2.068	2.225	2.665	3.258	2.944	2.056
4.552	4.113	3.981	4.528	4.795	4.212
2.613	2.420	2.817	2.104	2.648	3.232
3.353	3.312	3.273	3.512	3.737	4.292
6.948	5.830	6.399	6.829	6.360	5.606
2.466	2.967	3.063	2.336	3.226	1.567
2.696	2.077	2.779	2.678	3.147	1.413
1.635	2.325	2.374	2.061	1.913	3.129
4.418	3.285	2.586	3.909	3.641	2.650
4.766	3.048	3.219	2.884	4.216	2.930
5.786	4.993	5.342	6.621	5.165	4.113
3.194	1.733	3.148	2.537	3.142	0.929
1.609	2.050	2.891	1.928	1.185	4.014
4.058	5.443	4.814	4.238	4.963	3.490
4.538	5.088	4.445	4.159	4.758	3.355
4.164	3.184	4.222	3.543	3.232	1.727
2.613	1.889	2.404	1.839	2.819	2.910
5.566	4.679	4.800	5.138	5.334	4.785
4.153	3.850	4.164	3.958	4.082	2.276
6.240	4.115	5.345	6.311	5.688	5.834
2.053	2.535	2.326	3.204	2.579	2.665
3.075	2.437	3.264	3.948	2.601	1.109
3.400	3.988	4.006	5.245	5.034	6.746
1.756	1.759	1.226	1.894	1.655	2.666
3.716	2.233	3.142	3.754	3.405	3.545
3.680	1.922	1.983	2.983	2.316	2.699
3.344	2.859	2.484	3.462	3.336	2.451
2.731	4.367	3.781	3.739	3.405	3.593
3.101	3.456	3.385	3.280	2.629	1.295
5.696	5.687	5.657	5.406	5.812	6.098
3.002	3.933	2.748	2.892	2.697	2.054
2.528	2.203	2.539	2.011	1.982	2.457
2.393	1.831	1.945	2.735	2.614	6.772
1.983	2.537	3.302	3.712	4.277	6.541
2.940	2.989	2.028	2.766	2.545	2.983
4.554	2.789	3.669	4.410	3.048	4.715
3.875	2.846	3.642	3.172	3.675	4.127
2.549	2.799	2.893	1.969	2.743	3.786
3.586	4.341	3.662	3.247	3.280	2.605
2.726	3.565	3.140	2.371	3.210	1.608
3.849	2.781	3.394	2.735	3.610	3.600
3.262	3.488	3.399	2.957	3.803	3.786
3.338	4.124	3.752	3.366	3.521	3.119
4.376	4.498	4.773	3.817	4.709	2.718
1.965	2.219	2.504	2.242	2.301	1.600

4.064	5.089	4.542	4.704	4.233	4.570
7.257	5.762	6.501	6.992	6.112	4.019
4.702	3.109	4.240	4.516	3.888	2.532
2.517	2.670	2.427	3.284	2.471	2.458
1.899	1.645	2.036	1.850	2.276	3.637
4.219	3.156	2.617	4.641	3.226	5.161
2.316	2.857	2.680	2.882	2.347	4.183
5.097	4.426	4.416	6.206	4.366	6.374
2.739	2.043	2.332	2.996	3.059	3.162
3.344	3.384	3.833	4.200	4.026	5.101
3.553	4.239	4.140	3.684	3.590	3.429
2.502	2.546	2.228	2.489	2.651	2.828
4.558	4.727	3.806	3.755	3.507	2.980
3.741	2.890	2.724	2.970	3.809	2.160
2.174	3.571	4.401	1.782	2.017	2.306
5.767	6.584	6.071	5.790	6.331	5.434
1.998	2.324	1.893	2.207	2.504	2.180
2.548	2.574	2.224	2.957	2.619	3.340
2.486	2.771	2.919	2.538	2.811	2.924
3.364	2.808	3.206	4.029	2.241	5.013
3.849	2.268	2.534	3.074	2.345	1.876
2.343	2.349	2.662	3.027	2.032	1.992
2.624	2.133	2.876	2.579	2.345	3.085
2.589	2.127	2.395	2.756	2.733	3.973
3.151	2.932	2.781	3.600	3.457	3.499
3.384	4.317	3.235	2.849	2.837	2.560
3.095	2.756	3.483	3.276	3.710	2.729
3.175	2.376	2.693	3.221	3.075	3.656
4.167	4.502	4.202	4.589	4.753	5.021
2.080	1.905	2.165	2.080	1.883	2.585
6.607	6.055	6.208	7.012	6.465	6.496
3.819	2.732	2.206	3.839	2.794	2.401
2.324	2.801	2.600	2.738	2.899	3.420
4.158	4.676	4.587	3.843	4.054	3.386
5.195	4.073	4.172	5.371	4.186	5.371
3.786	1.667	2.906	3.217	2.376	1.659
4.909	5.254	4.981	5.223	5.858	5.848
2.413	2.347	2.465	3.521	2.907	4.004
2.606	3.457	2.522	3.337	2.897	3.739
3.551	3.189	4.023	2.584	3.668	3.910
2.061	2.748	3.055	2.759	2.560	2.671
4.448	3.169	3.647	4.003	4.075	5.384
2.592	2.869	2.926	2.767	2.956	2.711
1.926	2.450	2.121	2.516	1.443	2.960
2.243	2.617	1.810	1.654	2.099	2.355
4.220	4.492	4.144	4.047	4.033	4.010
3.278	3.868	2.779	3.716	3.268	1.992
3.884	3.559	3.560	3.928	3.173	3.100
2.423	4.109	3.170	2.522	3.600	1.718
5.357	5.904	5.577	4.908	4.912	3.279

3.134	3.398	2.665	3.284	3.533	2.256
3.680	3.621	3.016	3.229	3.538	2.487
2.525	2.500	1.810	2.814	2.256	2.892
2.244	2.965	2.683	3.299	3.029	4.440
7.723	8.130	7.900	7.819	7.287	8.357
3.759	3.832	3.523	3.094	3.943	2.605
1.483	1.671	2.174	1.998	1.617	1.852
3.968	2.971	3.223	3.724	3.115	3.551
2.579	4.383	3.687	2.654	3.247	3.634
1.660	1.891	1.972	1.935	1.444	2.508
2.597	2.702	2.005	2.350	2.479	2.413
2.844	2.870	2.717	3.283	3.517	3.083
3.188	3.005	2.106	3.565	2.774	3.097
6.587	6.652	5.966	6.903	6.335	6.970
2.444	3.071	3.424	4.472	3.988	5.432
4.396	3.284	3.411	3.350	3.401	3.469
1.579	1.393	1.301	1.287	1.514	2.087
3.573	3.255	3.365	3.798	3.543	1.766
4.042	2.705	3.613	3.359	4.303	1.154
2.117	2.329	3.007	2.407	2.687	2.743
4.187	4.348	4.559	4.041	4.673	4.512
2.288	2.157	2.384	1.854	2.746	2.621
3.155	2.973	2.772	3.550	3.251	3.084
2.575	2.326	2.625	2.834	2.288	3.029
4.297	2.616	2.528	3.418	3.310	2.843
2.826	3.084	3.179	2.821	2.880	1.051
2.977	3.473	3.439	3.815	3.025	1.962
3.830	3.230	3.351	3.621	2.721	1.643
3.044	2.377	2.840	3.668	2.482	0.864
6.553	6.298	6.855	6.034	6.124	3.542
3.808	4.004	3.960	3.559	4.250	2.959
3.962	4.718	4.578	4.285	5.097	5.936
3.452	3.072	3.785	4.175	3.577	3.628
3.613	2.947	3.738	3.865	2.418	4.246
4.154	3.633	4.323	4.354	2.091	1.044
1.992	2.333	2.089	1.876	1.828	2.383
2.408	2.133	2.767	3.230	2.234	2.828
2.768	2.238	2.715	3.338	2.749	2.900
3.797	4.266	4.086	3.937	4.425	4.828
3.886	2.679	2.834	1.900	1.884	2.330
3.802	3.862	4.049	4.140	4.600	2.701
4.880	5.475	5.071	4.842	4.769	3.694
3.211	3.234	3.652	2.193	2.433	1.082
3.418	2.047	3.496	4.018	2.880	2.008
4.766	5.184	4.897	4.438	4.410	5.193
3.539	2.937	3.390	3.793	3.728	4.432
2.722	1.886	2.602	2.718	2.338	2.989
7.434	6.333	6.544	7.221	6.873	7.777
3.587	3.133	3.651	4.059	3.679	3.245
2.750	2.859	2.445	3.048	2.754	2.052

3.049	1.999	2.405	3.008	2.402	2.853
2.951	3.553	3.171	2.535	3.385	4.324
3.123	2.938	2.219	3.182	2.924	3.073
3.372	3.402	2.813	3.308	2.830	4.237
3.469	2.653	2.655	2.996	2.421	1.989
3.575	3.864	3.873	3.861	3.121	4.049
3.514	3.623	3.582	3.996	3.957	4.406
3.003	3.081	2.537	2.616	3.208	2.428
7.841	7.418	7.088	7.936	7.729	7.788
2.202	2.452	2.301	3.762	3.297	3.293
2.430	4.171	3.689	2.486	3.211	0.861
4.850	4.788	5.085	5.042	4.239	2.090
7.271	6.367	6.875	7.266	6.985	8.041
2.029	2.023	2.279	2.400	1.991	2.840
3.917	4.237	3.007	3.769	4.120	5.122
3.472	3.516	3.832	3.935	4.555	6.266
3.187	3.508	3.079	2.912	2.894	2.441
1.321	1.662	1.462	1.230	1.373	1.850
3.063	2.915	3.544	3.187	3.619	4.499
3.193	2.945	2.574	2.395	2.803	2.329
5.645	5.995	5.819	5.767	6.122	2.949
3.853	3.429	4.186	4.943	4.438	4.066
7.651	7.107	7.486	7.626	6.823	8.416
2.319	2.598	2.167	2.511	2.894	3.359
3.130	1.688	3.389	2.567	1.680	1.586
2.074	3.005	2.389	1.786	2.775	2.406
3.639	3.648	3.868	3.552	3.759	2.217
2.829	2.638	4.090	3.255	2.792	3.313
2.714	2.291	3.134	2.230	2.293	2.512
2.892	3.094	3.810	2.649	2.741	3.036
2.447	2.523	2.688	2.307	2.507	2.358
2.528	2.485	3.165	2.802	3.370	2.638
1.566	2.008	2.161	1.923	2.291	3.882
3.893	3.777	2.355	3.733	3.717	2.808
3.126	3.237	3.289	3.526	2.885	2.824
3.663	3.605	3.926	3.060	3.902	3.082
2.562	2.078	2.546	3.005	2.124	3.143
3.472	3.290	2.596	3.319	2.985	3.302
3.765	2.888	2.784	3.521	3.041	2.665
2.762	2.032	3.435	2.752	2.679	3.016
4.620	3.948	4.886	4.720	4.864	3.162
2.621	4.841	3.126	2.226	3.022	3.859
4.392	4.188	4.200	4.637	4.472	4.645
5.834	5.408	5.689	5.825	5.873	5.017
7.097	7.279	7.364	7.711	7.859	5.792
2.471	2.323	2.539	2.035	2.738	1.886
9.325	9.581	9.352	9.491	9.280	9.892
3.327	3.707	3.380	3.209	3.272	3.645
2.829	2.682	2.047	2.568	3.090	3.022
6.716	7.280	7.023	6.573	6.356	6.758

8.057	8.006	8.030	7.911	7.807	8.573
3.333	3.435	2.968	4.222	3.795	4.548
5.840	5.568	5.461	6.284	5.757	7.030
4.771	4.718	5.310	5.359	4.960	5.132
6.348	6.562	6.231	6.100	5.856	5.204
4.774	4.982	4.983	4.765	5.309	3.195
2.935	3.208	3.663	3.444	2.859	3.565
3.489	3.637	3.633	3.410	3.520	4.307
2.435	2.719	2.369	2.372	2.429	2.905
5.350	5.304	5.397	5.152	5.209	4.003
4.781	4.970	5.105	4.910	4.998	4.235
2.884	3.671	3.573	3.986	3.913	3.139
3.738	4.242	4.620	4.477	4.108	3.065
3.980	3.475	4.174	4.050	3.836	1.191
4.845	4.884	4.978	4.920	4.512	5.404
3.246	2.623	2.919	3.366	2.970	2.629
7.566	7.351	7.397	7.337	7.223	8.015
2.769	3.406	2.506	2.127	2.606	3.528
8.878	9.492	9.103	8.683	8.729	9.395
10.272	10.717	10.585	10.249	10.325	10.619
5.078	4.860	4.753	5.222	5.340	4.993
3.157	3.102	3.663	3.903	4.221	3.019
10.131	9.791	10.071	9.825	9.749	10.361
8.011	7.643	8.275	7.601	8.020	8.648
4.698	3.689	3.808	3.947	4.156	4.865
5.541	5.615	6.216	5.555	5.307	4.984
6.820	6.783	6.745	6.904	6.576	6.004
4.606	5.052	4.555	4.843	4.556	3.858
1.681	2.581	1.679	2.188	1.839	1.273
2.089	1.803	1.498	2.137	2.030	2.440
4.119	4.678	4.124	4.702	4.518	4.189
2.014	1.994	2.143	1.941	2.373	3.580
5.425	5.768	5.552	5.599	5.902	5.284
6.259	6.046	5.986	5.528	6.202	6.508
5.971	6.053	6.195	5.517	5.651	7.120
4.498	4.412	4.399	4.179	4.727	5.137
2.534	2.454	2.831	3.493	3.492	2.569
6.013	6.730	6.111	6.068	6.215	5.804
6.526	6.454	6.525	6.278	6.365	5.889
2.609	2.811	2.965	2.938	3.207	4.649
4.694	4.622	4.972	5.171	5.695	4.504
3.169	3.248	2.812	3.905	3.752	4.021
5.418	4.956	5.207	4.922	4.941	4.744
5.204	3.769	1.484	0.961	3.044	4.380
2.595	3.192	2.554	3.162	3.265	2.494
5.743	6.131	5.675	5.834	5.933	5.553
4.032	3.457	3.796	3.934	3.707	3.835
5.800	5.852	5.979	6.068	5.935	5.594
2.381	2.284	2.879	2.651	2.589	2.396
5.732	5.483	5.826	5.678	5.391	2.677

4.571	4.422	4.877	4.106	4.568	1.699
5.578	5.686	5.778	5.695	5.913	5.418
7.875	7.814	7.701	7.925	7.836	7.137
4.528	4.796	4.812	4.999	5.433	6.483
3.168	3.483	2.474	2.783	2.166	1.338
8.430	8.593	8.482	8.443	8.288	7.139
5.191	5.441	5.532	5.105	4.986	3.673
9.731	9.678	9.733	9.767	9.564	9.050
4.319	5.010	4.343	4.331	4.127	3.794
4.298	3.627	3.476	3.884	3.642	2.877
6.246	6.267	6.214	5.452	6.003	3.684
3.337	2.662	3.385	2.889	3.289	1.707
2.359	2.818	3.278	2.459	2.913	1.996
2.245	2.704	3.010	2.670	3.038	3.737
3.082	3.726	3.333	2.921	3.261	2.329
5.026	5.040	4.954	5.437	5.443	6.681
6.128	6.442	6.029	6.079	6.128	5.260
9.330	8.974	9.014	8.979	8.995	7.159
5.050	5.487	5.401	4.976	5.284	3.437
2.423	2.256	1.987	1.878	1.734	3.334
5.561	5.182	5.473	5.043	5.210	3.914
2.677	2.246	2.326	2.160	2.472	3.256
4.583	4.041	4.544	4.403	4.393	3.561
7.566	7.305	7.336	7.180	7.429	6.420
6.078	6.572	6.244	6.106	6.391	5.431
2.827	4.452	4.233	3.916	4.509	2.420
5.077	6.031	6.450	6.216	6.149	0.899
3.821	3.507	3.685	4.022	3.800	4.658
8.024	7.857	7.921	8.136	7.988	5.018
5.764	6.168	5.869	6.077	5.885	7.077
3.312	3.428	3.596	3.474	3.679	3.916
2.468	2.556	2.195	2.016	2.328	3.450
3.464	3.257	3.845	3.092	3.685	3.603
5.567	3.023	2.388	5.107	2.086	1.840
4.057	4.382	4.507	3.682	3.492	4.156
2.031	2.593	3.100	1.871	2.613	2.208
4.406	4.084	4.546	3.951	3.992	4.744
5.320	5.342	4.415	4.881	4.181	3.344
5.353	5.316	5.252	4.764	3.816	3.225
8.470	8.330	8.765	8.756	9.025	10.974
3.658	3.252	3.095	3.997	3.583	4.323
4.394	4.376	4.373	3.970	4.658	5.746
7.838	7.957	7.894	8.063	7.993	6.953
3.636	3.910	4.157	3.768	3.796	3.117
5.329	5.011	5.419	5.316	5.305	6.865
5.489	5.556	5.363	5.482	5.796	6.629
3.721	3.637	2.516	4.006	3.375	4.171
4.827	4.257	4.542	4.933	4.591	5.258
3.083	3.661	2.909	2.486	2.647	3.751
6.366	6.336	6.405	6.691	6.504	6.008

3.245	2.977	3.942	2.761	3.624	3.409
4.291	4.382	4.241	4.171	4.479	5.739
3.837	3.267	3.912	3.213	3.670	3.466
3.254	3.412	3.395	3.456	3.749	3.833
3.931	4.105	4.836	4.488	4.410	5.514
7.129	7.070	7.500	7.366	7.102	7.538
3.010	1.995	3.006	2.596	2.814	3.162
2.827	2.875	2.874	3.350	2.793	3.178
2.603	2.533	3.216	2.677	3.210	2.746
7.697	7.291	7.210	7.731	7.584	8.673
2.742	3.423	4.062	3.031	2.993	4.266
8.251	7.897	7.352	7.260	7.405	7.214
6.735	6.619	6.687	6.702	6.770	5.436
8.160	7.837	8.117	8.122	8.061	5.626
3.649	4.106	2.690	3.273	3.006	2.622
4.991	5.478	5.084	4.415	5.173	3.564
8.053	8.103	8.120	8.093	8.040	7.389
4.315	4.362	4.164	4.049	4.231	4.289
2.729	2.888	2.183	3.461	3.728	7.712
5.469	5.510	5.315	4.751	4.433	5.242
7.108	7.526	7.227	7.164	7.119	5.340
8.240	8.656	8.402	8.430	8.244	7.493
5.274	5.441	5.231	4.873	5.048	6.481
3.308	3.767	3.568	3.419	3.720	4.683
8.816	9.043	8.967	8.867	8.817	7.499
6.648	7.604	7.555	6.396	7.100	7.471
2.267	1.549	1.328	1.779	1.681	1.588
7.970	7.452	7.466	8.171	7.993	8.453
6.230	6.051	6.262	5.756	4.970	5.765
1.649	2.588	2.782	2.771	1.811	2.620
5.288	5.281	4.987	5.403	4.783	5.073
3.618	3.497	3.323	4.066	4.443	4.052
5.303	4.890	5.641	4.919	4.700	3.576
5.568	5.286	5.099	5.550	5.321	4.546
5.009	4.616	4.083	4.624	4.239	4.274
5.907	5.274	5.350	5.947	6.337	6.200
6.401	7.241	6.136	7.983	5.533	6.898
8.768	7.196	7.379	8.460	7.488	5.931
7.018	7.153	6.782	7.109	6.723	6.499
7.364	6.895	6.999	7.369	6.581	6.950
4.155	4.505	3.633	4.450	3.797	4.416
1.957	2.596	2.609	2.394	2.937	2.969
6.604	6.752	6.993	7.110	7.014	8.008
9.245	9.654	9.284	9.133	9.792	11.331
6.918	6.890	7.097	6.883	7.249	7.014
5.313	6.158	5.685	5.669	5.489	5.214
8.676	8.940	8.735	8.801	8.898	8.093
5.740	5.719	5.550	5.754	5.845	4.769
2.082	3.705	4.438	2.117	3.252	3.397
4.081	5.903	5.493	4.215	3.488	5.908

3.876	4.363	3.384	3.965	4.006	3.182
3.204	2.629	2.616	2.546	2.747	2.102
2.107	1.521	1.449	2.389	2.836	2.510
8.159	8.577	8.398	8.390	8.574	6.313
7.800	7.749	7.609	7.796	8.022	6.046
8.543	8.095	8.572	8.484	8.473	5.716
7.925	7.475	7.955	7.991	8.041	6.140
7.135	6.731	7.307	7.092	6.750	6.972
7.630	7.435	7.854	7.294	7.387	5.455
5.842	5.869	5.456	5.361	5.707	4.098
3.203	3.270	3.672	3.343	2.921	2.766
8.694	8.901	8.526	8.752	8.701	6.416
7.414	7.561	7.411	7.441	7.277	5.445
3.442	3.783	3.976	3.728	2.542	3.663
3.420	3.061	3.769	4.429	3.298	4.027
2.787	2.002	3.244	2.304	2.167	3.124
4.879	5.464	5.343	4.843	5.053	3.073
3.446	3.277	3.450	3.130	2.910	2.547
3.138	2.621	2.472	2.313	2.836	2.915
3.415	2.530	2.763	2.859	3.407	4.414
3.446	2.202	2.258	3.152	2.085	2.239
6.897	6.743	7.242	7.138	7.118	6.072
8.308	6.641	6.223	8.177	7.916	8.983
5.184	5.229	4.638	4.790	4.754	4.592
4.486	5.021	4.407	4.087	4.433	3.122
3.554	3.899	3.531	3.531	3.581	4.213
4.033	3.693	4.784	3.251	2.839	4.227
5.429	4.806	4.303	5.720	5.509	5.163
3.895	4.055	4.594	5.257	4.074	5.544
1.037	4.121	4.819	5.536	5.483	5.457
3.231	3.854	4.677	5.026	4.009	4.526
2.705	3.274	3.736	4.583	3.218	4.733
5.128	4.951	4.973	5.121	5.437	4.724
2.930	2.051	2.930	3.423	2.273	1.453
5.876	4.926	5.515	5.195	5.509	4.925
3.536	3.776	3.945	3.867	3.659	1.722
4.058	3.065	3.342	2.918	3.580	3.283
4.533	4.372	4.522	4.313	4.667	5.617
4.049	4.331	4.271	4.081	4.004	4.851
5.810	5.926	6.647	6.166	6.190	7.126
2.978	2.747	3.916	3.990	3.950	4.614
5.705	5.713	5.588	6.129	6.408	6.893
2.254	2.132	3.075	2.688	2.694	3.533
4.244	3.403	3.806	3.389	4.023	3.180
2.772	2.013	2.463	2.365	2.248	3.577
3.538	1.493	2.521	2.391	1.602	3.288
7.000	7.406	6.941	7.074	6.892	6.282
5.304	5.215	5.375	5.080	5.420	4.207
4.117	3.975	4.569	3.126	4.064	3.223
8.513	8.650	8.773	8.510	8.803	7.630

5.477	5.136	5.639	5.291	5.866	3.785
2.890	5.383	1.138	3.343	1.710	2.043
5.012	4.284	4.224	4.790	4.691	5.184
4.318	4.012	4.359	4.110	3.448	5.631
4.052	4.017	4.219	3.307	3.862	5.842
5.300	5.593	5.818	5.950	5.693	4.848
5.092	5.496	5.272	5.755	6.408	7.833
2.611	2.750	2.730	2.015	3.288	2.932
6.466	5.997	5.868	6.087	5.990	6.303
4.169	4.314	4.087	4.211	4.292	5.349
4.438	5.321	5.035	5.931	5.589	4.240
3.498	2.987	3.286	2.356	3.508	2.219
3.209	4.339	3.500	3.024	3.332	3.343
3.766	3.455	3.849	3.525	4.224	4.308
4.460	4.705	4.305	4.412	4.704	5.329
4.835	4.670	4.758	4.848	5.123	6.659
5.699	5.156	5.309	5.319	5.443	4.780
3.306	4.043	3.461	2.969	4.023	3.319
6.068	5.879	5.977	5.905	6.135	6.690
4.339	4.439	4.801	4.221	4.933	3.322
5.434	4.958	4.970	5.376	5.663	6.742
6.489	6.617	6.691	6.368	6.711	4.418
2.805	3.171	3.448	3.375	2.700	1.836
7.165	7.180	6.866	7.367	7.203	7.445
7.317	7.613	7.467	7.391	7.357	7.066
5.622	6.113	5.982	5.610	6.055	4.947
6.369	6.755	6.447	6.258	6.254	7.175
3.290	3.285	3.675	3.954	3.644	5.571
5.056	5.689	5.581	5.572	5.564	6.407
4.819	5.173	4.746	4.901	5.477	6.623
6.579	6.085	6.744	6.725	6.622	5.887
2.502	2.920	2.468	1.867	2.350	2.590
10.587	10.486	10.330	10.571	10.421	8.734
6.235	6.728	6.733	7.097	7.096	7.692
6.718	5.697	6.523	6.354	6.296	4.906
5.503	5.853	5.567	5.607	5.606	5.156
6.416	6.692	6.756	6.490	6.402	6.546
5.198	5.786	5.251	5.763	5.315	5.187
4.884	4.477	4.833	5.073	4.943	4.358
2.009	2.620	2.279	1.779	1.539	1.895
3.143	2.650	2.724	2.705	2.720	3.227
5.976	6.269	6.339	6.400	6.563	7.457
5.350	4.923	4.979	5.314	5.281	4.316
1.859	2.368	2.909	1.408	1.910	1.983
4.083	5.406	5.232	3.227	3.930	4.136
2.108	2.230	2.396	1.837	2.168	2.204
5.070	5.512	5.361	4.689	5.222	5.536
4.659	5.216	4.900	4.587	4.263	5.726
3.569	2.899	3.003	2.987	3.617	3.573
5.957	6.219	5.903	5.680	5.984	6.007

3.337	2.967	2.859	2.746	4.025	2.643
3.945	1.724	2.519	3.003	4.007	2.293
2.987	3.303	2.137	2.019	2.840	3.393
7.299	7.782	8.298	7.787	7.709	4.237
6.029	6.292	5.801	5.204	5.596	5.436
4.972	4.322	4.952	4.101	4.434	4.802
9.321	9.775	9.762	9.621	9.531	8.338
1.654	1.780	2.176	1.674	1.865	4.256
3.242	4.403	4.013	3.950	4.286	3.314
2.915	2.779	3.287	3.179	3.719	4.237
6.437	7.137	6.735	6.359	6.674	3.990
7.532	7.293	7.302	7.275	6.963	6.640
4.991	4.891	4.620	3.959	4.990	3.714
5.196	5.072	5.072	5.582	5.289	3.789
5.136	5.198	5.245	5.796	5.186	4.923
4.774	4.722	4.797	4.556	4.591	3.642
7.460	7.023	7.210	7.185	7.371	6.630
7.619	7.757	7.610	7.558	7.793	6.787
5.903	5.749	6.127	5.786	5.742	6.956
5.006	5.050	4.627	2.978	3.571	3.791
3.031	3.400	3.960	3.298	3.407	4.125
3.894	4.476	4.237	4.273	4.261	5.050
5.518	3.368	3.922	5.573	4.901	5.255
2.310	2.370	2.091	2.017	2.046	3.002
2.307	2.597	2.783	2.921	1.862	2.170
6.527	6.572	6.085	6.194	6.249	7.002
4.381	4.764	4.547	4.562	4.965	2.729
7.246	6.973	7.231	7.275	7.180	6.416
5.252	5.488	5.133	5.809	5.659	6.733
2.506	3.579	2.896	3.560	4.025	5.009
4.999	4.909	5.336	4.975	4.700	4.008
5.990	4.585	5.058	4.765	4.673	4.548
3.721	3.072	2.553	2.723	3.242	3.587
5.765	5.519	5.599	5.328	5.371	4.656
5.741	5.813	5.501	5.766	5.404	5.179
7.355	7.280	7.146	6.935	7.449	6.242
8.457	8.938	8.900	8.598	8.876	7.344
2.761	3.287	3.528	2.940	2.509	2.383
3.776	3.867	3.879	2.913	3.758	2.851
4.083	4.724	4.612	4.359	4.647	5.474
9.359	9.411	9.379	9.384	9.345	8.520
4.621	4.527	4.677	4.256	4.531	4.962
1.823	2.620	2.497	2.566	2.520	2.741
2.410	3.081	3.136	2.575	2.892	2.951
3.759	3.589	3.829	4.063	4.366	4.880
3.758	3.258	2.392	3.697	3.837	4.567
3.895	3.662	3.895	3.895	4.120	4.384
3.125	3.682	2.912	2.841	2.909	2.937
8.498	8.597	8.413	8.642	8.542	6.467
5.150	4.684	4.996	4.655	5.059	5.129

2.942	2.700	3.048	2.965	2.425	1.559
2.595	2.349	2.682	2.232	2.285	3.515
3.153	3.868	3.257	4.607	4.616	6.001
5.876	5.869	6.091	6.044	6.129	3.493
3.539	3.147	2.185	3.531	3.177	1.886
2.448	2.127	2.089	1.699	1.795	2.182
7.072	6.500	6.697	6.733	6.535	4.306
4.850	4.770	4.138	4.558	4.678	4.766
5.738	5.702	5.396	5.105	5.465	5.595
8.490	8.401	8.566	8.469	8.326	5.409
4.365	4.426	4.491	5.256	4.829	6.000
3.472	3.346	3.576	4.083	3.399	2.728
2.561	2.764	3.530	3.029	3.194	4.084
2.923	2.708	2.264	2.621	2.144	1.920
3.280	3.717	3.823	3.398	3.506	3.042
5.855	5.516	5.081	5.172	5.663	8.099
5.268	4.456	5.098	4.928	5.015	4.019
6.010	5.506	5.939	5.777	5.655	3.930
4.351	4.668	4.695	4.372	4.560	5.133
3.799	4.102	3.779	3.757	3.943	4.254
3.714	4.221	3.958	3.600	4.300	5.501
3.801	3.780	4.201	3.830	3.616	2.446
3.431	3.597	3.198	3.650	3.227	4.249
4.231	4.021	3.847	4.284	4.372	5.817
5.886	5.845	6.022	5.605	5.887	4.759
4.210	4.521	4.625	3.619	4.773	2.536
5.434	4.966	5.165	5.173	4.995	4.984
5.814	5.554	5.578	5.411	5.598	6.400
4.048	4.918	4.761	3.922	4.362	4.737
3.262	2.298	2.335	2.184	2.588	2.644
2.861	3.538	2.637	2.265	2.618	3.365
2.497	2.859	3.767	2.748	3.110	3.501
2.805	3.029	3.463	3.638	3.339	5.014
4.019	4.034	3.873	3.006	3.957	3.955
5.144	5.030	4.448	4.751	5.175	3.145
3.607	3.226	2.841	4.066	3.985	4.651
6.856	6.658	6.739	6.695	6.693	6.094
7.646	7.019	7.233	7.400	7.215	6.417
8.577	8.221	8.281	8.596	8.214	7.531
3.396	3.152	2.996	3.161	3.279	3.453
4.682	4.495	4.678	4.723	5.014	5.991
4.483	4.505	4.351	4.607	4.566	3.627
1.976	2.796	2.191	2.812	2.885	1.764
5.757	5.610	5.959	5.730	5.825	4.737
3.196	3.822	3.274	3.023	3.427	2.136
2.450	2.112	1.706	1.845	1.930	2.129
5.841	5.728	6.082	5.707	5.732	6.868
4.153	4.577	4.494	3.736	4.185	4.454
6.681	6.774	7.037	7.289	7.331	8.531
4.138	4.124	4.755	3.730	3.416	3.235

5.851	6.498	6.208	6.342	6.057	5.332
3.275	3.604	3.731	3.710	3.303	3.459
3.736	3.237	3.718	4.127	3.155	3.185
3.748	4.131	3.512	2.703	3.653	4.036
5.772	5.797	5.394	5.348	5.734	6.582
8.094	8.012	8.001	7.997	8.035	5.987
3.978	2.997	3.278	3.301	3.663	2.749
2.896	2.907	2.955	3.129	3.016	3.607
4.815	4.145	3.776	4.650	5.231	5.956
6.257	3.010	3.343	4.113	5.482	2.028
5.444	5.292	4.192	4.933	5.575	3.550
9.011	7.089	6.807	9.026	7.667	10.399
6.382	5.676	6.036	6.383	5.993	5.723
3.072	3.106	3.706	2.891	3.081	3.802
5.186	4.799	5.080	4.730	5.126	4.099
3.942	2.219	2.129	2.178	3.259	1.656
5.083	5.279	5.245	4.797	5.189	4.037
4.369	4.320	5.009	4.101	4.408	4.236
4.106	4.150	4.060	3.453	4.257	5.207
4.261	4.261	4.913	5.096	4.585	4.516
5.160	4.964	4.817	5.132	5.170	4.371
4.888	4.272	4.419	5.283	4.747	6.338
5.981	6.579	6.115	6.059	6.060	5.182
5.123	6.831	6.084	5.767	5.734	7.400
10.350	10.227	10.180	9.860	10.021	4.830
2.417	2.857	3.194	2.849	3.099	2.026
2.735	2.982	2.935	2.302	2.724	3.019
5.500	5.918	5.720	5.739	5.834	5.149
6.808	7.097	7.048	6.852	6.843	4.741
10.362	10.598	9.966	10.469	10.262	9.450
3.781	4.033	3.815	3.215	3.797	4.571
4.264	3.701	3.617	3.275	3.322	2.539
2.853	2.126	2.261	3.127	2.503	1.315
4.888	5.179	4.889	5.426	5.456	6.301
3.825	3.203	2.635	2.783	3.323	4.001
2.842	3.199	3.102	2.794	3.646	3.483
4.324	2.656	3.335	3.366	3.500	4.054
3.429	3.948	3.086	3.672	2.455	2.338
2.330	2.508	3.069	2.943	3.040	2.844
9.224	10.079	9.660	9.917	9.915	9.526
5.882	5.497	5.778	5.344	5.868	6.515
3.751	3.099	3.298	3.415	3.347	5.339
4.936	4.205	5.165	4.921	5.101	5.927
7.400	7.687	7.376	7.571	7.688	5.959
4.381	4.679	4.590	4.858	4.793	6.620
5.833	6.252	6.409	5.999	6.270	5.025
3.230	4.306	4.066	4.048	3.844	3.416
6.982	6.700	6.725	6.869	6.620	6.586
4.321	4.663	5.089	4.276	4.680	3.579
5.611	5.743	5.930	5.254	5.692	3.818

8.566	8.379	8.387	8.250	8.384	7.199
7.446	7.614	7.639	7.519	7.669	7.115
5.799	4.906	4.988	5.459	5.625	6.714
4.661	5.245	5.112	4.423	4.098	4.318
6.043	6.281	6.294	6.315	6.349	5.992
5.939	6.129	5.794	5.631	5.777	5.863
8.739	9.344	8.942	8.942	9.054	8.206
5.901	6.688	6.170	6.281	6.366	5.173
6.379	6.630	6.589	6.460	6.539	6.055
6.551	6.869	6.697	6.715	6.951	6.151
3.613	3.609	2.942	3.917	4.174	4.637
3.776	4.064	3.801	5.024	4.431	2.018
5.889	5.877	6.877	6.067	6.323	2.528
4.479	3.461	4.510	3.920	4.312	2.698
5.322	5.173	4.791	5.352	5.492	6.218
3.702	4.884	4.337	3.915	4.468	1.566
2.099	2.556	2.396	2.203	2.269	4.383
5.253	5.411	5.239	4.945	5.231	4.569
4.154	4.367	4.189	3.608	4.013	4.870
4.471	4.233	4.734	3.931	3.932	5.069
5.541	4.114	5.111	4.811	4.770	4.463
6.466	6.373	6.246	6.015	6.044	4.725
7.643	7.809	7.732	7.389	7.530	4.737
4.818	5.610	5.174	4.963	5.263	3.551
3.640	3.865	3.687	3.938	3.812	4.535
3.672	3.776	3.603	3.266	3.641	4.082
4.779	4.603	4.568	4.610	4.463	2.862
5.423	6.124	5.905	5.632	5.663	4.858
7.682	8.064	7.823	7.792	7.703	7.108
6.491	6.522	6.357	6.074	6.259	5.911
5.507	5.745	5.627	6.125	6.372	7.508
4.425	5.019	5.322	5.174	5.366	3.501
6.341	6.696	6.149	6.312	6.448	5.782
4.402	4.453	4.459	4.801	4.894	5.766
6.910	6.883	6.747	6.943	6.851	6.358
4.455	4.745	4.511	4.583	4.428	5.543
3.843	4.804	4.490	4.722	4.974	6.456
5.933	4.923	5.455	4.963	5.315	4.818
8.083	8.602	8.412	8.320	8.672	6.603
5.981	5.993	5.927	5.971	5.821	4.526
2.817	3.103	2.647	2.572	3.220	2.811
5.268	5.028	5.370	5.674	5.276	4.859
5.808	6.465	5.721	5.692	5.891	5.074
2.448	1.856	2.984	2.253	2.002	2.952
5.361	5.518	5.334	5.272	5.600	4.862
8.835	9.027	8.625	9.229	8.902	9.640
4.088	4.165	4.701	4.398	4.720	5.015
4.587	4.381	3.907	4.872	3.786	2.972
5.881	6.355	6.759	5.926	6.795	7.127
3.242	2.847	2.787	3.970	3.558	3.047

5.112	4.767	4.840	4.996	4.507	4.643
2.191	3.412	3.138	3.223	2.946	2.383
6.283	6.458	6.944	6.346	6.604	4.878
5.598	4.904	4.978	5.417	5.401	6.652
6.260	6.340	6.128	6.184	5.785	5.439
5.929	5.761	5.804	4.909	5.743	4.903
3.743	4.096	4.192	3.074	4.089	2.959
4.582	4.650	4.553	4.313	4.147	5.034
3.627	3.526	3.638	3.039	2.909	4.737
6.519	6.529	6.463	6.512	6.294	5.744
3.250	3.477	3.283	2.889	3.499	4.262
7.527	7.878	7.755	8.169	8.258	9.875
7.500	7.334	7.196	7.423	7.549	8.955
2.879	4.067	4.224	2.985	3.196	4.433
5.082	5.021	4.901	5.151	5.030	3.067
3.041	3.648	4.151	4.166	4.464	5.806
6.053	5.765	5.830	5.828	5.940	4.206
4.476	4.001	4.870	4.035	4.314	5.387
4.421	2.750	3.846	4.164	4.529	2.932
1.690	2.123	1.612	1.536	1.558	3.512
8.443	8.048	8.095	8.421	8.121	5.249
6.212	6.314	6.413	6.269	6.212	5.332
2.948	2.344	2.529	2.419	2.622	3.520
5.788	5.525	5.557	5.448	5.610	4.921
8.796	8.740	8.321	8.599	8.626	9.544
3.585	4.351	3.516	4.300	4.300	4.241
6.728	6.559	6.569	6.456	6.520	5.710
4.426	4.890	4.750	4.802	4.725	3.983
4.013	4.134	4.009	3.564	3.122	3.821
2.702	2.946	1.973	2.360	2.134	2.639
1.844	2.902	3.358	2.535	2.171	2.719
4.895	4.998	4.197	5.518	5.577	7.583
3.202	2.425	2.129	1.853	3.077	2.052
3.801	3.853	5.096	3.987	3.811	3.811
1.797	1.766	2.211	1.550	1.920	3.469
8.988	9.025	8.762	9.093	9.313	8.695
6.578	6.184	6.661	5.956	6.157	6.776
3.780	3.040	3.322	3.772	3.590	3.010
6.730	7.301	6.995	6.931	7.085	4.981
8.308	8.490	8.480	8.335	8.738	6.273
4.760	3.315	2.932	3.616	3.939	2.153
1.504	1.506	1.477	1.772	1.538	5.196
4.990	6.797	5.120	5.433	6.015	5.379
6.316	5.779	5.808	6.044	5.918	2.810
4.510	4.810	4.683	4.865	5.090	7.200
4.610	4.528	4.529	4.714	4.983	3.846
7.482	7.280	7.169	7.713	7.957	8.336
6.156	6.628	6.745	6.356	6.461	5.768
6.294	5.845	5.801	5.521	5.292	5.385
2.271	2.447	1.798	2.986	2.086	1.652

7.167	7.433	8.218	7.358	7.640	6.943
5.016	5.082	4.871	4.988	4.803	4.265
1.592	1.926	1.831	1.907	1.553	2.678
4.725	4.736	4.760	4.879	5.344	3.053
5.901	5.655	5.920	6.352	6.107	4.505
8.123	7.735	8.109	7.996	7.919	4.874
3.534	3.407	3.859	3.403	3.689	3.748
4.017	4.143	4.073	4.018	3.874	4.494
5.661	5.854	5.769	5.425	5.648	4.877
2.384	2.780	2.361	2.411	2.955	1.746
7.270	7.788	7.702	6.935	7.342	7.568
3.728	4.364	3.732	3.691	3.650	2.361
9.932	10.426	10.037	9.816	10.151	8.518
6.742	6.776	6.770	6.383	6.460	5.697
7.239	7.395	7.315	7.005	7.247	6.036
5.471	5.446	5.407	5.371	5.912	3.745
5.971	6.316	6.359	6.148	6.175	7.487
7.092	7.081	7.070	6.739	7.018	5.842
5.335	5.446	5.340	5.749	5.916	6.832
6.547	6.708	6.973	6.708	6.686	5.228
4.166	3.912	3.651	3.877	3.636	3.537
4.594	4.658	4.669	4.388	4.546	5.567
5.716	5.573	5.540	5.716	5.548	6.738
4.787	4.395	4.740	4.422	4.727	2.988
4.068	4.649	4.294	4.286	4.402	1.233
2.248	1.517	1.482	2.487	1.946	3.020
3.514	3.647	3.275	3.262	3.932	4.026
1.817	1.822	2.220	1.965	1.376	2.916
5.216	6.212	5.980	5.773	6.134	5.707
6.712	6.604	6.831	6.381	6.580	4.934
6.803	6.374	6.857	6.781	6.738	5.687
4.778	2.957	3.269	4.337	3.450	3.788
6.088	6.115	6.081	5.559	6.120	5.695
7.763	7.258	7.669	7.623	7.523	6.888
6.643	6.817	6.809	6.469	6.864	4.306
5.361	5.672	5.654	5.111	5.496	2.345
6.786	6.460	6.357	6.466	6.307	5.396
6.150	5.317	5.487	5.665	5.408	5.001
2.699	5.209	4.998	2.807	3.524	2.109
4.416	3.062	2.760	4.016	3.720	1.987
6.094	6.732	6.371	6.471	6.373	5.693
6.939	7.094	6.733	7.749	6.740	8.540
1.778	1.921	0.934	1.893	1.478	2.221
5.965	5.477	5.091	5.841	5.790	7.445
4.233	3.327	3.249	3.504	2.384	3.006
6.999	7.376	7.155	7.055	7.111	6.174
3.694	3.561	3.436	4.110	2.883	3.879
3.873	3.534	4.032	3.951	4.105	5.124
7.640	7.918	7.683	7.790	7.730	7.205
5.745	6.552	6.054	6.078	6.453	5.177

2.142	2.444	2.893	2.868	3.000	2.005
4.013	3.019	3.017	3.677	3.705	2.605
3.364	4.218	4.158	3.943	3.892	3.587
2.472	1.301	2.162	2.183	1.494	3.671
2.958	3.021	2.449	3.025	3.001	4.314
4.908	5.295	5.637	5.319	5.618	4.522
3.935	3.829	4.299	4.087	4.507	3.381
4.222	3.984	4.310	4.009	4.051	5.064
4.757	4.304	3.935	5.072	4.470	6.011
5.903	5.468	5.332	5.730	5.140	7.027
4.305	4.711	4.473	4.233	4.435	3.627
7.534	8.090	7.768	7.685	7.918	6.270
5.180	5.148	5.061	5.188	4.801	5.020
5.587	4.330	4.921	5.503	4.755	5.430
3.024	2.786	2.847	2.848	2.937	2.315
5.191	4.472	4.553	4.463	4.337	4.038
5.296	5.308	5.329	5.194	5.237	4.064
7.006	6.999	6.789	6.650	6.907	7.629
11.503	11.661	11.714	11.554	11.830	10.112
3.051	2.917	2.937	2.676	3.444	1.796
4.468	4.527	4.677	4.628	4.412	3.669
8.812	8.731	8.720	8.833	8.866	7.067
4.481	4.807	4.866	4.043	4.830	5.341
4.231	3.921	4.484	3.548	4.419	4.737
5.005	4.985	5.487	3.793	5.368	5.859
3.556	3.698	4.329	3.862	4.254	4.879
3.549	3.317	3.871	2.971	2.623	2.702
1.282	2.373	3.828	2.943	0.981	3.461
3.780	4.150	4.325	3.836	3.049	3.935
2.441	1.861	3.106	2.558	2.353	2.672
1.516	0.754	2.322	1.155	1.439	2.182
7.443	7.870	7.705	7.494	7.527	6.769
3.819	3.235	3.794	3.934	4.056	2.526
8.386	8.521	8.659	8.362	8.587	6.181
6.336	6.436	6.196	6.398	6.424	6.417
4.241	4.257	3.966	3.886	4.010	4.280
4.244	3.710	3.918	4.283	3.769	3.894
4.325	3.809	3.509	3.580	3.675	4.095
3.667	3.599	3.489	3.471	3.532	3.808
2.305	1.605	3.135	2.994	2.772	3.517
6.314	5.400	5.712	6.100	6.150	5.648
3.918	4.595	3.984	3.830	3.895	4.253
8.932	8.726	8.812	8.577	8.798	7.882
5.387	5.918	5.225	5.687	4.924	6.149
8.494	9.567	8.118	9.916	8.341	9.131
3.152	2.340	3.472	3.677	2.057	3.741
4.540	5.235	4.046	4.734	4.267	4.827
5.665	5.917	5.102	5.713	4.545	5.465
6.367	5.984	6.295	6.327	5.524	6.365
3.488	3.440	3.881	3.651	3.884	3.881

3.634	2.979	3.181	4.121	3.706	4.537
6.470	6.237	6.004	6.409	5.876	6.052
9.012	8.765	8.396	8.514	7.905	8.186
7.971	7.545	7.306	8.106	9.273	8.565
4.773	5.247	5.016	4.905	5.229	4.633
3.523	3.743	3.595	3.916	3.643	4.083
3.818	3.346	3.166	3.523	2.931	3.380
1.340	2.113	2.191	2.315	4.360	1.681
1.682	1.508	1.663	2.770	1.596	2.165
7.717	7.342	6.747	8.126	6.784	8.388
8.675	9.125	8.367	8.941	8.490	9.142
3.646	4.340	3.730	4.142	3.331	3.658
2.742	3.727	2.690	3.242	2.156	3.252
1.898	2.313	2.473	1.954	2.271	3.012
6.785	7.502	6.773	7.671	7.422	8.015
4.258	3.235	3.099	4.585	3.993	4.794
2.817	1.657	1.531	2.912	2.649	2.937
7.283	8.192	7.685	8.264	7.073	9.773
7.322	6.455	5.893	6.866	6.341	7.673
7.371	5.775	6.092	6.984	5.723	6.733
10.053	9.847	9.921	10.138	10.077	10.813
10.852	10.381	10.645	10.662	10.188	10.973
8.196	7.335	7.388	8.491	7.335	8.649
4.032	4.076	4.374	4.208	4.141	4.840
8.179	8.572	7.953	8.907	8.096	8.804
6.250	6.521	6.388	6.643	6.202	5.933
4.756	5.271	4.505	4.869	4.507	5.399
3.513	2.839	3.916	2.979	3.437	4.559
8.197	7.927	7.368	8.210	7.040	8.924
5.656	5.866	6.081	6.060	5.746	6.667
6.603	6.505	6.525	6.364	6.460	6.354
3.762	2.889	3.017	3.405	2.591	3.971
9.606	9.052	8.901	9.465	9.202	9.159
2.371	2.789	1.994	3.043	2.471	3.820
3.297	2.091	2.632	2.298	2.832	2.710
3.431	2.392	2.089	3.195	2.174	4.250
3.662	3.252	3.749	3.734	3.765	3.750
5.020	4.853	4.587	5.246	4.714	4.871
7.260	7.906	7.235	7.131	6.952	6.945
5.310	5.359	4.526	4.674	4.633	5.674
5.622	5.981	5.546	6.038	5.066	5.667
6.759	6.586	6.046	6.494	5.263	6.092
3.550	2.531	2.843	3.153	3.281	3.353
6.115	3.966	4.805	5.574	5.051	5.555
9.474	6.172	8.396	9.236	7.911	9.381
8.051	7.728	7.535	7.970	7.819	8.557
4.948	4.822	4.379	5.091	4.588	6.065
3.684	3.611	3.663	3.912	3.763	3.847
3.017	1.862	3.040	2.253	2.818	3.520
5.896	6.124	6.355	6.141	6.180	7.267

5.454	6.039	5.488	5.537	5.625	5.812
2.898	3.505	3.518	4.116	3.544	3.563
5.942	5.102	5.483	6.063	5.258	6.277
5.101	4.735	5.070	4.619	4.809	4.905
5.632	5.674	5.459	5.405	5.124	5.439
7.786	7.825	7.520	7.376	7.116	8.011
8.404	7.909	8.560	7.804	10.104	9.912
2.709	3.052	3.254	3.326	2.990	3.202
5.298	5.413	5.564	5.928	4.940	6.311
7.203	6.587	7.141	7.688	6.263	6.009
5.412	5.025	4.826	5.095	4.928	4.804
5.991	5.288	5.349	5.962	5.305	4.195
5.237	4.514	5.001	5.536	4.437	3.639
6.632	5.993	6.509	6.641	6.095	5.727
3.496	3.084	3.089	2.942	2.293	1.413
5.443	4.156	5.128	4.567	4.360	3.036
5.342	3.754	5.412	5.218	4.134	3.621
9.364	8.512	9.439	9.283	8.643	7.535
3.879	2.369	1.458	3.349	2.376	1.199
5.308	3.322	4.268	4.998	3.901	2.414
10.054	9.542	9.761	9.856	9.498	8.009
4.529	3.725	4.107	4.528	3.114	3.016
5.703	5.541	6.064	6.012	5.267	4.967
6.148	5.428	6.293	6.322	5.414	4.535
5.562	4.277	4.782	5.764	5.062	4.469
5.453	4.699	5.096	5.062	5.230	4.045
4.567	4.691	4.757	5.198	4.780	3.704
5.195	1.766	1.933	5.126	4.464	2.989
6.872	6.166	6.807	6.733	5.975	4.937
6.141	5.632	6.434	5.584	5.146	3.890
4.375	3.031	3.660	3.626	2.303	0.840
5.663	4.994	5.890	5.375	4.837	2.257
5.934	4.950	6.045	5.914	5.344	3.034
5.006	3.990	4.386	5.070	4.254	4.856
7.948	7.260	7.953	7.572	7.282	5.445
9.895	9.241	9.979	9.781	9.194	8.145
3.852	2.123	3.579	4.560	2.385	4.132
4.158	3.276	4.020	4.014	3.424	2.976
2.704	3.278	2.773	3.647	2.561	2.698
1.788	3.909	4.930	2.771	2.096	1.279
4.568	2.618	2.794	4.294	3.084	3.387
6.074	5.373	6.227	6.100	5.206	3.857
11.226	11.041	10.976	11.095	10.819	10.122
6.734	6.050	6.109	6.348	5.799	4.442
4.659	4.316	4.233	4.731	3.949	3.105
10.645	10.490	10.420	10.707	10.143	9.389
11.134	11.001	10.821	11.103	10.618	10.208
3.150	2.870	3.210	3.125	2.481	1.764
8.794	8.495	8.182	8.346	7.668	7.031
8.252	8.080	7.667	7.685	7.380	6.414

4.783	4.221	4.807	4.327	3.497	1.940
9.530	8.942	8.907	9.044	8.390	7.229
7.657	7.408	7.001	7.544	6.882	5.895
6.678	5.763	6.225	6.113	5.262	3.739
6.065	5.913	6.323	6.382	5.413	4.368
11.736	11.587	11.514	11.768	11.514	10.787
5.061	4.879	5.131	5.382	4.452	3.439
3.448	2.633	2.724	3.137	2.294	1.452
11.344	11.129	11.080	11.210	10.971	10.031
5.185	4.385	4.583	5.416	4.356	4.623
11.591	11.465	11.407	11.604	11.272	10.567
11.587	11.480	11.367	11.595	11.184	10.558
11.502	11.367	11.245	11.460	11.316	10.633
3.654	3.545	4.288	3.663	3.103	2.057
8.001	7.911	8.041	8.135	7.611	6.738
9.696	9.812	9.632	9.416	8.834	8.774
3.435	1.657	3.061	3.683	1.706	1.863
7.437	7.437	7.254	7.337	6.695	6.970
7.264	7.082	7.173	7.066	6.493	6.212
6.173	4.508	5.456	5.537	4.613	5.158
5.623	4.397	4.344	5.258	4.314	3.404
7.006	6.351	6.802	6.633	6.374	4.706
5.800	5.443	5.699	5.919	5.491	3.235
7.623	7.239	7.218	7.476	7.083	5.778
7.112	6.412	6.638	7.090	6.429	4.922
7.795	7.296	7.662	7.706	7.359	7.686
7.954	7.521	7.966	7.920	7.513	7.946
5.821	5.523	5.202	5.421	5.466	5.500
4.095	2.642	3.486	3.604	3.946	4.673
2.231	2.188	2.043	1.433	2.614	2.749
2.540	2.605	2.162	3.357	2.133	3.556
2.869	2.869	3.159	2.455	2.559	1.461
4.755	4.665	4.689	4.904	4.259	4.407
3.805	2.960	3.347	3.113	3.527	4.348
4.977	4.747	3.743	5.120	4.516	5.650
7.172	7.063	7.003	6.482	6.173	6.838
4.024	3.353	3.991	4.189	3.865	4.090
4.870	4.175	4.743	4.477	4.038	5.671
8.786	8.324	8.395	8.824	8.123	8.771
6.775	6.292	6.085	6.599	6.242	7.230
2.912	2.127	2.385	2.630	2.817	3.052
4.744	4.782	3.364	3.980	4.870	3.392
5.665	5.261	5.749	5.201	5.166	5.276
1.387	1.492	1.070	1.474	1.254	1.399
3.409	2.888	2.792	3.283	2.897	1.925
4.461	4.176	3.770	3.940	4.304	3.803
4.286	4.511	4.193	4.352	3.871	4.878
4.281	4.259	3.947	3.849	2.807	4.133
7.436	7.289	7.527	7.636	7.285	7.499
5.434	5.551	5.723	5.632	5.244	5.367

8.473	8.410	8.529	8.332	8.316	8.471
5.306	4.749	4.812	5.287	4.732	5.841
5.027	4.442	4.122	4.555	4.394	5.271
2.177	1.419	1.584	2.286	2.948	2.712
4.447	4.002	3.945	3.494	3.805	4.043
5.685	5.511	5.342	5.100	5.225	5.118
4.490	3.547	3.829	3.757	3.864	3.326
6.488	5.811	6.213	5.832	5.624	6.315
8.354	8.048	8.081	8.146	7.691	8.393
4.837	6.262	4.676	5.866	4.594	5.638
5.089	5.016	5.060	4.244	5.341	5.439
5.010	4.397	4.773	5.093	4.307	4.530
2.012	2.429	2.641	2.515	2.966	3.231
2.985	2.583	3.231	2.268	2.660	2.574
11.117	11.113	11.326	10.926	10.464	11.208
2.687	2.468	1.167	2.549	2.395	2.048
8.393	8.183	8.657	7.935	7.374	8.293
2.405	2.626	2.287	2.056	2.123	2.276
2.138	2.126	2.358	2.420	1.541	1.885
4.786	4.440	4.487	3.525	3.622	3.990
3.675	3.586	3.126	3.852	3.190	3.392
2.391	1.397	2.274	2.399	2.333	1.906
8.175	7.560	7.962	7.845	7.241	7.099
6.041	5.810	5.692	5.544	5.418	5.414
4.071	3.159	3.842	3.861	3.569	3.751
7.179	6.342	7.139	6.588	6.786	7.052
4.814	4.690	4.234	4.847	3.331	3.900
7.440	7.453	7.086	7.524	7.215	7.507
3.252	2.078	2.222	3.374	3.672	2.807
0.918	1.072	1.764	1.238	1.861	2.464
7.368	7.065	7.160	6.996	7.011	5.683
6.192	5.595	5.457	6.076	5.479	5.206
10.526	10.353	9.870	10.113	9.838	9.929
6.096	5.619	5.712	6.336	6.232	6.371
6.796	6.928	7.134	6.738	6.972	5.670
7.530	7.298	7.220	6.940	7.213	4.472
7.606	7.414	7.491	7.830	7.277	6.570
5.303	5.600	5.331	5.555	5.722	6.577
5.492	6.002	5.483	5.885	5.966	4.481
4.311	4.622	4.593	4.565	4.048	3.793
3.802	4.084	4.192	4.336	4.141	5.259
1.741	2.273	2.306	1.827	1.138	2.217
3.610	3.366	4.041	3.688	4.192	4.832
6.375	6.693	6.496	6.508	6.791	7.954
5.196	4.628	4.805	3.734	4.621	2.646
4.682	4.547	4.768	4.281	4.908	4.927
6.198	7.169	6.652	6.686	6.991	6.948
6.286	5.812	6.010	6.010	5.558	5.832
7.046	6.860	6.989	6.903	6.996	6.414
7.619	6.032	5.825	5.811	7.102	4.941

4.997	5.574	5.567	5.603	5.232	6.112
6.853	6.882	7.083	6.817	6.809	6.005
3.921	4.090	4.096	3.440	4.038	3.509
4.145	4.335	4.561	3.529	4.563	3.748
6.880	6.910	6.807	6.723	6.490	5.843
3.667	3.374	3.168	3.870	3.795	2.416
6.502	5.883	6.024	6.525	6.382	4.586
6.737	6.162	6.389	6.236	6.231	4.001
6.213	5.975	6.360	6.224	6.197	5.242
3.727	2.264	2.983	2.535	2.423	4.148
4.330	4.396	5.116	4.346	4.275	2.070
6.150	5.607	5.537	5.306	5.321	4.182
4.910	5.684	4.913	4.747	5.451	2.998
2.323	2.403	2.354	2.337	2.352	2.632
3.775	3.847	3.480	2.993	3.382	2.522
3.917	3.598	4.195	4.343	4.521	5.736
4.161	3.528	3.606	3.616	4.187	4.627
2.796	2.043	1.919	2.940	2.033	2.385
3.518	3.630	3.430	3.769	3.739	4.808
5.923	5.582	5.563	5.891	5.700	5.285
4.970	4.555	4.995	5.224	5.524	6.640
8.427	7.258	7.867	7.943	8.054	8.193
2.648	1.799	2.337	2.271	2.989	3.591
6.626	6.208	5.878	6.024	6.679	6.983
4.310	4.333	4.374	4.231	4.104	4.343
4.158	4.621	4.867	4.108	4.650	5.055
3.345	3.762	4.001	3.574	4.595	3.174
1.340	1.228	1.432	1.153	1.150	1.387
4.378	3.652	3.794	4.305	4.020	3.072
2.009	2.528	1.867	2.175	2.011	2.035
2.813	2.479	1.671	3.044	2.460	2.560
5.661	5.476	5.067	5.307	5.510	7.629
5.291	5.494	5.420	5.127	5.185	6.593
2.691	2.957	2.852	2.880	2.744	4.013
2.121	2.240	2.499	1.989	2.177	2.041
3.418	3.723	3.691	3.346	3.182	3.326
3.234	3.312	3.343	2.451	2.533	3.045
2.387	1.862	3.479	3.419	2.286	2.313
2.609	2.032	2.521	1.958	2.579	1.883
4.315	4.570	4.162	4.856	4.415	5.743
2.824	3.864	3.391	4.323	3.969	4.900
4.135	3.715	4.015	4.090	3.549	3.075
5.006	5.291	5.587	5.347	5.158	4.881
4.876	5.390	5.051	5.065	4.952	3.682
4.263	3.188	2.895	4.133	4.118	4.358
2.439	2.789	1.821	2.519	3.206	2.499
3.490	3.669	3.595	3.669	3.629	3.307
6.264	6.773	6.470	6.077	6.851	3.859
4.568	4.497	4.725	4.637	4.695	5.170
8.958	9.074	9.153	8.947	9.121	8.603

5.208	5.338	5.185	5.118	5.240	4.810
1.628	2.589	2.013	1.917	2.014	2.125
6.718	6.566	6.538	6.739	6.385	5.001
4.654	4.419	4.672	4.985	4.895	2.723
1.533	2.356	2.232	2.272	2.745	2.885
2.103	1.730	1.943	1.676	1.680	2.482
6.988	7.331	7.053	7.397	7.312	6.776
6.965	6.974	6.872	6.991	7.251	6.929
5.844	6.059	5.845	6.159	6.258	6.896
6.722	6.314	5.896	6.959	6.721	7.739
9.606	10.065	11.094	9.798	9.907	11.053
2.859	3.129	2.299	2.321	2.856	2.405
4.973	4.397	5.541	4.397	4.495	3.953
5.089	5.173	4.680	4.963	4.849	4.604
5.196	5.078	5.860	5.126	5.535	3.210
2.416	1.813	1.813	1.212	1.782	1.685
5.345	4.687	5.071	4.457	4.371	3.904
4.714	4.255	4.938	4.342	4.812	3.719
5.195	4.951	5.260	4.927	4.954	4.663
7.343	7.519	7.585	7.664	7.493	6.554
6.108	6.328	6.018	6.254	6.413	5.616
5.389	5.272	5.158	5.441	5.059	4.872
6.762	6.628	6.454	6.518	6.614	5.120
5.783	5.983	5.989	5.893	5.812	4.498
5.937	6.666	6.308	6.488	6.414	7.582
4.921	3.514	3.936	4.738	4.643	5.165
5.206	5.719	5.336	5.669	5.266	5.890
4.950	5.683	4.992	5.805	5.687	2.419
2.851	3.314	2.908	3.114	3.711	3.900
6.221	6.795	6.440	6.247	6.255	7.340
3.836	4.495	4.417	3.624	4.124	4.138
7.306	7.896	7.468	6.860	7.023	7.833
3.092	2.502	2.760	2.841	2.079	2.567
3.607	4.183	4.060	4.189	4.151	5.604
5.724	5.488	5.349	5.295	5.693	6.579
6.762	6.634	6.740	6.950	6.434	7.633
6.177	6.175	6.053	5.980	5.913	7.830
9.045	8.221	8.298	7.607	7.940	7.547
1.749	1.740	1.715	1.963	2.307	2.560
2.844	3.240	2.837	2.897	2.999	3.967
7.582	7.798	7.744	7.725	7.888	7.143
6.317	5.886	5.935	6.170	5.851	4.817
7.786	7.958	8.016	8.133	7.862	7.747
4.602	4.630	4.114	4.443	4.139	6.721
3.095	2.485	2.607	2.744	2.935	3.204
5.176	5.145	4.768	4.991	4.924	4.144
6.469	6.418	6.402	6.589	6.232	3.878
3.945	4.595	3.994	4.370	3.931	3.241
5.288	5.584	5.558	5.228	5.057	4.727
5.249	5.026	5.170	5.066	5.004	4.496

5.823	5.611	5.828	5.234	5.670	3.924
3.029	2.512	2.887	2.118	2.575	2.803
6.489	6.556	6.584	6.406	6.525	7.089
4.177	4.363	4.504	4.385	4.472	5.139
5.860	6.235	6.238	5.867	6.174	5.191
4.442	3.845	4.424	3.607	3.791	3.480
6.035	7.872	6.813	6.425	7.044	6.248
5.278	4.502	5.167	4.181	4.168	2.867
6.302	6.295	6.672	5.828	6.317	3.946
2.984	2.851	2.938	3.240	2.977	1.864
2.946	3.300	2.492	2.280	3.397	1.242
6.036	6.093	6.127	5.860	5.948	3.505
4.153	4.292	4.571	5.249	5.474	7.254
4.720	4.748	4.644	4.428	4.670	3.800
4.395	4.067	4.325	4.264	4.299	3.379
2.755	2.451	2.970	2.211	2.614	3.518
3.145	3.915	3.955	3.463	3.264	4.765
4.631	4.594	4.680	5.326	4.865	3.698
3.901	3.169	2.754	2.758	3.348	5.179
5.191	5.580	5.062	5.595	5.472	6.300
6.117	5.802	6.054	6.122	5.918	1.967
7.283	7.639	7.354	7.331	7.267	6.438
8.396	8.251	8.403	8.426	8.033	5.773
4.420	4.820	4.705	4.361	4.406	4.203
7.298	6.993	7.262	6.823	7.109	4.997
2.573	3.356	3.834	3.084	3.428	3.339
4.967	4.286	5.159	3.943	4.160	4.720
5.969	6.150	5.570	6.155	6.048	3.133
2.548	3.026	2.453	2.819	1.892	3.591
5.701	5.805	6.066	6.163	5.808	4.858
3.990	3.710	4.036	3.872	4.144	2.195
4.788	4.928	5.015	4.786	4.959	3.985
4.076	3.417	3.389	3.813	3.570	3.989
5.669	5.539	5.671	5.528	5.384	6.407
6.879	6.608	6.501	6.353	6.507	5.881
3.548	3.938	3.470	3.689	3.634	3.603
4.897	5.211	4.854	5.503	5.291	6.201
7.026	7.790	7.663	7.524	7.999	6.900
5.439	5.364	5.330	5.436	5.538	6.516
8.073	8.287	8.140	8.032	8.323	8.511
5.993	5.983	6.221	6.227	6.050	3.205
4.771	4.677	4.673	4.550	4.515	3.754
6.624	7.132	7.080	6.921	6.824	5.841
2.979	4.567	4.871	1.913	3.544	1.593
2.587	2.730	2.531	2.812	2.549	2.729
1.662	2.745	2.768	2.766	2.794	2.043
7.115	7.185	7.261	6.667	6.903	5.308
3.944	4.600	4.428	2.867	3.284	5.610
2.974	2.848	2.615	3.142	3.724	2.419
4.602	4.820	4.536	4.383	4.447	4.689

3.680	4.193	4.356	3.892	4.295	3.249
4.796	4.315	4.199	4.312	4.194	4.938
5.060	5.151	5.104	5.015	5.162	4.380
4.120	3.750	4.422	4.007	3.969	6.904
3.672	4.167	4.013	3.890	3.770	5.477
10.105	10.587	10.597	10.348	10.377	8.911
7.277	6.772	7.250	7.397	7.364	6.264
6.200	6.624	6.447	6.481	6.447	7.213
8.648	8.432	8.261	8.138	8.249	8.527
5.044	4.975	5.071	4.847	5.127	4.412
2.069	2.150	1.843	1.777	2.189	5.421
6.106	5.836	6.029	6.467	5.755	4.083
6.657	6.649	6.631	6.597	6.519	6.122
3.434	3.470	3.448	3.169	3.397	2.711
6.608	6.709	6.789	6.642	6.673	5.071
4.130	4.041	3.458	4.770	3.974	3.370
5.347	4.787	5.521	5.136	5.416	4.107
8.026	7.972	7.727	7.932	8.212	7.139
3.013	2.849	2.730	2.210	3.064	3.003
5.450	5.571	5.716	5.774	6.196	3.481
9.288	9.382	9.334	9.566	9.481	7.819
5.674	5.292	5.493	5.209	5.078	6.231
5.195	5.483	5.258	5.056	5.203	5.618
10.941	10.862	10.974	10.951	10.820	9.304
2.843	3.920	3.425	2.580	3.200	3.871
6.423	6.679	6.384	6.811	6.725	7.642
7.744	7.826	7.749	7.761	7.720	6.351
3.235	2.955	3.596	3.157	2.922	2.124
8.676	8.499	8.574	8.840	8.430	6.729
5.308	5.157	4.963	5.294	5.406	5.132
5.137	5.292	5.020	4.800	4.816	2.120
5.590	5.603	5.602	5.563	5.512	6.099
7.111	7.509	7.642	7.488	7.536	6.680
5.295	5.375	5.179	5.818	5.505	6.073
5.995	6.279	5.851	6.218	5.818	5.307
4.285	4.234	4.380	4.484	5.072	4.114
2.516	2.166	2.569	2.474	2.713	3.126
3.192	2.125	2.380	2.766	1.855	1.456
4.556	5.244	5.217	4.838	4.595	5.090
7.566	7.878	7.704	7.665	7.799	5.595
6.165	5.000	4.914	6.479	5.716	6.924
5.836	6.080	5.110	5.930	5.980	6.194
2.877	3.521	3.021	3.346	2.348	3.414
6.542	6.603	6.382	6.140	6.494	6.236
5.687	6.101	5.801	5.784	5.567	4.842
3.435	3.261	3.207	3.297	4.110	5.392
2.552	3.388	3.225	2.688	2.828	4.278
6.164	6.186	5.778	6.112	6.076	4.780
4.949	5.305	5.032	5.180	5.710	4.426
3.760	3.970	3.517	3.425	3.356	3.057

8.777	8.902	8.838	8.872	9.124	7.405
9.334	9.630	9.581	9.383	9.470	6.566
5.879	6.469	6.041	6.122	6.695	5.811
3.169	3.655	3.816	3.803	4.422	3.605
4.241	4.084	3.730	3.791	4.366	5.539
6.719	7.185	7.251	6.887	7.380	4.673
5.789	5.367	5.779	5.837	5.750	4.497
4.225	3.970	3.684	3.429	4.016	2.677
3.175	3.106	3.375	2.775	2.555	3.844
4.595	4.903	4.817	4.652	4.692	4.014
2.621	3.605	3.060	2.899	2.690	4.335
9.401	9.361	9.469	9.073	9.109	6.808
9.216	8.974	9.070	9.051	8.800	6.493
5.656	5.567	5.575	5.903	5.359	3.809
2.131	2.995	2.364	2.444	2.721	2.698
7.602	7.338	7.423	7.706	7.361	6.523
2.658	3.384	3.515	3.395	3.825	4.573
6.805	6.399	6.438	6.417	6.463	4.971
6.748	6.380	6.508	6.214	6.538	4.984
5.416	5.963	5.534	5.935	5.835	4.349
5.871	5.524	6.108	5.663	6.014	6.863
6.197	6.454	6.521	6.222	6.661	5.029
8.311	8.684	8.591	8.393	8.519	6.876
6.182	6.241	6.160	6.556	6.735	8.485
1.646	1.688	2.036	2.540	1.942	3.190
5.813	4.442	4.985	5.088	5.093	4.283
6.074	5.767	6.086	5.951	6.065	6.190
3.591	4.342	3.878	4.044	3.787	2.591
6.349	6.683	6.433	6.663	6.340	5.341
6.004	6.614	6.231	6.126	6.417	5.082
5.801	6.056	5.815	5.948	6.174	7.685
6.987	6.883	7.169	7.341	7.081	4.317
5.417	4.328	4.912	4.893	5.100	3.410
3.748	3.406	3.517	2.935	3.550	2.646
6.206	6.570	6.189	6.039	5.817	3.936
6.320	5.888	6.492	6.526	6.245	5.583
6.639	6.304	6.587	6.183	6.182	3.349
7.021	6.675	7.156	6.492	6.636	5.384
6.834	7.312	6.299	6.735	7.334	6.758
5.158	5.234	4.894	4.900	5.242	3.915
9.006	9.561	9.567	9.434	9.421	7.942
7.307	6.869	7.170	6.943	7.302	5.085
2.779	3.371	3.606	2.850	3.576	3.397
6.459	6.297	6.043	6.050	5.944	2.301
5.765	5.660	6.095	6.119	5.756	5.059
3.669	3.319	4.150	3.515	4.012	4.181
3.998	3.024	2.798	4.054	3.583	4.833
4.403	4.783	4.948	4.506	4.359	3.199
2.357	3.040	3.243	2.848	3.932	3.313
3.757	4.813	4.036	3.688	4.207	3.555

3.434	3.518	3.590	3.044	3.668	1.578
3.835	4.071	3.906	3.861	3.816	2.158
4.571	4.740	4.319	4.750	4.781	3.640
3.861	4.139	3.836	3.943	4.049	3.615
3.389	2.502	3.727	2.679	3.023	2.975
2.212	3.350	2.452	2.704	2.007	2.620
4.858	5.459	5.385	5.325	5.352	4.567
6.758	6.487	6.197	6.963	6.765	6.302
6.055	5.885	6.016	5.875	6.135	4.833
4.613	5.022	5.282	4.915	4.955	4.111
2.528	3.402	2.784	1.858	2.988	3.807
5.247	4.157	3.721	4.537	4.944	4.600
2.405	2.273	2.072	1.639	2.342	2.377
7.922	8.242	8.136	7.923	8.017	6.200
2.164	2.430	2.714	1.607	1.758	3.523
4.652	4.275	4.628	4.461	4.632	5.487
5.360	5.440	5.233	5.315	5.454	7.056
8.051	7.908	7.998	7.805	7.964	7.334
4.310	4.433	4.569	3.637	4.543	4.186
4.510	4.592	4.779	3.897	4.591	4.408
3.190	3.184	3.135	3.093	3.140	4.117
5.383	5.220	4.854	5.666	5.637	5.585
2.975	2.726	3.379	4.308	2.757	3.024
3.306	2.787	3.268	4.039	3.423	4.471
4.776	3.458	4.458	4.838	3.815	5.457
2.748	2.402	2.380	3.323	2.401	2.748
2.934	2.667	2.461	3.402	2.574	3.663
5.306	4.543	4.991	5.287	4.551	5.324
3.991	2.858	3.138	4.025	4.023	4.412
4.720	3.212	4.097	4.916	4.361	4.827
4.827	5.399	5.408	5.471	5.750	7.111
4.635	5.156	4.888	5.056	5.364	5.501
5.793	5.800	6.060	5.821	5.932	5.779
6.519	6.668	6.848	6.418	6.451	4.777
4.326	4.349	4.366	4.340	4.345	4.369
6.321	6.359	6.656	6.188	6.638	5.109
5.641	5.637	5.571	5.432	5.662	5.903
6.571	7.061	6.768	6.704	6.811	5.278
5.372	4.813	5.162	5.156	5.074	4.340
5.130	5.440	5.661	5.654	5.577	4.303
6.169	5.826	5.490	5.907	5.877	1.652
5.958	6.210	5.716	6.177	6.047	5.071
2.760	2.259	2.310	2.347	2.552	2.272
4.805	3.369	3.060	3.396	4.093	2.536
3.821	3.835	3.281	3.739	3.890	3.236
2.662	3.029	2.823	2.396	2.715	3.797
5.551	5.494	5.553	5.600	5.466	5.572
3.613	3.300	2.996	3.203	3.959	3.734
5.784	5.953	6.427	5.862	5.666	5.020
4.428	3.558	3.866	4.127	3.278	4.070

3.514	3.789	3.461	3.211	3.028	3.470
2.546	1.898	1.794	2.260	2.248	3.035
5.167	4.720	4.715	4.321	4.599	2.946
6.226	5.908	6.085	6.391	6.054	7.117
1.993	3.024	2.270	2.500	2.651	3.017
6.009	5.638	5.894	5.988	6.045	7.169
3.910	4.417	4.268	3.771	3.878	3.955
3.952	3.715	3.335	3.999	3.731	3.882
3.972	3.638	3.916	4.528	2.833	2.981
5.262	5.511	5.319	5.408	5.561	4.660
4.744	5.182	4.925	5.112	5.310	6.453
2.897	2.269	2.487	2.317	2.494	2.284
2.616	2.243	2.878	3.210	3.451	2.782
2.055	2.136	2.982	1.637	3.131	3.651
2.157	3.272	2.681	2.786	3.027	1.207
2.500	2.621	2.464	2.661	2.792	2.500
5.842	6.105	6.191	5.683	5.951	5.425
4.991	5.046	4.960	4.948	5.048	4.861
3.314	3.962	3.642	3.028	3.577	2.959
2.965	2.952	3.157	2.668	2.993	2.486
3.483	3.812	3.753	3.893	4.145	4.191
5.166	5.321	4.702	4.995	4.379	4.081
6.267	5.622	5.600	5.510	5.208	5.139
2.344	1.656	2.399	1.596	1.707	1.651
2.274	1.860	1.994	1.920	2.052	1.824
2.748	1.914	2.620	2.660	2.108	2.699
3.822	2.763	4.180	5.682	2.318	5.430
5.405	5.113	5.231	5.425	5.531	6.439
6.408	6.379	6.472	6.579	6.587	5.338
6.305	6.129	6.109	6.424	6.161	4.335
6.138	5.888	6.064	5.917	6.105	5.532
2.750	1.822	2.302	2.294	1.819	3.109
4.332	4.146	4.279	3.862	4.029	4.918
5.787	5.346	4.954	5.055	5.069	4.787
3.303	3.794	3.303	3.480	3.533	3.625
3.051	2.216	1.819	1.700	2.570	3.138
4.410	4.505	4.363	4.050	4.059	2.582
4.525	4.490	4.323	4.745	4.946	6.081
8.564	8.166	8.379	8.077	8.278	2.149
5.831	5.988	5.743	5.980	5.792	5.253
1.730	1.857	1.965	1.898	2.278	2.083
4.259	4.794	4.584	4.993	4.970	2.592
4.496	4.390	4.067	4.197	4.686	4.112
2.938	2.803	2.993	2.553	2.907	3.666
7.874	8.791	8.087	8.863	9.353	11.232
4.848	5.586	5.626	5.615	5.622	5.215
1.713	1.733	2.042	1.402	1.973	2.129
2.235	2.240	2.598	1.988	2.330	2.501
7.344	7.665	7.826	7.600	8.142	6.554
3.243	3.738	3.419	3.195	3.731	2.078

6.500	7.437	5.738	6.463	6.508	5.701
3.600	3.272	3.712	4.104	4.279	5.454
4.995	4.686	4.874	4.368	4.763	5.649
4.727	3.882	3.840	3.530	3.865	3.619
7.589	7.515	7.638	7.195	7.293	7.089
1.979	1.355	1.746	2.279	1.558	2.248
4.288	3.929	4.587	4.790	4.403	5.266
3.839	3.835	3.621	2.623	3.323	2.862
5.672	6.513	5.818	5.996	5.828	6.767
6.112	6.121	5.760	5.688	5.709	6.027
5.007	4.508	4.797	4.036	4.721	4.090
2.838	2.526	2.596	2.478	2.499	2.903
6.219	5.428	6.241	6.106	5.961	5.505
4.570	4.953	5.305	4.201	4.919	3.826
2.888	3.241	2.943	2.693	3.781	3.523
4.418	4.996	4.247	4.840	5.020	4.464
4.239	2.821	2.949	3.154	3.456	3.163
6.205	4.853	5.051	4.843	5.265	4.723
5.820	6.306	5.444	6.202	6.409	7.518
4.243	4.139	4.512	4.292	4.715	5.996
4.242	3.841	4.346	4.322	4.642	4.146
2.260	2.451	2.810	3.313	2.865	2.119
4.311	4.452	4.178	4.215	4.341	4.746
3.133	3.909	2.974	4.248	4.447	4.362
2.543	2.011	3.757	2.986	3.015	1.851
7.010	6.800	7.162	6.964	6.852	4.366
4.067	2.869	3.009	4.490	2.971	2.452
4.428	4.289	4.206	3.966	4.277	2.835
3.916	3.979	3.572	3.897	4.195	3.451
4.438	4.521	4.480	4.516	4.516	4.135
4.317	4.387	4.447	4.480	4.556	3.688
5.625	5.998	5.775	5.657	5.593	4.779
5.340	5.873	5.820	6.259	5.912	6.359
5.113	4.647	4.924	4.950	4.807	2.262
3.176	4.005	3.788	3.024	4.495	3.145
2.417	3.435	3.647	2.974	2.928	3.342
2.675	3.023	3.440	2.852	3.120	3.599
6.423	6.307	6.921	6.700	6.403	6.136
4.370	4.444	4.140	5.181	5.235	5.662
5.217	5.144	4.525	5.063	5.470	4.743
6.744	6.256	5.894	5.596	5.465	6.306
7.120	7.170	7.060	6.848	7.193	7.794
7.725	7.561	6.873	7.025	7.130	7.774
3.579	4.378	3.681	4.667	4.226	5.900
7.156	6.422	6.879	6.204	7.436	6.589
6.449	6.691	6.872	6.859	6.516	5.689
4.243	4.049	4.625	4.837	4.962	5.960
6.105	5.646	5.406	5.744	5.759	8.198
2.897	3.399	3.717	2.731	2.854	3.049
2.607	3.392	3.083	2.388	2.485	2.832

5.799	5.478	5.714	5.315	5.388	5.052
5.000	5.022	4.439	4.650	4.252	4.544
4.015	4.450	5.087	4.295	4.529	4.261
2.206	2.559	3.046	3.394	2.996	3.528
6.104	5.984	5.883	5.936	5.932	7.107
7.013	6.940	6.917	6.932	6.786	6.216
3.767	4.200	3.726	3.495	4.094	3.888
3.339	4.140	3.819	2.755	2.925	4.606
5.205	5.029	4.882	4.387	4.534	4.836
2.986	3.942	4.511	3.176	3.474	4.115
5.956	4.910	5.253	5.828	5.106	7.061
4.935	4.586	4.553	3.993	4.577	4.370
2.749	3.252	2.781	2.220	2.296	2.748
2.677	3.483	3.962	2.640	3.699	4.411
1.661	1.968	1.900	1.293	1.732	1.345
4.411	4.692	4.933	4.720	4.983	4.517
7.748	6.733	6.381	7.044	6.568	6.054
4.694	3.980	3.764	4.032	3.274	3.400
6.088	6.483	6.162	6.293	6.511	5.167
2.654	3.382	3.703	2.428	2.866	3.775
4.127	4.287	4.025	4.242	4.239	5.190
1.918	2.068	2.244	2.194	1.973	2.324
3.617	4.409	4.018	4.381	4.452	5.376
5.424	5.960	5.628	6.103	6.725	7.815
3.023	3.231	3.588	2.660	2.554	3.528
4.525	4.153	5.420	4.639	4.513	4.312
2.861	2.863	3.521	2.789	3.005	3.431
4.440	4.738	4.846	4.987	4.811	6.305
2.944	2.289	2.620	1.789	2.413	2.699
3.207	2.918	3.507	3.561	3.709	4.348
6.703	6.997	6.688	6.644	6.797	6.004
6.095	6.421	6.476	6.556	6.381	5.313
4.431	4.371	4.101	4.361	5.044	5.637
2.769	3.495	3.785	3.686	3.586	4.463
4.265	3.403	3.993	3.194	3.302	1.860
2.918	3.520	3.840	2.950	3.248	4.261
2.344	1.939	2.510	2.616	2.140	2.925
8.389	8.379	8.296	8.037	8.062	6.692
6.500	6.659	6.409	6.613	6.535	4.164
5.637	5.440	5.436	5.417	5.406	2.781
4.104	4.399	4.046	4.140	3.926	4.951
1.434	1.480	1.564	1.898	1.955	4.221
3.241	3.512	3.135	3.133	3.397	5.000
4.019	4.427	4.647	4.042	4.342	5.938
5.316	5.833	5.532	6.725	6.563	7.706
5.350	5.587	5.390	5.683	5.699	4.271
7.524	7.795	7.727	7.550	7.591	7.143
7.071	7.770	7.445	7.464	7.581	6.831
4.918	5.427	5.331	5.307	5.259	6.334
2.898	2.613	2.683	3.095	2.670	1.657

5.708	6.082	5.675	5.079	5.226	2.349
3.383	2.889	3.637	2.519	3.483	3.484
4.714	4.566	5.216	4.569	4.302	3.386
5.498	5.145	5.193	4.783	5.273	4.264
5.705	6.165	5.925	5.767	5.803	6.371
6.714	6.551	5.864	6.862	6.907	8.492
5.154	5.707	4.906	5.461	5.480	5.885
2.670	3.868	3.488	3.774	3.724	2.898
2.875	3.423	3.152	3.752	3.240	2.910
6.384	6.438	6.448	6.182	6.207	5.444
2.791	2.733	2.724	2.505	2.502	2.885
3.152	3.499	3.325	2.738	3.264	4.782
4.564	5.332	5.281	4.261	4.174	5.897
5.150	5.528	5.314	5.530	5.386	4.780
7.141	6.755	6.614	6.705	6.632	5.403
4.526	4.404	5.068	4.702	5.081	3.768
3.470	3.782	3.912	3.688	4.512	3.281
4.411	4.801	4.398	4.418	4.508	5.473
7.356	6.958	7.291	7.264	7.062	6.426
6.397	6.573	6.824	6.807	6.555	5.289
4.999	5.908	5.149	4.823	5.074	4.125
6.214	6.293	6.059	5.827	6.280	4.355
3.816	3.753	3.530	3.777	3.669	2.760
4.681	4.677	5.039	4.708	5.139	5.500
6.445	5.975	5.821	5.923	5.905	4.738
3.059	3.236	3.469	2.725	3.355	2.706
4.056	5.117	5.165	4.188	4.733	3.868
3.885	4.026	4.319	4.429	4.782	6.320
3.386	3.566	3.708	3.599	3.958	4.633
3.578	3.318	3.816	3.030	3.348	2.612
3.373	3.943	3.936	4.199	3.708	4.348
3.476	3.499	3.672	3.145	3.206	2.589
4.649	3.978	4.777	4.696	4.302	3.750
2.841	3.265	3.236	3.373	3.676	3.676
5.157	4.779	5.052	4.854	4.817	3.587
4.603	5.148	5.250	4.979	4.955	3.341
6.031	6.139	6.477	6.219	6.146	5.523
5.295	5.147	5.094	5.005	5.141	4.231
2.556	2.823	3.212	2.178	2.228	2.694
4.724	5.229	5.617	4.664	5.047	7.387
3.120	3.285	3.345	2.810	3.828	3.407
5.300	5.184	5.702	5.754	5.050	4.698
2.639	1.952	1.833	2.655	3.172	2.952
6.189	6.796	6.956	6.378	6.112	5.166
5.702	5.680	5.824	5.917	5.975	4.864
4.701	4.831	4.903	5.216	4.823	5.868
4.356	3.844	3.493	3.940	3.319	3.500
2.635	2.538	3.013	2.627	2.877	3.976
2.688	2.463	2.606	2.373	2.057	2.769
2.373	2.466	3.058	2.350	2.819	2.156

5.808	5.618	5.638	5.983	5.690	5.616
7.957	7.572	7.615	7.434	7.540	6.125
4.721	5.007	5.502	5.190	5.065	4.527
1.901	1.913	2.225	1.694	2.233	1.805
6.052	6.395	6.170	6.882	6.814	9.235
6.324	6.357	5.987	6.309	6.379	5.749
7.414	7.942	7.511	7.250	7.467	6.641
4.878	4.419	4.873	5.121	4.375	4.324
3.233	3.889	3.651	3.360	3.225	3.065
2.076	2.182	2.203	1.614	1.855	2.144
2.735	3.095	2.806	2.981	3.658	2.795
4.102	3.490	4.247	4.347	4.247	3.260
3.642	4.216	3.627	3.526	2.716	2.675
6.293	6.560	6.318	6.688	6.698	7.625
2.810	3.430	3.216	3.245	2.833	3.709
5.604	5.846	5.528	5.664	6.065	7.405
5.496	5.241	5.121	6.180	6.514	7.477
4.298	3.434	3.937	4.165	4.031	5.783
3.345	3.701	3.974	3.975	3.434	4.565
7.910	7.828	7.747	8.076	7.642	7.599
9.462	9.572	9.503	9.651	9.435	8.338
3.104	3.694	3.786	2.032	2.192	3.230
3.850	3.603	2.848	3.523	3.817	4.030
4.696	5.146	5.411	5.042	5.093	5.673
4.028	4.167	4.438	3.257	4.358	4.293
2.462	0.919	3.440	3.090	3.341	2.350
7.084	6.694	6.682	6.572	6.476	5.572
5.084	5.555	5.356	5.879	6.140	7.128
7.053	7.278	7.369	7.140	6.955	6.660
5.368	5.741	5.333	6.357	6.166	6.940
6.565	6.896	6.601	6.679	6.418	5.771
7.830	8.273	8.021	8.199	8.324	7.171
3.889	3.801	3.382	4.334	4.104	6.929
3.861	3.931	4.188	3.481	4.214	4.447
4.969	4.643	3.984	3.879	4.063	3.955
3.853	3.426	3.506	3.717	3.633	3.138
1.333	1.855	1.978	1.687	1.830	2.048
4.607	4.617	4.821	4.157	4.398	2.345
4.457	4.978	4.876	4.681	4.466	4.068
3.742	3.347	2.892	3.050	2.944	2.969
3.507	2.551	3.686	2.896	2.739	3.271
3.484	3.598	3.883	3.488	3.675	2.876
4.292	4.294	4.679	3.945	4.115	3.349
5.859	5.136	4.703	4.219	4.884	5.516
2.631	2.907	1.815	1.855	2.078	3.404
3.416	3.392	3.441	2.982	3.641	3.178
5.008	4.965	4.613	4.465	4.724	5.279
6.266	6.242	6.202	5.314	5.635	6.043
7.214	7.138	7.134	6.935	7.288	8.213
4.238	3.424	3.754	4.130	4.002	2.910

3.531	5.406	4.473	4.192	4.093	4.635
4.056	3.441	3.828	3.791	3.037	3.504
2.607	2.431	2.246	2.072	2.670	2.157
1.832	2.815	2.426	1.559	1.826	2.351
6.860	6.915	7.342	7.077	7.116	7.221
2.490	1.907	2.080	1.529	1.625	2.274
3.339	2.757	3.562	2.920	3.341	2.233
3.652	3.134	2.500	3.953	3.222	1.656
4.316	3.856	3.971	4.356	4.505	6.256
3.974	3.468	3.926	3.744	4.086	4.867
4.811	4.347	4.659	4.726	4.626	5.472
4.043	4.154	4.166	4.368	4.166	3.546
4.146	4.376	4.427	4.678	4.471	3.767
4.328	4.203	4.229	4.555	4.440	3.797
4.110	4.295	4.291	4.596	4.607	3.782
3.619	3.844	3.985	4.168	4.197	4.156
4.951	4.263	4.497	4.624	4.523	4.860
1.728	2.813	2.249	2.471	2.695	3.106
6.508	6.399	6.729	6.378	6.412	5.323
5.858	5.541	5.695	5.324	5.957	6.746
7.025	7.336	7.130	6.938	7.117	6.294
7.936	7.737	7.695	7.543	7.508	6.116
5.249	4.915	5.158	4.560	4.645	3.949
7.016	7.177	7.219	7.140	7.271	6.467
6.656	7.296	7.121	6.777	7.056	6.359
7.783	7.962	8.143	8.284	8.270	6.371
3.958	4.560	4.317	4.384	4.580	4.328
4.321	4.993	4.788	4.075	4.840	4.574
3.167	3.224	3.431	3.453	3.679	3.608
3.266	4.280	3.601	3.783	3.687	4.453
6.035	6.127	6.356	6.432	6.980	8.065
4.463	4.490	4.540	4.372	3.875	4.212
3.675	4.161	3.641	3.722	3.949	5.013
2.852	3.063	3.099	3.012	3.412	2.648
2.937	3.123	3.057	3.080	4.193	5.110
5.549	5.128	5.111	5.798	5.457	6.535
6.146	6.087	5.896	6.038	6.258	6.185
3.206	3.710	3.054	2.960	3.472	2.449
4.324	4.529	5.046	4.371	4.805	4.008
4.238	3.452	2.915	4.038	3.648	3.462
7.184	7.503	7.160	7.041	6.873	5.642
9.350	9.112	9.418	9.401	9.683	8.996
6.762	6.497	6.834	6.512	7.760	8.664
8.364	8.367	8.519	8.342	8.212	7.138
2.592	2.765	2.939	2.690	2.891	3.065
3.792	2.469	1.709	2.377	3.228	1.628
1.693	1.814	2.079	1.589	3.010	1.830
5.049	5.249	4.943	4.381	4.794	4.771
7.056	6.710	5.945	6.235	6.550	7.873
6.036	6.084	6.417	6.030	5.514	4.588

3.177	4.285	2.930	3.458	4.113	4.141
3.280	3.917	4.208	3.759	3.258	1.436
3.786	4.166	4.130	3.775	3.904	3.841
3.631	4.169	3.702	3.079	3.172	4.197
2.150	2.313	1.957	2.058	1.931	2.856
3.837	4.163	4.035	3.999	3.824	3.046
4.195	4.530	4.567	4.416	4.732	5.464
2.460	2.121	2.540	2.867	2.453	2.921
2.491	2.090	2.012	1.964	1.888	2.247
2.658	3.274	2.225	2.258	1.190	3.458
6.790	6.587	6.714	7.044	7.023	7.931
1.836	2.660	2.114	1.501	2.423	2.183
2.337	2.374	3.199	2.709	2.745	3.100
6.508	6.031	5.921	6.597	6.101	5.216
5.124	5.359	5.359	5.374	5.397	4.984
3.505	2.794	3.200	2.243	2.361	2.802
7.598	8.092	7.862	7.908	7.901	6.780
3.400	3.043	3.606	2.783	2.824	2.819
4.182	3.027	3.950	3.478	3.225	3.680
3.276	3.042	2.892	3.083	3.832	3.276
5.416	6.018	5.680	5.618	5.860	3.763
2.969	3.328	2.996	2.483	2.338	2.146
1.626	1.833	1.777	1.219	1.592	1.983
2.988	2.916	4.059	2.335	2.828	3.026
3.143	2.915	2.836	3.058	2.594	2.828
3.013	3.611	3.452	3.703	3.434	3.583
2.431	2.626	2.635	2.258	3.252	2.946
2.487	2.777	2.119	2.871	2.740	2.858
3.045	2.483	3.108	2.130	2.948	2.585
4.258	4.440	4.754	3.789	4.420	5.397
1.714	2.044	1.888	1.345	1.808	2.352
5.224	5.000	5.513	5.428	5.070	3.430
1.236	1.813	1.487	1.902	1.712	1.369
0.820	3.163	2.963	3.875	2.792	2.507
4.026	6.327	5.142	6.532	4.422	5.436
1.730	2.210	2.327	1.976	2.351	1.969
2.872	2.436	2.731	2.766	3.169	3.237
6.607	6.742	6.848	6.704	6.775	3.117
3.966	4.027	4.829	4.949	4.315	2.113
5.155	4.262	4.293	4.259	3.982	3.950
5.553	5.224	4.656	5.739	5.711	6.999
3.888	3.267	3.856	3.773	3.107	2.112
2.452	3.070	3.341	2.952	4.594	3.079
5.220	5.004	5.309	4.842	5.672	6.198
6.728	6.934	6.983	6.789	6.856	6.121
4.887	5.331	5.371	4.824	5.038	4.308
4.353	4.714	4.923	4.144	4.476	3.750
8.962	9.538	9.278	9.250	9.231	7.833
4.737	4.842	4.324	3.688	3.998	5.205
4.831	4.463	5.141	4.148	4.767	4.537

3.385	3.088	2.758	2.543	2.872	2.927
5.352	5.138	5.376	5.482	5.504	5.323
4.298	4.620	4.625	4.611	4.518	5.670
2.961	3.152	3.115	4.339	3.907	4.687
3.163	2.429	3.376	1.903	2.541	3.403
4.060	3.584	3.559	4.462	4.404	5.131
4.611	4.840	4.743	4.515	4.549	5.084
3.300	3.301	2.413	3.715	3.851	5.052
3.627	3.680	4.208	3.728	4.125	4.662
2.885	3.573	2.875	3.048	2.406	4.255
7.271	6.935	6.819	7.473	7.034	6.379
6.835	6.468	6.628	6.743	6.437	5.683
2.686	2.703	2.734	2.462	2.930	2.825
4.969	4.704	4.403	4.371	4.666	4.131
4.721	4.513	4.946	4.215	4.968	5.836
3.820	4.507	4.011	3.756	4.042	3.483
5.153	5.030	5.529	4.904	5.253	4.661
4.019	4.673	4.382	4.154	4.580	4.340
5.226	5.780	5.940	5.083	5.378	4.369
4.149	3.392	4.090	4.023	3.957	3.183
2.251	2.937	2.979	2.930	2.642	3.981
4.712	4.932	4.796	3.926	4.794	2.917
5.569	5.859	5.633	5.489	5.935	3.998
3.023	3.519	3.123	2.768	3.297	2.160
6.482	6.904	6.234	6.951	7.163	8.220
4.086	4.365	3.921	3.508	3.596	4.164
2.859	3.309	3.134	3.642	3.863	4.678
6.093	5.684	5.876	6.064	5.933	7.056
1.534	1.557	2.638	2.768	2.897	4.900
5.790	6.232	6.075	5.833	6.056	4.830
6.364	7.107	6.907	6.597	6.844	6.278
5.595	5.436	5.557	5.551	5.634	4.748
5.223	4.813	4.849	4.400	4.467	4.000
6.672	7.102	6.865	7.030	6.652	5.578
4.293	3.744	3.973	3.655	3.985	5.225
3.134	3.335	2.585	3.530	2.682	3.879
2.036	2.493	1.982	1.897	2.279	2.880
4.837	5.797	5.232	5.534	5.078	5.772
5.391	5.362	5.059	5.478	4.835	4.348
3.151	2.983	3.089	2.389	3.440	3.263
2.257	2.823	3.153	2.888	2.729	3.277
8.751	8.267	8.659	8.950	8.848	5.727
4.632	4.361	4.180	4.266	4.090	3.219
4.225	4.634	4.551	4.787	4.984	6.566
5.693	5.644	5.708	5.071	5.411	4.507
3.806	2.892	3.463	3.420	3.773	3.419
4.583	4.959	3.918	3.861	4.425	3.779
5.663	5.868	6.152	5.413	5.479	6.047
5.870	5.961	5.788	5.880	5.910	5.137
4.882	5.087	4.746	4.932	4.799	5.273

7.866	7.993	7.857	8.246	7.995	6.534
2.191	2.026	1.602	1.721	1.742	3.519
4.638	5.344	4.453	4.598	4.838	4.193
2.867	3.290	3.573	3.575	2.915	3.994
3.636	3.866	4.741	3.582	4.544	3.653
8.306	8.350	8.383	8.227	8.040	6.564
8.368	8.247	8.325	8.126	8.047	7.065
8.386	8.227	8.548	8.457	8.632	7.490
5.254	5.156	5.551	5.557	5.568	4.216
3.006	2.294	2.112	3.185	2.746	2.492
3.190	2.354	1.482	3.579	3.697	3.987
5.175	5.231	5.165	5.779	5.766	6.861
4.023	4.494	4.726	4.046	4.377	3.168
5.639	5.996	5.905	5.751	5.943	5.032
8.035	8.343	8.018	8.001	8.300	7.254
4.544	3.850	3.835	4.111	4.151	5.411
6.448	6.900	6.515	6.616	6.766	6.309
6.461	6.157	6.635	6.551	6.343	6.099
3.510	3.225	3.320	4.005	3.686	3.417
3.678	3.799	3.190	3.268	3.494	3.339
4.082	3.611	3.935	3.966	3.655	4.116
2.914	2.351	2.549	2.546	2.230	2.177
6.868	6.752	6.676	6.940	6.919	5.526
2.089	2.473	2.279	2.631	2.334	1.912
4.741	4.473	4.951	4.222	4.533	5.140
3.403	3.295	3.152	3.409	3.446	3.442
5.087	5.618	5.229	5.507	5.136	4.736
5.467	6.291	5.736	5.878	5.706	4.663
5.485	5.768	5.532	5.474	5.100	5.214
2.582	2.360	2.582	2.503	2.904	2.740
3.903	3.990	4.206	4.288	4.030	2.913
4.848	4.586	4.751	4.616	4.451	5.173
7.044	6.656	6.819	6.752	6.536	5.801
6.302	6.431	6.570	6.421	6.552	5.162
4.730	4.875	4.800	4.524	4.512	1.750
5.012	5.780	5.248	5.164	5.370	5.042
2.973	3.071	3.133	3.479	2.762	4.360
5.130	5.308	4.779	5.261	5.157	6.268
7.589	7.756	7.605	7.880	7.807	8.044
4.039	4.102	3.949	4.791	4.861	5.950
3.341	3.348	3.282	3.704	3.636	5.363
3.804	3.053	3.675	3.850	3.378	6.998
5.194	4.471	4.496	5.043	4.215	5.214
3.806	2.661	3.355	4.030	3.472	3.980
4.631	4.835	5.087	5.266	5.500	4.584
4.895	5.471	2.594	2.790	3.344	2.867
3.337	1.436	2.570	3.908	3.468	2.283
6.738	1.474	6.498	6.599	6.738	1.384
5.117	5.020	5.105	5.256	5.426	6.071
6.418	6.644	6.531	7.191	7.334	8.618

7.286	6.966	6.918	7.099	7.050	7.055
3.068	2.379	2.910	1.895	2.105	1.537
2.038	2.451	2.132	2.305	3.041	2.237
2.214	2.548	2.093	2.099	2.045	2.611
4.758	4.670	4.616	4.428	4.049	2.918
2.342	1.632	2.310	1.893	1.922	2.216
7.056	7.719	7.163	6.634	6.963	7.193
4.144	5.242	4.731	3.961	4.341	5.343
4.853	4.499	3.887	4.610	4.002	4.544
7.290	7.188	7.587	7.270	7.237	6.281
5.364	4.583	4.575	3.870	4.863	3.463
4.906	5.519	5.088	4.679	5.311	4.322
6.895	6.797	6.886	6.900	6.980	5.125
4.315	4.544	4.033	4.340	4.581	3.812
3.809	2.881	4.097	3.520	3.252	4.663
5.549	1.692	5.212	5.580	5.362	1.537
5.217	1.913	4.925	5.394	5.134	1.549
5.833	6.181	6.007	5.703	5.960	5.625
8.357	7.299	6.906	7.678	7.679	8.961
4.258	4.307	4.464	4.187	4.311	4.841
3.761	4.389	3.958	4.178	4.258	3.730
2.251	3.432	2.509	3.870	3.887	4.535
7.458	7.641	7.774	6.412	7.067	8.095
2.590	2.817	2.817	3.250	2.931	2.546
4.794	4.645	5.117	4.963	4.805	4.231
2.872	2.569	2.533	3.056	3.158	2.169
4.641	4.217	4.087	4.477	4.652	5.578
6.178	5.900	6.110	6.075	6.005	5.152
3.704	4.493	3.769	3.948	4.052	4.883
3.608	4.116	3.816	3.806	4.005	5.244
5.897	5.240	5.899	5.774	6.352	5.418
5.624	5.327	5.209	5.159	5.396	5.512
6.120	6.051	5.300	5.998	5.578	7.298
6.241	6.043	5.978	6.170	6.208	5.686
6.258	6.852	6.579	6.590	6.078	5.408
6.969	7.239	6.956	7.095	7.208	5.982
3.203	2.043	3.610	3.439	2.382	2.748
7.201	7.450	7.299	6.966	7.384	4.833
5.238	5.538	5.465	5.203	5.629	3.358
4.986	4.930	4.969	4.858	5.170	4.014
1.732	2.834	1.420	2.085	2.758	2.526
7.018	7.455	7.459	7.471	7.416	7.017
4.127	3.759	4.103	4.020	3.737	3.451
8.183	8.104	8.152	7.775	8.143	7.383
8.004	8.041	8.069	8.031	8.041	7.463
3.509	3.956	3.887	3.692	3.706	3.759
5.143	4.695	5.267	4.811	4.906	4.661
3.038	3.050	3.576	3.070	3.102	3.190
5.545	5.651	5.513	5.504	5.547	4.672
7.756	7.576	7.805	7.699	7.795	7.106

5.327	5.986	5.509	5.485	5.518	4.942
3.814	2.960	3.324	3.955	3.823	3.519
2.643	3.334	3.321	1.731	2.452	3.708
6.323	6.279	6.151	6.784	6.738	6.986
6.271	6.545	6.625	6.181	6.454	5.221
3.507	2.153	2.708	2.993	2.784	4.226
4.918	4.654	4.996	5.302	4.703	3.961
7.205	8.124	7.942	8.540	8.491	10.514
7.022	7.532	6.962	6.857	7.177	6.000
5.890	5.623	5.780	5.504	5.299	4.471
3.843	3.395	3.518	4.356	3.684	3.951
3.801	4.785	4.371	4.655	5.095	5.955
2.021	2.458	2.409	3.624	2.395	2.469
4.306	4.859	4.622	4.657	5.214	6.159
8.299	8.045	8.187	8.286	8.222	5.196
7.837	8.187	7.856	7.761	8.121	5.040
2.890	2.904	3.245	3.318	3.360	4.271
6.050	5.881	5.933	5.682	5.767	2.532
5.633	5.399	5.805	5.323	5.292	6.862
5.296	5.156	5.404	5.526	5.610	6.419
2.617	1.995	2.327	2.635	2.413	2.845
6.357	6.453	6.512	6.343	6.664	3.644
7.207	6.859	7.168	7.284	7.013	5.947
7.911	7.607	7.796	7.969	7.731	6.459
3.013	4.019	3.695	3.566	3.640	4.178
3.457	3.801	3.916	4.255	3.811	4.289
6.130	6.775	6.817	6.520	6.307	5.342
4.592	4.458	4.406	4.213	3.639	3.277
3.669	4.644	4.292	4.340	4.083	4.140
4.694	3.984	4.302	4.196	4.225	3.913
8.974	9.334	9.175	9.224	9.285	8.409
4.885	5.019	5.430	5.189	5.232	4.016
4.336	4.730	4.316	4.522	4.748	4.112
6.538	7.296	7.159	6.797	6.952	6.487
2.328	2.035	2.042	2.627	2.383	3.982
8.058	8.533	8.271	8.284	8.353	7.298
5.462	4.523	4.475	4.420	4.735	4.589
5.543	4.620	4.556	4.520	4.724	4.560
5.397	4.431	4.433	4.197	4.529	4.236
5.367	4.612	4.666	4.482	4.394	4.717
7.459	7.270	7.282	7.505	7.438	6.296
4.569	4.538	4.775	4.844	4.714	3.165
5.244	4.776	4.963	5.350	4.672	4.423
7.539	8.154	7.859	7.875	7.985	7.865
5.212	3.555	4.151	4.368	4.158	3.141
6.388	6.735	6.736	6.933	6.579	6.076
4.487	4.820	4.777	5.090	4.822	5.986
3.673	4.111	3.707	4.043	4.233	2.827
6.508	6.410	5.802	6.220	6.202	6.939
6.987	6.733	6.531	7.078	7.161	8.522

7.134	2.122	7.021	7.269	7.148	2.233
3.046	2.893	3.422	3.291	3.414	2.949
5.652	5.971	6.065	5.693	5.640	5.154
6.297	6.242	5.956	6.186	6.356	7.453
1.527	1.354	1.315	1.467	1.669	1.553
4.842	0.535	4.260	4.962	4.453	0.891
4.176	3.742	3.580	4.136	3.296	4.136
8.327	8.814	8.208	8.303	8.773	8.430
8.542	8.155	8.536	8.482	8.439	7.811
6.807	6.988	6.696	7.196	7.098	8.090
6.906	7.203	6.800	6.799	6.900	6.301
4.881	4.465	5.022	4.347	4.260	4.902
3.660	3.878	4.205	3.387	3.362	4.611
4.333	4.298	3.898	4.297	4.059	5.579
4.384	5.008	4.778	4.817	5.137	5.814
5.032	4.998	4.913	4.833	5.167	5.500
5.799	5.639	5.636	5.295	5.709	7.050
7.163	7.838	7.575	7.608	7.818	8.328
3.503	4.385	4.438	3.475	3.904	4.715
3.001	3.185	2.735	3.042	3.498	2.757
2.314	2.405	2.515	2.405	3.308	2.677
5.474	3.612	3.142	4.583	4.558	5.294
5.943	5.334	5.621	6.259	5.805	7.612
1.198	1.132	0.767	1.048	1.244	1.203
6.714	6.071	5.888	6.153	6.072	6.062
6.813	7.167	7.316	7.285	7.064	6.427
4.610	4.914	4.623	4.628	4.624	4.109
3.447	3.767	3.234	3.708	3.310	3.221
9.025	9.273	9.130	9.254	9.125	8.676
4.963	4.308	4.837	4.581	4.988	4.005
2.689	3.433	3.894	2.671	2.961	3.195
5.297	5.589	5.684	5.794	5.765	6.684
4.506	5.395	5.486	4.779	5.522	4.086
2.443	2.354	2.594	2.458	2.245	2.883
5.942	5.567	5.714	5.336	5.561	5.874
4.277	4.257	4.725	3.769	3.883	4.771
5.390	4.576	4.934	5.326	5.679	2.765
4.839	5.361	5.197	5.088	5.314	2.529
3.230	4.057	3.979	4.094	3.905	5.126
2.351	2.031	2.202	2.061	2.289	2.180
4.321	4.551	4.561	4.815	4.342	4.572
2.410	1.740	1.848	2.306	1.639	1.511
2.636	1.672	2.111	2.772	2.391	1.849
6.351	6.252	6.155	6.242	6.390	5.798
7.229	6.992	7.139	7.251	7.046	5.806
4.295	4.737	4.687	4.754	4.893	4.268
5.565	6.358	5.961	5.749	5.808	5.402
9.189	9.569	9.573	9.303	9.445	8.637
6.857	6.662	7.094	7.143	6.772	6.218
2.887	2.334	2.645	2.555	2.932	3.036

4.109	3.949	3.844	3.965	3.983	3.519
3.010	3.291	3.137	4.116	3.502	5.155
3.070	4.130	2.688	3.380	2.941	2.016
5.901	5.361	6.503	4.607	5.025	5.687
4.097	4.230	4.483	4.100	4.303	2.266
8.547	8.374	8.280	8.098	8.306	4.615
5.822	5.797	5.392	5.535	5.482	2.083
6.621	6.573	6.811	6.357	6.973	6.033
6.343	6.202	6.559	6.019	6.405	5.262
3.136	3.562	3.327	3.066	3.698	2.762
2.181	2.084	2.586	2.676	2.375	2.586
7.349	7.502	7.604	7.437	7.516	6.776
4.378	3.028	4.365	4.454	2.391	4.870
2.109	1.160	1.673	1.550	1.840	1.586
4.016	3.091	3.565	2.447	2.827	4.315
4.073	2.562	3.140	2.673	2.735	3.625
4.345	5.052	4.613	3.957	4.846	5.526
4.625	3.929	4.594	4.889	4.311	4.373
4.967	5.236	5.820	4.876	5.319	5.492
1.550	1.442	1.382	1.522	1.394	2.076
3.578	3.879	3.966	4.540	4.302	2.514
3.699	3.353	3.837	3.855	3.679	3.135
3.959	3.524	4.067	4.122	3.836	3.333
5.519	5.553	5.898	5.817	5.949	4.202
4.554	4.969	5.455	5.126	5.410	2.593
5.463	5.990	5.880	5.497	5.522	5.990
5.020	5.246	5.477	5.626	5.355	5.030
4.149	4.104	4.483	3.069	3.796	4.588
7.847	7.919	7.836	7.715	7.573	5.954
7.735	7.651	7.603	7.424	7.593	8.865
2.488	2.466	3.048	3.174	3.055	3.061
4.905	5.871	4.502	6.288	5.902	6.327
4.742	5.527	5.192	5.517	5.129	6.052
8.140	8.761	8.423	8.378	8.386	7.429
2.957	2.619	1.956	2.886	2.052	1.841
7.071	6.859	6.843	7.080	7.139	7.417
2.834	2.421	2.544	3.030	3.025	3.435
6.110	5.884	5.998	5.550	5.769	5.962
7.011	7.396	7.383	7.202	7.317	4.234
5.911	5.826	6.095	6.273	5.955	7.402
4.626	5.472	4.989	4.713	5.018	5.547
2.581	2.614	2.842	2.361	2.556	3.967
3.735	4.410	4.386	4.068	4.399	3.735
6.160	6.086	6.294	6.278	6.205	7.696
3.606	3.626	3.061	3.437	2.938	4.316
3.963	3.745	3.971	3.893	4.019	2.947
7.720	7.671	7.464	7.640	7.853	6.451
6.494	6.346	6.646	6.768	6.526	6.127
5.963	6.584	6.405	6.138	6.329	5.828
6.892	6.976	6.839	7.006	6.949	6.584

7.143	7.086	7.006	6.802	6.971	5.931
1.962	1.747	2.319	1.691	1.690	2.648
8.337	8.599	8.540	8.519	8.579	7.234
5.168	4.754	4.926	4.790	4.966	7.614
4.289	3.775	4.289	4.536	4.023	4.362
4.859	4.767	4.836	4.654	4.917	3.774
3.675	4.268	4.309	4.311	4.346	4.999
3.070	3.348	2.517	2.582	2.740	2.629
1.824	2.083	2.352	2.048	2.288	1.950
8.223	8.399	8.392	8.105	8.261	7.549
2.899	2.579	2.955	2.729	2.665	2.389
5.166	4.873	5.242	5.133	4.978	4.971
3.251	3.620	3.361	3.314	3.324	3.427
2.977	2.142	2.594	2.363	2.668	2.860
5.253	5.397	5.616	5.373	5.876	3.674
3.412	3.723	2.963	4.096	4.021	4.077
5.811	5.949	5.986	6.165	5.561	3.485
3.508	3.584	3.905	3.881	3.612	5.307
6.700	6.315	6.652	6.493	6.414	5.881
6.426	6.190	6.254	6.134	5.974	4.160
5.673	6.336	6.041	6.113	6.277	6.720
1.918	1.901	1.701	2.132	2.231	2.358
6.060	6.204	6.062	5.941	6.329	7.043
7.955	7.920	7.736	8.021	8.128	8.749
5.974	5.888	6.032	5.764	5.968	5.499
4.826	5.082	4.944	4.864	4.828	4.502
4.569	4.702	4.924	4.591	4.402	3.030
4.158	4.310	4.033	4.291	3.577	3.039
3.010	2.999	2.985	2.633	2.670	2.967
4.022	3.576	3.863	3.721	3.691	4.049
2.606	3.380	3.368	2.965	2.970	2.227
8.077	7.788	7.777	8.547	8.328	9.520
4.644	4.799	4.690	5.127	4.957	5.733
4.149	4.208	3.969	3.685	3.607	3.024
5.599	5.559	5.414	5.224	5.268	5.857
4.103	3.324	3.443	3.563	3.704	4.676
5.768	5.878	6.923	6.187	7.189	6.280
6.199	6.341	6.225	5.517	6.063	5.679
1.435	1.927	1.951	1.983	1.737	2.877
1.910	1.743	1.719	1.727	1.560	3.472
0.994	9.007	1.357	1.278	1.495	9.677
5.425	5.263	5.741	5.398	5.265	3.910
5.320	6.249	5.834	5.552	5.865	4.249
6.042	6.407	6.567	6.152	6.721	5.596
4.268	4.212	4.516	4.692	4.638	3.981
4.155	3.051	3.555	4.324	3.705	3.846
2.673	2.643	3.273	2.266	2.976	1.573
2.128	2.469	2.628	2.511	2.521	2.674
3.776	3.044	4.001	3.564	3.579	2.595
4.963	5.611	5.375	5.860	5.571	6.308

7.979	8.048	7.877	7.840	8.036	7.547
4.068	4.585	4.679	4.380	4.672	4.760
3.520	3.936	4.090	3.536	3.513	2.670
4.922	4.488	4.670	4.815	4.721	4.691
4.251	4.172	4.412	4.603	4.050	4.275
1.889	2.956	2.391	2.659	2.254	2.717
4.234	4.289	2.986	4.979	3.592	4.927
3.413	3.912	4.004	3.193	3.193	3.390
4.094	3.099	3.358	3.997	3.344	3.467
1.954	1.820	1.963	1.244	2.235	3.384
5.322	4.766	4.692	4.793	4.691	5.307
5.248	5.094	5.416	5.271	5.141	4.765
7.450	7.558	7.645	7.381	7.417	8.375
5.147	4.497	3.906	4.764	5.414	4.473
3.100	2.507	2.383	1.568	2.474	1.987
4.859	5.616	5.149	5.574	5.737	5.626
4.213	4.477	4.374	4.359	4.287	4.819
6.817	6.945	7.018	7.025	7.206	6.814
7.734	7.999	7.891	7.635	7.991	7.433
5.201	4.789	4.513	4.924	5.123	3.796
3.607	4.058	3.526	3.511	3.761	2.733
3.591	2.954	2.673	2.375	2.792	3.060
2.115	3.049	2.769	3.199	2.515	2.388
3.703	4.384	3.939	3.898	3.862	3.238
4.546	4.396	4.884	3.988	4.350	3.091
8.050	7.789	8.185	8.160	8.023	7.144
4.598	5.135	4.949	4.772	4.391	3.630
6.931	7.016	6.922	6.537	6.942	6.323
7.408	7.656	7.470	7.383	7.238	6.715
10.335	10.387	10.284	10.415	10.503	9.109
5.717	5.281	5.392	5.402	5.392	4.431
3.150	2.800	2.829	3.503	2.704	3.166
6.304	6.418	6.083	6.162	6.105	5.766
5.077	5.216	4.872	4.662	4.824	5.332
5.197	4.687	5.140	5.415	4.841	1.552
3.932	3.819	4.301	4.040	4.181	4.311
4.172	4.519	4.723	4.379	4.837	2.621
2.949	2.723	2.873	3.331	2.588	3.445
3.132	1.968	2.162	3.748	2.723	4.003
5.849	5.861	5.664	5.804	5.749	5.978
7.247	7.067	7.040	7.029	7.151	5.823
5.681	5.439	5.311	5.796	5.797	7.244
4.464	4.555	3.977	4.440	3.772	3.507
5.480	6.311	6.100	6.178	6.064	5.935
5.248	5.450	5.793	5.137	5.343	4.691
5.156	5.197	5.100	5.382	5.588	6.558
4.320	4.573	4.668	4.991	4.506	4.633
4.196	2.900	3.673	3.600	2.606	4.096
7.362	7.462	7.355	7.374	7.080	6.974
5.445	5.853	5.597	5.643	5.557	5.209

3.987	4.467	4.160	3.901	3.588	4.198
4.058	3.556	3.450	3.579	3.890	2.950
3.765	4.156	3.997	4.409	4.364	3.464
7.025	7.282	7.177	7.173	7.037	6.349
3.554	3.539	3.257	3.248	3.839	3.415
6.138	6.164	6.399	6.041	6.172	4.906
5.708	5.275	5.789	5.700	4.855	4.808
5.808	5.948	6.653	5.888	5.973	7.210
4.823	5.730	5.839	5.191	5.859	4.365
5.849	5.859	6.346	6.137	6.077	7.170
3.662	3.913	3.985	4.276	3.944	4.740
6.758	7.100	6.849	7.078	6.845	5.744
3.423	3.266	3.958	3.160	3.297	3.671
3.449	3.617	3.462	2.210	3.609	3.436
4.713	5.382	5.298	5.152	5.122	4.501
6.050	5.792	5.826	5.847	5.742	7.016
6.126	6.373	6.509	6.573	6.312	7.482
3.377	3.406	3.135	3.775	3.923	4.699
7.206	7.630	7.306	7.330	7.432	6.731
5.112	4.406	5.150	4.752	4.940	4.174
4.739	5.087	5.233	4.624	4.319	2.418
3.615	3.040	3.908	3.652	3.759	2.531
7.273	7.814	7.844	8.112	8.247	9.137
2.143	2.569	2.289	2.282	2.587	2.722
8.026	8.355	8.118	7.886	8.036	7.018
5.569	6.083	5.501	5.786	5.734	4.840
6.725	6.543	6.317	6.441	6.560	7.200
4.832	4.846	5.070	4.817	5.322	5.939
6.621	6.869	6.782	6.581	6.830	5.889
4.250	3.997	4.854	4.541	4.703	4.821
3.749	4.886	4.620	4.459	4.754	4.254
9.045	7.010	7.059	6.196	8.007	6.670
4.187	3.436	3.461	3.492	3.461	4.554
6.853	5.175	5.012	4.871	6.057	5.994
5.172	6.098	5.472	5.543	5.482	3.978
5.395	5.671	5.333	5.569	5.349	4.979
4.391	3.898	3.928	4.530	4.287	3.057
3.588	4.090	4.181	3.536	4.173	2.348
3.350	1.963	3.867	3.782	3.385	1.180
6.079	6.033	6.456	6.987	6.861	7.921
3.596	3.254	2.655	2.626	2.611	3.203
3.200	3.580	3.142	2.912	3.251	2.599
7.553	7.719	7.586	7.734	7.574	6.288
5.601	4.743	4.651	5.603	5.661	6.220
6.092	4.564	5.101	4.710	6.057	4.724
4.769	4.055	3.793	3.900	4.425	3.756
5.014	5.365	4.635	5.058	5.070	4.385
3.582	4.519	4.451	4.215	4.599	3.951
5.123	5.414	5.232	5.391	5.413	5.080
3.831	4.854	4.388	4.985	4.333	4.446

5.272	4.969	5.198	5.680	5.501	7.399
7.778	7.906	7.743	7.747	7.800	7.282
6.673	6.381	6.330	6.643	6.701	5.231
5.726	5.918	5.747	5.350	5.519	5.905
2.217	2.217	1.977	2.146	2.476	1.524
2.291	2.330	2.323	2.658	2.711	3.304
7.050	6.355	6.760	6.787	6.631	6.554
5.509	5.475	5.163	5.469	5.887	5.675
3.817	4.094	4.317	4.367	3.890	4.325
4.607	4.341	4.726	5.050	3.832	5.130
3.827	4.176	3.918	4.021	4.091	4.372
5.772	6.222	6.094	5.797	6.237	5.633
6.274	6.208	6.341	6.083	6.466	6.634
4.563	4.653	4.179	4.129	5.123	5.336
3.596	4.032	3.371	4.084	3.651	2.659
4.849	4.664	4.698	4.765	5.101	4.030
5.116	4.735	4.896	5.021	4.946	4.638
6.682	6.296	6.710	6.360	6.170	5.470
3.340	3.325	3.344	2.693	3.118	3.571
6.046	5.842	5.734	6.193	5.962	5.911
4.507	3.902	4.525	4.303	4.662	4.309
3.706	3.687	3.561	3.418	4.093	4.011
4.317	4.916	4.599	4.186	4.358	1.808
2.995	2.414	3.874	1.343	2.433	4.167
2.580	2.375	3.354	2.578	2.638	1.586
3.406	3.825	3.418	3.513	3.624	3.685
2.667	3.363	3.530	2.895	3.051	3.117
4.787	5.027	4.400	4.276	4.522	4.023
4.225	4.365	3.932	4.611	4.503	4.527
3.354	4.073	3.749	4.146	3.680	3.332
4.781	5.447	5.350	5.293	5.411	5.248
3.394	5.077	4.342	4.154	4.829	4.560
3.651	4.027	4.969	4.319	4.716	4.448
2.415	4.133	4.284	3.376	4.117	2.544
2.783	4.121	3.374	2.894	3.398	2.692
2.137	2.087	2.764	3.154	2.726	1.898
4.221	4.352	4.339	4.522	4.509	4.400
2.834	3.323	4.099	3.156	3.009	3.211
6.250	6.026	6.328	6.551	6.317	5.888
8.408	8.910	8.474	8.768	8.681	7.544
4.436	5.218	4.917	4.546	4.606	4.062
4.603	4.542	4.398	4.247	4.295	4.610
4.507	4.930	4.054	4.584	4.213	3.893
2.873	3.203	3.623	3.130	3.019	3.584
3.860	4.340	3.754	4.151	4.090	4.794
5.461	6.134	5.988	5.648	5.982	4.858
6.181	5.941	6.019	6.189	5.887	5.750
5.196	4.759	5.157	4.310	4.482	5.322
2.104	3.035	2.170	2.734	2.610	2.237
3.282	2.505	2.792	3.462	3.382	3.737

4.042	4.816	4.923	4.619	4.369	4.250
3.023	3.298	3.173	3.173	3.173	3.767
3.334	3.575	3.379	3.428	3.863	4.460
2.435	3.050	3.396	2.725	2.970	3.479
5.620	5.815	6.166	5.782	5.941	5.632
4.777	5.389	5.032	4.724	4.618	4.407
1.665	2.052	1.812	1.997	2.038	2.367
4.765	4.380	4.047	4.491	4.380	4.409
4.745	5.077	4.665	4.772	4.898	4.794
2.640	3.721	3.323	3.867	2.989	2.700
6.226	5.729	6.199	6.205	6.293	6.762
3.907	4.487	4.171	4.365	4.596	4.194
3.397	3.653	3.756	3.248	3.806	2.201
3.826	3.788	3.571	3.863	3.972	4.562
2.342	3.202	2.266	2.704	3.092	1.631
3.669	4.359	4.730	4.374	4.275	4.625
2.444	2.981	2.921	2.456	3.156	3.242
3.382	3.323	3.624	3.713	3.147	2.267
5.981	6.743	6.235	6.032	6.431	5.511
4.924	4.614	4.943	5.197	5.015	4.462
4.425	5.409	4.680	4.124	4.938	4.938
6.233	6.539	6.234	5.987	6.344	6.636
5.521	5.218	5.845	5.763	5.831	2.812
2.770	2.595	3.023	2.856	2.816	3.633
4.004	3.619	4.203	4.348	3.869	3.636
3.557	4.095	4.412	3.886	4.208	3.934
4.747	4.991	5.001	4.888	4.836	6.061
3.332	4.011	2.918	4.195	3.018	3.554
4.897	5.126	5.576	4.959	5.560	6.035
4.035	4.090	4.090	4.353	4.330	4.760
3.636	3.707	4.123	4.268	4.252	3.710
5.376	5.632	5.999	5.541	5.234	5.623
6.079	6.345	6.537	6.298	6.415	5.856
4.145	5.061	4.462	5.083	4.831	4.728
5.317	5.192	5.903	5.010	5.674	5.044
4.892	5.834	5.450	5.688	5.774	4.638
3.933	4.784	4.494	3.895	4.888	3.835
3.309	2.808	2.338	2.900	2.588	2.439
4.594	5.180	4.419	5.240	5.381	4.507
6.330	5.799	6.554	6.458	5.884	6.394
5.245	5.937	5.411	5.272	5.587	4.596
3.818	4.506	4.066	3.819	3.973	3.374
5.539	5.445	5.293	6.047	5.476	7.023
4.806	5.717	5.767	5.762	5.822	4.816
3.396	2.387	2.387	2.224	2.716	2.193
3.869	4.310	4.796	4.953	4.964	5.294
6.805	6.852	6.618	6.479	6.469	5.563
2.123	3.418	3.464	3.418	3.917	3.942
4.912	5.027	4.803	5.050	4.806	4.305
3.223	3.238	3.832	3.588	3.854	4.521

2.762	3.381	1.916	3.797	4.135	4.094
3.363	3.312	3.470	2.876	3.481	3.402
5.496	5.687	5.620	5.733	5.697	5.183
6.770	6.683	6.802	6.829	6.763	6.723
5.244	4.759	4.840	5.028	5.074	6.205
5.713	5.329	5.306	4.941	5.221	4.731
5.019	5.740	5.102	5.359	4.781	3.616
4.537	5.024	5.254	5.392	5.132	6.075
2.723	2.843	3.081	3.460	3.261	2.837
2.178	2.827	3.202	2.558	2.451	2.461
5.097	5.894	5.545	5.995	6.351	7.646
3.848	4.296	4.415	4.522	4.353	4.001
5.362	4.632	5.035	5.413	4.763	4.632
3.935	4.107	4.709	3.994	3.204	4.125
4.344	5.115	4.792	5.214	5.333	4.346
3.767	4.843	4.340	4.772	4.595	4.409
4.375	4.803	4.622	4.737	4.460	3.549
2.842	2.933	2.762	3.068	1.923	2.535
4.012	4.973	4.393	4.737	4.763	3.845
3.704	4.131	4.263	4.099	4.291	3.981
3.310	3.970	3.954	3.229	2.584	3.123
2.135	3.016	2.550	2.597	2.250	3.371
3.580	4.200	4.479	3.731	3.897	4.200
4.215	4.371	3.578	4.442	4.190	3.843
3.399	4.061	3.775	3.798	3.318	3.207
4.527	4.751	4.390	4.683	5.114	4.366
3.251	4.021	3.700	4.052	4.372	3.891
4.985	4.607	5.191	5.122	5.287	5.273
1.613	2.102	2.453	2.891	3.014	3.072
5.419	6.036	5.674	5.356	5.723	5.397
4.923	5.570	5.343	5.165	5.504	5.355
4.045	4.981	4.474	4.695	4.286	4.420
4.076	3.608	4.090	4.291	4.799	3.741
4.722	5.003	4.625	4.594	4.886	4.072
2.896	2.829	3.227	3.627	2.818	2.498
3.626	4.382	3.661	3.814	4.177	4.189
4.276	4.805	4.002	4.550	4.452	4.098
5.080	4.069	4.206	5.071	4.366	5.496
4.196	4.571	4.314	4.504	4.590	5.088
5.984	6.388	6.229	6.689	6.650	7.573
5.394	5.877	5.658	6.175	5.875	5.015
4.683	5.376	5.312	5.143	5.266	4.526
7.539	7.826	8.147	7.669	8.114	7.512
4.209	4.408	4.538	4.493	4.154	3.617
3.879	3.534	3.742	3.654	4.161	3.451
3.419	3.941	3.996	2.986	3.860	2.150
4.676	4.496	4.885	5.024	4.973	3.241
3.208	3.385	3.902	3.299	3.601	2.559
2.219	2.390	2.386	2.281	2.328	3.402
2.630	2.851	3.090	2.983	2.636	3.586

4.366	4.315	3.790	3.854	3.863	3.576
3.482	4.389	3.975	4.437	4.615	4.110
4.223	4.176	4.310	2.750	3.779	2.173
4.854	4.739	4.113	4.649	4.014	4.799
2.669	2.686	2.863	2.950	2.267	2.975
5.395	5.995	5.791	5.544	5.750	4.522
3.649	3.798	2.708	2.697	3.763	4.097
4.401	4.034	4.366	4.684	4.186	4.729
4.864	5.108	4.820	5.248	5.022	5.318
2.974	3.232	3.092	2.749	3.588	3.320
4.883	4.519	4.461	4.226	4.871	5.258
3.996	3.331	3.274	2.337	2.988	2.721
4.024	3.221	3.245	4.007	3.616	2.948
3.729	3.308	3.420	3.745	3.962	3.803
3.284	3.880	3.507	3.733	3.283	2.654
7.189	7.062	7.057	7.264	6.995	6.089
3.206	3.507	2.945	3.081	3.341	2.454
4.456	4.794	4.141	4.684	4.859	3.789
5.840	5.914	5.785	5.952	5.771	5.189
4.301	4.807	4.920	5.154	4.574	4.643
6.201	6.037	6.276	6.283	6.318	6.093
4.152	3.494	4.343	4.164	4.164	3.962
5.052	5.407	4.932	5.197	5.719	6.131
5.002	5.143	5.427	6.297	5.821	4.522
3.794	4.454	4.423	4.145	3.439	3.139
3.544	3.143	3.470	3.726	2.735	3.857
3.394	2.734	3.300	3.337	3.696	3.676
6.835	6.993	6.615	6.839	6.968	6.622
4.878	4.891	5.431	4.785	5.435	2.734
5.143	5.445	5.302	5.459	5.963	3.247
2.937	1.795	2.650	2.443	1.394	2.883
4.032	3.949	3.790	4.156	4.029	4.139
3.760	4.222	3.565	4.133	4.105	3.559
4.886	5.576	4.981	5.240	5.336	4.038
2.347	3.868	4.133	3.859	3.843	4.082
2.130	2.570	2.822	2.997	2.581	2.326
4.872	4.708	4.601	5.284	4.999	5.727
4.140	4.230	3.890	3.774	3.435	2.067
4.248	4.022	4.038	4.447	3.936	3.887
4.786	5.416	5.281	5.248	5.316	4.994
2.165	2.190	2.071	2.088	2.122	1.757
4.201	4.114	4.817	4.344	4.385	4.498
3.173	3.751	3.842	3.129	3.958	4.566
2.889	3.054	4.002	3.759	3.028	2.888
6.333	6.025	6.124	6.263	6.370	5.490
3.864	4.211	3.688	3.858	4.129	3.839
4.359	4.460	4.078	4.169	3.708	4.006
4.048	3.725	4.316	4.813	4.312	4.438
3.697	4.305	3.739	3.580	3.432	4.223
3.990	4.045	3.889	4.484	4.239	4.194

3.109	3.981	3.335	3.650	3.794	3.211
3.380	2.977	2.022	3.281	3.585	4.529
2.975	2.990	3.025	3.034	2.914	2.737
3.944	3.048	3.555	3.796	3.789	2.765
3.870	4.043	3.616	4.622	4.060	4.604
3.975	3.832	4.004	3.816	3.809	4.416
3.702	3.861	4.121	3.932	4.223	3.536
3.055	3.501	3.407	3.395	3.685	3.990
4.588	4.948	5.013	4.794	4.611	4.597
3.851	4.473	4.566	4.469	4.368	3.032
4.186	3.600	4.166	4.607	4.209	4.218
6.324	5.886	6.533	6.305	6.530	6.501
8.843	9.110	8.908	8.764	8.983	7.758

14.CTL.FC.F	15.CTL.FC.M	16.CTL.FC.M	17.CTL.FC.M	18.CTL.FC.M	s.post	s.unscaled
4.245	4.286	3.635	3.760	2.663	0.506	0.606
7.770	6.741	7.752	6.303	7.967	0.503	0.606
4.578	4.290	5.237	3.450	4.219	0.458	0.606
3.175	1.924	2.667	2.789	2.859	0.459	0.606
5.492	6.417	5.335	6.595	6.010	0.447	0.606
3.667	4.047	4.074	3.719	4.529	0.420	0.606
4.508	4.789	3.991	4.941	5.368	0.357	0.606
7.588	7.928	6.613	8.038	8.182	0.406	0.606
6.174	6.356	5.707	6.726	6.770	0.370	0.606
7.028	6.387	7.292	5.739	6.078	0.466	0.606
6.615	5.833	7.114	5.644	5.314	0.444	0.606
5.795	4.750	6.109	4.674	4.714	0.612	0.606
2.614	2.003	1.224	2.853	2.866	0.447	0.606
2.103	1.832	3.358	3.034	3.176	0.495	0.606
9.724	8.961	10.335	7.900	7.800	0.548	0.606
7.002	6.448	6.210	6.904	7.038	0.404	0.606
6.818	5.879	7.584	3.764	4.175	1.083	0.606
7.707	6.633	9.304	5.423	5.870	0.747	0.606
6.089	4.334	5.436	4.316	5.733	0.611	0.606
5.645	5.080	5.261	5.149	5.269	0.353	0.606
2.238	1.572	2.147	1.679	1.851	0.404	0.606
3.742	2.030	1.921	1.779	1.640	0.862	0.606
5.006	4.636	5.466	4.045	4.929	0.470	0.606
6.841	6.436	6.510	7.055	6.680	0.362	0.606
4.441	4.813	4.185	4.784	5.088	0.466	0.606
5.347	6.828	6.654	5.630	5.739	0.552	0.606
5.466	5.486	3.293	6.158	6.681	0.691	0.606
4.531	4.766	5.129	4.460	3.772	0.377	0.606
5.396	5.904	6.715	5.899	5.640	0.391	0.606
6.388	5.022	5.707	5.383	6.264	0.553	0.606
4.914	5.520	3.185	5.658	5.363	0.618	0.606
2.556	3.853	3.345	3.922	4.233	0.517	0.606
5.679	6.235	5.699	6.317	6.303	0.320	0.606
3.532	3.721	4.170	3.492	3.584	0.466	0.606
1.506	2.494	2.042	1.838	1.947	0.354	0.606
2.922	2.845	2.676	1.998	2.398	0.425	0.606
6.660	6.710	7.866	5.955	6.307	0.475	0.606
4.643	4.452	5.550	4.310	4.370	0.457	0.606
5.054	5.472	6.463	4.760	5.129	0.516	0.606
5.010	4.380	5.869	3.993	4.436	0.450	0.606
5.701	7.057	4.753	7.175	6.912	0.602	0.606
4.222	4.757	4.731	3.127	4.320	0.581	0.606
3.991	3.822	5.936	3.842	4.441	0.667	0.606
7.422	7.129	7.940	6.785	6.820	0.336	0.606
6.231	6.005	3.828	6.478	6.474	0.635	0.606
6.700	5.670	5.208	5.769	5.875	0.426	0.606
3.137	3.427	2.534	3.895	4.031	0.492	0.606
4.401	4.388	4.307	4.501	4.840	0.387	0.606
3.499	4.221	3.972	4.222	4.132	0.393	0.606

6.794	4.157	6.277	4.541	4.544	0.657	0.606
5.274	3.955	4.670	3.922	4.625	0.471	0.606
3.800	3.926	5.158	3.352	4.037	0.479	0.606
2.226	2.241	2.692	2.640	2.163	0.430	0.606
3.030	4.349	3.861	4.223	3.796	0.514	0.606
3.872	2.086	2.087	3.294	4.350	0.538	0.606
5.761	5.124	6.240	5.052	5.059	0.421	0.606
2.453	1.773	3.398	1.964	1.570	0.538	0.606
1.228	1.921	1.777	1.976	1.406	0.577	0.606
6.045	3.793	3.301	4.394	4.103	0.818	0.606
3.168	3.344	2.821	3.575	3.684	0.470	0.606
4.111	5.098	4.484	4.597	4.182	0.443	0.606
3.652	3.250	4.820	3.491	3.367	0.424	0.606
2.521	2.749	2.858	3.030	2.984	0.432	0.606
4.431	4.423	5.673	4.127	3.444	0.569	0.606
2.025	3.139	1.771	2.931	3.008	0.470	0.606
1.858	1.414	2.283	1.272	1.587	0.506	0.606
2.900	2.249	3.216	3.011	3.386	0.451	0.606
5.809	4.899	4.641	5.236	5.935	0.583	0.606
4.041	3.629	4.469	3.576	3.593	0.469	0.606
3.798	2.867	3.502	3.035	2.532	0.482	0.606
3.462	3.290	3.830	2.856	2.991	0.460	0.606
2.313	1.964	2.553	2.582	1.692	0.450	0.606
3.093	0.998	5.435	1.227	0.613	1.176	0.606
3.039	2.573	2.698	3.099	3.470	0.464	0.606
2.305	1.904	2.242	1.835	2.307	0.399	0.606
3.360	3.797	3.054	3.729	2.829	0.425	0.606
2.320	2.407	2.272	3.669	3.293	0.517	0.606
1.886	1.410	1.712	1.521	2.597	0.390	0.606
3.612	3.114	2.616	3.383	4.368	0.451	0.606
2.242	1.224	3.573	1.720	2.044	0.797	0.606
2.991	4.973	3.116	4.257	4.855	0.593	0.606
3.745	3.097	2.423	3.484	3.797	0.440	0.606
3.145	2.570	4.049	2.822	2.816	0.465	0.606
1.545	2.990	1.825	3.505	2.916	0.479	0.606
3.324	2.043	4.005	1.374	1.648	0.718	0.606
6.185	5.308	6.284	4.473	4.757	0.536	0.606
4.212	3.542	3.433	4.287	3.335	0.440	0.606
1.860	1.873	1.664	2.224	2.513	0.456	0.606
3.335	3.433	2.274	3.267	2.984	0.429	0.606
2.197	2.147	2.521	2.433	2.902	0.498	0.606
3.067	2.290	3.439	1.831	1.808	0.456	0.606
4.860	2.406	4.492	2.947	2.112	0.872	0.606
1.979	2.453	2.145	2.797	2.373	0.411	0.606
2.341	3.362	3.632	2.019	2.214	0.541	0.606
0.457	4.422	2.481	2.603	2.178	0.956	0.606
2.931	3.032	3.227	3.821	3.482	0.428	0.606
1.847	3.960	3.005	4.252	4.088	0.610	0.606
2.163	2.868	1.624	4.007	2.630	0.476	0.606
1.087	3.812	1.492	4.095	4.445	0.845	0.606

3.423	4.133	3.890	3.477	3.701	0.436	0.606
1.709	2.650	2.184	1.946	1.984	0.448	0.606
3.590	3.505	4.597	2.971	3.471	0.413	0.606
2.573	2.286	2.573	2.573	3.833	0.490	0.606
2.935	2.728	2.659	2.858	2.754	0.506	0.606
3.245	3.857	4.073	3.831	2.580	0.501	0.606
3.674	4.107	2.716	4.296	4.156	0.473	0.606
3.024	2.543	3.628	2.814	2.093	0.423	0.606
1.969	2.815	2.428	2.018	2.480	0.392	0.606
2.766	3.564	1.585	4.366	3.388	0.522	0.606
3.162	2.390	1.691	2.402	2.458	0.393	0.606
2.916	2.234	2.215	3.163	2.798	0.395	0.606
0.607	2.160	0.798	2.574	1.844	0.475	0.606
5.305	5.907	4.851	6.354	6.327	0.543	0.606
2.975	1.814	3.446	1.957	1.611	0.535	0.606
5.461	4.258	4.405	3.769	3.986	0.586	0.606
3.435	2.793	3.497	3.619	3.218	0.433	0.606
2.342	2.654	3.100	3.033	2.917	0.488	0.606
2.371	2.341	1.889	2.256	3.651	0.418	0.606
1.277	4.371	0.838	3.561	3.775	0.765	0.606
3.083	1.968	2.228	1.896	2.146	0.479	0.606
5.224	5.196	6.052	5.831	5.976	0.335	0.606
3.242	2.886	4.499	2.793	2.800	0.508	0.606
6.228	5.270	6.872	5.821	5.342	0.402	0.606
1.376	2.570	1.691	3.396	3.494	0.562	0.606
2.072	2.599	2.688	2.626	1.831	0.416	0.606
2.016	2.068	1.774	2.269	3.078	0.485	0.606
3.992	2.132	1.948	2.487	2.317	0.886	0.606
2.962	4.103	2.859	3.325	2.442	0.454	0.606
1.137	2.559	0.920	3.104	2.588	0.566	0.606
2.521	3.055	2.475	2.662	2.779	0.503	0.606
2.297	2.530	1.460	2.844	2.280	0.503	0.606
4.845	4.661	3.305	5.148	5.100	0.555	0.606
2.484	4.038	3.411	4.401	3.622	0.525	0.606
1.445	2.382	3.453	2.816	1.832	0.670	0.606
3.846	3.392	4.616	3.195	3.647	0.464	0.606
1.352	2.425	6.266	2.433	2.673	1.012	0.606
6.727	5.489	5.442	6.359	5.955	0.611	0.606
4.098	2.924	3.205	3.090	2.570	0.545	0.606
5.578	4.947	5.730	4.557	4.785	0.393	0.606
2.414	2.141	1.898	1.239	1.721	0.464	0.606
4.097	4.618	4.218	5.147	5.596	0.523	0.606
6.284	5.332	5.556	5.606	5.720	0.489	0.606
6.974	6.300	6.227	6.987	7.240	0.467	0.606
4.278	3.613	3.656	3.137	3.254	0.368	0.606
4.157	4.993	3.916	4.155	4.780	0.439	0.606
6.487	4.544	4.018	4.514	4.390	0.577	0.606
3.970	4.733	3.329	4.343	5.565	0.623	0.606
3.026	3.600	3.441	3.367	3.640	0.470	0.606
9.237	8.097	7.211	7.837	8.020	0.465	0.606

4.297	5.371	4.137	5.055	5.502	0.437	0.606
4.335	3.579	3.632	3.718	3.868	0.412	0.606
2.189	1.921	2.529	2.092	2.219	0.434	0.606
2.764	3.581	4.287	2.203	2.707	0.757	0.606
5.494	4.657	5.146	4.293	4.773	0.462	0.606
3.709	3.478	3.331	3.164	3.804	0.346	0.606
7.091	7.001	8.099	5.674	5.731	0.633	0.606
7.683	7.770	6.956	7.962	7.609	0.346	0.606
2.456	2.374	1.911	3.178	3.072	0.431	0.606
5.071	5.428	5.303	4.591	6.209	0.426	0.606
3.946	3.724	2.408	4.189	4.571	0.501	0.606
3.874	3.946	4.031	3.319	3.732	0.460	0.606
5.708	5.342	6.197	5.694	5.470	0.401	0.606
5.226	5.805	5.914	5.980	6.256	0.415	0.606
2.255	3.457	1.881	2.801	3.081	0.481	0.606
2.545	3.468	3.133	2.672	2.987	0.655	0.606
3.284	4.195	2.730	4.212	4.519	0.458	0.606
4.080	3.681	5.103	3.423	3.473	0.420	0.606
4.982	5.824	3.329	5.698	5.938	0.661	0.606
3.256	4.649	2.924	4.920	4.470	0.646	0.606
6.242	6.614	6.574	6.359	6.134	0.552	0.606
6.713	6.307	6.282	6.650	6.613	0.340	0.606
6.313	6.867	5.443	6.466	6.514	0.400	0.606
5.868	6.283	4.836	6.952	6.951	0.573	0.606
5.605	5.668	5.742	5.957	6.191	0.330	0.606
3.307	2.983	3.406	4.252	3.894	0.412	0.606
4.957	6.057	4.605	6.025	5.823	0.440	0.606
4.211	5.225	5.155	5.851	5.892	0.497	0.606
4.725	5.283	4.395	5.413	5.484	0.347	0.606
3.436	3.649	3.091	2.533	3.498	0.421	0.606
5.122	3.714	4.058	3.991	3.537	0.749	0.606
5.027	4.782	6.132	4.746	4.740	0.427	0.606
4.709	5.851	3.324	6.061	6.053	0.624	0.606
3.944	3.199	3.697	3.599	3.155	0.428	0.606
4.203	4.040	4.738	3.200	3.746	0.409	0.606
4.398	4.621	4.429	5.098	5.154	0.363	0.606
4.062	3.941	4.114	3.721	4.242	0.451	0.606
5.192	4.689	4.565	4.809	4.984	0.518	0.606
7.251	7.827	6.643	8.096	8.197	0.412	0.606
8.123	8.247	5.459	8.926	9.058	0.799	0.606
6.468	5.446	6.583	4.093	4.968	0.734	0.606
3.375	1.855	3.262	3.227	3.095	0.507	0.606
2.426	2.629	2.864	2.201	2.485	0.374	0.606
5.676	5.740	5.525	4.754	5.093	0.486	0.606
3.061	1.742	3.927	1.610	1.977	0.638	0.606
2.498	3.050	3.388	1.762	2.669	0.507	0.606
1.528	2.127	2.184	2.078	2.569	0.672	0.606
1.589	2.345	2.134	3.639	2.999	0.511	0.606
3.115	3.521	2.989	4.301	3.655	0.483	0.606
5.125	1.188	6.524	2.073	1.984	1.209	0.606

3.055	3.038	3.457	3.857	2.881	0.428	0.606
3.469	3.513	1.850	4.038	3.189	0.548	0.606
2.009	3.289	2.326	3.995	3.866	0.591	0.606
5.886	5.624	6.911	3.598	4.110	0.744	0.606
3.573	2.578	2.647	3.017	3.301	0.385	0.606
3.312	2.690	2.742	2.708	2.716	0.544	0.606
4.239	4.900	3.364	5.379	4.723	0.494	0.606
5.557	7.145	6.226	5.291	5.581	0.591	0.606
3.952	4.560	3.936	4.683	5.038	0.544	0.606
4.644	4.046	3.672	4.254	3.629	0.411	0.606
4.502	3.876	5.866	4.062	4.560	0.534	0.606
1.728	2.418	2.487	1.724	1.991	0.562	0.606
2.206	3.036	3.050	2.612	2.737	0.437	0.606
5.475	5.212	5.865	4.421	4.998	0.553	0.606
5.593	5.769	4.585	6.146	6.031	0.427	0.606
1.954	1.568	1.817	1.631	1.554	0.477	0.606
3.644	3.381	3.190	3.709	4.010	0.404	0.606
6.819	9.150	5.169	9.121	8.988	0.875	0.606
4.653	4.307	5.699	3.708	4.121	0.440	0.606
4.813	4.091	5.213	4.364	3.996	0.461	0.606
6.816	6.129	5.852	6.420	6.111	0.426	0.606
5.062	5.757	4.597	5.896	5.715	0.485	0.606
4.326	3.658	2.014	3.650	3.415	0.685	0.606
7.253	6.257	5.634	6.538	6.341	0.483	0.606
6.658	7.040	3.705	7.523	7.357	0.741	0.606
2.228	2.931	2.606	3.270	2.433	0.484	0.606
1.869	1.828	2.674	2.323	1.639	0.750	0.606
5.862	5.166	7.000	4.934	4.865	0.477	0.606
4.453	4.860	4.619	4.221	4.736	0.380	0.606
3.924	5.436	4.319	4.801	3.355	0.701	0.606
4.619	4.257	4.233	4.310	4.573	0.433	0.606
3.370	5.192	3.775	5.106	4.208	0.481	0.606
4.635	4.961	5.496	3.715	4.721	0.564	0.606
4.313	4.714	2.967	5.060	5.213	0.534	0.606
3.581	4.236	2.276	4.871	4.420	0.574	0.606
3.819	4.000	2.403	3.651	3.244	0.484	0.606
3.797	3.689	2.302	4.015	3.182	0.527	0.606
2.800	3.627	2.007	3.768	2.798	0.542	0.606
4.174	5.897	3.829	6.611	6.373	0.674	0.606
6.259	6.997	6.069	7.235	7.087	0.386	0.606
7.452	8.431	6.199	8.365	8.578	0.548	0.606
4.172	3.827	4.494	4.039	3.874	0.360	0.606
3.831	3.280	3.586	2.605	2.915	0.475	0.606
3.107	3.441	3.742	3.588	2.699	0.416	0.606
9.187	9.600	8.369	9.606	9.618	0.413	0.606
4.067	3.651	3.687	3.225	3.892	0.527	0.606
4.244	3.112	3.121	3.789	3.396	0.523	0.606
3.395	2.840	2.781	2.984	2.758	0.483	0.606
3.855	3.576	4.325	5.132	4.464	0.481	0.606
6.891	6.670	4.119	7.194	6.931	0.843	0.606

5.656	5.950	4.857	6.373	6.328	0.406	0.606
4.139	4.558	5.522	3.685	3.941	0.416	0.606
7.183	7.657	7.283	7.965	7.754	0.327	0.606
5.125	4.483	5.503	4.139	4.752	0.489	0.606
3.933	2.476	4.029	3.571	2.675	0.471	0.606
5.498	7.851	3.722	8.127	7.848	0.944	0.606
2.672	3.821	2.766	3.213	3.639	0.414	0.606
6.674	5.707	6.607	5.196	5.981	0.480	0.606
2.729	2.757	2.253	3.286	3.183	0.411	0.606
4.217	4.792	2.462	5.315	5.489	0.605	0.606
2.139	1.460	1.374	2.543	2.500	0.454	0.606
5.536	5.710	5.650	5.463	5.603	0.434	0.606
6.983	7.188	7.352	7.299	7.685	0.292	0.606
5.326	5.777	5.053	6.071	6.078	0.351	0.606
5.725	5.835	5.420	5.693	6.477	0.406	0.606
3.423	3.499	2.742	3.515	4.010	0.411	0.606
3.289	2.048	4.145	1.422	1.484	0.621	0.606
7.295	6.627	7.532	5.977	5.560	0.589	0.606
5.782	4.598	6.054	4.199	3.660	0.629	0.606
7.228	6.551	7.228	6.055	6.647	0.462	0.606
4.159	4.290	5.383	2.667	3.625	0.545	0.606
6.287	5.921	6.742	6.006	6.645	0.432	0.606
7.603	6.018	3.390	6.292	6.344	0.815	0.606
6.152	6.990	7.780	6.453	6.737	0.467	0.606
5.037	4.285	5.158	4.303	4.546	0.418	0.606
5.230	6.560	5.969	3.877	5.259	0.802	0.606
5.554	6.932	6.372	3.762	5.726	0.900	0.606
5.602	6.971	6.340	3.765	5.643	0.879	0.606
3.891	3.879	4.143	2.927	2.413	0.469	0.606
5.274	4.513	4.372	5.177	4.834	0.461	0.606
1.756	1.964	1.972	1.205	2.018	0.456	0.606
1.393	3.589	1.075	3.993	3.592	0.696	0.606
1.495	2.497	1.141	2.695	2.390	0.551	0.606
6.468	7.072	6.494	7.401	7.162	0.516	0.606
7.877	8.970	8.763	9.238	9.193	0.519	0.606
7.984	8.218	8.003	8.625	8.700	0.320	0.606
6.862	6.250	3.960	6.575	6.320	0.647	0.606
2.735	2.495	3.695	2.634	2.421	0.429	0.606
2.295	4.652	1.415	5.643	5.461	0.922	0.606
4.270	3.996	4.385	3.385	3.736	0.427	0.606
3.741	2.174	2.627	3.708	3.413	0.487	0.606
3.735	3.579	3.712	3.962	3.756	0.466	0.606
2.215	2.693	2.356	2.585	1.830	0.433	0.606
7.783	8.118	7.855	8.216	8.316	0.358	0.606
3.838	2.669	3.070	3.598	2.949	0.510	0.606
3.182	2.386	1.323	3.448	2.760	0.578	0.606
5.265	4.413	5.247	4.419	4.193	0.350	0.606
6.202	6.598	5.492	6.335	6.433	0.370	0.606
3.138	2.958	4.275	2.030	4.999	1.021	0.606
7.129	6.184	7.821	6.034	6.058	0.467	0.606

7.277	6.430	7.669	5.648	6.574	0.529	0.606
4.902	4.507	4.251	4.457	5.017	0.460	0.606
4.070	3.424	3.339	3.352	2.820	0.480	0.606
3.752	2.621	2.144	2.755	2.476	0.456	0.606
6.803	6.641	7.314	5.865	6.705	0.390	0.606
4.688	5.072	4.389	4.775	5.408	0.484	0.606
4.388	3.835	3.543	4.136	4.867	0.407	0.606
2.289	2.721	3.195	3.610	3.575	0.503	0.606
2.658	2.344	2.168	2.049	2.239	0.609	0.606
4.758	4.811	4.557	5.372	5.251	0.367	0.606
2.912	1.957	3.472	1.732	1.822	0.535	0.606
1.592	3.117	1.263	2.433	3.272	0.563	0.606
3.930	3.067	2.615	3.736	3.281	0.403	0.606
2.266	3.131	2.210	3.164	2.967	0.824	0.606
4.683	5.115	5.141	2.902	4.526	0.698	0.606
6.433	6.974	7.942	4.968	6.072	0.778	0.606
3.413	4.039	4.900	2.243	3.559	0.649	0.606
4.970	5.417	5.211	4.928	5.815	0.467	0.606
5.716	6.197	5.597	6.434	6.323	0.317	0.606
3.742	2.208	3.716	2.126	1.885	0.510	0.606
1.980	2.385	3.193	2.707	2.309	0.457	0.606
2.826	2.814	2.814	3.385	3.619	0.445	0.606
6.515	7.012	8.194	6.104	6.299	0.463	0.606
3.632	4.472	2.578	3.853	3.380	0.441	0.606
2.311	1.769	2.120	1.595	2.440	0.471	0.606
6.201	6.614	3.714	6.313	6.748	0.719	0.606
2.212	2.253	2.370	3.694	3.241	0.449	0.606
5.918	6.653	6.654	6.550	6.847	0.319	0.606
4.519	4.866	5.718	3.661	3.944	0.461	0.606
7.053	8.121	4.430	8.615	8.407	0.964	0.606
3.288	3.855	4.587	3.084	3.960	0.473	0.606
5.182	5.627	5.245	5.618	5.733	0.335	0.606
3.138	2.321	2.227	2.190	2.407	0.505	0.606
3.837	4.191	3.661	3.175	3.510	0.466	0.606
5.263	5.671	5.972	5.764	5.626	0.346	0.606
3.575	2.536	4.260	2.598	2.812	0.564	0.606
5.588	6.917	5.899	6.306	5.897	0.710	0.606
1.701	2.619	3.556	2.897	3.588	0.488	0.606
5.246	5.838	5.544	3.683	4.151	0.562	0.606
4.904	6.628	6.929	6.103	4.912	0.731	0.606
3.106	3.351	3.453	3.481	3.135	0.505	0.606
6.165	5.220	2.789	5.440	5.642	0.787	0.606
9.293	9.326	9.071	9.455	9.338	0.297	0.606
8.465	8.205	7.989	8.276	8.467	0.356	0.606
3.788	2.936	2.212	2.667	3.679	0.463	0.606
5.907	4.209	4.702	4.389	4.683	0.504	0.606
7.293	6.870	7.561	6.770	6.790	0.320	0.606
2.348	2.658	2.569	2.357	2.496	0.439	0.606
5.424	6.147	5.354	6.097	6.285	0.377	0.606
6.459	5.724	7.079	5.579	5.706	0.469	0.606

6.296	5.518	6.852	5.066	5.468	0.609	0.606
6.540	5.915	7.139	5.469	5.655	0.435	0.606
6.504	7.004	6.837	6.978	7.070	0.325	0.606
6.002	6.260	5.933	6.301	6.298	0.334	0.606
4.393	4.167	2.839	4.413	4.956	0.650	0.606
3.308	4.261	3.383	4.037	4.372	0.505	0.606
3.645	4.192	3.507	4.466	4.703	0.489	0.606
6.621	6.724	5.265	7.268	7.016	0.503	0.606
6.886	6.887	5.499	7.451	7.252	0.532	0.606
3.861	4.296	3.305	3.860	4.594	0.692	0.606
4.353	3.194	1.664	4.636	4.727	1.111	0.606
1.132	1.844	3.010	1.412	1.644	0.615	0.606
3.997	5.317	4.792	5.738	5.371	0.517	0.606
5.539	5.578	5.358	6.060	5.853	0.359	0.606
2.922	3.133	1.721	2.848	4.010	0.689	0.606
6.358	5.876	4.070	6.342	6.681	0.752	0.606
3.621	3.274	0.859	3.252	4.284	0.796	0.606
4.116	4.902	4.101	5.615	5.420	0.489	0.606
2.820	3.761	2.758	4.177	4.415	0.583	0.606
3.314	4.626	2.947	5.047	4.881	0.529	0.606
3.573	1.615	2.032	2.546	2.041	0.540	0.606
4.194	4.942	2.199	5.763	4.805	0.638	0.606
6.927	7.124	7.232	7.004	5.885	0.481	0.606
4.792	5.651	4.995	5.848	4.715	0.636	0.606
8.826	8.114	9.115	7.871	7.771	0.373	0.606
4.918	4.993	3.358	5.589	5.577	0.533	0.606
6.210	5.368	6.571	5.131	5.310	0.432	0.606
3.393	2.802	4.406	1.893	2.385	0.598	0.606
3.860	4.661	4.806	5.210	5.221	0.397	0.606
6.324	5.997	5.445	6.149	6.400	0.424	0.606
4.579	3.325	2.870	3.808	3.600	0.444	0.606
6.182	7.016	5.725	7.409	7.196	0.482	0.606
4.723	4.510	5.487	4.238	3.842	0.444	0.606
9.539	8.211	10.130	7.333	6.590	0.697	0.606
4.133	6.717	2.492	6.689	6.690	0.943	0.606
6.847	6.121	6.465	5.996	6.538	0.494	0.606
6.728	7.044	5.525	7.287	7.807	0.477	0.606
7.467	7.542	7.892	7.049	7.561	0.419	0.606
4.555	3.815	5.766	3.949	3.973	0.480	0.606
4.451	4.274	5.224	3.485	4.364	0.375	0.606
1.969	1.776	2.299	1.830	2.316	0.620	0.606
6.228	5.138	6.183	5.461	5.605	0.428	0.606
4.750	4.480	4.742	4.828	4.857	0.326	0.606
3.614	4.496	4.073	3.503	4.413	0.543	0.606
3.977	5.316	4.079	5.391	5.567	0.457	0.606
2.431	1.913	2.724	1.361	1.925	0.654	0.606
7.364	6.244	6.350	6.089	7.178	0.451	0.606
2.935	1.713	2.328	2.415	1.973	0.384	0.606
2.144	2.124	3.428	1.836	2.325	0.432	0.606
4.620	4.552	4.386	4.617	5.328	0.521	0.606

6.710	6.289	7.410	5.476	6.123	0.432	0.606
8.327	7.883	8.313	6.894	7.622	0.437	0.606
9.342	9.525	7.218	9.549	9.641	0.524	0.606
4.861	4.519	4.539	4.106	4.750	0.443	0.606
1.913	2.884	2.682	1.934	2.581	0.486	0.606
2.564	2.360	4.745	1.755	2.132	0.643	0.606
2.443	3.272	1.913	2.768	2.707	0.382	0.606
4.644	3.033	4.369	4.549	4.655	0.528	0.606
2.575	3.457	2.930	3.069	1.898	0.528	0.606
5.312	5.571	7.467	4.590	4.632	0.666	0.606
3.277	2.417	3.020	1.481	1.447	0.578	0.606
2.184	2.837	1.441	1.884	2.114	0.540	0.606
4.036	2.434	4.364	3.160	3.055	0.480	0.606
3.988	3.882	3.861	3.977	3.870	0.439	0.606
3.082	3.008	3.316	2.747	2.544	0.427	0.606
4.123	3.341	5.005	1.727	2.879	0.757	0.606
4.229	5.134	3.721	5.004	5.326	0.509	0.606
5.169	4.711	4.530	5.875	4.979	0.451	0.606
3.987	3.488	4.212	3.852	2.332	0.521	0.606
5.384	6.331	4.628	6.387	6.169	0.455	0.606
5.073	4.787	6.004	4.485	5.382	0.453	0.606
1.861	2.260	1.862	2.569	3.406	0.442	0.606
6.395	6.170	3.785	6.526	6.394	0.595	0.606
5.921	6.337	5.441	6.674	6.783	0.378	0.606
3.506	3.195	3.306	3.741	3.931	0.349	0.606
6.065	6.215	6.284	6.151	6.533	0.313	0.606
7.425	7.345	5.841	7.727	7.589	0.506	0.606
4.836	4.662	5.055	3.663	3.770	0.687	0.606
6.069	5.533	6.515	5.735	6.250	0.512	0.606
6.608	7.114	6.960	7.318	7.116	0.319	0.606
5.601	4.873	5.016	4.815	4.749	0.523	0.606
5.932	6.799	6.273	4.158	4.335	0.800	0.606
9.327	9.156	9.911	8.487	8.263	0.426	0.606
3.930	4.339	3.976	2.065	3.883	0.741	0.606
2.962	2.093	3.422	2.962	3.130	0.527	0.606
3.145	3.654	3.306	2.498	3.049	0.379	0.606
2.897	2.375	2.758	2.726	2.504	0.393	0.606
3.775	4.281	4.354	2.544	3.128	0.854	0.606
3.770	3.587	4.378	2.928	2.734	0.491	0.606
9.027	8.377	9.444	7.991	8.266	0.401	0.606
3.896	3.895	4.384	3.788	4.210	0.294	0.606
4.213	4.609	3.834	4.333	4.888	0.424	0.606
1.705	1.982	2.225	1.603	1.450	0.511	0.606
4.966	5.370	5.395	4.201	5.147	0.483	0.606
6.088	6.075	4.613	6.431	6.311	0.495	0.606
7.730	7.917	8.374	8.198	8.280	0.329	0.606
5.880	5.309	6.425	5.405	5.415	0.401	0.606
4.660	5.640	5.240	4.727	4.872	0.411	0.606
7.230	5.939	7.410	3.518	2.671	1.072	0.606
4.710	5.330	4.911	5.726	5.425	0.397	0.606

4.235	4.733	5.705	3.673	4.465	0.580	0.606
3.380	2.305	2.881	2.628	2.432	0.503	0.606
2.594	4.434	2.317	4.580	3.891	0.948	0.606
4.141	2.798	3.407	3.286	3.276	0.405	0.606
5.363	5.147	5.854	4.826	4.790	0.350	0.606
6.339	6.110	7.415	5.791	6.099	0.405	0.606
3.120	4.240	3.414	4.045	3.945	0.471	0.606
5.667	6.413	5.187	6.554	6.552	0.388	0.606
7.026	7.456	6.892	7.623	7.495	0.314	0.606
5.083	5.417	3.768	6.252	5.360	0.575	0.606
7.849	6.803	6.976	6.586	7.077	0.422	0.606
1.945	1.774	2.408	1.462	1.241	0.503	0.606
5.982	5.262	6.693	5.040	5.239	0.429	0.606
4.419	4.027	3.641	3.945	4.541	0.404	0.606
4.839	5.531	2.961	6.072	5.877	0.685	0.606
4.336	3.554	4.964	3.035	2.859	0.504	0.606
4.134	2.387	3.871	3.092	2.053	0.509	0.606
6.962	6.485	7.622	5.449	5.565	0.498	0.606
5.274	4.271	4.724	3.862	3.649	0.460	0.606
3.357	3.132	3.461	2.972	3.108	0.505	0.606
6.448	7.242	6.380	7.468	7.337	0.344	0.606
3.998	3.721	4.669	4.736	4.201	0.633	0.606
2.317	2.597	3.406	3.043	2.947	0.410	0.606
2.516	2.770	2.389	3.029	1.938	0.501	0.606
2.596	2.583	3.641	2.156	2.048	0.501	0.606
7.555	8.685	6.825	8.861	8.646	0.513	0.606
8.586	8.504	8.094	8.775	8.708	0.328	0.606
6.873	3.566	2.673	3.521	3.257	1.025	0.606
3.790	3.780	4.036	3.342	3.585	0.367	0.606
5.976	6.608	3.941	6.908	6.400	0.670	0.606
3.693	3.664	4.157	3.448	3.560	0.386	0.606
2.767	3.570	3.715	2.745	3.609	0.417	0.606
5.748	6.801	4.338	6.631	5.884	0.665	0.606
3.430	3.006	4.189	3.497	2.918	0.469	0.606
5.126	4.791	5.768	3.888	3.719	0.460	0.606
6.025	5.788	6.667	5.347	5.269	0.381	0.606
6.687	6.327	5.113	6.650	6.669	0.430	0.606
7.631	8.192	7.315	8.581	8.518	0.351	0.606
3.454	3.101	3.075	2.067	3.380	0.433	0.606
1.543	2.262	2.615	2.370	1.794	0.444	0.606
5.928	7.387	5.055	7.771	7.181	0.631	0.606
5.208	4.963	3.376	5.612	5.351	0.504	0.606
3.834	4.873	4.028	4.720	5.084	0.429	0.606
3.570	3.685	3.050	3.835	3.931	0.429	0.606
6.636	4.961	6.742	4.174	4.719	0.584	0.606
4.838	4.187	4.949	3.705	4.138	0.391	0.606
2.732	2.127	2.241	2.331	2.871	0.440	0.606
7.695	6.978	7.960	6.949	7.200	0.396	0.606
2.526	2.522	2.180	3.419	3.464	0.476	0.606
7.683	8.554	6.515	8.848	8.834	0.526	0.606

4.666	5.074	5.277	4.718	4.533	0.444	0.606
6.083	7.033	6.422	7.331	7.178	0.457	0.606
6.314	6.809	6.298	7.050	6.812	0.341	0.606
4.567	5.853	4.818	5.522	5.881	0.402	0.606
3.294	4.938	3.453	5.651	5.371	0.636	0.606
6.593	7.245	4.377	7.416	6.810	0.681	0.606
2.413	3.927	2.609	4.436	4.447	0.672	0.606
4.932	5.531	4.066	6.002	6.052	0.574	0.606
10.229	10.669	9.929	10.761	11.062	0.319	0.606
2.361	1.687	1.646	2.143	2.479	0.666	0.606
1.914	1.703	1.651	1.577	1.599	0.420	0.606
4.772	5.218	4.111	5.396	5.810	0.475	0.606
6.065	6.641	5.776	6.543	6.493	0.324	0.606
7.785	8.451	7.536	8.862	8.498	0.393	0.606
5.208	6.552	5.340	6.907	6.556	0.437	0.606
3.890	5.202	2.677	5.966	5.607	0.671	0.606
4.299	4.727	5.074	3.861	4.978	0.502	0.606
5.234	5.044	4.077	5.641	5.308	0.413	0.606
4.656	6.212	3.574	6.749	6.537	0.723	0.606
5.525	5.171	4.485	5.629	5.263	0.402	0.606
5.564	5.860	7.209	5.261	4.212	0.617	0.606
7.570	8.166	9.319	7.300	7.529	0.576	0.606
6.587	6.741	8.961	6.387	6.769	0.613	0.606
4.695	3.717	4.475	2.797	4.841	0.517	0.606
3.498	2.990	2.314	1.369	3.165	0.522	0.606
5.461	5.420	4.711	5.068	5.808	0.454	0.606
2.841	3.163	2.203	3.177	2.757	0.396	0.606
3.977	4.315	3.831	4.781	4.885	0.377	0.606
2.709	5.082	2.839	4.674	5.154	0.658	0.606
6.540	6.253	6.872	5.924	6.523	0.383	0.606
3.554	2.558	3.408	2.212	2.597	0.552	0.606
5.257	4.858	5.132	4.760	4.907	0.323	0.606
1.389	2.461	1.422	2.671	2.039	0.430	0.606
5.082	5.016	5.534	4.651	4.667	0.383	0.606
6.241	6.076	6.443	6.570	6.537	0.462	0.606
2.537	3.030	2.712	3.806	3.918	0.412	0.606
6.577	6.513	6.099	6.634	6.786	0.321	0.606
4.556	4.126	3.920	4.138	4.280	0.440	0.606
5.012	5.706	2.304	5.954	5.970	0.795	0.606
5.495	5.560	4.886	5.884	5.667	0.414	0.606
7.902	6.814	9.605	5.445	6.843	0.761	0.606
4.553	4.936	4.216	5.619	4.983	0.473	0.606
4.613	5.619	3.739	6.454	5.730	0.600	0.606
6.234	6.354	4.456	6.323	6.841	0.542	0.606
7.113	7.787	6.387	8.116	8.006	0.452	0.606
5.575	5.088	6.545	5.515	5.055	0.436	0.606
4.822	5.098	4.697	5.958	5.499	0.374	0.606
4.102	4.499	3.527	4.680	4.634	0.367	0.606
7.883	8.641	6.162	8.874	8.590	0.727	0.606
5.038	3.277	6.722	4.138	4.677	0.693	0.606

3.393	4.138	4.711	3.413	3.063	0.631	0.606
3.744	3.742	3.939	3.550	4.309	0.424	0.606
6.162	5.615	7.147	5.215	5.631	0.423	0.606
3.374	3.034	2.736	2.799	3.260	0.439	0.606
6.163	6.246	7.099	5.864	6.109	0.350	0.606
5.557	5.666	5.437	6.389	5.882	0.449	0.606
6.969	6.840	6.787	7.058	7.130	0.322	0.606
6.848	6.406	7.957	6.274	6.410	0.427	0.606
5.683	6.366	5.910	6.339	6.547	0.352	0.606
3.055	3.308	3.602	2.733	2.535	0.411	0.606
6.341	6.589	6.130	7.007	6.903	0.403	0.606
4.280	5.039	3.875	5.488	5.563	0.436	0.606
6.493	6.729	7.390	6.131	6.295	0.407	0.606
6.985	6.042	7.455	5.808	5.903	0.423	0.606
8.405	8.215	7.774	8.344	8.494	0.320	0.606
1.799	2.470	3.382	1.710	2.321	0.563	0.606
6.889	5.279	7.737	4.351	5.117	0.819	0.606
6.802	6.644	8.060	5.785	5.873	0.475	0.606
5.657	5.064	5.395	4.315	4.838	0.385	0.606
8.103	7.681	6.364	7.639	7.798	0.428	0.606
6.121	5.422	6.311	5.998	6.045	0.511	0.606
1.954	1.950	1.934	2.897	2.429	0.371	0.606
4.969	5.222	4.617	5.650	5.782	0.332	0.606
4.277	4.063	5.282	3.740	3.983	0.432	0.606
5.893	5.623	3.219	5.668	5.238	0.796	0.606
10.190	10.042	9.883	10.001	10.263	0.321	0.606
8.648	9.310	8.413	9.351	9.245	0.333	0.606
1.873	2.494	2.672	2.494	2.642	0.441	0.606
7.085	9.039	6.486	9.517	9.451	0.709	0.606
6.643	7.795	4.047	7.820	8.100	0.857	0.606
5.141	5.908	2.022	5.908	6.170	0.989	0.606
7.616	8.257	6.833	8.415	8.244	0.381	0.606
2.409	2.193	1.858	2.912	2.663	0.457	0.606
3.089	2.241	1.824	1.358	1.700	0.509	0.606
8.787	9.046	7.951	8.928	9.199	0.329	0.606
10.282	10.813	9.787	10.828	10.933	0.344	0.606
5.209	5.527	5.202	5.880	5.756	0.349	0.606
3.183	3.469	3.150	3.262	3.605	0.521	0.606
9.105	9.366	8.680	9.534	9.457	0.346	0.606
9.728	10.124	9.239	10.217	9.971	0.333	0.606
9.445	9.069	9.865	8.566	9.235	0.371	0.606
8.255	8.431	6.518	8.723	8.651	0.520	0.606
8.810	9.245	7.832	9.394	9.547	0.422	0.606
8.058	8.889	7.519	9.315	9.314	0.459	0.606
5.561	5.581	5.045	6.057	5.865	0.321	0.606
6.925	7.895	6.217	8.152	7.973	0.461	0.606
4.118	4.259	2.883	4.857	4.471	0.480	0.606
9.363	8.849	9.888	9.200	8.956	0.372	0.606
8.003	7.545	4.437	8.118	8.090	0.924	0.606
3.604	4.648	3.670	4.670	4.860	0.571	0.606

4.561	3.965	4.742	3.434	3.746	0.497	0.606
6.949	6.808	6.406	6.814	7.326	0.306	0.606
5.812	5.366	5.374	5.405	5.451	0.340	0.606
7.064	7.413	7.031	7.543	7.558	0.323	0.606
7.304	9.689	6.907	10.250	10.215	0.852	0.606
3.618	2.634	2.743	3.378	3.321	0.505	0.606
7.348	7.726	5.921	8.077	7.898	0.512	0.606
8.142	8.698	7.782	8.942	8.960	0.372	0.606
2.149	2.056	2.478	2.498	1.921	0.481	0.606
3.337	3.196	4.189	3.395	2.755	0.489	0.606
6.849	7.252	6.052	7.356	7.298	0.342	0.606
5.575	5.846	5.419	6.237	5.928	0.487	0.606
2.787	3.400	3.553	2.193	3.170	0.608	0.606
7.730	7.536	7.851	6.589	7.249	0.380	0.606
4.546	4.600	3.677	5.201	4.508	0.405	0.606
5.050	5.565	4.074	6.637	6.853	0.751	0.606
7.104	7.904	7.224	8.346	8.112	0.504	0.606
6.644	5.531	7.556	5.719	5.783	0.546	0.606
6.935	7.149	5.457	7.265	7.309	0.439	0.606
2.291	2.710	2.150	4.245	3.706	0.539	0.606
4.557	4.312	5.365	4.124	4.243	0.430	0.606
5.254	6.429	2.541	6.709	6.195	0.790	0.606
2.183	2.069	2.457	3.133	2.251	0.436	0.606
4.235	4.258	4.577	3.966	4.133	0.415	0.606
7.501	7.787	6.763	7.824	7.872	0.347	0.606
6.638	7.487	3.438	7.723	7.706	0.876	0.606
3.688	4.780	3.328	4.001	3.568	0.451	0.606
2.328	3.486	3.209	1.682	2.209	0.573	0.606
1.924	3.024	3.235	3.289	2.303	0.459	0.606
2.462	2.635	2.341	1.972	2.917	0.403	0.606
8.125	7.680	8.878	7.154	7.029	0.495	0.606
4.220	4.156	4.161	3.528	3.247	0.465	0.606
3.684	3.166	3.218	2.442	2.330	0.485	0.606
2.676	2.034	2.790	2.259	2.190	0.449	0.606
4.387	4.781	5.349	5.540	5.311	0.434	0.606
5.724	5.478	5.997	5.075	4.905	0.575	0.606
4.856	5.824	4.535	6.073	5.899	0.405	0.606
5.486	5.635	3.664	5.978	5.671	0.616	0.606
4.954	6.386	4.703	6.501	6.092	0.488	0.606
7.301	8.368	7.565	8.768	8.833	0.494	0.606
4.379	6.106	4.248	6.225	5.871	0.580	0.606
4.359	4.184	3.423	5.296	4.581	0.448	0.606
2.859	3.230	3.243	3.748	3.849	0.449	0.606
6.108	6.620	4.407	6.435	5.725	0.634	0.606
6.101	5.556	7.343	4.915	5.293	0.514	0.606
5.680	7.025	5.575	7.003	6.675	0.446	0.606
4.206	4.446	4.643	3.894	3.750	0.415	0.606
1.379	1.382	1.090	1.193	1.044	0.404	0.606
8.833	9.897	6.484	10.146	9.925	0.776	0.606
3.320	3.338	4.017	3.011	3.626	0.464	0.606

4.180	3.780	4.233	4.003	3.962	0.424	0.606
4.811	4.596	5.869	3.928	4.508	0.438	0.606
8.013	7.164	8.456	7.160	7.155	0.387	0.606
4.008	3.434	3.642	3.407	3.640	0.426	0.606
4.938	5.706	6.207	5.749	6.122	0.456	0.606
7.071	7.403	6.580	7.589	7.382	0.376	0.606
6.195	5.824	6.884	5.564	5.271	0.386	0.606
6.569	6.411	7.547	6.772	6.722	0.441	0.606
3.995	3.625	4.003	3.876	3.701	0.389	0.606
7.403	6.449	7.320	5.482	5.807	0.482	0.606
4.324	5.009	3.783	5.147	4.764	0.416	0.606
6.666	6.182	5.328	6.030	6.262	0.456	0.606
2.270	3.102	2.551	3.556	3.136	0.373	0.606
3.622	3.057	4.238	3.081	2.774	0.450	0.606
4.026	5.708	2.863	6.416	6.109	0.813	0.606
2.769	4.422	1.978	4.254	5.523	0.739	0.606
6.503	5.934	7.124	6.156	6.220	0.375	0.606
2.097	3.963	2.912	2.694	3.014	0.716	0.606
3.352	4.130	4.044	3.332	4.057	0.409	0.606
7.070	8.158	8.450	7.713	7.402	0.473	0.606
4.549	3.064	1.988	3.277	2.704	0.527	0.606
5.391	6.272	5.506	6.855	6.250	0.419	0.606
4.357	3.437	4.626	3.714	4.070	0.396	0.606
1.916	3.060	1.862	2.078	2.379	0.560	0.606
5.911	4.989	5.916	4.687	5.061	0.452	0.606
4.122	4.262	5.040	4.371	3.980	0.429	0.606
4.759	4.581	4.423	4.929	5.257	0.355	0.606
4.694	3.687	4.952	3.393	3.962	0.419	0.606
4.231	3.301	4.261	3.010	3.064	0.449	0.606
3.421	3.318	2.838	4.414	3.820	0.418	0.606
2.502	3.379	1.728	3.717	2.952	0.560	0.606
2.692	3.113	1.774	3.346	3.071	0.464	0.606
4.639	5.119	3.945	5.753	5.153	0.465	0.606
6.734	6.418	5.348	7.028	7.055	0.559	0.606
7.709	6.901	7.853	4.407	5.174	0.805	0.606
2.587	3.890	3.244	1.815	2.592	0.674	0.606
9.071	9.239	6.707	9.620	9.514	0.699	0.606
4.892	5.312	4.266	5.495	5.206	0.470	0.606
5.677	5.183	3.601	5.475	5.814	0.565	0.606
6.170	7.399	7.609	5.873	6.538	0.509	0.606
2.829	3.399	2.368	3.558	3.340	0.508	0.606
5.844	6.290	5.450	7.274	6.484	0.456	0.606
2.380	3.395	2.255	3.105	3.272	0.431	0.606
2.910	3.367	3.410	3.296	3.042	0.352	0.606
6.649	7.351	6.424	7.300	7.177	0.411	0.606
8.503	7.864	8.384	7.413	7.444	0.387	0.606
3.307	2.740	2.783	2.647	2.832	0.379	0.606
3.529	4.571	3.988	5.091	5.348	0.529	0.606
4.854	5.157	5.098	4.336	4.999	0.515	0.606
3.315	4.259	5.072	3.423	3.513	0.565	0.606

2.052	2.913	2.919	2.723	2.494	0.430	0.606
3.550	2.945	4.552	1.958	2.388	0.707	0.606
4.981	3.600	4.609	3.229	4.038	0.790	0.606
3.909	4.866	4.642	4.201	4.119	0.399	0.606
7.307	6.496	7.046	6.675	6.887	0.391	0.606
3.254	3.569	2.198	3.779	4.179	0.523	0.606
2.865	4.208	3.800	4.473	5.544	0.655	0.606
1.416	1.506	1.239	3.298	4.655	1.053	0.606
3.812	3.843	3.140	4.047	5.374	0.639	0.606
4.369	3.901	5.768	4.068	4.019	0.510	0.606
4.961	4.682	5.328	4.627	4.370	0.378	0.606
2.906	3.351	4.585	3.229	2.843	0.547	0.606
4.603	5.408	3.935	5.774	5.370	0.468	0.606
9.094	9.116	8.260	9.435	9.481	0.328	0.606
6.732	6.049	6.047	6.355	6.412	0.349	0.606
4.456	3.502	3.441	3.545	4.543	0.545	0.606
3.392	2.857	3.785	2.211	2.512	0.622	0.606
2.996	2.877	3.467	2.278	2.503	0.499	0.606
4.125	3.382	3.917	4.139	3.565	0.407	0.606
7.292	6.799	8.766	5.555	5.219	0.667	0.606
3.929	4.357	4.045	5.150	4.748	0.454	0.606
7.133	7.465	6.801	7.709	7.598	0.320	0.606
6.686	6.695	6.311	6.936	7.313	0.325	0.606
2.505	2.822	2.249	2.197	2.732	0.451	0.606
2.296	2.047	1.975	1.765	1.830	0.325	0.606
7.008	7.877	5.809	8.117	8.039	0.547	0.606
5.260	6.856	4.654	7.256	6.934	0.641	0.606
7.174	6.927	5.852	7.373	7.231	0.474	0.606
4.204	4.593	3.947	5.187	4.936	0.366	0.606
4.793	5.568	5.386	5.841	5.522	0.379	0.606
7.349	7.560	6.437	7.730	7.279	0.416	0.606
5.623	5.998	5.254	6.459	6.379	0.367	0.606
5.346	7.279	3.715	7.808	7.099	0.861	0.606
2.121	3.292	1.617	3.476	3.206	0.474	0.606
2.862	2.970	3.576	1.967	2.829	0.445	0.606
5.137	6.055	5.397	4.396	5.312	0.461	0.606
5.618	6.377	5.516	6.646	6.607	0.380	0.606
5.154	4.801	3.258	4.948	4.625	0.534	0.606
2.783	2.365	3.183	1.673	1.609	0.433	0.606
3.061	3.032	2.245	2.626	2.493	0.461	0.606
3.578	2.589	2.508	2.576	2.157	0.562	0.606
6.885	6.842	7.632	7.415	6.954	0.372	0.606
4.492	4.962	4.434	5.513	4.916	0.411	0.606
5.227	4.459	6.359	4.444	4.882	0.484	0.606
6.164	6.585	5.286	7.007	6.963	0.421	0.606
7.062	7.622	7.914	6.637	6.590	0.462	0.606
2.996	3.507	4.432	2.824	3.024	0.459	0.606
3.632	4.206	4.887	3.463	3.910	0.592	0.606
7.671	7.965	7.188	8.198	8.081	0.322	0.606
6.800	6.579	8.078	5.255	5.531	0.611	0.606

3.161	2.462	4.573	2.305	2.500	0.574	0.606
4.166	5.141	2.635	5.567	5.418	0.605	0.606
3.079	4.617	3.256	4.523	4.852	0.487	0.606
4.528	6.989	4.410	5.384	4.930	0.788	0.606
4.638	2.698	4.679	1.226	1.667	0.828	0.606
4.936	3.834	5.182	4.080	4.228	0.475	0.606
3.211	2.191	2.897	2.479	2.481	0.464	0.606
4.765	3.812	5.761	2.117	3.080	0.731	0.606
3.008	3.356	3.480	2.580	3.646	0.439	0.606
2.607	1.888	1.333	2.795	2.303	0.487	0.606
4.287	4.027	4.270	3.234	3.553	0.540	0.606
4.616	4.106	5.124	3.582	3.638	0.480	0.606
5.320	5.997	5.274	5.928	5.841	0.428	0.606
5.625	4.208	6.136	3.381	3.813	0.677	0.606
5.217	4.943	5.972	5.043	4.756	0.413	0.606
3.687	2.994	5.492	3.467	3.167	0.531	0.606
4.406	3.221	5.881	2.395	3.031	0.708	0.606
3.792	2.612	2.678	2.769	3.485	0.540	0.606
3.748	3.530	3.422	3.942	4.579	0.400	0.606
4.316	4.855	3.401	4.893	4.764	0.402	0.606
4.588	7.551	1.463	7.702	7.457	1.378	0.606
2.942	1.278	1.006	0.925	0.941	0.748	0.606
2.849	1.828	2.123	2.193	2.238	0.495	0.606
2.070	3.429	0.822	2.374	2.645	0.637	0.606
6.027	6.463	6.375	6.735	6.807	0.302	0.606
6.122	6.527	6.222	6.825	6.748	0.394	0.606
4.768	5.142	4.347	5.533	5.263	0.337	0.606
4.257	4.239	4.295	4.466	4.296	0.385	0.606
3.634	2.238	2.739	2.400	2.296	0.605	0.606
4.706	4.286	6.025	4.667	4.760	0.402	0.606
6.092	6.756	4.543	6.882	6.448	0.479	0.606
2.551	3.219	2.250	3.673	3.623	0.426	0.606
4.026	4.417	3.511	4.672	4.721	0.378	0.606
1.536	4.182	2.843	4.564	4.833	0.749	0.606
6.232	5.375	4.652	5.792	6.340	0.517	0.606
2.888	4.166	1.808	4.044	4.298	0.647	0.606
3.445	3.320	3.522	3.372	3.697	0.382	0.606
4.301	3.721	3.651	3.287	2.937	0.370	0.606
6.608	6.455	5.299	6.342	6.483	0.487	0.606
4.542	5.268	4.170	5.831	5.840	0.478	0.606
5.301	4.747	4.587	5.312	5.305	0.467	0.606
5.747	6.262	5.916	6.486	6.378	0.346	0.606
7.211	7.778	6.627	7.893	7.883	0.360	0.606
4.845	5.676	4.767	5.784	5.956	0.369	0.606
7.269	7.690	7.714	7.992	7.518	0.444	0.606
5.166	6.216	4.801	6.530	6.347	0.471	0.606
3.799	4.287	4.612	3.968	4.379	0.427	0.606
4.063	2.934	2.512	3.093	3.236	0.393	0.606
4.283	4.893	4.435	5.522	5.254	0.406	0.606
2.835	4.052	2.300	3.895	4.019	0.509	0.606

2.756	2.412	2.691	2.535	2.554	0.394	0.606
4.658	5.179	4.351	5.986	5.124	0.512	0.606
3.186	3.622	2.773	4.074	3.974	0.432	0.606
5.801	6.514	6.610	5.384	5.721	0.431	0.606
5.817	5.905	5.335	6.114	6.392	0.404	0.606
2.653	3.643	2.124	4.157	3.779	0.557	0.606
5.491	5.460	4.725	4.991	5.524	0.452	0.606
3.598	3.680	3.612	4.227	3.968	0.380	0.606
6.084	6.371	5.605	6.728	6.705	0.349	0.606
3.888	3.087	2.194	3.940	3.022	0.585	0.606
4.566	4.416	5.632	3.594	3.536	0.506	0.606
4.192	4.360	3.747	4.884	4.082	0.350	0.606
3.878	2.857	2.949	2.645	2.754	0.509	0.606
3.553	4.422	3.232	4.372	4.070	0.441	0.606
2.235	2.178	1.530	1.791	2.261	0.545	0.606
2.848	2.814	2.848	2.559	2.267	0.406	0.606
3.765	4.798	5.760	3.563	3.838	0.675	0.606
3.616	3.320	4.203	2.825	3.274	0.521	0.606
2.396	2.253	2.913	3.456	2.871	0.448	0.606
1.906	3.225	2.035	2.045	2.885	0.488	0.606
3.846	4.280	5.158	4.363	3.799	0.432	0.606
1.851	2.632	2.541	2.624	2.530	0.431	0.606
6.410	6.058	7.219	5.647	5.982	0.470	0.606
1.404	2.895	2.418	2.145	2.721	0.561	0.606
4.016	7.759	3.966	8.189	7.614	1.020	0.606
3.295	1.394	1.125	1.137	0.953	0.720	0.606
4.626	5.917	5.863	3.933	4.744	0.794	0.606
5.579	4.007	3.692	3.124	3.294	0.855	0.606
3.417	2.993	2.659	3.204	3.037	0.492	0.606
5.854	5.611	7.007	4.914	5.081	0.493	0.606
5.489	5.891	5.053	6.329	6.454	0.440	0.606
3.628	4.212	3.005	4.742	4.489	0.444	0.606
3.345	1.935	2.273	2.769	3.568	0.457	0.606
2.441	2.795	3.234	2.440	2.093	0.405	0.606
3.014	3.867	2.337	3.178	3.156	0.464	0.606
5.438	4.931	3.098	4.727	5.131	0.541	0.606
4.376	4.255	4.350	3.722	4.488	0.372	0.606
6.355	5.013	5.347	5.379	5.860	0.505	0.606
3.910	3.865	3.864	3.670	4.079	0.475	0.606
4.099	4.392	4.426	4.103	4.242	0.360	0.606
3.523	1.835	1.588	1.590	1.405	0.843	0.606
2.001	2.116	1.753	2.084	2.508	0.442	0.606
4.204	5.049	3.391	4.584	4.083	0.430	0.606
2.592	3.904	4.068	2.397	2.951	0.706	0.606
5.928	7.278	7.484	4.630	6.124	0.831	0.606
6.454	7.557	7.779	5.134	6.516	0.764	0.606
4.559	4.425	3.873	3.531	3.085	0.576	0.606
4.780	6.067	4.352	6.243	5.932	0.576	0.606
5.053	7.943	2.670	8.994	8.807	1.343	0.606
1.727	2.187	2.012	2.111	1.933	0.532	0.606

4.896	5.857	2.955	5.898	6.217	0.748	0.606
3.289	2.653	4.443	3.223	3.367	0.490	0.606
2.285	2.141	2.096	2.385	2.407	0.425	0.606
3.389	4.382	3.307	2.858	3.190	0.532	0.606
3.588	4.559	3.559	3.096	3.709	0.468	0.606
4.294	5.387	4.693	4.406	5.171	0.495	0.606
2.993	4.073	3.642	3.090	3.736	0.468	0.606
3.082	4.168	3.761	3.167	3.810	0.477	0.606
3.077	4.096	3.739	3.128	3.787	0.461	0.606
3.231	4.206	3.824	3.056	3.886	0.456	0.606
3.068	4.154	3.665	2.867	3.509	0.544	0.606
3.491	3.171	3.559	3.198	2.859	0.416	0.606
3.202	2.833	2.416	2.596	2.754	0.428	0.606
2.710	4.177	1.837	4.877	4.020	0.662	0.606
2.205	2.153	2.232	2.398	3.373	0.394	0.606
2.722	2.809	2.798	4.424	3.997	0.548	0.606
5.542	2.509	4.857	1.543	1.180	1.190	0.606
2.775	2.611	4.264	2.379	2.357	0.471	0.606
4.398	3.656	4.768	2.599	3.288	0.535	0.606
4.228	3.910	3.688	3.921	3.909	0.367	0.606
5.058	4.204	6.563	3.246	3.746	0.667	0.606
1.760	3.711	1.578	4.081	4.306	0.683	0.606
4.888	5.064	4.701	5.589	5.060	0.448	0.606
4.082	3.220	3.961	3.164	3.040	0.433	0.606
3.596	2.669	3.434	3.438	2.472	0.545	0.606
6.715	6.475	6.926	6.506	6.433	0.324	0.606
3.354	3.906	3.292	3.484	4.255	0.408	0.606
6.831	7.218	6.592	7.358	7.547	0.333	0.606
2.983	3.406	2.553	4.240	3.620	0.495	0.606
3.490	2.262	2.647	2.720	2.561	0.410	0.606
3.261	3.137	2.424	4.214	4.289	0.520	0.606
5.904	5.614	6.735	4.219	5.089	0.490	0.606
5.029	5.063	4.991	5.732	5.344	0.340	0.606
6.057	6.040	5.506	6.067	5.623	0.406	0.606
4.574	6.254	2.814	6.407	6.747	0.941	0.606
3.788	3.295	4.174	3.177	2.977	0.535	0.606
3.081	2.864	3.433	2.250	2.309	0.411	0.606
8.654	8.605	9.501	6.269	8.180	0.727	0.606
2.973	3.688	3.953	2.835	3.241	0.541	0.606
5.253	5.599	4.987	5.993	5.881	0.400	0.606
5.498	5.602	5.767	5.473	5.749	0.400	0.606
2.555	2.128	2.190	2.784	2.430	0.342	0.606
4.400	3.767	4.615	3.698	3.586	0.436	0.606
4.598	4.546	5.516	4.509	4.438	0.394	0.606
2.365	3.278	2.231	3.505	2.967	0.428	0.606
2.395	6.057	1.459	6.276	6.568	1.178	0.606
7.457	6.301	5.837	4.172	5.041	0.706	0.606
7.777	6.618	6.120	4.414	5.276	0.722	0.606
7.652	6.498	5.986	4.421	5.219	0.712	0.606
7.780	6.617	6.096	4.447	5.305	0.723	0.606

7.811	6.647	6.151	4.389	5.276	0.733	0.606
7.828	6.635	6.119	4.347	5.276	0.740	0.606
7.828	6.683	6.071	4.441	5.347	0.719	0.606
6.956	5.880	5.354	4.054	4.795	0.645	0.606
7.104	5.990	5.505	4.111	4.729	0.663	0.606
1.654	1.634	1.561	2.131	2.268	0.449	0.606
4.169	4.317	5.193	3.495	3.933	0.422	0.606
2.809	2.403	3.358	2.627	2.886	0.497	0.606
3.129	4.197	2.484	4.346	3.602	0.654	0.606
4.051	3.116	5.045	3.725	3.681	0.499	0.606
2.871	3.583	5.143	3.164	3.309	1.064	0.606
2.168	3.813	3.389	1.336	2.258	0.655	0.606
6.124	6.068	5.979	6.344	6.608	0.366	0.606
2.372	3.546	2.679	2.432	3.019	0.967	0.606
4.617	5.431	5.921	5.598	5.219	0.456	0.606
2.154	2.346	1.228	2.440	2.329	0.376	0.606
3.378	2.451	2.335	2.497	2.970	0.394	0.606
4.666	4.985	3.521	4.906	4.559	0.682	0.606
3.696	3.405	3.494	3.475	3.440	0.457	0.606
3.241	2.606	3.077	3.045	2.333	0.431	0.606
5.492	2.692	5.188	2.426	2.747	0.827	0.606
7.115	7.362	6.226	7.491	7.528	0.353	0.606
5.699	5.832	5.652	6.092	6.210	0.357	0.606
5.141	5.312	4.197	5.481	6.323	0.644	0.606
3.676	3.352	1.898	3.392	4.022	0.623	0.606
5.207	5.608	5.109	5.720	5.989	0.417	0.606
2.412	2.147	2.141	1.960	1.547	0.530	0.606
8.228	8.435	8.916	7.621	7.882	0.368	0.606
6.604	7.045	7.452	6.290	6.356	0.381	0.606
7.056	7.480	7.804	6.573	6.774	0.386	0.606
6.823	7.176	7.512	6.472	6.638	0.326	0.606
6.839	7.343	7.635	6.319	6.583	0.404	0.606
6.728	7.109	7.504	6.427	6.628	0.325	0.606
4.096	3.652	4.062	4.260	4.724	0.423	0.606
4.655	3.767	5.339	4.187	4.875	0.526	0.606
3.763	4.271	3.592	3.897	4.915	0.626	0.606
2.203	2.986	3.202	2.303	2.349	0.463	0.606
2.226	1.547	1.204	1.311	0.940	0.541	0.606
5.494	5.727	4.655	6.777	6.112	0.435	0.606
3.653	3.341	4.527	3.076	2.960	0.506	0.606
3.322	3.299	2.168	3.354	3.785	0.439	0.606
2.720	2.736	2.286	2.717	2.580	0.410	0.606
1.321	1.302	1.762	1.856	1.622	0.934	0.606
2.355	2.298	3.774	2.343	3.472	0.450	0.606
2.035	3.640	3.001	2.941	3.045	0.493	0.606
5.882	5.437	6.570	5.109	5.494	0.400	0.606
3.617	3.885	3.383	2.560	3.201	0.601	0.606
5.599	5.713	6.311	5.662	6.247	0.318	0.606
2.964	2.936	2.800	2.742	2.144	0.397	0.606
3.803	3.388	4.403	3.629	3.733	0.457	0.606

3.383	2.282	1.940	1.694	2.211	0.895	0.606
3.238	3.716	1.784	4.419	3.980	0.616	0.606
5.428	5.217	5.695	4.722	4.757	0.354	0.606
2.304	2.074	3.235	2.697	1.982	0.479	0.606
3.386	4.427	2.440	4.565	3.903	0.498	0.606
3.792	2.734	4.236	3.090	2.403	0.464	0.606
4.300	5.240	4.276	5.668	5.176	0.404	0.606
4.172	3.923	3.578	4.355	4.675	0.434	0.606
5.006	4.025	5.377	4.006	4.080	0.423	0.606
4.510	5.498	5.147	5.851	6.020	0.442	0.606
2.335	2.772	2.380	1.896	2.280	0.399	0.606
4.101	4.614	4.671	3.596	3.980	0.352	0.606
5.440	7.001	4.978	7.418	7.124	0.580	0.606
6.630	7.548	5.416	8.196	7.880	0.588	0.606
7.035	9.297	6.851	9.342	9.001	0.765	0.606
3.202	4.010	3.410	3.824	3.911	0.365	0.606
5.420	4.071	5.109	2.327	4.009	0.809	0.606
7.242	5.791	7.794	5.643	5.722	0.550	0.606
2.698	4.495	2.725	4.251	3.450	0.643	0.606
5.015	5.108	4.819	4.455	5.155	0.596	0.606
4.602	4.789	4.735	5.431	4.702	0.484	0.606
1.789	4.738	1.462	4.693	4.585	0.868	0.606
5.552	4.967	6.313	5.737	5.089	0.438	0.606
5.283	5.552	5.419	5.699	5.536	0.420	0.606
6.514	6.580	4.715	6.881	7.408	0.654	0.606
5.112	5.294	4.462	6.094	5.757	0.415	0.606
2.987	5.216	2.236	6.051	5.797	0.819	0.606
5.991	2.810	3.173	3.471	3.344	0.772	0.606
5.570	5.651	6.323	5.646	5.597	0.383	0.606
6.187	7.203	4.820	7.561	7.208	0.635	0.606
5.551	6.291	2.512	6.418	6.232	0.749	0.606
4.573	5.660	3.146	5.984	5.847	0.575	0.606
1.838	2.838	2.899	1.752	2.278	0.445	0.606
5.388	5.738	4.746	6.288	5.914	0.456	0.606
5.685	6.732	2.783	7.243	6.739	0.958	0.606
3.055	3.852	1.774	4.185	3.411	0.517	0.606
4.013	3.804	3.170	4.111	3.922	0.495	0.606
3.999	5.399	2.552	5.982	4.964	0.717	0.606
7.208	7.827	7.081	8.222	8.102	0.419	0.606
6.453	5.615	2.888	5.762	5.635	0.724	0.606
6.020	6.933	4.674	7.355	7.156	0.623	0.606
6.662	7.513	4.559	7.724	7.638	0.672	0.606
5.693	6.804	3.089	7.404	7.253	0.821	0.606
3.730	5.084	2.709	5.149	4.856	0.552	0.606
6.407	7.877	5.369	8.324	8.163	0.636	0.606
6.809	7.443	3.804	7.795	7.799	0.979	0.606
7.413	9.591	4.395	10.083	9.853	1.178	0.606
6.024	6.031	6.053	6.004	5.617	0.456	0.606
2.157	2.563	2.955	2.342	2.305	0.523	0.606
3.906	5.381	1.527	5.672	5.598	0.898	0.606

7.856	7.573	7.083	8.232	8.062	0.436	0.606
8.531	8.821	4.406	9.097	9.065	0.875	0.606
5.577	5.957	4.089	6.228	6.195	0.498	0.606
8.231	8.155	5.058	8.504	8.412	0.732	0.606
5.696	7.880	4.831	7.892	7.932	0.770	0.606
5.570	4.922	6.530	4.425	4.562	0.473	0.606
7.005	7.459	3.234	7.585	6.944	0.967	0.606
9.543	6.661	2.303	7.393	6.928	1.411	0.606
5.831	4.969	5.489	4.796	5.164	0.505	0.606
2.600	4.376	2.201	3.197	3.905	0.489	0.606
3.187	4.000	4.232	3.645	4.004	0.391	0.606
4.789	5.132	3.519	4.906	5.112	0.398	0.606
5.968	5.845	3.050	6.041	6.090	0.747	0.606
4.601	4.981	3.326	5.076	4.750	0.448	0.606
6.522	7.905	5.596	8.441	8.310	0.613	0.606
5.448	7.235	1.270	7.635	7.661	1.295	0.606
7.132	10.497	5.590	10.683	10.509	1.133	0.606
7.214	8.356	5.651	8.596	8.252	0.677	0.606
6.405	6.875	6.355	7.199	6.962	0.336	0.606
7.833	7.798	5.973	7.625	7.982	0.529	0.606
7.676	8.426	7.743	8.772	8.611	0.462	0.606
6.804	6.076	3.526	5.753	5.895	0.758	0.606
4.748	6.674	3.956	7.319	7.441	0.820	0.606
4.791	6.381	3.785	6.711	6.682	0.687	0.606
5.392	7.946	5.308	7.728	7.671	0.719	0.606
5.242	8.027	3.211	8.171	7.692	1.133	0.606
8.778	8.919	8.280	9.333	9.317	0.341	0.606
3.567	4.262	2.292	4.679	4.000	0.543	0.606
7.874	7.646	6.716	7.943	8.150	0.387	0.606
7.026	7.912	6.352	7.898	7.734	0.461	0.606
6.791	6.870	6.419	7.237	7.286	0.347	0.606
8.095	9.655	5.956	9.729	9.624	0.800	0.606
2.598	2.832	2.707	2.067	2.974	0.420	0.606
2.886	2.392	3.905	1.785	2.019	0.517	0.606
7.962	6.719	8.205	4.963	5.601	0.738	0.606
6.916	6.479	5.799	6.597	6.730	0.371	0.606
5.733	4.260	5.209	3.540	3.990	0.601	0.606
4.571	3.990	3.786	4.253	4.313	0.412	0.606
3.061	2.111	2.708	1.994	2.551	0.487	0.606
4.662	4.305	5.177	4.103	4.456	0.351	0.606
8.052	6.383	8.347	5.281	5.616	0.810	0.606
5.537	6.045	2.337	7.348	5.448	1.055	0.606
2.694	2.609	1.659	2.725	2.959	0.549	0.606
4.126	4.656	4.560	4.665	4.249	0.475	0.606
3.169	3.419	3.543	3.913	4.183	0.543	0.606
7.947	7.564	7.233	8.054	7.966	0.353	0.606
6.457	6.496	4.297	6.837	6.697	0.621	0.606
1.777	1.768	2.124	1.582	2.096	0.450	0.606
2.810	2.540	3.632	2.806	3.249	0.411	0.606
2.207	2.199	2.667	1.641	2.768	0.473	0.606

1.541	2.021	2.114	1.386	1.647	0.421	0.606
3.855	4.284	3.642	6.182	5.896	0.680	0.606
6.617	6.768	8.164	6.406	6.609	0.444	0.606
2.346	2.274	3.441	2.853	2.649	0.399	0.606
4.433	3.892	3.894	3.850	3.818	0.446	0.606
4.512	4.822	4.574	4.477	4.449	0.419	0.606
7.503	7.409	8.601	6.835	6.981	0.395	0.606
4.123	3.018	1.888	2.998	2.972	0.556	0.606
4.385	7.308	3.004	7.546	7.375	1.145	0.606
2.862	3.287	3.491	3.436	3.522	0.440	0.606
3.338	5.532	2.039	5.828	6.276	0.893	0.606
5.767	5.928	6.920	5.139	5.461	0.428	0.606
2.025	3.088	3.083	3.772	3.163	0.460	0.606
2.224	2.774	1.141	2.274	2.558	0.448	0.606
6.131	7.048	6.142	7.150	6.975	0.382	0.606
4.324	3.501	4.274	3.099	3.584	0.374	0.606
1.628	4.533	1.709	2.701	4.410	0.731	0.606
4.592	4.453	5.076	4.974	4.945	0.444	0.606
2.600	2.886	2.060	2.943	2.829	0.455	0.606
5.912	5.811	4.857	6.145	6.351	0.372	0.606
4.031	4.624	3.871	3.585	4.428	0.485	0.606
5.506	4.524	5.732	4.364	4.347	0.613	0.606
2.223	3.043	1.678	4.327	3.245	0.579	0.606
5.674	5.884	4.371	5.852	6.514	0.456	0.606
2.959	1.937	2.033	2.418	1.844	0.535	0.606
3.831	3.238	4.266	2.819	2.947	0.540	0.606
5.417	5.752	5.449	6.040	6.224	0.333	0.606
2.178	2.467	2.318	2.532	2.234	0.446	0.606
3.381	3.226	4.137	1.720	2.258	0.617	0.606
6.600	5.886	6.337	6.275	5.967	0.420	0.606
6.215	5.293	5.691	5.592	5.611	0.487	0.606
4.561	2.702	4.956	5.070	4.790	0.711	0.606
4.948	2.894	4.929	5.143	4.817	0.799	0.606
6.349	5.768	7.060	6.634	6.125	0.476	0.606
5.036	4.391	4.106	4.448	4.460	0.408	0.606
2.235	2.681	1.810	4.017	3.567	0.538	0.606
5.140	5.214	4.564	5.769	5.572	0.314	0.606
3.552	3.926	3.782	3.871	4.026	0.513	0.606
5.206	3.543	4.305	3.373	3.773	0.463	0.606
4.265	3.346	3.922	3.041	3.107	0.421	0.606
3.919	2.133	2.961	2.236	3.022	0.489	0.606
3.582	3.706	3.266	2.289	3.245	0.531	0.606
3.725	2.890	2.374	3.442	2.958	0.465	0.606
2.739	2.370	2.168	2.651	2.457	0.569	0.606
4.067	5.474	2.217	5.806	5.271	0.768	0.606
3.403	1.978	2.796	2.326	2.618	0.500	0.606
4.909	5.117	2.921	5.003	4.731	0.553	0.606
2.796	2.243	2.188	2.326	1.856	0.551	0.606
3.599	5.400	3.430	5.711	5.355	0.614	0.606
4.540	4.459	4.038	4.972	4.556	0.416	0.606

3.435	2.855	3.090	3.769	2.883	0.487	0.606
4.401	3.021	2.953	4.351	3.922	0.545	0.606
2.597	3.531	2.217	3.715	3.788	0.478	0.606
1.934	1.617	1.613	1.285	1.475	0.637	0.606
3.616	2.818	2.253	3.053	3.195	0.425	0.606
3.765	2.974	2.874	3.181	2.896	0.454	0.606
7.966	8.430	7.334	8.777	8.556	0.369	0.606
1.829	1.322	1.430	1.586	1.462	0.521	0.606
3.810	3.730	2.044	4.445	3.950	0.529	0.606
2.025	2.543	1.387	2.506	2.353	0.380	0.606
2.240	2.680	1.869	3.745	3.063	0.444	0.606
4.183	3.491	2.913	3.136	3.617	0.496	0.606
4.260	4.570	4.366	4.317	4.736	0.447	0.606
4.191	4.200	3.662	4.076	4.030	0.492	0.606
3.350	3.571	3.726	2.806	3.378	0.393	0.606
4.000	3.662	4.398	2.736	3.948	0.541	0.606
2.757	1.460	2.456	1.466	1.720	0.498	0.606
4.717	6.345	4.684	6.441	6.044	0.482	0.606
3.908	4.363	3.942	4.869	4.628	0.413	0.606
2.632	2.418	3.189	3.741	3.617	0.516	0.606
2.112	4.692	2.154	5.700	5.115	0.836	0.606
2.626	4.300	2.414	5.103	4.742	0.574	0.606
2.494	3.064	2.750	2.824	3.109	0.531	0.606
4.702	4.902	6.566	4.981	3.846	1.030	0.606
2.301	2.150	2.398	1.746	1.788	0.619	0.606
2.396	3.453	2.997	2.951	2.291	0.561	0.606
1.911	2.217	1.645	1.381	1.582	0.632	0.606
3.313	1.533	3.225	2.868	2.806	0.586	0.606
4.173	3.223	3.195	3.411	4.114	0.443	0.606
4.107	4.712	1.638	5.076	4.579	0.710	0.606
3.726	3.953	5.160	4.082	3.893	0.603	0.606
3.008	2.656	1.924	3.627	3.400	0.419	0.606
2.905	1.941	2.821	1.977	1.475	0.472	0.606
4.771	4.951	4.925	3.844	4.738	0.475	0.606
3.484	4.180	3.075	3.615	4.060	0.476	0.606
6.413	6.253	5.835	6.480	6.470	0.306	0.606
4.235	4.235	3.391	4.669	4.935	0.420	0.606
5.450	3.935	6.157	3.299	4.113	0.694	0.606
2.671	3.914	2.881	3.469	3.276	0.440	0.606
3.026	4.293	3.416	4.406	4.209	0.401	0.606
6.194	6.251	7.118	6.057	6.143	0.408	0.606
1.612	2.971	1.900	3.156	2.685	0.483	0.606
4.709	4.734	3.991	5.032	4.742	0.423	0.606
8.103	7.495	8.825	7.646	7.431	0.439	0.606
3.215	3.635	3.546	2.572	3.371	0.705	0.606
2.600	2.446	3.114	2.763	2.748	0.420	0.606
3.611	5.000	4.171	4.800	4.809	0.433	0.606
5.432	6.316	6.034	6.557	6.389	0.336	0.606
3.032	3.888	1.791	4.340	4.283	0.605	0.606
3.499	3.651	4.228	3.062	3.699	0.455	0.606

3.542	3.572	4.162	3.444	3.953	0.492	0.606
5.217	6.438	6.778	4.287	5.595	0.818	0.606
4.779	4.747	4.139	4.434	4.635	0.416	0.606
3.820	6.841	6.328	3.832	5.553	1.160	0.606
7.675	6.993	8.020	6.632	6.843	0.415	0.606
2.827	3.309	4.259	4.068	3.654	0.865	0.606
2.739	3.079	3.694	3.592	4.235	1.108	0.606
4.250	4.694	4.282	3.324	4.498	0.775	0.606
7.678	8.686	7.021	8.915	8.650	0.486	0.606
1.954	2.069	1.477	1.439	3.014	0.554	0.606
7.574	6.458	9.018	4.912	5.647	0.784	0.606
3.851	3.211	3.450	2.622	2.618	0.626	0.606
5.233	3.811	3.150	4.284	3.481	0.706	0.606
3.425	3.300	2.653	3.783	4.199	0.450	0.606
3.301	4.483	4.295	3.269	3.856	0.705	0.606
3.043	3.867	3.330	2.362	2.751	0.515	0.606
2.756	2.311	2.779	1.364	1.828	0.514	0.606
2.645	3.097	3.606	2.217	2.695	0.536	0.606
6.003	5.299	5.660	4.822	6.329	0.563	0.606
5.052	5.226	6.265	3.161	4.596	0.676	0.606
3.734	4.188	5.476	3.143	3.740	0.655	0.606
3.194	3.395	3.696	2.890	3.153	0.463	0.606
4.083	3.121	3.853	3.526	3.259	0.419	0.606
5.867	5.228	6.563	2.692	2.749	0.966	0.606
2.814	2.263	4.987	1.841	1.596	0.821	0.606
0.999	1.468	1.451	1.135	1.237	0.460	0.606
2.986	2.368	2.586	2.590	2.270	0.443	0.606
5.597	6.360	6.857	5.196	5.934	0.504	0.606
4.052	3.641	4.114	3.561	3.463	0.350	0.606
5.731	5.888	5.511	5.184	5.284	0.309	0.606
7.969	8.495	8.744	6.385	7.846	0.660	0.606
1.292	1.648	1.548	1.067	0.841	0.751	0.606
5.923	5.856	5.732	3.143	4.776	0.651	0.606
3.107	3.207	3.481	3.052	2.749	0.296	0.606
3.988	4.240	5.231	3.213	3.205	0.458	0.606
6.484	5.662	4.246	5.984	6.158	0.468	0.606
3.008	3.096	3.475	2.008	2.790	0.727	0.606
4.508	4.887	5.636	3.165	4.068	0.737	0.606
3.712	4.202	4.944	2.891	3.258	0.465	0.606
4.748	5.150	5.449	3.661	5.285	0.536	0.606
5.246	4.449	5.498	3.803	4.082	0.565	0.606
8.669	8.367	9.064	7.122	8.050	0.562	0.606
3.533	3.588	3.727	3.097	3.638	0.407	0.606
4.683	4.992	4.710	5.235	5.189	0.332	0.606
5.171	3.834	6.264	3.613	3.609	0.784	0.606
5.047	6.549	5.530	6.704	6.494	0.452	0.606
5.360	4.879	5.660	3.301	3.785	0.530	0.606
4.120	3.715	5.663	2.957	3.289	0.531	0.606
5.752	5.458	5.934	5.419	5.354	0.379	0.606
2.874	3.857	3.628	3.785	3.982	0.323	0.606

4.619	5.977	4.610	5.789	6.128	0.593	0.606
2.896	3.625	5.071	3.083	3.543	0.541	0.606
3.146	2.873	3.082	3.139	2.448	0.482	0.606
4.501	3.259	3.105	3.621	3.839	0.613	0.606
6.341	7.623	4.634	8.060	7.962	0.689	0.606
3.931	3.946	1.616	4.710	5.040	0.715	0.606
6.600	7.956	4.730	8.092	8.040	0.790	0.606
5.512	6.107	2.765	6.505	6.490	0.804	0.606
5.194	5.436	6.964	3.477	3.674	0.884	0.606
7.144	6.369	7.510	6.403	6.698	0.432	0.606
4.131	5.816	2.559	6.295	6.077	0.749	0.606
3.095	2.955	3.738	2.879	2.421	0.430	0.606
2.660	3.894	2.642	3.528	4.080	0.587	0.606
4.193	4.193	4.108	4.975	5.067	0.419	0.606
6.053	6.076	5.635	6.150	6.723	0.431	0.606
5.196	4.917	2.367	5.331	5.680	0.680	0.606
6.959	7.606	6.521	8.255	7.735	0.582	0.606
3.168	6.146	1.803	6.485	6.856	1.110	0.606
5.250	5.091	3.667	5.116	4.465	0.495	0.606
8.056	8.897	5.962	9.396	9.347	0.697	0.606
6.784	5.161	7.039	4.209	4.057	0.707	0.606
3.511	4.717	4.362	4.124	4.174	0.427	0.606
4.624	5.629	3.680	5.872	5.189	0.471	0.606
4.047	4.190	4.952	3.996	3.626	0.511	0.606
6.446	7.123	5.549	7.296	7.190	0.426	0.606
5.880	6.997	4.030	7.143	6.935	0.759	0.606
5.129	5.431	3.989	5.901	5.603	0.571	0.606
5.646	4.631	6.434	4.878	4.680	0.443	0.606
4.793	4.699	4.528	5.491	5.280	0.347	0.606
3.203	3.696	3.807	4.672	4.727	0.453	0.606
5.431	6.049	5.354	6.106	6.135	0.397	0.606
6.595	8.167	6.862	8.443	8.095	0.473	0.606
3.468	3.913	4.103	3.519	3.497	0.593	0.606
5.582	4.776	6.398	3.883	3.778	0.628	0.606
5.433	5.451	4.946	5.779	5.631	0.327	0.606
2.562	2.948	2.767	1.985	2.763	0.420	0.606
2.022	2.862	3.878	2.343	2.684	0.467	0.606
5.289	5.791	4.489	5.911	5.754	0.452	0.606
2.393	2.440	1.756	2.813	2.464	0.394	0.606
3.305	2.633	3.232	2.900	2.868	0.473	0.606
6.629	7.344	5.577	7.628	7.935	0.499	0.606
4.232	4.096	3.596	4.589	4.325	0.392	0.606
5.254	5.366	3.566	5.927	5.615	0.507	0.606
5.908	5.087	6.390	4.146	4.572	0.492	0.606
3.834	2.859	4.149	3.597	3.552	0.437	0.606
6.180	6.599	2.808	6.830	6.972	0.862	0.606
5.157	6.723	4.642	7.068	6.738	0.584	0.606
3.872	5.688	3.454	6.246	5.866	0.718	0.606
4.479	3.903	4.632	3.940	3.929	0.476	0.606
3.981	4.722	4.011	4.381	4.116	0.424	0.606

5.156	6.813	6.476	5.628	5.648	0.599	0.606
4.363	3.470	4.312	2.756	3.753	0.504	0.606
5.153	3.789	5.523	3.607	3.416	0.578	0.606
3.704	4.260	3.007	4.911	4.519	0.485	0.606
3.445	3.033	4.215	3.599	2.926	0.473	0.606
6.747	8.186	6.980	8.817	8.354	0.469	0.606
4.566	5.820	2.460	6.191	5.263	0.723	0.606
7.189	7.928	4.111	8.476	7.908	0.902	0.606
5.456	6.501	3.139	7.306	6.702	0.796	0.606
3.566	4.704	2.796	4.664	4.553	0.504	0.606
4.335	4.777	3.005	4.727	6.269	0.965	0.606
6.341	6.555	4.850	7.165	7.032	0.564	0.606
5.681	4.972	5.774	4.968	4.590	0.387	0.606
4.629	5.242	5.258	5.230	5.833	0.392	0.606
4.283	4.624	3.959	4.850	4.886	0.387	0.606
3.798	4.115	3.939	4.458	4.270	0.360	0.606
3.771	4.165	2.630	4.367	4.062	0.457	0.606
5.428	6.240	6.028	6.319	5.975	0.381	0.606
5.551	6.288	4.304	6.270	6.063	0.520	0.606
4.852	5.477	3.817	6.439	5.858	0.584	0.606
6.531	5.839	7.265	6.202	6.507	0.456	0.606
3.359	3.125	3.364	3.876	3.473	0.412	0.606
4.245	4.302	3.785	4.557	4.327	0.360	0.606
5.554	5.321	6.651	5.155	5.296	0.433	0.606
6.960	7.108	7.826	7.062	7.061	0.368	0.606
7.886	6.622	7.983	4.786	5.080	0.715	0.606
2.353	2.249	1.517	3.328	3.029	0.508	0.606
4.833	4.667	5.713	3.327	3.444	0.520	0.606
6.885	8.055	6.731	8.690	8.304	0.470	0.606
2.150	2.821	2.604	2.331	3.162	0.526	0.606
2.616	2.853	3.072	2.797	3.698	0.462	0.606
5.785	5.797	6.962	5.403	5.532	0.505	0.606
2.937	3.699	2.896	2.963	4.037	0.515	0.606
3.180	4.418	4.231	3.851	3.763	0.608	0.606
3.473	4.469	4.405	3.894	5.580	0.778	0.606
2.950	3.223	2.734	1.986	2.799	0.608	0.606
2.578	3.192	2.798	3.640	3.666	0.357	0.606
6.253	6.018	7.173	5.349	5.743	0.410	0.606
4.830	4.285	5.355	3.870	4.390	0.397	0.606
1.889	2.176	1.917	2.367	1.965	0.429	0.606
2.824	3.061	4.105	2.862	3.310	0.533	0.606
2.838	2.200	3.458	2.127	2.127	0.428	0.606
4.530	5.056	3.450	5.592	5.278	0.480	0.606
3.938	4.143	4.483	3.019	3.721	0.745	0.606
4.819	4.755	3.190	5.281	4.705	0.625	0.606
3.930	3.925	3.036	3.822	4.725	0.625	0.606
5.324	4.043	5.898	3.183	2.504	0.770	0.606
4.509	4.073	3.390	3.967	4.713	0.437	0.606
3.666	4.738	2.635	4.519	4.618	0.489	0.606
2.607	1.471	1.406	1.692	1.614	0.537	0.606

4.050	4.763	4.939	4.664	4.680	0.445	0.606
2.506	2.708	2.509	3.121	3.140	0.405	0.606
6.419	5.799	6.793	5.394	5.186	0.417	0.606
7.021	7.393	5.142	7.778	7.481	0.591	0.606
6.099	7.377	2.277	7.787	7.179	1.080	0.606
6.659	5.089	6.690	4.683	4.749	0.531	0.606
3.236	2.377	2.515	2.221	2.386	0.521	0.606
2.066	2.625	1.659	1.679	2.058	0.519	0.606
2.301	2.267	2.331	2.293	2.255	0.526	0.606
5.848	6.684	2.416	6.966	6.490	0.976	0.606
5.470	5.038	2.078	5.817	5.572	0.835	0.606
6.201	10.091	5.284	6.344	5.911	1.265	0.606
2.514	5.412	3.385	2.791	2.600	1.159	0.606
6.194	6.246	5.655	6.889	6.628	0.350	0.606
1.556	2.463	2.231	2.178	2.796	0.543	0.606
6.765	5.955	7.676	6.000	5.628	0.444	0.606
6.757	7.448	6.016	7.999	7.856	0.432	0.606
4.202	4.702	3.931	4.932	5.085	0.400	0.606
6.170	6.846	5.834	7.079	7.013	0.374	0.606
4.674	5.849	4.412	6.290	6.145	0.497	0.606
4.484	6.583	4.185	6.838	6.910	0.670	0.606
3.869	2.052	1.863	1.466	1.738	0.557	0.606
3.001	3.838	3.187	3.236	3.955	0.390	0.606
4.285	4.003	6.145	4.010	4.199	0.453	0.606
2.507	4.154	2.339	4.218	4.058	0.528	0.606
4.250	6.536	4.822	6.806	6.959	0.830	0.606
1.615	4.654	2.300	4.371	4.455	0.758	0.606
5.731	3.813	2.693	4.303	4.621	0.657	0.606
2.461	4.679	2.536	5.362	5.319	0.744	0.606
2.761	4.729	2.384	4.279	4.719	0.578	0.606
2.884	1.537	1.058	2.215	2.148	1.370	0.606
2.577	2.564	1.753	2.870	2.479	0.537	0.606
2.950	4.240	2.405	3.134	2.774	0.453	0.606
2.578	3.611	2.419	3.383	3.332	0.407	0.606
3.517	2.879	1.925	3.347	4.123	0.639	0.606
5.242	5.190	3.023	5.251	5.458	0.613	0.606
4.650	6.113	4.258	6.416	5.807	0.602	0.606
3.229	4.262	3.634	4.337	4.034	0.371	0.606
3.175	3.183	3.529	2.790	3.173	0.400	0.606
6.218	6.863	5.790	7.213	7.143	0.417	0.606
6.035	5.936	7.672	5.023	5.028	0.626	0.606
6.122	5.366	6.809	3.786	4.409	0.645	0.606
3.751	2.771	4.517	3.296	2.121	0.586	0.606
2.812	4.325	2.425	4.855	4.627	0.649	0.606
6.858	5.814	6.916	5.278	5.155	0.546	0.606
4.753	4.690	3.281	5.092	5.311	0.463	0.606
4.911	4.874	4.324	3.819	4.285	0.386	0.606
2.237	3.372	2.249	3.354	3.439	0.397	0.606
3.490	3.973	4.159	3.421	3.702	0.435	0.606
3.647	5.045	3.879	4.794	4.835	0.460	0.606

4.960	4.091	5.554	3.266	3.743	0.593	0.606
4.401	4.135	5.064	3.835	3.567	0.525	0.606
2.604	4.074	3.921	3.839	3.868	0.462	0.606
8.422	7.975	7.731	7.588	8.325	0.472	0.606
6.540	7.122	6.296	7.448	7.324	0.343	0.606
4.291	4.330	3.536	4.712	4.715	0.418	0.606
2.074	2.600	2.467	2.630	1.833	0.532	0.606
5.570	6.642	3.825	6.725	6.353	0.614	0.606
4.024	3.634	2.374	3.646	3.557	0.450	0.606
4.868	6.099	3.941	6.471	6.382	0.671	0.606
5.142	5.046	4.013	5.518	5.341	0.454	0.606
1.680	2.518	2.625	2.185	2.229	0.504	0.606
7.174	6.968	2.254	7.401	7.262	1.082	0.606
5.966	4.522	4.625	4.771	4.850	0.467	0.606
5.844	3.065	6.035	3.209	2.779	0.873	0.606
2.784	3.337	2.657	3.373	2.908	0.400	0.606
5.474	4.820	6.280	4.400	4.862	0.543	0.606
7.912	7.999	8.042	8.150	8.026	0.308	0.606
5.623	6.394	6.464	7.219	7.429	0.485	0.606
5.630	5.701	6.976	3.395	3.558	0.730	0.606
3.013	3.329	4.248	2.830	2.652	0.408	0.606
2.182	2.590	1.992	2.767	2.688	0.381	0.606
2.403	2.542	2.234	2.345	2.666	0.473	0.606
3.154	2.898	3.038	3.569	2.929	0.398	0.606
3.239	3.171	3.957	2.798	3.150	0.429	0.606
8.777	8.518	9.607	7.029	7.475	0.558	0.606
3.503	3.093	3.091	2.942	2.980	0.598	0.606
3.800	3.285	2.324	3.287	3.241	0.549	0.606
3.795	4.059	3.739	3.268	4.343	0.610	0.606
3.112	2.678	2.993	2.469	2.578	0.409	0.606
6.998	7.097	4.246	6.855	7.360	0.713	0.606
2.213	2.500	1.971	2.509	2.297	0.420	0.606
4.038	1.547	4.905	1.101	1.819	0.874	0.606
5.192	4.308	4.699	2.551	3.401	0.745	0.606
2.720	2.437	3.233	2.068	2.063	0.548	0.606
4.182	3.308	2.160	4.051	3.846	0.573	0.606
5.490	5.975	6.064	5.140	5.947	0.403	0.606
5.503	6.071	6.140	5.160	6.001	0.417	0.606
5.503	6.019	6.064	5.123	5.961	0.408	0.606
4.509	4.926	5.129	4.738	4.911	0.341	0.606
4.761	5.031	5.112	4.331	4.865	0.348	0.606
4.271	4.141	3.032	4.289	5.760	0.672	0.606
1.532	1.751	1.686	1.908	1.819	0.370	0.606
8.418	8.753	9.406	7.051	7.463	0.533	0.606
2.914	2.896	2.327	2.770	3.593	0.578	0.606
6.220	5.800	7.150	4.458	4.457	0.620	0.606
4.749	4.393	3.528	4.078	4.713	0.584	0.606
5.458	5.720	6.110	5.269	4.902	0.391	0.606
4.131	3.746	3.581	3.755	3.957	0.448	0.606
4.635	4.281	4.941	3.678	3.844	0.408	0.606

6.652	7.176	6.838	7.773	7.654	0.365	0.606
9.021	9.652	8.243	9.606	9.630	0.387	0.606
6.894	7.437	3.572	7.609	7.510	0.938	0.606
6.453	6.597	3.374	6.788	6.639	0.799	0.606
6.844	6.725	5.588	7.033	6.677	0.393	0.606
4.070	3.279	4.558	2.669	3.405	0.446	0.606
3.750	2.517	1.964	3.614	3.325	0.603	0.606
4.519	5.343	2.550	5.709	5.223	0.676	0.606
6.390	7.534	4.301	7.780	7.968	0.867	0.606
5.890	7.135	3.760	7.327	7.563	0.915	0.606
2.735	2.534	2.003	2.609	2.797	0.441	0.606
6.627	6.662	5.641	6.778	6.858	0.380	0.606
4.631	4.259	5.206	3.464	4.349	0.511	0.606
3.982	4.379	5.386	3.764	4.254	0.450	0.606
6.966	5.374	7.891	4.034	5.097	0.860	0.606
3.458	3.599	3.735	2.504	3.501	0.598	0.606
4.617	4.243	4.029	4.422	4.711	0.370	0.606
10.765	9.923	10.237	7.593	8.487	0.791	0.606
5.844	6.359	6.289	6.772	6.846	0.382	0.606
3.530	3.161	1.658	2.979	3.014	0.702	0.606
2.594	3.167	1.467	4.239	2.817	0.590	0.606
4.557	5.513	2.951	5.382	5.276	0.569	0.606
3.235	3.609	2.968	3.038	3.766	0.384	0.606
6.641	8.737	6.005	9.256	9.069	0.743	0.606
4.713	3.890	6.115	3.480	3.992	0.549	0.606
3.965	5.298	3.526	4.691	4.670	0.645	0.606
4.816	5.800	6.327	4.205	4.634	0.583	0.606
7.049	7.712	7.956	6.831	6.970	0.467	0.606
4.870	5.734	2.686	6.148	6.046	0.731	0.606
4.761	4.410	6.378	3.582	3.886	0.691	0.606
10.175	9.444	10.621	7.872	8.650	0.610	0.606
6.839	7.813	3.486	8.540	8.062	0.927	0.606
6.853	7.780	3.055	8.419	7.906	0.972	0.606
5.125	5.989	4.871	6.056	6.181	0.379	0.606
5.719	6.749	5.181	7.123	6.661	0.484	0.606
7.210	6.811	7.456	7.260	7.365	0.465	0.606
8.356	7.150	9.296	6.449	6.389	0.640	0.606
5.895	6.560	6.161	7.339	6.742	0.592	0.606
4.491	5.816	3.083	6.081	6.211	0.767	0.606
3.217	4.336	2.005	5.445	5.407	0.685	0.606
3.160	2.521	1.570	2.816	3.218	0.549	0.606
7.491	7.889	5.448	7.867	7.839	0.555	0.606
7.901	8.341	5.561	8.757	8.518	0.682	0.606
4.471	4.201	4.633	4.695	4.551	0.368	0.606
8.190	7.020	8.648	6.480	6.531	0.592	0.606
6.626	7.470	5.281	8.019	8.132	0.707	0.606
3.567	4.737	3.827	4.755	4.977	0.488	0.606
4.607	4.755	4.208	4.782	4.971	0.375	0.606
3.707	3.745	2.670	3.208	3.579	0.549	0.606
6.984	7.326	6.445	7.474	7.539	0.412	0.606

5.931	6.322	6.488	6.683	6.878	0.350	0.606
1.863	2.142	1.799	3.371	1.838	0.413	0.606
3.907	4.251	2.590	4.813	4.899	0.550	0.606
3.426	2.182	2.523	2.412	2.319	0.704	0.606
1.796	1.871	1.383	1.313	2.126	0.401	0.606
5.157	3.833	4.650	4.038	4.032	0.483	0.606
4.905	4.295	4.977	2.963	3.840	0.508	0.606
2.666	3.013	1.646	3.052	2.978	0.404	0.606
2.844	3.584	3.164	2.987	3.276	0.402	0.606
3.175	3.272	2.922	3.169	4.303	0.536	0.606
2.531	3.269	2.225	3.870	3.644	0.471	0.606
3.620	3.425	2.219	3.293	3.055	0.554	0.606
4.455	5.701	3.912	5.482	5.110	0.500	0.606
2.346	2.081	2.102	2.799	2.994	0.354	0.606
3.173	3.028	2.400	2.177	2.765	0.548	0.606
2.346	2.145	1.725	2.241	2.221	0.514	0.606
3.346	4.710	3.569	3.519	3.465	0.613	0.606
3.022	4.565	3.693	3.423	3.642	0.508	0.606
1.944	1.220	1.813	1.924	1.732	0.448	0.606
7.722	7.654	7.479	8.026	8.109	0.321	0.606
5.872	4.299	6.795	3.271	3.688	0.768	0.606
3.322	5.637	3.146	6.033	5.894	0.689	0.606
3.149	3.711	3.551	3.206	3.766	0.453	0.606
2.501	2.679	2.294	2.405	3.065	0.425	0.606
1.916	1.755	1.269	1.539	1.664	0.460	0.606
3.162	2.018	3.301	2.307	2.049	0.475	0.606
3.124	2.936	2.665	2.643	2.672	0.458	0.606
4.526	4.035	4.302	2.897	3.428	0.447	0.606
4.167	3.821	5.025	3.094	3.425	0.434	0.606
5.280	5.080	5.672	4.499	5.124	0.562	0.606
5.483	6.431	6.554	5.674	5.922	0.402	0.606
3.728	3.380	3.573	3.765	3.410	0.498	0.606
4.599	4.656	4.400	4.580	4.737	0.331	0.606
3.663	3.682	4.168	3.438	3.019	0.350	0.606
5.822	5.564	4.605	5.033	5.247	0.425	0.606
6.273	5.980	5.922	6.748	6.535	0.331	0.606
3.396	4.010	4.053	3.450	4.052	0.459	0.606
5.298	5.602	5.316	6.015	6.003	0.402	0.606
3.839	4.044	3.411	3.991	4.246	0.402	0.606
5.520	6.571	5.762	6.018	5.902	0.667	0.606
4.553	4.031	1.206	4.075	3.521	0.750	0.606
4.940	5.418	3.075	5.520	5.479	0.537	0.606
7.412	6.755	6.843	5.891	6.562	0.414	0.606
5.200	5.673	5.424	5.785	6.075	0.305	0.606
5.662	6.121	5.242	6.453	6.266	0.368	0.606
4.646	5.196	4.976	4.999	5.289	0.403	0.606
2.368	2.321	1.926	2.222	1.991	0.436	0.606
5.845	6.771	5.678	6.936	7.448	0.584	0.606
4.926	6.664	7.382	3.886	5.807	0.855	0.606
2.207	1.289	2.326	1.452	1.674	0.660	0.606

5.074	3.534	6.385	2.917	3.501	1.019	0.606
5.356	4.707	7.025	3.244	4.038	0.731	0.606
7.511	6.772	7.980	6.783	7.037	0.410	0.606
6.326	6.675	5.222	7.127	7.224	0.600	0.606
4.400	3.773	3.922	3.515	4.225	0.435	0.606
5.827	5.966	3.347	5.786	5.942	0.633	0.606
3.799	4.369	1.645	4.995	5.007	0.720	0.606
3.915	4.481	3.005	4.238	4.463	0.521	0.606
2.679	1.911	2.718	2.394	1.420	0.505	0.606
5.537	4.858	7.098	4.485	4.338	0.656	0.606
7.815	7.478	8.615	6.519	6.798	0.449	0.606
4.486	6.251	2.171	7.051	6.909	1.043	0.606
4.018	6.172	3.360	6.626	6.258	0.744	0.606
3.570	4.076	2.849	4.384	3.563	0.480	0.606
4.306	5.544	4.063	5.432	5.281	0.454	0.606
4.996	5.049	2.542	4.997	4.833	0.667	0.606
2.364	1.643	2.570	2.433	2.254	0.432	0.606
6.414	6.034	7.679	5.283	5.265	0.546	0.606
1.806	2.473	3.238	1.473	2.338	0.581	0.606
7.086	6.641	7.733	5.781	6.089	0.562	0.606
4.296	3.573	4.544	3.186	3.336	0.410	0.606
6.145	4.104	5.706	3.397	4.098	0.756	0.606
6.538	6.869	6.250	6.473	6.648	0.403	0.606
5.041	4.773	5.474	3.952	5.204	0.499	0.606
3.113	1.932	2.744	2.281	2.692	0.601	0.606
2.017	2.405	1.887	2.405	2.952	0.547	0.606
4.561	3.094	4.378	2.629	2.478	0.629	0.606
2.735	3.094	2.919	1.610	2.700	0.640	0.606
6.104	3.923	3.550	4.441	5.455	0.721	0.606
2.463	2.765	1.903	2.788	3.447	0.483	0.606
4.596	3.637	4.669	3.527	3.461	0.401	0.606
4.814	5.144	4.942	4.618	4.886	0.328	0.606
4.509	4.329	5.173	3.961	4.149	0.362	0.606
3.227	3.321	2.610	4.560	3.244	0.462	0.606
2.161	2.539	2.570	2.346	1.902	0.410	0.606
4.064	3.439	5.293	2.801	3.288	0.559	0.606
4.365	5.374	6.010	5.341	5.381	0.416	0.606
4.926	7.647	2.763	7.913	7.891	1.216	0.606
5.457	5.405	5.693	5.699	5.276	0.351	0.606
4.313	1.885	1.629	3.279	2.134	0.699	0.606
6.716	6.353	7.107	5.617	6.141	0.542	0.606
4.234	4.884	5.078	5.794	5.175	0.398	0.606
4.138	4.367	3.020	4.530	4.008	0.482	0.606
3.857	3.515	4.323	4.127	3.535	0.413	0.606
4.507	4.324	6.634	4.211	3.885	0.683	0.606
5.611	5.455	6.076	5.083	4.429	0.537	0.606
4.143	5.118	3.144	5.694	5.415	0.659	0.606
4.810	5.822	4.286	5.754	5.822	0.447	0.606
4.914	2.047	2.136	1.528	2.134	0.835	0.606
3.070	3.260	3.492	3.377	3.052	0.465	0.606

4.330	4.791	4.251	4.657	5.186	0.368	0.606
4.959	5.014	4.434	5.315	5.043	0.372	0.606
7.109	6.829	7.240	6.553	7.069	0.326	0.606
5.255	6.679	4.923	6.873	6.552	0.528	0.606
5.836	5.022	6.441	5.447	4.691	0.421	0.606
4.811	5.768	4.907	5.777	5.554	0.422	0.606
8.187	7.599	8.416	6.845	6.905	0.458	0.606
2.492	2.495	1.189	2.878	3.613	0.691	0.606
6.667	5.617	7.094	5.045	5.257	0.506	0.606
3.633	3.523	4.003	2.923	3.287	0.403	0.606
6.886	5.667	6.361	5.242	5.837	0.481	0.606
2.319	7.056	2.946	6.993	6.916	1.180	0.606
5.442	4.852	6.070	4.854	4.765	0.409	0.606
2.891	1.654	2.588	1.802	1.974	0.499	0.606
3.633	3.205	4.330	2.605	2.843	0.425	0.606
7.460	7.776	8.147	6.016	7.058	0.583	0.606
4.763	4.457	5.414	2.993	3.795	0.596	0.606
4.154	4.097	4.831	2.872	3.073	0.545	0.606
3.005	6.486	2.615	2.920	3.107	0.887	0.606
2.903	3.003	3.069	1.732	2.276	0.613	0.606
5.474	6.591	4.160	6.919	6.822	0.625	0.606
6.383	6.463	4.898	7.135	6.884	0.620	0.606
4.068	4.152	4.094	3.796	3.823	0.512	0.606
3.611	4.628	4.238	4.919	5.183	0.565	0.606
3.437	3.640	4.171	3.298	3.429	0.411	0.606
3.125	3.927	2.640	3.796	4.003	0.400	0.606
5.527	5.892	5.152	6.402	6.022	0.429	0.606
4.426	5.117	4.791	4.591	3.849	0.465	0.606
5.521	5.811	5.470	6.259	5.986	0.319	0.606
6.206	7.663	5.586	8.038	8.048	0.659	0.606
4.906	5.244	4.691	5.495	5.143	0.342	0.606
2.006	2.079	1.303	1.986	2.068	0.461	0.606
4.128	3.746	3.547	3.635	3.553	0.614	0.606
4.952	5.304	5.433	5.741	5.756	0.352	0.606
2.475	2.353	1.782	2.984	3.392	0.592	0.606
3.967	3.578	4.025	3.717	3.411	0.421	0.606
2.550	1.854	2.575	2.375	2.664	0.464	0.606
3.920	3.351	2.842	3.375	3.680	0.505	0.606
3.410	3.108	3.483	3.416	3.272	0.339	0.606
2.852	2.006	2.599	1.893	1.472	0.556	0.606
2.853	1.556	2.508	1.964	1.496	0.499	0.606
7.873	6.710	7.627	6.861	6.733	0.387	0.606
2.542	1.900	3.685	2.492	2.201	0.411	0.606
0.933	2.765	0.781	4.122	3.362	0.730	0.606
5.392	6.324	6.268	4.429	5.466	0.649	0.606
1.925	1.733	1.224	3.442	2.860	0.586	0.606
4.160	2.629	2.910	2.568	3.097	0.380	0.606
3.176	2.983	2.138	2.319	3.209	0.496	0.606
3.014	4.564	2.713	4.256	3.668	0.528	0.606
1.907	1.973	1.509	2.221	2.306	0.399	0.606

3.439	3.189	3.632	2.152	3.066	0.496	0.606
3.254	3.381	3.322	3.356	3.496	0.448	0.606
1.998	3.217	1.933	3.870	3.104	0.508	0.606
2.393	2.818	2.260	3.190	3.911	0.573	0.606
3.510	2.426	3.845	2.841	2.797	0.590	0.606
2.098	3.721	2.334	3.816	3.889	0.481	0.606
1.145	3.019	1.402	3.097	3.295	0.542	0.606
2.730	2.536	3.106	2.386	3.113	0.526	0.606
0.920	2.497	0.790	3.318	3.603	0.742	0.606
1.433	2.138	1.457	3.117	3.221	0.518	0.606
1.876	2.416	2.075	1.603	2.445	0.435	0.606
4.030	4.870	3.629	4.777	4.990	0.462	0.606
3.497	2.888	4.705	2.898	3.115	0.565	0.606
3.073	4.460	2.538	4.973	4.968	0.667	0.606
4.128	3.360	3.445	3.325	2.743	0.624	0.606
2.218	3.415	1.973	2.571	2.141	0.520	0.606
3.282	3.575	3.497	3.164	3.332	0.606	0.606
3.339	2.957	2.673	3.494	2.963	0.560	0.606
4.798	2.638	4.771	3.502	3.034	0.580	0.606
2.889	2.662	3.046	3.289	2.667	0.486	0.606
2.744	1.930	5.515	1.185	1.561	0.940	0.606
2.555	2.949	2.634	3.427	3.200	0.454	0.606
3.720	4.560	4.230	4.035	4.514	0.361	0.606
2.879	2.423	2.888	2.248	2.938	0.495	0.606
2.930	2.430	2.797	1.734	2.262	0.469	0.606
2.896	2.235	2.110	2.270	1.900	0.439	0.606
3.769	3.590	3.496	3.882	3.424	0.442	0.606
3.607	3.750	2.909	5.188	4.775	0.539	0.606
1.112	1.773	1.252	1.838	1.267	0.482	0.606
4.209	5.343	2.636	5.597	5.483	0.743	0.606
1.728	2.287	2.551	2.354	2.564	0.375	0.606
5.189	5.770	4.582	5.418	5.607	0.392	0.606
3.834	2.276	3.602	2.230	2.391	0.463	0.606
3.125	3.860	3.271	2.941	3.918	0.410	0.606
2.689	3.246	3.125	3.628	4.042	0.443	0.606
2.351	3.105	2.333	4.250	3.618	0.531	0.606
2.269	2.545	3.819	2.361	1.900	0.574	0.606
4.543	3.608	4.545	3.321	4.168	0.485	0.606
3.089	3.098	2.636	3.666	3.510	0.391	0.606
2.237	2.357	1.960	2.128	2.496	0.394	0.606
2.279	2.128	3.339	2.598	2.271	0.420	0.606
4.090	4.104	4.961	2.802	3.169	0.482	0.606
2.690	2.219	1.907	2.376	3.377	0.400	0.606
4.190	4.137	4.424	4.940	4.939	0.506	0.606
2.444	3.030	2.453	3.714	3.209	0.450	0.606
2.887	2.827	2.480	2.572	3.017	0.455	0.606
3.605	3.520	4.044	2.667	3.145	0.376	0.606
4.531	4.286	3.297	3.853	4.393	0.445	0.606
6.579	4.763	4.648	5.129	5.173	0.641	0.606
4.209	4.898	4.300	4.278	4.942	0.386	0.606

6.950	7.217	6.007	7.153	7.146	0.409	0.606
6.312	6.372	7.229	5.645	5.849	0.376	0.606
2.717	3.941	2.645	4.130	3.858	0.441	0.606
7.578	5.806	8.172	4.124	4.834	0.867	0.606
4.246	4.454	3.881	5.200	4.695	0.552	0.606
4.479	4.086	4.801	3.208	3.529	0.397	0.606
5.557	5.139	6.077	4.928	5.123	0.421	0.606
4.491	5.033	5.206	3.731	5.018	0.654	0.606
3.727	3.448	4.363	3.523	3.677	0.452	0.606
3.239	3.789	2.861	4.172	4.065	0.447	0.606
3.980	3.392	4.047	3.403	3.485	0.386	0.606
5.770	5.743	5.459	4.571	6.203	0.459	0.606
5.598	6.029	6.211	4.088	5.788	0.593	0.606
2.895	2.065	3.044	2.142	1.986	0.464	0.606
2.670	1.999	2.336	1.516	2.427	0.492	0.606
6.052	5.647	5.584	4.563	5.655	0.442	0.606
5.054	7.068	3.815	7.052	6.337	0.736	0.606
3.934	3.650	1.207	4.726	4.179	0.717	0.606
2.325	4.970	1.564	5.906	5.773	0.979	0.606
4.740	3.530	3.780	2.746	3.306	0.526	0.606
5.818	4.778	5.227	3.991	4.669	0.503	0.606
7.296	7.665	7.282	7.928	7.710	0.350	0.606
2.850	4.778	1.881	5.714	5.008	0.794	0.606
5.807	6.166	6.260	6.661	6.634	0.329	0.606
5.191	5.194	5.678	5.601	5.746	0.435	0.606
6.586	7.596	4.919	7.738	7.369	0.598	0.606
4.954	4.019	4.486	2.586	5.111	0.927	0.606
2.378	4.170	2.609	1.633	1.591	0.907	0.606
2.277	3.188	1.929	1.482	3.318	0.612	0.606
3.141	2.861	3.180	3.152	3.326	0.457	0.606
6.326	9.132	3.984	9.228	9.470	1.191	0.606
5.910	5.857	6.040	4.300	5.451	0.566	0.606
2.535	3.655	2.975	2.465	1.896	0.617	0.606
4.118	4.194	3.858	3.593	4.049	0.471	0.606
1.069	2.454	0.984	1.084	1.295	0.647	0.606
2.323	2.887	2.103	2.180	2.580	0.680	0.606
6.235	6.961	6.957	5.544	5.725	0.635	0.606
2.134	2.139	2.478	1.357	1.714	0.545	0.606
4.071	3.587	2.940	3.070	3.140	0.574	0.606
3.373	1.186	4.229	2.226	2.968	0.893	0.606
2.848	1.874	1.998	2.024	2.146	0.498	0.606
5.440	4.658	5.291	5.429	4.985	0.492	0.606
4.341	4.241	2.347	4.908	4.754	0.545	0.606
2.771	2.039	2.252	2.021	1.849	0.367	0.606
2.623	3.057	2.102	2.840	3.377	0.456	0.606
6.879	7.114	8.228	6.473	6.936	0.409	0.606
3.982	3.255	4.137	2.845	3.494	0.454	0.606
6.927	6.550	6.749	5.840	6.448	0.355	0.606
4.018	4.901	4.681	3.534	4.005	0.595	0.606
7.160	7.323	7.725	5.048	6.384	0.882	0.606

7.181	6.684	8.175	7.091	7.748	0.464	0.606
7.213	8.041	6.652	8.283	8.311	0.437	0.606
5.802	5.779	3.366	5.853	5.804	0.593	0.606
3.590	5.299	3.717	5.015	5.771	0.726	0.606
4.729	4.615	6.001	4.808	4.764	0.436	0.606
4.353	3.659	3.280	3.724	3.006	0.396	0.606
1.602	3.263	2.112	3.250	2.200	0.493	0.606
7.520	6.605	8.077	5.306	5.518	0.634	0.606
2.008	4.128	3.256	3.429	4.102	0.721	0.606
2.895	2.601	2.321	3.335	1.946	0.573	0.606
3.677	3.455	2.539	3.867	3.370	0.389	0.606
4.986	3.964	5.684	3.584	3.751	0.496	0.606
4.128	3.210	3.831	3.129	3.406	0.442	0.606
5.446	6.733	4.529	6.845	6.412	0.529	0.606
4.937	4.675	3.837	3.743	4.723	0.526	0.606
3.709	2.675	2.423	4.123	3.665	0.826	0.606
2.604	2.238	3.149	1.281	0.843	0.885	0.606
5.121	5.885	2.706	6.142	6.419	0.873	0.606
6.212	5.417	8.176	4.911	5.527	0.690	0.606
5.775	4.309	4.324	4.494	3.901	0.623	0.606
3.017	2.837	1.472	3.614	2.629	0.500	0.606
5.981	5.457	6.628	4.863	5.146	0.423	0.606
2.632	3.297	2.439	3.541	3.665	0.359	0.606
3.691	4.201	3.672	2.539	3.659	0.569	0.606
1.982	1.996	2.112	1.940	1.999	0.529	0.606
5.064	4.184	5.772	2.635	3.657	0.685	0.606
3.716	3.565	3.881	3.393	4.543	0.501	0.606
3.155	3.577	3.475	3.573	3.099	0.401	0.606
8.169	6.457	8.826	5.741	5.969	0.746	0.606
4.759	4.550	3.886	5.928	5.421	0.529	0.606
4.000	3.462	3.520	3.492	4.259	0.442	0.606
5.065	4.228	3.450	4.959	4.437	0.491	0.606
1.838	2.276	1.259	3.190	3.077	0.464	0.606
3.103	3.581	3.506	3.189	4.229	0.413	0.606
5.506	6.594	4.241	7.019	6.453	0.585	0.606
5.753	7.141	6.585	7.178	6.984	0.465	0.606
6.569	2.359	4.710	2.788	1.322	1.306	0.606
6.552	7.564	5.848	8.092	7.785	0.545	0.606
4.189	3.192	4.686	3.390	3.215	0.438	0.606
2.185	2.325	1.976	1.197	2.010	0.448	0.606
2.507	2.288	1.626	2.808	2.176	0.643	0.606
4.195	3.791	3.925	3.331	3.461	0.416	0.606
4.813	3.064	2.493	3.503	2.949	0.527	0.606
5.055	4.852	5.015	4.423	4.493	0.350	0.606
5.361	5.387	3.989	5.806	5.411	0.447	0.606
6.103	6.696	5.690	6.586	6.189	0.416	0.606
5.065	3.642	3.704	3.279	4.051	0.474	0.606
2.732	2.731	2.380	1.878	2.713	0.422	0.606
2.418	1.853	3.748	1.907	2.595	0.539	0.606
5.329	6.338	4.687	6.734	6.813	0.497	0.606

6.079	6.149	4.717	6.517	6.484	0.546	0.606
3.267	2.343	2.512	2.700	2.605	0.433	0.606
3.086	2.796	1.727	2.527	3.493	0.509	0.606
3.772	3.879	3.783	4.216	4.114	0.476	0.606
5.005	5.221	5.231	5.799	5.832	0.357	0.606
2.249	2.542	2.013	2.444	3.111	0.417	0.606
5.422	6.082	5.501	6.082	5.829	0.375	0.606
2.550	3.186	3.222	3.307	3.967	0.425	0.606
3.992	4.672	5.024	3.966	4.765	0.428	0.606
4.319	4.581	5.218	4.971	4.724	0.370	0.606
2.858	3.754	2.293	3.840	3.166	0.474	0.606
3.351	4.284	3.785	4.488	4.665	0.406	0.606
4.774	4.714	4.730	4.022	4.429	0.371	0.606
4.775	4.706	4.859	4.008	4.447	0.367	0.606
3.895	3.449	4.494	3.005	3.101	0.476	0.606
7.314	7.710	7.362	7.334	5.655	0.657	0.606
1.237	7.181	2.359	7.347	6.758	1.341	0.606
3.766	2.656	4.269	2.874	3.191	0.603	0.606
7.231	6.994	7.601	5.846	5.927	0.464	0.606
7.103	6.876	7.491	5.730	5.826	0.462	0.606
6.972	6.798	7.504	5.662	5.790	0.468	0.606
4.835	4.639	5.430	3.762	3.874	0.452	0.606
5.910	5.525	5.494	4.919	5.486	0.489	0.606
7.532	7.849	7.399	8.134	8.058	0.307	0.606
4.669	5.428	5.056	5.460	5.418	0.338	0.606
2.178	2.101	2.620	2.040	2.394	0.485	0.606
2.462	2.793	4.158	2.561	2.243	0.627	0.606
3.010	2.782	3.021	2.618	2.508	0.424	0.606
1.878	2.968	3.001	2.550	2.406	0.489	0.606
3.385	3.985	4.207	2.287	4.106	0.521	0.606
2.670	3.298	2.567	3.868	3.655	0.390	0.606
4.928	5.477	3.348	5.686	5.697	0.569	0.606
5.253	4.881	5.241	4.913	4.278	0.367	0.606
5.601	5.338	5.821	5.942	5.349	1.779	0.606
4.477	4.686	4.361	4.272	4.492	0.470	0.606
4.067	3.562	3.616	3.139	3.646	0.498	0.606
3.355	3.084	3.718	2.920	3.369	0.370	0.606
5.012	5.646	5.117	5.831	5.657	0.504	0.606
3.248	3.544	4.168	2.696	2.002	0.626	0.606
1.705	3.322	4.768	1.984	4.129	0.900	0.606
1.690	3.313	4.759	2.088	4.139	0.910	0.606
4.703	4.317	3.686	4.844	4.719	0.383	0.606
2.348	3.286	3.468	2.177	2.932	0.527	0.606
4.962	4.626	4.686	3.627	3.946	0.458	0.606
2.653	2.804	2.660	2.385	2.667	0.413	0.606
5.239	5.284	5.447	4.580	4.638	0.493	0.606
8.432	7.784	8.952	7.317	7.575	0.386	0.606
7.075	7.330	6.187	7.495	7.576	0.406	0.606
5.284	5.362	3.942	5.680	5.750	0.433	0.606

5.845	5.476	4.930	6.233	6.101	0.384	0.606
2.723	2.483	5.020	2.809	2.162	0.572	0.606
5.747	5.151	6.632	5.242	5.568	0.379	0.606
2.133	3.623	1.681	2.169	2.209	0.845	0.606
3.917	3.386	4.395	4.186	3.568	0.369	0.606
4.974	5.161	4.931	5.453	5.199	0.404	0.606
5.833	5.533	7.229	4.423	4.628	0.546	0.606
2.478	3.683	2.324	3.283	3.302	0.378	0.606
2.021	3.225	1.168	4.461	3.617	0.675	0.606
1.025	1.868	1.388	1.382	1.438	0.480	0.606
4.563	5.448	5.143	5.246	5.145	0.424	0.606
3.051	4.899	2.104	5.563	5.217	0.911	0.606
3.651	4.946	4.416	5.345	5.099	0.471	0.606
5.180	6.953	4.628	7.444	7.469	0.721	0.606
5.988	6.580	5.154	6.730	6.975	0.435	0.606
6.576	6.394	5.983	6.823	6.437	0.330	0.606
4.942	5.453	3.965	6.055	5.892	0.536	0.606
5.511	6.476	5.076	6.782	6.746	0.602	0.606
4.824	2.631	2.462	2.843	2.489	0.616	0.606
4.886	6.578	4.540	6.987	6.611	0.566	0.606
10.323	9.248	10.409	8.944	8.793	0.482	0.606
7.248	6.832	7.367	5.804	5.633	0.506	0.606
1.640	2.163	1.912	2.316	2.421	0.466	0.606
4.773	5.432	3.298	5.098	5.615	0.493	0.606
5.062	6.209	6.118	5.297	5.614	0.520	0.606
2.521	3.702	2.479	2.538	2.672	0.479	0.606
4.992	5.356	5.290	6.457	6.056	0.434	0.606
4.934	4.631	4.753	5.358	5.589	0.395	0.606
5.590	5.539	4.611	6.478	5.764	0.418	0.606
5.322	5.550	5.137	5.700	5.697	0.366	0.606
1.952	2.378	1.442	3.006	1.771	0.432	0.606
5.900	5.397	6.587	5.040	4.989	0.421	0.606
2.913	1.113	3.089	1.886	2.786	0.583	0.606
8.811	8.329	9.515	7.678	7.983	0.416	0.606
5.379	7.120	4.001	7.078	7.260	0.731	0.606
5.696	8.027	4.152	8.229	8.090	0.851	0.606
5.960	3.533	2.415	3.055	3.342	0.689	0.606
4.940	5.426	3.161	5.530	4.995	0.511	0.606
4.015	4.540	4.584	4.243	4.466	0.440	0.606
3.490	3.232	4.675	3.609	2.659	0.518	0.606
3.781	1.828	1.266	1.281	1.700	0.869	0.606
2.106	2.527	3.785	2.284	2.364	0.522	0.606
5.581	5.472	5.898	6.054	6.200	0.458	0.606
4.192	3.891	4.951	2.787	2.346	0.861	0.606
3.835	4.372	2.901	5.068	4.830	0.476	0.606
7.128	7.012	6.197	7.186	7.257	0.336	0.606
6.325	6.519	6.184	6.882	7.163	0.327	0.606
2.840	2.067	2.202	2.176	2.204	0.498	0.606
2.680	2.397	4.291	1.833	2.358	0.513	0.606
2.730	2.773	2.729	2.616	2.496	0.599	0.606

3.372	3.398	2.478	3.973	3.794	0.500	0.606
6.925	7.043	6.874	7.564	7.389	0.404	0.606
5.518	6.584	3.883	6.764	6.644	0.661	0.606
6.400	7.499	4.540	7.771	7.368	0.681	0.606
6.360	6.328	7.038	5.850	5.726	0.357	0.606
3.652	6.026	3.445	6.641	6.571	0.749	0.606
4.170	4.308	4.855	3.807	4.028	0.431	0.606
2.379	2.429	3.337	2.744	2.744	0.518	0.606
1.240	2.274	1.145	3.183	3.002	0.592	0.606
1.850	1.947	2.428	1.677	1.467	0.430	0.606
3.576	5.179	1.973	5.420	5.501	0.840	0.606
3.008	5.498	1.614	5.775	5.780	0.889	0.606
6.433	7.232	5.856	7.650	7.407	0.469	0.606
4.891	4.907	6.673	4.795	4.486	0.500	0.606
4.582	4.790	3.831	4.634	3.747	0.475	0.606
2.026	4.546	2.180	4.604	4.900	0.766	0.606
2.187	2.582	1.640	2.110	2.956	0.410	0.606
2.294	3.829	2.398	4.126	3.964	0.515	0.606
2.120	3.392	2.451	3.371	2.824	0.437	0.606
1.025	1.602	1.805	2.827	2.937	0.456	0.606
3.227	1.844	2.810	1.681	1.487	0.726	0.606
6.160	6.593	6.719	6.929	6.840	0.325	0.606
2.440	2.629	1.896	3.054	3.372	0.428	0.606
2.641	2.691	4.493	2.329	2.008	0.502	0.606
3.499	4.107	2.724	4.126	3.612	0.346	0.606
2.827	3.382	2.120	3.137	3.029	0.353	0.606
7.290	8.448	4.973	8.530	8.496	0.734	0.606
7.933	7.696	6.304	8.161	8.241	0.435	0.606
4.045	3.482	5.094	3.912	3.534	0.429	0.606
4.903	3.259	2.518	3.839	4.288	0.927	0.606
3.866	2.960	3.028	2.885	2.264	0.478	0.606
4.917	5.144	3.623	5.637	5.522	0.487	0.606
3.282	1.702	1.576	1.897	1.878	0.904	0.606
2.557	2.398	2.828	1.856	1.829	0.380	0.606
3.021	2.088	2.031	1.460	0.838	0.606	0.606
3.026	1.977	1.761	2.515	2.285	0.571	0.606
3.392	2.253	2.454	2.637	2.544	0.454	0.606
3.562	3.639	2.410	4.200	3.715	0.548	0.606
4.298	4.823	3.285	5.059	5.110	0.567	0.606
4.376	2.944	2.842	3.084	3.021	0.673	0.606
2.911	2.265	2.289	2.572	2.776	0.505	0.606
8.294	8.856	9.191	8.967	8.687	0.437	0.606
7.624	7.868	7.115	8.089	8.027	0.315	0.606
6.351	5.678	6.615	6.073	5.970	0.457	0.606
6.938	7.194	8.462	8.059	7.389	0.649	0.606
5.418	5.244	5.164	5.713	5.908	0.364	0.606
5.332	5.011	6.403	5.052	4.644	0.385	0.606
6.498	6.617	5.855	6.763	6.628	0.379	0.606
5.058	5.094	5.613	5.272	5.628	0.320	0.606
5.489	5.440	5.566	5.702	5.494	0.356	0.606

4.101	4.167	3.697	4.211	4.468	0.368	0.606
9.715	9.784	9.441	9.727	9.738	0.327	0.606
3.485	4.091	2.984	4.007	4.445	0.402	0.606
2.911	2.659	3.072	1.978	2.660	0.437	0.606
3.759	2.687	3.866	3.519	3.421	0.442	0.606
3.262	2.738	3.616	2.821	2.659	0.418	0.606
2.378	1.854	2.654	1.929	1.510	0.737	0.606
2.657	3.311	3.527	3.294	2.294	0.424	0.606
8.939	8.585	8.981	7.928	7.973	0.391	0.606
9.279	10.226	4.907	10.150	10.163	1.165	0.606
6.064	6.278	5.411	6.537	6.607	0.403	0.606
3.879	4.383	3.429	4.813	4.701	0.466	0.606
2.534	3.617	3.029	4.267	3.272	0.553	0.606
3.023	3.546	3.023	2.983	2.513	0.548	0.606
5.534	4.773	6.183	4.524	4.675	0.438	0.606
8.905	8.378	8.240	8.598	8.353	0.348	0.606
3.676	2.784	4.231	2.849	2.690	0.546	0.606
6.016	7.436	4.058	7.774	7.586	0.931	0.606
6.399	6.574	3.721	6.259	6.349	0.619	0.606
8.348	7.444	8.566	7.014	7.160	0.415	0.606
9.263	7.969	9.751	7.346	7.495	0.574	0.606
5.577	5.264	5.577	5.018	5.204	0.431	0.606
4.899	4.404	5.359	3.587	4.305	0.518	0.606
7.493	8.224	7.418	8.460	8.075	0.384	0.606
7.705	6.420	8.759	5.638	5.753	0.654	0.606
4.829	4.603	4.913	2.911	3.954	0.614	0.606
2.002	2.283	2.646	2.253	2.113	0.336	0.606
1.657	1.973	1.814	2.306	1.776	0.426	0.606
2.136	3.150	2.812	2.345	2.573	0.486	0.606
4.435	4.384	2.731	5.286	4.987	0.541	0.606
5.585	6.033	5.038	6.412	5.956	0.415	0.606
7.412	7.633	2.923	7.980	7.860	1.101	0.606
5.167	5.292	3.911	5.208	5.551	0.411	0.606
2.632	2.418	2.474	3.444	2.944	0.619	0.606
3.718	5.152	3.470	5.380	4.968	0.530	0.606
4.832	4.101	4.680	3.967	4.061	0.383	0.606
3.451	3.275	3.835	2.824	4.070	0.499	0.606
3.027	2.604	4.153	3.710	2.996	0.510	0.606
5.822	6.527	5.482	6.971	6.928	0.493	0.606
3.861	4.601	3.294	5.821	5.755	0.575	0.606
2.261	2.135	2.520	1.884	2.274	0.503	0.606
1.765	2.818	2.353	3.003	3.713	0.503	0.606
7.482	7.679	6.282	8.272	8.068	0.459	0.606
6.037	6.205	5.957	6.719	6.822	0.331	0.606
1.998	1.552	3.521	1.639	2.039	0.572	0.606
2.407	2.670	2.168	2.681	2.307	0.465	0.606
2.015	1.318	1.867	2.177	2.385	0.531	0.606
3.268	4.031	2.071	5.070	5.360	0.766	0.606
2.518	2.198	2.547	2.442	2.442	0.346	0.606
2.890	3.500	1.871	4.143	3.893	0.613	0.606

7.062	6.526	7.937	6.092	6.068	0.508	0.606
4.675	3.792	5.237	2.373	2.147	0.901	0.606
7.113	6.308	6.847	6.171	5.920	0.431	0.606
6.742	5.197	6.752	4.524	4.890	0.579	0.606
1.493	4.461	2.283	5.428	4.565	0.879	0.606
2.675	1.924	1.557	3.254	1.922	0.571	0.606
4.603	6.463	3.268	6.834	6.682	1.030	0.606
3.940	4.618	5.016	3.556	4.234	0.553	0.606
6.024	5.584	5.252	6.304	5.878	0.504	0.606
7.348	6.141	7.816	6.201	6.390	0.468	0.606
3.542	4.036	3.170	3.585	5.015	0.465	0.606
3.811	3.506	3.466	4.512	5.936	0.978	0.606
6.535	6.598	6.283	6.785	6.811	0.351	0.606
3.269	1.165	1.657	1.029	1.186	0.931	0.606
8.927	8.959	8.731	8.336	8.992	0.344	0.606
5.149	5.198	4.257	5.435	5.497	0.395	0.606
3.816	3.009	3.949	3.128	3.052	0.386	0.606
4.266	3.954	4.962	3.800	4.147	0.465	0.606
4.957	5.597	5.451	4.497	5.221	0.407	0.606
7.119	5.552	7.581	6.370	6.249	0.518	0.606
1.922	2.295	3.767	1.820	2.079	0.498	0.606
7.800	7.769	6.265	7.115	6.654	0.513	0.606
5.869	5.512	6.557	4.662	4.964	0.470	0.606
5.157	5.943	4.487	6.014	5.858	0.510	0.606
3.722	3.078	3.598	2.920	3.208	0.434	0.606
6.246	5.797	6.855	5.328	5.129	0.412	0.606
4.376	5.389	3.846	6.550	5.890	0.625	0.606
3.966	4.159	3.302	4.632	3.687	0.478	0.606
6.668	7.188	6.107	7.323	7.206	0.358	0.606
6.256	6.618	5.806	6.780	6.480	0.383	0.606
2.918	3.399	2.333	3.350	3.169	0.478	0.606
5.053	5.091	5.836	5.379	4.776	0.340	0.606
4.730	5.842	4.238	6.092	5.500	0.554	0.606
4.822	4.666	4.858	4.911	5.674	0.594	0.606
4.440	4.195	3.440	4.676	4.533	0.664	0.606
8.084	7.810	6.797	8.036	8.122	0.416	0.606
5.776	5.784	4.973	5.825	5.862	0.339	0.606
4.281	3.959	4.119	4.750	4.124	0.446	0.606
2.816	2.436	2.830	2.623	2.578	0.377	0.606
5.104	4.733	5.413	4.350	4.275	0.393	0.606
7.288	7.341	6.147	7.912	7.888	0.415	0.606
7.135	6.848	5.802	7.110	7.070	0.410	0.606
7.485	7.631	7.078	7.988	8.084	0.348	0.606
3.206	3.927	3.368	4.116	4.492	0.401	0.606
4.597	2.836	2.836	2.746	2.789	0.604	0.606
2.417	1.811	1.869	2.307	2.081	0.528	0.606
4.220	1.851	1.946	1.893	1.767	1.035	0.606
2.435	2.976	3.618	1.557	2.344	0.593	0.606
4.396	3.412	5.474	3.370	3.370	0.536	0.606
6.822	7.420	7.249	7.398	7.365	0.385	0.606

7.199	7.107	7.079	7.137	7.183	0.376	0.606
5.773	5.843	6.127	5.344	5.707	0.362	0.606
5.667	2.498	2.000	2.843	2.865	0.797	0.606
2.430	2.497	2.423	2.322	2.404	0.501	0.606
3.155	2.872	2.799	2.517	3.901	0.644	0.606
4.409	5.769	6.546	3.247	3.152	1.480	0.606
4.137	4.950	3.856	3.891	5.379	0.578	0.606
4.231	4.716	3.769	4.644	5.022	0.400	0.606
4.255	3.544	5.657	3.046	3.373	0.730	0.606
9.923	9.137	10.501	8.715	8.940	0.460	0.606
6.916	6.201	6.617	5.990	7.084	0.772	0.606
3.792	4.105	3.615	4.088	3.920	0.494	0.606
3.494	4.573	3.670	3.831	4.289	0.532	0.606
8.509	6.171	7.108	6.698	6.636	0.711	0.606
8.289	8.129	5.106	8.193	8.502	0.789	0.606
3.647	3.795	3.954	2.396	3.232	0.478	0.606
3.201	2.809	2.764	3.960	3.659	0.408	0.606
8.507	8.316	8.719	7.820	8.165	0.327	0.606
5.367	5.675	5.991	4.891	4.940	0.456	0.606
2.620	2.137	2.121	2.939	2.299	0.650	0.606
3.298	1.925	1.756	2.349	2.295	0.762	0.606
4.112	3.952	3.521	4.323	3.903	0.308	0.606
4.782	5.044	4.306	5.588	5.101	0.436	0.606
8.765	8.035	8.515	7.241	7.620	0.523	0.606
4.340	4.537	4.155	4.040	4.611	0.352	0.606
2.600	1.572	2.218	2.516	2.640	0.561	0.606
3.322	3.353	2.341	4.239	4.064	0.484	0.606
5.331	5.384	6.527	3.794	4.224	0.508	0.606
5.898	6.280	4.780	5.997	6.020	0.425	0.606
4.662	4.826	5.680	4.101	3.842	0.607	0.606
2.444	3.145	3.313	2.804	2.495	0.457	0.606
5.698	5.965	3.220	6.516	6.282	0.717	0.606
4.562	5.849	3.509	6.552	6.012	0.723	0.606
4.269	5.941	4.537	6.252	6.295	0.728	0.606
4.637	6.108	2.741	6.588	5.939	0.898	0.606
7.526	8.328	6.576	8.565	8.765	0.468	0.606
7.699	7.427	6.372	7.581	7.462	0.391	0.606
5.766	4.386	6.417	4.172	4.012	0.602	0.606
2.215	2.814	2.668	2.556	2.866	0.367	0.606
3.870	3.128	2.363	2.845	2.727	0.515	0.606
6.144	5.620	5.734	6.574	6.250	0.525	0.606
5.676	5.205	4.162	5.552	5.347	0.545	0.606
3.233	4.912	3.035	4.618	4.978	0.654	0.606
1.910	3.289	2.178	2.888	3.597	0.451	0.606
6.741	6.742	5.736	7.124	7.138	0.393	0.606
3.201	3.632	3.757	3.100	3.050	0.451	0.606
4.808	5.884	5.019	5.105	5.383	0.383	0.606
5.998	7.427	5.891	7.913	7.446	0.613	0.606
4.882	4.634	4.887	3.908	4.597	0.443	0.606
4.545	2.768	3.031	2.904	3.280	0.576	0.606

4.381	4.502	4.177	5.215	5.256	0.397	0.606
3.310	4.319	3.199	3.678	4.248	0.458	0.606
5.633	6.232	5.697	6.146	6.129	0.313	0.606
4.395	4.145	4.444	4.200	3.741	1.031	0.606
7.772	7.994	7.687	8.258	8.586	0.314	0.606
5.387	4.988	6.532	3.705	4.845	0.606	0.606
2.620	3.171	2.625	3.166	3.126	0.382	0.606
3.748	4.421	4.414	3.896	3.880	0.593	0.606
6.270	5.956	4.759	6.192	6.709	0.516	0.606
7.927	7.575	4.987	8.308	8.235	0.984	0.606
5.816	5.919	2.246	6.593	5.973	0.835	0.606
7.927	7.579	8.634	7.075	7.237	0.395	0.606
2.302	2.360	3.050	1.545	2.528	0.511	0.606
4.263	4.546	3.497	4.869	4.966	0.485	0.606
3.248	3.347	2.940	3.986	3.941	0.353	0.606
4.253	6.265	3.580	6.187	5.594	0.636	0.606
4.327	3.551	3.900	3.175	4.295	0.436	0.606
8.094	8.653	8.921	8.407	8.276	0.459	0.606
8.164	7.578	8.640	6.250	6.246	0.566	0.606
7.550	7.656	3.789	7.997	8.087	0.849	0.606
3.356	3.780	2.975	4.487	4.118	0.464	0.606
2.875	2.521	2.706	2.826	2.435	0.402	0.606
7.783	6.768	8.488	5.226	5.637	0.723	0.606
6.430	7.005	7.098	6.370	7.022	0.450	0.606
7.194	6.997	4.388	7.873	7.651	0.796	0.606
7.018	6.748	7.625	5.819	6.085	0.470	0.606
5.854	6.220	5.818	5.910	6.116	0.357	0.606
2.953	2.850	3.187	2.875	2.440	0.468	0.606
6.989	6.621	7.496	6.255	6.442	0.367	0.606
2.687	3.681	3.646	4.882	4.320	0.553	0.606
5.668	5.991	6.443	5.757	6.036	0.441	0.606
3.238	3.938	4.824	4.562	4.271	0.428	0.606
2.475	3.522	3.387	2.639	2.539	0.454	0.606
6.168	4.606	4.733	3.013	4.355	0.789	0.606
2.553	3.584	3.939	3.602	3.044	0.476	0.606
5.680	5.653	7.077	5.946	5.428	0.391	0.606
4.957	4.881	5.026	4.366	4.395	0.350	0.606
4.490	4.706	3.772	5.472	5.494	0.412	0.606
4.854	5.159	3.833	5.643	5.990	0.483	0.606
4.610	5.409	5.469	4.132	4.572	0.682	0.606
4.496	4.891	4.814	4.120	4.533	0.380	0.606
6.434	7.584	6.909	6.031	6.544	0.491	0.606
1.334	1.653	2.214	0.950	1.604	0.673	0.606
1.671	1.463	3.431	1.445	1.180	0.715	0.606
1.354	2.950	1.735	3.250	3.285	0.497	0.606
3.112	5.327	5.882	5.972	6.168	0.881	0.606
1.987	3.911	4.886	4.355	3.492	0.672	0.606
4.038	7.240	2.168	7.575	7.269	1.152	0.606
7.653	6.752	8.166	6.231	6.629	0.454	0.606
5.751	5.440	5.841	4.389	6.324	0.449	0.606

5.176	4.385	4.242	4.764	5.021	0.699	0.606
4.686	4.099	4.050	3.735	4.271	0.486	0.606
5.189	5.692	4.875	4.672	5.141	0.410	0.606
2.094	2.739	2.122	2.114	2.238	0.414	0.606
4.676	5.134	3.710	5.161	5.312	0.449	0.606
3.270	2.961	2.507	2.044	3.167	0.469	0.606
6.786	5.277	6.884	4.727	4.774	0.626	0.606
4.052	2.993	3.791	2.528	3.412	0.554	0.606
10.635	9.380	11.003	8.191	8.609	0.652	0.606
6.811	4.454	7.193	3.108	3.493	0.973	0.606
7.763	7.366	8.632	7.280	7.454	0.457	0.606
6.162	6.937	6.442	7.467	7.812	0.478	0.606
6.851	5.586	8.356	4.303	5.081	0.866	0.606
3.630	3.380	3.843	3.019	3.440	0.374	0.606
3.658	3.368	3.941	3.326	3.289	0.461	0.606
5.562	6.204	2.307	6.170	6.331	0.874	0.606
3.340	2.753	3.990	2.549	2.613	0.426	0.606
3.067	3.114	2.903	3.403	2.353	0.478	0.606
3.662	3.269	4.175	3.144	3.570	0.421	0.606
6.316	6.666	5.018	6.789	6.888	0.432	0.606
3.855	4.472	3.941	3.975	4.898	0.401	0.606
6.295	6.914	5.255	7.800	6.996	0.701	0.606
1.647	2.246	3.574	2.427	2.001	0.589	0.606
3.537	3.783	4.207	3.959	3.988	0.516	0.606
7.432	7.070	5.407	7.449	7.422	0.507	0.606
2.513	2.705	1.620	3.047	3.059	0.406	0.606
3.420	2.711	3.294	3.227	3.280	0.395	0.606
4.142	4.468	3.546	5.458	4.734	0.457	0.606
5.491	7.598	3.445	8.317	7.693	0.909	0.606
5.893	6.479	4.349	6.972	6.651	0.560	0.606
2.601	3.296	1.336	3.238	3.487	0.542	0.606
5.117	5.824	2.967	6.249	6.628	0.690	0.606
4.353	4.197	3.120	4.790	4.566	0.404	0.606
4.135	7.078	3.242	7.130	7.036	0.881	0.606
6.745	6.192	7.084	6.025	6.518	0.416	0.606
4.514	3.160	3.611	3.247	3.525	0.430	0.606
3.827	6.029	2.836	6.432	6.339	0.822	0.606
4.557	2.925	4.662	3.553	3.315	0.499	0.606
3.784	4.207	2.407	4.342	4.256	0.459	0.606
6.203	5.644	7.039	5.204	5.713	0.405	0.606
3.408	3.376	3.024	2.458	3.027	0.402	0.606
3.193	3.097	3.297	3.062	3.375	0.428	0.606
6.805	7.364	6.791	7.697	7.374	0.423	0.606
5.368	5.631	5.751	5.089	5.683	0.487	0.606
2.556	1.894	2.128	1.814	2.610	0.571	0.606
5.144	4.433	4.814	4.513	4.426	0.380	0.606
8.457	7.864	9.024	7.273	7.577	0.423	0.606
6.598	5.502	7.497	4.114	4.636	0.755	0.606
7.183	7.665	7.121	8.039	7.964	0.389	0.606
5.459	6.080	3.450	6.967	6.372	0.664	0.606

1.983	2.293	1.725	3.007	2.609	0.448	0.606
3.500	3.849	4.947	3.755	3.841	0.416	0.606
4.516	3.999	4.692	3.280	4.039	0.497	0.606
4.555	5.535	2.400	6.233	6.291	0.890	0.606
7.314	7.296	7.323	7.747	7.693	0.346	0.606
3.589	3.010	3.364	3.277	3.332	0.327	0.606
5.610	4.954	5.620	4.760	5.390	0.467	0.606
9.175	8.230	9.880	6.754	7.395	0.669	0.606
5.328	6.229	5.175	6.144	6.274	0.476	0.606
3.121	1.059	2.176	2.637	3.003	0.540	0.606
4.678	4.376	3.222	5.203	5.410	0.556	0.606
3.712	4.510	2.242	4.393	4.762	0.548	0.606
3.814	3.533	4.133	3.215	4.570	0.379	0.606
7.933	7.480	8.182	7.619	8.617	0.682	0.606
7.310	8.310	7.729	8.196	8.354	0.425	0.606
4.206	4.483	3.600	4.361	5.030	0.384	0.606
5.855	6.235	6.182	5.735	6.014	0.389	0.606
4.849	3.601	6.382	2.642	3.306	0.736	0.606
3.868	3.876	4.724	2.512	3.060	0.553	0.606
5.146	5.450	4.593	6.215	6.182	0.446	0.606
5.473	6.191	5.950	6.337	6.329	0.397	0.606
5.979	6.261	6.754	6.517	6.082	0.333	0.606
6.151	6.450	6.138	6.004	6.249	0.470	0.606
6.454	6.622	5.552	6.950	6.854	0.386	0.606
2.923	4.447	1.975	4.456	4.182	0.588	0.606
5.345	5.678	4.427	5.734	5.428	0.404	0.606
4.422	4.648	4.413	5.541	5.588	0.496	0.606
4.755	4.619	5.072	5.087	5.075	0.469	0.606
4.613	3.373	4.859	3.679	3.678	0.462	0.606
4.537	5.357	4.768	4.198	5.026	0.500	0.606
4.486	5.867	5.882	4.950	6.976	0.909	0.606
3.545	3.913	3.973	3.640	3.311	0.440	0.606
3.655	1.837	1.712	2.163	1.769	0.757	0.606
6.221	7.886	6.931	6.456	6.844	0.628	0.606
3.531	3.686	4.379	2.895	3.424	0.759	0.606
1.801	3.217	1.815	3.037	2.876	0.464	0.606
8.676	7.934	7.920	7.874	8.769	0.502	0.606
8.052	8.211	7.536	8.355	8.248	0.330	0.606
8.258	6.488	8.895	4.994	5.350	0.818	0.606
5.668	5.247	6.044	5.086	5.115	0.438	0.606
7.455	7.475	4.163	7.260	7.322	0.747	0.606
5.579	4.300	4.064	4.709	4.831	0.524	0.606
5.307	6.275	5.021	6.553	6.468	0.480	0.606
4.649	4.533	4.287	3.334	3.850	0.403	0.606
3.784	2.534	2.177	3.511	3.522	0.517	0.606
3.037	3.888	3.739	3.938	3.631	0.377	0.606
6.882	6.579	7.698	5.110	5.874	0.587	0.606
7.562	7.823	7.078	7.920	7.820	0.332	0.606
4.478	5.668	4.907	5.558	5.117	0.454	0.606
6.649	7.292	5.300	7.468	7.382	0.537	0.606

5.157	4.995	4.961	4.998	4.912	0.493	0.606
3.446	3.288	3.261	3.062	3.069	0.428	0.606
6.957	5.458	6.619	6.538	6.476	0.641	0.606
2.832	4.431	5.184	4.993	4.205	0.466	0.606
2.758	3.449	3.332	4.217	2.939	0.531	0.606
3.358	3.222	3.550	2.417	2.952	0.398	0.606
5.083	4.941	5.854	4.312	4.639	0.369	0.606
6.613	6.869	5.489	7.024	6.708	0.391	0.606
3.351	3.876	2.771	3.090	3.009	0.453	0.606
7.500	8.094	7.250	8.622	8.505	0.425	0.606
3.427	3.343	3.550	2.666	3.826	0.442	0.606
4.845	3.932	5.447	3.102	3.244	0.538	0.606
5.875	6.544	5.157	7.160	6.906	0.437	0.606
4.081	5.539	5.435	4.781	4.517	0.512	0.606
4.503	5.258	5.336	3.505	4.993	0.570	0.606
5.729	5.167	4.693	5.092	5.066	0.388	0.606
4.161	5.111	4.158	5.186	4.952	0.395	0.606
4.421	4.504	3.268	4.980	4.654	0.492	0.606
5.685	5.791	4.316	6.241	6.051	0.474	0.606
5.104	5.601	5.261	6.417	5.748	0.383	0.606
4.389	3.958	4.661	3.990	4.129	0.454	0.606
4.890	5.285	2.380	5.870	5.056	0.753	0.606
2.939	2.731	2.472	3.797	3.163	0.451	0.606
6.379	6.624	4.624	7.278	6.895	0.625	0.606
6.035	5.146	4.255	6.171	5.697	0.621	0.606
5.832	6.493	5.720	6.797	6.713	0.351	0.606
4.134	5.727	4.021	6.777	5.987	0.648	0.606
2.083	2.990	1.722	3.082	3.065	0.495	0.606
4.641	7.521	4.001	7.598	7.584	0.935	0.606
2.635	4.586	2.307	4.246	3.803	0.567	0.606
7.175	7.735	7.258	7.843	7.894	0.344	0.606
6.068	6.814	4.489	7.186	6.782	0.584	0.606
8.910	9.389	8.916	9.886	9.900	0.398	0.606
2.814	3.377	2.722	2.383	2.180	0.512	0.606
6.257	5.686	6.861	5.525	5.457	0.411	0.606
2.727	2.750	2.446	2.292	2.265	0.378	0.606
3.480	2.407	2.347	2.348	2.107	0.500	0.606
6.457	6.816	7.102	7.299	7.199	0.376	0.606
2.458	2.613	2.556	2.976	2.987	0.484	0.606
3.032	2.510	3.311	2.682	2.270	0.445	0.606
5.100	5.299	4.713	5.536	5.520	0.371	0.606
3.889	5.275	3.737	5.733	5.075	0.466	0.606
7.120	6.414	7.261	5.877	5.466	0.663	0.606
4.193	3.089	2.994	3.292	3.441	0.473	0.606
6.035	5.261	6.864	5.631	5.733	0.421	0.606
4.059	4.414	4.869	3.642	3.730	0.445	0.606
4.176	4.261	4.804	4.123	4.979	0.412	0.606
2.408	2.755	1.339	4.440	3.350	0.568	0.606
2.374	3.311	1.563	3.697	3.768	0.517	0.606
4.301	4.039	5.177	2.983	3.538	0.514	0.606

4.764	4.592	5.358	4.508	5.008	0.324	0.606
5.262	5.102	5.783	5.501	5.927	0.366	0.606
2.832	2.813	2.961	2.817	3.473	0.473	0.606
6.930	7.628	6.243	7.418	7.432	0.369	0.606
5.206	5.650	5.430	4.736	4.745	0.533	0.606
3.211	4.757	3.077	5.174	4.894	0.662	0.606
4.700	5.511	3.865	5.264	4.861	0.413	0.606
3.222	3.629	3.406	3.259	2.578	0.583	0.606
5.200	4.709	4.472	5.054	5.498	0.384	0.606
4.339	1.733	1.492	1.074	2.319	0.995	0.606
6.389	6.907	5.938	7.152	7.212	0.371	0.606
5.478	4.576	5.463	4.934	4.555	0.420	0.606
5.740	6.527	6.291	7.023	6.601	0.407	0.606
4.262	4.092	5.225	3.601	3.978	0.471	0.606
4.718	3.436	5.639	2.684	2.829	0.608	0.606
2.503	2.955	2.277	3.355	2.877	0.441	0.606
4.526	4.203	4.383	2.680	3.102	0.549	0.606
1.902	2.181	2.709	1.255	2.431	0.615	0.606
2.907	3.237	2.947	4.228	2.936	0.444	0.606
3.937	3.151	3.638	3.950	3.811	0.598	0.606
5.076	4.287	3.539	4.665	5.334	0.498	0.606
2.452	2.579	1.774	1.786	3.180	0.719	0.606
3.012	2.659	2.388	2.675	3.666	0.627	0.606
2.364	2.735	2.770	2.376	2.835	0.510	0.606
3.144	3.082	3.585	2.297	3.082	0.434	0.606
4.807	5.148	4.946	4.574	5.900	0.641	0.606
4.641	4.508	3.233	4.371	4.503	0.514	0.606
3.622	2.780	2.959	3.010	3.313	0.366	0.606
1.890	2.644	1.605	2.211	2.508	0.436	0.606
3.110	3.017	4.156	2.986	4.641	0.657	0.606
6.564	7.637	6.184	8.384	8.075	0.491	0.606
5.105	5.168	3.040	5.300	5.084	0.575	0.606
6.381	5.492	6.703	4.222	4.533	0.563	0.606
5.544	6.060	3.609	6.617	6.133	0.595	0.606
4.871	1.968	4.997	5.183	4.830	0.950	0.606
5.074	4.958	4.465	4.447	3.784	0.610	0.606
4.079	1.925	4.529	3.263	2.901	0.610	0.606
5.909	6.314	4.866	6.580	6.575	0.481	0.606
4.756	4.027	4.465	4.423	3.632	0.493	0.606
4.444	3.799	4.845	3.989	4.225	0.457	0.606
4.895	5.198	2.815	5.884	5.520	0.612	0.606
3.988	5.113	3.249	5.584	6.118	0.580	0.606
2.325	3.104	1.029	4.151	2.893	0.673	0.606
1.752	2.968	2.067	3.365	2.504	0.502	0.606
4.309	6.077	3.600	6.363	6.424	0.689	0.606
4.062	4.490	5.313	3.923	4.322	0.451	0.606
4.100	4.226	3.884	2.973	4.058	0.427	0.606
2.454	2.375	2.375	1.470	2.131	0.435	0.606
2.689	2.277	2.102	1.888	2.429	0.470	0.606
4.943	4.788	4.514	4.918	5.068	0.449	0.606

4.478	3.132	3.264	3.740	4.207	0.482	0.606
3.723	3.199	2.553	2.675	4.029	0.705	0.606
4.518	4.431	4.336	4.269	3.903	0.343	0.606
6.521	7.420	6.831	7.820	7.816	0.414	0.606
3.077	1.969	2.335	1.779	3.231	0.563	0.606
3.543	3.140	3.876	3.977	3.399	0.431	0.606
2.473	2.992	2.700	2.889	2.505	0.461	0.606
3.562	3.827	3.390	3.736	2.967	0.479	0.606
2.477	2.667	2.000	2.383	2.460	0.482	0.606
5.671	5.055	6.496	4.640	4.829	0.467	0.606
2.958	2.524	2.786	2.926	2.962	0.435	0.606
3.397	2.371	1.875	2.698	2.854	0.480	0.606
2.846	2.663	3.776	3.184	3.312	0.443	0.606
8.102	7.433	8.986	7.171	7.561	0.431	0.606
4.521	2.221	4.915	3.454	3.466	0.592	0.606
4.439	5.654	3.705	5.714	6.098	0.562	0.606
5.712	6.178	5.497	6.381	5.629	0.454	0.606
4.808	5.045	4.917	5.812	4.853	0.448	0.606
7.561	7.640	6.786	7.645	7.790	0.396	0.606
3.679	4.878	5.533	4.415	4.401	0.646	0.606
5.576	5.451	5.374	5.956	5.883	0.309	0.606
5.171	5.055	4.963	5.617	5.704	0.375	0.606
7.139	6.400	7.383	6.200	6.514	0.410	0.606
6.238	6.807	5.155	7.430	7.368	0.583	0.606
5.677	5.455	7.044	5.636	5.862	0.447	0.606
1.255	2.578	1.421	2.520	2.597	0.507	0.606
6.084	6.850	3.995	7.385	6.939	0.775	0.606
5.541	4.524	6.431	3.563	4.432	0.601	0.606
5.977	5.448	2.621	5.441	5.350	0.672	0.606
4.310	4.006	4.414	4.178	4.457	0.403	0.606
3.941	3.599	3.315	3.004	3.592	0.431	0.606
4.862	4.865	4.079	4.820	5.448	0.387	0.606
2.491	4.052	2.251	3.284	3.513	0.496	0.606
2.733	2.170	2.507	2.638	2.804	0.513	0.606
6.270	6.350	5.785	6.701	6.225	0.353	0.606
3.186	3.928	2.452	3.772	3.582	0.396	0.606
6.397	8.989	4.396	8.885	8.614	0.961	0.606
6.309	6.824	5.776	7.329	7.300	0.387	0.606
3.842	3.789	2.995	3.945	3.996	0.504	0.606
3.622	5.058	3.780	5.234	5.238	0.525	0.606
5.249	6.143	4.552	6.485	6.124	0.454	0.606
6.749	7.000	6.556	7.650	7.432	0.336	0.606
6.545	5.905	7.185	6.076	5.967	0.374	0.606
3.725	3.488	4.102	3.806	3.470	0.524	0.606
5.096	5.892	4.267	6.266	6.323	0.520	0.606
5.708	5.159	4.291	5.509	5.070	0.382	0.606
4.588	4.979	4.588	5.265	5.186	0.423	0.606
5.519	6.117	4.952	5.971	5.690	0.429	0.606
6.193	6.769	6.419	7.063	6.785	0.327	0.606
5.880	6.229	5.975	6.410	6.650	0.345	0.606

6.293	6.789	5.736	7.231	6.781	0.403	0.606
8.121	6.767	8.005	7.525	6.842	0.462	0.606
5.956	6.513	5.028	7.081	6.798	0.482	0.606
4.534	5.980	4.260	6.586	5.969	0.558	0.606
1.598	3.420	2.540	3.956	4.294	0.624	0.606
4.502	5.960	3.254	6.508	6.025	0.665	0.606
5.074	5.128	3.936	5.428	5.299	0.416	0.606
4.588	5.718	4.974	5.743	5.674	0.375	0.606
3.619	4.395	4.631	4.504	4.779	0.411	0.606
7.862	7.561	8.772	7.253	7.350	0.400	0.606
5.682	5.351	5.264	5.717	6.233	0.396	0.606
7.158	8.693	5.911	9.259	8.969	0.661	0.606
2.992	2.884	1.722	2.747	2.556	0.512	0.606
2.541	3.125	3.373	2.664	2.466	0.615	0.606
6.425	7.767	7.543	4.451	6.239	0.973	0.606
3.241	3.190	4.293	2.677	2.156	1.030	0.606
5.367	4.656	5.612	5.053	5.453	0.906	0.606
3.877	3.884	4.236	3.675	3.643	0.512	0.606
3.064	3.886	4.054	2.732	3.301	0.639	0.606
2.057	1.695	1.964	1.051	1.476	0.688	0.606
4.057	4.280	4.550	3.344	3.956	0.367	0.606
2.783	2.477	2.802	2.797	2.204	0.475	0.606
6.404	5.610	6.270	5.065	5.352	0.452	0.606
3.091	3.398	2.616	3.225	3.200	0.384	0.606
6.992	7.808	7.567	8.070	7.789	0.339	0.606
5.635	4.005	5.092	3.892	4.242	0.567	0.606
3.829	4.121	4.875	3.622	3.747	0.545	0.606
1.864	5.561	4.027	5.665	5.939	0.900	0.606
1.804	2.668	3.050	2.442	1.262	0.606	0.606
1.692	4.465	1.785	4.723	3.614	0.745	0.606
4.297	5.108	3.744	5.203	5.176	0.421	0.606
3.908	3.605	3.932	3.324	3.747	0.478	0.606
2.412	1.729	2.708	2.659	1.688	0.494	0.606
3.193	3.648	3.229	3.233	3.888	0.402	0.606
4.814	5.335	4.977	6.090	6.061	0.443	0.606
4.556	3.786	5.229	2.802	3.569	0.509	0.606
3.529	3.190	2.189	3.809	3.396	0.546	0.606
4.668	4.929	5.205	4.348	4.302	0.497	0.606
4.986	5.528	2.743	6.135	6.261	0.833	0.606
4.688	4.699	2.192	5.142	5.501	0.650	0.606
6.130	7.448	4.296	8.000	7.720	0.806	0.606
4.139	2.963	3.508	1.997	3.120	0.540	0.606
4.447	4.994	3.386	5.086	4.565	0.606	0.606
3.652	3.616	2.960	3.926	3.332	0.481	0.606
2.964	3.208	2.342	3.086	3.289	0.397	0.606
2.345	3.632	2.214	3.828	3.688	0.439	0.606
1.694	1.332	1.649	1.768	1.459	0.629	0.606
4.953	5.283	1.862	5.490	6.105	0.817	0.606
1.651	1.927	1.606	2.092	1.835	0.606	0.606
4.287	3.721	3.988	3.679	3.380	0.297	0.606

5.166	5.101	6.373	5.304	4.889	0.546	0.606
7.395	6.660	7.882	6.080	5.871	0.541	0.606
6.743	5.872	5.575	6.000	6.425	0.544	0.606
6.327	4.789	7.150	5.035	5.420	0.612	0.606
3.478	3.524	3.535	2.728	2.995	0.372	0.606
5.841	6.011	6.011	6.328	6.599	0.371	0.606
3.431	2.754	2.902	2.939	2.867	0.484	0.606
2.327	3.530	2.487	4.523	4.012	0.570	0.606
4.116	3.865	4.379	3.357	3.729	0.433	0.606
5.223	4.565	4.446	5.333	4.778	0.647	0.606
6.869	6.525	4.800	7.278	6.957	0.577	0.606
4.877	5.052	5.600	5.010	5.275	0.373	0.606
2.864	2.835	3.425	2.864	2.617	0.323	0.606
6.139	6.673	6.072	7.074	6.977	0.363	0.606
3.938	3.696	4.459	4.167	4.253	0.410	0.606
3.839	3.979	3.094	4.032	4.205	0.452	0.606
2.844	1.804	2.178	1.944	2.463	0.375	0.606
5.152	5.424	4.087	5.543	5.851	0.483	0.606
4.316	4.686	4.628	4.388	4.606	0.359	0.606
4.019	4.895	3.651	5.176	4.849	0.445	0.606
3.378	3.742	2.168	4.431	4.267	0.563	0.606
6.785	6.798	7.396	6.918	6.914	0.477	0.606
4.910	5.170	5.924	4.901	4.911	0.556	0.606
3.881	3.226	3.874	3.430	3.777	0.430	0.606
9.702	8.519	9.461	8.660	8.791	0.388	0.606
5.532	5.129	5.221	5.221	5.326	0.444	0.606
4.311	3.073	4.149	4.338	3.334	0.471	0.606
3.786	3.390	3.310	2.584	3.737	0.431	0.606
7.169	6.718	7.011	6.350	6.245	0.335	0.606
1.678	2.268	1.467	2.236	1.532	0.441	0.606
4.966	5.604	4.554	6.688	6.219	0.527	0.606
4.014	2.803	3.887	3.014	2.509	0.532	0.606
4.030	5.272	2.833	5.440	5.277	0.643	0.606
1.053	2.811	1.615	3.190	2.884	0.597	0.606
3.838	3.766	5.087	3.096	3.542	0.516	0.606
2.233	2.965	2.994	1.693	2.943	0.433	0.606
1.808	2.516	3.118	2.559	3.100	0.360	0.606
2.089	2.611	3.045	1.867	2.440	0.462	0.606
1.550	2.896	1.804	3.273	2.965	0.481	0.606
2.262	2.824	2.326	2.068	2.517	0.391	0.606
2.462	1.943	2.277	2.267	2.829	0.401	0.606
3.125	3.155	3.548	2.644	2.878	0.423	0.606
3.775	3.047	4.119	3.481	3.395	0.431	0.606
4.346	5.522	2.481	5.961	5.171	0.657	0.606
3.490	4.419	1.531	4.542	4.703	0.714	0.606
3.263	3.004	2.755	3.546	3.078	0.456	0.606
4.510	5.160	5.189	5.043	4.924	0.444	0.606
2.356	2.671	2.730	3.028	2.730	0.480	0.606
3.110	3.330	2.079	2.527	2.687	0.411	0.606
3.047	2.966	2.135	3.664	3.618	0.425	0.606

5.311	5.787	5.659	5.021	5.383	0.409	0.606
6.764	5.655	6.479	5.515	5.477	0.414	0.606
5.979	5.677	6.754	4.200	4.582	0.607	0.606
4.699	5.501	4.475	6.958	6.538	0.664	0.606
4.284	4.983	3.902	5.626	5.786	0.631	0.606
6.611	6.741	6.303	6.791	7.041	0.451	0.606
4.171	3.094	1.496	3.006	3.252	0.525	0.606
3.812	3.931	3.949	4.167	4.239	0.371	0.606
3.045	2.473	3.086	1.780	2.129	0.394	0.606
5.109	5.748	3.626	5.973	5.781	0.519	0.606
10.249	9.239	10.494	8.785	8.769	0.475	0.606
5.708	4.809	6.105	4.809	5.409	0.447	0.606
2.551	2.922	2.167	2.509	2.639	0.497	0.606
3.361	2.431	3.953	2.111	2.310	0.613	0.606
3.702	3.155	3.996	2.734	2.610	0.514	0.606
2.909	4.092	2.730	3.610	5.143	0.665	0.606
7.079	7.620	6.386	6.973	7.342	0.489	0.606
8.640	7.349	8.698	7.127	7.192	0.475	0.606
7.075	6.214	6.537	6.727	6.675	0.370	0.606
6.308	6.252	6.830	6.312	6.186	0.349	0.606
5.904	6.788	4.480	6.331	6.498	0.635	0.606
5.105	4.570	3.005	5.117	5.234	0.523	0.606
5.991	5.442	3.280	6.664	5.799	0.717	0.606
5.706	6.404	6.507	6.583	6.278	0.353	0.606
6.568	6.160	7.280	5.754	6.027	0.387	0.606
7.333	7.133	5.777	6.913	7.312	0.421	0.606
7.610	6.195	8.607	3.888	5.781	0.952	0.606
2.670	2.589	3.679	1.740	2.413	0.465	0.606
2.103	1.278	2.103	0.855	2.154	0.497	0.606
2.411	2.788	2.781	2.917	4.175	0.522	0.606
5.955	6.969	5.589	5.749	4.132	0.653	0.606
4.136	3.911	3.031	3.878	4.341	0.575	0.606
3.338	2.434	2.911	3.666	1.540	0.530	0.606
5.315	6.064	5.656	6.413	5.998	0.600	0.606
1.985	1.559	1.342	1.858	1.338	0.413	0.606
5.339	5.358	5.004	5.107	6.656	0.522	0.606
2.640	2.800	2.501	3.110	3.458	0.595	0.606
4.639	4.274	3.393	5.671	4.527	0.733	0.606
3.379	3.513	3.411	2.897	3.468	0.553	0.606
2.713	2.027	2.049	3.915	3.470	0.542	0.606
3.281	3.597	3.374	3.310	3.788	0.528	0.606
3.870	3.883	3.246	4.347	4.453	0.429	0.606
4.947	4.563	4.009	3.548	4.799	0.480	0.606
4.322	4.703	4.330	5.099	5.463	0.472	0.606
1.003	6.805	5.320	7.164	7.111	1.343	0.606
3.325	3.116	3.521	3.109	2.918	0.548	0.606
7.143	6.346	8.146	6.507	6.229	0.449	0.606
6.145	5.641	6.733	5.671	5.392	0.463	0.606
3.574	2.537	3.035	3.255	2.933	0.497	0.606
3.733	3.393	3.087	4.014	3.339	0.495	0.606

3.138	3.162	2.851	3.159	3.583	0.437	0.606
2.969	5.635	5.272	3.070	4.724	0.930	0.606
2.787	1.023	1.718	1.818	1.510	0.819	0.606
3.480	4.780	1.866	5.180	4.967	0.651	0.606
4.316	4.689	4.258	4.917	4.558	0.452	0.606
4.324	3.646	4.085	3.394	3.726	0.521	0.606
3.437	3.097	3.728	3.230	4.462	0.509	0.606
2.527	5.120	1.458	5.926	5.507	1.001	0.606
3.539	2.521	4.221	3.777	2.485	0.460	0.606
3.235	2.483	4.698	2.879	2.719	0.486	0.606
5.903	4.277	6.654	3.784	4.092	0.640	0.606
2.342	3.556	3.354	2.456	2.480	0.448	0.606
3.387	2.491	3.003	1.892	2.670	0.598	0.606
3.364	2.796	2.080	3.360	2.725	0.456	0.606
4.616	7.177	4.268	6.367	5.760	0.690	0.606
4.389	3.227	3.386	2.494	2.352	0.705	0.606
5.217	3.738	4.097	3.635	3.657	0.479	0.606
5.067	7.803	2.142	8.308	8.013	1.302	0.606
6.915	8.166	5.312	8.683	8.203	0.715	0.606
3.912	4.335	3.659	4.523	4.111	0.314	0.606
7.417	7.317	5.687	7.893	7.623	0.458	0.606
3.992	3.921	3.271	4.974	4.463	0.439	0.606
1.899	2.578	1.976	2.183	2.492	0.475	0.606
8.314	7.226	8.978	6.974	7.396	0.481	0.606
6.256	5.113	4.309	5.905	7.354	0.814	0.606
6.240	5.993	4.855	6.377	6.076	0.501	0.606
4.175	4.982	4.514	4.391	5.028	0.334	0.606
3.861	4.423	4.389	4.006	3.888	0.445	0.606
4.639	4.827	1.899	5.211	5.767	0.711	0.606
6.366	5.910	6.304	6.472	6.432	0.465	0.606
10.713	10.738	11.443	10.123	10.782	0.336	0.606
5.174	4.965	5.096	4.785	5.253	0.487	0.606
5.163	4.795	5.353	4.211	4.711	0.425	0.606
2.394	2.366	2.524	2.139	1.865	0.406	0.606
2.559	3.429	1.974	2.191	3.112	0.411	0.606
5.410	5.782	5.831	5.334	6.294	0.397	0.606
3.834	4.082	4.589	3.093	4.009	0.422	0.606
6.644	6.449	6.568	6.037	6.556	0.346	0.606
3.781	2.856	4.293	3.627	3.067	0.500	0.606
4.657	4.119	4.914	4.064	4.044	0.445	0.606
5.280	6.622	5.071	6.566	6.482	0.617	0.606
4.909	5.089	6.056	3.470	4.072	0.663	0.606
6.061	5.613	6.094	5.063	5.170	0.361	0.606
2.159	2.285	2.540	2.334	2.456	0.448	0.606
6.462	7.397	5.627	8.168	8.099	0.531	0.606
4.719	5.231	4.597	4.840	5.052	0.483	0.606
3.573	3.798	4.923	3.250	3.126	0.411	0.606
3.954	2.660	3.007	3.086	3.849	0.621	0.606
4.062	3.300	4.618	4.366	4.457	0.656	0.606
4.955	4.839	5.804	5.443	5.580	0.405	0.606

6.915	7.143	6.423	7.359	7.367	0.338	0.606
8.028	7.606	7.856	7.104	7.797	0.383	0.606
6.346	5.861	6.157	5.514	5.848	0.298	0.606
6.430	5.523	7.434	5.330	5.341	0.494	0.606
5.435	4.566	5.898	3.904	4.034	0.483	0.606
3.347	3.157	3.481	2.388	3.027	0.469	0.606
6.310	6.589	5.933	6.837	6.928	0.422	0.606
8.174	8.747	7.544	8.750	8.709	0.410	0.606
8.652	9.200	8.012	9.283	9.278	0.423	0.606
8.256	8.754	7.614	8.808	8.820	0.411	0.606
8.043	8.594	7.382	8.649	8.672	0.413	0.606
8.242	8.768	7.614	8.775	8.799	0.407	0.606
7.676	8.215	6.976	8.182	8.288	0.419	0.606
7.750	9.714	4.187	9.849	9.602	1.199	0.606
6.173	5.312	6.559	5.597	5.357	0.377	0.606
6.035	8.329	3.059	8.948	8.962	1.293	0.606
3.880	5.915	4.505	6.600	6.303	0.649	0.606
6.747	7.655	4.488	8.041	7.925	0.825	0.606
4.715	7.870	2.847	8.392	8.215	1.182	0.606
4.055	7.011	2.446	7.262	6.678	1.027	0.606
5.859	7.070	5.695	7.352	7.369	0.510	0.606
4.753	8.113	2.489	8.378	8.475	1.258	0.606
4.103	4.881	3.705	5.550	5.332	0.520	0.606
3.551	6.501	2.196	6.748	6.906	1.070	0.606
3.827	3.011	2.161	2.209	2.778	0.518	0.606
7.289	6.626	8.013	7.529	7.919	0.492	0.606
8.032	8.894	3.375	9.410	9.250	1.319	0.606
2.852	3.511	1.895	3.983	3.629	0.560	0.606
6.725	3.469	2.302	3.372	2.640	0.976	0.606
4.427	5.500	3.336	5.852	6.190	0.764	0.606
6.476	7.251	3.157	7.400	7.589	1.076	0.606
6.291	8.267	8.747	6.781	6.515	0.952	0.606
6.456	8.151	7.081	7.485	6.744	0.640	0.606
3.793	4.510	4.111	5.592	3.773	0.935	0.606
4.159	2.503	1.828	2.307	2.482	0.566	0.606
3.205	3.627	3.575	2.690	2.882	0.533	0.606
3.645	4.464	3.179	4.176	4.089	0.379	0.606
2.375	2.426	2.496	1.887	2.401	0.461	0.606
2.675	3.313	2.751	3.564	3.488	0.449	0.606
5.712	6.248	4.978	6.573	6.484	0.411	0.606
1.927	2.649	1.048	2.873	2.879	0.448	0.606
7.099	7.340	7.670	4.461	5.884	0.738	0.606
5.484	5.659	3.377	5.359	5.080	0.548	0.606
4.279	5.410	4.509	5.554	5.253	0.466	0.606
2.890	2.824	4.195	2.694	3.147	0.434	0.606
5.900	4.812	4.915	3.427	3.855	0.592	0.606
5.710	4.932	6.180	4.584	4.859	0.430	0.606
5.444	6.422	3.443	6.726	6.337	0.756	0.606
2.339	3.436	1.192	3.405	3.935	0.649	0.606
2.119	2.480	2.891	2.603	2.639	0.427	0.606

5.729	4.588	6.199	4.703	5.142	0.443	0.606
3.542	3.191	4.319	2.536	2.770	0.636	0.606
7.439	8.403	5.304	8.661	8.247	0.771	0.606
3.528	1.917	3.672	2.053	2.604	0.727	0.606
5.478	3.953	4.486	5.044	4.481	0.588	0.606
5.677	5.831	5.617	6.018	5.823	0.313	0.606
5.963	6.501	5.899	7.064	6.732	0.450	0.606
2.412	2.754	2.381	2.846	2.835	0.587	0.606
4.671	4.086	4.981	4.127	4.107	0.404	0.606
3.149	2.923	2.849	2.780	2.356	0.520	0.606
2.232	2.296	2.444	1.461	1.649	0.424	0.606
3.689	4.437	2.681	4.342	4.332	0.550	0.606
7.192	8.293	8.166	8.322	8.239	0.386	0.606
6.748	6.446	5.847	6.636	6.563	0.346	0.606
5.737	5.194	4.973	4.619	5.172	0.449	0.606
2.813	3.378	3.564	2.044	2.403	0.616	0.606
4.336	5.599	4.204	3.429	4.799	0.807	0.606
3.765	4.689	2.296	2.502	3.494	0.941	0.606
4.380	4.736	4.282	4.457	4.590	0.575	0.606
3.588	4.173	3.245	2.981	5.939	0.793	0.606
3.053	2.409	2.004	2.367	2.772	0.410	0.606
5.788	5.721	6.628	4.999	5.533	0.514	0.606
7.455	7.602	7.258	7.938	8.038	0.323	0.606
4.924	5.109	4.792	4.855	5.231	0.425	0.606
3.198	3.267	2.328	3.756	3.431	0.409	0.606
1.999	4.234	1.873	4.570	4.891	0.760	0.606
3.112	3.534	2.919	3.625	4.039	0.357	0.606
6.272	6.595	6.399	7.459	6.851	0.424	0.606
2.832	8.341	3.981	8.532	8.586	1.497	0.606
6.825	6.950	6.063	7.507	7.547	0.433	0.606
3.688	4.944	2.098	4.972	5.319	0.676	0.606
6.524	6.145	6.272	6.420	6.785	0.396	0.606
7.015	6.944	7.159	6.316	6.329	0.348	0.606
3.651	4.624	4.862	5.072	4.594	0.393	0.606
6.383	5.666	5.961	6.131	5.971	0.431	0.606
3.093	2.330	2.548	2.013	2.214	0.568	0.606
1.597	2.835	1.681	3.090	3.082	0.546	0.606
6.489	6.730	5.834	6.769	6.868	0.436	0.606
5.193	4.495	5.367	4.146	4.773	0.390	0.606
1.876	2.007	2.170	2.305	1.986	0.477	0.606
3.890	3.155	4.162	2.370	2.722	0.505	0.606
4.200	4.025	3.994	4.368	4.679	0.441	0.606
5.088	4.556	5.554	3.550	4.749	0.428	0.606
2.080	3.105	1.472	2.664	2.090	0.416	0.606
3.810	3.354	3.066	3.045	3.524	0.462	0.606
11.010	10.574	10.849	8.892	8.901	0.577	0.606
5.976	5.699	5.835	6.058	5.968	0.339	0.606
3.722	4.991	3.149	5.799	5.423	0.641	0.606
1.938	3.256	1.976	3.776	3.020	0.508	0.606
3.759	4.738	2.886	5.234	4.899	0.546	0.606

3.797	5.138	4.252	4.882	4.924	0.561	0.606
5.622	5.158	5.174	5.299	5.167	0.487	0.606
3.187	5.132	1.846	5.558	5.540	0.841	0.606
5.983	6.101	5.235	6.152	5.957	0.344	0.606
4.210	5.092	3.634	5.478	4.760	0.427	0.606
3.733	3.676	2.817	3.369	3.622	0.340	0.606
3.030	1.678	3.396	2.072	2.223	0.494	0.606
3.939	3.833	3.800	2.248	3.798	0.671	0.606
1.904	2.185	2.631	2.380	3.701	0.496	0.606
8.628	9.093	8.058	9.143	9.379	0.369	0.606
2.244	2.542	1.668	2.510	2.538	0.432	0.606
3.298	2.914	4.040	3.311	3.689	0.376	0.606
4.528	4.770	5.058	4.048	4.751	0.467	0.606
3.763	3.377	3.809	2.698	3.902	0.461	0.606
4.125	4.649	4.557	3.738	5.175	0.548	0.606
3.742	3.995	4.334	2.670	3.473	0.541	0.606
4.475	4.170	4.175	3.294	4.029	0.419	0.606
3.225	2.605	3.961	3.554	2.397	0.578	0.606
6.737	7.257	7.085	7.836	7.166	0.404	0.606
8.748	8.466	9.007	8.424	9.042	0.555	0.606
2.752	3.218	3.199	1.895	2.381	0.493	0.606
4.687	3.149	5.041	1.993	2.758	0.766	0.606
3.000	2.644	2.188	3.161	4.856	0.799	0.606
4.990	4.812	1.468	5.262	6.024	0.988	0.606
3.546	3.944	2.487	4.157	4.219	0.519	0.606
5.312	5.169	6.059	3.893	3.829	0.574	0.606
2.344	1.758	2.592	2.019	2.603	0.734	0.606
3.902	3.836	2.246	4.350	4.409	0.507	0.606
6.113	4.631	5.191	4.743	5.328	0.464	0.606
3.768	2.541	3.949	3.184	3.580	0.513	0.606
4.641	5.235	5.180	4.972	4.759	0.512	0.606
3.177	2.655	3.307	2.643	3.022	0.461	0.606
2.838	2.968	1.855	3.632	3.347	0.495	0.606
2.798	3.276	2.786	3.754	3.672	0.341	0.606
4.037	5.682	4.116	4.888	5.278	0.519	0.606
7.919	6.863	8.129	5.241	6.043	0.694	0.606
3.131	3.089	2.921	3.654	3.234	0.444	0.606
3.875	4.137	4.243	4.510	4.468	0.357	0.606
5.278	4.561	6.026	4.890	4.882	0.433	0.606
3.859	3.316	4.509	3.310	2.924	0.466	0.606
6.803	6.974	6.075	7.779	7.502	0.544	0.606
4.684	4.865	5.469	4.680	4.806	0.611	0.606
5.426	5.294	6.455	4.123	4.632	0.576	0.606
4.271	4.317	4.721	4.568	4.000	0.484	0.606
3.498	4.296	3.271	4.693	4.812	0.428	0.606
3.525	2.382	1.927	2.629	2.441	0.431	0.606
1.621	4.207	1.972	3.917	5.040	0.802	0.606
8.264	2.960	1.421	3.385	3.023	1.526	0.606
6.035	4.375	1.954	5.351	4.985	0.844	0.606
5.976	6.634	1.544	7.552	7.034	1.215	0.606

5.558	6.094	3.131	6.588	6.549	0.845	0.606
3.841	5.009	4.504	4.961	4.670	0.627	0.606
4.561	4.588	4.241	5.099	5.456	0.368	0.606
6.676	7.671	5.672	8.000	7.678	0.519	0.606
5.752	6.547	3.294	6.230	6.410	0.809	0.606
5.743	5.531	3.837	6.411	6.426	0.630	0.606
9.078	7.540	9.707	7.061	7.053	0.622	0.606
6.852	6.600	6.895	6.384	6.717	0.315	0.606
6.098	5.201	6.669	5.117	5.513	0.473	0.606
2.218	3.094	1.987	2.625	3.607	0.434	0.606
8.116	8.675	8.553	7.844	7.146	0.524	0.606
3.181	2.096	2.355	2.674	3.033	0.737	0.606
6.103	5.874	6.543	5.400	5.509	0.365	0.606
5.912	6.772	6.726	4.799	6.125	0.718	0.606
4.196	4.245	4.234	3.456	3.787	0.510	0.606
5.688	3.857	4.278	3.619	3.949	0.624	0.606
6.819	7.107	6.728	7.450	7.431	0.324	0.606
4.985	4.926	5.239	4.475	4.242	0.356	0.606
5.914	5.818	6.983	5.563	5.882	0.402	0.606
5.013	4.773	4.462	5.555	5.223	0.550	0.606
3.040	4.048	3.444	3.242	4.103	0.606	0.606
4.252	4.735	4.728	3.788	4.316	0.375	0.606
3.386	2.868	3.306	3.808	2.972	0.393	0.606
4.479	5.051	2.194	5.327	5.536	0.684	0.606
9.935	10.506	9.847	10.394	10.416	0.329	0.606
3.085	3.655	3.153	3.891	3.825	0.454	0.606
4.250	4.788	4.696	4.649	4.745	0.393	0.606
5.061	5.907	2.780	5.662	6.022	0.740	0.606
3.902	3.285	2.909	4.419	4.098	0.437	0.606
5.540	4.345	5.563	3.967	4.893	0.431	0.606
3.552	4.896	2.971	5.274	4.676	0.640	0.606
3.322	2.393	2.277	3.622	3.560	0.433	0.606
8.787	9.640	6.022	9.629	9.596	0.865	0.606
5.427	4.608	4.326	4.453	4.463	0.488	0.606
3.590	3.713	3.685	3.151	3.137	0.452	0.606
5.122	5.732	5.924	4.834	5.118	0.490	0.606
6.019	6.166	5.600	6.397	6.338	0.382	0.606
2.835	2.613	2.964	2.169	2.187	0.510	0.606
4.956	5.487	4.842	6.120	6.092	0.426	0.606
4.355	4.763	4.532	5.449	4.904	0.359	0.606
5.539	5.673	5.121	6.138	6.324	0.350	0.606
2.907	3.766	2.964	3.633	3.756	0.399	0.606
2.267	2.341	1.671	3.336	3.070	0.550	0.606
3.308	3.700	3.367	2.362	3.151	0.480	0.606
2.085	1.560	1.574	2.238	1.933	0.487	0.606
2.206	3.341	3.607	4.143	4.058	0.728	0.606
5.965	5.013	5.423	5.329	5.268	0.390	0.606
5.837	4.754	5.187	5.045	5.029	0.404	0.606
4.651	4.780	3.721	4.999	4.560	0.539	0.606
1.953	3.416	2.024	3.512	2.443	0.591	0.606

7.386	7.080	6.881	6.977	7.183	0.377	0.606
7.457	7.053	6.936	6.978	7.197	0.385	0.606
4.779	2.722	3.585	3.137	3.527	0.445	0.606
3.520	3.091	2.981	3.231	2.063	0.510	0.606
3.504	3.166	3.092	3.126	1.839	0.574	0.606
6.003	4.841	5.597	5.175	4.594	0.391	0.606
7.115	5.505	6.652	5.713	5.757	0.436	0.606
6.539	5.255	6.223	5.409	5.124	0.393	0.606
6.719	5.195	6.537	5.563	5.538	0.405	0.606
4.140	3.468	3.574	3.077	2.461	0.589	0.606
7.900	7.556	8.221	7.285	7.724	0.344	0.606
5.751	6.164	6.610	6.671	6.917	0.377	0.606
7.256	7.309	7.604	7.974	7.708	0.346	0.606
3.580	4.030	3.941	3.724	3.681	0.421	0.606
9.107	9.311	7.586	9.434	9.235	0.524	0.606
7.997	8.186	7.508	8.295	8.356	0.312	0.606
5.620	5.016	5.274	5.611	6.071	0.393	0.606
6.000	6.392	6.253	6.754	6.705	0.343	0.606
4.918	5.285	5.251	5.348	5.979	0.329	0.606
5.294	5.254	5.596	5.906	5.838	0.366	0.606
3.703	3.872	3.531	4.139	4.584	0.431	0.606
2.402	1.962	2.361	1.936	1.504	0.384	0.606
2.394	2.328	1.800	2.041	2.378	0.540	0.606
4.928	4.708	5.479	5.258	5.025	0.356	0.606
6.179	5.668	5.397	5.537	5.822	0.428	0.606
5.324	5.395	5.945	5.461	5.467	0.465	0.606
6.416	6.721	6.441	6.393	7.185	0.435	0.606
4.561	2.841	3.486	2.696	2.844	0.687	0.606
6.600	7.477	6.111	7.754	7.395	0.409	0.606
8.787	9.096	7.439	9.307	9.140	0.446	0.606
6.086	5.112	7.450	3.722	3.396	0.869	0.606
5.134	4.740	3.725	5.419	5.252	0.519	0.606
4.348	5.229	3.819	4.724	4.699	0.726	0.606
3.621	3.010	2.694	2.998	2.176	0.445	0.606
4.295	3.435	3.745	3.487	3.294	0.352	0.606
5.859	5.670	5.896	6.161	6.382	0.325	0.606
3.884	5.819	3.231	5.976	5.810	0.703	0.606
3.063	3.828	2.799	3.727	3.596	0.410	0.606
6.541	5.600	6.515	6.029	6.131	0.425	0.606
2.932	3.139	2.492	3.235	3.061	0.489	0.606
2.716	2.436	2.731	1.856	2.309	0.459	0.606
2.926	2.978	2.985	2.672	2.257	0.427	0.606
5.842	6.069	5.903	6.086	6.057	0.385	0.606
2.741	2.272	2.589	1.874	2.187	0.364	0.606
2.122	2.809	2.245	2.313	2.840	0.526	0.606
3.576	2.275	1.369	2.716	3.307	0.594	0.606
1.543	2.818	2.049	2.993	3.432	0.467	0.606
3.721	3.702	2.956	5.159	4.526	0.605	0.606
6.315	7.131	5.392	7.258	7.261	0.510	0.606
7.749	8.338	5.608	8.675	8.369	0.657	0.606

5.787	6.798	4.324	7.039	6.712	0.657	0.606
3.349	2.711	4.233	3.925	3.166	0.621	0.606
3.479	3.119	2.639	3.398	3.368	0.473	0.606
3.055	3.636	2.758	3.615	3.480	0.369	0.606
6.020	5.966	6.309	6.261	6.254	0.412	0.606
4.516	6.173	5.964	3.587	4.044	0.855	0.606
2.182	3.722	3.020	3.373	3.152	0.507	0.606
5.041	6.417	4.630	7.520	6.726	0.658	0.606
4.096	6.069	1.873	6.781	6.823	1.177	0.606
4.077	5.998	1.753	6.514	5.752	0.882	0.606
4.153	3.973	3.097	3.990	3.852	0.507	0.606
6.104	5.430	6.412	4.373	5.199	0.667	0.606
9.489	8.430	10.523	7.267	7.830	0.701	0.606
7.116	6.721	6.664	6.421	6.804	0.510	0.606
4.155	4.703	4.510	3.440	4.164	0.556	0.606
2.779	3.549	1.943	2.375	3.129	0.440	0.606
3.671	4.415	3.407	5.073	4.336	0.445	0.606
7.320	8.133	7.815	7.122	7.308	0.371	0.606
2.892	4.267	3.480	4.611	3.622	0.477	0.606
3.026	3.473	2.174	4.234	4.270	0.521	0.606
4.007	3.091	4.918	2.235	2.553	0.588	0.606
2.870	4.896	1.719	5.562	5.168	0.801	0.606
3.720	4.255	5.042	3.270	3.477	0.606	0.606
3.986	5.949	2.000	6.530	5.914	0.924	0.606
4.412	4.425	3.108	5.261	5.230	0.631	0.606
3.781	4.181	4.501	4.664	4.542	0.510	0.606
3.896	6.328	6.245	7.134	6.719	0.846	0.606
6.283	6.910	5.329	6.741	6.683	0.442	0.606
5.073	5.663	4.449	5.246	5.612	0.484	0.606
1.865	2.431	2.117	2.008	1.876	0.587	0.606
10.155	9.587	10.730	9.128	9.380	0.412	0.606
4.235	5.337	3.343	6.129	5.874	0.646	0.606
6.896	6.285	7.712	6.134	6.195	0.403	0.606
3.092	2.762	3.152	2.369	2.809	0.450	0.606
5.646	6.131	6.124	5.331	5.769	0.367	0.606
4.993	4.611	4.940	4.313	4.419	0.375	0.606
4.149	3.724	2.351	3.370	4.270	0.681	0.606
1.603	1.910	1.707	2.049	2.273	0.551	0.606
6.784	7.426	5.049	7.624	7.475	0.565	0.606
5.324	4.747	5.915	5.270	5.580	0.481	0.606
2.967	2.830	3.229	2.430	3.499	0.680	0.606
3.677	3.742	3.295	4.337	3.742	0.428	0.606
2.669	4.849	2.390	4.848	4.617	0.642	0.606
4.958	4.151	4.708	3.244	3.470	0.529	0.606
6.104	6.979	8.122	5.685	5.968	0.598	0.606
4.708	4.456	2.329	5.030	4.549	0.608	0.606
2.005	1.716	1.278	2.937	3.353	0.509	0.606
2.634	2.727	1.431	1.992	1.860	0.873	0.606
5.600	6.059	3.093	6.869	6.699	0.768	0.606
6.536	7.975	6.954	8.445	8.309	0.517	0.606

6.535	7.269	6.320	7.952	8.010	0.471	0.606
7.575	7.367	6.769	7.892	7.835	0.370	0.606
5.255	4.536	4.336	4.894	4.980	0.399	0.606
5.202	5.839	3.576	6.184	6.526	0.677	0.606
6.502	6.612	4.989	7.485	7.096	0.617	0.606
5.549	5.953	4.781	5.714	5.597	0.397	0.606
5.944	6.686	4.748	6.702	6.390	0.431	0.606
7.782	8.780	2.432	9.030	8.881	1.350	0.606
4.887	7.773	3.110	7.988	7.768	0.972	0.606
5.953	8.437	3.821	9.076	8.652	1.134	0.606
4.813	5.218	3.354	5.256	5.633	0.490	0.606
4.875	4.656	3.372	5.166	5.473	0.456	0.606
3.554	3.434	2.558	4.339	3.827	0.457	0.606
6.795	6.514	6.244	6.985	6.962	0.322	0.606
4.759	6.036	2.890	6.239	6.136	0.701	0.606
1.681	3.615	1.931	4.806	4.064	0.720	0.606
8.222	8.366	9.332	7.851	8.195	0.417	0.606
6.184	8.213	4.958	8.858	8.287	0.883	0.606
3.822	3.797	1.753	4.487	4.291	0.566	0.606
2.578	2.418	2.451	3.165	2.782	0.380	0.606
6.842	6.724	6.787	6.130	6.307	0.454	0.606
5.577	5.823	4.979	6.428	6.033	0.487	0.606
3.582	4.492	2.182	5.292	4.788	0.668	0.606
1.652	1.515	2.069	2.162	1.727	0.343	0.606
3.321	2.471	3.282	3.132	2.688	0.468	0.606
2.938	2.957	2.935	2.430	2.379	0.556	0.606
1.926	1.994	1.933	2.834	1.481	0.425	0.606
1.478	2.874	1.843	2.983	2.762	0.493	0.606
2.368	2.306	2.693	2.621	3.387	0.376	0.606
6.597	6.914	6.360	7.192	6.863	0.374	0.606
4.388	4.873	4.439	5.160	5.088	0.335	0.606
6.644	5.859	6.860	4.774	4.972	0.534	0.606
3.946	3.766	3.783	3.473	4.294	0.374	0.606
6.768	6.907	6.342	6.894	7.041	0.363	0.606
5.124	4.203	5.166	4.793	4.845	0.509	0.606
2.421	2.358	2.406	2.251	1.713	0.486	0.606
4.802	5.359	4.012	5.507	5.565	0.478	0.606
3.912	4.098	3.327	4.506	4.196	0.418	0.606
5.566	4.244	7.014	4.623	4.013	0.896	0.606
2.536	2.628	5.305	5.736	6.121	1.034	0.606
4.759	5.395	3.821	4.641	3.775	1.092	0.606
4.875	5.182	4.200	4.290	4.252	0.829	0.606
5.700	5.754	5.913	6.430	6.015	0.300	0.606
6.075	6.458	6.933	6.219	6.464	0.415	0.606
5.334	4.597	5.824	4.420	4.870	0.414	0.606
5.727	6.242	6.352	6.081	6.255	0.358	0.606
5.524	6.013	5.524	6.182	6.183	0.329	0.606
3.673	3.046	4.493	3.507	3.516	0.465	0.606
1.630	1.466	2.145	2.043	1.695	0.485	0.606
5.996	6.407	3.983	6.977	6.807	0.719	0.606

6.541	6.739	6.581	7.372	7.071	0.440	0.606
8.433	9.241	7.073	9.559	9.483	0.593	0.606
5.951	6.438	6.430	6.367	6.595	0.438	0.606
3.941	4.163	2.816	5.410	4.760	0.567	0.606
5.247	4.826	5.283	3.951	4.535	0.410	0.606
6.132	5.323	5.943	5.519	5.423	0.448	0.606
1.805	2.716	2.571	1.799	1.562	0.478	0.606
4.718	4.328	5.442	3.179	4.243	0.604	0.606
4.937	4.119	6.156	4.121	4.521	0.627	0.606
7.765	7.689	8.645	6.753	6.853	0.483	0.606
7.416	7.234	8.357	6.363	6.431	0.493	0.606
4.851	4.409	4.735	3.532	4.473	0.532	0.606
3.782	4.260	3.420	4.902	4.205	0.395	0.606
8.714	8.381	9.440	7.679	7.723	0.488	0.606
2.900	2.711	1.860	2.680	3.289	0.441	0.606
5.048	4.815	5.468	4.416	4.838	0.453	0.606
5.344	4.050	4.778	3.475	4.042	0.624	0.606
1.789	2.444	2.491	1.642	2.126	0.410	0.606
4.904	5.141	4.985	4.477	4.385	0.402	0.606
2.866	2.858	2.760	3.191	2.663	0.553	0.606
3.943	4.835	5.302	3.300	4.256	0.496	0.606
5.316	5.807	6.565	5.390	5.370	0.364	0.606
4.565	5.271	4.586	5.204	5.300	0.359	0.606
8.397	9.370	9.476	7.879	8.278	0.479	0.606
3.095	3.314	3.428	2.399	3.344	0.427	0.606
2.663	2.625	2.877	2.442	1.844	0.452	0.606
5.942	6.357	6.131	6.255	6.611	0.317	0.606
7.485	6.583	7.631	6.682	6.939	0.356	0.606
3.282	2.470	3.968	1.820	2.148	0.511	0.606
6.648	9.098	7.810	5.551	6.523	1.086	0.606
4.734	5.096	2.290	3.458	3.871	0.698	0.606
8.329	6.852	9.053	4.873	5.217	0.911	0.606
5.495	6.307	1.414	6.676	6.312	1.114	0.606
2.208	2.931	2.152	3.254	2.871	0.410	0.606
2.788	3.758	1.485	3.861	4.125	0.602	0.606
3.805	4.192	4.949	4.126	4.396	0.387	0.606
3.282	3.028	3.207	3.094	2.463	0.394	0.606
3.650	3.876	4.336	3.582	3.813	0.386	0.606
5.136	5.428	5.872	4.106	4.573	0.561	0.606
2.127	2.016	2.475	1.754	2.060	0.470	0.606
5.263	4.412	5.822	4.024	3.831	0.488	0.606
2.771	2.337	2.353	2.370	2.770	0.422	0.606
2.648	2.624	2.298	2.935	2.873	0.481	0.606
3.651	2.748	2.809	2.935	2.597	0.415	0.606
5.629	6.140	5.505	6.809	6.794	0.459	0.606
4.126	4.161	3.454	4.105	4.852	0.373	0.606
3.965	3.820	4.197	4.711	3.968	0.551	0.606
3.000	2.217	2.898	1.721	1.779	0.424	0.606
3.800	1.734	3.395	2.096	1.837	0.586	0.606
4.171	1.664	3.805	2.405	1.985	0.618	0.606

4.231	1.843	3.645	2.465	2.066	0.611	0.606
3.967	2.825	3.640	3.774	2.563	0.493	0.606
3.761	4.361	4.115	3.549	2.703	0.525	0.606
3.704	3.934	4.111	3.288	2.657	0.461	0.606
4.974	5.511	5.603	3.516	4.853	0.649	0.606
4.825	5.711	6.509	4.102	4.671	0.668	0.606
6.945	8.977	2.920	9.461	9.263	1.388	0.606
3.687	3.872	2.921	3.696	3.775	0.395	0.606
3.847	3.478	2.388	3.633	4.094	0.561	0.606
3.877	4.102	2.999	3.006	3.842	0.461	0.606
3.821	3.809	2.946	2.142	3.854	0.506	0.606
4.740	4.337	4.256	3.439	4.476	0.478	0.606
3.647	3.258	2.667	4.154	3.637	0.470	0.606
3.933	4.363	4.457	3.185	3.606	0.687	0.606
5.573	5.471	6.412	5.012	5.087	0.382	0.606
3.552	3.940	4.024	3.556	3.305	0.347	0.606
4.940	5.230	4.530	5.214	5.518	0.361	0.606
7.769	7.944	7.663	8.345	8.300	0.326	0.606
6.533	6.406	7.279	5.886	5.831	0.394	0.606
5.339	4.626	4.622	3.947	4.542	0.384	0.606
4.448	5.611	4.036	5.640	5.803	0.474	0.606
5.348	6.215	3.332	6.977	6.827	0.778	0.606
2.443	2.752	1.779	4.169	3.495	0.622	0.606
7.001	7.086	7.385	7.361	7.157	0.371	0.606
8.327	6.570	8.142	6.130	7.799	0.567	0.606
2.593	2.637	2.913	2.424	1.781	0.429	0.606
2.238	2.022	1.804	2.226	1.653	0.367	0.606
1.706	2.261	2.176	1.908	2.049	0.345	0.606
5.237	5.014	4.602	5.187	5.661	0.432	0.606
7.655	6.857	7.998	6.562	6.621	0.460	0.606
4.248	4.121	4.747	3.046	3.309	0.507	0.606
7.565	7.928	7.329	8.134	8.055	0.310	0.606
5.784	4.335	5.640	4.294	4.574	0.620	0.606
3.425	3.419	4.239	3.229	3.712	0.354	0.606
5.892	6.319	5.301	6.584	6.810	0.506	0.606
6.369	6.969	6.910	7.443	7.163	0.433	0.606
4.719	4.567	4.183	4.168	4.407	0.440	0.606
3.459	4.105	3.358	2.695	3.594	0.468	0.606
6.008	6.470	5.447	6.749	6.242	0.427	0.606
3.991	4.419	3.758	4.459	4.895	0.463	0.606
6.155	4.937	5.883	4.836	5.108	0.550	0.606
4.042	2.860	3.287	1.542	1.949	0.615	0.606
2.600	2.569	2.581	2.311	2.638	0.360	0.606
2.752	4.757	3.000	3.855	4.611	0.577	0.606
7.971	6.344	7.840	5.398	5.244	0.710	0.606
6.686	5.399	7.286	4.442	5.395	0.693	0.606
3.628	3.309	4.332	3.238	2.959	0.439	0.606
3.946	4.259	3.957	5.061	4.664	0.418	0.606
4.921	4.546	5.952	4.743	4.689	0.439	0.606
6.214	5.632	6.369	5.599	5.196	0.407	0.606

8.325	8.282	8.758	8.296	8.578	0.385	0.606
2.670	3.571	2.693	2.457	1.841	0.699	0.606
2.647	3.219	1.868	3.129	1.793	0.750	0.606
4.585	4.662	5.089	4.504	4.662	0.548	0.606
4.836	4.375	4.496	5.168	4.218	0.431	0.606
5.437	7.438	6.913	6.946	4.819	0.857	0.606
7.376	6.410	8.276	5.519	6.078	0.544	0.606
6.508	5.443	6.727	5.379	5.343	0.508	0.606
3.120	2.961	4.500	2.732	2.428	0.500	0.606
10.348	9.486	10.602	8.863	9.047	0.449	0.606
6.036	6.387	6.012	6.789	6.438	0.388	0.606
4.881	5.878	5.383	5.058	4.917	0.443	0.606
7.961	8.483	8.317	7.847	8.105	0.435	0.606
6.297	6.863	7.720	6.103	6.276	0.419	0.606
3.469	5.664	5.038	4.477	4.719	0.679	0.606
1.935	1.798	4.034	1.979	2.264	0.622	0.606
4.627	5.093	6.740	3.437	4.845	0.788	0.606
2.110	4.215	1.877	2.515	2.186	0.706	0.606
2.846	4.785	4.897	3.666	3.319	0.594	0.606
3.503	4.861	4.286	2.216	3.001	0.762	0.606
2.863	3.272	4.943	2.250	3.544	0.571	0.606
3.589	6.587	4.792	3.307	3.652	1.019	0.606
2.554	4.339	2.909	3.813	2.475	0.773	0.606
5.061	6.496	7.032	5.551	5.783	0.527	0.606
4.030	6.409	2.818	5.353	5.394	0.907	0.606
3.049	2.366	4.231	2.963	2.480	0.661	0.606
1.351	2.784	1.996	1.280	2.057	0.539	0.606
3.841	4.669	2.453	4.733	4.353	0.681	0.606
4.103	2.853	5.437	3.012	2.179	0.867	0.606
2.955	4.838	4.541	4.441	3.593	0.684	0.606
3.982	5.500	4.310	1.984	2.966	1.129	0.606
3.887	4.758	4.222	4.648	3.191	0.489	0.606
5.413	5.921	4.431	6.566	5.374	0.591	0.606
3.921	4.238	6.629	3.939	3.967	0.619	0.606
8.456	9.562	8.528	9.425	9.650	0.405	0.606
2.650	3.561	4.431	1.975	2.999	0.685	0.606
6.632	8.331	7.663	6.355	7.230	0.644	0.606
9.525	9.516	9.284	8.667	9.378	0.330	0.606
6.089	6.750	7.112	5.980	6.232	0.387	0.606
4.419	5.320	7.472	5.577	5.126	0.579	0.606
4.607	5.589	6.968	3.992	4.724	0.565	0.606
3.703	4.133	4.697	3.112	3.983	0.534	0.606
4.159	6.118	5.478	4.542	5.190	0.598	0.606
5.527	4.588	5.067	3.494	3.752	0.511	0.606
5.240	6.126	8.008	6.392	5.980	0.619	0.606
4.055	5.069	2.728	3.508	4.202	0.689	0.606
4.821	5.170	4.630	5.643	5.834	0.369	0.606
7.727	9.031	7.630	9.314	9.228	0.533	0.606
7.231	7.474	5.549	7.442	7.340	0.462	0.606
4.366	5.643	5.427	3.964	3.491	0.731	0.606

4.098	4.223	4.542	3.311	4.008	0.425	0.606
5.156	5.335	5.263	4.055	5.365	0.394	0.606
7.101	7.274	7.147	6.122	6.954	0.459	0.606
7.117	7.293	6.931	5.968	7.199	0.411	0.606
4.025	4.021	3.651	3.861	4.946	0.475	0.606
6.640	6.004	6.067	4.835	6.204	0.847	0.606
8.436	8.723	8.570	7.465	8.420	0.415	0.606
7.140	7.090	7.155	5.693	7.086	0.459	0.606
8.259	8.245	8.385	6.920	8.268	0.436	0.606
8.573	8.153	8.080	6.947	8.422	0.430	0.606
5.835	4.904	5.230	5.030	5.235	0.409	0.606
7.566	7.852	7.737	6.596	7.710	0.415	0.606
7.135	7.481	7.380	6.197	7.352	0.409	0.606
7.176	7.171	6.769	5.714	7.186	0.428	0.606
6.875	7.250	7.076	6.117	7.066	0.381	0.606
7.150	7.310	6.742	5.683	7.276	0.446	0.606
5.220	4.952	5.408	4.614	4.717	0.508	0.606
5.249	4.798	5.240	3.825	4.403	0.574	0.606
5.222	4.950	5.384	3.990	4.584	0.556	0.606
5.457	4.987	5.314	3.660	4.637	0.607	0.606
5.424	4.979	5.241	3.692	4.709	0.582	0.606
5.482	5.014	5.455	3.534	4.674	0.638	0.606
5.468	5.020	5.385	3.521	4.678	0.631	0.606
5.436	4.718	5.209	3.458	4.422	0.676	0.606
5.422	4.718	5.210	3.458	4.397	0.672	0.606
4.590	3.926	4.363	2.897	3.688	0.650	0.606
4.623	4.536	4.874	4.077	3.866	0.498	0.606
4.299	4.012	4.474	2.996	3.632	0.537	0.606
2.808	2.632	3.188	1.660	2.303	0.519	0.606
4.393	3.977	4.391	2.964	3.586	0.549	0.606
3.158	2.595	3.059	1.930	2.387	0.542	0.606
4.924	4.415	5.131	3.198	4.343	0.594	0.606
4.916	4.399	5.121	3.204	4.202	0.598	0.606
4.054	3.444	4.210	2.311	3.122	0.580	0.606
5.185	4.598	5.369	3.318	4.303	0.619	0.606
3.914	3.208	4.114	2.047	3.057	0.632	0.606
7.552	6.718	7.446	5.342	6.485	0.610	0.606
5.579	4.673	5.489	3.574	4.565	0.603	0.606
6.634	5.866	6.567	4.791	5.698	0.578	0.606
6.629	5.890	6.609	4.593	5.698	0.594	0.606
3.087	2.853	3.449	1.824	3.164	0.544	0.606
2.380	2.425	1.623	1.779	1.605	0.590	0.606
1.869	2.172	2.252	1.881	1.686	0.462	0.606
2.075	2.477	2.490	1.681	1.724	0.497	0.606
4.360	4.619	4.758	3.462	4.343	0.745	0.606
6.192	4.783	5.344	3.858	4.329	0.806	0.606
5.673	3.952	4.801	3.209	3.755	0.835	0.606
3.584	3.296	3.206	2.194	2.830	0.610	0.606
4.386	3.850	4.220	2.716	3.234	0.608	0.606
5.007	4.483	4.856	3.204	3.810	0.627	0.606

4.442	3.856	4.023	2.764	3.231	0.594	0.606
4.672	4.116	4.281	2.977	3.428	0.593	0.606
4.676	4.044	4.193	2.876	3.308	0.618	0.606
5.326	4.754	4.785	3.765	4.132	0.565	0.606
4.456	4.322	4.170	4.833	3.454	0.559	0.606
3.434	3.336	2.734	3.144	2.353	0.520	0.606
2.655	2.812	2.860	2.641	1.734	0.431	0.606
2.394	2.429	2.724	2.420	1.556	0.420	0.606
2.340	2.323	2.431	2.600	1.911	0.416	0.606
2.434	2.299	2.332	2.560	1.907	0.422	0.606
2.463	2.311	2.457	2.373	1.717	0.450	0.606
7.130	8.376	8.283	4.245	6.750	0.967	0.606
9.181	9.105	9.351	6.254	8.127	0.717	0.606
9.212	9.165	9.378	6.244	8.143	0.721	0.606
8.798	8.747	8.986	5.935	7.809	0.713	0.606
9.077	8.991	9.217	6.048	7.939	0.727	0.606
1.192	7.439	1.246	1.350	1.028	2.657	0.606
3.577	4.195	3.823	2.733	2.977	0.522	0.606
3.567	4.335	4.059	3.062	3.000	0.538	0.606
7.054	6.674	6.527	5.177	5.595	0.540	0.606
3.288	2.318	2.714	2.301	2.482	1.184	0.606
6.257	6.004	5.335	4.297	5.225	0.549	0.606
8.173	7.666	7.192	5.759	6.535	0.616	0.606
4.920	6.872	6.804	4.268	6.096	0.997	0.606
4.041	6.214	6.005	3.787	4.253	1.133	0.606
1.219	7.682	1.547	1.261	1.462	2.631	0.606
2.159	1.919	2.266	1.727	1.717	0.362	0.606
7.726	8.435	8.293	6.989	8.233	0.412	0.606
7.777	8.427	8.354	7.028	8.221	0.407	0.606
4.978	5.304	5.009	4.319	5.068	0.331	0.606
4.880	5.056	4.585	3.861	4.863	0.385	0.606
5.031	5.033	4.679	4.104	4.843	0.348	0.606
4.993	5.079	4.551	3.998	4.823	0.378	0.606
3.839	4.019	3.740	3.392	3.705	0.311	0.606
3.468	3.924	3.657	3.047	3.600	0.320	0.606
3.225	3.639	3.350	2.897	3.317	0.318	0.606
4.980	5.341	5.112	5.198	4.726	0.478	0.606
5.919	8.737	5.228	9.213	8.818	0.899	0.606
1.931	2.279	2.195	2.548	2.511	0.352	0.606
2.285	1.482	2.652	2.419	1.909	0.515	0.606
5.977	6.405	5.341	6.208	6.585	0.359	0.606
4.138	3.855	4.576	3.372	3.565	0.370	0.606
6.298	6.271	4.991	6.294	6.601	0.501	0.606
2.334	3.181	4.155	2.567	3.037	0.486	0.606
3.941	4.301	4.308	3.514	3.860	0.409	0.606
7.626	7.404	6.534	7.494	7.648	0.464	0.606
7.320	6.394	5.769	6.246	6.591	0.531	0.606
4.095	4.087	4.630	3.544	4.012	0.463	0.606
2.620	2.990	4.568	2.619	2.916	0.518	0.606
4.194	3.909	4.029	3.703	4.006	0.397	0.606

4.629	4.270	5.032	4.060	3.625	0.489	0.606
4.540	5.209	5.692	4.371	4.898	0.540	0.606
4.072	4.371	2.693	4.732	4.662	0.493	0.606
4.011	3.341	4.209	2.185	2.496	0.541	0.606
2.938	2.626	1.841	2.457	2.509	0.453	0.606
5.902	5.606	4.240	5.767	5.688	0.434	0.606
7.140	5.476	7.311	3.782	4.280	0.853	0.606
3.987	4.202	5.235	4.160	3.971	0.423	0.606
3.497	2.578	3.444	3.955	4.287	0.668	0.606
4.248	4.416	4.630	4.631	4.779	0.350	0.606
6.195	5.968	7.021	5.954	6.008	0.458	0.606
2.387	1.562	3.608	2.304	2.092	0.512	0.606
5.674	7.366	5.629	7.916	7.548	0.596	0.606
4.812	4.374	4.629	3.979	3.493	0.439	0.606
3.666	5.756	1.640	6.336	5.876	1.118	0.606
4.442	2.407	2.493	2.068	1.685	0.667	0.606
2.935	1.863	1.874	1.581	1.740	0.437	0.606
1.973	2.034	2.164	1.804	1.783	0.516	0.606
2.123	2.618	2.217	2.740	2.478	0.469	0.606
5.787	1.463	1.401	1.900	1.400	1.082	0.606
6.304	1.383	1.721	1.509	1.145	1.055	0.606
7.408	2.937	2.178	2.099	2.409	1.158	0.606
4.500	2.769	2.305	2.248	2.556	0.563	0.606
3.699	2.199	2.327	2.778	2.523	0.470	0.606
4.706	1.943	2.265	2.232	2.721	0.713	0.606
3.610	1.165	1.452	1.453	1.217	0.629	0.606
3.517	2.585	2.176	1.898	1.398	0.592	0.606
6.078	1.542	1.388	1.429	1.815	0.969	0.606
3.557	2.206	2.196	2.368	2.048	0.535	0.606
2.194	1.515	1.360	1.263	1.751	0.443	0.606
2.256	1.690	1.746	2.757	2.182	0.598	0.606
3.015	2.851	2.424	2.516	2.492	0.603	0.606
3.269	1.667	1.898	1.863	1.763	0.519	0.606
1.540	2.054	1.668	2.064	1.982	0.511	0.606
2.323	1.589	1.604	1.329	1.718	0.333	0.606
2.219	2.782	2.659	1.969	2.494	0.392	0.606
1.201	1.470	1.731	1.052	1.330	0.342	0.606
1.326	1.709	1.652	1.286	1.249	0.489	0.606
2.204	3.438	3.571	2.753	2.514	0.453	0.606
4.715	3.119	4.373	1.744	2.207	0.694	0.606
2.394	2.200	2.085	2.038	2.272	0.697	0.606
4.512	2.172	2.132	1.461	2.073	0.709	0.606
3.475	1.697	2.880	2.157	1.671	0.640	0.606
4.798	2.427	2.215	2.819	2.175	0.650	0.606
1.855	2.295	1.993	2.163	1.648	0.439	0.606
1.485	2.545	1.660	2.329	1.660	1.494	0.606
4.186	4.134	2.921	4.133	3.323	0.388	0.606
5.432	7.496	4.620	7.519	6.833	0.663	0.606
1.746	1.941	1.612	1.921	1.909	0.416	0.606
3.638	3.457	3.304	2.894	3.578	0.772	0.606

3.814	3.344	4.837	2.763	3.029	0.478	0.606
3.484	3.491	5.052	2.155	2.734	0.538	0.606
6.654	6.519	5.517	7.098	7.080	0.471	0.606
7.052	7.026	7.597	7.006	7.198	0.394	0.606
5.534	5.277	6.771	4.934	5.561	0.430	0.606
1.965	2.503	2.249	2.872	2.808	0.412	0.606
2.473	3.378	3.092	2.322	3.336	0.562	0.606
5.922	3.605	4.961	4.194	4.433	0.712	0.606
2.948	3.370	3.001	3.781	4.166	0.435	0.606
3.668	3.226	3.485	4.493	4.261	0.444	0.606
4.795	4.731	4.861	4.877	5.252	0.521	0.606
3.167	4.238	3.528	4.689	4.445	0.459	0.606
4.470	5.727	2.205	5.972	5.494	0.782	0.606
1.186	3.023	1.839	2.677	2.797	0.426	0.606
5.914	6.062	5.137	6.355	6.500	0.369	0.606
2.450	4.308	2.037	4.480	4.096	0.624	0.606
3.344	2.363	1.738	2.792	3.659	0.710	0.606
2.133	3.171	2.032	3.537	2.674	0.521	0.606
6.413	5.424	5.521	4.936	5.233	0.567	0.606
3.676	3.969	2.957	4.862	4.067	0.450	0.606
4.252	3.276	2.327	3.620	4.064	0.488	0.606
6.638	8.259	4.337	8.561	8.296	0.860	0.606
4.536	4.054	4.986	4.775	5.410	0.491	0.606
4.914	5.887	3.904	5.729	5.644	0.463	0.606
2.283	3.396	1.862	4.058	2.541	0.512	0.606
3.691	3.624	4.437	3.354	3.873	0.495	0.606
5.223	5.181	5.595	4.956	5.684	0.398	0.606
5.395	5.238	5.340	4.644	4.410	0.359	0.606
2.008	1.847	2.106	2.248	2.081	0.403	0.606
3.819	4.398	4.103	4.086	4.273	0.358	0.606
3.867	3.223	4.541	3.619	3.795	0.485	0.606
3.815	3.734	4.125	2.386	1.946	0.633	0.606
3.356	3.150	2.887	2.897	3.727	0.427	0.606
5.519	5.352	5.129	4.559	4.616	0.409	0.606
3.446	2.997	2.885	2.540	3.186	0.450	0.606
2.983	4.178	3.344	3.150	3.247	0.395	0.606
7.728	7.471	6.148	8.081	7.748	0.455	0.606
5.380	5.349	4.801	5.878	6.022	0.429	0.606
3.660	4.846	4.284	3.836	4.674	0.480	0.606
3.707	4.655	2.738	4.790	4.606	0.574	0.606
4.944	5.230	4.141	5.314	5.520	0.450	0.606
3.844	5.831	5.950	6.370	5.224	0.662	0.606
3.466	2.470	3.110	2.013	2.650	0.608	0.606
7.748	8.040	7.236	8.311	8.166	0.542	0.606
2.873	2.317	2.028	2.039	2.688	0.387	0.606
2.681	3.385	2.465	3.576	3.734	0.460	0.606
4.483	4.793	4.333	5.024	5.051	0.357	0.606
10.080	10.773	11.763	10.490	10.074	0.518	0.606
10.145	10.905	11.837	10.581	10.173	0.515	0.606
9.563	10.310	11.404	10.040	9.659	0.548	0.606

9.275	10.016	10.984	9.816	9.374	0.538	0.606
9.765	10.516	11.414	10.299	9.831	0.516	0.606
9.475	10.270	11.074	9.956	9.510	0.511	0.606
9.363	10.106	11.035	9.892	9.342	0.547	0.606
9.296	10.072	11.108	9.903	9.269	0.564	0.606
4.064	4.418	5.270	4.516	4.872	0.440	0.606
4.247	4.824	5.428	4.624	5.099	0.440	0.606
9.452	8.562	10.281	7.514	7.405	0.665	0.606
7.541	7.450	7.092	8.318	8.053	0.486	0.606
9.572	9.700	9.251	9.439	9.466	0.451	0.606
2.768	2.760	3.502	3.003	2.337	0.663	0.606
1.641	1.316	2.695	2.378	1.716	0.708	0.606
8.918	9.487	10.303	9.337	8.913	0.622	0.606
1.104	3.889	1.479	3.828	4.142	0.763	0.606
5.411	4.452	4.297	4.014	4.852	0.527	0.606
3.220	2.261	2.394	1.757	2.264	0.394	0.606
4.345	4.793	5.064	4.505	4.656	0.418	0.606
3.721	3.944	3.869	3.524	3.917	0.366	0.606
8.758	9.483	9.277	9.989	9.489	0.596	0.606
5.337	5.809	6.915	4.989	5.353	0.546	0.606
3.785	5.236	1.786	6.053	5.755	0.896	0.606
3.735	4.046	2.133	4.427	4.856	0.652	0.606
2.998	5.467	1.847	5.846	5.989	0.917	0.606
3.124	3.486	1.450	4.132	4.246	0.615	0.606
6.021	8.560	4.379	9.120	8.870	0.969	0.606
7.079	2.698	2.018	4.003	3.823	1.071	0.606
1.753	4.722	3.086	5.573	5.511	0.843	0.606
3.340	4.058	3.165	4.708	4.282	0.437	0.606
5.101	5.903	3.280	6.636	5.941	0.796	0.606
3.384	5.768	2.288	5.807	5.566	0.784	0.606
2.518	2.619	2.295	2.225	2.103	0.416	0.606
2.897	5.092	2.481	5.869	5.276	0.713	0.606
3.275	3.535	4.089	3.023	2.788	0.440	0.606
3.242	2.813	2.957	2.808	2.813	0.479	0.606
4.152	2.861	3.150	3.459	2.979	0.450	0.606
4.526	4.295	4.969	3.372	3.765	0.507	0.606
4.859	4.389	4.926	3.992	4.128	0.397	0.606
3.408	3.380	3.986	3.278	3.079	0.416	0.606
3.776	4.551	2.144	4.810	4.934	0.576	0.606
5.155	5.150	4.339	5.628	5.460	0.347	0.606
4.362	5.017	4.766	3.217	4.050	0.559	0.606
5.534	5.628	5.359	5.220	6.036	0.471	0.606
2.640	2.272	2.508	2.326	3.347	0.450	0.606
1.867	5.481	1.757	5.701	5.284	0.949	0.606
5.674	5.945	6.270	6.190	6.142	0.352	0.606
5.848	5.139	5.236	3.672	4.321	0.499	0.606
6.012	5.848	6.195	4.578	6.146	0.581	0.606
3.213	4.473	3.876	4.064	3.737	0.455	0.606
3.911	3.944	4.491	3.337	3.864	0.550	0.606
6.511	5.682	6.082	5.780	5.710	0.430	0.606

3.461	3.981	3.036	3.828	3.986	0.339	0.606
4.495	3.723	3.980	3.979	4.435	0.353	0.606
8.697	9.339	7.720	9.482	9.371	0.410	0.606
4.366	4.589	3.960	3.981	3.631	0.367	0.606
4.337	4.182	4.006	3.705	4.557	0.582	0.606
4.275	4.366	4.045	3.442	4.706	0.626	0.606
4.746	4.746	4.456	3.734	5.022	0.599	0.606
4.733	5.325	5.205	5.488	5.799	0.393	0.606
3.325	5.235	4.579	5.877	5.207	0.652	0.606
3.118	4.037	3.376	4.163	3.356	0.656	0.606
7.230	7.069	7.761	5.862	6.643	0.477	0.606
3.723	3.200	3.542	3.054	3.555	0.400	0.606
4.739	6.386	6.041	3.199	5.291	0.988	0.606
4.406	5.162	4.470	3.470	4.064	0.502	0.606
4.335	2.934	3.687	2.668	3.705	0.553	0.606
4.421	3.264	4.339	3.109	4.150	0.648	0.606
7.670	8.096	7.703	7.913	8.617	0.791	0.606
3.708	2.822	3.488	2.773	3.430	0.419	0.606
4.844	5.232	5.360	5.504	5.453	0.506	0.606
4.528	4.243	5.656	3.802	4.340	0.456	0.606
4.073	4.091	4.182	3.453	4.151	0.614	0.606
6.212	7.333	6.967	5.823	7.626	0.563	0.606
7.640	8.551	7.229	5.945	7.012	0.700	0.606
4.008	5.686	4.923	3.090	4.341	0.516	0.606
1.977	3.099	3.145	2.155	2.388	0.512	0.606
2.233	4.224	3.784	2.853	2.946	0.580	0.606
2.649	4.409	4.321	2.897	3.099	0.645	0.606
3.139	4.996	4.807	3.420	3.610	0.649	0.606
3.049	4.614	3.888	2.753	3.303	0.555	0.606
2.485	3.017	3.505	3.621	2.970	0.438	0.606
5.939	5.173	5.945	4.837	5.099	0.404	0.606
2.505	3.466	4.065	3.096	3.096	0.415	0.606
5.938	6.311	5.352	6.458	6.382	0.349	0.606
0.878	3.199	2.180	3.209	3.532	0.637	0.606
6.853	6.177	7.704	6.745	6.603	0.400	0.606
4.178	2.365	3.850	2.268	2.231	0.654	0.606
4.380	3.372	4.219	2.977	3.451	0.423	0.606
3.034	3.715	2.632	4.142	3.815	0.398	0.606
2.641	2.397	2.864	2.862	2.418	0.405	0.606
2.487	2.152	2.880	2.599	2.980	0.412	0.606
2.565	3.030	2.879	3.288	3.991	0.487	0.606
3.327	5.397	4.215	5.066	4.467	0.701	0.606
4.164	5.858	3.658	5.200	5.285	0.871	0.606
5.967	7.120	6.822	6.638	6.437	0.586	0.606
3.888	4.231	3.549	4.051	4.393	0.599	0.606
7.873	8.302	8.237	7.021	8.025	0.400	0.606
1.995	3.281	2.449	2.714	3.354	0.513	0.606
3.122	3.858	2.739	4.676	4.114	0.476	0.606
3.977	4.428	4.090	3.150	3.023	0.506	0.606
3.328	2.872	4.645	4.565	2.910	0.545	0.606

4.722	4.509	4.049	5.142	4.732	0.524	0.606
2.515	2.736	2.450	1.830	2.647	0.406	0.606
2.848	2.531	2.867	3.191	2.023	0.556	0.606
2.194	2.826	2.982	3.543	2.812	0.493	0.606
2.375	3.771	4.574	1.927	1.808	0.908	0.606
1.816	2.384	5.168	2.407	2.543	0.674	0.606
2.119	2.228	1.780	1.818	2.957	0.512	0.606
2.622	3.192	3.826	2.157	1.849	0.526	0.606
6.535	6.400	6.065	6.721	6.691	0.335	0.606
3.496	3.269	4.776	3.475	2.224	0.590	0.606
2.320	2.144	3.043	2.673	1.883	0.496	0.606
2.398	2.260	2.588	1.946	2.273	0.505	0.606
1.171	3.353	2.539	2.219	3.014	0.537	0.606
2.481	2.481	3.533	2.580	2.829	0.549	0.606
2.055	3.094	3.421	2.630	2.162	0.432	0.606
4.615	4.448	4.286	5.196	4.159	0.457	0.606
3.387	3.312	3.002	2.576	3.217	0.529	0.606
1.735	2.815	2.784	2.428	2.772	0.637	0.606
6.307	4.501	3.240	4.890	5.406	0.784	0.606
1.758	2.944	3.944	2.620	3.405	0.697	0.606
3.108	3.643	3.752	2.860	3.720	0.573	0.606
3.173	3.141	4.050	3.342	2.956	0.451	0.606
3.957	3.823	3.654	3.977	3.873	0.420	0.606
2.183	2.832	2.192	3.505	3.237	0.457	0.606
2.073	3.179	2.244	2.955	2.852	0.611	0.606
1.395	1.797	1.306	1.257	1.250	0.603	0.606
3.853	2.974	4.203	2.618	2.848	0.503	0.606
5.562	4.994	6.250	5.214	5.113	0.431	0.606
5.233	6.498	5.109	6.696	6.440	0.467	0.606
4.261	5.109	3.345	5.052	5.278	0.442	0.606
4.016	4.117	4.228	2.977	4.324	0.624	0.606
3.673	4.824	4.121	5.095	5.162	0.431	0.606
2.224	2.353	2.472	2.297	2.644	0.452	0.606
7.654	7.219	7.348	7.472	7.285	0.334	0.606
5.618	5.399	6.106	4.655	4.928	0.383	0.606
2.982	3.417	3.313	1.748	2.946	0.479	0.606
3.023	3.057	3.023	2.530	2.917	0.440	0.606
3.837	3.925	4.975	3.377	3.222	0.473	0.606
3.964	4.292	5.409	3.386	3.562	0.559	0.606
4.480	4.736	5.179	4.158	4.499	0.412	0.606
2.015	1.673	1.701	0.718	1.783	0.517	0.606
4.077	3.981	5.014	3.928	3.593	0.441	0.606
1.898	1.771	1.944	1.516	1.797	0.366	0.606
1.213	4.062	1.717	4.348	4.448	0.770	0.606
6.704	6.330	6.787	4.760	5.756	0.522	0.606
3.048	3.109	3.308	2.680	2.903	0.341	0.606
4.381	3.744	4.583	4.086	4.324	0.451	0.606
6.998	6.854	7.376	5.862	6.598	0.527	0.606
4.777	4.219	5.133	4.196	4.585	0.413	0.606
3.989	4.308	4.731	2.962	3.971	0.602	0.606

4.422	5.054	3.736	3.685	4.453	0.451	0.606
3.161	2.590	2.587	3.740	3.156	0.454	0.606
1.438	1.921	1.331	1.752	1.861	0.485	0.606
2.539	2.913	2.867	2.001	2.414	0.599	0.606
3.728	4.507	4.525	4.139	4.108	0.528	0.606
1.901	3.997	2.600	2.585	2.349	0.755	0.606
3.183	3.632	2.894	3.625	3.259	0.436	0.606
6.678	6.700	7.692	6.989	6.841	0.448	0.606
1.081	1.359	0.913	2.581	2.701	0.518	0.606
3.611	3.775	4.176	4.577	3.974	0.771	0.606
1.966	1.390	2.107	1.768	1.926	0.398	0.606
2.555	2.358	2.111	1.690	1.530	0.445	0.606
4.032	3.601	1.994	3.095	2.492	0.790	0.606
2.016	2.092	2.494	1.974	1.901	0.586	0.606
2.718	2.821	2.695	2.394	2.646	0.532	0.606
1.822	2.369	1.723	2.103	1.682	0.364	0.606
3.325	4.339	3.959	3.574	4.852	0.543	0.606
3.812	4.695	2.608	4.791	4.516	0.633	0.606
4.004	3.694	3.344	3.611	4.087	0.446	0.606
4.148	4.238	4.015	4.034	4.339	0.456	0.606
4.937	5.733	5.657	4.110	5.586	0.576	0.606
4.940	5.336	5.044	4.802	5.402	0.332	0.606
2.689	2.547	3.567	2.483	2.595	0.504	0.606
5.059	6.166	5.236	6.608	6.200	0.565	0.606
6.317	6.795	6.298	7.012	7.296	0.385	0.606
6.274	5.370	5.671	5.306	5.993	0.365	0.606
3.489	2.879	2.572	3.939	3.452	0.379	0.606
9.745	9.682	4.183	10.137	10.276	1.369	0.606
5.772	5.386	6.715	5.045	5.189	0.422	0.606
2.565	2.477	2.192	2.177	2.213	0.376	0.606
1.768	1.906	1.576	2.578	3.628	0.551	0.606
5.154	4.494	3.497	4.141	4.419	0.453	0.606
6.135	6.468	6.623	6.740	6.573	0.357	0.606
4.619	4.125	4.249	4.511	4.319	0.397	0.606
7.265	7.714	6.138	7.957	8.072	0.419	0.606
4.239	3.884	3.120	4.764	4.431	0.396	0.606
6.617	7.097	6.601	7.429	7.330	0.321	0.606
6.070	5.822	6.271	4.829	5.154	0.487	0.606
5.052	5.267	3.848	5.094	4.908	0.436	0.606
8.419	7.537	6.595	8.056	7.631	0.437	0.606
2.865	3.240	2.396	3.551	3.098	0.384	0.606
6.008	5.576	5.503	5.528	5.514	0.335	0.606
1.917	1.968	2.027	1.936	2.250	0.393	0.606
7.827	6.786	7.030	6.357	6.732	0.454	0.606
5.726	6.435	5.711	6.406	6.428	0.423	0.606
2.568	2.782	3.448	3.310	3.767	0.469	0.606
2.484	2.417	1.884	3.533	3.719	0.690	0.606
4.408	5.417	4.128	4.490	4.514	0.411	0.606
5.004	5.154	5.902	5.611	5.508	0.338	0.606
6.689	6.989	6.393	6.999	7.041	0.318	0.606

5.206	4.581	6.579	4.040	3.111	0.717	0.606
3.964	6.999	3.992	7.365	7.318	0.874	0.606
5.493	5.531	5.705	5.870	5.892	0.336	0.606
6.932	6.337	7.352	6.079	5.981	0.386	0.606
4.567	3.852	4.560	4.408	4.092	0.367	0.606
7.276	7.361	6.726	7.646	7.544	0.366	0.606
3.951	3.783	3.034	4.293	4.269	0.682	0.606
5.871	4.482	4.309	5.173	5.137	0.667	0.606
6.990	6.271	5.712	6.710	6.554	0.399	0.606
6.849	5.712	5.308	6.444	6.173	0.521	0.606
4.844	3.952	3.020	4.425	4.420	0.525	0.606
3.692	3.562	2.441	2.740	3.731	0.534	0.606
4.263	3.556	2.982	3.917	4.242	0.443	0.606
1.785	3.588	2.009	0.948	2.955	0.614	0.606
5.655	5.345	4.502	4.910	4.885	0.460	0.606
3.468	3.665	2.107	1.195	3.423	0.644	0.606
9.797	8.525	8.053	9.006	8.893	0.448	0.606
3.650	3.421	3.626	2.479	2.998	0.476	0.606
9.117	7.853	7.834	8.414	8.447	0.443	0.606
3.693	3.448	2.604	4.224	3.619	0.509	0.606
8.367	7.022	6.630	7.430	7.649	0.477	0.606
10.299	9.160	8.700	9.498	9.532	0.447	0.606
6.817	5.402	5.546	6.301	6.331	0.437	0.606
9.726	8.529	8.142	9.155	9.030	0.436	0.606
8.349	7.265	7.079	7.601	7.538	0.450	0.606
6.753	5.436	5.324	5.663	5.300	0.529	0.606
8.159	7.206	6.831	7.677	7.522	0.470	0.606
9.379	8.167	7.936	8.573	8.688	0.461	0.606
8.174	6.901	6.531	7.361	7.605	0.433	0.606
7.202	6.188	6.222	6.698	6.693	0.399	0.606
8.553	7.073	7.004	7.915	7.899	0.450	0.606
6.706	5.556	5.487	5.879	5.219	0.529	0.606
5.860	5.439	4.867	5.282	5.584	0.414	0.606
3.599	4.433	3.919	4.651	3.926	0.393	0.606
7.920	7.331	8.794	6.279	7.114	0.478	0.606
2.915	2.282	2.637	2.554	2.631	0.492	0.606
7.335	8.093	6.498	8.051	7.951	0.421	0.606
5.073	5.869	3.339	6.218	6.007	0.591	0.606
4.897	5.358	2.285	5.680	5.636	0.786	0.606
4.090	3.810	3.272	4.535	4.570	0.488	0.606
4.831	4.648	4.267	4.232	4.069	0.348	0.606
3.621	3.738	4.163	3.988	4.038	0.394	0.606
4.764	5.545	5.369	4.517	4.814	0.517	0.606
3.420	3.479	4.437	2.822	3.626	0.458	0.606
6.781	6.862	6.146	7.085	7.295	0.369	0.606
3.394	2.842	3.368	3.011	3.025	0.492	0.606
6.297	6.086	7.342	5.617	5.662	0.482	0.606
3.091	3.352	3.107	3.050	2.572	0.380	0.606
4.572	5.511	4.077	4.040	4.897	0.585	0.606
5.408	5.893	6.046	4.799	4.796	0.402	0.606

3.301	3.016	2.384	2.893	2.084	0.476	0.606
5.109	5.334	4.796	5.820	5.030	0.379	0.606
3.727	3.993	2.845	4.373	3.473	0.445	0.606
6.730	7.513	7.381	7.827	7.494	0.458	0.606
3.681	1.697	2.144	1.556	1.230	0.548	0.606
2.814	1.950	1.364	1.385	1.595	0.414	0.606
4.321	2.925	2.606	3.248	2.860	0.487	0.606
3.374	2.909	2.488	1.822	1.997	0.455	0.606
4.218	4.365	3.845	4.498	4.428	0.302	0.606
5.838	5.447	5.477	5.940	6.035	0.357	0.606
1.652	3.288	3.158	2.261	1.416	0.627	0.606
2.576	3.303	2.036	3.191	3.386	0.401	0.606
1.440	3.353	1.896	4.110	2.736	0.570	0.606
3.911	3.908	1.655	3.059	3.640	0.581	0.606
7.975	6.673	7.454	6.661	6.684	0.381	0.606
4.586	4.233	4.008	4.584	5.024	0.371	0.606
7.605	8.321	7.127	8.391	8.313	0.383	0.606
2.574	3.154	3.501	3.154	2.298	0.426	0.606
4.950	3.858	5.974	2.908	3.343	0.639	0.606
1.600	1.549	1.812	2.145	1.903	0.451	0.606
6.412	5.193	7.935	3.888	4.699	0.825	0.606
5.427	5.264	3.512	5.454	5.369	0.457	0.606
3.023	2.472	1.905	2.837	2.991	0.494	0.606
3.675	3.860	3.903	3.554	3.406	0.413	0.606
4.779	4.471	4.632	4.249	4.280	0.338	0.606
3.911	4.375	2.839	4.418	4.642	0.575	0.606
4.347	5.293	4.469	6.316	5.919	0.534	0.606
3.520	2.966	4.808	2.701	2.708	0.678	0.606
4.623	4.291	5.330	3.922	4.334	0.534	0.606
8.993	8.640	9.012	8.329	8.578	0.316	0.606
5.365	3.195	5.300	2.758	3.681	0.759	0.606
4.429	3.997	4.790	3.472	3.895	0.378	0.606
3.235	3.564	3.575	2.512	2.504	0.538	0.606
2.155	2.388	2.159	2.611	1.601	0.425	0.606
3.347	3.601	3.250	2.610	3.246	0.589	0.606
4.662	4.432	3.800	3.143	3.904	0.474	0.606
6.311	7.060	6.505	4.636	5.530	0.557	0.606
5.378	4.650	5.335	4.786	4.597	0.411	0.606
8.076	7.287	8.942	7.477	7.529	0.463	0.606
4.299	3.550	4.306	3.658	4.476	0.673	0.606
2.609	3.115	2.844	2.483	2.528	0.731	0.606
6.690	5.745	5.864	5.922	6.928	0.441	0.606
1.879	4.699	1.701	5.429	4.954	0.866	0.606
8.310	7.495	8.508	6.955	6.546	0.458	0.606
4.373	2.097	4.184	2.663	2.300	0.611	0.606
4.658	5.441	4.979	5.066	4.666	0.497	0.606
5.343	8.210	4.403	8.355	8.181	0.891	0.606
1.776	3.268	1.094	3.284	2.910	0.654	0.606
7.122	6.840	6.283	6.657	7.182	0.335	0.606
3.844	3.782	3.415	2.361	3.973	0.463	0.606

2.297	2.242	3.122	2.177	1.936	0.426	0.606
1.930	2.745	2.391	3.141	3.090	0.511	0.606
4.022	4.284	4.442	3.440	4.480	0.388	0.606
7.930	8.065	7.124	8.265	8.348	0.380	0.606
4.681	3.521	5.815	3.665	3.370	0.535	0.606
3.788	4.010	3.528	4.306	3.897	0.459	0.606
3.379	4.116	4.333	2.664	3.057	0.615	0.606
5.597	6.150	3.538	6.804	6.456	0.659	0.606
7.696	7.358	8.364	6.407	6.478	0.465	0.606
3.483	3.762	3.723	4.197	4.185	0.387	0.606
2.896	3.196	1.863	3.874	3.024	0.501	0.606
4.216	5.720	3.858	5.618	5.600	0.591	0.606
1.728	2.005	2.102	1.795	2.087	0.428	0.606
6.263	7.018	3.138	7.289	7.192	0.909	0.606
4.476	5.910	2.884	6.077	5.849	0.696	0.606
5.390	4.891	6.036	5.042	4.814	0.464	0.606
7.342	7.645	6.810	6.330	6.418	0.485	0.606
5.503	5.056	5.834	3.934	4.277	0.477	0.606
7.114	7.052	6.814	6.675	6.838	0.399	0.606
4.958	6.453	3.695	6.734	6.761	0.647	0.606
5.277	4.764	5.957	4.414	4.717	0.465	0.606
5.000	4.748	4.557	4.954	5.139	0.394	0.606
3.740	3.399	3.207	2.772	2.822	0.441	0.606
5.152	2.275	4.256	2.029	2.952	0.769	0.606
5.508	6.161	4.734	5.799	5.914	0.377	0.606
1.179	3.638	1.261	2.789	3.003	0.589	0.606
4.771	3.444	4.369	3.567	3.917	0.528	0.606
1.640	3.050	1.646	3.004	3.385	0.485	0.606
5.070	4.716	5.528	5.050	5.417	0.336	0.606
6.093	6.252	5.773	5.935	6.340	0.382	0.606
4.745	5.181	4.160	5.248	4.975	0.445	0.606
2.676	3.625	2.950	3.950	4.202	0.479	0.606
5.925	5.980	2.536	6.479	6.331	0.952	0.606
7.341	7.996	5.214	8.387	8.300	0.744	0.606
5.443	5.921	3.188	6.498	6.191	0.616	0.606
5.042	5.930	2.813	6.707	6.208	0.914	0.606
2.266	2.765	1.825	2.777	3.515	0.625	0.606
5.422	6.656	5.812	6.992	6.788	0.477	0.606
4.563	5.476	4.236	5.600	5.693	0.483	0.606
3.566	4.491	2.000	5.111	5.116	0.623	0.606
4.017	5.150	3.129	5.031	5.658	0.582	0.606
5.166	7.101	4.002	7.535	7.351	0.814	0.606
5.017	4.918	1.355	5.973	5.245	0.896	0.606
4.428	5.538	2.250	5.892	5.776	0.857	0.606
5.874	7.577	2.371	8.227	8.034	1.250	0.606
5.221	4.460	2.727	5.363	4.479	0.586	0.606
3.491	3.591	3.843	3.175	2.750	0.548	0.606
7.627	7.563	5.684	7.991	7.785	0.559	0.606
6.706	7.488	6.916	8.003	7.879	0.563	0.606
1.682	2.571	2.618	3.658	2.852	0.533	0.606

2.975	2.315	1.895	2.874	2.451	0.407	0.606
3.797	4.125	4.526	3.988	3.521	0.703	0.606
2.713	5.463	3.617	5.592	5.370	0.684	0.606
3.383	2.665	1.459	3.301	2.624	0.530	0.606
1.979	4.386	2.122	5.251	4.948	0.778	0.606
5.762	8.216	6.273	8.702	8.385	0.842	0.606
2.899	3.516	3.365	4.442	4.767	0.533	0.606
4.444	3.462	2.512	3.530	2.866	0.499	0.606
3.068	6.542	2.122	6.730	6.772	1.028	0.606
1.414	3.117	1.666	2.929	4.107	0.554	0.606
4.878	5.964	2.833	6.407	6.407	0.847	0.606
5.213	6.724	2.888	7.358	7.111	0.922	0.606
7.092	5.856	7.137	4.515	5.915	0.762	0.606
5.323	5.159	3.854	4.887	5.235	0.479	0.606
4.283	5.905	2.997	5.987	5.919	0.682	0.606
5.137	6.369	5.391	7.387	6.718	0.583	0.606
4.811	5.895	3.043	6.448	6.287	0.875	0.606
4.819	5.719	3.549	6.837	6.552	0.944	0.606
9.217	7.457	8.837	7.796	7.533	0.530	0.606
3.105	4.174	3.047	4.179	3.980	0.420	0.606
2.262	3.178	2.435	2.880	2.686	0.391	0.606
6.748	4.413	3.054	4.648	5.105	0.852	0.606
7.068	6.150	7.903	5.838	5.971	0.484	0.606
5.133	3.951	6.146	4.140	3.905	0.612	0.606
6.025	7.651	4.778	8.013	7.990	0.747	0.606
2.379	6.514	2.606	6.441	5.873	0.946	0.606
2.892	2.247	2.531	2.328	2.454	0.449	0.606
4.513	6.633	1.651	7.469	7.003	1.150	0.606
2.258	3.073	2.866	3.073	3.000	0.464	0.606
5.874	7.290	5.885	7.256	7.181	0.488	0.606
3.786	4.155	3.009	4.678	4.247	0.434	0.606
3.238	4.066	2.620	3.794	3.211	0.486	0.606
1.716	2.238	2.709	1.675	2.099	0.425	0.606
2.664	2.198	2.421	2.929	2.321	0.402	0.606
3.353	3.816	2.818	4.102	4.636	0.480	0.606
6.412	5.252	3.280	5.934	5.342	0.672	0.606
2.623	3.693	2.711	3.235	3.990	0.420	0.606
3.840	3.630	4.157	3.127	2.948	0.405	0.606
4.478	3.892	2.613	4.658	4.689	0.593	0.606
5.924	7.069	4.998	7.439	7.434	0.604	0.606
3.141	3.098	3.303	2.522	3.105	0.435	0.606
3.601	4.089	3.327	4.619	4.503	0.392	0.606
5.554	7.493	8.404	6.696	6.197	0.589	0.606
4.648	6.292	3.520	6.453	6.213	0.746	0.606
6.988	5.974	6.886	6.405	6.344	0.542	0.606
2.613	2.488	3.430	3.501	3.095	0.426	0.606
3.300	2.556	3.042	2.975	2.726	0.423	0.606
3.293	2.104	2.694	1.971	2.666	0.534	0.606
5.089	3.180	5.544	2.759	2.484	0.719	0.606
4.688	6.083	3.132	6.415	6.144	0.714	0.606

4.198	5.768	1.942	6.240	5.772	0.993	0.606
6.387	7.474	4.463	7.875	7.834	0.678	0.606
3.031	5.991	2.318	6.610	6.659	0.971	0.606
2.097	3.961	1.490	4.563	4.807	0.754	0.606
2.401	5.487	2.287	6.426	6.271	0.977	0.606
3.214	5.828	2.510	6.164	5.930	0.919	0.606
4.663	6.609	2.552	6.566	6.392	0.869	0.606
4.416	5.131	4.001	5.658	5.718	0.617	0.606
1.535	6.294	1.502	6.486	6.117	1.205	0.606
4.446	6.115	4.720	6.575	6.446	0.535	0.606
4.270	4.065	4.439	3.768	4.002	0.375	0.606
6.899	7.031	7.415	6.102	7.045	0.374	0.606
4.497	6.860	3.657	7.461	7.049	0.934	0.606
7.550	7.412	7.819	6.519	6.980	0.375	0.606
3.944	3.896	4.989	4.627	4.749	0.429	0.606
3.938	4.271	5.249	4.756	4.379	0.473	0.606
5.669	5.482	6.291	5.557	5.852	1.830	0.606
5.857	5.521	6.521	5.288	5.216	0.461	0.606
4.771	4.470	4.028	4.684	6.224	0.684	0.606
5.196	4.601	5.762	2.957	3.708	0.626	0.606
4.704	4.704	1.429	5.191	5.781	1.083	0.606
5.400	5.080	4.499	6.055	6.075	0.605	0.606
5.498	6.594	5.246	7.401	7.089	0.674	0.606
4.874	6.401	5.710	7.301	6.784	0.539	0.606
4.573	4.294	5.434	4.306	4.742	0.379	0.606
3.336	4.609	3.553	3.758	3.879	0.572	0.606
4.730	4.334	3.856	4.194	4.291	0.360	0.606
4.757	4.635	5.221	4.521	4.401	0.378	0.606
5.138	5.591	3.223	5.951	6.166	0.742	0.606
6.966	6.719	7.767	5.956	6.254	0.410	0.606
8.036	8.246	7.608	8.525	8.484	0.313	0.606
7.726	8.507	5.695	8.875	8.549	0.657	0.606
5.973	6.255	5.138	6.852	6.654	0.410	0.606
4.894	3.774	5.042	4.096	3.949	0.441	0.606
5.061	5.311	4.467	5.996	6.170	0.507	0.606
3.734	4.098	3.187	4.822	4.477	0.421	0.606
3.940	4.307	4.545	4.526	4.518	0.441	0.606
6.088	4.040	3.532	4.919	4.491	0.529	0.606
6.602	7.815	3.621	8.165	7.627	0.927	0.606
7.858	8.908	7.862	9.020	8.932	0.435	0.606
6.161	5.627	6.481	6.281	6.008	0.489	0.606
2.742	6.715	2.710	6.848	6.304	1.029	0.606
4.892	5.645	3.418	5.998	5.835	0.586	0.606
7.807	8.209	8.120	8.447	8.373	0.354	0.606
5.691	6.279	4.239	6.575	6.392	0.626	0.606
7.664	6.904	8.758	6.895	6.629	0.481	0.606
6.762	7.261	5.561	7.594	7.337	0.519	0.606
4.659	3.984	4.240	3.406	4.092	0.422	0.606
3.950	4.270	3.058	4.673	4.589	0.412	0.606
4.957	5.679	4.758	6.144	5.916	0.472	0.606

6.265	7.464	5.109	7.444	7.399	0.545	0.606
6.916	6.929	6.777	7.292	7.053	0.298	0.606
2.506	2.948	1.992	3.670	3.459	0.455	0.606
3.127	3.315	1.736	3.005	3.470	0.500	0.606
5.314	5.966	4.499	6.835	6.333	0.556	0.606
6.177	8.327	3.241	8.570	8.138	1.006	0.606
6.340	6.725	6.142	7.026	7.213	0.398	0.606
8.754	7.925	9.277	7.245	7.626	0.458	0.606
3.205	3.314	4.346	2.842	3.187	0.448	0.606
4.375	5.702	2.233	6.131	5.486	0.741	0.606
4.120	4.769	3.446	4.940	4.854	0.398	0.606
1.703	1.966	1.428	2.420	2.541	0.416	0.606
5.632	4.178	6.304	3.566	3.517	0.636	0.606
3.690	3.986	3.165	4.802	4.399	0.420	0.606
6.488	5.613	7.224	5.966	5.518	0.433	0.606
5.685	6.684	3.246	7.098	6.800	0.803	0.606
7.902	6.544	8.647	6.049	6.140	0.555	0.606
2.393	4.106	2.064	3.958	3.977	0.512	0.606
3.379	4.891	5.052	3.355	3.225	0.576	0.606
8.148	7.288	8.869	6.753	6.798	0.506	0.606
8.464	7.566	7.030	7.981	8.111	0.405	0.606
5.404	6.017	4.841	6.677	6.009	0.499	0.606
8.578	8.509	7.565	8.863	8.748	0.431	0.606
6.793	7.706	6.749	7.763	8.033	0.389	0.606
7.608	8.001	5.616	7.979	7.806	0.590	0.606
3.130	3.286	3.005	4.197	2.974	0.473	0.606
2.116	3.144	2.721	2.761	3.151	0.420	0.606
9.860	10.825	8.215	11.106	10.822	0.701	0.606
9.227	8.713	9.935	8.554	8.660	0.341	0.606
9.111	9.307	9.380	9.531	9.204	0.306	0.606
4.145	3.431	5.462	3.451	2.939	0.544	0.606
8.909	8.945	8.058	9.292	9.442	0.407	0.606
3.179	4.962	3.454	5.225	4.698	0.564	0.606
1.762	1.846	2.113	2.113	1.968	0.422	0.606
5.604	5.924	6.398	6.337	5.714	0.457	0.606
5.680	6.535	5.907	7.124	7.201	0.493	0.606
4.172	6.694	5.334	7.134	7.295	0.675	0.606
2.401	2.747	1.718	3.085	3.200	0.453	0.606
2.993	3.126	4.009	3.261	3.097	0.480	0.606
6.242	6.639	5.070	6.752	6.818	0.482	0.606
4.459	5.089	5.254	5.390	5.095	0.399	0.606
6.731	6.559	6.131	7.323	7.390	0.361	0.606
4.964	4.533	5.859	4.181	3.871	0.522	0.606
6.432	5.102	4.561	4.685	4.859	0.696	0.606
6.020	5.543	6.784	5.080	5.711	0.376	0.606
3.842	2.504	2.747	2.279	2.976	0.529	0.606
6.966	6.541	6.718	6.237	6.322	0.339	0.606
5.857	4.537	5.767	4.160	3.848	0.575	0.606
3.301	3.128	1.986	3.876	3.782	0.490	0.606
6.122	7.152	7.256	7.196	6.730	0.452	0.606

7.537	8.591	7.179	8.578	8.616	0.424	0.606
4.447	3.493	2.378	3.078	3.275	0.508	0.606
4.704	5.261	3.161	4.747	5.290	0.483	0.606
5.926	3.102	2.105	2.885	3.325	0.871	0.606
6.222	6.556	5.842	7.054	6.780	0.351	0.606
4.032	2.988	2.064	3.510	3.067	0.423	0.606
4.917	4.929	5.412	5.605	5.667	0.371	0.606
4.861	5.970	5.434	6.195	5.743	0.381	0.606
6.956	7.852	8.090	8.105	7.750	0.351	0.606
4.104	5.051	3.223	5.491	4.993	0.589	0.606
6.096	6.293	8.259	6.143	6.011	0.507	0.606
5.137	4.949	5.367	5.168	5.260	0.301	0.606
4.194	3.888	3.507	4.038	4.391	0.580	0.606
3.461	3.264	4.349	2.679	2.598	0.657	0.606
2.877	1.961	2.891	2.815	2.735	0.422	0.606
3.031	4.194	3.366	3.460	3.285	0.474	0.606
9.963	8.061	10.540	6.410	6.574	0.939	0.606
5.478	6.171	3.458	6.290	5.564	0.767	0.606
3.371	2.933	3.074	3.109	2.786	0.451	0.606
3.556	2.582	3.704	1.437	1.603	0.612	0.606
5.517	5.457	3.724	5.426	5.315	0.555	0.606
0.752	2.537	1.099	3.142	2.914	0.628	0.606
3.944	5.084	4.075	4.338	4.418	0.375	0.606
6.734	7.141	6.449	7.484	7.063	0.367	0.606
6.079	6.474	6.182	6.717	6.771	0.345	0.606
4.920	5.276	4.586	5.774	5.875	0.354	0.606
5.887	6.395	5.902	6.425	6.759	0.317	0.606
1.859	2.272	2.272	1.948	1.975	0.506	0.606
3.437	3.628	3.557	3.928	3.934	0.452	0.606
2.487	2.367	1.547	2.534	2.210	0.558	0.606
3.378	2.727	3.016	3.009	3.429	0.572	0.606
6.144	6.159	3.153	7.148	7.207	0.905	0.606
1.965	1.991	1.307	1.272	1.456	0.517	0.606
3.964	3.483	3.180	3.716	3.716	0.397	0.606
4.057	2.807	2.865	2.946	3.375	0.494	0.606
3.537	4.109	2.942	4.015	4.346	0.489	0.606
2.398	2.390	2.177	2.590	1.983	0.427	0.606
3.758	3.798	4.311	3.557	3.699	0.311	0.606
6.100	6.590	4.537	6.662	6.741	0.587	0.606
2.398	2.688	2.375	2.072	1.802	0.537	0.606
6.452	6.888	2.666	7.310	6.968	0.958	0.606
3.180	3.567	3.492	3.624	4.245	0.397	0.606
5.303	5.386	5.933	5.553	5.146	0.351	0.606
2.053	2.715	1.647	2.203	2.219	0.472	0.606
3.390	2.333	4.461	1.728	2.171	0.655	0.606
5.598	6.939	7.776	5.205	4.991	0.639	0.606
3.123	4.533	4.814	3.046	3.179	0.636	0.606
4.447	3.290	4.283	3.564	3.696	0.409	0.606
2.893	4.594	2.374	5.023	4.966	0.710	0.606
5.444	4.795	4.818	4.747	5.073	0.330	0.606

1.875	2.671	3.088	3.349	3.785	0.528	0.606
5.058	4.865	4.880	5.247	5.406	0.376	0.606
2.741	3.032	2.237	1.764	2.946	0.410	0.606
8.042	7.494	8.602	6.970	7.327	0.375	0.606
8.757	8.611	9.662	7.773	8.105	0.420	0.606
9.094	7.940	4.128	8.665	8.331	1.194	0.606
5.884	6.226	5.733	6.721	6.494	0.376	0.606
6.596	6.785	6.719	7.370	7.122	0.351	0.606
3.174	3.097	2.720	4.110	3.283	0.412	0.606
5.724	5.553	5.987	4.736	5.710	0.421	0.606
8.239	9.379	9.607	6.638	7.880	0.803	0.606
5.371	6.213	4.742	6.695	6.178	0.443	0.606
5.417	4.299	5.211	4.285	4.389	0.436	0.606
5.022	4.847	5.244	4.641	4.514	0.386	0.606
4.919	4.389	5.259	4.352	4.644	0.355	0.606
4.634	3.713	5.074	2.806	3.665	0.459	0.606
1.752	2.395	2.636	2.584	1.422	0.451	0.606
5.004	4.944	5.114	4.987	4.664	0.375	0.606
2.060	2.694	2.543	2.364	2.492	0.455	0.606
2.032	3.247	2.566	3.939	2.480	0.640	0.606
4.752	5.001	4.617	5.316	5.490	0.339	0.606
3.120	3.011	3.262	3.048	3.028	0.405	0.606
5.862	6.617	6.648	5.124	5.842	0.587	0.606
6.102	6.195	5.915	5.935	6.352	0.383	0.606
2.825	2.256	2.581	1.830	2.332	0.579	0.606
3.399	5.674	3.910	6.538	6.691	0.725	0.606
4.891	4.093	5.741	2.779	2.811	0.825	0.606
4.673	5.267	4.539	5.520	5.045	0.452	0.606
6.755	5.320	5.548	4.827	5.500	0.531	0.606
2.053	2.390	2.053	1.343	1.785	0.399	0.606
6.335	5.307	6.160	4.434	5.123	0.434	0.606
2.913	1.552	2.200	2.238	1.738	0.453	0.606
5.564	3.921	5.998	3.020	3.674	0.654	0.606
6.973	7.211	7.064	7.293	6.948	0.342	0.606
7.391	6.512	7.541	6.278	6.174	0.433	0.606
3.924	3.334	4.533	3.193	2.968	0.487	0.606
4.685	5.341	4.282	4.610	4.714	0.401	0.606
3.562	3.223	3.487	2.924	3.107	0.401	0.606
6.359	5.461	5.667	5.312	4.929	0.387	0.606
1.827	3.396	2.402	2.806	3.262	0.469	0.606
7.271	6.479	7.136	4.922	4.922	0.633	0.606
4.341	4.345	4.402	3.733	4.502	0.519	0.606
2.699	2.680	3.411	2.288	1.974	0.504	0.606
6.576	7.222	6.097	7.890	7.654	0.474	0.606
7.683	8.103	6.709	8.794	8.587	0.549	0.606
5.991	5.332	3.692	5.402	5.672	0.590	0.606
5.825	6.784	5.981	6.327	6.570	0.444	0.606
6.071	4.990	6.583	5.667	6.145	0.465	0.606
6.531	6.149	8.674	4.458	4.453	0.903	0.606
5.105	1.992	2.463	2.343	2.581	0.707	0.606

2.997	3.486	3.386	3.575	3.881	0.389	0.606
3.597	3.850	3.896	3.253	3.666	0.412	0.606
4.813	5.436	2.768	5.303	5.075	0.574	0.606
3.500	3.939	2.641	4.891	4.520	0.519	0.606
5.999	5.517	6.591	5.634	6.038	0.365	0.606
2.951	6.522	5.750	6.894	7.405	1.113	0.606
2.641	2.056	2.625	3.205	2.752	0.489	0.606
3.324	3.453	3.873	3.491	3.122	0.433	0.606
1.871	3.760	2.283	4.176	4.256	0.551	0.606
5.100	5.250	5.490	4.726	5.691	0.356	0.606
7.275	6.928	8.505	6.955	7.049	0.454	0.606
2.419	3.428	3.100	1.986	2.754	0.587	0.606
4.083	3.268	1.930	2.234	2.696	0.518	0.606
3.741	2.781	5.095	1.783	2.613	0.763	0.606
1.742	2.957	2.019	1.861	2.551	0.659	0.606
2.956	2.922	2.956	2.084	2.346	0.559	0.606
3.711	3.310	4.241	2.721	3.685	0.472	0.606
4.983	4.500	5.228	3.977	4.371	0.390	0.606
2.331	3.092	3.281	2.331	2.912	0.558	0.606
6.737	6.204	3.766	6.266	6.321	0.626	0.606
5.094	5.327	5.642	4.097	4.095	0.489	0.606
3.052	2.762	2.536	2.332	2.721	0.419	0.606
3.759	4.646	4.033	3.432	3.724	0.429	0.606
3.375	4.660	2.840	5.427	5.308	0.565	0.606
3.820	2.904	4.244	2.968	2.831	0.554	0.606
4.795	5.333	3.672	5.743	5.441	0.598	0.606
6.521	6.080	3.252	6.888	6.590	0.844	0.606
5.362	6.003	4.802	5.916	5.988	0.386	0.606
3.800	3.625	3.585	3.350	3.554	0.472	0.606
1.976	3.834	3.462	3.619	2.356	0.618	0.606
4.611	3.866	2.700	4.241	4.263	0.462	0.606
4.122	3.702	3.490	3.057	3.955	0.417	0.606
3.597	2.626	2.382	2.165	3.898	0.782	0.606
2.042	2.534	1.182	1.892	2.382	0.522	0.606
4.082	3.765	3.652	4.401	4.538	0.362	0.606
6.821	6.913	6.411	7.204	6.939	0.380	0.606
2.593	2.410	2.085	3.326	2.558	0.465	0.606
3.044	2.474	2.909	2.785	2.802	0.428	0.606
2.318	2.764	3.183	2.853	2.629	0.451	0.606
5.109	4.607	6.606	3.822	4.098	0.570	0.606
3.256	0.906	1.757	0.845	0.756	0.849	0.606
1.073	2.546	1.269	1.590	1.488	0.490	0.606
3.470	1.591	2.054	1.990	1.336	1.072	0.606
2.462	3.218	3.695	2.384	3.130	0.440	0.606
3.911	3.257	4.947	2.440	2.791	0.570	0.606
4.079	2.448	3.641	2.442	2.601	0.586	0.606
1.570	2.847	1.663	4.989	3.675	0.713	0.606
2.466	1.671	3.636	2.243	2.310	0.507	0.606
3.509	6.049	3.729	6.152	6.422	0.717	0.606
1.174	2.383	0.915	2.440	2.298	0.452	0.606

2.336	1.118	1.719	1.846	1.886	0.481	0.606
2.079	1.257	1.363	3.557	3.210	0.586	0.606
5.145	5.617	5.759	5.860	6.286	0.412	0.606
3.398	3.147	4.196	3.051	3.297	0.392	0.606
3.964	4.252	4.373	3.581	4.492	0.440	0.606
4.319	3.267	4.451	4.137	3.862	0.461	0.606
2.098	3.031	1.823	2.942	2.793	0.439	0.606
3.468	3.251	4.041	3.427	3.735	0.710	0.606
3.713	2.632	3.346	2.898	2.772	0.464	0.606
2.026	2.365	2.293	2.267	2.807	0.442	0.606
4.263	3.435	1.927	4.075	5.141	1.101	0.606
2.859	3.426	3.522	2.812	3.675	0.438	0.606
0.994	5.424	1.042	5.985	6.281	1.222	0.606
2.824	2.798	3.888	3.023	4.036	0.435	0.606
2.587	2.594	3.595	1.906	2.428	0.425	0.606
3.689	3.004	2.818	2.827	2.930	0.457	0.606
2.299	3.665	2.241	2.817	4.043	0.528	0.606
5.267	5.720	2.965	6.013	5.892	0.700	0.606
3.619	4.922	1.712	5.159	4.806	0.860	0.606
4.570	5.239	3.139	5.757	4.996	0.637	0.606
5.385	4.022	6.159	3.698	3.116	0.819	0.606
5.529	3.258	6.384	2.261	1.832	0.986	0.606
2.230	2.754	2.263	4.091	3.498	0.661	0.606
1.947	4.082	1.854	4.138	2.866	0.602	0.606
5.617	5.740	3.094	7.084	6.466	0.955	0.606
5.895	5.707	5.489	6.068	6.142	0.311	0.606
6.663	5.623	5.864	6.653	6.444	0.569	0.606
2.866	1.613	2.596	1.219	2.868	0.620	0.606
5.365	3.541	5.824	1.637	1.868	0.957	0.606
7.417	7.440	7.395	7.813	7.856	0.357	0.606
2.119	3.244	1.990	3.517	3.479	0.439	0.606
3.573	3.929	3.496	4.628	4.658	0.388	0.606
2.540	3.741	3.587	3.646	3.580	0.441	0.606
4.870	5.481	5.470	5.649	5.706	0.364	0.606
1.845	1.565	1.808	1.670	1.307	0.444	0.606
2.771	2.483	2.929	2.845	2.445	0.405	0.606
4.913	3.690	4.142	4.383	4.455	0.420	0.606
3.417	2.302	4.868	2.897	2.648	0.792	0.606
4.239	4.523	2.683	4.561	4.664	0.609	0.606
3.782	3.076	3.742	3.218	3.430	0.564	0.606
3.070	2.294	4.663	1.894	1.875	0.580	0.606
2.459	2.784	2.470	2.056	1.776	0.428	0.606
4.348	3.785	4.723	4.319	4.557	0.340	0.606
5.407	4.774	5.913	4.852	4.904	0.432	0.606
3.952	4.072	2.872	4.363	3.871	0.442	0.606
2.746	1.828	1.436	1.884	1.984	0.447	0.606
7.507	7.246	7.352	7.139	6.967	0.353	0.606
4.140	4.140	4.847	4.895	4.447	0.408	0.606
3.279	5.595	1.613	5.229	5.963	0.884	0.606
2.462	3.175	3.175	2.562	3.163	0.479	0.606

3.370	1.990	4.390	1.674	2.890	0.569	0.606
3.237	2.572	4.389	1.896	2.730	0.638	0.606
2.909	4.874	3.554	5.006	5.209	0.592	0.606
4.560	5.428	4.513	5.503	5.737	0.415	0.606
2.361	3.171	1.643	3.484	3.090	0.476	0.606
5.498	5.662	6.081	5.683	5.464	0.320	0.606
4.439	4.439	4.668	4.428	4.407	0.442	0.606
3.983	4.197	3.406	3.655	3.687	0.457	0.606
4.966	4.945	5.340	4.850	5.507	0.542	0.606
3.072	4.576	3.944	4.353	4.320	0.412	0.606
4.732	5.447	6.007	5.709	5.412	0.631	0.606
5.761	4.412	6.098	3.259	4.625	0.640	0.606
5.285	3.978	4.702	5.312	4.431	0.487	0.606
5.507	5.171	5.669	4.744	4.184	0.487	0.606
7.488	7.114	8.361	6.345	6.492	0.457	0.606
7.198	6.754	8.082	6.148	6.692	0.422	0.606
7.243	6.939	8.082	6.095	6.634	0.431	0.606
7.315	7.050	8.188	6.375	6.805	0.409	0.606
4.289	5.062	5.283	4.141	5.157	0.541	0.606
1.495	5.570	1.899	5.248	5.319	1.010	0.606
3.605	2.833	2.040	3.753	3.681	0.466	0.606
2.873	2.231	3.413	2.315	1.889	0.468	0.606
2.251	3.098	2.601	2.033	2.060	0.492	0.606
4.487	4.247	6.360	3.627	3.289	0.646	0.606
7.111	6.573	7.946	6.220	6.518	0.416	0.606
3.203	3.174	4.241	3.489	3.208	0.474	0.606
4.765	2.523	4.341	2.283	2.939	0.830	0.606
5.635	5.338	5.699	4.203	5.365	0.530	0.606
4.271	4.460	4.844	5.050	5.440	0.500	0.606
7.298	6.029	7.680	5.273	5.463	0.610	0.606
5.343	5.957	2.140	5.657	6.032	0.850	0.606
3.415	3.793	2.943	4.293	4.340	0.522	0.606
5.576	7.039	3.207	7.730	6.866	0.919	0.606
3.512	2.773	3.575	2.194	2.970	0.461	0.606
3.950	4.028	2.524	4.771	4.626	0.481	0.606
2.898	2.961	4.000	2.288	1.924	0.540	0.606
2.375	3.188	1.582	3.404	3.760	0.484	0.606
3.197	3.187	4.184	3.024	3.870	0.458	0.606
3.150	2.743	3.727	3.539	2.325	0.574	0.606
4.648	5.165	5.026	5.383	5.025	0.339	0.606
4.451	4.148	2.545	4.133	4.680	0.561	0.606
3.546	1.950	2.707	1.594	1.956	0.511	0.606
2.107	3.622	1.644	4.117	4.028	0.660	0.606
3.619	2.185	3.044	2.487	2.449	0.502	0.606
3.162	4.086	2.333	4.433	4.385	0.534	0.606
1.172	2.573	1.447	2.747	3.629	0.600	0.606
4.627	4.794	2.723	5.069	5.226	0.574	0.606
2.027	2.824	1.790	2.065	2.373	0.466	0.606
2.065	1.683	1.440	1.926	1.735	0.417	0.606
3.856	2.557	4.893	2.863	3.908	0.693	0.606

1.061	2.712	0.822	2.597	2.145	0.568	0.606
4.155	3.395	4.874	3.125	3.111	0.474	0.606
4.804	3.442	6.131	4.002	4.212	0.874	0.606
2.802	3.353	2.718	3.988	4.033	0.456	0.606
4.781	4.365	5.913	4.041	3.551	0.721	0.606
5.690	4.726	5.541	5.219	5.282	0.443	0.606
3.277	1.817	2.065	1.954	1.921	0.563	0.606
1.880	1.632	1.195	1.370	1.295	1.354	0.606
4.415	3.358	5.229	2.742	3.146	0.629	0.606
6.463	5.378	5.884	6.546	5.700	0.479	0.606
5.465	4.929	4.302	5.802	5.584	0.515	0.606
1.478	2.577	1.072	2.605	3.513	0.615	0.606
0.913	4.889	1.363	5.730	4.519	1.066	0.606
3.095	4.934	3.730	5.263	4.751	0.499	0.606
5.252	6.231	3.010	6.824	6.350	0.868	0.606
4.797	1.449	1.845	2.363	1.665	0.877	0.606
7.486	6.780	8.663	6.323	6.497	0.524	0.606
6.697	5.549	7.385	4.122	4.318	0.772	0.606
3.706	3.264	1.902	1.372	3.462	0.799	0.606
6.996	6.380	7.978	5.030	5.526	0.605	0.606
6.579	6.629	7.928	6.050	7.133	0.576	0.606
2.950	2.442	1.748	2.361	1.714	0.445	0.606
6.966	6.331	7.737	5.314	5.374	0.560	0.606
3.867	3.417	4.198	2.656	3.360	0.459	0.606
2.004	1.727	2.293	1.734	1.073	0.561	0.606
7.135	7.307	6.543	7.467	7.664	0.334	0.606
7.364	7.986	7.203	8.198	8.160	0.396	0.606
4.254	4.271	3.807	3.851	3.949	0.456	0.606
5.509	5.581	5.075	5.983	6.007	0.341	0.606
4.068	4.447	5.134	3.656	3.891	0.390	0.606
3.759	4.239	3.833	4.150	4.317	0.497	0.606
4.508	6.004	4.472	6.376	6.303	0.566	0.606
4.892	6.889	5.276	7.619	7.291	0.644	0.606
4.175	5.537	3.546	5.845	5.608	0.559	0.606
2.799	4.032	2.898	2.946	3.474	0.578	0.606
3.527	3.206	4.558	3.002	2.948	0.506	0.606
6.613	7.942	5.686	8.212	7.878	0.606	0.606
5.364	6.280	3.968	6.135	6.073	0.575	0.606
2.447	2.436	1.848	2.660	2.094	0.492	0.606
5.649	6.016	4.720	5.787	6.371	0.511	0.606
3.836	6.520	3.368	6.942	6.420	0.827	0.606
3.764	3.964	2.790	3.440	3.912	0.465	0.606
2.252	1.603	1.825	2.160	1.547	0.395	0.606
3.571	3.106	4.072	3.174	3.527	0.482	0.606
0.910	2.110	1.171	2.232	3.394	0.607	0.606
4.404	3.628	4.844	4.210	3.267	0.430	0.606
1.352	3.382	2.187	4.348	4.032	0.721	0.606
1.935	1.768	2.923	2.010	1.718	0.427	0.606
3.691	2.444	2.919	1.772	1.376	0.600	0.606
5.582	3.894	4.913	4.473	4.452	0.532	0.606

1.796	3.254	1.860	2.733	3.048	0.510	0.606
3.794	3.418	3.385	3.334	3.262	0.437	0.606
1.607	1.657	3.445	1.916	2.257	0.619	0.606
3.032	3.176	3.113	2.659	2.826	0.350	0.606
4.018	3.549	3.958	3.015	3.467	0.481	0.606
2.616	2.735	2.500	2.489	2.643	0.463	0.606
2.168	2.617	1.713	2.953	2.896	0.362	0.606
1.355	2.625	1.795	2.224	3.047	0.509	0.606
5.694	5.280	5.604	5.643	5.404	0.405	0.606
3.880	3.729	4.075	3.973	3.755	0.530	0.606
1.885	1.428	1.589	1.599	1.994	0.317	0.606
4.160	2.166	5.151	2.231	3.514	0.738	0.606
5.122	2.520	3.943	2.955	2.885	0.816	0.606
2.811	3.692	3.025	3.854	3.908	0.400	0.606
3.540	3.227	4.306	2.972	3.211	0.503	0.606
4.835	4.831	4.188	4.933	5.242	0.405	0.606
2.389	2.740	2.156	4.173	3.127	0.430	0.606
3.055	2.739	3.626	2.665	2.239	0.430	0.606
3.112	4.269	2.072	4.213	3.561	0.521	0.606
3.223	2.045	2.158	2.746	3.402	0.500	0.606
3.257	3.948	2.470	4.175	3.318	0.456	0.606
3.138	3.323	2.342	3.424	3.301	0.433	0.606
3.388	3.130	2.158	3.707	3.815	0.534	0.606
1.961	2.409	2.232	3.174	3.410	0.471	0.606
3.209	2.431	2.690	2.472	2.019	0.402	0.606
2.960	3.855	4.095	4.254	3.661	0.403	0.606
1.970	1.811	1.627	1.334	1.379	0.380	0.606
2.690	2.523	3.268	1.597	1.958	0.507	0.606
3.788	2.875	3.773	3.159	2.626	0.460	0.606
4.725	3.871	4.657	3.503	4.101	0.476	0.606
2.061	4.013	2.728	4.534	4.172	0.606	0.606
3.130	2.204	3.494	2.695	2.823	0.528	0.606
4.013	3.096	4.105	4.175	4.182	0.476	0.606
3.345	2.983	2.799	4.034	4.004	0.390	0.606
3.238	3.550	5.190	3.394	3.954	0.536	0.606
4.171	3.255	4.032	3.503	3.368	0.508	0.606
2.214	3.194	1.907	3.740	3.894	0.530	0.606
4.409	2.698	4.097	2.716	3.252	0.615	0.606
5.225	4.501	5.492	4.339	3.848	0.537	0.606
3.218	1.642	1.604	3.086	2.668	0.491	0.606
3.103	3.515	3.664	4.423	4.340	0.453	0.606
3.202	3.412	2.410	3.721	3.562	0.525	0.606
8.027	7.736	8.532	7.132	7.727	0.391	0.606
2.624	3.521	2.520	4.064	3.272	0.479	0.606
1.762	2.337	2.852	2.782	3.316	0.449	0.606
4.596	4.019	5.382	5.051	4.562	0.458	0.606
3.479	3.030	1.749	3.530	3.364	0.489	0.606
5.026	3.742	3.747	3.415	3.596	0.455	0.606
1.432	1.913	1.309	2.173	3.295	0.456	0.606
3.020	1.739	2.733	2.329	2.588	0.488	0.606

4.171	3.361	4.677	3.462	3.945	0.408	0.606
4.026	4.081	3.642	4.303	3.258	0.494	0.606
5.319	5.898	6.250	4.836	5.631	0.407	0.606
1.777	1.986	1.855	1.846	2.118	0.418	0.606
3.554	4.580	2.320	4.563	4.399	0.556	0.606
5.076	5.222	4.644	5.434	5.052	0.447	0.606
8.699	7.175	8.498	7.419	7.385	0.477	0.606
8.650	7.053	8.439	7.352	7.305	0.492	0.606
5.780	4.396	5.751	4.097	3.149	0.842	0.606
4.101	3.808	5.882	3.695	4.062	0.500	0.606
5.332	5.169	6.168	4.283	4.387	0.505	0.606
4.985	5.358	6.134	4.413	4.505	0.476	0.606
5.108	5.321	6.105	3.555	4.111	0.571	0.606
6.202	5.498	7.147	5.308	5.345	0.533	0.606
4.260	4.871	5.600	2.756	3.981	0.596	0.606
2.989	4.431	6.686	2.664	4.784	0.983	0.606
2.161	2.748	2.543	2.889	3.200	0.424	0.606
4.698	3.638	5.160	2.927	3.327	0.562	0.606
5.329	5.069	5.105	5.370	5.289	0.456	0.606
3.344	4.752	3.871	3.642	4.459	0.471	0.606
8.227	9.761	9.676	9.912	8.209	0.646	0.606
3.887	4.900	4.273	5.360	5.425	0.447	0.606
4.399	4.111	4.342	4.785	4.501	0.372	0.606
3.726	3.326	2.630	3.936	3.095	0.431	0.606
4.311	4.631	3.548	3.132	5.157	0.526	0.606
3.593	3.443	1.428	4.660	4.048	0.616	0.606
3.508	3.256	4.701	3.071	3.246	0.468	0.606
4.226	4.765	3.140	4.659	4.561	0.508	0.606
3.972	4.637	5.738	4.610	4.269	0.505	0.606
4.025	4.304	4.541	5.047	4.261	0.410	0.606
3.326	3.347	3.163	3.005	3.341	0.377	0.606
3.571	3.542	4.024	3.155	2.996	0.430	0.606
3.577	2.602	2.638	3.908	3.467	0.435	0.606
5.320	5.399	5.028	5.881	5.703	0.358	0.606
2.032	2.638	2.902	3.300	2.719	0.341	0.606
1.855	2.037	2.528	2.208	2.987	0.446	0.606
2.934	2.764	3.019	2.200	2.250	0.440	0.606
1.801	1.866	1.872	1.827	1.657	0.369	0.606
2.196	2.749	3.209	1.438	2.691	0.749	0.606
4.901	2.539	2.019	1.727	2.827	1.029	0.606
2.913	2.426	3.046	2.932	2.623	0.466	0.606
3.862	4.140	3.862	4.136	4.400	0.489	0.606
2.426	2.283	3.966	3.082	2.800	0.415	0.606
4.493	3.756	3.912	4.103	4.045	0.435	0.606
2.532	1.874	3.256	2.230	2.326	0.479	0.606
2.670	2.205	3.951	1.994	2.444	0.507	0.606
2.146	2.199	3.406	2.404	2.480	0.501	0.606
3.210	2.760	2.427	2.721	3.076	0.407	0.606
3.727	3.619	3.532	4.380	3.919	0.368	0.606
3.587	2.259	3.445	2.886	3.087	0.498	0.606

4.048	3.272	3.817	3.459	3.481	0.461	0.606
3.145	2.718	2.962	2.440	3.076	0.595	0.606
4.133	2.955	4.564	3.299	3.007	0.505	0.606
3.855	4.021	3.445	4.336	3.939	0.475	0.606
2.891	3.444	3.935	3.404	3.420	0.440	0.606
4.254	4.071	6.577	3.348	3.858	0.644	0.606
3.594	4.086	1.228	4.651	4.632	0.737	0.606
3.590	2.929	4.221	2.817	3.129	0.442	0.606
2.520	1.968	2.638	2.758	1.793	0.632	0.606
2.065	2.262	2.780	2.308	2.466	0.410	0.606
3.740	1.781	2.373	3.393	2.779	0.472	0.606
3.608	3.689	5.009	2.799	2.740	0.523	0.606
2.957	1.459	1.698	3.184	2.517	0.492	0.606
3.050	2.651	2.579	1.794	3.121	0.575	0.606
4.841	5.152	4.477	5.915	5.456	0.449	0.606
5.445	4.868	6.365	4.752	4.943	0.421	0.606
4.860	4.616	5.910	5.537	5.447	0.520	0.606
3.937	2.657	3.999	2.570	2.892	0.464	0.606
2.241	4.843	1.590	4.926	5.009	0.826	0.606
5.996	5.867	6.254	6.029	6.096	0.515	0.606
1.843	2.519	1.910	2.914	1.954	0.617	0.606
3.508	2.640	3.143	2.767	2.720	0.414	0.606
4.484	3.783	4.835	3.355	3.691	0.514	0.606
2.020	2.153	2.345	2.300	1.836	0.406	0.606
3.426	2.542	5.586	2.875	3.315	0.704	0.606
1.825	1.449	2.678	0.914	1.074	0.650	0.606
4.622	2.670	2.185	3.189	3.137	0.586	0.606
3.566	3.392	4.610	3.120	3.159	0.437	0.606
5.731	2.665	6.358	2.202	1.652	1.072	0.606
2.623	5.901	1.929	5.269	5.218	0.801	0.606
3.767	4.312	5.351	4.507	3.875	0.585	0.606
3.447	2.797	3.042	3.307	3.740	0.426	0.606
3.124	2.138	2.636	2.814	2.631	0.509	0.606
3.906	4.709	4.152	4.203	4.302	0.521	0.606
2.572	2.782	6.011	2.775	3.939	0.792	0.606
3.772	3.437	4.510	3.282	3.335	0.435	0.606
3.942	4.506	4.237	4.127	4.666	0.452	0.606
2.673	2.625	1.769	2.788	2.183	0.410	0.606
3.321	3.279	3.737	3.146	3.036	0.382	0.606
3.558	3.089	4.222	1.599	2.575	0.560	0.606
3.122	2.418	3.953	2.662	2.444	0.481	0.606
6.244	5.317	6.563	5.963	5.623	0.420	0.606
4.220	4.494	3.993	5.639	5.243	0.524	0.606
2.148	2.293	1.482	2.442	1.960	0.434	0.606
4.323	3.605	4.505	3.573	3.291	0.522	0.606
4.603	5.100	2.466	5.738	5.186	0.627	0.606
3.258	3.715	4.425	3.639	3.745	0.406	0.606
2.627	2.682	2.968	2.115	2.456	0.477	0.606
2.837	3.535	3.522	1.726	2.701	0.499	0.606
3.295	3.133	2.431	3.289	3.049	0.606	0.606

2.789	2.791	3.846	2.312	2.277	0.703	0.606
4.338	4.396	5.457	3.611	3.742	0.464	0.606
2.819	1.126	2.954	2.875	1.596	0.728	0.606
3.725	3.228	4.692	3.382	2.809	0.461	0.606
4.132	3.202	5.356	2.529	2.714	0.604	0.606
3.679	2.498	3.898	2.814	2.937	0.427	0.606
4.571	6.874	3.812	7.253	6.777	0.797	0.606
4.204	6.311	2.992	6.893	6.215	0.818	0.606
3.977	3.086	5.906	3.479	4.449	0.645	0.606
4.721	5.150	5.397	5.481	5.884	0.463	0.606
4.489	4.746	5.438	3.630	4.753	0.480	0.606
8.224	7.821	8.752	7.123	7.716	0.420	0.606
2.133	2.798	2.478	2.652	2.542	0.442	0.606
5.463	3.667	5.193	4.556	4.235	0.575	0.606
3.034	2.252	4.737	1.781	2.148	0.609	0.606
3.088	2.861	1.679	3.286	3.460	0.631	0.606
4.304	3.398	4.453	3.496	3.454	0.641	0.606
3.486	2.204	3.803	2.362	2.364	0.453	0.606
1.563	2.658	1.753	3.309	3.046	0.441	0.606
2.880	1.915	2.025	1.362	2.116	0.498	0.606
2.008	3.525	4.130	2.908	3.307	0.505	0.606
3.487	4.703	2.901	4.662	4.850	0.539	0.606
3.591	3.645	4.811	3.555	3.907	0.509	0.606
4.109	3.901	3.944	4.584	3.630	0.423	0.606
4.189	3.961	4.403	3.213	3.578	0.386	0.606
3.882	3.309	4.574	3.206	2.744	0.512	0.606
1.641	2.193	2.443	1.976	1.414	0.379	0.606
3.779	3.234	5.491	3.307	3.663	0.584	0.606
6.291	6.429	6.867	5.783	6.496	0.431	0.606
1.565	3.919	1.213	3.075	4.212	0.631	0.606
1.270	1.696	1.765	2.437	2.511	0.420	0.606
3.090	3.465	3.619	2.970	2.801	0.446	0.606
4.245	2.731	4.000	3.630	3.910	0.486	0.606
4.284	3.742	5.255	4.239	3.989	0.526	0.606
4.722	4.936	6.456	4.937	5.149	0.534	0.606
3.096	2.526	3.683	1.674	2.658	0.729	0.606
1.288	1.355	1.259	2.549	1.641	0.415	0.606
6.714	5.953	6.674	5.228	5.900	0.577	0.606
2.623	3.230	2.112	3.040	3.276	0.405	0.606
2.259	2.667	1.816	2.398	1.849	0.434	0.606
2.008	1.700	1.216	3.221	3.078	0.682	0.606
4.068	3.552	4.808	3.105	3.377	0.682	0.606
3.201	2.887	2.250	2.874	3.136	0.489	0.606
4.605	5.160	4.671	5.101	5.241	0.371	0.606
3.822	3.805	2.491	4.239	4.653	0.603	0.606
3.135	3.879	5.064	2.840	3.016	0.806	0.606
3.161	2.836	4.007	3.369	3.361	0.439	0.606
3.953	3.878	3.686	3.812	5.096	0.665	0.606
4.687	6.213	3.943	6.796	7.035	0.687	0.606
1.343	4.262	1.575	4.357	4.268	0.769	0.606

2.694	3.289	3.126	2.780	2.966	0.489	0.606
3.041	3.156	3.672	2.372	2.719	0.457	0.606
0.970	2.047	1.130	2.872	3.216	0.584	0.606
2.296	2.009	1.089	2.144	2.402	0.492	0.606
3.580	3.441	4.742	2.757	3.432	0.425	0.606
2.304	1.537	2.670	2.709	2.081	0.440	0.606
1.830	3.036	3.517	3.921	3.138	0.555	0.606
4.134	3.219	4.133	3.656	3.427	0.440	0.606
3.094	2.331	3.225	2.079	2.409	0.507	0.606
4.538	5.409	6.036	4.070	4.414	0.755	0.606
2.168	2.291	3.089	2.250	2.243	0.512	0.606
1.320	1.298	1.336	0.779	3.279	0.667	0.606
2.547	2.495	3.499	1.942	1.814	0.502	0.606
3.780	1.105	1.834	3.247	3.133	0.919	0.606
3.308	2.187	1.058	2.834	1.443	0.678	0.606
2.690	2.586	3.521	2.397	3.088	0.422	0.606
5.427	3.277	6.060	2.304	3.125	0.979	0.606
2.565	3.541	2.566	2.139	2.581	0.518	0.606
1.603	2.520	0.503	3.465	2.353	0.570	0.606
2.657	1.528	4.112	1.714	1.417	0.649	0.606
2.923	2.546	3.308	2.953	3.139	0.412	0.606
1.950	2.533	1.466	2.370	2.281	0.388	0.606
2.644	3.001	2.443	3.170	3.151	0.396	0.606
2.312	2.709	2.847	2.051	1.657	0.440	0.606
2.015	3.953	1.295	4.929	4.748	0.825	0.606
3.601	3.287	4.940	2.311	2.476	0.585	0.606
1.591	3.362	0.992	4.217	4.078	0.752	0.606
6.700	6.343	6.931	5.531	6.515	0.443	0.606
3.206	3.198	2.491	3.350	3.419	0.457	0.606
2.823	3.174	3.050	4.043	3.690	0.491	0.606
3.096	2.682	3.065	2.562	2.692	0.417	0.606
3.600	4.131	4.125	4.880	4.788	0.361	0.606
1.734	3.548	2.058	3.557	3.701	0.660	0.606
3.194	3.838	4.426	2.626	2.601	0.455	0.606
2.671	2.786	1.886	2.671	2.488	0.505	0.606
3.522	3.174	3.229	2.994	3.077	0.488	0.606
4.008	4.220	4.937	3.828	3.922	0.386	0.606
2.168	1.992	2.240	2.237	1.846	0.588	0.606
3.107	2.875	3.044	2.620	3.047	0.407	0.606
2.762	1.564	3.479	1.064	1.668	0.504	0.606
1.691	2.438	1.417	2.988	2.815	0.476	0.606
2.915	2.602	2.737	2.057	2.270	0.720	0.606
3.908	4.824	4.992	4.168	4.829	0.617	0.606
5.857	5.818	6.469	4.885	5.951	0.423	0.606
5.841	5.822	6.559	4.888	5.978	0.443	0.606
7.132	7.023	7.900	6.433	6.911	0.382	0.606
4.813	4.806	4.599	2.720	5.038	0.687	0.606
2.797	2.669	1.932	2.428	2.914	0.368	0.606
3.186	2.344	4.545	2.200	3.178	0.537	0.606
3.905	3.512	4.007	2.623	2.252	0.535	0.606

4.420	4.406	5.197	4.096	4.149	0.692	0.606
2.129	1.535	2.232	1.803	2.069	0.391	0.606
3.002	1.622	1.504	1.836	1.417	0.613	0.606
4.462	4.186	5.139	4.287	3.600	0.492	0.606
5.251	2.704	5.510	2.660	1.918	0.902	0.606
4.113	2.745	5.304	2.384	3.245	0.764	0.606
1.988	1.771	2.348	2.113	1.486	0.516	0.606
3.635	3.336	4.255	4.288	4.781	0.638	0.606
2.071	2.348	2.387	2.483	2.779	0.703	0.606
7.420	7.543	7.695	6.161	7.491	0.556	0.606
7.725	7.383	8.348	6.623	7.271	0.440	0.606
2.328	2.939	3.300	2.163	2.288	0.386	0.606
3.003	4.243	3.103	4.229	3.560	0.529	0.606
2.505	2.412	1.583	2.891	2.841	0.393	0.606
1.300	1.968	3.502	2.941	3.125	0.691	0.606
2.015	3.695	2.932	3.349	3.309	0.580	0.606
1.913	1.534	2.389	2.557	1.857	0.429	0.606
1.041	3.251	1.895	3.467	2.773	0.589	0.606
3.130	2.936	4.242	3.148	3.158	0.564	0.606
4.329	3.573	4.156	2.820	2.654	0.645	0.606
4.913	4.006	4.242	3.422	4.255	0.615	0.606
1.862	1.442	3.006	1.833	2.046	0.463	0.606
2.970	2.343	2.970	2.213	2.871	0.412	0.606
2.432	2.327	2.625	3.098	2.825	0.378	0.606
1.721	2.746	1.493	4.065	2.354	0.566	0.606
1.508	2.505	1.730	2.482	2.675	0.511	0.606
3.001	3.967	3.811	4.630	4.372	0.470	0.606
3.592	2.468	4.173	1.925	2.049	0.529	0.606
1.759	3.092	1.718	3.761	4.276	0.636	0.606
3.506	4.140	4.353	4.557	4.044	0.381	0.606
3.071	2.910	3.186	2.864	2.392	0.479	0.606
3.027	2.530	1.994	2.044	3.184	0.420	0.606
2.623	2.329	2.909	2.679	2.451	0.426	0.606
4.968	4.813	5.367	4.741	4.640	0.465	0.606
3.618	4.926	5.025	4.218	4.925	0.435	0.606
4.280	6.396	2.412	5.666	5.765	1.011	0.606
4.527	3.097	5.153	2.999	3.206	0.721	0.606
2.838	2.203	3.504	2.546	2.019	0.461	0.606
3.622	2.691	3.388	3.189	3.570	0.435	0.606
3.951	3.088	3.471	2.854	3.058	0.510	0.606
7.044	6.392	7.049	6.058	5.608	0.663	0.606
4.499	2.904	4.886	3.303	3.151	0.541	0.606
4.506	3.144	5.836	1.939	2.172	0.907	0.606
4.750	4.219	3.936	3.150	3.497	0.518	0.606
3.203	2.885	3.873	3.307	2.917	0.544	0.606
8.458	8.598	8.260	8.817	8.882	0.344	0.606
5.347	6.035	7.910	4.268	4.703	0.709	0.606
4.204	4.355	5.177	3.980	3.789	0.438	0.606
1.987	2.739	3.326	2.854	2.736	0.411	0.606
4.372	3.427	5.101	3.534	4.475	0.438	0.606

3.916	3.561	4.615	3.811	3.506	0.428	0.606
3.475	1.816	3.454	2.406	1.903	0.551	0.606
4.155	3.998	4.813	4.015	4.723	0.438	0.606
2.046	1.981	1.009	2.276	2.899	0.448	0.606
5.412	4.716	3.612	5.456	5.757	0.559	0.606
2.413	3.181	2.169	2.809	3.518	0.440	0.606
1.252	3.152	1.185	2.899	2.664	0.574	0.606
1.728	2.053	1.938	2.408	3.231	0.475	0.606
4.041	2.846	4.491	3.254	3.724	0.462	0.606
2.475	2.664	1.852	2.081	2.071	0.390	0.606
4.490	4.888	3.593	4.577	5.062	0.471	0.606
2.888	2.857	3.376	2.837	2.295	0.539	0.606
2.780	3.244	4.213	3.992	3.357	0.524	0.606
3.422	4.565	6.361	3.366	6.178	1.141	0.606
2.360	3.503	2.168	4.629	3.815	0.613	0.606
4.715	2.366	2.951	2.959	3.228	0.532	0.606
2.583	2.219	3.010	2.704	2.538	0.644	0.606
2.657	2.682	3.452	2.483	2.489	0.473	0.606
4.035	3.299	5.171	3.240	3.605	0.564	0.606
1.974	3.097	2.064	3.004	3.578	0.499	0.606
2.168	1.808	1.822	1.651	2.042	0.514	0.606
2.654	2.180	2.140	2.082	1.997	0.406	0.606
3.892	2.795	2.522	2.502	3.297	0.462	0.606
1.043	2.521	1.100	1.321	2.842	0.476	0.606
4.495	5.498	3.093	5.793	5.886	0.663	0.606
2.873	2.653	2.330	2.718	2.755	0.459	0.606
2.444	2.280	1.804	3.498	3.326	0.552	0.606
2.257	2.669	2.127	1.689	2.195	0.607	0.606
2.668	2.970	2.769	3.033	4.102	0.424	0.606
4.495	3.690	2.854	4.449	3.813	0.417	0.606
2.117	1.784	3.215	1.643	1.841	0.458	0.606
4.588	4.203	3.816	4.500	4.181	0.503	0.606
1.366	3.056	2.492	2.702	2.397	0.478	0.606
3.354	3.816	2.365	4.578	4.147	0.525	0.606
2.346	2.694	2.537	3.662	3.578	0.435	0.606
4.821	3.703	3.836	3.905	4.313	0.452	0.606
3.095	3.435	6.483	2.668	2.164	0.886	0.606
2.889	2.810	5.914	2.004	2.104	0.724	0.606
2.564	2.670	2.652	3.794	3.414	0.494	0.606
1.281	2.384	1.467	1.544	1.756	0.796	0.606
4.279	4.514	4.690	6.359	5.030	0.649	0.606
2.709	2.480	2.589	2.589	3.631	0.453	0.606
3.103	3.223	4.888	2.543	2.600	0.623	0.606
4.262	3.605	4.791	2.962	3.663	0.517	0.606
2.840	2.983	3.301	2.929	2.417	0.436	0.606
1.074	6.109	2.293	5.913	5.657	1.106	0.606
2.633	3.157	4.115	1.642	3.202	0.683	0.606
6.467	5.001	6.788	4.771	4.882	0.548	0.606
4.547	3.970	4.641	4.561	4.605	0.428	0.606
2.964	2.456	2.291	3.171	3.347	0.457	0.606

3.004	2.335	3.295	2.175	2.259	0.462	0.606
4.566	4.412	5.343	4.437	4.270	0.462	0.606
3.355	2.248	3.933	2.670	2.055	0.564	0.606
1.989	1.917	2.185	1.290	2.173	0.510	0.606
5.400	5.906	5.229	6.470	5.833	0.723	0.606
4.067	4.228	4.821	4.479	4.438	0.365	0.606
2.756	2.685	3.381	2.284	3.647	0.507	0.606
5.452	5.105	5.366	5.942	5.721	0.399	0.606
3.044	2.931	3.167	3.522	3.451	0.441	0.606
5.334	5.440	5.670	5.475	4.961	0.440	0.606
3.650	3.499	2.736	3.195	3.980	0.468	0.606
3.058	2.410	3.772	2.069	2.280	0.496	0.606
4.194	4.890	3.238	3.551	3.868	0.850	0.606
3.736	2.994	3.363	2.276	3.342	0.633	0.606
7.873	7.619	7.513	8.062	7.861	0.342	0.606
4.192	3.893	4.564	3.962	4.576	0.464	0.606
2.072	3.651	2.173	3.589	3.799	0.518	0.606
4.335	4.615	5.580	3.304	3.522	0.526	0.606
4.315	4.339	4.972	4.789	4.842	0.395	0.606
3.432	2.669	3.582	1.479	2.476	0.461	0.606
2.096	2.183	2.173	3.140	1.975	0.486	0.606
1.430	2.536	1.736	3.235	2.199	0.526	0.606
3.638	4.128	3.524	4.583	4.122	0.423	0.606
2.866	3.408	3.093	3.681	2.187	0.434	0.606
4.695	4.168	4.944	3.926	3.987	0.629	0.606
4.666	5.072	2.060	6.105	5.652	0.756	0.606
1.643	2.091	1.634	1.731	1.309	0.401	0.606
3.068	2.077	3.582	1.924	2.880	0.492	0.606
1.789	3.261	1.533	4.312	3.676	0.640	0.606
2.736	3.517	2.321	4.386	4.280	0.513	0.606
3.877	5.217	2.309	6.005	5.558	0.804	0.606
1.807	2.927	2.919	1.736	2.404	0.448	0.606
4.237	3.724	3.534	4.251	4.386	0.426	0.606
4.982	4.087	5.719	4.901	4.679	0.428	0.606
3.411	2.552	4.103	2.435	2.902	0.527	0.606
2.056	3.105	1.756	2.178	2.479	0.536	0.606
4.810	5.333	5.579	4.203	5.017	0.467	0.606
3.213	3.159	3.179	3.413	2.768	0.434	0.606
2.547	2.536	3.393	3.127	2.815	0.413	0.606
3.891	4.768	4.058	3.930	3.430	0.628	0.606
5.665	2.919	5.093	5.899	4.150	0.694	0.606
3.964	1.817	4.477	2.407	1.200	0.900	0.606
2.555	2.952	2.855	2.517	2.437	0.483	0.606
2.069	1.520	1.645	1.551	1.427	0.498	0.606
3.668	4.878	4.080	5.593	5.447	0.996	0.606
3.677	3.696	3.793	2.855	1.951	0.608	0.606
3.256	3.054	4.983	3.739	3.054	0.524	0.606
4.501	2.381	3.007	1.971	2.211	0.623	0.606
2.344	3.478	2.790	3.105	3.253	0.448	0.606
2.443	2.442	2.916	2.718	2.231	0.368	0.606

1.883	3.255	1.902	3.052	2.767	0.422	0.606
4.313	5.121	4.102	6.233	5.503	0.682	0.606
1.794	3.490	2.055	2.279	2.983	0.505	0.606
5.521	5.117	5.186	4.807	5.192	0.469	0.606
3.976	2.655	4.307	2.582	2.132	0.656	0.606
3.293	3.938	3.476	3.850	3.048	0.535	0.606
5.266	4.838	6.130	5.140	5.165	0.488	0.606
1.861	2.207	1.835	1.949	2.024	0.445	0.606
2.671	2.424	2.159	2.291	1.591	0.528	0.606
2.107	2.790	2.588	3.573	2.817	0.484	0.606
2.749	3.267	3.305	2.509	3.260	0.405	0.606
2.220	4.740	0.932	3.943	4.475	0.915	0.606
4.554	3.488	5.032	3.409	4.091	0.668	0.606
2.771	2.113	2.029	1.756	2.279	0.492	0.606
1.611	1.956	2.042	3.324	2.226	0.451	0.606
6.885	6.449	7.271	5.070	6.777	0.627	0.606
6.464	5.805	6.814	4.760	6.173	0.627	0.606
4.222	2.980	4.321	2.878	3.882	0.584	0.606
4.028	3.524	3.455	4.185	3.668	0.408	0.606
6.583	7.003	6.833	7.258	6.839	0.517	0.606
10.203	9.130	9.879	9.159	9.028	0.391	0.606
2.209	3.706	3.086	2.162	2.357	0.599	0.606
4.155	3.274	2.869	3.749	2.859	0.637	0.606
3.909	3.964	3.264	3.785	4.273	0.429	0.606
1.997	1.959	2.400	1.790	2.123	0.419	0.606
4.129	4.418	3.941	4.480	3.668	0.376	0.606
2.792	3.918	3.132	2.171	1.318	1.197	0.606
2.120	3.025	3.033	3.546	3.460	0.512	0.606
4.550	4.185	4.807	5.099	5.399	0.627	0.606
5.323	4.296	5.272	3.382	3.466	0.894	0.606
2.243	2.728	2.209	2.007	2.573	0.462	0.606
5.056	3.413	5.987	3.687	3.292	0.609	0.606
4.863	4.250	5.349	4.860	5.076	0.398	0.606
5.408	5.514	5.518	5.241	5.968	0.486	0.606
6.173	5.546	6.348	5.526	5.988	0.581	0.606
4.130	1.946	2.438	1.955	1.862	0.776	0.606
2.010	2.828	2.505	3.109	2.197	0.474	0.606
2.106	3.064	1.694	2.935	2.736	0.475	0.606
5.178	5.626	5.470	5.431	5.096	0.362	0.606
2.933	3.781	1.550	4.007	4.136	0.598	0.606
2.764	4.565	4.556	3.942	4.387	0.460	0.606
4.868	5.347	3.472	4.516	4.305	0.924	0.606
4.010	3.061	2.429	2.637	2.539	0.905	0.606
2.760	2.504	2.994	1.716	2.748	0.425	0.606
5.062	5.169	5.096	5.792	5.765	0.409	0.606
5.897	4.829	6.499	5.479	5.994	0.675	0.606
6.051	6.266	5.583	6.066	6.436	0.551	0.606
8.822	8.824	9.669	9.526	8.662	0.474	0.606
0.841	2.656	0.998	3.618	3.972	0.721	0.606
9.696	9.413	8.024	9.812	9.698	0.422	0.606

2.955	3.762	1.770	3.270	3.837	0.482	0.606
6.176	6.264	5.304	6.454	6.334	0.497	0.606
5.142	3.476	4.074	5.002	4.142	0.570	0.606
2.979	3.101	2.349	3.541	3.692	0.378	0.606
3.077	3.212	1.732	2.976	2.797	0.522	0.606
3.687	4.235	4.448	4.443	4.275	0.442	0.606
2.119	2.915	1.617	3.990	3.090	0.530	0.606
5.115	2.362	3.691	2.416	2.424	0.729	0.606
5.538	6.063	6.095	5.426	5.643	0.453	0.606
2.670	2.193	2.645	2.328	1.644	0.520	0.606
2.033	2.947	1.678	4.096	2.798	0.601	0.606
1.816	1.860	1.847	1.931	2.868	0.420	0.606
2.954	3.788	2.252	4.899	4.290	0.625	0.606
3.889	4.282	3.467	3.458	3.804	0.534	0.606
2.904	3.320	3.517	2.708	3.243	0.371	0.606
3.576	2.989	3.232	3.101	3.101	0.435	0.606
2.980	3.065	2.615	2.912	3.680	0.358	0.606
4.164	4.627	4.217	4.918	4.652	0.395	0.606
3.462	3.501	3.313	3.790	4.176	0.392	0.606
2.043	1.094	2.310	1.691	1.879	0.618	0.606
2.219	2.912	3.347	2.470	3.197	0.492	0.606
4.238	4.822	3.442	4.987	4.426	0.420	0.606
5.105	5.539	5.505	5.748	5.650	0.409	0.606
8.271	8.712	6.747	9.351	9.069	0.541	0.606
2.048	2.264	2.067	2.397	2.152	0.378	0.606
2.776	2.598	2.670	2.038	2.108	0.377	0.606
4.185	4.121	4.360	3.277	3.341	0.506	0.606
3.855	3.858	3.874	3.376	3.447	0.394	0.606
8.888	8.078	9.661	6.973	7.352	0.572	0.606
2.386	2.837	1.247	2.837	4.564	0.720	0.606
3.381	3.096	5.487	3.565	3.594	0.651	0.606
3.554	2.987	4.323	2.355	3.315	0.605	0.606
3.005	2.124	2.978	1.810	2.309	0.536	0.606
7.261	6.716	7.702	5.483	6.253	0.589	0.606
5.509	5.751	5.753	5.061	5.136	0.357	0.606
6.763	6.693	4.850	7.061	6.943	0.547	0.606
2.836	4.001	2.440	3.616	2.121	0.529	0.606
7.301	7.096	7.864	7.408	7.475	0.320	0.606
7.295	7.985	6.430	8.098	7.850	0.420	0.606
5.380	6.897	4.018	7.255	7.278	0.793	0.606
7.514	7.435	6.888	7.726	7.823	0.343	0.606
3.012	3.836	3.142	3.927	3.537	0.577	0.606
3.079	2.587	3.229	2.947	3.055	0.392	0.606
4.573	3.227	4.393	3.771	3.731	0.520	0.606
5.300	5.165	7.133	4.703	4.661	0.589	0.606
5.099	5.741	2.269	6.003	5.434	0.874	0.606
5.913	7.950	3.726	8.390	8.235	0.969	0.606
4.389	6.101	3.873	6.633	6.144	0.820	0.606
5.195	5.201	5.511	3.621	5.128	0.493	0.606
2.049	1.667	1.982	1.233	1.287	0.622	0.606

3.675	3.316	3.888	2.161	2.305	0.522	0.606
3.638	3.776	3.182	5.063	4.273	0.538	0.606
3.191	4.800	2.297	5.432	5.110	0.701	0.606
6.048	6.977	3.548	7.369	7.137	0.815	0.606
2.888	4.214	3.271	3.034	3.484	0.577	0.606
3.301	3.626	5.115	3.641	3.730	0.458	0.606
4.701	4.459	4.918	3.650	4.047	0.370	0.606
5.071	5.354	5.222	5.247	5.186	0.349	0.606
5.467	6.326	5.292	6.624	6.603	0.421	0.606
7.019	7.006	6.647	7.356	7.306	0.362	0.606
8.014	6.684	9.472	4.007	4.588	1.046	0.606
5.103	5.093	6.453	5.259	4.965	0.570	0.606
4.718	5.459	3.521	5.428	5.241	0.482	0.606
4.549	4.150	5.343	4.109	4.018	0.455	0.606
5.242	5.256	4.059	5.778	5.900	0.417	0.606
5.848	5.486	7.387	4.550	4.726	0.587	0.606
2.571	4.573	2.761	4.748	3.784	0.625	0.606
6.310	6.549	5.842	7.172	7.154	0.403	0.606
3.262	4.562	2.596	5.621	4.518	0.664	0.606
3.274	3.117	3.689	3.856	3.399	0.437	0.606
2.194	2.617	2.919	1.687	1.854	0.526	0.606
3.893	5.039	4.296	3.930	4.540	0.639	0.606
6.435	6.245	7.377	6.698	6.037	0.500	0.606
5.783	4.340	5.018	5.131	4.707	0.634	0.606
3.436	3.246	3.764	2.702	3.331	0.464	0.606
4.962	4.634	4.786	4.967	5.443	0.456	0.606
1.825	2.682	1.832	3.086	2.421	0.417	0.606
3.895	4.620	3.437	4.654	4.960	0.468	0.606
4.253	3.963	3.096	4.264	3.973	0.439	0.606
4.008	5.239	3.639	5.905	5.668	0.638	0.606
5.493	5.816	5.750	5.851	5.976	0.430	0.606
2.935	1.798	1.810	1.372	1.556	0.592	0.606
4.155	2.560	2.691	2.811	3.219	0.584	0.606
4.462	4.272	3.253	4.658	4.932	0.416	0.606
6.957	7.001	6.065	7.462	7.156	0.399	0.606
1.024	2.161	1.093	3.687	3.580	0.731	0.606
4.029	4.258	2.821	4.163	4.061	0.609	0.606
5.070	5.821	5.211	6.032	5.862	0.356	0.606
4.931	5.237	4.076	5.253	5.633	0.419	0.606
4.966	3.608	5.793	2.150	2.780	0.786	0.606
0.995	1.716	1.755	1.754	2.677	0.509	0.606
5.375	7.631	6.339	7.901	7.903	0.820	0.606
2.658	2.365	2.406	2.046	2.079	0.373	0.606
3.965	4.877	2.262	4.763	4.468	0.653	0.606
5.763	7.189	6.903	7.622	7.443	0.556	0.606
2.742	1.512	1.205	2.039	1.934	0.658	0.606
4.405	3.907	5.393	3.264	4.653	0.511	0.606
5.202	5.399	4.216	5.853	5.816	0.401	0.606
3.847	4.964	3.552	5.261	4.704	0.480	0.606
5.149	5.966	5.876	6.025	6.198	0.494	0.606

5.467	5.054	5.173	5.755	5.304	0.477	0.606
4.741	4.575	3.638	5.247	4.797	0.429	0.606
4.803	5.540	3.352	6.122	6.193	0.636	0.606
5.119	5.969	3.762	6.489	5.985	0.610	0.606
7.496	8.567	8.460	9.145	8.796	0.598	0.606
6.650	7.418	5.889	8.330	8.049	0.570	0.606
5.483	7.217	4.274	7.639	7.603	0.719	0.606
3.462	4.512	3.172	4.824	4.952	0.576	0.606
3.888	5.300	3.035	5.358	5.057	0.562	0.606
4.310	4.067	4.688	3.938	3.935	0.382	0.606
3.076	2.578	4.024	2.855	3.263	0.457	0.606
2.760	2.505	2.668	2.024	2.801	0.423	0.606
4.110	3.765	4.096	3.252	4.577	0.422	0.606
7.999	6.793	7.036	5.827	6.049	0.516	0.606
3.590	3.872	3.908	2.754	3.215	0.461	0.606
3.538	3.659	3.822	2.607	3.001	0.470	0.606
3.496	3.650	3.777	2.750	3.010	0.470	0.606
3.420	3.574	3.645	2.698	2.877	0.496	0.606
3.305	3.488	3.625	2.582	2.936	0.466	0.606
3.400	3.550	3.734	2.613	2.905	0.485	0.606
1.569	2.379	2.431	1.888	2.181	0.510	0.606
5.424	5.106	6.056	4.416	5.424	0.413	0.606
2.942	3.491	3.124	3.130	3.519	0.389	0.606
5.797	5.359	5.593	5.248	4.982	0.350	0.606
3.860	4.268	3.628	4.103	4.568	0.578	0.606
5.761	5.752	6.115	5.069	4.968	0.410	0.606
3.571	3.028	4.577	2.090	3.013	1.218	0.606
6.205	6.039	6.845	6.124	6.387	0.433	0.606
2.246	1.633	3.366	1.541	1.677	0.705	0.606
2.470	2.043	2.254	2.593	3.069	0.570	0.606
5.207	5.006	6.023	5.348	4.975	0.488	0.606
5.821	6.216	4.759	6.891	7.045	0.536	0.606
7.737	5.978	6.348	6.537	6.145	0.525	0.606
3.598	4.427	2.912	3.880	4.398	0.466	0.606
3.606	4.469	2.467	4.373	4.137	0.524	0.606
4.466	4.283	4.589	3.400	4.556	0.433	0.606
6.458	6.142	7.269	4.801	5.973	0.741	0.606
1.109	3.794	2.009	4.609	4.716	0.789	0.606
2.881	3.182	4.007	2.661	3.005	0.478	0.606
3.752	3.770	3.781	3.169	3.735	0.373	0.606
4.901	6.072	5.702	4.983	5.194	0.560	0.606
4.510	4.574	3.208	4.421	4.228	0.419	0.606
4.540	4.037	3.753	4.164	4.290	0.530	0.606
5.916	6.195	5.534	6.269	6.399	0.388	0.606
1.061	2.970	0.661	3.509	2.959	0.702	0.606
4.988	4.963	5.346	5.392	4.749	0.360	0.606
4.395	4.579	3.240	5.185	4.766	0.438	0.606
3.985	3.854	4.713	3.645	4.085	0.446	0.606
3.289	3.091	4.046	2.531	3.015	0.527	0.606
4.676	7.269	6.618	6.484	7.110	0.834	0.606

4.439	4.949	4.440	3.686	4.366	0.847	0.606
3.281	3.927	3.299	4.484	4.297	0.359	0.606
2.838	5.561	2.791	6.156	6.042	0.807	0.606
5.153	4.337	5.386	3.984	3.738	0.422	0.606
4.804	5.626	4.554	6.010	5.712	0.522	0.606
4.123	4.044	3.429	3.945	4.194	0.381	0.606
3.522	2.082	2.009	2.544	2.344	0.651	0.606
7.960	1.609	2.063	2.203	2.101	1.286	0.606
2.919	3.001	2.362	3.322	3.271	0.359	0.606
2.664	3.237	2.140	3.712	3.326	0.403	0.606
2.444	3.544	2.658	3.225	2.862	0.452	0.606
6.784	7.281	6.010	7.305	7.074	0.388	0.606
1.838	2.203	2.075	2.291	3.048	0.399	0.606
4.314	4.417	4.333	4.459	4.618	0.517	0.606
3.317	3.476	3.281	3.415	2.804	0.391	0.606
1.620	2.979	3.152	3.023	2.703	0.543	0.606
4.762	3.892	5.267	3.684	3.945	0.575	0.606
8.891	7.768	9.362	4.989	6.407	0.900	0.606
7.672	7.495	5.793	7.389	7.308	0.431	0.606
5.890	6.030	4.003	6.122	5.977	0.524	0.606
4.217	4.821	2.473	5.751	3.710	0.699	0.606
6.770	7.099	6.809	6.987	6.749	0.350	0.606
3.472	2.362	2.227	3.457	3.629	0.406	0.606
4.418	4.412	3.857	4.377	4.558	0.353	0.606
6.136	5.632	7.450	5.770	5.928	0.422	0.606
3.721	2.440	3.464	3.059	3.128	0.589	0.606
6.536	6.631	5.283	7.012	7.069	0.478	0.606
2.808	2.514	3.283	2.213	2.627	0.438	0.606
5.926	5.791	6.784	5.186	5.531	0.412	0.606
1.623	1.742	1.761	1.773	1.366	0.393	0.606
8.650	8.766	10.028	6.715	7.189	0.663	0.606
2.998	6.549	2.579	6.425	6.683	0.995	0.606
9.970	9.033	9.765	9.069	9.302	0.383	0.606
3.482	3.590	2.835	2.622	2.954	0.532	0.606
3.233	3.756	2.664	4.312	4.381	0.527	0.606
2.349	2.067	2.865	1.821	2.025	0.364	0.606
2.993	3.085	3.867	2.748	2.403	0.423	0.606
5.542	5.031	5.568	5.417	5.183	0.357	0.606
5.140	6.178	5.260	6.973	6.345	0.587	0.606
7.924	8.580	8.511	9.010	8.690	0.359	0.606
3.317	2.970	3.256	2.481	3.659	0.490	0.606
5.110	5.959	5.075	6.183	5.942	0.539	0.606
7.622	6.639	9.023	5.967	6.038	0.625	0.606
4.556	4.698	4.839	3.531	4.368	0.459	0.606
4.845	4.556	4.003	4.763	4.337	0.364	0.606
4.052	4.284	4.222	4.202	4.511	0.429	0.606
5.406	5.967	5.025	5.986	6.299	0.363	0.606
6.345	7.061	6.632	6.668	6.176	0.461	0.606
9.052	9.090	7.800	9.296	9.130	0.415	0.606
3.387	3.499	3.951	3.579	3.596	0.598	0.606

9.123	9.082	6.644	9.503	9.324	0.695	0.606
7.710	8.468	7.085	8.767	8.616	0.467	0.606
4.775	4.843	4.140	5.831	5.134	0.461	0.606
5.820	5.906	5.542	6.156	5.668	0.400	0.606
5.359	6.458	4.817	6.693	6.365	0.458	0.606
4.253	4.105	5.190	3.303	3.819	0.454	0.606
3.648	3.936	3.966	3.565	3.686	0.397	0.606
3.912	4.307	3.423	3.756	3.591	0.381	0.606
3.786	4.142	5.733	3.186	4.027	0.886	0.606
4.774	4.435	5.414	4.272	4.320	0.682	0.606
5.333	5.497	2.710	5.615	5.871	0.683	0.606
7.828	7.223	7.650	6.654	6.726	0.340	0.606
2.303	2.268	2.449	2.140	2.229	0.432	0.606
4.787	5.453	4.754	5.198	5.355	0.427	0.606
8.829	8.061	9.490	7.591	7.614	0.457	0.606
8.246	7.313	8.651	7.294	7.158	0.405	0.606
6.963	6.609	5.281	7.411	7.115	0.458	0.606
6.235	5.331	6.458	5.308	5.000	0.442	0.606
8.647	7.813	9.298	7.464	7.393	0.453	0.606
8.035	7.585	2.725	7.978	8.011	1.238	0.606
4.527	4.149	4.840	4.335	4.693	0.362	0.606
6.227	5.983	4.393	6.131	6.365	0.465	0.606
3.162	2.597	1.482	2.785	2.564	0.609	0.606
3.470	4.918	3.070	4.750	4.980	0.522	0.606
5.107	4.975	4.625	5.339	5.171	0.324	0.606
2.666	2.897	2.666	2.859	2.285	0.432	0.606
4.050	3.651	4.242	4.439	4.664	0.398	0.606
4.905	4.332	3.333	3.640	3.730	0.495	0.606
6.443	6.614	4.364	6.946	6.741	0.602	0.606
6.433	6.516	5.394	6.664	6.796	0.400	0.606
6.426	5.984	6.696	5.463	5.808	0.360	0.606
4.338	4.593	4.705	4.158	4.359	0.371	0.606
5.136	5.354	3.911	5.237	5.643	0.436	0.606
3.835	3.777	4.207	3.840	3.466	0.467	0.606
5.195	5.846	4.906	5.924	6.420	0.374	0.606
2.820	2.368	2.320	2.290	2.758	0.474	0.606
3.538	4.225	1.970	3.616	4.022	0.525	0.606
5.241	4.379	6.187	4.255	4.119	0.638	0.606
3.497	5.447	2.522	5.132	5.239	0.721	0.606
8.020	7.294	7.705	7.631	7.462	0.338	0.606
6.785	6.483	7.566	5.887	6.217	0.405	0.606
2.975	2.903	2.300	2.494	1.892	0.450	0.606
6.215	6.396	4.936	6.811	6.599	0.434	0.606
5.907	6.305	4.796	6.086	6.116	0.378	0.606
1.478	4.871	1.100	5.476	6.102	1.072	0.606
4.182	3.924	4.524	3.193	3.885	0.585	0.606
6.759	6.507	3.738	6.594	6.173	0.735	0.606
7.522	8.675	7.471	8.880	8.562	0.484	0.606
4.804	5.273	3.822	5.512	5.487	0.441	0.606
3.854	4.165	3.781	4.475	4.889	0.485	0.606

7.732	7.515	8.444	7.460	7.274	0.421	0.606
3.697	5.018	3.043	5.026	4.888	0.492	0.606
2.862	2.708	3.383	1.839	1.967	0.474	0.606
3.341	3.044	2.911	2.898	2.844	0.320	0.606
3.061	3.522	2.602	1.787	3.482	0.594	0.606
5.935	5.994	6.690	5.635	5.063	0.383	0.606
2.113	1.596	2.721	2.221	2.452	0.465	0.606
4.421	5.345	4.692	6.046	5.490	0.488	0.606
3.864	3.914	3.807	3.251	3.617	0.378	0.606
4.038	4.086	3.788	4.820	4.014	0.410	0.606
5.119	5.473	5.077	5.858	5.878	0.366	0.606
3.937	3.090	5.031	2.514	3.802	0.614	0.606
6.282	4.173	5.849	2.981	3.171	0.912	0.606
5.430	5.699	4.776	5.729	5.327	0.351	0.606
7.153	6.110	7.472	6.189	6.226	0.381	0.606
5.953	6.266	6.039	6.585	6.657	0.322	0.606
6.609	7.127	7.240	6.975	7.473	0.453	0.606
3.505	3.761	1.628	4.248	4.095	0.706	0.606
4.918	3.467	3.377	2.456	3.308	0.570	0.606
4.648	5.670	4.180	6.269	5.530	0.513	0.606
5.438	4.854	5.042	5.280	5.464	0.397	0.606
2.209	3.068	2.873	1.934	2.034	0.758	0.606
5.663	5.174	3.120	5.464	5.720	0.629	0.606
6.551	6.963	5.603	7.143	7.280	0.463	0.606
2.841	4.077	2.335	3.135	4.783	0.574	0.606
3.938	4.790	2.853	4.914	4.686	0.543	0.606
0.904	5.569	1.006	5.651	6.285	1.235	0.606
7.188	7.081	7.361	6.419	6.866	0.361	0.606
5.274	5.829	4.991	5.871	6.419	0.414	0.606
4.506	4.569	5.027	2.985	3.645	0.662	0.606
4.266	5.329	4.783	5.006	5.339	0.426	0.606
5.013	4.596	5.089	3.677	4.024	0.428	0.606
4.404	4.513	5.186	4.300	4.721	0.363	0.606
5.436	6.164	4.215	6.477	6.756	0.611	0.606
2.907	2.638	2.407	3.000	3.191	0.489	0.606
5.087	5.336	4.678	5.549	5.553	0.357	0.606
3.085	2.358	2.453	1.887	2.438	0.440	0.606
5.213	5.356	3.661	5.658	5.534	0.467	0.606
7.512	7.550	6.201	7.825	8.074	0.454	0.606
3.995	3.428	2.316	3.935	4.210	0.491	0.606
3.880	4.344	4.471	3.969	4.864	0.414	0.606
3.702	4.067	4.445	3.760	3.981	0.485	0.606
6.595	5.827	6.980	5.651	5.913	0.378	0.606
5.374	5.834	3.828	5.705	5.782	0.444	0.606
6.634	6.647	6.193	6.879	7.132	0.312	0.606
5.422	6.468	5.072	6.884	6.668	0.454	0.606
2.450	2.637	2.307	2.806	2.708	0.442	0.606
5.433	6.017	5.563	6.058	5.793	0.311	0.606
2.234	4.139	2.423	2.168	3.158	0.857	0.606
3.028	5.874	3.249	6.220	6.218	0.740	0.606

6.480	7.510	6.491	7.533	7.387	0.378	0.606
7.368	6.674	2.782	8.044	6.633	1.277	0.606
5.740	5.357	4.014	5.827	5.766	0.525	0.606
5.314	4.378	3.075	4.879	5.024	0.558	0.606
8.161	6.852	8.799	6.992	7.141	0.513	0.606
4.974	3.752	4.922	3.336	3.693	0.497	0.606
5.253	4.186	3.667	3.502	5.065	0.547	0.606
3.329	2.489	2.342	2.801	3.337	0.526	0.606
0.829	5.031	1.256	4.606	4.074	0.960	0.606
2.330	2.944	2.944	3.103	3.609	0.470	0.606
4.947	5.184	3.582	5.151	5.052	0.505	0.606
5.080	6.020	5.548	6.688	5.349	0.668	0.606
5.189	5.405	4.696	5.482	5.633	0.333	0.606
6.496	5.899	6.701	5.462	5.828	0.488	0.606
3.137	3.802	3.956	3.910	3.629	0.494	0.606
2.770	2.475	2.361	2.886	3.430	0.449	0.606
4.808	5.308	5.875	5.324	5.795	0.366	0.606
3.758	4.437	4.167	4.477	4.420	0.401	0.606
3.766	3.072	4.201	2.946	3.309	0.419	0.606
4.581	4.483	5.772	4.500	4.553	0.418	0.606
3.749	3.256	3.524	3.569	3.681	0.344	0.606
6.035	6.180	6.088	6.240	6.280	0.350	0.606
8.293	7.814	8.752	7.565	8.268	0.431	0.606
2.463	4.451	4.296	3.414	4.941	0.559	0.606
1.930	2.751	2.023	3.011	3.057	0.370	0.606
5.119	5.636	5.542	5.704	5.745	0.371	0.606
3.898	2.786	4.234	2.236	2.240	0.653	0.606
3.859	4.129	3.170	4.974	4.890	0.556	0.606
3.740	3.329	3.398	3.564	4.679	0.501	0.606
1.737	2.380	2.728	1.838	2.141	0.566	0.606
2.927	3.254	3.452	2.758	2.607	0.455	0.606
4.199	3.495	3.873	3.171	3.923	0.446	0.606
4.069	4.529	3.497	3.963	4.093	0.399	0.606
5.999	7.976	4.451	8.396	7.909	0.808	0.606
3.043	3.676	3.686	2.887	2.941	0.412	0.606
4.249	3.914	3.146	4.192	3.902	0.419	0.606
4.221	4.841	4.634	4.135	4.082	0.355	0.606
5.687	6.522	6.171	6.638	6.356	0.362	0.606
4.607	4.706	4.066	4.968	5.188	0.478	0.606
3.699	6.523	2.404	6.703	6.296	0.864	0.606
2.934	0.994	1.959	2.512	2.438	0.520	0.606
3.277	3.006	4.079	2.049	1.788	0.581	0.606
3.575	3.113	4.180	3.988	3.404	0.472	0.606
5.080	4.246	6.426	3.454	3.651	0.662	0.606
1.562	2.050	2.286	1.514	0.945	0.591	0.606
5.314	4.960	4.997	4.940	5.261	0.419	0.606
2.452	2.573	3.441	3.505	3.473	0.483	0.606
5.077	5.768	5.127	6.042	5.653	0.361	0.606
4.515	3.597	6.538	3.608	3.859	0.788	0.606
6.181	5.207	6.492	4.896	5.407	0.447	0.606

3.175	4.432	3.550	4.510	4.647	0.400	0.606
4.480	4.109	5.529	3.823	4.630	0.411	0.606
3.907	5.010	4.406	4.826	5.490	0.666	0.606
4.497	5.028	4.901	4.111	4.573	0.388	0.606
3.674	3.350	4.496	3.464	3.933	0.429	0.606
3.629	4.515	4.340	4.504	4.417	0.415	0.606
3.663	4.521	4.220	4.531	4.439	0.415	0.606
3.270	5.520	3.557	5.770	5.496	0.643	0.606
4.922	3.861	5.150	2.668	3.594	0.533	0.606
3.313	2.765	3.274	2.825	3.362	0.423	0.606
6.407	6.531	6.233	6.772	6.913	0.319	0.606
5.022	5.610	4.660	5.734	5.988	0.451	0.606
7.210	6.895	7.321	6.277	5.665	0.431	0.606
5.884	6.482	6.302	5.552	5.799	0.395	0.606
4.549	4.471	5.227	3.621	4.432	0.443	0.606
4.116	2.430	3.858	2.817	2.277	0.598	0.606
3.202	2.108	2.329	3.174	2.169	0.451	0.606
2.225	2.701	2.704	2.840	2.680	0.445	0.606
3.419	3.433	2.300	3.003	3.063	0.572	0.606
5.015	5.889	4.956	5.273	5.358	0.360	0.606
4.231	4.133	4.930	3.626	3.915	0.726	0.606
2.341	2.336	2.603	2.977	2.874	0.461	0.606
2.515	1.433	2.728	1.646	2.564	0.542	0.606
2.738	3.683	4.002	3.222	3.597	0.469	0.606
3.957	1.685	3.538	2.765	2.809	0.737	0.606
2.758	3.624	3.584	4.484	2.680	0.646	0.606
4.010	4.877	4.477	3.850	3.962	0.552	0.606
4.709	6.419	3.041	6.687	6.965	0.858	0.606
3.447	2.814	5.079	3.862	3.816	0.816	0.606
3.026	3.973	4.676	3.280	3.673	0.533	0.606
3.667	4.116	4.711	2.145	2.512	0.604	0.606
3.071	2.723	1.825	2.614	1.994	0.553	0.606
3.594	3.800	3.411	3.431	3.098	0.523	0.606
2.659	2.872	3.746	2.403	3.029	0.439	0.606
4.769	4.804	5.303	3.698	4.528	0.454	0.606
4.357	2.546	4.875	3.779	3.967	0.692	0.606
6.892	5.649	6.808	6.717	6.533	0.524	0.606
2.387	3.737	3.046	3.559	3.619	0.513	0.606
3.086	3.191	2.054	3.867	3.383	0.485	0.606
2.082	2.231	1.636	3.057	3.350	0.449	0.606
2.521	3.331	3.137	2.880	2.901	0.437	0.606
1.710	2.949	1.824	1.889	2.034	0.590	0.606
2.430	3.094	2.159	2.436	2.708	0.600	0.606
3.970	2.337	4.360	3.056	2.785	0.696	0.606
3.404	3.205	2.698	3.022	2.092	0.470	0.606
4.099	3.907	3.736	4.128	4.363	0.388	0.606
1.850	2.288	3.574	1.486	2.187	0.863	0.606
5.223	4.367	6.247	4.249	4.588	0.499	0.606
2.395	2.718	4.995	2.937	4.528	0.729	0.606
2.621	3.038	2.615	3.629	2.797	0.475	0.606

4.501	3.610	4.191	3.877	3.823	0.433	0.606
3.252	2.402	2.089	1.992	2.667	0.502	0.606
4.608	3.672	4.445	3.444	3.455	0.428	0.606
4.306	3.574	3.839	3.334	2.891	0.480	0.606
2.546	2.490	2.513	2.897	2.012	0.565	0.606
5.023	4.746	4.709	5.150	5.601	0.589	0.606
2.292	4.360	2.769	2.748	2.282	0.684	0.606
5.531	3.889	7.673	3.673	4.250	0.862	0.606
3.410	2.507	5.086	2.850	2.790	0.554	0.606
2.846	1.858	3.006	2.655	2.389	0.446	0.606
3.031	3.187	2.577	3.412	3.605	0.392	0.606
3.265	2.932	5.182	3.007	3.708	0.578	0.606
1.741	1.495	1.289	2.491	1.743	0.422	0.606
1.115	1.794	1.881	2.913	2.117	0.523	0.606
2.537	2.782	1.838	2.841	3.340	0.487	0.606
1.255	2.435	4.526	2.290	2.701	0.678	0.606
3.308	3.853	4.703	4.520	4.109	0.637	0.606
1.944	3.736	2.648	2.632	2.007	0.464	0.606
4.962	3.642	5.356	3.656	3.830	0.673	0.606
3.047	2.700	3.314	3.299	3.169	0.429	0.606
2.833	2.597	2.098	2.744	1.436	0.623	0.606
3.645	2.765	3.645	3.718	3.697	0.632	0.606
3.079	2.209	4.039	2.319	2.049	0.485	0.606
3.368	5.408	4.260	3.632	3.864	0.600	0.606
1.382	0.566	1.349	0.946	1.350	0.595	0.606
3.383	3.656	3.102	2.826	3.604	0.430	0.606
2.056	3.633	2.388	2.662	3.458	0.563	0.606
2.767	2.036	3.968	3.032	2.721	0.586	0.606
3.742	2.792	3.065	3.016	3.074	0.454	0.606
4.547	4.361	4.504	4.689	4.734	0.424	0.606
2.917	3.792	2.622	3.168	4.032	0.443	0.606
2.720	2.821	1.742	3.089	3.139	0.585	0.606
2.895	2.927	3.551	3.611	3.985	0.508	0.606
3.360	3.452	3.168	3.814	3.360	0.454	0.606
2.107	3.574	2.507	3.819	3.769	0.605	0.606
2.521	2.532	2.580	2.313	2.896	0.478	0.606
3.473	3.663	3.257	4.076	3.727	0.478	0.606
3.941	4.489	4.478	4.484	4.253	0.477	0.606
3.570	3.835	4.692	3.485	2.536	0.505	0.606
3.365	3.768	3.835	3.960	4.104	0.521	0.606
2.813	3.779	3.315	3.117	2.892	0.631	0.606
3.606	5.569	4.592	5.122	3.954	0.529	0.606
0.923	3.073	1.653	1.857	2.408	0.503	0.606
3.360	3.272	3.064	3.198	2.734	0.621	0.606
4.085	3.099	4.060	3.608	3.262	0.528	0.606
3.567	4.880	4.104	4.254	3.921	0.389	0.606
3.199	2.086	4.545	2.365	2.543	0.585	0.606
3.587	3.788	2.887	3.420	3.556	0.442	0.606
1.807	2.956	2.818	2.826	3.534	0.514	0.606
3.057	3.228	4.151	3.498	2.634	0.523	0.606

2.417	2.779	2.798	2.812	2.790	0.468	0.606
4.708	5.385	5.888	5.808	5.457	0.445	0.606
2.154	2.444	1.619	1.791	2.177	0.501	0.606
2.657	2.003	1.910	2.324	2.242	0.423	0.606
4.057	3.721	3.868	3.516	3.003	0.394	0.606
2.930	1.952	2.440	2.923	1.999	0.506	0.606
5.044	3.286	4.119	3.083	2.958	0.553	0.606
5.485	5.243	5.420	4.330	4.720	0.698	0.606
4.117	3.759	4.433	4.031	4.214	0.510	0.606
1.809	1.598	3.151	3.628	1.691	0.742	0.606
2.995	2.123	2.370	1.725	2.530	0.543	0.606
2.829	2.957	2.770	2.908	2.776	0.410	0.606
2.331	2.650	2.668	2.245	1.988	0.466	0.606
3.840	2.883	3.700	3.689	3.981	0.531	0.606
1.977	1.494	1.913	1.356	1.980	0.461	0.606
6.242	5.280	5.537	5.924	5.377	0.535	0.606
3.178	4.041	3.670	3.469	3.156	0.505	0.606
3.412	2.454	2.535	2.478	1.586	0.677	0.606
1.896	4.316	3.390	2.808	2.215	0.747	0.606
3.221	2.927	3.549	2.936	2.278	0.525	0.606
1.083	3.082	1.844	1.810	1.916	0.744	0.606
2.158	2.914	2.979	3.177	2.124	0.507	0.606
2.444	2.368	2.672	3.272	2.231	0.443	0.606
3.644	3.103	3.775	2.313	3.827	0.557	0.606
4.514	3.213	1.701	4.224	3.910	0.618	0.606
1.398	1.795	2.254	1.412	1.784	0.486	0.606
5.158	4.563	5.566	4.664	4.390	0.538	0.606
6.280	4.038	3.280	4.635	3.655	0.862	0.606
4.051	1.735	3.658	1.861	1.112	0.769	0.606
6.478	5.886	6.084	6.548	6.400	0.497	0.606
6.429	6.078	6.317	6.125	5.833	0.682	0.606
2.981	3.652	2.720	2.587	3.477	0.546	0.606
4.127	3.820	3.353	4.214	3.890	0.455	0.606
2.866	2.981	4.197	3.952	3.380	0.432	0.606
3.095	2.444	2.006	3.129	1.706	0.517	0.606
3.924	3.605	3.464	3.924	4.095	0.471	0.606
2.867	3.010	2.616	2.764	1.734	0.593	0.606
2.022	2.818	1.181	3.025	3.789	0.661	0.606
2.356	3.111	1.572	4.318	3.957	0.575	0.606
3.045	2.382	1.969	2.940	2.269	0.557	0.606
4.290	3.734	5.150	2.869	2.392	0.646	0.606
2.737	3.817	3.280	3.554	2.580	0.736	0.606
0.888	3.198	3.238	3.773	3.257	0.710	0.606
1.931	3.295	2.470	2.382	2.176	0.637	0.606
6.362	5.785	7.090	6.045	5.783	0.436	0.606
3.639	4.042	1.470	3.647	2.009	0.886	0.606
3.856	4.806	4.991	5.184	4.698	0.417	0.606
7.542	6.588	7.500	5.908	6.533	0.570	0.606
3.497	3.628	3.674	3.195	3.214	0.438	0.606
3.833	3.469	4.369	3.017	4.028	0.622	0.606

1.844	2.235	3.386	1.777	3.031	0.618	0.606
1.076	2.603	2.787	1.561	2.269	0.519	0.606
3.408	2.703	3.060	3.592	2.607	0.567	0.606
0.864	1.569	1.508	1.037	1.093	0.609	0.606
4.143	5.249	5.235	4.560	4.377	0.401	0.606
5.161	4.253	5.063	3.707	3.773	0.554	0.606
3.049	3.119	3.827	2.633	2.766	0.492	0.606
2.758	2.770	3.170	2.448	3.150	0.560	0.606
3.960	4.942	3.080	5.395	5.170	0.495	0.606
1.496	1.755	1.140	2.064	1.966	0.627	0.606
2.853	3.119	2.546	2.810	2.810	0.574	0.606
3.134	3.986	4.402	3.844	3.262	0.684	0.606
3.839	3.376	4.310	3.029	3.444	0.810	0.606
2.475	3.153	3.362	1.507	1.925	0.706	0.606
3.758	3.495	4.376	3.960	3.207	0.432	0.606
2.771	3.232	3.587	3.553	3.926	0.589	0.606
2.678	2.954	2.643	2.166	2.134	0.398	0.606
2.075	2.525	2.409	1.309	1.968	0.540	0.606
2.358	2.291	2.763	2.099	2.635	0.469	0.606
3.465	2.354	2.798	2.593	2.163	0.456	0.606
2.317	2.749	4.082	2.973	2.595	0.681	0.606
1.253	1.940	1.943	1.999	2.174	0.548	0.606
4.265	4.286	4.625	4.272	3.997	0.437	0.606
2.736	2.662	2.764	3.173	2.748	0.417	0.606
3.679	2.857	3.969	3.777	3.289	0.470	0.606
4.013	3.278	4.149	3.745	3.007	0.505	0.606
4.825	3.156	1.816	1.623	2.853	0.801	0.606
4.608	2.661	3.597	2.358	3.221	0.703	0.606
4.531	2.610	3.620	2.245	3.029	0.667	0.606
3.612	2.164	2.250	2.673	1.868	0.519	0.606
6.239	4.968	7.277	4.852	3.865	0.814	0.606
3.146	2.091	2.641	3.233	3.401	0.480	0.606
4.197	1.909	2.610	3.359	4.121	0.610	0.606
2.154	2.167	2.649	2.969	2.752	0.465	0.606
5.700	6.112	7.195	6.521	6.549	0.567	0.606
4.270	3.576	4.795	4.198	3.941	0.438	0.606
3.096	2.296	2.657	2.795	3.394	0.459	0.606
4.625	4.533	5.003	4.423	4.794	0.418	0.606
6.095	4.534	5.310	4.296	4.348	0.616	0.606
4.366	2.043	3.671	1.648	1.611	0.712	0.606
4.407	4.300	5.397	3.459	3.924	0.679	0.606
2.163	2.560	1.831	2.643	2.174	0.411	0.606
3.591	2.264	2.752	2.345	3.581	0.496	0.606
4.422	3.643	4.292	3.815	3.657	0.449	0.606
5.332	6.264	4.298	6.613	6.378	0.593	0.606
3.804	2.691	3.882	2.847	2.800	0.557	0.606
3.327	2.552	2.051	2.300	2.525	0.634	0.606
2.821	2.521	2.963	3.234	3.304	0.562	0.606
1.928	3.252	1.797	4.209	4.144	0.586	0.606
2.723	4.229	4.663	3.756	3.407	0.742	0.606

1.867	3.370	1.777	1.789	2.650	0.563	0.606
5.380	2.977	5.778	3.178	3.346	0.711	0.606
3.558	4.075	4.258	3.833	4.686	0.601	0.606
3.597	3.677	3.294	3.288	2.725	0.516	0.606
4.885	5.017	5.118	4.624	4.373	0.401	0.606
2.282	1.720	1.739	2.083	2.053	0.589	0.606
1.916	2.375	1.015	2.644	3.584	0.659	0.606
3.155	1.669	4.132	3.174	2.882	0.601	0.606
2.617	3.090	1.910	2.235	2.056	0.469	0.606
4.692	4.241	4.736	3.361	3.843	0.488	0.606
2.197	2.551	3.159	2.377	2.926	0.474	0.606
4.244	6.201	3.577	6.571	6.059	0.777	0.606
3.761	3.621	3.393	4.125	2.812	0.478	0.606
2.490	4.028	3.528	4.193	4.013	0.447	0.606
1.236	1.830	2.341	1.781	1.006	0.556	0.606
2.639	2.338	2.316	2.150	2.392	0.422	0.606
4.653	5.195	2.499	6.030	5.907	0.973	0.606
2.539	2.227	2.840	1.286	3.432	0.684	0.606
3.339	2.678	3.038	1.568	3.514	0.602	0.606
3.758	3.733	4.502	3.362	4.228	0.472	0.606
1.639	2.625	2.106	2.970	2.950	0.639	0.606
5.629	4.652	5.607	5.296	4.817	0.411	0.606
4.292	3.725	4.415	3.101	3.352	0.434	0.606
1.879	2.771	2.498	0.431	3.102	0.688	0.606
2.809	2.374	2.425	3.025	2.809	0.442	0.606
3.472	2.614	3.969	2.572	2.349	0.425	0.606
6.254	6.651	6.101	7.130	7.050	0.325	0.606
7.133	9.496	6.650	9.763	9.663	0.778	0.606
4.980	5.799	6.430	5.794	5.497	0.591	0.606
6.083	6.792	6.421	6.718	7.001	0.320	0.606
3.939	3.539	5.084	3.187	3.053	0.512	0.606
3.734	4.642	3.232	4.818	5.120	0.471	0.606
8.343	8.389	8.515	8.046	8.664	0.339	0.606
2.260	4.887	2.520	5.451	5.012	0.961	0.606
4.624	3.869	4.612	3.739	4.624	0.518	0.606
8.736	9.184	6.248	9.390	9.397	0.677	0.606
5.847	6.376	6.074	6.695	6.464	0.340	0.606
3.780	4.248	3.742	3.543	3.930	0.462	0.606
3.684	4.302	4.020	4.457	4.480	0.342	0.606
5.964	5.795	6.205	5.067	4.759	0.395	0.606
6.308	7.885	7.103	7.757	7.546	0.478	0.606
5.457	4.467	4.907	4.918	4.840	0.536	0.606
1.874	2.209	2.001	2.300	2.220	0.790	0.606
3.805	2.806	2.659	2.972	2.606	0.362	0.606
4.055	4.552	5.061	3.260	3.667	0.723	0.606
5.862	6.379	5.686	6.956	6.882	0.435	0.606
3.282	4.495	3.535	4.926	4.403	0.456	0.606
4.370	3.327	3.831	3.613	3.911	0.497	0.606
4.294	4.954	2.549	5.729	5.425	0.735	0.606
2.342	2.253	2.655	1.879	1.712	0.498	0.606

5.509	5.204	6.182	4.788	4.451	0.427	0.606
3.099	2.942	2.616	3.611	4.014	0.482	0.606
1.555	4.913	5.393	1.992	2.918	0.912	0.606
2.297	2.980	2.224	2.301	2.714	0.630	0.606
2.919	2.295	3.382	2.195	2.479	0.432	0.606
4.449	5.109	3.887	5.183	4.866	0.510	0.606
3.889	4.184	3.741	3.081	3.685	0.558	0.606
4.801	4.923	4.316	4.759	5.058	0.443	0.606
6.909	7.289	5.192	7.382	7.537	0.512	0.606
7.236	7.549	7.741	7.325	7.100	0.351	0.606
7.940	6.741	8.937	6.888	6.328	0.588	0.606
3.205	2.183	3.084	1.814	1.906	0.528	0.606
8.925	7.269	9.448	5.735	6.451	0.837	0.606
8.163	8.682	8.161	8.726	8.835	0.316	0.606
4.224	4.453	3.075	4.508	4.742	0.518	0.606
8.922	7.807	9.940	5.923	6.350	0.854	0.606
9.004	7.808	10.052	5.963	6.330	0.862	0.606
3.843	4.166	4.499	3.428	4.318	0.451	0.606
3.232	2.547	2.157	2.903	3.591	0.472	0.606
5.043	4.404	4.937	4.624	4.482	0.405	0.606
4.585	3.642	5.535	4.136	3.809	0.458	0.606
3.014	3.306	3.038	3.387	3.542	0.403	0.606
2.005	3.345	2.058	3.475	3.251	0.447	0.606
3.740	4.958	3.275	5.439	5.089	0.519	0.606
4.362	4.521	4.575	5.361	5.300	0.493	0.606
6.691	5.707	7.366	4.892	5.652	0.541	0.606
2.522	5.430	6.854	5.554	6.562	0.908	0.606
7.091	7.800	6.744	7.942	8.049	0.402	0.606
4.515	4.079	4.023	3.427	4.953	0.601	0.606
4.824	4.680	5.481	5.372	5.197	0.338	0.606
5.364	3.988	4.037	4.242	4.298	0.443	0.606
7.109	7.437	6.402	7.962	8.009	0.403	0.606
3.742	5.070	2.756	5.135	5.389	0.599	0.606
4.206	6.822	3.196	6.898	6.793	0.965	0.606
4.696	4.133	2.730	4.492	4.502	0.504	0.606
2.396	2.807	2.631	2.504	2.231	0.433	0.606
2.342	1.974	2.278	2.806	2.683	1.125	0.606
2.806	3.225	2.763	2.045	3.141	0.580	0.606
3.607	3.838	3.418	3.302	3.506	0.380	0.606
5.525	5.611	2.410	6.206	5.722	0.790	0.606
2.660	4.929	4.250	4.993	5.968	0.710	0.606
2.408	4.717	4.080	4.770	5.728	0.722	0.606
3.869	3.927	4.514	2.970	3.427	0.482	0.606
3.416	4.033	3.248	3.898	4.474	0.445	0.606
6.929	7.172	7.212	7.891	7.606	0.344	0.606
4.091	4.336	4.508	4.771	4.187	0.420	0.606
4.116	3.126	2.552	3.182	3.602	0.435	0.606
5.255	5.046	4.704	4.305	4.870	0.479	0.606
6.266	6.196	6.855	5.901	7.079	0.462	0.606
4.112	4.023	3.747	4.932	4.372	0.401	0.606

6.644	6.368	5.741	6.793	7.218	0.370	0.606
6.239	6.093	6.229	6.369	6.775	0.312	0.606
5.513	5.966	5.424	5.901	6.268	0.432	0.606
6.103	6.939	6.111	6.827	6.994	0.395	0.606
4.602	4.691	4.687	4.880	5.112	0.377	0.606
6.315	6.496	6.141	6.567	6.713	0.320	0.606
5.765	5.986	5.953	6.524	5.977	0.341	0.606
6.063	6.929	6.594	7.574	7.472	0.414	0.606
3.604	4.162	3.809	4.467	4.151	0.380	0.606
5.202	5.719	4.731	5.623	5.422	0.340	0.606
9.483	9.974	9.096	10.019	10.331	0.332	0.606
6.167	7.001	6.732	7.445	7.382	0.353	0.606
4.333	4.051	5.349	4.243	4.071	0.442	0.606
2.763	3.262	2.321	3.192	3.331	0.391	0.606
4.261	4.437	4.495	4.933	4.258	0.506	0.606
5.504	5.911	5.623	5.764	6.301	0.358	0.606
5.162	5.243	4.161	5.530	5.498	0.400	0.606
6.296	6.477	6.964	5.842	6.119	0.406	0.606
3.235	2.447	4.722	2.332	2.429	0.705	0.606
2.254	2.626	3.158	0.809	2.841	0.644	0.606
3.163	3.270	4.035	2.248	2.600	0.608	0.606
4.811	5.038	5.868	2.814	4.270	0.728	0.606
5.660	5.787	6.238	6.134	6.229	0.311	0.606
6.147	6.234	5.868	6.644	6.460	0.368	0.606
3.248	2.676	3.049	2.383	2.430	0.407	0.606
4.036	3.538	3.892	2.982	3.032	0.448	0.606
7.446	6.421	7.157	6.811	6.928	0.408	0.606
2.054	3.403	2.205	2.868	2.849	0.542	0.606
7.651	7.269	6.928	6.378	7.259	0.522	0.606
8.952	8.731	8.862	7.266	8.012	0.485	0.606
1.972	1.705	2.355	1.607	1.794	0.444	0.606
4.215	3.173	2.972	4.464	4.202	0.567	0.606
3.108	1.902	2.075	1.838	2.044	0.408	0.606
2.360	2.428	4.046	2.525	2.287	0.638	0.606
3.209	2.540	3.007	3.046	3.149	0.436	0.606
2.836	6.062	3.662	3.312	3.650	1.103	0.606
6.327	6.572	7.155	6.386	5.798	0.446	0.606
4.132	3.320	4.928	4.885	4.594	1.024	0.606
7.659	4.484	7.520	7.101	6.397	0.878	0.606
6.323	5.607	6.974	7.112	6.155	0.801	0.606
1.654	3.074	3.075	2.825	2.035	0.762	0.606
2.998	4.632	2.339	2.821	3.533	0.632	0.606
6.803	8.591	8.050	7.543	7.001	0.573	0.606
9.126	9.593	10.124	9.263	8.385	0.503	0.606
6.610	6.733	6.964	6.418	5.655	0.440	0.606
3.460	4.253	3.605	4.902	4.552	0.392	0.606
4.510	4.658	4.581	4.520	4.796	0.345	0.606
4.253	4.089	3.763	4.380	4.173	0.446	0.606
4.548	4.993	4.601	4.580	5.357	0.379	0.606
2.971	3.626	2.548	3.668	3.920	0.667	0.606

3.446	2.798	3.910	2.178	2.186	0.465	0.606
4.198	4.514	4.824	3.098	3.634	0.541	0.606
4.431	4.045	4.801	3.888	3.828	0.446	0.606
9.326	7.691	9.490	7.394	7.367	0.571	0.606
4.117	4.879	4.932	3.863	4.516	0.491	0.606
6.324	7.002	6.368	7.052	6.952	0.322	0.606
7.345	7.732	7.275	7.645	7.621	0.302	0.606
6.950	6.742	7.116	7.005	7.005	0.349	0.606
6.100	6.074	6.339	6.221	6.631	0.440	0.606
5.534	5.680	5.255	5.701	5.785	0.350	0.606
3.498	3.378	3.876	2.575	2.971	0.493	0.606
4.445	4.838	5.106	5.044	4.552	0.349	0.606
6.543	6.401	6.120	6.804	6.620	0.318	0.606
3.978	4.023	3.457	4.076	4.157	0.389	0.606
3.664	3.036	4.553	3.014	4.146	0.570	0.606
6.674	6.517	6.827	5.999	6.066	0.431	0.606
6.998	6.069	7.629	6.192	6.332	0.420	0.606
7.883	7.370	7.741	7.574	7.357	0.353	0.606
10.317	10.499	9.900	9.455	9.456	0.452	0.606
4.243	3.630	3.786	5.057	4.518	1.036	0.606
7.561	7.397	6.893	6.976	4.581	0.883	0.606
7.842	7.820	7.270	7.795	7.857	0.562	0.606
6.048	5.387	5.611	6.072	5.800	1.058	0.606
6.890	6.628	6.607	6.546	7.214	0.711	0.606
9.690	9.052	9.212	9.474	9.463	0.571	0.606
8.010	8.507	6.618	7.150	7.404	0.640	0.606
4.035	4.076	2.610	3.965	3.481	0.611	0.606
8.288	8.910	8.218	7.263	7.996	0.656	0.606
6.461	6.319	6.093	6.047	6.394	0.863	0.606
7.860	7.738	7.440	7.524	7.327	0.566	0.606
9.777	9.440	9.265	9.320	9.355	0.498	0.606
3.152	3.991	3.715	2.650	4.780	0.691	0.606
5.089	4.701	4.960	5.082	5.262	0.555	0.606
6.084	5.413	6.861	4.658	5.005	0.485	0.606
4.058	4.715	2.403	5.530	5.267	0.738	0.606
6.054	6.197	5.574	6.645	6.935	0.415	0.606
3.190	3.157	2.985	2.400	2.871	0.397	0.606
4.163	2.879	2.571	3.308	4.004	0.571	0.606
3.754	5.567	4.050	7.020	5.630	0.922	0.606
4.666	3.711	5.634	4.082	4.222	0.453	0.606
7.485	6.234	7.580	5.308	5.697	0.547	0.606
5.618	5.390	5.124	4.571	5.175	0.463	0.606
4.917	4.111	4.744	3.591	3.395	0.525	0.606
5.441	5.937	5.804	3.551	4.635	0.653	0.606
3.035	2.550	3.460	2.280	2.662	0.524	0.606
5.650	6.036	6.618	6.579	6.366	0.527	0.606
6.110	5.742	3.662	5.449	6.322	0.499	0.606
4.065	4.294	4.030	3.312	3.752	0.473	0.606
2.508	2.325	2.792	2.606	3.426	0.419	0.606
1.516	1.985	2.899	2.005	1.930	0.499	0.606

2.178	2.970	2.967	2.847	2.993	0.501	0.606
8.275	8.491	7.366	8.943	8.827	0.402	0.606
4.401	3.426	5.314	4.123	3.863	0.463	0.606
2.439	3.773	3.020	2.039	3.119	0.423	0.606
7.180	7.586	5.311	7.892	7.742	0.577	0.606
5.299	6.111	5.434	5.450	5.559	0.697	0.606
5.534	4.642	5.835	4.540	4.637	0.408	0.606
3.491	3.668	3.855	2.778	3.256	0.433	0.606
2.875	2.808	3.627	2.069	2.389	0.584	0.606
3.431	2.617	2.382	2.575	2.651	0.459	0.606
5.753	6.246	5.509	5.753	6.211	0.682	0.606
5.612	5.639	6.530	5.950	5.698	0.362	0.606
4.933	4.922	5.501	4.087	3.511	0.518	0.606
3.622	2.897	2.181	2.023	2.883	0.560	0.606
3.784	3.652	2.528	4.864	3.726	0.588	0.606
4.893	4.065	4.669	3.387	3.631	0.479	0.606
4.476	5.238	3.576	5.719	5.676	0.483	0.606
3.885	3.012	2.719	2.579	2.476	0.987	0.606
4.556	6.221	4.409	6.634	6.541	0.609	0.606
5.576	5.012	5.245	4.571	5.747	0.377	0.606
7.443	6.392	8.475	5.181	5.409	0.715	0.606
6.957	6.362	7.838	5.251	5.447	0.547	0.606
4.911	5.023	5.955	4.070	4.417	0.573	0.606
2.148	3.868	2.911	1.980	2.360	0.866	0.606
8.774	9.238	7.969	9.536	9.457	0.442	0.606
3.141	4.687	3.662	4.547	4.470	0.512	0.606
4.474	3.294	4.189	2.879	3.245	0.571	0.606
7.291	7.508	6.967	7.670	7.677	0.303	0.606
7.755	7.080	8.219	6.039	6.212	0.506	0.606
5.057	5.326	5.489	3.979	5.220	0.585	0.606
5.314	4.078	5.425	4.147	4.219	0.505	0.606
3.051	3.634	2.566	5.014	4.442	0.576	0.606
2.341	2.519	3.202	2.108	2.491	0.439	0.606
3.087	4.021	2.795	4.420	4.407	0.477	0.606
7.250	6.422	7.540	5.014	5.116	0.612	0.606
5.091	5.891	4.374	6.341	6.208	0.474	0.606
6.807	6.748	6.818	7.087	7.168	0.323	0.606
6.293	7.870	3.908	8.553	8.241	0.963	0.606
3.565	4.789	3.412	4.690	4.660	0.442	0.606
4.779	5.715	5.276	5.547	5.696	0.422	0.606
6.672	5.662	6.768	5.571	5.525	0.541	0.606
1.811	3.960	1.658	2.929	2.615	0.703	0.606
6.286	6.675	5.936	6.916	6.785	0.351	0.606
4.504	3.695	3.452	4.234	4.430	0.485	0.606
3.886	3.862	3.652	3.578	3.237	0.425	0.606
3.767	3.870	4.119	3.737	3.499	0.400	0.606
6.593	5.859	6.725	5.025	4.685	0.499	0.606
4.378	3.421	4.364	4.747	2.891	0.559	0.606
7.483	6.721	7.511	6.637	6.248	0.385	0.606
5.003	5.188	5.047	5.346	5.629	0.340	0.606

3.993	2.765	2.720	3.697	3.517	0.573	0.606
5.341	5.169	5.281	4.303	4.856	0.415	0.606
4.705	4.715	5.317	3.818	3.942	0.404	0.606
4.588	5.370	4.030	5.675	5.855	0.462	0.606
5.816	5.984	7.214	5.273	5.858	0.609	0.606
6.751	7.936	7.662	6.841	7.824	0.711	0.606
7.540	8.156	5.691	8.578	8.684	0.681	0.606
7.830	8.175	6.684	8.663	8.430	0.508	0.606
8.705	8.100	5.983	8.360	8.543	0.613	0.606
3.834	3.923	4.958	3.597	3.572	0.417	0.606
8.415	8.890	5.542	9.381	9.417	0.883	0.606
6.581	7.012	7.648	7.451	7.269	0.351	0.606
8.201	8.069	7.398	8.415	8.444	0.354	0.606
4.016	5.406	4.788	2.094	2.894	0.970	0.606
6.096	5.582	6.974	5.092	5.282	0.427	0.606
3.588	4.848	3.247	4.783	3.992	0.438	0.606
7.412	8.021	6.342	7.852	7.970	0.466	0.606
7.005	6.028	6.442	6.072	5.955	0.444	0.606
4.007	5.364	3.966	5.792	5.427	0.509	0.606
2.952	3.146	2.900	1.779	2.460	0.449	0.606
3.417	3.011	2.917	2.248	3.261	0.421	0.606
2.873	2.532	2.707	2.331	1.583	0.499	0.606
7.611	8.206	6.325	8.593	8.471	0.591	0.606
5.918	5.190	5.820	5.065	5.241	0.479	0.606
8.001	7.149	8.061	7.227	7.407	0.339	0.606
7.721	6.899	7.703	7.102	7.157	0.332	0.606
5.961	5.338	5.963	5.214	5.282	0.441	0.606
6.218	5.899	6.995	6.586	6.480	0.357	0.606
3.841	2.091	4.787	1.704	2.364	0.737	0.606
7.905	6.437	8.076	7.635	7.221	0.544	0.606
6.836	5.161	6.800	5.588	6.263	0.548	0.606
8.620	7.630	8.449	7.888	7.836	0.363	0.606
5.181	4.665	6.019	5.754	5.784	0.584	0.606
4.519	3.244	4.551	3.382	3.637	0.478	0.606
3.069	3.196	2.857	4.588	3.808	0.419	0.606
8.797	8.744	9.169	8.824	8.754	0.315	0.606
6.725	7.268	7.140	7.493	7.355	0.589	0.606
5.197	5.278	6.287	4.398	4.741	0.425	0.606
5.668	6.267	5.326	6.188	6.229	0.307	0.606
6.856	7.572	5.987	7.437	7.400	0.503	0.606
5.902	7.163	3.612	7.469	7.400	0.763	0.606
3.880	3.418	4.664	3.629	2.983	0.633	0.606
4.104	5.498	4.567	3.471	4.396	0.657	0.606
4.217	4.400	4.561	3.509	4.169	0.564	0.606
3.694	3.140	4.361	2.811	3.011	0.504	0.606
6.426	7.153	6.675	7.446	7.277	0.373	0.606
9.135	9.650	8.756	10.095	9.881	0.359	0.606
5.264	5.514	5.629	3.559	4.796	0.714	0.606
6.452	4.815	6.528	4.070	4.750	0.589	0.606
4.444	2.281	3.928	1.943	2.017	0.759	0.606

5.945	6.514	5.247	6.414	6.445	0.374	0.606
2.044	3.223	3.504	2.559	2.341	0.943	0.606
6.362	6.399	6.213	7.149	6.560	0.440	0.606
7.862	7.855	7.673	7.911	7.997	0.382	0.606
4.556	5.306	1.556	5.518	5.592	0.785	0.606
2.646	2.372	2.724	2.212	2.868	0.629	0.606
5.002	4.870	4.848	5.139	4.838	1.265	0.606
7.978	8.547	5.074	8.614	8.505	0.771	0.606
8.646	8.293	8.462	7.847	7.713	0.441	0.606
8.279	7.129	9.077	5.974	6.237	0.633	0.606
7.802	8.133	7.151	8.585	8.305	0.365	0.606
7.266	7.699	7.011	7.773	8.014	0.331	0.606
5.749	7.425	6.452	7.452	7.447	0.452	0.606
4.655	4.562	4.118	4.408	4.611	0.370	0.606
3.834	2.774	1.329	2.784	3.104	0.590	0.606
5.164	5.279	5.600	5.544	5.740	0.453	0.606
6.083	5.615	7.106	4.854	5.118	0.481	0.606
6.689	6.018	7.128	6.261	6.308	0.421	0.606
7.673	7.808	6.963	8.201	8.262	0.412	0.606
8.128	8.254	5.903	8.280	8.426	0.598	0.606
6.985	6.442	6.607	6.024	6.059	0.337	0.606
4.296	5.566	2.451	6.274	6.115	0.816	0.606
4.111	2.031	1.884	2.369	2.780	0.565	0.606
4.675	4.679	4.251	4.981	4.653	0.324	0.606
1.782	3.959	2.144	4.023	3.813	0.707	0.606
7.012	7.709	7.147	7.789	7.705	0.307	0.606
3.630	3.637	4.324	3.899	4.236	0.399	0.606
3.254	4.099	3.543	4.522	4.575	0.408	0.606
5.249	5.766	5.276	5.857	5.675	0.308	0.606
4.693	5.416	4.423	5.313	6.045	0.416	0.606
9.933	9.254	10.039	8.362	7.502	0.700	0.606
2.572	2.531	4.324	3.762	3.554	0.641	0.606
2.996	2.829	2.169	2.996	3.312	0.450	0.606
7.167	8.577	8.070	8.864	8.788	0.526	0.606
5.604	6.516	3.984	7.029	6.939	0.712	0.606
5.004	5.105	3.913	5.100	4.587	0.426	0.606
7.942	7.758	6.178	8.083	8.136	0.503	0.606
5.516	5.051	5.989	4.735	4.919	0.340	0.606
5.240	5.084	5.966	4.732	4.974	0.363	0.606
6.745	7.850	5.272	8.263	7.889	0.658	0.606
10.297	7.951	3.947	8.348	9.021	1.405	0.606
11.438	10.932	5.266	10.943	11.164	1.404	0.606
11.075	9.851	6.639	10.290	10.663	1.120	0.606
7.375	8.108	6.513	8.524	8.270	0.526	0.606
1.886	2.025	1.875	3.458	2.426	0.468	0.606
3.109	3.985	2.341	4.697	4.415	0.608	0.606
5.092	4.111	5.926	3.230	3.790	0.654	0.606
3.368	2.649	3.453	2.581	2.701	0.587	0.606
6.696	5.854	7.088	5.924	6.160	0.387	0.606
4.982	5.259	3.230	5.729	5.968	0.762	0.606

5.423	7.377	3.468	7.715	7.381	0.841	0.606
5.447	5.254	6.223	4.531	5.129	0.365	0.606
5.253	4.477	5.441	4.267	4.906	0.413	0.606
6.173	5.549	6.230	5.839	5.613	0.375	0.606
5.594	7.462	3.502	7.834	8.006	0.942	0.606
6.374	7.715	5.542	7.998	7.637	0.685	0.606
4.639	5.711	3.142	6.010	5.575	0.613	0.606
2.366	3.766	2.866	3.354	3.030	0.437	0.606
5.003	5.805	4.706	5.772	5.158	0.366	0.606
1.516	2.563	1.127	2.028	2.782	0.484	0.606
3.116	5.566	2.075	6.442	5.947	0.948	0.606
1.932	5.736	1.741	6.784	6.499	1.213	0.606
6.221	6.919	6.175	6.961	7.051	0.354	0.606
3.578	3.679	3.940	2.844	3.170	0.383	0.606
4.189	4.020	4.208	3.025	3.731	0.396	0.606
4.270	4.017	3.994	3.735	3.830	0.435	0.606
3.274	2.596	3.320	3.203	2.950	0.371	0.606
6.679	6.609	7.503	5.996	6.601	0.372	0.606
2.469	1.870	2.813	1.085	1.421	0.508	0.606
2.505	2.378	2.599	1.406	2.066	0.577	0.606
4.355	5.819	5.950	5.918	4.885	0.777	0.606
2.456	3.841	3.701	3.092	3.115	0.394	0.606
6.570	7.279	7.182	6.829	6.400	0.447	0.606
3.187	3.684	3.229	3.614	3.697	0.392	0.606
4.440	4.328	4.451	3.884	4.334	0.394	0.606
4.052	3.763	2.339	4.284	3.475	0.470	0.606
3.863	6.077	3.767	6.170	5.879	0.684	0.606
3.016	4.172	4.047	2.512	2.492	0.561	0.606
8.950	9.483	8.179	9.711	9.613	0.374	0.606
4.276	4.670	3.891	4.622	3.978	0.430	0.606
2.946	2.850	3.079	3.234	3.591	0.362	0.606
5.663	5.427	5.148	5.064	5.483	0.463	0.606
4.845	4.807	3.110	5.024	5.030	0.471	0.606
4.795	3.776	4.044	4.083	3.829	0.388	0.606
3.268	3.830	2.243	4.340	4.066	0.495	0.606
5.960	7.131	6.007	7.028	6.999	0.386	0.606
4.215	4.524	4.269	3.940	4.646	0.463	0.606
3.246	3.548	4.472	2.884	3.496	0.392	0.606
4.055	5.412	4.184	5.757	5.495	0.471	0.606
4.612	5.216	5.214	4.906	4.880	0.422	0.606
4.775	3.795	5.274	2.881	3.470	0.505	0.606
4.813	4.240	4.634	3.172	3.996	0.429	0.606
5.196	5.454	5.202	5.936	5.634	0.373	0.606
4.354	6.060	3.621	6.395	6.746	0.718	0.606
7.511	7.081	8.739	6.938	6.947	0.426	0.606
5.099	3.439	5.834	2.594	2.057	0.886	0.606
6.034	6.253	5.116	6.157	6.206	0.389	0.606
4.544	4.977	2.520	4.862	5.436	0.720	0.606
4.106	4.909	4.819	4.321	4.278	0.376	0.606
8.631	8.201	9.518	7.839	7.764	0.461	0.606

5.621	5.744	6.266	6.454	6.270	0.424	0.606
2.456	2.615	3.068	2.749	2.458	0.415	0.606
6.146	4.947	6.427	4.500	4.533	0.492	0.606
2.414	3.192	2.788	3.077	2.479	0.437	0.606
4.599	4.666	4.277	5.347	4.862	0.310	0.606
4.321	4.599	3.769	5.228	4.733	0.399	0.606
6.591	5.165	6.601	4.578	4.834	0.626	0.606
2.907	3.394	3.726	3.052	2.606	0.460	0.606
2.724	2.448	2.039	2.108	2.205	0.350	0.606
7.623	6.721	8.533	4.982	5.090	0.724	0.606
4.442	4.954	4.299	4.923	5.169	0.357	0.606
5.879	6.244	5.011	6.499	6.287	0.437	0.606
3.331	3.040	2.485	2.189	1.827	0.456	0.606
8.464	9.956	8.804	10.050	10.164	0.474	0.606
3.261	3.089	3.248	2.284	2.510	0.365	0.606
3.680	2.906	2.949	2.987	3.003	0.471	0.606
2.527	2.675	2.092	2.581	2.674	0.424	0.606
3.846	3.812	4.309	2.865	3.364	0.519	0.606
4.881	4.430	5.128	3.406	3.411	0.522	0.606
3.968	3.796	2.308	4.842	4.304	0.589	0.606
4.099	3.367	3.418	3.941	3.794	0.537	0.606
3.040	3.493	2.835	2.631	3.399	0.438	0.606
7.426	8.577	4.373	8.758	8.754	0.955	0.606
3.277	3.752	4.383	4.371	4.357	0.394	0.606
4.533	4.838	4.228	5.272	4.824	0.351	0.606
2.266	2.183	2.103	3.017	2.492	0.496	0.606
5.550	6.520	3.489	6.942	6.784	0.843	0.606
2.543	1.966	2.044	2.482	2.141	0.624	0.606
7.238	8.318	7.078	8.258	8.251	0.370	0.606
5.754	6.469	6.149	5.915	6.155	0.379	0.606
3.946	3.717	4.056	3.878	3.769	0.441	0.606
1.686	2.180	1.717	1.958	1.329	0.428	0.606
3.740	4.648	4.360	3.878	4.282	0.413	0.606
5.897	6.972	4.437	7.673	7.224	0.637	0.606
4.352	4.741	4.246	4.517	4.878	0.410	0.606
7.615	7.428	7.005	7.940	7.845	0.342	0.606
9.573	9.619	9.071	10.117	9.953	0.331	0.606
9.556	9.545	9.005	10.117	9.929	0.339	0.606
4.163	3.811	4.407	3.396	3.794	0.460	0.606
4.161	4.919	3.461	4.764	5.121	0.416	0.606
2.367	3.060	1.360	3.612	2.480	0.540	0.606
3.774	2.816	3.576	2.943	4.247	0.480	0.606
3.479	2.455	2.783	2.948	3.314	0.450	0.606
6.710	6.095	7.128	5.921	5.969	0.414	0.606
5.363	4.148	6.261	5.690	5.308	0.529	0.606
6.722	6.135	7.194	6.762	6.606	0.385	0.606
4.700	3.738	4.066	4.055	4.104	0.477	0.606
4.166	5.047	2.832	6.157	7.075	1.230	0.606
5.109	5.683	4.617	5.772	5.427	0.392	0.606
3.781	4.433	2.668	3.991	4.469	0.507	0.606

2.391	2.502	2.216	2.536	2.393	0.436	0.606
3.126	2.948	3.117	2.343	2.101	0.517	0.606
2.779	2.795	1.657	3.134	2.482	0.413	0.606
6.036	5.186	5.986	5.225	5.289	0.422	0.606
2.013	1.710	3.137	1.423	1.897	0.467	0.606
8.320	7.256	8.828	6.291	6.599	0.548	0.606
8.276	7.973	9.156	6.869	7.643	0.537	0.606
3.939	3.376	3.949	3.452	3.818	0.358	0.606
3.380	2.433	2.710	2.534	2.787	0.497	0.606
9.633	8.477	9.277	8.509	8.320	0.396	0.606
8.894	7.137	8.784	7.631	7.598	0.515	0.606
7.910	7.174	7.354	6.519	6.578	0.419	0.606
9.225	7.982	8.889	8.118	8.000	0.414	0.606
9.684	8.537	9.340	8.552	8.468	0.393	0.606
9.517	8.265	9.215	8.441	8.302	0.391	0.606
5.827	5.461	7.127	4.918	4.523	0.570	0.606
8.031	6.964	7.807	7.124	6.920	0.378	0.606
5.548	4.923	6.104	4.684	4.739	0.497	0.606
4.688	5.652	3.863	5.908	5.562	0.600	0.606
2.798	3.709	2.998	3.659	3.775	0.403	0.606
3.169	3.497	4.140	3.484	3.615	0.501	0.606
2.514	4.547	3.217	3.021	2.588	0.592	0.606
6.088	5.123	4.992	5.714	5.620	0.803	0.606
2.970	2.250	2.294	3.576	3.575	0.486	0.606
8.727	9.234	7.215	9.393	9.436	0.538	0.606
6.113	7.666	3.191	8.054	7.589	0.929	0.606
4.525	7.364	7.771	5.865	5.998	1.043	0.606
5.066	6.619	4.124	6.632	6.783	0.682	0.606
2.335	2.699	2.772	2.765	2.507	0.321	0.606
4.310	5.019	3.745	3.972	4.668	0.623	0.606
4.553	5.369	3.913	6.584	6.240	0.843	0.606
4.224	4.492	2.648	4.762	4.627	0.552	0.606
2.640	2.982	2.077	3.817	3.266	0.480	0.606
5.188	6.013	6.242	5.212	6.309	0.479	0.606
5.550	4.928	3.497	5.988	5.438	0.622	0.606
6.257	7.124	5.760	7.351	7.080	0.480	0.606
6.028	6.732	6.046	6.855	6.859	0.351	0.606
1.345	4.969	4.543	5.955	5.785	1.015	0.606
3.998	4.864	4.065	5.154	5.046	0.366	0.606
2.663	2.801	1.208	3.184	2.854	0.447	0.606
5.464	5.917	4.843	5.942	5.377	0.402	0.606
5.362	6.123	5.672	6.602	6.374	0.380	0.606
4.337	3.724	3.225	3.998	3.304	0.495	0.606
4.773	4.190	4.034	4.174	4.170	0.651	0.606
3.924	3.402	2.503	3.232	2.314	0.725	0.606
5.604	5.808	5.648	6.264	6.143	0.447	0.606
6.601	6.014	6.794	5.488	5.393	0.434	0.606
8.504	8.769	7.732	9.054	8.986	0.360	0.606
2.627	3.494	2.316	3.233	4.214	0.422	0.606
5.498	4.272	2.595	4.501	4.754	0.752	0.606

4.602	2.694	2.566	3.034	2.834	0.517	0.606
3.436	2.016	1.400	2.309	2.878	0.554	0.606
4.046	2.231	2.012	2.617	1.969	0.462	0.606
5.079	5.615	4.683	5.896	5.760	0.365	0.606
4.226	3.278	2.476	4.234	4.110	0.521	0.606
4.458	9.603	5.168	9.247	9.276	1.261	0.606
5.422	4.679	5.327	5.268	5.202	0.447	0.606
6.339	6.403	2.537	6.304	6.506	0.893	0.606
7.950	9.776	6.115	9.927	10.400	0.868	0.606
5.481	6.141	4.679	6.450	6.442	0.544	0.606
5.367	3.964	3.890	4.148	4.392	0.385	0.606
5.544	6.379	3.602	6.764	6.649	0.725	0.606
5.177	6.055	4.082	6.189	6.278	0.578	0.606
7.371	8.126	6.091	8.564	8.431	0.624	0.606
7.255	7.445	5.127	7.730	7.666	0.548	0.606
4.380	4.540	4.351	4.661	2.617	0.650	0.606
9.284	9.113	6.233	9.368	9.492	0.743	0.606
9.174	9.399	6.774	9.615	9.668	0.718	0.606
3.963	3.677	1.384	3.520	3.267	0.538	0.606
6.715	7.850	5.514	7.863	7.595	0.512	0.606
4.978	4.919	6.310	5.363	3.759	0.539	0.606
4.646	4.473	4.333	3.943	3.934	0.379	0.606
3.810	4.110	2.623	4.562	4.752	0.625	0.606
6.430	7.232	6.047	7.461	6.761	0.424	0.606
7.159	6.034	7.346	5.635	6.578	0.458	0.606
5.416	6.899	4.218	6.776	6.770	0.556	0.606
4.779	3.795	4.319	4.477	4.615	0.474	0.606
2.352	2.970	3.106	2.514	2.609	0.479	0.606
4.886	3.300	3.340	3.401	3.442	0.479	0.606
2.390	1.626	1.621	2.314	1.625	0.469	0.606
4.810	6.218	5.152	6.335	6.627	0.498	0.606
4.999	5.473	3.687	6.112	5.993	0.514	0.606
4.993	4.653	4.718	4.727	4.341	0.444	0.606
6.174	5.582	6.621	5.569	6.436	0.499	0.606
6.440	7.899	6.715	8.106	7.683	0.486	0.606
2.681	3.279	3.786	1.872	2.411	0.562	0.606
2.584	3.660	3.965	3.422	3.005	0.389	0.606
4.254	5.148	3.322	4.800	4.716	0.482	0.606
5.005	6.476	3.190	7.102	6.470	0.907	0.606
3.028	2.582	3.612	2.244	2.794	0.433	0.606
3.591	3.725	3.641	2.676	3.613	0.369	0.606
4.472	4.601	4.510	4.378	4.662	0.422	0.606
8.299	8.460	8.068	8.848	8.753	0.303	0.606
9.668	9.598	8.561	10.000	9.686	0.376	0.606
2.295	3.328	2.893	3.587	3.214	0.521	0.606
2.216	3.391	2.070	3.015	3.072	0.433	0.606
2.442	2.987	2.088	2.771	2.684	0.488	0.606
5.045	5.146	4.926	5.433	5.279	0.387	0.606
4.928	5.668	4.100	5.814	5.386	0.449	0.606
5.946	6.179	6.452	6.194	6.504	0.400	0.606

6.953	6.822	7.549	5.217	5.908	0.596	0.606
2.741	3.499	1.872	3.268	3.465	0.608	0.606
1.879	2.552	2.562	2.732	2.245	0.466	0.606
3.773	3.828	3.915	3.707	3.431	0.462	0.606
4.704	3.948	4.220	5.136	4.175	0.534	0.606
2.834	4.486	1.367	5.233	5.206	0.830	0.606
2.159	1.982	2.377	1.681	2.794	0.458	0.606
7.299	6.919	8.147	6.739	6.905	0.426	0.606
5.684	5.663	4.446	5.996	6.522	0.485	0.606
3.653	3.437	2.009	3.813	3.764	0.583	0.606
3.662	5.003	3.814	5.232	4.922	0.543	0.606
2.636	3.842	3.303	3.521	3.213	0.379	0.606
4.030	4.431	3.038	5.350	4.874	0.543	0.606
4.656	5.379	2.075	5.277	5.127	0.826	0.606
4.128	3.980	2.933	3.833	3.736	0.460	0.606
2.417	1.401	1.924	2.111	2.304	0.425	0.606
3.193	3.973	3.429	4.376	4.652	0.440	0.606
4.927	4.336	5.566	4.473	4.489	0.448	0.606
3.679	2.418	3.301	2.896	2.947	0.455	0.606
4.155	3.535	3.946	2.990	3.930	0.467	0.606
3.259	2.779	2.839	2.380	3.045	0.441	0.606
1.878	2.294	2.060	1.744	2.042	0.420	0.606
5.369	5.632	5.096	4.663	5.334	0.467	0.606
2.641	2.968	2.687	2.534	2.077	0.478	0.606
2.888	4.007	2.602	3.939	4.109	0.628	0.606
2.924	4.160	2.885	3.687	4.441	0.520	0.606
3.661	3.810	2.340	3.530	3.787	0.476	0.606
2.911	1.733	3.134	1.841	2.581	0.428	0.606
2.942	3.474	2.690	3.109	3.321	0.429	0.606
6.970	6.985	5.954	7.455	7.430	0.383	0.606
5.664	5.407	4.818	5.631	5.691	0.411	0.606
3.428	3.305	2.612	2.101	2.619	0.521	0.606
7.943	7.011	4.196	7.025	7.201	0.843	0.606
8.570	7.999	8.799	8.051	8.298	0.371	0.606
3.426	2.973	3.381	4.888	6.264	0.919	0.606
4.888	5.569	5.163	5.549	5.859	0.337	0.606
5.853	6.431	5.648	6.324	6.452	0.320	0.606
4.529	5.804	3.679	6.360	5.893	0.569	0.606
7.171	5.919	5.829	7.149	7.008	0.567	0.606
4.336	5.674	2.354	6.392	6.404	0.903	0.606
1.730	3.840	2.812	2.626	3.242	0.543	0.606
2.262	2.411	2.461	3.001	2.794	0.517	0.606
3.106	2.816	1.731	2.678	3.870	0.599	0.606
3.357	4.018	4.028	2.771	4.265	0.454	0.606
3.570	3.100	4.266	2.626	3.664	0.513	0.606
7.011	5.720	7.807	5.971	5.968	0.580	0.606
5.548	4.778	6.712	4.466	4.246	0.528	0.606
5.324	5.400	6.243	3.593	4.067	0.747	0.606
3.025	3.133	2.262	3.397	5.179	0.840	0.606
7.204	6.908	7.428	6.995	7.386	0.488	0.606

6.936	7.370	6.696	7.415	7.318	0.303	0.606
7.634	7.311	7.991	6.159	6.684	0.733	0.606
6.115	7.794	4.775	8.256	8.248	0.790	0.606
4.501	5.036	3.145	5.606	5.388	0.584	0.606
4.480	4.818	2.663	5.635	5.450	0.721	0.606
3.586	3.605	3.371	3.330	2.778	0.476	0.606
3.162	5.464	3.582	5.796	5.998	0.648	0.606
2.719	3.099	2.306	3.207	3.136	0.439	0.606
2.495	4.458	3.407	4.634	4.766	0.610	0.606
3.014	4.191	1.809	4.359	4.737	0.585	0.606
4.388	4.974	3.596	4.825	5.031	0.468	0.606
3.710	4.217	2.297	4.093	3.966	0.508	0.606
1.164	5.066	1.130	5.933	5.809	1.160	0.606
1.490	1.351	1.889	1.827	1.641	0.461	0.606
3.641	2.737	3.472	3.117	2.698	0.443	0.606
3.997	2.680	2.837	3.251	2.829	0.490	0.606
4.978	4.637	4.792	5.886	4.966	0.513	0.606
6.560	5.788	5.390	6.108	5.725	0.687	0.606
4.259	3.142	2.705	3.187	3.033	0.707	0.606
2.891	2.489	3.166	2.630	3.086	0.517	0.606
4.301	3.328	2.756	4.061	3.347	0.730	0.606
5.263	4.403	3.802	4.849	4.156	0.625	0.606
3.720	3.183	1.367	2.927	2.364	0.590	0.606
3.444	3.369	2.918	3.301	2.327	0.465	0.606
3.494	2.999	4.027	2.620	2.338	0.604	0.606
4.190	3.485	6.186	1.275	1.661	0.990	0.606
3.913	3.569	4.931	2.296	2.819	0.732	0.606
1.770	3.106	2.268	2.328	2.226	0.561	0.606
3.323	3.461	3.090	3.502	2.723	0.437	0.606
3.572	2.118	4.400	0.431	1.322	0.910	0.606
2.597	3.740	3.432	3.498	3.096	0.465	0.606
3.217	3.350	3.614	2.468	3.067	0.405	0.606
5.634	5.530	5.153	6.194	6.050	0.366	0.606
5.811	6.152	5.798	6.285	6.413	0.294	0.606
3.278	3.853	1.631	3.396	3.779	0.487	0.606
4.701	4.681	5.259	4.047	3.820	0.401	0.606
2.731	3.312	2.230	3.589	3.488	0.443	0.606
6.409	6.210	7.739	6.306	6.346	0.478	0.606
4.447	5.885	4.769	6.277	6.079	0.500	0.606
6.439	6.195	5.036	6.904	6.547	0.450	0.606
2.254	3.098	3.653	1.633	2.293	0.511	0.606
4.272	4.698	3.751	4.967	4.884	0.393	0.606
3.074	3.488	4.605	3.420	3.603	0.426	0.606
2.990	3.097	3.102	2.309	3.138	0.417	0.606
3.702	4.069	4.950	4.091	4.567	0.470	0.606
1.475	2.097	1.323	1.918	1.405	0.432	0.606
4.508	5.584	5.493	4.189	4.845	0.724	0.606
7.607	8.092	8.232	8.268	8.318	0.398	0.606
1.571	2.716	2.522	3.366	3.105	0.498	0.606
1.671	3.380	1.745	3.806	4.228	0.592	0.606

2.852	4.344	3.216	3.358	3.303	0.510	0.606
2.298	3.442	2.493	3.332	3.167	0.436	0.606
4.924	5.426	4.613	5.580	5.767	0.399	0.606
6.564	6.445	7.002	6.292	6.126	0.330	0.606
7.480	6.709	7.891	6.080	6.039	0.470	0.606
3.700	4.162	3.168	3.759	4.109	0.434	0.606
2.602	2.745	3.326	2.618	2.461	0.413	0.606
7.554	8.206	6.967	8.149	8.381	0.392	0.606
3.297	2.337	2.850	2.010	2.336	0.521	0.606
1.987	2.756	1.307	3.046	3.360	0.533	0.606
6.340	7.079	5.479	7.854	7.594	0.559	0.606
7.464	8.351	7.118	8.867	8.735	0.569	0.606
1.932	4.248	2.919	3.591	2.959	0.633	0.606
4.085	4.327	3.317	3.420	4.067	0.571	0.606
2.638	2.480	2.932	2.222	2.225	0.423	0.606
1.659	2.548	2.337	2.105	2.034	0.382	0.606
2.837	2.461	3.738	2.690	2.818	0.429	0.606
4.278	4.010	2.697	3.932	4.088	0.448	0.606
1.847	3.960	1.358	2.798	2.592	0.765	0.606
7.137	6.129	8.486	6.023	5.838	0.575	0.606
4.710	4.895	5.900	5.086	5.629	0.522	0.606
6.014	5.138	6.137	4.644	6.095	0.793	0.606
4.942	5.144	5.303	3.784	4.752	0.783	0.606
4.459	4.153	4.093	5.093	4.937	0.412	0.606
6.293	7.278	8.133	7.192	6.601	0.656	0.606
2.402	2.115	3.097	1.592	2.709	0.487	0.606
2.966	2.321	0.536	0.499	2.712	0.554	0.606
3.371	3.661	0.936	4.965	3.467	0.802	0.606
4.049	3.968	5.008	3.677	3.762	0.477	0.606
3.896	4.030	3.284	4.475	4.466	0.423	0.606
3.510	2.935	4.643	2.537	2.760	0.516	0.606
6.674	5.783	6.466	5.185	5.245	0.410	0.606
6.855	7.766	3.210	7.890	7.993	1.000	0.606
5.509	5.102	6.225	4.407	4.127	0.520	0.606
8.924	7.564	9.000	6.408	6.690	0.689	0.606
8.943	8.829	8.471	9.216	9.114	0.304	0.606
4.746	4.483	3.038	4.935	4.583	0.448	0.606
4.930	4.639	5.206	4.493	4.422	0.342	0.606
1.689	2.568	1.525	2.293	2.387	0.407	0.606
5.960	5.861	6.097	5.677	5.888	0.441	0.606
8.879	8.266	10.181	7.007	7.602	0.617	0.606
6.892	8.458	5.850	8.482	8.578	0.617	0.606
6.275	5.917	7.104	5.707	5.541	0.411	0.606
6.416	6.990	4.721	7.280	7.200	0.793	0.606
4.377	6.734	3.839	7.139	7.075	0.954	0.606
4.408	3.715	4.614	3.339	3.845	0.397	0.606
5.389	5.389	5.080	5.220	5.189	0.361	0.606
5.201	4.585	4.528	4.520	5.439	0.433	0.606
7.224	7.280	5.545	7.405	7.607	0.435	0.606
5.712	4.852	4.805	4.639	4.859	0.481	0.606

2.294	3.105	2.477	3.745	2.653	0.384	0.606
4.760	6.094	4.121	6.373	6.034	0.504	0.606
5.816	5.547	2.732	5.175	5.458	0.776	0.606
5.720	5.827	5.716	6.219	5.994	0.310	0.606
3.740	3.225	3.462	3.404	3.735	0.454	0.606
3.924	2.340	1.777	1.784	2.234	0.608	0.606
4.014	5.045	4.699	4.860	4.894	0.458	0.606
6.549	5.947	7.567	5.198	5.365	0.540	0.606
4.685	4.743	4.443	3.848	5.335	0.505	0.606
8.899	7.518	8.918	6.701	6.645	0.619	0.606
5.378	4.409	6.527	4.208	4.266	0.514	0.606
5.895	4.915	5.677	5.420	5.111	0.352	0.606
5.563	4.470	5.146	4.550	4.714	0.384	0.606
5.133	5.758	4.155	5.339	5.086	0.506	0.606
2.926	2.011	1.531	2.218	1.697	0.418	0.606
3.534	3.988	4.439	4.930	3.852	0.508	0.606
5.336	5.665	5.109	5.669	5.628	0.376	0.606
5.726	6.503	5.188	7.025	6.929	0.450	0.606
7.957	9.134	4.113	9.379	8.940	1.026	0.606
4.594	3.783	4.862	3.850	4.657	0.432	0.606
5.780	5.568	5.731	4.346	5.421	0.472	0.606
4.549	5.052	4.389	5.482	5.230	0.387	0.606
7.580	7.276	8.390	5.820	6.830	0.533	0.606
4.630	4.678	4.773	3.949	4.591	0.388	0.606
2.687	3.277	3.301	3.459	3.771	0.449	0.606
1.608	4.601	1.716	4.632	5.111	0.837	0.606
3.185	3.055	3.849	2.535	2.401	0.474	0.606
3.884	1.979	1.914	2.217	1.654	0.498	0.606
3.705	3.004	2.658	2.487	2.841	0.509	0.606
3.508	0.841	1.615	1.599	1.702	0.572	0.606
2.742	1.862	2.382	2.968	2.200	0.524	0.606
3.815	2.981	3.353	2.619	3.250	0.535	0.606
4.775	4.485	4.640	4.539	4.527	0.412	0.606
4.438	4.002	5.129	4.422	4.367	0.518	0.606
5.857	3.637	4.761	3.562	3.342	0.582	0.606
5.234	4.144	5.128	4.798	5.094	0.403	0.606
4.333	2.757	3.439	3.587	3.600	0.431	0.606
4.352	3.855	2.941	3.439	2.988	0.453	0.606
7.132	5.898	7.294	5.549	5.762	0.482	0.606
5.031	5.736	5.240	6.066	5.648	0.352	0.606
6.891	7.831	7.763	8.154	8.212	0.450	0.606
6.170	5.276	3.128	5.855	5.719	1.001	0.606
5.411	4.029	4.638	4.536	4.630	0.501	0.606
5.054	5.270	5.186	5.536	5.573	0.396	0.606
5.416	5.661	3.160	6.569	6.158	0.724	0.606
1.962	2.687	2.133	3.145	3.078	0.450	0.606
6.541	6.629	4.315	7.127	7.193	0.596	0.606
4.490	7.034	2.865	7.006	7.108	1.061	0.606
9.026	8.568	9.998	8.610	8.713	0.414	0.606
5.521	6.389	5.782	6.041	6.217	0.442	0.606

4.595	6.064	5.237	6.279	5.918	0.501	0.606
3.670	3.816	3.930	4.496	4.386	0.455	0.606
4.804	5.442	5.399	6.128	5.967	0.461	0.606
4.817	4.875	4.794	4.419	4.837	0.444	0.606
3.641	4.109	4.648	3.252	3.763	0.520	0.606
4.933	5.146	4.293	4.642	5.224	0.426	0.606
4.030	4.071	2.769	3.722	4.374	0.443	0.606
2.674	4.231	4.318	5.117	4.384	0.497	0.606
4.613	5.539	5.436	5.373	5.708	0.657	0.606
3.926	5.148	4.598	4.833	5.509	0.649	0.606
3.579	4.414	4.257	4.399	4.118	0.534	0.606
2.597	2.443	1.930	2.317	2.951	0.417	0.606
3.136	3.457	4.097	4.276	4.304	0.461	0.606
4.541	3.736	5.378	4.125	4.008	0.465	0.606
5.423	5.643	4.199	5.653	5.842	0.446	0.606
2.772	3.359	3.162	2.100	2.994	0.540	0.606
2.326	2.532	2.553	1.918	1.762	0.428	0.606
6.201	6.956	3.318	7.405	7.312	0.868	0.606
6.586	6.351	5.838	6.967	6.625	0.332	0.606
6.808	7.648	6.663	7.773	7.754	0.383	0.606
3.205	3.680	2.947	3.305	3.450	0.431	0.606
1.720	2.046	1.749	1.891	1.768	0.400	0.606
1.910	1.433	1.442	3.022	1.644	0.456	0.606
3.893	3.471	2.452	3.624	4.867	0.756	0.606
6.795	6.459	2.784	6.505	6.771	0.959	0.606
4.604	6.381	2.292	6.833	6.594	0.982	0.606
6.712	6.637	5.012	6.473	6.677	0.455	0.606
7.182	7.815	3.829	8.160	8.328	1.031	0.606
6.071	5.176	6.448	3.466	3.528	0.720	0.606
2.107	1.678	1.394	2.354	1.903	0.433	0.606
6.814	7.023	6.314	7.140	7.330	0.329	0.606
4.772	5.217	4.364	4.951	5.269	0.383	0.606
6.537	6.445	6.964	6.847	6.918	0.327	0.606
3.486	2.823	3.836	2.343	3.137	0.621	0.606
3.969	3.969	3.949	4.375	3.859	0.443	0.606
5.787	4.874	5.446	5.371	4.866	0.683	0.606
5.711	5.593	5.483	5.679	5.034	0.352	0.606
5.524	5.985	5.411	6.510	6.462	0.555	0.606
2.141	2.546	2.071	3.450	2.650	0.426	0.606
5.196	6.989	7.381	7.332	7.271	0.766	0.606
6.084	7.205	4.476	7.617	7.564	0.688	0.606
5.755	5.362	6.959	4.861	4.809	0.532	0.606
3.066	6.077	4.428	6.437	5.928	0.765	0.606
4.517	3.922	4.483	4.368	4.458	0.362	0.606
4.953	4.759	4.208	5.235	5.448	0.454	0.606
5.177	5.468	4.088	5.665	5.596	0.453	0.606
5.507	5.052	6.100	5.052	4.922	0.373	0.606
3.833	4.050	3.398	4.406	4.308	0.400	0.606
4.380	3.310	3.190	3.286	4.279	0.555	0.606
6.385	4.827	4.882	4.073	3.570	0.601	0.606

4.913	5.152	5.425	4.412	5.106	0.388	0.606
7.071	6.796	5.604	7.270	7.108	0.425	0.606
7.202	5.887	7.775	5.281	5.869	0.538	0.606
6.711	7.284	6.740	7.256	7.494	0.391	0.606
5.598	4.390	5.439	4.713	4.694	0.389	0.606
4.772	4.497	3.659	4.969	4.600	0.431	0.606
5.453	2.606	1.414	4.087	3.702	0.860	0.606
6.548	6.158	7.217	6.131	6.040	0.492	0.606
4.340	4.427	4.417	4.416	4.317	0.524	0.606
4.797	4.950	6.562	3.995	4.178	0.846	0.606
6.187	4.650	6.258	5.178	5.367	0.544	0.606
5.931	6.146	6.823	5.123	5.421	0.471	0.606
3.895	4.276	3.942	4.573	4.398	0.372	0.606
5.917	6.454	5.539	6.744	6.188	0.515	0.606
5.969	6.823	4.568	6.840	7.075	0.549	0.606
7.309	7.336	9.068	6.083	6.979	0.588	0.606
4.838	5.008	3.619	3.733	5.548	0.638	0.606
4.561	4.104	4.424	3.577	3.528	0.385	0.606
3.089	2.723	3.283	2.362	2.424	0.509	0.606
5.164	4.268	4.689	4.221	4.448	0.371	0.606
3.512	4.978	2.841	5.820	5.509	0.613	0.606
4.666	4.954	5.148	5.391	5.136	0.382	0.606
7.553	8.126	7.349	8.256	8.039	0.342	0.606
3.648	4.197	4.102	2.839	3.329	0.511	0.606
6.271	6.168	6.859	6.154	5.883	0.418	0.606
7.555	8.208	7.363	8.718	8.592	0.418	0.606
2.825	2.749	3.192	2.039	3.499	0.455	0.606
6.554	7.473	5.564	7.508	7.465	0.525	0.606
1.503	1.983	1.581	2.145	1.957	0.569	0.606
5.100	6.246	5.658	6.279	6.129	0.402	0.606
5.781	7.324	4.731	7.431	7.084	0.614	0.606
4.366	2.562	3.064	4.178	3.220	0.467	0.606
3.740	5.173	3.553	5.642	5.564	0.554	0.606
4.541	2.826	3.022	3.560	3.761	0.442	0.606
9.232	7.973	7.312	7.966	8.111	0.527	0.606
7.510	6.771	5.746	7.284	7.406	0.532	0.606
4.641	4.461	3.459	4.624	4.835	0.496	0.606
6.499	6.602	7.506	6.483	6.575	0.382	0.606
5.038	5.689	4.666	5.558	5.612	0.355	0.606
4.274	2.113	1.388	2.000	2.033	0.707	0.606
5.703	6.186	6.355	6.023	5.391	0.490	0.606
4.523	3.923	3.010	4.122	3.991	0.456	0.606
6.235	5.935	6.468	5.794	6.073	0.445	0.606
2.504	2.212	2.609	1.820	2.047	0.387	0.606
6.639	7.042	6.443	7.110	7.082	0.330	0.606
3.649	3.811	4.112	3.373	4.029	0.404	0.606
5.065	5.355	5.715	5.758	5.388	0.380	0.606
8.129	7.716	8.631	7.605	7.841	0.354	0.606
4.302	3.310	3.761	3.635	3.437	0.454	0.606
4.654	5.282	4.746	5.012	5.367	0.370	0.606

5.710	5.929	5.201	5.310	5.550	0.469	0.606
7.252	7.330	7.366	6.377	6.418	0.442	0.606
4.580	4.711	3.208	4.755	4.931	0.401	0.606
7.941	7.920	6.359	8.201	7.499	0.444	0.606
8.551	8.278	8.781	7.633	8.004	0.355	0.606
7.424	7.862	7.148	8.342	8.100	0.334	0.606
4.944	6.115	6.062	4.449	3.930	0.670	0.606
3.362	4.058	3.570	3.646	4.021	0.427	0.606
4.747	5.281	4.402	5.429	5.059	0.396	0.606
6.942	6.764	6.834	7.397	6.850	0.386	0.606
3.313	4.315	4.450	4.051	4.786	0.467	0.606
3.721	3.663	4.251	3.786	4.221	0.386	0.606
5.547	5.478	5.489	5.744	5.668	0.297	0.606
6.075	6.967	5.171	7.502	6.986	0.535	0.606
4.356	4.557	4.993	4.234	4.329	0.418	0.606
5.405	6.075	6.252	5.725	6.004	0.471	0.606
6.083	7.635	3.800	7.923	7.640	0.859	0.606
3.517	2.889	2.610	2.161	2.802	0.436	0.606
6.068	5.854	5.061	6.168	6.233	0.414	0.606
5.871	5.767	5.881	5.445	5.516	0.319	0.606
3.478	5.673	2.433	5.837	5.397	0.793	0.606
6.959	6.427	7.436	6.013	6.426	0.368	0.606
6.862	6.418	7.026	6.648	6.053	0.353	0.606
5.298	5.897	5.910	5.224	4.765	0.382	0.606
2.136	1.598	1.853	1.933	1.411	0.360	0.606
6.115	6.318	5.993	6.494	6.492	0.328	0.606
5.874	5.277	6.335	5.068	5.025	0.407	0.606
5.403	4.568	5.032	4.709	4.929	0.399	0.606
5.873	5.018	5.992	4.878	5.039	0.406	0.606
3.970	3.616	3.140	2.711	3.097	0.500	0.606
4.217	3.996	5.490	3.273	3.683	0.453	0.606
4.938	4.569	5.539	3.237	3.215	0.536	0.606
2.325	2.538	1.890	2.763	3.040	0.556	0.606
8.718	7.954	9.376	7.708	7.824	0.426	0.606
6.464	6.784	5.994	7.287	7.098	0.411	0.606
3.834	3.860	3.906	4.931	4.583	0.397	0.606
4.878	4.863	4.985	4.436	5.206	0.343	0.606
5.438	5.069	4.219	5.405	5.656	0.381	0.606
5.077	5.022	5.575	5.101	5.615	0.370	0.606
5.088	3.374	4.156	3.865	3.359	0.534	0.606
6.291	8.177	3.428	8.222	8.050	0.928	0.606
8.065	7.446	8.391	7.658	7.520	0.459	0.606
4.577	4.467	5.316	4.098	4.319	0.430	0.606
2.833	4.858	1.993	3.025	3.026	1.171	0.606
5.261	3.674	5.575	2.537	3.108	0.692	0.606
7.292	7.566	6.651	7.652	7.750	0.352	0.606
5.338	4.137	4.385	4.276	4.337	0.509	0.606
3.497	3.525	3.670	2.769	3.773	0.427	0.606
7.992	9.011	6.976	9.229	8.958	0.568	0.606
6.776	7.843	5.657	7.286	6.965	0.504	0.606

8.033	9.128	6.727	8.690	8.626	0.570	0.606
5.518	6.403	4.043	6.678	5.952	0.626	0.606
3.685	3.867	2.322	3.856	3.314	0.418	0.606
4.659	4.808	5.575	4.047	4.277	0.463	0.606
6.081	5.547	8.086	3.962	4.054	0.894	0.606
3.736	3.316	3.554	2.833	2.952	0.507	0.606
4.032	4.194	4.463	3.530	3.498	0.498	0.606
4.002	4.001	4.991	3.879	4.075	0.436	0.606
6.990	5.804	6.915	5.670	5.999	0.477	0.606
6.266	5.710	6.612	5.624	5.963	0.455	0.606
7.788	7.223	7.639	6.577	6.907	0.381	0.606
5.310	5.568	4.181	5.081	5.549	0.521	0.606
2.719	2.315	1.494	1.560	2.291	0.414	0.606
4.963	5.510	5.605	4.307	4.856	0.578	0.606
6.819	7.058	6.754	7.296	7.521	0.332	0.606
6.271	6.605	6.302	6.769	6.796	0.317	0.606
4.816	5.370	5.090	3.976	4.978	0.651	0.606
8.422	8.790	8.387	9.469	9.091	0.341	0.606
5.260	6.079	5.167	6.268	5.866	0.362	0.606
5.795	5.201	4.830	5.694	5.076	0.356	0.606
4.004	3.927	4.015	2.915	3.322	0.419	0.606
2.273	2.223	1.709	1.859	2.453	0.417	0.606
2.678	2.411	2.912	2.350	2.094	0.440	0.606
4.800	5.680	5.770	5.173	5.271	0.571	0.606
4.202	5.149	3.238	4.958	4.818	0.439	0.606
2.818	2.349	3.722	1.976	2.029	0.463	0.606
8.958	8.610	10.025	7.459	7.626	0.552	0.606
8.563	8.960	8.080	9.172	8.983	0.340	0.606
6.268	7.178	5.886	7.390	6.913	0.421	0.606
5.189	5.375	3.025	6.081	5.479	0.582	0.606
6.595	8.044	5.983	8.105	7.708	0.532	0.606
4.504	4.217	4.303	4.263	4.496	0.489	0.606
3.350	3.142	4.175	3.807	3.591	0.491	0.606
6.413	4.185	5.106	3.108	4.215	0.853	0.606
6.510	7.042	6.433	7.254	7.121	0.340	0.606
5.423	5.918	3.784	6.036	5.794	0.512	0.606
4.257	5.203	3.525	5.001	4.964	0.440	0.606
4.052	6.575	2.192	6.794	6.513	0.967	0.606
1.667	2.062	2.299	2.792	2.069	0.413	0.606
3.279	3.422	4.915	3.749	3.926	0.445	0.606
5.881	6.230	5.560	6.560	6.041	0.377	0.606
8.505	8.697	8.127	8.847	8.970	0.352	0.606
2.810	3.956	3.524	4.458	3.993	0.421	0.606
6.598	7.144	5.668	7.413	7.400	0.586	0.606
5.069	5.505	5.022	6.107	6.053	0.409	0.606
3.949	5.333	3.098	5.638	5.562	0.587	0.606
3.023	4.488	2.910	5.183	5.772	0.980	0.606
9.050	8.044	9.340	7.889	7.655	0.437	0.606
3.105	3.458	3.815	3.064	4.403	0.426	0.606
5.013	5.877	4.727	5.524	5.755	0.479	0.606

8.380	7.744	9.454	7.109	7.275	0.484	0.606
4.034	4.852	4.661	4.395	5.300	0.488	0.606
6.050	5.531	5.975	4.738	5.201	0.496	0.606
4.244	3.639	4.311	3.169	2.716	0.500	0.606
5.240	5.340	4.799	4.924	5.994	0.691	0.606
4.194	3.091	1.421	3.517	4.205	0.634	0.606
3.804	5.336	4.430	2.919	3.431	0.734	0.606
3.964	4.937	4.490	4.789	5.113	0.407	0.606
6.003	5.333	5.743	4.798	5.861	0.462	0.606
1.947	2.052	2.006	2.119	2.229	0.323	0.606
4.430	4.471	4.259	3.514	3.873	0.506	0.606
3.390	3.243	4.129	2.971	3.632	0.521	0.606
6.999	9.227	6.452	9.511	9.388	0.721	0.606
1.984	2.653	1.440	3.331	3.630	0.526	0.606
4.971	4.591	4.674	3.355	3.969	0.524	0.606
5.062	4.993	5.048	4.839	4.732	0.321	0.606
7.275	6.220	4.958	6.893	6.765	0.539	0.606
4.173	3.887	4.242	3.839	3.784	0.443	0.606
6.597	6.017	6.050	5.235	5.195	0.481	0.606
5.528	5.550	5.452	5.626	5.721	0.325	0.606
4.663	4.518	4.708	3.134	3.968	0.592	0.606
4.099	6.167	3.297	6.228	6.405	0.879	0.606
7.597	7.027	8.186	6.276	6.319	0.474	0.606
4.657	5.318	4.192	5.452	5.297	0.550	0.606
6.849	6.030	3.939	6.080	5.927	0.541	0.606
6.690	5.911	7.897	5.165	5.182	0.586	0.606
5.715	5.368	5.635	4.076	4.659	0.484	0.606
6.788	6.603	5.728	6.962	6.656	0.357	0.606
3.696	3.024	3.646	2.315	3.260	0.657	0.606
6.321	5.102	5.958	5.591	5.532	0.433	0.606
6.132	5.783	5.559	5.808	5.668	0.305	0.606
3.734	4.450	4.635	2.942	4.085	0.676	0.606
6.383	7.406	6.806	7.391	7.218	0.387	0.606
2.805	3.150	2.858	2.959	2.915	0.535	0.606
7.013	7.007	5.790	7.431	7.238	0.442	0.606
3.622	2.874	2.889	3.203	3.768	0.440	0.606
3.949	2.793	2.954	3.874	3.918	0.481	0.606
6.106	5.101	6.492	4.483	4.848	0.489	0.606
4.414	4.455	4.916	3.776	3.478	0.390	0.606
7.135	5.851	6.988	5.728	5.595	0.552	0.606
2.811	2.859	2.346	2.444	2.272	0.415	0.606
5.259	4.425	5.707	3.708	3.849	0.494	0.606
3.514	3.902	2.700	4.562	4.010	0.441	0.606
8.294	6.553	8.624	4.645	5.642	0.863	0.606
5.747	4.032	3.830	4.234	4.392	0.464	0.606
7.833	8.472	7.215	8.638	8.441	0.384	0.606
5.959	6.357	5.701	6.691	6.382	0.337	0.606
5.775	5.638	5.300	5.010	5.378	0.369	0.606
4.083	3.687	4.400	3.395	3.976	0.649	0.606
2.879	3.348	3.834	3.006	3.139	0.477	0.606

6.099	6.059	7.273	4.709	4.623	0.606	0.606
3.749	3.128	3.376	3.439	3.362	0.417	0.606
3.502	6.005	2.564	6.725	6.237	0.857	0.606
4.114	4.253	4.720	3.894	3.653	0.626	0.606
2.243	2.645	2.765	4.196	3.164	0.472	0.606
8.563	8.470	9.538	6.412	7.447	0.684	0.606
2.465	2.573	1.824	2.584	2.561	0.683	0.606
6.731	6.734	7.821	6.855	6.451	0.465	0.606
6.087	6.668	6.877	6.854	6.616	0.439	0.606
5.564	5.243	6.727	4.597	4.470	0.491	0.606
4.902	5.808	5.166	4.030	4.925	0.483	0.606
3.365	3.701	2.802	4.036	4.201	0.471	0.606
4.794	5.112	5.863	4.280	4.756	0.632	0.606
5.950	6.387	7.003	6.019	6.433	0.401	0.606
7.584	8.259	7.808	6.971	7.815	0.462	0.606
2.051	3.133	4.295	2.707	3.335	0.623	0.606
5.703	6.244	5.167	6.089	6.034	0.391	0.606
9.173	8.843	9.900	8.242	8.267	0.408	0.606
5.691	5.099	6.064	5.333	5.192	0.438	0.606
5.722	6.328	5.220	6.794	5.959	0.437	0.606
6.091	6.825	4.867	7.238	7.130	0.571	0.606
5.144	4.090	4.373	3.963	3.886	0.377	0.606
5.668	5.724	3.302	6.189	6.107	0.616	0.606
6.736	5.756	6.985	5.348	5.306	0.445	0.606
6.061	6.025	6.265	5.795	5.852	0.315	0.606
5.826	4.915	6.322	3.868	4.340	0.572	0.606
5.688	5.633	5.678	4.819	4.848	0.387	0.606
10.214	9.685	10.269	8.659	9.018	0.460	0.606
6.857	6.221	7.825	5.468	5.943	0.499	0.606
5.634	6.144	5.445	6.023	6.163	0.461	0.606
5.509	5.914	5.947	5.208	5.486	0.432	0.606
3.819	4.193	4.967	4.075	4.415	0.404	0.606
3.659	3.416	4.165	2.699	2.429	0.545	0.606
3.856	4.737	2.611	4.707	4.166	0.519	0.606
4.698	5.262	3.910	5.095	5.168	0.406	0.606
1.833	2.984	3.435	3.322	3.676	0.486	0.606
7.994	8.135	7.650	8.727	8.620	0.333	0.606
7.130	7.582	5.133	7.858	7.799	0.584	0.606
5.216	5.170	3.133	5.377	5.926	0.693	0.606
3.338	4.021	2.680	4.237	4.066	0.446	0.606
3.863	4.333	3.206	4.597	4.608	0.447	0.606
3.856	4.342	3.214	4.608	4.658	0.443	0.606
3.601	4.481	2.956	4.198	4.420	0.435	0.606
3.254	4.489	3.009	4.308	4.419	0.524	0.606
6.602	6.996	4.840	7.156	7.120	0.547	0.606
5.519	5.608	3.434	5.812	5.826	0.624	0.606
3.174	3.843	2.049	3.427	3.700	0.575	0.606
4.404	4.983	4.847	4.080	4.646	0.435	0.606
4.701	4.216	5.829	4.428	4.339	0.427	0.606
3.760	3.558	3.657	2.887	4.841	0.535	0.606

3.377	2.935	2.876	2.629	2.160	0.419	0.606
7.397	6.754	6.124	6.852	7.054	0.404	0.606
2.485	3.282	4.045	2.997	2.708	0.394	0.606
3.420	3.407	3.617	2.948	3.029	0.350	0.606
6.929	7.082	6.641	7.250	7.023	0.342	0.606
5.409	5.506	6.484	4.861	5.156	0.421	0.606
5.044	4.859	4.958	4.881	4.787	0.340	0.606
6.711	7.236	6.052	7.484	7.218	0.400	0.606
5.864	5.807	5.725	6.045	6.225	0.359	0.606
5.000	5.523	5.228	5.657	5.984	0.359	0.606
1.770	2.130	2.124	1.553	2.486	0.475	0.606
1.936	3.633	2.369	2.919	2.361	0.477	0.606
1.368	2.564	2.590	2.695	2.607	0.418	0.606
1.478	1.693	1.531	1.707	1.727	0.608	0.606
6.595	6.121	6.681	6.175	5.760	0.363	0.606
6.739	7.293	6.434	7.743	7.441	0.392	0.606
3.422	3.964	3.756	3.851	3.110	0.458	0.606
3.285	1.861	2.765	2.178	2.323	0.489	0.606
4.413	4.960	5.252	5.595	5.103	0.396	0.606
5.496	6.094	5.260	6.175	6.335	0.377	0.606
2.160	2.863	2.070	2.951	3.607	0.721	0.606
3.166	2.941	2.862	2.826	2.862	0.507	0.606
2.470	3.156	2.461	3.855	3.676	0.456	0.606
6.280	6.127	6.851	6.297	6.516	0.370	0.606
4.241	1.999	2.119	2.237	2.010	0.770	0.606
3.805	3.820	3.974	3.210	3.362	0.466	0.606
2.852	2.179	2.810	1.942	2.233	0.470	0.606
5.525	6.476	5.615	6.720	6.460	0.387	0.606
3.275	4.196	2.885	4.954	4.566	0.500	0.606
0.861	1.889	0.916	3.646	1.934	0.576	0.606
2.623	3.757	3.771	3.713	2.754	0.758	0.606
2.792	3.113	3.768	3.819	3.020	0.449	0.606
3.982	2.848	4.756	2.006	2.519	0.602	0.606
7.401	6.674	8.552	5.277	5.699	0.618	0.606
6.815	5.811	7.562	5.479	5.143	0.508	0.606
3.608	3.359	3.542	3.919	3.926	0.317	0.606
3.226	3.555	3.326	4.164	4.169	0.398	0.606
2.866	3.286	2.328	3.764	3.593	0.387	0.606
4.174	4.956	4.530	5.144	5.029	0.381	0.606
1.849	1.281	2.053	1.929	2.462	1.117	0.606
7.120	6.820	8.021	5.926	6.641	0.509	0.606
3.979	3.802	3.115	4.337	4.603	0.880	0.606
7.196	7.730	6.584	8.147	8.295	0.472	0.606
4.356	4.964	2.697	5.042	4.634	0.539	0.606
4.823	5.488	2.863	5.942	5.638	0.698	0.606
6.461	6.864	6.674	7.584	7.357	0.377	0.606
5.067	4.644	6.147	4.364	4.194	0.434	0.606
2.170	1.153	2.662	2.661	2.118	0.545	0.606
3.710	3.128	2.959	3.118	3.701	0.408	0.606
3.252	1.778	3.077	2.445	1.883	0.621	0.606

3.194	1.781	3.468	3.058	3.789	0.485	0.606
6.292	6.874	6.356	7.269	6.807	0.459	0.606
3.422	3.839	3.728	4.242	4.395	0.367	0.606
3.696	3.424	3.894	4.020	3.446	0.443	0.606
7.435	7.061	6.455	7.529	7.298	0.395	0.606
5.613	5.455	4.862	5.643	5.893	0.362	0.606
7.273	6.874	6.290	7.352	7.386	0.378	0.606
6.924	6.820	6.296	7.287	7.099	0.376	0.606
6.269	6.383	5.816	6.390	6.478	0.316	0.606
7.898	7.390	6.972	7.770	7.574	0.391	0.606
6.298	6.464	5.814	6.517	6.460	0.330	0.606
5.495	5.121	3.759	6.048	6.114	0.706	0.606
3.793	3.895	3.461	4.508	4.542	0.431	0.606
7.000	6.707	6.088	7.137	6.880	0.359	0.606
6.890	6.879	6.411	7.242	7.252	0.343	0.606
9.082	8.775	7.963	8.922	8.924	0.371	0.606
8.674	8.279	7.689	8.684	8.402	0.428	0.606
8.437	7.952	7.100	8.238	8.229	0.394	0.606
8.273	7.987	7.286	8.295	8.082	0.389	0.606
6.368	6.051	5.926	6.397	6.632	0.392	0.606
3.178	4.262	1.880	4.934	4.546	0.634	0.606
4.885	5.468	2.915	5.305	5.665	0.611	0.606
6.525	6.638	6.829	7.147	7.070	0.338	0.606
2.088	3.239	1.953	3.290	2.921	0.462	0.606
6.508	7.766	7.388	7.911	7.899	0.404	0.606
5.043	5.459	4.962	6.065	5.569	0.351	0.606
6.454	6.863	6.028	7.124	7.189	0.370	0.606
6.290	6.829	6.381	7.363	7.087	0.335	0.606
5.536	5.713	4.706	6.195	6.096	0.407	0.606
3.262	3.533	3.798	3.453	3.317	0.452	0.606
6.513	5.449	7.142	3.396	4.040	0.785	0.606
4.622	4.606	2.897	5.192	4.888	0.714	0.606
3.929	4.545	4.135	4.045	3.886	0.512	0.606
6.810	7.948	7.690	8.507	7.818	0.461	0.606
4.156	4.357	4.602	3.600	4.100	0.443	0.606
4.275	4.323	4.665	3.726	4.138	0.404	0.606
4.122	4.305	4.609	3.607	4.107	0.430	0.606
5.414	5.066	5.559	4.953	5.110	0.385	0.606
4.165	4.253	4.567	3.588	4.058	0.444	0.606
4.851	5.764	4.945	6.097	5.641	0.497	0.606
5.050	5.513	5.403	5.819	6.073	0.490	0.606
3.563	2.406	4.614	2.988	3.393	0.443	0.606
3.477	3.879	3.950	3.313	3.509	0.464	0.606
2.403	2.580	3.749	1.633	2.251	0.476	0.606
6.665	7.053	6.864	7.174	6.772	0.436	0.606
6.777	6.732	7.014	7.121	7.069	0.311	0.606
6.553	7.841	6.592	7.946	7.718	0.426	0.606
6.782	7.325	5.382	7.312	7.532	0.651	0.606
5.712	3.448	6.070	3.886	3.004	0.857	0.606
5.219	5.210	6.272	4.734	4.961	0.453	0.606

4.014	3.263	3.933	3.067	3.009	0.380	0.606
6.236	6.621	5.700	6.938	6.854	0.351	0.606
6.639	7.245	6.507	7.249	7.183	0.326	0.606
8.021	9.701	7.297	9.834	9.863	0.636	0.606
6.787	7.330	5.813	7.535	7.399	0.433	0.606
3.717	3.201	3.762	2.940	3.189	0.333	0.606
8.101	8.970	7.271	9.129	9.185	0.521	0.606
2.767	1.970	2.708	2.357	1.913	0.501	0.606
5.408	5.869	3.491	6.641	6.190	0.589	0.606
1.730	2.234	3.235	3.087	2.973	0.486	0.606
6.041	5.991	4.911	6.013	6.544	0.428	0.606
6.351	5.741	6.653	5.708	6.041	0.463	0.606
5.049	4.175	5.000	4.006	4.261	0.444	0.606
3.517	3.317	3.595	2.976	2.829	0.584	0.606
3.838	3.911	3.363	4.358	4.715	0.451	0.606
3.433	3.378	2.028	4.319	3.959	0.638	0.606
4.901	4.693	3.298	4.367	4.650	0.447	0.606
2.853	3.635	3.722	3.074	3.075	0.514	0.606
5.367	5.763	5.298	5.849	6.109	0.334	0.606
3.852	3.998	3.446	4.499	5.085	0.455	0.606
5.755	6.481	5.915	6.666	6.475	0.322	0.606
5.724	5.407	5.240	6.073	6.132	0.361	0.606
5.023	3.762	4.096	4.005	4.459	0.445	0.606
2.643	2.507	2.251	2.512	2.710	0.403	0.606
3.658	4.930	3.687	5.448	5.206	0.464	0.606
5.014	4.453	4.115	5.338	5.770	0.710	0.606
5.443	6.583	5.629	6.835	6.697	0.389	0.606
9.319	9.495	8.584	9.654	9.886	0.388	0.606
7.439	7.482	8.385	6.338	6.378	0.455	0.606
2.427	2.222	2.085	1.541	2.143	0.535	0.606
0.853	1.059	1.017	1.369	1.828	1.105	0.606
5.225	6.591	4.515	7.115	6.948	0.610	0.606
6.356	7.632	5.607	7.991	7.280	0.485	0.606
3.778	1.887	3.041	2.312	3.315	0.496	0.606
5.759	4.948	6.036	3.282	3.191	0.755	0.606
5.886	6.811	5.009	6.997	6.969	0.569	0.606
7.979	7.670	8.093	8.021	8.334	0.299	0.606
2.721	3.175	2.359	3.890	3.803	0.490	0.606
5.826	6.582	3.616	6.958	6.513	0.675	0.606
2.518	3.127	2.964	4.189	2.989	0.472	0.606
6.045	7.165	4.772	7.676	7.867	0.624	0.606
4.895	6.734	4.091	6.434	6.346	0.584	0.606
3.614	3.308	2.925	2.947	3.462	0.416	0.606
4.670	5.485	3.508	6.012	5.457	0.546	0.606
2.875	6.978	1.907	7.042	7.098	1.248	0.606
5.998	6.570	5.698	6.877	7.034	0.388	0.606
5.068	4.544	5.629	4.007	4.179	0.497	0.606
3.739	2.845	4.943	2.270	3.032	0.537	0.606
8.315	7.966	8.271	7.361	7.541	0.332	0.606
4.803	4.278	4.268	4.067	4.270	0.314	0.606

4.271	4.347	5.515	4.111	4.260	0.458	0.606
3.318	4.070	3.292	4.787	4.834	0.499	0.606
4.533	3.412	6.178	2.754	3.542	0.750	0.606
4.002	2.408	3.676	2.267	2.159	0.835	0.606
3.411	3.788	3.399	3.530	3.495	0.706	0.606
2.673	1.292	1.662	1.726	1.194	0.576	0.606
4.243	3.616	3.871	4.735	4.435	0.426	0.606
3.992	5.969	3.008	6.118	6.054	0.714	0.606
4.001	3.985	4.088	3.381	4.431	0.464	0.606
4.599	4.780	4.031	3.944	4.775	0.481	0.606
2.511	2.041	3.341	2.348	2.429	0.746	0.606
2.672	3.955	2.079	4.188	4.020	0.653	0.606
1.684	2.088	3.023	2.178	2.690	0.381	0.606
4.087	5.047	4.049	4.799	5.169	0.463	0.606
7.075	6.999	6.151	7.228	7.125	0.357	0.606
4.228	1.636	1.435	1.304	1.030	0.833	0.606
4.810	4.078	2.383	4.527	4.708	0.644	0.606
6.269	7.809	6.560	7.875	7.873	0.518	0.606
2.923	3.205	3.684	2.470	2.906	0.442	0.606
3.585	3.745	2.948	3.192	3.954	0.404	0.606
5.497	5.322	7.273	4.239	4.542	0.645	0.606
4.759	5.751	4.389	5.249	4.979	0.493	0.606
4.646	4.282	5.140	4.490	4.158	0.438	0.606
4.114	3.546	4.977	2.594	2.314	0.577	0.606
6.662	6.048	7.689	5.482	5.730	0.482	0.606
3.306	4.034	1.388	5.093	4.439	0.724	0.606
2.861	3.146	3.900	3.460	2.905	0.648	0.606
3.610	4.548	3.620	4.526	4.682	0.441	0.606
3.788	4.657	3.798	4.599	4.412	0.404	0.606
3.803	4.799	3.868	4.773	4.612	0.408	0.606
4.131	5.678	2.907	5.661	5.321	0.639	0.606
4.981	4.657	4.205	5.573	5.681	0.528	0.606
4.186	4.037	4.608	3.764	3.425	0.445	0.606
3.639	4.527	5.398	4.343	4.962	0.458	0.606
2.694	3.386	2.069	3.726	3.522	0.471	0.606
2.007	2.861	2.548	3.570	2.552	0.404	0.606
1.885	1.573	2.380	2.308	2.195	0.460	0.606
2.764	2.795	2.463	2.685	2.736	0.398	0.606
6.764	6.103	7.561	5.918	6.293	0.419	0.606
4.718	3.872	5.003	3.020	3.772	0.713	0.606
3.201	2.832	2.100	3.119	2.894	0.861	0.606
4.796	6.854	3.593	6.452	6.469	0.689	0.606
7.335	6.291	6.858	7.099	6.812	0.581	0.606
3.645	2.244	3.231	1.958	2.098	0.473	0.606
5.641	4.838	6.573	4.383	4.793	0.473	0.606
4.806	4.880	4.850	4.338	4.680	0.339	0.606
4.825	5.506	5.286	4.278	4.901	0.419	0.606
4.783	5.479	5.299	4.244	4.874	0.428	0.606
3.813	3.524	3.231	2.558	3.163	0.363	0.606
2.466	2.634	2.458	2.228	2.367	0.306	0.606

2.875	2.916	2.724	2.251	2.682	0.326	0.606
3.314	3.406	3.011	2.279	3.163	0.356	0.606
3.693	3.604	3.382	2.682	3.376	0.326	0.606
3.490	3.322	3.112	2.342	3.153	0.345	0.606
7.505	7.384	6.796	7.911	8.030	0.358	0.606
3.306	3.466	4.737	3.072	3.270	0.475	0.606
5.499	5.340	5.735	5.620	6.023	0.345	0.606
4.433	5.051	5.905	4.642	4.950	0.611	0.606
5.742	6.375	5.757	6.245	6.286	0.395	0.606
4.863	4.544	6.076	4.407	4.171	0.462	0.606
3.230	3.555	3.529	3.091	3.074	0.483	0.606
3.535	3.810	4.553	2.456	3.361	0.590	0.606
7.233	7.110	7.568	6.680	6.788	0.360	0.606
5.613	4.848	5.373	5.347	4.835	0.431	0.606
4.113	2.917	2.674	4.085	3.684	0.477	0.606
5.356	5.729	4.554	5.849	5.437	0.453	0.606
3.573	3.593	3.749	3.886	3.991	0.425	0.606
3.742	4.384	2.401	4.473	5.158	0.599	0.606
2.996	2.858	3.614	2.693	2.755	0.438	0.606
7.059	7.137	6.102	7.583	7.630	0.407	0.606
2.949	1.619	1.999	3.723	3.567	0.826	0.606
8.410	7.842	8.613	7.533	8.254	0.362	0.606
2.741	1.985	3.385	1.550	2.141	0.554	0.606
3.106	2.530	2.389	3.408	3.425	0.472	0.606
4.198	4.163	3.300	4.228	4.214	0.370	0.606
4.888	5.850	4.792	6.539	5.938	0.449	0.606
3.194	2.908	2.477	3.317	3.627	0.427	0.606
6.470	6.971	5.666	7.245	7.281	0.454	0.606
4.087	3.434	3.937	2.549	3.487	0.596	0.606
2.608	4.641	2.567	4.300	4.653	0.611	0.606
3.829	3.591	3.905	3.385	3.515	0.358	0.606
2.050	3.250	1.489	4.246	3.757	0.641	0.606
4.441	7.432	4.452	7.499	7.551	0.793	0.606
5.761	5.464	6.630	4.440	4.838	0.538	0.606
5.080	6.206	4.233	6.324	5.953	0.538	0.606
4.838	5.467	4.341	4.556	5.271	0.399	0.606
7.986	8.345	8.027	8.315	8.834	0.386	0.606
5.286	6.260	4.649	5.146	5.846	0.475	0.606
3.063	3.315	3.425	2.937	3.243	0.432	0.606
4.489	4.962	4.798	4.288	4.862	0.410	0.606
3.818	3.059	1.386	2.965	3.177	0.623	0.606
3.080	4.921	1.612	5.495	5.192	0.782	0.606
5.810	6.438	6.070	7.075	6.782	0.400	0.606
6.010	7.076	3.108	7.498	7.102	0.956	0.606
4.351	4.748	4.832	3.675	4.444	0.489	0.606
5.463	5.310	5.861	3.608	4.515	0.653	0.606
3.459	3.212	3.181	3.092	2.582	0.397	0.606
8.202	7.608	8.657	7.811	7.735	0.397	0.606
4.930	4.046	6.286	3.850	4.970	0.507	0.606
2.138	3.220	1.454	2.754	3.087	0.433	0.606

4.286	3.490	4.126	2.561	2.270	0.638	0.606
6.521	6.725	7.286	7.061	6.865	0.347	0.606
7.029	7.249	7.753	7.418	7.470	0.344	0.606
7.714	7.628	6.595	7.407	7.727	0.387	0.606
6.342	6.551	3.238	6.726	6.647	0.764	0.606
6.356	6.769	3.426	7.292	7.015	0.782	0.606
4.572	6.035	3.640	6.083	6.032	0.601	0.606
3.505	4.741	2.048	5.295	4.972	0.742	0.606
6.442	7.275	3.783	7.847	7.458	0.923	0.606
5.704	4.755	5.512	3.921	4.530	0.461	0.606
6.989	6.746	7.594	5.841	6.782	0.396	0.606
2.851	2.843	3.752	3.393	2.229	0.470	0.606
5.451	6.325	6.101	6.622	6.667	0.354	0.606
5.146	4.901	4.851	5.573	5.714	0.371	0.606
4.057	4.465	4.958	4.207	4.334	0.413	0.606
4.282	4.249	4.481	4.392	5.028	0.323	0.606
5.183	5.484	5.983	5.550	5.895	0.429	0.606
3.937	3.695	1.880	3.316	4.107	0.717	0.606
5.915	5.687	5.621	6.127	5.953	0.392	0.606
3.152	4.049	3.188	3.272	3.559	0.357	0.606
6.230	5.926	3.792	6.515	6.794	0.756	0.606
1.699	2.797	1.229	2.925	2.236	0.451	0.606
9.369	8.753	7.705	9.300	9.483	0.486	0.606
8.834	8.166	7.041	8.766	9.032	0.522	0.606
4.213	5.283	5.916	4.797	4.609	0.451	0.606
5.409	5.318	5.782	5.316	5.042	0.367	0.606
3.928	3.413	3.749	3.055	3.375	0.459	0.606
9.016	7.940	9.643	7.315	7.302	0.538	0.606
4.128	3.853	4.443	3.373	3.691	0.336	0.606
2.728	4.062	3.475	1.134	3.192	0.762	0.606
3.443	3.597	3.856	3.592	2.967	0.388	0.606
4.593	4.226	4.540	3.652	3.688	0.373	0.606
4.086	4.861	4.970	4.388	4.484	0.614	0.606
5.849	4.185	4.179	4.887	4.632	0.761	0.606
4.352	3.423	4.456	3.067	2.988	0.446	0.606
1.845	2.008	2.870	2.820	2.154	0.512	0.606
9.316	8.135	9.083	7.929	7.676	0.436	0.606
9.484	8.309	9.814	8.158	8.222	0.469	0.606
4.021	4.253	3.256	4.813	4.472	0.415	0.606
5.928	4.708	3.883	5.047	4.902	0.488	0.606
4.030	3.847	4.582	4.152	4.162	0.502	0.606
5.510	5.586	6.466	5.555	5.940	0.418	0.606
5.567	5.048	5.160	4.439	4.996	0.380	0.606
4.950	5.180	6.451	4.752	4.830	0.422	0.606
7.112	8.940	6.943	9.215	9.088	0.600	0.606
4.452	3.385	4.320	2.734	3.678	0.611	0.606
2.775	2.403	1.965	2.901	2.533	0.410	0.606
6.916	7.959	7.118	7.966	7.915	0.355	0.606
7.872	8.279	7.977	8.117	8.002	0.323	0.606
4.102	4.463	3.530	4.818	4.392	0.432	0.606

3.126	3.830	3.874	3.266	3.302	0.412	0.606
4.973	5.475	2.985	6.345	5.818	0.668	0.606
4.027	3.893	4.487	3.353	3.370	0.449	0.606
4.960	5.170	4.462	5.279	5.129	0.314	0.606
4.867	5.676	3.701	6.548	5.368	0.637	0.606
3.906	3.448	4.334	3.728	3.892	0.457	0.606
6.462	7.062	2.852	7.863	7.464	0.970	0.606
5.413	5.763	5.653	5.630	5.527	0.348	0.606
3.420	2.549	3.616	2.529	3.403	0.456	0.606
3.400	3.853	3.628	3.137	3.749	0.357	0.606
2.379	2.899	1.684	2.567	3.429	0.405	0.606
4.364	5.157	4.032	5.937	4.607	0.557	0.606
5.738	6.577	5.889	6.802	6.618	0.407	0.606
6.299	6.879	3.860	6.987	6.865	0.698	0.606
6.354	5.883	2.632	5.037	5.630	0.924	0.606
5.630	6.082	2.571	6.640	6.467	0.860	0.606
6.095	5.054	4.861	5.552	5.270	0.476	0.606
7.677	7.511	7.152	7.818	7.941	0.286	0.606
3.503	3.412	3.233	4.028	4.100	0.390	0.606
3.480	3.453	2.298	2.951	3.737	0.391	0.606
4.373	5.442	5.021	5.575	5.305	0.374	0.606
1.009	1.828	1.264	1.402	1.067	0.795	0.606
6.833	6.463	7.096	7.178	7.100	0.314	0.606
9.103	9.177	8.895	9.520	9.692	0.314	0.606
8.780	9.523	6.531	9.572	9.334	0.641	0.606
5.252	5.126	6.762	4.650	4.948	0.753	0.606
5.054	5.345	3.617	5.763	5.794	0.587	0.606
4.535	4.654	3.235	4.439	4.281	0.483	0.606
4.010	4.673	3.880	4.702	4.866	0.405	0.606
7.741	7.712	7.434	7.603	7.951	0.384	0.606
4.713	4.159	5.109	3.995	4.286	0.478	0.606
4.605	5.656	5.001	5.852	5.426	0.367	0.606
6.034	5.727	6.108	6.037	6.227	0.463	0.606
4.751	4.408	4.161	4.723	4.970	0.364	0.606
4.516	4.969	4.107	5.089	4.726	0.343	0.606
3.617	2.689	2.918	2.779	2.706	0.412	0.606
4.125	3.297	4.613	3.133	3.782	0.395	0.606
3.551	3.769	2.658	4.187	3.810	0.402	0.606
6.184	7.038	6.021	6.982	7.377	0.383	0.606
7.668	7.557	8.497	6.940	6.255	0.454	0.606
5.518	5.757	3.471	6.089	6.224	0.640	0.606
4.905	4.949	4.962	4.915	4.564	0.364	0.606
5.123	4.602	4.914	3.271	5.322	0.620	0.606
4.025	3.904	4.452	4.345	4.134	0.361	0.606
4.931	3.814	3.714	3.586	4.456	0.666	0.606
7.318	7.167	8.046	8.066	8.860	0.612	0.606
6.234	6.851	6.445	7.373	7.045	0.387	0.606
3.780	3.294	4.629	3.252	3.530	0.472	0.606
5.848	6.810	3.766	6.841	6.664	0.653	0.606
5.619	6.579	3.104	6.904	6.526	0.801	0.606

7.505	7.958	7.260	8.515	8.446	0.368	0.606
3.472	4.542	3.742	4.837	4.959	0.444	0.606
6.954	7.553	6.986	8.188	7.787	0.384	0.606
6.885	8.862	7.245	8.936	8.692	0.699	0.606
2.963	3.398	1.454	3.217	3.377	0.508	0.606
3.767	5.055	2.021	5.477	5.180	0.703	0.606
6.587	6.481	5.633	7.262	7.210	0.397	0.606
3.089	4.028	3.245	4.565	4.095	0.423	0.606
4.376	5.629	3.803	6.266	5.755	0.613	0.606
2.992	2.972	2.906	2.762	2.422	0.439	0.606
5.131	4.312	4.487	4.330	4.590	0.314	0.606
4.575	5.722	4.879	3.794	4.105	0.526	0.606
6.649	7.410	6.933	7.591	7.541	0.310	0.606
5.190	4.865	4.943	5.274	5.157	0.373	0.606
3.660	4.191	3.844	3.320	2.719	0.513	0.606
4.804	5.203	4.752	4.267	4.176	0.419	0.606
5.248	5.096	4.617	4.959	4.626	0.430	0.606
7.906	7.599	7.178	6.976	6.488	0.465	0.606
5.438	5.397	5.024	4.791	4.542	0.408	0.606
6.534	6.642	5.763	6.377	5.854	0.441	0.606
5.972	6.465	5.561	6.159	5.576	0.433	0.606
8.014	7.857	7.434	7.320	6.505	0.477	0.606
6.072	6.457	5.692	5.765	4.834	0.430	0.606
5.847	5.941	5.559	5.231	5.231	0.382	0.606
7.062	6.888	5.999	6.491	6.148	0.491	0.606
4.121	4.189	4.504	4.424	3.250	0.610	0.606
4.168	3.857	4.067	4.194	3.195	0.491	0.606
6.391	6.663	6.113	6.236	5.626	0.475	0.606
3.967	5.268	4.727	4.919	3.870	0.453	0.606
6.751	7.292	5.860	7.709	7.654	0.417	0.606
4.026	3.811	4.532	2.855	3.301	0.479	0.606
5.141	8.265	6.564	7.749	7.202	0.732	0.606
4.925	6.689	3.753	6.661	6.336	0.701	0.606
5.146	5.427	4.649	5.866	5.511	0.431	0.606
4.528	4.147	4.859	3.902	3.730	0.494	0.606
6.776	8.820	7.190	8.801	8.567	0.582	0.606
1.506	2.109	2.383	2.482	2.141	0.447	0.606
4.414	6.340	3.506	6.689	6.762	0.732	0.606
2.092	3.100	2.051	3.627	3.410	0.445	0.606
6.622	4.537	7.290	4.309	4.152	0.759	0.606
3.494	2.995	3.148	3.168	2.900	0.539	0.606
4.048	3.598	3.341	3.627	3.854	0.384	0.606
3.081	3.278	2.891	4.362	3.593	0.506	0.606
6.002	6.353	4.505	6.697	5.939	0.470	0.606
2.876	5.306	2.834	5.270	4.594	0.612	0.606
4.407	4.458	4.007	3.628	3.158	0.466	0.606
3.006	2.765	3.301	3.101	2.472	0.413	0.606
10.062	8.960	10.177	7.021	7.623	0.685	0.606
3.923	3.362	4.000	3.317	4.571	0.417	0.606
6.699	6.166	7.279	5.543	5.515	0.453	0.606

6.058	5.141	5.812	5.080	5.576	0.362	0.606
3.849	1.523	1.870	1.886	2.610	0.628	0.606
6.765	7.457	6.842	7.684	7.548	0.345	0.606
6.186	7.020	6.785	7.358	7.285	0.380	0.606
6.140	5.896	5.925	5.365	6.046	0.482	0.606
5.730	5.621	6.534	4.766	5.195	0.385	0.606
5.779	6.981	3.448	7.438	7.613	0.906	0.606
5.754	6.243	4.865	6.919	6.647	0.485	0.606
4.190	6.706	2.778	7.202	6.816	0.991	0.606
4.507	3.995	4.641	4.473	4.607	0.460	0.606
2.037	2.677	1.291	3.320	3.518	0.531	0.606
4.785	4.379	4.970	3.498	4.731	0.570	0.606
3.520	3.730	4.111	3.438	3.510	0.402	0.606
2.454	2.457	2.920	2.337	2.209	0.395	0.606
5.376	4.931	5.732	5.576	5.306	0.367	0.606
2.221	2.482	2.921	2.551	2.441	0.518	0.606
5.869	4.402	4.049	4.760	5.257	0.450	0.606
3.632	2.664	3.043	2.719	2.802	0.355	0.606
5.853	7.288	3.911	7.376	7.116	0.749	0.606
3.514	3.329	4.452	1.917	2.207	0.786	0.606
3.563	3.251	3.727	2.719	3.467	0.479	0.606
8.674	8.865	8.018	8.909	9.172	0.396	0.606
5.881	4.366	5.556	4.031	4.244	0.510	0.606
4.674	4.775	4.572	5.378	5.508	0.332	0.606
2.838	3.607	3.299	3.045	2.967	0.380	0.606
2.818	3.498	3.102	3.935	3.951	0.418	0.606
2.738	2.633	3.500	2.401	2.909	0.557	0.606
1.684	3.517	1.976	3.680	2.260	0.546	0.606
3.732	4.039	3.645	3.658	4.281	0.501	0.606
6.732	5.700	7.329	4.971	5.527	0.517	0.606
4.202	4.153	3.470	3.225	3.611	0.396	0.606
5.502	5.887	5.847	5.417	5.895	0.409	0.606
3.424	3.409	2.472	3.897	3.994	0.500	0.606
6.620	6.529	4.607	6.668	6.522	0.493	0.606
7.706	7.348	8.709	6.117	6.382	0.553	0.606
3.339	3.674	3.940	3.236	2.954	0.358	0.606
8.001	8.239	7.903	8.671	8.787	0.318	0.606
5.765	5.907	6.120	5.325	5.618	0.442	0.606
5.453	5.941	6.263	5.178	5.385	0.397	0.606
7.133	6.174	7.137	5.564	6.551	0.485	0.606
5.742	5.768	3.567	5.624	5.774	0.577	0.606
6.090	6.912	3.990	6.838	7.072	0.821	0.606
3.834	2.866	3.705	2.748	2.776	0.438	0.606
4.056	4.436	4.721	4.555	4.440	0.411	0.606
7.563	8.039	7.895	8.371	8.437	0.357	0.606
4.656	4.084	2.808	4.546	4.862	0.452	0.606
5.402	3.306	1.829	3.853	4.035	0.721	0.606
7.066	6.712	6.965	6.298	6.650	0.326	0.606
4.697	5.565	4.572	5.735	4.957	0.412	0.606
4.646	4.613	4.037	4.348	4.527	0.444	0.606

5.684	4.881	5.908	4.497	4.256	0.452	0.606
3.943	4.468	3.810	4.491	4.385	0.343	0.606
4.465	5.541	5.306	4.648	4.219	0.461	0.606
4.846	6.492	2.207	6.344	6.597	0.935	0.606
5.324	5.315	5.824	5.546	5.933	0.429	0.606
2.688	3.866	2.989	4.423	4.064	0.561	0.606
8.285	7.634	9.618	7.460	8.108	0.520	0.606
3.172	3.044	3.963	3.487	3.439	0.389	0.606
4.638	4.427	4.534	4.317	4.446	0.382	0.606
5.968	5.620	6.524	5.400	5.368	0.418	0.606
4.793	4.950	4.190	5.518	5.361	0.437	0.606
7.949	7.373	9.206	7.515	7.639	0.534	0.606
3.820	3.159	3.650	3.250	3.389	0.445	0.606
3.919	3.981	4.315	4.043	3.340	0.397	0.606
3.488	3.930	3.504	3.795	3.596	0.348	0.606
8.542	8.813	8.498	9.085	9.106	0.307	0.606
7.541	7.810	7.485	8.081	8.091	0.311	0.606
7.213	7.455	7.165	7.728	7.767	0.310	0.606
2.488	5.103	2.492	4.312	4.782	0.749	0.606
5.914	7.502	6.991	4.198	3.280	0.892	0.606
3.873	3.368	4.138	2.718	3.093	0.605	0.606
5.578	5.350	6.114	4.935	5.039	0.430	0.606
2.942	4.255	3.016	4.279	3.715	0.449	0.606
3.423	4.820	4.386	3.967	4.010	0.780	0.606
4.415	4.581	3.533	4.637	5.028	0.431	0.606
4.749	5.356	5.255	4.401	4.823	0.522	0.606
6.600	6.458	5.902	6.163	6.340	0.588	0.606
2.522	3.516	3.454	3.108	3.485	0.369	0.606
2.581	1.146	1.058	1.733	1.795	0.396	0.606
5.193	7.863	2.872	8.020	8.218	1.143	0.606
5.326	5.215	3.134	5.842	5.369	0.594	0.606
6.100	7.126	4.358	7.450	7.302	0.809	0.606
6.952	8.161	3.784	8.593	8.495	1.039	0.606
4.730	4.526	2.537	5.297	4.542	0.672	0.606
4.816	4.385	5.888	4.016	4.075	0.521	0.606
7.251	7.118	6.034	7.516	7.273	0.372	0.606
2.659	2.857	3.529	2.605	3.018	0.406	0.606
5.247	5.222	6.260	4.666	4.880	0.536	0.606
3.433	3.996	2.889	4.657	3.816	0.494	0.606
5.081	4.165	5.669	2.449	3.239	0.730	0.606
4.142	4.517	3.989	5.143	4.517	0.458	0.606
1.856	2.556	1.918	2.844	2.966	0.357	0.606
1.652	2.877	1.353	2.905	3.375	0.521	0.606
8.996	9.029	9.363	8.475	8.324	0.384	0.606
4.923	6.272	4.497	6.727	6.473	0.544	0.606
3.989	4.095	3.035	4.485	4.172	0.428	0.606
3.298	2.571	4.052	1.787	1.977	0.601	0.606
4.490	5.576	5.093	5.389	5.865	0.461	0.606
7.685	7.854	7.761	8.253	8.045	0.303	0.606
5.720	5.163	6.138	4.406	4.709	0.431	0.606

4.734	5.107	3.873	4.678	4.434	0.577	0.606
2.564	2.718	2.042	1.981	1.941	0.495	0.606
2.486	3.321	1.892	2.794	3.175	0.545	0.606
3.743	3.481	3.074	4.225	4.391	0.453	0.606
5.394	6.222	3.629	6.533	6.305	0.625	0.606
2.860	2.715	1.951	3.166	2.967	0.493	0.606
2.412	2.974	3.732	3.246	2.985	0.481	0.606
6.094	6.342	3.701	6.765	6.527	0.728	0.606
6.114	7.082	4.198	7.230	7.252	0.708	0.606
5.964	7.092	3.882	7.430	7.316	0.755	0.606
5.449	8.124	3.929	8.400	8.358	0.966	0.606
3.782	5.476	2.702	5.806	5.296	0.682	0.606
2.649	2.824	1.953	3.156	3.071	0.443	0.606
2.840	3.210	3.436	1.946	2.376	0.552	0.606
4.686	4.722	5.037	5.021	5.122	0.334	0.606
5.091	4.789	5.613	5.223	5.330	0.363	0.606
3.298	3.242	3.107	3.685	3.630	0.410	0.606
4.969	4.980	2.417	5.515	5.752	0.635	0.606
5.177	5.700	4.252	5.510	5.482	0.483	0.606
2.403	3.639	2.375	2.877	2.297	0.589	0.606
6.892	6.984	7.135	7.596	7.199	0.354	0.606
7.462	7.551	7.124	7.757	7.836	0.341	0.606
2.665	3.125	3.282	2.982	2.600	0.434	0.606
4.684	4.703	4.943	4.049	4.437	0.379	0.606
5.504	5.435	4.998	5.792	5.888	0.349	0.606
6.263	7.207	7.290	6.979	7.019	0.346	0.606
10.766	10.818	10.573	10.435	10.407	0.284	0.606
5.946	4.131	3.626	4.546	4.534	0.520	0.606
11.225	10.473	11.189	11.545	10.808	0.383	0.606
4.747	3.871	4.255	4.022	3.015	0.599	0.606
0.974	1.499	1.570	1.993	4.255	0.668	0.606
5.544	6.051	6.426	5.786	5.891	0.446	0.606
2.581	2.255	2.614	2.884	1.800	0.397	0.606
4.483	1.707	3.721	2.193	1.582	0.810	0.606
4.242	3.952	4.800	3.229	2.676	0.506	0.606
3.339	1.597	4.355	2.433	3.085	0.707	0.606
2.596	2.569	3.098	2.548	2.649	0.448	0.606
2.496	2.703	2.788	2.590	2.510	0.413	0.606
5.147	5.647	5.868	5.094	5.188	0.465	0.606
2.510	2.435	2.594	2.794	2.510	0.403	0.606
3.769	1.672	2.876	2.967	1.808	0.938	0.606
4.032	3.566	4.299	4.508	4.327	0.490	0.606
1.855	1.433	2.479	1.753	2.708	0.408	0.606
2.288	2.809	2.148	3.111	2.890	0.472	0.606
3.639	2.778	3.341	1.714	2.702	0.563	0.606
4.977	3.343	6.252	1.991	2.161	0.975	0.606
4.477	3.175	2.488	4.539	4.416	0.681	0.606
5.648	5.855	6.346	5.874	5.759	0.396	0.606
5.482	3.541	6.212	2.694	4.212	0.845	0.606
3.130	2.920	3.456	2.719	2.275	0.442	0.606

1.592	3.820	3.001	3.234	2.319	0.644	0.606
4.160	3.866	4.483	4.043	4.810	0.451	0.606
2.924	2.725	2.446	3.077	3.404	0.572	0.606
4.115	3.665	4.930	4.381	4.663	0.486	0.606
0.804	3.715	3.271	2.105	0.899	0.770	0.606
3.416	3.617	2.810	2.374	3.312	0.733	0.606
2.242	3.551	2.118	2.864	2.319	0.552	0.606
3.261	3.195	3.370	3.172	2.584	0.587	0.606
3.174	4.352	2.860	4.854	4.714	0.644	0.606
2.902	4.955	3.196	4.103	2.538	0.699	0.606
2.349	3.117	3.374	3.005	2.030	0.571	0.606
2.765	2.605	2.640	2.994	3.006	0.508	0.606
3.415	2.519	3.096	2.135	2.929	0.569	0.606
5.699	5.676	5.939	5.356	5.660	0.349	0.606
0.532	3.449	1.899	1.989	1.852	0.616	0.606
2.036	2.385	2.245	2.708	2.452	0.658	0.606
5.082	4.641	5.691	4.719	5.464	0.574	0.606
2.814	2.557	2.778	2.842	2.619	0.778	0.606
3.705	3.825	2.165	4.268	3.889	0.593	0.606
3.758	3.486	4.231	4.102	3.790	0.400	0.606
3.233	2.630	3.358	3.154	2.358	0.341	0.606
1.385	1.959	1.750	2.210	1.579	0.605	0.606
2.168	2.106	1.424	3.190	3.180	0.608	0.606
2.848	3.305	3.102	2.921	2.791	0.485	0.606
4.211	5.571	4.980	5.111	4.375	0.670	0.606
2.065	2.035	2.675	2.348	2.256	0.430	0.606
2.001	3.858	3.246	5.717	5.433	0.969	0.606
4.576	3.892	5.325	3.787	3.922	0.530	0.606
8.985	10.061	9.822	9.146	9.299	0.423	0.606
8.505	9.295	9.083	8.454	8.608	0.410	0.606
1.426	2.198	1.323	3.001	2.366	0.558	0.606
2.142	2.053	1.672	2.534	2.257	0.443	0.606
4.772	2.499	3.890	3.891	3.308	0.665	0.606
1.595	2.553	2.787	1.391	2.132	0.584	0.606
4.797	3.584	5.031	3.469	3.294	0.604	0.606
2.102	2.745	2.883	3.001	2.378	0.439	0.606
2.740	2.331	2.224	2.530	3.323	0.467	0.606
3.310	2.610	3.784	2.582	3.084	0.611	0.606
3.727	2.731	3.588	3.636	2.743	0.512	0.606
2.993	2.015	2.174	3.326	3.336	0.536	0.606
5.112	2.973	5.689	3.869	3.383	0.794	0.606
2.909	1.562	1.564	2.526	2.370	0.464	0.606
3.001	2.484	3.261	2.726	2.781	0.547	0.606
2.757	1.877	3.885	2.903	2.739	0.823	0.606
2.365	2.783	1.389	3.620	4.079	0.758	0.606
4.925	4.228	4.633	4.190	4.244	0.463	0.606
2.415	1.825	2.232	2.926	1.607	0.565	0.606
1.859	2.959	1.239	2.971	2.689	0.643	0.606
2.720	2.044	2.582	2.565	2.047	0.598	0.606
3.474	3.239	4.446	2.578	3.130	0.520	0.606

4.250	2.331	3.181	2.998	2.505	0.468	0.606
2.760	3.953	1.830	3.477	3.087	0.621	0.606
3.439	2.722	3.461	2.495	2.845	0.432	0.606
3.754	1.639	2.436	1.368	2.319	0.644	0.606
3.281	2.814	4.678	3.308	2.531	0.557	0.606
3.127	1.852	4.530	2.598	3.346	0.630	0.606
3.875	4.867	5.029	4.198	4.252	0.513	0.606
4.996	4.238	4.794	4.106	4.257	0.530	0.606
2.951	3.371	3.438	3.660	2.974	0.585	0.606
1.805	2.150	3.019	2.933	2.595	0.533	0.606
1.529	1.499	2.300	1.643	2.152	0.631	0.606
2.394	3.539	3.250	3.466	4.470	0.624	0.606
1.501	3.951	2.355	3.960	2.460	0.661	0.606
4.071	3.329	4.299	3.620	3.329	0.555	0.606
2.321	2.944	2.376	3.365	1.195	0.583	0.606
5.045	4.869	4.402	4.492	4.695	0.463	0.606
3.652	3.905	4.020	3.748	2.921	0.490	0.606
3.661	3.224	3.285	4.485	4.148	0.713	0.606
1.958	2.039	2.263	1.978	3.093	0.499	0.606
3.660	1.785	3.302	1.851	1.900	0.643	0.606
2.165	2.284	2.739	2.737	1.603	0.435	0.606
4.150	3.992	4.313	4.266	3.847	0.409	0.606
4.206	3.728	4.212	2.845	4.117	0.613	0.606
2.321	2.335	2.050	2.515	2.515	0.485	0.606
2.483	3.432	2.944	2.946	2.433	0.458	0.606
2.447	3.129	3.630	4.051	2.513	0.493	0.606
2.465	1.786	1.905	2.945	1.413	0.519	0.606
1.966	2.100	2.258	3.250	2.820	0.551	0.606
2.331	3.059	2.704	2.810	3.326	0.501	0.606
4.945	4.620	3.869	5.482	5.388	0.441	0.606
2.211	2.205	1.765	3.495	3.164	0.777	0.606
4.540	3.819	3.818	3.104	3.413	0.515	0.606
2.823	1.739	2.714	2.090	1.971	0.483	0.606
2.333	2.917	2.442	2.300	2.309	0.572	0.606
2.891	3.049	2.658	2.153	2.234	0.617	0.606
4.748	6.712	6.011	5.592	5.963	0.687	0.606
3.484	2.430	3.199	3.088	3.351	0.418	0.606
2.649	5.168	4.455	4.641	4.477	0.597	0.606
6.234	5.870	6.776	4.174	5.250	0.535	0.606
4.934	6.337	6.897	3.207	4.836	1.324	0.606
2.821	3.160	3.492	2.241	3.722	1.123	0.606
5.643	6.011	5.686	3.541	5.116	0.766	0.606
4.351	4.335	4.294	3.860	4.577	0.417	0.606
5.424	5.265	6.002	4.571	4.756	0.466	0.606
4.650	5.554	3.803	6.111	5.904	0.715	0.606
8.848	6.868	6.891	6.726	7.030	0.533	0.606
3.083	3.411	2.792	3.847	3.867	0.481	0.606
8.450	8.782	8.119	9.186	8.750	0.305	0.606
6.257	6.632	6.257	7.065	6.669	0.331	0.606
4.147	4.583	3.034	4.782	4.673	0.455	0.606

2.534	2.035	2.047	1.969	1.959	0.412	0.606
3.574	3.371	3.977	3.577	3.053	0.615	0.606
4.568	3.734	6.203	2.442	3.334	0.780	0.606
4.139	5.116	5.057	4.906	5.116	0.475	0.606
3.371	3.173	2.223	3.107	3.581	0.442	0.606
8.473	7.438	8.591	7.324	7.413	0.403	0.606
7.367	6.820	8.087	6.145	6.283	0.417	0.606
3.319	2.199	4.072	2.623	3.549	0.557	0.606
2.998	3.520	3.650	3.020	3.711	0.421	0.606
6.331	5.966	7.142	5.629	5.684	0.410	0.606
2.598	4.309	2.108	5.194	4.782	0.714	0.606
4.714	5.329	4.550	4.601	5.201	0.529	0.606
5.758	6.385	5.686	6.576	6.649	0.325	0.606
5.078	5.191	5.655	5.471	5.130	0.512	0.606
4.485	5.019	4.027	5.419	4.947	0.396	0.606
7.718	7.812	6.307	7.991	8.084	0.459	0.606
4.307	5.453	2.065	5.561	5.392	0.744	0.606
4.875	5.729	2.694	6.224	5.678	0.782	0.606
4.570	4.284	3.658	4.865	4.864	0.417	0.606
1.909	2.831	1.871	2.411	3.289	0.578	0.606
6.321	6.626	6.254	6.905	6.690	0.338	0.606
5.513	5.884	5.169	6.243	5.924	0.367	0.606
3.195	3.379	2.262	3.222	3.149	0.406	0.606
6.941	5.938	7.018	5.333	5.689	0.449	0.606
4.234	2.617	3.845	4.127	3.320	0.489	0.606
5.531	6.016	5.214	6.534	6.446	0.405	0.606
5.424	4.312	4.517	4.650	4.052	0.575	0.606
4.824	3.937	4.484	4.866	4.518	0.558	0.606
6.114	5.202	6.924	5.672	4.440	0.511	0.606
2.970	2.199	3.816	2.172	3.243	0.445	0.606
6.957	7.379	6.315	7.479	7.269	0.348	0.606
2.917	3.730	2.133	3.878	3.947	0.499	0.606
3.535	3.988	3.782	3.273	3.473	0.465	0.606
6.096	7.111	6.307	7.554	7.383	0.373	0.606
5.254	4.875	4.398	5.419	5.016	0.340	0.606
6.119	6.558	5.319	6.560	6.692	0.414	0.606
5.828	5.760	5.949	6.351	6.150	0.319	0.606
6.376	6.256	6.911	5.295	5.650	0.390	0.606
5.998	6.325	5.282	6.303	6.624	0.404	0.606
4.349	4.887	4.295	5.029	4.990	0.354	0.606
7.363	6.832	7.226	7.507	7.591	0.394	0.606
4.410	4.185	5.007	3.874	4.021	0.361	0.606
2.972	5.164	3.981	3.987	3.516	0.706	0.606
4.454	5.282	3.678	5.671	5.253	0.773	0.606
9.837	7.931	9.463	8.951	7.884	0.652	0.606
2.643	3.000	4.735	2.524	2.275	0.764	0.606
1.284	2.278	1.862	1.614	3.293	0.708	0.606
1.941	2.573	2.315	1.563	2.550	0.538	0.606
2.233	2.095	2.878	2.102	2.273	0.397	0.606
5.327	5.087	6.261	5.524	4.799	0.445	0.606

4.107	4.538	2.964	4.359	4.687	0.555	0.606
3.440	3.378	3.382	3.953	3.602	0.468	0.606
3.818	4.488	4.575	4.951	3.807	0.420	0.606
2.761	2.521	1.918	0.847	2.506	0.637	0.606
3.901	3.332	5.437	4.938	4.759	0.529	0.606
3.534	3.225	4.439	3.657	3.229	0.591	0.606
1.273	2.350	2.720	2.540	2.159	0.394	0.606
3.988	3.526	4.587	4.466	4.470	0.504	0.606
3.679	2.511	3.690	2.638	3.224	0.465	0.606
4.732	4.576	4.760	4.394	4.174	0.424	0.606
6.306	5.080	5.144	5.992	5.471	0.491	0.606
2.151	3.026	2.981	4.013	4.546	0.532	0.606
4.015	4.159	1.759	4.556	3.633	0.715	0.606
7.079	6.877	7.311	6.330	6.719	0.521	0.606
5.354	4.921	3.586	5.056	5.238	0.986	0.606
3.648	2.909	3.203	3.272	2.788	0.475	0.606
3.373	3.305	3.280	1.812	1.593	0.602	0.606
2.502	2.170	3.172	2.527	2.482	0.459	0.606
1.988	0.828	1.990	1.186	0.674	0.648	0.606
3.645	3.345	4.684	3.271	3.098	0.420	0.606
1.404	3.315	2.257	2.538	2.402	0.484	0.606
2.895	3.228	3.519	2.450	3.044	0.659	0.606
1.728	2.683	2.683	3.492	2.614	0.472	0.606
2.864	1.458	2.168	1.798	1.248	0.634	0.606
7.636	7.142	8.107	7.319	6.524	0.500	0.606
5.042	3.788	4.667	4.831	4.214	0.469	0.606
4.244	3.854	4.455	4.886	4.061	0.418	0.606
4.154	3.751	4.568	3.154	4.008	0.556	0.606
3.180	2.308	3.004	2.911	3.096	0.466	0.606
3.503	1.934	3.611	2.790	2.974	0.572	0.606
3.824	2.864	3.734	2.298	2.478	0.522	0.606
4.896	4.809	5.248	4.378	3.697	0.529	0.606
3.576	2.547	3.571	3.381	2.241	0.470	0.606
2.093	1.965	2.899	2.564	2.369	0.542	0.606
4.662	3.145	4.985	3.455	2.172	0.679	0.606
6.228	5.146	6.190	5.165	5.212	0.480	0.606
3.117	1.330	2.048	1.836	2.078	0.488	0.606
4.004	3.828	4.323	3.576	2.532	0.576	0.606
9.958	9.820	9.637	9.636	9.235	0.340	0.606
6.374	4.837	7.001	4.983	4.795	0.699	0.606
3.435	1.883	4.433	1.558	2.272	0.848	0.606
4.203	2.351	3.980	1.245	2.283	0.780	0.606
3.000	4.529	5.298	3.990	4.088	0.615	0.606
1.940	4.153	2.904	2.066	3.360	1.090	0.606
7.231	7.868	8.586	7.131	6.458	0.783	0.606
5.037	5.408	5.914	4.740	4.646	0.443	0.606
2.196	2.009	1.909	2.517	1.534	0.726	0.606
0.959	1.568	1.411	1.417	2.361	0.496	0.606
5.769	5.068	6.080	5.539	4.884	0.502	0.606
1.918	1.401	2.268	2.272	2.321	0.692	0.606

2.787	1.582	2.939	0.604	2.181	0.716	0.606
6.281	4.257	7.180	3.347	2.237	1.217	0.606
6.125	4.310	5.807	4.380	4.209	0.619	0.606
4.298	5.022	5.019	4.607	4.604	0.364	0.606
3.730	3.086	2.032	1.830	0.901	0.782	0.606
4.264	1.600	2.671	2.541	3.138	0.772	0.606
3.916	2.920	4.245	3.006	1.527	0.676	0.606
5.523	2.830	4.964	4.036	4.718	0.605	0.606
2.597	2.678	2.757	2.700	3.333	0.516	0.606
3.904	4.296	4.172	3.991	4.226	0.426	0.606
2.838	2.734	2.474	3.345	2.019	0.472	0.606
2.901	3.256	2.768	3.804	2.620	0.444	0.606
2.266	2.305	2.724	3.124	2.514	0.492	0.606
3.059	3.239	3.905	2.609	2.524	0.627	0.606
4.680	4.226	5.039	4.998	4.932	0.615	0.606
2.135	1.802	2.017	1.705	2.246	0.440	0.606
3.138	3.562	3.987	3.214	2.694	0.589	0.606
2.981	2.551	1.995	3.199	2.485	0.502	0.606
4.934	5.102	5.429	4.541	4.978	0.572	0.606
3.635	2.750	4.405	2.240	1.274	0.746	0.606
1.902	1.451	2.196	1.414	1.926	0.481	0.606
4.334	3.445	4.612	2.078	3.314	0.710	0.606
5.015	2.397	3.388	3.828	2.618	0.774	0.606
7.114	6.360	7.652	6.759	6.225	0.406	0.606
3.375	3.879	3.815	3.385	2.680	0.438	0.606
4.743	3.999	4.347	3.483	4.023	0.494	0.606
3.969	2.794	3.101	2.765	3.312	0.707	0.606
2.400	2.094	3.064	1.936	2.236	0.715	0.606
1.841	1.664	2.564	1.010	1.585	0.478	0.606
3.874	1.764	4.654	3.645	2.836	0.698	0.606
2.900	2.222	2.285	2.755	3.038	0.568	0.606
4.739	2.790	3.957	3.044	3.797	0.707	0.606
3.336	2.494	3.724	1.162	2.519	0.646	0.606
2.671	2.679	2.409	3.137	2.800	0.464	0.606
1.877	3.094	2.927	1.821	2.582	0.600	0.606
4.365	3.706	3.849	3.404	3.854	0.502	0.606
4.215	3.410	4.241	3.350	3.471	0.496	0.606
4.306	4.220	2.786	4.979	4.884	0.572	0.606
3.960	3.680	2.588	4.625	4.361	0.527	0.606
3.957	3.875	2.665	4.690	4.644	0.534	0.606
3.907	2.376	3.702	1.919	2.901	0.826	0.606
3.235	2.531	4.076	2.323	2.016	0.558	0.606
3.384	3.294	3.373	2.843	3.251	0.578	0.606
3.750	3.204	3.700	3.558	3.659	0.538	0.606
1.821	3.690	2.520	4.707	3.030	0.735	0.606
3.141	2.693	2.417	2.697	2.714	0.429	0.606
3.830	4.275	3.404	4.046	3.680	0.420	0.606
3.178	2.434	3.479	2.382	1.811	0.584	0.606
3.152	2.363	4.128	2.008	2.635	0.590	0.606
3.422	3.233	3.385	3.039	3.067	0.416	0.606

4.127	3.155	3.872	3.957	3.719	0.602	0.606
2.996	2.654	2.803	1.491	1.732	0.598	0.606
4.177	2.053	3.524	4.319	4.251	0.768	0.606
2.956	2.254	2.729	2.853	2.267	0.589	0.606
1.752	1.090	1.442	0.577	1.118	0.616	0.606
2.306	2.385	2.837	2.433	2.127	0.455	0.606
4.199	3.462	3.839	3.901	4.390	0.554	0.606
2.547	2.213	3.553	2.895	1.543	0.468	0.606
3.643	4.436	4.095	5.967	5.307	0.724	0.606
1.933	2.550	3.478	3.119	2.837	0.434	0.606
0.247	1.911	1.572	0.761	1.253	0.572	0.606
3.096	2.748	3.161	3.451	3.468	0.457	0.606
3.162	2.932	3.544	3.451	2.939	0.569	0.606
3.240	3.016	3.410	2.571	3.031	0.699	0.606
2.554	2.159	1.769	2.036	2.178	0.626	0.606
3.410	3.211	1.429	3.793	3.983	0.720	0.606
2.811	2.968	3.082	4.668	4.547	0.759	0.606
2.421	2.167	2.133	2.804	1.261	0.492	0.606
2.905	2.891	2.887	3.388	1.937	0.449	0.606
4.997	4.062	5.229	3.545	4.414	0.609	0.606
5.246	5.338	5.510	5.169	4.872	0.372	0.606
2.838	2.194	3.035	2.491	1.895	0.496	0.606
2.596	2.509	2.354	2.794	2.269	0.536	0.606
4.256	3.101	3.268	3.430	3.335	0.515	0.606
2.747	2.777	4.042	3.226	1.626	0.585	0.606
4.703	3.500	4.868	2.938	3.852	0.563	0.606
2.912	3.008	3.004	2.007	3.395	0.460	0.606
3.488	3.534	3.349	2.449	2.984	0.414	0.606
2.286	1.750	3.027	2.228	2.729	0.500	0.606
6.612	3.194	6.373	3.362	4.514	1.176	0.606
1.522	2.070	1.507	1.776	2.245	0.422	0.606
4.962	1.649	2.566	2.907	2.764	0.650	0.606
2.673	2.621	3.830	4.933	3.421	0.644	0.606
5.005	3.178	4.651	2.849	2.477	0.735	0.606
3.716	4.401	4.489	4.772	3.738	0.449	0.606
3.288	2.648	2.503	2.908	3.134	0.526	0.606
4.694	4.538	5.350	3.938	4.641	0.572	0.606
4.999	4.328	5.270	4.264	4.616	0.502	0.606
4.073	4.266	2.244	4.118	3.522	0.954	0.606
6.319	4.251	6.461	3.920	4.044	0.782	0.606
2.584	1.506	3.074	2.719	1.984	0.745	0.606
4.923	2.887	3.917	3.489	3.579	0.534	0.606
3.763	0.525	1.974	2.864	2.237	0.760	0.606
6.151	5.393	6.480	4.862	5.893	0.476	0.606
6.194	4.445	5.743	4.211	4.604	0.670	0.606
2.489	1.918	1.409	2.509	2.666	0.393	0.606
3.860	3.255	3.455	3.710	3.759	0.480	0.606
5.005	3.978	4.769	4.706	4.177	0.612	0.606
2.947	2.682	3.793	3.106	2.708	0.574	0.606
2.733	1.356	2.611	2.750	2.459	0.549	0.606

4.915	4.661	5.784	5.633	4.728	0.510	0.606
3.197	2.322	3.003	2.161	2.224	0.485	0.606
3.444	2.164	3.288	3.368	2.538	0.584	0.606
3.882	3.039	3.628	4.058	3.204	0.587	0.606
4.759	3.926	5.413	4.067	3.343	0.504	0.606
3.345	2.725	2.168	2.851	2.725	0.553	0.606
2.125	1.649	2.126	1.919	1.869	0.616	0.606
2.984	3.836	4.054	3.049	3.132	0.502	0.606
3.086	2.053	3.849	3.629	3.032	0.506	0.606
3.416	3.315	3.703	1.894	2.154	0.744	0.606
3.551	1.905	3.390	1.789	3.187	0.785	0.606
3.189	2.626	3.415	3.529	2.744	0.445	0.606
1.927	3.193	3.027	2.292	1.710	0.571	0.606
3.277	2.365	2.519	1.913	3.019	0.655	0.606
3.184	1.637	3.161	2.584	2.571	0.626	0.606
5.787	4.850	6.196	5.861	5.288	0.529	0.606
2.155	1.765	2.300	2.281	2.252	0.587	0.606
3.437	4.007	3.658	4.171	4.013	0.421	0.606
5.238	4.695	3.521	6.506	6.061	0.715	0.606
2.325	1.678	2.471	1.739	2.398	0.544	0.606
3.216	2.992	4.206	3.767	3.246	0.405	0.606
1.643	1.987	2.845	1.928	2.956	0.572	0.606
5.132	4.689	5.544	4.161	3.378	0.552	0.606
3.865	2.132	2.291	2.544	2.531	0.794	0.606
4.899	5.504	3.926	5.653	5.051	0.673	0.606
4.476	2.382	3.602	2.908	3.055	0.767	0.606
4.792	4.383	4.576	3.514	4.658	0.473	0.606
2.808	2.669	2.748	2.719	2.510	0.645	0.606
5.008	3.171	3.949	4.406	3.307	0.580	0.606
5.745	4.180	5.654	4.373	4.993	0.507	0.606
3.822	4.031	3.506	3.385	3.395	0.604	0.606
3.093	2.193	2.862	3.841	1.450	0.618	0.606
1.514	2.064	0.495	2.633	3.282	0.796	0.606
3.097	2.821	2.695	1.833	2.108	0.590	0.606
2.537	2.419	1.966	4.171	3.402	0.832	0.606
4.016	2.934	3.300	3.138	3.210	0.521	0.606
2.664	1.848	1.521	3.169	1.934	0.479	0.606
5.515	4.710	5.598	2.626	2.880	0.794	0.606
3.125	3.863	4.325	4.678	4.107	0.506	0.606
2.982	2.365	2.287	2.655	2.339	0.460	0.606
3.105	3.165	3.713	2.276	2.015	0.577	0.606
3.431	3.407	3.639	3.605	2.510	0.446	0.606
2.766	1.681	2.110	2.192	2.224	0.520	0.606
2.959	3.781	2.937	3.832	2.805	0.563	0.606
4.286	2.858	4.886	2.899	2.556	0.836	0.606
1.380	2.071	2.962	3.829	3.522	0.779	0.606
5.134	3.700	5.223	4.750	3.827	0.553	0.606
1.582	1.212	1.613	2.706	1.977	0.489	0.606
3.381	1.896	3.873	0.464	1.296	0.874	0.606
4.455	2.208	4.423	3.308	3.110	0.875	0.606

2.610	2.862	3.484	3.654	3.032	0.440	0.606
2.167	2.549	2.570	3.520	1.802	0.471	0.606
1.803	2.887	2.342	2.469	1.923	0.484	0.606
7.375	6.283	6.517	6.641	6.771	0.507	0.606
4.142	3.217	3.903	4.148	4.000	0.678	0.606
2.796	2.470	3.090	2.267	2.323	0.428	0.606
3.817	3.347	4.238	4.083	4.380	0.502	0.606
3.008	2.000	2.545	2.413	2.434	0.512	0.606
6.430	5.741	5.842	6.200	6.049	0.459	0.606
4.220	5.010	4.330	3.205	4.496	0.487	0.606
2.962	3.269	2.175	2.852	3.383	0.514	0.606
2.850	1.694	1.580	2.320	2.204	0.423	0.606
3.151	2.735	2.765	3.182	2.585	0.442	0.606
2.662	1.362	2.557	0.843	1.757	0.701	0.606
1.325	1.334	1.879	1.551	2.174	0.622	0.606
1.192	5.295	1.111	6.230	5.215	1.251	0.606
2.089	2.030	3.370	2.022	1.689	0.489	0.606
3.774	1.985	3.285	1.925	2.355	0.706	0.606
4.398	2.030	5.548	4.017	2.729	0.785	0.606
3.213	2.353	3.168	2.714	3.389	0.493	0.606
2.987	2.430	2.133	2.169	2.359	0.560	0.606
4.584	3.902	4.596	3.988	3.726	0.505	0.606
4.410	2.585	2.939	2.283	2.269	0.761	0.606
2.825	2.764	2.709	3.589	2.413	0.550	0.606
4.086	2.814	4.410	3.032	2.729	0.589	0.606
2.713	2.239	2.061	2.398	1.957	0.537	0.606
2.381	2.662	3.187	1.973	1.413	0.636	0.606
2.296	2.404	1.645	1.524	1.596	0.560	0.606
5.343	6.389	5.586	5.238	5.063	0.567	0.606
4.920	6.041	5.046	4.846	4.737	0.519	0.606
2.137	2.460	4.349	2.810	2.342	0.473	0.606
2.494	2.274	1.505	1.411	2.276	0.486	0.606
2.155	2.788	1.814	1.645	1.845	0.454	0.606
2.425	1.427	1.309	1.365	1.491	0.647	0.606
1.985	2.385	2.994	1.976	2.236	0.422	0.606
1.728	1.794	2.420	1.965	1.035	0.557	0.606
5.535	5.032	4.576	4.204	2.760	0.707	0.606
3.598	5.216	4.537	5.180	5.000	0.669	0.606
6.194	6.854	5.178	7.053	7.404	0.497	0.606
0.773	3.169	0.553	0.450	1.973	0.911	0.606
6.395	7.253	6.247	7.604	7.548	0.425	0.606
3.522	4.415	3.989	3.562	3.662	0.380	0.606
4.625	3.194	2.291	4.582	4.231	0.872	0.606
2.793	2.646	2.292	2.998	2.631	0.536	0.606
5.158	8.149	5.826	8.690	8.766	0.857	0.606
1.415	3.865	1.478	4.647	4.679	0.784	0.606
3.954	2.276	3.503	1.737	2.541	0.537	0.606
3.734	2.733	2.523	2.723	3.014	0.426	0.606
4.434	4.093	4.124	4.557	5.026	0.362	0.606
2.263	2.138	1.751	1.930	2.412	0.387	0.606

2.999	3.788	3.742	4.610	3.974	0.536	0.606
3.924	4.431	4.552	3.872	3.709	0.449	0.606
4.655	2.968	4.520	2.544	3.000	0.548	0.606
3.828	2.628	3.386	3.813	4.078	0.574	0.606
2.359	2.918	3.737	1.658	1.953	0.439	0.606
2.538	2.659	2.170	3.845	2.505	0.503	0.606
4.757	4.109	5.313	3.887	3.866	0.455	0.606
4.388	4.265	5.830	4.432	4.281	0.522	0.606
4.059	3.840	5.039	3.872	3.886	0.424	0.606
3.075	2.965	4.041	2.422	3.462	0.520	0.606
3.433	3.087	4.897	2.787	4.000	0.599	0.606
3.160	3.361	5.399	3.899	3.173	0.679	0.606
1.555	2.232	1.509	1.029	1.389	0.835	0.606
7.166	5.367	8.220	3.168	4.069	1.020	0.606
6.254	5.360	6.221	3.655	4.647	0.750	0.606
2.268	1.879	2.176	1.986	2.432	0.392	0.606
5.469	2.436	3.591	2.547	2.748	0.806	0.606
4.241	3.049	3.538	3.780	3.405	0.609	0.606
2.846	1.822	2.908	3.501	2.709	0.443	0.606
1.866	1.873	2.818	2.402	1.556	0.687	0.606
4.299	3.831	4.938	3.409	3.148	0.461	0.606
4.296	3.476	2.194	4.409	4.373	0.612	0.606
3.788	2.577	3.102	1.889	2.013	0.524	0.606
3.200	4.661	4.052	3.530	4.098	0.539	0.606
2.625	2.377	2.422	3.020	2.934	0.472	0.606
2.897	2.582	3.856	2.417	2.746	0.456	0.606
2.937	3.239	4.269	2.495	2.457	0.474	0.606
3.090	3.318	3.428	3.145	2.966	0.419	0.606
2.982	2.388	2.458	2.859	2.292	0.442	0.606
2.232	2.259	3.260	2.564	1.868	0.547	0.606
2.470	2.068	2.968	1.824	1.903	0.538	0.606
3.313	2.966	4.601	3.885	3.408	0.792	0.606
3.437	3.024	3.178	2.913	2.747	0.408	0.606
6.266	6.225	5.912	6.766	6.829	0.395	0.606
2.591	1.879	2.253	2.110	2.332	0.636	0.606
3.240	3.575	1.000	4.804	4.139	0.829	0.606
3.869	1.234	3.216	2.567	2.266	0.601	0.606
7.807	7.924	8.001	6.438	7.279	0.429	0.606
3.576	2.583	3.920	3.093	2.123	0.542	0.606
1.714	2.031	1.565	3.510	3.049	0.473	0.606
2.228	3.141	2.323	3.048	3.630	0.444	0.606
2.748	4.565	5.043	3.996	4.675	0.682	0.606
2.562	2.041	2.719	2.144	1.872	0.383	0.606
3.184	3.775	3.747	3.195	3.586	0.411	0.606
2.381	1.144	2.266	1.038	1.246	0.703	0.606
3.276	2.938	3.086	2.756	2.304	0.454	0.606
1.201	3.029	2.337	4.399	4.141	0.697	0.606
1.849	2.125	1.546	2.998	2.144	0.487	0.606
2.115	2.383	0.693	2.118	3.115	0.532	0.606
2.390	4.832	2.429	4.742	4.697	0.650	0.606

2.724	2.618	3.709	2.288	2.318	0.527	0.606
2.823	1.885	2.198	2.754	2.181	0.429	0.606
3.400	4.401	6.500	4.476	4.334	0.607	0.606
2.288	2.333	1.909	3.730	3.972	0.527	0.606
3.412	2.211	1.812	3.084	2.847	0.465	0.606
3.746	2.579	2.914	1.968	2.903	0.645	0.606
2.048	2.805	2.179	1.847	2.456	0.487	0.606
4.102	3.461	3.398	2.441	2.302	0.461	0.606
2.960	2.095	1.951	2.440	2.086	0.446	0.606
4.538	3.769	4.736	4.140	4.051	0.430	0.606
1.728	4.229	2.166	4.216	3.855	0.653	0.606
2.237	3.829	2.680	3.077	2.511	0.454	0.606
2.904	3.225	4.000	2.337	2.908	0.453	0.606
2.820	2.064	2.458	1.786	2.012	0.458	0.606
2.243	2.644	3.578	1.610	2.706	0.558	0.606
1.920	2.701	2.168	1.605	3.473	0.501	0.606
2.628	2.705	1.935	2.559	2.712	0.420	0.606
4.434	4.089	4.451	2.781	3.507	0.609	0.606
1.846	3.571	1.887	3.935	3.656	0.590	0.606
3.342	3.299	4.236	3.204	3.101	0.451	0.606
4.171	4.307	4.445	4.168	5.379	0.438	0.606
4.155	4.082	4.492	3.744	4.690	0.489	0.606
4.612	2.199	5.201	1.890	1.360	0.920	0.606
3.911	4.835	5.006	2.925	4.202	0.538	0.606
4.988	4.348	4.523	4.166	3.975	0.373	0.606
3.475	2.861	2.429	2.630	2.743	0.469	0.606
2.354	2.442	2.984	2.662	2.548	0.492	0.606
4.658	4.800	1.590	4.690	4.886	0.994	0.606
2.578	2.011	3.760	2.818	2.819	0.476	0.606
2.004	2.266	2.876	3.491	2.046	0.639	0.606
4.387	3.282	4.396	3.943	3.303	0.581	0.606
2.493	4.443	2.964	0.936	4.872	0.902	0.606
2.005	2.486	2.868	2.372	2.663	0.460	0.606
2.266	3.165	1.805	3.336	3.112	0.409	0.606
2.440	1.401	3.289	1.587	2.264	0.620	0.606
3.571	3.783	2.898	4.404	3.821	0.402	0.606
4.221	3.756	4.260	4.137	3.680	0.591	0.606
8.205	7.326	8.662	6.086	6.411	0.618	0.606
4.873	4.722	4.787	5.277	4.630	0.443	0.606
4.470	3.907	4.983	3.759	4.231	0.424	0.606
3.572	2.166	4.021	1.813	2.094	0.646	0.606
6.897	6.380	8.356	4.689	5.049	0.730	0.606
2.393	3.526	2.287	2.968	2.879	0.439	0.606
3.019	2.976	2.678	2.908	2.077	0.512	0.606
3.906	4.315	2.531	5.134	4.384	0.526	0.606
3.152	2.636	2.521	3.216	3.023	0.467	0.606
1.784	3.351	1.976	4.089	4.098	0.617	0.606
3.537	2.443	3.470	2.604	2.209	0.559	0.606
1.668	2.517	1.179	3.451	3.334	0.602	0.606
2.771	2.458	2.654	2.704	2.168	0.498	0.606

1.655	3.125	1.950	2.443	2.783	0.505	0.606
3.750	3.781	4.070	3.433	3.676	0.418	0.606
2.551	4.402	2.164	4.739	5.033	0.753	0.606
5.088	3.444	6.032	2.428	3.064	0.760	0.606
3.324	3.312	1.713	4.693	4.657	0.655	0.606
4.175	3.784	4.467	2.711	2.896	0.505	0.606
2.501	3.902	2.865	3.973	4.078	0.522	0.606
4.454	4.916	3.337	4.101	4.622	0.463	0.606
3.028	1.847	2.669	2.046	2.632	0.594	0.606
2.890	1.936	2.790	1.923	2.386	0.523	0.606
7.524	7.368	8.238	2.809	3.553	1.558	0.606
2.352	2.968	3.196	3.608	2.015	0.489	0.606
4.378	3.794	3.843	4.189	3.893	0.468	0.606
2.389	1.724	1.106	2.811	1.662	0.636	0.606
4.868	4.083	4.308	3.974	4.305	0.416	0.606
2.752	4.158	4.170	4.585	3.266	0.592	0.606
1.008	2.380	1.664	3.129	3.224	0.590	0.606
3.716	3.327	2.588	3.213	2.646	0.530	0.606
1.818	2.824	1.494	2.471	3.280	0.496	0.606
4.978	4.388	5.447	5.529	5.306	0.465	0.606
2.133	2.084	2.875	2.093	2.819	0.371	0.606
2.722	3.199	4.385	3.681	3.276	0.487	0.606
2.612	3.527	1.784	4.453	3.303	0.536	0.606
4.472	3.935	4.659	4.132	4.281	0.414	0.606
2.198	1.915	4.011	2.182	3.154	0.693	0.606
2.313	3.384	1.733	3.184	3.640	0.535	0.606
3.863	2.663	4.397	2.454	1.837	0.627	0.606
4.645	3.755	4.206	4.254	4.703	0.468	0.606
4.066	2.661	2.802	2.116	2.060	0.576	0.606
3.681	3.383	4.536	4.252	4.577	0.527	0.606
5.154	3.402	4.544	3.649	3.986	0.682	0.606
3.748	2.022	3.556	3.507	3.325	0.519	0.606
2.635	1.969	2.421	2.299	2.524	0.521	0.606
5.194	4.420	6.175	4.350	4.565	0.468	0.606
2.675	2.377	3.256	3.281	2.638	0.527	0.606
2.836	2.048	3.292	2.505	1.968	0.456	0.606
2.442	5.263	2.730	4.714	5.145	0.754	0.606
0.589	2.577	1.224	3.064	2.737	0.534	0.606
3.047	3.571	2.055	3.173	4.052	0.479	0.606
4.614	4.629	3.991	4.631	4.541	0.393	0.606
3.979	3.175	3.976	4.187	3.858	0.475	0.606
3.835	2.696	4.899	3.279	4.324	0.701	0.606
2.664	1.608	3.481	2.420	2.631	0.512	0.606
2.737	2.687	2.731	3.650	2.801	0.435	0.606
3.080	3.735	3.436	3.294	3.594	0.379	0.606
3.397	3.136	3.669	3.818	4.348	0.421	0.606
3.751	2.638	3.566	3.790	3.056	0.471	0.606
1.473	3.185	3.278	1.639	3.076	0.752	0.606
2.284	4.456	3.791	3.000	4.180	0.727	0.606
3.535	5.916	5.493	4.258	5.668	0.816	0.606

3.087	2.669	2.115	3.420	2.743	0.491	0.606
4.975	4.380	5.549	3.821	4.077	0.442	0.606
2.023	2.374	2.582	3.053	2.404	0.457	0.606
2.622	2.312	3.315	2.417	2.325	0.433	0.606
1.602	2.043	1.027	3.321	2.330	0.512	0.606
4.120	3.542	4.175	4.376	4.741	0.427	0.606
1.491	1.972	2.088	2.352	2.100	0.382	0.606
2.087	3.129	3.130	3.486	2.289	0.506	0.606
1.706	2.567	3.097	3.643	2.642	0.660	0.606
1.701	2.906	2.994	3.777	2.839	0.686	0.606
1.994	2.618	2.982	3.590	3.851	0.502	0.606
4.404	3.726	5.248	3.364	3.908	0.477	0.606
4.554	4.767	4.880	4.585	4.286	0.502	0.606
2.931	3.519	3.464	3.118	2.831	0.399	0.606
3.000	6.523	1.767	2.321	3.636	1.098	0.606
3.491	2.470	4.268	3.247	2.813	0.535	0.606
4.833	4.491	3.888	5.433	5.055	0.660	0.606
3.759	3.191	3.691	3.385	3.258	0.436	0.606
1.593	0.928	1.566	2.009	1.436	0.811	0.606
2.847	2.536	3.348	2.514	2.274	0.484	0.606
3.192	2.593	1.955	2.091	2.008	0.408	0.606
1.730	2.944	1.785	3.237	3.188	0.466	0.606
2.967	2.279	2.038	2.386	2.643	0.408	0.606
1.876	2.577	2.465	1.988	2.024	0.397	0.606
5.654	4.375	5.988	4.021	4.344	0.693	0.606
6.711	7.011	6.900	7.627	7.291	0.390	0.606
4.879	2.304	3.656	2.000	3.092	0.867	0.606
1.575	2.209	1.768	3.417	2.440	0.460	0.606
3.741	2.750	3.527	2.113	3.279	0.526	0.606
1.989	2.737	3.602	2.930	3.261	0.489	0.606
3.320	2.841	4.234	3.508	2.453	0.482	0.606
3.894	3.408	3.423	2.788	3.729	0.569	0.606
3.354	3.398	3.079	2.805	2.474	0.369	0.606
4.835	3.701	4.127	3.169	3.546	0.537	0.606
4.375	4.612	3.419	4.674	4.752	0.378	0.606
2.027	2.256	2.144	1.400	2.198	0.416	0.606
2.055	3.345	2.589	2.467	2.243	0.443	0.606
2.807	2.403	4.466	2.313	1.567	0.631	0.606
1.799	2.053	2.073	3.330	3.444	0.471	0.606
3.452	3.104	2.293	2.217	3.102	0.440	0.606
3.965	2.408	3.602	2.571	1.470	0.657	0.606
5.080	4.509	5.171	4.246	4.510	0.450	0.606
5.165	4.518	5.612	4.878	4.119	0.460	0.606
1.768	3.074	2.195	3.287	2.686	0.540	0.606
2.859	3.192	3.775	3.005	2.112	0.445	0.606
2.582	2.407	1.398	1.906	1.986	0.401	0.606
2.939	3.586	2.867	3.745	3.451	0.405	0.606
3.522	4.249	3.409	4.579	4.101	0.624	0.606
4.249	2.570	4.085	2.913	2.157	0.739	0.606
3.129	1.877	3.022	2.689	2.315	0.414	0.606

4.989	5.406	3.605	4.875	5.516	0.512	0.606
3.006	2.315	3.357	1.790	2.083	0.550	0.606
2.176	3.357	2.014	3.554	3.643	0.656	0.606
1.882	2.494	5.622	1.749	1.620	0.763	0.606
2.894	3.365	2.165	3.104	3.590	0.400	0.606
3.075	1.780	1.483	2.505	2.598	0.474	0.606
4.186	4.077	4.733	4.449	4.416	0.453	0.606
2.960	2.316	2.912	1.886	2.338	0.412	0.606
3.831	3.247	4.568	3.521	3.597	0.361	0.606
6.252	6.171	6.125	6.524	6.749	0.430	0.606
1.978	2.347	2.771	1.985	3.055	0.444	0.606
1.822	3.035	1.568	4.130	3.085	0.593	0.606
2.534	2.416	2.502	1.881	1.591	0.534	0.606
2.374	3.132	2.620	3.416	3.358	0.448	0.606
3.475	3.856	3.105	4.356	5.349	0.667	0.606
5.096	4.734	4.128	5.468	4.618	0.712	0.606
0.612	1.999	0.719	2.699	2.547	0.567	0.606
3.174	2.349	2.860	1.323	2.578	0.652	0.606
3.687	5.259	4.060	4.094	4.680	0.472	0.606
4.247	4.263	3.405	4.506	4.294	0.451	0.606
2.013	3.388	2.398	4.038	3.849	0.549	0.606
2.062	1.988	2.648	2.316	2.207	0.478	0.606
4.269	4.737	4.352	4.096	4.412	0.470	0.606
2.903	3.378	2.518	4.383	4.242	0.525	0.606
6.005	4.777	3.233	5.633	5.721	0.843	0.606
2.758	1.484	3.546	2.478	2.491	0.505	0.606
1.201	2.710	1.464	4.173	3.810	0.707	0.606
7.013	4.232	7.429	3.465	4.400	0.904	0.606
2.772	1.380	3.396	0.995	1.934	0.614	0.606
3.574	2.748	3.213	3.786	2.342	0.478	0.606
1.776	1.508	1.358	3.335	2.064	0.554	0.606
2.748	2.391	1.849	3.438	3.766	0.517	0.606
3.960	4.028	4.161	3.873	2.999	0.449	0.606
1.893	2.720	1.219	3.608	2.269	0.511	0.606
5.668	5.583	4.746	4.716	5.770	0.472	0.606
1.592	2.425	1.621	2.285	3.586	0.475	0.606
1.735	3.035	2.521	2.571	2.617	0.419	0.606
5.125	2.374	6.091	1.726	2.026	1.148	0.606
4.991	2.512	3.641	2.639	4.542	1.033	0.606
2.690	2.388	3.962	2.488	2.348	0.542	0.606
4.029	2.671	3.162	2.903	3.534	0.629	0.606
3.895	3.696	4.105	3.693	3.227	0.391	0.606
3.180	3.108	2.943	3.107	3.307	0.396	0.606
3.278	3.690	2.296	3.956	3.585	0.451	0.606
1.406	2.205	1.214	2.698	3.302	0.490	0.606
3.431	3.583	3.932	3.280	3.394	0.458	0.606
3.871	3.452	3.803	3.395	3.607	0.430	0.606
2.886	4.291	4.038	3.935	3.456	0.530	0.606
2.147	4.208	1.744	3.978	4.171	0.631	0.606
2.457	2.541	2.840	3.673	2.130	0.417	0.606

3.824	4.722	4.094	3.606	4.812	0.523	0.606
5.015	5.767	5.193	6.165	6.708	0.608	0.606
2.641	3.914	2.007	4.848	4.263	0.659	0.606
2.618	2.626	2.040	1.967	2.850	0.423	0.606
2.579	2.625	1.754	2.031	2.457	0.465	0.606
4.962	3.632	5.412	3.650	3.142	0.687	0.606
4.864	1.352	2.841	2.390	2.494	0.738	0.606
6.299	4.515	5.758	5.579	5.116	0.674	0.606
3.275	1.959	3.170	2.690	2.529	0.492	0.606
4.351	3.805	3.765	3.776	3.357	0.437	0.606
2.870	4.404	2.382	3.513	3.738	0.540	0.606
3.147	1.568	2.626	2.055	2.375	0.562	0.606
4.063	3.560	2.134	3.762	4.122	0.700	0.606
3.205	3.234	1.903	3.934	3.192	0.582	0.606
2.212	2.960	2.517	2.120	1.594	0.663	0.606
5.286	6.197	5.594	5.809	6.165	0.425	0.606
2.747	2.260	2.756	2.355	2.814	0.425	0.606
4.100	2.651	4.949	2.546	2.290	0.583	0.606
2.705	2.889	2.706	2.771	2.384	0.426	0.606
4.279	3.466	3.560	3.784	2.048	0.776	0.606
1.519	2.786	2.016	2.701	3.081	0.458	0.606
1.447	2.655	2.057	3.232	2.780	0.453	0.606
3.137	2.641	3.734	2.337	2.579	0.447	0.606
3.512	2.599	4.806	2.346	2.392	0.578	0.606
3.469	3.085	3.673	3.392	2.841	0.446	0.606
2.830	3.378	2.665	3.340	3.750	0.422	0.606
2.482	2.560	2.670	2.653	3.133	0.351	0.606
3.504	2.705	2.840	3.672	3.010	0.492	0.606
5.630	4.332	5.685	4.571	4.231	0.538	0.606
1.533	2.503	3.121	2.212	1.969	0.426	0.606
6.837	5.186	6.063	6.429	6.373	0.452	0.606
3.152	1.850	2.785	3.351	2.802	0.710	0.606
3.524	2.658	4.993	2.616	2.951	0.569	0.606
3.785	3.717	4.424	4.286	4.228	0.370	0.606
4.803	4.251	4.602	5.055	4.129	0.454	0.606
2.400	2.518	2.006	2.951	2.874	0.506	0.606
5.416	5.235	5.743	5.294	5.100	0.435	0.606
3.151	3.590	3.587	2.743	3.381	0.543	0.606
3.724	2.922	3.501	2.639	1.482	0.613	0.606
3.184	2.942	3.551	3.328	4.042	0.417	0.606
2.083	2.620	2.318	2.872	3.181	0.605	0.606
5.129	3.426	4.895	3.252	4.078	0.586	0.606
1.166	1.943	3.143	3.327	2.422	0.445	0.606
1.728	2.193	2.792	2.118	1.596	0.561	0.606
2.175	2.149	2.676	1.484	2.175	0.482	0.606
3.581	3.456	3.373	4.361	3.768	0.326	0.606
2.448	2.978	1.375	2.653	3.534	0.483	0.606
3.300	2.943	3.628	2.994	3.429	0.420	0.606
1.637	2.884	1.545	2.952	2.426	0.501	0.606
4.820	5.576	4.003	4.934	5.206	0.582	0.606

2.256	2.699	2.330	3.527	2.990	0.400	0.606
2.050	3.268	2.230	3.672	3.460	0.479	0.606
4.504	2.116	4.081	2.556	1.877	0.632	0.606
3.898	2.814	4.853	2.651	2.123	0.623	0.606
7.867	8.315	8.957	7.496	7.957	0.382	0.606
2.268	3.575	2.479	3.747	4.275	0.523	0.606
1.808	2.199	1.809	1.835	1.950	0.386	0.606
2.901	3.841	3.408	3.947	3.487	0.426	0.606
3.763	3.656	3.462	2.885	2.988	0.562	0.606
2.294	2.165	1.573	2.176	2.126	0.540	0.606
2.418	3.044	2.860	2.150	2.469	0.425	0.606
3.755	3.070	3.279	3.134	3.616	0.371	0.606
2.310	2.081	1.762	3.039	3.544	0.585	0.606
6.951	5.551	6.858	6.257	5.788	0.452	0.606
5.323	3.304	5.773	3.533	2.813	0.797	0.606
3.381	3.350	4.066	3.350	3.339	0.462	0.606
1.415	1.017	1.372	1.517	1.202	0.522	0.606
2.597	3.603	2.206	4.911	4.041	0.623	0.606
1.074	3.518	2.757	4.144	3.983	0.733	0.606
3.717	2.299	2.834	2.784	2.849	0.503	0.606
4.337	4.638	3.916	4.222	4.654	0.421	0.606
1.919	2.365	1.682	2.588	1.577	0.439	0.606
2.818	3.627	1.934	3.405	3.450	0.478	0.606
2.944	3.171	2.392	3.090	2.710	0.479	0.606
3.060	2.975	3.259	1.861	2.984	0.522	0.606
1.283	2.274	1.055	3.608	3.357	0.584	0.606
1.975	2.659	0.936	2.941	3.886	0.610	0.606
1.819	2.656	1.926	3.661	4.395	0.594	0.606
0.843	2.848	0.928	2.822	2.819	0.598	0.606
4.001	6.253	1.598	6.932	6.292	1.010	0.606
3.734	3.187	2.849	4.378	4.351	0.466	0.606
4.872	3.880	5.217	4.404	4.436	0.617	0.606
4.165	2.919	3.396	3.197	2.809	0.486	0.606
4.186	4.166	3.636	3.620	3.163	0.639	0.606
0.976	1.473	1.919	4.489	4.393	0.863	0.606
2.722	2.218	2.671	1.712	1.600	0.446	0.606
3.057	2.268	3.091	2.597	2.206	0.410	0.606
3.446	3.099	1.105	4.004	3.523	0.662	0.606
4.298	3.807	5.813	4.215	4.243	0.536	0.606
2.426	2.342	2.101	2.739	2.853	0.473	0.606
3.659	3.666	3.890	3.134	4.779	0.460	0.606
4.430	5.086	3.418	6.290	5.774	0.689	0.606
1.445	2.492	0.732	3.681	3.101	0.651	0.606
2.568	2.423	2.749	3.529	3.602	0.549	0.606
4.805	4.813	5.685	4.623	5.269	0.444	0.606
4.272	2.643	3.331	3.400	3.371	0.509	0.606
3.479	2.611	2.693	2.519	2.698	0.462	0.606
7.623	6.800	7.667	7.171	6.953	0.404	0.606
3.203	2.381	2.817	3.094	3.054	0.504	0.606
3.217	1.865	1.708	2.706	2.918	0.455	0.606

2.590	2.125	1.952	2.282	2.687	0.487	0.606
3.563	2.953	3.715	3.212	3.339	0.440	0.606
3.146	2.564	4.051	2.919	3.277	0.469	0.606
3.365	2.985	3.939	3.473	3.369	0.429	0.606
2.246	2.033	1.379	3.081	2.720	0.464	0.606
3.842	2.576	3.482	3.906	3.592	0.470	0.606
4.365	4.487	5.479	3.413	3.862	0.449	0.606
2.995	2.782	2.745	2.476	3.259	0.444	0.606
7.325	7.094	7.399	7.226	7.392	0.448	0.606
4.084	2.335	3.071	2.402	2.393	0.528	0.606
1.176	2.890	1.007	3.390	3.023	0.664	0.606
2.781	4.300	2.274	5.049	5.069	0.694	0.606
7.485	6.673	7.767	7.033	6.313	0.466	0.606
2.107	2.414	2.570	2.716	2.306	0.366	0.606
4.294	4.219	4.825	3.791	4.057	0.499	0.606
6.230	3.034	6.706	3.303	3.531	0.879	0.606
3.122	2.784	3.718	2.572	3.629	0.506	0.606
2.169	1.378	1.159	1.418	1.317	0.656	0.606
4.005	3.728	3.882	2.657	3.633	0.429	0.606
3.061	3.135	5.480	2.919	2.571	0.609	0.606
3.783	5.730	3.503	6.147	5.732	0.733	0.606
4.365	4.168	2.374	4.606	4.760	0.637	0.606
7.887	6.975	8.235	7.555	7.888	0.463	0.606
2.582	1.852	3.242	2.647	2.450	0.389	0.606
2.147	1.408	1.182	2.584	1.947	0.481	0.606
2.660	2.245	2.389	3.226	2.716	0.476	0.606
3.270	3.403	2.918	3.359	3.875	0.450	0.606
3.058	2.746	3.298	4.377	4.044	0.446	0.606
2.620	2.918	3.659	2.818	2.818	0.423	0.606
2.117	3.336	4.221	2.688	2.883	0.503	0.606
2.255	2.470	3.136	2.992	2.393	0.475	0.606
2.532	3.001	2.316	2.783	2.690	0.421	0.606
2.377	2.566	2.953	1.853	1.769	0.477	0.606
2.422	3.146	1.716	3.203	3.054	0.553	0.606
2.782	3.491	1.838	3.568	2.962	0.548	0.606
3.388	3.953	2.446	3.646	4.210	0.436	0.606
2.962	2.954	3.735	3.584	3.001	0.446	0.606
3.091	3.541	3.705	2.730	3.791	0.520	0.606
1.859	2.684	2.733	4.529	3.621	0.594	0.606
2.586	2.682	4.150	3.667	3.277	0.449	0.606
3.244	4.654	3.501	4.727	4.810	0.495	0.606
3.293	3.699	1.840	2.074	2.252	0.836	0.606
4.378	4.359	4.529	4.284	4.285	0.448	0.606
4.890	5.153	3.872	5.953	5.749	0.535	0.606
6.543	7.579	4.335	7.514	7.495	0.791	0.606
1.927	2.012	3.391	2.724	2.901	0.429	0.606
9.294	9.736	10.006	9.005	9.452	0.314	0.606
2.897	3.966	3.758	3.516	3.178	0.415	0.606
2.658	2.728	2.992	2.455	2.201	0.401	0.606
6.589	7.109	7.734	6.661	6.688	0.372	0.606

8.450	8.100	8.451	7.611	8.229	0.319	0.606
3.877	2.908	4.562	3.119	2.833	0.518	0.606
6.083	5.504	6.317	5.641	5.715	0.524	0.606
4.312	3.965	4.569	5.239	4.482	0.553	0.606
5.688	5.409	5.155	6.221	5.866	0.505	0.606
3.708	3.293	2.740	4.875	4.762	0.664	0.606
3.326	3.437	3.947	3.491	3.785	0.358	0.606
3.932	4.065	3.931	3.370	3.686	0.356	0.606
2.726	3.242	3.159	2.609	2.914	0.325	0.606
4.079	5.070	4.477	5.504	5.251	0.441	0.606
4.179	4.590	4.362	5.098	5.141	0.333	0.606
3.043	3.009	3.244	3.984	3.381	0.427	0.606
3.238	3.476	3.404	4.620	3.620	0.379	0.606
2.619	3.841	2.092	4.118	3.735	0.652	0.606
3.620	5.673	5.836	4.531	4.978	0.530	0.606
3.311	2.950	4.074	3.246	2.608	0.457	0.606
7.775	8.061	8.679	7.164	7.422	0.388	0.606
2.863	3.043	4.303	2.434	2.579	0.535	0.606
8.776	9.737	9.760	8.671	8.897	0.406	0.606
10.353	10.939	10.812	9.885	10.542	0.361	0.606
5.209	5.039	5.835	4.975	5.539	0.444	0.606
3.481	3.106	3.165	3.200	3.765	0.406	0.606
10.065	10.103	10.679	9.761	10.094	0.295	0.606
8.381	8.646	8.947	8.087	8.197	0.326	0.606
4.605	4.363	5.204	4.601	4.095	0.444	0.606
4.941	5.401	4.251	5.808	5.814	0.592	0.606
6.091	6.430	6.077	6.679	6.673	0.307	0.606
4.812	4.531	4.790	4.660	4.610	0.425	0.606
1.516	2.402	2.369	3.080	2.459	0.642	0.606
2.986	2.089	1.704	2.584	1.282	0.456	0.606
4.235	4.577	3.664	5.018	4.467	0.408	0.606
1.967	2.247	3.425	1.962	1.974	0.566	0.606
5.410	5.626	4.981	5.530	5.707	0.373	0.606
6.147	6.223	6.051	5.520	6.212	0.396	0.606
6.941	6.571	7.094	5.580	5.789	0.438	0.606
4.747	4.324	5.346	4.793	4.408	0.364	0.606
2.638	2.012	1.543	2.621	2.639	0.417	0.606
5.795	5.887	6.095	6.061	6.347	0.410	0.606
5.367	6.226	5.500	6.515	6.292	0.464	0.606
3.853	2.791	3.457	1.800	2.503	0.747	0.606
3.996	5.242	3.685	5.776	5.528	0.568	0.606
4.140	3.677	4.446	3.524	3.420	0.381	0.606
5.418	5.049	4.417	5.411	4.959	0.460	0.606
3.789	2.896	5.310	4.272	1.420	1.172	0.606
2.746	2.508	1.960	2.558	2.893	0.509	0.606
5.862	5.744	5.667	5.724	5.770	0.386	0.606
4.780	3.067	2.906	3.091	3.832	0.564	0.606
5.661	5.594	4.525	5.997	6.239	0.379	0.606
4.100	2.222	2.255	2.393	2.566	0.723	0.606
3.616	5.525	3.010	5.769	6.170	0.753	0.606

3.053	3.705	1.430	4.005	3.862	0.613	0.606
5.397	5.360	4.977	5.923	5.677	0.371	0.606
7.181	7.627	6.080	7.883	7.898	0.441	0.606
6.516	6.002	7.625	4.806	4.731	0.586	0.606
1.561	2.295	1.203	3.604	3.589	0.599	0.606
7.750	7.974	6.911	8.669	8.425	0.454	0.606
4.737	5.410	3.061	5.492	5.477	0.621	0.606
9.314	9.586	9.023	9.686	9.548	0.320	0.606
4.158	4.190	4.480	4.788	4.832	0.371	0.606
2.885	4.052	2.619	4.252	3.910	0.450	0.606
3.551	6.232	2.508	6.615	6.079	0.838	0.606
1.384	3.105	1.858	4.279	3.649	0.629	0.606
1.441	2.940	0.864	3.260	2.920	0.582	0.606
3.282	2.746	3.225	1.882	2.977	0.468	0.606
2.711	3.610	2.300	3.257	3.433	0.458	0.606
6.313	5.534	6.785	4.876	4.784	0.538	0.606
5.307	6.162	5.111	6.623	6.206	0.410	0.606
8.002	9.095	6.357	9.229	9.027	0.637	0.606
4.951	5.096	3.553	5.756	5.455	0.564	0.606
2.619	1.671	3.032	1.695	2.121	0.561	0.606
4.572	5.128	3.691	5.496	5.316	0.454	0.606
2.450	2.350	2.924	2.029	2.121	0.393	0.606
4.086	4.529	2.782	4.439	4.382	0.406	0.606
6.827	7.341	6.753	7.351	7.346	0.357	0.606
5.749	6.017	4.650	6.261	6.221	0.419	0.606
3.706	3.767	2.741	3.843	4.352	0.588	0.606
1.193	4.968	2.379	5.668	5.674	1.055	0.606
4.878	4.221	4.202	4.195	4.876	0.335	0.606
6.589	7.491	5.186	8.067	7.854	0.812	0.606
6.798	5.967	6.444	5.907	5.702	0.419	0.606
3.899	3.694	4.122	3.070	3.252	0.383	0.606
3.438	2.514	2.332	2.088	2.214	0.425	0.606
3.469	4.012	3.706	2.453	3.336	0.537	0.606
3.308	5.664	5.730	3.561	5.190	1.378	0.606
4.227	4.681	4.228	3.506	3.955	0.444	0.606
1.826	2.591	1.991	0.963	1.746	0.575	0.606
4.489	4.734	4.026	4.347	4.323	0.326	0.606
3.584	6.198	5.423	4.001	5.042	0.996	0.606
4.214	6.265	5.589	4.471	5.243	1.072	0.606
10.317	9.233	10.828	7.860	8.604	0.637	0.606
4.185	3.223	4.694	3.454	3.604	0.448	0.606
5.826	5.280	6.895	4.108	3.882	0.610	0.606
7.635	7.479	7.315	8.006	7.937	0.341	0.606
2.889	3.818	3.686	4.160	3.978	0.385	0.606
6.440	6.201	7.206	5.255	5.781	0.414	0.606
6.762	5.817	5.289	6.090	5.925	0.446	0.606
4.720	2.931	4.203	3.215	3.934	0.507	0.606
5.253	4.941	5.065	5.060	4.719	0.333	0.606
3.055	3.523	3.401	3.333	2.722	0.470	0.606
5.690	6.171	5.778	6.404	6.578	0.379	0.606

3.190	3.186	2.692	3.290	3.605	0.416	0.606
5.641	4.518	5.095	4.443	3.957	0.434	0.606
3.976	2.685	2.126	3.475	3.385	0.475	0.606
3.866	3.022	3.845	3.158	3.597	0.368	0.606
4.533	4.348	4.928	3.691	4.667	0.500	0.606
7.390	7.761	8.692	7.391	7.513	0.396	0.606
3.500	2.573	4.433	3.158	2.655	0.505	0.606
3.474	3.349	3.144	3.232	2.576	0.463	0.606
3.631	2.828	1.987	3.329	2.396	0.470	0.606
8.327	7.134	8.682	7.623	7.448	0.410	0.606
3.778	4.173	4.286	3.027	3.323	0.532	0.606
6.929	7.728	7.606	7.350	7.139	0.467	0.606
5.509	6.488	5.683	6.865	6.760	0.406	0.606
4.992	8.014	6.457	8.333	8.197	0.728	0.606
2.238	3.620	2.156	3.958	3.785	0.550	0.606
4.103	5.214	2.983	5.466	5.190	0.612	0.606
7.502	7.789	7.444	8.422	8.227	0.324	0.606
4.052	4.764	4.851	3.767	4.082	0.483	0.606
4.634	2.962	5.380	3.057	1.925	1.047	0.606
5.290	5.520	6.149	5.242	5.470	0.442	0.606
5.442	7.123	5.313	7.368	7.066	0.518	0.606
8.024	8.189	7.409	8.382	8.565	0.357	0.606
6.358	5.846	6.188	5.159	5.247	0.423	0.606
4.393	4.005	4.579	3.501	3.718	0.412	0.606
8.268	8.793	6.843	9.080	8.973	0.514	0.606
6.654	7.349	5.758	6.082	6.052	0.517	0.606
1.684	2.025	2.012	1.361	2.566	0.387	0.606
9.145	7.961	9.107	8.251	7.584	0.459	0.606
5.679	6.002	6.454	5.937	5.660	0.601	0.606
2.418	2.456	1.452	1.800	2.594	0.641	0.606
5.977	5.365	6.222	5.406	5.190	0.415	0.606
5.037	3.390	5.358	3.195	2.593	0.681	0.606
4.312	5.240	4.905	5.327	5.667	0.521	0.606
5.679	5.097	5.240	4.843	4.901	0.468	0.606
4.388	4.271	4.557	4.729	4.189	0.546	0.606
6.685	5.692	6.106	5.959	5.430	0.507	0.606
6.567	5.851	6.739	6.243	6.409	0.632	0.606
8.681	8.740	6.364	8.083	7.794	1.012	0.606
7.470	7.317	7.362	7.127	6.997	0.355	0.606
7.905	6.965	7.574	7.154	7.230	0.438	0.606
4.344	3.171	4.272	3.550	3.784	0.521	0.606
3.258	1.977	2.319	2.116	2.492	0.474	0.606
7.804	6.942	8.325	7.056	6.934	0.414	0.606
11.103	10.263	11.022	9.388	9.123	0.512	0.606
6.959	6.850	6.392	6.730	6.930	0.455	0.606
6.720	5.745	3.497	5.678	5.362	0.674	0.606
8.078	8.810	6.571	8.811	9.005	0.531	0.606
5.616	5.317	4.564	5.719	6.010	0.432	0.606
3.833	3.790	4.462	3.140	3.146	0.702	0.606
5.497	5.159	6.428	3.674	4.863	0.874	0.606

3.129	3.523	3.182	3.151	3.834	0.393	0.606
2.066	2.854	2.389	2.898	2.836	0.341	0.606
3.273	1.454	2.838	2.263	2.737	0.595	0.606
7.635	7.899	4.151	8.372	8.420	0.904	0.606
7.061	7.766	5.718	7.656	8.043	0.571	0.606
6.642	7.799	3.228	8.415	8.492	1.034	0.606
6.521	7.732	4.606	7.769	8.030	0.769	0.606
7.122	6.801	4.624	7.766	7.186	0.644	0.606
5.765	7.236	2.738	7.907	7.412	0.953	0.606
5.703	5.104	3.146	5.327	5.716	0.691	0.606
5.612	3.283	1.650	3.734	3.830	0.823	0.606
7.161	8.471	4.026	8.772	8.602	1.056	0.606
6.192	7.222	2.815	7.209	7.281	1.143	0.606
5.590	3.087	3.015	3.376	3.734	0.599	0.606
3.469	3.458	4.292	3.568	3.505	0.449	0.606
3.280	2.420	3.722	3.314	1.906	0.494	0.606
3.245	4.782	0.778	4.855	5.230	0.938	0.606
3.130	3.032	2.261	2.914	4.105	0.427	0.606
3.469	2.869	2.344	2.647	2.723	0.510	0.606
3.690	2.884	3.104	3.189	3.244	0.469	0.606
2.984	3.638	2.757	2.852	1.816	0.671	0.606
6.575	6.831	6.361	6.850	7.088	0.338	0.606
7.981	6.919	8.239	6.960	8.092	0.690	0.606
4.352	4.700	4.839	4.766	4.640	0.423	0.606
3.431	4.069	2.968	4.216	4.656	0.433	0.606
3.531	2.949	4.089	3.380	2.969	0.446	0.606
4.125	4.721	4.492	3.903	4.025	0.581	0.606
4.496	5.354	5.795	5.184	5.134	0.619	0.606
4.362	4.416	5.348	4.657	4.590	0.579	0.606
4.625	3.079	5.688	5.673	5.124	1.277	0.606
4.440	4.440	4.692	3.797	4.440	0.534	0.606
3.373	3.348	4.528	3.822	4.207	0.564	0.606
4.451	4.590	5.299	5.206	5.569	0.403	0.606
1.707	1.774	1.016	2.302	2.318	0.441	0.606
5.553	5.348	5.156	5.793	5.541	0.588	0.606
3.106	2.680	1.368	4.164	3.536	0.596	0.606
2.962	3.204	2.940	2.779	3.108	0.442	0.606
4.693	4.678	5.416	3.913	4.929	0.432	0.606
4.221	4.124	5.070	4.078	4.526	0.360	0.606
6.953	6.245	7.190	6.303	6.193	0.384	0.606
3.653	3.311	3.412	2.166	2.819	0.693	0.606
6.773	5.669	7.599	5.420	5.747	0.650	0.606
3.808	2.207	1.871	1.868	3.206	0.575	0.606
2.582	3.645	2.671	3.589	3.295	0.358	0.606
2.565	2.466	2.094	3.747	3.173	0.441	0.606
2.866	2.224	2.687	2.031	2.381	0.664	0.606
6.660	6.915	5.870	7.131	6.904	0.352	0.606
3.722	5.090	4.413	5.175	4.911	0.496	0.606
3.331	4.437	4.180	3.244	2.986	0.776	0.606
7.913	8.493	7.531	8.817	8.805	0.430	0.606

4.955	5.141	4.303	6.058	5.299	0.523	0.606
2.841	4.220	1.618	1.668	5.262	1.430	0.606
4.373	4.595	5.121	4.630	4.911	0.459	0.606
4.186	5.318	6.035	3.952	3.999	0.600	0.606
5.311	4.488	5.868	4.243	4.384	0.568	0.606
4.787	5.319	4.156	4.849	5.960	0.435	0.606
7.164	6.655	8.215	4.183	5.265	0.789	0.606
3.281	3.397	4.654	2.157	2.122	0.608	0.606
6.805	5.738	6.645	6.433	6.370	0.453	0.606
4.483	3.582	4.885	4.299	3.728	0.508	0.606
5.034	5.266	4.893	4.842	4.419	0.413	0.606
2.694	4.085	2.849	3.359	2.975	0.482	0.606
2.588	3.318	3.109	2.663	3.738	0.459	0.606
3.896	4.420	4.683	4.023	3.621	0.387	0.606
4.930	4.935	5.781	4.537	4.282	0.383	0.606
6.281	5.460	6.869	4.613	4.627	0.567	0.606
4.823	5.578	4.055	5.986	5.571	0.470	0.606
3.713	3.351	2.710	3.391	3.466	0.448	0.606
7.026	6.281	6.792	5.959	5.904	0.384	0.606
3.829	4.862	3.872	5.039	4.832	0.460	0.606
6.528	5.678	7.274	5.335	5.491	0.512	0.606
5.292	6.505	3.817	6.705	6.480	0.619	0.606
2.211	2.871	2.306	3.071	3.143	0.404	0.606
7.317	7.094	7.484	7.557	7.242	0.351	0.606
6.930	7.207	7.306	7.437	7.445	0.310	0.606
5.044	5.632	5.132	5.673	5.735	0.317	0.606
6.663	6.921	6.479	6.275	6.468	0.355	0.606
5.512	4.386	6.046	3.944	3.539	0.592	0.606
6.050	5.837	6.672	4.735	5.486	0.439	0.606
6.560	5.454	6.648	4.403	4.669	0.581	0.606
5.429	6.089	5.680	6.778	6.638	0.372	0.606
2.491	2.888	2.407	2.022	2.407	0.435	0.606
9.414	10.400	7.696	10.530	10.496	0.639	0.606
7.691	7.151	8.358	5.867	6.907	0.488	0.606
5.283	6.937	3.859	6.801	6.096	0.676	0.606
5.693	5.599	4.442	5.509	5.693	0.360	0.606
6.263	6.815	6.271	6.594	6.473	0.313	0.606
5.476	5.390	5.520	5.674	5.460	0.345	0.606
4.495	5.028	3.840	5.011	4.697	0.363	0.606
1.448	2.635	3.936	3.072	2.991	0.675	0.606
3.059	3.100	2.827	2.991	2.757	0.339	0.606
7.246	6.787	8.114	6.146	6.555	0.448	0.606
4.878	5.000	4.630	5.365	5.291	0.369	0.606
2.221	2.892	3.047	1.311	1.842	0.714	0.606
2.421	5.770	5.464	1.381	2.429	1.115	0.606
2.389	1.972	1.709	1.725	1.470	0.548	0.606
4.846	5.776	5.809	3.681	4.867	0.596	0.606
5.312	5.583	5.681	4.738	4.665	0.374	0.606
3.262	3.535	3.175	3.282	3.865	0.556	0.606
5.757	5.887	6.569	5.812	5.534	0.432	0.606

3.461	3.261	4.124	3.067	3.381	0.534	0.606
1.986	2.338	2.350	2.287	3.690	0.740	0.606
3.150	5.693	3.826	3.614	2.228	1.151	0.606
5.593	8.001	6.187	8.135	7.688	0.995	0.606
5.723	6.181	4.934	5.043	5.606	0.445	0.606
4.447	4.654	6.125	4.388	4.801	0.686	0.606
8.338	9.366	8.614	9.681	9.463	0.506	0.606
3.324	2.091	3.187	1.239	1.894	0.661	0.606
3.561	3.544	3.958	3.464	3.872	0.428	0.606
4.433	3.562	5.085	3.407	3.586	0.495	0.606
4.419	6.773	2.433	7.055	6.709	0.938	0.606
6.342	7.076	7.267	7.535	7.550	0.383	0.606
3.666	4.800	4.028	4.880	4.871	0.516	0.606
4.244	4.753	4.139	5.391	5.135	0.449	0.606
4.914	4.905	5.753	5.445	5.473	0.363	0.606
4.976	4.454	3.062	4.744	5.101	0.533	0.606
7.017	7.178	5.005	7.756	7.486	0.562	0.606
7.485	7.583	5.415	7.502	7.622	0.580	0.606
6.799	6.121	6.542	6.052	6.240	0.342	0.606
2.401	4.629	3.792	3.226	2.736	0.874	0.606
4.177	3.658	5.338	2.660	3.718	0.559	0.606
4.838	4.721	5.027	4.283	4.251	0.390	0.606
2.789	4.642	6.905	5.099	5.207	0.819	0.606
2.411	1.990	2.062	2.090	2.308	0.411	0.606
3.283	2.764	2.332	3.023	3.065	0.828	0.606
6.606	6.893	7.393	6.047	6.213	0.388	0.606
4.139	4.141	1.910	3.836	4.984	0.591	0.606
6.883	6.962	6.568	7.087	7.163	0.335	0.606
6.561	6.939	7.871	5.397	5.380	0.614	0.606
5.022	4.312	5.754	2.357	2.800	0.834	0.606
3.504	5.058	3.478	5.323	5.268	0.495	0.606
4.625	5.309	4.623	5.346	4.502	0.408	0.606
3.154	3.177	3.177	3.098	3.067	0.401	0.606
5.627	5.774	3.250	6.122	5.752	0.713	0.606
5.975	5.588	3.111	5.814	5.611	0.588	0.606
6.726	7.289	5.852	7.468	6.930	0.442	0.606
7.805	8.599	7.110	8.546	8.790	0.493	0.606
2.445	3.259	2.969	3.126	2.897	0.389	0.606
2.446	4.263	2.678	3.825	3.682	0.482	0.606
4.557	4.555	6.136	4.033	4.339	0.512	0.606
8.804	8.938	7.020	9.728	9.451	0.507	0.606
5.261	4.910	5.194	4.637	4.383	0.330	0.606
1.669	3.314	2.564	2.102	2.702	0.450	0.606
3.679	2.790	2.908	2.493	2.921	0.423	0.606
4.657	4.342	5.685	4.129	3.966	0.401	0.606
4.656	3.764	4.571	3.441	3.510	0.505	0.606
3.822	4.267	2.682	3.497	3.845	0.575	0.606
2.166	3.783	3.287	3.271	2.945	0.464	0.606
7.115	8.408	3.739	8.677	8.453	0.889	0.606
5.592	5.780	6.295	4.307	4.754	0.440	0.606

1.449	2.854	1.466	2.561	3.130	0.440	0.606
2.896	2.662	3.481	2.293	2.615	0.395	0.606
5.954	4.748	6.913	2.963	3.978	0.786	0.606
5.107	5.828	2.139	6.167	6.112	0.875	0.606
2.156	3.102	2.496	3.311	3.584	0.478	0.606
1.785	1.881	2.169	2.614	1.624	0.388	0.606
5.204	6.726	4.438	7.145	6.913	0.724	0.606
5.061	5.124	6.020	3.936	4.667	0.438	0.606
5.033	5.777	5.358	5.071	5.372	0.386	0.606
6.488	8.411	5.291	8.488	8.454	0.824	0.606
6.114	5.351	7.603	4.254	4.974	0.649	0.606
3.437	3.718	2.673	3.916	3.992	0.490	0.606
3.618	3.725	5.006	3.096	2.979	0.554	0.606
3.030	2.676	2.748	1.861	3.168	0.432	0.606
2.782	3.592	3.113	3.146	3.435	0.405	0.606
8.008	5.589	3.889	5.870	6.581	0.839	0.606
4.095	5.006	5.113	5.209	4.956	0.454	0.606
4.540	6.060	3.531	6.357	5.909	0.647	0.606
5.400	5.332	5.876	4.450	4.425	0.444	0.606
3.840	4.059	5.099	3.389	3.505	0.402	0.606
5.520	4.507	6.316	4.229	4.605	0.470	0.606
3.074	3.556	2.883	4.026	3.776	0.411	0.606
3.642	3.149	2.412	4.181	3.615	0.479	0.606
5.518	4.811	6.158	3.411	4.028	0.604	0.606
5.319	5.977	5.534	6.292	6.023	0.430	0.606
3.342	4.483	1.723	4.135	4.446	0.718	0.606
4.271	5.361	4.209	5.123	4.991	0.526	0.606
6.182	6.014	6.979	5.789	5.643	0.381	0.606
4.518	5.666	6.158	3.811	4.434	0.751	0.606
1.918	1.872	2.251	2.022	2.069	0.426	0.606
2.947	2.814	3.550	1.580	2.316	0.759	0.606
2.725	4.046	3.708	2.792	2.723	0.722	0.606
4.169	4.169	4.551	2.853	3.612	0.565	0.606
3.307	4.193	4.509	1.918	3.089	0.802	0.606
4.633	5.531	4.572	5.015	4.161	0.669	0.606
4.381	3.440	5.340	2.798	3.431	0.651	0.606
6.454	6.441	5.471	6.837	6.391	0.363	0.606
6.523	7.152	6.041	7.467	7.557	0.417	0.606
7.371	8.148	7.630	8.510	8.105	0.346	0.606
3.781	3.720	4.210	3.327	3.046	0.482	0.606
5.984	5.408	6.537	4.617	4.736	0.447	0.606
4.426	4.352	3.799	4.692	4.652	0.333	0.606
1.683	2.495	1.616	2.908	2.643	0.416	0.606
4.954	5.316	4.558	5.974	5.628	0.363	0.606
2.539	2.719	1.982	4.144	3.458	0.477	0.606
1.761	2.876	1.680	2.144	2.171	0.438	0.606
6.502	5.890	5.755	5.945	5.809	0.342	0.606
4.488	4.755	5.755	3.284	3.774	0.583	0.606
8.228	7.372	9.146	6.868	6.833	0.544	0.606
3.619	4.253	4.951	2.399	2.853	0.901	0.606

6.161	6.291	5.327	6.345	6.178	0.398	0.606
5.872	3.794	2.270	4.182	3.680	1.040	0.606
3.609	2.764	2.465	4.046	3.929	0.455	0.606
3.330	5.148	5.502	2.420	2.960	1.038	0.606
5.903	5.634	6.001	4.897	5.066	0.416	0.606
7.423	8.042	2.546	8.055	7.893	1.258	0.606
3.171	3.739	2.707	4.418	4.142	0.472	0.606
3.548	3.193	4.031	2.995	3.028	0.358	0.606
5.079	4.880	6.430	4.007	4.605	0.676	0.606
2.715	2.489	2.419	3.480	5.299	1.081	0.606
5.046	4.445	2.650	4.774	4.840	0.650	0.606
8.844	8.437	10.033	8.252	7.743	0.881	0.606
5.807	5.788	5.516	6.157	6.525	0.460	0.606
3.887	3.619	3.744	2.782	3.207	0.389	0.606
4.391	4.620	4.062	4.954	5.078	0.390	0.606
1.810	1.895	2.192	1.319	3.473	0.661	0.606
4.002	4.893	3.141	5.426	5.505	0.526	0.606
5.152	5.821	5.231	4.249	3.998	0.636	0.606
3.639	4.143	4.541	3.923	4.180	0.441	0.606
4.126	4.988	3.997	4.488	5.380	0.576	0.606
4.267	4.804	4.304	5.210	5.222	0.465	0.606
4.617	5.031	6.261	4.720	5.548	0.561	0.606
5.889	5.979	5.769	6.293	6.134	0.419	0.606
7.457	5.954	2.280	6.390	6.705	1.197	0.606
3.565	10.082	4.205	10.128	9.742	1.470	0.606
2.156	2.674	2.171	3.021	2.589	0.399	0.606
4.721	2.620	2.456	3.307	1.925	0.740	0.606
4.942	5.281	5.283	5.647	5.709	0.449	0.606
6.178	6.807	5.247	7.160	7.068	0.560	0.606
9.602	9.899	8.351	9.787	10.404	0.500	0.606
3.834	4.121	5.441	3.159	3.891	0.616	0.606
3.306	3.682	1.626	3.869	3.000	0.537	0.606
1.092	2.035	1.129	2.953	2.895	0.507	0.606
5.991	5.303	6.534	5.228	5.190	0.445	0.606
4.581	3.202	2.915	3.256	4.178	0.487	0.606
3.266	3.368	2.588	2.853	2.540	0.452	0.606
4.181	2.419	3.278	2.967	3.277	0.537	0.606
2.447	3.002	2.303	3.608	3.378	0.492	0.606
3.012	3.159	3.096	2.272	2.861	0.488	0.606
9.458	9.450	10.071	9.573	9.897	0.337	0.606
5.707	5.371	4.753	5.847	5.749	0.529	0.606
4.191	3.464	4.096	3.800	3.698	0.573	0.606
6.455	5.487	6.052	5.300	5.112	0.405	0.606
6.575	7.000	5.020	7.808	7.596	0.599	0.606
6.337	4.867	6.477	4.076	4.689	0.631	0.606
5.793	6.041	4.741	6.499	6.204	0.420	0.606
3.285	3.221	3.126	3.507	3.736	0.385	0.606
6.426	6.202	6.485	6.480	6.766	0.498	0.606
4.345	5.167	4.327	4.698	4.428	0.589	0.606
3.907	5.489	3.720	5.758	4.861	0.513	0.606

7.738	8.208	7.689	8.513	8.242	0.366	0.606
7.231	7.507	7.015	7.971	7.771	0.348	0.606
6.361	5.595	6.769	5.367	5.482	0.480	0.606
4.286	4.108	4.286	4.693	4.911	0.670	0.606
5.737	5.777	5.835	6.251	6.276	0.390	0.606
5.521	6.068	6.573	5.627	5.919	0.467	0.606
8.301	8.876	7.879	9.175	9.143	0.373	0.606
5.768	5.937	5.234	5.973	6.184	0.469	0.606
6.206	6.406	5.864	6.801	6.532	0.344	0.606
6.296	6.396	5.886	6.667	6.661	0.309	0.606
4.160	3.996	6.021	4.190	3.438	0.660	0.606
2.443	3.853	2.034	3.883	4.424	0.586	0.606
2.061	5.731	2.476	6.469	6.126	0.985	0.606
3.014	3.283	2.434	4.241	4.400	0.548	0.606
5.996	5.694	6.173	5.002	5.151	0.371	0.606
2.795	3.984	1.099	4.261	4.236	0.720	0.606
4.505	2.006	2.864	2.238	2.059	0.654	0.606
5.032	5.324	4.446	4.963	5.381	0.382	0.606
4.891	4.579	4.442	3.560	4.006	0.460	0.606
4.969	5.322	6.008	4.490	4.733	0.455	0.606
5.739	4.811	2.810	4.936	4.408	0.682	0.606
5.152	5.985	4.920	6.586	6.532	0.604	0.606
6.068	7.545	4.383	7.886	7.637	0.781	0.606
3.842	5.233	3.464	4.878	5.161	0.823	0.606
3.680	4.277	4.801	4.141	4.334	0.354	0.606
3.750	4.502	4.045	3.402	3.382	0.563	0.606
3.089	4.242	2.152	4.348	4.702	0.550	0.606
4.956	5.462	6.112	5.999	5.916	0.389	0.606
7.350	7.631	7.081	7.597	8.009	0.360	0.606
5.914	5.994	6.097	5.999	6.532	0.407	0.606
7.864	6.505	8.905	5.512	5.849	0.682	0.606
5.587	5.471	3.192	5.169	5.249	0.709	0.606
5.510	6.119	5.475	6.050	6.348	0.399	0.606
5.554	4.934	5.370	4.250	4.941	0.453	0.606
6.610	6.695	6.462	7.042	7.004	0.313	0.606
4.601	4.702	5.796	4.210	4.944	0.448	0.606
5.760	5.726	7.022	3.611	3.978	0.709	0.606
3.969	6.091	4.818	5.074	5.128	0.512	0.606
6.986	8.004	6.483	7.910	8.084	0.441	0.606
5.546	5.698	4.120	6.080	5.622	0.486	0.606
3.099	2.545	3.464	2.538	2.784	0.565	0.606
4.763	5.308	4.143	5.784	5.287	0.418	0.606
5.976	5.414	5.027	5.720	5.705	0.394	0.606
2.706	2.252	3.019	2.257	1.987	0.536	0.606
4.993	5.307	4.815	5.341	5.297	0.392	0.606
8.885	8.802	7.847	8.756	9.105	0.404	0.606
4.679	4.894	5.256	4.126	4.419	0.397	0.606
3.228	3.497	2.702	4.846	4.504	0.509	0.606
7.054	6.957	6.821	5.412	6.160	0.520	0.606
4.238	2.319	2.311	1.734	3.730	0.668	0.606

4.828	4.276	4.103	4.846	4.893	0.358	0.606
3.097	2.834	2.144	3.006	2.645	0.465	0.606
5.327	6.304	4.472	6.443	6.522	0.521	0.606
6.236	5.719	6.995	5.522	5.391	0.435	0.606
5.528	6.050	5.093	6.129	6.237	0.391	0.606
4.427	5.917	5.451	5.570	5.217	0.741	0.606
3.288	3.981	3.917	4.100	3.696	0.419	0.606
4.523	4.488	3.854	4.525	4.659	0.361	0.606
4.329	3.387	4.906	3.166	3.635	0.518	0.606
6.125	6.055	4.930	6.481	6.560	0.398	0.606
3.348	3.859	3.267	3.145	3.714	0.400	0.606
9.642	8.663	10.142	7.655	8.021	0.542	0.606
7.849	7.778	8.715	6.938	7.716	0.449	0.606
3.685	4.069	3.800	3.429	3.862	0.480	0.606
3.951	4.354	1.953	4.718	4.734	0.617	0.606
5.528	5.079	6.648	2.616	3.454	0.852	0.606
5.052	5.810	3.819	6.279	6.197	0.560	0.606
5.121	4.398	5.355	4.167	4.417	0.420	0.606
2.812	2.776	2.201	3.545	4.203	0.801	0.606
3.455	1.855	1.838	1.700	1.802	0.678	0.606
6.180	8.042	3.461	8.438	8.442	1.135	0.606
5.702	6.097	5.521	6.557	6.392	0.348	0.606
3.333	3.197	3.744	2.906	3.074	0.342	0.606
5.832	5.390	4.098	5.863	5.299	0.455	0.606
8.745	8.219	7.214	8.116	8.668	0.548	0.606
4.487	3.716	4.639	3.630	3.214	0.552	0.606
5.646	6.181	5.269	6.603	6.365	0.361	0.606
4.599	4.935	3.209	4.915	4.693	0.467	0.606
3.638	3.942	4.014	3.479	3.804	0.406	0.606
2.366	2.118	2.749	2.217	2.251	0.400	0.606
2.755	1.921	2.555	2.001	2.373	0.497	0.606
7.852	6.597	8.282	4.533	4.848	0.815	0.606
1.510	1.821	1.577	1.669	3.213	0.541	0.606
4.274	4.168	3.892	3.811	4.000	0.393	0.606
7.162	1.388	1.479	1.562	1.211	1.474	0.606
8.929	9.013	8.420	9.045	9.221	0.432	0.606
7.166	6.260	4.114	6.118	6.241	0.720	0.606
2.771	3.531	3.217	3.565	4.408	0.539	0.606
6.421	6.900	4.545	6.653	6.699	0.860	0.606
6.707	8.108	4.329	8.261	8.284	0.835	0.606
3.034	2.868	2.623	3.100	4.968	0.688	0.606
8.120	1.331	1.704	1.249	2.035	1.666	0.606
6.028	6.479	4.573	5.059	5.037	0.635	0.606
3.727	6.085	1.642	6.244	5.990	0.966	0.606
7.138	5.679	7.213	5.098	4.560	0.614	0.606
4.918	5.147	2.477	4.764	4.481	0.740	0.606
7.532	7.020	7.573	7.066	7.702	0.402	0.606
6.878	6.832	4.789	6.488	6.393	0.571	0.606
3.321	5.808	5.402	5.199	5.632	0.821	0.606
3.628	2.967	1.479	3.039	2.370	0.492	0.606

7.461	8.040	7.954	7.558	7.606	0.485	0.606
4.867	4.822	3.865	4.960	4.988	0.434	0.606
2.480	1.793	2.284	1.540	1.818	0.453	0.606
3.692	4.699	3.087	5.257	4.998	0.620	0.606
5.161	5.670	3.961	5.932	5.945	0.485	0.606
5.790	7.946	4.757	8.157	7.810	0.840	0.606
4.197	3.688	3.029	4.595	4.289	0.440	0.606
4.248	4.668	4.515	3.629	3.737	0.370	0.606
5.676	5.626	4.044	5.573	5.931	0.472	0.606
2.643	2.733	1.532	3.034	2.996	0.496	0.606
7.070	7.251	7.801	6.601	6.699	0.410	0.606
3.541	3.710	1.902	3.982	3.128	0.515	0.606
9.325	9.900	8.246	10.219	10.151	0.494	0.606
6.682	6.266	3.628	7.000	6.681	0.695	0.606
7.507	7.105	5.368	7.673	7.481	0.543	0.606
4.721	5.341	3.660	6.266	5.822	0.638	0.606
7.130	6.971	7.736	5.626	6.000	0.515	0.606
6.251	6.817	5.464	7.251	7.315	0.467	0.606
6.890	5.799	7.640	4.831	5.682	0.550	0.606
4.286	6.468	5.724	5.897	6.290	0.561	0.606
3.350	3.622	3.332	3.284	4.884	0.480	0.606
5.315	4.859	5.661	3.777	4.658	0.483	0.606
6.921	5.802	7.258	5.696	5.641	0.414	0.606
4.133	5.317	4.008	4.737	4.631	0.567	0.606
2.067	3.221	1.886	4.060	3.996	0.696	0.606
2.974	1.581	3.301	1.974	1.559	0.664	0.606
4.082	3.635	4.512	3.300	4.281	0.382	0.606
1.726	2.113	1.987	1.796	2.190	0.414	0.606
5.702	5.962	6.976	5.126	5.587	0.544	0.606
5.333	6.409	4.413	6.406	6.293	0.547	0.606
6.648	6.420	3.620	6.941	6.826	0.755	0.606
4.266	3.502	1.895	4.006	4.293	0.606	0.606
6.503	5.752	3.683	5.711	5.704	0.613	0.606
6.915	7.503	7.570	7.783	7.760	0.483	0.606
5.558	6.637	4.613	6.973	7.164	0.663	0.606
3.675	5.347	2.300	5.957	5.663	0.844	0.606
5.207	5.907	4.840	6.277	6.878	0.488	0.606
4.613	5.328	3.706	5.607	5.809	0.555	0.606
2.296	2.730	1.887	3.400	2.300	0.602	0.606
1.992	3.933	2.426	3.307	4.826	1.357	0.606
5.586	6.133	5.923	6.375	6.396	0.319	0.606
7.863	7.492	8.443	6.751	6.886	0.469	0.606
4.388	2.179	3.229	1.303	1.705	0.685	0.606
6.302	6.317	7.382	5.231	5.846	0.508	0.606
3.225	3.606	2.534	3.605	3.621	0.540	0.606
6.428	6.603	6.041	7.015	6.872	0.305	0.606
4.432	3.269	3.698	3.850	3.735	0.448	0.606
4.923	4.247	4.361	3.672	4.258	0.390	0.606
7.266	7.813	7.606	7.906	7.859	0.351	0.606
6.585	5.655	2.678	6.340	5.603	0.853	0.606

3.468	2.325	1.266	2.950	2.678	0.549	0.606
1.746	3.254	2.116	3.123	3.533	0.618	0.606
3.895	4.421	3.978	4.707	3.802	0.467	0.606
3.158	2.840	4.417	2.200	1.321	0.662	0.606
3.881	3.232	5.731	2.358	2.551	0.665	0.606
4.688	5.452	4.924	5.420	5.786	0.371	0.606
3.140	4.389	2.292	4.482	4.395	0.551	0.606
4.439	4.035	4.570	4.375	3.973	0.381	0.606
5.974	5.028	6.766	4.815	4.706	0.584	0.606
6.170	5.714	6.269	5.594	5.747	0.452	0.606
2.989	4.026	2.958	5.012	4.585	0.476	0.606
6.989	7.073	4.975	7.611	7.946	0.572	0.606
4.806	4.666	3.683	5.277	4.713	0.371	0.606
5.225	4.514	4.785	4.933	4.887	0.421	0.606
2.450	3.400	2.851	2.577	1.736	0.436	0.606
2.850	4.080	4.256	5.024	5.164	0.549	0.606
4.828	5.479	3.771	5.673	5.252	0.460	0.606
7.514	7.044	8.500	6.682	7.012	0.400	0.606
10.707	11.647	7.799	11.804	11.649	1.005	0.606
1.854	2.286	1.849	3.072	2.957	0.402	0.606
3.529	4.355	3.781	4.593	4.505	0.335	0.606
8.024	8.605	5.687	8.902	8.792	0.702	0.606
4.953	4.850	4.412	4.788	4.667	0.664	0.606
4.971	4.419	4.087	4.355	3.681	0.718	0.606
5.747	5.191	5.033	5.309	4.904	0.933	0.606
4.659	4.080	3.984	4.152	3.773	0.646	0.606
3.767	3.124	3.299	3.725	3.179	0.586	0.606
4.199	2.409	3.483	4.835	2.337	1.004	0.606
4.511	3.481	4.058	3.629	3.986	0.378	0.606
3.121	3.159	2.732	2.704	2.262	0.406	0.606
2.094	1.430	1.222	2.305	1.377	0.751	0.606
6.737	7.105	6.483	7.523	7.215	0.329	0.606
3.534	3.148	3.371	3.600	4.188	0.441	0.606
7.171	8.343	4.505	8.467	8.587	0.913	0.606
7.530	5.823	4.090	6.303	6.360	0.711	0.606
3.833	4.476	4.819	4.357	4.178	0.502	0.606
4.138	3.972	4.097	4.329	4.064	0.392	0.606
3.873	4.192	4.181	3.611	3.704	0.543	0.606
3.530	4.292	4.097	3.820	3.832	0.428	0.606
4.004	3.106	4.933	1.713	2.936	0.611	0.606
7.338	5.617	3.602	5.272	6.296	0.849	0.606
4.013	3.942	4.381	4.081	3.911	0.358	0.606
7.629	8.557	7.142	8.536	8.687	0.449	0.606
5.887	5.413	6.449	5.233	5.425	0.525	0.606
8.879	8.200	9.228	8.283	9.408	0.518	0.606
3.897	3.666	3.825	2.808	3.239	0.551	0.606
4.613	4.070	4.784	3.800	4.530	0.728	0.606
5.326	5.119	7.153	5.689	6.049	0.652	0.606
6.469	5.759	5.962	6.310	5.812	0.555	0.606
3.753	3.344	4.049	3.766	3.546	0.391	0.606

3.874	3.784	4.923	3.913	3.584	0.556	0.606
5.900	6.201	6.091	6.105	6.032	0.330	0.606
9.174	8.562	9.157	8.574	8.458	0.412	0.606
8.837	7.793	8.570	7.787	7.749	0.478	0.606
5.457	5.094	5.240	4.833	4.796	0.434	0.606
3.301	4.389	4.029	3.992	3.989	0.422	0.606
3.416	3.904	3.699	3.626	3.362	0.483	0.606
2.724	1.395	2.228	2.115	2.055	0.764	0.606
3.039	1.325	2.901	1.608	1.839	0.509	0.606
8.908	7.389	8.380	7.073	6.260	0.636	0.606
9.001	8.830	9.300	8.693	8.569	0.322	0.606
5.465	3.247	2.875	3.570	3.443	0.826	0.606
3.356	3.213	3.431	2.048	2.680	0.537	0.606
2.477	1.868	1.759	2.315	2.889	0.548	0.606
8.509	7.460	7.869	7.292	7.476	0.429	0.606
4.845	4.178	4.831	3.751	3.461	0.559	0.606
3.998	2.782	3.429	1.941	1.579	0.774	0.606
9.437	6.250	8.549	6.179	6.829	0.838	0.606
8.814	6.765	7.016	6.661	6.613	0.628	0.606
7.485	6.278	6.858	6.613	5.482	0.636	0.606
10.939	10.502	11.193	10.057	10.135	0.328	0.606
10.726	11.033	11.064	10.433	10.491	0.296	0.606
9.662	7.972	9.415	8.171	7.629	0.595	0.606
5.040	4.058	4.859	3.590	4.004	0.405	0.606
8.505	7.798	8.693	8.043	8.442	0.393	0.606
6.047	6.476	6.548	6.095	5.920	0.330	0.606
5.710	5.487	5.497	4.778	4.821	0.472	0.606
4.418	3.706	4.390	4.251	3.354	0.458	0.606
8.818	8.253	8.777	7.803	7.218	0.535	0.606
6.300	6.302	6.688	5.663	6.013	0.393	0.606
6.514	6.163	6.558	6.475	6.172	0.319	0.606
3.440	2.694	2.936	3.279	3.626	0.465	0.606
9.846	9.414	9.569	9.267	9.322	0.320	0.606
3.091	1.864	3.862	2.785	2.296	0.543	0.606
2.232	3.088	3.761	3.144	2.055	0.462	0.606
3.820	1.620	4.334	1.422	2.501	0.792	0.606
3.653	4.040	3.886	2.281	3.530	0.534	0.606
5.266	5.009	5.810	4.920	5.539	0.350	0.606
7.762	7.795	7.889	6.829	6.746	0.482	0.606
5.752	5.083	5.896	4.949	5.235	0.385	0.606
6.099	5.307	5.931	5.453	5.577	0.453	0.606
6.059	5.820	6.780	6.349	5.847	0.636	0.606
3.362	3.315	3.068	3.261	2.196	0.440	0.606
6.631	4.713	6.395	5.742	4.394	0.785	0.606
10.150	8.551	8.600	8.865	7.938	0.813	0.606
8.292	8.391	8.349	7.646	7.583	0.363	0.606
6.218	4.795	5.822	4.942	4.299	0.558	0.606
3.937	3.656	4.053	3.833	3.788	0.386	0.606
2.664	2.900	3.714	3.173	2.646	0.500	0.606
5.988	7.160	7.284	6.036	6.037	0.477	0.606

5.610	5.330	6.140	4.828	5.277	0.433	0.606
3.916	3.122	3.534	3.111	3.952	0.484	0.606
6.771	5.423	6.321	5.727	5.305	0.573	0.606
4.718	4.922	4.902	5.072	4.836	0.316	0.606
5.534	5.178	6.034	5.106	5.044	0.333	0.606
8.052	7.883	8.406	7.405	7.480	0.363	0.606
9.817	8.705	9.136	9.284	9.348	0.630	0.606
3.195	2.656	3.680	2.426	3.326	0.432	0.606
6.149	5.667	6.790	5.171	5.209	0.494	0.606
6.485	6.729	4.753	7.101	6.802	0.667	0.606
5.471	5.068	6.547	4.977	5.221	0.433	0.606
6.296	4.925	2.074	4.788	6.102	1.031	0.606
5.140	4.117	2.137	4.503	4.952	0.769	0.606
6.981	5.962	4.010	6.212	6.260	0.707	0.606
2.835	2.344	1.391	2.426	3.015	0.542	0.606
4.182	4.085	2.075	3.445	5.025	0.960	0.606
3.980	4.936	2.146	4.915	4.511	0.704	0.606
9.324	9.023	6.129	9.178	9.720	0.877	0.606
3.482	1.952	0.934	1.973	2.149	0.720	0.606
4.360	3.201	1.375	3.816	4.407	0.911	0.606
9.479	9.663	7.153	9.886	9.830	0.682	0.606
5.024	2.069	1.028	2.945	3.896	0.878	0.606
6.373	5.664	3.293	5.736	5.704	0.659	0.606
5.729	5.895	2.905	5.980	5.798	0.787	0.606
5.522	2.769	1.984	4.836	5.196	0.969	0.606
5.145	4.838	1.734	4.613	5.195	0.860	0.606
5.075	3.830	1.314	4.443	4.664	0.958	0.606
4.926	4.467	1.001	4.615	5.735	1.310	0.606
6.463	6.519	3.188	6.550	6.645	0.825	0.606
5.243	5.730	2.196	6.075	5.628	0.851	0.606
3.419	2.303	0.707	3.704	3.618	0.816	0.606
4.525	5.753	0.809	5.666	5.718	1.211	0.606
5.328	5.603	1.995	5.999	5.514	0.949	0.606
4.015	4.294	4.544	4.910	3.684	0.463	0.606
7.034	7.479	3.343	7.762	7.865	0.992	0.606
9.513	9.609	6.709	9.594	9.754	0.753	0.606
4.468	3.151	1.505	3.140	4.035	0.815	0.606
4.877	3.188	1.777	3.022	3.985	0.874	0.606
2.693	2.765	1.288	1.303	2.151	0.644	0.606
2.959	2.015	1.419	4.475	2.654	0.997	0.606
4.598	3.701	1.935	3.069	4.123	0.906	0.606
5.742	5.884	1.383	6.088	6.153	1.050	0.606
11.030	10.869	9.154	10.852	11.051	0.494	0.606
5.465	6.456	2.537	6.395	6.232	0.875	0.606
4.034	4.461	2.164	4.825	4.507	0.658	0.606
10.416	10.339	7.945	10.317	10.521	0.624	0.606
11.038	10.913	8.714	10.741	10.949	0.581	0.606
2.568	2.770	1.909	2.774	3.141	0.589	0.606
8.373	8.509	6.350	8.285	8.477	0.698	0.606
8.104	7.777	5.842	7.772	7.688	0.736	0.606

3.679	4.151	1.191	2.248	4.795	0.896	0.606
8.906	8.756	5.960	8.983	9.121	0.980	0.606
7.344	7.317	4.661	7.161	7.500	0.763	0.606
5.340	5.809	2.421	6.069	5.476	0.883	0.606
5.283	5.750	2.002	6.012	5.862	0.930	0.606
11.564	11.480	9.604	11.511	11.650	0.513	0.606
4.390	4.718	2.248	4.867	5.181	0.727	0.606
2.415	2.746	1.039	2.756	3.044	0.631	0.606
11.062	10.916	8.647	11.004	11.224	0.594	0.606
5.581	4.189	1.868	4.208	5.035	0.938	0.606
11.476	11.305	9.413	11.301	11.482	0.519	0.606
11.456	11.296	9.389	11.296	11.495	0.527	0.606
11.378	11.250	9.512	11.242	11.343	0.483	0.606
2.572	3.384	1.360	1.976	3.495	0.661	0.606
8.214	8.047	7.448	8.194	7.804	0.474	0.606
9.889	9.334	9.182	9.239	9.289	0.415	0.606
2.253	2.030	1.965	2.005	1.824	0.531	0.606
7.573	7.768	7.027	7.078	7.221	0.412	0.606
7.721	3.040	5.882	6.761	6.906	1.159	0.606
5.048	5.026	4.140	5.089	4.993	0.518	0.606
4.555	4.139	3.280	4.901	4.721	0.609	0.606
6.410	6.520	5.571	6.970	6.802	0.567	0.606
5.297	5.791	4.361	5.277	5.346	0.620	0.606
7.243	7.148	6.390	7.641	7.553	0.564	0.606
6.768	6.145	5.142	6.545	6.917	0.608	0.606
8.310	8.122	8.403	7.722	7.832	0.305	0.606
8.407	8.338	8.562	7.923	7.967	0.304	0.606
6.095	5.402	6.079	5.578	5.258	0.396	0.606
4.452	4.817	6.097	3.328	2.362	0.734	0.606
2.520	2.238	3.656	2.353	2.645	0.470	0.606
2.851	2.128	3.825	2.177	2.831	0.603	0.606
2.864	2.872	3.170	3.108	2.592	0.485	0.606
4.781	5.006	5.028	4.217	5.116	0.416	0.606
3.568	3.865	4.494	3.153	3.139	0.419	0.606
5.695	4.107	5.919	4.690	4.685	0.570	0.606
6.905	7.158	6.026	5.681	6.853	0.432	0.606
3.887	5.006	4.902	3.463	4.501	0.553	0.606
4.858	3.704	4.856	4.359	4.100	0.622	0.606
9.191	8.931	9.054	8.438	8.326	0.332	0.606
7.434	6.943	7.860	6.620	6.020	0.491	0.606
1.444	2.791	3.741	1.639	2.817	0.623	0.606
3.642	4.623	4.859	3.342	4.811	0.655	0.606
5.754	5.632	5.748	5.368	5.440	0.404	0.606
1.945	1.343	2.242	1.349	1.400	0.446	0.606
3.307	1.608	1.338	2.972	2.187	0.628	0.606
3.850	4.070	4.151	3.972	4.105	0.426	0.606
4.710	4.356	5.328	4.422	4.340	0.374	0.606
4.222	4.534	3.253	3.106	4.109	0.537	0.606
8.142	8.379	8.312	8.299	6.839	0.490	0.606
5.783	5.568	5.487	5.137	5.512	0.368	0.606

9.047	8.799	9.114	8.781	8.809	0.297	0.606
5.473	4.953	6.004	5.448	5.018	0.351	0.606
5.590	4.197	4.494	3.445	4.699	0.476	0.606
2.008	1.738	1.923	1.210	2.125	0.647	0.606
4.182	4.343	4.599	3.760	4.626	0.395	0.606
5.712	5.355	6.433	4.678	5.146	0.409	0.606
4.197	3.921	3.498	3.309	3.777	0.458	0.606
6.798	6.389	6.478	6.182	6.257	0.345	0.606
9.044	8.364	9.027	8.065	7.790	0.370	0.606
5.367	4.905	5.434	4.764	4.679	0.560	0.606
5.363	5.719	5.112	5.172	5.557	0.512	0.606
5.081	4.881	4.711	5.056	4.577	0.404	0.606
2.464	2.561	3.923	3.015	2.832	0.460	0.606
2.831	2.897	2.012	2.679	3.065	0.403	0.606
11.149	11.066	11.359	9.392	10.766	0.429	0.606
1.986	1.964	3.826	1.943	2.625	0.609	0.606
8.483	8.707	8.858	7.870	7.784	0.411	0.606
2.361	2.288	1.818	1.159	1.710	0.542	0.606
2.230	2.500	2.328	1.864	2.386	0.494	0.606
4.770	4.572	5.571	4.320	4.425	0.518	0.606
3.652	3.868	3.675	3.279	3.806	0.468	0.606
3.151	2.572	2.661	1.538	2.133	0.416	0.606
8.218	8.047	7.697	7.808	7.895	0.364	0.606
6.460	6.069	6.022	5.970	6.173	0.410	0.606
3.395	3.035	2.318	3.878	3.236	0.548	0.606
7.511	7.567	7.838	7.260	6.705	0.381	0.606
4.718	4.298	4.757	3.846	4.387	0.462	0.606
8.368	8.031	8.229	7.600	7.593	0.356	0.606
2.332	2.075	2.342	3.130	2.342	0.499	0.606
1.983	2.090	2.029	1.983	1.025	0.517	0.606
6.384	7.353	3.411	7.530	6.893	0.803	0.606
5.889	5.518	3.362	5.766	5.912	0.636	0.606
9.739	9.594	9.207	10.423	10.383	0.431	0.606
6.238	6.080	6.391	6.268	6.225	0.451	0.606
5.551	6.843	6.424	7.097	7.075	0.471	0.606
5.642	6.972	5.522	7.472	7.276	0.658	0.606
7.280	6.812	5.643	7.669	7.704	0.591	0.606
6.531	5.645	6.719	5.321	5.660	0.391	0.606
5.113	5.452	2.644	6.136	5.788	0.664	0.606
4.706	4.067	4.019	4.744	4.257	0.402	0.606
4.933	4.011	5.520	3.984	4.171	0.454	0.606
2.023	2.765	2.386	1.801	1.684	0.537	0.606
4.932	3.771	5.134	3.490	3.549	0.531	0.606
7.642	6.761	7.973	6.422	6.886	0.483	0.606
3.253	4.735	1.738	4.246	4.586	0.687	0.606
4.705	5.037	4.503	5.016	4.355	0.338	0.606
6.403	6.786	6.560	6.545	6.747	0.475	0.606
5.920	5.832	6.332	5.677	5.875	0.405	0.606
6.576	6.816	6.506	6.960	7.232	0.336	0.606
4.436	6.823	5.253	4.670	4.839	0.866	0.606

5.894	5.236	6.610	5.646	5.674	0.410	0.606
5.834	7.024	6.447	7.232	6.833	0.374	0.606
3.550	4.324	3.757	3.369	3.482	0.518	0.606
3.984	3.972	3.359	2.494	4.361	0.470	0.606
6.144	6.545	5.768	7.080	6.816	0.414	0.606
2.256	4.026	2.129	3.202	3.407	0.481	0.606
5.409	6.130	3.839	6.666	6.452	0.619	0.606
4.314	6.187	3.685	6.381	6.439	0.705	0.606
5.682	5.929	4.997	6.189	6.178	0.429	0.606
2.786	1.981	3.708	3.951	2.776	0.634	0.606
2.474	4.372	2.050	4.471	4.407	0.649	0.606
4.141	5.867	4.933	6.408	5.902	0.603	0.606
2.595	4.962	2.485	4.912	5.233	0.703	0.606
3.108	2.885	3.457	1.778	2.485	0.433	0.606
1.785	2.834	2.777	3.801	3.852	0.511	0.606
5.803	5.891	6.667	4.439	4.331	0.521	0.606
4.644	3.797	4.028	3.792	4.132	0.363	0.606
3.163	2.363	2.487	2.144	2.235	0.540	0.606
4.453	3.789	5.303	3.568	3.892	0.440	0.606
4.748	5.363	4.431	5.626	5.726	0.385	0.606
6.512	5.510	7.145	5.438	5.185	0.535	0.606
7.915	7.606	7.727	7.705	8.111	0.376	0.606
2.756	2.622	3.196	2.150	2.330	0.474	0.606
6.869	6.650	7.086	6.098	6.402	0.341	0.606
4.512	4.393	4.707	3.625	4.452	0.434	0.606
4.844	5.000	5.797	3.463	4.448	0.526	0.606
3.489	4.050	3.051	4.882	4.333	0.488	0.606
3.200	1.392	0.847	1.286	1.282	0.693	0.606
3.498	4.025	2.513	4.516	3.639	0.511	0.606
3.404	2.025	2.277	2.537	1.980	0.619	0.606
3.394	2.686	2.982	2.138	2.148	0.500	0.606
7.484	5.408	6.703	5.333	6.067	0.526	0.606
6.449	5.923	7.168	4.886	5.489	0.508	0.606
3.692	3.791	4.173	2.854	2.972	0.455	0.606
2.863	1.977	1.503	1.797	1.946	0.507	0.606
4.615	2.816	2.380	2.758	3.495	0.728	0.606
2.646	3.396	2.818	3.036	2.530	0.425	0.606
1.872	3.063	2.885	2.509	2.946	0.474	0.606
2.183	2.511	2.089	3.697	2.926	0.463	0.606
5.040	4.705	6.052	3.889	4.375	0.500	0.606
4.014	3.747	4.473	3.307	3.356	0.534	0.606
4.032	3.580	3.243	4.674	4.226	0.495	0.606
4.932	5.026	4.709	5.244	5.027	0.337	0.606
4.220	4.498	3.488	5.288	5.168	0.444	0.606
3.651	3.220	4.323	3.377	2.770	0.556	0.606
2.853	2.929	3.229	2.679	3.045	0.534	0.606
3.278	3.868	2.944	3.734	3.649	0.436	0.606
5.854	6.154	3.600	6.179	6.524	0.874	0.606
4.707	5.135	5.654	4.558	5.033	0.451	0.606
8.502	9.016	8.451	9.147	8.936	0.294	0.606

4.886	4.884	4.059	5.404	5.408	0.426	0.606
2.324	2.955	3.105	2.291	2.537	0.411	0.606
6.287	6.183	4.765	6.507	6.434	0.497	0.606
4.139	4.933	2.991	5.250	4.928	0.656	0.606
3.458	3.088	4.703	2.361	2.650	0.663	0.606
2.035	2.348	1.544	2.035	1.708	0.410	0.606
6.563	7.506	7.866	7.057	7.145	0.370	0.606
7.208	7.011	4.928	6.804	6.716	0.475	0.606
6.956	6.451	7.670	5.992	6.186	0.416	0.606
7.106	6.155	7.640	6.408	6.884	0.523	0.606
11.116	11.254	11.521	9.406	9.136	0.636	0.606
2.802	3.245	2.818	3.271	3.426	0.443	0.606
4.549	5.043	5.059	5.585	4.384	0.510	0.606
4.212	5.241	3.843	5.372	5.062	0.404	0.606
3.229	5.599	2.569	5.578	5.277	0.787	0.606
1.241	2.346	1.899	1.535	1.729	0.465	0.606
3.672	4.570	3.546	5.356	5.214	0.523	0.606
4.708	4.614	2.902	5.300	4.849	0.556	0.606
5.220	5.165	3.912	5.698	5.294	0.438	0.606
6.855	7.441	5.738	7.412	7.518	0.416	0.606
5.703	6.207	5.579	6.359	6.424	0.343	0.606
5.548	5.011	2.935	5.496	5.456	0.606	0.606
5.590	6.277	3.406	6.641	6.452	0.688	0.606
5.310	5.805	3.732	6.178	5.897	0.534	0.606
7.123	6.608	8.391	6.042	6.332	0.495	0.606
3.890	3.840	4.310	3.688	4.817	0.675	0.606
5.135	5.748	5.609	5.001	5.340	0.379	0.606
4.317	4.965	1.564	5.285	5.413	0.833	0.606
1.987	2.111	4.005	2.626	3.231	0.573	0.606
7.735	6.417	7.306	6.243	6.217	0.568	0.606
4.314	4.679	4.569	3.224	4.151	0.644	0.606
7.665	7.494	7.565	6.914	7.325	0.397	0.606
1.933	2.559	2.468	2.080	3.353	0.549	0.606
5.343	4.577	4.982	3.947	3.774	0.463	0.606
6.313	5.454	6.426	5.417	4.970	0.438	0.606
7.521	7.005	7.888	6.783	6.846	0.338	0.606
7.325	6.396	7.736	6.192	5.920	0.523	0.606
7.370	9.239	9.096	7.883	8.015	0.643	0.606
1.972	3.316	2.012	2.179	2.836	0.478	0.606
3.989	2.951	4.660	3.252	2.803	0.441	0.606
7.410	7.775	7.226	7.891	7.843	0.336	0.606
5.153	5.996	5.077	6.256	6.161	0.406	0.606
7.789	7.920	7.981	8.099	8.213	0.294	0.606
5.265	5.071	6.666	3.672	4.695	0.664	0.606
2.623	3.037	2.699	2.574	2.676	0.358	0.606
4.358	4.604	3.004	4.896	4.824	0.513	0.606
4.185	5.882	2.455	6.506	6.467	0.852	0.606
3.692	3.910	2.892	4.472	4.034	0.400	0.606
5.100	5.535	5.446	5.758	5.369	0.408	0.606
4.708	4.801	4.106	5.457	5.150	0.336	0.606

4.819	6.046	3.984	5.895	5.362	0.562	0.606
3.012	2.433	2.525	2.688	2.530	0.423	0.606
6.790	6.649	7.843	6.471	6.612	0.345	0.606
4.927	4.634	5.024	4.481	4.376	0.365	0.606
5.920	6.477	5.213	5.955	6.329	0.387	0.606
4.563	3.646	5.026	4.108	4.096	0.466	0.606
9.072	7.182	3.482	6.787	6.875	1.105	0.606
3.750	4.928	2.167	5.249	4.582	0.662	0.606
5.494	5.587	3.048	6.591	6.185	0.759	0.606
2.648	1.985	1.258	3.631	3.353	0.546	0.606
1.467	3.179	1.315	3.017	2.870	0.550	0.606
4.206	5.646	3.573	6.124	6.295	0.656	0.606
7.215	5.570	7.673	4.126	4.331	0.828	0.606
3.643	4.541	4.103	4.250	4.731	0.393	0.606
4.130	4.203	3.634	4.690	4.418	0.411	0.606
4.151	2.158	2.750	2.634	2.790	0.488	0.606
3.665	4.136	4.295	3.192	4.111	0.423	0.606
3.564	4.316	4.239	4.613	4.856	0.513	0.606
4.784	3.292	3.404	2.740	2.428	0.657	0.606
6.167	5.617	6.999	5.276	5.299	0.452	0.606
3.480	6.022	1.763	6.337	6.057	1.000	0.606
6.486	7.405	6.645	7.504	7.390	0.369	0.606
6.803	8.112	4.450	8.670	8.442	0.998	0.606
4.740	4.713	4.510	4.589	4.842	0.471	0.606
5.572	7.166	3.878	7.493	7.116	0.759	0.606
4.108	3.287	3.086	3.399	3.493	0.515	0.606
4.133	5.083	5.355	3.742	4.808	0.748	0.606
3.711	5.811	3.305	5.709	6.118	0.726	0.606
3.477	2.719	3.575	3.042	2.351	0.440	0.606
4.825	5.920	4.468	5.869	5.920	0.437	0.606
3.260	3.336	1.962	3.541	3.949	0.498	0.606
4.840	5.222	4.486	5.029	4.868	0.358	0.606
4.103	3.409	3.494	3.910	3.616	0.402	0.606
6.043	5.947	6.882	5.664	5.487	0.388	0.606
6.825	6.007	5.311	6.297	6.561	0.483	0.606
3.803	2.778	3.094	3.239	3.462	0.448	0.606
6.086	5.139	6.199	4.710	5.116	0.448	0.606
7.322	7.440	6.889	7.088	7.368	0.429	0.606
6.551	5.979	6.588	5.127	5.334	0.453	0.606
8.478	8.601	8.730	7.741	8.006	0.343	0.606
4.239	5.978	2.377	6.074	6.443	0.857	0.606
4.022	4.676	3.963	4.583	4.835	0.419	0.606
6.286	6.566	5.652	6.841	6.902	0.365	0.606
3.551	5.136	3.682	3.754	2.653	1.016	0.606
2.498	3.057	2.914	2.663	2.797	0.594	0.606
3.361	2.548	2.517	2.315	2.178	0.409	0.606
5.404	6.750	4.251	7.358	7.059	0.640	0.606
4.704	4.265	4.468	3.904	3.978	0.647	0.606
1.706	2.133	2.108	1.594	2.846	0.566	0.606
5.558	3.949	5.478	4.441	5.078	0.682	0.606

3.168	4.200	3.854	3.631	4.224	0.361	0.606
5.019	4.505	5.246	3.923	4.217	0.460	0.606
4.287	4.986	4.120	5.038	5.215	0.363	0.606
6.690	4.160	6.076	4.900	4.273	0.683	0.606
5.072	3.798	3.987	3.720	3.801	0.468	0.606
10.061	9.981	5.854	10.012	10.182	1.034	0.606
7.108	6.902	5.112	6.862	6.912	0.476	0.606
5.289	6.532	7.065	6.054	5.892	0.595	0.606
8.215	8.173	8.553	7.918	8.284	0.340	0.606
4.893	5.027	4.232	5.240	5.211	0.400	0.606
4.093	1.948	3.401	2.019	2.640	0.683	0.606
4.484	5.739	5.143	5.879	6.504	0.566	0.606
6.177	6.616	6.310	6.778	6.569	0.325	0.606
2.925	3.184	3.344	3.221	2.992	0.298	0.606
5.134	6.253	4.811	6.707	6.711	0.493	0.606
3.557	3.616	3.059	3.989	3.766	0.362	0.606
4.389	5.193	3.380	5.733	5.438	0.535	0.606
7.229	7.818	5.156	7.838	7.893	0.649	0.606
2.507	3.078	2.991	2.362	2.348	0.401	0.606
3.939	5.809	3.984	5.519	5.265	0.531	0.606
9.032	9.039	5.258	9.442	9.479	0.999	0.606
5.871	5.440	5.764	5.315	5.210	0.341	0.606
5.931	5.516	5.988	4.711	5.132	0.357	0.606
10.129	10.742	8.126	10.816	10.805	0.621	0.606
3.172	3.847	4.028	2.807	3.381	0.535	0.606
7.527	6.863	7.708	6.432	6.612	0.403	0.606
6.731	7.182	5.773	7.946	7.616	0.520	0.606
2.517	3.176	1.830	2.721	3.262	0.399	0.606
6.987	8.067	5.432	8.512	8.402	0.653	0.606
4.859	5.337	5.219	5.424	5.513	0.333	0.606
3.754	5.007	1.277	5.774	5.217	0.870	0.606
5.736	5.633	6.573	5.276	5.748	0.455	0.606
6.810	7.323	6.874	7.496	7.544	0.328	0.606
5.335	5.496	5.490	4.556	5.278	0.380	0.606
5.895	5.817	5.134	6.068	6.027	0.362	0.606
3.905	4.378	3.646	3.968	4.261	0.446	0.606
1.347	2.498	2.628	2.814	2.718	0.482	0.606
1.804	2.449	2.251	1.480	2.140	0.426	0.606
4.763	5.102	5.468	3.945	5.482	0.451	0.606
6.843	7.576	2.924	7.780	7.818	1.031	0.606
6.133	5.250	5.860	5.260	5.443	0.529	0.606
7.204	6.534	7.603	5.312	5.854	0.488	0.606
3.135	3.093	3.554	3.312	2.788	0.452	0.606
5.976	6.403	5.935	6.452	6.176	0.320	0.606
5.841	5.504	3.809	6.148	5.900	0.528	0.606
6.622	3.330	3.285	3.689	3.436	0.757	0.606
3.594	2.903	3.635	3.017	3.078	0.540	0.606
5.708	5.720	2.641	6.321	6.092	0.752	0.606
4.354	4.927	3.805	4.928	5.178	0.413	0.606
2.704	3.641	2.953	3.798	3.737	0.405	0.606

8.307	8.960	5.679	9.040	8.939	0.729	0.606
7.073	9.238	5.200	9.458	9.332	1.020	0.606
8.235	6.073	2.421	5.835	6.878	1.170	0.606
4.472	4.220	4.681	3.140	3.458	0.491	0.606
4.435	3.672	3.834	4.084	4.469	0.416	0.606
5.526	7.278	3.301	7.027	7.190	0.823	0.606
5.091	5.612	2.734	6.271	6.062	0.725	0.606
3.308	3.457	2.180	3.801	3.688	0.546	0.606
2.799	3.392	3.436	3.341	3.063	0.372	0.606
3.265	4.620	3.965	4.712	4.749	0.474	0.606
4.106	3.896	4.351	2.873	3.674	0.538	0.606
8.050	9.385	4.867	9.412	9.219	1.019	0.606
8.098	8.881	3.672	9.304	9.025	1.142	0.606
5.043	5.430	2.898	5.922	5.932	0.715	0.606
3.478	3.215	2.573	2.579	2.669	0.473	0.606
7.121	7.034	5.361	7.573	7.510	0.485	0.606
4.528	3.957	5.212	3.183	3.510	0.494	0.606
5.569	6.497	4.577	6.796	6.291	0.493	0.606
5.577	6.555	4.560	6.725	6.327	0.490	0.606
5.059	5.598	4.482	5.464	5.370	0.376	0.606
7.198	6.679	8.043	5.277	5.993	0.571	0.606
6.102	6.201	2.730	6.470	6.397	0.788	0.606
7.688	8.062	6.658	8.549	8.523	0.463	0.606
8.191	6.729	8.164	6.637	5.730	0.633	0.606
3.020	1.649	4.044	1.828	2.574	0.559	0.606
4.213	5.264	3.272	5.836	5.559	0.554	0.606
5.781	5.951	6.187	6.196	5.796	0.396	0.606
1.516	3.244	1.080	3.864	3.564	0.658	0.606
6.082	6.658	4.657	6.623	6.445	0.544	0.606
5.419	6.582	3.845	5.731	5.743	0.498	0.606
7.398	6.385	7.853	6.041	5.709	0.537	0.606
5.119	6.631	2.875	7.177	7.061	0.901	0.606
4.911	4.372	4.778	4.704	4.469	0.621	0.606
3.045	2.751	1.933	3.561	3.280	0.425	0.606
4.446	6.162	2.717	6.453	6.141	0.800	0.606
5.728	5.660	5.703	6.323	6.002	0.422	0.606
4.600	6.110	2.947	6.454	6.461	0.832	0.606
6.282	6.835	5.513	6.978	7.045	0.460	0.606
8.010	5.668	2.323	6.027	7.231	1.354	0.606
5.493	4.826	2.446	5.288	5.108	0.646	0.606
8.620	8.775	3.051	9.565	9.324	1.287	0.606
5.693	7.188	4.493	7.164	7.075	0.699	0.606
4.004	2.493	2.920	2.712	2.747	0.498	0.606
3.726	5.634	2.512	6.163	6.233	0.876	0.606
5.158	5.513	4.790	5.656	5.923	0.377	0.606
4.073	3.769	4.718	2.893	3.306	0.665	0.606
4.086	3.487	4.828	4.454	3.641	0.556	0.606
5.309	4.527	1.514	5.033	5.042	0.892	0.606
3.760	2.472	3.340	2.707	3.474	0.481	0.606
4.082	4.234	3.575	3.776	4.252	0.464	0.606

5.572	3.180	1.546	3.124	3.803	0.881	0.606
2.414	3.845	1.712	4.591	4.681	0.785	0.606
4.479	4.553	3.551	4.625	4.761	0.406	0.606
4.652	3.906	2.975	3.961	4.096	0.431	0.606
2.695	4.141	3.023	3.427	3.303	0.486	0.606
4.239	3.479	4.235	2.518	3.386	0.544	0.606
4.606	5.180	4.904	5.524	5.060	0.354	0.606
5.885	6.108	6.177	6.334	6.144	0.386	0.606
5.117	5.500	4.667	6.065	6.088	0.406	0.606
4.328	4.865	4.956	5.181	5.280	0.357	0.606
3.484	3.167	3.172	2.479	2.249	0.628	0.606
4.173	5.136	4.651	3.973	4.543	0.571	0.606
2.365	3.143	2.337	2.245	2.340	0.435	0.606
7.135	7.908	5.474	8.083	8.008	0.694	0.606
3.255	2.614	3.001	2.804	2.542	0.391	0.606
5.439	4.986	5.728	4.300	4.474	0.382	0.606
7.054	5.953	7.313	4.766	5.292	0.541	0.606
7.804	7.712	7.372	8.289	7.928	0.330	0.606
4.005	4.773	4.228	3.705	4.086	0.387	0.606
4.263	4.976	4.416	3.935	4.263	0.379	0.606
3.954	3.140	3.607	2.890	3.563	0.362	0.606
5.106	4.527	4.678	4.596	4.833	0.530	0.606
3.141	1.998	3.164	3.198	2.877	0.481	0.606
4.378	2.978	4.093	3.097	3.201	0.566	0.606
5.552	3.779	5.051	3.619	4.485	0.707	0.606
4.016	2.748	3.122	1.980	2.239	0.485	0.606
4.214	2.647	3.397	1.894	2.575	0.617	0.606
5.570	4.517	5.065	5.077	5.168	0.444	0.606
4.416	3.719	4.246	3.006	3.531	0.584	0.606
4.602	4.183	3.855	5.130	4.689	0.515	0.606
6.960	5.952	7.396	5.011	5.318	0.590	0.606
5.955	5.836	5.965	4.286	4.994	0.510	0.606
5.852	5.792	5.454	6.139	6.078	0.384	0.606
5.648	6.553	4.428	6.617	6.421	0.584	0.606
4.473	4.092	4.699	4.075	4.140	0.424	0.606
5.633	6.579	3.936	6.412	6.436	0.642	0.606
5.791	5.297	4.125	5.687	5.844	0.488	0.606
6.358	6.514	4.757	6.457	6.735	0.474	0.606
4.556	5.272	3.682	4.873	5.237	0.440	0.606
4.729	5.346	4.524	5.975	5.741	0.425	0.606
1.585	6.006	2.015	5.914	6.152	1.094	0.606
5.125	5.686	5.371	5.922	5.566	0.314	0.606
2.554	3.356	2.652	3.292	2.609	0.449	0.606
2.134	2.832	2.848	3.403	3.413	0.472	0.606
3.570	4.081	3.196	3.637	3.056	0.451	0.606
3.217	3.221	4.031	2.145	2.648	0.511	0.606
5.196	4.267	2.828	5.576	4.955	0.840	0.606
3.977	2.852	3.499	2.934	3.025	0.394	0.606
5.299	5.834	4.489	5.521	5.884	0.435	0.606
4.119	3.940	4.741	3.955	3.983	0.405	0.606

4.182	2.573	3.425	4.456	3.357	0.496	0.606
2.949	2.351	2.444	2.094	2.587	0.350	0.606
3.748	5.031	3.399	5.749	5.079	0.620	0.606
7.051	6.355	7.785	6.273	6.365	0.370	0.606
2.534	3.076	2.960	2.551	2.311	0.419	0.606
6.418	6.108	7.445	6.163	6.299	0.390	0.606
3.986	4.532	4.898	3.892	4.285	0.360	0.606
3.973	3.441	3.093	3.004	4.191	0.389	0.606
3.430	4.042	4.542	4.843	4.097	0.538	0.606
5.131	4.981	4.970	5.538	5.539	0.319	0.606
6.086	5.610	7.051	4.928	5.021	0.483	0.606
3.400	2.580	2.223	2.489	2.404	0.434	0.606
4.359	2.175	1.978	3.027	3.418	0.698	0.606
2.097	2.207	2.589	1.923	2.298	0.496	0.606
1.016	2.021	1.049	2.076	3.081	0.483	0.606
3.085	2.065	2.171	2.773	2.031	0.519	0.606
6.124	5.410	5.348	5.432	6.041	0.387	0.606
5.124	4.721	3.751	5.293	5.167	0.421	0.606
2.493	3.710	2.604	3.227	2.862	0.510	0.606
2.620	2.874	1.763	2.784	2.357	0.447	0.606
4.250	3.597	3.498	3.827	3.909	0.367	0.606
4.010	5.219	4.060	5.347	4.731	0.428	0.606
5.413	5.281	5.201	5.594	6.086	0.439	0.606
3.161	2.213	1.576	2.525	1.682	0.820	0.606
2.651	2.366	1.593	2.202	1.789	0.409	0.606
2.758	2.646	2.915	2.530	2.071	0.438	0.606
5.123	4.687	5.429	4.487	3.362	0.856	0.606
6.142	5.237	6.192	4.910	5.306	0.395	0.606
6.437	6.063	4.529	6.438	6.500	0.572	0.606
5.020	6.057	2.949	6.690	6.108	0.813	0.606
5.652	5.985	3.488	6.502	6.274	0.606	0.606
2.354	2.128	2.115	1.975	1.727	0.494	0.606
4.818	4.742	5.206	3.761	4.113	0.441	0.606
6.012	5.479	5.257	5.713	5.024	0.404	0.606
2.210	3.525	1.642	3.517	3.400	0.596	0.606
2.712	2.084	2.101	1.976	2.773	0.513	0.606
2.788	4.399	2.887	4.548	3.714	0.563	0.606
5.941	5.483	6.477	4.559	4.622	0.496	0.606
2.456	8.212	2.269	8.560	8.443	1.525	0.606
5.332	5.617	5.638	5.636	5.819	0.315	0.606
2.535	1.704	1.328	1.787	1.269	0.456	0.606
2.818	3.732	3.806	4.467	4.109	0.546	0.606
4.169	4.238	4.553	4.476	4.084	0.302	0.606
3.335	2.765	3.070	2.654	2.649	0.349	0.606
10.882	9.662	10.848	8.109	8.217	0.744	0.606
4.771	5.366	5.154	5.781	5.456	0.406	0.606
2.151	1.818	2.138	1.859	1.544	0.372	0.606
2.554	2.627	2.109	2.140	2.349	0.426	0.606
6.718	7.643	7.322	7.946	7.746	0.399	0.606
2.020	3.961	1.879	3.621	3.429	0.554	0.606

5.705	5.897	5.507	5.959	6.494	0.439	0.606
4.760	4.671	6.006	3.336	3.852	0.554	0.606
5.464	5.276	5.738	4.366	4.947	0.436	0.606
4.196	4.826	4.068	3.783	4.859	0.523	0.606
6.097	7.190	6.979	7.168	7.432	0.509	0.606
2.237	1.762	1.946	2.232	2.255	0.510	0.606
5.495	4.696	6.573	3.894	4.353	0.641	0.606
3.126	3.192	3.308	2.374	3.454	0.560	0.606
6.278	5.909	6.592	5.495	6.071	0.514	0.606
5.928	6.464	6.191	5.530	6.492	0.427	0.606
4.036	4.586	2.666	5.069	4.777	0.569	0.606
2.684	2.643	3.017	2.493	2.281	0.364	0.606
4.715	6.035	5.362	4.621	6.496	0.566	0.606
4.025	4.682	4.554	4.962	5.329	0.456	0.606
3.715	3.164	4.991	3.525	3.202	0.541	0.606
3.989	3.994	4.698	3.974	4.623	0.434	0.606
2.460	3.551	3.496	2.569	3.118	0.623	0.606
4.486	5.950	4.744	4.654	4.503	0.666	0.606
7.494	6.248	7.252	5.579	6.194	0.497	0.606
5.743	4.284	4.166	4.176	4.706	0.492	0.606
4.526	4.628	3.973	4.179	4.170	0.424	0.606
2.166	2.660	2.386	2.245	3.114	0.412	0.606
4.838	5.074	6.376	3.224	3.661	0.698	0.606
4.570	3.803	5.168	3.221	3.679	0.645	0.606
2.386	3.147	3.014	3.006	3.064	0.486	0.606
4.524	6.926	4.291	7.581	7.018	0.751	0.606
2.374	3.071	1.829	3.607	3.291	0.588	0.606
2.905	3.748	3.597	4.234	4.284	0.434	0.606
3.031	3.306	3.265	2.668	4.187	0.463	0.606
3.848	3.979	4.206	4.585	4.283	0.340	0.606
4.398	4.306	1.732	4.884	4.710	0.671	0.606
5.115	5.706	4.785	5.795	5.530	0.373	0.606
6.258	5.828	6.515	5.795	6.296	0.416	0.606
2.874	4.716	2.954	5.293	4.689	0.637	0.606
2.403	3.387	3.134	3.307	3.729	0.478	0.606
3.254	2.866	3.937	3.540	3.887	0.388	0.606
3.312	3.499	3.307	2.186	3.245	0.472	0.606
6.462	6.920	5.942	6.889	6.823	0.442	0.606
4.931	4.060	4.988	4.305	4.702	0.457	0.606
4.630	5.140	4.587	5.646	5.202	0.343	0.606
5.614	6.525	5.446	4.951	6.021	0.511	0.606
7.989	7.292	7.888	6.715	6.834	0.380	0.606
7.962	7.374	6.725	6.690	7.088	0.399	0.606
5.264	4.651	4.268	4.326	4.652	0.711	0.606
6.124	6.850	7.345	6.680	6.172	0.538	0.606
5.594	6.628	5.888	6.817	6.860	0.418	0.606
6.091	4.893	6.916	4.205	4.362	0.597	0.606
6.834	6.113	7.837	5.186	5.933	0.612	0.606
2.401	3.046	2.988	3.357	3.521	0.428	0.606
2.316	3.458	2.713	2.371	2.444	0.444	0.606

5.339	5.526	4.516	5.965	5.695	0.372	0.606
5.239	4.763	4.781	4.348	4.689	0.350	0.606
3.356	3.803	3.421	4.424	4.224	0.389	0.606
3.504	4.159	4.663	2.447	3.300	0.592	0.606
6.977	6.621	7.263	5.866	6.005	0.390	0.606
6.190	6.935	6.440	6.963	6.802	0.318	0.606
4.110	4.242	4.795	3.062	3.868	0.641	0.606
3.513	3.843	4.203	3.020	3.593	0.639	0.606
4.448	5.283	5.794	4.189	3.880	0.757	0.606
4.007	4.092	5.005	2.846	4.132	0.643	0.606
5.906	5.198	6.591	5.597	6.230	0.573	0.606
3.801	4.867	5.687	3.777	4.690	0.618	0.606
3.407	2.803	3.715	3.015	2.877	0.498	0.606
4.175	3.530	5.129	2.529	3.605	0.678	0.606
1.517	3.146	2.338	1.912	1.696	0.624	0.606
4.316	4.718	4.044	5.019	4.664	0.384	0.606
6.327	6.490	6.386	6.334	7.194	0.471	0.606
4.093	3.413	3.156	3.553	4.135	0.439	0.606
6.057	5.948	5.543	6.061	6.061	0.445	0.606
3.542	3.915	3.706	2.019	2.696	0.561	0.606
4.810	4.453	5.290	3.667	4.360	0.404	0.606
1.659	2.237	1.738	1.922	2.356	0.360	0.606
5.769	5.008	6.706	3.573	3.913	0.661	0.606
8.098	6.675	8.371	5.296	5.854	0.740	0.606
3.046	3.449	3.602	2.232	2.868	0.491	0.606
3.921	4.639	4.288	5.078	4.885	0.423	0.606
3.437	2.681	3.999	3.289	3.313	0.426	0.606
6.130	5.330	6.981	4.568	4.666	0.526	0.606
2.601	1.982	3.386	2.324	2.217	0.451	0.606
3.948	3.470	3.913	3.282	3.389	0.399	0.606
6.343	6.688	5.800	6.749	6.754	0.413	0.606
6.335	6.051	6.734	6.263	6.131	0.395	0.606
5.535	4.805	5.913	4.285	4.360	0.447	0.606
4.417	4.119	4.675	3.451	4.078	0.431	0.606
2.400	3.625	2.404	3.894	3.827	0.514	0.606
3.675	3.982	4.639	3.443	3.770	0.571	0.606
2.861	2.674	3.217	1.744	2.154	0.439	0.606
7.792	8.020	3.281	8.343	7.989	1.093	0.606
4.845	6.265	4.570	6.871	6.634	0.619	0.606
3.479	5.298	2.810	5.833	5.495	0.701	0.606
4.564	4.354	4.108	4.273	4.193	0.511	0.606
4.000	2.926	4.919	1.488	1.681	0.750	0.606
4.770	4.206	5.248	3.605	3.121	0.607	0.606
5.585	6.244	8.347	3.718	4.504	0.902	0.606
7.465	6.207	8.070	5.307	6.028	0.686	0.606
5.497	5.249	4.301	5.762	5.477	0.515	0.606
7.571	7.525	6.597	7.652	7.556	0.348	0.606
5.679	7.210	5.787	7.268	7.666	0.526	0.606
6.436	5.836	7.095	5.292	5.151	0.476	0.606
1.724	2.715	1.460	3.942	2.539	0.539	0.606

1.551	5.214	1.316	5.781	5.477	1.001	0.606
3.891	3.326	4.612	3.221	3.255	0.574	0.606
3.815	5.080	3.530	4.968	4.773	0.600	0.606
4.896	5.695	4.338	5.161	4.976	0.439	0.606
5.891	5.467	4.858	6.012	5.532	0.427	0.606
8.259	6.869	9.074	5.905	6.312	0.711	0.606
5.762	5.299	5.831	5.058	5.190	0.460	0.606
3.635	3.535	2.880	4.127	4.254	0.519	0.606
3.161	2.929	2.348	3.116	3.491	0.356	0.606
5.490	6.294	5.828	6.728	6.388	0.370	0.606
3.115	2.690	2.940	1.916	3.125	0.470	0.606
3.894	3.615	3.831	3.938	2.919	0.435	0.606
5.546	5.702	6.260	4.579	4.724	0.469	0.606
4.796	5.063	4.001	5.176	5.337	0.370	0.606
6.183	6.697	4.450	7.107	6.902	0.628	0.606
4.887	4.797	3.784	4.886	5.282	0.471	0.606
3.776	4.050	3.308	4.146	4.329	0.368	0.606
4.938	4.738	5.960	3.870	4.756	0.458	0.606
6.686	7.252	6.678	7.462	7.203	0.351	0.606
6.264	6.469	5.856	6.877	6.675	0.420	0.606
4.335	5.322	4.342	5.466	5.007	0.431	0.606
5.612	6.241	3.550	6.634	6.717	0.818	0.606
3.109	4.107	3.087	4.533	3.850	0.426	0.606
5.742	5.146	6.942	4.619	4.904	0.486	0.606
5.017	5.751	4.799	5.997	6.013	0.419	0.606
2.509	2.680	2.800	3.637	3.356	0.391	0.606
3.955	4.548	5.002	4.323	5.058	0.517	0.606
5.734	4.323	6.394	4.112	3.954	0.667	0.606
4.114	3.333	3.427	3.549	3.814	0.408	0.606
2.594	3.686	1.976	3.390	3.732	0.571	0.606
4.387	3.546	5.085	4.284	4.273	0.385	0.606
2.312	3.109	2.294	3.524	2.958	0.464	0.606
4.168	4.318	3.746	4.546	4.505	0.448	0.606
4.839	3.721	5.239	3.079	2.849	0.563	0.606
3.504	5.026	2.845	5.336	4.696	0.674	0.606
3.811	5.169	3.656	4.704	5.007	0.484	0.606
5.635	6.274	5.418	6.238	6.413	0.342	0.606
4.796	5.166	4.014	5.078	5.192	0.404	0.606
2.612	1.978	3.312	1.817	2.202	0.486	0.606
8.333	5.338	6.324	4.824	4.832	0.737	0.606
3.020	3.582	3.217	3.961	3.054	0.413	0.606
5.145	5.041	5.230	5.988	6.025	0.476	0.606
1.834	2.424	1.542	2.497	2.264	0.693	0.606
6.425	6.272	3.485	6.845	6.519	0.831	0.606
5.409	5.722	4.392	6.177	5.900	0.479	0.606
5.511	4.946	6.130	4.631	4.882	0.417	0.606
3.533	4.365	2.177	3.360	3.490	0.449	0.606
3.995	3.383	4.686	2.785	3.246	0.482	0.606
3.176	2.733	4.099	2.589	2.281	0.441	0.606
2.875	3.222	2.810	2.840	2.448	0.462	0.606

5.287	5.638	5.601	5.956	5.913	0.291	0.606
6.919	7.787	5.174	7.948	7.608	0.696	0.606
4.439	4.852	4.721	5.264	5.193	0.353	0.606
1.895	1.909	2.062	1.440	1.766	0.392	0.606
8.635	7.339	9.394	5.736	6.255	0.781	0.606
6.214	6.221	4.192	6.514	6.398	0.498	0.606
6.330	7.409	6.392	7.584	7.318	0.382	0.606
4.659	4.522	3.708	4.364	4.862	0.423	0.606
3.133	3.443	3.112	3.907	3.724	0.355	0.606
2.203	1.559	2.087	1.872	1.740	0.488	0.606
3.107	2.631	2.023	2.714	3.568	0.469	0.606
3.262	3.946	1.875	3.908	3.992	0.572	0.606
2.662	3.603	3.385	3.811	4.245	0.453	0.606
7.562	6.787	8.254	6.342	6.623	0.459	0.606
4.204	2.717	3.915	3.026	3.382	0.615	0.606
6.765	6.675	7.727	5.381	5.082	0.609	0.606
6.673	6.645	8.063	4.778	5.755	0.687	0.606
5.361	3.922	6.311	3.898	3.578	0.640	0.606
4.274	3.539	4.616	3.818	3.468	0.460	0.606
7.349	7.728	6.718	8.318	8.068	0.435	0.606
8.768	9.222	7.835	9.532	9.463	0.422	0.606
2.261	3.246	1.668	2.396	2.387	0.530	0.606
4.972	3.274	3.850	3.861	3.485	0.648	0.606
5.146	5.348	5.674	3.991	4.895	0.415	0.606
3.756	3.831	5.462	3.799	3.175	0.528	0.606
3.799	2.765	3.903	3.090	3.198	0.748	0.606
6.329	6.480	5.423	6.690	6.688	0.379	0.606
6.857	6.222	7.736	4.910	5.502	0.601	0.606
6.398	6.938	6.621	7.223	7.316	0.322	0.606
6.843	6.454	6.803	5.173	5.732	0.587	0.606
5.844	6.267	6.082	6.640	6.817	0.376	0.606
7.896	7.533	6.318	8.026	8.199	0.426	0.606
5.773	5.615	6.287	2.416	3.539	0.864	0.606
4.348	4.180	3.445	3.759	3.948	0.428	0.606
4.444	5.619	5.049	3.902	3.847	0.655	0.606
3.875	3.410	4.660	3.513	2.998	0.464	0.606
2.034	2.111	1.707	1.961	2.240	0.376	0.606
3.000	5.003	2.525	4.839	4.626	0.648	0.606
4.229	5.067	3.642	4.940	4.289	0.448	0.606
2.634	2.733	3.320	2.782	3.221	0.443	0.606
3.271	3.139	3.908	4.055	3.521	0.454	0.606
2.797	3.453	2.866	4.203	3.768	0.429	0.606
3.174	3.631	3.627	3.234	4.725	0.515	0.606
5.397	5.421	4.354	4.347	4.415	0.581	0.606
2.966	2.049	2.433	2.296	1.306	0.585	0.606
3.445	3.741	2.681	3.446	3.328	0.526	0.606
4.860	5.255	5.651	4.587	4.946	0.370	0.606
5.780	7.129	7.514	4.853	5.778	0.751	0.606
7.838	7.263	8.297	7.263	6.931	0.540	0.606
3.038	3.498	2.493	4.371	3.698	0.462	0.606

5.026	4.578	4.680	3.461	5.019	0.662	0.606
3.641	3.048	3.617	3.317	3.410	0.490	0.606
2.488	2.566	2.944	2.730	2.426	0.379	0.606
2.318	2.179	2.988	1.709	1.905	0.567	0.606
7.246	7.225	7.461	7.301	7.103	0.346	0.606
1.526	2.516	1.302	2.041	2.116	0.499	0.606
1.916	3.139	2.011	2.700	3.367	0.423	0.606
1.810	3.333	1.871	2.753	2.921	0.477	0.606
6.314	4.530	7.333	3.970	3.783	0.752	0.606
5.226	4.056	5.161	3.455	3.793	0.468	0.606
5.375	4.782	4.960	5.178	5.144	0.310	0.606
4.328	3.409	3.269	3.453	4.480	0.406	0.606
4.593	4.184	3.481	3.847	4.925	0.440	0.606
4.662	3.718	3.879	4.014	4.713	0.395	0.606
4.722	3.825	4.077	4.254	4.886	0.427	0.606
4.224	3.280	3.532	3.926	4.407	0.419	0.606
4.722	4.871	6.357	4.841	4.792	0.436	0.606
3.313	2.096	1.968	2.192	2.546	0.470	0.606
5.642	6.487	5.548	6.611	6.492	0.380	0.606
6.145	5.505	3.828	6.195	5.693	0.568	0.606
6.632	7.100	5.917	6.957	7.165	0.372	0.606
6.950	7.524	5.722	7.725	7.882	0.499	0.606
4.561	4.832	3.826	4.723	4.964	0.364	0.606
6.910	6.800	6.330	7.011	7.032	0.309	0.606
6.432	6.824	6.516	6.985	6.801	0.358	0.606
5.974	7.697	6.138	7.770	7.911	0.504	0.606
4.153	4.192	2.348	4.254	4.375	0.512	0.606
4.356	5.133	5.222	4.103	4.662	0.470	0.606
3.299	3.579	4.103	3.403	3.327	0.462	0.606
4.510	4.209	5.002	3.159	3.915	0.461	0.606
8.502	7.902	8.975	5.814	6.122	0.672	0.606
4.410	4.433	5.020	4.830	4.867	0.411	0.606
5.067	4.391	6.030	3.242	3.154	0.643	0.606
2.679	2.955	1.750	3.620	3.441	0.488	0.606
4.053	3.081	2.570	3.719	3.619	0.557	0.606
6.309	5.386	6.497	5.507	5.549	0.424	0.606
5.524	5.338	6.585	6.228	6.615	0.418	0.606
2.771	3.678	3.095	2.632	3.809	0.475	0.606
3.965	4.785	3.979	4.571	3.922	0.452	0.606
3.984	3.601	3.681	4.263	3.862	0.437	0.606
6.116	7.093	5.888	7.223	7.238	0.489	0.606
9.545	9.702	9.855	9.216	9.292	0.349	0.606
8.615	7.313	8.632	5.965	6.329	0.675	0.606
7.792	8.248	6.503	8.412	8.324	0.467	0.606
4.095	3.533	3.579	2.431	2.613	0.453	0.606
2.183	2.884	2.520	2.447	2.424	0.609	0.606
1.613	2.456	2.296	1.889	2.079	0.415	0.606
4.490	5.007	4.425	4.834	4.378	0.498	0.606
6.786	7.051	7.694	6.810	6.784	0.453	0.606
5.304	5.934	5.269	6.625	6.576	0.646	0.606

3.839	2.960	4.401	2.851	3.774	0.482	0.606
2.377	3.733	1.884	3.531	3.530	0.571	0.606
4.080	4.535	4.838	3.252	3.842	0.461	0.606
4.586	4.266	6.019	3.535	3.564	0.565	0.606
2.549	2.413	2.841	1.716	2.273	0.479	0.606
3.169	3.968	2.977	4.225	3.623	0.369	0.606
5.152	5.065	6.394	4.252	4.700	0.451	0.606
2.778	2.319	2.510	2.078	2.675	0.345	0.606
2.697	2.021	2.884	1.925	2.858	0.520	0.606
2.428	3.915	3.580	3.926	1.336	0.888	0.606
8.072	6.724	8.214	7.029	7.038	0.421	0.606
2.462	1.945	2.909	1.763	2.232	0.446	0.606
3.033	2.707	2.902	2.199	3.075	0.334	0.606
5.991	5.509	3.368	6.222	6.159	0.625	0.606
4.753	4.704	5.111	5.022	5.627	0.435	0.606
2.353	2.644	3.265	2.146	2.896	0.477	0.606
6.866	7.334	6.640	7.796	7.914	0.423	0.606
3.073	3.305	3.202	3.144	2.870	0.492	0.606
3.250	3.831	3.650	3.146	2.914	0.503	0.606
3.622	3.997	4.464	3.991	2.422	0.672	0.606
4.359	5.855	4.066	5.859	5.854	0.639	0.606
2.641	1.772	2.770	2.344	2.940	0.480	0.606
2.446	1.997	1.691	1.668	3.270	0.547	0.606
2.574	2.806	4.282	2.333	2.642	0.626	0.606
3.352	2.998	2.124	2.771	2.852	0.470	0.606
2.866	3.597	3.974	3.461	4.012	0.625	0.606
3.101	2.405	3.032	3.669	3.335	0.448	0.606
2.971	1.944	2.383	1.827	2.381	0.506	0.606
2.461	2.987	2.819	2.627	2.485	0.519	0.606
5.011	4.740	5.816	3.346	4.239	0.648	0.606
2.858	2.812	2.141	1.854	1.719	0.520	0.606
4.510	5.176	3.440	5.811	5.737	0.600	0.606
1.416	1.622	2.064	1.577	1.610	0.652	0.606
1.559	2.765	3.469	0.779	4.211	1.055	0.606
2.595	4.360	5.780	2.806	6.515	1.260	0.606
1.823	2.354	2.223	2.110	2.931	0.463	0.606
4.752	2.965	2.545	3.058	2.815	0.458	0.606
3.550	6.423	3.037	7.073	6.571	0.905	0.606
2.557	4.041	2.439	5.084	4.644	0.657	0.606
4.322	4.633	4.413	3.836	4.531	0.422	0.606
6.897	4.825	5.700	5.208	5.824	0.605	0.606
3.101	3.793	2.574	3.778	3.763	0.494	0.606
3.874	3.574	3.626	3.095	3.537	0.490	0.606
5.113	4.809	5.784	4.538	4.898	0.461	0.606
5.981	6.745	6.635	7.136	6.956	0.349	0.606
4.107	5.224	4.152	5.579	5.228	0.434	0.606
4.470	4.291	4.104	4.814	4.800	0.394	0.606
8.387	8.781	6.810	9.047	9.086	0.521	0.606
4.618	4.683	5.433	3.540	4.752	0.557	0.606
4.301	5.430	4.933	4.542	3.807	0.483	0.606

2.996	2.951	3.043	2.802	2.592	0.359	0.606
5.188	5.188	6.117	5.808	5.713	0.356	0.606
4.844	4.902	5.623	4.296	4.514	0.384	0.606
4.868	3.521	6.194	2.645	3.317	0.701	0.606
3.872	2.593	2.534	3.226	3.361	0.503	0.606
5.185	4.306	6.259	4.797	4.315	0.477	0.606
4.649	4.989	5.151	4.267	4.683	0.430	0.606
4.748	2.947	4.538	3.259	3.318	0.579	0.606
4.305	3.231	3.006	3.360	3.953	0.527	0.606
2.760	3.552	4.048	3.608	3.790	0.562	0.606
6.644	6.890	6.624	7.240	7.119	0.350	0.606
6.146	6.516	5.276	6.948	6.569	0.389	0.606
3.263	2.730	3.524	2.373	3.220	0.364	0.606
4.480	4.360	4.528	4.494	4.053	0.389	0.606
5.327	4.889	5.269	3.675	4.802	0.497	0.606
3.068	3.866	3.997	3.362	4.032	0.468	0.606
4.981	5.411	4.544	5.622	5.498	0.385	0.606
4.418	4.251	4.407	5.066	5.044	0.384	0.606
4.961	5.485	3.234	5.782	5.889	0.620	0.606
3.830	3.957	2.994	3.685	3.969	0.367	0.606
3.720	2.916	1.525	2.352	2.381	0.565	0.606
4.152	4.288	2.000	4.492	4.350	0.576	0.606
5.891	5.555	1.839	5.904	6.014	0.973	0.606
2.003	2.509	1.150	2.359	3.107	0.438	0.606
7.774	6.312	6.812	7.006	7.153	0.580	0.606
3.881	4.164	3.586	3.323	3.731	0.426	0.606
4.935	3.964	4.726	2.776	3.340	0.607	0.606
6.685	6.054	6.782	6.064	6.088	0.368	0.606
3.719	3.157	4.523	1.813	2.237	0.733	0.606
5.035	5.679	4.825	5.722	5.858	0.373	0.606
6.656	7.010	6.025	6.888	6.988	0.346	0.606
4.580	5.487	4.997	5.566	5.592	0.358	0.606
3.846	4.599	4.027	4.483	5.046	0.376	0.606
6.060	6.604	5.532	6.875	6.807	0.507	0.606
4.533	4.662	5.804	3.600	3.714	0.477	0.606
3.443	3.388	4.287	3.254	3.017	0.402	0.606
2.373	1.725	1.984	2.249	2.096	0.590	0.606
5.444	4.815	4.387	5.682	5.203	0.395	0.606
4.447	5.373	4.271	5.737	4.773	0.407	0.606
2.907	3.566	2.995	2.807	3.258	0.447	0.606
3.361	2.801	3.925	2.846	3.018	0.425	0.606
7.660	8.420	5.259	9.238	8.569	0.925	0.606
2.847	4.089	3.350	4.875	4.176	0.491	0.606
5.909	5.284	7.045	3.897	4.219	0.681	0.606
5.058	5.539	5.000	5.826	5.509	0.397	0.606
4.111	3.179	4.205	3.446	4.145	0.493	0.606
4.896	3.801	2.561	4.033	4.004	0.563	0.606
5.725	5.947	5.885	6.040	5.741	0.345	0.606
4.931	5.811	5.505	6.069	6.086	0.362	0.606
4.989	4.578	5.202	4.883	4.903	0.378	0.606

7.223	7.798	6.158	8.054	8.378	0.501	0.606
3.544	2.172	4.336	1.973	1.478	0.638	0.606
4.125	4.412	3.721	4.383	4.276	0.329	0.606
3.060	3.995	3.602	3.667	3.253	0.447	0.606
3.723	4.344	3.582	4.474	4.341	0.411	0.606
7.540	8.244	5.840	8.306	8.123	0.550	0.606
7.510	7.935	6.669	8.266	8.156	0.427	0.606
8.067	8.514	7.544	8.769	8.674	0.395	0.606
5.248	5.500	5.053	5.444	5.303	0.370	0.606
3.199	2.525	2.423	2.924	2.229	0.460	0.606
4.066	2.859	3.010	2.703	3.186	0.586	0.606
5.826	6.089	7.135	5.190	5.655	0.536	0.606
3.292	4.425	2.585	4.821	3.875	0.562	0.606
5.289	5.955	5.194	6.178	5.759	0.379	0.606
7.853	7.927	7.005	8.048	8.292	0.369	0.606
5.220	3.890	4.255	3.858	4.033	0.498	0.606
6.064	6.169	5.414	6.486	6.507	0.346	0.606
6.150	6.385	6.231	6.533	6.419	0.324	0.606
3.855	3.027	3.344	3.432	3.816	0.413	0.606
3.850	3.688	2.762	3.475	3.771	0.469	0.606
3.817	3.454	3.052	4.060	3.991	0.427	0.606
2.539	3.174	2.080	2.389	3.290	0.455	0.606
5.824	6.412	5.472	6.704	6.834	0.389	0.606
3.198	1.815	2.814	2.097	1.568	0.408	0.606
4.828	4.866	5.463	4.464	4.122	0.430	0.606
3.626	3.081	1.390	3.462	3.024	0.480	0.606
5.534	5.360	3.214	5.212	5.441	0.536	0.606
4.828	5.154	3.744	5.747	5.786	0.456	0.606
5.087	5.517	5.016	6.466	5.642	0.440	0.606
2.813	2.141	2.258	2.820	2.884	0.414	0.606
3.970	3.945	3.381	3.914	3.808	0.370	0.606
5.231	5.044	6.088	4.044	4.590	0.454	0.606
6.123	6.549	5.929	6.688	6.760	0.354	0.606
5.755	6.284	1.676	6.730	6.640	0.958	0.606
2.999	4.447	2.182	4.834	4.414	0.684	0.606
4.856	5.062	5.267	5.492	5.345	0.386	0.606
3.678	2.756	4.228	2.180	3.071	0.528	0.606
6.280	5.547	6.535	5.064	5.084	0.445	0.606
8.293	7.446	8.882	7.589	7.524	0.413	0.606
6.359	4.019	6.028	4.540	4.581	0.633	0.606
5.178	4.141	5.262	3.685	3.970	0.511	0.606
5.175	3.198	4.686	4.039	2.834	0.924	0.606
4.728	4.614	3.890	4.644	4.770	0.494	0.606
4.346	3.483	4.571	3.695	4.059	0.665	0.606
5.204	4.747	4.408	4.963	5.343	0.372	0.606
2.368	3.195	2.700	3.681	3.847	0.787	0.606
4.154	3.569	3.881	3.644	3.345	0.762	0.606
6.346	6.353	6.465	6.650	6.769	1.918	0.606
5.733	5.064	5.819	4.937	5.261	0.376	0.606
8.516	7.733	9.549	6.389	6.490	0.645	0.606

6.726	7.094	5.824	7.232	7.174	0.439	0.606
2.072	3.149	1.690	2.286	1.724	0.458	0.606
3.268	2.238	2.186	3.244	3.765	0.527	0.606
2.400	2.462	2.433	1.799	2.400	0.535	0.606
3.660	3.863	1.845	4.808	4.809	0.625	0.606
2.274	2.924	1.886	2.550	2.034	0.762	0.606
7.403	7.640	6.459	6.444	6.789	0.425	0.606
4.482	5.093	4.781	3.527	4.375	0.478	0.606
4.350	4.734	3.897	3.736	4.330	0.536	0.606
6.518	7.376	6.567	7.695	7.473	0.405	0.606
3.533	4.346	2.580	4.811	4.265	0.585	0.606
4.956	5.153	4.242	5.215	5.142	0.363	0.606
6.118	6.574	4.237	6.685	6.934	0.597	0.606
3.854	4.435	3.404	4.601	4.315	0.454	0.606
3.530	3.296	3.745	3.640	4.045	0.522	0.606
5.692	4.821	5.076	5.060	5.239	1.638	0.606
5.641	4.649	4.905	4.833	5.060	1.532	0.606
5.440	5.845	5.337	5.959	6.067	0.365	0.606
7.778	8.028	10.363	7.412	8.938	0.633	0.606
4.524	4.690	4.995	4.502	3.818	0.394	0.606
4.149	4.616	4.156	4.066	4.205	0.344	0.606
4.524	3.691	5.292	1.916	2.906	0.759	0.606
7.616	8.320	8.673	6.042	7.378	0.704	0.606
2.596	2.656	2.296	3.012	3.092	0.499	0.606
4.306	4.626	4.430	5.206	4.723	0.328	0.606
2.415	2.916	2.368	3.393	2.835	0.365	0.606
5.359	4.478	5.703	3.711	4.689	0.483	0.606
5.547	6.130	4.713	6.124	5.831	0.491	0.606
4.274	4.372	5.281	4.109	3.639	0.437	0.606
4.638	3.951	4.719	2.886	4.154	0.584	0.606
5.127	5.778	4.988	5.811	5.957	0.402	0.606
5.746	5.870	6.083	4.820	5.153	0.453	0.606
6.210	6.923	5.296	5.751	5.522	0.503	0.606
5.927	5.626	6.120	6.408	6.013	0.342	0.606
6.078	6.081	6.052	6.589	6.471	0.392	0.606
6.836	7.179	5.722	7.149	7.069	0.418	0.606
2.842	3.156	2.410	4.175	2.745	0.579	0.606
6.628	7.304	3.418	7.509	7.186	0.904	0.606
4.566	5.251	3.441	5.827	5.557	0.623	0.606
4.179	4.626	4.157	5.118	4.895	0.363	0.606
3.087	2.409	3.769	1.643	2.512	0.527	0.606
6.801	7.136	7.003	7.404	7.635	0.367	0.606
3.582	3.836	2.983	4.166	4.021	0.354	0.606
7.597	8.194	7.771	8.178	8.088	0.334	0.606
7.572	7.764	7.625	7.870	8.003	0.330	0.606
4.131	4.181	4.170	3.991	3.723	0.382	0.606
4.983	5.167	3.239	5.475	5.256	0.515	0.606
4.128	2.628	2.393	3.457	3.504	0.460	0.606
4.534	5.394	5.197	5.490	5.431	0.480	0.606
7.574	7.431	6.153	7.796	7.689	0.428	0.606

5.149	5.553	5.009	5.546	5.615	0.335	0.606
3.974	3.502	4.210	4.056	3.362	0.429	0.606
2.856	3.660	4.049	2.410	3.615	0.643	0.606
6.354	5.805	6.793	6.421	6.792	0.482	0.606
5.677	6.108	5.685	6.306	6.443	0.446	0.606
3.443	3.298	4.797	2.993	2.940	0.533	0.606
4.218	4.846	4.222	4.910	4.701	0.349	0.606
10.257	8.836	10.753	7.507	7.848	0.745	0.606
6.536	6.947	6.046	7.033	7.203	0.404	0.606
5.483	5.749	4.769	5.439	5.506	0.380	0.606
3.955	3.885	3.346	3.640	3.550	0.451	0.606
5.673	4.548	4.795	4.834	4.166	0.487	0.606
4.155	2.885	1.743	2.319	1.541	0.575	0.606
6.085	4.639	5.058	4.788	4.358	0.506	0.606
6.309	8.395	3.689	8.463	8.398	0.990	0.606
6.990	7.251	3.115	7.744	7.651	1.018	0.606
3.926	4.181	4.630	3.112	2.926	0.509	0.606
4.043	5.634	2.615	6.243	5.932	0.843	0.606
6.864	6.185	7.552	5.173	5.722	0.477	0.606
6.108	5.778	6.891	5.278	5.478	0.439	0.606
2.942	2.209	3.040	1.720	2.127	0.457	0.606
5.168	6.525	3.279	6.961	6.650	0.849	0.606
6.452	6.815	5.237	7.336	7.156	0.543	0.606
7.274	7.448	4.435	7.742	7.723	0.733	0.606
3.739	4.299	4.019	3.351	3.695	0.416	0.606
3.838	4.134	4.479	3.364	3.426	0.389	0.606
5.682	6.390	5.880	6.753	6.461	0.400	0.606
2.764	3.875	3.471	5.127	4.201	0.480	0.606
3.962	3.785	3.635	4.458	3.923	0.382	0.606
3.958	3.539	3.863	4.290	4.059	0.378	0.606
8.655	8.974	8.578	9.129	9.266	0.298	0.606
4.701	4.873	3.974	5.397	5.632	0.419	0.606
4.227	4.573	3.471	4.243	4.561	0.356	0.606
6.620	6.622	6.009	7.038	6.982	0.443	0.606
3.506	3.073	4.199	2.398	2.864	0.607	0.606
7.779	8.198	7.089	8.140	8.317	0.352	0.606
5.232	3.966	4.358	4.596	4.514	0.459	0.606
5.137	4.044	4.457	4.656	4.551	0.464	0.606
5.210	4.302	4.269	4.431	4.407	0.463	0.606
5.263	3.838	3.223	4.474	4.057	0.507	0.606
6.315	7.005	6.513	7.271	7.366	0.350	0.606
4.746	4.554	2.879	4.871	4.981	0.565	0.606
4.639	4.952	4.580	5.639	5.034	0.409	0.606
7.920	7.810	8.198	7.860	7.913	0.327	0.606
4.065	4.450	3.357	3.151	3.153	0.626	0.606
6.202	6.335	6.340	6.664	6.812	0.302	0.606
5.556	4.664	6.143	4.804	4.692	0.429	0.606
3.183	4.135	2.274	4.283	4.038	0.512	0.606
6.840	6.112	6.249	6.020	6.355	0.400	0.606
8.454	7.450	8.968	6.829	6.985	0.496	0.606

6.379	6.481	6.548	7.215	7.028	1.930	0.606
2.614	3.861	3.422	3.385	3.675	0.426	0.606
5.244	6.033	5.888	6.129	6.038	0.367	0.606
7.063	5.930	6.707	5.967	6.254	0.398	0.606
3.094	1.748	2.078	1.346	1.267	0.770	0.606
4.952	4.542	5.314	3.902	4.138	1.714	0.606
4.299	3.690	3.218	4.950	3.565	0.507	0.606
9.308	8.090	7.061	8.394	8.882	0.613	0.606
7.921	8.674	7.477	8.160	8.550	0.329	0.606
7.646	7.424	8.515	6.504	6.766	0.462	0.606
6.421	6.450	6.135	6.798	7.028	0.425	0.606
4.641	5.035	5.250	4.303	5.010	0.375	0.606
4.047	4.247	4.625	3.807	4.136	0.590	0.606
5.307	4.386	4.789	4.034	4.434	0.410	0.606
6.140	5.421	5.931	4.946	4.753	0.415	0.606
5.939	5.541	5.960	5.151	5.108	0.399	0.606
6.733	5.768	6.010	5.756	5.538	0.404	0.606
9.329	7.255	6.335	7.288	7.413	0.656	0.606
5.033	4.944	4.819	3.512	4.272	0.553	0.606
3.255	2.319	2.007	2.870	3.230	0.390	0.606
1.820	2.995	4.080	2.713	2.940	0.489	0.606
4.522	3.525	4.619	3.493	5.286	0.640	0.606
6.730	6.092	7.927	5.873	6.030	0.554	0.606
1.294	1.126	1.666	1.372	1.305	0.475	0.606
6.241	5.967	5.509	5.884	6.233	0.478	0.606
6.465	6.819	6.473	7.366	7.414	0.366	0.606
4.152	4.438	4.307	5.071	4.694	0.378	0.606
3.430	3.494	2.298	3.153	4.063	0.514	0.606
9.044	9.000	8.195	9.080	9.327	0.334	0.606
4.925	6.382	5.224	6.527	4.187	0.884	0.606
2.806	2.965	3.351	3.076	3.286	0.439	0.606
6.556	6.011	7.229	5.639	6.065	0.380	0.606
5.204	5.646	3.532	5.323	5.473	0.644	0.606
3.913	2.758	2.325	2.394	2.732	0.435	0.606
5.007	5.589	5.249	5.065	5.723	0.384	0.606
4.139	4.457	4.010	3.630	4.264	0.522	0.606
4.679	5.140	2.639	5.753	5.607	0.728	0.606
2.670	5.123	3.243	5.385	5.409	0.700	0.606
4.834	4.099	5.373	3.332	4.286	0.479	0.606
1.704	2.543	2.756	2.018	2.066	0.393	0.606
4.271	4.609	4.374	4.354	4.473	0.320	0.606
2.253	2.706	3.172	1.961	2.320	0.781	0.606
1.890	2.360	2.265	2.461	2.451	0.735	0.606
6.153	6.217	5.873	6.013	6.234	0.402	0.606
6.189	6.658	5.291	7.168	7.021	0.434	0.606
4.361	4.525	4.223	4.533	4.758	0.382	0.606
5.068	5.573	5.174	5.567	5.868	0.374	0.606
8.993	9.299	8.743	9.478	9.549	0.332	0.606
6.342	6.580	6.106	6.940	7.058	0.378	0.606
3.015	3.056	1.594	3.008	2.905	0.494	0.606

3.805	3.691	3.397	3.673	4.188	0.369	0.606
4.467	4.097	6.252	2.998	3.378	0.619	0.606
2.497	2.838	1.921	3.173	3.952	0.504	0.606
4.974	7.031	7.141	4.782	5.970	0.952	0.606
3.235	4.254	0.822	4.134	4.474	0.816	0.606
6.764	8.466	3.352	8.679	8.405	1.226	0.606
3.509	5.014	1.165	5.794	5.874	0.975	0.606
6.607	6.365	6.750	6.540	6.658	0.360	0.606
5.008	6.311	2.558	6.211	6.254	0.785	0.606
3.657	2.721	2.143	3.238	3.882	0.437	0.606
2.796	2.439	2.855	2.728	2.886	0.426	0.606
6.859	7.362	7.119	7.356	7.671	0.349	0.606
5.474	3.344	3.631	3.886	3.439	0.875	0.606
1.829	1.720	1.988	1.382	1.286	0.491	0.606
4.595	2.728	2.734	3.307	2.990	0.696	0.606
4.749	2.740	4.348	3.312	4.026	0.825	0.606
5.244	5.536	6.346	3.681	3.995	0.574	0.606
4.506	3.500	2.877	3.843	4.184	0.618	0.606
6.325	5.812	5.158	4.812	4.838	0.479	0.606
2.928	1.583	1.080	1.544	1.562	0.477	0.606
4.867	3.983	3.147	2.939	3.537	0.535	0.606
3.827	3.745	2.086	4.028	3.340	0.485	0.606
4.005	3.911	2.486	4.425	3.675	0.491	0.606
5.079	5.540	3.260	5.790	5.753	0.539	0.606
4.049	5.053	2.540	5.588	5.684	0.735	0.606
5.724	6.004	5.324	4.816	6.207	0.454	0.606
4.843	5.230	5.476	5.040	5.523	0.342	0.606
4.091	4.749	4.679	3.822	4.376	0.487	0.606
6.115	7.576	6.213	7.780	7.697	0.501	0.606
8.324	8.043	9.082	7.332	7.712	0.415	0.606
3.127	2.898	2.678	2.621	3.200	0.413	0.606
4.979	6.125	5.761	5.193	5.521	0.607	0.606
5.970	5.080	6.108	4.894	5.513	0.414	0.606
7.826	8.234	7.688	8.433	8.612	0.382	0.606
1.732	1.779	1.967	2.039	1.705	0.398	0.606
7.302	7.051	7.103	6.947	6.905	0.300	0.606
2.428	1.928	3.442	2.471	2.289	0.502	0.606
6.172	6.187	6.976	6.049	5.736	0.339	0.606
5.278	7.169	2.902	7.359	6.966	0.979	0.606
6.694	6.246	7.594	6.082	6.459	0.415	0.606
5.400	5.269	5.334	5.338	4.985	0.387	0.606
3.679	3.075	3.836	2.362	2.506	0.471	0.606
3.350	4.514	2.943	4.096	4.668	0.486	0.606
6.725	5.938	6.425	5.976	6.237	0.450	0.606
4.283	2.924	3.228	3.693	3.301	0.537	0.606
4.782	3.478	3.004	4.216	3.880	0.805	0.606
6.474	7.175	5.290	7.577	7.646	0.569	0.606
6.192	6.321	5.880	6.613	6.499	0.364	0.606
5.845	5.809	5.573	6.275	6.215	0.365	0.606
6.643	6.997	6.278	7.055	7.087	0.367	0.606

6.445	6.769	5.788	6.985	7.062	0.379	0.606
3.676	2.363	3.384	2.241	2.241	0.644	0.606
7.792	8.440	7.337	8.772	8.614	0.455	0.606
6.846	5.048	6.331	4.935	5.028	0.626	0.606
4.575	4.229	4.036	4.366	4.392	0.406	0.606
4.111	4.696	3.515	4.490	4.623	0.374	0.606
4.763	4.238	6.021	4.372	4.451	0.477	0.606
3.702	2.251	1.777	2.612	2.839	0.796	0.606
2.690	2.608	1.378	2.999	2.603	0.476	0.606
7.722	8.193	7.459	8.287	8.111	0.321	0.606
2.635	2.568	2.805	2.789	3.042	0.430	0.606
4.903	4.858	4.705	5.256	5.328	0.379	0.606
5.013	3.019	3.871	3.578	2.928	0.884	0.606
3.032	2.304	2.777	2.508	2.796	0.331	0.606
4.203	5.269	3.853	5.879	5.649	0.516	0.606
4.943	3.988	5.323	3.728	3.262	0.581	0.606
3.537	4.848	1.092	6.053	5.995	1.009	0.606
4.588	4.208	6.053	3.063	3.483	0.621	0.606
6.077	6.769	5.815	6.819	6.617	0.338	0.606
3.646	6.331	3.293	6.263	6.370	0.713	0.606
6.338	6.489	6.672	5.528	6.283	0.384	0.606
2.051	2.258	2.000	2.582	2.116	0.417	0.606
6.369	5.946	5.692	5.526	6.316	0.365	0.606
8.778	8.120	9.093	7.934	7.806	0.354	0.606
5.184	5.841	4.002	6.017	5.834	0.551	0.606
5.126	4.934	4.528	5.263	5.330	0.407	0.606
3.442	4.402	2.431	4.925	4.534	0.539	0.606
3.409	4.010	2.831	3.673	4.626	0.564	0.606
3.288	2.941	2.929	2.605	2.646	0.315	0.606
3.807	4.062	3.888	4.432	3.710	0.529	0.606
3.449	3.386	2.970	2.775	3.269	0.485	0.606
9.282	8.605	9.593	7.613	8.099	0.514	0.606
5.925	5.297	6.178	5.122	5.025	0.380	0.606
2.542	3.668	1.989	4.476	4.001	0.505	0.606
5.758	5.125	5.166	5.292	5.507	0.314	0.606
3.982	3.280	4.084	3.381	3.576	0.389	0.606
6.816	6.037	5.615	5.888	6.564	0.524	0.606
5.822	6.083	6.006	6.225	6.157	0.409	0.606
2.051	1.722	1.849	2.077	1.658	0.446	0.606
2.494	2.178	1.795	1.776	1.748	0.533	0.606
1.330	1.308	1.433	1.150	1.209	3.327	0.606
4.059	5.121	3.274	5.044	5.476	0.493	0.606
3.949	5.387	2.235	5.663	5.892	0.710	0.606
5.268	6.534	3.515	6.629	6.469	0.656	0.606
4.254	4.196	3.393	4.830	4.216	0.412	0.606
3.632	3.387	2.782	4.109	3.500	0.514	0.606
2.753	2.631	1.467	3.234	2.296	0.476	0.606
2.401	2.696	3.015	2.618	2.613	0.373	0.606
2.664	2.890	2.715	3.575	3.767	0.448	0.606
5.949	5.691	6.851	4.907	5.735	0.434	0.606

7.622	7.691	6.873	8.223	8.214	0.335	0.606
3.834	4.759	5.219	3.895	4.525	0.461	0.606
3.190	3.064	1.887	3.597	3.983	0.450	0.606
5.722	5.354	4.750	4.787	5.594	0.443	0.606
4.475	3.978	5.338	4.512	4.213	0.417	0.606
3.591	2.387	3.382	2.403	2.884	0.407	0.606
4.005	3.657	4.738	3.886	2.038	0.692	0.606
3.154	4.267	3.844	3.099	3.919	0.492	0.606
3.059	3.654	3.400	3.344	2.810	0.475	0.606
3.260	1.917	2.538	3.356	2.448	0.437	0.606
4.979	4.531	5.020	4.617	4.734	0.336	0.606
4.958	4.715	5.032	5.608	5.182	0.344	0.606
8.022	8.223	8.615	7.396	7.385	0.364	0.606
3.702	4.237	4.835	4.019	3.998	0.573	0.606
1.746	1.835	2.274	1.949	2.222	0.505	0.606
4.844	5.775	4.972	4.788	5.523	0.436	0.606
4.903	4.548	5.015	3.837	4.114	0.360	0.606
6.800	6.971	7.551	7.202	7.074	0.360	0.606
7.437	7.831	7.544	8.146	7.904	0.346	0.606
4.111	4.959	4.125	5.142	5.052	0.484	0.606
3.469	3.932	3.633	3.517	3.611	0.408	0.606
3.758	2.307	2.518	4.559	4.214	0.641	0.606
3.311	2.651	2.953	3.183	3.440	0.437	0.606
3.255	4.522	4.375	4.259	3.884	0.620	0.606
3.140	4.948	3.673	4.524	4.469	0.491	0.606
7.508	8.413	7.973	8.386	8.372	0.370	0.606
4.027	4.817	4.309	4.279	4.731	0.430	0.606
6.533	7.101	6.796	6.459	6.700	0.373	0.606
7.057	7.139	6.771	7.510	7.464	0.334	0.606
10.174	10.355	8.479	10.297	10.402	0.516	0.606
5.002	5.357	4.242	5.330	5.412	0.429	0.606
3.270	3.035	3.512	3.199	2.896	0.500	0.606
5.442	6.153	5.944	6.766	6.649	0.368	0.606
4.716	5.167	4.845	4.656	5.072	0.433	0.606
3.381	4.687	1.141	4.216	5.014	1.082	0.606
4.262	4.144	5.399	4.516	4.645	0.351	0.606
2.956	4.552	1.173	5.049	4.673	0.751	0.606
3.882	3.203	4.046	3.567	2.866	0.402	0.606
3.154	2.523	4.314	3.307	2.518	0.717	0.606
5.022	5.746	6.259	5.858	5.186	0.567	0.606
5.853	6.804	5.892	7.415	6.947	0.411	0.606
6.810	5.577	7.334	5.542	5.922	0.480	0.606
4.210	3.183	5.877	3.294	4.192	0.730	0.606
5.809	6.002	5.812	6.192	6.194	0.373	0.606
4.534	5.097	4.752	5.170	5.242	0.339	0.606
6.232	5.301	6.755	5.066	5.501	0.433	0.606
4.903	4.821	5.158	4.672	4.541	0.425	0.606
4.656	2.637	3.736	3.894	2.857	0.534	0.606
7.079	7.331	7.475	7.661	7.301	0.320	0.606
4.908	5.116	5.646	5.261	5.737	0.360	0.606

4.608	4.804	5.712	4.314	4.006	0.411	0.606
3.715	3.640	2.959	4.539	3.662	0.427	0.606
3.228	3.982	3.716	3.875	4.659	0.403	0.606
6.741	6.634	6.060	7.338	7.243	0.392	0.606
3.859	3.895	4.147	3.718	3.137	0.378	0.606
5.582	5.957	4.615	6.613	6.122	0.438	0.606
5.438	5.829	4.388	6.124	5.860	0.478	0.606
6.969	5.902	6.756	5.581	6.329	0.460	0.606
6.588	6.049	2.342	6.404	5.808	0.972	0.606
7.288	6.495	7.578	5.887	5.700	0.440	0.606
4.374	4.184	4.652	3.394	3.691	0.388	0.606
6.329	6.460	4.999	7.098	7.086	0.483	0.606
4.949	3.482	4.680	4.606	3.773	0.553	0.606
3.715	3.830	4.077	3.010	3.021	0.437	0.606
4.812	5.312	4.571	5.491	4.988	0.453	0.606
6.861	6.292	7.071	6.604	5.890	0.375	0.606
7.891	7.258	8.978	6.521	6.661	0.547	0.606
4.077	3.586	6.297	3.628	3.422	0.682	0.606
6.563	7.013	5.850	7.538	7.487	0.414	0.606
4.014	4.640	3.236	5.057	4.632	0.436	0.606
2.785	4.898	2.199	5.411	5.154	0.739	0.606
3.318	3.510	2.331	3.703	3.762	0.504	0.606
8.885	8.291	9.532	7.683	7.779	0.467	0.606
2.738	2.469	3.743	1.815	2.123	0.422	0.606
7.616	8.176	7.408	7.984	7.884	0.337	0.606
5.173	5.282	5.211	5.771	5.764	0.357	0.606
6.543	6.832	7.007	6.518	6.629	0.315	0.606
6.172	5.503	5.258	4.355	5.087	0.417	0.606
6.138	6.677	6.441	6.666	7.194	0.415	0.606
4.578	3.961	5.246	4.961	4.781	0.460	0.606
4.301	4.372	4.357	4.793	4.780	0.378	0.606
6.267	7.852	7.240	6.199	5.689	0.743	0.606
4.093	3.893	4.384	3.491	3.658	0.389	0.606
5.564	5.717	6.408	4.694	4.570	0.593	0.606
3.729	4.482	3.501	5.580	5.712	0.646	0.606
5.179	5.440	4.633	5.737	5.806	0.391	0.606
3.373	3.602	3.685	4.154	4.270	0.437	0.606
3.466	4.472	2.492	3.121	3.195	0.502	0.606
3.273	3.535	3.251	2.799	4.014	0.740	0.606
7.434	6.439	7.950	5.811	6.525	0.560	0.606
2.408	2.405	2.328	2.468	2.883	0.466	0.606
2.861	3.069	2.290	3.376	3.167	0.496	0.606
7.142	7.241	6.634	7.679	7.640	0.390	0.606
5.791	5.554	6.832	5.142	5.255	0.484	0.606
4.825	4.693	5.541	4.188	4.839	0.504	0.606
3.419	3.822	3.992	4.721	4.581	0.443	0.606
3.622	4.637	4.709	5.137	5.174	0.450	0.606
3.687	4.159	3.396	4.208	4.602	0.386	0.606
5.436	5.220	5.099	5.324	5.613	0.346	0.606
4.408	3.688	4.313	4.249	4.837	0.518	0.606

5.890	4.971	6.702	5.220	5.213	0.641	0.606
7.318	7.623	7.027	7.998	7.977	0.321	0.606
5.108	6.089	3.289	6.471	6.640	0.657	0.606
5.574	6.291	6.508	4.678	5.524	0.445	0.606
3.343	1.928	2.217	2.004	1.929	0.498	0.606
2.926	2.070	2.683	2.673	2.548	0.364	0.606
6.841	6.952	6.528	7.216	7.017	0.327	0.606
4.947	4.716	5.096	5.414	5.837	0.467	0.606
3.411	3.433	3.802	3.967	3.565	0.480	0.606
4.617	4.558	5.914	4.808	4.819	0.446	0.606
3.999	3.832	4.344	4.678	3.828	0.388	0.606
5.266	5.848	5.825	6.186	5.893	0.350	0.606
6.634	6.576	6.636	6.925	6.377	0.385	0.606
4.292	4.517	4.893	3.846	4.443	0.436	0.606
2.584	3.173	3.953	3.307	3.213	0.525	0.606
4.094	4.569	4.073	5.115	4.953	0.406	0.606
4.361	4.720	5.121	5.350	4.721	0.430	0.606
5.163	6.213	5.241	6.806	6.687	0.496	0.606
2.406	2.834	2.692	3.001	3.164	0.418	0.606
6.009	5.777	6.341	6.632	5.779	0.379	0.606
3.475	4.302	5.712	4.343	4.724	0.548	0.606
3.458	3.465	3.265	3.000	4.272	0.377	0.606
3.564	4.335	1.551	4.589	4.650	0.726	0.606
3.845	2.254	4.425	2.336	1.489	0.775	0.606
2.007	2.018	1.087	3.271	2.904	0.479	0.606
4.031	3.251	4.061	3.176	3.368	0.389	0.606
2.970	3.637	3.602	3.275	3.264	0.441	0.606
4.318	3.920	3.924	4.614	4.651	0.443	0.606
4.224	4.224	4.696	4.791	4.665	0.491	0.606
3.188	3.958	3.852	3.816	4.173	0.391	0.606
5.101	5.088	5.542	5.477	5.441	0.411	0.606
4.250	4.200	4.329	4.450	5.087	0.489	0.606
3.878	3.998	3.327	4.607	4.756	0.433	0.606
3.339	2.993	2.745	3.997	2.977	0.558	0.606
2.952	3.740	3.028	3.127	3.136	0.521	0.606
2.128	2.142	1.724	2.687	2.905	0.421	0.606
4.289	4.089	5.139	4.234	4.251	0.427	0.606
2.894	3.484	2.790	3.834	3.200	0.478	0.606
6.024	6.196	5.811	6.577	6.517	0.384	0.606
7.840	8.104	7.196	8.403	8.590	0.477	0.606
4.671	4.779	5.249	4.817	5.291	0.419	0.606
4.022	3.944	3.927	4.355	3.950	0.458	0.606
4.293	4.578	3.954	4.612	4.586	0.530	0.606
3.185	3.302	3.689	2.978	2.848	0.375	0.606
4.657	3.889	5.029	4.169	3.900	0.381	0.606
5.401	5.783	4.487	6.366	6.010	0.511	0.606
5.665	5.568	5.835	5.833	6.495	0.460	0.606
5.588	4.892	6.109	5.236	5.365	0.468	0.606
2.563	2.600	3.533	2.340	2.664	0.406	0.606
2.095	2.747	3.648	3.500	2.837	0.483	0.606

4.187	4.529	4.240	4.704	5.047	0.589	0.606
2.722	2.944	3.358	2.916	2.669	0.544	0.606
4.636	3.495	5.191	4.037	3.778	0.410	0.606
3.495	2.269	3.129	2.440	2.651	0.413	0.606
5.623	6.153	6.655	6.005	6.078	0.414	0.606
3.851	4.475	4.283	5.072	5.145	0.406	0.606
2.057	2.025	3.006	1.762	1.384	0.454	0.606
4.443	3.561	5.445	3.857	4.644	0.439	0.606
4.846	4.826	5.162	5.112	4.138	0.368	0.606
3.458	3.436	3.194	3.420	3.778	0.495	0.606
6.649	6.399	7.620	6.348	6.231	0.374	0.606
3.879	4.010	3.818	4.144	4.050	0.370	0.606
2.723	3.783	2.481	3.679	4.334	0.536	0.606
4.150	4.024	4.565	3.764	3.810	0.403	0.606
2.402	1.595	2.174	2.605	3.186	0.478	0.606
3.872	3.900	4.057	3.941	4.442	0.409	0.606
1.982	3.312	3.051	3.235	2.967	0.426	0.606
2.917	3.607	3.280	3.231	3.462	0.348	0.606
5.847	6.034	5.495	6.338	6.349	0.363	0.606
4.269	4.546	3.898	4.868	4.877	0.378	0.606
4.469	4.030	4.559	4.267	4.247	0.402	0.606
6.748	6.207	5.306	6.007	6.016	0.366	0.606
3.609	5.385	1.329	5.987	5.509	0.900	0.606
2.640	2.239	1.203	3.172	3.101	0.473	0.606
3.390	3.887	2.562	4.979	4.422	0.487	0.606
3.565	4.446	4.245	4.224	4.153	0.441	0.606
5.330	4.655	6.051	5.060	4.786	0.439	0.606
3.501	3.337	2.985	4.276	3.085	0.461	0.606
5.118	4.764	5.964	5.297	5.166	0.394	0.606
4.038	4.317	5.376	4.294	4.893	0.373	0.606
2.980	3.448	3.504	3.677	4.160	0.410	0.606
5.075	5.782	5.973	5.794	5.964	0.327	0.606
5.227	6.375	6.149	6.575	6.343	0.453	0.606
4.713	4.936	5.135	4.476	5.102	0.535	0.606
3.869	5.682	5.136	5.668	5.518	0.472	0.606
5.216	5.214	4.654	5.396	5.615	0.452	0.606
4.181	4.268	4.084	4.376	4.569	0.435	0.606
2.103	1.868	2.965	3.583	2.480	0.571	0.606
4.480	4.349	4.353	4.719	5.080	0.562	0.606
6.111	6.211	6.273	7.055	6.676	0.403	0.606
4.411	5.389	5.395	5.297	5.809	0.406	0.606
3.207	3.649	3.286	4.041	4.287	0.366	0.606
6.986	5.712	6.892	5.726	5.934	0.428	0.606
5.310	5.847	4.512	5.795	6.156	0.484	0.606
2.176	2.416	2.362	2.514	3.217	0.424	0.606
4.638	4.259	5.750	4.164	5.844	0.534	0.606
6.004	6.437	5.753	6.699	6.783	0.427	0.606
2.752	3.564	3.635	3.597	4.465	0.556	0.606
4.061	4.268	3.444	5.094	5.068	0.515	0.606
4.899	4.156	5.403	3.417	2.690	0.577	0.606

3.565	3.150	4.342	3.307	3.939	0.545	0.606
3.196	3.495	2.307	3.421	3.438	0.395	0.606
5.268	5.479	5.286	5.896	5.718	0.295	0.606
6.504	6.736	6.824	6.586	6.883	0.343	0.606
5.458	4.737	6.148	4.814	4.916	0.493	0.606
4.988	5.359	5.412	5.544	5.372	0.387	0.606
3.602	4.886	3.818	5.567	5.143	0.492	0.606
6.344	4.932	6.086	5.434	5.220	0.399	0.606
3.171	3.065	2.691	2.647	3.496	0.432	0.606
1.730	2.144	3.308	2.542	2.940	0.466	0.606
7.155	6.133	8.075	5.491	5.719	0.637	0.606
3.697	4.345	3.874	4.603	4.647	0.335	0.606
5.421	4.754	4.632	5.339	5.378	0.400	0.606
3.495	3.834	4.385	4.634	3.843	0.515	0.606
4.067	4.350	3.546	3.868	4.521	0.428	0.606
4.998	4.235	4.659	4.066	5.002	0.487	0.606
4.201	3.987	4.992	4.666	4.312	0.398	0.606
2.229	3.418	2.623	2.713	1.217	0.644	0.606
3.806	4.433	3.605	4.948	4.411	0.417	0.606
4.642	4.192	4.627	4.146	4.503	0.424	0.606
3.933	3.360	3.704	2.823	4.016	0.499	0.606
3.791	2.503	3.665	2.040	2.018	0.513	0.606
3.622	4.477	4.356	4.067	4.420	0.537	0.606
4.130	3.679	4.614	3.460	3.872	0.406	0.606
3.279	3.596	3.773	3.844	3.522	0.345	0.606
4.327	4.329	3.949	4.604	4.684	0.454	0.606
3.584	3.841	4.199	4.377	4.364	0.432	0.606
4.992	4.723	6.015	5.339	5.292	0.445	0.606
2.927	2.119	2.322	2.137	2.804	0.424	0.606
4.983	6.021	5.592	6.003	6.077	0.368	0.606
5.234	5.132	5.740	5.705	5.426	0.375	0.606
4.038	4.623	4.586	4.119	4.125	0.385	0.606
4.007	3.212	3.822	4.227	3.968	0.501	0.606
3.998	4.620	4.817	4.659	4.926	0.401	0.606
2.532	3.312	2.976	2.857	3.221	0.478	0.606
3.588	3.681	4.285	3.745	4.026	0.412	0.606
3.746	4.016	4.417	4.406	3.891	0.384	0.606
5.373	3.942	5.506	3.558	4.142	0.637	0.606
4.380	4.361	5.917	4.438	4.634	0.435	0.606
7.093	7.073	8.634	6.544	6.316	0.479	0.606
5.078	5.205	5.301	5.451	5.750	0.440	0.606
4.693	4.973	4.942	5.363	5.158	0.348	0.606
7.279	7.426	8.094	8.454	7.939	0.357	0.606
3.794	3.164	4.964	4.044	4.223	0.443	0.606
3.235	3.540	4.276	4.293	3.550	0.427	0.606
3.003	2.342	2.981	2.464	2.822	0.443	0.606
4.208	3.766	4.028	4.567	4.722	0.445	0.606
3.319	3.109	3.084	3.767	3.453	0.429	0.606
3.740	2.613	3.298	2.534	2.518	0.421	0.606
3.772	2.963	3.786	2.476	3.219	0.450	0.606

3.200	3.923	2.684	4.052	4.304	0.416	0.606
3.809	4.004	3.945	4.162	4.301	0.433	0.606
2.849	2.944	2.574	4.670	4.214	0.599	0.606
4.449	4.881	6.497	3.965	4.419	0.668	0.606
3.383	2.992	3.373	3.033	2.988	0.349	0.606
[truncated: 503,348 more chars]
